# A Sight of the Diagnostic Value of Aberrant Cell-Free DNA Methylation in Lung Cancer

**DOI:** 10.1155/2022/9619357

**Published:** 2022-01-27

**Authors:** Hui Zhao, Hui Zhang, Wei Xu, Baiqing Wen, Yani Kang

**Affiliations:** ^1^School of Biomedical Engineering, Bio-ID Center, Shanghai Jiao Tong University, Shanghai 200240, China; ^2^Department of Thoracic Surgery, Shanghai Chest Hospital, Shanghai Lung Cancer Center, Shanghai Jiao Tong University, Shanghai 200030, China

## Abstract

**Background:**

Lung cancer is one of the most commonly diagnosed cancer worldwide. As one of the liquid biopsy analytes, alternations in cell-free DNA (cfDNA) methylation could function as promising biomarkers for lung cancer detection.

**Methods:**

In this study, differential methylation analysis was performed to identify candidate markers, and lasso regression with 10-fold cross-validation (CV) was used to establish the diagnostic marker panel. The performance of the binary classifier was evaluated using the receiver operating characteristic (ROC) curve and the precision-recall (PR) curve.

**Results:**

We identified 4072 differentially methylated regions (DMRs) based on cfDNA methylation data, and then a 10-DMR marker panel was established. The panel achieved an area under the ROC curve (AUROC) of 0.922 and an area under the PR curve (AUPR) of 0.899 in a cfDNA cohort containing 29 lung cancer and 74 normal samples, showing outstanding performance. Besides, the cfDNA-derived markers also performed well in primary tissue datasets, which were more robust than the tissue-derived markers.

**Conclusion:**

Our study suggested that the 10-DMR marker panel attained high accuracy and robustness and may function as a novel and promising target for lung cancer detection.

## 1. Introduction

Lung cancer is one of the leading causes of cancer death in the world [1]. Tissue biopsy is by far the gold standard to establish a diagnosis of cancer. However, conventional sampling methods bear some limitations, including procedural complications, lacking sufficient high-quality material, and sampling biases [2]. Compared to tissue-based approaches, liquid biopsy, which analyzes circulating tumor markers from peripheral blood, is noninvasive and has less difficulty in obtaining samples [3]. Therefore, increased attention has been attached to liquid biopsy over the past two decades.

Cell-free DNA (cfDNA), one of the liquid biopsy analytes, refers to extracellular DNA fragments mainly released from cells through apoptosis and necrosis [2]. Besides, cfDNA can be actively secreted by forming a cfDNA-lipoprotein complex in a homeostatic manner, and the complex can function as an intercellular messenger [4]. Various tumor-related alternations of cfDNA have been reported in previous research. Raised cfDNA levels in plasma were found in patients with cancer compared to healthy individuals [5]. Somatic mutations of circulating tumor DNA (ctDNA) are tumor-specific and could provide diagnostic information [6]. Besides, microsatellite instability and epigenetic changes were also detected [7, 8]. The quantitative and genomic information of cfDNA allows for detecting cancer and monitoring disease burden.

DNA methylation, a crucial epigenetic modification, plays an imperative role in the biological process [9]. Aberrant DNA methylation is a critical factor of tumorigenesis and tumor progression [10]. Therefore, alternations in DNA methylation have been heralded as promising diagnostic markers of cancer. Methylation patterns across a genomic region tend to be the same [11]. Chan et al. employed this feature to explore the genome-wide hypomethylation for cancer diagnosis [12]. Consistent methylation patterns were found in cfDNA compared to the cells they originated, suggesting cfDNA is promising diagnostic biomarkers [13]. Apart from owning the common characteristics shared by liquid biopsy analytes, cfDNA methylation alternations have relatively high sensitivity and specificity and are feasible for early detection of cancer [14]. A significant correlation between aberrant cfDNA methylation and cancer has been reported in several studies, in settings such as breast, prostate, and testicular [15–17]. However, the research taking advantage of real-time PCR could only focus on several genomic loci and provided a limited picture of genome-wide methylation patterns. Other studies leveraging next-generation sequencing (NGS) technology and/or methylation array tended to begin with tissue DNA methylation data, and thus, the features of cfDNA data were ignored, and further evaluation in cfDNA samples was required for tissue-derived markers [18].

In this study, we explored the specific cfDNA methylation markers that distinguish patients with lung cancer from healthy individuals significantly. Four independent cohorts were employed in the analytical pipeline, including two cfDNA datasets and two tissue datasets. Differential methylation analysis was performed to screen out candidate markers in the discovery phase, and then lasso regression with 10-fold CV was performed to identify diagnostic markers in the model construction phase. Analyses showed that tissue-derived markers did not perform as well in cfDNA. However, cfDNA-derived markers achieved an AUROC greater than 0.90 in both cfDNA and tissue datasets, implying the potential benefits offered by identifying markers directly from cfDNA samples.

## 2. Materials and Methods

### 2.1. Data Acquisition and Preprocessing

Four independent datasets were used in differential methylation analysis and model construction phase, two for tissue samples and the others for cfDNA samples. Tissue 450 k data of 121 NSCLC and 12 normal samples were obtained from GSE56044 to create tissue set 1, and low-quality samples were removed using mean detection *p* value < 0.01 as a cutoff. Poor performing probes with detection *p* value < 0.01 and those associated with SNPs or located on sex chromosomes were also filtered out. The level-3 DNA methylation data of the LUNG cohort were downloaded from the UCSC Xena platform to create tissue set 2, with sex chromosome and cross-reactive probes removed for downstream analysis. The cohort was profiled by the Illumina HumanMethylation450 platform and included 832 lung cancer and 75 normal samples. CfDNA set 1 was retrieved from GSE122126, comprised of MethylationEPIC data of 4 NSCLC and 12 normal samples. Raw data from IDAT files were read in, and the processing procedures were as described above for tissue set 1. Then, data were normalized using the preprocessFunnorm function. CfDNA set 2 was comprised of processed reduced representation bisulfite sequencing (RRBS) data from Cell-Free Epigenome Atlas (CFEA) database [19]. The RRBS data of 29 lung cancer and 74 nontumor samples were originally derived from GSE79277 and preprocessed using CFEA benchmarking pipelines.

The preprocessing procedures were conducted using the minfi package in R 4.0.2. The hg19 human reference genome was used for annotation. Differentially methylated regions or positions were determined using cfDNA, and tissue sets 1 and diagnostic models were finally trained on cfDNA and tissue sets 2.

### 2.2. Identification of DNA Methylation Markers of Tissue Samples

Beta-value of tissue sets was converted to *M*-value for differential analysis of methylation levels and model construction. Differentially methylated positions (DMPs) between lung cancer and nontumor samples were determined using empirical Bayesian methods in the limma package, with ∣log2FC | >2 and adjusted *p* value < 0.05 considered statistically significant. To improve detection reliability, the exact workflow was applied to both datasets, and the overlaps between DMPs of two tissue sets with the same alternation patterns were considered as potential biomarkers.

Methylation data of potential markers in tissue set 2 was then partitioned into 10 disjoint groups randomly. Nine subsets were used as the training set, and the remaining subset was used as the validation set. Lasso regression was then first trained on the training set and assessed on the validation set using the glmnet package. The procedure was repeated 10 times, with each of the 10 subsets used as the validation set, to improve the data efficiency.

The performance of tissue-derived markers was also evaluated in cfDNA samples. Considering the low coverage of RRBS data in cfDNA set 2, A-clustering algorithm was implemented to detect regions of coregulated CpG sites and merge *M*-value located in such regions separately. Parameters dist.thresh and bp.thres.clust were set to 1 and 300, referring to similarity distance threshold and maximum length between neighbor CpG sites. The procedure rendered tissue 450 k data and cfDNA RRBS data comparable. Multiple imputation was then performed to fill in missing values in RRBS data, and the diagnostic model was also built using lasso regression.

### 2.3. Identification of DNA Methylation Markers of cfDNA Samples

Differential methylation analysis was performed on cfDNA set 1 using the DMRcate package. Two crucial parameters lambda and C were set to 1000 and 2, referring to Gaussian kernel bandwidth and scaling factor for bandwidth. DMRs were determined with the thresholds of HMFDR < 0.01 and treated as a source of candidate markers. Then, cfDNA set 2 was imported following the strategy below. For CpG sites located in each DMR location, the methylated and unmethylated intensity values in RRBS data were merged to measure the average methylation. DMR locations where methylation data of more than 60% samples were missing were removed for low quality. Wilcoxon signed-rank test was performed on cfDNA set 2 between lung cancer and normal samples to select potential diagnostic markers. The strategy solved the problem caused by the low coverage of RRBS data and rendered the selection more robust to some extent. Multiple imputation was implemented to fill in missing values using the mice package.

Lasso regression with 10-fold CV was implemented to build cfDNA diagnostic models using the glmnet package. Parameters family and type.measure were set to “binomial” and “auc” to fit the model. Cross-validation could prevent overfitting and improve the generalization capability of the classification model. To figure out the robustness of makers identified, the cfDNA-derived diagnostic markers were validated in tissue samples. The genomic positions of cfDNA-derived markers were mapped to 450 k data from tissue set 2 using findOverlaps function in GenomicRanges packages. Then, lasso regression with 10-fold cross-validation was performed to construct the diagnostic model.

### 2.4. Model Evaluation Using ROC and PR Curves

ROC curve was used to evaluate the performance of marker panels derived from two different sources by plotting sensitivity against specificity at various thresholds. AUROC could reflect the probability that the rank of positive instance was higher than that of negative instance predicted by the classifier. Considering the imbalance of datasets in the model construction phase, PR curve and AUPR, an evaluation tool summarizing the trade-off between precision and recall, were used as an alternative evaluation metric. A higher AUPR value indicates better binary classifier performance, which is similar to AUROC.

XL-minimal hypergeometric (XL-mHG) test was also implemented to assess the discrimination ability of each marker. The method treated lung cancer and healthy group as two clusters and binarized methylation data of markers in a marker-specific and cluster-specific manner. Then, XL-mHG *p* value was calculated to assess the statistical significance of enrichment and the performance of markers in isolating lung cancer samples.

### 2.5. Functional Annotation of cfDNA-Derived DMRs

Functional enrichment analysis, based on the Gene Ontology (GO) knowledgebase and Kyoto Encyclopedia of Genes and Genomes (KEGG) database, was performed on the genes whose predefined promoters overlapped with DMRs identified. GO classifies functions in the aspect of molecular function, cellular component, and biological process, and KEGG provides the information of molecular interaction and reaction networks. GO terms or KEGG pathways with BH-adjusted *p* values <0.1 were considered to be enriched significantly using the hypergeometric test.

### 2.6. Validation of cfDNA-Derived Markers

To validate cfDNA-derived markers, methylation levels of randomly selected genes (*TRAF1*, *RPTOR*, and *SPON2*) were determined using data analytics in cfDNA and pyrosequencing in tissue. Whole genome bisulfite sequencing (WGBS) data of cfDNA was retrieved from the European Nucleotide Archive (ENA), including 5 lung cancer and 45 normal samples (PRJNA418597, PRJNA494975). The WGBS data was preprocessed in the same workflow for RRBS data. Then, Kolmogorov-Smirnov test (K-S test) was performed to validate that methylation levels were different between lung cancer and normal samples. Genomic DNA isolated from 3 lung cancer vs. 3 control tissue samples was bisulfite-converted using EZ DNA Methylation-Gold™ Kit. And the procedure was approved by the Ethics Committees on Human Research of Shanghai Chest Hospital (Approve ID: KS(Y)1987). Then, PCR was performed with biotinylated primers designed by PyroMark Assay Design Software 2.0 (Table S4). 1% agarose gel electrophoresis was carried out to check the PCR products. Finally, pyrosequencing reactions were performed in a PyroMark Q96 system (Qiagen), and methylation levels were then quantified.

## 3. Results

### 3.1. Dataset Information and Genomic Coverage Analysis

**Figure 1:**
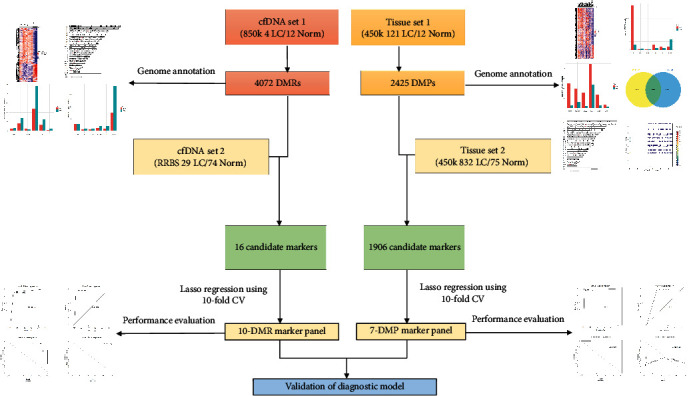
Graphic workflow of this study.

Four independent datasets were included in the study, among which cfDNA and tissue sets 1 were for differential methylation analysis and cfDNA and tissue sets 2 were for model construction. Detailed dataset information after data preprocessing was listed in Table 1, and the whole workflow was shown in Figure 1.

**Figure 2:**
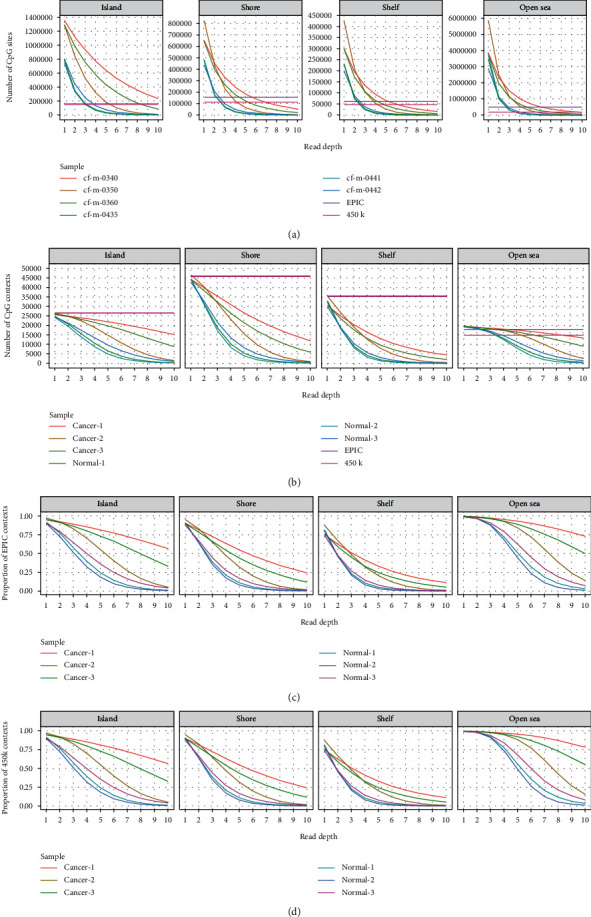
Genomic coverage analysis of RRBS libraries stratified by CpG resort context. (a) The number of CpG loci covered at different read depths. (b) Number of discrete CpG contexts covered at different read depths. (c) The proportion of the contexts covered by the Infinium EPIC array which were also detected in RRBS libraries. (d) The proportion of the contexts covered by the Infinium 450 k array which were also detected by RRBS libraries.

Considering that RRBS usually did not interrogate the same CpG loci as 450 k and EPIC array did, genomic coverage analysis was performed to test the concordance between the datasets using different detection techniques. 3 normal and 3 lung cancer samples in cfDNA set 2 were selected randomly to be examined in detail. All 6 RRBS libraries covered more CpG loci than EPIC and 450 k arrays did at each CpG resort context when read depth was greater than or equal to 2 (Figure 2(a)). From the perspective of the CpG resort context, RRBS libraries covered more discrete open sea regions at ≥2× but fewer other regions than the Infinium arrays did (Figure 2(b)). At least 70% of CpG islands and 95% of open seas identified by the EPIC array were also covered by all RRBS libraries at ≥2× (Figure 2(c)). However, the consistency of CpG shores and shelves detected by the two methods was relatively low. Similar results were observed in the comparison between RRBS and 450 k array stratified by CpG resort context as more than 90% of probes from 450 k array were included by EPIC array (Figure 2(d)).

### 3.2. Identification and Evaluation of Tissue-Derived Methylation Markers

**Figure 3:**
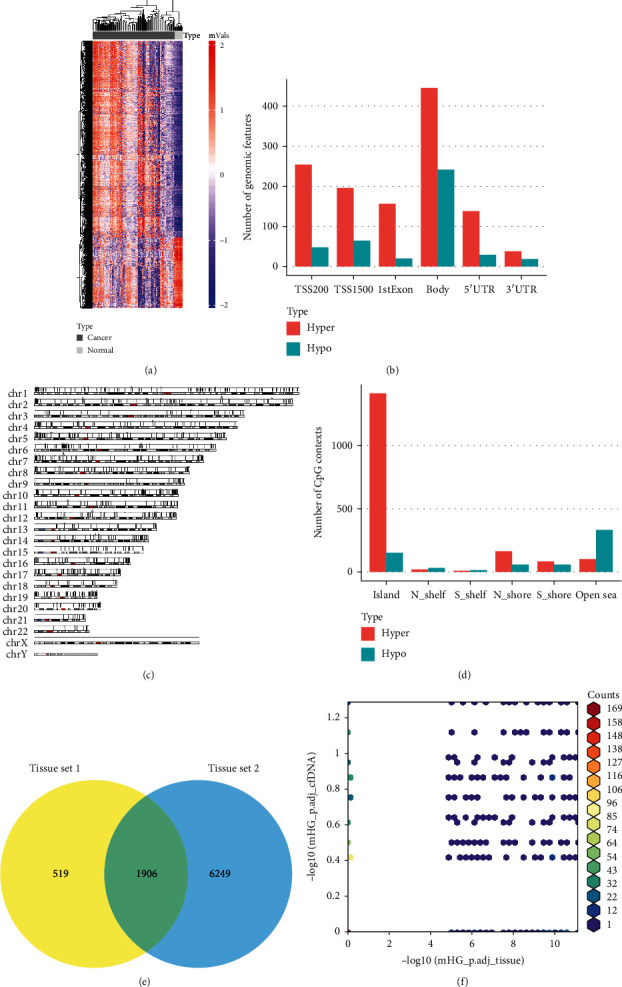
Differential methylation analysis of tissue samples. (a) The hierarchical clustering heat map of DMPs detected in tissue cohort. (b) Number of hypermethylated (red) and hypomethylated (blue) positions located in different genomic features. (c) Distribution of DMPs across the whole genome. (d) Number of hypermethylated (red) and hypomethylated (blue) positions located in different CpG resort contexts. (e) Overlap of DMPs detected in tissue set 1 and 2 separately. (f) Enrichment analysis of candidate markers in different sources using XL-mHG test.

**Figure 4:**
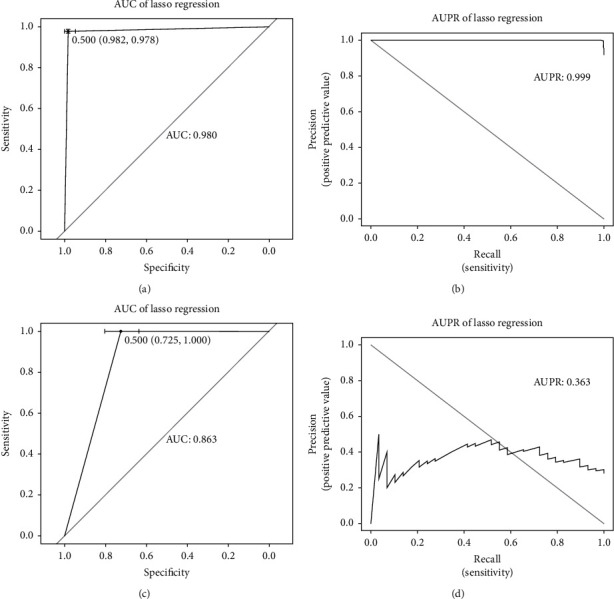
Evaluation of tissue-derived markers. (a) ROC curve and AUROC of marker panel in tissue dataset. (b) PR curve and AUPR of marker panel in tissue dataset. (c) ROC curve and AUROC of marker panel in cfDNA dataset. (d) PR curve and AUPR of marker panel in cfDNA dataset.

In the differential methylation analysis phase, 2425 DMPs were identified by empirical Bayes methods using the limma package, with a cut-off value of ∣log2FC | >2 and adjusted *p* value < 0.05. Supervised hierarchical clustering distinguished 121 lung cancer samples from 12 healthy samples significantly based on the *M*-value of DMPs (Figure 3(a)). More hypermethylated DMPs (red) were found in each genomic feature than hypomethylated DMPs (blue) (Figure 3(b)). Besides, the proportion of hypermethylated regions in promoters was relatively high. From the perspective of distribution in different chromosomes, the density of DMPs in chromosome 19 was the highest, and most DMPs were located in chromosome 1 (Figure 3(c)). Significant enrichment of hypermethylated CpG sites was found in CpG islands (Figure 3(d)). DNA methylation data of tissue set 2 were also processed using the same pipeline, and a total of 8155 CpG sites were identified as DMPs. Then, an overlap of 1906 DMPs between the two datasets was screened out to be candidate markers (Figure 3(e)). XL-mHG test revealed that the aberrant methylation status of the majority of candidate markers was significantly enriched in the cancer group (Figure 3(f)). However, the discrimination ability of the same CpG loci in cfDNA set 1 was relatively low, suggesting that tissue-derived markers were less robust in surrogate plasma cfDNA. Lasso regression was performed with 10-fold CV, and finally, 7 CpG sites were selected to fit a linear combination of weighted coefficients and *M*-value (Table 2). 6 of the 7 markers were found to be located in CpG islands and were significantly hypermethylated in lung cancer samples compared to the normal reference (Table S1). The coefficients of the 6 markers were positive, whose higher methylation levels suggest a higher probability of being diagnosed with lung cancer correspondently. The left marker, cg02443967, was located in the open sea and acted oppositely. The 7-DMP marker panel performed well in the tissue cohort, achieving an AUROC of 0.980 and an AUPR of 0.999 (Figures 4(a) and 4(b)). However, the false-negative rate of the classifier was significantly high in cfDNA set 2, which tended to divide samples into the healthy group (Figures 4(c) and 4(d)). Considering the low sensitivity of tissue-derived markers in the cfDNA cohort, markers based on methylation data derived directly from cfDNA samples were identified.

### 3.3. Identification and Evaluation of cfDNA-Derived Methylation Markers

**Figure 5:**
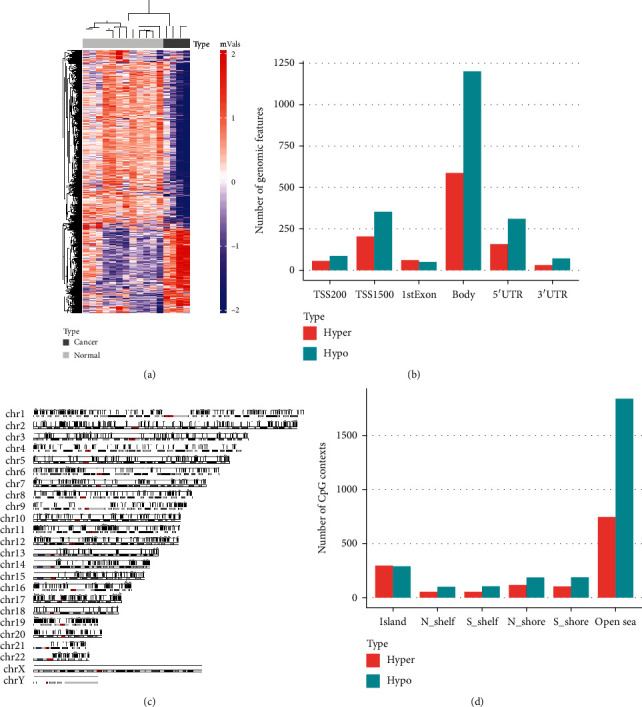
Differential methylation analysis of cfDNA samples. (a) The hierarchical clustering heat map of DMPs detected in tissue cohort. (b) Number of hypermethylated (red) and hypomethylated (blue) positions located in different genomic features. (c) Distribution of DMPs across the whole genome. (d) Number of hypermethylated (red) and hypomethylated (blue) positions located in different CpG resort contexts.

A total of 4072 DMRs were found in cfDNA set 1 using DMRcate package (HMFDR < 0.01), of which 2707 (66.48%) regions were hypomethylated and 1365 (33.5%) regions were hypermethylated. The methylation patterns of the 4072 DMRs were significantly different, according to which the normal and lung cancer samples were separated into two clusters (Figure 5(a)). Across the whole genomes, the ratios of hypomethylated (blue) and hypermethylated (red) regions in different genomic features were relatively consistent (Figure 5(b)), with the majority of DMRs located in the gene body. However, the absolute number of hypomethylated regions was larger than that of hypermethylated regions in each genomic feature except 1st Exon. The distribution of DMRs in discrete chromosomes in cfDNA data was similar to that in tissue data (Figure 5(c)). Furthermore, CpG annotations revealed that most DMRs, especially hypomethylated regions, were located in the open sea, referring to the genomic regions at least 4 kb away from CpG islands (Figure 5(d)). Meanwhile, a relatively high proportion of hypermethylated regions were found in CpG islands.

**Figure 6:**
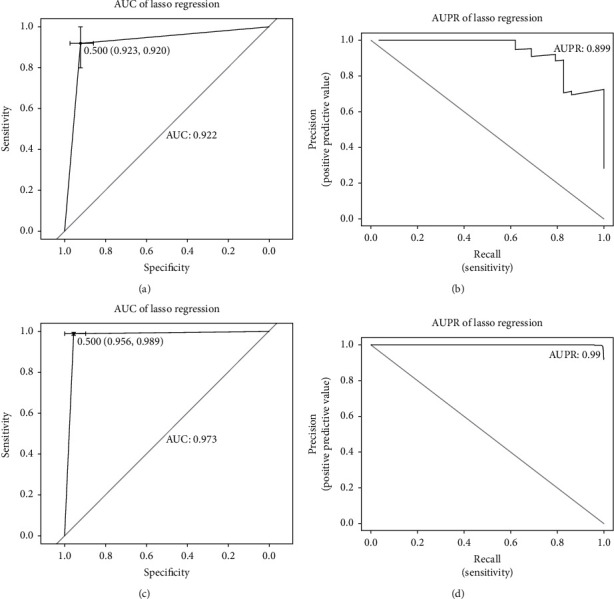
Evaluation of cfDNA-derived markers. (a) ROC curve and AUROC of marker panel in cfDNA dataset. (b) PR curve and AUPR of marker panel in cfDNA dataset. (c) ROC curve and AUROC of marker panel in tissue dataset. (d) PR curve and AUPR of marker panel in tissue dataset.

RRBS data of 29 lung cancer and 74 normal samples were extracted from GSE79277 and preprocessed using CFEA standard pipeline to identify diagnostic markers. We compared the genomic location between DNA methylation data from cfDNA sets 1 and 2. And the methylated and unmethylated RRBS reads were agglomerated if they were in the location of the same DMR identified in cfDNA set 1. Considering that *M*-value is more statistically valid, log2 ratios of methylated and unmethylated reads were calculated. Then, 16 candidate markers that distinguished between lung cancer and normal states were selected using the Wilcoxon test with *p* value < 0.001 and ∣log2FC | >1.2. Lasso regression was performed on the remaining candidate markers using the 10-fold CV technique. Finally, a 10-DMR diagnostic model was constructed in the form of a linear equation. The detailed information was listed in Table 3, and each regression coefficient represented the contribution of the corresponding DMR marker. Genome annotation revealed that 9 of 10 DMRs were mapped to known genes, including *LYPD8*, *VPS13D*, *AMPD3*, *RPTOR*, *TRPV2*, *VGLL4*, *SPON2, CXXC5*, and *TRAF1.* To assess the performance of the binary classifier, ROC and PR curves were created (Figures 6(a) and 6(b)). A 2000 stratified bootstrap analysis revealed that AUROC was 0.922, with a sensitivity of 0.920 (95% CI: 0.800-1.000) and a specificity of 0.923 (95% CI: 0.859-0.974). AUPR was calculated to be 0.899, indicating that the diagnostic model dealt well with skewed data. Methylation outliers of all the 10 markers showed significant enrichment in the lung cancer group by the XL-mHG test. In addition, 29 CpG sites in tissue set 2 that overlapped with the genomic locations of the 10 DMRs were retrieved, and then a 10-CpG marker panel was established. The panel showed an outstanding discrimination ability, with an AUROC of 0.973 and an AUPR of 0.990 (Figures 6(c) and 6(d)). In conclusion, the 10-DMR marker panel showed high accuracy and robustness both in cfDNA and tissue sets.

### 3.4. Overrepresentation Analysis of DMR-Related Genes

**Figure 7:**
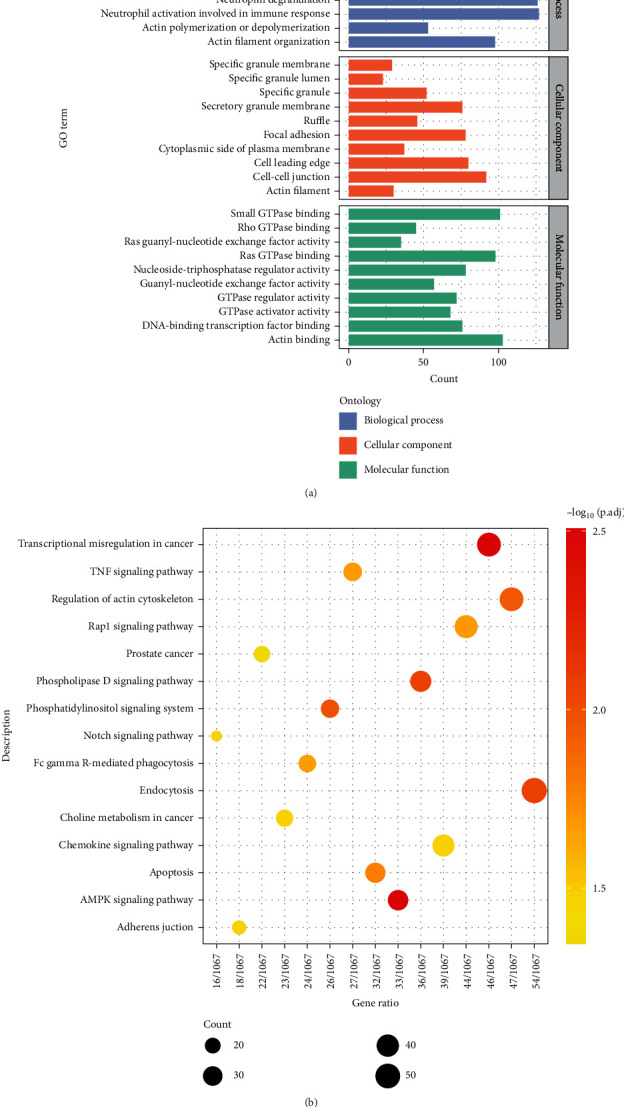
Overrepresentation analysis of cfDNA-derived DMGs. (a) GO analysis with genes whose promoters overlap with DMRs. (b) KEGG analysis with genes whose promoters overlap with DMRs.

To explore the biological functions associated with DNA methylation alternations, overrepresentation analysis was performed on the 3134 genes with their promoters overlapping with DMRs detected in cfDNA set 1. GO annotations revealed that differentially methylated genes (DMGs) are significantly enriched in biological processes related to energy metabolism and cell behavior, such as regulation of GTPase activity and cell-cell adhesion (Figure 7(a)). KEGG pathways overrepresented in DMGs also showed a high correlation with cellular polarization and actin reorganization, which played important roles in tumor migration and invasion (Figure 7(b)).

### 3.5. Validation of cfDNA-Derived Markers with Pyrosequencing

K-S test was performed on the processed WGBS data of *TRAF1*, *RPTOR*, and *SPON2* in cfDNA samples and normal controls (Table S6). Besides, pyrosequencing was also performed in 6 tissue samples, considering that methylations patterns of cfDNA are supposed to be highly correlated to tissues where they originate. Finally, lower methylation levels were found at all of the selected CpG sites in lung cancer samples compared to normal controls, showing a high consistency of methylation patterns at cfDNA-derived markers between cfDNA and tissue samples (Figure S3).

## 4. Discussion

It is well established that epigenetic abnormalities, including changes in DNA methylation patterns, contribute to the dysregulation of gene expression, which is related to the initiation and progression of tumorigenesis [20]. CfDNA methylation patterns are highly correlated to their originated cells. Besides, detection of cfDNA, one of the liquid analytes, is minimally invasive. Therefore, aberrant cfDNA methylation patterns are one of the most promising targets for cancer diagnosis [21]. However, previous studies tended to use methylation data from tissue as the source of candidate markers for cancer diagnosis, which may lose the unique features of cfDNA methylation patterns [11, 22, 23].

In this study, we identified 4072 DMRs (HMFDR < 0.01) and 3134 DMGs of patients with lung cancer compared to healthy individuals using the Infinium MethyaltionEPIC array data. The biological functions of DMGs were significantly enriched in physiological and pathological processes, including TNF signaling pathway, Notch signaling pathway, and Fc gamma R-mediated phagocytosis. Then, we merged the *M*-value of neighboring CpG sites in RRBS data as weighted averages based on the DMRs identified in cfDNA set 1 and regressed out the DMRs with poor discrimination ability using lasso regression with 10-fold cv. Finally, a 10-DMR marker panel was established, achieving an AUROC of 0.922 and AUPR of 0.899.

Inspired by single-cell sequencing data clustering, the XL-mHG test was introduced to evaluate the single marker considering the similarity between classifying patients and cell types. As a rank-based, nonparametric method, XL-mHG binarizes the *M*-value of DNA methylation data at a dynamic cutoff and introduces two parameters X and L to control the number of true positives and false positives [24]. Consequentially, the XL-mHG test is appropriate to reveal the robustness of our novel markers. As shown in Table 3, all of the 10 DMRs could function as independent diagnostic markers. DMR_7, which is located in chromosome 9: 123688715 – 123689193, owns the lowest *p* value. Based on hg19, DMR_7 was annotated as TNF Receptor Associated Factor 1 (*TRAF1*), a member of the TRAF family. *TRAF1* is crucial in activating the BRAF/MEK/ERK signaling pathway and nonsmall cell lung (NSCLC) carcinogenesis [25]. Research showed overexpression of *TRAF1* in human lung cancer cells, which is consistent with our analysis outcome. Knockdown of *TRAF1* could inhibit proliferation of NSCLC cells and induce cellular apoptosis, and thus, *TRAF1* was a promising diagnostic and therapeutic target for NSCLC.

We then performed lasso regression on the 36 CpG sites from tissue set 2 that was overlapping with DMRs of marker panel to assess the performance of cfDNA-derived markers in tissue samples. An AUROC of 0.973 and an AUPR of 0.990 were obtained, suggesting the robustness of 10 DMR markers. However, the cfDNA-derived panel may not be the optimal solution to the lasso regression based on the methylation levels of tissue samples, so it was different from the tissue-derived panel. Given that the majority of studies concerning the identification of cfDNA based markers start with primary tissue, we also evaluated tissue-derived marker panel in RRBS data. Although AUROC was calculated to be 0.863, AUPR was only 0.363 because of the highly imbalanced data. In fact, only one sample was predicted to be the patient in the dataset which included 29 lung cancer and 74 normal samples indeed. Therefore, our findings suggest that markers derived directly from cfDNA samples would be more robust than those derived from primary tissue.

Apart from epigenetic features, tumor-related elements have various forms in blood circulation [26]. Alterations in messenger RNA profiles were found in platelets in patients with cancer, which could function as biomarkers for detection [27]. Engineered immune cells were also leveraged for the early detection of cancer [28]. Combining these elements comprehensively may contribute to a diagnostic classifier with greater accuracy. Krug et al. found that a combination of ctDNA and exosomal RNA could increase the sensitivity of detecting *EGFR* mutation in plasma [29]. Protein markers incorporating with mutations in ctDNA were superior to any single element in the early diagnosis of pancreatic cancer [30]. Cancer detection may also benefit from an integrative analysis of methylation alternations of cfDNA and other features, such as cfDNA fragmentation landscapes, SNVs, and SCNAs, which need further investigations [31].

We also acknowledged several limitations in our study. First, the cfDNA set 2 is sparse, and the distribution of detected CpG sites has a low consistency across samples. Second, the sample size of the cfDNA dataset is relatively small, which requires future studies in a larger independent cfDNA database to validate our findings and improve the generalization ability of the cfDNA marker panel. Finally, RRBS and the Infinium array both have limited coverage of CpG sites considering that around 28.3 million CpG sites are present in the human genome, and thus, more accurate detection techniques with high coverage for DNA methylation are needed [32].

In summary, our study performed a genome-wide methylation analysis on cfDNA methylation data directly. A novel 10-DMR marker panel was constructed with high sensitivity and specificity both in cfDNA and primary tissue datasets, which could be heralded as promising diagnostic markers in lung cancer.

## Figures and Tables

**Table 1:** Summary of four datasets after preprocessing.

Datasets	Sample sizes	Detection techniques	CpG sites
cfDNA set 1 [13]	4 LC/12 norm	Infinium EPIC array	752843
cfDNA set 2 [33]	29 LC/74 norm	RRBS	/
Tissue set 1 [34]	121 LC/12 norm	Infinium 450 K array	431802
Tissue set 2	832 LC/75 norm	Infinium 450 K array	337278

LC: lung cancer group; Norm: normal group. Detailed clinical information of participants is provided in the corresponding datasets.

**Table 2:** Diagnostic marker panel derived from tissue samples.

DMP marker	Coefficient	Genomic location	Gene
cg16732616	0.07184664	chr1: 50886782	*DMRTA2*
cg06962177	0.05050583	chr1: 63785946	*/*
cg22167515	0.09985845	chr1: 91192466	*/*
cg03964958	0.01627892	chr2: 176964720	*HOXD12*
cg16768018	0.11208871	chr3: 147108843	*ZIC4*
cg26521404	0.29924966	chr7: 27204981	*HOXA9*
cg02443967	-0.19001655	chr10: 98129902	*TLL2*

DMP markers are made up of methylation values of CpG sites.

**Table 3:** Diagnostic marker panel derived from cfDNA samples.

DMR marker	Coefficient	Genomic location	Gene	XL-mHG *p* value
DMR_1	-0.007012253	chr1: 12493605-12494280	*VPS13D*	0.012529923
DMR_2	-0.293540674	chr1: 36983119-36983235	*/*	1.21307E-05
DMR_3	0.145449335	chr1: 248902767-248904167	*LYPD8*	0.0010886
DMR_4	-0.180719422	chr3: 11651536-11651990	*VGLL4*	0.001477124
DMR_5	0.3167126	chr4: 1195845-1196179	*SPON2*	0.00018493
DMR_6	0.194881649	chr5: 139043443-139044215	*CXXC5*	6.8938E-05
DMR_7	0.235620277	chr9: 123688715-123689193	*TRAF1*	2.2204E-16
DMR_8	0.29712874	chr11: 10476242-10477461	*AMPD3*	0.00183147
DMR_9	0.341871604	chr17: 16319861-16320257	*TRPV2*	6.4709E-04
DMR_10	0.236108117	chr17: 78753273-78754372	*RPTOR*	0.001784

DMR markers are made up of mean methylation values of CpG sites in the corresponding DMRs.

## Data Availability

The datasets analyzed in this study are available in the GEO database [http://www.ncbi.nlm.nih.gov/geo/] and the TCGA database [https://portal.gdc.cancer.gov/].

## Abbreviations

AUPR: Area under the PR curve
AUROC: Area under the ROC curve
cfDNA: Cell-free DNA
ctDNA: Circulating tumor DNA
CV: Cross-validation
DMG: Differentially methylated genes
DMP: Differentially methylated position
DMR: Differentially methylated region
GO: Gene Ontology
KEGG: Kyoto Encyclopedia of Genes and Genomes
PR: Precision-recall
ROC: Receiver operating characteristic
RRBS: Reduced representation bisulfite sequencing
*TRAF1*: TNF receptor-associated factor 1
XL-mHG: XL-minimal hypergeometric.

## Additional Points

*Code Availability*. Code will be available on reasonable request from the corresponding author.

## Supplementary Materials

Supplementary 1 Figure S1: methylation levels of the 10 cfDNA-derived markers.

Supplementary 2 Figure S2: assessment of the 10 cfDNA-derived markers using XL-mHG test.

Supplementary 3 Figure S3: validation of selected CpG sites.

Supplementary 4 Table S1: differently methylated positions detected in tissue samples.

Supplementary 5 Table S2: differently methylated regions detected in cfDNA samples.

Supplementary 6 Table S3: GO analysis of DMGs in cfDNA samples.

Supplementary 7 Table S4: KEGG analysis of DMGs in cfDNA samples.

Supplementary 8 Table S5: primer sequences for pyrosequencing.

Supplementary 9 Table S6: two-sample Kolmogorov-Smirnov test in cfDNA samples.
