# Supplementary figures and images for: A Sight of the Diagnostic Value of Aberrant Cell-Free DNA Methylation in Lung Cancer
Source: Dis Markers. 2022 Jan 27;2022:9619357. doi: 10.1155/2022/9619357 (PMC8814721; doi:10.1155/2022/9619357)

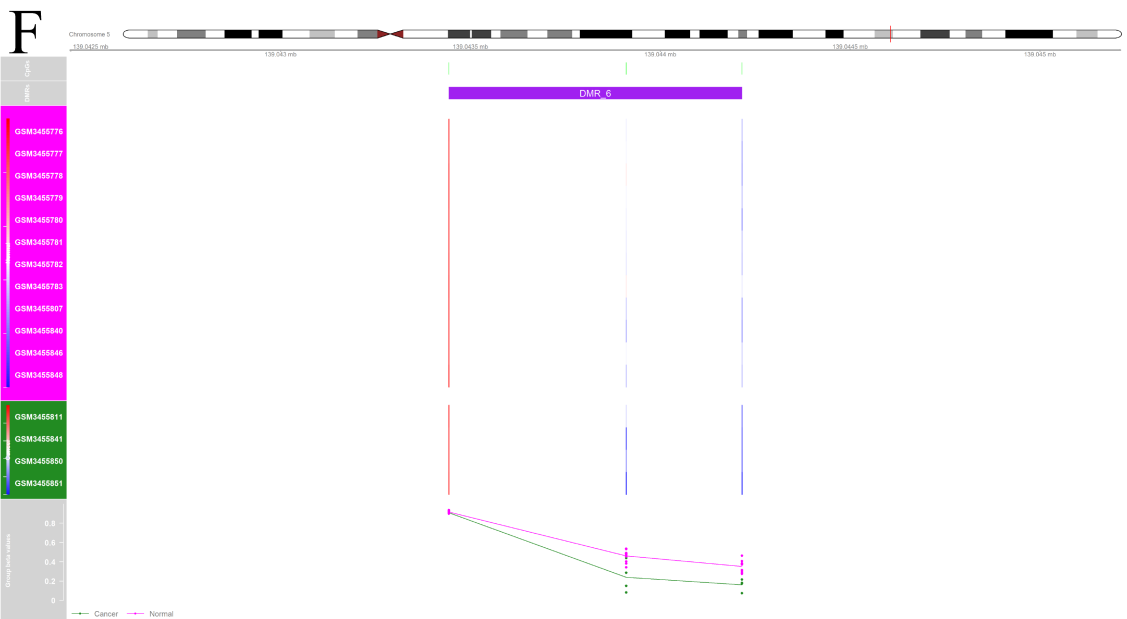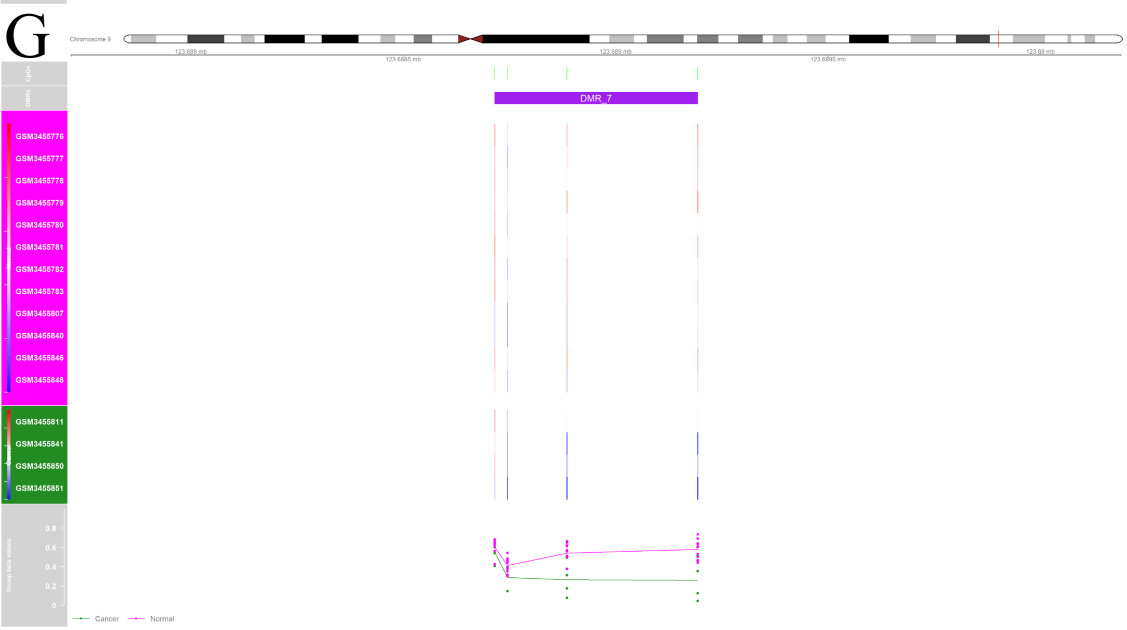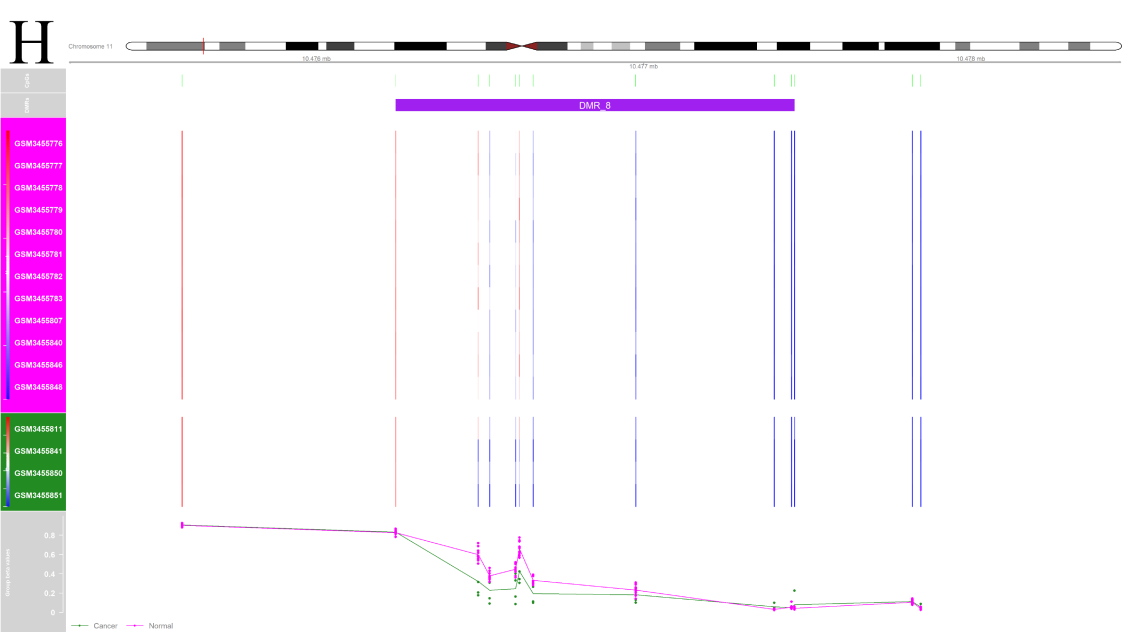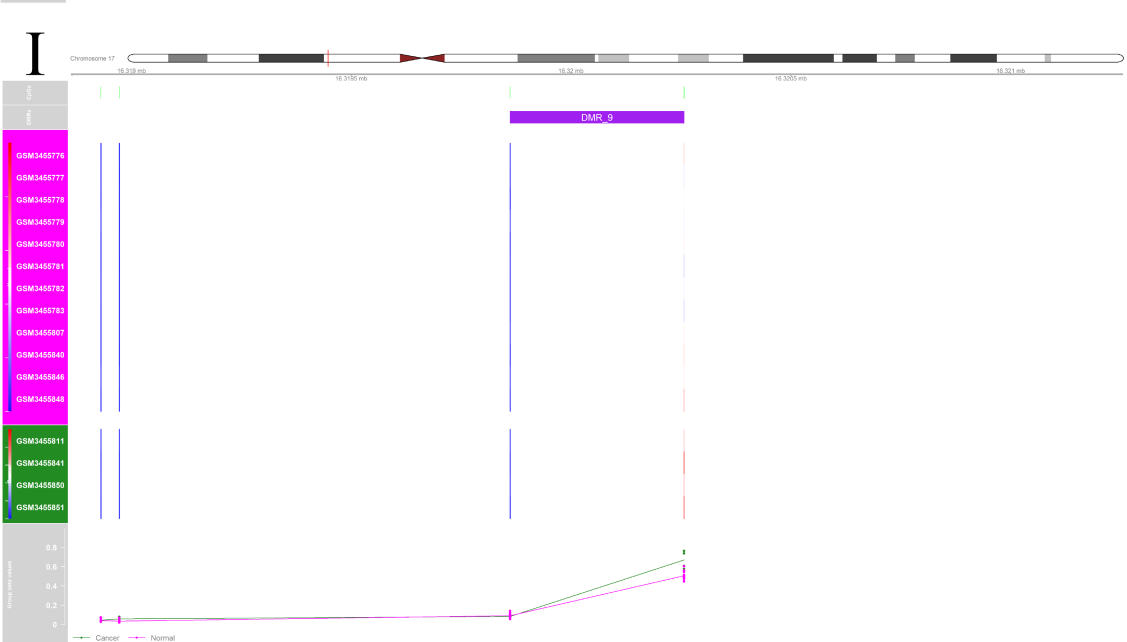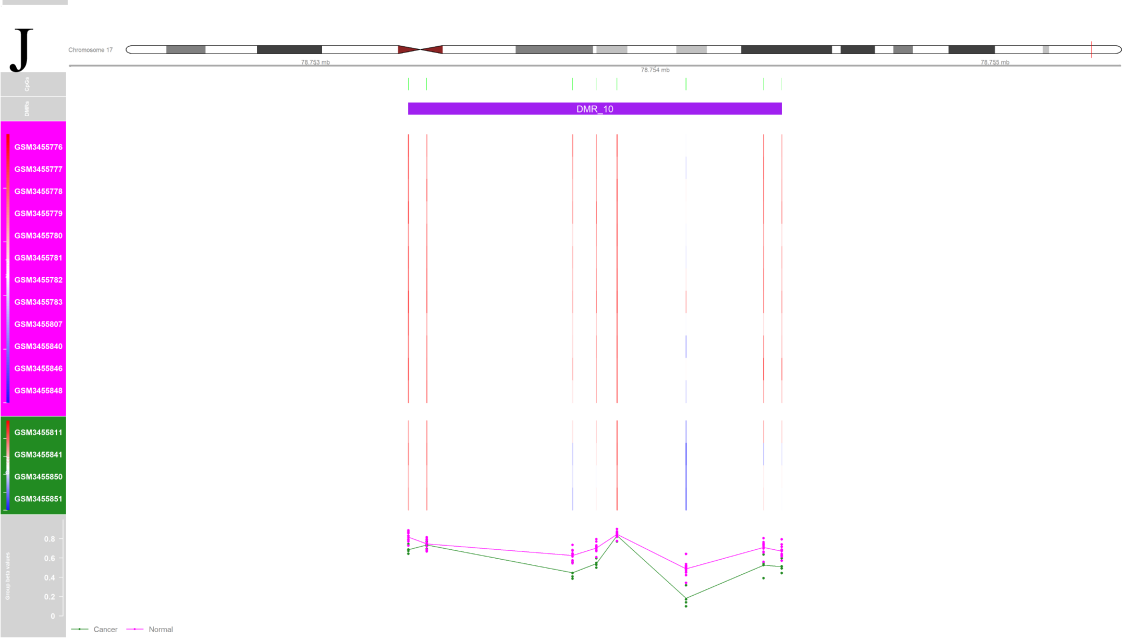

Supplement: Supplementary 1 — Figure S1: methylation levels of the 10 cfDNA-derived markers. [file 9619357.f1.pdf]

A

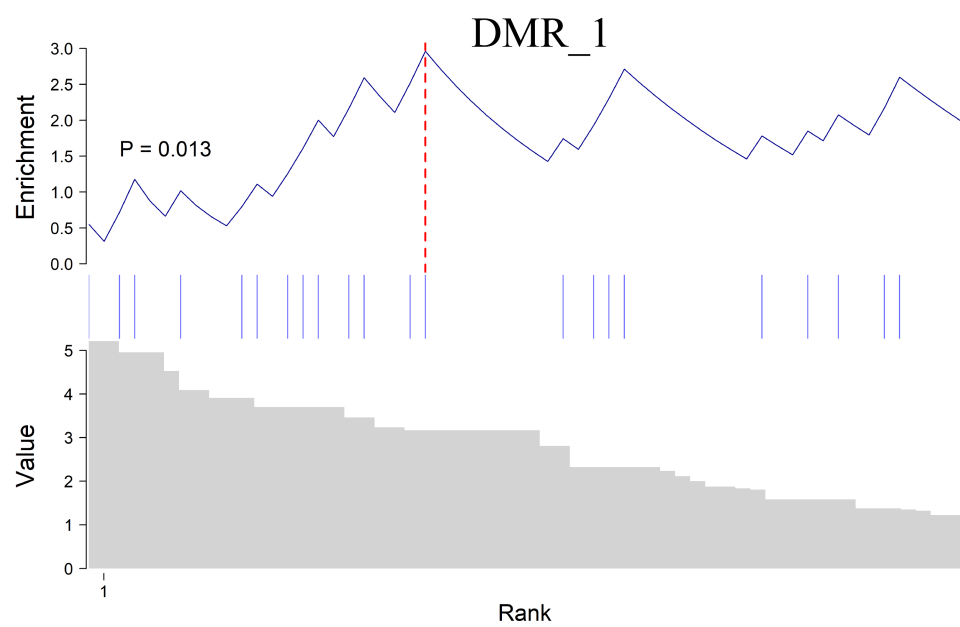

B

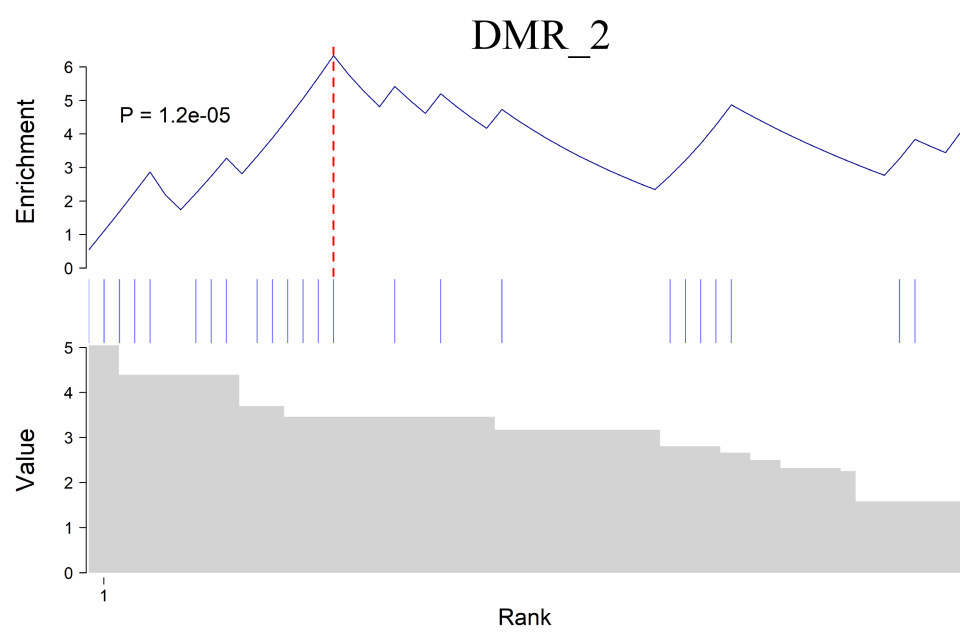

C

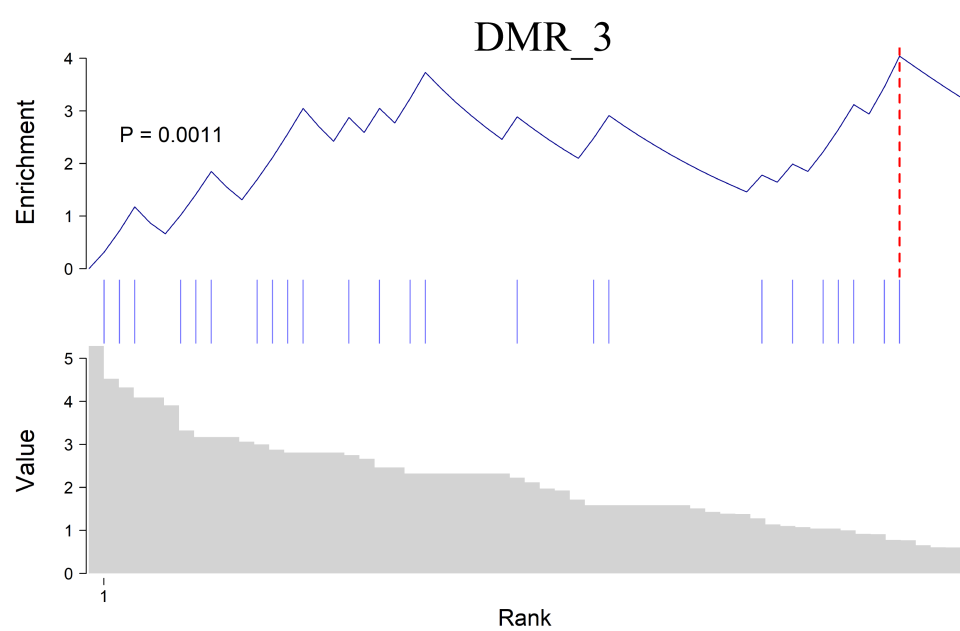

D

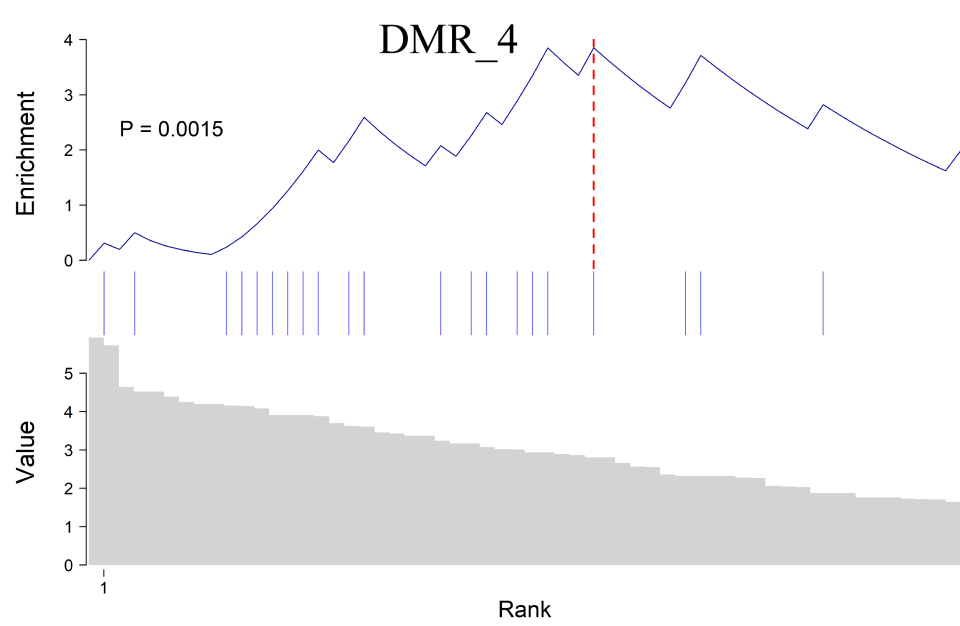

E

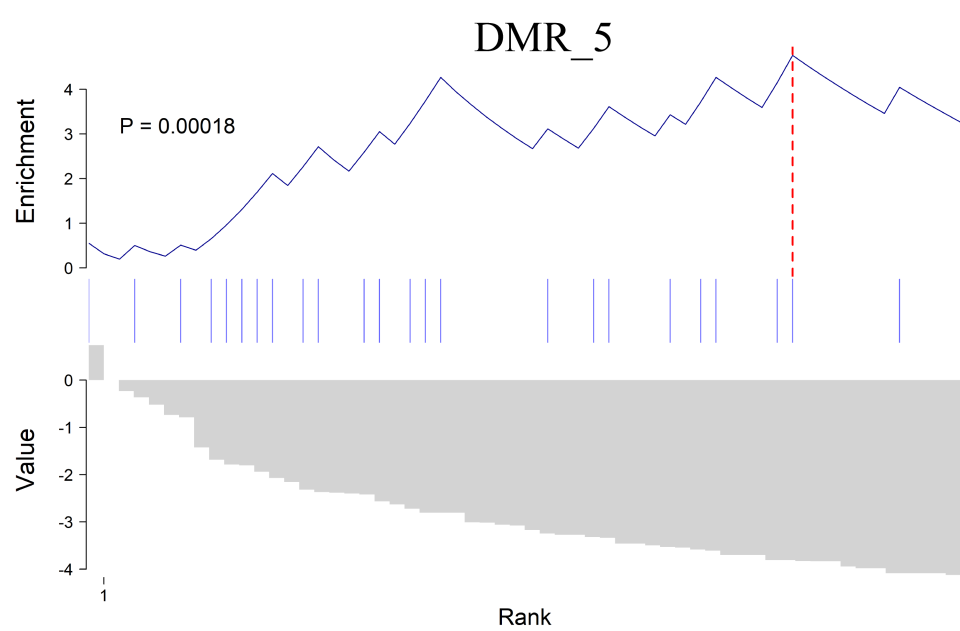

F

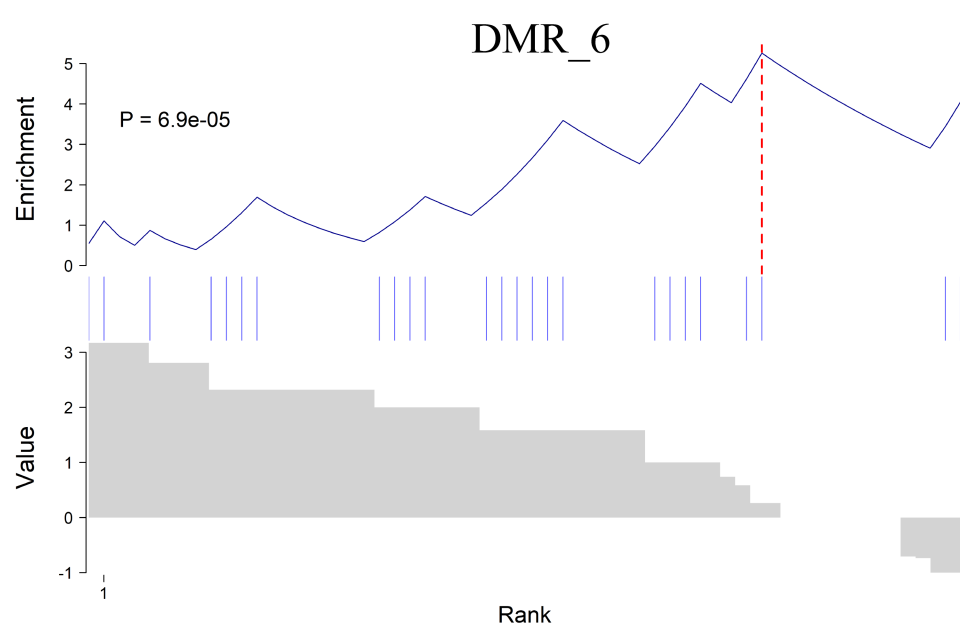

G

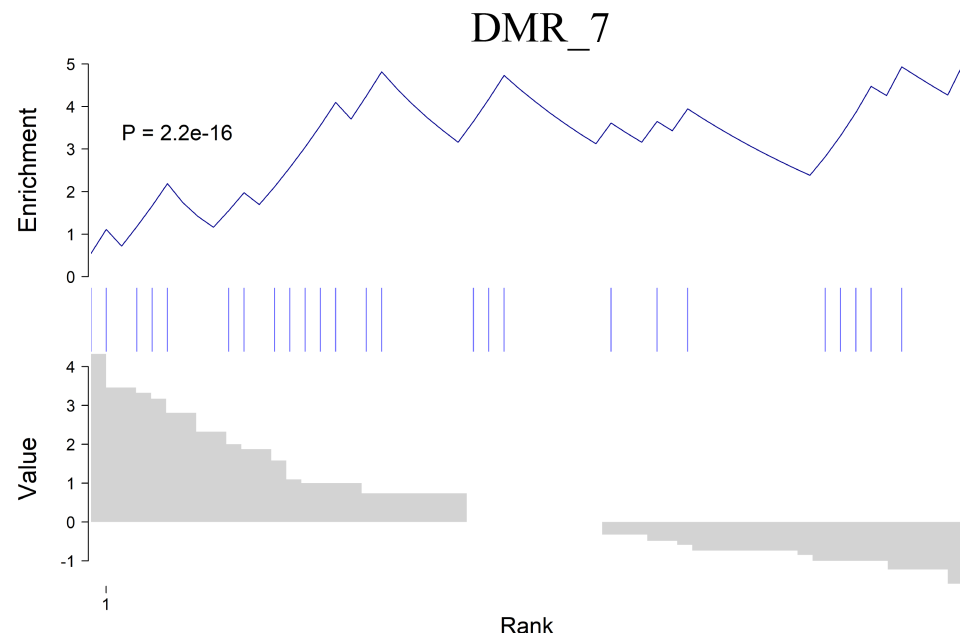

H

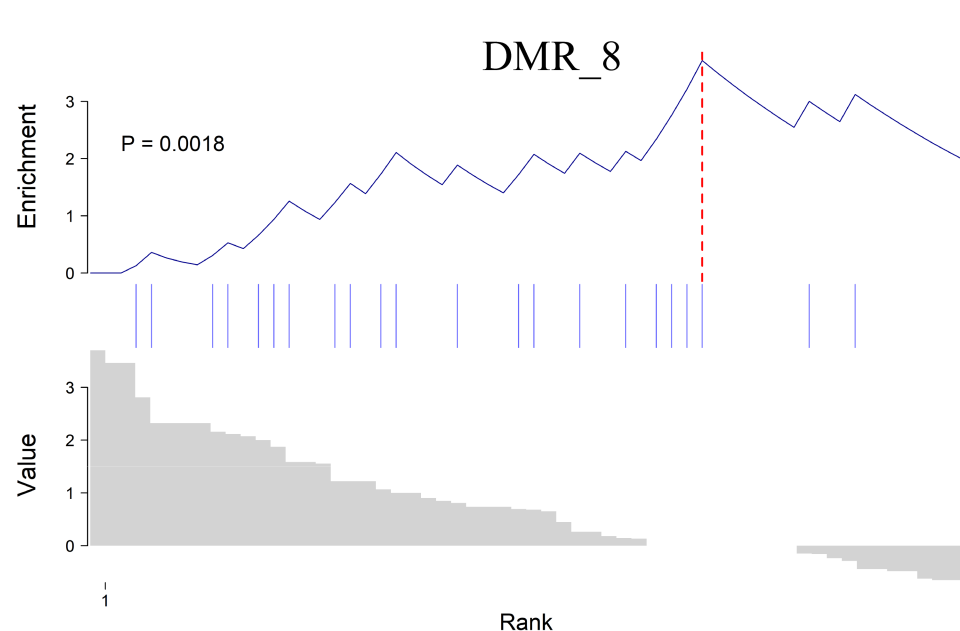

I

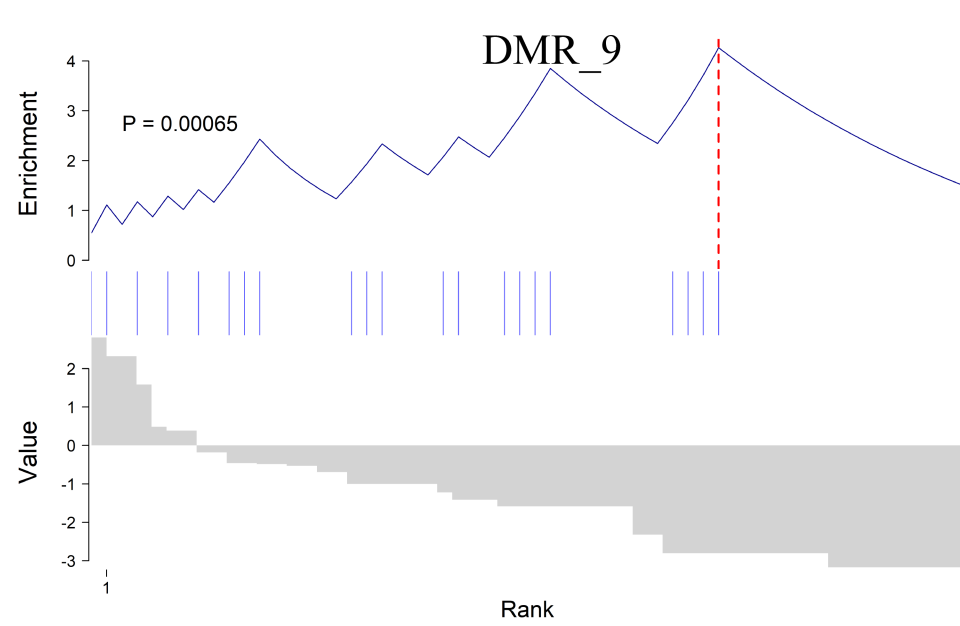

J

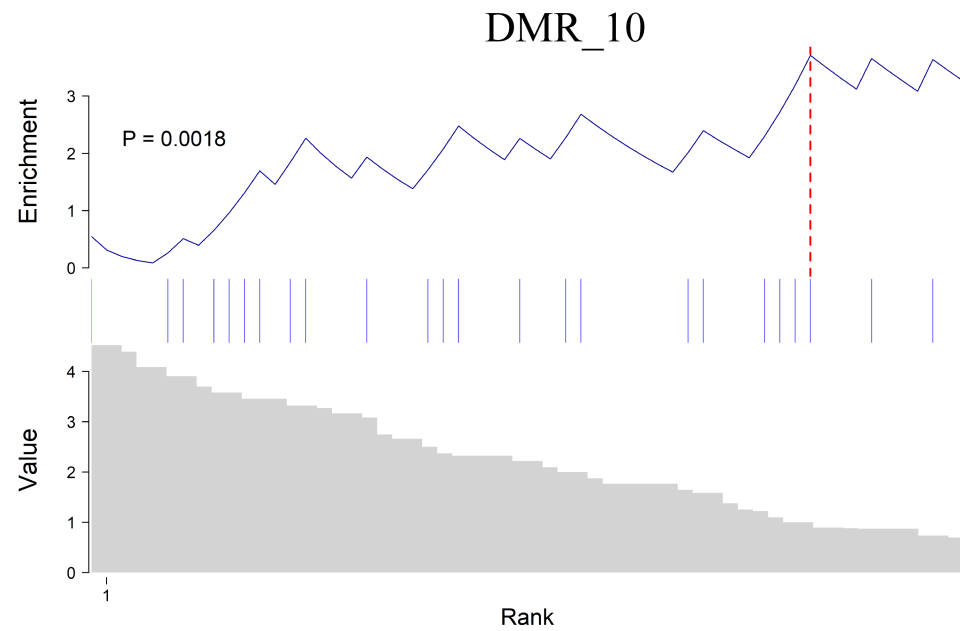

Supplement: Supplementary 2 — Figure S2: assessment of the 10 cfDNA-derived markers using XL-mHG test. [file 9619357.f2.pdf]

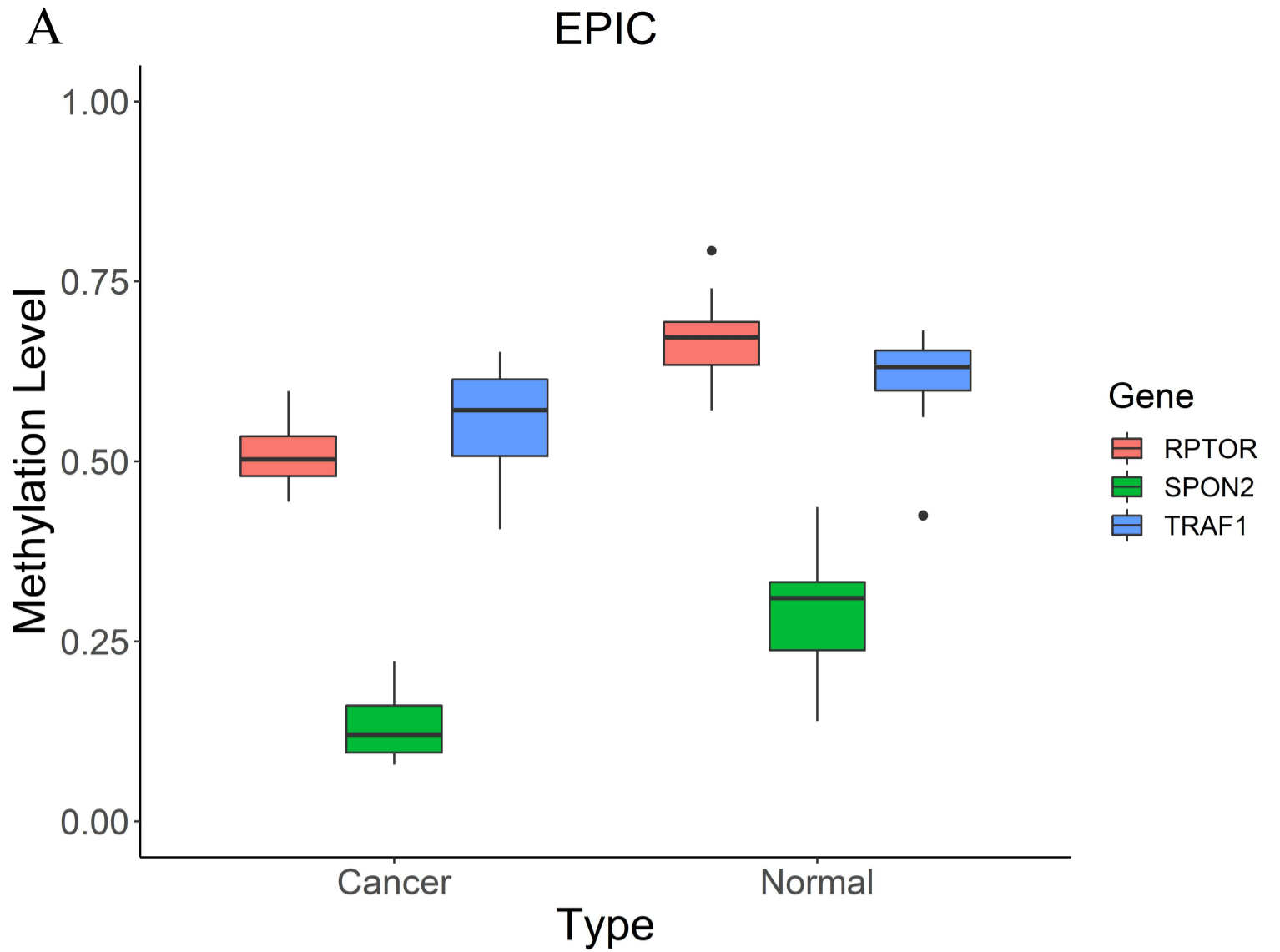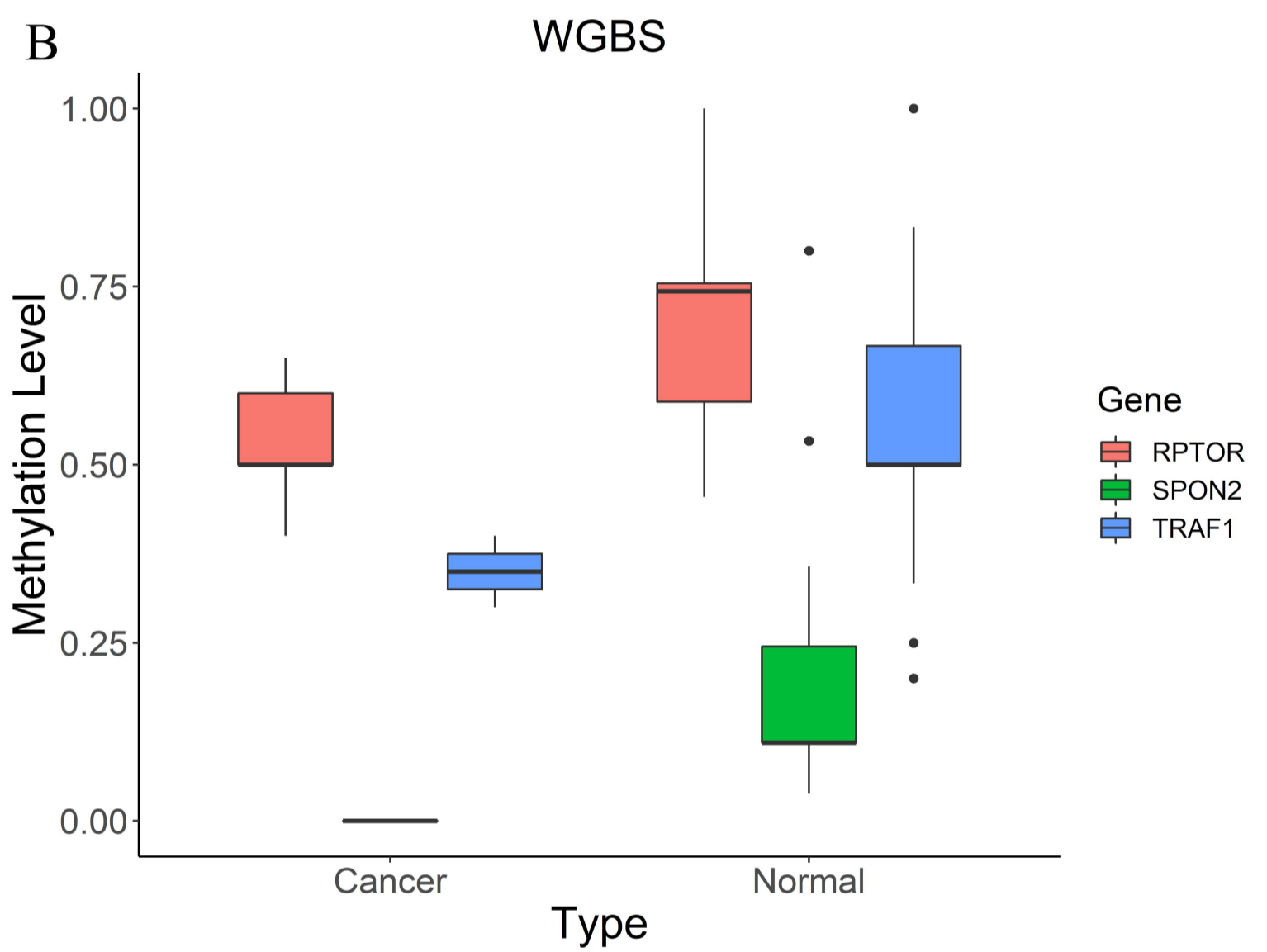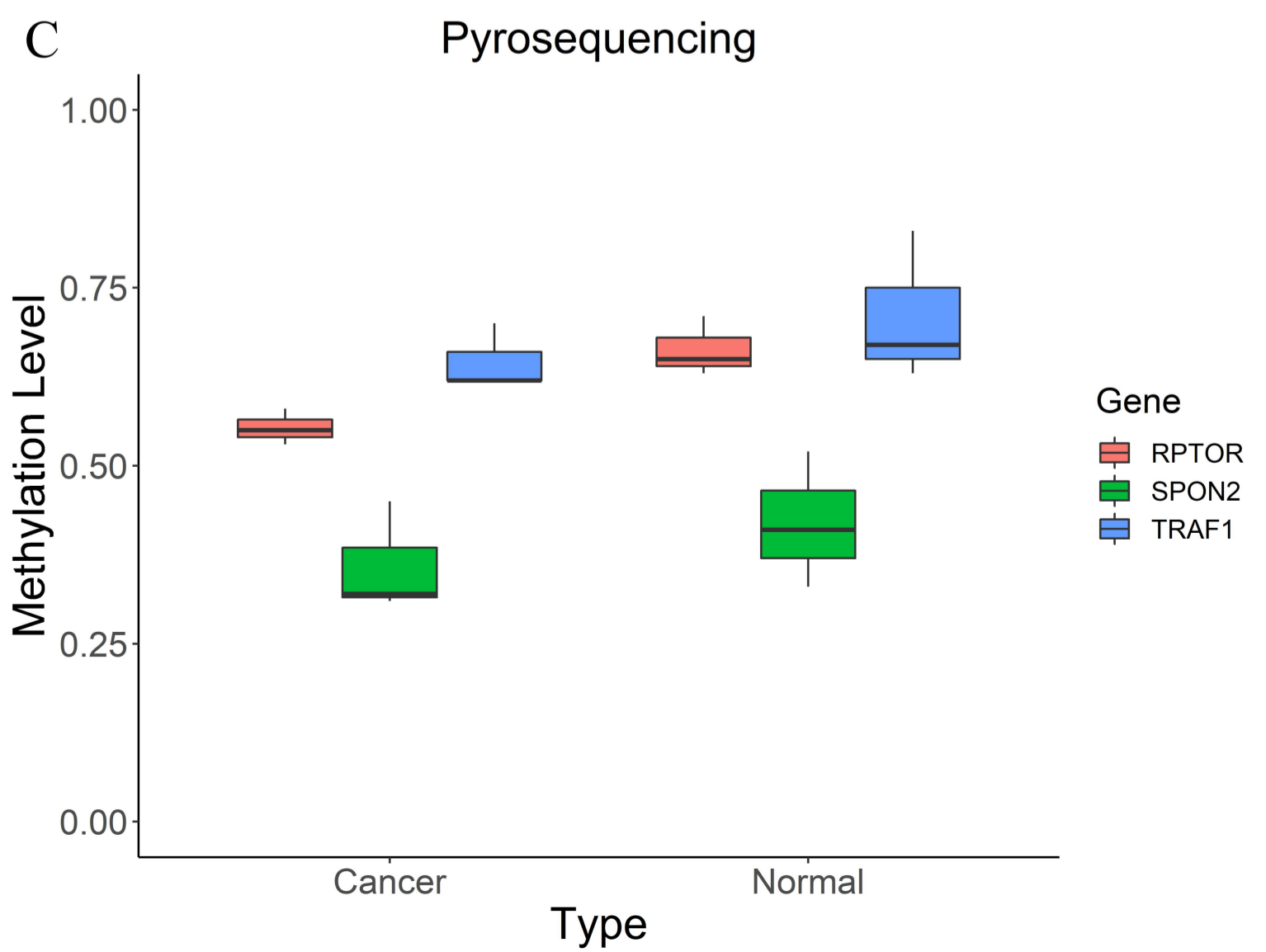

Supplement: Supplementary 3 — Figure S3: validation of selected CpG sites. [file 9619357.f3.pdf]
