# Supplementary material for: A Sight of the Diagnostic Value of Aberrant Cell-Free DNA Methylation in Lung Cancer
Source: Dis Markers. 2022 Jan 27;2022:9619357. doi: 10.1155/2022/9619357 (PMC8814721; doi:10.1155/2022/9619357)
Supplement: Supplementary 5 — Table S2: differently methylated regions detected in cfDNA samples. [file 9619357.f5.pdf]

Table S2. Differently methylated regions detected in cfDNA samples

seqnames	start	end	width	strand	no.cpgs	min_smoothed_fdr	Stouffer	HMFDR	Fisher	maxdiff	meandiff	overlapping.genes
chr8	57471162	57473034	1873	*	7	8.40313E-89	9.23481E-12	5.54156E-05	7.47857E-12	-0.391331342	-0.178883561	LINC00968
chr17	504991	505019	29	*	2	2.91964E-43	1.01497E-06	7.43082E-05	1.85744E-06	-0.232243338	-0.196953154	VPS53
chr18	9782387	9782637	251	*	2	2.45161E-37	2.99906E-05	7.52076E-05	2.32338E-05	-0.271725761	-0.190481921	RAB31
chr1	94454318	94454989	672	*	2	8.47897E-40	2.75162E-07	7.7036E-05	6.20377E-07	-0.326708972	-0.310175341	
chr4	83383646	83384006	361	*	2	6.45012E-32	0.003155768	8.23353E-05	0.000255977	-0.250398387	-0.142990446	
chr16	54169984	54170217	234	*	2	1.51675E-37	5.04986E-08	8.24423E-05	1.36785E-07	-0.342251445	-0.312857032	
chr16	4463520	4464427	908	*	2	5.92942E-36	0.001087205	8.67423E-05	0.000179234	0.21258547	0.095964797	CORO7-PAM16, CORO7
chr12	109538736	109539174	439	*	2	3.13612E-31	0.004303464	8.67469E-05	0.00029294	-0.440756	-0.232337826	UNG
chr1	235820710	235821511	802	*	2	4.81932E-32	0.014384457	8.67487E-05	0.000389906	-0.413198424	-0.211867023	
chr11	117280371	117280644	274	*	2	8.77541E-31	0.017749666	8.67488E-05	0.000404066	-0.402358353	-0.194543836	CEP164
chr11	122080867	122081755	889	*	2	3.39013E-30	0.000619107	8.71915E-05	0.000140308	-0.323398309	-0.182725774	RP11-820L6.1
chr1	38316406	38317368	963	*	2	1.89717E-29	0.006331677	8.8212E-05	0.000330229	-0.316193226	-0.164761978	MTF1
chr2	174025831	174025843	13	*	2	6.74789E-39	5.68876E-08	8.84365E-05	1.53811E-07	-0.433807194	-0.41848839	MLTK
chr8	28974423	28974428	6	*	2	1.44154E-33	9.13782E-08	0.000104661	2.42268E-07	-0.283814671	-0.283354015	CTD-2647L4.1, KIF13B
chr13	30434282	30434395	114	*	2	8.83767E-33	1.15334E-07	0.000107984	3.00858E-07	-0.422321225	-0.324964369	
chr12	113799660	113799663	4	*	2	6.16977E-33	9.90588E-08	0.000108915	2.62064E-07	-0.336335245	-0.294330622	PLBD2
chr6	75898357	75899060	704	*	2	7.72875E-29	0.001510008	0.000111457	0.000243551	-0.36447455	-0.192547998	COL12A1
chr3	66492298	66492992	695	*	2	4.83367E-30	0.001813104	0.000111458	0.000261927	-0.257396131	-0.134962959	LRIG1
chr20	31070190	31070849	660	*	2	2.161E-30	0.003508408	0.000111462	0.000332105	-0.250011334	-0.128785914	C20orf112
chr2	223709146	223709253	108	*	2	8.37516E-28	2.73953E-06	0.000111861	4.64659E-06	-0.274429318	-0.239650376	
chr6	90031402	90031462	61	*	2	8.13792E-24	0.000198669	0.000113046	9.11839E-05	-0.245690253	-0.170690332	
chr15	57317829	57317980	152	*	2	1.16876E-23	0.000902323	0.000113075	0.000197254	-0.361225285	-0.19844657	TCF12
chr8	37650291	37650963	673	*	2	4.04142E-27	0.02567203	0.00011309	0.000534636	-0.292334459	-0.150953084	GPR124
chr1	6142608	6142719	112	*	2	3.34284E-34	9.6925E-08	0.000113614	2.58113E-07	-0.407014115	-0.381117377	KCNAB2
chr8	38024119	38024747	629	*	3	1.3262E-44	3.23794E-06	0.000116612	1.73791E-06	-0.314520334	-0.165418273	LSM1
chr7	72856462	72856835	374	*	2	6.13983E-31	1.23942E-07	0.000126564	3.27406E-07	-0.414164772	-0.327603627	BAZ1B
chr9	107953895	107954218	324	*	2	3.36877E-31	1.16819E-07	0.000127286	3.10299E-07	-0.390716199	-0.383794865	

chr2	31454631	31455457	827	*	3	7.41631E-40	2.45086E-05	0.000128983	9.93095E-06	-0.307077976	-0.153079483	CAPN14
chr5	177756270	177757945	1676	*	3	2.66183E-34	0.014035516	0.000130751	0.000392059	-0.37109686	-0.152200546	COL23A1
chr9	486351	486354	4	*	2	1.04124E-27	5.17309E-07	0.000135445	1.19287E-06	-0.331904086	-0.286868535	KANK1, RP11-165F24.3
chr14	87179265	87179368	104	*	2	7.40412E-25	7.62667E-06	0.000138196	1.10841E-05	-0.383728689	-0.239798958	
chr2	106814630	106814741	112	*	2	1.53306E-26	1.59486E-06	0.000138868	3.15136E-06	-0.380480571	-0.277157543	
chr14	21089025	21089395	371	*	2	3.00864E-25	4.57808E-05	0.000138882	4.05778E-05	-0.270931049	-0.09563883	
chr1	198901839	198902062	224	*	2	1.01095E-28	1.93104E-07	0.000145682	4.99244E-07	-0.349324738	-0.317608782	MIR181A1HG
chr11	10457727	10458246	520	*	2	9.38715E-24	0.004088238	0.00014602	0.000426649	-0.401919611	-0.211161603	AMPD3
chr10	52157789	52158244	456	*	2	3.26474E-23	0.004847679	0.000146021	0.000450836	-0.337462962	-0.188831645	SGMS1
chr11	8385712	8385767	56	*	2	5.1828E-24	5.6421E-06	0.0001506	9.00432E-06	-0.296732385	-0.265147643	
chr11	32610465	32611002	538	*	2	2.87478E-23	0.018079986	0.00015207	0.000647845	-0.344043681	-0.178454647	EIF3M
chr11	112193244	112193518	275	*	2	3.93274E-21	0.045408201	0.000152561	0.000740377	-0.431413956	-0.214511156	RP11-356J5.12
chr2	102771739	102772623	885	*	4	2.93989E-44	0.001599886	0.000154038	1.01073E-05	-0.356838194	-0.155641089	IL1R1
chr13	37028408	37028767	360	*	2	3.26748E-21	0.211915551	0.00015726	0.000818794	-0.305817057	-0.151625119	
chr21	16868337	16869510	1174	*	5	4.68331E-53	2.62269E-06	0.000160515	2.30467E-07	-0.381334398	-0.113047362	AJ009632.3
chr16	1934273	1935058	786	*	4	1.39974E-37	0.000992466	0.000161253	0.000133522	-0.240560077	-0.068960077	MEIOB
chr5	93905343	93906294	952	*	4	5.99298E-42	7.32384E-09	0.00016296	1.72402E-08	0.444885696	0.244372194	RP11-461G12.2, KIAA0825
chr5	55442750	55444106	1357	*	5	1.04787E-54	6.43549E-08	0.000165931	1.00887E-08	-0.317064896	-0.168319446	ANKRD55
chr5	176329146	176329724	579	*	2	2.638E-26	3.16554E-07	0.000170282	7.97632E-07	-0.400481592	-0.317106798	
chr12	14537885	14538000	116	*	2	4.03616E-19	0.101592921	0.000171074	0.000866677	-0.112204199	-0.055273505	ATF7IP
chr2	70681570	70682536	967	*	3	1.69895E-35	7.68645E-08	0.000175026	1.43574E-07	-0.403805281	-0.234908488	TGFA
chr5	34001680	34001718	39	*	2	3.05681E-22	1.09243E-05	0.000175591	1.57963E-05	-0.427720866	-0.323879142	AMACR, RP11-1084J3.4
chr8	134094692	134095284	593	*	2	5.68937E-23	0.000221469	0.000176733	0.000123579	-0.265784462	-0.105665234	TG, SLA
chr11	119486443	119486923	481	*	2	1.22286E-22	0.00301843	0.000176838	0.000440343	-0.312475239	-0.167839099	RP11-196E1.3
chr3	152215049	152215571	523	*	2	9.37843E-24	0.007369812	0.000176848	0.000591334	-0.410793017	-0.220488342	
chr11	35366721	35367036	316	*	2	1.085E-19	0.017860411	0.000176853	0.000731874	-0.272453358	-0.144037724	SLC1A2
chr4	22509445	22510091	647	*	2	3.03976E-22	0.019533321	0.000176853	0.000744506	-0.267135985	-0.123982362	GPR125
chr10	14596405	14597004	600	*	2	6.40421E-26	0.069277028	0.000176856	0.000873331	-0.200889064	-0.102956238	FAM107B
chr14	23348291	23348949	659	*	2	3.0941E-24	0.081900169	0.000176857	0.000882841	-0.242785952	-0.120508079	LRP10

chr4	3228567	3228890	324	*	2	1.50756E-20	0.031325301	0.000179522	0.000814383	-0.321148114	-0.157477297	HTT
chr4	40211657	40212592	936	*	2	4.89927E-22	6.95799E-07	0.000179561	1.62475E-06	-0.372951424	-0.298550646	RHOH
chr12	109234959	109235071	113	*	3	3.39148E-34	4.39595E-08	0.000181033	1.02236E-07	-0.377111228	-0.239789341	SSH1
chr18	74113813	74114728	916	*	4	7.43561E-47	5.53218E-07	0.000182451	9.23926E-09	-0.396755229	-0.260231017	ZNF516
chr16	85815505	85815851	347	*	3	6.8023E-40	3.75624E-10	0.000182851	2.43385E-09	-0.380396562	-0.304213004	EMC8, RNU1-103P
chr19	4991580	4991633	54	*	2	2.1054E-27	2.62114E-07	0.000185751	6.79383E-07	-0.411203547	-0.383610722	KDM4B
chr19	40650939	40651007	69	*	2	2.54107E-19	0.000218075	0.000186721	0.000126045	0.262754266	0.17598811	
chr21	39649500	39649958	459	*	3	2.09953E-35	2.66998E-09	0.000187784	1.4359E-08	-0.31713365	-0.258652553	KCNJ15
chr3	72987896	72988159	264	*	2	4.56246E-19	0.002819375	0.000187865	0.00044779	-0.271198192	-0.146360325	GXYLT2
chr11	124405865	124406358	494	*	2	8.87238E-21	0.004082993	0.00018787	0.000512883	-0.363819847	-0.194060111	
chr9	91919480	91919723	244	*	2	1.26296E-18	0.00732296	0.000187877	0.000618234	-0.290490711	-0.137535384	
chr5	7803175	7803274	100	*	2	7.05451E-26	3.31486E-07	0.000189791	8.43516E-07	-0.324757028	-0.272284946	ADCY2
chr17	14219881	14219963	83	*	2	2.85056E-25	4.6391E-07	0.000191035	1.14354E-06	-0.285038038	-0.24379659	HS3ST3B1
chr4	146685656	146686236	581	*	2	1.06892E-22	7.25633E-05	0.000191406	6.3697E-05	-0.282783048	-0.104389624	C4orf51, ZNF827
chr14	31560390	31560529	140	*	2	1.0221E-22	2.9448E-06	0.00019292	5.68377E-06	-0.230300475	-0.175087684	AP4S1
chr18	21596915	21597505	591	*	3	4.48916E-24	0.001391918	0.000197802	0.000361894	-0.199749631	-0.046107115	TTC39C
chr15	93790914	93791068	155	*	2	2.27546E-25	4.2168E-07	0.000198014	1.05464E-06	-0.354147553	-0.340020287	
chr6	151701556	151701920	365	*	2	9.12738E-20	0.000828766	0.000198116	0.000270178	0.275030418	0.120173008	ZBTB2
chr9	133473444	133473888	445	*	2	1.7907E-21	0.007159872	0.000198168	0.000639637	-0.314386425	-0.163765513	FUBP3
chr6	91113506	91113687	182	*	2	3.21501E-24	1.07896E-06	0.000199801	2.43225E-06	-0.376559989	-0.267696616	
chr10	80697460	80698358	899	*	2	9.61834E-21	2.73301E-05	0.000203557	3.30303E-05	-0.149275514	-0.029868829	
chr14	21359737	21359943	207	*	2	8.51919E-25	5.51024E-07	0.000205775	1.34878E-06	-0.322760614	-0.273267103	RNASE3
chr12	114298934	114299076	143	*	2	8.24052E-26	3.08509E-07	0.000206163	7.98232E-07	-0.385479862	-0.379126298	RBM19
chr17	3738423	3738968	546	*	2	7.85924E-24	3.06569E-07	0.000207836	7.94407E-07	-0.336059761	-0.330517594	C17orf85
chr4	57594308	57594984	677	*	3	1.01411E-26	0.000144875	0.00020795	5.96117E-05	-0.213080775	-0.101355351	
chr17	80346039	80346912	874	*	4	2.58438E-33	0.032037138	0.000209882	3.52342E-05	-0.281603536	-0.105012497	
chr9	126959556	126960331	776	*	2	2.00008E-21	3.15041E-05	0.000210417	3.708E-05	0.22367885	0.158884317	
chr1	40425741	40426487	747	*	2	3.15209E-24	8.30389E-07	0.00021263	1.95316E-06	-0.242775058	-0.193393029	MFSD2A
chr2	149823115	149823755	641	*	2	2.58983E-20	0.089457391	0.000214149	0.001050875	-0.3375026	-0.16956081	KIF5C

chr9	36169175	36169949	775	*	3	2.28182E-29	0.021810948	0.000216453	0.00094645	-0.383495949	-0.098601173	CCIN
chr3	38029323	38030079	757	*	5	5.37795E-56	1.11678E-13	0.000218573	2.16429E-12	-0.395496463	-0.278889674	VILL
chr1	229114792	229115634	843	*	3	5.23856E-23	0.031835064	0.000218958	0.000967261	-0.238721607	-0.085887884	
chr9	764094	764329	236	*	2	1.30225E-17	0.004997411	0.000219826	0.000616447	-0.190713015	-0.116015097	
chr7	36097189	36097877	689	*	2	3.29476E-20	0.018516599	0.000219841	0.000880597	0.276575554	0.146375166	
chr3	40271029	40271748	720	*	2	2.665E-22	5.68392E-07	0.000221886	1.40192E-06	-0.274423844	-0.069291529	MYRIP
chr12	78392336	78392948	613	*	2	2.51999E-21	0.000133124	0.000222592	0.000101508	-0.298328928	-0.174266181	NAV3
chr21	40124792	40125580	789	*	3	6.29096E-28	0.040470137	0.000225693	0.002055468	-0.284692596	-0.091290642	
chr10	75335352	75336540	1189	*	4	6.24411E-25	0.070708911	0.000226114	0.003015011	-0.300454758	-0.095725242	USP54
chr12	26676779	26676849	71	*	2	3.79115E-25	3.54937E-07	0.000226428	9.17398E-07	-0.378121259	-0.347646594	ITPR2
chr2	208461657	208461998	342	*	2	5.42681E-26	7.58424E-07	0.000227308	1.82122E-06	-0.297922054	-0.297529269	CREB1, METTL21A
chr13	29279713	29280140	428	*	2	2.62587E-19	0.000812268	0.000229513	0.000292419	-0.309328606	-0.179009433	SLC46A3
chr2	43635340	43635730	391	*	2	1.30425E-20	0.00084008	0.000229516	0.00029745	-0.346279127	-0.212377129	RNU6-958P, THADA
chr5	174938424	174939135	712	*	2	6.56065E-20	0.029457489	0.000229602	0.00099292	-0.411819767	-0.201808715	SFXN1
chr16	27873237	27874129	893	*	3	1.76517E-22	0.023481134	0.000230143	0.00168076	-0.435596612	-0.133636991	GSG1L
chr15	94642624	94643799	1176	*	5	5.28475E-39	0.011130944	0.000230604	3.91562E-05	-0.394445769	-0.160525065	CTD-2643K12.3
chr20	48385019	48385709	691	*	2	2.42419E-22	6.34775E-07	0.000233098	1.55901E-06	-0.216319379	-0.20738829	
chr11	65141519	65142032	514	*	2	2.01667E-19	0.003678066	0.00023776	0.00058454	-0.367683861	-0.174320889	RP11-867O8.5
chr13	50796845	50797125	281	*	2	4.17495E-24	7.11348E-07	0.00023831	1.73213E-06	-0.381751674	-0.33587134	DLEU1
chr8	130898833	130899478	646	*	2	1.28267E-19	0.003571223	0.000239486	0.000581041	0.286147692	0.149839862	FAM49B
chr4	54367835	54367958	124	*	2	2.81098E-25	6.81935E-07	0.000240253	1.66977E-06	-0.3025525	-0.243615305	FIP1L1, LNX1-AS1, LNX1
chr11	44612458	44614255	1798	*	4	3.93406E-37	5.84636E-12	0.000243447	7.60611E-11	-0.469309486	-0.29051129	CD82
chr6	139656042	139656762	721	*	2	1.23307E-21	8.65331E-07	0.000244827	2.07165E-06	-0.280961721	-0.262972263	
chr14	78031892	78032088	197	*	2	1.30174E-23	4.50564E-07	0.000245233	1.15133E-06	-0.401993461	-0.370054459	SPTLC2
chr14	101587161	101587855	695	*	2	2.16733E-21	0.041716377	0.000248082	0.001114162	-0.12006463	-0.061076353	RP11-8L8.2
chr3	59032572	59033261	690	*	2	6.5808E-22	0.009519473	0.000248354	0.000825212	-0.286199513	-0.145523228	C3orf67
chr2	239343317	239343799	483	*	2	1.72572E-24	0.026134259	0.000249759	0.001043937	-0.302751064	-0.155447139	ASB1
chr6	143718452	143719125	674	*	3	1.18092E-21	0.237408712	0.000254253	0.003649954	-0.38550817	-0.128717754	
chr17	56359527	56359578	52	*	2	1.40827E-25	1.20659E-06	0.000254785	2.79893E-06	-0.186639395	-0.173687018	

chr17	28671936	28672726	791	*	3	3.6439E-30	2.7343E-05	0.000255341	2.97991E-06	-0.262892452	-0.147297311	
chr2	43241407	43241873	467	*	2	5.01034E-23	7.43358E-07	0.000255366	1.81993E-06	-0.391577691	-0.362957653	
chr14	92939857	92940131	275	*	2	6.23958E-22	2.47469E-06	0.000255974	5.22769E-06	-0.279219909	-0.217682159	SLC24A4
chr2	174023580	174024669	1090	*	3	2.20039E-30	2.34321E-06	0.000262771	4.99705E-06	-0.294585477	-0.171822903	MLTK
chr4	40239725	40240766	1042	*	3	1.36858E-26	0.000498414	0.00026299	5.23785E-06	-0.36736085	-0.173574435	RHOH
chr8	96240914	96241358	445	*	2	5.03491E-18	0.004157492	0.00026447	0.000659672	0.206914922	0.122863806	KB-1047C11.2
chr2	62515892	62515978	87	*	2	1.29668E-15	0.010255735	0.000264491	0.000884359	0.216106289	0.104190163	
chr1	33246019	33246431	413	*	2	3.97511E-19	0.053951604	0.000264507	0.001213009	-0.239005374	-0.120245938	YARS
chr16	48532923	48533930	1008	*	3	5.24062E-21	0.005977225	0.000265044	0.000558262	-0.235271058	-0.087785181	
chr2	36741839	36742484	646	*	2	3.82498E-20	0.056624016	0.000267748	0.001232007	-0.30326349	-0.152782601	CRIM1
chr19	41782099	41782183	85	*	3	2.31299E-28	2.34486E-07	0.000267906	4.08825E-07	-0.382432383	-0.249757063	HNRNPUL1
chr2	25873029	25873395	367	*	3	5.56496E-35	1.42838E-09	0.000268525	8.67002E-09	-0.379144103	-0.319726863	DTNB
chr3	189111648	189111683	36	*	2	2.56024E-17	0.000362947	0.000268597	0.00020598	-0.232571818	-0.139965701	
chr1	6553227	6553510	284	*	2	5.63483E-19	0.000943146	0.00026872	0.000346791	0.214713901	0.129304495	PLEKHG5
chr19	52715638	52715929	292	*	2	1.8839E-16	0.084951795	0.000268842	0.001279631	-0.379271242	-0.18815328	PPP2R1A
chr1	23911837	23912230	394	*	3	4.13083E-20	0.03768048	0.000269212	0.001774285	-0.316841652	-0.088485508	MDS2
chr1	8278295	8278817	523	*	2	1.0808E-19	0.031978692	0.000272672	0.001161578	-0.344811688	-0.175406863	RP11-431K24.4
chr8	8231531	8232204	674	*	2	1.2869E-18	0.007979523	0.000272764	0.000841358	-0.25912521	-0.133434887	SGK223
chr17	25858091	25860000	1910	*	5	8.17894E-41	1.82581E-13	0.000278897	4.62973E-12	-0.323409185	-0.214759571	KSR1
chr1	206922233	206922738	506	*	3	3.17347E-23	0.000484069	0.000279077	6.83599E-05	-0.337920304	-0.153274277	
chr20	30700869	30701083	215	*	2	3.57776E-24	5.45479E-07	0.000281292	1.39342E-06	-0.289805914	-0.289684546	TM9SF4
chr1	230289718	230290089	372	*	3	1.2787E-31	6.25469E-09	0.000281859	3.14857E-08	-0.351802466	-0.322578577	GALNT2
chr15	101973616	101974405	790	*	2	1.70351E-20	0.000438158	0.000284405	0.000236783	-0.33910567	-0.179703721	PCSK6
chr16	88686083	88686428	346	*	2	4.25702E-18	0.000146952	0.000284988	0.000121476	-0.375034734	-0.169165893	ZC3H18
chr1	175136705	175137236	532	*	2	3.55352E-19	0.018242606	0.000285265	0.001083554	-0.267357994	-0.12671386	KIAA0040
chr9	134127575	134128240	666	*	2	9.18833E-19	0.000598056	0.000285349	0.000282385	-0.304439762	-0.164483307	
chr11	117406287	117406767	481	*	2	1.48658E-17	0.010431582	0.000285525	0.000942379	-0.196902696	-0.084042991	DSCAML1
chr12	48294379	48295148	770	*	2	6.64824E-20	4.3557E-06	0.000286117	8.64613E-06	-0.424616597	-0.327570263	RP11-89H19.1, VDR
chr8	103780068	103780382	315	*	2	1.84672E-16	0.003800996	0.000286563	0.0006747	-0.112985819	-0.034965742	

chr16	81582954	81583089	136	*	2	2.33505E-18	2.26019E-05	0.000286654	3.23705E-05	-0.304250015	-0.191798251	CMIP
chr9	93959671	93960152	482	*	3	2.68491E-31	3.25486E-09	0.000287718	1.81077E-08	-0.367774238	-0.205850338	
chr6	14733316	14733832	517	*	2	1.64767E-17	0.015992896	0.000289185	0.001062971	0.2370054	0.123759334	
chr10	114781270	114781546	277	*	2	9.41099E-17	0.033667857	0.000289194	0.001230101	-0.354562251	-0.17922929	TCF7L2
chr1	116519323	116520317	995	*	4	9.38947E-35	0.000759444	0.000289242	9.94536E-05	-0.340365346	-0.093095756	SLC22A15
chr6	12001569	12002135	567	*	3	1.99773E-28	2.11844E-05	0.000289953	3.45334E-06	-0.366449082	-0.235990287	
chr12	64646546	64646917	372	*	3	2.25828E-24	4.81299E-06	0.000292251	7.35934E-06	-0.360906437	-0.200055745	RPS11P6
chr21	36616969	36616995	27	*	2	2.8929E-21	1.26853E-06	0.000293144	2.99325E-06	-0.34244936	-0.34038309	RUNX1
chr7	151503515	151503608	94	*	4	8.69679E-40	1.6949E-10	0.000294812	1.36611E-09	-0.250391474	-0.187028612	PRKAG2
chr7	47560215	47560370	156	*	2	1.75507E-20	2.77288E-06	0.000295117	5.93208E-06	-0.320195281	-0.256617852	TNS3
chr5	95260542	95260620	79	*	2	1.00634E-15	0.000865641	0.000296351	0.000351708	-0.318791682	-0.179175764	ELL2
chr13	41718870	41719542	673	*	2	6.41083E-18	0.014344999	0.000296499	0.001055921	-0.359979045	-0.195521812	
chr14	91827651	91828035	385	*	2	6.62263E-22	7.93977E-07	0.000297036	1.97206E-06	-0.372273992	-0.329796011	CCDC88C
chr15	25650611	25651282	672	*	3	1.81747E-20	0.198369267	0.000297228	0.003793042	-0.324022109	-0.110827561	SNHG14, UBE3A
chr18	51882163	51882189	27	*	2	1.83314E-20	0.000159236	0.000297267	0.000130498	-0.301809953	-0.223136998	STARD6
chr8	19535747	19536244	498	*	3	9.05861E-21	0.119721927	0.000299648	0.003575788	-0.381625926	-0.132817116	RP11-1105O14.1, CSGALNACT1
chr1	233004866	233005032	167	*	2	2.37201E-15	0.003246623	0.000300001	0.000654425	-0.313910649	-0.162775402	
chr10	129794994	129795003	10	*	2	1.0674E-22	8.08267E-07	0.000300152	2.00689E-06	-0.316207044	-0.258950954	PTPRE
chr8	6794872	6796618	1747	*	8	9.80516E-66	5.76016E-18	0.00030472	3.64182E-16	-0.395533074	-0.224844054	DEFA4
chr22	39792498	39792959	462	*	2	3.06129E-21	1.34914E-06	0.000306138	3.18315E-06	0.168118959	0.135164724	
chr14	91111400	91112135	736	*	3	2.46505E-25	0.001678773	0.000306589	0.000471674	-0.289134468	-0.128137736	RP11-1078H9.5, TTC7B
chr20	22665210	22666782	1573	*	7	3.63019E-58	3.99996E-06	0.000311115	1.88078E-09	-0.284249079	-0.149230837	RP11-359G22.2
chr1	167732969	167733310	342	*	2	6.44739E-17	0.028505387	0.000312118	0.00127454	-0.3108103	-0.159644116	MPZL1
chr19	17219073	17219214	142	*	2	1.63176E-21	2.12713E-06	0.000312169	4.76659E-06	-0.305744436	-0.282934954	MYO9B
chr8	37661612	37662390	779	*	2	3.64499E-18	0.000159079	0.000313832	0.000133727	-0.334468103	-0.12558928	GPR124
chr1	224957573	224958213	641	*	2	1.34334E-17	0.005830837	0.000314458	0.000843165	-0.163084528	-0.087374494	RP11-449J1.1
chr15	75079350	75080115	766	*	2	4.51546E-19	0.010432682	0.000314476	0.001014645	0.24243485	0.117557496	CSK
chr12	89777963	89778485	523	*	2	1.25579E-20	3.2309E-06	0.000315966	6.85703E-06	-0.242090191	-0.240125254	
chr6	28874479	28875354	876	*	5	7.02426E-51	5.29663E-12	0.000316234	8.05814E-11	-0.309892594	-0.241153573	TRIM27

chr8	142389397	142391497	2101	*	5	6.622E-29	0.020282391	0.000316993	3.62625E-05	-0.300134517	-0.095704143	
chr14	89925446	89925468	23	*	2	1.90489E-21	7.24213E-07	0.000316997	1.82999E-06	-0.255222107	-0.251582165	FOXN3, RP11-33N16.3
chr19	5983451	5983811	361	*	2	3.14279E-18	4.57435E-05	0.000317455	5.67587E-05	-0.101388224	-0.089322299	CTC-232P5.1
chr16	84860766	84860918	153	*	3	1.03786E-29	7.49192E-06	0.000318921	2.70306E-06	-0.482204603	-0.312630705	CRISPLD2
chr11	34393106	34393828	723	*	3	3.47028E-21	7.41508E-06	0.000320178	2.9164E-06	-0.391313008	-0.208360826	
chr18	2857042	2857947	906	*	2	2.27135E-17	1.54219E-06	0.000321135	3.61102E-06	-0.323748033	-0.213790217	EMILIN2
chr5	177913434	177913485	52	*	3	1.147E-30	3.20071E-09	0.000321715	1.85062E-08	-0.470589063	-0.326534205	COL23A1
chr22	24536471	24536572	102	*	2	1.69143E-18	1.05948E-05	0.000322221	1.85136E-05	-0.08724934	-0.085972716	CABIN1
chr17	27264093	27264560	468	*	2	8.93159E-18	0.004211029	0.000322235	0.000761607	-0.415666576	-0.22391414	RP11-20B24.5, PHF12
chr12	8043600	8044199	600	*	4	7.17468E-28	0.00636446	0.000322362	2.28075E-05	-0.330145487	-0.124197749	SLC2A14
chr5	17436371	17436741	371	*	6	2.4995E-41	1.97288E-06	0.000322425	4.20894E-08	-0.37448417	-0.164606015	RP11-321E2.3
chr22	23153695	23154645	951	*	4	2.58916E-37	2.5244E-10	0.000322431	1.70149E-09	-0.327851123	-0.22280292	IGLV3-10
chr12	93344946	93345531	586	*	2	2.4246E-17	0.00642954	0.000324031	0.000891202	-0.261297387	-0.136145996	
chr12	10336861	10337070	210	*	2	1.62803E-19	4.49837E-06	0.000324168	9.13661E-06	-0.341302309	-0.271706116	TMEM52B
chr11	121330348	121330401	54	*	3	1.76634E-31	3.46192E-09	0.000326225	1.9945E-08	-0.333786199	-0.271444581	SORL1
chr2	111790599	111791007	409	*	2	3.53807E-18	5.86809E-05	0.000327393	6.87608E-05	-0.273882289	-0.100127605	ACOXL
chr12	64960957	64961489	533	*	2	1.6019E-20	1.45011E-06	0.000328688	3.43188E-06	-0.275620337	-0.038287379	
chr1	116560364	116560807	444	*	2	5.91992E-18	0.007018032	0.000328991	0.000928094	-0.3541622	-0.195116955	SLC22A15
chr20	33723949	33724170	222	*	2	9.07224E-15	0.011500501	0.000329317	0.001080512	-0.255525266	-0.132322912	EDEM2
chr9	93822926	93823465	540	*	2	8.85823E-17	0.014589929	0.000330073	0.001153479	-0.236798929	-0.111181405	
chr11	117873636	117874181	546	*	2	1.56917E-17	0.000482563	0.000331223	0.00027169	-0.253511638	-0.104840535	
chr3	171282985	171283606	622	*	2	2.27152E-19	0.002353735	0.000331453	0.000610646	-0.252679272	-0.109049209	
chr11	69150514	69152387	1874	*	5	4.0187E-33	3.13723E-08	0.000331519	1.61061E-08	0.20061598	0.138763515	MYEOV
chr16	30953496	30953591	96	*	2	3.048E-14	0.009969179	0.000331525	0.001042254	0.064451651	0.033592246	FBXL19
chr14	78488583	78488668	86	*	2	3.74615E-14	0.011877073	0.000331924	0.001096869	-0.32420702	-0.149647262	
chr2	233981788	233981885	98	*	2	1.67528E-20	1.65182E-06	0.000332318	3.85848E-06	-0.32778667	-0.318852438	INPP5D
chr17	6830903	6831506	604	*	2	7.7932E-17	0.056935107	0.00033488	0.001494201	-0.263126713	-0.130985299	ALOX12P2, AC027763.2, RP11-530N7.2
chr18	51787171	51787310	140	*	2	2.00008E-21	1.06009E-06	0.000335076	2.60121E-06	-0.252340296	-0.244226508	
chr10	28700186	28700847	662	*	2	5.16176E-17	0.015020326	0.000335758	0.001177638	-0.253284754	-0.130754553	

chr18	9494893	9495499	607	*	2	1.72101E-17	0.000638427	0.000336624	0.000321116	-0.23608192	-0.168665796	RALBP1
chr14	93652505	93652954	450	*	2	4.71015E-18	0.08250378	0.000337508	0.001557779	-0.243411675	-0.118851118	TMEM251, RP11-371E8.4
chr10	11879646	11880373	728	*	2	6.15352E-18	6.14993E-05	0.000338101	7.20026E-05	-0.323537888	-0.125085324	PROSER2
chr6	14596742	14596806	65	*	2	8.02194E-22	1.16933E-06	0.000338734	2.84501E-06	-0.337260915	-0.219786769	
chr1	147101752	147101904	153	*	2	3.59718E-16	0.000111952	0.000338748	0.000109494	-0.417957391	-0.246445852	ACP6
chr1	32721065	32721190	126	*	2	7.50156E-22	8.21038E-07	0.000339099	2.06829E-06	-0.375447378	-0.306232488	LCK
chr20	24899180	24899794	615	*	2	5.58618E-21	0.000720861	0.000339554	0.000344994	-0.252166723	-0.144267939	
chr15	39602982	39603094	113	*	2	4.01175E-15	0.000794578	0.000339573	0.000363705	-0.250702309	-0.105995629	RP11-624L4.1, RP11-462P6.1
chr3	52235276	52235959	684	*	2	3.19324E-17	0.00738876	0.000339774	0.000966644	-0.255828159	-0.144488962	ALAS1
chr12	64299398	64299607	210	*	2	1.24749E-14	0.015834722	0.000339797	0.001204521	-0.345954415	-0.175593026	SRGAP1
chr4	77198037	77198262	226	*	2	1.3048E-14	0.019741496	0.000339802	0.00126865	-0.250592991	-0.128493363	FAM47E, FAM47E-STBD1, FAM47E-STBD1
chr15	70632081	70632371	291	*	2	7.44025E-15	0.027233683	0.000339807	0.001355395	-0.288701893	-0.150455646	
chr9	132337542	132337640	99	*	2	5.22526E-14	0.05895429	0.000339816	0.00151886	-0.278209384	-0.141664813	
chr5	32390541	32391135	595	*	2	1.70684E-16	0.047623746	0.000340516	0.001483423	-0.299727184	-0.152755518	ZFR
chr11	12216962	12217666	705	*	3	1.52849E-23	9.66491E-05	0.000340944	5.07373E-05	0.2139141	0.09965271	MICAL2
chr13	50858812	50859451	640	*	2	1.61701E-19	1.27531E-05	0.000341719	2.18419E-05	-0.338220162	-0.268990363	DLEU1
chr17	79128827	79129078	252	*	5	1.50987E-43	6.9778E-11	0.000342324	1.96787E-10	-0.359941429	-0.268532014	AATK
chr13	99094983	99096365	1383	*	4	3.51216E-32	0.051116592	0.000342413	0.003751951	-0.407094859	-0.113623094	FARP1
chr12	121677269	121677522	254	*	2	1.49741E-15	0.22253978	0.000343359	0.001651135	-0.289417538	-0.146419921	CAMKK2
chr3	52879316	52879742	427	*	2	1.11334E-16	0.000549453	0.000343844	0.00029861	-0.357387348	-0.211762528	TMEM110-MUSTN1, TMEM110
chr22	50586299	50586605	307	*	2	6.45932E-31	9.50185E-07	0.000344432	2.36562E-06	-0.424164072	-0.33407678	MOV10L1
chr6	24686728	24687380	653	*	3	1.33902E-25	5.14785E-05	0.000344996	9.02247E-06	-0.368558033	-0.195890046	ACOT13
chr5	73938574	73939283	710	*	2	3.80661E-24	9.4316E-07	0.000346228	2.35142E-06	-0.312072023	-0.278821623	HEXB
chr7	36022276	36022841	566	*	4	1.19725E-23	0.000910024	0.000346712	0.000120058	-0.369134058	-0.154340543	
chr13	25079332	25080267	936	*	3	6.55584E-21	0.042486359	0.000347464	0.000919443	-0.234702109	-0.098145685	TPTE2P6, PARP4
chr10	31146954	31147843	890	*	3	2.33632E-26	0.000157678	0.000348315	6.86982E-06	-0.359058873	-0.212654948	ZNF438
chr8	121094884	121095224	341	*	2	6.42822E-15	0.027579582	0.000348486	0.001386968	-0.189918838	-0.099043532	COL14A1
chr1	43000462	43001072	611	*	2	9.95823E-17	0.005096282	0.000349365	0.000866118	-0.341021156	-0.191867316	CCDC30
chr9	133574171	133575011	841	*	3	1.81716E-24	4.9676E-06	0.000350609	2.8035E-06	0.239605508	0.138262191	EXOSC2

chr13	95133236	95133339	104	*	2	9.30533E-21	1.25747E-05	0.000351132	2.17603E-05	-0.302476726	-0.206113966	
chr14	103870751	103871452	702	*	5	1.48111E-34	1.16736E-07	0.000352195	1.22923E-08	-0.438988741	-0.243889583	MARK3
chr7	77096927	77096967	41	*	2	8.55855E-22	1.32568E-06	0.000352827	3.2023E-06	-0.328947635	-0.306059089	
chr9	98619666	98619894	229	*	2	2.33945E-14	0.036657651	0.000352962	0.001472464	-0.364614968	-0.186460976	LINC00476
chr12	92453691	92453837	147	*	2	8.90093E-14	0.018606118	0.000355814	0.001298443	-0.233344854	-0.108237372	C12orf79
chr22	32347971	32348805	835	*	3	1.50023E-26	2.10433E-05	0.000356305	4.64669E-06	-0.239382365	-0.158185838	YWHAH
chr17	1372403	1372751	349	*	3	6.20195E-20	0.231393554	0.000356631	0.004555056	-0.253255078	-0.082786579	MYO1C
chr2	197583266	197583934	669	*	4	1.96077E-29	7.92429E-06	0.00035692	1.39008E-05	-0.424847832	-0.154617948	CCDC150
chr22	29107974	29108527	554	*	3	2.18474E-17	0.285822643	0.000357774	0.004004938	-0.294760213	-0.104828349	CHEK2
chr2	109883024	109883620	597	*	2	2.1987E-18	0.004140705	0.000357928	0.000813866	-0.261402075	-0.119452883	SH3RF3
chr8	37740548	37741280	733	*	2	5.99934E-20	7.65846E-06	0.000358053	1.45918E-05	-0.218319704	-0.074031991	RAB11FIP1
chr2	37416839	37416883	45	*	2	4.11751E-22	4.44442E-06	0.00035909	9.24495E-06	-0.308516573	-0.214581956	SULT6B1
chr4	89445475	89446409	935	*	3	5.19366E-29	8.22755E-07	0.000360628	1.18403E-06	-0.372456438	-0.258408004	HERC3
chr13	110324345	110324798	454	*	2	1.2073E-16	0.000331211	0.000361616	0.00022746	-0.379750619	-0.20605849	
chr7	75580813	75581161	349	*	2	2.01779E-15	0.002013571	0.000362022	0.000602373	-0.373784211	-0.219182226	POR
chr11	6222458	6222973	516	*	3	1.91765E-29	3.75959E-09	0.000363674	2.19478E-08	-0.353977472	-0.308495915	
chr7	98724624	98724797	174	*	2	1.69565E-14	0.001359374	0.000364103	0.000500568	-0.207764607	-0.139520879	SMURF1
chr19	15980239	15980980	742	*	3	7.05357E-25	0.000308843	0.000366577	1.38811E-05	-0.307668679	-0.205989424	
chr22	38685847	38686149	303	*	2	9.85838E-15	0.005560666	0.000367397	0.00092675	-0.283767986	-0.15683864	
chr1	235010536	235010674	139	*	2	6.79282E-17	3.57875E-05	0.000367692	4.98628E-05	-0.201770303	-0.054273023	
chr6	35569471	35570573	1103	*	3	1.24244E-23	1.2804E-06	0.000369869	2.5476E-06	-0.325348548	-0.140112426	FKBP5
chr19	8367077	8367151	75	*	2	1.46362E-14	0.006243326	0.000370727	0.000972309	-0.281792428	-0.159129294	CD320
chr4	140655478	140656096	619	*	2	3.10826E-18	0.009609555	0.000371581	0.001123144	-0.316844309	-0.157138229	MGST2, MAML3
chr15	71099856	71100339	484	*	2	1.08028E-15	0.005355529	0.000371705	0.000921638	-0.242402187	-0.125739623	
chr13	111007189	111007387	199	*	2	1.33701E-14	0.006588593	0.000371716	0.000992725	0.174762114	0.081256039	COL4A2
chr8	67042176	67042666	491	*	2	1.32789E-15	0.012614673	0.000371743	0.001216711	-0.299311959	-0.152533132	TRIM55
chr6	26439594	26440101	508	*	2	2.0576E-19	0.000763159	0.000372234	0.000374827	-0.208041385	-0.086550103	
chr17	79228937	79229385	449	*	3	2.16499E-26	8.94149E-08	0.000373785	3.37204E-07	-0.165183426	-0.150712549	SLC38A10
chr19	39143287	39143850	564	*	2	8.00881E-20	0.000259133	0.00037461	0.000198743	-0.091012621	-0.081528386	ACTN4

chr5	177814702	177815693	992	*	3	1.83196E-20	0.003123861	0.000378118	0.000466807	-0.412452594	-0.158792558	COL23A1
chr10	73149975	73150448	474	*	2	2.66126E-15	0.04552951	0.000378255	0.001611538	0.203940526	0.104935551	
chr12	26624359	26625047	689	*	2	2.87796E-18	9.94927E-07	0.000378355	2.4965E-06	-0.27412654	-0.242420865	ITPR2
chr5	54197311	54197508	198	*	2	3.24909E-20	1.08969E-06	0.000379788	2.71221E-06	-0.197210919	-0.174816284	
chr17	73310148	73310221	74	*	2	7.06403E-20	2.67038E-06	0.000379812	6.02114E-06	-0.214842014	-0.207927943	
chr10	111659687	111659903	217	*	2	1.04538E-13	0.027469216	0.000380238	0.001488245	-0.316347449	-0.163867221	XPNPEP1
chr10	73083123	73083223	101	*	2	1.90523E-21	7.52551E-06	0.000382779	1.46133E-05	-0.37474319	-0.227141741	SLC29A3
chr20	3745817	3746315	499	*	2	2.61932E-21	1.13667E-06	0.000383001	2.82081E-06	-0.33108915	-0.282565197	C20orf27
chr9	7844737	7844790	54	*	2	4.2003E-20	1.02987E-06	0.000383635	2.58042E-06	-0.3595772	-0.351724056	TMEM261
chr8	128403369	128403642	274	*	2	4.57562E-14	0.014337379	0.000383752	0.001291075	-0.301114088	-0.163037602	RP11-382A18.2, CASC8
chr22	42304331	42304580	250	*	2	9.96539E-15	0.004239243	0.000383857	0.000861983	-0.206478418	-0.083385245	
chr2	176326848	176326854	7	*	2	4.85552E-20	1.08167E-06	0.000383966	2.69799E-06	-0.374084912	-0.338839207	
chr3	30820620	30820664	45	*	2	8.27066E-22	1.04289E-06	0.000384397	2.61062E-06	-0.383519339	-0.37810186	GADL1
chr4	77518782	77519000	219	*	2	8.02198E-20	1.3934E-06	0.000384683	3.39046E-06	-0.303617003	-0.212665182	SHROOM3
chr3	127455234	127456204	971	*	4	3.61693E-34	7.55393E-07	0.000385061	5.404E-08	0.243839768	0.163921076	MGLL
chr12	94676369	94676940	572	*	6	1.04691E-44	2.53886E-09	0.000385681	9.23898E-10	-0.296668073	-0.169988353	PLXNC1, RP11-1105G2.3
chr22	24861698	24861780	83	*	2	5.72196E-17	7.57884E-05	0.000385907	8.80085E-05	0.237302883	0.163174181	ADORA2A-AS1
chr7	41045529	41046414	886	*	2	8.91318E-16	1.2334E-06	0.000386282	3.04E-06	-0.225136288	-0.222820217	
chr12	8171633	8172018	386	*	2	3.18351E-16	0.003285736	0.000386338	0.00078026	-0.096667263	-0.04027395	
chr12	117039927	117040247	321	*	2	3.52931E-16	0.000177183	0.000387445	0.000157974	-0.330052298	-0.179642818	RP11-497G19.3
chr8	126649807	126650226	420	*	2	2.33389E-20	2.44828E-06	0.000388476	5.60176E-06	-0.308368761	-0.206587993	
chr15	63126894	63128725	1832	*	7	7.41491E-45	0.000438434	0.000389552	1.19518E-06	-0.426155238	-0.131075736	TLN2, RP11-1069G10.1
chr2	169939873	169939876	4	*	2	1.94318E-19	1.73261E-06	0.000389868	4.12774E-06	-0.304481198	-0.29149894	DHRS9
chr16	11330882	11330934	53	*	2	3.90202E-20	1.03307E-06	0.000392246	2.59501E-06	-0.26870292	-0.23946768	
chr2	39355435	39356767	1333	*	3	1.20553E-23	0.014210042	0.000392589	1.81326E-05	-0.307174134	-0.036406325	Y_RNA
chr11	34477595	34477922	328	*	2	3.05792E-14	0.019026674	0.000392927	0.001412075	-0.266808202	-0.138647946	CAT
chr2	102233707	102234069	363	*	2	4.25728E-16	0.012811859	0.000394219	0.001278188	-0.303427776	-0.128198182	
chr6	136724406	136724451	46	*	2	5.35219E-18	5.99049E-06	0.000395827	1.21605E-05	-0.260819553	-0.209138312	MAP7
chr17	37791589	37791687	99	*	2	1.10689E-22	1.22866E-06	0.00039583	3.03946E-06	-0.394407085	-0.355581378	PPP1R1B

chr8	96060571	96060712	142	*	2	1.35204E-17	1.7267E-05	0.000396776	2.90267E-05	-0.296405293	-0.252711456	NDUFAF6
chr20	30793018	30794065	1048	*	3	2.0674E-25	0.000957853	0.00039735	1.08203E-05	-0.367798901	-0.236185681	PLAGL2
chr10	5333615	5334280	666	*	3	6.53644E-28	4.34295E-09	0.000397534	2.53091E-08	-0.267237376	-0.225492249	
chr17	53847116	53847554	439	*	2	7.74733E-16	0.004984024	0.000398326	0.000941734	-0.239167068	-0.126145418	PCTP
chr1	2081984	2082848	865	*	6	9.91225E-53	3.48834E-12	0.000398571	3.44111E-12	-0.395487356	-0.235812297	PRKCZ
chr14	65183532	65183677	146	*	3	3.86732E-24	4.12345E-06	0.000399518	2.78053E-06	-0.202211867	-0.139608552	PLEKHG3
chr4	37867680	37868470	791	*	2	1.04336E-16	4.16104E-05	0.000400396	5.75062E-05	-0.404728568	-0.119454719	
chr16	11443268	11445004	1737	*	6	3.82964E-38	0.000157049	0.000403291	7.13441E-06	-0.322972384	-0.094354499	RMI2
chr1	44423187	44423424	238	*	2	4.3317E-18	2.97031E-05	0.000406089	4.47111E-05	-0.33266392	-0.21661471	IPO13
chr4	185754633	185755032	400	*	3	1.25743E-28	4.93132E-09	0.000406547	2.86273E-08	-0.299144476	-0.23139707	
chr11	33308269	33308345	77	*	2	1.65379E-19	1.35072E-06	0.000409325	3.32549E-06	-0.425013181	-0.334881432	HIPK3
chr4	147029689	147030199	511	*	3	4.03209E-18	0.22716995	0.000409355	0.005461719	-0.278575544	-0.095099635	
chr7	137661683	137662133	451	*	2	2.5916E-15	0.014706531	0.000409508	0.001366818	-0.271652647	-0.131166997	CREB3L2
chr3	13087700	13088006	307	*	2	5.17567E-14	0.010539748	0.000411677	0.001247509	0.245828891	0.116722781	IQSEC1
chr17	4064737	4065331	595	*	2	6.48103E-21	1.13437E-06	0.000411975	2.84242E-06	-0.32911971	-0.264209438	CYB5D2
chr12	47481988	47482450	463	*	2	4.10975E-15	0.005096654	0.00041305	0.000974139	0.212517925	0.098278915	PCED1B
chr17	67497778	67497879	102	*	2	1.99615E-19	1.42329E-06	0.000413265	3.49056E-06	-0.323589521	-0.315708903	MAP2K6, RP1-193H18.3
chr16	15796887	15797297	411	*	2	9.5734E-21	1.13526E-06	0.000414279	2.84646E-06	-0.260739092	-0.240417308	NDE1, MYH11
chr16	50718807	50719042	236	*	2	1.42529E-19	6.14908E-06	0.000414711	1.25593E-05	0.146162606	0.128065198	
chr8	126588554	126588891	338	*	2	3.32615E-16	8.45771E-05	0.000414928	9.78997E-05	-0.433479504	-0.262098129	
chr20	1550119	1550240	122	*	2	2.08454E-19	1.45826E-06	0.000415346	3.57011E-06	-0.275120663	-0.265824024	RP4-576H24.4, SIRPB1
chr1	9338002	9338423	422	*	3	2.1764E-24	0.000283596	0.000416794	1.33554E-05	-0.276744495	-0.166176038	
chr19	56130797	56131083	287	*	2	1.52372E-17	1.12893E-05	0.000417075	2.08672E-05	-0.136582862	-0.091786564	
chr1	232735381	232735450	70	*	2	1.22348E-17	2.16073E-05	0.000417267	3.51835E-05	-0.297690094	-0.204122326	
chr19	2943570	2943999	430	*	2	3.20664E-15	0.00933923	0.000419212	0.001217659	-0.19131161	-0.07811557	ZNF77
chr12	122245655	122246256	602	*	2	1.44291E-16	0.012227781	0.00041944	0.001322324	-0.253671716	-0.132661858	SETD1B
chr2	26231776	26232329	554	*	2	1.78482E-15	0.003575198	0.000419483	0.000854165	-0.085062147	-0.052478806	
chr1	235922479	235922666	188	*	2	9.94803E-17	1.88422E-05	0.000420632	3.16404E-05	-0.325905621	-0.198609351	LYST
chr2	102978461	102978605	145	*	3	7.47372E-15	0.03758336	0.000421014	0.003067897	-0.257113359	-0.096462635	IL18R1

chr16	70597619	70597967	349	*	3	4.5916E-22	4.65352E-06	0.000421082	8.70886E-06	-0.258153903	-0.115005514	SF3B3
chr15	49785386	49785618	233	*	2	1.74627E-14	0.00053353	0.000421897	0.000327497	-0.398510393	-0.20849603	FAM227B
chr4	40588553	40589108	556	*	2	1.2243E-20	5.12075E-05	0.000422114	6.83971E-05	-0.145366769	-0.104902246	RBM47
chr2	223670481	223670610	130	*	2	2.14347E-19	1.3277E-06	0.000422368	3.28813E-06	-0.385645627	-0.378728901	
chr4	106080109	106080663	555	*	2	3.20703E-15	0.014065802	0.000422422	0.001383087	-0.099553595	-0.053917517	TET2
chr3	122487700	122488513	814	*	2	4.5761E-16	9.63333E-05	0.000422428	0.000108119	-0.254003737	-0.168593054	HSPBAP1
chr6	15901093	15901433	341	*	2	6.41005E-14	0.023841521	0.00042244	0.001574202	-0.28826776	-0.150027409	
chr11	6225537	6226437	901	*	5	1.06645E-26	0.001083989	0.000422588	3.70558E-05	-0.376091888	-0.107605474	
chr3	4765914	4766210	297	*	2	1.20446E-14	0.007356579	0.000424522	0.001135188	-0.204195416	-0.082809421	ITPR1
chr8	96099956	96100187	232	*	3	4.48378E-18	0.001631735	0.000424684	0.000249313	-0.156454132	-0.078374757	NDUFAF6, RP11-320N21.2
chr2	171600867	171600960	94	*	3	1.39267E-24	1.1329E-07	0.000425104	4.21185E-07	-0.387412649	-0.302150987	AC007405.4
chr5	39101019	39101380	362	*	2	5.76265E-14	0.029753865	0.000426197	0.001658648	-0.240336165	-0.130187837	
chr5	132160489	132161127	639	*	3	2.7881E-21	0.2445477	0.000426902	0.005761709	-0.2804897	-0.095152133	SHROOM1
chr12	10324843	10325581	739	*	7	2.399E-39	0.003663736	0.000427351	1.11584E-05	-0.421900065	-0.130940387	TMEM52B
chr12	104063217	104064350	1134	*	4	1.05114E-21	0.013539853	0.000428046	0.000433349	-0.311285058	-0.060918261	STAB2
chr16	4912632	4912779	148	*	2	1.6542E-18	2.91424E-06	0.000428527	6.63813E-06	-0.312226189	-0.303830274	UBN1
chr11	821723	822402	680	*	5	3.22062E-45	5.95697E-13	0.00042891	1.44842E-11	-0.272359914	-0.203822199	PNPLA2
chr22	39570107	39570729	623	*	2	4.27772E-17	0.000924652	0.000429962	0.00045146	-0.263577344	-0.143782866	
chr22	23763821	23764189	369	*	2	6.80343E-14	0.117233872	0.000430893	0.001974735	-0.320072564	-0.16037654	
chr18	46202798	46203533	736	*	2	5.00032E-17	0.000355722	0.000431683	0.000259556	-0.237181212	-0.144456591	CTIF, RP11-426J5.2
chr2	8438973	8439217	245	*	2	2.61434E-19	1.37111E-06	0.000431697	3.3949E-06	-0.232721041	-0.198749081	LINC00299
chr4	105978652	105979020	369	*	2	3.32119E-19	1.26398E-06	0.000432449	3.15476E-06	-0.330076473	-0.29522132	RP11-556I14.1
chr12	56029543	56030242	700	*	2	1.02111E-18	0.003896064	0.000432459	0.000903528	-0.305871407	-0.14607016	
chr1	95581522	95581952	431	*	2	1.15211E-18	0.02468157	0.000432474	0.001616109	-0.069601217	-0.043725273	
chr18	74764374	74765155	782	*	3	5.06313E-20	0.003267252	0.000433534	0.000191898	-0.122557212	-0.068862896	MBP
chr2	207987538	207987951	414	*	2	3.34696E-17	0.063182677	0.000433785	0.001886213	-0.3360309	-0.155947866	KLF7
chr17	33884702	33885742	1041	*	3	4.68837E-25	0.000475706	0.00043409	1.78702E-05	0.253278901	0.114376894	SLFN14
chr21	39810663	39811743	1081	*	4	1.36194E-21	3.99164E-05	0.000434531	1.51751E-06	-0.387650233	-0.19755239	ERG
chr10	43468702	43469164	463	*	2	1.38263E-14	0.011040396	0.000435304	0.001319281	-0.318204096	-0.163932608	

chr12	116718617	116719189	573	*	2	4.48408E-15	0.050788916	0.000435352	0.001841707	-0.351571817	-0.178053511	
chr11	59822727	59824161	1435	*	9	4.58847E-62	3.33179E-19	0.000435802	5.54707E-17	-0.38460699	-0.206804112	MS4A3
chr4	185923877	185924141	265	*	3	2.22564E-22	9.20111E-06	0.000436028	4.41766E-06	-0.265416704	-0.182088999	RP11-386B13.4
chr6	152950812	152950866	55	*	2	3.90228E-20	1.25889E-06	0.000436081	3.14661E-06	-0.332136989	-0.327023333	SYNE1
chr1	207509316	207509334	19	*	2	2.06835E-19	1.47472E-06	0.000436221	3.63064E-06	-0.344178113	-0.296874737	CD55
chr15	76431162	76431489	328	*	2	1.73289E-15	0.019152676	0.000436423	0.001536465	-0.303252921	-0.155243444	C15orf27
chr10	5734571	5735349	779	*	4	3.17646E-19	0.054454141	0.000439426	0.004056894	-0.210860643	-0.066091269	FAM208B
chr14	105751629	105751711	83	*	2	6.60854E-15	0.006392961	0.000441051	0.00111001	-0.338723455	-0.17908169	BRF1
chr15	89939252	89939709	458	*	3	2.49621E-20	0.016639818	0.000441821	8.6404E-05	-0.237555305	-0.1173031	LINC00925
chr16	88907184	88907370	187	*	2	9.07527E-21	1.29617E-06	0.00044197	3.23651E-06	-0.412993063	-0.328145832	GALNS
chr5	107978715	107979173	459	*	2	2.46389E-14	0.08324713	0.000442012	0.001970159	0.222871996	0.113254723	
chr1	31873665	31874123	459	*	2	2.19495E-14	0.022956959	0.00044211	0.001619109	-0.298574018	-0.16189494	
chr20	2085157	2085344	188	*	2	6.53011E-15	0.000209665	0.000442397	0.000187379	-0.340876967	-0.228065327	STK35
chr1	116270626	116271272	647	*	4	1.43982E-27	4.88603E-07	0.000443515	9.07001E-07	-0.321537378	-0.185518216	CASQ2
chr3	141005905	141006210	306	*	2	2.16929E-13	0.030568134	0.000443679	0.00172278	-0.318320717	-0.156096054	ACPL2, RP11-438D8.2
chr7	139108026	139108111	86	*	2	3.65132E-13	0.073765475	0.000443799	0.001955046	-0.348055421	-0.175541981	C7orf55-LUC7L2
chr11	70432884	70433293	410	*	3	5.61801E-16	0.06629329	0.000444593	0.003012028	-0.35094326	-0.124438509	SHANK2
chr5	154172209	154172825	617	*	2	7.06868E-21	5.24229E-05	0.000444857	7.09015E-05	-0.285140389	-0.197707293	LARP1
chr6	166649636	166650404	769	*	4	3.05852E-32	4.07057E-07	0.000445189	2.05437E-07	-0.318347469	-0.128948544	
chr3	48557198	48557236	39	*	2	3.52492E-19	3.62563E-06	0.000445387	8.09024E-06	0.201391096	0.200526653	PFKFB4
chr17	78753273	78754372	1100	*	8	2.47617E-52	2.2083E-09	0.000446441	1.09133E-10	-0.314623698	-0.147390096	RPTOR
chr12	32638773	32638839	67	*	3	5.75395E-25	3.16068E-07	0.00044688	1.12008E-06	-0.237501008	-0.210307069	FGD4
chr6	138055931	138056102	172	*	2	1.28188E-17	1.12316E-05	0.000447037	2.1144E-05	-0.302682419	-0.236648616	RP11-356I2.1
chr2	216370262	216370291	30	*	2	1.51174E-18	2.17024E-06	0.000448354	5.15279E-06	-0.282966487	-0.265067339	
chr6	138195986	138196387	402	*	3	1.04104E-23	0.000672963	0.000449127	2.73343E-05	-0.39821153	-0.248804209	TNFAIP3
chr19	54584636	54585167	532	*	5	1.53687E-32	5.9127E-05	0.000450289	8.35781E-07	-0.414313203	-0.200219758	
chr22	23730033	23730059	27	*	2	3.30977E-18	5.60381E-06	0.000450827	1.18109E-05	0.177067217	0.12233289	
chr4	155469629	155471134	1506	*	6	1.71237E-24	0.214059695	0.000450829	0.007640897	-0.34275045	-0.04765539	PLRG1
chr22	40685946	40686295	350	*	2	9.57862E-18	7.38322E-06	0.000451271	1.49323E-05	-0.206746193	-0.18994967	TNRC6B

chr1	12774815	12775608	794	*	4	1.97299E-27	0.018243072	0.000451653	2.67734E-05	-0.40012032	-0.178392242	
chr2	239463246	239463810	565	*	5	2.6707E-33	7.79234E-06	0.000451922	1.12129E-06	-0.350588458	-0.158575729	LINC01107
chr6	140160925	140161193	269	*	2	4.11642E-19	1.35833E-06	0.000452745	3.38725E-06	-0.310189838	-0.286480608	
chr18	9215747	9215818	72	*	2	7.8708E-22	2.07545E-06	0.00045282	4.9591E-06	-0.288224721	-0.256841918	RP11-21J18.1, ANKRD12
chr1	182097885	182098716	832	*	4	3.77283E-27	0.000110674	0.000452863	1.28958E-05	-0.403803168	-0.194975839	GS1-122H1.2
chr6	152639235	152639548	314	*	2	7.06005E-15	0.045494089	0.000455368	0.001882995	-0.246783396	-0.120334963	SYNE1
chr6	20907308	20907817	510	*	2	1.41107E-14	0.011086651	0.000456809	0.001369513	-0.259594736	-0.140950408	CDKAL1
chr6	22113460	22113516	57	*	2	2.01062E-17	5.3728E-06	0.000456852	1.14232E-05	-0.280099023	-0.261852685	CASC15
chr17	650113	650835	723	*	6	1.39586E-23	0.768852991	0.000457479	0.040860109	-0.125932358	-0.024234043	GEMIN4
chr7	151442351	151442967	617	*	4	6.12027E-33	1.54278E-06	0.000458126	1.33928E-07	-0.379213757	-0.234650922	PRKAG2
chr11	103930498	103930844	347	*	2	4.10806E-14	0.001833945	0.000458278	0.000667617	-0.342176759	-0.182802681	PDGFD
chr6	161790605	161790663	59	*	2	2.41594E-17	8.67301E-06	0.000459222	1.71644E-05	-0.361719912	-0.267574106	PARK2
chr3	186982752	186982969	218	*	2	1.26951E-14	0.000186507	0.000460816	0.000176565	0.124759203	0.088592966	MASP1
chr13	113343376	113343878	503	*	2	4.60551E-18	0.032558901	0.000462213	0.001802621	-0.178615135	-0.090913028	
chr9	71459366	71459620	255	*	2	2.69172E-20	1.42774E-06	0.00046237	3.55324E-06	-0.338635873	-0.270475872	PIP5K1B
chr15	58818581	58819005	425	*	2	8.42426E-19	1.87097E-06	0.000462811	4.53403E-06	-0.34535072	-0.308768034	LIPC
chr18	77551953	77552568	616	*	3	9.14319E-22	0.000116148	0.000463684	5.93551E-05	-0.345869394	-0.16990026	
chr8	82042993	82043566	574	*	2	1.20467E-14	0.083090138	0.000463844	0.002053621	-0.225035306	-0.110842023	
chr7	50726586	50727932	1347	*	5	2.1764E-24	0.003743341	0.000465495	0.000141242	-0.342682588	-0.10082496	GRB10
chr11	122612819	122612858	40	*	2	2.17955E-16	1.46309E-05	0.000466242	2.65441E-05	-0.195746912	-0.159657292	UBASH3B
chr6	89759594	89760139	546	*	2	2.1713E-15	0.000558837	0.000467339	0.000355042	-0.255747326	-0.168361509	
chr14	61965603	61966327	725	*	2	3.33559E-16	0.000727622	0.000467466	0.000413812	-0.29876951	-0.161598095	PRKCH
chr5	16455785	16455898	114	*	3	1.19948E-20	4.91894E-05	0.000467658	1.56552E-05	-0.306761422	-0.162434448	ZNF622
chr5	172280684	172280876	193	*	2	9.25353E-17	0.000257209	0.000469999	0.000220131	-0.248020861	-0.173104516	ERGIC1
chr5	133837030	133837273	244	*	2	8.39699E-14	0.009882334	0.000471218	0.001351765	0.179833063	0.085239426	
chr3	155570275	155570809	535	*	3	3.49503E-19	0.046367175	0.000471555	0.003077794	-0.26622949	-0.108157445	SLC33A1
chr4	1939509	1939928	420	*	3	1.29445E-15	0.044691031	0.000471594	0.003550496	-0.31756944	-0.114788158	WHSC1
chr2	20706515	20707110	596	*	3	2.69485E-17	0.07181327	0.000471597	0.003827063	0.22735859	0.0716873	
chr17	465530	466113	584	*	2	1.44983E-14	0.212452047	0.000474532	0.002200233	-0.213113312	-0.106436117	VPS53

chr12	7901661	7902153	493	*	2	2.16065E-21	2.58332E-06	0.000474994	6.06978E-06	0.184584789	0.151926561	CLEC4C
chr12	105042855	105043367	513	*	2	1.26525E-16	3.52169E-05	0.000475056	5.35976E-05	-0.328253533	-0.201981356	CHST11
chr2	102890416	102890476	61	*	2	1.48497E-13	0.000437677	0.000475686	0.000309383	-0.323079105	-0.177301168	
chr10	352348	352668	321	*	2	3.14557E-13	0.015521083	0.000476523	0.001558636	-0.369369432	-0.191635988	DIP2C
chr20	24961060	24961593	534	*	2	2.04378E-14	0.018152697	0.000476531	0.001624212	-0.19261808	-0.087831699	APMAP
chr3	52877048	52877766	719	*	6	1.22857E-42	1.56654E-09	0.000477351	9.02367E-10	-0.418780669	-0.218771917	TMEM110-MUSTN1, TMEM110
chr16	474271	474937	667	*	5	2.18989E-43	1.03688E-08	0.000477386	1.98891E-09	-0.264016764	-0.179908684	
chr8	134369245	134369320	76	*	2	6.92001E-14	0.00111174	0.000478305	0.000529797	-0.276166418	-0.152995602	
chr15	101469838	101470215	378	*	2	3.48876E-15	0.001830487	0.000480205	0.000686325	0.273138012	0.148565514	LRRK1
chr16	85539811	85540297	487	*	2	3.06444E-16	0.006017179	0.000480393	0.001152799	-0.327040763	-0.166707771	
chr2	152220410	152220529	120	*	2	2.60552E-12	0.011533847	0.000481779	0.001442438	-0.280843142	-0.147042389	TNFAIP6
chr11	3660884	3661319	436	*	2	2.08331E-15	0.009175708	0.000481792	0.001341115	-0.306015719	-0.147404708	ART5
chr7	47846057	47846424	368	*	2	2.87638E-18	1.21939E-05	0.000483639	2.30712E-05	-0.310224386	-0.209114623	C7orf69, HUS1, PKD1L1
chr13	24854061	24854830	770	*	2	2.65346E-16	0.04956337	0.00048497	0.002009921	-0.383804008	-0.189108418	RP11-307N16.6, SPATA13
chr2	42325942	42326070	129	*	2	6.7922E-17	3.79299E-05	0.000486183	5.71724E-05	-0.29070267	-0.205837778	
chr12	2393671	2393684	14	*	2	9.65184E-19	1.56068E-06	0.000487478	3.87786E-06	-0.251423935	-0.234790459	CACNA1C, CACNA1C-IT3
chr15	56703433	56703763	331	*	2	1.77988E-13	0.007959862	0.00048833	0.001290526	0.219379515	0.102246878	TEX9
chr14	65098936	65099398	463	*	2	2.79047E-15	0.000198208	0.000488747	0.000188715	-0.339770814	-0.121366152	
chr17	66301429	66301554	126	*	2	3.92207E-12	0.03134133	0.000488875	0.001873325	-0.297181903	-0.157202508	ARSG
chr5	36679892	36680436	545	*	2	3.52319E-14	0.098135716	0.000488983	0.002179269	-0.248717257	-0.122601245	SLC1A3, CTD-2353F22.1
chr6	46096318	46096367	50	*	2	1.46509E-20	9.94135E-06	0.000490782	1.95379E-05	-0.378095374	-0.260808537	RP1-8B1.4
chr2	28601340	28601410	71	*	2	1.48429E-15	2.45382E-05	0.00049132	4.08143E-05	-0.151298888	-0.121368003	
chr2	42542416	42543153	738	*	2	2.54982E-15	0.000176472	0.000491546	0.000174956	-0.248590517	-0.167569159	EML4
chr22	38326590	38327168	579	*	2	1.94516E-15	0.121342377	0.000491581	0.002220698	-0.215284737	-0.107818483	MICALL1
chr20	34229823	34230453	631	*	2	7.85975E-16	0.000355715	0.000492081	0.00027667	-0.380814959	-0.200205509	CPNE1, RP1-309K20.6
chr22	26992969	26993176	208	*	3	2.94816E-18	0.000981453	0.000493613	8.02519E-05	0.197234281	0.108697605	
chr5	59558467	59559236	770	*	5	2.3813E-38	1.55252E-12	0.000495644	3.06789E-11	-0.41116339	-0.260773822	PDE4D
chr9	73195793	73196319	527	*	2	5.25884E-14	0.439424597	0.000496204	0.002307157	-0.337878823	-0.166909416	TRPM3
chr15	94791949	94792045	97	*	2	9.24617E-13	0.001421637	0.000496851	0.000616512	-0.218183536	-0.134372971	MCTP2

chr16	85470485	85470674	190	*	3	1.11664E-13	0.289243692	0.000497216	0.00573106	0.248586211	0.091878724	
chr1	232644166	232644483	318	*	2	7.64914E-13	0.038067639	0.000498064	0.001971138	-0.390459313	-0.198200653	SIPA1L2
chr7	42942369	42942901	533	*	4	1.4546E-24	3.29891E-06	0.000498811	6.0302E-06	-0.356517416	-0.17802766	
chr18	5463618	5463975	358	*	2	1.10698E-13	0.001692622	0.000501221	0.000677423	-0.373972963	-0.211097124	RP11-286N3.1, EPB41L3
chr11	76353433	76353533	101	*	2	5.35558E-12	0.014678323	0.000501526	0.001595999	0.1630309	0.076374643	
chr22	50227466	50227722	257	*	2	1.95434E-12	0.040567119	0.000501569	0.002004088	-0.37061014	-0.189160984	RP3-522J7.6
chr21	37501846	37502003	158	*	3	1.97098E-22	4.48359E-06	0.000501846	4.43505E-06	-0.268567876	-0.179476106	
chr10	134170262	134170702	441	*	2	2.08421E-17	6.27568E-06	0.000503259	1.33065E-05	-0.340422486	-0.229655516	LRRC27
chr12	1769331	1769824	494	*	2	2.88098E-18	0.000217135	0.000504257	0.000203334	-0.15329851	-0.037413084	MIR3649
chr19	4548354	4548585	232	*	3	8.96962E-19	1.66086E-05	0.000504377	2.80698E-05	-0.210007008	-0.129852256	SEMA6B
chr6	158507063	158508543	1481	*	6	2.72533E-42	6.68211E-06	0.00050454	1.02375E-08	-0.315616205	-0.190759063	SYNJ2
chr21	43977332	43977377	46	*	2	7.76437E-12	0.015799031	0.000505969	0.001639987	0.227316984	0.117548603	SLC37A1
chr2	65593761	65595186	1426	*	10	3.93368E-52	2.04316E-07	0.00050789	3.95068E-10	-0.246751041	-0.112988356	SPRED2
chr4	48637066	48637722	657	*	3	2.05686E-19	0.000494184	0.000508686	0.000253372	0.213083967	0.097196057	FRYL
chr11	86384670	86384908	239	*	3	5.24599E-21	0.008710958	0.000508887	0.00102621	-0.337771345	-0.120973711	
chr11	45757906	45758932	1027	*	3	1.06857E-17	0.014458793	0.000508911	0.001139572	-0.364309376	-0.140173349	CTD-2210P24.1
chr19	3870806	3871442	637	*	3	1.47593E-17	0.002016755	0.000509013	0.000737126	0.248801558	0.141009478	FTLP5
chr17	29700948	29701389	442	*	2	4.28901E-14	0.001550942	0.000509126	0.000654114	-0.362090939	-0.199696197	NF1
chr16	67206722	67207722	1001	*	4	3.40282E-21	0.00807437	0.000509609	0.00136574	-0.239171394	-0.049110097	NOL3
chr12	112074044	112074158	115	*	3	2.58549E-23	1.14707E-07	0.000510111	4.8742E-07	0.214565199	0.164290091	
chr22	47015531	47016623	1093	*	6	1.36557E-38	3.15383E-05	0.000510655	9.85528E-08	-0.352133081	-0.132621438	GRAMD4
chr3	14864757	14865571	815	*	4	1.36325E-23	0.013040015	0.000511354	5.42595E-05	-0.360076784	-0.15484189	FGD5
chr11	73115015	73116329	1315	*	6	1.92133E-38	2.9009E-10	0.000511358	3.52825E-11	-0.299403025	-0.214471052	FAM168A
chr11	119551071	119551662	592	*	3	1.13972E-22	3.38866E-05	0.000512002	1.03547E-05	-0.156733293	-0.095772954	PVRL1
chr6	16753080	16753571	492	*	3	6.83928E-17	0.068400727	0.000512167	0.00171806	-0.382182085	-0.139483934	ATXN1
chr7	45243040	45243365	326	*	2	6.15631E-14	0.026527135	0.000512728	0.001881384	0.207991401	0.101448011	
chr3	72135680	72135799	120	*	2	1.01348E-15	2.49973E-05	0.000513459	4.19491E-05	-0.230176477	-0.149345244	LINC00877
chr9	72783051	72783091	41	*	2	3.26477E-14	9.33501E-05	0.000514449	0.000114148	-0.308494007	-0.225692294	MAMDC2, MAMDC2-AS1
chr19	6234327	6234625	299	*	2	2.89971E-13	0.001753517	0.000514585	0.00070054	-0.224297153	-0.124344365	MLLT1

chr11	72926963	72927832	870	*	5	1.57905E-39	7.69006E-13	0.000516958	1.90033E-11	-0.325454813	-0.288570719		
chr12	106620860	106621388	529	*	2	7.65373E-14	0.026496884	0.000518583	0.00189809	-0.174957975	-0.089582902		
chr17	66339916	66339947	32	*	2	6.46229E-18	4.19334E-06	0.000518707	9.43811E-06	-0.240068811	-0.159402049		ARSG
chr17	3623472	3623819	348	*	2	1.50252E-15	4.37668E-05	0.000519085	6.5174E-05	-0.292209863	-0.177245725		ITGAE
chr18	28786267	28786898	632	*	2	1.44425E-15	7.97806E-05	0.000520546	0.000102286	-0.389132349	-0.217417976		
chr1	211725112	211725933	822	*	2	7.50717E-15	7.31614E-05	0.000521564	9.60515E-05	-0.240806452	-0.077075807		
chr14	65641106	65641415	310	*	2	6.68073E-13	0.005341951	0.000522029	0.001166399	0.171257166	0.106293696		
chr5	17401890	17402750	861	*	3	6.49256E-25	0.00025258	0.000522304	1.46862E-05	-0.337853549	-0.219073206		
chr1	15738732	15739283	552	*	3	5.85837E-15	0.115729497	0.000522529	0.003930242	-0.228897096	-0.088810685		EFHD2
chr16	69223771	69224882	1112	*	4	2.10223E-30	3.49513E-06	0.000523442	1.63616E-07	-0.384650005	-0.210567646		CIRH1A, SNTB2
chr12	95115217	95115543	327	*	2	1.30278E-12	0.145507439	0.000523886	0.002370627	0.206694208	0.10322839		
chr14	73928420	73929106	687	*	2	1.40997E-16	0.002895846	0.000524596	0.000901612	-0.277544921	-0.123912379		NUMB
chr1	118150234	118150813	580	*	2	2.95756E-16	0.016952197	0.000524797	0.001719221	-0.192409576	-0.094653152		FAM46C
chr9	114706631	114706971	341	*	2	1.02408E-12	0.035371958	0.000524842	0.002030841	-0.385211566	-0.196916223		
chr10	112117365	112117449	85	*	2	2.13714E-16	2.08358E-05	0.000527634	3.65361E-05	0.149345889	0.143799429		
chr12	56914153	56914766	614	*	3	5.84265E-17	0.021192875	0.000529705	0.002706468	-0.297577195	-0.082827127		
chr4	160188727	160189254	528	*	3	1.32267E-20	0.003618423	0.000531404	0.000468743	-0.341041905	-0.09026469		RAPGEF2
chr9	101572656	101573412	757	*	2	5.31946E-17	2.21263E-06	0.000531414	5.37434E-06	0.239037277	0.188082566		GALNT12
chr2	58335008	58335178	171	*	3	1.76395E-25	1.69773E-08	0.000532762	9.07193E-08	-0.33180021	-0.306256387		VRK2
chr19	8749178	8749571	394	*	2	6.07703E-13	0.060565538	0.000532818	0.002235094	-0.285252412	-0.144670099		
chr22	20019674	20020377	704	*	4	6.36358E-29	1.30853E-06	0.000534698	1.17519E-06	-0.388852883	-0.193918057		TANGO2
chr5	141345793	141346577	785	*	4	4.48195E-17	0.052868853	0.000535054	0.00463425	-0.284365122	-0.090973265		RNF14, PCDH12
chr1	35915171	35915735	565	*	2	8.50278E-17	0.000116322	0.000536607	0.000135811	-0.4266424	-0.263597376		KIAA0319L
chr5	72131020	72131603	584	*	2	1.06851E-16	7.74653E-06	0.000537175	1.61442E-05	-0.37088122	-0.116051409		TNPO1
chr3	194867333	194868507	1175	*	6	4.86076E-35	0.002003188	0.00053721	4.53728E-05	0.161438093	0.034875709		XXYLT1, RN7SL36P
chr8	128048285	128048403	119	*	2	4.39694E-12	0.004511275	0.000537788	0.00111153	-0.400479351	-0.206711949		
chr1	27157564	27158887	1324	*	5	1.80112E-34	0.000648772	0.000540498	6.28375E-07	-0.241111323	-0.128143864		ZDHHC18
chr18	77442923	77443784	862	*	3	4.95374E-21	9.96295E-08	0.000541318	3.71591E-07	-0.267235668	-0.196794284		CTDP1
chr8	97632763	97632845	83	*	2	3.09917E-12	0.048679043	0.000542167	0.002200535	-0.30299816	-0.148596193		

chr17	38261988	38262482	495	*	2	1.35433E-13	0.009593271	0.00054518	0.001489487	-0.349322953	-0.164400125	
chr19	4950746	4950888	143	*	2	3.93108E-14	0.000103522	0.000546031	0.000125793	-0.372095082	-0.222427592	UHRF1
chr4	84071170	84071797	628	*	3	2.52448E-24	4.28296E-08	0.000546281	1.98576E-07	-0.31955592	-0.226425188	
chr13	110350436	110350537	102	*	2	4.81142E-18	2.0183E-06	0.000546415	4.96623E-06	0.205278652	0.20212268	
chr3	141994724	141996258	1535	*	3	1.04008E-17	0.000471813	0.000547764	7.58647E-05	-0.217384923	-0.1026466	
chr5	149318703	149320352	1650	*	6	2.3107E-34	6.26999E-08	0.000548789	2.1272E-08	-0.362270472	-0.170564856	PDE6A, Y_RNA
chr16	54114317	54115019	703	*	3	7.10097E-16	0.036986704	0.000550704	0.002892086	-0.306570463	-0.120894667	FTO
chr14	104393559	104393771	213	*	3	8.71954E-16	0.111740717	0.000550708	0.004218918	0.165727635	0.050051511	C14orf2
chr3	197272311	197272328	18	*	2	4.71384E-18	2.01433E-06	0.000554178	4.96669E-06	-0.316323063	-0.294588096	BDH1
chr8	108343968	108344200	233	*	2	5.62239E-18	2.03802E-06	0.000554318	5.01956E-06	-0.31312115	-0.22587896	ANGPT1
chr15	42022754	42023104	351	*	2	3.52708E-16	4.56802E-05	0.00055458	6.88022E-05	-0.263207641	-0.194995319	MGA
chr17	14651102	14651898	797	*	2	3.37669E-14	2.01306E-06	0.000557059	4.96723E-06	-0.306719322	-0.305350736	
chr3	10247112	10247487	376	*	2	2.83238E-13	0.02086011	0.000557074	0.001899932	-0.247702666	-0.131271278	IRAK2
chr9	84228185	84228672	488	*	2	1.56218E-13	0.047559935	0.000557111	0.002242963	-0.267700948	-0.130087855	TLE1
chr2	111751981	111752234	254	*	2	5.28426E-12	0.034237435	0.000557208	0.002119316	-0.216552154	-0.103601037	ACOXL
chr8	144437314	144438377	1064	*	6	4.86519E-30	1.40589E-05	0.000557882	1.45655E-05	-0.280170493	-0.109189404	TOP1MT
chr9	81760592	81761043	452	*	3	1.78803E-21	0.000278962	0.000558319	1.6E-05	-0.384269519	-0.241207136	
chr18	60119693	60119756	64	*	3	5.78738E-27	1.24566E-08	0.000559261	6.96275E-08	-0.270723852	-0.210765886	
chr16	85946351	85947866	1516	*	8	2.28536E-38	8.93196E-10	0.000560185	7.58923E-10	-0.300881671	-0.12182413	IRF8
chr2	44334601	44335362	762	*	2	4.39288E-15	3.08659E-05	0.000560366	5.0853E-05	-0.248641894	-0.067478287	
chr11	19298660	19298668	9	*	2	5.50227E-18	2.13569E-06	0.000561032	5.24456E-06	-0.275504951	-0.244397019	RP11-428C19.4
chr3	52502445	52502766	322	*	2	1.46027E-14	0.00052872	0.000561107	0.000377097	-0.251303149	-0.140251118	NISCH
chr2	231582109	231582349	241	*	2	7.05566E-13	0.005452598	0.000562078	0.00123692	-0.236091184	-0.146056007	CAB39
chr19	5669474	5669909	436	*	2	3.29504E-13	0.006820725	0.000562111	0.001348736	-0.385757963	-0.201345795	
chr2	66723102	66723348	247	*	2	3.09603E-12	0.066832336	0.000562263	0.002367617	0.175590206	0.083481497	MEIS1
chr6	28884599	28888395	3797	*	15	3.99816E-41	0.002909852	0.000564572	3.37453E-09	-0.369042976	-0.102730527	TRIM27
chr3	12392024	12393021	998	*	4	3.87924E-25	2.17315E-06	0.000564798	3.35869E-07	-0.329889218	-0.226315734	PPARG
chr22	50980910	50981406	497	*	4	4.29988E-34	8.50236E-11	0.000564931	1.05467E-09	-0.349215186	-0.314620297	CTA-384D8.34, CTA-384D8.35
chr12	11840863	11841125	263	*	2	2.43268E-12	0.004940316	0.000566432	0.001194879	0.138904761	0.081450176	ETV6

chr10	75676660	75677011	352	*	3	2.27606E-25	1.81187E-08	0.00056804	9.69467E-08	-0.223975072	-0.18782708	PLAU, C10orf55
chr18	24269574	24270091	518	*	5	2.98948E-26	0.002053315	0.000568703	7.42004E-05	-0.307231045	-0.125388751	AQP4-AS1, RP11-17A19.2, U3
chr7	148317765	148318131	367	*	2	1.8044E-12	0.173874522	0.000569224	0.002569198	0.172327129	0.086758105	
chr12	125201256	125201577	322	*	3	1.23586E-20	7.42096E-05	0.000569627	1.29588E-05	-0.238249005	-0.155886662	
chr9	131463631	131463936	306	*	2	8.57296E-15	0.003402077	0.000570867	0.001023737	-0.127876985	-0.072428273	
chr15	67125242	67125879	638	*	2	3.16479E-15	6.18811E-05	0.000571403	8.75368E-05	-0.170672123	-0.133393672	
chr21	40694070	40694407	338	*	2	3.19252E-13	0.008980775	0.000572563	0.001508925	-0.310707553	-0.144995146	BRWD1-AS1
chr10	104216121	104216266	146	*	3	3.80996E-20	1.33702E-05	0.000572749	8.15488E-06	-0.358571345	-0.181831352	RP11-18I14.10
chr20	39135432	39135472	41	*	3	3.23071E-20	1.83624E-06	0.000572833	4.03028E-06	-0.11208028	-0.065919648	
chr17	591717	591826	110	*	2	4.94991E-13	0.000320678	0.000574341	0.000278555	-0.319545379	-0.174374677	VPS53
chr1	56973115	56974060	946	*	2	1.87064E-15	0.024942685	0.000574673	0.002032118	-0.400113059	-0.197315436	PPAP2B
chr3	69130853	69130918	66	*	2	1.55129E-17	1.09135E-05	0.000575341	2.19043E-05	0.199506238	0.150160871	
chr13	31256334	31256790	457	*	3	6.21287E-14	0.112990359	0.000575553	0.005774674	-0.295747127	-0.111242731	
chr18	60710837	60710970	134	*	2	6.18768E-12	0.004655517	0.000575926	0.001179051	-0.328895391	-0.15592132	
chr8	38259567	38259904	338	*	2	2.37151E-15	0.01120858	0.00057605	0.001632073	-0.357281628	-0.182717316	LETM2
chr2	69811375	69811847	473	*	3	1.86924E-19	3.5441E-06	0.000576071	8.72654E-06	-0.312704907	-0.207827584	AAK1
chr12	4698603	4699618	1016	*	4	1.13866E-29	5.11917E-07	0.000576955	1.26895E-07	-0.418543361	-0.252445033	DYRK4
chr2	32489693	32491644	1952	*	10	4.16848E-61	1.02648E-11	0.000578765	1.06557E-11	-0.457100137	-0.186044438	NLRC4
chr7	151620691	151620888	198	*	2	1.26205E-12	0.000958804	0.000578792	0.000543284	-0.249384062	-0.141921281	
chr8	74224899	74225117	219	*	2	7.96843E-13	0.00074079	0.000579479	0.000468824	-0.311390265	-0.189267526	RDH10, RP11-434I12.2
chr7	39740448	39740780	333	*	2	2.95977E-13	0.023847258	0.000579528	0.002024366	-0.272440834	-0.141419491	RALA, AC004837.5
chr18	9658035	9658170	136	*	2	1.404E-11	0.009124997	0.000580183	0.001531761	-0.405760926	-0.211892201	
chr17	36571512	36572011	500	*	3	1.09282E-16	0.000369717	0.000582338	0.000133582	-0.273604061	-0.133204362	
chr5	100115478	100115829	352	*	2	9.91743E-13	0.003760045	0.000584736	0.001086925	-0.346702739	-0.181605715	
chr9	134207932	134208328	397	*	2	1.59694E-12	0.078001829	0.000587016	0.002498741	0.223167657	0.110735712	
chr14	90440238	90441059	822	*	3	4.93364E-23	6.64527E-05	0.000587019	1.46559E-05	-0.362710552	-0.232372055	TDP1
chr16	56892074	56892460	387	*	2	6.38232E-13	0.016380151	0.000587554	0.001856985	-0.326700625	-0.154497411	MIR138-2
chr9	130603515	130603995	481	*	3	1.75612E-14	0.007750095	0.000588569	0.00169197	0.179804094	0.073850413	ENG
chr6	341180	341625	446	*	3	3.64861E-19	0.000594918	0.000589959	5.28402E-05	-0.281343057	-0.08469842	DUSP22

chr16	84377124	84377912	789	*	3	6.6328E-23	1.23557E-07	0.000592166	4.95965E-07	-0.390185471	-0.189654635	
chr5	142527658	142528481	824	*	2	1.22181E-14	5.07047E-06	0.000593659	1.14102E-05	-0.257545869	-0.205953434	ARHGAP26
chr2	64450285	64450561	277	*	2	9.1623E-16	1.50776E-05	0.000593943	2.89075E-05	-0.348718745	-0.277210601	AC074289.1
chr11	58991197	58991803	607	*	2	2.24211E-14	0.013519758	0.000594003	0.001770398	-0.342950513	-0.163092089	
chr10	13812071	13812323	253	*	4	1.21956E-22	0.001067407	0.000594117	1.64881E-05	-0.282478717	-0.160523893	FRMD4A
chr4	15080890	15081163	274	*	2	3.37724E-17	3.52956E-06	0.000594771	8.29276E-06	-0.230876176	-0.212755286	RP11-665G4.1
chr5	54916621	54917216	596	*	2	2.27008E-16	2.6017E-06	0.000594989	6.31486E-06	-0.29766841	-0.250031915	
chr9	126065063	126065710	648	*	4	9.96069E-29	2.39944E-05	0.000595236	4.31692E-07	0.18335103	0.1065747	
chr9	139795523	139796368	846	*	5	1.44155E-29	0.000953738	0.000596213	2.64953E-05	-0.289012078	-0.086337285	TRAF2
chr5	86707074	86707303	230	*	2	1.15474E-12	0.006718433	0.000596307	0.001397442	-0.372659325	-0.211157167	CCNH
chr11	47517145	47517381	237	*	2	1.84398E-13	0.015932407	0.00059641	0.001863584	-0.392584147	-0.18492754	CELF1
chr9	127117328	127117813	486	*	2	3.97586E-13	0.063464297	0.000596484	0.00247332	-0.415024297	-0.208594188	PSMB7
chr17	9802783	9803389	607	*	4	6.70627E-33	4.27261E-10	0.000597566	4.0311E-09	-0.344447032	-0.241632077	RCVRN
chr5	158654243	158654655	413	*	2	2.07401E-14	0.169834361	0.0005997	0.002687489	-0.343860488	-0.171350234	CTB-11I22.1
chr7	104771546	104771554	9	*	2	2.61883E-17	7.2028E-06	0.00059998	1.5508E-05	0.162599856	0.142125949	SRPK2
chr13	114263342	114264438	1097	*	4	1.40221E-28	4.83731E-07	0.00060019	3.42943E-07	-0.353426083	-0.174339793	TFDP1
chr13	99969116	99969273	158	*	2	1.10607E-11	0.010028827	0.000600715	0.001622203	-0.28229362	-0.133253123	UBAC2
chr1	155620312	155620728	417	*	2	8.92339E-13	0.007914865	0.000601244	0.001493938	-0.297626125	-0.153725734	MSTO1, MSTO2P, RP11-29H23.5
chr15	67492223	67492541	319	*	2	8.11915E-13	0.045368296	0.000601388	0.002371882	-0.25544116	-0.124643502	
chr14	77417199	77417243	45	*	2	5.40141E-15	2.10488E-05	0.000603128	3.81734E-05	-0.223299315	-0.183883422	
chr14	89774031	89774948	918	*	3	7.81306E-17	1.25356E-06	0.000604788	2.7654E-06	-0.197135684	0.018449807	FOXN3
chr1	2076496	2076778	283	*	2	1.17725E-13	0.205628112	0.000606584	0.002732159	-0.204820217	-0.101735967	PRKCZ, RP5-892K4.1
chr10	126359887	126360266	380	*	2	3.25162E-15	4.41537E-05	0.000607189	6.89647E-05	-0.360423494	-0.215775641	RP11-12J10.3, FAM53B
chr1	6419608	6419767	160	*	2	6.56401E-19	0.000230898	0.000607442	0.00022985	0.16758379	0.128878192	ACOT7
chr8	37967091	37967201	111	*	2	1.74036E-19	2.5035E-06	0.0006081	6.11824E-06	-0.315053291	-0.294320493	ASH2L
chr4	108749402	108749432	31	*	2	3.08456E-11	0.009228116	0.000608442	0.001591212	-0.345062718	-0.192888443	SGMS2
chr17	80832328	80834228	1901	*	9	6.57646E-52	9.43065E-08	0.000608934	1.7169E-10	-0.374256953	-0.179564081	TBCD
chr7	100885251	100885706	456	*	2	3.59067E-14	0.032263905	0.000608985	0.002250719	-0.193712044	-0.102774291	FIS1
chr20	44597685	44598468	784	*	2	1.39684E-15	0.009345983	0.000609521	0.001600271	-0.290411551	-0.133524518	ZNF335

chr12	132274349	132274355	7	*	2	1.08685E-11	0.013817863	0.000609567	0.001816916	-0.302553515	-0.164985633	SFSWAP
chr17	62168737	62169667	931	*	2	2.20836E-12	2.39212E-06	0.000609633	5.87424E-06	-0.348997483	-0.109671531	ERN1
chr2	206853238	206853388	151	*	2	3.84634E-12	0.019011754	0.000609827	0.001990003	-0.200465492	-0.103319167	
chr15	29285352	29285727	376	*	2	2.20657E-12	0.034319906	0.000610602	0.002283643	0.14800283	0.070238183	APBA2
chr20	49041703	49042109	407	*	2	2.18357E-12	0.109727715	0.000610654	0.00266467	-0.233492287	-0.115885343	
chr7	50252529	50253138	610	*	2	6.19336E-16	3.68683E-05	0.000611976	6.0041E-05	-0.145017229	-0.102227558	
chr7	37298864	37299382	519	*	4	9.59116E-27	1.84346E-05	0.000613135	1.27454E-06	-0.185700057	-0.109968391	ELMO1
chr8	21769695	21771059	1365	*	5	3.06605E-29	0.002849256	0.000613868	0.000127153	0.194560246	0.069328065	DOK2
chr7	137775104	137776547	1444	*	5	6.07441E-27	0.011558665	0.000613913	9.3966E-05	-0.282844635	-0.102855086	AKR1D1, RN7SKP223
chr9	112889386	112890072	687	*	3	1.81812E-21	1.33506E-06	0.000614643	3.59786E-06	-0.317063512	-0.205299271	PALM2-AKAP2, AKAP2
chr13	46679079	46679242	164	*	3	3.85144E-13	0.048845219	0.00061465	0.003641228	-0.319154188	-0.114182803	CPB2-AS1, CPB2
chr5	39215190	39215507	318	*	2	4.49477E-14	0.000117491	0.000617182	0.000144363	-0.289035273	-0.076091802	FYB
chr1	114429815	114430786	972	*	5	4.1702E-27	1.95306E-05	0.000619155	1.93208E-05	-0.310554457	-0.107752418	AP4B1-AS1, BCL2L15
chr3	186742897	186743256	360	*	2	1.82484E-14	0.00208585	0.000620415	0.000855082	0.111339972	0.015363399	ST6GAL1
chr8	38205729	38206041	313	*	2	3.83937E-12	0.135303141	0.000620683	0.002739799	-0.352775692	-0.175736795	WHSC1L1
chr2	37422644	37422917	274	*	2	1.26784E-16	0.006918549	0.000620739	0.001453121	-0.334494425	-0.148665724	SULT6B1
chr7	139865374	139865794	421	*	2	2.05741E-12	0.063133071	0.000620929	0.002556832	-0.323126343	-0.163854212	KDM7A
chr17	25886248	25887224	977	*	6	3.76097E-35	1.10317E-10	0.000621744	1.85302E-10	-0.327358808	-0.190332088	KSR1
chr12	1949503	1949586	84	*	2	5.22348E-14	0.00120297	0.000621911	0.000641286	-0.155821634	-0.09302951	CACNA2D4
chr10	80059603	80059610	8	*	2	5.45168E-14	5.37645E-05	0.000622038	8.0903E-05	-0.129239746	-0.124124226	LINC00856, LINC00595, RP11-90J7.2
chr9	2156196	2157105	910	*	3	1.46845E-17	0.000583792	0.0006229	0.000213621	-0.304203684	-0.154887654	SMARCA2
chr20	43882990	43884219	1230	*	8	7.64094E-50	4.70545E-12	0.000624008	1.60769E-12	-0.330036056	-0.148006992	SLPI
chr16	2043558	2044230	673	*	3	2.19466E-15	0.016131874	0.00062484	0.002056776	-0.164323551	-0.063341711	SYNGR3
chr13	37011756	37011966	211	*	2	8.42414E-13	0.025496335	0.000624971	0.0021825	-0.390565898	-0.187530255	CCNA1
chr4	77137900	77138258	359	*	3	1.20632E-17	3.24506E-05	0.000627254	4.49147E-05	-0.20111571	-0.059074445	FAM47E
chr15	72468522	72469006	485	*	3	5.35538E-17	0.000696522	0.000627359	0.000442912	0.220216906	0.102328227	GRAMD2
chr3	195106412	195106547	136	*	2	2.3181E-16	5.77086E-06	0.000627768	1.29025E-05	-0.197399352	-0.19493602	ACAP2
chr1	44798857	44799317	461	*	4	1.29371E-15	0.111418895	0.000628379	0.006049683	-0.302222407	-0.086245986	ERI3
chr2	10860366	10860952	587	*	2	1.04098E-16	0.22997362	0.000628547	0.002827444	-0.134365783	-0.066746393	AC092687.3

chr12	15409818	15409872	55	*	2	4.43876E-13	0.000652143	0.000628604	0.00045338	-0.320402624	-0.18463492	RERG
chr1	243641182	243641454	273	*	2	9.96768E-12	0.012815012	0.000629804	0.001818903	-0.3154842	-0.164237345	SDCCAG8
chr5	151132418	151132702	285	*	2	1.44375E-15	0.002869026	0.00063137	0.00100918	-0.398161635	-0.209817391	ATOX1
chr11	44896984	44897089	106	*	2	1.11619E-17	2.61818E-06	0.000631427	6.40267E-06	-0.273371398	-0.214096198	TSPAN18
chr1	150277456	150278094	639	*	3	3.19965E-14	0.013783536	0.000633209	0.00250051	-0.166589797	-0.046882322	MRPS21
chr2	36757828	36758130	303	*	3	2.29452E-13	0.020129182	0.000633293	0.003079493	-0.281227598	-0.100596391	CRIM1
chr4	1294432	1295078	647	*	8	7.13516E-58	6.24199E-16	0.000634088	9.68886E-15	-0.372407198	-0.27575369	MAEA
chr14	25045028	25046267	1240	*	7	3.09669E-43	2.05313E-09	0.000634891	1.98577E-10	-0.365403015	-0.226756856	CTSG
chr17	56352875	56352895	21	*	2	3.52122E-17	2.92151E-06	0.000635751	7.07295E-06	-0.29440368	-0.276414311	MPO
chr19	15698550	15699028	479	*	2	2.70575E-15	2.95292E-05	0.000637591	5.09356E-05	-0.301174981	-0.237790002	
chr11	128333168	128333573	406	*	2	3.01025E-13	0.008566116	0.000640156	0.001607071	0.17464549	0.094286111	ETS1
chr1	197433283	197433347	65	*	2	2.84703E-17	3.12561E-06	0.000641483	7.52426E-06	-0.272391736	-0.231690285	CRB1
chr14	56264322	56264651	330	*	3	2.30973E-20	3.99512E-06	0.000642403	5.64365E-06	0.219589599	0.091809804	
chr14	71608659	71609158	500	*	2	1.3507E-16	3.04587E-06	0.000645057	7.35763E-06	-0.255420811	-0.195121622	RP6-91H8.3
chr14	64933763	64933964	202	*	2	3.96832E-14	0.000270577	0.000647876	0.000262949	-0.365967043	-0.238008429	AKAP5, ZBTB25
chr21	34431089	34431807	719	*	4	6.04775E-22	0.021090836	0.000648771	7.95986E-05	-0.314446176	-0.154020246	LINC00945, AP000282.2
chr8	37730745	37730793	49	*	2	1.15256E-11	0.007542616	0.000650079	0.001550381	-0.19341037	-0.108450832	RAB11FIP1
chr14	88457175	88457365	191	*	2	1.17323E-14	0.000213996	0.000652003	0.000224939	-0.294588364	-0.163317386	GALC
chr3	183260220	183260375	156	*	2	1.05035E-12	0.0003274	0.000653028	0.000299387	0.198402884	0.130257551	KLHL6
chr10	45958759	45959309	551	*	4	3.15106E-33	7.47781E-11	0.000653654	9.73419E-10	-0.353178206	-0.292299774	MARCH8
chr1	95260703	95261186	484	*	2	1.70449E-12	0.060030248	0.000655496	0.002658039	-0.34570322	-0.174055312	LINC01057
chr1	48224130	48225077	948	*	3	2.0198E-15	0.153623286	0.000655863	0.00349742	-0.332089819	-0.101149155	
chr10	126790026	126790644	619	*	3	4.02141E-15	0.018455648	0.000656207	0.000879737	-0.402914134	-0.15733246	CTBP2
chr6	14758733	14759269	537	*	3	4.08317E-21	2.15944E-07	0.000656524	7.28155E-07	-0.257455885	-0.18017861	
chr16	67439320	67439511	192	*	2	3.32174E-14	0.005000083	0.000656619	0.001325836	-0.297299658	-0.156072383	ZDHHC1
chr14	102676957	102677307	351	*	4	1.94218E-32	1.36383E-10	0.000656988	1.63329E-09	-0.442862536	-0.32113466	WDR20
chr7	10980278	10981034	757	*	4	1.66394E-25	0.082426075	0.000657909	0.006879472	0.116880114	0.030933199	RP5-855F16.1
chr7	44145110	44145333	224	*	2	1.57417E-13	0.026403428	0.000658233	0.002292221	-0.090875876	-0.030910951	AEBP1
chr22	27671465	27672129	665	*	4	8.96468E-25	0.000905224	0.000659064	4.56529E-05	-0.27258887	-0.108489164	

chr8	123859056	123859953	898	*	4	7.3355E-18	0.056906344	0.000659254	0.002643974	-0.28399669	-0.087967556	ZHX2
chr1	76250734	76250742	9	*	2	2.01536E-16	0.000582455	0.00066061	0.000433989	-0.371283991	-0.218873366	ACADM
chr17	75537017	75537444	428	*	3	1.35026E-19	0.004427355	0.000660889	3.49934E-05	0.187365512	0.112570177	
chr1	35920054	35920411	358	*	3	1.41574E-21	4.38415E-07	0.000661282	1.31541E-06	-0.378617435	-0.239401685	KIAA0319L
chr8	71128925	71129750	826	*	3	4.03509E-14	0.078138117	0.000661448	0.005547658	-0.126967428	-0.048492235	NCOA2
chr7	107202516	107203448	933	*	3	7.08369E-17	0.037262805	0.000662841	0.004073192	-0.319071511	-0.113149586	COG5
chr19	40728255	40728617	363	*	2	1.61118E-14	0.061653464	0.000663292	0.002694304	0.196370062	0.101597922	CNTD2
chr15	65167336	65168143	808	*	2	7.69834E-14	9.94321E-05	0.00066428	0.000131365	0.186727741	0.040290163	AC069368.3
chr5	49739158	49739641	484	*	2	2.64001E-14	0.000251157	0.000664467	0.000252864	-0.213692249	-0.167395299	
chr11	34663205	34663681	477	*	3	8.53571E-15	0.009695531	0.000664775	0.001701875	-0.283248589	-0.108561148	EHF
chr22	24992578	24992604	27	*	2	7.7179E-17	3.36883E-06	0.00066561	8.08928E-06	-0.194412463	-0.185952008	SNRPD3, GGT1
chr9	95497984	95498022	39	*	2	5.34519E-12	0.000637934	0.000665632	0.000460509	0.150366102	0.111537896	BICD2
chr11	13319763	13320237	475	*	2	1.5061E-12	0.010877148	0.000666553	0.001797715	-0.203037654	-0.106057623	ARNTL
chr2	129199624	129200109	486	*	2	7.18304E-13	0.019373387	0.000667092	0.002142916	0.221878948	0.099384862	
chr8	144623533	144625381	1849	*	10	3.4963E-23	0.391103871	0.000668484	0.005851074	-0.258839281	-0.009600957	7SK, ZC3H3, RP11-661A12.5
chr8	101315424	101316253	830	*	8	5.54525E-39	0.000293636	0.000670237	2.2521E-07	-0.356903199	-0.125669836	RNF19A
chr11	74974504	74975244	741	*	4	3.73408E-23	0.000272183	0.000670991	1.68423E-05	-0.311130523	-0.161321825	ARRB1
chr8	37757546	37758453	908	*	3	5.90347E-25	7.17769E-07	0.000671078	2.42495E-06	-0.217836169	-0.161504226	
chr2	169893315	169893745	431	*	2	1.14431E-12	0.181527063	0.000671306	0.002975275	-0.29349321	-0.147420439	
chr3	194979346	194980397	1052	*	4	1.35923E-19	0.002393302	0.000672572	0.000433111	-0.403343014	-0.139688684	XXYLT1
chr16	11682634	11682916	283	*	2	2.98717E-15	0.000603052	0.000672598	0.000447306	-0.113272846	-0.081342567	LITAF
chr5	124687070	124688089	1020	*	3	1.89428E-16	0.036418894	0.00067281	0.00319372	-0.254848888	-0.104932111	
chr7	150436665	150437232	568	*	2	1.57947E-13	0.001002321	0.000673134	0.000604547	-0.341883174	-0.15244357	GIMAP5
chr20	45946271	45947895	1625	*	9	3.15644E-42	0.000114832	0.000675518	7.37312E-06	-0.180743019	-0.072720879	AL031666.2, ZMYND8
chr1	12240593	12241073	481	*	2	1.98616E-16	3.0501E-06	0.000676969	7.41626E-06	-0.272791377	-0.250671022	TNFRSF1B
chr4	56962740	56963276	537	*	2	1.09438E-12	0.013210641	0.000680481	0.0019445	0.187419265	0.087792145	
chr15	49355609	49356663	1055	*	3	2.22649E-19	9.19198E-06	0.00068303	1.1816E-05	-0.351880156	-0.155716242	
chr8	74331668	74332829	1162	*	9	8.74986E-36	0.035415218	0.000683587	6.98997E-05	-0.24712768	-0.067559272	STAU2-AS1, STAU2
chr1	182764125	182765260	1136	*	3	1.861E-19	8.13121E-05	0.000684556	1.8256E-05	-0.245093767	-0.141867621	NPL

chr17	71190894	71191193	300	*	2	8.01999E-13	0.000454169	0.000684653	0.000378014	-0.211841795	-0.129588998	RP11-143K11.5, COG1
chr10	82362642	82363434	793	*	6	2.66306E-19	0.201149028	0.000685102	0.002467965	-0.155053815	-0.0523126	SH2D4B
chr15	90368716	90369040	325	*	2	3.83024E-17	1.11859E-05	0.000685152	2.32231E-05	-0.216743837	-0.186246931	
chr8	28908912	28909794	883	*	3	3.47061E-17	4.26311E-06	0.000686578	6.74571E-06	0.161663325	0.069510698	HMBOX1
chr11	116684684	116685586	903	*	5	1.0243E-25	0.000119917	0.000686679	2.23945E-05	-0.38986147	-0.130539479	AP006216.5
chr2	42165157	42165504	348	*	2	8.83627E-13	0.032285168	0.000686768	0.002479083	0.225249345	0.109370681	C2orf91
chr6	3025064	3026244	1181	*	3	2.76368E-15	1.34766E-07	0.000686888	5.52099E-07	-0.200219864	-0.15346357	RP1-90J20.11
chr10	99168386	99168651	266	*	3	2.87637E-25	1.55555E-08	0.000691556	8.82653E-08	-0.331550221	-0.292278621	RP11-452K12.7
chr2	47197798	47198044	247	*	2	8.6008E-15	2.35282E-05	0.00069182	4.33091E-05	-0.284477812	-0.198671542	TTC7A
chr6	135516912	135518423	1512	*	6	3.79944E-21	3.26382E-05	0.00069215	2.68281E-06	-0.33558866	-0.152437941	MYB, MYB-AS1
chr17	7529693	7530477	785	*	2	3.79792E-14	0.084873675	0.00069232	0.002902052	-0.278485559	-0.137600122	SHBG, SAT2
chr5	149996672	149997694	1023	*	7	2.08797E-20	0.204537247	0.000692856	0.025680288	-0.30176075	-0.047105571	SYNPO
chr4	174411279	174411723	445	*	2	7.16197E-18	3.4428E-06	0.000692943	8.29558E-06	-0.237394088	-0.209224434	
chr5	138718812	138719470	659	*	5	5.59248E-16	0.370701638	0.000693315	0.027353836	-0.253168258	-0.05549869	SLC23A1
chr14	105792505	105792853	349	*	3	2.94358E-15	0.004069778	0.000693436	0.00124855	-0.385401233	-0.143287228	PACS2
chr6	44004565	44004688	124	*	2	5.91145E-16	1.041E-05	0.000695395	2.19076E-05	-0.262619261	-0.205521495	RP5-1120P11.1
chr11	134125564	134126504	941	*	6	2.81587E-39	7.06031E-08	0.000696217	5.02953E-09	-0.373956341	-0.232558943	ACAD8
chr16	57864721	57865112	392	*	4	1.72172E-13	0.137465934	0.000697279	0.010690448	-0.161350459	-0.046332821	KIFC3
chr7	1063891	1064100	210	*	2	1.42006E-15	0.000148472	0.000697588	0.000178944	-0.229948408	-0.160123565	C7orf50
chr8	117049539	117050063	525	*	2	1.10099E-12	0.006525794	0.000697965	0.001539983	-0.289172541	-0.127435408	LINC00536
chr14	65140250	65140623	374	*	2	1.9163E-13	0.000229995	0.000698856	0.000243307	-0.239719636	-0.153321982	
chr8	19539991	19540809	819	*	11	8.9835E-71	1.74911E-17	0.000702317	2.10068E-16	-0.363142128	-0.199998094	RP11-1105O14.1, CSGALNACT1
chr22	24039722	24040094	373	*	4	1.12693E-22	2.24233E-05	0.000702463	5.18443E-06	-0.308133004	-0.167394761	RGL4, GUSBP11, KB-1572G7.2, AP000347.2
chr5	32538579	32539013	435	*	2	4.40535E-12	0.070472074	0.000703161	0.002879304	-0.255491611	-0.125094245	SUB1
chr10	118974438	118974916	479	*	2	7.57876E-14	0.027255056	0.000703718	0.002434145	-0.318439623	-0.167464979	
chr3	184297380	184297522	143	*	3	9.86857E-14	0.001701244	0.000704417	0.000757232	-0.285695576	-0.135998192	EIF2B5, EPHB3
chr11	110296837	110297121	285	*	3	5.08104E-15	0.001063275	0.000706013	0.000368412	-0.436108845	-0.175142436	
chr5	54275155	54275198	44	*	2	1.86026E-12	0.000239623	0.000706147	0.000251328	-0.27528564	-0.18747858	ESM1
chr6	168495097	168495103	7	*	2	1.39451E-10	0.045367454	0.000708839	0.002715534	-0.13539721	-0.064486677	

chr2	46294020	46294133	114	*	2	2.65456E-11	0.180936095	0.000708882	0.00312067	0.213124021	0.107485899	PRKCE
chr15	101746686	101748973	2288	*	5	8.95096E-18	3.42254E-05	0.000709817	7.59604E-06	-0.414657091	-0.077340675	CHSY1
chr12	69401330	69401704	375	*	2	2.90885E-12	0.005052153	0.000709899	0.001402392	-0.104871016	-0.062411278	
chr17	74666659	74667276	618	*	3	3.48573E-22	1.46836E-05	0.000712076	1.06176E-05	-0.233027496	-0.113633247	
chr21	45507885	45508464	580	*	4	4.36374E-22	0.000270627	0.000713519	2.27825E-05	-0.376202931	-0.182804111	TRAPPC10
chr5	14325937	14326531	595	*	5	3.11169E-28	3.82304E-08	0.000714192	1.38563E-07	-0.318228062	-0.18236578	TRIO
chr18	77195886	77196450	565	*	3	1.32141E-18	0.000118836	0.000714201	0.000125192	0.211592754	0.112209564	NFATC1
chr8	37992045	37992521	477	*	2	6.2085E-13	0.00108907	0.000714793	0.000654636	-0.262216344	-0.140688623	ASH2L
chr14	24704981	24705400	420	*	2	2.63359E-14	0.136034376	0.000715148	0.003100812	0.18224157	0.093378052	GMPR2
chr6	7402594	7403054	461	*	5	5.40989E-17	0.326752426	0.000715958	0.019452857	-0.289227779	-0.061333703	RIOK1
chr6	21856149	21857295	1147	*	4	3.32854E-26	5.17583E-10	0.000716778	5.34824E-09	-0.265820689	-0.218297172	CASC15
chr11	78000703	78001599	897	*	3	3.72635E-17	6.92778E-06	0.000717471	1.56503E-05	-0.263500745	-0.212168753	GAB2
chr3	128710390	128710863	474	*	3	2.31236E-20	3.87192E-06	0.000717615	5.31283E-06	-0.379602797	-0.220986814	KIAA1257
chr8	48556931	48557420	490	*	4	1.62051E-24	5.40426E-05	0.000717744	2.2948E-06	-0.379887763	-0.193668048	SPIDR
chr2	239478365	239478514	150	*	3	8.03338E-20	1.98016E-06	0.000718662	3.56918E-06	-0.245368906	-0.142125107	
chr2	103038171	103038531	361	*	2	2.45893E-12	0.015016643	0.000718757	0.00210716	-0.113970729	-0.049711	IL18RAP
chr4	7647940	7649606	1667	*	6	7.96145E-29	4.055E-05	0.000718844	1.94044E-05	-0.302178476	-0.107817337	SORCS2
chr16	85577361	85578516	1156	*	4	1.36207E-24	8.73135E-05	0.000720069	8.00511E-06	-0.327826434	-0.196001266	
chr2	242801252	242802192	941	*	6	7.57196E-30	3.10887E-05	0.000720301	1.88127E-06	0.182725552	0.093212873	
chr9	130910995	130911792	798	*	5	2.44919E-32	2.11811E-05	0.000721161	6.67897E-07	-0.240703238	-0.119716669	LCN2
chr17	1586965	1587888	924	*	3	6.59373E-21	0.001557035	0.000721185	0.000782083	-0.191315325	-0.070674478	PRPF8
chr11	63618574	63619042	469	*	3	4.34798E-14	0.195258059	0.000722551	0.00732811	0.229320036	0.073228253	MARK2
chr17	48285819	48286851	1033	*	3	8.51745E-16	0.048093216	0.000722685	0.003483493	0.16187549	0.04926519	RP11-893F2.5
chr11	3147616	3148731	1116	*	5	2.5886E-32	5.35979E-06	0.00072331	1.77825E-07	-0.291188161	-0.158304891	OSBPL5
chr3	127831261	127831673	413	*	3	1.40344E-12	0.148416758	0.000723704	0.007406056	0.204789691	0.062147263	RUVBL1
chr21	34383810	34383987	178	*	2	1.26539E-10	0.107700416	0.000723817	0.003081912	-0.29833008	-0.147521251	AP000282.2
chr7	38695932	38696726	795	*	2	7.06262E-14	2.31843E-05	0.000726073	4.33135E-05	-0.139271494	-0.133994247	KRT8P20
chr16	8943122	8943436	315	*	2	5.20368E-14	0.00791317	0.000727639	0.001707593	-0.331642724	-0.149111545	PMM2, RP11-77H9.2
chr1	35913782	35914081	300	*	3	1.75972E-22	9.77713E-08	0.000728302	4.50205E-07	-0.333592553	-0.216681597	KIAA0319L

chr16	2576082	2576370	289	*	3	5.93841E-21	9.73381E-07	0.00072966	2.68533E-06	-0.210406322	-0.170213054	ATP6C, AMDHD2
chr4	124476694	124477103	410	*	3	4.43388E-16	0.000248377	0.000729818	0.000226919	-0.147579381	-0.093100587	
chr17	80260410	80261212	803	*	3	3.52058E-18	0.000103378	0.000730387	5.74088E-05	0.217646522	0.106649949	
chr2	231731972	231732669	698	*	3	9.47024E-16	0.002703843	0.000731562	0.000950905	-0.174967051	-0.088419616	ITM2C
chr2	237657127	237657200	74	*	2	2.60504E-17	4.52493E-06	0.000731771	1.06756E-05	-0.264111403	-0.229856681	AC011286.1
chr20	33222426	33222860	435	*	3	3.86225E-20	5.28664E-05	0.000731994	1.90129E-05	-0.371249906	-0.245735873	PIGU
chr5	172202805	172203421	617	*	3	6.20667E-17	0.001759999	0.000732297	4.50951E-05	-0.263379767	-0.160630235	RP11-779O18.3
chr12	57893726	57894138	413	*	2	6.50712E-12	0.010294973	0.000732313	0.001887752	-0.30281951	-0.165887505	MARS
chr21	39682577	39683147	571	*	3	6.43804E-15	0.020485036	0.000732353	0.002313648	-0.328229706	-0.110229674	
chr19	33781539	33781998	460	*	3	4.85173E-13	0.112090805	0.000733169	0.006554209	-0.31872651	-0.09786554	
chr6	131893378	131894023	646	*	4	9.38944E-24	0.001386316	0.000734876	6.42306E-05	-0.387679233	-0.203708439	
chr1	154237531	154238859	1329	*	6	4.77158E-22	0.018867905	0.000735213	0.003132423	0.348798184	0.055162625	UBAP2L
chr21	30449989	30450130	142	*	2	1.04287E-16	4.37759E-06	0.000735354	1.03728E-05	-0.326968647	-0.289957714	MAP3K7CL
chr1	25439789	25440431	643	*	4	9.64625E-19	0.00024355	0.000735807	3.72508E-05	-0.210016606	-0.10038668	
chr2	190522601	190523706	1106	*	4	1.5924E-18	0.000168656	0.000736878	0.000162152	0.177291904	0.045085942	
chr1	109935710	109936194	485	*	2	6.83978E-15	0.008552198	0.000737557	0.00177468	-0.291931361	-0.172195081	SORT1
chr5	177777391	177777704	314	*	3	2.96875E-19	0.000102661	0.000738429	1.94123E-05	-0.210363059	-0.124468753	COL23A1
chr5	93758545	93758649	105	*	2	9.78129E-11	0.006987027	0.000741096	0.001648217	-0.288935235	-0.171027711	KIAA0825
chr11	112263480	112263709	230	*	3	7.21987E-19	1.65901E-05	0.00074145	1.33975E-05	-0.241887703	-0.176477629	
chr1	77999560	77999814	255	*	2	2.07484E-11	0.347249376	0.000742279	0.003296746	-0.303545808	-0.152530626	AK5
chr2	208734940	208735200	261	*	2	6.41282E-13	0.000201237	0.000742749	0.000227422	-0.350727657	-0.248030848	PLEKHM3
chr16	28517278	28518347	1070	*	7	2.32504E-43	2.20876E-13	0.000743623	3.30239E-12	-0.320602306	-0.166571778	IL27
chr2	7150625	7150719	95	*	2	3.4171E-16	4.71179E-06	0.000743992	1.10949E-05	-0.290879353	-0.252637116	RNF144A
chr6	7165647	7167468	1822	*	4	3.91544E-22	3.19513E-10	0.000745124	3.56606E-09	-0.307394601	-0.124921826	RREB1
chr16	84118667	84118790	124	*	3	6.20197E-18	6.95324E-06	0.000745933	1.68638E-05	-0.180630329	-0.131840132	MBTPS1
chr22	21258874	21259095	222	*	2	1.01607E-11	0.001239959	0.000747204	0.00072159	-0.329045415	-0.185331406	
chr5	43533981	43534107	127	*	2	2.03576E-10	0.054273345	0.000747847	0.002923105	0.23492191	0.123399665	PAIP1
chr5	78184094	78184639	546	*	2	1.96536E-12	0.012131937	0.000748379	0.002027968	-0.375956537	-0.191583431	ARSB
chr7	1012599	1012935	337	*	2	4.87831E-16	6.66427E-05	0.000748613	0.000101245	-0.242194	-0.207494892	COX19

chr17	76384259	76384516	258	*	2	7.54471E-11	0.094689094	0.000749014	0.003137848	-0.167801284	-0.082945038	PGS1
chr6	138426712	138427309	598	*	3	7.04489E-15	0.001091221	0.000749547	0.000601555	-0.213783711	-0.128696134	PERP
chr8	23405208	23405296	89	*	2	1.60428E-10	0.012666427	0.000749741	0.002059769	-0.210147075	-0.11462595	SLC25A37
chr6	25965330	25966684	1355	*	4	2.59359E-24	2.06627E-06	0.000750894	2.94354E-07	-0.283190174	-0.212432679	TRIM38
chr3	46504469	46505093	625	*	3	1.43044E-16	0.058534958	0.000751814	0.004205762	-0.213529796	-0.057478362	LTF
chr16	1988449	1988798	350	*	2	2.65137E-16	3.9563E-06	0.000752597	9.50726E-06	-0.338274861	-0.289716966	MSRB1
chr1	180579182	180579844	663	*	3	7.30336E-17	1.54333E-05	0.000752878	1.39285E-05	-0.348364937	-0.066995379	
chr10	3933423	3933940	518	*	3	1.33378E-13	0.02194119	0.000754338	0.00263033	-0.275743234	-0.100468029	
chr20	33491767	33492100	334	*	2	1.28333E-11	0.004871052	0.00075589	0.001438812	0.255173835	0.12040007	ACSS2
chr10	3507176	3507462	287	*	2	6.94534E-12	0.00967138	0.000757567	0.001891488	-0.24363255	-0.143901834	
chr15	91490436	91490804	369	*	2	1.98169E-12	0.009489829	0.000760771	0.001884228	0.11281909	0.07122839	UNC45A
chr3	52320096	52321832	1737	*	14	1.00802E-41	0.006860792	0.000761296	0.000310106	-0.381784988	-0.069125989	GLYCTK, WDR82
chr2	129491004	129491398	395	*	2	1.16014E-12	0.021620826	0.000761666	0.002443273	-0.267734254	-0.129853524	
chr1	236109168	236109875	708	*	3	4.33607E-20	1.24274E-05	0.000761676	1.42936E-05	-0.405042316	-0.250644313	
chr1	1689012	1690389	1378	*	7	3.18387E-23	0.412601043	0.000762065	0.01055152	-0.189847289	-0.040213272	NADK
chr14	77542043	77543192	1150	*	8	5.51459E-31	0.000226185	0.000762398	4.87995E-06	-0.347635104	-0.102734947	RP11-7F17.4, RP11-7F17.3
chr7	4774448	4775278	831	*	3	2.79523E-14	0.00032733	0.000762902	0.00018494	0.11297161	0.073372586	FOXK1
chr1	12094651	12095619	969	*	2	1.50143E-12	9.45884E-06	0.000762948	2.05426E-05	-0.27186069	-0.223161953	
chr10	72326192	72326780	589	*	2	6.03619E-13	0.003877578	0.000765722	0.00131073	-0.243176635	-0.127532459	PALD1
chr6	167493383	167493614	232	*	2	1.74724E-11	0.009224622	0.000766006	0.00187395	0.109044121	0.068759438	RP11-517H2.6
chr2	48373026	48373252	227	*	2	7.7251E-11	0.013867613	0.000766089	0.002154778	-0.230990196	-0.107517842	AC079807.4
chr17	35467087	35467482	396	*	2	1.57065E-11	0.028647451	0.000766187	0.002633179	-0.188214638	-0.097869173	ACACA
chr11	64267330	64269071	1742	*	5	6.71785E-24	8.37537E-05	0.000766289	3.57068E-07	0.16750087	0.017691971	AP005273.1
chr12	108054373	108054842	470	*	3	9.526E-15	0.016422763	0.000766691	0.001303571	-0.275892134	-0.056048643	
chr14	20978481	20978830	350	*	5	1.8863E-21	0.019151921	0.00076704	0.000189367	-0.262563095	-0.094520893	RNASE10
chr8	126365081	126365454	374	*	2	3.43907E-12	0.004934748	0.000769511	0.001463929	-0.273571939	-0.168215584	NSMCE2
chr16	68280921	68281979	1059	*	4	2.76492E-28	1.14847E-09	0.000769861	1.01968E-08	0.200282761	0.143861781	PLA2G15
chr17	56357994	56358504	511	*	3	8.71659E-27	1.05471E-07	0.000772614	4.73123E-07	-0.224410549	-0.199588674	MPO
chr20	23107102	23107233	132	*	2	9.69462E-14	8.47419E-05	0.000774306	0.000122955	-0.094464383	-0.085847643	LINC00656

chr10	82251396	82251784	389	*	2	7.31479E-16	6.38366E-06	0.000774605	1.46114E-05	-0.368276545	-0.251806786	TSPAN14
chr22	31618985	31620013	1029	*	5	9.59215E-25	4.01124E-06	0.000776737	2.12107E-07	-0.288077562	-0.059275421	LIMK2
chr19	39142011	39142260	250	*	2	1.18835E-12	0.001671363	0.000776976	0.000869058	-0.32005765	-0.195977672	ACTN4
chr1	24523702	24524234	533	*	2	5.11095E-16	0.000172941	0.00077718	0.000208029	-0.389551547	-0.22522548	
chr15	92399195	92399897	703	*	2	2.38198E-14	0.001135649	0.00077852	0.000701899	0.132820724	0.023074894	SLCO3A1
chr4	87882342	87883021	680	*	2	5.49584E-15	6.45507E-05	0.000778695	0.000100026	-0.252208607	-0.149910441	AFF1
chr20	3087359	3088152	794	*	4	5.34466E-22	1.11944E-05	0.000779115	8.06007E-06	-0.226751821	-0.106735166	UBOX5-AS1
chr20	1800881	1801483	603	*	2	4.17016E-13	0.001160761	0.000780587	0.000711738	-0.3395626	-0.144178205	
chr17	79244158	79244962	805	*	4	6.70057E-22	0.004842081	0.000781783	8.81398E-05	-0.360255732	-0.13669236	SLC38A10
chr2	47073778	47074152	375	*	2	1.40202E-14	0.209427455	0.000781943	0.003419071	-0.304685859	-0.151975936	LINC01119
chr6	157942421	157942704	284	*	2	5.60748E-16	5.48186E-06	0.000782212	1.27903E-05	-0.2910894	-0.227884905	ZDHHC14
chr22	30612521	30612925	405	*	2	1.59725E-12	0.006357765	0.000783111	0.001647572	0.219057177	0.124201911	
chr20	43984001	43984247	247	*	2	2.09346E-11	0.00228509	0.000785308	0.001030389	0.168766408	0.073453179	
chr20	48383802	48383974	173	*	2	5.60213E-18	3.93359E-06	0.00078605	9.51581E-06	-0.26790732	-0.25255388	
chr8	23397998	23399610	1613	*	6	1.21164E-31	3.23448E-05	0.000786319	6.60586E-08	-0.268768748	-0.137782167	SLC25A37
chr17	4092306	4093344	1039	*	4	1.07195E-16	0.014272975	0.000788567	8.39376E-05	-0.228876984	-0.083744319	ANKFY1
chr15	86171055	86171721	667	*	3	8.47418E-19	2.42513E-06	0.000788859	4.57865E-06	-0.281092136	-0.170732095	AKAP13, RP11-815J21.4
chr21	35016787	35016873	87	*	2	2.7617E-15	3.76435E-05	0.000788983	6.56355E-05	-0.339368495	-0.218123114	ITSN1, AP000304.12
chr10	105792531	105793358	828	*	3	1.48477E-20	6.32629E-08	0.0007892	3.04497E-07	-0.310517516	-0.215843382	COL17A1
chr18	61344467	61345309	843	*	3	9.5716E-20	2.6647E-06	0.000789848	5.20413E-06	-0.308923012	-0.21081341	SERPINB11
chr3	182929476	182930505	1030	*	3	1.74269E-18	4.54664E-06	0.000789971	7.78739E-06	-0.247055251	-0.189710257	MCF2L2
chr19	4374239	4374567	329	*	2	3.32917E-12	0.002695254	0.000793238	0.001126398	-0.280874369	-0.148583641	SH3GL1
chr11	10482235	10482612	378	*	3	5.7523E-24	8.82275E-08	0.000795592	4.21334E-07	-0.412822456	-0.312566309	AMPD3
chr9	35042014	35042395	382	*	3	1.85257E-16	0.002514057	0.000795842	0.000241749	-0.165240888	-0.098932762	C9orf131, FLJ00273
chr16	17555588	17555963	376	*	2	3.04517E-11	0.091849251	0.000796022	0.003295393	-0.220768072	-0.111144092	XYLT1
chr7	127035858	127036361	504	*	2	2.29172E-12	0.002874543	0.000796168	0.001165329	-0.307099485	-0.172318292	ZNF800
chr3	5133902	5134159	258	*	2	8.1068E-14	4.90799E-05	0.000796439	8.12968E-05	-0.310511759	-0.18406345	
chr16	84218197	84219629	1433	*	7	4.11429E-38	6.78669E-06	0.000796599	2.71402E-08	0.229220627	0.124383051	TAF1C
chr15	91094168	91095069	902	*	3	1.97655E-16	0.001640999	0.000799015	0.000315	-0.368238042	-0.168351288	CRTC3

chr12	122249869	122251166	1298	*	7	4.70494E-21	0.196242237	0.000799231	0.000951461	-0.24066205	-0.026532345	SETD1B
chr15	50570103	50570473	371	*	2	6.38526E-16	5.15299E-06	0.000800045	1.21476E-05	-0.29775837	-0.225231686	GABPB1
chr5	176766085	176766954	870	*	3	2.50941E-19	0.000222139	0.000800154	4.82752E-05	-0.14462769	-0.018748587	LMAN2
chr2	40599929	40599951	23	*	2	1.6328E-16	6.70078E-06	0.000800365	1.53314E-05	-0.160794054	-0.151904271	SLC8A1
chr17	56315750	56316162	413	*	4	2.66524E-21	9.76883E-05	0.000801582	1.68099E-05	-0.330831392	-0.123194237	LPO
chr14	89626932	89626995	64	*	2	2.05638E-11	0.001906122	0.000801643	0.000948979	-0.169749024	-0.092826502	FOXN3
chr10	43366622	43367193	572	*	3	1.22167E-12	0.062856088	0.000803767	0.005851032	-0.284221446	-0.094200444	RP11-124O11.1
chr7	141646270	141647505	1236	*	4	3.18567E-22	0.002139771	0.000804216	5.26533E-05	-0.345937869	-0.150421771	MGAM, CLEC5A
chr16	54209183	54209779	597	*	4	1.2451E-21	0.002791078	0.000805999	0.000177873	-0.244183438	-0.101587742	
chr11	121466430	121466629	200	*	3	3.83179E-12	0.104290799	0.000806583	0.006613424	-0.25327075	-0.085799724	SORL1
chr18	21163172	21163549	378	*	2	1.62559E-11	0.007672862	0.000808363	0.00181461	-0.332213919	-0.17074486	NPC1
chr16	90023526	90024450	925	*	4	2.35912E-30	5.23223E-09	0.000809216	3.00841E-08	-0.29153104	-0.139128642	DEF8
chr8	22131448	22132434	987	*	4	1.63842E-29	0.000151627	0.000809761	2.16129E-05	-0.311105848	-0.131753097	CTD-2530N21.4
chr6	6928298	6928585	288	*	2	8.74263E-11	0.03743771	0.000813354	0.002927798	-0.317541762	-0.168575741	
chr10	90611604	90612228	625	*	6	2.04186E-37	3.08553E-11	0.000814072	5.80529E-10	-0.315533527	-0.172027016	
chr1	153337977	153338215	239	*	2	7.71513E-15	1.46237E-05	0.000814369	3.02421E-05	-0.206294578	-0.20310989	
chr22	24894704	24895105	402	*	2	2.06061E-11	0.020920031	0.00081474	0.002549641	-0.148270848	-0.064810725	UPB1
chr12	10283049	10283763	715	*	3	1.62215E-14	0.017370683	0.000815411	0.001635041	-0.273506949	-0.130057113	
chr17	15141116	15142509	1394	*	5	3.31223E-23	0.001630072	0.000818398	0.000133559	-0.262576054	-0.113577989	PMP22, snoU13
chr6	34308310	34309455	1146	*	3	4.35602E-17	0.007092874	0.000818679	0.000250269	-0.295637901	-0.059499259	NUDT3, RPS10-NUDT3
chr9	96929106	96929332	227	*	2	6.0818E-12	0.00055318	0.000819237	0.000466378	0.052352412	0.019032117	RP11-2B6.3
chr7	94185174	94185539	366	*	2	4.30123E-11	0.110784326	0.00081947	0.003437324	-0.294477953	-0.150135776	CASD1
chr21	18823448	18823803	356	*	2	2.66622E-12	0.000651873	0.000819708	0.000517195	-0.245467838	-0.175066302	
chr12	12620523	12620789	267	*	2	1.83268E-12	0.000303284	0.000820455	0.00031442	-0.315871905	-0.173719152	
chr18	77554583	77554951	369	*	2	3.06815E-13	0.059885749	0.000820803	0.003205479	-0.165963856	-0.075703776	RP11-154H12.3
chr11	63858820	63858880	61	*	2	3.30534E-16	4.25262E-06	0.000821317	1.02687E-05	-0.315542963	-0.278069899	RP11-21A7A.2, MACROD1
chr8	121797718	121798364	647	*	3	9.96878E-14	0.004792379	0.000821703	0.001678225	-0.282573385	-0.12231339	SNTB1
chr6	32967930	32968406	477	*	2	1.21115E-11	0.07606679	0.000823538	0.003320855	-0.382207775	-0.19719142	
chr4	1227091	1227400	310	*	2	2.6508E-12	0.004169582	0.000824597	0.001420187	0.17976445	0.09984265	CTBP1

chr14	105860954	105861260	307	*	3	3.48129E-18	0.000137696	0.00082523	3.69683E-05	-0.281985969	-0.055109048	PACS2
chr19	718875	719951	1077	*	5	5.60717E-20	0.002545847	0.000826474	0.00130796	-0.162001244	-0.061484567	PALM
chr12	113330370	113330801	432	*	2	2.95993E-12	0.024604284	0.000827582	0.002694146	-0.241697921	-0.12436021	RPH3A
chr12	10320327	10320770	444	*	4	3.94532E-24	6.52762E-08	0.000828221	3.45666E-07	-0.298681383	-0.21723394	OLR1
chr5	139043443	139044215	773	*	3	9.19784E-20	0.000119236	0.000828981	2.94142E-05	-0.230835469	-0.145481538	CXXC5
chr9	7976529	7977013	485	*	2	1.25005E-11	0.337930589	0.00083059	0.003640734	-0.312731322	-0.156922858	
chr6	13408158	13408527	370	*	3	8.83988E-14	0.067138613	0.000831293	0.006129727	-0.338647271	-0.109015036	GFOD1
chr17	78793479	78793807	329	*	2	4.07821E-14	0.000125683	0.000831307	0.000169193	-0.249459662	-0.173722438	RPTOR
chr1	36272405	36273317	913	*	3	9.1963E-18	0.000446136	0.000832834	0.000123518	-0.141458326	-0.066880911	
chr6	90271152	90271380	229	*	3	1.21496E-14	0.067497725	0.00083308	0.006192436	-0.284097333	-0.094623065	ANKRD6, RP11-16C18.3
chr7	50791421	50791508	88	*	2	1.06087E-11	0.000437472	0.000833326	0.000404118	-0.247184438	-0.161109348	GRB10
chr3	194826197	194827291	1095	*	6	1.78916E-29	2.35199E-05	0.000833457	7.13343E-07	-0.380393021	-0.165402894	XXYLT1
chr12	50481901	50482541	641	*	3	1.93245E-13	0.007296656	0.000833606	0.00149772	-0.363467543	-0.07565283	SMARCD1
chr16	56817295	56817478	184	*	3	7.41523E-14	0.016391216	0.000834297	0.000657457	-0.331336408	-0.160147833	NUP93
chr8	130692949	130693960	1012	*	3	1.13369E-14	0.112745728	0.000834644	0.007009907	-0.251053478	-0.083352158	RP11-419K12.1
chr16	4395425	4396298	874	*	4	5.50531E-23	2.15686E-05	0.000835315	8.5215E-06	-0.281219262	-0.157000306	PAM16, CORO7-PAM16
chr3	14859299	14859913	615	*	3	1.21414E-15	0.0239945	0.00083554	0.003952555	0.186134421	0.054059751	
chr3	128134323	128134845	523	*	2	1.29188E-14	2.45817E-05	0.000835945	4.70498E-05	-0.169738585	-0.01218542	
chr4	7830035	7830788	754	*	4	1.01805E-22	4.14733E-05	0.000837149	1.01861E-05	-0.343027393	-0.128898156	AFAP1
chr16	87228728	87228921	194	*	2	5.14666E-13	0.001213289	0.000837381	0.000757924	0.240840401	0.136886164	C16orf95
chr2	106438120	106438173	54	*	2	1.97152E-14	1.66391E-05	0.000840946	3.39744E-05	-0.382769113	-0.242399161	NCK2
chr2	38101511	38101852	342	*	2	2.46991E-13	0.000270221	0.000842233	0.000293757	0.197611745	0.133771276	LINC00211
chr1	13949753	13950049	297	*	3	4.68964E-12	0.114658268	0.000843715	0.006174129	-0.290777565	-0.117326657	RNA5SP41
chr11	32915497	32916120	624	*	4	4.26919E-15	0.194862533	0.000843896	0.008938127	-0.14034842	-0.034112494	QSER1
chr19	51843601	51843854	254	*	2	4.40981E-15	1.18894E-05	0.000844058	2.55131E-05	0.109941259	0.0678845	VSIG10L
chr20	35943580	35943761	182	*	2	6.45343E-14	4.5616E-05	0.000844274	7.79997E-05	0.211470109	0.147584463	MANBAL
chr9	110310795	110311041	247	*	3	6.93795E-20	9.19645E-07	0.000846364	2.85097E-06	-0.233841801	-0.181077705	
chr5	148799955	148800342	388	*	2	1.84269E-12	0.01409534	0.000846436	0.002330321	-0.250734854	-0.119972494	MIR143HG
chr4	55198258	55198664	407	*	3	2.54678E-14	0.018672801	0.000846599	0.003520049	-0.400461918	-0.141955471	

chr19	32895186	32895911	726	*	3	3.26881E-22	0.000116801	0.000846916	2.95895E-05	-0.277752389	-0.156310996	AC007773.2
chr14	70098523	70099253	731	*	2	4.368E-14	3.85206E-05	0.000847253	6.817E-05	-0.250954041	-0.174945556	KIAA0247
chr9	80851623	80852403	781	*	2	1.24055E-14	0.000811051	0.00084767	0.000601383	-0.31786917	-0.146354447	CEP78
chr14	102746504	102747018	515	*	2	5.55977E-14	5.43652E-05	0.00084795	8.97612E-05	-0.31143683	-0.206156288	MOK
chr12	49071788	49072098	311	*	2	2.27216E-12	0.000355499	0.000848251	0.000355035	-0.311152892	-0.121771693	KANSL2
chr2	85944732	85945326	595	*	2	3.40579E-15	4.92614E-05	0.000848294	8.3034E-05	-0.246634338	-0.052123954	
chr11	62344463	62344975	513	*	4	1.6238E-13	0.210741395	0.000848623	0.015361904	-0.29685363	-0.080985922	MIR3654, TUT1
chr19	33764550	33765020	471	*	2	3.53872E-14	0.029799082	0.000849186	0.002881386	-0.334060187	-0.160131106	
chr6	2798624	2798912	289	*	2	1.17513E-10	0.074901887	0.000849268	0.003401424	-0.291188616	-0.147571039	
chr2	120191616	120192027	412	*	2	7.29208E-12	0.086144963	0.000849275	0.003458285	-0.276015045	-0.136434058	TMEM37
chr15	93571988	93572609	622	*	2	3.13769E-15	0.000158448	0.000850534	0.000202134	-0.105477956	-0.079776585	
chr7	2153671	2154175	505	*	5	9.43758E-16	0.605630578	0.000850706	0.037998929	-0.212556649	-0.040287066	MAD1L1
chr5	171807791	171808623	833	*	3	7.0326E-19	0.004280129	0.000851052	4.77099E-05	-0.338866279	-0.160782562	SH3PXD2B
chr1	65381300	65381747	448	*	2	6.93826E-13	0.002355604	0.000851767	0.001097803	0.156367133	0.091381127	JAK1
chr1	17956219	17956700	482	*	2	8.8824E-12	0.012938417	0.000851824	0.002275876	-0.228986342	-0.098081297	ARHGEF10L
chr1	182538701	182539141	441	*	2	6.17278E-12	0.038869397	0.000852004	0.00306227	0.232190478	0.120139328	
chr9	127114588	127114914	327	*	2	2.59173E-13	0.093471771	0.000853736	0.003504093	-0.33737917	-0.164752042	NEK6
chr9	97545514	97545827	314	*	3	2.30286E-18	0.000523961	0.000853736	3.98542E-05	-0.376318811	-0.150520858	C9orf3
chr5	10335254	10336062	809	*	3	4.50627E-16	0.00020028	0.000854567	0.000139498	-0.339197383	-0.174394863	
chr22	17955274	17956641	1368	*	10	1.12836E-45	2.55903E-09	0.000854867	3.15782E-10	-0.260161521	-0.099379224	CECR2
chr16	81347052	81347858	807	*	5	7.66901E-27	3.5207E-06	0.000854924	3.81943E-06	0.203942814	0.095392219	
chr6	134956367	134956701	335	*	3	3.48518E-14	0.007960738	0.000855024	0.001139671	-0.253089946	-0.10296238	RP11-557H15.4
chr16	53120120	53120404	285	*	2	9.84725E-11	0.014283634	0.000855878	0.002359416	0.253223233	0.120864603	CHD9
chr3	14265164	14265984	821	*	2	3.52376E-13	1.31607E-05	0.000858726	2.79363E-05	0.16691481	0.130388251	
chr3	123531610	123532834	1225	*	4	1.46396E-19	6.73806E-06	0.00085999	1.13072E-05	0.186420423	0.128338206	MYLK
chr10	98679920	98679962	43	*	2	4.71658E-10	0.016045581	0.000862321	0.00246169	0.23158299	0.124712681	LCOR
chr3	159726855	159726973	119	*	3	6.64677E-22	6.6884E-08	0.000862632	3.38099E-07	-0.247334153	-0.212791684	IL12A-AS1
chr11	12158816	12159950	1135	*	6	6.18232E-20	0.031282343	0.000865016	0.00442184	-0.329396432	-0.048403793	MICAL2
chr7	17409912	17410340	429	*	3	4.71811E-14	0.001337768	0.000866378	0.000619392	-0.163275797	-0.053037864	

chr12	129304569	129305480	912	*	4	1.8659E-18	0.000110537	0.000866992	0.000116878	-0.385253218	-0.1537512	SLC15A4
chr8	97597118	97597395	278	*	2	2.52384E-13	0.000249294	0.000867018	0.000281114	-0.252496456	-0.164281549	SDC2
chr11	44730975	44731693	719	*	2	2.81172E-13	7.92016E-05	0.000867215	0.000121165	-0.134354616	-0.112199255	
chr20	31036723	31037515	793	*	4	8.90923E-26	2.59952E-05	0.000867715	8.44148E-07	-0.203393227	-0.132832324	C20orf112
chr4	54928819	54928822	4	*	2	1.44272E-16	4.90619E-06	0.000867919	1.17649E-05	-0.298337629	-0.297244163	FIP1L1, CHIC2
chr9	116377482	116377632	151	*	2	6.19287E-15	1.07135E-05	0.000868378	2.34413E-05	0.199918058	0.192430143	
chr1	221338314	221338400	87	*	2	1.08129E-10	0.0022246	0.000869464	0.001078602	-0.121287382	-0.051419789	
chr17	41172092	41174035	1944	*	9	1.53737E-36	4.07719E-06	0.000870188	2.78329E-09	-0.282053982	-0.115684169	VAT1
chr3	37948694	37949403	710	*	2	2.36413E-13	7.12771E-05	0.000870247	0.000111813	0.174716656	0.152405025	CTDSPL
chr8	131454686	131456284	1599	*	10	1.39125E-29	0.043597687	0.000871264	0.00285257	-0.151902876	-0.012720363	ASAP1
chr3	154688152	154689271	1120	*	4	1.93562E-14	0.159786789	0.00087138	0.014222408	-0.167716771	-0.049952929	
chr17	1510080	1510666	587	*	2	5.0223E-16	0.003165805	0.000872266	0.001291404	-0.199704686	-0.112394879	SLC43A2
chr2	231299880	231300356	477	*	2	2.03883E-11	0.096064407	0.000873139	0.003581726	-0.302587558	-0.144249912	SP100
chr15	74927688	74928240	553	*	2	8.07625E-13	0.000452493	0.000874391	0.000422217	-0.179833092	-0.106333211	CLK3, EDC3
chr20	62297697	62297748	52	*	2	4.79947E-15	8.99365E-06	0.000875237	2.01545E-05	-0.117278092	-0.110685065	RTEL1, RTEL1-TNFRSF6B
chr4	113192453	113192515	63	*	2	5.94696E-16	5.00452E-06	0.000875928	1.19916E-05	-0.331857172	-0.328915568	
chr22	43490246	43490400	155	*	2	1.93297E-10	0.039651525	0.000876426	0.003145214	-0.145953614	-0.063515069	
chr1	236016383	236017325	943	*	4	1.32808E-18	1.40098E-06	0.000876657	3.59926E-06	-0.277019131	-0.146505672	LYST
chr5	79799656	79799917	262	*	2	3.27189E-11	0.00190175	0.000876765	0.000997497	-0.272188207	-0.15551029	FAM151B
chr2	74358223	74358815	593	*	2	3.39293E-16	0.040038655	0.000878383	0.003157044	0.120021114	0.063610997	
chr10	6441379	6442501	1123	*	3	6.67662E-13	0.000546324	0.000878438	0.000157362	-0.337590869	-0.166667121	
chr1	55753454	55753555	102	*	2	4.34992E-11	0.0743187	0.000878972	0.003497595	0.199116025	0.106517673	
chr8	622011	622993	983	*	4	2.78673E-17	0.011030914	0.000880746	0.003166601	-0.291812653	-0.049220267	ERICH1
chr4	159727112	159727390	279	*	2	8.53913E-11	0.006748786	0.000882558	0.001829398	-0.184842208	-0.1138552	FNIP2
chr8	1896437	1898438	2002	*	11	1.62596E-52	3.5412E-12	0.000883228	5.632E-12	-0.322139388	-0.1406582	ARHGEF10
chr4	88140261	88140994	734	*	3	8.50086E-16	0.014938266	0.000883495	0.002886037	-0.229199988	-0.051508608	KLHL8
chr21	43919316	43919853	538	*	3	3.66224E-13	0.074491868	0.000883591	0.0064033	0.184063091	0.076865432	SLC37A1
chr10	129925561	129925760	200	*	2	2.84877E-14	0.0381941	0.000884353	0.003143731	-0.211921699	-0.108876696	
chr16	85935556	85936666	1111	*	9	8.74911E-23	0.107657641	0.000884582	0.007483206	-0.269272238	-0.013075306	IRF8

chr16	85063738	85063787	50	*	3	2.21378E-21	6.54828E-07	0.000884838	1.93241E-06	-0.372444416	-0.285752086	KIAA0513
chr19	8568012	8569300	1289	*	4	1.93801E-25	6.00053E-07	0.000885442	1.12082E-06	-0.372951084	-0.183890022	
chr17	80199608	80201299	1692	*	7	9.23394E-26	6.02929E-05	0.000886871	1.27132E-06	-0.309977238	-0.112320094	SLC16A3, CSNK1D
chr16	72965762	72966213	452	*	2	3.5713E-14	3.60559E-05	0.000888118	6.5423E-05	-0.18463303	-0.134826063	ZFHX3
chr1	214151615	214152076	462	*	2	4.0331E-14	0.133018772	0.000888384	0.003737019	0.170330938	0.09064782	PROX1-AS1
chr8	61495559	61497168	1610	*	6	6.37475E-29	1.05177E-05	0.000888403	2.9947E-06	-0.372218328	-0.162102708	RAB2A
chr20	5668835	5669604	770	*	2	2.18484E-12	0.073087106	0.000888982	0.003523304	-0.418847656	-0.213124709	
chr7	138803555	138803912	358	*	2	4.49658E-13	0.000118268	0.000890956	0.000165699	-0.330162021	-0.090207097	
chr17	773023	773283	261	*	3	4.63454E-12	0.053039979	0.000891869	0.002446453	0.127707211	0.032895138	NXN
chr1	109582945	109583681	737	*	2	1.57668E-13	0.001288542	0.000892587	0.000811683	-0.203863222	-0.114223402	WDR47
chr16	89048704	89049838	1135	*	5	2.08523E-17	0.269954399	0.000896099	0.015418586	-0.194729017	-0.061208507	
chr17	14641538	14642137	600	*	3	1.5264E-16	0.003785256	0.00089741	0.00013702	-0.327270748	-0.17820652	
chr16	56443155	56443295	141	*	2	2.05682E-15	6.41322E-06	0.000897612	1.50176E-05	-0.280324499	-0.199169085	RP11-413H22.2, AMFR
chr9	4850209	4851340	1132	*	4	1.44815E-20	0.000125204	0.000898566	4.55747E-05	0.214154824	0.020527188	RCL1, MIR101-2, RP11-125K10.5
chr22	38819702	38820214	513	*	4	3.28746E-22	0.005917676	0.00089901	0.00010859	-0.296154683	-0.154354635	
chr16	11400403	11401781	1379	*	4	8.27803E-14	0.000478425	0.000899305	4.29465E-05	-0.353210394	-0.161223872	RMI2
chr3	122043799	122044172	374	*	5	3.27746E-31	5.09203E-10	0.000900052	3.51243E-09	-0.343105542	-0.216329222	CSTA
chr10	125769668	125770239	572	*	5	1.72161E-21	0.002751207	0.000900781	0.00012397	-0.30823637	-0.096247889	CHST15
chr5	142983747	142983808	62	*	2	9.15563E-17	6.65262E-06	0.000900906	1.55233E-05	-0.171332393	-0.16982816	
chr22	43369117	43369590	474	*	3	2.26473E-15	8.30788E-05	0.000901228	8.53286E-05	-0.304276892	-0.127015047	PACSIN2
chr5	17444061	17444953	893	*	6	5.34302E-40	5.83984E-11	0.000901558	1.23923E-10	-0.353578265	-0.266068928	RP11-321E2.4
chr12	1922058	1922255	198	*	3	2.4703E-20	2.09717E-06	0.000901582	5.06342E-06	-0.279593638	-0.203280494	CACNA2D4
chr13	80354114	80354532	419	*	2	3.55422E-11	0.01616612	0.000901904	0.002550561	-0.324410921	-0.167071948	
chr17	30675814	30676722	909	*	3	6.08732E-20	0.004159176	0.000902374	7.78811E-05	0.207339679	0.119348392	RP11-227G15.3, C17orf75
chr3	177166686	177167606	921	*	3	8.59677E-19	2.72436E-05	0.00090319	1.61856E-05	-0.31601002	-0.179327631	LINC00578
chr14	106405869	106406341	473	*	4	1.29843E-19	0.000718	0.000903467	5.47302E-05	0.234711379	0.126735333	IGHV6-1
chr14	72943461	72943540	80	*	2	1.97239E-12	0.000150537	0.000904616	0.000199173	-0.238818242	-0.154311452	RGS6
chr17	74479764	74480528	765	*	3	3.06212E-19	0.000987669	0.00090465	4.79908E-05	-0.360500101	-0.221422372	RHBDF2
chr4	6694021	6695698	1678	*	11	7.86618E-55	8.71304E-15	0.000904812	1.78454E-14	-0.322091272	-0.152155228	S100P, AC093323.1

chr10	81708991	81709191	201	*	2	1.72354E-15	2.05754E-05	0.000910057	4.1351E-05	-0.326125368	-0.240076872	MBL1P, SFTPD
chr17	3704471	3707364	2894	*	15	5.68252E-57	4.92518E-25	0.000911919	2.22773E-22	-0.428628425	-0.226606553	ITGAE, CTD-3195I5.5
chr17	79239572	79239903	332	*	3	1.02394E-11	0.393984147	0.000912574	0.011138023	-0.280175702	-0.093352331	SLC38A10
chr1	200112467	200113062	596	*	3	1.14424E-11	0.540153535	0.000912598	0.011866614	-0.326828	-0.112318659	NR5A2
chr12	6288571	6288946	376	*	2	4.32992E-11	0.077172526	0.000913311	0.003629369	0.152597268	0.079542202	
chr5	40485321	40485667	347	*	2	8.47498E-11	0.033797903	0.000913721	0.003142217	-0.293276548	-0.154149311	
chr20	33463466	33463803	338	*	3	1.8427E-12	0.210385992	0.000914131	0.009666617	-0.097298961	-0.029425553	ACSS2
chr5	176747268	176748043	776	*	2	5.7581E-13	5.54479E-05	0.000914257	9.31845E-05	-0.204283089	-0.145712414	
chr11	19263864	19264434	571	*	4	3.01003E-16	0.093374244	0.000914383	0.002490771	-0.166971165	-0.042045843	RP11-428C19.4
chr3	98312651	98313993	1343	*	7	2.01697E-25	0.005185245	0.000916388	0.000800189	-0.243373322	-0.033257492	
chr10	121011103	121011644	542	*	2	1.44434E-12	0.000678459	0.000917004	0.000559749	0.137005039	0.122072357	GRK5
chr11	111636425	111636595	171	*	2	8.1545E-12	0.026738236	0.000917951	0.002981551	0.03428834	0.015636449	PPP2R1B
chr1	116191397	116191657	261	*	2	2.41389E-15	6.83428E-06	0.000921841	1.59555E-05	0.187208828	0.178871552	VANGL1
chr8	125827396	125828298	903	*	3	3.4696E-21	1.06235E-06	0.000922376	2.84931E-06	-0.238745729	-0.172582489	RP11-1082L8.4
chr16	11875806	11877212	1407	*	9	1.03245E-37	0.000133354	0.000927272	7.5365E-07	0.195191671	0.078764022	ZC3H7A
chr11	64163330	64164279	950	*	6	1.20481E-29	7.77675E-06	0.000928576	7.75204E-07	-0.188361312	-0.080665687	
chr4	184773853	184774543	691	*	5	2.02911E-26	0.000429554	0.000929098	5.56153E-06	0.194448328	0.113263619	
chr9	71409257	71409959	703	*	3	1.68031E-16	0.000111704	0.000929361	0.000134783	-0.293278358	-0.060307885	PIP5K1B
chr12	133239966	133241537	1572	*	3	3.00804E-13	5.86996E-07	0.000929554	2.12464E-06	-0.352130149	-0.252487735	POLE
chr11	10625668	10626235	568	*	4	4.38664E-14	0.012585449	0.00092964	0.001959067	-0.378611941	-0.106404972	LYVE1, MRVI1
chr6	111912751	111913206	456	*	2	3.61663E-11	0.032788733	0.000929804	0.003163872	-0.341844217	-0.180172731	TRAF3IP2-AS1, TRAF3IP2
chr2	46621477	46622721	1245	*	4	1.58099E-21	0.000682108	0.000930032	6.64969E-05	-0.293542101	-0.136556234	
chr17	71064722	71065768	1047	*	3	2.00491E-19	4.01212E-08	0.000930659	2.17295E-07	-0.278165429	-0.178104969	SLC39A11
chr5	73399136	73399693	558	*	4	8.94919E-22	0.000147227	0.000931081	7.68298E-06	-0.176355296	-0.097277135	
chr2	179395885	179396688	804	*	5	7.46171E-30	1.56981E-05	0.000931382	8.67579E-07	-0.225007356	-0.140222461	TTN-AS1, TTN
chr17	60972789	60973151	363	*	3	4.41709E-21	1.53714E-07	0.000933735	6.81119E-07	-0.41605788	-0.251458159	
chr19	55385010	55385662	653	*	5	1.86682E-27	5.76661E-05	0.000934024	2.40792E-06	-0.350120374	-0.180789971	
chr6	44019598	44020777	1180	*	3	1.50056E-15	0.002282487	0.000939081	0.001133944	-0.160412167	-0.077272571	RP5-1120P11.1
chr15	64456062	64456365	304	*	3	2.05339E-14	0.010421185	0.000940281	0.002723575	-0.252014851	-0.067486033	

chr8	10236366	10236882	517	*	2	1.68732E-13	7.19612E-05	0.000940567	0.000115361	0.19182367	0.155554886	MSRA
chr16	3014320	3015092	773	*	4	8.16776E-20	0.003518503	0.000940721	0.001419495	-0.044273954	-0.002341344	KREMEN2
chr1	184377004	184377525	522	*	3	2.00687E-12	0.056279451	0.000942088	0.005758217	-0.245486414	-0.08856252	C1orf21, GS1-115G20.1
chr16	89299756	89300741	986	*	3	2.3518E-17	9.97596E-06	0.000942297	1.61783E-05	-0.372552477	-0.189025522	
chr12	27174164	27174721	558	*	5	2.89627E-22	0.004862971	0.000942299	0.000220503	-0.274188082	-0.093546407	
chr12	124718401	124718939	539	*	3	1.22927E-14	0.001821717	0.000944965	0.00100376	-0.310152755	-0.139842059	FAM101A
chr15	52122389	52122580	192	*	3	4.57837E-15	0.006047162	0.000945344	0.002014192	-0.046799226	-0.015192529	TMOD3
chr11	63331821	63332343	523	*	2	7.0191E-13	0.015013969	0.000946464	0.002580612	0.138929464	0.077040057	
chr8	74582345	74582660	316	*	4	2.51024E-12	0.111332019	0.000946605	0.009612345	-0.271322918	-0.08216731	STAU2
chr7	4752170	4753002	833	*	4	3.51333E-30	4.18164E-06	0.000946621	6.62818E-07	-0.348199796	-0.221837474	FOXK1
chr2	86387377	86388045	669	*	4	1.49217E-13	0.078522171	0.000947657	0.007084594	-0.243686328	-0.075351891	IMMT
chr22	30639611	30639979	369	*	4	1.73559E-12	0.310338819	0.000948429	0.020260861	0.164088623	0.040498578	LIF
chr8	29914021	29914060	40	*	2	2.25114E-15	5.7978E-06	0.000948786	1.38373E-05	-0.33706981	-0.29629683	
chr19	9907044	9907569	526	*	3	4.60702E-16	0.000158692	0.00094906	6.9462E-05	-0.231835212	-0.148474619	CTD-2623N2.5
chr17	38077703	38077870	168	*	2	1.39301E-14	1.2675E-05	0.000949062	2.75687E-05	-0.249717615	-0.17787657	ORMDL3
chr16	30976186	30976273	88	*	2	3.74782E-11	0.002065303	0.000949437	0.001090747	-0.246968866	-0.134243391	SETD1A
chr14	21510056	21510384	329	*	4	3.85521E-13	0.519929579	0.00095034	0.024441091	-0.172773328	-0.044702478	NDRG2
chr2	9427798	9428037	240	*	2	3.81011E-12	0.015863677	0.000950687	0.002635434	0.159480634	0.052089148	ASAP2
chr5	33229162	33229692	531	*	2	1.30701E-11	0.063759544	0.000950952	0.003655768	0.20149339	0.093790929	CTD-2066L21.3
chr3	71151690	71151744	55	*	2	2.90501E-14	1.89381E-05	0.0009514	3.89212E-05	-0.319836806	-0.252020235	FOXP1
chr14	21372793	21373109	317	*	2	9.20292E-12	0.000603782	0.000952519	0.000529283	-0.24730977	-0.141364437	RP11-219E7.4
chr21	44473496	44474019	524	*	5	8.39883E-22	3.87769E-05	0.000953489	2.9975E-05	-0.394784983	-0.163770248	CBS
chr2	26835353	26835566	214	*	2	6.36043E-10	0.179532299	0.000953514	0.004043213	-0.255475398	-0.126282087	CIB4
chr11	1776289	1776688	400	*	2	2.11161E-14	4.70377E-05	0.000953714	8.26779E-05	-0.212128682	-0.180751753	RP11-295K3.1, CTSD
chr3	114936863	114937219	357	*	2	1.2406E-10	0.028334887	0.000954637	0.003118644	-0.358773065	-0.170116051	
chr16	11199143	11199192	50	*	2	8.09338E-11	0.000986156	0.000955282	0.000718684	-0.070221193	-0.043238677	CLEC16A
chr4	47466696	47467252	557	*	4	5.25089E-16	0.054004894	0.000955701	0.002628135	-0.30574256	-0.102046018	
chr10	75530708	75530718	11	*	2	3.61728E-12	0.006544354	0.000957421	0.001906708	-0.155864106	-0.091620987	SEC24C
chr1	203561580	203562914	1335	*	4	1.98188E-22	1.11499E-05	0.000959571	9.23722E-06	-0.379570605	-0.195333377	

chr1	16292047	16292746	700	*	2	8.6529E-13	0.000161627	0.000960926	0.000214464	-0.360022397	-0.223899522	ZBTB17
chr8	29624720	29624883	164	*	2	5.77478E-10	0.015069676	0.000962104	0.002614757	-0.166388976	-0.094495607	AC145110.1
chr2	9430153	9430274	122	*	2	2.16786E-10	0.003760348	0.000962989	0.001491119	0.225175419	0.100262953	ASAP2
chr14	76880315	76880784	470	*	2	2.08501E-11	0.006064422	0.000963184	0.001853244	0.043004902	0.038352829	ESRRB
chr1	31544059	31544293	235	*	2	6.0451E-11	0.001662009	0.000964238	0.000977089	-0.18558169	-0.099094542	
chr6	20483859	20484204	346	*	2	5.66725E-15	8.93936E-06	0.000964405	2.03786E-05	-0.330029322	-0.267168991	E2F3
chr8	29230547	29230998	452	*	3	1.00363E-13	0.013467726	0.000967699	0.003286183	-0.159828002	-0.070902989	
chr22	37428975	37429269	295	*	2	3.11615E-15	6.58377E-06	0.000967973	1.55476E-05	-0.22445875	-0.211507164	
chr6	37024066	37024255	190	*	2	2.48642E-10	0.06154573	0.000968575	0.003691643	-0.189794244	-0.090343302	
chr3	184292880	184294106	1227	*	3	1.56613E-15	0.000314107	0.00096968	4.69938E-05	-0.262616948	-0.155388033	EIF2B5, EPHB3
chr7	971027	971767	741	*	4	1.1442E-21	4.90442E-05	0.000970102	7.4078E-06	-0.306893664	-0.176426225	ADAP1, COX19
chr21	22400862	22401770	909	*	2	6.00281E-12	8.82551E-06	0.000970517	2.01705E-05	-0.169446661	-0.159781909	NCAM2
chr19	35465641	35466896	1256	*	3	5.92935E-17	3.80117E-07	0.000970961	1.55111E-06	-0.334483855	-0.202832077	
chr16	68826954	68827444	491	*	2	2.14933E-11	0.007961807	0.00097447	0.002091239	-0.321413669	-0.168227917	CDH1
chr19	38760771	38761503	733	*	2	8.11668E-15	8.16899E-06	0.00097708	1.88597E-05	0.214631128	0.203671766	SPINT2
chr6	34663155	34664215	1061	*	3	2.3748E-13	0.000339286	0.00097754	0.000344696	-0.088625599	-0.028326881	RP11-140K17.3, C6orf106
chr12	6948846	6949472	627	*	5	2.32166E-19	0.001601984	0.000978238	0.001095303	0.158173638	0.057908216	LEPREL2, GNB3
chr2	157262176	157262380	205	*	2	3.55615E-14	1.79686E-05	0.000978376	3.74356E-05	-0.322603347	-0.236080687	
chr10	51487545	51487759	215	*	5	2.94434E-13	0.086841812	0.000980184	0.007465662	-0.16549682	-0.044173764	
chr7	155150015	155151427	1413	*	6	1.38132E-30	0.000818548	0.000980403	4.27506E-06	-0.286304801	-0.148920569	BLACE
chr6	17930180	17930519	340	*	2	4.86947E-15	8.62585E-06	0.000980473	1.98001E-05	-0.242413844	-0.18483982	KIF13A
chr2	8799821	8800129	309	*	2	1.22527E-11	0.002962343	0.000981198	0.001341194	-0.165213225	-0.064690031	
chr1	28449602	28450028	427	*	2	1.23237E-11	0.001552308	0.000981469	0.000949513	-0.337353602	-0.13845353	
chr16	19589790	19590554	765	*	5	5.2113E-20	0.014663983	0.000982147	0.000492972	-0.200425405	-0.074165107	C16orf62
chr10	92680914	92681505	592	*	3	1.69109E-12	0.049938871	0.000982447	0.00590787	-0.203495514	-0.049135156	ANKRD1
chr10	98899484	98900495	1012	*	3	1.53308E-14	0.468829476	0.000984013	0.012457497	-0.262325175	-0.086990633	SLIT1, ARHGAP19-SLIT1
chr12	7046277	7047250	974	*	4	7.06829E-15	0.01372439	0.00098517	0.003906665	0.041426758	0.001147312	ATN1
chr19	43856494	43857874	1381	*	8	4.42731E-32	1.81638E-05	0.000986138	4.03628E-06	-0.340254206	-0.099005339	CD177
chr19	574762	575412	651	*	5	3.35024E-32	5.69027E-09	0.000986418	1.51934E-08	0.213586474	0.137752572	BSG

chr11	44976423	44977160	738	*	2	4.50431E-13	3.18702E-05	0.000987177	6.07109E-05	-0.315129665	-0.206477094	
chr1	2090814	2090849	36	*	2	8.87797E-14	0.000168445	0.000987382	0.000223242	-0.244310854	-0.224464045	PRKCZ
chr8	1807993	1808475	483	*	2	6.24939E-13	0.06586434	0.000988121	0.00379214	0.160807935	0.079328784	ARHGEF10
chr15	56201496	56201688	193	*	2	8.50158E-10	0.569116462	0.00098819	0.004256638	-0.265388785	-0.132767479	NEDD4
chr1	59510738	59511602	865	*	2	4.68579E-12	0.000146379	0.000989233	0.000201416	0.152235239	0.116619243	
chr5	76028910	76029522	613	*	3	7.76835E-13	0.02811182	0.000990147	0.00376486	0.098290817	0.036374422	F2R
chr10	50594340	50595181	842	*	2	5.86921E-13	8.7771E-06	0.000990951	2.01408E-05	-0.310955787	-0.097253421	DRGX
chr17	60142491	60142985	495	*	3	1.39824E-16	0.004728985	0.000991212	0.000714401	0.030321824	0.001254695	MED13
chr11	450652	451101	450	*	3	8.11729E-15	0.088473038	0.000991217	0.007664415	0.043546429	0.016761581	PTDSS2
chr1	116460862	116460865	4	*	2	1.07943E-11	0.000217517	0.000991464	0.000269103	-0.161137128	-0.124519604	
chr11	133995070	133995787	718	*	2	9.62158E-13	0.001030485	0.000991613	0.00075198	0.243239333	0.155122455	JAM3
chr19	54566838	54567279	442	*	6	4.16233E-15	0.12756884	0.000993321	0.024854388	-0.377301013	-0.072421659	VSTM1
chr3	31728749	31729018	270	*	2	1.17742E-10	0.003451256	0.000993401	0.001457816	-0.299146267	-0.157917082	OSBPL10
chr3	39254882	39255676	795	*	6	6.86923E-15	0.575573815	0.000993598	0.048540153	-0.274690477	-0.046521827	
chr16	28020418	28020812	395	*	2	5.10907E-11	0.005967472	0.000993808	0.001878396	-0.225387478	-0.097682106	GSG1L
chr8	134143217	134143581	365	*	2	4.45307E-11	0.117192624	0.000994625	0.004078136	-0.237707282	-0.11905458	TG
chr1	169600039	169600704	666	*	4	3.80125E-15	0.200931354	0.000994936	0.00369077	0.142413285	0.058500571	
chr16	86756180	86756774	595	*	3	4.31474E-13	0.061316053	0.000996679	0.00662385	-0.139589125	-0.043006325	CTD-2015G9.2
chr3	52220172	52220370	199	*	2	3.8457E-10	0.007699909	0.000997721	0.002096243	-0.260882365	-0.147858464	
chr12	720995	721502	508	*	4	4.76913E-23	1.42911E-06	0.000997993	6.78361E-07	-0.316261457	-0.19066666	NINJ2
chr3	186692377	186692770	394	*	2	6.23104E-12	0.00253127	0.000998539	0.001250125	-0.182149222	-0.054474732	ST6GAL1
chr15	40623714	40624103	390	*	4	9.37215E-19	0.001684513	0.001001436	7.16967E-05	0.172789583	0.084458208	C15orf52, RNA5SP392
chr3	171964315	171965350	1036	*	4	2.58459E-13	0.305020306	0.001001789	0.019092967	-0.356243685	-0.076105598	FNDC3B
chr12	47477069	47477223	155	*	2	2.43958E-14	1.40703E-05	0.001001823	3.04831E-05	-0.257259625	-0.085550845	PCED1B
chr17	4081325	4082271	947	*	5	1.95428E-24	2.08145E-06	0.001001871	9.90815E-07	-0.425364375	-0.161800912	CYB5D2, ANKFY1
chr17	45943858	45944921	1064	*	4	5.08694E-13	0.19177627	0.001002184	0.013650727	0.111725633	0.027174664	
chr16	87470083	87471211	1129	*	6	1.18716E-23	0.000124613	0.00100299	0.000106956	0.210156361	0.084315001	ZCCHC14
chr11	6631411	6632072	662	*	3	2.35766E-13	0.198376735	0.001003109	0.007872983	0.220294301	0.085322567	ILK, TAF10
chr17	78681826	78683457	1632	*	7	4.7952E-26	0.000102872	0.00100382	7.13145E-07	-0.332539628	-0.138286555	RPTOR

chr2	60755743	60756422	680	*	2	6.53684E-14	2.4222E-05	0.00100501	4.84897E-05	-0.163138399	-0.148625415	BCL11A
chr2	106010000	106010271	272	*	2	3.56039E-10	0.023195865	0.001005087	0.003077485	0.156809068	0.082871128	FHL2
chr15	91304710	91306100	1391	*	7	1.41578E-37	3.03742E-07	0.001007134	1.28793E-09	-0.272582965	-0.165181776	BLM
chr13	99174312	99175893	1582	*	11	4.14688E-19	0.008409634	0.001009016	0.000660231	0.171010539	0.070709352	STK24
chr17	34931841	34932199	359	*	2	5.25884E-12	0.025050878	0.001009328	0.003152986	0.143247338	0.076875672	GGNBP2
chr7	158497997	158498010	14	*	2	1.87118E-10	0.008102155	0.001010583	0.002158382	0.02763409	0.016404817	
chr4	7636841	7638222	1382	*	6	2.1004E-33	0.000485642	0.00101093	2.49935E-07	-0.297776017	-0.14150494	SORCS2
chr3	195973977	195974349	373	*	4	2.18357E-12	0.333935672	0.001011139	0.021636606	-0.397015733	-0.098070748	PCYT1A
chr2	64680053	64681012	960	*	3	2.55375E-16	0.015273044	0.001011387	0.003567782	-0.210459455	-0.068165739	AC008074.3
chr2	219222315	219223002	688	*	4	1.69022E-21	0.001920497	0.001011599	8.01596E-05	-0.330601807	-0.131264535	C2orf62, AC021016.8
chr1	174924851	174924874	24	*	2	2.50748E-10	0.016876533	0.001011833	0.002813569	-0.227270819	-0.123680066	RABGAP1L
chr9	136892128	136892236	109	*	2	7.0739E-11	0.189281803	0.00101236	0.004270124	-0.132274115	-0.064985789	LINC00094
chr2	174888959	174890570	1612	*	9	1.40516E-31	2.93551E-07	0.001012697	1.0561E-07	-0.296898457	-0.114001852	RP11-394I13.1
chr17	17311855	17311866	12	*	2	2.30828E-15	6.46349E-06	0.001013104	1.53949E-05	-0.314222501	-0.302243131	
chr17	7379681	7380112	432	*	2	5.95388E-11	0.033112529	0.001013747	0.00339384	0.201855606	0.104678119	ZBTB4
chr10	114816211	114816714	504	*	2	5.77341E-13	0.000145964	0.001014031	0.000202739	-0.130464088	-0.012028	TCF7L2
chr2	152213504	152214177	674	*	4	1.89119E-20	0.016743066	0.001014385	0.000193373	-0.293211042	-0.149458946	TNFAIP6
chr17	39653832	39654883	1052	*	5	8.25238E-28	4.92224E-06	0.001016343	7.88647E-07	-0.375122523	-0.179413573	
chr19	11349627	11350165	539	*	7	1.57064E-28	4.3447E-05	0.001016753	3.81605E-06	-0.22103908	-0.087223305	C19orf80, DOCK6
chr2	16059290	16059540	251	*	2	2.41742E-11	0.028215383	0.001017405	0.003272679	0.119065373	0.052992773	
chr16	24989622	24989925	304	*	3	9.88604E-12	0.015645139	0.001018663	0.003465027	0.181342571	0.074773111	ARHGAP17
chr3	66500294	66500703	410	*	2	1.32996E-10	0.064703379	0.001020763	0.003885048	-0.168778596	-0.081256946	LRIG1
chr2	208939532	208939842	311	*	2	1.30056E-12	0.000401358	0.001021462	0.000417345	-0.146770884	-0.11045619	
chr9	100130105	100130170	66	*	2	6.11354E-14	2.12971E-05	0.001022161	4.36541E-05	0.173457842	0.149864185	CCDC180, RP11-23J9.4
chr22	39788527	39788876	350	*	2	2.47974E-10	0.043284838	0.001025348	0.003630892	-0.251321109	-0.13180182	
chr2	160068293	160068902	610	*	3	3.21775E-18	0.000324436	0.001025601	4.23529E-05	0.150514838	0.095803028	TANC1
chr12	15125458	15126020	563	*	3	1.50747E-15	0.001829492	0.001026268	0.001072693	0.161100273	0.031363401	PDE6H
chr7	101460955	101461188	234	*	2	5.34254E-13	0.000231752	0.001026927	0.000285401	-0.135308101	-0.087686716	CUX1
chr14	105157570	105157713	144	*	4	2.51999E-21	1.29587E-06	0.001026983	4.68763E-06	0.161365922	0.131348969	INF2, RP11-982M15.5

chr3	188149132	188150137	1006	*	3	9.55123E-13	0.261649457	0.00102894	0.011323979	-0.289652089	-0.102281254	LPP
chr3	171611793	171612219	427	*	4	2.37944E-14	0.01728483	0.001030936	0.001458499	-0.359506448	-0.113753888	TMEM212, TMEM212-AS1
chr4	140098382	140099401	1020	*	7	4.52121E-33	7.05747E-07	0.001031003	2.52986E-07	-0.088685911	-0.033814202	
chr7	44674851	44675025	175	*	2	3.35716E-12	0.000128289	0.001033092	0.00018524	0.177875727	0.177627082	OGDH
chr16	58914220	58914345	126	*	3	1.38131E-16	8.78603E-05	0.001036552	2.98635E-05	-0.2736314	-0.187251078	RP11-410D17.2
chr12	13248548	13248988	441	*	3	8.38636E-15	0.005217996	0.001036971	0.001190617	-0.336282275	-0.086914555	KIAA1467, GSG1
chr8	125951226	125951852	627	*	2	3.43654E-14	7.92015E-05	0.00103825	0.000128097	0.236915438	0.197388112	LINC00964, RP11-1082L8.2
chr19	11406819	11406993	175	*	2	1.87186E-14	0.000139281	0.00103866	0.000197399	-0.125363538	-0.102992417	TSPAN16
chr18	19383644	19383957	314	*	2	1.66909E-12	0.000129504	0.001039209	0.000186932	-0.267919927	-0.204384913	MIB1
chr1	167520506	167520923	418	*	2	1.22704E-12	0.001154099	0.001039885	0.000824331	-0.236220408	-0.137590989	CREG1
chr22	21076017	21076342	326	*	2	1.02161E-11	0.010718502	0.00104	0.002453319	-0.356620726	-0.173442616	PI4KA
chr11	46258039	46258826	788	*	3	1.8781E-22	2.47019E-07	0.001040405	1.04402E-06	-0.177607383	-0.155686761	
chr9	3256732	3257397	666	*	3	1.58358E-15	0.000264815	0.001041096	9.81677E-05	-0.347363794	-0.195884202	RFX3
chr1	178252770	178253267	498	*	2	1.4555E-13	4.17188E-05	0.001042015	7.67762E-05	-0.340192824	-0.218549164	RASAL2
chr15	90769027	90769260	234	*	2	5.10618E-11	0.095500484	0.001042883	0.004162107	-0.163572748	-0.082610235	SEMA4B
chr15	39746976	39747399	424	*	2	2.84975E-12	0.001368289	0.001043308	0.000912597	-0.260290559	-0.148813376	
chr2	113012287	113013820	1534	*	10	1.05688E-26	0.13813795	0.001045163	0.000691348	-0.31178184	-0.040818893	ZC3H8
chr11	47428611	47429159	549	*	2	3.67624E-14	0.013042892	0.001045553	0.002643607	-0.236058686	-0.121569983	SLC39A13, RP11-750H9.5
chr5	177889945	177890603	659	*	3	4.95893E-16	0.009350946	0.001045703	0.000639079	-0.277089417	-0.122524516	COL23A1
chr15	64208035	64208452	418	*	2	2.11952E-11	0.009044591	0.001045802	0.002307835	0.147044304	0.097481688	DAPK2
chr3	99832772	99833293	522	*	3	1.87607E-16	9.27807E-05	0.001046703	0.000128062	-0.242055737	-0.088036677	CMSS1, FILIP1L
chr12	121091775	121091847	73	*	2	3.04592E-10	0.016725819	0.001050336	0.002883309	0.141322662	0.073654238	CABP1
chr8	130695235	130696901	1667	*	7	2.4481E-41	6.6231E-12	0.001050395	2.52551E-11	-0.316415567	-0.217705851	RP11-419K12.1
chr7	151504844	151505116	273	*	3	1.12443E-13	0.004291022	0.001053573	0.001821927	-0.330406165	-0.130231221	RP13-452N2.1, PRKAG2
chr1	12493605	12494280	676	*	3	2.38295E-12	0.005640585	0.001053857	0.002153202	0.203747434	0.106295456	VPS13D
chr10	519360	519456	97	*	2	1.86261E-15	4.21247E-05	0.001054475	7.76202E-05	0.180038875	0.171654427	DIP2C
chr10	81030244	81030727	484	*	2	1.86023E-12	0.000251965	0.001055048	0.000306108	-0.279932104	-0.104458803	ZMIZ1
chr21	44588272	44588747	476	*	5	6.0778E-17	0.370540074	0.001055176	0.036194878	-0.153915245	-0.037588104	
chr3	52001426	52002969	1544	*	14	1.75294E-30	0.000998838	0.001056861	0.000119793	-0.295335517	-0.03432891	PCBP4, RP11-155D18.14, RP11-155D18.12, ABHD14B

chr10	125187143	125187216	74	*	2	8.51455E-15	2.02048E-05	0.001058704	4.20484E-05	-0.255480747	-0.194735821	RP11-282I1.1, RP11-338O1.2
chr17	64298763	64299562	800	*	5	2.01948E-16	0.003850948	0.00106049	0.001836931	0.033002668	0.006366543	PRKCA
chr8	124890314	124890680	367	*	3	2.67491E-11	0.022612573	0.001063583	0.003902301	-0.313117423	-0.106926223	FER1L6
chr16	85028515	85028831	317	*	2	1.16952E-13	0.000223589	0.001064314	0.000281953	-0.154759247	-0.112295317	ZDHHC7
chr11	107710989	107711373	385	*	2	1.86661E-12	0.058031393	0.001064369	0.003951306	-0.299498995	-0.147478631	SLC35F2
chr10	106081758	106082244	487	*	2	1.33441E-12	0.000177163	0.001064872	0.000238059	-0.215158634	-0.157840397	ITPRIP
chr7	2184839	2186344	1506	*	7	1.3443E-27	0.062562333	0.001064903	0.000441282	-0.3611717	-0.07887463	MAD1L1
chr5	148421136	148422395	1260	*	5	5.4016E-26	5.17567E-06	0.001065578	2.01542E-07	-0.232496806	0.0605412	SH3TC2
chr17	79124325	79125029	705	*	2	3.41834E-13	1.41541E-05	0.001070389	3.10172E-05	-0.305805513	-0.239422383	AATK
chr11	88078288	88078629	342	*	2	8.53893E-11	0.002898842	0.001071323	0.001397052	-0.232500459	-0.162374078	
chr22	29139874	29140725	852	*	4	1.08913E-18	0.000588995	0.001071588	0.000165338	-0.196880788	-0.00189053	HSCB
chr13	52973203	52973481	279	*	2	2.28985E-10	0.153175159	0.001072486	0.004438902	-0.276225322	-0.138758396	THSD1
chr11	129912137	129912579	443	*	5	3.22795E-14	0.132747166	0.001073775	0.010538251	-0.283974396	-0.069483466	
chr15	52711232	52711819	588	*	2	2.7318E-12	0.000757372	0.00107407	0.000646832	-0.22019953	-0.133676374	MYO5A
chr2	61407270	61407414	145	*	2	2.91843E-16	7.65699E-06	0.001074293	1.80651E-05	0.1756956	0.136465812	AHSA2
chr12	96389412	96391252	1841	*	14	7.13516E-58	1.67102E-05	0.00107432	2.51365E-08	-0.37152102	-0.10395138	RP11-256L6.3, HAL
chr17	2937203	2937884	682	*	4	1.99215E-12	0.13491016	0.001074922	0.014463015	0.210346494	0.057505909	RAP1GAP2
chr19	42706353	42706800	448	*	2	1.04996E-13	3.29148E-05	0.001076711	6.3658E-05	-0.227012441	-0.17697192	DEDD2
chr2	201593074	201593511	438	*	2	1.28917E-11	0.013005052	0.001077079	0.002697229	-0.283638395	-0.15130654	AOX3P, AOX2P, AC007163.3
chr12	65014995	65015547	553	*	3	1.59472E-12	0.081038468	0.001077167	0.007143363	-0.296665684	-0.110180059	RASSF3
chr15	66947167	66947617	451	*	6	8.0568E-29	3.61296E-08	0.001078818	1.03765E-07	0.264741835	0.181133457	RP11-321F6.1
chr1	1695247	1695585	339	*	3	8.36341E-17	2.43454E-05	0.001080055	3.8884E-05	-0.211092994	-0.093774555	NADK
chr6	30296689	30297941	1253	*	12	4.14858E-68	2.08727E-14	0.001080713	2.14025E-15	-0.358649479	-0.189321134	TRIM39, TRIM39-RPP21
chr2	85629084	85629337	254	*	2	2.4784E-14	1.30441E-05	0.001081009	2.89395E-05	-0.241173081	-0.199923598	CAPG
chr1	20914028	20916179	2152	*	11	1.33699E-31	2.55215E-06	0.001081298	3.93801E-09	-0.329023843	-0.082725578	CDA
chr7	2874522	2874595	74	*	2	2.05867E-09	0.0213264	0.001081916	0.00317581	-0.248633811	-0.133311231	GNA12
chr20	61582596	61582921	326	*	2	6.39698E-13	0.045189183	0.001082851	0.003826209	-0.354968409	-0.173668478	
chr1	113464716	113465091	376	*	2	2.18118E-13	0.006702664	0.00108481	0.00209118	0.175101336	0.0798895	SLC16A1
chr1	19407878	19409764	1887	*	7	2.1147E-26	8.08286E-06	0.001087048	1.45596E-06	-0.270710957	-0.102730663	UBR4

chr20	4172792	4173535	744	*	4	1.12934E-20	6.93955E-07	0.001090358	8.32883E-07	0.211653015	0.045142616	
chr18	9849810	9850143	334	*	2	3.2675E-10	0.033014413	0.001090526	0.003590623	-0.183961287	-0.099562118	RAB31
chr20	49067744	49068130	387	*	2	2.76876E-14	2.16259E-05	0.00109114	4.48333E-05	-0.199533718	-0.143248527	
chr16	48222834	48222917	84	*	2	1.99835E-09	0.079319195	0.001091239	0.004227761	-0.212949576	-0.109981022	ABCC11
chr15	75319403	75320365	963	*	3	4.49361E-16	0.005632046	0.001091808	0.002008424	0.228875915	0.084099034	PPCDC
chr13	53601944	53603286	1343	*	7	8.4375E-23	0.072986758	0.001093374	0.000906256	-0.314712376	-0.075596642	OLFM4
chr10	73497514	73499076	1563	*	8	1.3977E-18	0.000308104	0.001093781	2.97793E-05	-0.313429618	-0.092823886	CDH23, C10orf105
chr1	226848569	226849798	1230	*	5	8.93698E-24	5.45306E-05	0.001094249	1.03338E-05	-0.18406372	-0.026233425	ITPKB, ITPKB-IT1
chr12	90234041	90234391	351	*	2	1.17474E-10	0.004013708	0.001094944	0.001664708	-0.253041595	-0.151150059	
chr10	43476985	43477278	294	*	4	1.50236E-13	0.001733909	0.001095624	0.001005283	0.162336106	0.073999372	
chr9	135035970	135036868	899	*	4	1.85148E-22	4.55363E-05	0.001096341	2.54286E-05	-0.257268102	-0.090105567	
chr4	3404372	3404905	534	*	2	3.00084E-12	0.000334785	0.001097659	0.000379744	0.160769437	0.117034969	RGS12
chr3	31252037	31252039	3	*	2	1.04961E-14	8.22817E-06	0.00110072	1.93313E-05	-0.333094909	-0.331143167	
chr5	78279424	78279841	418	*	2	5.91454E-13	0.043561386	0.001101765	0.003850762	-0.221934085	-0.115720994	ARSB
chr7	99953465	99956107	2643	*	10	8.55372E-32	2.96796E-05	0.001102326	1.40453E-07	-0.359359623	-0.107105717	STAG3L5P-PVRIG2P-PILRB, PILRB
chr15	38963907	38963972	66	*	3	2.09804E-13	0.001023664	0.001102402	0.000744105	-0.213537756	-0.131244069	
chr14	74296334	74297416	1083	*	5	2.8591E-22	0.00135081	0.001104441	5.24646E-05	-0.24443311	-0.089487266	RP5-1021I20.2
chr11	4469737	4470183	447	*	2	4.57845E-11	0.014134027	0.001104784	0.002827424	-0.304608223	-0.145046554	
chr11	65291370	65291709	340	*	3	1.03802E-12	0.055710702	0.001104975	0.006052827	0.146761309	0.063916479	
chr8	6758616	6758788	173	*	2	1.76002E-09	0.028695147	0.001105016	0.003502865	-0.214488717	-0.099314964	
chr8	127832164	127832509	346	*	2	4.56235E-10	0.058386737	0.001105151	0.004078874	-0.289090071	-0.148345635	
chr21	35967898	35968217	320	*	2	6.24914E-10	0.094170887	0.001105204	0.004364398	-0.299909168	-0.148872906	RCAN1
chr2	100106981	100107672	692	*	3	4.74904E-18	0.000260417	0.001109154	4.48432E-05	-0.281911974	-0.185301534	
chr19	34965973	34966155	183	*	2	2.20965E-09	0.151766958	0.001112028	0.004577167	-0.29178442	-0.147124492	
chr10	104813866	104814269	404	*	2	4.6747E-11	0.006739196	0.001113216	0.002131793	0.208580271	0.13546173	CNNM2
chr11	67251154	67251939	786	*	3	1.04013E-17	7.28166E-05	0.001113919	9.46897E-05	0.166131724	0.122848433	AIP
chr21	36118936	36119409	474	*	2	7.63011E-14	0.021821071	0.00111587	0.003272006	-0.168015854	-0.080051145	AP000330.8
chr10	114574152	114574959	808	*	3	3.0072E-18	1.20337E-07	0.001118089	5.85626E-07	-0.259852538	-0.176806839	VTI1A
chr19	7538084	7538423	340	*	2	4.02414E-10	0.022960399	0.001121609	0.003333526	-0.2302003	-0.107301761	

chr17	64536954	64537735	782	*	2	1.17166E-11	7.95668E-06	0.001122626	1.88156E-05	0.168034324	0.149909292	PRKCA
chr5	176980153	176980309	157	*	2	1.2038E-10	0.018993109	0.00112279	0.003152059	-0.313826543	-0.146272795	FAM193B
chr9	112951179	112951406	228	*	2	1.45799E-09	0.043260728	0.001123006	0.003904263	-0.161437194	-0.073925783	
chr15	76604101	76604817	717	*	4	8.4249E-18	0.774558191	0.001123821	0.030691648	0.11497126	0.037932744	
chr6	151246387	151246895	509	*	2	3.11974E-14	9.52313E-06	0.001123881	2.20834E-05	-0.261025443	-0.242182398	MTHFD1L
chr2	74708388	74709216	829	*	4	8.99243E-19	0.000617981	0.00112434	0.000437263	-0.305035748	-0.077337488	CCDC142
chr18	13375087	13376599	1513	*	5	1.21079E-15	0.000221433	0.001124873	2.10144E-05	0.195813855	0.094350022	LDLRAD4
chr1	43402591	43402937	347	*	2	5.31768E-10	0.179316389	0.00112541	0.004669929	-0.159455038	-0.080328452	SLC2A1
chr8	21859910	21860740	831	*	3	9.67467E-19	0.037799068	0.001125823	0.000131426	-0.322076681	-0.184572823	XPO7
chr14	55035889	55036043	155	*	2	2.00026E-14	0.000119862	0.00112595	0.000180998	0.19437411	0.124386434	SAMD4A
chr20	50112307	50113484	1178	*	5	6.75244E-24	0.000515106	0.001128026	4.33677E-05	-0.294381449	-0.097682748	NFATC2
chr4	6635119	6635485	367	*	2	1.51993E-14	8.43712E-06	0.001129633	1.98445E-05	-0.219707269	-0.183783654	
chr12	117477387	117478480	1094	*	5	2.08954E-24	0.005226805	0.001129972	0.000104555	-0.19515634	-0.093160236	TESC
chr6	113787498	113787865	368	*	2	3.75509E-10	0.077409493	0.001130656	0.0043404	-0.302304651	-0.152636109	
chr15	52201998	52202509	512	*	2	3.80659E-12	0.000446126	0.001131384	0.000468252	-0.208659676	-0.078781421	TMOD3
chr14	23284518	23285302	785	*	4	9.95767E-16	0.001974006	0.001131591	0.000780015	-0.313140442	-0.11467594	SLC7A7
chr15	101738274	101738616	343	*	4	1.10758E-11	0.136050295	0.00113192	0.013031334	0.177441326	0.066368027	CHSY1
chr3	139393960	139394779	820	*	2	2.22248E-13	1.62055E-05	0.001131973	3.52417E-05	0.20749217	0.145135059	NMNAT3
chr6	41840102	41840188	87	*	2	3.87006E-10	0.007730508	0.001133446	0.002286281	-0.325384333	-0.170811158	USP49
chr1	236118040	236118450	411	*	3	5.03425E-15	4.14314E-05	0.001133537	7.90537E-05	-0.393247694	-0.182598765	
chr19	1423273	1423967	695	*	4	3.05034E-15	0.005194753	0.001134597	0.000878522	-0.305423273	-0.092275576	DAZAP1
chr2	169402526	169402543	18	*	2	2.14397E-13	3.47628E-05	0.001134686	6.74401E-05	-0.144829109	-0.139193583	CERS6
chr6	138030371	138030763	393	*	2	1.07055E-10	0.004403228	0.001136561	0.001780693	-0.32697571	-0.173293716	
chr18	456806	457306	501	*	3	3.97508E-14	0.000144779	0.00113739	0.000188998	-0.223841755	-0.130564597	COLEC12
chr10	99095883	99095890	8	*	2	2.89773E-15	9.70941E-06	0.00114332	2.25258E-05	-0.234738292	-0.207385102	RP11-452K12.4
chr14	55840839	55841074	236	*	2	1.57354E-09	0.051794838	0.001144195	0.00410683	0.185815076	0.100981582	ATG14
chr5	131999237	132000721	1485	*	5	1.67503E-20	0.000407573	0.001145082	6.5466E-05	0.151436834	0.060395481	AC004041.2
chr8	22852120	22853394	1275	*	6	2.05669E-26	0.045282245	0.001145285	0.000437201	-0.290431956	-0.079029069	RHOBTB2, PEBP4, RP11-875O11.1
chr19	1177518	1177575	58	*	2	1.22477E-13	1.97635E-05	0.001145912	4.19207E-05	-0.219990799	-0.194062063	

chr9	33446442	33447032	591	*	2	8.04124E-14	0.010157288	0.001146517	0.002571154	-0.183758335	-0.112758794	AQP3
chr11	34202237	34202256	20	*	2	3.59171E-09	0.029869011	0.001146531	0.003641079	-0.262733175	-0.138881889	ABTB2
chr8	102507382	102507837	456	*	3	2.05059E-13	0.003756901	0.001147844	0.001040856	-0.211622137	-0.101513796	GRHL2
chr14	93116574	93117002	429	*	2	2.72143E-13	4.38066E-05	0.001148515	8.18875E-05	-0.180291287	-0.146516768	RIN3
chr19	19702165	19702508	344	*	2	3.48833E-10	0.011796395	0.001149308	0.002725425	-0.144976127	-0.063631702	PBX4
chr2	113345365	113346635	1271	*	4	1.09452E-16	4.59945E-05	0.001149372	3.53621E-05	-0.304741291	-0.147755261	CHCHD5, AC012442.5
chr2	218785909	218786102	194	*	2	8.25331E-14	7.27213E-05	0.001150911	0.000123416	0.154370898	0.152111765	TNS1
chr21	37535372	37536685	1314	*	6	1.3863E-28	2.34047E-05	0.001151174	2.34287E-06	-0.241890012	-0.113498327	DOPEY2
chr10	35703143	35703322	180	*	3	2.63402E-10	0.310628159	0.001151603	0.010570071	-0.34594245	-0.12088849	CCNY
chr19	7525528	7525578	51	*	2	5.36591E-10	0.00881596	0.001152867	0.002440367	-0.108764565	-0.059224534	CTD-2207O23.3, ARHGEF18
chr14	105899248	105899515	268	*	4	4.85428E-14	0.001312343	0.001153944	0.000588326	-0.159387548	-0.037422198	TEX22, MTA1
chr22	27067406	27067721	316	*	3	2.93855E-17	0.000288939	0.001158058	4.89606E-05	-0.219649126	-0.110083023	MIAT, CTA-373H7.7
chr22	24938255	24938711	457	*	2	4.60338E-14	1.36638E-05	0.001159887	3.05096E-05	-0.273805493	-0.187817159	GUCD1
chr9	124009589	124009784	196	*	3	5.66921E-21	7.12733E-08	0.001159979	3.78089E-07	-0.337521591	-0.296816522	GSN
chr19	51626812	51627334	523	*	4	1.17871E-19	7.1693E-06	0.001160135	9.87326E-06	-0.225692168	-0.110054817	
chr6	42984291	42984695	405	*	4	1.53946E-11	0.378795684	0.001161268	0.022296798	0.166990947	0.047640545	KLHDC3
chr22	18112640	18112710	71	*	2	9.8477E-13	0.071124893	0.001161484	0.004384863	-0.28854825	-0.142033184	BCL2L13
chr3	15519117	15519578	462	*	3	8.391E-17	1.16016E-05	0.00116183	2.24572E-05	-0.282599108	-0.202202649	COLQ
chr14	75736090	75736811	722	*	3	1.16546E-14	4.95639E-05	0.001163805	8.3741E-05	-0.246840921	-0.095831449	RP11-293M10.2
chr2	127822551	127822822	272	*	4	1.04338E-16	9.66489E-05	0.001163968	0.00011432	-0.07041012	-0.041975709	BIN1
chr14	31859505	31859515	11	*	2	8.1581E-15	8.53774E-06	0.001166121	2.01494E-05	0.185238992	0.157883831	HEATR5A, RP11-176H8.1
chr19	928287	929119	833	*	3	4.07908E-13	0.002547671	0.001166786	0.000399869	-0.218694397	-0.12311136	ARID3A, AC005391.2
chr17	76850052	76850277	226	*	6	2.85297E-33	1.50141E-08	0.00116685	4.06502E-09	-0.274251823	-0.152773775	TIMP2
chr4	184916766	184917058	293	*	2	2.66933E-10	0.031113182	0.001166904	0.003729372	-0.246824199	-0.126228664	STOX2
chr19	4901791	4902899	1109	*	7	1.64066E-23	9.51182E-06	0.001167216	2.10481E-06	-0.308013601	-0.124292894	ARRDC5
chr22	30714890	30714999	110	*	2	9.98988E-14	1.81778E-05	0.001168841	3.91565E-05	-0.169525121	-0.15564423	TBC1D10A
chr8	59185164	59185425	262	*	2	7.9455E-10	0.010899696	0.001169369	0.002677747	-0.20493539	-0.113192512	
chr9	75200681	75200832	152	*	2	3.05054E-09	0.038944027	0.001169851	0.003942137	-0.279423301	-0.152970269	TMC1
chr17	80696071	80696228	158	*	2	4.76767E-10	0.007811482	0.001170087	0.002345165	0.176101773	0.096613736	FN3K

chr3	177159641	177159881	241	*	4	7.08373E-16	0.003114738	0.00117091	0.000227375	-0.187627436	-0.002011583	LINC00578
chr3	136440459	136440469	11	*	2	3.02224E-11	0.000878023	0.001172016	0.000740015	-0.067802327	-0.063468543	STAG1
chr21	35899585	35900624	1040	*	4	2.3686E-16	0.000370425	0.001175053	0.000246614	-0.356386374	-0.114290508	RCAN1
chr8	9472057	9472784	728	*	3	3.39846E-17	4.8112E-07	0.001181949	1.95692E-06	-0.248029574	-0.173247358	TNKS
chr16	11306333	11306502	170	*	2	8.53398E-10	0.004984883	0.001181995	0.001934551	-0.297262917	-0.157660389	RP11-396B14.2
chr3	50658081	50658472	392	*	2	4.53429E-10	0.094959496	0.001183266	0.004627116	-0.153206967	-0.077309961	MAPKAPK3
chr20	59928451	59928453	3	*	2	1.93892E-10	0.001801322	0.001183643	0.001142525	-0.229081189	-0.156231571	CDH4
chr11	113958057	113958706	650	*	3	6.60138E-14	0.000213799	0.001184347	8.06845E-05	-0.209766482	-0.088297967	ZBTB16
chr2	29149895	29150512	618	*	4	4.76665E-19	3.4511E-06	0.001186789	2.90011E-06	-0.225001313	-0.163479293	WDR43, SNORD53, Y_RNA
chr7	75505977	75505981	5	*	2	1.02172E-14	8.6329E-06	0.00118692	2.04022E-05	-0.337118106	-0.319850179	RHBDD2
chr5	33997223	33997484	262	*	3	2.10671E-12	0.043282313	0.001187599	0.000928967	-0.26448258	-0.136453412	AMACR, RP11-1084J3.4, RP11-1084J3.3
chr19	840737	841082	346	*	7	8.31842E-44	3.8925E-14	0.001188991	2.75084E-12	-0.214244732	-0.154386568	PRTN3
chr1	184580432	184580661	230	*	2	1.53473E-09	0.210078179	0.00118997	0.004937723	0.217314639	0.109958198	C1orf21
chr16	646175	646579	405	*	3	3.17686E-13	0.00072246	0.001190415	0.000466208	-0.185370406	-0.128192125	RAB40C
chr2	40740063	40740436	374	*	2	4.55405E-13	0.147883239	0.001192996	0.004854383	-0.229193855	-0.113572613	SLC8A1
chr1	111770111	111770718	608	*	4	2.9014E-14	0.127483935	0.001194202	0.005078577	-0.248461467	-0.081691146	CHI3L2
chr7	1082103	1082392	290	*	2	2.40884E-15	0.00082865	0.001195112	0.000720139	0.174659957	0.125899214	AC073957.15, C7orf50
chr4	40814531	40814675	145	*	3	1.03765E-18	6.32649E-07	0.001195492	2.25458E-06	-0.249290291	-0.203815142	APBB2
chr20	47393755	47393961	207	*	2	2.00817E-11	0.001604208	0.001196304	0.001075288	-0.186028887	-0.117185534	PREX1
chr12	120692709	120692870	162	*	2	2.29043E-12	0.000211461	0.001198557	0.000282869	0.171692757	0.131931801	PXN
chr1	32782332	32782341	10	*	2	2.3978E-11	0.000203712	0.001198698	0.00027523	0.129028538	0.121079132	HDAC1
chr7	1220356	1221192	837	*	4	1.35968E-15	0.069714982	0.001198721	0.001675575	-0.235113395	-0.099421403	
chr16	67361154	67361968	815	*	4	1.5264E-16	0.017127201	0.001198907	0.003145375	0.191621236	0.079297789	LRRC36
chr12	18963829	18964297	469	*	4	9.45901E-19	0.000839439	0.001200929	8.87632E-05	-0.266693312	-0.103090059	
chr7	127231392	127231698	307	*	2	3.14794E-10	0.125190638	0.001201641	0.004821106	-0.184940182	-0.092919362	ARF5, FSCN3, GCC1
chr14	70263811	70265306	1496	*	7	5.34395E-25	9.83094E-06	0.001202325	1.47015E-06	-0.278481624	-0.109710226	SLC10A1
chr20	31669279	31669392	114	*	2	6.1038E-10	0.057965772	0.001203436	0.004366694	-0.255350622	-0.13050389	BPIFB4
chr2	242174625	242175569	945	*	4	1.07423E-19	0.000186471	0.001205142	6.46762E-05	-0.276589203	-0.106857082	HDLBP
chr5	135394043	135394767	725	*	5	1.52895E-21	4.34685E-05	0.001205923	1.69106E-05	-0.204321828	-0.093920717	TGFBI

chr21	45772566	45774664	2099	*	11	5.98452E-51	1.37733E-11	0.001206264	1.63351E-12	-0.378648016	-0.12016222	TRPM2
chr22	37667522	37667917	396	*	2	4.37884E-10	0.039044986	0.001207341	0.004043607	-0.120566551	-0.062417541	
chr19	2137773	2137818	46	*	3	1.85782E-11	0.043080311	0.001208166	0.006393807	-0.130898521	-0.049479381	AP3D1
chr14	35855812	35856002	191	*	2	2.03397E-10	0.024369522	0.001208341	0.003586162	0.19390839	0.10043481	
chr17	7017474	7019284	1811	*	9	1.25156E-28	0.029232635	0.001209009	0.000283357	-0.195215535	-0.045766568	ASGR2
chr2	232458914	232459434	521	*	2	3.76696E-11	0.002710651	0.001210506	0.001447564	0.173012428	0.076561926	C2orf57
chr14	89951037	89951777	741	*	2	3.47511E-12	9.26066E-06	0.001211312	2.17862E-05	-0.243652109	-0.240107751	FOXN3, RP11-33N16.3
chr2	242941132	242941490	359	*	2	6.45216E-11	0.001238095	0.001211807	0.000929096	0.157315904	0.068851809	AC131097.3
chr19	40006192	40006625	434	*	4	2.38914E-17	0.032190052	0.001212115	0.002879155	0.065349902	0.018809222	SELV
chr6	144018187	144018564	378	*	2	4.59817E-12	0.000179109	0.001213541	0.000251301	-0.292833734	-0.231453892	PHACTR2
chr18	72706612	72707209	598	*	2	6.39267E-14	7.03129E-05	0.001213696	0.000121945	0.181725594	0.125392166	ZNF407
chr15	52393162	52393233	72	*	2	5.13336E-10	0.009061179	0.001214136	0.002554981	-0.127235515	-0.039823345	
chr4	141047768	141048293	526	*	3	8.60294E-18	0.00090274	0.00121555	0.000115464	0.136687769	0.086496645	MAML3
chr15	92934441	92934809	369	*	2	3.96355E-10	0.024493311	0.001215689	0.003607763	-0.238499406	-0.1221903	
chr20	47887009	47887476	468	*	3	2.91829E-11	0.033533284	0.001216053	0.005846534	-0.240771707	-0.079922803	ZNFX1
chr7	139251131	139251615	485	*	3	7.78244E-11	0.056932023	0.001216658	0.007364976	0.161948276	0.044771179	HIPK2
chr17	75399899	75400156	258	*	2	4.53195E-10	0.039178532	0.001217058	0.004072324	-0.197510382	-0.092454588	SEPT9
chr16	3017495	3017955	461	*	4	3.60526E-14	0.036325866	0.001217168	0.007469435	0.097001171	0.022109099	KREMEN2
chr3	195171693	195171852	160	*	2	2.97823E-12	7.75317E-05	0.001217641	0.000131954	-0.259313183	-0.241103774	
chr11	107993298	107993467	170	*	2	8.53687E-14	0.260498143	0.001218686	0.005078036	-0.191630495	-0.095252841	ACAT1
chr19	56148037	56148120	84	*	3	3.08916E-13	0.000465477	0.001220945	0.000404715	0.1599693	0.080078455	ZNF580, ZNF581
chr10	71097327	71097735	409	*	6	1.53335E-26	1.35072E-07	0.001223661	2.287E-07	-0.381870532	-0.165974425	HK1
chr19	42259081	42259395	315	*	4	9.21609E-20	4.82487E-05	0.001223836	1.08124E-05	-0.296214311	-0.15522395	CEA, CEACAM6
chr16	57157313	57157904	592	*	3	5.24967E-20	1.76843E-07	0.001223867	8.5228E-07	-0.175327768	-0.069428271	CPNE2
chr6	13414079	13414265	187	*	2	1.10583E-09	0.020688518	0.001228776	0.003459087	0.197176364	0.095242619	GFOD1
chr19	3398778	3399015	238	*	3	6.70461E-17	3.29372E-05	0.00122899	2.67903E-05	-0.241912653	-0.165977454	NFIC
chr19	42613359	42613682	324	*	2	5.01659E-10	0.00745313	0.001230942	0.002377062	0.189965243	0.114810927	POU2F2
chr3	193964618	193965305	688	*	6	1.38795E-23	0.001670626	0.001231706	5.16647E-05	-0.382395498	-0.13218284	RP11-513G11.4
chr14	23912889	23913135	247	*	2	7.65378E-11	0.011641147	0.001232543	0.002846881	0.176893177	0.098204678	

chr1	9444934	9445612	679	*	3	1.90849E-17	6.98701E-05	0.001233518	3.49315E-05	0.179344885	0.127983033	
chr3	184880078	184880944	867	*	7	2.45506E-14	0.07118859	0.001234046	0.015991425	-0.14105987	-0.028772462	EHHADH-AS1
chr4	38859587	38859770	184	*	4	2.27374E-23	9.91122E-08	0.001234722	2.86284E-07	-0.328709954	-0.211044798	
chr20	1146798	1146889	92	*	3	1.96163E-12	0.001913304	0.00123571	0.001182417	-0.234186947	-0.111253509	PSMF1
chr2	220384302	220384841	540	*	2	3.62971E-12	0.000391074	0.001236639	0.00044398	0.192547209	0.156489644	ASIC4
chr16	17448296	17448516	221	*	3	3.31997E-13	0.002922023	0.001237178	0.00036897	-0.294968064	-0.160820839	XYLT1
chr3	119498870	119500686	1817	*	5	3.49371E-22	8.69365E-08	0.00123848	3.57281E-07	-0.320614348	-0.161848549	NR1I2
chr1	38947929	38948122	194	*	2	2.50642E-11	0.000252287	0.001239542	0.000325681	-0.165582018	-0.116827787	
chr5	14405920	14407367	1448	*	9	1.16722E-26	0.002835376	0.00123987	7.98309E-06	0.18789515	0.068650581	TRIO
chr9	123346214	123346320	107	*	2	6.48628E-09	0.102660495	0.001242478	0.004864126	0.244394534	0.125690728	
chr22	38301066	38301577	512	*	3	5.31446E-13	0.072458141	0.001244834	0.008431286	-0.197430047	-0.045370456	
chr18	11994896	11994976	81	*	2	2.77411E-10	0.00270338	0.001245049	0.001468806	-0.212331062	-0.129431979	IMPA2, RP11-820I16.3
chr15	52405464	52405647	184	*	2	5.59235E-12	0.000765879	0.001245108	0.000698025	-0.122345124	-0.072174274	
chr5	53816023	53816563	541	*	2	1.32475E-11	0.002680328	0.001245466	0.001462389	-0.123012424	-0.105959859	SNX18
chr17	3602004	3602036	33	*	2	1.89747E-14	1.00699E-05	0.001246168	2.3574E-05	-0.257667955	-0.225326881	
chr20	24968599	24969842	1244	*	3	1.61232E-11	1.06555E-05	0.001246497	1.45905E-05	-0.306749066	-0.158631973	APMAP
chr7	130372167	130372419	253	*	2	3.14307E-10	0.003807104	0.001246776	0.00175431	-0.234016405	-0.15217679	TSGA13
chr9	37936386	37937279	894	*	3	1.76097E-14	4.06639E-05	0.001249632	7.32815E-05	0.191038127	0.136842683	RP11-613M10.9, SHB, RNU7-124P
chr16	89519917	89520128	212	*	2	1.00902E-12	9.98294E-05	0.00125033	0.000162326	-0.222124778	-0.181844559	ANKRD11
chr7	139432152	139432178	27	*	2	3.44976E-14	2.33842E-05	0.001251113	4.92744E-05	-0.18389469	-0.175316675	HIPK2
chr17	57502922	57503086	165	*	2	3.72153E-11	0.000738615	0.001251736	0.000683562	0.210864156	0.143641992	
chr4	42645776	42646654	879	*	3	3.30171E-13	5.87286E-05	0.001251804	4.96204E-05	0.099503855	0.087095478	ATP8A1
chr14	23015657	23016326	670	*	5	3.95164E-24	6.53312E-05	0.001251963	2.93361E-06	0.224884903	0.111158005	
chr2	223313524	223313861	338	*	2	6.12206E-11	0.005720843	0.001251966	0.002137497	-0.134643596	-0.091959083	SGPP2
chr22	23592850	23593559	710	*	3	3.84491E-13	0.005162965	0.001252369	0.001267725	-0.346292031	-0.147351413	BCR
chr14	69395234	69395636	403	*	2	2.89415E-11	0.002008296	0.001252404	0.001252366	-0.079718651	-0.053502756	ACTN1
chr10	30780298	30780854	557	*	2	1.01906E-11	0.000495809	0.001253092	0.000525271	-0.23271801	-0.160243437	
chr3	140997743	140998267	525	*	3	1.09333E-13	0.001591026	0.001253694	0.000578602	-0.205969587	-0.09384967	ACPL2, RP11-438D8.2
chr1	31956270	31956405	136	*	3	1.21911E-17	1.89198E-06	0.00125395	5.0533E-06	-0.263786346	-0.145682041	

chr1	36004549	36004559	11	*	2	2.12099E-14	1.12648E-05	0.001254821	2.60717E-05	0.128497683	0.121517825	KIAA0319L
chr1	15348161	15348264	104	*	2	2.82744E-09	0.070691561	0.001255598	0.004671643	0.127645451	0.066125762	KAZN
chr9	7961014	7961264	251	*	2	5.9315E-11	0.001163337	0.001255835	0.000910792	-0.360081329	-0.201142712	RP11-29B9.2
chr13	108975215	108975920	706	*	2	1.74373E-12	3.29399E-05	0.001256267	6.59734E-05	-0.264807281	-0.246728284	
chr22	47021746	47022654	909	*	8	4.22287E-17	0.410144714	0.00125656	0.044145978	-0.315736864	-0.037121873	GRAMD4
chr19	707610	708469	860	*	3	4.70578E-14	0.059507621	0.001256666	0.007329161	-0.184462054	-0.077824272	
chr1	17614144	17614280	137	*	2	6.06452E-14	1.43329E-05	0.001257837	3.22576E-05	-0.294752692	-0.240466595	
chr16	57700944	57702239	1296	*	9	3.26233E-51	1.69807E-14	0.001258969	1.23489E-13	-0.282417474	-0.176208409	GPR97
chr15	39627235	39627548	314	*	2	3.96827E-15	9.78268E-06	0.001259444	2.30099E-05	-0.225901609	-0.217722407	RP11-624L4.1
chr2	27850964	27852421	1458	*	15	1.12966E-36	0.018305597	0.001259531	0.00551983	-0.212894074	-0.032226738	ZNF512, RP11-158I13.2, GPN1, CCDC121
chr2	65088881	65092221	3341	*	7	3.54993E-20	3.26254E-09	0.001260412	7.63071E-09	-0.267780874	-0.136879194	AC007880.1
chr7	45128053	45128199	147	*	2	9.14937E-12	0.021784005	0.001260478	0.0035808	0.150803185	0.070829251	NACAD
chr2	38827187	38827464	278	*	2	2.85454E-10	0.003880061	0.001260806	0.001782873	-0.225534179	-0.094882904	HNRNPLL
chr22	45123972	45125218	1247	*	4	7.35223E-17	0.023073589	0.001261009	0.006120773	-0.155417904	-0.06622513	PRR5, PRR5-ARHGAP8, ARHGAP8
chr12	58011837	58012601	765	*	4	7.12171E-16	0.010017192	0.001261588	0.001298434	0.164202419	0.085628846	ARHGEF25, AC025165.8
chr1	174756086	174756146	61	*	2	8.17012E-11	0.000367427	0.001261884	0.00042858	0.129440702	0.086186543	RABGAP1L
chr9	139926494	139927646	1153	*	5	6.74119E-24	4.98449E-09	0.001264046	4.87558E-08	-0.235615361	-0.142701736	C9orf139, FUT7
chr16	88547533	88547861	329	*	4	4.89562E-18	0.000387816	0.001264186	5.39092E-05	0.187960652	0.091802175	ZFPM1
chr17	47464627	47465656	1030	*	4	9.50424E-20	1.37307E-06	0.001265169	2.95301E-06	0.19810636	0.154393436	RP11-81K2.1
chr11	36475297	36475492	196	*	2	1.28904E-12	0.001362351	0.001265262	0.001005598	-0.104365851	-0.024477575	PRR5L
chr8	37378276	37379119	844	*	5	8.35827E-20	0.00016399	0.001268236	2.43042E-05	-0.292900494	-0.045264962	RP11-150O12.1
chr16	11299313	11299766	454	*	2	1.19575E-10	0.003952888	0.001268262	0.001805809	-0.20077152	-0.125856513	RP11-396B14.2
chr4	680656	681509	854	*	6	6.78184E-23	0.001465597	0.001268845	0.000105802	-0.257564839	-0.080441768	MFSD7
chr6	36857023	36857605	583	*	2	3.25981E-13	2.04297E-05	0.001268881	4.39888E-05	-0.20728592	-0.009818683	C6orf89
chr11	104905889	104906152	264	*	2	6.1254E-10	0.040505977	0.001270001	0.004242797	-0.248555766	-0.12590317	CASP1
chr19	42946985	42947497	513	*	5	9.45693E-17	0.017781694	0.001270022	0.000759201	-0.176476929	-0.068940011	LIPE-AS1, CXCL17
chr15	83419719	83420281	563	*	2	8.12419E-14	0.036592617	0.001273238	0.004152377	-0.320306412	-0.162395766	RP11-752G15.6
chr22	18386258	18386798	541	*	3	1.39902E-10	0.0586688	0.001274697	0.006645847	0.157518815	0.058789919	MICAL3
chr6	11778902	11779078	177	*	3	2.80891E-11	0.079617846	0.001276751	0.007425575	-0.211623637	-0.060381922	ADTRP

chr17	19174172	19174605	434	*	2	1.62725E-11	0.000442971	0.001277554	0.00049036	0.137865083	0.085900565	EPN2
chr10	134511545	134512224	680	*	3	4.72268E-12	0.150935793	0.001278448	0.005475412	0.160526091	0.061665213	INPP5A
chr11	73721165	73721374	210	*	3	2.34812E-17	0.001308881	0.001278508	7.34461E-05	0.203461286	0.123184275	
chr3	171528573	171529610	1038	*	7	1.30665E-28	0.000251297	0.001279786	2.06939E-05	-0.32406068	-0.126412162	PLD1
chr5	67730071	67731643	1573	*	6	1.05502E-15	0.002553146	0.001280026	0.000144171	-0.149460035	0.019419389	CTC-537E7.3
chr3	149241941	149242458	518	*	2	2.26838E-11	0.001049842	0.001281376	0.000863555	-0.150873063	-0.129720603	WWTR1
chr10	29976889	29977335	447	*	3	4.03842E-14	0.000703885	0.001281993	0.000555325	-0.328962986	-0.152785286	SVIL
chr17	9923497	9924869	1373	*	6	4.10616E-18	5.24185E-08	0.001284954	6.48011E-08	-0.279343622	-0.131244314	GAS7
chr14	70186289	70186639	351	*	4	1.81873E-21	1.5721E-07	0.001287405	6.47216E-07	-0.313690328	-0.225352078	
chr19	42816045	42816803	759	*	2	2.88282E-12	0.063973827	0.001288519	0.00469564	0.151480632	0.07299224	
chr17	70974212	70974552	341	*	2	1.33526E-10	0.00144649	0.001292883	0.001053635	-0.208691886	-0.069863691	SLC39A11
chr8	128805414	128806271	858	*	4	1.6731E-13	0.091228856	0.001294631	0.010286577	-0.336135558	-0.072773338	
chr5	122765476	122765825	350	*	2	5.44755E-15	1.26774E-05	0.00129486	2.90894E-05	-0.196651924	-0.172287295	
chr11	3177622	3178322	701	*	7	1.47004E-34	2.22575E-08	0.001296715	5.09102E-09	-0.277965861	-0.19293461	OSBPL5
chr14	23583133	23584303	1171	*	3	4.73767E-11	6.17316E-05	0.001296917	4.22223E-05	-0.250901437	-0.132635205	
chr11	66979798	66980547	750	*	2	3.4735E-12	1.57646E-05	0.001302561	3.52743E-05	-0.274726703	-0.226439352	KDM2A
chr7	7557590	7557741	152	*	2	6.75426E-09	0.083048877	0.001302838	0.004930833	-0.25653345	-0.130242433	COL28A1
chr9	137301117	137302231	1115	*	6	1.96786E-31	1.2926E-08	0.001306241	1.68601E-08	-0.314211385	-0.139533554	RXRA
chr6	147091024	147092242	1219	*	6	2.73499E-25	5.85954E-05	0.001307689	1.2699E-05	-0.291849255	-0.100006339	ADGB
chr17	3861144	3861634	491	*	5	2.26118E-11	0.291276847	0.001307844	0.02951444	-0.251471776	-0.047662915	ATP2A3
chr11	46720913	46721921	1009	*	3	3.21762E-17	0.02986198	0.001308014	0.005719509	-0.231317947	-0.082869573	ARHGAP1
chr12	53610295	53610772	478	*	2	7.43197E-12	0.004966479	0.001308885	0.002057063	0.132839847	0.090821979	RARG
chr12	111655037	111655507	471	*	4	3.47049E-11	0.384340683	0.001309704	0.022540526	-0.134654175	-0.041001471	CUX2
chr16	87588559	87588893	335	*	3	2.33064E-11	0.063102208	0.001310669	0.003437974	-0.186831708	-0.042624031	
chr3	117158482	117158774	293	*	2	2.2754E-09	0.057573089	0.001311889	0.004677477	-0.343831998	-0.177550271	LSAMP
chr17	1690794	1691057	264	*	2	1.91153E-09	0.015537495	0.001311971	0.003302453	0.202988569	0.095206184	SMYD4
chr19	15390948	15392457	1510	*	9	1.15361E-39	1.88465E-08	0.001312159	2.78071E-09	-0.348922278	-0.108087387	BRD4
chr10	5932700	5933311	612	*	3	1.6182E-12	0.039409209	0.001312593	0.003767479	0.115848804	0.036693573	FBXO18
chr1	156465749	156467188	1440	*	5	3.71797E-17	1.11698E-06	0.001314282	2.42516E-06	0.161561398	0.121699112	MEF2D

chr10	104381522	104381651	130	*	2	4.27424E-15	1.07083E-05	0.001314556	2.50996E-05	0.186588725	0.182203374	SUFU
chr6	75982940	75983278	339	*	3	2.54677E-18	1.09501E-06	0.001316983	3.67414E-06	-0.350655187	-0.21897597	TMEM30A
chr6	134537845	134538515	671	*	2	1.12994E-12	1.36691E-05	0.001317633	3.1177E-05	-0.293129787	-0.251607081	SGK1
chr2	219609824	219610606	783	*	3	1.45165E-12	0.001708147	0.00131883	0.001119643	-0.33412503	-0.107882648	TTLL4
chr3	56625176	56625471	296	*	2	8.73316E-12	0.000154767	0.001320655	0.00023163	-0.275764802	-0.16894881	CCDC66
chr1	159046391	159047177	787	*	8	2.57537E-32	7.75639E-06	0.001321191	2.91787E-07	-0.220088282	-0.086423585	AIM2
chr7	36776674	36776989	316	*	2	2.62207E-10	0.010397403	0.001321238	0.002857983	-0.205772002	-0.124767487	
chr17	48367746	48368059	314	*	2	1.62917E-09	0.030675516	0.001321584	0.004088588	-0.101994589	-0.036562904	
chr12	76955696	76956058	363	*	2	1.80742E-11	0.002324422	0.001321638	0.001399058	-0.158041414	-0.122408001	
chr13	99223336	99223562	227	*	2	5.49181E-10	0.010324662	0.001323281	0.002853025	0.184630115	0.101088029	STK24
chr10	100074395	100074568	174	*	2	5.45086E-10	0.007116321	0.001323531	0.002442325	-0.226799218	-0.085052571	
chr14	21898688	21899093	406	*	3	1.2621E-12	0.023468572	0.001324696	0.005103007	-0.268129235	-0.094546948	CHD8
chr4	41983325	41984148	824	*	6	2.08075E-21	0.002964929	0.001325899	0.000141271	-0.280475827	-0.09912103	DCAF4L1
chr8	59067871	59068143	273	*	2	7.64196E-12	0.000133321	0.001326266	0.000206919	-0.163466587	-0.135673417	FAM110B
chr10	11682351	11682423	73	*	2	9.9651E-13	3.77389E-05	0.00132688	7.48623E-05	-0.34994417	-0.280067369	
chr12	132280296	132280704	409	*	3	3.47677E-13	0.001401528	0.001326897	0.001019435	-0.292388551	-0.148937491	SFSWAP
chr17	400410	400920	511	*	3	1.6182E-12	0.011736702	0.001327529	0.001687713	0.210272879	0.090639062	
chr2	43767347	43768564	1218	*	4	1.59738E-18	1.19593E-07	0.00132866	5.93352E-07	-0.269901258	-0.189706979	THADA
chr10	5593559	5593926	368	*	2	3.4048E-10	0.017419969	0.001330178	0.003467947	0.140885041	0.078342962	
chr9	130007437	130007838	402	*	4	4.21349E-11	0.037908961	0.001330668	0.006887489	0.035721646	0.010849396	GARNL3
chr12	50066533	50068610	2078	*	6	2.68059E-21	7.66365E-09	0.001331513	4.11895E-08	0.20352587	0.117819726	FMNL3
chr17	56354594	56355431	838	*	4	2.25261E-24	1.62519E-09	0.001332189	1.7773E-08	-0.24473065	-0.205925405	MPO
chr1	24796905	24797333	429	*	4	4.91628E-18	0.013951803	0.001332678	0.000268051	-0.252561205	-0.124051045	NIPAL3
chr4	140810584	140813287	2704	*	11	6.87816E-32	0.000213282	0.001332764	7.49984E-08	-0.304433514	-0.045673694	MAML3
chr12	96199743	96199841	99	*	2	4.10572E-09	0.009045421	0.001334053	0.00271903	0.178321034	0.105232024	RP11-536G4.1, RP11-536G4.2
chr5	150480839	150480993	155	*	2	3.06271E-15	1.09347E-05	0.001334456	2.56289E-05	0.161535008	0.155751611	ANXA6
chr21	39600121	39601779	1659	*	8	3.44989E-22	0.000828574	0.001338342	2.449E-07	-0.322173687	-0.144724336	KCNJ15
chr3	15446547	15446747	201	*	2	1.05263E-10	0.000503531	0.001340794	0.000546179	-0.307282581	-0.171484016	METTL6
chr8	28233035	28234388	1354	*	5	4.90511E-15	4.47706E-05	0.001340924	7.42346E-05	-0.357119794	-0.104273592	ZNF395, FBXO16

chr12	112215328	112215782	455	*	2	1.33076E-13	1.09369E-05	0.001341271	2.56528E-05	-0.319132669	-0.232948889	RP11-162P23.2, ALDH2
chr19	827429	828170	742	*	10	3.11657E-57	7.58615E-19	0.001343025	3.82412E-16	-0.307190906	-0.176619292	AZU1
chr17	73304723	73305193	471	*	3	9.38087E-20	1.00916E-07	0.001344745	5.3033E-07	-0.290552793	-0.240776928	
chr9	139886304	139887683	1380	*	10	9.12114E-25	0.022574986	0.001345828	0.001696247	-0.086105293	-0.001971951	C9orf142
chr11	47608310	47609357	1048	*	4	8.42652E-14	0.056475387	0.001346477	0.007899421	-0.231533291	-0.057683335	FAM180B
chr2	236522903	236523362	460	*	3	7.8976E-16	0.000282204	0.001346962	8.69678E-05	0.174161583	0.088578763	AGAP1
chr2	28304009	28304502	494	*	3	7.91079E-12	0.006548427	0.00134701	0.001902193	-0.233085655	-0.052159785	BRE
chr10	7226492	7226892	401	*	2	1.19506E-10	0.044629196	0.001348299	0.004544043	-0.180718442	-0.086565208	SFMBT2
chr15	63823236	63824532	1297	*	3	1.03245E-13	0.001298005	0.001348471	9.60589E-05	-0.296527218	-0.154582624	USP3
chr17	39092846	39093054	209	*	2	4.10676E-11	0.032441453	0.001349531	0.004215702	-0.239391767	-0.111247189	AC004231.2, KRT23
chr21	17054876	17055183	308	*	2	6.91835E-10	0.004654987	0.001352125	0.002033935	-0.32067955	-0.197397724	
chr1	28269763	28270137	375	*	2	5.96095E-14	1.14198E-05	0.001358657	2.67106E-05	-0.241522381	-0.176842186	SMPDL3B, RP11-460I13.2
chr15	39803760	39803814	55	*	2	4.5782E-10	0.000922561	0.001359292	0.000818962	0.189735765	0.110953131	
chr7	2606716	2607424	709	*	7	1.84836E-24	0.008080558	0.001360362	8.62436E-05	-0.34092759	-0.059200033	IQCE
chr12	76338627	76338898	272	*	2	2.94489E-09	0.031702177	0.001360732	0.004217164	0.158848852	0.082270448	RP11-114H23.1
chr6	32945234	32946446	1213	*	10	8.01673E-16	0.990746211	0.001361517	0.389811229	-0.352622932	-0.033467253	BRD2
chr9	131007914	131007980	67	*	2	4.55153E-10	0.003534725	0.001362033	0.001779906	-0.224065332	-0.123855569	DNM1
chr11	71846733	71846788	56	*	2	3.20631E-14	1.40375E-05	0.001362776	3.20908E-05	-0.263529725	-0.244506068	FOLR3
chr22	38430277	38430796	520	*	3	5.869E-17	3.90649E-05	0.001364427	3.20064E-05	-0.121640548	-0.042852588	POLR2F
chr1	19217472	19218212	741	*	3	1.20636E-18	0.0077393	0.001367303	0.000202699	-0.249983227	-0.13041382	RP13-279N23.2, ALDH4A1
chr10	126453317	126454007	691	*	3	2.16055E-13	0.000330518	0.001368583	0.000395865	-0.361643683	-0.171029791	RP11-12J10.3, METTL10
chr11	96065844	96065862	19	*	2	6.18953E-14	1.18036E-05	0.001368814	2.75389E-05	-0.27334014	-0.252393785	MAML2
chr17	41376588	41376758	171	*	3	2.93648E-10	0.01221562	0.001369717	0.003703942	-0.298711005	-0.133580986	LINC00854
chr11	3188566	3189207	642	*	3	4.9302E-19	0.001636091	0.001370054	8.47427E-05	-0.296589688	-0.188854675	
chr7	99076604	99077294	691	*	3	9.90724E-17	3.27947E-06	0.001370227	1.09018E-05	0.166124022	0.139401488	ZNF789
chr2	102759368	102759782	415	*	3	1.9009E-13	0.004572276	0.001371417	0.002093624	-0.169987544	-0.066969174	IL1R1
chr17	76340915	76341204	290	*	2	1.77661E-09	0.012995062	0.001371784	0.003194985	-0.217625026	-0.092716062	AC061992.2
chr19	56608297	56609064	768	*	5	4.37778E-23	5.21455E-07	0.001374131	7.00428E-07	0.211112237	0.125411946	ZNF787
chr10	134020204	134020998	795	*	3	3.26685E-13	0.059727773	0.001374858	0.006441541	0.232138171	0.097374012	STK32C

chr3	42631429	42632121	693	*	4	2.48262E-18	0.004821656	0.001375802	0.000568012	-0.300162285	-0.101166419	SS18L2, SEC22C
chr1	153366428	153366607	180	*	2	4.49151E-14	1.14844E-05	0.001376128	2.68963E-05	-0.268714015	-0.241729324	
chr7	97923799	97923834	36	*	2	2.72728E-14	5.35729E-05	0.001376145	0.00010103	-0.157687952	-0.131232895	BRI3, BAIAP2L1
chr7	1982333	1983984	1652	*	7	4.47594E-22	0.00012033	0.001377406	2.70004E-05	-0.418639852	-0.075069437	MAD1L1
chr16	85207349	85208316	968	*	4	5.84974E-16	0.000379266	0.001377539	0.00020113	0.236083456	0.114986002	CTC-786C10.1
chr13	114834683	114834922	240	*	2	1.65146E-10	0.06062222	0.00137813	0.004913375	-0.243995819	-0.131287222	RASA3
chr9	129893312	129894158	847	*	5	4.00011E-14	0.044725232	0.001380942	0.00692168	-0.286918678	-0.066752404	RALGPS1
chr1	108622363	108622429	67	*	2	3.46033E-11	0.000182975	0.001382782	0.000266965	0.050559437	0.049192462	
chr16	88679600	88679919	320	*	4	1.75603E-10	0.180637131	0.001383065	0.020095614	-0.24204615	-0.074214014	ZC3H18
chr12	125420722	125420914	193	*	3	3.54409E-15	1.00644E-05	0.001383922	2.75074E-05	-0.296387788	-0.164817943	
chr8	52873103	52874692	1590	*	7	9.46859E-30	4.86539E-05	0.001384995	1.79841E-06	-0.223054772	-0.093752598	RP11-546K22.1
chr7	37023984	37024713	730	*	3	2.1156E-15	2.95778E-05	0.001387121	6.53643E-05	-0.186508473	-0.120439921	ELMO1
chr6	159240283	159241461	1179	*	10	5.39056E-25	0.525100006	0.001387631	0.011227836	-0.387991249	-0.06628422	EZR-AS1, EZR
chr7	101671556	101672056	501	*	2	1.82222E-12	7.22759E-05	0.001387677	0.000129276	-0.275214086	-0.200405417	CUX1
chr9	32565609	32566125	517	*	2	8.36521E-12	0.015226864	0.001387842	0.003412332	0.204475927	0.092409453	TOPORS-AS1, NDUFB6
chr3	141524149	141524333	185	*	2	4.7218E-09	0.015841844	0.00138787	0.003460328	0.199233278	0.111232829	GRK7
chr1	33804213	33804514	302	*	2	6.36864E-10	0.066182913	0.001388457	0.00501926	-0.317919441	-0.163376675	PHC2
chr2	33359059	33359688	630	*	11	3.3052E-48	3.16389E-13	0.001392641	3.62308E-12	-0.254251485	-0.122699606	LTBP1
chr5	176836472	176837920	1449	*	6	3.76303E-14	0.013685051	0.001392717	0.00034965	-0.279242659	-0.101629871	GRK6, F12
chr16	67560245	67560556	312	*	2	1.47586E-11	0.000574418	0.001394492	0.000607217	0.144650297	0.103823556	FAM65A
chr1	3507069	3507151	83	*	2	1.22394E-10	0.000548887	0.001394805	0.000588876	-0.154225314	-0.111251878	MEGF6
chr6	108878861	108879314	454	*	2	5.36665E-13	0.000980496	0.001394839	0.000861597	0.034662862	0.007447329	
chr16	88228257	88228780	524	*	5	1.63688E-15	0.025980119	0.001397882	0.001390133	-0.261022535	-0.089763906	LA16c-444G7.2
chr16	4729905	4730465	561	*	4	8.24061E-25	5.67453E-09	0.001398192	5.24994E-08	-0.259951916	-0.237443505	MGRN1
chr17	74593349	74594038	690	*	3	1.3109E-14	0.00886749	0.001398243	0.000614029	-0.301987141	-0.148681102	
chr11	67050236	67053829	3594	*	10	3.44759E-34	5.44619E-14	0.001399747	3.58714E-12	-0.337930452	-0.211412755	ADRBK1
chr1	2171253	2172659	1407	*	5	1.12779E-24	0.000850026	0.001401847	5.33791E-06	-0.239727125	-0.130441537	SKI
chr7	41741320	41741671	352	*	3	8.48388E-10	0.493267149	0.001403826	0.015897352	0.20417697	0.073346824	INHBA-AS1, AC005027.3, INHBA
chr17	29151924	29151953	30	*	2	1.23783E-07	0.07447057	0.001406997	0.005171679	0.024928999	0.013229613	

chr1	249110746	249111789	1044	*	6	2.0757E-28	4.267E-07	0.001408426	3.1378E-07	-0.346350763	-0.157549447	SH3BP5L
chr2	106360196	106361676	1481	*	6	9.74973E-22	0.002079541	0.001409574	0.000102563	0.102021411	-0.011596153	NCK2
chr19	3521010	3521502	493	*	5	2.20223E-27	4.16666E-09	0.001410012	3.63685E-08	-0.193270561	0.010072213	FZR1, SNORD38
chr13	41929843	41930111	269	*	2	1.8952E-10	0.029162364	0.001411547	0.004240426	-0.249249663	-0.135207536	NAA16
chr6	116691382	116692074	693	*	6	6.68626E-25	4.59874E-07	0.001412982	1.2349E-06	-0.139979309	-0.066336631	DSE, RP1-93H18.1
chr2	242139599	242139924	326	*	3	1.04208E-16	5.88514E-05	0.001414503	8.24902E-05	-0.306653639	-0.149199908	ANO7
chr2	166982292	166983534	1243	*	4	2.16479E-18	0.007394163	0.001414655	0.000348045	-0.269618209	-0.094910236	AC010127.3, SCN1A
chr16	880206	880371	166	*	2	6.0896E-09	0.199279601	0.0014151	0.0057259	0.15132605	0.069586351	
chr21	47038433	47038905	473	*	3	6.03463E-18	1.61794E-06	0.001416183	5.07507E-06	-0.283818371	-0.166805738	
chr17	44248233	44248641	409	*	3	2.97079E-20	7.67463E-06	0.001416758	1.39879E-05	-0.300875287	-0.17841993	KANSL1
chr12	15103680	15104916	1237	*	4	1.63525E-22	0.000206376	0.001419041	6.21691E-06	0.197892596	0.109054535	ARHGDIB
chr11	12146343	12147260	918	*	3	5.5978E-12	0.007913154	0.001421005	0.003086665	-0.194926244	-0.05937319	MICAL2
chr7	2653651	2654420	770	*	6	1.26824E-26	5.55141E-05	0.001422296	1.06878E-06	0.297900456	0.074816802	IQCE
chr19	16254282	16254516	235	*	5	2.22122E-18	0.000619216	0.001423277	4.36828E-05	-0.16918701	-0.064410316	HSH2D
chr14	100900810	100900999	190	*	2	1.29784E-13	3.76754E-05	0.001426322	7.59563E-05	-0.259696749	-0.258644607	WDR25
chr5	133841762	133842146	385	*	4	6.78445E-16	3.3195E-05	0.001426332	6.55311E-05	0.167117296	0.09026487	
chr17	79967022	79967042	21	*	3	5.49517E-10	0.152206159	0.001427854	0.012158294	0.145309646	0.061303705	ASPSCR1
chr1	224545104	224545736	633	*	4	8.1319E-17	0.060892878	0.001430724	0.003958264	-0.311029031	-0.091479295	CNIH4
chr1	92951594	92953279	1686	*	26	1.71508E-72	8.37802E-05	0.001432717	1.79702E-09	-0.360694708	-0.059967885	GFI1
chr7	90794392	90794769	378	*	3	8.72952E-15	0.00649082	0.00143325	0.000206609	-0.25568342	-0.144510292	CDK14
chr6	16684297	16684836	540	*	2	7.06934E-13	2.80911E-05	0.001433638	5.92364E-05	-0.116992499	-0.115219228	ATXN1
chr1	8273352	8274044	693	*	4	2.31983E-22	1.29789E-05	0.001434896	1.71455E-06	-0.272744764	-0.189263343	RP11-431K24.3
chr5	169727643	169727897	255	*	2	4.88768E-11	0.000308257	0.001435858	0.000397615	-0.2754453	-0.184824234	
chr1	236094805	236096728	1924	*	5	7.53475E-20	2.81698E-09	0.001436242	2.65042E-08	-0.357007482	-0.201054781	
chr21	46332181	46332291	111	*	2	3.25449E-10	0.004964471	0.001436999	0.002177517	-0.229610313	-0.130218099	ITGB2
chr19	15751297	15751949	653	*	2	2.18247E-14	0.000338925	0.001437098	0.000425815	-0.219531004	-0.15452378	CYP4F3
chr7	158892077	158892397	321	*	2	2.32347E-09	0.055283173	0.00143869	0.005000004	0.153550742	0.078925582	VIPR2
chr1	47051416	47053057	1642	*	5	9.91522E-11	0.203898489	0.001438932	0.001300192	-0.317781241	-0.126146021	MKNK1
chr21	44166975	44167104	130	*	2	2.50181E-10	0.002524216	0.001439037	0.001534299	-0.352845254	-0.254907276	PDE9A

chr14	74862615	74862957	343	*	2	3.29753E-11	0.000322615	0.001439699	0.00041129	0.177806794	0.130963342	
chr11	64532839	64533623	785	*	7	1.2753E-18	0.010993293	0.001441467	0.000590201	0.146558585	0.045764825	SF1
chr15	90547692	90548061	370	*	4	2.9206E-21	3.60103E-07	0.00144203	1.43725E-06	-0.326683419	-0.200982401	ZNF710
chr12	124069475	124070499	1025	*	8	4.45595E-22	0.104462689	0.001442455	0.004386576	-0.31926608	-0.070895836	TMED2, RP11-486O12.2
chr8	129061048	129061665	618	*	5	5.08523E-15	0.280392644	0.001442557	0.030499975	-0.103027027	-0.016486627	PVT1, MIR1207
chr2	9894165	9894734	570	*	4	2.79475E-20	2.4972E-06	0.001442838	6.86036E-06	0.130272735	0.094864466	
chr1	234905686	234906223	538	*	3	2.56172E-15	7.45585E-05	0.001443911	5.65952E-05	0.187643714	0.124788407	
chr6	32060681	32061478	798	*	6	1.79175E-22	0.005259389	0.001447534	0.000226048	0.210298817	0.077411683	TNXB
chr1	204110366	204111253	888	*	6	6.79503E-13	0.512023617	0.001449136	0.046614528	0.148272436	0.029957861	RP11-74C13.3, ETNK2
chr22	25799070	25800078	1009	*	4	2.75661E-19	1.84278E-06	0.001449421	4.24054E-06	-0.158164118	-0.106880225	LRP5L
chr1	25235737	25235862	126	*	2	1.90385E-10	0.036188561	0.001449557	0.004579302	0.18794612	0.091529505	RUNX3
chr7	127997529	127998600	1072	*	3	1.0578E-14	5.71065E-07	0.001450037	2.43439E-06	-0.212564781	-0.055054749	PRRT4
chr4	154001159	154001197	39	*	2	9.47983E-11	0.000277537	0.001452543	0.000369991	-0.140062823	-0.100982293	RP11-285C1.2
chr17	8761973	8762140	168	*	3	1.39648E-16	3.94333E-06	0.001454108	1.22161E-05	-0.183247202	-0.151451376	PIK3R6
chr20	48837617	48838080	464	*	2	2.46593E-13	2.12156E-05	0.001455012	4.66085E-05	-0.249087647	-0.224113005	
chr14	24539335	24540773	1439	*	13	4.18471E-48	1.66156E-10	0.001456273	1.21306E-10	-0.355686011	-0.125700277	CPNE6
chr2	27665711	27666036	326	*	2	4.71735E-12	0.017997988	0.001457003	0.003743158	0.14894172	0.081719612	KRTCAP3
chr19	47122109	47122445	337	*	2	1.03479E-09	0.022578526	0.001457147	0.004029017	-0.056510275	-0.033320455	
chr10	75534560	75534675	116	*	2	2.09893E-10	0.007495754	0.001457304	0.002658557	-0.09545695	-0.035805438	FUT11
chr22	37960396	37960426	31	*	2	1.01573E-12	5.78123E-05	0.001458668	0.000109111	0.189909415	0.145658398	CDC42EP1
chr17	2264242	2265510	1269	*	7	1.81344E-20	0.067764869	0.001459048	0.002551652	-0.16537219	-0.044609827	SGSM2
chr2	102927278	102927898	621	*	5	8.46937E-12	0.118351853	0.001459081	0.014906153	-0.194014608	-0.06062135	
chr15	90291404	90291815	412	*	3	1.23101E-13	0.018473951	0.001459163	0.001234499	-0.215755217	-0.105611379	
chr22	42336869	42337222	354	*	2	1.77031E-09	0.076770892	0.001460766	0.005358602	-0.204867304	-0.098356494	CENPM
chr16	89461734	89462661	928	*	6	3.35916E-26	2.04493E-05	0.001461433	1.1748E-05	-0.33960222	-0.072383113	ANKRD11, RP1-168P16.2
chr1	9133353	9133414	62	*	2	1.65076E-13	2.33059E-05	0.001462514	5.06196E-05	-0.326004544	-0.310103064	SLC2A5
chr8	116462301	116462556	256	*	2	8.07311E-10	0.002996267	0.001462541	0.001699667	-0.098467688	-0.061059819	TRPS1
chr9	126232182	126232853	672	*	3	6.7301E-18	9.38322E-07	0.001463075	3.76486E-06	-0.258074211	-0.082993679	DENND1A
chr17	76818733	76820132	1400	*	6	1.25875E-21	0.010613152	0.001463173	0.000145546	-0.285330319	-0.086173951	USP36

chr16	50815179	50815307	129	*	2	1.50216E-08	0.144582231	0.001463756	0.005774773	0.202172115	0.101710898	CYLD
chr2	70188512	70189903	1392	*	7	2.3666E-30	3.48312E-05	0.001463848	2.39804E-06	-0.379949763	-0.11799295	ASPRV1, PCBP1-AS1
chr17	78796363	78797061	699	*	4	4.14813E-19	0.00031923	0.001464141	8.88554E-05	-0.218594011	-0.123466765	RPTOR
chr17	79054073	79054476	404	*	2	3.62109E-11	0.003555324	0.001464266	0.001861805	0.162319465	0.101964445	BAIAP2
chr10	89503133	89503153	21	*	2	1.76752E-09	0.004351968	0.001464706	0.002064433	-0.236522213	-0.124867398	PAPSS2
chr7	5258357	5258485	129	*	3	8.94407E-14	0.000266967	0.00146924	0.000336969	-0.28764359	-0.153956038	WIPI2
chr1	40029385	40030697	1313	*	9	4.51984E-30	0.000440968	0.001475692	1.61353E-05	-0.216024473	-0.058402871	PABPC4
chr3	195946195	195947062	868	*	7	4.21174E-28	9.20572E-06	0.001476461	6.3946E-07	-0.391783307	-0.15286873	SLC51A, PCYT1A
chr12	95034515	95034650	136	*	2	1.37396E-13	1.46732E-05	0.001476778	3.37987E-05	-0.302297389	-0.283186117	TMCC3
chr17	16344368	16344574	207	*	3	4.00217E-15	4.21091E-05	0.001476853	6.45981E-05	-0.229486894	-0.152536658	FAM211A-AS1
chr1	155940889	155941006	118	*	2	3.00864E-09	0.021770254	0.001478208	0.004025427	-0.107907962	-0.061554599	ARHGEF2
chr10	11937059	11937255	197	*	3	2.12627E-20	2.04129E-07	0.001479862	1.00485E-06	0.187586901	0.141796282	
chr11	64483118	64483691	574	*	2	2.52593E-13	0.00249177	0.001479878	0.001546855	0.164181629	0.116628969	NRXN2
chr16	995827	996172	346	*	2	2.26408E-09	0.026258496	0.001479969	0.004265194	-0.341045747	-0.176678479	LMF1
chr3	107942749	107943092	344	*	2	2.7471E-13	0.000394877	0.001480097	0.000480142	-0.182586242	-0.129225809	
chr1	26880207	26881684	1478	*	11	2.11172E-37	9.1996E-05	0.001480135	6.13794E-07	-0.326942125	-0.109896178	RPS6KA1, MIR1976
chr16	86011615	86012840	1226	*	8	1.29828E-32	9.54433E-05	0.001480615	1.60197E-06	-0.239214035	-0.081460638	
chr9	132572181	132572194	14	*	2	7.31798E-09	0.022037198	0.001482751	0.004049996	-0.181636133	-0.095213142	TOR1B
chr2	86397937	86398455	519	*	2	2.14165E-11	0.002583016	0.00148915	0.001583396	-0.287864395	-0.167917824	IMMT
chr22	33158075	33158236	162	*	2	5.12004E-12	0.000109335	0.001489633	0.00018356	0.182861665	0.139125588	SYN3
chr2	197106919	197107109	191	*	2	1.18552E-08	0.249046171	0.001493123	0.006054357	-0.107808426	-0.053237022	HECW2
chr1	145523488	145523592	105	*	2	9.78572E-09	0.017069219	0.001493167	0.00374067	0.132848386	0.063721538	PEX11B
chr15	90605618	90608429	2812	*	7	1.35381E-14	3.96625E-08	0.001494264	1.68396E-07	0.162677234	0.009851565	ZNF710
chr10	85933300	85933798	499	*	3	6.43276E-14	0.038158568	0.001496249	0.001169836	0.162972979	0.083641347	C10orf99
chr7	101361395	101361745	351	*	3	2.4496E-19	1.87638E-07	0.00149727	9.34687E-07	-0.242534029	-0.217877192	
chr8	142219329	142220329	1001	*	4	2.95224E-20	4.65625E-06	0.001498002	2.04757E-06	-0.287114471	-0.157369505	SLC45A4
chr4	24975717	24975754	38	*	2	2.20725E-13	1.70696E-05	0.00149861	3.8722E-05	-0.223547406	-0.207279744	CCDC149
chr9	138029495	138029591	97	*	2	2.49632E-11	0.000133923	0.001504303	0.00021587	0.145985543	0.134642707	
chr8	142030202	142030569	368	*	3	3.50662E-13	0.001923586	0.001507925	0.000537571	0.184280741	0.069978155	

chr21	46327720	46328106	387	*	2	4.74361E-10	0.005612805	0.001509567	0.002380318	-0.054577625	-0.045904902	ITGB2
chr4	10075904	10076009	106	*	2	1.64414E-10	0.001753471	0.001509653	0.001278231	0.1967202	0.078539025	WDR1
chr3	48408093	48408202	110	*	3	3.53256E-17	1.27341E-06	0.001512475	4.66252E-06	-0.369189068	-0.260967558	
chr3	45688760	45689110	351	*	2	1.14266E-11	0.0001481	0.001513172	0.000233837	-0.147046791	-0.115807495	LIMD1
chr5	141551033	141551276	244	*	2	2.34156E-09	0.050331676	0.001514709	0.005112037	-0.094664914	-0.040512152	
chr5	75377860	75379515	1656	*	9	3.14684E-25	6.74587E-07	0.001515224	4.71056E-06	-0.037855709	0.011777002	SV2C, CTC-235G5.3
chr7	36698354	36698863	510	*	2	2.86315E-13	1.80469E-05	0.001516432	4.07458E-05	-0.202327957	-0.190940628	AOAH
chr1	229206074	229206273	200	*	2	2.8495E-13	1.94974E-05	0.001517801	4.36137E-05	-0.219538254	-0.188690263	
chr2	46227654	46227968	315	*	3	6.16444E-10	0.407648509	0.00151886	0.016555746	0.223458381	0.076452383	PRKCE
chr16	89003641	89004908	1268	*	5	2.55682E-19	0.015188519	0.00151942	0.001153225	-0.1402555	-0.060903066	CBFA2T3, RP11-830F9.7
chr11	62229913	62231081	1169	*	6	2.78415E-18	0.001058108	0.001519813	0.000145581	0.211895177	0.090872044	AHNAK
chr20	62632981	62633123	143	*	3	1.072E-09	0.021596831	0.001521675	0.004752204	0.155448202	0.060597182	PRPF6, ZNF512B
chr20	30300972	30301179	208	*	2	5.00344E-09	0.049867854	0.001521941	0.0051214	-0.134579146	-0.06377485	RP11-243J16.7, BCL2L1
chr11	47410526	47410707	182	*	2	2.18074E-13	4.12251E-05	0.001521971	8.31108E-05	-0.223795186	-0.189041176	RP11-750H9.5
chr7	128083590	128083732	143	*	2	3.11033E-09	0.076134983	0.001522493	0.005536694	-0.10644942	-0.055741498	
chr4	6754822	6755303	482	*	4	1.81392E-16	1.91627E-05	0.001525067	4.21337E-05	0.159738024	0.098147576	
chr8	19555033	19555369	337	*	2	4.50871E-10	0.072515056	0.001528503	0.005511522	-0.187259897	-0.09910448	CSGALNACT1
chr5	179031306	179031448	143	*	2	1.47989E-09	0.048581124	0.001529398	0.005112722	0.166931779	0.087014199	RUFY1
chr8	105424058	105424730	673	*	5	1.58631E-28	5.46501E-10	0.00152966	7.43823E-09	-0.241769173	-0.202500786	DPYS
chr17	79415682	79416550	869	*	3	5.61515E-12	0.036783219	0.001529854	0.005275254	-0.28277559	-0.068512573	RP11-1055B8.7
chr3	182977036	182977408	373	*	2	2.89189E-12	0.000876447	0.001530331	0.000836862	-0.359820354	-0.213715977	B3GNT5, MCF2L2
chr12	10182372	10184080	1709	*	9	3.3157E-31	4.68779E-05	0.001532361	2.09021E-06	-0.223097278	-0.066482876	CLEC9A, RP11-133L14.5
chr1	158604822	158605286	465	*	2	5.70381E-11	0.002190108	0.00153322	0.001466096	-0.176493616	-0.099855752	SPTA1
chr19	16986822	16988083	1262	*	4	6.02068E-14	0.000113145	0.001535605	3.25957E-06	0.181732468	0.099688964	SIN3B
chr17	74557494	74558202	709	*	4	2.63332E-22	1.28625E-05	0.001536076	2.0071E-06	-0.224193042	-0.147038299	SNHG16
chr5	137203424	137203640	217	*	2	3.68097E-10	0.006536198	0.001537357	0.00258545	0.152763085	0.090088999	MYOT, RP11-381K20.2
chr16	27654256	27654375	120	*	3	1.36794E-14	0.000347256	0.00153915	7.83869E-05	-0.232633195	-0.125607119	KIAA0556
chr11	64642144	64643272	1129	*	7	9.96673E-33	2.12512E-11	0.001541736	6.20626E-10	-0.187905651	-0.051423589	EHD1
chr22	39895842	39896232	391	*	2	1.51696E-11	0.029769698	0.001542384	0.004559899	0.206585344	0.099961023	MIEF1

chr5	1477719	1478509	791	*	3	8.03555E-19	7.80467E-07	0.001542592	3.08815E-06	-0.332422154	-0.266760891	LPCAT1
chr7	966081	967834	1754	*	11	1.68946E-21	0.010423899	0.001542643	0.001659911	-0.232408513	-0.038478276	ADAP1, COX19
chr7	141395696	141395853	158	*	2	1.64216E-08	0.081592149	0.0015433	0.005658254	-0.092577347	-0.044184655	KIAA1147
chr11	57299541	57299709	169	*	2	3.31986E-10	0.027296771	0.001543678	0.004451445	0.149563688	0.067925194	
chr1	153321198	153321421	224	*	2	1.07852E-10	0.046116244	0.001543929	0.005092718	-0.231815621	-0.108409325	PGLYRP4
chr2	71779926	71780175	250	*	2	4.4443E-09	0.037150035	0.00154477	0.004841086	-0.193938721	-0.086166357	DYSF
chr10	101609768	101610732	965	*	3	5.10414E-15	5.52891E-05	0.001546235	5.78129E-05	-0.276865034	-0.203173636	ABCC2
chr4	39481907	39482781	875	*	8	4.01572E-33	0.000382592	0.001550631	1.6843E-06	-0.339672754	-0.119871262	RP11-472B18.1
chr12	121419635	121419771	137	*	2	6.64574E-12	0.00019575	0.001558122	0.000292285	0.194045444	0.162773957	HNF1A
chr5	66253919	66255772	1854	*	10	4.3643E-34	1.7359E-07	0.001559413	1.01962E-07	-0.386192898	-0.079054207	MAST4
chr7	150146890	150148073	1184	*	11	5.56682E-19	0.579409968	0.001560085	0.061515711	-0.113777365	-0.013620098	GIMAP8
chr14	99501573	99502466	894	*	3	1.35035E-16	7.93433E-06	0.001561027	1.806E-05	-0.248853414	-0.161864008	
chr10	1088372	1088692	321	*	4	1.66526E-20	2.34218E-06	0.00156257	2.33123E-06	-0.294808353	-0.212305011	IDI2-AS1, IDI1
chr21	43316274	43316705	432	*	3	1.21791E-19	2.73244E-06	0.001565965	7.60897E-06	-0.221963029	-0.17896118	C2CD2
chr6	30704809	30704998	190	*	3	6.02045E-10	0.1069876	0.001567515	0.011471219	0.132670574	0.066750336	FLOT1
chr5	94027110	94027888	779	*	4	1.62262E-19	2.67619E-06	0.00156774	5.10135E-06	-0.290742167	-0.187676119	ANKRD32
chr3	172041893	172042282	390	*	2	4.08404E-11	0.001048225	0.00156866	0.000949751	-0.250257547	-0.14959362	FNDC3B
chr15	86315115	86315211	97	*	3	1.76342E-18	1.23101E-06	0.001569852	4.82752E-06	-0.235600679	-0.172510781	KLHL25
chr1	26490540	26490628	89	*	2	2.01769E-09	0.043260115	0.001571242	0.005088768	0.110685661	0.067799164	
chr10	75654257	75654563	307	*	2	7.68963E-11	0.000372189	0.00157414	0.000471351	0.188761999	0.139193205	
chr1	9714280	9714845	566	*	7	1.92763E-27	3.70127E-06	0.001577394	8.7557E-07	-0.290691568	-0.120118676	PIK3CD, C1orf200
chr9	21032451	21032989	539	*	2	1.53517E-14	1.92607E-05	0.001579044	4.34355E-05	0.147283486	0.144301328	
chr6	6885899	6886806	908	*	3	6.8978E-14	7.80671E-05	0.00157932	9.84264E-05	-0.309792608	-0.180104576	
chr2	144019668	144020044	377	*	2	1.09843E-09	0.017828198	0.001580181	0.003953395	-0.285912351	-0.133132045	ARHGAP15
chr18	61557687	61557735	49	*	2	5.54942E-14	2.24293E-05	0.001583743	4.96615E-05	-0.290112525	-0.235450734	SERPINB2
chr7	99970448	99972461	2014	*	8	5.57774E-29	7.58653E-09	0.001585655	2.44558E-09	-0.292020401	-0.111570993	PILRA
chr12	58210661	58211237	577	*	5	2.64621E-15	0.047902328	0.001586618	0.002804933	-0.236634807	-0.061333766	AVIL
chr11	65059447	65060386	940	*	3	1.69756E-15	0.000560923	0.001587348	0.000108492	-0.260463322	-0.124765889	POLA2
chr15	39727283	39727923	641	*	3	5.79769E-16	0.000266842	0.001590277	0.000157072	0.215437836	0.127441385	

chr2	232880298	232880480	183	*	2	3.26669E-10	0.013737313	0.001592318	0.003616133	-0.165018275	-0.086847186	DIS3L2, AC105461.1
chr21	43652704	43653234	531	*	2	9.39954E-13	1.53509E-05	0.001593176	3.55928E-05	-0.280771284	-0.233085994	ABCG1
chr1	53856140	53856531	392	*	2	1.36288E-09	0.009408064	0.001596263	0.003111598	-0.1049518	-0.057874758	
chr2	20306539	20306827	289	*	2	3.41282E-13	3.78385E-05	0.001596563	7.80746E-05	0.160477531	0.150716726	AC098828.2
chr13	43354421	43354782	362	*	4	1.35589E-18	2.37575E-05	0.001597232	2.80735E-05	-0.326061159	-0.190794268	
chr18	46333108	46333236	129	*	2	2.41979E-08	0.139956859	0.001601898	0.006218948	0.154076934	0.076475916	CTIF
chr3	64177129	64177237	109	*	2	4.3166E-09	0.003565516	0.001603425	0.001964267	-0.225639834	-0.121009514	PRICKLE2-AS3, PRICKLE2
chr13	114783597	114783665	69	*	2	1.36897E-10	0.00140265	0.001604634	0.001152119	0.169179308	0.115428587	RASA3
chr10	97514686	97515753	1068	*	7	7.35401E-31	0.000166973	0.001604906	8.85247E-07	-0.319191425	-0.155255932	ENTPD1, ENTPD1-AS1
chr16	73071300	73072333	1034	*	3	1.13387E-13	0.000301469	0.001605695	0.000395226	0.215382957	0.12072987	ZFHX3
chr7	6631231	6631520	290	*	4	1.27638E-14	0.027076517	0.001605913	0.000807143	-0.37847144	-0.129336168	C7orf26
chr1	27634683	27634872	190	*	3	2.05426E-09	0.080412989	0.001606221	0.008005682	-0.063645286	-0.023404921	WDTC1
chr2	64559272	64559457	186	*	2	2.22349E-09	0.045924461	0.001606774	0.005249825	0.166457458	0.073311179	
chr16	14051717	14051856	140	*	2	6.62963E-09	0.019918149	0.001609751	0.004160019	-0.170995645	-0.096115304	
chr17	4387045	4387100	56	*	2	3.40175E-10	0.00381796	0.001609792	0.002040671	-0.227309538	-0.13730852	SPNS3, RP13-580F15.2
chr19	13053719	13054718	1000	*	5	3.91012E-28	6.43057E-09	0.001611779	2.67247E-08	-0.365484674	-0.221042927	CALR
chr17	8629735	8629854	120	*	2	2.12057E-08	0.041991234	0.001612934	0.00515745	-0.246065607	-0.131254217	
chr2	231662312	231662582	271	*	2	1.08449E-10	0.001102629	0.001613047	0.000993678	-0.197370434	-0.127687238	CAB39
chr6	31656126	31658789	2664	*	9	8.98957E-23	0.000556865	0.00161548	8.77143E-06	0.214666697	0.07764464	ABHD16A, XXbac-BPG32J3.20
chr10	90638291	90638765	475	*	2	7.1244E-11	0.050493799	0.001616044	0.005384625	-0.325632472	-0.165603339	
chr5	173285610	173286145	536	*	3	1.01179E-15	3.51324E-06	0.001616784	1.16106E-05	-0.331648006	-0.143859083	
chr1	247581408	247582066	659	*	7	3.78749E-30	0.002318721	0.001618508	7.55353E-06	-0.350039458	-0.105616661	NLRP3
chr15	40632399	40633816	1418	*	11	1.31839E-32	0.000100384	0.001619959	3.03263E-06	-0.229184495	-0.058497236	C15orf52
chr17	40838861	40839022	162	*	3	2.64396E-11	0.016710566	0.001621336	0.00413096	0.073335224	0.037435181	CNTNAP1, CTD-3193K9.3
chr9	2044055	2044541	487	*	2	6.92647E-11	0.001197777	0.001621861	0.001049752	0.087453732	0.057299496	SMARCA2, RP11-264I13.2
chr10	134504552	134504666	115	*	2	3.93457E-09	0.068256732	0.00162205	0.005725715	0.130056238	0.070764015	INPP5A
chr4	157713603	157713817	215	*	2	1.09944E-09	0.01551521	0.001622993	0.003834033	0.221751961	0.085046097	PDGFC
chr5	148442740	148444109	1370	*	7	1.89222E-16	5.58315E-05	0.001623263	3.74281E-05	0.211934209	0.097959458	CTC-529P8.1
chr12	8210810	8211391	582	*	4	1.94328E-20	8.98849E-08	0.001623484	5.62078E-07	-0.302527304	-0.230774106	C3AR1

chr7	16461579	16461798	220	*	2	5.44985E-11	0.040847497	0.001623685	0.005149896	-0.13741113	-0.061496431	
chr13	29291375	29291701	327	*	2	5.34524E-11	0.180850911	0.001624715	0.00641582	-0.184213332	-0.093498715	SLC46A3
chr7	5447079	5447353	275	*	2	5.93565E-09	0.338519774	0.001627378	0.006576777	-0.290699301	-0.144883291	TNRC18
chr12	96633384	96633723	340	*	2	9.94726E-11	0.003705517	0.00162853	0.002022373	0.18822697	0.087106377	ELK3
chr16	69776039	69776499	461	*	2	1.14504E-12	0.000477541	0.001631412	0.000570326	-0.147273335	-0.083334121	CTD-2033A16.3, NOB1
chr12	52603300	52605004	1705	*	6	2.61331E-17	0.004146307	0.001637971	0.000415734	-0.249493309	-0.047501338	LINC00592, C12orf80
chr15	40398307	40399723	1417	*	10	7.56056E-31	0.001475142	0.001638363	1.18382E-05	-0.192475543	-0.064331757	BMF
chr16	84390897	84391112	216	*	2	6.86216E-12	7.18369E-05	0.00163954	0.00013425	-0.117381812	-0.077741947	
chr9	271757	273074	1318	*	7	2.39251E-23	0.011475921	0.001640253	0.000315511	-0.22789251	-0.069210836	DOCK8, RP11-59O6.3
chr3	178815437	178815496	60	*	2	7.21006E-12	0.000577026	0.001640313	0.000651916	-0.22934996	-0.177341441	
chr16	84676858	84677351	494	*	2	4.2074E-12	7.21717E-05	0.001640992	0.000134792	-0.371430442	-0.136948828	
chr6	43274373	43275674	1302	*	4	2.00683E-11	0.009927854	0.001641284	0.000899908	0.2353537	0.035257173	CRIP3, ZNF318
chr12	25706982	25707667	686	*	4	2.6487E-17	0.000375664	0.001645804	2.6571E-05	-0.251756672	-0.135142165	IFLTD1
chr21	43983837	43983971	135	*	2	1.76757E-08	0.046869811	0.001646252	0.005375268	-0.16342042	-0.076503507	SLC37A1
chr12	53267981	53268997	1017	*	5	1.25471E-12	0.04320711	0.00165017	0.010572443	-0.06385471	-0.018325213	
chr19	50335849	50336107	259	*	2	1.21734E-10	0.006697304	0.001650407	0.002733234	-0.188461834	-0.107312086	MED25
chr12	107300707	107300784	78	*	2	2.32056E-10	0.000457219	0.001652935	0.00055599	-0.119291505	-0.08960128	
chr17	42871894	42872309	416	*	2	2.34923E-11	0.001350805	0.001652974	0.001141967	0.184923886	0.075344008	CTC-296K1.4
chr6	105725878	105726842	965	*	4	1.56162E-14	0.000192662	0.00165454	0.000259669	0.15299021	0.065898246	PREP
chr9	19925171	19925583	413	*	2	2.38494E-11	0.000831183	0.001658692	0.000837882	0.101853141	0.006906008	
chr7	106061801	106062067	267	*	2	1.18764E-08	0.221681234	0.001659181	0.006604269	-0.249332702	-0.125475289	CTB-111H14.1
chr1	36983119	36983235	117	*	2	4.37069E-12	0.000351191	0.001659658	0.000461046	0.101842666	0.087879105	
chr7	2078036	2078988	953	*	4	3.2778E-11	0.312992875	0.001660126	0.029087598	0.156748642	0.044108311	MAD1L1
chr11	112037134	112038104	971	*	9	4.82885E-19	0.025777809	0.001662802	0.008002993	-0.170185039	-0.040583753	SDHD, TEX12, RP11-356J5.4
chr12	6332834	6335277	2444	*	8	2.89674E-17	6.49779E-06	0.001664684	2.81275E-07	0.226654787	0.089794435	CD9
chr11	45257323	45257480	158	*	2	7.03716E-09	0.016801121	0.001665477	0.004018052	-0.122322903	-0.04501375	
chr10	73612154	73612360	207	*	2	9.33865E-11	0.05435398	0.00166595	0.005601812	-0.292341349	-0.148642802	
chr19	1513513	1514425	913	*	4	6.95214E-17	0.005013087	0.001666279	0.000792827	0.091869193	0.051063955	ADAMTSL5
chr3	120401058	120402723	1666	*	7	1.46189E-19	0.002523991	0.001666921	5.41091E-06	0.239492478	0.014778371	HGD

chr4	119991548	119991634	87	*	2	8.76327E-11	0.000422811	0.001667021	0.000527712	-0.262558349	-0.1689951	
chr11	69414593	69414923	331	*	2	2.38461E-11	0.000242427	0.001672572	0.000351682	-0.304848723	-0.096093277	
chr12	77426239	77426479	241	*	2	6.49134E-13	2.43843E-05	0.001672827	5.39323E-05	-0.249556524	-0.249223302	E2F7
chr5	115962086	115962584	499	*	3	1.99748E-11	0.001108164	0.001673113	0.000986986	-0.264629029	-0.130382769	
chr3	64083605	64083787	183	*	2	9.95574E-14	9.44601E-05	0.00167409	0.000168674	0.218413703	0.169231758	RP11-129B22.1, PRICKLE2
chr17	57444078	57444356	279	*	2	3.66785E-10	0.284823585	0.001677507	0.006727466	0.151830497	0.0766632	YPEL2
chr1	9129487	9130738	1252	*	6	2.72554E-31	1.83098E-06	0.001678763	1.18895E-07	-0.24658853	-0.125435351	SLC2A5
chr11	47736132	47737105	974	*	10	4.97603E-15	0.676884871	0.001681988	0.025757993	-0.129630245	-0.0239916	AGBL2
chr13	114802548	114803271	724	*	5	1.95388E-13	0.019043334	0.001682398	0.004583025	0.165335737	0.052351472	RASA3
chr19	51981608	51981718	111	*	2	2.63876E-11	0.00371333	0.001684282	0.002063879	0.19907742	0.115087696	CEACAM18
chr15	73074167	73075587	1421	*	8	1.911E-17	0.00035294	0.001686685	0.000201613	-0.238112759	-0.025247088	ADPGK-AS1, ADPGK
chr15	85358533	85359250	718	*	4	2.43492E-14	0.003919376	0.001687012	0.000884608	-0.235223355	-0.113506992	
chr10	94448532	94448702	171	*	2	8.92688E-09	0.03754101	0.001688658	0.00519761	-0.287270773	-0.136855273	HHEX
chr12	29376421	29376872	452	*	4	3.66599E-20	4.16837E-06	0.001689272	2.47741E-06	-0.229493682	-0.142459546	FAR2
chr7	24324976	24325371	396	*	4	2.41453E-10	0.094842559	0.001689727	0.015740437	0.069288127	0.02023605	NPY
chr7	23489752	23489930	179	*	2	5.9472E-09	0.014554208	0.001690173	0.003850769	-0.242224301	-0.096171551	IGF2BP3
chr10	82036111	82036320	210	*	2	5.67395E-09	0.007810158	0.001690904	0.002977241	0.196205209	0.093836383	MAT1A
chr8	1940413	1940523	111	*	2	3.75307E-12	4.7385E-05	0.001693539	9.56556E-05	-0.172841536	-0.128168792	KBTBD11
chr20	1842141	1842208	68	*	2	1.38989E-12	3.02773E-05	0.001693783	6.52383E-05	-0.212875106	-0.131687575	
chr17	55122538	55122747	210	*	2	7.75938E-09	0.077324995	0.001694137	0.006057314	-0.281068433	-0.134540688	
chr10	103698992	103699807	816	*	3	1.30385E-16	0.002674357	0.001695229	0.000133761	0.182438269	0.112831099	C10orf76
chr2	100135414	100135460	47	*	2	1.91459E-12	3.40653E-05	0.001695982	7.22342E-05	-0.360866757	-0.259962184	
chr7	127720696	127720983	288	*	2	1.72644E-09	0.032926169	0.001699214	0.005044066	0.186741736	0.08900077	SND1
chr18	59768649	59769538	890	*	2	1.69539E-10	3.65365E-05	0.001699266	7.67365E-05	-0.254145707	-0.249010312	PIGN
chr22	50499398	50499580	183	*	2	7.57609E-11	0.000302154	0.001699775	0.000416613	-0.19943186	-0.181017243	MLC1
chr1	219047954	219048260	307	*	2	9.21908E-11	0.000334112	0.001703197	0.00044884	-0.325390076	-0.200570252	
chr20	31123895	31123990	96	*	2	1.13791E-09	0.014025724	0.001704756	0.003819559	0.114478603	0.051607478	C20orf112
chr7	140732689	140732738	50	*	2	4.27747E-11	0.000133997	0.001710441	0.000224323	-0.035025737	-0.030368154	
chr16	3188215	3189015	801	*	5	3.67627E-19	0.000316602	0.001713386	7.37522E-05	0.230902705	0.084627483	ZNF213

chr11	73681281	73681337	57	*	2	1.74795E-13	2.47697E-05	0.001715143	5.49138E-05	0.148169196	0.110443021	DNAJB13, RP11-167N4.2
chr14	101308921	101309386	466	*	3	2.22887E-14	8.20894E-05	0.001719234	0.000113886	-0.219898071	-0.119591519	MEG3, RP11-123M6.2
chr2	135149599	135150189	591	*	3	3.26411E-16	1.04506E-05	0.001721917	2.12881E-05	-0.246857506	-0.15178683	MGAT5
chr8	145064570	145064874	305	*	3	2.94439E-10	0.204171163	0.001723842	0.013637088	-0.065310365	-0.021251775	GRINA, PARP10
chr8	20060877	20061561	685	*	3	3.78715E-15	0.002840552	0.001724508	0.000187393	-0.370828347	-0.223451533	ATP6V1B2
chr2	85955624	85957232	1609	*	5	1.88334E-15	2.00184E-05	0.001724608	2.00237E-06	-0.260321236	-0.108727655	
chr17	29379458	29379641	184	*	2	8.56684E-09	0.026018897	0.001725006	0.004764804	0.185421067	0.089770168	RP11-848P1.9, RP11-271K11.5
chr1	115293662	115294759	1098	*	3	7.18361E-11	9.43096E-05	0.001725318	8.72749E-05	-0.332882251	-0.099440871	CSDE1
chr22	32873526	32873961	436	*	4	9.06358E-16	4.2137E-05	0.00172598	4.52142E-05	0.152809906	0.080581801	FBXO7
chr15	63623836	63624154	319	*	3	5.44003E-11	0.0875681	0.001726973	0.011060631	0.194429367	0.07782652	CA12
chr8	142222147	142223071	925	*	5	1.9305E-13	0.152230251	0.001727146	0.012144468	-0.268360512	-0.068869048	SLC45A4
chr2	69026797	69027039	243	*	2	1.66364E-09	0.002039484	0.001727782	0.001497465	0.152401979	0.088794176	ARHGAP25
chr17	35870381	35870940	560	*	5	2.26144E-17	0.005716256	0.001728428	0.000412925	0.132189235	0.059277256	DUSP14
chr12	110815848	110816408	561	*	2	3.07238E-13	0.018667651	0.001728535	0.004281007	-0.291446933	-0.149001491	ANAPC7
chr3	186646931	186647026	96	*	4	1.43045E-21	0.000160487	0.00172976	3.43738E-06	0.189667767	0.138196372	
chr11	19265869	19265989	121	*	2	2.82102E-10	0.000575389	0.001731732	0.00066519	-0.220876609	-0.140073572	RP11-428C19.4
chr22	23478054	23478672	619	*	4	2.37192E-20	1.45473E-05	0.001735263	6.95703E-06	0.198565578	0.121406776	RTDR1
chr6	109777642	109778682	1041	*	5	1.95046E-20	0.000209986	0.001736087	3.16485E-05	0.148967341	0.074762054	MICAL1
chr8	117664615	117664855	241	*	2	1.58594E-08	0.047687261	0.001737024	0.00562795	-0.15671568	-0.082430665	EIF3H
chr2	55320612	55320703	92	*	2	2.02587E-12	3.42828E-05	0.001738577	7.29788E-05	-0.325064155	-0.265689604	RTN4
chr5	96293389	96294344	956	*	4	2.64849E-20	7.43032E-06	0.001739538	4.28148E-06	-0.293332457	-0.138366402	LNPEP
chr1	229194510	229195169	660	*	2	9.20112E-12	4.01698E-05	0.001742775	8.3638E-05	-0.119391005	-0.11023109	
chr14	24032626	24033309	684	*	4	4.47851E-13	0.011994667	0.001742843	0.002089369	-0.272817662	-0.080874583	RP11-66N24.3, AP1G2
chr4	83803228	83804018	791	*	4	4.40465E-10	0.344341182	0.001742891	0.030693949	-0.157253617	-0.035310681	SEC31A
chr6	13462275	13462406	132	*	2	1.48185E-12	8.77431E-05	0.001746378	0.000160636	-0.265486918	-0.235101406	GFOD1
chr14	75282966	75283416	451	*	2	4.05634E-10	0.005774386	0.001748445	0.00263917	-0.280708379	-0.149998682	YLPM1
chr20	49232141	49232374	234	*	2	5.50268E-09	0.006004489	0.001748543	0.002689608	0.124641673	0.070736871	FAM65C
chr6	36734511	36734921	411	*	3	2.68933E-12	0.004239166	0.001748707	0.000838959	-0.184906464	-0.103356107	CPNE5
chr7	2486036	2486525	490	*	3	6.77137E-12	0.005281741	0.001749684	0.001254778	0.130108183	0.065830634	AC004840.9

chr2	241585833	241585888	56	*	3	4.55873E-09	0.147839564	0.001750515	0.013884942	-0.12783921	-0.026127017	
chr2	225434838	225435435	598	*	4	2.37513E-16	0.000451195	0.001750683	0.000103412	-0.295857781	-0.118072153	CUL3
chr5	90478716	90479831	1116	*	3	8.46169E-10	0.000141662	0.001751281	6.95706E-05	-0.266987775	-0.155794192	
chr2	169927922	169928251	330	*	3	7.95851E-12	0.005185486	0.001752742	0.001360991	-0.192713987	-0.108572233	DHRS9
chr5	172331733	172332685	953	*	7	2.54049E-18	0.144164376	0.001753288	0.002806155	-0.182549934	-0.037675901	ERGIC1
chr9	100292179	100292291	113	*	2	4.10184E-08	0.220143075	0.001754432	0.006928481	0.172867889	0.088994129	TMOD1
chr14	94856217	94858066	1850	*	8	6.39998E-35	2.70393E-06	0.001754489	7.3156E-08	-0.250213665	-0.107122914	SERPINA1
chr3	177209040	177209049	10	*	2	4.20892E-13	1.85256E-05	0.001758355	4.26946E-05	0.206144375	0.171138101	LINC00578
chr21	45939800	45940127	328	*	2	1.22881E-09	0.003752388	0.001761377	0.002128759	0.163107274	0.074393926	C21orf90, TSPEAR
chr2	230745792	230745848	57	*	2	1.89546E-10	0.019900799	0.00176147	0.004435546	-0.133324323	-0.072600164	TRIP12
chr1	28422834	28423646	813	*	6	2.80841E-15	0.018293201	0.001762333	0.000789121	-0.195900994	0.003973251	SPCS2P4
chr2	38150028	38150157	130	*	2	1.08869E-08	0.010077501	0.001762682	0.003418378	0.230400522	0.133844602	
chr2	120974080	120974289	210	*	4	9.15991E-11	0.329502874	0.001763391	0.013561121	0.155079217	0.055710429	MTND5P28, AC012363.4
chr9	132346133	132346274	142	*	2	4.22849E-09	0.011092685	0.001764035	0.003560343	-0.219153621	-0.113621612	RP11-492E3.2
chr11	503270	504301	1032	*	7	5.64907E-38	6.47834E-09	0.00176418	6.49968E-10	-0.300987969	-0.156792008	RNH1
chr12	13214320	13215070	751	*	5	6.73555E-14	0.482918869	0.001764687	0.014983508	0.155652465	0.039378351	KIAA1467
chr3	42894502	42895364	863	*	2	3.61442E-11	6.04591E-05	0.001765252	0.000118452	0.212224352	0.174228734	ACKR2, RP11-141M3.5, KRBOX1, KRBOX1
chr21	43965974	43965993	20	*	2	2.97671E-09	0.049902042	0.00176709	0.005762421	0.143787352	0.068756128	SLC37A1
chr16	79454227	79454280	54	*	2	2.46217E-08	0.149392889	0.00176743	0.006798975	0.035595754	0.017553754	
chr21	42795929	42796537	609	*	2	6.48379E-11	0.098178338	0.001768071	0.006498456	0.19352054	0.098931923	MX1
chr22	51109146	51109466	321	*	2	2.47718E-09	0.007192666	0.001768857	0.002949897	0.144998796	0.086573922	
chr19	2091161	2091406	246	*	2	8.02087E-09	0.009933838	0.001772615	0.003410052	-0.115336183	-0.069194996	MOB3A
chr16	72822033	72822362	330	*	2	8.09208E-11	0.064481825	0.001774364	0.006092512	-0.27321346	-0.127107512	ZFHX3
chr8	142244625	142245835	1211	*	4	4.77545E-15	2.42143E-06	0.001776505	5.85691E-06	-0.296616757	-0.142536843	SLC45A4
chr19	41254683	41255444	762	*	3	3.40549E-14	0.018219993	0.001777309	0.000957072	-0.252544371	-0.040686011	C19orf54
chr3	186083375	186083922	548	*	2	1.83207E-11	0.000147528	0.001777473	0.000244705	-0.177468805	-0.052249051	
chr17	8323278	8323910	633	*	3	9.19822E-10	0.076419689	0.001780367	0.009601816	-0.229834383	-0.063637936	NDEL1
chr2	242702120	242703672	1553	*	6	2.62162E-18	4.47397E-05	0.001781661	1.16236E-05	-0.36059273	-0.112696691	D2HGDH
chr7	151169166	151170083	918	*	3	4.52701E-16	1.20705E-05	0.001782874	2.48004E-05	-0.177735987	-0.035015798	RHEB

chr6	32904074	32905320	1247	*	12	5.79979E-43	9.7184E-12	0.001783707	2.2503E-12	-0.372907524	-0.173930458	HLA-DMB, AL645941.1, XXbac-BPG181M17.5
chr1	94198190	94198243	54	*	2	9.3758E-09	0.016517383	0.00178511	0.004191663	0.139967686	0.053543381	BCAR3
chr5	149568693	149569501	809	*	3	7.86108E-12	0.01378194	0.001785924	0.004646121	0.108038895	0.043508455	
chr7	97755534	97755792	259	*	6	6.28206E-31	8.19827E-11	0.001787728	1.57625E-09	-0.328736903	-0.215725856	LMTK2
chr10	102107266	102107584	319	*	2	7.54951E-11	0.080144471	0.001787964	0.006366334	0.016922737	0.011950184	SCD
chr6	14232235	14232335	101	*	2	3.30214E-08	0.0058693	0.001789659	0.002697912	0.075728708	0.032526311	
chr14	45744024	45744380	357	*	3	4.93781E-10	0.006216287	0.00179264	0.002938642	-0.110084599	-0.053203906	
chr12	132180381	132180564	184	*	2	7.93084E-09	0.005530985	0.001794396	0.002624837	-0.149607004	-0.055535901	
chr5	99381840	99382144	305	*	4	4.90565E-11	0.024703748	0.001794692	0.003836014	0.25112518	0.089901664	RP11-475J5.7
chr2	216489226	216489343	118	*	2	4.37436E-08	0.099564068	0.001795134	0.00659309	-0.324539014	-0.163915893	AC012668.2, LINC00607
chr2	180137570	180139789	2220	*	7	9.53434E-14	0.000265191	0.001797319	6.44314E-06	-0.298383357	0.001071933	AC093911.1
chr6	80580112	80580937	826	*	5	2.98512E-12	0.183031912	0.001799327	0.029521861	-0.326798989	-0.068478164	
chr17	3844820	3845067	248	*	2	3.63912E-10	0.005976538	0.001800958	0.002732112	0.181466985	0.104562963	ATP2A3
chr4	185352181	185352359	179	*	2	2.56103E-09	0.129031592	0.001802596	0.006819678	-0.273489445	-0.137582925	IRF2
chr19	19574754	19576220	1467	*	8	4.2844E-18	0.000424558	0.001802868	1.66722E-05	-0.217875288	-0.05217884	GATAD2A
chr5	131763112	131763756	645	*	3	1.72941E-16	4.88555E-06	0.001803456	1.58991E-05	-0.301300846	-0.172845748	C5orf56
chr18	55250568	55250579	12	*	2	4.78003E-11	0.000130506	0.00180713	0.000223156	0.162006125	0.138992763	FECH
chr13	113305704	113305901	198	*	2	6.62415E-11	0.000560686	0.001807261	0.000664621	0.155380668	0.095136846	C13orf35
chr6	134498979	134499589	611	*	8	9.76102E-14	0.28053927	0.001809039	0.052158611	-0.086546731	-0.015765672	SGK1
chr5	173235343	173235445	103	*	2	4.04112E-11	0.001091635	0.001809257	0.001041006	-0.29690623	-0.190852602	CTB-43E15.4
chr3	195454591	195454921	331	*	2	2.38075E-10	0.000618598	0.001811336	0.000712246	0.234889743	0.165016722	LINC00969, MUC20
chr7	129360448	129360579	132	*	2	5.29155E-13	1.94669E-05	0.001813488	4.48254E-05	-0.244016352	-0.197933108	NRF1
chr18	22039496	22040005	510	*	5	1.29574E-23	1.25325E-06	0.00181409	1.09339E-06	-0.327863016	-0.195406866	
chr11	35088518	35088821	304	*	2	6.18205E-09	0.010845917	0.001815748	0.003595102	0.160583545	0.088695028	
chr11	35101356	35101445	90	*	2	3.79554E-09	0.00190022	0.001816511	0.001472992	-0.155575483	-0.102191688	
chr4	56859639	56860292	654	*	2	2.1181E-11	8.82222E-05	0.001818721	0.000163022	0.17528039	0.131569035	CEP135
chr1	2165579	2166155	577	*	3	6.78859E-13	0.002401622	0.001819969	0.000492042	-0.236119921	-0.111975711	SKI
chr16	19132987	19133370	384	*	3	5.70249E-11	0.02237269	0.001820253	0.005387067	-0.302792757	-0.104905474	CTD-2349B8.1
chr16	23869600	23869974	375	*	2	1.93927E-11	0.000372843	0.00182068	0.000498139	-0.075970574	-0.015761971	PRKCB

chr15	72511937	72512069	133	*	2	8.1537E-09	0.004259315	0.001820758	0.002318976	0.197378445	0.132055791	PKM
chr17	30130951	30131482	532	*	3	1.53188E-11	0.031840557	0.001826065	0.00251556	-0.251357887	-0.104352213	
chr16	2660277	2660876	600	*	3	3.39098E-15	0.011765919	0.001827642	0.000373768	-0.253192161	-0.146409192	AC141586.5
chr1	2450741	2451511	771	*	4	7.10273E-12	0.541714181	0.001827897	0.032792001	0.179907391	0.043434269	PANK4
chr16	85111583	85112625	1043	*	5	1.26491E-22	5.19856E-07	0.001828146	1.28177E-06	-0.319169507	-0.119521863	KIAA0513
chr7	30954522	30954882	361	*	2	1.2535E-10	0.000420922	0.001829215	0.000544825	0.187057197	0.153638515	AQP1, AQP1
chr8	101476032	101476716	685	*	3	1.00816E-11	0.005806052	0.001829474	0.000231438	0.132294986	0.07477339	
chr2	240077557	240077990	434	*	2	6.60164E-11	0.009750965	0.001829494	0.003453145	-0.337145493	-0.175687852	HDAC4
chr8	48647583	48649767	2185	*	7	4.55729E-25	3.74778E-08	0.001830197	3.39649E-08	-0.244582465	-0.114910948	SPIDR, CEBPD
chr2	99181318	99181779	462	*	4	1.67146E-15	0.000313276	0.001832637	0.000147748	-0.243874334	-0.117640822	INPP4A
chr14	24550395	24551212	818	*	7	1.19185E-11	0.370402085	0.001833133	0.090072725	0.167282746	0.033099274	NRL
chr13	99088196	99088723	528	*	4	1.96437E-14	0.001276799	0.001835024	0.000399902	0.224465772	0.089788906	FARP1
chr19	16546817	16547001	185	*	2	7.54471E-11	0.000183062	0.001839498	0.000292629	0.21241582	0.156186231	EPS15L1
chr13	24440615	24441476	862	*	2	2.87491E-10	2.39931E-05	0.001839571	5.40365E-05	0.206683735	0.182063465	MIPEP
chr6	157274188	157274454	267	*	2	4.3021E-10	0.000647418	0.001840317	0.000739759	-0.181979015	-0.130640824	ARID1B
chr1	224023297	224023540	244	*	2	6.61793E-09	0.016828446	0.001841759	0.00431308	-0.20057169	-0.090828631	TP53BP2
chr8	7113417	7113462	46	*	2	4.85109E-08	0.03655369	0.001842519	0.005516498	-0.030618117	-0.0270777	
chr11	34331548	34331908	361	*	2	3.09129E-10	0.001535782	0.001843555	0.001303377	-0.293579446	-0.130338779	ABTB2
chr3	131219430	131219470	41	*	3	3.9485E-17	2.04906E-06	0.00184417	7.72869E-06	-0.275456096	-0.180336402	MRPL3
chr20	37512948	37513080	133	*	2	3.49082E-08	0.029085985	0.001848199	0.005184827	-0.126116808	-0.054603697	PPP1R16B
chr16	88554846	88555715	870	*	3	1.00171E-17	3.39972E-07	0.001849312	1.64366E-06	0.190401675	0.166587548	ZFPM1
chr22	19166301	19167309	1009	*	12	3.1636E-20	0.225281597	0.00185067	0.018896738	0.038348846	0.0091298	SLC25A1, CLTCL1
chr2	128051925	128052889	965	*	4	3.18505E-23	1.32302E-06	0.001852344	3.18099E-06	-0.323391912	-0.189073168	
chr3	112165478	112165653	176	*	2	2.7704E-08	0.02699502	0.001853241	0.005079651	-0.302908744	-0.156149418	
chr6	22043778	22043967	190	*	2	3.19412E-10	0.000410891	0.001857496	0.000538482	0.210763133	0.144554406	CASC15
chr20	814736	816362	1627	*	6	1.97249E-13	0.00563326	0.001859886	0.001044851	0.181329247	0.044589787	FAM110A
chr13	30962973	30964034	1062	*	5	1.48013E-14	0.004899066	0.001860414	7.33104E-05	-0.331152025	-0.13170684	
chr2	216769058	216769199	142	*	2	2.17681E-08	0.361553594	0.001861197	0.007402535	-0.310018144	-0.154808876	
chr8	101442975	101443981	1007	*	5	2.61298E-16	0.004041017	0.001863082	0.000360458	0.186452205	0.06960718	KB-173C10.1

chr15	55574912	55575701	790	*	3	3.08949E-12	0.000140455	0.00186369	0.000141292	-0.236112158	-0.170533976	RAB27A
chr17	998432	999650	1219	*	5	3.8332E-25	6.32939E-10	0.001863771	1.00179E-08	-0.433139401	-0.262269612	ABR
chr19	50069106	50069228	123	*	2	5.11184E-08	0.093701474	0.001863845	0.006744529	0.162213915	0.077898026	NOSIP
chr12	54197762	54198103	342	*	2	2.82578E-09	0.005039913	0.001864813	0.002563677	0.127506145	0.08900792	
chr3	171873675	171874327	653	*	3	1.85965E-15	0.011015865	0.001865031	0.000162613	-0.254012686	-0.167083405	FNDC3B
chr19	1236736	1237242	507	*	3	4.59974E-13	0.010979728	0.001865396	0.003920075	0.148082557	0.041774712	C19orf26
chr2	39931192	39931201	10	*	2	6.47197E-10	0.000482577	0.001866006	0.000605405	-0.105523019	-0.092081488	TMEM178A
chr2	29319869	29319945	77	*	2	5.82994E-09	0.016561097	0.001867096	0.004328579	-0.132587968	-0.054182775	AC105398.3
chr4	8202280	8202708	429	*	4	9.18413E-14	0.001223718	0.001867365	0.000671999	-0.123521782	-0.061575763	SH3TC1
chr16	30721139	30721715	577	*	4	2.94721E-20	2.3279E-07	0.001867459	1.23217E-06	-0.305876349	-0.16917186	SRCAP
chr2	134975778	134977141	1364	*	3	2.24242E-13	5.25555E-07	0.001868609	2.40418E-06	-0.304139423	-0.244915119	MGAT5
chr19	41200462	41200618	157	*	2	3.20702E-08	0.048015309	0.00186889	0.005968212	0.167642105	0.095637352	ADCK4
chr12	65048810	65049149	340	*	4	3.80543E-18	1.23565E-05	0.001872156	1.17812E-05	-0.27728719	-0.13572446	RASSF3
chr19	52263842	52266043	2202	*	9	1.08438E-21	0.000962955	0.001873133	4.29359E-05	-0.309604105	-0.066663653	FPR2, FPR1
chr10	72997126	72997755	630	*	2	1.24933E-11	5.24756E-05	0.001873171	0.000106544	0.142520358	0.127086369	UNC5B
chr7	65590173	65590707	535	*	2	1.02157E-11	7.66284E-05	0.001874981	0.000146279	-0.256862142	-0.155644362	AC068533.7, CRCP, RP5-1132H15.1
chr2	179387456	179387931	476	*	4	7.87077E-11	0.112409388	0.00187543	0.008889614	-0.235406168	-0.087900361	TTN-AS1
chr13	114897925	114898515	591	*	12	1.13263E-14	0.487738181	0.001880215	0.129009373	-0.093787071	-0.015748485	RASA3
chr1	6303793	6305348	1556	*	8	5.37626E-16	0.190597218	0.001883021	0.017551021	0.109722316	0.03666267	HES3
chr9	100531857	100531996	140	*	2	1.98708E-08	0.010088459	0.001883363	0.003570664	0.118097809	0.086634686	
chr17	41844017	41844416	400	*	3	7.45157E-11	0.011227153	0.001883416	0.002901224	-0.266446833	-0.1074971	DUSP3
chr3	58512299	58512535	237	*	2	3.26147E-09	0.002544694	0.001887191	0.001783074	-0.223610261	-0.120433502	ACOX2
chr5	150677795	150678334	540	*	3	4.78539E-11	0.015130414	0.001889077	0.002176254	-0.245523494	-0.131123448	SLC36A3
chr16	89431242	89431720	479	*	3	1.77628E-14	0.000370898	0.001889244	0.00025376	-0.232096756	-0.087197399	ANKRD11
chr1	183559527	183560294	768	*	2	2.74225E-11	0.00014594	0.001889395	0.000246944	-0.206861186	-0.053252252	SMG7, NCF2
chr14	21483525	21483641	117	*	3	3.74024E-15	6.72582E-05	0.001889483	6.17506E-05	-0.206407202	-0.151666731	
chr6	32179862	32180641	780	*	6	3.34215E-16	0.13950181	0.001889651	0.002905219	-0.369169546	-0.085349275	NOTCH4
chr5	158636444	158637684	1241	*	15	3.35302E-52	2.75602E-07	0.00189061	3.42968E-10	-0.317652994	-0.08145382	CTB-11I22.1, RNF145
chr11	66276414	66277055	642	*	2	3.52295E-13	0.001679152	0.001890697	0.001394043	-0.185743151	-0.125212408	DPP3, CTD-3074O7.11

chr1	212291510	212291760	251	*	2	2.39933E-08	0.082139097	0.001891318	0.006691338	0.151478965	0.07322198	
chr13	114270609	114271629	1021	*	6	3.46577E-21	3.40061E-06	0.001896021	3.85474E-06	-0.308804283	-0.146585917	TFDP1
chr3	169866747	169867567	821	*	4	1.57682E-14	0.129487287	0.001898148	0.001090711	-0.3244742	-0.13786987	PHC3
chr2	42331922	42332600	679	*	2	6.94617E-12	4.68248E-05	0.001898415	9.69891E-05	-0.330576427	-0.265294603	
chr17	75314602	75317185	2584	*	19	3.23827E-47	8.27497E-06	0.001900188	6.19001E-08	-0.339235057	-0.073836384	SEPT9
chr15	40339900	40340786	887	*	3	2.61923E-15	3.81294E-05	0.001905821	5.32298E-05	-0.137861358	-0.10233054	SRP14-AS1
chr3	111314102	111314495	394	*	3	1.06192E-10	0.037792445	0.001906284	0.007552903	-0.226341785	-0.083894377	CD96, ZBED2
chr17	80246233	80246403	171	*	2	2.45561E-10	0.002948219	0.001910368	0.001951341	0.140384102	0.106446319	
chr4	185733358	185734598	1241	*	9	2.59564E-21	0.000257358	0.001912919	1.14952E-06	-0.303193025	-0.100217241	ACSL1
chr16	69966641	69967766	1126	*	8	9.71824E-29	2.91243E-05	0.00191491	9.59419E-07	-0.342384157	-0.119502611	WWP2, MIR140
chr12	121714889	121715406	518	*	2	4.05426E-12	3.966E-05	0.001915198	8.42781E-05	-0.303927292	-0.238302374	CAMKK2
chr8	19614946	19616304	1359	*	8	2.08879E-19	0.000394451	0.001916751	9.98647E-05	-0.213698342	-0.064989786	CSGALNACT1
chr7	156930564	156930922	359	*	2	1.81347E-11	0.001809297	0.001917154	0.001469039	-0.334914468	-0.150677901	
chr4	157907601	157907848	248	*	2	1.89612E-08	0.051774111	0.001917898	0.00619507	0.193301257	0.093800484	RPPH1-3P
chr8	126301474	126302234	761	*	3	9.30313E-15	1.09912E-05	0.001918277	2.74437E-05	0.170750201	0.140921485	NSMCE2
chr18	24040860	24041183	324	*	2	1.56814E-09	0.208847651	0.00191869	0.007464032	0.187015045	0.094044471	KCTD1
chr14	102499390	102499720	331	*	2	1.06196E-09	0.071357916	0.001918766	0.006610096	-0.347945848	-0.175536104	DYNC1H1
chr22	40405832	40406049	218	*	3	2.82925E-11	0.006931391	0.001923837	0.002863134	-0.205541714	-0.104586382	FAM83F
chr20	35236199	35236543	345	*	2	3.47985E-12	4.89109E-05	0.001924635	0.00010094	-0.290298557	-0.084966561	TGIF2-C20orf24, C20orf24
chr11	818331	819513	1183	*	9	3.92554E-34	5.61507E-05	0.001926148	2.75992E-07	-0.28136755	-0.099157786	PNPLA2
chr7	2138722	2138813	92	*	2	6.91127E-09	0.305525867	0.001928963	0.007610453	0.180687132	0.090521154	MAD1L1
chr5	53966914	53967333	420	*	2	3.57507E-09	0.051359072	0.001931503	0.006218261	-0.278623851	-0.143801985	
chr16	3646322	3646442	121	*	2	2.9722E-09	0.109534324	0.001932203	0.007095109	0.182558709	0.092732408	SLX4
chr9	126985646	126985794	149	*	2	4.58591E-11	0.000122434	0.001933737	0.000216049	-0.247463749	-0.207820295	
chr11	47437783	47438068	286	*	2	4.90454E-10	0.006984126	0.001934341	0.003075061	-0.198879429	-0.113842082	SLC39A13
chr20	2292148	2292333	186	*	2	9.4418E-09	0.013951534	0.001935134	0.004154837	-0.327740966	-0.170391433	TGM3
chr3	119030023	119030451	429	*	2	1.68125E-12	2.4696E-05	0.001935385	5.58892E-05	-0.180845316	-0.179172083	ARHGAP31
chr2	241807539	241808595	1057	*	8	2.35765E-19	0.066749294	0.001938324	0.000885602	-0.238236873	-0.052531771	AGXT
chr20	3026904	3027028	125	*	2	3.65498E-09	0.004726676	0.001940592	0.002539276	0.027742005	0.006300652	MRPS26

chr13	27883202	27883302	101	*	2	2.61081E-09	0.001316036	0.001941909	0.001212464	-0.173767277	-0.030424751	
chr17	28442480	28443042	563	*	2	5.14385E-12	0.038334137	0.001942043	0.005814199	-0.294511649	-0.142934704	NSRP1, RP11-1148O4.2
chr1	221066688	221066979	292	*	2	1.17677E-10	0.048507139	0.001942211	0.006164253	0.16594851	0.077053486	
chr22	42830856	42831015	160	*	2	1.73563E-08	0.099162358	0.001942551	0.00703419	-0.254482693	-0.130946689	
chr6	82459006	82459960	955	*	3	4.55862E-14	0.001678744	0.001943263	0.000428617	-0.149491699	-0.100280194	FAM46A
chr5	3059042	3059243	202	*	3	4.35573E-09	0.037276098	0.001943607	0.006868578	0.129870396	0.060552464	
chr14	102438447	102439006	560	*	3	5.01914E-14	1.96707E-05	0.001945567	4.04531E-05	-0.214433037	-0.09088662	DYNC1H1
chr6	159488631	159488668	38	*	2	7.35158E-10	0.00343095	0.001946671	0.00214467	-0.306125874	-0.198125283	
chr6	34789052	34789117	66	*	3	5.93207E-09	0.232708821	0.001947723	0.007439157	-0.271351956	-0.099397383	UHRF1BP1
chr12	90256714	90257533	820	*	3	6.4586E-15	0.00925904	0.001948438	0.000174378	0.080709302	0.052703359	
chr6	109780058	109780119	62	*	2	9.48355E-09	0.005949644	0.001952706	0.002862196	-0.273745344	-0.168728945	MICAL1
chr17	75260857	75260964	108	*	3	2.78297E-13	3.27839E-05	0.001953295	7.81948E-05	-0.24078829	-0.148399306	RP11-285E9.6, RP11-285E9.5
chr2	149017993	149018451	459	*	2	7.65588E-10	0.021192594	0.00195391	0.004878955	0.109343848	0.050234266	MBD5, SNORA48
chr6	149803087	149803206	120	*	3	1.73776E-09	0.07370228	0.001954676	0.004709693	-0.156879858	-0.064605905	ZC3H12D
chr12	104194588	104194724	137	*	2	2.7777E-08	0.127566557	0.0019551	0.007292936	-0.164717537	-0.080104595	NT5DC3
chr16	30689847	30689953	107	*	2	2.74348E-08	0.218659228	0.001955126	0.007604119	-0.185697092	-0.092830111	
chr2	119731566	119731815	250	*	2	6.485E-10	0.000674283	0.001955715	0.000779903	-0.22304778	-0.074114154	MARCO
chr9	109683834	109684453	620	*	4	4.65575E-14	0.002854645	0.001956995	0.001607511	0.223669639	0.039571116	ZNF462
chr17	4620700	4621252	553	*	4	2.73366E-12	0.008418944	0.001958979	0.001718529	-0.314633967	-0.107888887	ARRB2
chr1	203489668	203489725	58	*	2	1.76511E-13	3.0778E-05	0.001959394	6.79342E-05	-0.146592472	-0.133965003	
chr17	79798629	79801920	3292	*	18	1.10878E-36	3.59044E-10	0.001959868	3.11047E-10	-0.267466569	-0.101003566	RP11-498C9.2, P4HB
chr16	57713544	57714235	692	*	4	2.05132E-15	0.00304094	0.001965838	0.000421896	-0.123189048	-0.04824934	GPR97
chr1	40861387	40863166	1780	*	5	4.84732E-19	9.06763E-07	0.001967178	3.42572E-07	-0.324166008	-0.228566208	SMAP2
chr11	115371789	115372348	560	*	2	1.19655E-10	0.153326283	0.001968485	0.007466442	-0.062943447	-0.031958357	CADM1
chr14	103432390	103432476	87	*	2	7.37382E-09	0.031480608	0.001968915	0.00556191	-0.240640261	-0.126601891	CDC42BPB
chr6	20688798	20689477	680	*	3	1.94393E-09	0.231476546	0.001970099	0.01643478	0.103878511	0.036756757	CDKAL1
chr16	56886303	56886386	84	*	2	1.68329E-09	0.001859522	0.001971442	0.001514431	-0.146147762	-0.086842158	
chr11	63513622	63513812	191	*	2	3.36672E-11	0.000108288	0.001973841	0.00019671	0.171115109	0.157637769	RTN3
chr14	23007634	23008246	613	*	4	1.16956E-11	0.06241078	0.001974619	0.005417033	-0.253092233	-0.072664024	TRAJ6

chr12	4324216	4324382	167	*	2	1.51132E-08	0.00633554	0.001974934	0.002971751	-0.202454775	-0.172577666	
chr22	46471129	46471442	314	*	2	5.0193E-12	0.000253127	0.001975045	0.000383894	-0.136349916	-0.012325296	FLJ27365
chr13	111090433	111091294	862	*	8	6.41886E-17	0.051024915	0.001975981	0.002161027	0.148430728	0.041854805	COL4A2
chr15	92874272	92874307	36	*	2	3.24009E-08	0.015907515	0.001977067	0.004435261	0.121349633	0.073027764	
chr2	74693332	74693876	545	*	4	3.98503E-18	0.000849101	0.001978745	0.000276819	-0.384549116	-0.165003515	
chr11	59191289	59191513	225	*	3	5.5227E-09	0.675250028	0.001980131	0.013013987	-0.27799749	-0.09784547	
chr6	149805995	149806659	665	*	11	4.13691E-27	0.000719609	0.001980495	9.76973E-05	0.243479416	0.05021266	ZC3H12D
chr17	80415420	80416454	1035	*	15	8.17894E-41	0.017156828	0.001985696	4.29758E-05	-0.309826748	-0.042251213	NARF, RP13-20L14.6
chr19	10213271	10213961	691	*	8	6.87259E-16	0.029244899	0.001986712	0.007058477	0.174988305	-0.019749679	ANGPTL6
chr16	31077385	31077750	366	*	2	4.35895E-10	0.004301656	0.001987101	0.002450433	-0.216861126	-0.11552799	AC135050.5, ZNF668
chr10	12126673	12126715	43	*	2	1.72069E-11	6.99211E-05	0.001987499	0.000137402	-0.333399165	-0.248365713	DHTKD1
chr2	119699603	119700029	427	*	4	2.26321E-15	0.041228262	0.001987674	0.00039989	-0.254067959	-0.117476365	MARCO
chr13	100007116	100007277	162	*	2	2.63698E-14	3.79271E-05	0.001989979	8.168E-05	0.17694976	0.029146054	UBAC2
chr15	75314761	75314907	147	*	2	2.05983E-15	0.000411072	0.001991556	0.000552579	0.129850345	0.077935052	
chr7	149191957	149192381	425	*	2	8.84172E-12	0.000298654	0.001992057	0.000436261	-0.205666165	-0.145104537	ZNF746
chr22	32365941	32366276	336	*	4	1.96748E-09	0.255050806	0.001993444	0.028176898	0.165412606	0.055479779	CTA-342B11.1
chr22	28073992	28074146	155	*	4	5.66442E-13	0.004440803	0.00199431	0.00054406	0.172350834	0.077556496	RP1-213J1P__B.1
chr3	47124414	47125793	1380	*	5	2.92064E-17	0.000817196	0.001996021	1.06697E-05	-0.374142243	-0.205059715	SETD2
chr5	58652602	58653738	1137	*	8	5.84273E-30	5.90196E-07	0.0019974	2.09236E-07	-0.320448754	-0.089739213	PDE4D
chr10	126826304	126826410	107	*	2	2.79965E-09	0.017030001	0.001997787	0.004583105	0.195118948	0.107633247	CTBP2
chr17	72276181	72276388	208	*	2	2.39369E-08	0.086884294	0.001999499	0.007064613	0.191780588	0.094071216	DNAI2
chr1	28447740	28447896	157	*	2	1.78312E-08	0.006909379	0.00199953	0.003122039	-0.09253332	-0.030486992	RP5-1053E7.3
chr12	58287143	58288081	939	*	4	6.18548E-21	9.28849E-05	0.00199964	4.60205E-06	-0.230476896	-0.11810335	RP11-620J15.2
chr11	72454696	72455648	953	*	2	1.03183E-09	4.4701E-05	0.001999862	9.41887E-05	0.200286003	0.176845705	ARAP1
chr11	3852056	3852978	923	*	4	1.30087E-16	3.90754E-06	0.002000967	1.26951E-05	-0.150067645	-0.108617354	RHOG
chr20	30125302	30126382	1081	*	4	4.00711E-13	0.000367921	0.0020018	0.000129565	-0.314047133	-0.131801793	HM13
chr19	11517436	11517953	518	*	3	1.97408E-10	0.031217243	0.002002901	0.006511819	-0.303911131	-0.092767491	RGL3
chr1	10271191	10272185	995	*	7	1.34626E-18	0.063270508	0.002007616	0.002477749	-0.251179844	-0.046299386	KIF1B
chr10	104392124	104392151	28	*	2	2.62465E-08	0.205107791	0.002010296	0.007761279	-0.128748387	-0.061472406	SUFU

chr7	2110826	2111060	235	*	2	1.20836E-08	0.030522998	0.002010753	0.005598173	-0.103559351	-0.053914126	MAD1L1
chr11	67034249	67034540	292	*	3	1.94049E-10	0.101717312	0.002011874	0.010817023	0.038725286	0.012339464	ADRBK1
chr15	93347048	93347244	197	*	2	1.22557E-11	6.63443E-05	0.002014437	0.000131886	-0.24327957	-0.19061295	FAM174B
chr19	10201790	10201834	45	*	2	2.00306E-10	0.000538739	0.002014514	0.000674228	-0.136051198	-0.126019191	C19orf66
chr6	170194529	170195479	951	*	3	3.51812E-16	9.19366E-07	0.002019423	3.88014E-06	-0.253113822	-0.184502317	LINC00574, LINC00242
chr1	248902767	248904167	1401	*	9	1.16534E-34	7.34443E-09	0.002019889	2.07653E-09	-0.330923678	-0.167189148	LYPD8
chr10	72026887	72027576	690	*	4	7.77814E-11	0.174597622	0.002022726	0.011676074	-0.26836477	-0.044353401	NPFFR1
chr15	101597093	101597169	77	*	2	4.03381E-09	0.003373352	0.002026166	0.002171763	-0.183772897	-0.113258529	LRRK1, RP11-505E24.2
chr10	45849898	45850164	267	*	2	2.36739E-09	0.001722569	0.00202628	0.001464631	0.158122286	0.10801659	
chr19	49385356	49385429	74	*	2	1.99112E-12	2.90351E-05	0.002027151	6.49309E-05	-0.244588837	-0.16873653	TULP2
chr19	850975	853540	2566	*	14	7.28682E-40	1.10086E-16	0.002028401	9.35243E-15	-0.243906436	-0.13078616	ELANE
chr1	203198757	203199023	267	*	3	1.38831E-15	6.58822E-05	0.002030612	0.00010041	-0.290104253	-0.167824966	CHIT1
chr11	118094830	118096372	1543	*	6	1.94208E-24	1.4431E-06	0.002030924	1.59415E-06	-0.286570932	-0.127335103	AMICA1
chr1	238025414	238025587	174	*	4	1.6833E-13	0.002518884	0.00203192	0.000370121	-0.274818269	-0.132611351	RP11-193H5.1
chr6	24489671	24490221	551	*	6	8.16711E-24	0.000782996	0.002032374	2.22675E-05	-0.160925092	-0.075014741	GPLD1
chr4	26206080	26206130	51	*	2	3.38699E-11	9.19571E-05	0.002033437	0.000173388	-0.086613121	-0.084155469	RBPJ
chr16	85622137	85622276	140	*	3	4.03181E-13	0.001497048	0.002034942	0.000203016	0.201322204	0.12702499	
chr17	27418559	27419266	708	*	6	7.90264E-23	4.64794E-05	0.002034981	2.90436E-05	0.172730426	0.083937893	MYO18A
chr15	93383226	93383415	190	*	2	2.84096E-08	0.01412779	0.002035292	0.004319949	-0.255794476	-0.136267486	
chr1	235092611	235092860	250	*	4	1.22216E-15	9.55487E-05	0.002036887	0.000196775	-0.341306195	-0.250675381	
chr18	60903834	60904328	495	*	3	4.00926E-12	0.011144608	0.002037237	0.002398937	-0.078979982	-0.058391637	BCL2
chr9	140942546	140942584	39	*	2	1.60865E-07	0.021056611	0.002037717	0.005015288	-0.222631548	-0.101125293	CACNA1B
chr20	1614192	1614462	271	*	2	2.96727E-09	0.00734168	0.002040415	0.003253547	-0.287845305	-0.157164665	RP11-77C3.3, SIRPG
chr16	65159613	65160241	629	*	3	1.22623E-09	0.040549635	0.002041007	0.008894771	-0.193338571	-0.05146291	CDH11
chr8	669161	670293	1133	*	7	3.09678E-17	0.003604159	0.00204184	0.002459077	0.107643051	0.031308877	ERICH1
chr11	62473858	62474118	261	*	7	1.22002E-10	0.769614174	0.002045198	0.133387933	0.110215558	0.01511228	BSCL2, HNRNPUL2-BSCL2
chr21	30722759	30722924	166	*	2	1.5642E-08	0.113681181	0.002045909	0.007475032	0.216618124	0.111223951	BACH1
chr20	48891434	48892664	1231	*	4	5.04661E-18	0.000447753	0.00204982	7.20863E-05	-0.267598637	-0.13461442	RP11-290F20.3
chr19	1154276	1155738	1463	*	7	2.07877E-31	5.25651E-07	0.00205056	2.53775E-08	-0.252711993	-0.156366199	SBNO2

chr8	128231103	128232015	913	*	5	4.77015E-15	0.033659779	0.002051251	0.001153636	-0.200108139	-0.087165815	CCAT1
chr12	57386819	57387400	582	*	6	5.53019E-24	3.40189E-06	0.002053328	4.80506E-06	-0.303693427	-0.122976951	
chr6	170364239	170365361	1123	*	4	4.73502E-13	9.23367E-05	0.002054267	1.66305E-05	-0.26560326	-0.124143259	
chr16	28997459	28999707	2249	*	8	3.48202E-26	1.51654E-09	0.002054891	5.44725E-09	0.227488653	0.092502596	RP11-264B17.3, LAT
chr7	127861992	127862224	233	*	2	2.96165E-08	0.022515464	0.002054946	0.005162566	-0.098826093	-0.063889571	
chr9	125693565	125694300	736	*	5	3.45039E-10	0.611016819	0.002057097	0.051218555	-0.069548378	-0.016124507	ZBTB26
chr17	14206572	14207241	670	*	9	5.14082E-13	0.401527061	0.002061394	0.092012364	-0.122993565	0.005339215	HS3ST3B1, RP11-214O1.2
chr1	158800880	158801271	392	*	3	2.79206E-14	1.90035E-05	0.002061439	5.01153E-05	-0.213891826	-0.13076742	MNDA
chr11	134118834	134118974	141	*	2	2.21237E-08	0.126862248	0.002062304	0.007622045	0.179403469	0.087458468	THYN1
chr19	2430539	2431468	930	*	5	1.60745E-20	0.00250046	0.002064065	0.000207454	-0.186451957	-0.052305993	LMNB2
chr1	153362927	153364020	1094	*	9	1.42846E-39	1.05804E-11	0.002064531	4.56807E-11	-0.311210904	-0.174524387	S100A8
chr17	8815262	8816266	1005	*	7	7.05493E-18	0.028967629	0.002064556	0.002038157	0.164144453	0.043048128	PIK3R5
chr22	37782781	37782857	77	*	3	2.44499E-12	0.000134704	0.00206544	0.000237163	0.177423128	0.116473479	RP1-63G5.5, ELFN2
chr1	160708987	160709239	253	*	3	4.26857E-13	5.04063E-05	0.002065761	0.000108087	-0.190249324	-0.089831732	SLAMF7
chr21	35190531	35190823	293	*	3	3.55219E-09	0.104584489	0.002066786	0.009437028	-0.28685601	-0.083964625	ITSN1, AP000304.12
chr12	133248929	133249308	380	*	6	5.53342E-19	0.00098251	0.002068019	0.000156996	-0.379875833	-0.06554354	POLE
chr5	57786008	57786449	442	*	5	1.64328E-10	0.091926485	0.002070299	0.02173831	-0.24731394	-0.037662294	
chr2	145170401	145170848	448	*	3	2.30854E-13	3.20009E-05	0.002073431	6.52386E-05	-0.230595488	-0.048813043	ZEB2
chr2	144995448	144996219	772	*	3	9.37026E-11	0.098619482	0.002075022	0.012289522	-0.192300275	-0.047641031	GTDC1
chr14	51027861	51028564	704	*	3	6.69247E-13	0.014402435	0.002076594	0.00524438	-0.204576236	-0.070299367	ATL1
chr12	132303409	132303880	472	*	5	1.38406E-22	1.85135E-06	0.002077838	1.81957E-06	-0.340158223	-0.185885551	
chr4	183838736	183839473	738	*	12	1.49752E-19	0.101807242	0.002078047	0.011864849	0.15139611	0.025063507	DCTD
chr11	67803715	67804112	398	*	5	6.37406E-16	0.056600023	0.002079999	0.000588353	-0.207169404	-0.078301997	NDUFS8
chr10	49647801	49648060	260	*	2	8.57752E-10	0.001958802	0.002081617	0.001605589	-0.186453329	-0.131404319	
chr5	149494527	149494751	225	*	2	2.27025E-09	0.016539131	0.002081929	0.00466347	0.181756713	0.113986431	PDGFRB
chr11	68694943	68695532	590	*	2	2.46599E-11	8.69302E-05	0.002082304	0.000166473	-0.210779985	-0.1854795	IGHMBP2
chr17	56345257	56345839	583	*	2	4.69651E-11	0.000166044	0.002082854	0.000281221	-0.339498741	-0.250823484	LPO
chr19	4518598	4519155	558	*	4	1.75609E-18	4.14575E-05	0.002083038	6.37481E-05	-0.160644076	-0.114726453	
chr2	9562849	9565042	2194	*	20	5.10295E-34	0.000429137	0.002084966	7.473E-06	-0.372800325	-0.034543609	CPSF3, ITGB1BP1

chr22	37317797	37320126	2330	*	9	2.53757E-19	7.5228E-06	0.002086508	5.94183E-07	-0.29285051	-0.083664643	CSF2RB
chr2	8767362	8767381	20	*	2	4.0353E-09	0.003940355	0.002088048	0.002404154	-0.200678391	-0.112559616	
chr7	139366500	139366758	259	*	2	7.33049E-09	0.052757739	0.002089341	0.006652021	0.180976706	0.095981427	HIPK2
chr3	49866227	49866346	120	*	2	1.08687E-12	2.68616E-05	0.002091982	6.09475E-05	0.22277722	0.216945405	TRAIP
chr8	144577050	144577094	45	*	2	1.84312E-07	0.099795633	0.002092986	0.007487033	0.071194486	0.034449349	ZC3H3
chr18	688285	689054	770	*	3	5.03256E-14	0.004164669	0.002097341	0.000235126	-0.159647013	-0.096143201	ENOSF1
chr16	49672617	49672687	71	*	2	1.21216E-07	0.187190636	0.002098348	0.008005446	-0.159056494	-0.080193854	ZNF423
chr16	27767814	27767978	165	*	2	3.63025E-08	0.059987879	0.002099354	0.006866387	-0.146070318	-0.074542034	KIAA0556
chr2	240290984	240291958	975	*	7	8.9087E-19	0.042960418	0.0020996	0.001914873	-0.285314532	-0.067629572	HDAC4
chr18	74725828	74726002	175	*	2	1.19756E-08	0.004446283	0.002099625	0.002572779	-0.23873378	-0.144387068	MBP
chr17	48608677	48609332	656	*	4	7.34719E-11	0.033637846	0.0020997	0.009963542	-0.302108141	-0.079153493	MYCBPAP
chr2	38405469	38406306	838	*	2	1.53913E-10	7.01616E-05	0.002099993	0.000139505	-0.135801878	-0.131736936	CYP1B1-AS1
chr2	208100660	208101334	675	*	3	6.2085E-13	0.001279919	0.002101593	0.000158503	-0.322974025	-0.206249716	AC007879.2, AC007879.3
chr2	230989183	230989623	441	*	3	4.58025E-16	0.000392382	0.002101641	0.000140994	-0.217792185	-0.128018175	
chr2	242954018	242954430	413	*	3	5.25289E-11	0.135182749	0.00210207	0.01528316	-0.357675121	-0.117808957	AC131097.3
chr4	89378894	89379578	685	*	5	2.54054E-14	0.229734721	0.002104112	0.01530448	-0.320477244	-0.071239726	HERC5
chr1	95064909	95064974	66	*	2	2.18102E-09	0.001162277	0.002106865	0.001161338	-0.164439753	-0.11895509	
chr9	131355186	131355483	298	*	2	1.83975E-08	0.023520027	0.002107088	0.005333612	0.179425322	0.093967139	SPTAN1
chr20	35422495	35423080	586	*	3	4.0655E-10	0.02472184	0.002110288	0.006637436	0.166176694	0.069019449	SOGA1
chr8	141572044	141572142	99	*	2	9.11333E-12	0.000232819	0.002111795	0.000367783	-0.193424105	-0.126702332	AGO2
chr22	31608519	31609244	726	*	4	2.25392E-19	0.000721905	0.002112841	0.00047747	-0.170175701	-0.051076888	LIMK2
chr5	171432751	171433005	255	*	2	6.50099E-11	0.153865335	0.002115603	0.007938605	0.017083864	0.007899306	FBXW11
chr12	54783416	54784180	765	*	2	7.85827E-11	0.001175221	0.002115751	0.001171978	0.16362867	0.062117957	RP11-753H16.3, RP11-753H16.5, ZNF385A
chr1	23343326	23343542	217	*	2	3.25434E-08	0.069707748	0.002117207	0.00712273	-0.205264454	-0.111164661	
chr3	71295335	71295684	350	*	5	2.14081E-14	0.015596649	0.002122907	0.000562039	0.215523425	0.086697926	FOXP1
chr7	154777894	154778512	619	*	5	3.30791E-10	0.325500797	0.002124902	0.037137822	-0.170064003	-0.033110986	PAXIP1
chr21	48054897	48055715	819	*	13	1.59702E-30	0.00740656	0.002127566	4.91596E-06	-0.048385351	0.003359352	PRMT2
chr9	101010744	101011718	975	*	4	3.24463E-16	1.28325E-05	0.002128578	1.54703E-05	-0.230593442	-0.139825595	TBC1D2
chr14	75301206	75301411	206	*	2	4.90694E-13	2.74727E-05	0.00212903	6.2344E-05	0.169522067	0.163492604	YLPM1

chr11	36448470	36448722	253	*	2	1.26953E-09	0.00212787	0.002131355	0.001707967	-0.176646852	-0.105517097	PRR5L
chr12	132397677	132398533	857	*	5	1.03439E-23	9.11372E-05	0.002133873	2.14474E-06	-0.162924447	-0.081785583	ULK1
chr2	33516023	33516791	769	*	3	5.02836E-15	3.65546E-05	0.002134208	5.26482E-05	0.156563782	0.121354687	LTBP1
chr19	19496104	19496697	594	*	8	3.64078E-15	0.097108849	0.002135868	0.005548445	0.023619979	0.008184703	GATAD2A
chr4	3322285	3322357	73	*	2	3.47222E-08	0.014420002	0.002135926	0.004498761	0.160355551	0.101382529	RGS12
chr14	24801970	24802150	181	*	2	8.44457E-10	0.002389078	0.002135936	0.001832246	-0.068268735	-0.047390283	ADCY4, RP11-934B9.3
chr12	66061959	66062166	208	*	2	9.75837E-09	0.025415088	0.002136797	0.005527389	-0.174466854	-0.10428222	
chr17	73631460	73631785	326	*	3	8.95263E-16	0.000184391	0.002136953	9.66642E-05	0.203157331	0.100914983	SMIM5, RECQL5
chr13	27843068	27843690	623	*	4	8.22329E-11	0.047503265	0.002137666	0.010694779	0.187746496	0.043268719	
chr13	45390049	45390159	111	*	2	1.06488E-08	0.00641462	0.002138921	0.003136571	-0.155472526	-0.092900348	
chr11	65770645	65771070	426	*	3	1.07027E-12	0.114801444	0.002141939	0.014049343	0.137727252	0.057771484	BANF1
chr16	3354260	3355079	820	*	4	8.67787E-16	0.033484137	0.00214307	0.005490951	-0.20444337	-0.035796925	TIGD7
chr10	22725204	22726060	857	*	6	7.56471E-20	0.000107432	0.002143358	4.60966E-05	0.191278091	0.090089062	SPAG6
chr18	74214646	74214729	84	*	2	3.54918E-12	3.45739E-05	0.002143641	7.63766E-05	-0.310386837	-0.310142562	
chr20	32668426	32668826	401	*	3	6.89012E-09	0.344691105	0.002148164	0.020380335	0.098779973	0.039013407	RALY
chr21	36261693	36261702	10	*	2	6.15894E-10	0.196341702	0.002151673	0.008204671	0.02422309	0.012102793	RUNX1
chr11	62366806	62367988	1183	*	6	2.16312E-18	0.056624805	0.00215257	0.001427112	0.223427961	0.002059361	MTA2
chr10	71867147	71868555	1409	*	5	3.12189E-17	0.055787279	0.002153261	0.00065137	0.19237726	0.074472836	H2AFY2, AIFM2
chr5	123919649	123920010	362	*	2	4.77411E-10	0.000792898	0.002153566	0.000907027	0.208358405	0.133025951	
chr17	79047681	79048599	919	*	5	1.60424E-17	0.002759202	0.002154004	0.000137242	-0.285716688	-0.116633452	BAIAP2
chr1	17424524	17425160	637	*	2	8.23171E-12	3.01095E-05	0.002155959	6.77247E-05	-0.186952645	-0.142751252	PADI2
chr11	22850866	22851502	637	*	11	1.50017E-13	0.176788995	0.002161339	0.053893249	0.036545799	0.003844807	RP11-17A1.3, SVIP
chr3	147089362	147089629	268	*	2	5.47867E-09	0.171033416	0.002161589	0.008156492	0.146074356	0.074194344	
chr22	35806330	35807232	903	*	5	4.43773E-14	0.001211626	0.002162307	5.7583E-05	-0.176147717	0.004879048	MCM5
chr5	88589895	88590117	223	*	2	1.23581E-08	0.00464837	0.002162974	0.002678705	-0.176322306	-0.109130546	MEF2C-AS1
chr1	59041407	59044110	2704	*	16	7.7148E-34	3.83858E-06	0.002164632	5.38808E-09	-0.353022496	-0.105829975	TACSTD2
chr2	37642493	37642646	154	*	2	5.70003E-09	0.001747313	0.002167754	0.001526454	0.185214775	0.133590535	
chr8	68287227	68287355	129	*	2	3.10645E-08	0.007523733	0.002169485	0.003417616	-0.266281841	-0.148274807	
chr6	6588089	6589075	987	*	7	7.72939E-17	0.006016364	0.00217739	0.000492008	0.166708043	0.037606361	LY86, LY86-AS1

chr2	59504985	59505167	183	*	2	4.73858E-08	0.019877213	0.002178583	0.005150459	-0.345425253	-0.177908355	AC007131.1, RP11-444A22.1, AC007131.2
chr9	127024180	127025282	1103	*	5	1.50918E-14	0.024563334	0.002179176	0.001865802	-0.255545472	-0.082208402	NEK6, RP11-121A14.3
chr22	39868756	39869399	644	*	2	2.84192E-12	4.01457E-05	0.002182298	8.72652E-05	0.116897574	0.012883196	MGAT3
chr10	121297031	121297227	197	*	2	8.8514E-09	0.011188529	0.002184285	0.004109483	0.198520177	0.111048106	RGS10
chr5	39470228	39470464	237	*	2	4.21752E-12	3.79617E-05	0.002189745	8.3173E-05	-0.27615586	-0.215774693	
chr19	54809989	54810355	367	*	5	1.16684E-12	0.003036225	0.002192528	0.001600753	-0.296720894	-0.093869015	
chr1	66859276	66859345	70	*	2	7.04182E-12	4.38865E-05	0.002194224	9.43621E-05	-0.217000562	-0.19946003	
chr10	100011569	100011724	156	*	4	6.32417E-10	0.078758449	0.002196215	0.012877495	0.160580302	0.047337245	LOXL4
chr17	42983032	42983267	236	*	2	2.14324E-11	0.001258821	0.002197612	0.001246726	0.159991328	0.119449556	GFAP
chr7	65791335	65791671	337	*	2	1.73565E-08	0.053139898	0.002198727	0.006933259	-0.307255033	-0.164902923	TPST1
chr5	173219000	173219205	206	*	2	6.72974E-09	0.029350792	0.002198733	0.005907651	0.117729525	0.055249408	CTB-43E15.4
chr4	140968835	140969039	205	*	2	1.43322E-08	0.008247887	0.002201403	0.00360117	0.138830337	0.092617981	MAML3
chr11	10924151	10924478	328	*	2	5.73282E-10	0.000591759	0.00220286	0.000745883	0.153614641	0.012020777	ZBED5-AS1
chr16	81642149	81642691	543	*	3	7.65009E-15	3.85708E-05	0.002203335	9.37013E-05	-0.268725499	-0.134866084	CMIP
chr1	203155737	203156784	1048	*	8	6.37993E-22	7.98539E-06	0.002207341	2.90494E-06	-0.315134232	-0.118569724	CHI3L1
chr21	44010866	44010869	4	*	2	2.40967E-12	2.89069E-05	0.002208555	6.55908E-05	-0.11983094	-0.11167185	
chr2	217280995	217281424	430	*	2	9.82279E-11	0.000871016	0.002208757	0.000977436	0.191825597	0.118198078	SMARCAL1
chr12	6752881	6753626	746	*	6	5.51699E-26	2.48395E-06	0.00221032	4.36477E-07	-0.332934352	-0.17212245	ACRBP
chr22	39347307	39348639	1333	*	6	4.86696E-22	8.39025E-06	0.002210422	1.82517E-06	-0.192758143	-0.052194517	
chr22	31642860	31644558	1699	*	12	5.14501E-31	4.69165E-06	0.002216652	2.77376E-07	0.238259335	0.003684533	LIMK2
chr19	4539943	4544095	4153	*	14	3.89129E-35	3.21683E-15	0.002219369	1.17665E-14	-0.297901596	-0.139167598	CTB-50L17.14, LRG1, SEMA6B
chr22	23449278	23449985	708	*	2	1.24749E-10	2.91987E-05	0.002222968	6.62434E-05	0.197639166	0.184390676	GNAZ, RTDR1
chr4	142142166	142142438	273	*	3	1.85575E-08	0.036023909	0.002223444	0.006805373	0.02978077	0.012093756	ZNF330
chr14	107079290	107079472	183	*	2	1.63043E-08	0.072361506	0.002225641	0.007465446	0.13642791	0.065096153	
chr11	34229970	34230092	123	*	2	6.26488E-08	0.017886726	0.002227078	0.005030587	-0.271142981	-0.143180002	ABTB2
chr22	37256352	37257594	1243	*	7	4.88395E-23	7.98376E-05	0.00222796	1.09978E-05	-0.221854309	-0.070306972	NCF4, CTA-833B7.2
chr13	52732648	52734432	1785	*	21	1.19199E-26	0.027598596	0.002230084	0.005160465	-0.32644284	-0.003611682	NEK3
chr3	101503735	101504046	312	*	3	9.40809E-13	0.003514264	0.0022323	0.000675251	-0.278894798	-0.151653402	NXPE3
chr2	75145273	75145387	115	*	2	7.23566E-09	0.016425789	0.00223585	0.004883055	0.086706807	0.051943659	AC104135.3

chr14	88469009	88469180	172	*	2	2.35832E-08	0.025224084	0.002237958	0.005699994	-0.263170972	-0.122344796	
chr22	17703558	17703776	219	*	4	5.5177E-12	0.093047893	0.00223799	0.011050764	-0.178657951	-0.050674165	
chr14	62036609	62037555	947	*	6	6.16686E-21	1.4613E-05	0.00223822	7.31989E-06	-0.109495644	-0.064597691	RP11-47I22.4, RP11-47I22.3, RP11-47I22.2
chr7	210075	210429	355	*	2	1.81838E-11	0.020773455	0.002240147	0.005335381	0.024507297	0.01716268	FAM20C
chr11	33744823	33745446	624	*	4	2.45497E-16	0.01724673	0.002246783	0.000770493	-0.295549747	-0.086920321	CD59
chr5	67520858	67522532	1675	*	10	2.42353E-20	0.003216117	0.002247123	1.02145E-05	0.17509623	0.018766439	PIK3R1
chr8	28195955	28197671	1717	*	10	6.10361E-32	0.002433848	0.002247227	6.01874E-05	-0.326445724	-0.087341989	PNOC, RP11-380I10.4
chr13	115026464	115027249	786	*	3	4.47918E-13	0.003384332	0.002247332	0.000210136	-0.176077428	-0.031424009	CDC16
chr13	24758029	24758155	127	*	2	4.62466E-10	0.001028706	0.002248279	0.001102011	-0.089024886	-0.061890017	RP11-307N16.6, SPATA13
chr11	95374762	95374866	105	*	2	4.6935E-10	0.000509636	0.002248353	0.00067522	-0.288584889	-0.162286205	
chr10	129949483	129949871	389	*	2	9.47976E-13	0.000612925	0.002250777	0.000771081	0.119672768	0.085605199	
chr21	35883571	35884679	1109	*	7	2.7589E-27	4.09162E-08	0.00225302	1.92521E-07	-0.247623724	-0.133548539	KCNE1
chr11	63258712	63259705	994	*	10	1.08727E-19	0.235632135	0.002253273	0.0054192	-0.205834858	-0.045184518	
chr12	56537247	56537425	179	*	2	3.46301E-08	0.08656573	0.002253723	0.007782992	-0.233715572	-0.112729603	ESYT1
chr15	90371358	90371981	624	*	4	8.18548E-14	0.006825812	0.002253947	0.00053552	-0.291102927	-0.109663244	
chr13	115085283	115085339	57	*	2	3.93248E-12	3.42445E-05	0.002255027	7.63953E-05	0.143968448	0.139229577	CHAMP1
chr8	62602522	62602643	122	*	2	8.96442E-08	0.028437966	0.002255478	0.005959108	0.171812575	0.072259827	ASPH
chr2	54198660	54199039	380	*	2	2.24848E-11	0.03472632	0.002255742	0.006331236	0.125649127	0.06476873	ACYP2
chr10	72983319	72983571	253	*	2	1.62819E-08	0.018582095	0.002255951	0.005148185	0.175304166	0.094263607	UNC5B
chr1	193074497	193075640	1144	*	12	8.50527E-26	0.117330432	0.002258214	0.004001195	-0.277724529	-0.042002148	GLRX2
chr22	29513985	29514072	88	*	2	3.72524E-10	0.00025666	0.002258739	0.00040488	-0.150375245	-0.140931157	KREMEN1
chr3	194411417	194411443	27	*	2	1.37074E-09	0.000856171	0.002258755	0.000975011	0.188689492	0.126576779	
chr17	16200441	16200581	141	*	2	3.42906E-11	0.00013618	0.002259436	0.000245347	-0.206380572	-0.035339304	PIGL
chr3	15328890	15329313	424	*	3	6.36945E-15	3.27176E-05	0.002259724	4.70442E-05	-0.277985475	-0.145293948	SH3BP5
chr10	81202867	81203244	378	*	2	5.67803E-10	0.0019779	0.002261243	0.001681938	-0.216472574	-0.077071692	ZCCHC24
chr14	77382482	77382822	341	*	2	1.93694E-09	0.001958612	0.00226605	0.001673556	-0.271899675	-0.091677117	
chr19	41854950	41855775	826	*	4	9.12673E-11	0.030422567	0.002266765	0.006637431	0.146143899	0.062264779	TGFB1
chr19	2421956	2422172	217	*	2	1.83604E-09	0.002234499	0.002267031	0.001813931	0.128179325	0.075847846	TMPRSS9
chr6	38123146	38123312	167	*	2	2.67509E-11	7.82774E-05	0.002269468	0.000155577	-0.133369749	-0.126439311	

chr10	125805554	125805592	39	*	2	2.13449E-09	0.012271304	0.002269858	0.004382906	0.090752762	0.059134216	CHST15
chr14	101034794	101035262	469	*	3	9.71764E-12	0.001553809	0.002270212	0.00143931	-0.050941936	0.000774942	BEGAIN
chr2	231191876	231192294	419	*	6	2.50509E-12	0.571314708	0.002271022	0.038683076	-0.257608367	-0.042859094	SP140, SP140L
chr2	71291297	71292691	1395	*	6	1.6952E-24	1.54452E-08	0.00227288	6.05446E-08	-0.328687348	-0.143437397	NAGK, AC007040.8
chr7	55602109	55602372	264	*	2	1.74053E-13	3.00276E-05	0.002274111	6.81471E-05	-0.273350188	-0.229556349	VOPP1
chr8	1692726	1694654	1929	*	6	8.97571E-22	6.61413E-06	0.002274749	4.60074E-06	0.161804819	0.021602251	
chr4	2915985	2916624	640	*	4	1.44924E-11	0.004502957	0.002275056	0.002205701	-0.277996656	-0.106357851	ADD1
chr8	37658755	37659174	420	*	2	6.89561E-12	0.000909588	0.002285309	0.001021102	0.149460672	0.131496499	GPR124
chr12	2416089	2416339	251	*	2	1.37435E-08	0.014836239	0.002286771	0.00476297	-0.238956428	-0.101272996	CACNA1C
chr11	67255424	67255752	329	*	3	2.65408E-11	0.020201537	0.002287749	0.002647524	-0.145846094	-0.037731389	AIP
chr7	36639726	36640882	1157	*	4	2.72071E-15	0.002487953	0.002290807	0.000335434	-0.278605004	-0.124850802	AOAH, AOAH-IT1
chr14	51710280	51711237	958	*	4	2.51598E-14	0.002397352	0.00229289	0.000282948	-0.21063123	-0.013160657	TMX1, SNORA70
chr9	19103992	19104016	25	*	3	2.58394E-14	0.000400365	0.002293846	0.000299154	-0.157908257	-0.089613204	
chr10	44407865	44408207	343	*	3	4.22557E-11	0.003104983	0.002294241	0.00226407	-0.20030323	-0.076489455	RP11-168P8.3
chr1	226841990	226842455	466	*	2	8.91726E-12	0.000179141	0.002294702	0.000306974	-0.142254951	-0.015767258	ITPKB
chr3	16765888	16766180	293	*	2	1.00728E-08	0.004622488	0.002296211	0.002762467	0.190985761	0.076020146	
chr1	145726754	145727016	263	*	3	8.51934E-10	0.057947293	0.002297295	0.011444504	0.186894655	0.076476102	PDZK1
chr1	5942634	5943388	755	*	6	1.57187E-10	0.14779567	0.002298772	0.031811098	0.160693801	0.028886429	NPHP4
chr12	14988018	14988123	106	*	2	1.35462E-08	0.002546378	0.002301155	0.001976158	0.222841792	0.141716181	C12orf60, ART4
chr8	145578911	145580093	1183	*	6	8.99819E-19	0.124348989	0.0023019	0.002765251	-0.219330661	-0.059654082	SLC52A2, GS1-393G12.13, FBXL6
chr9	137263193	137263731	539	*	5	6.72722E-20	0.002683399	0.002303273	4.32615E-05	-0.283367783	-0.147128611	RXRA
chr2	28497547	28497868	322	*	3	2.78762E-08	0.812197377	0.002304546	0.025652792	-0.320699497	-0.107814333	BRE
chr13	103423896	103424762	867	*	3	2.36181E-13	0.004362134	0.00230498	0.000403773	-0.242700092	-0.11723249	TEX30
chr17	1371153	1371321	169	*	2	2.72769E-08	0.185563445	0.002306937	0.008673519	-0.16651864	-0.082654764	MYO1C
chr14	23586582	23589419	2838	*	10	1.86035E-29	5.61427E-15	0.002308068	4.12374E-13	-0.334395383	-0.169142844	CEBPE
chr17	17179362	17179426	65	*	3	8.24851E-10	0.00611076	0.002308617	0.002019568	-0.214819981	-0.114159824	COPS3
chr16	2081194	2081446	253	*	3	7.87946E-13	0.000384816	0.002309137	0.000264236	0.255724853	0.166994344	SLC9A3R2
chr7	4754502	4755415	914	*	9	2.58137E-46	8.27681E-17	0.00230968	2.50791E-14	-0.287816252	-0.200234428	FOXK1
chr10	106053665	106054220	556	*	3	1.53918E-09	0.178231659	0.002314179	0.017996943	0.165209215	0.060030653	GSTO2

chr4	175205044	175206750	1707	*	11	4.01927E-21	0.118729646	0.002314845	0.020148512	-0.369473425	-0.040612586	CEP44, FBXO8
chr19	6886467	6887544	1078	*	6	4.31879E-14	0.006482307	0.002314962	0.000201757	-0.224834632	-0.074193162	
chr13	21578684	21579100	417	*	5	2.07876E-15	0.001020875	0.002317775	0.000297843	-0.20073421	-0.096680517	LATS2
chr5	73181670	73181701	32	*	2	3.44389E-08	0.008974662	0.002318453	0.003867524	-0.231203592	-0.144481676	ARHGEF28
chr7	30952730	30953124	395	*	3	1.06402E-09	0.024812753	0.002320085	0.007599048	0.159637342	0.075140332	AQP1, AQP1
chr1	24239771	24239820	50	*	2	5.76463E-08	0.001313855	0.002320746	0.001313673	-0.106449363	-0.078218388	CNR2
chr4	4323012	4323632	621	*	4	3.08367E-11	0.136044357	0.002321631	0.004143982	0.207097808	0.096643073	ZBTB49
chr10	134399880	134401153	1274	*	8	1.7067E-21	8.84774E-07	0.002323774	2.39685E-06	-0.299583706	-0.021945884	INPP5A
chr1	100543333	100543434	102	*	2	9.16181E-08	0.019762327	0.002324162	0.005376035	0.113677887	0.066790364	HIAT1, RP4-714D9.2
chr1	26395442	26395951	510	*	3	2.23065E-09	0.076941283	0.002324399	0.01242417	0.159293253	0.057398627	
chr22	38965411	38967436	2026	*	13	1.13382E-15	0.111397934	0.002329569	0.002464779	-0.241056485	-0.002269729	DMC1
chr19	38812379	38812803	425	*	3	7.43377E-13	0.000573621	0.00232969	0.00039779	0.172749325	0.125486124	KCNK6
chr1	8931135	8932359	1225	*	7	2.94308E-26	1.1437E-08	0.002329981	2.0551E-08	-0.379019416	-0.153568222	ENO1
chr14	65204509	65204625	117	*	3	6.47816E-10	0.015046675	0.002330532	0.00255224	-0.115135613	-0.070287366	PLEKHG3
chr19	16423883	16424005	123	*	2	1.25547E-07	0.045907564	0.002331266	0.00700155	-0.232358583	-0.117937419	CTD-2562J15.6
chr22	41487569	41487761	193	*	4	9.68649E-10	0.06297828	0.002332353	0.010927004	0.030146807	0.015831194	
chr16	4102293	4105170	2878	*	15	6.45612E-22	0.00096388	0.002332418	7.59613E-06	-0.304866315	-0.098929461	ADCY9
chr20	55949676	55949957	282	*	3	1.56562E-15	4.00825E-06	0.002333367	1.32215E-05	-0.231075199	-0.126434291	RAE1
chr17	80180987	80181015	29	*	2	7.09034E-14	3.34759E-05	0.00233545	7.53223E-05	-0.311441217	-0.290906975	
chr15	77280925	77281418	494	*	3	6.49568E-13	0.000204882	0.002336663	0.000209988	-0.184510074	-0.111455618	
chr4	737453	738199	747	*	5	3.0959E-13	0.026638264	0.002336891	0.003311496	-0.240405422	-0.08015487	PCGF3
chr5	148807759	148810203	2445	*	12	3.57101E-18	0.00027477	0.002337863	2.44221E-06	-0.18927771	-0.07889999	MIR143HG, MIR143, MIR145
chr19	14785593	14785849	257	*	5	9.97355E-17	0.000421394	0.002338127	5.13489E-05	-0.058209119	-0.03258666	EMR3
chr16	85551478	85552230	753	*	4	6.18558E-19	3.54535E-05	0.002339793	1.12624E-05	0.210984873	0.141328001	
chr7	137621009	137621198	190	*	2	2.81694E-08	0.06025782	0.002341436	0.007489284	-0.247475087	-0.119812243	CREB3L2
chr14	77249068	77249073	6	*	2	5.47674E-10	0.002134847	0.002342295	0.001793025	0.170550019	0.102535575	VASH1, RP11-488C13.5
chr6	33389072	33389696	625	*	4	2.67898E-16	0.003039451	0.002343583	0.000364329	0.203738147	0.102681745	SYNGAP1
chr8	142179603	142181089	1487	*	11	1.94823E-25	0.000447246	0.00234565	9.01548E-05	-0.343998093	-0.03282086	DENND3
chr3	141056124	141056238	115	*	2	7.64132E-12	4.34082E-05	0.002351134	9.46522E-05	0.185396975	0.17090612	ZBTB38, RP11-438D8.2

chr2	7171869	7172724	856	*	9	9.45489E-37	1.22843E-09	0.002352021	1.48938E-09	-0.32087044	-0.123422823	RNF144A
chr8	33397227	33397536	310	*	4	5.61259E-15	0.000446668	0.002354757	0.000199414	0.142740487	0.040521335	
chr16	3843012	3843415	404	*	5	7.5707E-14	0.035816123	0.002356599	0.001334615	0.218646796	0.087021793	CREBBP
chr15	22920060	22920168	109	*	2	2.24921E-08	0.067930022	0.002358217	0.007722156	0.162525601	0.079612952	CYFIP1
chr5	177929808	177929948	141	*	2	7.49466E-08	0.016201682	0.00236182	0.00504045	-0.171156888	-0.070972524	COL23A1
chr17	517799	518485	687	*	2	9.65101E-11	7.05884E-05	0.002364131	0.000143846	-0.235851775	-0.165630976	VPS53
chr22	43327098	43327600	503	*	3	1.55097E-13	0.002178596	0.002367594	0.000315088	-0.193401182	-0.123239228	PACSIN2
chr22	18452721	18453180	460	*	2	8.84961E-12	3.31952E-05	0.002370841	7.49484E-05	0.168162692	0.148754925	MICAL3
chr22	51042356	51042761	406	*	2	5.795E-11	0.001546581	0.002371126	0.001474029	0.079307292	0.064275678	MAPK8IP2
chr15	38987310	38988560	1251	*	5	3.45088E-18	0.081269341	0.002372059	0.002171207	0.215060101	0.059998017	
chr6	2784576	2784588	13	*	2	3.92581E-13	3.53391E-05	0.002372166	7.91992E-05	0.090143716	0.088549388	WRNIP1
chr8	23351369	23351994	626	*	5	3.57944E-22	3.86876E-08	0.002377594	2.61639E-07	0.178951148	0.136242324	CTC-756D1.3
chr16	4819304	4819632	329	*	2	8.32079E-09	0.047155708	0.00237873	0.007158591	-0.199851589	-0.105216604	
chr21	36398056	36398338	283	*	2	1.4557E-14	3.39306E-05	0.002382653	7.64707E-05	-0.177122938	-0.142581398	RUNX1
chr8	123629859	123630361	503	*	2	3.12229E-11	8.62101E-05	0.002383477	0.000170539	-0.27898584	-0.084712223	
chr2	235949554	235949598	45	*	2	1.34911E-08	0.006600734	0.002385831	0.003394833	-0.146555462	-0.093532559	SH3BP4
chr15	81317493	81317622	130	*	2	2.7444E-08	0.007466407	0.002389602	0.003610679	-0.264635431	-0.146678061	C15orf26
chr17	57410172	57410687	516	*	2	1.59744E-11	0.002264819	0.00239138	0.001877761	-0.09007609	-0.058285555	YPEL2
chr12	125188554	125189190	637	*	2	2.34798E-10	0.000334311	0.00239383	0.000504835	-0.35194921	-0.195457636	
chr15	58430391	58430682	292	*	4	4.24024E-09	0.615482512	0.002393999	0.046212535	-0.351136083	-0.083732058	AQP9, ALDH1A2
chr5	139040546	139041014	469	*	5	2.25045E-16	1.1464E-05	0.002397329	3.84725E-05	-0.08595207	-0.062020305	CXXC5
chr10	5987662	5988276	615	*	7	2.77668E-23	7.04282E-07	0.002397438	8.1736E-07	-0.275416726	-0.089434452	RP11-536K7.3
chr2	219030123	219031719	1597	*	6	3.16378E-12	0.030530183	0.002397825	0.001027243	-0.21481021	-0.080169153	CXCR1
chr1	235100100	235100790	691	*	3	4.11007E-12	0.001041095	0.002401999	0.001015275	0.192408179	0.122803914	RP11-443B7.1
chr5	124063914	124064481	568	*	4	2.74873E-09	0.220422062	0.002406444	0.027298096	0.162013111	0.038700377	ZNF608
chr11	70256556	70258111	1556	*	7	7.6472E-21	0.000166158	0.002406742	2.03328E-05	0.214695891	0.050057224	CTTN
chr3	148446876	148448256	1381	*	7	4.88602E-14	0.040284176	0.002407414	0.004483948	-0.167436141	-0.047247374	AGTR1
chr8	144654887	144656271	1385	*	13	5.57616E-36	0.000173894	0.00240855	1.53242E-06	-0.304911952	-0.102633408	RP11-661A12.9, MROH6
chr11	109952914	109953349	436	*	2	3.41062E-11	9.69406E-05	0.002410238	0.000188571	-0.223480277	-0.035625687	

chr5	127194101	127194369	269	*	3	1.95492E-09	0.019002251	0.002410784	0.006033353	-0.148802424	-0.035444357	CTC-228N24.1
chr16	84223350	84223841	492	*	3	6.26334E-14	0.001786807	0.002412115	0.001633534	-0.212127297	-0.094458234	
chr10	51574776	51575388	613	*	2	2.63377E-11	0.161146485	0.002413164	0.008908393	-0.265162547	-0.134560578	NCOA4
chr20	47340424	47341147	724	*	3	4.24731E-15	0.006059088	0.002413886	0.00023932	-0.245160631	-0.135934999	PREX1
chr1	162287718	162288210	493	*	3	6.09867E-14	0.000336797	0.002414445	0.000186862	-0.23506492	-0.139603923	NOS1AP
chr11	10476242	10477461	1220	*	10	4.68769E-37	2.42204E-06	0.002416716	1.97657E-08	-0.285275141	-0.102560444	AMPD3
chr12	1212226	1212255	30	*	2	3.86787E-13	6.2051E-05	0.002416945	0.000129481	-0.22927808	-0.183731881	ERC1
chr3	52124234	52124791	558	*	3	1.16559E-13	0.000803188	0.002417647	0.000255662	0.22045367	0.135402251	POC1A
chr6	35993792	35994020	229	*	2	1.10309E-08	0.086464745	0.002421026	0.008245995	-0.149475756	-0.072792496	
chr2	238990085	238990731	647	*	8	4.37447E-19	0.023096685	0.002423454	0.001234879	0.146172787	0.040285727	UBE2F-SCLY, SCLY
chr3	171845173	171845883	711	*	2	1.30215E-10	4.94868E-05	0.00242628	0.000106656	-0.186776549	-0.155953462	FNDC3B
chr1	6662478	6663269	792	*	6	1.48099E-15	0.008608143	0.002431193	0.002196381	0.044115119	0.00975601	KLHL21
chr9	100991448	100991672	225	*	3	1.5083E-08	0.204731834	0.002431846	0.019373529	-0.310056098	-0.090542531	TBC1D2
chr7	642127	643155	1029	*	6	2.37226E-25	3.33259E-05	0.002434651	5.78613E-06	0.194170383	0.095054988	AC147651.4, PRKAR1B
chr2	25427064	25427652	589	*	6	1.21263E-11	0.09608336	0.002435289	0.018884641	-0.147166396	-0.033742629	AC012457.2
chr6	29705436	29706377	942	*	5	1.84952E-17	0.004262447	0.002440797	0.000176823	-0.261742736	-0.079952213	HLA-F, HLA-F-AS1
chr21	45140424	45140931	508	*	3	6.33243E-11	0.002075752	0.002446843	0.001767152	-0.104355494	-0.060280955	PDXK
chr7	135368563	135369001	439	*	2	1.87165E-10	0.000278703	0.002449456	0.000442436	-0.282784825	-0.067798671	C7orf73, SLC13A4
chr10	129172498	129172573	76	*	3	7.35719E-16	1.69553E-06	0.002452796	7.14372E-06	0.17324093	0.148168651	DOCK1
chr17	80266884	80267099	216	*	4	8.8427E-15	0.000646694	0.002452821	9.60009E-05	0.181451752	0.109505348	
chr9	139592589	139593588	1000	*	3	1.04087E-10	0.002018356	0.002454013	0.000551067	-0.21219557	-0.10329193	
chr17	57889637	57889850	214	*	2	1.38438E-08	0.004518776	0.002456681	0.002833715	-0.258961282	-0.160913458	VMP1
chr6	140371491	140371628	138	*	2	2.80953E-08	0.00569761	0.002458827	0.00320587	0.165679017	0.025539202	
chr9	33473445	33474350	906	*	12	2.69489E-19	0.091967334	0.002460525	0.008245766	-0.029059023	0.000170429	NOL6
chr8	71381070	71381216	147	*	3	2.95555E-11	0.007595453	0.002461915	0.001309803	-0.243143846	-0.146150606	
chr16	3173975	3174915	941	*	8	5.15761E-19	0.02588937	0.0024622	0.000105359	0.0238005	0.002823596	RP11-473M20.14
chr16	1537775	1539318	1544	*	11	7.59406E-32	0.006273047	0.002468015	6.34183E-06	-0.290105776	-0.10505748	PTX4
chr14	91862813	91862907	95	*	4	1.8257E-08	0.241920841	0.002469723	0.022977542	-0.21090882	-0.062757023	CCDC88C
chr15	101714939	101715819	881	*	6	2.44479E-11	0.215168449	0.002469832	0.02202208	-0.205395608	-0.045255044	

chr9	127273296	127273395	100	*	2	9.05875E-08	0.006258034	0.002471797	0.00337426	0.167286781	0.069094057	
chr19	41920834	41921140	307	*	4	1.42578E-11	0.002687355	0.002471946	0.002217375	-0.284157411	-0.099283821	CTC-435M10.3, BCKDHA
chr3	10334546	10335844	1299	*	8	1.5924E-18	0.00384367	0.002472678	0.000939635	-0.214006035	-0.049903112	GHRLOS, GHRL, SEC13
chr1	161059411	161060622	1212	*	8	1.25873E-22	4.76508E-05	0.002472843	5.54677E-05	-0.246616477	-0.010295605	
chr5	176972593	176972809	217	*	2	3.23279E-09	0.000998585	0.002480403	0.001125859	-0.186163725	-0.111017368	FAM193B
chr17	42410950	42411197	248	*	2	3.19965E-12	4.00461E-05	0.002481451	8.905E-05	-0.231249938	-0.199295925	
chr5	67562753	67563114	362	*	2	4.65798E-10	0.000426097	0.002481691	0.000613166	-0.223546684	-0.173506534	PIK3R1
chr11	120811322	120811938	617	*	2	1.32591E-10	0.000158046	0.002482649	0.000283665	0.14102213	0.116477261	GRIK4
chr19	3179364	3180035	672	*	5	1.58957E-13	0.028659219	0.002483472	0.004134228	-0.114385071	-0.038865735	S1PR4
chr15	101566796	101567894	1099	*	3	4.80762E-13	0.001962533	0.002484873	0.001612868	0.090556298	0.051534659	LRRK1
chr17	19290353	19291768	1416	*	10	2.32977E-31	1.20976E-07	0.002485378	8.2686E-08	-0.154608144	-0.064397084	MFAP4
chr2	235878176	235878276	101	*	2	1.26829E-09	0.001031044	0.002485571	0.001151611	0.104246148	0.089392455	SH3BP4
chr2	187420032	187420141	110	*	2	8.6922E-08	0.012783866	0.002487463	0.004733307	0.155536748	0.094767823	
chr20	18490820	18491545	726	*	6	3.40275E-22	0.00022031	0.002490113	2.56136E-05	-0.282197999	-0.132366351	SEC23B
chr12	122234980	122235560	581	*	5	3.58208E-16	0.000350557	0.002493744	0.000129233	0.214494291	0.095245543	RP11-347I19.8, RHOF, AC084018.1
chr2	191885762	191886753	992	*	5	2.05592E-12	0.018607583	0.002497986	0.005148167	-0.210679053	-0.047522377	AC067945.4
chr15	51659419	51659538	120	*	3	1.91879E-08	0.445080448	0.0025002	0.020967573	-0.243922931	-0.083954159	GLDN
chr8	134306801	134307728	928	*	4	2.33303E-18	1.89428E-05	0.002501204	7.99434E-06	-0.255086213	-0.152335273	NDRG1
chr4	38138950	38139045	96	*	2	3.63276E-08	0.005632699	0.002501596	0.003218527	-0.177352879	-0.098170504	TBC1D1
chr8	56684606	56684895	290	*	3	1.37071E-13	0.001201346	0.002505126	0.001025661	0.12330408	0.061191822	TMEM68
chr11	64481018	64481499	482	*	4	6.8343E-13	0.00120765	0.002509437	0.001286536	0.044266358	0.009922119	NRXN2
chr12	117174227	117175042	816	*	4	9.99816E-15	0.01137701	0.002511229	0.00114421	-0.183438291	-0.037370935	C12orf49
chr20	48658164	48658258	95	*	4	1.28904E-14	0.000446627	0.00251175	0.000125429	-0.256278106	-0.130584997	
chr7	157092682	157093534	853	*	5	1.62566E-18	0.000822562	0.002513863	5.53062E-05	-0.160301081	-0.061441499	
chr17	34207332	34208154	823	*	7	1.17393E-18	0.000102622	0.002514206	3.21885E-05	0.135370468	0.082417525	AC015849.2, CCL5
chr17	16319861	16320257	397	*	2	1.20511E-08	0.054439205	0.00251762	0.007744419	0.168873173	0.08020155	TRPV2
chr17	78444511	78444702	192	*	3	3.59888E-08	0.037924678	0.002518726	0.009789946	0.117062372	0.053367822	NPTX1
chr19	10792615	10793130	516	*	6	5.31727E-14	0.088770276	0.002519024	0.006091997	-0.165249628	-0.019430849	ILF3
chr14	52333121	52333365	245	*	2	1.59158E-08	0.003824915	0.002519461	0.002620392	-0.261716775	-0.111890251	GNG2

chr4	84407534	84407894	361	*	2	6.73086E-10	0.001314482	0.002519907	0.001362876	-0.144292545	-0.08403512	FAM175A
chr20	61492204	61492669	466	*	2	5.2338E-11	0.002791119	0.002524373	0.002186884	0.056558348	0.041900587	TCFL5
chr3	108800810	108800886	77	*	2	7.42659E-08	0.008733753	0.002524724	0.004024781	0.119074699	0.084597825	MORC1
chr15	55513621	55513769	149	*	2	1.32503E-10	0.000139992	0.002524964	0.000258179	-0.132678219	-0.100933019	RAB27A
chr20	48410517	48411590	1074	*	4	1.33143E-10	0.021908838	0.00253236	0.00056261	-0.237581048	-0.114701966	RNU6-919P
chr7	97912149	97912641	493	*	2	2.73443E-10	0.001704014	0.002533709	0.001616886	-0.127734646	-0.099805848	BRI3
chr9	139590572	139590683	112	*	2	8.69845E-08	0.019784113	0.002535891	0.005711502	-0.161706433	-0.076232553	
chr12	121123557	121124624	1068	*	6	9.91728E-17	0.000644688	0.00253898	0.000240703	-0.164169466	-0.012726181	
chr2	240142589	240147161	4573	*	12	1.29523E-25	3.91311E-13	0.002545123	6.70586E-13	-0.281716717	-0.1186558	HDAC4
chr2	262323	262646	324	*	2	5.4428E-11	0.000320036	0.00255104	0.000498391	0.125954186	0.112055175	SH3YL1
chr9	126061965	126062469	505	*	2	4.74361E-10	0.000535038	0.002555328	0.000732839	0.163665338	0.122025432	
chr16	85449409	85449611	203	*	2	5.36754E-11	9.83653E-05	0.00255915	0.000193503	0.175458085	0.158421089	
chr2	113884734	113885277	544	*	3	3.60982E-14	0.000649766	0.002561345	0.000397131	-0.241552172	-0.101654504	IL1RN
chr1	159172927	159174610	1684	*	7	3.62937E-25	4.79088E-06	0.00256317	4.48602E-07	-0.291353434	0.025217577	CADM3, DARC, CTA-134P22.2
chr10	104403435	104405343	1909	*	9	7.11056E-26	2.61953E-05	0.002563666	7.25564E-07	0.033645205	0.012603454	TRIM8, RP11-47A8.5
chr17	57440029	57440226	198	*	2	4.55345E-11	8.79317E-05	0.002567353	0.000176251	0.199241711	0.18397394	YPEL2
chr20	11898851	11900410	1560	*	6	4.08584E-19	0.000441396	0.00256955	2.24625E-05	0.145484419	0.080204306	BTBD3, RP4-742J24.2
chr9	100881995	100883593	1599	*	5	2.57358E-18	8.49718E-05	0.002570052	1.21623E-05	-0.213641379	-0.124608278	CORO2A
chr14	69448184	69448387	204	*	2	3.53466E-10	0.00063165	0.002571834	0.000828568	0.181903625	0.115814053	ACTN1-AS1
chr2	60441042	60441246	205	*	2	1.06393E-11	5.42005E-05	0.002574656	0.000116661	-0.200408189	-0.1186917	
chr15	43531947	43532243	297	*	3	3.52707E-15	9.08437E-06	0.002574715	2.97235E-05	-0.169126965	-0.117968477	TGM5
chr2	15317374	15317393	20	*	2	1.99111E-08	0.002295282	0.002576985	0.00196373	0.090228952	0.080624164	NBAS
chr18	55317649	55317652	4	*	2	4.18512E-13	7.61831E-05	0.002577387	0.000156246	-0.149606457	-0.139889692	RP11-35G9.3, RP11-35G9.5, ATP8B1
chr16	86018680	86018936	257	*	2	3.26684E-10	0.000289185	0.002577516	0.000462442	-0.201690553	-0.122324435	
chr5	150476928	150477744	817	*	2	3.37863E-10	3.91868E-05	0.002577894	8.79253E-05	-0.299139045	-0.220586388	
chr1	33515863	33516627	765	*	4	7.31326E-11	0.005389956	0.002580133	0.002790804	-0.340294915	-0.085137283	AK2
chr2	169925400	169926103	704	*	5	1.47411E-16	0.000743168	0.002581367	0.000255152	-0.296878941	-0.11243022	DHRS9
chr7	75947745	75948532	788	*	4	4.78169E-16	0.00177709	0.002587384	0.000744192	0.18067342	0.016342253	
chr15	80189128	80190631	1504	*	14	2.81069E-29	0.001183826	0.002587573	0.000163049	-0.254060396	-0.058005759	MTHFS, ST20-MTHFS

chr7	134853645	134855206	1562	*	10	1.26301E-33	5.84774E-05	0.002587988	2.4205E-06	-0.273337735	-0.068892891	RP11-134L10.1, C7orf49
chr4	15691786	15693171	1386	*	5	4.03837E-13	6.7415E-06	0.002588913	4.535E-06	-0.222523004	-0.08242654	FAM200B
chr8	134203235	134203841	607	*	7	6.96313E-26	7.50854E-07	0.002589378	9.31338E-07	0.219982018	0.095823313	WISP1
chr2	171783942	171785822	1881	*	19	4.16814E-28	0.007037505	0.002591211	0.001542237	-0.368284205	-0.042373717	GORASP2
chr11	16809233	16809606	374	*	2	3.94714E-10	0.001135991	0.002591771	0.001251498	-0.162450654	-0.042945829	PLEKHA7
chr16	30775137	30775658	522	*	3	2.75563E-11	0.05334644	0.002593034	0.011552632	-0.218215088	-0.080341612	RNF40
chr1	156478347	156478934	588	*	3	9.24372E-15	0.003565898	0.002595141	0.000261337	-0.134531865	0.0019279	
chr15	63050387	63051004	618	*	5	2.57814E-11	0.014399819	0.002597383	0.00562634	-0.239061317	-0.043939857	TLN2
chr1	156119420	156119773	354	*	5	1.47511E-17	0.000598493	0.002597714	6.40574E-05	-0.261854525	-0.101463359	SEMA4A
chr16	1608794	1609908	1115	*	8	9.51402E-20	0.007625333	0.002598242	0.001215751	-0.371166863	-0.058304046	IFT140
chr1	28856281	28857093	813	*	4	2.66052E-16	1.20943E-05	0.002599063	4.0126E-05	-0.258684271	-0.01001039	RCC1
chr6	37224216	37225644	1429	*	11	4.54732E-27	0.008848831	0.002601655	0.000590446	-0.222402266	-0.048948867	TBC1D22B, TMEM217
chr1	85742205	85742698	494	*	5	2.70575E-15	0.002365544	0.002604277	0.001119795	0.027236025	0.004709826	RP11-131L23.1, BCL10
chr13	95254690	95255258	569	*	2	1.07876E-12	0.001112269	0.002612864	0.001238	-0.190346319	-0.082497725	GPR180
chr1	206970717	206971646	930	*	4	4.24568E-17	0.000238362	0.002613618	9.42494E-05	-0.363860574	-0.124764594	
chr14	92324826	92325159	334	*	2	1.25286E-11	4.84025E-05	0.002615159	0.000106041	0.157630916	0.040347818	TC2N
chr2	40658891	40658918	28	*	2	5.73183E-10	0.000874188	0.002623196	0.001050945	-0.226262965	-0.1378293	SLC8A1
chr1	150939564	150939602	39	*	2	4.74198E-08	0.102585506	0.00262467	0.009049376	0.153029388	0.07543082	CERS2
chr2	169770161	169770254	94	*	2	7.22657E-09	0.100568137	0.002624828	0.009022427	-0.174635404	-0.090975367	
chr16	82096585	82096624	40	*	3	1.5172E-13	8.18445E-05	0.002625382	9.59649E-05	-0.193836678	-0.131082743	HSD17B2, RP11-510J16.5
chr2	239195537	239195602	66	*	2	2.07816E-08	0.009655351	0.002627877	0.00432396	-0.127264097	-0.074476439	PER2
chr12	133340907	133340965	59	*	2	4.11738E-12	6.3595E-05	0.002628042	0.000134422	0.118544782	0.090950265	
chr6	42891866	42892367	502	*	2	2.11264E-14	0.000128196	0.002630951	0.000242648	0.164447218	0.14263751	PTCRA
chr9	34374990	34375053	64	*	2	6.55428E-08	0.050236125	0.002632427	0.007859028	-0.18995149	-0.088028236	KIAA1161
chr12	54324491	54324895	405	*	5	8.33998E-11	0.010216775	0.002638224	0.004990027	0.065068437	0.026850645	
chr13	114912269	114912327	59	*	2	3.2015E-11	0.000818058	0.002639732	0.001005776	0.093290885	0.080983969	
chr3	123411782	123412055	274	*	3	2.42494E-12	0.015105484	0.002642961	0.000878449	0.155153248	0.098878836	MYLK
chr19	51728379	51728586	208	*	2	1.99948E-09	0.06223945	0.002643962	0.008294678	-0.056230371	-0.025215461	CD33
chr3	127473259	127473936	678	*	4	1.03577E-14	0.000273734	0.002643966	5.40498E-05	-0.275175391	-0.000937649	MGLL

chr7	97663876	97663940	65	*	3	3.59621E-12	6.06844E-05	0.002648465	0.000143003	0.202159631	0.160557274	
chr13	113832355	113832963	609	*	6	7.37949E-10	0.303896195	0.002650207	0.036731834	0.129042921	0.020215726	PCID2
chr7	101722855	101723947	1093	*	7	7.40005E-15	0.001411602	0.00265215	0.00087327	0.181110328	0.064062263	CUX1
chr14	24781881	24782128	248	*	4	1.67121E-10	0.095101085	0.002652645	0.020918216	0.116736963	0.024018338	LTB4R
chr2	135674925	135675644	720	*	4	7.01941E-18	0.000803857	0.002658632	0.000117335	-0.200835696	-0.088898008	CCNT2-AS1
chr9	136720870	136721646	777	*	3	1.84941E-11	0.000718732	0.002662736	0.000860268	-0.259646142	-0.119987612	VAV2
chr4	154462667	154462759	93	*	2	7.35363E-08	0.037929644	0.002663586	0.007347345	0.155997801	0.089587102	KIAA0922
chr15	41836440	41837601	1162	*	13	2.96669E-21	0.200248238	0.002664004	0.025555435	-0.228529794	-0.023050936	RPAP1
chr4	7800971	7801337	367	*	4	1.30519E-14	0.016498469	0.002665393	0.000529638	0.166218862	0.072598845	AFAP1
chr6	167060990	167062226	1237	*	4	2.14696E-11	5.29341E-05	0.002665895	5.64612E-05	0.206429508	0.116202364	RPS6KA2
chr16	85658239	85658855	617	*	3	2.0161E-11	0.012203767	0.00266688	0.002562406	0.171646945	0.073468988	GSE1
chr1	90228362	90229631	1270	*	9	4.74376E-21	0.002187367	0.002667208	0.000226799	0.235911445	0.026099668	LRRC8C, RP11-302M6.4
chr2	60618524	60619220	697	*	6	3.30368E-17	0.002168399	0.002667886	0.000265698	0.165045606	0.066458905	
chr3	71591429	71592784	1356	*	6	8.01784E-25	1.30032E-09	0.002671642	2.18694E-08	-0.335652801	-0.179838874	FOXP1
chr7	38356729	38357684	956	*	5	3.50662E-13	0.019415525	0.002673043	0.001112657	-0.135432703	-0.078562342	TRGV9
chr3	122281881	122282157	277	*	3	5.05835E-10	0.147422427	0.002673152	0.018231049	0.185991745	0.067654718	PARP9
chr2	74779799	74780617	819	*	9	8.78108E-25	0.000497813	0.002674756	3.7067E-05	0.287567751	0.088601755	DOK1, LOXL3
chr3	184052473	184052664	192	*	3	4.27417E-09	0.304495431	0.002675082	0.023483218	0.126703648	0.03897023	EIF2B5, EIF4G1
chr22	37309130	37309658	529	*	3	8.7752E-09	0.507489859	0.002675285	0.021751354	-0.140906256	-0.041928495	
chr1	203255843	203256204	362	*	4	6.65727E-11	0.067901591	0.002675627	0.012780311	-0.346647465	-0.106834236	
chr20	30618874	30619846	973	*	10	2.64045E-23	0.007923277	0.002676168	8.96275E-05	-0.262238828	-0.064760827	CCM2L, RP1-310O13.7
chr16	84400620	84401247	628	*	3	3.99373E-12	0.033650075	0.002677089	0.004245036	-0.157966708	-0.086431836	
chr12	56425731	56425944	214	*	2	2.73585E-08	0.004334396	0.002677812	0.002903596	-0.310968535	-0.171354491	IKZF4, RP11-603J24.4
chr4	154387984	154388535	552	*	4	1.34883E-15	0.000932627	0.00267958	0.000538907	-0.163224988	-0.039837046	KIAA0922
chr16	69762026	69762563	538	*	3	8.262E-09	0.339588834	0.002681149	0.020021382	-0.285077479	-0.096684927	CTD-2033A16.3
chr16	88972466	88973351	886	*	6	1.58079E-19	0.000421458	0.002685379	6.97356E-05	0.261180182	0.095308104	CBFA2T3
chr12	132899234	132899503	270	*	2	1.09295E-09	0.003215518	0.002685602	0.002453167	0.172751127	0.119705626	GALNT9
chr20	34193349	34193408	60	*	2	2.29355E-08	0.145433338	0.002688696	0.009656371	0.106084913	0.054381455	FER1L4
chr1	211757109	211757434	326	*	3	8.91463E-14	0.002194942	0.002694045	0.000379544	-0.231843849	-0.135875306	

chr11	134103727	134104882	1156	*	6	2.94738E-20	0.002366319	0.002695231	0.0001716	0.186166687	0.056716603	VPS26B
chr3	197675996	197676032	37	*	2	8.69572E-12	0.000721488	0.002695731	0.000927659	0.172738126	0.108105448	IQCG
chr22	24915037	24916084	1048	*	5	4.2756E-16	0.068073441	0.002695859	0.001370125	-0.234066676	-0.082358616	UPB1
chr1	110345967	110346588	622	*	2	8.7573E-11	4.87247E-05	0.002698495	0.000107241	0.163675807	0.154552308	
chr8	126448033	126448338	306	*	2	1.35483E-09	0.014833949	0.002698709	0.005314616	-0.245609444	-0.150049276	TRIB1
chr11	2532782	2532836	55	*	2	1.1574E-08	0.001432778	0.002709942	0.001489331	-0.297951743	-0.195151879	KCNQ1
chr9	116792216	116793036	821	*	4	4.01711E-16	2.49366E-05	0.002712386	4.20434E-05	0.180051767	0.125783755	ZNF618
chr20	49201542	49202225	684	*	3	8.56687E-15	0.002703184	0.00271387	0.000331251	-0.256243389	-0.131063121	
chr5	133585406	133585491	86	*	2	3.5892E-08	0.0036695	0.002719569	0.002665348	-0.278376477	-0.124378415	CDKL3, CTD-2410N18.4
chr5	126853924	126853954	31	*	2	2.0879E-11	0.008650632	0.002719683	0.004191978	0.026180712	0.017977429	PRRC1
chr2	2686097	2686793	697	*	2	9.08124E-11	0.000441335	0.002722744	0.000649585	0.15826628	0.114874879	
chr9	111175602	111175675	74	*	2	1.466E-07	0.014279892	0.00272759	0.005267044	-0.241178217	-0.137640645	
chr19	12997418	12998927	1510	*	9	3.30175E-38	4.93573E-11	0.00273149	2.07138E-10	0.196184505	0.128261951	KLF1
chr21	27512686	27514071	1386	*	7	2.61572E-16	0.001386943	0.002733596	0.000172347	-0.24889273	-0.087206352	APP
chr1	246588503	246588723	221	*	2	2.75076E-12	5.45678E-05	0.002733873	0.00011863	0.174356104	0.161037765	SMYD3
chr6	41253757	41255090	1334	*	9	1.07647E-26	0.000181319	0.002742141	7.09081E-06	-0.316755251	-0.121676271	TREM1
chr1	179333987	179334176	190	*	2	1.21644E-08	0.019428445	0.002745055	0.005987806	-0.208171336	-0.108696346	
chr18	72067689	72067761	73	*	2	1.77061E-12	5.82801E-05	0.002749719	0.000125733	-0.13993445	-0.139806074	
chr6	37504404	37505024	621	*	4	7.39564E-10	0.047970239	0.002750043	0.009770537	0.101493015	0.006366348	
chr6	27247586	27247663	78	*	2	3.68376E-08	0.021703652	0.002751861	0.006253273	0.029885445	0.02839639	
chr1	160066344	160066587	244	*	2	7.71848E-11	0.000323265	0.002759312	0.000514712	-0.122902072	-0.094185011	IGSF8
chr13	99964340	99964882	543	*	4	2.02132E-16	0.000419174	0.002760194	6.99393E-05	-0.172810733	-0.101692558	UBAC2
chr17	73265381	73267556	2176	*	13	2.85437E-18	0.00729431	0.00276096	0.000462586	-0.322782214	-0.021687716	RP11-649A18.12, MIF4GD
chr3	15382789	15383715	927	*	5	5.41691E-17	0.003063829	0.002763949	0.000530064	0.161460393	0.050675856	SH3BP5
chr10	32627475	32627605	131	*	2	4.01112E-11	7.3849E-05	0.00276782	0.000154361	-0.262772281	-0.071178013	EPC1
chr17	47468118	47468455	338	*	2	7.11054E-09	0.01252133	0.002769001	0.005025878	-0.189329429	-0.09043428	RP11-81K2.1
chr12	53902690	53903067	378	*	4	2.23275E-15	1.89461E-05	0.002770563	3.88326E-05	-0.297814072	-0.092771082	RP11-793H13.10, ATF7
chr19	7413482	7414062	581	*	6	6.60933E-19	2.40226E-05	0.002771154	4.68058E-05	-0.291969279	-0.095639671	CTB-133G6.1
chr8	23396373	23396887	515	*	3	2.37372E-14	1.03731E-05	0.002772002	3.22576E-05	-0.277864235	-0.201633369	SLC25A37

chr1	167570711	167570890	180	*	2	3.61248E-12	4.92814E-05	0.00277336	0.000108821	-0.266216222	-0.233131489	
chr20	35093928	35094679	752	*	3	5.18562E-12	0.001172919	0.002775089	0.000399012	0.138718125	0.08558696	DLGAP4
chr17	62408907	62409584	678	*	2	8.71348E-11	5.42037E-05	0.002777508	0.000118274	-0.245800567	-0.174724846	
chr7	2115808	2117171	1364	*	7	3.78013E-28	5.5232E-05	0.002777571	7.04392E-07	-0.30763439	-0.123107846	MAD1L1
chr15	63233142	63233164	23	*	2	1.287E-11	4.52569E-05	0.002782881	0.000101052	0.149937612	0.148765738	
chr17	18853473	18853737	265	*	2	1.6914E-10	0.005908588	0.002783084	0.003506776	0.167613424	0.074873233	SLC5A10
chr6	34245268	34245341	74	*	2	5.41424E-10	0.000389127	0.00278405	0.000594996	0.171765323	0.136823281	
chr18	74124270	74124445	176	*	2	1.0618E-08	0.006164038	0.002789744	0.003590215	-0.146629427	-0.080776078	ZNF516
chr8	142131465	142131725	261	*	2	1.4196E-11	0.000100875	0.002789894	0.00020145	-0.176233608	-0.167544814	DENND3
chr13	50216117	50216366	250	*	2	1.91258E-08	0.005517907	0.002793189	0.003388957	-0.165724024	-0.100876419	
chr14	24804750	24805389	640	*	3	2.88938E-14	0.000608775	0.002798054	0.00059473	-0.190861932	-0.100246157	RP11-934B9.3, RIPK3
chr10	34062018	34062234	217	*	3	5.25174E-10	0.011243859	0.002802622	0.004370541	-0.075925741	-0.042629548	
chr11	60634051	60634092	42	*	2	1.5234E-08	0.012345954	0.00280268	0.005033333	0.11702723	0.068687316	
chr10	6245036	6245271	236	*	4	3.53689E-10	0.041887937	0.002804249	0.012144609	0.029431822	0.010491434	PFKFB3
chr20	57581903	57583709	1807	*	26	1.71508E-72	1.08729E-09	0.002804463	1.75019E-12	-0.339184352	-0.067183844	CTSZ
chr9	130116144	130116418	275	*	2	5.64283E-10	0.002847312	0.00280478	0.002333337	-0.208372423	-0.070497398	GARNL3
chr14	91853682	91853728	47	*	2	4.72912E-09	0.003570234	0.002805725	0.002666678	0.126811128	0.07254499	CCDC88C
chr20	39800368	39801027	660	*	3	3.63961E-09	0.035643322	0.002808754	0.007222045	0.164685591	0.050002214	PLCG1
chr17	45337985	45338019	35	*	2	2.21637E-09	0.00068944	0.002808822	0.000911844	0.148902476	0.129251745	ITGB3, ITGB3
chr5	158381069	158381268	200	*	2	1.75313E-08	0.004147219	0.002810206	0.002907259	-0.148044279	-0.125664946	EBF1
chr2	36922916	36924820	1905	*	5	1.28074E-14	5.51001E-07	0.002811099	1.12141E-06	-0.218892681	-0.079651628	VIT
chr1	165823725	165823772	48	*	2	1.08873E-09	0.000320927	0.002812886	0.000514861	-0.226693285	-0.147773959	UCK2
chr4	2942362	2942425	64	*	2	2.37818E-09	0.006090568	0.002813283	0.003584929	0.1946867	0.121021293	NOP14-AS1, NOP14
chr1	6978618	6978798	181	*	2	7.62552E-11	9.87922E-05	0.002816225	0.000198346	-0.124624852	-0.123719501	CAMTA1
chr6	30045041	30045280	240	*	3	2.04153E-13	0.002923167	0.00281641	0.0007314	0.159537683	0.082263081	
chr11	129723947	129723987	41	*	2	7.0188E-13	4.76952E-05	0.00281674	0.000106027	-0.133554803	-0.112707066	TMEM45B
chr12	25103173	25103643	471	*	3	1.66982E-15	6.06513E-05	0.002817769	0.000135264	0.164243865	0.101758311	RP11-662I13.2
chr19	44304559	44304770	212	*	3	7.85932E-10	0.004525979	0.002818539	0.002502025	0.126242034	0.079560384	LYPD5
chr8	24242024	24242109	86	*	2	8.18832E-08	0.06107615	0.002819184	0.008682279	-0.197096896	-0.103957599	ADAMDEC1, RP11-624C23.1

chr1	249157502	249157575	74	*	2	4.03915E-10	0.000197661	0.002826722	0.00035161	-0.057474195	-0.020616668	AL672294.1
chr3	194844174	194844358	185	*	2	5.78592E-08	0.044363226	0.002827185	0.008028747	0.120660835	0.065839418	XXYLT1
chr22	19919974	19920275	302	*	3	4.77854E-09	0.123050302	0.002831034	0.015035402	-0.226004837	-0.082423127	TXNRD2
chr14	57274550	57275282	733	*	8	1.80105E-12	0.142243547	0.002833258	0.018171211	0.079652289	0.022398283	OTX2
chr10	80757927	80758294	368	*	2	4.16534E-09	0.001529312	0.002834006	0.001585547	-0.169457652	-0.110384716	ZMIZ1-AS1
chr2	43440068	43440321	254	*	2	6.73028E-10	0.000368552	0.002836052	0.000574214	-0.145742039	-0.120706616	THADA
chr11	68846972	68847839	868	*	3	9.49373E-13	0.002126749	0.00283849	0.001465072	-0.241170356	-0.052533586	TPCN2
chr11	70173450	70173985	536	*	4	7.29295E-16	0.003258035	0.00283868	0.000333452	-0.197808333	-0.071267148	PPFIA1, AP000487.6
chr6	29601705	29602390	686	*	3	5.35356E-13	0.004231773	0.002839318	0.002691754	-0.190596355	-0.019869976	GABBR1
chr7	535789	535820	32	*	2	5.66855E-08	0.010057607	0.002839332	0.004622526	0.155019615	0.106216836	
chr2	60579767	60581381	1615	*	6	3.34963E-12	0.063384686	0.00284189	0.002609665	-0.349034292	-0.075026404	AC007381.3
chr1	19248952	19249872	921	*	3	5.07865E-11	0.000760966	0.002842112	0.000492819	-0.230756417	-0.117986081	IFFO2
chr17	25659609	25659820	212	*	3	8.5545E-09	0.030081379	0.002842263	0.009257183	-0.097412124	-0.031501721	
chr20	19954588	19955868	1281	*	5	6.89692E-16	1.89585E-05	0.002844006	2.83375E-06	-0.274815416	-0.157557871	RIN2
chr14	22889758	22891383	1626	*	6	5.42901E-16	2.01154E-06	0.002847358	2.09257E-06	-0.270546256	-0.134140684	TRDV2, AE000661.37
chr19	38791235	38791472	238	*	2	4.41368E-11	8.40247E-05	0.002848154	0.000173334	0.117892398	0.091436031	CTB-102L5.4
chr2	219246070	219247055	986	*	10	3.49247E-29	0.000518507	0.002849136	4.09662E-06	-0.245939852	-0.077132294	SLC11A1
chr6	7468364	7469052	689	*	6	8.50975E-15	0.000570663	0.002849863	0.000309495	0.162676281	0.085721	
chr20	3761916	3762520	605	*	8	1.05607E-11	0.043446388	0.002854034	0.017720253	-0.090103326	-0.016180884	SPEF1
chr14	59813888	59814285	398	*	3	1.23046E-10	0.00217328	0.002863377	0.00201038	-0.307601944	-0.124753561	DAAM1
chr2	105924245	105925965	1721	*	7	1.36869E-16	6.26236E-06	0.002868707	8.40031E-06	-0.21247371	-0.015631468	TGFBRAP1
chr17	70693956	70694204	249	*	2	1.04633E-10	0.000133708	0.002869489	0.000256488	0.149546087	0.128830829	SLC39A11
chr14	23762832	23763073	242	*	3	1.21904E-09	0.002275568	0.00287039	0.001998175	0.162237021	0.048125312	RP11-124D2.7, HOMEZ
chr20	47846707	47847532	826	*	2	1.54454E-09	8.5027E-05	0.002874017	0.000175415	-0.112961003	-0.007118232	DDX27
chr2	175545838	175547747	1910	*	10	2.20479E-16	6.72126E-05	0.002877835	1.94743E-05	-0.344813713	-0.042987388	AC018890.6, WIPF1
chr3	11651536	11651990	455	*	4	2.69286E-15	0.00022104	0.002881588	5.85746E-05	0.121048224	0.065195456	VGLL4
chr15	39874413	39874776	364	*	2	1.91189E-14	5.43127E-05	0.00288582	0.000119278	0.120686592	0.096522275	THBS1
chr17	40463425	40464318	894	*	4	8.90067E-16	2.8593E-05	0.002887362	6.61967E-05	-0.226594269	-0.106761727	STAT5A
chr13	78090644	78090688	45	*	2	2.89224E-11	6.04737E-05	0.002893962	0.000131035	-0.261758879	-0.243455194	

chr12	27856714	27856865	152	*	2	2.94903E-08	0.113298786	0.00289502	0.009948473	-0.162084789	-0.077850104	RP11-1060J15.4
chr16	89785686	89787000	1315	*	9	8.27151E-21	0.003638454	0.002895263	0.000264025	-0.109908494	-0.015073099	ZNF276, VPS9D1
chr17	1637068	1637391	324	*	4	4.9337E-10	0.010603545	0.002895924	0.005170328	0.034255224	0.016155087	WDR81, RP11-961A15.1
chr11	124709084	124709245	162	*	3	6.19332E-10	0.047258476	0.002896888	0.006995238	0.050774383	0.024072339	
chr1	161183762	161184528	767	*	5	4.60551E-18	0.004517167	0.002909078	0.000187323	-0.34422256	-0.147441268	NDUFS2
chr11	2542580	2543051	472	*	4	3.6465E-09	0.269204461	0.002913197	0.028045579	-0.236749182	-0.057943074	KCNQ1
chr4	114262093	114262363	271	*	2	4.52785E-09	0.014280645	0.002913735	0.005497616	0.13662821	0.053452182	ANK2
chr8	128755270	128755294	25	*	2	1.6474E-11	4.83122E-05	0.00291466	0.000107841	-0.1902202	-0.133243783	
chr11	71956145	71957134	990	*	6	4.10692E-20	3.65737E-06	0.002917167	1.57273E-05	0.116012996	0.08604347	PHOX2A
chr4	185781167	185781248	82	*	2	7.7982E-11	9.08981E-05	0.002919108	0.000186257	-0.275120248	-0.180500104	
chr22	24488507	24489132	626	*	3	6.86449E-13	0.002191807	0.002920013	0.000469125	0.138118206	0.087036066	CABIN1
chr6	108395545	108395859	315	*	2	9.54996E-11	0.007602211	0.00292027	0.00410428	0.029784522	0.013091061	OSTM1
chr14	25064779	25065130	352	*	3	8.67512E-11	0.034311517	0.002921371	0.00154526	-0.186479079	-0.093653905	RP11-104E19.1
chr15	83736925	83736960	36	*	2	3.20996E-11	0.038909516	0.002922994	0.007928154	0.182518358	0.080134964	RP11-382A20.5, RP11-382A20.6
chr4	10022984	10023201	218	*	4	1.99741E-13	7.22811E-05	0.002923083	0.0001597	-0.177150922	-0.078284489	SLC2A9
chr14	23266481	23266959	479	*	3	1.72612E-10	0.015733376	0.002924864	0.00250863	-0.293513327	-0.069666214	SLC7A7
chr2	239358563	239358907	345	*	3	1.06714E-10	0.014054472	0.002925097	0.001893698	-0.189280103	-0.099956547	ASB1
chr3	170019329	170019826	498	*	3	1.1041E-10	0.006024292	0.00292593	0.001619656	-0.275790762	-0.129846206	PRKCI
chr11	45070638	45070822	185	*	3	3.22603E-13	2.07283E-05	0.002927744	5.98443E-05	0.16696594	0.131532214	
chr17	48456071	48456169	99	*	2	5.0003E-12	5.73826E-05	0.002929623	0.000125464	0.138756805	0.138306157	EME1, LRRC59
chr1	246271686	246271874	189	*	3	5.37352E-10	0.001216483	0.002931531	0.001179608	0.113898815	0.075678341	SMYD3
chr17	16189554	16190567	1014	*	5	8.86399E-19	2.94559E-05	0.002939407	1.09894E-05	0.165943133	0.097283039	PIGL
chr3	171094025	171094370	346	*	2	1.49631E-11	4.90949E-05	0.002941443	0.000109539	-0.143811396	-0.105113082	TNIK
chr19	10399805	10401361	1557	*	9	5.45654E-14	0.014419105	0.002943249	0.004960062	-0.065638853	-0.023306882	ICAM5
chr10	134621538	134622602	1065	*	5	1.36535E-15	0.000255123	0.00294374	0.000260827	0.179986888	0.084988125	TTC40
chr1	168194826	168195398	573	*	11	1.88968E-13	0.495259604	0.002944582	0.068367364	0.203528109	0.011732958	SFT2D2
chr2	232256981	232257271	291	*	2	2.21337E-08	0.134853803	0.002946982	0.010331447	-0.266155834	-0.137459897	AC017104.6
chr20	3804771	3805186	416	*	2	3.68979E-10	0.000387666	0.002950682	0.000604409	0.11032593	0.078258441	AP5S1
chr17	9939895	9940970	1076	*	7	1.59248E-24	7.03622E-06	0.002952412	2.23036E-06	-0.29687629	-0.13743748	GAS7

chr13	28674885	28675510	626	*	6	4.99576E-13	0.017296859	0.002954264	0.006819344	-0.217396448	-0.056012411	
chr16	85334190	85334289	100	*	2	2.40686E-08	0.009402093	0.00295449	0.004587336	0.122286386	0.052106255	
chr12	7066563	7066938	376	*	5	8.65736E-16	0.003343125	0.002954814	0.000192337	0.195877382	0.08516894	PTPN6
chr1	236027847	236028011	165	*	2	1.65934E-11	6.87431E-05	0.002954823	0.000146972	-0.236919164	-0.19903631	LYST
chr19	45250754	45251551	798	*	5	1.87796E-12	0.054748872	0.002958171	0.009045651	-0.061742588	0.003442779	BCL3
chr7	96745340	96746356	1017	*	5	5.98223E-16	0.013750019	0.002958311	0.000894836	-0.148144518	0.001282055	ACN9
chr15	29232842	29233610	769	*	3	8.67158E-11	0.000324721	0.002959778	0.000338894	0.211137878	0.103354269	APBA2
chr17	47480679	47481492	814	*	5	1.28668E-14	0.000849548	0.002961974	0.000395247	-0.244479858	-0.078942377	RP11-81K2.1, RP11-1079K10.4, PHB
chr9	123688715	123689193	479	*	4	7.76404E-17	4.35467E-05	0.00296316	3.45611E-05	-0.33204493	-0.202974269	TRAF1
chr6	139794360	139795527	1168	*	5	7.2484E-16	2.95101E-06	0.002963407	1.08703E-06	-0.155602391	-0.095676843	
chr1	212587188	212588848	1661	*	14	4.28556E-19	0.04658377	0.002968013	0.004433933	-0.048590866	-0.00459845	TMEM206
chr19	58918743	58919549	807	*	4	2.12379E-17	1.51524E-05	0.002970245	2.00507E-05	0.188363053	0.096448371	ZNF584, CTD-2619J13.14
chr2	3522506	3523935	1430	*	12	1.16859E-18	0.014228531	0.002970446	0.004522604	-0.046416646	0.002716665	AC142528.1, ADI1
chr1	110302332	110302560	229	*	5	1.54743E-14	0.005647213	0.002977663	0.000506628	-0.138106748	-0.040058568	GSTM5, RP4-735C1.4, EPS8L3
chr2	11917623	11917787	165	*	2	3.63964E-08	0.184234521	0.00297876	0.010763113	-0.181991115	-0.088465506	LPIN1
chr17	4875559	4876237	679	*	5	1.93096E-14	0.016528898	0.002979337	0.000893794	0.160152157	0.067592684	RP5-1050D4.2, CAMTA2
chr5	1128038	1128391	354	*	3	1.15121E-10	0.008601574	0.002981971	0.001491799	0.133041199	0.085697238	
chr14	92507291	92507800	510	*	4	1.97798E-14	0.083033217	0.002986833	0.001341892	0.144406868	0.058825762	
chr20	62669102	62670570	1469	*	9	5.11569E-22	1.13357E-06	0.002986961	3.47632E-06	-0.084132034	-0.009939616	LINC00176, ZNF512B
chr10	115478900	115479248	349	*	3	2.95285E-10	0.001827524	0.002990434	0.001749036	-0.154557929	-0.061623325	CASP7, RP11-211N11.5
chr22	47081903	47082260	358	*	3	6.56461E-10	0.176150497	0.002991446	0.016872727	-0.318855852	-0.124062312	CERK
chr19	47287778	47288915	1138	*	8	8.11077E-22	0.000187898	0.002991796	1.48088E-05	-0.060452409	-0.016554891	SLC1A5
chr2	216948982	216949469	488	*	2	9.48492E-10	0.000586143	0.002994822	0.000828933	0.115755245	0.099518434	TMEM169
chr20	5159145	5159437	293	*	3	8.71715E-08	0.118282206	0.002996013	0.018170289	0.169926963	0.049798042	CDS2
chr13	47471264	47472429	1166	*	14	2.0203E-41	6.68395E-11	0.002998135	3.81021E-10	0.152517045	0.08985598	
chr10	98405486	98406291	806	*	2	1.52632E-09	5.12608E-05	0.003007422	0.000114189	-0.20988332	-0.201284705	PIK3AP1
chr16	56390024	56390429	406	*	2	6.78421E-11	0.003490082	0.003008789	0.002728031	0.191583394	0.083475263	GNAO1
chr11	134132696	134132814	119	*	2	3.47857E-10	0.000175865	0.003009907	0.000325077	0.168870219	0.144109422	ACAD8
chr16	30545962	30546843	882	*	14	1.1601E-14	0.342920893	0.003011417	0.051369718	0.034002972	0.005640643	AC002310.12, ZNF747, ZNF747, AC002310.13

chr1	212412767	212413038	272	*	2	3.433E-09	0.000817205	0.003015583	0.001057851	0.194927222	0.15618583	
chr1	45007988	45009401	1414	*	4	4.67834E-14	2.27199E-08	0.003020747	2.20961E-07	0.147598353	-0.041273519	RNF220
chr16	68734216	68734735	520	*	2	2.38759E-10	0.000194531	0.003020896	0.000353211	-0.24273917	-0.069047258	CDH3
chr20	30195969	30196714	746	*	5	5.72357E-18	0.000293162	0.003021572	0.00012589	-0.179063448	-0.059097085	
chr6	44197180	44197198	19	*	2	1.3994E-07	0.02376531	0.003023423	0.006898883	-0.118319036	-0.069484812	SLC29A1
chr5	169409132	169409235	104	*	2	5.92701E-08	0.019256662	0.003026131	0.006373	0.136586915	0.090286858	DOCK2
chr15	45248086	45248932	847	*	7	8.80955E-12	0.137394419	0.003027404	0.020799726	0.104248805	0.021921341	C15orf43
chr18	72920877	72921969	1093	*	10	1.59256E-13	0.165526991	0.003029466	0.028066468	0.041572354	0.008163706	ZADH2
chr4	140770576	140770596	21	*	2	6.46782E-11	0.000146731	0.003031355	0.000280607	0.190100505	0.174178054	MAML3
chr10	14598044	14598289	246	*	4	1.38778E-10	0.228556638	0.003032465	0.035877533	-0.244563734	-0.047139399	FAM107B
chr7	127761751	127762091	341	*	2	2.76488E-09	0.000761144	0.003037862	0.001008066	-0.152172998	-0.134053413	
chr17	77966750	77967813	1064	*	7	1.09244E-24	3.94224E-05	0.003038551	7.93144E-06	-0.235157299	-0.098966905	TBC1D16
chr1	231799060	231799423	364	*	2	2.99492E-11	5.45755E-05	0.003044019	0.000120881	-0.205025364	-0.190689079	TSNAX-DISC1, DISC1
chr5	56718310	56718809	500	*	4	9.30883E-14	0.08480056	0.003045626	0.000950288	0.103149012	0.009879894	CTD-2023N9.1, SALL4P1
chr15	70432922	70434018	1097	*	3	1.06045E-09	0.000156562	0.00305161	0.000154428	-0.267969713	-0.153484916	
chr11	1319648	1321213	1566	*	10	1.15279E-24	2.92534E-06	0.003054954	1.28804E-06	-0.18655082	-0.005926325	TOLLIP
chr5	179194913	179196144	1232	*	3	2.4427E-14	1.27272E-06	0.003056004	5.82862E-06	-0.214559343	-0.182788402	MAML1
chr19	54368958	54369311	354	*	5	1.44948E-12	0.010494766	0.003059195	0.004608323	-0.134376935	-0.04123737	AC008440.10, AC008753.6
chr17	1614268	1614953	686	*	2	2.25166E-12	0.00219639	0.003061545	0.002073993	0.146447056	0.052804633	MIR22HG
chr1	46915458	46916419	962	*	5	2.22362E-12	0.006648276	0.003065536	0.000762381	0.128090363	0.057170152	
chr14	50506033	50507529	1497	*	8	4.28809E-21	0.003889887	0.003066563	0.000404419	-0.360147575	-0.081963691	RP11-58E21.3, RP11-58E21.5
chr22	40341342	40343102	1761	*	6	2.89933E-13	0.000706315	0.003067104	3.30668E-05	0.156043725	0.055554524	GRAP2
chr7	50626272	50626298	27	*	2	4.12904E-11	6.76924E-05	0.003067614	0.000146029	0.18659028	0.169686037	DDC
chr5	442385	443220	836	*	9	7.79961E-14	0.096409	0.003069828	0.031071314	0.054355328	0.00896684	C5orf55
chr1	182760143	182761262	1120	*	4	2.50711E-14	1.8596E-05	0.003071851	6.43448E-05	0.127770599	0.099836476	NPL
chr1	36946448	36949518	3071	*	10	1.64805E-16	5.34313E-05	0.003074545	3.46713E-06	-0.291060947	-0.091010599	CSF3R
chr1	203187208	203187773	566	*	3	1.06564E-10	0.014240747	0.003078701	0.001142578	-0.216310413	-0.051262999	CHIT1
chr17	48619037	48619308	272	*	2	4.47016E-08	0.031025549	0.003091912	0.007691386	-0.081865578	-0.028461332	EPN3
chr16	85095535	85097151	1617	*	11	3.10193E-21	4.05404E-05	0.003097177	1.83772E-06	0.183174281	0.064262878	KIAA0513

chr17	8222055	8222359	305	*	5	1.53514E-10	0.011617849	0.003097196	0.00486309	0.17955663	0.060990796	ARHGEF15
chr17	38490228	38490520	293	*	2	7.80882E-10	0.000361881	0.003097468	0.000581919	-0.232090496	-0.140094863	RARA
chr15	93579369	93580846	1478	*	9	7.59368E-34	4.71631E-11	0.003099766	4.11436E-10	-0.250065751	-0.133483948	
chr21	34395548	34396221	674	*	3	2.49491E-10	0.015245972	0.003099816	0.006950295	0.056635542	0.017498561	AP000282.2
chr19	15122135	15122224	90	*	3	2.64886E-12	0.001212792	0.00310116	0.001272416	0.050965805	0.03654958	CCDC105, SLC1A6
chr18	2845398	2848065	2668	*	15	4.52649E-24	5.37879E-06	0.003103837	8.39728E-07	0.148543724	0.020088561	EMILIN2
chr2	234359654	234359783	130	*	3	6.9423E-11	0.002365252	0.003105707	0.001297926	-0.190116335	-0.118517317	DGKD
chr12	109059679	109059806	128	*	2	1.06023E-08	0.003044238	0.003105935	0.00255533	-0.312713485	-0.169302932	CORO1C
chr11	65559109	65559280	172	*	3	3.67624E-14	0.000836094	0.00310755	0.000412263	0.108919327	0.068255885	OVOL1
chr11	94276072	94279068	2997	*	21	1.15181E-55	8.44334E-13	0.003110893	2.25442E-14	-0.276717667	-0.093746889	PIWIL4, FUT4, RP11-867G2.8
chr2	99871738	99872952	1215	*	5	1.35758E-19	0.001622766	0.003115219	7.00498E-05	-0.314124225	-0.091418297	C2orf15, LYG2
chr3	5239311	5239540	230	*	2	5.07807E-12	5.4841E-05	0.003116893	0.000121879	-0.213888044	-0.171714847	EDEM1
chr5	179207291	179207533	243	*	3	2.01346E-09	0.006771275	0.003117249	0.002757192	-0.276208082	-0.113907126	MAML1
chr5	78203170	78203311	142	*	2	3.14092E-08	0.011593647	0.003118495	0.005232116	-0.220257215	-0.122067759	ARSB
chr5	1386150	1386887	738	*	6	9.23597E-11	0.787055549	0.003119842	0.051480505	0.035874474	0.010116018	
chr17	78747934	78748494	561	*	6	3.76159E-21	2.53912E-05	0.003120096	7.9073E-06	-0.310626568	-0.131415626	RPTOR
chr3	149191167	149191624	458	*	4	1.71548E-12	0.073426548	0.003124391	0.003713576	-0.180125211	-0.08471051	
chr1	220964827	220965460	634	*	3	1.70634E-13	0.013352649	0.00312663	0.0003849	-0.146797865	-0.094569989	MARC1
chr19	1854549	1854819	271	*	4	1.31645E-17	6.98748E-06	0.003130406	1.03726E-05	0.151189987	0.074335506	KLF16
chr9	92041302	92042058	757	*	2	6.41595E-10	0.000259372	0.003133894	0.000449487	0.102083107	0.075309916	SEMA4D
chr13	28931547	28932217	671	*	3	1.93807E-12	0.01041395	0.003133909	0.000804896	0.200020996	0.106730997	FLT1
chr2	85933069	85933851	783	*	3	2.33966E-12	7.94362E-05	0.003135024	0.000141872	-0.199912387	-0.066499026	
chr16	84627507	84629008	1502	*	8	4.08605E-15	0.000111046	0.003137813	2.60469E-05	-0.185279477	0.005176513	RP11-61F12.1, COTL1
chr5	180301107	180301165	59	*	2	9.17125E-11	9.10038E-05	0.003137917	0.000189194	-0.151984565	-0.105129739	
chr7	4727742	4727998	257	*	2	1.32976E-11	0.000110628	0.003138727	0.000223339	0.177208854	0.129819617	FOXK1
chr6	109611667	109611834	168	*	2	2.15646E-09	0.00399464	0.003140477	0.003017053	-0.222390693	-0.146602687	PTCHD3P3
chr17	76408337	76409193	857	*	3	6.05356E-11	0.004181802	0.003142927	0.000476437	-0.260153816	-0.16281911	PGS1
chr17	78261378	78261792	415	*	2	1.34128E-09	0.000993352	0.003153101	0.001235702	-0.144730095	-0.134178873	RNF213
chr11	6629445	6629459	15	*	2	2.63297E-12	0.000116312	0.003159398	0.000233308	0.217924391	0.143585558	ILK, TAF10

chr11	126275374	126276079	706	*	6	1.36037E-15	0.007688213	0.003160511	0.001227392	-0.155761001	-0.041217727	ST3GAL4
chr3	50275112	50276265	1154	*	8	5.68465E-20	0.000212807	0.003168543	0.000170754	-0.270343382	-0.040833134	GNAI2
chr7	27200671	27202797	2127	*	7	2.6959E-11	0.027569942	0.003168563	0.002186749	-0.139287097	-0.023991849	HOXA9
chr9	137809628	137810490	863	*	5	2.60597E-13	0.007624592	0.003172297	0.000550502	-0.273046046	-0.119734402	FCN1
chr22	36011405	36012221	817	*	5	2.0386E-09	0.309104685	0.003173057	0.044704951	-0.183750022	-0.029192322	MB
chr20	20248523	20248696	174	*	3	1.321E-08	0.010730336	0.003178062	0.004986062	0.130949549	0.06664837	C20orf26
chr8	144213544	144213766	223	*	2	6.69402E-09	0.117301358	0.003181946	0.010800751	-0.283864023	-0.143733949	
chr1	44430285	44430318	34	*	2	3.38365E-09	0.001313823	0.003181981	0.001504973	-0.181262153	-0.133878622	IPO13
chr12	6442329	6443282	954	*	9	5.27896E-21	0.002890982	0.00318739	0.000208788	-0.224095361	-0.05738965	TNFRSF1A
chr14	39643268	39644110	843	*	4	3.68061E-14	0.030415487	0.003189791	0.009630668	0.154067593	0.033841599	RP11-407N17.4
chr2	36780691	36780705	15	*	3	4.57917E-12	0.000118463	0.003190814	0.000218584	-0.19295841	-0.153421871	FEZ2
chr5	10353763	10354563	801	*	6	1.22368E-12	0.244179165	0.003191982	0.032810051	0.03761846	0.003576932	MARCH6
chr16	3559384	3559696	313	*	4	2.6081E-13	0.003868895	0.003192383	0.000534526	-0.197053348	-0.072408969	LA16c-306E5.3, CLUAP1
chr2	134883195	134884342	1148	*	3	2.13595E-10	3.48578E-05	0.003193903	7.56137E-05	-0.270718619	-0.038057796	MGAT5
chr3	11623526	11624533	1008	*	7	3.54777E-16	0.045929673	0.003193908	0.001710917	-0.170243147	-0.050593693	VGLL4
chr12	69021282	69022158	877	*	4	2.60597E-13	2.64725E-06	0.003194717	1.05841E-05	0.178571831	0.029493822	RAP1B
chr7	2158094	2158623	530	*	4	5.55103E-12	0.000906014	0.003195274	0.001050052	0.123942654	0.080856223	MAD1L1
chr2	240387066	240387439	374	*	3	6.02311E-10	0.002018187	0.00319593	0.00194994	-0.280725734	-0.093195733	
chr17	80251443	80251697	255	*	5	2.00179E-12	0.006427999	0.003198802	0.002357787	0.135353439	0.053200848	RP13-516M14.1
chr11	119232198	119232263	66	*	2	4.48857E-08	0.010502913	0.003198923	0.005075144	0.216350943	0.132872492	USP2
chr1	8271918	8272277	360	*	5	4.6863E-13	0.007876568	0.003203202	0.001491355	-0.226842156	-0.076645052	RP11-431K24.3
chr14	62547769	62548443	675	*	5	1.1134E-11	0.004737166	0.003206632	0.002310484	0.096324546	0.039700722	SYT16, RP11-355I22.5
chr9	101469203	101469285	83	*	2	1.34979E-08	0.010221284	0.003206677	0.005017338	0.096857047	0.05497229	GABBR2
chr8	131325625	131326139	515	*	2	2.50009E-10	0.000169574	0.003208071	0.000320507	-0.087542024	-0.086971274	ASAP1
chr14	91544928	91544974	47	*	2	1.58352E-10	0.000111491	0.003208934	0.000225867	0.130231218	0.127774558	C14orf159
chr4	2932265	2932447	183	*	2	7.06562E-08	0.030942532	0.003210603	0.007893183	-0.241238838	-0.11073982	MFSD10
chr17	17616093	17616235	143	*	2	1.99934E-08	0.003167209	0.003211883	0.002660884	-0.145137214	-0.085059648	RAI1
chr2	85730887	85731028	142	*	2	3.69578E-08	0.003941533	0.003211951	0.003028797	0.138638498	0.081974779	
chr3	128838350	128838576	227	*	2	4.09405E-10	0.000437947	0.003212905	0.000681654	-0.227000381	-0.13791544	RAB43, ISY1-RAB43

chr1	43854136	43855122	987	*	5	5.60071E-18	0.095017309	0.003216154	0.001892045	-0.237683594	-0.089916307	RP1-92O14.6, MED8
chr19	10340639	10340858	220	*	3	1.02654E-09	0.003034753	0.003222548	0.002585633	0.18464375	0.095224947	DNMT1, S1PR2
chr16	88597301	88599035	1735	*	8	6.39612E-15	5.6576E-05	0.003227599	6.66337E-05	0.20862663	0.079694072	ZFPM1, RP11-21B21.4
chr1	154978318	154979211	894	*	3	2.48359E-12	0.004608353	0.003230547	0.000373901	-0.189040492	-0.099927904	ZBTB7B
chr18	72187462	72187476	15	*	2	3.65509E-11	6.08463E-05	0.003234572	0.000134328	0.17727986	0.14838811	CNDP2
chr17	3824384	3824599	216	*	3	2.07147E-14	6.89445E-06	0.003238724	2.26068E-05	0.130804952	0.097978487	
chr11	77530668	77532226	1559	*	15	1.05203E-25	0.005506853	0.003245366	0.00135724	-0.08717082	-0.01285506	AAMDC, RSF1
chr12	117480333	117480973	641	*	2	3.34015E-10	8.18907E-05	0.003250307	0.000174002	0.221846808	0.191882464	TESC
chr16	88536306	88538355	2050	*	8	9.79757E-31	7.7484E-12	0.003254559	3.78659E-10	0.152651333	0.105376235	ZFPM1
chr2	70213021	70213304	284	*	2	2.43972E-10	0.000156785	0.003254745	0.00030144	-0.217077204	-0.181126918	PCBP1-AS1
chr2	208014177	208014670	494	*	3	3.27482E-10	0.001692843	0.003257663	0.001553298	0.165370142	0.102467257	KLF7
chr19	11691153	11691168	16	*	2	2.14422E-12	8.92839E-05	0.003259439	0.000187525	-0.173721017	-0.166256887	ZNF627
chr1	36906600	36907439	840	*	5	3.11218E-15	0.000202366	0.003259595	0.000145974	-0.134660593	-0.058202307	OSCP1
chr3	43821497	43821635	139	*	2	6.8955E-10	0.000446587	0.003263496	0.000695348	0.213676591	0.148476791	
chr1	158110575	158110665	91	*	3	8.38348E-10	0.00215457	0.003270825	0.002109667	0.180942304	0.088941703	
chr14	50810773	50810863	91	*	2	1.3903E-11	8.13637E-05	0.003273225	0.000173265	0.111672721	0.091898114	CDKL1
chr19	55173997	55174624	628	*	6	3.43105E-15	0.005675898	0.00327568	0.000617274	0.144462516	0.054967281	LILRB4
chr1	184899584	184899589	6	*	2	7.43511E-08	0.003553413	0.003276533	0.002878954	-0.153170269	-0.117009394	FAM129A
chr4	105827658	105828553	896	*	5	4.39603E-19	1.41019E-07	0.003281565	8.43666E-07	0.167394178	0.112500998	RP11-556I14.1, RP11-556I14.2
chr6	31846769	31847633	865	*	9	1.39483E-20	0.000866344	0.003284563	0.000142195	0.175356369	0.070860605	SLC44A4, EHMT2
chr2	231089641	231091055	1415	*	9	2.14755E-23	0.000349359	0.003285126	2.14512E-05	-0.211827716	-0.046474041	SP140, SP110
chr3	141144475	141145231	757	*	4	4.19975E-13	0.049872161	0.003286146	0.001725794	-0.250688738	-0.084501104	ZBTB38
chr10	87676015	87676527	513	*	3	3.4219E-08	0.03896134	0.003287712	0.01093838	0.148419052	0.041325342	GRID1
chr5	177915909	177916440	532	*	2	3.36171E-10	0.000409378	0.003293514	0.000652279	-0.361693035	-0.249556573	COL23A1
chr1	870791	871980	1190	*	8	8.60076E-17	0.001030534	0.003298194	0.000615459	0.156209096	0.110661593	SAMD11
chr14	58905592	58905740	149	*	3	1.14179E-15	1.76097E-06	0.003301407	7.82421E-06	-0.237243175	-0.208111721	KIAA0586
chr2	228678005	228678693	689	*	6	1.51989E-15	0.040616919	0.003302257	0.0008254	-0.255201085	-0.094559103	CCL20
chr3	128778575	128780916	2342	*	15	6.38562E-28	1.60063E-08	0.003305604	2.09295E-08	0.248162938	0.058739608	GP9
chr12	129294939	129295649	711	*	4	1.10339E-14	6.17368E-05	0.003307385	0.000110047	0.177126442	0.085153018	SLC15A4

chr12	118797525	118797962	438	*	4	1.81564E-11	0.00689429	0.003308578	0.001795475	-0.189874385	-0.081405874	TAOK3
chr3	127329123	127329458	336	*	2	2.55618E-09	0.000669593	0.003316184	0.000947647	-0.229682039	-0.160811659	MCM2
chr8	21541227	21541551	325	*	3	6.80031E-10	0.034479022	0.003316591	0.002715121	-0.126344828	-0.065832423	
chr16	53088123	53089351	1229	*	12	3.64019E-17	0.002853895	0.003319548	0.002270746	-0.069516065	-0.000637898	CHD9
chr20	1294814	1294858	45	*	2	5.9942E-08	0.018164458	0.003333795	0.006638808	-0.160076258	-0.065541757	SDCBP2
chr1	21896305	21896750	446	*	2	2.04094E-12	6.30674E-05	0.003338222	0.000139352	0.149566556	0.14735372	ALPL
chr16	66442374	66442791	418	*	2	1.58797E-11	9.74253E-05	0.003339063	0.000203035	0.23895818	0.173727815	LINC00920
chr7	5523576	5523691	116	*	3	1.28809E-09	0.003673473	0.003340339	0.001888834	-0.195416592	-0.104096968	FBXL18
chr20	62133588	62133626	39	*	3	2.41397E-11	0.000211367	0.0033459	0.00040776	0.137307134	0.099543778	
chr10	134544617	134545286	670	*	3	4.24782E-12	0.007563353	0.003348206	0.002273372	0.109074205	0.062458916	INPP5A
chr5	148533711	148533875	165	*	2	2.08561E-10	0.000127654	0.003348861	0.000255517	0.177794509	0.034188319	ABLIM3
chr10	63807168	63809170	2003	*	17	1.11719E-40	1.25447E-14	0.003352575	9.67774E-13	0.208018243	0.087946113	ARID5B
chr16	21168523	21171470	2948	*	20	1.15647E-33	2.02657E-05	0.003357309	9.03033E-07	-0.298961098	-0.042157179	TMEM159, DNAH3
chr22	24856594	24856877	284	*	2	3.24957E-08	0.001849249	0.003360668	0.001936285	0.195062538	0.116882541	ADORA2A-AS1
chr2	24715094	24715213	120	*	2	1.06811E-09	0.000799615	0.003362179	0.001084288	-0.045388138	-0.001906779	NCOA1
chr17	79297435	79298502	1068	*	5	8.52664E-19	6.63627E-07	0.003365407	1.71512E-06	-0.244846862	-0.159268179	TMEM105
chr1	226349343	226349364	22	*	2	1.32252E-08	0.001349631	0.003367125	0.001569031	-0.185495162	-0.120248009	ACBD3
chr4	2801717	2802612	896	*	3	2.621E-15	1.82373E-06	0.00337002	8.16086E-06	-0.221851123	-0.186552466	SH3BP2
chr2	75184528	75185645	1118	*	10	1.68822E-15	0.101373337	0.003370371	0.020423913	-0.263981982	-0.034086318	POLE4
chr20	44986386	44988023	1638	*	4	2.72856E-13	3.30051E-07	0.003373231	2.08023E-06	-0.149891756	-0.131647745	SLC35C2
chr13	113355060	113355356	297	*	3	1.43791E-08	0.07938824	0.00337651	0.01428801	0.149450248	0.059466594	ATP11A
chr6	36665240	36665620	381	*	7	1.49121E-20	1.30388E-06	0.003379034	5.67706E-06	-0.125395487	-0.07924868	
chr1	156549200	156549533	334	*	5	3.96937E-11	0.02721384	0.003381079	0.003923027	0.190270103	0.077999614	TTC24
chr1	57015477	57015717	241	*	2	3.49734E-08	0.003027898	0.003381925	0.002655478	0.151414547	0.096089469	PPAP2B
chr16	89363811	89364225	415	*	7	2.05703E-10	0.337237514	0.003383769	0.054460585	-0.154264143	-0.035898924	AC137932.5, ANKRD11
chr16	47048004	47048265	262	*	4	3.01765E-09	0.033856146	0.003387313	0.010764374	-0.135715931	-0.05624997	RP11-169E6.4, RP11-169E6.3
chr19	22700869	22701260	392	*	2	1.82224E-11	0.000123805	0.003387697	0.000249625	-0.163039041	-0.152063179	ZNF98
chr14	101194145	101194267	123	*	2	2.29802E-09	0.012222623	0.003394002	0.005649766	-0.249592624	-0.155951234	DLK1
chr19	823662	823841	180	*	2	1.04442E-12	6.48261E-05	0.003394485	0.000143151	-0.195722585	-0.167683729	

chr14	92979104	92979709	606	*	5	2.69447E-13	0.096565102	0.003395793	0.014446756	0.150684991	0.031117664	
chr17	73089282	73089425	144	*	2	4.91199E-09	0.002115788	0.003399643	0.00212463	-0.236134472	-0.098427204	SLC16A5
chr19	51875451	51876788	1338	*	6	5.70529E-13	0.01723555	0.003399976	0.000465109	-0.261200548	-0.090251278	NKG7
chr1	117544206	117544416	211	*	2	1.40911E-10	0.000234337	0.003402705	0.000423569	-0.235648058	-0.185242078	CD101
chr13	113242739	113243542	804	*	8	1.63294E-19	0.016365075	0.003405944	0.001294201	-0.244355749	-0.06796142	
chr11	72505386	72505607	222	*	2	3.62769E-10	0.023367321	0.003406808	0.007439844	-0.116243187	-0.06728081	
chr1	33799703	33800886	1184	*	3	2.36868E-10	1.37379E-05	0.003407406	4.17029E-05	-0.227571484	-0.110299795	PHC2
chr18	60645905	60646671	767	*	3	1.32791E-13	1.59855E-05	0.003408291	4.49501E-05	-0.305642713	-0.141301301	PHLPP1
chr8	144410701	144410972	272	*	3	2.61185E-11	0.013416406	0.003420595	0.001233654	-0.238707493	-0.126779162	TOP1MT
chr5	173293229	173294009	781	*	4	1.92268E-15	0.000140598	0.003422934	7.86306E-05	-0.190704317	-0.133741997	
chr17	32740467	32741121	655	*	3	2.48205E-11	0.002392772	0.003423552	0.000659682	0.16348633	0.097526723	
chr1	19549300	19550005	706	*	4	1.94774E-08	0.141778477	0.003424728	0.030398531	-0.097857548	-0.030279052	RP1-43E13.2, EMC1
chr7	124571116	124571363	248	*	3	1.02262E-08	0.053518415	0.003429239	0.00924615	0.155376639	0.076646674	POT1-AS1
chr10	11284548	11284602	55	*	2	1.34291E-08	0.000992287	0.003433	0.001275925	0.141172345	0.098036075	CELF2
chr17	4852013	4852795	783	*	7	1.11573E-15	0.001146113	0.003437172	0.000661897	-0.061679559	-0.00988053	ENO3, PFN1
chr3	133970647	133971155	509	*	3	2.57468E-09	0.142575102	0.003438762	0.021517363	0.152258602	0.053382434	
chr3	101406347	101406600	254	*	3	5.06319E-09	0.045180121	0.003439522	0.009237929	-0.315478528	-0.115252665	
chr17	42466567	42467307	741	*	4	8.33662E-13	0.000430086	0.003440393	0.00018644	0.18025693	0.019329446	ITGA2B
chr3	194897309	194897437	129	*	2	1.04642E-10	0.000403514	0.003443571	0.000653886	0.136022372	0.098952311	XXYLT1
chr3	12395554	12395914	361	*	2	2.53131E-10	0.000155049	0.003446004	0.000302625	-0.197058321	-0.129706607	PPARG
chr17	455014	455636	623	*	3	7.50397E-12	0.022872469	0.00345063	0.001144664	0.139784421	0.084340012	VPS53
chr17	78734663	78735596	934	*	7	1.13143E-16	2.6995E-06	0.003450797	6.15247E-06	-0.125924194	-0.082003381	RPTOR
chr3	42529630	42529667	38	*	2	6.94503E-08	0.038671712	0.003453739	0.00893928	0.128476705	0.070746595	
chr3	11266965	11267627	663	*	8	1.15353E-19	0.007068968	0.003453927	0.00048308	-0.329015503	-0.089831873	HRH1
chr17	73056681	73056797	117	*	2	6.06586E-11	0.00119932	0.003463002	0.001462768	-0.136372796	-0.096263073	KCTD2, RP11-1112G13.3
chr4	1729045	1729116	72	*	2	4.98977E-10	0.000450545	0.003463162	0.000713252	-0.109231896	-0.079943146	TACC3
chr2	137068792	137068818	27	*	2	4.1779E-09	0.000493048	0.003463747	0.000764281	-0.148858207	-0.136294999	
chr6	136847105	136847410	306	*	4	3.03776E-10	0.029633147	0.003465477	0.008750698	-0.11673897	-0.052148072	MAP7
chr5	1514077	1514608	532	*	4	4.17281E-14	0.001746181	0.003466051	0.000503402	-0.242621198	-0.108030029	LPCAT1

chr12	93854885	93854963	79	*	2	5.50607E-11	6.79461E-05	0.003466485	0.000149693	-0.168741685	-0.149355029	
chr10	99188343	99188478	136	*	2	2.23813E-10	0.000150958	0.003466729	0.000296349	-0.218706126	-0.193353476	PGAM1
chr17	79459400	79459563	164	*	4	1.48467E-08	0.085905801	0.003467484	0.014573242	-0.488272719	-0.106622022	
chr15	100889886	100891814	1929	*	16	1.77749E-33	0.004468049	0.003467549	1.36636E-05	-0.293726104	-0.045924479	SPATA41
chr3	48228826	48230398	1573	*	12	5.4637E-22	0.003034781	0.003473343	0.000373977	0.071288223	0.015334515	CDC25A
chr20	44538288	44540278	1991	*	15	7.54773E-23	0.002369884	0.003476319	9.3864E-05	-0.227670725	-0.026898042	PLTP
chr5	14664248	14664662	415	*	6	2.54821E-12	0.006983152	0.003477435	0.005296414	-0.044807123	-0.009321763	CTD-2165H16.4
chr4	973154	973177	24	*	2	1.3179E-09	0.000279456	0.003483382	0.000491101	-0.235065997	-0.212391559	DGKQ, SLC26A1
chr19	51104400	51105087	688	*	4	1.75127E-11	0.003261582	0.003484594	0.001480791	0.208098965	0.083867209	
chr1	243447766	243448000	235	*	2	8.78161E-08	0.010851706	0.003490357	0.005434881	-0.104908252	-0.069431319	SDCCAG8
chr18	9474143	9474234	92	*	2	3.4396E-09	0.014210957	0.00349184	0.006157842	-0.18068021	-0.101582172	
chr2	207999044	207999734	691	*	5	6.16654E-16	0.028638389	0.003495127	0.00175191	-0.223161463	-0.080508916	KLF7
chr15	64748093	64748145	53	*	2	2.0782E-08	0.001369136	0.003496396	0.001609158	-0.103415913	-0.101551015	
chr5	149887008	149887787	780	*	7	7.62963E-18	0.000478316	0.003499969	0.000148876	-0.121233601	-0.057964631	NDST1
chr6	32050905	32051108	204	*	5	6.29733E-14	0.031164062	0.003501843	0.001906867	0.100397847	0.054511956	TNXB
chr4	1075561	1076636	1076	*	6	2.42838E-14	0.00078159	0.003504378	0.000626768	-0.130322267	-0.058360846	RNF212
chr11	69294736	69295162	427	*	5	4.07422E-12	0.007438358	0.003510067	0.001620097	0.209264958	0.065023168	
chr5	66597184	66597522	339	*	2	3.64703E-11	0.000162643	0.003512766	0.000316304	-0.173276446	-0.130070517	
chr22	27017087	27017910	824	*	7	7.76606E-23	5.7441E-05	0.00351413	1.09602E-05	0.154388103	0.082633616	
chr1	160765919	160765923	5	*	2	1.57031E-11	0.000181665	0.003516202	0.00034677	0.17104087	0.14715204	LY9
chr6	44039484	44039689	206	*	2	2.99409E-08	0.004977376	0.003517359	0.003630825	0.141164597	0.097196407	RP5-1120P11.1
chr22	31668350	31668786	437	*	3	1.42245E-08	0.018388628	0.003517586	0.005929374	-0.315774267	-0.105169389	LIMK2
chr17	3863976	3864217	242	*	2	6.61793E-09	0.000834794	0.003517772	0.001137243	-0.137516808	-0.110265881	ATP2A3
chr12	109553359	109554634	1276	*	8	3.16353E-17	0.031636921	0.003518023	0.001106901	-0.229345633	-0.066557554	RP11-968O1.5, ACACB
chr15	29405983	29406153	171	*	5	5.85136E-09	0.240542294	0.003522603	0.022861903	0.135096888	0.028394377	APBA2
chr20	31099338	31099384	47	*	2	2.13654E-08	0.290107934	0.003524075	0.012772203	-0.189018779	-0.094664394	C20orf112
chr1	115628940	115629264	325	*	2	1.52468E-08	0.003641899	0.003524877	0.00303043	0.192344257	0.106604518	TSPAN2
chr6	131455796	131456001	206	*	2	8.41258E-08	0.100957689	0.003528393	0.011465201	0.13960477	0.068646353	
chr1	147245168	147246154	987	*	6	1.75908E-19	0.00035081	0.003530439	3.85644E-05	0.103991371	0.048405434	GJA5

chr1	209847618	209849006	1389	*	12	8.46554E-16	0.13974188	0.003532805	0.012555897	-0.153855156	-0.011595518	G0S2, RP1-28O10.1
chr17	27048223	27048708	486	*	4	1.14344E-12	0.032903446	0.003534802	0.007703359	-0.233923754	-0.040069498	RPL23A, AC010761.8
chr1	181056127	181056912	786	*	2	1.49298E-10	0.000169761	0.00354192	0.000328396	-0.31810405	-0.207535577	
chr11	60930188	60930380	193	*	2	5.77236E-11	0.001863329	0.003542333	0.001991332	-0.292199948	-0.172698941	
chr10	17496239	17496989	751	*	5	9.56727E-10	0.082801069	0.003542336	0.019113692	-0.056564749	-0.018386063	ST8SIA6
chr1	24053350	24053982	633	*	2	6.94701E-10	7.14341E-05	0.003550006	0.000157025	-0.24472648	-0.204939122	
chr12	81332684	81332744	61	*	2	1.1683E-07	0.018981536	0.00355047	0.007045435	-0.181110858	-0.105031799	ACSS3
chr3	101707435	101707603	169	*	2	1.71474E-08	0.001279795	0.003555365	0.001546408	-0.185017486	-0.110016609	RP11-221J22.1, RP11-221J22.2
chr2	47206681	47206965	285	*	2	1.95583E-11	0.00012287	0.003555537	0.000250564	-0.117411763	-0.097175996	TTC7A
chr5	108166523	108166624	102	*	2	8.91622E-10	0.000226182	0.003561706	0.000416407	0.100808373	0.083488644	FER
chr3	142839022	142840240	1219	*	7	1.17395E-17	0.00022051	0.003562118	0.000160964	0.050543299	0.026086913	CHST2
chr15	89920887	89921845	959	*	12	2.69313E-13	0.128931065	0.003565098	0.055135352	0.164308334	0.026946014	LINC00925
chr19	45866874	45867461	588	*	6	1.97127E-10	0.073441735	0.003565192	0.018156111	0.116956628	0.017945318	ERCC2
chr17	1463484	1464048	565	*	3	1.01092E-12	0.000288584	0.003568565	0.000343796	0.156272331	0.109151397	PITPNA
chr3	52260090	52260965	876	*	8	3.94684E-20	6.30447E-05	0.003569463	4.95752E-05	-0.206152346	-0.063806631	TLR9, TLR9
chr10	7221504	7221713	210	*	4	1.56729E-11	0.000367364	0.003572369	0.000640854	0.13977218	0.095642488	SFMBT2
chr12	25204113	25204249	137	*	2	4.78799E-08	0.005829466	0.003577295	0.004001904	0.116373957	0.044354266	LRMP
chr3	58200021	58200599	579	*	4	9.12359E-12	0.005189177	0.003581455	0.002214844	-0.314891729	-0.103708105	DNASE1L3
chr1	161087200	161088678	1479	*	17	1.36529E-19	0.065361309	0.00358769	0.016054947	0.049546724	0.008097083	NIT1, PFDN2
chr9	139439761	139439826	66	*	2	6.55314E-10	0.002689826	0.003589131	0.002538387	0.018511747	0.01772793	NOTCH1
chr19	46095477	46095562	86	*	2	6.9962E-09	0.006585886	0.003594376	0.004288013	-0.165335893	-0.101955456	OPA3, GPR4
chr7	156836773	156837580	808	*	4	1.3429E-11	0.01970239	0.003595782	0.000985224	-0.21759428	-0.013542392	
chr17	3906640	3906858	219	*	3	4.59018E-10	0.027759942	0.003596428	0.007104404	0.165772634	0.063058511	
chr8	99437999	99438016	18	*	2	1.07302E-10	0.000728463	0.003596923	0.001037455	-0.180801288	-0.117164576	STK3
chr15	74212306	74212896	591	*	3	1.27384E-10	0.000312527	0.003597039	0.000503219	-0.351637069	-0.151926932	LOXL1-AS1
chr11	71259251	71259881	631	*	5	9.39947E-13	0.027606663	0.003597945	0.002330108	-0.21204612	-0.057013971	KRTAP5-9
chr9	139652617	139653003	387	*	5	3.58588E-14	0.001000167	0.003605565	0.000655129	0.163605069	0.066875691	LCN8
chr11	121460778	121461566	789	*	5	1.7213E-13	0.001972861	0.003614191	0.000963557	-0.146747819	-0.064329506	SORL1
chr15	45473810	45474074	265	*	3	4.53305E-11	0.000306124	0.003614194	0.000552937	-0.165871798	-0.121971283	RP11-519G16.2, SHF

chr14	60671024	60671041	18	*	2	2.16468E-09	0.000508571	0.003618226	0.000793516	-0.197062421	-0.155749479	
chr5	137577858	137578003	146	*	3	2.06189E-09	0.002865427	0.003621366	0.001984435	0.04116325	0.012044374	
chr19	55838021	55838191	171	*	2	1.09604E-11	0.000244192	0.003628115	0.000445265	-0.206362069	-0.159904084	TMEM150B
chr15	77285359	77287906	2548	*	13	6.23529E-16	0.00124765	0.003628794	6.95903E-06	-0.414214366	-0.09465274	PSTPIP1
chr11	128392102	128393063	962	*	7	2.84716E-12	0.016840801	0.003629556	0.009740921	-0.026292192	-0.004304844	ETS1
chr2	218937172	218937635	464	*	5	3.79503E-15	0.000763327	0.003638424	0.000539107	-0.222230903	-0.053202935	RUFY4
chr5	140044315	140044696	382	*	4	7.83276E-13	0.002053745	0.003641525	0.001126351	-0.027624795	-0.000717391	WDR55
chr12	117131523	117131988	466	*	4	4.02907E-11	0.005095131	0.003643026	0.002306658	0.127081822	0.070040001	RP11-497G19.1
chr1	151634388	151634697	310	*	5	5.86846E-17	8.77975E-05	0.003648573	2.75924E-05	-0.274258308	-0.138567544	SNX27
chr2	28939458	28940400	943	*	2	3.98953E-08	7.93056E-05	0.003652061	0.000172861	-0.232055245	-0.168774968	AC097724.3
chr11	67069814	67071064	1251	*	13	2.99975E-26	0.00410225	0.003655973	0.000211446	-0.16648789	-0.042619057	ANKRD13D, SSH3
chr3	181421548	181421703	156	*	2	3.06688E-08	0.042037656	0.003660049	0.009566549	0.057647928	0.044717788	SOX2-OT
chr14	69438267	69438358	92	*	2	7.89184E-11	7.6989E-05	0.003660519	0.00016852	0.162971496	0.142613987	ACTN1
chr12	133020942	133021135	194	*	2	2.08202E-08	0.020481227	0.003663514	0.007418601	0.197572579	0.103945667	
chr16	30062955	30063148	194	*	3	1.04766E-12	0.000607237	0.003664273	0.000328291	0.21215209	0.112401542	FAM57B
chr10	126390317	126390659	343	*	2	1.47682E-09	0.007529818	0.00366637	0.004655944	-0.148031877	-0.06018922	RP11-12J10.3, FAM53B
chr11	75901632	75902270	639	*	3	5.41593E-11	0.000230024	0.003667774	0.000299879	0.096201153	-0.016297066	RP11-619A14.2, WNT11
chr1	160545145	160546979	1835	*	4	2.6348E-09	2.36015E-05	0.003670039	4.17515E-05	0.145982501	0.07790163	CD84
chr1	116371802	116371871	70	*	2	1.15272E-08	0.047828511	0.003673693	0.009964956	-0.136675674	-0.075007139	
chr22	38730793	38731297	505	*	3	4.27658E-11	0.001408049	0.003674373	0.001154163	-0.166302049	-0.08428746	CSNK1E
chr14	24563095	24564438	1344	*	16	2.90166E-24	0.059405113	0.003676359	0.0044443	-0.03775199	-0.005309954	PCK2, NRL
chr2	51256078	51256146	69	*	2	3.45281E-11	0.000414193	0.003680033	0.000680742	-0.180528069	-0.149613654	NRXN1
chr1	145433592	145433673	82	*	2	5.89777E-08	0.002408571	0.003682497	0.002394646	-0.211790654	-0.125571115	
chr7	47554427	47554571	145	*	2	2.40977E-08	0.056491128	0.003684154	0.010451787	-0.256547717	-0.130977854	TNS3
chr19	40336878	40337541	664	*	8	9.61241E-14	0.043279827	0.003687765	0.015210874	0.144756152	0.020704978	FBL
chr14	75765352	75766014	663	*	4	9.58318E-17	5.41458E-06	0.003695734	1.03404E-05	-0.312272425	-0.189608864	
chr2	10588232	10589956	1725	*	13	6.36302E-26	0.015387345	0.003700296	0.000114802	0.163742692	0.021361795	RP11-320M2.1, ODC1
chr10	135101975	135102171	197	*	2	3.36221E-08	0.02848478	0.00370088	0.008472587	-0.044467561	-0.026674101	TUBGCP2
chr11	1783832	1784413	582	*	2	1.00518E-09	0.020482938	0.003703958	0.007472907	-0.117062604	-0.075291559	CTSD, AC068580.5

chr16	28160523	28161100	578	*	2	4.9571E-10	0.000132253	0.003708289	0.000269033	-0.181567305	-0.114747427	XPO6
chr8	6735510	6735628	119	*	2	2.82303E-10	0.000382493	0.003708382	0.000641061	-0.228765442	-0.168579812	GS1-24F4.2, DEFB1
chr6	31660252	31661178	927	*	5	5.83686E-17	0.000100928	0.003713236	0.000111469	0.172249567	0.088423836	ABHD16A, XXbac-BPG32J3.20
chr1	155271423	155271762	340	*	2	2.8007E-11	0.000711019	0.003713704	0.001030316	-0.173713775	-0.046278396	
chr1	54852036	54852233	198	*	2	9.12586E-08	0.027149385	0.003718964	0.008354014	0.13285133	0.075225025	SSBP3
chr5	34042521	34044058	1538	*	9	1.09244E-24	1.73369E-05	0.00372051	8.99799E-07	-0.15918963	-0.058373363	RP11-1084J3.4, C1QTNF3
chr3	156530417	156530658	242	*	3	9.95448E-08	0.042859254	0.00372103	0.012972567	-0.100515474	-0.06193037	LINC00886
chr9	138697196	138697959	764	*	2	1.24895E-09	7.79837E-05	0.003723951	0.000170925	-0.212490781	-0.036549746	
chr13	111838017	111839335	1319	*	8	1.05619E-20	3.87368E-05	0.003725323	2.1788E-05	0.174746895	0.053459733	ARHGEF7
chr2	239197607	239197782	176	*	2	2.46217E-08	0.00189	0.003725386	0.002054524	0.033631582	0.022164576	PER2
chr12	133336962	133337116	155	*	4	5.3599E-09	0.070883131	0.003728974	0.009846746	0.164830213	0.078631835	ANKLE2
chr1	179050298	179051893	1596	*	17	3.39998E-13	0.616753872	0.003731738	0.044713931	0.034934094	0.001208496	TOR3A
chr19	3831806	3831859	54	*	2	1.24913E-07	0.101799817	0.003732045	0.012009035	0.200137116	0.096973595	ZFR2
chr7	75507337	75508496	1160	*	12	2.79574E-14	0.595756201	0.003733684	0.061575115	0.064264571	0.009411321	RHBDD2
chr5	118741167	118741229	63	*	2	1.54657E-08	0.0009452	0.003734077	0.001271645	0.094530146	0.086359286	
chr5	176966237	176966387	151	*	2	1.84083E-08	0.005696664	0.00373523	0.004044263	-0.3589327	-0.1482218	FAM193B
chr4	128701638	128704651	3014	*	14	1.53946E-11	0.031385704	0.003735245	0.000240119	-0.242281572	-0.028263535	HSPA4L
chr22	38484884	38485757	874	*	5	5.05893E-11	0.01903744	0.003735937	0.011357226	0.086361827	0.053880281	BAIAP2L2
chr13	77030645	77030775	131	*	2	2.44317E-09	0.000541605	0.003737018	0.000841165	0.113333443	0.111707167	
chr22	45608345	45610231	1887	*	15	3.18684E-36	0.008861313	0.003748048	2.37218E-05	-0.278354972	-0.031384525	KIAA0930
chr1	16840801	16840816	16	*	2	1.21691E-09	0.000240587	0.003749643	0.00044361	0.063572316	0.054471139	
chr10	82296191	82296516	326	*	2	1.40981E-09	0.012765153	0.003754881	0.006135147	-0.159392508	-0.099417922	
chr16	72041376	72042695	1320	*	10	1.1866E-16	0.392040225	0.003756702	0.015257033	-0.426829212	-0.044877438	DHODH
chr20	30001044	30001697	654	*	3	6.09253E-10	0.052381628	0.003757008	0.014027091	0.200995777	0.057541182	
chr19	35485361	35485586	226	*	5	2.05627E-11	0.090841624	0.003763743	0.003913067	-0.133114298	-0.054464674	
chr4	40464575	40464664	90	*	3	7.93457E-11	0.00026393	0.003768526	0.000394606	-0.252038186	-0.139626677	RBM47
chr19	49954994	49955555	562	*	5	1.81047E-15	0.003372173	0.003770448	0.002710961	0.095090884	0.026297366	PIH1D1
chr15	58624344	58624534	191	*	2	1.65917E-08	0.086989882	0.003772015	0.01175848	-0.050787117	-0.030110303	ALDH1A2
chr16	3630187	3631153	967	*	5	4.1199E-14	0.004495295	0.003774857	0.002025417	-0.208719584	-0.058794614	

chr8	38830814	38832335	1522	*	12	3.91255E-17	0.005791573	0.003775306	0.001111159	-0.17657747	-0.056542506	PLEKHA2, HTRA4
chr8	17014337	17014845	509	*	3	5.88535E-13	8.13965E-05	0.003779711	0.000159645	-0.054820516	0.001135376	ZDHHC2
chr5	168218017	168218392	376	*	2	5.39624E-10	0.003587375	0.00378615	0.003111118	-0.175293591	-0.126958897	SLIT3
chr2	26243148	26243460	313	*	2	3.01078E-09	0.000526873	0.003790822	0.000827356	0.130477161	0.010814459	
chr16	30751795	30751899	105	*	2	2.47849E-08	0.007870186	0.003796071	0.004859783	0.118544167	0.03691208	SRCAP
chr6	36724036	36725742	1707	*	5	1.26938E-13	0.000110886	0.003800145	2.56129E-05	0.203048004	0.096072466	CPNE5
chr20	35972070	35972536	467	*	4	1.37309E-12	0.001225784	0.003814508	0.001006305	0.130993285	0.066644174	
chr19	39217856	39218661	806	*	6	2.04797E-11	0.162784835	0.003817331	0.017350781	0.106661987	0.042298158	ACTN4
chr17	43306622	43306802	181	*	2	1.09422E-10	8.38719E-05	0.003827706	0.000182996	-0.179538948	-0.170632798	FMNL1
chr4	8242386	8242745	360	*	3	1.23798E-11	0.008931641	0.003831844	0.000601033	-0.084788233	-0.058334091	SH3TC1
chr7	99102011	99103043	1033	*	16	1.06849E-17	0.007695414	0.003835829	0.010885235	-0.041811257	-0.009957732	ZKSCAN5
chr13	111317935	111318640	706	*	5	1.86873E-13	0.000109336	0.003840103	0.000168861	-0.25481615	-0.100810325	CARS2
chr11	66046770	66047229	460	*	2	4.71211E-15	0.000307618	0.003842019	0.000544598	0.113427862	0.110244229	CNIH2
chr3	185543470	185544216	747	*	5	6.31336E-16	0.00144162	0.003842217	0.000174744	0.130258938	0.00974389	
chr5	71776744	71776849	106	*	2	4.6499E-11	8.60232E-05	0.003844781	0.000187223	-0.14966774	-0.14432398	ZNF366
chr8	29939956	29941333	1378	*	12	1.51899E-17	0.051263861	0.003846953	0.004553724	0.049250067	0.003050357	RP11-489E7.4, TMEM66
chr16	87339553	87339877	325	*	4	2.40503E-16	3.58151E-06	0.00385995	1.15702E-05	0.174217116	0.132261857	C16orf95, RP11-178L8.4
chr12	120105885	120106292	408	*	4	3.77476E-09	0.070587782	0.003862038	0.019719877	-0.044132152	-0.00777475	PRKAB1
chr10	12648032	12648526	495	*	4	1.92779E-11	0.001158234	0.003865628	0.0007591	-0.185579744	-0.089881136	CAMK1D
chr8	23605543	23605601	59	*	3	4.27564E-09	0.001788673	0.003868919	0.001881782	0.047460731	0.043239514	RP11-175E9.1
chr5	180630287	180630434	148	*	2	9.40861E-08	0.188641238	0.003871736	0.013438925	-0.217606258	-0.109803623	TRIM7
chr14	102315629	102316107	479	*	2	8.07691E-10	0.000247287	0.003872073	0.000457335	0.181719048	0.050213909	PPP2R5C
chr17	3594952	3595341	390	*	3	9.51203E-09	0.073525211	0.003874206	0.00771898	-0.084114122	-0.010330478	P2RX5-TAX1BP3, P2RX5
chr16	31488852	31489209	358	*	3	2.77926E-11	0.00075287	0.003875049	0.000761036	-0.241840571	-0.113444194	TGFB1I1
chr4	88452108	88452259	152	*	3	4.95392E-13	0.000336801	0.00387551	0.000382867	-0.164043028	-0.015873949	SPARCL1
chr16	85568220	85569506	1287	*	5	2.05847E-12	1.69838E-05	0.003877694	5.55001E-06	0.148413652	0.004402605	
chr19	18315067	18315256	190	*	2	1.1912E-07	0.001095473	0.003882836	0.001434396	-0.102399966	-0.061357293	
chr17	78755379	78755841	463	*	5	3.14883E-20	0.000228353	0.003883617	4.92242E-05	-0.175059429	-0.070901239	RPTOR
chr4	75858510	75858589	80	*	2	1.47955E-08	0.032919278	0.003883729	0.009220282	0.012059601	0.00878647	PARM1

chr3	194906043	194906452	410	*	3	1.10607E-11	0.001266223	0.003887582	0.000789973	-0.282517054	-0.144302677	XXYLT1
chr10	5938186	5938657	472	*	2	1.38377E-09	0.000402467	0.003900565	0.000677061	0.169257667	0.122387524	FBXO18
chr10	103826480	103826705	226	*	2	4.23545E-08	0.009201554	0.003906928	0.005355639	0.11639628	0.051314781	HPS6
chr2	241501875	241502462	588	*	2	1.45239E-11	0.000146482	0.003912731	0.000296654	0.142733898	0.118480011	DUSP28, ANKMY1
chr2	48795994	48796213	220	*	3	3.98513E-09	0.013083343	0.003917505	0.004461549	-0.164959362	-0.077742715	STON1, STON1-GTF2A1L
chr7	36700744	36701016	273	*	4	1.60095E-09	0.00741488	0.00391811	0.00363081	-0.152800642	-0.047553487	AOAH
chr11	67266128	67266176	49	*	2	9.95545E-11	8.60102E-05	0.003921858	0.000187849	0.15311142	0.120053229	PITPNM1
chr8	134310304	134310718	415	*	4	3.68575E-14	0.001423003	0.003921862	0.000534083	-0.137707504	-0.070158506	NDRG1
chr16	85676292	85676861	570	*	5	2.51481E-16	0.000240485	0.003926753	7.99746E-05	0.200953583	0.088706845	GSE1
chr6	18155101	18155916	816	*	9	3.98747E-16	0.001409401	0.00394163	0.000862897	-0.103339748	-0.025270192	KDM1B, TPMT
chr19	45999794	46000819	1026	*	9	6.97016E-13	0.050162673	0.003948969	0.010219399	-0.091244124	-0.017320853	PPM1N, RTN2
chr13	114871422	114872102	681	*	4	1.28546E-14	0.000404759	0.003949483	0.00023771	0.167928553	0.105153368	RASA3
chr1	116653093	116653561	469	*	3	1.04441E-13	0.005816388	0.003950857	0.000524792	-0.179355944	-0.09230878	
chr2	138721303	138721868	566	*	8	1.22767E-11	0.166198055	0.003951084	0.018252761	0.245216356	0.046790789	HNMT
chr10	106088702	106089307	606	*	6	6.35986E-22	3.93383E-06	0.003952588	1.43343E-06	-0.20252448	-0.067517382	ITPRIP
chr14	88459216	88461489	2274	*	19	5.06763E-27	0.00848266	0.003952743	2.28819E-05	-0.390772581	-0.041319693	GALC
chr10	11191659	11191891	233	*	3	2.2059E-10	0.009847423	0.003959745	0.002130306	-0.131612688	-0.077677286	CELF2
chr5	176931315	176931422	108	*	3	8.2808E-08	0.085225145	0.003963302	0.016160429	-0.073822375	-0.030224273	DOK3
chr2	64863301	64864645	1345	*	6	8.01316E-19	9.23864E-08	0.003965142	6.9847E-07	-0.173548071	-0.096725379	SERTAD2
chr16	88539861	88540992	1132	*	7	6.5205E-16	1.35746E-05	0.003966617	4.08123E-05	0.24311488	0.125910404	ZFPM1
chr15	40802888	40803733	846	*	4	2.74926E-10	0.015767586	0.003966782	0.006650618	-0.23040703	-0.074127658	RP11-111A22.1
chr2	145188144	145189636	1493	*	3	1.49638E-10	7.26215E-06	0.003967035	2.61231E-05	0.190068125	0.136469832	ZEB2
chr10	116286411	116286911	501	*	6	2.50385E-11	0.03965288	0.003970028	0.008927847	-0.039956192	-0.010504086	ABLIM1
chr17	46712052	46712086	35	*	2	7.87417E-12	0.000170549	0.003972769	0.000338314	0.2124597	0.187665106	HOXB-AS4
chr17	74639731	74640078	348	*	6	7.6599E-21	5.16057E-06	0.003973801	2.41085E-06	0.18292951	0.108745301	ST6GALNAC1
chr2	242699863	242699892	30	*	2	1.89754E-08	0.006649011	0.003974308	0.004554974	-0.17372284	-0.096110231	D2HGDH
chr13	28371000	28371547	548	*	4	4.36473E-11	0.004137936	0.003975269	0.002750992	0.074699934	0.03991321	
chr8	21776623	21777941	1319	*	10	3.22195E-15	0.03129419	0.003976047	0.003425672	-0.082121687	-0.018553867	XPO7
chr13	114828264	114829833	1570	*	6	1.13874E-11	0.001563306	0.003988739	0.000464101	-0.244550379	-0.079003049	RASA3

chr3	46131480	46131862	383	*	2	5.00575E-11	0.000136484	0.003990095	0.00028052	-0.235799778	-0.067655332	
chr17	79824551	79825218	668	*	3	1.17158E-13	5.30673E-05	0.003995984	0.000106601	0.135069838	-0.021279345	
chr2	68348223	68348450	228	*	2	1.49732E-08	0.002141769	0.003998046	0.002301237	0.103205569	0.088139678	
chr10	130338213	130339475	1263	*	11	9.123E-15	0.259862626	0.003998107	0.036878474	-0.052725987	0.009401297	
chr4	6781565	6782128	564	*	3	4.67667E-12	0.000810385	0.003998691	0.000718767	-0.130426356	0.007112167	
chr7	55621099	55621712	614	*	5	6.25067E-14	0.01351159	0.003999341	0.006273056	0.137547104	0.05592347	VOPP1
chr5	77882104	77882454	351	*	3	6.45008E-11	0.034782043	0.004005681	0.001126479	-0.151728043	-0.090426628	LHFPL2
chr4	183728479	183729461	983	*	6	4.61381E-12	0.011042969	0.004011455	0.006051185	-0.18647503	-0.087113301	
chr8	11720161	11720363	203	*	3	3.06247E-10	0.001253933	0.004017225	0.001334353	-0.066009685	-0.064362741	CTSB
chr11	36432567	36433057	491	*	2	7.8564E-11	0.000467507	0.004022503	0.000768201	-0.14819404	-0.12708016	PRR5L
chr15	52030001	52031467	1467	*	9	1.26824E-26	6.84826E-06	0.004023701	3.76178E-06	0.17950159	0.056831715	LYSMD2
chr20	62085851	62086790	940	*	4	3.15231E-13	1.98895E-05	0.004024519	6.40222E-05	0.128437917	0.106060111	KCNQ2
chr1	32739049	32739493	445	*	2	9.99482E-11	0.12481923	0.004025403	0.01319586	0.088170264	0.041082179	LCK
chr2	102798651	102798966	316	*	4	5.48819E-11	0.007888429	0.004028254	0.001922823	-0.054156335	-0.017462997	
chr22	44575455	44576268	814	*	4	7.10815E-10	0.015762703	0.004031882	0.002626051	-0.189014446	-0.08343858	PARVG
chr2	234261725	234262993	1269	*	12	5.53555E-19	0.009409338	0.004033267	0.002361741	0.151054977	0.023096189	RP11-400N9.1
chr10	73472315	73472531	217	*	3	1.90246E-09	0.008860435	0.004033676	0.005290452	-0.144053024	-0.077211785	CDH23, C10orf105
chr10	11943616	11944468	853	*	3	1.01131E-10	7.08252E-05	0.004034139	0.000123849	0.213859045	0.13210539	
chr16	2054944	2055684	741	*	5	7.11429E-12	0.026666238	0.004037138	0.00322797	-0.116217424	-0.063692795	ZNF598
chr10	28550201	28550572	372	*	3	1.14171E-09	0.006931409	0.004044963	0.002328482	0.197551551	0.096148382	MPP7
chr16	11473477	11473669	193	*	2	9.80489E-09	0.000755325	0.004045889	0.001109654	-0.159075182	-0.025812046	CTD-3088G3.8
chr12	6657744	6659770	2027	*	13	1.30282E-25	1.1486E-07	0.00404899	6.01203E-09	-0.222042779	-0.073526254	IFFO1
chr20	1745259	1745769	511	*	3	6.94771E-09	0.006493925	0.004049538	0.004559442	-0.149743572	-0.032814003	
chr2	114384894	114385169	276	*	3	1.05313E-08	0.007316381	0.004051579	0.004614247	0.027285801	0.015778935	RABL2A
chr16	85435739	85435753	15	*	2	4.40453E-08	0.001460956	0.004059914	0.001787352	0.183654894	0.147130403	
chr4	152608012	152608770	759	*	5	2.83353E-13	0.014863788	0.004060043	0.001847212	0.127269918	0.054225415	PET112
chr12	1058210	1059459	1250	*	12	9.61038E-26	0.000109598	0.00406022	8.86364E-06	-0.046488262	0.003064985	RAD52
chr1	24120017	24120115	99	*	3	1.36193E-07	0.208682442	0.004064412	0.027000149	-0.16400458	-0.058652314	LYPLA2
chr5	175982402	175982645	244	*	3	1.06398E-10	0.001634263	0.004066523	0.000719604	-0.236775953	-0.09639342	CDHR2

chr19	46031516	46033285	1770	*	6	6.88043E-16	2.74788E-05	0.004071253	7.11626E-06	0.205498417	-0.014479765	OPA3
chr20	6035902	6035997	96	*	2	1.68324E-13	0.000106865	0.004076983	0.000228384	0.179987325	0.164004185	
chr5	175116770	175117129	360	*	2	6.94315E-09	0.001657028	0.004084983	0.001954308	-0.280011871	-0.168999223	
chr21	37757070	37757240	171	*	3	1.77345E-11	0.011636971	0.004090185	0.002262036	0.138543833	0.076133803	MORC3
chr19	847943	848026	84	*	3	4.03355E-13	1.3851E-05	0.004096754	4.70429E-05	-0.193566741	-0.163890839	PRTN3
chr17	76170799	76171208	410	*	3	4.04636E-10	0.150875402	0.004098412	0.024359369	-0.20535982	-0.055632325	TK1
chr16	87871160	87871219	60	*	2	6.76123E-10	0.000553859	0.004100993	0.000881077	0.237608391	0.166626027	RP4-536B24.2, SLC7A5
chr12	116586872	116587387	516	*	4	3.31446E-13	0.002077541	0.004101279	0.000415018	0.199267575	0.09438035	MED13L
chr8	141046436	141046852	417	*	5	1.59604E-14	0.000153265	0.00410412	0.000166231	0.124572827	0.078477893	TRAPPC9
chr8	110356640	110357011	372	*	3	6.75929E-12	0.001528137	0.004107249	0.00044525	0.189753801	0.129748329	ENY2
chr3	128368930	128370463	1534	*	14	3.48965E-28	1.06725E-06	0.004110608	5.79968E-07	-0.324560097	-0.038376673	RPN1
chr13	48890413	48890459	47	*	2	2.03511E-08	0.00212295	0.00411264	0.002316033	0.168513076	0.149322419	RB1
chr2	9340871	9341171	301	*	3	2.97386E-12	0.000139448	0.004116221	0.000182196	0.177819601	0.123614953	
chr3	122829800	122830175	376	*	2	2.29378E-10	0.000100494	0.004117034	0.000216929	0.161208014	0.152443586	PDIA5
chr1	206945034	206946595	1562	*	9	3.88057E-27	7.99245E-08	0.004119599	2.18408E-07	-0.248719714	-0.103958239	IL10
chr17	38084119	38085536	1418	*	7	1.2344E-12	0.004926405	0.004128324	0.001635058	0.212043763	0.089707712	RP11-387H17.4
chr4	154449090	154449177	88	*	2	3.05632E-10	0.000121877	0.004129215	0.00025638	-0.292304517	-0.249626801	KIAA0922
chr13	30938773	30939971	1199	*	4	9.96521E-16	0.000338452	0.004138732	0.000191942	0.150700412	0.09172	LINC00426
chr10	3938517	3939127	611	*	3	7.02853E-14	0.000539371	0.004140294	0.000377217	-0.309494947	-0.204847028	
chr9	130524573	130526319	1747	*	11	1.62E-14	0.004955549	0.00414391	0.00081463	0.173240886	-0.000229885	SH2D3C
chr22	31479561	31479615	55	*	2	9.41832E-08	0.141083248	0.004144403	0.01375213	0.144221454	0.075671991	SMTN
chr8	142106211	142106775	565	*	2	3.32265E-10	0.000100105	0.004145896	0.000216472	-0.173972393	-0.142367729	
chr20	60769534	60769785	252	*	2	1.59844E-10	0.000223026	0.004146968	0.00042719	-0.24074703	-0.188279289	MTG2
chr1	150971753	150971889	137	*	2	9.68873E-11	0.000102678	0.004147248	0.000221305	-0.10866293	-0.08045306	FAM63A
chr22	43484984	43485702	719	*	13	1.04196E-15	0.169362227	0.004151682	0.021410601	-0.16552449	-0.005468255	TTLL1
chr3	5137549	5137953	405	*	4	1.48809E-10	0.005538297	0.00415355	0.003064809	0.05941417	0.036198144	
chr13	29156897	29157266	370	*	2	7.65812E-09	0.004167936	0.004154785	0.00356532	-0.082707715	-0.054200424	
chr17	4854754	4854972	219	*	3	2.45345E-08	0.014530838	0.004155016	0.007291231	0.169556702	0.088184636	ENO3
chr5	86176688	86177200	513	*	2	2.37539E-09	0.000448303	0.004158969	0.000750754	0.127537028	0.089163443	

chr17	6616351	6617470	1120	*	14	8.19354E-15	0.20167395	0.004164156	0.006259857	0.109195834	0.015744559	SLC13A5
chr15	75917396	75919788	2393	*	20	2.76298E-15	0.047422477	0.004164393	0.002100731	-0.341259863	-0.013530197	CTD-2026K11.3, SNUPN
chr13	41136994	41137894	901	*	5	4.60419E-13	0.104820984	0.004165139	0.004673514	-0.193990072	-0.053820585	FOXO1
chr12	120114103	120114603	501	*	2	3.19131E-10	0.000106265	0.004166395	0.000228178	0.142547426	0.116579353	PRKAB1
chr19	46170845	46171507	663	*	8	3.64499E-18	0.016213392	0.00416642	0.000747848	-0.21937649	-0.043443119	GIPR
chr19	54877455	54878038	584	*	5	2.45696E-16	0.002269506	0.004168718	0.00127379	-0.327306203	-0.101262431	LAIR1
chr2	232044866	232045255	390	*	2	9.59721E-09	0.001218247	0.004173605	0.001589954	-0.202500375	-0.088654463	
chr8	38275151	38275192	42	*	2	4.18747E-10	0.000182063	0.004175626	0.000361303	-0.114497162	-0.082594259	FGFR1
chr1	202975059	202975221	163	*	2	2.2414E-08	0.005378671	0.004187749	0.004158398	-0.27235782	-0.16542033	
chr7	44180931	44181092	162	*	3	5.3691E-08	0.04298574	0.004193766	0.010491821	0.129580599	0.062152745	MYL7
chr17	43224158	43224934	777	*	10	1.22233E-15	0.084243759	0.004193824	0.00928743	-0.231303217	-0.012740786	HEXIM1
chr17	28704799	28707461	2663	*	14	6.60728E-16	0.001481386	0.004197763	0.000105316	-0.141787483	-0.010998263	CPD
chr10	43932149	43932570	422	*	7	4.61665E-10	0.069970553	0.004199479	0.021404413	0.025451723	0.006690258	ZNF487
chr6	6915600	6916301	702	*	3	1.82029E-09	0.002321934	0.004199521	0.002516339	-0.259415941	-0.115565417	RP3-429O6.1
chr11	128636211	128636703	493	*	2	3.19668E-09	0.000558388	0.004201982	0.000893313	0.125688483	0.104025289	FLI1
chr11	18719308	18719488	181	*	2	1.41705E-08	0.042833838	0.004207315	0.010644153	-0.211953213	-0.10873254	TMEM86A
chr19	5074224	5074758	535	*	4	4.6183E-15	7.5678E-06	0.004209989	2.5851E-05	-0.182750366	-0.109185136	KDM4B
chr2	99280007	99280963	957	*	6	1.6002E-17	0.001079832	0.004211999	2.55435E-05	-0.321879618	-0.076731897	MGAT4A
chr6	3068257	3069545	1289	*	5	1.17553E-11	0.000104092	0.004231899	0.000165001	0.050100599	-0.008282386	RIPK1, RP1-40E16.12
chr16	357654	358591	938	*	5	3.17662E-12	0.008689879	0.004235635	0.000522204	0.144929578	0.061244731	AXIN1
chr8	144432274	144432323	50	*	2	5.6481E-11	0.000175247	0.004239031	0.000351045	-0.091561437	-0.086749167	TOP1MT
chr22	44568203	44568812	610	*	9	7.91346E-30	6.90933E-10	0.004241731	9.59271E-09	-0.282794128	-0.123266649	PARVB
chr22	23933841	23933959	119	*	2	1.16006E-09	0.00051582	0.004242986	0.000842632	-0.164898678	-0.145823053	
chr1	35660486	35660588	103	*	2	2.57615E-08	0.012678438	0.004247869	0.006592043	-0.137101506	-0.044547704	
chr2	10429878	10430135	258	*	3	3.84504E-11	0.002410823	0.004247977	0.000511412	0.147885099	0.095208899	
chr19	8579985	8580509	525	*	5	7.49014E-16	1.8521E-05	0.00425038	5.99672E-05	-0.259682741	-0.060674454	
chr1	145045085	145045138	54	*	2	1.91875E-09	0.000429105	0.004254985	0.000730111	0.170438265	0.138319838	PDE4DIP
chr14	23623480	23624377	898	*	7	1.90943E-13	0.015125691	0.004255661	0.004844418	0.171549833	0.049829218	SLC7A8
chr20	61542163	61542613	451	*	5	2.16608E-08	0.155245148	0.004256804	0.025396875	0.038833993	0.013753941	DIDO1

chr15	99256874	99257110	237	*	2	4.00582E-10	0.000139192	0.004257957	0.000289004	-0.207879371	-0.031759391	IGF1R
chr19	4968104	4968614	511	*	5	6.39641E-16	0.004210928	0.004268443	0.000343819	-0.216940297	-0.042362812	
chr8	27466501	27466508	8	*	2	1.67416E-10	0.000115672	0.004269123	0.000246645	0.139107111	0.128802183	CLU
chr16	89286751	89287425	675	*	4	5.46307E-14	0.000197701	0.004270059	0.000173149	0.185895478	0.103573003	ZNF778
chr19	567046	568176	1131	*	3	3.40532E-09	7.85633E-05	0.004272457	0.000135383	-0.273625563	-0.186599871	AC009005.2
chr19	55594963	55595022	60	*	2	8.95226E-10	0.000334929	0.004276269	0.000600004	0.128228943	0.1170629	EPS8L1
chr12	77156168	77158144	1977	*	14	2.2951E-20	0.027119654	0.004276549	0.001229441	0.147276985	0.022113545	ZDHHC17
chr7	100655589	100655601	13	*	2	5.64266E-11	0.000101634	0.00427833	0.000220567	-0.152229546	-0.148169905	MUC12
chr10	80516517	80516893	377	*	2	9.68694E-11	0.0001104	0.004284895	0.000237053	-0.163629723	-0.1435075	
chr14	94491958	94493296	1339	*	13	2.62972E-22	0.001323735	0.004285061	0.000197844	-0.330489463	-0.033753535	OTUB2
chr8	142238452	142239055	604	*	8	4.51011E-14	0.160026452	0.004289687	0.008189868	-0.259168381	-0.053764412	SLC45A4
chr19	48759881	48760233	353	*	4	2.00084E-09	0.030343762	0.004295271	0.008224552	-0.157928302	-0.088095741	ZNF114, CTC-241F20.3
chr2	73519034	73519364	331	*	3	9.77875E-10	0.005560617	0.004297565	0.003847494	0.074301748	0.038686396	EGR4
chr9	85881935	85881968	34	*	2	5.22859E-10	0.000256574	0.004299188	0.000483665	0.134005841	0.109318957	FRMD3
chr11	114100017	114100689	673	*	2	4.12255E-11	0.000101859	0.004300295	0.000221193	-0.17310696	-0.053958755	ZBTB16, RP11-64D24.2
chr6	33254385	33254927	543	*	9	6.90106E-21	0.001385527	0.004300338	0.000468545	-0.328138734	-0.074020879	WDR46
chr16	89831123	89831437	315	*	2	1.04629E-09	0.000233935	0.004311862	0.0004485	-0.174951147	-0.11518425	FANCA
chr16	23562225	23562343	119	*	3	1.24239E-07	0.009267156	0.004313512	0.006026512	-0.031810558	-0.02139531	EARS2
chr19	58629522	58630612	1091	*	11	5.68411E-26	0.000114938	0.004314429	3.0378E-05	-0.258881505	-0.018765433	ZSCAN18
chr19	51898727	51898904	178	*	2	5.47026E-10	0.000316423	0.004314605	0.000574419	-0.244346564	-0.164945995	CTD-2616J11.14
chr5	169784957	169785032	76	*	2	3.38258E-10	0.000121753	0.004317778	0.000258335	-0.160201116	-0.134131293	KCNIP1
chr2	64712535	64713105	571	*	3	9.87621E-12	0.008252655	0.004320083	0.005770848	-0.215664189	-0.065119821	
chr16	68702501	68702627	127	*	2	5.32618E-08	0.001747648	0.004332781	0.002076199	-0.12814591	-0.07371034	CDH3
chr1	204231300	204231408	109	*	2	1.12492E-10	0.000420856	0.004337484	0.000722903	0.109566053	0.09034472	PLEKHA6
chr20	34541013	34543081	2069	*	13	2.79574E-14	0.033647075	0.004341512	0.000697208	-0.19188387	-0.016112669	SCAND1
chr22	50173724	50174297	574	*	8	1.05437E-17	0.00024338	0.00434182	0.000133916	-0.201855796	-0.069253881	BRD1
chr1	45097216	45097499	284	*	3	1.44128E-11	0.002050438	0.004348349	0.000983371	0.222274471	0.130329941	RNF220
chr17	3794211	3796782	2572	*	11	1.31463E-13	0.000794401	0.00434935	0.000196734	0.1714983	0.024809602	CAMKK1
chr7	101798913	101799218	306	*	3	1.9133E-11	0.003295835	0.004349554	0.000735536	0.133307014	0.091273628	CUX1

chr1	243958196	243958259	64	*	3	7.94972E-12	0.000111928	0.004350625	0.000169149	0.188654944	0.125629211	AKT3
chr20	52199520	52199778	259	*	5	3.98681E-19	3.27507E-07	0.004355348	1.98561E-06	-0.244719606	-0.166460425	ZNF217
chr9	123546098	123546374	277	*	2	2.75812E-10	0.000111145	0.00435625	0.000239153	-0.167606231	-0.124733649	FBXW2
chr10	64563185	64565772	2588	*	18	6.21607E-21	0.008428198	0.004358541	0.002088503	-0.221052193	0.004086849	ADO, RP11-436D10.3
chr12	27395963	27397422	1460	*	14	3.82627E-29	0.000571041	0.004360527	2.58464E-05	-0.322901853	-0.08029044	STK38L
chr10	126213758	126213886	129	*	2	2.92856E-08	0.032345411	0.004361547	0.009935169	0.146643285	0.078780129	LHPP
chr11	45710875	45711290	416	*	4	1.45607E-11	0.01406373	0.004363699	0.002983448	0.192196503	0.072617661	
chr1	228333578	228333668	91	*	2	2.26802E-09	0.001290046	0.004366367	0.001684608	0.145148633	0.095088382	GUK1
chr9	131902135	131902471	337	*	4	6.63459E-11	0.001283964	0.004366495	0.001156499	-0.160141118	-0.082818434	PPP2R4
chr19	3123192	3123291	100	*	2	3.51946E-10	0.000555837	0.004373734	0.000901131	-0.164350978	-0.01846032	GNA11
chr21	34100988	34101413	426	*	3	3.06247E-10	0.297128923	0.004374402	0.021613756	-0.196030916	-0.058058276	PAXBP1-AS1
chr1	27884179	27884618	440	*	3	1.1224E-08	0.003845269	0.004376718	0.003578659	-0.132514299	-0.010747053	AHDC1
chr17	73451648	73452755	1108	*	11	2.26193E-15	0.066500158	0.004382688	0.007330629	-0.244961332	-0.02430179	KIAA0195
chr17	4834179	4835041	863	*	4	2.70008E-13	0.003178221	0.004396622	0.001133851	0.205274153	0.085783539	
chr11	5246823	5246944	122	*	2	3.04047E-11	0.000276777	0.0043979	0.000517563	0.137537604	0.124419606	HBB
chr6	47246556	47247047	492	*	2	5.90595E-10	0.000121947	0.00440707	0.000259681	-0.237625512	-0.232833979	TNFRSF21
chr11	118978103	118978508	406	*	4	5.46051E-11	0.082955045	0.004409533	0.024653583	0.021739798	0.000469566	C2CD2L, DPAGT1
chr14	105857176	105857250	75	*	3	8.42307E-09	0.021163886	0.004418217	0.005852256	0.136969183	0.072677374	PACS2
chr19	41035368	41035836	469	*	2	1.22479E-07	0.245077764	0.004418401	0.015332448	0.019178572	0.011421832	SPTBN4
chr2	242710665	242711046	382	*	5	1.64292E-09	0.047338431	0.00442446	0.014861818	-0.19508264	-0.058992715	
chr5	138860810	138861241	432	*	3	1.49107E-12	0.000365285	0.004425511	0.000470879	-0.106050759	-0.066596543	TMEM173
chr2	27993847	27994018	172	*	2	1.531E-09	0.052134192	0.004426902	0.011720533	-0.175554534	-0.076062328	
chr8	97157453	97157756	304	*	2	1.35657E-07	0.007322497	0.004432787	0.005096026	0.026461921	0.01655114	GDF6
chr2	175463829	175464253	425	*	2	5.02613E-09	0.009278152	0.004436587	0.005785064	0.113595793	0.071294915	AC018890.6, WIPF1
chr1	1710453	1712885	2433	*	15	2.05258E-31	6.17537E-08	0.004437865	2.61776E-08	-0.227703049	-0.079192366	NADK
chr6	10585683	10585889	207	*	3	1.92227E-09	0.002542888	0.00444805	0.002683162	0.228034477	0.098721323	GCNT2
chr7	151403110	151403164	55	*	2	1.81788E-10	0.000176937	0.004452344	0.000357617	-0.207901792	-0.15088861	PRKAG2
chr1	201251911	201252974	1064	*	12	9.96297E-24	0.001094276	0.004452548	0.000104387	0.074334015	0.024610984	PKP1
chr8	42009223	42009622	400	*	2	1.25334E-09	0.072316163	0.004454379	0.012842828	-0.184378856	-0.08724818	RP11-589C21.5

chr5	173198447	173198932	486	*	4	3.11715E-13	0.002936292	0.004460739	0.000365626	0.146718222	0.086241133	
chr7	129254604	129254787	184	*	2	1.10886E-08	0.000739191	0.004466801	0.001128298	-0.173577048	-0.143133109	NRF1
chr14	72053158	72053361	204	*	3	2.99826E-08	0.0708767	0.004468039	0.014331165	-0.158506412	-0.082254767	SIPA1L1
chr12	111801136	111801794	659	*	6	8.57485E-10	0.096761319	0.00446811	0.019832218	-0.096866968	-0.026244434	FAM109A
chr8	102149430	102150072	643	*	7	5.44013E-16	0.021507585	0.004468943	0.00258322	-0.154194714	-0.048335571	
chr12	57631687	57632706	1020	*	6	1.97861E-12	0.036380502	0.004470903	0.004407232	0.082920336	0.022086071	NDUFA4L2
chr14	104570754	104571173	420	*	5	4.24952E-09	0.050826122	0.004472874	0.015694696	0.145580805	0.05675175	ASPG
chr18	47815407	47815588	182	*	5	9.41322E-08	0.642105005	0.004474609	0.106675554	-0.223797718	-0.042149694	
chr12	14413090	14413185	96	*	2	3.72721E-08	0.004036164	0.004479992	0.00362809	-0.198336006	-0.142814069	
chr10	14614164	14614830	667	*	6	3.37737E-17	0.00386887	0.004485515	0.000131061	-0.287017546	-0.144782899	FAM107B
chr1	161146978	161148880	1903	*	18	4.68354E-24	0.130146975	0.004488477	0.001662309	0.206060826	0.024740941	PPOX, B4GALT3
chr10	129947756	129947858	103	*	3	1.22274E-07	0.106890636	0.004490059	0.013989774	0.123224328	0.05786012	
chr16	68033509	68034002	494	*	3	1.36E-08	0.01440067	0.004491977	0.004185068	-0.160235613	-0.07228808	DUS2, DPEP2
chr19	41882069	41882741	673	*	13	1.65999E-25	0.039244585	0.004493964	0.000115383	-0.205219366	-0.0442638	CTC-435M10.3, TMEM91
chr5	1474995	1475732	738	*	3	2.55164E-10	0.034274856	0.004498107	0.007714814	-0.270751335	-0.099034638	LPCAT1
chr3	14185508	14186122	615	*	3	1.23982E-12	0.015946363	0.004504756	0.001638963	-0.124489322	-0.078127435	RP11-434D12.1
chr11	133372412	133372672	261	*	3	1.01189E-07	0.066836491	0.004506364	0.007859064	-0.12336576	-0.020572122	OPCML
chr19	839537	839689	153	*	3	4.21602E-18	4.92843E-06	0.004510543	2.04689E-05	-0.193113233	-0.176606442	
chr15	48484282	48484469	188	*	4	2.11252E-08	0.061290169	0.004513396	0.010980682	0.111376639	0.053201835	CTXN2, SLC12A1, RP11-605F22.1
chr18	28743074	28743186	113	*	3	3.52425E-09	0.002067123	0.004514171	0.002126209	0.132589274	0.085585419	
chr3	32474566	32474793	228	*	2	5.44047E-09	0.000488469	0.00451609	0.000822488	-0.161775435	-0.135518666	CMTM7
chr17	65516746	65516777	32	*	2	4.07801E-08	0.004631183	0.004519511	0.003959552	-0.09267668	-0.056352289	PITPNC1
chr6	159549155	159549723	569	*	4	8.20049E-17	9.03429E-06	0.004519741	1.89533E-05	0.19248011	0.118973745	
chr8	30600962	30601401	440	*	4	1.6917E-11	0.075508444	0.004533084	0.024226428	0.104426798	0.023925649	UBXN8
chr7	102065448	102065866	419	*	2	3.2778E-11	0.000113107	0.004536832	0.000244584	-0.253063508	-0.190411533	PRKRIP1
chr17	46190702	46190705	4	*	2	8.07691E-10	0.00017235	0.004539617	0.000351173	-0.271769494	-0.253968835	SNX11
chr10	75415704	75416206	503	*	6	2.09252E-15	0.001182081	0.004547278	0.000156733	0.120485277	0.057975779	RP11-464F9.21, SYNPO2L
chr4	68334016	68334446	431	*	3	4.58272E-12	0.000319972	0.004547809	0.00029208	-0.234385727	-0.03429129	
chr2	105940276	105940459	184	*	2	3.68182E-08	0.002905277	0.004549701	0.002972914	0.144089514	0.05703283	TGFBRAP1

chr11	67796808	67797936	1129	*	12	4.70893E-26	0.001658777	0.004555937	0.000184141	-0.233429302	-0.04512982	RP5-901A4.1
chr9	84270635	84271042	408	*	2	1.74477E-09	0.000303027	0.004556008	0.000562272	-0.187261077	-0.141195747	TLE1
chr11	66688584	66688628	45	*	2	1.20501E-10	0.000186459	0.004559152	0.000375684	-0.115683445	-0.081978589	PC
chr5	131605459	131605617	159	*	2	1.204E-07	0.008079494	0.004560458	0.005459872	0.141226981	0.061139012	PDLIM4, P4HA2
chr6	166756767	166756786	20	*	2	3.17208E-08	0.00730334	0.004563159	0.005169373	-0.052627359	-0.040120955	
chr17	73901394	73902746	1353	*	4	3.5796E-13	0.00040093	0.004563693	0.000177172	-0.161123531	-0.084997883	MRPL38, RP11-552F3.12
chr7	66119076	66119479	404	*	3	1.45637E-13	7.75167E-05	0.0045666	0.000198081	0.019898874	0.009660225	KCTD7
chr19	46526100	46527546	1447	*	16	2.15486E-25	0.022552424	0.004576388	0.000807059	-0.176709963	-0.049126102	PGLYRP1
chr1	110009509	110009770	262	*	2	2.95062E-08	0.161408402	0.004577655	0.015179934	0.03466487	0.016623508	SYPL2
chr17	71361857	71361901	45	*	3	1.74216E-08	0.024423227	0.004578098	0.003949373	0.265972942	0.177235413	SDK2
chr5	145966494	145966653	160	*	3	4.49177E-09	0.013626357	0.00458331	0.004276664	-0.231973127	-0.083958304	CTB-99A3.1
chr4	159690352	159690372	21	*	2	6.87751E-08	0.063753206	0.004584486	0.012715971	0.011992741	0.007011809	FNIP2
chr16	19191723	19191796	74	*	2	9.17334E-08	0.003029992	0.004585074	0.003065029	0.11259436	0.045987641	SYT17
chr15	37387304	37388127	824	*	6	2.29841E-14	0.006935737	0.004585883	0.001276859	0.084553308	0.041068915	MEIS2
chr11	64108241	64109823	1583	*	9	9.7713E-21	0.005282642	0.004588464	0.000796558	-0.325986073	-0.083392062	CCDC88B
chr17	36665826	36666642	817	*	6	3.26144E-09	0.138775361	0.004594552	0.042925885	-0.117960308	-0.028824553	ARHGAP23
chr18	46458754	46459473	720	*	2	6.22367E-09	0.000115067	0.00459817	0.000248856	-0.160161605	-5.78641E-05	SMAD7
chr11	64097027	64097101	75	*	3	5.04371E-11	0.000340955	0.004598205	0.000658807	0.183001964	0.123808881	
chr1	154405251	154405736	486	*	2	1.3683E-09	0.000735241	0.004600674	0.001134524	-0.13534207	-0.104661151	IL6R
chr7	23720549	23721400	852	*	4	7.63075E-14	0.01038716	0.0046029	0.002690906	-0.26176757	-0.053136194	FAM221A
chr17	17722503	17723462	960	*	4	1.93722E-11	0.030360533	0.004603612	0.006742361	-0.253762155	-0.07308341	SREBF1
chr2	71221537	71222556	1020	*	15	8.85734E-16	0.319995951	0.004614515	0.037930454	-0.031927532	0.002311826	AC007040.6, AC007040.11, TEX261
chr19	2273706	2273779	74	*	2	4.41989E-08	0.053915542	0.00461717	0.012206592	0.029263009	0.005789984	
chr7	92047156	92047388	233	*	3	7.61577E-10	0.001579593	0.004622096	0.001188113	0.189189867	0.10087526	TMBIM7P
chr22	40814878	40814966	89	*	2	1.38919E-07	0.157220641	0.004623006	0.015252692	0.089351018	0.048172867	MKL1
chr1	14029478	14030141	664	*	5	3.89674E-15	0.00519235	0.004645418	0.00095499	-0.292062686	-0.116217458	PRDM2
chr7	157198482	157198869	388	*	3	1.49525E-11	0.000508394	0.004648616	0.000812689	0.15370995	0.091649097	DNAJB6
chr16	16169888	16170435	548	*	4	3.07749E-18	1.4504E-07	0.004655112	1.23548E-06	-0.192298847	-0.171959657	ABCC1
chr2	207847270	207848321	1052	*	5	2.88055E-14	0.003316905	0.004663318	0.000808049	-0.236353191	-0.069074081	

chr1	65363870	65363974	105	*	2	6.84547E-10	0.000166153	0.004663558	0.000342289	0.218142164	0.171950689	JAK1
chr17	73082678	73084329	1652	*	15	1.80402E-20	0.011758535	0.00466429	0.001712722	0.15639744	0.02690832	SLC16A5
chr17	79004850	79006166	1317	*	12	2.95571E-27	1.95482E-08	0.004670752	8.06067E-08	-0.228568526	-0.105091215	BAIAP2-AS1
chr10	93861385	93861824	440	*	2	5.95476E-10	0.000433324	0.004670836	0.000755253	0.162464459	0.159922666	CPEB3
chr16	51185407	51186266	860	*	7	6.7125E-14	0.361536347	0.00467212	0.015997262	0.03164568	0.003177539	
chr1	44819838	44821812	1975	*	17	2.79683E-18	0.013517897	0.004674036	0.000831283	-0.197039026	-0.02399021	ERI3
chr10	52287323	52287340	18	*	2	3.75894E-09	0.000333478	0.00467739	0.000611934	0.118440637	0.105223977	SGMS1
chr5	619736	619994	259	*	3	7.78174E-08	0.042436504	0.004685463	0.008887509	-0.213717322	-0.091000621	CEP72
chr20	4070556	4071004	449	*	2	4.13502E-10	0.000147029	0.004686032	0.000308601	-0.289810472	-0.26327808	
chr6	131520697	131521103	407	*	5	1.32816E-08	0.0481246	0.00468773	0.018482181	0.1589541	0.014098927	AKAP7
chr2	11490628	11490636	9	*	2	8.68313E-08	0.010793641	0.00468949	0.006454702	0.186266648	0.102322906	
chr4	7301176	7301529	354	*	2	6.26299E-09	0.002224693	0.004689844	0.002527244	0.145753663	0.126706572	SORCS2
chr12	4676482	4676791	310	*	3	7.36369E-09	0.00699175	0.004695685	0.004089041	-0.191027711	-0.002085263	DYRK4
chr12	56638534	56638705	172	*	3	4.49068E-08	0.101706045	0.004700087	0.018120186	0.11641553	0.047079898	ANKRD52
chr7	30196739	30197130	392	*	6	2.86066E-14	0.00068755	0.004700257	0.000308376	0.167643973	0.071232676	MTURN
chr3	62364315	62365402	1088	*	9	1.16314E-12	0.019978001	0.004702478	0.013598217	0.064460998	0.028444391	
chr12	49971316	49972129	814	*	4	1.26887E-09	0.011946927	0.004702889	0.004752431	0.194140385	0.066852134	PRPF40B
chr7	4762742	4763655	914	*	5	4.74291E-12	0.048566478	0.004707926	0.008347476	0.155177613	0.060366742	FOXK1
chr1	59509582	59509687	106	*	2	2.44936E-10	0.000177265	0.004714363	0.000362461	-0.182577574	-0.148055666	RP4-794H19.4
chr11	7534028	7534677	650	*	4	2.30592E-12	0.000666463	0.004718211	0.000996562	-0.067745454	0.0011208	PPFIBP2
chr16	66555859	66556133	275	*	2	3.33593E-10	0.000123693	0.004722181	0.000266233	-0.133959365	-0.12604763	TK2
chr1	156123979	156124654	676	*	4	3.73158E-15	3.25811E-05	0.004725396	6.93295E-05	-0.23174876	-0.114933891	SEMA4A
chr1	1196457	1198269	1813	*	8	1.63497E-11	0.011170191	0.004739757	0.000271505	-0.136782646	0.016471318	UBE2J2
chr22	26137524	26138429	906	*	11	4.86519E-30	1.61956E-05	0.004745752	4.19492E-07	0.182039883	0.085225609	MYO18B
chr17	43368729	43368748	20	*	2	2.17666E-08	0.001064532	0.004748917	0.001511782	-0.169471063	-0.121291181	MAP3K14
chr11	64338639	64339197	559	*	3	5.17481E-11	0.000377741	0.004750741	0.000555867	-0.177409434	-0.082308732	SLC22A11
chr9	137692717	137693277	561	*	3	2.20033E-09	0.004957314	0.004753005	0.002767832	0.158341844	0.077803851	COL5A1
chr1	43231631	43232666	1036	*	4	4.29029E-17	4.29173E-05	0.004754685	9.32551E-05	0.027488959	0.013110292	LEPRE1
chr7	102576079	102576477	399	*	4	3.68375E-10	0.002321364	0.00476227	0.00134764	-0.155091367	-0.064913691	LRRC17, FBXL13

chr19	19738054	19738592	539	*	2	3.89402E-08	0.002543033	0.004762352	0.002780981	0.111459568	0.027346432	LPAR2
chr5	131808798	131808970	173	*	3	4.27051E-08	0.007846736	0.004765946	0.005874163	0.161092463	0.096225131	C5orf56
chr1	52831953	52832660	708	*	9	4.00576E-10	0.385265967	0.004780277	0.079847395	-0.04876555	0.000745755	
chr13	46948804	46949210	407	*	3	5.67676E-12	0.000352831	0.004782427	0.000442595	-0.294490485	-0.170957931	KIAA0226L, PPP1R2P4
chr19	12890029	12890523	495	*	2	2.556E-09	0.000693017	0.004785114	0.001098071	-0.168533146	-0.130480483	HOOK2
chr14	22886075	22886796	722	*	3	5.04989E-10	0.001196536	0.004786825	0.000514131	-0.118376885	-0.017817946	AE000661.37
chr4	54458246	54459009	764	*	5	4.01613E-12	0.090517429	0.004786988	0.010232738	-0.142468129	-0.062911043	FIP1L1, LNX1
chr5	71146767	71146779	13	*	2	1.7985E-07	0.003039539	0.004788054	0.003131925	0.114887494	0.032671504	
chr3	147077127	147077774	648	*	7	7.21992E-11	0.018415529	0.004788997	0.005940299	0.064065741	0.022160821	RP11-649A16.1
chr9	130859347	130860839	1493	*	12	1.63413E-26	3.4296E-06	0.004801828	1.69684E-07	-0.196135152	-0.032952579	SLC25A25
chr2	20601663	20601928	266	*	2	1.19057E-08	0.002646633	0.004804741	0.002866214	-0.142165874	-0.098783553	
chr7	105088656	105088822	167	*	2	3.74522E-08	0.001255517	0.004807343	0.001712334	-0.238381111	-0.150795627	PUS7
chr8	134706823	134706976	154	*	2	2.64474E-09	0.000291326	0.004810536	0.00055169	-0.099660513	-0.067358847	
chr8	134162674	134163056	383	*	3	3.72361E-11	0.000160695	0.00481291	0.000229005	-0.239191465	-0.168547145	
chr1	25198759	25199304	546	*	2	9.63256E-10	0.000126561	0.004819597	0.000272577	-0.111342687	-0.095409603	
chr4	125504	126058	555	*	2	7.72139E-09	0.005535052	0.004826177	0.004547751	0.15641053	0.130119964	ZNF718
chr12	124941307	124942176	870	*	8	9.00003E-26	1.53904E-06	0.004833542	7.01142E-07	0.151848819	0.04477185	NCOR2
chr15	83477608	83478411	804	*	11	3.02715E-24	0.00027018	0.004833909	6.23301E-05	0.197751193	0.037146449	WHAMM
chr7	2903855	2904037	183	*	2	6.50724E-10	0.000667449	0.004836379	0.001070331	-0.209507312	-0.167244885	
chr14	64194118	64194567	450	*	3	1.74752E-11	0.001713572	0.004840383	0.002005419	0.028399873	0.020708559	SGPP1
chr3	48936775	48936979	205	*	2	2.4485E-09	0.001042009	0.004854329	0.001499626	0.130797995	0.090743238	
chr6	100039303	100039476	174	*	3	3.85516E-08	0.062284306	0.004854819	0.007532011	-0.036047229	-0.001207018	
chr1	110155535	110156682	1148	*	6	1.27712E-13	0.000761238	0.00485977	0.000279624	0.153357719	0.080303978	GNAT2
chr1	1363280	1363291	12	*	2	9.64939E-09	0.004517057	0.004861016	0.004042738	0.025079612	0.02025357	
chr19	55416788	55417647	860	*	7	1.33047E-15	0.018630286	0.004861755	0.000476832	0.235084237	0.102399365	NCR1
chr15	39877764	39877954	191	*	2	4.92537E-10	0.000138399	0.004862307	0.000294986	0.160329667	0.150289245	THBS1
chr1	203737534	203738799	1266	*	8	4.96973E-16	4.24127E-05	0.004869067	2.48914E-05	-0.193021745	-0.055422346	LAX1
chr14	88490135	88490333	199	*	2	3.65502E-08	0.01280831	0.004884179	0.007200129	0.201500253	0.114336138	RP11-300J18.1
chr11	62572592	62574403	1812	*	17	3.14118E-31	3.54598E-06	0.00489015	2.57993E-07	-0.309224928	-0.025075047	NXF1, STX5

chr5	10626811	10626937	127	*	2	3.79273E-09	0.000335639	0.004890512	0.000622095	-0.296185747	-0.223596324	ANKRD33B
chr9	138951648	138952311	664	*	3	1.14699E-11	7.11923E-05	0.004891716	0.000196954	-0.192468865	-0.11620978	NACC2
chr1	221055097	221055964	868	*	7	2.24854E-15	0.087512483	0.004893769	0.004356239	-0.213824283	-0.04710486	HLX
chr5	39203007	39203057	51	*	2	1.40953E-09	0.0003847	0.00489407	0.000695107	-0.261478113	-0.229329663	FYB
chr1	3129958	3130550	593	*	5	1.44426E-14	0.011200026	0.004896454	0.001800136	0.172459703	0.060645966	PRDM16
chr12	48287192	48287729	538	*	3	6.88979E-12	0.000354091	0.004902135	0.00035465	0.161691467	0.086043223	RP11-89H19.1, VDR
chr18	77218378	77219240	863	*	5	5.22736E-16	0.000326426	0.004904649	8.52139E-05	0.214820017	0.120172502	NFATC1
chr3	18933367	18933400	34	*	2	3.14323E-09	0.000293483	0.004907862	0.000557719	-0.109153102	-0.077294996	AC144521.1
chr8	143319094	143319259	166	*	2	7.22643E-08	0.333736883	0.004911739	0.017047142	-0.06943846	-0.034748993	TSNARE1
chr21	44061403	44061597	195	*	2	7.61877E-08	0.007510991	0.00491179	0.005462486	0.167134828	0.105669261	
chr4	18022372	18022498	127	*	3	9.75107E-09	0.405950475	0.004913002	0.022148751	0.019568171	0.009188165	LCORL
chr22	46403218	46403316	99	*	2	5.47905E-10	0.001716158	0.004913318	0.002155917	-0.144980756	-0.094041586	
chr8	129162178	129162406	229	*	2	5.52749E-09	0.001383028	0.004915094	0.001850805	-0.12221061	-0.012083279	MIR1208
chr22	47169227	47169976	750	*	10	4.68497E-12	0.142205434	0.004918256	0.023378778	-0.216104495	-0.056805815	TBC1D22A
chr6	41747367	41748543	1177	*	10	3.34427E-23	0.008915555	0.004921028	6.21565E-05	-0.094698509	-0.024633994	PRICKLE4, FRS3
chr11	36422377	36422615	239	*	5	4.29817E-21	1.26721E-07	0.004931424	9.63495E-07	-0.27172554	-0.161353298	PRR5L
chr5	40797688	40798723	1036	*	14	3.38353E-12	0.626402853	0.00493353	0.15421216	-0.041827272	-0.006152181	PRKAA1
chr17	18149890	18150954	1065	*	5	1.04275E-14	0.005113615	0.004937518	0.003271161	0.125098963	0.064387438	FLII
chr5	150050273	150051052	780	*	3	6.38911E-12	3.86035E-05	0.004938004	0.000101851	0.084604673	0.036356811	MYOZ3, CTC-345K18.2
chr2	240422121	240423096	976	*	4	1.46581E-14	2.49535E-06	0.004946168	1.1688E-05	-0.223406148	-0.171032004	
chr1	11900652	11903897	3246	*	10	4.97057E-17	4.91501E-07	0.004946801	1.52882E-07	-0.318025869	-0.041324144	CLCN6, NPPA-AS1
chr15	91382476	91382972	497	*	5	1.30261E-14	0.000833973	0.004952361	0.000182032	-0.127464746	-0.061224678	CTD-3094K11.1
chr19	24097411	24097753	343	*	3	1.20304E-12	0.000251176	0.004956854	0.000402934	0.100302254	0.076147405	ZNF726
chr6	31544694	31545473	780	*	7	1.85847E-23	0.000279493	0.004961604	2.51725E-05	0.20661561	0.105159828	TNF
chr6	45447935	45448233	299	*	2	9.49611E-09	0.000658313	0.004965024	0.001067538	-0.129890879	-0.089071078	RUNX2
chr5	153872690	153873149	460	*	3	6.18043E-10	0.001354872	0.004969798	0.001717848	0.214431678	0.090960833	CTB-158E9.2
chr16	57077322	57077666	345	*	2	1.14526E-08	0.000815626	0.004970258	0.001258392	-0.164899243	-0.09316826	NLRC5
chr11	64645556	64647924	2369	*	21	5.95297E-22	0.000660032	0.004971681	4.6302E-05	0.194863175	-0.009435995	EHD1
chr1	234781700	234781920	221	*	3	9.93468E-10	0.00555909	0.004975687	0.001671618	-0.28823396	-0.152756134	RP4-781K5.9

chr15	67439378	67441067	1690	*	8	9.60201E-14	2.15385E-05	0.004981883	1.41281E-05	-0.195815555	-0.075296206	SMAD3
chr3	124775999	124776038	40	*	2	1.1041E-10	0.000238698	0.004987833	0.000471476	-0.088266242	-0.088233891	
chr19	8008274	8009199	926	*	12	3.1826E-15	0.002907138	0.004992844	0.002865367	-0.054090418	-0.009753469	TIMM44
chr19	2620967	2621851	885	*	2	5.75373E-09	0.000333733	0.004997155	0.000622528	-0.131720794	-0.08021007	GNG7
chr11	63752561	63754305	1745	*	17	5.44917E-18	0.019683743	0.004997379	0.002222278	-0.296926555	-0.018746956	AP000721.4, OTUB1
chr12	54779077	54780377	1301	*	7	1.04966E-12	0.000276564	0.005000393	0.000198343	0.153343948	0.012800178	RP11-753H16.3, RP11-753H16.5, ZNF385A
chr13	28953821	28953831	11	*	2	7.49983E-10	0.000153194	0.00500274	0.000323699	0.140629173	0.131205453	FLT1
chr11	94373920	94374424	505	*	3	2.46655E-11	0.000786058	0.005004023	0.000595696	-0.147371934	-0.080347881	RP11-867G2.5, RP11-867G2.8
chr10	98415822	98416848	1027	*	5	1.25604E-15	1.04589E-06	0.00500649	7.36474E-06	-0.244598889	-0.124490074	PIK3AP1
chr2	11514876	11515248	373	*	4	9.91177E-14	0.000122304	0.005008156	7.77152E-05	0.146456599	0.105621588	
chr11	48041783	48042101	319	*	2	1.28801E-10	0.000207761	0.005013081	0.000419962	-0.2662338	-0.175944186	PTPRJ
chr20	25025765	25026187	423	*	2	2.42043E-10	0.000146791	0.005020511	0.000312211	-0.191789939	-0.035322087	ACSS1
chr2	11605539	11606277	739	*	5	7.48557E-16	0.01628348	0.005026443	0.002403531	-0.050077074	-0.018588954	AC099344.1, E2F6
chr6	35228319	35228552	234	*	2	7.85266E-08	0.044536519	0.005031566	0.012250218	-0.151926808	-0.093977308	ZNF76
chr19	45943663	45943865	203	*	2	8.68262E-09	0.006151163	0.005032224	0.004940413	0.153742364	0.104151728	ERCC1
chr17	25571675	25572026	352	*	2	2.03986E-11	0.000137696	0.005033032	0.000295546	-0.219620526	-0.203641724	
chr1	44443270	44444305	1036	*	6	2.90758E-15	0.022023242	0.005033212	0.003088662	-0.213135681	-0.071642449	ATP6V0B
chr13	111819380	111820230	851	*	3	2.27492E-12	4.34983E-06	0.005034324	1.8984E-05	0.124996523	0.117755994	ARHGEF7
chr18	11179065	11179360	296	*	2	8.66472E-10	0.000168332	0.005042524	0.000351514	-0.174617573	-0.112282831	
chr5	176942521	176942953	433	*	3	1.59209E-11	0.007905903	0.005044534	0.003721943	0.131193074	0.060834271	DDX41
chr4	141348167	141349725	1559	*	18	8.86051E-25	0.002079691	0.005050039	0.000278277	-0.191068639	-0.031385195	CLGN
chr16	4582488	4582555	68	*	2	5.89378E-09	0.000390785	0.00505462	0.000709899	0.147159651	0.142947687	CDIP1
chr1	6530205	6531727	1523	*	7	2.56114E-15	0.053099676	0.005059006	0.000817271	-0.101561003	-0.038045396	PLEKHG5
chr2	217639206	217639532	327	*	2	7.1981E-10	0.000142039	0.005067158	0.000303973	0.145357007	0.127246794	AC007563.5
chr9	135861399	135861738	340	*	3	8.80761E-10	0.001311416	0.005079348	0.001307612	0.148627805	0.106783827	GFI1B
chr1	27952048	27953763	1716	*	8	2.43707E-20	0.000666813	0.005080673	2.45579E-05	-0.316717119	-0.100586	FGR
chr7	5271480	5272846	1367	*	8	3.02802E-19	8.67117E-05	0.005089016	5.09803E-05	0.143695288	0.077529022	WIPI2
chr1	45814217	45814632	416	*	2	4.80931E-09	0.000473212	0.005089509	0.000829465	0.219897706	0.13725573	TESK2
chr7	134836211	134836467	257	*	2	4.7119E-08	0.005780766	0.005095032	0.004795078	-0.112538389	-0.081276638	TMEM140, C7orf49

chr22	45071340	45071884	545	*	3	4.38399E-14	0.000445711	0.005095247	0.000768671	0.147611568	0.121601711	PRR5
chr11	6258935	6259853	919	*	5	1.6656E-12	0.001425505	0.00509644	0.000902053	0.219753803	0.067059971	CNGA4
chr8	141360555	141361266	712	*	5	1.18205E-15	0.001061685	0.0051045	0.000130582	-0.292938758	-0.127599996	TRAPPC9
chr11	5841482	5841688	207	*	3	1.1534E-10	0.001051508	0.00510531	0.000675665	0.184568922	0.119386816	OR52N2, TRIM5
chr1	155991941	155992141	201	*	2	1.3827E-08	0.013223995	0.005105398	0.007507896	-0.183091996	-0.101489579	
chr5	76256017	76256334	318	*	2	9.50245E-10	0.000203756	0.005108526	0.000414725	0.132034349	0.118421212	CRHBP
chr16	70472477	70473006	530	*	5	1.59642E-10	0.445736056	0.005112786	0.039980182	-0.068293133	-0.019891654	ST3GAL2
chr5	207590	208042	453	*	2	8.9774E-09	0.000978786	0.00511401	0.001457199	0.206639446	0.131321894	CCDC127
chr10	114437411	114438072	662	*	5	1.02565E-11	0.02522634	0.005133344	0.002416825	0.139628035	0.04770912	VTI1A, RP11-25C19.3
chr10	44274107	44274960	854	*	6	1.56375E-14	0.004289565	0.005137521	0.00137294	0.157080922	0.049412749	RP11-272J7.4
chr1	19000539	19000726	188	*	2	1.14368E-09	0.000185987	0.005141195	0.000384261	0.213233194	0.170494038	PAX7
chr7	148843026	148844604	1579	*	10	5.05475E-12	0.003183558	0.005141532	0.001154206	-0.302550153	-0.018379856	ZNF398
chr7	942748	942874	127	*	3	6.73254E-08	0.01570693	0.005147039	0.007726034	0.15603637	0.072027159	ADAP1, COX19
chr10	121095770	121096263	494	*	3	4.10116E-11	0.002721349	0.005149717	0.002106029	0.121787305	0.074397435	GRK5
chr1	32264286	32264703	418	*	7	3.89186E-11	0.05017047	0.005150797	0.008540786	0.160086272	0.067315994	SPOCD1
chr1	16483255	16483658	404	*	3	8.51733E-13	0.040414219	0.005154746	0.002222541	-0.170689698	-0.085801174	RP11-276H7.2
chr12	24374824	24375526	703	*	3	3.72135E-11	2.70198E-05	0.005157434	7.89589E-05	-0.157489655	-0.066549559	RP11-444D3.1
chr17	7742126	7743032	907	*	6	1.19599E-13	0.001563027	0.005158883	0.001753038	-0.159292236	-0.088636705	
chr6	33256950	33258581	1632	*	32	1.5607E-17	0.291317654	0.00516299	0.175561372	-0.2119266	-0.015772627	PFDN6, WDR46
chr1	24144067	24144422	356	*	2	2.73364E-08	0.003695114	0.005167853	0.003673883	0.183708747	0.109459804	HMGCL
chr22	36752926	36754002	1077	*	4	1.2191E-11	0.001358377	0.005169749	0.00012912	0.134860739	0.089591982	MYH9
chr7	157089894	157090728	835	*	6	4.02107E-17	0.038867608	0.005176717	0.000849291	-0.21953467	-0.040657058	
chr1	6520772	6520777	6	*	2	4.53458E-09	0.000427002	0.005183127	0.000767586	0.055323195	0.050027074	ESPN
chr21	30554251	30554264	14	*	2	1.82573E-10	0.000180843	0.005185823	0.00037581	0.164008991	0.130383012	
chr1	2230601	2230923	323	*	5	1.84941E-11	0.001251377	0.005187677	0.001223884	0.167286742	0.101113274	SKI
chr1	11797908	11798278	371	*	2	7.15081E-09	0.000691373	0.005191495	0.001123901	-0.167840384	-0.127615978	AGTRAP
chr10	85954622	85954958	337	*	2	2.55868E-08	0.002641753	0.005199268	0.002961837	0.05905488	0.05309514	CDHR1
chr6	88106888	88106922	35	*	2	2.65155E-08	0.000817483	0.005200805	0.001278992	-0.117272037	-0.087117899	RP1-102H19.8, C6orf164
chr22	37641093	37641970	878	*	4	1.76923E-15	6.25538E-06	0.00520099	1.77849E-05	-0.126808824	-0.086435721	

chr3	124504020	124504100	81	*	2	8.17815E-10	0.00015399	0.005204422	0.000327559	0.180359638	0.158696411	ITGB5-AS1, ITGB5
chr1	16693215	16694269	1055	*	14	1.50193E-20	0.007222329	0.005208356	0.000269383	0.075966502	-0.004316609	SZRD1
chr1	205601084	205601417	334	*	5	2.1147E-08	0.209427836	0.005210554	0.030485265	-0.025800592	-0.000940466	ELK4
chr3	45928007	45928018	12	*	2	6.24691E-10	0.00017162	0.005226596	0.000359878	0.138495222	0.115359027	CCR9, LZTFL1
chr7	123388340	123389622	1283	*	12	1.07795E-18	0.001858186	0.005228555	0.000355534	0.029334278	0.00541456	RP11-390E23.6, WASL
chr12	54555643	54555649	7	*	2	4.2376E-11	0.000151845	0.005237524	0.000323992	0.160249768	0.145809438	
chr16	30042330	30042541	212	*	4	6.3358E-09	0.042138113	0.005242766	0.01583111	0.033552417	0.014673839	FAM57B
chr11	66886118	66886765	648	*	5	8.99069E-13	0.001083523	0.005258006	0.000557127	-0.069331536	-0.024840166	KDM2A
chr12	1756074	1756191	118	*	2	3.56348E-10	0.000616302	0.005261571	0.001031787	-0.162931279	-0.136400339	WNT5B
chr3	46446998	46447254	257	*	2	1.86668E-08	0.217386418	0.005263431	0.017584129	-0.142749443	-0.073094779	RP11-24F11.2
chr9	107666012	107666037	26	*	2	1.64434E-08	0.00062001	0.005266392	0.001036921	0.136027147	0.102653042	ABCA1
chr2	114359188	114359736	549	*	3	1.5304E-10	0.001018854	0.005272733	0.00097114	0.112690625	0.104492096	
chr19	30573088	30573118	31	*	2	2.24579E-10	0.000150304	0.005277647	0.000321595	-0.191814508	-0.184143976	
chr1	10917292	10917391	100	*	3	1.81624E-09	0.001433177	0.005281207	0.001923609	0.080838261	0.068500965	
chr14	104338788	104338807	20	*	2	4.08994E-09	0.000567771	0.005284939	0.000968591	0.139464543	0.10479601	CTD-2134A5.4
chr11	72982929	72983405	477	*	5	7.18482E-14	0.000432296	0.005288943	0.000354365	-0.15902084	0.023659114	P2RY6
chr12	123956152	123956471	320	*	2	1.24439E-08	0.001533543	0.005295054	0.002048884	-0.106086839	-0.087577463	SNRNP35, RILPL1
chr6	30720203	30720484	282	*	4	1.79851E-09	0.096276869	0.005297847	0.02099248	-0.146109703	-0.059538927	
chr4	1742776	1742950	175	*	3	1.52935E-09	0.002382471	0.005300304	0.002737954	0.162806327	0.030171013	TACC3, AC016773.1
chr10	30720010	30720817	808	*	5	6.65096E-17	5.90262E-06	0.005308273	1.74819E-05	0.245385909	0.154086618	
chr17	924473	924476	4	*	2	7.61529E-11	0.000469799	0.00531446	0.000834372	0.148105624	0.122310608	ABR
chr17	80112660	80113111	452	*	4	6.05203E-12	0.006345755	0.005328149	0.001158805	0.304535737	0.141024616	RP11-1376P16.2, CCDC57
chr18	9707031	9708454	1424	*	10	2.55717E-15	0.000722576	0.0053306	0.000985082	-0.238705232	-0.029692608	RAB31
chr10	134236118	134236975	858	*	3	8.52788E-10	0.001211578	0.005334352	0.001650545	0.074418811	-0.018802293	
chr7	66204797	66206258	1462	*	6	2.69104E-13	0.0007358	0.005334623	9.72461E-05	-0.194010109	-0.038655365	KCTD7, RABGEF1, RP11-792A8.4
chr12	49449334	49449697	364	*	2	1.35147E-08	0.000300512	0.005336331	0.000580035	0.109581767	0.022840382	KMT2D
chr13	77564706	77565320	615	*	3	2.86381E-11	0.020586521	0.005343849	0.0100419	0.276439926	0.116537032	CLN5
chr2	30453758	30455589	1832	*	13	4.94824E-24	0.000355309	0.005347464	3.93473E-05	-0.061461885	-0.004285606	LBH
chr4	25033126	25033520	395	*	4	5.07542E-12	0.05048545	0.005349198	0.001916964	0.149886255	0.060687616	

chr16	8726527	8726753	227	*	2	3.18356E-08	0.001200216	0.005350221	0.001721542	-0.201687928	-0.131191144	METTL22
chr2	220134120	220134324	205	*	2	2.8566E-08	0.000967184	0.005354016	0.001466411	0.148478203	0.142901412	TUBA4B, TUBA4A
chr11	119558088	119558768	681	*	4	9.58934E-16	8.05567E-07	0.005357591	5.44306E-06	-0.154791744	-0.128245747	PVRL1
chr18	3251477	3252175	699	*	4	2.40903E-13	1.87073E-05	0.005359903	4.89861E-05	0.131374277	0.090079765	MYL12A
chr3	132035647	132036382	736	*	4	5.22437E-10	0.01362335	0.00536025	0.004650442	-0.348514519	-0.100373368	ACPP
chr17	42402347	42402882	536	*	6	1.39985E-08	0.276401274	0.005362589	0.018804679	-0.01425685	-0.003593722	
chr11	33277265	33279136	1872	*	16	1.93698E-24	0.056531985	0.005365008	0.002684019	-0.227835726	-0.037772218	HIPK3
chr19	10305125	10305305	181	*	2	1.24012E-08	0.004342213	0.00536689	0.004137747	0.024160443	0.015961218	DNMT1
chr16	56459026	56459217	192	*	3	1.43814E-07	0.04252516	0.005369143	0.016537199	0.033889857	0.015168348	AMFR
chr7	29523006	29523410	405	*	6	5.5623E-12	0.041327188	0.005375273	0.005712206	-0.285981337	-0.088027633	CHN2
chr8	67563906	67564402	497	*	3	6.13253E-10	0.003922567	0.005376991	0.00104965	0.117203975	0.016494848	VCPIP1
chr16	11835468	11837547	2080	*	18	1.28535E-22	0.006009656	0.005388741	0.000266732	-0.12555755	-0.020326874	RP11-490O6.2, TXNDC11
chr7	1004997	1005089	93	*	2	5.27696E-08	0.001141936	0.005389239	0.001663623	-0.162865516	-0.119108539	COX19
chr1	45504888	45504925	38	*	2	3.58449E-10	0.000273547	0.005392294	0.000537632	0.11711737	0.111707676	ZSWIM5
chr19	58978543	58978849	307	*	3	9.84257E-10	0.096209856	0.005392662	0.024030711	0.013768683	0.000688511	ZNF324
chr17	47865453	47866462	1010	*	12	2.23957E-13	0.015425655	0.005393728	0.011617872	-0.078910854	-0.002108465	KAT7, FAM117A
chr17	35850482	35851459	978	*	6	1.05092E-19	0.000195576	0.005394789	1.20825E-05	-0.312801755	-0.147255383	DUSP14
chr17	62308897	62309293	397	*	3	1.20014E-09	0.001252997	0.005395957	0.00169918	0.1206209	0.061705278	TEX2
chr22	30901707	30902896	1190	*	5	4.68068E-15	0.007742152	0.005406196	0.001258881	0.158200251	0.06076271	
chr16	57180054	57180175	122	*	3	1.39099E-08	0.013549825	0.005409031	0.005710665	0.120786031	0.018157971	CPNE2
chr19	39832561	39832795	235	*	3	8.275E-10	0.055205204	0.005412479	0.010845062	-0.024164022	-0.001372375	GMFG, CTC-246B18.10
chr12	14436575	14436900	326	*	2	2.96663E-10	0.000706116	0.005426749	0.001157799	0.181917796	0.140047573	
chr10	73516760	73517463	704	*	5	4.20726E-16	0.00015019	0.005430027	0.000174749	-0.155084503	-0.018544803	CDH23, C10orf54
chr11	67141686	67142562	877	*	7	6.80728E-20	4.13858E-05	0.005434149	5.48675E-05	-0.141855881	-0.049561292	
chr1	151805241	151805661	421	*	4	2.09435E-11	0.02057158	0.005437039	0.003181507	-0.219131197	-0.081429925	
chr8	134262768	134263111	344	*	2	7.87362E-11	0.000401259	0.005437259	0.00073893	-0.179139496	-0.123329781	NDRG1
chr16	28608125	28608894	770	*	2	4.85342E-08	0.000241516	0.005439273	0.000485179	0.153234495	0.144635022	SULT1A2
chr22	24950379	24951987	1609	*	21	8.51639E-25	0.002922289	0.00544456	0.000249687	-0.039040853	0.005714412	SNRPD3, GUCD1
chr17	75539901	75539921	21	*	3	1.98509E-13	1.23986E-05	0.005448361	4.58155E-05	0.190103821	0.164903593	

chr1	36184400	36185939	1540	*	11	1.1485E-13	0.035049943	0.005455053	0.002265251	-0.174577585	-0.012226515	C1orf216, CLSPN
chr17	1490023	1490557	535	*	3	2.03915E-12	0.000529804	0.005459366	0.000445415	-0.287529336	-0.155313078	SLC43A2
chr13	36920987	36921853	867	*	6	7.93776E-18	0.427401135	0.00546376	0.008706342	-0.215846314	-0.046202381	SPG20OS, SPG20
chr12	47219626	47221046	1421	*	14	2.63497E-38	3.87465E-11	0.005470358	2.19731E-10	0.369841297	0.133193921	SLC38A4
chr14	21785459	21785911	453	*	4	3.57665E-10	0.010910727	0.005483262	0.002631499	0.117423875	0.001256931	RPGRIP1
chr3	14319984	14320523	540	*	2	2.28683E-09	0.000162334	0.00548566	0.000346175	-0.218189701	-0.201370819	RP11-536I6.2
chr2	120413205	120413260	56	*	2	1.12588E-07	0.00156445	0.005487503	0.002106137	0.143585605	0.109509042	PCDP1
chr4	10020360	10021355	996	*	6	3.41523E-14	0.000810726	0.005490737	0.000415107	-0.132779516	-0.057020891	SLC2A9
chr20	32011218	32011533	316	*	2	1.8178E-08	0.000857618	0.005494437	0.001349889	0.179074603	0.114924274	SNTA1
chr11	78003296	78003665	370	*	3	1.25881E-10	0.004517464	0.005503797	0.001340769	-0.285543499	-0.170739407	GAB2
chr10	82115447	82116392	946	*	8	8.22876E-13	0.060169275	0.005503942	0.012288963	0.159107835	0.043552416	DYDC2, DYDC1
chr2	24161954	24162667	714	*	6	2.09693E-15	0.054826336	0.00550751	0.00325179	-0.329837895	-0.100124461	UBXN2A
chr19	50269083	50270616	1534	*	14	3.60095E-28	5.35707E-08	0.005510808	1.19404E-07	-0.185348237	-0.030608312	AP2A1
chr17	63053929	63053996	68	*	2	3.89541E-12	0.000211526	0.005514325	0.000434857	0.147770309	0.123806951	
chr16	69975149	69976228	1080	*	6	2.361E-14	0.00028342	0.00551685	0.00023999	-0.1254998	-0.040353061	WWP2
chr15	81586571	81589273	2703	*	14	1.93051E-23	1.28801E-05	0.005517985	6.09582E-07	-0.21849619	-0.071655223	IL16
chr2	202654941	202655138	198	*	5	1.588E-11	0.002404166	0.00552724	0.001107246	0.181989431	0.058486898	
chr20	36405608	36405675	68	*	2	9.09197E-08	0.003302556	0.005527406	0.003523478	-0.198692124	-0.132085248	CTNNBL1
chr8	97778709	97779220	512	*	2	2.46631E-08	0.00039514	0.005529434	0.000732814	-0.14997683	-0.141445987	CPQ
chr1	151253959	151255071	1113	*	11	3.19294E-13	0.033945678	0.005533857	0.006662811	0.035690091	-0.003448902	ZNF687, RP11-126K1.2
chr3	108180778	108181336	559	*	4	3.15814E-14	0.000174162	0.005540668	0.000123654	0.174414273	0.118610085	MYH15
chr3	51428079	51430288	2210	*	17	7.53146E-12	0.032240068	0.005547087	0.005884487	-0.085475396	-0.010648353	RBM15B
chr20	36153186	36153947	762	*	6	1.57296E-11	0.042098515	0.005547846	0.00854931	0.160782448	0.058404628	BLCAP
chr9	136284455	136284475	21	*	2	1.71168E-10	0.000372201	0.005553398	0.000698567	-0.160212	-0.023207657	ADAMTS13
chr13	41634374	41635251	878	*	6	9.71223E-14	0.004863042	0.005556253	0.003509509	-0.081819389	-0.006678136	ELF1
chr1	222816858	222817647	790	*	5	6.00233E-14	0.00095717	0.005557534	0.000260793	0.152567131	0.055685342	MIA3
chr12	52638005	52638158	154	*	3	2.38589E-08	0.034731736	0.005561705	0.009454841	-0.107962396	-0.046192261	KRT7
chr7	139425741	139426030	290	*	2	2.25783E-09	0.000258333	0.005565811	0.000516015	-0.221873623	-0.046579434	HIPK2
chr17	43517764	43517795	32	*	2	1.45161E-08	0.000923204	0.005576083	0.001434528	-0.115613842	-0.088198074	PLEKHM1

chr17	75149960	75150033	74	*	2	2.34457E-09	0.000481786	0.005577048	0.000862418	-0.199231008	-0.134428406	SEC14L1
chr19	37177434	37178489	1056	*	8	2.20607E-15	0.027659634	0.005581448	0.002312443	-0.123026094	-0.0215132	AC074138.3
chr13	24843347	24844938	1592	*	11	4.7779E-26	0.00011815	0.00559183	6.68087E-05	-0.348489654	-0.065953474	RP11-307N16.6, SPATA13
chr2	74611500	74612812	1313	*	9	7.81673E-29	4.16632E-08	0.005593541	1.66379E-08	0.147276095	0.055724039	DCTN1-AS1, DCTN1
chr10	80848009	80848143	135	*	2	1.41944E-09	0.000187388	0.00559384	0.00039317	-0.157910507	-0.117309429	ZMIZ1
chr13	32889023	32889994	972	*	10	1.19634E-25	2.42171E-06	0.005601767	3.55348E-06	-0.350563113	-0.062417429	BRCA2, ZAR1L
chr14	105953300	105954118	819	*	5	1.17004E-08	0.004885642	0.0056065	0.005042738	0.02679826	0.015447998	CRIP1, CRIP1
chr10	25242304	25242747	444	*	2	1.93008E-10	0.000179036	0.005607687	0.000378233	0.192741154	0.175984298	RP11-165A20.3
chr9	77502231	77503686	1456	*	12	9.31775E-23	0.000568193	0.005610704	0.000124676	-0.310611804	-0.054469817	TRPM6
chr9	74383817	74384969	1153	*	8	1.65742E-14	0.018784595	0.00562232	0.00148316	0.031604695	0.001111744	TMEM2
chr6	41472657	41473517	861	*	5	9.35302E-11	0.023447357	0.005629109	0.009338893	0.024996719	0.013891907	RP11-328M4.3, RP11-328M4.2
chr16	89608196	89608497	302	*	5	2.75397E-16	4.50815E-05	0.005633011	3.48585E-05	0.163581313	0.089807579	SPG7
chr1	2517410	2518505	1096	*	13	8.06382E-14	0.0172017	0.005633472	0.010125295	-0.059933235	-0.021012499	FAM213B
chr11	65430511	65430610	100	*	3	5.95909E-08	0.015762306	0.005636253	0.007881742	-0.065234113	-0.024923329	RELA
chr12	100866177	100866802	626	*	4	7.12412E-13	0.006139684	0.005637466	0.000786027	-0.176736834	-0.054096995	
chr13	47371412	47371987	576	*	10	2.84192E-12	0.382539025	0.005639234	0.12691355	-0.213595081	-0.021611591	
chr11	58980377	58981095	719	*	5	1.02625E-11	0.001604199	0.005645633	0.001395283	-0.162324012	-0.039954406	MPEG1
chr2	27282579	27282584	6	*	2	2.5246E-09	0.000450479	0.005648048	0.000819599	0.190928021	0.179584152	AGBL5
chr14	90167459	90168307	849	*	7	3.09165E-11	0.01707796	0.005663268	0.013684036	0.085250887	0.025262105	RP11-33N16.3
chr16	2094109	2094700	592	*	5	7.39941E-12	0.000401136	0.005668531	0.000757881	0.141504909	0.074911514	NTHL1
chr20	47345118	47345204	87	*	2	1.84688E-07	0.002580528	0.005691523	0.003028682	-0.207940435	-0.123052977	PREX1
chr15	92490606	92490956	351	*	2	7.00617E-09	0.000608239	0.005693272	0.001044484	-0.098004585	-0.073988482	SLCO3A1
chr3	13060741	13060974	234	*	4	3.74519E-12	0.002241691	0.005701839	0.000587657	0.160247689	0.095679532	IQSEC1
chr20	42187103	42187837	735	*	8	9.3011E-15	0.186100339	0.005705568	0.008129961	-0.22278827	-0.072595911	SGK2
chr7	46972570	46972700	131	*	2	1.02385E-09	0.000177509	0.005707281	0.000376689	-0.154117199	-0.117917154	AC004901.1
chr1	22979387	22979739	353	*	5	1.81511E-10	0.026163112	0.005716274	0.00674054	0.098328979	0.043271324	C1QB
chr19	45567390	45567904	515	*	3	1.06599E-08	0.046608028	0.005716881	0.01668493	0.143460274	0.039977642	CLASRP
chr22	51019849	51019923	75	*	3	1.43035E-08	0.016752982	0.005719496	0.005822852	-0.149791198	-0.057240107	CHKB-CPT1B, CHKB
chr19	30432102	30432971	870	*	6	6.68117E-21	6.76099E-07	0.00572072	4.07857E-06	0.105407645	0.050109617	URI1

chr6	32906460	32906526	67	*	2	4.88864E-08	0.002737722	0.005722941	0.003159181	-0.207229355	-0.114143892	HLA-DMB, XXbac-BPG181M17.5
chr13	76207486	76207568	83	*	2	6.7213E-10	0.000248329	0.00572754	0.000502155	0.126091882	0.125335783	RP11-29G8.3, LMO7, RP11-173B14.5
chr12	125019756	125020479	724	*	8	1.12707E-13	0.031100325	0.005731352	0.00298103	-0.103606157	-0.018873282	NCOR2
chr3	128417740	128417935	196	*	2	5.48124E-09	0.000915788	0.005732421	0.001438361	-0.103474741	-0.047472176	
chr6	26020944	26021147	204	*	2	5.64954E-11	0.005836635	0.005733587	0.005116278	0.055369669	0.024015936	HIST1H3A
chr4	1221838	1221935	98	*	2	1.7749E-09	0.00030515	0.005734175	0.000597184	-0.188339974	-0.171090461	CTBP1
chr7	45038259	45039616	1358	*	10	1.12487E-21	2.19399E-05	0.005736323	4.33724E-05	0.195005705	0.004019005	CCM2
chr7	75582881	75583421	541	*	4	2.75669E-11	0.001308181	0.005737896	0.000846963	-0.288961427	-0.135289313	POR
chr1	161179001	161179293	293	*	4	9.57049E-10	0.00687655	0.005740998	0.005002648	-0.063742478	-0.024333173	NDUFS2
chr6	153451812	153453173	1362	*	8	4.57325E-15	0.005550345	0.005744047	0.00070812	-0.140060942	-0.016160707	RGS17
chr11	71725245	71725590	346	*	4	8.19108E-13	0.006558885	0.005747691	0.001189336	-0.1524566	-0.069687101	RP11-849H4.4, NUMA1
chr19	836606	836716	111	*	3	8.10449E-11	0.001529206	0.005754726	0.00076883	-0.118508519	-0.066348548	
chr8	77528430	77528456	27	*	2	1.87415E-09	0.000413635	0.005755185	0.000768304	0.196953366	0.177771166	ZFHX4-AS1
chr13	114918456	114918702	247	*	3	7.10721E-10	0.014158916	0.005762688	0.003774224	0.145551983	0.086613377	
chr5	180017195	180017227	33	*	2	8.1134E-08	0.040093895	0.005770763	0.013013843	0.032694465	0.021900685	SCGB3A1
chr9	139554979	139555972	994	*	4	2.34169E-13	5.6519E-06	0.005778149	2.88018E-05	-0.157038515	-0.10859163	EGFL7
chr19	49568106	49568962	857	*	5	3.87314E-11	0.229781189	0.005780291	0.019470552	0.165436104	0.052136185	CTB-60B18.18, NTF4
chr13	113356896	113357667	772	*	5	5.01084E-16	0.000293699	0.005780711	0.000137211	-0.233985163	-0.123105712	ATP11A
chr5	90485840	90486010	171	*	2	5.30686E-10	0.000262667	0.005782521	0.000527591	-0.223275066	-0.182397625	
chr6	155054535	155055468	934	*	6	2.30394E-13	0.00601756	0.005791812	0.002860175	0.035008403	-0.000419894	SCAF8
chr7	161652	162052	401	*	4	5.65997E-08	0.11213197	0.005791867	0.015142328	-0.229840268	-0.065318979	RP11-90P13.1
chr5	124057499	124057960	462	*	3	2.44128E-10	0.002494701	0.005792389	0.001462624	0.132315853	0.033071699	ZNF608
chr19	41827759	41827894	136	*	2	8.10583E-10	0.00018308	0.005797479	0.000387993	0.151847077	0.139184403	CCDC97, TGFB1
chr18	67624071	67625835	1765	*	9	9.35052E-15	0.002588852	0.005824003	0.000168233	0.194569171	0.086701965	CD226
chr12	2985293	2986037	745	*	6	5.45801E-13	0.09784835	0.005830323	0.003999936	-0.216444008	-0.04386024	RHNO1, FOXM1
chr16	3306524	3307326	803	*	5	6.69045E-17	9.88731E-06	0.00583434	2.18478E-05	-0.305419614	-0.130395974	MEFV
chr14	31698967	31699236	270	*	2	1.69917E-08	0.00069836	0.005834783	0.001172702	-0.095968317	-0.071479472	
chr5	131723239	131724046	808	*	3	3.75078E-11	0.001551096	0.005839692	0.001285841	0.17777926	-0.00206388	SLC22A5
chr6	6657296	6657777	482	*	2	8.18845E-10	0.001329324	0.005843431	0.001913905	0.110481236	0.073772432	

chr22	37677244	37678241	998	*	7	6.50476E-16	0.07865186	0.00584735	0.003460274	0.233609027	0.037258888	CYTH4
chr8	22933021	22933496	476	*	2	9.90164E-10	0.000650655	0.005856973	0.001110467	-0.207765943	-0.125912513	RP11-875O11.2
chr10	17428519	17428890	372	*	5	8.2646E-12	0.001773676	0.005867372	0.001163304	0.13012438	0.077808371	ST8SIA6
chr15	78368276	78369232	957	*	2	3.92317E-08	0.001755352	0.005868753	0.002344833	-0.157333688	-0.11540452	TBC1D2B
chr19	57874988	57875295	308	*	6	1.29793E-10	0.342341252	0.005875039	0.021062784	-0.053056934	-0.010793206	ZNF547, TRAPPC2P1, AC003002.4
chr4	2938941	2939149	209	*	2	4.75469E-08	0.040610622	0.005881039	0.013244985	0.092005674	0.037411824	NOP14-AS1
chr12	6750437	6750943	507	*	6	9.37054E-16	0.006608877	0.005881536	0.000230227	0.150958072	0.0658669	ACRBP
chr10	13390377	13390774	398	*	3	1.01598E-09	0.01182661	0.005884244	0.007375917	-0.031228133	-0.00405884	
chr1	150943828	150944300	473	*	2	6.54985E-09	0.235501726	0.005885243	0.019430403	-0.176382632	-0.088827544	CERS2
chr21	36207798	36208014	217	*	2	6.03619E-10	0.00053894	0.005892311	0.000957559	-0.194101883	-0.137194382	RUNX1
chr1	19440010	19440269	260	*	2	3.95054E-08	0.005436587	0.005892538	0.004968108	0.129522136	0.110203304	UBR4
chr16	69140530	69140892	363	*	3	1.10895E-08	0.187827042	0.005892606	0.023784904	0.031648168	0.005465533	HAS3
chr17	913998	914053	56	*	2	9.64365E-08	0.002204841	0.005908931	0.002760436	0.146672662	0.125926121	ABR
chr2	46524557	46525678	1122	*	5	2.94082E-14	0.008454246	0.005916013	0.002973624	0.040844064	0.013243911	EPAS1
chr10	119134612	119134712	101	*	2	4.1301E-08	0.003734702	0.005918476	0.003933751	0.029776932	0.011108526	PDZD8
chr2	26225713	26226017	305	*	3	1.17429E-10	0.000246057	0.005919432	0.000469385	-0.13496298	-0.098320242	
chr5	76369467	76369633	167	*	2	1.87367E-08	0.003291162	0.005919793	0.00362275	0.12902249	0.019675946	ZBED3
chr16	2262293	2265528	3236	*	17	2.61331E-17	0.004540733	0.005920786	8.67409E-05	-0.150824243	-0.012540835	RP11-304L19.8, PGP
chr4	40194771	40195318	548	*	4	2.80075E-10	0.013434416	0.005922992	0.004131458	-0.1623559	-0.071035892	RHOH
chr4	100737138	100738139	1002	*	7	1.91414E-14	0.012778022	0.005942547	0.001931132	-0.199920073	-0.049292153	DAPP1
chr10	73846858	73849480	2623	*	20	2.06367E-36	0.001583539	0.00595287	1.47706E-07	0.094123339	-0.004330269	SPOCK2
chr20	1310451	1310884	434	*	3	2.71887E-10	0.000552236	0.005956022	0.000975273	0.11454086	0.076523784	SDCBP2-AS1
chr19	16556362	16556657	296	*	2	3.78377E-08	0.053270038	0.005968684	0.014652942	-0.226102983	-0.120585777	EPS15L1
chr9	34662282	34663048	767	*	5	5.15174E-10	0.076848355	0.005974206	0.014966517	-0.129804369	-0.013764105	CCL27, RP11-195F19.30
chr6	40567553	40567579	27	*	2	5.70701E-08	0.004930813	0.005977547	0.004711426	0.094226495	0.087138104	
chr12	41868018	41868324	307	*	2	3.72391E-09	0.000297586	0.005979799	0.000590238	0.163337691	0.121340985	PDZRN4
chr3	119318411	119318672	262	*	3	1.06842E-08	0.003082362	0.005982213	0.002802982	0.142315866	0.058152312	PLA1A
chr10	81034149	81034736	588	*	4	4.21819E-12	0.001199406	0.005986308	0.001070095	-0.197207878	-0.119164571	ZMIZ1
chr12	58175385	58176175	791	*	3	2.98986E-11	0.008057914	0.005987092	0.002760117	0.123109648	0.016218069	METTL21B, RP11-571M6.15

chr5	150460100	150460163	64	*	2	1.11746E-07	0.098192787	0.005987608	0.017285021	0.017939456	0.00696831	TNIP1
chr3	12219504	12219682	179	*	2	5.34591E-09	0.000367092	0.005988881	0.000703244	-0.153492927	-0.131260246	SYN2
chr2	102577397	102577876	480	*	2	1.02352E-08	0.000637641	0.005991111	0.001099876	-0.217106905	-0.167414317	
chr22	39639003	39639859	857	*	4	4.10706E-10	0.001362198	0.00600521	0.001219939	0.046813503	0.014986526	PDGFB
chr6	170532633	170532835	203	*	4	1.72987E-11	0.003325412	0.006008156	0.001134636	-0.068055108	-0.047553554	
chr8	24769895	24771645	1751	*	15	7.54968E-18	0.000657277	0.006008305	0.000167327	0.09694678	0.026719931	NEFM, GS1-72M22.1
chr5	124081066	124081950	885	*	5	2.38568E-10	0.095813717	0.006023851	0.015067377	-0.127215247	-0.045502977	ZNF608
chr7	47306613	47306668	56	*	2	2.95601E-09	0.000470075	0.00603292	0.000862806	0.193263207	0.154820849	
chr6	109010947	109011288	342	*	4	2.14434E-10	0.051255483	0.006034908	0.003446607	-0.182262346	-0.077493746	
chr16	2136269	2136968	700	*	7	4.89354E-11	0.007301212	0.006035754	0.00457922	-0.046859519	0.0048761	TSC2
chr12	117042854	117042895	42	*	2	3.147E-08	0.000757181	0.00603916	0.001261909	-0.152033493	-0.125560897	
chr1	54618655	54619895	1241	*	11	5.36835E-19	0.000644523	0.006045457	0.000353582	-0.279480148	-0.025393168	CDCP2, RP11-446E24.4
chr10	99093461	99094391	931	*	4	4.07979E-10	0.064395333	0.006045771	0.001932738	0.028770698	0.011859311	RP11-452K12.4, FRAT2
chr4	148653439	148653917	479	*	7	2.14617E-11	0.019584922	0.00604628	0.008060066	0.033481559	0.001580384	ARHGAP10
chr11	69259247	69261044	1798	*	9	1.67528E-20	2.76662E-06	0.00605327	3.96547E-06	-0.270560867	-0.119422371	AP000439.2
chr17	16367146	16367232	87	*	2	2.65643E-09	0.000914873	0.006060331	0.001462534	0.187845647	0.139659507	FAM211A-AS1, FAM211A
chr20	48728064	48728642	579	*	3	9.21866E-11	0.000327749	0.006061862	0.00070543	0.153587942	0.097007017	UBE2V1, TMEM189-UBE2V1, TMEM189
chr17	26899172	26899686	515	*	5	5.33007E-10	0.057724811	0.00607242	0.024937674	0.161356466	0.065363479	RP11-192H23.4
chr1	61547969	61548526	558	*	8	7.40439E-09	0.522918665	0.006072809	0.116680776	0.024896387	-0.004501696	NFIA
chr12	56327037	56327217	181	*	2	2.66964E-10	0.000766535	0.006088783	0.001277133	-0.130503321	-0.109947731	DGKA
chr1	198648220	198649066	847	*	4	6.42318E-10	0.007742359	0.006091477	0.000945834	-0.20245591	-0.114963625	PTPRC, PEBP1P3
chr9	37485478	37485592	115	*	2	4.64851E-09	0.00042148	0.00609246	0.000791214	0.136950783	0.134040455	
chr14	69526817	69526840	24	*	2	2.21574E-08	0.000622643	0.00609869	0.001084646	0.111089044	0.102730997	DCAF5
chr17	36858826	36859078	253	*	2	5.94271E-09	0.013203504	0.0061005	0.008320434	0.015588704	0.009936984	CTB-58E17.1
chr1	170043489	170044179	691	*	8	1.12084E-13	0.0338779	0.006101906	0.004137589	-0.059547802	-0.01538543	KIFAP3
chr21	47738771	47738804	34	*	2	4.28659E-10	0.000302888	0.006105875	0.000601779	-0.150385381	-0.130119164	C21orf58
chr2	135471997	135472528	532	*	3	7.87399E-11	0.000116785	0.00610719	0.000313588	0.150912998	0.062661463	TMEM163
chr6	1389146	1389367	222	*	3	1.61177E-08	0.036955907	0.006114361	0.016304978	0.054876448	0.025411372	
chr11	66113995	66114776	782	*	4	1.06307E-10	0.001046144	0.006121002	0.001268364	0.117859767	0.034437861	B3GNT1

chr22	50154174	50155070	897	*	3	2.96277E-08	0.003911776	0.006125905	0.000974397	-0.162514516	-0.113214165	
chr3	10374067	10375226	1160	*	3	1.1928E-09	2.16714E-05	0.006146966	7.39532E-05	0.149205777	0.051472983	ATP2B2
chr2	219536975	219537816	842	*	4	1.53317E-13	0.005363983	0.00615449	0.002584827	-0.153350416	-0.025353205	STK36, RNF25
chr15	41191138	41191151	14	*	2	1.32084E-09	0.000357778	0.006157349	0.000692868	-0.155296457	-0.151810737	VPS18
chr3	129146731	129147397	667	*	3	1.81927E-08	0.023237324	0.006165706	0.004383777	-0.063811249	-0.028053924	EFCAB12
chr2	225809244	225809808	565	*	4	1.17353E-09	0.012418028	0.006170523	0.001835591	-0.216461362	-0.017488157	DOCK10
chr17	76886835	76887311	477	*	3	3.55879E-10	0.00575883	0.006176065	0.002703047	-0.142214733	-0.059324588	TIMP2, DDC8
chr4	178229651	178231443	1793	*	15	1.12167E-27	6.58421E-06	0.006176664	1.54461E-05	-0.306903847	-0.039594915	NEIL3
chr15	90735063	90735422	360	*	3	2.15839E-12	5.48693E-05	0.006182073	0.000144642	0.196380732	0.132034055	SEMA4B
chr12	57923710	57924237	528	*	2	2.89968E-10	0.000209553	0.006184709	0.000441165	-0.184452529	-0.034069598	MBD6, DCTN2
chr17	61915833	61916730	898	*	5	7.49066E-14	0.045178931	0.006185948	0.002119491	-0.195422276	-0.090979051	SMARCD2
chr4	7780490	7781082	593	*	3	1.0016E-10	0.000196509	0.006202016	0.000352053	0.132960077	0.08315326	AFAP1-AS1, AFAP1
chr17	3807247	3807312	66	*	2	5.36446E-08	0.002158242	0.006210677	0.002773093	-0.180489617	-0.11041623	P2RX1
chr5	65735649	65736106	458	*	2	9.05655E-09	0.000483152	0.006218191	0.000889043	-0.13846247	-0.006372074	
chr20	20348331	20348687	357	*	3	1.33247E-10	0.018484707	0.006222425	0.005171762	0.032176239	0.014221054	
chr19	35629576	35630651	1076	*	14	3.39379E-31	0.001304817	0.006231205	7.32587E-06	-0.208151097	-0.061894145	FXYD1, CTD-2527I21.4, LGI4, AC020907.2
chr7	157405010	157405170	161	*	2	9.58539E-09	0.006225123	0.006231381	0.005540676	0.134632678	0.098200146	PTPRN2
chr15	75660047	75660780	734	*	6	4.8783E-12	0.00552427	0.006235336	0.005650083	0.051678853	0.014556974	RP11-817O13.8, MAN2C1
chr8	42400563	42400586	24	*	2	8.19446E-10	0.000400001	0.006236899	0.000762175	0.115591807	0.10185732	SMIM19
chr8	103803399	103803666	268	*	2	5.92916E-09	0.00035784	0.00623806	0.000695037	-0.092902365	-0.079040896	
chr7	39989471	39990544	1074	*	11	3.08327E-13	0.22931717	0.00624664	0.034056027	0.035194581	0.00348593	CDK13
chr8	144634355	144634524	170	*	3	4.697E-13	0.000349415	0.006252597	0.000736295	-0.086631623	-0.05637091	
chr2	9770727	9771671	945	*	9	4.88057E-13	0.020449269	0.006256753	0.00781993	-0.12022887	-0.002997884	YWHAQ
chr1	226830923	226832000	1078	*	3	4.11937E-12	2.97946E-05	0.006269656	9.63385E-05	-0.223083532	-0.12901899	ITPKB
chr12	118745017	118745504	488	*	3	6.58409E-09	0.001295453	0.006275541	0.001745144	-0.134601868	-0.076638218	TAOK3
chr14	72064773	72065164	392	*	3	1.14468E-10	0.003308039	0.006294518	0.001322992	-0.13425073	-0.063050357	SIPA1L1
chr22	19929557	19930802	1246	*	6	5.84409E-13	0.000110813	0.006295305	0.000123894	-0.258772601	-0.070469457	COMT
chr6	157890509	157890698	190	*	2	1.01238E-07	0.001617134	0.006310236	0.00226975	-0.131878951	-0.08657177	ZDHHC14
chr6	154689546	154689719	174	*	2	2.36667E-09	0.000213896	0.006319682	0.000450854	-0.277596118	-0.194119822	

chr10	16741464	16741619	156	*	3	8.85537E-08	0.004900599	0.00632345	0.00478208	0.103740841	0.081774963	RSU1
chr3	30654871	30656010	1140	*	5	1.33254E-10	0.000216818	0.006325772	9.65461E-05	-0.214089228	-0.067350566	TGFBR2
chr16	88568237	88569490	1254	*	9	3.9534E-22	2.39256E-06	0.006326539	2.91254E-06	0.225917219	0.090398604	ZFPM1
chr1	45190519	45190912	394	*	2	3.04542E-08	0.634949137	0.006346554	0.021475356	-0.180522616	-0.085425614	C1orf228
chr1	90032648	90032655	8	*	2	8.8914E-08	0.001562754	0.006360771	0.00222014	-0.099186939	-0.069895784	LRRC8B
chr3	43732993	43733831	839	*	4	6.47582E-11	0.004344141	0.006361698	0.004363391	-0.239155	-0.052319731	ABHD5, ANO10
chr11	75898195	75898203	9	*	2	2.3314E-10	0.000222233	0.006368928	0.000466607	0.169373356	0.143883448	WNT11
chr20	30060822	30061679	858	*	7	1.10138E-15	0.002493862	0.006369314	0.000315291	0.149036933	0.065465034	DEFB124
chr15	41851964	41852193	230	*	3	8.62272E-10	0.013303114	0.006383176	0.006680069	-0.064634297	-0.038342743	TYRO3
chr22	23161339	23161379	41	*	3	5.73587E-09	0.001757746	0.006388736	0.002329099	0.141769345	0.069910398	
chr1	108506812	108507910	1099	*	11	8.01014E-13	0.152835511	0.006396052	0.039745497	-0.049412957	-0.002640764	VAV3-AS1, VAV3
chr3	128792713	128792776	64	*	3	1.19739E-10	0.000412777	0.006397085	0.000815173	0.102656447	0.072989926	
chr1	211779938	211780386	449	*	2	2.86137E-09	0.000243319	0.006397348	0.00050473	-0.170058284	-0.148155419	
chr1	110610995	110612044	1050	*	6	6.17924E-14	0.013269724	0.006397407	0.002747202	0.066890166	0.033922685	STRIP1, ALX3
chr16	67466260	67466470	211	*	2	7.39658E-08	0.058311342	0.006405792	0.015854215	0.127748172	0.057121871	HSD11B2
chr22	21983606	21984483	878	*	7	1.94267E-09	0.078143174	0.006408149	0.030150343	-0.019731736	-0.00209434	YDJC
chr8	67975874	67976534	661	*	6	4.75233E-17	0.001078092	0.006414969	0.000660941	-0.335880437	-0.132863281	CSPP1, COPS5
chr4	10458049	10458667	619	*	5	1.08811E-11	0.008419819	0.006420221	0.005613077	0.193713656	0.052820975	ZNF518B
chr19	49126940	49127660	721	*	6	1.46316E-14	0.000150825	0.006425862	0.0003688	0.158804877	0.033559272	SPHK2, AC022154.7
chr2	161127677	161127827	151	*	2	1.8178E-07	0.025400108	0.006429124	0.011689225	0.096224796	0.042974439	ITGB6
chr6	31683051	31683352	302	*	6	7.33868E-16	2.76974E-05	0.006439975	6.14544E-05	0.180751507	0.103521821	LY6G6F, MEGT1, LY6G6D
chr12	68042072	68043561	1490	*	8	2.1189E-14	0.001339855	0.006440128	0.000717741	-0.091895788	-0.025902225	DYRK2
chr2	232530555	232531087	533	*	2	1.29537E-09	0.000218759	0.006441465	0.000461317	0.107727997	0.028478734	
chr11	3175007	3175636	630	*	3	1.19028E-12	1.12863E-05	0.006442384	4.53016E-05	-0.138770614	-0.099345041	OSBPL5
chr11	117150669	117150989	321	*	3	3.47585E-10	0.001000152	0.0064431	0.00096636	0.179309878	0.112444883	RNF214
chr19	39902727	39903392	666	*	5	3.30054E-10	0.194896413	0.006446657	0.046522294	0.031171163	0.001899207	PLEKHG2
chr6	170535321	170536124	804	*	8	9.9337E-12	0.042907417	0.006449657	0.001856151	-0.225967352	-0.072027528	
chr12	7260403	7261227	825	*	12	8.42799E-13	0.113297398	0.006450147	0.039189591	-0.236038056	-0.00845408	C1RL-AS1, C1RL
chr11	110299342	110301128	1787	*	11	2.31059E-18	0.012095739	0.006450589	0.000413373	-0.13164189	-0.029175604	FDX1

chr16	86017231	86017380	150	*	2	1.77989E-08	0.009292801	0.006457795	0.007116032	-0.144063839	-0.092255941	RP11-542M13.2
chr11	16834800	16834902	103	*	2	2.79907E-09	0.000227106	0.006461462	0.000476692	-0.152964774	-0.139116792	PLEKHA7
chr16	69362128	69363236	1109	*	6	3.62553E-14	0.012818988	0.00646718	0.000792356	-0.136347429	-0.058017587	COG8, PDF, RP11-343C2.12
chr20	31072454	31072777	324	*	3	7.69827E-09	0.012964862	0.006473041	0.006307876	-0.165171763	-0.059240401	C20orf112
chr1	45277159	45277354	196	*	2	1.68912E-09	0.002209143	0.006474505	0.002864515	0.184718181	0.116380005	BTBD19
chr5	1168528	1169632	1105	*	5	8.03375E-13	0.016907669	0.006479193	0.002833407	-0.193908537	-0.066712217	
chr20	48807167	48808334	1168	*	9	1.00339E-11	0.05027479	0.006485246	0.004449067	-0.068068423	-0.003280496	CEBPB
chr8	86290450	86291400	951	*	4	3.70029E-10	0.015433229	0.006488987	0.002118826	0.123700403	0.014776166	CA3, CA1
chr16	2801009	2802372	1364	*	14	2.48807E-17	0.160750333	0.006489989	0.007981341	0.138603159	0.021076195	SRRM2, SRRM2-AS1
chr9	35658010	35658919	910	*	5	1.73942E-12	0.006197615	0.006490025	0.001685449	-0.061165012	-0.021071581	CCDC107, RMRP, ARHGEF39
chr2	96990480	96991791	1312	*	13	5.11579E-19	0.000336549	0.006498806	0.000310351	-0.058673041	0.005301131	ITPRIPL1
chr17	46687312	46687865	554	*	3	3.90343E-10	0.098660062	0.006500064	0.025333689	0.039032476	0.014335603	HOXB7
chr13	111159111	111160844	1734	*	7	2.11637E-16	0.000290235	0.006509881	1.99479E-05	0.160296649	0.080089617	COL4A2, COL4A2-AS1
chr11	118476352	118476517	166	*	2	4.44267E-08	0.018498892	0.006510013	0.010205954	-0.175032095	-0.078509611	
chr11	44626708	44628259	1552	*	7	6.77329E-17	0.004428743	0.006513296	0.000361653	-0.230636044	-0.045374637	CD82, RP11-58K22.5, RP11-58K22.4
chr15	85923174	85924342	1169	*	12	2.42424E-18	0.007611032	0.006515214	0.001647071	0.031798437	0.005425485	AKAP13
chr11	126326411	126326696	286	*	2	5.12359E-09	0.000364184	0.006515487	0.000712266	0.121198161	0.0993166	KIRREL3
chr4	1195845	1196179	335	*	3	8.0997E-14	2.12404E-05	0.006517706	7.76297E-05	-0.161080671	-0.118951523	SPON2
chr1	52608409	52608614	206	*	3	3.45542E-10	0.068002001	0.00653296	0.020032748	0.020549754	0.002054049	ZFYVE9
chr12	102090101	102091773	1673	*	13	8.68026E-23	0.015598063	0.006535028	0.000559337	-0.236561547	-0.026331585	CHPT1
chr13	107214319	107214359	41	*	2	2.72275E-08	0.001418125	0.006539829	0.002087023	0.17796244	0.167104416	ARGLU1
chr6	15662707	15664231	1525	*	15	5.71102E-14	0.060564114	0.006541231	0.015101407	0.169100669	0.010692131	DTNBP1
chr16	70733234	70734327	1094	*	7	4.819E-21	3.03736E-06	0.006542799	2.54463E-06	-0.207083242	-0.101920707	VAC14
chr4	186124639	186124759	121	*	2	1.88332E-09	0.038242841	0.006544824	0.01394166	0.026971542	0.014987762	KIAA1430
chr3	46989208	46989655	448	*	2	2.14326E-09	0.001127228	0.006546675	0.001757415	-0.171799738	-0.033366601	CCDC12
chr13	108919855	108921244	1390	*	5	1.01646E-13	2.92109E-05	0.006557644	4.92045E-05	-0.229269031	-0.119057125	TNFSF13B
chr19	44009237	44009549	313	*	4	1.06581E-08	0.032021253	0.006559154	0.014868404	0.164677349	0.067948593	
chr22	20747254	20747837	584	*	4	1.13245E-09	0.06263392	0.00655972	0.026247287	-0.278262546	-0.071144194	
chr8	144679690	144680433	744	*	9	6.00441E-12	0.142493597	0.006561512	0.040182576	0.032916419	0.005967874	TIGD5, EEF1D

chr2	206628088	206629314	1227	*	12	1.00032E-31	7.42092E-09	0.006563566	2.98426E-08	0.201750187	0.105288314	NRP2, AC007362.3
chr1	156818592	156819393	802	*	6	2.38243E-11	0.008481421	0.006566325	0.002082844	-0.113332841	0.01599327	NTRK1, INSRR
chr5	1103325	1103453	129	*	2	6.31302E-08	0.052606238	0.006567619	0.015622371	0.115894291	0.066974869	SLC12A7
chr5	169739625	169740386	762	*	4	8.95487E-12	0.002519287	0.006573496	0.001220431	-0.27859326	-0.107609398	CTB-114C7.4
chr8	30299680	30300306	627	*	7	5.98365E-13	0.008176537	0.006580332	0.002823948	0.167378204	0.045612663	RBPMS
chr19	14062697	14063471	775	*	9	5.68423E-19	0.003018624	0.006581089	0.000119843	0.036545698	0.004511442	DCAF15, PODNL1
chr2	178256731	178257342	612	*	9	2.69056E-16	0.019734174	0.006583068	0.009717985	-0.317378822	-0.04176924	NFE2L2, AC074286.1
chr19	18110560	18111886	1327	*	10	1.50289E-17	0.000108382	0.006585271	3.24836E-05	0.166756992	0.050644585	KCNN1
chr19	1622280	1623075	796	*	6	2.77658E-15	0.015563929	0.006589556	0.002408989	-0.283345008	-0.079595614	TCF3
chr2	160589097	160589392	296	*	3	6.23896E-09	0.147991226	0.006593428	0.003873481	-0.201825108	-0.103637383	MARCH7
chr12	110464779	110465221	443	*	3	3.35029E-11	0.002263142	0.006600944	0.000938187	-0.234365814	-0.118158663	ANKRD13A
chr4	185671960	185672248	289	*	3	7.56444E-12	8.56191E-05	0.00660738	0.000226377	0.134581966	0.069054382	
chr10	11615505	11615853	349	*	3	5.96527E-10	0.000796239	0.006607615	0.001239354	-0.143048224	-0.070023806	USP6NL
chr20	17854235	17854245	11	*	2	6.28976E-09	0.000509109	0.006621526	0.000942493	-0.183112427	-0.14248622	
chr1	172112722	172114419	1698	*	10	8.30321E-13	0.020829209	0.006628466	0.002525567	0.179028147	0.053621476	DNM3, DNM3OS, MIR199A2
chr11	93271083	93271677	595	*	5	2.87725E-15	0.00435794	0.006633972	0.000115074	-0.151297416	-0.093287941	SMCO4
chr21	34773372	34775045	1674	*	11	3.15549E-24	0.000291856	0.006640624	2.9833E-05	-0.357833497	-0.098935949	
chr6	42951711	42952586	876	*	14	3.89134E-17	0.008425853	0.006653892	0.001829297	-0.075500644	-0.009938113	PPP2R5D
chr5	150473002	150473165	164	*	2	6.94957E-09	0.000470222	0.006655217	0.000884308	-0.058000928	-0.057643719	TNIP1
chr3	39322541	39323729	1189	*	10	1.11672E-13	0.190982923	0.006659076	0.021493701	-0.120747503	0.000962817	CX3CR1
chr11	125757328	125757446	119	*	4	2.85443E-08	0.010940741	0.006667298	0.007898673	0.0224709	0.007333595	HYLS1
chr22	45636109	45636477	369	*	3	2.99542E-09	0.037084375	0.006672316	0.017423554	0.019947554	0.004081637	KIAA0930
chr16	11589604	11589683	80	*	2	1.91654E-08	0.000677451	0.006679348	0.001189301	0.116572902	0.09208576	CTD-3088G3.8
chr12	45970012	45970082	71	*	2	6.94138E-09	0.000362096	0.006679377	0.000712795	-0.120814086	-0.110734112	RP11-352M15.1
chr16	54325129	54325319	191	*	2	3.91021E-08	0.056395515	0.006680691	0.016169604	0.067585416	0.045810803	
chr21	46954058	46954883	826	*	4	6.48426E-13	0.000598792	0.006680912	0.000490756	-0.150143074	-0.096090089	SLC19A1
chr14	104027293	104028127	835	*	2	2.06129E-09	0.03308861	0.006685903	0.013375086	0.116860876	0.051111694	RP11-894P9.2, BAG5
chr12	123520574	123520699	126	*	2	2.66073E-09	0.000381052	0.006687988	0.000744028	0.149416988	0.144685017	PITPNM2
chr17	954410	954490	81	*	2	2.11759E-08	0.001745831	0.006689328	0.002450344	0.169615903	0.132832854	ABR

chr16	71843250	71844250	1001	*	7	2.01792E-22	0.000711063	0.006692447	0.000110319	-0.246907554	-0.027727245	
chr17	52938144	52938163	20	*	2	1.45329E-08	0.001074399	0.006692804	0.001706188	0.133832616	0.114209405	
chr20	52492209	52492248	40	*	2	7.9904E-10	0.000316209	0.006698519	0.000636492	-0.305431563	-0.270038774	
chr2	208393204	208394839	1636	*	11	1.67134E-17	8.62636E-05	0.006699792	5.98928E-05	0.182250277	-0.002918397	CREB1, AC007879.5
chr1	51730449	51730576	128	*	2	5.83696E-08	0.005767543	0.006701959	0.00547993	0.160242994	0.115828616	RNF11
chr3	52316234	52316296	63	*	2	1.98743E-07	0.001948364	0.006714952	0.002656151	-0.173215424	-0.143774149	WDR82
chr7	73669010	73669227	218	*	4	4.70126E-08	0.008235513	0.006721863	0.00614818	-0.01904712	-0.006701819	
chr14	64909105	64909251	147	*	3	3.59337E-09	0.001735166	0.006727055	0.001931438	0.09969482	0.079033903	MTHFD1, CTD-2555O16.2
chr13	51289696	51289912	217	*	4	7.37203E-17	1.06263E-06	0.006728739	7.03768E-06	-0.26331883	-0.192870548	DLEU1, DLEU7
chr14	104355829	104356172	344	*	4	7.39975E-09	0.010828539	0.006732271	0.005803953	0.187112171	0.072896076	
chr12	6304557	6304747	191	*	4	4.55778E-09	0.00354325	0.006736163	0.004157562	0.087375694	0.056996925	
chr6	64515937	64516331	395	*	4	5.491E-08	0.017213136	0.006743801	0.010355801	-0.211467893	-0.075154016	EYS
chr1	205782522	205783009	488	*	5	3.3402E-09	0.103275895	0.006749374	0.03350282	0.017401899	-0.00013402	SLC41A1
chr14	52118241	52119097	857	*	7	4.7373E-13	0.02517628	0.006752011	0.006490544	0.027303933	0.001088787	FRMD6
chr19	54377682	54377836	155	*	2	8.33775E-08	0.040509649	0.006755898	0.014552092	-0.200577062	-0.095165709	MYADM
chr16	74775045	74776130	1086	*	6	5.3012E-11	0.115148022	0.006759789	0.003212684	0.194930607	0.077688422	FA2H
chr16	58417718	58418628	911	*	6	3.29161E-11	0.023113139	0.006766139	0.002076788	-0.213818346	-0.029428193	GINS3, RNU6-1110P
chr22	50746891	50747878	988	*	6	1.07744E-14	0.003360659	0.006776549	0.000437563	0.205039594	0.111160685	DENND6B
chr2	445069	445430	362	*	2	4.0937E-09	0.009357692	0.006777047	0.007328341	0.167930389	0.067815905	
chr2	183902764	183903412	649	*	8	2.18665E-16	0.013215079	0.006782007	0.001993902	0.04703907	0.006971244	NCKAP1
chr20	62505169	62505188	20	*	2	8.70703E-09	0.000360641	0.006782857	0.000712812	-0.159294083	-0.151835142	TPD52L2
chr11	8736732	8737458	727	*	3	5.86156E-09	0.00161796	0.00678532	0.002307312	-0.192370237	-0.07075232	ST5
chr19	4402502	4403593	1092	*	8	6.41527E-13	0.060193297	0.006785748	0.009911335	0.025075088	0.011289685	CHAF1A
chr19	1075595	1077244	1650	*	7	7.00535E-18	9.8181E-08	0.006793136	5.31852E-07	0.218642532	0.054563299	HMHA1
chr17	74442338	74443080	743	*	3	8.7412E-10	7.37862E-05	0.006803346	0.000187241	-0.127914103	0.017261383	UBE2O
chr15	45461444	45461636	193	*	2	1.23286E-09	0.000246852	0.006806332	0.000517177	0.110081148	0.104628924	SHF
chr2	60983440	60983838	399	*	3	4.66823E-09	0.042150487	0.006809934	0.018829493	0.016997669	0.010518769	PAPOLG
chr11	14666247	14666512	266	*	3	2.96081E-09	0.013327309	0.006815473	0.00706115	0.02946768	0.012107078	PDE3B
chr12	56367210	56368226	1017	*	17	6.93277E-25	1.93424E-05	0.00681566	1.31126E-05	-0.098767758	-0.02391309	RAB5B

chr6	116832940	116833441	502	*	2	7.26074E-09	0.000404651	0.006817962	0.000785607	0.156644376	0.132048959	FAM26E, TRAPPC3L
chr14	56533845	56533858	14	*	2	3.20761E-08	0.001915186	0.006820776	0.002638633	0.116372743	0.073116753	
chr3	196351327	196352455	1129	*	6	3.91134E-16	6.1272E-05	0.006826731	9.80847E-05	-0.208077801	-0.09341561	
chr6	30168380	30168773	394	*	3	3.20677E-08	0.292076083	0.006836484	0.034822361	-0.164444079	-0.058087127	TRIM26
chr4	6927183	6928241	1059	*	5	2.91566E-12	0.002290059	0.006844096	0.00149939	-0.211216291	-0.080333823	TBC1D14
chr14	50863674	50865194	1521	*	12	2.56116E-29	1.46577E-06	0.006846576	5.34874E-07	-0.193958112	-0.082988978	RP11-247L20.3, CDKL1
chr13	103053409	103053652	244	*	3	7.05272E-09	0.137202917	0.00684705	0.030460067	0.026495507	0.011328725	FGF14
chr1	54638137	54638490	354	*	4	5.88642E-13	0.012490938	0.006849101	0.00103204	-0.195422101	-0.084063717	RP11-446E24.4, CYB5RL
chr7	44083518	44084618	1101	*	14	3.5326E-12	0.286295169	0.006851328	0.041903014	0.151378038	0.015020033	DBNL
chr17	54669616	54669896	281	*	2	1.05449E-07	0.004513839	0.006852174	0.004746009	0.024720298	0.002319107	
chr22	39919067	39919237	171	*	2	3.04744E-09	0.000357239	0.006854829	0.000708806	0.107822994	0.102931514	
chr17	26925417	26926868	1452	*	16	2.00502E-18	0.145564262	0.006861119	0.00602112	0.070001322	0.005830612	SPAG5-AS1, RP11-192H23.4, SPAG5
chr7	127033083	127033519	437	*	4	8.82581E-12	0.004660878	0.006864192	0.002163387	0.032191892	0.007725201	ZNF800
chr4	10006885	10007994	1110	*	5	1.29609E-09	0.047053037	0.006864271	0.00181272	-0.200967721	-0.073623044	SLC2A9
chr1	160833130	160834267	1138	*	6	2.31265E-14	0.001366971	0.006864351	0.000750147	-0.158838474	0.009008662	
chr18	77821978	77822187	210	*	2	1.43567E-08	0.011007139	0.006872124	0.008077577	-0.088578309	-0.055667857	RP11-795F19.5, RBFADN
chr21	33895894	33896417	524	*	2	9.41446E-09	0.000297941	0.006881624	0.00060861	0.09693056	0.08537544	
chr10	21822787	21824447	1661	*	10	4.6067E-19	3.13317E-05	0.006882544	1.37537E-05	-0.065850761	-0.029922878	MLLT10
chr17	77302056	77302162	107	*	2	7.2548E-08	0.003918872	0.006891847	0.004340784	0.166882066	0.14203274	RBFOX3
chr11	63580553	63580733	181	*	5	1.07209E-08	0.031700657	0.006892152	0.019854142	0.018715849	0.007934838	
chr13	30169482	30170129	648	*	6	8.58251E-10	0.015021691	0.006893919	0.013884869	0.033190086	0.00398876	SLC7A1
chr22	50623687	50624276	590	*	5	2.74673E-09	0.123789137	0.00690465	0.030163316	0.041493493	0.017912763	
chr11	236908	237407	500	*	6	9.328E-11	0.303054171	0.006910606	0.030698014	0.021726272	0.004398018	PSMD13, SIRT3
chr19	17530035	17531370	1336	*	16	2.0618E-13	0.076362444	0.006918171	0.016290241	0.064412808	0.009160334	MVB12A, CTD-2521M24.6, CTD-2521M24.8
chr11	43941935	43942682	748	*	6	6.53857E-12	0.006351782	0.006924943	0.00242936	-0.170810941	-0.055466795	RP11-613D13.4, ALKBH3-AS1
chr1	155293541	155295093	1553	*	14	2.3063E-18	8.20602E-05	0.006933769	9.96308E-05	-0.072590759	-0.008115644	RUSC1, RUSC1-AS1
chr11	77899047	77900023	977	*	11	1.10126E-12	0.252457881	0.006934391	0.035758887	0.043306275	0.007811132	USP35, KCTD21
chr19	40872142	40872286	145	*	2	1.89039E-08	0.00303815	0.006936923	0.003670921	-0.125593543	-0.045547571	PLD3
chr4	129349106	129349141	36	*	2	7.8006E-08	0.00065846	0.00693723	0.001174485	0.155008822	0.129814008	RP11-420A23.1

chr10	119296756	119296942	187	*	2	1.7063E-11	0.0025478	0.006939425	0.003251781	-0.045056962	-0.014851068	EMX2OS
chr7	138144067	138145031	965	*	6	9.70352E-14	0.001388705	0.006945328	0.001079983	-0.13800993	-0.043482018	
chr1	35245523	35247084	1562	*	11	1.36385E-10	0.004138171	0.006947148	0.001729349	0.10041702	0.030926432	GJB3, SMIM12, RP1-34M23.5
chr2	75061219	75063068	1850	*	11	4.71175E-18	0.008672148	0.006948846	0.000910705	-0.072579154	-0.003283789	HK2
chr19	17445012	17445073	62	*	2	2.06068E-09	0.027042918	0.006952653	0.012621033	0.11887305	0.06875404	ANO8
chr6	41195891	41196139	249	*	2	6.41584E-10	0.000431368	0.006970469	0.000832657	-0.238071381	-0.223132405	TREML4
chr7	26007848	26008481	634	*	2	2.26239E-08	0.000293985	0.006974243	0.000603366	0.096950708	0.093494201	
chr6	90788825	90788857	33	*	2	5.54216E-09	0.000289918	0.006975158	0.000596267	0.12705596	0.118415618	BACH2
chr12	51664226	51664655	430	*	10	4.43457E-09	0.553185898	0.006978156	0.135055068	0.023958619	0.002763259	DAZAP2, SMAGP
chr11	75142449	75142651	203	*	2	9.51237E-09	0.23892537	0.006978528	0.022404688	0.120686086	0.059926465	
chr3	15356739	15356881	143	*	3	4.59412E-11	7.58004E-05	0.006978537	0.000208954	-0.269714357	-0.187830461	SH3BP5
chr15	91140952	91141539	588	*	2	4.7051E-09	0.0002565	0.0069918	0.000537235	0.142327411	0.031692125	CRTC3
chr5	38403622	38404322	701	*	4	2.95327E-14	0.000724369	0.007001045	0.000195294	0.154033755	0.099626722	EGFLAM
chr11	62432494	62433815	1322	*	12	4.33237E-15	0.02817269	0.007001305	0.014661253	0.030457735	0.001136032	METTL12, SNORA57, RP11-831H9.11, C11orf48
chr20	36661238	36662003	766	*	7	2.05132E-10	0.044062666	0.007001886	0.006045646	-0.174959796	-0.0349548	RPRD1B, TTI1
chr11	129991445	129992368	924	*	6	5.5602E-18	7.71177E-08	0.007015819	9.03299E-07	0.142496463	0.095712338	APLP2
chr11	117695372	117696822	1451	*	8	1.10819E-19	4.31349E-06	0.007021318	2.23404E-06	-0.210921246	-0.072824568	FXYD2, FXYD6-FXYD2
chr1	200983238	200983313	76	*	2	9.9626E-10	0.000261208	0.007024606	0.000546174	-0.21375252	-0.174229805	KIF21B
chr9	95895956	95896749	794	*	5	1.43187E-16	0.000471244	0.007026422	0.000149077	-0.078382329	-0.03446762	NINJ1
chr8	49647703	49648519	817	*	12	4.81818E-25	0.012083259	0.00702951	0.000598204	-0.25233046	-0.046941063	EFCAB1
chr2	135808369	135808617	249	*	4	2.93843E-12	0.000176502	0.007031799	0.000284189	0.198680764	0.12408169	
chr8	1954777	1955214	438	*	5	2.606E-17	3.17711E-07	0.00703292	2.90535E-06	-0.234924039	-0.139446022	KBTBD11
chr15	75493593	75494077	485	*	8	8.19825E-13	0.127259366	0.007041121	0.014330177	-0.099903792	-0.009005533	C15orf39
chr7	27702416	27704065	1650	*	14	2.28238E-15	0.014246291	0.007042334	0.000660216	0.214361797	0.010590792	HIBADH
chr13	28194657	28195822	1166	*	13	4.09101E-10	0.311721572	0.007048401	0.067708635	0.032265427	0.003808379	POLR1D
chr1	226889496	226890287	792	*	2	2.37736E-08	0.001147222	0.007054954	0.001823781	0.160504712	0.105804368	ITPKB
chr18	74207388	74207551	164	*	4	6.83238E-10	0.031618749	0.007056859	0.012089255	0.021330398	0.011349927	RP11-17M16.1
chr17	7461260	7462249	990	*	13	3.09008E-15	0.076874087	0.00705725	0.005355803	-0.100809367	-0.02668442	TNFSF12, TNFSF12-TNFSF13, TNFSF13
chr12	51718559	51719349	791	*	4	4.89655E-13	0.175571077	0.007060135	0.008235069	-0.263735081	-0.103549539	

chr19	2549886	2550027	142	*	2	2.43548E-08	0.000596255	0.007066077	0.001089379	-0.158646635	-0.117896525	GNG7
chr8	142167262	142167457	196	*	2	3.02881E-08	0.003711373	0.007072845	0.004234523	0.131196995	0.055189843	DENND3
chr8	101964947	101966860	1914	*	17	2.43006E-17	0.010129685	0.007077828	0.0020712	-0.245099915	-0.031820619	YWHAZ
chr6	30302939	30303126	188	*	2	1.08732E-07	0.065978493	0.007081863	0.017692585	-0.278224289	-0.147647062	TRIM39, TRIM39-RPP21
chr2	174829142	174830453	1312	*	7	8.12177E-17	0.010388898	0.007082187	0.000782059	-0.144467435	-0.015388626	SP3
chr8	103249036	103249853	818	*	3	7.27186E-12	4.03961E-05	0.007091572	0.000129689	-0.253073026	-0.160730197	RRM2B
chr6	6724955	6725495	541	*	4	5.69444E-10	0.026410376	0.00710201	0.014059843	-0.281662907	-0.078395284	
chr17	8056492	8057058	567	*	5	6.55435E-12	0.045125899	0.007103485	0.00253485	-0.109954641	-0.040043973	PER1, RP11-599B13.6
chr10	22828670	22829118	449	*	4	1.03138E-13	0.002820534	0.007109899	0.000163379	0.148079151	0.097631363	PIP4K2A
chr10	99478572	99478719	148	*	2	6.6423E-08	0.025975788	0.007109918	0.012579298	-0.086720717	-0.057284292	
chr6	32099450	32099564	115	*	2	2.38294E-10	0.000286095	0.007111496	0.000591828	0.173404969	0.146371651	
chr10	120354627	120355756	1130	*	10	8.84013E-13	0.417880075	0.007122068	0.021216849	0.096429102	0.038444809	PRLHR
chr8	134073176	134073884	709	*	4	4.53387E-12	0.000528193	0.007123472	0.001037511	0.164683151	0.065290004	TG, SLA
chr11	1299151	1299791	641	*	5	9.60123E-09	0.07139688	0.007133402	0.030192675	0.109115094	0.041880273	TOLLIP
chr9	119311603	119311706	104	*	2	9.08663E-08	0.001606384	0.00713674	0.002358244	-0.267065005	-0.206667925	RP11-264C15.2, ASTN2
chr4	2812455	2812744	290	*	2	1.23967E-07	0.087583786	0.007142042	0.019242351	-0.065868874	-0.042167595	SH3BP2
chr8	22446484	22446737	254	*	2	5.99248E-09	0.001830056	0.007150749	0.002596902	0.193819526	0.139115231	PDLIM2
chr15	41101870	41102374	505	*	3	6.00062E-10	0.011197364	0.007164992	0.001823483	0.156394577	0.090462861	ZFYVE19
chr10	134539818	134540383	566	*	5	1.84049E-12	0.15549458	0.007167185	0.006217307	0.14192441	0.059903487	INPP5A
chr18	72165429	72166704	1276	*	6	1.43707E-14	0.006230279	0.007171922	0.001163413	-0.10954825	-0.051568474	CNDP2
chr17	32266	32503	238	*	4	1.45511E-12	0.000174553	0.007176738	0.00037829	-0.23557375	-0.144175559	
chr3	171024571	171024841	271	*	3	5.41094E-10	0.001350703	0.007183864	0.001198286	0.151854595	0.070755345	TNIK
chr15	97037828	97038051	224	*	2	3.11506E-08	0.000714809	0.00718569	0.001266206	-0.113224553	-0.07252282	
chr6	90025136	90026078	943	*	6	1.79874E-13	0.000382709	0.007188812	0.000158	0.105177124	0.035232583	
chr14	77783299	77783447	149	*	2	7.06334E-09	0.000322533	0.00718886	0.000656829	-0.138105771	-0.121707355	POMT2
chr3	176919547	176919713	167	*	3	1.94784E-11	5.40904E-05	0.00719428	0.000157467	-0.20291075	-0.17842263	
chr12	124903636	124903668	33	*	2	5.3685E-09	0.001152902	0.007199023	0.001842277	0.134818599	0.093976598	NCOR2
chr1	179923514	179924316	803	*	11	3.25853E-10	0.2484846	0.007199178	0.090582318	0.029656867	0.004072614	CEP350, RP11-533E19.5
chr19	18698825	18699234	410	*	5	5.42394E-10	0.022446565	0.007200384	0.010225677	-0.17246279	-0.085481501	CRLF1

chr11	57252275	57252371	97	*	2	3.60173E-08	0.003649834	0.007200397	0.00421949	-0.23781534	-0.153607076	SLC43A1
chr1	160160587	160160766	180	*	4	8.51876E-09	0.221661471	0.007219093	0.013159273	0.166568727	0.062365805	CASQ1
chr2	179149472	179149605	134	*	3	1.82766E-11	6.41551E-05	0.007222627	0.000202475	0.138648531	0.129662543	OSBPL6
chr12	113013215	113013494	280	*	4	9.4769E-08	0.005367735	0.007226365	0.005918033	0.120425272	0.06215293	RPH3A
chr2	39453847	39453876	30	*	2	4.25536E-08	0.000767465	0.007229291	0.001342283	0.141148412	0.105362398	CDKL4
chr6	30002671	30002763	93	*	3	1.74897E-10	0.000212854	0.007232889	0.000533881	-0.17943513	-0.129307545	ZNRD1-AS1
chr6	30127760	30129346	1587	*	12	3.88377E-24	5.44756E-05	0.007242104	4.18309E-06	0.209437371	0.079490127	TRIM10
chr15	52643585	52643727	143	*	2	6.20571E-08	0.001607673	0.007243781	0.002371696	-0.111291767	-0.085494781	MYO5A
chr17	25798180	25799447	1268	*	11	9.79117E-22	2.48979E-05	0.00724452	1.73071E-05	0.124955686	0.054279719	KSR1
chr9	140135453	140136534	1082	*	8	7.33386E-14	0.000802761	0.007244626	0.000138848	0.066104372	0.017056338	TUBB4B
chr16	2984407	2984768	362	*	5	4.78547E-09	0.267004077	0.007256554	0.019140506	-0.214586357	-0.064878825	FLYWCH1
chr6	41123086	41123396	311	*	3	1.09545E-08	0.009833996	0.007264595	0.00834087	0.09648392	0.083914707	
chr19	2256468	2257204	737	*	7	9.87462E-19	0.001124471	0.007267734	0.000136187	0.123661528	0.065288938	JSRP1
chr9	132388503	132389032	530	*	3	3.97914E-10	0.168851982	0.007281509	0.012309129	0.030138044	0.011785671	NTMT1
chr19	10928172	10928696	525	*	9	8.63264E-21	2.99305E-05	0.007282256	2.16503E-05	0.126021112	0.065644873	DNM2, MIR199A1
chr1	161052851	161054092	1242	*	5	2.2523E-12	0.001256549	0.007283632	0.001776401	-0.206533711	-0.044231301	PVRL4
chr10	96122379	96122890	512	*	5	2.15824E-09	0.185659395	0.007285143	0.010997028	0.038991821	0.009458421	NOC3L
chr3	58523313	58523649	337	*	4	4.00313E-10	0.005647789	0.007286249	0.003027112	-0.236610575	-0.098919174	
chr1	29572478	29572561	84	*	2	1.49642E-07	0.017091574	0.007303925	0.010513795	-0.081038746	-0.060088549	PTPRU
chr3	18786729	18787223	495	*	3	3.77781E-10	0.000364093	0.007305391	0.000799636	-0.235935818	-0.132678496	AC144521.1
chr6	34359501	34361621	2121	*	11	1.37743E-14	0.003677519	0.007314255	0.000195568	-0.261102531	-0.020097243	NUDT3, RPS10-NUDT3
chr11	1942404	1943014	611	*	7	4.95374E-21	0.000303523	0.007316826	5.90047E-05	0.144498416	0.07368419	TNNT3
chr10	129099107	129099456	350	*	3	8.68808E-09	0.004720729	0.007324171	0.003105332	0.157046788	0.064182822	DOCK1
chr5	139138880	139139201	322	*	3	1.29456E-11	4.02357E-05	0.007336622	0.000133704	-0.240194727	-0.207141625	
chr8	29211863	29211949	87	*	2	5.61569E-10	0.00047062	0.007344506	0.000905868	-0.213478403	-0.164836855	
chr10	134310836	134311114	279	*	3	9.33361E-09	0.073822884	0.007347474	0.003591023	-0.118896522	-0.065725633	
chr13	30982826	30982971	146	*	2	1.18443E-08	0.000810661	0.007347752	0.001408263	-0.20932188	-0.0966077	
chr14	24778911	24779959	1049	*	6	5.73324E-16	0.000265512	0.007348043	0.000349219	-0.130404765	-0.048670777	LTB4R2, LTB4R2, CIDEB
chr20	30168680	30168797	118	*	3	1.21921E-07	0.014496752	0.007354303	0.010370536	-0.134319481	-0.063354647	

chr19	36100919	36101191	273	*	2	1.34814E-08	0.005361019	0.007354651	0.005468774	-0.122999041	-0.041685094	AC002115.9
chr1	1549615	1550648	1034	*	7	8.03494E-18	0.000617895	0.007358275	0.000138591	-0.180105005	-0.052723173	RP11-345P4.9
chr1	7238945	7238998	54	*	2	2.85034E-08	0.000887898	0.007362004	0.001514089	0.185987469	0.143126814	CAMTA1
chr1	109102050	109103095	1046	*	6	9.5715E-14	7.75684E-05	0.007362618	6.54391E-05	0.180705326	0.03193681	FAM102B
chr19	13209470	13211261	1792	*	8	9.39221E-14	2.4663E-05	0.007369757	4.02915E-05	-0.07189493	-0.029645579	NFIX, LYL1
chr12	53607916	53608405	490	*	3	1.9159E-10	0.000596965	0.007370154	0.00096455	0.145992029	0.087185096	RARG
chr5	159625135	159626172	1038	*	8	1.37303E-14	0.002483973	0.007377769	0.001318931	0.150125736	0.06190793	FABP6, CTB-127C13.1
chr13	52585283	52586563	1281	*	17	1.92702E-14	0.023051345	0.007379474	0.007563786	0.055408471	0.009907659	ALG11, ATP7B
chr7	150728263	150728338	76	*	2	3.91149E-08	0.002819127	0.007379903	0.003574359	-0.132674771	-0.111651443	ABCB8
chr19	47248003	47249219	1217	*	8	1.93844E-14	0.000840265	0.007384412	0.000565387	0.12980369	0.000529356	STRN4
chr17	75368902	75370611	1710	*	14	1.76489E-18	0.003008781	0.007384698	0.001723538	0.062609791	0.00973616	SEPT9, RP11-936I5.1
chr7	631862	633202	1341	*	7	1.80719E-12	0.001143793	0.007386269	0.000432555	0.160609017	0.071309357	PRKAR1B
chr5	137774392	137774585	194	*	5	2.67423E-08	0.009018941	0.007389542	0.009587742	0.193766727	0.046424264	
chr22	46373530	46373951	422	*	3	5.57106E-10	0.073936776	0.007393929	0.013887916	0.061505356	0.041384881	
chr19	8565406	8565520	115	*	3	3.98585E-10	0.002834246	0.007394808	0.002973962	-0.171315402	-0.087566977	PRAM1
chr11	12136405	12136467	63	*	2	7.99283E-09	0.000329472	0.00739588	0.000672694	0.153386195	0.15268535	MICAL2
chr16	4987201	4987927	727	*	10	3.29341E-11	0.531641222	0.007397048	0.10460225	-0.088961082	-0.011008283	PPL
chr20	55017557	55017932	376	*	2	1.4886E-08	0.000997476	0.007408123	0.001661585	-0.081583184	-0.074701535	CASS4
chr6	108572926	108573461	536	*	4	1.71444E-13	5.30575E-05	0.007423394	0.000149762	0.155246359	0.103955653	RP1-128O3.5, SNX3
chr14	24455908	24456378	471	*	2	1.70414E-09	0.000786558	0.007428187	0.001379012	-0.163124318	-0.150120594	DHRS4L2, DHRS4-AS1
chr8	73998208	73998859	652	*	3	8.4445E-09	0.01023616	0.007428376	0.001717367	0.130371905	0.082854898	SBSPON
chr11	113846918	113847135	218	*	5	4.84409E-14	0.021181105	0.007429962	0.000626632	-0.284701023	-0.103988937	HTR3A
chr5	138941366	138941688	323	*	5	8.34341E-10	0.028700511	0.007445454	0.016268869	0.020266471	0.006581797	UBE2D2
chr9	6779450	6779489	40	*	2	7.49546E-09	0.000474116	0.007448946	0.000914425	-0.185495328	-0.185028741	KDM4C
chr8	11415543	11416365	823	*	5	1.01878E-10	0.008157305	0.007456857	0.000766952	-0.100648323	-0.03179101	BLK, RP11-148O21.2
chr11	64127791	64128339	549	*	3	1.67202E-11	7.17279E-05	0.007457897	0.000223353	-0.152161326	-0.104051975	RPS6KA4
chr3	49823664	49824475	812	*	17	5.6952E-11	0.759291544	0.007459032	0.203113764	-0.048376161	-0.001935612	IP6K1
chr9	124587471	124587820	350	*	3	2.59282E-10	0.00302189	0.007460573	0.001245478	0.117418849	0.08165902	TTLL11
chr1	228890820	228891306	487	*	4	2.16843E-09	0.002003417	0.007463806	0.002815279	-0.188396541	-0.112845883	

chr1	70820512	70820798	287	*	10	3.61685E-11	0.416495803	0.007469401	0.172158925	0.042217613	0.003167011	HHLA3
chr1	225997649	225998232	584	*	4	7.26722E-11	0.019529804	0.007478493	0.002653871	0.02603481	0.001103377	EPHX1
chr14	21571844	21572766	923	*	6	3.30426E-09	0.066136581	0.007479626	0.016364833	0.017415414	0.003188592	TMEM253, ZNF219
chr16	81591788	81591792	5	*	2	4.98073E-10	0.000352135	0.007482127	0.000713422	0.075032901	0.068694491	CMIP
chr17	7482456	7483518	1063	*	6	1.62037E-11	0.012418365	0.007485391	0.004362602	-0.069660329	-0.021164942	SNORA67, CD68
chr6	167506553	167508537	1985	*	12	3.7227E-12	0.004876317	0.007497721	0.000241074	-0.119379695	-0.01079865	RP11-517H2.6
chr8	142182536	142183860	1325	*	8	1.6055E-12	0.000236513	0.007498424	0.00013956	0.18753737	-0.027930737	DENND3
chr3	71631744	71632188	445	*	3	9.4387E-11	0.098487653	0.007508432	0.004249171	0.019563014	0.008978135	FOXP1
chr1	26857284	26857774	491	*	4	1.0947E-11	0.001936864	0.007521761	0.000920308	-0.174470047	-0.086916229	RPS6KA1
chr17	40306700	40307717	1018	*	11	1.47537E-22	2.06781E-05	0.007525145	1.15623E-05	0.081946004	0.002174269	RP11-358B23.1, CTD-2132N18.3, RAB5C
chr6	31645677	31646377	701	*	6	7.37928E-12	0.026659093	0.007534282	0.005399249	0.134513549	0.053681707	LY6G5C
chr5	179779115	179779664	550	*	4	1.7332E-10	0.017269964	0.007534594	0.003870378	0.097550252	0.055740304	GFPT2
chr13	113706662	113707929	1268	*	8	3.50491E-15	0.001842995	0.007537678	0.000660132	0.157675631	0.058022425	MCF2L
chr17	7123415	7123994	580	*	8	1.12774E-08	0.139267268	0.007556958	0.073673229	0.024064841	0.007241268	ACADVL
chr7	2149259	2151788	2530	*	12	4.388E-18	6.18168E-05	0.007558643	1.00161E-05	0.171453706	0.076775043	MAD1L1
chr3	4793370	4794082	713	*	5	3.46927E-13	1.57875E-05	0.007562904	7.70698E-05	-0.060788318	-0.045042282	ITPR1
chr16	622502	622992	491	*	4	6.62874E-11	0.019508763	0.007571875	0.002455371	0.128955327	0.058784919	PIGQ
chr12	48213040	48214620	1581	*	11	1.60209E-18	1.85213E-06	0.007572514	2.95252E-06	-0.209805533	-0.03554432	HDAC7
chr12	51403056	51403474	419	*	5	3.16103E-15	0.000161108	0.007579782	8.43961E-05	-0.13729908	-0.08124062	SLC11A2
chr19	43099795	43099845	51	*	2	1.10369E-07	0.001428097	0.007580691	0.002203816	-0.192044706	-0.15448252	LIPE-AS1
chr2	242641476	242641489	14	*	2	1.5295E-07	0.026884724	0.007580826	0.013295313	0.034304506	0.015038056	ING5
chr17	5137892	5138696	805	*	8	3.65373E-15	0.032845367	0.007582003	0.00034612	-0.143568315	-0.051592317	RP11-333E1.1, SCIMP
chr18	77441381	77441810	430	*	3	5.6105E-10	0.112120541	0.007591171	0.028754459	-0.174904167	-0.07119787	CTDP1
chr11	12108103	12108286	184	*	3	1.00463E-08	0.002239354	0.007592323	0.003175957	0.199846199	0.130831079	
chr16	66865015	66865112	98	*	4	1.60807E-07	0.120933797	0.007592502	0.021866475	0.032247144	-0.003293227	NAE1
chr19	17211623	17211772	150	*	2	7.05279E-11	0.000421885	0.007600309	0.000833453	0.1652178	0.151960906	MYO9B
chr5	177036766	177037104	339	*	3	3.06766E-11	0.000226432	0.00762233	0.000484722	-0.21094186	-0.150340472	B4GALT7
chr11	64878218	64878267	50	*	2	1.56988E-08	0.057169971	0.007623649	0.01783427	-0.116352455	-0.051906196	VPS51, AP003068.9
chr3	11732893	11733395	503	*	3	6.88437E-10	0.001365668	0.007628646	0.001389674	0.135048857	0.088604638	VGLL4

chr10	99343311	99344164	854	*	7	2.83729E-14	0.004755667	0.007632199	0.001985887	0.167661547	0.044544376	ANKRD2, HOGA1, PI4K2A, PI4K2A
chr7	23387365	23387862	498	*	5	1.9089E-11	0.000615124	0.007639311	0.000879569	-0.149608242	-0.036447925	IGF2BP3
chr8	146024438	146024786	349	*	4	7.51825E-10	0.006588096	0.007639345	0.004685776	0.024680301	0.00527378	ZNF517
chr2	239988967	239989132	166	*	2	1.56168E-08	0.001024664	0.007644395	0.001712539	0.126070174	0.113646803	HDAC4
chr16	57643645	57644411	767	*	4	9.69094E-12	0.005078178	0.007657182	0.001011637	0.175842174	0.109817983	
chr19	35760554	35760861	308	*	3	7.28623E-09	0.003489382	0.007659133	0.004170422	0.049532679	0.030404288	USF2
chr8	142161840	142161988	149	*	2	4.3439E-09	0.014372486	0.007671924	0.009897679	-0.213015602	-0.115791517	DENND3
chr12	45685719	45686426	708	*	5	4.37878E-11	0.053337875	0.007672218	0.004833519	0.205085673	0.064787718	ANO6
chr6	116707933	116708668	736	*	3	3.04141E-10	0.000138804	0.007674133	0.000381524	-0.15551604	-0.043117835	DSE
chr9	71819562	71820047	486	*	5	8.50519E-19	3.29228E-07	0.007688172	2.96095E-06	0.167357811	0.124112711	TJP2
chr5	88120826	88121200	375	*	2	4.2107E-08	0.002044792	0.007689629	0.002889149	-0.1435702	-0.091300721	MEF2C
chr16	15149275	15149995	721	*	3	1.1764E-10	0.014762003	0.007691983	0.009473536	0.025856263	0.008866645	PDXDC1, NTAN1
chr17	48201129	48201233	105	*	2	2.00572E-08	0.001424289	0.007699245	0.002210458	0.141580827	0.105021178	SAMD14
chr16	54085289	54085398	110	*	2	2.34004E-08	0.000758945	0.007702468	0.00135337	-0.159726581	-0.106116837	FTO, RP11-357N13.1
chr19	47839132	47840131	1000	*	4	7.23566E-09	7.06117E-05	0.007702806	0.000167611	-0.170703593	-0.046652941	C5AR2
chr16	30584674	30584961	288	*	3	1.04859E-08	0.184233815	0.00770956	0.016160438	0.15570699	0.061541564	AC002310.7
chr14	69424756	69425084	329	*	2	7.80417E-09	0.000594255	0.007717649	0.001110677	0.109471825	0.092812368	ACTN1
chr10	72200612	72201741	1130	*	13	5.35039E-14	0.031671933	0.007728773	0.016801789	0.076029477	0.032438919	NODAL
chr11	67219241	67220318	1078	*	11	7.08024E-13	0.05464961	0.007738229	0.016737485	0.181511927	0.046656812	CABP4, GPR152
chr4	1319719	1320674	956	*	5	1.54747E-14	0.003008285	0.007741667	0.000661147	0.1415601	0.071400345	MAEA
chr4	8291937	8292801	865	*	4	1.86003E-09	0.014840248	0.007743478	0.004392311	-0.218052008	-0.068899584	HTRA3
chr5	149466066	149466715	650	*	7	5.16027E-20	0.000101456	0.007743839	3.37909E-05	-0.179449554	-0.075773908	CSF1R
chr14	69281297	69282256	960	*	3	1.02551E-11	5.36264E-05	0.007745039	0.000167183	0.090499494	0.084028872	
chr20	3693158	3693221	64	*	3	3.54573E-10	0.00024274	0.007749802	0.000605288	-0.162546755	-0.106379722	
chr21	45575559	45575832	274	*	3	6.6135E-09	0.001553842	0.00775257	0.002174257	0.10938214	0.075755514	
chr2	106761185	106761805	621	*	4	1.98229E-12	0.00029359	0.007756478	0.000670466	0.102384205	0.081678644	UXS1
chr16	85146088	85146768	681	*	6	4.2481E-14	0.001233848	0.007767254	0.000430862	0.090009024	0.048027205	FAM92B
chr2	64370690	64371680	991	*	9	1.15553E-11	0.09421836	0.007777738	0.028327381	0.026667197	0.001230728	AC074289.1, PELI1
chr15	50412191	50412411	221	*	3	2.47718E-09	0.001007354	0.00778122	0.001793339	-0.149556124	-0.128796391	ATP8B4

chr2	43446987	43447375	389	*	3	1.65436E-08	0.005342772	0.007792717	0.003156683	-0.043177972	-0.011764595	THADA
chr15	43512998	43513563	566	*	5	1.86556E-09	0.00904579	0.007794129	0.006060589	0.149540322	0.072554854	EPB42
chr6	108280315	108280406	92	*	2	1.37571E-09	0.020629113	0.007795406	0.011975132	0.143624811	0.051745005	RP1-191J18.66
chr7	128049980	128050616	637	*	9	9.97674E-12	0.533165557	0.007797844	0.041108292	0.031620917	0.00653421	IMPDH1
chr7	32931884	32932444	561	*	6	5.65577E-10	0.008160906	0.007798372	0.006822706	-0.038728523	-0.002181877	AVL9, KBTBD2
chr7	99036293	99037189	897	*	14	5.92367E-15	0.081435751	0.00779895	0.009088692	0.047684973	0.004329053	CPSF4, PTCD1, ATP5J2-PTCD1
chr19	4638933	4640159	1227	*	16	3.13912E-15	0.007757173	0.007802961	0.005763428	-0.054774682	0.001462807	TNFAIP8L1
chr2	97171002	97171449	448	*	5	4.98375E-10	0.002776791	0.00780762	0.002848814	0.165474909	0.064719124	NEURL3
chr14	64009472	64010574	1103	*	12	6.28489E-18	0.004718765	0.007810213	0.001827788	0.040568366	0.004294421	CTD-2302E22.4, PPP2R5E
chr2	169652805	169653384	580	*	4	8.86491E-13	0.006604113	0.007817472	0.000366205	0.182700891	0.122812687	NOSTRIN
chr19	49843565	49843922	358	*	4	6.50072E-09	0.006126233	0.007821746	0.004967932	0.130079624	0.086150732	CD37, CTC-301O7.4, TEAD2
chr6	86353447	86354018	572	*	5	3.53036E-09	0.027884171	0.007824879	0.01286065	-0.039082981	-0.011542321	SYNCRIP
chr3	42699933	42701042	1110	*	11	6.48646E-24	7.20593E-05	0.007831689	3.96278E-05	-0.265016717	-0.063848319	ZBTB47
chr1	28201862	28202173	312	*	3	4.85132E-09	0.007119875	0.007832551	0.001979514	-0.228265177	-0.108481392	THEMIS2
chr20	8638302	8639258	957	*	5	2.95454E-12	0.002004111	0.007836381	0.001348203	-0.340617601	-0.122395699	PLCB1
chr1	45805948	45807214	1267	*	12	3.67473E-17	0.046099988	0.007836468	0.008040792	-0.199476717	-0.014478334	TOE1, MUTYH
chr9	134619599	134619980	382	*	2	2.20343E-09	0.000347677	0.007836666	0.000712467	0.170103042	0.003057246	
chr1	3425603	3425761	159	*	2	1.75583E-07	0.009699413	0.007839074	0.008068001	-0.116265971	-0.082527565	MEGF6
chr11	116740987	116741639	653	*	3	1.96506E-11	0.00044226	0.007851866	0.00072135	-0.199497026	-0.133484213	SIK3
chr18	3066514	3067624	1111	*	6	7.1116E-12	0.012315286	0.007855128	0.001904739	-0.259003765	-0.004754494	MYOM1
chr21	33670510	33672713	2204	*	7	4.93006E-13	0.001410015	0.007858673	0.000222232	0.182145152	0.053514292	MRAP
chr2	28974102	28974942	841	*	9	8.89147E-13	0.001166373	0.00786099	0.001442089	0.027598623	-0.004687435	PPP1CB
chr13	114880761	114880889	129	*	2	4.33832E-08	0.059539302	0.007861766	0.01846776	0.166713804	0.071125256	RASA3
chr19	13946770	13947914	1145	*	11	6.20405E-18	0.00466659	0.007870319	0.000199514	-0.179445624	-0.049294278	MIR24-2, MIR27A, MIR23A
chr6	4889428	4890656	1229	*	9	6.9247E-19	0.000153372	0.007876068	0.000153538	-0.166462574	-0.069534296	CDYL
chr1	12508973	12509707	735	*	4	2.35259E-15	1.17635E-06	0.007881174	8.47031E-06	0.202574411	0.15778676	VPS13D
chr8	17980057	17980148	92	*	2	9.70577E-08	0.005813518	0.00788901	0.005944462	0.172483733	0.102184916	
chr4	71704501	71705074	574	*	7	1.74076E-08	0.753043242	0.007891901	0.228671892	-0.068381496	-0.013769102	GRSF1
chr9	116341874	116342094	221	*	3	2.557E-09	0.002427898	0.00789856	0.002097509	-0.098069971	-0.052207278	RGS3, RP11-168K11.2

chr3	72151345	72151536	192	*	2	2.43555E-08	0.000842503	0.007903581	0.001481471	-0.134596447	-0.120622597	LINC00877
chr16	67424195	67425219	1025	*	5	1.29015E-10	0.024212309	0.007929694	0.002910685	-0.212823239	-0.073712838	TPPP3, RNU1-123P
chr1	246843900	246844422	523	*	3	2.27065E-11	7.57265E-05	0.007936299	0.000212908	0.12369762	0.088771025	
chr9	124073197	124073313	117	*	3	5.30551E-08	0.007264906	0.007942331	0.003990651	0.095476059	0.044767726	GSN
chr17	80192565	80194552	1988	*	14	5.50413E-17	0.005397154	0.007950863	0.000352415	0.138160728	-0.027207541	SLC16A3
chr11	66725504	66726268	765	*	9	1.91034E-10	0.041801477	0.00797268	0.014385302	-0.128494369	-0.010962735	PC
chr20	1926131	1927911	1781	*	8	3.27553E-12	0.002058618	0.007976472	8.95381E-05	-0.321378052	-0.065360816	RP4-684O24.5
chr9	91933073	91933906	834	*	10	3.99373E-12	0.016975758	0.007977889	0.007274364	-0.054389864	-0.013116631	SECISBP2
chr16	11449467	11449661	195	*	2	6.58409E-09	0.000695366	0.007983086	0.001273005	0.10738787	0.100430129	
chr15	89181732	89182208	477	*	6	5.04454E-09	0.091868683	0.007985141	0.023180949	-0.117142733	-0.009894456	ISG20
chr19	42380725	42381258	534	*	4	2.36504E-12	0.003019469	0.007985772	0.000668904	-0.109167217	-0.062054608	CD79A
chr14	54954596	54955579	984	*	4	2.35953E-11	0.002697302	0.007987341	0.000850854	0.149811576	0.050337094	GMFB
chr11	73308991	73310242	1252	*	14	5.43023E-14	0.02775642	0.007989248	0.011023922	-0.274765733	-0.027665278	FAM168A
chr3	114012316	114012912	597	*	5	1.24567E-14	0.014448066	0.007989288	0.00042669	0.162580914	0.06356193	TIGIT
chr19	825035	825052	18	*	3	1.26826E-12	2.59309E-05	0.007993663	9.75078E-05	-0.179331332	-0.162347717	
chr22	23523942	23524136	195	*	2	1.07777E-07	0.010761882	0.007994772	0.008647172	-0.038942848	-0.031350439	BCR
chr14	74317981	74318494	514	*	11	1.53851E-15	0.065870695	0.007999165	0.011099271	-0.206491952	-0.04184882	
chr12	121022424	121022843	420	*	4	3.37202E-10	0.004233477	0.008009271	0.005165273	0.054525135	0.02831176	
chr1	208085455	208085889	435	*	2	1.33346E-09	0.006624489	0.008020461	0.006499657	-0.158312904	-0.113701665	
chr1	153513810	153514482	673	*	4	6.58932E-11	0.001206907	0.008021132	0.001450116	0.143855879	0.091397105	S100A5
chr11	64836948	64837316	369	*	3	3.62011E-09	0.008066347	0.008021227	0.003450513	-0.078061933	-0.050934119	CDCA5
chr1	235091106	235091608	503	*	4	4.05604E-13	0.000467541	0.008024715	0.000883078	-0.168957147	-0.080902387	
chr8	6565029	6566322	1294	*	15	1.17743E-12	0.135669377	0.008030682	0.041517971	-0.037960061	0.003825532	AGPAT5, CTD-2541M15.1
chr1	95583406	95583636	231	*	2	2.91518E-09	0.009875391	0.008030919	0.008253713	0.040866459	0.023092153	TMEM56, TMEM56-RWDD3
chr12	116996359	116997022	664	*	6	2.76529E-12	0.01529437	0.008044475	0.005267574	0.192594351	0.059781188	
chr7	128579876	128580792	917	*	9	2.4743E-22	8.42458E-08	0.008046012	5.24937E-07	-0.179995445	-0.072141091	IRF5
chr2	129251945	129252477	533	*	2	1.63327E-09	0.000341473	0.008049479	0.000705381	0.13181505	0.106383501	
chr19	846179	846354	176	*	3	6.06836E-11	8.0298E-05	0.008057211	0.000226539	-0.195860542	-0.158758807	PRTN3
chr19	34287017	34287540	524	*	5	1.47687E-11	0.006820344	0.008062879	0.001951376	-0.069979428	-0.021890463	KCTD15

chr3	151102860	151102879	20	*	2	1.56851E-07	0.002502575	0.00806514	0.003400342	-0.234704922	-0.177721749	MED12L
chr1	184775205	184775699	495	*	3	4.04524E-10	0.000623996	0.008072786	0.001090703	-0.201372236	-0.119725679	FAM129A
chr16	89724306	89724915	610	*	5	4.17537E-10	0.039628014	0.008072827	0.017460063	0.025136591	0.01071338	SPATA33
chr1	54356260	54356568	309	*	4	2.17638E-12	0.251584365	0.008078031	0.005147172	-0.2550664	-0.08977877	YIPF1
chr3	69249276	69250885	1610	*	7	6.51183E-11	0.000545833	0.008086948	0.000324134	0.174068471	0.048710693	FRMD4B
chr2	85817259	85817956	698	*	3	9.0096E-10	0.000207531	0.008089272	0.000522882	-0.232444172	-0.070654947	VAMP5
chr7	148801711	148802155	445	*	3	1.6573E-11	7.56665E-05	0.008089794	0.000224104	0.14178414	0.117758844	ZNF425
chr1	209798124	209798721	598	*	2	1.02568E-08	0.009014849	0.008096741	0.007861224	-0.219778922	-0.081205819	LAMB3
chr2	182756909	182757482	574	*	4	4.18965E-10	0.038869804	0.008122292	0.009614675	0.017296697	9.23599E-06	SSFA2
chr8	125685871	125685873	3	*	2	1.12668E-08	0.000358307	0.008132756	0.00073643	0.1103386	0.088523144	MTSS1
chr12	114403667	114405061	1395	*	12	1.89802E-12	0.060185813	0.008136842	0.007385091	-0.23090409	-0.012434961	RBM19
chr8	102216949	102217924	976	*	10	1.90115E-11	0.298161452	0.008139039	0.087875433	-0.047148121	-0.014984193	ZNF706
chr22	27006126	27006498	373	*	2	4.71191E-09	0.000340195	0.008141053	0.000704694	0.1696524	0.151546687	CRYBB1
chr12	122238038	122239035	998	*	7	3.25621E-10	0.119115223	0.008141068	0.01710199	-0.084879771	-0.009340003	RP11-347I19.8, RHOF, AC084018.1
chr19	36485959	36486149	191	*	5	1.12743E-07	0.227741284	0.008143136	0.052855369	-0.025078183	-0.00838532	SDHAF1
chr22	18601993	18602121	129	*	2	5.29686E-08	0.000941017	0.008145454	0.001631004	0.090901909	0.085069032	PEX26, TUBA8
chr7	56120127	56120290	164	*	2	7.24732E-08	0.032988997	0.008150413	0.015197089	-0.053789583	-0.042135249	CCT6A
chr17	26799289	26799509	221	*	4	7.19343E-09	0.028516073	0.008150566	0.006964197	0.115681996	0.032932008	RP11-192H23.4
chr3	50387780	50388823	1044	*	19	6.16609E-14	0.12318568	0.008154094	0.055627426	0.035182703	0.004959323	CYB561D2, XXcos-LUCA11.5, NPRL2
chr7	2800436	2800791	356	*	3	1.3322E-08	0.094507935	0.008157569	0.019146373	0.141255507	0.058431247	AMZ1, GNA12
chr14	23479311	23479801	491	*	7	2.11552E-12	0.009113396	0.008158587	0.00233554	0.090265755	0.031770071	C14orf93
chr14	105219617	105220026	410	*	3	2.00036E-09	0.053544728	0.00817807	0.014514064	0.01436944	0.008250156	SIVA1
chr5	134208721	134209038	318	*	3	7.66219E-10	0.001840098	0.008204797	0.002500802	0.158779077	0.033623586	
chr13	45150990	45151437	448	*	2	7.97118E-12	0.000741014	0.008205961	0.00134974	-0.094285005	-0.037585754	TSC22D1
chr1	3801660	3801728	69	*	3	3.80835E-09	0.000711149	0.00821203	0.00109849	0.104016422	0.087979635	DFFB
chr17	48970334	48970357	24	*	2	1.90241E-09	0.000427317	0.008220817	0.000856854	-0.253880401	-0.215677059	TOB1-AS1
chr10	3868080	3868493	414	*	3	1.51521E-09	0.003191598	0.008221169	0.003304102	0.123372457	0.074139351	
chr11	68606654	68607257	604	*	4	1.23903E-09	0.019694611	0.008224532	0.008140001	-0.035934221	-0.000171976	CPT1A
chr12	65671664	65672174	511	*	5	2.12845E-13	0.003645707	0.008224772	0.001873259	-0.189203887	-0.076194698	

chr3	138657429	138658243	815	*	4	3.68218E-09	0.180530152	0.008225312	0.056295805	0.04115314	0.001862438	RP11-548O1.3
chr20	23113228	23113870	643	*	4	1.62228E-14	2.66333E-06	0.008244062	1.66447E-05	-0.24134371	-0.108255234	LINC00656
chr19	45457673	45459103	1431	*	20	9.4207E-21	0.029323308	0.008245119	0.001154483	-0.06446077	-0.00061471	CLPTM1
chr2	24306908	24308660	1753	*	18	2.28367E-16	0.061065549	0.008247118	0.0055219	-0.082367055	-0.003087008	FAM228B, TP53I3
chr20	61568856	61569754	899	*	8	8.74354E-11	0.023234573	0.00824801	0.016821732	-0.023630128	0.00020085	GID8, DIDO1
chr7	112120730	112121259	530	*	9	2.04804E-17	0.010596457	0.008271189	0.00021599	0.192446268	0.059661367	IFRD1, LSMEM1
chr4	468196	468201	6	*	2	1.85009E-07	0.00039613	0.008273077	0.000804762	-0.04850124	-0.046134442	ZNF721
chr11	70048796	70049600	805	*	14	1.90784E-13	0.226907211	0.00827312	0.026831117	-0.231798766	-0.023126885	FADD
chr10	112127385	112127915	531	*	2	1.71637E-08	0.000498437	0.008285346	0.0009766	0.116024113	0.102400112	
chr2	60610208	60611170	963	*	5	2.69081E-12	0.003483273	0.008287963	0.001375242	-0.113682783	-0.019496242	AC007381.2
chr16	50743027	50743296	270	*	2	1.22829E-08	0.001412233	0.008291969	0.002248659	-0.136498753	-0.103499137	NOD2
chr9	35538212	35539509	1298	*	7	4.28463E-16	0.009256351	0.008298033	0.001197424	-0.199139647	-0.067142452	RUSC2
chr2	60687132	60687823	692	*	4	2.4487E-13	5.20865E-05	0.00829837	0.000138446	-0.243267239	-0.105747446	BCL11A
chr10	23384047	23385979	1933	*	8	4.13528E-15	0.000538132	0.008312053	4.56641E-05	-0.217927583	-0.029097973	MSRB2
chr17	27918761	27920934	2174	*	15	2.3291E-17	3.83712E-05	0.008312691	0.000129501	0.046690586	0.000489165	RP11-68I3.2, ANKRD13B, GIT1, RP11-68I3.7
chr19	46915776	46916588	813	*	4	4.0544E-15	3.66924E-05	0.008328087	0.000129711	0.179162232	0.095459391	CCDC8
chr8	145180747	145180933	187	*	2	1.54019E-07	0.011098035	0.008331248	0.008990329	-0.066244394	-0.050341174	
chr11	66055170	66055683	514	*	4	1.37691E-15	3.39709E-05	0.008348838	8.86643E-05	-0.121404085	-0.110448537	YIF1A
chr5	126312517	126312773	257	*	2	1.34878E-08	0.000388524	0.008361893	0.000793361	0.176098205	0.155049016	MARCH3
chr2	241507374	241508108	735	*	8	3.01587E-12	0.009657629	0.008363944	0.002394089	-0.055204667	-0.006769628	RNPEPL1, ANKMY1
chr7	45151048	45152241	1194	*	14	3.19672E-13	0.304125297	0.008366659	0.038401338	-0.160775964	-0.013437649	TBRG4
chr5	156696185	156696520	336	*	4	6.72644E-15	4.86498E-06	0.008367289	2.89859E-05	-0.220103229	-0.175194315	CYFIP2
chr7	156833137	156833196	60	*	2	4.77557E-08	0.008940455	0.008371325	0.007954883	0.128749173	0.079092939	
chr2	237780629	237780791	163	*	2	4.56511E-09	0.000399664	0.008373387	0.000812853	-0.275945181	-0.272939472	
chr6	156885358	156886090	733	*	5	2.77922E-11	0.008953789	0.008382903	0.001484198	0.082188811	0.045289965	
chr1	51984744	51985215	472	*	7	6.37555E-14	0.014100789	0.008383916	0.004146305	0.03338447	0.013676031	RP11-191G24.1, EPS15
chr5	158758490	158758705	216	*	3	1.22661E-07	0.006999828	0.008384011	0.005587935	0.019074882	0.013662887	AC008697.1
chr20	62491759	62492820	1062	*	8	2.35481E-14	0.001071523	0.008393212	0.001227404	-0.241665761	-0.024498503	ABHD16B
chr10	73488971	73489276	306	*	3	2.19916E-10	0.000624841	0.008393637	0.000874172	-0.201118863	-0.109205413	CDH23, C10orf105

chr19	58816220	58816722	503	*	5	9.42774E-11	0.074947118	0.008403816	0.004939532	0.069480715	0.014446622	AC010642.1, ERVK3-1
chr1	23907333	23907905	573	*	4	1.15472E-11	0.001511918	0.008407338	0.000697873	0.166501822	0.094190151	
chr12	3836653	3838094	1442	*	7	4.46077E-15	6.72347E-07	0.008437618	4.69786E-06	-0.30119632	-0.103138459	EFCAB4B
chr14	23790225	23791411	1187	*	11	3.22167E-18	0.003783961	0.008440187	0.000389871	-0.038398403	-0.009311543	BCL2L2-PABPN1, PABPN1, AL049829.1
chr7	158263204	158263984	781	*	4	1.6272E-12	0.00015169	0.008453297	0.000305118	-0.224399543	-0.138645229	PTPRN2
chr3	46778871	46779226	356	*	3	3.63191E-10	0.003383045	0.008460354	0.001456132	0.143603927	0.073846807	PRSS50
chr16	72206241	72206655	415	*	5	3.50482E-09	0.398565522	0.008463817	0.017819134	-0.1982697	-0.064119259	PMFBP1
chr12	76424649	76424973	325	*	4	9.99423E-10	0.010147992	0.008467417	0.008328965	0.032290343	0.002451308	RP11-290L1.3, PHLDA1
chr11	13485254	13485556	303	*	3	4.53993E-09	0.270211888	0.008493805	0.039361128	-0.243913823	-0.082653399	
chr15	79229592	79229985	394	*	5	6.11354E-14	0.000232739	0.008498996	0.00013842	-0.219637174	-0.021611618	CTSH
chr16	55794282	55794910	629	*	5	1.48915E-11	0.012113708	0.008503218	0.001496213	-0.192048608	-0.11806469	CES1P1
chr5	134210233	134210859	627	*	9	1.60169E-09	0.2242503	0.008509964	0.066670803	0.066507243	0.01596955	TXNDC15
chr13	99739316	99740388	1073	*	8	2.98776E-13	0.007576314	0.008513702	0.001013947	0.09226936	0.022687414	DOCK9-AS2
chr2	201450323	201451026	704	*	13	6.0959E-09	0.331564618	0.008517998	0.167833011	0.112905167	0.004873838	AOX1
chr4	102734752	102734940	189	*	3	6.57614E-08	0.00267611	0.008519072	0.003592971	0.114532716	0.087415786	BANK1
chr12	114886059	114886561	503	*	4	5.38721E-08	0.135702798	0.00852132	0.026079483	0.100028073	0.02481024	
chr12	125258948	125259401	454	*	4	3.35256E-12	3.04408E-05	0.008523771	0.00012152	-0.31196405	-0.120641451	
chr2	232790231	232791964	1734	*	15	1.3661E-13	0.018638643	0.008527355	0.004050001	0.020822124	0.007385825	NPPC
chr1	201709135	201709390	256	*	3	3.61713E-08	0.980408187	0.008527983	0.068561961	-0.057858177	-0.019366279	NAV1, IPO9-AS1
chr16	202482	202565	84	*	3	3.22908E-11	5.91864E-05	0.008533516	0.000188958	0.206584488	0.141301626	
chr21	46334192	46334322	131	*	3	4.71408E-09	0.00523643	0.008537227	0.001756703	-0.114534317	-0.062802819	ITGB2
chr9	92219187	92220617	1431	*	10	4.34413E-13	0.0687557	0.008550205	0.004751711	-0.068017986	-0.009658125	GADD45G
chr12	123632825	123634384	1560	*	6	1.64632E-12	7.28418E-05	0.008560614	0.0001198	0.200026825	0.101485204	PITPNM2
chr17	61698753	61700755	2003	*	13	7.95137E-22	1.90766E-06	0.008562048	1.29375E-06	-0.084325835	-0.012799544	MAP3K3
chr2	11527246	11527344	99	*	3	1.06084E-11	2.58549E-05	0.008568056	9.8205E-05	0.11282003	0.102360557	
chr14	55850710	55850801	92	*	2	1.33747E-09	0.000447429	0.008572148	0.000898658	0.110250975	0.094173641	ATG14
chr17	39780836	39781997	1162	*	4	1.50001E-11	2.53931E-05	0.008572809	9.2661E-05	-0.197223912	-0.111060041	KRT17, JUP
chr16	21288548	21288557	10	*	2	5.08935E-08	0.003610157	0.008577355	0.004501369	0.117220462	0.115445174	CRYM
chr19	50848091	50848114	24	*	2	1.42973E-08	0.000377255	0.008586079	0.000777852	0.149747721	0.112756145	NR1H2

chr13	76123086	76124267	1182	*	15	1.00976E-14	0.197832662	0.008587169	0.029368241	0.031947357	0.006712294	UCHL3, RP11-29G8.3, COMMD6
chr14	20945034	20945319	286	*	2	1.68695E-08	0.000914249	0.008598181	0.001617817	0.099708938	0.089281837	PNP, RP11-203M5.8
chr15	80452147	80452305	159	*	4	2.63307E-08	0.006044845	0.008598247	0.00668945	0.121457847	0.070006993	FAH
chr17	2095079	2095179	101	*	2	1.41288E-08	0.000565381	0.008604643	0.001093905	-0.194186341	-0.147525912	SMG6
chr16	57392173	57392764	592	*	5	4.24302E-10	0.223261015	0.008608576	0.01971589	-0.149683686	-0.049837633	CCL22
chr2	219849782	219850123	342	*	2	1.23158E-08	0.000723938	0.00863049	0.001341462	-0.032857163	-0.000979929	FEV
chr1	172419004	172419269	266	*	2	5.33052E-09	0.00041053	0.008634743	0.000836807	-0.188887231	-0.113222894	C1orf105
chr1	223316927	223317548	622	*	6	3.96506E-11	0.189819776	0.00864251	0.011642595	0.109239396	0.037035859	RP11-239E10.2
chr22	23909175	23909219	45	*	3	3.47704E-08	0.02058135	0.008652194	0.005821548	0.106143275	0.052225156	
chr9	139333297	139333683	387	*	2	1.925E-11	0.000701029	0.0086542	0.001307618	-0.032667165	-0.023038041	INPP5E
chr2	208575645	208576794	1150	*	14	7.05908E-13	0.090276133	0.008656335	0.020465167	-0.073402726	-0.009390209	CCNYL1
chr10	32217547	32218391	845	*	12	1.06031E-14	0.094510169	0.008658303	0.007141325	0.031829551	-0.002170384	ARHGAP12
chr6	40554379	40555076	698	*	8	2.78216E-09	0.320421812	0.00865846	0.059655838	0.043545956	0.004096044	LRFN2
chr3	46394241	46395542	1302	*	7	6.88104E-16	4.08309E-06	0.008664503	1.16918E-05	0.181398567	0.043220275	CCR2
chr2	166809971	166811308	1338	*	12	3.46855E-13	0.018870581	0.008666918	0.002496297	-0.154078718	-0.029451849	TTC21B
chr21	40310499	40310774	276	*	3	2.81059E-09	0.001866814	0.008674831	0.002952739	0.121731661	0.067683528	AF064858.6
chr1	26086818	26087303	486	*	2	6.46849E-09	0.013961114	0.008681977	0.010433214	0.199776205	0.121722564	MAN1C1
chr10	106013534	106014624	1091	*	13	7.12171E-16	0.061766163	0.008685577	0.002597601	0.136403506	0.000895508	GSTO1
chr6	31237824	31238388	565	*	3	1.09178E-13	2.29656E-05	0.008685956	8.9633E-05	-0.22241722	-0.060768737	HLA-C
chr5	176936872	176938522	1651	*	11	3.88724E-23	4.3587E-05	0.008691197	1.04375E-05	-0.23972192	-0.084776694	DOK3
chr17	80860956	80861380	425	*	4	1.00937E-08	0.024586718	0.008699313	0.007804532	-0.09800795	-0.036731877	TBCD
chr2	9919440	9919730	291	*	3	4.87071E-09	0.001974192	0.008704294	0.002519158	0.141477628	0.054838392	
chr16	89469541	89469729	189	*	2	3.60261E-08	0.001369774	0.00870591	0.002229937	-0.200669067	-0.146579549	ANKRD11, RP1-168P16.2
chr17	43208740	43209292	553	*	4	8.79035E-09	0.018139278	0.008708464	0.010618578	-0.173421512	-0.036581894	PLCD3
chr11	47470768	47471789	1022	*	10	1.15598E-16	0.092504761	0.008710019	0.008203484	-0.3538143	-0.07724055	
chr2	9458925	9459596	672	*	7	9.05156E-14	0.009162087	0.008710986	0.0007833	0.10695684	0.04571035	ASAP2
chr1	100816943	100817300	358	*	7	4.04052E-12	0.024749417	0.008713538	0.006093704	-0.034310145	-0.010074334	CDC14A
chr19	42894425	42894930	506	*	8	5.47663E-13	0.001000647	0.008715744	0.00137393	0.165368906	0.07445406	CNFN
chr20	30104637	30104660	24	*	2	1.51803E-07	0.002523668	0.008722473	0.003520787	-0.23956725	-0.142658493	HM13

chr5	32395298	32395364	67	*	2	5.26996E-09	0.000398148	0.008723151	0.000816986	-0.187261761	-0.176347019	ZFR
chr12	120632764	120633105	342	*	5	5.04664E-09	0.131528052	0.008741827	0.01344097	-0.098894894	-0.015673952	
chr8	141074510	141074581	72	*	2	1.69347E-08	0.000411818	0.008753582	0.00084137	0.14054157	0.12027132	TRAPPC9
chr18	5579335	5579338	4	*	2	6.80641E-09	0.000390631	0.008759211	0.00080449	-0.154817342	-0.154790624	EPB41L3
chr2	178116991	178117066	76	*	2	9.397E-08	0.000951518	0.00876161	0.00167877	-0.22637464	-0.174080996	NFE2L2
chr4	185771716	185772619	904	*	3	4.5747E-10	4.73384E-05	0.008765416	0.000165099	-0.145730578	-0.134366173	RP11-701P16.5, MIR3945
chr10	75173179	75174496	1318	*	15	5.6913E-13	0.07932039	0.008770189	0.018553506	-0.041421161	-0.005006999	ANXA7
chr17	79479952	79481801	1850	*	10	1.00558E-12	0.00119526	0.00877462	0.000950616	-0.064068857	-0.010508823	RP13-766D20.1, RP13-766D20.2, ACTG1
chr11	70253427	70253499	73	*	3	7.47387E-11	6.73414E-05	0.008775856	0.000208532	0.152901275	0.11852739	CTTN
chr11	70961596	70962357	762	*	6	5.67971E-09	0.047066608	0.008787586	0.015785956	0.041287473	0.019039659	SHANK2
chr10	103824106	103824819	714	*	3	8.55966E-12	0.000561982	0.008792236	0.000783076	0.152255866	0.076929144	
chr12	8834114	8834151	38	*	3	8.937E-08	0.027625443	0.00879644	0.00761953	-0.155443723	-0.086044256	
chr8	28258603	28259252	650	*	3	2.97502E-11	0.000222003	0.008810476	0.000574412	0.064957531	0.035144853	ZNF395, FBXO16
chr6	10722915	10723588	674	*	11	1.89774E-09	0.225974143	0.008818418	0.065755482	0.01550722	0.005628145	TMEM14C
chr8	35093901	35094053	153	*	3	1.10476E-08	0.331424205	0.008823577	0.050536492	0.023233905	0.013020798	UNC5D
chr21	33783915	33785063	1149	*	13	8.3232E-14	0.008830969	0.008829025	0.009901966	0.035417962	0.006083985	EVA1C
chr6	18386873	18387809	937	*	12	9.46183E-17	0.095658773	0.008829354	0.002141984	0.041703915	0.015415019	RNF144B
chr9	117156701	117157157	457	*	4	8.2417E-10	0.015385518	0.00883025	0.003593541	-0.321643129	-0.130530554	
chr6	170462469	170462947	479	*	3	3.20721E-08	0.010188951	0.008834236	0.005412953	0.10808149	0.070114322	
chr2	11531606	11531852	247	*	2	1.65088E-09	0.000563872	0.008836784	0.001098115	0.158099304	0.140015724	
chr19	35491265	35491296	32	*	2	8.69511E-08	0.004835766	0.0088411	0.005551303	0.029489953	0.020892495	GRAMD1A
chr11	68039143	68039669	527	*	6	1.6249E-08	0.014572799	0.008842556	0.015941625	0.025035415	0.00377607	C11orf24
chr11	47968764	47968995	232	*	2	4.82312E-08	0.000838771	0.008851293	0.001521304	-0.139904619	-0.114348123	
chr2	172290183	172291397	1215	*	13	1.9784E-11	0.185359335	0.00885918	0.028550465	-0.042763763	0.004084865	DCAF17, METTL8
chr7	33944924	33944927	4	*	2	2.08217E-09	0.000400903	0.008863595	0.000824426	-0.050397857	-0.041227549	BMPER
chr9	117373787	117374308	522	*	3	1.18558E-08	0.052814128	0.008874306	0.019230254	0.02992192	0.007132379	C9orf91
chr12	739312	740338	1027	*	6	1.85789E-19	5.33082E-08	0.008882888	8.2308E-07	-0.335420313	-0.241626985	RP11-218M22.1, NINJ2
chr1	2250136	2250525	390	*	3	1.02272E-08	0.007136045	0.008900224	0.007471271	0.188429451	0.08725198	
chr17	9862147	9862446	300	*	4	2.42675E-09	0.067119526	0.008908343	0.011571878	-0.089815453	-0.033588122	GAS7

chr3	169378675	169379010	336	*	6	2.02032E-09	0.095534369	0.008914633	0.019393174	0.052807959	0.023236211	MECOM
chr9	131085470	131085780	311	*	3	3.61762E-09	0.022772911	0.00891808	0.013377205	-0.040931349	-0.001666391	COQ4
chr12	32832461	32832968	508	*	3	5.3816E-10	0.001763234	0.00892565	0.002782614	-0.044871116	-0.007533387	DNM1L
chr15	41184874	41185399	526	*	2	5.39453E-10	0.00040574	0.008930671	0.000834147	-0.206641561	-0.153085814	
chr2	106795252	106795369	118	*	2	1.01955E-07	0.001573391	0.008956971	0.002501813	0.165013113	0.138199403	UXS1
chr3	15797850	15798041	192	*	2	1.40418E-09	0.000827087	0.008957953	0.001508809	0.162380933	0.127461186	ANKRD28
chr16	85603371	85603643	273	*	2	6.38634E-08	0.005993245	0.008958348	0.006421058	-0.061954948	-0.041005917	
chr4	39172219	39172243	25	*	2	7.56555E-08	0.000899908	0.008959567	0.001615102	-0.221893707	-0.163598442	
chr2	110084578	110084755	178	*	2	6.98611E-08	0.00173798	0.008961265	0.002698374	0.163104088	0.136805402	SH3RF3
chr14	39816220	39816502	283	*	2	2.04958E-08	0.000508582	0.008961945	0.001010503	-0.149362089	-0.04114883	RP11-407N17.3, CTAGE5
chr14	67655758	67656241	484	*	4	8.1359E-13	4.03889E-05	0.008968444	0.000159527	0.116832011	0.058936931	FAM71D
chr17	42425721	42426506	786	*	4	5.63147E-11	0.004975163	0.008972513	0.001215512	-0.115296766	-0.058038145	GRN
chr22	39897580	39897884	305	*	3	6.32603E-11	0.01021847	0.008989151	0.006817396	0.14617891	0.064458755	MIEF1
chr5	43313384	43314056	673	*	12	2.06427E-14	0.006901812	0.008998396	0.006062754	-0.067526749	-0.011157976	HMGCS1
chr2	177356020	177356448	429	*	3	7.11134E-10	0.019760087	0.009010424	0.003020258	0.120908924	0.069064556	
chr14	61108227	61108807	581	*	3	8.02886E-10	0.001876302	0.009038668	0.002884192	0.114173501	0.072251475	RP11-1042B17.5
chr11	59951557	59952153	597	*	6	3.39626E-17	0.005969021	0.009062943	0.000468565	-0.150484609	-0.060132917	MS4A6A
chr12	57939823	57940980	1158	*	8	4.17693E-12	0.018380352	0.009081702	0.010040979	-0.207023608	-0.031020077	DCTN2
chr2	239046879	239047774	896	*	8	5.73097E-14	0.034069454	0.009083281	0.003426045	0.185238252	0.04451238	KLHL30
chr18	51795309	51796014	706	*	10	7.62761E-10	0.33849279	0.009084949	0.057004303	-0.060311155	-0.008494189	POLI
chr4	967094	967541	448	*	9	6.14562E-13	0.046148247	0.0090905	0.010514025	-0.067711534	-0.011697716	DGKQ
chr16	67224199	67224839	641	*	5	5.35755E-10	0.080129373	0.009107353	0.006028294	0.139524935	0.061785509	
chr3	197260526	197261031	506	*	2	3.97025E-08	0.000518807	0.009117096	0.001031355	-0.170579572	-0.132625927	BDH1
chr16	1742281	1742458	178	*	2	3.27031E-08	0.00061783	0.009122675	0.001193361	0.141164702	0.110816249	HN1L
chr7	116730466	116731139	674	*	6	9.82732E-12	0.013887796	0.009142146	0.003052041	0.158348902	0.031708297	ST7, ST7-OT4, ST7-AS2
chr15	98196234	98196333	100	*	3	4.09901E-08	0.018558416	0.00917121	0.013894818	0.086079991	0.077407068	
chr2	199239878	199240201	324	*	4	3.75162E-10	0.004926271	0.009173516	0.001949716	0.191001806	0.097828389	PLCL1
chr3	183414508	183415433	926	*	6	2.90006E-10	0.017031675	0.009180145	0.007818907	0.190497589	0.049992238	
chr1	207277450	207277581	132	*	4	5.25818E-09	0.002921077	0.009181423	0.002183739	-0.12071794	-0.088465252	

chr20	2673604	2674697	1094	*	6	6.88839E-12	0.000562828	0.009194889	0.000446014	0.042474382	0.025376576	EBF4
chr8	126320054	126320143	90	*	2	2.3632E-08	0.000466546	0.009194893	0.000944747	-0.198942581	-0.154481431	NSMCE2
chr3	121311783	121312383	601	*	6	2.38381E-12	0.000116181	0.009214191	0.000429568	0.148656016	0.099034015	FBXO40
chr15	76136129	76136475	347	*	4	8.80754E-12	0.00080804	0.009216148	0.000890119	-0.033000808	-0.000308604	UBE2Q2
chr11	12263722	12263892	171	*	3	3.00703E-08	0.00518132	0.009221528	0.00392416	-0.232135324	-0.111237801	MICAL2
chr3	187387555	187388737	1183	*	13	8.20468E-14	0.295012645	0.009236809	0.02862049	0.138555255	0.033099173	SST
chr10	31288287	31288997	711	*	5	2.65527E-14	0.023945163	0.009248446	0.000944734	-0.20075171	-0.10734769	ZNF438
chr15	80443908	80445874	1967	*	14	2.63713E-14	0.004029126	0.009248967	0.000328258	0.147651058	0.02762303	FAH
chr21	36281988	36282425	438	*	2	3.07649E-08	0.000456999	0.009251726	0.000929522	-0.13163031	-0.13108485	RUNX1
chr2	240196171	240197864	1694	*	7	1.21256E-20	2.11502E-05	0.009274208	4.93956E-06	-0.212026482	-0.101668118	HDAC4
chr12	124433888	124434452	565	*	4	6.34615E-11	0.00032195	0.009274601	0.000531545	0.187948546	0.103214607	CCDC92
chr4	95683604	95683637	34	*	2	2.67576E-08	0.000487345	0.009278061	0.000982076	0.113373357	0.107425218	BMPR1B
chr12	89749718	89749822	105	*	2	2.84626E-08	0.005506181	0.009279477	0.006173693	0.069813303	0.058088289	
chr1	63249197	63249213	17	*	4	7.94227E-09	0.005828962	0.009297237	0.004574185	0.155918141	0.072615392	
chr8	141354521	141354668	148	*	2	1.11354E-07	0.001074143	0.009320536	0.001880325	0.094812874	0.075900634	TRAPPC9
chr3	99790859	99790878	20	*	2	2.34059E-08	0.000462948	0.009322765	0.000941245	-0.119866368	-0.103670119	CMSS1, FILIP1L
chr22	29729206	29729347	142	*	4	4.83353E-12	0.000147926	0.009324484	0.000343531	-0.116903401	-0.062848018	AP1B1, SNORD125
chr6	4728383	4728467	85	*	2	1.84142E-07	0.001288408	0.0093357	0.002171142	0.126340004	0.098094301	CDYL
chr19	2323913	2324957	1045	*	6	1.96723E-11	0.00066554	0.009340217	0.000646751	0.139418782	0.082120506	LSM7
chr1	26616481	26616534	54	*	2	3.36707E-08	0.00077939	0.009366519	0.001453897	-0.227674062	-0.166637843	UBXN11
chr1	1092940	1093212	273	*	2	1.76042E-07	0.002419161	0.009376465	0.003503494	-0.065524361	-0.054185234	
chr22	19949873	19950733	861	*	8	1.40557E-12	0.046079266	0.009380299	0.005732288	0.131343265	0.015925799	COMT
chr16	89181807	89182492	686	*	7	1.29109E-11	0.003155001	0.009385295	0.002777365	-0.069587608	-0.023422047	ACSF3
chr1	87794525	87794589	65	*	2	5.31462E-08	0.021139288	0.009400835	0.013529836	0.021630321	0.01879128	LMO4
chr3	52739927	52740483	557	*	10	7.68151E-09	0.678054591	0.009406248	0.263147159	0.020365029	0.002295009	SPCS1, GLT8D1
chr14	103822206	103822211	6	*	2	3.18796E-08	0.000771603	0.009425795	0.001444248	0.159973478	0.129328765	
chr11	128563027	128564756	1730	*	11	1.56767E-23	1.79546E-05	0.009458502	4.37154E-06	-0.094735804	-0.022480587	FLI1, SENCR
chr7	2609648	2609875	228	*	3	3.6527E-10	0.00148686	0.009459225	0.001699469	0.240279821	0.141509978	IQCE
chr2	48755207	48757722	2516	*	16	8.78736E-14	0.00080974	0.009477997	0.000100369	-0.20493134	-0.006089899	STON1, STON1-GTF2A1L

chr14	61103439	61104571	1133	*	6	2.77188E-15	0.000420127	0.009482926	0.000141096	0.070603938	0.036618507	
chr11	93465336	93466424	1089	*	5	3.777E-12	0.00921776	0.009496814	0.003675813	-0.271185426	-0.053463349	TAF1D, SNORA8, SNORD5
chr16	420490	420755	266	*	4	1.26883E-08	0.077413095	0.009502804	0.004226285	-0.059450031	-0.028995233	MRPL28
chr8	91657390	91657772	383	*	2	1.42121E-09	0.000485711	0.009508569	0.000984301	0.043336151	0.030808298	TMEM64
chr5	37208976	37209440	465	*	3	1.8598E-11	3.27044E-05	0.009510612	0.000123416	-0.222607166	-0.132136472	C5orf42
chr6	170597494	170598912	1419	*	6	1.88122E-18	0.000864726	0.009514193	0.000159866	0.124077812	0.032372077	DLL1
chr6	30636602	30636608	7	*	2	1.8489E-07	0.001242755	0.00952435	0.00212281	0.141118343	0.111279526	DHX16
chr8	22435728	22437249	1522	*	15	1.2755E-21	0.001244665	0.009526676	0.000116173	-0.132971254	0.00825006	PDLIM2
chr14	64228585	64228599	15	*	2	3.38689E-08	0.000533567	0.009531939	0.001066105	-0.190560866	-0.181839918	
chr19	53140973	53141372	400	*	2	2.65441E-10	0.006435436	0.009532778	0.006910799	0.013634012	0.010818764	ZNF83
chr12	7168012	7168545	534	*	4	1.68605E-13	0.004725461	0.009538531	0.000793464	0.178987669	0.097669472	C1S
chr1	51702423	51702659	237	*	2	1.54992E-08	0.018390774	0.00954555	0.012719637	0.017799903	0.011864593	RNF11
chr6	109280380	109280598	219	*	3	1.62654E-08	0.013431142	0.009545863	0.003738672	-0.184402705	-0.087445909	ARMC2
chr3	172241791	172241975	185	*	2	7.11895E-08	0.007568029	0.009566961	0.007668468	-0.180355965	-0.103596735	
chr11	75826133	75826760	628	*	4	2.82346E-11	0.00010745	0.009569968	0.000252837	0.12379175	0.084289974	UVRAG
chr19	16629806	16630140	335	*	4	8.23694E-08	0.019590766	0.009573352	0.006564088	0.122897407	0.042936026	C19orf44, CTD-3222D19.2, CHERP
chr12	6874709	6874964	256	*	2	2.43646E-08	0.001493602	0.009574661	0.002453716	0.068240731	0.063670802	PTMS, MLF2
chr17	5389134	5390301	1168	*	19	1.95783E-22	0.000279819	0.009579832	0.000211651	-0.086404564	-0.010171183	MIS12, DERL2
chr6	58778937	58779139	203	*	3	2.84464E-11	3.63469E-05	0.009584397	0.000135477	0.105533391	0.082527961	
chr22	26908008	26908989	982	*	17	3.47351E-11	0.378854414	0.009585392	0.167172639	0.046258644	-0.003254515	CTA-445C9.14, TFIP11
chr12	6880611	6881629	1019	*	9	4.33065E-13	0.043652836	0.009592081	0.006878284	-0.16684745	-0.041686291	
chr16	25226421	25227013	593	*	5	2.01378E-12	0.008361141	0.009595769	0.000389878	0.125005869	0.017307017	
chr10	74079772	74080805	1034	*	5	2.61858E-10	0.000824223	0.009597085	0.000349235	-0.070971967	-0.014603963	
chr5	58335804	58336072	269	*	4	9.84921E-09	0.031046135	0.009608381	0.021072745	-0.119816161	-0.035680137	RP11-266N13.2, PDE4D
chr7	136641675	136641740	66	*	2	2.54253E-08	0.000465816	0.009614768	0.00095215	-0.221476305	-0.170748528	CHRM2, hsa-mir-490
chr11	628318	628677	360	*	3	5.65659E-11	0.010095797	0.009622585	0.002820024	0.15123824	0.077808993	
chr2	224808641	224809074	434	*	3	5.3496E-09	0.013130028	0.009632044	0.004100101	-0.148382662	-0.067688629	WDFY1
chr7	1084162	1084791	630	*	5	4.94235E-09	0.113863341	0.0096397	0.018925167	0.041158535	0.007776108	GPR146, C7orf50
chr1	36348216	36349271	1056	*	11	5.28062E-18	2.07287E-05	0.009640923	4.90426E-05	0.143371285	0.032320318	AGO1

chr10	134258421	134258819	399	*	3	4.18031E-09	0.000837142	0.009644656	0.001026666	-0.120841026	-0.074400557	C10orf91
chr12	124875481	124876101	621	*	3	2.22755E-10	0.000788223	0.009647197	0.001590957	-0.081210032	-0.046036688	NCOR2
chr20	1246093	1246779	687	*	7	2.23471E-16	0.037474435	0.009658908	0.002374188	0.136333863	0.04342573	RAD21L1
chr10	134454161	134454481	321	*	3	4.0148E-09	0.002541875	0.009667908	0.001740035	0.102807779	0.06742111	INPP5A
chr11	74303296	74304091	796	*	10	6.82451E-12	0.043970306	0.009674567	0.012482974	0.030163463	0.000312074	POLD3
chr17	48736189	48736199	11	*	2	4.69115E-08	0.000949544	0.009675362	0.001720687	0.148282016	0.111177108	ABCC3
chr9	100459634	100459861	228	*	4	2.57131E-08	0.095969874	0.009681998	0.014397814	0.042422054	0.005281378	XPA
chr17	179551	179611	61	*	2	1.74985E-07	0.009615687	0.009682763	0.008926575	-0.14411433	-0.085055955	RPH3AL
chr10	102509510	102510568	1059	*	5	8.10854E-11	0.000139481	0.009685287	0.000318963	0.07809283	0.054729619	PAX2
chr3	32822789	32823185	397	*	3	2.50938E-09	0.002007712	0.009686281	0.002788223	-0.085878748	-0.021300342	
chr12	11911896	11912232	337	*	3	3.60496E-10	0.000634555	0.009698479	0.001379007	0.169600014	0.122922799	ETV6
chr4	2934188	2934614	427	*	2	5.31771E-10	0.001122109	0.009698967	0.001968138	-0.190775445	-0.172606738	MFSD10
chr17	7083015	7083064	50	*	2	1.36532E-07	0.000474026	0.00970899	0.000968301	-0.134343792	-0.124349001	
chr9	123630545	123630782	238	*	2	1.07924E-09	0.001077143	0.00971838	0.00190609	0.127949797	0.033816316	PHF19
chr12	122984780	122985592	813	*	10	1.00645E-11	0.008556726	0.009744292	0.011880013	0.027492196	0.001502772	ZCCHC8
chr22	46507241	46508604	1364	*	8	1.43506E-17	1.20687E-05	0.009758774	8.79157E-06	0.174172427	0.097606274	FLJ27365
chr12	69725435	69725444	10	*	2	1.4138E-07	0.000665641	0.009771796	0.001289661	-0.172201168	-0.137409931	
chr17	79202120	79202505	386	*	5	2.63785E-10	0.04385455	0.009771841	0.005770592	-0.163541781	-0.070511872	ENTHD2
chr1	1310123	1310496	374	*	4	7.14759E-11	0.024753533	0.009778008	0.00932951	-0.063223136	-0.018136539	AURKAIP1
chr22	50979643	50979665	23	*	2	1.27006E-08	0.001305721	0.009791489	0.002224372	-0.199167195	-0.191770607	
chr1	28878155	28880147	1993	*	13	1.34115E-19	1.22484E-05	0.009791519	7.3349E-06	0.087699131	-0.00080135	TRNAU1AP
chr19	3961086	3962194	1109	*	13	6.76643E-13	0.154882779	0.009794595	0.028224163	0.144819414	0.034018018	DAPK3, MIR637
chr17	6927329	6927424	96	*	2	7.86657E-09	0.002453577	0.009797548	0.00359459	0.140031178	0.116078669	BCL6B
chr14	65169664	65170728	1065	*	7	3.05424E-12	0.011632985	0.009804538	0.010453332	-0.075457585	-0.007641563	
chr7	96632635	96633060	426	*	4	1.92205E-08	0.190456067	0.009820027	0.029134739	0.05143642	0.023664569	DLX6-AS1
chr17	73042519	73043736	1218	*	13	1.91347E-12	0.204008163	0.009827041	0.029764348	-0.06769751	-0.007575858	KCTD2, ATP5H
chr1	117284260	117284802	543	*	7	1.32325E-08	0.146883	0.00983201	0.025639416	-0.030613665	-0.000942812	NEFHP1
chr17	8868480	8870355	1876	*	15	1.9532E-17	0.000672691	0.009840172	0.000160656	-0.28961963	-0.02017912	CTB-41I6.1, PIK3R5
chr1	173836636	173838081	1446	*	24	4.32601E-19	0.015129571	0.009850929	0.00233915	-0.067815717	-0.003813251	ZBTB37, GAS5

chr17	76371406	76371879	474	*	3	1.03479E-09	0.000297107	0.009857777	0.000689876	-0.151097202	-0.082564115	
chr17	38171455	38171697	243	*	5	2.60025E-12	0.00176723	0.009865371	0.000814364	-0.15474918	-0.091957415	CSF3
chr8	124193667	124194847	1181	*	11	2.37234E-18	0.013553537	0.009865874	0.000467959	0.155323792	0.036637262	FAM83A, RP11-539E17.5
chr5	87973899	87974547	649	*	8	1.70908E-14	0.000663007	0.009872892	0.001004591	0.073104322	0.041643043	CTC-467M3.1, LINC00461
chr2	71114785	71115994	1210	*	9	6.0292E-14	0.317881302	0.009881408	0.015188091	0.043598311	0.015348712	LINC01143
chr1	226896665	226897499	835	*	4	1.82819E-13	1.50766E-05	0.009898914	7.63705E-05	0.19919599	0.130623087	ITPKB
chr14	23845870	23847052	1183	*	8	2.49108E-16	0.084141424	0.009904042	0.002664097	0.153408173	0.061971594	CMTM5
chr2	131850237	131850909	673	*	5	9.32559E-10	0.347384087	0.00991626	0.06705947	0.016527731	-0.00159426	FAM168B
chr11	82746478	82746732	255	*	3	7.97314E-09	0.034829012	0.009919532	0.003888571	0.115818852	0.075970721	RP11-659G9.3, RAB30
chr15	43802612	43803877	1266	*	15	2.53981E-16	0.028722529	0.009962161	0.002658698	-0.03494127	0.006491509	MAP1A, TP53BP1
chr20	824609	825268	660	*	8	1.14848E-18	0.017590634	0.009963507	0.000906666	0.155877562	0.050325566	FAM110A
chr11	76493379	76493489	111	*	2	1.194E-07	0.425688696	0.009976521	0.031285657	-0.132990597	-0.066530356	TSKU
chr10	121485184	121485763	580	*	3	5.98093E-09	0.011668927	0.009981858	0.010143928	0.039271401	0.015225546	INPP5F
chr15	55610624	55610640	17	*	2	1.10876E-10	0.000715574	0.009982272	0.001376085	-0.062224409	-0.049048334	RAB27A, RP11-139H15.1
chr12	110136015	110136044	30	*	3	9.46398E-10	0.000237925	0.010000082	0.000544147	0.16758871	0.133708024	
chr12	4917740	4918391	652	*	8	1.3613E-11	0.028781369	0.010005617	0.01011354	-0.082793539	-0.022222515	GALNT8, KCNA6, RP11-234B24.4
chr12	12937632	12938465	834	*	8	7.93963E-17	0.000105442	0.010020797	8.84869E-05	0.209011776	0.090493567	APOLD1
chr7	138916732	138917062	331	*	3	7.16666E-08	0.011654941	0.0100418	0.010896847	0.019599106	0.012721502	UBN2
chr20	54966092	54967718	1627	*	19	1.65931E-23	0.001025473	0.010041916	3.35565E-05	-0.127634612	-0.01019733	CSTF1, AURKA
chr16	88557453	88558237	785	*	5	5.75581E-13	0.013546342	0.010045774	0.00213717	0.20553994	0.086228231	ZFPM1
chr6	151203416	151203458	43	*	2	3.30339E-08	0.000508626	0.010075414	0.001035755	0.164503217	0.144059991	MTHFD1L
chr19	5903868	5905215	1348	*	17	1.64472E-15	0.136373782	0.010078833	0.011053452	0.040497838	0.001823105	AC104532.2, VMAC, NDUFA11
chr17	7791319	7792134	816	*	6	1.06236E-09	0.627416659	0.010083637	0.038381524	-0.147387296	-0.009900929	CHD3
chr12	77456884	77456902	19	*	2	3.6855E-08	0.000535918	0.010098617	0.00108316	0.105304815	0.09698907	E2F7
chr8	82191575	82192945	1371	*	14	1.66893E-13	0.017619252	0.010105734	0.013751127	-0.31658714	-0.036698294	FABP5, RP11-363E6.3
chr2	113544177	113545053	877	*	5	1.56953E-08	0.030496241	0.010119826	0.003897487	0.146846753	0.009304834	
chr16	81129991	81130802	812	*	9	1.44495E-08	0.194222337	0.01012956	0.070948117	-0.062864093	-0.008249119	GCSH
chr16	81684572	81684618	47	*	2	9.67321E-09	0.00051748	0.010148916	0.001052591	0.165481686	0.142762086	CMIP
chr6	14518881	14518907	27	*	2	1.22477E-08	0.00052004	0.010160508	0.001057253	0.155180175	0.144584766	

chr14	50802227	50802253	27	*	2	1.0504E-08	0.000517904	0.010175614	0.001053888	0.16974399	0.153769079	ATP5S, CDKL1
chr12	108079287	108079950	664	*	13	1.24492E-09	0.344121751	0.010176919	0.120862464	0.022266972	0.003269677	PWP1
chr1	89521035	89521065	31	*	3	1.26566E-08	0.005927109	0.010188188	0.002542068	0.09561914	0.054562536	GBP1
chr12	56349110	56349394	285	*	4	7.45437E-09	0.049190571	0.010195894	0.008012408	0.144750003	0.069866546	PMEL
chr19	52004630	52005651	1022	*	9	2.03971E-14	0.060551595	0.010205238	0.004029271	-0.091887647	-0.027363855	SIGLEC12
chr1	25290947	25292412	1466	*	16	1.19994E-17	0.013637795	0.010219603	0.004320632	0.141638673	0.048257304	RUNX3
chr19	1465060	1466162	1103	*	5	6.47506E-14	0.004509451	0.010220194	0.000690737	0.189836711	0.077949303	APC2, C19orf25
chr3	112329157	112329355	199	*	2	4.21446E-08	0.000576748	0.010229816	0.001155542	-0.171206356	-0.05487827	CCDC80
chr2	241458886	241460002	1117	*	7	1.46768E-18	5.96038E-08	0.010230847	8.76841E-07	0.206609197	0.113399908	ANKMY1
chr5	88119579	88119740	162	*	4	1.99429E-13	1.63116E-05	0.010233163	7.08181E-05	-0.271831956	-0.148912442	MEF2C
chr11	67372519	67374847	2329	*	20	5.52629E-19	0.000247114	0.010236514	0.000225293	0.193601763	0.028463823	RP11-655M14.12, NDUFV1, C11orf72
chr16	1578619	1578807	189	*	3	1.49129E-10	0.00097916	0.010255836	0.001305941	0.146216031	0.127452108	TMEM204, IFT140
chr1	201123225	201124117	893	*	13	3.30141E-17	0.000567039	0.010265189	0.000651869	-0.081352291	-0.027006471	TMEM9
chr11	33758505	33759043	539	*	3	7.47193E-10	0.196012987	0.010269818	0.047277772	0.152094965	0.065362927	
chr1	144995682	144996395	714	*	5	6.58548E-12	0.135062825	0.01029123	0.009399349	0.155442094	0.059033793	PDE4DIP
chr22	29950244	29950274	31	*	2	9.70577E-08	0.109966077	0.010294928	0.02677901	0.107055758	0.047750257	THOC5
chr16	31141403	31142148	746	*	3	1.12954E-12	5.43033E-05	0.010305203	0.000192055	-0.087121862	-0.072059479	KAT8
chr10	134095646	134095998	353	*	3	5.90737E-08	0.023884487	0.010313268	0.01515624	-0.10640679	-0.007730172	STK32C
chr14	76045663	76046018	356	*	2	1.9254E-08	0.052969601	0.010323578	0.021398228	-0.117534472	-0.067519284	FLVCR2, AC007182.6
chr5	54527320	54527671	352	*	3	8.10301E-11	0.003085515	0.010337191	0.001859297	0.041338205	0.037456468	CCNO
chr18	77169782	77170635	854	*	6	4.79062E-09	0.043218074	0.010357181	0.024478532	0.134157798	0.001750732	NFATC1
chr11	9595544	9596475	932	*	8	7.77215E-11	0.010290906	0.01036061	0.014790254	0.017881715	0.01019952	WEE1
chr13	112770729	112770897	169	*	3	2.16737E-08	0.003916517	0.01037485	0.005312379	-0.216641241	-0.050870427	
chr14	69415588	69416134	547	*	2	1.14258E-07	0.000538126	0.010381644	0.001093154	0.108912169	0.108427017	ACTN1
chr20	61282847	61283288	442	*	2	2.06719E-08	0.000810484	0.010394671	0.001539621	-0.168388698	-0.122481966	SLCO4A1
chr13	52367810	52368291	482	*	3	2.84338E-08	0.002868648	0.010396375	0.001955221	-0.107138352	-0.061089138	DHRS12
chr22	21921060	21922038	979	*	10	1.27507E-11	0.064906501	0.010408755	0.009671255	-0.091812826	0.000461865	UBE2L3
chr2	169102866	169103738	873	*	4	3.33813E-09	0.007916191	0.010449811	0.007676286	0.029714726	0.017121968	STK39
chr15	91458121	91460071	1951	*	9	5.44765E-20	1.45659E-05	0.010462484	5.32074E-06	-0.248406926	-0.101667563	MAN2A2

chr6	109288488	109288592	105	*	2	5.68413E-08	0.000832683	0.010473182	0.001577049	-0.059403265	-0.05078783	ARMC2
chr7	4778839	4779342	504	*	6	2.62626E-10	0.017580593	0.010478347	0.005241068	0.238154176	0.072763829	FOXK1
chr1	37947276	37947371	96	*	2	4.22006E-08	0.000558745	0.010497827	0.001131117	-0.192775032	-0.147376945	ZC3H12A
chr4	2733908	2733970	63	*	2	1.21908E-07	0.000551685	0.010501055	0.001119075	-0.154498722	-0.128508394	FAM193A
chr19	55013549	55013954	406	*	6	1.43574E-11	0.00658842	0.010509884	0.002432022	-0.190156329	-0.105880549	LAIR2
chr2	113569828	113570123	296	*	3	3.34667E-10	0.000243746	0.010517136	0.000551088	0.153305092	0.112156251	
chr12	132515664	132515752	89	*	2	9.97919E-08	0.002305793	0.010525064	0.003517286	0.091172212	0.081090174	EP400
chr5	134375057	134377058	2002	*	11	6.0359E-20	3.37563E-06	0.010527071	6.12636E-06	-0.107220471	0.030706962	C5orf66, CTC-276P9.1
chr8	641466	641497	32	*	3	2.66943E-09	0.000432555	0.010535385	0.00101314	0.220192745	0.13297027	ERICH1
chr8	20880199	20880673	475	*	4	1.28101E-10	0.000934961	0.010536747	0.001455165	0.146753279	0.083506877	
chr12	118810436	118810441	6	*	2	1.07845E-08	0.000571857	0.010544197	0.001154583	-0.067962826	-0.060292699	TAOK3
chr3	71084623	71084634	12	*	2	4.25925E-08	0.00055525	0.010544253	0.001126147	-0.157034826	-0.153136121	FOXP1
chr22	24178181	24179356	1176	*	5	1.85154E-08	0.002641678	0.010547198	0.00150649	0.148237642	0.06400803	DERL3
chr14	21058360	21058768	409	*	4	1.58029E-10	0.110680369	0.010552808	0.006998512	-0.245532213	-0.097377084	RP11-14J7.6, RNASE11, RNASE11, RNASE12
chr1	28471208	28471395	188	*	2	7.04227E-08	0.001398435	0.010555685	0.002398459	-0.147542748	-0.053689261	
chr2	42588233	42588967	735	*	14	7.54814E-17	0.001528666	0.010557763	0.00117379	-0.042837981	-0.012436897	COX7A2L
chr1	156595164	156595461	298	*	3	3.0808E-09	0.00978962	0.010564241	0.009894764	0.055424902	0.013916083	HAPLN2
chr17	42989137	42989323	187	*	3	8.94551E-08	0.134752942	0.010568308	0.010228611	0.137773739	0.069831365	GFAP
chr7	116138462	116138591	130	*	2	1.43395E-10	0.00113308	0.010569254	0.002029952	0.134837309	0.110914409	CAV2, AC002066.1
chr3	47014128	47014492	365	*	2	2.73843E-08	0.000590126	0.010570053	0.001186266	0.171913367	0.13893344	CCDC12
chr12	57118908	57119835	928	*	15	8.02977E-20	0.072543759	0.010571312	0.001946231	0.096618404	0.008228336	NACA
chr7	99006392	99007560	1169	*	10	3.38878E-11	0.21973954	0.010600894	0.01403488	-0.108093488	-0.006360135	BUD31, PDAP1
chr1	234914480	234915028	549	*	2	1.14333E-07	0.00057212	0.010605314	0.001156419	-0.110166878	-0.004397751	
chr17	70710154	70711001	848	*	5	4.50468E-12	0.009152329	0.010605547	0.001494992	-0.286369393	-0.098705654	SLC39A11, AC080037.1
chr17	74099707	74100277	571	*	15	5.71786E-11	0.416383074	0.010614174	0.082592257	-0.303611642	-0.031905884	EXOC7
chr16	72088241	72088622	382	*	3	4.18444E-12	8.46631E-05	0.010627603	0.000274323	-0.190726437	-0.142481738	HP, HPR, TXNL4B
chr6	106582669	106583218	550	*	4	2.13998E-10	0.000938522	0.010630083	0.001728881	0.105124098	0.070167239	
chr11	70268504	70269613	1110	*	5	1.87708E-10	0.001528447	0.010667465	0.000316487	0.130931501	0.062463745	CTTN
chr14	25042782	25043625	844	*	3	2.44977E-08	0.000783411	0.010667555	0.0013986	-0.161169688	-0.107369934	CTSG

chr3	196527614	196528060	447	*	3	6.5009E-09	0.003435851	0.010681075	0.002118091	0.125056471	0.069419573	PAK2
chr22	18390778	18390828	51	*	2	3.56855E-09	0.000825922	0.010700925	0.001574534	-0.120617375	-0.099634386	MICAL3
chr6	29598417	29600206	1790	*	19	5.33978E-33	0.000143823	0.010709284	6.82563E-06	0.221145087	0.047112078	GABBR1
chr17	41002433	41002900	468	*	2	1.29155E-09	0.000587662	0.010711538	0.001185378	0.101676901	0.094589661	AOC2
chr12	57081186	57081572	387	*	3	1.87934E-08	0.006703058	0.010716507	0.006134827	-0.046638206	-0.010423306	PTGES3
chr1	150487740	150488444	705	*	6	1.13089E-09	0.063075172	0.010719482	0.016099463	0.029076574	0.005036859	LINC00568
chr9	128509729	128509870	142	*	4	1.03133E-09	0.023107351	0.010720199	0.00539169	0.021900161	0.00989585	PBX3
chr19	34662636	34663206	571	*	10	1.3659E-15	0.10231876	0.010732322	0.008321711	0.098187726	0.014660869	
chr2	219773416	219773473	58	*	2	1.28788E-07	0.003384327	0.010732464	0.004689334	0.134243965	0.096940213	AC073128.10
chr12	124908112	124908931	820	*	7	2.72998E-14	0.000231822	0.010732957	0.000452591	0.155304338	0.057591614	NCOR2
chr19	11449933	11450645	713	*	8	8.33653E-13	0.001213928	0.010733295	0.001639181	0.059594595	0.014682739	RAB3D
chr6	36253157	36253720	564	*	2	7.77013E-09	0.011858384	0.010740935	0.010637631	0.137217266	0.084494099	PNPLA1
chr16	85981336	85981947	612	*	5	2.52823E-09	0.002187276	0.010744743	0.002930854	-0.202271025	-0.023045878	
chr2	28582676	28584004	1329	*	4	6.80527E-12	1.72893E-05	0.010768291	8.16848E-05	0.218155025	-0.029417405	
chr11	10715175	10716164	990	*	14	3.23248E-16	0.000980107	0.010782454	0.000719686	0.188377279	0.076759687	MRVI1
chr8	42195726	42196371	646	*	7	1.25057E-09	0.05259535	0.010791478	0.024610584	0.022253343	0.003576623	POLB
chr10	76585791	76586650	860	*	10	1.26273E-18	7.10431E-05	0.010798738	9.7014E-05	-0.087720884	-0.013937976	KAT6B
chr12	13132534	13133131	598	*	7	2.28356E-13	0.015752556	0.010801085	0.003657917	0.113478235	0.041837591	RP11-392P7.6, HEBP1
chr1	27480106	27480849	744	*	5	8.28581E-14	6.57918E-05	0.010806157	0.000179783	0.146506397	0.095722514	SLC9A1
chr2	97454042	97454334	293	*	5	1.22372E-11	0.001297369	0.010807809	0.000768743	0.157556241	0.09394251	CNNM4
chr10	5275884	5276318	435	*	4	2.65515E-09	0.002069799	0.010810517	0.002975728	-0.170442687	-0.087287828	
chr11	67777664	67777952	289	*	6	3.28886E-08	0.430653021	0.010816023	0.031243014	-0.124331945	-0.013059687	ALDH3B1
chr12	81102486	81102749	264	*	3	2.77959E-09	0.005445724	0.010818268	0.003343999	-0.071771539	-0.005433434	MYF6
chr13	21750473	21751373	901	*	10	6.38905E-11	0.136118257	0.01082379	0.046887651	-0.047686209	0.00053851	MRP63, SKA3
chr11	61334859	61335600	742	*	5	2.98115E-10	0.014916127	0.010840268	0.004060326	0.094679766	0.04397593	SYT7
chr14	91162832	91163190	359	*	2	6.27897E-08	0.01006354	0.010842625	0.009693651	0.152810682	0.040720276	TTC7B
chr17	56604947	56606542	1596	*	6	6.25257E-13	8.64645E-05	0.010842776	0.000122614	0.178414101	0.106372088	RP11-112H10.4, SEPT4
chr1	154164951	154164994	44	*	2	9.77902E-08	0.004773939	0.010846596	0.005990897	-0.184707193	-0.119307668	TPM3
chr10	75541506	75541698	193	*	5	3.24984E-09	0.012674544	0.010847659	0.005695473	0.025324387	0.012335237	

chr20	61315208	61315831	624	*	3	1.76204E-10	0.001385905	0.010865831	0.001548831	-0.248396629	-0.118464843	SLCO4A1
chr3	4943647	4944056	410	*	2	6.86039E-08	0.000591631	0.010866374	0.001195706	0.085727417	0.071187173	BHLHE40-AS1
chr12	4140624	4141193	570	*	5	5.60414E-09	0.020165495	0.010888018	0.003940066	-0.089179852	-0.007668862	
chr14	70826572	70827442	871	*	6	2.99042E-15	0.003669327	0.010889762	0.001396701	-0.160058778	-0.067566735	SYNJ2BP-COX16
chr8	130691264	130691356	93	*	2	4.43219E-08	0.000627963	0.010895173	0.001258096	0.147112096	0.035306652	CCDC26
chr9	132401827	132402804	978	*	5	4.33641E-11	0.003202902	0.010903018	0.001101436	-0.196512397	-0.069377724	RP11-483H20.4, ASB6
chr3	12800802	12801712	911	*	8	6.88208E-17	0.002606761	0.010903376	0.00021293	0.165649565	0.071760101	TMEM40
chr7	2287330	2287495	166	*	2	1.22606E-08	0.00062325	0.010906784	0.001250415	0.167082073	0.15416855	NUDT1
chr1	236229267	236229607	341	*	2	1.21402E-08	0.007150594	0.01092717	0.007859549	0.186183708	0.139530104	
chr4	159593054	159593775	722	*	15	1.35217E-10	0.195510656	0.010929895	0.072580957	-0.037273604	-0.002205162	ETFDH, C4orf46
chr3	44157590	44157927	338	*	2	4.95576E-08	0.000697917	0.010930172	0.001375691	0.097712775	0.089582792	
chr12	49177075	49177153	79	*	2	1.62384E-08	0.004681376	0.010942932	0.005931989	0.120540944	0.104262752	ADCY6
chr11	334251	334833	583	*	6	7.14871E-13	0.027977877	0.010946965	0.001651213	-0.15002365	-0.055118609	
chr1	112032288	112032495	208	*	2	1.58045E-08	0.000802736	0.010956677	0.001546439	-0.131716334	-0.101509423	ADORA3
chr6	157912969	157913024	56	*	2	7.04727E-08	0.000689195	0.010970407	0.001362384	-0.157114524	-0.135316855	ZDHHC14
chr11	17229115	17230914	1800	*	9	1.82152E-10	0.006466683	0.010976014	0.001644305	-0.10706959	-0.012446346	NUCB2, PIK3C2A
chr11	914329	915440	1112	*	12	7.51122E-21	0.000573733	0.010977591	5.70301E-05	0.152276032	0.064644532	CHID1
chr3	187454786	187455953	1168	*	7	3.31907E-12	0.01147233	0.010979319	0.003393191	-0.199897426	-0.011173488	BCL6
chr12	122287619	122288010	392	*	6	9.68645E-16	5.68441E-05	0.010979963	4.33044E-05	-0.246019361	-0.162884669	HPD
chr13	114855198	114855429	232	*	2	2.52151E-08	0.00064596	0.010983257	0.001290593	0.223845612	0.146877498	RASA3
chr1	15735965	15736719	755	*	9	3.34398E-09	0.235094199	0.010987674	0.057735032	-0.044807512	-0.003486622	EFHD2
chr10	45948576	45949022	447	*	5	2.83052E-09	0.038883559	0.010996521	0.008316107	-0.23306796	-0.094152307	
chr1	27216367	27217013	647	*	12	2.81787E-10	0.406047823	0.011012598	0.078352532	0.023119169	-0.004058071	GPN2, GPATCH3
chr6	74354232	74354305	74	*	3	7.40553E-08	0.003760612	0.011016891	0.004756296	-0.157039337	-0.084978221	SLC17A5
chr14	35885253	35885344	92	*	2	7.04872E-08	0.001557174	0.011020644	0.002642042	0.08425614	0.075992443	
chr7	101559134	101560291	1158	*	11	5.04144E-15	0.070947964	0.011024991	0.001553382	0.181080602	0.051144356	CUX1
chr2	219923682	219924408	727	*	5	1.60233E-11	0.015884769	0.011025515	0.003050003	0.074248403	0.042635483	IHH
chr1	42234586	42234721	136	*	2	5.4363E-08	0.000603708	0.011028324	0.001220055	-0.101794619	-0.090792048	HIVEP3
chr19	49976438	49976497	60	*	2	4.78388E-12	0.000604323	0.011028629	0.001221114	0.143727144	0.132772096	CTD-3148I10.9

chr18	43620112	43620428	317	*	2	9.42749E-09	0.000833892	0.011045963	0.001598932	0.129374226	0.120849923	PSTPIP2
chr18	157716	158838	1123	*	11	2.90981E-11	0.148797973	0.011047646	0.02687453	-0.098711754	-0.007321108	USP14
chr14	101146259	101146675	417	*	4	5.00737E-10	0.000378115	0.011053507	0.000878263	0.238944879	0.127848474	
chr19	55549414	55550348	935	*	11	2.51076E-23	2.29888E-06	0.011063931	2.2977E-06	0.174160918	0.098971459	CTC-550B14.7, GP6, CTC-550B14.6
chr22	41939981	41941494	1514	*	15	4.79708E-25	7.91049E-06	0.011070028	5.33932E-06	-0.077944692	-0.019564917	POLR3H
chr7	647622	647637	16	*	2	1.75141E-07	0.001091682	0.011072302	0.001994019	0.108538529	0.105714343	AC147651.4, PRKAR1B
chr19	10679308	10680229	922	*	11	1.15887E-15	0.000316627	0.011072892	0.00039443	0.044273239	-0.000936979	CDKN2D
chr17	74844962	74845143	182	*	3	8.78194E-09	0.000650519	0.011081035	0.00147168	0.143190016	0.105623844	
chr17	34222748	34223153	406	*	2	7.82056E-08	0.000610373	0.01109248	0.001232919	-0.180500592	-0.130243575	AC015849.16
chr9	139924651	139924709	59	*	2	2.42409E-10	0.000649689	0.011108023	0.001299983	-0.122972561	-0.102862103	C9orf139, FUT7
chr2	106054990	106055507	518	*	7	6.63803E-15	0.007858054	0.01111983	0.000970247	0.202190578	0.060678295	
chr17	37775764	37775851	88	*	3	8.71539E-10	0.02465478	0.011122658	0.0030777	-0.212957469	-0.112041531	
chr11	92500085	92500136	52	*	2	3.67737E-08	0.00082627	0.011135335	0.001589815	-0.228153756	-0.149607402	FAT3
chr1	212965581	212966209	629	*	4	1.47181E-11	0.006403099	0.011138044	0.00718803	0.184573005	0.050411746	TATDN3
chr10	90640622	90640720	99	*	2	2.3981E-08	0.007296263	0.01114514	0.00803244	-0.023090767	-0.003042747	STAMBPL1
chr5	482407	482948	542	*	2	2.26981E-08	0.02733567	0.011148613	0.016948631	0.155523308	0.070625636	SLC9A3
chr2	58656064	58656105	42	*	2	1.50574E-10	0.000757018	0.011150866	0.001478833	0.018100576	0.013536322	LINC01122
chr9	139258402	139258524	123	*	5	1.7477E-07	0.210482592	0.011155945	0.013712056	0.030725886	0.016103709	CARD9
chr18	47003170	47003177	8	*	2	1.13472E-07	0.000855382	0.011163081	0.00163692	-0.065009283	-0.059809118	
chr22	32146690	32146705	16	*	2	2.41725E-09	0.000937661	0.011174845	0.001765822	0.133384967	0.107941399	
chr19	4276778	4277656	879	*	4	1.53259E-09	0.006721021	0.01119672	0.002678148	0.146632492	0.075557021	
chr3	12199836	12200427	592	*	4	1.87593E-08	0.033873249	0.011203777	0.019568528	-0.243618482	-0.013346703	SYN2, TIMP4
chr13	25085301	25086777	1477	*	6	5.62918E-09	0.000237553	0.011214792	0.000227281	-0.144555667	-0.052429434	TPTE2P6, PARP4
chr9	79328522	79328644	123	*	2	1.8355E-07	0.001111766	0.011222277	0.002030621	0.11173201	0.106470923	PRUNE2
chr4	102267366	102269374	2009	*	15	3.69769E-17	0.00654663	0.011227526	0.000265238	-0.191679797	-0.024206529	AP001816.1, PPP3CA
chr4	86850108	86850721	614	*	4	9.85521E-12	0.003989303	0.011236073	0.00176555	-0.16142204	-0.067525117	ARHGAP24
chr8	19674470	19675432	963	*	13	1.81192E-12	0.101763448	0.011240337	0.025856681	-0.044652568	-0.004534546	INTS10
chr8	126443767	126443772	6	*	2	1.00977E-07	0.059702852	0.011266378	0.02371326	0.014160867	0.008312857	TRIB1
chr3	155422092	155423168	1077	*	7	1.17732E-10	0.001495435	0.01129898	0.001295667	-0.128383873	-0.061951617	PLCH1

chr2	89035483	89035504	22	*	2	6.46566E-08	0.000646352	0.011307777	0.001299235	0.163292737	0.15983235	RPIA
chr11	61722539	61722937	399	*	4	1.23182E-08	0.032233741	0.011315533	0.008969908	-0.184214195	-0.0553625	BEST1
chr15	75248496	75249380	885	*	7	7.03699E-12	0.010029048	0.011315794	0.005284606	0.060815757	0.014554934	RPP25
chr5	112042650	112043716	1067	*	14	7.08992E-18	0.001489144	0.011322384	0.00041838	0.125028569	0.029459674	APC
chr4	90228685	90229039	355	*	5	1.15657E-07	0.067599749	0.01132673	0.025566341	0.022902042	0.011029021	GPRIN3
chr20	47443694	47444241	548	*	3	9.50619E-10	0.002331382	0.011331512	0.003684714	-0.054332393	-0.000878147	PREX1
chr12	111016163	111016566	404	*	2	4.2048E-08	0.000658486	0.011335515	0.001320455	-0.110190407	-0.109487481	PPTC7
chr6	38325960	38326204	245	*	2	3.94273E-08	0.000801185	0.011341838	0.001556231	0.120439353	0.09454885	BTBD9
chr2	25565215	25565841	627	*	13	7.08872E-13	0.140251815	0.011344966	0.01605206	0.058568199	-0.004804363	DNMT3A
chr2	10182878	10184650	1773	*	19	2.06822E-18	0.002368402	0.011348196	0.000815638	0.116852702	0.006820992	KLF11
chr17	684490	685509	1020	*	12	1.47654E-15	0.001425451	0.011358683	0.001760982	0.179197888	0.019087412	GLOD4
chr4	1686288	1687333	1046	*	6	6.88566E-11	0.03622223	0.011361819	0.007508283	-0.06314095	-0.019902543	
chr2	30369105	30370399	1295	*	13	3.70529E-20	0.002325228	0.011362733	0.000188977	-0.063709216	-0.018984208	YPEL5
chr1	224370155	224371490	1336	*	13	1.57444E-13	0.024472226	0.011363516	0.011910331	-0.300936973	-0.025916452	DEGS1
chr14	93672722	93673792	1071	*	11	1.47933E-18	4.18722E-06	0.01136553	1.9416E-05	-0.183431063	-0.024950175	RP11-371E8.4, UBR7, C14orf142
chr11	457742	458052	311	*	2	9.17218E-11	0.000739154	0.011375006	0.001456076	0.12852766	0.118580275	PTDSS2
chr2	157184816	157184978	163	*	3	5.34874E-09	0.016757217	0.011390186	0.006806977	-0.217325942	-0.138233952	NR4A2
chr5	80596198	80597291	1094	*	7	5.48746E-15	0.000442334	0.011403895	0.000316246	-0.248010226	-0.032777205	CKMT2-AS1
chr6	133035150	133035379	230	*	4	2.66448E-08	0.036845931	0.011405862	0.009924118	-0.194936848	-0.069509627	VNN1
chr1	26348325	26348484	160	*	2	1.50954E-08	0.001094143	0.011406516	0.00201284	0.129554276	0.104651466	EXTL1
chr1	7913067	7914017	951	*	9	1.16582E-23	4.53892E-08	0.011418351	3.98186E-07	0.141136972	0.094494774	UTS2
chr6	135602552	135602559	8	*	2	6.93154E-08	0.000662602	0.01141918	0.001329472	-0.129060484	-0.122715838	
chr12	54656228	54656343	116	*	2	4.90773E-08	0.000643791	0.011423202	0.001297644	-0.1097136	-0.098858822	CBX5
chr2	86667850	86669446	1597	*	18	2.05614E-14	0.021698637	0.011431039	0.003702518	-0.041806824	-0.005447072	KDM3A
chr3	32463113	32464005	893	*	3	5.70626E-10	0.00027348	0.011440096	0.000638696	-0.178850205	-0.111848377	CMTM7
chr17	16118138	16118251	114	*	2	5.84223E-09	0.001379613	0.011449113	0.002427218	0.030478617	0.022992014	NCOR1
chr21	43823604	43824262	659	*	10	6.48246E-19	2.43759E-06	0.011451944	1.47866E-05	0.276688069	0.108713786	UBASH3A
chr2	118981748	118982679	932	*	8	1.1898E-13	0.00090732	0.0114526	0.001424517	0.053356615	0.010536659	
chr17	47286445	47287974	1530	*	14	5.3505E-17	0.001084311	0.011453584	0.000378982	0.145458409	0.042086218	ABI3, GNGT2

chr17	80376872	80376884	13	*	2	2.53112E-08	0.003031669	0.011457946	0.004432942	0.020352729	0.000205243	HEXDC
chr8	41654331	41656940	2610	*	14	5.0495E-13	1.90315E-05	0.011463672	1.03111E-05	-0.188586832	0.040136376	ANK1
chr1	11795409	11796573	1165	*	14	9.51593E-21	0.002178105	0.01147021	0.000153988	-0.206293049	-0.039144427	AGTRAP
chr6	31547704	31550241	2538	*	32	8.08473E-25	3.41524E-06	0.011475457	2.44702E-06	0.219580864	0.022871609	LTB
chr14	55594613	55595320	708	*	3	5.70197E-11	0.000566392	0.011484161	0.001176487	-0.205711404	-0.166622632	LGALS3
chr8	8930709	8930785	77	*	2	1.72562E-08	0.001143722	0.011510493	0.00209123	0.108628071	0.106887872	ERI1
chr5	59188112	59189724	1613	*	8	2.32066E-12	0.000126487	0.011526302	0.000333547	0.122589571	0.049173292	PDE4D
chr1	26855423	26855926	504	*	5	1.76581E-08	0.144608194	0.011532032	0.049626504	-0.126654942	-0.026581109	
chr4	144436129	144436344	216	*	3	3.72965E-09	0.010497945	0.011533357	0.006543639	0.168262843	0.093557579	SMARCA5
chr1	40532772	40532873	102	*	2	6.73088E-08	0.001938398	0.011538709	0.003174726	-0.138075694	-0.045713549	CAP1
chr20	55967503	55968294	792	*	5	8.59109E-12	0.000496795	0.011570394	0.000491854	0.185147829	0.109179081	RBM38, RP4-800J21.3
chr2	27593896	27594150	255	*	4	7.62092E-11	0.057070944	0.011595251	0.009939528	0.02118395	0.009482365	SNX17
chr8	119124103	119124790	688	*	4	7.33864E-12	0.000430227	0.011595803	0.001065809	-0.230812797	-0.055148284	
chr2	62080815	62081471	657	*	8	1.39902E-10	0.014032663	0.011607685	0.015388892	0.02909794	-0.000596627	FAM161A
chr10	131567980	131568379	400	*	4	1.29901E-08	0.096665162	0.011639639	0.006131743	-0.184285694	-0.077360111	
chr6	23177275	23178052	778	*	5	3.39143E-09	0.028566959	0.011648142	0.002594242	0.166571339	0.08886101	
chr6	11737639	11737776	138	*	2	1.09375E-07	0.023768421	0.011655318	0.016236974	0.134231436	0.063358972	ADTRP
chr2	28617552	28618402	851	*	7	2.75635E-13	9.48587E-05	0.011660837	0.000227795	-0.105429189	-0.047566089	FOSL2
chr17	56437800	56437930	131	*	2	1.04799E-07	0.00080979	0.011679616	0.001580569	0.112320636	0.097767581	BZRAP1-AS1, RNF43
chr5	95159127	95159639	513	*	5	4.02538E-12	0.005735392	0.011680707	0.002411456	-0.192880084	-0.062712143	RHOBTB3
chr12	8129632	8130007	376	*	2	3.44501E-08	0.00068804	0.01169937	0.001379409	-0.14211768	-0.021730625	
chr20	45179226	45179413	188	*	5	9.42086E-13	5.28316E-05	0.011699539	0.000236201	-0.143785241	-0.076716282	
chr15	100048210	100048500	291	*	4	1.24129E-08	0.002782803	0.011699609	0.004479538	0.179516618	0.091552939	MEF2A
chr20	3190112	3190600	489	*	5	1.37646E-12	0.009792982	0.01170031	0.004244182	-0.038805276	0.000902038	ITPA
chr14	21152498	21153451	954	*	8	3.71053E-15	0.05087367	0.011701619	0.010210149	-0.1914659	-0.036997336	RNASE4, ANG, AL163636.6
chr10	114133235	114134550	1316	*	8	1.36929E-18	5.2013E-05	0.011707616	9.30671E-06	0.152188626	0.009923442	ACSL5
chr16	87736388	87737549	1162	*	6	1.25641E-14	0.00279598	0.011709808	0.000556997	-0.164924775	-0.084763057	KLHDC4, FLJ00104
chr1	90456081	90456818	738	*	3	2.29872E-08	0.001779181	0.011711801	0.001941763	0.126859817	0.088577019	
chr8	103252525	103252842	318	*	2	3.91478E-09	0.002296451	0.011732383	0.003632573	-0.119672189	-0.079554389	KB-431C1.4

chr1	42500967	42501967	1001	*	7	6.67914E-12	0.0013405	0.011734642	0.000984341	0.035408253	0.00952566	HIVEP3
chr20	61427684	61428215	532	*	8	2.35186E-11	0.122065282	0.011748971	0.014999591	-0.061906644	-0.02421067	MRGBP
chr2	44395262	44396058	797	*	8	5.04684E-15	0.000410729	0.011753209	0.000321093	-0.055548121	-0.0254432	PPM1B, RP11-559M23.1
chr16	1521617	1522109	493	*	4	2.96889E-10	0.000813333	0.011760906	0.001131467	-0.115403551	-0.075192846	CLCN7, LA16c-390E6.3
chr14	103494686	103494823	138	*	2	7.83571E-08	0.000688595	0.011776852	0.001382271	-0.131827399	-0.000510981	CDC42BPB
chr13	34184663	34185311	649	*	8	1.43274E-13	0.027355104	0.011780404	0.0026134	0.117154179	0.034305707	RP11-37L2.1, RP11-141M1.3
chr11	126872615	126873181	567	*	6	4.02707E-10	0.045697151	0.011795231	0.011017204	-0.146184188	-0.053668813	KIRREL3-AS3, KIRREL3
chr7	143088472	143089459	988	*	7	2.24593E-18	7.58397E-08	0.011827701	1.01414E-06	0.172955005	0.112873238	EPHA1
chr14	105390934	105391118	185	*	4	5.23608E-08	0.014367302	0.011837875	0.006220967	-0.164579512	-0.086223042	
chr11	32913903	32913998	96	*	3	3.76658E-10	0.02418309	0.011859169	0.004432157	0.024412253	0.016179353	
chr17	1479213	1479834	622	*	2	8.12018E-08	0.000878236	0.011865798	0.001696883	-0.145393182	-0.119919809	SLC43A2
chr2	85919996	85922172	2177	*	10	8.1609E-12	0.008190094	0.011868605	7.55906E-05	-0.184757915	0.02409209	GNLY
chr16	67270593	67271014	422	*	4	1.49447E-11	0.00053345	0.011870488	0.000843923	0.144621791	0.045272382	FHOD1
chr20	43588633	43590115	1483	*	13	1.25035E-13	0.000101918	0.011879207	8.81437E-05	0.207107377	0.022404538	TOMM34
chr14	74181035	74181552	518	*	5	2.19565E-09	0.017047511	0.011894156	0.010803751	0.038374138	0.01567067	PNMA1
chr7	4872926	4873549	624	*	5	1.53517E-09	0.065628825	0.011894899	0.008932945	0.092301733	0.047247727	RADIL
chr7	23052972	23054418	1447	*	13	1.81598E-16	0.036847146	0.011898123	0.001878985	-0.11044712	-0.021829596	FAM126A
chr15	90357202	90357521	320	*	2	1.29725E-08	0.004990632	0.011899342	0.006409035	-0.122719016	-0.074209838	ANPEP
chr14	73602718	73603844	1127	*	17	1.70831E-13	0.071350125	0.011916646	0.031989813	0.064743669	-0.001951785	PSEN1
chr8	61565814	61566439	626	*	7	8.83044E-13	0.024297051	0.011916806	0.00463206	0.02081998	0.009131681	RP11-91I20.2
chr4	84049907	84050796	890	*	3	3.16965E-10	0.000107925	0.011919847	0.000348709	-0.146364075	-0.097726702	PLAC8
chr5	42951711	42952369	659	*	4	1.14181E-12	5.82879E-05	0.011957319	0.000204942	0.07332904	0.058599564	
chr2	97523237	97523931	695	*	10	1.69481E-12	0.077810437	0.011959057	0.008432216	-0.065262709	-0.006306089	ANKRD23, ANKRD39
chr4	6919718	6920113	396	*	3	6.77018E-11	0.000119844	0.01196008	0.000387968	-0.122970742	-0.079753257	TBC1D14
chr4	3390813	3391495	683	*	5	8.45419E-09	0.03364922	0.011998514	0.017336001	0.096988498	0.006653459	RGS12
chr5	1113320	1113627	308	*	5	2.11513E-14	0.000151871	0.012006501	0.000205785	-0.161545277	-0.081963219	
chr7	100860016	100861373	1358	*	16	5.64683E-13	0.052507393	0.01201781	0.039460486	-0.054722745	-0.00452364	ZNHIT1, PLOD3
chr3	148708091	148709111	1021	*	7	1.26056E-14	0.003970718	0.012023213	0.001133452	-0.144567456	-0.046658248	
chr11	64490494	64490871	378	*	8	2.1037E-08	0.344829414	0.012047861	0.052621791	0.028261312	0.006028424	NRXN2

chr2	231067424	231068246	823	*	6	3.46452E-10	0.137851336	0.012051822	0.011413288	-0.240873279	-0.064009583	SP140, SP110
chr5	132112449	132113201	753	*	14	9.40779E-11	0.83853038	0.012053375	0.255115533	0.03010628	0.005238569	SEPT8
chr12	56981517	56982283	767	*	5	3.82852E-12	6.58157E-05	0.012064306	0.000253611	0.151557967	0.007716688	RBMS2
chr12	58160843	58160989	147	*	3	7.14062E-08	0.070791432	0.012065027	0.023427001	-0.145820237	-0.034993247	CYP27B1
chr13	33001751	33002464	714	*	11	2.29653E-10	0.041127668	0.012084889	0.028159523	-0.065935043	-0.009369532	N4BP2L1, RP11-298P3.4
chr8	27219512	27219812	301	*	2	1.13499E-07	0.000918037	0.012108502	0.001768574	-0.13437276	-0.025331873	PTK2B
chr10	43633258	43634212	955	*	9	1.70806E-11	0.031721194	0.012121234	0.013256032	0.016034131	0.004970598	CSGALNACT2, RP11-351D16.3
chr4	185377809	185378132	324	*	3	2.52449E-10	0.001398696	0.012136704	0.001644635	0.131226245	0.082361617	IRF2
chr9	73216095	73216478	384	*	3	4.84432E-10	0.000116612	0.01215609	0.000381136	0.143419567	0.121111588	TRPM3
chr3	151986340	151987978	1639	*	17	1.21716E-17	0.027387828	0.012165219	0.002824809	0.023464106	0.002186048	MBNL1, MBNL1-AS1
chr11	65658459	65659772	1314	*	5	2.10752E-11	0.002844636	0.012165924	0.001002338	0.122519004	0.04050543	CCDC85B, FOSL1
chr1	235667507	235668421	915	*	10	3.08977E-09	0.038191453	0.012175719	0.026391716	-0.034780983	-0.006457039	B3GALNT2
chr14	93170710	93171393	684	*	4	3.21283E-10	0.00068068	0.012186501	0.001125454	-0.198502915	-0.036175928	LGMN
chr11	9335544	9336845	1302	*	18	1.66987E-17	0.01377154	0.012187816	0.00305124	-0.058862985	-0.006015372	TMEM41B
chr17	4166847	4168654	1808	*	16	3.45678E-24	0.000440105	0.012188671	4.97802E-05	0.123465887	0.006268905	ANKFY1
chr2	97202143	97202475	333	*	7	2.24731E-09	0.070167541	0.012195969	0.0209998	0.049535422	0.002891478	
chr6	24774713	24775047	335	*	5	2.1087E-09	0.019521681	0.01219948	0.01085234	-0.074592624	-0.01879506	
chr11	74173518	74173933	416	*	3	1.07297E-08	0.003866842	0.012217424	0.004429343	-0.277235909	-0.12353314	RP11-702H23.4, KCNE3
chr10	13569995	13571273	1279	*	11	1.67284E-16	0.003674971	0.01222235	0.000771183	0.044710119	0.008204962	RP11-214D15.2, BEND7
chr6	108882119	108882205	87	*	3	4.91017E-08	0.001368043	0.01222764	0.002672576	-0.140301884	-0.052263679	FOXO3
chr10	115438867	115439884	1018	*	15	1.06266E-11	0.101638323	0.012244298	0.044966875	-0.05831796	-0.010724514	CASP7
chr17	30668751	30669189	439	*	4	1.7508E-09	0.010275373	0.012249331	0.010407776	-0.036215548	-0.012441611	RP11-227G15.3, C17orf75
chr19	52703857	52704819	963	*	4	4.07738E-11	0.000443812	0.012249973	0.000659574	0.10149105	0.072482486	PPP2R1A
chr12	16499963	16500185	223	*	3	1.69639E-08	0.019937175	0.012253174	0.004128084	-0.223806087	-0.125141642	MGST1
chr2	103035835	103035968	134	*	2	3.58514E-08	0.001017021	0.012279019	0.001930818	-0.143519795	-0.134404272	IL18RAP
chr5	177876427	177876930	504	*	4	4.08237E-09	0.033213061	0.012280249	0.00573097	-0.192486241	-0.097979985	COL23A1
chr8	9762716	9762850	135	*	2	7.01307E-09	0.006526675	0.012286823	0.007788944	0.020143844	0.017518381	LINC00599
chr12	4385029	4386203	1175	*	7	1.40021E-12	0.027325036	0.012297053	0.010816343	0.132713724	0.033218496	CCND2, CCND2-AS1
chr2	227700114	227701050	937	*	13	6.99075E-22	0.002448057	0.012316979	0.000344716	0.155982349	0.043690113	RHBDD1

chr4	184367267	184367379	113	*	2	8.81867E-08	0.001713899	0.012325617	0.002941297	-0.154495089	-0.09851105	CDKN2AIP
chr1	246952118	246954479	2362	*	15	6.70895E-11	0.003864448	0.012329132	0.001595773	0.13610104	0.022481701	RP11-439E19.3, KIF28P
chr18	21033072	21033823	752	*	6	1.92784E-11	0.017148907	0.012340789	0.00315268	0.051814862	0.014684335	RIOK3
chr1	223354216	223354607	392	*	5	1.83139E-10	0.001710346	0.012364445	0.001724407	-0.179849173	-0.049657004	RP11-239E10.3
chr8	42623718	42623946	229	*	6	3.38926E-11	0.01436164	0.012371472	0.001933951	-0.159837475	-0.074927159	CHRNA6
chr11	8833689	8833973	285	*	4	1.42664E-12	0.006352055	0.012377941	0.000708837	-0.127423297	-0.06874442	ST5
chr3	71730474	71731211	738	*	3	5.4219E-09	0.000213161	0.012380812	0.000604999	-0.193905576	-0.103605069	EIF4E3
chr15	42693448	42694406	959	*	7	1.70848E-13	0.000701148	0.01238538	0.001049969	0.200723113	0.032642233	CAPN3, RP11-164J13.1
chr17	4844136	4844722	587	*	3	4.59979E-08	0.260340329	0.012423779	0.056827855	0.076214161	0.029176272	RNF167
chr8	41504887	41505625	739	*	8	2.54473E-09	0.091111598	0.012426738	0.048133714	0.119153302	0.039877483	NKX6-3
chr16	28834065	28835277	1213	*	8	1.33701E-14	0.023562943	0.012439116	0.003881034	-0.212856528	-0.05121604	ATXN2L
chr19	2235103	2237081	1979	*	19	1.87785E-17	1.2686E-06	0.012454408	9.60199E-06	-0.255827337	-0.012801199	SF3A2, PLEKHJ1
chr17	36955626	36955717	92	*	2	5.81214E-08	0.004461647	0.012458598	0.006033532	0.019471308	0.005605482	PIP4K2B
chr7	156803122	156803578	457	*	5	6.48298E-08	0.0795629	0.012480487	0.029051504	-0.053369513	-0.009098165	MNX1-AS1, MNX1
chr8	104152750	104154149	1400	*	12	4.66347E-17	0.001148036	0.012483793	0.000588565	-0.07025237	0.000315566	BAALC, C8orf56
chr17	81060149	81060259	111	*	3	5.88154E-10	0.000135434	0.012487793	0.000416736	0.241955929	0.184722935	
chr6	32044163	32044959	797	*	8	2.73977E-13	0.152003219	0.012488765	0.007760818	0.159030922	-0.011086144	TNXB
chr6	132128915	132129021	107	*	6	1.57661E-07	0.034145057	0.012505946	0.008783349	-0.05085538	-0.007322101	
chr2	27346079	27346508	430	*	5	5.08585E-08	0.160779043	0.012527166	0.028825822	-0.070010429	-0.039504745	
chr15	49461632	49462514	883	*	10	2.35209E-11	0.085940378	0.012555431	0.017068743	0.121060143	0.029253224	GALK2
chr3	129345793	129346101	309	*	3	4.38697E-08	0.002223992	0.012557534	0.003394455	0.026433847	0.007044549	
chr17	74565757	74565795	39	*	2	1.79349E-07	0.003643126	0.012564048	0.005235397	-0.139030029	-0.102731521	ST6GALNAC2
chr17	29248641	29248848	208	*	4	7.88655E-08	0.017592261	0.012588428	0.012664919	-0.065603759	-0.029473739	ADAP2
chr18	14748250	14748439	190	*	4	1.46028E-07	0.009212751	0.012594574	0.011428196	-0.090740713	-0.073840798	ANKRD30B
chr14	103565144	103566588	1445	*	9	1.75128E-15	2.56514E-06	0.012595878	1.39553E-05	0.181552855	0.125321556	RP11-736N17.8, EXOC3L4
chr5	39219235	39220149	915	*	5	1.90348E-14	7.79964E-05	0.012598735	6.71145E-05	-0.179259553	-0.075882618	FYB
chr14	21488952	21491111	2160	*	10	3.69861E-13	0.003418804	0.012603104	0.000374181	0.127819294	0.019004075	NDRG2
chr12	56862504	56863389	886	*	10	2.581E-19	0.000485573	0.012635709	0.000490971	-0.146916886	-0.044143481	SPRYD4, MIP
chr17	19880691	19881339	649	*	6	5.52688E-12	0.003466648	0.012642395	0.002554528	0.045587301	0.014778209	AKAP10

chr12	54690656	54690833	178	*	4	8.75304E-10	0.000650816	0.012656177	0.001615453	-0.091005477	-0.058206743	RP11-968A15.8, NFE2
chr17	7155896	7156125	230	*	3	4.17004E-09	0.006872562	0.012670007	0.007815429	0.023019755	-0.004149606	ELP5, RP1-4G17.5
chr7	72992005	72993765	1761	*	16	3.30336E-23	1.74747E-05	0.012685093	1.30298E-05	0.105751608	-0.005890788	TBL2
chr12	109896495	109897234	740	*	5	5.48542E-14	0.000186648	0.012688554	0.00031268	0.080445381	0.05557123	KCTD10
chr14	102963178	102964872	1695	*	10	1.35968E-15	1.44047E-07	0.012690912	2.69543E-06	0.218613965	0.095084373	TECPR2
chr8	125740425	125741566	1142	*	13	4.71645E-16	0.01343837	0.012693144	0.004316885	0.122233913	0.032439271	MTSS1
chr16	1195373	1195491	119	*	2	8.47311E-08	0.001788161	0.012701635	0.003067106	0.204037856	0.134057801	
chr20	21085983	21086336	354	*	4	1.18223E-09	0.011262925	0.012715768	0.007235572	0.03385296	0.021496281	LINC00237
chr1	226925082	226926318	1237	*	6	4.13063E-11	0.01136366	0.012717949	0.009818225	-0.025066571	0.00204478	ITPKB
chr20	34823091	34824487	1397	*	13	1.01891E-14	0.06899416	0.012722111	0.004679121	0.151616935	0.044467498	AAR2
chr7	150086034	150086169	136	*	2	7.55373E-08	0.000846786	0.012725047	0.001672121	-0.148392819	-0.129501066	ZNF775
chr20	32274191	32274911	721	*	7	1.63125E-11	0.094144476	0.012726744	0.010069157	0.127293223	0.014682242	E2F1
chr17	8287126	8287763	638	*	4	1.30814E-09	0.033098623	0.012737325	0.025412208	0.048569672	0.030419635	
chr4	110480700	110481823	1124	*	10	3.47466E-10	0.410417565	0.012748774	0.060143258	0.032182692	0.000533342	CCDC109B
chr10	102671221	102672937	1717	*	15	5.39928E-19	0.000554896	0.012767459	0.000170952	0.216833702	-0.007900224	FAM178A, RP11-179B2.2
chr17	22020759	22020985	227	*	4	2.24657E-11	6.26869E-05	0.012776098	0.000224393	0.121748758	0.088906476	
chr9	131486216	131487427	1212	*	11	2.42972E-16	0.000967517	0.012778604	0.000321707	0.138729568	0.021063009	RP11-545E17.3, ZDHHC12
chr12	116985510	116985884	375	*	3	2.576E-09	0.001655165	0.012795181	0.003013197	-0.097288236	-0.074447711	
chr12	22778485	22778766	282	*	3	1.06203E-09	0.004008077	0.01280028	0.005958135	-0.023636646	0.002501478	ETNK1
chr5	95997186	95998008	823	*	18	3.86413E-10	0.797273504	0.012800955	0.29284704	0.03492167	0.00261651	CAST
chr9	132815532	132816865	1334	*	13	3.78994E-16	0.031934065	0.012809667	0.00494951	0.02492824	0.010763028	GPR107
chr1	204380802	204381404	603	*	10	1.67614E-11	0.015609887	0.012816964	0.012374693	0.067229004	-0.007275777	RP11-739N20.2, PPP1R15B
chr1	15271760	15272567	808	*	11	1.81579E-18	1.70583E-05	0.012851118	2.71967E-05	0.139744566	0.078736267	KAZN
chr16	83762345	83762355	11	*	2	5.60915E-08	0.000804445	0.012855404	0.001605219	-0.143067617	-0.139214036	CDH13, RP11-298D21.1
chr9	139366472	139367397	926	*	5	1.73026E-11	1.27371E-05	0.012863097	7.38398E-05	0.153387711	0.115420582	SEC16A
chr13	113435678	113435942	265	*	3	4.85727E-10	0.066352101	0.012871306	0.004213071	0.105364448	0.063720883	ATP11A
chr17	3584925	3585166	242	*	3	8.75537E-09	0.000613985	0.012871706	0.001213058	0.11200202	0.089662686	P2RX5-TAX1BP3, P2RX5
chr6	41437676	41438063	388	*	3	3.56893E-08	0.003741353	0.012884124	0.00444214	0.066404208	0.035070057	
chr7	102714667	102715565	899	*	14	3.06614E-13	0.106105709	0.012903736	0.01993983	-0.039555796	0.001768947	ARMC10, FBXL13

chr17	57970021	57970985	965	*	16	2.76407E-12	0.072618789	0.012903932	0.040329115	-0.04875837	-0.009538696	RPS6KB1, TUBD1
chr8	145158241	145160216	1976	*	20	4.09205E-19	0.000539422	0.012915568	0.000393383	0.140033445	0.00688707	MAF1, SHARPIN
chr15	45028083	45028161	79	*	3	7.71423E-08	0.008527613	0.01291634	0.007969613	0.173985174	0.088474808	TRIM69
chr1	161168073	161172056	3984	*	28	3.4461E-22	0.000138735	0.012920267	1.14283E-06	-0.167244281	0.014684222	NDUFS2, ADAMTS4
chr13	31479752	31481184	1433	*	11	2.75493E-21	1.62745E-06	0.012932744	9.55855E-06	0.151105752	0.042407328	MEDAG, TEX26-AS1
chr22	46432208	46432835	628	*	3	3.56504E-10	0.00030617	0.012936522	0.000848555	0.029096204	0.026507953	
chr7	155174508	155175340	833	*	7	1.35239E-09	0.3141444	0.012970614	0.021611717	-0.253828359	-0.028404338	AC008060.7
chr4	3773891	3773996	106	*	4	1.54695E-08	0.022770024	0.012972904	0.004854771	0.091201064	0.047117647	
chr14	20247183	20247919	737	*	3	8.22626E-09	0.000447061	0.012985664	0.001136142	-0.126988269	-0.09817083	OR4N2
chr19	7733642	7734203	562	*	6	1.10734E-18	4.6927E-07	0.012986055	4.54979E-06	-0.191211009	-0.13303267	RETN
chr18	19179948	19180362	415	*	2	1.24305E-11	0.001115685	0.012990042	0.002111674	-0.047872913	-0.005733471	ESCO1
chr17	64575563	64575592	30	*	3	1.40229E-07	0.005557955	0.013003817	0.006978639	-0.14038847	-0.07894342	PRKCA
chr7	158250911	158250978	68	*	2	2.04481E-09	0.000955926	0.013006458	0.001859092	-0.218393665	-0.163985509	PTPRN2
chr20	35503983	35504553	571	*	8	8.24308E-16	1.47127E-05	0.013007318	6.3948E-05	0.200274079	0.083997278	TLDC2
chr20	61309080	61310117	1038	*	5	3.29827E-13	1.696E-05	0.013020168	8.21628E-05	-0.17677852	-0.119310256	SLCO4A1
chr17	17006819	17007127	309	*	2	3.80865E-08	0.000833063	0.013032149	0.001657922	0.100742864	0.098049647	MPRIP
chr17	201273	201737	465	*	3	1.55034E-10	0.000351121	0.01304002	0.00093495	0.10069663	0.087241298	RPH3AL
chr4	170928131	170928419	289	*	2	1.54719E-07	0.000916507	0.01304146	0.001796115	0.097169694	0.006609108	MFAP3L
chr11	72929553	72929983	431	*	3	4.61796E-10	0.021402693	0.013041771	0.014857659	-0.193828789	-0.076181871	P2RY2
chr1	27669563	27669932	370	*	3	8.38073E-12	0.000113648	0.013043693	0.000381824	-0.151993596	-0.095285253	SYTL1
chr14	91281552	91281948	397	*	3	1.38512E-08	0.002334496	0.013065798	0.004041928	0.159943716	0.10581687	TTC7B
chr8	142189535	142189806	272	*	6	4.87967E-10	0.019590975	0.013067369	0.007859005	0.15924797	0.054903343	DENND3
chr12	103310839	103311500	662	*	8	2.37741E-12	0.025239823	0.013082073	0.008356599	0.098809748	0.024231098	PAH
chr22	46028579	46028727	149	*	2	1.67722E-07	0.000941595	0.013089958	0.001838523	0.136480843	0.101126005	
chr3	135915333	135915556	224	*	2	8.33909E-10	0.002758418	0.01310237	0.00432292	-0.053029417	-0.01854821	MSL2
chr2	43037868	43038067	200	*	2	3.48608E-08	0.423761034	0.013114185	0.039287449	0.025069116	0.013203113	
chr1	111742914	111744503	1590	*	13	1.05466E-12	0.000257577	0.013141104	0.000271022	-0.208707847	-0.036959008	CHI3L2, DENND2D
chr11	73694157	73695045	889	*	8	9.5198E-15	0.001349378	0.013144853	0.001388426	0.099362581	0.014371328	UCP2
chr7	144531951	144533283	1333	*	12	2.23516E-11	0.207831526	0.013176419	0.021717538	0.064648999	0.00636254	TPK1

chr7	2671535	2671838	304	*	3	3.42798E-09	0.248357381	0.013183174	0.057674989	0.029175571	0.007209468	TTYH3
chr13	78271382	78273164	1783	*	20	4.87613E-19	0.001782058	0.013200712	0.000852707	-0.030646343	0.001852899	SLAIN1, MIR3665
chr6	15505949	15506085	137	*	2	4.22647E-08	0.001705183	0.013201539	0.002986369	0.138417397	0.105203739	JARID2
chr17	27187966	27188437	472	*	2	9.5231E-09	0.004917157	0.01320307	0.006604889	0.090650799	0.069297732	ERAL1, MIR451B
chr11	47398575	47400931	2357	*	14	6.34242E-19	1.76915E-06	0.013208464	2.01283E-06	-0.190877084	-0.057290843	SPI1
chr4	48832037	48832156	120	*	2	1.13921E-08	0.091632583	0.013220767	0.030561293	-0.123623027	-0.064214224	OCIAD1
chr14	95651866	95652850	985	*	7	6.11497E-16	0.000453029	0.013235187	0.000241966	-0.074707303	-0.041333873	CLMN, CTD-2240H23.2
chr16	70759095	70760142	1048	*	6	8.35309E-15	3.09331E-05	0.013239736	9.16197E-05	0.129698786	0.065488751	VAC14
chr7	148936039	148937111	1073	*	12	3.962E-14	0.009592506	0.013240242	0.001857521	-0.217151157	-0.033456685	ZNF212
chr11	63996766	63997982	1217	*	10	7.22765E-16	0.116363128	0.013256457	0.01100996	0.192581741	0.044004783	NUDT22, DNAJC4, RP11-783K16.14
chr17	21116851	21117780	930	*	8	6.21856E-14	0.007826816	0.013259998	0.002071138	0.032014042	0.013642949	AC087294.2, TMEM11
chr1	6475317	6475535	219	*	3	2.15573E-09	0.000338831	0.013268549	0.000900646	-0.108300183	-0.079209789	HES2
chr9	38069796	38069959	164	*	3	3.24808E-09	0.003388233	0.013271737	0.002689179	0.022095903	0.008313982	
chr11	75109830	75111245	1416	*	13	8.61714E-14	0.032521527	0.013284187	0.010771749	-0.059024319	-0.004068424	RPS3
chr11	46638289	46638919	631	*	10	2.61731E-09	0.17684582	0.013285163	0.043093719	-0.03941763	-0.001229501	ATG13, HARBI1
chr1	153329460	153330776	1317	*	11	1.68775E-16	0.000766345	0.013292657	2.34892E-05	-0.154031256	-0.060024038	S100A9
chr16	3639688	3639839	152	*	2	1.41285E-07	0.001430891	0.013312392	0.002601466	-0.096977407	-0.092419713	SLX4
chr3	53289400	53290482	1083	*	14	9.04631E-12	0.118831314	0.013314435	0.055046174	-0.078045632	-0.005851618	TKT
chr13	50509902	50510427	526	*	6	3.63219E-10	0.085332644	0.013316433	0.03639534	-0.055937747	-0.013207107	SPRYD7
chr5	150018831	150020288	1458	*	7	6.19543E-09	0.003570013	0.013344568	0.000484359	-0.198481362	-0.073689821	SYNPO
chr8	145643030	145644123	1094	*	6	1.58374E-13	0.005338527	0.013346705	0.001292565	0.179670529	0.09245413	
chr9	84386662	84386734	73	*	2	1.75708E-07	0.000935752	0.013357067	0.001837267	-0.15902092	-0.143804556	RP11-154D17.1
chr16	4357055	4357936	882	*	7	1.34363E-11	0.012680158	0.013358909	0.00178586	-0.126279549	-0.027360645	
chr2	239330108	239330202	95	*	3	7.72139E-09	0.000638917	0.013359737	0.001223474	0.154326905	0.115680332	
chr19	44123486	44124896	1411	*	15	4.37273E-12	0.120597857	0.013365115	0.018760533	0.115409346	0.018167499	ZNF428
chr1	6052166	6052785	620	*	14	1.00655E-14	0.012271065	0.01336871	0.003742222	0.019438906	0.005413793	KCNAB2, NPHP4
chr1	239550177	239550578	402	*	4	1.11386E-08	0.036113074	0.013378544	0.018946073	0.024563355	0.013079667	CHRM3
chr10	102891568	102891879	312	*	2	2.96339E-08	0.019703321	0.013382852	0.015884977	0.039852639	0.030994486	TLX1
chr2	242887053	242887391	339	*	3	6.57738E-09	0.004775871	0.01339276	0.003462228	0.176473016	0.101526119	AC131097.3

chr8	144896176	144896694	519	*	4	3.90041E-13	0.001167872	0.013395348	0.00096509	-0.189654473	-0.105199085	SCRIB
chr8	145639652	145639880	229	*	3	9.43732E-08	0.017096647	0.013400892	0.014195027	0.132057791	0.047655673	SLC39A4
chr3	50273096	50273340	245	*	4	1.14098E-09	0.007239101	0.013417081	0.007188525	-0.065503844	-0.054225042	GNAI2
chr19	5477638	5478484	847	*	4	1.21682E-09	0.000298684	0.013425327	0.000449451	0.153049418	0.088199149	
chr12	104851172	104851316	145	*	2	1.96568E-09	0.051798062	0.013438086	0.025241564	0.026645525	0.009851337	CHST11
chr11	113930430	113931305	876	*	8	1.75977E-11	0.033226738	0.013446641	0.004607911	-0.084167407	-0.009681567	ZBTB16
chr1	226109692	226110727	1036	*	3	6.8055E-09	0.000147563	0.013449118	0.0004726	0.155109992	-0.002733179	RP4-559A3.7, PYCR2
chr12	57881856	57883315	1460	*	8	7.01439E-15	0.13912516	0.013461348	0.001108545	0.115366804	0.022656893	MARS, ARHGAP9
chr7	95064015	95064738	724	*	12	7.25351E-11	0.105246703	0.013490787	0.040457964	-0.06176947	-0.00264242	PON2
chr3	46943819	46943940	122	*	2	1.73006E-07	0.000926934	0.0134929	0.001826865	-0.131503641	-0.089401776	PTH1R
chr10	65280473	65281299	827	*	11	3.05149E-16	0.003235908	0.013504029	0.000285372	-0.270703839	-0.029529497	REEP3
chr12	54069773	54071168	1396	*	20	1.27067E-16	0.034024659	0.013512965	0.017611937	0.043214744	0.00772215	ATP5G2
chr21	47808810	47809310	501	*	5	8.02808E-12	7.57198E-05	0.013519827	0.000310203	0.140346354	0.08904761	PCNT
chr11	118307464	118307782	319	*	4	2.22437E-09	0.030416046	0.013526449	0.006580269	-0.261889411	-0.077597022	KMT2A
chr17	1665119	1665303	185	*	6	1.08717E-08	0.034922842	0.013526911	0.01202734	-0.166507314	-0.06206751	SERPINF1
chr12	58158537	58159051	515	*	3	8.30909E-10	0.002852171	0.013551055	0.002810263	0.078634666	0.039659995	CYP27B1
chr19	13048623	13051573	2951	*	17	1.12433E-13	0.03714024	0.013562656	0.00017283	-0.169084481	-0.018843075	CALR
chr5	96078430	96078834	405	*	4	3.08102E-09	0.018399678	0.013615129	0.007921762	-0.221512446	-0.129998334	CAST
chr18	74845706	74846035	330	*	3	1.30876E-08	0.002987544	0.01365672	0.004388246	-0.132439573	-0.04603504	
chr14	102198157	102198604	448	*	3	2.86559E-10	7.11183E-05	0.013723109	0.000262498	-0.125612367	-0.115156762	RP11-796G6.2
chr2	96068327	96068732	406	*	8	4.63945E-09	0.096937182	0.013728509	0.031078607	0.018667775	0.006788021	FAHD2A
chr6	32095148	32096917	1770	*	36	6.06524E-25	0.003901909	0.013734355	0.007053728	0.068852339	0.005892287	ATF6B, FKBPL
chr6	33215649	33215685	37	*	2	8.15696E-08	0.080518007	0.013754663	0.03015642	-0.16563458	-0.077577036	
chr17	41919393	41919603	211	*	3	7.66335E-09	0.004251455	0.013787388	0.004146017	0.225569714	0.108902661	
chr10	5455556	5455710	155	*	3	4.64376E-08	0.003354875	0.013800593	0.005334223	-0.094500803	-0.077112388	NET1
chr2	136757496	136757908	413	*	2	2.4597E-08	0.001394134	0.01381174	0.002571357	0.164559342	0.13230437	AC093391.2
chr13	114929471	114929474	4	*	2	1.12097E-08	0.000923317	0.013813711	0.001830258	0.120716276	0.102959768	
chr16	2770211	2770429	219	*	5	9.1614E-08	0.06519721	0.013831866	0.035606399	0.183226267	0.059154505	PRSS27
chr14	21943848	21944110	263	*	2	8.34244E-10	0.004827665	0.013832625	0.006635619	0.054498721	0.01695509	RAB2B

chr8	20831094	20831946	853	*	6	6.85973E-11	0.000468114	0.013835267	0.000957116	0.114060997	0.063541809	RP11-421P23.1, RP11-369E15.3
chr16	88846234	88846292	59	*	2	5.3138E-09	0.001044525	0.013843527	0.002029693	0.121213871	0.106504882	PIEZO1
chr5	1886828	1888218	1391	*	19	4.22631E-18	0.075754194	0.01385117	0.002763729	0.071758518	0.019864618	CTD-2194D22.4, IRX4
chr3	122514443	122514814	372	*	6	4.73947E-12	0.096601089	0.013854226	0.003114034	-0.201403206	-0.062799102	DIRC2
chr21	39871287	39871503	217	*	4	4.05929E-10	0.000466885	0.013870347	0.001157364	-0.204251041	-0.09917043	ERG
chr17	77810269	77810912	644	*	3	8.5288E-09	0.001366628	0.0138773	0.00213138	-0.105142847	-0.058693173	CBX4
chr5	32312181	32313186	1006	*	5	3.91307E-10	0.405251424	0.013878819	0.044754743	0.07355023	0.008979904	MTMR12
chr16	87350553	87351569	1017	*	11	4.61316E-18	0.000446354	0.013915774	0.000124725	-0.050625044	0.004010943	RP11-178L8.7, C16orf95, RP11-178L8.4
chr22	29977162	29977949	788	*	8	1.43515E-08	0.355591861	0.013931494	0.062058251	0.117138961	0.020300268	NIPSNAP1
chr14	51297334	51297571	238	*	6	8.00179E-11	0.035779937	0.013932055	0.010589705	-0.061861333	-0.018285924	NIN
chr22	29783861	29785355	1495	*	12	1.11075E-12	0.023415355	0.013975552	0.002136504	0.093365116	0.020575412	AP1B1
chr12	66581539	66583431	1893	*	13	7.40573E-15	0.001244062	0.013989934	0.000754904	-0.236885415	-0.033499149	IRAK3
chr9	35755286	35755346	61	*	2	3.59745E-08	0.001047222	0.01401858	0.002039838	0.138330656	0.132964106	RGP1, MSMP
chr11	36396962	36397458	497	*	4	9.71978E-11	0.010482074	0.014024966	0.004641972	0.10226565	0.069903652	PRR5L
chr16	731417	732033	617	*	7	3.97907E-09	0.321763944	0.014027971	0.127053859	0.204955589	0.038790707	STUB1, JMJD8
chr16	23607392	23607835	444	*	9	4.76734E-13	0.01072774	0.01403279	0.005655775	0.031748602	0.004837898	NDUFAB1
chr18	77710649	77710866	218	*	3	2.16489E-09	0.002356682	0.014043943	0.002674778	-0.053228699	-0.007078488	PQLC1
chr20	58629939	58631038	1100	*	8	5.29744E-18	1.25669E-05	0.014068004	2.83037E-05	-0.333963102	-0.071859494	C20orf197
chr6	159458115	159458149	35	*	3	1.13666E-08	0.003860695	0.01407405	0.005892998	-0.168288929	-0.097346448	RP1-111C20.4, TAGAP
chr3	169530027	169530817	791	*	11	2.31642E-10	0.363099856	0.01407478	0.081587783	-0.153059983	-0.018854269	LRRC34
chr19	9731681	9732024	344	*	7	1.38961E-09	0.039041443	0.014091524	0.018954358	0.01793146	0.012062761	C19orf82, ZNF561
chr10	75570314	75571738	1425	*	8	1.90118E-08	0.017457034	0.014099772	0.006171808	-0.224961347	-0.03840881	RP11-574K11.31, NDST2
chr15	78835700	78835713	14	*	2	1.15737E-07	0.00122465	0.01410012	0.002324811	-0.075882177	-0.071370041	PSMA4
chr19	35521622	35521772	151	*	3	2.59763E-08	0.009042659	0.014106913	0.010359788	0.016434089	0.009360614	SCN1B
chr2	16804409	16805111	703	*	5	6.61606E-10	0.051771503	0.014110972	0.002374642	-0.162784411	-0.057873291	FAM49A
chr3	13922426	13922921	496	*	2	1.60158E-08	0.015457764	0.014134023	0.014250131	0.160246555	0.09679339	
chr3	52720031	52720522	492	*	7	1.05074E-07	0.277444678	0.014138331	0.04778745	-0.191676176	-0.029948354	GNL3
chr3	188862579	188862727	149	*	3	9.73209E-08	0.040917355	0.014143914	0.004977501	-0.135681832	-0.071335513	TPRG1
chr7	134142981	134143249	269	*	2	2.08526E-08	0.08788753	0.014159299	0.031661654	0.144412996	0.073509417	AKR1B1

chr17	42081594	42082647	1054	*	12	7.70359E-25	2.75599E-07	0.014162209	1.09918E-06	0.187239101	0.069178788	NAGS, PYY
chr6	154677796	154678593	798	*	5	6.25416E-12	0.000198782	0.014175314	0.00015863	-0.155037269	-0.08772447	IPCEF1
chr15	48874772	48874903	132	*	2	2.09261E-07	0.000984139	0.014182209	0.001941817	0.13236829	0.099346307	FBN1
chr2	222384636	222384727	92	*	3	1.99855E-08	0.00172178	0.014183254	0.002141001	0.093431628	0.073172719	EPHA4
chr16	67880254	67880962	709	*	8	6.49061E-12	0.028882605	0.014200586	0.009585369	0.040348006	0.01634504	NUTF2, CENPT
chr8	54755419	54755598	180	*	6	1.03374E-07	0.466206494	0.014208708	0.121008994	0.033691437	0.007243066	ATP6V1H
chr21	46234991	46235052	62	*	2	1.30024E-07	0.000973133	0.014231782	0.001925087	-0.124888272	-0.116085884	SUMO3
chr3	169939691	169940839	1149	*	13	1.27927E-17	0.000263325	0.014245625	0.000236114	-0.093293493	-0.025004859	PRKCI
chr1	109419967	109420278	312	*	3	6.75752E-08	0.025536165	0.014258537	0.013227229	-0.093220906	-0.03142803	GPSM2, AKNAD1
chr13	46757395	46757637	243	*	4	2.82458E-11	5.5824E-05	0.014262839	0.000245421	0.180355463	0.108801935	LCP1
chr20	33297742	33298052	311	*	2	2.31667E-08	0.001859657	0.014305931	0.003270504	0.035413195	0.029776238	TP53INP2, NCOA6
chr2	128568574	128569513	940	*	12	1.39949E-12	0.048699788	0.014308646	0.008758329	-0.115383304	-0.00718776	WDR33
chr6	126239565	126240163	599	*	4	3.40132E-11	0.000672604	0.014311861	0.001163024	-0.082178784	-0.02552607	NCOA7
chr14	61122327	61122911	585	*	3	2.08942E-09	0.014161772	0.014324337	0.01330114	0.038872716	0.012749589	SIX1
chr16	67907176	67907310	135	*	3	7.28249E-08	0.040897918	0.014326073	0.026274049	0.019659829	0.008496839	EDC4
chr2	139260299	139260866	568	*	2	6.82634E-09	0.003113727	0.014327192	0.00487493	-0.143419388	-0.102436217	SPOPL
chr1	201139713	201140721	1009	*	6	1.4888E-14	0.000372444	0.01432793	0.000767839	0.079150279	0.025131638	TMEM9
chr20	55836268	55836676	409	*	2	6.1301E-10	0.001304201	0.014336935	0.002457843	0.169654702	0.145348133	BMP7
chr7	1601923	1602273	351	*	5	6.74755E-08	0.210714537	0.014361655	0.017599012	0.102504717	0.035745297	
chr19	57050312	57050834	523	*	6	3.75878E-09	0.031070212	0.014368645	0.011410325	-0.04115196	-0.02090496	ZFP28
chr16	89177266	89177293	28	*	2	3.54084E-08	0.001008486	0.014378977	0.001987918	0.106095668	0.083547094	ACSF3
chr3	27770852	27771053	202	*	3	7.3887E-08	0.03157393	0.014381147	0.010823394	-0.069099929	-0.011973429	
chr9	133884862	133885058	197	*	3	7.93641E-09	0.006914833	0.014381208	0.006430982	0.084679803	0.01369806	LAMC3
chr7	139477474	139478495	1022	*	11	5.28756E-12	0.042427163	0.014382022	0.01588794	0.053896688	0.006061648	TBXAS1, HIPK2
chr14	53618732	53619311	580	*	3	4.26844E-09	0.008484493	0.014386364	0.008400657	-0.024946771	-0.007885617	DDHD1
chr11	46143434	46143461	28	*	2	4.33595E-08	0.036601221	0.014398039	0.022656899	0.020023766	0.012489751	
chr3	197024963	197025640	678	*	8	1.59136E-12	0.01197981	0.014413637	0.006816657	-0.03151227	-0.008733644	DLG1-AS1, DLG1
chr19	54768850	54768927	78	*	2	8.08867E-08	0.001718217	0.01441983	0.003077249	-0.148952055	-0.112389155	
chr6	170602911	170602968	58	*	2	1.14182E-07	0.003131282	0.014427233	0.004906653	0.106735571	0.018694459	FAM120B

chr10	104153568	104154605	1038	*	18	3.1647E-13	0.10940106	0.014427612	0.050320756	-0.195592888	-0.016263519	NFKB2
chr11	33562503	33563946	1444	*	9	2.60071E-17	0.000156102	0.014428596	7.80102E-05	-0.214103499	-0.06775083	KIAA1549L
chr17	53827579	53827880	302	*	4	3.19319E-09	0.016040147	0.014434281	0.015355514	-0.137303401	-0.05697193	
chr3	155524335	155524983	649	*	4	9.59514E-11	0.000289611	0.014440408	0.000707635	-0.128651028	-0.067551151	
chr12	110435360	110435418	59	*	2	2.08172E-09	0.001001203	0.014449073	0.001978005	-0.200854154	-0.170626952	
chr4	54424764	54425349	586	*	5	3.11943E-09	0.004570281	0.014457737	0.003432063	-0.175062313	0.000528	FIP1L1, LNX1
chr1	17304887	17305562	676	*	6	5.99938E-09	0.047694841	0.014460971	0.01747468	-0.119246839	-0.042558662	RP1-37C10.3, MFAP2
chr2	114646429	114648348	1920	*	17	2.89143E-13	0.045442328	0.014461662	0.003346971	0.165239125	-0.001286308	ACTR3, RP11-141B14.1
chr8	6484293	6484687	395	*	4	5.65895E-11	9.69668E-05	0.014485417	0.000374479	0.099340374	0.074686577	MCPH1, CTD-2541M15.1
chr13	45914478	45916557	2080	*	17	1.34825E-10	0.106570572	0.014498597	0.01357292	-0.113164542	-0.009007661	RP11-290D2.6, TPT1-AS1, TPT1
chr1	154540535	154540904	370	*	2	2.63494E-08	0.002056715	0.014525281	0.00355661	-0.090861437	-0.035397872	CHRNB2
chr5	131629910	131631610	1701	*	19	1.61263E-21	0.000891244	0.014529377	7.11569E-05	-0.166125844	-0.028573507	SLC22A4, P4HA2
chr11	133826329	133827076	748	*	6	1.9461E-12	0.00248459	0.014539684	0.00235165	0.044539809	0.019708558	IGSF9B, AP000911.1
chr6	135506834	135507123	290	*	2	3.83345E-08	0.001072265	0.014544897	0.002097655	-0.163332311	-0.119061487	MYB
chr3	11766272	11766469	198	*	2	9.44809E-08	0.001026584	0.014546466	0.002022843	-0.194868168	-0.023903236	AC090939.1
chr20	34892811	34892819	9	*	2	1.56712E-07	0.003532678	0.014547661	0.00538455	0.10928056	0.102617891	
chr16	57661327	57662541	1215	*	13	2.93194E-12	0.26436493	0.01454906	0.049812692	0.17905622	0.0421488	GPR56, RP11-405F3.5
chr5	131745958	131746256	299	*	4	2.92181E-08	0.116986227	0.014550868	0.014343315	0.040471519	0.011885845	
chr16	3661771	3663072	1302	*	7	2.95664E-10	0.003511142	0.014550983	0.000867711	-0.17747554	-0.026089656	DNASE1
chr19	16178030	16178570	541	*	12	4.62231E-14	0.068949227	0.014555361	0.005803004	0.190226505	0.067983678	TPM4
chr19	7166458	7167821	1364	*	6	6.44854E-09	0.000331407	0.014557543	0.000367557	0.153705988	0.104724642	INSR
chr11	62371423	62371596	174	*	3	8.86084E-09	0.035096108	0.014572122	0.005201229	0.121028394	0.074709642	EML3
chr1	110673082	110673319	238	*	3	5.09243E-08	0.035120778	0.014574095	0.016552403	0.06592424	0.036989796	
chr22	22292442	22293793	1352	*	11	5.06245E-15	0.001368149	0.014592851	0.000130579	-0.258580738	-0.021520971	LL22NC03-86G7.1, PPM1F
chr5	539420	539998	579	*	4	3.40853E-12	0.000136566	0.014616255	0.0002972	0.112845608	0.018314696	
chr11	10952789	10953109	321	*	3	2.32329E-08	0.026166032	0.014641212	0.010729391	-0.022894162	-0.004437613	
chr1	244531718	244531920	203	*	3	2.54249E-08	0.003474488	0.014643768	0.002934635	-0.176707066	-0.126416124	C1orf100
chr1	22213376	22214106	731	*	5	3.50431E-11	0.009384544	0.014659559	0.002426081	0.091094829	0.034110011	HSPG2
chr1	172608566	172608704	139	*	3	7.31788E-10	0.000127529	0.014668162	0.000429337	0.124064267	0.099009557	

chr6	40445150	40445699	550	*	3	2.64805E-09	0.000222919	0.014679287	0.000674852	0.173357384	0.139962708	LRFN2
chr10	25350761	25351314	554	*	4	1.80986E-11	0.00126862	0.014702105	0.001339629	0.162118592	0.107399907	
chr16	48592404	48592694	291	*	3	4.96354E-09	0.000593953	0.014707477	0.001365381	-0.250278746	-0.158452515	N4BP1
chr2	54900910	54901055	146	*	3	7.10104E-08	0.002351006	0.014712157	0.004226554	-0.119945037	-0.077785453	
chr6	692465	692947	483	*	2	8.5903E-09	0.015528032	0.014719919	0.014580454	0.017793988	0.011791473	EXOC2
chr18	9885685	9886064	380	*	4	2.41428E-09	0.007468395	0.014743773	0.004433601	0.107293277	0.047123961	TXNDC2
chr11	64620640	64621560	921	*	5	8.58967E-14	2.63616E-05	0.014747012	0.00010969	-0.234152944	-0.123788381	EHD1
chr7	139498041	139498312	272	*	3	8.00157E-10	0.000117469	0.014748501	0.000406183	0.12191838	0.114234842	TBXAS1
chr1	152008878	152011357	2480	*	18	1.50644E-18	8.94754E-07	0.014750543	1.00886E-05	-0.293443551	-0.035555376	NBPF18P, S100A11
chr7	2611716	2612101	386	*	4	1.15697E-10	0.001293746	0.014801656	0.000978377	0.118368846	0.076288757	IQCE
chr16	58031389	58032920	1532	*	5	9.87006E-12	6.58911E-05	0.014803433	0.00032815	-0.235144212	-0.083461712	ZNF319
chr17	694393	695156	764	*	3	2.80039E-10	0.00327408	0.014817062	0.00303167	-0.127873111	-0.010067095	RNMTL1
chr17	80056046	80056900	855	*	7	4.44021E-14	0.006242286	0.014826514	0.000670776	-0.038161096	-0.000471634	FASN
chr4	75960115	75960138	24	*	2	9.53189E-08	0.001349981	0.014842532	0.002549573	0.080183773	0.078525291	PARM1
chr4	110624351	110625181	831	*	12	1.66246E-15	0.014304432	0.014865869	0.00367876	-0.094553862	-0.028703344	CASP6
chr20	60860733	60860984	252	*	3	2.47503E-08	0.001612018	0.014882935	0.002244631	0.13800797	0.089401156	OSBPL2
chr2	227771209	227771954	746	*	4	1.91814E-10	0.000191309	0.014883698	0.000506339	0.16451846	0.095550513	RHBDD1
chr12	6664536	6664872	337	*	2	3.3528E-08	0.016341331	0.014890947	0.015108844	0.025437054	0.018228065	IFFO1
chr15	81517541	81517758	218	*	5	6.45834E-10	0.01031956	0.014893041	0.0023324	0.172330892	0.080375604	IL16
chr17	80969109	80969749	641	*	5	1.09262E-12	0.00131443	0.014913502	0.001293449	-0.189173079	-0.085270352	B3GNTL1
chr6	31025759	31026287	529	*	7	3.83177E-10	0.152236885	0.014916719	0.015236629	-0.111692371	-0.004706148	HCG22
chr7	75957040	75958048	1009	*	8	2.08933E-11	0.010615035	0.014923551	0.001307375	0.13506591	0.00033768	YWHAG
chr3	50379334	50380958	1625	*	7	1.88059E-13	1.43474E-05	0.014943404	5.76459E-05	-0.155782382	-0.019907366	ZMYND10-AS1, ZMYND10
chr18	13610632	13611576	945	*	13	7.93147E-14	0.215011516	0.014948855	0.032726995	0.162110015	0.04547079	LDLRAD4, MIR4526
chr10	114911413	114912080	668	*	6	1.27505E-13	0.00436296	0.01495992	0.001594254	-0.161988914	-0.060967691	TCF7L2
chr1	59249218	59250203	986	*	10	5.19076E-17	0.014815853	0.014985824	0.001065452	-0.040205998	-0.007159047	JUN
chr10	98589334	98589349	16	*	2	2.66441E-08	0.001216469	0.01498688	0.002345302	0.139090677	0.132416062	
chr1	202995853	202996541	689	*	5	9.21507E-10	0.004919113	0.014990188	0.005053867	0.084961212	0.030741714	PPFIA4
chr4	10686300	10686610	311	*	7	3.94278E-08	0.149667285	0.014990228	0.033170956	-0.162915707	-0.043041214	CLNK

chr12	6960295	6961946	1652	*	22	6.19074E-16	0.182785997	0.014992076	0.016351951	-0.117418118	-0.007662075	USP5, CDCA3
chr6	31689459	31693656	4198	*	39	1.82717E-26	2.26959E-07	0.015001815	7.83798E-08	0.190942336	0.064189465	C6orf25, LY6G6C
chr17	37349785	37351198	1414	*	7	6.09804E-13	5.30537E-05	0.015011214	4.19448E-05	0.153806396	0.075595959	CACNB1
chr4	90033921	90033974	54	*	2	3.81762E-08	0.001248195	0.015012147	0.002396706	0.13718651	0.126602118	TIGD2
chr17	40558061	40558245	185	*	3	3.85969E-08	0.093327602	0.015036689	0.013103911	-0.192925906	-0.10167084	PTRF
chr21	40717570	40717775	206	*	3	9.56776E-09	0.001747386	0.015053819	0.002488621	0.081216698	0.065758412	HMGN1
chr12	33591336	33591505	170	*	2	1.22962E-08	0.001270236	0.0150573	0.002433304	0.108454325	0.097880373	SYT10
chr9	86594091	86594226	136	*	3	2.58387E-08	0.011929922	0.015085184	0.013320139	-0.017996696	-0.003582672	HNRNPK
chr6	30068587	30071295	2709	*	40	1.2915E-31	0.007272695	0.015092985	0.001152499	-0.289036243	-0.016444961	TRIM31
chr3	152016806	152017819	1014	*	6	4.74194E-11	0.288965095	0.015094383	0.027525519	-0.10558238	-0.047066358	MBNL1
chr1	161128567	161129745	1179	*	16	4.19116E-11	0.365324093	0.015095573	0.11568813	0.05769414	0.011418132	UFC1, USP21, RP11-297K8.2
chr20	57607406	57608039	634	*	11	2.28841E-08	0.168082337	0.0151281	0.112658154	0.066470637	0.010322641	ATP5E
chr6	138820503	138820840	338	*	4	2.94564E-09	0.000895721	0.015129828	0.001764118	-0.06124985	-0.040694794	NHSL1
chr22	24236101	24237672	1572	*	16	2.5909E-15	0.014057435	0.015136926	0.002736173	-0.049445983	0.003046994	AP000350.10, MIF, AP000350.4
chr14	20922685	20923814	1130	*	18	4.17001E-20	0.00049109	0.015160525	0.000301728	-0.035658843	0.001832015	APEX1, OSGEP
chr1	54562121	54562543	423	*	4	1.06148E-08	0.004965605	0.015199966	0.005589357	-0.161596256	-0.087475303	TCEANC2
chr11	46298891	46299204	314	*	7	6.33324E-08	0.292784147	0.015201171	0.068933465	0.126952954	0.024561347	
chr11	5265992	5266120	129	*	2	8.29149E-09	0.001107569	0.015201195	0.002176359	0.167702866	0.1439985	CTD-2643I7.1
chr16	85256083	85256305	223	*	3	6.32921E-08	0.00218281	0.015208183	0.00362198	0.098402203	0.071554934	
chr13	114005848	114006027	180	*	5	4.31401E-12	6.60965E-05	0.015220761	0.000290361	0.128197291	0.070610174	GRTP1-AS1, GRTP1
chr10	135202522	135203200	679	*	7	1.76261E-15	4.20177E-06	0.015328458	3.64943E-05	-0.157565782	-0.112631135	PAOX
chr1	44114346	44116508	2163	*	17	1.10339E-14	0.000421249	0.015331696	9.49459E-05	-0.170895131	-0.021009378	KDM4A
chr12	101603174	101604314	1141	*	16	7.31006E-11	0.306803457	0.015352961	0.058960686	0.09631736	0.030607927	SLC5A8
chr5	70882722	70883447	726	*	11	3.24731E-11	0.022173513	0.015360583	0.011905328	0.078467218	0.012719829	MCCC2
chr14	23445909	23446246	338	*	4	1.1007E-10	0.00107035	0.01536311	0.001448338	-0.136539484	-0.078764874	RP11-298I3.5, AJUBA
chr12	53692108	53692645	538	*	3	5.16563E-10	0.002959621	0.015364236	0.003717214	-0.154050316	-0.086231107	PFDN5, RP11-680A11.5
chr8	23382950	23383218	269	*	2	7.15626E-08	0.001783286	0.015368161	0.003224097	-0.13342549	-0.128037805	
chr11	68227638	68228078	441	*	8	2.50675E-09	0.048681417	0.015370463	0.01910412	-0.027845926	0.001230904	
chr15	85523010	85525019	2010	*	14	5.02877E-15	0.000889421	0.015373244	0.001179754	-0.04145176	-0.004071702	PDE8A

chr3	150803295	150804490	1196	*	8	1.62544E-11	0.141388125	0.01537651	0.019161035	-0.09487486	-0.025128676	MED12L
chr3	142607280	142607715	436	*	4	5.05721E-08	0.251962146	0.015393386	0.081832792	-0.058024539	-0.011759368	PCOLCE2
chr5	891843	892529	687	*	5	1.04274E-10	0.009142037	0.015408881	0.006685969	-0.121413645	-0.049009125	BRD9
chr14	104171259	104171695	437	*	3	3.87808E-08	0.001315349	0.015424989	0.002746786	-0.154233306	0.01174068	XRCC3
chr2	9142741	9143163	423	*	3	1.42916E-09	0.001194967	0.015440506	0.002319519	-0.133344889	-0.090601497	MBOAT2
chr1	111682392	111683302	911	*	10	3.35514E-16	0.017250627	0.015450078	0.001850081	-0.082042205	-0.025665496	CEPT1, DRAM2
chr8	77586264	77586439	176	*	3	7.35407E-08	0.004555033	0.015468322	0.006964826	0.051737202	0.034316045	ZFHX4-AS1
chr13	114065669	114066074	406	*	3	4.78563E-08	0.059759419	0.015470803	0.021642712	-0.194473623	-0.028584934	
chr9	128003255	128004142	888	*	11	1.58982E-12	0.005440506	0.015473536	0.003718973	-0.045278532	-0.002932998	RP11-65N13.8, HSPA5
chr17	54990662	54990833	172	*	2	1.73343E-10	0.003233808	0.01547632	0.005142095	0.025302084	0.021423423	TRIM25
chr18	5296331	5298430	2100	*	17	4.7847E-13	0.034668322	0.015483468	0.002535523	-0.275339351	-0.009457748	ZBTB14
chr11	1374851	1375657	807	*	5	3.00879E-10	0.002037034	0.015484061	0.001955731	0.103350902	0.033457969	
chr19	11592764	11592958	195	*	3	5.42205E-08	0.006216159	0.015498879	0.006116791	0.039842376	0.021978661	CTC-398G3.6
chr9	136241865	136242381	517	*	3	1.34762E-09	0.00357909	0.015503537	0.003575562	-0.182827022	-0.059522013	SURF4
chr14	105155327	105155825	499	*	6	4.41065E-09	0.111954506	0.015533906	0.032414882	-0.068898217	-0.033923036	
chr2	208104158	208104372	215	*	2	7.2275E-09	0.001165808	0.015543544	0.002282688	-0.111987875	-0.080463161	
chr10	101945124	101946761	1638	*	16	3.46886E-18	0.00878762	0.0155646	0.001486993	-0.133499113	-0.000303508	ERLIN1
chr2	231789465	231789987	523	*	3	4.11814E-09	0.00208141	0.015602503	0.003177049	-0.213077466	-0.088748487	GPR55
chr2	172750661	172751245	585	*	12	3.11022E-13	0.009360798	0.015614782	0.002973819	0.036800888	0.01067349	SLC25A12
chr6	114291911	114292802	892	*	9	1.41541E-09	0.289101461	0.015626629	0.071350546	0.042684415	0.007294919	RP3-399L15.3, HDAC2
chr17	42977401	42977986	586	*	4	1.11285E-11	0.031278197	0.015628065	0.010857754	-0.210137522	-0.043364585	CCDC103, FAM187A
chr2	438948	439337	390	*	2	2.02526E-09	0.001910364	0.015647194	0.003423262	0.161856993	0.107760795	
chr1	29587088	29587490	403	*	5	4.86284E-08	0.028975521	0.015658423	0.023436312	0.125750906	0.040283876	PTPRU
chr2	28112817	28114462	1646	*	24	7.22617E-16	0.003885642	0.015683189	0.001752574	-0.104293537	-0.008432896	MRPL33, BRE, RBKS
chr13	98959770	98959799	30	*	2	5.52836E-08	0.001362194	0.01569577	0.002602924	0.118928947	0.098932576	FARP1
chr20	57765315	57765489	175	*	3	1.33517E-08	0.003324211	0.015698208	0.003233434	0.136923873	0.054755668	
chr12	110486280	110486573	294	*	6	5.48874E-08	0.012178985	0.015708542	0.01057496	-0.042150773	-0.013651033	C12orf76
chr3	10377872	10378056	185	*	4	2.28017E-08	0.030113451	0.015724323	0.00543198	0.114863924	0.055935143	ATP2B2
chr16	30381677	30382569	893	*	6	3.45257E-10	0.001200604	0.015735457	0.001887706	0.026496509	0.004491758	MYLPF

chr1	228271035	228271368	334	*	3	5.55929E-09	0.059730902	0.015738244	0.00803533	0.034983926	0.016185747	ARF1
chr2	200775554	200776533	980	*	7	1.13231E-10	0.003970605	0.015747784	0.002239648	-0.050908165	0.000815245	C2orf69, AC073043.1
chr1	155159820	155160365	546	*	3	3.42165E-09	0.000501457	0.015774879	0.001212522	0.158129755	0.113927918	MUC1
chr12	15782193	15782294	102	*	2	1.33823E-07	0.001183224	0.015787052	0.002319099	0.135973738	0.124281881	EPS8
chr12	112819476	112819495	20	*	2	1.33941E-07	0.058453554	0.015787748	0.029405768	0.024409714	0.004665581	HECTD4
chr7	6212481	6213211	731	*	6	1.39162E-12	0.005479276	0.015812024	0.001390134	-0.136151822	0.00567603	CYTH3
chr12	113376260	113376681	422	*	6	1.08298E-08	0.158903026	0.015816281	0.018490157	0.032171399	0.011442007	OAS3, RP1-71H24.1
chr2	11123476	11123616	141	*	3	7.36941E-08	0.001478807	0.015816953	0.002594553	0.184870183	0.109944842	
chr14	58862146	58863362	1217	*	14	1.83166E-20	0.004091036	0.015817965	0.000100983	0.141125474	0.027468245	TOMM20L, RP11-517O13.3
chr5	175200401	175200576	176	*	4	2.09855E-10	0.001306405	0.015825204	0.000955955	0.116821905	0.069174543	
chr18	19756582	19757468	887	*	5	1.2112E-10	0.012467423	0.015845313	0.003573719	-0.072127766	-0.041469906	GATA6, RP11-627G18.2
chr1	147142184	147142420	237	*	4	2.62312E-09	0.017031598	0.015858282	0.004704954	0.024558445	0.012461282	ACP6
chr9	114423570	114423680	111	*	5	1.06344E-07	0.022334446	0.015869735	0.014705014	0.018514852	0.011987114	DNAJC25, DNAJC25-GNG10, GNG10
chr12	6487052	6487113	62	*	4	4.78077E-09	0.033482889	0.015880474	0.004538838	0.122016016	0.079023413	LTBR
chr11	47416886	47417832	947	*	6	1.2589E-12	0.002814443	0.015884524	0.002457863	-0.207101678	-0.055653554	RP11-750H9.5
chr5	14870749	14871921	1173	*	9	1.06215E-12	0.111948497	0.015901989	0.013354023	0.14335898	0.014406484	ANKH
chr2	103352296	103352342	47	*	2	1.01179E-07	0.002289182	0.015909137	0.003970061	-0.141672227	-0.114004441	MFSD9
chr2	111879378	111879843	466	*	3	1.0266E-11	0.014399374	0.015909185	0.005291352	0.041191763	0.027541239	BCL2L11
chr16	81812336	81812765	430	*	7	5.64894E-10	0.005977347	0.015911104	0.006690899	0.071466432	0.020725497	PLCG2
chr19	55866087	55866677	591	*	10	2.52306E-08	0.345901501	0.015913255	0.077566899	0.060817764	0.011869688	COX6B2, CTD-2105E13.6, FAM71E2
chr2	198364115	198365671	1557	*	24	2.9521E-14	0.199659033	0.015950886	0.085484176	-0.132961409	-0.018560225	HSPE1, HSPE1-MOB4, HSPD1
chr17	43049442	43049895	454	*	2	5.0789E-08	0.001208158	0.015956434	0.00236539	0.099110906	0.097530533	
chr16	81678497	81678968	472	*	7	2.27273E-12	0.01854252	0.015963993	0.003169966	0.158702099	0.05350953	CMIP
chr3	132378605	132380190	1586	*	17	9.97924E-14	0.102647847	0.015973657	0.004450502	-0.264812118	-0.014621148	UBA5, ACAD11, NPHP3
chr16	4012936	4014295	1360	*	8	2.12242E-13	0.006999341	0.015981939	0.001379287	0.15890497	0.051713375	ADCY9
chr9	139640053	139640661	609	*	4	7.03249E-09	0.05325921	0.015993321	0.009441517	0.14817225	0.071214031	LCN6, LCN6
chr1	179851994	179852419	426	*	3	3.00455E-08	0.00869491	0.016015125	0.01035232	0.021739312	0.014995713	TOR1AIP1
chr11	62312720	62313983	1264	*	6	1.61377E-10	0.000894325	0.016019623	0.001471482	-0.056875401	9.24272E-05	RP11-864I4.4, AHNAK
chr4	47839218	47839283	66	*	2	2.29973E-08	0.002279742	0.016025329	0.003965233	0.017620744	0.016463845	CORIN

chr17	3871303	3872569	1267	*	4	5.69782E-08	6.52642E-05	0.016027104	0.000266589	0.118840896	0.05645291	
chr10	23003092	23003653	562	*	5	1.66835E-11	0.003823291	0.016028579	0.00304798	0.029276288	0.008518524	PIP4K2A
chr14	52781184	52781800	617	*	3	1.33769E-10	0.001533124	0.016030206	0.002978067	0.041402208	0.023230537	PTGER2
chr16	3332644	3333497	854	*	12	1.88867E-13	0.012558861	0.016033725	0.001993907	0.019103267	0.000951701	ZNF263
chr3	160167317	160167507	191	*	2	6.98434E-08	0.352961836	0.016034327	0.045834557	-0.060574582	-0.03103934	RP11-432B6.3, TRIM59
chr2	109223537	109223691	155	*	2	1.70437E-09	0.001241565	0.016041891	0.002422633	0.186467804	0.124977248	LIMS1
chr4	39978799	39980114	1316	*	9	1.12335E-12	0.000215014	0.016074961	0.000299215	-0.068307993	-0.005908324	PDS5A
chr13	45885329	45885891	563	*	6	1.68001E-09	0.001790453	0.016077762	0.003731366	0.021920359	0.007628937	
chr14	50087420	50088260	841	*	7	2.78453E-11	0.009144305	0.016082413	0.007728686	0.020594995	-0.000137147	MGAT2, RP11-649E7.5
chr17	72731453	72732432	980	*	4	1.74604E-11	0.000886854	0.016128649	0.001011728	-0.174730924	-0.095033523	RAB37
chr1	203764137	203766628	2492	*	15	3.17475E-12	0.000112717	0.016134448	0.000213112	-0.215808407	-0.035251456	ZC3H11A, ZBED6
chr17	39769012	39769294	283	*	5	1.89976E-08	0.019505074	0.016139117	0.008975145	-0.121668815	-0.074234578	KRT16
chr12	117468061	117468529	469	*	4	1.01521E-09	0.003565618	0.016139483	0.003476494	0.149905015	0.082045481	FBXW8
chr20	33734406	33735232	827	*	10	2.03561E-10	0.126119585	0.016139812	0.051474173	-0.058125547	-0.005612467	EDEM2
chr5	137089872	137090202	331	*	7	9.11738E-08	0.539395686	0.016148425	0.140297599	-0.069991139	-0.020144838	HNRNPA0
chr2	99124870	99125292	423	*	4	2.00359E-09	0.016800064	0.016180845	0.002012903	0.11660408	-0.011912696	INPP4A
chr10	5727416	5727477	62	*	2	6.71799E-08	0.004804413	0.016193872	0.007003136	-0.031891408	-0.003136567	FAM208B
chr15	70994404	70995014	611	*	8	1.359E-14	0.00544608	0.016218395	0.000463952	0.095015208	0.044271691	UACA
chr15	42840643	42842330	1688	*	15	4.46554E-14	0.004500553	0.016230347	0.002069345	-0.205064373	-0.018878977	HAUS2, LRRC57
chr9	139922545	139922595	51	*	2	5.48685E-09	0.002278232	0.016246159	0.003978319	0.039598034	0.029200568	C9orf139, ABCA2
chr17	58155376	58155868	493	*	2	2.49649E-09	0.001257582	0.016255753	0.002455911	-0.045963807	-0.010668044	HEATR6
chr1	1208426	1208573	148	*	2	2.20674E-08	0.002884632	0.016259695	0.004784697	0.031802176	0.025472976	UBE2J2
chr12	46776356	46776835	480	*	4	7.58857E-09	0.232861771	0.016259903	0.018084039	0.116631721	0.042221462	
chr1	159823687	159825173	1487	*	10	2.96172E-14	0.002271914	0.01627783	0.000450039	-0.109652595	-0.028268734	C1orf204, VSIG8
chr6	82957093	82958324	1232	*	14	3.95128E-24	5.0728E-06	0.016308429	5.20195E-06	-0.194151322	-0.025544155	IBTK
chr2	127412692	127414108	1417	*	13	3.50782E-23	4.79649E-05	0.016316685	1.71378E-05	-0.185098809	-0.05130152	GYPC
chr2	220107847	220108496	650	*	8	6.5935E-14	9.98691E-05	0.016316935	0.00043238	-0.252540383	-0.130174547	GLB1L
chr7	112089544	112091280	1737	*	16	5.53858E-18	0.000235202	0.016346116	0.000176481	0.169078104	0.003701639	IFRD1
chr7	139875917	139876578	662	*	4	6.6827E-12	0.000773885	0.016371976	0.000856497	0.04247244	0.007227546	KDM7A

chr15	91565312	91565459	148	*	3	4.8742E-08	0.06665669	0.016375765	0.029374185	0.02291919	0.012030089	VPS33B
chr11	118763674	118764491	818	*	9	1.15812E-12	0.051716226	0.016376081	0.006132987	0.144622353	0.036397561	CXCR5
chr18	67136810	67137182	373	*	4	1.5698E-11	0.000151691	0.0164014	0.000444661	0.13419822	0.091673044	DOK6, RP11-465I4.3
chr1	19043507	19043598	92	*	3	2.46083E-08	0.000856664	0.016409944	0.001757722	0.072897728	0.04101587	PAX7
chr7	100701511	100701574	64	*	3	2.81664E-08	0.006079031	0.016425102	0.004792871	0.117026048	0.076604196	MUC17
chr1	212780243	212780517	275	*	2	5.14996E-08	0.002762714	0.016425552	0.004641476	-0.044593298	-0.012194855	ATF3
chr14	21979151	21980058	908	*	11	3.48467E-11	0.115412837	0.016474281	0.02535025	0.041722092	-0.007682801	METTL3
chr1	26685715	26685827	113	*	2	3.75586E-08	0.001291972	0.016483374	0.002519534	-0.124744344	-0.117813906	
chr6	151258337	151258520	184	*	3	2.97972E-08	0.001360693	0.016485222	0.002777233	-0.174776623	-0.137977769	MTHFD1L
chr6	36237828	36238410	583	*	7	1.96419E-10	0.008027678	0.016485876	0.006990656	0.129517174	0.066034514	PNPLA1
chr14	64854366	64855287	922	*	14	3.71576E-18	0.073604631	0.016488468	0.004094814	0.035322338	0.003620625	MTHFD1
chr12	127212034	127212528	495	*	4	5.92101E-09	0.005403565	0.016491974	0.004171083	0.112626123	0.044799476	LINC00943
chr6	52225658	52227260	1603	*	11	1.19315E-15	0.00084682	0.016515164	0.000603491	-0.16526927	-0.016891697	PAQR8
chr2	220252215	220252856	642	*	8	1.88258E-10	0.019784242	0.016516244	0.012585383	-0.081171036	-0.013184256	DNPEP
chr12	96587734	96588210	477	*	8	3.25716E-08	0.267035284	0.016520773	0.041068767	-0.071626361	-0.017149825	ELK3
chr16	85963932	85964550	619	*	4	3.7505E-09	0.007132757	0.016538554	0.004473402	-0.219505819	-0.11088762	
chr15	92005137	92005193	57	*	3	1.55945E-07	0.003718908	0.016557293	0.003859277	0.103698617	0.071261816	RP11-661P17.1
chr10	90031307	90031655	349	*	3	1.78241E-08	0.000708228	0.016560432	0.001684827	0.118973189	0.052655824	
chr10	30331680	30331911	232	*	3	8.07736E-09	0.00977737	0.016561149	0.004696704	0.132534958	0.075446027	KIAA1462
chr4	1593537	1595726	2190	*	11	4.00824E-12	1.43766E-05	0.016583083	2.64781E-05	0.133023086	0.070976414	
chr20	61436671	61437268	598	*	9	5.49871E-10	0.243537696	0.016596913	0.039863321	-0.14456653	-0.00733619	OGFR, OGFR-AS1
chr11	70265910	70266299	390	*	6	2.02865E-13	0.000119318	0.016612199	0.000187361	0.106598093	0.077558426	CTTN
chr5	134787465	134788128	664	*	4	3.28847E-11	0.002628339	0.016636928	0.001783756	-0.121981324	-0.044772563	CTB-138E5.1, TIFAB
chr11	124615853	124616925	1073	*	8	8.55135E-13	0.001256603	0.016638962	0.000450195	-0.127943695	-0.035841635	NRGN, RP11-677M14.2
chr12	132269563	132270218	656	*	6	2.41111E-15	0.001278572	0.016648757	0.000342038	0.129559791	0.076218747	SFSWAP
chr1	2162682	2162931	250	*	3	3.4235E-08	0.016001117	0.01665459	0.010804987	0.182238719	0.115246044	SKI
chr6	167188846	167189543	698	*	5	3.04118E-10	0.003138502	0.016657828	0.003473391	0.104231161	0.037258622	RPS6KA2
chr1	150130379	150131579	1201	*	6	5.71083E-09	0.001456816	0.016662342	0.000696749	0.137204293	0.07631741	PLEKHO1
chr1	33219158	33220450	1293	*	10	1.56825E-10	0.034459398	0.016662634	0.005643061	-0.176014404	-0.006798544	KIAA1522

chr3	27763266	27764052	787	*	8	1.20125E-08	0.531763139	0.016667765	0.110809172	0.018800566	0.004793818	EOMES
chr16	79633542	79633911	370	*	5	4.59863E-13	9.07397E-05	0.016685441	0.000432965	0.038408996	0.014538911	MAF
chr12	122230972	122232994	2023	*	13	4.03898E-18	0.000208955	0.016689621	9.51965E-05	-0.064260315	-0.000830567	RHOF
chr16	15766017	15766418	402	*	4	4.3094E-10	0.003756839	0.016695404	0.002727278	-0.104370779	-0.064278912	NDE1
chr9	71393662	71393959	298	*	3	6.56389E-12	0.000197569	0.016699393	0.000638123	0.112571943	0.077243726	PIP5K1B
chr12	6745057	6746030	974	*	14	2.5544E-28	1.99058E-10	0.016701722	1.77873E-08	0.204196475	0.097961164	LPAR5
chr17	79428036	79428142	107	*	2	4.39111E-08	0.001490265	0.016727512	0.002845292	-0.194961962	-0.184523639	RP11-1055B8.7, RP11-1055B8.8
chr14	95942049	95942666	618	*	5	1.17007E-09	0.04405284	0.016759113	0.004152194	-0.117556193	-0.051109961	SYNE3
chr17	643533	643676	144	*	3	1.00697E-07	0.006462136	0.016759588	0.004267661	-0.147856108	-0.087942315	FAM57A
chr19	49576955	49577303	349	*	5	1.5583E-10	0.164124589	0.016767164	0.006613739	0.158757691	0.081739806	
chr14	105902718	105903354	637	*	3	6.62599E-10	0.000229784	0.016767438	0.000731604	0.176957359	0.102733915	TEX22, MTA1
chr7	6629452	6630190	739	*	6	1.7471E-11	0.011578232	0.016767819	0.006073436	0.02964428	0.009478148	C7orf26
chr10	135193212	135193704	493	*	6	2.53394E-10	0.110813484	0.016777412	0.015375399	0.121176312	0.046578989	PAOX
chr8	95906921	95908119	1199	*	11	7.83495E-11	0.034520256	0.016790726	0.007576752	-0.070464565	-0.009230554	NDUFAF6, CCNE2
chr17	7757933	7761357	3425	*	35	1.14721E-21	0.013472525	0.016792526	2.3589E-05	0.13689174	0.019686809	KDM6B, TMEM88, CYB5D1, LSMD1
chr1	227127636	227128537	902	*	7	4.17392E-11	0.019890691	0.016797335	0.003429132	0.041777526	0.015019424	ADCK3
chr12	57472074	57472922	849	*	12	2.85134E-21	6.24443E-05	0.016797677	2.43424E-05	-0.048831029	-0.025730632	TMEM194A
chr6	12956438	12956489	52	*	2	1.19893E-07	0.001678994	0.016811334	0.003141281	0.105835149	0.101843266	PHACTR1
chr3	188286217	188287006	790	*	6	1.58116E-09	0.142475142	0.016822612	0.02109727	-0.155992569	-0.037908225	LPP, LPP-AS1
chr1	247171234	247171886	653	*	11	1.14332E-11	0.043173544	0.016826369	0.00643471	-0.097522963	-0.017682102	ZNF695, ZNF670
chr7	135346500	135347238	739	*	9	7.5511E-09	0.579477321	0.016829004	0.139985429	-0.106604785	-0.012386258	
chr1	154947991	154948144	154	*	2	1.06465E-08	0.001525391	0.01683348	0.00290472	-0.040849084	-0.011252655	CKS1B
chr15	43621948	43622988	1041	*	16	1.15365E-11	0.101506082	0.01685894	0.065782151	0.038559574	0.004913078	ADAL, LCMT2
chr19	41595975	41596066	92	*	3	1.95643E-08	0.000716775	0.016860073	0.001804046	0.048939235	0.039972761	CYP2A13
chr7	157475548	157475737	190	*	3	2.906E-10	0.02723731	0.01686744	0.005848667	0.11132337	0.045120193	PTPRN2
chr3	179040370	179041226	857	*	7	9.93316E-10	0.127089225	0.01688094	0.006461091	-0.362840595	-0.046418726	ZNF639
chr16	50730221	50730737	517	*	3	4.33243E-11	0.001885386	0.016884176	0.002652919	-0.09455682	-0.039014063	NOD2
chr6	15090163	15090384	222	*	4	7.89255E-09	0.00911909	0.016908407	0.004590066	-0.244180814	-0.123507988	RP11-146I2.1
chr19	53030705	53031511	807	*	9	2.68258E-11	0.013697052	0.016920417	0.005372776	0.056662473	0.013290673	ZNF808

chr3	194877106	194877369	264	*	3	1.1675E-07	0.009318971	0.016930299	0.011301933	0.145161173	0.07318766	XXYLT1
chr17	12921492	12921796	305	*	4	1.27024E-07	0.112656506	0.016939216	0.0138296	-0.093513414	-0.032055862	ELAC2
chr6	29973407	29974022	616	*	3	7.21175E-10	0.003065974	0.016952291	0.004607617	-0.154726367	-0.098637665	ZNRD1-AS1, HCG4P3
chr5	142782415	142784982	2568	*	24	1.8494E-19	9.24364E-05	0.016960445	4.49702E-05	-0.109683328	-0.019437402	NR3C1
chr15	63414256	63415064	809	*	5	2.1515E-10	0.00750957	0.01696068	0.006530316	0.0694832	-0.002507269	LACTB
chr10	72136860	72137020	161	*	3	1.33863E-08	0.001824788	0.016989807	0.00286476	0.121175545	0.101395652	LRRC20
chr16	67891976	67892349	374	*	2	7.73089E-08	0.031366945	0.016993291	0.023142781	0.067596447	0.056952221	NUTF2
chr6	31939280	31941422	2143	*	52	3.90354E-25	0.012733419	0.016995851	0.005626356	0.187654316	0.010510168	STK19, DXO
chr1	160068178	160069109	932	*	19	1.14887E-16	0.195033464	0.017014049	0.023599959	-0.084215135	-0.022961994	IGSF8
chr1	40419558	40419793	236	*	2	1.75086E-11	0.001372254	0.017015137	0.002668081	0.08074356	0.069562835	
chr20	35917127	35917924	798	*	6	1.11815E-09	0.005785051	0.017030551	0.004203444	-0.165917906	-0.035630969	
chr8	17767590	17768115	526	*	7	1.53877E-10	0.007608345	0.017036221	0.004801281	-0.104524537	-0.052217836	FGL1
chr1	27668058	27668556	499	*	8	5.06763E-17	2.21862E-06	0.017041599	2.9603E-05	-0.181948497	-0.062192111	SYTL1
chr16	11890465	11890746	282	*	3	1.3985E-08	0.003101746	0.017055151	0.005553476	-0.046380646	-0.025734975	ZC3H7A
chr14	51561293	51561618	326	*	4	7.78159E-09	0.221687595	0.017055444	0.015247733	0.046316122	0.014459426	TRIM9
chr22	24407474	24407907	434	*	7	6.31917E-08	0.07585263	0.017068734	0.049594875	0.044068321	0.008168744	CABIN1
chr6	30103459	30105198	1740	*	13	2.21443E-17	0.001821348	0.017143448	0.000114428	0.171838407	0.055545548	TRIM40
chr19	54604098	54604200	103	*	4	6.52927E-08	0.032880335	0.017192311	0.008370655	-0.128998135	-0.053781633	OSCAR
chr16	56333966	56334484	519	*	5	3.55747E-08	0.019993221	0.017290981	0.012335883	0.141416279	0.060747199	GNAO1
chr12	12503186	12504089	904	*	10	7.26944E-13	0.021472905	0.017296771	0.002791606	-0.090310283	-0.012791914	MANSC1
chr5	59995354	59995837	484	*	4	2.05545E-11	0.001950956	0.017297422	0.001703234	0.034631986	0.019633787	DEPDC1B
chr12	121890311	121890503	193	*	3	7.46401E-08	0.003027249	0.017335146	0.005111117	0.114195995	0.089778806	KDM2B
chr14	67878535	67879385	851	*	13	1.87607E-16	0.014051234	0.017344709	0.001558899	0.233172591	0.047501303	PLEK2
chr18	59854373	59855274	902	*	7	1.43177E-10	0.04920131	0.017359137	0.010348224	-0.030662356	-0.007490919	KIAA1468
chr19	4152810	4153713	904	*	8	3.96871E-13	0.002076823	0.017361495	0.001828902	0.156382455	0.044358918	CREB3L3
chr6	97371949	97372970	1022	*	10	2.70192E-11	0.173689429	0.017369201	0.014509668	0.073671499	0.008883866	KLHL32
chr11	31846819	31846849	31	*	3	2.003E-07	0.01170274	0.017376323	0.009802754	0.040524239	0.03046815	RCN1
chr2	190524924	190526769	1846	*	15	1.74354E-15	0.009780583	0.017382132	0.000658337	0.089526509	0.003375995	ASNSD1
chr3	48884952	48885189	238	*	2	1.91989E-07	0.049493695	0.017397021	0.029144481	0.029581066	0.017341598	PRKAR2A-AS1, PRKAR2A

chr17	76274577	76274856	280	*	4	6.54721E-10	0.000479459	0.017447553	0.001106981	-0.150775575	-0.109044409	
chr6	132811238	132811784	547	*	4	1.00588E-10	0.00119379	0.01749304	0.001186218	0.200773033	0.070164382	STX7
chr2	119915368	119915729	362	*	3	1.01077E-07	0.00447377	0.017509844	0.007198228	0.078482887	0.053406743	C1QL2
chr12	114232702	114232937	236	*	4	7.24124E-12	2.42656E-05	0.017511939	0.000137605	0.150385381	0.13096388	
chr10	48416391	48417399	1009	*	12	2.74079E-18	0.007175516	0.017520297	0.000372296	0.194155048	0.063232291	GDF2
chr5	82373222	82373944	723	*	13	1.11565E-13	0.065257337	0.017556012	0.016822668	-0.050869501	-0.008251352	XRCC4, TMEM167A
chr2	225792082	225792829	748	*	4	6.65708E-10	0.002393175	0.017571359	0.001129912	-0.201101133	0.018482429	DOCK10
chr8	1708438	1708447	10	*	2	5.32878E-08	0.001550053	0.017577147	0.002973447	0.114009247	0.113445864	CLN8
chr19	6529614	6530704	1091	*	4	4.96662E-10	0.000104004	0.017600342	0.000399315	0.101058407	0.064943809	
chr4	57684399	57685107	709	*	5	1.7134E-11	0.000165036	0.017602197	0.00028048	0.120744472	0.040518094	SPINK2, Y_RNA
chr9	35675387	35676166	780	*	8	4.5391E-09	0.013151721	0.017610574	0.009846805	0.105382352	0.036335987	CA9, ARHGEF39
chr4	41936041	41937393	1353	*	10	4.6545E-14	0.042103793	0.017616039	0.010756255	-0.24603613	-0.049393455	TMEM33
chr7	100025489	100029306	3818	*	27	2.46062E-12	0.003975666	0.017627562	0.000257797	-0.292109232	-0.022580173	MEPCE, ZCWPW1
chr13	41052081	41053032	952	*	3	1.60505E-08	0.020755055	0.017635633	0.011277085	0.135197485	0.09339068	LINC00598
chr6	33171765	33173501	1737	*	28	1.60151E-18	0.04934977	0.017636709	0.00892821	-0.147679787	0.010664633	SLC39A7, HSD17B8
chr2	198379881	198381500	1620	*	25	4.50077E-27	0.002481828	0.017642624	0.000209384	-0.033643634	-0.004383324	HSPE1-MOB4, MOB4, HSPD1
chr16	967350	967377	28	*	2	6.81743E-08	0.002146347	0.017658216	0.00388019	-0.258199707	-0.104916502	LMF1
chr10	121301765	121302268	504	*	3	6.88261E-09	0.004743617	0.01772228	0.007622639	-0.096463152	-0.018678427	RGS10
chr4	52709356	52709358	3	*	2	2.12575E-07	0.002016043	0.017728142	0.00369314	-0.036042816	-0.029117036	DCUN1D4
chr9	136726359	136726575	217	*	3	3.44205E-10	0.000255939	0.017734599	0.000802161	0.139666095	0.116812391	VAV2
chr7	90224886	90225365	480	*	5	3.68245E-09	0.04517545	0.017740567	0.006098776	-0.053245362	-0.03480667	CDK14, AC002456.2
chr6	30484334	30484429	96	*	2	9.25357E-09	0.002054785	0.017759658	0.003752027	-0.061093482	-0.055271429	XXbac-BPG249D20.9
chr22	31626484	31626752	269	*	3	2.41603E-08	0.004412967	0.017760045	0.004022336	-0.090678741	-0.053727058	LIMK2
chr5	140998344	140998928	585	*	10	2.54277E-11	0.064240138	0.017777565	0.024136866	-0.041390711	-0.010266417	AC008781.7, DIAPH1
chr10	89420170	89420794	625	*	5	2.13045E-10	0.004074152	0.017787063	0.005313028	0.179625035	0.079898785	PAPSS2
chr1	55353327	55353706	380	*	3	1.75979E-08	0.031227543	0.017800351	0.016467961	-0.180742556	-0.041226101	RP11-67L3.5, RP11-67L3.4
chr11	114270563	114271474	912	*	15	4.17521E-09	0.400474916	0.017837609	0.126334363	0.033989016	7.7935E-05	RBM7, RP11-212D19.4, C11orf71
chr13	50699716	50699887	172	*	6	8.30646E-08	0.025476062	0.017896359	0.027254595	-0.067879001	-0.027215518	DLEU1, DLEU2
chr21	44312947	44313427	481	*	9	1.38069E-08	0.033463733	0.017913332	0.029064042	0.0240741	0.007450557	NDUFV3

chr1	215255572	215255716	145	*	2	4.3551E-08	0.008069726	0.017913832	0.010490539	0.068451839	0.052312986	KCNK2
chr5	79553147	79553606	460	*	4	1.62101E-09	0.004666189	0.017922666	0.002354646	-0.138910877	-0.049913437	
chr17	5290658	5290775	118	*	3	6.23659E-08	0.001189998	0.017959911	0.002645567	-0.201982935	-0.096363274	NUP88
chr2	74757529	74757563	35	*	2	1.23331E-07	0.001909298	0.017964773	0.003545747	0.024647308	0.007648177	HTRA2
chr5	10761669	10762626	958	*	5	4.02945E-11	0.000134719	0.017968816	0.000179585	-0.113448481	-0.048737802	CTD-2154B17.4
chr6	110721305	110722253	949	*	4	1.91585E-08	0.001402655	0.017971337	0.00131314	-0.201184051	-0.063838798	DDO
chr7	3083333	3083721	389	*	3	6.03002E-10	0.000317044	0.018019022	0.000963017	0.044420611	0.03030388	CARD11
chr5	52095950	52096100	151	*	3	2.68124E-08	0.001162634	0.018022216	0.002608628	0.015377643	0.012242613	ITGA1, PELO
chr2	121009176	121010281	1106	*	10	2.98635E-13	0.174559217	0.018023639	0.007148502	-0.175777573	-0.013734719	RALB
chr1	46049433	46050509	1077	*	9	2.31935E-11	0.39668426	0.018028358	0.043968209	0.024143662	0.001027495	NASP
chr19	37957601	37957760	160	*	3	1.33961E-08	0.001088419	0.018050835	0.002566184	-0.069360388	-0.029397492	ZNF569
chr1	18956762	18957375	614	*	5	3.65777E-09	0.00927205	0.018069945	0.0032395	0.030221323	0.022293157	
chr17	62339926	62340143	218	*	4	6.19503E-08	0.111914587	0.018081828	0.021316973	-0.120955828	-0.03981979	TEX2
chr6	32805398	32812953	7556	*	103	9.28823E-23	0.189259565	0.018082522	0.006368808	0.206236598	0.006063317	TAPSAR1, PSMB9, TAP2, TAP2, PSMB8
chr19	17000585	17001305	721	*	4	1.40574E-08	0.008343266	0.018094316	0.007823	0.130131964	0.072579333	F2RL3
chr2	20550205	20552116	1912	*	13	9.51287E-13	0.000547132	0.0181011	0.000546389	0.123440496	-0.002076635	PUM2
chr19	50180204	50180436	233	*	7	1.37923E-07	0.062381273	0.018109526	0.026792476	-0.056281626	-0.006263791	PRMT1
chr9	92097137	92097516	380	*	3	1.27852E-08	0.002852006	0.018144974	0.003402265	0.081798266	0.054133607	SEMA4D
chr8	56903606	56904259	654	*	3	7.96147E-09	0.000226403	0.018157254	0.000738339	-0.143869728	-0.120536536	LYN
chr9	90589798	90589971	174	*	7	1.13642E-07	0.010935426	0.018161529	0.019426031	0.069488449	0.03927819	
chr13	95355117	95355281	165	*	3	1.46028E-08	0.002015983	0.018166576	0.003903195	0.098815014	0.069036386	
chr10	134602228	134602530	303	*	3	1.04182E-07	0.002827048	0.018185239	0.003407671	0.052105371	0.042940413	
chr2	128458240	128458845	606	*	11	8.91486E-19	8.2448E-05	0.018185397	8.14977E-05	0.084704211	0.034144865	SFT2D3, WDR33
chr6	32054561	32055738	1178	*	25	2.35948E-14	0.154617436	0.018188176	0.067137596	0.170327112	0.037463678	TNXB
chr5	107005999	107006413	415	*	3	8.2603E-09	0.00715033	0.018195224	0.006415411	0.042384074	0.025822478	EFNA5
chr12	109569059	109569180	122	*	5	3.17054E-09	0.008927602	0.018211527	0.003914446	0.124100309	0.072444462	ACACB
chr16	3493133	3494155	1023	*	15	4.31391E-21	4.2029E-06	0.018212285	3.17079E-05	-0.178712467	-0.100404713	NAA60, NAA60, ZNF597
chr1	109849854	109850837	984	*	6	6.74672E-10	0.026073537	0.018217208	0.006636714	0.202280052	0.075297241	
chr20	61669194	61669380	187	*	5	1.55197E-09	0.031613058	0.018222788	0.007023926	0.1104171	0.051311537	RP11-305P22.9, LINC01056

chr12	120884387	120884702	316	*	7	1.07017E-07	0.043580614	0.018237033	0.030565111	0.019774845	0.006938304	AL021546.6, GATC
chr3	107574971	107575062	92	*	2	1.63618E-07	0.001549243	0.018238075	0.002997642	-0.149091711	-0.138150406	LINC00635
chr15	65053596	65054317	722	*	4	8.22393E-10	0.032552467	0.018252022	0.003888021	0.089104266	0.066902308	RBPMS2
chr19	40324187	40324762	576	*	6	1.69802E-09	0.042660622	0.018261072	0.024342808	-0.032720373	0.001271896	DYRK1B
chr8	101322518	101323246	729	*	7	2.99704E-11	0.127752149	0.018267303	0.013158196	0.164642561	0.04935228	RNF19A
chr6	151814565	151815241	677	*	11	1.46899E-13	0.019899856	0.018270342	0.006473345	-0.297168929	-0.014306416	CCDC170
chr2	85805621	85805631	11	*	2	1.78335E-08	0.00178404	0.018274546	0.00336885	-0.044197803	-0.036188482	VAMP8
chr12	32686638	32686645	8	*	2	8.37183E-08	0.001588663	0.018286552	0.003062526	-0.064875674	-0.052865978	FGD4
chr13	114825082	114825608	527	*	4	1.24394E-08	0.034435737	0.018295449	0.024293688	0.138243793	0.071140072	RASA3
chr10	38264940	38265215	276	*	2	6.2701E-08	0.050651412	0.01831041	0.030384648	-0.035746616	-0.021354371	ZNF25
chr12	98909434	98910308	875	*	9	7.22765E-16	0.000195045	0.018316501	0.000322227	-0.062089702	-0.018536737	TMPO, TMPO-AS1
chr14	95829909	95830175	267	*	5	1.39957E-09	0.001533788	0.018334472	0.00232611	0.137813934	0.074174428	
chr5	55148279	55148974	696	*	4	9.89399E-09	0.005429142	0.01835092	0.003693455	-0.194812235	-0.118629147	IL31RA
chr20	43024441	43024748	308	*	3	5.28737E-10	0.000396876	0.018351379	0.001107286	0.158213488	0.113570538	HNF4A
chr11	67006774	67007811	1038	*	17	1.43474E-14	0.103758364	0.018355745	0.01047402	-0.054970349	-0.017580139	KDM2A
chr11	1912178	1912495	318	*	4	1.6714E-10	0.000710588	0.018361524	0.001550793	-0.087408198	-0.067493614	LSP1
chr6	33235504	33236353	850	*	13	7.3197E-14	0.341878965	0.018390631	0.031177658	0.180503093	0.025568306	VPS52
chr17	151914	152350	437	*	5	2.84696E-09	0.033061797	0.018392818	0.004237727	0.089690709	0.0610081	RPH3AL
chr20	10653164	10654831	1668	*	11	1.18E-11	0.001596985	0.018397449	0.000615323	0.027574232	0.009069636	RP11-103J8.1, JAG1
chr13	36044768	36045352	585	*	6	4.02053E-11	0.010776597	0.018439428	0.002324122	0.047972676	0.032791413	NBEA
chr1	33463340	33463882	543	*	3	2.36385E-09	0.000328487	0.01844922	0.000964874	-0.133074723	-0.099733974	RP1-117O3.2
chr6	150285026	150285475	450	*	5	8.11964E-09	0.012616562	0.018468677	0.007092892	-0.060726641	-0.002771763	ULBP1
chr16	19777410	19777742	333	*	3	3.49193E-08	0.001042581	0.018509456	0.002203222	0.328834181	0.182758763	IQCK, CTD-2380F24.1
chr3	127542959	127543047	89	*	2	2.62742E-09	0.001617126	0.018510899	0.003116612	0.13712616	0.110794733	
chr16	29819361	29819572	212	*	5	3.63526E-08	0.209830775	0.018552101	0.014502687	-0.110050218	-0.044305247	MAZ
chr9	139237366	139237889	524	*	4	6.41223E-09	0.005559907	0.018571567	0.00352697	0.170560441	0.014866654	GPSM1
chr8	114448998	114449487	490	*	9	1.85446E-09	0.039017526	0.01860805	0.02500782	0.045999632	0.008148185	CSMD3
chr13	23500752	23500838	87	*	2	1.39381E-07	0.005599424	0.018610767	0.008221354	-0.068253735	-0.045462764	
chr6	6006770	6008642	1873	*	18	3.98713E-17	0.022609952	0.018622192	0.002508029	0.057019191	0.011921061	NRN1

chr17	7553760	7554513	754	*	10	3.78806E-11	0.017671904	0.018623997	0.018772899	0.052961376	0.004445953	ATP1B2
chr17	38229424	38229533	110	*	2	9.72274E-08	0.001711429	0.018652637	0.003271714	0.106530156	0.101104065	THRA
chr1	65432346	65432742	397	*	9	1.97588E-12	0.025360724	0.018657307	0.004504049	0.033310211	0.000394004	
chr11	118481025	118481577	553	*	3	2.93752E-09	0.002481402	0.018658917	0.004370052	0.036272574	0.027478913	PHLDB1
chr11	8703871	8704374	504	*	9	3.48615E-09	0.162336137	0.01869856	0.082172246	0.036035583	0.005802388	RPL27A
chr6	111927123	111927484	362	*	4	6.09994E-10	0.004666851	0.018714338	0.004325371	-0.055986569	-0.030752941	TRAF3IP2
chr1	165414189	165414379	191	*	3	1.0653E-08	0.001298267	0.018718423	0.002408763	0.03309455	0.027250666	RXRG
chr9	96720819	96721102	284	*	2	1.07864E-08	0.001800154	0.018737213	0.003414234	-0.050866913	-0.050229605	RP11-231K24.2
chr11	63655748	63656342	595	*	8	1.00317E-14	0.045332353	0.018737748	0.001406921	-0.14958791	-0.055232429	MARK2
chr12	2026496	2027076	581	*	4	3.76123E-12	0.000102753	0.018740378	0.000413318	-0.106268953	-0.040928779	CACNA2D4
chr19	7767229	7767584	356	*	3	2.84111E-08	0.598779747	0.018746467	0.107308079	-0.124318142	-0.041772848	
chr17	36715074	36715953	880	*	4	1.76899E-10	2.26244E-05	0.018764456	0.000133087	-0.114917296	-0.072536567	SRCIN1
chr8	70982867	70983348	482	*	3	5.05046E-08	0.308875851	0.018806925	0.086718378	0.048211247	0.016220869	PRDM14
chr16	57297286	57297444	159	*	3	2.99867E-09	0.000287192	0.018833983	0.000890562	0.160562203	0.109152	PLLP
chr22	38071001	38072101	1101	*	14	8.31371E-17	0.002669584	0.018885175	0.001015347	0.07260364	0.01657295	LGALS1
chr1	93297424	93298275	852	*	8	2.37092E-10	0.01858153	0.018896403	0.010093281	0.028552607	-0.000484718	RPL5
chr1	21588448	21589450	1003	*	4	2.22456E-09	0.000123363	0.018917225	0.000471502	0.149861903	0.028064712	ECE1
chr14	105713608	105716436	2829	*	25	9.55214E-14	0.032230001	0.018931118	0.004145635	0.150701228	0.021070712	BTBD6, BRF1
chr10	135149648	135150139	492	*	3	3.36217E-10	0.004535593	0.018964696	0.006841051	0.092069478	0.031096128	ZNF511, CALY
chr5	56112222	56112786	565	*	3	7.33448E-08	0.014650928	0.018967839	0.010563383	-0.044011466	-0.003646694	MAP3K1
chr2	98280029	98280810	782	*	10	7.88548E-12	0.025710863	0.018977426	0.015813684	-0.037953224	-0.007855268	LINC01125, ACTR1B
chr12	3601306	3602546	1241	*	10	2.08626E-10	0.049398042	0.018989386	0.02301902	0.054350899	0.013793783	PRMT8
chr5	133406542	133407209	668	*	4	3.65831E-09	0.00218427	0.01900322	0.002329825	0.09088139	0.041204581	
chr1	156094519	156094952	434	*	4	1.29836E-08	0.00271426	0.019010749	0.004393748	0.149646876	0.097909766	LMNA
chr7	1126903	1127767	865	*	6	1.96014E-11	0.006147305	0.01902093	0.00489263	-0.117063514	0.019949857	GPER1, C7orf50
chr8	124428770	124429792	1023	*	8	8.72355E-11	0.172208937	0.019047575	0.105730226	-0.066831309	-0.024501205	WDYHV1
chr7	6199980	6200140	161	*	3	4.84042E-08	0.002292852	0.019088154	0.004637261	0.099272037	0.061914259	USP42
chr2	179315623	179316706	1084	*	16	7.85584E-13	0.082584541	0.01912899	0.047376203	-0.047961244	-0.007186305	DFNB59, PRKRA
chr2	201728833	201730276	1444	*	17	3.79322E-11	0.592997124	0.019149773	0.077530366	0.105507286	0.009731284	CLK1

chr3	58222756	58223527	772	*	9	1.31383E-09	0.063735367	0.019150046	0.021478888	-0.061214965	-0.020607827	ABHD6
chr3	173113376	173113519	144	*	3	5.33938E-08	0.002023012	0.019160548	0.00408051	-0.059907709	-0.006242952	
chr7	26415606	26416987	1382	*	12	5.8182E-14	0.003666176	0.019164184	0.000445924	-0.066240253	-0.005749338	AC004540.4
chr1	249132477	249132838	362	*	4	2.01329E-08	0.002348797	0.019174084	0.002748563	0.059260712	0.023056338	ZNF672
chr19	3698157	3698223	67	*	2	3.27685E-08	0.001738178	0.019212596	0.003336865	0.150432202	0.110644026	PIP5K1C
chr17	8026413	8026777	365	*	2	3.12146E-10	0.312384266	0.019218401	0.052442164	0.040521288	0.018525135	HES7
chr2	240152334	240152476	143	*	2	4.81106E-08	0.001712534	0.019222486	0.003296543	-0.05268785	-0.050786068	HDAC4
chr11	20409647	20409780	134	*	2	1.45413E-07	0.008527118	0.019234913	0.011206984	0.033264108	0.023893672	PRMT3
chr1	235107109	235107121	13	*	2	9.38384E-08	0.001867628	0.019251461	0.003541796	0.125569309	0.118701049	
chr9	95527179	95527975	797	*	5	4.83597E-09	0.002506542	0.019253652	0.003135658	-0.05100331	-0.015027277	
chr5	53605192	53606129	938	*	3	2.19362E-09	0.172499648	0.019277946	0.048198489	0.186965093	0.054842222	ARL15
chr17	79819298	79819480	183	*	3	1.4746E-08	0.004820677	0.019306185	0.005229115	0.036394399	0.017272439	
chr11	47198535	47199922	1388	*	15	2.74793E-11	0.067507843	0.019311678	0.016462479	0.132219523	-0.009021879	ARFGAP2, PACSIN3
chr8	9413989	9414115	127	*	3	2.23079E-08	0.012090093	0.019316325	0.013459392	0.025819983	0.016681145	TNKS, RP11-375N15.2
chr19	6424783	6425207	425	*	4	1.34776E-08	0.043506144	0.01936275	0.008453531	-0.044503185	-0.02047591	KHSRP
chr1	184006496	184007117	622	*	6	1.23908E-13	0.001509674	0.01936742	0.000486618	-0.161866607	-0.064462685	COLGALT2
chr7	27778939	27780234	1296	*	19	1.21522E-13	0.103229019	0.019372595	0.037612115	-0.050138647	-0.010865813	TAX1BP1, AC004549.6
chr1	67519155	67519814	660	*	6	2.69271E-11	0.034420518	0.019375592	0.012261065	-0.101245403	-0.010286628	SLC35D1
chr1	86861147	86862273	1127	*	14	1.94873E-12	0.091294925	0.019381441	0.028965208	0.050705453	0.006005511	ODF2L
chr15	101458112	101458783	672	*	5	4.64976E-12	0.016137165	0.019385092	0.004004441	-0.196604598	-0.073872312	RP11-66B24.4
chr19	8509809	8510764	956	*	5	2.24601E-11	0.000741661	0.019394442	0.001934527	-0.030359172	-0.00088973	HNRNPM
chr12	48111841	48112358	518	*	4	4.74138E-10	0.000588732	0.019395447	0.00131625	0.158815435	0.083814559	RP1-197B17.3, ENDOU
chr15	75743136	75744976	1841	*	14	1.26512E-16	0.002302741	0.019397035	0.000354457	-0.053755183	-0.011316152	SIN3A
chr6	146863357	146864390	1034	*	12	1.26453E-14	0.046087857	0.01940199	0.017337826	-0.102839743	-0.009495975	
chr16	88769443	88769970	528	*	5	2.60813E-10	0.002862124	0.019402362	0.004912267	0.118013567	0.049465628	RNF166
chr1	17409889	17409984	96	*	4	1.1885E-10	0.000138411	0.019404713	0.00051192	-0.136127038	-0.084918864	PADI2
chr16	2478353	2478800	448	*	4	9.02218E-10	0.014889872	0.019407976	0.005582982	0.063068108	0.027706484	
chr3	158288695	158289160	466	*	12	9.30897E-11	0.033019961	0.019409354	0.024069774	-0.057464286	-0.005103899	MLF1, RP11-538P18.2
chr20	24930041	24930541	501	*	3	1.93388E-08	0.001479422	0.019421575	0.003219034	-0.141301136	-0.0732846	CST7

chr1	109968772	109969933	1162	*	17	1.31151E-11	0.584210493	0.019423391	0.092309418	-0.045181478	-0.014175914	PSMA5
chr2	51260176	51260399	224	*	2	4.1589E-08	0.077366523	0.019448787	0.037580145	0.141446551	0.087825722	AC007682.1
chr8	42129353	42129644	292	*	2	4.50675E-09	0.001748782	0.019464337	0.003363842	-0.118667633	-0.077730645	IKBKB
chr11	66636291	66636369	79	*	3	9.89646E-08	0.001970042	0.019491735	0.003234343	-0.15683502	-0.033530839	PC
chr17	17684010	17684042	33	*	2	1.19736E-07	0.001980244	0.019493408	0.003727032	0.100582236	0.099698429	RAI1
chr2	98261952	98262923	972	*	14	9.69534E-16	0.012137926	0.019499811	0.002777482	-0.067961947	-0.021211407	COX5B
chr10	23216182	23216299	118	*	2	8.50428E-09	0.001836492	0.019512138	0.003504385	0.162891453	0.153812283	
chr17	32690127	32690732	606	*	7	3.89337E-13	0.000116012	0.019514106	0.000419393	-0.131321782	-0.03179496	CCL1
chr13	79979614	79980098	485	*	6	5.0712E-09	0.022765933	0.019529008	0.021630502	0.021404357	0.004500793	RBM26
chr20	17206946	17207562	617	*	5	5.35792E-09	0.006791744	0.019547892	0.004367303	0.040085652	0.021583248	PCSK2
chr17	74498083	74498760	678	*	4	1.99803E-11	0.00045068	0.019573953	0.00085798	-0.105292998	-0.08536734	
chr1	17745990	17746597	608	*	7	1.56006E-12	0.000173209	0.019579569	0.000575333	0.209276168	0.066931371	RCC2
chr6	3848218	3850106	1889	*	33	4.92393E-29	0.1262448	0.019601261	0.000837383	-0.234935796	-0.053609093	RP11-420L9.4, FAM50B
chr18	25757202	25757555	354	*	4	5.90387E-09	0.019183121	0.019622296	0.007094167	0.032746578	0.016476358	CDH2
chr1	110881368	110882527	1160	*	11	4.12711E-11	0.03960624	0.019634865	0.02086146	-0.030257498	0.000141691	RBM15, RP5-1074L1.1
chr2	8422236	8422332	97	*	3	1.14172E-07	0.002085772	0.019639099	0.003910203	0.115893214	0.069848524	LINC00299
chr17	42289769	42290419	651	*	5	4.84856E-08	0.021833253	0.019663723	0.014678066	0.095814122	0.050415242	CTB-175E5.7, UBTF
chr2	106682209	106682640	432	*	3	2.56502E-08	0.011516816	0.019665185	0.014773656	-0.063991509	0.015937154	C2orf40
chr8	125550836	125551626	791	*	15	4.11383E-10	0.342897422	0.019677005	0.088988432	0.023412825	0.003627892	NDUFB9, TATDN1
chr9	6681415	6681608	194	*	3	6.65809E-10	0.003670079	0.019679315	0.004535132	0.035313413	0.01458098	
chr4	89512872	89513664	793	*	9	8.32859E-10	0.761165507	0.019684663	0.123643041	-0.05696799	-0.003919762	HERC3
chr2	208489307	208491029	1723	*	16	8.41832E-13	0.01654542	0.019720292	0.00171162	-0.066536033	-0.000591885	METTL21A
chr2	196520930	196522552	1623	*	19	5.15387E-17	0.08397993	0.019728054	0.003900085	-0.296016203	-0.037701653	SLC39A10
chr7	19157650	19158041	392	*	9	3.52868E-09	0.06408848	0.019734433	0.04596064	0.050650588	0.018861314	
chr10	6185502	6186794	1293	*	11	6.13911E-18	0.018276358	0.019740963	0.00113817	-0.168565675	-0.031024201	
chr6	10555682	10556523	842	*	9	4.30379E-15	0.004883024	0.019776826	0.001072525	0.161780487	0.070703304	GCNT2
chr22	46645903	46646882	980	*	8	8.93258E-09	0.058073011	0.019779569	0.030507462	-0.039697235	-0.002859018	CDPF1
chr16	596670	597434	765	*	5	5.02051E-12	0.000311386	0.019786371	0.001086225	-0.145172546	-0.105343177	CAPN15, LA16c-366D1.3
chr3	49760915	49761251	337	*	5	9.14415E-10	0.009306289	0.019786923	0.010315428	0.015039921	0.004216198	AMIGO3, GMPPB

chr6	10505886	10505955	70	*	3	1.61893E-07	0.00136604	0.019821997	0.003055689	-0.214074856	-0.152369656	GCNT2
chr21	38377938	38379036	1099	*	8	1.30208E-11	0.007731965	0.019822565	0.003647907	0.076334694	0.030398395	RIPPLY3
chr5	130500762	130501079	318	*	4	1.02251E-07	0.008646997	0.019829543	0.011040939	-0.042765657	-0.00200904	HINT1
chr7	2769681	2769865	185	*	2	7.23408E-08	0.002163523	0.019859024	0.004024345	-0.107722602	-0.102906493	AMZ1, GNA12
chr17	66596999	66597007	9	*	2	3.21057E-08	0.001879917	0.019886132	0.003588505	0.037384526	0.034172415	RP11-118B18.1, FAM20A
chr18	47087723	47088780	1058	*	11	7.31418E-14	0.000903574	0.019891103	0.001280745	0.035551884	0.01619461	LIPG
chr16	2256121	2256716	596	*	5	5.90201E-12	0.016501206	0.019909575	0.005855216	-0.10113303	-0.036935732	MLST8
chr19	36004994	36005395	402	*	3	2.37558E-09	0.002040166	0.019937899	0.004200888	0.076892252	0.0738583	
chr11	124647244	124647347	104	*	3	6.54987E-08	0.003008148	0.019976826	0.003933787	0.105593291	0.064517896	MSANTD2
chr6	135502390	135502875	486	*	4	1.01873E-10	0.005172922	0.019997547	0.008181036	-0.047168673	-0.015279711	MYB
chr17	1986479	1987136	658	*	4	1.41138E-10	0.00054114	0.020007092	0.001376752	-0.185707542	-0.086166871	SMG6
chr13	114261869	114261986	118	*	3	1.85458E-09	0.000215027	0.020015505	0.000730296	-0.114379045	-0.104789652	TFDP1
chr1	11119628	11120633	1006	*	8	2.35706E-09	0.010488404	0.020019727	0.004489317	0.025715492	0.003289058	SRM
chr2	27545550	27546456	907	*	13	2.00791E-12	0.030644141	0.020025703	0.007847782	-0.034336051	-0.0036128	MPV17
chr11	1325718	1326704	987	*	6	1.6489E-13	0.000160708	0.020030378	0.000186065	0.165138842	0.070862689	TOLLIP
chr17	60758710	60758801	92	*	3	4.71168E-08	0.001337875	0.020063592	0.003132452	0.117461358	0.07087322	MRC2
chr1	152083276	152083292	17	*	2	7.92644E-08	0.001900736	0.020075475	0.003629298	0.05465529	0.04631859	TCHH
chr17	78800767	78800806	40	*	3	3.58189E-10	0.00024451	0.020078626	0.00080818	-0.124208304	-0.112743266	RPTOR
chr2	100824336	100824930	595	*	5	6.2614E-10	0.000877442	0.020103789	0.001220743	0.158269205	0.074470293	LINC01104
chr2	163199311	163200671	1361	*	13	4.66798E-17	0.000398289	0.02015046	0.000160045	-0.278375855	-0.06842974	GCA
chr15	71407484	71408080	597	*	5	8.15909E-10	0.098391499	0.020217474	0.03313145	0.052865451	0.01692655	THSD4, CT62
chr5	159933856	159934412	557	*	3	2.92572E-09	0.000235979	0.020224116	0.000786367	0.155047948	0.10554404	
chr2	223176167	223177008	842	*	8	2.39908E-08	0.331114371	0.020268206	0.083438821	0.052994695	0.017544349	
chr14	102430437	102430709	273	*	3	2.07113E-08	0.000407827	0.020280743	0.001214874	0.085147744	0.043637724	
chr22	31891748	31892562	815	*	15	4.48781E-12	0.077910712	0.020288309	0.03343144	0.081296416	0.001475768	DRG1, SFI1, EIF4ENIF1
chr20	61810348	61810902	555	*	5	6.73703E-10	0.038382273	0.020310647	0.01294913	-0.070988094	-0.002793041	RP5-963E22.4
chr22	42765138	42765436	299	*	5	3.73733E-09	0.015740849	0.020353739	0.011330916	0.031309038	0.003473015	Z83851.1
chr2	24232161	24233341	1181	*	12	3.92426E-11	0.026058108	0.020374614	0.024262805	0.28415789	0.011594673	MFSD2B
chr19	18632488	18633523	1036	*	12	4.64376E-12	0.002968116	0.020394324	0.004557621	-0.056168758	-0.01101016	ELL

chr3	5229596	5230189	594	*	7	3.69614E-09	0.47941968	0.020416796	0.090444656	0.031578357	0.005507297	EDEM1, AC026202.1
chr5	68530331	68531229	899	*	16	1.15138E-14	0.009907667	0.020423631	0.006459974	-0.054913523	-0.014721741	CDK7
chr15	74902534	74903214	681	*	3	6.82496E-09	0.000242027	0.020452719	0.000807099	0.10292435	0.089432006	CLK3
chr1	47081524	47083518	1995	*	14	1.31119E-12	0.000228872	0.020463576	0.000148022	-0.164891197	-0.00980837	MKNK1, MOB3C
chr12	70759866	70760024	159	*	5	2.46881E-08	0.217747526	0.0204877	0.024994593	-0.067367329	-0.007975022	
chr1	6480167	6480594	428	*	3	2.95361E-09	0.00238757	0.020493761	0.004911903	0.072267262	0.044277166	HES2
chr16	1545088	1545405	318	*	3	6.20896E-08	0.011484931	0.020506908	0.006485812	0.106331409	0.07250336	TELO2
chr12	27934708	27934973	266	*	3	5.04142E-08	0.003513292	0.020517446	0.0042572	0.120744322	0.076681629	KLHL42
chr3	187009575	187009702	128	*	2	1.11571E-07	0.004937381	0.020523482	0.007745999	0.134574533	0.107888489	MASP1
chr6	150183516	150184138	623	*	4	4.63041E-09	0.009770516	0.020591157	0.007144588	0.151527066	0.054482545	RP11-244K5.8, LRP11
chr17	7218050	7219319	1270	*	12	1.99155E-12	0.024622865	0.020645887	0.012060546	-0.032904292	0.003589087	GPS2, RP11-542C16.2, NEURL4
chr3	119812813	119814693	1881	*	13	2.15155E-14	4.85914E-05	0.020649761	0.000160982	0.026892406	0.009008667	RP11-18H7.1, GSK3B
chr9	139636616	139637895	1280	*	6	1.04014E-10	0.000428086	0.020662465	0.000666545	0.152088867	0.048031321	LCN10, LCN6
chr11	57093322	57093618	297	*	3	2.20739E-08	0.050769465	0.020674686	0.008862182	0.158797734	0.092339944	RP11-872D17.4, SSRP1
chr10	1704858	1705085	228	*	3	2.38808E-08	0.001391446	0.020682998	0.002803723	0.103802584	0.073893241	ADARB2
chr6	117803953	117804230	278	*	2	5.83076E-08	0.002113879	0.020686371	0.003988635	-0.075787988	-0.018607429	DCBLD1, GOPC
chr14	24711390	24713132	1743	*	15	4.04616E-14	0.000133664	0.020693438	0.00018871	0.124806995	0.007863407	TINF2
chr16	30771976	30773936	1961	*	21	6.7505E-24	0.009002152	0.020698257	0.000185699	-0.101578653	-0.000119457	PHKG2, RNF40, C16orf93
chr4	184826953	184827754	802	*	4	8.02024E-09	0.002404039	0.020717697	0.0037812	-0.046308057	0.000204511	STOX2
chr5	180673096	180674133	1038	*	11	1.51112E-10	0.024353275	0.020724119	0.011612551	-0.072209894	0.005508439	CTC-338M12.4, GNB2L1
chr4	39448464	39449130	667	*	5	4.49334E-10	0.001920771	0.020732418	0.003586461	0.136168387	0.082536726	KLB
chr2	24582653	24582688	36	*	2	1.03156E-07	0.003045079	0.020746887	0.005355198	-0.040409182	-0.009783452	ITSN2
chr1	153918286	153918709	424	*	2	5.28109E-08	0.003516701	0.020774515	0.005998734	0.022802167	0.003479532	DENND4B
chr2	68479620	68480222	603	*	4	6.56693E-09	0.002215135	0.020785961	0.003324008	0.028420506	0.012825884	RP11-474G23.3, RP11-474G23.1, PPP3R1
chr19	50143224	50143951	728	*	11	4.88578E-19	1.19407E-05	0.020797138	2.52534E-05	-0.058636145	-0.019952574	RRAS
chr5	102201825	102202139	315	*	4	2.4916E-08	0.435811301	0.020801844	0.068113971	-0.044332051	-0.015464388	PAM
chr20	36155925	36157405	1481	*	44	1.58959E-19	0.467129716	0.020840559	0.093769713	-0.047687605	0.000537018	BLCAP
chr1	45792006	45792733	728	*	10	6.49822E-09	0.173751167	0.020849354	0.028076743	0.08790309	0.018494197	HPDL
chr16	75498408	75498625	218	*	6	8.50904E-09	0.007267676	0.020872101	0.010346387	0.02091584	0.000349093	RP11-77K12.1, TMEM170A

chr1	167189623	167190631	1009	*	11	1.05353E-10	0.20877054	0.020892637	0.026597739	-0.076617798	-0.014214694	POU2F1, RP11-277B15.3
chr1	181074635	181074790	156	*	3	2.27695E-08	0.023788997	0.020898618	0.00868201	0.028944912	0.014021431	
chr19	47128970	47129637	668	*	5	1.28326E-10	0.000496283	0.020900516	0.001359314	0.167097386	0.118146197	
chr18	12702185	12703040	856	*	15	1.17166E-11	0.120799407	0.020922968	0.037040757	-0.101533481	-0.007316402	PSMG2, CEP76
chr11	31821298	31822341	1044	*	9	7.38677E-10	0.000991385	0.020931426	0.002677567	0.112510553	0.031747311	PAX6
chr21	43771120	43771664	545	*	6	1.06992E-13	0.000142338	0.020948961	0.000221681	0.161817117	0.109379796	TFF2
chr8	70746128	70748063	1936	*	22	4.57348E-13	0.131099029	0.020951649	0.016173635	-0.142916054	-0.003933465	RP11-159H10.3, SLCO5A1
chr19	917326	917813	488	*	5	3.43431E-09	0.032588253	0.02096191	0.030040127	0.039896992	0.010494975	KISS1R
chr13	25946838	25947459	622	*	4	2.28191E-11	0.000508715	0.02096457	0.001255672	-0.170572366	-0.025951943	ATP8A2
chr1	2574031	2574885	855	*	7	3.91938E-11	0.000989289	0.02099936	0.001669361	-0.066934614	-0.007488454	TTC34
chr15	85259471	85259484	14	*	3	1.83466E-07	0.025597834	0.021031384	0.023534381	0.024435161	0.014246959	SEC11A
chr12	14133667	14133887	221	*	3	3.36497E-08	0.003460635	0.021038601	0.006531734	0.035444157	0.025240684	
chr2	38302487	38302674	188	*	3	1.48631E-07	0.06542238	0.021050152	0.043735136	0.088962069	0.0152624	CYP1B1
chr1	234795783	234796042	260	*	4	1.53435E-07	0.008469734	0.021106371	0.005963655	0.148926703	0.076092044	RP4-781K5.4, RP4-781K5.5
chr11	117855870	117857990	2121	*	13	2.66851E-09	0.041119705	0.021108572	0.004785941	-0.11366595	0.003158206	IL10RA
chr5	95768418	95769305	888	*	11	6.85247E-10	0.086547027	0.021135114	0.069139655	0.051683631	0.014594368	CTD-2337A12.1, PCSK1
chr2	227834295	227834762	468	*	2	3.06259E-08	0.079399661	0.021170444	0.040124581	0.095570695	0.032284476	RHBDD1
chr10	14880332	14881096	765	*	7	1.258E-11	0.004790013	0.021189267	0.006408497	-0.065277514	-0.023387991	HSPA14, CDNF
chr17	7311030	7311868	839	*	6	3.13372E-12	0.008723903	0.021201216	0.006583176	-0.125612074	-0.047536043	NLGN2
chr12	111126844	111127287	444	*	7	2.54626E-08	0.064970363	0.021223086	0.025859684	-0.069573793	-0.023794489	HVCN1
chr12	7054989	7055829	841	*	11	1.76096E-10	0.078262553	0.021226983	0.032986049	-0.208127094	-0.033043398	C12orf57, PTPN6
chr10	76968350	76970516	2167	*	20	3.68358E-15	0.004182759	0.021227571	0.000402584	0.10006609	0.009395035	VDAC2
chr19	49991271	49991389	119	*	2	1.19122E-07	0.040901082	0.021228228	0.03000032	0.022611887	0.006722935	RPL13A
chr11	20620002	20621341	1340	*	11	7.40696E-15	0.007079644	0.021248235	0.002535237	0.101971811	0.03942091	SLC6A5
chr17	46692008	46692168	161	*	3	1.35998E-07	0.004301044	0.021293019	0.007243131	0.029225782	0.023391245	HOXB7, HOXB8
chr16	89766660	89767958	1299	*	8	1.24546E-16	1.7668E-05	0.02133723	1.96857E-05	0.058976558	0.003291706	SPATA2L
chr5	96143578	96144364	787	*	14	1.45941E-12	0.034619866	0.021348262	0.017018144	-0.098805154	-0.004252083	ERAP1
chr3	134203672	134204409	738	*	7	1.85699E-10	0.105094374	0.021356688	0.018398701	-0.039322796	0.000867578	ANAPC13
chr15	51988940	51989637	698	*	4	3.09227E-10	9.20909E-05	0.021387261	0.000422365	0.129513493	0.097767489	SCG3

chr19	4455239	4455957	719	*	7	4.03928E-11	0.001327575	0.021394101	0.003013144	0.227327544	0.099440065	CTB-50L17.9, UBXN6
chr3	49506547	49508289	1743	*	10	8.37876E-13	9.31754E-05	0.021415709	0.000144203	0.132551457	0.011542922	DAG1
chr6	64281439	64281996	558	*	9	4.57817E-15	0.004328936	0.021433454	0.000978211	-0.144874279	-0.057138346	PTP4A1, RP5-1148A21.3
chr8	144363043	144363930	888	*	7	4.8733E-12	0.011184686	0.021436682	0.003913709	-0.146727317	-0.035262908	RP13-582O9.5
chr13	99130655	99130967	313	*	3	5.50146E-08	0.000890828	0.021437228	0.00224175	0.085901335	0.066072366	STK24
chr3	3168755	3169230	476	*	3	4.47228E-09	0.000450762	0.021437784	0.001347447	0.053734706	0.035588185	TRNT1
chr12	49627010	49627934	925	*	4	5.68629E-12	5.18979E-05	0.021464135	0.000272184	-0.105784771	-0.074617179	TUBA1C, RP11-977B10.2
chr6	83902926	83904067	1142	*	21	5.37892E-12	0.079413294	0.021472372	0.076327419	-0.054380549	-0.008385516	RWDD2A, PGM3
chr4	71858619	71859713	1095	*	15	3.23012E-12	0.010327633	0.021480804	0.01053761	0.093142521	0.014735078	MOB1B, DCK
chr9	139743122	139743802	681	*	9	2.80356E-12	0.00182205	0.02148533	0.002896715	-0.046396562	-0.003191701	PHPT1
chr2	173940430	173940639	210	*	5	5.131E-08	0.139635037	0.021487149	0.05499246	0.022897883	0.003182887	MLTK
chr4	56814925	56815073	149	*	6	1.07847E-08	0.02434041	0.021490254	0.01678594	0.016872912	0.006894888	CEP135
chr11	65149587	65151509	1923	*	15	9.73735E-18	0.037508921	0.021502407	0.003421414	0.106924216	0.005866623	SLC25A45
chr17	13971618	13972896	1279	*	10	2.47682E-12	0.048192614	0.021508697	0.029591279	0.140530982	0.026051804	COX10, COX10-AS1
chr21	34638009	34638188	180	*	3	9.27616E-09	0.003776008	0.021517764	0.007021024	-0.108194336	-0.038416602	AP000295.9, IL10RB-AS1
chr8	64081095	64081408	314	*	8	6.31971E-08	0.611308283	0.021526416	0.074534782	0.069517419	0.009576608	YTHDF3
chr11	45826170	45826537	368	*	4	1.73341E-08	0.005286709	0.021526809	0.00915729	0.027469605	0.012244589	SLC35C1
chr7	87849017	87849336	320	*	5	2.3398E-08	0.133042424	0.021528763	0.052095271	0.018196781	0.007865077	AC003991.3, SRI
chr14	60386705	60387117	413	*	4	6.72501E-10	0.014122931	0.021573695	0.003788519	-0.152011727	-0.06284686	LRRC9, RP11-62H20.1
chr2	157190747	157190958	212	*	2	3.60173E-08	0.036870765	0.021619662	0.028765359	0.021230854	0.014938545	NR4A2
chr5	42992128	42992998	871	*	4	2.4811E-10	0.000669954	0.021631486	0.001522795	0.041361085	0.028108662	
chr2	96873994	96874823	830	*	14	2.60902E-09	0.515526433	0.021669793	0.131493104	0.019961893	0.002626883	STARD7-AS1, STARD7
chr13	20702922	20702970	49	*	2	1.53578E-07	0.013718838	0.021683861	0.016182176	0.011876166	0.008821283	
chr16	49732224	49732623	400	*	4	2.26473E-11	4.88111E-05	0.02174199	0.000260488	0.181212724	0.153410995	ZNF423
chr6	163149320	163149453	134	*	2	1.76558E-07	0.021279704	0.021747586	0.021278983	-0.129379819	-0.10394104	PACRG
chr11	1953661	1953846	186	*	2	4.36545E-08	0.002297003	0.021764017	0.004322044	0.178345882	0.13665217	TNNT3
chr19	44506373	44507379	1007	*	14	4.5939E-14	0.007197229	0.021798215	0.002063874	0.093847838	0.000468048	ZNF230, RP11-15A1.7
chr3	136580640	136581035	396	*	8	7.64403E-11	0.02444831	0.021847385	0.008614315	0.023810304	0.010335832	RP11-85F14.5
chr6	26757644	26758395	752	*	5	3.06737E-08	0.002146112	0.021849144	0.003699314	0.08433808	0.050322819	

chr1	14075362	14076293	932	*	13	3.32391E-15	0.007063225	0.021863761	0.001364572	-0.029399603	-0.002125721	PRDM2
chr2	45231782	45232117	336	*	3	1.873E-08	0.004829284	0.021874105	0.00670041	0.03485589	0.022779503	
chr19	48112407	48112503	97	*	3	1.25819E-07	0.003841827	0.021875303	0.00649065	0.020715675	0.015131545	CTD-2571L23.8, GLTSCR1
chr3	149529930	149530987	1058	*	15	1.46125E-16	0.01119332	0.021882072	0.002236295	0.168427447	0.03497743	RNF13, ANKUB1
chr1	3528355	3529108	754	*	5	7.32237E-10	0.015915314	0.021882482	0.008241203	0.067048017	0.007214411	
chr20	3775618	3776988	1371	*	17	1.0283E-14	0.01948187	0.021902893	0.00572557	0.068151153	0.008410236	CDC25B
chr19	4066100	4067604	1505	*	15	6.52185E-13	0.117022739	0.021910719	0.004450162	-0.061616451	-0.023100553	ZBTB7A
chr2	169312172	169312933	762	*	10	1.57369E-10	0.259506893	0.021980746	0.015249118	0.048097372	0.000450422	CERS6
chr19	48547158	48547362	205	*	4	8.22719E-09	0.002911331	0.021988111	0.005823983	0.127581955	0.099306226	CABP5
chr1	249152821	249154285	1465	*	13	2.68751E-15	0.007534935	0.021991702	0.000834259	-0.144278193	-0.024835586	AL672294.1, ZNF692
chr19	4471492	4472531	1040	*	11	1.83205E-13	0.004255234	0.02201035	0.00338323	-0.040908287	-0.01116238	HDGFRP2
chr3	14443428	14443694	267	*	5	4.75739E-10	0.002106358	0.022026434	0.004860334	0.103597074	0.057545332	
chr8	57359908	57360226	319	*	4	3.04377E-09	0.031322001	0.022030234	0.013809526	0.132782435	0.079471087	RP11-17A4.2
chr3	39233997	39234148	152	*	3	9.04121E-09	0.000610695	0.022043745	0.001748646	0.105162887	0.077048255	XIRP1
chr9	139871632	139872395	764	*	6	3.86363E-08	0.091772828	0.022051736	0.042239368	-0.185648551	-0.031222214	PTGDS
chr17	74379499	74381784	2286	*	16	2.56329E-13	0.021386253	0.022054308	0.001759653	-0.132253049	-0.012360809	SPHK1, PRPSAP1
chr19	19516893	19517029	137	*	4	1.32243E-08	0.001806067	0.022055463	0.00349315	0.036284455	0.008011907	GATAD2A
chr9	33263879	33265144	1266	*	15	3.47657E-14	0.173743756	0.022067733	0.009135341	-0.096016081	-0.020642405	CHMP5, BAG1
chr4	15375625	15375866	242	*	3	9.26476E-08	0.024191649	0.022090463	0.013550565	0.076721608	0.049863666	C1QTNF7, RP11-665G4.1, RP11-484O2.1
chr22	19701734	19702664	931	*	12	9.96161E-11	0.072302223	0.022093934	0.01943577	0.04038289	7.13499E-07	SEPT5
chr4	2935996	2937381	1386	*	18	1.37548E-17	0.057285869	0.022124668	0.002983451	0.027506675	-0.000444907	NOP14-AS1, MFSD10
chr22	38349045	38349943	899	*	16	4.34942E-10	0.465599445	0.022133175	0.154322798	-0.029104037	-0.000576148	POLR2F, C22orf23
chr19	17566260	17566626	367	*	4	1.68232E-08	0.000642347	0.022153249	0.002033723	0.074151754	0.062476717	CTD-2521M24.10, NXNL1
chr14	54976505	54977199	695	*	8	9.88371E-11	0.103419443	0.022186708	0.019775567	-0.064946245	-0.002008549	CGRRF1
chr5	132165899	132166725	827	*	6	1.98277E-10	0.002477694	0.02221314	0.004137331	0.214672668	0.044611836	SHROOM1
chr5	96298581	96299144	564	*	4	4.38804E-10	0.000278048	0.022222548	0.000806343	0.137203726	0.090522656	LNPEP
chr11	118084192	118085281	1090	*	5	5.33899E-10	0.042861625	0.022235535	0.014925569	-0.104186555	-0.043657972	AMICA1
chr17	5342414	5343211	798	*	9	5.44869E-10	0.05197559	0.022257926	0.040463839	0.065540278	0.01498656	C1QBP
chr20	30406052	30407098	1047	*	9	1.33598E-13	0.016655976	0.022274066	0.002776288	-0.208333576	-0.057478219	

chr7	36428656	36429476	821	*	6	2.6931E-10	0.046806839	0.022317557	0.031395163	-0.061049854	-0.021119007	ANLN, KIAA0895
chr15	90437671	90437891	221	*	5	4.22187E-08	0.0525688	0.022318567	0.010029561	0.050253227	0.014332816	C15orf38-AP3S2
chr10	3827200	3827321	122	*	2	1.70684E-07	0.007547951	0.022326337	0.010884776	0.039834492	0.025033078	KLF6
chr5	86415878	86415900	23	*	2	1.24978E-07	0.00398399	0.022346482	0.006752659	0.099872487	0.077682675	RP11-72L22.1
chr12	105626788	105627446	659	*	5	6.28021E-08	0.009900814	0.022374207	0.00956949	0.076952164	0.039739634	APPL2
chr8	128519635	128520078	444	*	3	4.93181E-09	0.00025813	0.022374667	0.000877304	0.107566574	0.091447309	
chr17	76126032	76126886	855	*	7	3.84499E-14	9.6848E-05	0.022387304	0.000289679	-0.109218962	-0.012311075	TMC8, TMC6
chr16	71894168	71894738	571	*	5	1.65895E-09	0.223625037	0.022409933	0.028539842	0.111444585	0.044426628	ATXN1L, IST1, ZNF821
chr15	41061384	41062224	841	*	10	1.68762E-22	7.10873E-09	0.022414689	4.0368E-07	-0.201281578	-0.100315948	C15orf62, DNAJC17
chr11	108092276	108094111	1836	*	39	8.38568E-19	0.272192588	0.022417049	0.077847265	-0.059922475	-0.008232167	ATM, NPAT
chr6	167040440	167041649	1210	*	10	5.5406E-16	4.57123E-05	0.022425329	0.000142579	-0.108770777	-0.017104325	RPS6KA2
chr7	148725440	148726390	951	*	9	8.02613E-11	0.001678849	0.022436716	0.002528744	0.028882725	0.005231752	PDIA4
chr13	41239125	41239152	28	*	2	8.7729E-08	0.051423995	0.022483882	0.034632138	-0.012530163	-0.009960941	FOXO1
chr17	37322028	37322540	513	*	5	5.74596E-10	0.001426712	0.022500308	0.002452297	-0.204118446	-0.105185867	ARL5C
chr7	21467390	21467723	334	*	4	3.76863E-09	0.005909909	0.022501824	0.004271429	-0.073848732	-0.011445807	SP4
chr11	93582929	93583973	1045	*	10	1.58411E-09	0.102324198	0.022525049	0.017817322	0.09722615	0.028319048	VSTM5
chr11	13299888	13300784	897	*	5	2.52384E-09	0.005747879	0.022536198	0.008186544	0.046321898	0.026368211	ARNTL
chr22	38516736	38516895	160	*	3	1.46102E-08	0.000762558	0.022544755	0.001961278	0.113742404	0.065086189	PLA2G6
chr1	159893182	159894416	1235	*	12	5.96694E-11	0.12526144	0.022558562	0.043056215	-0.069875705	-0.01955373	TAGLN2
chr14	105955879	105958196	2318	*	22	1.09627E-19	0.057513648	0.022596501	0.002387519	-0.037147756	-0.002100638	CRIP1, C14orf80
chr22	31477504	31477754	251	*	5	5.33738E-09	0.062968038	0.022619318	0.022859923	0.047780446	0.022796213	SMTN
chr16	75656489	75656847	359	*	3	3.59308E-08	0.018380314	0.02262581	0.016230853	0.024516789	0.011919566	ADAT1
chr1	155829430	155829652	223	*	3	9.63804E-09	0.008266941	0.02262881	0.006908293	-0.097891932	-0.023361105	SYT11
chr11	108422663	108423325	663	*	8	1.98509E-13	0.001149407	0.022645263	0.000671549	0.142495851	0.062296313	EXPH5
chr17	62207605	62208434	830	*	7	2.16669E-13	0.01067868	0.022650901	0.001873382	0.139102131	0.043571015	ERN1
chr11	82444798	82445384	587	*	8	1.10384E-09	0.025798984	0.022710254	0.014889944	0.112010143	0.033558615	FAM181B
chr3	51422103	51423030	928	*	13	2.3807E-11	0.143423265	0.02271464	0.021296416	-0.034242412	-0.002199982	MANF
chr1	10459573	10459695	123	*	2	4.14192E-08	0.003215557	0.022721056	0.00573244	0.018037313	0.016602153	PGD
chr1	3585225	3585587	363	*	5	4.27798E-11	0.000209305	0.022744707	0.000530753	-0.068544523	-0.058655136	TP73, RP5-1092A11.5

chr2	11533716	11533970	255	*	5	1.35991E-09	0.000711659	0.022770151	0.001700359	0.147944943	0.090818168	
chr11	64322994	64323456	463	*	7	3.97679E-11	0.004451849	0.022776976	0.002219682	0.143447026	0.067435751	SLC22A11
chr21	46340351	46341918	1568	*	12	4.53695E-18	1.14917E-05	0.02279585	6.57876E-05	-0.195127263	-0.081563881	ITGB2-AS1, ITGB2
chr5	150603302	150605144	1843	*	14	4.54189E-09	0.061438262	0.022802633	0.006473626	-0.113173447	-0.005930931	GM2A, CCDC69
chr1	161474586	161475240	655	*	5	1.39415E-09	0.003664709	0.022817781	0.003048621	-0.104653416	-0.015039835	FCGR2A
chr13	73301012	73301231	220	*	5	7.35006E-09	0.01086408	0.022830153	0.016557492	0.022558007	0.0112136	MZT1
chr6	5004148	5004599	452	*	8	4.08979E-08	0.164892763	0.02283057	0.038977267	-0.020782838	0.003921563	RPP40
chr14	61655727	61655919	193	*	4	1.76158E-09	0.000298957	0.022838505	0.001124829	-0.08849398	-0.002441393	PRKCH
chr19	10362061	10363328	1268	*	12	6.80092E-12	0.000813032	0.022851764	0.003441009	-0.032216535	-0.008289126	MRPL4, CTD-2369P2.5
chr2	27434612	27435915	1304	*	21	3.19102E-16	0.130666821	0.02286611	0.020598944	-0.043477484	-0.00842561	ATRAID, SLC5A6
chr19	56904442	56905587	1146	*	14	2.27338E-16	0.003421588	0.02287242	0.00122193	0.109107936	0.005001592	ZNF582-AS1, ZNF582
chr17	75437512	75438188	677	*	5	9.38478E-11	0.000377647	0.022880125	0.001045466	-0.184199918	-0.073171618	SEPT9
chr19	6503884	6504030	147	*	3	7.32209E-10	0.000343957	0.022923886	0.001114533	0.149708825	0.100282599	
chr17	66196975	66197219	245	*	4	2.3172E-09	0.000492288	0.022990811	0.001417402	0.02598743	0.017774875	
chr1	153606178	153606818	641	*	10	5.81329E-09	0.041999755	0.022997441	0.0335267	-0.046212444	-0.01430369	CHTOP, S100A13, RP1-178F15.5, RP1-178F15.4
chr12	10095902	10096794	893	*	6	1.22792E-13	3.84775E-06	0.022997467	4.21336E-05	-0.207362199	-0.159289841	AC091814.2
chr7	87505059	87506111	1053	*	15	1.11823E-14	0.025321317	0.023023382	0.005865508	-0.036665617	-0.000234721	DBF4, SLC25A40
chr17	54857463	54858161	699	*	6	5.55122E-09	0.005862167	0.023023543	0.007118251	-0.035086678	-0.002175167	
chr19	2427407	2428350	944	*	8	1.96785E-09	0.158428481	0.023027879	0.028692579	-0.02086862	-0.001451516	TIMM13, LMNB2
chr5	75919188	75919481	294	*	5	2.71604E-09	0.00983761	0.023050092	0.005031331	-0.087663925	-0.049032676	IQGAP2, F2RL2
chr7	129410417	129411088	672	*	7	4.70851E-12	0.000142768	0.023060536	0.000645902	0.114775581	0.074489486	
chr6	44309765	44310331	567	*	8	8.05969E-10	0.016162862	0.023081391	0.016246106	-0.078815508	-0.026914356	RP11-444E17.6
chr15	75940069	75940778	710	*	8	5.45124E-09	0.113582242	0.023100573	0.052388912	0.025419017	-0.000437189	SNX33, IMP3
chr2	38831166	38831576	411	*	2	1.64051E-08	0.00636175	0.023131986	0.009732105	-0.169991368	-0.114380284	
chr4	77870047	77870605	559	*	3	1.96173E-08	0.03182976	0.02315509	0.013016219	-0.056712965	-0.015475698	
chr6	53409368	53410182	815	*	11	1.05263E-10	0.130593097	0.023169436	0.031124484	0.058832106	0.009325454	GCLC
chr16	57831745	57832760	1016	*	11	2.34535E-18	3.5675E-05	0.023187187	3.02607E-05	0.144311558	0.0904957	CTD-2600O9.1, KIFC3
chr8	142137922	142139341	1420	*	12	3.8273E-12	0.001313332	0.023188509	0.000638336	-0.031020574	0.004086644	DENND3, RP11-809O17.1
chr17	79848761	79850075	1315	*	17	7.37665E-13	0.163810327	0.023210287	0.027012706	0.036631482	0.002559322	ANAPC11, ALYREF

chr2	110371114	110371903	790	*	13	2.96426E-10	0.292315448	0.023245716	0.079814094	-0.054490065	-0.006324762	SEPT10
chr1	202775609	202776513	905	*	7	1.99046E-12	0.003665549	0.023257169	0.001177815	-0.043820393	-0.014582006	KDM5B
chr6	99275622	99275960	339	*	3	1.06603E-08	0.034547648	0.02327116	0.029366418	0.037571864	0.026393767	
chr3	127311038	127311169	132	*	3	3.96663E-09	0.002083592	0.023275862	0.004031004	-0.090769179	-0.073098093	TPRA1
chr1	41156520	41157111	592	*	7	2.5895E-10	0.171283141	0.023290969	0.029186232	0.141828331	0.045554629	NFYC-AS1
chr1	150459835	150459957	123	*	3	1.87012E-07	0.015957642	0.02330037	0.008477952	-0.05155371	-0.03144056	TARS2
chr7	47344579	47344750	172	*	4	7.65549E-09	0.001225401	0.023301995	0.002466457	0.153576073	0.088367928	TNS3
chr12	58145750	58146835	1086	*	20	9.85468E-12	0.064304733	0.023305582	0.05307899	-0.040994532	0.002231368	CDK4
chr3	49057935	49059487	1553	*	19	1.45802E-15	0.047816808	0.023311016	0.014725299	-0.038570893	-0.002843187	NDUFAF3, DALRD3, MIR191
chr4	141071988	141072046	59	*	2	7.6462E-08	0.002709778	0.023325316	0.005030081	-0.028578853	-0.009191132	MAML3
chr9	110045901	110046355	455	*	5	9.67088E-10	0.01577423	0.023326426	0.005658569	0.023813859	0.013591615	RAD23B
chr11	123984775	123984852	78	*	2	3.56515E-08	0.004741574	0.023336342	0.007824146	0.092363899	0.09066528	
chr8	144639260	144640556	1297	*	13	1.03451E-14	0.001797543	0.023342147	0.000158507	-0.133890565	-0.017391294	GSDMD
chr17	3433157	3433451	295	*	3	9.89019E-08	0.05168758	0.023362776	0.010274601	0.1445982	0.09285362	TRPV3
chr16	2015448	2015450	3	*	2	9.8024E-08	0.002882369	0.023368907	0.005290317	-0.078861582	-0.063306023	SNHG9
chr12	12869398	12871110	1713	*	19	2.21788E-17	0.018058468	0.023370395	0.003268663	-0.11819539	-0.015027604	CDKN1B
chr6	154799632	154799716	85	*	3	1.78593E-07	0.002216123	0.023371593	0.00382612	-0.127894654	-0.078544119	CNKSR3
chr6	11093513	11094883	1371	*	15	7.03388E-16	0.000246011	0.023380265	0.000783952	0.032237273	0.004059966	SMIM13
chr3	185270308	185270558	251	*	4	5.89423E-10	0.003897228	0.023436042	0.002340467	0.14084731	0.08590591	LIPH
chr19	7983606	7984171	566	*	7	1.1858E-09	0.029066746	0.0234515	0.01270221	0.14422192	0.041948025	TGFBR3L, CTD-3193O13.1
chr17	73874443	73874790	348	*	7	6.28497E-10	0.027365883	0.023456086	0.010183533	0.037639668	0.015227967	RP11-552F3.9, TRIM47
chr6	43737555	43738026	472	*	6	4.62849E-11	0.010415346	0.023464782	0.007624799	-0.066359131	-0.004384041	VEGFA
chr1	18902086	18902229	144	*	3	7.06919E-08	0.000729337	0.023495751	0.002028699	0.24698913	0.215953295	
chr14	65230287	65231319	1033	*	5	2.38012E-10	0.000612994	0.023521224	0.000673587	-0.116179715	-0.071692481	SPTB
chr1	100503189	100504499	1311	*	13	1.56444E-13	0.020626483	0.023563562	0.008853013	0.040530378	0.008699753	HIAT1
chr16	71523144	71524135	992	*	11	2.89599E-19	1.30447E-05	0.02356675	2.27884E-05	0.169742659	0.073303852	AC010547.9, ZNF19
chr6	110011968	110012723	756	*	15	1.01612E-09	0.484068325	0.023603511	0.181127755	-0.057762279	-0.010243121	FIG4, AK9
chr15	62352174	62353290	1117	*	13	2.97063E-12	0.018361579	0.023607076	0.008798955	-0.030595939	0.005034178	RP11-643M14.1, VPS13C
chr6	28557823	28557883	61	*	3	1.75058E-07	0.004021155	0.023629022	0.00629217	0.043064806	0.032068635	RP5-1186N24.3, SCAND3

chr21	46830529	46830534	6	*	2	2.29121E-08	0.002926875	0.023709095	0.005376024	0.10078887	0.098986156	COL18A1
chr12	4488749	4489779	1031	*	9	3.57591E-15	0.00010097	0.023724341	0.000212998	0.188500259	0.085492396	FGF23
chr7	148581708	148582347	640	*	5	8.02549E-11	0.00839821	0.023744801	0.002963276	0.092560121	-0.001013329	
chr13	114126424	114126476	53	*	3	9.54406E-08	0.024230823	0.023769344	0.009439084	-0.072432988	-0.037576939	DCUN1D2-AS, DCUN1D2
chr20	23343673	23344170	498	*	4	3.22469E-08	0.003912056	0.023832173	0.004395823	0.072720899	0.050285735	GZF1
chr17	79196677	79196758	82	*	4	1.81611E-07	0.00359214	0.0238549	0.005504041	0.034970461	0.025223967	AZI1
chr2	101925327	101925483	157	*	5	1.46646E-07	0.01198981	0.023869998	0.013768307	-0.021567822	0.004142858	
chr16	23652268	23653174	907	*	12	3.62099E-09	0.144627595	0.023909552	0.053529011	0.031628646	0.002611964	DCTN5, PALB2
chr17	41977951	41978218	268	*	7	5.90059E-08	0.02462521	0.023931787	0.016908672	-0.035001874	-0.004822823	MPP2
chr17	56356470	56356963	494	*	3	1.48048E-10	0.001425774	0.023963127	0.003433733	-0.151805068	-0.089119543	MPO
chr1	26735846	26736154	309	*	6	1.04419E-07	0.162323018	0.02396347	0.061067095	0.018488754	0.012364373	
chr22	41215939	41216528	590	*	5	1.36768E-10	0.005471274	0.023974819	0.002784569	-0.101914746	-0.020013395	
chr17	78417789	78418426	638	*	7	1.70663E-11	0.015924222	0.024006054	0.002598635	0.144080611	0.08381934	
chr11	62104220	62105152	933	*	8	4.47304E-12	0.05177045	0.024007257	0.00866878	-0.174298593	-0.03803784	ASRGL1, RP11-703H8.7
chr4	139937227	139937474	248	*	4	3.4805E-09	0.034445725	0.02401299	0.012928519	0.033113557	0.016928789	CCRN4L
chr6	31782374	31783468	1095	*	23	9.14557E-12	0.655717864	0.02401369	0.280395924	-0.046378345	-0.00362978	HSPA1A, HSPA1L
chr2	26100811	26101831	1021	*	11	6.291E-12	0.028351102	0.024026202	0.007170627	0.060702257	0.014008049	ASXL2
chr19	38825649	38827205	1557	*	12	4.08563E-16	0.000840244	0.024031773	0.00016447	-0.114444891	-0.028428339	CATSPERG
chr2	27712243	27713158	916	*	16	5.98749E-14	0.008997483	0.024060836	0.006272686	0.158796643	0.029873775	IFT172
chr8	135725535	135726252	718	*	5	7.28358E-11	0.000948348	0.024066884	0.001671157	0.173133378	0.035103369	
chr19	10449942	10450472	531	*	8	1.99123E-10	0.429124972	0.024098551	0.070375302	-0.189154995	-0.026171371	ICAM3
chr2	219745647	219745748	102	*	2	1.19009E-08	0.002924951	0.024172093	0.005399722	0.082097852	0.05120285	WNT10A
chr10	17270232	17271079	848	*	11	2.09487E-10	0.14459587	0.024196218	0.037112819	-0.053290212	-0.011342293	VIM, VIM-AS1
chr16	71660734	71660786	53	*	2	6.88201E-08	0.00272583	0.024213674	0.005101513	0.046935443	0.030804625	MARVELD3
chr4	77226417	77227162	746	*	6	6.29284E-11	0.002249164	0.024252653	0.003670458	-0.039861977	-0.023460532	FAM47E-STBD1, FAM47E-STBD1
chr5	179233770	179233875	106	*	4	1.94941E-07	0.166538963	0.024257346	0.060369663	-0.028828468	-0.008757199	SQSTM1, MGAT4B
chr16	70780647	70780947	301	*	4	2.40699E-10	9.50213E-05	0.02427347	0.000447445	-0.129250172	-0.086037181	VAC14
chr6	33280390	33282624	2235	*	43	5.92421E-19	0.194222064	0.024273681	0.060830133	-0.042519794	-0.002217683	TAPBP, ZBTB22
chr5	139078511	139078529	19	*	2	9.55132E-08	0.003127012	0.024291579	0.005705943	0.092430085	0.086671628	CTB-35F21.1

chr5	176560594	176561193	600	*	4	6.76036E-09	0.147112596	0.024303999	0.031737687	-0.011050409	-0.002091442	NSD1
chr16	30485296	30485966	671	*	7	9.15503E-13	0.000755489	0.024328743	0.001049025	-0.243240746	-0.019724695	ITGAL
chr12	54120876	54122179	1304	*	14	4.42951E-13	0.011602992	0.024330173	0.009258067	-0.064614374	-0.008363049	CALCOCO1
chr1	22109860	22111043	1184	*	8	8.43926E-11	0.001455775	0.024340094	0.004529895	0.188077006	0.021849748	USP48
chr8	11659727	11660968	1242	*	18	3.80724E-14	0.021870823	0.024360716	0.015009845	0.032819808	0.001754507	FDFT1, RP11-297N6.4
chr17	74366059	74366578	520	*	3	2.19065E-08	0.000369507	0.024390581	0.001201974	0.089480476	0.082766835	PRPSAP1
chr12	109250445	109250988	544	*	5	9.13459E-09	0.005027874	0.024497151	0.009083934	-0.05995918	-0.027345204	SSH1
chr5	176738672	176739172	501	*	6	5.01954E-11	0.022760579	0.02451327	0.00454269	0.045513426	-0.001027129	MXD3
chr1	207242569	207242586	18	*	2	2.64164E-08	0.003723269	0.024516668	0.006574889	-0.146143397	-0.13994855	PFKFB2
chr4	106816089	106816136	48	*	2	7.08178E-08	0.005297003	0.02453493	0.008647623	-0.075069972	-0.017527704	NPNT, INTS12
chr7	43909588	43909721	134	*	3	1.74898E-08	0.015260616	0.02456378	0.019215252	-0.034960417	-0.015133035	URGCP-MRPS24
chr3	183902771	183903567	797	*	6	1.38456E-09	0.045819829	0.024597993	0.035198266	0.105014689	0.048926941	EIF2B5
chr1	150995449	150995772	324	*	3	1.85443E-08	0.000491051	0.024601262	0.001510342	0.099576019	0.072575167	PRUNE
chr9	116355097	116355621	525	*	4	3.41581E-09	0.000585779	0.024602538	0.001990699	0.093309106	0.053436904	RGS3
chr17	46811492	46811527	36	*	2	3.41782E-08	0.009241323	0.024611272	0.013030747	0.024160526	0.021954609	
chr12	113909234	113909718	485	*	3	2.62169E-08	0.020315747	0.024620166	0.021698115	0.076319808	0.03374434	LHX5
chr1	31769247	31770205	959	*	14	2.55337E-12	0.03979762	0.024636878	0.007711961	0.031309741	-0.002467296	ZCCHC17, SNRNP40
chr17	66031745	66031871	127	*	8	1.11426E-07	0.048216962	0.024640421	0.028484716	-0.059774493	-0.00060633	KPNA2
chr4	54929912	54931210	1299	*	9	5.53609E-13	0.0005949	0.024664331	0.000721681	-0.05673859	-0.006745628	FIP1L1, CHIC2
chr17	38443649	38443899	251	*	5	4.477E-08	0.088102485	0.024666453	0.037785713	-0.053887645	-0.013080944	CDC6
chr9	127961717	127961907	191	*	3	3.34057E-08	0.006409341	0.024668896	0.008755906	0.122678875	0.090455051	
chr5	176816539	176816828	290	*	4	1.20817E-07	0.003669869	0.024697451	0.007189937	0.090811509	0.061065875	SLC34A1
chr11	18034355	18035136	782	*	13	7.45144E-08	0.585693722	0.024798683	0.140245121	0.042107412	-0.0018771	SERGEF, RP1-59M18.2
chr18	12883603	12884567	965	*	10	1.3873E-14	0.000668411	0.024817922	0.001539894	0.024320598	0.003385986	PTPN2
chr12	124117938	124119250	1313	*	18	1.9238E-11	0.322689719	0.024887277	0.064871768	-0.147893561	-0.004803416	GTF2H3, EIF2B1
chr19	10735474	10736448	975	*	10	6.10457E-15	0.008024115	0.024893859	0.001698156	-0.129607833	-0.040603107	SLC44A2
chr10	126479779	126480372	594	*	4	1.08804E-08	0.013632323	0.024938407	0.013285512	-0.052553027	-0.018061817	RP11-12J10.3, METTL10
chr6	10881782	10881949	168	*	4	7.24552E-08	0.063687907	0.02497657	0.011469754	0.040913199	0.025945046	RP11-637O19.3, SYCP2L, GCM2
chr6	35887678	35888885	1208	*	8	6.88131E-13	0.007352203	0.025005644	0.005112391	-0.049048658	-0.016081649	SRPK1

chr19	38041156	38041600	445	*	3	4.176E-09	0.003678982	0.025031102	0.007093354	-0.043813533	-0.010276261	ZNF571-AS1
chr12	22696567	22697784	1218	*	15	1.10335E-16	5.08431E-05	0.025038955	0.00021106	0.101800094	0.017228507	C2CD5
chr10	28821963	28822500	538	*	11	4.25707E-10	0.009974891	0.025083249	0.016760689	-0.0845419	-0.010308483	WAC
chr2	219433334	219434220	887	*	10	1.23556E-09	0.020337237	0.025087361	0.014533743	0.023626543	0.003891392	RQCD1
chr3	184870137	184871070	934	*	11	2.86945E-14	0.018031621	0.025087632	0.003977866	-0.05795972	-0.011013325	C3orf70
chr7	150777171	150778724	1554	*	14	2.44887E-16	0.005177379	0.025100704	0.001420491	0.079197238	-0.003791485	FASTK, TMUB1
chr2	220093858	220095334	1477	*	21	8.54832E-16	0.02094002	0.025118419	0.007306338	-0.077191219	-0.001798693	ANKZF1, ATG9A
chr13	60737991	60738538	548	*	13	1.23247E-08	0.281460718	0.025136414	0.139108863	0.027995202	0.002163232	DIAPH3
chr11	15962841	15963167	327	*	5	8.36794E-09	0.020604266	0.025148701	0.005800442	0.14413943	0.075084839	
chr6	30709428	30713026	3599	*	71	2.96531E-32	0.001096091	0.025156901	0.000654411	0.066262125	-0.002142382	XXbac-BPG252P9.10, FLOT1, IER3
chr14	24610792	24611268	477	*	11	2.83107E-08	0.100206006	0.02516501	0.063778077	0.029493465	0.006031591	EMC9
chr16	87886582	87886933	352	*	4	3.61525E-08	0.007854986	0.025171546	0.006742015	0.114540422	0.017968467	SLC7A5
chr1	1850263	1851910	1648	*	21	9.55684E-15	0.000635747	0.025189136	0.001464493	-0.102544116	-0.017420832	TMEM52
chr6	2876395	2877211	817	*	6	1.54368E-10	0.003267818	0.025207997	0.002776268	0.090827025	-0.000533369	RP11-420G6.4
chr22	31835691	31836332	642	*	5	1.01669E-09	0.007882668	0.025212685	0.00359531	0.138328372	0.076536628	DRG1, EIF4ENIF1
chr1	207992084	207992469	386	*	3	9.59224E-08	0.025869387	0.025257753	0.024474885	0.121138182	0.056121386	C1orf132
chr19	10514249	10514734	486	*	8	9.62932E-10	0.037272799	0.025336481	0.020827709	-0.026231357	0.000547615	CDC37
chr6	28543508	28543693	186	*	4	7.04036E-09	0.002480062	0.025356572	0.004329635	0.101480649	0.066539671	SCAND3
chr8	124214953	124215152	200	*	4	1.00188E-07	0.00495038	0.025389045	0.008045769	0.153665688	0.090756372	FAM83A, FAM83A-AS1
chr18	9334488	9335102	615	*	8	5.6059E-11	0.001962924	0.0254068	0.003692781	-0.082381622	-0.042777685	TWSG1
chr2	95537197	95537475	279	*	4	1.10725E-08	0.013199588	0.02547257	0.007604622	0.148951596	0.097312246	TEKT4, AC097374.2
chr2	68693636	68695071	1436	*	12	1.25269E-12	0.001595318	0.025493278	0.00207833	-0.208218855	-0.027881099	APLF, FBXO48
chr2	87018585	87019492	908	*	8	7.34809E-11	0.013657001	0.025496821	0.008596603	0.128671179	0.052248415	CD8A
chr17	57918262	57918682	421	*	5	7.16167E-11	0.000105785	0.02550976	0.000437771	0.094341342	0.075689693	VMP1, MIR21
chr11	85375464	85375937	474	*	3	2.64106E-08	0.033610969	0.025510194	0.018342117	0.03307017	0.016708574	CREBZF
chr14	99736462	99737609	1148	*	8	6.14686E-11	0.097072197	0.025517404	0.013516601	0.047606812	0.014181093	BCL11B
chr16	11723091	11724177	1087	*	5	1.75909E-09	0.000113792	0.02552022	0.000514225	-0.16255214	-0.100451894	LITAF
chr5	122180749	122181105	357	*	8	9.40292E-08	0.171441885	0.025543494	0.064971147	0.017987753	0.001711305	SNX24
chr3	71113568	71114771	1204	*	9	1.18058E-13	0.055827208	0.025577354	0.004623401	-0.13796132	-0.008414491	FOXP1

chr10	127512596	127512887	292	*	2	7.89289E-08	0.036342838	0.025600272	0.031308488	0.023408209	0.02041401	BCCIP
chr7	30027454	30028890	1437	*	7	1.06266E-11	0.002964566	0.025611191	0.001950979	-0.093215554	-0.036365125	AC007285.6, SCRN1
chr4	668354	668877	524	*	3	6.78251E-09	0.289003573	0.025616162	0.037769908	0.057169922	0.03074097	MYL5
chr6	30749279	30749628	350	*	4	4.65005E-09	0.00193116	0.025627426	0.002228577	-0.048322603	-0.034274332	HCG20
chr12	122617356	122617908	553	*	6	8.79403E-09	0.179444315	0.025633511	0.026745657	0.140907937	0.042437628	MLXIP
chr19	39340777	39341449	673	*	5	4.5921E-10	0.06272604	0.02564914	0.026398832	0.020020536	0.007653758	HNRNPL
chr3	53925831	53925981	151	*	3	1.39178E-07	0.073078436	0.025691462	0.039820026	0.031709527	0.005144164	SELK
chr17	38135812	38137489	1678	*	16	9.01377E-18	0.000257884	0.025694794	0.000165491	0.16455383	0.009833856	PSMD3
chr11	789849	790293	445	*	8	8.21059E-12	0.002826678	0.025705606	0.003036346	0.111837415	0.02591746	CEND1
chr5	55290639	55291546	908	*	13	2.19793E-13	0.012736353	0.025710361	0.005787347	-0.12142629	-0.016149417	CTD-2031P19.3, IL6ST
chr12	49372162	49373263	1102	*	8	2.33614E-11	0.001516492	0.025715742	0.003343057	0.060135544	0.025096068	WNT1
chr7	130130122	130132453	2332	*	50	8.6222E-40	6.43748E-05	0.025725114	8.98621E-06	-0.173755356	-0.05705639	MEST
chr16	4673918	4674018	101	*	4	8.8782E-08	0.008611555	0.025758655	0.013296663	-0.24890174	-0.097764889	MGRN1
chr16	9055493	9056115	623	*	7	1.5933E-09	0.147434133	0.025772178	0.018445902	0.135209345	0.036580376	USP7
chr14	102605829	102606609	781	*	12	1.53914E-15	0.001839803	0.025782753	0.001662914	-0.035380741	0.000693272	WDR20, HSP90AA1
chr4	76649539	76650351	813	*	11	1.46712E-11	0.094331001	0.025791787	0.022790274	-0.064581732	-0.008200262	USO1, G3BP2
chr6	24494605	24494793	189	*	4	3.9479E-08	0.026014865	0.025792214	0.016622663	0.077308802	0.048068932	GPLD1
chr6	32135129	32138839	3711	*	41	1.5381E-21	0.097872682	0.025799146	0.001793589	0.178859369	0.028384312	PPT2-EGFL8, EGFL8, AGPAT1
chr4	123653918	123654127	210	*	4	6.5311E-09	0.07160461	0.025817072	0.018091048	-0.043396084	-0.005019976	BBS12
chr17	75473344	75474070	727	*	6	5.7695E-12	0.000189732	0.025818133	0.000455396	0.119153216	0.079179633	SEPT9
chr10	125650957	125651726	770	*	11	9.80745E-10	0.031719731	0.025829304	0.027317846	-0.090134454	0.00089398	CPXM2
chr2	38602911	38603784	874	*	8	3.85903E-13	0.003934092	0.025862621	0.002507737	-0.045905535	0.0009408	ATL2
chr17	73257138	73257286	149	*	3	5.03109E-08	0.0186481	0.025880147	0.014754104	0.024519527	0.013965988	GGA3
chr6	28248578	28249704	1127	*	13	1.78628E-10	0.177406858	0.025881154	0.038428607	-0.041630973	0.004763624	PGBD1
chr1	94057247	94057587	341	*	4	4.89496E-09	0.000579353	0.025905569	0.001802151	-0.211965808	-0.083102718	BCAR3
chr16	1047460	1048154	695	*	6	3.33936E-11	0.003426631	0.025909289	0.0020138	0.098395068	0.066096342	RP11-161M6.3
chr3	101394920	101396021	1102	*	11	2.93808E-13	0.042699182	0.025916778	0.016066077	-0.051956385	-0.002667598	ZBTB11-AS1, ZBTB11
chr7	157646656	157647847	1192	*	8	2.38125E-15	0.000379083	0.025918221	0.000262546	-0.201335222	-0.106434273	AC011899.9, PTPRN2
chr17	8125848	8126456	609	*	4	9.89399E-09	0.005977874	0.02593137	0.007183531	0.028038417	0.014446964	LINC00324

chr16	88564247	88565080	834	*	4	1.08348E-10	0.00103869	0.025931371	0.00225575	0.119704911	0.103026549	ZFPM1
chr20	49573966	49575492	1527	*	16	2.49964E-11	0.006612205	0.025936185	0.003063529	-0.145921242	-0.019345269	MOCS3, DPM1
chr11	66529657	66530027	371	*	4	3.45212E-08	0.001074288	0.025983372	0.003218783	-0.099610886	-0.046881416	C11orf80, RP11-658F2.3
chr11	19733794	19735276	1483	*	13	2.30894E-20	0.000136271	0.025989595	6.03437E-05	0.021978649	0.008669499	NAV2, RP11-359E10.1
chr20	32582025	32582236	212	*	3	3.54886E-08	0.005732714	0.025991308	0.009532014	0.025196232	0.01467015	RALY, RP5-1125A11.1
chr11	1357820	1358151	332	*	2	6.3404E-08	0.005815053	0.025997722	0.009453072	0.030258421	0.006992906	
chr19	47951302	47951619	318	*	4	1.13446E-08	0.004115318	0.026005852	0.00419381	-0.120286442	-0.011192606	SLC8A2
chr1	43290363	43291390	1028	*	7	7.4867E-13	0.009585679	0.026028667	0.002133259	0.196261236	0.092347488	ERMAP
chr17	1552970	1553661	692	*	8	6.19158E-11	0.01647134	0.026048752	0.004930582	0.147271929	0.033369898	RILP
chr4	15004942	15006499	1558	*	11	6.72036E-13	0.001002969	0.026091365	0.001530856	-0.152368126	-0.021806807	CPEB2
chr3	194096808	194097221	414	*	4	2.29517E-09	0.001469532	0.026116698	0.001939559	0.110753615	0.077450505	
chr7	155436277	155437243	967	*	13	6.0388E-11	0.046246106	0.026133023	0.028070026	0.058985099	0.004205692	RBM33, AC009403.2
chr3	158519176	158520176	1001	*	13	6.54635E-10	0.20976489	0.026160311	0.098001824	-0.035869307	0.00407991	MFSD1, RP11-379F4.9
chr9	102582694	102584806	2113	*	10	5.41174E-11	0.000318449	0.026182821	0.000454074	-0.048746821	-0.007561901	NR4A3
chr1	214158727	214159589	863	*	5	1.14932E-09	0.003100942	0.026229598	0.00546698	0.076028857	0.033361856	PROX1, PROX1-AS1
chr1	16532990	16533537	548	*	5	1.00652E-09	0.043167065	0.026316727	0.016927739	-0.066420979	-0.019417436	ARHGEF19
chr6	41130718	41131213	496	*	5	2.31641E-10	0.01729451	0.026333554	0.005329513	-0.137877778	-0.069828212	TREM2
chr12	58013915	58013942	28	*	2	1.48271E-07	0.291499807	0.026349697	0.066669227	0.04374804	0.017456535	SLC26A10
chr11	34127281	34127291	11	*	2	1.82346E-07	0.00389289	0.026386832	0.006949602	0.016036716	0.011750606	NAT10
chr19	10676186	10678015	1830	*	12	3.29593E-17	4.82922E-07	0.026423866	3.20657E-06	0.15434004	0.025432529	KRI1, CDKN2D
chr18	13640835	13641894	1060	*	7	2.40607E-09	0.000627983	0.02643025	0.001047787	0.133346478	-0.037553808	LDLRAD4
chr10	99257608	99258985	1378	*	15	2.29374E-13	0.03588119	0.02643963	0.009900792	-0.09509433	-0.014308574	UBTD1, MMS19
chr7	4814154	4814294	141	*	2	6.8274E-08	0.003475041	0.026491254	0.006352788	0.100030271	0.085075363	
chr4	3742095	3742357	263	*	4	7.95197E-09	0.003381422	0.02649858	0.005131226	0.118237674	0.079620172	
chr1	154151169	154151980	812	*	5	4.4076E-12	4.44421E-05	0.026499929	0.000274348	0.175602388	0.108332701	TPM3
chr14	75594752	75594775	24	*	2	1.12235E-08	0.003165294	0.026579822	0.005895936	-0.149547688	-0.137331786	RP11-950C14.7
chr9	130497263	130498316	1054	*	10	3.10865E-16	0.001311709	0.026579907	0.00030808	0.083127455	-0.004861212	TOR2A
chr12	67663678	67664046	369	*	3	2.73165E-08	0.028240596	0.026583321	0.030331424	-0.033996174	-0.009573122	CAND1
chr10	103875961	103875969	9	*	2	1.87541E-07	0.003357883	0.026604592	0.006186325	-0.108421935	-0.102602947	LDB1

chr3	52864659	52864907	249	*	4	4.08144E-08	0.0288209	0.026608114	0.01096122	0.143523245	0.075474477	ITIH4, RP5-966M1.6
chr19	34111524	34112991	1468	*	12	4.88744E-16	0.00744559	0.026635552	0.001336122	0.146635098	0.063958491	CHST8
chr19	52097276	52097905	630	*	7	1.38151E-11	0.001907438	0.026653885	0.001682886	-0.042801083	0.002349043	AC018755.16, AC018755.1
chr14	97058964	97059467	504	*	6	2.695E-10	0.001662251	0.02665729	0.002513379	0.120722833	0.037543154	RP11-433J8.1
chr6	135375451	135375929	479	*	4	3.40745E-09	0.036699392	0.026662202	0.015252544	-0.047525343	-0.000265593	HBS1L
chr12	51631858	51633050	1193	*	21	1.15442E-24	2.02681E-05	0.026691133	2.60128E-05	-0.068410501	-0.007136493	DAZAP2
chr4	111532679	111533547	869	*	7	9.31766E-12	0.000885733	0.026698711	0.002371832	0.077380735	0.056366299	RP11-380D23.2
chr19	10444429	10444917	489	*	6	2.10696E-10	0.020243886	0.026732765	0.002716791	0.110134173	0.032668249	ICAM3
chr14	78227161	78227346	186	*	4	1.50347E-07	0.077163853	0.026749948	0.042427153	0.02781697	0.009219741	SLIRP, C14orf178, SNW1
chr14	95786621	95786663	43	*	2	5.84708E-08	0.011369034	0.026752323	0.015551433	0.114615519	0.076488465	
chr12	118572829	118573745	917	*	8	4.45261E-09	0.088739881	0.026755188	0.006186586	-0.034820674	-0.006535146	PEBP1, VSIG10
chr12	51784414	51784989	576	*	9	5.84887E-11	0.223487041	0.026757529	0.041632941	-0.127722732	-0.019655773	GALNT6
chr3	48470799	48471995	1197	*	11	6.93936E-14	0.000766229	0.026783044	0.000802401	-0.133489099	-0.034547589	PLXNB1
chr17	78851149	78851503	355	*	4	5.2164E-09	0.000419135	0.026791554	0.001509352	0.150339818	0.092397755	RPTOR
chr20	18447291	18448030	740	*	9	4.38796E-09	0.072737085	0.026793694	0.022729813	0.034071663	0.007959377	POLR3F, DZANK1
chr10	49892433	49893549	1117	*	16	3.1069E-20	0.000103026	0.02680823	7.76706E-05	-0.124729914	-0.042867935	WDFY4
chr11	44960854	44961359	506	*	3	4.29467E-08	0.000843736	0.02685169	0.002365349	-0.144496141	-0.108535105	TP53I11
chr11	48037488	48037919	432	*	3	1.25859E-07	0.00106239	0.026879542	0.0027222	-0.101193307	-0.038657568	PTPRJ
chr1	36042986	36043401	416	*	6	1.44644E-08	0.00804081	0.026904082	0.010054114	0.038472995	0.018251087	TFAP2E, RP4-728D4.2
chr14	59729610	59730159	550	*	7	2.44033E-14	1.69701E-05	0.026930801	6.40829E-05	0.162363401	0.090528678	DAAM1
chr6	31673800	31674897	1098	*	7	5.70237E-13	8.29462E-06	0.026933967	8.40557E-05	0.167826615	0.083557341	LY6G6F, MEGT1, XXbac-BPG32J3.20
chr17	7475958	7476167	210	*	6	3.97553E-08	0.355965192	0.026972157	0.045820865	0.020958082	0.010579426	SENP3-EIF4A1, EIF4A1, SNORA67
chr6	10383084	10385320	2237	*	14	3.96361E-11	0.000529104	0.026974134	0.000454631	-0.138658223	0.01910061	
chr19	18718675	18719091	417	*	4	3.91799E-10	0.003928351	0.026982487	0.005110111	0.070834864	0.037055366	TMEM59L
chr8	57905925	57906543	619	*	4	2.80308E-10	0.041881918	0.027037762	0.015044988	-0.053473326	0.000186642	IMPAD1
chr19	5719823	5720952	1130	*	17	9.38322E-12	0.246807537	0.027045906	0.047438961	0.066750044	0.008337256	CATSPERD, LONP1
chr14	21270407	21271485	1079	*	12	1.72259E-11	0.189250212	0.027053383	0.021343035	0.18466949	0.053650053	RNASE1
chr1	11322191	11322899	709	*	10	1.80936E-10	0.076099904	0.027062267	0.017915101	-0.050254996	-0.002220128	MTOR
chr19	45656942	45657270	329	*	4	2.08714E-09	0.000871439	0.027082516	0.002541954	-0.068964466	-0.014405124	MARK4, NKPD1

chr12	6716081	6716597	517	*	5	1.32131E-11	0.001014193	0.027087279	0.001567779	-0.119701413	-0.07213495	CHD4
chr6	42531521	42531902	382	*	11	1.17917E-10	0.145853949	0.027090396	0.024043222	-0.053401725	-0.017253136	UBR2
chr3	142682652	142683329	678	*	10	4.02166E-09	0.15619808	0.027092902	0.047793465	0.101950265	0.021397613	RP11-372E1.6
chr13	28554471	28555387	917	*	4	2.52848E-09	0.0002075	0.02714918	0.00086891	0.195592085	0.125408927	URAD
chr19	33166019	33166844	826	*	11	1.30809E-09	0.035702713	0.027158467	0.017231124	0.074668226	0.015543416	RGS9BP, ANKRD27
chr5	152871549	152871803	255	*	6	4.50429E-08	0.179622513	0.027167694	0.026430716	-0.166513854	-0.05384692	GRIA1
chr1	92012408	92012736	329	*	3	5.52084E-10	0.000449566	0.027177342	0.001460536	-0.150259018	-0.078909002	
chr4	778827	779173	347	*	3	3.02382E-08	0.000893637	0.027200203	0.002454186	0.202496084	0.140590211	CPLX1
chr1	153746588	153748120	1533	*	14	3.76382E-15	0.000217358	0.027240965	0.000734269	0.096637054	0.029592912	SLC27A3
chr2	67624207	67624930	724	*	10	2.96596E-10	0.099076912	0.027278686	0.021702477	-0.025895903	-0.000399832	ETAA1
chr6	142622429	142623767	1339	*	12	1.52816E-16	0.000320996	0.027281712	0.000195622	-0.097632659	-0.019315215	GPR126
chr1	1262717	1263239	523	*	4	3.19874E-09	0.00052005	0.027289019	0.001867783	0.092263008	-0.010339739	GLTPD1
chr1	20959826	20960434	609	*	9	2.18325E-08	0.186192721	0.027314326	0.068640596	0.036260705	0.008067724	PINK1
chr21	34497409	34497493	85	*	3	4.88394E-08	0.000512924	0.027381579	0.001627839	-0.139965042	-0.106872095	
chr6	33239127	33240471	1345	*	35	5.84094E-10	0.829085204	0.027422023	0.527998057	0.043537417	0.002710963	RPS18, VPS52
chr10	111767584	111768423	840	*	7	1.14287E-10	0.000917392	0.027439337	0.002025083	-0.06338921	-0.036712514	ADD3
chr16	89033692	89034292	601	*	5	5.43875E-10	0.001713834	0.027464989	0.003298485	-0.185718403	-0.118945603	CBFA2T3
chr19	41769376	41770635	1260	*	12	4.30656E-11	0.363697852	0.027468754	0.044142346	0.026357501	0.006082394	HNRNPUL1
chr12	94853345	94854469	1125	*	14	5.51745E-11	0.19924451	0.027511734	0.022735863	-0.069217992	-0.012332839	CCDC41
chr17	79790705	79791653	949	*	12	1.44672E-13	0.006604318	0.027512761	0.002348383	0.061496615	0.014770332	FAM195B, PPP1R27
chr9	34956991	34957576	586	*	5	2.55543E-09	0.00253754	0.027515616	0.006191649	-0.182980563	-0.070368519	KIAA1045
chr7	1545522	1546068	547	*	5	2.12084E-12	1.99179E-05	0.027524047	0.000158245	-0.147182026	-0.108789926	
chr6	44280781	44280859	79	*	2	1.17612E-07	0.003765732	0.027595521	0.00684889	-0.098787469	-0.036361992	RP11-444E17.6, AARS2
chr20	35374068	35374156	89	*	2	3.40239E-08	0.032590741	0.027622676	0.030685226	0.047375153	0.030957606	NDRG3
chr8	54935605	54936189	585	*	4	1.68058E-08	0.069664992	0.027681661	0.022409671	-0.106134301	-0.029746652	
chr19	39368345	39369067	723	*	4	9.96483E-11	0.351770704	0.027698019	0.027544883	0.074779286	0.035944289	RINL
chr11	88070661	88071409	749	*	10	3.57392E-09	0.175310441	0.027706418	0.043584543	0.017974784	0.004445079	CTSC
chr15	63340085	63342033	1949	*	20	2.64074E-16	0.00024198	0.02771229	0.000253756	0.062111259	0.007151545	TPM1, RP11-244F12.3
chr7	37389785	37390106	322	*	3	6.89237E-08	0.000605448	0.027745972	0.001866719	-0.081570921	-0.0571999	ELMO1

chr16	30365840	30367279	1440	*	17	2.78161E-13	0.30828235	0.027801469	0.032608684	-0.055435776	-0.001891281	RP11-347C12.10, CD2BP2
chr6	36515007	36515856	850	*	8	1.39808E-09	0.423645517	0.027804119	0.031577122	-0.128742538	-0.035671098	STK38
chr11	65625153	65628378	3226	*	28	1.5923E-17	0.001229287	0.027806984	0.000674399	-0.080235607	-0.004781444	MUS81, CFL1
chr17	1928740	1929555	816	*	5	4.81465E-10	0.000366747	0.027810091	0.001295984	-0.030785447	0.015164166	
chr7	150548765	150549472	708	*	8	4.67363E-11	0.001572761	0.027861317	0.002354041	-0.152836768	-0.062608378	AOC1
chr17	3847872	3848324	453	*	4	1.02568E-08	0.013716723	0.027865739	0.005497534	0.121219964	0.083482628	ATP2A3
chr7	93519855	93520566	712	*	16	2.67031E-10	0.034916495	0.027885975	0.050121674	-0.09321751	-0.016024895	GNGT1, AC002076.10, TFPI2
chr13	113951295	113951297	3	*	2	1.37549E-07	0.00365967	0.027905983	0.006712843	-0.045946156	-0.0372839	
chr1	110161760	110163645	1886	*	23	7.72377E-17	0.009985234	0.027915819	0.002252364	0.041890554	0.002861892	AMPD2
chr2	8723906	8724060	155	*	4	4.62978E-08	0.007200503	0.028032982	0.008046899	-0.077916033	-0.043278537	AC011747.4
chr12	132862024	132862110	87	*	4	1.37402E-07	0.003059607	0.028060999	0.005889419	0.146577936	0.084299305	GALNT9
chr10	102989551	102990026	476	*	4	2.67198E-08	0.018653734	0.02806453	0.016610384	0.031171379	0.020154322	LBX1-AS1, LBX1
chr10	27149349	27150662	1314	*	15	3.82769E-11	0.008806719	0.028084195	0.005078255	-0.191238904	-0.02060891	ABI1
chr4	100815442	100815781	340	*	6	4.22851E-09	0.005032293	0.028128883	0.00552126	-0.037247473	0.003773447	LAMTOR3
chr8	140715018	140715802	785	*	7	5.63839E-10	0.054093552	0.028136015	0.011674984	0.039039123	0.011250606	KCNK9
chr7	123264700	123265073	374	*	6	4.38411E-11	0.003386814	0.028140748	0.002249568	0.182133369	0.097948515	ASB15, RP11-390E23.3
chr6	133036545	133036651	107	*	3	2.14604E-08	0.000755119	0.028156997	0.002212063	0.069134804	0.055726557	
chr6	43214396	43214850	455	*	4	6.41791E-09	0.01924935	0.028191213	0.003319354	0.15089695	0.086658614	TTBK1
chr2	97425517	97426274	758	*	4	7.77991E-11	0.025833369	0.028196233	0.019039029	0.136881904	0.052506696	
chr9	124132714	124133556	843	*	9	1.42406E-12	0.067110241	0.028200205	0.047509101	0.119089269	0.035003815	
chr1	2460621	2461929	1309	*	13	2.76257E-16	0.000260335	0.028256553	0.000509724	-0.063376493	0.013184094	HES5
chr5	137667509	137668198	690	*	12	2.43452E-08	0.117557903	0.028341079	0.073760028	0.059269589	0.00759963	FAM53C, CDC25C
chr20	2853149	2853464	316	*	3	1.20657E-08	0.072976538	0.028346187	0.051455769	0.015966533	0.008308996	PTPRA
chr19	55770481	55771041	561	*	7	2.88379E-09	0.044960951	0.028364589	0.034414786	-0.083932255	-0.014079001	
chr11	107728952	107730039	1088	*	11	3.18251E-14	0.010041498	0.028367494	0.00192875	-0.034818073	-0.004080824	SLC35F2
chr16	28874307	28875484	1178	*	15	2.85559E-12	0.092811825	0.02836986	0.048244609	-0.076095759	-0.008018017	SH2B1, RP11-22P6.2
chr9	71161608	71162063	456	*	3	1.12792E-07	0.000699673	0.028371759	0.00208675	-0.134221724	-0.083904358	RP11-274B18.4
chr5	133984553	133984612	60	*	2	9.17521E-08	0.137727184	0.028373188	0.059241088	0.023090407	0.009904223	SEC24A, SAR1B
chr4	926135	926530	396	*	12	2.0469E-08	0.134166834	0.028404789	0.082370357	0.02935261	0.002037032	TMEM175, GAK

chr1	22140859	22141014	156	*	2	1.63627E-07	0.005321983	0.028431576	0.009074875	0.011577407	0.010269664	LDLRAD2
chr22	47158239	47159009	771	*	11	2.15768E-08	0.213489895	0.028488239	0.168566681	0.049167313	0.003752718	TBC1D22A, CTA-29F11.1
chr18	46987786	46988046	261	*	2	1.6347E-07	0.004718477	0.028490674	0.008264832	-0.08386339	-0.077703965	
chr9	116326448	116327278	831	*	8	4.64671E-14	1.37492E-05	0.028503425	0.000100796	-0.182529135	-0.09796667	RGS3
chr2	113341214	113342738	1525	*	16	2.17474E-13	0.057078247	0.028512926	0.015841925	0.084038449	0.013123548	CHCHD5
chr17	10600667	10601181	515	*	12	1.0111E-10	0.041014201	0.028520363	0.027415453	0.030242301	0.007790109	ADPRM, SCO1
chr4	15779364	15781661	2298	*	15	2.12833E-09	0.040793835	0.028535865	0.007236796	-0.160906061	-0.02505567	CD38
chr15	72522949	72524237	1289	*	14	3.60722E-16	0.001275017	0.028552059	0.000921238	0.032680106	0.010572548	PKM
chr16	89386798	89388396	1599	*	15	9.8638E-12	0.009192503	0.028594425	0.002011448	0.141857938	0.055340247	AC137932.6, ANKRD11
chr18	29673246	29673278	33	*	2	1.15165E-08	0.003594701	0.028643924	0.006660653	-0.023759217	-0.00350605	RP11-53I6.2, RNF138
chr7	65541029	65541747	719	*	3	4.03632E-08	0.016633126	0.028653687	0.019541389	-0.045143492	-0.017508918	ASL
chr20	18567851	18569196	1346	*	15	3.71551E-17	0.01432485	0.028686906	0.001914737	-0.110545241	-0.013282521	DTD1
chr8	17940941	17941088	148	*	3	1.70764E-08	0.029117367	0.028719472	0.012733597	-0.025608705	0.000779571	ASAH1
chr19	45504242	45505298	1057	*	9	2.38932E-13	0.002478051	0.028758458	0.001113463	0.060077116	0.010842161	RELB
chr20	1447723	1447874	152	*	3	1.03447E-08	0.197730029	0.028771233	0.01827972	-0.040458749	-0.023089923	NSFL1C
chr10	102996254	102998762	2509	*	18	5.90981E-14	5.52335E-05	0.028848863	0.000100124	0.110700162	0.035791403	LBX1-AS1
chr3	167967220	167967462	243	*	3	4.86151E-08	0.021868792	0.028849188	0.013570117	-0.194055896	-0.052141268	EGFEM1P
chr3	48646722	48647426	705	*	11	7.83113E-11	0.059885307	0.028858579	0.014790961	0.107189641	0.025142212	UQCRC1
chr6	164006905	164007415	511	*	4	1.43693E-08	0.000594053	0.028892632	0.001843591	0.124780744	0.096496883	
chr10	96990543	96991505	963	*	8	1.10543E-13	0.000293168	0.028923493	0.000655185	-0.184470088	-0.065823621	RP11-310E22.4
chr19	36393344	36393696	353	*	3	2.13455E-08	0.016864176	0.0289531	0.012177394	-0.104881091	-0.04500502	HCST
chr19	42788848	42788966	119	*	2	1.36131E-07	0.012830196	0.028963641	0.017438315	-0.026982847	-0.01051647	CIC
chr1	204403606	204403697	92	*	3	5.29579E-08	0.000564309	0.029003607	0.001787619	0.120820385	0.051748017	RP11-739N20.2, PIK3C2B
chr18	47017829	47018647	819	*	7	4.59919E-10	0.499271175	0.029048392	0.115045625	0.056733128	0.017459937	MIR1539, RPL17-C18orf32, RPL17, SNORD58B
chr12	92538613	92539873	1261	*	13	9.04942E-16	0.009440776	0.029055784	0.002821405	0.035570099	0.009202504	RP11-796E2.4, BTG1
chr11	64879330	64880626	1297	*	10	1.4133E-16	0.012726405	0.029061625	0.002956718	-0.033907124	0.00460419	VPS51, TM7SF2
chr6	32077744	32078624	881	*	11	9.20505E-14	0.008682195	0.029104841	0.005713393	0.125927735	0.055617096	TNXB, ATF6B
chr12	8234613	8235314	702	*	10	3.17409E-09	0.032344317	0.029109948	0.044071132	0.044570344	-0.003603403	NECAP1
chr5	174870544	174871289	746	*	4	2.72827E-09	0.018451488	0.029138927	0.022895341	0.068430376	0.048111641	DRD1

chr8	145024637	145025610	974	*	8	1.32768E-11	0.000790002	0.029145144	0.002256764	0.127434789	0.00286263	PLEC
chr1	65533358	65533502	145	*	4	1.43545E-07	0.012808468	0.029152155	0.01243743	-0.041130423	0.005977667	RP4-535B20.1
chr11	134093731	134095386	1656	*	19	7.8755E-22	0.000141544	0.02916146	8.0989E-05	0.031018107	0.006248595	VPS26B, NCAPD3
chr16	68771763	68772469	707	*	5	6.33948E-09	0.014489904	0.029169065	0.020017761	0.160981665	0.05361746	CDH1
chr16	67926962	67927632	671	*	6	1.53381E-08	0.012337221	0.029176781	0.010753463	-0.054215843	-0.009559438	PSKH1
chr6	43596764	43597312	549	*	11	7.51481E-10	0.190181045	0.029257757	0.057810439	-0.078745813	-0.002451718	MAD2L1BP, GTPBP2
chr20	57594082	57594545	464	*	6	4.95088E-10	0.030720168	0.029260073	0.004732097	0.14839191	0.062266091	TUBB1
chr15	68871574	68871866	293	*	3	5.84285E-08	0.006348138	0.029284891	0.011294012	0.024045724	0.009000206	CORO2B
chr7	44162801	44163975	1175	*	13	1.30887E-14	0.01285423	0.029285466	0.002621671	0.064656971	0.004428861	POLD2
chr2	220196530	220196755	226	*	3	7.13605E-08	0.002621578	0.029306375	0.005714663	0.128041193	0.079052426	RESP18
chr16	81039227	81041398	2172	*	25	1.58381E-11	0.044432421	0.029352859	0.007449284	0.150185044	0.006874818	CENPN, CMC2
chr20	44717989	44718168	180	*	2	5.12733E-08	0.013261166	0.029445631	0.017955436	0.020630391	0.014718865	NCOA5
chr2	53994269	53995338	1070	*	11	7.41608E-11	0.036299104	0.029482497	0.01531844	-0.14883556	-0.020960071	CHAC2, GPR75-ASB3, ASB3
chr7	20370159	20370742	584	*	6	1.50284E-08	0.060401229	0.029487002	0.028050036	0.021423141	0.009165374	ITGB8, CTA-293F17.1
chr11	2398327	2399360	1034	*	18	8.86312E-14	0.140213447	0.029555623	0.04931261	0.039974257	0.005487801	CD81, CD81-AS1
chr16	66585425	66586745	1321	*	15	2.55429E-14	0.006178289	0.029566437	0.002059529	0.127151719	0.009452822	CKLF, CKLF-CMTM1, TK2
chr2	86830411	86830855	445	*	2	3.46767E-08	0.213749609	0.029590048	0.068729826	0.085216221	0.039415333	AC015971.2, CHMP3, RNF103-CHMP3, RNF103
chr1	151371944	151372652	709	*	9	9.57523E-12	0.000488318	0.029631087	0.001827901	0.04883126	0.001827259	PSMB4
chr2	317257	317938	682	*	7	1.61175E-10	0.04456825	0.029643533	0.0033267	0.135065075	0.042626888	AC079779.6
chr12	122241139	122242756	1618	*	15	4.96859E-16	0.04493657	0.029647697	0.008321371	-0.065415548	-0.014590717	SETD1B, RHOF, AC084018.1
chr3	16554619	16555789	1171	*	12	2.84431E-09	0.082225806	0.029648165	0.039258553	-0.056815034	-0.014207286	RFTN1
chr17	782910	783451	542	*	4	2.18012E-08	0.001495321	0.029707167	0.003208943	0.200258628	0.119269216	RP11-676J12.6, NXN
chr4	177116464	177116771	308	*	5	5.03714E-09	0.168225766	0.029723166	0.054368039	0.036884021	0.008433657	SPATA4
chr9	115141761	115141763	3	*	2	1.45024E-07	0.013256024	0.029729376	0.018018152	-0.023347863	-0.018187105	
chr21	47648535	47649127	593	*	11	3.94459E-10	0.028949508	0.029730836	0.035100897	-0.051683671	-0.018960583	LSS
chr1	23670246	23671499	1254	*	13	2.64692E-13	0.011352302	0.029769666	0.004488316	-0.037025917	-0.001204655	HNRNPR
chr8	96036568	96037660	1093	*	11	2.00577E-12	0.007386078	0.029784068	0.000824315	-0.132502646	-0.013578141	NDUFAF6
chr19	18284045	18284600	556	*	11	2.37932E-09	0.621681597	0.029824805	0.058952999	-0.09094283	-0.015848756	PIK3R2, IFI30
chr1	228699886	228700583	698	*	8	7.00245E-10	0.089008246	0.029859246	0.012166234	0.109415689	0.039603362	BTNL10

chr6	31587680	31590870	3191	*	41	3.7927E-20	0.000148877	0.029904947	6.09635E-05	-0.208884453	-0.022193947	PRRC2A, SNORA38
chr1	7122541	7122970	430	*	5	2.03397E-08	0.014685419	0.02994041	0.007751938	0.170939144	-0.013431449	CAMTA1
chr9	2016890	2017576	687	*	3	2.55321E-11	0.001445789	0.030004538	0.003675755	-0.025244188	-0.007174637	SMARCA2
chr2	176993211	176993841	631	*	5	1.73566E-09	0.002345995	0.030057391	0.003809404	-0.050894727	0.002941854	HOXD-AS2
chr3	50125982	50126711	730	*	13	5.39795E-15	0.012314859	0.03006527	0.001715066	0.110318392	0.015748392	RBM6, RBM5
chr2	33172442	33172444	3	*	2	4.67617E-08	0.00441007	0.030077678	0.007950787	0.023452483	0.020551949	LTBP1
chr13	113862899	113864409	1511	*	17	8.54902E-12	0.012817226	0.030086255	0.003111638	-0.049121264	-0.002113062	CUL4A, PCID2
chr3	133464234	133465469	1236	*	14	8.09151E-12	0.149834864	0.030089316	0.013591671	0.111598082	0.024116443	TFP1, TF
chr5	176734343	176735073	731	*	5	2.42894E-10	0.000101866	0.030098184	0.000484018	-0.138407094	-0.100707234	MXD3
chr4	106395029	106395648	620	*	13	3.77689E-08	0.424785689	0.030108168	0.168605752	-0.060033998	-0.006079528	PPA2
chr5	110847826	110849104	1279	*	14	6.97763E-13	0.021032382	0.030120088	0.01355137	-0.129905652	-0.01755894	STARD4-AS1, STARD4
chr7	151216931	151217961	1031	*	15	9.82851E-17	0.024848685	0.030145348	0.002527302	-0.110541647	-0.011482888	RHEB
chr11	61109711	61110630	920	*	6	2.1626E-11	0.005498708	0.030201118	0.002517036	-0.110861912	-0.036439444	DAK, DDB1
chr17	33390736	33390854	119	*	3	2.89809E-08	0.000645446	0.03022949	0.002019805	-0.049008724	-0.043840681	RFFL, RAD51L3-RFFL
chr7	26437681	26438304	624	*	6	1.65984E-08	0.005653269	0.030297901	0.008932189	-0.021324419	0.006197424	AC004540.5
chr1	171226225	171226702	478	*	5	3.81699E-09	0.004342629	0.030299085	0.005153272	-0.078527312	-0.04134736	FMO1
chr7	81399308	81399747	440	*	6	6.7417E-09	0.004779173	0.030324884	0.006613328	-0.166926593	-0.062508284	HGF
chr5	169816472	169816960	489	*	5	2.33974E-08	0.003462669	0.030326211	0.008051083	0.176360595	0.065524161	KCNIP1, CTD-2270F17.1, KCNMB1
chr17	40896484	40897182	699	*	9	9.91829E-11	0.015121872	0.030326663	0.015301934	-0.125910269	-0.022572112	EZH1
chr16	69344779	69346037	1259	*	12	2.23835E-11	0.004408193	0.030345301	0.004440585	0.073444202	0.004558994	RP11-343C2.11, VPS4A
chr9	91924749	91926624	1876	*	12	3.69691E-11	0.004335139	0.030387473	0.001523581	0.110333979	0.00226255	CKS2
chr4	84256364	84257282	919	*	8	2.23924E-11	0.099021814	0.030412607	0.032521226	-0.147102727	-0.049580845	
chr6	27775346	27776524	1179	*	9	3.44799E-13	0.000643833	0.030417702	0.000640225	-0.126430271	-0.030229008	HIST1H2AI, HIST1H2BL
chr7	100240094	100240548	455	*	6	3.90359E-09	0.007091637	0.030449468	0.008402318	0.117552633	0.063625242	TFR2
chr20	6104215	6104886	672	*	7	1.12328E-09	0.002523992	0.030452293	0.004170451	0.19585411	0.013041591	
chr5	113698445	113698664	220	*	3	1.16651E-07	0.007234857	0.030460671	0.01280452	0.060466342	0.044662519	KCNN2
chr5	94955756	94956900	1145	*	8	2.05599E-10	0.007143189	0.030473692	0.004562797	0.030125949	0.011122692	GPR150
chr4	140477492	140478540	1049	*	11	2.32062E-12	0.005238214	0.030546232	0.002073989	0.025881755	0.007800861	RP11-342I1.2, SETD7
chr17	3183418	3183548	131	*	4	1.44052E-08	0.000516992	0.030568392	0.001961235	-0.200329007	-0.109590851	RP11-64J4.2

chr14	65381025	65381608	584	*	12	1.86384E-08	0.230630389	0.030587881	0.075963184	0.0240346	0.007614944	CHURC1, CHURC1-FNTB, FNTB
chr1	6644416	6645171	756	*	4	2.95138E-08	0.011989843	0.030614243	0.008186011	0.125525607	0.055432031	ZBTB48
chr1	59247425	59247603	179	*	4	1.23966E-07	0.006770902	0.030615229	0.008537847	-0.045353319	-0.03874339	JUN
chr10	46089769	46090807	1039	*	13	4.65135E-14	0.005182749	0.030615806	0.002763571	0.054829143	0.014798004	MARCH8
chr8	90770129	90770456	328	*	4	2.22349E-09	0.003885488	0.03062159	0.006160647	0.019490041	0.003323188	RIPK2
chr19	908894	909533	640	*	4	3.01307E-09	0.000265832	0.030629291	0.00114362	-0.146913345	-0.090843788	R3HDM4
chr19	58715251	58715677	427	*	4	1.07136E-09	0.000540895	0.030650894	0.002035001	0.152500224	0.098332548	ZNF274
chr12	54379466	54379493	28	*	3	1.41594E-07	0.078975354	0.030662488	0.057930272	0.104615565	0.038967585	HOXC10
chr8	20054372	20055012	641	*	14	1.51174E-08	0.356053579	0.030701069	0.099918661	0.055267839	0.006975449	ATP6V1B2
chr13	114820807	114821832	1026	*	7	1.10546E-12	0.000338515	0.03074018	0.000476463	0.133819128	0.077011718	RASA3
chr13	52026439	52027945	1507	*	17	3.63055E-15	0.040699334	0.030752862	0.011775292	-0.078793027	-0.009585229	INTS6-AS1, INTS6
chr16	31482846	31484722	1877	*	20	4.59856E-14	0.008603219	0.030761067	0.003188667	0.176625194	0.042120384	TGFB1I1
chr12	6797663	6798801	1139	*	13	5.55753E-15	0.072308685	0.030795784	0.015636909	-0.131424735	-0.02695549	ZNF384
chr15	78631512	78633662	2151	*	24	3.5505E-17	0.03035806	0.030822107	0.003061614	0.131577805	0.027188045	CRABP1
chr19	12476683	12477259	577	*	4	2.82081E-08	0.152370386	0.030859997	0.039297849	0.050047422	0.017698281	ZNF442
chr7	115850376	115850450	75	*	5	1.95311E-08	0.007418052	0.030877115	0.005095056	0.036795751	0.016252935	
chr21	34143691	34145043	1353	*	13	6.6861E-13	0.004287388	0.030903007	0.00515859	-0.065668201	-0.016357951	C21orf49, PAXBP1
chr13	88328009	88329884	1876	*	11	4.5664E-11	2.3369E-05	0.03090702	0.000174005	-0.164125551	-0.093818764	SLITRK5
chr20	7999698	8001076	1379	*	14	3.86839E-14	0.05670665	0.03090727	0.004437214	-0.046875419	-0.001735678	RP5-971N18.3, TMX4
chr4	122871735	122873523	1789	*	13	2.81946E-16	3.82022E-05	0.030932161	9.54469E-05	-0.041171047	0.0101159	TRPC3
chr17	48278778	48279265	488	*	9	3.83617E-17	3.10037E-06	0.030950715	2.74976E-05	0.088691584	0.050040577	COL1A1
chr1	97186897	97187516	620	*	7	4.05934E-08	0.288248903	0.030966155	0.078186398	-0.02798768	-0.005765403	PTBP2
chr17	30469748	30470448	701	*	7	4.49172E-09	0.079109808	0.030980858	0.022718292	-0.075681904	-0.019738451	RHOT1, AC090616.2
chr3	182982918	182983386	469	*	3	4.1171E-08	0.000642475	0.030983192	0.002030215	-0.079669081	-0.070353002	B3GNT5, MCF2L2
chr20	44562842	44563668	827	*	9	1.34593E-09	0.016841426	0.030997053	0.009351751	-0.033504786	0.00350569	PCIF1
chr13	42614071	42614179	109	*	5	2.81295E-10	0.000920195	0.031063269	0.00228268	-0.040365856	-0.026094816	DGKH
chr19	42772446	42772892	447	*	4	1.92201E-08	0.027058831	0.031063295	0.009115314	0.016293931	0.004112578	CIC
chr13	112547341	112548306	966	*	8	8.70465E-14	5.9622E-05	0.031067605	0.000107295	0.143574899	0.080494958	
chr19	1885142	1885717	576	*	6	1.1599E-10	0.000316624	0.031105488	0.001532186	-0.061280353	-0.034123597	ABHD17A

chr16	81772546	81773196	651	*	8	6.78033E-12	0.001675094	0.031112886	0.001495138	-0.075826128	-0.048456543	RP11-960L18.1, PLCG2
chr1	236958163	236958982	820	*	16	1.58963E-09	0.543756866	0.031211252	0.177271185	-0.031152471	-0.001008674	MTR
chr7	99213802	99214727	926	*	13	1.4593E-14	0.000973113	0.031234233	0.001560028	-0.11760229	-0.007508526	ZSCAN25
chr1	153538406	153538964	559	*	4	3.95948E-08	0.028003282	0.031235507	0.011664493	0.083446939	0.004514183	S100A2
chr2	69000590	69002111	1522	*	14	6.20405E-18	0.001484133	0.031240402	0.002137468	0.120938844	0.025832496	ARHGAP25
chr2	98329337	98330493	1157	*	10	1.66175E-13	0.005487231	0.031246745	0.001280358	0.125523599	0.06039818	ZAP70
chr16	66913527	66915214	1688	*	14	7.99348E-12	0.005880126	0.031268792	0.004983988	0.1132951	0.004201496	PDP2
chr14	39735211	39735496	286	*	2	8.17596E-08	0.009504864	0.031274873	0.014491533	-0.080987687	-0.058501485	RP11-407N17.3, CTAGE5, RP11-407N17.5
chr17	28804287	28804679	393	*	5	6.322E-08	0.050752508	0.031280842	0.031041932	-0.033962838	-0.005326915	GOSR1
chr6	31554829	31555016	188	*	4	2.00112E-08	0.01803198	0.031294853	0.007707389	-0.129996841	-0.046162577	LST1
chr5	75012816	75013682	867	*	11	5.77894E-13	0.000991664	0.031335113	0.002051586	0.104784068	-0.011827653	POC5
chr4	74718978	74719306	329	*	6	1.11012E-10	0.000287147	0.031348009	0.000986919	-0.140278184	-0.084573976	PF4V1
chr1	3649250	3649971	722	*	7	9.14811E-10	0.192914448	0.031373959	0.011270972	0.214837185	0.057128371	TP73
chr17	46667587	46667812	226	*	5	3.4996E-10	0.000937163	0.031375913	0.001436333	-0.089547517	-0.061325553	HOXB-AS3, HOXB3
chr8	104512506	104513083	578	*	5	2.82395E-08	0.019286148	0.03147893	0.008231198	-0.078254709	0.005657437	RIMS2, RP11-1C8.4
chr15	41245254	41245706	453	*	9	2.18012E-08	0.408017972	0.031514066	0.083450263	0.021106769	0.006296725	CHAC1
chr14	39901328	39901899	572	*	7	2.26606E-08	0.10228739	0.031542009	0.031471969	-0.032225527	-0.004683075	FBXO33
chr5	150399890	150400180	291	*	6	1.12953E-07	0.088357735	0.031547777	0.012764713	-0.040865757	-0.003924653	GPX3
chr5	175875380	175875427	48	*	3	1.05988E-07	0.006393027	0.031561619	0.008839282	-0.02477625	-0.007740248	FAF2
chr9	136214605	136215687	1083	*	12	7.52239E-15	0.001955515	0.031561959	0.000721091	-0.070662336	-0.015484162	RPL7A, MED22
chr6	31695027	31698899	3873	*	68	6.91136E-25	0.106521289	0.031566641	0.001166869	0.178585736	0.010881596	DDAH2, CLIC1
chr10	28965584	28966475	892	*	6	1.16514E-10	0.002688302	0.031575492	0.004621722	-0.107809832	0.000163669	BAMBI
chr13	61989893	61990025	133	*	2	1.2494E-07	0.075910536	0.031608954	0.050717995	0.114922789	0.041967869	PCDH20
chr12	89747474	89748300	827	*	4	1.40088E-10	0.010091824	0.031628221	0.014826203	0.040914814	0.006335213	
chr12	54388411	54389982	1572	*	9	2.56403E-10	9.46095E-05	0.031637596	0.000333	0.065478384	0.012122583	RP11-834C11.12, HOXC6, HOXC9, HOXC-AS2
chr17	7982773	7983254	482	*	6	6.34153E-10	0.001606472	0.031645901	0.002570176	0.023138801	0.012596345	AC129492.6, ALOX12B
chr2	24149416	24150622	1207	*	9	8.31728E-15	1.45328E-05	0.031706856	8.61921E-05	-0.0454985	-0.004142076	UBXN2A, ATAD2B
chr2	166649910	166650805	896	*	5	8.28521E-11	0.004918236	0.031707881	0.003822364	-0.032596366	0.00178942	GALNT3
chr1	85666272	85667233	962	*	7	1.7063E-11	0.010946687	0.031708901	0.004606187	0.029457542	0.013723723	SYDE2

chr11	2720810	2722713	1904	*	32	3.85384E-37	1.80293E-05	0.031720804	2.17676E-06	-0.151681033	-0.060851387	KCNQ1, KCNQ1OT1
chr11	65405760	65407734	1975	*	11	7.70573E-12	0.025181946	0.031724951	0.001837644	-0.185096025	-0.040682431	SIPA1
chr2	55844203	55844929	727	*	7	6.73589E-11	0.019897491	0.031745295	0.005262731	0.022584773	-0.00275171	SMEK2
chr10	70167214	70167341	128	*	5	1.79081E-08	0.007459216	0.031763525	0.008426542	-0.043714463	-0.011161747	
chr2	97405048	97406466	1419	*	15	2.68154E-08	0.425576144	0.03177178	0.083305973	0.170091408	-0.001472379	LMAN2L
chr15	90293883	90294776	894	*	11	3.35862E-13	0.018732447	0.031809078	0.005489824	0.060488029	0.014446634	MESP1
chr9	4299221	4299245	25	*	2	1.84995E-07	0.011529203	0.031839764	0.016766975	0.015731118	0.015459373	GLIS3
chr2	47596073	47596787	715	*	9	8.1004E-12	0.018766697	0.031841487	0.006164823	-0.076401956	-0.010413905	EPCAM
chr11	2321770	2323459	1690	*	30	6.94326E-23	0.000248993	0.031847234	0.000183886	-0.085159882	-0.024240971	TSPAN32, C11orf21
chr9	132565255	132566166	912	*	9	1.287E-10	0.000790981	0.031870192	0.001120827	-0.108106211	-0.018697003	TOR1B
chr11	59383068	59383888	821	*	9	3.24542E-10	0.316378273	0.031892522	0.063194635	-0.049132095	-0.00421542	OSBP
chr1	226410871	226411715	845	*	10	7.52509E-11	0.119238625	0.031904771	0.023212418	0.191790263	0.03917369	MIXL1
chr2	173291908	173293811	1904	*	23	4.58446E-17	0.001070974	0.031926155	0.00023262	0.073361799	0.003034296	ITGA6, AC078883.4
chr19	2050859	2050866	8	*	2	1.53229E-07	0.650570751	0.031930404	0.082850317	0.032009022	0.015920777	MKNK2
chr1	182361453	182362088	636	*	6	5.72339E-08	0.766846063	0.031981611	0.194917617	-0.02987393	-0.010057543	
chr3	138328184	138328334	151	*	3	2.37503E-08	0.006859818	0.032035616	0.011586223	-0.034975448	-0.027860919	FAIM
chr9	131872005	131874186	2182	*	18	2.15192E-16	4.62858E-06	0.032064837	3.08674E-05	-0.075953679	-0.02002946	PPP2R4, CRAT
chr4	113152836	113153655	820	*	6	6.24017E-10	0.187405172	0.032095941	0.04610957	-0.056971864	-0.014742175	AP1AR
chr7	132766817	132767313	497	*	11	8.8669E-09	0.287530101	0.032097868	0.09052195	-0.062019466	-0.007456936	CHCHD3
chr13	84455525	84455742	218	*	4	7.60561E-09	0.010713437	0.032189449	0.010289985	0.092869424	0.03391413	SLITRK1
chr1	45956256	45957060	805	*	10	2.51755E-12	0.015272425	0.032191226	0.007054314	-0.057080456	-0.010395162	TESK2
chr17	58677051	58677844	794	*	9	9.23723E-10	0.037633059	0.032239669	0.019228698	0.022786066	-0.001965734	PPM1D
chr3	48593844	48594968	1125	*	11	3.25287E-12	0.001769598	0.032288602	0.004052912	-0.05669824	-0.007323074	PFKFB4
chr3	51975220	51977255	2036	*	18	3.71066E-14	0.001116637	0.032323542	0.001027134	0.141220849	0.005130591	PARP3, RRP9
chr15	89630886	89632159	1274	*	13	1.11688E-10	0.038890106	0.032330485	0.0160966	-0.047316745	-0.006764176	ABHD2, RP11-326A19.3
chr12	117627286	117628187	902	*	7	8.46438E-12	0.011301455	0.032375686	0.005535628	0.061790356	0.02182529	FBXO21
chr17	73150004	73151410	1407	*	14	5.1394E-17	0.002973455	0.032406792	0.0007572	0.046832442	0.009220954	HN1
chr5	146832518	146833665	1148	*	6	1.42575E-11	0.004902927	0.032507578	0.005970069	0.020527853	0.008093799	DPYSL3
chr1	169555674	169556312	639	*	9	2.87259E-09	0.003036999	0.032522257	0.007322289	0.123389317	0.035584849	F5

chr6	34203887	34204913	1027	*	8	5.10907E-11	0.01618935	0.032524369	0.006297132	-0.057685596	-0.022701116	HMGA1
chr3	155587952	155588386	435	*	9	8.82163E-09	0.043475555	0.032537948	0.040905885	0.024389399	0.000828638	GMPS
chr21	43916100	43917047	948	*	12	3.52639E-09	0.141729447	0.032630697	0.050473949	0.022893368	0.007282585	SLC37A1, AP001625.4, RSPH1
chr20	60717299	60718270	972	*	6	2.21071E-10	0.005905021	0.032665231	0.010645945	-0.045630452	-0.006691461	PSMA7
chr17	56408688	56408804	117	*	4	1.31353E-07	0.139069077	0.032679779	0.054224364	-0.059219129	-0.007848635	BZRAP1-AS1, MIR142
chr10	105727248	105727425	178	*	3	6.92563E-08	0.253351611	0.032685827	0.06093876	0.014564468	0.006408949	SLK
chr14	52455552	52456445	894	*	11	7.94564E-10	0.227900064	0.032707019	0.036071465	-0.237029206	-0.040276465	C14orf166
chr1	119531093	119532773	1681	*	16	7.27445E-14	0.001429433	0.032717106	0.000849644	0.071915897	0.036648308	TBX15
chr17	39992344	39992697	354	*	7	1.06086E-08	0.013184301	0.032759715	0.011590597	0.213395574	0.02062344	KLHL10, NT5C3B
chr5	114938002	114938439	438	*	9	5.70666E-08	0.302665901	0.032769331	0.080630186	-0.061888596	-0.012063724	AC010226.4, TMED7-TICAM2, TICAM2
chr4	83718952	83719566	615	*	4	1.38721E-11	0.000870121	0.032813248	0.002456394	-0.049674214	0.004096365	SCD5
chr12	98986733	98986887	155	*	3	6.94589E-09	0.002768918	0.032880514	0.005706642	-0.126667058	0.003245073	
chr17	76183078	76183641	564	*	13	2.10937E-12	0.043639722	0.032894387	0.011839692	0.045409052	0.00535245	AFMID, TK1
chr1	90097496	90097981	486	*	6	2.62726E-10	0.050898374	0.032908275	0.008577772	0.098187114	0.04504493	RP11-413E1.4, RP5-1007M22.2
chr4	188916496	188917251	756	*	12	2.30829E-10	0.052270532	0.032944431	0.023192323	0.110897624	0.030602466	ZFP42
chr22	31794522	31796123	1602	*	12	9.82178E-10	0.002374697	0.032972331	0.004124266	-0.10125992	-0.021398095	DRG1
chr11	75379223	75379867	645	*	11	1.24583E-10	0.025465791	0.03299268	0.030206789	0.03501249	0.008097944	MAP6
chr16	57495650	57496971	1322	*	13	1.37186E-13	0.006166832	0.033027831	0.003275968	0.131888784	0.027052289	AC009052.12, POLR2C
chr11	111782282	111783355	1074	*	10	4.49688E-10	0.134455616	0.033078527	0.036795859	0.112937004	0.045540956	HSPB2, CRYAB
chr17	28256424	28257543	1120	*	14	1.84258E-12	0.015706549	0.033091616	0.005739248	-0.084211301	-0.01092257	EFCAB5, SSH2
chr17	76037035	76037562	528	*	6	2.46E-10	0.000410442	0.033137034	0.001507574	-0.161477394	-0.102742624	TNRC6C
chr16	85342729	85343650	922	*	5	2.2019E-10	0.000412027	0.033147725	0.001165116	-0.062885466	-0.045549319	
chr7	128695016	128695548	533	*	12	4.04535E-09	0.119559156	0.033151611	0.059973842	-0.044059869	-0.004768324	TNPO3
chr10	64575798	64578186	2389	*	17	2.89903E-10	0.017089548	0.033155739	0.00442414	-0.055760485	0.003507579	EGR2
chr14	23341133	23342291	1159	*	7	7.87582E-10	0.005331818	0.033159036	0.008390534	0.147572244	0.02089736	LRP10
chr3	127346859	127348828	1970	*	13	2.62112E-09	0.015854632	0.033160651	0.003064477	0.133959645	0.025172899	PODXL2
chr19	39109076	39110397	1322	*	12	1.0883E-10	0.004320627	0.033179078	0.003997277	0.063959832	-0.004947586	EIF3K, MAP4K1
chr19	1904288	1905417	1130	*	11	1.1732E-09	0.002090578	0.033183352	0.00470632	-0.074983592	-0.008451142	SCAMP4, ADAT3
chr17	40823420	40823930	511	*	3	5.80083E-09	0.012289015	0.033206984	0.012926653	0.18727026	0.132365311	PLEKHH3

chr20	60877067	60877648	582	*	4	8.37941E-11	0.00262575	0.033225612	0.005417665	-0.057162754	-0.020555882	ADRM1
chr7	999981	999994	14	*	2	1.4573E-07	0.004723574	0.033227487	0.008624036	0.086870912	0.08355648	COX19
chr17	40539702	40540865	1164	*	14	1.85069E-11	0.099310749	0.033244513	0.018701835	-0.049731236	-0.006401042	STAT3
chr21	34863496	34865077	1582	*	20	5.08911E-17	0.029660153	0.033244615	0.00116317	-0.111772952	-0.011772975	DNAJC28
chr2	64751054	64751596	543	*	8	2.23001E-08	0.059137436	0.033294293	0.042757913	0.021839033	0.00689794	AFTPH, RP11-568N6.1
chr11	128776112	128776548	437	*	3	2.7921E-09	0.006602858	0.033320617	0.009246824	0.106668877	0.017530058	KCNJ5
chr17	6898315	6901744	3430	*	24	3.08469E-20	3.79233E-06	0.033364627	5.64904E-06	-0.206250654	-0.027842432	ALOX12, AC027763.2, RP11-589P10.7, RP11-589P10.5
chr19	50935676	50936172	497	*	7	2.07046E-11	0.003220952	0.033381466	0.001849954	0.122497824	0.031372177	MYBPC2
chr1	24513672	24515560	1889	*	16	7.49745E-11	0.002704344	0.033385344	0.00266386	0.142855326	0.004757206	IFNLR1
chr6	7106947	7107129	183	*	4	3.11057E-10	0.001084312	0.033409137	0.003139641	-0.0571261	-0.038232763	
chr3	25824257	25825855	1599	*	15	2.10327E-10	0.121220694	0.033434031	0.019839139	-0.148939855	-0.00930718	OXSM, NGLY1
chr3	38080925	38081287	363	*	3	1.76531E-08	0.004039611	0.033437867	0.008675321	0.038340739	0.019809579	DLEC1
chr8	86089007	86089337	331	*	6	3.1213E-09	0.10138425	0.033439394	0.015498138	0.097326167	0.023603761	RP11-219B4.7
chr17	42634716	42634955	240	*	2	1.68833E-08	0.004885313	0.033470984	0.00887697	-0.086426869	-0.078469699	FZD2
chr19	18303328	18303893	566	*	7	2.20922E-08	0.025585413	0.033514416	0.018909217	0.022070986	0.002139906	
chr4	76439140	76439704	565	*	14	6.96233E-10	0.361861325	0.033520112	0.123370495	-0.040829557	-0.008326977	THAP6, RCHY1
chr16	4897216	4897716	501	*	4	7.22435E-09	0.017454129	0.033534543	0.021450659	-0.032965957	-0.016658677	UBN1, GLYR1
chr1	86621931	86623101	1171	*	8	8.90612E-11	0.002313648	0.033538163	0.003366709	-0.05585043	-0.019524183	COL24A1
chr19	17622109	17622568	460	*	7	8.27504E-09	0.046376576	0.033556626	0.020213962	-0.061594617	-0.017063301	PGLS, CTD-3131K8.2
chr1	156390220	156390418	199	*	4	4.56795E-08	0.002820564	0.033587985	0.004909145	0.089790587	0.050466307	MIR9-1, C1orf61
chr6	31648146	31649728	1583	*	34	8.81152E-19	0.001947255	0.033592086	0.003241412	0.135295714	0.00827958	LY6G5C
chr4	25235416	25235765	350	*	9	1.59065E-08	0.175320351	0.033597342	0.033480615	-0.105459595	-0.006551591	PI4K2B
chr5	61601128	61601647	520	*	5	1.52037E-10	0.007014863	0.033628608	0.00257236	0.149734757	0.027077869	
chr8	56852148	56852290	143	*	4	1.83781E-07	0.012196194	0.03364255	0.018887424	-0.054485411	-0.03331484	LYN
chr6	34759890	34760183	294	*	3	1.26981E-09	0.029202971	0.033654896	0.0254082	0.028696978	-0.003170359	UHRF1BP1
chr5	150631895	150632733	839	*	11	2.65588E-11	0.051511706	0.033697868	0.012851752	-0.233851675	-0.028268638	GM2A
chr16	89940311	89940631	321	*	3	1.79012E-09	0.002077596	0.033719579	0.004869537	-0.049297399	-0.035695464	TCF25
chr5	43042726	43043360	635	*	4	1.59191E-08	0.016069996	0.033724015	0.011213189	-0.045329556	-0.005400343	AC025171.1, CTD-2201E18.3, ANXA2R
chr7	142659349	142659757	409	*	4	3.49288E-08	0.001363802	0.033742203	0.003744493	0.121194347	0.065596396	KEL

chr3	105085991	105087718	1728	*	10	1.09694E-12	0.000484168	0.033791796	0.001488447	-0.052001045	-0.003739937	ALCAM
chr10	99052468	99053150	683	*	10	1.16879E-11	0.046041814	0.033815504	0.019732696	0.153089558	0.022851434	
chr12	6876188	6877080	893	*	7	2.97056E-10	0.013686996	0.033822207	0.004050729	0.075572145	0.024716041	PTMS, MLF2
chr16	70414267	70414816	550	*	3	1.21536E-07	0.001376166	0.033890484	0.003705592	-0.047407764	-0.024185809	RP11-529K1.4, ST3GAL2
chr14	56777076	56778402	1327	*	9	8.76348E-13	8.64284E-05	0.033900309	0.000262039	-0.174176518	-0.012239864	RP11-930O11.1, RP11-930O11.2
chr12	102455204	102455923	720	*	8	1.31628E-09	0.011206965	0.03396283	0.014568362	0.033391733	0.007347376	CCDC53
chr19	49622373	49623726	1354	*	8	8.40782E-13	0.002969368	0.033962885	0.002006129	-0.142624462	-0.017103058	PPFIA3, C19orf73
chr6	31627511	31629199	1689	*	28	3.30936E-17	0.007367878	0.033993709	0.001556063	0.187658953	0.02080208	C6orf47-AS1, C6orf47, GPANK1
chr1	36396054	36396445	392	*	8	1.13622E-08	0.045336243	0.034088749	0.033296789	-0.102293378	-0.029073553	AGO3
chr19	6736979	6738762	1784	*	19	4.25705E-15	0.002893177	0.034142765	0.004923629	-0.050198291	-0.01295482	TRIP10, GPR108
chr21	35746798	35747831	1034	*	11	1.61304E-11	0.099144857	0.034170831	0.024345208	-0.087815598	-0.012727915	SMIM11, AP000320.6
chr1	68150731	68152310	1580	*	10	5.7674E-09	0.015972264	0.034208205	0.012848957	0.055874675	0.006527445	GADD45A
chr6	27280506	27280722	217	*	3	4.37971E-08	0.031922274	0.034269752	0.027821194	-0.034262747	-0.004701889	
chr6	134217242	134217401	160	*	2	1.25716E-07	0.005022754	0.034322934	0.009136766	0.051265947	0.033268767	
chr17	78193187	78193423	237	*	4	1.04022E-07	0.019598014	0.034324905	0.018846002	-0.032958876	-0.004444764	SGSH
chr4	128886005	128886867	863	*	17	5.88457E-09	0.704388507	0.034355295	0.150215477	-0.025371338	-0.002227249	C4orf29, MFSD8
chr16	88844969	88845204	236	*	4	4.31437E-09	0.002627852	0.034368043	0.00424172	0.15228282	0.080376976	PIEZO1
chr2	128785194	128786246	1053	*	9	1.90972E-09	0.072119224	0.034439033	0.023884647	0.020306309	0.007051363	SAP130
chr5	109024217	109026041	1825	*	11	5.14739E-11	0.000510661	0.034443365	0.000302618	-0.054604849	-0.005805385	MAN2A1
chr1	231472815	231474257	1443	*	20	5.72112E-13	0.024531099	0.034486863	0.026975588	-0.028792229	-0.000752484	SPRTN, EXOC8
chr16	89023389	89023633	245	*	3	1.16451E-08	0.001066592	0.034527291	0.003125518	0.166573353	0.129445499	CBFA2T3
chr13	112721712	112721950	239	*	7	8.34173E-09	0.012190209	0.034547096	0.01099827	0.10744	0.059253965	SOX1
chr6	31867757	31870990	3234	*	43	4.59864E-13	0.011309859	0.034579518	0.002778303	0.138703598	0.005146363	C2, ZBTB12
chr11	18813191	18813556	366	*	7	2.89839E-08	0.12363838	0.034607688	0.030965806	-0.073465088	-0.01853549	PTPN5
chr15	45740221	45741008	788	*	9	7.11047E-13	0.002093514	0.034608458	0.001807154	-0.149920494	-0.043601479	C15orf48, RP11-519G16.3
chr5	141487482	141488201	720	*	10	5.19721E-12	0.050634762	0.034644593	0.010755549	0.122028605	0.048285212	NDFIP1
chr4	77996840	77996859	20	*	2	9.11907E-08	0.005278338	0.034664806	0.009531583	0.008777263	0.008589863	CCNI
chr2	55746825	55747270	446	*	3	5.74614E-09	0.09208803	0.034667294	0.044142515	-0.038683958	-0.00851206	CCDC104
chr4	149363450	149363912	463	*	9	5.84175E-11	0.004711951	0.034691508	0.005130838	0.024503355	0.008701351	NR3C2

chr17	81041756	81042023	268	*	4	8.11391E-08	0.041129193	0.0346988	0.015373821	-0.110606581	-0.059655329	METRNL
chr3	127872092	127873114	1023	*	12	3.69371E-10	0.071092035	0.03472145	0.016798491	0.023200813	0.005982844	EEFSEC, RUVBL1
chr3	150420821	150421424	604	*	6	9.07124E-10	0.166727534	0.034747093	0.047103163	0.111079131	0.040406038	RP11-103G8.2, FAM194A
chr9	90113281	90114156	876	*	8	5.38032E-11	0.037069345	0.03475981	0.003397949	-0.070067194	-0.024651857	DAPK1
chr1	45476118	45476762	645	*	6	8.99891E-09	0.142759034	0.034769181	0.044503131	0.042940429	0.014231479	HECTD3
chr10	38146040	38147196	1157	*	20	1.52921E-13	0.009129655	0.034777649	0.009679606	-0.047419096	0.000184713	RP11-162G10.5, ZNF248
chr12	96252937	96253460	524	*	4	5.75714E-09	0.015685032	0.034793156	0.018281991	0.029764562	0.014011685	SNRPF
chr4	25915260	25916067	808	*	8	3.70381E-10	0.009892722	0.034804074	0.006733997	-0.053504996	-0.000755807	SMIM20
chr3	124302724	124303745	1022	*	12	7.24926E-12	0.05977718	0.034808913	0.009553395	0.122254366	0.035268054	KALRN
chr11	126173810	126174329	520	*	7	9.8121E-10	0.01368803	0.034864353	0.009398911	-0.049829877	-0.00301801	DCPS, RP11-712L6.5
chr9	2014957	2015056	100	*	2	1.8461E-07	0.013058301	0.034881478	0.018944074	-0.042793542	-0.031584128	
chr19	13263341	13264324	984	*	6	8.85336E-10	0.013358379	0.03488265	0.009993605	0.025170911	0.004485	IER2, CTC-250I14.6
chr7	101459283	101459954	672	*	7	1.42376E-09	0.01152923	0.034884204	0.011274232	0.018607783	0.004997719	CUX1
chr2	179343803	179344387	585	*	3	2.51948E-09	0.003470325	0.034885627	0.00747865	-0.08261221	-0.045083813	
chr13	80915122	80915525	404	*	7	2.39076E-09	0.012466686	0.034900555	0.007629885	-0.090477944	-0.007279569	
chr12	50016493	50016798	306	*	6	1.06627E-08	0.049460108	0.034927248	0.027020931	0.024420814	0.005994524	PRPF40B
chr1	26947282	26947893	612	*	6	7.19035E-11	0.000354349	0.034935541	0.000817422	-0.040156136	-0.011155776	
chr13	100633154	100633502	349	*	4	1.55127E-08	0.017091468	0.035045968	0.022011253	0.029056305	0.020894284	
chr16	67977865	67978450	586	*	10	2.6087E-14	0.000293659	0.035078296	0.001243481	0.177330078	0.050440594	LCAT, SLC12A4, CTC-479C5.17
chr16	1822289	1822795	507	*	4	2.43631E-08	0.071022237	0.035078681	0.048535205	0.023030821	0.015436656	MRPS34
chr12	107487485	107487874	390	*	6	5.18072E-08	0.06896739	0.035078922	0.051038446	-0.058316036	-0.006686295	RP11-797M17.1, CRY1
chr15	90727363	90728157	795	*	11	7.07429E-16	0.014979562	0.035103341	0.001795641	-0.139709274	0.025817994	SEMA4B
chr1	165599603	165600573	971	*	13	5.27754E-13	0.012080015	0.035130657	0.005638842	0.036488327	0.006543691	MGST3
chr19	859065	859983	919	*	8	2.7822E-09	0.135662363	0.035159531	0.016958711	-0.11676956	-0.047376514	CFD
chr17	40168882	40170049	1168	*	19	9.27556E-13	0.754573985	0.035173791	0.085223401	0.022493346	0.001080222	NKIRAS2, DNAJC7
chr4	38524849	38526047	1199	*	7	8.36754E-10	0.000757823	0.035278454	0.000686177	-0.14574039	0.056337593	
chr8	103818636	103818919	284	*	4	3.61697E-09	0.001644023	0.0353529	0.003956017	-0.051592296	-0.002434153	KB-1732A1.1
chr1	55680726	55681841	1116	*	10	2.72556E-14	0.000630352	0.035372584	0.000405523	-0.056250221	-0.010675188	USP24
chr12	56709155	56709880	726	*	7	2.93594E-11	0.005171493	0.035452057	0.002508928	0.02574291	0.011249141	RP11-977G19.10, CNPY2

chr19	5622997	5623459	463	*	12	4.56494E-10	0.237082107	0.035470033	0.057336903	0.02089245	0.003364999	SAFB, SAFB2
chr20	61953801	61954258	458	*	4	1.39552E-07	0.001925348	0.035515821	0.005564296	0.081059364	0.072820036	COL20A1
chr7	77165728	77167726	1999	*	20	1.46075E-17	3.89484E-05	0.035521771	0.000550223	-0.190860116	-0.027420363	PTPN12
chr15	90118406	90119427	1022	*	12	1.22778E-13	0.002917522	0.035538249	0.002306223	-0.045864364	-0.000575181	RP11-429B14.1, TICRR
chr6	37467396	37467799	404	*	8	1.00893E-11	0.000767421	0.035571334	0.002293403	0.037400161	0.013735165	CCDC167
chr1	146643600	146644500	901	*	11	2.95892E-11	0.020867548	0.035597571	0.009823618	0.024379689	0.010468752	RP11-337C18.8, PRKAB2
chr15	74988326	74988803	478	*	11	3.61257E-08	0.0698219	0.03564341	0.045350061	-0.04693237	-0.000606768	EDC3
chr17	27037982	27039132	1151	*	14	9.5299E-17	0.002024603	0.035695217	0.000954916	-0.06308737	-0.002075663	PROCA1
chr19	49658548	49659299	752	*	6	5.76494E-09	0.002239767	0.035744136	0.004318188	0.119891684	0.069628772	HRC
chr16	3988694	3988700	7	*	2	5.90202E-08	0.005601485	0.035744253	0.010076378	-0.178975268	-0.173394899	RP11-462G12.2
chr5	180229544	180230767	1224	*	12	1.66157E-13	0.134931989	0.035792831	0.011724557	-0.044255817	-0.002982215	MGAT1
chr4	103421931	103422974	1044	*	10	8.1141E-13	0.005459471	0.035795709	0.004463064	0.029272652	0.003005989	NFKB1, AF213884.2
chr8	61428991	61429827	837	*	12	8.67126E-10	0.267987799	0.03584629	0.03024487	-0.043434341	-0.003767024	RAB2A, RP11-163N6.2
chr17	41322915	41323562	648	*	10	3.66833E-09	0.31260764	0.035848894	0.070837723	-0.098215162	-0.023525734	NBR1
chr1	205290353	205290930	578	*	7	2.22219E-08	0.055500571	0.035863181	0.055160166	0.023402499	0.004846942	NUAK2
chr1	51442861	51443457	597	*	6	1.93463E-09	0.004274546	0.035907773	0.009552781	0.022056915	0.008699264	
chr5	1800782	1801904	1123	*	8	2.04696E-09	0.068960823	0.035918507	0.01689066	-0.04256349	-0.000278562	NDUFS6, MRPL36
chr1	119548852	119550484	1633	*	9	1.6161E-13	8.46756E-06	0.035957063	7.63216E-05	0.079497343	0.055823688	
chr17	79884562	79886116	1555	*	15	7.79166E-17	0.000718606	0.035969273	0.00041291	-0.079803373	-0.005765617	MAFG-AS1, MAFG
chr17	65372852	65374598	1747	*	17	1.53946E-11	0.045549187	0.03606777	0.010526669	-0.045404077	-0.007160633	PITPNC1
chr6	46620240	46620935	696	*	14	8.93068E-09	0.279509947	0.036118591	0.083570013	0.025486266	0.003589076	SLC25A27, CYP39A1
chr2	176958174	176958552	379	*	2	1.50001E-07	0.006534964	0.03612083	0.011413956	0.038777461	0.034299074	HOXD13
chr19	45737208	45738115	908	*	10	1.93917E-10	0.051264051	0.036206333	0.014147878	-0.143429123	-0.051390875	MARK4, EXOC3L2
chr8	143781204	143781985	782	*	11	7.83468E-16	0.000963648	0.036213386	0.000776056	-0.156754201	-0.048980171	LY6K
chr21	45502757	45503189	433	*	6	1.1484E-09	0.002293456	0.036238487	0.003224987	0.128341003	0.059854297	TRAPPC10
chr11	63953100	63953512	413	*	7	5.90914E-09	0.012486361	0.036258975	0.025319351	-0.04822089	-0.008868403	STIP1
chr21	34392029	34392373	345	*	4	9.20146E-08	0.002126662	0.036332741	0.005246228	0.041425235	0.021910599	AP000282.2
chr10	86087566	86089017	1452	*	13	2.6242E-11	0.017732176	0.036364079	0.008336913	-0.163086613	-0.031225964	CCSER2
chr1	38260810	38261401	592	*	7	1.46806E-09	0.088973206	0.036421319	0.021510636	0.156295267	0.060885023	MANEAL

chr5	180650106	180651008	903	*	10	3.55499E-10	0.009103115	0.036477666	0.014443232	-0.049950645	-0.008418378	TRIM41
chr1	25070672	25072216	1545	*	16	1.90948E-12	0.532045126	0.036493493	0.120102998	-0.056673463	-0.010822214	CLIC4
chr14	93798983	93799226	244	*	4	1.026E-08	0.035357831	0.036498541	0.014574971	-0.046667115	-0.023457239	BTBD7
chr19	52692431	52693623	1193	*	15	2.2171E-12	0.106032365	0.036508769	0.016585887	0.043048107	0.011348758	PPP2R1A
chr3	50193049	50193465	417	*	3	2.35805E-09	0.001735207	0.036524898	0.004537314	0.065029513	0.038524305	SEMA3F, RP11-493K19.3
chr3	143690741	143692356	1616	*	19	5.04595E-15	0.012190105	0.036530308	0.009442604	0.039928477	0.003143248	C3orf58
chr19	41220765	41221198	434	*	8	2.54463E-08	0.030635298	0.036602455	0.0230262	-0.03731362	-0.007656442	ADCK4
chr16	57837309	57837563	255	*	3	8.42434E-08	0.018068464	0.036625519	0.022687314	0.131653817	0.074340237	CTD-2600O9.1, KIFC3
chr17	71228270	71229370	1101	*	17	5.10425E-12	0.014547313	0.036663604	0.032658198	-0.06893824	-0.011346474	C17orf80, FAM104A
chr6	64345196	64345855	660	*	6	4.7316E-10	0.00474203	0.036671063	0.007283027	0.04402043	-0.008083469	PHF3
chr6	52284632	52285530	899	*	12	2.36104E-11	0.057991183	0.036694954	0.018913913	-0.039074708	0.001227289	EFHC1
chr17	27369764	27370117	354	*	6	1.30999E-08	0.058473964	0.036726957	0.020263438	0.121934526	0.052880369	PIPOX
chr9	34611951	34612674	724	*	6	1.95414E-09	0.002312144	0.036733381	0.004321175	-0.06013087	-0.014317108	RPP25L
chr11	14522100	14522617	518	*	3	1.74602E-07	0.025540672	0.036734231	0.031516667	0.082046034	0.005301789	PSMA1, PSMA1
chr6	28834254	28834520	267	*	8	3.09192E-08	0.034404358	0.036743667	0.02352499	-0.116805303	-0.046059682	
chr6	24911545	24912385	841	*	7	5.67676E-12	0.001942827	0.036750488	0.003829747	0.1612209	0.042216453	FAM65B
chr10	134599783	134600998	1216	*	25	1.13925E-14	0.133095416	0.036770948	0.065055046	0.096389753	0.002327041	
chr12	50560469	50561215	747	*	7	6.78281E-10	0.003491658	0.0367899	0.003614515	-0.065919745	-0.018720297	CERS5
chr6	138725556	138725872	317	*	3	1.15186E-07	0.011057517	0.036807171	0.016937408	-0.065146571	-0.029184452	HEBP2
chr2	152954970	152956745	1776	*	17	1.93321E-11	0.063904034	0.036814254	0.012397103	0.103281395	0.005529508	AC079790.2, CACNB4
chr7	36764613	36765149	537	*	3	5.66855E-08	0.02516009	0.036981299	0.032789128	0.111396067	-0.011937844	
chr8	22550720	22551655	936	*	10	5.39472E-10	0.577352557	0.036987933	0.120229006	0.02886096	-0.004909519	RP11-459E5.1, EGR3
chr19	33182526	33183187	662	*	6	3.73887E-10	0.00157009	0.037003084	0.001458837	0.042887222	0.006412931	NUDT19, CTD-2538C1.2
chr11	67275245	67276572	1328	*	18	8.51762E-14	0.047476246	0.03701889	0.007618374	-0.10922185	-0.018866396	CDK2AP2
chr20	44441124	44442019	896	*	13	8.79959E-13	0.014308214	0.037020247	0.010028366	0.132211775	0.009338202	UBE2C
chr17	54230560	54230996	437	*	8	8.14841E-09	0.015194029	0.037088637	0.013662088	0.084354872	0.032976995	ANKFN1
chr1	231114438	231115458	1021	*	14	9.29589E-15	0.003138752	0.037094013	0.002029178	-0.033538142	0.003279488	ARV1, TTC13
chr11	1781505	1781710	206	*	5	8.63577E-10	0.000350223	0.037134048	0.001436205	-0.092991261	-0.069896896	AC068580.6, CTSD
chr16	81526954	81527989	1036	*	8	3.39989E-09	0.023649569	0.03713912	0.020630613	0.12224415	0.053207211	CMIP

chr14	91579689	91580129	441	*	4	4.90637E-08	0.066493439	0.037163188	0.041913152	0.161502899	0.063463195	C14orf159
chr6	30312580	30313517	938	*	30	2.24706E-12	0.90283418	0.037182754	0.536082389	-0.055235869	-0.004334799	TRIM39-RPP21, RPP21
chr19	39137911	39138334	424	*	11	2.99977E-10	0.03652857	0.037203618	0.026351812	-0.05047179	-0.014475488	ACTN4
chr3	37284959	37285071	113	*	3	8.06294E-09	0.028069234	0.037218694	0.021994918	0.01819335	0.005585106	GOLGA4, RP11-259K5.2, AC097359.1
chr9	104295918	104296699	782	*	8	1.54067E-08	0.027480975	0.03723006	0.031037409	-0.054377683	0.002073455	RNF20
chr16	30007834	30008249	416	*	5	3.38477E-09	0.007570316	0.037244729	0.008056356	-0.090121089	-0.00950215	INO80E
chr15	43662581	43663795	1215	*	10	1.17501E-08	0.023769692	0.037255661	0.011593381	-0.05961519	-0.005844545	TUBGCP4, ZSCAN29
chr15	40762407	40763763	1357	*	12	8.8666E-13	0.004704676	0.037301872	0.001587711	0.0832924	-0.006741194	CHST14
chr20	19915682	19916159	478	*	8	2.73018E-11	0.003222211	0.0373187	0.002755074	0.130753813	0.046953109	RIN2
chr19	1605171	1606356	1186	*	12	7.41767E-17	1.43758E-05	0.037346924	3.60824E-05	0.032550033	0.003843715	UQCR11, UQCR11
chr15	91497863	91498269	407	*	8	3.25958E-09	0.166483633	0.037351239	0.022108092	-0.062272379	-0.001272774	RCCD1, AC068831.6
chr16	85645571	85647010	1440	*	13	8.23336E-14	0.182907792	0.037370224	0.013294268	-0.038383613	0.004131831	GSE1
chr1	176175820	176177166	1347	*	13	1.48032E-10	0.135757748	0.037375663	0.021779262	0.03015269	0.002958462	RP11-195C7.1, RFWD2
chr15	44092166	44093429	1264	*	17	1.82648E-14	0.303771706	0.037417322	0.010642757	-0.092991291	-0.020829538	SERF2, HYPK, SERINC4
chr6	97345342	97346406	1065	*	14	9.3822E-13	0.001666553	0.037432563	0.00541273	-0.058800241	-0.008856473	NDUFAF4
chr1	38478221	38479228	1008	*	13	3.92172E-10	0.032303855	0.037432698	0.028665254	0.05213961	0.001449589	UTP11L
chr21	43815685	43816355	671	*	7	6.54355E-09	0.017359568	0.037496413	0.013326498	0.124480167	0.046360017	TMPRSS3
chr16	69373666	69373701	36	*	5	1.20233E-07	0.011190687	0.037581454	0.01638964	0.020163349	0.001693463	NIP7, RP11-343C2.9, RP11-343C2.7
chr6	28367070	28367885	816	*	9	1.54633E-11	0.000560001	0.037603972	0.00216016	0.104992908	0.04481878	ZSCAN12
chr2	191208002	191208694	693	*	9	6.97112E-10	0.033571974	0.037607801	0.011746387	-0.098237816	-0.001016029	INPP1, HIBCH
chr1	6845033	6845440	408	*	11	2.32491E-08	0.037613851	0.037609736	0.057041503	-0.1517133	-0.021031722	CAMTA1
chr1	11713643	11714269	627	*	6	6.5464E-09	0.003367919	0.037640046	0.004280027	0.208323544	0.071477724	FBXO2
chr3	185655795	185656291	497	*	9	3.6134E-11	0.0167531	0.037640841	0.007013189	-0.161303961	-0.038046358	TRA2B
chr3	179754239	179755696	1458	*	11	3.2081E-10	0.002111754	0.037642762	0.002595007	-0.152860144	-0.004171912	PEX5L
chr2	120770681	120771169	489	*	7	2.94513E-09	0.111984589	0.037675003	0.031746827	0.031128259	0.003947777	EPB41L5
chr18	19749138	19749540	403	*	6	2.76222E-08	0.012335282	0.037694252	0.013771342	0.029791168	0.00575174	GATA6
chr1	200271765	200272215	451	*	4	5.12148E-09	0.022466914	0.037697274	0.018475961	0.199433425	0.104402601	
chr16	66582592	66583237	646	*	5	2.73665E-09	0.001221344	0.037786824	0.003399989	0.11915563	0.026637944	Y_RNA, TK2
chr16	8738240	8738608	369	*	6	8.75786E-11	0.00017478	0.037794133	0.000735247	-0.104613259	-0.078204609	METTL22

chr1	46806025	46807522	1498	*	17	1.53749E-14	0.003500478	0.037810141	0.003214777	-0.121382452	-0.014024159	NSUN4
chr4	56412099	56412880	782	*	8	5.3816E-10	0.006541396	0.037851745	0.004601685	-0.042620725	-0.003365076	CLOCK
chr16	31105482	31106703	1222	*	11	1.76647E-09	0.057929495	0.037881207	0.015761658	-0.054282592	-0.006148999	RP11-196G11.1, VKORC1
chr22	42466345	42467252	908	*	11	4.67055E-13	0.003459801	0.037934465	0.002968748	-0.071940721	-0.011065921	NAGA
chr11	94963991	94964817	827	*	10	5.05583E-09	0.031131386	0.037960387	0.028766309	-0.110544361	-0.017207495	RP11-712B9.2, SESN3
chr1	26869340	26869637	298	*	4	6.00175E-09	0.000501318	0.038059824	0.001943376	-0.08033882	-0.058057215	RPS6KA1
chr12	118541585	118542254	670	*	7	3.69227E-09	0.006535319	0.038087789	0.006132103	-0.056453053	-0.007799888	VSIG10
chr1	3663832	3664084	253	*	5	9.0071E-08	0.026827944	0.038095283	0.030604221	0.034877313	0.008424853	TP73-AS1
chr14	67707842	67708475	634	*	9	7.49961E-10	0.099228038	0.038101557	0.031324048	-0.064591177	-0.015120368	MPP5
chr22	19709548	19710386	839	*	8	1.99607E-09	0.012438045	0.038108491	0.01348825	0.23914199	0.095478976	SEPT5
chr16	2932470	2932616	147	*	4	1.4928E-08	0.011513917	0.038131592	0.016305691	-0.061892127	-0.01160315	
chr9	95431707	95432460	754	*	4	1.10542E-08	0.055600793	0.038167841	0.033874715	0.027448797	0.010807581	IPPK
chr9	111695968	111696342	375	*	2	3.28544E-08	0.038437158	0.038216106	0.039705825	0.020744937	0.012794228	IKBKAP
chr4	6642252	6643729	1478	*	11	1.29819E-14	7.52168E-05	0.038229494	0.000526756	-0.04801105	-0.008463787	MRFAP1
chr2	38152187	38152364	178	*	7	1.09744E-07	0.055606222	0.038240933	0.042555694	-0.048080554	-0.003015688	RMDN2
chr7	65419185	65419261	77	*	6	1.5638E-11	8.5585E-05	0.038258778	0.000585688	0.142627543	0.114846688	VKORC1L1
chr10	38299179	38299886	708	*	11	2.3418E-10	0.073619792	0.038313048	0.015160617	-0.065640694	-0.006334431	ZNF33A
chr4	129732568	129733057	490	*	7	1.5521E-08	0.126502954	0.038354799	0.048623223	-0.04237184	0.001881397	JADE1
chr7	27190235	27191564	1330	*	12	2.01831E-10	0.064875323	0.038392371	0.009377004	0.057099054	0.023340448	HOXA-AS3, HOXA3, RP1-170O19.22, RP1-170O19.23
chr22	36682843	36683020	178	*	5	9.85487E-09	0.001160346	0.038421187	0.003706336	0.083300572	0.046076986	MYH9
chr12	123463762	123464655	894	*	10	1.36041E-11	0.045121218	0.038439855	0.012247056	0.076250171	0.030167774	OGFOD2, RP11-197N18.2, ARL6IP4, ABCB9
chr15	51633816	51633821	6	*	2	2.02198E-07	0.116881999	0.038448886	0.068558652	0.107395941	0.063302535	
chr17	27053969	27054058	90	*	4	1.91001E-07	0.005809205	0.03853779	0.012259406	0.021442607	0.015455074	NEK8, TLCD1
chr1	150981652	150981947	296	*	2	4.52385E-08	0.403292523	0.038542359	0.093577563	0.133960865	0.065750834	PRUNE
chr7	50823767	50823908	142	*	3	7.7638E-08	0.002451277	0.038564052	0.005912266	0.102111549	0.080277337	GRB10
chr1	145039193	145040057	865	*	8	2.51942E-11	0.000699332	0.038607235	0.001986741	0.037005839	0.008624969	PDE4DIP
chr15	44719213	44719665	453	*	6	2.05931E-08	0.020356331	0.038618621	0.011175744	-0.078237279	-0.034898904	CTDSPL2, RP11-516C1.1
chr3	125802485	125803306	822	*	9	2.63974E-09	0.012271526	0.03877887	0.011767693	-0.026728512	-0.002808021	RP11-124N2.1, SLC41A3
chr11	58909927	58910583	657	*	18	3.55702E-10	0.276312356	0.038781854	0.070528486	0.036736756	-0.002799283	FAM111A, AP001258.4, AP001258.5

chr5	149379650	149380356	707	*	14	8.88884E-10	0.080219728	0.03878334	0.051062161	-0.041095667	-0.003768274	HMGXB3, TIGD6
chr10	121355708	121357240	1533	*	13	3.22822E-16	0.005672815	0.038840954	0.001055671	-0.055651092	-0.000448166	TIAL1
chr16	46781713	46782760	1048	*	9	8.38538E-11	0.002696617	0.038844883	0.006027507	0.182650898	0.069145177	MYLK3
chr2	162016459	162017589	1131	*	12	8.84555E-12	0.021671746	0.038868817	0.00548194	-0.072331984	-0.006696479	TANK, AC009313.1
chr9	35689475	35690395	921	*	8	7.27186E-12	0.004031134	0.038916462	0.001641889	0.187294263	0.048394526	TPM2
chr6	29910755	29911558	804	*	9	1.00503E-10	0.017994365	0.03900205	0.004078773	0.224863359	0.049437587	HLA-A, HCG4P5
chr11	124622317	124622775	459	*	5	9.43809E-08	0.020687923	0.039019595	0.011965914	0.107039759	0.029645437	ESAM
chr6	112041092	112041335	244	*	4	4.4549E-08	0.003912546	0.039082557	0.00846162	0.049623004	0.043939117	FYN
chr1	45240400	45241824	1425	*	14	2.83836E-14	0.039325472	0.039119889	0.003924041	-0.109074761	-0.01487493	RPS8, SNORD55, RP11-269F19.2
chr20	62886751	62887006	256	*	6	2.59983E-08	0.049834058	0.039173679	0.01569806	-0.099241413	-0.016720272	
chr5	115152326	115153252	927	*	13	9.47043E-15	0.010803638	0.039261733	0.002074453	0.067351364	0.018872262	CDO1
chr8	97273336	97274070	735	*	11	1.32663E-09	0.10790394	0.039274045	0.053679092	-0.052139583	0.000655733	PTDSS1, MTERFD1
chr13	111364847	111368529	3683	*	33	1.31786E-15	0.000331726	0.039286302	0.001167503	-0.067346039	-0.017775523	ING1, CARS2
chr19	46273153	46273434	282	*	3	6.60019E-09	0.006395759	0.039325712	0.012647677	-0.02485655	0.001216686	AC074212.6, DMPK
chr22	42896426	42897250	825	*	7	3.82777E-10	0.031348068	0.039348731	0.01766868	0.070777222	0.018532144	SERHL
chr19	38039469	38039666	198	*	2	8.64362E-08	0.006499019	0.039414299	0.011644079	-0.164700642	-0.164273173	ZNF793, CTD-3064H18.4
chr7	100463206	100464145	940	*	7	1.18302E-09	0.002351723	0.039425898	0.006681661	-0.107528541	-0.062240413	SLC12A9
chr12	133066471	133067405	935	*	14	9.45183E-13	0.007665879	0.039507377	0.014023047	-0.080319359	-0.03236126	FBRSL1
chr1	234349253	234350515	1263	*	9	7.43546E-13	7.08666E-06	0.039517861	0.000107592	-0.112942064	-0.02791948	SLC35F3, RP4-799P18.2
chr2	62932096	62933278	1183	*	11	5.15723E-13	0.000540612	0.039518962	0.001819025	-0.115066334	-0.032593367	EHBP1
chr2	203130903	203130955	53	*	2	1.25605E-07	0.19054898	0.039524913	0.082289635	-0.018160471	-0.00087079	NOP58
chr1	226012500	226013212	713	*	7	6.3975E-11	0.019318682	0.039567514	0.004649596	0.121159934	0.050990618	EPHX1
chr11	32454718	32455025	308	*	7	6.03792E-08	0.007993948	0.039568565	0.017782989	0.08328982	0.034572814	WT1
chr1	154973402	154973945	544	*	4	3.15067E-08	0.002741536	0.039597887	0.006400075	0.020835285	0.008130385	
chr1	75596930	75597304	375	*	2	7.69382E-08	0.011775264	0.039611164	0.018385642	0.023349272	0.017420072	LHX8, RP11-510C10.2, RP11-510C10.3
chr11	69489591	69490221	631	*	7	5.35545E-09	0.805610465	0.039641164	0.139460053	0.024837348	0.005894348	ORAOV1
chr17	7209803	7211307	1505	*	19	1.43586E-17	0.000922095	0.039641534	0.00067371	-0.12285127	-0.022509225	EIF5A
chr6	119255801	119256453	653	*	5	7.07977E-09	0.010534055	0.03967358	0.013718637	-0.052764337	-0.000851336	RP11-351A11.1, MCM9
chr4	83955829	83956648	820	*	13	2.26751E-10	0.021874711	0.039679217	0.0245203	-0.032724374	-0.005483294	COPS4

chr17	61778366	61778813	448	*	4	1.37347E-09	0.000313666	0.039770232	0.001460034	0.070694473	0.051559855	LIMD2
chr6	39082812	39083205	394	*	9	3.3887E-08	0.075294098	0.039801732	0.0325553	0.080279027	0.024336725	SAYSD1
chr10	114709137	114710261	1125	*	10	3.38398E-10	0.023489697	0.039818563	0.020464753	-0.255586627	-0.040981067	TCF7L2
chr5	79783132	79784454	1323	*	13	1.0586E-14	0.012412569	0.039898354	0.003994984	-0.084161691	-0.011310612	FAM151B, CTD-2015H6.3
chr8	26148993	26149300	308	*	4	1.51544E-07	0.011678284	0.039903288	0.015103306	0.01546114	0.003856399	PPP2R2A
chr11	61128853	61130361	1509	*	22	8.6516E-13	0.116909438	0.039953239	0.017739505	0.058814906	0.005529882	TMEM138, CYB561A3
chr3	158390329	158391040	712	*	12	3.40476E-09	0.058744475	0.039996225	0.03556307	-0.040006235	-0.019602842	GFM1, LXN
chr17	79283028	79283959	932	*	11	9.29473E-11	0.008912164	0.040034617	0.013834177	-0.132685376	-0.047257153	LINC00482
chr5	76932526	76933163	638	*	4	1.29641E-08	0.002274472	0.040044992	0.005734552	0.042881254	0.015072365	OTP
chr15	41708714	41709524	811	*	9	6.06167E-10	0.011403001	0.040047814	0.012291455	0.031475747	0.012866565	RTF1
chr1	24017943	24018676	734	*	11	4.69732E-09	0.017935242	0.040047937	0.026565128	0.037927016	0.005517795	RPL11
chr2	179059440	179059452	13	*	2	7.02082E-08	0.007360877	0.040050501	0.012903176	0.02012736	0.017178805	OSBPL6
chr6	30611822	30611830	9	*	2	1.81416E-07	0.039706965	0.040064279	0.041416883	0.069780575	0.049462487	ATAT1
chr7	100209537	100210548	1012	*	16	8.58024E-13	0.019564846	0.040072841	0.008383758	-0.060493047	-0.012626728	MOSPD3
chr14	91976141	91976961	821	*	16	8.08768E-12	0.063749917	0.040143292	0.022811368	-0.068147197	-0.010263119	SMEK1
chr1	153700021	153700650	630	*	9	2.24641E-11	0.000534463	0.040169217	0.001901986	-0.043582438	-0.005626701	INTS3
chr3	52812361	52813920	1560	*	13	1.24152E-10	0.014904655	0.040178077	0.002989133	-0.138380313	-0.0373427	ITIH1
chr10	74856286	74856747	462	*	6	3.04038E-08	0.00205145	0.04027261	0.006838159	-0.032644687	0.005120092	P4HA1
chr17	38573776	38575162	1387	*	19	5.49873E-12	0.062487147	0.040322723	0.038364274	-0.041193466	-0.011407944	TOP2A
chr8	23562918	23563970	1053	*	7	4.53301E-12	0.02380541	0.040334822	0.020735893	0.114527806	0.042763348	NKX2-6
chr1	144931754	144932609	856	*	14	1.02717E-09	0.202922755	0.040377265	0.053589356	0.016918312	0.004058195	PDE4DIP
chr19	384036	384408	373	*	4	1.69411E-08	0.000384473	0.040409264	0.001702723	0.13081048	0.081058449	
chr19	13905866	13906241	376	*	6	5.2621E-10	0.000391917	0.04042751	0.001643002	-0.044111559	-0.011832012	
chr7	72972103	72972737	635	*	13	9.64545E-10	0.415481897	0.040455871	0.05696582	0.162112597	0.040355472	BCL7B
chr6	31275148	31276669	1522	*	25	4.54748E-17	0.03757281	0.040518321	0.012900888	0.135838643	0.058405882	XXbac-BPG248L24.10
chr7	66309627	66310092	466	*	8	5.78569E-09	0.037155281	0.040529041	0.014598457	0.017149263	0.008035106	GTF2IRD1P1
chr19	2785107	2785891	785	*	10	6.03619E-10	0.004255457	0.040559555	0.009268731	-0.05762004	-0.000513083	THOP1
chr4	184426031	184427252	1222	*	10	8.77973E-14	0.006620161	0.040575576	0.002514846	-0.047655221	-0.004941876	ING2
chr3	14219389	14220835	1447	*	22	4.7934E-10	0.180993462	0.040583626	0.073805172	-0.133063243	-0.006980922	LSM3, XPC

chr8	101225028	101225902	875	*	8	3.38986E-09	0.005312493	0.040600518	0.006476006	0.030783848	0.004363938	SPAG1
chr1	201798408	201798459	52	*	3	1.38721E-07	0.003521936	0.040634962	0.007759487	-0.01871956	-0.007355857	IPO9, IPO9-AS1
chr19	46998786	46999840	1055	*	15	1.18833E-09	0.075854835	0.040636273	0.059184181	0.144850766	0.029368169	AC011484.1, PPP5D1, PNMAL2
chr18	18691651	18692338	688	*	13	2.25953E-09	0.153389906	0.040671112	0.036454641	-0.055006692	-0.016335439	ROCK1
chr12	53835350	53836318	969	*	8	1.37792E-12	0.012552144	0.040694605	0.004446977	-0.116176763	-0.027070436	PRR13, PCBP2, RP11-793H13.8
chr2	62422633	62423716	1084	*	9	1.47092E-11	0.00150902	0.040696739	0.001006769	-0.04558989	-0.007686829	B3GNT2
chr13	110790566	110791045	480	*	4	7.12078E-08	0.064243325	0.040742049	0.037839277	-0.045291368	-0.006476786	
chr17	40760955	40761818	864	*	21	1.51694E-19	0.002867318	0.040762729	0.001148976	0.027626307	-0.000913805	TUBG1, FAM134C
chr1	226069919	226071128	1210	*	15	6.7966E-13	0.009132597	0.04077084	0.012513409	-0.132459712	-0.009345386	TMEM63A
chr10	81106469	81107211	743	*	10	8.10946E-09	0.209360802	0.040809051	0.060552039	0.145694039	0.019651365	
chr16	73082051	73082535	485	*	6	4.50223E-10	0.001319714	0.040819108	0.004261063	0.029441356	0.01445465	ZFHX3
chr19	14247862	14248428	567	*	5	6.5698E-09	0.015426462	0.04082242	0.019364151	-0.035096803	-0.012430358	CTB-55O6.12
chr11	61658919	61659716	798	*	10	3.71339E-10	0.020906407	0.040896375	0.006506066	0.07678432	0.009767994	FADS3
chr14	23388339	23388846	508	*	10	2.46837E-08	0.225367086	0.040966094	0.052470921	0.038787941	0.004837894	PRMT5-AS1, RBM23
chr4	1796142	1796226	85	*	2	4.39592E-08	0.012701369	0.041027616	0.019671438	-0.017981241	-0.013121474	FGFR3
chr18	60987425	60987446	22	*	4	3.78643E-08	0.327730289	0.041047012	0.167766482	0.023152813	0.005378091	
chr9	6757347	6758935	1589	*	18	1.31524E-18	0.006992661	0.041092768	0.001051167	-0.038869791	0.003734748	KDM4C
chr17	37353483	37353657	175	*	2	6.26561E-08	0.008358185	0.041125428	0.014356407	-0.082394042	-0.06157833	CACNB1
chr3	119298195	119298802	608	*	10	2.47076E-08	0.065894467	0.041137868	0.039446257	-0.061169027	0.004128791	ADPRH, RP11-190C22.9
chr19	38146906	38147309	404	*	5	3.93457E-08	0.014684035	0.04114706	0.017265285	0.051430247	0.016306403	ZFP30
chr5	43603643	43603786	144	*	2	3.46961E-08	0.043408727	0.041203286	0.044215702	-0.251403668	-0.131118498	NNT
chr1	245026373	245027404	1032	*	8	1.19035E-09	0.049357334	0.041207187	0.03363411	-0.083742722	-0.018661233	HNRNPU
chr8	28243821	28244474	654	*	12	2.64925E-09	0.205592248	0.041255276	0.053273523	-0.040295058	-0.005213623	ZNF395, FBXO16
chr16	29801824	29802448	625	*	12	3.2981E-08	0.301590002	0.041282665	0.096288953	-0.03377748	-0.0120238	KIF22
chr10	131935156	131935477	322	*	3	2.16063E-08	0.042607798	0.04131779	0.033730276	0.045874039	0.015754922	GLRX3
chr10	75758046	75758501	456	*	4	1.10791E-10	0.000430592	0.041329876	0.001846681	0.035313924	0.010594267	VCL
chr14	24615978	24616620	643	*	7	2.72657E-09	0.003963885	0.041335084	0.009424198	-0.047863357	-0.00623091	RNF31, PSME2
chr2	62115351	62116013	663	*	11	1.26421E-11	0.003851171	0.041341711	0.003737604	0.018374941	0.006975754	COMMD1, CCT4
chr12	54891213	54891655	443	*	5	1.13346E-09	0.000378714	0.041384211	0.001818882	-0.078950294	-0.038605692	RP11-753H16.3, NCKAP1L

chr11	46615999	46616039	41	*	2	9.27326E-08	0.59593492	0.041425207	0.102006108	-0.096148262	-0.048599406		
chr13	31735875	31736727	853	*	16	2.99244E-13	0.006690755	0.041469588	0.008284553	0.043986868	0.002659409		HSPH1
chr14	35008661	35009852	1192	*	11	8.16584E-11	0.00142182	0.041475235	0.003451233	0.141814847	0.024306907		EAPP
chr5	16465883	16466296	414	*	7	4.30548E-08	0.006642849	0.041525252	0.011004034	0.05376709	0.007174609		ZNF622
chr19	50433262	50433949	688	*	4	1.25248E-09	0.003431528	0.041526886	0.006324899	0.085303081	0.028824189		ATF5, CTC-326K19.6
chr15	68522224	68522275	52	*	3	1.95698E-07	0.047285096	0.041533319	0.029676564	-0.049620926	-0.014427109		CLN6
chr19	1940184	1940655	472	*	5	7.63046E-08	0.022121054	0.041534288	0.010988879	0.02280799	0.007172837		
chr10	98479703	98480746	1044	*	11	3.29438E-12	0.026522941	0.041535084	0.012263631	0.024436503	0.012302466		PIK3AP1
chr11	66383678	66384451	774	*	10	4.04901E-11	0.002919344	0.041550151	0.004306361	-0.052972582	0.000348148		RBM14, RBM4, RBM14-RBM4
chr2	209119250	209120308	1059	*	11	4.62181E-13	0.001597118	0.041564107	0.002606959	-0.05400604	-0.002624972		IDH1-AS1, IDH1
chr1	180600890	180601918	1029	*	10	1.03E-12	0.000781435	0.041673829	0.002368108	-0.081501908	-0.012387048		XPR1
chr10	14051445	14051679	235	*	4	4.7893E-09	0.005364634	0.041677437	0.012249352	0.26342692	0.199479126		FRMD4A
chr2	208030666	208032061	1396	*	14	4.37384E-14	0.003993817	0.041707393	0.003296655	0.034459041	0.001295389		KLF7
chr12	112450556	112451600	1045	*	14	9.30496E-09	0.011023952	0.041713834	0.021942594	-0.026238222	0.001262107		ERP29, TMEM116
chr12	113622691	113623594	904	*	11	1.52597E-09	0.234827669	0.04173945	0.040021376	0.029367339	0.003191507		RITA1, DDX54, RP11-545P7.4
chr10	104263472	104263948	477	*	9	4.59508E-08	0.034791553	0.041740122	0.029388131	-0.038999888	-0.001687965		SUFU
chr6	127836731	127837030	300	*	2	4.07548E-09	0.085803011	0.041755922	0.063334129	-0.095532243	-0.04284665		SOGA3, SOGA3
chr11	18548210	18549289	1080	*	16	2.93023E-11	0.031938481	0.041782613	0.02838896	0.076492194	0.003144305		TSG101
chr12	51157902	51158154	253	*	3	2.07499E-08	0.002525714	0.041866348	0.006381778	-0.043683476	-0.012611616		ATF1
chr1	64058180	64059385	1206	*	19	1.41603E-13	0.112311932	0.041900388	0.019942382	-0.08481482	-0.014075182		PGM1, ITGB3BP
chr12	53661219	53661577	359	*	2	6.8188E-08	0.063291935	0.041956694	0.054787374	-0.074801596	-0.055750046		
chr6	3064047	3064805	759	*	7	5.21332E-09	0.007857652	0.041958904	0.008766936	0.03141731	0.009109017		RIPK1
chr6	130339410	130339826	417	*	9	3.50642E-08	0.018417118	0.041993017	0.027684324	0.023221338	0.006013943		L3MBTL3
chr17	27181725	27182137	413	*	8	3.84911E-08	0.486543095	0.042059154	0.100053963	-0.052726608	-0.013577053		ERAL1, FAM222B
chr12	6419136	6419784	649	*	11	2.88034E-12	0.140788013	0.042096351	0.02440555	0.191779954	0.051622024		PLEKHG6
chr19	49223814	49224165	352	*	5	2.08259E-08	0.002966716	0.042097211	0.00677379	-0.107585969	-0.078011588		RASIP1
chr1	167905194	167906565	1372	*	20	4.19174E-17	0.011534697	0.042105105	0.008021552	-0.054819855	-0.003752307		DCAF6, MPC2
chr2	182321269	182322898	1630	*	16	5.78665E-09	0.041493413	0.042134349	0.007988959	-0.110101678	-0.008336564		ITGA4
chr6	25992641	25992679	39	*	2	1.01101E-07	0.019962271	0.042146649	0.02737412	-0.043231917	-0.039370651		U91328.21

chr1	202130344	202131184	841	*	5	1.05445E-11	0.000166333	0.042151295	0.000988595	-0.07383885	-0.053898216	PTPN7
chr3	39093076	39093977	902	*	12	1.40691E-10	0.064950694	0.04215152	0.010675934	-0.070796938	-0.006379083	WDR48
chr1	16087752	16088002	251	*	3	7.03474E-08	0.003045806	0.042217752	0.007324562	0.10736107	0.075270293	FBLIM1
chr12	54346462	54346859	398	*	4	6.55831E-09	0.000493604	0.042262889	0.002078974	0.096968599	0.054439366	
chr21	44496043	44497077	1035	*	12	2.58762E-12	0.02713619	0.042264795	0.011838688	0.087307037	0.01788731	CBS
chr3	33759422	33760473	1052	*	15	4.57402E-12	0.052314606	0.04234247	0.026244988	-0.145184195	-0.019082607	CLASP2
chr2	175351471	175352427	957	*	13	1.57098E-10	0.005174459	0.042343304	0.013493009	0.085793411	-0.016334801	AC010894.3, GPR155
chr2	71295019	71295731	713	*	14	3.43445E-08	0.196028257	0.042360819	0.147475474	-0.07426748	0.000668239	NAGK, RP11-467P9.1
chr7	138347950	138348023	74	*	2	7.15263E-08	0.007815662	0.042406158	0.013745351	0.102543214	0.075174422	SVOPL
chr12	53644994	53646162	1169	*	14	2.16087E-14	0.007005371	0.042423721	0.00453761	0.089951681	0.020605566	MFSD5
chr11	3186095	3187032	938	*	9	1.99741E-13	0.001914527	0.042446401	0.001026174	-0.088078799	0.013835904	OSBPL5
chr11	2482223	2482929	707	*	13	5.26641E-09	0.164972997	0.04247596	0.104965201	0.136886057	0.044833088	KCNQ1
chr14	69619846	69619916	71	*	3	1.1912E-07	0.03952714	0.042487169	0.045064509	-0.032454766	-0.001614312	DCAF5
chr17	34135721	34136959	1239	*	14	4.09007E-11	0.140591948	0.042506796	0.031460863	-0.045448213	-0.002563621	TAF15, AC015849.12
chr6	33287941	33288372	432	*	8	9.93653E-09	0.156803337	0.042510404	0.056738871	-0.139552895	0.015817567	DAXX
chr19	50168129	50168328	200	*	4	1.44008E-07	0.021978653	0.042614239	0.024429903	-0.049215898	-0.012819579	IRF3
chr6	30227294	30228431	1138	*	27	6.61626E-20	0.049387317	0.042621788	0.006947391	0.135216597	0.012748518	HLA-L, HCG17
chr1	41174793	41175199	407	*	6	2.68872E-08	0.127569649	0.042657806	0.027201423	0.129621307	0.048187188	NFYC
chr19	36545155	36546357	1203	*	15	4.00154E-10	0.131277919	0.042729264	0.039032138	-0.023952723	-0.001064475	WDR62, THAP8
chr11	64850581	64851957	1377	*	17	1.64813E-09	0.160755836	0.042738637	0.063726219	-0.1546878	-0.009472878	ZFPL1, CDCA5
chr6	29690766	29692281	1516	*	28	1.95388E-13	0.11823075	0.042758086	0.091243762	0.119675947	0.001086102	HLA-F, HCG4P11
chr4	6202091	6202609	519	*	7	3.18504E-08	0.060883877	0.042822881	0.021971034	-0.038376139	0.002026482	RP11-586D19.1, JAKMIP1
chr1	206679959	206680748	790	*	10	8.91074E-11	0.057157362	0.042869713	0.016265749	0.063333845	0.015887163	
chr7	100547176	100547182	7	*	2	8.30731E-08	0.01220313	0.042940056	0.019395133	0.073579269	0.06110591	
chr2	157291759	157292214	456	*	7	4.11618E-08	0.077989703	0.043041323	0.038678208	-0.115847454	-0.040522619	GPD2
chr11	65343118	65344604	1487	*	10	6.66554E-10	0.002507464	0.043118762	0.001842444	0.145523789	0.027239561	EHBP1L1
chr10	112630726	112632370	1645	*	18	3.10278E-13	0.0011882	0.043181901	0.001283705	0.169158357	0.008486903	RP11-313D6.3, PDCD4, PDCD4-AS1
chr1	65885364	65886416	1053	*	16	3.95761E-13	0.088157817	0.043190587	0.026382531	-0.06425145	-0.020623454	LEPR, LEPROT
chr5	175788536	175789734	1199	*	16	3.44799E-13	0.070379813	0.043195005	0.012734542	0.107369646	0.013999615	KIAA1191

chr20	30795258	30795840	583	*	11	7.81152E-09	0.14454098	0.043243795	0.031917879	-0.050293237	0.002335474	POFUT1, PLAGL2
chr12	14721288	14721386	99	*	2	8.72166E-08	0.234912857	0.043276842	0.092802829	0.132287316	0.072265627	RP11-695J4.2
chr16	85722306	85722814	509	*	8	4.03291E-08	0.040952979	0.043277339	0.022962576	0.022142165	0.007230855	GINS2
chr5	5422589	5423457	869	*	8	3.77417E-10	0.105318589	0.043280135	0.011417461	-0.159663764	-0.018142223	KIAA0947
chr4	74486080	74486675	596	*	11	1.93884E-12	0.076244845	0.043280538	0.008197534	-0.113866095	-0.003640241	RASSF6
chr14	101908561	101909681	1121	*	6	4.23812E-10	0.000681461	0.043342568	0.001163237	0.107942616	0.056595021	RP11-168L7.3
chr2	241525653	241526190	538	*	4	3.68315E-08	0.221406265	0.043353611	0.056524117	-0.020202272	-0.007057659	CAPN10, CAPN10-AS1
chr6	6004686	6005349	664	*	6	2.69429E-09	0.003519202	0.043378852	0.005954586	0.084042669	0.027148091	NRN1
chr12	56120970	56123223	2254	*	27	1.72017E-16	0.025316721	0.043393661	0.001708919	-0.066117287	-0.014247383	RP11-644F5.11, CD63
chr2	136499099	136499517	419	*	6	2.61842E-08	0.003083628	0.043473208	0.006418894	-0.020250338	-8.40472E-06	UBXN4
chr16	22103791	22103855	65	*	3	2.09257E-07	0.294862429	0.043476398	0.120594526	0.109395867	0.047005306	
chr15	34260433	34261172	740	*	8	4.55388E-10	0.003339235	0.043482972	0.006528999	0.167671823	0.049082515	CHRM5, AVEN
chr4	40517938	40518143	206	*	6	1.19295E-07	0.027185826	0.043492389	0.02612205	0.124270661	0.019827286	RBM47
chr8	144241809	144242448	640	*	8	8.09288E-08	0.392199942	0.043582016	0.033540368	0.056567604	0.009143993	LY6H
chr2	198062260	198062750	491	*	4	7.29641E-08	0.007796974	0.043646897	0.012733223	-0.250917471	-0.124242516	AC013264.2, ANKRD44
chr10	119303831	119306025	2195	*	17	1.74287E-13	0.021235557	0.04366266	0.010970911	0.072023647	0.017975777	EMX2, EMX2OS
chr2	238499456	238500389	934	*	9	2.29029E-09	0.00988325	0.043692105	0.005411949	0.100095111	0.038585831	AC104667.3, RAB17
chr1	29063669	29064149	481	*	3	2.04398E-08	0.003811714	0.043718683	0.009098293	0.030930556	0.023758314	YTHDF2
chr16	75096418	75097037	620	*	4	2.44941E-08	0.001122419	0.043743886	0.003923895	0.092453996	0.000344642	ZNRF1
chr19	17958505	17959281	777	*	13	1.68498E-09	0.126824641	0.043801324	0.049404626	0.048868082	0.021775903	JAK3
chr19	57018614	57019069	456	*	7	6.54812E-08	0.129370722	0.043802015	0.033112423	0.084976402	0.003071144	
chr5	78531912	78532560	649	*	7	1.87461E-10	0.001765975	0.043826781	0.004077176	0.033116724	0.010889864	JMY
chr5	74632092	74633655	1564	*	16	4.20372E-10	0.065866695	0.043860798	0.018234761	-0.077012344	-0.001331297	HMGCR, CTD-2235C13.2
chr18	11688008	11688350	343	*	3	5.25792E-08	0.025664551	0.043861269	0.021102774	-0.057295439	-0.040286825	
chr3	100119718	100120793	1076	*	15	3.35029E-11	0.034240212	0.04386353	0.012173802	-0.039644024	0.001164299	LNP1, TOMM70A
chr2	106013556	106014017	462	*	3	2.15956E-08	0.009067357	0.043873093	0.015870127	0.095438597	0.033166182	FHL2
chr21	38337889	38338458	570	*	3	3.98471E-08	0.013968103	0.043873766	0.023231945	-0.090070729	-0.019756971	HLCS
chr4	39640740	39640920	181	*	4	4.73431E-08	0.014457226	0.043897926	0.019199459	-0.047246721	-0.013067678	RP11-539G18.2
chr3	10028155	10028530	376	*	5	1.24439E-08	0.023516902	0.043933253	0.027976151	-0.015945823	0.003967947	EMC3

chr2	192712530	192712975	446	*	4	1.42672E-08	0.005595578	0.04394231	0.010876341	0.134950199	0.089898232	AC098617.1
chr12	121147730	121148294	565	*	3	6.13716E-08	0.004920055	0.043963431	0.010021343	0.09953755	0.048765755	UNC119B
chr12	122825944	122826219	276	*	4	9.36381E-09	0.000741174	0.044046991	0.002865419	0.067320235	0.055881765	CLIP1
chr1	33646902	33647869	968	*	10	1.11653E-14	0.007380696	0.044106715	0.001962396	-0.05840797	-0.003965641	TRIM62
chr8	27167110	27169256	2147	*	16	2.32699E-09	0.010523353	0.044125165	0.005088656	-0.159025697	-0.013370984	PTK2B, TRIM35
chr7	1980117	1980892	776	*	6	1.48613E-09	0.001317275	0.044163471	0.003727676	-0.127188433	-0.076452945	MAD1L1
chr3	149094653	149096029	1377	*	11	7.35367E-12	0.000286523	0.044231771	0.000827878	-0.227640357	-0.096858642	TM4SF1-AS1, TM4SF1
chr12	109027870	109028610	741	*	4	1.23726E-08	0.002338686	0.044234335	0.007171382	0.094793688	-0.002425158	RP11-689B22.2
chr1	225964937	225965777	841	*	13	3.17654E-13	0.001621644	0.044275666	0.001910471	-0.111843405	-0.016925816	SRP9
chr8	87521102	87521456	355	*	8	1.24812E-08	0.161231273	0.044298986	0.017263782	-0.120377501	-0.02611215	CPNE3, RMDN1
chr1	16301562	16302813	1252	*	11	2.53096E-12	0.000883521	0.044336901	0.002049274	0.11826892	0.000787455	ZBTB17
chr9	130921736	130922050	315	*	5	9.72927E-08	0.015465941	0.044363119	0.016890759	-0.135057136	-0.039077523	
chr15	49170244	49170509	266	*	6	1.26939E-07	0.074440326	0.044411599	0.042091232	-0.032396868	0.003210269	EID1, AC012379.1, SHC4
chr4	57301897	57301998	102	*	4	4.6665E-08	0.044410756	0.044461757	0.04257961	0.020897572	0.009488907	PAICS
chr2	20425649	20425680	32	*	2	7.23649E-08	0.020185132	0.044464045	0.028168583	0.055162952	0.046418559	
chr2	74776815	74777409	595	*	8	1.68826E-10	0.107043613	0.044469603	0.030233055	0.095042068	0.017890313	DOK1, LOXL3
chr17	3539210	3539728	519	*	9	3.1445E-09	0.04124993	0.044539284	0.0229989	-0.030744534	-0.00202226	SHPK, SHPK
chr9	21993972	21994965	994	*	7	3.26071E-11	0.000163812	0.044553112	0.000806255	-0.091690153	-0.030284887	RP11-145E5.5, CDKN2B-AS1, CDKN2A
chr5	81573780	81575188	1409	*	15	4.57647E-13	0.023605072	0.044608414	0.002201706	-0.137781225	-0.030881142	RPS23
chr3	14165843	14167165	1323	*	16	1.4013E-16	0.000501906	0.044608918	0.00082647	-0.03813755	-0.001800167	TMEM43, CHCHD4
chr11	67806118	67806526	409	*	7	2.08546E-08	0.024416557	0.044625322	0.028762573	0.104458266	0.021964355	TCIRG1
chr11	3818695	3819539	845	*	18	2.99965E-14	0.012042556	0.044642998	0.006739254	-0.082910928	-0.0032056	PGAP2, NUP98
chr20	54580070	54580196	127	*	4	1.92984E-08	0.003132875	0.04465789	0.007341407	0.060116211	0.044128718	CBLN4
chr1	26797576	26799365	1790	*	15	1.49574E-10	0.023875993	0.044752696	0.004461755	-0.129648972	-0.028606578	DHDDS, HMGN2
chr16	3238423	3239638	1216	*	6	6.7213E-10	0.000656871	0.044755087	0.00171064	0.034690777	0.021310919	AJ003147.9
chr16	47007486	47008028	543	*	7	4.41362E-08	0.121860468	0.044814898	0.061725445	0.025917348	0.010524577	RP11-169E6.1, DNAJA2
chr7	16793256	16794078	823	*	8	1.51411E-11	0.004981956	0.044831118	0.009113404	0.02716377	0.009747485	TSPAN13
chr8	11626495	11628346	1852	*	14	8.45179E-16	1.16888E-05	0.044902882	0.000117902	0.09474854	-0.010081226	NEIL2
chr3	53915801	53916644	844	*	10	2.99191E-10	0.077317023	0.044984016	0.018417335	0.028359365	-0.005640455	ACTR8, AC012467.1

chr11	2290615	2292004	1390	*	10	4.17339E-12	0.004935818	0.045029465	0.004791218	0.067075362	0.019088856	ASCL2
chr8	38243907	38244679	773	*	11	6.33117E-10	0.019545143	0.045043194	0.024495617	-0.129801507	-0.012881278	LETM2
chr19	42636625	42637137	513	*	7	9.71342E-10	0.011406219	0.045056854	0.005874474	0.048480872	0.023945008	CTC-378H22.1, POU2F2
chr4	6988322	6989584	1263	*	16	5.38773E-14	0.011758035	0.045057253	0.002833469	-0.112167151	-0.011178228	TBC1D14
chr6	108485043	108487820	2778	*	20	1.12245E-11	0.001221567	0.045177124	0.000752218	0.08230674	0.013568924	NR2E1, OSTM1
chr4	56261740	56262107	368	*	7	8.42307E-09	0.061074654	0.045223458	0.0178589	0.016176085	0.003817489	SRD5A3-AS1
chr12	31477192	31477737	546	*	5	6.42318E-10	0.000395001	0.045267995	0.001811212	-0.051779756	-0.018555755	AC024940.1, FAM60A
chr16	68057150	68057911	762	*	9	3.35876E-10	0.018815625	0.045291414	0.042495251	0.105483421	0.005310477	DUS2, DDX28
chr8	63951472	63951879	408	*	8	2.27523E-08	0.014346962	0.045381411	0.021733488	0.016609106	0.006365666	GGH
chr17	74721824	74722995	1172	*	16	6.18117E-11	0.295805653	0.045407806	0.052325654	-0.09313573	-0.011704198	METTL23, JMJD6
chr8	81399093	81399518	426	*	3	2.82727E-08	0.005821031	0.045488976	0.012009596	-0.074629978	-0.040717679	ZBTB10
chr16	29673933	29674487	555	*	5	3.70265E-08	0.092684212	0.045512244	0.027098503	-0.058768247	-0.025185506	SPN
chr11	71159708	71159905	198	*	5	6.66757E-08	0.03178931	0.045547232	0.021263883	0.02732741	0.012432561	RP11-660L16.2, DHCR7
chr3	121553940	121554459	520	*	10	1.82733E-09	0.403873607	0.045559387	0.077411339	-0.057261773	-0.02029049	EAF2
chr16	4322750	4323571	822	*	11	1.50954E-10	0.10904747	0.04562894	0.015491067	-0.054641373	-0.017008638	TFAP4
chr9	36135760	36135812	53	*	2	1.93922E-07	0.01377631	0.045642259	0.021653325	0.156839462	0.125745762	
chr22	41777658	41778188	531	*	9	3.35866E-09	0.056374679	0.045654284	0.028721087	0.028415909	0.005922473	TEF
chr19	12661732	12663228	1497	*	14	6.98238E-10	0.021850092	0.045656887	0.012848826	-0.060873335	-0.01147618	ZNF709, ZNF564, CTD-2192J16.20
chr15	89148310	89148933	624	*	9	1.17872E-08	0.098456279	0.045658136	0.049160333	0.056433838	0.022304221	RP11-97O12.2
chr11	133938586	133939265	680	*	9	5.15178E-10	0.015051356	0.04571995	0.013612721	-0.042430709	0.00304648	JAM3
chr19	42746143	42746319	177	*	5	8.54581E-08	0.043524914	0.045862551	0.020796933	0.025063075	0.002510753	GSK3A, AC006486.9
chr11	57103414	57103951	538	*	10	2.69611E-10	0.014580481	0.045900555	0.009247809	-0.133009407	-0.005547508	
chr11	406492	407183	692	*	6	1.11015E-09	0.002942988	0.045918356	0.006674705	-0.072039902	0.000646296	SIGIRR
chr1	201475451	201476371	921	*	11	1.04204E-10	0.041090177	0.045927852	0.015017643	0.068902462	0.002948058	RP11-134G8.7, CSRP1
chr10	71930420	71931119	700	*	6	5.53713E-09	0.010720586	0.045944631	0.019286421	-0.055972664	-0.004197267	
chr2	114513914	114514751	838	*	13	7.4986E-15	0.000141316	0.045966167	0.00046257	-0.071957875	-0.023666741	SLC35F5
chr19	14142201	14142963	763	*	12	2.08217E-09	0.211161099	0.045976354	0.031524052	0.08279734	0.01302024	IL27RA, CTB-55O6.4
chr17	72931569	72932651	1083	*	13	1.40494E-12	0.003665833	0.04599711	0.003230519	0.138087578	0.045017692	OTOP3
chr8	57026301	57027352	1052	*	8	3.50328E-11	0.00025425	0.045998882	0.000921794	-0.12124348	-0.04590734	MOS

chr10	118928510	118928758	249	*	4	9.13558E-08	0.007088125	0.046000836	0.012474391	0.054662987	0.029429791	RP11-501J20.2
chr12	113229033	113229647	615	*	13	8.32637E-11	0.006621178	0.046038753	0.019750508	0.168496829	0.024323216	RPH3A
chr22	39714752	39715635	884	*	9	6.77833E-13	0.001732399	0.046193585	0.003079289	-0.038972975	0.003298789	RPL3, SNORD43
chr4	8442090	8443089	1000	*	13	2.58779E-11	0.113425584	0.046206298	0.028482731	0.026340065	0.004414899	TRMT44, ACOX3
chr1	180198528	180199980	1453	*	8	4.13077E-08	0.030060925	0.046242776	0.015011188	-0.033653949	0.000182064	LHX4
chr19	44331823	44332311	489	*	4	4.1967E-09	0.034038095	0.046255785	0.018690686	-0.125941431	-0.062920331	ZNF283
chr16	29822365	29822997	633	*	8	7.41244E-10	0.00268237	0.046259536	0.008813943	0.030294762	0.008329389	MAZ, AC009133.14, AC009133.20
chr12	111537057	111537211	155	*	4	1.5075E-07	0.001245218	0.046340657	0.004434641	-0.060535521	-0.045526994	CUX2
chr11	62359415	62359899	485	*	6	9.69728E-11	0.043583681	0.046344015	0.017215816	0.089203822	0.019143108	TUT1
chr19	58789674	58791159	1486	*	13	1.05798E-13	0.001548728	0.04638512	0.00233032	-0.046641903	-0.006073732	CTD-3138B18.4, ZNF8, ZNF8, AC010642.1, CTD-3138B18.5
chr12	125399097	125399716	620	*	8	1.21795E-09	0.006921751	0.046410561	0.011796404	-0.033046918	-0.00420371	UBC
chr4	89618533	89619549	1017	*	15	3.18544E-10	0.084914285	0.046414495	0.072937274	-0.094042349	-0.021524827	HERC3, NAP1L5
chr1	32478846	32479956	1111	*	11	1.8964E-10	0.09654803	0.046428984	0.032035171	-0.031420879	-0.005127985	KHDRBS1
chr1	55446131	55446960	830	*	7	7.69676E-10	0.001514693	0.046467951	0.004586797	0.105132371	0.027731733	TMEM61, RP11-12C17.2
chr11	47207435	47208367	933	*	9	1.35386E-09	0.054604612	0.046552758	0.013418428	0.036194675	0.008791553	PACSIN3
chr4	4859772	4862240	2469	*	32	3.32116E-12	0.030975375	0.04656354	0.0166186	0.099808354	0.012842672	MSX1
chr1	13839650	13840712	1063	*	13	4.27831E-13	0.003678675	0.046578908	0.003134343	0.091461304	0.013723234	RP4-597A16.2, LRRC38
chr22	26877690	26880401	2712	*	15	1.76548E-11	5.63964E-06	0.046579974	6.00818E-05	0.148504522	0.036079034	SRRD, HPS4
chr17	35078070	35078659	590	*	5	8.51876E-09	0.000951543	0.046580585	0.002733986	-0.079688807	-0.025996917	
chr15	85291082	85291317	236	*	4	3.45648E-08	0.044542288	0.046847649	0.020877508	-0.020991312	-0.005311986	
chr11	74178462	74179549	1088	*	11	5.52582E-09	0.022416662	0.046893131	0.015192198	0.107442585	0.019590388	RP11-702H23.4, KCNE3
chr7	77427242	77428849	1608	*	28	6.14073E-15	0.015379848	0.046898944	0.009001666	-0.080714386	-0.004972992	PHTF2, TMEM60
chr6	17393365	17393825	461	*	9	5.15338E-11	0.016307024	0.046914172	0.006796248	0.075149392	0.037863062	CAP2
chr19	16683254	16683989	736	*	10	1.04918E-09	0.032542372	0.046928487	0.023689482	0.031993444	0.003645948	CTD-3222D19.2
chr2	111435449	111435943	495	*	8	2.04181E-08	0.052064114	0.046930874	0.022286	-0.051060927	-0.018245132	BUB1
chr16	71928489	71930048	1560	*	19	2.1397E-10	0.043048804	0.04698509	0.031194889	-0.059149091	-0.000312997	IST1, ZNF821
chr19	9938028	9939038	1011	*	13	1.21784E-10	0.021227123	0.047079387	0.011654839	-0.132635136	-0.030072312	UBL5, FBXL12
chr17	17109239	17109936	698	*	9	1.16501E-09	0.004030726	0.047079832	0.006873701	-0.147785613	-0.024981989	MPRIP, PLD6, RP11-45M22.4
chr5	111093199	111094068	870	*	14	1.30061E-10	0.107599695	0.047085202	0.03608095	-0.041162154	-0.001610068	NREP

chr17	907643	907886	244	*	4	5.75546E-08	0.001403524	0.04709571	0.004638768	0.186339885	0.100519228	ABR
chr19	36266235	36266840	606	*	9	1.84579E-08	0.08919729	0.047112134	0.043615674	-0.056115982	-0.016690982	ARHGAP33
chr10	123922506	123923881	1376	*	14	1.69879E-13	0.001968286	0.047118871	0.005083967	0.070121292	0.033734104	TACC2
chr1	33283105	33284208	1104	*	15	4.22025E-12	0.426306222	0.047144495	0.038828309	0.043577211	0.005196031	S100PBP, YARS
chr6	157099227	157099924	698	*	4	3.55145E-08	0.003345872	0.047193844	0.008328014	0.03269588	-0.002354911	ARID1B
chr12	113913887	113914611	725	*	6	1.26077E-08	0.005023251	0.047247381	0.007831755	0.043493742	0.028071109	RP11-82C23.2
chr14	61201602	61201977	376	*	3	7.98556E-08	0.008187763	0.047256825	0.015194714	0.029040787	0.003900334	MNAT1
chr19	45393621	45394624	1004	*	12	6.55074E-13	0.000239201	0.047269771	0.001232508	-0.038342596	0.000107826	TOMM40, CTB-129P6.4
chr6	32921521	32921805	285	*	3	3.0111E-08	0.057097779	0.047333003	0.050857054	0.103713406	0.0392962	HLA-DMA
chr2	233390699	233391265	567	*	7	4.63622E-10	0.002285418	0.047335152	0.00458339	0.133794031	0.076801499	CHRND
chr11	1892038	1892888	851	*	9	6.94771E-09	0.043860737	0.047349031	0.017717558	0.119611143	0.047345056	LSP1
chr1	150208865	150209464	600	*	7	2.38589E-08	0.172909987	0.047372455	0.051179405	-0.134149179	-0.023503862	RNU2-17P
chr20	49253364	49253710	347	*	5	1.00214E-07	0.012494378	0.047388372	0.01170581	0.137129274	0.055161887	FAM65C
chr2	232572668	232573281	614	*	8	1.19391E-09	0.008470174	0.047430292	0.009915317	-0.11784995	-0.018730453	PTMA
chr7	27207996	27209350	1355	*	20	3.29096E-14	0.214910447	0.047444766	0.059669143	-0.105652584	-0.004040747	HOXA10-AS, HOXA9, RP1-170O19.20, MIR196B
chr14	77228497	77229233	737	*	7	4.72413E-09	0.003457632	0.047514405	0.006928843	0.078602874	0.028299465	VASH1
chr8	57123418	57124749	1332	*	10	1.44673E-10	0.001687368	0.047533425	0.002691539	-0.075444423	-0.007621804	CHCHD7, PLAG1
chr4	57975815	57977040	1226	*	14	3.0675E-10	0.02060732	0.047682867	0.021390073	-0.057043378	-0.003770116	IGFBP7-AS1, IGFBP7
chr6	30292743	30294255	1513	*	21	2.85906E-14	0.011769909	0.047694698	0.023171531	-0.067966169	0.002864265	HCG17, HCG18
chr12	65563159	65564395	1237	*	7	2.85347E-12	4.62382E-05	0.047802744	0.000361446	-0.052486525	0.002942221	LEMD3
chr5	72111910	72112729	820	*	13	1.64355E-09	0.20035457	0.047814021	0.041216842	-0.040672311	0.000550312	TNPO1, CTD-2631K10.1
chr6	150186269	150186488	220	*	5	3.8804E-09	0.026153919	0.047869531	0.014521505	-0.10045274	-0.057665283	RP11-244K5.8
chr22	24822802	24823554	753	*	10	6.90655E-11	0.004025677	0.047884272	0.005175721	-0.13428525	-0.019677323	SPECC1L-ADORA2A, ADORA2A
chr1	29508764	29509335	572	*	5	6.6659E-08	0.024517526	0.047930772	0.005877256	-0.052674175	-0.013389841	
chr19	37861449	37862272	824	*	12	4.60787E-09	0.044953279	0.04793349	0.025644018	-0.051334441	-0.011982277	ZNF527
chr12	63544175	63544430	256	*	3	1.79447E-08	0.002179541	0.047942808	0.006152807	0.056500973	0.039935682	AVPR1A
chr2	95831790	95831943	154	*	3	8.42585E-08	0.011654032	0.048021595	0.021115742	-0.029057185	-0.009553138	ZNF2
chr7	100424945	100425827	883	*	10	9.84347E-13	0.002528871	0.048064696	0.001464979	-0.07049241	-0.007364324	SLC12A9, EPHB4
chr17	14204310	14204478	169	*	3	1.60392E-07	0.024661497	0.048212665	0.030971842	0.023120453	0.011410849	HS3ST3B1

chr10	121651781	121652237	457	*	9	1.26853E-08	0.020236306	0.04825206	0.02531794	-0.030003066	-0.003179123	SEC23IP, MCMBP
chr20	43743736	43744110	375	*	7	6.14077E-09	0.007245318	0.04826183	0.01207038	0.125616442	0.049929421	WFDC5
chr1	154531393	154531564	172	*	6	3.55398E-09	0.072874486	0.048274354	0.011842927	0.049213237	0.014378764	UBE2Q1
chr19	51486901	51487968	1068	*	15	1.54554E-15	0.010875693	0.048297953	0.002864519	0.150418683	0.06088416	CTB-147C22.9, KLK7
chr2	178129336	178130312	977	*	11	1.95226E-10	0.017898103	0.048318411	0.01165806	-0.053904325	-0.000588836	AC079305.10, NFE2L2
chr11	66360740	66361081	342	*	5	2.29508E-09	0.001007907	0.048350707	0.002952629	-0.114533045	-0.071992578	CCS
chr20	39656786	39658073	1288	*	11	7.93151E-13	0.0007789	0.04835188	0.001602781	-0.040247776	-0.000957893	TOP1
chr17	79268614	79268763	150	*	2	9.5233E-08	0.08092839	0.048381386	0.067103594	0.018343306	0.013011728	SLC38A10
chr20	57617810	57618246	437	*	6	8.70787E-08	0.012450315	0.048467659	0.016831807	0.041150001	0.018799763	SLMO2
chr5	149339858	149340861	1004	*	13	9.14195E-10	0.32179773	0.04849255	0.038822409	-0.054593625	0.002830913	SLC26A2
chr1	232765331	232765605	275	*	3	5.07367E-08	0.002944278	0.048540456	0.007796158	-0.201282734	-0.052995011	
chr8	42397376	42397764	389	*	5	4.23911E-09	0.025135162	0.048575765	0.014247125	0.140954804	0.069018997	SMIM19
chr1	193090988	193091399	412	*	7	1.97033E-09	0.033789578	0.048593211	0.013836329	-0.073479512	5.13591E-05	CDC73
chr1	172501480	172502226	747	*	9	4.06548E-09	0.212182059	0.048750743	0.025798157	-0.056584328	-0.01191592	SUCO
chr16	82659960	82660873	914	*	15	9.7015E-11	0.091884582	0.048752178	0.040425728	0.094935573	0.021267133	CDH13
chr14	101512216	101514051	1836	*	14	2.16075E-11	0.005890102	0.048757043	0.001948645	0.146593304	0.054846193	MIR381HG, MIR381, MIR487B, MIR539
chr1	100598159	100599169	1011	*	14	8.06239E-10	0.051040594	0.048852894	0.04385493	-0.033099971	-0.002787828	TRMT13, SASS6
chr2	74756234	74756297	64	*	3	1.83837E-07	0.008271286	0.048869704	0.01536341	0.059628085	0.024182251	AUP1
chr16	69220400	69220726	327	*	3	3.77656E-08	0.00588448	0.048877694	0.01228721	-0.049317649	-0.013699217	CIRH1A
chr3	178978849	178979013	165	*	5	6.04293E-08	0.001092245	0.048922008	0.004516829	0.168156537	0.106315293	KCNMB3, LRRFIP1P1
chr7	92464881	92465342	462	*	8	4.5403E-08	0.092338326	0.048949386	0.050970616	-0.04914281	0.003671938	CDK6
chr3	185825749	185827776	2028	*	17	9.98538E-16	0.001248257	0.048953867	0.000519014	-0.054123891	-0.022579742	ETV5, DGKG
chr6	32145373	32146779	1407	*	28	9.68711E-19	0.246929937	0.049005437	0.039621416	-0.062003557	-0.006835537	RNF5, AGPAT1
chr19	50379397	50380022	626	*	9	1.03883E-10	0.002499956	0.049026268	0.009706321	-0.049704317	-0.010071089	AKT1S1
chr1	193028138	193029428	1291	*	20	4.98459E-21	0.0032525	0.049103269	0.000454289	-0.064216684	-0.008649	TROVE2, UCHL5
chr6	31631638	31634237	2600	*	56	6.76247E-32	0.00012117	0.049121894	0.000129419	0.147026315	0.011150589	CSNK2B, CSNK2B-LY6G5B-1181, GPANK1
chr10	95326974	95327325	352	*	5	3.01509E-10	0.003194344	0.049130862	0.008898365	0.055633053	-0.010760826	FFAR4
chr17	41131552	41133424	1873	*	22	1.90931E-21	7.89124E-06	0.049133971	3.81513E-05	0.061570257	-0.011081476	RUNDC1, PTGES3L-AARSD1, PTGES3L
chr9	20621203	20622658	1456	*	16	8.12584E-13	0.02494706	0.049136174	0.008942084	-0.063223906	-0.018766989	MLLT3

chr5	77590494	77591002	509	*	12	1.71708E-09	0.042992233	0.049160997	0.040195381	-0.051849335	-0.003677017	AP3B1
chr14	69446137	69446981	845	*	10	1.03681E-10	0.05688812	0.049169201	0.02374012	-0.076693824	-0.01661626	ACTN1-AS1, ACTN1
chr11	10471371	10471687	317	*	3	2.8351E-08	0.018445938	0.049286129	0.021391952	-0.158693544	-0.076680157	AMPD3
chr19	57351440	57352807	1368	*	22	1.18119E-14	0.050724801	0.049386854	0.024277352	-0.13350223	-0.025661047	MIMT1, ZIM2, PEG3
chr1	16543684	16544950	1267	*	9	1.10953E-10	0.000382858	0.049428222	0.001315958	0.145968943	0.020418373	ANO7P1
chr6	35435693	35436891	1199	*	11	3.53126E-11	0.026165945	0.049563765	0.005547066	-0.071597544	0.003184109	RPL10A
chr19	52391078	52391304	227	*	7	1.23134E-07	0.027649994	0.049597269	0.009494775	0.123934606	0.073762276	CTC-429C10.2, ZNF577
chr8	67624233	67625280	1048	*	12	1.08649E-12	0.021533694	0.049634846	0.006378647	-0.050750065	-0.006804783	C8orf44-SGK3, SGK3
chr5	67583598	67584297	700	*	12	3.11892E-09	0.049168673	0.049756268	0.038021842	0.055585419	0.009441677	PIK3R1
chr1	153939684	153941904	2221	*	24	9.5882E-11	0.001174494	0.049788688	0.00187877	0.2063835	0.013819731	CREB3L4, SLC39A1
chr2	16082022	16082927	906	*	7	2.4056E-09	0.162750965	0.049835256	0.021468528	-0.129076888	-0.029387267	MYCN, MYCNOS
chr22	37172204	37172423	220	*	5	1.61362E-07	0.009357442	0.049918209	0.009889388	-0.044777083	-0.013281912	IFT27
chr15	48623100	48624389	1290	*	20	2.28251E-15	0.002215036	0.049972828	0.001611624	-0.059126465	-0.010709649	DUT, RP11-154J22.1
chr19	36980364	36980975	612	*	21	3.87127E-14	0.049237451	0.049994404	0.032848309	-0.046134876	-0.009963135	CTD-2630F21.1, ZNF566
chr17	79816559	79817633	1075	*	6	1.00027E-10	0.00041496	0.050000713	0.001689022	-0.129223785	-0.091319044	P4HB
chr5	172483116	172484594	1479	*	15	2.93212E-12	0.000360052	0.05005244	0.001314501	-0.045697724	-0.005932814	CREBRF
chr17	73105356	73106517	1162	*	12	2.04416E-09	0.046286885	0.050057592	0.014429261	0.038169211	0.005724083	ARMC7
chr11	95522512	95524042	1531	*	18	6.83325E-14	0.002097111	0.050071885	0.004369054	-0.039926223	-0.013041131	CEP57, FAM76B
chr9	103191365	103191817	453	*	6	4.74361E-10	0.000455214	0.050089579	0.001481843	0.100356475	0.070106587	MSANTD3
chr10	26727173	26728036	864	*	8	5.60127E-10	0.040000904	0.050114228	0.035340494	0.053170912	0.01168639	APBB1IP
chr5	86563580	86564119	540	*	10	2.74799E-09	0.012137655	0.050152185	0.022757667	-0.064171084	-0.010005571	RASA1
chr19	49403382	49403969	588	*	7	5.50834E-08	0.348614481	0.0501624	0.032470509	-0.063108209	-0.02277686	NUCB1
chr2	102608155	102608349	195	*	5	1.8523E-07	0.001910339	0.050176496	0.006868628	-0.082526874	-0.071827508	IL1R2
chr15	44117116	44117184	69	*	4	1.95627E-07	0.001996644	0.050202137	0.006260687	0.066928442	0.054541188	
chr8	132916222	132916947	726	*	4	4.37886E-10	0.002039015	0.050213199	0.005507631	0.040008601	0.022263262	EFR3A
chr20	655888	656558	671	*	5	6.49224E-08	0.029193704	0.050250081	0.026010881	-0.062721878	0.00133121	RP5-850E9.3, SCRT2
chr1	47799302	47799834	533	*	7	4.49142E-09	0.034261458	0.050293559	0.012596206	-0.03084063	-0.001024201	CMPK1
chr7	87563649	87564345	697	*	8	1.48499E-08	0.044799426	0.050302015	0.032119186	-0.025704693	0.003662684	ADAM22
chr19	10397031	10398215	1185	*	8	2.4487E-09	0.00177015	0.050306016	0.003306579	-0.095365111	-0.026091859	ICAM1, ICAM4, CTD-2369P2.8

chr19	54663286	54664576	1291	*	14	1.46077E-09	0.044331386	0.05045653	0.045940633	0.047415249	0.003688372	LENG1, TMC4
chr20	45318448	45319455	1008	*	8	4.01383E-11	0.027059278	0.050550136	0.016017808	-0.089021821	-0.024142182	RP1-28H20.3
chr16	68482463	68483194	732	*	11	5.08141E-11	0.011022944	0.050608308	0.012208234	0.101529984	0.026769426	SMPD3
chr1	227915846	227916787	942	*	14	1.83293E-11	0.008239721	0.050672376	0.011026809	-0.082192238	-0.005126681	SNAP47
chr1	213124368	213124896	529	*	5	2.51877E-08	0.003238005	0.050689612	0.007545445	0.026726686	0.018670254	VASH2
chr5	1003897	1004495	599	*	5	1.26662E-08	0.020728411	0.050697829	0.014180906	0.129820199	0.077654141	AC116351.2
chr20	39766360	39766794	435	*	6	1.54772E-09	0.001300664	0.050746996	0.004162367	0.034372621	0.015894447	PLCG1, RP1-1J6.2
chr12	93964231	93965732	1502	*	23	3.15961E-12	0.056046681	0.050749672	0.029448457	0.030283125	0.003738748	SOCS2, SOCS2-AS1
chr11	64545585	64546660	1076	*	16	2.99491E-12	0.185251238	0.050781017	0.026144726	-0.063561066	-0.01081079	AP001462.6, SF1
chr18	60381593	60382764	1172	*	15	2.61284E-14	0.042206324	0.0508744	0.002059576	-0.139590909	-0.005702519	PHLPP1
chr4	48781911	48782840	930	*	12	1.40534E-09	0.05443118	0.05087486	0.034909578	-0.046738493	-0.016394027	FRYL
chr7	150974273	150974839	567	*	9	1.41932E-09	0.033246253	0.050903016	0.015411135	0.035301887	0.01106884	SMARCD3
chr1	6673671	6673734	64	*	4	8.17183E-08	0.003623927	0.050943888	0.007681721	-0.095124673	-0.024055857	KLHL21
chr19	41858816	41860095	1280	*	11	7.37034E-13	0.003173547	0.05095287	0.004065837	-0.059923719	-0.02946966	CTC-435M10.3, TMEM91, TGFB1
chr11	10829582	10830972	1391	*	19	5.64705E-17	0.018285905	0.05096161	0.005637077	-0.041413862	-0.000526384	RP11-685M7.3, EIF4G2
chr15	101419243	101419518	276	*	8	1.20043E-07	0.036400223	0.051142759	0.025589072	-0.06069548	-0.015363286	RP11-66B24.8, ALDH1A3
chr2	207629824	207630694	871	*	18	1.45337E-10	0.060070959	0.051178767	0.062713788	0.033173144	-0.002580466	FASTKD2, MDH1B
chr12	54812606	54813531	926	*	11	5.25935E-11	0.102349805	0.051203402	0.018965385	-0.062001148	-0.017448873	RP11-753H16.3, RP11-753H16.5, ITGA5
chr16	68676555	68677020	466	*	6	2.12006E-08	0.140221421	0.051337201	0.031605144	0.055977158	0.023076733	CDH3
chr6	99962757	99964114	1358	*	14	2.20933E-11	0.004070231	0.051345322	0.009500474	0.125098183	0.013778095	USP45
chr1	47009446	47010223	778	*	7	1.89032E-10	0.002994375	0.051359259	0.002667064	0.081436559	0.038371637	MKNK1-AS1
chr1	161067663	161068715	1053	*	12	7.70901E-11	0.016611555	0.051392167	0.01185709	0.061105882	0.012780456	KLHDC9
chr12	26985412	26986520	1109	*	12	5.40023E-10	0.066038304	0.051400587	0.025111045	0.02266297	0.005302712	ITPR2
chr19	10380576	10381577	1002	*	11	2.54355E-11	0.017257841	0.051410141	0.010978944	0.034244898	0.006213409	ICAM1, CTD-2369P2.5
chr1	229478617	229479495	879	*	9	3.28862E-09	0.077481481	0.051416656	0.017177713	-0.037834495	-0.003944912	CCSAP
chr16	57220573	57220662	90	*	2	5.54534E-08	0.018840727	0.051503926	0.02835642	-0.052802446	-0.042862916	RSPRY1
chr7	44240380	44240862	483	*	8	2.42826E-09	0.004868719	0.051517878	0.004824281	0.031665348	0.009950688	YKT6
chr5	179105622	179105869	248	*	4	4.99585E-08	0.000928853	0.051565809	0.003640382	0.01783636	0.0126502	CANX, CBY3
chr8	95961492	95962463	972	*	15	2.2739E-21	7.06422E-09	0.051567636	1.3732E-06	0.137227723	0.041941427	NDUFAF6, RP11-347C18.3, TP53INP1

chr18	42259024	42260451	1428	*	14	4.64398E-10	0.002096361	0.051656691	0.004159174	-0.052074714	0.007681312	SETBP1, RP11-456K23.1
chr10	70091418	70092729	1312	*	13	6.69173E-14	0.000502138	0.051737882	0.000897699	-0.084438661	-0.004614706	HNRNPH3, PBLD
chr1	1655838	1655942	105	*	4	9.6432E-08	0.001943054	0.051775355	0.005930911	0.05393292	0.032905396	RP1-283E3.8
chr5	140026925	140027949	1025	*	17	5.90796E-11	0.095696579	0.051779405	0.040825562	0.045961662	0.008109651	IK, MIR3655, NDUFA2
chr7	2646782	2648289	1508	*	10	7.87873E-11	0.007009078	0.051827816	0.017876315	0.160698309	0.073154252	IQCE
chr17	55162855	55163212	358	*	7	2.84329E-08	0.11555823	0.051839685	0.055830447	0.031753798	0.00019399	AKAP1
chr2	233414677	233415810	1134	*	19	3.28976E-11	0.15095696	0.05187943	0.05599848	0.126859831	0.015830008	EIF4E2, TIGD1, MIR5001
chr3	50374304	50376000	1697	*	25	1.66535E-15	0.199060131	0.051907495	0.016029262	-0.063279374	-0.001861885	RASSF1
chr2	70780723	70781770	1048	*	14	5.27073E-13	0.008238251	0.051916041	0.004278125	-0.049431432	0.002660378	TGFA
chr2	176980837	176981654	818	*	9	5.05935E-09	0.129907377	0.051967034	0.018658383	0.054389264	0.028501775	HOXD10
chr7	96654548	96654832	285	*	7	1.02214E-07	0.020821899	0.052032447	0.01862629	0.055425178	0.028977694	
chr17	61819073	61819520	448	*	8	4.68435E-08	0.081779629	0.052038525	0.041953788	0.031587808	0.001468991	STRADA, RP11-51F16.8
chr9	36276879	36277154	276	*	5	1.42125E-07	0.001628263	0.052077072	0.005960786	-0.228631375	-0.149508311	CLTA, GNE
chr22	41697229	41698001	773	*	8	1.64996E-10	0.003315021	0.052123629	0.003784061	-0.064834754	-0.023503088	ZC3H7B
chr20	61463757	61464168	412	*	4	8.21631E-08	0.002458296	0.052146366	0.007010582	-0.092344008	-0.062433448	COL9A3
chr3	181428046	181430485	2440	*	28	2.82874E-18	5.3687E-06	0.052173274	1.16332E-05	-0.078387847	0.017414084	SOX2-OT, SOX2
chr14	70654824	70655056	233	*	2	7.51028E-09	0.012030603	0.052209863	0.020418422	-0.095440442	-0.061835907	RP11-486O13.2, SLC8A3
chr2	232348334	232348794	461	*	5	1.7461E-08	0.002616043	0.052252952	0.005206868	-0.109537909	-0.080538048	NCL
chr5	72740460	72740748	289	*	5	2.64157E-08	0.022192264	0.052268522	0.016343597	0.090344269	0.044317831	FOXD1
chr8	72756058	72757004	947	*	13	6.37552E-10	0.053549051	0.052283193	0.037450559	0.111614107	0.01266688	RP11-383H13.1, MSC
chr10	15901712	15902731	1020	*	13	2.47556E-10	0.410410227	0.052320167	0.097966678	-0.053415346	-0.002510039	FAM188A
chr3	53164314	53165031	718	*	10	1.88249E-09	0.047357872	0.052335403	0.025062735	-0.053944333	-0.00990415	RFT1
chr20	42219091	42219578	488	*	6	1.08046E-08	0.004771122	0.052335723	0.011142949	-0.054761036	-0.025633372	IFT52
chr17	79859809	79859999	191	*	8	3.72848E-09	0.018829685	0.05234345	0.01128136	0.067620263	0.032618513	NPB, PCYT2
chr20	21494547	21495476	930	*	11	1.15068E-08	0.260718989	0.052371811	0.043382387	0.047270694	0.010729526	NKX2-2
chr4	42399792	42400116	325	*	6	1.21454E-07	0.032220196	0.052435422	0.047413955	0.029755196	0.016553921	SHISA3
chr11	796069	796607	539	*	8	9.53816E-10	0.011012484	0.052477594	0.010201628	0.0319034	0.006585457	SLC25A22
chr19	54693250	54694922	1673	*	27	6.4382E-13	0.020234153	0.052528761	0.02244793	-0.065723248	-0.015234008	TSEN34, MBOAT7
chr2	200320750	200322034	1285	*	12	9.52945E-10	0.019520056	0.052597506	0.008865587	-0.036975828	0.007044188	SATB2

chr6	29795501	29796614	1114	*	14	5.75588E-14	0.000984791	0.052769321	0.003021375	0.11300819	0.042729028	HLA-G, HCG4P8
chr17	43044987	43045386	400	*	4	7.12116E-08	0.06781225	0.052783819	0.043952623	0.055621852	0.037072197	C1QL1
chr19	18942465	18942619	155	*	5	1.19552E-07	0.010904221	0.05284059	0.012829846	-0.023770123	-0.010848123	
chr1	45138784	45140869	2086	*	17	1.56017E-12	0.034912886	0.052941066	0.003219887	-0.072878297	0.001762121	C1orf228, TMEM53
chr19	40971204	40972683	1480	*	17	1.04196E-15	0.000547005	0.052967276	0.000963736	0.128382018	0.011754924	SPTBN4, BLVRB
chr5	52285480	52285560	81	*	2	3.51307E-08	0.099150824	0.053053511	0.077950175	-0.043533283	-0.029494526	ITGA2, CTD-2175A23.1
chr2	61765360	61765778	419	*	7	1.51842E-08	0.179862646	0.053070471	0.030338637	0.031773032	0.011010158	XPO1
chr2	162093969	162095662	1694	*	10	1.19518E-10	8.03861E-05	0.053165174	0.000363791	0.242777004	0.046716764	AC009299.2
chr8	21882104	21882942	839	*	7	7.23118E-10	0.01068302	0.053192113	0.011149856	0.138225794	0.047090862	NPM2
chr6	28889682	28891917	2236	*	47	4.84514E-19	0.372089932	0.053200162	0.035265526	-0.134386766	-0.005574765	TRIM27
chr19	14639450	14640867	1418	*	19	4.87346E-12	0.203658658	0.053289471	0.058102773	-0.12520792	-0.009072464	TECR, MIR639, DNAJB1
chr15	41408316	41409215	900	*	11	2.13126E-10	0.005536955	0.053333354	0.01101252	0.032570008	0.00811727	INO80
chr10	134362678	134362785	108	*	3	8.05939E-09	0.002647385	0.053425191	0.007457872	0.082026923	0.06319328	INPP5A
chr14	55737596	55738545	950	*	13	4.10204E-12	0.047734386	0.053470174	0.023885815	0.070387069	0.013664822	FBXO34, RP11-665C16.6
chr10	12391591	12391835	245	*	5	4.09568E-08	0.028659524	0.053509375	0.010450619	-0.052004933	0.008236411	CAMK1D
chr19	36630543	36631318	776	*	12	1.69155E-09	0.007991051	0.053603436	0.016775722	0.023543732	0.001647623	CAPNS1
chr7	129781152	129781681	530	*	5	6.54015E-08	0.002748656	0.05361511	0.007843347	-0.067567927	-0.030862561	RP11-775D22.2
chr6	8064622	8064764	143	*	4	5.98471E-08	0.001414379	0.053638192	0.005105191	-0.073489375	-0.025048397	BLOC1S5, EEF1E1-BLOC1S5
chr1	38455263	38455987	725	*	12	3.28914E-10	0.014509691	0.053653691	0.013413265	-0.061729119	-0.00714384	SF3A3
chr6	28603161	28603779	619	*	11	1.11114E-09	0.18929402	0.053674254	0.125669783	0.030466514	0.015799235	
chr19	2096331	2096346	16	*	3	1.88086E-07	0.342207599	0.053684075	0.142136426	-0.007635128	-0.004872552	MOB3A
chr1	224622417	224622855	439	*	7	3.10071E-08	0.333784829	0.053714098	0.105811136	0.030397579	0.000392403	CNIH3, WDR26
chr8	145700190	145701245	1056	*	7	5.81851E-10	0.000948199	0.053719821	0.003292693	0.155482464	0.050754914	FOXH1
chr17	74136758	74137769	1012	*	8	3.2517E-09	0.01380937	0.053822485	0.005638493	-0.058755581	-0.012254424	RNF157-AS1, FOXJ1
chr16	54963754	54964029	276	*	2	1.88487E-07	0.01316582	0.05382476	0.022080523	-0.037836873	-0.007400797	
chr1	38413252	38413374	123	*	3	1.90497E-08	0.003565103	0.05386185	0.009285703	0.086974081	0.077169067	
chr1	32110078	32111281	1204	*	19	1.28504E-11	0.135197412	0.053904465	0.034720972	0.10266787	0.00690026	RP11-73M7.6, RP11-73M7.9, PEF1
chr19	45349207	45349903	697	*	9	1.1645E-10	0.002338494	0.053948554	0.006158264	-0.064455491	-0.031646025	PVRL2
chr1	113615644	113616366	723	*	9	7.29258E-09	0.040194	0.05395806	0.020468093	-0.069235912	-0.000865385	LRIG2, RP11-31F15.2

chr15	68498457	68498589	133	*	2	1.23749E-07	0.032193401	0.054037662	0.041726685	-0.071241157	-0.05116681	RP11-315D16.2
chr20	19866743	19867423	681	*	9	1.62343E-09	0.033714617	0.054460951	0.013740967	0.145888511	0.061328948	RIN2
chr1	202317604	202318195	592	*	8	2.24754E-10	0.003511056	0.054469748	0.002695682	-0.040698082	-0.002069474	PPP1R12B
chr1	48937016	48937445	430	*	4	1.50121E-08	0.009777335	0.054577036	0.011844345	-0.070731195	-0.033830401	SPATA6
chr2	173420763	173421844	1082	*	14	5.2441E-13	0.001539016	0.05458516	0.002354127	-0.158465908	-0.008660503	PDK1, AC093818.1
chr4	99181856	99182242	387	*	4	5.729E-08	0.124598942	0.0545917	0.106453093	0.023913882	0.000369231	RP11-323J4.1
chr21	45758354	45758395	42	*	3	7.97184E-08	0.009957015	0.054660286	0.019348715	-0.048388732	-0.044010881	C21orf2
chr6	152489392	152489702	311	*	5	2.43693E-08	0.000542042	0.054668325	0.00287234	0.124747541	0.085101574	SYNE1
chr17	73390116	73390197	82	*	2	9.83412E-08	0.017567632	0.054728359	0.027537689	0.039377271	0.008712724	GRB2
chr16	75600378	75600717	340	*	5	2.73301E-08	0.001979649	0.054780307	0.006156724	0.025864728	0.01144922	GABARAPL2, RP11-77K12.8
chr22	29137385	29138453	1069	*	15	7.12507E-10	0.02257283	0.054839122	0.01909709	0.065442047	0.004509603	HSCB, CHEK2
chr2	172947914	172949870	1957	*	12	2.64327E-10	0.000327279	0.054855833	0.001471181	0.103164498	0.022203036	DLX1
chr1	111506233	111507160	928	*	16	2.47008E-13	0.015374993	0.054863724	0.010626049	0.100220407	-0.009920134	RP11-96K19.5, LRIF1
chr10	134350658	134351765	1108	*	10	3.42413E-12	0.000966625	0.054910369	0.001932013	-0.062021119	-0.022532514	INPP5A
chr21	37528663	37528972	310	*	4	4.94788E-08	0.001263669	0.054933369	0.004844096	-0.052626447	-0.030545104	
chr8	10697017	10697698	682	*	9	2.50235E-10	0.041982772	0.05494425	0.0149146	0.036073133	0.010073828	SOX7, SOX7, PINX1
chr12	80328163	80328999	837	*	6	3.81799E-09	0.079569513	0.054963445	0.049509752	-0.057191582	-0.006735465	RP11-84G21.1, PPP1R12A
chr5	132201652	132203292	1641	*	20	1.93755E-12	0.014932129	0.05498591	0.003790739	0.081043835	-5.75143E-05	UQCRQ, GDF9
chr10	22633916	22635028	1113	*	14	5.65732E-12	0.014902885	0.055009583	0.011817755	0.076555837	0.031193428	SPAG6
chr3	169381055	169381522	468	*	5	9.38401E-09	0.157477846	0.055082799	0.027008285	-0.060106535	-0.014794512	MECOM
chr11	68670746	68671486	741	*	9	8.73598E-10	0.022177121	0.055132627	0.007796962	0.111214412	0.031824252	IGHMBP2, MRPL21
chr20	43935283	43935551	269	*	9	1.0876E-07	0.016657163	0.055297629	0.03814846	0.081177246	0.042797609	RBPJL, MATN4
chr6	13272885	13273147	263	*	4	4.11596E-09	0.000864055	0.055341227	0.003650471	-0.069508735	-0.061018656	PHACTR1, RP1-257A7.4, TBC1D7
chr15	101084507	101085388	882	*	11	7.30506E-13	0.000130686	0.055368292	0.00098936	0.204199586	0.114481148	CERS3
chr8	21645807	21646309	503	*	6	3.02605E-08	0.033259489	0.055448345	0.022238485	0.038890442	0.01800915	GFRA2
chr4	107237103	107238654	1552	*	14	8.7768E-12	0.023177517	0.055464189	0.002905636	-0.054640919	-0.006998442	AIMP1, TBCK
chr10	27443031	27444140	1110	*	12	1.08339E-11	0.018733649	0.055474173	0.01009279	-0.030966238	-0.006254969	MASTL, YME1L1
chr10	5406543	5407119	577	*	9	3.77656E-08	0.327011043	0.055492966	0.057619108	0.121531812	0.036207839	UCN3
chr9	35665143	35665465	323	*	4	1.68784E-07	0.081250802	0.055494667	0.051040494	0.013820847	0.008472147	ARHGEF39

chr4	2795105	2795705	601	*	7	1.95794E-09	0.001290289	0.055496437	0.00458169	-0.090736843	-0.025408103	SH3BP2
chr1	8484004	8484703	700	*	11	1.1336E-09	0.008178304	0.055500147	0.013766511	-0.130587854	-0.036633168	RERE
chr5	133512410	133513291	882	*	11	1.71575E-09	0.041290171	0.055527486	0.034651486	-0.082032246	-0.011363669	SKP1, CTD-2410N18.5
chr6	90538851	90539804	954	*	11	6.46444E-11	0.01621992	0.055556279	0.011728729	-0.027554657	-0.004449177	CASP8AP2
chr19	7745377	7745524	148	*	3	7.20793E-08	0.004413233	0.055575228	0.010920646	0.028178188	0.005925079	CTD-3214H19.16
chr7	75677497	75677766	270	*	3	6.77713E-08	0.243892878	0.055665145	0.140396744	0.018415561	0.005547352	MDH2
chr1	118471375	118473064	1690	*	20	1.08897E-15	0.000350631	0.055703474	0.000720605	-0.074342928	-0.015726336	WDR3, GDAP2
chr2	239008705	239009246	542	*	8	2.11408E-09	0.004412976	0.055773248	0.010062026	0.16200158	0.074910462	ESPNL
chr18	35145353	35147276	1924	*	30	2.16359E-19	0.016158405	0.055796495	0.001535986	0.049266882	0.008511724	CELF4
chr15	52970418	52971596	1179	*	9	1.14269E-10	0.000623612	0.055813155	0.00267611	0.057872446	-0.021333126	FAM214A
chr1	153755803	153756397	595	*	7	1.35846E-09	0.005847422	0.055830413	0.003515708	-0.093237216	-0.028190874	
chr19	33071683	33072230	548	*	6	3.86396E-08	0.092239682	0.055830803	0.044909636	-0.097573026	-0.0173334	PDCD5
chr1	113932889	113934059	1171	*	14	6.08109E-11	0.003875186	0.055906104	0.006381297	-0.042797326	0.00648974	MAGI3
chr15	76628725	76629771	1047	*	8	9.41589E-09	0.030621241	0.055949592	0.0121793	0.064540557	0.011522503	ISL2
chr17	36719518	36719773	256	*	5	6.43543E-08	0.002161313	0.055986295	0.00629238	0.068158185	0.037381186	SRCIN1
chr8	143581070	143581481	412	*	2	6.56042E-08	0.020022308	0.056064319	0.030536946	0.137671899	0.113655006	BAI1
chr15	45670406	45671028	623	*	10	1.26208E-08	0.309681491	0.056203709	0.104348238	0.017545747	0.003564309	GATM
chr1	111746640	111748268	1629	*	14	7.56077E-17	0.00073278	0.056211587	0.000741663	-0.118325405	-0.036855766	CHI3L2, DENND2D
chr10	96161300	96162988	1689	*	14	2.05284E-13	0.000444491	0.056228813	0.001628305	-0.099129819	0.002107927	TBC1D12
chr1	180471735	180472686	952	*	9	1.83659E-08	0.064527824	0.056374076	0.086585842	-0.097229371	-0.021628892	ACBD6
chr20	3749406	3749756	351	*	4	2.47453E-08	0.001430017	0.056395501	0.005370905	0.048035771	0.012978961	
chr5	31531945	31532116	172	*	3	2.19183E-08	0.027720391	0.056423201	0.030337371	0.015366549	0.012223134	DROSHA
chr3	56835282	56836259	978	*	7	6.41859E-09	0.014765776	0.056495459	0.010953419	0.148747699	0.029132862	ARHGEF3
chr1	109203901	109204654	754	*	10	3.03979E-10	0.049765355	0.056556046	0.026699198	0.076505621	0.016831061	HENMT1
chr1	197743842	197745295	1454	*	20	2.4362E-12	0.034510775	0.056601719	0.012365218	-0.123340877	-0.005528616	DENND1B
chr12	9102068	9103184	1117	*	14	1.28687E-12	0.016625492	0.056636377	0.004444205	-0.054768683	-0.001614391	KLRG1, M6PR
chr7	145813413	145813946	534	*	8	5.10036E-09	0.021483144	0.056641326	0.009185065	0.052482158	0.011330677	CNTNAP2
chr22	44287485	44288659	1175	*	15	3.18097E-13	0.166672618	0.056678149	0.008970953	0.120320582	0.024454966	PNPLA5
chr1	202113040	202114133	1094	*	12	3.93741E-12	0.0062907	0.056738623	0.003238514	-0.092744271	0.000193124	ARL8A

chr19	18747598	18748017	420	*	8	3.43792E-09	0.01467467	0.056819811	0.018701402	-0.056045729	-0.028723419	KLHL26
chr2	136288315	136289456	1142	*	14	1.39244E-15	0.026033929	0.056838941	0.002976845	-0.049473857	-0.016556566	R3HDM1, ZRANB3
chr2	208989209	208990238	1030	*	12	7.1415E-11	0.04565386	0.056935659	0.022379094	-0.143635121	-0.026931031	CRYGD
chr5	153825063	153826017	955	*	10	1.65162E-09	0.024407186	0.057124018	0.009223347	-0.130435018	-0.019673585	SAP30L, SAP30L-AS1
chr6	24645875	24646551	677	*	13	1.25281E-09	0.453680149	0.05716618	0.082839774	-0.038863584	0.002914736	KIAA0319
chr2	47403193	47403831	639	*	5	2.40816E-11	0.015222439	0.05718356	0.020820644	-0.078090942	-0.047472721	RP11-761B3.1, CALM2
chr12	64615340	64616385	1046	*	14	1.0736E-12	0.002516343	0.0571962	0.002133879	-0.048571954	0.00535235	RPS11P6, C12orf66
chr17	27621110	27621314	205	*	7	1.73985E-07	0.005073739	0.057305733	0.009722521	-0.045895934	-0.007925309	NUFIP2
chr2	109402797	109403293	497	*	11	1.7424E-08	0.174358613	0.05733492	0.053336994	-0.030562945	-0.003698549	CCDC138
chr2	43822965	43823287	323	*	7	4.65095E-08	0.023541088	0.057336349	0.029206361	0.02472765	0.007520961	THADA
chr8	53852101	53852661	561	*	7	1.31281E-08	0.002238972	0.057396944	0.00725582	-0.043628772	-0.0085365	NPBWR1
chr20	34287144	34288088	945	*	8	8.35193E-09	0.055048731	0.057435016	0.040880204	0.104785622	0.01597044	ROMO1, NFS1
chr7	65669728	65670474	747	*	10	1.30534E-08	0.162400882	0.057495208	0.057487335	-0.051285526	-0.003473869	TPST1
chr6	33284839	33286244	1406	*	15	6.30915E-11	0.008644405	0.0574969	0.015294751	0.112792081	0.004661335	ZBTB22
chr1	213188705	213189823	1119	*	16	9.53259E-11	0.160810071	0.057648483	0.039123031	-0.121542489	-0.013470509	ANGEL2
chr14	103058097	103059453	1357	*	14	5.46975E-11	0.037912046	0.057772274	0.017332474	-0.042802796	-0.003280121	RCOR1
chr10	135050619	135052004	1386	*	24	5.59936E-18	0.001531892	0.05783031	0.00557839	0.137565511	0.034029831	VENTX
chr11	8986094	8987427	1334	*	16	7.23432E-09	0.127300907	0.057843435	0.039000589	-0.119080427	-0.002121497	TMEM9B-AS1, TMEM9B
chr17	63557609	63558518	910	*	9	1.85098E-09	0.000588341	0.057883301	0.002988342	0.113004967	0.03114343	AXIN2, CTD-2535L24.2
chr2	73460340	73461528	1189	*	16	5.70278E-11	0.032595876	0.057908329	0.016940827	-0.021625219	-6.89144E-06	CCT7, PRADC1
chr15	58357874	58357989	116	*	7	1.37136E-07	0.146665498	0.057937665	0.058887499	0.083795591	0.028275476	CTD-2330J20.2, ALDH1A2
chr16	57405979	57406074	96	*	3	1.60462E-07	0.024824406	0.057961868	0.029381662	0.12598325	0.074553235	
chr1	203733914	203734559	646	*	9	2.84368E-09	0.016245102	0.058020919	0.014974039	0.097086833	0.046641823	LAX1
chr1	28969288	28970259	972	*	14	9.11758E-10	0.05433977	0.058051618	0.031688551	-0.036331825	-0.004967769	RP11-442N24__B.1, TAF12
chr1	185286441	185286607	167	*	4	3.67532E-08	0.003837246	0.05808501	0.01042639	-0.088423313	-0.024627729	IVNS1ABP
chr2	220042748	220043530	783	*	9	1.47682E-09	0.002564789	0.058097326	0.006524102	0.011873373	0.002910323	FAM134A, CNPPD1
chr15	51057417	51058141	725	*	11	4.22255E-10	0.028798315	0.058195052	0.014422096	-0.042550485	-0.011501954	SPPL2A
chr1	211306365	211307563	1199	*	11	4.32052E-09	0.01322313	0.058216397	0.013671786	-0.075482368	0.0059712	KCNH1, KCNH1-IT1
chr2	74405770	74406553	784	*	12	3.39417E-09	0.027829473	0.058283549	0.020400358	0.099540693	-0.003882469	MOB1A

chr18	12377377	12378044	668	*	9	7.20671E-13	0.013366754	0.058377887	0.007436375	-0.073511844	-0.025866681	
chr20	35580059	35580258	200	*	6	2.5244E-08	0.014465406	0.05841215	0.011605673	0.018803921	0.00809168	SAMHD1
chr17	75136729	75137495	767	*	9	1.76963E-10	0.003744802	0.058441926	0.005197459	-0.07373521	-0.034577997	SEC14L1
chr12	14996143	14996776	634	*	10	3.70639E-11	0.02210493	0.058487804	0.007976796	0.15769546	0.06272403	C12orf60, RP11-233G1.4, ART4
chr3	14692357	14693598	1242	*	10	2.51357E-11	0.000561507	0.058600068	0.002086893	0.06425881	0.01762038	CCDC174
chr12	112204136	112204761	626	*	10	2.878E-08	0.071726933	0.058615155	0.036850271	0.044056573	0.013058421	RP11-162P23.2, ALDH2
chr2	21022431	21023042	612	*	11	6.11431E-10	0.016563963	0.058631786	0.014705564	0.022181589	0.008196884	C2orf43
chr3	48723293	48724305	1013	*	7	4.26957E-08	0.009854937	0.058643702	0.012796609	0.100126848	0.017576535	NCKIPSD
chr16	19566400	19567590	1191	*	15	3.50912E-12	0.01065315	0.058683618	0.006187429	0.065698908	0.009391362	C16orf62
chr4	960520	961658	1139	*	7	4.27977E-10	0.004893042	0.058699393	0.00565593	-0.191671232	-0.058330381	DGKQ
chr1	34632402	34632588	187	*	5	9.00159E-08	0.021600902	0.058732021	0.016858874	0.09599694	0.026963343	C1orf94
chr19	18450967	18451650	684	*	15	1.68498E-09	0.061452837	0.058757295	0.053714281	-0.052702378	0.004880211	PGPEP1
chr1	167522981	167523555	575	*	9	4.1015E-08	0.024686902	0.058799816	0.023939	-0.047067422	-0.006959549	CREG1
chr7	113722758	113724153	1396	*	10	2.23463E-10	0.000399882	0.058827317	0.001803971	0.099082255	0.023204832	
chr12	122326914	122327438	525	*	5	1.1757E-09	0.006728696	0.058838609	0.011886525	0.054336408	0.019072597	PSMD9, RP11-87C12.2
chr6	16238547	16239574	1028	*	9	7.07849E-12	0.037636258	0.058840957	0.027076439	-0.053775712	-0.003797273	GMPR
chr1	6453427	6454543	1117	*	16	1.42049E-12	0.019288242	0.058877133	0.015914594	0.067951296	0.001641062	RP1-202O8.3, ACOT7
chr6	24126060	24126497	438	*	10	4.38752E-10	0.095718805	0.058917173	0.01864072	0.067193633	0.025540685	NRSN1
chr19	18529969	18529997	29	*	4	1.37139E-07	0.008947999	0.058950956	0.014351294	-0.068996405	-0.042273618	SSBP4
chr4	56501718	56502116	399	*	4	9.0961E-09	0.038674153	0.058952782	0.024798076	0.0511804	0.01804917	NMU
chr1	156560925	156561646	722	*	11	8.93945E-10	0.074725199	0.059006365	0.019843049	0.116344815	0.004201496	APOA1BP
chr17	40687226	40687943	718	*	10	1.50624E-11	0.01682941	0.05905917	0.011722123	0.117335542	0.041562281	
chr5	176433461	176434079	619	*	11	2.45838E-09	0.058431223	0.059132715	0.022299107	-0.056598156	-0.006556268	UIMC1
chr3	196668656	196669655	1000	*	14	1.45863E-12	0.037920955	0.059205166	0.021475734	-0.065474912	-0.00627979	NCBP2-AS1, NCBP2-AS2, NCBP2
chr5	156569391	156570357	967	*	12	1.14024E-09	0.040998227	0.059218736	0.046220412	-0.043449776	-0.010989103	ITK, HAVCR2, MED7
chr20	46130313	46131144	832	*	14	1.84155E-09	0.03969069	0.059230176	0.044545651	-0.031751329	0.000553758	NCOA3
chr2	74648306	74648964	659	*	8	7.22719E-08	0.084114495	0.059322819	0.040434569	-0.0266487	-0.003727375	WDR54, C2orf81
chr2	43453251	43454950	1700	*	27	6.19654E-13	0.087109622	0.059440453	0.055912887	-0.103896839	-0.011819423	THADA, ZFP36L2
chr10	71211210	71211838	629	*	8	2.10923E-08	0.010785355	0.05944142	0.013613963	0.064498497	0.021097969	TSPAN15

chr22	19466632	19467810	1179	*	18	2.80275E-12	0.014365966	0.059471895	0.026780776	-0.04878712	-0.015627944	CDC45, UFD1L
chr1	33502512	33503118	607	*	11	2.7853E-08	0.357979898	0.059478521	0.093599329	-0.060871459	-0.011958504	AK2
chr1	112297799	112298991	1193	*	16	9.06188E-11	0.056225685	0.059483313	0.008007631	-0.034433992	0.005372797	DDX20, FAM212B
chr16	2563036	2564146	1111	*	17	9.08391E-10	0.149582926	0.059523023	0.094909325	0.099083303	0.012562298	RP11-20I23.1, ATP6V0C, ATP6C
chr7	150102268	150103902	1635	*	10	4.56344E-11	0.002507403	0.059562246	0.004093045	0.069110534	0.017045413	ZNF775
chr3	88108136	88108324	189	*	7	1.44031E-07	0.064772773	0.059588678	0.037665713	-0.046989464	-0.00727132	CGGBP1
chr10	90342899	90343435	537	*	11	6.93173E-09	0.061682538	0.05959793	0.035282953	0.099096238	0.028585524	RNLS
chr19	54057208	54058085	878	*	9	1.52765E-10	0.002484837	0.059636413	0.003209694	-0.127957769	-0.081729032	ZNF331
chr11	1769152	1770066	915	*	9	6.62012E-13	5.29692E-05	0.059680431	0.000640176	0.208970042	0.106542514	IFITM10, RP11-295K3.1
chr15	74658981	74660563	1583	*	12	6.58358E-11	0.027148584	0.059705502	0.010815709	0.118415393	0.036438834	CTD-2311M21.3, CYP11A1
chr11	101981004	101981422	419	*	8	3.5809E-08	0.159728169	0.059734408	0.055113734	-0.292573286	-0.049177208	YAP1
chr12	56694541	56694932	392	*	4	2.5987E-08	0.007505443	0.059825117	0.013023848	0.125627872	0.084476526	RP11-977G19.11, RP11-977G19.10
chr12	120555025	120555510	486	*	5	9.04701E-08	0.044917108	0.059919132	0.028796857	-0.030278905	0.00131872	RAB35
chr11	46354271	46354803	533	*	10	1.83136E-08	0.209918557	0.059920501	0.095017956	0.047463335	0.011710065	DGKZ
chr1	186649354	186649985	632	*	12	2.68238E-08	0.038125758	0.059945151	0.034319864	-0.049824399	-0.015078453	RP5-973M2.2, PTGS2
chr1	50886393	50887477	1085	*	8	6.05176E-12	6.64786E-05	0.060004674	0.000820964	0.079799285	0.051202523	DMRTA2
chr5	54528989	54530114	1126	*	12	7.38775E-10	0.012771048	0.060022612	0.017424845	-0.040217696	0.002797295	RP11-506H20.1, CCNO
chr10	1095317	1095578	262	*	6	8.0328E-08	0.012711476	0.060183633	0.019223596	-0.045889282	-0.013740579	WDR37
chr14	78083170	78083524	355	*	5	6.08858E-08	0.009313767	0.060210187	0.010039464	-0.022583403	-0.002032049	
chr5	102594123	102594617	495	*	6	8.25778E-09	0.026368826	0.060213157	0.009412934	-0.039394622	-0.005630504	C5orf30
chr5	7868703	7869652	950	*	15	3.02423E-16	0.001999192	0.060236654	0.001363387	-0.040410817	0.002533421	MTRR, FASTKD3
chr1	101004934	101005509	576	*	5	2.72852E-08	0.001063113	0.060237226	0.004776847	0.051388804	0.021910093	GPR88
chr15	82338222	82339233	1012	*	12	2.36943E-09	0.097207638	0.060273891	0.035932126	-0.117445146	-0.012136609	MEX3B
chr1	114301549	114302602	1054	*	15	4.22594E-10	0.462845686	0.060316437	0.11318723	0.027566156	0.007148207	PHTF1
chr6	27805693	27806393	701	*	10	7.7487E-10	0.022415703	0.060377124	0.007090888	-0.095286938	-0.017511293	HIST1H2BN, HIST1H2AK
chr6	29759947	29761288	1342	*	23	6.23995E-22	0.000622343	0.060401018	0.000410894	0.068203145	0.027616893	HLA-V, HCG4
chr6	12011930	12013002	1073	*	11	1.01864E-09	0.023739654	0.060402711	0.027533405	-0.050591784	-0.022227003	HIVEP1
chr8	124084365	124085299	935	*	13	2.79668E-11	0.02965215	0.060409324	0.015173398	-0.10202822	-0.020848484	TBC1D31
chr11	34074141	34074400	260	*	4	3.29884E-08	0.002837992	0.060533921	0.009042879	0.032206712	0.018061048	CAPRIN1

chr17	36908879	36909310	432	*	8	4.24258E-08	0.085985416	0.060712186	0.054641319	-0.042385768	-0.007169083	PSMB3
chr14	75469136	75469881	746	*	12	5.73287E-10	0.020866121	0.060716024	0.015947685	-0.038108206	0.003939638	EIF2B2
chr6	30538523	30540994	2472	*	35	4.11648E-12	0.124120073	0.06073241	0.028429947	-0.115188331	0.000278429	ABCF1
chr20	44746392	44747351	960	*	9	9.78043E-09	0.081614361	0.060835832	0.018421902	-0.085631128	0.004384429	CD40
chr2	61293079	61293725	647	*	9	7.36384E-09	0.103095106	0.06090766	0.050432441	-0.044872541	-0.000106872	KIAA1841
chr1	45987459	45987978	520	*	8	6.15721E-08	0.028999247	0.061036804	0.054202081	-0.080409599	-0.022820723	PRDX1
chr19	1490631	1491289	659	*	7	2.51677E-09	0.014957142	0.06104537	0.014560538	-0.077272192	-0.003632791	REEP6, PCSK4
chr1	94883469	94884189	721	*	10	1.10734E-08	0.016952436	0.061145019	0.016333332	0.03475071	0.012791157	ABCD3
chr2	25015152	25016582	1431	*	19	4.02815E-16	0.001634783	0.061216728	0.003250507	-0.12169291	-0.013137557	CENPO, PTRHD1
chr17	46178618	46179468	851	*	11	2.81387E-10	0.013307337	0.061281797	0.019699524	0.07752575	0.00375137	CBX1
chr11	65029414	65030075	662	*	7	2.05044E-08	0.021079536	0.061351863	0.020101221	0.02329704	0.007217283	POLA2
chr9	93563351	93564149	799	*	12	1.15128E-08	0.177790602	0.061365062	0.069536841	-0.140794092	-0.001507003	SYK
chr1	16553267	16553549	283	*	5	7.73345E-08	0.002226021	0.061387121	0.006840005	-0.073624307	0.027868432	ANO7P1
chr3	42845813	42846188	376	*	8	4.79416E-09	0.079868562	0.061389478	0.030401804	-0.098754074	-0.016523746	HIGD1A
chr18	33708920	33710235	1316	*	20	1.16475E-11	0.087480635	0.061443298	0.038162909	0.038294427	9.81564E-05	ELP2, SLC39A6
chr6	160209868	160210888	1021	*	13	5.60027E-13	0.001365244	0.061510424	0.003013261	-0.073332169	-0.004342416	MRPL18, TCP1
chr5	54603874	54603891	18	*	2	1.71651E-07	0.019917462	0.061532987	0.031427185	-0.081098449	-0.065943573	SKIV2L2
chr22	39101190	39102110	921	*	15	9.58198E-12	0.017624646	0.061535675	0.012769015	-0.053829636	-0.012536509	GTPBP1
chr3	118864895	118865255	361	*	8	8.83378E-08	0.054158297	0.061572509	0.041538158	0.156315317	0.055059161	C3orf30, IGSF11
chr17	46125498	46126298	801	*	9	8.26972E-10	0.001062609	0.061587355	0.004628674	-0.046850269	-0.009679636	NFE2L1
chr12	103889166	103890011	846	*	12	5.26414E-09	0.048903456	0.061634427	0.020881373	-0.056455464	-0.002046106	C12orf42
chr6	111279446	111280279	834	*	18	5.05325E-10	0.196717158	0.061659664	0.080735836	-0.045378493	-0.004731552	GTF3C6
chr4	1399938	1400189	252	*	2	1.13566E-07	0.158793314	0.061743322	0.104488545	0.033167854	0.021688277	NKX1-1
chr14	96000874	96001369	496	*	12	1.11352E-09	0.044612946	0.061777222	0.033752884	0.028349056	0.001346073	GLRX5, SNHG10
chr7	107384245	107384783	539	*	6	1.2739E-07	0.113055801	0.061835438	0.068899606	-0.044066039	0.002929357	CBLL1, AC002467.7
chr19	3572410	3572957	548	*	10	3.98599E-08	0.149778531	0.061878782	0.04727734	0.031463192	0.002643097	HMG20B, MFSD12
chr16	31519926	31520645	720	*	7	3.10071E-08	0.08331844	0.062002673	0.034103714	0.092411235	0.019388913	RP11-452L6.7, C16orf58
chr6	33395005	33395936	932	*	9	2.90694E-09	0.030392491	0.062068989	0.022191579	-0.042290453	-0.014419922	SYNGAP1
chr20	62168764	62169334	571	*	8	1.60556E-08	0.009953664	0.062074204	0.016065463	0.065179944	0.01183376	

chr2	47629743	47630845	1103	*	13	2.96092E-11	0.019743367	0.062089818	0.009873274	-0.038521797	4.12015E-05	MSH2
chr3	160282993	160283655	663	*	11	3.36216E-08	0.049429039	0.062169322	0.050808449	-0.03619045	-0.002506253	KRT8P12, KPNA4
chr14	59654921	59656055	1135	*	12	1.01017E-09	0.029692931	0.062175727	0.020783706	0.016649321	0.00505528	DAAM1
chr12	110150904	110151678	775	*	5	1.3953E-09	0.011883722	0.062178864	0.016963308	0.133759508	0.052767814	
chr9	115512278	115513756	1479	*	16	1.02892E-09	0.019653855	0.062286345	0.009200903	0.06837077	0.008904892	SNX30, RP11-276E15.4
chr12	14518526	14518974	449	*	7	2.17515E-08	0.002749632	0.062309321	0.005617175	-0.065115452	-0.020859011	ATF7IP
chr17	16283541	16284362	822	*	9	7.30156E-11	0.010146449	0.06233166	0.007778531	-0.056883295	-0.010074119	UBB
chr8	65286244	65286632	389	*	6	3.44113E-08	0.013691374	0.062345455	0.032553645	0.042812168	0.022660081	LINC00966
chr2	170684197	170684455	259	*	2	4.85447E-08	0.018198944	0.062429434	0.029544951	0.036231386	0.014456735	UBR3
chr13	23310464	23310675	212	*	3	1.6437E-07	0.0052326	0.062542748	0.01326796	-0.14759776	-0.121614559	
chr1	16010601	16010732	132	*	3	4.35187E-08	0.006710593	0.06258558	0.015897777	-0.069918741	-0.051343735	
chr6	41039639	41041678	2040	*	22	1.422E-21	3.1483E-05	0.062628553	3.96387E-05	-0.065146045	-0.013534486	NFYA, OARD1
chr8	38088736	38089439	704	*	11	5.6802E-09	0.536969467	0.062639797	0.126215639	-0.054761146	-0.012393591	DDHD2
chr12	109124574	109125017	444	*	7	1.66949E-08	0.056530924	0.062689462	0.022796692	-0.090385747	-0.039488	CORO1C
chr17	11900221	11901614	1394	*	13	1.22315E-12	0.00025431	0.062745067	0.002353656	0.078781559	0.009304422	ZNF18
chr3	145879084	145879710	627	*	11	9.45058E-11	0.018574788	0.062821694	0.011672424	-0.194876675	-0.04484927	PLOD2
chr3	130745160	130745959	800	*	19	3.82105E-10	0.063903266	0.062878418	0.047180127	0.038989999	0.002542488	NEK11, ASTE1
chr11	75525525	75526473	949	*	15	8.17862E-13	0.002672769	0.06290885	0.005960353	-0.052144528	-0.007137214	UVRAG, RP11-535A19.2
chr5	39074123	39075183	1061	*	13	6.64896E-11	0.052596497	0.06295245	0.02416152	0.022119257	0.002922944	RICTOR
chr11	63448356	63448992	637	*	11	1.35666E-12	0.046458772	0.062982494	0.006053788	-0.086492374	-0.002510294	RTN3
chr1	53392650	53393560	911	*	12	7.73089E-10	0.371546836	0.063099102	0.176889835	-0.058754834	-0.000394958	SCP2, ECHDC2
chr14	105531855	105532252	398	*	8	1.58805E-08	0.011047353	0.063102938	0.016232747	-0.050625312	-0.019166531	
chr6	30522620	30525447	2828	*	60	6.2238E-16	0.558610777	0.063132246	0.070090805	0.136986238	-0.00102119	PRR3, GNL1
chr3	44380113	44380138	26	*	3	1.59745E-07	0.501192259	0.06313946	0.055687947	0.018994376	0.010757422	TCAIM
chr12	25055214	25056670	1457	*	17	1.00167E-15	0.00251281	0.063191099	0.001225776	-0.047852022	-0.000275214	AC026310.1, BCAT1
chr10	102899577	102899983	407	*	5	7.22245E-08	0.005334452	0.063220574	0.009675457	0.097559416	0.003346359	
chr13	96328888	96329629	742	*	10	2.95656E-10	0.006129914	0.063233796	0.006999643	-0.091941365	-0.014225879	DNAJC3, DNAJC3-AS1
chr1	173445917	173446516	600	*	11	1.35622E-10	0.008648764	0.06325327	0.01258978	-0.078937975	-0.007898698	PRDX6
chr1	1369616	1370722	1107	*	9	1.94574E-09	0.0967982	0.063253632	0.064592936	-0.058005347	-0.020354879	VWA1, RP4-758J18.10

chr2	84686507	84687236	730	*	9	2.93644E-09	0.045926635	0.063379436	0.019441739	-0.101040149	-0.02737604	SUCLG1
chr7	150755180	150755981	802	*	18	4.90527E-12	0.096903541	0.063394114	0.043150278	0.150267454	0.007317626	SLC4A2, CDK5
chr5	50265407	50265924	518	*	7	4.37364E-09	0.012825577	0.063400356	0.013543657	-0.055390342	0.012744548	CTD-2089N3.3, CTD-2089N3.2
chr20	33145456	33147017	1562	*	12	1.7213E-13	0.00120329	0.063445194	0.001765673	0.120487264	0.025709134	MAP1LC3A
chr11	537287	537737	451	*	11	3.06939E-08	0.070353854	0.063509008	0.059884564	0.06022041	0.017979162	LRRC56, HRAS
chr9	125795283	125795935	653	*	7	4.37617E-11	0.000274122	0.063566479	0.002041079	-0.08813787	-0.041679164	RABGAP1
chr12	111180513	111180884	372	*	5	7.32472E-08	0.004134031	0.063580826	0.010495064	0.01602912	0.008844671	PPP1CC
chr11	73881204	73882184	981	*	15	5.11197E-10	0.226971326	0.06386772	0.113850424	-0.076445374	-0.001428741	PPME1, C2CD3
chr17	37761216	37761316	101	*	2	3.62954E-08	0.171690989	0.06389673	0.110016429	0.066201199	0.037046005	NEUROD2
chr7	2884124	2884448	325	*	7	2.56433E-08	0.023052357	0.063924869	0.01893566	-0.104060522	-0.041960047	
chr15	79164980	79165973	994	*	11	2.16988E-08	0.543489372	0.063950715	0.104766488	-0.055429049	-0.005436335	MORF4L1
chr16	27413786	27414281	496	*	5	4.06065E-08	0.002917317	0.063989445	0.008649167	0.102134494	0.042542172	IL21R
chr6	30130819	30132715	1897	*	27	8.627E-16	0.002008947	0.064043866	0.001101331	0.162074656	-0.004039121	TRIM15
chr11	62439250	62439866	617	*	10	1.0707E-10	0.025641654	0.064211229	0.025385503	0.019977316	-0.000304916	C11orf83, C11orf48
chr14	65005951	65007512	1562	*	30	3.93211E-19	0.005934199	0.064226916	0.000711754	0.166293814	0.00301828	HSPA2, RP11-973N13.4
chr16	88772210	88772686	477	*	6	2.70501E-08	0.002542355	0.064233068	0.008567519	0.037680184	0.018199891	RNF166
chr14	23235667	23236155	489	*	10	8.48581E-09	0.150803584	0.064266948	0.076474673	0.021374968	0.001584427	OXA1L, CTD-2555K7.2
chr12	133000028	133000830	803	*	8	3.17621E-10	0.000158425	0.064554794	0.001424273	-0.411666883	0.011192876	
chr18	48723744	48724109	366	*	4	1.01742E-07	0.041898731	0.06456533	0.059192887	0.012075037	0.009150218	MEX3C
chr14	90420875	90422339	1465	*	14	1.00316E-12	0.017317272	0.064643997	0.012787448	-0.052360112	-0.00433202	TDP1, RP11-33N16.3, EFCAB11
chr10	7829471	7830258	788	*	14	5.71576E-12	0.017911037	0.064656278	0.012552689	-0.032259438	0.005427096	ATP5C1, KIN
chr4	78079025	78079628	604	*	7	4.26811E-08	0.006906485	0.064658481	0.015517345	0.026704608	0.006825817	CCNG2
chr3	42694783	42695143	361	*	5	4.47888E-08	0.002175561	0.064703437	0.00811966	-0.089290946	-0.073386442	RP4-613B23.1
chr19	40596516	40597063	548	*	10	4.24011E-09	0.005817512	0.064722827	0.014727541	-0.029842403	-0.002264278	AC005614.3, AC005614.5, ZNF780A
chr10	45869189	45869816	628	*	10	5.44299E-09	0.164664044	0.064801184	0.042642453	0.05716234	0.005724073	ALOX5
chr6	31583458	31584223	766	*	5	3.79224E-08	0.001851922	0.064869719	0.007225637	-0.062091842	-0.042066803	AIF1
chr5	64919809	64920006	198	*	3	8.30137E-08	0.193362215	0.064904519	0.06768277	0.00780009	0.004910101	TRIM23
chr4	40632362	40633095	734	*	9	1.3305E-09	0.041403452	0.064926402	0.029761955	-0.10155021	-0.039108221	RBM47
chr5	134181226	134182149	924	*	14	2.85113E-13	0.005951832	0.064957069	0.003281151	-0.040587666	-0.009737198	DDX46, C5orf24

chr6	33245128	33246488	1361	*	41	2.53762E-15	0.126761566	0.064991491	0.08437723	0.134086355	0.020264618	B3GALT4
chr16	67571354	67571821	468	*	4	1.07428E-07	0.768620557	0.065088547	0.275669123	0.021817371	0.003225246	FAM65A
chr1	77684965	77685555	591	*	10	2.06842E-09	0.071244485	0.065099074	0.027544248	0.02157569	0.006889255	PIGK
chr22	38901968	38902252	285	*	2	1.30369E-08	0.02892519	0.065321738	0.041912403	-0.031228481	-0.007795585	DDX17
chr12	48151524	48152931	1408	*	18	7.29459E-12	0.011335043	0.065554929	0.027061659	-0.107850123	-0.020681694	SLC48A1, RAPGEF3
chr15	65281804	65282678	875	*	9	5.89423E-10	0.004924277	0.065588774	0.006221076	-0.046119272	-0.010952172	SPG21
chr22	24890045	24891666	1622	*	19	1.82513E-13	0.007303637	0.065715394	0.004971451	0.16348722	0.044656642	UPB1, ADORA2A-AS1
chr14	23563715	23565052	1338	*	22	1.20343E-16	0.000687348	0.065725475	0.000731207	-0.071201643	-0.012689775	C14orf119, ACIN1
chr5	118603908	118604540	633	*	10	1.07846E-08	0.205301961	0.065745086	0.060035406	-0.096446054	-0.013804968	TNFAIP8
chr1	228296788	228297275	488	*	11	1.58588E-08	0.126165298	0.065877463	0.062002458	-0.078874053	-0.016011499	MRPL55
chr6	143266406	143266788	383	*	3	4.38348E-08	0.540520697	0.065890755	0.227717554	0.021683378	0.002452451	
chr22	32341191	32341689	499	*	10	6.75411E-08	0.035444519	0.065961584	0.039383268	0.020850589	-0.001218156	YWHAH, C22orf24
chr5	176037325	176037510	186	*	3	2.69024E-08	0.031913832	0.065969646	0.037517177	0.031007362	0.023069833	
chr8	74659085	74659490	406	*	2	3.27576E-08	0.153020394	0.066031699	0.107428855	-0.044055849	-0.023633138	STAU2, RP11-463D19.2
chr17	42200988	42201211	224	*	8	7.87799E-08	0.176749326	0.066168063	0.033496007	-0.086000838	-0.030460587	HDAC5
chr2	70056602	70057052	451	*	7	2.68838E-08	0.021609501	0.066255824	0.041793654	0.026190839	0.003077097	GMCL1
chr20	62338600	62339243	644	*	13	6.59331E-09	0.295545591	0.066370749	0.109388808	-0.038968186	-0.002202464	ZGPAT, ARFRP1
chr12	65218019	65218869	851	*	14	1.8964E-10	0.074738177	0.066516516	0.047450827	0.049040463	0.010687331	TBC1D30
chr12	104679896	104680923	1028	*	8	8.93863E-10	0.00231463	0.066571122	0.00681603	0.138553556	1.71807E-06	TXNRD1
chr5	125936419	125936881	463	*	8	4.19288E-08	0.004848947	0.066578121	0.015542873	0.05743092	0.008856257	PHAX
chr2	11485384	11485784	401	*	5	8.30473E-08	0.003256447	0.066623711	0.008543265	-0.097656428	-0.031961821	ROCK2
chr5	133862209	133862682	474	*	2	5.11287E-08	0.06183525	0.066759781	0.068857735	0.019688356	0.01447792	JADE2
chr11	108368743	108369406	664	*	6	2.08259E-08	0.049688095	0.066766019	0.069425658	0.029649743	0.004989229	KDELC2
chr12	57853396	57854236	841	*	8	2.16675E-09	0.007061204	0.066802852	0.007647224	0.02498058	0.00846126	GLI1
chr6	111804044	111805608	1565	*	16	1.40395E-09	0.007489988	0.06703748	0.008238656	-0.046614692	-0.002465909	TRAF3IP2-AS1, REV3L
chr4	46391159	46392253	1095	*	13	6.43268E-12	0.108949318	0.067169896	0.022523874	0.064111782	0.015395475	GABRA2
chr1	110527112	110527798	687	*	10	7.10895E-09	0.33521876	0.067337459	0.12869089	0.018080405	-0.00023989	AHCYL1
chr16	9185438	9186674	1237	*	11	1.70535E-12	0.013474551	0.067366221	0.003829592	-0.041487239	0.005220731	C16orf72
chr18	71958904	71959407	504	*	10	3.95025E-09	0.026611156	0.06742314	0.024398938	-0.070197106	0.002241843	CYB5A

chr18	47807692	47808184	493	*	10	1.39605E-10	0.012406942	0.067453542	0.013006087	-0.060109571	-0.014158424	MBD1
chr1	115300440	115301208	769	*	12	1.57734E-10	0.014535432	0.067454741	0.013441419	-0.040618557	-0.018766845	CSDE1
chr2	30669385	30670170	786	*	11	8.72948E-10	0.004966041	0.067457424	0.014975129	-0.17128932	-0.043137452	LCLAT1
chr6	70576740	70577606	867	*	9	1.17939E-09	0.007387111	0.067474971	0.014007185	0.054166841	0.010136437	COL19A1
chr16	67260661	67261425	765	*	15	2.53525E-09	0.045328509	0.067510781	0.04436053	-0.049201792	-0.011979811	TMEM208, LRRC29, AC040160.1
chr3	63263752	63264335	584	*	9	7.14972E-08	0.053279774	0.067573685	0.031504993	0.179183181	0.045227383	SYNPR
chr6	123316988	123317212	225	*	7	1.58923E-07	0.122619648	0.06759946	0.124322485	0.084242457	0.007568433	CLVS2
chr11	62521055	62522365	1311	*	14	5.99035E-12	0.012190734	0.06783274	0.004729682	0.067395379	-0.001863579	ZBTB3
chr1	120190378	120190989	612	*	7	3.83834E-09	0.018786776	0.067879345	0.017883904	-0.078359086	-0.01021993	ZNF697
chr11	71823292	71823843	552	*	11	1.44061E-08	0.110415055	0.0679494	0.063020208	-0.05385879	-0.01376073	ANAPC15
chr4	81187575	81187610	36	*	5	2.57393E-08	0.001154804	0.06798945	0.005332976	-0.032663573	0.001964838	
chr11	61735136	61735531	396	*	8	5.33247E-08	0.011190198	0.067992393	0.019410686	-0.015156708	-0.000723123	AP003733.1
chr7	27281216	27284348	3133	*	34	3.07918E-14	2.29414E-05	0.068130559	0.00032496	0.082610151	0.034468456	EVX1, EVX1-AS
chr15	43477090	43478176	1087	*	16	2.18916E-10	0.071377101	0.06818942	0.02781402	-0.114874219	0.001873069	TMEM62, CCNDBP1, EPB42, RP11-473C18.3
chr11	6704824	6704982	159	*	5	1.02518E-07	0.032331305	0.068253299	0.011873554	0.015317624	0.001547055	
chr6	34113887	34113943	57	*	7	1.30824E-07	0.010172918	0.068371475	0.02443114	0.090599593	0.060085771	GRM4
chr7	4901337	4902257	921	*	11	3.76223E-12	0.000405891	0.068387146	0.00262041	0.130092699	0.06130858	RADIL, PAPOLB
chr1	205180527	205181581	1055	*	13	1.92317E-10	0.015626156	0.06839842	0.0223328	-0.040352408	-0.017458818	DSTYK
chr16	68014338	68014699	362	*	8	3.29457E-08	0.016390966	0.068419458	0.01423368	0.109257969	0.071405387	DPEP3
chr19	39389904	39390960	1057	*	20	5.66874E-14	0.120081326	0.06845289	0.025812188	-0.105724807	-0.006125991	NFKBIB, SIRT2
chr22	44350860	44351685	826	*	12	3.21758E-09	0.079234898	0.068484333	0.036323945	0.029395435	0.007499702	PNPLA3, SAMM50
chr3	49044175	49045655	1481	*	18	1.22825E-10	0.002681524	0.068584894	0.010201064	0.104388608	-0.001223483	P4HTM, WDR6
chr21	35013970	35015742	1773	*	19	2.11408E-09	0.035282873	0.068636109	0.020753995	-0.06589835	-0.010241349	ITSN1, AP000304.12, CRYZL1
chr16	3451006	3451666	661	*	10	2.5911E-08	0.084098324	0.068690809	0.052929275	-0.038515517	-0.002759974	NAA60, ZNF174, ZSCAN32
chr1	52456341	52456918	578	*	13	9.04546E-09	0.078313891	0.06878328	0.039508248	0.083102304	0.021725041	RAB3B
chr2	24345949	24346867	919	*	10	2.35232E-09	0.152824505	0.068989927	0.039145425	0.025395595	-0.002618315	FAM228B, PFN4
chr16	31044110	31045464	1355	*	20	7.76132E-10	0.29467339	0.06901874	0.066100801	-0.057037914	-0.004177087	STX4
chr6	42981424	42982050	627	*	14	2.55521E-11	0.010983203	0.069064498	0.00915607	-0.059693664	-0.01203615	KLHDC3, MEA1
chr19	47778045	47778150	106	*	5	8.6379E-08	0.006188852	0.06906674	0.017008827	-0.080737081	-0.053046944	PRR24

chr11	45868398	45869488	1091	*	15	2.06193E-12	0.022181914	0.069084787	0.00605901	0.098520111	0.020272139	CRY2
chr18	9913006	9914102	1097	*	13	1.21444E-12	0.005169058	0.069111378	0.007006212	0.027308536	0.00954694	VAPA
chr3	111805225	111805408	184	*	6	1.90258E-07	0.305454557	0.069111768	0.116042002	-0.054603035	-0.013049856	C3orf52
chr1	28240714	28241681	968	*	14	1.21211E-11	0.028692322	0.069123105	0.018800964	-0.050159835	0.006488526	RPA2
chr10	104503149	104503860	712	*	9	2.78945E-08	0.02053391	0.069142455	0.0191957	-0.054195679	-0.021507625	SFXN2, WBP1L
chr1	67896128	67896741	614	*	5	5.32788E-09	0.025205283	0.069177246	0.039899718	0.01433864	0.004715079	
chr6	31164776	31165512	737	*	14	1.45267E-11	0.038616149	0.069274552	0.03694419	0.092643209	0.034234292	XXbac-BPG299F13.17
chr14	75348249	75348400	152	*	5	1.42888E-07	0.003107758	0.069290718	0.010255896	-0.028548555	0.000124084	
chr19	1174154	1174419	266	*	7	9.4808E-08	0.018558394	0.069332536	0.024592475	-0.07216772	-0.023925992	SBNO2
chr1	156046778	156047143	366	*	5	8.82057E-08	0.041443828	0.069363545	0.054946742	0.121406126	0.055201212	MEX3A
chr19	3699951	3700773	823	*	8	6.84586E-08	0.013024009	0.069443813	0.01037524	-0.031466328	-0.006256977	PIP5K1C
chr2	198317568	198318740	1173	*	19	1.06715E-16	0.000272906	0.069516865	0.000645669	-0.080885453	-0.016696774	COQ10B
chr17	32483560	32484259	700	*	13	1.83075E-09	0.082193041	0.069528077	0.036202754	0.061356484	0.022930827	ASIC2
chr11	64948214	64949502	1289	*	19	2.19952E-12	0.037309756	0.069536657	0.02945701	-0.065584149	0.001502804	CAPN1, AP003068.23
chr12	132568535	132569020	486	*	10	2.04186E-10	0.002164298	0.069549064	0.006036563	-0.065248443	-0.002770918	EP400NL
chr16	2009320	2009675	356	*	7	2.51204E-08	0.009956516	0.069578229	0.016801385	0.014601151	0.003179946	NDUFB10
chr16	2040863	2042103	1241	*	11	1.71169E-09	0.006785121	0.069680602	0.011245964	0.07602016	0.030655931	SYNGR3
chr5	133860141	133861078	938	*	5	1.14769E-09	0.021731416	0.069776709	0.033998166	-0.036967524	-0.009933595	JADE2
chr6	10694545	10695351	807	*	15	7.53413E-11	0.153071718	0.069817355	0.02471799	0.030119459	-0.005159423	PAK1IP1, C6orf52
chr4	37828046	37828219	174	*	6	8.95908E-08	0.002353584	0.069872304	0.00852976	0.019203679	0.002742673	
chr4	38665594	38666663	1070	*	12	1.21365E-11	0.0091803	0.069923145	0.005759578	-0.097031723	-0.029334914	KLF3, RP11-617D20.1, AC021860.1
chr21	47062953	47063665	713	*	8	7.38674E-11	0.000781176	0.070031641	0.00291618	-0.058268106	-0.018622822	PCBP3
chr16	23567971	23569487	1517	*	23	9.84147E-13	0.075978304	0.0700771	0.01302766	0.117814978	0.004823439	UBFD1, EARS2
chr22	28314999	28315683	685	*	10	3.50517E-09	0.023413618	0.070129035	0.013695496	-0.032878638	0.002481303	TTC28-AS1, PITPNB
chr2	180725622	180726697	1076	*	14	3.7184E-09	0.020368449	0.07014344	0.014327761	0.034718027	0.010740396	ZNF385B, MIR1258
chr11	118271932	118272803	872	*	14	1.24164E-09	0.072761693	0.07018967	0.040197438	-0.026731002	-0.009232625	ATP5L, RP11-770J1.5
chr4	81104695	81105823	1129	*	8	6.08615E-09	0.005903861	0.0702614	0.0091666	0.062725165	0.016754074	PRDM8, RP11-377G16.2
chr16	777273	777724	452	*	4	4.30389E-08	0.014058914	0.070306971	0.031057516	-0.052116747	-0.025528207	HAGHL
chr6	27835191	27835460	270	*	7	1.10988E-07	0.013351647	0.07040702	0.018705679	-0.028777003	4.6836E-05	HIST1H1B

chr17	72199366	72200159	794	*	13	2.43734E-11	0.003282019	0.070471954	0.006888707	-0.050400554	-0.014043314	RPL38
chr2	28615209	28615925	717	*	10	1.03549E-08	0.073133098	0.070542059	0.034626024	-0.037068776	0.000532516	FOSL2, AC104695.3
chr5	148205612	148206412	801	*	10	3.06689E-09	0.011008465	0.070807539	0.014278396	-0.035051235	-0.00037932	ADRB2
chr14	101292306	101292680	375	*	5	6.37631E-08	0.012581525	0.070835292	0.027757564	-0.123619296	-0.091288144	MEG3
chr1	9256341	9257421	1081	*	8	2.26606E-08	0.038900326	0.070860598	0.018900813	-0.149853942	-0.021789767	RP3-510D11.1
chr19	39420815	39421614	800	*	15	5.21546E-09	0.423826647	0.0708641	0.204371213	0.016017507	0.000601606	MRPS12, SARS2, CTC-360G5.8
chr5	134734307	134735104	798	*	9	1.59349E-11	0.027251407	0.070969513	0.009985418	-0.047456959	-0.020730821	CTC-203F4.2, H2AFY
chr19	10828564	10829423	860	*	19	1.47502E-08	0.868993695	0.071022525	0.291718165	0.024279026	0.002528957	DNM2, MIR638
chr2	131862633	131863063	431	*	6	7.00735E-08	0.034964531	0.071156286	0.031525693	-0.087052087	-0.034607761	PLEKHB2
chr1	212003502	212004642	1141	*	15	5.96761E-11	0.058115918	0.071220101	0.032281652	-0.037503663	0.000986476	RP11-552D8.1, LPGAT1
chr12	52462420	52463368	949	*	8	2.72289E-10	0.001275557	0.07130901	0.004774607	0.12657931	0.052333349	C12orf44
chr16	2272577	2273300	724	*	5	2.21211E-08	0.030792586	0.071401792	0.043241074	0.085236568	0.027005466	
chr19	39881393	39882194	802	*	12	7.86752E-10	0.005310375	0.071402922	0.017145076	-0.065357551	-0.012838107	MED29, PAF1
chr2	201171120	201171404	285	*	7	3.36522E-08	0.069543164	0.071512966	0.037628829	-0.074430727	-0.037676775	SPATS2L
chr6	45389535	45390293	759	*	14	2.20303E-08	0.026604584	0.071607338	0.05417563	-0.075336524	-0.023460652	RUNX2, RP1-244F24.1
chr19	42363855	42364034	180	*	5	3.92266E-08	0.005084798	0.071981966	0.014127259	-0.084008068	-0.03688144	RPS19
chr6	97285024	97285954	931	*	10	6.42149E-11	0.001873707	0.072039131	0.00565224	0.057101652	0.01215609	GPR63
chr9	36258437	36258971	535	*	10	9.24375E-09	0.020537641	0.072168517	0.033925829	-0.044699555	0.004158794	CLTA, GNE
chr19	40732305	40732902	598	*	11	3.32993E-09	0.158446078	0.072185961	0.025929854	0.105697083	0.027812835	CNTD2
chr15	72667883	72669149	1267	*	18	7.66098E-14	0.002895519	0.072285641	0.00657551	-0.055396793	0.004221161	HEXA-AS1, RP11-106M3.2, HEXA
chr1	207627372	207627656	285	*	8	9.03192E-08	0.031760539	0.072292363	0.029613698	0.033395879	0.003822363	CR2
chr8	120867626	120868354	729	*	9	8.00651E-12	0.005993872	0.072355354	0.00789672	0.014788533	0.006997357	DSCC1
chr6	30654410	30659692	5283	*	69	2.24802E-15	0.3009122	0.072409153	0.022876216	0.172601446	0.014935345	PPP1R18, NRM
chr16	57769092	57769885	794	*	10	1.40787E-08	0.059771972	0.072451989	0.031130846	-0.056587679	-0.001175792	KATNB1
chr5	127420015	127420219	205	*	4	3.46301E-08	0.002698334	0.072510516	0.009611176	-0.083250238	-0.028106203	SLC12A2
chr17	73780373	73781307	935	*	17	3.07587E-11	0.130564627	0.072580205	0.053167158	0.040340229	-0.000196854	UNK, H3F3B, MIR4738
chr7	107530905	107532014	1110	*	14	1.87805E-11	0.00834433	0.072669393	0.004784632	0.103903096	0.001255555	DLD
chr15	69222895	69223018	124	*	5	8.92406E-08	0.00843009	0.072708504	0.01458688	-0.171830481	-0.104102591	SPESP1, RP11-809H16.2, NOX5
chr14	76127164	76128003	840	*	14	4.17218E-10	0.103448536	0.072782593	0.023479091	-0.044327066	-0.006225386	FLVCR2, TTLL5, C14orf1

chr6	30624395	30624769	375	*	8	1.16698E-08	0.002908169	0.072850479	0.008037821	-0.13122683	-0.061737313	DHX16
chr2	60781524	60781678	155	*	4	1.67482E-07	0.012109543	0.072907719	0.027666771	-0.051974792	-0.01852587	
chr19	38746749	38747796	1048	*	13	1.19441E-09	0.123881727	0.072915222	0.06051255	-0.053423316	0.006722419	SPINT2, PPP1R14A
chr12	104359324	104359732	409	*	8	9.18322E-08	0.010544692	0.072951002	0.014886993	-0.110792027	-0.01870195	TDG, C12orf73
chr1	155231766	155232677	912	*	15	5.15393E-10	0.19017156	0.072960571	0.050623768	-0.106755442	-0.01444533	SCAMP3, CLK2
chr4	5021111	5021778	668	*	7	2.33708E-08	0.016590762	0.072997787	0.017143795	0.083780609	0.021111492	CYTL1
chr3	40428713	40429472	760	*	7	3.24368E-09	0.002946191	0.07314306	0.007513093	-0.104458044	-0.010374134	ENTPD3, ENTPD3-AS1
chr15	93447445	93447586	142	*	5	1.05323E-07	0.027283479	0.073199949	0.024103848	0.015883068	0.009336397	CHD2
chr11	5617367	5618023	657	*	7	9.77147E-09	0.00096029	0.073252056	0.004370371	-0.068705078	-0.039509839	TRIM6, TRIM6-TRIM34, HBG2, AC015691.13
chr22	39190118	39190571	454	*	9	1.6618E-08	0.006513891	0.073340582	0.013729365	-0.041207246	-0.015517737	SUN2, DNAL4
chr3	45729807	45731405	1599	*	26	7.50013E-12	0.151334226	0.07335853	0.050110363	-0.045265609	-0.000758621	SACM1L, LIMD1-AS1
chr10	11206221	11207015	795	*	9	3.7076E-08	0.026019472	0.073430057	0.03579344	-0.044732173	-0.006901966	CELF2
chr8	22462455	22463191	737	*	8	2.09341E-09	0.070628386	0.073563165	0.029316487	0.058750511	-0.008418378	CCAR2
chr3	113160183	113161177	995	*	13	2.0154E-09	0.017266709	0.073588104	0.019315416	-0.140018986	-0.050662196	WDR52
chr14	37125914	37125997	84	*	2	1.54549E-07	0.02907492	0.073632589	0.043998889	0.030789424	0.024021846	RP11-964E11.2
chr2	175198952	175200286	1335	*	13	4.14358E-10	0.055294903	0.07364705	0.019236811	-0.057938387	0.014122129	SP9
chr10	81966990	81967666	677	*	8	6.79792E-10	0.001347148	0.073726912	0.004743826	-0.166222421	-0.090351401	LINC00857
chr7	94023308	94024254	947	*	11	5.12148E-09	0.023373563	0.073929128	0.026777347	-0.071209857	0.026812175	COL1A2
chr10	72362292	72362866	575	*	9	3.64525E-09	0.003705279	0.074086463	0.01191699	0.089047852	0.055466846	PRF1
chr6	24720710	24720818	109	*	3	8.04199E-08	0.073312198	0.074114441	0.088518136	0.010728811	0.009503837	C6orf62
chr19	12776902	12777903	1002	*	20	3.78838E-13	0.009257941	0.074212068	0.013115044	-0.059050142	-0.001544076	WDR83, MAN2B1, CTD-2192J16.24
chr10	112678594	112679649	1056	*	19	3.0626E-11	0.041045458	0.074288724	0.031887868	-0.072241968	-0.009505643	SHOC2, BBIP1
chr4	154709756	154710796	1041	*	17	1.15902E-12	0.013387973	0.074475251	0.014050802	0.05290088	0.003536665	SFRP2
chr3	101498065	101498416	352	*	7	2.55851E-08	0.109614149	0.07458319	0.052084461	-0.039833617	-0.004623193	NXPE3
chr6	90062465	90062885	421	*	9	6.69677E-08	0.016548471	0.07470388	0.031925918	0.024970138	0.001178644	UBE2J1
chr14	35451467	35452441	975	*	15	4.80547E-13	0.020170437	0.07476041	0.010754362	-0.03484894	0.000154565	SRP54, RP11-85K15.2
chr20	5485144	5485511	368	*	8	3.12744E-09	0.022977493	0.074783247	0.011187242	-0.119919349	-0.07285199	LINC00654
chr7	25019751	25020404	654	*	7	9.15492E-09	0.003074746	0.074811077	0.011142201	-0.041006114	-0.015748904	OSBPL3
chr11	110583549	110584091	543	*	10	5.22173E-08	0.106053342	0.074856672	0.059258865	-0.183874673	-0.012735858	ARHGAP20

chr17	35305470	35306345	876	*	11	2.8292E-11	0.003161231	0.074858866	0.006302137	0.023854939	0.007714171	AATF
chr3	57261306	57261829	524	*	8	2.48345E-10	0.002660408	0.074942512	0.004004035	-0.033292769	-0.009179936	APPL1
chr11	66034681	66036614	1934	*	56	1.74727E-16	0.517421334	0.074993914	0.188592392	0.079599642	-0.005076155	KLC2, RAB1B, RP11-867G23.2
chr5	108084635	108084930	296	*	4	1.1512E-07	0.036565831	0.075109863	0.058843199	-0.04181994	-0.021299077	FER
chr2	38977957	38978678	722	*	6	9.77532E-09	0.011843855	0.075190551	0.024310646	-0.043145458	-0.004199408	GEMIN6, SRSF7
chr2	33824170	33824684	515	*	11	4.90024E-08	0.309405267	0.075453132	0.149998509	-0.020638033	-0.004484578	FAM98A
chr11	93517122	93517837	716	*	13	1.90748E-10	0.038703638	0.075722549	0.02790991	-0.097377104	-0.020135564	MED17, TAF1D
chr20	45035173	45036183	1011	*	13	1.5663E-10	0.004164883	0.07573169	0.010178157	0.155997693	-0.002418224	ELMO2
chr5	149736866	149737143	278	*	4	1.40695E-07	0.383891839	0.075903269	0.097842226	-0.075514965	-0.034381199	
chr6	41513733	41514112	380	*	7	5.03156E-08	0.004069249	0.075937113	0.010122252	-0.031621797	-0.006414641	RP11-328M4.2
chr2	74730047	74730166	120	*	3	7.09041E-08	0.19871402	0.076113235	0.065523969	-0.103002314	-0.020354561	LBX2-AS1, LBX2
chr1	33115769	33116824	1056	*	17	1.86023E-12	0.043226465	0.076151953	0.024855976	-0.104080808	-0.011206322	RBBP4, ZBTB8OS
chr16	75681490	75682551	1062	*	13	1.93195E-10	0.009395959	0.076362467	0.019037119	-0.029945898	0.002590083	TERF2IP, KARS
chr11	16759744	16760677	934	*	15	6.82775E-09	0.036490405	0.076620037	0.043866174	-0.070765368	-0.009783637	C11orf58, SOX6
chr6	166795816	166797429	1614	*	32	6.24114E-17	0.048653137	0.076658222	0.029376239	-0.175390651	-0.007128739	RP1-168L15.5, MPC1
chr14	60976115	60976285	171	*	2	5.02818E-08	0.02308663	0.076660924	0.037738981	0.040474086	0.025521072	SIX6, C14orf39
chr12	51419900	51420538	639	*	12	1.25008E-08	0.038689821	0.076664069	0.039783341	-0.029115175	0.000566026	SLC11A2
chr1	224803364	224804226	863	*	9	1.35245E-10	0.000398285	0.076681406	0.001939712	0.059634592	0.017253959	CNIH3, RP11-100E13.1
chr1	235291861	235293080	1220	*	12	5.0243E-10	0.023079128	0.076808652	0.016405321	0.105500279	0.003494101	TOMM20
chr11	61594965	61595983	1019	*	14	1.81272E-12	0.021580904	0.077007127	0.015440613	-0.09141846	-0.010885332	FADS2, FADS1
chr7	111201994	111202900	907	*	13	6.75808E-09	0.093491043	0.077132753	0.027668975	-0.051638186	-0.008785644	IMMP2L
chr5	95297236	95298307	1072	*	12	2.16709E-10	0.024771113	0.077147282	0.023345655	-0.052116984	-0.011768386	CTD-2337A12.1, ELL2
chr6	32014148	32015773	1626	*	23	1.93674E-11	0.157827107	0.077182566	0.037988481	0.125672595	0.036380421	TNXB
chr12	49182837	49182908	72	*	4	2.05993E-07	0.139838962	0.077257687	0.08083898	-0.076860646	-0.031360169	
chr5	131347650	131347999	350	*	9	1.40755E-07	0.11109881	0.077283372	0.068515977	0.101392637	0.028132212	AC034228.2, ACSL6
chr4	185747061	185748017	957	*	12	1.30617E-09	0.029414761	0.077473986	0.040949552	0.028360894	0.005624553	ACSL1
chr2	172544044	172544336	293	*	7	1.2951E-07	0.056546868	0.077552137	0.067399958	-0.049440604	-0.013924923	DYNC1I2
chr8	146017580	146018604	1025	*	12	6.48629E-12	0.009698549	0.07756735	0.006314323	-0.041957534	-0.010473866	RPL8
chr16	18812258	18813117	860	*	10	5.11111E-10	0.036399839	0.077622936	0.0088525	-0.100264042	-0.021541913	RP11-1035H13.3, ARL6IP1

chr1	33816020	33816758	739	*	6	1.96236E-08	0.032794989	0.07768026	0.025782937	-0.140418122	-0.019670679	RP11-415J8.5, PHC2
chr6	30853569	30854551	983	*	10	1.53053E-08	0.058533476	0.077681981	0.052857729	0.118890151	0.056804745	DDR1
chr11	65546988	65547886	899	*	7	1.02926E-09	0.001554614	0.077706506	0.008168328	-0.148898593	-0.065870239	AP5B1, AP001266.1
chr14	24583182	24584561	1380	*	20	1.68954E-12	0.001846554	0.077879741	0.004493397	-0.048584265	-0.010745965	DCAF11, NRL
chr3	32859377	32859587	211	*	6	1.3563E-07	0.109644561	0.077899294	0.032788949	0.018668666	0.00563325	TRIM71
chr3	9438726	9440081	1356	*	14	8.40431E-12	0.001051652	0.077900098	0.002591815	-0.061603117	-0.02158734	SETD5, SETD5-AS1
chr14	69262818	69263504	687	*	9	1.13885E-09	0.004663432	0.077927707	0.009723809	0.083012723	0.018559725	ZFP36L1
chr17	62502038	62503014	977	*	12	6.34006E-12	0.016775464	0.077942623	0.012891955	-0.045979477	-0.007866603	CEP95, DDX5
chr3	52489323	52489874	552	*	9	7.8531E-08	0.007130873	0.077943725	0.020035886	0.038622277	-0.001011732	NISCH
chr1	44883990	44884264	275	*	7	2.66082E-09	0.001876017	0.078024803	0.005294805	-0.139602006	-0.042137095	RNF220
chr3	108307933	108308545	613	*	17	1.31451E-08	0.373555891	0.078222699	0.153547081	-0.254702792	-0.021212715	DZIP3, KIAA1524
chr16	68344213	68344683	471	*	5	4.30938E-08	0.002624958	0.078240475	0.010433401	0.03107766	0.007221902	SLC7A6OS
chr15	81292926	81293687	762	*	9	6.03498E-11	0.003230226	0.078384858	0.007835329	-0.027983686	0.000148659	MESDC1
chr6	111136363	111136979	617	*	11	3.24195E-11	0.021364951	0.078386813	0.018009679	-0.04731474	-0.007931608	CDK19
chr14	55368924	55370231	1308	*	11	7.72868E-10	0.160475244	0.078392081	0.048957882	0.029601412	0.003431053	GCH1
chr4	37687247	37688741	1495	*	11	2.02573E-09	0.002374818	0.078392353	0.004648657	-0.055315632	-0.018883044	RELL1
chr1	182808025	182808991	967	*	14	1.91625E-11	0.014344806	0.07843053	0.006363454	0.057387189	0.009911041	DHX9
chr11	2919689	2920564	876	*	13	6.54501E-09	0.050144912	0.078579261	0.054583513	0.096595785	-0.013728984	SLC22A18AS
chr12	25403680	25405317	1638	*	26	2.25997E-13	0.027591271	0.078599346	0.007731033	0.076862426	-0.009381159	KRAS
chr16	4289807	4289924	118	*	3	1.64217E-07	0.058245965	0.078604681	0.053925877	0.086295478	0.060817236	SRL
chr7	140396886	140397380	495	*	10	1.73284E-08	0.004813796	0.078607947	0.02308639	-0.052307706	-0.015774986	NDUFB2
chr19	51870364	51871466	1103	*	7	1.29617E-08	0.001748643	0.07871705	0.006118073	-0.063542483	-0.027477633	CTD-2616J11.11, CLDND2
chr11	129244505	129244523	19	*	2	1.74589E-07	0.029076131	0.078745519	0.045084507	0.022683816	0.01704655	
chr12	132851518	132851967	450	*	5	4.18762E-08	0.001813519	0.078806819	0.008006757	0.094484987	0.082861029	GALNT9
chr7	86848751	86849303	553	*	10	2.41885E-08	0.048095722	0.079163878	0.041084246	-0.097219351	-0.020261192	TMEM243
chr11	117049400	117050102	703	*	11	9.78211E-09	0.032689798	0.079877177	0.027850064	0.035283415	0.006999009	SIDT2
chr22	38054183	38054892	710	*	9	1.12473E-08	0.013737839	0.079913121	0.012521779	-0.059038372	-0.011985688	SH3BP1, PDXP, Z83844.1
chr7	86974351	86974744	394	*	2	1.40369E-08	0.113238703	0.079956984	0.105014788	0.012091728	0.009393794	TP53TG1
chr14	73392648	73393301	654	*	13	5.1032E-09	0.159691552	0.080042149	0.066721532	0.094403329	0.005862938	DCAF4

chr1	94374370	94374966	597	*	5	1.73965E-08	0.030978721	0.080086245	0.036988174	0.024715086	0.0133694	GCLM
chr2	220110385	220110639	255	*	5	8.84866E-08	0.147305303	0.080198554	0.097807853	0.071946179	0.016585999	STK16
chr17	48171720	48172668	949	*	18	1.01488E-13	0.078398623	0.080202374	0.042107846	0.035138057	-0.004339858	PDK2
chr1	31190863	31191746	884	*	9	3.11858E-10	0.049204199	0.080321026	0.014017202	-0.091392814	-0.030410652	MATN1-AS1, MATN1
chr5	39424930	39425549	620	*	11	2.70265E-08	0.015268392	0.080347825	0.027907913	-0.050581047	-0.025029581	C9, DAB2
chr5	151150029	151152154	2126	*	21	1.02178E-13	0.014988649	0.080725077	0.002744292	0.097100556	0.00076499	G3BP1, ATOX1
chr11	57479604	57480041	438	*	14	5.6413E-10	0.037846837	0.080772381	0.0337646	-0.046332245	-0.014125209	MED19
chr17	2303788	2305255	1468	*	13	3.27236E-09	0.015740931	0.080779723	0.023203446	-0.073185195	-0.002836537	MNT
chr1	15943511	15944060	550	*	7	2.99628E-08	0.030528583	0.081036113	0.045094193	-0.043641501	-0.010494531	DDI2
chr19	40030677	40031046	370	*	9	1.10359E-08	0.007494456	0.081075913	0.028827695	-0.036385102	-0.004520085	EID2
chr4	89079587	89080686	1100	*	12	1.40923E-09	0.002996983	0.081085539	0.012852872	-0.023152014	0.002931049	ABCG2
chr6	139349120	139349771	652	*	10	3.797E-09	0.010534672	0.081112761	0.018301365	-0.060841944	-0.014432812	
chr10	70660528	70661472	945	*	12	2.17773E-09	0.002860596	0.081327745	0.017020416	-0.044881191	-0.012520882	DDX50
chr5	154238120	154238496	377	*	6	2.28102E-09	0.031155004	0.081355028	0.055077759	-0.035883929	-0.025107154	CNOT8, FAXDC2
chr1	235490951	235492429	1479	*	19	4.37514E-12	0.02706925	0.08142486	0.032165552	-0.113851004	-0.022851958	GGPS1, ARID4B
chr4	89205337	89206201	865	*	14	3.12626E-08	0.076889887	0.081483474	0.072002755	-0.044766233	-0.012585905	RP11-10L7.1, PPM1K
chr10	81205166	81205554	389	*	5	1.80879E-07	0.0024244	0.081652976	0.010260136	-0.056835441	-0.016597769	ZCCHC24
chr2	677101	677585	485	*	7	3.14697E-08	0.16044272	0.081748587	0.059180997	-0.042904842	-0.015117166	AC092159.2, TMEM18
chr10	101491598	101492713	1116	*	19	3.221E-12	0.176106983	0.081864156	0.036065994	-0.05499396	4.47264E-05	CUTC, COX15
chr4	44450358	44451077	720	*	9	1.336E-09	0.004741451	0.081954091	0.01021921	-0.037309359	0.005048855	KCTD8
chr5	139780509	139782041	1533	*	17	1.73277E-10	0.094771193	0.082045856	0.035599267	-0.036099577	0.003746232	ANKHD1, ANKHD1-EIF4EBP3, CTC-329D1.2
chr11	2019079	2020560	1482	*	35	5.08315E-21	0.059172337	0.082230826	0.01354483	-0.134408174	-0.046524286	H19
chr1	50893067	50893469	403	*	4	9.94225E-09	0.004257422	0.082310447	0.014257204	0.070392124	0.042684799	
chr9	95857577	95859189	1613	*	17	2.67575E-10	0.003803078	0.082314445	0.007520729	-0.097668123	-0.025464861	C9orf89, RP11-274J16.5
chr16	1832094	1832551	458	*	3	7.08831E-08	0.295600977	0.082337426	0.107906654	-0.017406463	-0.00874012	SPSB3
chr1	91486951	91487865	915	*	10	9.99957E-10	0.003736105	0.082345287	0.010613268	-0.04545727	-0.013793776	ZNF644
chr3	129693370	129693791	422	*	6	4.05847E-08	0.052373784	0.082345925	0.086256709	0.064544091	0.030233258	TRH
chr15	37393009	37394166	1158	*	12	1.05108E-11	0.005825392	0.082420195	0.012430194	0.048357331	0.012828663	MEIS2
chr3	69788137	69788878	742	*	10	1.5597E-10	0.000924547	0.082712078	0.004263882	-0.088181594	-0.032524737	MITF

chr4	78978133	78979207	1075	*	12	1.97853E-08	0.087262653	0.082786232	0.046504936	0.040443731	0.013277032	FRAS1
chr3	156272685	156273486	802	*	15	1.33289E-10	0.023857599	0.082902153	0.026377128	0.165516198	0.029745096	SSR3
chr8	74207183	74207199	17	*	3	9.48875E-08	0.015902284	0.082942598	0.033293726	0.024838872	0.004937674	RDH10, RPL7
chr19	7553700	7554359	660	*	12	4.22012E-10	0.022488948	0.08296011	0.018228504	0.091894966	0.015194397	PEX11G
chr16	46722642	46724037	1396	*	15	1.1148E-13	0.001329131	0.082984003	0.002617575	0.024098878	0.003944684	ORC6, VPS35
chr1	34642386	34643124	739	*	12	2.02487E-09	0.022027653	0.083211192	0.016832862	0.118581228	0.038849	C1orf94
chr6	29716186	29717656	1471	*	24	2.23451E-11	0.067886891	0.083274026	0.049578735	-0.064795527	-0.001215724	HCG9P5, HLA-F-AS1, MICE
chr22	43505892	43507369	1478	*	28	8.46261E-15	0.180370862	0.083317259	0.073498498	0.059219103	0.00099326	BIK
chr15	75871478	75872043	566	*	11	3.12199E-08	0.015757863	0.083434511	0.039225738	-0.041869845	-0.007825022	PTPN9, CTD-2323K18.1
chr2	45878852	45878977	126	*	4	1.82346E-07	0.013409426	0.083465006	0.024378943	0.008954659	0.001179065	PRKCE
chr17	44928851	44929215	365	*	6	3.69841E-08	0.019003959	0.0835427	0.034310714	0.012543509	0.002807921	WNT9B
chr1	67390349	67391144	796	*	15	2.10286E-12	0.007171989	0.083578569	0.009189612	-0.109236792	-0.003341447	MIER1, WDR78
chr6	127664193	127664850	658	*	9	8.3216E-09	0.021418911	0.083640581	0.018612733	0.035983224	0.012126181	ECHDC1
chr4	4291695	4292567	873	*	16	3.06386E-10	0.056422849	0.083650882	0.046189812	-0.040893227	-0.00564089	ZBTB49, LYAR
chr1	224517614	224518531	918	*	17	1.09664E-09	0.120683234	0.083660355	0.062636201	-0.064163695	-0.010335396	NVL
chr10	94050649	94051938	1290	*	17	2.32757E-09	0.048740037	0.083833958	0.025713051	-0.065740661	-0.002832817	MARCH5, CPEB3
chr19	54618544	54619166	623	*	14	9.45771E-10	0.113739635	0.083966648	0.048583908	-0.058162722	-0.008829111	PRPF31, TFPT
chr17	34890851	34891014	164	*	4	9.02016E-08	0.079839754	0.08416729	0.07143235	0.023418041	-0.002852175	PIGW, MYO19
chr6	27356414	27357040	627	*	8	6.8974E-09	0.001234595	0.084265212	0.0071236	-0.041653526	0.004985001	ZNF391
chr11	68815449	68816453	1005	*	13	2.30367E-12	0.000867349	0.084325575	0.002229107	0.118086916	-0.006002042	TPCN2
chr6	139694674	139695867	1194	*	18	2.06581E-14	0.007771516	0.08441733	0.015807736	-0.054214846	-0.003501651	CITED2
chr22	42016856	42018038	1183	*	23	6.75425E-11	0.388437036	0.084604148	0.156139139	-0.056308695	-0.010107343	XRCC6, DESI1
chr3	167452449	167453518	1070	*	16	1.05294E-12	0.015314038	0.084715244	0.008091264	-0.059585837	-0.007734047	SERPINI1, PDCD10
chr2	176964456	176964720	265	*	6	6.02901E-08	0.010708599	0.084725162	0.024003051	0.067068794	0.052531687	HOXD12
chr12	53613892	53614500	609	*	13	1.54043E-10	0.010651712	0.084758429	0.01351044	-0.038997487	-0.004338571	RARG
chr19	38865020	38865513	494	*	12	4.57422E-08	0.035745566	0.084774872	0.049489026	-0.043721551	-0.003016883	PSMD8
chr12	31881969	31882626	658	*	11	1.61339E-08	0.049291069	0.084913777	0.034230412	-0.095319281	-0.022449919	AMN1
chr17	65241476	65242096	621	*	5	1.03683E-08	0.049607904	0.085203293	0.074190087	0.023934757	-0.000526258	RP11-401F2.3, HELZ
chr6	29720315	29721015	701	*	20	5.15559E-09	0.200711557	0.085259905	0.12696872	-0.054572308	-0.004575042	

chr19	54959757	54960304	548	*	10	1.27377E-08	0.013941438	0.085279067	0.026245397	-0.039217571	-0.004527074	LENG8, LENG8-AS1
chr19	39402823	39403047	225	*	6	1.74598E-07	0.00928367	0.085427611	0.017239518	0.152064697	0.079830791	
chr21	34914246	34915592	1347	*	23	2.0426E-13	0.014706631	0.0854521	0.013416417	-0.0484993	-7.95327E-05	SON, GART
chr17	46620895	46621638	744	*	8	1.96274E-09	0.014733207	0.085453634	0.01311688	-0.053167362	0.004577741	HOXB-AS1, HOXB2
chr6	15245667	15246159	493	*	10	1.63823E-08	0.006112339	0.085527373	0.022953269	-0.02498415	-0.005059273	
chr11	33795595	33796289	695	*	12	3.3476E-09	0.010469249	0.085551242	0.018517984	-0.129593929	-0.019626851	RP11-646J21.5, FBXO3
chr19	11289087	11289376	290	*	8	1.31648E-08	0.008148544	0.085577038	0.012009905	0.165094997	0.054140381	KANK2
chr2	70314274	70315091	818	*	9	7.72958E-09	0.020896905	0.085806178	0.023596586	-0.099410037	-0.015543524	PCBP1, PCBP1-AS1
chr13	37453429	37454023	595	*	8	2.96686E-08	0.03383718	0.085932868	0.035037977	0.116768197	0.042092494	SMAD9
chr15	79103718	79104136	419	*	6	2.96165E-08	0.011714119	0.086046823	0.020949557	0.052240678	0.018994022	MORF4L1, ADAMTS7
chr13	50017825	50019081	1257	*	18	3.03895E-08	0.04383701	0.086174379	0.052147268	-0.066276683	-0.008677334	SETDB2, CAB39L, AL136218.1
chr11	6625141	6625673	533	*	6	7.05864E-08	0.007490537	0.086176131	0.020441933	-0.039676219	-0.015701414	ILK, RP11-732A19.8
chr3	197475824	197477097	1274	*	18	2.31871E-12	0.019696844	0.086246021	0.020030763	-0.035744711	-0.006060777	FYTTD1, KIAA0226
chr16	89557182	89557420	239	*	6	1.07908E-07	0.013163204	0.086386648	0.03283452	-0.032048348	-0.01363798	SPG7
chr2	163174891	163175335	445	*	8	1.04444E-07	0.013103949	0.086387103	0.029838143	-0.073919278	-0.005879856	IFIH1
chr12	89745673	89746328	656	*	5	1.23634E-08	0.065683494	0.086403209	0.108350184	-0.033965732	-0.01203645	DUSP6
chr4	151935870	151936810	941	*	12	4.54769E-10	0.004423631	0.086536112	0.010609477	-0.027172433	0.006039864	LRBA
chr5	98108744	98109470	727	*	11	4.22285E-09	0.013324861	0.086559818	0.01605006	-0.087595749	-0.007921939	RGMB, RGMB-AS1
chr4	68566961	68567439	479	*	10	1.74812E-08	0.010910483	0.086593612	0.026151402	-0.044773587	-0.017287912	UBA6-AS1
chr12	4757717	4758503	787	*	13	1.03453E-08	0.051485186	0.086795853	0.036601355	-0.026185328	0.00429633	RP11-500M8.7, NDUFA9, AKAP3
chr6	154359967	154360590	624	*	9	4.62354E-08	0.01186312	0.087046371	0.023858486	0.116131537	0.035403978	OPRM1
chr14	57856758	57857666	909	*	14	1.14751E-09	0.084090131	0.087079809	0.042457124	-0.106688057	-0.011423892	NAA30
chr12	57039784	57040486	703	*	11	1.55623E-08	0.045901152	0.087095144	0.036835751	-0.066310235	-0.013666827	ATP5B
chr19	35772657	35773718	1062	*	9	9.67297E-09	0.001106955	0.087120862	0.005400968	0.101070828	-0.005274059	HAMP
chr1	93644951	93646783	1833	*	27	6.16349E-14	0.026405906	0.08719506	0.022018514	-0.043044221	0.001666213	CCDC18, TMED5
chr19	34012536	34013247	712	*	12	3.54526E-09	0.056022409	0.087386222	0.029962678	-0.052669926	-0.006393888	PEPD
chr20	47835194	47836128	935	*	12	4.70345E-09	0.012839634	0.087653627	0.026470908	0.054759459	0.001129185	DDX27
chr10	106027915	106028628	714	*	11	3.36781E-09	0.088455474	0.087669831	0.053286662	0.070131698	0.01459212	MIR4482-1
chr10	104211296	104211761	466	*	9	4.26683E-08	0.05663951	0.088087272	0.023960823	-0.057449676	-0.029150239	RP11-18I14.10, C10orf95

chr17	72889708	72890209	502	*	11	1.75895E-08	0.006228009	0.088094352	0.021536419	0.125637803	0.014729819	FADS6
chr1	117664076	117665053	978	*	10	3.51112E-09	0.031892	0.088129621	0.020327184	0.064871764	0.00123353	TRIM45
chr1	114354442	114355622	1181	*	16	4.73232E-11	0.016567603	0.088195111	0.017995332	0.103896674	-0.009230813	RP5-1073O3.2, RSBN1
chr6	26538188	26538671	484	*	8	3.17576E-08	0.026488555	0.088228761	0.03195953	-0.061704505	-0.028927666	HMGN4
chr13	26796192	26796831	640	*	9	6.51451E-09	0.212756853	0.088288963	0.092850902	0.091247362	0.014098341	RNF6
chr6	29595299	29595661	363	*	9	1.65027E-08	0.039841572	0.08852488	0.032536194	0.123606019	0.042809802	GABBR1
chr17	47439400	47440217	818	*	10	9.40261E-08	0.038105629	0.088893337	0.050452307	-0.05371649	-0.014944982	RP11-1079K10.3, ZNF652
chr6	116891948	116892775	828	*	14	3.30532E-10	0.127639489	0.089016541	0.034967274	-0.045271882	0.003760077	RWDD1
chr3	48541379	48542288	910	*	10	4.39862E-09	0.035751058	0.089468195	0.013948946	-0.097793738	0.000750728	SHISA5
chr1	25256369	25257599	1231	*	15	4.78225E-11	0.071664182	0.089477115	0.031439791	0.11128301	0.027103139	RUNX3
chr5	175815445	175815846	402	*	10	5.61545E-08	0.214757233	0.089490269	0.079189948	-0.030861416	-0.002051708	ARL10, HIGD2A, NOP16
chr1	231376203	231377187	985	*	16	3.06611E-10	0.028903815	0.089505882	0.033668882	-0.033329835	0.002517539	GNPAT, C1orf131
chr19	4867785	4868496	712	*	9	5.08348E-08	0.087408294	0.090089259	0.039059283	0.0641028	0.001585898	
chr21	47604291	47604898	608	*	4	2.23376E-08	0.005582833	0.090112403	0.017743107	-0.223103689	-0.135374836	SPATC1L
chr1	165667743	165668349	607	*	12	8.47684E-10	0.11938232	0.090164436	0.083935506	-0.035510296	-0.0123666	RP11-466F5.6, ALDH9A1
chr3	49027117	49028001	885	*	18	7.61337E-11	0.015391502	0.090280746	0.040418723	0.026932675	-0.001328675	P4HTM, RP13-131K19.2
chr3	186287958	186288771	814	*	13	1.00226E-08	0.038862431	0.090533315	0.031989303	-0.035345026	-0.001778393	DNAJB11, TBCCD1
chr1	173793075	173793936	862	*	17	4.85621E-09	0.144616218	0.090553176	0.118255328	-0.044958677	-0.009254769	DARS2, CENPL
chr19	44079778	44079985	208	*	7	3.58513E-08	0.036725545	0.090604927	0.066301232	-0.044380049	-0.014660446	XRCC1, L34079.2
chr12	123717050	123718706	1657	*	22	5.84974E-16	0.000112326	0.090767322	0.001365142	-0.055437091	-0.003615565	C12orf65, MPHOSPH9
chr7	5464498	5464875	378	*	4	1.56197E-07	0.012142499	0.090783885	0.031284639	-0.051824665	-0.005019734	TNRC18
chr2	202645530	202646449	920	*	14	4.06518E-09	0.036097484	0.090787265	0.028369482	-0.078194789	-0.01089966	ALS2
chr5	33936171	33936597	427	*	11	2.32444E-08	0.020541883	0.090970832	0.035343031	0.026121449	0.011352902	RXFP3
chr3	50336314	50337494	1181	*	23	1.47183E-11	0.099204631	0.091003245	0.058390223	-0.05061819	-0.016708273	HYAL3, NAT6, HYAL1
chr17	37843591	37844223	633	*	9	2.55535E-09	0.021451405	0.091261825	0.026083812	0.101458172	0.028439577	ERBB2, PGAP3
chr6	24403062	24403712	651	*	9	3.28333E-08	0.020140978	0.091480155	0.020812152	-0.039386924	0.004229379	MRS2
chr15	59396749	59397510	762	*	12	6.78696E-09	0.170844376	0.091558539	0.045154341	-0.06199126	-0.020390287	CCNB2
chr6	30594037	30594749	713	*	15	2.03871E-10	0.206885707	0.091782034	0.091058626	-0.095138662	0.012210105	MRPS18B, ATAT1
chr16	66877873	66878575	703	*	9	9.42406E-09	0.049877167	0.091833138	0.061880506	0.148410277	0.027683288	CA7, NAE1, RP11-61A14.1

chr3	12598043	12598824	782	*	8	7.17927E-10	0.001121073	0.091865422	0.006852269	-0.042721537	-0.011270215	MKRN2, C3orf83
chr19	11494980	11495077	98	*	2	1.03726E-07	0.423976619	0.091922914	0.182688597	0.018733455	0.008117261	EPOR, RGL3
chr12	57633980	57635339	1360	*	12	1.99465E-09	0.027559832	0.091979284	0.015677783	-0.068179057	-0.007869473	NDUFA4L2
chr3	15106787	15107309	523	*	10	4.88597E-08	0.18211664	0.091980743	0.11209305	-0.072255296	-0.022064886	MRPS25
chr16	31085157	31086340	1184	*	18	1.06425E-10	0.0245203	0.091981239	0.022840713	-0.041540681	-0.006954687	ZNF646, ZNF668
chr11	60673398	60674370	973	*	12	6.13019E-09	0.00305277	0.091986894	0.013594813	-0.036039981	-0.002710842	RP11-881M11.2, PRPF19
chr3	19988132	19988887	756	*	9	3.41499E-09	0.00404064	0.092037165	0.008735769	-0.084874323	-0.022041064	RAB5A, EFHB
chr1	156252187	156253073	887	*	12	1.25614E-09	0.006675612	0.092264372	0.014373814	0.060260118	-0.012471847	TMEM79, SMG5
chr16	20817639	20817951	313	*	7	1.56144E-07	0.004918308	0.092350575	0.017434627	-0.020565773	-0.004682951	AC004381.6, ERI2
chr1	3566577	3568245	1669	*	32	1.64334E-15	0.013638365	0.092572493	0.010376979	-0.107234331	0.020081089	WRAP73
chr10	115933849	115934100	252	*	7	1.03675E-07	0.096961922	0.092903599	0.060048906	0.030137594	0.008558584	C10orf118, MIR2110
chr2	63815556	63816428	873	*	22	9.03804E-11	0.232032288	0.09290881	0.088453441	-0.078206585	-0.009854449	MDH1, WDPCP
chr6	31509147	31510735	1589	*	32	8.43301E-12	0.181454233	0.092971889	0.065686869	-0.082766609	-0.009506002	DDX39B-AS1, DDX39B, ATP6V1G2-DDX39B
chr7	157179773	157180126	354	*	7	1.01279E-07	0.002191954	0.093019224	0.00934104	0.123148082	0.049521917	DNAJB6
chr9	34988588	34989493	906	*	6	6.11893E-09	0.165492871	0.093031353	0.064467839	0.079755901	0.017818419	
chr10	22624094	22625015	922	*	7	1.07137E-08	0.019955006	0.093067369	0.024179476	0.07285757	0.033853807	
chr1	151762906	151763866	961	*	13	1.10456E-10	0.016226109	0.093084115	0.021949903	-0.091905353	-0.033555669	RP11-98D18.9, TDRKH
chr5	131132739	131133339	601	*	11	6.8124E-08	0.048425672	0.093173821	0.066570947	0.022509099	0.00725221	
chr3	186524859	186525224	366	*	3	1.39388E-07	0.064614563	0.093181431	0.069727272	0.086127714	0.042135223	
chr1	211432139	211434045	1907	*	18	2.13394E-11	0.106582865	0.093668298	0.070302448	-0.082293115	-0.020697903	RCOR3
chr15	75135060	75136324	1265	*	12	6.45459E-10	0.00019631	0.093687875	0.001800098	0.120048903	0.026618155	ULK3, SCAMP2
chr13	38923870	38924477	608	*	13	1.11889E-09	0.018535888	0.093747298	0.023235147	-0.029368016	-0.001972557	UFM1
chr20	2821310	2822144	835	*	17	1.16891E-09	0.447783125	0.093895602	0.22230816	0.05735173	0.009355518	VPS16, PCED1A
chr7	35293080	35293759	680	*	8	2.00478E-08	0.008560465	0.094056979	0.015446724	0.063394384	0.030318151	TBX20
chr20	25604462	25605178	717	*	16	7.10119E-10	0.128118619	0.094193526	0.066736979	0.154283701	0.013633829	ZNF337-AS1, NANP
chr5	76114373	76115672	1300	*	12	1.28392E-09	0.1978588	0.094376444	0.055382663	0.022423143	0.007764218	F2RL1
chr9	130829320	130830096	777	*	7	5.9329E-09	0.005845229	0.094547567	0.012516561	-0.067602681	-0.012029588	NAIF1
chr12	53773602	53773854	253	*	6	1.24549E-07	0.00910044	0.094751842	0.025945148	-0.041002343	-0.016438998	
chr12	50794485	50795366	882	*	12	9.77697E-11	0.030773525	0.094774764	0.022084717	-0.07867182	-0.006271108	LARP4

chr17	56084468	56085409	942	*	13	1.27597E-09	0.007193721	0.095164686	0.026765191	-0.086302434	-0.005513226	SRSF1
chr14	99947545	99948289	745	*	9	2.60874E-09	0.073675936	0.095175977	0.031600544	0.02457776	0.005697093	CCNK
chr1	26560657	26561325	669	*	6	2.81239E-08	0.058264409	0.095223091	0.05575101	-0.066412688	-0.00911577	CEP85
chr1	226249300	226249852	553	*	7	7.14624E-09	0.009068074	0.0953823	0.02292738	-0.138017538	-0.0164287	H3F3A, RP11-396C23.4
chr10	22609629	22610125	497	*	8	2.39317E-08	0.012432728	0.095437201	0.033593449	-0.049714976	-0.014567909	COMMD3-BMI1
chr8	144328500	144329544	1045	*	12	2.96991E-09	0.040453417	0.095546225	0.052751361	-0.110211404	-0.028090382	ZFP41, ZFP41
chr8	124780013	124781210	1198	*	15	1.3021E-09	0.011422899	0.0957506	0.022639974	0.042415522	0.000296407	FAM91A1
chr7	156742224	156742617	394	*	5	4.02025E-08	0.043829444	0.095811653	0.063154788	0.022982206	0.004474329	NOM1
chr20	2451474	2452132	659	*	9	1.43492E-08	0.005231917	0.095831493	0.011671195	0.09329405	0.004965378	SNRPB, RP4-734P14.4
chr16	67700461	67701378	918	*	12	8.83941E-09	0.038095206	0.095831649	0.041947042	0.038722312	-0.006628553	C16orf86, ENKD1
chr2	98612331	98613076	746	*	11	7.51657E-09	0.059655606	0.095892387	0.036607735	-0.072758933	0.004671498	TMEM131
chr19	37063768	37064886	1119	*	17	3.31574E-09	0.011911809	0.096041439	0.018374993	-0.07548781	-0.00612035	AC092295.7, ZNF529
chr1	230777617	230778283	667	*	11	2.47263E-08	0.062936753	0.096069074	0.076569598	0.141315975	0.028521843	COG2
chr22	50608218	50608509	292	*	3	1.02741E-07	0.700483516	0.096080148	0.294951618	-0.012389737	-0.006238881	
chr6	33159547	33160869	1323	*	19	1.94418E-15	0.002699093	0.09613731	0.00500209	0.066676464	0.010216885	COL11A2
chr11	47447566	47448534	969	*	13	8.12986E-09	0.015532894	0.096164586	0.019740051	0.122103044	0.008729982	PSMC3
chr14	59950705	59951720	1016	*	17	1.97507E-10	0.050819883	0.096203486	0.029939807	-0.0315915	0.000741207	JKAMP, L3HYPDH, RP11-701B16.2
chr19	36235836	36236844	1009	*	14	1.13983E-09	0.041870252	0.096293218	0.020760037	0.039124398	-0.001224128	PSENEN, AC002398.9, AD000671.6, U2AF1L4
chr14	94547496	94547988	493	*	12	7.87976E-09	0.384911012	0.096296859	0.070326298	-0.071884402	-0.010759252	IFI27L1, DDX24
chr18	3247654	3248148	495	*	5	3.0181E-08	0.033993911	0.096322122	0.030915955	-0.015381558	0.004313435	MYL12A
chr1	37979729	37980592	864	*	12	3.946E-10	0.010132421	0.096378756	0.023940187	-0.09872427	-0.022394977	MEAF6
chr8	128747564	128748337	774	*	18	4.67677E-09	0.179017966	0.096471782	0.128196525	-0.05126444	-0.003812024	MYC
chr4	57332795	57333267	473	*	8	2.22729E-08	0.10838364	0.096480412	0.027917354	0.078356908	0.02637058	SRP72
chr2	211035257	211035960	704	*	7	4.21238E-09	0.023216526	0.096584971	0.019661532	0.030221435	-0.000850737	KANSL1L
chr14	58711498	58711972	475	*	8	4.47356E-08	0.002354958	0.096593994	0.011416667	-0.029611411	-0.003665479	PSMA3, C14orf37
chr22	39548724	39549099	376	*	5	7.43758E-08	0.694067533	0.096742232	0.349664917	-0.037089693	-0.008211392	
chr5	37371047	37371931	885	*	14	1.33718E-11	0.007077903	0.096803734	0.011406751	0.130815739	0.020955333	NUP155
chr3	48956740	48957001	262	*	6	2.73812E-08	0.094295634	0.096820434	0.084380928	-0.030190323	-0.001414498	ARIH2, ARIH2OS
chr2	47142555	47144128	1574	*	16	2.14871E-10	0.074743791	0.097017339	0.017765055	0.108724218	0.000221393	TTC7A, MCFD2

chr12	123380523	123381231	709	*	11	1.42049E-08	0.048083934	0.09727608	0.030809729	0.115606643	0.029339724	VPS37B
chr11	130184488	130185670	1183	*	16	4.90352E-12	0.027686258	0.097351383	0.014375716	-0.152860261	-0.025862561	RP11-121M22.1, ZBTB44
chr17	72772340	72773085	746	*	14	4.88629E-09	0.009915491	0.097499322	0.029547631	-0.311608896	-0.037270015	TMEM104, NAT9
chr19	36238931	36239797	867	*	11	4.73013E-09	0.08994397	0.097591106	0.04338749	-0.023517178	0.001784469	AC002398.9, LIN37, AC002398.11
chr17	35969138	35969956	819	*	16	7.52861E-09	0.115814012	0.097832098	0.070712201	0.06948802	0.00479728	RP11-697E22.1, SYNRG, DDX52
chr21	37432039	37433546	1508	*	22	2.10467E-11	0.274235483	0.098011615	0.064834278	0.064656099	0.009402024	AP000688.11, SETD4
chr12	49658188	49659154	967	*	10	4.00489E-10	0.009568847	0.098075889	0.009680644	-0.0542173	-0.002116531	TUBA1C, RP11-977B10.2, RP11-161H23.5
chr8	21998797	21999976	1180	*	15	3.07881E-10	0.009948684	0.098147683	0.017045677	0.044293328	0.014367223	REEP4
chr8	23385905	23386623	719	*	13	1.17004E-08	0.029370348	0.09843685	0.045637192	0.050906142	-0.007049575	SLC25A37
chr6	32936520	32937207	688	*	5	9.74991E-10	0.004992641	0.098513485	0.019022818	-0.092021888	-0.046554671	BRD2, HLA-DMA
chr15	70388682	70389744	1063	*	13	2.33448E-10	0.127090743	0.098548108	0.090790368	-0.040906469	-0.004164807	TLE3
chr6	28983835	28985069	1235	*	23	5.66018E-12	0.073111719	0.098655495	0.032186892	0.119028885	0.010217263	
chr22	32870150	32871233	1084	*	15	4.63572E-10	0.058041016	0.098706993	0.039080877	-0.037430973	-0.000249287	FBXO7
chr5	16617252	16617354	103	*	2	1.41987E-07	0.080893833	0.098802756	0.097436408	0.01638855	0.005281336	RP11-260E18.1, CTC-461F20.1
chr16	72127419	72128225	807	*	17	4.65637E-09	0.104431452	0.09898698	0.095528259	-0.047206416	-0.007995136	DHX38, TXNL4B
chr1	40505597	40506858	1262	*	18	1.0575E-10	0.014405688	0.099099342	0.013519234	-0.03671761	-0.006644458	CAP1
chr19	49496473	49497177	705	*	13	4.42589E-10	0.008646197	0.099118136	0.014716507	-0.032230776	0.001689956	RUVBL2, GYS1
chr19	13055829	13056485	657	*	10	1.43868E-10	0.00384132	0.099318585	0.011120857	-0.061665905	-0.025387084	
chr17	39968505	39969397	893	*	17	2.6992E-14	0.004949422	0.099431753	0.006738849	0.084446568	0.01544371	FKBP10, LEPREL4
chr1	151020868	151021033	166	*	3	1.37557E-07	0.125387387	0.099487476	0.110250755	-0.025267029	-0.004907213	C1orf56
chr10	123356041	123356219	179	*	3	1.27004E-07	0.106570942	0.099602523	0.124529001	0.067318997	0.062453176	FGFR2
chr3	131100199	131100713	515	*	9	6.62447E-08	0.005239933	0.099625998	0.02289124	-0.045251617	0.001902637	NUDT16, RP11-933H2.4
chr11	118901039	118901842	804	*	12	4.94834E-10	0.005598205	0.099688108	0.009835362	-0.049708432	-0.012419194	SLC37A4
chr6	109415607	109417087	1481	*	18	1.63664E-11	0.001265045	0.099715059	0.006449709	-0.029214488	-0.005358786	CEP57L1, SESN1
chr19	17581060	17581966	907	*	11	4.94072E-11	0.00013684	0.099725731	0.002298633	-0.032401564	-0.019765978	SLC27A1
chr16	56485177	56485501	325	*	10	5.73107E-09	0.032348075	0.099873109	0.023376472	-0.075212263	-0.028208727	OGFOD1, NUDT21
chr8	144691562	144691748	187	*	2	3.77467E-08	0.078321421	0.100182142	0.096193338	-0.022996043	-0.006649802	PYCRL
chr15	41805868	41806588	721	*	12	2.02799E-09	0.007785989	0.100332038	0.019708084	0.132353086	0.034554386	LTK
chr11	118401666	118402342	677	*	11	9.26728E-09	0.039198012	0.100457913	0.040319442	-0.070969007	-0.01340616	TTC36, TMEM25, RP11-770J1.3

chr20	57462978	57467268	4291	*	65	9.88089E-20	0.00028398	0.10083522	0.000536576	-0.178301996	-0.036440815	GNAS, RP1-309F20.3
chr5	96270961	96271822	862	*	11	1.06486E-08	0.031152634	0.100995561	0.036868999	-0.047293738	-0.017896104	LNPEP, CTD-2260A17.2
chr1	26145578	26147252	1675	*	14	3.12523E-11	0.067390534	0.101004719	0.042920449	-0.047212853	-0.001169786	RP1-317E23.6, MTFR1L, RP1-317E23.3, AL020996.1
chr6	27839850	27840257	408	*	9	8.64083E-09	0.037760471	0.101105086	0.021773206	-0.066592083	-0.023043916	HIST1H3I
chr6	122720467	122720929	463	*	11	4.76208E-08	0.086839986	0.101363706	0.044229855	0.026198901	0.004617227	HSF2
chr1	42384474	42384647	174	*	4	1.82567E-07	0.041427988	0.101459215	0.053749862	0.129328423	0.092965898	HIVEP3
chr3	138067053	138067500	448	*	10	4.25088E-08	0.166334932	0.101540331	0.060343692	-0.05777968	-0.006869593	MRAS
chr8	25316023	25317051	1029	*	20	3.93224E-13	0.001074338	0.101544142	0.006234187	-0.083938782	-0.005922543	CDCA2
chr6	32939192	32943025	3834	*	71	3.17127E-15	0.11119937	0.101598609	0.056874143	-0.160626105	0.00146622	BRD2, XXbac-BPG181M17.6
chr14	81686781	81688076	1296	*	18	5.34536E-10	0.006650965	0.101626136	0.016209208	0.113995611	-0.001711169	GTF2A1
chr3	32611876	32613083	1208	*	15	1.92834E-09	0.023651305	0.101641928	0.029439799	0.056236591	-0.000127712	DYNC1LI1
chr18	904243	904963	721	*	11	1.14763E-08	0.641642554	0.101643077	0.221125191	0.089541456	0.029113015	ADCYAP1, RP11-672L10.3, RP11-672L10.2
chr5	140070872	140071988	1117	*	18	8.6685E-12	0.033454873	0.101668204	0.024075943	0.128153778	0.003262606	HARS2, HARS
chr19	42721812	42722910	1099	*	14	2.28194E-08	0.014837051	0.101919016	0.02388988	-0.04535175	-0.009978625	DEDD2
chr17	41623744	41624234	491	*	11	2.7441E-08	0.019084784	0.10205292	0.03639239	-0.036803962	0.006657588	RP11-392O1.4, ETV4
chr6	88299463	88300299	837	*	16	5.94385E-09	0.080992073	0.10217779	0.063214582	-0.031837399	0.004116924	ORC3, RARS2
chr1	10003059	10003752	694	*	12	3.24806E-09	0.037137658	0.102194911	0.03861375	-0.046496115	-0.003834501	RP11-84A14.4, NMNAT1, LZIC
chr8	140717297	140717689	393	*	4	4.65602E-08	0.151314306	0.102484297	0.116568428	0.104682126	0.033832308	
chr2	240322401	240323028	628	*	6	2.7854E-08	0.033112574	0.102587573	0.054215462	0.015202843	-0.003369937	HDAC4
chr20	57226445	57227438	994	*	11	4.84764E-10	0.041153596	0.102673135	0.026721649	-0.04845791	-0.009958388	STX16, STX16-NPEPL1
chr16	56641777	56642761	985	*	11	2.35109E-09	0.046526778	0.102772588	0.016398984	0.120774838	0.01046814	MT2A
chr17	1531579	1531887	309	*	7	9.21282E-08	0.059455295	0.103211862	0.055242741	-0.021398988	-0.00691069	SLC43A2
chr13	103532159	103532435	277	*	8	5.18962E-09	0.000970781	0.10341211	0.005812277	0.103669693	0.063044869	
chr22	41985886	41986335	450	*	9	1.91345E-08	0.005121766	0.103424409	0.014214586	-0.078806221	-0.040157523	PMM1
chr3	49711033	49712339	1307	*	18	1.23807E-11	0.027324478	0.103529963	0.01928246	-0.072184251	-0.027314661	APEH
chr12	82752376	82752887	512	*	10	2.9942E-08	0.046353653	0.104118684	0.05782619	-0.035085491	-0.002499257	METTL25, CCDC59
chr19	46388787	46390155	1369	*	11	8.26169E-10	0.003496871	0.104393115	0.009812926	0.027770503	0.009556405	IRF2BP1
chr19	52900469	52901124	656	*	11	2.45117E-08	0.068772059	0.104457404	0.051642827	0.052416261	-0.007016377	ZNF528, CTD-3018O17.3
chr1	185125656	185126416	761	*	15	8.48026E-08	0.171617014	0.104604823	0.101687084	-0.072944013	-0.007530838	SWT1, TRMT1L

chr7	137686767	137687260	494	*	12	5.79101E-09	0.007563872	0.104724233	0.025939043	-0.032566099	-0.003413805	AKR1D1, CREB3L2
chr3	127770634	127771325	692	*	9	3.89068E-08	0.154843214	0.10476688	0.049693127	-0.070774473	-0.00181178	SEC61A1
chr17	4046031	4047033	1003	*	14	4.70267E-11	0.000621935	0.104868532	0.005012787	-0.073044254	-0.005663051	CYB5D2, ZZEF1
chr2	99771257	99771627	371	*	7	7.21044E-08	0.219989137	0.105071009	0.093088465	0.011826766	0.005570559	C2orf15, LIPT1, MRPL30, TSGA10
chr1	228327428	228327881	454	*	13	5.62338E-09	0.041264705	0.105115162	0.037665763	-0.096601642	-0.029374461	GUK1
chr4	174254507	174255944	1438	*	21	3.13238E-13	0.022166917	0.105437835	0.031028205	-0.06396651	-0.014538532	HMGB2
chr6	32820249	32822346	2098	*	53	1.01003E-15	0.533464804	0.105446938	0.177871397	-0.108296264	-0.005469914	PSMB9, TAP1
chr13	111522222	111522314	93	*	3	1.11816E-07	0.017637762	0.105540521	0.03967395	0.144292108	0.115039145	
chr15	90776873	90777598	726	*	9	8.45806E-08	0.523128357	0.10579194	0.182513191	0.020864157	0.006879823	GDPGP1, CIB1
chr17	8152333	8152823	491	*	11	3.27133E-08	0.017663697	0.105801594	0.032336315	-0.05279043	-0.00161971	PFAS
chr15	42787797	42788175	379	*	6	6.12989E-08	0.010952299	0.105865973	0.031183456	-0.036954393	0.007803271	SNAP23
chr10	51572203	51572718	516	*	9	4.94257E-08	0.005025095	0.106053934	0.017363057	-0.04541885	-0.021470544	NCOA4
chr8	37887424	37887635	212	*	2	9.39433E-08	0.047093153	0.106073135	0.070787899	-0.050503421	-0.010743392	
chr6	83775339	83775943	605	*	13	6.39542E-09	0.047138815	0.106115486	0.039998082	0.03046011	0.006721495	UBE3D
chr11	35160827	35160892	66	*	3	1.89129E-07	0.10376943	0.106132414	0.129544451	-0.039755328	-0.002559535	CD44
chr2	153573883	153575050	1168	*	12	8.4575E-09	0.249636644	0.106311994	0.100409926	-0.134606737	-0.010113588	ARL6IP6, PRPF40A
chr11	125439578	125439882	305	*	3	4.21793E-08	0.01872676	0.106318545	0.041456666	0.023737877	0.015708198	EI24
chr12	30907191	30907923	733	*	11	8.31948E-09	0.033498124	0.106321216	0.025923357	-0.072577948	-0.010759831	CAPRIN2
chr5	145562010	145562627	618	*	14	7.81114E-09	0.096548888	0.106347718	0.054537603	-0.044879882	-0.009081588	LARS
chr14	69725831	69727860	2030	*	24	2.20428E-11	0.004477526	0.106358769	0.009307768	0.052655538	0.01019756	GALNT16, RP11-363J20.2
chr2	70141705	70142523	819	*	11	3.72367E-09	0.03265412	0.106383156	0.032269232	-0.041312877	-0.014168719	MXD1
chr20	30192898	30193601	704	*	11	5.52124E-09	0.066393823	0.106532317	0.020599174	0.020663185	0.005604079	ID1
chr12	85306323	85307131	809	*	13	3.92476E-08	0.041817466	0.106913069	0.05874272	-0.081470782	-0.000738062	SLC6A15
chr1	168148299	168148540	242	*	3	1.44447E-07	0.099204245	0.106957637	0.112134691	0.01854704	0.011864151	TIPRL
chr3	13520959	13521915	957	*	24	3.98936E-10	0.421552333	0.107156355	0.1587128	0.024871368	0.003150811	HDAC11, HDAC11-AS1
chr10	101088508	101089149	642	*	11	6.7525E-10	0.005807466	0.107175894	0.023659418	0.074632734	0.02615276	CNNM1
chr2	47168440	47169026	587	*	8	3.21433E-08	0.271830498	0.107517829	0.129399355	0.026085337	-0.0012034	TTC7A, MCFD2
chr1	24285274	24285721	448	*	7	5.18428E-08	0.067390821	0.10759969	0.051880222	0.022989313	-0.001405662	PNRC2, CNR2
chr12	44229417	44229512	96	*	2	1.60172E-07	0.055955576	0.107813078	0.080148324	-0.020735679	-0.003919937	

chr14	69260653	69261160	508	*	7	9.56178E-09	0.019265084	0.107954997	0.024829602	0.062466985	0.008779875	ZFP36L1
chr1	174967663	174968509	847	*	11	1.25954E-08	0.168081312	0.107965012	0.103056528	0.123502739	0.026652971	CACYBP
chr3	172428426	172429421	996	*	14	3.53719E-10	0.100096589	0.107989097	0.026208645	-0.072073355	-0.008025302	NCEH1
chr11	62495049	62495467	419	*	5	1.40585E-07	0.1251258	0.108072467	0.130305265	0.030357594	0.004218582	
chr5	68462459	68462783	325	*	7	4.56024E-08	0.002885736	0.10814362	0.015362119	0.070932104	0.036242553	
chr3	13114441	13114803	363	*	7	8.07172E-08	0.009734466	0.108260652	0.025605134	0.116363361	0.056573663	IQSEC1
chr1	10092629	10093824	1196	*	17	1.9331E-11	0.005798818	0.108291653	0.018412872	-0.041139105	0.00346589	UBE4B
chr1	200011273	200011988	716	*	13	1.82808E-09	0.006876909	0.108534196	0.020658598	0.051430934	0.013765678	NR5A2
chr2	145274125	145274441	317	*	3	1.74708E-08	0.01897499	0.108766148	0.042343297	0.03398773	0.016297605	ZEB2
chr6	31669485	31671391	1907	*	40	8.62582E-18	0.006130401	0.10924257	0.009950952	0.127871446	-0.006830323	ABHD16A, XXbac-BPG32J3.20
chr14	64970743	64972179	1437	*	16	2.76789E-09	0.228518564	0.109295519	0.089299302	-0.039548364	-0.003288225	ZBTB1, ZBTB25
chr8	118532625	118533254	630	*	11	4.42761E-09	0.005198563	0.109358054	0.0161987	-0.052844336	-0.016362349	MED30
chr11	44331893	44333192	1300	*	38	5.65147E-18	0.051643198	0.109484762	0.02106776	0.093622145	0.019983766	
chr11	59522428	59523399	972	*	12	8.45618E-09	0.034163583	0.109608675	0.038562036	-0.026999045	-0.000233953	STX3, AP000640.10
chr6	32121055	32123034	1980	*	49	6.33827E-20	0.043174793	0.109686037	0.037029542	0.15375517	0.017176859	PPT2, PPT2-EGFL8, PRRT1
chr7	95951129	95951808	680	*	12	2.5233E-08	0.120528725	0.109689191	0.070120202	0.036070767	0.007778335	SLC25A13
chr3	38065951	38066270	320	*	3	7.99368E-08	0.017147454	0.109977684	0.039850034	0.033506207	0.022960713	PLCD1
chr22	22221878	22222305	428	*	8	6.84646E-08	0.007026813	0.11009115	0.0180028	-0.034540268	-0.009409656	MAPK1
chr1	150947566	150947665	100	*	5	2.00221E-07	0.027051893	0.110134678	0.06379377	0.019985838	0.011761676	RP11-316M1.3
chr22	41681654	41682824	1171	*	18	6.20033E-10	0.389285823	0.110255644	0.069837758	-0.09367737	-0.004592322	RANGAP1
chr6	146350041	146350618	578	*	10	1.01309E-07	0.075284617	0.110277394	0.102865565	0.054737447	0.010692742	GRM1
chr17	4613328	4613992	665	*	10	8.05001E-09	0.008341989	0.110505527	0.025730183	-0.068804417	-0.01876546	ARRB2
chr17	26662236	26662842	607	*	13	9.35536E-08	0.025704108	0.11077619	0.066056716	-0.021032825	0.000881856	TNFAIP1, IFT20
chr12	56521892	56522453	562	*	9	7.04878E-09	0.008229955	0.111114623	0.027661584	-0.024969151	-0.010860368	ESYT1, RP11-603J24.5
chr11	125461928	125462736	809	*	10	1.87111E-08	0.099717378	0.111270696	0.06063511	-0.026554782	0.001871856	STT3A, STT3A-AS1
chr3	33482134	33482401	268	*	6	9.33465E-08	0.07934335	0.111516679	0.087147188	0.011544105	0.002951791	UBP1
chr12	27091095	27091484	390	*	10	4.36656E-09	0.011268451	0.111829253	0.017269389	-0.082886956	-0.011563024	FGFR1OP2, ASUN
chr2	201753695	201754268	574	*	12	2.14719E-08	0.011087138	0.11217699	0.02947579	-0.071398295	-0.015892888	NIF3L1, PPIL3
chr6	151560577	151561366	790	*	11	5.24433E-09	0.01187977	0.112493456	0.017824845	0.084953982	0.031334298	AKAP12

chr19	19754386	19754909	524	*	10	3.28537E-08	0.020038003	0.112512257	0.029000659	0.028574631	0.001979018	GMIP
chr14	77563968	77564635	668	*	11	3.61347E-08	0.012484139	0.112759154	0.035060826	0.020921171	0.004554941	CIPC
chr17	45726624	45727226	603	*	11	1.54273E-08	0.024711519	0.112885394	0.034823792	-0.061325971	-0.019791951	KPNB1, RP11-580I16.2
chr15	45879275	45879998	724	*	15	2.10175E-09	0.042438561	0.112976857	0.029471743	-0.042971792	-0.000954655	BLOC1S6, RP11-96O20.4
chr2	216946279	216946473	195	*	5	1.37372E-07	0.19222316	0.11298265	0.118380514	-0.044568404	-0.005304057	PECR
chr17	5322575	5323521	947	*	19	1.93634E-10	0.368971552	0.113030851	0.106935171	-0.041362225	-0.006615122	RPAIN, NUP88
chr19	52872727	52873277	551	*	12	8.95451E-08	0.011258343	0.113572612	0.036179091	0.101003432	0.029992753	ZNF880
chr2	26568235	26569594	1360	*	16	3.09184E-10	0.062889079	0.113643667	0.028497929	-0.049469705	-8.34883E-05	EPT1, GPR113
chr10	102746998	102747346	349	*	11	1.335E-07	0.191585893	0.113668656	0.10480248	-0.041916482	-0.00554782	C10orf2, MRPL43
chr19	45680969	45682354	1386	*	18	6.79451E-12	0.002532621	0.113738933	0.014439487	-0.068071525	-0.011318608	MARK4, BLOC1S3, TRAPPC6A
chr19	16186840	16187364	525	*	7	9.18203E-08	0.016219769	0.113960305	0.045885508	-0.057227629	5.90675E-05	TPM4
chr2	228028931	228029924	994	*	11	2.10573E-10	0.018424311	0.114067569	0.028586942	0.058992966	0.012926378	COL4A3
chr1	212208516	212209225	710	*	15	4.44977E-09	0.00880104	0.114630801	0.037903689	0.059050064	-0.002268756	DTL, INTS7
chr7	72936186	72937171	986	*	13	1.25766E-08	0.025956678	0.114999644	0.054750198	-0.024570699	0.004451749	BAZ1B
chr1	35544630	35544839	210	*	5	1.8611E-07	0.034754122	0.115108187	0.067321509	0.013258066	0.009018346	ZMYM1
chr13	41885260	41885657	398	*	6	5.21847E-08	0.859676613	0.115169471	0.495712034	0.032861654	0.00383193	NAA16
chr6	31148332	31148748	417	*	15	2.18346E-09	0.003868159	0.115253472	0.025246514	0.11353095	0.062225214	POU5F1
chr17	41277059	41277541	483	*	15	8.88682E-09	0.081341933	0.11618692	0.070423354	-0.057994235	-0.016158457	BRCA1
chr22	51020960	51021833	874	*	14	2.30146E-08	0.031704546	0.116285552	0.050342355	-0.030355898	0.001587375	CHKB-AS1, CHKB-CPT1B, CHKB
chr1	207494472	207495363	892	*	14	3.49457E-09	0.012349633	0.116388227	0.027015976	-0.037835425	-0.011424964	CD55
chr10	89621419	89623157	1739	*	45	7.31228E-15	0.099476401	0.116441193	0.093575305	-0.04685471	-0.003888637	PTEN, KLLN
chr17	46018654	46019006	353	*	10	8.52284E-08	0.013133394	0.116801493	0.030586871	-0.052489113	-0.007788472	PNPO, AC003665.1
chr6	31125930	31127527	1598	*	35	8.87897E-12	0.394223992	0.116841706	0.116973156	0.111840282	0.00896514	TCF19, CCHCR1
chr12	31743367	31743464	98	*	3	2.47815E-08	0.175973772	0.11687691	0.132875459	-0.031164165	-0.014882044	DENND5B-AS1, DENND5B
chr19	18433592	18433643	52	*	2	1.23807E-07	0.055571488	0.116980483	0.082420917	-0.019176929	-0.003906326	LSM4
chr16	67694647	67695984	1338	*	20	8.17137E-10	0.255879418	0.117019386	0.143901228	0.088543883	0.013341721	PARD6A, ACD
chr2	29092871	29093594	724	*	12	1.43804E-08	0.006582375	0.117120431	0.017441746	-0.021692142	0.003039539	TRMT61B
chr12	132195279	132195732	454	*	8	1.17325E-07	0.030186158	0.117693457	0.055461912	0.019077993	0.004148286	SFSWAP
chr15	83316640	83316962	323	*	6	1.14449E-08	0.215776895	0.117749036	0.22577501	0.072172525	0.020037679	RP11-752G15.3, CPEB1

chr2	63274857	63277327	2471	*	34	8.64229E-14	0.06243625	0.117877427	0.037166692	0.058048813	0.009982889	OTX1, AC009501.4
chr17	7307207	7307701	495	*	10	1.0942E-07	0.057242438	0.118110937	0.062872599	-0.041053158	-0.011548206	TMEM256-PLSCR3, C17orf61-PLSCR3, TMEM256
chr6	79787561	79788336	776	*	11	6.25856E-08	0.121537308	0.11813874	0.175400348	-0.033308011	-0.005177507	PHIP
chr13	29292888	29293848	961	*	12	2.21489E-09	0.008237667	0.118385151	0.020696823	-0.038626182	-0.002404152	SLC46A3
chr18	48555713	48556386	674	*	6	6.93328E-09	0.01379661	0.118472596	0.032469549	0.083480791	0.025709057	RP11-729L2.2, SMAD4
chr19	19030094	19030860	767	*	13	5.74513E-10	0.008714266	0.118545881	0.017176959	-0.09737292	-0.009341391	DDX49, COPE, AC002985.3
chr17	61904790	61905527	738	*	14	4.8329E-09	0.1910248	0.118546033	0.110325565	0.055694666	0.006179017	PSMC5, FTSJ3
chr21	35831871	35832364	494	*	13	1.4134E-09	0.006434242	0.118642506	0.024071104	0.173763626	0.08402464	KCNE1
chr7	16685273	16685823	551	*	11	8.54164E-09	0.016404616	0.119366269	0.035583301	-0.055408418	-0.001670759	BZW2, ANKMY2
chr17	38256932	38257323	392	*	10	8.12478E-09	0.011131451	0.119587411	0.023524006	-0.05573219	-0.010389337	NR1D1
chr1	155145555	155146720	1166	*	16	1.02395E-09	0.018216001	0.119704671	0.040433249	-0.093773112	-0.004450874	TRIM46, KRTCAP2, RP11-201K10.3
chr11	46402051	46402535	485	*	6	1.46352E-07	0.091569383	0.119952566	0.117637704	-0.038102092	-0.008997826	DGKZ, MDK
chr11	105947912	105949106	1195	*	20	1.95033E-10	0.142496932	0.120061592	0.057379494	-0.044291329	0.000163939	AASDHPPT, KBTBD3
chr1	114447334	114447798	465	*	10	9.52577E-09	0.009610526	0.120109604	0.028544671	-0.100765862	-0.026404237	DCLRE1B, AP4B1
chr10	71332744	71333638	895	*	16	1.089E-08	0.136983551	0.120405059	0.145043677	-0.031931929	0.00636144	NEUROG3
chr19	16770718	16771394	677	*	9	3.9306E-09	0.012917332	0.120634032	0.037895469	-0.071703837	-0.005056442	CTC-429P9.4, SMIM7
chr20	35402123	35402523	401	*	13	8.84963E-08	0.133482785	0.120721831	0.065999347	-0.050061916	-0.021597531	DSN1
chr3	147126206	147126444	239	*	5	8.86249E-08	0.068417885	0.12108636	0.106433594	0.037379276	0.028744599	ZIC1
chr11	71935199	71935353	155	*	2	4.31935E-08	0.049116172	0.121090512	0.076633698	-0.033288765	-0.009767306	INPPL1
chr11	57282256	57283278	1023	*	14	2.0718E-10	0.007835175	0.121149992	0.021726088	-0.041429696	-0.010714445	SLC43A1
chr17	7834607	7836318	1712	*	19	4.64477E-09	0.065523422	0.121177999	0.073663108	0.04747273	0.004298133	CNTROB, TRAPPC1
chr12	115121047	115122101	1055	*	8	1.49218E-09	0.021131027	0.121395818	0.063309836	0.023619069	0.004898692	TBX3
chr6	30034178	30035234	1057	*	19	7.7396E-12	0.005456934	0.121533695	0.016203054	-0.048122775	-0.014919079	PPP1R11
chr2	170550423	170550468	46	*	2	1.93249E-07	0.065375292	0.121589876	0.093413573	-0.079219191	-0.008398967	CCDC173
chr6	30640154	30641220	1067	*	40	5.17648E-10	0.774538205	0.121595424	0.594345206	-0.064529188	0.001936973	DHX16
chr11	117747934	117748236	303	*	8	1.28996E-07	0.005602227	0.121934197	0.026748893	0.126285703	0.07967961	FXYD6
chr16	66967867	66968030	164	*	2	1.92111E-07	0.093536948	0.121951742	0.117401874	0.019656011	0.006381127	FAM96B
chr7	129592601	129592850	250	*	3	1.77504E-07	0.264009908	0.12203915	0.219004074	0.010686152	0.003651271	UBE2H
chr1	2457999	2458506	508	*	16	2.82253E-09	0.072730169	0.122130977	0.044315614	-0.067150021	-0.013438553	PANK4

chr1	18433794	18434118	325	*	4	6.88393E-08	0.032238623	0.122367873	0.06591658	0.034902735	0.022605032	
chr6	30684736	30686368	1633	*	31	2.60385E-15	0.206363682	0.122529697	0.084079744	0.049056895	0.00129684	MDC1
chr7	27224700	27226148	1449	*	28	1.3083E-14	0.053155789	0.122649572	0.044669707	0.107702768	0.034224668	HOXA11-AS, HOXA11
chr6	30584720	30585650	931	*	26	7.95391E-11	0.064333739	0.122842378	0.086736313	-0.059270564	-0.00048938	MRPS18B, PPP1R10
chr6	43138798	43139692	895	*	12	1.09252E-09	0.197881476	0.122994103	0.079194034	-0.040953312	-0.007502742	SRF
chr2	225266263	225266656	394	*	5	1.58669E-07	0.02819917	0.123456116	0.065768518	-0.050587664	-0.032157159	FAM124B
chr14	58764383	58765087	705	*	9	9.36992E-09	0.162739225	0.123585902	0.045994283	-0.050878244	-0.017572965	C14orf37, RP11-349A22.5
chr3	9834426	9834755	330	*	12	1.13723E-07	0.081232917	0.123724158	0.08462471	0.028007685	0.006923147	ARPC4, TADA3
chr22	19435145	19436224	1080	*	12	2.56475E-09	0.001589073	0.12488915	0.013779673	0.051748234	-0.015407648	AC000068.5, HIRA, C22orf39
chr2	202507362	202507823	462	*	11	1.70594E-08	0.033471395	0.12509215	0.080758316	0.025084124	0.005270695	TMEM237
chr17	18162315	18162372	58	*	3	1.87104E-07	0.071463415	0.125095136	0.109613625	0.032132141	0.022164122	
chr1	150336613	150337482	870	*	15	5.03465E-09	0.046237699	0.125359869	0.043376182	-0.042731668	-0.008931725	RPRD2
chr17	48637104	48638103	1000	*	21	5.3153E-10	0.129820949	0.125472006	0.091186978	0.102654723	0.008549038	CACNA1G-AS1
chr1	40782458	40783264	807	*	11	3.49624E-09	0.005420858	0.125573444	0.019164045	0.116609053	0.044978877	COL9A2
chr6	30614632	30615000	369	*	10	1.67513E-08	0.071245218	0.125719955	0.092467393	0.033564522	-1.46452E-06	C6orf136
chr19	50528391	50529510	1120	*	17	6.84306E-10	0.016975601	0.1258033	0.023374207	-0.042195819	-0.002642245	ZNF473, VRK3
chr7	99155595	99156469	875	*	15	1.85258E-09	0.065915631	0.126418923	0.068789001	-0.05482989	-0.006187936	ZNF655, GS1-259H13.10, FAM200A
chr11	78285742	78286346	605	*	11	1.82946E-08	0.004136693	0.126460619	0.019973385	-0.068762967	-0.004350954	NARS2
chr2	88354927	88355481	555	*	13	5.18532E-08	0.012509969	0.12668464	0.033453154	-0.043225183	0.00016784	KRCC1
chr12	56551390	56552132	743	*	15	2.42857E-08	0.048496417	0.126813049	0.055226182	-0.056830461	-0.012553875	MYL6B, MYL6, RP11-603J24.14
chr19	49140995	49141364	370	*	7	9.77152E-08	0.1230874	0.126893542	0.11114869	-0.044030524	-0.015271015	SEC1P, CA11
chr1	9970042	9971115	1074	*	16	1.81077E-09	0.016828393	0.127895831	0.051092304	-0.102119522	-0.024145563	CTNNBIP1
chr3	49977118	49977988	871	*	16	3.79825E-09	0.01652001	0.127980792	0.047548682	0.027675731	0.004397948	RBM6
chr7	127983655	127984063	409	*	8	1.24245E-07	0.03997001	0.128989449	0.078519695	-0.038582367	-0.0001033	RBM28
chr6	30881112	30882384	1273	*	38	2.38412E-14	0.36140145	0.12923955	0.16733962	0.101574874	0.01580552	GTF2H4, VARS2
chr1	160231971	160233057	1087	*	17	7.84064E-09	0.031642586	0.129431615	0.04367656	-0.076546798	-0.010532761	RP11-574F21.2, DCAF8, DCAF8
chr16	11349372	11350371	1000	*	13	1.48523E-08	0.070641159	0.129470788	0.055317558	-0.065876724	-0.016368234	RMI2, SOCS1
chr17	8021572	8022404	833	*	14	1.07655E-09	0.034266342	0.129489324	0.03480994	-0.026112001	-0.001902428	ALOXE3
chr6	33290408	33291111	704	*	24	1.25394E-11	0.23295503	0.129563682	0.116994613	-0.106079423	-0.019789606	DAXX

chr18	2571349	2572167	819	*	13	7.99088E-10	0.171214095	0.129580947	0.100835231	-0.045059813	0.001779243	NDC80, METTL4
chr15	64648051	64648224	174	*	2	1.86326E-07	0.077721135	0.12972332	0.107523441	-0.06847561	-0.02895056	CSNK1G1, CTD-2116N17.1
chr20	25062447	25063170	724	*	11	3.55516E-09	0.001618318	0.130102628	0.010809505	0.111772966	0.04191779	VSX1
chr14	24024869	24025569	701	*	15	8.66304E-08	0.195720949	0.130149263	0.210026217	-0.02759103	0.000578043	RP11-66N24.4, THTPA, ZFHX2
chr14	92572856	92573175	320	*	9	7.24582E-08	0.013626781	0.130492346	0.043301046	0.035835662	0.007590703	ATXN3
chr12	45609527	45610283	757	*	11	1.07025E-08	0.120699143	0.130557433	0.094400687	-0.055525609	-0.017133534	ANO6, PLEKHA8P1
chr5	148930374	148931484	1111	*	17	1.12722E-08	0.087241206	0.130630649	0.090504686	-0.04177304	-0.003107448	CSNK1A1
chr11	111956827	111957872	1046	*	23	2.75366E-08	0.300806453	0.130713153	0.135811293	-0.048212831	-0.00051182	SDHD, SDHD, TIMM8B
chr6	32862050	32862445	396	*	14	1.19521E-07	0.519679629	0.130793516	0.27780697	-0.044296506	-8.68275E-05	
chr3	134513993	134514472	480	*	11	1.5157E-08	0.004465998	0.131319874	0.022008664	-0.082348813	-0.022397224	EPHB1
chr6	30180688	30182518	1831	*	47	4.19441E-14	0.09917825	0.131723339	0.110923789	0.112095243	0.002545766	TRIM26
chr20	982991	983130	140	*	5	1.28765E-07	0.036419197	0.132058958	0.074419924	0.013793921	0.004871038	
chr10	105155729	105156690	962	*	21	8.78752E-11	0.070699428	0.132153637	0.049511608	-0.062231901	-0.008755047	PDCD11, USMG5
chr12	57623725	57624530	806	*	12	2.33612E-08	0.031029466	0.132644226	0.033168531	0.076347368	0.01421038	SHMT2
chr15	72612125	72612853	729	*	14	1.41076E-08	0.023248194	0.133047422	0.058803763	0.040543782	0.005848186	RP11-106M3.3, CELF6, CELF6, RP11-106M3.2
chr22	18120948	18121431	484	*	13	1.03511E-07	0.07232874	0.133295203	0.064799963	-0.049063891	-0.013703801	BCL2L13
chr1	19577645	19578612	968	*	16	2.29209E-09	0.01292408	0.133325904	0.046115929	-0.025348313	0.00162231	MRTO4, EMC1
chr12	133464074	133465188	1115	*	28	1.70194E-09	0.437946387	0.133555361	0.25926794	0.126817173	-4.23069E-05	RP11-46H11.12, CHFR
chr1	23495375	23495393	19	*	2	1.67313E-07	0.087989406	0.134335434	0.118173396	0.008462847	0.002466949	LUZP1
chr6	33385056	33386556	1501	*	50	2.87846E-13	0.427217748	0.134660612	0.216894569	0.075664635	0.007711844	CUTA
chr11	506809	507555	747	*	16	4.06333E-08	0.163064321	0.134708343	0.155177069	-0.041392491	-0.008268064	RNH1
chr1	26232706	26232895	190	*	6	1.19436E-07	0.274939791	0.134778932	0.224814653	-0.036498799	-0.014744798	STMN1, MIR3917
chr6	31620354	31621299	946	*	25	3.40756E-11	0.343876576	0.135309826	0.141072523	-0.078528888	-0.004619115	APOM, BAG6
chr6	32163810	32165321	1512	*	34	6.01639E-14	0.115409852	0.135834432	0.06131549	0.082570338	0.00828511	NOTCH4
chr19	55897101	55897819	719	*	13	1.43355E-08	0.067473941	0.1361054	0.086120832	0.025538146	-0.002986303	RPL28
chr10	25464059	25464719	661	*	12	1.23248E-09	0.003169675	0.136199013	0.024894085	0.051418106	0.015514805	GPR158, GPR158-AS1
chr3	157823708	157824217	510	*	9	7.00193E-08	0.262006058	0.137258959	0.12807416	0.052984008	0.016612005	RSRC1, SHOX2
chr1	155107785	155108588	804	*	12	6.69267E-09	0.070053882	0.137884431	0.03874821	-0.065549345	-0.030433415	SLC50A1
chr6	33265886	33267886	2001	*	53	2.94609E-12	0.842772932	0.138494963	0.546161634	-0.141868505	-0.003558181	PFDN6, RGL2, TAPBP

chr2	264695	265084	390	*	11	2.14576E-08	0.038719206	0.138839926	0.07908986	-0.02661838	-0.004282029	ACP1, SH3YL1
chr15	69706318	69706619	302	*	8	7.66868E-08	0.144071278	0.140092918	0.119610636	0.083900836	0.017707174	KIF23, RP11-253M7.1
chr17	42263806	42264871	1066	*	19	3.51E-09	0.102931273	0.140161756	0.108976317	-0.03706444	0.001508787	TMUB2, ASB16-AS1
chr1	53067637	53068579	943	*	10	5.14212E-09	0.058431913	0.140649203	0.082457956	-0.055938645	0.003078747	GPX7
chr17	6543784	6544486	703	*	14	5.48399E-09	0.122344538	0.140695164	0.089472113	-0.053613902	-0.002078924	TXNDC17, KIAA0753
chr3	37033980	37035399	1420	*	35	5.9411E-12	0.243946191	0.141216624	0.258931702	-0.072549027	-0.004374619	MLH1, EPM2AIP1
chr3	129158884	129159190	307	*	10	9.61205E-08	0.114575202	0.141461989	0.080878487	-0.041774893	-0.005582764	IFT122
chr11	63438791	63439440	650	*	14	1.89341E-08	0.03919686	0.141592399	0.06979716	0.016930787	0.001022803	ATL3
chr15	74219307	74219948	642	*	4	2.64667E-08	0.124876405	0.141963915	0.10186879	-0.025151632	-0.001329397	LOXL1, LOXL1-AS1
chr20	42142005	42143211	1207	*	25	1.07941E-10	0.513247698	0.142145762	0.203376577	-0.115885831	-0.037866015	L3MBTL1
chr12	45269992	45270476	485	*	10	4.664E-09	0.022985762	0.142719086	0.032401882	0.024483503	0.009411997	NELL2
chr17	46655164	46656093	930	*	18	1.78606E-08	0.064943155	0.142726931	0.084598045	-0.055142706	0.001415284	HOXB-AS3, HOXB3, HOXB4
chr1	149900628	149900705	78	*	3	1.25674E-07	0.047002093	0.143076373	0.089591844	0.090918258	0.06047276	MTMR11
chr20	49126382	49126905	524	*	11	8.7097E-08	0.1510693	0.14384541	0.11061255	0.063594623	0.001252749	PTPN1
chr19	52643011	52643487	477	*	10	4.98039E-08	0.002991059	0.144167625	0.021394552	-0.045571373	-0.010069832	ZNF616, CTC-471J1.8
chr2	25142101	25142523	423	*	6	8.80287E-08	0.095082448	0.144354687	0.086275413	0.058441654	0.010823346	ADCY3
chr1	11865920	11866529	610	*	15	3.73446E-08	0.140613303	0.144447674	0.161470736	-0.045761932	-0.00982829	CLCN6, MTHFR
chr20	36147549	36149750	2202	*	42	4.71942E-16	0.163144168	0.145188255	0.083972744	-0.10131827	-0.030423896	NNAT, BLCAP
chr4	85402397	85402870	474	*	3	1.15048E-07	0.04486806	0.145241454	0.086227872	0.034765268	0.02390704	
chr17	61920084	61920732	649	*	10	1.04182E-07	0.167226185	0.145253671	0.095229523	-0.036889653	-0.011616136	SMARCD2
chr19	4769001	4769688	688	*	13	4.64355E-08	0.016328435	0.146086183	0.049959898	0.124181713	0.037982236	MIR7-3HG
chr11	47270041	47270344	304	*	7	1.31248E-07	0.152925032	0.146700491	0.119586392	-0.04799838	-0.003402615	NR1H3, ACP2
chr1	173684086	173684474	389	*	3	7.77282E-08	0.075821562	0.147807172	0.117309866	-0.06443943	-0.02343313	KLHL20
chr6	2764512	2765204	693	*	9	2.73146E-08	0.006564184	0.148454597	0.035022659	0.079634723	0.002852028	
chr3	197518634	197518711	78	*	2	1.82513E-07	0.123605559	0.149290153	0.152525354	-0.035407442	-0.010379338	LRCH3
chr10	30722981	30723526	546	*	6	7.39601E-08	0.266973921	0.149892554	0.150903449	-0.007383537	-0.002568134	MAP3K8
chr8	95565682	95566223	542	*	9	5.31799E-08	0.019720621	0.149944643	0.049511776	-0.023035832	0.002650129	RP11-267M23.4, KIAA1429
chr9	127630689	127630854	166	*	2	1.11213E-07	0.072224871	0.150071188	0.10878149	-0.06055883	-0.025666114	ARPC5L
chr11	2421572	2422730	1159	*	26	5.9762E-10	0.246629941	0.150217722	0.142745569	-0.065217338	-0.000494801	TSSC4

chr6	33167661	33169106	1446	*	31	3.91488E-10	0.505687015	0.150309174	0.361950915	-0.062768386	-0.003019555	SLC39A7, RXRB
chr19	11546103	11547126	1024	*	20	3.86484E-09	0.936379391	0.152799292	0.558746809	0.042232497	0.002684463	PRKCSH, CCDC151
chr4	84457116	84457579	464	*	14	5.0826E-08	0.094337632	0.153142188	0.150800857	-0.025869127	0.006627033	AGPAT9
chr16	55689477	55689534	58	*	3	2.09837E-07	0.041980627	0.154060604	0.086086096	0.094427104	0.066133062	SLC6A2
chr11	2906667	2908116	1450	*	29	2.43722E-11	0.036658527	0.154278381	0.055174645	0.133006461	0.006451012	CDKN1C
chr11	28129499	28130018	520	*	15	3.91478E-09	0.003884896	0.155950245	0.026704998	-0.04807015	0.001747133	METTL15, KIF18A
chr13	48877957	48878466	510	*	3	7.29955E-08	0.453623453	0.15631383	0.194986207	-0.042664723	-0.026650894	RB1
chr1	43638094	43638119	26	*	2	1.72807E-07	0.12173732	0.156872036	0.154821267	-0.03104706	-0.022424098	WDR65, EBNA1BP2
chr1	151694320	151694582	263	*	4	1.19139E-07	0.023915358	0.157147408	0.066177011	0.069623158	0.04991422	AL589765.1, RIIAD1
chr3	47323804	47325421	1618	*	22	4.69574E-10	0.003326985	0.157652826	0.023199896	0.105561627	-0.007140909	KLHL18, KIF9
chr12	48690974	48691036	63	*	2	1.37823E-07	0.081820582	0.1589078	0.121109035	0.075254773	0.063792291	
chr13	78492916	78493878	963	*	31	3.06578E-10	0.331157777	0.160444815	0.277712979	-0.04612181	-0.000966032	RNF219-AS1, EDNRB
chr7	151722222	151722814	593	*	12	4.34969E-08	0.007750543	0.161741614	0.044049444	0.090031328	0.017783941	GALNT11
chr6	33421704	33423122	1419	*	38	5.5106E-14	0.142594846	0.163351984	0.155463762	0.101204435	0.00454309	ZBTB9
chr4	114681840	114681880	41	*	2	6.9542E-08	0.306165142	0.163472675	0.248532769	-0.029285503	-0.016927756	CAMK2D
chr17	72919480	72919704	225	*	4	9.31664E-09	0.037987878	0.168672385	0.091224175	0.026563293	0.01717289	
chr11	61583560	61584116	557	*	12	7.14527E-08	0.083225782	0.169396215	0.127195863	-0.101191405	-0.014056419	FADS2, FADS1
chr7	116593263	116594199	937	*	14	2.4606E-09	0.007102763	0.171397601	0.049351823	-0.043233367	-0.011724233	ST7, ST7-OT4, ST7-AS1
chr12	123459241	123459749	509	*	11	3.68336E-08	0.565073249	0.171970738	0.291503179	-0.018846715	-0.003055796	OGFOD2, ABCB9
chr15	74284600	74284603	4	*	2	2.01668E-07	0.125071307	0.172288545	0.164475857	0.012002965	0.009324772	STOML1
chr17	79935490	79935872	383	*	4	4.54408E-08	0.311262867	0.173233127	0.259767338	0.007035079	0.000331893	ASPSCR1
chr6	32063874	32064810	937	*	28	1.52018E-10	0.215935052	0.17431316	0.255596703	-0.14927709	-0.056933782	TNXB
chr2	44588434	44589279	846	*	16	5.20228E-08	0.050856893	0.175392177	0.105850945	-0.027110594	-0.008536468	CAMKMT, PREPL
chr6	128841761	128841771	11	*	2	2.11737E-07	0.094032202	0.175976897	0.13858043	0.012571518	0.001837638	PTPRK
chr11	64889310	64889881	572	*	14	6.26571E-08	0.286740328	0.178033638	0.183643944	0.017016161	0.003989082	MRPL49, FAU, SYVN1
chr15	89010209	89010921	713	*	17	1.37903E-09	0.002294981	0.179271554	0.030113704	-0.041349757	-0.008004053	MRPS11, MRPL46, RP11-97O12.7
chr1	183605108	183605490	383	*	12	9.82312E-08	0.037408258	0.180419538	0.081036605	-0.052028229	-0.01967688	RGL1
chr6	30028257	30029370	1114	*	31	3.97994E-11	0.252657521	0.180619139	0.247021064	-0.044969028	-0.004387738	ZNRD1, ZNRD1-AS1
chr20	61447442	61448534	1093	*	27	4.279E-11	0.021971159	0.182440512	0.066405097	0.059612191	0.011334103	COL9A3

chr11	2890258	2891495	1238	*	30	2.57631E-13	0.163694221	0.187945105	0.10262147	0.144220942	0.049425682	KCNQ1DN
chr7	11013361	11013816	456	*	10	1.08306E-07	0.05725905	0.189855462	0.124768507	-0.025679188	-0.000477882	PHF14
chr17	77770879	77771036	158	*	2	1.42856E-07	0.303462979	0.194392238	0.275934363	-0.01075811	-0.003217863	CBX8
chr2	201980638	201981146	509	*	13	9.88707E-08	0.048098386	0.194964916	0.119932365	-0.052777995	-0.021404336	CFLAR
chr1	3447729	3447740	12	*	2	1.85663E-07	0.290811494	0.195084492	0.271754811	0.015187103	0.009929084	MEGF6
chr4	2470786	2471043	258	*	6	9.29762E-08	0.230729922	0.1956801	0.237127608	-0.07544477	-0.027488361	RNF4
chr7	143012820	143012960	141	*	2	5.39979E-08	0.432223319	0.197557119	0.315200369	-0.020765419	-0.01186308	
chr7	94284629	94287242	2614	*	73	2.8292E-11	0.110937093	0.197815661	0.288828966	-0.120030954	-0.020549613	PEG10, SGCE
chr17	17398264	17398771	508	*	2	1.15921E-07	0.15366277	0.20059205	0.199867907	0.038567252	0.037864331	RASD1
chr7	87229552	87229722	171	*	3	1.44841E-07	0.078901	0.200993557	0.147493762	0.023706095	0.018199522	ABCB1
chr5	122759147	122759164	18	*	2	2.14504E-07	0.297778241	0.20433521	0.282229903	-0.005135048	-0.003553856	CEP120
chr1	52343884	52344471	588	*	10	2.29336E-08	0.038955355	0.207748841	0.125752256	-0.028901097	-0.006931155	NRD1
chr8	30890632	30891583	952	*	15	5.88765E-09	0.21056063	0.210277522	0.273956824	-0.043595153	-0.004558705	WRN, PURG
chr5	50679147	50679290	144	*	4	1.27991E-07	0.178895126	0.212824535	0.267696742	0.02789422	0.005351449	ISL1, CTD-2314G24.2
chr12	72057638	72057807	170	*	9	1.80414E-07	0.164932293	0.214447018	0.245493215	-0.081319795	-0.01900428	THAP2, ZFC3H1
chr10	92979413	92979600	188	*	2	3.62814E-08	0.832575385	0.214831957	0.373902365	0.047847625	0.023705357	
chr17	1956958	1958235	1278	*	42	1.53759E-11	0.181484951	0.217832979	0.255083829	-0.086862116	-0.002885466	HIC1
chr3	9791984	9792140	157	*	2	1.20063E-07	0.312672346	0.224052505	0.304468614	0.010350079	0.00688838	OGG1
chr6	31650735	31651158	424	*	13	2.26881E-08	0.087766769	0.22504178	0.133863016	-0.128554584	-0.081316349	LY6G5C
chr6	80656888	80656973	86	*	2	1.60304E-07	0.391904131	0.226596615	0.334403005	0.007486377	0.002832558	ELOVL4
chr15	60884119	60884637	519	*	4	5.55862E-08	0.376925807	0.241156847	0.300192484	0.027136086	0.009359468	RP11-219B17.1, RORA
chr1	54518349	54518906	558	*	5	3.1361E-08	0.219213442	0.246793619	0.304066504	-0.031590901	-0.004656062	TMEM59
chr8	11560851	11561283	433	*	2	7.94832E-08	0.638428965	0.256980864	0.414698484	0.036980673	0.017550351	GATA4
chr16	22217447	22218046	600	*	6	9.26673E-08	0.532280963	0.257636212	0.47496555	0.017568162	0.002536631	EEF2K
chr21	27107628	27107814	187	*	5	1.11105E-07	0.603156769	0.2578845	0.522534565	-0.043720062	-0.004982861	GABPA, ATP5J
chr1	41445105	41445556	452	*	6	9.07056E-09	0.52514084	0.258117455	0.579717884	0.013745855	0.006113687	CTPS1
chr2	46843544	46844136	593	*	4	2.80521E-08	0.279442255	0.258410356	0.333685629	-0.041402795	-0.008663915	CRIPT, PIGF
chr6	101847318	101847349	32	*	2	2.00785E-07	0.185159397	0.26011238	0.252791521	0.036126717	0.033806713	GRIK2
chr1	35324844	35325189	346	*	3	1.37709E-07	0.722995874	0.266854315	0.519568461	0.016998515	0.002421319	SMIM12

chr1	3688916	3688921	6	*	2	1.64822E-07	0.193073538	0.268012411	0.262866562	-0.007509352	-0.006590526	
chr5	147162432	147162461	30	*	2	1.88924E-07	0.19527611	0.271492908	0.26627908	0.028999423	0.017938127	
chr11	60680165	60680515	351	*	2	4.00439E-08	0.334666865	0.281471682	0.358884881	0.055830838	0.014276638	
chr16	2198949	2199120	172	*	2	1.22738E-07	0.24554329	0.28281715	0.3091465	0.013578927	0.009727567	RAB26
chr4	2243925	2243942	18	*	2	1.82787E-07	0.261575226	0.28838257	0.322962126	-0.039365768	-0.025117467	
chr22	18507401	18507404	4	*	2	1.88646E-07	0.255272796	0.289080104	0.319256375	-0.021095319	-0.020456771	
chr22	26875858	26876075	218	*	2	8.16108E-08	0.412976343	0.299561241	0.406364231	-0.028542729	-0.022601263	HPS4
chr20	57225100	57225195	96	*	2	9.0373E-08	0.284300649	0.304665825	0.347207945	0.039556392	0.023575669	
chr15	25684578	25684818	241	*	4	1.89978E-07	0.224866196	0.312087766	0.358865365	-0.04225885	-0.015326112	
chr8	98656243	98656671	429	*	8	1.06558E-07	0.195808073	0.316211179	0.398784886	-0.062675717	-0.025439484	MTDH
chr17	48797139	48797403	265	*	2	1.74103E-07	0.315420473	0.341430341	0.389235053	-0.021647004	-0.007097205	LUC7L3
chr2	10442879	10443138	260	*	4	1.00824E-07	0.600396048	0.352330928	0.580661616	-0.017969165	-0.008138237	HPCAL1
chr6	33377795	33378130	336	*	7	1.58192E-07	0.696980425	0.369988388	0.681228276	-0.043361524	0.000285004	
chr12	56545925	56546207	283	*	6	1.84814E-07	0.711371235	0.376155861	0.729862307	-0.031175405	-0.010119742	MYL6B, RP11-603J24.14
chr6	32048632	32049053	422	*	3	3.9696E-08	0.372163499	0.421593143	0.524367629	0.046276765	0.042020025	TNXB
chr17	76227508	76227681	174	*	2	7.98735E-08	0.412666308	0.437934812	0.508546424	0.041630461	0.036972349	TMEM235
chr8	41347837	41348205	369	*	2	9.02455E-08	0.846315649	0.442438776	0.635845817	-0.012098062	-0.00755966	GOLGA7
chr1	7622519	7622560	42	*	2	1.52929E-07	0.781492959	0.471528891	0.656953247	0.01777275	0.010279266	CAMTA1
chr1	204485540	204485559	20	*	2	1.87399E-07	0.46756798	0.473551028	0.56206555	0.010712004	0.003978101	MDM4
chr1	38156178	38156235	58	*	4	1.8699E-07	0.875801128	0.494997872	0.81169718	0.02416294	0.003446001	C1orf109
chr5	134872029	134872124	96	*	3	1.80859E-07	0.843895422	0.498463606	0.720269913	-0.041535686	-0.005829581	
chr12	50222430	50222472	43	*	3	2.11683E-07	0.711183454	0.498682554	0.723211729	-0.008540616	-0.001447516	BCDIN3D-AS1, NCKAP5L
chr16	89044523	89044646	124	*	2	6.67907E-08	0.887533886	0.80282184	0.92790144	-0.020572505	-0.018786831	
chr1	154975574	154975779	206	*	2	1.27221E-07	0.968394141	0.867687253	0.968795882	0.003879233	0.001874176	ZBTB7B
