# Supplementary material for: A Sight of the Diagnostic Value of Aberrant Cell-Free DNA Methylation in Lung Cancer
Source: Dis Markers. 2022 Jan 27;2022:9619357. doi: 10.1155/2022/9619357 (PMC8814721; doi:10.1155/2022/9619357)
Supplement: Supplementary 7 — Table S4: KEGG analysis of DMGs in cfDNA samples. [file 9619357.f7.pdf]

Table S4. KEGG analysis of DMGs in cfDNA samples

ID	Description	GeneRatio	BgRatio	pvalue	p.adjust	qvalue	Count	geneID
hsa04071	Sphingolipid signaling pathway	34/1067	119/8105	6.18E-06	0.002007575	0.001677585	34	5590/5293/9517/4363/5295/9846/5578/5337/7124/7186/253782/1509/10000/2771/7132/56848/259230/5527/5579/5518/23236/166929/5529/5528/81537/130367/596/9294/5519/5581/2768/8698/5970/29956
hsa04152	AMPK signaling pathway	33/1067	120/8105	2.03E-05	0.003032855	0.002534337	33	57521/51422/5468/5293/1385/8408/5862/5210/5295/6009/10645/10000/5564/3480/5527/51719/32/5518/64764/5529/5528/8900/31/5519/23216/5106/7249/2308/6319/6720/1374/5209/5562
hsa05202	Transcriptional misregulation in cancer	46/1067	192/8105	2.80E-05	0.003032855	0.002534337	46	1669/1991/1053/6256/4353/5328/5468/2078/861/2313/1436/7185/4066/7704/3728/7048/4299/5218/7709/171558/3065/3480/2321/5081/5914/4298/860/2120/54738/8864/4914/8900/3205/4211/6929/4208/1051/2308/10912/598/3087/5970/79058/2005/7468/8350
hsa04072	Phospholipase D signaling pathway	36/1067	148/8105	0.000146822	0.009941583	0.00830746	36	108/5293/8395/115/5295/146850/9846/5578/5337/1785/6009/23533/10000/56848/1609/80310/3577/5155/1606/8527/23236/1759/196883/5898/26052/9170/2768/27128/2149/56034/185/7249/5159/5335/55326/8613
hsa04144	Endocytosis	54/1067	252/8105	0.000152947	0.009941583	0.00830746	54	5590/10938/160/64744/8395/5878/23527/5869/4088/80223/408/7456/5337/1785/11031/2352/116987/3107/116985/7048/274/28964/8724/112936/3134/3480/58513/50807/2870/8976/22905/57154/2869/9922/8853/3577/3800/6455/5584/409/1759/26052/27128/131890/2060/4734/381/80230/55040/93343/8218/9372/156/23111
hsa04070	Phosphatidylinositol signaling system	26/1067	97/8105	0.000235494	0.01275595	0.010659223	26	8871/5293/3709/3632/8395/3635/5295/3707/5578/3708/3631/5305/1609/5579/1606/8527/56623/23236/3613/55361/5297/5335/22876/113026/8760/9807
hsa05135	Yersinia infection	33/1067	137/8105	0.000331542	0.014124399	0.011802736	33	58484/27040/3586/6195/5293/9844/3932/5608/8395/1793/114548/5295/391/8874/7456/4210/7124/7186/4773/6196/4772/10458/10000/5829/8976/7410/71/64283/834/5335/5970/10451/2533
hsa04810	Regulation of actin cytoskeleton	47/1067	218/8105	0.000347678	0.014124399	0.011802736	47	3682/54434/3985/2934/5293/4629/8395/1793/5295/4638/8874/26999/10627/221178/4627/28964/10458/5305/87/3674/81/54961/5829/3693/8976/2260/5216/80310/7410/3690/71/1129/5155/5500/3689/10787/3645/9170/2768/2149/56034/5159/23191/58498/7430/3694/10451
hsa04210	Apoptosis	32/1067	136/8105	0.000629517	0.022732545	0.018995941	32	1522/1439/5293/3709/2081/5295/7124/7186/7185/3708/1509/1512/8737/10000/84823/7132/143/71/1677/1508/6708/7277/9020/51807/840/4914/596/10912/598/6709/5970/8772
hsa05131	Shigellosis	50/1067	246/8105	0.000955707	0.029388148	0.024557546	50	58484/57521/3098/5293/3709/83660/9844/1793/114548/3554/5295/2017/7124/7186/6352/10627/26100/3708/8737/87/10000/81/7132/3611/5829/8976/5216/7335/10318/22863/3099/71/8915/23236/596/5581/27128/9181/834/2308/598/5335/5970/23291/58498/113026/7322/10801/340061/8350
hsa04015	Rap1 signaling pathway	44/1067	210/8105	0.000994676	0.029388148	0.024557546	44	5590/27040/108/5293/83660/5608/284/115/5295/5908/5578/1436/2357/7057/10000/2771/3674/3480/2321/2260/9693/5579/5216/80310/7410/3690/71/5155/26037/23236/5584/3689/196883/57568/5898/999/2775/9170/2149/56034/5159/5335/10451/2533
hsa04668	TNF signaling pathway	27/1067	112/8105	0.001102242	0.029852401	0.024945488	27	5293/1385/5608/7133/5295/7124/7186/7128/6352/7185/8737/10000/7132/8986/11035/6364/64764/9020/840/64127/10059/8809/1051/5970/602/3976/8772
hsa04725	Cholinergic synapse	27/1067	113/8105	0.001271154	0.030146757	0.02519146	27	108/5293/3709/1385/775/115/5295/146850/5578/3785/3708/23533/10000/2771/5579/2788/2767/1129/2784/23236/64764/3784/196883/596/54331/2775/774
hsa04931	Insulin resistance	26/1067	108/8105	0.001380558	0.030146757	0.02519146	26	5590/51422/6195/5293/1385/5295/7124/6196/10000/7132/5564/5579/7376/32/64764/5500/5581/9945/5106/6513/2308/6720/5970/1374/5562/5524
hsa04666	Fc gamma R-mediated phagocytosis	24/1067	97/8105	0.001391389	0.030146757	0.02519146	24	27040/3985/2934/5293/8395/3635/1793/5295/9846/5578/5337/1785/274/10000/50807/56848/5579/8853/7410/5788/5581/5335/10451/8613

hsa04211	Longevity regulating pathway	22/1067	89/8105	0.002190755	0.043801265	0.036601543	22	57521/51422/5468/108/5293/1385/8408/115/5295/6009/10645/10919/10000/5564/3480/64764/847/196883/7249/2308/5970/5562
hsa04928	Parathyroid hormone synthesis, secretion and action	25/1067	106/8105	0.002291143	0.043801265	0.036601543	25	5144/6256/108/3709/1385/4209/11214/115/7421/408/5578/5337/3708/2771/2260/5579/2767/860/23236/64764/409/196883/596/4208/2768
hsa04330	Notch signaling pathway	16/1067	59/8105	0.003152857	0.050041088	0.041815711	16	55534/9612/9794/84441/6310/28514/3516/171558/3065/7088/1487/1488/8650/1387/4851/4855
hsa04728	Dopaminergic synapse	29/1067	132/8105	0.003346749	0.050041088	0.041815711	29	3709/1385/84152/775/408/5578/3708/1312/1644/10000/2771/6323/5527/5579/2788/3800/2784/5518/23236/64764/5500/409/406/5529/5528/54331/2775/5519/774
hsa04062	Chemokine signaling pathway	39/1067	192/8105	0.003357024	0.050041088	0.041815711	39	5590/108/5293/9844/115/5295/5908/729230/146850/408/2268/6352/23533/10000/2771/57580/5829/2870/10803/5579/2869/2788/6364/7410/3577/2784/23236/409/196883/54331/131890/1794/5335/5970/10850/6367/1524/10451/156
hsa05231	Choline metabolism in cancer	23/1067	98/8105	0.003595681	0.050041088	0.041815711	23	5130/5293/8395/5295/5578/5337/6009/80736/10000/1609/5579/56994/80310/5155/1606/6584/8527/56034/7249/5159/1120/5335/8613
hsa04919	Thyroid hormone signaling pathway	27/1067	121/8105	0.00362484	0.050041088	0.041815711	27	6256/5293/486/5295/5578/6009/10000/1827/3065/23389/5579/9969/3690/71/8648/489/1387/23236/4851/4855/116931/7249/6513/2308/10499/5335/113026
hsa05220	Chronic myeloid leukemia	19/1067	76/8105	0.003723515	0.050041088	0.041815711	19	5293/861/5295/4088/9846/1871/6776/7048/10000/3065/7040/1487/1488/613/5925/10912/598/5970/2122
hsa04520	Adherens junction	18/1067	71/8105	0.003993452	0.050041088	0.041815711	18	4088/7048/10458/87/5777/81/6934/3480/8976/2260/2241/5795/4008/71/1387/999/5818/81607
hsa05163	Human cytomegalovirus infection	44/1067	225/8105	0.004052037	0.050041088	0.041815711	44	811/108/5293/3709/1385/5608/11214/115/3554/5295/5578/1871/6009/7124/7186/6352/4773/3708/4772/3107/8737/10000/2771/3134/7132/5829/3716/5579/2788/2767/3690/2784/3570/23236/64764/196883/5925/54331/2775/2768/7249/5970/8772/340061
hsa05166	Human T-cell leukemia virus 1 infection	43/1067	219/8105	0.004135133	0.050041088	0.041815711	43	3109/811/8379/108/5293/83660/4215/1385/3932/55697/115/3554/5295/4088/1871/7124/4773/6776/4772/3107/7048/10000/3134/7132/8881/64784/3716/7040/1387/64764/9020/2113/3689/196883/8900/5925/6929/11200/51434/6513/598/5970/2005
hsa04380	Osteoclast differentiation	28/1067	128/8105	0.00415726	0.050041088	0.041815711	28	5468/5293/1385/3932/5608/10326/3554/5295/4689/9846/7124/7186/1436/4773/4772/7048/10000/7132/4688/3716/7040/11006/3690/9020/2274/55423/1540/5970
hsa04710	Circadian rhythm	10/1067	31/8105	0.004862528	0.056440057	0.047162866	10	51422/1453/1385/5564/1454/406/8864/5187/23291/5562
hsa05215	Prostate cancer	22/1067	97/8105	0.006687979	0.074102302	0.061921924	22	5328/7039/5293/2078/1385/5295/1871/10000/6934/3480/2260/80310/5155/1387/64764/3645/5925/596/56034/5159/2308/5970
hsa04920	Adipocytokine signaling pathway	17/1067	69/8105	0.006898088	0.074102302	0.061921924	17	51422/6256/2180/7133/7124/7186/10645/10000/7132/5564/32/5106/6513/5970/1374/4852/5562

hsa04390	Hippo signaling pathway	32/1067	157/8105	0.00706822	0.074102302	0.061921924	32	5590/1453/7533/23418/4088/7048/6934/8994/4092/26524/7481/7040/8312/25937/71/658/81029/6788/1454/5518/5500/5584/3689/7534/999/5519/7159/8463/392255/122786/10971/23291
hsa05132	Salmonella infection	46/1067	249/8105	0.009861299	0.10015382	0.08369129	46	58484/1639/5293/9844/399/5608/114548/5878/391/5869/1785/7124/7186/26999/1778/10627/54106/10383/79026/8737/10000/6934/7132/8976/10540/11035/5216/57617/5788/71/3800/9842/112574/7277/64746/51807/840/10787/5898/596/27128/834/23191/5970/58498/8772
hsa04371	Apelin signaling pathway	28/1067	137/8105	0.010855156	0.104901012	0.087658173	28	9759/51422/108/3709/4209/115/4638/146850/4088/6546/4899/3708/23533/10000/2771/5564/56848/2788/2784/23236/196883/999/54331/4208/5581/185/5140/5562
hsa00532	Glycosaminoglycan biosynthesis - chondroitin sulfate / dermatan sulfate	7/1067	20/8105	0.01097426	0.104901012	0.087658173	7	55790/29940/22856/50515/51363/64131/11285
hsa04218	Cellular senescence	31/1067	156/8105	0.011449085	0.106312934	0.088838014	31	5293/3709/10114/5608/5295/4088/1871/3364/6009/4773/28996/3708/4772/3107/7048/10000/3134/993/7040/5500/2113/8900/5925/10758/2305/11200/7249/2308/10912/5970/23291
hsa05032	Morphine addiction	20/1067	91/8105	0.013372511	0.12072406	0.100880348	20	5144/108/115/408/5578/2771/2870/5579/2869/2788/2784/409/196883/54331/2550/2775/774/9568/5140/156
hsa04929	GnRH secretion	15/1067	64/8105	0.017054969	0.148253446	0.123884661	15	5293/3709/775/5295/408/5578/3780/3708/10000/5579/2767/23236/409/2550/9568
hsa04961	Endocrine and other factor-regulated calcium reabsorption	13/1067	53/8105	0.017620851	0.148253446	0.123884661	13	160/486/115/7421/6546/5578/1785/491/5579/23236/1759/26052/8218
hsa05212	Pancreatic cancer	17/1067	76/8105	0.018216545	0.148253446	0.123884661	17	7039/5293/675/5295/4088/1871/5337/7048/10000/10928/3716/7040/5898/5925/10912/598/5970
hsa04611	Platelet activation	25/1067	124/8105	0.018246578	0.148253446	0.123884661	25	5590/2815/108/5293/3709/83660/115/5295/5908/4638/146850/10627/3708/23533/10000/2771/3674/3690/71/23236/5500/5584/196883/5023/2149
hsa04933	AGE-RAGE signaling pathway in diabetic complications	21/1067	100/8105	0.018874269	0.149613104	0.125020829	21	5590/5293/5295/4088/5578/1284/7124/6776/4772/7048/10000/7040/5579/23236/596/5581/185/2308/5335/5970/113026
hsa04510	Focal adhesion	37/1067	201/8105	0.020269833	0.155751674	0.130150386	37	29780/5293/83660/8395/1793/5295/5908/4638/5578/1284/10627/7057/87/10000/3674/81/7148/3611/5829/3480/2321/3693/5579/80310/7410/3690/71/5155/5500/596/64098/56034/5159/3914/58498/3694/10451
hsa05100	Bacterial invasion of epithelial cells	17/1067	77/8105	0.020607145	0.155751674	0.130150386	17	5293/9844/1793/5295/391/2017/1785/3611/5829/8976/71/1759/999/26052/79658/8218/10801
hsa04151	PI3K-Akt signaling pathway	60/1067	354/8105	0.021639817	0.15983956	0.133566337	60	57521/6256/7039/5293/1385/4602/1441/7533/284/118788/5295/146850/5578/1284/6009/6446/1436/23239/7057/23533/10000/3674/7148/3480/2321/3693/3716/2260/5527/2788/80310/1440/3690/1129/2784/3570/5155/5518/64764/5529/5528/7534/4914/596/54331/9170/5519/2149/56034/5106/7249/5159/598/10971/3914/5970/10110/3694/4909/5562
hsa05221	Acute myeloid leukemia	15/1067	67/8105	0.025399008	0.178311528	0.149002022	15	1053/4353/5293/861/5295/1436/6776/7704/3728/10000/6934/5914/8864/8900/5970

hsa00562	Inositol phosphate metabolism	16/1067	73/8105	0.02584893	0.178311528	0.149002022	16	8871/5293/3632/8395/3635/3707/3631/5305/56623/23236/3613/55361/5297/5335/22876/113026
hsa01521	EGFR tyrosine kinase inhibitor resistance	17/1067	79/8105	0.026098304	0.178311528	0.149002022	17	7039/5293/5295/5578/10000/3480/3716/5579/4763/80310/3570/5155/596/56034/5159/598/5335
hsa04142	Lysosome	25/1067	128/8105	0.026335241	0.178311528	0.149002022	25	1522/1511/23659/3074/2588/6556/11154/22901/2581/1509/4891/1512/162/9516/1508/6272/4864/411/8906/533/968/8943/1203/8218/527
hsa04213	Longevity regulating pathway - multiple species	14/1067	62/8105	0.028182113	0.183140389	0.153037151	14	57521/51422/108/5293/115/5295/10000/5564/3065/3480/847/196883/2308/5562
hsa04064	NF-kappa B signaling pathway	21/1067	104/8105	0.028372378	0.183140389	0.153037151	21	27040/5328/3932/3554/7124/7186/7128/7185/10673/23085/8737/7132/5579/8915/9020/596/10912/598/1540/5335/5970
hsa04750	Inflammatory mediator regulation of TRP channels	20/1067	98/8105	0.028738953	0.183140389	0.153037151	20	108/5293/3709/5608/115/3554/5295/5578/3708/5583/55515/3269/5579/23236/5500/196883/4914/5581/5335/51393
hsa04150	mTOR signaling pathway	29/1067	155/8105	0.030291131	0.188087103	0.157170761	29	57521/6195/5293/8408/5295/5578/6009/7124/6446/6196/2887/10000/526/7132/3480/7481/5579/55615/8140/6396/51719/81029/57600/7249/9675/23175/10641/5562/528
hsa05225	Hepatocellular carcinoma	31/1067	168/8105	0.030672666	0.188087103	0.157170761	31	7039/5293/5295/4088/5578/1871/7048/10000/6934/6595/3480/7481/7040/5579/2949/8312/57492/71/81029/4258/6602/4780/23401/6603/5925/9446/10912/598/5335/10587/119391
hsa00310	Lysine degradation	14/1067	63/8105	0.032004848	0.188897216	0.157847714	14	29072/217/64754/55526/10919/8085/23067/7799/9739/63976/38/2122/54904/7468
hsa04110	Cell cycle	24/1067	124/8105	0.032091853	0.188897216	0.157847714	24	51343/7027/8379/7533/4088/1871/4174/8881/7709/3065/993/7040/10274/4171/1387/902/7534/8900/5925/11200/51434/10912/8556/10971
hsa00072	Synthesis and degradation of ketone bodies	4/1067	10/8105	0.032548443	0.188897216	0.157847714	4	622/3155/38/3157
hsa00630	Glyoxylate and dicarboxylate metabolism	8/1067	30/8105	0.035936042	0.200840756	0.167828065	8	283871/132158/84532/189/847/55902/112817/38
hsa04540	Gap junction	18/1067	88/8105	0.035961604	0.200840756	0.167828065	18	108/3709/1453/115/5578/3708/10383/2771/5579/80310/2767/5155/23236/7277/51807/196883/56034/5159
hsa05230	Central carbon metabolism in cancer	15/1067	70/8105	0.036460322	0.200840756	0.167828065	15	3098/5293/9123/5295/6510/10000/5223/2260/8140/3099/4914/5315/5159/6513/23410
hsa05130	Pathogenic Escherichia coli infection	35/1067	197/8105	0.037723393	0.201894856	0.1687089	35	4629/114548/3554/7456/2017/7124/7186/26999/8440/55971/4627/10458/10383/8737/5777/7132/8976/4644/71/7277/51807/840/9632/10787/9170/2768/27128/2149/9181/834/4641/23191/5970/7430/8772

hsa05165	Human papillomavirus infection	55/1067	331/8105	0.037894111	0.201894856	0.1687089	55	5590/55534/5293/23352/1385/9794/5295/1284/6009/7124/84441/3107/7057/10000/3516/3134/3674/526/6934/7132/7148/3065/5829/7481/3693/3716/5527/8312/3690/81029/5518/1387/64764/5584/5529/5528/8900/5925/5315/4851/4855/5519/533/7337/7249/5159/2308/4599/3914/5970/3694/390992/8772/527/528
hsa05418	Fluid shear stress and atherosclerosis	26/1067	139/8105	0.038810818	0.203443805	0.170003244	26	5590/5293/5608/3554/5295/7124/10000/3674/7132/4688/6383/2949/3690/71/658/4258/5155/4780/596/9446/4208/9181/4880/5970/119391/5562
hsa05142	Chagas disease	20/1067	102/8105	0.042077596	0.217066965	0.181387132	20	811/3586/5293/5295/7124/6352/54106/7048/10000/2771/7132/7040/2767/5518/23236/2775/5519/713/5970/8772
hsa00520	Amino sugar and nucleotide sugar metabolism	11/1067	48/8105	0.044013386	0.222283865	0.185746517	11	55577/3098/3074/51005/80896/1118/80146/3099/606495/5373/9945
hsa04022	cGMP-PKG signaling pathway	30/1067	167/8105	0.045356893	0.222283865	0.185746517	30	108/3709/1385/486/4209/775/115/4638/146850/6546/4773/491/3708/4772/23533/10000/2771/2767/489/23236/64764/5500/196883/4208/5581/2768/4880/185/5140/10335
hsa04730	Long-term depression	13/1067	60/8105	0.045582519	0.222283865	0.185746517	13	3709/5578/2781/3708/2771/3480/5579/2767/5518/23236/2775/5519/2768
hsa04010	MAPK signaling pathway	49/1067	294/8105	0.045824674	0.222283865	0.185746517	49	7039/6195/4215/5608/775/284/93589/3554/5908/408/5578/7124/7186/1436/6196/4772/7048/10000/7132/8986/3480/2321/2260/7040/9693/5579/4763/80310/6788/5155/8569/23542/9020/409/51347/4914/4208/1845/2768/56034/7867/5159/10912/774/5970/2122/4909/2005/51776
hsa00600	Sphingolipid metabolism	11/1067	49/8105	0.050277084	0.240294887	0.200797023	11	9517/2581/253782/56848/259230/166929/81537/130367/29956/64781/8613
hsa00564	Glycerophospholipid metabolism	19/1067	98/8105	0.051559637	0.242853362	0.202934955	19	23659/5130/79888/5337/9489/1609/56994/1606/8527/51365/23646/1120/81490/23175/8760/11313/55326/55224/8613
hsa04261	Adrenergic signaling in cardiomyocytes	27/1067	150/8105	0.054258507	0.251914496	0.210506688	27	3753/108/1385/486/775/93589/115/146850/6546/5578/491/23533/10000/2771/5527/489/5518/23236/64764/5500/3784/5529/5528/196883/596/5519/185
hsa00601	Glycosphingolipid biosynthesis - lacto and neolacto series	7/1067	27/8105	0.055491838	0.253210386	0.21158957	7	2526/2529/84002/6484/8703/2651/10678
hsa04962	Vasopressin-regulated water reabsorption	10/1067	44/8105	0.056095839	0.253210386	0.21158957	10	1639/1385/397/115/5878/5869/1778/10540/64764/360
hsa04340	Hedgehog signaling pathway	12/1067	56/8105	0.057477305	0.25589211	0.213830492	12	23295/23288/1453/408/51684/8452/57154/1454/409/596/23291/156
hsa03410	Base excision repair	8/1067	33/8105	0.059902472	0.261196289	0.21826281	8	5426/55247/7374/4913/143/4595/10714/56655
hsa04935	Growth hormone synthesis, secretion and action	22/1067	119/8105	0.060276067	0.261196289	0.21826281	22	108/5293/3709/1385/5608/775/115/5295/5578/6776/3708/10000/2771/5579/2767/51738/1387/23236/64764/196883/5335/6750

hsa05170	Human immunodeficiency virus 1 infection	36/1067	212/8105	0.062659029	0.267949796	0.223906226	36	811/3985/5293/3709/5608/7133/5295/5578/7124/7186/4773/3708/4772/3107/8737/10000/2771/3134/7132/5829/162/5579/2788/85363/2767/2784/8906/596/54331/2775/598/5335/5970/23291/8772/340061
hsa04922	Glucagon signaling pathway	20/1067	107/8105	0.064452889	0.268629243	0.224473991	20	51422/108/3709/1385/3708/10000/5564/5223/32/1387/23236/64764/5315/31/5106/6513/2308/5140/1374/5562
hsa04923	Regulation of lipolysis in adipocytes	12/1067	57/8105	0.064471018	0.268629243	0.224473991	12	57104/11343/108/5293/115/5295/10000/2771/196883/51099/5140/4852
hsa04662	B cell receptor signaling pathway	16/1067	82/8105	0.066621613	0.270328638	0.225894052	16	5293/3635/118788/5295/4773/4772/10000/5777/5579/11006/973/7410/8915/27071/5970/10451
hsa04670	Leukocyte transendothelial migration	21/1067	114/8105	0.067374214	0.270328638	0.225894052	21	5293/399/5295/5908/4689/5578/10627/87/2771/81/4688/5829/5579/83700/7410/71/3689/5335/58498/7430/10451
hsa04724	Glutamatergic synapse	21/1067	114/8105	0.067374214	0.270328638	0.225894052	21	108/3709/775/115/5578/5337/3708/2771/2900/5579/2788/6506/2784/6511/23236/196883/6507/54331/2775/22941/156
hsa04971	Gastric acid secretion	15/1067	76/8105	0.068253202	0.270515739	0.226050399	15	3772/108/3709/115/4638/5578/3708/2771/5579/71/23236/3784/196883/7430/6750
hsa04921	Oxytocin signaling pathway	27/1067	154/8105	0.070967264	0.277417126	0.231817388	27	7226/51422/108/3709/775/93589/115/4638/146850/5578/4773/3708/10645/4772/23533/2771/5564/1827/5579/57118/71/23236/5500/196883/2775/4208/5562
hsa05146	Amoebiasis	19/1067	102/8105	0.072185287	0.277417126	0.231817388	19	1511/3586/5293/3554/5295/5878/5869/5578/1284/7124/87/81/7040/5579/2767/23236/3689/3914/5970
hsa04930	Type II diabetes mellitus	10/1067	46/8105	0.072555248	0.277417126	0.231817388	10	5590/3098/5293/775/5295/7124/3099/5315/5581/774
hsa04066	HIF-1 signaling pathway	20/1067	109/8105	0.075354161	0.284768633	0.237960509	20	2023/3098/5293/284/5295/5578/10000/3480/2321/5579/2027/3099/3570/8569/1387/596/6513/5335/5970/5209
hsa04392	Hippo signaling pathway - multiple species	7/1067	29/8105	0.077467654	0.289390661	0.241822804	7	8994/26524/25937/6788/1454/8463/122786
hsa00620	Pyruvate metabolism	10/1067	47/8105	0.081811622	0.298750303	0.249643978	10	217/84532/5091/32/55902/5315/31/5106/38/98
hsa04973	Carbohydrate digestion and absorption	10/1067	47/8105	0.081811622	0.298750303	0.249643978	10	6518/3098/5293/486/5295/8972/10000/5579/3099/23236
hsa04660	T cell receptor signaling pathway	19/1067	104/8105	0.084335906	0.301420097	0.251874931	19	27040/3586/5293/3932/5295/7124/9402/8440/4773/4772/10000/5777/7410/5788/8915/9020/5335/5970/10451

hsa05223	Non-small cell lung cancer	14/1067	72/8105	0.084397627	0.301420097	0.251874931	14	6256/7039/5293/5295/5578/1871/6776/10000/27436/5579/3800/5925/10912/5335
hsa04910	Insulin signaling pathway	24/1067	137/8105	0.085468172	0.301925609	0.252297351	24	5590/57521/51422/3098/5293/3632/5575/5295/6009/10000/5564/3099/32/8569/5500/5584/31/5106/7249/2308/6720/5140/10211/5562
hsa03015	mRNA surveillance pathway	18/1067	98/8105	0.087514994	0.30402827	0.254054392	18	10482/81608/51692/8761/9887/80335/100529063/8106/5527/26528/23293/5518/5500/5529/5528/5519/10898/64895
hsa04014	Ras signaling pathway	38/1067	232/8105	0.08793433	0.30402827	0.254054392	38	8844/27040/7039/5293/284/5295/5908/5878/5869/9846/5578/5337/1436/9462/10000/22821/3480/10928/2321/2260/8831/5579/2788/4763/80310/2784/5155/2113/5898/4914/54331/51365/56034/5159/598/5335/5970/4909
hsa04915	Estrogen signaling pathway	24/1067	138/8105	0.091170199	0.309617245	0.258724694	24	7039/108/5293/3709/1385/2289/115/5295/3708/1509/3872/10000/2771/5914/8648/23236/64764/196883/596/2550/2775/25984/9568/10499
hsa00280	Valine, leucine and isoleucine degradation	10/1067	48/8105	0.091758526	0.309617245	0.258724694	10	27034/217/34/11112/593/197322/3155/38/3157/316
hsa01524	Platinum drug resistance	14/1067	73/8105	0.092408839	0.309617245	0.258724694	14	5293/5295/1244/10000/2949/4258/596/9446/2067/598/540/7507/119391/8772
hsa04145	Phagosome	26/1067	152/8105	0.094872362	0.314627733	0.262911595	26	3109/4353/811/4973/4689/5878/5869/1778/6441/3107/7057/10383/3134/526/81035/4688/3693/8685/3690/71/7277/51807/3689/533/527/528
hsa00533	Glycosaminoglycan biosynthesis - keratan sulfate	4/1067	14/8105	0.101392484	0.330663726	0.276311713	4	9435/8703/6483/10678
hsa04914	Progesterone-mediated oocyte maturation	18/1067	100/8105	0.101742685	0.330663726	0.276311713	18	51343/6195/8379/108/5293/115/5295/6196/10000/2771/8881/3480/993/22849/196883/8900/51434/5140
hsa01523	Antifolate resistance	7/1067	31/8105	0.103833715	0.333164564	0.278401482	7	4363/7124/2352/1244/6573/8714/5970
hsa04960	Aldosterone-regulated sodium reabsorption	8/1067	37/8105	0.104562417	0.333164564	0.278401482	8	5293/486/5295/5578/6446/9351/5579/3291
hsa04350	TGF-beta signaling pathway	17/1067	94/8105	0.105882613	0.334095625	0.279179502	17	4052/7027/4088/7124/7048/7057/4092/7040/57154/658/5518/1387/5519/392255/3624/4838/9372
hsa05214	Glioma	14/1067	75/8105	0.109804919	0.340515535	0.284544156	14	7039/5293/5295/5578/1871/10000/3480/5579/57118/5155/5925/5159/10912/5335
hsa04024	cAMP signaling pathway	35/1067	216/8105	0.110012711	0.340515535	0.284544156	35	5144/108/5293/1385/486/84152/775/5348/115/5295/5908/5337/491/4772/10000/2771/2696/7410/1262/51738/1129/489/1387/64764/5500/196883/2550/2149/7434/9568/5140/5970/4852/6750/10451

hsa05110	Vibrio cholerae infection	10/1067	50/8105	0.113714673	0.345395036	0.288621601	10	9414/115/5578/526/71/3784/533/5335/527/528
hsa05144	Malaria	10/1067	50/8105	0.113714673	0.345395036	0.288621601	10	3586/7124/54106/7057/6383/7040/3043/1440/3689/2532
hsa04530	Tight junction	28/1067	169/8105	0.115560499	0.347751502	0.290590729	28	5590/51422/4629/9414/861/2017/10627/123720/4627/87/81/5564/9693/83700/71/5518/7277/5584/51807/23370/5519/5581/55114/9181/4734/7430/11346/5562
hsa04114	Oocyte meiosis	22/1067	129/8105	0.119772718	0.353873941	0.295706807	22	6195/8379/108/3709/7533/115/6196/3708/8881/3480/5527/22849/5518/5500/5529/5528/196883/7534/5519/51434/10971/23291
hsa04926	Relaxin signaling pathway	22/1067	129/8105	0.119772718	0.353873941	0.295706807	22	5590/108/5293/1385/115/5295/408/5578/1284/7048/10000/2771/7040/2788/2784/23236/64764/409/196883/54331/2775/5970
hsa00240	Pyrimidine metabolism	11/1067	57/8105	0.121859466	0.356795735	0.298148339	11	978/1807/953/50484/7084/7371/51733/4860/1635/1723/7083
hsa04020	Calcium signaling pathway	38/1067	240/8105	0.127395166	0.369673474	0.308909332	38	108/845/3709/775/115/3707/4638/6546/5578/491/3708/2321/56413/2260/56848/3269/5579/80310/57118/2767/1129/489/5155/23236/219931/196883/4914/5023/2149/56034/185/5159/774/444/5335/5026/113026/844
hsa04713	Circadian entrainment	17/1067	97/8105	0.131277801	0.374648289	0.313066425	17	108/1385/775/115/5578/3708/2771/9722/5579/2788/2784/23236/196883/8864/5187/54331/2775
hsa00524	Neomycin, kanamycin and gentamicin biosynthesis	2/1067	5/8105	0.131975119	0.374648289	0.313066425	2	3098/3099
hsa00770	Pantothenate and CoA biosynthesis	5/1067	21/8105	0.132567856	0.374648289	0.313066425	5	1807/339896/217/51733/60490
hsa04625	C-type lectin receptor signaling pathway	18/1067	104/8105	0.134291272	0.376247098	0.314402433	18	8844/3586/5293/3709/114548/5295/7124/4773/3708/4772/10000/8915/9020/834/1540/5970/602/6367
hsa04927	Cortisol synthesis and secretion	12/1067	65/8105	0.140023593	0.388954426	0.325021027	12	108/3709/1385/775/115/3708/56246/2767/23236/64764/196883/185
hsa05417	Lipid and atherosclerosis	34/1067	215/8105	0.144504093	0.394780924	0.329889808	34	6256/5468/4973/5293/5608/2081/114548/5295/5908/4689/5578/7124/7186/6352/9619/4773/3708/4772/10000/57534/7132/4688/7410/19/23236/4780/840/5452/596/834/598/5335/5970/10451
hsa05218	Melanoma	13/1067	72/8105	0.145454618	0.394780924	0.329889808	13	5293/5295/1871/10000/3480/2260/80310/5155/999/5925/56034/5159/10912
hsa04658	Th1 and Th2 cell differentiation	16/1067	92/8105	0.146979975	0.394780924	0.329889808	16	3109/27040/55534/3932/9794/84441/4773/6776/4772/28514/3516/3716/4851/864/5335/5970

hsa05222	Small cell lung cancer	16/1067	92/8105	0.146979975	0.394780924	0.329889808	16	6256/5293/5295/1284/1871/7186/7185/10000/3674/7709/5925/596/10912/598/3914/5970
hsa00640	Propanoate metabolism	7/1067	34/8105	0.151212011	0.402818883	0.336606548	7	84532/32/55902/593/31/38/79611
hsa04360	Axon guidance	29/1067	182/8105	0.156863805	0.414477534	0.346348838	29	10501/5590/10154/54434/3985/5293/5295/5578/8440/4773/2049/10627/64218/219699/10509/2771/54961/3611/22885/10507/658/81029/57522/5998/6586/5335/3983/6585/137970
hsa00983	Drug metabolism - other enzymes	14/1067	80/8105	0.161165124	0.422408592	0.352976248	14	978/1807/4353/50484/7084/2949/7371/7172/4258/51733/9446/119391/7083/3614
hsa05161	Hepatitis B	26/1067	162/8105	0.163117033	0.424104285	0.354393216	26	5293/6554/1385/5608/5295/4088/5578/1871/7124/4773/6776/4772/7048/10000/3716/7040/5579/1387/64764/7534/8900/5925/596/10971/5970/8772
hsa05203	Viral carcinogenesis	32/1067	204/8105	0.164522872	0.424364551	0.354610702	32	9759/2934/8379/5293/23352/1385/55697/51564/7533/5295/7186/7185/6776/3107/87/3516/3134/81/3065/5829/3716/1387/64764/7534/8900/5925/5315/6672/7337/27044/10971/5970
hsa00430	Taurine and hypotaurine metabolism	3/1067	11/8105	0.167641091	0.429002791	0.358486543	3	339896/2678/84890
hsa00561	Glycerolipid metabolism	11/1067	61/8105	0.171950129	0.436592125	0.364828399	11	57104/11343/3990/217/132158/1609/1606/8527/23175/55326/8613
hsa00450	Selenocompound metabolism	4/1067	17/8105	0.176486383	0.442761952	0.369984076	4	51540/9060/10587/4141
hsa04664	Fc epsilon RI signaling pathway	12/1067	68/8105	0.177104781	0.442761952	0.369984076	12	27040/5293/5608/3635/5295/9846/5578/7124/10000/7410/5335/10451
hsa00440	Phosphonate and phosphinate metabolism	2/1067	6/8105	0.181247573	0.442995784	0.370179473	2	5130/56994
hsa00750	Vitamin B6 metabolism	2/1067	6/8105	0.181247573	0.442995784	0.370179473	2	8566/316
hsa04918	Thyroid hormone synthesis	13/1067	75/8105	0.181287505	0.442995784	0.370179473	13	108/3709/1385/486/115/5578/3708/7038/433/5579/23236/64764/196883
hsa04140	Autophagy - animal	22/1067	137/8105	0.186656934	0.444715122	0.371616199	22	57521/5293/8408/2081/5295/6009/26100/3708/1509/10645/10000/7405/3480/22863/1508/23604/596/81671/7249/598/1613/5562
hsa05167	Kaposi sarcoma-associated herpesvirus infection	30/1067	193/8105	0.187310317	0.444715122	0.371616199	30	5293/3709/1385/5608/5295/146850/1871/7186/4773/3708/4772/3107/23533/10000/3134/6934/7132/57580/1827/3716/2788/22863/2784/5155/1387/5925/54331/5335/5970/8772

hsa04727	GABAergic synapse	15/1067	89/8105	0.187464528	0.444715122	0.371616199	15	108/775/115/5578/2771/5579/2788/2784/196883/5334/54331/2550/2775/774/9568
hsa05235	PD-L1 expression and PD-1 checkpoint pathway in cancer	15/1067	89/8105	0.187464528	0.444715122	0.371616199	15	27040/5293/4215/3932/5608/5295/4773/54106/4772/10000/27436/5777/3716/5335/5970
hsa03320	PPAR signaling pathway	13/1067	76/8105	0.194101779	0.457123754	0.381985194	13	6256/5468/4973/2180/5360/3611/34/2172/8309/5106/3157/6319/1374
hsa05410	Hypertrophic cardiomyopathy	15/1067	90/8105	0.199395664	0.463568974	0.387370997	15	51422/7273/775/93589/6546/7124/3674/5564/3693/7040/3690/71/489/3694/5562
hsa05120	Epithelial cell signaling in Helicobacter pylori infection	12/1067	70/8105	0.204192496	0.463568974	0.387370997	12	6352/28964/526/83700/1445/3577/9020/533/5335/5970/527/528
hsa04621	NOD-like receptor signaling pathway	28/1067	181/8105	0.204569771	0.463568974	0.387370997	28	1669/7226/58484/9447/3709/9051/114548/4210/7124/7186/7128/6352/3708/8737/3716/11035/1508/23236/64127/596/7158/10059/834/598/51393/597 0/8772/340061
hsa00061	Fatty acid biosynthesis	4/1067	18/8105	0.204803064	0.463568974	0.387370997	4	2180/32/31/197322
hsa00510	N-Glycan biosynthesis	9/1067	50/8105	0.205024239	0.463568974	0.387370997	9	6184/4249/11320/4248/6480/8703/440138/57134/1798
hsa05143	African trypanosomiasis	7/1067	37/8105	0.206823081	0.463568974	0.387370997	7	3586/5578/7124/54106/5579/3043/23236
hsa05216	Thyroid cancer	7/1067	37/8105	0.206823081	0.463568974	0.387370997	7	6256/5468/6934/4914/999/10912/8031
hsa04925	Aldosterone synthesis and secretion	16/1067	98/8105	0.213123455	0.47441865	0.396437285	16	108/3709/1385/775/115/5578/491/3708/5579/57118/2767/23236/64764/196883/5581/185
hsa04721	Synaptic vesicle cycle	13/1067	78/8105	0.220925001	0.488439629	0.408153601	13	160/1785/526/6506/6511/1759/6507/26052/533/774/8218/527/528
hsa04610	Complement and coagulation cascades	14/1067	85/8105	0.222847624	0.489361336	0.408923804	14	5265/5328/719/1604/5055/5648/1191/2161/966/716/3689/1361/2149/713
hsa02010	ABC transporters	8/1067	45/8105	0.234260876	0.51097171	0.426982028	8	4363/9619/1244/1672/19/8714/11194/85320
hsa04061	Viral protein interaction with cytokine and cytokine receptor	16/1067	100/8105	0.237750432	0.512300518	0.428092416	16	3586/7133/729230/7124/6352/1436/7132/10803/6364/3577/3570/8807/8809/10850/6367/1524

hsa04659	Th17 cell differentiation	17/1067	107/8105	0.238022702	0.512300518	0.428092416	17	3109/27040/6256/3932/861/3554/4088/4773/6776/4772/7048/3716/7040/5914/3570/5335/5970
hsa04141	Protein processing in endoplasmic reticulum	26/1067	171/8105	0.242745737	0.516852313	0.431896022	26	5034/811/6184/2081/267/8720/10483/7186/10960/9695/51009/22824/118424/6396/55741/4780/9632/596/165324/57134/7322/7993/22872/258010/10299/5071
hsa04370	VEGF signaling pathway	10/1067	59/8105	0.243318166	0.516852313	0.431896022	10	5293/5295/5578/4773/10000/5829/56848/5579/7867/5335
hsa04115	p53 signaling pathway	12/1067	73/8105	0.247921276	0.523210486	0.437209086	12	3732/7057/50484/64065/84883/596/11200/7249/10912/598/9540/10572
hsa00563	Glycosylphosphatidylinositol (GPI)-anchor biosynthesis	5/1067	26/8105	0.251617766	0.527585639	0.440865085	5	9487/128869/2822/23556/9091
hsa00010	Glycolysis / Gluconeogenesis	11/1067	67/8105	0.262087404	0.539171438	0.450546497	11	2023/3098/217/83440/5223/84532/2027/3099/55902/5315/5106
hsa04720	Long-term potentiation	11/1067	67/8105	0.262087404	0.539171438	0.450546497	11	6195/3709/775/5908/5578/6196/3708/5579/1387/23236/5500
hsa00051	Fructose and mannose metabolism	6/1067	33/8105	0.262120268	0.539171438	0.450546497	6	3098/5210/55556/3099/5373/5209
hsa00670	One carbon pool by folate	4/1067	20/8105	0.264756459	0.541168862	0.4522156	4	25902/10588/100528021/4522
hsa04068	FoxO signaling pathway	20/1067	131/8105	0.271781493	0.551350033	0.460723266	20	51422/3586/5293/5295/4088/6446/7048/10000/5564/3480/7040/1454/1387/847/8698/5106/2308/10912/10110/5562
hsa00514	Other types of O-glycan biosynthesis	8/1067	47/8105	0.273130324	0.551350033	0.460723266	8	2590/152002/79695/727936/29954/6480/8703/50614
hsa04714	Thermogenesis	34/1067	232/8105	0.275015409	0.551728444	0.461039477	34	57104/57521/51422/9658/11343/5468/6195/108/2180/1385/90639/115/6009/137682/6196/4720/6595/5564/2260/4728/57492/71/64764/6602/63976/196883/6603/4712/56901/7249/6392/1374/5562/10476
hsa05414	Dilated cardiomyopathy	15/1067	96/8105	0.277599186	0.553495309	0.462515918	15	108/7273/775/93589/115/6546/7124/3674/3693/7040/3690/71/489/196883/3694
hsa03440	Homologous recombination	7/1067	41/8105	0.290506351	0.575698562	0.481069568	7	641/675/5893/146956/10714/84142/9577
hsa05211	Renal cell carcinoma	11/1067	69/8105	0.295188947	0.57866089	0.483544971	11	7039/5293/5295/5908/10000/7040/5155/1387/2113/2034/6513

hsa04270	Vascular smooth muscle contraction	20/1067	133/8105	0.295562178	0.57866089	0.483544971	20	108/3709/4629/775/115/4638/5578/3708/4627/5583/5579/2767/23236/5500/196883/5581/2768/4880/185/10335
hsa04934	Cushing syndrome	23/1067	155/8105	0.300335251	0.584484769	0.488411564	23	108/3709/1385/775/9070/115/5908/1871/3708/9049/2771/6934/56246/7481/8312/8085/2767/81029/23236/64764/196883/5925/185
hsa00604	Glycosphingolipid biosynthesis - ganglio series	3/1067	15/8105	0.315479476	0.610302558	0.509985619	3	3074/9197/6483
hsa04640	Hematopoietic cell lineage	15/1067	99/8105	0.320105175	0.615586875	0.51440134	15	3109/2815/928/1441/1604/3554/7124/1436/3674/966/1440/3690/3570/951/945
hsa00340	Histidine metabolism	4/1067	22/8105	0.327375184	0.625864322	0.522989457	4	3034/217/55748/3176
hsa03030	DNA replication	6/1067	36/8105	0.334609185	0.634326093	0.530060347	6	5426/4174/23649/4171/10714/56655
hsa05152	Tuberculosis	26/1067	180/8105	0.335849867	0.634326093	0.530060347	26	3109/8844/3586/1385/7421/5878/5869/7124/54106/1509/10000/7132/3716/56848/7040/8915/1387/3689/3656/64127/596/533/1051/5970/8772/527
hsa05323	Rheumatoid arthritis	14/1067	93/8105	0.337656659	0.634326093	0.530060347	14	3109/284/7124/6352/10673/526/2321/7040/6364/3689/533/8741/527/528
hsa04911	Insulin secretion	13/1067	86/8105	0.340688019	0.636342564	0.531745366	13	108/1385/486/775/115/5578/3780/5579/2767/23236/64764/196883/6513
hsa04310	Wnt signaling pathway	24/1067	166/8105	0.342719961	0.636479928	0.53186015	24	4088/81839/5578/4773/29964/4772/166336/6934/7481/5579/8312/7088/1487/1488/81029/1454/1387/23236/23401/23002/57680/8607/23291/8840
hsa00230	Purine metabolism	19/1067	130/8105	0.348564709	0.642066121	0.536528127	19	5144/272/5145/108/953/115/50484/5149/5152/4860/2987/196883/9060/5315/204/51292/26289/5140/3614
hsa05321	Inflammatory bowel disease	10/1067	65/8105	0.349679087	0.642066121	0.536528127	10	3109/3586/4088/7124/4772/7040/8807/64127/8809/5970
hsa00730	Thiamine metabolism	3/1067	16/8105	0.35375056	0.643140875	0.537426221	3	249/204/26289
hsa01200	Carbon metabolism	17/1067	116/8105	0.356108103	0.643140875	0.537426221	17	2023/3098/283871/4967/83440/5223/132158/84532/5091/2027/189/3099/847/55902/5315/38/6392
hsa00515	Mannose type O-glycan biosynthesis	4/1067	23/8105	0.359067804	0.643140875	0.537426221	4	2526/29954/8703/11041

hsa04614	Renin-angiotensin system	4/1067	23/8105	0.359067804	0.643140875	0.537426221	4	1511/4012/5550/185
hsa00410	beta-Alanine metabolism	5/1067	30/8105	0.36015889	0.643140875	0.537426221	5	1807/339896/217/55748/51733
hsa04972	Pancreatic secretion	15/1067	102/8105	0.364117147	0.644461226	0.538529542	15	108/3709/486/115/5908/5578/491/3708/5579/489/23236/219931/3784/196883/1361
hsa04613	Neutrophil extracellular trap formation	27/1067	190/8105	0.364864202	0.644461226	0.538529542	27	566/1991/9759/1511/4353/5293/51564/5295/4689/5578/2358/2357/10000/3674/4688/3065/5579/3690/71/23236/3689/834/5335/5970/366/55506/8350
hsa04974	Protein digestion and absorption	15/1067	103/8105	0.379017281	0.665841169	0.556395211	15	91522/1308/486/6510/6546/1284/1303/9056/7373/3784/285641/1289/1361/23428/340267
hsa00534	Glycosaminoglycan biosynthesis - heparan sulfate / heparin	4/1067	24/8105	0.390720623	0.677202743	0.565889256	4	9953/3340/64131/11285
hsa00120	Primary bile acid biosynthesis	3/1067	17/8105	0.39173574	0.677202743	0.565889256	3	23600/6718/8309
hsa00910	Nitrogen metabolism	3/1067	17/8105	0.39173574	0.677202743	0.565889256	3	761/759/771
hsa05145	Toxoplasmosis	16/1067	112/8105	0.40337483	0.686841131	0.573943358	16	3109/3586/5608/146850/7124/23533/10000/2771/7132/3716/7040/596/2775/598/3914/5970
hsa04514	Cell adhesion molecules	21/1067	149/8105	0.403688824	0.686841131	0.573943358	21	3109/57863/4685/3107/10666/3134/6383/1001/201633/9378/83700/5788/1002/9019/9379/3689/999/8506/23705/5818/64115
hsa04976	Bile secretion	13/1067	90/8105	0.405028304	0.686841131	0.573943358	13	6256/200931/108/6554/486/115/1244/358/8714/196883/2052/6513/366
hsa00513	Various types of N-glycan biosynthesis	6/1067	39/8105	0.409016653	0.686841131	0.573943358	6	6184/3074/11320/8703/440138/57134
hsa05169	Epstein-Barr virus infection	28/1067	202/8105	0.414952164	0.686841131	0.573943358	28	3109/811/9612/5293/953/5608/5295/1871/7124/7186/7128/3107/8737/10000/3516/3134/3065/3716/9020/8900/5925/596/4734/864/10912/5970/8772/5719
hsa00512	Mucin type O-glycan biosynthesis	5/1067	32/8105	0.415390504	0.686841131	0.573943358	5	2590/55808/79695/50614/6483
hsa04215	Apoptosis - multiple species	5/1067	32/8105	0.415390504	0.686841131	0.573943358	5	7132/840/596/598/8772

hsa04932	Non-alcoholic fatty liver disease	21/1067	150/8105	0.416315349	0.686841131	0.573943358	21	51422/6256/5293/2081/5295/7124/7186/10000/4720/7132/5564/7040/4728/3570/840/4712/56901/6720/5970/6392/5562
hsa05133	Pertussis	11/1067	76/8105	0.417367383	0.686841131	0.573943358	11	3394/3586/114548/7124/2771/716/840/3689/834/713/5970
hsa03460	Fanconi anemia pathway	8/1067	54/8105	0.418444751	0.686841131	0.573943358	8	641/675/116028/146956/2175/2067/84464/11201
hsa05017	Spinocerebellar ataxia	20/1067	143/8105	0.421916037	0.688378503	0.575228028	20	5293/3709/8408/2081/5295/5578/7186/26100/6310/3708/10000/3516/5579/22863/489/6511/23236/5695/2259/5719
hsa04924	Renin secretion	10/1067	69/8105	0.424171231	0.688378503	0.575228028	10	3709/1385/775/3708/2771/358/1508/23236/185/5140
hsa03420	Nucleotide excision repair	7/1067	47/8105	0.425735628	0.688378503	0.575228028	7	5426/902/2067/10714/7507/2068/56655
hsa05140	Leishmaniasis	11/1067	77/8105	0.435122038	0.696623952	0.582118153	11	3109/3586/4689/7124/5777/4688/3716/7040/5579/3689/5970
hsa05412	Arrhythmogenic right ventricular cardiomyopathy	11/1067	77/8105	0.435122038	0.696623952	0.582118153	11	775/93589/6546/3728/3674/6934/3693/3690/71/489/3694
hsa05205	Proteoglycans in cancer	28/1067	205/8105	0.447760728	0.713344298	0.596090134	28	5328/5293/3709/5295/5578/2017/7124/3708/7057/10000/5777/6383/5829/3480/7481/3693/2260/7040/5579/7410/3690/71/81029/5500/287/5335/7430/10451
hsa04623	Cytosolic DNA-sensing pathway	9/1067	63/8105	0.451239393	0.715379525	0.597790826	9	9447/5435/6352/8737/11035/834/5970/51082/340061
hsa04912	GnRH signaling pathway	13/1067	93/8105	0.453745272	0.715860259	0.59819254	13	108/3709/4215/5608/775/115/5578/5337/3708/5579/2767/23236/196883
hsa05219	Bladder cancer	6/1067	41/8105	0.458317506	0.718137604	0.600095552	6	1871/7057/999/23604/5925/1613
hsa05210	Colorectal cancer	12/1067	86/8105	0.461803078	0.718137604	0.600095552	12	7039/5293/5295/4088/7048/10000/6934/7040/8312/5898/596/10912
hsa04916	Melanogenesis	14/1067	101/8105	0.461817721	0.718137604	0.600095552	14	108/1385/115/5578/2771/6934/7481/5579/81029/1387/23236/64764/196883/2775
hsa00531	Glycosaminoglycan degradation	3/1067	19/8105	0.465648348	0.720646252	0.602191848	3	3074/2588/411

hsa00565	Ether lipid metabolism	7/1067	49/8105	0.470820287	0.721776383	0.603136216	7	79888/5337/56994/23646/387521/387522/8613
hsa04672	Intestinal immune network for IgA production	7/1067	49/8105	0.470820287	0.721776383	0.603136216	7	3109/3586/10673/10803/7040/9020/8741
hsa01212	Fatty acid metabolism	8/1067	57/8105	0.481297089	0.730987268	0.610833086	8	2180/34/31/197322/38/6319/1374/37
hsa00380	Tryptophan metabolism	6/1067	42/8105	0.482606145	0.730987268	0.610833086	6	217/55526/1644/847/38/316
hsa04966	Collecting duct acid secretion	4/1067	27/8105	0.483576193	0.730987268	0.610833086	4	526/10723/527/528
hsa04723	Retrograde endocannabinoid signaling	20/1067	148/8105	0.486757134	0.732389206	0.612004583	20	11343/108/3709/775/115/5578/3708/2771/4720/5579/4728/2788/2784/23236/196883/54331/2775/4712/56901/774
hsa04979	Cholesterol metabolism	7/1067	50/8105	0.493044117	0.738244079	0.616897077	7	114879/3990/5360/19/6272/4864/55908
hsa00760	Nicotinate and nicotinamide metabolism	5/1067	35/8105	0.496427465	0.738244079	0.616897077	5	65220/349565/4860/23410/316
hsa05213	Endometrial cancer	8/1067	58/8105	0.501900201	0.738244079	0.616897077	8	5293/5295/10000/6934/3611/8312/999/10912
hsa05150	Staphylococcus aureus infection	13/1067	96/8105	0.502044529	0.738244079	0.616897077	13	1669/3109/3586/719/2358/2357/3872/5648/1672/716/3689/25984/713
hsa05164	Influenza A	23/1067	172/8105	0.502271529	0.738244079	0.616897077	23	3109/10482/11100/5293/114548/5295/8480/5578/7124/6352/10000/7132/3716/100529063/8106/5579/71/1387/834/4599/5970/10898/8772
hsa00471	D-Glutamine and D-glutamate metabolism	1/1067	5/8105	0.506371815	0.738244079	0.616897077	1	80017
hsa00071	Fatty acid degradation	6/1067	43/8105	0.506549014	0.738244079	0.616897077	6	2180/217/34/38/1374/37
hsa00650	Butanoate metabolism	4/1067	28/8105	0.513310914	0.743852945	0.621583999	4	622/3155/38/3157
hsa00330	Arginine and proline metabolism	7/1067	51/8105	0.514975116	0.743852945	0.621583999	7	217/4953/55748/8659/112817/112483/8974

hsa04550	Signaling pathways regulating pluripotency of stem cells	19/1067	143/8105	0.520551251	0.74483042	0.622400805	19	5293/5295/4088/463/10000/3480/7481/3716/2260/8312/658/81029/2103/4211/6929/10336/3624/4838/3976
hsa04978	Mineral absorption	8/1067	59/8105	0.522247631	0.74483042	0.622400805	8	486/7421/6546/491/4891/140803/475/540
hsa00350	Tyrosine metabolism	5/1067	36/8105	0.522527187	0.74483042	0.622400805	5	3081/1312/1644/2184/316
hsa01522	Endocrine resistance	13/1067	98/8105	0.533681161	0.757407761	0.632910777	13	108/5293/115/5295/1871/28514/10000/3480/196883/5925/596/4851/4855
hsa04744	Phototransduction	4/1067	29/8105	0.542230866	0.766195789	0.640254295	4	5957/5145/2780/131890
hsa04726	Serotonergic synapse	15/1067	115/8105	0.557605365	0.784509713	0.655557914	15	3709/775/5578/3708/1644/2771/351/5579/2788/3359/2784/23236/54331/2775/774
hsa04650	Natural killer cell mediated cytotoxicity	17/1067	131/8105	0.565116442	0.788632469	0.659003003	17	27040/5293/3932/6452/5295/5578/7124/4773/4772/3107/5777/9437/5579/7410/3689/5335/10451
hsa05031	Amphetamine addiction	9/1067	69/8105	0.565974533	0.788632469	0.659003003	9	1385/84152/775/5578/1644/3065/5579/64764/5500
hsa00900	Terpenoid backbone biosynthesis	3/1067	22/8105	0.568137827	0.788632469	0.659003003	3	3422/38/3157
hsa00020	Citrate cycle (TCA cycle)	4/1067	30/8105	0.570241939	0.788632469	0.659003003	4	4967/5091/5106/6392
hsa05330	Allograft rejection	5/1067	38/8105	0.572850081	0.788882527	0.659211958	5	3109/3586/7124/3107/3134
hsa04120	Ubiquitin mediated proteolysis	18/1067	140/8105	0.581546214	0.797478986	0.666395395	18	51343/23295/11060/8916/8452/63893/8881/118424/23221/57154/92912/7337/4734/51434/9320/23291/7322/5071
hsa05415	Diabetic cardiomyopathy	26/1067	203/8105	0.592561374	0.809169943	0.676164681	26	5590/5293/5295/4689/4088/5578/1509/7048/10000/4720/4688/7040/5579/4728/489/23236/5500/4712/9945/56901/185/6513/5970/6392/10476/5524
hsa05310	Asthma	4/1067	31/8105	0.597268241	0.811820655	0.678379689	4	3109/3586/6037/7124
hsa04964	Proximal tubule bicarbonate reclamation	3/1067	23/8105	0.59949833	0.811820655	0.678379689	3	486/358/5106

hsa00603	Glycosphingolipid biosynthesis - globo and isoglobo series	2/1067	15/8105	0.60619597	0.817484191	0.683112296	2	3074/6483
hsa04722	Neurotrophin signaling pathway	15/1067	119/8105	0.613499317	0.823914372	0.688485532	15	6195/5293/4215/397/5295/5908/6196/10000/6272/3656/4914/596/5335/5970/4909
hsa00260	Glycine, serine and threonine metabolism	5/1067	40/8105	0.620255154	0.829559363	0.693202642	5	875/5223/132158/189/211
hsa04977	Vitamin digestion and absorption	3/1067	24/8105	0.629323717	0.838238557	0.700455215	3	4363/6573/9963
hsa00480	Glutathione metabolism	7/1067	57/8105	0.637725249	0.841529218	0.703204982	7	2678/4953/50484/2949/4258/9446/119391
hsa05134	Legionellosis	7/1067	57/8105	0.637725249	0.841529218	0.703204982	7	58484/7124/840/3689/834/23786/5970
hsa05030	Cocaine addiction	6/1067	49/8105	0.639562206	0.841529218	0.703204982	6	1385/84152/1644/2771/64764/5970
hsa05224	Breast cancer	18/1067	147/8105	0.667219238	0.87438005	0.730656042	18	5293/675/5295/1871/28514/10000/6934/3480/7481/2260/8312/81029/8648/23401/5925/4851/4855/10912
hsa04950	Maturity onset diabetes of the young	3/1067	26/8105	0.684242088	0.888770041	0.742680715	3	6927/3087/2494
hsa04940	Type I diabetes mellitus	5/1067	43/8105	0.685155249	0.888770041	0.742680715	5	3109/7124/3107/3134/5799
hsa05226	Gastric cancer	18/1067	149/8105	0.689895502	0.888770041	0.742680715	18	6256/5293/5295/4088/1871/3728/7048/10000/6934/7481/7040/8312/81029/23401/999/5925/596/10912
hsa05160	Hepatitis C	19/1067	157/8105	0.68995895	0.888770041	0.742680715	19	6256/5293/7533/5295/1871/7124/7186/8737/10000/7132/3716/5518/7534/5925/5519/4599/10971/5970/8772
hsa05416	Viral myocarditis	7/1067	60/8105	0.691873294	0.888770041	0.742680715	7	3109/1604/3107/3134/71/3689/1981
hsa04970	Salivary secretion	11/1067	93/8105	0.695094094	0.889392049	0.743200481	11	108/3709/486/115/4025/5578/491/3708/5579/23236/196883
hsa00511	Other glycan degradation	2/1067	18/8105	0.706480344	0.899749089	0.751855109	2	3074/4123

hsa04657	IL-17 signaling pathway	11/1067	94/8105	0.708725436	0.899749089	0.751855109	11	6279/3934/7124/7186/7128/1440/6364/10758/1051/5970/8772
hsa04620	Toll-like receptor signaling pathway	12/1067	104/8105	0.731807706	0.925437761	0.77332127	12	3663/5293/54472/5608/5295/7124/6352/54106/8737/10000/5970/8772
hsa04512	ECM-receptor interaction	10/1067	88/8105	0.738486373	0.930263842	0.777354076	10	2815/22987/1284/7057/3674/7148/3693/3690/3914/3694
hsa04630	JAK-STAT signaling pathway	19/1067	162/8105	0.741413189	0.930344735	0.777421673	19	3586/1439/5293/1441/5295/6776/10000/5777/3716/1440/3570/5155/2670/1387/596/5159/598/3976/316
hsa05340	Primary immunodeficiency	4/1067	38/8105	0.75569058	0.941933767	0.787105788	4	3932/7374/973/5788
hsa00920	Sulfur metabolism	1/1067	10/8105	0.756445272	0.941933767	0.787105788	1	9060
hsa01240	Biosynthesis of cofactors	18/1067	156/8105	0.761959255	0.945178465	0.789817146	18	65220/25902/217/349565/249/2235/211/5498/5373/8566/4522/60490/5315/204/4143/26289/124454/1723
hsa04146	Peroxisome	9/1067	82/8105	0.769115526	0.950427931	0.794203745	9	2180/55711/23600/189/847/55670/8309/3155/8799
hsa04060	Cytokine-cytokine receptor interaction	35/1067	295/8105	0.77402397	0.952870418	0.796244754	35	246778/3586/1439/3603/1441/7133/3554/729230/7124/6352/1436/10673/7048/7132/27242/10803/7040/3557/284340/1440/6364/658/3577/3570/8807/8809/392255/8742/8741/10850/3624/4838/6367/3976/1524
hsa05016	Huntington disease	36/1067	306/8105	0.793437547	0.970641449	0.811094717	36	1639/160/5468/55567/1385/8408/2081/7186/5435/4899/26100/3708/10383/4720/3065/10540/4728/6506/3064/22863/3800/1387/23236/64764/7277/51807/6507/5695/4712/56901/774/64446/6392/8218/5719/10476
hsa00052	Galactose metabolism	3/1067	31/8105	0.794432694	0.970641449	0.811094717	3	3098/8972/3099
hsa04012	ErbB signaling pathway	9/1067	85/8105	0.804676084	0.978533359	0.817689415	9	7039/5293/5295/5578/8440/6776/10000/5579/5335
hsa04216	Ferroptosis	4/1067	41/8105	0.806913663	0.978533359	0.817689415	4	2180/4891/112483/8031
hsa05206	MicroRNAs in cancer	36/1067	310/8105	0.817687326	0.9820693	0.820644144	36	9759/57521/27086/5328/5293/4363/406935/406937/5295/90427/5578/1871/406976/9839/406894/7057/407018/407010/7148/3065/993/5579/3690/5155/1387/596/406977/4851/1786/4855/5581/113130/5159/5335/7430/6541
hsa04913	Ovarian steroidogenesis	5/1067	51/8105	0.81953847	0.9820693	0.820644144	5	108/115/3294/3480/196883

hsa00860	Porphyrin and chlorophyll metabolism	4/1067	42/8105	0.82191646	0.9820693	0.820644144	4	2235/211/5498/124454
hsa05332	Graft-versus-host disease	4/1067	42/8105	0.82191646	0.9820693	0.820644144	4	3109/7124/3107/3134
hsa04130	SNARE interactions in vesicular transport	3/1067	33/8105	0.828540682	0.986357955	0.824227862	3	6811/143187/10791
hsa04622	RIG-I-like receptor signaling pathway	7/1067	70/8105	0.832209802	0.987110167	0.824856431	7	7124/7186/8737/1540/5970/8772/340061
hsa05320	Autoimmune thyroid disease	5/1067	53/8105	0.844757421	0.998349679	0.834248477	5	3109/3586/3107/7038/3134
hsa05217	Basal cell carcinoma	6/1067	63/8105	0.853838068	1	0.83562753	6	51684/6934/7481/8312/81029/10912
hsa05034	Alcoholism	20/1067	187/8105	0.870391863	1	0.83562753	20	9759/1385/51564/84152/55315/10645/1644/2771/84254/3065/2788/2784/64764/5500/54331/2775/2030/4852/55506/8350
hsa00500	Starch and sucrose metabolism	3/1067	36/8105	0.87055084	1	0.83562753	3	3098/8972/3099
hsa01230	Biosynthesis of amino acids	7/1067	75/8105	0.880433437	1	0.83562753	7	2023/875/5223/5091/2027/5315/4143
hsa00250	Alanine, aspartate and glutamate metabolism	3/1067	37/8105	0.882383778	1	0.83562753	3	8659/189/9945
hsa01040	Biosynthesis of unsaturated fatty acids	2/1067	27/8105	0.88773709	1	0.83562753	2	11332/6319
hsa04137	Mitophagy - animal	6/1067	68/8105	0.899013701	1	0.83562753	6	8408/51024/23786/598/5970/5071
hsa04612	Antigen processing and presentation	7/1067	78/8105	0.903393975	1	0.83562753	7	3109/811/1385/7124/3107/3134/1508
hsa00360	Phenylalanine metabolism	1/1067	17/8105	0.909483091	1	0.83562753	1	1644
hsa03018	RNA degradation	7/1067	79/8105	0.910162483	1	0.83562753	7	2023/27257/23404/8761/80153/51690/2027

hsa00270	Cysteine and methionine metabolism	4/1067	50/8105	0.910284311	1	0.83562753	4	875/1786/4143/55256
hsa03008	Ribosome biogenesis in eukaryotes	10/1067	110/8105	0.927495857	1	0.83562753	10	10482/23160/10171/28987/6023/65083/2091/83732/780851/84916
hsa03020	RNA polymerase	2/1067	31/8105	0.928684545	1	0.83562753	2	5435/51082
hsa05162	Measles	13/1067	139/8105	0.93449488	1	0.83562753	13	5293/5295/7128/54106/6776/10000/3716/596/598/8667/4599/5970/8772
hsa04975	Fat digestion and absorption	3/1067	43/8105	0.935131713	1	0.83562753	3	19/38/8613
hsa05014	Amyotrophic lateral sclerosis	39/1067	364/8105	0.935612095	1	0.83562753	39	1639/10482/55567/8408/5608/2081/7133/8480/7124/7186/26100/10383/4741/4720/7132/10540/4728/9688/5216/6506/22863/6396/71/3800/847/7277/51807/596/5695/4712/834/56901/598/64446/6392/310/5719/10476/5071
hsa04136	Autophagy - other	2/1067	32/8105	0.936454104	1	0.83562753	2	57521/26100
hsa04217	Necroptosis	15/1067	159/8105	0.941641298	1	0.83562753	15	114548/7124/7186/7128/6776/8737/7132/3716/11035/596/10059/834/1540/8772/55506
hsa00970	Aminoacyl-tRNA biosynthesis	5/1067	66/8105	0.946678636	1	0.83562753	5	79587/124454/4141/5188/8565
hsa04742	Taste transduction	7/1067	86/8105	0.947061497	1	0.83562753	7	775/3359/2784/23236/196883/2550/9568
hsa03022	Basal transcription factors	3/1067	45/8105	0.947160255	1	0.83562753	3	6881/902/2068
hsa05010	Alzheimer disease	39/1067	369/8105	0.947237067	1	0.83562753	39	5293/3709/8408/775/2081/5295/7124/7186/26100/57142/3708/10383/10000/351/4720/10313/7132/7481/8312/4728/22863/81029/3800/489/1454/23236/7277/51807/840/23401/5695/4712/56901/5970/6392/8883/8772/5719/10476
hsa04260	Cardiac muscle contraction	7/1067	87/8105	0.95104895	1	0.83562753	7	845/486/775/93589/6546/489/444
hsa03050	Proteasome	3/1067	46/8105	0.952363986	1	0.83562753	3	9491/5695/5719
hsa00830	Retinol metabolism	5/1067	68/8105	0.955301643	1	0.83562753	5	10170/157506/317749/8854/316

hsa05322	Systemic lupus erythematosus	12/1067	136/8105	0.95561083	1	0.83562753	12	1991/3109/1511/3586/6634/7124/87/81/716/713/55506/8350
hsa05022	Pathways of neurodegeneration - multiple diseases	51/1067	475/8105	0.956738288	1	0.83562753	51	1639/55567/3709/8408/5608/775/2081/7133/5578/7124/7186/26100/6310/3708/10383/4741/351/4720/7132/118424/7481/10540/5579/8312/4728/3064/22863/81029/3800/489/1454/23236/847/7277/51807/840/23401/596/5695/4712/56901/774/598/64446/5335/5970/6392/8772/5719/10476/5071
hsa03060	Protein export	1/1067	23/8105	0.961278316	1	0.83562753	1	28972
hsa03430	Mismatch repair	1/1067	23/8105	0.961278316	1	0.83562753	1	10714
hsa05020	Prion disease	27/1067	273/8105	0.961350625	1	0.83562753	27	5293/3709/1385/775/5295/4689/4685/7124/6352/3708/10383/4720/4688/4728/3800/64764/7277/51807/5695/4851/4712/56901/774/713/6392/5719/10476
hsa04917	Prolactin signaling pathway	5/1067	70/8105	0.962633933	1	0.83562753	5	5293/5295/6776/10000/5970
hsa05171	Coronavirus disease - COVID-19	22/1067	232/8105	0.967153314	1	0.83562753	22	719/5293/114548/5295/5578/7124/5648/100529239/7132/3716/5579/716/1440/3570/834/185/4599/713/5335/6147/5970/340061
hsa00140	Steroid hormone biosynthesis	4/1067	61/8105	0.968086166	1	0.83562753	4	6718/3294/1312/3291
hsa00982	Drug metabolism - cytochrome P450	5/1067	72/8105	0.96884601	1	0.83562753	5	2949/4258/9446/119391/316
hsa05204	Chemical carcinogenesis	6/1067	83/8105	0.970343497	1	0.83562753	6	6799/2949/4258/9446/2052/119391
hsa00190	Oxidative phosphorylation	11/1067	133/8105	0.97141461	1	0.83562753	11	4720/526/4728/533/4712/56901/6392/64077/10476/527/528
hsa00790	Folate biosynthesis	1/1067	26/8105	0.974680438	1	0.83562753	1	249
hsa00062	Fatty acid elongation	1/1067	27/8105	0.978024412	1	0.83562753	1	11332
hsa00980	Metabolism of xenobiotics by cytochrome P450	5/1067	78/8105	0.982208085	1	0.83562753	5	2949/4258/9446/2052/119391
hsa03040	Spliceosome	12/1067	151/8105	0.984285445	1	0.83562753	12	6634/23450/51690/100534599/10594/151903/8449/56259/24148/25766/10523/5356

hsa00030	Pentose phosphate pathway	1/1067	30/8105	0.985633721	1	0.83562753	1	132158
hsa00053	Ascorbate and aldarate metabolism	1/1067	30/8105	0.985633721	1	0.83562753	1	217
hsa03013	RNA transport	15/1067	186/8105	0.989683059	1	0.83562753	15	10482/8480/8761/26999/8893/9688/10460/6396/10073/10605/8667/23191/1981/50628/780851
hsa00590	Arachidonic acid metabolism	3/1067	61/8105	0.990659627	1	0.83562753	3	2678/4051/127281
hsa05012	Parkinson disease	21/1067	249/8105	0.993031024	1	0.83562753	21	3709/2081/3708/10383/2771/4720/118424/4728/3800/7277/51807/4780/5695/4712/56901/598/5335/6392/5719/10476/5071
hsa05033	Nicotine addiction	1/1067	40/8105	0.996520706	1	0.83562753	1	774
hsa04080	Neuroactive ligand-receptor interaction	28/1067	341/8105	0.998684025	1	0.83562753	28	1511/719/2358/2357/2900/56413/5031/3269/2696/51738/1129/1269/9294/2550/5023/9170/2149/8698/185/7434/9568/5026/2894/64106/4852/1241/2834/6750
hsa05168	Herpes simplex virus 1 infection	45/1067	498/8105	0.998689135	1	0.83562753	45	3109/29992/811/29990/10482/5293/5295/201514/6009/7124/7186/6352/8893/54106/3107/10000/197320/3134/199692/7132/155054/148198/3716/730087/155061/170960/3690/57541/58492/5500/5452/596/6672/255403/7249/340385/598/55769/5970/163071/284306/25799/8772/340061/5818
hsa03010	Ribosome	5/1067	158/8105	0.999997219	1	0.83562753	5	11222/100529239/54460/10573/6147
hsa04740	Olfactory transduction	8/1067	443/8105	1	1	0.83562753	8	123041/6546/408/2788/390077/1262/409/156
