# Supplementary material for: A Sight of the Diagnostic Value of Aberrant Cell-Free DNA Methylation in Lung Cancer
Source: Dis Markers. 2022 Jan 27;2022:9619357. doi: 10.1155/2022/9619357 (PMC8814721; doi:10.1155/2022/9619357)
Supplement: Supplementary 8 — Table S5: primer sequences for pyrosequencing. [file 9619357.f8.pdf]

Table S5. Primer sequences for pyrosequencing

Gene	Primer	Sequence
TRAF1	Fwd	GTGTTATAGATTTTTAGAAAGTATAGTGAGA
	Rev	AAACACAAACCAAAAACCAACTATA
	Seq	TGGTTGAGAATAGTAGGTTGT
RPTOR	Fwd	AAGGGGATTAGTGGAGGATATT
	Rev	AAATAAAAAATATACRTATCACAAATCAC
	Seq	GGTAGGGTTGTTGTAGG
SPON2	Fwd	TTTYGTTTGGAGGTTAAGGTTG
	Rev	ACTTATAAAACAAACCCACCCTC
	Seq	CAAACCCACCCTCCAAC
