# Supplementary material for: A Sight of the Diagnostic Value of Aberrant Cell-Free DNA Methylation in Lung Cancer
Source: Dis Markers. 2022 Jan 27;2022:9619357. doi: 10.1155/2022/9619357 (PMC8814721; doi:10.1155/2022/9619357)
Supplement: Supplementary 9 — Table S6: two-sample Kolmogorov-Smirnov test in cfDNA samples. [file 9619357.f9.pdf]

Table S6. Two-sample Kolmogorov-Smirnov test in cfDNA samples
alternative hypothesis: the CDF of cancer samples lies above that of normal samples

Gene	D^+	p value
TRAF1	0.60976	0.03637
RPTOR	0.65854	0.02096
SPON2	0.78049	0.004385
