# Supplementary material for: A Sight of the Diagnostic Value of Aberrant Cell-Free DNA Methylation in Lung Cancer
Source: Dis Markers. 2022 Jan 27;2022:9619357. doi: 10.1155/2022/9619357 (PMC8814721; doi:10.1155/2022/9619357)
Supplement: Supplementary 4 — Table S1: differently methylated positions detected in tissue samples. [file 9619357.f4.pdf]

Table S1. Differently methylated positions detected in tissue samples

Probe	chr	pos	strand	UCSC_RefGene_Name	UCSC_RefGene_Group	logFC	AveExpr	t	P.Value	adj.P.Val	B
cg24334634	chr13	114126676	+	DCUN1D2	Body	1.8316338	2.3777878	13.864607	1.31E-27	5.64E-22	51.631342
cg00781839	chr13	114127083	+	DCUN1D2	Body	2.0325022	2.6812476	13.297138	3.36E-26	2.28E-21	48.509955
cg08328160	chr6	32151394	-	AGER;AGER	Body;Body	1.4998317	1.8104797	13.320912	2.93E-26	2.28E-21	48.641258
cg10154122	chr19	38754855	+	SPINT2;SPINT2	TSS1500;TSS1500	-0.988469	-3.287854	-13.28025	3.70E-26	2.28E-21	48.416652
cg14823851	chr17	59532306	-	TBX4	TSS1500	1.7036785	0.082957	13.449401	1.40E-26	2.28E-21	49.350127
cg20568402	chr13	114126476	+	DCUN1D2	Body	1.7323431	2.2055425	13.350628	2.85E-26	2.28E-21	48.666472
cg26521404	chr7	27204981	+	HOXA9	1stExon	3.2849987	-0.19617	13.334288	2.72E-26	2.28E-21	48.715116
cg15628956	chr12	53448234	-	TENC1;TENC1;TENC1	Body;Body;Body	-1.844035	-3.33524	-13.24741	4.47E-26	2.41E-21	48.235184
cg01839430	chr2	216177098	-	ATIC	Body	-1.520528	-2.663246	-13.14258	8.17E-26	3.92E-21	47.655295
cg01772014	chr20	30606095	+	C20orf160	Body	-1.496706	-2.401886	-13.1141	9.63E-26	4.16E-21	47.497615
cg11114465	chr3	194360708	-			1.5294312	2.5749403	13.043431	1.45E-25	5.68E-21	47.106077
cg02627286	chr16	89590583	-	SPG7;SPG7	Body;Body	2.5052392	3.7302278	12.849235	4.43E-25	1.59E-20	46.028411
cg23209302	chr1	1326152	-	CCNL2;CCNL2;CCNL2;CCNL2	Body;3'UTR;3'UTR;3'UTR	1.4165773	1.8964285	12.748924	7.91E-25	2.63E-20	45.470777
cg13394216	chr6	1635808	-	GMDS	Body	1.9656966	2.6303025	12.71417	9.67E-25	2.98E-20	45.277436
cg01399319	chr1	1326204	-	CCNL2;CCNL2;CCNL2;CCNL2	Body;3'UTR;3'UTR;3'UTR	1.4565457	2.3184145	12.701523	1.04E-24	2.99E-20	45.207058
cg08149422	chr22	21983606	-	YDJC	Body	-1.424014	-3.898793	-12.68037	1.18E-24	3.17E-20	45.089342
cg08722200	chr20	55960520	-			2.8086708	3.9277653	12.548224	2.52E-24	6.41E-20	44.353244
cg23413697	chr7	5602639	-			-2.170332	-3.206723	-12.52047	2.96E-24	7.11E-20	44.198537

cg20133191	chr7	2638016	+	IQCE;IQCE	Body;Body	2.0170901	3.2507759	12.435257	4.85E-24	1.10E-19	43.723224
cg26084949	chr14	75039090	+	LTBP2	Body	-1.954573	-3.282092	-12.40412	5.81E-24	1.25E-19	43.549462
cg26026748	chr1	164629875	+	PBX1	Body	-2.261821	-2.957223	-12.33924	8.46E-24	1.74E-19	43.187244
cg24407065	chr1	169335802	+	BLZF1;NME7;NME7	TSS1500;Body;5'UTR	1.5645311	2.5449658	12.302114	1.05E-23	2.06E-19	42.979846
cg21931656	chr11	72462963	+	ARAP1	5'UTR	-1.322586	-3.33528	-12.2691	1.27E-23	2.38E-19	42.795368
cg00129232	chr17	37814104	-	STARD3;STARD3;STARD3	Body;Body;Body	3.0277661	4.1026745	12.28063	1.33E-23	2.40E-19	42.748026
cg08943107	chr1	39943966	+	MACF1;MACF1	Body;Body	1.7861386	2.7376654	12.232589	1.57E-23	2.71E-19	42.591327
cg15427886	chr8	55379663	+			2.3619074	0.0824732	12.154899	2.46E-23	4.09E-19	42.156928
cg01367005	chr17	38518966	-	GJD3;GJD3	1stExon;3'UTR	-1.245969	-2.646154	-12.0933	3.52E-23	5.63E-19	41.812298
cg05945615	chr22	38035456	-	SH3BP1	TSS1500	-2.036706	-4.400326	-12.05514	4.39E-23	6.78E-19	41.59875
cg03915012	chr4	902074	+	GAK	Body	1.6852049	2.0829929	12.028962	5.11E-23	7.36E-19	41.452242
cg07195577	chr17	27052828	-	TLCD1;TLCD1	Body;Body	-2.038218	-3.234887	-12.03332	4.99E-23	7.36E-19	41.476623
cg06854487	chr11	71791011	-	NUMA1;LRTOMT;LRTOMT;LRTOMT;LRTOMT;LRTOMT;LRTOMT	5'UTR;TSS1500;TSS1500;TSS1500;TSS1500;TSS1500;TSS1500	-0.835913	-3.508408	-11.99591	6.20E-23	8.63E-19	41.267196
cg18432877	chr17	65190638	-	HELZ	Body	1.7059727	2.3939603	11.978068	6.87E-23	9.27E-19	41.16729
cg06569947	chr13	114126889	+	DCUN1D2	Body	1.6274567	2.6291553	11.968023	7.28E-23	9.53E-19	41.111036
cg06783737	chr6	26752689	-			3.1890137	0.5404547	11.921614	9.54E-23	1.21E-18	40.851098
cg03177025	chr2	28112917	-	BRE;BRE;BRE;BRE;RBKS;BRE;LOC100302650	TSS1500;TSS1500;TSS1500;TSS1500;Body;TSS1500;Body	-1.47207	-2.636811	-11.90477	1.05E-22	1.28E-18	40.756741
cg12606391	chr13	26852712	+	CDK8	Body	1.5375528	2.8051579	11.902782	1.06E-22	1.28E-18	40.745604
cg13531667	chr5	112825132	+	MCC	TSS1500	-2.145891	-2.883067	-11.87119	1.28E-22	1.49E-18	40.568597
cg14754787	chr17	35299600	+	LHX1	Body	2.6347415	0.4003708	11.830406	1.62E-22	1.84E-18	40.340048

cg06376214	chr1	26633439	-	UBXN11;UBXN11;UBXN11	5'UTR;TSS1500;TSS1500	-1.1604	-3.622289	-11.82321	1.69E-22	1.87E-18	40.299718
cg07216425	chr12	53297819	+	KRT8	Body	-1.56708	-3.626258	-11.79762	1.96E-22	2.12E-18	40.156253
cg03286774	chr16	89590357	-	SPG7;SPG7	Body;Body	1.6527386	2.7253168	11.842398	2.32E-22	2.44E-18	39.992523
cg07495357	chr6	43021181	-	CUL7;CUL7	Body;5'UTR	-1.321822	-3.811216	-11.74905	2.60E-22	2.61E-18	39.883995
cg13153865	chr8	90914101	+	OSGIN2;OSGIN2;OSGIN2	5'UTR;1stExon;TSS1500	-1.231441	-2.784718	-11.75165	2.56E-22	2.61E-18	39.898541
cg09496762	chr20	42286524	-			-1.617725	-3.53884	-11.74444	2.67E-22	2.62E-18	39.858138
cg06422947	chr17	41167707	-	VAT1	3'UTR	1.4308635	2.0716869	11.690985	3.64E-22	3.44E-18	39.558387
cg10189882	chr8	665934	-	ERICH1	Body	1.4576595	1.8708366	11.68472	3.78E-22	3.44E-18	39.52325
cg10725623	chr6	32131571	-	EGFL8	TSS1500	1.5277937	2.7694714	11.682482	3.83E-22	3.44E-18	39.510699
cg24251800	chr17	47675686	-			1.5739224	2.0819419	11.688143	3.70E-22	3.44E-18	39.542448
cg16341737	chr13	113634407	-	MCF2L;MCF2L	Body;Body	2.2492386	2.5758883	11.67693	3.95E-22	3.48E-18	39.479561
cg00300822	chr16	30075511	-	ALDOA;ALDOA;ALDOA	TSS200;TSS1500;5'UTR	-0.977938	-3.563863	-11.66592	4.21E-22	3.51E-18	39.417815
cg09179046	chr16	14164850	+	MKL2	TSS1500	-1.209589	-3.363397	-11.66553	4.22E-22	3.51E-18	39.415607
cg16768018	chr3	147108843	-	ZIC4;ZIC4;ZIC4;ZIC4;ZIC4	Body;Body;Body;Body;Body	2.3585464	0.1298958	11.671711	4.07E-22	3.51E-18	39.450289
cg25602159	chr19	41222284	-	ADCK4;ITPKC;ADCK4	5'UTR;TSS1500;TSS1500	-1.467453	-3.303363	-11.65155	4.58E-22	3.73E-18	39.337236
cg00129196	chr2	20190016	+	WDR35;WDR35	TSS200;TSS200	-0.954623	-2.994105	-11.64555	4.74E-22	3.79E-18	39.303531
cg06643271	chr17	76128170	+	TMC8;TMC6	Body;5'UTR	-1.81376	-2.638331	-11.62957	5.20E-22	4.08E-18	39.213908
cg19002941	chr8	641497	+	ERICH1	Body	1.597207	2.0905317	11.624385	5.36E-22	4.13E-18	39.184833
cg25774643	chr11	627175	-	SCT	TSS200	3.3344399	0.5124017	11.595529	6.34E-22	4.80E-18	39.022953
cg02174232	chr3	49712164	+	APEH	Body	-1.570057	-2.733056	-11.60661	6.57E-22	4.89E-18	38.987335

cg04009429	chr7	100883125	+	FIS1	Body	1.3437621	1.8790987	11.549811	8.27E-22	5.95E-18	38.766443
cg23582919	chr16	23568380	-	UBFD1;EARS2;EARS2	TSS1500;Body;Body	-1.021399	-3.62833	-11.55002	8.26E-22	5.95E-18	38.767587
cg14836636	chr12	120106292	+	PRKAB1	Body	-2.103535	-4.569349	-11.51398	1.02E-21	7.21E-18	38.5654
cg11936643	chr10	116286411	-	ABLIM1;ABLIM1;ABLIM1;ABLIM1	Body;Body;5'UTR;Body	-1.910195	-2.777904	-11.47662	1.27E-21	8.81E-18	38.355722
cg16110314	chr1	246203683	-	SMYD3;SMYD3	Body;Body	1.491548	2.553193	11.473262	1.29E-21	8.84E-18	38.336873
cg15986164	chr4	901785	+	GAK	Body	1.3750724	2.3571508	11.425313	1.71E-21	1.15E-17	38.06775
cg00249511	chr11	627152	-	SCT;SCT	5'UTR;1stExon	3.5954477	-0.056135	11.416407	1.80E-21	1.17E-17	38.017762
cg03964958	chr2	176964720	+	HOXD12	1stExon	2.3687753	-0.105317	11.418484	1.77E-21	1.17E-17	38.029425
cg06767326	chr13	114126798	+	DCUN1D2	Body	1.9350871	2.8072585	11.369372	2.36E-21	1.52E-17	37.753746
cg07452625	chr16	89598568	-	SPG7;SPG7	Body;Body	1.3904903	1.7928557	11.351357	2.62E-21	1.66E-17	37.652614
cg09325711	chr7	39662764	+	RALA	TSS1500	-2.177679	-3.439099	-11.29106	3.72E-21	2.33E-17	37.314102
cg06430102	chr19	1151960	+	SBNO2	Body	1.4666304	1.7140161	11.282697	3.91E-21	2.39E-17	37.267167
cg07351192	chr3	5137773	+			1.9737965	-0.567756	11.28177	3.93E-21	2.39E-17	37.261962
cg17760049	chr7	4774448	+	FOXK1	Body	2.3752489	3.2242988	11.256705	4.54E-21	2.72E-17	37.121242
cg08171483	chr3	38010391	-	CTDSPL;CTDSPL;MIR26A1	Body;Body;TSS1500	1.5250673	1.4700119	11.235327	5.14E-21	3.04E-17	37.001217
cg05696153	chr16	81732038	-	CMIP;CMIP	Body;Body	1.6677777	2.6215637	11.219854	5.63E-21	3.28E-17	36.914344
cg05780177	chr1	197744757	+	DENND1B;DENND1B	TSS200;TSS200	-0.991712	-2.785418	-11.21137	5.91E-21	3.40E-17	36.866717
cg21768042	chr5	141059890	+	ARAP3	Body	1.1264077	1.5842165	11.209015	5.99E-21	3.41E-17	36.853494
cg19358397	chr11	1794018	+			-1.440715	-2.528154	-11.19053	6.67E-21	3.74E-17	36.749691
cg19586165	chr17	37814072	-	STARD3;STARD3;STARD3	Body;Body;Body	1.6476806	2.7446507	11.184469	6.91E-21	3.83E-17	36.715682

cg02166383	chr14	107252649	+			-2.326712	-1.864657	-11.16908	7.56E-21	4.13E-17	36.629306
cg07893916	chr2	203155462	+	SNORD11B;NOP58	TSS1500;Body	1.4103326	2.2510513	11.157111	8.11E-21	4.37E-17	36.562081
cg12260990	chr22	47371122	-	TBC1D22A	Body	1.8680883	3.6364094	11.158436	8.81E-21	4.67E-17	36.481406
cg18139800	chr6	32151517	+	AGER;AGER	Body;Body	1.1581828	1.0544314	11.139992	8.95E-21	4.67E-17	36.465967
cg25287474	chr9	14693538	+	ZDHHC21	TSS200	-2.188418	-3.643314	-11.13761	9.08E-21	4.67E-17	36.452602
cg26993251	chr11	63934014	+	MACROD1	TSS1500	-2.370595	-3.938179	-11.22034	8.98E-21	4.67E-17	36.464792
cg14381919	chr11	12701969	+	TEAD1	5'UTR	-2.26009	-3.403126	-11.11331	1.05E-20	5.31E-17	36.316157
cg25390506	chr7	135347085	-	PL-5283	TSS200	-1.924491	-5.361329	-11.09404	1.17E-20	5.87E-17	36.207986
cg04864807	chr2	121412139	-			2.4900922	1.384365	11.089161	1.20E-20	5.90E-17	36.180588
cg27616751	chr7	100883077	+	FIS1	3'UTR	1.9778628	3.0834478	11.089136	1.20E-20	5.90E-17	36.180446
cg02412969	chr7	5602052	-			-0.924857	-3.816987	-11.0836	1.24E-20	6.03E-17	36.149386
cg07031551	chr11	72463331	-	ARAP1	5'UTR	-1.119571	-3.871482	-11.08056	1.26E-20	6.07E-17	36.13231
cg25889465	chr17	76203084	-	AFMID;AFMID	3'UTR;3'UTR	1.2762996	2.5584596	11.07038	1.47E-20	6.96E-17	35.988886
cg24393699	chr1	197744782	+	DENND1B;DENND1B	TSS200;TSS200	-0.988142	-3.570466	-11.03606	1.64E-20	7.69E-17	35.882448
cg02576092	chr1	27668556	-	SYTL1;SYTL1	1stExon;5'UTR	-1.000612	-3.514646	-11.03316	1.67E-20	7.73E-17	35.866174
cg22167515	chr1	91192466	-			2.8261169	-0.463628	11.025379	1.74E-20	8.00E-17	35.822506
cg16186001	chr5	159448398	+	TTC1	Body	1.2021651	2.2182199	11.014408	1.86E-20	8.44E-17	35.760916
cg20308684	chr15	67000898	+	SMAD6;SMAD6;SMAD6	Body;Body;TSS200	1.2441864	2.3960338	11.006238	1.95E-20	8.76E-17	35.715052
cg27252696	chr6	100912940	-	SIM1	TSS1500	3.0450697	-0.069137	10.977712	2.30E-20	1.02E-16	35.554916
cg06491116	chr11	118122715	-	MPZL3	Body	-1.523533	-3.384564	-10.97543	2.33E-20	1.03E-16	35.542102

cg27235726	chr7	750819	+	PRKAR1B;PRKAR1B;PRKAR1B;PRKAR1B;PRKAR1B;PRKAR1B	Body;Body;Body;Body;Body;Body	0.9557026	1.2475902	10.973373	2.36E-20	1.03E-16	35.530555
cg06439269	chr4	2256756	+	MXD4	Body	1.4521409	1.9916101	10.970204	2.40E-20	1.04E-16	35.512769
cg07714812	chr6	1635611	+	GMDS	Body	1.4729667	1.6207847	10.958256	2.57E-20	1.08E-16	35.445697
cg09841704	chr15	81295984	+	MESDC1;MESDC1	1stExon;3'UTR	1.5982936	2.0296433	10.958821	2.57E-20	1.08E-16	35.448873
cg27418434	chr7	751009	-	PRKAR1B;PRKAR1B;PRKAR1B;PRKAR1B;PRKAR1B;PRKAR1B	Body;Body;Body;Body;Body;Body	1.8946825	2.4028503	10.957965	2.58E-20	1.08E-16	35.444064
cg17701921	chr19	537170	-	CDC34	Body	2.4058695	4.0382013	10.986223	2.61E-20	1.08E-16	35.433731
cg04155747	chr4	74128032	+			1.1736981	2.7499196	10.949565	2.71E-20	1.11E-16	35.396913
cg07938459	chr12	7055586	-	PTPN6	TSS200	-1.262232	-2.482144	-10.9343	2.96E-20	1.20E-16	35.311225
cg19558628	chr14	24801616	-	ADCY4	Body	-1.293303	-3.106898	-10.92237	3.17E-20	1.28E-16	35.244271
cg13539545	chr14	60973823	+			3.022226	0.0295596	10.913684	3.33E-20	1.33E-16	35.195512
cg17724627	chr12	50041367	+	FMNL3;FMNL3	Body;Body	1.6664313	2.4472661	10.880418	4.04E-20	1.60E-16	35.008804
cg11207652	chr4	24860210	+	CCDC149;CCDC149	Body;Body	1.2871598	2.515391	10.877528	4.11E-20	1.61E-16	34.992584
cg03859915	chr2	28112817	-	BRE;BRE;BRE;BRE;RBKS;BRE;LOC100302650	TSS1500;TSS1500;TSS1500;TSS1500;Body;TSS1500;Body	-1.450839	-2.884303	-10.87179	4.25E-20	1.65E-16	34.960391
cg15698065	chr13	114127206	-	DCUN1D2	Body	1.3831809	1.8983975	10.861972	4.50E-20	1.74E-16	34.905282
cg07961632	chr1	27668421	-	SYTL1	TSS200	-1.541214	-2.459119	-10.85786	4.61E-20	1.75E-16	34.882225
cg13327911	chr6	55965977	+	COL21A1	Body	-1.878607	-2.814524	-10.85866	4.59E-20	1.75E-16	34.886699
cg03283569	chr9	127963181	-	RABEPK;RABEPK	1stExon;5'UTR	-1.220297	-3.69903	-10.85254	4.76E-20	1.79E-16	34.852372
cg12362629	chr22	36716469	+	MYH9	Body	1.3537506	1.4209525	10.84783	4.89E-20	1.82E-16	34.825918
cg09618197	chr6	5152468	-	LYRM4;LYRM4	Body;Body	1.4943637	2.4212262	10.832687	5.34E-20	1.97E-16	34.740936
cg06766367	chr16	67062687	-	CBFB;CBFB	TSS1500;TSS1500	-1.400825	-2.641423	-10.82973	5.43E-20	1.99E-16	34.724343

cg19069882	chr11	118123243	+	MPZL3	TSS1500	-1.671357	-3.263578	-10.81526	5.90E-20	2.12E-16	34.643167
cg20935317	chr1	10739184	+	CASZ1;CASZ1	Body;Body	1.2350686	2.0858335	10.816071	5.88E-20	2.12E-16	34.647698
cg11786839	chr6	1635578	+	GMDS	Body	1.2619485	1.3372396	10.806869	6.20E-20	2.21E-16	34.59606
cg16620283	chr2	203155849	+	SNORD11B;NOP58	TSS200;Body	1.5898382	1.808823	10.800116	6.45E-20	2.28E-16	34.558169
cg17723299	chr1	197744929	-	DENND1B;DENND1B	TSS1500;TSS1500	-1.564644	-2.671276	-10.79617	6.60E-20	2.32E-16	34.536051
cg21341586	chr4	99851281	-	EIF4E;EIF4E;EIF4E;EIF4E;EIF4E	5'UTR;1stExon;5'UTR;TSS1500;1stExon	-2.293873	-2.706908	-10.78137	7.19E-20	2.50E-16	34.453012
cg11855693	chr12	96632551	-	ELK3	Body	1.5225422	2.3322487	10.776392	7.40E-20	2.56E-16	34.425057
cg06525280	chr2	216177303	-	ATIC	Body	-1.582654	-2.19662	-10.76823	7.76E-20	2.64E-16	34.379258
cg26422458	chr1	79472452	+	ELTD1;ELTD1	5'UTR;1stExon	2.239611	0.4018302	10.769489	7.70E-20	2.64E-16	34.38633
cg02262727	chr11	43707755	+	HSD17B12	Body	1.5227735	2.5461033	10.761771	8.05E-20	2.72E-16	34.343028
cg03724759	chr1	26633534	+	UBXN11;UBXN11;UBXN11	5'UTR;TSS1500;TSS1500	-1.220778	-3.681123	-10.757	8.28E-20	2.77E-16	34.316277
cg23929284	chr20	30605916	+	C20orf160	Body	-2.623986	-3.80239	-10.76861	8.41E-20	2.79E-16	34.301374
cg02330500	chr14	95731916	+	CLMN	Body	-1.753822	-2.953553	-10.71052	1.08E-19	3.57E-16	34.055521
cg00251824	chr8	641466	-	ERICH1	Body	1.1877312	2.2120073	10.70354	1.13E-19	3.69E-16	34.016367
cg08937613	chr4	881914	+	GAK	Body	1.2999382	2.2460832	10.682537	1.28E-19	4.14E-16	33.898569
cg13595913	chr9	5786038	-	ERMP1	3'UTR	1.1049884	1.4642164	10.672374	1.35E-19	4.36E-16	33.841569
cg01280080	chr2	216177276	-	ATIC	Body	-1.769042	-2.356749	-10.68084	1.40E-19	4.47E-16	33.810784
cg14468280	chr20	47862976	-	ZNFX1	3'UTR	1.6531455	1.7099988	10.665267	1.41E-19	4.48E-16	33.801711
cg15626828	chr1	198651141	-	PTPRC;PTPRC;PTPRC;PTPRC	Body;Body;Body;Body	-2.015686	-2.979004	-10.66907	1.50E-19	4.71E-16	33.745004
cg05457373	chr15	67000902	+	SMAD6;SMAD6;SMAD6	Body;Body;TSS200	1.2778098	2.8452121	10.651486	1.53E-19	4.78E-16	33.724431

cg01383890	chr15	62358982	+	C2CD4A	TSS200	-1.431038	-3.275361	-10.6405	1.63E-19	5.06E-16	33.662841
cg17566936	chr4	48271107	-	TEC	5'UTR	-1.201434	-3.670166	-10.61712	1.86E-19	5.75E-16	33.531732
cg06651376	chr17	1690794	-	SMYD4	Body	2.1787004	3.5482405	10.613326	1.91E-19	5.84E-16	33.510461
cg16966315	chr7	149112318	+			2.6811441	-0.340126	10.6212	1.97E-19	6.00E-16	33.478331
cg02580005	chr4	151504187	+	LRBA;MAB21L2	Body;1stExon	1.5420522	1.5891212	10.602859	2.02E-19	6.11E-16	33.451774
cg24035245	chr1	119535928	+			2.2588936	0.4673768	10.588453	2.20E-19	6.60E-16	33.371016
cg03143486	chr7	811491	-	HEATR2	Body	1.5798873	1.7263625	10.580572	2.30E-19	6.86E-16	33.326834
cg16433074	chr4	41050961	-	APBB2;APBB2;APBB2	5'UTR;5'UTR;5'UTR	1.7665516	2.4605586	10.591109	2.35E-19	6.94E-16	33.309454
cg11441693	chr17	48194635	-	SAMD14	Body	-1.738424	-2.392941	-10.56513	2.52E-19	7.40E-16	33.240287
cg02421543	chr1	93810826	-	DR1	TSS1500	-1.281926	-4.177954	-10.56021	2.59E-19	7.57E-16	33.212708
cg16659510	chr16	87996870	+	BANP;BANP	5'UTR;5'UTR	1.7836437	3.4594347	10.55192	2.72E-19	7.88E-16	33.166233
cg06029935	chr8	145049044	+	PLEC1;PLEC1	Body;TSS1500	-1.610964	-2.433527	-10.54907	2.77E-19	7.96E-16	33.150239
cg26052635	chr2	29339307	-	CLIP4	5'UTR	-1.55725	-2.53264	-10.54472	2.84E-19	8.11E-16	33.125889
cg22698604	chr10	116286502	+	ABLIM1;ABLIM1;ABLIM1;ABLIM1;ABLIM1	Body;1stExon;Body;5'UTR;Body	-1.312866	-3.825469	-10.53667	2.97E-19	8.44E-16	33.080747
cg05516390	chr16	73088677	-	ZFHX3	5'UTR	-1.889777	-2.381889	-10.50402	3.59E-19	1.01E-15	32.89779
cg09464735	chr5	172175855	+			1.4990382	1.9710229	10.501923	3.64E-19	1.02E-15	32.886049
cg08331427	chr5	138842004	-	ECSCR	Body	1.3960587	2.4506035	10.498831	3.70E-19	1.03E-15	32.868725
cg26229879	chr1	156571314	-	GPATCH4;GPATCH4	TSS200;TSS200	-0.88473	-4.267887	-10.48855	3.93E-19	1.09E-15	32.811095
cg18886436	chr12	54785171	-	ZNF385A;ZNF385A	TSS200;TSS200	-2.541855	-3.462329	-10.48649	4.29E-19	1.17E-15	32.725938
cg26942532	chr7	75678159	-	MDH2;STYXL1	Body;TSS1500	-0.836109	-3.691223	-10.47427	4.27E-19	1.17E-15	32.731108

cg12099279	chr19	40732518	+	CNTD2	1stExon	-1.87308	-2.738031	-10.48206	4.40E-19	1.20E-15	32.701206
cg09248007	chr16	14164860	+	MKL2	TSS1500	-1.037562	-3.410308	-10.45377	4.80E-19	1.30E-15	32.616279
cg18116174	chr5	142311682	+	ARHGAP26;ARHGAP26	Body;Body	1.1287095	1.503453	10.451379	4.87E-19	1.31E-15	32.602888
cg26421308	chr11	86663040	+	FZD4	Body	1.3172501	1.9724976	10.444856	5.06E-19	1.35E-15	32.566349
cg03239279	chr7	100883248	+	FIS1	Body	1.1514582	2.4538769	10.422458	5.76E-19	1.52E-15	32.44091
cg18710162	chr17	33390808	-	RFFL;RFFL	TSS200;5'UTR	-1.84412	-3.798656	-10.4226	5.76E-19	1.52E-15	32.441692
cg09934056	chr19	8400203	-	KANK3	Body	-1.917335	-2.682096	-10.42079	5.82E-19	1.52E-15	32.431591
cg01778114	chr2	216877947	+	MREG	Body	-1.650446	-4.740902	-10.41461	6.03E-19	1.57E-15	32.396983
cg14287742	chr1	169335773	+	BLZF1;NME7;NME7	TSS1500;Body;5'UTR	1.2638133	1.9611611	10.410111	6.19E-19	1.60E-15	32.371765
cg06801994	chr13	113834082	-	PCID2;PCID2;PCID2	Body;Body;Body	2.3089366	3.8349444	10.398522	6.62E-19	1.70E-15	32.306871
cg04312358	chr19	41197166	-	NUMBL	TSS1500	-1.739757	-3.796368	-10.41892	6.85E-19	1.75E-15	32.274952
cg23023126	chr19	308808	-	MIER2	Body	2.640622	3.4381892	10.391814	6.88E-19	1.75E-15	32.269311
cg04549287	chr13	52952319	+	THSD1;THSD1	Body;Body	1.3095774	1.9214524	10.389094	6.99E-19	1.76E-15	32.254084
cg13028471	chr4	139163535	-	SLC7A11	TSS200	-1.448065	-4.291826	-10.38279	7.25E-19	1.82E-15	32.218789
cg06024289	chr12	53342595	-	KRT18;KRT18	TSS200;TSS1500	-1.674076	-3.631436	-10.37729	7.48E-19	1.86E-15	32.18799
cg08539620	chr17	40118030	+	CNP	TSS1500	-1.37117	-3.05466	-10.37731	7.48E-19	1.86E-15	32.1881
cg18565103	chr1	26633265	-	UBXN11;UBXN11;UBXN11	5'UTR;TSS200;TSS200	-1.296333	-4.332711	-10.37413	7.62E-19	1.88E-15	32.170281
cg14138050	chr9	14693504	+	ZDHHC21	TSS200	-1.919752	-5.101309	-10.36357	8.10E-19	1.99E-15	32.111218
cg01552919	chr4	901847	-	GAK	Body	1.7169575	2.1488555	10.361444	8.20E-19	2.00E-15	32.099292
cg09494609	chr12	53448180	+	TENC1;TENC1;TENC1	Body;Body;Body	-1.627407	-2.382507	-10.35467	8.53E-19	2.07E-15	32.061378

cg27665659	chr7	100797595	-	AP1S1	TSS200	-1.141704	-3.599625	-10.35364	8.58E-19	2.07E-15	32.055594
cg27587645	chr19	2015311	+	BTBD2	1stExon	-1.322421	-3.434308	-10.34957	8.78E-19	2.11E-15	32.032845
cg01748627	chr11	67272202	+	PITPNM1;PITPNM1	5'UTR;5'UTR	-1.408681	-3.456974	-10.33821	9.38E-19	2.24E-15	31.969255
cg22478261	chr20	49348806	-	PARD6B	Body	-1.580185	-3.391026	-10.33735	9.43E-19	2.24E-15	31.964457
cg00280758	chr4	140221967	-	NAA15	TSS1500	-1.832781	-4.385008	-10.33046	9.81E-19	2.32E-15	31.925865
cg24278165	chr8	23083551	+	TNFRSF10A	TSS1500	-3.515143	1.371386	-10.33472	1.03E-18	2.42E-15	31.878989
cg20440575	chr5	68462711	+	CCNB1	TSS1500	-1.533103	-5.111534	-10.31968	1.04E-18	2.44E-15	31.865522
cg05027258	chr2	9459293	-	ASAP2;ASAP2	Body;Body	1.6307149	1.9583449	10.316509	1.06E-18	2.47E-15	31.847802
cg14415844	chr14	91884659	+	CCDC88C	TSS1500	-1.07446	-3.021529	-10.31319	1.08E-18	2.50E-15	31.829219
cg06704466	chr2	237010257	-	AGAP1;AGAP1	Body;Body	2.3741086	4.3746512	10.324273	1.09E-18	2.51E-15	31.820048
cg13203973	chr8	675274	+	ERICH1	Body	1.6351936	4.0050438	10.30277	1.15E-18	2.63E-15	31.77093
cg22871253	chr6	159238744	-	EZR;EZR	Body;Body	-1.745585	-2.615917	-10.30062	1.17E-18	2.65E-15	31.758913
cg19718686	chr12	115141551	+			-1.594077	-3.169399	-10.29585	1.20E-18	2.71E-15	31.732192
cg11324957	chr15	53086857	-			2.5306299	-1.105359	10.298109	1.27E-18	2.83E-15	31.674156
cg13625187	chr12	27736287	+	PPFIBP1;PPFIBP1	5'UTR;5'UTR	1.5031279	1.8743183	10.324417	1.27E-18	2.83E-15	31.676895
cg14369455	chr4	113300768	+	ALPK1;ALPK1	Body;Body	1.3992792	2.008031	10.286275	1.27E-18	2.83E-15	31.678645
cg17523415	chr10	126765380	-	CTBP2;CTBP2	5'UTR;5'UTR	1.2469851	1.3419816	10.284338	1.28E-18	2.84E-15	31.66781
cg00356723	chr11	63705697	+	NAA40	TSS1500	-1.53838	-3.21808	-10.32574	1.36E-18	3.00E-15	31.610719
cg10843210	chr3	195613589	-	TNK2;TNK2	Body;Body	1.0790505	1.6493409	10.270668	1.39E-18	3.04E-15	31.591338
cg11302791	chr8	10635419	-	PINX1	Body	1.5452641	1.8699011	10.268776	1.40E-18	3.06E-15	31.580756

cg26080154	chr11	1314228	-	TOLLIP	Body	1.2452477	1.8571189	10.255874	1.51E-18	3.28E-15	31.508593
cg12986611	chr5	172274118	-	ERGIC1	Body	1.3248524	2.7435507	10.252869	1.54E-18	3.32E-15	31.491784
cg15103195	chr7	100797603	-	AP1S1	TSS200	-0.9369	-2.718413	-10.24581	1.60E-18	3.44E-15	31.45233
cg26575445	chr3	169756530	+	GPR160	5'UTR	-1.074487	-3.567743	-10.24094	1.65E-18	3.52E-15	31.42505
cg02075410	chr4	151504138	+	LRBA;MAB21L2;MAB21L2	Body;1stExon;5'UTR	2.571467	3.3255297	10.239815	1.66E-18	3.53E-15	31.418785
cg25384043	chr15	81295608	+	MESDC1	1stExon	1.4019569	2.0452346	10.23536	1.70E-18	3.60E-15	31.39387
cg13705888	chr6	32131722	-	EGFL8	TSS1500	1.4966206	2.4761886	10.230657	1.75E-18	3.68E-15	31.367571
cg07883124	chr13	113634042	-	MCF2L;MCF2L	Body;1stExon	1.7298256	2.1640852	10.228779	1.77E-18	3.70E-15	31.357072
cg23079522	chr3	160569628	-	PPM1L	Body	1.308931	2.2749931	10.227347	1.78E-18	3.72E-15	31.349067
cg18613031	chr10	45967496	+	MARCH8;MARCH8;MARCH8	Body;Body;Body	1.1233031	2.1851813	10.225071	1.80E-18	3.75E-15	31.336338
cg15711404	chr17	80803949	+	TBCD	Body	1.1504508	1.5376928	10.222146	1.84E-18	3.78E-15	31.319982
cg25974626	chr7	32977142	+	RP9P	Body	1.2851168	2.4892478	10.222096	1.84E-18	3.78E-15	31.319702
cg13699808	chr20	45985339	-	ZMYND8;ZMYND8;ZMYND8	Body;Body;Body	-1.696871	-2.223666	-10.22066	1.85E-18	3.79E-15	31.311689
cg17343374	chr4	901981	-	GAK	Body	1.1054681	1.0306998	10.212488	1.94E-18	3.95E-15	31.265988
cg01881046	chr4	186124759	+	KIAA1430	5'UTR	-1.375492	-3.806034	-10.20939	1.98E-18	3.99E-15	31.248691
cg12597389	chr7	8482235	-	NXPH1	Body	2.1435149	-0.211246	10.210161	1.97E-18	3.99E-15	31.252977
cg26351001	chr4	184644413	+			-1.358911	-2.132383	-10.20569	2.02E-18	4.05E-15	31.227988
cg25671716	chr12	96441714	-			-1.339672	-4.066294	-10.20095	2.08E-18	4.15E-15	31.201485
cg16622514	chr5	74772303	-	COL4A3BP;COL4A3BP;COL4A3BP	Body;Body;Body	1.2268883	2.87641	10.198392	2.11E-18	4.19E-15	31.187188
cg16577132	chr14	31408344	+	STRN3;STRN3	Body;Body	1.301412	2.7822416	10.195381	2.14E-18	4.24E-15	31.170352

cg16423756	chr11	122526190	-	UBASH3B	TSS1500	-1.042266	-3.562711	-10.18685	2.25E-18	4.44E-15	31.122696
cg00492309	chr16	2221491	-	TRAF7	Body	0.9930804	2.0184785	10.182107	2.31E-18	4.54E-15	31.096161
cg10677959	chr6	32151346	-	AGER;AGER	Body;Body	1.1092549	1.5416361	10.177124	2.38E-18	4.63E-15	31.068308
cg15333769	chr22	26983348	-	TPST2	5'UTR	0.922292	2.0936963	10.177347	2.38E-18	4.63E-15	31.069555
cg11446240	chr2	136633306	+	MCM6	Body	-1.548412	-4.16735	-10.18308	2.47E-18	4.78E-15	31.033768
cg06962177	chr1	63785946	-			3.2188748	0.0794885	10.167578	2.52E-18	4.85E-15	31.01496
cg16753167	chr13	113642487	-	MCF2L;MCF2L	Body;Body	2.3971738	3.8467686	10.158427	2.65E-18	5.09E-15	30.963827
cg20146489	chr1	169847977	-	SCYL3;SCYL3	Body;Body	1.5168125	2.6488651	10.162969	2.77E-18	5.30E-15	30.921097
cg04834502	chr16	48644360	+	N4BP1	TSS1500	-1.635023	-3.316614	-10.14309	2.90E-18	5.47E-15	30.878104
cg15138339	chr17	40715222	+	COASY;COASY;COASY;COASY;COASY	1stExon;Body;Body;Body;5'UTR	-2.480984	0.5519031	-10.14296	2.90E-18	5.47E-15	30.877432
cg18456803	chr13	41593519	+	ELF1	TSS200	-2.173519	-2.127504	-10.14339	2.89E-18	5.47E-15	30.879787
cg16732616	chr1	50886782	-	DMRTA2	Body	4.4507966	-0.654633	10.138978	2.97E-18	5.57E-15	30.855157
cg14156751	chr10	50976571	+			1.6373065	0.3582868	10.132652	3.08E-18	5.74E-15	30.819816
cg16881676	chr12	49659619	-	TUBA1C	Body	-1.638509	-2.939611	-10.13227	3.09E-18	5.74E-15	30.817699
cg22538338	chr13	42748498	-	DGKH;DGKH	Body;Body	1.4216425	2.1455587	10.124273	3.23E-18	5.99E-15	30.773014
cg09506661	chr19	1152022	+	SBNO2	Body	1.7553176	2.0785182	10.118972	3.33E-18	6.15E-15	30.743401
cg05239956	chr14	24036509	+	AP1G2	Body	-0.868438	-3.932431	-10.10295	3.65E-18	6.72E-15	30.653918
cg10170214	chr10	102765227	-	LZTS2	Body	1.8794477	3.1171937	10.096087	3.80E-18	6.93E-15	30.615593
cg10272731	chr16	85614015	-			1.5666107	2.2623588	10.096672	3.79E-18	6.93E-15	30.618856
cg23303108	chr8	23083578	-	TNFRSF10A	TSS1500	-2.769415	1.0868106	-10.09357	3.86E-18	7.00E-15	30.601553

cg19519747	chr2	177014555	+	MIR10B	TSS1500	1.7456592	0.1882853	10.089866	3.94E-18	7.12E-15	30.580854
cg21870038	chr17	33390736	-	RFFL;RFFL;RFFL	1stExon;5'UTR;5'UTR	-1.67045	-3.260852	-10.08226	4.12E-18	7.41E-15	30.538411
cg26908825	chr6	1635818	-	GMDS	Body	1.4721344	1.8385465	10.078469	4.21E-18	7.54E-15	30.517217
cg17102069	chr6	37139542	+	PIM1	Body	-2.330852	-4.920675	-10.07442	4.31E-18	7.69E-15	30.494608
cg13538637	chr7	7759221	+	RPA3	TSS1500	-2.098487	-1.727592	-10.07057	4.41E-18	7.83E-15	30.473091
cg09479241	chr17	27052676	+	TLCD1;TLCD1	Body;Body	-2.1347	-2.778719	-10.06776	4.48E-18	7.89E-15	30.457426
cg10707788	chr6	1635847	-	GMDS	Body	2.3183035	2.8525226	10.068407	4.46E-18	7.89E-15	30.461039
cg23605961	chr7	751331	-	PRKAR1B;PRKAR1B;PRKAR1B;PRKAR1B;PRKAR1B;PRKAR1B	5'UTR;5'UTR;5'UTR;5'UTR;5'UTR;5'UTR	2.0970322	0.2062917	10.066949	4.50E-18	7.90E-15	30.452904
cg06518233	chr10	102765350	-	LZTS2	Body	1.0825898	1.7207087	10.06472	4.56E-18	7.97E-15	30.440458
cg09142117	chr16	2079658	-	SLC9A3R2;SLC9A3R2	Body;Body	1.0450014	1.9783417	10.032195	5.50E-18	9.53E-15	30.258921
cg13654884	chr10	94594240	+	EXOC6	TSS1500	1.3677262	2.0996009	10.0325	5.49E-18	9.53E-15	30.260623
cg05036846	chr1	24648984	+	GRHL3;GRHL3;GRHL3	TSS1500;Body;TSS1500	-1.60327	-2.88971	-10.02774	5.64E-18	9.74E-15	30.23407
cg04214370	chr1	246203659	-	SMYD3;SMYD3	Body;Body	1.3102843	2.4600286	10.019745	5.91E-18	1.02E-14	30.189454
cg11872373	chr7	151833893	-	MLL3	3'UTR	1.1110725	1.779187	10.019235	5.92E-18	1.02E-14	30.186607
cg01405040	chr2	176964137	-	HOXD12	TSS1500	2.7224943	-0.139004	10.011736	6.19E-18	1.06E-14	30.144769
cg13795336	chr16	81711865	-	CMIP;CMIP	Body;Body	1.2070131	2.4930846	10.00943	6.27E-18	1.07E-14	30.131902
cg19172487	chr22	44394856	-	PARVB	TSS1500	1.5751744	2.1940068	10.008749	6.29E-18	1.07E-14	30.128106
cg15084543	chr1	79472408	-	ELTD1;ELTD1	5'UTR;1stExon	2.1688339	1.1410242	10.007692	6.33E-18	1.07E-14	30.122204
cg06671340	chr1	197744787	+	DENND1B;DENND1B	TSS200;TSS200	-1.505366	-3.505683	-10.0028	6.51E-18	1.09E-14	30.094889
cg01525669	chr13	107211833	-	ARGLU1	Body	1.9404912	4.4844428	10.012	6.61E-18	1.11E-14	30.080968

cg05049448	chr7	2321355	+	SNX8	Body	1.3020232	0.6052095	9.9945292	6.83E-18	1.14E-14	30.048779
cg24441911	chr12	7280988	+	RBP5	Body	0.9971616	2.1943462	9.9929434	6.89E-18	1.15E-14	30.039933
cg05373863	chr4	139163429	-	SLC7A11;SLC7A11	1stExon;5'UTR	-1.216682	-3.542819	-9.992173	6.93E-18	1.15E-14	30.035637
cg19643921	chr19	41197106	+	NUMBL	TSS1500	-1.768812	-3.444126	-9.990228	7.00E-18	1.15E-14	30.024787
cg04762412	chr20	44691505	-	NCOA5	Body	1.7618137	2.9513314	9.9889467	7.06E-18	1.16E-14	30.017641
cg07691684	chr4	16773677	+	LDB2;LDB2	Body;Body	-1.902686	-0.13643	-9.985027	7.22E-18	1.18E-14	29.995781
cg25750259	chr20	43104962	-	TTPAL;TTPAL	5'UTR;5'UTR	-1.49488	-3.288536	-9.984896	7.22E-18	1.18E-14	29.99505
cg19760241	chr17	35300010	+	LHX1	Body	2.4117415	0.3069464	9.9839525	7.26E-18	1.18E-14	29.989786
cg01106881	chr2	220227422	-			-1.918704	-3.626343	-9.97039	7.85E-18	1.27E-14	29.914149
cg08301307	chr14	103599204	-	TNFAIP2	Body	1.0503554	1.3436814	9.9697301	7.88E-18	1.27E-14	29.91047
cg23049737	chr1	8412697	+	RERE;RERE;RERE	3'UTR;3'UTR;3'UTR	2.2944114	2.9972619	9.9697916	7.88E-18	1.27E-14	29.910813
cg15946904	chr17	957248	-	ABR;ABR;ABR	Body;Body;Body	1.967232	3.8802355	9.9897386	8.04E-18	1.29E-14	29.892259
cg10853830	chr11	67266176	-	PITPNM1;PITPNM1	Body;Body	2.229259	3.7273814	9.9591513	8.38E-18	1.33E-14	29.851483
cg05032848	chr17	33390854	+	RFFL;RFFL	TSS200;5'UTR	-1.767874	-3.707148	-9.955855	8.54E-18	1.36E-14	29.833106
cg06215569	chr1	110611465	-	ALX3	Body	2.6552804	-0.801073	9.9533001	8.67E-18	1.37E-14	29.81886
cg15010544	chr3	195613641	+	TNK2;TNK2	Body;Body	0.9368672	1.7530906	9.9515568	8.75E-18	1.38E-14	29.809141
cg05543456	chr4	2256532	-	MXD4	Body	1.1324167	2.3478321	9.9452683	9.08E-18	1.42E-14	29.774083
cg16310077	chr14	105554214	+			-1.542723	-3.730873	-9.945434	9.07E-18	1.42E-14	29.775007
cg23235154	chr6	159290589	-			-1.15094	-3.728492	-9.935353	9.61E-18	1.50E-14	29.718814
cg04317651	chr19	3768001	-			1.1168545	2.1169172	9.9416607	9.90E-18	1.54E-14	29.690005

cg22455529	chr17	73495353	+	KIAA0195	Body	1.139698	2.1220229	9.9260202	1.01E-17	1.57E-14	29.666793
cg27450010	chr4	16639181	-	LDB2;LDB2	Body;Body	-2.027989	-1.363803	-9.921495	1.04E-17	1.61E-14	29.641571
cg03169557	chr16	89598950	+	SPG7;SPG7	Body;Body	2.7367446	3.5969519	9.9085616	1.12E-17	1.72E-14	29.569501
cg25466805	chr11	72463704	-	ARAP1	TSS1500	-0.952304	-2.787163	-9.907136	1.13E-17	1.73E-14	29.561554
cg02744699	chr17	80197898	-			2.7357233	5.1215359	9.9366288	1.17E-17	1.78E-14	29.532831
cg08443563	chr2	112456811	+			-2.216112	-2.296669	-9.894113	1.22E-17	1.85E-14	29.488997
cg15727424	chr6	32016100	-	TNXB	Body	-1.159903	-3.761416	-9.890437	1.24E-17	1.89E-14	29.468518
cg04917391	chr4	40859259	+	APBB2;APBB2;APBB2;APBB2;APBB2;APBB2;APBB2	Body;TSS200;TSS200;Body;TSS200;Body;TSS200	-1.661268	-1.251839	-9.885465	1.28E-17	1.93E-14	29.440821
cg25418484	chr11	71709762	+	IL18BP;IL18BP;IL18BP;IL18BP;IL18BP	TSS200;TSS1500;TSS200;TSS1500;TSS1500	1.2905914	2.377759	9.8806685	1.32E-17	1.98E-14	29.414104
cg09492252	chr21	43369331	-	C2CD2	Body	1.1408099	1.836123	9.8753235	1.36E-17	2.04E-14	29.384332
cg26553736	chr15	49491609	+	GALK2;GALK2	Body;Body	1.3116439	2.9127395	9.8685422	1.41E-17	2.11E-14	29.346563
cg13014333	chr6	31762776	+	VARS	Body	-1.827602	-3.202619	-9.86303	1.46E-17	2.17E-14	29.315866
cg20434178	chr2	172968684	-	DLX2	TSS1500	-1.345691	-3.790292	-9.856629	1.51E-17	2.24E-14	29.28022
cg14791193	chr1	16702476	-	C1orf144;C1orf144	Body;Body	1.4289281	1.9564858	9.8547092	1.53E-17	2.25E-14	29.269531
cg18196506	chr14	107281450	-			-1.625948	-1.154737	-9.85462	1.53E-17	2.25E-14	29.269036
cg25015277	chr16	2918180	-			-0.904845	-4.419702	-9.854253	1.53E-17	2.25E-14	29.266993
cg05252161	chr14	105956199	+	C14orf80;C14orf80;C14orf80	TSS1500;TSS1500;TSS1500	-2.015606	-5.822172	-9.895005	1.58E-17	2.32E-14	29.238605
cg25351606	chr6	100917427	+			1.9388848	1.0137207	9.8464519	1.60E-17	2.34E-14	29.223555
cg16483490	chr2	111879711	+	BCL2L11;BCL2L11;BCL2L11	5'UTR;5'UTR;5'UTR	-0.635198	-2.77445	-9.83335	1.73E-17	2.51E-14	29.150614
cg16581308	chr4	16862080	-	LDB2;LDB2	Body;Body	-1.967213	-0.245077	-9.833731	1.73E-17	2.51E-14	29.152737

cg11117099	chr17	43325657	+	LOC100133991	Body	-1.348878	-2.325957	-9.832479	1.74E-17	2.51E-14	29.145764
cg20827629	chr1	231500113	+	EGLN1	3'UTR	1.1633859	2.9729433	9.8426014	1.75E-17	2.52E-14	29.139965
cg15759721	chr17	57918630	+	MIR21	Body	-1.797259	-2.054019	-9.841861	1.76E-17	2.52E-14	29.135856
cg23595396	chr6	31164914	-	HCG27	TSS1500	-1.784597	-3.026236	-9.827754	1.79E-17	2.55E-14	29.119465
cg13151135	chr1	26560306	-	CCDC21	TSS1500	-1.161048	-3.283743	-9.824631	1.82E-17	2.59E-14	29.102081
cg02443967	chr10	98129902	-	TLL2	Body	-4.259304	2.1581699	-9.838284	1.91E-17	2.72E-14	29.054386
cg00983637	chr1	26663115	+	AIM1L	Body	-1.363935	-4.852625	-9.814452	1.93E-17	2.73E-14	29.045429
cg14410016	chr2	176986659	+	HOXD9	TSS1500	1.5339865	0.520098	9.8128968	1.94E-17	2.74E-14	29.036774
cg08786822	chr4	3109268	+	HTT	Body	1.1430549	2.3905998	9.8100724	1.98E-17	2.78E-14	29.021056
cg23281340	chr19	6210918	-	MLLT1	3'UTR	1.0241274	1.9176596	9.801408	2.08E-17	2.91E-14	28.972843
cg10997479	chr3	62362924	+			1.5309256	-0.564361	9.7964551	2.14E-17	2.99E-14	28.945285
cg03896661	chr2	197036775	+	STK17B	TSS1500	-0.905557	-4.283488	-9.795301	2.15E-17	3.00E-14	28.938865
cg08337166	chr12	108686134	+	CMKLR1;CMKLR1;CMKLR1;CMKLR1	Body;Body;Body;Body	-1.401373	-0.823804	-9.793184	2.18E-17	3.02E-14	28.927086
cg16887264	chr4	147561775	+	POU4F2	Body	3.276298	-1.211536	9.7909095	2.21E-17	3.05E-14	28.914431
cg18910243	chr10	45967498	+	MARCH8;MARCH8;MARCH8	Body;Body;Body	1.5439011	2.7505077	9.7783367	2.37E-17	3.27E-14	28.84449
cg17380661	chr6	100912946	-	SIM1	TSS1500	2.7835878	-0.356295	9.7765565	2.40E-17	3.30E-14	28.834588
cg20779964	chr20	54580070	-	CBLN4	TSS200	1.6708796	0.1882163	9.7704448	2.48E-17	3.40E-14	28.800595
cg16073378	chr2	468413	-			2.4659787	-0.558276	9.7776511	2.54E-17	3.47E-14	28.780241
cg14171343	chr9	114219932	+	KIAA0368	Body	1.1139281	2.5104631	9.7660133	2.55E-17	3.47E-14	28.775948
cg07004744	chr8	641479	-	ERICH1	Body	1.8435226	2.5262549	9.7649424	2.56E-17	3.48E-14	28.769993

cg20765267	chr8	104428676	+	DCAF13;SLC25A32	Body;TSS1500	-1.636566	-4.319468	-9.756422	2.69E-17	3.63E-14	28.722608
cg23488578	chr14	95585023	-	DICER1;DICER1	Body;Body	1.1583763	2.1631815	9.7567322	2.69E-17	3.63E-14	28.724336
cg00799748	chr16	1611052	+	IFT140	Body	1.7857752	3.0062056	9.7640627	2.74E-17	3.68E-14	28.704371
cg23342625	chr6	20877340	+	CDKAL1	Body	-2.265047	-0.107639	-9.753201	2.74E-17	3.68E-14	28.704702
cg04227922	chr2	119615496	-			2.0274354	-0.038908	9.7506374	2.78E-17	3.72E-14	28.690446
cg13432294	chr1	8937640	-	ENO1	5'UTR	-1.650204	-3.322949	-9.74924	2.80E-17	3.74E-14	28.682675
cg15888569	chr4	15033042	+	CPEB2;CPEB2	Body;Body	1.9100171	1.1244212	9.7467403	2.84E-17	3.78E-14	28.668778
cg24722073	chr4	41882163	+			2.4518121	-0.636164	9.7883422	2.90E-17	3.84E-14	28.628596
cg05958294	chr13	113834216	-	PCID2;PCID2;PCID2	Body;Body;Body	1.2509167	1.5378367	9.7425364	2.91E-17	3.85E-14	28.645405
cg08188368	chr2	233680285	-	GIGYF2;GIGYF2;GIGYF2;GIGYF2	Body;Body;Body;Body	1.2628558	2.5009159	9.740374	2.95E-17	3.88E-14	28.633384
cg12710531	chr11	88032774	+	CTSC	Body	1.5435371	2.7564198	9.7401566	2.95E-17	3.88E-14	28.632175
cg14488715	chr9	139407288	+	NOTCH1	Body	1.6130844	2.3789586	9.7349862	3.04E-17	3.98E-14	28.603432
cg14264571	chr9	35754691	-	MSMP	TSS1500	1.0695356	1.9052864	9.7139984	3.43E-17	4.48E-14	28.486781
cg10168149	chr2	200335558	+	FLJ32063	Body	2.9688366	-0.377258	9.7092452	3.53E-17	4.59E-14	28.460367
cg23974819	chr12	7055829	-	PTPN6;PTPN6	1stExon;5'UTR	-1.177248	-2.453806	-9.705194	3.61E-17	4.68E-14	28.437859
cg16636692	chr6	27115249	+	HIST1H2BK;HIST1H2AH	TSS1500;1stExon	-1.044606	-3.013579	-9.704638	3.62E-17	4.68E-14	28.434768
cg04925599	chr1	19923108	+	C1orf151	TSS1500	-0.701734	-4.104239	-9.703561	3.65E-17	4.69E-14	28.428781
cg05631399	chr11	66481581	+	SPTBN2	Body	1.1788267	1.5362845	9.7033084	3.65E-17	4.69E-14	28.42738
cg21355828	chr7	149389814	+			2.0022137	0.41284	9.7132701	3.67E-17	4.70E-14	28.422916
cg06083330	chr4	147561203	+	POU4F2	Body	2.822172	-0.413712	9.6954554	3.82E-17	4.85E-14	28.383749

cg16676676	chr1	228558987	+	OBSCN	Body	-2.724457	1.0607087	-9.696249	3.80E-17	4.85E-14	28.388158
cg18253799	chr17	78069191	+	CCDC40	Body	1.884072	3.9903628	9.6957576	3.81E-17	4.85E-14	28.385428
cg01439753	chr16	81726721	-	CMIP;CMIP	Body;Body	1.1147327	1.7418107	9.6889937	3.96E-17	5.02E-14	28.347851
cg08979319	chr6	100896055	-	SIM1	Body	1.4735612	0.2541891	9.6860871	4.03E-17	5.09E-14	28.331706
cg01354473	chr7	27204803	+	HOXA9	1stExon	1.4944145	-0.214038	9.6827684	4.11E-17	5.17E-14	28.313271
cg09040896	chr16	1560040	-	TELO2	3'UTR	1.1109845	1.5271283	9.6811945	4.15E-17	5.20E-14	28.304529
cg06460883	chr5	154137632	-	LARP1;LARP1	Body;Body	-0.951576	-3.69512	-9.678693	4.21E-17	5.26E-14	28.290633
cg10549973	chr14	58863097	-	TOMM20L	Body	-1.357372	-2.600075	-9.67796	4.22E-17	5.27E-14	28.286566
cg25021051	chr12	53448178	+	TENC1;TENC1;TENC1	Body;Body;Body	-1.567193	-2.177074	-9.676264	4.26E-17	5.31E-14	28.277142
cg10696787	chr17	65242740	-	HELZ	TSS1500	-1.343054	-2.413969	-9.674579	4.31E-17	5.33E-14	28.267788
cg13020466	chr17	76416705	+	PGS1	3'UTR	1.1498435	2.0631598	9.6851366	4.31E-17	5.33E-14	28.267625
cg26064895	chr4	48271121	-	TEC	5'UTR	-1.526992	-2.527033	-9.684002	4.34E-17	5.35E-14	28.233608
cg19294311	chr15	62358920	+	C2CD4A	TSS1500	-1.337835	-2.429281	-9.667346	4.49E-17	5.52E-14	28.227616
cg18110444	chr2	136743460	+	DARS	TSS1500	-2.533516	-3.76816	-9.670938	4.68E-17	5.74E-14	28.189011
cg04306817	chr16	30075547	+	ALDOA;ALDOA;ALDOA;ALDOA	TSS200;TSS1500;TSS1500;5'UTR	-1.439109	-4.715943	-9.656043	4.79E-17	5.86E-14	28.164852
cg03373115	chr1	64058883	+	PGM1	TSS200	-1.28634	-3.580541	-9.650168	4.95E-17	6.04E-14	28.132238
cg04305653	chr22	47272422	+	TBC1D22A	Body	1.0186979	1.7005343	9.6473175	5.03E-17	6.12E-14	28.116412
cg16509851	chr4	174430614	-			2.7776233	-0.5672	9.6397966	5.26E-17	6.38E-14	28.074662
cg10819733	chr22	24237672	-			-1.709268	-2.961702	-9.636321	5.36E-17	6.49E-14	28.055369
cg18598029	chr6	31164921	-	HCG27	TSS1500	-2.498117	-3.252543	-9.663645	5.52E-17	6.66E-14	28.028854

cg25588852	chr2	216877276	-	MREG	Body	-1.464168	-2.701294	-9.619646	5.90E-17	7.10E-14	27.96283
cg16391955	chr1	50881185	+			2.3899015	-0.135577	9.6189343	5.92E-17	7.11E-14	27.958879
cg04271792	chr6	28831393	+			-1.206411	-3.415718	-9.615626	6.04E-17	7.20E-14	27.940523
cg13316407	chr11	71709725	+	IL18BP;IL18BP;IL18BP;IL18BP;IL18BP	TSS1500;TSS1500;TSS1500;TSS1500;TSS1500	1.3702194	3.1961539	9.6159866	6.03E-17	7.20E-14	27.942523
cg01429449	chr2	176936397	-			1.6680625	-0.130933	9.6144322	6.08E-17	7.23E-14	27.933898
cg16108230	chr6	117584815	-			1.7609277	0.2266813	9.6099283	6.24E-17	7.38E-14	27.908909
cg19866866	chr1	182026044	-	ZNF648	Body	2.2575294	0.0948524	9.609921	6.24E-17	7.38E-14	27.908868
cg16489193	chr6	33240059	-	VPS52;RPS18	TSS1500;Body	-1.553764	-3.292493	-9.618743	6.30E-17	7.44E-14	27.899682
cg02700845	chr2	135003128	-			-1.310604	-3.192886	-9.60372	6.46E-17	7.61E-14	27.874464
cg01440489	chr13	41593385	-	ELF1;ELF1	5'UTR;1stExon	-1.839869	-1.819586	-9.602304	6.52E-17	7.65E-14	27.866613
cg16634108	chr13	25254440	+	ATP12A	TSS1500	-1.14026	-3.193049	-9.597892	6.68E-17	7.82E-14	27.842137
cg02596427	chr2	47295058	-	TTC7A	Body	1.4988124	1.8402295	9.5950232	6.80E-17	7.93E-14	27.826224
cg10583119	chr19	54237755	+	MIR518D	TSS1500	-1.639746	-1.934437	-9.59305	6.87E-17	8.00E-14	27.815281
cg21303763	chr12	3309708	-	TSPAN9;TSPAN9	5'UTR;5'UTR	-1.767979	-3.129987	-9.592371	6.90E-17	8.01E-14	27.811512
cg04244171	chr3	43331949	-	SNRK;SNRK	5'UTR;5'UTR	1.1090203	1.9474252	9.589819	7.00E-17	8.10E-14	27.797358
cg18115040	chr2	176981328	-	HOXD10	TSS200	2.0269421	0.1180663	9.5982616	7.08E-17	8.16E-14	27.78641
cg24888989	chr3	44803291	-	KIF15;KIF15;KIAA1143	1stExon;5'UTR;TSS200	-2.291735	-4.712122	-9.587823	7.08E-17	8.16E-14	27.78629
cg02632314	chr13	41593416	+	ELF1;ELF1	5'UTR;1stExon	-2.002223	-1.979288	-9.581051	7.36E-17	8.44E-14	27.748733
cg22531371	chr1	155162756	+	MUC1;MUC1;MUC1;MUC1;MUC1;MUC1;MUC1	TSS200;TSS200;TSS200;TSS200;TSS200;TSS200;TSS200	-1.382972	-2.157731	-9.580847	7.37E-17	8.44E-14	27.747599
cg24762359	chr13	25254447	+	ATP12A	TSS1500	-1.173737	-2.726474	-9.579653	7.42E-17	8.48E-14	27.740979

cg12433486	chr15	72520804	+	PKM2;PKM2;PKM2	5'UTR;5'UTR;5'UTR	-1.860489	-2.898965	-9.578481	7.47E-17	8.51E-14	27.734481
cg21718627	chr11	87035156	+	TMEM135;TMEM135;TMEM135	3'UTR;Body;3'UTR	1.3397317	2.7994915	9.5757435	7.59E-17	8.62E-14	27.719301
cg22186315	chr17	79259963	+	SLC38A10;SLC38A10	Body;Body	1.2571563	2.2390238	9.5726658	7.72E-17	8.75E-14	27.702235
cg11563680	chr6	100917397	+			1.8129897	0.5216169	9.5714459	7.78E-17	8.79E-14	27.695471
cg14205001	chr9	139406515	-	NOTCH1	Body	1.0463096	1.501459	9.5700548	7.84E-17	8.84E-14	27.687758
cg19897172	chr7	135347071	-	PL-5283	TSS200	-1.069338	-3.490846	-9.565101	8.07E-17	9.07E-14	27.660294
cg25799994	chr2	166650699	+	GALNT3;GALNT3	5'UTR;1stExon	-0.696781	-3.697994	-9.56326	8.15E-17	9.14E-14	27.650084
cg22618571	chr12	42719574	-	PPHLN1;PPHLN1;PPHLN1;PPHLN1;ZCRB1;PPHLN1;PPHLN1;PPHLN1;PPHLN1	TSS1500;TSS1500;TSS1500;TSS1500;5'UTR;TSS1500;TSS1500;TSS1500;TSS1500	-1.075313	-3.63134	-9.560042	8.30E-17	9.29E-14	27.632249
cg10020892	chr1	147016901	-	BCL9	5'UTR	-2.403986	-2.457795	-9.55944	8.33E-17	9.30E-14	27.628912
cg08461949	chr19	52104632	+			2.0304489	-0.030891	9.5652593	8.56E-17	9.52E-14	27.604456
cg00866814	chr19	49017364	+	LMTK3	TSS1500	-0.919058	-2.968713	-9.550877	8.75E-17	9.71E-14	27.581445
cg02350627	chr7	94138909	-	CASD1	TSS1500	-0.907987	-3.499271	-9.547799	8.91E-17	9.86E-14	27.564383
cg13562122	chr1	21587009	-	ECE1;ECE1;ECE1;ECE1	Body;Body;Body;Body	1.4869378	1.8371828	9.5468214	8.96E-17	9.89E-14	27.558967
cg13283952	chr2	121412005	+			2.5804858	1.4690832	9.5437982	9.11E-17	1.00E-13	27.542212
cg03503758	chr19	2614104	+	GNG7	5'UTR	-1.815078	-3.371156	-9.543318	9.14E-17	1.00E-13	27.53955
cg02299586	chr1	26633131	+	UBXN11;UBXN11;UBXN11;UBXN11;UBXN11	5'UTR;1stExon;5'UTR;1stExon;5'UTR	-0.839151	-4.290454	-9.53776	9.43E-17	1.03E-13	27.508752
cg13301014	chr19	38754768	-	SPINT2;SPINT2	TSS1500;TSS1500	-1.121525	-2.87522	-9.537365	9.46E-17	1.03E-13	27.506558
cg07394299	chr5	36745618	+			-1.482546	-3.625556	-9.5337	9.66E-17	1.05E-13	27.486252
cg13505279	chr15	55611881	-	PIGB	Body	-1.374805	-2.140297	-9.543414	9.69E-17	1.05E-13	27.483269
cg17276002	chr10	75172806	-	ANXA7;ANXA7	5'UTR;5'UTR	-2.531232	-3.370623	-9.533451	9.67E-17	1.05E-13	27.484871

cg18592384	chr2	9459318	-	ASAP2;ASAP2	Body;Body	1.372987	1.5026164	9.5324257	9.73E-17	1.05E-13	27.479191
cg06884882	chr1	45187946	-	C1orf228	Body	-1.170537	-1.783181	-9.536864	1.01E-16	1.09E-13	27.447087
cg19742736	chr20	439960	+	TBC1D20	Body	1.4000082	2.4115044	9.5311437	1.04E-16	1.12E-13	27.415961
cg03517024	chr16	2221057	-	TRAF7	Body	2.0624429	2.8486542	9.5405393	1.05E-16	1.12E-13	27.409993
cg13376158	chr17	73128023	-	NT5C	TSS200	-0.772225	-3.513815	-9.515832	1.07E-16	1.15E-13	27.387258
cg01302240	chr9	116263669	-	RGS3;RGS3;RGS3	TSS200;Body;Body	1.4324923	1.6935587	9.5127451	1.09E-16	1.16E-13	27.370162
cg13412545	chr5	172175765	+			1.3292857	-0.031435	9.5043621	1.14E-16	1.22E-13	27.323732
cg02909991	chr17	76127829	-	TMC8;TMC6	Body;5'UTR	-1.305868	-2.531091	-9.503416	1.15E-16	1.22E-13	27.31849
cg24898753	chr11	61735917	+	FTH1	TSS1500	-1.277702	-2.535442	-9.50341	1.15E-16	1.22E-13	27.318456
cg19847601	chr15	99670282	+	SYNM;SYNM	Body;Body	1.1388075	1.7531151	9.4969372	1.19E-16	1.26E-13	27.282614
cg08686960	chr19	36642720	-	COX7A1	Body	1.2848874	1.1336403	9.4957817	1.20E-16	1.27E-13	27.276215
cg18267049	chr10	101875028	+			2.9240416	-0.342156	9.4938725	1.21E-16	1.28E-13	27.265644
cg08766099	chr4	734746	+	PCGF3	Body	1.2196293	1.7802092	9.4934042	1.22E-16	1.28E-13	27.263051
cg06158650	chr1	119522637	+	TBX15	5'UTR	1.9571907	-0.6342	9.4914002	1.23E-16	1.28E-13	27.251955
cg11755933	chr11	67266128	-	PITPNM1;PITPNM1	Body;Body	2.2802259	3.2590975	9.4918638	1.23E-16	1.28E-13	27.254521
cg23804620	chr4	6690894	+			-2.438158	-3.916146	-9.491378	1.23E-16	1.28E-13	27.251834
cg06424065	chr4	6247640	-			2.1256561	0.3546445	9.4883613	1.25E-16	1.30E-13	27.235129
cg15938260	chr17	80804078	+	TBCD	Body	2.1439731	2.6820299	9.4838965	1.28E-16	1.33E-13	27.21041
cg21876145	chr2	20243393	-	LAPTM4A	Body	1.2664418	2.5265202	9.483752	1.29E-16	1.33E-13	27.20961
cg03013422	chr12	125550350	-	AACS	Body	-1.144128	-4.007341	-9.48159	1.30E-16	1.34E-13	27.197639

cg10755235	chr14	60974039	+			2.5304662	-0.798501	9.5007906	1.31E-16	1.35E-13	27.191668
cg02994246	chr1	21587174	-	ECE1;ECE1;ECE1;ECE1	Body;Body;Body;Body	1.5918288	2.2709888	9.4842948	1.36E-16	1.40E-13	27.156828
cg11145643	chr14	100839818	-	WARS;WARS;WARS;WARS	5'UTR;5'UTR;5'UTR;5'UTR	1.2569991	2.6391026	9.4693238	1.40E-16	1.43E-13	27.129744
cg15219320	chr5	139090356	-			-1.082657	-4.045795	-9.465383	1.43E-16	1.46E-13	27.107934
cg11014794	chr10	93999688	+	CPEB3	Body	-1.491767	-1.958315	-9.473655	1.44E-16	1.47E-13	27.098574
cg12279138	chr1	224370322	+	DEGS1;DEGS1	TSS1500;TSS1500	-1.815723	-2.272687	-9.461835	1.46E-16	1.48E-13	27.088298
cg09317554	chr4	151505084	+	LRBA;MAB21L2	Body;1stExon	1.6136153	1.5432199	9.4612135	1.46E-16	1.49E-13	27.084859
cg10773016	chr4	6690812	-			-1.963842	-3.086004	-9.457211	1.50E-16	1.52E-13	27.062712
cg01942790	chr4	1211419	-	CTBP1;CTBP1	Body;Body	2.0856742	3.5013099	9.4552309	1.51E-16	1.53E-13	27.051754
cg07264904	chr13	113633855	+	MCF2L;MCF2L;MCF2L	Body;5'UTR;1stExon	1.5552653	2.5983968	9.4526257	1.54E-16	1.55E-13	27.037339
cg00325866	chr15	62359130	+	C2CD4A	TSS200	-0.988041	-4.307276	-9.449362	1.56E-16	1.57E-13	27.019279
cg07339393	chr10	3789537	-			2.4169194	3.3894101	9.4389476	1.66E-16	1.66E-13	26.961667
cg08438705	chr19	1152189	+	SBNO2	Body	1.808651	2.8216468	9.438947	1.66E-16	1.66E-13	26.961663
cg22305258	chr17	4643367	-	CXCL16;ZMYND15;ZMYND15;CXCL16	TSS200;5'UTR;5'UTR;TSS200	-2.342311	-3.328664	-9.439562	1.65E-16	1.66E-13	26.965065
cg10788213	chr22	45719176	-	FAM118A;FAM118A	Body;Body	1.5576771	4.0106783	9.4345196	1.70E-16	1.70E-13	26.937173
cg24586978	chr13	99612723	-	DOCK9;DOCK9;DOCK9;DOCK9	Body;Body;Body;Body	1.5320771	1.1833954	9.433173	1.72E-16	1.71E-13	26.929726
cg20614671	chr7	2321431	-	SNX8	Body	1.0861442	0.5002909	9.4310779	1.74E-16	1.72E-13	26.918138
cg02694427	chr2	176964512	-	HOXD12	TSS200	2.1674983	-0.016534	9.427179	1.78E-16	1.76E-13	26.896574
cg07181702	chr17	57918682	-	MIR21	Body	-1.820525	-2.015487	-9.423057	1.82E-16	1.80E-13	26.87378
cg23759826	chr7	35735287	+	HERPUD2	TSS1500	-1.33029	-3.828955	-9.406677	2.00E-16	1.97E-13	26.783211

cg25151688	chr15	66585534	+	DIS3L;DIS3L	TSS200;TSS1500	-1.16063	-3.401077	-9.404545	2.02E-16	1.99E-13	26.771427
cg00754027	chr1	200590459	-	KIF14	TSS1500	-1.255167	-3.518171	-9.402432	2.05E-16	2.01E-13	26.759743
cg05068777	chr7	127426100	+	SND1	Body	1.0441211	2.8735167	9.3980391	2.10E-16	2.05E-13	26.735462
cg00454770	chr10	116286974	+	ABLIM1;ABLIM1;ABLIM1;ABLIM1	Body;Body;TSS1500;Body	-1.568517	-2.203245	-9.406006	2.12E-16	2.07E-13	26.725502
cg13944175	chr19	52222698	-	HAS1	Body	1.9662852	-0.184565	9.3939875	2.15E-16	2.09E-13	26.713069
cg05685297	chr8	96070313	+	C8orf38	3'UTR	0.9611901	1.4265833	9.3908073	2.19E-16	2.12E-13	26.695492
cg14594876	chr20	32031951	-	SNTA1	TSS1500	-0.910889	-3.79943	-9.390692	2.19E-16	2.12E-13	26.694855
cg13771753	chr6	32016070	-	TNXB	Body	-1.194853	-3.686995	-9.39024	2.19E-16	2.12E-13	26.692355
cg18483459	chr2	203294144	-	BMPR2	Body	1.6233081	1.0806011	9.3887615	2.21E-16	2.14E-13	26.684186
cg25788793	chr4	10034580	+	SLC2A9	5'UTR	-1.602227	-1.903514	-9.388509	2.22E-16	2.14E-13	26.682791
cg26319363	chr4	753903	-	PCGF3	Body	1.4426607	1.91495	9.3873128	2.23E-16	2.14E-13	26.67618
cg27374758	chr3	121675259	+			1.2962023	1.6019063	9.3845868	2.27E-16	2.17E-13	26.661116
cg22508782	chr3	49028594	-	P4HTM;P4HTM	Body;Body	-1.313518	-3.306589	-9.383327	2.28E-16	2.18E-13	26.654156
cg15633390	chr4	99851211	-	EIF4E;EIF4E;EIF4E;EIF4E;EIF4E	5'UTR;1stExon;5'UTR;TSS1500;1stExon	-1.956987	-2.381708	-9.382623	2.29E-16	2.19E-13	26.650264
cg12303084	chr20	45985741	+	ZMYND8;ZMYND8;ZMYND8	TSS1500;TSS1500;TSS1500	-1.166641	-3.035593	-9.381419	2.31E-16	2.20E-13	26.643609
cg09621958	chr3	112395854	+			-1.877472	-3.524522	-9.378973	2.34E-16	2.22E-13	26.630093
cg04276626	chr17	57918500	+	MIR21	TSS200	-2.135198	-2.054867	-9.37792	2.35E-16	2.23E-13	26.624278
cg04871778	chr1	55267125	-	TTC22;TTC22	TSS200;TSS200	-1.190499	-2.832768	-9.37472	2.40E-16	2.27E-13	26.606599
cg09622121	chr6	31164916	-	HCG27	TSS1500	-2.717987	-3.739097	-9.383144	2.42E-16	2.28E-13	26.599092
cg02216727	chr17	38520653	-	GJD3;GJD3	1stExon;5'UTR	1.3476776	1.4086631	9.3812652	2.44E-16	2.30E-13	26.588745

cg06365535	chr17	59534102	+	TBX4	Body	1.7242172	0.5494589	9.3709728	2.45E-16	2.30E-13	26.585896
cg19993316	chr15	69452462	+	MIR548H4;GLCE	Body;TSS1500	-1.570859	-3.680654	-9.370558	2.45E-16	2.30E-13	26.583601
cg02379560	chr4	40859280	+	APBB2;APBB2;APBB2;APBB2;APBB2;APBB2;APBB2	Body;TSS200;TSS200;Body;TSS200;Body;TSS200	-2.274253	-2.383189	-9.370157	2.46E-16	2.30E-13	26.581389
cg18471993	chr6	100917416	+			1.7402503	0.3056784	9.3692562	2.47E-16	2.31E-13	26.576412
cg10519271	chr1	28286837	+	XKR8	1stExon	-0.717392	-3.255343	-9.367828	2.49E-16	2.32E-13	26.568521
cg15958034	chr13	97889396	+	MBNL2;MBNL2	5'UTR;5'UTR	1.1360455	1.1414082	9.3770749	2.50E-16	2.33E-13	26.565663
cg17858500	chr20	3734725	+	C20orf27	Body	2.3419119	3.1964945	9.3662687	2.52E-16	2.34E-13	26.559909
cg25426203	chr10	88600793	+	BMPR1A	5'UTR	1.3610323	1.8207081	9.3553589	2.68E-16	2.48E-13	26.499649
cg18410627	chr9	129677882	-	RALGPS1	5'UTR	-0.979555	-3.746553	-9.353978	2.70E-16	2.49E-13	26.492024
cg25406779	chr19	40732712	+	CNTD2	TSS200	-1.388449	-2.592779	-9.353634	2.70E-16	2.49E-13	26.490121
cg17094249	chr1	24867657	+			-2.075706	-2.339359	-9.350836	2.75E-16	2.53E-13	26.474674
cg25718696	chr4	151503588	-	LRBA;MAB21L2;MAB21L2	Body;1stExon;5'UTR	1.1461256	2.5864467	9.3485461	2.78E-16	2.56E-13	26.462026
cg10011746	chr16	89865162	-	FANCA;FANCA	3'UTR;Body	1.0081456	2.1997262	9.346455	2.82E-16	2.58E-13	26.450479
cg03738025	chr6	105388694	+			2.2971615	0.8708336	9.3309855	3.08E-16	2.81E-13	26.365072
cg05393086	chr16	5092012	-			-1.788913	-0.807411	-9.328781	3.12E-16	2.84E-13	26.352903
cg22270027	chr4	81128234	-			1.4951406	1.1226072	9.3287497	3.12E-16	2.84E-13	26.35273
cg22399133	chr2	208989248	-	CRYGD;CRYGD	1stExon;5'UTR	3.8265649	0.1187113	9.3339132	3.20E-16	2.91E-13	26.328444
cg14231297	chr19	58629901	+	ZSCAN18;ZSCAN18	TSS200;TSS200	2.0130438	0.1365483	9.3189356	3.29E-16	2.98E-13	26.298563
cg14822966	chr4	1714979	+	SLBP	TSS1500	-1.165003	-3.605028	-9.319013	3.29E-16	2.98E-13	26.298992
cg07507218	chr6	32085535	-	ATF6B;ATF6B	Body;Body	0.9175846	1.7363914	9.3133116	3.40E-16	3.07E-13	26.267527

cg09882144	chr12	125339904	+	SCARB1;SCARB1	Body;Body	1.2075546	2.0722443	9.3113291	3.44E-16	3.10E-13	26.256587
cg09887059	chr12	113916664	+			2.3698087	0.3827784	9.308802	3.49E-16	3.14E-13	26.242643
cg04312967	chr16	1597521	-	IFT140;TMEM204	Body;Body	1.5842745	3.7173478	9.3585008	3.50E-16	3.14E-13	26.24419
cg25681774	chr17	957287	+	ABR;ABR;ABR	Body;Body;Body	0.9821698	1.921561	9.3051467	3.56E-16	3.19E-13	26.222475
cg16221879	chr18	11982241	+	IMPA2	Body	-0.964429	-3.899328	-9.302364	3.62E-16	3.24E-13	26.207124
cg12595013	chr3	147128123	+	ZIC1	1stExon	1.9024297	-0.285981	9.2998893	3.67E-16	3.28E-13	26.19347
cg18786873	chr1	110610899	-	ALX3	Body	2.9405962	-0.679071	9.2912768	3.86E-16	3.43E-13	26.145962
cg10981894	chr6	32085379	-	ATF6B;ATF6B	Body;Body	0.9396516	1.6913666	9.2858266	3.98E-16	3.52E-13	26.115902
cg19433091	chr15	43426498	-	TMEM62	Body	-1.292057	-2.271019	-9.286043	3.97E-16	3.52E-13	26.117094
cg25076980	chr6	31638016	+	LY6G5B	TSS1500	1.1454719	1.403866	9.2862997	3.97E-16	3.52E-13	26.118511
cg04830146	chr17	31203787	+	MYO1D	Body	-0.809971	-4.361737	-9.274681	4.24E-16	3.74E-13	26.054439
cg25227672	chr11	124805912	-	HEPACAM;HEPACAM	5'UTR;1stExon	-1.570823	-0.606146	-9.25844	4.65E-16	4.10E-13	25.964904
cg09575756	chr5	138842776	-	ECSCR	TSS1500	1.124059	1.4639665	9.253315	4.79E-16	4.21E-13	25.936658
cg01082057	chr7	750720	+	PRKAR1B;PRKAR1B;PRKAR1B;PRKAR1B;PRKAR1B;PRKAR1B	Body;Body;Body;Body;Body;Body	1.1331957	2.3896282	9.2504285	4.87E-16	4.27E-13	25.92075
cg24186398	chr11	1321530	-	TOLLIP	Body	1.1257069	2.0804017	9.2484068	4.93E-16	4.31E-13	25.909608
cg03030317	chr6	31164924	-	HCG27	TSS1500	-2.309568	-3.224557	-9.247975	4.94E-16	4.32E-13	25.90723
cg16956686	chr7	4304779	+	SDK1;SDK1	Body;Body	1.9259173	0.9865597	9.2423761	5.10E-16	4.44E-13	25.876376
cg27069285	chr12	110427408	+	GIT2;GIT2;GIT2;GIT2;GIT2;GIT2	Body;Body;Body;Body;Body;Body	1.1360279	2.2106457	9.2425297	5.09E-16	4.44E-13	25.877223
cg10216414	chr2	101868516	+	C2orf29	TSS1500	-1.404016	-2.094891	-9.239009	5.20E-16	4.51E-13	25.857825
cg12513140	chr3	150160121	-	TSC22D2	Body	1.3223833	2.3754117	9.2365808	5.27E-16	4.57E-13	25.844446

cg20575277	chr15	90728569	+	SEMA4B	5'UTR	-1.660569	-2.891573	-9.234311	5.34E-16	4.62E-13	25.831941
cg01943585	chr1	112981693	-	CTTNBP2NL	Body	1.331976	1.7661188	9.2292482	5.49E-16	4.74E-13	25.804051
cg09820244	chr1	54790722	+	SSBP3;SSBP3;SSBP3	Body;Body;Body	1.4410244	1.0172496	9.2287481	5.51E-16	4.75E-13	25.801296
cg05368724	chr2	97561669	-	FAM178B;FAM178B	5'UTR;Body	-1.950181	-0.308756	-9.223226	5.68E-16	4.89E-13	25.770876
cg15834072	chr4	155411603	+	DCHS2;DCHS2	1stExon;1stExon	1.7787333	-0.110937	9.2125155	6.04E-16	5.19E-13	25.711894
cg07954423	chr9	130741881	+	FAM102A	Body	-0.952537	-3.208643	-9.208419	6.18E-16	5.30E-13	25.68934
cg17138769	chr10	118892211	+	VAX1	Body	2.8891946	-0.590712	9.2033306	6.37E-16	5.44E-13	25.661322
cg07860213	chr8	70982039	-	PRDM14	Body	2.7682165	-0.080429	9.1965985	6.61E-16	5.63E-13	25.624262
cg09999582	chr2	103352711	-	MFSD9	Body	-1.314964	-2.661507	-9.196806	6.61E-16	5.63E-13	25.625402
cg22500132	chr1	155162752	+	MUC1;MUC1;MUC1;MUC1;MUC1;MUC1;MUC1	TSS200;TSS200;TSS200;TSS200;TSS200;TSS200;TSS200	-1.946709	-1.777273	-9.196428	6.62E-16	5.63E-13	25.623324
cg14084456	chr1	204486034	+	MDM4;MDM4	Body;5'UTR	-0.72542	-3.48082	-9.194885	6.68E-16	5.67E-13	25.614828
cg21684012	chr6	100912906	+	SIM1	TSS1500	2.397128	-0.700577	9.191731	6.80E-16	5.76E-13	25.597469
cg03407567	chr12	56545570	+	MYL6B	TSS1500	-1.510461	-2.431447	-9.19017	6.86E-16	5.79E-13	25.588876
cg08549335	chr7	30387954	-	ZNRF2	Body	-2.275478	-2.627636	-9.198947	6.88E-16	5.79E-13	25.586626
cg24988684	chr17	76128556	+	TMC8;TMC6	Body;TSS200	-1.59409	-2.138804	-9.199096	6.88E-16	5.79E-13	25.587442
cg23285459	chr7	2802560	+	GNA12	Body	1.6508657	1.2772671	9.1848163	7.07E-16	5.94E-13	25.559413
cg14737484	chr6	32054561	+	TNXB	Body	-2.205163	-2.126613	-9.182043	7.19E-16	6.02E-13	25.544152
cg10101680	chr14	93770551	-	BTBD7;BTBD7	5'UTR;5'UTR	1.1999404	2.6348857	9.1733719	7.55E-16	6.32E-13	25.49644
cg04751009	chr14	76123777	+	C14orf1	Body	1.0732147	2.6368382	9.1691655	7.73E-16	6.46E-13	25.473297
cg18135132	chr11	10814092	-			-1.422915	-3.333003	-9.166889	7.83E-16	6.53E-13	25.460773

cg27009703	chr7	27204894	+	HOXA9	1stExon	1.5644792	0.3263435	9.1627526	8.02E-16	6.67E-13	25.438021
cg12551635	chr1	27994779	+	IFI6;IFI6;IFI6	Body;Body;Body	1.6526161	4.5328064	9.1690908	8.15E-16	6.77E-13	25.422428
cg03331300	chr2	169102866	+	STK39	Body	-0.907513	-3.340284	-9.153277	8.46E-16	7.01E-13	25.385907
cg07155724	chr14	105165730	+	INF2;INF2;INF2	5'UTR;5'UTR;5'UTR	1.0402222	0.4646043	9.1469867	8.77E-16	7.25E-13	25.351315
cg05400252	chr12	57394816	+	ZBTB39	3'UTR	1.1785046	2.5441003	9.1550826	8.83E-16	7.29E-13	25.345608
cg11671688	chr6	110301075	+	GPR6	1stExon	1.6346001	0.4677312	9.1410877	9.07E-16	7.47E-13	25.318881
cg13023623	chr14	57276257	-	OTX2	5'UTR	2.5004475	0.7479833	9.1404194	9.10E-16	7.47E-13	25.315206
cg25622366	chr2	63281139	+	OTX1	Body	3.5370935	-0.482579	9.140702	9.09E-16	7.47E-13	25.31676
cg23746497	chr6	105388668	+			1.7551643	1.0906626	9.1391033	9.17E-16	7.51E-13	25.307971
cg02319613	chr2	216176467	+	ATIC	TSS1500	-0.895179	-4.368126	-9.137803	9.24E-16	7.56E-13	25.300825
cg24933605	chr2	216177085	-	ATIC	Body	-1.649298	-3.309402	-9.13715	9.27E-16	7.57E-13	25.297233
cg06766541	chr7	128556540	-			1.0814737	0.4327423	9.133695	9.46E-16	7.71E-13	25.27824
cg12611860	chr11	17191617	-	PIK3C2A	TSS1500	1.1862996	2.3012482	9.1324907	9.52E-16	7.74E-13	25.27162
cg13314310	chr2	20190065	-	WDR35;WDR35	TSS200;TSS200	-0.932972	-3.270612	-9.137115	9.77E-16	7.93E-13	25.247478
cg22620221	chr7	153584416	+	DPP6	TSS200	2.1172995	-0.071921	9.1257502	9.89E-16	8.02E-13	25.234572
cg20942867	chr1	27426144	+	SLC9A1	3'UTR	-1.75272	-2.736079	-9.12532	9.92E-16	8.02E-13	25.232209
cg11724984	chr12	121890864	-	KDM2B;KDM2B	Body;Body	1.8474686	3.2548127	9.1199726	1.02E-15	8.25E-13	25.202821
cg00702638	chr3	44803293	-	KIF15;KIF15;KIAA1143	1stExon;5'UTR;TSS200	-2.384861	-4.619439	-9.119532	1.02E-15	8.26E-13	25.200402
cg11856711	chr12	54785309	-	ZNF385A;ZNF385A	TSS1500;TSS1500	-1.761259	-3.206264	-9.147093	1.03E-15	8.27E-13	25.200696
cg02597246	chr16	81711959	+	CMIP;CMIP	Body;Body	1.4916723	1.8749037	9.1157218	1.05E-15	8.41E-13	25.179463

cg01645729	chr2	131582240	-			-1.198597	-3.935473	-9.110046	1.08E-15	8.67E-13	25.148278
cg23060384	chr3	194206773	+			-0.817841	-4.235599	-9.106361	1.10E-15	8.83E-13	25.128032
cg16326402	chr2	158698856	-	ACVR1;ACVR1	5'UTR;5'UTR	1.4325274	1.0683659	9.1039779	1.12E-15	8.94E-13	25.114943
cg01931840	chr4	129192374	+	PGRMC2	3'UTR	0.9852867	2.6720011	9.1028075	1.13E-15	8.98E-13	25.108513
cg18866529	chr15	62359103	+	C2CD4A	TSS200	-1.011824	-4.274391	-9.096391	1.17E-15	9.29E-13	25.073272
cg02196651	chr4	54457842	+	LNX1	TSS200	-1.727217	-2.567621	-9.095403	1.18E-15	9.33E-13	25.067845
cg18901278	chr17	76128531	+	TMC8;TMC6	Body;TSS200	-1.249568	-2.197547	-9.092869	1.19E-15	9.45E-13	25.053928
cg04416734	chr16	30075192	-	ALDOA;ALDOA;ALDOA	TSS1500;TSS1500;5'UTR	-1.661372	-1.868094	-9.090799	1.21E-15	9.54E-13	25.042559
cg24110966	chr6	2968851	-	SERPINB6	5'UTR	1.2228221	2.2495984	9.0884453	1.22E-15	9.65E-13	25.029634
cg27663938	chr12	103696381	+	C12orf42;C12orf42	Body;Body	3.0654748	0.1338073	9.0969204	1.29E-15	1.02E-12	24.957385
cg09915444	chr15	62359275	+	C2CD4A;C2CD4A	1stExon;5'UTR	-0.853245	-3.647395	-9.076828	1.31E-15	1.03E-12	24.96585
cg25588480	chr17	4763240	-	MINK1;MINK1;MINK1;MINK1	Body;Body;Body;Body	2.2699915	3.8314939	9.0729945	1.33E-15	1.05E-12	24.944806
cg20430656	chr13	113642457	+	MCF2L;MCF2L	Body;Body	1.8453366	2.9563366	9.0721446	1.34E-15	1.05E-12	24.940141
cg23735712	chr4	40858984	+	APBB2;APBB2;APBB2;APBB2;APBB2;APBB2;APBB2	Body;5'UTR;5'UTR;5'UTR;Body;5'UTR;Body	-2.369297	-3.212765	-9.070318	1.36E-15	1.06E-12	24.930112
cg15958839	chr2	54843164	+	SPTBN1;SPTBN1	Body;Body	1.3753044	1.8227126	9.0661073	1.39E-15	1.08E-12	24.907004
cg08837858	chr19	42720994	+	DEDD2	Body	-0.612899	-3.825967	-9.064601	1.40E-15	1.09E-12	24.898737
cg05545808	chr16	81712102	+	CMIP;CMIP	Body;Body	1.2275774	1.6590012	9.0634752	1.41E-15	1.10E-12	24.892558
cg01182690	chr12	42719544	+	PPHLN1;PPHLN1;PPHLN1;PPHLN1;ZCRB1;PPHLN1;PPHLN1;PPHLN1;PPHLN1	TSS1500;TSS1500;TSS1500;TSS1500;5'UTR;TSS1500;TSS1500;TSS1500;TSS1500	-1.493987	-3.668396	-9.062261	1.42E-15	1.10E-12	24.885895
cg24663845	chr3	125820497	+	SLC41A3	TSS200	1.0049817	1.058399	9.0606932	1.43E-15	1.11E-12	24.877291
cg09660894	chr5	14358043	-	TRIO	Body	1.3957217	1.8595978	9.0580446	1.45E-15	1.12E-12	24.862757

cg02827029	chr12	121890907	+	KDM2B;KDM2B	Body;Body	1.3397583	5.3791394	9.0684314	1.52E-15	1.17E-12	24.821953
cg09765089	chr7	27291346	+			2.7971454	-0.11111	9.0504701	1.52E-15	1.17E-12	24.821198
cg08342886	chr6	33240066	-	VPS52;RPS18	TSS1500;Body	-1.257776	-3.526911	-9.067125	1.53E-15	1.18E-12	24.814457
cg04892766	chr3	72570318	-			-1.501108	-0.71216	-9.056143	1.55E-15	1.19E-12	24.804062
cg09639151	chr5	140810920	-	PCDHGA4;PCDHGA11;PCDHGA11;PCDHGA9;PCDHGA1;PCDHGB1;PCDHGB6;PCDHGA12;PCDHGB3;PCDHGB7;PCDHGA6;PCDHGA8;PCDHGA10;PCDHGA12;PCDHGA5;PCDHGB4;PCDHGA	Body;Body;Body;Body;Body;Body;Body;1stExon;Body;Body;Body;Body;1stExon;Body;Body;Body;Body;Body;Body;Body	1.9158511	0.0889477	9.0471844	1.55E-15	1.19E-12	24.803173
cg11213690	chr7	149112402	+			2.5196558	0.1842072	9.0460943	1.55E-15	1.19E-12	24.797192
cg18191540	chr14	107281437	-			-1.759985	-1.584362	-9.044808	1.57E-15	1.19E-12	24.790137
cg24932241	chr11	442077	+	ANO9	TSS200	-0.933135	-2.963859	-9.044882	1.57E-15	1.19E-12	24.790539
cg11191040	chr1	28907634	+	SNHG12;SNORA44;SNORA16A;SNORA61	Body;TSS1500;TSS200;TSS1500	-0.955371	-4.61379	-9.042963	1.58E-15	1.21E-12	24.780013
cg00700039	chr18	2659555	-	SMCHD1	Body	-1.549798	-1.989813	-9.056358	1.63E-15	1.24E-12	24.75644
cg02555944	chr8	143644107	-			-1.833109	-0.813237	-9.046557	1.63E-15	1.24E-12	24.751276
cg15541630	chr8	87081770	+	PSKH2	1stExon	1.6443256	0.3839009	9.0347299	1.66E-15	1.26E-12	24.734859
cg15239304	chr2	224810907	-	WDFY1	TSS1500	1.3777499	2.2938098	9.0310536	1.69E-15	1.28E-12	24.714699
cg07300534	chr16	81726931	-	CMIP;CMIP	Body;Body	1.1183312	1.647397	9.0306226	1.70E-15	1.28E-12	24.712335
cg11667101	chr10	105127581	-	TAF5	TSS200	-1.048129	-4.595112	-9.029702	1.71E-15	1.29E-12	24.707288
cg14987309	chr2	236843038	-	AGAP1;AGAP1	Body;Body	1.0734366	1.3740164	9.0271037	1.73E-15	1.30E-12	24.69304
cg18505593	chr14	100059479	-	CCDC85C	Body	2.5506022	3.8967572	9.0262591	1.74E-15	1.30E-12	24.688408
cg19178151	chr4	184938359	+	STOX2	Body	1.3929703	1.6562898	9.0264894	1.74E-15	1.30E-12	24.689671
cg14136781	chr9	124087974	-	GSN;GSN;GSN;GSN;GSN;GSN;GSN;GSN	Body;Body;Body;Body;Body;Body;Body;Body	1.0937686	1.9669641	9.0244213	1.76E-15	1.32E-12	24.678332
cg26626042	chr6	32151594	+	AGER;AGER	Body;Body	0.8383032	2.1257888	9.0162131	1.84E-15	1.38E-12	24.633332

cg01709975	chr4	25847472	+	SEL1L3	Body	0.8114169	1.7386397	9.0153637	1.85E-15	1.38E-12	24.628676
cg17897352	chr10	121011644	+	GRK5	Body	1.2031505	0.9469381	9.0142523	1.86E-15	1.39E-12	24.622584
cg01797704	chr16	81577049	-	CMIP;CMIP	Body;Body	1.0691341	1.808379	9.0113988	1.89E-15	1.40E-12	24.606943
cg03849279	chr16	67751821	-	GFOD2;GFOD2;GFOD2	5'UTR;Body;Body	1.4087731	2.2994673	9.0114763	1.89E-15	1.40E-12	24.607368
cg21697851	chr21	38076869	-	SIM2;SIM2	Body;Body	2.5103259	-0.66238	9.0178631	1.92E-15	1.42E-12	24.594383
cg09175833	chr2	1598245	-			-1.51392	-0.279227	-9.002509	1.99E-15	1.47E-12	24.55822
cg25386676	chr5	172175721	+			2.1702519	2.4523728	9.0001604	2.02E-15	1.49E-12	24.545353
cg23221540	chr5	1092417	-	SLC12A7	Body	0.9835909	2.1378438	8.9985991	2.03E-15	1.50E-12	24.536798
cg25135457	chr17	40715244	+	COASY;COASY;COASY;COASY;COASY	1stExon;Body;Body;Body;5'UTR	-2.651506	0.9760205	-8.998154	2.04E-15	1.50E-12	24.534358
cg11807829	chr4	54458109	-	LNX1	TSS1500	-1.437485	-1.342462	-8.997547	2.05E-15	1.50E-12	24.531031
cg08220084	chr14	23426592	-	HAUS4;HAUS4;HAUS4	TSS1500;TSS1500;TSS1500	-0.904282	-3.371679	-8.99579	2.07E-15	1.52E-12	24.521404
cg13495850	chr18	20715040	+	CABLES1;CABLES1	TSS1500;Body	-1.422819	-3.588801	-8.990997	2.12E-15	1.55E-12	24.495147
cg06920324	chr7	149264011	+	ZNF767;ZNF767	Body;Body	1.1923039	2.6065263	8.9888458	2.15E-15	1.57E-12	24.483363
cg19588399	chr1	27668445	+	SYTL1	TSS200	-1.736951	-2.216224	-8.987941	2.16E-15	1.58E-12	24.478405
cg20502215	chr7	16793139	-	TSPAN13	TSS1500	-0.662706	-3.465268	-8.984655	2.20E-15	1.60E-12	24.460407
cg22954906	chr13	103531058	-	LOC121952	TSS1500	1.2466601	1.6162283	8.9690383	2.41E-15	1.75E-12	24.374884
cg25471234	chr6	32082643	+			1.2478273	1.3162346	8.9665628	2.44E-15	1.77E-12	24.36133
cg25743481	chr3	44803289	-	KIF15;KIF15;KIAA1143	1stExon;5'UTR;TSS200	-1.814303	-3.690385	-8.965426	2.46E-15	1.78E-12	24.355104
cg07303143	chr3	44803452	-	KIAA1143;KIF15	TSS1500;Body	-1.580948	-2.125733	-8.959561	2.54E-15	1.84E-12	24.322996
cg06485940	chr4	40859293	+	APBB2;APBB2;APBB2;APBB2;APBB2;APBB2;APBB2	Body;TSS200;TSS200;Body;TSS200;Body;TSS200	-1.734383	-2.334771	-8.954046	2.62E-15	1.89E-12	24.29281

cg19591623	chr19	311981	-	MIER2	Body	1.8929342	3.3177078	8.9496691	2.68E-15	1.93E-12	24.268857
cg18077971	chr2	223164867	+	PAX3;PAX3;PAX3;PAX3;PAX3;CCDC140;PAX3;PAX3;PAX3	TSS1500;TSS1500;TSS1500;TSS1500;TSS1500;5'UTR;TSS1500;TSS1500;TSS1500	2.7228236	0.2922879	8.9446782	2.76E-15	1.99E-12	24.241546
cg15488143	chr7	128924101	+	AHCYL2;AHCYL2	Body;Body	1.1455744	1.7286026	8.9418549	2.81E-15	2.02E-12	24.226097
cg09481404	chr15	53087316	-			2.3873615	-0.605751	8.9415161	2.81E-15	2.02E-12	24.224243
cg06550177	chr7	5469535	-			-2.372158	-3.506731	-8.939888	2.84E-15	2.03E-12	24.215333
cg25748441	chr2	202122587	+	CASP8;CASP8;CASP8;CASP8;CASP8	5'UTR;5'UTR;5'UTR;5'UTR;TSS200	-1.191841	-3.483587	-8.930703	2.99E-15	2.14E-12	24.165088
cg18236877	chr1	10733511	+	CASZ1;CASZ1	Body;Body	2.2993682	2.2014402	8.9389426	3.00E-15	2.14E-12	24.163754
cg11174855	chr10	134598352	-	NKX6-2	3'UTR	2.3912376	0.0355962	8.9292527	3.01E-15	2.15E-12	24.157155
cg00503302	chr3	62355319	-			1.7724873	-0.048712	8.925964	3.07E-15	2.18E-12	24.139167
cg12131862	chr1	203613877	+	ATP2B4;ATP2B4	5'UTR;5'UTR	1.5115834	1.6052069	8.9259904	3.07E-15	2.18E-12	24.139311
cg21647227	chr1	119527111	+	TBX15	5'UTR	1.7646705	-0.521908	8.9263094	3.06E-15	2.18E-12	24.141056
cg02131853	chr4	39034637	+	TMEM156	TSS1500	-1.559451	-1.285734	-8.923845	3.11E-15	2.20E-12	24.127579
cg18316386	chr12	58335867	-	XRCC6BP1	Body	-1.002768	-3.658344	-8.922684	3.13E-15	2.21E-12	24.12123
cg12141030	chr3	44803447	-	KIAA1143;KIF15	TSS1500;Body	-1.323674	-2.314248	-8.922281	3.13E-15	2.21E-12	24.119023
cg20644981	chr4	152020400	-	RPS3A	TSS1500	-1.361673	-2.095925	-8.917904	3.21E-15	2.26E-12	24.095089
cg03874199	chr2	176964456	-	HOXD12	TSS200	1.8257881	-0.885032	8.9163753	3.24E-15	2.28E-12	24.08673
cg08404702	chr16	2133388	-	TSC2;TSC2;TSC2	Body;Body;Body	1.9206518	3.0419118	8.9158104	3.25E-15	2.28E-12	24.083641
cg02686681	chr2	241853954	+			-1.060111	-4.00117	-8.914436	3.28E-15	2.30E-12	24.076128
cg03607117	chr3	53080440	+	SFMBT1;SFMBT1;SFMBT1	TSS1500;TSS1500;TSS1500	-1.153545	-3.560867	-8.912363	3.31E-15	2.32E-12	24.064791
cg07545037	chr12	54423920	-	HOXC4;HOXC5;HOXC6;HOXC6	5'UTR;Body;3'UTR;3'UTR	1.4121722	1.6659415	8.9087999	3.38E-15	2.36E-12	24.045312

cg08139247	chr14	38725312	-	CLEC14A;CLEC14A	1stExon;5'UTR	1.5803637	0.427618	8.9063492	3.43E-15	2.39E-12	24.031915
cg16736054	chr1	100758252	+	RTCD1;RTCD1	3'UTR;3'UTR	1.0523217	2.2324356	8.9027727	3.50E-15	2.44E-12	24.012365
cg27478167	chr7	817139	+	HEATR2	Body	1.1503103	3.3770687	8.9016547	3.52E-15	2.45E-12	24.006254
cg06745704	chr1	27668325	+	SYTL1	TSS200	-1.682774	-2.096786	-8.900806	3.54E-15	2.46E-12	24.001616
cg14789818	chr1	227748712	-			2.0753701	1.4489231	8.8995709	3.56E-15	2.47E-12	23.994864
cg21368161	chr7	1596136	-	TMEM184A	TSS200	-1.542124	-1.949904	-8.894802	3.66E-15	2.53E-12	23.968803
cg12509499	chr17	2095179	+	SMG6;SMG6	Body;Body	1.443052	1.6115425	8.8925528	3.71E-15	2.56E-12	23.95651
cg18862481	chr3	129693370	+	TRH;TRH	5'UTR;1stExon	2.9358128	-0.225314	8.8926138	3.71E-15	2.56E-12	23.956843
cg15222899	chr18	55095249	+			2.2994445	-0.05543	8.8903452	3.75E-15	2.58E-12	23.944447
cg09476997	chr16	2087932	+	SLC9A3R2;SLC9A3R2	Body;Body	2.0623193	2.4847852	8.9068588	3.77E-15	2.59E-12	23.942053
cg01602690	chr2	176987161	+	HOXD9	TSS1500	1.6825145	-0.488058	8.8881325	3.80E-15	2.61E-12	23.932357
cg07000334	chr12	54785286	-	ZNF385A;ZNF385A	TSS1500;TSS1500	-1.64393	-2.714879	-8.88807	3.80E-15	2.61E-12	23.932015
cg12215739	chr2	177014966	-	HOXD4;MIR10B	TSS1500;TSS200	1.8833353	-0.01761	8.8873365	3.82E-15	2.61E-12	23.928007
cg24023453	chr6	31164952	+	HCG27	TSS1500	-1.249296	-3.634707	-8.887258	3.82E-15	2.61E-12	23.927579
cg24354624	chr19	40732728	+	CNTD2	TSS200	-1.063767	-2.243588	-8.882681	3.92E-15	2.67E-12	23.902572
cg07455975	chr2	175112849	-	OLA1;OLA1	5'UTR;5'UTR	-0.771359	-3.559416	-8.881879	3.94E-15	2.68E-12	23.89819
cg27122213	chr14	57274763	+	OTX2	5'UTR	3.2748592	-0.753319	8.8807766	3.96E-15	2.69E-12	23.892169
cg09307985	chr19	36643070	-	COX7A1	Body	1.5113865	1.5420271	8.8795206	3.99E-15	2.71E-12	23.885307
cg14214165	chr2	218777482	-	TNS1	5'UTR	1.4958585	0.7138693	8.8787223	4.01E-15	2.72E-12	23.880947
cg18297437	chr3	147131332	-	ZIC1	Body	2.0223694	-0.307981	8.8707822	4.19E-15	2.84E-12	23.837578

cg09417568	chr9	99328811	-	CDC14B;CDC14B;CDC14B	Body;Body;Body	-0.864552	-4.650859	-8.865337	4.32E-15	2.92E-12	23.807843
cg00446235	chr1	161008644	+	TSTD1;TSTD1;TSTD1	Body;Body;Body	-1.548413	-2.761549	-8.863107	4.38E-15	2.95E-12	23.795666
cg11493223	chr17	76128522	+	TMC8;TMC6	Body;TSS200	-2.147243	-2.172206	-8.861713	4.41E-15	2.97E-12	23.788053
cg24327132	chr15	72520632	+	PKM2;PKM2;PKM2	5'UTR;5'UTR;5'UTR	-1.866266	-1.914972	-8.860806	4.43E-15	2.98E-12	23.783102
cg24056954	chr11	67926217	-	SUV420H1	Body	1.309662	2.8010706	8.8559236	4.56E-15	3.06E-12	23.756445
cg17562299	chr11	63674850	+	MARK2;MARK2;MARK2;MARK2;MARK2	Body;Body;Body;Body;Body	0.9134767	1.4789153	8.8516058	4.67E-15	3.13E-12	23.732875
cg05104283	chr17	75463860	-	SEPT9;SEPT9;SEPT9;SEPT9;SEPT9;SEPT9	Body;5'UTR;Body;Body;Body;Body	1.9348151	4.2202816	8.8679545	4.69E-15	3.14E-12	23.713893
cg06594404	chr4	380396	-			2.1582491	0.0303268	8.8500625	4.71E-15	3.15E-12	23.724451
cg10976673	chr4	139937474	-	CCRN4L	Body	-0.617808	-4.426538	-8.848757	4.75E-15	3.17E-12	23.717324
cg07319315	chr2	135149599	-	MGAT5	Body	-2.397741	0.2898081	-8.848292	4.76E-15	3.17E-12	23.714786
cg07702853	chr1	150254280	-	C1orf51	TSS1500	-1.694437	-3.90006	-8.870884	4.85E-15	3.22E-12	23.699918
cg11075346	chr7	35735308	+	HERPUD2	TSS1500	-1.103315	-3.740942	-8.84419	4.87E-15	3.23E-12	23.692401
cg15126975	chr2	175208605	-			3.2276462	-0.706671	8.8443767	4.86E-15	3.23E-12	23.693418
cg15034393	chr3	152856773	-			-1.696811	-2.427264	-8.842784	4.91E-15	3.25E-12	23.684725
cg10752158	chr4	81185022	-			2.0998664	-0.422787	8.8420223	4.93E-15	3.26E-12	23.680569
cg21122466	chr6	99323548	-	FBXL4	Body	1.0847067	1.8272995	8.8413759	4.95E-15	3.27E-12	23.677042
cg14935716	chr8	133805351	+	PHF20L1;PHF20L1;PHF20L1	Body;Body;Body	1.4731631	2.0400565	8.8399712	4.99E-15	3.29E-12	23.669376
cg10659886	chr19	58629719	-	ZSCAN18;ZSCAN18	Body;1stExon	1.3841694	0.1586347	8.8381627	5.04E-15	3.32E-12	23.659508
cg22920873	chr7	139025153	-	C7orf55	TSS1500	-1.748335	-5.40416	-8.846333	5.05E-15	3.32E-12	23.658825
cg00097146	chr2	45171818	+	SIX3	Body	2.8092371	-0.564685	8.8447817	5.09E-15	3.34E-12	23.65068

cg12556823	chr6	21023485	+	CDKAL1	Body	-2.58333	0.4595166	-8.835336	5.12E-15	3.35E-12	23.644084
cg17221941	chr1	10730684	+	CASZ1;CASZ1	Body;Body	1.4697475	1.6587348	8.8348555	5.13E-15	3.36E-12	23.641463
cg05902503	chr16	30133175	-	MAPK3;MAPK3;MAPK3	Body;Body;Body	1.2630229	1.6153626	8.8280543	5.33E-15	3.48E-12	23.604358
cg19868593	chr6	31744816	+	C6orf27	5'UTR	-1.363635	-2.605441	-8.82414	5.45E-15	3.56E-12	23.583005
cg15031753	chr4	184938291	-	STOX2	Body	1.3061286	1.7817944	8.8215686	5.53E-15	3.60E-12	23.568981
cg10606834	chr17	59532197	-			1.9141989	-0.326982	8.8182482	5.64E-15	3.67E-12	23.550872
cg05348708	chr17	79520615	-	C17orf70;C17orf70	TSS1500;TSS1500	-1.012286	-3.915742	-8.81651	5.69E-15	3.70E-12	23.541394
cg05750962	chr18	45535166	-			-1.050545	-3.135207	-8.804228	6.10E-15	3.96E-12	23.474424
cg04604142	chr17	48796515	-	LUC7L3;LUC7L3	TSS1500;TSS1500	-1.967343	-0.524952	-8.798664	6.29E-15	4.07E-12	23.444094
cg04797570	chr11	47587672	-	PTPMT1;PTPMT1	Body;Body	-0.959589	-2.894784	-8.796265	6.38E-15	4.12E-12	23.431022
cg13637552	chr11	1534712	+	HCCA2	Body	1.0177429	1.8849924	8.7955242	6.41E-15	4.14E-12	23.426982
cg20957164	chr3	12965323	+	IQSEC1;IQSEC1	Body;Body	0.8047878	1.4082731	8.7950804	6.42E-15	4.14E-12	23.424563
cg23167235	chr17	70532667	+			1.1386059	2.5909788	8.7949634	6.43E-15	4.14E-12	23.423926
cg06100227	chr6	36098581	+	MAPK13	Body	-1.175582	-4.155577	-8.802298	6.46E-15	4.15E-12	23.419319
cg19409133	chr1	28843744	+	SNHG3-RCC1;RCC1;SNHG3-RCC1;RCC1;SNHG3-RCC1;RCC1	Body;TSS1500;Body;TSS1500;5'UTR;TSS1500	-1.612882	-1.637826	-8.79373	6.47E-15	4.15E-12	23.417202
cg11009596	chr17	8113758	+	AURKB	5'UTR	-0.782758	-3.905747	-8.793245	6.49E-15	4.16E-12	23.414559
cg16620032	chr19	46097075	-	GPR4	5'UTR	-1.368029	-2.98273	-8.788038	6.68E-15	4.27E-12	23.386185
cg10512408	chr6	110504754	+	CDC40	Body	1.210109	2.1934184	8.7854949	6.78E-15	4.33E-12	23.372328
cg21179088	chr7	54612333	-	VSTM2A	Body	2.0928179	-0.104077	8.7851581	6.79E-15	4.33E-12	23.370493
cg03114584	chr4	1534311	+			-1.450139	-1.371822	-8.783263	6.86E-15	4.37E-12	23.360169

cg22584911	chr15	52176689	+	TMOD3	Body	1.352966	2.4636368	8.7830008	6.87E-15	4.37E-12	23.358739
cg01393234	chr1	226085079	+			-1.803341	-3.102509	-8.782676	6.89E-15	4.37E-12	23.35697
cg00546117	chr1	8445545	-	RERE;RERE;RERE	5'UTR;Body;Body	1.2947842	1.343328	8.7818794	6.92E-15	4.39E-12	23.352629
cg02652344	chr9	123553595	+	FBXW2	5'UTR	1.6045578	1.5442254	8.7810774	6.95E-15	4.40E-12	23.34826
cg25191628	chr1	50886949	-	DMRTA2	Body	2.9462166	-0.86501	8.7802112	6.98E-15	4.42E-12	23.343541
cg00433654	chr2	74684330	-	WBP1;INO80B	TSS1500;Body	-1.228194	-2.762016	-8.779534	7.01E-15	4.42E-12	23.339854
cg18563999	chr12	54785003	-	ZNF385A;ZNF385A	Body;Body	-2.2521	-2.896794	-8.779364	7.02E-15	4.42E-12	23.338924
cg19622755	chr2	47169299	-	MCFD2;TTC7A;MCFD2	TSS1500;Body;TSS1500	-2.105358	-4.190694	-8.779623	7.01E-15	4.42E-12	23.340339
cg04352272	chr17	3289798	-			1.9592325	-0.076095	8.7782416	7.06E-15	4.44E-12	23.332811
cg17390562	chr6	159238463	-	EZR;EZR	Body;Body	-0.997526	-3.177952	-8.775102	7.19E-15	4.51E-12	23.315711
cg20019985	chr11	32008961	+			1.574482	-0.218624	8.7828234	7.21E-15	4.52E-12	23.313772
cg11326574	chr12	59271109	-	LRIG3;LRIG3	Body;Body	-1.522322	-2.327171	-8.773782	7.24E-15	4.53E-12	23.308517
cg20510600	chr1	54820824	+	SSBP3;SSBP3;SSBP3	Body;Body;Body	1.2675484	2.0401829	8.7719846	7.31E-15	4.57E-12	23.298729
cg24161057	chr1	161008811	+	TSTD1;TSTD1;TSTD1	TSS200;TSS200;TSS200	-2.296726	-2.292921	-8.771855	7.32E-15	4.57E-12	23.298021
cg19400926	chr6	28889996	-	TRIM27	Body	-1.270742	-1.818794	-8.77142	7.34E-15	4.57E-12	23.295653
cg04415798	chr14	37128586	+	PAX9	5'UTR	2.77166	0.2125908	8.7778376	7.42E-15	4.61E-12	23.286686
cg21496803	chr6	167029080	+	RPS6KA2;RPS6KA2	Body;Body	2.0405859	3.5547459	8.7736414	7.59E-15	4.72E-12	23.263609
cg15092343	chr4	4860061	+	MSX1	TSS1500	2.2352497	0.0022025	8.7761272	7.85E-15	4.87E-12	23.232717
cg01409693	chr4	1542171	-			-1.435992	0.4670712	-8.756724	7.97E-15	4.94E-12	23.215629
cg02297801	chr1	1710663	-	NADK	TSS1500	-1.367281	-1.901715	-8.751885	8.19E-15	5.07E-12	23.189284

cg06655097	chr4	177455600	-			-2.035416	-0.228562	-8.751137	8.23E-15	5.08E-12	23.185211
cg24030037	chr8	27457282	+	CLU;CLU;CLU	Body;Body;Body	2.4715966	3.6229019	8.7475617	8.39E-15	5.18E-12	23.165753
cg01643628	chr8	59323755	+	UBXN2B	TSS200	-0.676302	-4.290885	-8.7472	8.41E-15	5.18E-12	23.163785
cg26764198	chr16	87469002	+	ZCCHC14	Body	0.8298389	1.5693515	8.7457635	8.48E-15	5.21E-12	23.155966
cg18832348	chr5	94844511	-	TTC37	Body	1.1893927	1.4884745	8.7454429	8.49E-15	5.22E-12	23.154221
cg27388911	chr3	24537277	+	THRB;THRB;THRB	TSS1500;TSS1500;TSS1500	-1.123812	-1.490227	-8.74003	8.76E-15	5.37E-12	23.124762
cg10422067	chr11	44963330	+	TP53I11;TP53I11	5'UTR;5'UTR	1.3563486	2.2142881	8.7386351	8.82E-15	5.40E-12	23.117173
cg12455762	chr4	40859095	+	APBB2;APBB2;APBB2;APBB2;APBB2;APBB2;APBB2;APBB2;APBB2;APBB2;APBB2	Body;5'UTR;5'UTR;5'UTR;1stExon;1stExon;5'UTR;Body;1stExon;Body;1stExon	-1.629068	-1.585102	-8.734094	9.05E-15	5.54E-12	23.092466
cg05171693	chr7	1117775	-	C7orf50;C7orf50;C7orf50	Body;Body;Body	1.3754285	2.4558527	8.7310745	9.21E-15	5.62E-12	23.076036
cg17941807	chr6	15509823	-	JARID2	Body	1.1080998	2.3025391	8.730434	9.24E-15	5.64E-12	23.072552
cg18817426	chr15	75230435	-	COX5A;COX5A	5'UTR;1stExon	1.4868378	-1.770696	8.7296783	9.28E-15	5.65E-12	23.068441
cg07318287	chr1	154377429	+	IL6R;IL6R	TSS1500;TSS1500	-1.124836	-2.805233	-8.727433	9.40E-15	5.72E-12	23.056226
cg13382100	chr12	132414849	-	PUS1;PUS1;PUS1	Body;Body;Body	-1.23225	-2.836399	-8.726613	9.44E-15	5.73E-12	23.051766
cg15105326	chr3	147108916	-	ZIC4;ZIC4;ZIC4;ZIC4;ZIC4	Body;Body;Body;Body;Body	1.4041645	0.0901593	8.7266269	9.44E-15	5.73E-12	23.051842
cg10184289	chr2	64870490	-	SERTAD2	5'UTR	-1.437302	-1.375238	-8.725768	9.49E-15	5.75E-12	23.047172
cg06629130	chr18	44787585	+			2.4557165	-0.528838	8.7252673	9.51E-15	5.75E-12	23.044447
cg06447424	chr13	20875743	-			1.764717	0.2275219	8.7211776	9.73E-15	5.88E-12	23.022202
cg12814535	chr12	70688893	-	CNOT2	Body	1.1021725	1.7317317	8.7207531	9.76E-15	5.88E-12	23.019894
cg00310375	chr2	85906657	+			-1.801061	-3.618092	-8.719448	9.83E-15	5.92E-12	23.012797
cg00719668	chr20	34541013	+			-1.301692	-1.842217	-8.718388	9.89E-15	5.93E-12	23.007032

cg02624332	chr3	101504465	-	FAM55C;FAM55C	Body;Body	0.9600785	2.2657283	8.7181462	9.90E-15	5.93E-12	23.005716
cg03724882	chr14	54864244	+	CDKN3;CDKN3	Body;Body	-0.953648	-3.904611	-8.718549	9.88E-15	5.93E-12	23.007905
cg14519917	chr1	111747302	+			-1.09477	-2.9333	-8.718288	9.89E-15	5.93E-12	23.006486
cg20968743	chr11	44883965	+	TSPAN18	5'UTR	-2.66782	-2.906543	-8.717097	9.96E-15	5.96E-12	23.000013
cg02725370	chr4	111551829	-	PITX2;PITX2	Body;Body	2.6704966	-1.132313	8.7163407	1.00E-14	5.97E-12	22.995897
cg15961044	chr18	59992280	-	TNFRSF11A	TSS1500	-0.950601	-3.123797	-8.715852	1.00E-14	5.98E-12	22.99324
cg13741211	chr7	98607297	+	TRRAP	Body	1.0426381	1.444928	8.7122573	1.02E-14	6.10E-12	22.973694
cg13435464	chr9	91927197	+	CKS2	Body	-0.835672	-3.898065	-8.708798	1.04E-14	6.21E-12	22.954886
cg12178431	chr3	14860764	+	FGD5	1stExon	-1.403707	0.6975902	-8.713096	1.07E-14	6.33E-12	22.935307
cg11586448	chr12	123383399	+			-1.808138	-2.301336	-8.704505	1.07E-14	6.34E-12	22.931548
cg15124109	chr1	156711332	+	MRPL24;MRPL24	TSS1500;TSS1500	-1.297805	-3.594385	-8.703505	1.08E-14	6.37E-12	22.926111
cg02386822	chr1	155162871	-	MUC1;MUC1;MUC1;MUC1;MUC1;MUC1;MUC1	TSS200;TSS200;TSS200;TSS200;TSS200;TSS200;TSS200	-1.030507	-1.710274	-8.698414	1.11E-14	6.54E-12	22.898439
cg01114217	chr6	150922145	+	PLEKHG1	5'UTR	-0.672063	-4.116344	-8.698015	1.11E-14	6.55E-12	22.896269
cg12374721	chr17	46799640	+	C17orf93;PRAC	TSS1500;Body	2.9758607	0.0980652	8.710987	1.13E-14	6.66E-12	22.880205
cg10824354	chr17	47049956	-			-1.388515	-2.007904	-8.691888	1.15E-14	6.76E-12	22.862973
cg03424058	chr1	215791864	-	KCTD3	Body	1.0571017	2.3977708	8.6888455	1.17E-14	6.87E-12	22.84644
cg27615388	chr5	63257092	-	HTR1A	1stExon	2.0576813	-0.283199	8.6872252	1.18E-14	6.92E-12	22.837636
cg08118034	chr22	29706595	+	GAS2L1;GAS2L1;GAS2L1	Body;Body;Body	-1.704549	1.0529724	-8.685508	1.19E-14	6.98E-12	22.828307
cg09990600	chr7	1596103	-	TMEM184A	TSS200	-1.100943	-2.892641	-8.684973	1.19E-14	6.99E-12	22.825399
cg20801751	chr1	228366494	-	C1orf69	3'UTR	1.5978021	2.353287	8.6844229	1.20E-14	7.00E-12	22.822411

cg04426588	chr17	54915771	-	DGKE	Body	1.1470334	2.6640328	8.6828387	1.21E-14	7.05E-12	22.813805
cg12670943	chr22	43006541	-	POLDIP3;POLDIP3	Body;Body	1.3539335	1.5796783	8.6810858	1.22E-14	7.12E-12	22.804282
cg12503394	chr7	1596118	-	TMEM184A	TSS200	-1.395999	-1.338695	-8.680128	1.23E-14	7.14E-12	22.799078
cg10639933	chr3	39426101	-	SLC25A38	Body	-1.325928	-1.806537	-8.679792	1.23E-14	7.15E-12	22.797252
cg11912239	chr17	76220608	+	BIRC5;BIRC5;BIRC5;EPR1	3'UTR;3'UTR;3'UTR;Body	1.4177651	2.5177953	8.6775617	1.24E-14	7.23E-12	22.785139
cg02859866	chr10	13760165	+	FRMD4A	Body	-1.056617	-1.779523	-8.677259	1.25E-14	7.23E-12	22.783493
cg04877966	chr15	75135169	-	ULK3	Body	-0.557037	-4.093332	-8.672355	1.28E-14	7.42E-12	22.756858
cg15692229	chr7	55257946	-	EGFR	Body	1.2864011	2.343404	8.6696742	1.30E-14	7.52E-12	22.742302
cg16619193	chr1	206643301	-	IKBKE	TSS1500	-1.074472	-2.115855	-8.668803	1.31E-14	7.55E-12	22.737571
cg20267580	chr1	85220130	+			2.0277972	3.163357	8.6753872	1.32E-14	7.60E-12	22.730948
cg02095937	chr3	160166877	+	TRIM59	5'UTR	-1.620588	-3.70146	-8.664392	1.34E-14	7.72E-12	22.713617
cg16640855	chr2	182543233	-	NEUROD1	Body	2.7604962	-0.45916	8.6635514	1.35E-14	7.75E-12	22.709054
cg18988498	chr11	31824262	+	PAX6;PAX6;PAX6	Body;Body;Body	1.6458214	0.1672906	8.6615621	1.36E-14	7.82E-12	22.698254
cg07044458	chr7	4751984	+	FOXK1	Body	-2.579462	-0.267763	-8.659973	1.37E-14	7.88E-12	22.689629
cg27515052	chr8	8513104	-			1.2530536	1.8942675	8.662023	1.42E-14	8.14E-12	22.658575
cg02560808	chr16	70835289	-	VAC14	TSS1500	-0.82538	-4.057052	-8.652357	1.43E-14	8.20E-12	22.648285
cg02388453	chr15	37084662	-	C15orf41;C15orf41	Body;Body	2.100536	1.7906244	8.6512078	1.44E-14	8.25E-12	22.642048
cg26244738	chr4	40859344	-	APBB2;APBB2;APBB2;APBB2;APBB2;APBB2;APBB2	Body;TSS200;TSS200;Body;TSS200;Body;TSS200	-1.433058	-3.280341	-8.646304	1.48E-14	8.47E-12	22.615435
cg22731271	chr2	66667059	-	MEIS1	Body	2.6555339	0.1524774	8.6521501	1.50E-14	8.56E-12	22.604874
cg25969122	chr11	119630362	+			-1.659344	-2.089828	-8.643667	1.50E-14	8.57E-12	22.601127

cg27023597	chr17	57918262	+	MIR21	TSS1500	-1.830679	-2.991544	-8.641232	1.52E-14	8.68E-12	22.587915
cg27260772	chr6	50791202	+	TFAP2B	Body	3.4347522	0.343616	8.6480682	1.53E-14	8.72E-12	22.583035
cg06381455	chr17	73472178	+	KIAA0195	Body	1.060123	1.1240658	8.6373868	1.56E-14	8.83E-12	22.56705
cg07915921	chr12	54321502	+			2.9588293	0.0515436	8.6375594	1.56E-14	8.83E-12	22.567986
cg14401320	chr14	105796811	-	PACS2;PACS2	Body;Body	1.2634805	2.4093212	8.6361932	1.57E-14	8.88E-12	22.560575
cg11338156	chr10	82423899	-			-1.516236	-1.446316	-8.631371	1.61E-14	9.11E-12	22.534417
cg15174623	chr7	35494345	+			1.2089052	-0.108148	8.6301774	1.62E-14	9.16E-12	22.527942
cg25324047	chr10	22765840	-			2.555464	-0.147835	8.6280343	1.64E-14	9.26E-12	22.516318
cg20765408	chr13	25085669	+	PARP4	5'UTR	-1.821391	-2.355194	-8.626683	1.65E-14	9.31E-12	22.508989
cg21953025	chr2	216877750	-	MREG	Body	-0.918801	-3.191418	-8.625065	1.67E-14	9.39E-12	22.500216
cg13564459	chr14	62004353	-	PRKCH	Body	-1.732399	-2.865798	-8.6237	1.68E-14	9.43E-12	22.49281
cg25092838	chr14	57279115	-	OTX2OS1	TSS1500	2.7110433	-0.183514	8.6317035	1.68E-14	9.43E-12	22.49424
cg02158567	chr2	169672780	-	NOSTRIN;NOSTRIN;NOSTRIN;NOSTRIN	5'UTR;Body;Body;Body	1.1419759	1.561864	8.6232435	1.69E-14	9.44E-12	22.490337
cg03091551	chr14	57275967	-	OTX2	5'UTR	1.9529722	-0.392075	8.6231216	1.69E-14	9.44E-12	22.489675
cg03679755	chr10	101875035	+			2.6776266	-0.489135	8.6221408	1.70E-14	9.48E-12	22.484357
cg14864572	chr8	129505584	-			1.2737159	2.7213894	8.6174848	1.74E-14	9.72E-12	22.459111
cg08155625	chr21	44851244	+			-2.098708	-0.603525	-8.615756	1.76E-14	9.79E-12	22.449736
cg15545021	chr2	30802226	+	LCLAT1;LCLAT1	Body;Body	1.0469652	2.6769171	8.6156919	1.76E-14	9.79E-12	22.44939
cg10783149	chr14	55707866	+			1.45841	1.6594581	8.6138	1.78E-14	9.88E-12	22.439133
cg24945701	chr3	62362783	+			1.9578623	-0.142455	8.6120604	1.80E-14	9.97E-12	22.429702

cg06972019	chr1	8937448	+	ENO1	5'UTR	-1.603945	-2.36477	-8.610414	1.81E-14	1.00E-11	22.420775
cg17467525	chr1	9686523	-			-1.598979	-2.166163	-8.606868	1.85E-14	1.02E-11	22.401559
cg10896774	chr7	142636840	+	C7orf34	1stExon	-1.296146	-1.956145	-8.602368	1.90E-14	1.05E-11	22.377167
cg08431536	chr2	223161894	-	PAX3;PAX3;PAX3;CCDC140;PAX3;PAX3;PAX3;PAX3;PAX3	Body;Body;Body;TSS1500;Body;Body;Body;Body;Body	2.8285183	-0.904205	8.6018395	1.90E-14	1.05E-11	22.374304
cg26595643	chr10	118899291	-	VAX1;VAX1	TSS1500;TSS1500	1.9578864	-0.577167	8.6089139	1.91E-14	1.05E-11	22.357332
cg03751906	chr12	129307800	+	SLC15A4	Body	-1.105182	-3.024132	-8.608273	1.92E-14	1.06E-11	22.36779
cg26077811	chr11	119232263	-	USP2;USP2	Body;Body	-1.344816	-2.359223	-8.598281	1.94E-14	1.07E-11	22.35502
cg10026317	chr1	47694919	-	TAL1;TAL1	5'UTR;1stExon	1.6729155	0.5315281	8.5972014	1.95E-14	1.07E-11	22.34917
cg07450698	chr17	80804126	+	TBCD	Body	1.0407427	0.0912428	8.5961922	1.96E-14	1.08E-11	22.343702
cg05894734	chr4	40859249	+	APBB2;APBB2;APBB2;APBB2;APBB2;APBB2;APBB2	Body;TSS200;TSS200;Body;TSS200;Body;TSS200	-2.17669	-3.253994	-8.595012	1.98E-14	1.08E-11	22.337307
cg18872604	chr11	64002754	-	VEGFB	Body	-1.029108	-2.188015	-8.594831	1.98E-14	1.08E-11	22.336327
cg04097131	chr6	15298461	+	JARID2	Body	1.388745	1.9284821	8.5937697	1.99E-14	1.09E-11	22.330577
cg05065989	chr7	27205200	+	HOXA9	TSS200	2.9942533	-2.069584	8.5926192	2.00E-14	1.09E-11	22.324343
cg15617155	chr1	63785321	+			2.4332382	-0.306528	8.5992433	2.02E-14	1.10E-11	22.318742
cg07484727	chr16	1587309	+	IFT140;TMEM204	Body;Body	1.06582	1.6378012	8.5896987	2.04E-14	1.11E-11	22.308522
cg04429812	chr17	37814043	+	STARD3;STARD3;STARD3	Body;Body;Body	1.1175884	3.3656162	8.5890034	2.04E-14	1.11E-11	22.304755
cg18343437	chr8	142528415	+			2.1280882	-0.410837	8.5850638	2.09E-14	1.13E-11	22.283415
cg04360793	chr1	79472361	-	ELTD1;ELTD1	5'UTR;1stExon	2.1956159	0.9207769	8.5898331	2.12E-14	1.15E-11	22.268137
cg19250790	chr19	2614086	+	GNG7	5'UTR	-2.268709	-4.573439	-8.577107	2.18E-14	1.18E-11	22.240323
cg23683800	chr10	115086103	+			-2.100786	-2.962408	-8.575333	2.21E-14	1.19E-11	22.230717

cg18074954	chr18	44787492	+			2.844519	-0.927434	8.5736864	2.23E-14	1.20E-11	22.221801
cg08087868	chr16	51188129	+			2.1392465	-0.335289	8.5678327	2.30E-14	1.24E-11	22.190108
cg26086029	chr14	92591610	+	CPSF2	5'UTR	1.1498967	1.8703525	8.5679759	2.30E-14	1.24E-11	22.190884
cg14415770	chr9	99637871	-			-0.612323	-2.942912	-8.566376	2.32E-14	1.25E-11	22.182225
cg03314644	chr14	31890001	+			-0.821076	-4.646555	-8.566004	2.32E-14	1.25E-11	22.180208
cg17176423	chr15	85278581	-			-1.059276	-2.084773	-8.565785	2.33E-14	1.25E-11	22.179024
cg09570682	chr6	106429443	+			3.2952886	-0.779953	8.5653616	2.33E-14	1.25E-11	22.176731
cg26351132	chr17	47049890	+			-1.445935	-2.342784	-8.571398	2.36E-14	1.26E-11	22.1551
cg19087971	chr7	751233	+	PRKAR1B;PRKAR1B;PRKAR1B;PRKAR1B;PRKAR1B;PRKAR1B	5'UTR;5'UTR;5'UTR;5'UTR;5'UTR;5'UTR	1.1556416	0.607883	8.5583759	2.43E-14	1.30E-11	22.138921
cg03532926	chr7	153584839	-	DPP6	Body	2.3454155	-0.055798	8.5731481	2.44E-14	1.30E-11	22.136203
cg01012445	chr4	151504920	+	LRBA;MAB21L2	Body;1stExon	1.4101017	1.3842089	8.5549154	2.47E-14	1.32E-11	22.120194
cg05570654	chr2	240259075	-	HDAC4	Body	0.9535624	1.5575768	8.5533254	2.49E-14	1.33E-11	22.11159
cg09145391	chr17	7338890	+	TMEM102;TMEM102	5'UTR;1stExon	-1.297219	-4.336608	-8.55232	2.51E-14	1.33E-11	22.106151
cg19533977	chr17	57719682	+	CLTC	Body	-2.541882	-2.882932	-8.552623	2.50E-14	1.33E-11	22.107792
cg22220165	chr17	18908242	+	SLC5A10;SLC5A10;FAM83G	Body;Body;TSS200	-1.191267	-4.296587	-8.55222	2.51E-14	1.33E-11	22.10561
cg22931402	chr4	113207418	-	TIFA	TSS1500	-1.339634	-3.219494	-8.552846	2.50E-14	1.33E-11	22.108995
cg24496614	chr11	61735970	+	FTH1	TSS1500	-1.410314	-2.173662	-8.549534	2.55E-14	1.35E-11	22.091077
cg10529845	chr16	49522337	-			-1.38718	-1.024324	-8.546412	2.59E-14	1.37E-11	22.074188
cg26698460	chr19	58716004	-	ZNF274;ZNF274;ZNF274	Body;Body;5'UTR	1.8657595	1.3921411	8.5454799	2.61E-14	1.38E-11	22.069143
cg21908886	chr1	166039444	+	FAM78B	3'UTR	-1.802684	-0.611146	-8.544332	2.62E-14	1.38E-11	22.062932

cg21831931	chr3	5137953	+			1.9894967	-0.727355	8.5419013	2.66E-14	1.40E-11	22.049785
cg25647234	chr13	108870281	-	ABHD13;LIG4	TSS1500;5'UTR	-0.755163	-4.197741	-8.539628	2.69E-14	1.42E-11	22.037487
cg26365299	chr7	27205381	-	HOXA9	TSS1500	3.1568252	-1.927709	8.5471106	2.70E-14	1.42E-11	22.037478
cg02983090	chr16	27437892	-	IL21R;IL21R;IL21R	TSS1500;5'UTR;5'UTR	-1.709426	-3.214733	-8.538341	2.71E-14	1.43E-11	22.030526
cg04608356	chr1	27668433	-	SYTL1	TSS200	-1.507008	-1.695224	-8.536627	2.74E-14	1.44E-11	22.021258
cg14585967	chr9	118135710	+	1-Dec	5'UTR	-1.83372	-0.630572	-8.534299	2.77E-14	1.45E-11	22.00867
cg06215107	chr4	151504725	-	LRBA;MAB21L2	Body;1stExon	1.5988718	0.9306174	8.5330354	2.79E-14	1.46E-11	22.001836
cg21052656	chr11	67250046	-	AIP	TSS1500	-1.624314	-1.568351	-8.53236	2.81E-14	1.47E-11	21.998182
cg25270670	chr11	61735789	-	FTH1	TSS1500	-1.376576	-4.018174	-8.531376	2.82E-14	1.47E-11	21.992865
cg04858834	chr1	54231196	-	TMEM48;TMEM48;TMEM48	3'UTR;3'UTR;Body	1.2754117	2.0577667	8.5258255	2.91E-14	1.52E-11	21.962852
cg13324546	chr8	23564031	-	NKX2-6	TSS200	2.8365597	-1.370968	8.5244759	2.93E-14	1.53E-11	21.955556
cg06821992	chr5	56790874	-			-1.501149	-1.959785	-8.524026	2.94E-14	1.53E-11	21.953123
cg20499290	chr10	38015579	+			1.1454728	0.5989703	8.5369816	2.98E-14	1.55E-11	21.941808
cg14314744	chr6	100897445	-	SIM1	Body	1.9156506	-0.318714	8.5277987	3.00E-14	1.56E-11	21.93309
cg19893751	chr2	45029285	+			2.2301393	0.0940449	8.5190902	3.02E-14	1.57E-11	21.926443
cg26289788	chr3	8818056	+			-1.444217	-0.541524	-8.518137	3.04E-14	1.57E-11	21.921294
cg02342906	chr1	10733858	-	CASZ1;CASZ1	Body;Body	1.2676508	2.0768449	8.5176292	3.05E-14	1.58E-11	21.918546
cg24041917	chr1	119535986	+			1.6809379	-1.137891	8.5140687	3.11E-14	1.60E-11	21.899304
cg17555825	chr5	76924190	+			1.8769756	-0.152875	8.5132707	3.12E-14	1.61E-11	21.894991
cg16470877	chr16	68877286	+	TMCO7	TSS1500	-0.859447	-3.962729	-8.510367	3.17E-14	1.63E-11	21.879299

cg01674274	chr16	692585	-	FAM195A	Body	-0.608359	-3.783404	-8.509748	3.18E-14	1.64E-11	21.875953
cg07052251	chr21	47392592	+			-1.486259	-3.544267	-8.507616	3.22E-14	1.66E-11	21.864434
cg13906823	chr1	161008824	+	TSTD1;TSTD1;TSTD1	TSS200;TSS200;TSS200	-2.289979	-1.961869	-8.505339	3.26E-14	1.68E-11	21.852133
cg08219218	chr4	151503501	+	LRBA;MAB21L2;MAB21L2	Body;1stExon;5'UTR	1.5633434	3.8836692	8.5176197	3.32E-14	1.70E-11	21.837697
cg24027320	chr20	13620031	+	TASP1	TSS1500	-1.396394	-2.497186	-8.501425	3.33E-14	1.71E-11	21.830987
cg06155414	chr13	97893311	-	MBNL2;MBNL2	5'UTR;5'UTR	1.3862931	1.4854928	8.4987468	3.38E-14	1.73E-11	21.816521
cg22588546	chr17	8382941	+	MYH10	Body	1.3311345	1.5992048	8.4952812	3.45E-14	1.76E-11	21.797802
cg13261883	chr7	3988772	-	SDK1	Body	1.9545614	1.4722501	8.5101134	3.46E-14	1.77E-11	21.797122
cg19939077	chr10	81108060	+	PPIF	Body	-1.295012	-2.157722	-8.494665	3.46E-14	1.77E-11	21.794474
cg26495711	chr2	240169280	-	HDAC4	Body	-2.097471	0.3636379	-8.492184	3.51E-14	1.79E-11	21.781075
cg10241809	chr17	80197360	+	SLC16A3;SLC16A3;SLC16A3	3'UTR;3'UTR;3'UTR	-1.132463	-2.631641	-8.491709	3.52E-14	1.79E-11	21.778511
cg18353563	chr3	127390935	+	ABTB1;PODXL2;ABTB1;ABTB1	TSS1500;Body;TSS1500;TSS1500	-1.241573	-3.177344	-8.491593	3.52E-14	1.79E-11	21.777882
cg17680767	chr5	73623208	-			-1.880051	-0.75869	-8.491354	3.53E-14	1.79E-11	21.776594
cg13856643	chr2	238500389	-	RAB17	TSS1500	-1.633338	-1.551124	-8.490781	3.54E-14	1.79E-11	21.773501
cg26985471	chr6	32085577	-	ATF6B;ATF6B	Body;Body	1.0717659	3.0466808	8.4980807	3.54E-14	1.79E-11	21.77293
cg14263345	chr9	138902595	-			1.6221969	2.7674134	8.4860119	3.63E-14	1.84E-11	21.747747
cg24292761	chr6	117584665	-			1.7446333	0.218884	8.4848821	3.66E-14	1.85E-11	21.741647
cg07907386	chr4	41749443	-	PHOX2B	Body	2.5886125	-0.511528	8.482834	3.70E-14	1.86E-11	21.730589
cg15666143	chr3	130061073	+			-1.863654	-1.344209	-8.482934	3.70E-14	1.86E-11	21.731131
cg16385763	chr1	21586831	-	ECE1;ECE1;ECE1;ECE1	Body;Body;Body;Body	1.8283728	2.772197	8.4832339	3.69E-14	1.86E-11	21.732748

cg24546446	chr4	54457928	-	LNX1	TSS1500	-1.338641	-2.337336	-8.482631	3.70E-14	1.86E-11	21.729494
cg19047265	chr2	177025998	-			2.0235626	0.3211742	8.4823697	3.71E-14	1.86E-11	21.728083
cg09960553	chr16	87840374	-			-0.939057	-3.515336	-8.480602	3.75E-14	1.88E-11	21.718542
cg14065115	chr6	100916959	+			1.8888676	-0.605887	8.478255	3.79E-14	1.90E-11	21.70587
cg14558529	chr6	27648341	-			2.2971288	0.2949246	8.4783231	3.79E-14	1.90E-11	21.706238
cg18443359	chr5	134374693	+			2.4115583	-0.842152	8.4772367	3.82E-14	1.91E-11	21.700374
cg16489192	chr7	75678113	-	MDH2;STYXL1	Body;TSS1500	-1.002771	-3.355414	-8.476992	3.82E-14	1.91E-11	21.699051
cg17514088	chr17	76503074	-	DNAH17	Body	1.1542827	1.8399153	8.4764762	3.83E-14	1.91E-11	21.696269
cg23806894	chr4	111535528	-			2.5974223	-0.423445	8.4744552	3.88E-14	1.93E-11	21.685361
cg12067736	chr1	8412849	-	RERE;RERE;RERE	3'UTR;3'UTR;3'UTR	1.5621732	0.5582491	8.4724202	3.92E-14	1.95E-11	21.674378
cg17934775	chr15	93450129	-	CHD2;CHD2	Body;Body	-1.354002	-1.821606	-8.479263	3.94E-14	1.96E-11	21.671592
cg12492087	chr15	42749885	+	ZFP106	TSS200	1.7538301	0.6133596	8.4713249	3.94E-14	1.96E-11	21.668467
cg05477514	chr8	23564008	-	NKX2-6	TSS200	1.8865011	-0.701311	8.4707896	3.96E-14	1.96E-11	21.665578
cg25375029	chr1	244820484	-	PPPDE1	Body	1.5047827	2.8430593	8.4700766	3.97E-14	1.97E-11	21.66173
cg05455680	chr22	44394758	+	PARVB	TSS1500	0.9146854	2.6546467	8.4693509	3.99E-14	1.97E-11	21.657814
cg24961583	chr3	27765409	+			2.5844046	-1.644112	8.4694912	3.98E-14	1.97E-11	21.658571
cg12408295	chr20	42286743	+			-1.237532	-3.202457	-8.468826	4.00E-14	1.97E-11	21.654982
cg13019491	chr14	60977856	-	SIX6	Body	2.678255	-0.320975	8.4754978	4.02E-14	1.98E-11	21.651538
cg07987843	chr12	3309495	-	TSPAN9;TSPAN9	5'UTR;5'UTR	-2.342513	-4.269206	-8.467107	4.04E-14	1.99E-11	21.645704
cg25013852	chr1	64058941	-	PGM1	TSS200	-0.475503	-3.648065	-8.466739	4.05E-14	1.99E-11	21.643721

cg26091021	chr2	177021826	-			2.8494823	-0.413078	8.4657929	4.07E-14	2.00E-11	21.638615
cg20716932	chr20	31331814	-	COMMD7;COMMD7	TSS200;TSS200	-0.866365	-2.322945	-8.463898	4.11E-14	2.02E-11	21.628392
cg23430664	chr17	48423021	-	XYLT2	TSS1500	-0.854128	-3.391266	-8.463197	4.13E-14	2.02E-11	21.624607
cg22720790	chr19	58716032	-	ZNF274;ZNF274;ZNF274	Body;Body;5'UTR	1.8520104	1.2087304	8.4613353	4.17E-14	2.04E-11	21.614565
cg09464206	chr2	202122309	+	CASP8;CASP8;CASP8;CASP8;CASP8	5'UTR;5'UTR;5'UTR;TSS1500;5'UTR	-1.377563	-2.724651	-8.459186	4.22E-14	2.06E-11	21.602969
cg11360546	chr7	1094263	+	C7orf50;C7orf50;C7orf50	Body;Body;Body	1.4814907	1.4873793	8.4591502	4.22E-14	2.06E-11	21.602777
cg12111309	chr4	57624039	-			-1.915434	-2.984164	-8.459328	4.22E-14	2.06E-11	21.603735
cg02515217	chr17	57918600	-	MIR21	TSS200	-1.991939	-2.275539	-8.456438	4.29E-14	2.09E-11	21.588146
cg07359518	chr12	132562495	+	EP400	3'UTR	1.1856901	2.1429967	8.4564655	4.29E-14	2.09E-11	21.588295
cg06345789	chr17	43325791	-	LOC100133991	Body	-1.408094	-1.763254	-8.454208	4.34E-14	2.11E-11	21.576119
cg11811610	chr6	10390811	+			1.2968248	-0.758406	8.4540055	4.34E-14	2.11E-11	21.575027
cg14162940	chr20	30605868	+	C20orf160	Body	-1.988737	-2.400127	-8.451897	4.40E-14	2.13E-11	21.563656
cg23497016	chr2	66666684	+	MEIS1	Body	2.1121689	-0.712429	8.44957	4.45E-14	2.16E-11	21.551105
cg03339247	chr16	73059888	+	ZFHX3;ZFHX3	5'UTR;5'UTR	1.3309619	1.3837546	8.4491158	4.46E-14	2.16E-11	21.548655
cg09058748	chr2	87035698	-	CD8A	TSS200	-0.966965	-3.679801	-8.448145	4.49E-14	2.17E-11	21.543421
cg15540371	chr14	105553520	+			-1.127699	-3.661547	-8.446673	4.53E-14	2.19E-11	21.535483
cg02861178	chr20	10492319	+	C20orf94	Body	-1.771075	-3.397716	-8.445306	4.56E-14	2.20E-11	21.528114
cg03946762	chr12	54785043	-	ZNF385A;ZNF385A;ZNF385A;ZNF385A	5'UTR;1stExon;1stExon;5'UTR	-1.709146	-2.262606	-8.443792	4.60E-14	2.22E-11	21.519946
cg21039708	chr14	57278729	-	OTX2OS1	TSS1500	3.2958453	0.4422867	8.4416324	4.65E-14	2.24E-11	21.508305
cg08491487	chr6	30039130	+	RNF39;RNF39	Body;Body	1.784203	0.2042203	8.4403689	4.69E-14	2.25E-11	21.501493

cg11642106	chr3	27772805	-			2.2869562	0.0462787	8.4399797	4.70E-14	2.25E-11	21.499395
cg15672768	chr5	140741220	-	PCDHGA4;PCDHGA2;PCDHGB2;PCDHGA1;PCDHGB1;PCDHGA3;PCDHGB2	Body;Body;1stExon;Body;Body;Body;1stExon	1.4177589	0.5332015	8.4399792	4.70E-14	2.25E-11	21.499392
cg22879834	chr2	239169591	+	PER2	Body	1.8584321	2.4431126	8.4391304	4.72E-14	2.26E-11	21.494817
cg23474190	chr21	38937109	+			-1.698968	-2.872433	-8.437406	4.77E-14	2.28E-11	21.48552
cg26690592	chr11	19261879	-	E2F8	5'UTR	-1.126009	-2.933561	-8.431421	4.93E-14	2.36E-11	21.453261
cg16425726	chr4	83680145	+	SCD5;SCD5	Body;Body	1.7011765	3.3953426	8.4459694	4.94E-14	2.36E-11	21.453185
cg24217704	chr2	166650930	+	GALNT3	TSS200	-1.073746	-4.128771	-8.437797	4.96E-14	2.36E-11	21.448515
cg25729585	chr1	203056475	+	MYOG	TSS1500	2.0603438	3.7940248	8.430023	4.97E-14	2.37E-11	21.445727
cg03444940	chr16	2133637	+	TSC2;TSC2;TSC2	Body;Body;Body	0.979228	1.3111932	8.4297535	4.97E-14	2.37E-11	21.444275
cg14615927	chr2	240259213	-	HDAC4	Body	1.5111568	1.9396242	8.4292726	4.99E-14	2.37E-11	21.441683
cg13756932	chr2	136737707	-	DARS	Body	1.0968292	1.4193398	8.4277301	5.03E-14	2.39E-11	21.433371
cg11561737	chr17	46796908	+			3.3039421	-1.745495	8.4342013	5.06E-14	2.40E-11	21.429186
cg23627350	chr1	183095301	-	LAMC1	Body	0.923412	2.8976861	8.4261848	5.07E-14	2.40E-11	21.425044
cg00458878	chr7	27260285	+			2.0642092	-0.695701	8.4257515	5.09E-14	2.41E-11	21.422709
cg15665400	chr2	133428652	+	LYPD1;LYPD1	TSS200;5'UTR	-1.771193	-2.98757	-8.423572	5.15E-14	2.43E-11	21.410963
cg13189020	chr17	4444924	+	MYBBP1A;MYBBP1A	Body;Body	1.0244764	1.1909091	8.4382556	5.16E-14	2.44E-11	21.411616
cg05617798	chr17	8113714	-	AURKB	5'UTR	-0.700371	-3.403194	-8.422958	5.16E-14	2.44E-11	21.407656
cg12204897	chr9	131486753	+	ZDHHC12	TSS1500	-1.07987	-3.857311	-8.420195	5.24E-14	2.47E-11	21.392771
cg22582113	chr9	130742348	+	FAM102A	1stExon	-0.766413	-3.975407	-8.420161	5.25E-14	2.47E-11	21.392585
cg26831119	chr4	111550830	-	PITX2;PITX2	Body;Body	2.4796412	0.0382406	8.4165324	5.35E-14	2.52E-11	21.37304

cg15007228	chr1	2210335	+	SKI	Body	1.1694549	1.1091383	8.4144125	5.42E-14	2.55E-11	21.361621
cg08478871	chr20	30606493	-	C20orf160	Body	-1.033925	-3.799933	-8.413402	5.45E-14	2.56E-11	21.356176
cg11247674	chr1	55267266	-	TTC22;TTC22	TSS1500;TSS1500	-1.308731	-3.115156	-8.413129	5.46E-14	2.56E-11	21.354708
cg11624060	chr5	1316038	+			-1.333671	-2.782535	-8.412788	5.47E-14	2.56E-11	21.352869
cg26692873	chr6	25417079	+	LRRC16A	Body	1.1238369	1.359254	8.4125746	5.47E-14	2.56E-11	21.351722
cg03074946	chr12	120687694	+	PXN;PXN;PXN;PXN	5'UTR;Body;1stExon;Body	1.1877833	0.625584	8.4110374	5.52E-14	2.58E-11	21.343442
cg18447772	chr7	27205224	+	HOXA9	TSS200	3.339512	-0.684275	8.4107975	5.53E-14	2.58E-11	21.34215
cg12019475	chr6	45700739	+			1.1401377	1.6894121	8.4104716	5.54E-14	2.58E-11	21.340396
cg04309212	chr2	223183932	-			-0.614783	-3.693945	-8.407396	5.63E-14	2.62E-11	21.323833
cg24504361	chr12	53297987	-	KRT8	Body	-1.398285	-1.197384	-8.405603	5.69E-14	2.65E-11	21.314175
cg00718470	chr15	98964982	-			-1.48049	-3.416124	-8.405004	5.71E-14	2.65E-11	21.310952
cg03354554	chr11	9781412	+			-2.351755	-1.650957	-8.403687	5.75E-14	2.67E-11	21.30386
cg10109215	chr20	30605810	+	C20orf160	Body	-1.076986	-2.040538	-8.401372	5.82E-14	2.70E-11	21.291396
cg17243289	chr18	45458021	+	SMAD2;SMAD2;SMAD2	TSS1500;TSS1500;TSS1500	-1.257448	-1.579074	-8.391268	6.16E-14	2.85E-11	21.237007
cg26530341	chr8	23083353	-	TNFRSF10A	TSS1500	-1.966263	-0.139429	-8.391168	6.16E-14	2.85E-11	21.236469
cg11536474	chr2	63286049	+			2.9134393	0.2107968	8.3899018	6.21E-14	2.87E-11	21.229653
cg11224624	chr8	145049280	+	PLEC1	Body	-2.320371	-4.292554	-8.396176	6.25E-14	2.88E-11	21.224914
cg14917100	chr2	127528627	+			-2.077731	0.237479	-8.38755	6.29E-14	2.90E-11	21.216999
cg22817650	chr1	193191405	+	CDC73	Body	1.9333961	2.8151357	8.3945206	6.30E-14	2.90E-11	21.216217
cg12502079	chr22	51169028	-	SHANK3	Body	1.1049579	0.8083097	8.3868957	6.31E-14	2.91E-11	21.213475

cg01381586	chr2	10587627	-	ODC1;SNORA80B	5'UTR;TSS1500	-1.393967	-3.429837	-8.386643	6.32E-14	2.91E-11	21.212114
cg09558427	chr20	45985705	+	ZMYND8;ZMYND8;ZMYND8	TSS1500;TSS1500;TSS1500	-2.016357	-4.996874	-8.386159	6.34E-14	2.91E-11	21.209509
cg07180538	chr7	4786899	-	FOXK1	Body	2.5934222	3.1754988	8.3841439	6.41E-14	2.94E-11	21.198668
cg09341491	chr16	50707795	+	SNX20;SNX20;SNX20	Body;Body;Body	1.2348249	1.2459177	8.3841468	6.41E-14	2.94E-11	21.198684
cg19416570	chr19	58715677	-	ZNF274;ZNF274;ZNF274	Body;Body;5'UTR	1.4944647	0.3931302	8.3837225	6.43E-14	2.94E-11	21.1964
cg27319917	chr16	48592694	+	N4BP1	Body	2.211559	0.0473613	8.3833995	6.44E-14	2.94E-11	21.194663
cg21686287	chr2	73021348	-	EXOC6B	Body	1.8699659	0.5579239	8.3805329	6.54E-14	2.99E-11	21.179239
cg00615919	chr2	240043915	+	HDAC4	Body	0.974281	2.0828366	8.3797365	6.57E-14	3.00E-11	21.174955
cg02377544	chr15	37388359	+	MEIS2;MEIS2;MEIS2;MEIS2;MEIS2;MEIS2;MEIS2	Body;Body;Body;Body;Body;Body;Body	1.107413	1.5021631	8.3922961	6.65E-14	3.03E-11	21.165378
cg12744634	chr1	26560303	-	CCDC21	TSS1500	-1.123298	-2.862139	-8.372793	6.83E-14	3.11E-11	21.137604
cg17655624	chr12	54785320	-	ZNF385A;ZNF385A	TSS1500;TSS1500	-2.045484	-3.432147	-8.387663	6.82E-14	3.11E-11	21.140375
cg24578966	chr4	84457523	+	AGPAT9	TSS200	-0.531913	-3.782899	-8.371662	6.87E-14	3.12E-11	21.13152
cg05973398	chr21	36420164	-	RUNX1	Body	-1.670906	-1.619765	-8.370139	6.93E-14	3.15E-11	21.12333
cg25585523	chr3	186524693	+	RFC4;RFC4	TSS1500;TSS1500	-0.781387	-3.874712	-8.366161	7.08E-14	3.21E-11	21.101935
cg12122737	chr22	42065726	-			1.3156568	1.5237191	8.3600122	7.33E-14	3.32E-11	21.068876
cg16665506	chr3	129326828	+	PLXND1	TSS1500	1.4697637	2.3331752	8.3591833	7.36E-14	3.33E-11	21.064419
cg15948504	chr1	151285561	+	PI4KB	Body	0.8303619	1.7025297	8.3581764	7.41E-14	3.35E-11	21.059006
cg17980283	chr2	45156642	+			0.9439937	0.0862573	8.3573888	7.44E-14	3.36E-11	21.054773
cg00831931	chr10	114823094	+	TCF7L2;TCF7L2;TCF7L2;TCF7L2;TCF7L2;TCF7L2	Body;Body;Body;Body;Body;Body	1.1784799	1.5577636	8.3559573	7.50E-14	3.38E-11	21.047077
cg16458866	chr1	40598779	+			1.7450118	0.0385172	8.3551453	7.53E-14	3.39E-11	21.042712

cg11737230	chr10	88159580	+			-0.986011	-3.703107	-8.354589	7.56E-14	3.40E-11	21.039724
cg17154218	chr12	124912182	+	NCOR2;NCOR2	Body;Body	1.069628	1.1864525	8.3588918	7.68E-14	3.45E-11	21.02506
cg25252598	chr12	6641602	-			-1.51262	-2.783053	-8.351464	7.69E-14	3.45E-11	21.022926
cg24411043	chr1	161008297	-	TSTD1;TSTD1;TSTD1	Body;Body;Body	-1.368984	-1.927095	-8.350167	7.74E-14	3.48E-11	21.015953
cg04102163	chr4	41881185	-			1.5890988	0.7514769	8.3482462	7.83E-14	3.51E-11	21.005632
cg11288999	chr19	41222328	-	ADCK4;ITPKC;ADCK4	5'UTR;TSS1500;TSS1500	-0.547316	-4.141441	-8.346857	7.89E-14	3.53E-11	20.998167
cg07792858	chr4	882175	+	GAK	Body	1.1085021	4.5448041	8.3466065	7.90E-14	3.53E-11	20.99682
cg12128828	chr14	91164042	+	TTC7B	Body	-0.96094	-3.433921	-8.346202	7.92E-14	3.54E-11	20.994646
cg15701111	chr12	52685334	-	KRT81	TSS200	1.3372885	1.169003	8.3519664	7.98E-14	3.56E-11	20.987931
cg11201447	chr8	128808063	+	MIR1204;PVT1	TSS200;Body	-2.624149	-1.683095	-8.343862	8.02E-14	3.58E-11	20.982073
cg13668646	chr6	46849146	-	GPR116;GPR116	Body;Body	1.3191112	3.0921182	8.3550019	8.17E-14	3.64E-11	20.955133
cg16919569	chr4	174452835	+	NBLA00301;HAND2	Body;TSS1500	1.6812385	0.6055091	8.3389509	8.24E-14	3.67E-11	20.955687
cg02362103	chr1	119532773	+	TBX15	TSS1500	1.8791134	-0.003641	8.3447715	8.31E-14	3.69E-11	20.949366
cg07405182	chr1	40598455	+			1.8492057	0.2559325	8.3367484	8.34E-14	3.71E-11	20.943855
cg05809668	chr7	35301188	-			2.5753649	-0.38478	8.3310549	8.61E-14	3.82E-11	20.913274
cg07506510	chr11	2951911	-	PHLDA2	TSS1500	-1.276554	-2.672533	-8.330195	8.65E-14	3.84E-11	20.908658
cg06663644	chr19	40971204	-	BLVRB	Body	-1.304457	-2.852383	-8.329873	8.67E-14	3.84E-11	20.906928
cg21940708	chr19	50191683	+	PRMT1;C19orf76;PRMT1;PRMT1	3'UTR;TSS1500;3'UTR;3'UTR	1.0658247	2.3820336	8.3298139	8.67E-14	3.84E-11	20.906609
cg07061500	chr6	159290520	-			-1.266774	-3.536433	-8.326463	8.83E-14	3.90E-11	20.888614
cg18623658	chr2	62900278	+	EHBP1	TSS1500	1.6687362	2.0108046	8.3261047	8.85E-14	3.91E-11	20.88669

cg26388152	chr17	27944453	-	CORO6	Body	-1.741854	-3.204997	-8.325889	8.86E-14	3.91E-11	20.885534
cg06936155	chr1	91192451	-			1.9090111	-1.054414	8.3252408	8.89E-14	3.92E-11	20.882051
cg22356324	chr10	71077947	-	HK1;HK1;HK1;HK1;HK1	Body;TSS1500;Body;Body;Body	-1.388311	-3.235821	-8.323553	8.98E-14	3.95E-11	20.872989
cg02742969	chr5	172175604	-			0.9595461	0.444931	8.3219036	9.06E-14	3.98E-11	20.864133
cg09622982	chr2	133428523	+	LYPD1;LYPD1	TSS200;5'UTR	-1.31035	-2.905722	-8.320977	9.11E-14	4.00E-11	20.859158
cg12558755	chr7	26332043	-	SNX10	5'UTR	-1.035712	-3.375004	-8.320637	9.12E-14	4.00E-11	20.857335
cg06012483	chr16	86388853	+			-1.638938	-0.765627	-8.320064	9.15E-14	4.01E-11	20.854256
cg03046346	chr11	61658150	+	FADS3	Body	-0.669298	-3.929092	-8.318721	9.22E-14	4.04E-11	20.847046
cg13127741	chr20	31331821	+	COMMD7;COMMD7	TSS200;TSS200	-1.341942	-1.92359	-8.316814	9.32E-14	4.08E-11	20.836808
cg27642428	chr11	63705693	-	NAA40	TSS1500	-1.978317	-4.891015	-8.323013	9.37E-14	4.09E-11	20.832625
cg17611742	chr1	197745238	-	DENND1B;DENND1B	TSS1500;TSS1500	-1.375332	-1.52762	-8.311801	9.58E-14	4.17E-11	20.8099
cg19291355	chr6	44281188	-	AARS2	TSS200	-1.778342	-4.394765	-8.311796	9.58E-14	4.17E-11	20.809876
cg25247520	chr8	128808017	+	MIR1204;PVT1	TSS200;Body	-2.207871	-0.910474	-8.311915	9.58E-14	4.17E-11	20.810515
cg07189157	chr7	149119407	-			-0.781612	-3.424599	-8.310327	9.66E-14	4.20E-11	20.801991
cg01417339	chr8	135813202	-	MIR30B	TSS1500	1.7204928	3.3311823	8.31586	9.75E-14	4.24E-11	20.794324
cg04650899	chr17	48194835	-	SAMD14	Body	-2.282093	-3.627078	-8.307237	9.83E-14	4.27E-11	20.785407
cg10430625	chr1	59320924	-			1.8725126	1.7167637	8.3054498	9.93E-14	4.31E-11	20.775819
cg24680129	chr12	13318625	-			-1.327419	-2.891739	-8.304553	9.97E-14	4.32E-11	20.771006
cg09904358	chr10	24446911	-	KIAA1217	5'UTR	-1.939044	0.1242085	-8.302872	1.01E-13	4.36E-11	20.761988
cg06186150	chr1	2458298	+	PANK4	TSS1500	-1.107696	-2.916787	-8.30104	1.02E-13	4.40E-11	20.752161

cg01606773	chr6	74414015	+	CD109;CD109;CD109	Body;Body;Body	-2.223066	-0.198661	-8.297745	1.04E-13	4.48E-11	20.734484
cg17086579	chr11	67266089	-	PITPNM1;PITPNM1	Body;Body	1.7910604	3.7414476	8.2949503	1.05E-13	4.54E-11	20.719491
cg04147642	chr6	32054659	-	TNXB	Body	-1.267063	-2.411467	-8.289918	1.08E-13	4.67E-11	20.692499
cg19618897	chr17	65242892	-			-1.348642	-1.441636	-8.287881	1.09E-13	4.71E-11	20.681579
cg23067085	chr3	71180032	-	FOXP1	Body	-1.81807	-3.390372	-8.287997	1.09E-13	4.71E-11	20.682199
cg01798589	chr3	36421572	-	STAC	TSS1500	-1.26928	-2.430072	-8.286895	1.10E-13	4.73E-11	20.676289
cg01295392	chr12	108169569	+	ASCL4;ASCL4	1stExon;3'UTR	1.7741715	-0.179684	8.2857971	1.11E-13	4.75E-11	20.670404
cg22670033	chr5	180646608	-			1.2028265	1.1708179	8.2859323	1.11E-13	4.75E-11	20.671129
cg27391093	chr2	105372650	-			-1.166939	-0.353969	-8.283959	1.12E-13	4.79E-11	20.660547
cg10420161	chr10	102899983	-			1.7231897	-0.015367	8.280787	1.14E-13	4.87E-11	20.643541
cg00746143	chr3	194959645	+	C3orf21	Body	1.3269879	1.2123957	8.2791561	1.15E-13	4.91E-11	20.634799
cg04013090	chr8	67342485	+	RRS1;RRS1	1stExon;3'UTR	-1.419829	-4.088338	-8.27825	1.15E-13	4.93E-11	20.629942
cg12064195	chr22	47367756	+	TBC1D22A	Body	0.8526435	1.3535698	8.281591	1.18E-13	5.03E-11	20.610972
cg12003230	chr21	44899139	-	C21orf84	TSS1500	1.2149406	2.1839263	8.2740838	1.18E-13	5.04E-11	20.60761
cg12161848	chr7	22254120	-	RAPGEF5	Body	1.3038914	2.0575134	8.272757	1.19E-13	5.07E-11	20.600499
cg06245711	chr17	74260776	-	FAM100B	TSS1500	-1.647798	-1.717178	-8.271348	1.20E-13	5.11E-11	20.592946
cg23095743	chr10	104000831	+	PITX3	5'UTR	2.1916939	-0.368168	8.270878	1.20E-13	5.11E-11	20.590429
cg11379081	chr1	209405050	+			1.8404768	0.4365386	8.2691581	1.21E-13	5.16E-11	20.581213
cg07724304	chr17	73509955	-	CASKIN2	5'UTR	1.1538229	1.6273049	8.2685937	1.22E-13	5.17E-11	20.578188
cg01409343	chr17	57915740	-	TMEM49	Body	-1.568319	-2.305422	-8.267737	1.22E-13	5.18E-11	20.573598

cg23440816	chr1	223948910	-	CAPN2;CAPN2	Body;Body	2.003375	1.8408466	8.2677639	1.22E-13	5.18E-11	20.573741
cg13747967	chr16	57392618	-	CCL22	TSS200	-1.36732	-1.563001	-8.266527	1.23E-13	5.21E-11	20.567116
cg02935904	chr1	55267293	+	TTC22;TTC22	TSS1500;TSS1500	-1.463107	-2.274709	-8.272765	1.24E-13	5.23E-11	20.563951
cg21783847	chr1	167522422	+	CREG1	Body	-1.193002	-2.331655	-8.268721	1.27E-13	5.35E-11	20.54217
cg01449818	chr10	116286605	-	ABLIM1;ABLIM1;ABLIM1;ABLIM1;ABLIM1	Body;1stExon;Body;5'UTR;Body	-0.707948	-3.877625	-8.260509	1.27E-13	5.37E-11	20.534873
cg07959094	chr19	15347074	+			0.8312442	1.9028713	8.2675638	1.27E-13	5.37E-11	20.536149
cg25539759	chr13	107211856	-	ARGLU1	Body	0.8226988	2.1536061	8.2604337	1.27E-13	5.37E-11	20.534468
cg13148085	chr17	65242711	-	HELZ	TSS1500	-1.087668	-2.369046	-8.258787	1.29E-13	5.41E-11	20.525647
cg20906291	chr6	10382103	-			1.5087559	0.7118301	8.2586618	1.29E-13	5.41E-11	20.524976
cg19618279	chr17	40715228	+	COASY;COASY;COASY;COASY;COASY	1stExon;Body;Body;Body;5'UTR	-2.090731	0.674254	-8.257564	1.29E-13	5.43E-11	20.519098
cg27038724	chr15	99670386	-	SYNM;SYNM	Body;Body	1.1339889	2.4534652	8.2574824	1.29E-13	5.43E-11	20.518659
cg16536855	chr10	71038317	-	HK1;HK1;HK1	5'UTR;5'UTR;5'UTR	1.5694606	1.0039934	8.256876	1.30E-13	5.45E-11	20.515411
cg24528523	chr11	2923808	+	SLC22A18AS;SLC22A18;SLC22A18	5'UTR;5'UTR;5'UTR	-0.839254	-3.779101	-8.254685	1.32E-13	5.51E-11	20.503676
cg04705435	chr11	17411270	+	KCNJ11;KCNJ11	TSS1500;TSS1500	-1.677713	-3.756876	-8.260463	1.32E-13	5.54E-11	20.498202
cg01381846	chr7	27204785	+	HOXA9	1stExon	2.1389469	0.0922644	8.2601442	1.33E-13	5.54E-11	20.496338
cg23565821	chr6	33385056	-	CUTA;CUTA;CUTA;CUTA;CUTA	Body;Body;Body;Body;Body	-1.564372	-2.878312	-8.250675	1.34E-13	5.61E-11	20.4822
cg09975715	chr17	8379050	+	MYH10	3'UTR	1.2291398	1.8561531	8.2499856	1.35E-13	5.63E-11	20.478508
cg11199046	chr12	115136308	-			1.3910315	-0.365676	8.2489778	1.36E-13	5.66E-11	20.473111
cg00736201	chr12	108643267	-	WSCD2	3'UTR	-1.667086	-0.153946	-8.247472	1.37E-13	5.70E-11	20.465047
cg13352750	chr7	27225123	-	HOXA11AS;HOXA11	Body;TSS1500	2.5726924	-0.834055	8.2448168	1.39E-13	5.77E-11	20.450832

cg16364495	chr4	40859251	+	APBB2;APBB2;APBB2;APBB2;APBB2;APBB2;APBB2	Body;TSS200;TSS200;Body;TSS200;Body;TSS200	-2.256966	-2.70855	-8.2447	1.39E-13	5.77E-11	20.450208
cg16888658	chr2	25142117	+	ADCY3	TSS200	-1.802602	-1.50522	-8.244872	1.39E-13	5.77E-11	20.451128
cg04402948	chr12	117613954	+	FBXO21;FBXO21	Body;Body	1.1068585	1.7173644	8.2444664	1.39E-13	5.77E-11	20.448956
cg02883503	chr16	75423900	-	CFDP1	Body	1.3180499	1.7024725	8.2413398	1.42E-13	5.87E-11	20.432218
cg20961591	chr19	38755588	-	SPINT2;SPINT2	1stExon;1stExon	-1.568008	-3.950686	-8.240273	1.42E-13	5.90E-11	20.426509
cg21747271	chr11	67250052	-	AIP	TSS1500	-1.550422	-1.767138	-8.237573	1.45E-13	5.98E-11	20.412056
cg07382920	chr1	3567646	-	TP73;WDR8	TSS1500;TSS1500	2.0132354	-0.430071	8.2370813	1.45E-13	5.99E-11	20.409423
cg10140240	chr17	76220672	-	BIRC5;BIRC5;BIRC5;EPR1	3'UTR;3'UTR;3'UTR;Body	1.2749145	2.9043143	8.2364723	1.45E-13	6.00E-11	20.406164
cg15506609	chr7	27206073	-	HOXA9	TSS1500	1.9049143	1.0217976	8.2353705	1.46E-13	6.04E-11	20.400267
cg26931862	chr12	54349169	+	HOXC12	1stExon	2.0819002	-0.532968	8.2351097	1.47E-13	6.04E-11	20.39887
cg02739870	chr5	1113320	+	SLC12A7	TSS1500	-1.649886	-0.932663	-8.234436	1.47E-13	6.05E-11	20.395267
cg12231340	chr12	52685221	+	KRT81	1stExon	1.1742074	1.06826	8.2340566	1.47E-13	6.06E-11	20.393234
cg19498319	chr7	16793126	-	TSPAN13	TSS1500	-0.571076	-3.741116	-8.23408	1.47E-13	6.06E-11	20.393357
cg01442005	chr17	47179278	-			-1.978656	-0.227919	-8.233151	1.48E-13	6.08E-11	20.388389
cg03410115	chr16	29989187	-	TAOK2;TAOK2	Body;Body	0.7890691	1.6194554	8.2331207	1.48E-13	6.08E-11	20.388226
cg13761949	chr10	35098359	-	PARD3	Body	-1.638392	-2.716454	-8.230871	1.50E-13	6.15E-11	20.376186
cg01891252	chr1	50881219	+			1.7716418	-0.91474	8.2303237	1.50E-13	6.16E-11	20.373258
cg27125965	chr19	11707246	-	ZNF627	TSS1500	1.2691843	1.8044279	8.2294398	1.51E-13	6.18E-11	20.368529
cg06993413	chr15	65810204	+	DPP8;DPP8;DPP8;DPP8	TSS200;TSS200;TSS1500;TSS200	-0.94431	-3.597397	-8.228713	1.52E-13	6.20E-11	20.364641
cg09010802	chr1	156776671	-	SH2D2A;SH2D2A;SH2D2A;SH2D2A	3'UTR;3'UTR;3'UTR;3'UTR	1.1649682	2.3638706	8.2265902	1.54E-13	6.27E-11	20.353281

cg06074958	chr12	57118908	+	NACA;NACA;NACA;NACA;NACA;NACA	1stExon;5'UTR;5'UTR;1stExon;5'UTR;5'UTR	-0.598294	-4.015067	-8.230853	1.56E-13	6.36E-11	20.33992
cg26708781	chr4	87921477	+	AFF1	Body	1.5745143	1.4073654	8.2210207	1.58E-13	6.45E-11	20.323486
cg19295314	chr6	1635640	+	GMDS	Body	1.0291439	0.3119676	8.2192719	1.60E-13	6.51E-11	20.314132
cg14032089	chr17	57917589	-	TMEM49;MIR21	3'UTR;TSS1500	-1.74083	-2.495774	-8.218396	1.61E-13	6.54E-11	20.309448
cg11303839	chr7	75405967	+	CCL26	5'UTR	-2.336143	-1.697637	-8.224679	1.61E-13	6.54E-11	20.30697
cg17941572	chr1	63784454	+			2.0631037	0.4761943	8.217523	1.62E-13	6.54E-11	20.304778
cg23339656	chr2	234159882	-	ATG16L1;ATG16L1	TSS1500;TSS1500	-1.411785	-1.260013	-8.217942	1.61E-13	6.54E-11	20.307018
cg26080331	chr13	113613318	+			1.9783676	3.3113275	8.2687791	1.61E-13	6.54E-11	20.305158
cg23817096	chr6	1620687	-			2.1804339	0.2597296	8.2169397	1.62E-13	6.56E-11	20.301658
cg27556193	chr2	63277930	+	OTX1	TSS200	-0.945351	-4.489039	-8.215968	1.63E-13	6.59E-11	20.296462
cg17093088	chr7	6544329	+	GRID2IP	Body	-1.18084	-2.923925	-8.214615	1.64E-13	6.63E-11	20.289224
cg02971793	chr7	23222205	+	NUPL2	Body	-1.462963	-4.830242	-8.227876	1.65E-13	6.65E-11	20.28764
cg09853702	chr12	47665230	-			-1.953192	-2.000616	-8.211637	1.67E-13	6.73E-11	20.273298
cg07973435	chr6	100905458	-	SIM1	Body	2.0812737	0.0765622	8.2101025	1.68E-13	6.78E-11	20.265095
cg07775417	chr4	84469563	+	AGPAT9	Body	-1.819413	-0.853775	-8.208816	1.69E-13	6.82E-11	20.258214
cg12127282	chr2	177014685	-	HOXD4;MIR10B	TSS1500;TSS1500	1.7820499	-0.315377	8.2086433	1.70E-13	6.82E-11	20.257293
cg22871653	chr1	119535817	-			1.9435466	-0.703528	8.2070204	1.71E-13	6.88E-11	20.248616
cg27025738	chr14	72006683	+	SIPA1L1	5'UTR	1.4371054	1.6882477	8.2121606	1.73E-13	6.94E-11	20.24019
cg05716350	chr4	79592882	-			1.2124849	1.8130883	8.2036711	1.74E-13	6.99E-11	20.230711
cg05964373	chr15	56657478	-	TEX9	TSS200	-0.701869	-4.202845	-8.201936	1.76E-13	7.04E-11	20.221436

cg11731300	chr1	161008542	+	TSTD1;TSTD1;TSTD1	Body;Body;Body	-2.01062	-2.842317	-8.202053	1.76E-13	7.04E-11	20.222061
cg26253134	chr2	70751721	+	TGFA;TGFA	Body;Body	-1.244465	-2.059796	-8.201875	1.76E-13	7.04E-11	20.221111
cg04016383	chr2	198513476	+	RFTN2	Body	1.0352568	1.2468295	8.2014369	1.77E-13	7.05E-11	20.218768
cg01877606	chr4	54457746	+	LNX1	TSS200	-1.067122	-1.608911	-8.201048	1.77E-13	7.06E-11	20.216692
cg02037307	chr5	134363562	-	PITX1	3'UTR	1.9763904	0.4261959	8.2149389	1.77E-13	7.06E-11	20.218796
cg01834614	chr1	147143012	-	ACP6	TSS1500	-1.059421	-2.83359	-8.200662	1.77E-13	7.06E-11	20.214627
cg13358636	chr2	124782263	-	CNTNAP5	TSS1500	2.2632994	-0.764315	8.1983644	1.80E-13	7.15E-11	20.202346
cg27295042	chr1	10738569	+	CASZ1;CASZ1	Body;Body	1.3973959	1.9660081	8.1951659	1.83E-13	7.27E-11	20.185252
cg19343530	chr6	28831388	+			-1.173891	-3.484084	-8.193373	1.85E-13	7.33E-11	20.175673
cg26365545	chr5	76932016	+	OTP	Body	1.6420124	0.2094181	8.1929476	1.85E-13	7.34E-11	20.173398
cg24390871	chr12	14996508	-	ART4	TSS200	1.5266302	1.4543314	8.1921728	1.86E-13	7.37E-11	20.169258
cg27032232	chr7	153584609	-	DPP6;DPP6	1stExon;5'UTR	2.3898477	0.1382369	8.2023104	1.90E-13	7.51E-11	20.14266
cg05859264	chr6	36097692	+	MAPK13	TSS1500	-1.279214	-0.835156	-8.194097	1.91E-13	7.56E-11	20.143879
cg00066816	chr5	158758353	+	IL12B	TSS1500	-0.992153	-1.829754	-8.185787	1.92E-13	7.61E-11	20.135141
cg00460776	chr12	120731287	+			-0.969275	-3.781977	-8.185671	1.93E-13	7.61E-11	20.134522
cg04620946	chr1	94144259	+	BCAR3	5'UTR	1.3782815	2.6932557	8.1852347	1.93E-13	7.62E-11	20.132191
cg25783892	chr10	85898649	+	GHITM	TSS1500	-1.977938	0.1932984	-8.184824	1.94E-13	7.63E-11	20.129994
cg19911880	chr8	131353969	-	ASAP1	Body	1.0776959	1.5080317	8.1839971	1.94E-13	7.66E-11	20.125579
cg18440069	chr14	101488391	-	MIR411;MIR379	TSS1500;TSS200	-1.504833	-0.107343	-8.183731	1.95E-13	7.66E-11	20.12416
cg09848693	chr3	65394690	+	MAGI1;MAGI1;MAGI1	Body;Body;Body	0.9818202	2.2691679	8.1833574	1.95E-13	7.67E-11	20.122163

cg01334972	chr11	72463230	+	ARAP1	5'UTR	-0.665602	-4.479377	-8.182132	1.96E-13	7.71E-11	20.115618
cg11868356	chr17	41621625	+	ETV4;ETV4	Body;Body	-1.45837	-2.301916	-8.181962	1.97E-13	7.71E-11	20.114712
cg13488011	chr15	81466841	-			-1.733162	-0.889321	-8.18186	1.97E-13	7.71E-11	20.114166
cg03195795	chr7	4786768	+	FOXK1	Body	1.3838073	1.3510642	8.1813261	1.97E-13	7.73E-11	20.111312
cg18888403	chr1	24152774	+	HMGCL;HMGCL	TSS1500;TSS1500	-2.174171	0.2350433	-8.180429	1.98E-13	7.76E-11	20.10652
cg00014998	chr2	177014962	-	HOXD4;MIR10B	TSS1500;TSS200	1.7638375	0.4508545	8.1801729	1.99E-13	7.77E-11	20.105154
cg24016624	chr19	45504513	-	RELB	TSS200	-0.584095	-4.027024	-8.184696	2.01E-13	7.86E-11	20.093929
cg17029193	chr1	8510607	-	RERE;RERE	Body;Body	1.9248872	2.115458	8.1839722	2.02E-13	7.88E-11	20.089927
cg04947157	chr17	76128481	+	TMC8;TMC6;TMC6	Body;5'UTR;1stExon	-1.772586	-1.843237	-8.175618	2.04E-13	7.94E-11	20.080831
cg08663890	chr1	111746277	-			-1.466149	-1.607862	-8.17492	2.04E-13	7.97E-11	20.077102
cg03613662	chr8	70404585	+	SULF1;SULF1;SULF1;SULF1	5'UTR;TSS1500;TSS1500;5'UTR	-1.31297	-1.763974	-8.174513	2.05E-13	7.98E-11	20.074929
cg04503570	chr1	226897117	+	ITPKB	Body	1.0237176	1.2706291	8.1805329	2.06E-13	8.01E-11	20.071604
cg11422521	chr2	171830314	+			-0.944469	-5.301355	-8.172897	2.07E-13	8.03E-11	20.066297
cg12346967	chr21	32185331	+	KRTAP8-1;KRTAP8-1	1stExon;3'UTR	-1.692423	0.0257706	-8.171687	2.08E-13	8.08E-11	20.05984
cg21076680	chr2	69135655	-			-1.704502	-2.19317	-8.171246	2.09E-13	8.09E-11	20.057485
cg00781795	chr18	55815677	+	NEDD4L;NEDD4L;NEDD4L;NEDD4L;NEDD4L;NEDD4L;NEDD4L	5'UTR;TSS1500;TSS1500;Body;TSS1500;Body;TSS1500	1.0381594	0.8371983	8.1770191	2.10E-13	8.13E-11	20.053029
cg12932613	chr15	53086630	-			1.4262456	1.4028945	8.1694536	2.11E-13	8.16E-11	20.047915
cg16785213	chr16	75599104	+	GABARAPL2	TSS1500	-1.046937	-3.362179	-8.166832	2.14E-13	8.27E-11	20.03392
cg10628699	chr13	103531040	-	LOC121952	TSS1500	1.3953004	1.7070292	8.1664018	2.14E-13	8.28E-11	20.031623
cg01313497	chr2	63278100	-	OTX1	5'UTR	-0.509883	-4.121141	-8.166236	2.14E-13	8.28E-11	20.030736

cg02114728	chr2	169102929	+	STK39	Body	-0.767569	-4.361543	-8.164569	2.16E-13	8.34E-11	20.021841
cg07462756	chr4	4859985	-	MSX1	TSS1500	1.5521871	-0.414526	8.1647289	2.16E-13	8.34E-11	20.022693
cg23244790	chr5	140811102	+	PCDHGA4;PCDHGA11;PCDHGA11;PCDHGA9;PCDHGA1;PCDHGB1;PCDHGB6;PCDHGA12;PCDHGB3;PCDHGB7;PCDHGA6;PCDHGA8;PCDHGA10;PCDHGA12;PCDHGA5;PCDHGB4;PCDHGA	Body;Body;Body;Body;Body;Body;Body;1stExon;Body;Body;Body;Body;Body;1stExon;Body;Body;Body;Body;Body;Body	2.1846868	-0.626122	8.1640957	2.17E-13	8.36E-11	20.019314
cg00766964	chr13	24693844	+			1.4341257	0.8072307	8.1624649	2.19E-13	8.43E-11	20.01061
cg06821075	chr10	99933209	+	C10orf28	5'UTR	0.8004139	2.6243303	8.1620208	2.19E-13	8.44E-11	20.008239
cg23193870	chr12	7055657	-	PTPN6	TSS200	-1.208052	-1.838426	-8.161801	2.20E-13	8.44E-11	20.007066
cg17603689	chr1	50883013	-			1.5112628	0.1877056	8.1602684	2.22E-13	8.51E-11	19.998887
cg08364561	chr11	31846844	+			2.8747938	0.3098985	8.1585979	2.24E-13	8.58E-11	19.989972
cg04907244	chr7	22894795	+	SNORD93	TSS1500	-1.908351	-2.196761	-8.157637	2.25E-13	8.62E-11	19.984843
cg07160746	chr20	43726765	-	KCNS1	Body	1.6699909	0.1037374	8.1564597	2.26E-13	8.66E-11	19.978562
cg27448161	chr12	53472792	-	SPRYD3	Body	-1.011922	-2.630874	-8.153413	2.30E-13	8.80E-11	19.962304
cg11028872	chr2	204486921	+			1.4755529	1.4794975	8.1524242	2.31E-13	8.84E-11	19.95703
cg07999201	chr4	16587121	+	LDB2;LDB2	Body;Body	-1.892674	0.1120402	-8.151642	2.32E-13	8.87E-11	19.952859
cg04688351	chr2	223154140	-	PAX3;PAX3;PAX3;PAX3;PAX3;PAX3	Body;Body;Body;Body;Body;Body	2.6855778	-0.615763	8.1574315	2.34E-13	8.91E-11	19.948593
cg07514159	chr10	80982257	-	ZMIZ1	Body	1.0011765	1.6028775	8.1501105	2.34E-13	8.93E-11	19.944687
cg12756396	chr1	50886969	-	DMRTA2	Body	2.103203	-0.371423	8.1496754	2.35E-13	8.95E-11	19.942366
cg03597347	chr6	28058909	-	ZSCAN12L1	TSS200	1.8344016	-0.223937	8.1451476	2.41E-13	9.16E-11	19.918214
cg21834063	chr6	32085541	+	ATF6B;ATF6B	Body;Body	0.8013127	1.2446613	8.145045	2.41E-13	9.16E-11	19.917667
cg20072171	chr3	62356962	-	FEZF2	Body	3.4740711	-0.878698	8.1434144	2.43E-13	9.24E-11	19.90897
cg02036261	chr14	57276057	-	OTX2	5'UTR	2.287658	-0.122035	8.138637	2.50E-13	9.47E-11	19.883493

cg16000989	chr4	41983716	+	DCAF4L1;DCAF4L1	5'UTR;1stExon	1.6086034	2.961213	8.1372688	2.52E-13	9.54E-11	19.876198
cg08833577	chr10	118899288	-	VAX1;VAX1	TSS1500;TSS1500	2.0386134	-0.037989	8.1413989	2.55E-13	9.67E-11	19.863286
cg24930915	chr16	2199120	+	RAB26	Body	-0.753072	-3.368649	-8.134004	2.56E-13	9.69E-11	19.858792
cg12401425	chr1	242011513	-	EXO1;EXO1;EXO1;EXO1	TSS1500;5'UTR;1stExon;TSS1500	-1.179103	-4.730753	-8.131033	2.60E-13	9.85E-11	19.84295
cg22445742	chr2	60680630	+	BCL11A;BCL11A	Body;Body	1.2596336	1.1484868	8.1302497	2.62E-13	9.88E-11	19.838777
cg03757387	chr4	109770908	+	COL25A1;COL25A1	Body;Body	-1.49799	-1.824601	-8.13008	2.62E-13	9.88E-11	19.837875
cg03025825	chr17	1975245	-	SMG6;SMG6	Body;Body	1.2818965	1.4082994	8.1293884	2.63E-13	9.91E-11	19.834186
cg07284476	chr17	65546480	+	PITPNC1;PITPNC1	Body;Body	1.152928	2.4358804	8.129272	2.63E-13	9.91E-11	19.833565
cg19780563	chr10	94594494	+	EXOC6;EXOC6	5'UTR;1stExon	1.0703306	2.2570861	8.1288611	2.64E-13	9.91E-11	19.831375
cg26474124	chr2	70368457	+			-1.493752	-1.342602	-8.128992	2.63E-13	9.91E-11	19.832072
cg07649862	chr16	88897202	+	GALNS	Body	1.7278033	3.8027735	8.1418781	2.64E-13	9.94E-11	19.830481
cg05024961	chr16	88498528	+	ZNF469	Body	-1.686629	0.3085619	-8.128071	2.65E-13	9.94E-11	19.827164
cg19017553	chr10	34817409	-	PARD3	Body	-1.531009	-1.054741	-8.126857	2.66E-13	1.00E-10	19.820693
cg13482308	chr9	37002685	-	PAX5	Body	3.1487658	-1.220004	8.1263613	2.67E-13	1.00E-10	19.818051
cg03408619	chr8	30300142	-	RBPMS;RBPMS;RBPMS;RBPMS	Body;Body;Body;Body	1.5456466	1.9316207	8.1259427	2.68E-13	1.00E-10	19.81582
cg21650861	chr3	127299468	-	TPRA1;TPRA1;TPRA1	5'UTR;5'UTR;TSS200	0.9239433	0.7188419	8.125554	2.68E-13	1.00E-10	19.813749
cg03147185	chr2	97008030	+	NCAPH	Body	-0.955007	-2.00384	-8.124668	2.70E-13	1.01E-10	19.809029
cg17039236	chr18	77548049	-			2.6706473	-0.980756	8.1237638	2.71E-13	1.01E-10	19.804208
cg00294025	chr12	7276360	-	RBP5	3'UTR	0.9238118	2.3918702	8.122468	2.73E-13	1.02E-10	19.797302
cg12052661	chr9	140772545	+	CACNA1B	1stExon	0.9681886	-0.183892	8.1218523	2.74E-13	1.02E-10	19.794021

cg15554023	chr1	144008937	+			1.1783134	0.9545915	8.1286194	2.74E-13	1.02E-10	19.795326
cg21862081	chr14	77769687	-	POMT2	Body	-1.632321	-0.645964	-8.122128	2.73E-13	1.02E-10	19.795488
cg24301620	chr6	101846872	+	GRIK2;GRIK2;GRIK2;GRIK2;GRIK2;GRIK2	1stExon;1stExon;5'UTR;1stExon;5'UTR;5'UTR	1.6135678	0.1276818	8.1208549	2.75E-13	1.02E-10	19.788707
cg01919567	chr2	127533123	+			-1.136522	-0.906613	-8.125515	2.79E-13	1.04E-10	19.77882
cg05610278	chr12	30908244	+	CAPRIN2;CAPRIN2;CAPRIN2	TSS1500;TSS1500;TSS1500	-0.757631	-4.646944	-8.11858	2.79E-13	1.04E-10	19.776587
cg08191854	chr21	45773294	-	TRPM2	TSS200	-1.812921	-2.491357	-8.117232	2.81E-13	1.04E-10	19.769402
cg03290748	chr15	68370885	-	PIAS1	Body	1.4099542	2.2124432	8.1168007	2.82E-13	1.04E-10	19.767105
cg12265623	chr7	8481994	-	NXPH1	Body	1.4696033	-0.532463	8.1166045	2.82E-13	1.04E-10	19.76606
cg16783744	chr8	105478855	-	DPYS	Body	2.1025601	-0.407894	8.108233	2.95E-13	1.09E-10	19.721466
cg03773809	chr4	120038219	+			1.2960524	1.4184223	8.1074338	2.97E-13	1.10E-10	19.71721
cg11202871	chr7	134851177	-	C7orf49;C7orf49;C7orf49	Body;Body;3'UTR	0.9371225	0.9118387	8.1074873	2.96E-13	1.10E-10	19.717495
cg22730029	chr13	41593582	-	ELF1	TSS200	-1.489604	-2.112511	-8.106598	2.98E-13	1.10E-10	19.712758
cg26476852	chr7	27204728	-	HOXA9	1stExon	2.2115171	-0.057034	8.1060221	2.99E-13	1.10E-10	19.709692
cg00500428	chr16	3074502	+	HCFC1R1;THOC6;HCFC1R1;HCFC1R1;THOC6	TSS1500;Body;TSS1500;TSS1500;Body	-0.69688	-2.535692	-8.104468	3.01E-13	1.11E-10	19.701418
cg23838005	chr17	74868604	-	MGAT5B;MGAT5B	Body;TSS200	-1.870993	-1.770592	-8.104483	3.01E-13	1.11E-10	19.701497
cg02853152	chr4	6690702	+			-1.740159	-2.924412	-8.100798	3.08E-13	1.13E-10	19.681873
cg07441518	chr11	31846849	+			2.0170184	0.1318109	8.1008476	3.07E-13	1.13E-10	19.682138
cg03362667	chr15	85928046	+	AKAP13;AKAP13	5'UTR;5'UTR	1.0783496	2.0010461	8.1006286	3.08E-13	1.13E-10	19.680972
cg26568372	chr1	91190173	-			1.9289496	-0.409955	8.096842	3.14E-13	1.15E-10	19.660813
cg26099134	chr6	100897309	+	SIM1	Body	1.3803211	-0.152487	8.10299	3.15E-13	1.16E-10	19.659134

cg03433048	chr17	4806453	+	CHRNE	TSS200	-1.241796	-2.181808	-8.095984	3.16E-13	1.16E-10	19.656247
cg11601663	chr10	15666820	+	ITGA8	Body	-1.593413	-0.63259	-8.094506	3.18E-13	1.17E-10	19.64838
cg19130973	chr3	12979917	-	IQSEC1;IQSEC1	Body;Body	1.1603625	1.1566942	8.0942034	3.19E-13	1.17E-10	19.646767
cg02177648	chr6	31744918	+	C6orf27;C6orf27	1stExon;5'UTR	-0.841652	-3.338255	-8.093626	3.20E-13	1.17E-10	19.643694
cg10299448	chr17	10018532	+	GAS7	Body	-0.678829	-4.501808	-8.093767	3.20E-13	1.17E-10	19.644445
cg00264754	chr11	40136810	-	LRRC4C	Body	-1.96907	0.2259562	-8.08935	3.28E-13	1.19E-10	19.620933
cg01765174	chr9	100880960	-	TRIM14;TRIM14;TRIM14;TRIM14	Body;Body;Body;Body	-1.509488	-0.550466	-8.088237	3.30E-13	1.20E-10	19.615012
cg26925980	chr10	19002068	+			-1.417246	-1.678333	-8.087629	3.31E-13	1.20E-10	19.611776
cg26444528	chr13	58207859	-	PCDH17	1stExon	2.1624985	0.0755615	8.087398	3.31E-13	1.20E-10	19.610547
cg07496545	chr5	140683737	+	SLC25A2	TSS200	1.9139675	2.5985549	8.0907337	3.37E-13	1.23E-10	19.594054
cg00224911	chr4	74486142	-	RASSF6;RASSF6	Body;TSS200	-1.277851	-3.860904	-8.083138	3.39E-13	1.23E-10	19.58788
cg20820543	chr6	135503889	-	MYB;MYB;MYB;MYB;MYB;MYB;MYB;MYB	Body;Body;Body;Body;Body;Body;Body;Body	-0.638283	-3.628401	-8.083236	3.39E-13	1.23E-10	19.588401
cg17676428	chr11	1314042	-	TOLLIP	Body	1.4920173	2.160946	8.0819532	3.41E-13	1.24E-10	19.581576
cg23213217	chr1	224370155	+	DEGS1;DEGS1	TSS1500;TSS1500	-1.459338	-0.598367	-8.080637	3.44E-13	1.24E-10	19.574574
cg11372831	chr4	57303157	+	PAICS;PAICS;PAICS;PPAT	Body;Body;Body;TSS1500	-0.653467	-4.009473	-8.07846	3.48E-13	1.26E-10	19.562993
cg17670263	chr19	54210233	+	MIR520C	TSS1500	-1.605219	-1.031303	-8.077158	3.50E-13	1.27E-10	19.556068
cg00602245	chr8	953031	+			-2.123175	1.2426589	-8.076624	3.51E-13	1.27E-10	19.553225
cg13289869	chr2	63277195	+	OTX1	TSS1500	-0.891875	-3.600322	-8.075603	3.53E-13	1.27E-10	19.547795
cg17927313	chr15	65151592	-	PLEKHO2	Body	1.1857708	0.9119504	8.0757672	3.53E-13	1.27E-10	19.548668
cg24000908	chr5	1113358	+	SLC12A7	TSS1500	-1.668151	-0.8559	-8.075652	3.53E-13	1.27E-10	19.548054

cg22884656	chr2	45157296	-			3.25851	-0.849021	8.0817492	3.54E-13	1.28E-10	19.546367
cg05287314	chr20	822198	+	FAM110A;FAM110A	5'UTR;5'UTR	-1.008004	-2.211799	-8.073155	3.58E-13	1.29E-10	19.534775
cg10881937	chr2	135064869	-	MGAT5	Body	1.197785	2.0607287	8.0720249	3.60E-13	1.30E-10	19.528765
cg25271892	chr11	62690462	-	CHRM1	TSS1500	-1.381838	-1.850483	-8.070878	3.63E-13	1.30E-10	19.522665
cg08042538	chr20	821854	-	FAM110A;FAM110A	5'UTR;5'UTR	-1.042047	-1.865541	-8.069938	3.65E-13	1.31E-10	19.517664
cg00706570	chr18	74692116	-	MBP;MBP;MBP;MBP;MBP	3'UTR;3'UTR;3'UTR;3'UTR;3'UTR	1.7269019	2.3383262	8.0694849	3.65E-13	1.31E-10	19.515257
cg00403955	chr16	11680241	+	LITAF;LITAF;LITAF;LITAF	5'UTR;TSS200;5'UTR;Body	-0.93754	-4.072118	-8.066241	3.72E-13	1.33E-10	19.498011
cg15444358	chr14	102051071	+			-1.650672	-1.281058	-8.066298	3.72E-13	1.33E-10	19.49831
cg01361351	chr22	29467363	-			-1.944833	-3.488501	-8.065371	3.74E-13	1.34E-10	19.493383
cg04662609	chr5	180646976	-			1.0532612	0.9667114	8.0648143	3.75E-13	1.34E-10	19.490423
cg13962372	chr1	22415149	+	CDC42;CDC42;CDC42	Body;Body;Body	1.4010059	0.6707748	8.0634454	3.78E-13	1.35E-10	19.483145
cg09717333	chr4	140222144	-	NAA15	TSS1500	-1.127515	-3.420565	-8.062534	3.80E-13	1.35E-10	19.478299
cg18160135	chr4	99851115	+	EIF4E;EIF4E;EIF4E;EIF4E;EIF4E	5'UTR;1stExon;5'UTR;TSS1500;1stExon	-1.307231	-2.917904	-8.068742	3.80E-13	1.36E-10	19.477359
cg00563824	chr1	209405064	+			1.9286186	0.2652939	8.0608759	3.83E-13	1.36E-10	19.469485
cg08812889	chr4	174439925	-			1.9445148	-0.070773	8.0609556	3.83E-13	1.36E-10	19.469909
cg07319375	chr1	6169384	+	CHD5	Body	-0.945193	-2.555985	-8.058678	3.88E-13	1.38E-10	19.457801
cg00037457	chr17	59531744	-			1.960376	0.5936461	8.0585189	3.88E-13	1.38E-10	19.456956
cg14035860	chr14	55707715	-			2.2278931	1.3198328	8.0580457	3.89E-13	1.38E-10	19.454441
cg01932361	chr16	86985993	+			-1.79619	-0.26696	-8.056465	3.93E-13	1.39E-10	19.446042
cg01062126	chr16	104539	+	SNRNP25;POLR3K	Body;TSS1500	-1.970769	1.022554	-8.054811	3.96E-13	1.40E-10	19.437249

cg08357990	chr2	33359550	-	LTBP1;LTBP1;LTBP1;LTBP1;LTBP1	TSS200;TSS200;Body;TSS200;TSS200	-1.294722	-2.378289	-8.054695	3.96E-13	1.40E-10	19.436635
cg10042106	chr14	57274739	+	OTX2	5'UTR	3.047783	-1.627654	8.054153	3.98E-13	1.41E-10	19.433753
cg05330497	chr16	58147704	-	C16orf80	3'UTR	1.1131876	2.7749806	8.053516	3.99E-13	1.41E-10	19.430367
cg02150262	chr11	124761914	+	ROBO4	Body	-1.573484	-0.558907	-8.052316	4.02E-13	1.42E-10	19.423989
cg02856338	chr8	101822108	-			1.8582734	-0.170022	8.0500637	4.07E-13	1.43E-10	19.412022
cg12892506	chr3	147113918	-	ZIC4;ZIC4;ZIC4	Body;Body;Body	2.6655424	-0.49761	8.0501854	4.06E-13	1.43E-10	19.412669
cg25172185	chr17	31689788	-	ACCN1	Body	-1.873899	-0.738454	-8.049625	4.08E-13	1.44E-10	19.409689
cg03592903	chr5	33737936	+	ADAMTS12	Body	-2.098269	-0.995055	-8.048178	4.11E-13	1.45E-10	19.402001
cg21040360	chr2	3383326	-	TTC15	TSS200	-1.166055	-3.977768	-8.047361	4.13E-13	1.45E-10	19.39766
cg09423283	chr6	146755301	+	GRM1;GRM1	3'UTR;Body	1.6605951	-0.552388	8.0470781	4.13E-13	1.45E-10	19.39616
cg01445578	chr10	22970764	+	PIP4K2A	Body	-1.222862	-1.264709	-8.046439	4.15E-13	1.46E-10	19.392762
cg00298065	chr19	58715577	-	ZNF274;ZNF274;ZNF274	Body;Body;5'UTR	1.4867367	0.9679533	8.046114	4.16E-13	1.46E-10	19.391038
cg13909405	chr2	37903481	-			-1.854212	-0.479688	-8.045145	4.18E-13	1.47E-10	19.385888
cg03663556	chr7	50900556	-			-2.039209	-2.554368	-8.051305	4.19E-13	1.47E-10	19.384903
cg22400703	chr2	68545995	+	CNRIP1;CNRIP1	Body;Body	2.0171048	0.5327104	8.0511132	4.19E-13	1.47E-10	19.384003
cg07345515	chr6	32054771	+	TNXB	Body	-1.474688	-0.925817	-8.044155	4.20E-13	1.47E-10	19.380632
cg12630938	chr10	120282778	+			-1.364907	-1.290893	-8.043483	4.22E-13	1.47E-10	19.377062
cg14347878	chr8	80977652	-	TPD52;TPD52;TPD52	Body;Body;Body	0.8749263	1.9120702	8.0430324	4.23E-13	1.48E-10	19.374667
cg04298224	chr13	113707471	-	MCF2L;MCF2L	Body;Body	0.9719508	1.092137	8.0412774	4.27E-13	1.49E-10	19.365345
cg07211684	chr4	160211221	+	RAPGEF2	Body	1.1101308	1.5904732	8.0393713	4.31E-13	1.50E-10	19.355221

cg15811515	chr16	31580989	+	CSDAP1	TSS200	2.7942243	0.1558001	8.0392869	4.31E-13	1.50E-10	19.354773
cg27563985	chr20	54580677	+	CBLN4	TSS1500	1.8670768	0.0402992	8.0435518	4.37E-13	1.52E-10	19.34393
cg14349667	chr4	81189706	-	FGF5;FGF5	Body;Body	2.2406092	-0.509988	8.042891	4.38E-13	1.53E-10	19.340315
cg19574488	chr3	61236547	+	FHIT;FHIT	5'UTR;5'UTR	-0.91394	-3.55933	-8.033826	4.45E-13	1.55E-10	19.325774
cg00683332	chr16	51188697	-			2.4102989	0.1891736	8.0336485	4.45E-13	1.55E-10	19.32483
cg23033158	chr3	117573066	+			-1.874442	-0.209918	-8.033313	4.46E-13	1.55E-10	19.323046
cg11772799	chr7	158799715	-	LOC154822	TSS1500	-1.515502	-0.603803	-8.032334	4.48E-13	1.55E-10	19.317851
cg05433391	chr6	71022175	+			-1.636078	-2.100653	-8.031716	4.50E-13	1.56E-10	19.31457
cg10429262	chr1	9189507	+	GPR157	TSS1500	-0.680348	-3.476561	-8.030447	4.53E-13	1.57E-10	19.30783
cg25859045	chr11	64809369	-	SAC3D1	Body	-0.899787	-4.335279	-8.036066	4.55E-13	1.57E-10	19.30416
cg21130374	chr21	42734266	-	MX2	5'UTR	-2.00838	-1.644188	-8.026039	4.64E-13	1.60E-10	19.284429
cg02637537	chr8	22447181	-	PDLIM2;PDLIM2;PDLIM2	Body;Body;Body	1.7623427	1.8697607	8.0255014	4.65E-13	1.61E-10	19.281577
cg20607293	chr1	147112703	+			-0.993488	-2.425668	-8.024906	4.67E-13	1.61E-10	19.278416
cg23994043	chr2	200326725	-	SATB2	TSS1500	1.8973083	-0.060384	8.0380665	4.67E-13	1.61E-10	19.280938
cg06102127	chr3	65924259	-	MAGI1;MAGI1;MAGI1	Body;Body;Body	1.0030616	2.1454175	8.0231384	4.71E-13	1.62E-10	19.269034
cg23829102	chr4	124476694	+			-1.439364	-2.201729	-8.02127	4.76E-13	1.64E-10	19.259118
cg09513990	chr6	27235822	+			2.9367276	-0.49722	8.0207264	4.78E-13	1.64E-10	19.256233
cg18175809	chr3	62355525	+	FEZF2	3'UTR	1.8093077	-0.633246	8.0207962	4.77E-13	1.64E-10	19.256604
cg13279673	chr6	50674522	+			2.4306393	-0.895381	8.0195348	4.81E-13	1.65E-10	19.249909
cg07967677	chr1	161008977	-	TSTD1;TSTD1;TSTD1	TSS1500;TSS1500;TSS1500	-1.418531	-1.817758	-8.023924	4.86E-13	1.67E-10	19.239856

cg14776518	chr15	63892607	-	FBXL22	Body	-1.327618	-2.192753	-8.017541	4.86E-13	1.67E-10	19.239327
cg10023272	chr1	110611823	-	ALX3	Body	2.2323309	-1.644486	8.0170904	4.87E-13	1.67E-10	19.236938
cg20699586	chr1	227748719	-			2.1359469	1.5819259	8.0146459	4.94E-13	1.69E-10	19.223968
cg25596287	chr1	28843736	+	SNHG3-RCC1;RCC1;SNHG3-RCC1;RCC1;SNHG3-RCC1;RCC1	Body;TSS1500;Body;TSS1500;5'UTR;TSS1500	-1.531244	-1.281579	-8.012719	4.99E-13	1.71E-10	19.213743
cg06516865	chr12	11883921	+	ETV6	Body	-1.493508	-1.863219	-8.010816	5.04E-13	1.72E-10	19.203649
cg13563728	chr9	101019042	-	TBC1D2	TSS1500	-1.168119	-2.38056	-8.010736	5.05E-13	1.72E-10	19.203223
cg01304894	chr16	70715140	-	MTSS1L	Body	0.9574468	1.4631576	8.0100559	5.06E-13	1.73E-10	19.199617
cg22274539	chr12	103696209	-	C12orf42;C12orf42	Body;Body	2.3250253	-0.112665	8.0286475	5.10E-13	1.74E-10	19.189898
cg22827250	chr5	134363823	-	PITX1	3'UTR	1.2714026	-0.004782	8.0089522	5.10E-13	1.74E-10	19.193762
cg04414349	chr7	129419676	+			-0.582943	-3.412314	-8.007569	5.13E-13	1.75E-10	19.186423
cg08684361	chr6	30852823	+	DDR1;DDR1	5'UTR;5'UTR	-1.364769	-2.802568	-8.012848	5.17E-13	1.76E-10	19.174403
cg22622834	chr1	204367853	-			-0.880595	-3.595508	-8.005263	5.20E-13	1.77E-10	19.174196
cg18356785	chr11	47611780	-	C1QTNF4	Body	1.6568313	1.2137303	8.0048261	5.21E-13	1.77E-10	19.171877
cg00063111	chr3	39448952	-	SNORA6;RPSA;RPSA	TSS1500;5'UTR;TSS200	-1.243254	-2.865481	-8.004153	5.23E-13	1.78E-10	19.168307
cg04075990	chr3	106176366	-			-1.258405	-3.128947	-8.010423	5.24E-13	1.78E-10	19.168397
cg09062462	chr1	85220242	-			1.27908	2.4660996	8.0104981	5.23E-13	1.78E-10	19.168794
cg18986651	chr3	145860909	-	PLOD2;PLOD2	Body;Body	1.1704823	0.9682928	8.0026261	5.28E-13	1.79E-10	19.16021
cg11732134	chr1	10732912	+	CASZ1;CASZ1	Body;Body	1.6732956	3.4063745	7.997725	5.42E-13	1.83E-10	19.134221
cg14640149	chr2	216546226	+			-1.691117	-2.157451	-7.997661	5.42E-13	1.83E-10	19.133884
cg26213458	chr12	5918191	-	ANO2	Body	-1.766508	-0.605622	-7.997217	5.43E-13	1.84E-10	19.131526

cg01258488	chr12	71116333	+	PTPRR;PTPRR	Body;Body	1.182447	2.4749919	7.9948277	5.51E-13	1.86E-10	19.118861
cg12054453	chr17	57915717	+	TMEM49	Body	-1.241989	-3.690837	-7.994593	5.51E-13	1.86E-10	19.117618
cg21158087	chr17	58216415	+			1.8590888	-0.345792	7.9934246	5.55E-13	1.87E-10	19.111423
cg00091064	chr6	41376604	+			1.0787172	1.5318471	7.9928049	5.57E-13	1.87E-10	19.108137
cg21054521	chr13	112759386	+			2.1591377	-0.506638	7.9992201	5.57E-13	1.87E-10	19.109234
cg03287111	chr2	121625634	+	GLI2	Body	1.7740037	-1.025941	7.991492	5.61E-13	1.89E-10	19.101178
cg07435237	chr16	31469562	-	ARMC5;ARMC5	TSS1500;TSS1500	-1.121127	-3.352201	-7.990717	5.63E-13	1.89E-10	19.09707
cg01295399	chr10	50820278	-	SLC18A3;CHAT;CHAT;CHAT	1stExon;TSS1500;5'UTR;TSS1500	0.9585878	0.5379813	7.9900498	5.65E-13	1.90E-10	19.093534
cg06829299	chr8	65286632	-			1.8358298	-0.421383	7.9890093	5.68E-13	1.91E-10	19.088019
cg13141289	chr15	49339044	-	SECISBP2L	TSS1500	-1.021496	-3.324713	-7.988849	5.69E-13	1.91E-10	19.087168
cg12494529	chr10	1082432	+	C10orf110;C10orf110;C10orf110;C10orf110	Body;Body;Body;Body	-1.805855	-0.046378	-7.98633	5.77E-13	1.93E-10	19.073821
cg06099244	chr3	112739834	-	C3orf17;C3orf17;C3orf17	TSS1500;TSS1500;TSS1500	0.9284771	2.4912642	7.983715	5.85E-13	1.96E-10	19.059962
cg01799521	chr1	39305163	-	RRAGC	3'UTR	1.1255179	1.7351725	7.9834512	5.86E-13	1.96E-10	19.058564
cg03729251	chr4	151501035	+	LRBA	Body	1.5999053	2.4205868	7.9870765	5.95E-13	1.99E-10	19.044918
cg08309687	chr21	35320596	+			-1.456592	-1.104463	-7.979418	5.99E-13	2.00E-10	19.037195
cg12706330	chr10	85898700	+	GHITM	TSS1500	-1.979959	0.0302846	-7.978216	6.03E-13	2.01E-10	19.030829
cg16726435	chr2	219128715	+			1.4992846	2.6973897	7.9843438	6.04E-13	2.01E-10	19.030473
cg05629774	chr12	132546836	+	EP400	Body	1.000571	1.3725509	7.9747836	6.15E-13	2.05E-10	19.012644
cg08731543	chr21	32202092	+	KRTAP7-1	TSS200	-1.802506	0.0377016	-7.973528	6.19E-13	2.06E-10	19.005995
cg01492656	chr4	40057241	-	N4BP2;LOC344967	TSS1500;Body	-1.890133	-4.2708	-7.972713	6.22E-13	2.07E-10	19.00168

cg11028923	chr5	43557644	-	PAIP1;PAIP1;PAIP1	TSS1500;TSS200;TSS1500	-1.283139	-2.590082	-7.972564	6.22E-13	2.07E-10	19.00089
cg15452970	chr1	91190620	+			1.3152581	0.5636242	7.9721492	6.23E-13	2.07E-10	18.998691
cg00739667	chr16	81731648	+	CMIP;CMIP	Body;Body	1.2726753	1.3817814	7.9710115	6.27E-13	2.08E-10	18.992666
cg21143896	chr7	2802374	+	GNA12	Body	1.4802785	1.1091099	7.9711598	6.27E-13	2.08E-10	18.993451
cg23709476	chr15	53088156	+			1.1012578	0.2453516	7.9710944	6.27E-13	2.08E-10	18.993105
cg11196788	chr11	71737549	-	NUMA1	Body	0.9795393	1.1089742	7.9695557	6.32E-13	2.09E-10	18.984956
cg17901038	chr17	74385781	-	UBE2O	3'UTR	2.3035844	4.2191237	7.9750197	6.36E-13	2.10E-10	18.981197
cg05318186	chr16	86392808	+			-1.20339	-1.123788	-7.968382	6.36E-13	2.10E-10	18.978743
cg19361800	chr2	208014177	+	KLF7	Body	1.2975423	0.9282794	7.9658245	6.45E-13	2.13E-10	18.965198
cg19414591	chr1	91190582	-			1.8631711	-0.958677	7.9655259	6.46E-13	2.13E-10	18.963617
cg20624041	chr11	74204975	-	LIPT2	TSS1500	-0.704086	-3.881207	-7.964867	6.49E-13	2.14E-10	18.960128
cg20754261	chr2	468330	+			2.1021678	-1.119292	7.9646517	6.50E-13	2.14E-10	18.958988
cg02459042	chr17	821621	+	NXN	Body	1.6001069	2.0433155	8.0470269	6.56E-13	2.16E-10	18.944748
cg19034132	chr10	75692227	+			-1.413719	-1.736965	-7.961988	6.59E-13	2.17E-10	18.944886
cg12771717	chr6	31164947	-	HCG27	TSS1500	-1.997683	-3.021185	-7.966067	6.67E-13	2.20E-10	18.9339
cg16061498	chr18	55095886	+			1.7099058	-0.435129	7.9595392	6.68E-13	2.20E-10	18.931922
cg15336438	chr10	76993893	-	COMTD1	Body	-1.271551	-2.063405	-7.958776	6.71E-13	2.20E-10	18.927881
cg20114732	chr6	166579135	+	T	Body	3.0837659	-0.769102	7.9585914	6.72E-13	2.20E-10	18.926906
cg14521746	chr11	77185492	+	PAK1;PAK1	TSS1500;TSS1500	-0.656474	-3.674244	-7.955491	6.83E-13	2.24E-10	18.910496
cg00579520	chr12	128752586	+	TMEM132C	Body	1.5904679	-0.874111	7.9539799	6.89E-13	2.26E-10	18.902498

cg11053466	chr12	6648770	-	IFFO1;IFFO1;IFFO1	3'UTR;3'UTR;3'UTR	-1.438182	-0.38649	-7.960164	6.89E-13	2.26E-10	18.902823
cg20785796	chr2	200326721	-	SATB2	TSS1500	1.9344685	0.5450237	7.9532966	6.91E-13	2.26E-10	18.898882
cg14933439	chr16	49407794	-	C16orf78	TSS200	-1.495055	-0.369008	-7.952004	6.96E-13	2.28E-10	18.892042
cg13169065	chr2	166650947	+	GALNT3	TSS200	-0.771609	-3.41938	-7.951595	6.98E-13	2.28E-10	18.889879
cg16104915	chr7	27205217	+	HOXA9	TSS200	2.8967664	-1.240558	7.9517074	6.97E-13	2.28E-10	18.890472
cg23972738	chr2	66667039	-	MEIS1	Body	2.0764876	0.0101923	7.9497656	7.05E-13	2.30E-10	18.880197
cg18451817	chr5	2132336	+			-1.714107	-0.378662	-7.949323	7.06E-13	2.30E-10	18.877854
cg11171221	chr10	124902844	-			2.2715037	-0.66607	7.9488329	7.08E-13	2.31E-10	18.875262
cg03791074	chr7	1117852	-	C7orf50;C7orf50;C7orf50	Body;Body;Body	1.5466553	2.1411226	7.9481472	7.11E-13	2.31E-10	18.871634
cg18202412	chr1	89406505	+	CCBL2;CCBL2	Body;Body	1.0107552	1.7245684	7.9477658	7.12E-13	2.32E-10	18.869616
cg20113824	chr8	70946946	-			2.1846532	-0.605087	7.9453737	7.22E-13	2.35E-10	18.85696
cg19815634	chr12	6280998	+			1.0171187	1.7432866	7.9446425	7.25E-13	2.35E-10	18.853092
cg11027217	chr4	113300650	+	ALPK1;ALPK1	Body;Body	1.6394094	2.4457166	7.9439167	7.28E-13	2.36E-10	18.849253
cg13327545	chr10	22623548	-			1.1137944	0.665339	7.9433514	7.30E-13	2.37E-10	18.846263
cg01697163	chr22	38035301	-	SH3BP1	TSS1500	-1.418954	-2.201187	-7.942973	7.31E-13	2.37E-10	18.844259
cg09841898	chr14	50886416	-	MAP4K5;MAP4K5	3'UTR;3'UTR	1.1086878	2.0924824	7.9428671	7.32E-13	2.37E-10	18.843701
cg15503081	chr19	12992867	+	DNASE2	TSS1500	-1.427673	-1.127906	-7.94263	7.33E-13	2.37E-10	18.842447
cg03267139	chr2	133036194	-			-1.1318	-0.015378	-7.946884	7.41E-13	2.40E-10	18.832626
cg08202226	chr1	153896590	+	GATAD2B	TSS1500	-1.950152	-1.276296	-7.940082	7.43E-13	2.40E-10	18.828967
cg21793127	chr17	73506038	-	CASKIN2;CASKIN2	Body;TSS200	1.2639115	1.541086	7.940064	7.43E-13	2.40E-10	18.828874

cg07621239	chr2	1598287	+			-1.791705	-0.10032	-7.938249	7.51E-13	2.42E-10	18.819274
cg12527684	chr4	99851156	-	EIF4E;EIF4E;EIF4E;EIF4E;EIF4E	5'UTR;1stExon;5'UTR;TSS1500;1stExon	-1.463766	-3.009019	-7.938189	7.51E-13	2.42E-10	18.818956
cg02229757	chr16	15685126	+			-1.018228	-3.903363	-7.937415	7.54E-13	2.43E-10	18.814862
cg14565725	chr1	119532044	-	TBX15;TBX15	1stExon;5'UTR	1.6911704	0.2149486	7.9410483	7.65E-13	2.46E-10	18.801829
cg03585912	chr6	10385776	+			2.1007087	0.1522757	7.9343514	7.67E-13	2.47E-10	18.798663
cg10903903	chr6	27647843	-			3.3159535	-0.105206	7.9402825	7.68E-13	2.47E-10	18.797789
cg05068848	chr4	23335733	-			-1.469143	-2.714508	-7.933285	7.71E-13	2.48E-10	18.793027
cg01000615	chr1	145562817	-	ANKRD35	Body	1.8521109	-0.544877	7.9321736	7.76E-13	2.49E-10	18.787148
cg08190858	chr12	54321346	-			3.0658076	-0.678394	7.9322815	7.75E-13	2.49E-10	18.787719
cg02521750	chr16	81712054	-	CMIP;CMIP	Body;Body	1.6290314	2.4155968	7.9316546	7.78E-13	2.49E-10	18.784404
cg06051311	chr6	30131001	+	TRIM15;TRIM15	5'UTR;1stExon	-2.218532	-1.297453	-7.931616	7.78E-13	2.49E-10	18.7842
cg22089890	chr17	48708077	-			-0.750242	-2.963992	-7.929081	7.89E-13	2.52E-10	18.7708
cg22488797	chr5	134363324	-			2.2863859	-0.554725	7.9289072	7.90E-13	2.52E-10	18.769879
cg17602167	chr6	36561558	-	SFRS3	TSS1500	-1.038657	-3.472697	-7.928127	7.93E-13	2.53E-10	18.765753
cg05903386	chr2	217555456	-	IGFBP5	Body	-1.511842	-3.454836	-7.927013	7.98E-13	2.55E-10	18.759864
cg25344265	chr5	38273388	-	EGFLAM	Body	-1.964159	-0.078435	-7.926972	7.98E-13	2.55E-10	18.759651
cg14063191	chr9	131486742	+	ZDHHC12	TSS1500	-0.989756	-3.918194	-7.924089	8.11E-13	2.58E-10	18.744409
cg24165439	chr10	45967484	+	MARCH8;MARCH8;MARCH8	Body;Body;Body	1.0394787	3.2237512	7.923556	8.13E-13	2.59E-10	18.741593
cg13220457	chr6	135504074	+	MYB;MYB;MYB;MYB;MYB;MYB;MYB;MYB	Body;Body;Body;Body;Body;Body;Body;Body	-1.007546	-3.363092	-7.923369	8.14E-13	2.59E-10	18.740603
cg12062220	chr15	53087232	+			1.9439313	-0.09849	7.9224053	8.19E-13	2.60E-10	18.735511

cg01771479	chr8	144198810	+			-1.170613	-1.832502	-7.921891	8.21E-13	2.61E-10	18.732792
cg08065657	chr19	52223099	-	HAS1	Body	1.6009004	-0.573786	7.9212767	8.24E-13	2.61E-10	18.729547
cg27559724	chr3	186524679	+	RFC4;RFC4	TSS200;TSS1500	-1.10027	-3.256124	-7.921045	8.25E-13	2.62E-10	18.728323
cg03063277	chr7	937827	-	ADAP1	3'UTR	2.0388879	2.9155307	7.9200838	8.29E-13	2.63E-10	18.723243
cg15089620	chr5	142417269	+	ARHGAP26;ARHGAP26	Body;Body	1.0363184	2.5654028	7.9180622	8.38E-13	2.66E-10	18.71256
cg23138413	chr8	49320596	-			-1.80762	-0.735559	-7.91621	8.47E-13	2.68E-10	18.702774
cg16823083	chr1	3567412	+	WDR8	TSS1500	2.0130732	-0.095098	7.913977	8.57E-13	2.71E-10	18.690975
cg00448814	chr6	28227482	-	NKAPL	1stExon	1.0774652	1.4035902	7.913688	8.58E-13	2.71E-10	18.689449
cg11066209	chr7	2282252	-	FTSJ2;NUDT1;NUDT1;NUDT1;NUDT1;NUDT1;NUDT1;NUDT1	TSS1500;5'UTR;5'UTR;5'UTR;TSS1500;5'UTR;TSS1500;5'UTR	-1.227888	-2.460125	-7.913478	8.59E-13	2.71E-10	18.688339
cg25471443	chr1	27668058	+	SYTL1	TSS1500	-1.07973	-1.692955	-7.911758	8.68E-13	2.74E-10	18.679253
cg01298102	chr16	21199840	-			-1.297993	-1.558975	-7.910475	8.74E-13	2.76E-10	18.672474
cg25741487	chr3	62363076	+			1.9202249	-0.616102	7.9091815	8.80E-13	2.77E-10	18.665643
cg24113409	chr6	10884314	+			1.5051737	0.1000682	7.9072907	8.89E-13	2.80E-10	18.655656
cg12446953	chr1	20960867	+	PINK1	Body	-1.25065	-2.999135	-7.905886	8.96E-13	2.82E-10	18.648235
cg18502142	chr7	96622709	+	DLX6AS	Body	2.8761329	0.552613	7.9058884	8.96E-13	2.82E-10	18.64825
cg26192520	chr8	101960390	-	YWHAZ;YWHAZ;YWHAZ;YWHAZ;YWHAZ;YWHAZ	Body;Body;Body;Body;Body;Body	-1.568577	-0.948483	-7.903667	9.07E-13	2.85E-10	18.636518
cg11069071	chr14	101525465	-	MIR496;MIR154	TSS1500;TSS1500	-1.541446	-0.965164	-7.902756	9.11E-13	2.86E-10	18.631709
cg14266527	chr4	151501298	+	LRBA	Body	1.2886777	1.351265	7.9089422	9.12E-13	2.86E-10	18.632631
cg20095656	chr5	176827793	-	PFN3	TSS200	1.6806716	1.4253334	7.9018906	9.16E-13	2.87E-10	18.627138
cg02885007	chr2	176987605	-	HOXD9	1stExon	1.6283694	-0.429735	7.9007896	9.21E-13	2.89E-10	18.621325

cg24159514	chr19	11785248	+	ZNF833	Body	1.8358586	1.6918076	7.9005878	9.22E-13	2.89E-10	18.620259
cg24496475	chr4	1400189	-			1.6550106	0.0618357	7.9005108	9.23E-13	2.89E-10	18.619853
cg05775492	chr16	86258717	+			-1.73643	-0.042514	-7.899759	9.26E-13	2.90E-10	18.615884
cg27176870	chr19	38754930	+	SPINT2;SPINT2	TSS200;TSS200	-1.810526	-4.61835	-7.989172	9.29E-13	2.90E-10	18.622219
cg01378515	chr3	124773106	-	HEG1	Body	1.3128616	0.9249018	7.898527	9.33E-13	2.91E-10	18.609379
cg01377200	chr4	184938316	-	STOX2	Body	1.5617042	2.2067105	7.8982418	9.34E-13	2.91E-10	18.607873
cg26541233	chr4	184941395	-			1.7170766	1.9737494	7.8966281	9.42E-13	2.94E-10	18.599354
cg27288226	chr19	57618436	+			1.8981459	0.1088829	7.8966568	9.42E-13	2.94E-10	18.599505
cg03865604	chr12	121717886	+	CAMKK2;CAMKK2;CAMKK2;CAMKK2;CAMKK2;CAMKK2;CAMKK2	5'UTR;5'UTR;5'UTR;5'UTR;5'UTR;5'UTR;5'UTR	0.9646895	1.7741159	7.8957548	9.47E-13	2.95E-10	18.594744
cg06653141	chr5	36745541	+			-1.00036	-3.252722	-7.894188	9.55E-13	2.97E-10	18.586474
cg14988680	chr1	212688417	+			1.4681853	0.6716723	7.8938818	9.57E-13	2.97E-10	18.584857
cg00366850	chr2	45232417	-	SIX2	3'UTR	2.1535441	0.1133686	7.8935684	9.58E-13	2.98E-10	18.583203
cg11866303	chr19	40732691	+	CNTD2	TSS200	-1.47688	-2.38912	-7.893201	9.60E-13	2.98E-10	18.581262
cg05833158	chr3	9993731	-	PRRT3	5'UTR	-1.199684	-3.779678	-7.89305	9.61E-13	2.98E-10	18.580466
cg24613080	chr17	32484035	-	ACCN1	TSS1500	2.396904	-0.294371	7.8926494	9.63E-13	2.99E-10	18.578352
cg10550308	chr3	184969158	+	EHHADH;EHHADH	5'UTR;Body	1.8931496	3.0064284	7.892317	9.65E-13	2.99E-10	18.576597
cg08362273	chr17	46719577	+			1.2700333	-0.042311	7.8904125	9.75E-13	3.02E-10	18.566546
cg27294813	chr2	109791599	+	SH3RF3	Body	-1.653914	-0.345347	-7.888567	9.85E-13	3.05E-10	18.556804
cg01304145	chr16	22296770	-	EEF2K	3'UTR	1.2208206	1.9313491	7.8879236	9.88E-13	3.05E-10	18.553411
cg07661904	chr10	71930999	-	SAR1A;SAR1A	TSS1500;TSS1500	-2.290051	0.4060576	-7.893326	9.93E-13	3.07E-10	18.550374

cg09462924	chr2	66666470	-	MEIS1	Body	2.593789	-0.446647	7.8929644	9.95E-13	3.07E-10	18.548391
cg24185397	chr17	25659609	+			-1.09313	-2.641038	-7.886745	9.95E-13	3.07E-10	18.547189
cg06665333	chr16	87873837	+	SLC7A5	Body	1.5691422	3.3409273	7.8925661	9.97E-13	3.07E-10	18.546294
cg19095981	chr5	53516701	+	ARL15	Body	1.2402814	2.0776505	7.8858847	9.99E-13	3.08E-10	18.542652
cg27321175	chr10	15689009	-	ITGA8	Body	-1.83196	-0.540994	-7.885628	1.00E-12	3.08E-10	18.541298
cg24475598	chr2	221712289	+			-1.566534	0.1318091	-7.890128	1.01E-12	3.11E-10	18.533455
cg04521004	chr3	157821262	-	SHOX2;SHOX2;SHOX2	Body;Body;Body	1.5812766	-0.28822	7.8815455	1.02E-12	3.14E-10	18.519757
cg19329160	chr13	79182095	-			1.985658	-0.631722	7.8798803	1.03E-12	3.17E-10	18.510973
cg04623270	chr16	58150017	+	C16orf80	Body	1.0543151	0.7509467	7.8793926	1.04E-12	3.18E-10	18.5084
cg03356595	chr16	51187807	+			2.4040683	-0.239949	7.8915333	1.04E-12	3.18E-10	18.508957
cg07974511	chr2	63283013	+	OTX1	Body	2.6187816	0.1888575	7.8760415	1.05E-12	3.23E-10	18.490724
cg20816447	chr4	15480781	-	CC2D2A;CC2D2A;CC2D2A	Body;Body;Body	-1.168477	-2.831617	-7.875077	1.06E-12	3.25E-10	18.485638
cg10659805	chr7	96631680	+	DLX6AS	Body	2.1292601	-0.13036	7.8731352	1.07E-12	3.28E-10	18.475396
cg04420878	chr8	56987290	+	RPS20;SNORD54;RPS20	TSS200;TSS1500;TSS200	-0.635557	-4.263747	-7.872172	1.08E-12	3.29E-10	18.470317
cg08726667	chr16	28573379	-	CCDC101	5'UTR	1.5058115	0.4468192	7.8713707	1.08E-12	3.31E-10	18.466091
cg17031967	chr6	25297487	-	LRRC16A	Body	1.3596272	1.1217061	7.8811178	1.10E-12	3.35E-10	18.454185
cg00981877	chr20	50366841	+	ATP9A	Body	-1.1365	-3.936757	-7.873476	1.11E-12	3.37E-10	18.445897
cg05982686	chr17	59564954	-			1.0345445	1.4776009	7.8720586	1.11E-12	3.40E-10	18.438366
cg10471113	chr16	1587842	+	IFT140;TMEM204	Body;Body	1.4123034	2.0921085	7.8622975	1.14E-12	3.46E-10	18.418256
cg12050175	chr10	44754714	+			-1.517747	-1.320591	-7.860924	1.14E-12	3.49E-10	18.411016

cg14255426	chr21	45773290	-	TRPM2	TSS200	-1.374401	-2.884415	-7.860578	1.15E-12	3.49E-10	18.409194
cg06809252	chr1	110612044	-	ALX3	Body	2.3165884	0.2340414	7.8592406	1.16E-12	3.51E-10	18.402143
cg25683810	chr2	204547817	+			1.3522228	1.4751139	7.8582202	1.16E-12	3.53E-10	18.396765
cg14911690	chr19	19729395	-	PBX4	1stExon	-1.687313	-3.231449	-7.857647	1.17E-12	3.54E-10	18.393746
cg03184290	chr4	111550666	-	PITX2;PITX2	Body;Body	2.6387129	-0.339767	7.8571328	1.17E-12	3.55E-10	18.391035
cg17061156	chr4	184941494	-			2.2350647	2.0661753	7.856643	1.17E-12	3.55E-10	18.388454
cg05800758	chr8	101225800	+	SPAG1;SPAG1	Body;Body	-0.689937	-3.69303	-7.854552	1.19E-12	3.59E-10	18.377434
cg23248920	chr6	159291177	+			-0.591629	-3.828368	-7.853462	1.19E-12	3.61E-10	18.37169
cg26581449	chr15	89285805	+			-1.325324	-0.802034	-7.853511	1.19E-12	3.61E-10	18.371951
cg14906690	chr10	76573506	+			1.0646586	1.526383	7.8502117	1.21E-12	3.67E-10	18.354567
cg06620200	chr19	54190235	+	MIR1283-1	TSS1500	-2.087664	-0.183184	-7.847866	1.23E-12	3.72E-10	18.342212
cg01413522	chr16	86519105	+			-1.397942	-0.702029	-7.847037	1.24E-12	3.73E-10	18.337842
cg10441424	chr5	1316636	-			-0.844834	-4.236384	-7.846775	1.24E-12	3.73E-10	18.336461
cg04472592	chr12	52585786	-	KRT80;KRT80	TSS200;TSS200	-1.197528	-0.88428	-7.846644	1.24E-12	3.73E-10	18.335773
cg21090298	chr13	113622686	+	MCF2L	TSS200	-1.008737	-1.642835	-7.84524	1.25E-12	3.76E-10	18.328378
cg05864433	chr7	2738728	-	AMZ1	5'UTR	-1.329047	-2.930973	-7.844178	1.25E-12	3.78E-10	18.322785
cg17998964	chr11	63655903	-	MARK2;MARK2;MARK2;MARK2;MARK2	Body;TSS200;Body;Body;Body	-1.114438	-2.413161	-7.842936	1.26E-12	3.80E-10	18.31624
cg01242619	chr8	145049472	+	PLEC1	Body	-1.219501	-2.734238	-7.842351	1.27E-12	3.81E-10	18.313163
cg21990700	chr12	7260776	-	LOC283314;C1RL	TSS200;Body	-1.450355	-2.547983	-7.852998	1.28E-12	3.84E-10	18.306735
cg24685134	chr1	55267152	-	TTC22;TTC22	TSS1500;TSS1500	-1.66103	-3.03033	-7.858814	1.28E-12	3.85E-10	18.305381

cg08113562	chr17	43508428	+	SH3D20	5'UTR	-1.845112	-3.306245	-7.839447	1.29E-12	3.86E-10	18.29787
cg05898092	chr17	80022616	+	DUS1L	Body	-1.194192	-0.661484	-7.838273	1.30E-12	3.88E-10	18.291686
cg04539111	chr16	67997858	-	SLC12A4;SLC12A4;SLC12A4;SLC12A4;SLC12A4	Body;Body;1stExon;Body;Body	1.3541993	1.0392407	7.8374998	1.30E-12	3.90E-10	18.287616
cg20934096	chr7	1117418	-	C7orf50;C7orf50;C7orf50	Body;Body;Body	1.7696008	1.9206031	7.8369639	1.30E-12	3.91E-10	18.284794
cg08404513	chr7	72868821	+	BAZ1B	Body	1.4427571	1.4756493	7.8363857	1.31E-12	3.92E-10	18.28175
cg07551060	chr10	121075316	+	GRK5	Body	-2.221948	-1.215369	-7.835545	1.31E-12	3.93E-10	18.277322
cg09656389	chr11	31839790	+	PAX6	TSS1500	1.8526896	0.176361	7.8337555	1.33E-12	3.97E-10	18.267903
cg14317046	chr19	18234076	+	MAST3	Body	0.9809848	1.4048918	7.832541	1.34E-12	3.99E-10	18.261509
cg05600126	chr17	950540	-	ABR;ABR;ABR	Body;Body;Body	0.9281904	0.8516009	7.8315781	1.34E-12	4.01E-10	18.25644
cg02460652	chr15	76431858	+	C15orf27	Body	-0.898628	-2.534359	-7.831243	1.35E-12	4.01E-10	18.254677
cg07214164	chr2	105480254	-			1.1778723	0.0168689	7.8311824	1.35E-12	4.01E-10	18.254358
cg23832497	chr6	121655383	-	C6orf170	Body	-1.45857	-2.339736	-7.83053	1.35E-12	4.02E-10	18.250924
cg11790673	chr1	212845129	-			-1.426251	-1.770036	-7.827952	1.37E-12	4.08E-10	18.237355
cg18733230	chr7	4763182	+	FOXK1	Body	1.4921031	3.1441825	7.8276706	1.37E-12	4.08E-10	18.235874
cg00840990	chr3	177308016	-			-1.818696	-2.211167	-7.826085	1.38E-12	4.11E-10	18.227529
cg05651778	chr17	60729731	-	MRC2	Body	-2.354165	-4.059027	-7.826144	1.38E-12	4.11E-10	18.227841
cg23244488	chr7	19146032	+			2.5563845	-0.683589	7.8262548	1.38E-12	4.11E-10	18.228423
cg20767356	chr1	150693100	-	HORMAD1	5'UTR	1.4776973	4.3846265	7.8239832	1.40E-12	4.15E-10	18.216469
cg26926612	chr1	214153294	+			2.3586613	-0.642584	7.8237327	1.40E-12	4.16E-10	18.215151
cg17934365	chr17	75180216	-	SEC14L1;SEC14L1;SEC14L1;SEC14L1;SEC14L1	Body;Body;TSS1500;Body;Body	0.8797276	2.9003168	7.8229994	1.41E-12	4.17E-10	18.211292

cg05154546	chr4	3373555	+	RGS12;RGS12;RGS12	Body;Body;Body	1.7903631	2.982054	7.8225794	1.41E-12	4.18E-10	18.209082
cg19777067	chr4	174440103	+			2.4991008	-0.253697	7.8223044	1.41E-12	4.18E-10	18.207635
cg19590511	chr17	957402	-	ABR;ABR;ABR	Body;Body;Body	2.0648285	5.0638697	7.8470147	1.41E-12	4.18E-10	18.211431
cg02926033	chr7	154540580	+	DPP6;DPP6;DPP6	Body;Body;Body	-1.412874	-0.235295	-7.820111	1.43E-12	4.22E-10	18.196095
cg09919570	chr16	49311782	+			2.0992409	-0.455211	7.819005	1.44E-12	4.25E-10	18.190276
cg10591607	chr6	101846916	+	GRIK2;GRIK2;GRIK2;GRIK2;GRIK2;GRIK2	1stExon;1stExon;5'UTR;1stExon;5'UTR;5'UTR	1.7195837	-0.86765	7.8175789	1.45E-12	4.27E-10	18.182774
cg18143317	chr11	2037124	+			-1.044478	-1.091172	-7.817563	1.45E-12	4.27E-10	18.182691
cg07035659	chr4	4859975	-	MSX1	TSS1500	1.3393916	-1.063898	7.817282	1.45E-12	4.28E-10	18.181212
cg20788020	chr8	105677501	-			-2.009492	-0.663632	-7.81661	1.46E-12	4.29E-10	18.177678
cg10191399	chr16	81727056	+	CMIP;CMIP	Body;Body	1.0759468	2.8001849	7.8162711	1.46E-12	4.30E-10	18.175894
cg08149193	chr11	44333067	+	ALX4	TSS1500	2.5496198	-0.532412	7.8158658	1.46E-12	4.30E-10	18.173762
cg02156723	chr1	12100631	+			-1.559948	-1.535288	-7.815021	1.47E-12	4.31E-10	18.169321
cg19211880	chr3	134088357	+	AMOTL2	Body	1.7600518	2.4198025	7.8337108	1.47E-12	4.31E-10	18.169116
cg27083614	chr19	3778090	+	MATK;MATK;MATK	3'UTR;3'UTR;3'UTR	-1.086979	-0.201273	-7.815067	1.47E-12	4.31E-10	18.169561
cg04359418	chr10	76573396	-			1.5130127	1.8171082	7.8142113	1.48E-12	4.33E-10	18.165059
cg22674699	chr2	176987918	-	HOXD9	1stExon	3.21124	-0.130517	7.8122402	1.49E-12	4.37E-10	18.154693
cg03688818	chr3	49711147	-	APEH	TSS1500	-0.841396	-3.790316	-7.811731	1.50E-12	4.38E-10	18.152015
cg07274716	chr5	134363973	+	PITX1	3'UTR	2.0162467	0.2015825	7.8101364	1.51E-12	4.41E-10	18.143629
cg21097354	chr7	27179161	+			1.755817	1.0998773	7.8100889	1.51E-12	4.41E-10	18.143379
cg25336332	chr3	24537309	+	THRB;THRB;THRB	TSS1500;TSS1500;TSS1500	-2.255092	-3.022123	-7.828787	1.51E-12	4.41E-10	18.148249

cg27364741	chr2	63281069	-	OTX1	Body	2.5245047	-0.187691	7.8153157	1.52E-12	4.43E-10	18.140244
cg05132999	chr1	51796987	+	TTC39A	Body	-1.745716	0.1241566	-7.807753	1.53E-12	4.46E-10	18.131096
cg12337525	chr4	81185228	-			1.7135113	-0.422504	7.8077676	1.53E-12	4.46E-10	18.131172
cg26147279	chr3	13977886	+	TPRXL	TSS1500	-1.045576	-0.463908	-7.807532	1.53E-12	4.46E-10	18.129933
cg10830649	chr2	166650933	+	GALNT3	TSS200	-1.547263	-4.693708	-7.80685	1.54E-12	4.48E-10	18.12635
cg00792015	chr2	218466469	-	DIRC3	Body	-1.216877	-1.106036	-7.806187	1.54E-12	4.49E-10	18.122863
cg06188545	chr1	156863643	-	PEAR1	5'UTR	2.0078209	0.7591496	7.8045747	1.56E-12	4.53E-10	18.114385
cg19265300	chr15	69702702	+			-1.063437	-0.998904	-7.809341	1.57E-12	4.55E-10	18.108961
cg24452128	chr6	10390919	-			3.1003907	-0.766254	7.8032636	1.57E-12	4.55E-10	18.107492
cg12426196	chr1	161008826	+	TSTD1;TSTD1;TSTD1	TSS200;TSS200;TSS200	-1.976391	-2.187547	-7.81471	1.57E-12	4.57E-10	18.106151
cg19772907	chr20	45985566	+	ZMYND8;ZMYND8;ZMYND8	TSS200;TSS200;TSS200	-1.566059	-4.467632	-7.801588	1.58E-12	4.59E-10	18.098683
cg20438306	chr4	41747092	-	PHOX2B	3'UTR	2.3521783	-1.029281	7.8195269	1.59E-12	4.60E-10	18.095046
cg04548856	chr14	57284528	-	OTX2OS1	Body	2.0518877	-0.88512	7.8007286	1.59E-12	4.60E-10	18.094166
cg00336320	chr6	36253099	+	PNPLA1;PNPLA1;PNPLA1	5'UTR;Body;5'UTR	1.9577877	-0.688782	7.8126915	1.59E-12	4.60E-10	18.095583
cg10517990	chr11	1661619	-	HCCA2	Body	-1.01603	-1.858297	-7.799964	1.60E-12	4.62E-10	18.090149
cg20009461	chr17	80560634	+	FOXK2	3'UTR	2.117592	3.1167267	7.7990048	1.60E-12	4.64E-10	18.085105
cg24809269	chr17	74260692	+	FAM100B	TSS1500	-1.811065	-1.01591	-7.798587	1.61E-12	4.64E-10	18.082909
cg14778622	chr2	136743698	+	DARS	TSS1500	-1.314552	-2.792971	-7.797984	1.61E-12	4.66E-10	18.07974
cg10511988	chr10	118891706	+	VAX1	3'UTR	1.9888926	-0.411069	7.802766	1.62E-12	4.68E-10	18.074411
cg23156348	chr11	124981869	+			-1.327464	-4.120374	-7.79684	1.62E-12	4.68E-10	18.073729

cg15358549	chr5	127872074	+	FBN2	Body	1.5991022	1.7349139	7.7936139	1.65E-12	4.76E-10	18.056773
cg26708319	chr4	111552040	-	PITX2;PITX2	Body;Body	2.1380513	-0.632704	7.7929337	1.66E-12	4.77E-10	18.053199
cg02828785	chr12	12846546	-	GPR19	5'UTR	1.3085279	1.5333482	7.7923813	1.66E-12	4.78E-10	18.050296
cg25889581	chr4	16708336	+	LDB2;LDB2	Body;Body	-1.630813	-0.221328	-7.792372	1.66E-12	4.78E-10	18.05025
cg24989534	chr2	216878510	-	MREG	TSS200	-0.622233	-3.869048	-7.791818	1.67E-12	4.79E-10	18.047337
cg20603543	chr6	155584968	+	TFB1M;CLDN20	Body;TSS200	0.9442094	2.2342512	7.7916751	1.67E-12	4.79E-10	18.046586
cg08006309	chr16	1587810	-	IFT140;TMEM204	Body;Body	1.1812946	1.3130641	7.79134	1.67E-12	4.80E-10	18.044825
cg14765959	chr6	106429316	-			2.7763777	-0.543828	7.7911052	1.67E-12	4.80E-10	18.043592
cg27180868	chr1	161008750	+	TSTD1;TSTD1;TSTD1;TSTD1;TSTD1;TSTD1	5'UTR;1stExon;5'UTR;1stExon;1stExon;5'UTR	-1.843355	-2.090605	-7.796824	1.68E-12	4.81E-10	18.038795
cg13027458	chr16	59789219	+	LOC644649	TSS200	-1.701105	-0.566073	-7.790634	1.68E-12	4.81E-10	18.041115
cg19273773	chr7	102790112	+	NAPEPLD;NAPEPLD	TSS1500;TSS1500	-1.567575	-3.490519	-7.79022	1.68E-12	4.81E-10	18.03894
cg16555417	chr1	40598772	-			1.4122508	-0.603621	7.7875997	1.71E-12	4.88E-10	18.025174
cg17468543	chr8	131358890	-	ASAP1	Body	1.0042582	2.8832487	7.7868246	1.71E-12	4.90E-10	18.021103
cg24530460	chr14	37053689	+			1.2221729	-1.002877	7.7864505	1.72E-12	4.90E-10	18.019138
cg15733114	chr12	14996515	-	ART4	TSS200	1.405606	1.0566116	7.7862228	1.72E-12	4.91E-10	18.017941
cg21796547	chr14	73705457	+	PAPLN	5'UTR	-1.133505	-3.01924	-7.785669	1.72E-12	4.92E-10	18.015032
cg27145042	chr16	25026956	-	ARHGAP17;ARHGAP17	TSS1500;TSS1500	-0.71869	-3.85714	-7.785071	1.73E-12	4.93E-10	18.011891
cg01176748	chr14	23426575	+	HAUS4;HAUS4;HAUS4	TSS1500;TSS1500;TSS1500	-1.306095	-4.430468	-7.78991	1.74E-12	4.96E-10	18.007067
cg05754905	chr1	160992352	-	F11R	TSS1500	-2.33801	0.5530238	-7.782723	1.75E-12	4.97E-10	17.999556
cg07489502	chr5	140811642	+	PCDHGA4;PCDHGA11;PCDHGA11;PCDHGA9;PCDHGA1;PCDHGB1;PCDHGB6;PCDHGA12;PCDHGB3;PCDHGB7;PCDHGA6;PCDHGA8;PCDHGA10;PCDHGA12;PCDHGA5;PCDHGB4;PCDHGA	Body;Body;Body;Body;Body;Body;Body;1stExon;Body;Body;Body;Body;Body;1stExon;Body;Body;Body;Body;Body;Body;Body	2.550445	-0.352074	7.801514	1.75E-12	4.97E-10	18.005846

cg08783253	chr17	40996565	+	AOC2;AOC2	TSS200;TSS200	1.3452375	0.8823446	7.782808	1.75E-12	4.97E-10	18.000005
cg12670347	chr12	114847578	-	TBX5;TBX5	TSS1500;TSS1500	1.7599221	0.0529127	7.7829542	1.75E-12	4.97E-10	18.000773
cg20170777	chr17	13972099	+	COX10	TSS1500	-1.514812	-0.726929	-7.783016	1.75E-12	4.97E-10	18.001098
cg21974506	chr6	33141291	-	COL11A2;COL11A2;COL11A2	Body;Body;Body	-2.094576	-0.275618	-7.788722	1.75E-12	4.97E-10	18.000785
cg09004581	chr6	74171029	-	MTO1;MTO1;MTO1	TSS1500;TSS1500;TSS1500	-1.411207	-3.001521	-7.781355	1.77E-12	5.01E-10	17.992373
cg13557031	chr9	131909876	-	PPP2R4;PPP2R4;PPP2R4;PPP2R4	3'UTR;3'UTR;3'UTR;3'UTR	1.4294409	4.0553198	7.7933147	1.77E-12	5.01E-10	17.994148
cg16986578	chr1	201979567	+	ELF3;ELF3	TSS200;TSS200	-1.35451	-0.954478	-7.780844	1.77E-12	5.01E-10	17.989691
cg08658787	chr12	113916646	+			1.4335629	1.4319117	7.7866308	1.77E-12	5.02E-10	17.989881
cg25742464	chr1	201978971	-	ELF3;ELF3	TSS1500;TSS1500	-1.132695	-2.337536	-7.780219	1.78E-12	5.02E-10	17.98641
cg16227072	chr11	77320503	+	AQP11	3'UTR	1.0439743	2.062048	7.7793623	1.78E-12	5.04E-10	17.981909
cg04411307	chr2	69391395	+	ANTXR1;ANTXR1	Body;Body	1.2303017	2.0468604	7.7787528	1.79E-12	5.05E-10	17.978708
cg12589298	chr19	50828905	-	KCNC3	Body	-1.752392	-3.64507	-7.784871	1.79E-12	5.05E-10	17.9806
cg12589486	chr5	149903908	-	NDST1	Body	0.9849158	2.5640338	7.7787013	1.79E-12	5.05E-10	17.978438
cg13080379	chr1	119527156	-	TBX15	5'UTR	1.574088	-0.603145	7.778058	1.80E-12	5.07E-10	17.975059
cg20935165	chr2	172972840	-			2.9774762	-0.812761	7.7769418	1.81E-12	5.09E-10	17.969198
cg17780246	chr19	54481467	-	CACNG8	Body	1.3350031	0.2270987	7.7755925	1.82E-12	5.13E-10	17.962114
cg03943218	chr10	102419600	+			2.554728	-0.530691	7.7748877	1.83E-12	5.14E-10	17.958413
cg24373187	chr1	54666401	-	CYB5RL;MRPL37	TSS1500;Body	-1.125959	-3.033756	-7.773744	1.84E-12	5.17E-10	17.952409
cg24347994	chr1	91192657	+			1.30533	-0.3061	7.7735272	1.84E-12	5.18E-10	17.951271
cg08197797	chr6	138428995	+	PERP	TSS1500	-0.984319	-3.273717	-7.773304	1.84E-12	5.18E-10	17.950101

cg08821811	chr16	11391517	+			-1.139003	-3.66504	-7.771324	1.86E-12	5.23E-10	17.939706
cg19565757	chr5	43557653	-	PAIP1;PAIP1;PAIP1	TSS1500;TSS200;TSS1500	-0.930233	-2.924295	-7.771335	1.86E-12	5.23E-10	17.93976
cg00223767	chr11	75831715	+	UVRAG	Body	1.851013	0.8897264	7.7708303	1.87E-12	5.24E-10	17.937113
cg05187155	chr4	160280029	+	RAPGEF2	3'UTR	0.9688699	2.7407195	7.7690376	1.89E-12	5.28E-10	17.927703
cg16774755	chr16	86421946	-			-1.732591	0.0261124	-7.769079	1.89E-12	5.28E-10	17.927921
cg26791464	chr1	40769744	-	COL9A2	Body	1.1122303	1.2952126	7.7689588	1.89E-12	5.28E-10	17.927289
cg15975802	chr12	7055458	-	PTPN6	TSS1500	-1.003529	-1.39219	-7.768649	1.89E-12	5.29E-10	17.925662
cg25719851	chr12	7055452	-	PTPN6	TSS1500	-1.162131	-1.229445	-7.76854	1.89E-12	5.29E-10	17.925092
cg02090418	chr4	113333269	+	ALPK1;ALPK1	Body;Body	1.5585933	3.0312716	7.7669815	1.91E-12	5.33E-10	17.916911
cg02561482	chr6	50813551	-	TFAP2B	3'UTR	2.0538068	-0.280008	7.7662826	1.92E-12	5.35E-10	17.913243
cg03506452	chr12	133458949	+	CHFR;CHFR;CHFR;CHFR;CHFR	Body;Body;Body;Body;Body	1.1750261	2.300056	7.7646437	1.93E-12	5.39E-10	17.904643
cg26799474	chr2	202098951	-	CASP8;CASP8;CASP8;CASP8	5'UTR;5'UTR;5'UTR;5'UTR	-2.203519	-0.654873	-7.764065	1.94E-12	5.40E-10	17.901606
cg09627520	chr3	58410836	-	PXK	3'UTR	1.3376554	0.6194764	7.7619695	1.96E-12	5.46E-10	17.89061
cg13713922	chr2	121625577	-	GLI2	Body	1.7519797	-0.506269	7.7734582	1.97E-12	5.47E-10	17.89036
cg23290344	chr8	24771466	+	NEFM;NEFM	TSS1500;1stExon	2.6065557	-0.467258	7.7674566	1.97E-12	5.47E-10	17.88944
cg05979020	chr2	176981336	-	HOXD10	TSS200	1.2432033	0.2111407	7.7610154	1.97E-12	5.48E-10	17.885604
cg05467676	chr3	72226965	+			-1.748065	-2.929269	-7.75962	1.99E-12	5.52E-10	17.878282
cg04271289	chr19	1152147	-	SBNO2	Body	1.7154522	2.295051	7.7650857	1.99E-12	5.53E-10	17.877026
cg17775765	chr13	95354021	+			1.1576246	0.5872715	7.7590823	1.99E-12	5.53E-10	17.875461
cg01294808	chr5	3599686	-	IRX1	Body	1.5639648	1.1058917	7.758726	2.00E-12	5.53E-10	17.873592

cg06580318	chr2	169747119	-	SPC25	TSS200	-1.17355	-3.531213	-7.758797	2.00E-12	5.53E-10	17.873966
cg15277906	chr8	97170364	-	GDF6	Body	1.422288	-0.589014	7.7587133	2.00E-12	5.53E-10	17.873525
cg10432422	chr1	17475819	-			1.3473524	0.152062	7.7674324	2.03E-12	5.61E-10	17.858991
cg17504641	chr10	44458167	+			-1.70717	0.1721186	-7.75552	2.03E-12	5.61E-10	17.856772
cg26110281	chr11	123885695	+	OR10G4	TSS1500	-1.812116	-0.310023	-7.755669	2.03E-12	5.61E-10	17.857554
cg27277463	chr12	62585031	+	FAM19A2	5'UTR	3.5723606	-1.048114	7.7552227	2.03E-12	5.62E-10	17.855214
cg08412188	chr7	74074356	-	GTF2I;GTF2I;GTF2I;GTF2I;GTF2I	5'UTR;5'UTR;5'UTR;5'UTR;5'UTR	-1.621705	-3.293	-7.754622	2.04E-12	5.63E-10	17.852065
cg09473613	chr1	24152604	-	HMGCL;HMGCL	TSS1500;TSS1500	-2.072027	-0.29968	-7.753219	2.06E-12	5.67E-10	17.844706
cg19683800	chr17	61518354	+	CYB561;CYB561;CYB561	TSS200;5'UTR;5'UTR	1.3246717	2.3737342	7.7526993	2.06E-12	5.68E-10	17.841978
cg24411961	chr1	34642396	-	C1orf94;C1orf94	5'UTR;TSS200	1.9041533	1.1507646	7.7522735	2.07E-12	5.69E-10	17.839745
cg21393163	chr1	12217629	-			-1.442582	-2.382791	-7.751836	2.07E-12	5.70E-10	17.837451
cg26601431	chr1	235054899	+			2.1456257	3.9778868	7.7516914	2.07E-12	5.70E-10	17.836692
cg19555331	chr4	147561900	+	POU4F2	Body	1.7494244	-1.112455	7.750822	2.08E-12	5.73E-10	17.832133
cg05686445	chr7	127636396	-	C7orf54;SND1	TSS1500;Body	1.1564868	2.5503918	7.7506575	2.09E-12	5.73E-10	17.83127
cg14189141	chr9	1042605	-			2.8839478	-0.7851	7.7504122	2.09E-12	5.73E-10	17.829983
cg10154971	chr16	59270585	-			-2.147684	0.4491847	-7.755538	2.10E-12	5.75E-10	17.827053
cg04787317	chr3	38009934	-	CTDSPL;CTDSPL;MIR26A1	Body;Body;TSS1500	0.8827872	2.0825203	7.7488933	2.11E-12	5.77E-10	17.822018
cg02164225	chr2	176977773	+			2.0298601	-0.977657	7.748198	2.11E-12	5.79E-10	17.818372
cg22697517	chr17	42785378	+	DBF4B;DBF4B	TSS1500;TSS1500	-1.086211	-1.301857	-7.747678	2.12E-12	5.80E-10	17.815647
cg08182501	chr2	177003406	-			1.3579575	0.9832081	7.7470026	2.13E-12	5.82E-10	17.812104

cg16054278	chr1	212620748	+			0.8780731	3.0183374	7.7466984	2.13E-12	5.82E-10	17.810509
cg18635552	chr5	3599719	+	IRX1	Body	1.7794349	-2.11493	7.7466935	2.13E-12	5.82E-10	17.810483
cg19620724	chr6	50810852	-	TFAP2B	Body	1.63446	-0.666778	7.7464897	2.13E-12	5.83E-10	17.809415
cg07329360	chr11	31841768	+			1.9094367	-0.179139	7.7461375	2.14E-12	5.83E-10	17.807568
cg12417815	chr5	148830633	+			1.1550073	1.5511612	7.7450488	2.15E-12	5.87E-10	17.801859
cg04645070	chr11	34393106	+			-1.213818	-2.877889	-7.7439	2.16E-12	5.90E-10	17.795836
cg19910937	chr4	128983807	+	LARP1B;LARP1B;LARP1B	5'UTR;5'UTR;5'UTR	-1.117553	-2.267174	-7.743478	2.17E-12	5.91E-10	17.793622
cg06117072	chr6	50791385	-	TFAP2B	Body	1.5351149	-0.389103	7.7422189	2.18E-12	5.94E-10	17.787024
cg22349489	chr6	27114943	-	HIST1H2BK;HIST1H2AH	TSS1500;1stExon	-1.3052	-3.834743	-7.741896	2.19E-12	5.95E-10	17.785331
cg16655338	chr6	72892901	-	RIMS1	Body	-1.571503	-3.589822	-7.745687	2.21E-12	6.02E-10	17.775509
cg13424940	chr9	136844397	-	VAV2;VAV2	Body;Body	1.136029	1.4212978	7.7391881	2.22E-12	6.03E-10	17.771136
cg18675097	chr6	28227127	+	NKAPL;NKAPL	5'UTR;1stExon	1.8582148	1.2174096	7.7385608	2.23E-12	6.05E-10	17.767848
cg11435087	chr19	38397154	-	SIPA1L3;WDR87;WDR87	TSS1500;1stExon;5'UTR	-1.072569	-3.698543	-7.737854	2.24E-12	6.06E-10	17.764142
cg20181079	chr11	59270409	+	OR4D11	TSS1500	-1.884327	0.0971661	-7.737911	2.24E-12	6.06E-10	17.764441
cg18143701	chr3	176862885	+	TBL1XR1	5'UTR	-1.414752	-2.89872	-7.737311	2.24E-12	6.08E-10	17.7613
cg09859564	chr10	281489	+	ZMYND11;ZMYND11;ZMYND11	Body;Body;Body	1.0373792	2.3088454	7.7367485	2.25E-12	6.09E-10	17.75835
cg10723962	chr6	26240782	+	HIST1H4F	1stExon	3.3328984	-1.633424	7.7487836	2.25E-12	6.09E-10	17.76152
cg17399143	chr7	42909134	-			-1.496466	-1.011554	-7.742609	2.25E-12	6.09E-10	17.759411
cg04816803	chr10	44426803	+			-2.072749	-1.101646	-7.736573	2.25E-12	6.09E-10	17.75743
cg22018329	chr19	3116716	-	GNA11	Body	1.6129634	5.4773704	7.73509	2.27E-12	6.13E-10	17.749658

cg19321018	chr12	15942969	+	EPS8	TSS1500	-1.230402	-2.420337	-7.734295	2.28E-12	6.16E-10	17.745491
cg03213374	chr12	8112512	+			-1.746764	-2.777333	-7.733605	2.29E-12	6.17E-10	17.741874
cg00660272	chr1	24231353	+	CNR2	5'UTR	-1.003595	-2.597852	-7.732702	2.30E-12	6.20E-10	17.737146
cg04228042	chr12	14996527	+	ART4	TSS200	1.4450345	1.7080847	7.7323064	2.30E-12	6.21E-10	17.735071
cg12308675	chr2	239169537	-	PER2	Body	1.3657428	1.9640117	7.7312497	2.32E-12	6.24E-10	17.729534
cg11899535	chr7	70598282	-	WBSCR17	Body	1.8587769	-0.647725	7.7304046	2.33E-12	6.27E-10	17.725107
cg08383929	chr5	180646754	+			0.9376022	1.2439546	7.7302534	2.33E-12	6.27E-10	17.724315
cg00870166	chr8	74660057	-	STAU2;STAU2;STAU2;STAU2;STAU2;STAU2;STAU2	TSS1500;TSS1500;TSS200;TSS1500;TSS1500;TSS1500;TSS1500	-1.294501	-3.104484	-7.729968	2.33E-12	6.27E-10	17.722817
cg19430780	chr19	38397004	-	SIPA1L3;WDR87	TSS1500;5'UTR	-0.982619	-1.952389	-7.729514	2.34E-12	6.29E-10	17.720439
cg17913068	chr11	70116114	-	PPFIA1;PPFIA1	TSS1500;TSS1500	-0.600023	-4.140632	-7.727872	2.36E-12	6.34E-10	17.71184
cg25510201	chr5	175980531	-	PCDH24	5'UTR	-0.965434	-1.479886	-7.723442	2.42E-12	6.49E-10	17.688634
cg21734996	chr4	16760829	+	LDB2;LDB2	Body;Body	-1.972827	0.7021827	-7.72175	2.44E-12	6.54E-10	17.679773
cg08682723	chr16	81713770	-	CMIP;CMIP	Body;Body	1.3847421	2.3217937	7.7213342	2.45E-12	6.55E-10	17.677594
cg23130254	chr2	176964588	-	HOXD12	1stExon	1.6998532	-1.122472	7.7200032	2.46E-12	6.60E-10	17.670624
cg12188526	chr14	71896386	+			1.1480819	2.2317879	7.7172852	2.50E-12	6.69E-10	17.656391
cg22235877	chr17	79791587	+	FAM195B;FAM195B;DYSFIP1	TSS1500;TSS1500;3'UTR	-0.90586	-2.639989	-7.717359	2.50E-12	6.69E-10	17.656776
cg03132824	chr4	3373006	-	RGS12;RGS12;RGS12	Body;Body;Body	2.3685568	1.2127295	7.7286619	2.51E-12	6.70E-10	17.656653
cg00396667	chr5	134364387	-	PITX1	3'UTR	2.1476988	0.0731331	7.7155509	2.52E-12	6.74E-10	17.64731
cg02098786	chr14	24801794	-	ADCY4	Body	-1.425423	-1.29233	-7.71467	2.54E-12	6.76E-10	17.642697
cg06769423	chr4	73920648	-	COX18	3'UTR	1.1512604	2.1489861	7.7148421	2.53E-12	6.76E-10	17.643599

cg08005122	chr11	110779637	-			-1.187607	-2.563914	-7.71464	2.54E-12	6.76E-10	17.642541
cg18582260	chr13	25085301	-	PARP4	5'UTR	-1.782907	-1.583877	-7.738598	2.54E-12	6.76E-10	17.647368
cg22274829	chr4	82393723	-	RASGEF1B	TSS1500	-1.565301	-2.0921	-7.725971	2.54E-12	6.77E-10	17.642576
cg06245037	chr11	44332938	+	ALX4	TSS1500	2.3185463	-0.871788	7.7136698	2.55E-12	6.79E-10	17.637462
cg08677210	chr17	55550613	+	MSI2;MSI2	Body;Body	1.0449619	1.0442095	7.7131133	2.56E-12	6.80E-10	17.634548
cg03719032	chr19	19374710	-	HAPLN4	TSS1500	-1.423966	-2.040996	-7.712361	2.57E-12	6.83E-10	17.630609
cg19978181	chr5	76923988	-			3.3350976	-1.646287	7.711583	2.58E-12	6.85E-10	17.626537
cg03599078	chr10	8078314	-			1.7518313	-0.108958	7.7104948	2.59E-12	6.89E-10	17.620841
cg08001899	chr17	47751234	-	SPOP;SPOP;SPOP;SPOP;SPOP;SPOP	5'UTR;5'UTR;5'UTR;5'UTR;5'UTR;5'UTR	1.0761786	2.1106501	7.7095721	2.61E-12	6.91E-10	17.616011
cg18809600	chr6	160389711	+	IGF2R	TSS1500	-0.73639	-4.115878	-7.709576	2.61E-12	6.91E-10	17.616031
cg25722212	chr1	111747324	+			-1.194558	-4.051918	-7.709265	2.61E-12	6.92E-10	17.614406
cg21591742	chr2	176980998	-	HOXD10	TSS1500	2.0479651	-0.5958	7.708491	2.62E-12	6.94E-10	17.610353
cg20575761	chr14	57275166	+	OTX2	5'UTR	1.7843282	0.0946232	7.7082488	2.62E-12	6.95E-10	17.609085
cg14523847	chr7	153584597	-	DPP6;DPP6	1stExon;5'UTR	2.2793125	0.1066567	7.7079571	2.63E-12	6.96E-10	17.607558
cg21449597	chr4	154036828	-			-1.406506	-1.510994	-7.713583	2.63E-12	6.96E-10	17.607705
cg06385324	chr16	2014621	+	SNHG9;SNORA78;RPS2	TSS1500;TSS1500;Body	-1.142015	-3.406849	-7.707631	2.63E-12	6.96E-10	17.605853
cg21166347	chr6	33240215	-	VPS52;RPS18	TSS1500;Body	-0.833963	-4.410439	-7.707513	2.64E-12	6.96E-10	17.605233
cg00225067	chr3	45647141	+	LIMD1	Body	0.9808345	2.5506418	7.7124057	2.65E-12	6.98E-10	17.601557
cg08136772	chr13	53420933	-	PCDH8;PCDH8	1stExon;1stExon	1.5846675	-0.129165	7.706615	2.65E-12	6.98E-10	17.600534
cg12764034	chr1	75602846	+	LHX8	Body	2.1172259	-0.488348	7.706565	2.65E-12	6.98E-10	17.600273

cg13466383	chr1	161008705	+	TSTD1;TSTD1;TSTD1;TSTD1;TSTD1;TSTD1	5'UTR;1stExon;5'UTR;1stExon;1stExon;5'UTR	-1.674722	-1.674547	-7.706696	2.65E-12	6.98E-10	17.600959
cg09327253	chr7	31147318	+			-1.582674	-0.436661	-7.705197	2.67E-12	7.03E-10	17.593112
cg19441717	chr4	85422453	+			1.5727863	-0.888387	7.7109231	2.67E-12	7.03E-10	17.593774
cg09807874	chr16	81510145	-	CMIP	Body	1.0586118	2.8585058	7.704644	2.68E-12	7.04E-10	17.59022
cg12003483	chr17	41624164	+	ETV4;ETV4	TSS1500;TSS1500	-0.617906	-3.471939	-7.704412	2.68E-12	7.04E-10	17.589004
cg23352146	chr11	12701929	+	TEAD1	5'UTR	-1.196624	-1.636291	-7.704416	2.68E-12	7.04E-10	17.589025
cg16209664	chr3	147131146	-	ZIC1	Body	1.231721	-0.774843	7.7033004	2.70E-12	7.08E-10	17.583189
cg21808448	chr1	39859609	+	MACF1;MACF1	Body;Body	1.0001374	1.6072706	7.703036	2.70E-12	7.08E-10	17.581805
cg23161999	chr2	162283189	-			2.8969683	-0.531562	7.707529	2.72E-12	7.13E-10	17.576091
cg24173049	chr17	6939795	-	SLC16A13	1stExon	-0.823666	-3.725781	-7.700615	2.74E-12	7.17E-10	17.569138
cg10688500	chr6	100016774	+	CCNC;CCNC	TSS200;TSS200	-0.690719	-4.21348	-7.699619	2.75E-12	7.20E-10	17.563927
cg09108394	chr16	23850106	-	PRKCB;PRKCB	Body;Body	-1.661729	-0.661022	-7.698337	2.77E-12	7.25E-10	17.557219
cg20051772	chr12	56325015	+	DGKA;DGKA;DGKA;DGKA;DGKA	TSS1500;TSS1500;TSS1500;5'UTR;1stExon	-1.351783	-2.037567	-7.69784	2.78E-12	7.26E-10	17.55462
cg16397638	chr1	228559083	+	OBSCN	Body	-1.623225	0.9676637	-7.696705	2.79E-12	7.30E-10	17.548681
cg06640630	chr1	211652276	-	RD3;RD3	3'UTR;3'UTR	1.2494507	0.9176794	7.6949433	2.82E-12	7.37E-10	17.539468
cg20786131	chr18	20714496	+	CABLES1;CABLES1	TSS1500;TSS200	-1.338793	-2.487293	-7.694866	2.82E-12	7.37E-10	17.539062
cg10449091	chr7	1783609	+	ELFN1	5'UTR	-0.997655	-0.932489	-7.693303	2.85E-12	7.43E-10	17.530888
cg05942459	chr6	101846805	-	GRIK2;GRIK2;GRIK2	TSS200;TSS200;TSS200	2.4171117	-0.54708	7.6927317	2.86E-12	7.44E-10	17.527901
cg13677149	chr7	27284789	-	EVX1	Body	2.1938603	-0.347262	7.6926828	2.86E-12	7.44E-10	17.527645
cg25448856	chr12	93172875	-	EEA1	Body	1.2649382	1.9066353	7.6925589	2.86E-12	7.44E-10	17.526997

cg08128264	chr16	893139	-			-1.354305	0.3707976	-7.692116	2.86E-12	7.45E-10	17.524681
cg14318249	chr9	80851623	+	CEP78;CEP78	Body;Body	-0.554056	-3.657145	-7.692144	2.86E-12	7.45E-10	17.524829
cg06007282	chr2	45231467	-			1.8627381	-0.300577	7.6973703	2.87E-12	7.47E-10	17.523061
cg00007076	chr8	67342600	+	RRS1;RRS1	1stExon;3'UTR	-1.551291	-1.925777	-7.690759	2.89E-12	7.50E-10	17.517584
cg14308254	chr8	124219699	+	FAM83A;FAM83A	Body;Body	1.330007	3.0023275	7.6888416	2.92E-12	7.57E-10	17.507558
cg00089798	chr12	62585204	-	FAM19A2;FAM19A2	5'UTR;1stExon	2.5671843	-1.106968	7.6876451	2.93E-12	7.62E-10	17.501301
cg02995055	chr13	42535448	+	KIAA0564;KIAA0564	TSS1500;TSS1500	-0.966062	-4.068388	-7.686691	2.95E-12	7.64E-10	17.496314
cg07454556	chr2	54754901	+	RPL23AP32;SPTBN1	TSS1500;Body	1.1080252	1.6243068	7.6865363	2.95E-12	7.64E-10	17.495504
cg21207003	chr3	113798887	-	QTRTD1	Body	1.4633324	1.7933568	7.6867602	2.95E-12	7.64E-10	17.496675
cg23963802	chr10	101874887	+			1.519432	-0.512421	7.6866406	2.95E-12	7.64E-10	17.496049
cg03696599	chr2	124782261	-	CNTNAP5	TSS1500	1.7643709	-0.775693	7.6862747	2.96E-12	7.65E-10	17.494136
cg10530889	chr10	93805590	-			0.8013386	0.6884695	7.6852435	2.97E-12	7.69E-10	17.488745
cg06003834	chr5	66354967	-	MAST4;MAST4	Body;Body	1.0396166	2.206504	7.6833928	3.00E-12	7.76E-10	17.47907
cg10460946	chr17	76247467	+			-1.61667	-2.242052	-7.683341	3.00E-12	7.76E-10	17.4788
cg17195127	chr15	65579557	-	PARP16	TSS1500	-0.81902	-2.355301	-7.683284	3.00E-12	7.76E-10	17.478502
cg24672624	chr1	24239771	-	CNR2;CNR2	5'UTR;1stExon	-1.477209	-0.798803	-7.682339	3.02E-12	7.79E-10	17.473563
cg02204261	chr4	134787901	-			-2.088607	0.6475463	-7.678589	3.08E-12	7.95E-10	17.453962
cg00425918	chr8	23563146	+	NKX2-6	Body	1.5473838	-0.553889	7.6780657	3.09E-12	7.96E-10	17.451225
cg08684565	chr7	135347028	-	PL-5283	TSS200	-0.73936	-4.464827	-7.676558	3.12E-12	8.02E-10	17.443347
cg06471491	chr17	57917974	-	MIR21	TSS1500	-1.32312	-2.011973	-7.676394	3.12E-12	8.03E-10	17.442486

cg18838433	chr15	99670476	-	SYNM;SYNM	Body;Body	1.0807382	1.6772671	7.6762415	3.12E-12	8.03E-10	17.441692
cg01015662	chr1	40598394	-			1.5698692	2.2622852	7.6756672	3.13E-12	8.05E-10	17.438691
cg23665216	chr14	105916422	+	MTA1	Body	1.1871262	2.3610397	7.6756177	3.13E-12	8.05E-10	17.438432
cg17104824	chr2	177014959	-	HOXD4;MIR10B	TSS1500;TSS200	1.7456313	0.3395944	7.6734895	3.17E-12	8.13E-10	17.427311
cg02805871	chr17	6939695	-	SLC16A13;SLC16A13	5'UTR;1stExon	-0.544689	-3.5289	-7.673277	3.17E-12	8.14E-10	17.426202
cg11931646	chr6	31804172	+	C6orf48;C6orf48;SNORD52	5'UTR;5'UTR;TSS1500	-1.518203	-3.227777	-7.678629	3.18E-12	8.15E-10	17.42526
cg01789499	chr4	81109888	-	PRDM8	5'UTR	1.3175658	1.2832502	7.6716186	3.20E-12	8.20E-10	17.417536
cg26661640	chr11	64458846	-	NRXN2;NRXN2	Body;Body	-0.965922	-0.70872	-7.671478	3.20E-12	8.20E-10	17.416799
cg23502378	chr6	157877218	+	ZDHHC14;ZDHHC14	Body;Body	0.7630267	1.8438985	7.6712169	3.21E-12	8.21E-10	17.415437
cg06486088	chr2	237010222	-	AGAP1;AGAP1	Body;Body	0.9895296	1.8641092	7.669833	3.23E-12	8.27E-10	17.408207
cg01419831	chr2	162283705	-			3.3800834	-1.143732	7.6690484	3.25E-12	8.30E-10	17.404108
cg07546078	chr2	69742449	-	AAK1	Body	1.1508176	2.1083507	7.6688137	3.25E-12	8.30E-10	17.402882
cg13501446	chr9	138900624	+			1.9940565	3.581294	7.6740556	3.26E-12	8.32E-10	17.40145
cg03019112	chr14	101518520	-	MIR487A	TSS1500	-1.712932	0.0797036	-7.667877	3.27E-12	8.33E-10	17.397991
cg14106085	chr1	47902269	-	FOXD2;FOXD2	1stExon;5'UTR	-0.863011	-3.892963	-7.667271	3.28E-12	8.36E-10	17.394826
cg10857221	chr17	75282140	+	9-Sep	Body	-1.123416	-1.500445	-7.666725	3.29E-12	8.38E-10	17.391973
cg17315844	chr5	10249113	-	FAM173B;CCT5	Body;TSS1500	-2.243078	0.1104314	-7.663308	3.35E-12	8.53E-10	17.374123
cg10432620	chr16	74808963	+	FA2H	TSS1500	-0.598959	-3.482249	-7.66268	3.36E-12	8.55E-10	17.370843
cg19536401	chr19	6504797	-			-1.430341	-0.520819	-7.662746	3.36E-12	8.55E-10	17.37119
cg24119944	chr14	91262410	+	TTC7B	Body	1.0080811	2.1062568	7.6682153	3.36E-12	8.55E-10	17.371008

cg21113740	chr1	79472343	-	ELTD1;ELTD1	5'UTR;1stExon	1.6671488	0.1715235	7.662139	3.37E-12	8.56E-10	17.368019
cg13200730	chr17	25827857	+	KSR1	5'UTR	1.0326348	2.3075176	7.6614544	3.38E-12	8.59E-10	17.364444
cg23510240	chr2	132345990	+			-1.854865	-0.695625	-7.660056	3.41E-12	8.65E-10	17.357141
cg09520126	chr7	158802720	-	LOC154822	Body	1.919131	3.3462183	7.6703498	3.43E-12	8.70E-10	17.352982
cg02480199	chr5	145725913	+			1.5833298	-0.36902	7.6567184	3.47E-12	8.79E-10	17.339715
cg14528147	chr1	113001201	+	CTTNBP2NL	3'UTR	0.8979252	2.3755652	7.6566693	3.47E-12	8.79E-10	17.339459
cg19426522	chr3	27766009	-			1.4027114	0.4358617	7.6559469	3.48E-12	8.82E-10	17.335687
cg22827063	chr2	66809119	+			1.8265248	-0.430006	7.6552301	3.50E-12	8.85E-10	17.331945
cg10600644	chr2	46530198	+	EPAS1	Body	1.18216	2.4030737	7.6543788	3.51E-12	8.89E-10	17.327501
cg18322569	chr1	91182777	+	BARHL2;BARHL2	5'UTR;1stExon	2.8588285	0.1434798	7.6522401	3.55E-12	8.98E-10	17.316337
cg27080119	chr4	120987409	+	MAD2L1	Body	-1.728489	-3.178407	-7.652335	3.55E-12	8.98E-10	17.316834
cg01946784	chr2	121991815	+	TFCP2L1	Body	1.0688998	1.3771497	7.6494411	3.61E-12	9.11E-10	17.301728
cg20967139	chr12	54785055	-	ZNF385A;ZNF385A;ZNF385A;ZNF385A	5'UTR;1stExon;1stExon;5'UTR	-2.07718	-2.588484	-7.646566	3.66E-12	9.25E-10	17.286725
cg21472506	chr2	63283967	+	OTX1	3'UTR	3.5589595	-0.437486	7.6518137	3.67E-12	9.27E-10	17.285562
cg03731268	chr1	62660861	-	L1TD1;L1TD1	5'UTR;5'UTR	1.9891346	-0.247474	7.6455498	3.68E-12	9.28E-10	17.281421
cg21604615	chr1	27668610	+	SYTL1;SYTL1	1stExon;5'UTR	-1.449912	-2.117561	-7.645628	3.68E-12	9.28E-10	17.281829
cg17115419	chr10	5593926	+			-1.970084	-0.535292	-7.644842	3.70E-12	9.31E-10	17.277729
cg02283366	chr1	63785544	+			2.580801	-0.818783	7.6444637	3.71E-12	9.33E-10	17.275754
cg12355110	chr19	57617754	-			1.4162589	-0.589888	7.6441743	3.71E-12	9.33E-10	17.274244
cg12378187	chr3	62355443	-	FEZF2	3'UTR	2.1988031	-0.117227	7.6498294	3.71E-12	9.33E-10	17.275229

cg01122755	chr6	44281249	+	AARS2	TSS200	-0.868023	-3.739965	-7.643623	3.72E-12	9.35E-10	17.271367
cg11405655	chr1	999599	+			-0.975955	-1.982082	-7.643088	3.73E-12	9.37E-10	17.268576
cg13702421	chr2	172955759	+			1.2780376	-0.74446	7.6431834	3.73E-12	9.37E-10	17.269074
cg23767994	chr7	96621323	+	DLX6AS	Body	1.6765873	0.2898502	7.6429341	3.74E-12	9.37E-10	17.267773
cg10635645	chr12	12938397	+	APOLD1;APOLD1	Body;TSS200	1.1243902	2.3884509	7.6476654	3.76E-12	9.41E-10	17.263961
cg02530321	chr20	43103795	+	TTPAL;TTPAL	TSS1500;TSS1500	-1.197037	-1.882688	-7.641126	3.77E-12	9.45E-10	17.258342
cg05386769	chr2	218681558	-	TNS1	Body	1.2245334	0.7005556	7.6404861	3.79E-12	9.48E-10	17.255002
cg16925459	chr7	18548581	-	HDAC9;HDAC9;HDAC9;HDAC9;HDAC9	Body;Body;Body;Body;Body	-1.624451	-1.40771	-7.638044	3.84E-12	9.60E-10	17.242262
cg14304515	chr20	822499	+	FAM110A;FAM110A	5'UTR;5'UTR	-0.716406	-1.128234	-7.636912	3.86E-12	9.65E-10	17.236356
cg16529477	chr2	223164854	+	PAX3;PAX3;PAX3;PAX3;PAX3;CCDC140;PAX3;PAX3;PAX3	TSS1500;TSS1500;TSS1500;TSS1500;TSS1500;5'UTR;<br>TSS1500;TSS1500;TSS1500	2.0525785	0.0526421	7.6366329	3.87E-12	9.66E-10	17.234903
cg03421657	chr1	166039754	-	FAM78B	Body	-1.530377	-0.961253	-7.634559	3.91E-12	9.76E-10	17.224089
cg02447304	chr6	74024628	+			1.7981268	0.4194835	7.6343022	3.92E-12	9.77E-10	17.222747
cg20924425	chr2	240121297	+	HDAC4	Body	1.2145579	1.4029293	7.6343405	3.91E-12	9.77E-10	17.222947
cg19816354	chr15	27519506	+	GABRG3	Body	-1.186863	-1.109003	-7.63173	3.97E-12	9.90E-10	17.209334
cg01720920	chr14	104185151	+	ZFYVE21	Body	0.9732857	0.6602495	7.6310062	3.99E-12	9.93E-10	17.205559
cg08282819	chr16	27437900	+	IL21R;IL21R;IL21R	TSS1500;5'UTR;5'UTR	-1.689094	-3.084268	-7.629929	4.01E-12	9.98E-10	17.199945
cg19456540	chr14	60976285	+	SIX6	1stExon	3.1845979	-1.113733	7.6279794	4.05E-12	1.01E-09	17.189778
cg08296903	chr16	14029456	+	ERCC4	Body	1.0904153	1.3925113	7.6276966	4.06E-12	1.01E-09	17.188303
cg12600174	chr7	27205230	+	HOXA9	TSS200	3.1337382	-0.531679	7.6327358	4.07E-12	1.01E-09	17.186234
cg16509777	chr2	45172265	-	SIX3	3'UTR	1.4176896	-0.352906	7.6263226	4.09E-12	1.02E-09	17.181141

cg23893897	chr14	50990170	-	MAP4K5;MAP4K5	Body;Body	1.1810724	2.0910247	7.6262997	4.09E-12	1.02E-09	17.181021
cg14764357	chr7	1093718	+	C7orf50;C7orf50;C7orf50	Body;Body;Body	1.4602475	2.2374357	7.624442	4.13E-12	1.02E-09	17.171337
cg05514299	chr17	74868670	-	MGAT5B;MGAT5B	Body;TSS200	-1.983051	-1.605397	-7.624013	4.14E-12	1.03E-09	17.169102
cg04528771	chr17	27893087	-	ABHD15	Body	-1.157308	-1.867674	-7.623766	4.14E-12	1.03E-09	17.167814
cg24865270	chr6	10422447	+			1.4281221	-0.687498	7.6222008	4.18E-12	1.04E-09	17.159655
cg09806671	chr1	165324679	+	LMX1A	Body	1.2416012	-0.174209	7.6219838	4.18E-12	1.04E-09	17.158524
cg01884549	chr12	116997308	+	MAP1LC3B2	5'UTR	-1.948291	-2.31952	-7.621492	4.20E-12	1.04E-09	17.155959
cg04242070	chr5	1749101	+			-1.569552	-0.747635	-7.621374	4.20E-12	1.04E-09	17.155345
cg25964534	chr6	31731300	-	C6orf26	Body	0.7739171	2.0957358	7.621021	4.21E-12	1.04E-09	17.153506
cg18324583	chr5	142975083	+			-1.625552	-0.11402	-7.61969	4.24E-12	1.05E-09	17.146567
cg18818531	chr11	65668277	+	FOSL1	TSS1500	-1.278348	-1.828743	-7.618551	4.26E-12	1.05E-09	17.140633
cg01887435	chr6	151402973	+	MTHFD1L	Body	1.541418	1.9021314	7.6178053	4.28E-12	1.06E-09	17.136748
cg24736989	chr14	60973904	+			1.8139847	-1.067534	7.614704	4.35E-12	1.07E-09	17.120589
cg04002220	chr16	89598305	-	SPG7;SPG7	Body;Body	0.9156394	1.3419425	7.612235	4.41E-12	1.09E-09	17.107726
cg25942990	chr2	177014996	+	HOXD4;MIR10B	TSS1500;TSS200	2.3776471	-0.691815	7.6177707	4.41E-12	1.09E-09	17.108406
cg25562958	chr14	101490676	-	MIR1197;MIR380;MIR323	TSS1500;TSS1500;TSS1500	-1.603573	-0.235217	-7.61166	4.42E-12	1.09E-09	17.104731
cg02053092	chr6	119220428	+	ASF1A	Body	1.213421	1.8305183	7.611102	4.44E-12	1.09E-09	17.101824
cg14408412	chr7	111776496	-	DOCK4	Body	1.1067691	1.7630132	7.6109358	4.44E-12	1.09E-09	17.100958
cg03726556	chr2	119607379	-			1.4994328	-0.585403	7.610433	4.45E-12	1.09E-09	17.098339
cg05314679	chr8	101821998	+			1.7834146	-0.908578	7.6083374	4.50E-12	1.11E-09	17.087423

cg23411036	chr13	115085339	+	ZNF828;ZNF828;ZNF828	5'UTR;5'UTR;5'UTR	0.9781007	0.2425581	7.6084	4.50E-12	1.11E-09	17.087749
cg19981568	chr14	60977300	-	SIX6	Body	2.8087672	-1.206178	7.611574	4.56E-12	1.12E-09	17.076196
cg20146541	chr1	248020697	-	TRIM58	1stExon	3.2162508	-0.67679	7.605619	4.57E-12	1.12E-09	17.073266
cg23906454	chr6	108785671	+	LACE1	Body	1.0251682	2.0795658	7.6048847	4.59E-12	1.12E-09	17.069442
cg07039560	chr5	140683681	+	SLC25A2	TSS200	1.2958156	1.0411022	7.6040971	4.61E-12	1.13E-09	17.06534
cg19106968	chr2	135149666	+	MGAT5	Body	-1.546441	-0.067954	-7.603656	4.62E-12	1.13E-09	17.063044
cg07704934	chr11	44332940	+	ALX4	TSS1500	1.937163	-0.506608	7.602905	4.64E-12	1.13E-09	17.059133
cg21146170	chr8	1266962	+			-1.526313	-0.007731	-7.602571	4.65E-12	1.14E-09	17.057392
cg21405799	chr16	84002843	+	NECAB2	Body	2.2932961	-0.528728	7.6026668	4.64E-12	1.14E-09	17.057893
cg01871214	chr4	41881417	+			2.1041559	-0.395859	7.6018691	4.66E-12	1.14E-09	17.053739
cg24951100	chr10	13760075	-	FRMD4A	Body	-1.324493	-2.170553	-7.600585	4.70E-12	1.15E-09	17.047052
cg06092869	chr1	225642746	-			-1.263931	-1.613793	-7.600388	4.70E-12	1.15E-09	17.046029
cg04242728	chr16	2536153	-	TBC1D24	5'UTR	1.0723349	1.1013578	7.599583	4.72E-12	1.15E-09	17.041836
cg24909548	chr19	36736272	-			2.2586985	-0.665164	7.5988815	4.74E-12	1.15E-09	17.038184
cg16551849	chr6	38563655	-	BTBD9;BTBD9;BTBD9;BTBD9	Body;5'UTR;1stExon;Body	1.0352481	2.0253494	7.5978848	4.76E-12	1.16E-09	17.032995
cg17032848	chr2	238647755	-	LRRFIP1;LRRFIP1;LRRFIP1;LRRFIP1;LRRFIP1	Body;Body;Body;Body;Body	1.0559639	1.4689487	7.5979148	4.76E-12	1.16E-09	17.033152
cg12273284	chr10	12490844	+	CAMK1D;CAMK1D	Body;Body	-2.632541	-2.418232	-7.597753	4.77E-12	1.16E-09	17.03231
cg17049418	chr6	3722453	+			1.3831289	0.7719831	7.5976161	4.77E-12	1.16E-09	17.031597
cg17124583	chr10	8097641	-	GATA3;GATA3	Body;Body	1.590987	-0.242172	7.5972447	4.78E-12	1.16E-09	17.029664
cg20298015	chr3	33316001	+			1.0601625	1.980229	7.5965669	4.80E-12	1.17E-09	17.026135

cg02477677	chr17	38512309	-	RARA;RARA;RARA;RARA	Body;Body;Body;Body	0.8773695	1.2968851	7.5963617	4.80E-12	1.17E-09	17.025067
cg06218688	chr11	19799429	+	NAV2;NAV2;NAV2	Body;Body;Body	-1.30926	-2.496378	-7.59555	4.83E-12	1.17E-09	17.020843
cg08363794	chr2	176986460	-	HOXD9	TSS1500	1.482113	-0.415265	7.5951596	4.84E-12	1.17E-09	17.01881
cg20801476	chr7	27281465	-	EVX1	TSS1500	1.2674468	-0.437091	7.5947584	4.85E-12	1.17E-09	17.016722
cg02013838	chr2	105460715	-			2.1842015	-0.643803	7.593766	4.87E-12	1.18E-09	17.011557
cg04021697	chr1	3567303	-	WDR8	TSS1500	2.9771851	-0.465946	7.5934611	4.88E-12	1.18E-09	17.00997
cg14516287	chr9	119987070	+	ASTN2;ASTN2;ASTN2	Body;Body;Body	-1.341061	-1.084382	-7.593343	4.88E-12	1.18E-09	17.009355
cg20971488	chr1	228786295	-	DUSP5P	Body	1.3703015	2.4664795	7.5934202	4.88E-12	1.18E-09	17.009757
cg21232956	chr9	129677782	-	RALGPS1	5'UTR	-0.657977	-3.159829	-7.609124	4.92E-12	1.19E-09	17.004399
cg14472366	chr13	20875915	+			1.4393643	0.7940282	7.5973668	4.92E-12	1.19E-09	17.002402
cg15978039	chr2	66667065	+	MEIS1	Body	1.9262618	-0.659858	7.5918451	4.92E-12	1.19E-09	17.001559
cg16894439	chr1	228634806	-			0.9986441	1.2720145	7.5914473	4.93E-12	1.19E-09	16.999489
cg02371119	chr3	171527346	+	PLD1;PLD1	5'UTR;5'UTR	-1.731837	-3.944137	-7.590474	4.96E-12	1.19E-09	16.994425
cg04978408	chr1	26632539	-	UBXN11;UBXN11;UBXN11	5'UTR;5'UTR;5'UTR	-0.785176	-2.888499	-7.590433	4.96E-12	1.19E-09	16.994209
cg05010865	chr8	145049189	+	PLEC1;PLEC1	Body;TSS1500	-2.081678	-4.699283	-7.596004	4.96E-12	1.19E-09	16.995326
cg19940482	chr11	125190793	-	PKNOX2	5'UTR	-1.285627	-0.83857	-7.590292	4.96E-12	1.19E-09	16.993477
cg02821464	chr11	1945607	+	TNNT3;TNNT3;TNNT3;TNNT3	Body;Body;Body;Body	-1.154401	-0.316164	-7.589517	4.98E-12	1.20E-09	16.989446
cg09624466	chr14	57278710	-	OTX2OS1	TSS1500	2.2270816	-0.100419	7.589607	4.98E-12	1.20E-09	16.989913
cg15995695	chr1	179334675	+	C1orf125;C1orf125	TSS1500;TSS200	-1.101012	-3.134233	-7.589508	4.98E-12	1.20E-09	16.989398
cg13480465	chr4	16795757	+	LDB2;LDB2	Body;Body	-1.718727	-0.510575	-7.588567	5.01E-12	1.20E-09	16.984503

cg14113046	chr19	54184536	-	MIR520F	TSS1500	-1.738394	0.260111	-7.594124	5.01E-12	1.20E-09	16.985547
cg02286008	chr16	30133247	-	MAPK3;MAPK3;MAPK3	Body;Body;Body	1.7494365	2.1991973	7.5884043	5.01E-12	1.20E-09	16.983654
cg09295202	chr14	21535394	-			-1.725381	-0.766818	-7.588013	5.02E-12	1.20E-09	16.981616
cg15285250	chr7	8482107	+	NXPH1	Body	2.0968641	-0.956974	7.5875027	5.04E-12	1.21E-09	16.978963
cg00566164	chr1	228559156	-	OBSCN	Body	-1.366304	1.0005562	-7.586395	5.07E-12	1.21E-09	16.973201
cg18391127	chr15	89462418	+			-1.600008	-0.062196	-7.585829	5.08E-12	1.22E-09	16.970257
cg20273260	chr2	133429169	+	LYPD1;LYPD1	TSS1500;TSS200	-1.434682	-1.352224	-7.583834	5.14E-12	1.23E-09	16.959879
cg09943716	chr20	55202236	-			2.153463	-1.130189	7.5822359	5.18E-12	1.24E-09	16.951563
cg01132653	chr14	101489649	-	MIR299;MIR411	TSS1500;TSS200	-1.64379	0.3506635	-7.581596	5.20E-12	1.24E-09	16.948235
cg23229261	chr2	63284066	+	OTX1	3'UTR	2.7138768	-0.777632	7.5816061	5.20E-12	1.24E-09	16.948287
cg14601053	chr9	130271989	-	FAM129B;FAM129B	Body;Body	1.1580577	1.4124041	7.5814363	5.21E-12	1.24E-09	16.947404
cg18326783	chr10	102765364	-	LZTS2	Body	1.0199217	1.6612031	7.5806019	5.23E-12	1.25E-09	16.943064
cg25247290	chr11	32461212	-	WIT1	Body	2.2030947	-0.438502	7.6034199	5.23E-12	1.25E-09	16.947859
cg24972297	chr4	153857113	-			-0.939678	-3.528145	-7.579732	5.25E-12	1.25E-09	16.938542
cg07533148	chr1	248020812	-	TRIM58	1stExon	3.5906354	-1.129641	7.5841397	5.29E-12	1.26E-09	16.933727
cg12663656	chr12	120427667	+	CCDC64;CCDC64	1stExon;5'UTR	-1.203529	-4.392506	-7.579383	5.42E-12	1.29E-09	16.909028
cg19368031	chr10	76688843	+	MYST4	Body	1.3414679	1.3629933	7.5738828	5.42E-12	1.29E-09	16.908122
cg10426658	chr14	105031243	-			-1.516932	0.2601363	-7.57276	5.46E-12	1.30E-09	16.902282
cg12616097	chr1	17051629	-			1.4710815	2.2997123	7.5710584	5.51E-12	1.31E-09	16.893438
cg21532801	chr20	56881949	+	PPP4R1L	Body	-1.553822	-1.71052	-7.571196	5.50E-12	1.31E-09	16.894155

cg23514340	chr8	94948185	+			-0.879341	-2.87061	-7.571084	5.50E-12	1.31E-09	16.893572
cg04487296	chr7	149389669	-			2.1282059	1.501797	7.575899	5.53E-12	1.31E-09	16.890953
cg18105139	chr6	28058856	+	ZSCAN12L1	TSS200	1.3660492	0.5167915	7.5702701	5.53E-12	1.31E-09	16.88934
cg18719939	chr3	48545308	-			-1.227996	-2.968012	-7.570385	5.53E-12	1.31E-09	16.889939
cg01493685	chr7	111375789	+	DOCK4	Body	-1.059995	-3.486891	-7.568405	5.58E-12	1.32E-09	16.879646
cg18131582	chr1	183912305	-	GLT25D2	Body	-2.110784	-0.566692	-7.566391	5.65E-12	1.34E-09	16.869176
cg18969232	chr2	45156937	-			1.7578592	-0.381329	7.566343	5.65E-12	1.34E-09	16.868927
cg22620090	chr6	105400993	-			2.751635	0.9349283	7.5718214	5.65E-12	1.34E-09	16.8698
cg00751332	chr18	20714332	-	CABLES1;CABLES1	TSS1500;TSS200	-1.566645	-1.439328	-7.563073	5.75E-12	1.36E-09	16.851935
cg14565439	chr5	107702185	-	FBXL17	Body	1.0620442	2.4529147	7.5627411	5.76E-12	1.36E-09	16.850208
cg24753760	chr6	101846767	-	GRIK2;GRIK2;GRIK2	TSS200;TSS200;TSS200	1.8199909	-0.714971	7.5627943	5.76E-12	1.36E-09	16.850484
cg11725972	chr7	155191845	+			-2.193654	0.969073	-7.561493	5.80E-12	1.37E-09	16.843723
cg07314298	chr2	11723111	+	GREB1;GREB1;GREB1	Body;Body;Body	-1.516542	-2.24997	-7.559527	5.86E-12	1.38E-09	16.833507
cg21446981	chr7	37534909	-			-1.25709	-2.453415	-7.558871	5.88E-12	1.39E-09	16.830097
cg24422900	chr19	19753746	+	GMIP	Body	-1.00079	-3.856406	-7.558672	5.88E-12	1.39E-09	16.829063
cg24463664	chr8	38160704	-	WHSC1L1	Body	1.2077616	2.0339584	7.5583728	5.89E-12	1.39E-09	16.827511
cg18694169	chr6	28227079	-	NKAPL	TSS200	1.691414	-0.372279	7.5577818	5.91E-12	1.39E-09	16.82444
cg24152098	chr13	113707579	-	MCF2L;MCF2L	Body;Body	1.0671316	2.1520285	7.5577948	5.91E-12	1.39E-09	16.824508
cg21195185	chr4	76555782	+	CDKL2	TSS200	-1.107226	-3.646616	-7.562795	5.93E-12	1.39E-09	16.822991
cg09361748	chr7	71682306	-	CALN1;CALN1	Body;Body	-1.38268	-0.941857	-7.555652	5.98E-12	1.41E-09	16.813377

cg11697427	chr10	102590168	-			1.8804135	-0.749217	7.5609922	5.99E-12	1.41E-09	16.813646
cg01261503	chr17	62493599	-	POLG2	TSS1500	-1.485484	-3.183896	-7.555066	6.00E-12	1.41E-09	16.81033
cg04549583	chr2	12146219	-			-1.190766	-3.365351	-7.554723	6.01E-12	1.41E-09	16.808549
cg24539599	chr5	112824920	-	MCC	TSS1500	-1.224632	-1.330978	-7.554473	6.02E-12	1.41E-09	16.807251
cg22747480	chr10	1166867	-	WDR37	Body	1.7157033	3.4678108	7.5707857	6.04E-12	1.41E-09	16.808264
cg00446697	chr11	1542313	-	HCCA2	Body	1.0868746	1.4463798	7.5532535	6.06E-12	1.42E-09	16.800918
cg08215811	chr5	139742900	-	SLC4A9	Body	-0.862733	-3.031107	-7.552373	6.09E-12	1.42E-09	16.796344
cg17514558	chr5	140621375	-	PCDHB19P	Body	1.9894303	0.2379437	7.5523769	6.09E-12	1.42E-09	16.796364
cg26245607	chr7	42981031	-			-1.409102	-3.908506	-7.551835	6.10E-12	1.43E-09	16.793551
cg02772121	chr6	30130881	+	TRIM15	TSS200	-2.512916	-0.323126	-7.551611	6.11E-12	1.43E-09	16.792386
cg13858803	chr9	33140269	+	B4GALT1	Body	1.2531669	1.900844	7.5516234	6.11E-12	1.43E-09	16.792451
cg06710785	chr10	83683829	-	NRG3;NRG3;NRG3	Body;Body;Body	-1.908614	0.0756104	-7.551307	6.12E-12	1.43E-09	16.790809
cg14145667	chr19	909533	-	C19orf22	Body	-1.392537	-1.492827	-7.551192	6.13E-12	1.43E-09	16.790211
cg18990050	chr7	1163642	+	C7orf50;C7orf50;C7orf50	Body;Body;Body	1.4387449	1.4970713	7.5556554	6.16E-12	1.44E-09	16.785983
cg24714561	chr14	90001131	-	FOXN3	5'UTR	1.4350784	2.3932028	7.5500088	6.17E-12	1.44E-09	16.784065
cg17117459	chr13	25085405	-	PARP4	5'UTR	-1.470407	-1.68472	-7.549754	6.17E-12	1.44E-09	16.782743
cg22176324	chr1	24648851	+	GRHL3;GRHL3;GRHL3	TSS1500;Body;TSS1500	-1.238223	-3.433549	-7.549762	6.17E-12	1.44E-09	16.782786
cg16190225	chr7	1163456	-	C7orf50;C7orf50;C7orf50	Body;Body;Body	1.0401922	0.8971602	7.5495839	6.18E-12	1.44E-09	16.781859
cg04625975	chr10	44285864	+	HNRNPA3P1	Body	-1.844128	0.3159795	-7.549272	6.19E-12	1.44E-09	16.780238
cg19272238	chr17	76220898	+	BIRC5;EPR1;BIRC5;BIRC5	3'UTR;TSS200;3'UTR;3'UTR	1.2661567	1.7844491	7.5488992	6.20E-12	1.44E-09	16.778304

cg03224650	chr1	228559090	+	OBSCN	Body	-1.585429	0.6384828	-7.548533	6.21E-12	1.44E-09	16.776402
cg05493356	chr17	79981909	-	STRA13;LRRC45	TSS1500;Body	-1.187349	-2.799394	-7.548378	6.22E-12	1.44E-09	16.775597
cg19221841	chr19	41197049	-	NUMBL	TSS1500	-2.229886	-5.911555	-7.582824	6.22E-12	1.44E-09	16.783066
cg04291025	chr15	69746140	+	RPLP1;RPLP1	Body;Body	-1.350737	-2.901488	-7.547765	6.24E-12	1.45E-09	16.772412
cg20874052	chr11	118437133	-	C11orf60;C11orf60	TSS1500;TSS1500	-1.365911	-1.247325	-7.547352	6.25E-12	1.45E-09	16.770272
cg03985459	chr6	37786245	+	ZFAND3	TSS1500	-0.882448	-4.515368	-7.546545	6.28E-12	1.45E-09	16.766078
cg14956201	chr5	14358153	+	TRIO	Body	0.9951783	1.4902853	7.5501652	6.34E-12	1.47E-09	16.757534
cg20725239	chr11	124805963	-	HEPACAM;HEPACAM	5'UTR;1stExon	-1.528568	-0.341836	-7.543753	6.38E-12	1.48E-09	16.751582
cg25037394	chr1	24152592	+	HMGCL;HMGCL	TSS1500;TSS1500	-2.335969	0.0547364	-7.54362	6.38E-12	1.48E-09	16.750892
cg12496211	chr12	2193060	+	CACNA1C;CACNA1C;CACNA1C;CACNA1C;CACNA1C;CACNA1C;CACNA1C;CACNA1C;CACNA1C;CACNA1C;CACNA1C;CACNA1C:CACNA1C:CACNA1C:CACNA1C:CACNA1C:CACNA1C:CACNA	Body;Body;Body;Body;Body;Body;Body;Body;Body;Body;Body;Body;Body;Body;Body;Body;Body;Body;Body:Body:Body:Body:Body	-1.530121	-1.1874	-7.543205	6.39E-12	1.48E-09	16.748738
cg22804528	chr3	124780157	+			1.2173657	0.6592908	7.5430172	6.40E-12	1.48E-09	16.747764
cg06192866	chr12	89748300	-			-0.843859	-3.756755	-7.542463	6.42E-12	1.48E-09	16.744885
cg06376426	chr1	24648203	-	GRHL3;GRHL3;GRHL3	TSS1500;Body;TSS1500	-1.3192	-0.546645	-7.541865	6.44E-12	1.49E-09	16.741783
cg10383447	chr16	58535093	+	NDRG4;NDRG4;NDRG4	Body;Body;Body	-2.359233	-3.243946	-7.541817	6.44E-12	1.49E-09	16.741534
cg16167060	chr2	235934945	-	SH3BP4	5'UTR	-2.002133	0.5105046	-7.540976	6.47E-12	1.49E-09	16.73717
cg03622664	chr13	21371628	-	XPO4	Body	1.2917622	1.4579591	7.5406348	6.48E-12	1.49E-09	16.735397
cg09159452	chr7	2609861	-	IQCE;IQCE	Body;Body	2.407892	2.3493563	7.5405458	6.49E-12	1.49E-09	16.734935
cg04066265	chr8	19460243	+	CSGALNACT1;CSGALNACT1;CSGALNACT1	TSS200;Body;5'UTR	1.459589	0.7392718	7.5388124	6.55E-12	1.51E-09	16.725938
cg05247341	chr15	74666916	+			-0.428653	-3.399577	-7.5381	6.57E-12	1.51E-09	16.72224
cg22508831	chr22	40573204	-	TNRC6B;TNRC6B;TNRC6B	Body;TSS1500;TSS1500	-0.779361	-3.597288	-7.538135	6.57E-12	1.51E-09	16.722422

cg23077820	chr2	223154176	+	PAX3;PAX3;PAX3;PAX3;PAX3;PAX3	Body;Body;Body;Body;Body;Body	1.3183207	1.0740387	7.5381142	6.57E-12	1.51E-09	16.722314
cg10791422	chr11	68748528	-	MRGPRD	TSS200	-1.237945	-0.233134	-7.543452	6.58E-12	1.51E-09	16.722759
cg22720029	chr22	32058579	+			-0.97431	-2.44098	-7.537403	6.60E-12	1.51E-09	16.718624
cg16755214	chr14	54720346	+			-1.553185	-1.110427	-7.537128	6.61E-12	1.52E-09	16.717195
cg07105285	chr11	92702663	-	MTNR1B	TSS200	1.1637809	-0.357465	7.5368379	6.62E-12	1.52E-09	16.71569
cg00945108	chr1	12806027	+	C1orf158	TSS200	-1.277339	-1.748986	-7.536692	6.62E-12	1.52E-09	16.714933
cg25339566	chr1	67218165	-	TCTEX1D1;TCTEX1D1	5'UTR;1stExon	3.1146186	-0.690424	7.5354041	6.67E-12	1.53E-09	16.70825
cg07078225	chr12	52652377	-			3.4408118	-0.863804	7.5348615	6.69E-12	1.53E-09	16.705434
cg09391066	chr6	3163814	+			-1.486824	-2.205627	-7.534389	6.70E-12	1.53E-09	16.702982
cg19278744	chr4	78079628	-	CCNG2	5'UTR	-0.85653	-2.467579	-7.533861	6.72E-12	1.54E-09	16.700241
cg22790839	chr5	157883933	-			-1.569638	-1.903914	-7.531868	6.80E-12	1.55E-09	16.689903
cg27641532	chr6	27648317	-			1.615942	0.2811489	7.5298326	6.87E-12	1.57E-09	16.679341
cg13575139	chr3	58456976	-			-1.197975	-1.684069	-7.529652	6.88E-12	1.57E-09	16.678407
cg17720286	chr19	1513941	+	ADAMTSL5	TSS1500	-1.485426	-3.508175	-7.529412	6.89E-12	1.57E-09	16.677162
cg07967632	chr15	101601131	-	LRRK1	Body	2.0797887	2.9557863	7.5341984	6.91E-12	1.58E-09	16.67484
cg01577083	chr16	5486268	+			-1.746351	-0.284032	-7.528513	6.92E-12	1.58E-09	16.672498
cg05021029	chr10	76947956	-			-1.536968	-0.605	-7.528519	6.92E-12	1.58E-09	16.672525
cg03956606	chr1	11249057	-	MTOR;ANGPTL7	Body;TSS1500	0.7331434	3.0397853	7.5281513	6.93E-12	1.58E-09	16.670619
cg21379639	chr3	49056420	-	DALRD3;DALRD3;NDUFAF3	5'UTR;TSS1500;TSS1500	-1.047775	-2.225892	-7.528193	6.93E-12	1.58E-09	16.670837
cg13477354	chr1	101705539	+	S1PR1	Body	1.4071682	1.6891518	7.5269066	6.98E-12	1.59E-09	16.664163

cg24505307	chr12	53448429	-	TENC1;TENC1;TENC1	Body;Body;Body	-0.723222	-3.784928	-7.526786	6.98E-12	1.59E-09	16.663538
cg12353688	chr7	27265004	-			1.5953446	-0.010061	7.5264316	7.00E-12	1.59E-09	16.661699
cg25026529	chr1	91183051	-	BARHL2	TSS1500	2.7282867	-0.844359	7.5262152	7.01E-12	1.59E-09	16.660577
cg04819760	chr10	22765645	+			1.6525983	-0.813724	7.5259196	7.02E-12	1.59E-09	16.659044
cg05447916	chr6	31744831	+	C6orf27	5'UTR	-0.977249	-2.846184	-7.525591	7.03E-12	1.59E-09	16.657337
cg12444411	chr7	2802554	+	GNA12	Body	1.438275	1.1179563	7.5311257	7.03E-12	1.59E-09	16.658936
cg06736663	chr2	219754761	+	WNT10A	Body	1.1510772	2.4810555	7.5253324	7.04E-12	1.60E-09	16.655998
cg03575245	chr3	194785559	+			1.0425424	0.8942311	7.5250164	7.05E-12	1.60E-09	16.654359
cg01401534	chr6	4079342	-	C6orf201;C6orf146;C6orf146	TSS200;5'UTR;1stExon	0.9778256	0.8604912	7.5217483	7.18E-12	1.62E-09	16.637411
cg22244192	chr6	33943837	-			1.6331934	-0.49468	7.521792	7.17E-12	1.62E-09	16.637637
cg01266985	chr5	1092066	+	SLC12A7	Body	0.877876	0.8551533	7.5211767	7.20E-12	1.63E-09	16.634447
cg04194947	chr2	177012191	-			1.7235176	1.0107741	7.5208935	7.21E-12	1.63E-09	16.632978
cg12776902	chr4	144452479	+	SMARCA5	Body	1.4224804	2.3136051	7.5268072	7.19E-12	1.63E-09	16.636584
cg13633270	chr18	55095555	-			1.51643	-0.385787	7.5208217	7.21E-12	1.63E-09	16.632605
cg18542639	chr15	41275117	+	INO80	Body	0.9919965	1.4614249	7.5209929	7.20E-12	1.63E-09	16.633494
cg22344631	chr2	177012133	+			1.569895	-0.788146	7.5210423	7.20E-12	1.63E-09	16.63375
cg27160395	chr2	177014949	-	HOXD4;MIR10B	TSS1500;TSS200	1.6223498	0.3886333	7.5211383	7.20E-12	1.63E-09	16.634248
cg19324997	chr2	240169161	-	HDAC4	Body	-3.465757	1.6882641	-7.519766	7.25E-12	1.63E-09	16.627132
cg01022345	chr14	101317819	+	MIR770;MEG3;MEG3;MEG3	TSS1500;Body;Body;Body	-1.350856	-0.313763	-7.51902	7.28E-12	1.64E-09	16.623263
cg13057055	chr17	36572011	-			-0.985081	-2.503786	-7.518741	7.29E-12	1.64E-09	16.621818

cg12325536	chr5	87968658	-	LOC645323;LOC645323;LOC645323	Body;Body;Body	1.5075395	-0.256913	7.5186023	7.30E-12	1.64E-09	16.621098
cg17583957	chr17	67536299	-	MAP2K6	Body	-2.117476	0.0695072	-7.518178	7.31E-12	1.65E-09	16.618898
cg14228146	chr2	119607395	-			1.5192553	-0.349558	7.516945	7.36E-12	1.66E-09	16.612505
cg22282405	chr6	50810682	+	TFAP2B	Body	2.4544178	-0.125095	7.5198171	7.47E-12	1.68E-09	16.600415
cg16234557	chr10	119292062	-	EMX2OS	Body	3.0720367	-1.223707	7.5191309	7.49E-12	1.68E-09	16.596865
cg09969277	chr15	89922328	+	LOC254559	Body	2.437961	-0.787621	7.5188577	7.50E-12	1.68E-09	16.595452
cg05437823	chr6	50813676	-	TFAP2B	3'UTR	2.3524536	-0.889445	7.5130144	7.52E-12	1.69E-09	16.59213
cg24598993	chr6	4923095	+	CDYL;CDYL;CDYL;CDYL	Body;Body;Body;Body	0.8699637	2.5376498	7.5130301	7.52E-12	1.69E-09	16.592211
cg01081168	chr1	212278486	-			2.0218849	1.2431306	7.511463	7.58E-12	1.70E-09	16.584089
cg02245378	chr2	223161771	-	PAX3;PAX3;PAX3;CCDC140;PAX3;PAX3;PAX3;PAX3;PAX3	Body;Body;Body;TSS1500;Body;Body;Body;Body;Body	1.6609016	-0.831181	7.5101908	7.63E-12	1.71E-09	16.577496
cg23172086	chr10	98129751	-	TLL2	Body	-2.091356	0.8686258	-7.510044	7.64E-12	1.71E-09	16.576733
cg14090487	chr22	38003190	-	GGA1;GGA1;GGA1	TSS1500;TSS1500;TSS1500	-0.605204	-4.294023	-7.508852	7.69E-12	1.72E-09	16.570555
cg22419414	chr13	46093146	+	COG3	Body	1.692142	2.4073686	7.5083192	7.71E-12	1.72E-09	16.567797
cg16546442	chr4	111539907	-	PITX2;PITX2;PITX2	Body;Body;Body	1.0679211	-0.74715	7.5075452	7.74E-12	1.73E-09	16.563786
cg06878108	chr14	104866636	+			-1.607293	-0.485365	-7.506781	7.78E-12	1.74E-09	16.559827
cg03287940	chr3	24537338	+	THRB;THRB;THRB	TSS1500;TSS1500;TSS1500	-1.896777	-3.469347	-7.511874	7.79E-12	1.74E-09	16.559331
cg09803262	chr7	96622608	-	DLX6AS	Body	2.0282015	-0.217974	7.5166164	7.82E-12	1.75E-09	16.556589
cg22991506	chr14	50468241	+	C14orf182	Body	-1.192332	-1.222193	-7.505207	7.84E-12	1.75E-09	16.551669
cg02911077	chr17	76128621	-	TMC8;TMC6	Body;TSS200	-1.141162	-2.075304	-7.505005	7.85E-12	1.75E-09	16.550626
cg26766064	chr17	79099711	-	MIR657;AATK;MIR338	TSS1500;Body;Body	-1.445807	-0.7337	-7.503314	7.92E-12	1.77E-09	16.54186

cg00084798	chr8	142841965	+			-2.101763	0.7629481	-7.502419	7.96E-12	1.77E-09	16.537225
cg06495961	chr7	153584582	-	DPP6;DPP6	1stExon;5'UTR	1.9340257	-0.117687	7.5005169	8.04E-12	1.79E-09	16.527373
cg09549949	chr2	240240119	-	HDAC4	Body	1.1587657	4.1094884	7.5003343	8.05E-12	1.79E-09	16.526427
cg16146033	chr11	62767323	-	SLC22A8	Body	-1.247994	-1.62146	-7.500177	8.06E-12	1.79E-09	16.525614
cg18120111	chr21	43917047	-	RSPH1	TSS1500	-0.697588	-2.52424	-7.504763	8.09E-12	1.80E-09	16.522567
cg26824709	chr5	159740383	-	CCNJL	TSS1500	-1.142492	-2.18491	-7.499119	8.10E-12	1.80E-09	16.520132
cg13187820	chr13	98960562	-	FARP1	Body	1.2641607	2.6876796	7.4987747	8.12E-12	1.80E-09	16.518349
cg02189785	chr9	139891008	-	CLIC3;CLIC3	1stExon;5'UTR	-1.12255	-3.235673	-7.496931	8.20E-12	1.82E-09	16.508802
cg00752195	chr4	1399516	+			1.3042153	-0.720559	7.4967248	8.21E-12	1.82E-09	16.507732
cg18193094	chr6	101846905	+	GRIK2;GRIK2;GRIK2;GRIK2;GRIK2;GRIK2	1stExon;1stExon;5'UTR;1stExon;5'UTR;5'UTR	1.8020293	-0.763954	7.4958408	8.25E-12	1.83E-09	16.503154
cg00897799	chr13	47367897	+	ESD	5'UTR	1.1439185	2.0051124	7.4999242	8.30E-12	1.84E-09	16.496246
cg20265661	chr2	133428502	+	LYPD1;LYPD1	TSS200;5'UTR	-2.167782	-3.656526	-7.504904	8.33E-12	1.84E-09	16.496164
cg20672981	chr7	27242161	+			1.0432824	0.5567053	7.4940472	8.33E-12	1.84E-09	16.493866
cg11754318	chr2	176964506	-	HOXD12	TSS200	1.2289757	-0.208965	7.4936696	8.34E-12	1.84E-09	16.491911
cg20972294	chr11	133005919	-	OPCML	Body	-1.902362	-0.445794	-7.493596	8.35E-12	1.84E-09	16.491528
cg23371746	chr1	119532925	-	TBX15	TSS1500	1.7825928	-0.061508	7.4936242	8.34E-12	1.84E-09	16.491676
cg01631684	chr10	65280961	+	REEP3	TSS200	-0.502196	-3.915164	-7.493249	8.36E-12	1.85E-09	16.489732
cg09645138	chr5	82155751	-			1.1539498	1.6791831	7.4923888	8.40E-12	1.85E-09	16.485279
cg22101249	chr6	32131615	-	EGFL8	TSS1500	1.0475132	1.2423066	7.4924541	8.40E-12	1.85E-09	16.485617
cg24947764	chr4	41881359	+			1.819443	-0.503502	7.4924759	8.40E-12	1.85E-09	16.48573

cg20892260	chr8	23564025	-	NKX2-6	TSS200	2.2457174	-0.62772	7.4918109	8.43E-12	1.86E-09	16.482287
cg10202693	chr1	26142709	+	SEPN1;SEPN1	3'UTR;3'UTR	0.9451071	1.4737488	7.4904948	8.49E-12	1.87E-09	16.475473
cg11794215	chr17	35423816	-			1.0696689	0.7642762	7.4899417	8.51E-12	1.87E-09	16.472609
cg13521620	chr17	57410687	-	YPEL2	5'UTR	1.5478482	1.4170495	7.4898092	8.52E-12	1.87E-09	16.471923
cg26116560	chr2	86565259	-	REEP1;REEP1;REEP1;REEP1	TSS200;TSS1500;TSS1500;TSS1500	-1.041402	-3.319792	-7.48924	8.54E-12	1.88E-09	16.468979
cg21069814	chr3	47891476	-	DHX30;DHX30	Body;Body	1.5918189	5.2849186	7.505159	8.57E-12	1.88E-09	16.470017
cg27155939	chr15	40347163	-			-1.243707	-0.782064	-7.488032	8.60E-12	1.89E-09	16.462725
cg21060085	chr4	40721181	-			-1.051134	-3.03749	-7.48737	8.63E-12	1.89E-09	16.459298
cg26954138	chr11	118437129	-	C11orf60;C11orf60	TSS1500;TSS1500	-1.355081	-1.600947	-7.487354	8.63E-12	1.89E-09	16.459216
cg03730428	chr11	32009004	+			1.920016	-0.602692	7.4912002	8.70E-12	1.91E-09	16.452477
cg16797777	chr6	160389717	+	IGF2R	TSS1500	-0.765382	-3.223863	-7.485895	8.70E-12	1.91E-09	16.45166
cg09164726	chr16	86121040	-			1.5988466	1.221241	7.4854419	8.72E-12	1.91E-09	16.449316
cg15470344	chr1	203056504	+	MYOG	TSS1500	1.0316553	2.0863266	7.484578	8.76E-12	1.92E-09	16.444845
cg10253847	chr6	28227085	-	NKAPL	TSS200	2.0459394	-0.617303	7.4841665	8.78E-12	1.92E-09	16.442716
cg07388969	chr15	38509709	+			-1.684592	-2.268856	-7.483332	8.82E-12	1.93E-09	16.438397
cg05845376	chr5	140683632	+	SLC25A2	TSS200	2.1474801	2.6083976	7.504609	8.85E-12	1.94E-09	16.439395
cg26735478	chr17	79694557	+			-1.822429	-0.518945	-7.482125	8.87E-12	1.94E-09	16.432153
cg00204802	chr13	113496682	-	ATP11A;ATP11A	Body;Body	1.5237867	2.410334	7.486788	8.91E-12	1.95E-09	16.429687
cg15557996	chr10	93999904	-	CPEB3	Body	-0.927819	-3.545351	-7.486795	8.91E-12	1.95E-09	16.429731
cg18387099	chr12	70132341	-	RAB3IP;RAB3IP;RAB3IP;RAB3IP	TSS1500;TSS1500;TSS1500;TSS1500	-0.633224	-3.750278	-7.481347	8.91E-12	1.95E-09	16.428125

cg20202078	chr7	129845466	-	TMEM209	TSS200	-0.961929	-4.287433	-7.480304	8.96E-12	1.96E-09	16.422726
cg08313880	chr17	4643401	-	CXCL16;ZMYND15;ZMYND15;CXCL16	TSS200;5'UTR;5'UTR;TSS200	-1.289566	-1.987391	-7.47968	8.99E-12	1.96E-09	16.419501
cg18845417	chr2	95745976	-			-1.451527	-0.324094	-7.479488	9.00E-12	1.96E-09	16.418504
cg24704593	chr7	20257178	+	MACC1	TSS200	-1.203726	-1.939272	-7.479386	9.01E-12	1.96E-09	16.417978
cg13502125	chr1	147826191	+			1.8950094	1.509715	7.4785604	9.05E-12	1.97E-09	16.413705
cg22489321	chr13	100643388	+			1.760422	-0.147553	7.478281	9.06E-12	1.97E-09	16.41226
cg07792478	chr8	65290484	+	MIR124-2	TSS1500	2.5237238	0.3173138	7.4780925	9.07E-12	1.97E-09	16.411284
cg23264682	chr12	4829115	-	GALNT8	TSS1500	-1.626133	0.0591611	-7.477781	9.08E-12	1.97E-09	16.409675
cg23758305	chr8	70982867	+	PRDM14	5'UTR	2.0776846	-1.13007	7.4778155	9.08E-12	1.97E-09	16.409851
cg15572436	chr11	62767169	+	SLC22A8	Body	-1.088959	-1.353025	-7.477185	9.11E-12	1.98E-09	16.406588
cg11729934	chr6	1601693	-			1.7167728	-0.677497	7.4769963	9.12E-12	1.98E-09	16.405613
cg21026830	chr14	60973517	+			2.9423281	-0.41363	7.4767643	9.13E-12	1.98E-09	16.404413
cg00166750	chr8	102138594	-			-0.766828	-3.775176	-7.475178	9.21E-12	2.00E-09	16.396208
cg02803819	chr7	27141067	+	HOXA2	Body	1.9561111	1.0459967	7.4744526	9.25E-12	2.00E-09	16.392454
cg01047586	chr2	176933407	+			2.0894554	-0.549845	7.4741621	9.26E-12	2.01E-09	16.390952
cg15822346	chr6	111408761	-	SLC16A10	TSS200	-0.753589	-3.536608	-7.47421	9.26E-12	2.01E-09	16.3912
cg08878567	chr16	13716635	+			-1.523852	-0.225253	-7.473403	9.30E-12	2.01E-09	16.387026
cg09301232	chr14	100681129	-			-0.931762	-3.157457	-7.478108	9.33E-12	2.02E-09	16.384878
cg23889730	chr14	94254131	-	PRIMA1	5'UTR	1.2719292	-0.083623	7.4712642	9.41E-12	2.03E-09	16.375962
cg04738965	chr3	147127662	-	ZIC1;ZIC1	1stExon;5'UTR	2.1712999	-0.060455	7.4696171	9.49E-12	2.05E-09	16.367444

cg04868238	chr8	22501100	+	FLJ14107;BIN3	TSS1500;Body	1.1276994	0.6716797	7.4695592	9.49E-12	2.05E-09	16.367144
cg10192893	chr4	41747895	-	PHOX2B	Body	2.7609828	-0.526763	7.4684854	9.55E-12	2.06E-09	16.361591
cg08419450	chr10	90013989	+			-1.722389	-0.297476	-7.466476	9.65E-12	2.08E-09	16.351201
cg06335867	chr7	8482325	+	NXPH1	Body	2.0117738	0.6864197	7.4655141	9.70E-12	2.09E-09	16.346228
cg20431250	chr6	108492653	+	NR2E1	Body	1.8224431	-0.572883	7.4655197	9.70E-12	2.09E-09	16.346257
cg12267021	chr11	71790932	-	NUMA1;LRTOMT;LRTOMT;LRTOMT;LRTOMT;LRTOMT;LRTOMT	5'UTR;TSS1500;TSS1500;TSS1500;TSS1500;TSS1500;TSS1500	-1.532493	-3.960793	-7.463069	9.83E-12	2.12E-09	16.333585
cg22445447	chr15	41712591	+	RTF1	Body	1.2953411	2.0000597	7.4617023	9.90E-12	2.13E-09	16.326521
cg00298919	chr16	86421796	+			-1.67313	0.1248028	-7.461518	9.91E-12	2.13E-09	16.32557
cg00058879	chr22	40082173	+	CACNA1I;CACNA1I	Body;Body	1.6862143	-0.850676	7.4600559	9.99E-12	2.15E-09	16.318011
cg21151287	chr5	14488003	-	TRIO	Body	0.8501784	2.9810983	7.4598622	1.00E-11	2.15E-09	16.31701
cg07647822	chr3	24537349	+	THRB;THRB;THRB	TSS1500;TSS1500;TSS1500	-1.19944	-2.756299	-7.458429	1.01E-11	2.17E-09	16.309603
cg14871932	chr7	44185323	-	GCK;GCK;GCK	Body;Body;Body	2.1173689	-0.725497	7.4581561	1.01E-11	2.17E-09	16.308191
cg03781262	chr19	58879871	-	ZNF837;ZNF837	Body;Body	1.1334563	1.9903203	7.4576474	1.01E-11	2.17E-09	16.305562
cg21092177	chr17	73128031	-	NT5C	TSS200	-0.609855	-3.921786	-7.455767	1.02E-11	2.19E-09	16.295846
cg05977992	chr6	151186411	+	MTHFD1L	TSS1500	-0.86778	-2.158313	-7.454611	1.03E-11	2.21E-09	16.289868
cg16178603	chr2	66667101	-	MEIS1	Body	2.587742	-0.781866	7.454517	1.03E-11	2.21E-09	16.289385
cg19323289	chr11	19463903	+	NAV2	Body	-1.259891	-1.223561	-7.458107	1.04E-11	2.23E-09	16.281672
cg00175487	chr14	101490186	+	MIR380;MIR299	TSS1500;Body	-1.51996	-0.377215	-7.451376	1.05E-11	2.24E-09	16.273154
cg06153192	chr16	1560142	-	TELO2	3'UTR	0.8761199	1.549073	7.4511967	1.05E-11	2.24E-09	16.272229
cg18094366	chr22	24237343	+	MIF	3'UTR	-1.687536	-4.917906	-7.450918	1.05E-11	2.24E-09	16.270791

cg06498232	chr3	72227063	+			-1.1763	-2.324187	-7.450719	1.05E-11	2.24E-09	16.269763
cg27037013	chr21	35320667	-			-1.350532	-0.865449	-7.450728	1.05E-11	2.24E-09	16.269806
cg13546414	chr14	37053664	+			1.5075219	0.3824081	7.4498426	1.05E-11	2.25E-09	16.265234
cg06592987	chr7	149112142	+			2.252588	-0.819484	7.4542377	1.06E-11	2.26E-09	16.261721
cg20524128	chr17	77965982	+	TBC1D16	Body	-1.391748	-3.294204	-7.448847	1.06E-11	2.26E-09	16.260088
cg24935332	chr11	31846819	-			1.542803	0.0711772	7.448307	1.06E-11	2.27E-09	16.257301
cg02925039	chr12	43945213	-	ADAMTS20	Body	1.423227	-0.436606	7.4462448	1.08E-11	2.29E-09	16.246649
cg27221631	chr4	77703381	-	SHROOM3	3'UTR	-1.978992	0.2186601	-7.446199	1.08E-11	2.29E-09	16.246413
cg06777472	chr20	57873033	-			-2.140549	0.3934103	-7.44578	1.08E-11	2.30E-09	16.244249
cg14486338	chr8	99440279	-	KCNS2	Body	2.2692877	0.3639033	7.4456384	1.08E-11	2.30E-09	16.243517
cg12190994	chr7	4305061	+	SDK1;SDK1	Body;3'UTR	1.444079	1.4157104	7.4445763	1.08E-11	2.31E-09	16.238031
cg03925294	chr4	6247629	-			1.0590021	-0.610172	7.4441346	1.09E-11	2.31E-09	16.23575
cg01198033	chr7	155302342	-	CNPY1	5'UTR	1.8493002	0.2083243	7.4440365	1.09E-11	2.31E-09	16.235243
cg20666917	chr2	99439848	+	C2orf55	Body	1.2524242	0.8049679	7.4432766	1.09E-11	2.32E-09	16.231319
cg16777057	chr2	219774048	-			-0.629324	-3.911597	-7.442958	1.09E-11	2.32E-09	16.229673
cg18451814	chr14	57278188	-	OTX2	TSS1500	2.1016556	0.0665473	7.4427692	1.10E-11	2.32E-09	16.228698
cg21068610	chr17	7790179	+	CHD3	Body	-0.684978	-3.560286	-7.44282	1.10E-11	2.32E-09	16.228962
cg19990182	chr2	20190080	-	WDR35;WDR35	TSS200;TSS200	-1.521367	-4.586993	-7.442518	1.10E-11	2.33E-09	16.227403
cg23712018	chr6	30039132	+	RNF39;RNF39	Body;Body	1.0735751	-0.332157	7.4415089	1.10E-11	2.34E-09	16.22219
cg17541025	chr3	72978020	-	GXYLT2	Body	1.3023519	3.38099	7.4461014	1.11E-11	2.35E-09	16.219796

cg15377283	chr8	55371369	-	SOX17	Body	1.8372841	-0.103504	7.4404427	1.11E-11	2.35E-09	16.216685
cg05514256	chr2	45231891	-			2.1480877	-0.425142	7.4447734	1.11E-11	2.36E-09	16.212213
cg14196790	chr5	131705035	-	SLC22A5	TSS1500	-1.204606	-2.824633	-7.444165	1.12E-11	2.37E-09	16.209816
cg22812770	chr1	52195547	-	OSBPL9;OSBPL9;OSBPL9;OSBPL9;OSBPL9;OSBPL9;OSBPL9;OSBPL9;OSBPL9;OSBPL9	5'UTR;1stExon;Body;5'UTR;Body;Body;1stExon;5'UTR;1stExon;5'UTR	-1.119489	-3.838087	-7.438621	1.12E-11	2.37E-09	16.207277
cg17527195	chr13	49403484	-			-1.457318	-0.510873	-7.438505	1.12E-11	2.37E-09	16.20668
cg00369677	chr2	237010048	-	AGAP1;AGAP1	Body;Body	1.0508252	2.8711049	7.4378817	1.12E-11	2.37E-09	16.203462
cg07604732	chr16	19295632	-	LOC728276	TSS1500	-1.16576	-1.508748	-7.437959	1.12E-11	2.37E-09	16.203859
cg06903075	chr11	71709751	+	IL18BP;IL18BP;IL18BP;IL18BP;IL18BP	TSS1500;TSS1500;TSS1500;TSS1500;TSS1500	1.0753019	2.1787903	7.4377314	1.13E-11	2.38E-09	16.202686
cg00178897	chr2	175208588	-			2.1418951	-1.17714	7.4376247	1.13E-11	2.38E-09	16.202135
cg15854847	chr8	23563970	+	NKX2-6	TSS200	2.452491	-1.015874	7.4372521	1.13E-11	2.38E-09	16.200212
cg19867917	chr2	3642629	-	COLEC11;COLEC11	TSS200;TSS200	1.6768052	0.9952378	7.4373377	1.13E-11	2.38E-09	16.200654
cg27655706	chr7	96044476	-			-1.255918	-1.903919	-7.437128	1.13E-11	2.38E-09	16.199569
cg25352836	chr19	19625264	-	TSSK6;TSSK6	3'UTR;1stExon	1.4998228	0.2835767	7.4363623	1.13E-11	2.39E-09	16.195618
cg17438030	chr5	72526379	-			1.6587647	0.2820459	7.4354949	1.14E-11	2.40E-09	16.19114
cg27496339	chr7	43803803	+	BLVRA	5'UTR	-1.392147	-1.854278	-7.435158	1.14E-11	2.40E-09	16.189401
cg03625953	chr17	1975218	-	SMG6;SMG6	Body;Body	1.1632256	0.5593765	7.4347002	1.14E-11	2.40E-09	16.187038
cg11344927	chr19	2739684	+	SLC39A3;SLC39A3	5'UTR;5'UTR	-0.701452	-3.548875	-7.439522	1.15E-11	2.41E-09	16.185896
cg13869484	chr1	9140563	-	SLC2A5	Body	-2.030124	-0.690219	-7.434271	1.15E-11	2.41E-09	16.184821
cg10761843	chr2	172411765	+	CYBRD1;CYBRD1	3'UTR;3'UTR	1.1779828	1.8729726	7.432359	1.16E-11	2.43E-09	16.174954
cg17849569	chr6	28058911	+	ZSCAN12L1	TSS200	1.2439607	0.5411776	7.4323866	1.16E-11	2.43E-09	16.175096

cg13896402	chr10	120840893	+	EIF3A	TSS1500	-0.809457	-4.906359	-7.431766	1.16E-11	2.44E-09	16.171891
cg17408527	chr19	58629967	-	ZSCAN18;ZSCAN18	TSS200;TSS200	1.4167326	-0.188221	7.4315849	1.16E-11	2.44E-09	16.170958
cg20975682	chr12	50019676	+	PRPF40B	5'UTR	1.0182642	0.8193474	7.431599	1.16E-11	2.44E-09	16.171031
cg23661183	chr10	116286593	+	ABLIM1;ABLIM1;ABLIM1;ABLIM1;ABLIM1	Body;1stExon;Body;5'UTR;Body	-0.855157	-4.464919	-7.431841	1.16E-11	2.44E-09	16.172279
cg16014770	chr12	31882421	+	AMN1;AMN1	TSS1500;TSS1500	-1.589812	-4.11524	-7.431206	1.17E-11	2.44E-09	16.169005
cg22115706	chr7	130125719	-	MEST	TSS1500	-2.189917	0.781713	-7.430848	1.17E-11	2.44E-09	16.167154
cg18269493	chr1	8772984	+	RERE;RERE	5'UTR;5'UTR	-1.032858	-2.825501	-7.430121	1.17E-11	2.45E-09	16.163404
cg08610862	chr1	197888469	+	LHX9;LHX9	Body;Body	2.2017921	-0.097123	7.4292353	1.18E-11	2.46E-09	16.158833
cg17436134	chr1	119536288	+			2.2721562	0.4543891	7.4288724	1.18E-11	2.47E-09	16.15696
cg00687073	chr8	954552	-			-1.362067	-0.699825	-7.427441	1.19E-11	2.48E-09	16.149576
cg05184377	chr10	50976646	+			1.1577108	-0.254346	7.4273594	1.19E-11	2.48E-09	16.149153
cg15890103	chr11	86663160	+	FZD4	Body	1.276923	2.110012	7.4275225	1.19E-11	2.48E-09	16.149994
cg24390913	chr10	118899788	+			2.4174447	-1.182826	7.4267851	1.19E-11	2.49E-09	16.14619
cg11455040	chr1	205649941	+	SLC45A3	TSS1500	-0.928198	-4.012774	-7.426195	1.20E-11	2.49E-09	16.143147
cg07757096	chr5	77591498	+	AP3B1	TSS1500	-1.357224	-4.138825	-7.425955	1.20E-11	2.50E-09	16.141908
cg19273683	chr1	21656047	-	ECE1	Body	-1.234669	-1.524369	-7.425932	1.20E-11	2.50E-09	16.141786
cg16578085	chr4	127771714	-			-1.621609	-1.117448	-7.424395	1.21E-11	2.51E-09	16.133858
cg07022048	chr12	52638592	-	KRT7	Body	-1.046186	-0.563255	-7.424062	1.21E-11	2.52E-09	16.132142
cg07914075	chr10	13482572	-	BEND7	Body	-0.794205	-3.776795	-7.422662	1.22E-11	2.54E-09	16.12492
cg07996838	chr7	111382084	+	DOCK4	Body	-1.891114	-1.969825	-7.422162	1.22E-11	2.54E-09	16.122338

cg08619385	chr4	144438035	+	SMARCA5	Body	1.0629543	2.4982656	7.422005	1.22E-11	2.54E-09	16.121528
cg05951296	chr10	130602126	+			-1.786235	-0.037881	-7.42097	1.23E-11	2.56E-09	16.11619
cg04165933	chr16	49833847	+	ZNF423	Body	-1.846587	0.3855357	-7.420711	1.23E-11	2.56E-09	16.114856
cg16411216	chr1	9340507	+			-0.997455	-4.017328	-7.42068	1.23E-11	2.56E-09	16.114696
cg07835424	chr2	200327334	+			2.7127757	-0.626298	7.4205476	1.23E-11	2.56E-09	16.114011
cg22622937	chr7	48129444	+	UPP1;UPP1	5'UTR;5'UTR	-0.876824	-3.115194	-7.420048	1.24E-11	2.56E-09	16.111434
cg05936441	chr20	37356844	+	SLC32A1	Body	1.3291426	-1.117753	7.4193693	1.24E-11	2.57E-09	16.107934
cg23654174	chr1	47779667	+	STIL;STIL	5'UTR;5'UTR	-0.762964	-3.689997	-7.419259	1.24E-11	2.57E-09	16.107363
cg18406033	chr2	223159869	-	PAX3;PAX3;PAX3;PAX3;PAX3;PAX3;PAX3;PAX3	Body;Body;Body;Body;Body;Body;Body;Body	2.044339	-0.460944	7.4190034	1.24E-11	2.57E-09	16.106046
cg11912765	chr2	223159838	-	PAX3;PAX3;PAX3;PAX3;PAX3;PAX3;PAX3;PAX3	Body;Body;Body;Body;Body;Body;Body;Body	2.6372924	-2.480347	7.4240901	1.24E-11	2.57E-09	16.10641
cg11984971	chr4	54765826	+			1.2670487	1.4610661	7.4234492	1.25E-11	2.58E-09	16.103131
cg04299439	chr2	182547599	+			1.5633394	-0.787741	7.4179919	1.25E-11	2.58E-09	16.10083
cg06635797	chr19	4915394	+	UHRF1;UHRF1	Body;Body	-1.532474	-2.164029	-7.417729	1.25E-11	2.59E-09	16.099475
cg21534578	chr1	197745159	-	DENND1B;DENND1B	TSS1500;TSS1500	-1.013287	-1.318437	-7.41756	1.25E-11	2.59E-09	16.098601
cg08904363	chr7	102790119	+	NAPEPLD;NAPEPLD	TSS1500;TSS1500	-1.099134	-3.009808	-7.417383	1.25E-11	2.59E-09	16.09769
cg07536910	chr2	176948105	-	EVX2	1stExon	2.2131379	-0.228332	7.4170412	1.26E-11	2.59E-09	16.095927
cg04834794	chr3	128400154	+			-0.837987	-4.177625	-7.427148	1.26E-11	2.60E-09	16.095947
cg13080565	chr6	101846797	-	GRIK2;GRIK2;GRIK2	TSS200;TSS200;TSS200	2.0303185	0.1057512	7.416583	1.26E-11	2.60E-09	16.093564
cg23679662	chr1	26633288	-	UBXN11;UBXN11;UBXN11	5'UTR;TSS200;TSS200	-1.483051	-5.245668	-7.426843	1.26E-11	2.60E-09	16.09436
cg22967396	chr14	57283942	+	OTX2OS1	Body	1.4961556	-0.945536	7.4149098	1.27E-11	2.62E-09	16.084936

cg15540820	chr3	27765283	+	EOMES	TSS1500	1.7950648	-0.73607	7.4134395	1.28E-11	2.64E-09	16.077355
cg07375978	chr12	52214320	-	FIGNL2	1stExon	-1.180253	-1.590987	-7.413039	1.28E-11	2.64E-09	16.075289
cg24856726	chr12	108169333	+	ASCL4	1stExon	2.1466288	-1.053525	7.4127158	1.29E-11	2.64E-09	16.073623
cg22467301	chr3	50265698	-			-1.005115	-1.711656	-7.41216	1.29E-11	2.65E-09	16.070759
cg17872886	chr2	3642732	-	COLEC11;COLEC11;COLEC11;COLEC11	1stExon;5'UTR;1stExon;5'UTR	1.3089785	0.5164817	7.4119963	1.29E-11	2.65E-09	16.069914
cg12474824	chr4	1227400	-	CTBP1;CTBP1	Body;Body	0.9963877	1.3490185	7.4112121	1.30E-11	2.66E-09	16.065871
cg26132774	chr13	79170146	-			2.8418163	-0.504785	7.4112759	1.30E-11	2.66E-09	16.0662
cg03503297	chr16	30441811	+	DCTPP1	TSS1500	-0.717117	-3.135533	-7.410814	1.30E-11	2.66E-09	16.063816
cg25428846	chr4	141107931	-			-1.857973	0.5822506	-7.410378	1.30E-11	2.67E-09	16.06157
cg09041678	chr11	31839947	+	PAX6	TSS1500	1.4796437	-0.465133	7.4100279	1.30E-11	2.67E-09	16.059766
cg22418337	chr5	2115107	+			-2.052469	-0.11881	-7.409517	1.31E-11	2.68E-09	16.057131
cg11940285	chr3	129693385	+	TRH;TRH	5'UTR;1stExon	2.6471497	-0.363034	7.4075254	1.32E-11	2.71E-09	16.046867
cg15964018	chr1	19490180	-	UBR4	Body	0.8698769	1.557118	7.4068094	1.33E-11	2.72E-09	16.043176
cg23090870	chr19	38755753	+	SPINT2;SPINT2	Body;Body	-1.085364	-1.081404	-7.406615	1.33E-11	2.72E-09	16.042174
cg18705773	chr5	170743564	-			2.3048678	-0.543924	7.4056625	1.34E-11	2.73E-09	16.037265
cg21657087	chr3	147139485	-			1.3143091	-0.63302	7.4054582	1.34E-11	2.73E-09	16.036212
cg00321901	chr8	10512459	-	RP1L1;RP1L1	1stExon;5'UTR	-0.920042	-1.669571	-7.404818	1.34E-11	2.74E-09	16.032911
cg03161803	chr6	27649120	+			3.0781213	0.5290521	7.4039418	1.35E-11	2.75E-09	16.028397
cg00130275	chr11	20409780	+	PRMT3;PRMT3;PRMT3	5'UTR;Body;Body	-0.597197	-3.664915	-7.403095	1.35E-11	2.76E-09	16.024035
cg11707035	chr2	240253573	-	HDAC4	Body	1.5181751	3.1023727	7.4017607	1.36E-11	2.78E-09	16.017158

cg20994118	chr1	209780966	-	CAMK1G	Body	-1.347932	-1.039363	-7.401207	1.37E-11	2.79E-09	16.014303
cg00489219	chr3	40566141	+	ZNF621;ZNF621	TSS1500;TSS1500	-0.594291	-3.198159	-7.400318	1.37E-11	2.80E-09	16.009726
cg22650099	chr4	17144496	+			-1.909558	0.2454864	-7.398996	1.38E-11	2.82E-09	16.002912
cg08293531	chr15	101601180	+	LRRK1	Body	1.9672596	2.8196771	7.4084814	1.39E-11	2.83E-09	15.999702
cg05354479	chr14	71276874	-	MAP3K9	TSS1500	-0.945771	-1.866992	-7.401478	1.40E-11	2.86E-09	15.990084
cg07679219	chr12	77417738	+	E2F7	3'UTR	1.6536686	3.0324802	7.3958307	1.41E-11	2.86E-09	15.986607
cg18076767	chr11	86453358	-			-1.762912	0.3881609	-7.395347	1.41E-11	2.87E-09	15.984118
cg00671600	chr20	61792443	+			-1.697644	-0.21667	-7.395066	1.41E-11	2.87E-09	15.982667
cg13977486	chr10	104211296	+	C10orf95;C10orf95	5'UTR;1stExon	-1.046356	-1.769261	-7.39512	1.41E-11	2.87E-09	15.982947
cg15225844	chr2	2189201	-	MYT1L	5'UTR	-2.060327	0.5161551	-7.3942	1.42E-11	2.88E-09	15.978208
cg20995304	chr12	48196167	+	HDAC7;HDAC7	Body;Body	1.3312891	1.4144631	7.3931668	1.43E-11	2.90E-09	15.972886
cg05785488	chr16	23568401	-	UBFD1;EARS2;EARS2	TSS1500;Body;Body	-1.354617	-6.243465	-7.392868	1.43E-11	2.90E-09	15.971348
cg15903956	chr15	74676231	+			-1.52132	-0.485083	-7.391853	1.44E-11	2.91E-09	15.966121
cg20016631	chr4	38871320	-	FAM114A1	5'UTR	1.5966714	0.4715203	7.3918385	1.44E-11	2.91E-09	15.966045
cg04351905	chr21	34441330	-	OLIG1	TSS1500	-1.34947	-0.47992	-7.390877	1.45E-11	2.93E-09	15.961093
cg10198943	chr5	75668478	+			-1.44567	0.1798348	-7.390166	1.45E-11	2.94E-09	15.957434
cg14564076	chr7	153584873	+	DPP6	Body	1.8492785	-0.828322	7.3900934	1.45E-11	2.94E-09	15.957058
cg17231999	chr4	41882564	+			3.2707103	-0.568386	7.3899933	1.45E-11	2.94E-09	15.956543
cg18944010	chr1	119522855	+	TBX15	5'UTR	1.7587816	-0.513031	7.3895519	1.46E-11	2.94E-09	15.95427
cg09423312	chr7	1163549	+	C7orf50;C7orf50;C7orf50	Body;Body;Body	1.4261895	0.6778471	7.3893775	1.46E-11	2.94E-09	15.953372

cg24522654	chr12	19535154	-			-1.692713	-1.058322	-7.388369	1.46E-11	2.96E-09	15.948181
cg00747477	chr17	31340147	-	ACCN1;ACCN1	3'UTR;3'UTR	-1.601532	-0.202378	-7.388089	1.47E-11	2.96E-09	15.94674
cg09234283	chr4	113207277	+	TIFA	TSS1500	-0.486671	-4.056073	-7.392958	1.47E-11	2.96E-09	15.946288
cg04349420	chr8	49414279	-			-1.380145	-2.734435	-7.387588	1.47E-11	2.97E-09	15.944159
cg07085895	chr11	19262124	-	E2F8;E2F8	1stExon;5'UTR	-0.695784	-3.634998	-7.386383	1.48E-11	2.98E-09	15.937952
cg15387123	chr9	139891058	-	CLIC3	TSS200	-1.119187	-1.803404	-7.38644	1.48E-11	2.98E-09	15.938247
cg27149973	chr17	74868725	-	MGAT5B;MGAT5B	Body;TSS200	-1.694037	-1.935198	-7.386549	1.48E-11	2.98E-09	15.93881
cg07038243	chr5	149868855	+			1.0824531	-0.539179	7.3860181	1.48E-11	2.98E-09	15.936075
cg06794543	chr15	42749748	-	ZFP106	TSS200	1.6345286	0.6265867	7.3854782	1.49E-11	2.99E-09	15.933296
cg10324998	chr1	92338262	+	TGFBR3	5'UTR	-1.044631	-2.768799	-7.385491	1.49E-11	2.99E-09	15.933363
cg24858591	chr3	44803638	-	KIAA1143;KIF15	TSS1500;Body	-0.788361	-4.274902	-7.385156	1.49E-11	2.99E-09	15.931638
cg00290373	chr5	68462622	-	CCNB1	TSS1500	-0.962523	-2.826804	-7.384974	1.49E-11	3.00E-09	15.930702
cg15298173	chr15	29903900	-			-0.97547	-0.977809	-7.384844	1.49E-11	3.00E-09	15.930031
cg00251358	chr5	81931501	-			-1.330929	-0.670631	-7.389714	1.50E-11	3.00E-09	15.929648
cg06240854	chr2	99771011	-	LIPT1;LIPT1;LIPT1;LIPT1;TSGA10;LIPT1	TSS1500;TSS1500;TSS1500;TSS1500;5'UTR;TSS1500	-1.123889	-1.444763	-7.384549	1.49E-11	3.00E-09	15.928512
cg12711755	chr15	41779622	+			1.4559487	1.7334192	7.3896773	1.50E-11	3.00E-09	15.929458
cg19174643	chr18	13136426	-			-1.380106	-2.451719	-7.384556	1.49E-11	3.00E-09	15.928546
cg10778862	chr22	38453075	-	PICK1;PICK1;PICK1	TSS200;TSS200;TSS200	-0.622269	-4.006775	-7.38366	1.50E-11	3.01E-09	15.923938
cg27060294	chr4	16839404	-	LDB2;LDB2	Body;Body	-2.044447	0.7037026	-7.38344	1.50E-11	3.01E-09	15.922804
cg00510447	chr16	3071608	+	TNFRSF12A	Body	-1.03744	-2.881079	-7.382966	1.51E-11	3.02E-09	15.920365

cg16570314	chr5	1003099	-			-2.607171	1.0516003	-7.381502	1.52E-11	3.04E-09	15.912826
cg23179181	chr17	27945461	-	CORO6	Body	-1.100523	-1.485519	-7.381564	1.52E-11	3.04E-09	15.913145
cg18490614	chr15	27213362	+			1.3803578	0.39657	7.3812824	1.52E-11	3.04E-09	15.911697
cg22969666	chr14	97688014	-			-1.879161	0.2681565	-7.381291	1.52E-11	3.04E-09	15.911744
cg08482501	chr20	5282952	+	PROKR2	Body	-1.968803	0.4292532	-7.379903	1.53E-11	3.06E-09	15.904597
cg09338032	chr12	113916609	+			1.5655798	-0.166139	7.3845934	1.54E-11	3.07E-09	15.903342
cg17888086	chr1	37945057	+	ZC3H12A	Body	-1.465681	-3.168406	-7.378928	1.54E-11	3.07E-09	15.899578
cg10734581	chr14	101520078	+	MIR134;MIR382	TSS1500;TSS1500	-1.026853	-0.983734	-7.378493	1.54E-11	3.08E-09	15.897343
cg12424383	chr1	27990967	-			-1.707523	0.4803026	-7.37827	1.55E-11	3.08E-09	15.896195
cg06565641	chr8	141359674	-	TRAPPC9;TRAPPC9	Body;Body	1.1438905	1.3775251	7.377652	1.55E-11	3.09E-09	15.893014
cg16081281	chr8	23559839	-			1.5026979	-0.862038	7.3775008	1.55E-11	3.09E-09	15.892236
cg13655674	chr1	119522386	-	TBX15	5'UTR	1.9851591	-0.893394	7.3772184	1.55E-11	3.09E-09	15.890783
cg13761284	chr9	130708393	+	FAM102A;FAM102A	Body;Body	0.7915755	1.5821704	7.3748431	1.57E-11	3.13E-09	15.87856
cg23057567	chr19	47017629	-			-1.547474	-1.227076	-7.374766	1.57E-11	3.13E-09	15.878163
cg16291880	chr6	50674399	+			2.0562864	-1.05575	7.3739053	1.58E-11	3.14E-09	15.873735
cg25168494	chr2	63286355	-			1.4238545	0.190661	7.3732628	1.59E-11	3.15E-09	15.87043
cg00201257	chr12	57390405	+	GPR182	3'UTR	0.911919	2.1127235	7.372996	1.59E-11	3.16E-09	15.869057
cg01030757	chr15	73866328	+	NPTN;NPTN;NPTN;NPTN	Body;Body;Body;Body	1.4351609	2.5545605	7.3721142	1.60E-11	3.17E-09	15.864521
cg03478444	chr16	49407465	+	C16orf78	TSS1500	-1.676878	0.2046471	-7.369278	1.62E-11	3.22E-09	15.849933
cg06152383	chr7	2884448	-	GNA12	TSS1500	-0.638754	-4.169941	-7.369273	1.62E-11	3.22E-09	15.849903

cg07599819	chr19	57308239	+	ZIM2;ZIM2;ZIM2	Body;Body;Body	-1.976494	0.2090197	-7.36903	1.62E-11	3.22E-09	15.848657
cg23958533	chr1	204446308	-	PIK3C2B	5'UTR	0.9941572	1.9856207	7.3688667	1.63E-11	3.22E-09	15.847816
cg17159699	chr8	53081140	-	ST18	Body	1.7333778	2.5546682	7.3938112	1.64E-11	3.25E-09	15.845469
cg00947599	chr7	2802697	+	GNA12	Body	1.1606784	0.5845453	7.3667427	1.64E-11	3.25E-09	15.836892
cg08455275	chr8	145047979	+	PLEC1;PLEC1	Body;TSS1500	-1.865132	-3.272679	-7.36623	1.65E-11	3.26E-09	15.834253
cg19848629	chr8	65286244	-			1.7715493	-0.775214	7.3650059	1.66E-11	3.28E-09	15.82796
cg12635937	chr4	81184925	+			0.9914343	0.0969514	7.3642248	1.67E-11	3.29E-09	15.823944
cg22567473	chr15	63902661	+	HERC1	Body	-1.111487	-3.981616	-7.363784	1.67E-11	3.30E-09	15.821675
cg27404351	chr11	123847741	+	OR10S1	1stExon	-1.120111	-0.739616	-7.363443	1.67E-11	3.30E-09	15.819921
cg09296270	chr2	65087288	+			-1.097751	-2.206746	-7.363195	1.68E-11	3.31E-09	15.81865
cg17113029	chr1	222135195	-			-1.568793	-2.265281	-7.363006	1.68E-11	3.31E-09	15.817678
cg00876153	chr22	29977322	-	NIPSNAP1	TSS200	-0.77001	-4.481093	-7.360802	1.70E-11	3.34E-09	15.806344
cg21273275	chr1	24648328	-	GRHL3;GRHL3;GRHL3	TSS1500;Body;TSS1500	-1.962323	-1.887837	-7.359969	1.70E-11	3.36E-09	15.802061
cg01701649	chr16	48589954	+	N4BP1	Body	1.8159607	0.3827337	7.3593304	1.71E-11	3.37E-09	15.798779
cg26736812	chr17	48042353	+			1.8565517	-1.122927	7.3593786	1.71E-11	3.37E-09	15.799027
cg27553372	chr12	120731091	+			-0.57187	-4.010718	-7.358539	1.72E-11	3.38E-09	15.794713
cg24929966	chr1	10726865	+	CASZ1;CASZ1	Body;Body	1.5486684	2.8131849	7.357938	1.72E-11	3.39E-09	15.791621
cg17578639	chr3	176862949	-	TBL1XR1	5'UTR	-1.680541	-2.011236	-7.362952	1.72E-11	3.39E-09	15.792219
cg24495585	chr4	56659985	+			2.9192382	-0.666664	7.3577104	1.72E-11	3.39E-09	15.790452
cg18184153	chr6	33385417	-	CUTA;CUTA;CUTA;CUTA;CUTA	Body;Body;Body;1stExon;Body	-0.633704	-4.49575	-7.357301	1.73E-11	3.40E-09	15.788348

cg06986158	chr6	30567633	+			1.1854586	1.8200293	7.3560767	1.74E-11	3.42E-09	15.782054
cg14898824	chr12	64851143	-	TBK1	Body	1.0912497	2.8834832	7.354572	1.75E-11	3.44E-09	15.774321
cg24240626	chr2	79386720	-	REG3A;REG3A;REG3A;REG3A	5'UTR;5'UTR;5'UTR;1stExon	-1.626438	-0.438637	-7.354256	1.76E-11	3.45E-09	15.772698
cg27461254	chr20	39135472	-			-1.248253	-1.452005	-7.35881	1.76E-11	3.46E-09	15.771
cg26959257	chr11	124711848	-			-1.250191	-1.862313	-7.352363	1.77E-11	3.48E-09	15.762971
cg13676800	chr9	89722432	-			-1.773848	-0.214191	-7.356528	1.78E-11	3.49E-09	15.759299
cg17484944	chr8	122736417	+			-1.554865	-0.743878	-7.351175	1.79E-11	3.50E-09	15.756862
cg15347097	chr12	110909554	+	C12orf24	Body	0.7099512	2.8129794	7.35071	1.79E-11	3.50E-09	15.754474
cg23037321	chr1	181003203	-	MR1	1stExon	-1.792728	-3.495733	-7.350607	1.79E-11	3.50E-09	15.753943
cg08688773	chr10	23461301	-			1.4039858	-1.671437	7.3499415	1.80E-11	3.52E-09	15.750526
cg12978582	chr7	44150888	+	AEBP1	Body	-1.270172	-0.188547	-7.349387	1.80E-11	3.52E-09	15.747677
cg00442282	chr17	38471064	+	RARA	5'UTR	-0.666378	-3.157985	-7.348608	1.81E-11	3.54E-09	15.743674
cg18461347	chr7	78947094	-	MAGI2	Body	-1.437445	-0.735175	-7.348414	1.81E-11	3.54E-09	15.742679
cg03568858	chr7	100465221	+	TRIP6	1stExon	-1.075584	-2.970326	-7.347665	1.82E-11	3.55E-09	15.738832
cg23429510	chr2	176936942	+			1.6128073	-0.69687	7.3474682	1.82E-11	3.55E-09	15.737819
cg04452534	chr5	56133370	-	MAP3K1	Body	1.2886513	1.9609794	7.3468339	1.83E-11	3.56E-09	15.73456
cg10804656	chr10	22623460	+			1.4060437	1.0621116	7.3461843	1.83E-11	3.57E-09	15.731223
cg26924967	chr19	3136097	-	GNA15	TSS200	-1.583849	-2.144609	-7.345521	1.84E-11	3.59E-09	15.727814
cg25755851	chr1	9335794	+			-1.58011	-2.557737	-7.344572	1.85E-11	3.60E-09	15.722943
cg23718606	chr6	27219812	+	PRSS16	Body	-0.781346	-2.382594	-7.344043	1.85E-11	3.61E-09	15.720222

cg06571387	chr2	176964101	+	HOXD12	TSS1500	1.3336015	-0.210968	7.3428162	1.87E-11	3.63E-09	15.713923
cg04654530	chr2	63282702	+	OTX1	Body	1.753498	-0.062173	7.3426211	1.87E-11	3.64E-09	15.71292
cg06100608	chr21	32013856	+	KRTAP20-3	TSS1500	-2.111978	0.5563544	-7.342414	1.87E-11	3.64E-09	15.711856
cg09361958	chr3	128947790	-			1.1705799	0.6184274	7.3418799	1.88E-11	3.65E-09	15.709114
cg08435780	chr16	49749891	+	ZNF423	Body	-1.14499	-0.387642	-7.341656	1.88E-11	3.65E-09	15.707967
cg20330126	chr22	36125053	+	APOL5	3'UTR	-1.576509	0.0936257	-7.341592	1.88E-11	3.65E-09	15.707634
cg23244540	chr2	127532388	-			-2.041539	0.5899087	-7.340866	1.89E-11	3.66E-09	15.703909
cg15972949	chr7	44240380	+	YKT6	TSS200	-0.584016	-3.802575	-7.339615	1.90E-11	3.68E-09	15.697482
cg27353899	chr3	195488922	+	MUC4;MUC4;MUC4	Body;Body;Body	1.3166072	0.6599686	7.3394443	1.90E-11	3.69E-09	15.696606
cg13755873	chr17	70113187	+			1.8314041	-1.436847	7.3390203	1.90E-11	3.69E-09	15.694429
cg22779330	chr11	69061911	+	MYEOV;MYEOV	5'UTR;1stExon	-1.799725	0.1033929	-7.33796	1.92E-11	3.71E-09	15.688985
cg07570113	chr16	59790180	-	LOC644649	TSS1500	-1.773531	-0.727678	-7.337854	1.92E-11	3.71E-09	15.688438
cg09537620	chr11	31826574	+	PAX6;PAX6;PAX6	Body;Body;Body	2.3645205	-0.750685	7.3426396	1.92E-11	3.72E-09	15.688097
cg04540406	chr16	46655675	-	SHCBP1	TSS1500	-1.244381	-2.061969	-7.337401	1.92E-11	3.72E-09	15.686116
cg20158248	chr16	16042956	+	ABCC1;ABCC1;ABCC1;ABCC1;ABCC1	TSS1500;TSS1500;TSS1500;TSS1500;TSS1500	-0.760201	-2.571137	-7.336388	1.93E-11	3.74E-09	15.680915
cg03022541	chr3	128186859	-	DNAJB8	TSS1500	-1.232381	-0.266178	-7.336155	1.93E-11	3.74E-09	15.679716
cg00793935	chr2	234847769	+	TRPM8	Body	2.7945731	-0.416117	7.3357538	1.94E-11	3.74E-09	15.677657
cg10278102	chr11	20410216	+	PRMT3;PRMT3;PRMT3	5'UTR;Body;Body	-1.353646	-1.426008	-7.335429	1.94E-11	3.75E-09	15.675987
cg18384778	chr19	57618136	+			2.4020524	-0.346737	7.335478	1.94E-11	3.75E-09	15.676241
cg26811898	chr4	125824919	-			-1.339646	-1.817892	-7.33513	1.94E-11	3.75E-09	15.674457

cg26469608	chr12	113913695	+			1.3919985	-0.34065	7.3348863	1.95E-11	3.76E-09	15.673203
cg07585427	chr1	11917550	-	NPPB	3'UTR	-1.439825	-1.00089	-7.334009	1.96E-11	3.77E-09	15.668699
cg01586851	chr4	1556595	+			-1.484298	-0.594482	-7.333042	1.97E-11	3.79E-09	15.663734
cg25607249	chr19	47288039	-	SLC1A5;SLC1A5;SLC1A5;SLC1A5	1stExon;5'UTR;Body;5'UTR	-1.311774	-3.16517	-7.332975	1.97E-11	3.79E-09	15.663389
cg02086467	chr14	57276527	-	OTX2	5'UTR	1.874913	-0.831412	7.3379763	1.97E-11	3.79E-09	15.664203
cg21038156	chr7	158936739	+	VIPR2	Body	2.8457996	-1.968273	7.3326014	1.97E-11	3.79E-09	15.661474
cg15247434	chr13	95201296	+			-0.737364	-3.647963	-7.331817	1.98E-11	3.81E-09	15.657445
cg02723856	chr15	53086726	+			1.6855457	-0.9415	7.3310129	1.99E-11	3.82E-09	15.65332
cg14240993	chr12	132929352	+			-1.414702	-0.10663	-7.330523	1.99E-11	3.83E-09	15.650807
cg12635314	chr10	2815004	-			-1.56921	0.083067	-7.330159	2.00E-11	3.84E-09	15.64894
cg25597797	chr19	3589762	-	GIPC3	Body	-1.400918	-1.170862	-7.329997	2.00E-11	3.84E-09	15.648109
cg07173635	chr9	971480	-			1.9570062	-0.422884	7.3296056	2.00E-11	3.84E-09	15.646097
cg19150852	chr3	43732102	+	ABHD5	TSS1500	-0.921708	-3.485674	-7.329683	2.00E-11	3.84E-09	15.646492
cg18525486	chr3	62357176	-	FEZF2	Body	1.5322004	-0.411528	7.3294994	2.00E-11	3.84E-09	15.645552
cg18293662	chr3	27766054	+			1.5272121	-0.677812	7.3284812	2.01E-11	3.86E-09	15.640327
cg24884703	chr1	91185422	-			2.5963445	-0.76253	7.3280547	2.02E-11	3.87E-09	15.638138
cg26172211	chr17	79170494	+	AZI1;AZI1	Body;Body	0.7123383	2.6661326	7.3267697	2.03E-11	3.89E-09	15.631544
cg15810758	chr6	99290349	+			1.9744112	-0.804309	7.3263016	2.04E-11	3.90E-09	15.629142
cg16466270	chr4	122305076	+			-1.780714	0.295574	-7.326047	2.04E-11	3.90E-09	15.627835
cg23893898	chr4	6247571	-			1.6728963	-0.493273	7.3257963	2.04E-11	3.91E-09	15.626549

cg21546184	chr8	65283649	-			1.9832242	-1.158892	7.3255007	2.05E-11	3.91E-09	15.625033
cg11468193	chr17	75282040	-	9-Sep	Body	-1.089409	-1.80139	-7.323624	2.07E-11	3.95E-09	15.615406
cg25764534	chr8	22485721	+	BIN3	Body	1.0355261	1.7880846	7.3236406	2.07E-11	3.95E-09	15.615489
cg13525877	chr9	135681852	-	C9orf98	Body	1.142995	2.0406969	7.3285186	2.07E-11	3.95E-09	15.615761
cg00348389	chr16	31235706	-	TRIM72	Body	1.1638707	1.0755782	7.3225427	2.08E-11	3.97E-09	15.609856
cg07150404	chr8	144416886	+	TOP1MT	Body	-0.568558	-4.083224	-7.321629	2.09E-11	3.99E-09	15.60517
cg07547765	chr17	46685448	+	HOXB7	Body	1.6318379	1.1951348	7.3199893	2.11E-11	4.02E-09	15.596757
cg23669081	chr17	46685353	-	HOXB7	Body	2.2077048	2.0274697	7.3245426	2.11E-11	4.03E-09	15.595404
cg01518607	chr6	27235891	+			2.6697438	0.5791552	7.3193067	2.12E-11	4.03E-09	15.593256
cg07318983	chr1	91185922	+			1.2966668	-0.18663	7.318917	2.12E-11	4.03E-09	15.591257
cg25850044	chr15	31507705	+			-1.129656	-2.870189	-7.318941	2.12E-11	4.03E-09	15.591381
cg22306155	chr18	45552771	+			1.0724543	2.7443037	7.3180885	2.13E-11	4.05E-09	15.587008
cg01850449	chr2	133428510	+	LYPD1;LYPD1	TSS200;5'UTR	-1.150931	-2.805413	-7.317841	2.13E-11	4.05E-09	15.58574
cg07868273	chr2	87035758	+	CD8A	TSS1500	-0.765365	-4.269117	-7.317548	2.14E-11	4.06E-09	15.584235
cg16705697	chr11	134731585	+			-2.468521	0.9993401	-7.338028	2.14E-11	4.06E-09	15.588228
cg19117070	chr6	3723112	-	C6orf145	3'UTR	1.0556061	1.8308262	7.3223276	2.14E-11	4.06E-09	15.584065
cg24085344	chr17	47485676	-	PHB	Body	-0.905188	-2.654855	-7.317312	2.14E-11	4.06E-09	15.583027
cg06988897	chr16	88803803	-	FAM38A	Body	-1.15998	-2.437524	-7.317036	2.14E-11	4.06E-09	15.581611
cg10627429	chr10	115511135	+	C10orf81	TSS200	-1.848706	0.3441696	-7.316038	2.15E-11	4.08E-09	15.576491
cg20216139	chr11	16632509	-			1.7747479	-0.224466	7.3151692	2.16E-11	4.10E-09	15.572036

cg25151532	chr1	43920103	-	HYI	TSS200	-1.009145	-2.151312	-7.314019	2.18E-11	4.12E-09	15.566136
cg20649017	chr2	176980837	-	HOXD10	TSS1500	2.3098828	-0.555858	7.3136918	2.18E-11	4.13E-09	15.564461
cg23811057	chr1	234908514	-			-1.382289	-2.712988	-7.313484	2.18E-11	4.13E-09	15.563396
cg14832317	chr4	13526597	+			1.0633607	-0.382453	7.3133148	2.18E-11	4.13E-09	15.562527
cg15871797	chr14	101139695	-			-1.082879	-2.286726	-7.313144	2.19E-11	4.13E-09	15.561652
cg18546419	chr5	176433891	+	UIMC1	TSS1500	-0.588748	-4.468773	-7.312849	2.19E-11	4.14E-09	15.560137
cg27313642	chr8	65291542	+	MIR124-2	TSS200	1.5661281	-1.325983	7.3127022	2.19E-11	4.14E-09	15.559386
cg12690127	chr12	7055683	-	PTPN6	TSS200	-1.536584	-1.901773	-7.312085	2.20E-11	4.15E-09	15.556222
cg16555388	chr3	172241216	-	TNFSF10;TNFSF10	5'UTR;1stExon	-0.780622	-3.757095	-7.312153	2.20E-11	4.15E-09	15.55657
cg02493544	chr11	125361336	-	FEZ1;FEZ1	5'UTR;5'UTR	-1.256092	-0.789337	-7.311994	2.20E-11	4.15E-09	15.555754
cg06250720	chr10	5593949	+			-1.67768	-0.693475	-7.311461	2.21E-11	4.16E-09	15.553025
cg25691553	chr3	49057884	+	NDUFAF3;DALRD3;NDUFAF3;MIR425;NDUFAF3;NDUFAF3	TSS200;5'UTR;TSS1500;TSS1500;TSS1500;TSS1500	-1.688268	-2.370893	-7.31014	2.22E-11	4.19E-09	15.546252
cg10754794	chr10	93916288	+	CPEB3	Body	1.1375748	0.8153715	7.3093199	2.23E-11	4.20E-09	15.542046
cg08278554	chr15	45722919	+	C15orf48;C15orf48;C15orf48	5'UTR;5'UTR;1stExon	-1.466545	-3.156867	-7.308921	2.24E-11	4.21E-09	15.540002
cg22052672	chr2	74663416	-	RTKN;RTKN;RTKN	Body;5'UTR;Body	1.0785275	2.4963223	7.3137164	2.24E-11	4.22E-09	15.539996
cg01465169	chr17	27944849	+	CORO6	Body	-2.432194	-3.777161	-7.308065	2.25E-11	4.23E-09	15.535613
cg03309308	chr16	68525329	+			-0.990864	-1.788557	-7.307618	2.25E-11	4.24E-09	15.533319
cg23474501	chr10	134902168	+	GPR123	5'UTR	1.8990123	-0.667885	7.3065389	2.26E-11	4.26E-09	15.52779
cg18698884	chr11	16632724	+			1.6981476	-0.090164	7.3044317	2.29E-11	4.30E-09	15.51699
cg08237521	chr16	66360304	+			-1.338917	-0.267825	-7.303927	2.30E-11	4.31E-09	15.514405

cg01977413	chr3	170224495	-	SLC7A14	Body	-1.432287	-2.924623	-7.303843	2.30E-11	4.31E-09	15.513971
cg17306740	chr20	56195434	-	ZBP1;ZBP1;ZBP1;ZBP1;ZBP1;ZBP1;ZBP1;ZBP1	1stExon;1stExon;1stExon;5'UTR;5'UTR;5'UTR;1stExon;5'UTR	-1.360249	-2.684116	-7.302054	2.32E-11	4.35E-09	15.504807
cg11627823	chr7	129424539	+			1.351674	0.1398274	7.3005608	2.34E-11	4.38E-09	15.497154
cg15797286	chr8	18067416	-	NAT1;NAT1;NAT1;NAT1;NAT1;NAT1;NAT1;NAT1	TSS1500;TSS1500;TSS1500;TSS1500;TSS1500;TSS1500;TSS1500;5'UTR	-1.19608	-1.43133	-7.300561	2.34E-11	4.38E-09	15.497155
cg16162122	chr20	62090270	+	KCNQ2;KCNQ2;KCNQ2;KCNQ2;KCNQ2	Body;Body;Body;Body;Body	-1.495444	-0.631192	-7.300602	2.34E-11	4.38E-09	15.497366
cg03900143	chr3	147111660	+	ZIC4;ZIC4;ZIC4;ZIC4;ZIC4	Body;TSS1500;Body;Body;TSS1500	2.2630653	-0.235686	7.3004453	2.34E-11	4.38E-09	15.496562
cg25422797	chr17	73506046	-	CASKIN2;CASKIN2	Body;TSS200	1.1122859	1.6712578	7.2994716	2.35E-11	4.40E-09	15.491573
cg07632771	chr7	148611823	+			-1.395473	-0.479422	-7.298689	2.36E-11	4.42E-09	15.487561
cg03983270	chr16	81731249	-	CMIP;CMIP	Body;Body	1.4901167	2.0425402	7.2981611	2.37E-11	4.43E-09	15.484859
cg21871883	chr21	34145043	-	C21orf49;C21orf66;C21orf66;C21orf66;C21orf49	Body;TSS1500;TSS1500;TSS1500;Body	-1.387248	-3.000499	-7.297793	2.37E-11	4.44E-09	15.482976
cg02064267	chr10	112220471	+			-1.416346	-1.765871	-7.297612	2.37E-11	4.44E-09	15.482044
cg23147597	chr19	45174681	+	CEACAM19;CEACAM19	TSS200;TSS200	-2.03493	1.0513612	-7.297584	2.37E-11	4.44E-09	15.481903
cg09298313	chr14	55569959	-			-1.490343	-1.819458	-7.296974	2.38E-11	4.45E-09	15.478775
cg26537478	chr2	66668012	-	MEIS1	Body	1.5735887	0.0029562	7.2965597	2.39E-11	4.46E-09	15.476656
cg26717066	chr5	132581310	+	FSTL4	Body	-1.566218	0.4043157	-7.295711	2.40E-11	4.48E-09	15.472309
cg25985721	chr8	143645672	+			-1.307756	-1.080187	-7.295475	2.40E-11	4.48E-09	15.471099
cg04546186	chr16	86555150	-			-1.559157	-0.039707	-7.294968	2.41E-11	4.49E-09	15.468501
cg07867517	chr16	75520165	-	CHST6	5'UTR	-2.279685	0.5315147	-7.293464	2.43E-11	4.52E-09	15.460797
cg22989843	chr2	223154201	+	PAX3;PAX3;PAX3;PAX3;PAX3;PAX3	Body;Body;Body;Body;Body;Body	1.9480206	-0.975602	7.2921227	2.44E-11	4.55E-09	15.453929
cg09487931	chr17	27230297	+	DHRS13	TSS1500	-0.617043	-3.471077	-7.290729	2.46E-11	4.59E-09	15.446792

cg12544191	chr5	172671754	+			1.5736033	-0.189555	7.2955675	2.46E-11	4.59E-09	15.447142
cg06183338	chr5	178004204	+	COL23A1	Body	2.2862224	-0.134014	7.3005261	2.47E-11	4.59E-09	15.447751
cg26232945	chr2	208798601	+	PLEKHM3	Body	1.0901825	1.7063283	7.2894348	2.48E-11	4.61E-09	15.440164
cg22310062	chr18	19181025	+	ESCO1	TSS1500	-0.904484	-3.75641	-7.288823	2.49E-11	4.62E-09	15.437029
cg05287372	chr3	69058876	+	C3orf64	Body	1.2039885	1.905107	7.2871802	2.51E-11	4.66E-09	15.42862
cg23510807	chr7	2567893	+	LFNG;LFNG;LFNG;LFNG	3'UTR;3'UTR;3'UTR;Body	-0.967338	-0.883169	-7.287237	2.51E-11	4.66E-09	15.42891
cg06961429	chr14	101493361	-	MIR329-2	TSS200	-1.580505	-0.440192	-7.285967	2.52E-11	4.69E-09	15.422409
cg00495860	chr21	38065524	+			2.3675489	-0.431341	7.2841077	2.55E-11	4.73E-09	15.41289
cg25776343	chr10	72336097	+			-1.216152	-0.480897	-7.283659	2.56E-11	4.74E-09	15.410595
cg02317955	chr17	79099481	+	MIR657;AATK	TSS1500;Body	-1.84776	0.3831028	-7.282389	2.57E-11	4.77E-09	15.404091
cg22368204	chr20	62055252	-	KCNQ2;KCNQ2;KCNQ2;KCNQ2	Body;Body;Body;Body	-1.706622	-0.332432	-7.281593	2.58E-11	4.79E-09	15.400016
cg14359329	chr2	126350052	-			-1.632269	0.0315837	-7.281427	2.59E-11	4.79E-09	15.399167
cg26787199	chr16	2044042	-	SYNGR3	3'UTR	-0.873549	-2.411959	-7.280642	2.60E-11	4.81E-09	15.395149
cg24884142	chr1	119532056	+	TBX15;TBX15	1stExon;5'UTR	1.8316524	-0.088122	7.2802564	2.60E-11	4.82E-09	15.393178
cg05139788	chr6	50690647	-	TFAP2D	Body	1.791921	-0.259191	7.280122	2.60E-11	4.82E-09	15.39249
cg18094693	chr19	56599082	+	ZNF787	3'UTR	0.779367	2.4613933	7.2792137	2.62E-11	4.84E-09	15.387841
cg10364040	chr2	176981064	+	HOXD10	TSS1500	2.6411865	-0.263513	7.277396	2.64E-11	4.89E-09	15.378539
cg10481740	chr4	1857103	+	LETM1	Body	-0.724467	-3.209574	-7.277209	2.64E-11	4.89E-09	15.37758
cg05389236	chr6	36635087	-			-1.539593	-0.743382	-7.276709	2.65E-11	4.90E-09	15.375026
cg11809107	chr2	3826782	-			-1.695597	-1.413514	-7.276417	2.66E-11	4.91E-09	15.373532

cg05349159	chr1	247695328	+	OR2C3;LOC148824	Body;TSS1500	-1.59915	0.0057574	-7.275948	2.66E-11	4.92E-09	15.371132
cg25296222	chr11	2037173	-			-1.208542	-0.043763	-7.27571	2.67E-11	4.92E-09	15.369914
cg00522935	chr12	1964603	+	CACNA2D4	Body	-1.18829	-0.608398	-7.274881	2.68E-11	4.94E-09	15.365673
cg24973591	chr4	10079421	-	WDR1;WDR1	Body;Body	1.4833595	2.7158341	7.2746331	2.68E-11	4.94E-09	15.364402
cg10416668	chr16	27437730	+	IL21R;IL21R;IL21R	TSS1500;5'UTR;5'UTR	-1.573606	-0.411198	-7.274099	2.69E-11	4.96E-09	15.361669
cg19912142	chr10	119494671	-			2.5544958	-0.854828	7.2739391	2.69E-11	4.96E-09	15.360852
cg26885268	chr7	149389941	+			1.6519955	-0.270599	7.2738697	2.69E-11	4.96E-09	15.360497
cg08513547	chr5	150323558	+	LOC134466	Body	-1.750238	-0.159944	-7.27366	2.70E-11	4.96E-09	15.359425
cg05036656	chr4	41875470	-			1.8680687	-0.406587	7.2733789	2.70E-11	4.96E-09	15.357986
cg05821464	chr22	47057502	-	GRAMD4	Body	-1.225723	-0.44539	-7.273306	2.70E-11	4.96E-09	15.357611
cg15232971	chr10	61667009	-	CCDC6	TSS200	-0.917723	-2.041786	-7.273257	2.70E-11	4.96E-09	15.357363
cg21270243	chr2	216176248	-	ATIC	TSS1500	-2.198334	-3.119995	-7.356994	2.71E-11	4.98E-09	15.375233
cg10073091	chr1	55352301	-	DHCR24	Body	-0.651345	-3.3686	-7.272548	2.71E-11	4.98E-09	15.353734
cg18073240	chr15	40731970	-	BAHD1	TSS1500	-1.040009	-1.822236	-7.27256	2.71E-11	4.98E-09	15.353799
cg22657492	chr5	137593488	-	GFRA3	Body	1.4473602	2.2214225	7.2720096	2.72E-11	4.99E-09	15.350981
cg26509691	chr5	134363516	-	PITX1	3'UTR	1.5759435	-0.817647	7.2719	2.72E-11	4.99E-09	15.35042
cg22068093	chr5	171284818	+			0.9715645	1.1129389	7.271645	2.72E-11	4.99E-09	15.349116
cg04223606	chr1	10532886	-	DFFA;DFFA	TSS1500;TSS1500	-1.179424	-3.679651	-7.27131	2.73E-11	5.00E-09	15.347403
cg01282174	chr11	119630144	+			-1.679054	-1.1434	-7.270339	2.74E-11	5.03E-09	15.342433
cg10765397	chr8	1283671	+			-1.362338	-0.118277	-7.26984	2.75E-11	5.04E-09	15.339885

cg12027068	chr3	159176057	-	SCHIP1	Body	-1.418728	-1.299807	-7.274501	2.75E-11	5.04E-09	15.340577
cg19924619	chr1	165323692	-	LMX1A	Body	2.4836402	-1.048559	7.2691041	2.76E-11	5.05E-09	15.336119
cg01955533	chr6	36650733	-	CDKN1A;CDKN1A	5'UTR;5'UTR	-1.22136	-1.210677	-7.268304	2.77E-11	5.07E-09	15.332025
cg04085768	chr4	113333052	+	ALPK1;ALPK1	Body;Body	1.1960697	2.2227332	7.2683643	2.77E-11	5.07E-09	15.332335
cg19459804	chr20	32032443	+	SNTA1	TSS1500	-0.640791	-3.611118	-7.27178	2.79E-11	5.11E-09	15.325635
cg04003903	chr4	8601549	-	CPZ;CPZ;CPZ	5'UTR;Body;Body	-1.071139	-0.687727	-7.266321	2.80E-11	5.12E-09	15.321887
cg13784235	chr6	108495865	+	NR2E1	Body	1.9128742	-0.631584	7.2662408	2.80E-11	5.12E-09	15.321476
cg18266783	chr1	156466729	+	MEF2D	5'UTR	1.4634888	1.2119404	7.2710555	2.81E-11	5.12E-09	15.32199
cg04548336	chr17	75282102	-	9-Sep	Body	-1.459112	-0.984645	-7.269952	2.82E-11	5.15E-09	15.316307
cg24069602	chr17	62045522	-	SCN4A	Body	-1.095994	-0.169017	-7.264714	2.83E-11	5.15E-09	15.313668
cg24527881	chr1	45187630	-	C1orf228	Body	-1.379614	-2.247637	-7.264458	2.83E-11	5.16E-09	15.312361
cg23082657	chr19	15226044	+	ILVBL	3'UTR	1.1338093	0.72722	7.262325	2.86E-11	5.21E-09	15.301453
cg14440205	chr14	101487928	+	MIR379	TSS1500	-1.342292	-0.148339	-7.26196	2.87E-11	5.22E-09	15.299587
cg22465736	chr17	26926841	+	SPAG5	TSS1500	-1.196628	-3.904216	-7.261984	2.87E-11	5.22E-09	15.299711
cg04179940	chr20	33871836	+	EIF6;EIF6;EIF6	Body;Body;Body	-0.866322	-3.438889	-7.260387	2.89E-11	5.26E-09	15.291547
cg08621441	chr1	1267745	-	TAS1R3	Body	-0.759249	-0.155081	-7.26041	2.89E-11	5.26E-09	15.291663
cg05454961	chr16	62745680	+			-1.789851	0.4428182	-7.260057	2.90E-11	5.27E-09	15.28986
cg17250863	chr20	33451272	+	GGT7	Body	1.8811142	2.9719387	7.259586	2.90E-11	5.28E-09	15.287451
cg02488942	chr2	105480687	-			1.4338217	-0.449401	7.2588215	2.92E-11	5.30E-09	15.283543
cg18780603	chr2	235947655	-	SH3BP4	Body	0.9795116	2.3455213	7.2587257	2.92E-11	5.30E-09	15.283053

cg19523667	chr7	44795780	+	ZMIZ2;ZMIZ2	TSS200;5'UTR	1.0155969	0.7859237	7.2583202	2.92E-11	5.31E-09	15.280981
cg07778029	chr7	27205114	+	HOXA9;HOXA9	1stExon;5'UTR	1.9367375	-1.15094	7.2570952	2.94E-11	5.34E-09	15.274719
cg02877575	chr11	131411235	-	NTM	Body	-1.999433	-0.602471	-7.256693	2.95E-11	5.35E-09	15.272663
cg07440826	chr16	89882328	-	FANCA;FANCA	Body;Body	-1.057364	-3.131818	-7.26157	2.95E-11	5.35E-09	15.27359
cg14444710	chr16	2587080	+	PDPK1;PDPK1	TSS1500;TSS1500	-1.631987	0.26299	-7.25635	2.95E-11	5.35E-09	15.270911
cg11155489	chr17	32613298	+	CCL11	Body	-1.697608	-0.645103	-7.260945	2.96E-11	5.36E-09	15.2704
cg01505556	chr6	30174125	+	TRIM26	5'UTR	-1.030942	-0.147654	-7.255898	2.96E-11	5.36E-09	15.268602
cg06275642	chr2	68546038	+	CNRIP1;CNRIP1	Body;Body	1.218864	-0.402784	7.255012	2.97E-11	5.38E-09	15.264072
cg19583967	chr14	64805944	+	ESR2	TSS1500	-0.81697	-2.206008	-7.25403	2.99E-11	5.41E-09	15.259052
cg12589098	chr10	130860205	-			-2.044601	0.5888211	-7.253666	3.00E-11	5.42E-09	15.257192
cg11949335	chr1	55247408	+	TTC22	Body	1.3043562	2.2019496	7.2519247	3.02E-11	5.47E-09	15.248296
cg17297328	chr1	108743728	-	SLC25A24	TSS1500	-1.256879	-3.062089	-7.249667	3.06E-11	5.53E-09	15.236761
cg21711862	chr2	554363	+			-2.154368	0.1022716	-7.249695	3.06E-11	5.53E-09	15.236904
cg06521562	chr8	143740009	-	JRK;JRK	3'UTR;3'UTR	0.9543963	1.2732007	7.2489206	3.07E-11	5.55E-09	15.232947
cg07790085	chr13	29597447	-	MTUS2	TSS1500	-1.796447	0.380219	-7.248128	3.09E-11	5.57E-09	15.228898
cg15513620	chr1	120174873	-	ZNF697	5'UTR	1.2141321	-1.110038	7.2480069	3.09E-11	5.57E-09	15.22828
cg03078239	chr8	9040449	-			1.0105146	2.1726264	7.2478341	3.09E-11	5.57E-09	15.227397
cg09197075	chr16	11769691	-	SNN	5'UTR	-1.773267	0.2743917	-7.247521	3.10E-11	5.58E-09	15.225799
cg19619405	chr12	43945892	+	ADAMTS20	TSS200	2.7481753	-0.278442	7.2474846	3.10E-11	5.58E-09	15.225612
cg25594100	chr7	4786943	+	FOXK1	Body	2.3468029	2.2388683	7.2472277	3.10E-11	5.58E-09	15.224299

cg03422527	chr6	32154568	+	PBX2	Body	0.7240836	1.2123116	7.246966	3.10E-11	5.59E-09	15.222963
cg06191357	chr1	172714273	+			-1.68585	-3.78923	-7.24608	3.12E-11	5.61E-09	15.218436
cg17331950	chr1	181075666	-			0.9320384	1.0377502	7.2460488	3.12E-11	5.61E-09	15.218278
cg07366188	chr17	76220929	+	BIRC5;EPR1;BIRC5;BIRC5	3'UTR;TSS200;3'UTR;3'UTR	1.437708	2.157608	7.2457549	3.12E-11	5.62E-09	15.216776
cg20208398	chr7	43804002	-	BLVRA	5'UTR	-1.256147	-2.14727	-7.244713	3.14E-11	5.65E-09	15.211454
cg06659338	chr1	54562040	+	C1orf83	Body	1.0950371	0.911745	7.2443339	3.15E-11	5.65E-09	15.209519
cg20457796	chr10	50819500	-	SLC18A3;CHAT	1stExon;5'UTR	1.6270202	-0.889432	7.2481897	3.17E-11	5.68E-09	15.205358
cg10574843	chr11	1994902	+			-1.565597	0.7877765	-7.242862	3.17E-11	5.69E-09	15.202
cg16268214	chr12	49351537	-	ARF3	TSS1500	-0.657297	-3.645175	-7.242275	3.18E-11	5.71E-09	15.199004
cg14519125	chr3	49893811	-	TRAIP	1stExon	-0.621288	-4.226799	-7.242135	3.18E-11	5.71E-09	15.198287
cg13409704	chr22	22012495	-			-0.88457	-2.787525	-7.241605	3.19E-11	5.72E-09	15.195585
cg07325168	chr7	38949140	+	VPS41;VPS41	TSS1500;TSS1500	-1.253779	-2.064979	-7.240674	3.21E-11	5.75E-09	15.19083
cg23159250	chr11	134783504	-			-1.406562	-0.278731	-7.240721	3.21E-11	5.75E-09	15.191067
cg16928066	chr2	73147814	-	EMX1	Body	1.4721016	-0.256296	7.2404889	3.21E-11	5.75E-09	15.189883
cg15151059	chr1	150936875	-	SETDB1;SETDB1	3'UTR;3'UTR	1.1593305	1.3140284	7.2403752	3.21E-11	5.75E-09	15.189302
cg07416383	chr7	155302899	+	CNPY1	5'UTR	2.5368542	-1.940072	7.2386229	3.24E-11	5.80E-09	15.180355
cg07145683	chr12	53297443	-	KRT8	Body	-1.019637	-2.892496	-7.238327	3.25E-11	5.81E-09	15.178844
cg09127044	chr5	54519159	-			1.4456183	-0.93146	7.2380696	3.25E-11	5.82E-09	15.177531
cg10726660	chr10	118153200	+			-2.153508	1.6179976	-7.23737	3.27E-11	5.83E-09	15.173958
cg22677715	chr2	162284644	-			1.6021577	-0.211058	7.2371182	3.27E-11	5.84E-09	15.172674

cg16579438	chr3	24537301	-	THRB;THRB;THRB	TSS1500;TSS1500;TSS1500	-1.267847	-1.58927	-7.241642	3.28E-11	5.85E-09	15.171932
cg10423771	chr10	50604499	-			1.5634588	-0.550481	7.2362068	3.29E-11	5.86E-09	15.168021
cg10500084	chr5	174172044	+			-1.187311	-0.812184	-7.235904	3.29E-11	5.87E-09	15.166476
cg01342926	chr4	128887246	+	MFSD8;C4orf29	TSS200;5'UTR	-0.642286	-3.67393	-7.233428	3.33E-11	5.95E-09	15.153838
cg04274288	chr6	27648004	+			1.4485104	-0.688636	7.2333394	3.34E-11	5.95E-09	15.153384
cg10945079	chr2	162283856	+			1.63258	-1.117315	7.2329902	3.34E-11	5.95E-09	15.151602
cg12293095	chr3	132324571	-	ACAD11	Body	0.9486189	2.2234337	7.2328931	3.34E-11	5.95E-09	15.151106
cg06042156	chr7	55661735	+			-1.251843	-0.531889	-7.228119	3.43E-11	6.10E-09	15.126741
cg04339692	chr15	48666418	+			-2.52275	1.6702071	-7.23765	3.44E-11	6.11E-09	15.127507
cg12526997	chr2	242974096	+			-2.16799	-2.048081	-7.232505	3.44E-11	6.12E-09	15.125386
cg21692450	chr1	46940325	-			1.6485372	-0.429497	7.2272476	3.45E-11	6.13E-09	15.122296
cg00115458	chr6	33141305	-	COL11A2;COL11A2;COL11A2	Body;Body;Body	-1.861878	-1.686816	-7.231962	3.45E-11	6.13E-09	15.122677
cg22917314	chr8	1136982	-			-1.378328	-0.167228	-7.226606	3.46E-11	6.14E-09	15.119022
cg21618333	chr15	62359139	+	C2CD4A	TSS200	-0.584542	-3.742743	-7.22571	3.47E-11	6.17E-09	15.11445
cg25953239	chr2	176982342	-	HOXD10	Body	2.1298684	-0.035746	7.2256381	3.47E-11	6.17E-09	15.114085
cg21459713	chr8	623688	+	ERICH1	Body	1.0692986	2.2325234	7.2246888	3.49E-11	6.20E-09	15.109242
cg20449483	chr6	37139433	+	PIM1	Body	-0.664389	-4.079999	-7.224278	3.50E-11	6.21E-09	15.107146
cg14879645	chr22	41048474	+			-1.310782	0.3081553	-7.224082	3.50E-11	6.21E-09	15.106146
cg23913421	chr6	50674663	-			2.7976083	-0.018952	7.2289553	3.50E-11	6.21E-09	15.107368
cg10520594	chr17	73127197	-	NT5C	Body	-0.960457	-4.014465	-7.222994	3.52E-11	6.24E-09	15.100595

cg14375496	chr17	440392	-	VPS53;VPS53	Body;Body	1.0728127	1.3459332	7.2279631	3.52E-11	6.24E-09	15.102316
cg17518215	chr2	172973241	+			1.7753032	0.0474889	7.2278628	3.52E-11	6.24E-09	15.101746
cg01795955	chr2	200468626	+			1.1307631	0.5242228	7.2224973	3.53E-11	6.25E-09	15.098063
cg21415385	chr3	11604056	-	VGLL4;VGLL4;VGLL4;VGLL4	Body;Body;Body;Body	0.8062338	0.4205731	7.2216733	3.55E-11	6.27E-09	15.09386
cg16336751	chr5	35618055	-	SPEF2;SPEF2;SPEF2;SPEF2	5'UTR;5'UTR;1stExon;1stExon	-1.716465	-3.04061	-7.221496	3.55E-11	6.28E-09	15.092954
cg10843343	chr12	53298027	-	KRT8	Body	-1.07558	-0.453063	-7.220914	3.56E-11	6.29E-09	15.089986
cg17945323	chr11	62767406	-	SLC22A8	Body	-1.427655	-0.908778	-7.220618	3.57E-11	6.30E-09	15.08848
cg10407113	chr16	71843647	-	AP1G1;AP1G1	TSS1500;TSS1500	-1.039323	-1.886307	-7.220011	3.58E-11	6.32E-09	15.08538
cg09238180	chr2	176993632	+	HOXD8	TSS1500	2.894664	-0.996707	7.2198587	3.58E-11	6.32E-09	15.084605
cg10020373	chr2	63277944	+	OTX1	TSS200	-0.693224	-4.820151	-7.219292	3.59E-11	6.34E-09	15.081714
cg15219228	chr6	35479628	+	TULP1	Body	2.8650039	-0.212504	7.2831066	3.60E-11	6.35E-09	15.095726
cg26578156	chr16	49875061	+			-1.619854	-0.296669	-7.218362	3.61E-11	6.36E-09	15.07697
cg10399099	chr16	51169072	+			1.7687406	0.7859011	7.2180854	3.62E-11	6.37E-09	15.075562
cg21427956	chr20	30619244	+	C20orf160	3'UTR	-1.184627	-1.524831	-7.217372	3.63E-11	6.39E-09	15.071921
cg13058623	chr1	233040477	+			-1.984862	-2.29438	-7.216802	3.64E-11	6.41E-09	15.069019
cg25785639	chr6	33385083	+	CUTA;CUTA;CUTA;CUTA;CUTA	Body;Body;Body;Body;Body	-0.703356	-4.910378	-7.215689	3.66E-11	6.44E-09	15.063341
cg15698244	chr6	76073316	+	FILIP1	Body	-1.515845	-0.50718	-7.215531	3.67E-11	6.45E-09	15.062535
cg07803375	chr7	811206	+	HEATR2	Body	1.7106867	1.4722418	7.2136208	3.70E-11	6.51E-09	15.052797
cg20111980	chr4	6676796	-	LOC93622	Body	-1.357913	-2.047976	-7.213338	3.71E-11	6.52E-09	15.051356
cg13184448	chr2	238647857	-	LRRFIP1;LRRFIP1;LRRFIP1;LRRFIP1;LRRFIP1	Body;Body;Body;Body;Body	1.3466818	1.5570466	7.2125903	3.72E-11	6.54E-09	15.047544

cg20687616	chr1	56883736	-			1.3465542	2.1350867	7.2125181	3.72E-11	6.54E-09	15.047176
cg14103263	chr19	2727148	+			-1.886798	-4.404144	-7.217144	3.73E-11	6.55E-09	15.047248
cg11286405	chr15	25451255	+	SNORD115-15;SNORD115-20;SNORD115-21	TSS200;TSS200;TSS200	-0.844299	-0.451314	-7.212181	3.73E-11	6.55E-09	15.045455
cg01004382	chr2	192015996	+	STAT4	TSS200	-1.146365	-1.05736	-7.21153	3.74E-11	6.57E-09	15.04214
cg02531277	chr7	101558597	-	CUX1;CUX1;CUX1	Body;Body;Body	0.7535048	3.0225438	7.2114492	3.75E-11	6.57E-09	15.041726
cg03970899	chr22	19710091	+	GP1BB;SEPT5	TSS1500;3'UTR	1.394374	2.0031015	7.2112789	3.75E-11	6.57E-09	15.040859
cg15514918	chr1	236087201	-			-1.828762	-0.625714	-7.209902	3.78E-11	6.61E-09	15.033839
cg00854817	chr12	113013393	-			1.5358361	-1.307146	7.2081809	3.81E-11	6.67E-09	15.025068
cg08717751	chr13	37881008	+			-2.340741	-0.899803	-7.207566	3.82E-11	6.69E-09	15.021933
cg11993173	chr8	54857695	-	RGS20;RGS20	Body;Body	-1.551	-1.888171	-7.207452	3.83E-11	6.69E-09	15.021356
cg12938814	chr5	154253549	-	CNOT8	Body	1.3852731	2.4349609	7.2123745	3.82E-11	6.69E-09	15.022922
cg21711876	chr19	312214	+	MIER2	Body	1.1154974	1.8742116	7.2072038	3.83E-11	6.70E-09	15.020088
cg04798684	chr17	74309056	-	PRPSAP1	Body	0.9317447	1.0294254	7.2066106	3.84E-11	6.71E-09	15.017065
cg18177414	chr7	149389929	+			2.5313369	-0.531756	7.206659	3.84E-11	6.71E-09	15.017312
cg13177786	chr11	10472099	-	AMPD3	TSS200	-1.522336	-4.192837	-7.206147	3.85E-11	6.72E-09	15.014701
cg24683222	chr4	41880440	+			1.6760871	1.1372773	7.206171	3.85E-11	6.72E-09	15.014825
cg06065125	chr4	85402870	+			2.0176985	-0.906392	7.2108495	3.85E-11	6.73E-09	15.0168
cg03071808	chr2	242908131	+			-2.290064	-3.051788	-7.205847	3.86E-11	6.73E-09	15.013173
cg16465768	chr2	136287945	+	R3HDM1;ZRANB3	TSS1500;5'UTR	-0.775191	-4.033711	-7.203987	3.90E-11	6.79E-09	15.003695
cg03816707	chr20	43726544	-	KCNS1	Body	1.5020919	0.167853	7.2036433	3.90E-11	6.80E-09	15.001945

cg07154254	chr1	201979128	+	ELF3;ELF3	TSS1500;TSS1500	-0.922111	-2.407583	-7.202494	3.93E-11	6.84E-09	14.996089
cg19504702	chr5	153862394	-			2.7777721	-1.446145	7.2170568	3.93E-11	6.84E-09	15.000357
cg27596415	chr6	75440995	-			-1.530324	-2.054401	-7.201802	3.94E-11	6.86E-09	14.992563
cg18637486	chr17	79099777	-	MIR657;AATK;MIR338	TSS1500;Body;TSS200	-2.273234	1.1907667	-7.201347	3.95E-11	6.87E-09	14.990247
cg11966450	chr18	59992157	-	TNFRSF11A	TSS1500	-1.585181	-3.026189	-7.200284	3.97E-11	6.91E-09	14.984829
cg00050496	chr12	110211701	-	MGC14436;MGC14436	TSS1500;TSS1500	-1.378288	-2.069433	-7.19937	3.99E-11	6.94E-09	14.980174
cg02027945	chr11	20618230	-			3.5946013	-0.376532	7.1984466	4.01E-11	6.97E-09	14.975471
cg18698788	chr15	89952499	-			3.0800722	-0.623657	7.1979112	4.02E-11	6.99E-09	14.972744
cg11114141	chr2	206633633	+	NRP2;NRP2;NRP2;NRP2;NRP2	Body;Body;Body;Body;Body	-1.59581	-0.165905	-7.196273	4.06E-11	7.05E-09	14.964402
cg10836101	chr19	30020886	+	VSTM2B	Body	2.0673243	-0.836663	7.1998993	4.08E-11	7.09E-09	14.9613
cg16697850	chr7	141946928	+	MOXD2	TSS200	-1.16864	-1.175491	-7.193778	4.11E-11	7.13E-09	14.951692
cg27212428	chr4	22031559	+			-1.415971	-0.079271	-7.192435	4.14E-11	7.18E-09	14.944856
cg19871597	chr13	59592287	+			-1.943089	-0.217815	-7.191803	4.15E-11	7.20E-09	14.94164
cg14265823	chr2	223163326	+	PAX3;PAX3;CCDC140;PAX3;PAX3;PAX3;PAX3;PAX3;PAX3	1stExon;1stExon;5'UTR;1stExon;1stExon;1stExon;1stExon;1stExon;1stExon	1.6961408	-0.547273	7.1916321	4.16E-11	7.21E-09	14.940768
cg05191839	chr13	50819265	-			-1.611167	-1.396933	-7.191193	4.17E-11	7.22E-09	14.938531
cg12268637	chr2	176964502	-	HOXD12	TSS200	1.3646782	-0.293865	7.1894106	4.21E-11	7.28E-09	14.929458
cg14526874	chr4	17348908	+			-1.813327	0.3762631	-7.189431	4.21E-11	7.28E-09	14.929561
cg13413955	chr9	842914	+	DMRT1	Body	1.7591143	0.0790987	7.1890989	4.21E-11	7.29E-09	14.927871
cg03295274	chr13	50190584	-			1.3433228	1.3337983	7.1889605	4.22E-11	7.29E-09	14.927166
cg00963378	chr13	20716638	-	GJA3	Body	2.0447801	-0.13852	7.1886987	4.22E-11	7.30E-09	14.925833

cg12082609	chr2	66671727	-	MEIS1	Body	1.4223332	0.1106032	7.1931447	4.23E-11	7.31E-09	14.925222
cg11328436	chr12	54360511	+	HOTAIR	Body	1.5791772	-0.365289	7.1876859	4.25E-11	7.33E-09	14.920678
cg19250113	chr17	29298832	-	RNF135;RNF135	Body;Body	-1.902678	0.131272	-7.187362	4.25E-11	7.34E-09	14.919031
cg10318258	chr14	24809759	-	RIPK3	TSS1500	-1.287086	-0.377667	-7.186109	4.28E-11	7.39E-09	14.912652
cg03731202	chr1	36574795	-			-1.446579	-1.574549	-7.185191	4.30E-11	7.42E-09	14.907981
cg04510420	chr16	88102758	-	BANP;BANP	Body;Body	1.4502458	4.7792021	7.1847736	4.31E-11	7.43E-09	14.905854
cg24539848	chr2	241458974	+	ANKMY1;ANKMY1	Body;Body	1.2369143	0.7115268	7.1847694	4.31E-11	7.43E-09	14.905833
cg08482531	chr10	8078127	-			1.3743445	-0.851136	7.1843276	4.32E-11	7.45E-09	14.903584
cg04674428	chr16	58956301	-			-2.018611	0.4629272	-7.182784	4.36E-11	7.50E-09	14.895727
cg03130910	chr1	234908226	+			-1.743971	-3.874304	-7.181746	4.38E-11	7.54E-09	14.890448
cg03301058	chr6	90007856	-	GABRR2	Body	-2.472794	-2.381068	-7.181693	4.38E-11	7.54E-09	14.890178
cg12547166	chr4	206877	-	ZNF876P	Body	1.461873	-0.435556	7.1814555	4.39E-11	7.55E-09	14.888969
cg11335171	chr6	33173333	+	HSD17B8	Body	-0.934177	-1.095356	-7.180569	4.41E-11	7.58E-09	14.88446
cg12215478	chr6	35887710	+	SRPK1	Body	-0.899335	-4.209285	-7.180316	4.41E-11	7.58E-09	14.88317
cg15012282	chr3	152059421	-	TMEM14E;MBNL1;MBNL1;MBNL1;MBNL1;MBNL1;MBNL1;MBNL1	TSS1500;Body;Body;Body;Body;Body;Body;Body	1.5817158	1.3212194	7.1803792	4.41E-11	7.58E-09	14.883492
cg25630588	chr3	50357322	+	HYAL2;HYAL2	Body;Body	1.292633	1.7094361	7.1797188	4.43E-11	7.61E-09	14.880132
cg12910906	chr2	131673894	+	ARHGEF4;ARHGEF4	TSS1500;TSS1500	1.2084122	-0.686879	7.1788438	4.45E-11	7.64E-09	14.87568
cg08143349	chr7	27180888	+			1.4685201	1.7231939	7.1787232	4.45E-11	7.64E-09	14.875066
cg03081173	chr6	31166502	+	HCG27	Body	-2.186118	-0.255466	-7.178286	4.46E-11	7.65E-09	14.872842
cg04927931	chr6	100911701	+	SIM1	TSS200	2.9732486	-0.408964	7.1782195	4.46E-11	7.65E-09	14.872504

cg11365072	chr5	154392274	+	KIF4B	TSS1500	-1.355884	-0.507995	-7.178104	4.47E-11	7.66E-09	14.871915
cg22797031	chr1	170630070	+			2.5283865	0.7697501	7.1778643	4.47E-11	7.66E-09	14.870697
cg14461974	chr2	3318039	-	TSSC1	Body	1.4011203	2.7455821	7.1772935	4.49E-11	7.68E-09	14.867793
cg13079949	chr19	3763242	-	MRPL54	Body	-1.083799	-3.817788	-7.17721	4.49E-11	7.68E-09	14.867367
cg00116092	chr1	217313044	-			1.6766918	-0.280177	7.1763907	4.51E-11	7.71E-09	14.8632
cg27041304	chr5	141693865	-	SPRY4;SPRY4	Body;Body	1.394551	3.6655982	7.179785	4.54E-11	7.77E-09	14.857298
cg09209510	chr14	100288389	+	EML1;EML1	Body;Body	-1.752597	-1.452423	-7.174765	4.55E-11	7.77E-09	14.854933
cg14679444	chr22	50470101	+	TTLL8	Body	-2.143065	0.8639036	-7.172864	4.59E-11	7.85E-09	14.845261
cg14429436	chr13	48876603	-	RB1	TSS1500	0.7945323	2.0996474	7.1719666	4.61E-11	7.88E-09	14.840698
cg02650767	chr1	247615210	+	OR2B11	1stExon	-1.286266	-0.978597	-7.171275	4.63E-11	7.91E-09	14.83718
cg03237166	chr9	38393112	-	ALDH1B1	5'UTR	-1.072554	-2.94832	-7.17063	4.65E-11	7.93E-09	14.833903
cg03035704	chr12	52301970	-	ACVRL1	5'UTR	1.0390453	1.084206	7.1693779	4.68E-11	7.98E-09	14.827534
cg07539798	chr5	140811312	+	PCDHGA4;PCDHGA11;PCDHGA11;PCDHGA9;PCDHGA1;PCDHGB1;PCDHGB6;PCDHGA12;PCDHGB3;PCDHGB7;PCDHGA6;PCDHGA8;PCDHGA10;PCDHGA12;PCDHGA5;PCDHGB4;PCDHGA	Body;Body;Body;Body;Body;Body;Body;1stExon;Body;Body;Body;Body;1stExon;Body;Body;Body;Body;Body;Body;Body	1.1504	0.5296735	7.1691889	4.68E-11	7.98E-09	14.826573
cg16860392	chr6	112005207	-	FYN;FYN;FYN	Body;Body;Body	1.2988169	1.7559377	7.1692736	4.68E-11	7.98E-09	14.827004
cg22158650	chr7	27291388	+			2.3984449	-0.499382	7.1691975	4.68E-11	7.98E-09	14.826617
cg01525538	chr1	228785987	-	DUSP5P	Body	1.3168886	0.4641451	7.1689964	4.69E-11	7.98E-09	14.825594
cg10258385	chr11	17410380	+	KCNJ11;KCNJ11	TSS200;5'UTR	-0.764811	-2.434711	-7.169033	4.68E-11	7.98E-09	14.825778
cg05369857	chr14	101923702	-			2.8154922	-0.929988	7.1731701	4.70E-11	8.00E-09	14.825681
cg13644317	chr18	70536461	-			1.7419711	-0.482779	7.1683577	4.70E-11	8.00E-09	14.822347
cg08766762	chr6	37521698	-			-1.739006	-0.534764	-7.168239	4.70E-11	8.00E-09	14.821743

cg23633856	chr1	170636363	-	PRRX1;PRRX1	Body;Body	1.3324381	0.2455866	7.1681809	4.71E-11	8.00E-09	14.821448
cg12743031	chr8	27219512	-	PTK2B;PTK2B;PTK2B;PTK2B	5'UTR;5'UTR;5'UTR;5'UTR	-1.106232	-0.618054	-7.167948	4.71E-11	8.01E-09	14.820265
cg00455386	chr7	140104086	+	RAB19	5'UTR	-1.082617	-1.87125	-7.17229	4.72E-11	8.02E-09	14.819255
cg05501996	chr5	50673730	-			1.7103647	-0.13265	7.1675309	4.72E-11	8.02E-09	14.818143
cg12564012	chr7	3989045	-	SDK1	Body	1.1169668	1.0772284	7.1659902	4.76E-11	8.08E-09	14.81031
cg22152446	chr4	48754953	+	FRYL	5'UTR	1.1224068	1.2854042	7.1658255	4.76E-11	8.09E-09	14.809473
cg20530056	chr1	206643377	+	IKBKE	TSS1500	-1.039549	-0.863918	-7.163675	4.82E-11	8.18E-09	14.798543
cg18747039	chr13	100650031	-			1.2085007	-0.861165	7.1615609	4.87E-11	8.26E-09	14.787796
cg11207307	chr12	8025593	-	SLC2A14	TSS200	1.546922	0.203651	7.1601451	4.91E-11	8.32E-09	14.7806
cg17107572	chr2	66667590	+	MEIS1	Body	1.9573236	-0.786542	7.1596739	4.92E-11	8.34E-09	14.778206
cg26516532	chr1	17475832	+			1.2687687	0.3168754	7.1594334	4.93E-11	8.35E-09	14.776984
cg26859016	chr1	206643375	-	IKBKE	TSS1500	-0.933558	-0.938858	-7.159059	4.94E-11	8.36E-09	14.775083
cg13830081	chr6	10404363	-	TFAP2A;TFAP2A;TFAP2A	Body;Body;Body	-0.774796	-4.062294	-7.157503	4.98E-11	8.43E-09	14.767175
cg01503516	chr15	85202406	-	NMB;NMB	TSS1500;TSS1500	-1.218398	-0.454177	-7.156625	5.00E-11	8.46E-09	14.762714
cg25388528	chr21	31987978	+	KRTAP20-1	TSS1500	-1.667106	-0.243114	-7.155861	5.02E-11	8.49E-09	14.758829
cg08363345	chr7	4752063	+	FOXK1	Body	-1.982642	-0.81798	-7.154315	5.06E-11	8.56E-09	14.750976
cg17183362	chr6	31804159	-	C6orf48;C6orf48;SNORD52	5'UTR;5'UTR;TSS1500	-0.769089	-3.073173	-7.153882	5.07E-11	8.57E-09	14.748775
cg06700275	chr17	77041801	+	C1QTNF1;C1QTNF1;C1QTNF1	5'UTR;Body;Body	-1.130816	-0.928554	-7.152623	5.11E-11	8.63E-09	14.742383
cg25720804	chr5	170736389	+	TLX3	1stExon	2.3673313	-0.47801	7.1519486	5.13E-11	8.66E-09	14.738956
cg15961744	chr5	140228182	-	PCDHA6;PCDHA2;PCDHA1;PCDHA7;PCDHA1;PCDHA6;PCDHA5;PCDHA9;PCDHA9;PCDHA3;PCDHA4;PCDHA8	Body;Body;Body;Body;Body;Body;Body;1stExon;1stExon;Body;Body;Body	1.2092009	-0.067026	7.1513421	5.14E-11	8.68E-09	14.735876

cg07791011	chr3	62363922	-			1.5880995	0.0534076	7.1511836	5.15E-11	8.68E-09	14.73507
cg24005685	chr4	111549880	-	PITX2;PITX2	Body;Body	1.7727548	-0.836203	7.1500052	5.18E-11	8.73E-09	14.729085
cg09719124	chr3	8570902	-	LMCD1	Body	1.2698294	1.2665865	7.1493964	5.19E-11	8.76E-09	14.725993
cg04943065	chr16	58896860	+			-1.710595	-0.077633	-7.149079	5.20E-11	8.77E-09	14.724383
cg15054725	chr2	223176606	-			2.4241868	-0.598679	7.1490641	5.20E-11	8.77E-09	14.724306
cg01558241	chr9	99637671	-			-1.149297	-2.627562	-7.14889	5.21E-11	8.77E-09	14.723423
cg00160027	chr16	49583602	-	ZNF423	Body	-1.000304	0.9369081	-7.148357	5.22E-11	8.79E-09	14.720717
cg00480389	chr20	54580133	+	CBLN4	TSS200	1.6879982	-1.027055	7.1484154	5.22E-11	8.79E-09	14.721011
cg14996220	chr12	85673270	+	ALX1	TSS1500	2.8557457	-1.966242	7.1520669	5.25E-11	8.83E-09	14.716682
cg20055426	chr12	127211421	-			1.6928874	-0.672416	7.1473033	5.25E-11	8.83E-09	14.715364
cg26209169	chr5	1316264	-			-1.33783	-2.216163	-7.146961	5.26E-11	8.84E-09	14.713623
cg11366178	chr4	54457737	+	LNX1	TSS200	-1.265509	-1.408469	-7.14674	5.27E-11	8.85E-09	14.712502
cg01656853	chr19	49199172	+	FUT2;FUT2	TSS200;TSS200	-0.961598	-1.562675	-7.146231	5.28E-11	8.87E-09	14.709918
cg06756164	chr1	109490978	+	CLCC1;CLCC1	Body;Body	1.5361622	0.3394437	7.1461369	5.28E-11	8.87E-09	14.709441
cg19214408	chr10	111981687	-	MXI1;MXI1	Body;5'UTR	-1.21049	-3.495499	-7.145473	5.30E-11	8.90E-09	14.706072
cg07886914	chr8	28198639	-	PNOC	3'UTR	-1.475207	0.2572504	-7.144979	5.32E-11	8.92E-09	14.703563
cg11970252	chr13	42188629	-	KIAA0564	Body	1.5509752	0.5714322	7.1435913	5.36E-11	8.98E-09	14.696516
cg11356950	chr4	1219707	+	CTBP1;CTBP1	Body;Body	0.7506242	2.3480255	7.1410572	5.43E-11	9.10E-09	14.683652
cg10996039	chr7	27178861	+			1.555622	0.6976072	7.1409616	5.43E-11	9.10E-09	14.683166
cg21063722	chr6	100911709	+	SIM1	TSS200	2.9683801	-0.267376	7.1497496	5.45E-11	9.13E-09	14.684126

cg24501177	chr15	70726696	+			1.2569732	1.81595	7.1402309	5.45E-11	9.13E-09	14.679458
cg05241025	chr22	24237256	+	MIF	Body	-0.573648	-4.047528	-7.139689	5.47E-11	9.15E-09	14.676705
cg13816466	chr9	35909466	+	LOC158376	TSS200	1.0454377	0.4184059	7.1391567	5.48E-11	9.17E-09	14.674005
cg04348265	chr4	186733060	+	SORBS2;SORBS2;SORBS2;SORBS2;SORBS2;SORBS2;SORBS2	5'UTR;5'UTR;5'UTR;1stExon;TSS1500;1stExon;TSS1500	-1.241428	-1.763487	-7.138249	5.51E-11	9.21E-09	14.669399
cg27543230	chr5	133562604	+	PPP2CA	TSS1500	-0.685323	-3.607433	-7.137846	5.52E-11	9.23E-09	14.667353
cg16031528	chr8	102449857	+			-1.502956	-0.021939	-7.137222	5.54E-11	9.25E-09	14.664185
cg16659728	chr11	68748506	+	MRGPRD	TSS200	-1.583207	-0.05918	-7.137209	5.54E-11	9.25E-09	14.664121
cg14427009	chr13	58207058	-	PCDH17	1stExon	1.3458228	-0.228701	7.1346015	5.62E-11	9.38E-09	14.650888
cg22076311	chr3	71180052	+	FOXP1	Body	-1.597924	-2.673844	-7.134114	5.63E-11	9.40E-09	14.648414
cg08690859	chr2	200333729	+	FLJ32063	Body	1.5029534	-0.438684	7.1335646	5.65E-11	9.42E-09	14.645627
cg11998307	chr17	1549434	+	SCARF1;SCARF1;SCARF1;SCARF1;SCARF1	TSS1500;TSS1500;TSS1500;TSS1500;TSS1500	0.8838215	1.4333363	7.1333615	5.65E-11	9.43E-09	14.644596
cg22825360	chr7	111866277	-	ZNF277	Body	1.0292363	2.7647597	7.132952	5.66E-11	9.44E-09	14.642519
cg26752663	chr2	25142016	+	ADCY3;ADCY3	5'UTR;1stExon	-1.363958	-0.716349	-7.137498	5.67E-11	9.45E-09	14.642946
cg04458776	chr11	62753944	-	SLC22A6;SLC22A6;SLC22A6;SLC22A6	TSS1500;TSS1500;TSS1500;TSS1500	-1.677745	-0.58405	-7.132304	5.68E-11	9.47E-09	14.639232
cg00790835	chr14	73029443	-	RGS6	3'UTR	-1.655216	0.1438533	-7.131871	5.70E-11	9.48E-09	14.637036
cg19320820	chr4	87966326	+	AFF1;AFF1	Body;Body	2.0800494	0.3492705	7.1413794	5.70E-11	9.48E-09	14.639562
cg12447832	chr2	3383257	-	TTC15	TSS200	-1.090743	-1.278767	-7.131564	5.71E-11	9.49E-09	14.635478
cg17863076	chr2	79312664	-	REG1B	Body	-1.227642	-0.969199	-7.130493	5.74E-11	9.54E-09	14.630044
cg04742334	chr20	37850248	-	LOC339568	Body	-1.655232	-0.481399	-7.130409	5.74E-11	9.54E-09	14.629616
cg24699146	chr1	24152579	-	HMGCL;HMGCL	TSS1500;TSS1500	-1.792378	-0.569938	-7.129776	5.76E-11	9.57E-09	14.626407

cg12290311	chr5	39424119	+	DAB2	5'UTR	1.6648688	0.8302608	7.1296102	5.76E-11	9.57E-09	14.625565
cg18413830	chr17	81031536	-			-1.598022	-0.388563	-7.164274	5.76E-11	9.57E-09	14.634654
cg21836903	chr3	71180071	+	FOXP1	Body	-1.683505	-2.727465	-7.133911	5.78E-11	9.59E-09	14.624705
cg05442717	chr17	38511950	-	RARA;RARA;RARA;RARA	Body;Body;Body;Body	0.9197975	0.7879837	7.1288449	5.79E-11	9.60E-09	14.621683
cg04452311	chr2	176936377	-			1.3077978	-0.73953	7.1284432	5.80E-11	9.62E-09	14.619646
cg00806704	chr13	28539042	+	CDX2	Body	1.7679886	0.009907	7.1281248	5.81E-11	9.63E-09	14.618031
cg00187686	chr11	59634218	-	TCN1	TSS200	-1.641588	-0.535961	-7.127769	5.82E-11	9.64E-09	14.616229
cg26231761	chr1	4744796	-	AJAP1;AJAP1	Body;Body	-1.480426	-0.34479	-7.12782	5.82E-11	9.64E-09	14.616484
cg17758721	chr20	2451208	+	SNRPB;SNRPB	Body;Body	-0.685347	-3.457206	-7.127562	5.83E-11	9.65E-09	14.615174
cg19328711	chr11	63655895	-	MARK2;MARK2;MARK2;MARK2;MARK2	Body;TSS200;Body;Body;Body	-0.955593	-3.194878	-7.127207	5.84E-11	9.67E-09	14.613374
cg03763769	chr1	197744884	+	DENND1B;DENND1B	TSS1500;TSS1500	-0.495944	-3.046537	-7.126666	5.85E-11	9.69E-09	14.610629
cg16473797	chr20	61755045	-			-1.313366	0.1274969	-7.126554	5.86E-11	9.69E-09	14.610063
cg06223018	chr3	49557076	-	DAG1;DAG1	Body;Body	1.0976299	2.0983543	7.1261815	5.87E-11	9.71E-09	14.608175
cg05516004	chr21	46496180	-	ADARB1;ADARB1;ADARB1;ADARB1;ADARB1;ADARB1;ADARB1	5'UTR;5'UTR;5'UTR;Body;Body;5'UTR;Body	0.8275341	1.7934581	7.125866	5.88E-11	9.71E-09	14.606575
cg10062919	chr17	38503802	+	RARA;RARA;RARA;RARA	Body;Body;Body;Body	0.9793072	-0.771233	7.1260031	5.87E-11	9.71E-09	14.60727
cg19814400	chr7	158936632	+	VIPR2	Body	3.4784357	-2.764005	7.1259319	5.88E-11	9.71E-09	14.606909
cg24227481	chr7	73417185	+			-1.588975	-2.594139	-7.130631	5.88E-11	9.71E-09	14.608174
cg13788117	chr19	35324528	+			-1.425219	-0.992599	-7.125572	5.89E-11	9.72E-09	14.605085
cg26531231	chr4	40336255	-	CHRNA9	TSS1500	-2.087002	0.3851073	-7.125272	5.90E-11	9.73E-09	14.603564
cg07719617	chr2	239169696	+	PER2	Body	1.1806399	1.1113801	7.1246348	5.92E-11	9.76E-09	14.600331

cg11601252	chr15	68122139	-	LBXCOR1	Body	3.5589239	-1.588835	7.1237475	5.94E-11	9.80E-09	14.595832
cg00785482	chr1	208058986	-			1.1502866	1.1094872	7.1230341	5.97E-11	9.83E-09	14.592214
cg19603100	chr14	101489985	-	MIR299;MIR380	TSS200;TSS1500	-1.551107	0.3341737	-7.122967	5.97E-11	9.83E-09	14.591876
cg15931949	chr3	138662315	-			1.9333449	-0.185875	7.1223413	5.99E-11	9.86E-09	14.588702
cg01090433	chr16	82673506	+	CDH13	Body	-1.307855	-2.265448	-7.121781	6.01E-11	9.89E-09	14.585861
cg03184472	chr2	39174738	-	LOC100271715	Body	-2.157665	0.4030306	-7.12131	6.02E-11	9.91E-09	14.58347
cg23819092	chr1	156047143	+	MEX3A	Body	0.979991	1.4501534	7.1208418	6.04E-11	9.93E-09	14.581099
cg07283896	chr6	11855283	+			-1.637228	-2.926016	-7.119799	6.07E-11	9.98E-09	14.575814
cg04885881	chr1	11123118	+			-1.264337	-1.350261	-7.118025	6.13E-11	1.01E-08	14.566818
cg06883496	chr13	28503235	-			1.601573	-0.451825	7.1181176	6.12E-11	1.01E-08	14.567288
cg19111287	chr4	3372915	-	RGS12;RGS12;RGS12	Body;Body;Body	1.5318092	1.179677	7.1180146	6.13E-11	1.01E-08	14.566766
cg25763542	chr19	310512	-	MIER2	Body	1.2798295	1.7511346	7.1178994	6.13E-11	1.01E-08	14.566182
cg00430895	chr18	13136340	+			-1.215873	-2.301918	-7.117779	6.13E-11	1.01E-08	14.565573
cg17410660	chr11	124806508	-	HEPACAM	TSS1500	-1.459777	-0.440324	-7.117738	6.14E-11	1.01E-08	14.565362
cg09601175	chr2	177025608	+			1.4117869	-0.880475	7.1175683	6.14E-11	1.01E-08	14.564504
cg08390877	chr2	119615998	+			1.7032784	-0.957657	7.1167806	6.17E-11	1.01E-08	14.560511
cg15068180	chr3	194017703	+			1.270605	1.9021047	7.1157909	6.20E-11	1.02E-08	14.555495
cg23928726	chr1	2344998	+	PEX10;PEX10	TSS1500;TSS1500	-1.092445	-1.330638	-7.115478	6.21E-11	1.02E-08	14.553907
cg16419054	chr14	105804208	-	PACS2;PACS2	Body;Body	0.8442611	0.9695489	7.1149358	6.23E-11	1.02E-08	14.551161
cg25416125	chr12	56329615	-	DGKA;DGKA;DGKA;DGKA	5'UTR;5'UTR;5'UTR;5'UTR	1.0643106	0.4922954	7.1139832	6.26E-11	1.02E-08	14.546334

cg04026926	chr11	2323395	+	TSPAN32;C11orf21;C11orf21	1stExon;TSS1500;TSS1500	-1.136355	-2.182474	-7.113275	6.28E-11	1.03E-08	14.542745
cg02052915	chr17	46797568	+			1.2938847	0.4340031	7.1125871	6.30E-11	1.03E-08	14.539259
cg07966265	chr1	10733477	-	CASZ1;CASZ1	Body;Body	1.031434	2.5527189	7.1173245	6.30E-11	1.03E-08	14.540762
cg19828416	chr17	56232302	+	OR4D1	TSS1500	-1.837432	0.6386369	-7.11227	6.31E-11	1.03E-08	14.537653
cg12861170	chr19	19639553	-	YJEFN3	TSS200	1.0711253	0.8667414	7.1118219	6.33E-11	1.03E-08	14.535381
cg19107469	chr20	55200445	+			1.5809587	-0.947523	7.111524	6.34E-11	1.04E-08	14.533872
cg09992746	chr15	60285821	+			1.3107281	1.0022848	7.1111053	6.35E-11	1.04E-08	14.53175
cg17501395	chr6	149806331	-	ZC3H12D	TSS200	-1.837876	-2.104707	-7.110898	6.36E-11	1.04E-08	14.530698
cg01350680	chr10	124903314	+			1.4188401	-0.667184	7.1154567	6.36E-11	1.04E-08	14.531396
cg07984133	chr13	28544592	-	CDX2	TSS1500	2.3446724	-0.778478	7.1104776	6.37E-11	1.04E-08	14.52857
cg03111498	chr20	25062860	-	VSX1;VSX1	TSS200;TSS200	1.9953704	-1.212698	7.1094915	6.41E-11	1.04E-08	14.523574
cg20777829	chr12	116549969	-	MED13L	Body	1.1986314	0.4797187	7.1094681	6.41E-11	1.04E-08	14.523455
cg21299542	chr4	111550407	+	PITX2;PITX2	Body;Body	1.4671911	-0.876826	7.1094628	6.41E-11	1.04E-08	14.523428
cg12637615	chr15	57332446	+	TCF12;TCF12;TCF12;TCF12	Body;Body;Body;Body	0.97894	2.4712355	7.1092352	6.42E-11	1.04E-08	14.522275
cg13897627	chr16	49378497	+			-1.481539	0.0409407	-7.118313	6.43E-11	1.05E-08	14.522967
cg08402365	chr2	172945274	-	MAP1D	3'UTR	2.4137243	-0.003864	7.1086811	6.43E-11	1.05E-08	14.519468
cg00956199	chr10	8078325	+			1.9363383	-0.557067	7.1073597	6.48E-11	1.05E-08	14.512774
cg20184732	chr9	100565953	-			-0.463341	-3.515104	-7.107289	6.48E-11	1.05E-08	14.512414
cg07375150	chr3	151046450	-	P2RY13;MED12L	Body;Body	-1.766764	-1.927957	-7.106731	6.50E-11	1.06E-08	14.509591
cg22396555	chr13	28502461	-			2.0051911	-0.212888	7.1060032	6.53E-11	1.06E-08	14.505903

cg07904475	chr4	151500931	+	LRBA	Body	1.217359	1.6402358	7.1056665	6.54E-11	1.06E-08	14.504197
cg23019717	chr6	43603423	-	MAD2L1BP;MAD2L1BP	TSS200;Body	-0.763457	-4.649734	-7.105322	6.55E-11	1.06E-08	14.502451
cg02531437	chr10	120355260	+	PRLHR	TSS200	1.4464795	-0.764882	7.1050425	6.56E-11	1.06E-08	14.501037
cg02886591	chr17	45773570	-	TBKBP1	1stExon	0.9775265	1.3065916	7.1050464	6.56E-11	1.06E-08	14.501056
cg22041712	chr1	214834360	+	CENPF	Body	1.1151106	1.767336	7.105128	6.56E-11	1.06E-08	14.50147
cg06772221	chr8	134062192	-	SLA;TG;SLA;SLA	Body;Body;Body;Body	-1.483986	-0.594925	-7.104484	6.58E-11	1.07E-08	14.498207
cg16578387	chr11	33744043	+	CD59;CD59;CD59;CD59;CD59;CD59;CD59;CD59;CD59	5'UTR;1stExon;5'UTR;5'UTR;5'UTR;5'UTR;5'UTR;5'UTR;5'UTR	0.9627241	1.6597926	7.1041841	6.59E-11	1.07E-08	14.496689
cg21812277	chr13	25085776	+	PARP4	5'UTR	-1.402071	-3.95823	-7.10414	6.59E-11	1.07E-08	14.496467
cg02662828	chr4	48492848	+	ZAR1	1stExon	0.8867096	0.6519907	7.1026498	6.64E-11	1.08E-08	14.488919
cg09016610	chr1	246203591	+	SMYD3;SMYD3	Body;Body	1.0727988	1.6074256	7.1072493	6.64E-11	1.08E-08	14.489898
cg06655810	chr7	27260102	+			1.3616035	-0.424191	7.1021789	6.66E-11	1.08E-08	14.486534
cg01629435	chr2	74153317	-	DGUOK;DGUOK	TSS1500;TSS1500	-2.109538	0.4605612	-7.101966	6.67E-11	1.08E-08	14.485455
cg16926102	chr10	23489850	+			1.0982701	-0.002911	7.1008576	6.70E-11	1.08E-08	14.479843
cg21195256	chr14	29244278	-	C14orf23;C14orf23	Body;Body	2.3517172	-0.109525	7.1005214	6.72E-11	1.09E-08	14.478141
cg14225678	chr7	54612325	-	VSTM2A	Body	2.4280029	-1.613876	7.1001708	6.73E-11	1.09E-08	14.476365
cg10782218	chr17	61518489	+	CYB561;CYB561;CYB561	TSS1500;5'UTR;5'UTR	0.7139558	2.221467	7.0993072	6.76E-11	1.09E-08	14.471992
cg11042505	chr2	15950244	+			-1.1098	-0.180783	-7.099079	6.77E-11	1.09E-08	14.470837
cg22795586	chr1	170629555	-			2.0036228	0.6445809	7.0989274	6.77E-11	1.09E-08	14.47007
cg20443254	chr12	108169020	-	ASCL4	1stExon	2.9488573	-0.602016	7.098706	6.78E-11	1.09E-08	14.468949
cg02286091	chr5	78407678	+	BHMT;BHMT	5'UTR;1stExon	2.0079712	-0.216633	7.1079316	6.79E-11	1.09E-08	14.470663

cg18908419	chr10	75800004	-	VCL;VCL	Body;Body	1.4942905	0.4250032	7.0983308	6.79E-11	1.10E-08	14.467049
cg16462648	chr5	55385250	+			-1.868226	-0.13043	-7.09816	6.80E-11	1.10E-08	14.466184
cg03405909	chr11	31845639	-			1.9447486	0.2780264	7.1025877	6.81E-11	1.10E-08	14.466337
cg07027613	chr12	7260608	-	C1RL;LOC283314	Body;TSS1500	-1.330842	-2.641116	-7.097982	6.81E-11	1.10E-08	14.465284
cg03846249	chr12	130621750	+			-1.380819	-2.100196	-7.097382	6.83E-11	1.10E-08	14.462247
cg01882866	chr14	103686900	-			1.7813506	-1.171544	7.096165	6.87E-11	1.11E-08	14.456085
cg22215392	chr3	12973168	+	IQSEC1;IQSEC1	Body;Body	0.7408645	1.1241131	7.0958736	6.88E-11	1.11E-08	14.454609
cg16833264	chr17	75052603	-			-0.968408	-4.104299	-7.098267	6.96E-11	1.12E-08	14.447264
cg08141659	chr13	113622633	+	MCF2L	TSS200	-0.885317	-1.824973	-7.093355	6.97E-11	1.12E-08	14.44186
cg25986240	chr4	9926439	-	SLC2A9;SLC2A9	Body;Body	-1.088106	-0.523573	-7.0933	6.98E-11	1.12E-08	14.441585
cg22903848	chr3	194924418	-	C3orf21	Body	0.7078379	1.2789174	7.0928196	6.99E-11	1.12E-08	14.439151
cg15990972	chr2	177012372	-			1.4106179	-0.118496	7.0925054	7.00E-11	1.12E-08	14.437561
cg25718402	chr17	7165909	+	CLDN7	TSS200	-0.452185	-3.972475	-7.09148	7.04E-11	1.13E-08	14.432369
cg10660256	chr5	78407683	+	BHMT;BHMT	5'UTR;1stExon	1.4941405	-0.573348	7.0913665	7.05E-11	1.13E-08	14.431797
cg04910970	chr6	100053930	+	PRDM13	TSS1500	1.3531801	0.6232432	7.090897	7.06E-11	1.13E-08	14.429421
cg10187674	chr17	67323922	+	ABCA5	TSS1500	-1.18412	-2.371988	-7.090042	7.10E-11	1.14E-08	14.425095
cg10403161	chr10	93805870	+			1.1077969	0.7787586	7.088904	7.14E-11	1.14E-08	14.419336
cg03551243	chr19	5718912	-	LONP1	Body	-0.838412	-3.712027	-7.088318	7.16E-11	1.15E-08	14.416373
cg20335735	chr6	150068006	-	NUP43	TSS1500	-1.284458	-0.184621	-7.088095	7.17E-11	1.15E-08	14.415241
cg07027075	chr4	39461721	-	LIAS;RPL9;LIAS	Body;TSS1500;Body	-1.087596	-2.839376	-7.086911	7.21E-11	1.15E-08	14.40925

cg01708273	chr2	176974060	+	HOXD11	3'UTR	1.8503689	0.0125864	7.0913912	7.22E-11	1.15E-08	14.409688
cg19631472	chr11	18415641	+	LDHA;LDHA;LDHA;LDHA;LDHA	TSS1500;TSS1500;TSS1500;TSS1500;TSS1500	-1.114396	-1.831709	-7.086807	7.22E-11	1.15E-08	14.408728
cg04349084	chr8	23602677	+			-1.967885	-1.727372	-7.086175	7.24E-11	1.16E-08	14.405526
cg10057614	chr16	56841060	-	NUP93	Body	1.059525	2.1512579	7.0907193	7.24E-11	1.16E-08	14.406295
cg16671079	chr17	74868561	+	MGAT5B;MGAT5B	Body;TSS200	-1.347882	-0.630444	-7.085637	7.26E-11	1.16E-08	14.402807
cg21831898	chr1	150254439	+	C1orf51	TSS1500	-2.363701	-5.644922	-7.094927	7.26E-11	1.16E-08	14.405085
cg01409460	chr7	34106680	-	BMPER	Body	-1.349181	-0.397397	-7.084619	7.30E-11	1.17E-08	14.397659
cg04035828	chr16	12405425	-	SNX29	Body	1.0369781	0.7993507	7.0841874	7.32E-11	1.17E-08	14.395474
cg14599380	chr4	17310680	-			-1.402353	-0.269057	-7.083344	7.35E-11	1.17E-08	14.391208
cg22550229	chr18	13138034	-			-1.385657	-1.837504	-7.081928	7.40E-11	1.18E-08	14.384047
cg04223420	chr5	134363877	-	PITX1	3'UTR	1.9110742	-0.075236	7.0818407	7.41E-11	1.18E-08	14.383604
cg12435059	chr3	37291314	+	GOLGA4	Body	0.8392203	2.0377016	7.0816465	7.42E-11	1.18E-08	14.382622
cg20841748	chr17	40838078	+	CNTNAP1	Body	1.0786874	0.8376134	7.0815744	7.42E-11	1.18E-08	14.382257
cg09924998	chr12	122751933	+	VPS33A	TSS1500	-1.061527	-1.448818	-7.080939	7.44E-11	1.19E-08	14.379041
cg12970171	chr5	6766613	+			-1.199042	-1.053157	-7.080694	7.45E-11	1.19E-08	14.377804
cg15007448	chr4	1970207	+	WHSC1;WHSC1;WHSC1;WHSC1	Body;Body;Body;Body	0.9069808	1.9949249	7.0806461	7.45E-11	1.19E-08	14.377562
cg16599983	chr6	32168969	-	NOTCH4	Body	-1.173532	-0.30269	-7.080556	7.46E-11	1.19E-08	14.377108
cg09044294	chr2	50234533	-	NRXN1;NRXN1;NRXN1	Body;Body;Body	-1.706989	0.3570767	-7.079425	7.50E-11	1.19E-08	14.371385
cg18477949	chr15	69760994	-			-0.977564	-1.043969	-7.079463	7.50E-11	1.19E-08	14.37158
cg05731122	chr12	48100286	-	RPAP3;RPAP3;RPAP3	TSS1500;TSS1500;TSS1500	-1.119466	-4.148624	-7.078279	7.55E-11	1.20E-08	14.365593

cg21011133	chr2	25142198	+	ADCY3	TSS200	-1.370576	-3.003677	-7.087623	7.55E-11	1.20E-08	14.368192
cg15000966	chr17	73128123	+	NT5C	TSS1500	-1.155498	-2.379544	-7.082656	7.56E-11	1.20E-08	14.365673
cg07788369	chr7	96619068	+	DLX6AS	Body	1.9629406	-0.224858	7.0775634	7.58E-11	1.20E-08	14.361973
cg27650726	chr4	6676596	+	LOC93622	Body	-0.855705	-4.110578	-7.076866	7.60E-11	1.21E-08	14.358448
cg05726109	chr22	19709755	+	SEPT5;GP1BB	Body;TSS1500	1.3261423	0.9682828	7.0761277	7.63E-11	1.21E-08	14.354714
cg09970569	chr8	109094032	-	RSPO2	Body	1.9595313	-1.141387	7.0761354	7.63E-11	1.21E-08	14.354753
cg21581504	chr2	68545960	+	CNRIP1;CNRIP1	Body;Body	1.5495088	0.9712832	7.0762795	7.63E-11	1.21E-08	14.355482
cg23741006	chr11	59634191	+	TCN1	TSS200	-1.402472	-1.55133	-7.076203	7.63E-11	1.21E-08	14.355096
cg00852783	chr1	26633632	-	UBXN11;UBXN11;UBXN11	5'UTR;TSS1500;TSS1500	-1.513762	-2.164955	-7.074769	7.69E-11	1.22E-08	14.347846
cg26396370	chr2	10194752	-	KLF11	3'UTR	1.1750821	1.9060759	7.0745491	7.70E-11	1.22E-08	14.346733
cg19973659	chr3	129612905	-	TMCC1;TMCC1	TSS1500;TSS1500	-0.923103	-3.945201	-7.073945	7.72E-11	1.22E-08	14.343677
cg17614703	chr5	179218853	-			0.9425528	1.2713732	7.0736109	7.73E-11	1.22E-08	14.34199
cg00316839	chr4	85414135	-			1.8488388	-0.136298	7.0733519	7.74E-11	1.22E-08	14.340681
cg04559779	chr8	65291730	-	MIR124-2	Body	1.8424194	-0.588587	7.0732314	7.75E-11	1.22E-08	14.340072
cg03649047	chr19	11529133	-	RGL3;RGL3	Body;Body	-0.535314	-3.018757	-7.073019	7.76E-11	1.23E-08	14.338997
cg22400015	chr2	220196714	-	RESP18	Body	1.2184049	0.2405869	7.0727362	7.77E-11	1.23E-08	14.337569
cg15022671	chr2	204502198	+			0.7053219	0.9513316	7.0769115	7.79E-11	1.23E-08	14.336684
cg25830182	chr4	85417077	+	NKX6-1	Body	2.1796745	-1.091879	7.072216	7.79E-11	1.23E-08	14.334939
cg14794735	chr7	129415968	+	MIR183;MIR96	TSS1500;TSS1500	0.8472018	1.4336115	7.0721009	7.80E-11	1.23E-08	14.334358
cg18650367	chr10	133909949	-			-1.624806	-2.406792	-7.075831	7.83E-11	1.24E-08	14.331235

cg26450106	chr20	24929607	-	CST7	TSS1500	-1.952689	-1.723149	-7.070168	7.87E-11	1.24E-08	14.32459
cg01658421	chr2	176945899	+	EVX2	Body	1.5327016	-0.450738	7.0696464	7.90E-11	1.24E-08	14.321952
cg03168749	chr11	124413574	+	OR8B12	TSS200	-1.829396	0.4251257	-7.069879	7.89E-11	1.24E-08	14.323129
cg07187521	chr16	25422897	+			-1.224564	-2.216608	-7.069576	7.90E-11	1.24E-08	14.321595
cg11744021	chr6	151342906	-	MTHFD1L	Body	1.2310104	1.0505777	7.0698254	7.89E-11	1.24E-08	14.322857
cg21205663	chr3	31269011	+			-1.879327	-1.045005	-7.069744	7.89E-11	1.24E-08	14.322443
cg26054057	chr3	38010715	+	CTDSPL;MIR26A1;CTDSPL	Body;TSS200;Body	1.0571101	0.8702038	7.0696428	7.90E-11	1.24E-08	14.321934
cg02556649	chr11	1945564	-	TNNT3;TNNT3;TNNT3;TNNT3	Body;Body;Body;Body	-1.711235	0.3957971	-7.078222	7.93E-11	1.25E-08	14.323647
cg23180938	chr5	115152485	+	CDO1	TSS200	3.5672924	-1.541538	7.0680111	7.96E-11	1.25E-08	14.313688
cg16497718	chr21	43242321	-	PRDM15;PRDM15	Body;Body	0.8760332	1.6265671	7.0677623	7.97E-11	1.25E-08	14.312431
cg06580196	chr3	50366021	-	TUSC2	TSS1500	-0.642172	-2.987764	-7.066891	8.01E-11	1.26E-08	14.308026
cg10155026	chr12	4553029	-	FGF6	Body	-0.987325	-1.172048	-7.066699	8.02E-11	1.26E-08	14.307057
cg10216717	chr12	128899290	-	TMEM132C	Body	-1.794948	-0.326413	-7.066662	8.02E-11	1.26E-08	14.30687
cg22491141	chr18	56932314	-			1.4810728	-0.267613	7.0667598	8.02E-11	1.26E-08	14.307364
cg02794779	chr13	100150711	+			-1.248949	-2.181508	-7.070572	8.05E-11	1.26E-08	14.304703
cg05371578	chr3	147128157	+	ZIC1	1stExon	1.3395663	-0.077935	7.0652307	8.08E-11	1.27E-08	14.299639
cg10678630	chr2	172864614	-	MAP1D	TSS200	-0.73955	-4.017064	-7.06427	8.12E-11	1.27E-08	14.294787
cg14559259	chr2	223168575	-	CCDC140	5'UTR	2.140782	-0.846159	7.0643376	8.12E-11	1.27E-08	14.295126
cg13386070	chr1	175046885	-	TNN	Body	-1.424365	-0.40717	-7.063368	8.16E-11	1.28E-08	14.290227
cg02039404	chr2	70368784	+			-1.400306	-0.643493	-7.06308	8.17E-11	1.28E-08	14.288771

cg10055566	chr6	28227220	+	NKAPL	1stExon	1.0310915	0.0206993	7.0630731	8.17E-11	1.28E-08	14.288738
cg06771126	chr4	57547699	-	HOPX;HOPX;HOPX;HOPX;HOPX;HOPX	5'UTR;1stExon;1stExon;5'UTR;5'UTR;1stExon	-1.328315	-1.976456	-7.06209	8.22E-11	1.29E-08	14.28377
cg03662014	chr16	29086271	-	RRN3P2	Body	2.2399743	-0.642708	7.0617452	8.23E-11	1.29E-08	14.28203
cg12467749	chr7	35294430	-	TBX20;TBX20	TSS1500;TSS1500	1.7692294	-1.547076	7.0614737	8.24E-11	1.29E-08	14.280659
cg15508809	chr14	29231309	+			1.450351	-0.582391	7.0614983	8.24E-11	1.29E-08	14.280783
cg19190762	chr13	44806055	-			-1.181735	-1.030017	-7.060717	8.27E-11	1.29E-08	14.276838
cg10168086	chr16	26499966	-			-2.030885	-0.726997	-7.060241	8.29E-11	1.30E-08	14.27443
cg07443529	chr17	36953613	-	PIP4K2B	Body	0.9331738	1.0548288	7.0596881	8.32E-11	1.30E-08	14.271639
cg11417029	chr11	74204987	+	LIPT2	TSS1500	-0.870891	-3.353337	-7.059494	8.33E-11	1.30E-08	14.27066
cg11311053	chr12	124912049	+	NCOR2;NCOR2	Body;Body	0.9928262	0.9452078	7.0591035	8.34E-11	1.30E-08	14.268687
cg06942701	chr2	162280009	-	TBR1	Body	1.0966807	-0.0709	7.0590205	8.35E-11	1.30E-08	14.268268
cg17451419	chr1	86047227	+	CYR61	Body	-1.124004	-3.046302	-7.058647	8.36E-11	1.30E-08	14.266381
cg22318806	chr6	31540411	-	LTA;LTA	5'UTR;5'UTR	-0.980827	-0.968349	-7.0587	8.36E-11	1.30E-08	14.266648
cg12433691	chr2	109065314	+	GCC2;GCC2	TSS1500;TSS1500	-0.65783	-3.966514	-7.058083	8.39E-11	1.31E-08	14.26353
cg08166631	chr1	40599011	+			0.9817032	1.2396113	7.0576919	8.41E-11	1.31E-08	14.261558
cg23260554	chr1	2934497	-			-1.131561	-0.916963	-7.056892	8.44E-11	1.31E-08	14.257518
cg00486340	chr4	4860026	+	MSX1	TSS1500	1.3745206	-1.027175	7.0552202	8.52E-11	1.33E-08	14.249076
cg20639948	chr8	82645116	+	CHMP4C	Body	-1.04801	-2.806281	-7.054908	8.53E-11	1.33E-08	14.247499
cg19010490	chr15	37388127	-	MEIS2;MEIS2;MEIS2;MEIS2;MEIS2;MEIS2;MEIS2	Body;Body;Body;Body;Body;Body;Body	1.2589021	2.1693391	7.0544727	8.55E-11	1.33E-08	14.245302
cg01959730	chr1	119548825	-			2.335273	-0.466989	7.0532041	8.61E-11	1.34E-08	14.238897

cg06779253	chr1	9375627	+	SPSB1	5'UTR	-1.044845	-1.325193	-7.052556	8.64E-11	1.34E-08	14.235626
cg16801374	chr12	102872708	-	IGF1;IGF1;IGF1;IGF1	Body;Body;Body;TSS1500	-0.903425	-3.668413	-7.052374	8.64E-11	1.34E-08	14.234708
cg13998945	chr7	45035665	-			-1.888707	0.1387075	-7.052007	8.66E-11	1.34E-08	14.232854
cg18728264	chr11	118766491	+	CXCR5;CXCR5;CXCR5	1stExon;3'UTR;3'UTR	-1.292963	-0.569753	-7.056321	8.67E-11	1.35E-08	14.232765
cg02456804	chr1	166039858	+	FAM78B	Body	-1.763069	0.1408899	-7.051448	8.69E-11	1.35E-08	14.23003
cg24892139	chr5	35618383	+	SPEF2;SPEF2	Body;Body	-1.919892	-3.073997	-7.050441	8.73E-11	1.35E-08	14.224951
cg22747746	chr8	23564193	+	NKX2-6	TSS1500	2.4297742	-0.92955	7.0547407	8.74E-11	1.36E-08	14.224897
cg12520549	chr2	162280741	-	TBR1	3'UTR	1.5712772	-1.118225	7.049908	8.76E-11	1.36E-08	14.222258
cg13612447	chr17	2653188	-			-0.880198	-3.692206	-7.049411	8.78E-11	1.36E-08	14.219748
cg12631000	chr1	10729216	+	CASZ1;CASZ1	Body;Body	1.0939446	1.5322931	7.0488569	8.80E-11	1.36E-08	14.216952
cg05617980	chr4	3373405	-	RGS12;RGS12;RGS12	Body;Body;Body	1.7878141	1.5885341	7.0533468	8.81E-11	1.36E-08	14.21778
cg14333454	chr1	27189298	-	SFN	TSS1500	-1.274487	-0.538525	-7.04848	8.82E-11	1.37E-08	14.215049
cg03092551	chr2	58273420	+	VRK2;VRK2;VRK2;VRK2;VRK2;VRK2	TSS1500;TSS1500;TSS1500;TSS1500;TSS1500;TSS1500	-0.845111	-1.637409	-7.047835	8.85E-11	1.37E-08	14.211796
cg12393275	chr2	79312224	+	REG1B	3'UTR	-2.23546	0.8585894	-7.047341	8.87E-11	1.37E-08	14.209303
cg23318812	chr12	66123127	+			2.5533426	-0.868369	7.0470014	8.89E-11	1.37E-08	14.207588
cg22443762	chr1	197890608	+	LHX9;LHX9	Body;Body	1.5061771	0.0188307	7.0451308	8.98E-11	1.39E-08	14.198148
cg04571189	chr2	10638072	-			-1.252427	-1.012667	-7.045009	8.98E-11	1.39E-08	14.197533
cg03362882	chr11	56945579	-			-1.333352	-0.429847	-7.044645	9.00E-11	1.39E-08	14.195698
cg23946364	chr2	235936477	+	SH3BP4	5'UTR	-1.729047	0.2713293	-7.044492	9.01E-11	1.39E-08	14.194922
cg16385330	chr4	151503878	+	LRBA;MAB21L2;MAB21L2	Body;1stExon;5'UTR	1.0112801	2.5169935	7.0441688	9.02E-11	1.39E-08	14.193293

cg20945085	chr2	74875227	-	C2orf65	TSS200	1.569577	1.2000297	7.0440222	9.03E-11	1.39E-08	14.192554
cg16523148	chr11	64901065	+	SYVN1;SYVN1	Body;Body	-0.931556	-1.619126	-7.047808	9.07E-11	1.40E-08	14.189971
cg21239660	chr17	45006500	-	GOSR2;GOSR2;GOSR2	Body;Body;Body	1.1534591	1.5623033	7.0429406	9.08E-11	1.40E-08	14.187097
cg16758800	chr7	96631902	-	DLX6AS	Body	1.1999986	-0.44218	7.0427684	9.09E-11	1.40E-08	14.186227
cg20192747	chr18	44774846	+			1.2118898	0.3988444	7.042433	9.10E-11	1.40E-08	14.184536
cg00886954	chr8	145047713	+	PLEC1;PLEC1	Body;TSS200	-2.223058	-3.323753	-7.041995	9.13E-11	1.41E-08	14.182324
cg18795133	chr5	141130629	+			-1.347324	-0.64029	-7.046444	9.13E-11	1.41E-08	14.186371
cg21439896	chr1	36108233	-	PSMB2	TSS1500	-1.115612	-0.515672	-7.04595	9.15E-11	1.41E-08	14.180519
cg03078363	chr12	54408664	+			2.7770822	-1.254032	7.0409352	9.18E-11	1.41E-08	14.176979
cg26847503	chr7	143056341	+	FAM131B;FAM131B	Body;Body	-1.080569	-0.802073	-7.039672	9.24E-11	1.42E-08	14.170605
cg03352106	chr12	21926476	-	KCNJ8	Body	2.2858349	0.8315037	7.0395388	9.24E-11	1.42E-08	14.169935
cg24088250	chr17	75252179	+			-0.888986	-0.797749	-7.039153	9.26E-11	1.42E-08	14.167988
cg03623878	chr13	113655560	+	MCF2L;MCF2L	Body;Body	-1.32517	-2.606801	-7.038594	9.29E-11	1.43E-08	14.165167
cg22396979	chr8	143915919	+	GML	TSS1500	-1.5435	0.7237195	-7.037478	9.34E-11	1.43E-08	14.15954
cg02079551	chr10	107023377	-	SORCS3	3'UTR	-1.971858	0.7409201	-7.037288	9.35E-11	1.44E-08	14.15858
cg19366835	chr6	79577801	+	IRAK1BP1	Body	-0.654303	-4.27219	-7.035761	9.43E-11	1.45E-08	14.150879
cg05156901	chr22	51016646	-	CPT1B;CPT1B;CPT1B;CHKB-CPT1B;CPT1B;CPT1B;CPT1B;CPT1B	5'UTR;5'UTR;TSS200;Body;5'UTR;5'UTR;5'UTR;5'UTR	1.5439355	1.2428334	7.0349767	9.47E-11	1.45E-08	14.146925
cg05539265	chr2	102717438	+			-1.632032	-0.470286	-7.033985	9.52E-11	1.46E-08	14.141923
cg16257051	chr5	153862183	+			2.9277489	-1.274002	7.0338666	9.52E-11	1.46E-08	14.141327
cg11667086	chr11	31848554	+			2.1092349	-1.163756	7.0335893	9.54E-11	1.46E-08	14.139929

cg02422997	chr15	100277091	+			0.8560279	2.2029272	7.0325106	9.59E-11	1.47E-08	14.13449
cg11517269	chr6	28058789	-	ZSCAN12L1	TSS200	1.5075469	0.3618629	7.0323884	9.60E-11	1.47E-08	14.133874
cg08993103	chr4	25776626	+	SEL1L3	Body	0.897584	2.0008077	7.0321906	9.61E-11	1.47E-08	14.132876
cg23193759	chr10	71389896	+	C10orf35	TSS200	-1.230966	-1.995886	-7.032065	9.61E-11	1.47E-08	14.132241
cg26728422	chr16	1429015	-	UNKL	5'UTR	-1.31627	-2.967954	-7.031152	9.66E-11	1.48E-08	14.127642
cg12279419	chr6	33943662	+			1.531103	-0.343476	7.0310807	9.66E-11	1.48E-08	14.12728
cg16679650	chr1	24239820	-	CNR2	TSS200	-1.082871	-1.744366	-7.029648	9.73E-11	1.49E-08	14.12006
cg03199926	chr20	60860733	-	OSBPL2;OSBPL2	Body;Body	1.0313872	1.8837028	7.0290527	9.76E-11	1.49E-08	14.117057
cg22551065	chr5	126565698	+			-1.076384	-1.977144	-7.029118	9.76E-11	1.49E-08	14.117387
cg04477010	chr8	38175544	+	WHSC1L1;WHSC1L1	Body;Body	1.6026908	2.9014412	7.0288588	9.77E-11	1.49E-08	14.116079
cg19783761	chr19	3136091	-	GNA15	TSS200	-1.157574	-1.600145	-7.028663	9.78E-11	1.49E-08	14.115094
cg15825116	chr2	223169586	+	CCDC140	3'UTR	2.107467	-2.229283	7.0376629	9.79E-11	1.49E-08	14.116854
cg21473142	chr3	27762095	+	EOMES	Body	1.7504028	0.0632053	7.0274572	9.85E-11	1.50E-08	14.109014
cg22695379	chr5	137593551	-	GFRA3	Body	0.8395301	1.589393	7.0272016	9.86E-11	1.50E-08	14.107726
cg00329300	chr1	161008462	-	TSTD1;TSTD1;TSTD1	Body;Body;Body	-1.276609	-1.359163	-7.026141	9.91E-11	1.51E-08	14.102382
cg06488443	chr2	162280341	+	TBR1	Body	1.298114	-0.859843	7.02581	9.93E-11	1.51E-08	14.100712
cg13661519	chr7	27141088	+	HOXA2	Body	1.5583119	1.1106182	7.0342558	9.97E-11	1.52E-08	14.102883
cg05057720	chr14	38724675	-	CLEC14A	1stExon	2.3924704	-0.103922	7.0246884	9.99E-11	1.52E-08	14.095059
cg25496678	chr10	118899407	+			1.9366242	-1.001685	7.0236894	1.00E-10	1.53E-08	14.090025
cg11229513	chr8	70984559	-	PRDM14	TSS1500	2.0188499	-0.264748	7.027863	1.01E-10	1.53E-08	14.089584

cg27107970	chr17	79520734	-	C17orf70;C17orf70	TSS1500;TSS1500	-1.06267	-2.855986	-7.023144	1.01E-10	1.53E-08	14.087275
cg18394527	chr20	43594843	+	STK4	TSS1500	-0.723862	-3.975002	-7.022436	1.01E-10	1.54E-08	14.083709
cg19786983	chr15	88336651	-			-1.264266	-0.77257	-7.022367	1.01E-10	1.54E-08	14.083363
cg22541254	chr6	101846779	-	GRIK2;GRIK2;GRIK2	TSS200;TSS200;TSS200	1.5399424	-0.807331	7.0223957	1.01E-10	1.54E-08	14.083506
cg26188212	chr1	228967124	+			-1.906185	-1.643299	-7.022432	1.01E-10	1.54E-08	14.083687
cg06999762	chr15	53093007	-			1.2569713	0.2772845	7.0221333	1.01E-10	1.54E-08	14.082184
cg05396044	chr1	173836727	-	GAS5;SNORD76;SNORD75;SNORD77;ZBTB37;ZBTB37	Body;TSS1500;TSS1500;TSS1500;TSS1500;TSS1500	-0.71353	-3.939308	-7.022003	1.01E-10	1.54E-08	14.081529
cg08596532	chr5	5033993	-	LOC340094	TSS1500	-1.530273	-1.090517	-7.021762	1.01E-10	1.54E-08	14.080311
cg16297011	chr4	13539023	+			2.5692443	-0.971547	7.0308104	1.01E-10	1.54E-08	14.082639
cg12159259	chr13	113642515	-	MCF2L;MCF2L	Body;Body	1.6961899	2.8034118	7.0216024	1.02E-10	1.54E-08	14.079509
cg16055026	chr5	87974547	+	LOC645323;LOC645323	Body;TSS1500	2.3449659	-1.523402	7.0215364	1.02E-10	1.54E-08	14.079176
cg09663494	chr16	67223733	+	EXOC3L	5'UTR	1.0255761	1.4722253	7.0214175	1.02E-10	1.54E-08	14.078577
cg03799713	chr7	54764119	+			-1.365932	-1.803224	-7.019951	1.02E-10	1.55E-08	14.07119
cg00467652	chr3	50357644	-	HYAL2;HYAL2	Body;Body	1.2645485	1.15511	7.0196249	1.03E-10	1.55E-08	14.069545
cg13122532	chr19	54210147	-	MIR520C	TSS1500	-1.08362	-0.176029	-7.019076	1.03E-10	1.56E-08	14.066782
cg05973132	chr2	240451894	-			-1.374353	-0.307537	-7.018797	1.03E-10	1.56E-08	14.065372
cg24880701	chr15	68121563	-	LBXCOR1	Body	2.3419845	-0.975541	7.0174077	1.04E-10	1.57E-08	14.058376
cg06894134	chr6	85476921	+			1.1462618	-0.705946	7.0168223	1.04E-10	1.57E-08	14.055427
cg24777710	chr7	127636733	+	C7orf54;SND1	TSS1500;Body	0.7465211	2.7034418	7.0148181	1.05E-10	1.59E-08	14.045333
cg24437070	chr20	62417813	+	ZBTB46	Body	-0.645669	-4.480287	-7.018458	1.06E-10	1.60E-08	14.042287

cg24700823	chr15	26447291	+			-1.505563	-0.07942	-7.013401	1.06E-10	1.60E-08	14.038195
cg11943883	chr12	108792983	-			-1.544463	-0.510488	-7.013237	1.06E-10	1.60E-08	14.03737
cg02450832	chr16	80308154	+			-1.794229	0.3385732	-7.012898	1.06E-10	1.60E-08	14.035661
cg05202804	chr19	11484815	+	C19orf39	TSS1500	-1.317919	-2.3783	-7.012659	1.06E-10	1.60E-08	14.03446
cg06580770	chr6	32054790	-	TNXB	Body	-1.42003	-0.540661	-7.01236	1.07E-10	1.61E-08	14.032953
cg03398865	chr5	1879554	+	IRX4	Body	1.1076005	-0.592283	7.0116085	1.07E-10	1.61E-08	14.029169
cg13942922	chr2	238383665	-			-1.644013	-2.749328	-7.011661	1.07E-10	1.61E-08	14.029432
cg24065328	chr6	31744735	+	C6orf27	5'UTR	-1.047115	-2.251949	-7.016086	1.07E-10	1.61E-08	14.030266
cg24691336	chr10	62492301	+			-1.127879	-2.743972	-7.011287	1.07E-10	1.61E-08	14.027552
cg22522688	chr12	109221232	+	SSH1;SSH1;SSH1;SSH1	1stExon;5'UTR;Body;Body	1.5600535	1.728029	7.0108314	1.07E-10	1.62E-08	14.025256
cg03441770	chr4	81128845	+			1.228295	1.2599657	7.0105488	1.08E-10	1.62E-08	14.023833
cg06012804	chr3	193849318	+			-1.131674	-1.572063	-7.010398	1.08E-10	1.62E-08	14.023072
cg01791371	chr6	50817193	-			2.1122997	-0.516167	7.0191189	1.08E-10	1.62E-08	14.023861
cg13073589	chr1	172517922	+	C1orf9;C1orf9	Body;Body	0.8485939	2.5903587	7.0092289	1.08E-10	1.63E-08	14.017188
cg24021846	chr7	103556529	+	RELN;RELN	Body;Body	-1.487287	-1.077496	-7.008521	1.09E-10	1.63E-08	14.013625
cg02853616	chr4	94755787	-			1.7454445	-0.48333	7.0173854	1.09E-10	1.63E-08	14.015165
cg08844325	chr2	9459368	+	ASAP2;ASAP2	Body;Body	1.5773289	2.4397292	7.0083041	1.09E-10	1.63E-08	14.012532
cg08251131	chr1	227915846	+	LOC100130093;LOC100130093	TSS1500;TSS1500	-0.40955	-3.283035	-7.007492	1.09E-10	1.64E-08	14.008443
cg10789956	chr8	26467541	+	DPYSL2	Body	1.1780914	0.5377482	7.0074154	1.09E-10	1.64E-08	14.008058
cg23517116	chr8	65499841	-			1.5013209	-0.587568	7.0074244	1.09E-10	1.64E-08	14.008103

cg18639142	chr19	1489759	-	PCSK4;REEP6	Body;TSS1500	-1.126765	-2.306835	-7.011357	1.10E-10	1.64E-08	14.006597
cg03889700	chr4	2869818	-	ADD1;ADD1;ADD1;ADD1	5'UTR;5'UTR;5'UTR;5'UTR	1.3534532	1.1622256	7.0064424	1.10E-10	1.65E-08	14.00316
cg23242052	chr2	176947786	-	EVX2	Body	1.7347283	-1.247463	7.0062737	1.10E-10	1.65E-08	14.002311
cg25631489	chr3	169900020	-	PHC3	TSS1500	-1.390756	-1.687824	-7.00578	1.10E-10	1.65E-08	13.999824
cg08202494	chr8	99961545	+	OSR2;OSR2	Body;Body	1.2890256	-0.076144	7.0054657	1.10E-10	1.65E-08	13.998244
cg01997272	chr14	103740476	+			1.7708898	-0.605905	7.0050126	1.11E-10	1.66E-08	13.995963
cg03312427	chr1	226778381	-	C1orf95	Body	-0.965749	-0.79879	-7.004655	1.11E-10	1.66E-08	13.994161
cg02128308	chr13	114126436	-	DCUN1D2	Body	0.9423873	2.0456226	7.004508	1.11E-10	1.66E-08	13.993423
cg06382344	chr2	162280519	+	TBR1	Body	2.9440994	-1.102917	7.0088041	1.11E-10	1.66E-08	13.997037
cg25130381	chr1	27440721	+	SLC9A1	Body	0.9864304	1.5235701	7.0043425	1.11E-10	1.66E-08	13.99259
cg17271223	chr7	132957775	+	EXOC4;EXOC4	Body;Body	1.1836785	2.3237172	7.0036756	1.11E-10	1.67E-08	13.989234
cg06907356	chr21	45664138	-			-1.92839	-1.2778	-7.003333	1.12E-10	1.67E-08	13.987512
cg03252499	chr11	124324477	+			-1.125231	-0.345105	-7.002587	1.12E-10	1.67E-08	13.983757
cg06873880	chr14	37123678	+			1.371182	-0.575482	7.0008127	1.13E-10	1.69E-08	13.974827
cg17654419	chr2	102012904	-			-1.687203	-0.239096	-7.000617	1.13E-10	1.69E-08	13.973843
cg18892668	chr14	101487503	+	MIR379	TSS1500	-1.045428	-0.322112	-6.999932	1.14E-10	1.70E-08	13.970397
cg25829425	chr6	6004897	-	NRN1	Body	1.2932429	-1.064963	6.9994302	1.14E-10	1.70E-08	13.96787
cg23316253	chr10	8076277	-			2.1006671	-0.925175	6.9991107	1.14E-10	1.70E-08	13.966263
cg26219092	chr8	134388022	+			-1.243333	-2.173755	-6.998498	1.15E-10	1.71E-08	13.963181
cg25316305	chr12	4361135	+			-1.99597	0.574146	-6.998346	1.15E-10	1.71E-08	13.962415

cg00121884	chr20	3799732	-	C20orf29	TSS1500	1.1158974	1.9059243	6.9978105	1.15E-10	1.71E-08	13.959721
cg15126179	chr13	79181338	+			1.4050671	0.022159	7.0020521	1.15E-10	1.71E-08	13.959857
cg09631769	chr22	49376886	+			-1.425763	-0.527006	-6.997312	1.15E-10	1.72E-08	13.957212
cg11961175	chr1	205649956	+	SLC45A3	TSS1500	-0.953081	-3.336231	-6.997133	1.15E-10	1.72E-08	13.956312
cg25763393	chr19	52956832	-	ZNF578;ZNF578	1stExon;5'UTR	2.0869634	-0.617789	6.9969735	1.15E-10	1.72E-08	13.95551
cg24663971	chr14	106410668	+			-1.996107	0.6104944	-6.995565	1.16E-10	1.73E-08	13.948425
cg00028935	chr3	147114406	-	ZIC4;ZIC4;ZIC4	Body;Body;Body	1.9007325	-0.957035	6.9945778	1.17E-10	1.74E-08	13.943459
cg18034637	chr11	111846087	-	DIXDC1	Body	1.5764672	1.1895097	6.9986846	1.17E-10	1.74E-08	13.942843
cg00663686	chr18	33553117	-	C18orf21	Body	-0.683825	-4.278062	-6.993729	1.17E-10	1.74E-08	13.93919
cg09094037	chr16	76342620	+	CNTNAP4;CNTNAP4	Body;TSS1500	-1.791598	0.4600605	-6.992714	1.18E-10	1.75E-08	13.934083
cg09281154	chr6	30852777	+	DDR1;DDR1	5'UTR;5'UTR	-0.57367	-4.287896	-6.99222	1.18E-10	1.76E-08	13.931601
cg11465213	chr15	90880158	+			-1.568515	-1.147628	-6.991678	1.19E-10	1.76E-08	13.928875
cg01759672	chr5	137593418	-	GFRA3	Body	1.396882	3.358475	7.0051957	1.19E-10	1.76E-08	13.932269
cg17470251	chr8	19459996	+	CSGALNACT1;CSGALNACT1;CSGALNACT1;CSGALNACT1	1stExon;5'UTR;Body;5'UTR	2.007417	0.4787147	6.9914727	1.19E-10	1.76E-08	13.927842
cg00335591	chr7	1596260	-	TMEM184A	TSS200	-1.924101	-0.37659	-6.99054	1.19E-10	1.77E-08	13.923152
cg06097704	chr17	820939	+	NXN	Body	1.8030285	2.1125462	6.9950197	1.19E-10	1.77E-08	13.924549
cg26738880	chr7	154429032	-	DPP6;DPP6;DPP6	Body;Body;Body	-1.936885	0.0588732	-6.990423	1.19E-10	1.77E-08	13.922564
cg16784745	chr12	54731086	-	MIR148B;COPZ1	Body;Body	1.0359211	1.9066174	6.9888849	1.20E-10	1.78E-08	13.91483
cg11827629	chr12	115822424	-			-1.74567	0.0889117	-6.988393	1.21E-10	1.79E-08	13.912356
cg04361749	chr2	240980455	+			-1.003621	-1.161746	-6.988212	1.21E-10	1.79E-08	13.911446

cg06645952	chr11	14402556	-			-1.178754	-1.868068	-6.987858	1.21E-10	1.79E-08	13.909667
cg12422154	chr6	30130819	-	TRIM15	TSS200	-1.88037	-0.50386	-6.986269	1.22E-10	1.81E-08	13.901677
cg24554061	chr11	63705760	-	NAA40	TSS1500	-1.265789	-4.506858	-6.990586	1.22E-10	1.81E-08	13.902192
cg23900681	chr14	96752271	+	ATG2B	Body	1.1998591	2.5532497	6.9902934	1.22E-10	1.81E-08	13.900722
cg12048674	chr1	205649805	+	SLC45A3	TSS200	-0.730058	-3.977087	-6.985591	1.23E-10	1.81E-08	13.89827
cg02919936	chr8	70982285	+	PRDM14	5'UTR	2.3237519	-2.116547	6.9854675	1.23E-10	1.81E-08	13.897649
cg05939312	chr8	144717687	-	ZNF623	TSS1500	-1.024435	-4.269089	-6.985242	1.23E-10	1.81E-08	13.896517
cg09435411	chr16	49056674	-			-1.495289	0.0533669	-6.98512	1.23E-10	1.81E-08	13.895904
cg14038391	chr10	102900130	+			3.162106	-1.487456	6.9849568	1.23E-10	1.81E-08	13.895081
cg01517500	chr2	10637974	-			-1.394663	0.0974372	-6.984446	1.23E-10	1.82E-08	13.892513
cg01978237	chr6	100896803	+	SIM1	Body	1.7348162	-0.469985	6.9839828	1.24E-10	1.82E-08	13.890186
cg09685096	chr1	86157111	+	ZNHIT6;ZNHIT6	Body;Body	1.3875852	1.4742693	6.9839404	1.24E-10	1.82E-08	13.889972
cg26238975	chr14	101520662	+	MIR134;MIR668;MIR382;MIR485	TSS1500;TSS1500;Body;TSS1500	-2.974198	1.4727635	-6.983493	1.24E-10	1.82E-08	13.887722
cg19265143	chr1	28907637	+	SNHG12;SNORA44;SNORA16A;SNORA61	Body;TSS1500;TSS200;TSS1500	-0.67494	-4.613993	-6.983357	1.24E-10	1.83E-08	13.887038
cg00017271	chr17	76220743	-	BIRC5;BIRC5;BIRC5;EPR1	3'UTR;3'UTR;3'UTR;Body	1.1183767	1.6616582	6.9828094	1.24E-10	1.83E-08	13.884288
cg04042828	chr4	152328900	+	FAM160A1	TSS1500	-1.174462	-2.22272	-6.982697	1.24E-10	1.83E-08	13.883722
cg04951371	chr2	3317860	+	TSSC1	Body	1.0581154	1.5495754	6.9820224	1.25E-10	1.84E-08	13.880332
cg26505596	chr6	32150656	-	AGER;AGER	Body;Body	1.1457071	4.0663111	6.985532	1.25E-10	1.84E-08	13.876938
cg14553377	chr11	2139449	-			-1.157405	-1.330709	-6.980856	1.26E-10	1.85E-08	13.874469
cg08235161	chr14	57262049	+			1.9330349	-0.674488	6.9850565	1.26E-10	1.85E-08	13.874553

cg00455327	chr18	20714217	-	CABLES1	TSS1500	-1.511929	-1.839957	-6.980182	1.26E-10	1.85E-08	13.871082
cg25946646	chr10	88628344	-	BMPR1A	5'UTR	1.2088264	2.2519472	6.98837	1.27E-10	1.86E-08	13.869638
cg10237148	chr14	104803942	-			-1.743849	0.1541831	-6.978901	1.27E-10	1.86E-08	13.864648
cg02865033	chr16	31235535	-	TRIM72	Body	0.8790946	1.1413984	6.97865	1.27E-10	1.86E-08	13.863385
cg23295629	chr8	128888486	-	PVT1	Body	-1.207834	-1.39138	-6.978322	1.27E-10	1.87E-08	13.861734
cg21554861	chr4	99851060	+	EIF4E;EIF4E;EIF4E;EIF4E;EIF4E	5'UTR;1stExon;5'UTR;TSS1500;1stExon	-1.003527	-2.744568	-6.977134	1.28E-10	1.88E-08	13.855767
cg15556672	chr5	68513082	-	MRPS36	TSS1500	-1.278781	-0.79295	-6.976328	1.29E-10	1.89E-08	13.85172
cg06409153	chr17	67323953	+	ABCA5	TSS1500	-1.140393	-2.543444	-6.97613	1.29E-10	1.89E-08	13.850722
cg16118387	chr1	210407755	+	C1orf133;SERTAD4	TSS1500;5'UTR	-0.923987	-1.745557	-6.975844	1.29E-10	1.89E-08	13.849287
cg08537847	chr5	148810203	-	MIR145;LOC728264	TSS200;Body	1.1192743	1.5257038	6.9753637	1.29E-10	1.89E-08	13.846874
cg12258785	chr3	24537407	+	THRB;THRB;THRB	TSS1500;TSS1500;TSS1500	-1.359404	-2.261396	-6.979606	1.29E-10	1.89E-08	13.847217
cg08097817	chr21	32255313	+	KRTAP11-1	TSS1500	-1.93737	0.55857	-6.97499	1.29E-10	1.89E-08	13.844996
cg15946312	chr5	180646880	-			0.9379693	1.5355571	6.9746475	1.30E-10	1.90E-08	13.843276
cg21995304	chr6	10391237	+			2.2354119	0.1870596	6.9741724	1.30E-10	1.90E-08	13.840889
cg01150683	chr10	111216646	+			1.2225138	-0.397271	6.9739346	1.30E-10	1.90E-08	13.839694
cg05455720	chr8	65291703	+	MIR124-2	TSS200	1.6137996	-0.864081	6.9729132	1.31E-10	1.91E-08	13.834564
cg07865607	chr19	3136076	+	GNA15	TSS200	-1.617135	-1.759767	-6.972945	1.31E-10	1.91E-08	13.834723
cg21399510	chr3	193922180	+			-1.922992	-3.410393	-6.972131	1.31E-10	1.92E-08	13.830633
cg11961746	chr7	5602219	-			-0.567072	-4.692812	-6.971968	1.32E-10	1.92E-08	13.829815
cg06966811	chr14	57276789	-	OTX2	5'UTR	1.4825553	0.3867898	6.971177	1.32E-10	1.93E-08	13.825844

cg08098964	chr19	7680443	-	KIAA1543;KIAA1543	Body;Body	1.305291	3.0932719	6.9706844	1.32E-10	1.93E-08	13.82337
cg01135648	chr4	41983611	-	DCAF4L1	TSS200	1.0227586	1.5784341	6.970349	1.33E-10	1.93E-08	13.821685
cg05090106	chr17	18908220	+	SLC5A10;SLC5A10;FAM83G	Body;Body;TSS200	-0.534832	-4.399638	-6.970109	1.33E-10	1.94E-08	13.820481
cg04467618	chr6	134210946	+	TCF21;TCF21	1stExon;1stExon	1.4449412	-0.344635	6.9695649	1.33E-10	1.94E-08	13.817748
cg09656363	chr13	53775035	+			1.0423621	-0.698251	6.9686904	1.34E-10	1.95E-08	13.813357
cg20950167	chr17	35300448	+	LHX1	3'UTR	1.7607334	0.626754	6.9683973	1.34E-10	1.95E-08	13.811885
cg06391932	chr2	45159894	+			1.2057941	-0.318843	6.9676519	1.35E-10	1.96E-08	13.808142
cg12099459	chr1	147826902	+			1.170093	1.2015131	6.9676131	1.35E-10	1.96E-08	13.807947
cg25609301	chr14	63508497	-	KCNH5;KCNH5;KCNH5	Body;5'UTR;Body	-1.69802	0.2700421	-6.967252	1.35E-10	1.96E-08	13.806131
cg15979932	chr17	55980107	+	CUEDC1	5'UTR	-1.437224	-0.640613	-6.967161	1.35E-10	1.96E-08	13.805677
cg06530983	chr16	9141884	-			-1.108122	-3.09969	-6.967084	1.35E-10	1.96E-08	13.805292
cg12480176	chr6	122774497	-	SERINC1	Body	0.9986272	1.7390305	6.9668237	1.35E-10	1.96E-08	13.803983
cg12671632	chr6	422935	-			-1.548096	-1.564519	-6.965991	1.36E-10	1.97E-08	13.799801
cg08029287	chr4	1503238	-			-0.966157	-0.913922	-6.965919	1.36E-10	1.97E-08	13.799442
cg05787556	chr5	170735186	-	TLX3	TSS1500	1.181517	-0.728196	6.9655945	1.36E-10	1.97E-08	13.797812
cg03292284	chr1	241804036	-	OPN3	TSS1500	-1.27846	-0.396376	-6.965433	1.36E-10	1.98E-08	13.797001
cg14319235	chr1	79472282	-	ELTD1	Body	1.4742307	-0.278873	6.9653254	1.36E-10	1.98E-08	13.796461
cg05210615	chr17	47512894	-			-0.902523	-1.443539	-6.965058	1.36E-10	1.98E-08	13.795117
cg25224048	chr5	38467248	-			1.5045658	1.8466022	6.9649082	1.36E-10	1.98E-08	13.794367
cg05030680	chr4	111542366	+	PITX2;PITX2;PITX2	Body;Body;Body	2.251775	-0.920211	6.9644396	1.37E-10	1.98E-08	13.792014

cg19579005	chr6	30782272	-			-1.753243	-2.959387	-6.973191	1.37E-10	1.98E-08	13.793773
cg19913582	chr5	167567540	-	ODZ2	Body	-1.701598	0.1386785	-6.963959	1.37E-10	1.99E-08	13.789604
cg08688659	chr11	35249965	+	CD44;CD44;CD44;CD44;CD44	Body;Body;Body;Body;Body	1.1364199	1.3270418	6.9635352	1.37E-10	1.99E-08	13.787474
cg12995090	chr18	8367691	-	LOC100192426;PTPRM;PTPRM	TSS1500;Body;Body	-1.242378	-1.85024	-6.963347	1.38E-10	1.99E-08	13.786527
cg02971381	chr12	6641751	-			-0.83133	-2.761309	-6.962509	1.38E-10	2.00E-08	13.782323
cg06299284	chr11	636659	-	DRD4	TSS1500	2.1307262	0.3764315	6.9623671	1.38E-10	2.00E-08	13.78161
cg12740337	chr6	28058973	+	ZSCAN12L1	Body	1.044096	-0.059789	6.9621206	1.38E-10	2.00E-08	13.780373
cg07178994	chr8	816998	-			-1.112368	-1.211319	-6.962054	1.38E-10	2.00E-08	13.780039
cg15608397	chr2	223163573	+	PAX3;PAX3;PAX3;PAX3;PAX3;PAX3;PAX3;PAX3;PAX3;CCDC140;PAX3;PAX3;PAX3;PAX3;PAX3;PAX3;PAX3	1stExon;5'UTR;1stExon;1stExon;5'UTR;1stExon;5'UTR;1stExon;5'UTR;5'UTR;1stExon;5'UTR;5'UTR;1stExon;5'UTR;1stExon;5'UTR	1.1927837	-0.307969	6.9613321	1.39E-10	2.01E-08	13.776415
cg23683588	chr14	37136283	+	PAX9	Body	2.0626134	-0.313915	6.9613148	1.39E-10	2.01E-08	13.776328
cg26770907	chr16	48844808	-			1.3679682	0.3449667	6.9609996	1.39E-10	2.01E-08	13.774747
cg08060501	chr16	4389056	+	GLIS2	3'UTR	0.9969043	1.2378114	6.9647247	1.40E-10	2.02E-08	13.772625
cg15458504	chr1	24828534	-	RCAN3	TSS1500	-1.248991	-2.886702	-6.95967	1.40E-10	2.02E-08	13.768074
cg19134568	chr4	147561339	-	POU4F2	Body	1.0504911	-0.505642	6.9591124	1.41E-10	2.03E-08	13.765275
cg08263416	chr14	72720835	-	RGS6	Body	-1.545409	-0.722478	-6.958421	1.41E-10	2.04E-08	13.761804
cg05021643	chr2	177029608	+	HOXD3	5'UTR	1.7188222	-0.113369	6.9627244	1.41E-10	2.04E-08	13.762605
cg08774207	chr16	86322930	+			-1.350564	-1.611688	-6.957173	1.42E-10	2.05E-08	13.755544
cg27213509	chr2	176947228	-	EVX2	Body	1.1307172	-0.616165	6.9571236	1.42E-10	2.05E-08	13.755295
cg00835279	chr2	3642710	-	COLEC11;COLEC11;COLEC11;COLEC11	1stExon;5'UTR;1stExon;5'UTR	1.3173322	-0.192193	6.956759	1.42E-10	2.05E-08	13.753466
cg05899103	chr15	65394570	-	LOC390595	Body	0.7634842	1.569624	6.9567597	1.42E-10	2.05E-08	13.753469

cg24693160	chr11	124438770	-	OR8A1	TSS1500	-1.725224	0.1528422	-6.956779	1.42E-10	2.05E-08	13.753567
cg16370398	chr12	54448913	+	HOXC4;HOXC4	Body;Body	2.2474315	0.981533	6.9649702	1.43E-10	2.06E-08	13.752552
cg06979550	chr5	68462676	-	CCNB1	TSS1500	-0.44417	-3.996094	-6.955804	1.43E-10	2.06E-08	13.748676
cg03697308	chr13	28545566	-			1.251398	0.7004662	6.9590845	1.44E-10	2.07E-08	13.744373
cg22853713	chr1	10732872	-	CASZ1;CASZ1	Body;Body	1.7076301	2.6445778	6.9526685	1.45E-10	2.09E-08	13.732944
cg11256152	chr8	141359786	+	TRAPPC9;TRAPPC9	Body;Body	1.0864367	1.3550807	6.952402	1.46E-10	2.09E-08	13.731608
cg00983904	chr12	6665288	-	IFFO1;IFFO1	TSS200;TSS200	2.1732317	-0.289106	6.9522631	1.46E-10	2.09E-08	13.730911
cg01055462	chr12	21590246	-	PYROXD1	TSS1500	-1.95011	-0.287969	-6.951751	1.46E-10	2.10E-08	13.72834
cg14511698	chr14	60976898	+	SIX6	Body	1.5222802	-0.632087	6.9501323	1.47E-10	2.12E-08	13.720223
cg07526656	chr12	7260660	-	C1RL;LOC283314	Body;TSS1500	-0.947646	-3.092346	-6.949944	1.47E-10	2.12E-08	13.71928
cg12624721	chr8	143511575	-			-1.807121	0.961565	-6.949303	1.48E-10	2.12E-08	13.716063
cg19476622	chr18	20714485	-	CABLES1;CABLES1	TSS1500;TSS200	-0.961441	-2.576669	-6.949195	1.48E-10	2.12E-08	13.715524
cg02954056	chr17	46827591	-			1.083739	-0.611331	6.9484208	1.49E-10	2.13E-08	13.71164
cg04883026	chr11	66487938	-	SPTBN2	Body	-1.529894	-1.243552	-6.961553	1.49E-10	2.13E-08	13.714269
cg05937737	chr3	170303540	+	SLC7A14	5'UTR	1.6716545	0.3869758	6.9525458	1.49E-10	2.13E-08	13.711634
cg02550950	chr2	197122104	-	HECW2	Body	-1.421939	-1.341458	-6.947991	1.49E-10	2.13E-08	13.709482
cg01284289	chr12	66025635	-			-1.529447	-1.309254	-6.947203	1.50E-10	2.14E-08	13.705532
cg07448060	chr7	79083753	+	MAGI2	TSS1500	1.7685616	-0.678557	6.9515379	1.50E-10	2.14E-08	13.706475
cg04707519	chr10	21799314	+			1.5902552	0.9243837	6.9469744	1.50E-10	2.14E-08	13.704386
cg13224161	chr6	33141279	-	COL11A2;COL11A2;COL11A2	Body;Body;Body	-1.772304	-0.262331	-6.951418	1.50E-10	2.14E-08	13.705873

cg20881888	chr3	50383079	-	ZMYND10;ZMYND10	1stExon;5'UTR	-1.515042	-2.198373	-6.945843	1.51E-10	2.15E-08	13.698713
cg16319535	chr5	153599583	-	GALNT10	Body	0.8561968	2.225869	6.9456609	1.51E-10	2.16E-08	13.6978
cg01091514	chr4	184644613	+			-1.282428	-1.05506	-6.945232	1.51E-10	2.16E-08	13.695648
cg05138957	chr14	101528123	-	MIR377	TSS1500	-1.347302	-0.301536	-6.945099	1.51E-10	2.16E-08	13.694984
cg07532089	chr2	71018354	-	FIGLA	TSS1500	0.9170676	3.1789997	6.9584199	1.51E-10	2.16E-08	13.69853
cg09255514	chr16	16181832	-	ABCC1;ABCC1;ABCC1;ABCC1;ABCC1	Body;Body;Body;Body;Body	1.2428043	0.9647544	6.944299	1.52E-10	2.17E-08	13.690972
cg12500879	chr11	31848649	-			3.3608434	-1.077255	6.94853	1.52E-10	2.17E-08	13.691534
cg22442352	chr5	149227361	-			1.4445077	2.5070644	6.9439992	1.52E-10	2.17E-08	13.689469
cg17707274	chr11	102401523	-	MMP7	TSS200	-1.414528	-2.675182	-6.943239	1.53E-10	2.18E-08	13.685656
cg15588215	chr3	27766045	+			1.2543023	-0.648953	6.9431592	1.53E-10	2.18E-08	13.685258
cg07533239	chr12	6422131	-	PLEKHG6;PLEKHG6;PLEKHG6;PLEKHG6	1stExon;Body;5'UTR;Body	-1.422778	-2.039858	-6.942038	1.54E-10	2.19E-08	13.679635
cg17385448	chr1	15911702	-	AGMAT	TSS200	-0.966867	-1.595616	-6.941517	1.54E-10	2.20E-08	13.677025
cg07068674	chr14	37123778	+			1.7950234	-0.212242	6.941198	1.54E-10	2.20E-08	13.675427
cg00144335	chr4	107176	-	ZNF718	Body	1.1424071	0.6218108	6.9408705	1.55E-10	2.20E-08	13.673785
cg22908679	chr14	79746212	-	NRXN3;NRXN3;NRXN3;NRXN3;NRXN3	Body;1stExon;1stExon;5'UTR;5'UTR	-1.487018	-3.33184	-6.940417	1.55E-10	2.21E-08	13.671514
cg11762968	chr13	95354190	-			1.2228909	-0.311789	6.9403149	1.55E-10	2.21E-08	13.671
cg22635491	chr12	115109934	-	TBX3;TBX3	Body;Body	2.2298219	-0.254236	6.9402543	1.55E-10	2.21E-08	13.670697
cg27399481	chr17	78069245	-	CCDC40	Body	0.9454151	2.7452937	6.9385701	1.56E-10	2.23E-08	13.662255
cg02649597	chr2	45171621	-	SIX3	Body	1.5517716	-1.141745	6.9382682	1.57E-10	2.23E-08	13.660743
cg22358236	chr11	62691452	-			-1.493165	-2.679934	-6.938325	1.57E-10	2.23E-08	13.661026

cg05323533	chr4	41750362	-	PHOX2B	Body	1.5430216	-0.163105	6.9377426	1.57E-10	2.23E-08	13.658108
cg15959252	chr12	48578724	-	C12orf68;C12orf68	3'UTR;1stExon	1.6135415	1.2550553	6.9419492	1.57E-10	2.23E-08	13.658491
cg21610839	chr3	23861312	-	UBE2E1;UBE2E1	Body;Body	0.9042518	0.9598106	6.9373398	1.57E-10	2.24E-08	13.65609
cg08752433	chr12	111016566	-	PPTC7	Body	-1.711066	-1.310199	-6.937241	1.58E-10	2.24E-08	13.655595
cg22130262	chr8	57026502	-	MOS	1stExon	2.052109	-0.979371	6.9366557	1.58E-10	2.24E-08	13.652662
cg23429240	chr12	109040983	+	CORO1C	3'UTR	1.1920413	0.801692	6.9357476	1.59E-10	2.25E-08	13.648112
cg13678641	chr17	8113909	+	AURKB	TSS200	-1.210967	-5.885009	-6.939994	1.59E-10	2.25E-08	13.648824
cg06616245	chr12	57854236	-	GLI1;GLI1;GLI1	5'UTR;5'UTR;5'UTR	-1.232127	-2.407413	-6.935453	1.59E-10	2.25E-08	13.646635
cg16143804	chr6	31706344	-	MSH5;MSH5;MSH5;MSH5	TSS1500;TSS1500;TSS1500;TSS1500	-1.127344	-2.29831	-6.935415	1.59E-10	2.25E-08	13.646445
cg08864645	chr6	33943929	+			1.6032656	0.1241907	6.9352888	1.59E-10	2.25E-08	13.645813
cg26375057	chr3	147127898	+	ZIC1;ZIC1	1stExon;5'UTR	1.2906571	-0.59096	6.9349742	1.59E-10	2.26E-08	13.644236
cg14526718	chr2	176932840	+			1.9645366	-0.865662	6.9387247	1.60E-10	2.26E-08	13.642478
cg24846030	chr6	30884683	+	VARS2;VARS2;VARS2	Body;Body;Body	1.1612644	0.7953611	6.9323472	1.62E-10	2.29E-08	13.631075
cg18407955	chr7	158110685	-	PTPRN2;PTPRN2;PTPRN2	Body;Body;Body	1.5802542	-0.397817	6.9307817	1.63E-10	2.31E-08	13.623233
cg02829688	chr1	119527008	+	TBX15	5'UTR	1.312789	-0.556241	6.9307171	1.63E-10	2.31E-08	13.622909
cg01007345	chr4	48271072	-	TEC	5'UTR	-1.071356	-2.226741	-6.930373	1.63E-10	2.31E-08	13.621185
cg19352038	chr2	223164869	+	PAX3;PAX3;PAX3;PAX3;PAX3;CCDC140;PAX3;PAX3;PAX3	TSS1500;TSS1500;TSS1500;TSS1500;TSS1500;5'UTR;<br>TSS1500;TSS1500;TSS1500	1.4841078	-0.099308	6.9299771	1.64E-10	2.31E-08	13.619203
cg12651953	chr17	31861187	-	ACCN1	Body	-1.864793	0.506421	-6.928873	1.65E-10	2.33E-08	13.613675
cg12847240	chr6	10382088	-			1.0747022	0.1143711	6.9288052	1.65E-10	2.33E-08	13.613333
cg18198306	chr6	153303350	-	FBXO5;FBXO5	5'UTR;Body	-1.5366	-2.652532	-6.928334	1.65E-10	2.33E-08	13.610975

cg10592734	chr7	139570596	-	TBXAS1;TBXAS1;TBXAS1;TBXAS1;TBXAS1;TBXAS1	Body;Body;Body;Body;Body;5'UTR	-1.835622	0.2354886	-6.927948	1.65E-10	2.33E-08	13.60904
cg25033990	chr17	32484014	-	ACCN1	TSS200	1.6650954	-1.315914	6.9278645	1.65E-10	2.33E-08	13.608622
cg09959790	chr13	59616937	-			-1.814595	0.3457799	-6.92744	1.66E-10	2.34E-08	13.606497
cg14571813	chr3	129062831	-			-1.74165	-2.274869	-6.927282	1.66E-10	2.34E-08	13.605705
cg16058274	chr14	94179847	-			-1.758295	-0.016247	-6.926409	1.67E-10	2.35E-08	13.601331
cg26286365	chr14	104648347	+			-1.502259	0.2215704	-6.930358	1.67E-10	2.35E-08	13.600643
cg24406391	chr1	155938911	+	ARHGEF2;ARHGEF2;ARHGEF2	Body;Body;Body	0.9299873	0.3948883	6.9254876	1.67E-10	2.36E-08	13.596719
cg09504320	chr1	157164796	-			1.3354878	0.5465585	6.9253587	1.68E-10	2.36E-08	13.596074
cg22494876	chr1	247618161	+			-1.927008	-0.390546	-6.925256	1.68E-10	2.36E-08	13.595559
cg21907107	chr5	76932062	+	OTP	Body	1.5109767	-0.119496	6.9251797	1.68E-10	2.36E-08	13.595177
cg00411097	chr7	1596050	-	TMEM184A;TMEM184A	1stExon;5'UTR	-1.009117	-1.619148	-6.924482	1.68E-10	2.37E-08	13.591682
cg26169845	chr17	1382768	-	MYO1C;MYO1C;MYO1C	Body;Body;Body	0.6734627	3.1184218	6.9331977	1.68E-10	2.37E-08	13.593969
cg09628877	chr3	195613739	-	TNK2;TNK2	Body;Body	1.0980321	4.1190989	6.928019	1.69E-10	2.38E-08	13.588834
cg05873085	chr2	237010312	+	AGAP1;AGAP1	Body;Body	1.011912	2.5198546	6.9235995	1.69E-10	2.38E-08	13.587265
cg03635766	chr17	38607865	+	IGFBP4	Body	1.3780259	0.9428385	6.9228323	1.70E-10	2.39E-08	13.583424
cg12233363	chr6	100913030	-	SIM1	TSS1500	1.4843392	-0.361022	6.9222059	1.70E-10	2.39E-08	13.580288
cg23740491	chr6	27228267	+			1.277468	-0.508479	6.9217759	1.71E-10	2.40E-08	13.578136
cg02892286	chr1	214153377	+			2.1328838	-0.898753	6.9215345	1.71E-10	2.40E-08	13.576927
cg07089942	chr16	49666504	+	ZNF423	Body	-1.27716	-0.546291	-6.921221	1.71E-10	2.40E-08	13.575359
cg14632902	chr9	139017648	+			-1.339015	0.1015317	-6.921132	1.71E-10	2.40E-08	13.574912

cg17643864	chr8	56852148	-	LYN;LYN	5'UTR;5'UTR	-1.613347	-2.98948	-6.9211	1.71E-10	2.40E-08	13.574751
cg26101485	chr8	142264769	+			1.6389758	1.9903485	6.9210745	1.71E-10	2.40E-08	13.574624
cg10458446	chr2	238500325	+	RAB17	TSS1500	-1.210295	-1.362117	-6.920805	1.72E-10	2.40E-08	13.573276
cg02640104	chr13	30476615	-			-1.213691	-3.546486	-6.920461	1.72E-10	2.41E-08	13.571552
cg12419685	chr20	33451367	-	GGT7	Body	1.4979651	2.6865149	6.9205099	1.72E-10	2.41E-08	13.571798
cg17804348	chr1	3649473	+	TP73;TP73;TP73;TP73	Body;Body;3'UTR;3'UTR	1.172999	0.6317948	6.9204848	1.72E-10	2.41E-08	13.571673
cg10180367	chr19	34808158	-	KIAA0355	Body	0.8082796	1.4457157	6.9199621	1.72E-10	2.41E-08	13.569056
cg24003866	chr4	184938470	-	STOX2	3'UTR	1.0400296	0.7913725	6.9198304	1.72E-10	2.41E-08	13.568397
cg08207707	chr11	124630919	-	ESAM	Body	1.4173334	1.0300587	6.9196355	1.73E-10	2.41E-08	13.567422
cg18498871	chr14	92785734	+			-1.50575	0.0739675	-6.919411	1.73E-10	2.42E-08	13.566298
cg02602455	chr9	36739781	+			1.5080827	-1.477057	6.9191411	1.73E-10	2.42E-08	13.564947
cg19870370	chr13	21010223	-	CRYL1	Body	0.8403303	0.9848726	6.9184445	1.74E-10	2.43E-08	13.56146
cg06786372	chr7	27141388	-	HOXA2	Body	1.6607017	0.5646873	6.9183332	1.74E-10	2.43E-08	13.560903
cg00272971	chr10	102419564	-			1.5516772	-0.93153	6.9166262	1.75E-10	2.45E-08	13.55236
cg10173774	chr2	186603441	+			1.2393912	-0.27054	6.9164056	1.76E-10	2.45E-08	13.551256
cg01561869	chr5	140725147	+	PCDHGA2;PCDHGA3;PCDHGA3;PCDHGA1	Body;1stExon;1stExon;Body	1.2700059	0.6487798	6.9160961	1.76E-10	2.45E-08	13.549707
cg09173325	chr12	53458408	-	SPRYD3	3'UTR	0.8841219	1.0950459	6.9154308	1.76E-10	2.46E-08	13.546378
cg18556455	chr2	45178474	-			1.0622348	1.6806847	6.914364	1.77E-10	2.47E-08	13.54104
cg26801037	chr7	157294357	-			-1.549858	-0.437565	-6.914224	1.78E-10	2.48E-08	13.540338
cg25900085	chr8	65291513	+	MIR124-2	TSS200	3.1068692	-1.568339	6.9184231	1.78E-10	2.48E-08	13.540887

cg22454022	chr8	69646661	+	C8orf34	Body	-1.269584	-2.421142	-6.913386	1.78E-10	2.48E-08	13.536144
cg07140497	chr12	113550113	+	RASAL1	Body	1.5214354	1.6399095	6.9128348	1.79E-10	2.49E-08	13.533389
cg21902327	chr12	4554786	-	FGF6	TSS200	-1.176066	0.285597	-6.912197	1.79E-10	2.50E-08	13.530198
cg09364733	chr13	33163175	+	PDS5B	5'UTR	1.2506822	1.3321637	6.9120549	1.80E-10	2.50E-08	13.529487
cg12626956	chr9	79634509	-	FOXB2	TSS200	1.4443169	-0.689289	6.9162858	1.80E-10	2.50E-08	13.530212
cg07470207	chr8	132828746	+			-2.153179	-1.890289	-6.916049	1.80E-10	2.50E-08	13.529148
cg12315997	chr4	39461668	-	LIAS;RPL9;RPL9;LIAS	Body;TSS1500;TSS1500;Body	-0.835489	-3.834049	-6.911622	1.80E-10	2.50E-08	13.527324
cg00097800	chr2	46593258	-	EPAS1	Body	1.8115782	2.0245601	6.9115084	1.80E-10	2.50E-08	13.526753
cg01055868	chr3	71307284	+	FOXP1;FOXP1	5'UTR;5'UTR	-1.7961	0.3951967	-6.910787	1.81E-10	2.51E-08	13.523146
cg10610428	chr12	5884295	-	ANO2	Body	-1.30326	-1.002102	-6.909822	1.82E-10	2.52E-08	13.518319
cg04377850	chr16	88677643	-	ZC3H18	Body	0.8170441	1.8114902	6.9095707	1.82E-10	2.53E-08	13.51706
cg12395420	chr19	54202584	-	MIR518F	TSS1500	-1.334347	0.1052506	-6.909097	1.82E-10	2.53E-08	13.514693
cg08607018	chr19	31842873	+			2.7204662	-0.399961	6.9081091	1.83E-10	2.54E-08	13.509749
cg07898303	chr13	44454458	-	C13orf31;C13orf31;CCDC122	5'UTR;5'UTR;TSS1500	-0.813874	-3.353631	-6.90795	1.83E-10	2.55E-08	13.508955
cg01027937	chr4	74486080	+	RASSF6;RASSF6;RASSF6	1stExon;5'UTR;Body	-0.849086	-2.826794	-6.907857	1.84E-10	2.55E-08	13.508487
cg21213593	chr5	123985368	-	ZNF608	Body	-1.567878	-2.601663	-6.907425	1.84E-10	2.55E-08	13.50633
cg11618529	chr19	58952108	-	ZNF132	TSS1500	1.2368227	-0.670925	6.907165	1.84E-10	2.55E-08	13.505027
cg00397274	chr2	240240189	+	HDAC4	Body	1.077294	2.754139	6.9070701	1.84E-10	2.55E-08	13.504553
cg19447686	chr12	53448085	+	TENC1;TENC1;TENC1	Body;Body;Body	-1.425931	-1.826079	-6.90692	1.84E-10	2.55E-08	13.503803
cg02931398	chr1	156637105	+			0.9020978	1.7561183	6.9058816	1.85E-10	2.57E-08	13.498609

cg01087419	chr12	14959261	-	C12orf69;C12orf60	Body;5'UTR	0.6754707	2.5338648	6.9056719	1.86E-10	2.57E-08	13.497561
cg03099208	chr7	8474544	-	NXPH1	5'UTR	1.6764826	-0.74331	6.9100067	1.86E-10	2.57E-08	13.498981
cg18247055	chr10	22634226	-	SPAG6;SPAG6	TSS200;TSS200	1.6807055	-0.305543	6.9054897	1.86E-10	2.57E-08	13.49665
cg14050824	chr9	140683797	-	EHMT1	Body	2.1244907	2.6113308	6.9132545	1.87E-10	2.58E-08	13.494407
cg10981651	chr5	153853545	+			1.7836762	-0.88809	6.9032211	1.88E-10	2.60E-08	13.485306
cg26267388	chr2	119591627	-			1.8297493	-0.907075	6.9031805	1.88E-10	2.60E-08	13.485103
cg12801030	chr1	203297311	+			-1.212293	-1.883908	-6.902368	1.89E-10	2.61E-08	13.481043
cg23982678	chr1	207224227	-	YOD1	1stExon	-1.139475	-3.288352	-6.901895	1.89E-10	2.61E-08	13.478675
cg07972322	chr7	134832939	-	TMEM140;TMEM140	1stExon;5'UTR	-0.766746	-4.063664	-6.901728	1.89E-10	2.62E-08	13.477839
cg03825810	chr3	38888170	+	SCN11A	3'UTR	-1.307498	-0.3592	-6.90159	1.90E-10	2.62E-08	13.477149
cg08243849	chr1	31539007	-	PUM1;PUM1	TSS1500;TSS1500	-0.682441	-2.081728	-6.905855	1.90E-10	2.62E-08	13.47826
cg06718763	chr1	24828586	-	RCAN3	TSS1500	-1.340015	-3.29798	-6.901054	1.90E-10	2.62E-08	13.474471
cg10317380	chr11	122934257	+	HSPA8;HSPA8	TSS1500;TSS1500	-1.010085	-3.13937	-6.901021	1.90E-10	2.62E-08	13.474307
cg17877704	chr12	127940483	-			1.9253362	-1.137124	6.9049755	1.90E-10	2.63E-08	13.473869
cg12502815	chr12	57623997	-	SHMT2;SHMT2;SHMT2;SHMT2;SHMT2;SHMT2;SHMT2;SHMT2;SHMT2	5'UTR;Body;1stExon;TSS200;Body;TSS200;Body;TSS200;Body	-0.677427	-4.346738	-6.900368	1.91E-10	2.63E-08	13.471043
cg23915111	chr1	200589553	-	KIF14;KIF14	1stExon;5'UTR	-0.583866	-3.981991	-6.899716	1.91E-10	2.64E-08	13.467783
cg18417954	chr19	55672513	+	C19orf51	Body	1.815453	-0.024887	6.8992844	1.92E-10	2.64E-08	13.465625
cg03713585	chr2	175693153	+	CHN1;CHN1	Body;Body	1.0639305	2.1282154	6.8986545	1.92E-10	2.65E-08	13.462477
cg09877744	chr22	51042356	+	MAPK8IP2;MAPK8IP2	Body;Body	1.2830618	0.2052206	6.8987062	1.92E-10	2.65E-08	13.462735
cg19802477	chr19	54850444	-	LILRA4	TSS200	-1.201022	-0.31203	-6.898456	1.93E-10	2.65E-08	13.461486

cg14317609	chr9	36986006	+	PAX5	Body	2.467554	-1.807934	6.8983033	1.93E-10	2.65E-08	13.460721
cg21241477	chr9	136223965	+	SURF1;SURF2	TSS1500;Body	-0.785386	-2.130382	-6.897559	1.94E-10	2.66E-08	13.456999
cg27578381	chr17	76814686	-	USP36	Body	1.0126474	1.3261086	6.8955143	1.96E-10	2.69E-08	13.446782
cg03380342	chr4	178400255	+			1.0077776	2.4254557	6.8994299	1.96E-10	2.69E-08	13.446076
cg26624021	chr16	56995739	-	CETP	TSS200	1.180326	1.6405204	6.8942012	1.97E-10	2.71E-08	13.440221
cg06142621	chr11	124805981	-	HEPACAM;HEPACAM	5'UTR;1stExon	-1.409032	-0.029544	-6.893939	1.97E-10	2.71E-08	13.438912
cg18445088	chr22	40081812	+	CACNA1I;CACNA1I	Body;Body	1.235743	-0.465799	6.8977108	1.98E-10	2.72E-08	13.437624
cg00087368	chr6	100896392	-	SIM1	Body	1.5750394	-0.405049	6.8930397	1.98E-10	2.72E-08	13.434417
cg04043150	chr1	110673319	+			1.1242252	-0.244713	6.8928775	1.98E-10	2.72E-08	13.433607
cg06587435	chr16	865125	+			-1.363789	0.150044	-6.8929	1.98E-10	2.72E-08	13.433717
cg10933003	chr4	174448341	-	HAND2	3'UTR	1.2778149	0.4578033	6.8925133	1.99E-10	2.72E-08	13.431787
cg00674679	chr2	176931680	+			2.0240364	-0.30266	6.8918767	1.99E-10	2.73E-08	13.428606
cg12655921	chr2	2019937	+	MYT1L	5'UTR	-1.653415	-0.163235	-6.891918	1.99E-10	2.73E-08	13.428814
cg11454459	chr10	30472048	-			-1.507981	0.1837028	-6.891344	2.00E-10	2.74E-08	13.425946
cg04835811	chr4	174431922	+			1.2458767	0.4833293	6.8910353	2.00E-10	2.74E-08	13.424403
cg00423453	chr11	5372944	-	OR51B6	1stExon	-1.509789	-0.271193	-6.890168	2.01E-10	2.75E-08	13.420069
cg06429887	chr14	24600500	+	FITM1	TSS200	1.1160848	0.9205608	6.8901061	2.01E-10	2.75E-08	13.41976
cg21342392	chr7	111203008	+	IMMP2L	TSS1500	-2.527037	0.5194913	-6.890014	2.01E-10	2.75E-08	13.419299
cg08455778	chr5	87975798	+	LOC645323	Body	1.888275	-1.476161	6.8899048	2.01E-10	2.75E-08	13.418755
cg20026939	chr18	44788898	+			1.6561844	-0.444411	6.8891455	2.02E-10	2.76E-08	13.414962

cg13909305	chr22	18229583	+	BID;BID;BID	Body;5'UTR;Body	0.827814	2.9135603	6.8888903	2.02E-10	2.77E-08	13.413687
cg06055873	chr7	27142204	+	HOXA2;HOXA2	1stExon;5'UTR	1.3168147	-0.342952	6.888546	2.03E-10	2.77E-08	13.411967
cg12685548	chr3	180644451	+	FXR1;FXR1;FXR1	Body;5'UTR;Body	1.2564602	1.2198273	6.8865331	2.05E-10	2.80E-08	13.401914
cg21707187	chr12	54345862	-			2.6573207	-0.970803	6.8900874	2.06E-10	2.81E-08	13.399483
cg11033271	chr7	21984339	-	CDCA7L;CDCA7L;CDCA7L	Body;Body;5'UTR	-0.945134	-4.745953	-6.894227	2.06E-10	2.81E-08	13.399771
cg22367191	chr6	10425648	-			1.9142038	-0.161168	6.8896014	2.06E-10	2.81E-08	13.397186
cg16151261	chr18	75691297	-			-1.63961	0.3514986	-6.885076	2.07E-10	2.82E-08	13.394636
cg23054189	chr1	248020692	-	TRIM58	1stExon	2.8652174	-0.518335	6.8845893	2.07E-10	2.82E-08	13.392206
cg27105634	chr11	5255241	+	HBD	Body	-1.576928	-0.390318	-6.884227	2.07E-10	2.83E-08	13.390399
cg10539274	chr4	74486446	-	RASSF6;RASSF6	TSS200;TSS1500	-1.18703	-2.559695	-6.884137	2.08E-10	2.83E-08	13.38995
cg06438577	chr14	105796713	-	PACS2;PACS2	Body;Body	0.7503983	1.3158454	6.8840146	2.08E-10	2.83E-08	13.389337
cg05066503	chr6	169978311	-	WDR27	Body	1.0142486	2.2723571	6.8880586	2.08E-10	2.83E-08	13.389368
cg05722504	chr4	41749361	+	PHOX2B	Body	1.0589512	-0.275899	6.8835378	2.08E-10	2.83E-08	13.386956
cg07953015	chr19	48285322	+	SEPW1	3'UTR	1.9052453	0.658097	6.8826893	2.09E-10	2.85E-08	13.382719
cg19894382	chr10	60086891	-			1.211066	1.3980699	6.8822781	2.10E-10	2.85E-08	13.380666
cg08465346	chr12	54441458	-	HOXC4	5'UTR	1.6809063	0.6511278	6.8861244	2.10E-10	2.86E-08	13.379854
cg11634248	chr11	67926133	+	SUV420H1	Body	0.9346597	1.7596626	6.8815784	2.10E-10	2.86E-08	13.377172
cg18516557	chr2	177004015	-			1.4247618	0.2877027	6.8815194	2.10E-10	2.86E-08	13.376878
cg06958537	chr21	40176950	-	ETS2	TSS1500	-1.115975	-2.976803	-6.881268	2.11E-10	2.86E-08	13.375622
cg09550083	chr2	66672337	-	MEIS1	Body	1.6605684	0.3503978	6.8813042	2.11E-10	2.86E-08	13.375803

cg26588061	chr10	12086036	-	UPF2;UPF2	TSS1500;TSS1500	-1.633344	-0.394303	-6.881364	2.11E-10	2.86E-08	13.376101
cg01031101	chr6	28227091	+	NKAPL	TSS200	1.7972523	0.1544917	6.8810768	2.11E-10	2.86E-08	13.374668
cg02486096	chr19	57373324	-			-1.189554	-1.377718	-6.880819	2.11E-10	2.87E-08	13.373381
cg14224444	chr6	118587190	+	SLC35F1	Body	-1.843482	-0.318852	-6.880797	2.11E-10	2.87E-08	13.373271
cg12265959	chr8	11324687	+	FAM167A	TSS1500	-0.844237	-3.440945	-6.880583	2.11E-10	2.87E-08	13.372202
cg23822732	chr2	2692854	+			-2.058042	0.4535484	-6.880474	2.11E-10	2.87E-08	13.371657
cg07355926	chr19	1852127	-			1.5456965	0.0498218	6.8803967	2.12E-10	2.87E-08	13.371273
cg22579504	chr7	5601919	-			-0.493142	-3.473934	-6.879884	2.12E-10	2.88E-08	13.368715
cg09881545	chr3	5137630	-			1.1818974	-0.314499	6.8787244	2.13E-10	2.89E-08	13.362925
cg08279097	chr1	114455336	-	DCLRE1B	3'UTR	1.102677	1.6207987	6.8785364	2.14E-10	2.89E-08	13.361986
cg11222217	chr1	10732519	-	CASZ1;CASZ1	Body;Body	1.5948578	2.0255585	6.8785069	2.14E-10	2.89E-08	13.361839
cg07921625	chr13	58208350	-	PCDH17	1stExon	1.5085423	-1.192468	6.8784122	2.14E-10	2.89E-08	13.361367
cg17107156	chr17	7350244	+	CHRNB1	Body	1.1617898	-0.5741	6.8782132	2.14E-10	2.90E-08	13.360373
cg09672383	chr2	25390540	-	POMC;POMC	5'UTR;5'UTR	1.2075429	-0.009398	6.878104	2.14E-10	2.90E-08	13.359828
cg17895254	chr10	62492157	-			-1.149329	-2.205641	-6.877906	2.14E-10	2.90E-08	13.358841
cg08049853	chr4	41875784	+			1.7534666	-0.414929	6.8776196	2.15E-10	2.90E-08	13.35741
cg13502540	chr6	31166512	+	HCG27	Body	-1.580096	-0.736032	-6.877296	2.15E-10	2.91E-08	13.355797
cg04462652	chr2	133429088	-	LYPD1;LYPD1	TSS1500;TSS200	-1.212687	-2.956406	-6.87714	2.15E-10	2.91E-08	13.355016
cg14041094	chr14	89148974	+	EML5	Body	1.269653	0.7971186	6.8769753	2.15E-10	2.91E-08	13.354194
cg07326016	chr16	88846234	+	FAM38A	Body	0.8347573	1.4945948	6.8768821	2.15E-10	2.91E-08	13.353729

cg11172423	chr1	43205623	-	CLDN19;CLDN19	1stExon;1stExon	0.8780881	1.3266336	6.8767149	2.16E-10	2.91E-08	13.352895
cg19125370	chr6	10390532	+			1.7465549	0.2543177	6.8764696	2.16E-10	2.91E-08	13.35167
cg15712304	chr11	125227489	+	PKNOX2	Body	-1.962169	0.3631769	-6.875412	2.17E-10	2.93E-08	13.346392
cg27536559	chr22	36465149	-			-1.317234	-1.771429	-6.873849	2.19E-10	2.95E-08	13.338594
cg17556527	chr11	67859023	-	CHKA;CHKA	Body;Body	1.4848942	1.8464946	6.873757	2.19E-10	2.95E-08	13.338133
cg14357535	chr2	25389040	+	POMC;POMC	5'UTR;5'UTR	-1.062215	-2.070038	-6.873613	2.19E-10	2.95E-08	13.337413
cg02707176	chr5	140798758	+	PCDHGA4;PCDHGA9;PCDHGA1;PCDHGB1;PCDHGB7;PCDHGB6;PCDHGB3;PCDHGA6;PCDHGA8;PCDHGA10;PCDHGA5;PCDHGB4;PCDHGB7;PCDHGA3;PCDHGA2;PCDHGB2;PCDHGA7;P[illegible]	Body;Body;Body;Body;1stExon;Body;Body;Body;Body;Body;Body;1stExon;Body;Body;Body;Body;Body	1.579802	0.8900495	6.8775292	2.20E-10	2.96E-08	13.336898
cg00348621	chr5	90680542	+	LOC100129716;ARRDC3	Body;TSS1500	-1.03321	-1.772839	-6.87252	2.20E-10	2.97E-08	13.331963
cg02576915	chr1	40769669	-	COL9A2	Body	1.0297941	1.3303368	6.8723211	2.21E-10	2.97E-08	13.330968
cg04606830	chr16	26999476	+			-1.721077	-0.071074	-6.872241	2.21E-10	2.97E-08	13.330568
cg18252309	chr7	153583867	-	DPP6	TSS1500	1.8463942	-0.855256	6.8722663	2.21E-10	2.97E-08	13.330695
cg08137085	chr7	1117540	-	C7orf50;C7orf50;C7orf50	Body;Body;Body	1.4439317	1.8348199	6.8717527	2.21E-10	2.98E-08	13.328132
cg26299169	chr13	28498956	+	PDX1	3'UTR	2.4522894	-0.405297	6.8715622	2.21E-10	2.98E-08	13.327182
cg15382538	chr5	72678193	+			2.1951069	-1.172557	6.8705399	2.23E-10	2.99E-08	13.322081
cg11334165	chr5	145758782	+			1.3995181	0.1418429	6.8702851	2.23E-10	3.00E-08	13.32081
cg11037477	chr4	99851008	-	EIF4E;EIF4E;EIF4E;EIF4E;EIF4E	5'UTR;1stExon;5'UTR;TSS1500;1stExon	-1.113079	-2.435765	-6.869057	2.24E-10	3.01E-08	13.314683
cg00743788	chr22	50701157	+	MAPK12	TSS1500	-1.41849	-1.494337	-6.867917	2.26E-10	3.03E-08	13.308999
cg01881322	chr6	53660854	-	LRRC1	Body	-1.006014	-2.217167	-6.867765	2.26E-10	3.03E-08	13.30824
cg23546474	chr2	223163033	+	PAX3;PAX3;PAX3;CCDC140;CCDC140;PAX3;PAX3;PAX3;PAX3;PAX3	Body;Body;Body;5'UTR;1stExon;Body;Body;Body;Body;Body	1.6543711	0.3739673	6.8671108	2.27E-10	3.04E-08	13.304975
cg15205441	chr10	126782357	-	CTBP2;CTBP2	5'UTR;5'UTR	1.2672797	1.4359211	6.865367	2.29E-10	3.07E-08	13.296278

cg23447996	chr9	93564603	-	SYK;SYK;SYK	Body;5'UTR;5'UTR	-1.081941	-2.301363	-6.865063	2.29E-10	3.07E-08	13.294765
cg12541454	chr4	174437915	-			1.4893504	0.386378	6.8637549	2.31E-10	3.09E-08	13.288239
cg09007236	chr17	78228820	-			-0.908534	-4.038177	-6.863203	2.31E-10	3.10E-08	13.285489
cg02264229	chr14	95240560	+			1.2087604	0.798346	6.862363	2.32E-10	3.11E-08	13.281298
cg13891768	chr4	111536024	-			1.9021773	-0.449827	6.8615785	2.33E-10	3.12E-08	13.277387
cg14118850	chr2	10447890	+	HPCAL1;HPCAL1	5'UTR;5'UTR	1.3760189	0.3413663	6.861038	2.34E-10	3.13E-08	13.274692
cg08649068	chr17	38512297	+	RARA;RARA;RARA;RARA	Body;Body;Body;Body	0.9090189	1.0157957	6.8600757	2.35E-10	3.15E-08	13.269894
cg22203219	chr12	6665335	+	IFFO1;IFFO1	TSS200;TSS200	1.5097787	-0.637116	6.8600317	2.35E-10	3.15E-08	13.269675
cg08661678	chr4	185381476	+	IRF2	5'UTR	0.8650179	2.5123292	6.8597779	2.35E-10	3.15E-08	13.268409
cg26612727	chr17	38024636	+	ZPBP2;ZPBP2	1stExon;1stExon	1.2081921	0.4962903	6.8597259	2.35E-10	3.15E-08	13.26815
cg20534702	chr7	4752170	-	FOXK1	Body	-1.928295	-0.04174	-6.859598	2.36E-10	3.15E-08	13.267515
cg23615741	chr10	101297642	+			1.938789	0.1612735	6.8593073	2.36E-10	3.15E-08	13.266064
cg03228555	chr19	45174912	-	CEACAM19;CEACAM19;CEACAM19;CEACAM19	5'UTR;5'UTR;1stExon;1stExon	-1.436943	-0.903639	-6.858872	2.37E-10	3.16E-08	13.263895
cg24302529	chr10	45923083	-	ALOX5	Body	1.1201592	0.336228	6.8581675	2.37E-10	3.17E-08	13.260381
cg14428146	chr8	23563925	+	NKX2-6	TSS200	2.7543718	-1.132236	6.8579363	2.38E-10	3.17E-08	13.259229
cg20194314	chr13	28503060	-			2.0365194	-1.164091	6.8575982	2.38E-10	3.18E-08	13.257544
cg19532939	chr12	14996525	+	ART4	TSS200	1.2315281	0.8766672	6.856699	2.39E-10	3.19E-08	13.253062
cg24599709	chr2	26578508	+	SELI	Body	-1.977355	0.4363647	-6.856699	2.39E-10	3.19E-08	13.253061
cg18303242	chr17	25879250	-	KSR1	5'UTR	-2.086117	0.721155	-6.856125	2.40E-10	3.20E-08	13.250202
cg05772104	chr2	98340425	+	ZAP70	5'UTR	0.9077097	0.5037525	6.8556673	2.40E-10	3.21E-08	13.247919

cg03692651	chr19	22444593	-			2.7415452	-0.025201	6.8552896	2.41E-10	3.21E-08	13.246037
cg23680787	chr14	104824167	-			-1.240459	-0.112613	-6.8554	2.41E-10	3.21E-08	13.246585
cg26316946	chr6	101846967	-	GRIK2;GRIK2;GRIK2;GRIK2;GRIK2;GRIK2	1stExon;1stExon;5'UTR;1stExon;5'UTR;5'UTR	1.8779074	-1.35687	6.8552829	2.41E-10	3.21E-08	13.246004
cg14188840	chr7	27220398	+	HOXA10	TSS1500	1.4788241	1.8061516	6.8549107	2.41E-10	3.22E-08	13.244149
cg19908611	chr7	1333912	+			-1.566532	0.1819981	-6.853558	2.43E-10	3.24E-08	13.23741
cg22162757	chr1	214699233	+	PTPN14	5'UTR	-2.067982	-0.649382	-6.852801	2.44E-10	3.25E-08	13.233638
cg00238818	chr5	176880261	+	PRR7	5'UTR	1.1920846	1.9456681	6.8525436	2.44E-10	3.25E-08	13.232353
cg07519259	chr2	31520167	-			1.2016802	1.5690792	6.852298	2.45E-10	3.25E-08	13.231129
cg06544405	chr18	70536807	-			1.6225711	-0.842857	6.8508441	2.47E-10	3.28E-08	13.223885
cg11484413	chr11	127645018	+			-1.86461	-0.461573	-6.850817	2.47E-10	3.28E-08	13.223752
cg20634266	chr1	91190493	+			1.3556263	-0.075953	6.8507477	2.47E-10	3.28E-08	13.223405
cg02042310	chr1	46940420	-			0.80115	-1.007698	6.8501841	2.47E-10	3.29E-08	13.220597
cg04165508	chr1	94167631	+			-1.687446	0.6654032	-6.849558	2.48E-10	3.30E-08	13.217478
cg17991843	chr8	51553739	-	SNTG1	Body	-1.693589	-0.014598	-6.849109	2.49E-10	3.30E-08	13.215242
cg26210721	chr17	74309004	-	PRPSAP1	Body	0.7842507	1.7674987	6.8491117	2.49E-10	3.30E-08	13.215254
cg02311152	chr14	71276396	+	MAP3K9	TSS1500	-0.579803	-3.442892	-6.84824	2.50E-10	3.32E-08	13.210911
cg14848772	chr6	27099813	+	HIST1H2AG	TSS1500	-1.718703	-3.330944	-6.860637	2.50E-10	3.32E-08	13.212393
cg00945234	chr1	50886411	+	DMRTA2	Body	1.7541082	-1.260871	6.8477247	2.51E-10	3.32E-08	13.208345
cg01279413	chr20	62030494	-			-1.408567	-1.609227	-6.847596	2.51E-10	3.32E-08	13.207703
cg03457729	chr15	89953295	-			2.1958709	-0.340667	6.8474774	2.51E-10	3.32E-08	13.207113

cg19837174	chr10	6389707	-			-1.338329	-0.777571	-6.847352	2.51E-10	3.33E-08	13.206488
cg16703762	chr5	76932047	+	OTP	Body	1.6409514	0.0819008	6.8468712	2.52E-10	3.33E-08	13.204094
cg05380759	chr3	45579354	+	LARS2	Body	0.9931111	0.8775991	6.8466916	2.52E-10	3.34E-08	13.203199
cg08918845	chr20	31595432	-	BPIL1;BPIL1	5'UTR;1stExon	-1.580037	0.1859365	-6.846255	2.52E-10	3.34E-08	13.201025
cg00230183	chr16	88882565	-	GALNS	Body	-1.094644	-1.333148	-6.850192	2.53E-10	3.34E-08	13.206142
cg15513743	chr1	119528688	-	TBX15	5'UTR	1.4707983	-0.316723	6.8460307	2.53E-10	3.34E-08	13.199907
cg03026306	chr18	21718767	+	CABYR;CABYR;CABYR;CABYR;CABYR;CABYR	TSS200;TSS200;TSS200;TSS200;TSS1500;TSS200	-1.291778	-2.838263	-6.845457	2.53E-10	3.35E-08	13.197052
cg06563089	chr12	75601157	-	KCNC2;KCNC2;KCNC2	Body;Body;Body	1.4206607	0.0300604	6.845261	2.54E-10	3.35E-08	13.196074
cg18423469	chr18	3726858	+	DLGAP1;DLGAP1	Body;Body	-1.744984	0.1200481	-6.845165	2.54E-10	3.36E-08	13.195593
cg20876010	chr1	64971752	-	CACHD1	Body	-1.030492	-1.641326	-6.844995	2.54E-10	3.36E-08	13.194749
cg25161912	chr12	66220771	-	HMGA2;RPSAP52;HMGA2	Body;TSS200;Body	-1.456151	-0.989652	-6.844429	2.55E-10	3.37E-08	13.19193
cg00917251	chr1	24829987	-	RCAN3	5'UTR	-1.039084	-2.643663	-6.844289	2.55E-10	3.37E-08	13.191233
cg11665991	chr1	50893469	+			1.6220675	-0.491426	6.8437834	2.56E-10	3.38E-08	13.188715
cg20629021	chr11	68580041	-	CPT1A;CPT1A	Body;Body	0.9693202	2.2609806	6.8436503	2.56E-10	3.38E-08	13.188052
cg06666093	chr6	159291020	-			-0.563156	-4.277935	-6.847765	2.56E-10	3.38E-08	13.18891
cg21524155	chr1	51762608	+	TTC39A;TTC39A	Body;Body	-0.616131	-4.49846	-6.841891	2.58E-10	3.41E-08	13.179294
cg15032166	chr7	155263349	-			1.1432988	-0.448566	6.8413325	2.59E-10	3.41E-08	13.176511
cg00110654	chr6	10385489	-			2.1843888	-1.089295	6.8411634	2.59E-10	3.42E-08	13.175669
cg09706133	chr15	68659758	-	ITGA11	Body	-0.983202	-1.505924	-6.840947	2.59E-10	3.42E-08	13.174589
cg09519218	chr10	126390317	+	FAM53B	Body	1.1302985	0.0060107	6.8407748	2.60E-10	3.42E-08	13.173734

cg00544674	chr11	39689583	+			-1.948902	0.0013779	-6.839751	2.61E-10	3.44E-08	13.168639
cg21714809	chr11	72463184	+	ARAP1	5'UTR	-0.809778	-3.423187	-6.83937	2.62E-10	3.44E-08	13.16674
cg20741169	chr7	27205159	-	HOXA9	TSS200	1.7777736	-1.422025	6.8380983	2.63E-10	3.47E-08	13.160409
cg14101959	chr9	128229793	-	MAPKAP1;MAPKAP1;MAPKAP1;MAPKAP1;MAPKAP1	Body;Body;Body;Body;Body	1.3109895	1.7090569	6.8377614	2.64E-10	3.47E-08	13.158732
cg25022915	chr17	49243257	-	NME2;NME2;NME1-NME2;NME2;NME2	TSS1500;TSS1500;Body;5'UTR;TSS1500	-1.147356	-1.813569	-6.835711	2.67E-10	3.51E-08	13.148524
cg20810997	chr20	29978353	+	DEFB119;DEFB119;DEFB119;DEFB119;DEFB119;DEFB119	5'UTR;1stExon;1stExon;5'UTR;5'UTR;1stExon	-1.116012	-1.334921	-6.835476	2.67E-10	3.51E-08	13.147354
cg04881622	chr16	86317741	+			-1.245674	-0.842303	-6.834646	2.68E-10	3.52E-08	13.143225
cg04904318	chr17	46607828	-	HOXB1	1stExon	2.6480415	3.1898701	6.8388252	2.68E-10	3.52E-08	13.144482
cg24748769	chr14	57271015	-	OTX2;OTX2	Body;Body	2.093607	-0.815113	6.8343975	2.68E-10	3.53E-08	13.141989
cg10651537	chr2	177134737	+	MTX2;MTX2	Body;Body	-0.575592	-4.726755	-6.834192	2.69E-10	3.53E-08	13.140965
cg24887265	chr2	45233528	-	SIX2	Body	2.3428933	0.2772638	6.832813	2.71E-10	3.55E-08	13.134104
cg27660490	chr12	3049788	-	TULP3;TULP3	Body;3'UTR	0.7321002	0.092831	6.8322158	2.71E-10	3.56E-08	13.131132
cg13212186	chr6	31166504	+	HCG27	Body	-1.597871	-0.293196	-6.836328	2.72E-10	3.56E-08	13.132079
cg00674896	chr2	102804576	-	IL1RL2	Body	-1.419686	-2.373291	-6.831388	2.73E-10	3.58E-08	13.127014
cg18270760	chr10	43691112	+	RASGEF1A	3'UTR	1.0254403	2.1784587	6.835284	2.73E-10	3.58E-08	13.126754
cg10743390	chr19	30020728	-	VSTM2B	Body	1.1432714	0.1469229	6.8305858	2.74E-10	3.59E-08	13.123022
cg24875595	chr2	79363761	-	REG1P	Body	-1.653352	-0.389836	-6.830644	2.74E-10	3.59E-08	13.123309
cg23467079	chr8	124219425	+	FAM83A;FAM83A	Body;Body	1.3900363	1.9523515	6.8303489	2.74E-10	3.59E-08	13.121843
cg11036833	chr5	115152494	+	CDO1	TSS200	3.0528441	-1.442432	6.8300358	2.74E-10	3.60E-08	13.120285
cg02857760	chr2	177012562	+			1.5382817	-0.245007	6.8296193	2.75E-10	3.60E-08	13.118213

cg27634876	chr14	52735485	+	PTGDR	Body	1.9190826	-1.853053	6.8292805	2.76E-10	3.61E-08	13.116528
cg19430694	chr7	811390	-	HEATR2	Body	1.0252174	-0.4059	6.8291188	2.76E-10	3.61E-08	13.115723
cg16321280	chr11	44332972	+	ALX4	TSS1500	1.6609336	-0.639247	6.8290395	2.76E-10	3.61E-08	13.115329
cg23318967	chr7	4751873	+	FOXK1	Body	-2.144587	0.226644	-6.828798	2.76E-10	3.61E-08	13.11413
cg02082273	chr11	10473038	-	AMPD3	Body	-1.058609	-0.891865	-6.828622	2.77E-10	3.61E-08	13.113249
cg05735180	chr18	55095177	+			2.1987889	-0.301259	6.8286247	2.77E-10	3.61E-08	13.113265
cg05860723	chr12	81102486	-	MYF6	Body	1.7262427	-0.349379	6.8286722	2.76E-10	3.61E-08	13.113502
cg16026549	chr6	32800519	+	TAP2;TAP2	Body;Body	2.1469918	3.5103423	6.8411867	2.77E-10	3.61E-08	13.12138
cg08092105	chr16	58535384	-	NDRG4;NDRG4;NDRG4	Body;Body;Body	-1.843333	-2.085455	-6.827802	2.78E-10	3.63E-08	13.109175
cg06807993	chr17	79099785	-	MIR657;AATK;MIR338	TSS1500;Body;TSS200	-2.280874	0.4763814	-6.827664	2.78E-10	3.63E-08	13.108485
cg10855978	chr8	23780241	+			-1.702292	-1.123714	-6.827011	2.79E-10	3.64E-08	13.105236
cg18268547	chr17	79615552	+	TSPAN10	3'UTR	1.6399923	0.5023201	6.8305783	2.80E-10	3.65E-08	13.103384
cg05940452	chr11	118842484	+	FOXR1;FOXR1	5'UTR;1stExon	1.3931243	0.9920965	6.8565065	2.81E-10	3.66E-08	13.113251
cg10081811	chr12	57390700	+			1.0754635	1.5062679	6.8242923	2.83E-10	3.69E-08	13.091716
cg05631737	chr8	49780701	-			-1.25713	-1.093731	-6.828215	2.83E-10	3.69E-08	13.091787
cg07645844	chr8	26722635	+	ADRA1A;ADRA1A;ADRA1A;ADRA1A;ADRA1A;ADRA1A;ADRA1A;ADRA1A	1stExon;5'UTR;1stExon;1stExon;5'UTR;5'UTR;5'UTR;1stExon	1.143057	-0.440479	6.8409014	2.83E-10	3.69E-08	13.094624
cg16384849	chr1	34642386	-	C1orf94;C1orf94	5'UTR;TSS200	1.260576	0.2076553	6.8234345	2.84E-10	3.70E-08	13.08745
cg23715830	chr3	13974614	+			2.2413629	-0.52322	6.8267768	2.85E-10	3.71E-08	13.090037
cg00806644	chr10	126889815	+			-2.147225	-1.44095	-6.822395	2.86E-10	3.72E-08	13.082279
cg06133145	chr14	94254351	+	PRIMA1	5'UTR	1.79275	-1.490321	6.8222977	2.86E-10	3.72E-08	13.081797

cg09880291	chr7	27184109	+	HOXA5	TSS1500	1.486165	0.9378617	6.8218189	2.86E-10	3.73E-08	13.079416
cg13921444	chr4	186733024	+	SORBS2;SORBS2;SORBS2;SORBS2;SORBS2;SORBS2;SORBS2	5'UTR;5'UTR;5'UTR;1stExon;TSS1500;1stExon;TSS1500	-1.230439	-0.89376	-6.821701	2.87E-10	3.73E-08	13.07883
cg27559399	chr1	201450997	+			-1.107133	-1.958679	-6.825829	2.87E-10	3.73E-08	13.07994
cg09974661	chr2	3642634	+	COLEC11;COLEC11	TSS200;TSS200	1.2471812	0.7454234	6.8213767	2.87E-10	3.73E-08	13.077217
cg08782899	chr17	79652389	-	ARL16;HGS	TSS1500;Body	-1.233955	0.2288926	-6.828629	2.89E-10	3.75E-08	13.079457
cg12368612	chr12	115135999	+			1.6841241	-0.386773	6.823423	2.90E-10	3.77E-08	13.067862
cg07014174	chr21	32253760	-	KRTAP11-1	1stExon	-1.573771	-0.118791	-6.818915	2.91E-10	3.78E-08	13.064979
cg11312408	chr13	58207013	+	PCDH17	1stExon	1.6320993	-1.092203	6.8187822	2.91E-10	3.78E-08	13.064318
cg05943655	chr6	43636172	+	RSPH9	Body	-1.470138	-1.857404	-6.818614	2.91E-10	3.78E-08	13.063484
cg09797337	chr13	112709538	+			1.848664	-0.338866	6.8226009	2.91E-10	3.78E-08	13.06392
cg17862981	chr2	149869265	+	KIF5C	3'UTR	1.27323	2.141754	6.8184698	2.91E-10	3.78E-08	13.062765
cg22218512	chr12	52308278	-	ACVRL1;ACVRL1	Body;Body	1.9987861	3.1835354	6.8308055	2.92E-10	3.79E-08	13.070119
cg18588811	chr14	101527144	+	MIR377	TSS1500	-1.598055	0.3113489	-6.817763	2.92E-10	3.79E-08	13.05925
cg17272838	chr4	152597659	+	PET112L	Body	-1.434225	0.3500039	-6.817587	2.93E-10	3.79E-08	13.058377
cg22596016	chr17	61711557	-	MAP3K3;MAP3K3	Body;Body	1.3566061	0.4844267	6.8170585	2.94E-10	3.80E-08	13.055749
cg09746494	chr4	147568715	+			1.289084	-1.155417	6.8166826	2.94E-10	3.81E-08	13.053881
cg22869030	chr4	85414147	-			1.9161296	-1.029839	6.8162475	2.95E-10	3.82E-08	13.051718
cg26530045	chr1	23487055	-	LUZP1;LUZP1	5'UTR;5'UTR	1.4910178	3.3163353	6.8157263	2.96E-10	3.83E-08	13.049128
cg20232986	chr6	100915767	+			1.9643056	-0.744256	6.8154381	2.96E-10	3.83E-08	13.047695
cg07059052	chr16	2828302	+	TCEB2;TCEB2	TSS1500;TSS1500	-1.089735	-3.088625	-6.814984	2.97E-10	3.83E-08	13.045439

cg13398470	chr8	27862840	+			-1.091181	-2.052048	-6.814997	2.97E-10	3.83E-08	13.045503
cg21653149	chr17	4108148	+	ANKFY1;ANKFY1	Body;Body	1.3496071	1.206585	6.8150189	2.97E-10	3.83E-08	13.045611
cg24659328	chr3	58563666	-	FAM107A;FAM107A	TSS200;TSS200	1.1023822	1.6808263	6.8151144	2.96E-10	3.83E-08	13.046086
cg08668316	chr4	380157	-			1.4798911	-0.20297	6.8147239	2.97E-10	3.84E-08	13.044145
cg11391201	chr11	44333070	-	ALX4	TSS1500	1.2995892	-0.882568	6.8147306	2.97E-10	3.84E-08	13.044179
cg22162847	chr1	150534779	-			-0.519765	-4.141163	-6.81891	2.97E-10	3.84E-08	13.045603
cg01770232	chr7	22766155	+	IL6	TSS1500	-1.277161	-2.586853	-6.822631	2.98E-10	3.84E-08	13.044161
cg05865548	chr14	101498206	+	MIR543	TSS200	-1.427451	-0.228758	-6.81412	2.98E-10	3.85E-08	13.041142
cg27143082	chr6	31744628	+	C6orf27	5'UTR	-0.766232	-2.351283	-6.813883	2.98E-10	3.85E-08	13.039969
cg02142926	chr6	134994674	+			-1.352928	-1.74192	-6.813751	2.99E-10	3.85E-08	13.039313
cg20637609	chr17	46825011	-			1.2934871	-0.403127	6.8132467	2.99E-10	3.86E-08	13.036804
cg10366797	chr12	54441509	+	HOXC4	5'UTR	1.1061622	0.5948274	6.8128608	3.00E-10	3.87E-08	13.034887
cg03364683	chr4	169402298	+	DDX60L	TSS1500	-1.294927	-3.751374	-6.812676	3.00E-10	3.87E-08	13.033968
cg19459207	chr5	49737773	+	EMB	TSS1500	-1.409339	-0.938948	-6.812569	3.00E-10	3.87E-08	13.033435
cg24938752	chr3	128294769	+	C3orf27;C3orf27	1stExon;5'UTR	1.8861757	3.3167429	6.8163379	3.01E-10	3.88E-08	13.032844
cg08465307	chr1	3827081	-	LOC100133612	Body	-2.092457	-0.621127	-6.814852	3.03E-10	3.90E-08	13.025475
cg11338339	chr3	194823909	+	C3orf21	Body	1.122928	2.2206099	6.8107107	3.03E-10	3.90E-08	13.024203
cg13435326	chr3	178907655	+	PIK3CA	5'UTR	1.3760244	0.8149261	6.8098946	3.05E-10	3.92E-08	13.020148
cg24750513	chr2	127819455	-	BIN1;BIN1;BIN1;BIN1;BIN1;BIN1;BIN1;BIN1;BIN1;BIN1	Body;Body;Body;Body;Body;Body;Body;Body;Body;Body	1.1966112	2.4525035	6.8096826	3.05E-10	3.92E-08	13.019095
cg24211976	chr15	60285744	+			1.6882319	1.2510234	6.8132221	3.06E-10	3.93E-08	13.01725

cg07811054	chr15	97319911	+			-1.447998	-0.16978	-6.808631	3.07E-10	3.94E-08	13.01387
cg10424681	chr6	4079350	-	C6orf201;C6orf146;C6orf146	TSS200;5'UTR;1stExon	1.1428439	1.1732778	6.8082609	3.07E-10	3.95E-08	13.012032
cg08198187	chr12	31882596	+	AMN1;AMN1	TSS1500;TSS1500	-1.444634	-0.623581	-6.808175	3.07E-10	3.95E-08	13.011607
cg27072683	chr10	102289885	-	NDUFB8	TSS1500	-0.909786	-2.702765	-6.807991	3.08E-10	3.95E-08	13.010689
cg01582066	chr22	32057065	-			-1.221676	-1.907124	-6.807046	3.09E-10	3.97E-08	13.005996
cg13892386	chr9	120508122	-			-1.594697	0.5299264	-6.806888	3.09E-10	3.97E-08	13.00521
cg22561329	chr19	57346735	-	PEG3;PEG3;ZIM2;PEG3;PEG3;ZIM2;ZIM2	5'UTR;5'UTR;5'UTR;5'UTR;5'UTR;5'UTR;5'UTR	-0.87437	-1.370781	-6.805705	3.11E-10	3.99E-08	12.999336
cg11671363	chr5	148810177	+	MIR145;LOC728264	TSS200;Body	1.5009958	1.6482712	6.8048271	3.13E-10	4.01E-08	12.994975
cg20309677	chr1	116925753	+	ATP1A1;ATP1A1;ATP1A1	TSS1500;Body;Body	1.5226708	1.7603347	6.8038674	3.14E-10	4.03E-08	12.990209
cg02877240	chr7	112089798	-	IFRD1;IFRD1	TSS1500;5'UTR	-1.244225	-1.965534	-6.802505	3.16E-10	4.06E-08	12.983441
cg21837189	chr1	226085836	-			-1.021883	-0.604982	-6.802376	3.17E-10	4.06E-08	12.982801
cg26066361	chr12	10282621	-	CLEC7A;CLEC7A;CLEC7A;CLEC7A;CLEC7A;CLEC7A	1stExon;1stExon;1stExon;1stExon;1stExon;1stExon	-1.86445	-0.219926	-6.802098	3.17E-10	4.06E-08	12.981424
cg06890747	chr7	39649290	-	LOC646999	Body	2.0847822	0.0678099	6.8018598	3.17E-10	4.07E-08	12.980239
cg06122871	chr6	85476210	-			2.0653051	-0.280039	6.801303	3.18E-10	4.08E-08	12.977475
cg17770886	chr10	135121331	+	TUBGCP2;ZNF511	5'UTR;TSS1500	-1.027379	-2.132427	-6.801319	3.18E-10	4.08E-08	12.977553
cg01412022	chr6	33150892	+	COL11A2;COL11A2;COL11A2	Body;Body;Body	0.7430369	1.8443094	6.8011497	3.19E-10	4.08E-08	12.976714
cg03661324	chr2	79411917	-			-1.427944	-1.320974	-6.800986	3.19E-10	4.08E-08	12.975902
cg14813258	chr11	27935243	+			-1.468094	-0.098515	-6.800574	3.20E-10	4.09E-08	12.973854
cg01522425	chr3	16525848	+	RFTN1	Body	1.136686	1.925676	6.8003146	3.20E-10	4.09E-08	12.972567
cg22688399	chr3	61234515	+	FHIT;FHIT	5'UTR;5'UTR	0.9574095	2.0686259	6.8043333	3.20E-10	4.09E-08	12.973176

cg27425784	chr2	98340511	-	ZAP70	Body	1.0788642	1.2396912	6.7994753	3.21E-10	4.11E-08	12.9684
cg26537568	chr20	32304371	-	PXMP4;PXMP4	Body;Body	0.8476194	0.6386287	6.7993658	3.22E-10	4.11E-08	12.967857
cg21439672	chr12	7260546	-	C1RL;LOC283314	Body;TSS1500	-1.258045	-1.38704	-6.798079	3.24E-10	4.13E-08	12.961467
cg10765655	chr8	58188909	-			-1.93619	0.015522	-6.797551	3.25E-10	4.14E-08	12.958848
cg00043364	chr5	133916105	-	PHF15	3'UTR	0.8439611	1.6057252	6.7971367	3.25E-10	4.15E-08	12.95679
cg15183991	chr8	145047566	+	PLEC1;PLEC1	Body;Body	-1.242386	-2.348011	-6.797041	3.25E-10	4.15E-08	12.956316
cg09071280	chr1	39541952	+			-1.532486	0.6530468	-6.796792	3.26E-10	4.16E-08	12.955079
cg06552160	chr2	1926724	-	MYT1L	Body	-1.645315	-0.518445	-6.796006	3.27E-10	4.17E-08	12.95118
cg23732024	chr8	74903801	-	LY96	Body	-2.274624	-1.434398	-6.7957	3.28E-10	4.18E-08	12.949659
cg10333400	chr16	882746	-			-1.556456	0.4808639	-6.795571	3.28E-10	4.18E-08	12.949022
cg03384920	chr2	79587308	-			-1.597311	-0.494084	-6.795429	3.28E-10	4.18E-08	12.948313
cg05211068	chr5	90575141	-			-2.034105	1.4693844	-6.795432	3.28E-10	4.18E-08	12.94833
cg10851763	chr21	43545792	-	UMODL1;UMODL1	Body;Body	-1.697248	-1.561257	-6.799379	3.28E-10	4.18E-08	12.948767
cg15312264	chr14	55907299	-	TBPL2	TSS200	1.8773162	0.0375526	6.799311	3.29E-10	4.18E-08	12.948286
cg14795752	chr1	91185441	-			1.6232521	-1.373709	6.7947836	3.29E-10	4.19E-08	12.945111
cg06135480	chr16	85644718	-	KIAA0182	TSS1500	-1.003848	-2.951698	-6.794241	3.30E-10	4.20E-08	12.942417
cg09976051	chr4	178362394	-	AGA	Body	-1.376023	-0.945145	-6.794172	3.30E-10	4.20E-08	12.942076
cg23291886	chr4	174440681	+			1.636111	-0.160576	6.7941576	3.30E-10	4.20E-08	12.942004
cg00469473	chr19	11357722	+	DOCK6	Body	0.7038915	1.8816265	6.7938769	3.31E-10	4.20E-08	12.940611
cg25063165	chr6	30737436	-			-1.199423	-1.469691	-6.79384	3.31E-10	4.20E-08	12.940429

cg10805850	chr13	98794470	+	FARP1;FARP1	TSS1500;TSS1500	-1.705247	-1.9609	-6.79767	3.31E-10	4.21E-08	12.940157
cg26249245	chr7	101915942	-	CUX1;CUX1	Body;Body	-0.745877	-0.66886	-6.793142	3.32E-10	4.22E-08	12.936962
cg17718401	chr4	119199352	-	SNORA24;SNHG8	TSS1500;TSS1500	-0.867577	-0.602288	-6.792914	3.32E-10	4.22E-08	12.935833
cg25689193	chr15	53086968	+			1.8152764	-0.813647	6.7929256	3.32E-10	4.22E-08	12.93589
cg12021814	chr2	172973195	-			1.6208342	-0.454421	6.7928112	3.33E-10	4.22E-08	12.935323
cg10724867	chr7	27218867	-	HOXA10	Body	1.2625546	1.7327974	6.7967995	3.33E-10	4.22E-08	12.935986
cg17384380	chr10	102419209	+			1.6909345	-1.125965	6.791873	3.34E-10	4.24E-08	12.930667
cg21918786	chr6	109611834	+			1.5143623	2.5622929	6.7916364	3.35E-10	4.24E-08	12.929493
cg23477460	chr3	66848765	-			1.4825778	-1.248172	6.7911661	3.35E-10	4.25E-08	12.927159
cg18082788	chr6	149806339	-	ZC3H12D	TSS200	-2.433472	-2.452399	-6.7945	3.37E-10	4.27E-08	12.924451
cg14462321	chr9	137232372	-	RXRA	Body	0.6115823	1.1529366	6.7901829	3.37E-10	4.27E-08	12.922281
cg21341391	chr17	51832493	+			-1.596457	-0.1107	-6.790227	3.37E-10	4.27E-08	12.922497
cg22455694	chr2	176974001	-	HOXD11	3'UTR	1.1947353	-1.026998	6.7901443	3.37E-10	4.27E-08	12.922089
cg01610632	chr3	147127579	-	ZIC1;ZIC1	1stExon;5'UTR	1.6273487	-0.264533	6.7898904	3.38E-10	4.27E-08	12.92083
cg24180759	chr5	167551234	-	ODZ2	Body	-1.564352	-0.156568	-6.789927	3.38E-10	4.27E-08	12.921012
cg00529567	chr3	124772735	+	HEG1	Body	0.8785763	2.4351699	6.7892034	3.39E-10	4.28E-08	12.917421
cg21199093	chr1	35836009	-	ZMYM4	Body	1.4346211	0.8938433	6.7932995	3.39E-10	4.28E-08	12.923665
cg00575896	chr3	50357171	+	HYAL2;HYAL2	Body;Body	1.0608109	0.6766549	6.7928346	3.40E-10	4.29E-08	12.916347
cg22941668	chr5	148810180	+	MIR145;LOC728264	TSS200;Body	1.432034	1.7553962	6.7878566	3.41E-10	4.31E-08	12.91074
cg05033952	chr16	34255535	+			-1.803413	0.426888	-6.787657	3.42E-10	4.31E-08	12.909751

cg09776772	chr7	2150534	+	MAD1L1;MAD1L1;MAD1L1	Body;Body;Body	1.8837677	2.7011576	6.7870712	3.43E-10	4.33E-08	12.906844
cg27130630	chr12	7260666	-	C1RL;LOC283314	Body;TSS1500	-1.370609	-3.053028	-6.786973	3.43E-10	4.33E-08	12.906355
cg11816081	chr17	47539827	-			-1.365993	-0.440113	-6.786804	3.43E-10	4.33E-08	12.90552
cg08516516	chr5	115152492	+	CDO1	TSS200	3.5882671	-2.161736	6.7867418	3.43E-10	4.33E-08	12.90521
cg13163930	chr6	27647896	-			2.1923227	-0.689821	6.7864336	3.44E-10	4.34E-08	12.903681
cg12309668	chr4	186732837	-	SORBS2;SORBS2;SORBS2;SORBS2;SORBS2;SORBS2;SORBS2	5'UTR;5'UTR;5'UTR;1stExon;TSS1500;1stExon;TSS1500	-1.343906	-1.259792	-6.786353	3.44E-10	4.34E-08	12.903279
cg02936593	chr11	45734486	+			-1.443351	-0.692695	-6.786053	3.44E-10	4.34E-08	12.901793
cg23003085	chr11	64084321	-	PRDX5;PRDX5;TRMT112;PRDX5	TSS1500;TSS1500;3'UTR;TSS1500	-1.077222	-2.166254	-6.78608	3.44E-10	4.34E-08	12.901927
cg06605803	chr10	118547802	-			-0.559517	-3.452307	-6.784932	3.46E-10	4.36E-08	12.896231
cg07279070	chr2	73148229	+	EMX1	Body	1.3766803	-0.524913	6.7848275	3.47E-10	4.36E-08	12.895715
cg13449377	chr7	96631089	+	DLX6AS	Body	1.1453953	1.6991395	6.7881541	3.48E-10	4.38E-08	12.893025
cg07594833	chr7	73919889	-	GTF2IRD1;GTF2IRD1	5'UTR;5'UTR	0.7485929	1.0410331	6.7837978	3.48E-10	4.39E-08	12.890608
cg13492245	chr9	130566157	+	FPGS;FPGS	Body;5'UTR	-1.238637	-2.597837	-6.783709	3.49E-10	4.39E-08	12.890167
cg04670802	chr6	100050812	-			2.5474297	-1.979025	6.7831179	3.50E-10	4.40E-08	12.887236
cg24114556	chr17	44897431	-	WNT3	TSS1500	2.3878533	-0.635025	6.7831169	3.50E-10	4.40E-08	12.887231
cg09404516	chr2	54832147	+	SPTBN1;SPTBN1	Body;Body	1.0125979	0.3108443	6.7827005	3.50E-10	4.40E-08	12.885167
cg12691004	chr2	88352949	-	KRCC1	5'UTR	1.8175241	-0.808291	6.7826438	3.50E-10	4.40E-08	12.884885
cg17353412	chr7	27141774	-	HOXA2	1stExon	1.8174753	0.7639085	6.7826821	3.50E-10	4.40E-08	12.885075
cg14569837	chr11	55798206	-	OR5AS1	1stExon	-1.667571	0.0957284	-6.78203	3.52E-10	4.42E-08	12.88184
cg24939196	chr6	169588809	+			-1.9501	-1.728885	-6.78451	3.54E-10	4.45E-08	12.880217

cg04425920	chr10	50604518	-			2.2521707	-0.153296	6.7797096	3.56E-10	4.47E-08	12.870336
cg09225373	chr6	52217264	+			-1.059071	-1.945547	-6.783799	3.56E-10	4.47E-08	12.871609
cg01729491	chr7	27225772	+	HOXA11AS;HOXA11	Body;TSS1500	1.1791133	-0.388343	6.7789971	3.57E-10	4.48E-08	12.866804
cg11160908	chr3	122712271	-	SEMA5B	5'UTR	-1.206501	-2.128224	-6.778914	3.57E-10	4.48E-08	12.866391
cg16851417	chr21	38077042	-	SIM2;SIM2	Body;Body	1.7620918	-0.318316	6.7789538	3.57E-10	4.48E-08	12.866589
cg21488279	chr5	122434178	+	PRDM6	Body	0.9071706	0.9093382	6.7915402	3.57E-10	4.48E-08	12.870764
cg13137032	chr4	5904897	+			-1.927715	0.4275243	-6.777637	3.60E-10	4.51E-08	12.86006
cg05247661	chr2	30472410	+	LBH	Body	1.2074561	1.3890615	6.7772667	3.60E-10	4.51E-08	12.858226
cg25617328	chr5	76939333	-			1.0301644	-0.591871	6.7763325	3.62E-10	4.53E-08	12.853595
cg14162417	chr1	226315857	+			-1.195382	-1.026167	-6.776253	3.62E-10	4.54E-08	12.853202
cg13371501	chr10	5935533	-	FBXO18;FBXO18	Body;TSS1500	1.2523783	2.3699162	6.776164	3.62E-10	4.54E-08	12.85276
cg13924554	chr13	50710952	-			1.0777002	2.7439364	6.7760564	3.63E-10	4.54E-08	12.852227
cg15231462	chr1	247769251	-	OR2G3	1stExon	-1.798268	-0.00699	-6.77603	3.63E-10	4.54E-08	12.852094
cg04715572	chr11	2033791	+			-1.084235	0.202141	-6.775159	3.64E-10	4.56E-08	12.847778
cg04739647	chr2	176987462	-	HOXD9;HOXD9	5'UTR;1stExon	2.1585285	-0.761081	6.7751146	3.64E-10	4.56E-08	12.847558
cg03742808	chr17	76128634	-	TMC8;TMC6	Body;TSS200	-0.959156	-1.497183	-6.775014	3.64E-10	4.56E-08	12.847061
cg18265162	chr1	210407588	-	C1orf133;SERTAD4	TSS200;5'UTR	-0.863843	-3.421726	-6.774449	3.66E-10	4.57E-08	12.844262
cg16997642	chr7	27184159	-	HOXA5	TSS1500	1.8256385	0.7338711	6.7742187	3.66E-10	4.57E-08	12.843118
cg22694818	chr1	75595970	+	LHX8	5'UTR	1.567053	0.1212323	6.7741634	3.66E-10	4.57E-08	12.842844
cg10371155	chr17	37561101	-	MED1	3'UTR	-1.678472	-0.87222	-6.773764	3.67E-10	4.58E-08	12.840867

cg21762788	chr8	9756501	-			1.9911144	-0.37718	6.7736892	3.67E-10	4.58E-08	12.840494
cg00252781	chr1	179334658	+	C1orf125;C1orf125	TSS1500;TSS200	-0.898579	-2.466499	-6.773603	3.67E-10	4.58E-08	12.840066
cg02687786	chr4	16971135	-			-1.582945	0.3632675	-6.773058	3.68E-10	4.59E-08	12.837368
cg06372887	chr7	44795754	-	ZMIZ2;ZMIZ2	TSS200;5'UTR	0.7885515	0.8541463	6.7765662	3.69E-10	4.61E-08	12.835821
cg13307880	chr5	140744367	-	PCDHGA4;PCDHGA2;PCDHGA5;PCDHGB2;PCDHGA1;PCDHGB1;PCDHGA3;PCDHGA5	Body;Body;1stExon;Body;Body;Body;Body;1stExon	1.4413389	-0.521884	6.7723942	3.69E-10	4.61E-08	12.834077
cg06440348	chr4	81111527	+	PRDM8	5'UTR	1.1988618	1.4501584	6.7721755	3.70E-10	4.61E-08	12.832993
cg00557947	chr10	102419245	+			2.8471695	-1.392231	6.7801127	3.70E-10	4.61E-08	12.833869
cg03589751	chr15	81134721	-	KIAA1199;MIR549	5'UTR;TSS1500	-1.415784	-0.188568	-6.771865	3.70E-10	4.61E-08	12.831455
cg27504738	chr12	66564266	+	TMBIM4	TSS1500	-1.70761	0.1740823	-6.771789	3.71E-10	4.62E-08	12.831079
cg04786142	chr1	234908381	+			-2.224478	-2.565795	-6.775593	3.71E-10	4.62E-08	12.830861
cg21463349	chr2	119600948	-	EN1	Body	2.73012	-2.174636	6.771252	3.72E-10	4.63E-08	12.828417
cg14094983	chr7	8482099	+	NXPH1	Body	2.2307597	-1.057251	6.7704238	3.73E-10	4.64E-08	12.824314
cg03148184	chr4	111562513	+			1.5437767	-0.779354	6.7703163	3.73E-10	4.64E-08	12.82378
cg16990168	chr1	119526060	+	TBX15	5'UTR	1.5691381	0.2005296	6.7702763	3.73E-10	4.64E-08	12.823582
cg23906261	chr10	118030848	+	GFRA1;GFRA1;GFRA1	Body;Body;Body	1.6655315	-0.992986	6.7697834	3.74E-10	4.65E-08	12.82114
cg08245989	chr12	9280923	-			-1.276016	0.1817207	-6.769595	3.75E-10	4.66E-08	12.820208
cg24444059	chr9	35909412	+	LOC158376	TSS200	1.1143312	0.4934998	6.7694016	3.75E-10	4.66E-08	12.819249
cg12691651	chr15	73075087	+	ADPGK;ADPGK;ADPGK	Body;Body;Body	-1.10681	-2.263076	-6.769123	3.76E-10	4.67E-08	12.817871
cg02470871	chr3	119278637	-	CD80	TSS200	-1.859741	-2.039059	-6.781276	3.76E-10	4.67E-08	12.820301
cg14804000	chr17	29298605	+	RNF135;RNF135	Body;Body	-1.152435	-3.525437	-6.768345	3.77E-10	4.68E-08	12.814012

cg05480110	chr3	195534854	-	MUC4;MUC4;MUC4	Body;Body;Body	-1.914477	1.2285476	-6.768069	3.78E-10	4.69E-08	12.812649
cg05877497	chr2	66667946	-	MEIS1	Body	1.9472127	0.2150043	6.7678421	3.78E-10	4.69E-08	12.811523
cg00924143	chr12	114881676	+			1.2198137	-1.235593	6.7663899	3.81E-10	4.73E-08	12.804329
cg16500018	chr19	7702178	-	STXBP2;STXBP2	Body;Body	-0.657304	-4.309339	-6.765651	3.82E-10	4.74E-08	12.800669
cg19161124	chr7	153583563	+	DPP6	TSS1500	1.3102295	0.0021146	6.7656399	3.82E-10	4.74E-08	12.800614
cg21077957	chr8	142505227	+	FLJ43860	Body	-1.229605	-0.329214	-6.765677	3.82E-10	4.74E-08	12.8008
cg26419880	chr1	6515624	+	ESPN	Body	2.0480396	-0.528685	6.7657222	3.82E-10	4.74E-08	12.801022
cg25440497	chr20	61757848	+			-1.334893	-0.510294	-6.765458	3.83E-10	4.74E-08	12.799712
cg25881804	chr20	21081901	-			3.0851895	-0.765547	6.7695272	3.83E-10	4.74E-08	12.801007
cg23708209	chr15	88843899	+			-1.388415	-0.672869	-6.765202	3.83E-10	4.75E-08	12.798445
cg24720571	chr6	6004328	-	NRN1	Body	3.0655835	-1.992912	6.7688828	3.84E-10	4.75E-08	12.797672
cg04750421	chr14	97208333	+			1.0712974	0.1621861	6.7638545	3.86E-10	4.78E-08	12.791772
cg03371669	chr2	176965435	-	HOXD12	Body	0.9764864	-0.45407	6.7634644	3.87E-10	4.78E-08	12.78984
cg12992240	chr11	64216638	+			-0.696906	-3.050752	-6.7632	3.87E-10	4.79E-08	12.788529
cg25483003	chr9	139948856	-	ENTPD2;ENTPD2	TSS1500;TSS1500	-1.353955	-2.210889	-6.762924	3.88E-10	4.79E-08	12.787166
cg09303701	chr2	20190082	-	WDR35;WDR35	TSS200;TSS200	-1.357535	-5.237507	-6.762744	3.88E-10	4.80E-08	12.786271
cg27628839	chr1	155534509	-			-1.503519	-0.184004	-6.762516	3.89E-10	4.80E-08	12.785146
cg14426510	chr17	48796535	+	LUC7L3;LUC7L3	TSS1500;TSS1500	-1.090628	-3.593367	-6.762434	3.89E-10	4.80E-08	12.784737
cg21888059	chr6	112042632	-	FYN;FYN;FYN	5'UTR;5'UTR;TSS1500	0.9754779	2.143432	6.7623443	3.89E-10	4.80E-08	12.784293
cg03283990	chr15	35129641	-			-1.267823	-0.734483	-6.76228	3.89E-10	4.80E-08	12.783976

cg14008999	chr17	750766	+	NXN	Body	1.5177292	-0.146967	6.7619912	3.90E-10	4.81E-08	12.782545
cg13291704	chr10	15761498	-	ITGA8	Body	2.6133024	-1.89626	6.7617415	3.90E-10	4.81E-08	12.781309
cg05752356	chr22	49373145	-			-1.302848	0.2916145	-6.76152	3.91E-10	4.82E-08	12.780211
cg10122865	chr2	63284132	+	OTX1	3'UTR	2.6964156	0.0145112	6.7614596	3.91E-10	4.82E-08	12.779913
cg02226239	chr8	143999155	-	CYP11B2	1stExon	-1.006615	-0.266518	-6.761074	3.92E-10	4.83E-08	12.778002
cg10055501	chr4	111540657	+	PITX2;PITX2;PITX2	Body;Body;Body	1.9659031	-0.307935	6.7602352	3.93E-10	4.85E-08	12.77385
cg26172812	chr19	48837548	+	TMEM143	Body	-1.081286	-3.297705	-6.760149	3.93E-10	4.85E-08	12.773425
cg25390658	chr7	134576216	-	CALD1;CALD1;CALD1;CALD1;CALD1;CALD1;CALD1	Body;1stExon;5'UTR;1stExon;5'UTR;Body;Body	-1.195728	-2.747166	-6.759748	3.94E-10	4.86E-08	12.771436
cg14688579	chr2	223168453	+	CCDC140	5'UTR	2.2365673	-1.377636	6.7592365	3.95E-10	4.87E-08	12.768906
cg07368796	chr10	43800133	+			-0.765999	-1.693084	-6.758852	3.96E-10	4.87E-08	12.767001
cg02127965	chr19	45175197	+	CEACAM19;CEACAM19;CEACAM19;CEACAM19	5'UTR;5'UTR;1stExon;1stExon	-1.506377	0.4142194	-6.758553	3.97E-10	4.88E-08	12.765524
cg19723715	chr7	1125817	-	GPER;C7orf50;C7orf50;GPER;C7orf50	TSS1500;Body;Body;TSS1500;Body	1.0078623	1.636886	6.7584479	3.97E-10	4.88E-08	12.765002
cg23097402	chr1	50883394	+	DMRTA2	3'UTR	2.0018328	0.0842507	6.7584907	3.97E-10	4.88E-08	12.765214
cg09336899	chr2	233766002	+	NGEF;NGEF	Body;Body	1.7078228	1.9147177	6.7576809	3.98E-10	4.90E-08	12.761205
cg21143441	chr17	8124011	+	C17orf44	Body	-1.398341	-0.549902	-6.757673	3.98E-10	4.90E-08	12.761164
cg12685539	chr11	10472001	-	AMPD3	TSS1500	-1.079301	-2.640671	-6.757223	3.99E-10	4.91E-08	12.75894
cg00754604	chr2	161230046	-	RBMS1;RBMS1	Body;Body	1.5442326	1.2962013	6.7569097	4.00E-10	4.91E-08	12.757387
cg16239218	chr10	117715809	-			-1.872733	0.2480186	-6.756664	4.00E-10	4.92E-08	12.756171
cg06821999	chr14	95238156	-			1.2854656	-0.085767	6.7558804	4.02E-10	4.94E-08	12.752293
cg13790426	chr1	109506963	-	CLCC1;CLCC1	TSS1500;TSS1500	-1.188134	-2.646271	-6.755388	4.03E-10	4.95E-08	12.749856

cg26875135	chr7	1221192	-			-1.166596	-1.883988	-6.75507	4.04E-10	4.95E-08	12.748283
cg25513635	chr19	18981378	+	GDF1;LASS1	5'UTR;3'UTR	1.5154355	-0.474413	6.7536983	4.07E-10	4.99E-08	12.741493
cg22032532	chr12	88974665	+	KITLG;KITLG	TSS1500;TSS1500	-0.996175	-2.52391	-6.753447	4.07E-10	4.99E-08	12.740249
cg13289553	chr5	32585524	-	SUB1	TSS200	-0.892891	-3.872365	-6.753079	4.08E-10	5.00E-08	12.738431
cg02104049	chr14	104665750	+			-1.080402	-0.169151	-6.753003	4.08E-10	5.00E-08	12.738052
cg13187990	chr8	1423775	-			-1.118114	-0.506692	-6.752925	4.08E-10	5.00E-08	12.737668
cg04096519	chr11	60620343	-	GPR44	Body	1.1696899	0.9742072	6.7567392	4.09E-10	5.01E-08	12.737804
cg11433622	chr2	66667549	-	MEIS1	Body	2.6629147	-0.720678	6.7521562	4.10E-10	5.02E-08	12.733862
cg20174696	chr6	167765215	+			-1.092082	-3.506927	-6.752193	4.10E-10	5.02E-08	12.734046
cg02711608	chr19	47287964	+	SLC1A5;SLC1A5;SLC1A5;SLC1A5	1stExon;5'UTR;Body;5'UTR	-0.8265	-2.544148	-6.751961	4.10E-10	5.02E-08	12.732897
cg19165390	chr18	56932082	-			1.2975887	-0.071738	6.7519471	4.10E-10	5.02E-08	12.732827
cg03936663	chr1	218098422	+			1.5978593	-0.173154	6.7517662	4.11E-10	5.02E-08	12.731932
cg12965599	chr3	147125924	-	ZIC1	TSS1500	1.7508471	-0.573482	6.7514026	4.11E-10	5.03E-08	12.730133
cg02051616	chr5	172661132	+	NKX2-5;NKX2-5;NKX2-5	Body;Body;Body	1.2608973	-0.406427	6.7511757	4.12E-10	5.04E-08	12.72901
cg18280830	chr14	57265875	-			1.5639148	0.5241342	6.7551763	4.12E-10	5.04E-08	12.730083
cg12769118	chr1	247712377	-	C1orf150	TSS200	-1.72393	-1.323937	-6.750742	4.13E-10	5.04E-08	12.726862
cg14283802	chr8	87684132	+	CNGB3	Body	-1.864244	0.0452008	-6.750625	4.13E-10	5.05E-08	12.726286
cg07759903	chr5	180545879	-			-1.484778	-0.036583	-6.750566	4.13E-10	5.05E-08	12.725993
cg10633906	chr7	1595602	+	TMEM184A	5'UTR	-0.972445	-0.852927	-6.750332	4.14E-10	5.05E-08	12.724836
cg06497848	chr10	118896363	+	VAX1;VAX1	Body;Body	1.2062218	-0.43623	6.7498569	4.15E-10	5.06E-08	12.722485

cg02773086	chr2	177027440	+	HOXD3	TSS1500	1.9412514	0.1430783	6.7619906	4.15E-10	5.07E-08	12.731233
cg27427514	chr3	193922037	+			-2.236489	-2.514431	-6.7486	4.17E-10	5.09E-08	12.716264
cg14015441	chr8	105479318	-	DPYS	TSS200	1.6749107	-0.324961	6.7485063	4.18E-10	5.09E-08	12.715803
cg07480608	chr3	15352493	-	SH3BP5;SH3BP5	Body;5'UTR	-1.199356	-0.563963	-6.7479	4.19E-10	5.11E-08	12.712804
cg13021333	chr6	6004207	-	NRN1	Body	2.656155	-0.630826	6.7475634	4.20E-10	5.11E-08	12.711139
cg07340241	chr16	22358051	-	CDR2	3'UTR	0.6741084	1.0693544	6.7473591	4.20E-10	5.12E-08	12.710128
cg26783057	chr10	118891670	+	VAX1	3'UTR	2.8997335	-1.294471	6.7472398	4.20E-10	5.12E-08	12.709538
cg08958294	chr6	146350131	-	GRM1;GRM1	5'UTR;5'UTR	2.2982948	-0.555496	6.7468722	4.21E-10	5.13E-08	12.70772
cg12515635	chr15	31660558	-	KLF13	Body	1.3030758	0.2408889	6.74558	4.24E-10	5.16E-08	12.701328
cg26345105	chr1	228634265	-			1.4577248	0.802771	6.7453042	4.25E-10	5.17E-08	12.699964
cg10059484	chr4	183839326	+	DCTD;DCTD	TSS1500;TSS1500	-1.031697	-2.593778	-6.745248	4.25E-10	5.17E-08	12.699686
cg20171999	chr8	67343066	-			-1.451192	-1.113113	-6.745041	4.25E-10	5.17E-08	12.698662
cg06654956	chr13	79161082	+			1.4003243	-0.4529	6.7440919	4.27E-10	5.19E-08	12.693969
cg13823169	chr9	139776893	-			-0.987894	-0.744979	-6.743961	4.27E-10	5.20E-08	12.693319
cg01187997	chr11	1594830	-	HCCA2;LOC338651	Body;Body	-1.096738	-2.91801	-6.743619	4.28E-10	5.20E-08	12.691631
cg07194984	chr11	2128771	+			-1.618282	4.59E-05	-6.74356	4.28E-10	5.20E-08	12.69134
cg23065927	chr12	3309816	-	TSPAN9;TSPAN9	5'UTR;5'UTR	-2.02608	-3.592521	-6.747193	4.29E-10	5.21E-08	12.690658
cg06648780	chr3	105133817	+	ALCAM	Body	1.2424849	1.2307962	6.7429782	4.30E-10	5.22E-08	12.688461
cg13316404	chr10	6191076	+	PFKFB3	Body	-1.288069	-0.231533	-6.74699	4.30E-10	5.22E-08	12.689655
cg00792513	chr6	100066698	-			1.4515903	0.5743112	6.7424947	4.31E-10	5.23E-08	12.68607

cg09986292	chr4	13525605	+			1.4475216	-0.998302	6.7411553	4.34E-10	5.26E-08	12.679447
cg19589494	chr11	9648370	-			1.2248665	1.993443	6.741124	4.34E-10	5.26E-08	12.679292
cg05273533	chr16	86470722	-			-1.557234	0.046832	-6.740816	4.34E-10	5.27E-08	12.677772
cg21875802	chr2	45231382	-			2.7446664	-1.358985	6.7403546	4.35E-10	5.28E-08	12.675488
cg02399464	chr12	50482541	+	SMARCD1;SMARCD1	Body;Body	-0.921806	-2.070226	-6.740263	4.36E-10	5.28E-08	12.675038
cg05693204	chr5	1860135	-			-1.080909	-1.229345	-6.738806	4.39E-10	5.32E-08	12.66783
cg05923687	chr1	63795711	+			2.5570606	-1.636204	6.7383272	4.40E-10	5.33E-08	12.665465
cg07554986	chr7	4751906	+	FOXK1	Body	-2.493068	0.4826089	-6.738228	4.40E-10	5.33E-08	12.664977
cg13315345	chr2	102803985	+	IL1RL2	5'UTR	-0.97858	-1.4154	-6.738102	4.41E-10	5.33E-08	12.664351
cg16674116	chr11	1319689	-	TOLLIP	Body	0.7862138	2.1782595	6.7379785	4.41E-10	5.33E-08	12.663741
cg16653620	chr2	162284135	-			1.9085267	-0.833389	6.7377852	4.41E-10	5.34E-08	12.662786
cg13242558	chr3	129062937	-			-1.408366	-1.534443	-6.737154	4.43E-10	5.35E-08	12.659667
cg22094599	chr4	139964398	+	CCRN4L	Body	1.1732928	0.5040869	6.7372085	4.43E-10	5.35E-08	12.659935
cg17840501	chr17	75385086	+	SEPT9;SEPT9;SEPT9;SEPT9;SEPT9	5'UTR;5'UTR;Body;Body;Body	1.0686706	0.0483397	6.7364473	4.44E-10	5.37E-08	12.656173
cg12866769	chr15	67041828	+	SMAD6;SMAD6;SMAD6	Body;Body;Body	0.999182	2.6551559	6.7404004	4.44E-10	5.37E-08	12.657133
cg23370512	chr11	61334385	+	SYT7	Body	-1.1052	-0.828042	-6.735932	4.45E-10	5.38E-08	12.653624
cg06708634	chr6	100906895	+	SIM1	Body	1.5719029	0.3604867	6.7397153	4.46E-10	5.39E-08	12.653753
cg19370653	chr4	111550040	+	PITX2;PITX2	Body;Body	1.9346379	-1.27613	6.7356326	4.46E-10	5.39E-08	12.652146
cg01591830	chr16	89666000	-			-1.127086	-1.674636	-6.735493	4.46E-10	5.39E-08	12.651454
cg22710661	chr11	17411071	-	KCNJ11;KCNJ11	TSS1500;TSS200	-0.633062	-4.589102	-6.735347	4.47E-10	5.39E-08	12.650737

cg20326647	chr8	144820709	-			-2.243518	0.8770991	-6.738304	4.49E-10	5.42E-08	12.646789
cg19043574	chr2	99439533	-	C2orf55	Body	1.9817992	-0.579601	6.7338246	4.50E-10	5.43E-08	12.643211
cg03893339	chr3	132438643	-	NPHP3	Body	0.9406367	2.2993932	6.7333125	4.51E-10	5.44E-08	12.64068
cg07015014	chr14	91770418	+	CCDC88C	Body	0.8090078	1.5441776	6.7331801	4.52E-10	5.45E-08	12.640026
cg13800769	chr12	4916913	-	KCNA6	TSS1500	-1.293334	-1.360867	-6.732843	4.53E-10	5.45E-08	12.63836
cg03087880	chr16	49666012	-	ZNF423	Body	-1.410694	-0.311955	-6.732375	4.54E-10	5.47E-08	12.636047
cg09967479	chr12	133010684	-			-1.16868	-0.324494	-6.731812	4.55E-10	5.48E-08	12.633266
cg04247717	chr2	131797702	+	ARHGEF4;ARHGEF4	Body;Body	0.8628778	-0.53845	6.7355418	4.56E-10	5.49E-08	12.633163
cg01792666	chr16	30710912	+	SRCAP	5'UTR	-0.601758	-3.264888	-6.731254	4.56E-10	5.49E-08	12.630509
cg06766579	chr1	150242418	+	APH1A;APH1A	TSS1500;TSS1500	0.8208083	1.6862046	6.7309977	4.57E-10	5.50E-08	12.629242
cg02671880	chr13	95355117	-			2.2523208	-0.862439	6.7348873	4.57E-10	5.50E-08	12.635416
cg22526226	chr12	46169000	+	ARID2	Body	0.8180541	1.792212	6.7306136	4.58E-10	5.51E-08	12.627344
cg23989673	chr15	53098257	-			1.9420317	-1.086375	6.7305473	4.58E-10	5.51E-08	12.627017
cg16426215	chr16	56706854	+			-1.037249	-1.737582	-6.730373	4.58E-10	5.51E-08	12.626154
cg04665046	chr9	123691674	+			-0.978133	-2.487586	-6.729401	4.61E-10	5.54E-08	12.621354
cg12682870	chr6	43766804	-			-1.233576	-1.443857	-6.729286	4.61E-10	5.54E-08	12.620786
cg16322014	chr11	6518559	-	DNHD1;DNHD1;DNHD1;DNHD1	5'UTR;1stExon;5'UTR;1stExon	1.1095317	1.1785643	6.7285733	4.63E-10	5.55E-08	12.617264
cg21831481	chr4	41880604	-			1.1938847	-0.840535	6.7285767	4.63E-10	5.55E-08	12.617281
cg26684776	chr6	90085550	-	RRAGD	Body	-1.382471	-1.237294	-6.728279	4.63E-10	5.56E-08	12.615813
cg22603555	chr10	105375201	-	SH3PXD2A	Body	0.9294824	1.425282	6.7280999	4.64E-10	5.57E-08	12.614926

cg13676215	chr7	17337976	-	AHR	TSS1500	-1.069744	-1.255034	-6.732027	4.64E-10	5.57E-08	12.615829
cg06793562	chr6	85482570	-			1.8153346	-0.649311	6.7278492	4.64E-10	5.57E-08	12.613687
cg07586956	chr2	70336043	-			-1.521235	-1.353664	-6.727493	4.65E-10	5.58E-08	12.61193
cg02100819	chr19	29284511	+			1.2825379	-0.906442	6.7270503	4.66E-10	5.59E-08	12.609741
cg09321400	chr16	55690443	-	SLC6A2	TSS200	2.1523126	-0.737168	6.7306128	4.67E-10	5.60E-08	12.608697
cg15368455	chr5	37835958	-	GDNF;GDNF;GDNF	TSS1500;5'UTR;TSS1500	1.4010032	0.2935347	6.7259571	4.69E-10	5.62E-08	12.604341
cg17033086	chr11	1937474	+			-1.483837	-0.202821	-6.725436	4.70E-10	5.63E-08	12.601767
cg23818246	chr18	22711612	-	ZNF521	Body	-2.039164	0.4557007	-6.724733	4.72E-10	5.65E-08	12.598295
cg03731131	chr19	44278274	-	KCNN4	Body	1.2359657	0.5111487	6.7240385	4.73E-10	5.67E-08	12.594866
cg16879247	chr6	36561383	-	SFRS3	TSS1500	-1.063194	-3.664369	-6.723885	4.74E-10	5.67E-08	12.594106
cg00152946	chr10	130338781	+			1.4776937	-0.41799	6.7237645	4.74E-10	5.67E-08	12.593513
cg13438893	chr13	79170303	+			2.470867	-0.846232	6.7236186	4.74E-10	5.68E-08	12.592793
cg16150752	chr5	16180072	-	11-Mar	TSS200	3.4233152	-0.741589	6.7271047	4.76E-10	5.69E-08	12.597886
cg18428542	chr2	91787196	-			-0.819907	-0.876115	-6.722505	4.77E-10	5.70E-08	12.587294
cg26133068	chr22	24199008	+	SLC2A11;SLC2A11;SLC2A11	TSS200;TSS1500;TSS200	-1.170028	-2.312831	-6.721949	4.79E-10	5.72E-08	12.584549
cg22890130	chr3	171856961	-	FNDC3B;FNDC3B	Body;Body	-1.349488	-2.709879	-6.721705	4.79E-10	5.73E-08	12.583345
cg13645811	chr6	158403520	+	SYNJ2	Body	-0.76048	-1.592921	-6.721522	4.80E-10	5.73E-08	12.582438
cg09132909	chr16	20055221	+	GPR139	Body	-1.356411	-0.308694	-6.720929	4.81E-10	5.74E-08	12.579513
cg02578087	chr3	8671361	+	C3orf32	Body	-1.757721	-1.477053	-6.720653	4.82E-10	5.75E-08	12.578149
cg04943797	chr20	61671429	+			-1.347358	-0.915768	-6.719941	4.84E-10	5.77E-08	12.574636

cg13753460	chr14	101520989	+	MIR134;MIR668;MIR485	TSS200;TSS1500;TSS1500	-1.127353	-0.400093	-6.71947	4.85E-10	5.78E-08	12.572307
cg10373607	chr20	51592177	+	TSHZ2	Body	-1.897635	0.1322913	-6.719067	4.86E-10	5.79E-08	12.570319
cg25139877	chr1	247691111	-	LOC148824	Body	-1.969768	0.6079826	-6.718866	4.86E-10	5.80E-08	12.569328
cg07435350	chr11	14539217	-	PSMA1;PSMA1;PSMA1	Body;Body;Body	1.0345541	2.9453214	6.7187402	4.87E-10	5.80E-08	12.568706
cg18080509	chr6	74009455	+	C6orf147	Body	1.0925923	0.6398741	6.7262243	4.88E-10	5.82E-08	12.568381
cg26848718	chr11	32454975	-	WT1;WT1;WT1;WT1	Body;Body;Body;Body	2.1357162	-1.518994	6.7300044	4.89E-10	5.82E-08	12.568032
cg02508743	chr8	56903623	-	LYN;LYN	Body;Body	-1.482127	-0.300948	-6.717527	4.90E-10	5.83E-08	12.562718
cg07236912	chr4	95429383	+	PDLIM5;PDLIM5;PDLIM5;PDLIM5;PDLIM5	Body;Body;Body;Body;Body	1.1768119	1.3755872	6.7167275	4.92E-10	5.85E-08	12.558771
cg13974464	chr19	49224165	-	MAMSTR;RASIP1	TSS1500;Body	-1.831056	-2.004454	-6.715577	4.94E-10	5.89E-08	12.553093
cg21589273	chr3	113416452	-	KIAA2018	TSS1500	-0.555335	-4.01177	-6.714961	4.96E-10	5.90E-08	12.550051
cg11178293	chr3	62355505	+	FEZF2	3'UTR	1.1964862	0.0686339	6.714684	4.97E-10	5.91E-08	12.548686
cg07119829	chr2	223168487	+	CCDC140	5'UTR	1.2990449	-0.878771	6.7137994	4.99E-10	5.94E-08	12.54432
cg00820405	chr2	97213642	+	ARID5A	Body	1.2429341	3.3695527	6.7135122	5.00E-10	5.94E-08	12.542902
cg05465955	chr17	40715236	+	COASY;COASY;COASY;COASY;COASY	1stExon;Body;Body;Body;5'UTR	-1.77804	0.1370848	-6.713495	5.00E-10	5.94E-08	12.542818
cg11256214	chr12	110211642	+	MGC14436;MGC14436	TSS1500;TSS1500	-1.142473	-2.094413	-6.713206	5.00E-10	5.95E-08	12.541394
cg19701577	chr7	27181418	-	HOXA5	3'UTR	1.5163718	1.1250304	6.713199	5.01E-10	5.95E-08	12.541357
cg16145825	chr1	228363521	+	C1orf69	3'UTR	0.5907834	2.0516972	6.7130853	5.01E-10	5.95E-08	12.540796
cg13904520	chr1	160783054	-	LY9	Body	-1.785129	-0.023503	-6.712377	5.03E-10	5.97E-08	12.5373
cg24171239	chr11	108627073	+	DDX10	Body	1.132309	2.406431	6.712038	5.03E-10	5.98E-08	12.535628
cg03329976	chr8	55370283	+	SOX17	TSS1500	1.6381815	-0.462002	6.7115162	5.05E-10	5.99E-08	12.533054

cg05157098	chr12	72059293	+	THAP2	Body	-1.174552	-1.896218	-6.7111	5.06E-10	6.00E-08	12.531
cg15212369	chr20	62274085	+	STMN3	Body	-1.172456	-0.96514	-6.710543	5.07E-10	6.02E-08	12.528252
cg08305799	chr8	30580804	+	GSR	Body	-1.863152	-2.731781	-6.71043	5.08E-10	6.02E-08	12.527694
cg19112186	chr22	51016638	-	CPT1B;CPT1B;CPT1B;CHKB-CPT1B;CPT1B;CPT1B;CPT1B;CPT1B	5'UTR;5'UTR;TSS200;Body;5'UTR;5'UTR;5'UTR;5'UTR	1.583546	1.2953434	6.7103411	5.08E-10	6.02E-08	12.527256
cg01036779	chr19	6768251	+	SH2D3A	TSS1500	-0.77663	-2.216473	-6.71009	5.09E-10	6.03E-08	12.526016
cg22609522	chr22	46403218	-	LOC730668	Body	-0.786861	-2.574799	-6.709908	5.09E-10	6.03E-08	12.525118
cg08672410	chr17	74497109	+	RHBDF2;RHBDF2	5'UTR;5'UTR	-0.671422	-3.639405	-6.709622	5.10E-10	6.04E-08	12.523708
cg23556108	chr2	60680616	+	BCL11A;BCL11A	Body;Body	1.2830145	2.5405202	6.7093294	5.11E-10	6.05E-08	12.522265
cg25567054	chr14	104824020	+			-0.887027	-0.75604	-6.709004	5.11E-10	6.05E-08	12.520659
cg11358713	chr7	41188092	-			-1.890361	0.5659026	-6.708946	5.12E-10	6.05E-08	12.520372
cg22637867	chr19	12624466	+	ZNF709	5'UTR	1.5997456	-0.009268	6.7126677	5.12E-10	6.06E-08	12.520421
cg04569082	chr12	127940323	-			2.7976727	-1.190115	6.7078041	5.15E-10	6.09E-08	12.514741
cg14218108	chr4	10658271	+	CLNK	Body	-1.855865	0.7593829	-6.707464	5.15E-10	6.10E-08	12.513061
cg09422450	chr16	79623618	+			2.0443758	0.2602193	6.7069241	5.17E-10	6.11E-08	12.5104
cg22836535	chr14	105956176	-	C14orf80;C14orf80;C14orf80	TSS1500;TSS1500;TSS1500	-1.157154	-5.738945	-6.723218	5.17E-10	6.11E-08	12.515758
cg14022137	chr6	150285634	+	ULBP1	Body	2.4015984	-3.277839	6.70941	5.21E-10	6.15E-08	12.504379
cg08065231	chr19	9473684	+	ZNF177	TSS200	2.9401178	-0.689766	6.7052968	5.21E-10	6.16E-08	12.502374
cg18065686	chr8	23411171	-	SLC25A37	Body	0.9376457	2.3414731	6.705206	5.21E-10	6.16E-08	12.501926
cg11823511	chr1	91183697	+	BARHL2	TSS1500	2.0933385	-0.22142	6.7047666	5.23E-10	6.17E-08	12.499759
cg06280210	chr7	26331247	-	SNX10	TSS1500	-0.852979	-3.447285	-6.703448	5.26E-10	6.21E-08	12.493255

cg08526825	chr16	2802229	+	SRRM2;LOC100128788	TSS200;Body	-0.998528	-5.025353	-6.702999	5.27E-10	6.22E-08	12.491041
cg10682957	chr11	117756485	+			1.8397032	0.3991608	6.7029774	5.27E-10	6.22E-08	12.490937
cg09238992	chr10	23484228	+			1.3949911	-1.129474	6.7026978	5.28E-10	6.23E-08	12.489558
cg01644481	chr6	106809170	+			-0.601774	-3.87169	-6.702234	5.29E-10	6.24E-08	12.487269
cg19694978	chr1	40420603	-	MFSD2A;MFSD2A	TSS200;TSS200	-0.902913	-5.030439	-6.706184	5.29E-10	6.24E-08	12.488499
cg25276892	chr22	40573076	-	TNRC6B;TNRC6B;TNRC6B	Body;TSS1500;TSS1500	-1.420086	-1.696201	-6.710186	5.30E-10	6.24E-08	12.489682
cg25748958	chr2	43672652	+	THADA;THADA	Body;Body	1.0570304	1.469889	6.7022431	5.29E-10	6.24E-08	12.487316
cg23912266	chr6	33139581	+	COL11A2;COL11A2;COL11A2	Body;Body;Body	-1.362574	-0.477464	-6.70183	5.31E-10	6.25E-08	12.485278
cg08750504	chr2	172946193	+			1.4094484	0.3735484	6.7011197	5.32E-10	6.27E-08	12.481777
cg10472711	chr7	797592	+	HEATR2	Body	1.060584	1.0830755	6.7005213	5.34E-10	6.29E-08	12.478827
cg06817917	chr10	88515527	-	BMPR1A	TSS1500	-0.536695	-3.698009	-6.699511	5.37E-10	6.32E-08	12.473847
cg01859340	chr18	44775576	+			1.4817222	-1.499371	6.6991968	5.38E-10	6.33E-08	12.472298
cg00872354	chr14	54080601	-			-1.229116	-1.283257	-6.699054	5.38E-10	6.33E-08	12.471596
cg24027996	chr12	111834727	+			-0.522073	-3.674897	-6.698388	5.40E-10	6.35E-08	12.46831
cg05259881	chr16	49631268	+	ZNF423	Body	-1.690912	0.8655368	-6.697647	5.42E-10	6.37E-08	12.46466
cg23833033	chr2	206544411	-			-1.325708	-0.300762	-6.697276	5.43E-10	6.38E-08	12.46283
cg04490211	chr10	110946662	-			-1.500674	-0.015518	-6.696861	5.44E-10	6.39E-08	12.460785
cg22522066	chr19	38754726	-	SPINT2;SPINT2	TSS1500;TSS1500	-0.788581	-3.403404	-6.696129	5.46E-10	6.42E-08	12.457177
cg08155116	chr17	79027207	-	BAIAP2;BAIAP2;BAIAP2;BAIAP2	Body;Body;Body;Body	1.4915783	1.7110795	6.6953388	5.48E-10	6.44E-08	12.453282
cg27539060	chr6	137112464	+	MAP3K5	Body	-0.898013	-3.767826	-6.69506	5.49E-10	6.45E-08	12.451906

cg01119374	chr2	109742964	+			-1.331699	-0.24699	-6.694508	5.51E-10	6.46E-08	12.449189
cg25384473	chr3	105082495	-			0.7754788	3.183235	6.6940878	5.52E-10	6.48E-08	12.447117
cg05050657	chr1	46957105	-			2.0057561	-0.496662	6.6937223	5.53E-10	6.49E-08	12.445316
cg09854003	chr5	170743459	+			1.6849195	-0.764298	6.6935665	5.53E-10	6.49E-08	12.444548
cg14831020	chr12	131418404	+			-1.446593	0.5199044	-6.693318	5.54E-10	6.50E-08	12.443323
cg14035521	chr9	37486469	-	POLR1E	Body	-1.114604	-2.488216	-6.705298	5.54E-10	6.50E-08	12.446794
cg08512167	chr19	45901567	-	PPP1R13L;PPP1R13L	Body;Body	1.1108254	0.0919243	6.6930095	5.55E-10	6.50E-08	12.441804
cg08258782	chr6	29987727	+	NCRNA00171	Body	0.6769573	2.0661587	6.7091598	5.55E-10	6.50E-08	12.44658
cg08069931	chr11	130731865	-			-0.720173	-3.154084	-6.69235	5.57E-10	6.52E-08	12.438553
cg03645522	chr4	141391200	+			0.8809267	0.5679794	6.696172	5.57E-10	6.52E-08	12.439231
cg02918093	chr16	10717941	+			0.8605923	1.9380221	6.6921088	5.58E-10	6.53E-08	12.437365
cg07628717	chr7	4752018	-	FOXK1	Body	-2.010346	-0.260987	-6.69171	5.59E-10	6.53E-08	12.4354
cg16472050	chr2	172945698	+			1.358897	0.0658144	6.6957017	5.59E-10	6.53E-08	12.436755
cg16596889	chr1	156891327	+	C1orf92	Body	-1.077944	-0.540408	-6.691767	5.59E-10	6.53E-08	12.435682
cg21182694	chr3	139062771	-	MRPS22	TSS200	-0.747708	-3.264833	-6.691712	5.59E-10	6.53E-08	12.435409
cg26492368	chr10	22634733	-	SPAG6;SPAG6	Body;Body	1.7331237	0.025813	6.6918353	5.58E-10	6.53E-08	12.436018
cg26790247	chr3	147108512	-	ZIC4;ZIC4;ZIC4;ZIC4;ZIC4	Body;Body;Body;Body;Body	1.8341373	-0.296425	6.6913346	5.60E-10	6.54E-08	12.433551
cg19261426	chr1	228560439	+	OBSCN	Body	-0.878771	-1.356932	-6.691042	5.61E-10	6.55E-08	12.432108
cg10721834	chr19	22990066	-			1.6635216	0.8324069	6.6904909	5.62E-10	6.57E-08	12.429394
cg25792439	chr17	78163268	-	CARD14;CARD14	Body;Body	1.1785417	1.841556	6.6943334	5.62E-10	6.57E-08	12.430187

cg09464990	chr17	70724035	-	SLC39A11;SLC39A11	Body;Body	-0.816745	-3.389968	-6.690156	5.63E-10	6.57E-08	12.427746
cg17444738	chr4	85417969	-	NKX6-1	Body	1.2024501	-0.729872	6.6894523	5.65E-10	6.60E-08	12.424278
cg21006727	chr21	46349059	-	ITGB2	TSS1500	-1.19626	0.1442946	-6.688833	5.67E-10	6.61E-08	12.421225
cg06405820	chr16	86951397	-			-1.665924	0.5193599	-6.688318	5.68E-10	6.63E-08	12.418692
cg14001023	chr19	46526321	-	PGLYRP1;PGLYRP1	1stExon;5'UTR	1.6570106	0.3214809	6.6881361	5.69E-10	6.63E-08	12.417794
cg05024715	chr11	123849197	-	OR10S1	TSS1500	-1.869459	0.1219348	-6.68771	5.70E-10	6.65E-08	12.415698
cg17241310	chr1	91182856	-	BARHL2	TSS200	1.430162	0.4009008	6.6876661	5.70E-10	6.65E-08	12.41548
cg12739119	chr1	43770523	-	TIE1	Body	1.519761	2.1717937	6.6860946	5.75E-10	6.70E-08	12.407739
cg02949991	chr22	29977262	+	NIPSNAP1	TSS200	-0.632994	-4.009216	-6.685683	5.76E-10	6.71E-08	12.405712
cg01771737	chr2	240199147	+	HDAC4	Body	0.9578323	1.7406311	6.6852827	5.77E-10	6.72E-08	12.403741
cg02892388	chr7	155260310	-			1.5347272	-0.560733	6.685192	5.78E-10	6.72E-08	12.403294
cg24965187	chr14	104824475	-			-1.359548	0.2468733	-6.683667	5.82E-10	6.78E-08	12.395784
cg22961237	chr12	92817091	+	CLLU1;CLLU1;CLLU1OS;CLLU1	Body;Body;Body;TSS1500	0.9885516	2.2440184	6.6868588	5.84E-10	6.80E-08	12.393434
cg01782132	chr16	28936054	-	RABEP2	Body	-0.371814	-3.51898	-6.682775	5.85E-10	6.80E-08	12.391392
cg11570011	chr4	16362340	-			-1.422736	0.3143759	-6.681805	5.88E-10	6.83E-08	12.386615
cg05058103	chr7	99051500	-	CPSF4;CPSF4	Body;Body	1.1278067	4.8900424	6.688835	5.90E-10	6.86E-08	12.384669
cg05171197	chr2	240230850	+	HDAC4	Body	1.7623747	2.0102197	6.6809127	5.90E-10	6.86E-08	12.382224
cg13154880	chr4	48486472	+	SLC10A4	Body	1.3783111	-0.879839	6.6810048	5.90E-10	6.86E-08	12.382677
cg13562266	chr8	1340351	-			-1.425122	-0.235472	-6.680676	5.91E-10	6.87E-08	12.38106
cg11006442	chr6	149721145	+	MAP3K7IP2;SUMO4	Body;TSS1500	1.1119235	2.7408943	6.6800339	5.93E-10	6.89E-08	12.377898

cg04548563	chr17	76356910	+	SOCS3	TSS1500	-0.61346	-4.338478	-6.679792	5.94E-10	6.89E-08	12.376705
cg13197551	chr20	60709957	-	LSM14B	3'UTR	0.8830036	1.6372385	6.679625	5.94E-10	6.90E-08	12.375885
cg24409566	chr11	2933967	+	SLC22A18;SLC22A18	Body;Body	-1.153403	-1.049153	-6.679381	5.95E-10	6.90E-08	12.374686
cg16367511	chr2	74425523	+	MTHFD2;MTHFD2	TSS200;TSS200	-1.327202	-3.636835	-6.679121	5.96E-10	6.91E-08	12.373405
cg20148334	chr1	209815452	-	LAMB3;LAMB3;LAMB3	Body;Body;Body	1.204879	0.13769	6.6788799	5.97E-10	6.92E-08	12.372217
cg16054018	chr10	104562697	-	C10orf26;C10orf26	Body;Body	1.0332509	2.1319069	6.6784923	5.98E-10	6.93E-08	12.370309
cg07211472	chr7	4304846	-	SDK1;SDK1	Body;Body	1.0130313	1.1944383	6.6782658	5.98E-10	6.94E-08	12.369195
cg07480446	chr2	239069576	-			0.9729988	1.0467702	6.6782915	5.98E-10	6.94E-08	12.369321
cg21223423	chr9	136472927	+			-1.195659	-1.309342	-6.676634	6.03E-10	6.99E-08	12.361162
cg08850461	chr5	87990082	-			2.2797153	-0.484127	6.6764956	6.04E-10	6.99E-08	12.360482
cg23322933	chr14	61109207	-			1.9138115	0.3311028	6.6761718	6.05E-10	7.00E-08	12.358888
cg23558337	chr20	10492394	-	C20orf94	Body	-0.978015	-4.126843	-6.676008	6.05E-10	7.01E-08	12.358082
cg24032710	chr14	104770459	+			-1.104658	-0.357689	-6.675926	6.06E-10	7.01E-08	12.35768
cg24154839	chr4	46995741	+	GABRA4	TSS200	2.0029276	-1.017531	6.6755973	6.07E-10	7.02E-08	12.356061
cg14338073	chr10	44359161	-			-1.82689	-0.136406	-6.675539	6.07E-10	7.02E-08	12.355774
cg05350798	chr14	104856667	+			-1.408321	-0.009246	-6.675298	6.08E-10	7.03E-08	12.354586
cg13385016	chr4	111538826	-	PITX2;PITX2;PITX2	3'UTR;3'UTR;3'UTR	1.5634653	0.3649351	6.6751675	6.08E-10	7.03E-08	12.353946
cg20532972	chr3	171179043	-	TNIK;TNIK;TNIK;TNIK;TNIK;TNIK;TNIK;TNIK;TNIK	TSS1500;TSS1500;TSS1500;TSS1500;TSS1500;TSS1500;TSS1500;TSS1500;TSS1500	-0.898597	-3.704608	-6.674981	6.09E-10	7.03E-08	12.353028
cg20197130	chr12	127256717	-			-2.294492	-0.29101	-6.690808	6.09E-10	7.04E-08	12.356811
cg05653018	chr1	201979533	+	ELF3;ELF3	TSS200;TSS200	-1.237938	-1.009048	-6.674665	6.10E-10	7.04E-08	12.351473

cg21164303	chr15	79603277	-	TMED3	TSS1500	-0.690339	-4.727299	-6.674467	6.10E-10	7.05E-08	12.350497
cg19194373	chr20	57875703	-	EDN3;EDN3;EDN3;EDN3;EDN3;EDN3;EDN3;EDN3	5'UTR;1stExon;1stExon;5'UTR;5'UTR;1stExon;1stExon;5'UTR	1.9595332	-0.618403	6.6743981	6.10E-10	7.05E-08	12.35016
cg00547530	chr16	3559687	+	CLUAP1;CLUAP1	Body;TSS1500	-1.042926	-1.124126	-6.674339	6.11E-10	7.05E-08	12.34987
cg14608903	chr10	116287249	+	ABLIM1;ABLIM1;ABLIM1;ABLIM1	Body;Body;TSS1500;Body	-1.305064	-0.705752	-6.673927	6.12E-10	7.06E-08	12.347842
cg14082413	chr1	26633267	-	UBXN11;UBXN11;UBXN11	5'UTR;TSS200;TSS200	-1.422074	-5.717805	-6.673103	6.14E-10	7.09E-08	12.343789
cg06604467	chr7	1232088	-			-1.279432	-3.279285	-6.672838	6.15E-10	7.10E-08	12.342483
cg10437722	chr17	35087542	+			-1.679967	0.0253717	-6.672753	6.16E-10	7.10E-08	12.342065
cg06880420	chr1	200119987	-	NR5A2;NR5A2	Body;Body	-1.525433	-0.999393	-6.676509	6.16E-10	7.10E-08	12.342411
cg03857553	chr22	29976613	+	NIPSNAP1	Body	-1.036451	-1.569521	-6.672242	6.17E-10	7.11E-08	12.339553
cg13884879	chr2	9540759	+	ASAP2;ASAP2	Body;Body	-1.635846	0.1418184	-6.672286	6.17E-10	7.11E-08	12.339769
cg18103859	chr14	61110002	+			2.5593773	-0.196694	6.6720875	6.18E-10	7.11E-08	12.338791
cg18573884	chr12	51639577	-	SMAGP;SMAGP;DAZAP2	3'UTR;3'UTR;Body	0.6170129	2.8362662	6.6720573	6.18E-10	7.11E-08	12.338643
cg10582871	chr1	10725332	+	CASZ1;CASZ1	Body;Body	1.3198333	3.043757	6.6756982	6.19E-10	7.12E-08	12.338595
cg22759823	chr19	57610870	+			2.704384	-0.758009	6.6789757	6.21E-10	7.14E-08	12.336477
cg06953130	chr10	11912410	-	C10orf47	3'UTR	1.0665211	1.4862337	6.6709595	6.21E-10	7.15E-08	12.333242
cg19300924	chr1	119536064	-			1.5972783	-1.409304	6.6702224	6.24E-10	7.17E-08	12.329615
cg18421840	chr7	31099814	+	ADCYAP1R1	5'UTR	-1.059689	-0.816296	-6.669518	6.26E-10	7.19E-08	12.326152
cg23774016	chr1	159284082	+	OR10J3	1stExon	-1.531889	-0.132742	-6.669498	6.26E-10	7.19E-08	12.326051
cg20600338	chr14	71786811	-			-0.649765	-4.078269	-6.669421	6.26E-10	7.20E-08	12.325674
cg24423088	chr21	32185365	+	KRTAP8-1	1stExon	-1.552636	0.5491477	-6.669246	6.27E-10	7.20E-08	12.324811

cg15668490	chr19	16254489	+	HSH2D	5'UTR	-1.01159	-1.995399	-6.669083	6.27E-10	7.20E-08	12.32401
cg11423556	chr2	69728114	-	AAK1	Body	1.0427911	1.3462521	6.668561	6.29E-10	7.22E-08	12.321443
cg03474926	chr9	136023407	-	RALGDS	Body	-0.633937	-1.681828	-6.668204	6.30E-10	7.23E-08	12.319687
cg15808426	chr19	57154302	+			1.4336583	-0.61757	6.6679718	6.31E-10	7.24E-08	12.318545
cg24335984	chr3	50361554	-	HYAL2	TSS1500	0.9755487	1.1113006	6.667773	6.31E-10	7.24E-08	12.317567
cg00154357	chr3	147111280	-	ZIC4;ZIC4;ZIC4;ZIC4;ZIC4	Body;TSS1500;Body;Body;TSS1500	1.2104677	0.3113459	6.6673031	6.33E-10	7.26E-08	12.315256
cg00254133	chr20	61340542	+	NTSR1;NTSR1	5'UTR;1stExon	2.0490248	-1.031003	6.6705654	6.35E-10	7.28E-08	12.313389
cg27462160	chr16	89768630	+	SPATA2L	TSS1500	-0.620674	-3.132652	-6.666623	6.35E-10	7.28E-08	12.311913
cg25507121	chr17	62051767	+	SCN4A	TSS1500	-1.453643	-0.453416	-6.666434	6.36E-10	7.29E-08	12.310984
cg23590660	chr12	8205602	-	FOXJ2	3'UTR	1.1018208	0.9153645	6.666192	6.36E-10	7.29E-08	12.309792
cg06014401	chr3	27765232	-	EOMES	TSS1500	2.085151	-1.783891	6.6658791	6.38E-10	7.30E-08	12.308253
cg15205636	chr13	21520468	-			2.6591365	-1.29644	6.6657326	6.38E-10	7.31E-08	12.307533
cg08152445	chr19	42734313	+			0.7535931	1.9410932	6.6655102	6.39E-10	7.31E-08	12.306439
cg08913410	chr7	30726859	+			-1.36425	-0.609333	-6.665112	6.40E-10	7.33E-08	12.30448
cg07489048	chr14	29236898	-	FOXG1	1stExon	1.7444683	0.3011284	6.6648916	6.41E-10	7.33E-08	12.303397
cg10583941	chr1	16770233	-	NECAP2;NECAP2;NECAP2	Body;Body;Body	0.7622924	2.1213317	6.6646876	6.41E-10	7.34E-08	12.302394
cg03554749	chr4	85405132	+			1.7463854	-0.963783	6.6646081	6.42E-10	7.34E-08	12.302003
cg01032851	chr16	68994912	-	TMCO7	Body	1.1694687	1.5517592	6.664119	6.43E-10	7.36E-08	12.299599
cg24283836	chr13	111061780	+	COL4A2	Body	0.9141216	1.6448153	6.6638444	6.44E-10	7.36E-08	12.298248
cg26034341	chr17	477314	+	VPS53;VPS53	Body;Body	1.5843815	1.7597515	6.6634931	6.45E-10	7.38E-08	12.296521

cg07626482	chr19	47289503	+	SLC1A5;SLC1A5;SLC1A5	TSS1500;Body;5'UTR	-0.93311	-2.547929	-6.66318	6.46E-10	7.39E-08	12.294979
cg03531853	chr9	86535572	+	KIF27	5'UTR	-0.889256	-3.642652	-6.661569	6.52E-10	7.44E-08	12.287063
cg06483505	chr6	4607680	+			-1.332548	-1.321841	-6.660314	6.56E-10	7.49E-08	12.280891
cg07837260	chr9	19788564	-			1.8967725	-0.82243	6.659967	6.57E-10	7.50E-08	12.279186
cg24555822	chr6	138428189	-	PERP	Body	-0.714696	-3.240451	-6.659246	6.59E-10	7.53E-08	12.27564
cg04956511	chr12	7056008	-	PTPN6	Body	-1.220202	-1.169188	-6.66282	6.61E-10	7.53E-08	12.275201
cg05226729	chr8	11839780	+	DEFB135	TSS200	-1.544198	0.4439845	-6.658993	6.60E-10	7.53E-08	12.274398
cg16700364	chr2	157178889	-			2.0211205	0.4296222	6.6589555	6.60E-10	7.53E-08	12.274214
cg18612627	chr12	14135203	+			1.2322586	-0.548676	6.6583971	6.62E-10	7.55E-08	12.27147
cg18096722	chr8	70947218	-			1.8004979	-1.315397	6.6582364	6.63E-10	7.56E-08	12.27068
cg13390867	chr9	86717438	-			2.1567941	-0.206623	6.6580839	6.63E-10	7.56E-08	12.26993
cg25225070	chr11	9587743	-			-1.234156	-2.805729	-6.657713	6.65E-10	7.57E-08	12.268105
cg13997788	chr2	46593223	-	EPAS1	Body	1.7819355	1.35872	6.6613847	6.65E-10	7.58E-08	12.268159
cg14183013	chr20	45985553	+	ZMYND8;ZMYND8;ZMYND8	TSS200;TSS200;TSS200	-1.52169	-4.066569	-6.657188	6.66E-10	7.59E-08	12.265525
cg26221992	chr6	74024572	+			1.6413653	-0.937571	6.6567919	6.68E-10	7.60E-08	12.263581
cg00068750	chr16	2535955	+	TBC1D24	5'UTR	1.1119878	1.5769328	6.6553506	6.73E-10	7.66E-08	12.256498
cg03262802	chr2	234118754	-			-1.384292	0.9792746	-6.658972	6.74E-10	7.66E-08	12.256319
cg15065340	chr3	195632915	+	TNK2	5'UTR	-1.341169	-1.18251	-6.658753	6.74E-10	7.67E-08	12.255245
cg00157572	chr14	60977961	+	SIX6	Body	1.4356191	-0.399754	6.6541847	6.77E-10	7.70E-08	12.250769
cg06580419	chr2	215276156	+	VWC2L	TSS1500	-1.322514	-0.639983	-6.653838	6.78E-10	7.71E-08	12.249064

cg16400238	chr17	27237364	-	PHF12	Body	0.8026529	2.2645725	6.6538602	6.78E-10	7.71E-08	12.249175
cg24221648	chr13	79170283	+			2.3506847	-0.140618	6.6533872	6.79E-10	7.72E-08	12.246851
cg07419011	chr12	120688060	+	PXN;PXN;PXN	Body;TSS200;Body	1.2223361	0.7998189	6.6532968	6.80E-10	7.72E-08	12.246406
cg18184218	chr10	24755438	+	KIAA1217;KIAA1217;KIAA1217	Body;Body;Body	-1.416208	-1.411958	-6.653204	6.80E-10	7.72E-08	12.245949
cg15528437	chr5	134386159	-			1.6574907	-0.738002	6.6526632	6.82E-10	7.74E-08	12.243294
cg23676302	chr2	177025859	-			1.7148058	-0.310043	6.6524808	6.83E-10	7.75E-08	12.242398
cg06369992	chr10	119494494	-			1.8346884	-1.743334	6.6523942	6.83E-10	7.75E-08	12.241972
cg09362969	chr1	3827065	-	LOC100133612	Body	-1.209186	0.0866828	-6.652165	6.84E-10	7.76E-08	12.240848
cg16570669	chr11	126923106	+			-1.593153	0.2934219	-6.652053	6.84E-10	7.76E-08	12.240296
cg16678111	chr1	2918724	+			-2.731722	0.8520357	-6.651982	6.84E-10	7.76E-08	12.239945
cg21000072	chr1	3567408	-	WDR8	TSS1500	1.6529061	-1.060082	6.6519855	6.84E-10	7.76E-08	12.239964
cg12111295	chr21	32253584	+	KRTAP11-1	1stExon	-1.262378	1.0504915	-6.655356	6.86E-10	7.78E-08	12.238752
cg23064082	chr11	30428652	+	MPPED2	Body	-1.881679	0.1619272	-6.651377	6.86E-10	7.78E-08	12.236974
cg17566874	chr5	131411272	-	CSF2	Body	-0.855928	-2.430486	-6.651173	6.87E-10	7.78E-08	12.235975
cg13485320	chr11	2920128	-	SLC22A18AS;SLC22A18	Body;TSS1500	-1.05678	-3.221658	-6.650867	6.88E-10	7.79E-08	12.234468
cg12986110	chr19	48551504	-	PLA2G4C;PLA2G4C;PLA2G4C	3'UTR;3'UTR;3'UTR	-1.623492	-0.407127	-6.650814	6.88E-10	7.79E-08	12.234209
cg19133883	chr20	1447874	-	NSFL1C;NSFL1C;NSFL1C;NSFL1C;NSFL1C;NSFL1C	5'UTR;5'UTR;1stExon;1stExon;1stExon;5'UTR	-0.549979	-3.983728	-6.650361	6.90E-10	7.81E-08	12.231984
cg19526659	chr19	57154862	-			1.1945352	-1.031786	6.6538506	6.91E-10	7.82E-08	12.231371
cg06536629	chr2	136634208	-	MCM6	TSS200	-0.746784	-5.288112	-6.648352	6.97E-10	7.89E-08	12.222118
cg14612417	chr2	95688557	-			-1.078914	-1.655177	-6.647958	6.99E-10	7.90E-08	12.220182

cg19801120	chr6	53530570	-	KLHL31	TSS200	1.0870726	0.6631664	6.6466208	7.03E-10	7.95E-08	12.213615
cg08189989	chr2	105459164	+			3.122246	-1.284504	6.6465206	7.04E-10	7.95E-08	12.213122
cg17922998	chr8	61863697	-			-1.931536	-0.835985	-6.646363	7.04E-10	7.96E-08	12.212347
cg04654288	chr6	10390247	+			1.2044538	-0.577388	6.6461084	7.05E-10	7.96E-08	12.211098
cg15707869	chr8	88884985	-	DCAF4L2;DCAF4L2	3'UTR;1stExon	-1.466567	-0.180816	-6.646135	7.05E-10	7.96E-08	12.21123
cg07417348	chr12	13332726	-			-1.550891	-2.509192	-6.645742	7.06E-10	7.98E-08	12.209301
cg20444218	chr11	73024037	+	ARHGEF17	Body	0.9539633	0.9642584	6.6457251	7.07E-10	7.98E-08	12.209216
cg01642521	chr11	20618250	-			2.8990789	-0.91057	6.6494449	7.07E-10	7.98E-08	12.209598
cg04951797	chr3	20226964	+	SGOL1;SGOL1;SGOL1;SGOL1;SGOL1;SGOL1	5'UTR;5'UTR;5'UTR;5'UTR;5'UTR;5'UTR	-1.230966	-2.704903	-6.64555	7.07E-10	7.98E-08	12.208355
cg10222027	chr16	31580590	-	CSDAP1	Body	2.2876238	-1.3482	6.645627	7.07E-10	7.98E-08	12.208734
cg15344659	chr11	30870939	+			-1.898913	0.2714972	-6.645147	7.09E-10	7.99E-08	12.206377
cg21026469	chr8	144820683	-			-1.623111	1.154235	-6.644601	7.11E-10	8.01E-08	12.203698
cg18025275	chr8	22498768	+	BIN3;FLJ14107	Body;Body	0.9096719	1.0521335	6.6444327	7.11E-10	8.02E-08	12.20287
cg01998856	chr11	1365592	+			-1.39186	-0.766662	-6.644345	7.12E-10	8.02E-08	12.202438
cg11636504	chr2	99439883	+	C2orf55	Body	1.2433469	0.1540493	6.6442206	7.12E-10	8.02E-08	12.201829
cg16971991	chr11	1146470	-			-1.243198	-1.99973	-6.644259	7.12E-10	8.02E-08	12.202019
cg16428357	chr20	34190258	-	FER1L4	Body	1.7550651	2.6740576	6.6440761	7.12E-10	8.02E-08	12.201119
cg21497990	chr7	155255122	+	EN2	Body	2.0467244	-0.628567	6.6436808	7.14E-10	8.04E-08	12.199179
cg02352240	chr16	51188372	+			1.1936406	0.0851156	6.6434259	7.15E-10	8.04E-08	12.197927
cg22360146	chr1	115301208	+	CSDE1;CSDE1;CSDE1	TSS1500;TSS1500;TSS1500	-1.757534	0.2676992	-6.643136	7.16E-10	8.05E-08	12.196505

cg14473102	chr2	176994448	+	HOXD8	TSS200	2.3920301	-0.319575	6.643078	7.16E-10	8.05E-08	12.196219
cg26102295	chr7	1423622	+			-1.965527	0.9404654	-6.642155	7.20E-10	8.09E-08	12.191688
cg26175448	chr17	39072167	+			-1.589303	-2.42828	-6.641906	7.20E-10	8.10E-08	12.190465
cg19505827	chr11	2323459	-	TSPAN32;C11orf21;C11orf21	Body;TSS1500;TSS1500	-1.286776	-1.757628	-6.641825	7.21E-10	8.10E-08	12.190068
cg26501671	chr11	124406358	-			-1.214251	-0.603945	-6.641426	7.22E-10	8.11E-08	12.188111
cg04323313	chr11	95566388	+	MTMR2;MTMR2;MTMR2;MTMR2	Body;3'UTR;3'UTR;3'UTR	0.7628983	2.8462005	6.641265	7.23E-10	8.12E-08	12.187319
cg02809746	chr16	49312033	-			3.0478807	-2.123889	6.6405168	7.26E-10	8.15E-08	12.183646
cg22485298	chr6	30853333	-	DDR1;DDR1	5'UTR;5'UTR	-1.245496	-1.797485	-6.640412	7.26E-10	8.15E-08	12.183131
cg11236429	chr4	174459412	+	NBLA00301	Body	2.3895443	-1.694894	6.6397277	7.28E-10	8.17E-08	12.179773
cg20300500	chr22	48606762	+			-0.800428	-0.979053	-6.639704	7.29E-10	8.17E-08	12.179659
cg26687173	chr19	33623091	+	WDR88	1stExon	1.01563	0.5766337	6.6397184	7.28E-10	8.17E-08	12.179728
cg23717441	chr3	117999270	-			-1.715495	-0.494479	-6.639551	7.29E-10	8.18E-08	12.178907
cg05165940	chr2	200329654	-			1.5990442	-0.585695	6.6393158	7.30E-10	8.19E-08	12.177752
cg20178976	chr20	52357117	+			-1.824262	-1.368755	-6.63906	7.31E-10	8.19E-08	12.176495
cg02288684	chr16	893259	-			-1.353743	-0.007364	-6.638662	7.32E-10	8.21E-08	12.174541
cg27434368	chr19	19650978	-	CILP2	Body	1.1709386	0.2608713	6.63849	7.33E-10	8.21E-08	12.173699
cg00364357	chr15	64443650	+	SNX22	TSS1500	-0.612741	-3.591171	-6.638334	7.34E-10	8.22E-08	12.172931
cg02691745	chr14	101488488	-	MIR411	TSS1500	-1.641724	0.4416084	-6.637886	7.35E-10	8.23E-08	12.170732
cg25999578	chr7	27205514	+	HOXA9	TSS1500	1.598628	-0.280216	6.6377792	7.36E-10	8.24E-08	12.17021
cg07964848	chr10	28035894	-	MKX	TSS1500	1.7919758	-1.053656	6.6372068	7.38E-10	8.26E-08	12.167401

cg25738714	chr1	50893658	-			2.3755871	-0.735294	6.6372089	7.38E-10	8.26E-08	12.167411
cg02478828	chr2	74153195	-	DGUOK;DGUOK	TSS1500;TSS1500	-2.421602	0.8500877	-6.637093	7.38E-10	8.26E-08	12.166845
cg09225449	chr5	138728367	-	LOC389333	1stExon	-1.398672	-3.288382	-6.636717	7.40E-10	8.27E-08	12.164998
cg13044675	chr11	65314161	+	LTBP3;LTBP3;LTBP3	Body;Body;Body	-1.77903	-2.208484	-6.640412	7.40E-10	8.28E-08	12.165502
cg11248896	chr2	177003747	-			1.6328662	-0.486591	6.6359063	7.43E-10	8.30E-08	12.16102
cg24124145	chr4	139936112	-	CCRN4L	TSS1500	-0.778903	-1.38964	-6.634526	7.48E-10	8.36E-08	12.154246
cg00760935	chr4	155411351	+	DCHS2;DCHS2	1stExon;1stExon	0.9795608	-0.716419	6.6342686	7.49E-10	8.37E-08	12.152984
cg22542222	chr10	132386640	+			-1.379949	0.0498348	-6.632244	7.57E-10	8.45E-08	12.143053
cg08438529	chr16	1052939	-			-1.559607	-1.697947	-6.631866	7.58E-10	8.47E-08	12.141199
cg24331475	chr18	21596915	+	TTC39C;TTC39C;TTC39C	Body;5'UTR;Body	-1.679345	-0.30785	-6.63183	7.58E-10	8.47E-08	12.141022
cg19942495	chr17	32484027	-	ACCN1	TSS1500	1.3677066	-0.384622	6.6316335	7.59E-10	8.47E-08	12.140057
cg03284102	chr7	10980944	+	NDUFA4	TSS1500	-2.010064	0.5716509	-6.631444	7.60E-10	8.48E-08	12.139128
cg14172846	chr1	112960391	+	CTTNBP2NL	Body	1.1556382	1.4803113	6.6310629	7.61E-10	8.49E-08	12.137258
cg01461514	chr7	55177182	-	EGFR;EGFR;EGFR;EGFR	Body;Body;Body;Body	-1.291016	-0.801344	-6.630454	7.64E-10	8.52E-08	12.13427
cg12670305	chr6	31762630	+	VARS	Body	-1.52718	-2.197725	-6.633999	7.65E-10	8.53E-08	12.133918
cg22143352	chr14	75897841	-	JDP2;JDP2;JDP2;JDP2	5'UTR;5'UTR;5'UTR;TSS1500	1.235104	0.5758943	6.6301353	7.65E-10	8.53E-08	12.132709
cg01609214	chr8	135817625	+	MIR30D	TSS1500	1.1941954	2.1638139	6.6296694	7.67E-10	8.54E-08	12.130424
cg25397945	chr19	37096148	-	ZNF529;ZNF529;ZNF529;ZNF382;ZNF529;ZNF529	Body;1stExon;5'UTR;TSS200;1stExon;5'UTR	3.2127586	-0.69889	6.6293069	7.68E-10	8.56E-08	12.128645
cg27342801	chr2	79387273	+	REG3A;REG3A;REG3A	TSS1500;TSS1500;TSS1500	-1.566686	0.4768131	-6.628912	7.70E-10	8.57E-08	12.126708
cg12500811	chr4	177648950	-	VEGFC	Body	-1.680077	0.2184453	-6.628702	7.71E-10	8.58E-08	12.125678

cg03052794	chr19	908894	-	C19orf22	Body	-1.398691	-1.324493	-6.628138	7.73E-10	8.60E-08	12.122911
cg05331856	chr13	40516584	+			-0.964472	-1.475648	-6.62775	7.74E-10	8.62E-08	12.121009
cg09174162	chr7	2150845	+	MAD1L1;MAD1L1;MAD1L1	Body;Body;Body	1.0135531	1.5693924	6.627443	7.75E-10	8.63E-08	12.119505
cg01395254	chr2	2336428	-	MYT1L	TSS1500	-1.896605	-0.004972	-6.627024	7.77E-10	8.64E-08	12.117449
cg02231066	chr3	52567130	+	NT5DC2;NT5DC2	Body;Body	-0.978292	-4.197878	-6.627071	7.77E-10	8.64E-08	12.117682
cg22055891	chr2	138829973	-			-2.303489	0.1526169	-6.626611	7.79E-10	8.66E-08	12.115426
cg20399871	chr7	27204663	-	HOXA9	1stExon	1.4192199	-0.873387	6.6259675	7.81E-10	8.68E-08	12.11227
cg08517455	chr2	99439997	+	C2orf55	Body	0.8513632	1.5080068	6.6255455	7.83E-10	8.70E-08	12.110201
cg13541527	chr6	31804078	+	C6orf48;C6orf48;SNORD52	5'UTR;5'UTR;TSS1500	-1.28108	-1.842319	-6.625536	7.83E-10	8.70E-08	12.110152
cg19801921	chr1	63784864	+			1.8752274	-0.405054	6.6254822	7.83E-10	8.70E-08	12.10989
cg05982271	chr4	76555948	+	CDKL2	TSS1500	-1.139426	-1.819008	-6.625335	7.84E-10	8.70E-08	12.109171
cg05138082	chr14	54392087	+			-1.479254	0.0836858	-6.624908	7.86E-10	8.72E-08	12.107075
cg10925829	chr20	60860984	+	OSBPL2;OSBPL2	Body;Body	0.6829861	0.8222445	6.6287413	7.86E-10	8.72E-08	12.108351
cg16053677	chr18	9261071	-	ANKRD12;ANKRD12	Body;Body	1.0808987	1.5975349	6.6244404	7.87E-10	8.74E-08	12.104783
cg15060438	chr2	109921842	-	SH3RF3	Body	-1.894987	0.5991275	-6.624367	7.88E-10	8.74E-08	12.104421
cg06341701	chr2	176986261	-	HOXD9	TSS1500	1.4392622	-0.204888	6.6235674	7.91E-10	8.77E-08	12.100503
cg15521572	chr18	74802637	-	MBP;MBP	Body;Body	1.0092761	1.7790936	6.6230776	7.93E-10	8.79E-08	12.098102
cg26796341	chr6	100905691	-	SIM1	Body	0.9605067	0.0418875	6.6226965	7.94E-10	8.81E-08	12.096234
cg02749784	chr8	120219927	+	MAL2	TSS1500	-1.780538	-0.226756	-6.622608	7.95E-10	8.81E-08	12.095797
cg19712979	chr6	33173307	-	HSD17B8	Body	-0.98013	-1.204443	-6.622375	7.96E-10	8.82E-08	12.094655

cg17384889	chr6	28227068	-	NKAPL	TSS200	1.7399682	-0.484631	6.6219854	7.97E-10	8.83E-08	12.092748
cg09644153	chr16	3345646	-			1.0902176	0.3177101	6.6257025	7.98E-10	8.83E-08	12.093478
cg13912311	chr9	127265348	+	NR5A1	Body	1.3660925	0.5362933	6.6216268	7.99E-10	8.84E-08	12.09099
cg07178825	chr1	3649574	+	TP73;TP73;TP73;TP73	Body;Body;3'UTR;3'UTR	1.0143164	-0.327604	6.6202153	8.05E-10	8.90E-08	12.084072
cg22598885	chr8	38175609	-	WHSC1L1;WHSC1L1	Body;Body	1.3502631	1.9207942	6.6200238	8.05E-10	8.91E-08	12.083133
cg03137889	chr6	108454859	-			-1.332972	-2.809484	-6.61986	8.06E-10	8.91E-08	12.082332
cg00469602	chr2	172955930	+			1.6610475	-0.17938	6.6195286	8.07E-10	8.93E-08	12.080706
cg09170112	chr12	132102188	-			-2.858035	0.8616236	-6.619509	8.07E-10	8.93E-08	12.08061
cg17454831	chr2	66667073	+	MEIS1	Body	1.7600598	-0.940614	6.6188954	8.10E-10	8.95E-08	12.077603
cg17051543	chr10	135092241	+			-1.235134	-1.51972	-6.618781	8.10E-10	8.95E-08	12.07704
cg10211062	chr2	47915862	-			-1.430398	-0.221049	-6.618703	8.11E-10	8.96E-08	12.076662
cg14248715	chr14	57284096	-	OTX2OS1	Body	2.8593655	-1.117278	6.6182882	8.12E-10	8.97E-08	12.074628
cg02832697	chr4	154126481	+	TRIM2;TRIM2	Body;5'UTR	-0.987498	-1.60031	-6.618119	8.13E-10	8.98E-08	12.073799
cg03655683	chr2	63281383	+	OTX1	Body	1.9803475	-0.673059	6.6176886	8.15E-10	9.00E-08	12.071689
cg19797376	chr1	47695475	+	TAL1	TSS200	1.4255652	-0.455083	6.6176218	8.15E-10	9.00E-08	12.071362
cg16519587	chr6	26614649	+			2.8210864	-1.072391	6.6173522	8.16E-10	9.01E-08	12.070041
cg09086835	chr15	89922345	+	LOC254559	Body	1.6341402	-0.698392	6.6170882	8.17E-10	9.02E-08	12.068747
cg09744759	chr7	5469255	+			-0.876817	-2.199489	-6.617012	8.18E-10	9.02E-08	12.068373
cg13134763	chr2	983306	+	SNTG2	Body	-2.625305	1.7061619	-6.616758	8.19E-10	9.03E-08	12.067132
cg06505619	chr16	698072	-	WDR90;FAM195A	TSS1500;Body	-1.487013	0.2810024	-6.616659	8.19E-10	9.03E-08	12.066642

cg13526642	chr9	112877160	-	AKAP2;PALM2-AKAP2;PALM2-AKAP2	Body;Body;Body	1.0753063	0.7605872	6.6166351	8.19E-10	9.03E-08	12.066527
cg13891985	chr10	13482679	+	BEND7	Body	-1.580706	-4.519276	-6.616133	8.21E-10	9.05E-08	12.064066
cg25528121	chr3	14860793	-	FGD5	1stExon	-2.156607	0.6203075	-6.623868	8.21E-10	9.05E-08	12.066626
cg04993267	chr5	148724716	-	GRPEL2	TSS1500	-0.8265	-1.97296	-6.615933	8.22E-10	9.05E-08	12.063089
cg26069745	chr7	27142200	+	HOXA2;HOXA2	1stExon;5'UTR	1.2909227	-0.525753	6.6153757	8.25E-10	9.08E-08	12.060357
cg20559943	chr4	185820756	-			-0.660284	-2.041523	-6.615279	8.25E-10	9.08E-08	12.059883
cg04368796	chr17	40171968	+	NKIRAS2;NKIRAS2;NKIRAS2;NKIRAS2;NKIRAS2	TSS200;TSS200;TSS200;TSS200;5'UTR	-1.482967	-4.245038	-6.614882	8.27E-10	9.09E-08	12.057939
cg10703826	chr1	119532116	+	TBX15;TBX15	1stExon;5'UTR	2.0954403	-0.274411	6.6185675	8.27E-10	9.10E-08	12.064967
cg18397073	chr19	42600278	-	POU2F2	Body	2.0953379	1.6936004	6.6185099	8.28E-10	9.10E-08	12.058111
cg10535143	chr2	216176526	-	ATIC	TSS200	-0.582243	-5.16363	-6.614485	8.28E-10	9.11E-08	12.055995
cg05798126	chr6	3089496	+	RIPK1	Body	0.7837503	2.0195343	6.6143522	8.29E-10	9.11E-08	12.055343
cg06458238	chr17	73506140	-	CASKIN2;CASKIN2	Body;TSS200	1.114868	1.1843274	6.6141094	8.30E-10	9.12E-08	12.054153
cg05708073	chr1	109637162	-	TMEM167B	3'UTR	1.0944297	0.9274608	6.6138381	8.31E-10	9.13E-08	12.052824
cg02357637	chr15	100268975	-	LYSMD4	3'UTR	0.5542751	1.879867	6.6134032	8.33E-10	9.15E-08	12.050693
cg12374506	chr12	49728243	-	C1QL4	Body	1.2900784	0.4532678	6.6126088	8.36E-10	9.18E-08	12.046802
cg04623172	chr10	31607306	+	ZEB1;ZEB1;ZEB1;LOC220930;ZEB1	TSS1500;TSS200;TSS1500;Body;TSS1500	-0.480854	-3.62118	-6.611413	8.41E-10	9.24E-08	12.040944
cg00593404	chr6	52377740	+	TRAM2	Body	1.161873	1.5162693	6.6108949	8.44E-10	9.26E-08	12.038407
cg13213009	chr8	74903761	-	LY96	1stExon	-2.057205	-2.36693	-6.610655	8.45E-10	9.27E-08	12.037231
cg09216236	chr1	65719627	+			-1.496991	-2.661001	-6.618184	8.45E-10	9.27E-08	12.04621
cg03935060	chr16	10272021	-	GRIN2A;GRIN2A;GRIN2A	Body;Body;Body	-1.875802	-0.124967	-6.608971	8.52E-10	9.34E-08	12.028984

cg11376147	chr11	57261198	-	SLC43A1	Body	-0.630521	-2.18491	-6.608995	8.52E-10	9.34E-08	12.029104
cg01829163	chr16	87871160	-	SLC7A5	Body	1.3816726	3.0783012	6.608783	8.53E-10	9.35E-08	12.028065
cg13396821	chr1	154812952	-	KCNN3;KCNN3	Body;Body	1.2516963	2.0196784	6.6074066	8.59E-10	9.41E-08	12.021325
cg17339234	chr4	48018954	-	NIPAL1	Body	-0.526391	-4.158579	-6.606742	8.62E-10	9.44E-08	12.018071
cg16348470	chr1	34642609	-	C1orf94;C1orf94;C1orf94	5'UTR;1stExon;5'UTR	2.4321095	-0.849697	6.6052716	8.68E-10	9.51E-08	12.010872
cg24377133	chr8	144170375	+			-1.185309	-2.710848	-6.604997	8.69E-10	9.52E-08	12.009528
cg02955989	chr19	30944506	+	ZNF536	Body	-1.543484	0.0069835	-6.604545	8.71E-10	9.54E-08	12.007313
cg04521510	chr7	27242044	+			2.7086743	-1.607921	6.6029726	8.78E-10	9.60E-08	11.999618
cg14014033	chr7	736792	-	PRKAR1B;PRKAR1B;PRKAR1B;PRKAR1B;PRKAR1B;PRKAR1B	Body;Body;Body;Body;Body;Body	1.0432036	1.2384471	6.6029729	8.78E-10	9.60E-08	11.999619
cg14709481	chr5	176325836	-	HK3	5'UTR	-1.20472	0.0686221	-6.603023	8.78E-10	9.60E-08	11.999866
cg27269149	chr8	1130775	-			-1.405378	0.0650259	-6.602973	8.78E-10	9.60E-08	11.999622
cg00233633	chr7	29153560	+	CPVL;CPVL	Body;Body	-1.448019	-1.785164	-6.602779	8.79E-10	9.61E-08	11.998672
cg11028075	chr10	97200911	+	SORBS1;SORBS1;SORBS1;SORBS1;SORBS1;SORBS1;SORBS1;SORBS1;SORBS1;SORBS1;SORBS1;SORBS1	1stExon;5'UTR;5'UTR;5'UTR;1stExon;5'UTR;5'UTR;1stExon;5'UTR;5'UTR;1stExon;1stExon	-1.434265	-0.565352	-6.602735	8.79E-10	9.61E-08	11.998457
cg12457909	chr7	39649359	+	LOC646999	Body	2.6875064	-1.683287	6.6024626	8.81E-10	9.62E-08	11.997121
cg00971110	chr7	84814140	-			1.0703707	1.2440362	6.6024128	8.81E-10	9.62E-08	11.996878
cg24154951	chr2	177001426	+			1.8083288	-0.384628	6.6021856	8.82E-10	9.63E-08	11.995766
cg06894334	chr4	1398599	+			1.3106912	-0.559232	6.6021274	8.82E-10	9.63E-08	11.995481
cg17326555	chr1	119535693	-			1.1828445	0.7332412	6.6019493	8.83E-10	9.64E-08	11.994609
cg18827787	chr18	61817320	-	C18orf20	TSS1500	-1.495569	0.2444997	-6.60162	8.84E-10	9.65E-08	11.993
cg04768606	chr4	1505838	+			-1.521166	0.6917379	-6.601431	8.85E-10	9.65E-08	11.99207

cg10351418	chr19	57303997	-	ZIM2;ZIM2;ZIM2	Body;Body;Body	-1.96508	0.7418143	-6.601451	8.85E-10	9.65E-08	11.992169
cg05039548	chr1	91190366	+			1.5884355	-0.844191	6.6011682	8.86E-10	9.67E-08	11.990787
cg26994225	chr2	119616769	-			1.5725047	-1.090973	6.6010226	8.87E-10	9.67E-08	11.990074
cg13261971	chr2	45231226	-			1.892843	-0.446583	6.6046873	8.88E-10	9.67E-08	11.99818
cg04021014	chr22	49502115	+			-1.09561	-0.611235	-6.600505	8.89E-10	9.69E-08	11.987543
cg22055728	chr7	27205658	+	HOXA9	TSS1500	1.6909092	-1.135095	6.6002906	8.90E-10	9.70E-08	11.986491
cg10982364	chr2	200329670	+			2.1591695	-0.545698	6.6077309	8.91E-10	9.71E-08	11.987862
cg03707599	chr7	75956931	+	YWHAG	3'UTR	1.4353703	-0.419752	6.5994039	8.94E-10	9.74E-08	11.982152
cg17493727	chr6	108169872	-			-0.94227	-3.215022	-6.599312	8.95E-10	9.74E-08	11.981702
cg00011482	chr14	37128790	-	PAX9	5'UTR	1.3803406	-1.05313	6.5989645	8.96E-10	9.75E-08	11.980002
cg12723011	chr4	15965102	+	FGFBP2	TSS1500	-1.100395	-2.076331	-6.598422	8.99E-10	9.78E-08	11.977346
cg08647446	chr4	74486298	-	RASSF6;RASSF6;RASSF6	1stExon;5'UTR;TSS200	-0.860265	-2.701554	-6.598164	9.00E-10	9.79E-08	11.976083
cg23359363	chr5	33764247	-	ADAMTS12	Body	-2.096241	0.2084088	-6.597894	9.01E-10	9.80E-08	11.974764
cg26828664	chr7	736945	+	PRKAR1B;PRKAR1B;PRKAR1B;PRKAR1B;PRKAR1B;PRKAR1B	Body;Body;Body;Body;Body;Body	1.0005734	1.0641906	6.6094239	9.02E-10	9.80E-08	11.978337
cg04071270	chr5	140457553	+			1.2997757	1.0839831	6.5976722	9.02E-10	9.81E-08	11.973679
cg02235562	chr13	36729137	-			2.0924516	-0.358741	6.597408	9.03E-10	9.81E-08	11.972386
cg13065683	chr7	39760443	-			-1.133068	-0.899052	-6.597421	9.03E-10	9.81E-08	11.97245
cg13278105	chr14	101488423	+	MIR411;MIR379	TSS1500;Body	-1.577267	0.8634363	-6.596881	9.06E-10	9.84E-08	11.969808
cg03130962	chr6	31111088	-	CCHCR1;CCHCR1;CCHCR1	Body;Body;Body	1.2820988	1.341514	6.5965992	9.07E-10	9.85E-08	11.968429
cg19336198	chr7	4746462	-	FOXK1	Body	1.1301387	1.3121687	6.6004331	9.07E-10	9.85E-08	11.969751

cg18297705	chr11	1994927	+			-0.973492	0.058682	-6.596177	9.09E-10	9.87E-08	11.966365
cg01016847	chr14	23303413	+	MRPL52;MRPL52;MRPL52;MRPL52;MRPL52;MRPL52	Body;Body;Body;Body;Body;Body	0.8271588	1.518954	6.5961161	9.09E-10	9.87E-08	11.966066
cg22800233	chr10	93805441	-			0.969646	0.1676688	6.5956159	9.12E-10	9.89E-08	11.963619
cg22157027	chr2	10638113	-			-1.441103	-0.279177	-6.595513	9.12E-10	9.89E-08	11.963113
cg13420848	chr1	197888894	-	LHX9;LHX9	Body;Body	2.3127129	-1.745583	6.5953352	9.13E-10	9.90E-08	11.962245
cg00779313	chr10	1166994	-	WDR37	Body	1.0104793	2.4323598	6.5952296	9.14E-10	9.90E-08	11.961728
cg00621646	chr11	123849141	-	OR10S1	TSS1500	-1.347044	-0.706705	-6.594803	9.16E-10	9.92E-08	11.959643
cg18929842	chr17	39804040	+			-0.869588	-1.579277	-6.59477	9.16E-10	9.92E-08	11.959482
cg08130663	chr17	1549896	+	SCARF1;RILP;SCARF1;SCARF1;SCARF1;SCARF1	TSS1500;Body;TSS1500;TSS1500;TSS1500;TSS1500	0.7334333	1.2786237	6.5946598	9.16E-10	9.92E-08	11.958941
cg08911152	chr8	134105078	-	TG;SLA;SLA	Body;Body;5'UTR	-1.378939	-2.535395	-6.594569	9.17E-10	9.92E-08	11.958499
cg09887220	chr13	95356202	+			1.2434518	0.2571894	6.5944436	9.17E-10	9.93E-08	11.957884
cg00775695	chr9	14693648	+	ZDHHC21	TSS200	-0.568638	-4.271356	-6.594351	9.18E-10	9.93E-08	11.957429
cg05255522	chr6	50682710	+	TFAP2D	Body	2.232595	-1.255512	6.5934751	9.22E-10	9.97E-08	11.953146
cg10303487	chr8	105479058	+	DPYS	1stExon	2.8251046	-1.337481	6.5935178	9.22E-10	9.97E-08	11.953355
cg13562284	chr11	32110323	-			-1.022754	-1.047715	-6.593443	9.22E-10	9.97E-08	11.952987
cg14535980	chr2	106681937	+	C2orf40	TSS200	1.4854517	-0.24657	6.5970443	9.23E-10	9.98E-08	11.953199
cg25124816	chr6	31750141	-	VARS	Body	0.9619195	4.3331216	6.5964268	9.26E-10	1.00E-07	11.950184
cg02244204	chr5	625213	-	CEP72	Body	1.0533882	4.1385962	6.5924225	9.27E-10	1.00E-07	11.947998
cg04951739	chr22	24093724	-	ZNF70	TSS1500	-0.544935	-3.355954	-6.592468	9.26E-10	1.00E-07	11.94822
cg16700282	chr17	46796298	+			1.3633635	-0.668887	6.595994	9.28E-10	1.00E-07	11.955797

cg22691524	chr3	185300576	-			0.830982	1.4974347	6.5921753	9.28E-10	1.00E-07	11.946789
cg12010198	chr8	145939499	+			1.073	0.4330659	6.5920857	9.28E-10	1.00E-07	11.94635
cg18716912	chr4	57861927	-	POLR2B	Body	1.3182451	1.3508877	6.5919579	9.29E-10	1.00E-07	11.945726
cg08931404	chr13	29227083	+			0.9678613	1.7690665	6.5915189	9.31E-10	1.00E-07	11.943579
cg12304937	chr7	680013	-	PRKAR1B;PRKAR1B;PRKAR1B;PRKAR1B;PRKAR1B;PRKAR1B	Body;Body;Body;Body;Body;Body	-1.358726	-1.005813	-6.591295	9.32E-10	1.01E-07	11.942485
cg26721627	chr11	1319713	-	TOLLIP	Body	0.8098583	1.4034559	6.590324	9.37E-10	1.01E-07	11.937735
cg09146872	chr17	820881	+	NXN	Body	1.298832	1.8917521	6.5894441	9.41E-10	1.01E-07	11.933432
cg23268290	chr17	6390502	+	PITPNM3;PITPNM3	Body;Body	0.8313436	0.9490846	6.5887908	9.44E-10	1.02E-07	11.930238
cg00573835	chr16	51188762	+			1.7231671	-1.254262	6.588627	9.45E-10	1.02E-07	11.929437
cg14792548	chr17	1970514	+	SMG6;SMG6	Body;Body	0.9962331	1.3323883	6.5881536	9.47E-10	1.02E-07	11.927122
cg03661929	chr3	116503715	-			-1.737116	-0.468998	-6.588004	9.48E-10	1.02E-07	11.926393
cg15440688	chr14	95237637	-	GSC	TSS1500	1.7233085	-0.311467	6.5876104	9.50E-10	1.02E-07	11.924467
cg13400168	chr19	54481620	+	CACNG8	Body	1.3300115	-1.189213	6.5873793	9.51E-10	1.02E-07	11.923337
cg16006349	chr12	54360460	-	HOTAIR	Body	1.6817347	-0.238964	6.586877	9.53E-10	1.03E-07	11.920881
cg06394247	chr9	36986788	+	PAX5	Body	1.6255867	-1.457986	6.5856473	9.59E-10	1.03E-07	11.91487
cg07862488	chr14	60977054	-	SIX6	Body	1.3482808	-0.745849	6.5856252	9.59E-10	1.03E-07	11.914762
cg12504882	chr2	176977374	-			1.3969945	-0.294872	6.5855487	9.60E-10	1.03E-07	11.914387
cg24543939	chr5	53952507	-			1.4534261	1.1600233	6.5852959	9.61E-10	1.03E-07	11.913152
cg12379452	chr17	54987827	+	TRIM25	Body	1.5475389	0.690547	6.5889227	9.62E-10	1.03E-07	11.913731
cg18410316	chr6	31207788	+			-1.648858	0.0058308	-6.584907	9.63E-10	1.03E-07	11.911252

cg13703871	chr19	9473674	-	ZNF177	TSS200	2.7626348	-0.651311	6.5846501	9.64E-10	1.04E-07	11.909995
cg05194102	chr16	2088960	+	SLC9A3R2;SLC9A3R2	3'UTR;3'UTR	1.7187972	2.0076699	6.583797	9.68E-10	1.04E-07	11.905825
cg05492975	chr20	60119508	-	CDH4	Body	-2.018277	0.2345249	-6.583454	9.70E-10	1.04E-07	11.904147
cg19025113	chr2	45155201	-			1.6256794	-0.032251	6.5833521	9.70E-10	1.04E-07	11.903651
cg13975303	chr22	36850894	-			-1.099685	-3.173012	-6.583115	9.71E-10	1.04E-07	11.902491
cg18372607	chr11	31827084	+	PAX6;PAX6;PAX6	Body;Body;Body	2.3089271	-0.217871	6.58266	9.74E-10	1.04E-07	11.900268
cg20516074	chr4	74486179	-	RASSF6;RASSF6	1stExon;TSS200	-1.068004	-5.684413	-6.582709	9.73E-10	1.04E-07	11.900506
cg16732787	chr1	228785992	-	DUSP5P	Body	1.2695306	0.7122935	6.5823563	9.75E-10	1.05E-07	11.898784
cg02735829	chr5	180167566	-	OR2Y1	TSS1500	-1.376318	-0.180941	-6.58221	9.76E-10	1.05E-07	11.898069
cg27623948	chr17	77208211	-	HRNBP3	5'UTR	-1.262991	0.3950845	-6.581686	9.79E-10	1.05E-07	11.895507
cg01056615	chr5	1140096	-			-1.152774	-0.652876	-6.58127	9.81E-10	1.05E-07	11.893474
cg07479030	chr13	113496719	+	ATP11A;ATP11A	Body;Body	1.0794503	1.1641416	6.5812483	9.81E-10	1.05E-07	11.893369
cg11294750	chr13	94957721	-	GPC6	Body	-1.518895	-0.489505	-6.581336	9.80E-10	1.05E-07	11.893796
cg14264194	chr1	211652549	-	RD3;RD3	Body;Body	1.1378828	0.0867675	6.5805495	9.84E-10	1.05E-07	11.889954
cg24556382	chr4	174173455	-	GALNT7	Body	-1.793086	0.1415404	-6.580095	9.86E-10	1.06E-07	11.887734
cg25546651	chr4	25910053	+			-1.71197	0.5667665	-6.57864	9.94E-10	1.06E-07	11.880623
cg17473765	chr5	139016709	-			-1.145097	-2.78406	-6.578519	9.94E-10	1.06E-07	11.880034
cg01556466	chr7	31146260	+	ADCYAP1R1	Body	-1.393553	-0.62414	-6.57821	9.96E-10	1.07E-07	11.878524
cg01827426	chr5	1752668	-			-1.30109	-0.458422	-6.577572	9.99E-10	1.07E-07	11.875408
cg10759648	chr11	132374480	+	OPCML;OPCML	Body;Body	-1.111823	-1.124	-6.580686	1.00E-09	1.07E-07	11.873544

cg19916364	chr1	68961838	+	DEPDC1;DEPDC1	Body;Body	-1.202248	-1.719835	-6.576786	1.00E-09	1.07E-07	11.871564
cg14343733	chr9	130703735	-	FAM102A;FAM102A	3'UTR;3'UTR	1.1319584	1.0691669	6.5763168	1.01E-09	1.07E-07	11.869274
cg13514050	chr20	33460585	-	GGT7	1stExon	-0.944016	-4.161193	-6.575413	1.01E-09	1.08E-07	11.864862
cg18569734	chr6	108496460	+	NR2E1	Body	1.2561823	-0.841954	6.5750293	1.01E-09	1.08E-07	11.862985
cg27168114	chr7	594586	-	PRKAR1B;PRKAR1B;PRKAR1B;PRKAR1B;PRKAR1B;PRKAR1B	Body;Body;Body;Body;Body;Body	-1.258072	0.2940351	-6.578438	1.01E-09	1.08E-07	11.862581
cg04982781	chr22	39714193	-	RPL3;RPL3;RNU86	Body;Body;TSS1500	-1.131985	-0.84084	-6.574354	1.02E-09	1.08E-07	11.859687
cg06787397	chr12	13530144	+	C12orf36	TSS1500	-1.100025	-0.071544	-6.573235	1.02E-09	1.09E-07	11.854221
cg24052338	chr19	58879059	-	ZNF837;ZNF837	3'UTR;3'UTR	1.0181663	1.9843466	6.5731838	1.02E-09	1.09E-07	11.853971
cg10029186	chr4	174430771	-			1.5474563	-1.218338	6.5729875	1.02E-09	1.09E-07	11.853013
cg24314549	chr17	1533570	-	SLC43A2	TSS1500	-0.472766	-3.024024	-6.572867	1.02E-09	1.09E-07	11.852425
cg22393461	chr1	10010658	+	NMNAT1	5'UTR	-0.891959	-2.847822	-6.572817	1.02E-09	1.09E-07	11.852179
cg15421305	chr5	134385984	+			0.8953164	-0.413389	6.5723825	1.03E-09	1.09E-07	11.850058
cg26745038	chr16	86371833	+	LOC732275	Body	-1.591397	0.3788903	-6.572277	1.03E-09	1.09E-07	11.849545
cg17928869	chr17	39822542	+			-1.0051	-3.044647	-6.571849	1.03E-09	1.10E-07	11.847451
cg22908616	chr11	6518577	-	DNHD1;DNHD1	5'UTR;5'UTR	0.9315789	0.8600602	6.5718677	1.03E-09	1.10E-07	11.847544
cg21399203	chr4	57623913	-			-1.829921	-1.52186	-6.571714	1.03E-09	1.10E-07	11.846791
cg10217445	chr21	34397784	+	OLIG2	TSS1500	1.3799487	-1.392097	6.5711453	1.03E-09	1.10E-07	11.844017
cg18231054	chr1	1858037	-			-2.337758	0.5822807	-6.571105	1.03E-09	1.10E-07	11.843821
cg21846790	chr16	4666047	+	FAM100A	TSS1500	-0.899175	-1.679027	-6.571184	1.03E-09	1.10E-07	11.844204
cg03388786	chr8	56852253	+	LYN;LYN	5'UTR;5'UTR	-1.404193	-2.180669	-6.570642	1.03E-09	1.10E-07	11.841558

cg03304718	chr8	9793923	+			-1.125869	-0.385066	-6.570449	1.04E-09	1.10E-07	11.840616
cg24363020	chr3	72706849	+			-1.092131	-1.323378	-6.570435	1.04E-09	1.10E-07	11.840548
cg21635870	chr6	101847058	-	GRIK2;GRIK2;GRIK2;GRIK2;GRIK2;GRIK2	1stExon;1stExon;5'UTR;1stExon;5'UTR;5'UTR	1.9375481	-0.83057	6.5699054	1.04E-09	1.10E-07	11.837963
cg09968620	chr2	176987383	-	HOXD9	TSS200	2.0420607	-1.598626	6.5696614	1.04E-09	1.11E-07	11.836771
cg03962217	chr7	154796431	+	LOC202781	Body	-1.421051	-0.08504	-6.569335	1.04E-09	1.11E-07	11.835179
cg17203063	chr12	75601711	-	KCNC2;KCNC2;KCNC2	Body;Body;Body	2.2973061	-1.071821	6.5693963	1.04E-09	1.11E-07	11.835477
cg24554839	chr1	166124903	+	MIR921;FAM78B	TSS1500;Body	-1.829954	-0.14839	-6.569303	1.04E-09	1.11E-07	11.835022
cg05270938	chr2	119616568	+			1.549812	-0.637002	6.568811	1.04E-09	1.11E-07	11.832619
cg14028400	chr6	28793656	-			1.4700999	0.0453997	6.5685409	1.05E-09	1.11E-07	11.8313
cg08701047	chr10	130085199	-			2.81155	-2.13916	6.5681716	1.05E-09	1.11E-07	11.829498
cg10392428	chr14	89782402	+	FOXN3;FOXN3	Body;Body	1.2537417	1.7176845	6.5680209	1.05E-09	1.11E-07	11.828762
cg16596317	chr1	85755348	+			-1.864635	-0.653493	-6.567132	1.05E-09	1.12E-07	11.824421
cg20597403	chr7	4305042	+	SDK1;SDK1	Body;3'UTR	1.0904035	1.2116908	6.5669672	1.05E-09	1.12E-07	11.823618
cg16065899	chr11	44332958	+	ALX4	TSS1500	1.1965223	-0.079193	6.5668697	1.05E-09	1.12E-07	11.823142
cg13972366	chr7	30265567	-			-1.116237	-1.645739	-6.566762	1.06E-09	1.12E-07	11.822619
cg11348165	chr1	27189270	-	SFN	TSS1500	-1.20602	-0.662193	-6.570125	1.06E-09	1.12E-07	11.822054
cg04768697	chr19	15122135	+	CCDC105	1stExon	1.664654	-0.744974	6.5653935	1.06E-09	1.13E-07	11.815936
cg07137606	chr1	242687018	-	PLD5	5'UTR	1.5647946	-0.066092	6.5651411	1.06E-09	1.13E-07	11.814704
cg13587449	chr10	72240883	+	KIAA1274	5'UTR	1.1225822	1.4286261	6.5651367	1.06E-09	1.13E-07	11.814683
cg02423064	chr17	32484008	-	ACCN1	TSS200	1.5511377	-1.322838	6.5650452	1.06E-09	1.13E-07	11.814237

cg10021364	chr19	54369015	-	MYADM	TSS1500	-1.072682	-1.502894	-6.565027	1.06E-09	1.13E-07	11.814147
cg24119284	chr3	58344724	+	PXK	Body	0.9132956	2.0137632	6.5647003	1.07E-09	1.13E-07	11.812553
cg26879578	chr1	95700272	+	RWDD3;RWDD3	Body;Body	-0.566553	-4.606715	-6.564375	1.07E-09	1.13E-07	11.810963
cg19635505	chr14	20903555	+	KLHL33	5'UTR	1.4111782	0.9922949	6.5642672	1.07E-09	1.13E-07	11.810439
cg14398243	chr2	46385031	-	PRKCE	Body	0.7901668	0.4319662	6.5640564	1.07E-09	1.13E-07	11.80941
cg01801603	chr8	123795433	-	ZHX2	5'UTR	-1.31125	-2.086782	-6.563917	1.07E-09	1.13E-07	11.808732
cg19267252	chr2	105459317	-			1.8905226	-0.821281	6.5638618	1.07E-09	1.13E-07	11.808461
cg02512902	chr17	25799447	+	KSR1	5'UTR	-1.349907	-0.717518	-6.563306	1.07E-09	1.14E-07	11.805751
cg05895091	chr11	57412323	-			-0.660959	-3.416805	-6.563269	1.07E-09	1.14E-07	11.805566
cg09922895	chr10	97038583	+	PDLIM1	Body	0.8141308	1.8623738	6.5631849	1.07E-09	1.14E-07	11.805157
cg26309929	chr3	24083947	+			1.6943682	0.97653	6.569923	1.08E-09	1.14E-07	11.803882
cg18801825	chr19	2648560	+	GNG7	5'UTR	-1.36857	-1.671291	-6.561507	1.08E-09	1.14E-07	11.796971
cg07637213	chr10	50820604	-	CHAT;SLC18A3;CHAT;CHAT;CHAT;CHAT;CHAT;CHAT;SLC18A3	TSS1500;1stExon;TSS1500;TSS1500;TSS1500;TSS1500;5'UTR;TSS1500;3'UTR	0.9902219	-0.640133	6.561329	1.08E-09	1.14E-07	11.796101
cg19440264	chr17	7350413	-	CHRNB1	Body	1.1420119	0.6718929	6.5613139	1.09E-09	1.14E-07	11.796028
cg24851084	chr17	62600789	-	SMURF2	Body	0.8901786	2.2945125	6.561242	1.09E-09	1.14E-07	11.795677
cg25412641	chr6	157877120	-	ZDHHC14;ZDHHC14	Body;Body	1.3607121	2.4687555	6.5650108	1.09E-09	1.14E-07	11.79695
cg07780348	chr11	118842413	+	FOXR1	TSS200	1.1496018	0.8618893	6.5608314	1.09E-09	1.15E-07	11.793673
cg20653075	chr8	65291682	+	MIR124-2	TSS200	1.4370789	-1.022167	6.5606373	1.09E-09	1.15E-07	11.792726
cg24335895	chr19	36643771	+	COX7A1	TSS200	1.2484616	0.2851127	6.5603986	1.09E-09	1.15E-07	11.791561
cg04427003	chr5	63257499	+	HTR1A	1stExon	1.1531049	-1.588506	6.5603122	1.09E-09	1.15E-07	11.79114

cg16765268	chr16	11229200	+	CLEC16A	Body	1.1454416	3.4961308	6.5601247	1.09E-09	1.15E-07	11.790225
cg27431877	chr12	124911924	+	NCOR2;NCOR2	Body;Body	0.9217847	1.1097196	6.5601211	1.09E-09	1.15E-07	11.790208
cg26296781	chr7	20259736	-			-1.672878	-2.027847	-6.560022	1.09E-09	1.15E-07	11.789725
cg02072400	chr12	127630550	+			1.959148	-1.123606	6.5597426	1.09E-09	1.15E-07	11.788361
cg19643211	chr14	104314557	+	PPP1R13B	TSS1500	-1.359179	-3.582585	-6.559257	1.10E-09	1.15E-07	11.785992
cg00550955	chr22	22012657	-			-1.159664	-0.230315	-6.559208	1.10E-09	1.15E-07	11.785755
cg14856585	chr14	94425764	-			-1.675029	-3.715632	-6.559047	1.10E-09	1.15E-07	11.784968
cg15160263	chr5	76923943	-			2.1076346	-1.149582	6.5589704	1.10E-09	1.15E-07	11.784593
cg22513691	chr1	10738664	+	CASZ1;CASZ1	Body;Body	1.0195925	2.0659014	6.5588393	1.10E-09	1.16E-07	11.783954
cg17966560	chr1	156357871	-			1.7918395	0.3728444	6.5583552	1.10E-09	1.16E-07	11.781592
cg02483101	chr8	130365373	+			-1.391983	-1.006302	-6.557491	1.11E-09	1.16E-07	11.777375
cg11219400	chr3	36949966	+	TRANK1	Body	-1.036766	-3.788414	-6.55701	1.11E-09	1.17E-07	11.775033
cg09533178	chr19	55630336	+	PPP1R12C	TSS1500	1.5428053	1.7372015	6.5604673	1.11E-09	1.17E-07	11.782877
cg12896170	chr6	28890069	+	TRIM27	Body	-1.416776	-0.24339	-6.556754	1.11E-09	1.17E-07	11.77378
cg23989821	chr14	60952405	+	C14orf39	5'UTR	2.7344838	-0.818192	6.5600819	1.11E-09	1.17E-07	11.773129
cg04401986	chr4	81189927	+	FGF5;FGF5	Body;Body	2.4934794	-1.218005	6.5557034	1.12E-09	1.17E-07	11.768657
cg07760161	chr17	7588378	-	TP53;WRAP53;TP53;TP53;TP53	5'UTR;TSS1500;5'UTR;5'UTR;5'UTR	-1.377711	-0.151273	-6.55567	1.12E-09	1.17E-07	11.768496
cg12143789	chr5	72594461	+			2.37753	-1.086982	6.5556806	1.12E-09	1.17E-07	11.768546
cg19449594	chr11	122292868	+			-1.835985	0.1358678	-6.555254	1.12E-09	1.17E-07	11.766464
cg05053440	chr1	227748442	-			1.3149916	0.0640946	6.5550832	1.12E-09	1.17E-07	11.765632

cg11954355	chr20	57393844	+	MIR298;MIR296	TSS1500;TSS1500	-1.149743	-1.505761	-6.555047	1.12E-09	1.17E-07	11.765454
cg01452911	chr16	86512095	+			-1.616398	0.5006708	-6.554279	1.12E-09	1.18E-07	11.761712
cg12709692	chr4	44425078	-	KCTD8	Body	-1.366114	-0.862506	-6.554369	1.12E-09	1.18E-07	11.76215
cg17831440	chr13	113699526	-	MCF2L;MCF2L	Body;Body	1.1283218	1.4839402	6.5543161	1.12E-09	1.18E-07	11.761891
cg16438397	chr5	77591635	+	AP3B1	TSS1500	-0.8179	-4.156798	-6.554039	1.13E-09	1.18E-07	11.760541
cg01950958	chr1	145589783	-	NUDT17	TSS1500	-0.946488	-2.77408	-6.553954	1.13E-09	1.18E-07	11.760127
cg07575466	chr1	40769708	-	COL9A2	Body	1.7415242	3.1686415	6.5575639	1.13E-09	1.18E-07	11.76068
cg05667197	chr7	142828903	+	PIP	TSS1500	-1.336573	-0.995508	-6.552999	1.13E-09	1.18E-07	11.755469
cg02628680	chr6	32015618	-	TNXB	Body	-1.167919	-0.676087	-6.552603	1.13E-09	1.19E-07	11.753537
cg25901381	chr7	27225485	-	HOXA11AS;HOXA11	Body;TSS1500	1.6206057	-1.343319	6.5525337	1.13E-09	1.19E-07	11.7532
cg08013557	chr3	147115043	-	ZIC4;ZIC4;ZIC4	Body;Body;Body	1.9120892	-1.199189	6.5522889	1.14E-09	1.19E-07	11.752006
cg16928869	chr20	60397766	+	CDH4	Body	-1.37961	-0.190593	-6.552037	1.14E-09	1.19E-07	11.750778
cg10439765	chr20	44657838	-	SLC12A5;SLC12A5;SLC12A5	1stExon;5'UTR;Body	2.3996648	-0.730978	6.5626155	1.14E-09	1.19E-07	11.750621
cg09558195	chr6	146350585	-	GRM1;GRM1	5'UTR;5'UTR	2.1463075	-1.47193	6.5504485	1.15E-09	1.20E-07	11.743033
cg02868123	chr2	79382913	+			-1.443698	0.1342491	-6.550354	1.15E-09	1.20E-07	11.742574
cg07136054	chr4	74486177	-	RASSF6;RASSF6	1stExon;TSS200	-1.300686	-3.934644	-6.549711	1.15E-09	1.20E-07	11.739437
cg07663765	chr16	84539641	+	KIAA1609	TSS1500	-1.075696	0.7469495	-6.549217	1.15E-09	1.20E-07	11.737028
cg10329928	chr2	20414196	+	SDC1;SDC1	Body;Body	-0.976435	-0.871503	-6.548706	1.16E-09	1.21E-07	11.734537
cg20002523	chr1	54665230	+	CYB5RL;MRPL37	5'UTR;TSS1500	-0.619384	-3.626663	-6.548487	1.16E-09	1.21E-07	11.733472
cg21766592	chr19	47288066	-	SLC1A5;SLC1A5;SLC1A5;SLC1A5	1stExon;5'UTR;Body;5'UTR	-1.349959	-3.705731	-6.548502	1.16E-09	1.21E-07	11.733543

cg07712663	chr2	16789846	+	FAM49A	5'UTR	-1.323519	-0.979408	-6.548277	1.16E-09	1.21E-07	11.732448
cg14055896	chr19	54481859	-	CACNG8	Body	2.2841028	-1.266082	6.559482	1.16E-09	1.21E-07	11.735609
cg03468671	chr14	39737849	+	CTAGE5;CTAGE5;CTAGE5;CTAGE5	Body;Body;Body;5'UTR	-0.756975	-3.484966	-6.547702	1.16E-09	1.21E-07	11.729643
cg01832325	chr11	5265215	+	HBBP1	TSS1500	-1.675945	-0.663084	-6.547389	1.16E-09	1.21E-07	11.728119
cg10048215	chr12	12938383	+	APOLD1;APOLD1	Body;TSS200	0.903313	1.3904534	6.5473711	1.16E-09	1.21E-07	11.728031
cg19763461	chr2	63284557	+			2.1250508	-0.524373	6.5474461	1.16E-09	1.21E-07	11.728397
cg18504632	chr3	71732622	+	EIF4E3;EIF4E3;EIF4E3;EIF4E3	3'UTR;3'UTR;3'UTR;3'UTR	1.1887067	1.7898741	6.5470389	1.17E-09	1.22E-07	11.726412
cg05058758	chr2	143784246	-	KYNU	Body	1.5095533	-0.261198	6.5467818	1.17E-09	1.22E-07	11.725159
cg21915313	chr5	140774426	+	PCDHGA4;PCDHGA6;PCDHGA1;PCDHGA5;PCDHGB1;PCDHGB4;PCDHGA3;PCDHGA2;PCDHGA8;PCDHGA7;PCDHGB2;PCDHGA8;PCDHGB3	Body;Body;Body;Body;Body;Body;Body;Body;1stExon;Body;Body;1stExon;Body	1.4211992	1.2552264	6.5464613	1.17E-09	1.22E-07	11.723597
cg22475859	chr2	365090	+			-1.564837	0.4613197	-6.545942	1.17E-09	1.22E-07	11.721067
cg14185229	chr19	46098784	+	GPR4	5'UTR	-1.418028	-0.27871	-6.54585	1.17E-09	1.22E-07	11.720618
cg02215357	chr13	53191046	-	HNRNPA1L2;HNRNPA1L2	TSS1500;TSS1500	-1.412429	-2.480756	-6.545533	1.18E-09	1.22E-07	11.719071
cg18166376	chr3	71180064	+	FOXP1	Body	-1.185917	-2.593931	-6.545296	1.18E-09	1.22E-07	11.717919
cg16731240	chr19	52391250	+	ZNF577;ZNF577;ZNF577	TSS200;TSS200;TSS200	2.0631291	-0.194967	6.5452096	1.18E-09	1.22E-07	11.717497
cg16589830	chr1	212284214	+			1.5736619	-0.112932	6.5451004	1.18E-09	1.22E-07	11.716964
cg18693673	chr19	58629975	-	ZSCAN18;ZSCAN18	TSS200;TSS200	1.5395909	-0.538316	6.5443807	1.18E-09	1.23E-07	11.713457
cg04760448	chr21	46890849	-	COL18A1;COL18A1;COL18A1	Body;Body;Body	-1.641973	-0.761892	-6.544293	1.18E-09	1.23E-07	11.71303
cg00174718	chr6	47136110	-			-1.057836	-2.0724	-6.544137	1.18E-09	1.23E-07	11.71227
cg15748507	chr10	120354627	-	PRLHR	Body	1.1037556	-0.330152	6.5441733	1.18E-09	1.23E-07	11.712446
cg06285439	chr2	206613589	+	NRP2;NRP2;NRP2;NRP2;NRP2	Body;Body;Body;Body;Body	-1.156902	-0.367448	-6.544036	1.18E-09	1.23E-07	11.711778

cg16042259	chr6	100917206	-			1.7473825	-0.681332	6.5433134	1.19E-09	1.23E-07	11.708256
cg02621130	chr6	166267729	-			2.3751769	-1.593352	6.5432062	1.19E-09	1.23E-07	11.707734
cg01406381	chr19	47288263	+	SLC1A5;SLC1A5;SLC1A5	TSS200;Body;5'UTR	-1.200585	-3.826616	-6.542996	1.19E-09	1.24E-07	11.706708
cg04214938	chr2	119496171	+			-2.422323	0.1418039	-6.550466	1.19E-09	1.24E-07	11.709312
cg00505045	chr4	147561669	-	POU4F2	Body	1.2881083	-0.823644	6.5428916	1.19E-09	1.24E-07	11.706201
cg17744293	chr1	50890952	+			1.30931	-0.723026	6.5428816	1.19E-09	1.24E-07	11.706152
cg14972143	chr4	99851003	-	EIF4E;EIF4E;EIF4E;EIF4E;EIF4E	5'UTR;1stExon;5'UTR;TSS1500;1stExon	-1.190749	-2.884595	-6.542816	1.19E-09	1.24E-07	11.705835
cg21553182	chr19	52956821	-	ZNF578	TSS200	1.5484231	-1.323378	6.5424589	1.19E-09	1.24E-07	11.704093
cg12109968	chr20	50383676	-	ATP9A	Body	-0.918192	-2.56044	-6.54202	1.20E-09	1.24E-07	11.701955
cg01132893	chr17	46712086	-			1.4032682	0.9882971	6.5417646	1.20E-09	1.24E-07	11.70071
cg04769392	chr6	80579458	-			-0.759554	-3.540624	-6.553031	1.20E-09	1.24E-07	11.712185
cg10417567	chr4	85418371	+	NKX6-1	Body	1.5652784	-1.12792	6.5417386	1.20E-09	1.24E-07	11.700583
cg11066580	chr7	4786879	-	FOXK1	Body	1.236278	2.7339421	6.5453428	1.20E-09	1.24E-07	11.701203
cg01244520	chr10	111178609	-			-1.533432	-0.610641	-6.540881	1.20E-09	1.25E-07	11.696406
cg04999999	chr20	62029358	+			-1.828764	1.3373918	-6.540827	1.20E-09	1.25E-07	11.696142
cg04856629	chr14	69105434	+			0.5853821	1.8224983	6.5403839	1.21E-09	1.25E-07	11.693983
cg18736063	chr20	21378560	-	NKX2-4	TSS1500	1.6198042	-0.970277	6.5404159	1.21E-09	1.25E-07	11.694139
cg06041479	chr11	134731610	+			-1.736086	0.8167967	-6.540265	1.21E-09	1.25E-07	11.693405
cg14575739	chr19	55098069	+	LILRA2;LILRA2	Body;Body	-1.550161	0.1330574	-6.539651	1.21E-09	1.25E-07	11.690415
cg10974219	chr17	12669028	-	MYOCD;MYOCD	3'UTR;3'UTR	-1.164974	-1.628698	-6.539099	1.21E-09	1.25E-07	11.687722

cg03464573	chr7	27205504	+	HOXA9	TSS1500	1.6486025	-1.005088	6.5389548	1.22E-09	1.26E-07	11.687022
cg06173663	chr7	101579929	-	CUX1;CUX1;CUX1	Body;Body;Body	1.8632185	1.2042138	6.5388343	1.22E-09	1.26E-07	11.686435
cg00151728	chr3	169478456	+			-1.450006	0.116822	-6.538474	1.22E-09	1.26E-07	11.684681
cg09298289	chr5	68485754	+	CENPH	Body	-0.490127	-4.40369	-6.538543	1.22E-09	1.26E-07	11.685016
cg21907579	chr12	114845868	+	TBX5;TBX5;TBX5;TBX5	1stExon;1stExon;5'UTR;5'UTR	1.8177708	-0.877117	6.5384994	1.22E-09	1.26E-07	11.684803
cg11775828	chr2	169100915	+	STK39	Body	-1.246428	-1.335626	-6.538064	1.22E-09	1.26E-07	11.682682
cg27093944	chr2	242908218	-			-2.349016	-2.198215	-6.537863	1.22E-09	1.26E-07	11.681704
cg10552126	chr7	154002064	-	DPP6;DPP6;DPP6	TSS1500;Body;Body	1.7665855	-1.005568	6.5375845	1.22E-09	1.26E-07	11.680347
cg00503383	chr1	91301651	+			2.1931731	-0.47207	6.5372431	1.23E-09	1.26E-07	11.678685
cg03363161	chr3	147139184	+			1.5489959	0.0296966	6.5371533	1.23E-09	1.26E-07	11.678247
cg05133853	chr11	5265008	+	HBBP1	TSS200	-1.502516	-1.498511	-6.536897	1.23E-09	1.27E-07	11.676997
cg25859099	chr7	20818767	-			1.3981844	-0.187855	6.5367426	1.23E-09	1.27E-07	11.676247
cg12778103	chr22	24237347	+	MIF	3'UTR	-0.771212	-4.048101	-6.540202	1.23E-09	1.27E-07	11.676393
cg26785346	chr4	186732936	-	SORBS2;SORBS2;SORBS2;SORBS2;SORBS2;SORBS2;SORBS2	5'UTR;5'UTR;5'UTR;1stExon;TSS1500;1stExon;TSS1500	-0.960998	-0.883658	-6.536482	1.23E-09	1.27E-07	11.67498
cg07184578	chr6	99291343	+			2.4784024	-0.200643	6.536345	1.23E-09	1.27E-07	11.674311
cg25641145	chr8	101572366	-	ANKRD46	TSS1500	-1.123378	-2.140633	-6.536073	1.23E-09	1.27E-07	11.672987
cg07873590	chr19	17858298	-	FCHO1;FCHO1	TSS1500;TSS1500	-1.187226	-3.899207	-6.535898	1.23E-09	1.27E-07	11.672135
cg13488501	chr1	46805584	+	NSUN4	TSS1500	-1.885831	0.6581912	-6.535717	1.24E-09	1.27E-07	11.671253
cg09156207	chr6	28058724	-	ZSCAN12L1	TSS1500	1.4678074	2.116493	6.535591	1.24E-09	1.27E-07	11.670639
cg07447260	chr10	103600544	-	KCNIP2;KCNIP2;KCNIP2;KCNIP2;KCNIP2;KCNIP2;KCNIP2	Body;Body;TSS1500;Body;Body;Body;Body	-0.592271	-4.048769	-6.534844	1.24E-09	1.28E-07	11.667002

cg20367304	chr4	154391944	-	KIAA0922;KIAA0922	Body;Body	-1.626558	0.1825558	-6.53467	1.24E-09	1.28E-07	11.666156
cg14963724	chr18	72166303	-	CNDP2;CNDP2	TSS1500;5'UTR	-1.340459	-2.04783	-6.538213	1.24E-09	1.28E-07	11.666531
cg22338152	chr19	57642143	+	USP29	Body	-1.757284	-0.024421	-6.53438	1.24E-09	1.28E-07	11.664742
cg00767010	chr9	99482094	-			1.7638213	-0.460789	6.5342006	1.24E-09	1.28E-07	11.663868
cg04362586	chr2	45231782	+			2.2295604	-0.538383	6.5341221	1.25E-09	1.28E-07	11.663486
cg13987885	chr9	37591782	-	TOMM5;TOMM5;TOMM5	Body;3'UTR;Body	-1.109285	-1.471375	-6.53398	1.25E-09	1.28E-07	11.662795
cg08514736	chr5	54529368	-	CCNO;CCNO	5'UTR;1stExon	-0.724742	-3.616986	-6.533235	1.25E-09	1.28E-07	11.659165
cg20850873	chr3	10149803	-	C3orf24;C3orf24;C3orf24;C3orf24	1stExon;1stExon;5'UTR;5'UTR	1.0364587	2.7102039	6.5367756	1.25E-09	1.29E-07	11.659733
cg22352800	chr2	177014467	-	MIR10B	TSS1500	1.3635921	0.2160519	6.5329151	1.25E-09	1.29E-07	11.657609
cg27648556	chr8	130641349	-			-1.278759	-0.38665	-6.532703	1.25E-09	1.29E-07	11.656574
cg11864327	chr5	178339172	+	ZFP2	5'UTR	-1.260731	0.2115106	-6.532643	1.25E-09	1.29E-07	11.656283
cg17799287	chr13	92001764	+	MIR19A;MIR17HG;MIR17HG;MIR17;MIR18A	TSS1500;Body;Body;TSS1500;TSS1500	-1.576588	-1.975256	-6.532261	1.26E-09	1.29E-07	11.654426
cg04534765	chr18	74962369	+	GALR1;GALR1	1stExon;5'UTR	2.5583365	-0.942678	6.5318345	1.26E-09	1.29E-07	11.652348
cg14945086	chr18	2657780	-	SMCHD1	Body	-1.414123	-0.300326	-6.531821	1.26E-09	1.29E-07	11.652281
cg11783264	chr19	1905046	-	SCAMP4;ADAT3	TSS1500;TSS1500	-0.767719	-3.684118	-6.531433	1.26E-09	1.29E-07	11.650391
cg15670990	chr6	32016115	+	TNXB	Body	-0.4303	-3.79796	-6.531286	1.26E-09	1.29E-07	11.649676
cg03460001	chr7	101460955	-	CUX1;CUX1;CUX1	Body;Body;Body	-1.02044	-3.349025	-6.531077	1.26E-09	1.30E-07	11.648659
cg05638493	chr5	140683367	-	SLC25A2	1stExon	1.1265739	0.0480766	6.5310501	1.26E-09	1.30E-07	11.648529
cg02067946	chr5	2149520	+			-0.900623	-1.04065	-6.530892	1.27E-09	1.30E-07	11.647758
cg24702069	chr13	109352806	-	MYO16	Body	-1.720473	-1.520111	-6.530913	1.27E-09	1.30E-07	11.647861

cg10110335	chr3	12197630	-	SYN2;TIMP4;SYN2	Body;Body;Body	-1.590246	0.0628694	-6.530232	1.27E-09	1.30E-07	11.644548
cg15233131	chr19	817832	+	LPPR3	Body	-0.969765	-0.224739	-6.529821	1.27E-09	1.30E-07	11.642547
cg03928812	chr11	59634276	-	TCN1	TSS1500	-1.731681	-0.883495	-6.52969	1.27E-09	1.30E-07	11.64191
cg26164712	chr11	118754565	+	CXCR5;CXCR5	5'UTR;1stExon	-1.73515	0.6456122	-6.529393	1.28E-09	1.30E-07	11.640464
cg06328750	chr7	157322599	-			-1.613869	-1.094072	-6.529307	1.28E-09	1.30E-07	11.640045
cg15616511	chr11	45887415	+	CRY2;CRY2	Body;Body	0.7769317	0.2662925	6.5286839	1.28E-09	1.31E-07	11.637011
cg09126279	chr4	40675171	+			-1.771404	-2.224585	-6.543573	1.28E-09	1.31E-07	11.640896
cg16661000	chr15	89437710	+	HAPLN3	5'UTR	-1.187512	-3.333763	-6.53147	1.29E-09	1.31E-07	11.633946
cg05043733	chr22	43605465	-	SCUBE1	Body	-1.198247	0.4679748	-6.527636	1.29E-09	1.31E-07	11.631909
cg12050271	chr1	2980399	-	FLJ42875	Body	1.4109788	0.6798963	6.5272491	1.29E-09	1.32E-07	11.630028
cg20850016	chr3	5165254	+	ARL8B	Body	-1.161992	-3.140219	-6.526738	1.29E-09	1.32E-07	11.627541
cg23213887	chr5	127872767	+	FBN2	Body	1.3772053	-0.425102	6.5267766	1.29E-09	1.32E-07	11.627729
cg01636354	chr3	134078647	-	AMOTL2	Body	1.2591976	1.1840472	6.5302622	1.29E-09	1.32E-07	11.627885
cg21266502	chr4	155663225	-			2.1358828	-0.58569	6.5256613	1.30E-09	1.33E-07	11.622301
cg01647693	chr8	143513441	-			-1.021073	-0.919501	-6.52548	1.30E-09	1.33E-07	11.621418
cg03078269	chr12	54354974	+			2.3558303	-0.25295	6.5248924	1.30E-09	1.33E-07	11.61856
cg14689479	chr8	53326618	+			1.3176584	0.7967651	6.5249018	1.30E-09	1.33E-07	11.618606
cg04499015	chr6	33141696	+	COL11A2;COL11A2;COL11A2	Body;Body;Body	-1.069627	-0.980275	-6.524515	1.31E-09	1.33E-07	11.616724
cg06383163	chr8	139509073	-	FAM135B	TSS200	1.2496761	-0.356284	6.5244965	1.31E-09	1.33E-07	11.616634
cg07076109	chr7	4747261	-	FOXK1	Body	1.6156453	2.6370144	6.5242689	1.31E-09	1.33E-07	11.615526

cg24301690	chr3	128712676	-	KIAA1257	5'UTR	-1.243941	-2.404199	-6.524074	1.31E-09	1.33E-07	11.614579
cg22929099	chr5	137593583	+	GFRA3	Body	0.8286265	1.8686473	6.5230078	1.32E-09	1.34E-07	11.609391
cg00872726	chr14	97499908	+			1.6152052	-0.5439	6.5226282	1.32E-09	1.34E-07	11.607544
cg15678052	chr1	9844683	-	CLSTN1;CLSTN1	Body;Body	-1.412593	0.0869408	-6.52262	1.32E-09	1.34E-07	11.607503
cg21200539	chr4	147562073	-	POU4F2	3'UTR	1.9999921	-1.616477	6.5216828	1.33E-09	1.35E-07	11.602945
cg11661281	chr11	57798863	-	OR6Q1;OR9Q1	1stExon;5'UTR	-1.36059	-1.227271	-6.524956	1.33E-09	1.35E-07	11.60211
cg08369295	chr22	25759225	-	LRP5L;LRP5L	TSS1500;5'UTR	1.5243601	3.0188658	6.5244333	1.33E-09	1.35E-07	11.607963
cg24127719	chr21	38068660	+			1.9629104	-0.623637	6.520731	1.33E-09	1.35E-07	11.598315
cg01191058	chr16	89048433	+			-2.143515	1.2710275	-6.520428	1.33E-09	1.36E-07	11.596842
cg11207983	chr1	150254477	-	C1orf51	TSS1500	-1.77301	-3.524138	-6.520429	1.33E-09	1.36E-07	11.596846
cg07450633	chr5	172175321	-			0.9810321	0.2358224	6.5194412	1.34E-09	1.36E-07	11.592041
cg02524954	chr11	32454964	-	WT1;WT1;WT1;WT1	Body;Body;Body;Body	2.3646214	-3.017207	6.5190645	1.34E-09	1.36E-07	11.590209
cg12486493	chr15	99417267	-	IGF1R	Body	-1.473768	-1.041427	-6.519103	1.34E-09	1.36E-07	11.590397
cg11115235	chr4	6247215	-			1.8083631	-1.078869	6.5184228	1.35E-09	1.37E-07	11.587089
cg13106156	chr6	33140934	+	COL11A2;COL11A2;COL11A2	Body;Body;Body	-1.253033	-0.249935	-6.518235	1.35E-09	1.37E-07	11.586177
cg25033030	chr16	89768881	+	SPATA2L	TSS1500	-0.956116	-3.208409	-6.518004	1.35E-09	1.37E-07	11.585053
cg07550921	chr8	122651520	+	HAS2;HAS2AS	5'UTR;TSS200	-1.197992	-3.28146	-6.517135	1.36E-09	1.38E-07	11.580825
cg03960562	chr11	193021	-	SCGB1C1;LOC653486	TSS200;TSS200	-0.601364	-0.358448	-6.516709	1.36E-09	1.38E-07	11.578754
cg08602008	chr19	48076841	-			1.2578121	0.1368168	6.5166163	1.36E-09	1.38E-07	11.578303
cg01890568	chr17	31437649	-	ACCN1;ACCN1	Body;Body	-2.034428	-0.063617	-6.51649	1.36E-09	1.38E-07	11.577689

cg13720362	chr6	108492271	-	NR2E1	Body	0.9816016	-0.425305	6.5161802	1.36E-09	1.38E-07	11.576183
cg20415811	chr2	74700246	+	CCDC142;MRPL53	3'UTR;TSS1500	-1.102326	-2.262286	-6.515949	1.36E-09	1.38E-07	11.57506
cg08803500	chr12	54338824	+	HOXC13	Body	2.8510265	-2.818286	6.5231763	1.37E-09	1.38E-07	11.576701
cg22341848	chr1	24106359	+	C1orf128	Body	-1.103337	-0.882476	-6.515446	1.37E-09	1.39E-07	11.572615
cg09177248	chr15	67000790	+	SMAD6;SMAD6;SMAD6	Body;Body;TSS200	1.169548	2.621265	6.5186928	1.37E-09	1.39E-07	11.57189
cg22346765	chr6	41007522	+	UNC5CL	TSS1500	-1.41249	0.0063845	-6.518297	1.37E-09	1.39E-07	11.569775
cg03224572	chr8	97172012	+	GDF6	Body	2.4331425	-0.942076	6.5145376	1.37E-09	1.39E-07	11.568196
cg01809854	chr13	24118951	-			0.9837119	1.3882019	6.513265	1.38E-09	1.40E-07	11.562009
cg01898698	chr2	105472759	-	POU3F3	1stExon	1.283556	-0.087339	6.5132127	1.38E-09	1.40E-07	11.561755
cg26192720	chr11	62557528	+	TMEM179B	3'UTR	1.2208261	1.6446576	6.5123619	1.39E-09	1.41E-07	11.557619
cg00504075	chr1	155827556	+	GON4L;GON4L	TSS1500;TSS1500	-1.575067	-0.715166	-6.511978	1.39E-09	1.41E-07	11.555752
cg10647833	chr6	167047842	+	RPS6KA2	Body	1.4868776	2.6544712	6.5119421	1.39E-09	1.41E-07	11.555578
cg12472483	chr7	157996945	+	PTPRN2;PTPRN2;PTPRN2	Body;Body;Body	1.4340985	-0.482987	6.512025	1.39E-09	1.41E-07	11.555981
cg04215950	chr1	7125137	+	CAMTA1	Body	-1.217064	-0.464443	-6.511185	1.40E-09	1.41E-07	11.551897
cg01423643	chr1	160992358	-	F11R	TSS1500	-2.288954	1.1737219	-6.511137	1.40E-09	1.41E-07	11.551666
cg01558212	chr2	200327336	+			1.6472365	-1.101431	6.5110615	1.40E-09	1.41E-07	11.551298
cg00350432	chr15	67065190	-	SMAD6;SMAD6;SMAD6	Body;Body;Body	1.1565127	1.4213421	6.5109085	1.40E-09	1.41E-07	11.550555
cg00400448	chr15	60285691	-			1.5672636	0.9260685	6.5142954	1.40E-09	1.42E-07	11.550351
cg22321318	chr7	157294388	-			-1.52716	-0.811464	-6.51064	1.40E-09	1.42E-07	11.549251
cg06346307	chr22	19949965	-	COMT;COMT;COMT;COMT	5'UTR;5'UTR;5'UTR;TSS200	-0.993631	-1.76071	-6.510568	1.40E-09	1.42E-07	11.548901

cg07281879	chr11	32459626	+	WIT1	Body	1.9102824	-0.924087	6.5104859	1.40E-09	1.42E-07	11.548501
cg17786215	chr20	49349030	+	PARD6B	Body	-0.790988	-2.520515	-6.510206	1.41E-09	1.42E-07	11.547142
cg06425919	chr11	17741925	+	MYOD1	1stExon	2.0663557	-1.045579	6.509384	1.41E-09	1.42E-07	11.543145
cg26949694	chr11	27742060	-	BDNF;BDNF;BDNF;BDNF;BDNF;BDNF;BDNF	Body;5'UTR;TSS1500;1stExon;5'UTR;1stExon;5'UTR	1.4125171	-0.726263	6.5094221	1.41E-09	1.42E-07	11.54333
cg27485075	chr2	47054963	-	LOC100134259	TSS200	-1.162999	0.3091573	-6.508731	1.42E-09	1.43E-07	11.539971
cg13046832	chr14	29254680	+	C14orf23	Body	2.5087003	-1.184894	6.5117348	1.42E-09	1.43E-07	11.538122
cg04034583	chr2	119603108	+	EN1	Body	0.9734687	0.18291	6.5152253	1.42E-09	1.43E-07	11.538173
cg02607059	chr15	101632053	-			1.2197794	1.8154397	6.5077979	1.42E-09	1.43E-07	11.535437
cg26090619	chr17	80330167	+			1.1075924	0.8126817	6.5070924	1.43E-09	1.44E-07	11.532009
cg09347824	chr6	44262755	+	TCTE1	5'UTR	-0.850413	-1.16227	-6.506912	1.43E-09	1.44E-07	11.531131
cg10764068	chr7	27289829	-			1.0284704	-0.502134	6.5069338	1.43E-09	1.44E-07	11.531238
cg07815836	chr19	58715351	+	ZNF274;ZNF274;ZNF274	Body;Body;5'UTR	0.9971591	0.4996574	6.5066571	1.43E-09	1.44E-07	11.529894
cg07109115	chr10	28030398	-	MKX	Body	1.2732325	-0.328379	6.5064765	1.43E-09	1.44E-07	11.529016
cg13728131	chr11	19799633	-	NAV2;NAV2;NAV2	Body;Body;Body	-1.162541	-2.258254	-6.50616	1.43E-09	1.44E-07	11.52748
cg25096745	chr11	35195513	-	CD44;CD44;CD44;CD44;CD44	Body;Body;Body;Body;Body	-1.328436	-1.020255	-6.505882	1.44E-09	1.44E-07	11.526128
cg24803687	chr11	5462916	-	OR51I1	TSS200	-1.643839	-0.36778	-6.50579	1.44E-09	1.44E-07	11.525682
cg16977711	chr19	56598916	+	ZNF787	3'UTR	0.6528004	2.6682004	6.5056625	1.44E-09	1.45E-07	11.525061
cg17217654	chr3	49058043	-	DALRD3;NDUFAF3;MIR425;NDUFAF3;NDUFAF3;NDUFAF3	5'UTR;TSS1500;TSS1500;TSS1500;5'UTR;TSS1500	-0.929288	-3.889014	-6.505447	1.44E-09	1.45E-07	11.524013
cg07641807	chr13	92001959	+	MIR19A;MIR17HG;MIR17HG;MIR20A;MIR17;MIR19B1;MIR18A	TSS1500;Body;Body;TSS1500;TSS1500;TSS1500;TSS1500	-1.4936	-2.19293	-6.505205	1.44E-09	1.45E-07	11.522839
cg23643813	chr1	1270995	+	DVL1	3'UTR	0.8570773	1.7574259	6.5046989	1.44E-09	1.45E-07	11.52038

cg26106948	chr3	27766284	-			1.4400922	-0.802418	6.5047184	1.44E-09	1.45E-07	11.520475
cg23043143	chr7	2612101	+	IQCE;IQCE	Body;Body	-1.399547	-0.725573	-6.504161	1.45E-09	1.45E-07	11.517769
cg08523865	chr2	124782253	-	CNTNAP5	TSS1500	1.3003926	-0.646381	6.5040379	1.45E-09	1.46E-07	11.517169
cg12899421	chr1	151512592	-	TUFT1;TUFT1	TSS200;TSS200	-0.730965	-2.974428	-6.503268	1.46E-09	1.46E-07	11.513431
cg14085673	chr10	95256679	+	CEP55;CEP55;CEP55	1stExon;5'UTR;5'UTR	-0.537602	-3.862066	-6.503115	1.46E-09	1.46E-07	11.512687
cg08884571	chr19	45901453	+	PPP1R13L;PPP1R13L	Body;Body	2.1526313	-0.349876	6.5028542	1.46E-09	1.46E-07	11.511419
cg01535630	chr1	3689683	+	LOC388588	5'UTR	-0.715832	-1.928456	-6.502701	1.46E-09	1.46E-07	11.510676
cg24760961	chr11	130782216	+	SNX19	Body	0.8995824	2.864849	6.5019501	1.46E-09	1.47E-07	11.507027
cg04088697	chr7	96627050	+	DLX6AS	Body	1.5808984	-0.953012	6.5018413	1.47E-09	1.47E-07	11.506499
cg03419151	chr17	6658728	+	XAF1;XAF1	TSS1500;TSS1500	0.9741152	1.1467031	6.5014976	1.47E-09	1.47E-07	11.504829
cg01959541	chr6	159291228	-			-0.693607	-4.036728	-6.501103	1.47E-09	1.47E-07	11.502913
cg05174942	chr5	140098392	-	VTRNA1-2	TSS200	-2.268217	0.230657	-6.50078	1.47E-09	1.48E-07	11.501345
cg05002837	chr22	47057371	-	GRAMD4	Body	-1.761755	0.8296571	-6.500335	1.48E-09	1.48E-07	11.499181
cg13820205	chr9	132448040	-	PRRX2	Body	-1.354682	-0.728688	-6.500097	1.48E-09	1.48E-07	11.498026
cg15645660	chr1	55247356	+	TTC22	Body	1.6219057	1.9055867	6.4999818	1.48E-09	1.48E-07	11.497468
cg03770410	chr20	57766332	+	ZNF831	1stExon	-1.298664	-0.310784	-6.499882	1.48E-09	1.48E-07	11.496985
cg26074603	chr12	75603068	-	KCNC2;KCNC2;KCNC2;KCNC2;KCNC2;KCNC2	1stExon;1stExon;5'UTR;5'UTR;1stExon;5'UTR	2.1464809	-0.684994	6.503441	1.48E-09	1.48E-07	11.506285
cg16104371	chr4	1538525	+			-2.197315	0.5884385	-6.499702	1.48E-09	1.48E-07	11.496108
cg23253053	chr12	111558736	-	CUX2	Body	-1.360684	-1.003733	-6.499682	1.48E-09	1.48E-07	11.496011
cg27581028	chr2	63277327	-	OTX1	TSS1500	-0.529745	-4.436495	-6.499647	1.48E-09	1.48E-07	11.495844

cg25278144	chr17	77962240	-	TBC1D16	Body	-1.241245	-0.65822	-6.499442	1.48E-09	1.48E-07	11.494845
cg19749224	chr1	43296467	-	ERMAP;ERMAP	Body;Body	0.855299	0.7596218	6.4987388	1.49E-09	1.49E-07	11.491432
cg19925849	chr19	54445377	+	CACNG7	Body	1.5985101	-0.598648	6.4986964	1.49E-09	1.49E-07	11.491225
cg04868764	chr15	41137019	+	SPINT1;SPINT1;SPINT1	Body;Body;1stExon	-0.567483	-3.486704	-6.498649	1.49E-09	1.49E-07	11.490995
cg07392449	chr8	11324666	+	FAM167A	TSS1500	-2.061122	-5.236994	-6.501729	1.49E-09	1.49E-07	11.489593
cg03290560	chr3	117774686	-			-1.757153	-0.017218	-6.497839	1.50E-09	1.49E-07	11.487062
cg08305942	chr16	79692354	-			-1.61759	-0.514901	-6.497833	1.50E-09	1.49E-07	11.487032
cg18604419	chr14	101513037	+	MIR889;MIR539	TSS1500;TSS1500	-1.995713	1.4636273	-6.497622	1.50E-09	1.49E-07	11.486011
cg03646740	chr13	114813015	-	RASA3	Body	-1.574864	0.3739107	-6.497172	1.50E-09	1.50E-07	11.483823
cg10431747	chr1	34642383	-	C1orf94;C1orf94	5'UTR;TSS200	1.3793595	0.2257476	6.4969655	1.50E-09	1.50E-07	11.482821
cg01014615	chr2	177027621	+	HOXD3	TSS1500	1.5361929	0.076056	6.4966349	1.50E-09	1.50E-07	11.481216
cg25951981	chr4	46995743	+	GABRA4	TSS200	2.2180763	-1.507608	6.4958547	1.51E-09	1.51E-07	11.477428
cg13396738	chr6	30850718	+	DDR1	TSS1500	-1.04024	-2.342572	-6.495733	1.51E-09	1.51E-07	11.476836
cg01346158	chr16	2088997	+	SLC9A3R2;SLC9A3R2	3'UTR;3'UTR	1.1440941	1.3516742	6.4991169	1.51E-09	1.51E-07	11.476933
cg27619796	chr16	86756180	+			-1.193157	-0.538757	-6.495436	1.51E-09	1.51E-07	11.475394
cg23683254	chr14	99584667	-			1.5881717	-0.531428	6.4989877	1.51E-09	1.51E-07	11.476306
cg16118089	chr12	125550542	+	AACS	Body	-1.049954	-2.359896	-6.495297	1.51E-09	1.51E-07	11.474723
cg19654882	chr11	3829219	-	PGAP2;PGAP2;PGAP2;PGAP2;PGAP2;PGAP2;PGAP2;PGAP2	Body;Body;Body;TSS1500;Body;TSS200;TSS1500;Body	-0.931191	-1.774729	-6.494634	1.52E-09	1.51E-07	11.471503
cg17218270	chr8	37749412	-	RAB11FIP1;RAB11FIP1	Body;Body	-1.488755	-1.364074	-6.494546	1.52E-09	1.51E-07	11.471074
cg00849713	chr6	166267744	-			2.0215553	-1.232725	6.4943106	1.52E-09	1.52E-07	11.469932

cg09177577	chr8	22503577	+	BIN3	Body	1.2466293	2.3192964	6.4940773	1.52E-09	1.52E-07	11.4688
cg21961694	chr11	62947565	-	SLC22A25	Body	-1.821975	-0.126691	-6.494008	1.52E-09	1.52E-07	11.468461
cg23321140	chr5	150410007	-	TNIP1	3'UTR	1.5033726	1.1201926	6.4939311	1.53E-09	1.52E-07	11.46809
cg03112033	chr12	31515551	-			-1.432005	-0.360054	-6.492903	1.53E-09	1.52E-07	11.463099
cg20095398	chr4	1571649	-			-1.021906	-0.392866	-6.492891	1.53E-09	1.52E-07	11.463043
cg26460366	chr4	17068050	+			-1.404067	-0.411637	-6.492845	1.53E-09	1.52E-07	11.462819
cg25515317	chr7	116164108	-	CAV1	TSS1500	1.3059066	2.2682353	6.491627	1.54E-09	1.53E-07	11.456907
cg20569588	chr1	55267285	+	TTC22;TTC22	TSS1500;TSS1500	-0.847015	-3.01347	-6.491445	1.54E-09	1.53E-07	11.456025
cg07739369	chr17	80241456	+			-1.54383	-1.803159	-6.491319	1.55E-09	1.54E-07	11.455411
cg03956042	chr3	194206497	-			-1.451845	-3.806165	-6.491245	1.55E-09	1.54E-07	11.455054
cg15108890	chr17	14111644	+	COX10	3'UTR	0.8714641	1.3551698	6.4911232	1.55E-09	1.54E-07	11.454462
cg24155668	chr6	36098398	+	MAPK13	1stExon	-0.803181	-4.505632	-6.490328	1.55E-09	1.54E-07	11.450601
cg08815081	chr10	28035731	-	MKX	TSS1500	1.53197	-0.755463	6.4901475	1.55E-09	1.54E-07	11.449727
cg02245566	chr1	158670617	+	OR6K2	TSS200	-2.074607	0.4277404	-6.490032	1.56E-09	1.54E-07	11.449164
cg14082893	chr15	75400931	+			-1.617008	-0.605143	-6.489971	1.56E-09	1.54E-07	11.448872
cg24585453	chr1	64058672	+	PGM1	TSS1500	-1.065721	-2.465223	-6.489652	1.56E-09	1.55E-07	11.447321
cg25581222	chr15	64539239	-	CSNK1G1	Body	-1.860511	-0.713169	-6.489694	1.56E-09	1.55E-07	11.447525
cg08584759	chr10	11912126	+	C10orf47	Body	1.3312409	1.2555889	6.4890444	1.56E-09	1.55E-07	11.444374
cg02385728	chr9	133803377	+	FIBCD1;FIBCD1	Body;Body	-1.441469	0.1899592	-6.48886	1.56E-09	1.55E-07	11.443479
cg09685182	chr3	130236037	+			2.0419107	-1.562712	6.4884673	1.57E-09	1.55E-07	11.441574

cg10440524	chr10	83728257	-	NRG3;NRG3;NRG3	Body;Body;Body	-1.350425	-0.712242	-6.492108	1.57E-09	1.55E-07	11.44277
cg25584668	chr6	31095908	-	PSORS1C1	5'UTR	-0.843228	-1.775793	-6.492109	1.57E-09	1.55E-07	11.442776
cg05614408	chr5	94901279	-	ARSK	Body	0.8761901	1.8084217	6.4883405	1.57E-09	1.55E-07	11.440959
cg10661615	chr10	120355262	+	PRLHR	TSS200	1.0869601	-0.762905	6.4882228	1.57E-09	1.55E-07	11.440388
cg00989505	chr14	101489496	+	MIR299;MIR411	TSS1500;TSS200	-1.58201	0.8357676	-6.487934	1.57E-09	1.56E-07	11.438985
cg10299383	chr8	54798735	+	RGS20;RGS20	Body;Body	-1.384459	-1.010969	-6.487858	1.57E-09	1.56E-07	11.438617
cg23860325	chr7	54271152	-	HPVC1	TSS1500	-1.378245	-0.956097	-6.487678	1.57E-09	1.56E-07	11.437746
cg04007890	chr7	976706	-	ADAP1	Body	-1.446851	0.4649944	-6.487615	1.57E-09	1.56E-07	11.43744
cg10926330	chr17	76268981	-			-0.983347	-2.336255	-6.48732	1.58E-09	1.56E-07	11.436007
cg25288068	chr6	28793681	-			1.8049224	0.5663272	6.4907884	1.58E-09	1.56E-07	11.436577
cg12663811	chr1	901685	-	PLEKHN1;PLEKHN1	TSS200;TSS200	-1.111725	-1.987799	-6.486535	1.58E-09	1.56E-07	11.432201
cg24468890	chr1	32757294	-	HDAC1	TSS1500	-0.959166	-2.140241	-6.486576	1.58E-09	1.56E-07	11.432399
cg03292206	chr2	111424914	+	BUB1	Body	1.1728931	1.7015102	6.486461	1.58E-09	1.56E-07	11.431841
cg09127909	chr8	1400265	+			-1.351472	0.7680458	-6.486193	1.59E-09	1.57E-07	11.430539
cg15971157	chr13	24464376	+	PCOTH;MIPEP;PCOTH	5'UTR;TSS1500;5'UTR	-1.030925	-1.951914	-6.485879	1.59E-09	1.57E-07	11.429019
cg01270241	chr14	97673030	-			-1.605335	-0.238073	-6.485661	1.59E-09	1.57E-07	11.427961
cg01695620	chr8	58122850	-			-2.351094	1.8197704	-6.489304	1.59E-09	1.57E-07	11.429387
cg21605781	chr12	105630720	+	APPL2	TSS1500	-1.150833	-1.543333	-6.489049	1.59E-09	1.57E-07	11.42795
cg08886711	chr12	48399180	-	COL2A1;COL2A1	TSS1500;TSS1500	1.2102135	0.1133901	6.4853046	1.59E-09	1.57E-07	11.426231
cg06208270	chr1	156046344	-	MEX3A	3'UTR	1.1115003	0.4489015	6.4851819	1.59E-09	1.57E-07	11.425635

cg03336167	chr11	2930995	-	SLC22A18;SLC22A18	Body;Body	1.9125252	-0.193768	6.4885855	1.60E-09	1.57E-07	11.425907
cg02583247	chr12	4555311	-	FGF6	TSS1500	-1.479518	0.4511025	-6.4847	1.60E-09	1.58E-07	11.423297
cg25579180	chr7	71098623	+	WBSCR17	Body	-1.209191	-3.215649	-6.484205	1.60E-09	1.58E-07	11.420895
cg05038216	chr2	29339091	+	CLIP4	5'UTR	-1.189441	-3.783659	-6.483629	1.61E-09	1.58E-07	11.418104
cg08066129	chr21	38077258	+	SIM2;SIM2	Body;Body	1.4474184	-0.43342	6.48637	1.61E-09	1.59E-07	11.414977
cg16708174	chr3	158430962	-	RARRES1;RARRES1	Body;Body	1.0918949	1.6142217	6.4827536	1.61E-09	1.59E-07	11.413858
cg02130107	chr16	29828598	-	C16orf53	Body	-1.003816	-2.506457	-6.482544	1.62E-09	1.59E-07	11.412843
cg13933174	chr4	184644321	+			-1.161826	-4.051769	-6.482366	1.62E-09	1.59E-07	11.411975
cg05380734	chr17	76814805	+	USP36	Body	0.8060347	0.1603926	6.482069	1.62E-09	1.59E-07	11.410537
cg20969194	chr2	223163175	-	PAX3;PAX3;PAX3;CCDC140;PAX3;PAX3;PAX3;PAX3;PAX3	Body;Body;Body;5'UTR;Body;Body;Body;Body;Body	1.304225	-0.847667	6.4820475	1.62E-09	1.59E-07	11.410433
cg17087669	chr6	36099123	-	MAPK13	Body	-1.009251	-1.541296	-6.481034	1.63E-09	1.60E-07	11.405518
cg13955931	chr2	92162790	-			0.9391217	0.6631941	6.480958	1.63E-09	1.60E-07	11.40515
cg08765597	chr11	114490767	-			-1.533298	-0.114089	-6.480841	1.63E-09	1.60E-07	11.404583
cg18395355	chr17	46796556	-			1.2106742	-0.835228	6.4805969	1.63E-09	1.60E-07	11.403399
cg19895047	chr8	19460197	-	CSGALNACT1;CSGALNACT1;CSGALNACT1	TSS200;Body;5'UTR	1.2250553	0.8404531	6.4802745	1.63E-09	1.61E-07	11.401836
cg21680327	chr1	36108225	-	PSMB2	TSS1500	-1.425527	-1.64113	-6.479919	1.64E-09	1.61E-07	11.40011
cg11396628	chr6	31095913	+	PSORS1C1	5'UTR	-0.76679	-1.598248	-6.479805	1.64E-09	1.61E-07	11.399561
cg26104752	chr1	119527377	-	TBX15	5'UTR	2.0300566	-2.071917	6.4797143	1.64E-09	1.61E-07	11.399119
cg27269561	chr16	3072713	+	HCFC1R1;HCFC1R1;HCFC1R1;THOC6;THOC6	3'UTR;3'UTR;3'UTR;TSS1500;TSS1500	-0.762351	-2.890554	-6.479561	1.64E-09	1.61E-07	11.398375
cg11232245	chr10	101284535	+			1.3092787	0.9460654	6.479252	1.64E-09	1.61E-07	11.396878

cg17945976	chr2	66667433	-	MEIS1	Body	1.8826276	-0.74852	6.4792151	1.64E-09	1.61E-07	11.396698
cg20370678	chr17	58216297	-			1.882582	-0.325592	6.4825989	1.64E-09	1.61E-07	11.39692
cg24387380	chr15	27159158	-	GABRA5;GABRA5	Body;Body	-1.380872	0.0964732	-6.47891	1.65E-09	1.61E-07	11.395219
cg06142740	chr2	28973577	+	PPP1CB;PPP1CB	TSS1500;TSS1500	-0.85315	-2.010789	-6.47854	1.65E-09	1.62E-07	11.393425
cg21078966	chr18	68699782	-			-1.46464	0.4679639	-6.478506	1.65E-09	1.62E-07	11.393261
cg01255417	chr6	10422636	+			1.3584153	-0.373071	6.4783165	1.65E-09	1.62E-07	11.392342
cg16142855	chr4	379900	-			1.3919882	0.0093322	6.4778295	1.65E-09	1.62E-07	11.389981
cg14546128	chr10	134491509	-	INPP5A	Body	1.3976361	3.4515079	6.4884526	1.66E-09	1.62E-07	11.40027
cg23001905	chr19	1265302	-			-1.453212	1.202846	-6.47617	1.67E-09	1.63E-07	11.381935
cg07086295	chr20	41814797	-	PTPRT;PTPRT	Body;Body	-1.22586	-0.705545	-6.476087	1.67E-09	1.63E-07	11.381534
cg20543651	chr8	55918395	-			-1.537259	-0.997763	-6.475922	1.67E-09	1.64E-07	11.380734
cg26860935	chr4	1407989	-			1.9863264	-0.558275	6.4758172	1.67E-09	1.64E-07	11.380226
cg24626659	chr10	134754286	+	C10orf93	Body	-1.742388	-0.769226	-6.475626	1.67E-09	1.64E-07	11.379297
cg04028616	chr16	84424282	-	ATP2C2	Body	0.8756645	1.7733813	6.4754316	1.67E-09	1.64E-07	11.378357
cg12432236	chr13	58209133	+	PCDH17	1stExon	1.8426741	-0.697867	6.4750296	1.68E-09	1.64E-07	11.376409
cg18849102	chr4	4859935	-	MSX1	TSS1500	1.0153463	-0.65062	6.4750573	1.68E-09	1.64E-07	11.376543
cg08462055	chr15	64944023	-	ZNF609	Body	-1.14243	-1.146658	-6.474818	1.68E-09	1.64E-07	11.375384
cg27029518	chr18	72166324	+	CNDP2;CNDP2	TSS1500;5'UTR	-1.144689	-1.981152	-6.474472	1.68E-09	1.65E-07	11.373706
cg01716252	chr21	46552605	+	ADARB1;ADARB1;ADARB1;ADARB1;ADARB1;ADARB1;ADARB1	5'UTR;5'UTR;5'UTR;Body;Body;5'UTR;Body	0.9444687	1.6448966	6.4743734	1.68E-09	1.65E-07	11.373228
cg07199337	chr11	20408536	+	PRMT3;PRMT3;PRMT3	TSS1500;TSS1500;TSS1500	-0.829826	-3.058381	-6.474385	1.68E-09	1.65E-07	11.373285

cg03797660	chr16	89035228	-	CBFA2T3	Body	-1.825708	1.6670703	-6.473867	1.69E-09	1.65E-07	11.370773
cg15538767	chr5	149870395	+			1.0650818	0.1548535	6.4771832	1.69E-09	1.65E-07	11.378198
cg04911280	chr6	44281184	-	AARS2	TSS200	-0.953242	-3.316669	-6.473523	1.69E-09	1.65E-07	11.369108
cg16629469	chr6	108440603	-			3.2538459	-1.206671	6.4733686	1.69E-09	1.65E-07	11.368359
cg02832905	chr18	75591983	-			-1.649881	0.4288786	-6.472843	1.70E-09	1.65E-07	11.365813
cg06247406	chr6	101846791	-	GRIK2;GRIK2;GRIK2	TSS200;TSS200;TSS200	1.4656252	-0.797585	6.4729096	1.70E-09	1.65E-07	11.366134
cg24842086	chr1	119531857	-	TBX15	5'UTR	1.5815859	-0.73408	6.4728755	1.70E-09	1.65E-07	11.365969
cg27226238	chr2	79365613	+	REG1P	TSS200	-1.82614	0.5335255	-6.472964	1.70E-09	1.65E-07	11.366398
cg00322212	chr22	28072555	-			-1.496405	0.0925914	-6.472475	1.70E-09	1.66E-07	11.36403
cg06659209	chr11	86662968	-	FZD4	Body	0.9518615	0.7383071	6.4724689	1.70E-09	1.66E-07	11.363999
cg25493335	chr21	31964676	+	KRTAP6-3	TSS200	-1.872761	1.1071081	-6.472454	1.70E-09	1.66E-07	11.363925
cg13234062	chr11	65668299	+	FOSL1	TSS1500	-0.883765	-2.668468	-6.471312	1.71E-09	1.67E-07	11.358391
cg18483289	chr20	644897	+	SCRT2	Body	0.9397915	-0.968171	6.4712873	1.71E-09	1.67E-07	11.358273
cg21151432	chr2	25142229	+	ADCY3	TSS200	-1.197189	-3.079205	-6.471104	1.71E-09	1.67E-07	11.357386
cg00804803	chr1	10601595	-	PEX14	Body	2.0252709	3.2801602	6.4708946	1.71E-09	1.67E-07	11.35637
cg23746050	chr11	69063559	+	MYEOV	Body	-1.371842	0.785023	-6.470412	1.72E-09	1.67E-07	11.354034
cg12973941	chr4	13545639	+	NKX3-2	1stExon	1.9100874	-1.116658	6.4698722	1.72E-09	1.68E-07	11.351417
cg11683764	chr1	201915757	+	LMOD1	TSS200	-0.938611	-3.495632	-6.469695	1.72E-09	1.68E-07	11.350559
cg20985269	chr1	158576809	-	OR10Z1	1stExon	-1.533091	-0.214537	-6.469501	1.73E-09	1.68E-07	11.34962
cg21336434	chr7	54271282	+	HPVC1	TSS1500	-1.65484	-0.326051	-6.468726	1.73E-09	1.68E-07	11.345863

cg24900563	chr11	10320161	-			-1.797237	-2.713243	-6.468721	1.73E-09	1.68E-07	11.345837
cg26425256	chr2	119611362	+			1.7544717	-1.228767	6.4687315	1.73E-09	1.68E-07	11.34589
cg18268562	chr11	118842392	+	FOXR1	TSS200	1.0875491	0.6055188	6.4684978	1.73E-09	1.69E-07	11.344758
cg06898823	chr11	121665583	+			-2.403539	1.8940198	-6.468338	1.74E-09	1.69E-07	11.343984
cg03548645	chr14	103369816	-	TRAF3;TRAF3;TRAF3	Body;Body;Body	1.1158566	4.7309382	6.4789254	1.74E-09	1.69E-07	11.345799
cg13235825	chr11	107574836	+			-1.653739	-0.018678	-6.467762	1.74E-09	1.69E-07	11.341194
cg00870279	chr6	33256222	-	PFDN6;WDR46;WDR46	TSS1500;Body;Body	-0.878185	-2.030962	-6.467694	1.74E-09	1.69E-07	11.340866
cg03045323	chr19	11484448	-	C19orf39	TSS1500	-1.072692	-2.627242	-6.46764	1.74E-09	1.69E-07	11.340601
cg03799378	chr10	44498046	+			-1.65931	1.263553	-6.466879	1.75E-09	1.70E-07	11.336915
cg07333792	chr14	26674164	-			1.7919249	-0.82066	6.4667853	1.75E-09	1.70E-07	11.336463
cg22660609	chr4	132984188	+			-2.47025	2.4780709	-6.469941	1.75E-09	1.70E-07	11.335677
cg15833353	chr5	33807278	-	ADAMTS12	Body	-1.439807	-1.230773	-6.46623	1.75E-09	1.70E-07	11.333771
cg18555440	chr11	17741687	+	MYOD1	1stExon	1.0795653	-1.314249	6.4658424	1.76E-09	1.70E-07	11.331896
cg19371121	chr1	175050479	-	TNN	Body	-1.960815	-0.26381	-6.465132	1.76E-09	1.71E-07	11.328456
cg21161526	chr9	123691672	+			-1.009779	-1.525692	-6.465153	1.76E-09	1.71E-07	11.328554
cg14395844	chr9	139980277	+	LOC100289341;MAN1B1	Body;TSS1500	-0.960724	-3.760539	-6.465012	1.76E-09	1.71E-07	11.327874
cg07892597	chr1	119532093	+	TBX15;TBX15	1stExon;5'UTR	1.1675351	0.0532738	6.4647396	1.77E-09	1.71E-07	11.326555
cg24811864	chr18	55019711	-	ST8SIA3	TSS200	1.4262551	-0.957515	6.4646838	1.77E-09	1.71E-07	11.326284
cg17298275	chr17	934652	+	ABR;ABR;ABR	Body;Body;Body	1.3453639	1.8049253	6.4643005	1.77E-09	1.72E-07	11.324428
cg08543028	chr12	31902503	+			-1.436239	-2.061741	-6.46411	1.77E-09	1.72E-07	11.323507

cg11888982	chr8	116231420	+			-2.067188	-0.301536	-6.464075	1.77E-09	1.72E-07	11.323336
cg14976447	chr8	24151874	+	ADAM28;ADAM28	Body;Body	-1.823843	-0.716705	-6.46671	1.78E-09	1.72E-07	11.320054
cg08857729	chr1	67217826	+	TCTEX1D1	TSS1500	2.3070497	-0.856318	6.4630123	1.78E-09	1.73E-07	11.31819
cg24159547	chr17	73128072	+	NT5C	TSS200	-1.460344	-4.995756	-6.462636	1.79E-09	1.73E-07	11.31637
cg27322846	chr2	162284638	-			1.2195664	-0.773135	6.4625704	1.79E-09	1.73E-07	11.31605
cg05036991	chr2	155089854	+	GALNT13	Body	-1.447577	-1.259102	-6.465261	1.79E-09	1.74E-07	11.312841
cg24974704	chr8	144577238	+	ZC3H3	Body	1.6536661	3.4408958	6.4615655	1.80E-09	1.74E-07	11.311185
cg17136011	chr10	123908897	+	TACC2;TACC2	Body;Body	0.879625	-0.763944	6.4610008	1.80E-09	1.74E-07	11.30845
cg14459021	chr9	140683212	-	EHMT1	Body	1.4652806	2.6774237	6.4607195	1.80E-09	1.74E-07	11.307089
cg06200982	chr4	113168556	+	AP1AR;AP1AR	Body;Body	0.9330183	2.4313963	6.4602367	1.81E-09	1.75E-07	11.304751
cg13114497	chr22	18986775	+			1.3667155	0.102507	6.4601488	1.81E-09	1.75E-07	11.304326
cg07525077	chr14	21359943	+	RNASE3	Body	-1.620381	-0.742605	-6.459806	1.81E-09	1.75E-07	11.302664
cg04366343	chr4	16819676	-	LDB2;LDB2	Body;Body	-1.900947	1.0353213	-6.459672	1.81E-09	1.75E-07	11.302019
cg11071207	chr1	110611218	-	ALX3	Body	1.6077078	-0.887245	6.4594674	1.81E-09	1.75E-07	11.301027
cg08907118	chr16	27482516	+	GTF3C1	Body	0.8868067	0.7813155	6.4593951	1.82E-09	1.75E-07	11.300677
cg05546044	chr22	22222597	+	MAPK1;MAPK1	TSS1500;TSS1500	-1.057632	-2.099034	-6.459293	1.82E-09	1.75E-07	11.300183
cg11464297	chr10	70302998	+			-1.437548	-0.600783	-6.462642	1.82E-09	1.75E-07	11.300385
cg02580917	chr5	68628240	-			-1.070516	-1.310723	-6.458952	1.82E-09	1.75E-07	11.298531
cg21295088	chr1	119531625	+	TBX15	5'UTR	1.4991774	-1.879445	6.4624496	1.82E-09	1.76E-07	11.299457
cg03377767	chr2	17997138	+	MSGN1	TSS1500	-1.338079	-0.395476	-6.458761	1.82E-09	1.76E-07	11.297606

cg07797805	chr20	25387977	-	GINS1	TSS1500	-0.826343	-2.947806	-6.458443	1.82E-09	1.76E-07	11.29607
cg03252770	chr4	154354354	+			-1.607168	-1.275839	-6.458383	1.82E-09	1.76E-07	11.295776
cg11556846	chr2	200468728	-			1.0310706	-0.170938	6.4581645	1.83E-09	1.76E-07	11.29472
cg21477176	chr19	54445888	+	CACNG7	3'UTR	1.4927211	-0.171671	6.4579721	1.83E-09	1.76E-07	11.293789
cg16550282	chr14	24657526	-	IPO4	Body	-0.808849	-4.214468	-6.465054	1.83E-09	1.76E-07	11.295604
cg02732509	chr12	58241200	-	CTDSP2	TSS1500	-1.058384	-1.417575	-6.457654	1.83E-09	1.76E-07	11.292247
cg25536739	chr5	54518883	+			1.9064721	-0.956032	6.457598	1.83E-09	1.76E-07	11.291978
cg19819837	chr5	145722517	+			1.2429773	-1.147622	6.4571926	1.84E-09	1.77E-07	11.290016
cg02802514	chr6	33173134	-	HSD17B8	Body	-0.94565	-1.983134	-6.457001	1.84E-09	1.77E-07	11.289089
cg09226692	chr6	43422490	+	DLK2;DLK2	Body;Body	-0.557634	-2.808006	-6.45694	1.84E-09	1.77E-07	11.288794
cg25626375	chr8	133072269	-	OC90	TSS1500	-1.755705	-1.134603	-6.456665	1.84E-09	1.77E-07	11.287464
cg09364980	chr19	29455913	+	LOC148145	TSS200	-1.832747	0.55796	-6.456428	1.84E-09	1.77E-07	11.286317
cg08641706	chr2	105069200	-	LOC150568	Body	-1.827265	-0.032755	-6.456274	1.84E-09	1.77E-07	11.285572
cg06097423	chr12	15955024	+			-1.735566	0.2655593	-6.455639	1.85E-09	1.78E-07	11.282495
cg05667348	chr10	118892581	+	VAX1	Body	2.2892336	-0.830492	6.4590564	1.85E-09	1.78E-07	11.283059
cg25113157	chr12	111537104	-	CUX2	Body	-1.38626	-1.526272	-6.45548	1.85E-09	1.78E-07	11.281725
cg16438182	chr17	78916567	-	RPTOR;RPTOR	Body;Body	1.3437525	1.5463112	6.4776429	1.85E-09	1.78E-07	11.28843
cg16924822	chr12	3309264	+	TSPAN9;TSPAN9	5'UTR;5'UTR	-1.38502	-2.528661	-6.455037	1.86E-09	1.78E-07	11.279584
cg16425489	chr5	169805583	-	KCNIP1;KCNMB1	Body;3'UTR	-1.652065	0.4299624	-6.454753	1.86E-09	1.78E-07	11.278207
cg27168573	chr12	120700929	-	PXN;PXN	Body;Body	0.9903647	0.6008177	6.4547153	1.86E-09	1.78E-07	11.278027

cg14602530	chr4	4859772	+			1.836039	-1.191103	6.454639	1.86E-09	1.78E-07	11.277658
cg17610169	chr11	10750973	-			1.1363461	2.2883653	6.4543444	1.86E-09	1.79E-07	11.276232
cg02272968	chr6	32015773	-	TNXB	Body	-1.547225	-1.102666	-6.457704	1.86E-09	1.79E-07	11.276524
cg04264908	chr17	35303330	-			3.3535929	-2.395743	6.4541674	1.86E-09	1.79E-07	11.275376
cg00225006	chr15	83477872	+	WHAMM	TSS200	-1.481699	-0.610931	-6.454038	1.86E-09	1.79E-07	11.274747
cg02657654	chr2	45154029	-			1.7063595	0.1082556	6.4540545	1.86E-09	1.79E-07	11.274829
cg17855264	chr15	37180636	+			1.6277356	-0.313151	6.4537685	1.87E-09	1.79E-07	11.273445
cg25195987	chr7	31125925	-	ADCYAP1R1	Body	-1.612998	0.1909235	-6.453547	1.87E-09	1.79E-07	11.272376
cg24923516	chr2	127955156	+	CYP27C1	Body	-2.263145	-0.213291	-6.457014	1.87E-09	1.79E-07	11.273194
cg08528417	chr7	1740507	+			-1.250746	0.1075707	-6.453274	1.87E-09	1.79E-07	11.271052
cg01312762	chr22	42222192	+	CCDC134	3'UTR	0.7773806	0.4620055	6.4528964	1.88E-09	1.80E-07	11.269225
cg10833551	chr11	30649517	+			-1.789126	-0.337793	-6.452827	1.88E-09	1.80E-07	11.268892
cg11679455	chr10	8100761	+	GATA3;GATA3	Body;Body	1.6655804	2.0703377	6.4526626	1.88E-09	1.80E-07	11.268094
cg02332766	chr14	81649681	-	GTF2A1;GTF2A1	Body;Body	0.9521246	1.3469767	6.4519821	1.88E-09	1.80E-07	11.264802
cg14307304	chr9	97221786	-	HIATL1	3'UTR	0.6936914	2.9263984	6.4519235	1.88E-09	1.80E-07	11.264518
cg26980789	chr12	40014292	-	ABCD2	TSS1500	-0.830738	-3.679779	-6.451427	1.89E-09	1.81E-07	11.262115
cg25141264	chr22	47560578	+	TBC1D22A	Body	0.9527819	1.2629384	6.4511792	1.89E-09	1.81E-07	11.260917
cg05167251	chr2	176987465	-	HOXD9;HOXD9	5'UTR;1stExon	2.159108	-0.345611	6.4505007	1.90E-09	1.81E-07	11.257635
cg03998066	chr16	31482846	-	TGFB1I1;TGFB1I1	TSS1500;TSS1500	1.3464318	3.2121288	6.4573197	1.90E-09	1.82E-07	11.258505
cg16501892	chr18	44140574	+	LOXHD1;LOXHD1	TSS1500;Body	-1.724412	0.2146727	-6.449526	1.91E-09	1.82E-07	11.252918

cg26593521	chr5	156594725	-	FAM71B	TSS1500	-1.352938	-0.092509	-6.449127	1.91E-09	1.83E-07	11.25099
cg05471169	chr17	80255419	-			-1.694428	-2.196331	-6.449059	1.91E-09	1.83E-07	11.250661
cg20559736	chr19	45174671	+	CEACAM19;CEACAM19	TSS200;TSS200	-0.747906	-0.188372	-6.448673	1.92E-09	1.83E-07	11.248795
cg21666367	chr8	27450279	+			-1.199263	-2.448324	-6.448598	1.92E-09	1.83E-07	11.248431
cg01733284	chr1	158391119	-	OR10K2	TSS1500	-2.236608	0.5461293	-6.448183	1.92E-09	1.83E-07	11.246426
cg05984317	chr7	20818928	-			1.2789335	0.2010694	6.4514494	1.92E-09	1.84E-07	11.246314
cg23953820	chr6	30851051	+	DDR1;DDR1	TSS1500;TSS1500	-0.910629	-0.213181	-6.447227	1.93E-09	1.84E-07	11.241799
cg23855715	chr22	33402686	-	SYN3;SYN3;SYN3;SYN3;SYN3	1stExon;5'UTR;5'UTR;5'UTR;1stExon	-1.247927	-0.702964	-6.450555	1.93E-09	1.84E-07	11.241996
cg04115307	chr14	101539612	+			-0.826868	-0.383263	-6.44682	1.93E-09	1.84E-07	11.239834
cg15931471	chr15	67033330	-	SMAD6;SMAD6;SMAD6	Body;Body;Body	1.4019977	1.6436132	6.4467338	1.93E-09	1.84E-07	11.239416
cg17356718	chr2	240230587	+	HDAC4	Body	1.1423943	0.8034568	6.4464458	1.94E-09	1.85E-07	11.238023
cg05200250	chr16	75264574	+	BCAR1;BCAR1;BCAR1;BCAR1;BCAR1;BCAR1;BCAR1;BCAR1;BCAR1	Body;Body;Body;Body;Body;Body;Body;Body;Body	1.0202786	1.7996244	6.4463934	1.94E-09	1.85E-07	11.237769
cg02875879	chr4	113569394	-	MIR302A;MIR302D;LARP7;LARP7;MIR367	Body;TSS200;Body;Body;TSS1500	0.8987127	1.5217194	6.4458829	1.94E-09	1.85E-07	11.235301
cg26499363	chr17	46796119	-			1.4608304	-0.975844	6.4458273	1.94E-09	1.85E-07	11.235032
cg02034689	chr5	54518136	-			1.5339181	0.5711669	6.4457365	1.94E-09	1.85E-07	11.234593
cg04970570	chr5	87990145	-			1.9998172	-0.558165	6.445648	1.95E-09	1.85E-07	11.234165
cg27180994	chr7	43929080	+	URGCP;URGCP;URGCP	5'UTR;Body;Body	0.8357486	1.8854376	6.4452011	1.95E-09	1.86E-07	11.232004
cg07152216	chr3	147141219	-			1.943361	-0.341361	6.444958	1.95E-09	1.86E-07	11.230828
cg24389585	chr7	27184125	+	HOXA5	TSS1500	1.3125124	0.7923355	6.4444711	1.96E-09	1.86E-07	11.228474
cg00312508	chr10	85322997	+			-1.243353	0.5462137	-6.444229	1.96E-09	1.86E-07	11.227301

cg04960065	chr4	185189300	+			-1.627738	-0.78498	-6.444156	1.96E-09	1.86E-07	11.226951
cg26894523	chr4	107725	-	ZNF718	Body	1.6590928	-1.351722	6.4436441	1.96E-09	1.87E-07	11.224476
cg05188959	chr17	77174625	-	HRNBP3	5'UTR	-1.322409	-0.286401	-6.443404	1.97E-09	1.87E-07	11.223313
cg17870569	chr2	84513930	+			-1.5293	-0.507069	-6.443439	1.97E-09	1.87E-07	11.223482
cg10430941	chr10	47656773	-	ANTXRL	TSS1500	-1.117054	0.0280325	-6.443064	1.97E-09	1.87E-07	11.221671
cg25749318	chr6	170103058	+	C6orf120;WDR27	1stExon;TSS1500	-0.831359	-3.640228	-6.442917	1.97E-09	1.87E-07	11.220961
cg03487935	chr7	51925284	+			-1.014483	-1.654395	-6.442614	1.97E-09	1.87E-07	11.219496
cg03507241	chr18	12307649	+	TUBB6	TSS1500	2.5163907	-1.553943	6.4427109	1.97E-09	1.87E-07	11.219964
cg12917718	chr13	70682019	-	KLHL1;KLHL1;ATXN8OS	1stExon;5'UTR;Body	1.7408806	-0.8868	6.4426224	1.97E-09	1.87E-07	11.219536
cg16340963	chr5	117002269	-			-1.69751	0.5224437	-6.442639	1.97E-09	1.87E-07	11.219617
cg18063733	chr5	50265443	-			3.0366594	-1.966181	6.4463212	1.97E-09	1.87E-07	11.221347
cg13941801	chr9	98585586	+	C9orf130	Body	-1.400265	0.6914032	-6.442532	1.98E-09	1.87E-07	11.219099
cg05405914	chr16	8972233	+			-1.062228	-1.23913	-6.442445	1.98E-09	1.88E-07	11.218679
cg13021619	chr8	23563859	+	NKX2-6	1stExon	1.8975884	-1.636893	6.4422528	1.98E-09	1.88E-07	11.217749
cg21956614	chr4	129439073	+			-1.483639	-2.565838	-6.442159	1.98E-09	1.88E-07	11.217298
cg26512045	chr6	31111158	+	CCHCR1;CCHCR1;CCHCR1	Body;Body;Body	1.1994883	0.8180188	6.441965	1.98E-09	1.88E-07	11.216358
cg22271457	chr8	118939185	-	EXT1	Body	0.7476531	2.3088304	6.4412452	1.99E-09	1.89E-07	11.212878
cg03323462	chr17	35300677	-			1.9368582	-0.715075	6.4407617	1.99E-09	1.89E-07	11.210541
cg27175093	chr14	57265910	-			1.7873057	-0.50023	6.4403978	2.00E-09	1.89E-07	11.208782
cg16987754	chr7	155242911	+			2.3783945	-1.227581	6.440054	2.00E-09	1.89E-07	11.207121

cg24212471	chr11	75180067	-	GDPD5	Body	0.8572933	0.9697643	6.4400685	2.00E-09	1.89E-07	11.207191
cg19317715	chr17	40996578	+	AOC2;AOC2	TSS200;TSS200	1.0337003	-0.029427	6.4398325	2.00E-09	1.90E-07	11.20605
cg03226000	chr16	55690378	-	SLC6A2	TSS200	1.7652688	-0.973254	6.4394871	2.01E-09	1.90E-07	11.20438
cg26021007	chr17	45283040	-			-1.250718	-2.327716	-6.4394	2.01E-09	1.90E-07	11.203958
cg06479254	chr2	176983285	-	HOXD10	Body	1.1619782	1.5313389	6.4393115	2.01E-09	1.90E-07	11.203532
cg08764914	chr5	79553422	+			-0.818637	-1.684973	-6.439175	2.01E-09	1.90E-07	11.202874
cg20248516	chr2	119602901	-	EN1	Body	1.476026	-0.26824	6.4391769	2.01E-09	1.90E-07	11.202881
cg05682319	chr5	1003069	+			-2.402149	0.5971283	-6.438883	2.01E-09	1.90E-07	11.201462
cg02807446	chr10	106682909	+	SORCS3	Body	-1.533565	0.1521039	-6.438674	2.01E-09	1.90E-07	11.200449
cg27275023	chr3	107676222	-			1.673134	0.074988	6.438699	2.01E-09	1.90E-07	11.200571
cg17838420	chr5	1877906	-	IRX4	3'UTR	1.3660567	-0.297048	6.4384255	2.02E-09	1.91E-07	11.19925
cg15746620	chr19	14785849	+	EMR3	TSS200	-1.172218	-2.094731	-6.437763	2.02E-09	1.91E-07	11.196048
cg07964219	chr21	46847898	-	COL18A1	Body	-1.228641	-0.779261	-6.437702	2.02E-09	1.91E-07	11.195752
cg14273502	chr12	113013494	+			1.1591914	0.2909269	6.4375062	2.03E-09	1.91E-07	11.194807
cg21542223	chr1	91190005	-			1.3435925	-0.755556	6.4366244	2.04E-09	1.92E-07	11.190546
cg10602537	chr5	172260815	+	ERGIC1	TSS1500	-0.892046	-1.767098	-6.436434	2.04E-09	1.92E-07	11.189627
cg00966482	chr6	11111926	+	HERV-FRD;LOC221710;HERV-FRD	1stExon;Body;5'UTR	0.9847901	1.6023695	6.4362206	2.04E-09	1.92E-07	11.188595
cg22169874	chr2	19273596	-			-1.293982	0.3547099	-6.436256	2.04E-09	1.92E-07	11.188765
cg07959986	chr22	49068771	-	FAM19A5;FAM19A5	Body;Body	-1.291408	-0.304966	-6.435494	2.05E-09	1.93E-07	11.185085
cg22275864	chr8	37822562	-	ADRB3	Body	1.7168707	-0.233993	6.4424531	2.05E-09	1.93E-07	11.186434

cg26425555	chr5	16616459	+	FAM134B	Body	-1.033869	-2.533032	-6.43478	2.05E-09	1.94E-07	11.181637
cg16135698	chr17	46825179	+			1.4366461	-1.110442	6.4346596	2.06E-09	1.94E-07	11.181052
cg11168293	chr22	49067022	+	FAM19A5;FAM19A5	Body;Body	-1.406618	0.4491373	-6.434504	2.06E-09	1.94E-07	11.180301
cg12964741	chr18	77160221	-	NFATC1;NFATC1;NFATC1;NFATC1;NFATC1	Body;TSS200;5'UTR;TSS200;Body	0.8410461	-1.717318	6.4344231	2.06E-09	1.94E-07	11.17991
cg17160439	chr11	64815744	+	NAALADL1	Body	1.2503557	0.9288422	6.4333873	2.07E-09	1.95E-07	11.174906
cg19188855	chr12	54355476	-			1.6170338	-0.729532	6.4332785	2.07E-09	1.95E-07	11.17438
cg08886154	chr7	127256676	-	PAX4	TSS1500	-1.22625	-0.535344	-6.432408	2.08E-09	1.96E-07	11.170177
cg09187933	chr12	127630812	+			1.5939877	-1.217534	6.4323592	2.08E-09	1.96E-07	11.16994
cg12111714	chr13	26043472	+	ATP8A2	Body	1.6270195	0.3300344	6.4320802	2.08E-09	1.96E-07	11.168592
cg07315493	chr16	1608794	+	IFT140	Body	1.1087084	3.4073347	6.4308246	2.10E-09	1.97E-07	11.162527
cg14031054	chr1	164681652	-	PBX1	Body	1.4392953	-0.351842	6.430739	2.10E-09	1.97E-07	11.162113
cg14212038	chr11	66738758	-			-1.333621	-1.374552	-6.430736	2.10E-09	1.97E-07	11.162099
cg23497644	chr2	170654835	+	SSB	TSS1500	-1.396835	-1.126402	-6.430307	2.10E-09	1.98E-07	11.160026
cg03320492	chr1	45187810	-	C1orf228	Body	-1.492354	-1.639565	-6.430211	2.10E-09	1.98E-07	11.159563
cg10249375	chr1	63795934	+			3.5283818	-1.572647	6.4333857	2.11E-09	1.98E-07	11.166969
cg26808687	chr1	201979499	+	ELF3;ELF3	TSS200;TSS200	-1.159151	-0.774039	-6.429781	2.11E-09	1.98E-07	11.157486
cg15934958	chr3	37212084	+	LRRFIP2;LRRFIP2;LRRFIP2	5'UTR;5'UTR;5'UTR	1.2101876	1.5512507	6.4296375	2.11E-09	1.98E-07	11.156794
cg01945793	chr7	10979586	+	NDUFA4	Body	-1.104567	-4.536565	-6.433028	2.11E-09	1.98E-07	11.157212
cg02221347	chr2	136743422	-	DARS	TSS1500	-0.780472	-4.921425	-6.429341	2.11E-09	1.98E-07	11.15536
cg04455430	chr1	50886920	+	DMRTA2	Body	1.3446434	-0.7359	6.4293324	2.11E-09	1.98E-07	11.15532

cg07513950	chr10	2371032	+			-1.920076	0.7617025	-6.429255	2.11E-09	1.98E-07	11.154944
cg09050058	chr5	2186856	+			-1.873625	-0.716686	-6.429141	2.11E-09	1.98E-07	11.154394
cg05221167	chr19	9609397	-	ZNF560	TSS200	1.5450587	-0.722732	6.4290872	2.11E-09	1.98E-07	11.154136
cg21219903	chr1	43920090	-	HYI	TSS200	-1.214604	-2.424039	-6.429037	2.11E-09	1.98E-07	11.153895
cg08414570	chr9	19788757	+			1.3619639	-1.034546	6.4288113	2.12E-09	1.99E-07	11.152804
cg01514353	chr22	46259643	-			-0.882338	-1.807068	-6.428014	2.13E-09	1.99E-07	11.148955
cg02118776	chr6	44281222	-	AARS2	TSS200	-0.889173	-3.860594	-6.438769	2.13E-09	1.99E-07	11.152481
cg08496162	chr16	88702312	+			-1.169665	1.3064522	-6.42788	2.13E-09	1.99E-07	11.148307
cg14768785	chr3	172166517	+	GHSR;GHSR	TSS1500;TSS1500	2.8724608	-1.539811	6.4278336	2.13E-09	1.99E-07	11.148082
cg24174608	chr15	38856094	+	RASGRP1;RASGRP1	Body;Body	-0.821589	-1.862599	-6.427624	2.13E-09	2.00E-07	11.14707
cg01146614	chr6	159235211	+	EZR;EZR	Body;Body	-1.437457	-2.887572	-6.427104	2.13E-09	2.00E-07	11.144558
cg02975060	chr14	101510016	-	MIR1185-2	TSS1500	-1.630103	0.6004022	-6.426875	2.14E-09	2.00E-07	11.143453
cg02318784	chr15	27213174	+			1.3405406	-0.227393	6.4265794	2.14E-09	2.00E-07	11.142026
cg13636096	chr10	2815015	-			-2.172775	1.5161526	-6.426411	2.14E-09	2.01E-07	11.141213
cg17284384	chr1	205649861	+	SLC45A3	TSS1500	-1.622889	-5.701795	-6.433458	2.14E-09	2.01E-07	11.143545
cg23049458	chr1	62660624	-	L1TD1;L1TD1	5'UTR;5'UTR	2.5113107	-0.624403	6.4261862	2.14E-09	2.01E-07	11.140128
cg00107772	chr14	57261886	+			1.679282	-0.95995	6.4260149	2.15E-09	2.01E-07	11.139301
cg06629999	chr22	49720192	+			-1.213708	-1.009187	-6.425754	2.15E-09	2.01E-07	11.138039
cg06222397	chr16	86953019	-			-1.029081	-1.357012	-6.424413	2.16E-09	2.02E-07	11.131567
cg10685128	chr4	141648570	+	TBC1D9	Body	-1.314461	0.0923477	-6.423573	2.17E-09	2.03E-07	11.127514

cg12510512	chr7	1717187	+			-1.25265	0.528001	-6.423595	2.17E-09	2.03E-07	11.12762
cg07824824	chr18	12254976	-	CIDEA;CIDEA	Body;Body	1.4450738	0.1377905	6.4230292	2.18E-09	2.04E-07	11.124888
cg17340519	chr1	224805852	+	CNIH3	Body	1.2957889	-0.427934	6.4230059	2.18E-09	2.04E-07	11.124775
cg17494199	chr13	106834942	+			-1.257682	-0.702448	-6.422883	2.18E-09	2.04E-07	11.124184
cg00393263	chr1	228336474	-	GJC2;GUK1;GUK1;GUK1	TSS1500;3'UTR;3'UTR;3'UTR	1.0572107	1.3524422	6.4226936	2.18E-09	2.04E-07	11.123268
cg01800345	chr3	156009151	-	KCNAB1;KCNAB1;KCNAB1;KCNAB1	Body;1stExon;5'UTR;Body	1.5511001	-1.445634	6.4226002	2.18E-09	2.04E-07	11.122817
cg04332534	chr19	37096487	-	ZNF382;ZNF529;ZNF529;ZNF529	5'UTR;TSS1500;TSS1500;TSS1500	3.014014	-1.295903	6.4225684	2.18E-09	2.04E-07	11.122664
cg20308204	chr22	32750782	+	RFPL3	TSS200	1.4284881	3.7243988	6.4221481	2.19E-09	2.04E-07	11.120635
cg20403557	chr5	115299088	+	LVRN	Body	1.7371186	-0.552098	6.4219513	2.19E-09	2.04E-07	11.119685
cg10378667	chr12	122326303	+	PSMD9	TSS1500	-0.848758	-2.072878	-6.42182	2.19E-09	2.05E-07	11.119053
cg24731702	chr19	8399563	+	KANK3	Body	-1.080488	-1.519195	-6.421696	2.19E-09	2.05E-07	11.118455
cg05394210	chr11	31130488	+			-1.751215	-0.210714	-6.421486	2.20E-09	2.05E-07	11.117438
cg26845297	chr4	85422409	-			1.3149265	-1.201394	6.4214138	2.20E-09	2.05E-07	11.117091
cg08445802	chr6	85482612	-			2.1147754	0.4622863	6.4248051	2.20E-09	2.05E-07	11.117572
cg03502002	chr18	74962133	-	GALR1;GALR1	1stExon;5'UTR	2.8240448	-0.403864	6.4241954	2.20E-09	2.05E-07	11.114846
cg06730183	chr5	76005699	+			-1.428398	0.4928009	-6.420667	2.20E-09	2.05E-07	11.113487
cg26413691	chr1	7119074	-	CAMTA1	Body	-1.448118	-0.485871	-6.420751	2.20E-09	2.05E-07	11.11389
cg00927594	chr15	101514299	-	LRRK1	Body	1.3143543	1.9131785	6.4202798	2.21E-09	2.06E-07	11.111618
cg19709355	chr17	38504102	+	RARA;RARA;RARA;RARA	Body;Body;Body;Body	1.4356252	-0.866984	6.4198414	2.21E-09	2.06E-07	11.109503
cg10443049	chr7	8481537	-	NXPH1	Body	1.9671182	-1.124275	6.4196834	2.22E-09	2.06E-07	11.10874

cg17200137	chr10	48416896	+	GDF2	TSS200	1.2873029	1.0685624	6.4192379	2.22E-09	2.07E-07	11.10659
cg20945261	chr3	13460618	-	NUP210	Body	-0.964725	-1.331902	-6.419194	2.22E-09	2.07E-07	11.106377
cg01503065	chr4	155411806	-	DCHS2;DCHS2	1stExon;1stExon	1.355341	-1.072617	6.419058	2.22E-09	2.07E-07	11.105722
cg26631454	chr13	114065669	+			-1.191462	0.1420895	-6.41894	2.22E-09	2.07E-07	11.105155
cg18264219	chr17	34346433	-	CCL23;CCL23	TSS1500;TSS1500	-1.580479	0.3709452	-6.418828	2.23E-09	2.07E-07	11.104614
cg04540442	chr12	34445809	-			-1.976104	1.1263371	-6.418627	2.23E-09	2.07E-07	11.103643
cg15416877	chr6	108492769	+	NR2E1	Body	1.1426731	-0.291385	6.4186334	2.23E-09	2.07E-07	11.103674
cg05921260	chr6	28484915	-	GPX6	TSS1500	-1.554766	-0.078189	-6.41851	2.23E-09	2.07E-07	11.103077
cg06961473	chr15	65369801	-	LOC390594	1stExon	0.9876819	1.1296333	6.4184731	2.23E-09	2.07E-07	11.1029
cg26348902	chr4	1399938	+			1.4916505	-1.36975	6.4185096	2.23E-09	2.07E-07	11.103076
cg01573140	chr7	44687078	-	OGDH;OGDH;OGDH	Body;Body;Body	0.7624558	2.0569827	6.4183106	2.23E-09	2.07E-07	11.102116
cg17936783	chr1	182027546	+	ZNF648	5'UTR	-1.244404	-0.299372	-6.41818	2.23E-09	2.07E-07	11.101488
cg06248767	chr8	56852290	-	LYN;LYN	5'UTR;5'UTR	-1.790069	-3.190236	-6.418135	2.23E-09	2.07E-07	11.101268
cg00593008	chr5	75465603	+	SV2C	Body	-1.357169	0.2496191	-6.418057	2.23E-09	2.07E-07	11.100895
cg00459623	chr2	176936581	+			3.2782034	-1.042093	6.4250058	2.24E-09	2.07E-07	11.102658
cg25021530	chr5	44333003	+	FGF10	Body	-1.222288	-1.189296	-6.417583	2.24E-09	2.08E-07	11.098607
cg02790305	chr4	186732932	+	SORBS2;SORBS2;SORBS2;SORBS2;SORBS2;SORBS2;SORBS2	5'UTR;5'UTR;5'UTR;1stExon;TSS1500;1stExon;TSS1500	-1.118448	-0.941338	-6.417074	2.24E-09	2.08E-07	11.09615
cg15545942	chr2	9526703	+	ASAP2;ASAP2	Body;Body	-1.678138	0.2260953	-6.417086	2.24E-09	2.08E-07	11.096207
cg19447221	chr10	23985722	+	KIAA1217	5'UTR	-1.524093	-0.06377	-6.416848	2.25E-09	2.08E-07	11.095059
cg04321240	chr15	87516203	+	AGBL1	Body	-1.476318	-0.254489	-6.416406	2.25E-09	2.09E-07	11.092927

cg06609496	chr5	1174426	+			-1.748154	0.0957333	-6.416218	2.25E-09	2.09E-07	11.092019
cg19994384	chr10	44436485	-			-1.236297	-1.533667	-6.416196	2.25E-09	2.09E-07	11.091914
cg23840027	chr1	29225533	-	EPB41;EPB41;EPB41;EPB41;EPB41	5'UTR;5'UTR;5'UTR;5'UTR;5'UTR	1.0838286	1.1911777	6.416057	2.26E-09	2.09E-07	11.091243
cg01991625	chr16	54404353	-			0.7797942	1.7523913	6.4154689	2.26E-09	2.10E-07	11.088406
cg16051195	chr6	166268183	-			1.42141	-0.58233	6.4152138	2.27E-09	2.10E-07	11.087176
cg03632704	chr5	87973439	-	LOC645323;LOC645323	Body;Body	1.4784526	-0.660154	6.414878	2.27E-09	2.10E-07	11.085556
cg18507379	chr5	140787507	-	PCDHGA4;PCDHGA6;PCDHGA9;PCDHGA1;PCDHGA8;PCDHGA5;PCDHGB1;PCDHGB6;PCDHGB4;PCDHGA3;PCDHGA2;PCDHGA7;PCDHGB2;PCDHGB6;PCDHGB5;PCDHGB3	Body;Body;Body;Body;Body;Body;Body;TSS1500;Body;Body;Body;Body;Body;TSS1500;Body;Body	3.1240022	-1.585774	6.4148095	2.27E-09	2.10E-07	11.085225
cg07149609	chr1	170630734	-			2.2766495	-0.281226	6.4146118	2.27E-09	2.10E-07	11.084272
cg08313757	chr2	97428708	+	CNNM4	Body	-0.726291	-2.025189	-6.414494	2.27E-09	2.10E-07	11.083702
cg09447895	chr5	165460007	-			-1.870643	0.6006961	-6.414477	2.27E-09	2.10E-07	11.083623
cg02081006	chr5	122430434	+	PRDM6	Body	2.1216575	-0.307541	6.4177986	2.28E-09	2.11E-07	11.083817
cg25781936	chr6	138750887	-	NHSL1;NHSL1	Body;Body	1.2030089	0.6688028	6.4141938	2.28E-09	2.11E-07	11.082256
cg16508627	chr16	22926601	-	HS3ST2	Body	-1.604712	0.6622839	-6.413738	2.28E-09	2.11E-07	11.080057
cg19449565	chr2	240230892	+	HDAC4	Body	1.4262646	1.4386585	6.4134072	2.29E-09	2.11E-07	11.078462
cg23894086	chr7	27128046	+			1.5504884	-0.611556	6.4133935	2.29E-09	2.11E-07	11.078396
cg05771518	chr12	54339288	-	HOXC13	3'UTR	1.7591467	-1.639053	6.4132153	2.29E-09	2.11E-07	11.077536
cg19730691	chr1	119526437	+	TBX15	5'UTR	1.0894486	0.8427531	6.4130167	2.29E-09	2.12E-07	11.076578
cg24009737	chr10	126781559	+	CTBP2;CTBP2	5'UTR;5'UTR	1.3606055	4.2887789	6.4162033	2.29E-09	2.12E-07	11.085523
cg07136635	chr5	52096641	+	ITGA1;PELO	Body;Body	-1.241803	-3.267046	-6.412272	2.30E-09	2.12E-07	11.072986
cg14537825	chr2	98344873	+	ZAP70	Body	-0.886818	-1.453265	-6.412252	2.30E-09	2.12E-07	11.072889

cg20334313	chr8	50822381	+			1.2522285	-0.889078	6.4113483	2.31E-09	2.13E-07	11.068532
cg02582957	chr2	182547955	+			1.6283526	-1.203809	6.4112338	2.31E-09	2.13E-07	11.06798
cg05148385	chr2	213950646	+	IKZF2;IKZF2	Body;Body	0.9169461	2.2330832	6.411137	2.31E-09	2.13E-07	11.067513
cg01157951	chr6	31540399	-	LTA;LTA	5'UTR;5'UTR	-0.84705	-0.935844	-6.410945	2.31E-09	2.14E-07	11.066586
cg15552461	chr19	55581114	+	RDH13	TSS1500	-1.075558	-2.315782	-6.414392	2.32E-09	2.14E-07	11.067414
cg04578894	chr5	33842297	-	ADAMTS12	Body	-2.132729	0.0887016	-6.410834	2.32E-09	2.14E-07	11.066052
cg12154110	chr19	49254220	+	FUT1	Body	0.6561425	0.926087	6.4103828	2.32E-09	2.14E-07	11.063876
cg07395055	chr2	118569210	+			-1.658561	0.3886581	-6.409109	2.34E-09	2.15E-07	11.057736
cg06148812	chr22	19746368	+	TBX1;TBX1;TBX1	5'UTR;5'UTR;5'UTR	1.4007083	-0.073946	6.4114384	2.35E-09	2.17E-07	11.053407
cg12788855	chr22	29977253	-	NIPSNAP1	TSS200	-0.509333	-3.424272	-6.407767	2.35E-09	2.17E-07	11.051264
cg11075693	chr6	82461261	+	FAM46A	Body	-0.972071	-2.73132	-6.407676	2.35E-09	2.17E-07	11.050826
cg25403721	chr12	114885372	-			1.9797996	-0.920219	6.4072324	2.36E-09	2.17E-07	11.048687
cg19675142	chr1	116107015	-			1.3121701	0.9483254	6.4069064	2.36E-09	2.17E-07	11.047116
cg19681842	chr14	93672832	+	UBR7;UBR7;C14orf142	TSS1500;TSS1500;Body	-1.294313	-1.819454	-6.406614	2.37E-09	2.18E-07	11.045706
cg26767897	chr2	31637348	+	XDH	Body	-2.215807	1.0747849	-6.406062	2.37E-09	2.18E-07	11.043045
cg14803306	chr2	47499716	+			-1.073354	-4.580665	-6.405875	2.37E-09	2.18E-07	11.042146
cg21991633	chr19	9515211	-			-1.687315	-0.076044	-6.405583	2.38E-09	2.19E-07	11.040735
cg09615505	chr2	176977285	-			1.5352009	-0.2594	6.4053715	2.38E-09	2.19E-07	11.039717
cg10907582	chr14	96411400	-			-1.620993	0.3669062	-6.405386	2.38E-09	2.19E-07	11.039786
cg17885226	chr6	105388731	+			1.5469504	0.1261263	6.4052565	2.38E-09	2.19E-07	11.039163

cg09263516	chr12	115107338	+			1.5557141	-0.398103	6.4047931	2.39E-09	2.19E-07	11.036929
cg25329318	chr5	38253946	-			-2.127256	0.2678979	-6.404769	2.39E-09	2.19E-07	11.036811
cg18594963	chr17	1375253	+	MYO1C;MYO1C;MYO1C	Body;Body;Body	0.7678591	1.1331036	6.4042487	2.39E-09	2.20E-07	11.034305
cg16725552	chr14	26338282	-			-1.670564	-0.305274	-6.404124	2.40E-09	2.20E-07	11.033702
cg13502741	chr11	31848488	-			2.0539614	-0.877694	6.4040766	2.40E-09	2.20E-07	11.033476
cg06685737	chr7	35301730	+			1.5312108	-0.332092	6.4039889	2.40E-09	2.20E-07	11.033053
cg02023728	chr11	77925099	-	USP35	3'UTR	0.7715984	1.6036398	6.4039046	2.40E-09	2.20E-07	11.032647
cg25731428	chr13	91830940	-			-1.49517	0.3094624	-6.407033	2.40E-09	2.20E-07	11.032202
cg09647108	chr17	41934923	-	CD300LG;CD300LG;CD300LG;CD300LG	Body;Body;3'UTR;Body	-1.105673	-0.4534	-6.403427	2.40E-09	2.21E-07	11.030343
cg27404023	chr2	162283994	-			1.701273	-0.857118	6.403336	2.40E-09	2.21E-07	11.029906
cg22829917	chr12	54357408	+	HOTAIR	Body	1.45687	-0.800773	6.4031508	2.41E-09	2.21E-07	11.029014
cg23813867	chr11	61735531	+	FTH1	TSS1500	-0.451507	-3.890416	-6.403101	2.41E-09	2.21E-07	11.028772
cg15362894	chr11	44702663	+			-1.344459	-0.955219	-6.402935	2.41E-09	2.21E-07	11.027973
cg21091985	chr6	31706222	-			-0.973588	-2.770243	-6.406193	2.41E-09	2.21E-07	11.028161
cg08371140	chr10	36738505	-			-1.372418	-1.806627	-6.402492	2.42E-09	2.21E-07	11.025838
cg04692403	chr6	134210812	+	TCF21;TCF21	1stExon;1stExon	1.3183817	-0.523446	6.4020096	2.42E-09	2.22E-07	11.023515
cg21700772	chr7	1644443	-			-1.106366	-1.228077	-6.401617	2.43E-09	2.22E-07	11.021625
cg17006281	chr7	154589595	+	DPP6;DPP6;DPP6	Body;Body;Body	-1.436527	-0.316887	-6.401391	2.43E-09	2.22E-07	11.020535
cg27328179	chr16	1040373	+			-0.98486	0.0924003	-6.401386	2.43E-09	2.22E-07	11.020507
cg24023258	chr8	143781297	+	LY6K;LY6K;LY6K	TSS1500;TSS1500;TSS1500	-1.421834	-1.985026	-6.401236	2.43E-09	2.22E-07	11.019786

cg21101743	chr7	39649254	-	LOC646999	Body	1.9898045	-0.973075	6.4007751	2.44E-09	2.23E-07	11.017567
cg07679948	chr12	56329641	-	DGKA;DGKA;DGKA;DGKA	5'UTR;5'UTR;5'UTR;5'UTR	1.5743855	1.4806337	6.4006649	2.44E-09	2.23E-07	11.017036
cg16889659	chr15	67043450	+	SMAD6;SMAD6;SMAD6	Body;Body;Body	0.9325454	1.7903793	6.4001117	2.44E-09	2.24E-07	11.01437
cg14424407	chr17	36734838	-	SRCIN1	Body	1.3070028	-0.709006	6.3989963	2.46E-09	2.25E-07	11.008996
cg22039357	chr1	184388675	-	C1orf21	5'UTR	-1.427449	-0.971965	-6.398692	2.46E-09	2.25E-07	11.007528
cg01190800	chr4	1539702	+			-1.145797	-0.486727	-6.398604	2.46E-09	2.25E-07	11.007108
cg10265740	chr3	44622865	+	ZNF167	Body	0.9524682	2.1584371	6.3984892	2.46E-09	2.25E-07	11.006553
cg10423607	chr8	68418480	+	CPA6;CPA6	Body;Body	-1.808071	-2.16664	-6.398458	2.46E-09	2.25E-07	11.006401
cg22278106	chr2	169746720	-	SPC25	5'UTR	-1.203731	-3.690305	-6.398063	2.47E-09	2.26E-07	11.004501
cg03079818	chr13	110324798	-			1.1651622	2.2976607	6.3979134	2.47E-09	2.26E-07	11.00378
cg11204562	chr10	115511153	+	C10orf81	TSS200	-1.913634	0.7318033	-6.397915	2.47E-09	2.26E-07	11.003788
cg01889237	chr6	137816623	+	OLIG3	TSS1500	1.2178022	-0.501549	6.3975537	2.48E-09	2.26E-07	11.002047
cg05346286	chr1	2980598	-	FLJ42875	Body	1.1183459	-0.425474	6.3974729	2.48E-09	2.26E-07	11.001658
cg18148375	chr2	1926535	-	MYT1L	Body	-1.40788	0.2150522	-6.397459	2.48E-09	2.26E-07	11.001591
cg06800565	chr1	161124156	+	UFC1	Body	-0.618909	-4.975572	-6.39739	2.48E-09	2.26E-07	11.001257
cg06585566	chr19	57276794	-			1.8925354	0.4781087	6.3969742	2.48E-09	2.26E-07	10.999256
cg16166968	chr1	120439036	-	ADAM30;ADAM30	1stExon;5'UTR	1.1685557	0.4447641	6.3964615	2.49E-09	2.27E-07	10.996786
cg09403666	chr10	23461493	+			1.9617214	-0.238883	6.3960149	2.49E-09	2.27E-07	10.994635
cg03262887	chr20	29978114	+	DEFB119;DEFB119;DEFB119	Body;Body;Body	-0.963366	-1.897525	-6.395697	2.50E-09	2.28E-07	10.993106
cg14887116	chr11	124293907	-	OR8B4	1stExon	-1.740411	0.5192982	-6.395671	2.50E-09	2.28E-07	10.992979

cg27629782	chr11	125073726	+	PKNOX2	5'UTR	-1.523847	0.0916737	-6.395578	2.50E-09	2.28E-07	10.992532
cg05901196	chr10	61664954	+	CCDC6	Body	-1.430341	-1.577598	-6.395473	2.50E-09	2.28E-07	10.992024
cg16389399	chr11	43603991	+			1.4490024	-0.643518	6.3945861	2.51E-09	2.29E-07	10.987754
cg13126206	chr8	22930189	+			-1.065494	0.6102361	-6.401498	2.51E-09	2.29E-07	10.989473
cg10785263	chr1	63785867	-			1.9718982	-0.720147	6.3943932	2.52E-09	2.29E-07	10.986825
cg03457195	chr2	162284206	-			1.2341852	-0.063742	6.3942489	2.52E-09	2.29E-07	10.98613
cg06288198	chr2	1203839	+	SNTG2	Body	-1.271275	-0.214726	-6.39422	2.52E-09	2.29E-07	10.985993
cg21545862	chr5	174159294	-			1.9612673	-0.810147	6.3940248	2.52E-09	2.29E-07	10.985051
cg23398173	chr7	135662345	+	MTPN;LUZP6	TSS200;TSS200	-0.906897	-3.378986	-6.393787	2.52E-09	2.29E-07	10.983908
cg16208657	chr15	68119293	+	LBXCOR1	Body	1.199914	-1.266338	6.3936051	2.53E-09	2.30E-07	10.98303
cg13893543	chr1	224805631	+	CNIH3	Body	1.547939	-1.564189	6.3934938	2.53E-09	2.30E-07	10.982494
cg22047672	chr1	150275877	-	MRPS21;MRPS21	Body;Body	1.0849401	2.0906819	6.3933689	2.53E-09	2.30E-07	10.981892
cg21865845	chr3	79815848	-	ROBO1	5'UTR	1.2071509	-0.251686	6.3932451	2.53E-09	2.30E-07	10.981296
cg19550914	chr3	52084505	+	DUSP7	3'UTR	0.8439466	0.6716774	6.3931777	2.53E-09	2.30E-07	10.980972
cg03049445	chr6	21277954	+			-1.519522	0.0484184	-6.392551	2.54E-09	2.31E-07	10.977953
cg25365544	chr2	238707589	-	RBM44	5'UTR	1.1760288	4.6725497	6.4030844	2.54E-09	2.31E-07	10.981197
cg16050028	chr17	6068454	-			-1.381806	-0.139551	-6.392136	2.54E-09	2.31E-07	10.975956
cg23774988	chr11	128419199	+	ETS1	Body	1.9706758	0.0232586	6.4177336	2.54E-09	2.31E-07	10.985529
cg24536349	chr8	143781340	-	LY6K;LY6K;LY6K	TSS200;TSS200;TSS200	-1.734106	-0.96007	-6.392053	2.54E-09	2.31E-07	10.975556
cg05948372	chr18	19283268	-	ABHD3	Body	-1.219754	-2.479599	-6.391904	2.55E-09	2.31E-07	10.974838

cg10103266	chr2	176977458	-			1.5280203	-0.933958	6.3916987	2.55E-09	2.31E-07	10.973851
cg02578944	chr6	28227392	+	NKAPL	1stExon	1.3192755	2.2660528	6.3950141	2.55E-09	2.31E-07	10.974181
cg23283423	chr17	821546	-	NXN	Body	1.4125904	1.4610354	6.3915889	2.55E-09	2.31E-07	10.973322
cg25763127	chr10	33527263	-	NRP1;NRP1;NRP1	Body;Body;Body	-2.033013	1.1099792	-6.391576	2.55E-09	2.31E-07	10.973261
cg24720355	chr1	119526255	+	TBX15	5'UTR	1.4003887	0.8382969	6.3910155	2.56E-09	2.32E-07	10.970561
cg23460578	chr2	177014009	-	MIR10B	TSS1500	1.237547	0.0353495	6.3909336	2.56E-09	2.32E-07	10.970167
cg14016236	chr3	171792545	-	FNDC3B;FNDC3B	5'UTR;5'UTR	1.5181436	0.5484143	6.3906572	2.56E-09	2.32E-07	10.968836
cg20298668	chr2	240230663	+	HDAC4	Body	1.3349512	1.031337	6.3903934	2.57E-09	2.32E-07	10.967566
cg16083044	chr6	168788815	+			1.5408657	3.1066529	6.3902129	2.57E-09	2.33E-07	10.966697
cg02020675	chr14	92786270	+			-1.718954	0.2157969	-6.400679	2.57E-09	2.33E-07	10.979126
cg00779056	chr16	1052756	-			-2.059586	0.8847253	-6.389569	2.58E-09	2.33E-07	10.963596
cg12883523	chr8	55372392	+	SOX17	Body	1.2910173	-0.738764	6.3893968	2.58E-09	2.33E-07	10.962769
cg17276590	chr10	119304487	-	EMX2OS;EMX2;EMX2	Body;Body;Body	2.7339347	-0.86057	6.3885179	2.59E-09	2.34E-07	10.958538
cg15000841	chr11	64815642	+	NAALADL1	Body	1.5886233	-1.467306	6.3884049	2.59E-09	2.35E-07	10.957994
cg18722841	chr11	71954982	-	PHOX2A	1stExon	1.7529575	-1.175554	6.3882177	2.59E-09	2.35E-07	10.957093
cg15015340	chr11	61486220	+	DAGLA	5'UTR	1.3541791	-0.628494	6.3875464	2.60E-09	2.35E-07	10.953862
cg14568830	chr10	23463243	-			1.8689349	-0.863168	6.3874726	2.60E-09	2.35E-07	10.953507
cg01287000	chr8	145938564	-			1.2403376	0.6107958	6.3873511	2.61E-09	2.36E-07	10.952922
cg25564800	chr3	122234134	+	KPNA1;KPNA1	TSS1500;TSS1500	-1.096397	-2.720938	-6.386905	2.61E-09	2.36E-07	10.950775
cg05029100	chr5	126054138	+			0.9945552	1.5196415	6.3863565	2.62E-09	2.37E-07	10.948135

cg08692733	chr10	112404738	-	RBM20	Body	1.3404285	-0.801045	6.3862028	2.62E-09	2.37E-07	10.947395
cg25749512	chr12	52301580	-	ACVRL1	5'UTR	0.9420492	0.6090775	6.3854043	2.63E-09	2.38E-07	10.943553
cg01954585	chr15	86270435	+	AKAP13;AKAP13;AKAP13	Body;Body;Body	1.2531823	1.145335	6.3849105	2.64E-09	2.38E-07	10.941177
cg18161890	chr12	52995250	-	KRT72;KRT72;KRT72;KRT72;KRT72;KRT72	1stExon;5'UTR;1stExon;5'UTR;1stExon;5'UTR	1.3665821	0.3979622	6.3844351	2.64E-09	2.39E-07	10.938889
cg05533037	chr21	32202010	-	KRTAP7-1	1stExon	-1.61765	0.0591341	-6.383589	2.65E-09	2.40E-07	10.934819
cg11020333	chr7	2861855	-	GNA12	Body	-1.827237	1.8604135	-6.383582	2.65E-09	2.40E-07	10.934782
cg20872981	chr2	54710221	+	SPTBN1	5'UTR	0.8775133	0.0623868	6.3836405	2.65E-09	2.40E-07	10.935066
cg16365445	chr10	33230686	+	ITGB1;ITGB1	5'UTR;5'UTR	1.157637	1.0536932	6.3834204	2.66E-09	2.40E-07	10.934007
cg02455346	chr1	221055401	+	HLX	Body	1.3889165	1.4075041	6.3830548	2.66E-09	2.40E-07	10.932248
cg24617504	chr7	155164762	+			1.0385727	-0.545826	6.383033	2.66E-09	2.40E-07	10.932143
cg08124372	chr17	21223255	+			-0.854323	0.2534517	-6.382461	2.67E-09	2.41E-07	10.929392
cg16565394	chr6	100896674	-	SIM1	Body	1.347515	-0.54072	6.382177	2.67E-09	2.41E-07	10.928024
cg07046870	chr3	122803153	+	PDIA5;PDIA5	Body;Body	1.1639578	1.7972067	6.381043	2.69E-09	2.42E-07	10.922569
cg21723707	chr8	1411812	-			-1.459987	0.6084371	-6.380771	2.69E-09	2.43E-07	10.921262
cg21402832	chr3	172241069	-	TNFSF10	1stExon	-1.061047	-3.624657	-6.380151	2.70E-09	2.43E-07	10.918277
cg21500126	chr8	119372894	+	SAMD12	Body	-1.557365	0.0591906	-6.383341	2.70E-09	2.44E-07	10.918309
cg11684549	chr17	79122243	+	AATK	Body	-1.614251	-0.52722	-6.379139	2.71E-09	2.44E-07	10.913411
cg25389463	chr1	235325040	+	RBM34;RBM34;RBM34	TSS1500;TSS1500;TSS1500	-1.035413	-3.110311	-6.378786	2.72E-09	2.45E-07	10.911713
cg16692735	chr10	135259860	+			1.2142789	1.397803	6.3787214	2.72E-09	2.45E-07	10.911402
cg18883209	chr22	44394601	-	PARVB	TSS1500	0.7594056	2.1734889	6.3818795	2.72E-09	2.45E-07	10.91129

cg24000437	chr1	152538857	-	LCE3E	5'UTR	-1.535716	-0.619032	-6.37779	2.73E-09	2.46E-07	10.906921
cg13492331	chr10	14779377	-	FAM107B	Body	-1.075214	-0.631682	-6.377633	2.74E-09	2.46E-07	10.906169
cg18884368	chr20	31331841	+	COMMD7;COMMD7	TSS200;TSS200	-0.641012	-4.387027	-6.377404	2.74E-09	2.46E-07	10.905065
cg19592637	chr20	25063052	+	VSX1;VSX1	TSS1500;TSS1500	1.4739996	1.1671306	6.377394	2.74E-09	2.46E-07	10.905018
cg03202564	chr10	50977130	-			1.1357453	0.7206306	6.3771742	2.74E-09	2.46E-07	10.903961
cg18536148	chr17	59533844	-	TBX4;TBX4	5'UTR;1stExon	1.2915555	-0.092154	6.3772078	2.74E-09	2.46E-07	10.904123
cg05471495	chr2	101369330	-			-1.448275	-3.278211	-6.376859	2.75E-09	2.47E-07	10.902447
cg15876968	chr1	85086322	-			-1.529286	-1.762338	-6.376382	2.75E-09	2.47E-07	10.900149
cg07099331	chr10	102419617	+			1.829651	-0.350997	6.3760847	2.76E-09	2.48E-07	10.898722
cg18894648	chr5	87974446	+	LOC645323;LOC645323	Body;TSS200	2.0937608	-1.15341	6.375568	2.76E-09	2.48E-07	10.896238
cg19303682	chr19	4229214	+	EBI3	TSS1500	-0.708905	-3.073729	-6.375439	2.77E-09	2.48E-07	10.895618
cg20749059	chr3	57094404	-	ARHGEF3;SPATA12	5'UTR;TSS200	-1.053519	-1.57676	-6.412634	2.77E-09	2.48E-07	10.92492
cg19267254	chr1	15828075	-	CASP9;CASP9	Body;Body	-1.342895	-1.692304	-6.375329	2.77E-09	2.48E-07	10.895088
cg04421523	chr6	12083035	+	HIVEP1	Body	0.7576377	2.5520223	6.3747023	2.78E-09	2.49E-07	10.892075
cg08753469	chr7	152842263	+			-1.746118	0.5685929	-6.374079	2.78E-09	2.50E-07	10.88908
cg05137680	chr5	138729068	-	LOC389333	1stExon	-1.154389	-3.222647	-6.373727	2.79E-09	2.50E-07	10.887387
cg18235734	chr1	91301731	+			2.1723494	-0.188525	6.3737685	2.79E-09	2.50E-07	10.887585
cg13982529	chr5	87974398	+	LOC645323;LOC645323	Body;TSS200	2.0541845	-0.883162	6.3877377	2.79E-09	2.50E-07	10.891728
cg01518889	chr3	128202493	-	GATA2;GATA2;GATA2	Body;Body;Body	2.5683654	-0.689277	6.3769262	2.79E-09	2.50E-07	10.887508
cg23235989	chr2	79428894	-			-1.246905	-0.657116	-6.373316	2.79E-09	2.50E-07	10.885409

cg22297934	chr17	76421671	-	DNAH17	Body	1.0806074	1.6356926	6.3731907	2.80E-09	2.51E-07	10.884808
cg10445315	chr21	38066047	+			2.0255568	-1.493982	6.3730104	2.80E-09	2.51E-07	10.883941
cg20091405	chr11	124370625	-			-1.702265	0.3746985	-6.373036	2.80E-09	2.51E-07	10.884064
cg02138218	chr1	175035502	+	TNN	TSS1500	-1.5947	0.1911248	-6.372705	2.80E-09	2.51E-07	10.882473
cg04392082	chr4	186732926	+	SORBS2;SORBS2;SORBS2;SORBS2;SORBS2;SORBS2;SORBS2	5'UTR;5'UTR;5'UTR;1stExon;TSS1500;1stExon;TSS1500	-1.626697	-2.169742	-6.372734	2.80E-09	2.51E-07	10.882612
cg02216780	chr13	46497736	-			-0.91423	-0.748825	-6.372661	2.80E-09	2.51E-07	10.882263
cg17952824	chr10	44287023	-	HNRNPA3P1	TSS1500	-1.454775	-0.816738	-6.372503	2.81E-09	2.51E-07	10.8815
cg10624054	chr12	53297547	-	KRT8	Body	-1.815485	-4.484846	-6.379053	2.81E-09	2.52E-07	10.882021
cg24879415	chr17	74534700	-	CYGB;PRCD	TSS1500;TSS1500	0.9188447	0.8969437	6.3719003	2.81E-09	2.52E-07	10.878605
cg26134090	chr4	48909057	-	OCIAD2;OCIAD2	TSS1500;TSS1500	-0.980405	-0.272655	-6.371435	2.82E-09	2.52E-07	10.876366
cg14945937	chr19	30162771	-	PLEKHF1	5'UTR	-1.277834	-1.513909	-6.371232	2.82E-09	2.53E-07	10.875394
cg24052359	chr7	156813574	-			1.4986387	-1.075273	6.3710265	2.83E-09	2.53E-07	10.874405
cg25405318	chr17	78354680	+	RNF213;LOC100294362	Body;Body	0.8738365	2.7337179	6.3707919	2.83E-09	2.53E-07	10.873277
cg00159682	chr8	6386150	+	ANGPT2;ANGPT2;ANGPT2;MCPH1	Body;Body;Body;Body	0.7828711	1.6656808	6.3698903	2.84E-09	2.54E-07	10.868944
cg13920090	chr1	27872303	+	AHDC1	3'UTR	1.0859523	0.8473067	6.3698247	2.84E-09	2.54E-07	10.868628
cg00718513	chr2	89215272	-			-1.180674	-0.770716	-6.369673	2.85E-09	2.54E-07	10.867898
cg15642292	chr1	150253842	-	C1orf51	TSS1500	-1.227566	-3.58208	-6.369508	2.85E-09	2.54E-07	10.867108
cg15974062	chr12	6833012	-	COPS7A;COPS7A;COPS7A;COPS7A	TSS200;TSS200;TSS1500;TSS1500	-0.574257	-3.742996	-6.369478	2.85E-09	2.54E-07	10.866963
cg02542748	chr10	131034863	-			-1.430317	0.1069389	-6.36919	2.85E-09	2.55E-07	10.865577
cg22961402	chr17	27913793	+	GIT1;GIT1	Body;Body	0.9122632	1.670849	6.3686634	2.86E-09	2.55E-07	10.863047

cg04560225	chr1	91195336	+			2.0300767	-0.198785	6.3684176	2.86E-09	2.56E-07	10.861866
cg16749093	chr11	65352605	-	EHBP1L1	Body	1.128611	2.7675586	6.3683202	2.87E-09	2.56E-07	10.861398
cg00619126	chr2	105459775	-			2.2014508	-1.00144	6.3681613	2.87E-09	2.56E-07	10.860635
cg14142007	chr2	176987225	-	HOXD9	TSS200	2.2205727	-1.405652	6.3715841	2.87E-09	2.56E-07	10.86187
cg25391023	chr6	32374754	-	BTNL2	Body	-1.510383	-0.596342	-6.368098	2.87E-09	2.56E-07	10.860329
cg05756029	chr11	18230754	-	LOC494141;LOC494141;LOC494141	Body;TSS200;Body	1.5098509	0.8115649	6.3674454	2.88E-09	2.57E-07	10.857195
cg17004025	chr19	840795	-	PRTN3	TSS200	-1.014307	-1.405094	-6.367225	2.88E-09	2.57E-07	10.856133
cg16222802	chr3	50295474	+	GNAI2;GNAI2	3'UTR;3'UTR	1.2158252	1.9890333	6.3671265	2.88E-09	2.57E-07	10.855662
cg01363572	chr16	9203216	+	C16orf72	Body	1.2106993	3.7207576	6.3664347	2.89E-09	2.58E-07	10.852338
cg10172584	chr19	19639581	-	YJEFN3	TSS200	1.2989319	2.2354348	6.3661705	2.90E-09	2.58E-07	10.851068
cg19022697	chr1	55247140	+	TTC22	Body	1.3827978	0.980913	6.3693162	2.90E-09	2.58E-07	10.850989
cg00190206	chr2	176999353	+			1.220631	1.0460308	6.3657887	2.90E-09	2.58E-07	10.849234
cg09029902	chr20	52199594	-	ZNF217;ZNF217	5'UTR;1stExon	-1.962713	-2.941148	-6.365776	2.90E-09	2.58E-07	10.849172
cg14051544	chr3	170303286	-	SLC7A14	5'UTR	1.8877004	-0.856954	6.3687323	2.91E-09	2.59E-07	10.847966
cg01284193	chr9	27109111	+	TEK	TSS200	1.1470318	1.2643305	6.3651896	2.91E-09	2.59E-07	10.846356
cg05875410	chr16	51187572	-			1.4945024	-0.667978	6.3647089	2.92E-09	2.60E-07	10.844047
cg01722932	chr11	92702653	-	MTNR1B	TSS200	0.6101142	0.2796314	6.3645125	2.92E-09	2.60E-07	10.843103
cg16325502	chr2	223166435	+	CCDC140	5'UTR	1.4423225	-1.037981	6.364528	2.92E-09	2.60E-07	10.843178
cg21232935	chr7	51113700	+	COBL	Body	-0.998775	-0.545635	-6.364526	2.92E-09	2.60E-07	10.843166
cg06294384	chr16	18057597	-			-1.387068	0.0584723	-6.364441	2.92E-09	2.60E-07	10.84276

cg05602648	chr1	230778078	-	COG2;COG2	TSS200;TSS200	-0.508039	-4.160909	-6.36432	2.92E-09	2.60E-07	10.842179
cg02196730	chr15	80188777	-	MTHFS	Body	-1.070454	-3.283601	-6.363801	2.93E-09	2.60E-07	10.839687
cg19417526	chr11	131895599	-	NTM;NTM;NTM;NTM	Body;Body;Body;Body	-1.722166	0.0090261	-6.363703	2.93E-09	2.60E-07	10.839214
cg06038133	chr17	27944585	-	CORO6	Body	-1.320374	-1.806163	-6.363263	2.94E-09	2.61E-07	10.8371
cg01004918	chr1	2731370	-			-1.113472	-0.081575	-6.363034	2.94E-09	2.61E-07	10.836002
cg22815118	chr11	1529654	+	HCCA2	Body	0.7812101	1.8334737	6.363007	2.94E-09	2.61E-07	10.835871
cg12041266	chr11	75050147	+	ARRB1;ARRB1	Body;Body	1.1648818	1.3913653	6.3628919	2.94E-09	2.61E-07	10.835318
cg27183679	chr4	111562545	-			2.1730665	-2.24485	6.3627159	2.95E-09	2.61E-07	10.834473
cg19314136	chr18	59004270	+			-1.718953	0.3681081	-6.362538	2.95E-09	2.62E-07	10.833618
cg14885748	chr12	127211262	-			1.9383824	-0.762276	6.3624882	2.95E-09	2.62E-07	10.833379
cg00259019	chr2	236611999	-	AGAP1;AGAP1	Body;Body	-1.71428	0.9673225	-6.362327	2.95E-09	2.62E-07	10.832603
cg14104252	chr9	116263568	-	RGS3;RGS3;RGS3	TSS200;Body;Body	0.8813492	1.9649298	6.365821	2.95E-09	2.62E-07	10.834225
cg25318976	chr1	5727352	+			-1.262487	-0.865395	-6.362324	2.95E-09	2.62E-07	10.832591
cg04630810	chr9	4662735	-	C9orf68;PPAPDC2	Body;1stExon	-0.609977	-3.959497	-6.361699	2.96E-09	2.62E-07	10.829589
cg06454380	chr22	50067053	-			-0.648216	-2.706825	-6.361651	2.96E-09	2.62E-07	10.82936
cg09395443	chr7	4746437	-	FOXK1	Body	1.1395045	0.5729386	6.3612854	2.97E-09	2.63E-07	10.827602
cg19718882	chr11	32458714	-	WIT1	Body	1.5800439	-1.034963	6.3608184	2.97E-09	2.63E-07	10.825359
cg02674465	chr7	4786986	+	FOXK1	Body	1.395522	2.4788682	6.3599688	2.99E-09	2.65E-07	10.821279
cg26363634	chr1	110162485	+	AMPD2;AMPD2;AMPD2	1stExon;TSS1500;5'UTR	-1.156961	-4.780076	-6.359819	2.99E-09	2.65E-07	10.820561
cg07011913	chr2	177014626	-	HOXD4;MIR10B	TSS1500;TSS1500	1.6131028	-0.556837	6.3597284	2.99E-09	2.65E-07	10.820125

cg24995678	chr2	235936394	-	SH3BP4	5'UTR	-2.461472	0.3310762	-6.359696	2.99E-09	2.65E-07	10.819968
cg21258596	chr21	38338458	+	HLCS	5'UTR	-0.546594	-3.413678	-6.359472	2.99E-09	2.65E-07	10.818895
cg09633579	chr11	61395464	+	RPLP0P2	Body	-0.958838	-0.566254	-6.35922	3.00E-09	2.65E-07	10.817684
cg15141467	chr1	155035375	-	EFNA4;EFNA4;EFNA4	TSS1500;TSS1500;TSS1500	-2.380794	-3.856904	-6.358913	3.00E-09	2.66E-07	10.816207
cg17657737	chr3	45224950	+			-0.736869	-1.736192	-6.358925	3.00E-09	2.66E-07	10.816267
cg10699171	chr6	24936965	+			-1.39018	-1.365532	-6.35855	3.01E-09	2.66E-07	10.814465
cg14031473	chr9	134206000	+			-1.250958	-0.597455	-6.358368	3.01E-09	2.66E-07	10.813591
cg20058148	chr4	156298427	-	MAP9	TSS1500	-1.272268	-2.360133	-6.358031	3.02E-09	2.67E-07	10.811976
cg11996592	chr8	144577094	+	ZC3H3	Body	0.7458927	1.7215986	6.3578934	3.02E-09	2.67E-07	10.811314
cg13492692	chr9	126777673	+	LHX2	Body	2.0960324	-1.353258	6.3576088	3.02E-09	2.67E-07	10.809947
cg26509328	chr18	44787728	-			1.1751393	-0.897582	6.357419	3.03E-09	2.67E-07	10.809036
cg27539214	chr16	67997921	+	SLC12A4;SLC12A4;SLC12A4;SLC12A4;SLC12A4	Body;Body;1stExon;Body;Body	0.9032865	0.8813392	6.3574037	3.03E-09	2.67E-07	10.808962
cg04304130	chr6	11111894	+	LOC221710;HERV-FRD	Body;5'UTR	0.8362824	1.5210713	6.3605965	3.03E-09	2.67E-07	10.809174
cg18140268	chr1	147781827	+			1.5614379	0.5581283	6.3568371	3.03E-09	2.68E-07	10.806242
cg14603098	chr17	32484259	-	ACCN1	TSS1500	2.8235361	-1.385721	6.3565612	3.04E-09	2.68E-07	10.804917
cg17712694	chr5	16180068	-	11-Mar	TSS200	2.9454962	-0.66834	6.3564998	3.04E-09	2.68E-07	10.804623
cg21051815	chr11	1542394	+	HCCA2	Body	1.0712291	0.92723	6.3592844	3.05E-09	2.69E-07	10.80266
cg03806038	chr6	149639041	-	MAP3K7IP2	TSS200	0.915566	-2.79174	6.3588684	3.06E-09	2.69E-07	10.80089
cg18523886	chr20	39126551	-			-1.844869	-0.736777	-6.355251	3.06E-09	2.70E-07	10.798629
cg18693985	chr5	173351052	-	CPEB4	Body	0.9986394	1.2320455	6.3548135	3.07E-09	2.70E-07	10.796528

cg00526711	chr15	91477898	+	UNC45A;UNC45A	5'UTR;TSS1500	-0.848605	-2.085436	-6.354643	3.07E-09	2.70E-07	10.795709
cg23888423	chr2	242254170	-	SEPT2;SEPT2;HDLBP;SEPT2;SEPT2	TSS1500;TSS1500;5'UTR;TSS1500;TSS1500	-0.484182	-4.575364	-6.35444	3.07E-09	2.71E-07	10.794734
cg11033617	chr5	179562118	-	RASGEF1C	Body	-1.267439	-2.141161	-6.354378	3.07E-09	2.71E-07	10.794437
cg24098008	chr10	132877018	-			-1.130788	-0.350011	-6.354313	3.07E-09	2.71E-07	10.794125
cg21902394	chr17	19883326	-			1.324669	1.8475672	6.3541761	3.07E-09	2.71E-07	10.793468
cg27652490	chr19	18761402	+	KLHL26	Body	1.0790502	1.2466193	6.3540209	3.08E-09	2.71E-07	10.792723
cg22257374	chr3	15139756	-	ZFYVE20	5'UTR	-0.605411	-1.416159	-6.353936	3.08E-09	2.71E-07	10.792317
cg03446636	chr1	63784592	-			0.9043539	0.6547863	6.3602562	3.09E-09	2.72E-07	10.792235
cg12024564	chr12	62546633	+	FAM19A2	5'UTR	-1.457601	-0.610165	-6.353161	3.09E-09	2.72E-07	10.788596
cg27526774	chr1	235099089	-			-2.436963	-2.791412	-6.353155	3.09E-09	2.72E-07	10.788566
cg00978051	chr16	59786279	+			-1.554711	0.2928295	-6.352138	3.11E-09	2.73E-07	10.783688
cg23364373	chr20	61167926	+	C20orf166	3'UTR	-1.508684	-0.749534	-6.352064	3.11E-09	2.73E-07	10.783331
cg27095222	chr11	88090861	-			-1.481161	-1.65711	-6.352103	3.11E-09	2.73E-07	10.78352
cg08918811	chr11	19262251	-	E2F8;E2F8	1stExon;5'UTR	-0.614947	-5.113482	-6.352005	3.11E-09	2.73E-07	10.783048
cg05800416	chr8	19460097	-	CSGALNACT1;CSGALNACT1;CSGALNACT1	TSS200;Body;5'UTR	1.1051004	1.0541931	6.3514194	3.12E-09	2.74E-07	10.780238
cg00092992	chr1	33596100	+			-0.867787	-1.237937	-6.351331	3.12E-09	2.74E-07	10.779811
cg11392689	chr1	52522762	-	BTF3L4;BTF3L4;BTF3L4	Body;5'UTR;5'UTR	-0.656484	-4.181049	-6.350726	3.13E-09	2.75E-07	10.776913
cg13647892	chr9	97317179	+			-1.902996	0.5204354	-6.350704	3.13E-09	2.75E-07	10.776806
cg08679180	chr11	73034459	+	ARHGEF17	Body	0.9648671	-0.638587	6.3505664	3.13E-09	2.75E-07	10.776144
cg06002066	chr2	173337636	-	ITGA6;ITGA6	Body;Body	0.8330224	2.0549251	6.3504403	3.13E-09	2.75E-07	10.77554

cg26139062	chr2	175260842	+	CIR1;SCRN3	TSS1500;5'UTR	-0.803625	-4.270484	-6.350215	3.14E-09	2.75E-07	10.774457
cg05167973	chr11	2949546	+	PHLDA2	3'UTR	-1.223429	-2.082093	-6.349446	3.15E-09	2.76E-07	10.770766
cg06952873	chr6	28059208	+	ZSCAN12L1	Body	1.201969	0.4263272	6.3493719	3.15E-09	2.76E-07	10.770413
cg16404157	chr14	38724648	-	CLEC14A	1stExon	1.7121618	-0.443441	6.3492925	3.15E-09	2.76E-07	10.770032
cg03760839	chr1	119531718	+	TBX15	5'UTR	1.1336005	0.3736272	6.3491997	3.15E-09	2.76E-07	10.769587
cg07871456	chr17	48796501	-	LUC7L3;LUC7L3	TSS1500;TSS1500	-1.180361	-1.325308	-6.349062	3.15E-09	2.77E-07	10.768927
cg02039214	chr15	88521695	+	NTRK3;NTRK3;NTRK3	Body;Body;3'UTR	-1.404847	0.1504215	-6.348746	3.16E-09	2.77E-07	10.767408
cg01205011	chr6	35262113	-	ZNF76	Body	-1.010961	-1.49251	-6.348249	3.17E-09	2.78E-07	10.765025
cg17007574	chr2	238571511	-	LRRFIP1	Body	-1.004256	-2.010283	-6.358374	3.17E-09	2.78E-07	10.76728
cg26960939	chr17	38717206	-	CCR7	Body	-1.233699	-1.05533	-6.347659	3.18E-09	2.78E-07	10.762195
cg18796287	chr5	138730886	+	LOC389333	TSS200	1.2348456	0.7270645	6.3502987	3.19E-09	2.79E-07	10.759603
cg09809932	chr1	6515597	-	ESPN	Body	1.9251732	-0.009158	6.3468409	3.19E-09	2.79E-07	10.758271
cg20825022	chr2	124920645	-	CNTNAP5	Body	-1.746925	-0.62947	-6.34668	3.19E-09	2.80E-07	10.7575
cg08846327	chr5	14359614	-	TRIO	Body	0.640045	1.8634307	6.3498827	3.20E-09	2.80E-07	10.757611
cg21901718	chr5	16180076	-	11-Mar	TSS200	3.5027124	-1.67114	6.3463733	3.20E-09	2.80E-07	10.756028
cg16436377	chr1	10725254	+	CASZ1;CASZ1	Body;Body	0.5914697	2.4774274	6.3462285	3.20E-09	2.80E-07	10.755333
cg11152384	chr11	68934300	-			-1.697452	-2.244321	-6.345875	3.20E-09	2.80E-07	10.753635
cg18911817	chr5	1010925	+	NKD2	Body	-1.420231	-0.895864	-6.345806	3.21E-09	2.80E-07	10.753306
cg26587228	chr19	57641846	+	USP29	Body	-1.236554	-1.115151	-6.345753	3.21E-09	2.80E-07	10.753054
cg00891886	chr7	73417264	+			-0.887553	-2.223714	-6.345287	3.21E-09	2.81E-07	10.750815

cg24245418	chr7	24324976	+	NPY	Body	3.1570244	-1.065566	6.344875	3.22E-09	2.82E-07	10.748841
cg01812571	chr12	132970724	-			-1.081784	0.6564162	-6.344772	3.22E-09	2.82E-07	10.748347
cg26189283	chr1	155109378	+	RAG1AP1;RAG1AP1;RAG1AP1	Body;Body;Body	-1.450873	-0.694855	-6.344552	3.23E-09	2.82E-07	10.747293
cg19793904	chr12	128943277	-	TMEM132C	Body	-1.434281	-0.286289	-6.347831	3.23E-09	2.82E-07	10.747785
cg09672200	chr7	1117916	-	C7orf50;C7orf50;C7orf50	Body;Body;Body	0.8643148	2.3403571	6.3441534	3.23E-09	2.82E-07	10.74538
cg17780682	chr17	913998	-	ABR;ABR;ABR	Body;Body;Body	0.9600028	1.2602084	6.343952	3.24E-09	2.83E-07	10.744415
cg18096962	chr5	72526232	-			1.1360209	-0.266667	6.3429543	3.25E-09	2.84E-07	10.73963
cg19098790	chr22	24921764	+	UPB1	3'UTR	-0.781073	-2.414296	-6.342938	3.25E-09	2.84E-07	10.739554
cg03475509	chr4	85402143	-			1.2558187	1.0880995	6.3426338	3.26E-09	2.84E-07	10.738093
cg15074033	chr12	52652462	-			1.986725	-1.00059	6.3418776	3.27E-09	2.85E-07	10.734467
cg06757585	chr5	140753531	+	PCDHGA4;PCDHGA1;PCDHGA6;PCDHGA5;PCDHGB1;PCDHGA3;PCDHGA2;PCDHGA6;PCDHGB2;PCDHGB3	Body;Body;TSS200;Body;Body;Body;Body;TSS200;Body;Body	1.3134412	-0.012043	6.3414515	3.28E-09	2.86E-07	10.732423
cg05020685	chr4	40858965	+	APBB2;APBB2;APBB2;APBB2;APBB2;APBB2;APBB2	Body;5'UTR;5'UTR;5'UTR;Body;5'UTR;Body	-1.304744	-1.797091	-6.340902	3.29E-09	2.87E-07	10.729789
cg23314514	chr14	104852775	+			-1.45752	0.3583913	-6.340895	3.29E-09	2.87E-07	10.729756
cg00282706	chr12	6055430	+	ANO2	TSS200	-1.594467	-1.096603	-6.340617	3.29E-09	2.87E-07	10.728423
cg13428516	chr19	49224033	-	MAMSTR;RASIP1	TSS1500;3'UTR	-1.278818	-1.892418	-6.340468	3.29E-09	2.87E-07	10.727708
cg18713028	chr12	54329935	+			1.4845518	-0.507117	6.342773	3.31E-09	2.89E-07	10.723793
cg05365532	chr19	55128350	+	LILRB1	TSS1500	-1.209718	-1.323702	-6.339244	3.31E-09	2.89E-07	10.721839
cg02753187	chr17	46919180	-	CALCOCO2	Body	2.0921471	-0.91137	6.3383825	3.33E-09	2.90E-07	10.71771
cg00999988	chr2	166649910	-	GALNT3	5'UTR	-1.100941	-1.924927	-6.338342	3.33E-09	2.90E-07	10.717514
cg22517388	chr5	71014918	-	CARTPT	TSS200	0.8231731	-0.020283	6.3380254	3.33E-09	2.90E-07	10.715998

cg14143580	chr20	29960982	-	DEFB118	3'UTR	-1.691293	-0.097414	-6.337373	3.34E-09	2.91E-07	10.712873
cg20046777	chr12	61961255	-			-1.905884	0.4301608	-6.337279	3.35E-09	2.91E-07	10.712419
cg04106201	chr7	39332739	-	POU6F2;POU6F2	Body;Body	0.8654302	1.4899219	6.3370325	3.35E-09	2.92E-07	10.711239
cg26169081	chr10	12648338	-	CAMK1D;CAMK1D	Body;Body	-0.981577	-0.3419	-6.336572	3.36E-09	2.92E-07	10.709033
cg11323650	chr11	55905246	+	OR8J3	TSS200	-1.501042	-0.718266	-6.336436	3.36E-09	2.92E-07	10.708378
cg12722217	chr15	44487545	-	FRMD5	TSS200	-0.832129	-4.804836	-6.336367	3.36E-09	2.92E-07	10.708051
cg04213384	chr13	98960589	-	FARP1	Body	1.5165461	1.7098944	6.3362668	3.36E-09	2.92E-07	10.707569
cg12986453	chr22	49137906	-	FAM19A5;FAM19A5	Body;Body	-1.478834	-0.080664	-6.335814	3.37E-09	2.93E-07	10.705398
cg08670181	chr16	48846942	+			-1.488362	0.0513289	-6.335691	3.37E-09	2.93E-07	10.704809
cg19789466	chr12	113344923	-	OAS1;OAS1;OAS1	1stExon;1stExon;1stExon	-1.223706	-2.664633	-6.335637	3.37E-09	2.93E-07	10.704553
cg11142389	chr3	147125765	+	ZIC4;ZIC1	TSS1500;TSS1500	1.4472443	-0.270699	6.3352519	3.38E-09	2.94E-07	10.702705
cg05595134	chr8	55047255	-	MRPL15	TSS1500	-1.753236	0.1601779	-6.334716	3.39E-09	2.94E-07	10.700139
cg23172828	chr15	66998998	+	SMAD6;SMAD6	Body;Body	1.6645043	2.217475	6.3345761	3.39E-09	2.95E-07	10.699466
cg22640982	chr10	97197695	+	SORBS1;SORBS1;SORBS1;SORBS1;SORBS1;SORBS1;SORBS1	Body;Body;Body;Body;Body;Body;Body	0.9646318	2.1296815	6.3343896	3.39E-09	2.95E-07	10.698573
cg04433322	chr17	42346894	+	SLC4A1	TSS1500	-1.1365	-0.781868	-6.334234	3.40E-09	2.95E-07	10.697826
cg20113820	chr8	1765074	+	MIR596	TSS1500	-1.153324	-1.87901	-6.334283	3.40E-09	2.95E-07	10.698064
cg27320005	chr5	140683767	+	SLC25A2	TSS200	1.3432505	2.4556267	6.3376151	3.40E-09	2.95E-07	10.698881
cg07746882	chr10	49866792	+			-0.816048	-0.762011	-6.33396	3.40E-09	2.95E-07	10.696512
cg16701059	chr15	101513798	-	LRRK1	Body	1.9241965	-1.242364	6.3337253	3.41E-09	2.95E-07	10.69539
cg10340048	chr8	74258684	+			-1.882284	-0.61921	-6.333473	3.41E-09	2.96E-07	10.694179

cg04154138	chr19	53758521	-	ZNF677	TSS1500	2.5458523	-1.803162	6.3332924	3.41E-09	2.96E-07	10.693315
cg16531181	chr10	130185387	+			-1.760524	0.383466	-6.333042	3.42E-09	2.96E-07	10.692117
cg08250187	chr2	17482455	+			-1.77623	-0.18862	-6.332347	3.43E-09	2.97E-07	10.688784
cg08022717	chr2	127955187	-	CYP27C1	Body	-1.722794	0.3916279	-6.332097	3.43E-09	2.98E-07	10.687587
cg21932416	chr2	176997707	+			1.8278756	-0.156124	6.3320362	3.43E-09	2.98E-07	10.687296
cg15194251	chr11	2239813	-			-1.099017	-0.732196	-6.331966	3.43E-09	2.98E-07	10.686961
cg02083214	chr8	871677	+			-1.197646	0.4288606	-6.331765	3.44E-09	2.98E-07	10.685998
cg16996281	chr11	119217884	-	MFRP;C1QTNF5	TSS1500;TSS1500	-1.319112	-0.478965	-6.335096	3.44E-09	2.98E-07	10.686828
cg05104447	chr6	41739545	+	FRS3	Body	0.6940895	2.1838605	6.3339001	3.46E-09	3.00E-07	10.691242
cg24903144	chr10	102509268	-	PAX2;PAX2;PAX2;PAX2;PAX2	Body;Body;Body;Body;Body	0.9855531	0.3707277	6.3302817	3.46E-09	3.00E-07	10.678891
cg00460268	chr3	50383175	-	ZMYND10	TSS200	-1.160734	-2.027242	-6.330208	3.47E-09	3.00E-07	10.678538
cg07267550	chr17	74536269	+	PRCD	1stExon	1.7853377	2.4831536	6.3331659	3.47E-09	3.00E-07	10.677595
cg22249789	chr2	63284574	-			2.0219838	-0.988335	6.3331903	3.47E-09	3.00E-07	10.677712
cg23619365	chr13	112712009	+			2.4179592	-0.550492	6.3330705	3.47E-09	3.01E-07	10.677367
cg24079381	chr17	77115473	+	HRNBP3	5'UTR	-1.100501	-1.295263	-6.329572	3.48E-09	3.01E-07	10.67549
cg07093324	chr2	114652143	-	ACTR3	Body	-1.898713	-2.030457	-6.329266	3.48E-09	3.01E-07	10.674025
cg16936953	chr17	57915665	+	TMEM49	Body	-1.031318	-3.150719	-6.329121	3.48E-09	3.01E-07	10.673332
cg01376829	chr2	102804405	+	IL1RL2	Body	-1.376022	-2.267697	-6.327844	3.51E-09	3.03E-07	10.667215
cg04221877	chr1	25257515	+	RUNX3;RUNX3	TSS1500;Body	1.3166823	1.286101	6.3278586	3.51E-09	3.03E-07	10.667284
cg05558615	chr17	5820161	-			-1.394595	-0.58099	-6.32791	3.51E-09	3.03E-07	10.667529

cg01463644	chr16	48115640	+			-1.404682	0.0508283	-6.327575	3.51E-09	3.03E-07	10.665925
cg26603524	chr1	145713802	+	CD160	5'UTR	-0.974848	-5.548141	-6.327564	3.51E-09	3.03E-07	10.665874
cg15829088	chr1	247802935	-			1.6954078	-0.233899	6.3308009	3.51E-09	3.03E-07	10.666284
cg18162670	chr14	103686567	-			0.9970401	0.9930289	6.3341687	3.51E-09	3.03E-07	10.667304
cg26688315	chr2	218027591	+			-1.34013	0.2610781	-6.327259	3.52E-09	3.04E-07	10.664411
cg10264791	chr14	104800976	+			-0.934382	-0.080697	-6.326834	3.52E-09	3.04E-07	10.662376
cg22417733	chr6	153303409	-	FBXO5;FBXO5	5'UTR;Body	-1.717969	-2.501572	-6.330231	3.52E-09	3.04E-07	10.673727
cg16780562	chr14	102033251	+			-1.263413	0.3114609	-6.326526	3.53E-09	3.04E-07	10.660901
cg08790487	chr5	179135308	+	CANX;CANX	Body;Body	0.941637	2.0105142	6.3260448	3.54E-09	3.05E-07	10.658598
cg20400904	chr11	59624274	+	TCN1	Body	-1.59432	0.2352012	-6.332602	3.54E-09	3.05E-07	10.659823
cg01653727	chr7	5527669	+	FBXL18	Body	-1.069133	-0.800242	-6.3253	3.55E-09	3.06E-07	10.65503
cg00197058	chr17	59554672	-	TBX4	Body	0.9536454	0.1178505	6.3250026	3.56E-09	3.07E-07	10.653607
cg01275887	chr7	4747216	-	FOXK1	Body	2.1462416	3.3379484	6.3238759	3.58E-09	3.08E-07	10.648213
cg23633330	chr1	207510679	-	CD55;CD55	Body;Body	-1.001919	-1.670922	-6.323655	3.58E-09	3.08E-07	10.647156
cg07092880	chr11	85601106	-	CCDC83	Body	-1.625321	0.9219149	-6.323284	3.59E-09	3.09E-07	10.645377
cg14556808	chr11	130752510	-	SNX19	Body	0.6621665	2.985325	6.3231394	3.59E-09	3.09E-07	10.644686
cg14739664	chr5	140683467	+	SLC25A2;SLC25A2	1stExon;5'UTR	1.2342058	2.6956126	6.3265138	3.59E-09	3.09E-07	10.645785
cg06485521	chr6	53530600	-	KLHL31	TSS200	0.9465037	0.7394815	6.3227473	3.60E-09	3.10E-07	10.642809
cg23358740	chr14	102198157	-	C14orf72	Body	-0.974707	-1.853843	-6.322072	3.61E-09	3.11E-07	10.639576
cg12483476	chr5	178421711	+	GRM6	1stExon	2.2550138	-1.096492	6.3215281	3.62E-09	3.11E-07	10.636973

cg16357969	chr4	3646912	+			-0.840289	-0.95554	-6.327913	3.63E-09	3.12E-07	10.637441
cg20070768	chr15	74285128	+	STOML1	TSS1500	1.1292521	1.9985194	6.3209512	3.63E-09	3.12E-07	10.634212
cg26809635	chr12	54355087	+			2.7116572	-1.029979	6.3209012	3.63E-09	3.12E-07	10.633972
cg18253802	chr6	135501780	+	MYB;MYB;MYB;MYB;MYB;MYB;MYB;MYB	TSS1500;TSS1500;TSS1500;TSS1500;TSS1500;TSS1500;TSS1500;TSS1500	-0.791214	-3.938997	-6.324109	3.63E-09	3.12E-07	10.634519
cg22947965	chr11	123775188	+			-1.793869	0.3336326	-6.320764	3.63E-09	3.12E-07	10.633317
cg24111137	chr8	59324179	+	UBXN2B	Body	-0.379968	-4.367146	-6.320748	3.63E-09	3.12E-07	10.633238
cg22304522	chr8	131914037	+	ADCY8	Body	-1.786177	-0.410734	-6.320646	3.63E-09	3.12E-07	10.632753
cg18621852	chr3	10150065	+	C3orf24;C3orf24	TSS200;TSS200	0.8660578	1.4931189	6.32047	3.64E-09	3.12E-07	10.631908
cg06322432	chr10	112289853	-			-0.944174	-2.752294	-6.320329	3.64E-09	3.13E-07	10.631232
cg16794471	chr2	153271840	-	FMNL2	Body	-1.134461	-0.747858	-6.320279	3.64E-09	3.13E-07	10.630993
cg21169267	chr9	130298299	-	FAM129B;FAM129B	Body;Body	0.7480976	1.2214254	6.3202406	3.64E-09	3.13E-07	10.63081
cg18721420	chr19	15121913	-	CCDC105	1stExon	2.0992269	-1.482596	6.3201047	3.64E-09	3.13E-07	10.630159
cg12858577	chr17	75385278	-	SEPT9;SEPT9;SEPT9;SEPT9;SEPT9	5'UTR;5'UTR;Body;Body;Body	1.5430101	0.3089611	6.3232505	3.65E-09	3.13E-07	10.640418
cg13688474	chr1	9600443	+	SLC25A33	Body	-0.873274	-2.517661	-6.318898	3.67E-09	3.15E-07	10.624382
cg11687036	chr3	147139563	+			1.3003526	-0.009041	6.3188399	3.67E-09	3.15E-07	10.624106
cg23082003	chr13	21713913	+	SAP18	TSS1500	-1.434958	0.1250611	-6.318628	3.67E-09	3.15E-07	10.62309
cg14043049	chr19	19748575	-	GMIP	Body	0.7637582	2.6912616	6.3181989	3.68E-09	3.15E-07	10.621039
cg17241776	chr13	51417469	+	DLEU7	1stExon	1.9084073	-0.160448	6.318082	3.68E-09	3.16E-07	10.620479
cg11891393	chr6	100911727	+	SIM1	TSS200	1.6624379	-0.622757	6.3178705	3.68E-09	3.16E-07	10.619467
cg19716643	chr14	52735026	+	PTGDR	1stExon	1.5935121	-0.868832	6.3178468	3.69E-09	3.16E-07	10.619354

cg14184780	chr9	95475751	+	BICD2;BICD2	Body;3'UTR	0.8026072	0.7654538	6.3176945	3.69E-09	3.16E-07	10.618625
cg00164189	chr22	30821186	-	MTP18;MTP18;SEC14L2	TSS1500;TSS1500;3'UTR	-1.025588	-1.179446	-6.317569	3.69E-09	3.16E-07	10.618024
cg13852116	chr10	122403903	-			-1.671256	-0.104719	-6.31746	3.69E-09	3.16E-07	10.617502
cg22743884	chr8	11839844	-	DEFB135	1stExon	-1.669439	-0.134749	-6.317005	3.70E-09	3.17E-07	10.615327
cg00806009	chr17	80739852	-	TBCD	Body	-0.73372	-3.207449	-6.316831	3.70E-09	3.17E-07	10.614494
cg01560676	chr5	172260799	-	ERGIC1	TSS1500	-1.010737	-1.592004	-6.316771	3.70E-09	3.17E-07	10.614204
cg09578475	chr19	9473688	+	ZNF177	TSS200	2.3146994	0.1773196	6.3166237	3.71E-09	3.17E-07	10.613501
cg17725129	chr12	4482799	+	FGF23	Body	-1.306182	-0.076054	-6.316633	3.71E-09	3.17E-07	10.613546
cg16929739	chr3	11178593	+	HRH1	TSS200	-1.561904	-0.619445	-6.316545	3.71E-09	3.17E-07	10.613126
cg00267142	chr6	167104601	-	RPS6KA2	Body	-1.695166	1.0969887	-6.31641	3.71E-09	3.17E-07	10.612476
cg05858889	chr7	39649224	-	LOC646999	Body	2.0817419	-1.263549	6.3164447	3.71E-09	3.17E-07	10.612645
cg20959866	chr1	4714008	-	AJAP1;AJAP1	TSS1500;TSS1500	1.5282502	-1.317003	6.31577	3.72E-09	3.18E-07	10.609416
cg23291301	chr2	105472469	+	POU3F3	1stExon	2.3123209	-1.423095	6.3156645	3.73E-09	3.18E-07	10.608911
cg13156014	chr2	219917079	-			-1.346455	-0.407484	-6.315608	3.73E-09	3.19E-07	10.60864
cg00731458	chr16	71463001	+			-1.454956	0.1517157	-6.31545	3.73E-09	3.19E-07	10.607887
cg01911239	chr5	140744076	-	PCDHGA4;PCDHGA2;PCDHGA5;PCDHGB2;PCDHGA1;PCDHGB1;PCDHGA3;PCDHGA5	Body;Body;1stExon;Body;Body;Body;Body;1stExon	0.9848949	-0.106611	6.3150608	3.74E-09	3.19E-07	10.606023
cg05128414	chr1	197882836	+	LHX9	Body	1.4713187	-1.340936	6.3148464	3.74E-09	3.20E-07	10.604997
cg14298200	chr8	65498812	+			1.3321862	0.0828106	6.3180477	3.74E-09	3.20E-07	10.605327
cg00585901	chr1	155035388	-	EFNA4;EFNA4;EFNA4	TSS1500;TSS1500;TSS1500	-2.540766	-3.387243	-6.314338	3.75E-09	3.20E-07	10.602563
cg07168810	chr11	128712280	+	KCNJ1;KCNJ1;KCNJ1;KCNJ1;KCNJ1	5'UTR;5'UTR;5'UTR;1stExon;5'UTR	-1.615743	0.1642637	-6.314222	3.75E-09	3.20E-07	10.602008

cg10319857	chr16	69653345	-	NFAT5;NFAT5;NFAT5;NFAT5;NFAT5	5'UTR;Body;Body;5'UTR;Body	1.0250952	2.2325526	6.3141107	3.75E-09	3.20E-07	10.601478
cg20952257	chr5	171074407	+			-1.049747	-1.077377	-6.314083	3.75E-09	3.20E-07	10.601344
cg18770579	chr19	50191342	+	C19orf76;PRMT1;PRMT1;PRMT1	TSS1500;Body;Body;Body	1.3051194	2.2913444	6.3140281	3.76E-09	3.20E-07	10.601083
cg22215453	chr19	55852665	+	SUV420H2	5'UTR	-0.615333	-3.586492	-6.31372	3.76E-09	3.21E-07	10.599609
cg08473330	chr2	63282883	+	OTX1	Body	1.245865	-0.317364	6.313426	3.77E-09	3.21E-07	10.598202
cg00073837	chr2	223177008	-			1.5535886	-0.675145	6.3127377	3.78E-09	3.22E-07	10.594909
cg04288999	chr2	66667852	-	MEIS1	Body	1.5954272	-0.898988	6.3126431	3.78E-09	3.22E-07	10.594457
cg01733928	chr14	88625398	-			-0.994247	0.5856125	-6.315781	3.79E-09	3.23E-07	10.594731
cg06534022	chr19	1028780	+	CNN2;CNN2	Body;Body	-0.347932	-3.67432	-6.312407	3.79E-09	3.23E-07	10.593329
cg09119757	chr16	49668262	-	ZNF423	Body	-1.348745	0.7446085	-6.312234	3.79E-09	3.23E-07	10.592499
cg06633061	chr14	57261601	-			1.4899672	-0.952127	6.3121176	3.79E-09	3.23E-07	10.591943
cg07339137	chr3	87276419	+	CHMP2B;CHMP2B	1stExon;5'UTR	0.9348873	-3.251309	6.3120542	3.79E-09	3.23E-07	10.59164
cg24956176	chr17	33542219	-			-1.224116	-1.259271	-6.311784	3.80E-09	3.23E-07	10.590346
cg03593578	chr2	45028225	+			1.6137743	-0.932564	6.3110513	3.81E-09	3.24E-07	10.586843
cg08475953	chr5	178004091	+	COL23A1	Body	2.1349402	-0.799823	6.3143904	3.81E-09	3.24E-07	10.587857
cg05207465	chr5	154318039	-	GEMIN5	TSS1500	-1.072287	-0.47023	-6.310713	3.82E-09	3.25E-07	10.585228
cg22553301	chr19	3839231	+	ZFR2	Body	-0.929064	-0.861041	-6.310422	3.82E-09	3.25E-07	10.583835
cg04576707	chr4	71811504	-	MOBKL1A	Body	0.9400273	1.6387065	6.3102694	3.83E-09	3.26E-07	10.583104
cg06354543	chr10	130376572	-			-1.54938	-0.219834	-6.31008	3.83E-09	3.26E-07	10.582198
cg26216724	chr5	1749217	+			-1.036157	-1.421792	-6.310112	3.83E-09	3.26E-07	10.582353

cg16666329	chr5	76932526	+	OTP	Body	1.352047	-1.219153	6.3098507	3.83E-09	3.26E-07	10.581101
cg00829269	chr20	44065852	-			-0.821424	-3.842607	-6.309221	3.85E-09	3.27E-07	10.578092
cg11838237	chr5	1752864	+			-1.537557	-0.958134	-6.30926	3.85E-09	3.27E-07	10.578277
cg15810386	chr5	31362491	-			-1.602904	-0.969729	-6.308855	3.85E-09	3.27E-07	10.576341
cg07616471	chr3	46413711	+	CCR5;CCR5	5'UTR;5'UTR	-0.969267	0.0863585	-6.308728	3.86E-09	3.28E-07	10.575731
cg02490942	chr13	113491766	+	ATP11A;ATP11A	Body;Body	2.4410138	3.6640991	6.3116602	3.86E-09	3.28E-07	10.575052
cg12095547	chr22	49697195	-			-1.078667	0.0415974	-6.307955	3.87E-09	3.29E-07	10.572039
cg04472001	chr3	126398566	-			-0.95685	-2.269436	-6.307866	3.87E-09	3.29E-07	10.571612
cg00735218	chr17	76421569	-	DNAH17	Body	0.9991405	1.1207293	6.3076931	3.88E-09	3.29E-07	10.570784
cg08097614	chr19	46388787	-	IRF2BP1	1stExon	-1.342365	-4.307273	-6.314155	3.88E-09	3.29E-07	10.571815
cg11012412	chr5	77147141	-			1.4769706	0.1340556	6.3141574	3.88E-09	3.29E-07	10.571828
cg16865446	chr11	31822302	+	PAX6;PAX6;PAX6	Body;Body;Body	2.5137448	-0.602396	6.3073903	3.88E-09	3.29E-07	10.569337
cg22651416	chr8	10643634	-	PINX1	Body	0.9906737	2.3244455	6.3072259	3.89E-09	3.30E-07	10.56855
cg11821795	chr22	47611604	-			-1.374819	-1.139077	-6.307017	3.89E-09	3.30E-07	10.567552
cg01499197	chr13	28544814	+	CDX2	TSS1500	1.4744493	-0.778501	6.3065515	3.90E-09	3.30E-07	10.565326
cg01329690	chr21	38580129	+	DSCR9	TSS1500	-1.18926	-1.49031	-6.306256	3.90E-09	3.31E-07	10.563915
cg20553124	chr6	36253157	-	PNPLA1;PNPLA1;PNPLA1	5'UTR;Body;5'UTR	1.5084876	-1.155551	6.3056721	3.92E-09	3.32E-07	10.561122
cg02577387	chr2	231855524	+			1.1847139	0.6458814	6.3052476	3.92E-09	3.32E-07	10.559093
cg12623302	chr6	28058802	-	ZSCAN12L1	TSS200	1.0924188	0.2848918	6.3048757	3.93E-09	3.33E-07	10.557315
cg01813327	chr16	883720	+			-1.129643	-1.104156	-6.304342	3.94E-09	3.34E-07	10.554764

cg16830930	chr8	55371392	+	SOX17	Body	1.1083117	0.2874612	6.3040917	3.95E-09	3.34E-07	10.553567
cg23913077	chr10	111018447	-			-1.557859	0.0283378	-6.303792	3.95E-09	3.35E-07	10.552135
cg01370541	chr4	41882812	+			1.7646556	-1.265329	6.3020104	3.99E-09	3.37E-07	10.54362
cg02589353	chr11	124471596	-			-1.233898	-0.938906	-6.302046	3.99E-09	3.37E-07	10.543792
cg19806642	chr5	178422140	+	GRM6	TSS200	1.9326471	-0.674426	6.3018081	3.99E-09	3.38E-07	10.542653
cg21230493	chr8	26722496	-	ADRA1A;ADRA1A;ADRA1A;ADRA1A;ADRA1A;ADRA1A;ADRA1A;ADRA1A	1stExon;5'UTR;1stExon;1stExon;5'UTR;5'UTR;5'UTR;1stExon	1.5034773	-1.218906	6.3016223	3.99E-09	3.38E-07	10.541765
cg17177602	chr1	1196457	+	UBE2J2;UBE2J2;LOC100128842;UBE2J2;UBE2J2	Body;Body;Body;Body;Body	0.9228461	0.9438474	6.3013	4.00E-09	3.38E-07	10.540225
cg24275315	chr4	85417312	-	NKX6-1	Body	1.2997675	-0.484105	6.301241	4.00E-09	3.38E-07	10.539943
cg07857469	chr4	81107185	+	PRDM8	5'UTR	1.0047193	-0.01561	6.3011733	4.00E-09	3.39E-07	10.53962
cg22135561	chr5	1865501	+			-1.63923	-0.223012	-6.301045	4.01E-09	3.39E-07	10.539007
cg05311410	chr7	27225523	+	HOXA11AS;HOXA11	Body;TSS1500	3.0063446	-1.148595	6.3008129	4.01E-09	3.39E-07	10.537897
cg08464190	chr16	49315302	+	CBLN1	1stExon	2.524469	-0.996749	6.3038989	4.02E-09	3.39E-07	10.537774
cg04747180	chr4	39116218	+	KLHL5;KLHL5;KLHL5;KLHL5	Body;Body;Body;Body	1.1175419	0.7711601	6.3002179	4.02E-09	3.40E-07	10.535054
cg09186818	chr11	119217331	+	C1QTNF5;C1QTNF5;MFRP;MFRP	1stExon;5'UTR;1stExon;5'UTR	-0.778223	-0.842967	-6.300063	4.03E-09	3.40E-07	10.534313
cg03776506	chr16	30581384	+	ZNF688;ZNF688	Body;Body	0.8955361	4.5880232	6.3134293	4.03E-09	3.41E-07	10.537722
cg04061411	chr15	78385733	-	SH2D7	Body	-0.961021	-1.883738	-6.299648	4.03E-09	3.41E-07	10.532331
cg24061208	chr18	14748250	-	ANKRD30B;ANKRD30B	1stExon;5'UTR	1.57419	-0.646474	6.2994532	4.04E-09	3.41E-07	10.5314
cg07930539	chr2	177025975	-			1.4612157	-0.082471	6.302616	4.04E-09	3.41E-07	10.531653
cg16812893	chr21	31813075	-	KRTAP15-1;KRTAP15-1	3'UTR;1stExon	-1.551983	0.0202057	-6.299283	4.04E-09	3.41E-07	10.530585
cg09151754	chr17	5019310	-	ZNF232	5'UTR	1.0758101	-0.256092	6.2991912	4.04E-09	3.41E-07	10.530148

cg07307078	chr18	12307630	+	TUBB6	TSS1500	1.9049408	-1.305574	6.2991189	4.04E-09	3.41E-07	10.529803
cg01461847	chr6	27648057	-			1.7574047	-0.933858	6.3022892	4.05E-09	3.41E-07	10.540512
cg02400433	chr6	100054987	+	PRDM13	1stExon	1.1854585	-1.061195	6.2990155	4.05E-09	3.41E-07	10.529309
cg04300873	chr17	42254647	-	ASB16;C17orf65	Body;5'UTR	0.6604961	2.1548456	6.2990373	4.05E-09	3.41E-07	10.529413
cg04431331	chr10	2267590	+			-1.428081	-0.615143	-6.298979	4.05E-09	3.41E-07	10.529137
cg07649045	chr11	7260764	-			-1.602926	-0.13759	-6.298879	4.05E-09	3.41E-07	10.528655
cg07762181	chr4	56660143	+			1.1916963	0.2348044	6.3019887	4.05E-09	3.42E-07	10.528894
cg26980729	chr6	105379117	-			-1.376371	-0.681654	-6.298344	4.06E-09	3.42E-07	10.526101
cg23035209	chr5	72795181	+	BTF3;BTF3	Body;Body	-0.91612	-2.65307	-6.297543	4.08E-09	3.43E-07	10.522275
cg19971716	chr4	122301740	-	QRFPR	1stExon	3.233138	-1.79915	6.3007687	4.08E-09	3.43E-07	10.533272
cg26839284	chr1	110162246	-	AMPD2;AMPD2	TSS1500;TSS1500	-0.933074	-4.773953	-6.297126	4.08E-09	3.44E-07	10.520283
cg14121103	chr3	43731973	-	ABHD5	TSS1500	-0.641228	-3.029595	-6.297067	4.09E-09	3.44E-07	10.52
cg10630085	chr11	73108402	+	RELT;RELT	3'UTR;3'UTR	1.1495065	1.7132256	6.2968367	4.09E-09	3.44E-07	10.5189
cg22142858	chr6	6004804	+	NRN1	Body	1.2303028	-1.109054	6.2967453	4.09E-09	3.44E-07	10.518463
cg26830108	chr7	100813299	-			-1.426634	-3.70087	-6.300028	4.09E-09	3.44E-07	10.519538
cg05740045	chr8	65499926	+			1.3181297	0.1326249	6.296595	4.10E-09	3.44E-07	10.517745
cg27519145	chr1	166039635	+	FAM78B	Body	-1.587448	0.4542866	-6.296443	4.10E-09	3.45E-07	10.51702
cg09248934	chr3	8661519	-	C3orf32	3'UTR	-1.191622	-0.124437	-6.295879	4.11E-09	3.46E-07	10.514324
cg12947833	chr11	111783499	+	HSPB2;CRYAB;HSPB2	5'UTR;TSS1500;1stExon	1.5001294	-0.257377	6.2954837	4.12E-09	3.46E-07	10.512437
cg09452312	chr16	79101802	+	WWOX	Body	-1.413811	0.0815748	-6.29542	4.12E-09	3.46E-07	10.512134

cg22799691	chr14	103821330	+			-1.575763	-0.279442	-6.295262	4.12E-09	3.46E-07	10.511378
cg04115702	chr17	77386380	+	HRNBP3	5'UTR	-1.374122	-0.389387	-6.295202	4.12E-09	3.46E-07	10.51109
cg19880542	chr3	11678156	-	VGLL4;VGLL4	Body;Body	1.1215829	1.5863253	6.2949126	4.13E-09	3.47E-07	10.509709
cg24670000	chr17	72370729	+			-1.478239	0.0020498	-6.294867	4.13E-09	3.47E-07	10.509489
cg05919685	chr1	159556997	+	APCS	TSS1500	-1.721755	-1.080709	-6.294575	4.14E-09	3.47E-07	10.508097
cg14334441	chr6	27235820	-			1.7206081	-0.479481	6.3008929	4.15E-09	3.48E-07	10.508397
cg17799946	chr1	91301962	+			1.8783305	-0.651638	6.2941424	4.15E-09	3.48E-07	10.506031
cg24387198	chr3	133381208	+	TOPBP1	TSS1500	-0.498733	-3.45039	-6.294208	4.14E-09	3.48E-07	10.506343
cg20315995	chr12	7280971	+	RBP5	Body	1.4104381	4.2611042	6.2939215	4.15E-09	3.48E-07	10.504976
cg06968859	chr2	80724209	+	CTNNA2;CTNNA2	Body;Body	-2.009643	-1.103352	-6.293877	4.15E-09	3.48E-07	10.504764
cg02291010	chr5	96079433	+	CAST;CAST;CAST;CAST;CAST;CAST;CAST;CAST;CAST;CAST;CAST	Body;Body;Body;Body;Body;Body;1stExon;Body;Body;Body;Body	0.9193072	1.8450035	6.2937595	4.15E-09	3.48E-07	10.504202
cg09179249	chr1	10732900	-	CASZ1;CASZ1	Body;Body	1.4046587	2.1567956	6.2935573	4.16E-09	3.49E-07	10.503236
cg04851742	chr13	28503508	-			1.3116527	-1.679909	6.2934383	4.16E-09	3.49E-07	10.502668
cg17278072	chr7	155258411	+			1.6784778	0.4248014	6.2929547	4.17E-09	3.50E-07	10.500358
cg07983575	chr16	48993133	+			-1.168528	-0.595255	-6.292909	4.17E-09	3.50E-07	10.500139
cg07452312	chr5	153775466	-	GALNT10	Body	-1.069958	-1.147744	-6.292571	4.18E-09	3.50E-07	10.498528
cg05474265	chr4	81128398	+			1.0742959	0.1683093	6.2923518	4.18E-09	3.50E-07	10.497479
cg06448603	chr5	33659084	+	ADAMTS12	Body	-2.026534	0.1057346	-6.292337	4.18E-09	3.50E-07	10.497408
cg05570237	chr21	27934499	-	CYYR1	Body	-1.767282	0.1371867	-6.295536	4.19E-09	3.51E-07	10.497884
cg01243072	chr2	43398171	+			1.4946559	2.6505282	6.2920717	4.19E-09	3.51E-07	10.496142

cg27034836	chr3	33139098	-	GLB1;TMPPE;TMPPE;GLB1;GLB1	TSS1500;TSS1500;TSS1500;TSS1500;TSS1500	-0.611824	-2.401909	-6.291738	4.20E-09	3.51E-07	10.494547
cg00014260	chr8	10383071	+	T-SP1	TSS200	-1.160503	-0.320289	-6.291631	4.20E-09	3.51E-07	10.494038
cg14360405	chr11	87912414	-			-1.187276	0.1690242	-6.291286	4.21E-09	3.52E-07	10.492392
cg24643393	chr7	73417261	+			-1.001969	-1.794303	-6.294236	4.21E-09	3.52E-07	10.491687
cg05422029	chr6	27235843	+			2.2756303	-0.50055	6.2907548	4.22E-09	3.53E-07	10.489854
cg09947611	chr11	1542360	+	HCCA2	Body	1.0634295	1.1190352	6.2907682	4.22E-09	3.53E-07	10.489918
cg20151301	chr10	1366415	-	ADARB2	Body	-1.549624	-0.658155	-6.290581	4.22E-09	3.53E-07	10.489024
cg18171097	chr3	169532134	-			-2.611871	2.5031354	-6.290364	4.22E-09	3.53E-07	10.487987
cg12606911	chr2	87036626	-	CD8A	TSS1500	2.1541195	0.615632	6.2897539	4.24E-09	3.54E-07	10.485075
cg17642708	chr5	8217322	-			-2.219695	1.2031488	-6.289724	4.24E-09	3.54E-07	10.48493
cg09890930	chr12	56149396	-	SARNP;SARNP	Body;Body	0.8497647	1.0001485	6.2896608	4.24E-09	3.54E-07	10.48463
cg18902641	chr6	27648411	+			0.9593569	-0.216499	6.289639	4.24E-09	3.54E-07	10.484526
cg14750948	chr3	147130477	+	ZIC1	Body	1.7851198	-0.165132	6.2929132	4.24E-09	3.54E-07	10.485614
cg05332306	chr17	9548278	-	USP43	TSS1500	-0.432054	-3.878338	-6.28913	4.25E-09	3.55E-07	10.482099
cg16086579	chr12	132562626	-	EP400	3'UTR	0.884379	2.1295555	6.2889494	4.25E-09	3.55E-07	10.481234
cg09483043	chr2	224810846	-	WDFY1	TSS1500	1.0639168	2.299985	6.2887878	4.26E-09	3.55E-07	10.480463
cg25130336	chr5	1772638	-			-1.277155	-1.321885	-6.288767	4.26E-09	3.55E-07	10.480366
cg10788210	chr13	60738032	+	DIAPH3;DIAPH3	5'UTR;1stExon	-1.397288	-5.769983	-6.291852	4.26E-09	3.56E-07	10.480321
cg21758962	chr11	30929224	-			-1.678249	-0.064428	-6.288284	4.27E-09	3.56E-07	10.47806
cg14780466	chr2	20870812	-	GDF7	Body	1.1775171	0.6919104	6.2880783	4.27E-09	3.56E-07	10.477076

cg16747567	chr14	71373858	+	PCNX	TSS1500	-0.711283	-4.269646	-6.287839	4.28E-09	3.57E-07	10.475932
cg24595606	chr6	108492358	+	NR2E1	Body	2.0726208	-0.062834	6.2872876	4.29E-09	3.58E-07	10.473302
cg09389824	chr10	31883099	+			-1.321373	-0.861201	-6.287046	4.29E-09	3.58E-07	10.472148
cg10177465	chr17	77536811	+			-1.18383	-0.712645	-6.287051	4.29E-09	3.58E-07	10.472171
cg09528494	chr8	11338675	-			-1.244098	-0.525898	-6.286678	4.30E-09	3.58E-07	10.470394
cg20485058	chr2	36157995	-			-2.014307	0.9026491	-6.2867	4.30E-09	3.58E-07	10.470496
cg07318145	chr1	247614654	+	OR2B11	1stExon	-1.204413	-0.533724	-6.289904	4.30E-09	3.58E-07	10.471035
cg00231728	chr4	113442709	+			1.395621	-1.296505	6.2861571	4.31E-09	3.59E-07	10.467906
cg18876826	chr1	38396334	+	INPP5B	Body	-1.257894	-1.265154	-6.286041	4.32E-09	3.59E-07	10.467352
cg11761200	chr2	79349970	-	REG1A	Body	-1.777647	0.423123	-6.285969	4.32E-09	3.59E-07	10.467009
cg16258229	chr1	5956985	+	NPHP4	Body	0.952686	1.9909274	6.2857137	4.32E-09	3.60E-07	10.46579
cg11980129	chr3	79815639	-	ROBO1	5'UTR	1.5581616	-1.400303	6.2855024	4.33E-09	3.60E-07	10.464781
cg01475377	chr6	109611718	-			1.0563879	1.0587652	6.2854155	4.33E-09	3.60E-07	10.464367
cg21113746	chr1	59039556	+			-1.047996	-1.038764	-6.285388	4.33E-09	3.60E-07	10.464236
cg15226029	chr6	32151731	+	AGER;AGER	Body;Body	0.7744748	1.2890993	6.2851841	4.33E-09	3.60E-07	10.463262
cg17863139	chr1	201366493	-	LAD1	Body	-0.929537	-1.26891	-6.285201	4.33E-09	3.60E-07	10.463342
cg11066780	chr1	10725571	-	CASZ1;CASZ1	Body;Body	1.441955	2.714779	6.2851061	4.34E-09	3.60E-07	10.46289
cg04436469	chr10	134598496	-	NKX6-2	Body	0.9425346	-0.487244	6.2847834	4.34E-09	3.61E-07	10.46135
cg04726200	chr11	2930485	+	SLC22A18;SLC22A18	Body;Body	0.9279564	0.9080694	6.2847687	4.34E-09	3.61E-07	10.46128
cg04155830	chr3	120314864	-	NDUFB4;NDUFB4	TSS1500;TSS1500	-0.942676	-3.743993	-6.284624	4.35E-09	3.61E-07	10.460588

cg22546775	chr12	66697281	-	HELB	Body	-1.386615	0.5906585	-6.284626	4.35E-09	3.61E-07	10.460598
cg20068510	chr15	95397535	-			-1.731028	0.7514805	-6.284529	4.35E-09	3.61E-07	10.460135
cg21646392	chr3	23710303	+			-1.307615	-0.354167	-6.28421	4.36E-09	3.62E-07	10.458615
cg27606499	chr3	147111120	+	ZIC4;ZIC4;ZIC4;ZIC4;ZIC4	Body;TSS1500;Body;Body;TSS1500	1.6507187	-0.617434	6.2841523	4.36E-09	3.62E-07	10.458339
cg20416037	chr12	57984712	-	PIP4K2C;PIP4K2C;PIP4K2C;PIP4K2C	TSS1500;TSS1500;TSS1500;TSS1500	-0.831839	-2.958888	-6.283981	4.36E-09	3.62E-07	10.457521
cg19791637	chr2	30633168	+			-1.233576	-0.93981	-6.283941	4.36E-09	3.62E-07	10.457329
cg04723401	chr14	29229249	+			2.5026859	-1.685827	6.2836367	4.37E-09	3.62E-07	10.455878
cg04788987	chr14	48096041	+	MDGA2	Body	-2.264599	2.0645941	-6.283634	4.37E-09	3.62E-07	10.455867
cg10991752	chr6	155845314	-			-1.508029	0.1744981	-6.2837	4.37E-09	3.62E-07	10.45618
cg10874111	chr7	158799748	-	LOC154822	TSS1500	-1.902029	0.9238433	-6.283593	4.37E-09	3.62E-07	10.45567
cg02253701	chr1	31627614	-			-0.842418	-2.911987	-6.283224	4.38E-09	3.63E-07	10.453907
cg07806552	chr16	81437124	+			-0.731203	-2.86155	-6.282928	4.38E-09	3.63E-07	10.452499
cg22858288	chr11	2930322	+	SLC22A18;SLC22A18	Body;Body	0.9929898	0.946156	6.2828384	4.39E-09	3.63E-07	10.452069
cg15471388	chr14	49360792	-			-1.649702	-1.026455	-6.282423	4.39E-09	3.64E-07	10.450089
cg08242636	chr16	67062389	-	CBFB;CBFB	TSS1500;TSS1500	-1.170766	-1.152275	-6.281965	4.40E-09	3.65E-07	10.447902
cg05245416	chr2	105087010	-	LOC150568	Body	-1.884762	0.6143646	-6.281624	4.41E-09	3.65E-07	10.446276
cg18143296	chr3	157812763	+			1.4711967	-0.568318	6.2813729	4.42E-09	3.66E-07	10.445077
cg18942579	chr17	57915773	-	TMEM49	Body	-1.199365	-2.899811	-6.28102	4.43E-09	3.66E-07	10.443393
cg05241355	chr14	57278351	-	OTX2	TSS1500	2.3070636	-0.922579	6.2840002	4.43E-09	3.67E-07	10.443141
cg08439518	chr16	86305423	+			-1.178263	-0.976631	-6.280386	4.44E-09	3.67E-07	10.440371

cg01581084	chr8	99961874	+	OSR2;OSR2	Body;Body	1.68351	0.0272466	6.2799975	4.45E-09	3.68E-07	10.438516
cg13445796	chr11	20181725	-	DBX1	1stExon	2.5755805	-0.93641	6.2798779	4.45E-09	3.68E-07	10.437945
cg06136199	chr11	9597577	-	WEE1;WEE1	Body;Body	-1.38242	-1.849174	-6.279395	4.46E-09	3.69E-07	10.435643
cg22963629	chr7	31147341	+			-1.650952	-0.416754	-6.278954	4.47E-09	3.70E-07	10.433538
cg26340700	chr1	231663886	+	TSNAX-DISC1;TSNAX-DISC1;TSNAX-DISC1;TSNAX;TSNAX-DISC1;TSNAX-DISC1;TSNAX-DISC1;TSNAX-DISC1;TSNAX-DISC1	TSS1500;TSS1500;TSS1500;TSS1500;TSS1500;TSS1500;TSS1500;TSS1500;TSS1500	-0.931899	-2.59109	-6.278752	4.48E-09	3.70E-07	10.432575
cg03319497	chr5	145722730	+			1.2794704	-1.148468	6.2784868	4.48E-09	3.70E-07	10.43131
cg21997510	chr8	10381792	+	T-SP1	TSS1500	-1.635784	0.30464	-6.281145	4.50E-09	3.71E-07	10.429304
cg24086040	chr2	241458939	+	ANKMY1;ANKMY1	Body;Body	0.8855989	0.3530018	6.2778328	4.50E-09	3.71E-07	10.428191
cg26221111	chr5	10310759	-			-1.772248	0.1051508	-6.277873	4.49E-09	3.71E-07	10.42838
cg27153327	chr16	1920815	-	C16orf73;C16orf73	5'UTR;5'UTR	-1.372541	-0.458081	-6.277814	4.50E-09	3.71E-07	10.4281
cg08125821	chr8	114445779	-	CSMD3;CSMD3	Body;Body	1.6008947	-1.621776	6.2774732	4.50E-09	3.72E-07	10.426476
cg17431079	chr1	39607710	-	MACF1	Body	1.1539849	1.8228201	6.2807396	4.50E-09	3.72E-07	10.427375
cg24244374	chr1	214153472	+			1.4550599	-0.488363	6.2773679	4.51E-09	3.72E-07	10.425974
cg00305585	chr16	1610898	+	IFT140	Body	0.8432914	0.7919686	6.2771415	4.51E-09	3.72E-07	10.424894
cg10965178	chr1	43766752	-	TIE1	1stExon	1.2325289	2.2220563	6.2771554	4.51E-09	3.72E-07	10.42496
cg26785702	chr11	118846877	+	FOXR1	Body	-1.246046	-0.582109	-6.276701	4.52E-09	3.73E-07	10.422791
cg15232539	chr8	19459672	+	CSGALNACT1;CSGALNACT1;CSGALNACT1;CSGALNACT1	1stExon;5'UTR;Body;5'UTR	2.3036463	-0.179237	6.2765942	4.52E-09	3.73E-07	10.422284
cg11238352	chr1	59609170	+	LOC729467	Body	1.2209178	2.6081176	6.2796954	4.53E-09	3.73E-07	10.422639
cg15371566	chr3	170187722	-	SLC7A14	Body	-1.351517	-0.75506	-6.276139	4.53E-09	3.74E-07	10.420115
cg11823159	chr2	128611859	-	POLR2D	Body	0.9713887	1.1341248	6.2757135	4.54E-09	3.75E-07	10.418084

cg13411015	chr14	57274900	-	OTX2	5'UTR	1.7537339	-1.564376	6.275411	4.55E-09	3.75E-07	10.416642
cg11471772	chr6	100911687	+	SIM1	TSS200	1.8942148	-0.757533	6.2884435	4.56E-09	3.76E-07	10.428171
cg06905901	chr19	14639450	-	MIR639;TECR	TSS1500;TSS1500	-1.302352	1.2613997	-6.274682	4.57E-09	3.76E-07	10.413164
cg20558612	chr7	19050135	-			-1.555021	0.330893	-6.274524	4.57E-09	3.76E-07	10.412414
cg07554011	chr11	537737	-	LRRC56	5'UTR	-0.756071	-1.275077	-6.274336	4.57E-09	3.77E-07	10.411514
cg13077031	chr6	15266782	-	JARID2	Body	1.1630835	1.1359316	6.2741405	4.58E-09	3.77E-07	10.410584
cg09305161	chr6	106551032	+	PRDM1;PRDM1	Body;Body	1.3780019	-0.275575	6.2739969	4.58E-09	3.77E-07	10.409899
cg17912835	chr2	105478804	-			1.5649576	0.3479865	6.2740272	4.58E-09	3.77E-07	10.410044
cg22103414	chr8	6638082	+			-1.279214	0.1385554	-6.274071	4.58E-09	3.77E-07	10.410253
cg22152605	chr19	52253373	-	FPR1	5'UTR	-1.325283	-0.548326	-6.27363	4.59E-09	3.78E-07	10.40815
cg25133016	chr12	57984719	+	PIP4K2C;PIP4K2C;PIP4K2C;PIP4K2C	TSS1500;TSS1500;TSS1500;TSS1500	-0.917944	-2.89576	-6.273658	4.59E-09	3.78E-07	10.408282
cg24949488	chr10	98064362	-	DNTT;DNTT	1stExon;1stExon	-1.389358	0.116691	-6.273404	4.60E-09	3.78E-07	10.407074
cg04562217	chr3	79815718	-	ROBO1	5'UTR	1.8270785	-1.200596	6.272772	4.61E-09	3.79E-07	10.404059
cg14326989	chr17	46799479	-	C17orf93;PRAC	TSS1500;Body	1.4134323	-0.093201	6.2723613	4.62E-09	3.80E-07	10.402101
cg13615402	chr15	25469586	-	SNORD115-15;SNORD115-21;SNORD115-30	Body;Body;TSS1500	-1.516616	0.1108998	-6.272091	4.63E-09	3.80E-07	10.400811
cg06765514	chr9	130689862	+	PIP5KL1;PIP5KL1	Body;TSS200	-0.639042	-4.11888	-6.272047	4.63E-09	3.80E-07	10.400601
cg24533720	chr7	6099233	-	EIF2AK1;EIF2AK1	TSS1500;TSS1500	-0.948483	-2.258308	-6.271853	4.63E-09	3.81E-07	10.399677
cg04416302	chr11	31143518	+			-1.6423	-0.242018	-6.271714	4.63E-09	3.81E-07	10.399018
cg14507337	chr14	60978161	+	SIX6	3'UTR	2.3014996	-0.609715	6.2717299	4.63E-09	3.81E-07	10.399092
cg01750611	chr17	27072015	-	TRAF4	Body	-0.571626	-3.703477	-6.271508	4.64E-09	3.81E-07	10.398033

cg06382559	chr10	102896640	-	TLX1	Body	1.4051307	-0.85533	6.2715686	4.64E-09	3.81E-07	10.398322
cg14830371	chr8	25991602	+			-1.269664	-3.17055	-6.271524	4.64E-09	3.81E-07	10.398108
cg19372975	chr2	242212901	-	HDLBP;HDLBP	5'UTR;TSS1500	1.92209	3.2569555	6.2712287	4.65E-09	3.81E-07	10.396702
cg00017489	chr7	153583318	-	DPP6	TSS1500	1.5060647	-0.41202	6.2710818	4.65E-09	3.81E-07	10.396002
cg05517233	chr5	135468041	+	SMAD5;SMAD5;SMAD5OS;SMAD5	TSS1500;TSS1500;Body;TSS1500	-1.025513	-2.621652	-6.270961	4.65E-09	3.82E-07	10.395426
cg06562888	chr5	148799955	+	LOC728264	Body	0.853706	0.4544989	6.2708641	4.65E-09	3.82E-07	10.394965
cg21851351	chr13	28368087	+	GSX1	3'UTR	2.0821033	-1.465704	6.2703638	4.67E-09	3.83E-07	10.39258
cg19488093	chr7	134576255	+	CALD1;CALD1;CALD1;CALD1;CALD1;CALD1;CALD1	Body;1stExon;5'UTR;1stExon;5'UTR;Body;Body	-1.321592	-2.627355	-6.270212	4.67E-09	3.83E-07	10.391857
cg00720839	chr6	32800486	+	TAP2;TAP2	Body;Body	1.7604052	3.1939918	6.2802188	4.67E-09	3.83E-07	10.395452
cg17080423	chr4	81189774	+	FGF5;FGF5	Body;Body	1.2748869	-1.18224	6.2694289	4.69E-09	3.84E-07	10.388124
cg01229452	chr17	7350282	-	CHRNB1	Body	0.9563828	-0.975121	6.2692493	4.69E-09	3.84E-07	10.387268
cg11971662	chr8	99499993	-	STK3	Body	0.8735492	1.612489	6.2692508	4.69E-09	3.84E-07	10.387275
cg21188820	chr3	27765546	+			1.669412	-1.507349	6.2692526	4.69E-09	3.84E-07	10.387284
cg18181201	chr1	242686709	-	PLD5	5'UTR	2.0631523	-0.944035	6.269028	4.70E-09	3.85E-07	10.386213
cg21220965	chr15	25442571	+			-1.026559	-0.408207	-6.268983	4.70E-09	3.85E-07	10.385997
cg26381313	chr10	102279455	+	SEC31B	5'UTR	0.8877544	-0.739604	6.268874	4.70E-09	3.85E-07	10.385479
cg26697065	chr16	30456379	+	SEPHS2	1stExon	-1.834983	0.6016471	-6.268312	4.71E-09	3.86E-07	10.382802
cg10213353	chr20	60860905	+	OSBPL2;OSBPL2	Body;Body	0.5381624	-0.080459	6.2682217	4.71E-09	3.86E-07	10.382371
cg13447099	chr1	21606051	-	ECE1;ECE1;ECE1;ECE1;ECE1	Body;Body;5'UTR;Body;1stExon	1.1019452	1.4295148	6.267882	4.72E-09	3.86E-07	10.380752
cg10164393	chr6	119032202	+			-1.581142	0.121142	-6.267803	4.72E-09	3.87E-07	10.380377

cg15067015	chr1	175671657	+	TNR	5'UTR	-1.440392	-0.794588	-6.266223	4.76E-09	3.90E-07	10.372845
cg22164238	chr1	110162488	+	AMPD2;AMPD2;AMPD2	1stExon;TSS1500;5'UTR	-0.693125	-4.162276	-6.265659	4.78E-09	3.90E-07	10.370161
cg23786580	chr7	43204506	+	HECW1	5'UTR	-1.66958	-1.893337	-6.265644	4.78E-09	3.90E-07	10.370088
cg17518550	chr1	247712383	-	C1orf150	TSS200	-1.946959	-1.968226	-6.265404	4.78E-09	3.91E-07	10.368945
cg16422098	chr10	15761881	-	ITGA8	TSS200	2.7281322	-1.487738	6.2651589	4.79E-09	3.91E-07	10.367777
cg19335412	chr10	90694875	+	ACTA2;ACTA2	3'UTR;3'UTR	-1.163328	-1.131276	-6.264744	4.80E-09	3.92E-07	10.365801
cg09281979	chr2	172289850	+	DCAF17;METTL8;DCAF17;DCAF17	TSS1500;5'UTR;TSS1500;TSS1500	-1.163207	-1.427534	-6.263917	4.82E-09	3.93E-07	10.36186
cg19678828	chr21	27945413	-	CYYR1;CYYR1	5'UTR;1stExon	1.2357598	-0.875897	6.2639244	4.82E-09	3.93E-07	10.361896
cg15809077	chr2	112462079	-			-1.720332	1.5515478	-6.263814	4.82E-09	3.94E-07	10.361369
cg19870978	chr5	137673245	+	FAM53C;FAM53C;FAM53C	1stExon;5'UTR;TSS1500	-0.663116	-1.657911	-6.263472	4.83E-09	3.94E-07	10.359742
cg27243685	chr21	43642366	+	ABCG1;ABCG1;ABCG1;ABCG1;ABCG1;ABCG1	Body;Body;5'UTR;Body;Body;Body	1.0984113	1.5364262	6.2632153	4.83E-09	3.95E-07	10.358518
cg21254883	chr4	671543	-	MYL5	TSS200	-1.12453	-0.225708	-6.26253	4.85E-09	3.96E-07	10.355252
cg15699524	chr5	170845610	-	FGF18	TSS1500	-1.13804	-0.195384	-6.262216	4.86E-09	3.96E-07	10.353757
cg20416769	chr1	209824825	+	LAMB3;LAMB3;LAMB3	5'UTR;5'UTR;TSS200	-1.155481	-2.123581	-6.262238	4.86E-09	3.96E-07	10.353863
cg02745847	chr17	47075880	-	IGF2BP1;IGF2BP1	Body;Body	2.1092996	0.398979	6.2651858	4.86E-09	3.97E-07	10.353351
cg12062672	chr19	643555	+	FGF22	Body	-2.176328	1.7828711	-6.261844	4.87E-09	3.97E-07	10.351985
cg03049917	chr15	88752391	+	NTRK3;NTRK3;NTRK3	Body;Body;Body	-1.517726	-0.011392	-6.261199	4.88E-09	3.98E-07	10.348915
cg10082525	chr14	101490145	+	MIR380;MIR299	TSS1500;Body	-1.636371	0.9425088	-6.260943	4.89E-09	3.99E-07	10.347694
cg07345999	chr11	16632636	+			1.3801189	0.1094549	6.2608722	4.89E-09	3.99E-07	10.347358
cg15900540	chr7	73050757	+			-0.776014	-3.176177	-6.260399	4.90E-09	3.99E-07	10.345105

cg09578568	chr4	113432770	+			1.0343802	-0.5092	6.2635799	4.90E-09	4.00E-07	10.345713
cg02902946	chr20	57617168	+	SLMO2	Body	-1.412516	-0.885356	-6.259489	4.92E-09	4.01E-07	10.340772
cg03714619	chr2	45160445	-			2.3610774	-0.616652	6.2591312	4.93E-09	4.02E-07	10.339067
cg12597983	chr17	59531696	+			1.49113	-1.733135	6.2589962	4.94E-09	4.02E-07	10.338425
cg17240760	chr1	111417994	-	CD53;CD53	5'UTR;5'UTR	-1.102812	-1.805541	-6.259011	4.94E-09	4.02E-07	10.338496
cg00088183	chr1	91185168	-			1.5223154	-0.786802	6.2586373	4.94E-09	4.03E-07	10.336716
cg01937809	chr1	37941854	-	ZC3H12A	Body	-1.317194	-1.411462	-6.258484	4.95E-09	4.03E-07	10.335984
cg02397479	chr13	99631309	+	DOCK9;DOCK9;DOCK9;DOCK9	Body;TSS1500;TSS1500;Body	0.9951949	2.2449775	6.2616603	4.95E-09	4.03E-07	10.336586
cg05828624	chr2	79347699	+	REG1A	5'UTR	-1.813777	0.6025894	-6.258317	4.95E-09	4.03E-07	10.33519
cg02184281	chr18	46447797	-	SMAD7	Body	0.8077754	1.4377749	6.2581803	4.96E-09	4.03E-07	10.33454
cg14482684	chr18	30051176	-	FAM59A	TSS1500	-1.383995	-5.361284	-6.26469	4.96E-09	4.03E-07	10.336497
cg18947553	chr1	227747468	-			1.3803502	1.2240529	6.2581045	4.96E-09	4.03E-07	10.334179
cg19241878	chr17	48846189	-	C17orf73	TSS1500	-1.467586	-0.697555	-6.258092	4.96E-09	4.03E-07	10.334121
cg02391713	chr6	105400985	-			2.3496554	0.8381491	6.2580341	4.96E-09	4.03E-07	10.333843
cg00650641	chr7	27127752	-			0.9094983	0.5628051	6.2577927	4.96E-09	4.04E-07	10.332694
cg06112335	chr7	119694495	+			-1.638728	1.1014935	-6.25762	4.97E-09	4.04E-07	10.331872
cg24368848	chr19	58545160	+	ZSCAN1	TSS1500	2.7452275	-0.392654	6.2571683	4.98E-09	4.05E-07	10.329722
cg11592230	chr2	26981306	-			-1.254583	0.1393862	-6.256948	4.99E-09	4.05E-07	10.328674
cg18005337	chr2	98340655	+	ZAP70	Body	1.5300423	2.4160228	6.2569184	4.99E-09	4.05E-07	10.328531
cg22175873	chr5	140801482	-	PCDHGA4;PCDHGA9;PCDHGA1;PCDHGB1;PCDHGB6;PCDHGB3;PCDHGA11;PCDHGB7;PCDHGA6;PCDHGA8;PCDHGA11;PCDHGA10;PCDHGA5;PCDHGB4;PCDHGA3;PCDHGA11;PCDHGA	Body;Body;Body;Body;Body;Body;1stExon;Body;Body;Body;1stExon;Body;Body;Body;Body;1stExon;Body;Body;Body;Body	1.1773801	-0.308212	6.2569105	4.99E-09	4.05E-07	10.328494

cg02345417	chr11	111631732	-	PPP2R1B;PPP2R1B	Body;Body	0.4945407	1.6602076	6.2564205	5.00E-09	4.06E-07	10.326161
cg09962925	chr16	86228639	+			2.1799534	3.2727432	6.2564264	5.00E-09	4.06E-07	10.32619
cg24465935	chr6	153938192	+			-2.021302	-0.178278	-6.256292	5.00E-09	4.06E-07	10.325549
cg04481096	chr7	27142100	-	HOXA2	1stExon	1.9067256	-0.319789	6.2561723	5.00E-09	4.06E-07	10.32498
cg16413030	chr2	176937704	-			2.0937619	-1.696971	6.2561458	5.01E-09	4.06E-07	10.324854
cg13840445	chr6	53530628	-	KLHL31	TSS200	1.5077713	1.084472	6.2557531	5.02E-09	4.07E-07	10.322984
cg13453288	chr1	53166996	+			-0.384473	0.6714158	-6.255541	5.02E-09	4.07E-07	10.321976
cg23546343	chr14	101347284	-	RTL1;MIR431;MIR433	1stExon;TSS200;TSS1500	-1.636149	0.5991007	-6.255311	5.03E-09	4.08E-07	10.32088
cg18081515	chr14	104630064	+	KIF26A	Body	-1.204649	0.0306808	-6.255101	5.03E-09	4.08E-07	10.319881
cg12455309	chr6	582493	+	EXOC2	Body	0.8999771	2.0277167	6.254907	5.04E-09	4.08E-07	10.318956
cg18903398	chr4	64214697	-			-1.072965	0.023278	-6.258158	5.04E-09	4.08E-07	10.319938
cg00702719	chr6	74025019	+			1.2225299	0.2820793	6.2548221	5.04E-09	4.08E-07	10.318552
cg15099397	chr13	110761577	+			-1.560801	0.6117318	-6.254346	5.05E-09	4.09E-07	10.316285
cg22512438	chr3	129693489	+	TRH;TRH	5'UTR;1stExon	3.1017971	-2.027765	6.2539637	5.06E-09	4.10E-07	10.314467
cg03822198	chr12	116997535	-	MAP1LC3B2	5'UTR	-1.39063	-1.804763	-6.253892	5.06E-09	4.10E-07	10.314123
cg09778596	chr2	162282922	-			1.2556989	0.8302453	6.2534877	5.07E-09	4.11E-07	10.312201
cg15837212	chr1	63795970	-			1.4278198	-0.867067	6.2565545	5.08E-09	4.11E-07	10.312556
cg13604166	chr3	133811326	+			1.2269885	0.7820558	6.2529797	5.08E-09	4.11E-07	10.309783
cg01750157	chr17	72426840	+	GPRC5C	TSS1500	-0.919714	-3.105732	-6.252584	5.09E-09	4.12E-07	10.307901
cg10993460	chr12	7276482	-	RBP5	3'UTR	0.8513043	1.311016	6.2525221	5.10E-09	4.12E-07	10.307606

cg21500354	chr11	6518331	+	DNHD1;DNHD1	TSS200;TSS200	0.9855522	1.4669872	6.2523536	5.10E-09	4.13E-07	10.306803
cg08862366	chr16	86152925	-			-1.450442	-0.20022	-6.252227	5.10E-09	4.13E-07	10.306199
cg04079301	chr3	147106635	+	ZIC4;ZIC4;ZIC4;ZIC4;ZIC4	3'UTR;Body;3'UTR;Body;3'UTR	1.3863527	-1.253088	6.252042	5.11E-09	4.13E-07	10.305321
cg15547672	chr17	78768940	-	RPTOR;RPTOR	Body;Body	0.7114292	2.0408672	6.2515891	5.12E-09	4.14E-07	10.303165
cg04895166	chr12	133010900	+			-1.183551	-0.189896	-6.251184	5.13E-09	4.15E-07	10.301237
cg01737026	chr6	101847656	+	GRIK2;GRIK2;GRIK2	Body;Body;Body	1.2464973	-0.617312	6.2507884	5.14E-09	4.15E-07	10.299355
cg13408605	chr20	1639816	-	SIRPG;SIRPG;SIRPG	TSS1500;TSS1500;TSS1500	-1.502161	-0.388704	-6.250692	5.14E-09	4.15E-07	10.298897
cg02265056	chr19	49016928	+	LMTK3	TSS1500	-0.424788	-3.943525	-6.250354	5.15E-09	4.16E-07	10.297287
cg01348086	chr5	176785296	-	RGS14	Body	-1.154779	-1.013245	-6.250176	5.16E-09	4.16E-07	10.29644
cg25314624	chr10	95256917	+	CEP55;CEP55	5'UTR;5'UTR	-1.589762	0.5166543	-6.250168	5.16E-09	4.16E-07	10.296403
cg16068263	chr13	36729231	+			2.0729018	-0.74843	6.2500568	5.16E-09	4.16E-07	10.295874
cg14631438	chr11	128732986	+	KCNJ1;KCNJ1;KCNJ1;KCNJ1	5'UTR;5'UTR;5'UTR;5'UTR	-1.687792	0.0824208	-6.249921	5.16E-09	4.17E-07	10.29523
cg05711235	chr8	65498612	+			1.5293304	0.1208911	6.2494728	5.17E-09	4.17E-07	10.293096
cg10279535	chr2	129250911	+			2.0647608	4.824691	6.2524879	5.18E-09	4.18E-07	10.292991
cg22924840	chr2	127646404	-			-1.338881	0.1545676	-6.249149	5.18E-09	4.18E-07	10.291553
cg12630983	chr5	127872200	+	FBN2	Body	1.1723112	2.344655	6.249106	5.18E-09	4.18E-07	10.29135
cg16384355	chr7	157320516	+			-2.25776	1.2056558	-6.24901	5.19E-09	4.18E-07	10.290895
cg00709979	chr16	85866348	-			-2.294777	1.7727929	-6.248879	5.19E-09	4.18E-07	10.290271
cg09978734	chr17	74330308	+	PRPSAP1	Body	0.9193583	2.1503214	6.248913	5.19E-09	4.18E-07	10.290432
cg13555050	chr4	111985640	+			-1.399086	0.2052912	-6.248623	5.20E-09	4.19E-07	10.28905

cg10730165	chr7	142364023	+			-2.294582	1.133192	-6.2485	5.20E-09	4.19E-07	10.288466
cg18687085	chr18	56067462	+	NEDD4L;NEDD4L;NEDD4L;NEDD4L;NEDD4L;NEDD4L;NEDD4L;NEDD4L;NEDD4L	3'UTR;3'UTR;3'UTR;3'UTR;3'UTR;3'UTR;3'UTR;3'UTR;3'UTR	-1.237772	0.0194492	-6.248404	5.20E-09	4.19E-07	10.288012
cg00840341	chr16	17609453	+			-1.971702	0.3403245	-6.248321	5.20E-09	4.19E-07	10.287615
cg01832036	chr17	48050274	-	DLX4;DLX4;DLX4	Body;5'UTR;1stExon	2.6460933	-1.032354	6.2480821	5.21E-09	4.20E-07	10.286479
cg10276869	chr16	85336004	-			-1.290393	-0.143169	-6.247883	5.21E-09	4.20E-07	10.285534
cg00311883	chr1	235099120	+			-2.664274	-3.189037	-6.25108	5.22E-09	4.20E-07	10.286304
cg13402865	chr1	206671408	-			0.8507887	2.8101899	6.2477812	5.22E-09	4.20E-07	10.285048
cg15484532	chr1	4714171	+	AJAP1;AJAP1	TSS1500;TSS1500	1.9170904	-1.000124	6.2476217	5.22E-09	4.20E-07	10.284289
cg00748911	chr10	44450977	+			-1.716208	0.5825232	-6.247465	5.23E-09	4.20E-07	10.283545
cg05098512	chr10	134861083	+			-1.264157	-0.044132	-6.246747	5.24E-09	4.22E-07	10.280127
cg16530437	chr17	53315783	+			-1.511022	-4.830389	-6.246499	5.25E-09	4.22E-07	10.278949
cg26140475	chr8	126525558	-			-1.61648	-2.759285	-6.246445	5.25E-09	4.22E-07	10.278691
cg24272559	chr1	166572716	+	FMO9P	TSS1500	-2.079624	0.6636256	-6.246398	5.25E-09	4.22E-07	10.27847
cg25668093	chr15	95399279	+			-1.615459	0.1548828	-6.246318	5.26E-09	4.22E-07	10.27809
cg24414363	chr22	42336273	-	CENPM;CENPM;CENPM	TSS200;Body;Body	-1.524306	-0.465037	-6.246271	5.26E-09	4.22E-07	10.277862
cg19672873	chr1	222910534	+	FAM177B	TSS200	-1.055304	-0.96106	-6.24605	5.26E-09	4.23E-07	10.276814
cg00377916	chr20	30008986	-	DEFB122	TSS1500	-0.925727	-0.907754	-6.245592	5.27E-09	4.24E-07	10.274633
cg20627174	chr2	119599744	+			2.0207053	-1.377662	6.245483	5.28E-09	4.24E-07	10.274116
cg26816907	chr1	197890812	-	LHX9;LHX9	Body;Body	1.1579789	0.3551493	6.2454743	5.28E-09	4.24E-07	10.274075
cg19861741	chr5	170742608	-			2.2302272	-1.515347	6.2453727	5.28E-09	4.24E-07	10.273592

cg24519413	chr1	26490540	+			-1.073182	-2.631034	-6.245215	5.28E-09	4.24E-07	10.272841
cg20127496	chr8	126676825	-			-1.704439	0.756721	-6.244991	5.29E-09	4.25E-07	10.271777
cg13226597	chr7	6412718	+	RAC1;RAC1	TSS1500;TSS1500	-1.549839	1.4255309	-6.244942	5.29E-09	4.25E-07	10.271543
cg21670717	chr13	30733388	+			-1.407204	-0.014878	-6.244914	5.29E-09	4.25E-07	10.271412
cg13418839	chr17	37798877	-	STARD3;STARD3;STARD3	5'UTR;5'UTR;5'UTR	1.1955245	2.2058603	6.2447609	5.30E-09	4.25E-07	10.270682
cg18090390	chr1	56880606	-			-1.37451	0.2357469	-6.244427	5.30E-09	4.25E-07	10.269092
cg19038027	chr10	134306852	+			-1.17195	-0.307768	-6.247239	5.32E-09	4.26E-07	10.268059
cg07000713	chr12	62463441	-	FAM19A2	5'UTR	-1.784559	-1.021284	-6.243454	5.33E-09	4.27E-07	10.264466
cg08520746	chr2	172945932	+			1.1343456	-0.58605	6.243282	5.33E-09	4.28E-07	10.263649
cg19363785	chr8	95092340	+			-1.981343	0.1888065	-6.243273	5.33E-09	4.28E-07	10.263605
cg15949277	chr1	37939724	-	ZC3H12A	TSS1500	-0.610939	-4.034955	-6.243092	5.34E-09	4.28E-07	10.262748
cg13420089	chr9	92253441	+	LOC100129066	TSS1500	-0.652083	-0.892256	-6.242633	5.35E-09	4.29E-07	10.260564
cg22724854	chr11	55737072	-	OR10AG1	TSS1500	-1.900567	0.0634149	-6.245231	5.37E-09	4.30E-07	10.258525
cg23619936	chr2	866258	-			-1.230229	-1.220014	-6.241624	5.38E-09	4.31E-07	10.255767
cg18904380	chr10	2544596	-			-1.418333	0.2567539	-6.240818	5.40E-09	4.32E-07	10.251935
cg19652416	chr10	134697862	+			-1.035632	-0.824825	-6.240855	5.40E-09	4.32E-07	10.252107
cg05904366	chr11	442072	+	ANO9	TSS200	-0.935098	-4.300075	-6.243685	5.41E-09	4.33E-07	10.251427
cg02773601	chr12	57623994	-	SHMT2;SHMT2;SHMT2;SHMT2;SHMT2;SHMT2;SHMT2;SHMT2;SHMT2	5'UTR;Body;1stExon;TSS200;Body;TSS200;Body;TSS200;Body	-0.408261	-4.172712	-6.240131	5.42E-09	4.34E-07	10.248666
cg22396119	chr8	122419020	+			-1.69286	-0.907693	-6.240056	5.42E-09	4.34E-07	10.248309
cg09550909	chr3	36949957	-	TRANK1	Body	-1.222077	-4.66623	-6.239876	5.43E-09	4.34E-07	10.247453

cg09535924	chr2	66671659	+	MEIS1	Body	1.2793963	0.3803148	6.2396447	5.43E-09	4.34E-07	10.246355
cg11268834	chr2	224824500	-	MRPL44	Body	1.7900254	-0.268717	6.2394664	5.44E-09	4.35E-07	10.245507
cg08122831	chr2	213697579	+			0.8422244	1.5170948	6.242347	5.45E-09	4.35E-07	10.245077
cg26467949	chr2	73441162	-	SMYD5	TSS1500	-1.153283	-1.497266	-6.238993	5.45E-09	4.36E-07	10.243259
cg06840491	chr10	48416966	-	GDF2	TSS200	1.134461	0.7184515	6.2420379	5.45E-09	4.36E-07	10.243366
cg14200127	chr1	97604182	+	DPYD	Body	-1.470635	-1.207761	-6.238419	5.46E-09	4.37E-07	10.240528
cg08330217	chr20	57982969	+			-1.637081	-0.341491	-6.237639	5.49E-09	4.38E-07	10.236823
cg16723381	chr2	127590178	-			-1.232621	-1.179472	-6.237321	5.49E-09	4.39E-07	10.235309
cg04496906	chr8	145101182	+	SPATC1;SPATC1	Body;Body	-1.020627	-1.778981	-6.240492	5.50E-09	4.39E-07	10.236028
cg18568589	chr19	9609395	-	ZNF560	TSS200	1.3618156	-0.103163	6.2370801	5.50E-09	4.39E-07	10.234165
cg05392323	chr1	23698143	+	C1orf213;C1orf213;C1orf213	3'UTR;1stExon;3'UTR	1.0744682	1.1601655	6.2369163	5.51E-09	4.39E-07	10.233386
cg19220586	chr1	167522470	-	CREG1	Body	-0.807911	-3.872492	-6.235566	5.54E-09	4.42E-07	10.226968
cg16729283	chr11	134681766	+			-2.050251	0.3643239	-6.235383	5.55E-09	4.43E-07	10.2261
cg25482755	chr2	242759635	+			-1.072469	-1.17483	-6.241918	5.55E-09	4.43E-07	10.228488
cg13719746	chr9	100747559	-	ANP32B	Body	-1.039911	-4.695165	-6.235306	5.55E-09	4.43E-07	10.225735
cg05653707	chr10	119304938	-	EMX2;EMX2;EMX2OS	Body;Body;TSS1500	2.0792096	-0.826646	6.2350007	5.56E-09	4.43E-07	10.224283
cg13333954	chr12	1098663	+			1.3183836	0.0907031	6.2347334	5.56E-09	4.44E-07	10.223013
cg00636809	chr10	64577607	-	EGR2;EGR2;EGR2;EGR2	5'UTR;TSS1500;TSS1500;TSS1500	-0.503382	-3.129501	-6.233044	5.61E-09	4.47E-07	10.214986
cg06666008	chr11	31819464	-	PAX6;PAX6;PAX6	Body;Body;Body	1.9949913	-1.293902	6.2329295	5.61E-09	4.48E-07	10.214442
cg02749105	chr19	58879056	-	ZNF837;ZNF837	3'UTR;3'UTR	1.1521041	1.2244909	6.2328097	5.62E-09	4.48E-07	10.213873

cg03704503	chr10	76994576	+	COMTD1	Body	-0.69605	-3.606615	-6.232692	5.62E-09	4.48E-07	10.213315
cg20810478	chr1	248020632	-	TRIM58	1stExon	2.6593929	-0.81214	6.2325518	5.63E-09	4.48E-07	10.212647
cg10603004	chr8	23564294	+	NKX2-6	TSS1500	1.6312527	-0.989107	6.2324605	5.63E-09	4.48E-07	10.212214
cg02462868	chr17	46712052	-			1.1468085	1.0583182	6.2315089	5.65E-09	4.50E-07	10.207693
cg05157251	chr18	57566460	-	PMAIP1	TSS1500	-1.134667	-3.724783	-6.231176	5.66E-09	4.51E-07	10.206111
cg00939432	chr22	29977677	+	NIPSNAP1	TSS1500	-1.421849	-0.193397	-6.230806	5.67E-09	4.52E-07	10.204355
cg22407942	chr7	22894828	+	SNORD93	TSS1500	-1.578045	-1.335456	-6.230682	5.68E-09	4.52E-07	10.203764
cg08873616	chr16	4526240	+	HMOX2;HMOX2;HMOX2;NMRAL1	TSS200;TSS200;5'UTR;TSS1500	-0.428251	-4.272501	-6.230484	5.68E-09	4.52E-07	10.202827
cg14974749	chr7	27181480	-	HOXA5	Body	1.650692	1.2556307	6.233668	5.68E-09	4.52E-07	10.203651
cg02054457	chr1	175683971	-	TNR	5'UTR	-1.474016	0.1629216	-6.23033	5.69E-09	4.52E-07	10.202091
cg27407147	chr15	89920824	-	LOC254559	TSS1500	1.7178739	0.2932307	6.2299956	5.70E-09	4.53E-07	10.200504
cg12506013	chr19	14991752	-	OR7A17	1stExon	-0.782201	-1.730201	-6.229296	5.72E-09	4.55E-07	10.197182
cg08614301	chr19	19729716	-	PBX4	TSS1500	-0.942641	-3.09839	-6.229229	5.72E-09	4.55E-07	10.196864
cg17774559	chr5	1879698	+	IRX4	Body	2.2501387	0.2314783	6.2292158	5.72E-09	4.55E-07	10.196801
cg08214029	chr17	34391478	-	CCL18	TSS200	-1.013815	-1.307013	-6.229133	5.72E-09	4.55E-07	10.196409
cg08180884	chr19	30939155	+	ZNF536	Body	-1.690933	0.5328408	-6.228765	5.73E-09	4.55E-07	10.194659
cg05477444	chr10	134527851	+	INPP5A	Body	0.8502977	1.3648438	6.2286757	5.73E-09	4.56E-07	10.194236
cg02689863	chr13	95357832	-			1.3785152	-0.424584	6.2285988	5.74E-09	4.56E-07	10.19387
cg03171770	chr10	43393728	-			1.626282	-0.186549	6.2282918	5.74E-09	4.56E-07	10.192412
cg05428701	chr11	129150329	-			-1.801392	-0.378775	-6.228242	5.75E-09	4.56E-07	10.192175

cg14270857	chr10	100996097	+	HPSE2;HPSE2;HPSE2;HPSE2	TSS1500;TSS1500;TSS1500;TSS1500	2.0904489	-1.765129	6.2282091	5.75E-09	4.56E-07	10.19202
cg00623161	chr8	1502766	+	DLGAP2	Body	-1.655072	0.9554837	-6.228056	5.75E-09	4.57E-07	10.191293
cg07880727	chr7	47846424	+	C7orf69;PKD1L1	Body;Body	1.0935223	0.3307334	6.2279096	5.76E-09	4.57E-07	10.190597
cg12377816	chr12	58758818	-			-1.123657	-2.21461	-6.227919	5.76E-09	4.57E-07	10.190643
cg19768898	chr19	56153481	+	ZNF580;ZNF580;ZNF580;ZNF580;ZNF580	1stExon;5'UTR;5'UTR;5'UTR;1stExon	-1.188029	-3.950684	-6.227894	5.76E-09	4.57E-07	10.190521
cg12389770	chr6	101847706	-	GRIK2;GRIK2;GRIK2	Body;Body;Body	1.4883242	-0.549391	6.2274511	5.77E-09	4.58E-07	10.18842
cg01921432	chr1	91182534	-	BARHL2	1stExon	1.7315694	-0.462434	6.2273098	5.77E-09	4.58E-07	10.187749
cg00185336	chr7	73153699	+	ABHD11;ABHD11;ABHD11;ABHD11;ABHD11;ABHD11;ABHD1<br>1	TSS1500;TSS1500;TSS1500;TSS1500;TSS1500;TSS15<br>00;TSS1500	-0.545951	-3.107232	-6.227171	5.78E-09	4.58E-07	10.187092
cg08474859	chr4	85414016	+			1.7579926	-0.554296	6.2271193	5.78E-09	4.58E-07	10.186844
cg01572891	chr7	4184354	-	SDK1;SDK1	Body;Body	1.7580628	-0.772176	6.2334471	5.78E-09	4.58E-07	10.188366
cg02619205	chr9	117157157	+	AKNA	TSS1500	0.9887273	-1.066031	6.2263767	5.80E-09	4.60E-07	10.183318
cg02631126	chr6	28058918	+	ZSCAN12L1	TSS200	1.3288884	0.74586	6.226218	5.80E-09	4.60E-07	10.182564
cg03694713	chr8	23564156	+	NKX2-6	TSS1500	1.9376691	-1.260921	6.2294156	5.81E-09	4.60E-07	10.183731
cg21840976	chr13	79181285	+			1.2081033	-0.683783	6.2358936	5.81E-09	4.60E-07	10.185773
cg17347178	chr5	140052701	-	DND1	Body	0.7916619	1.3364565	6.2321951	5.82E-09	4.61E-07	10.182439
cg26495526	chr16	49407968	-	C16orf78	1stExon	-1.461394	0.8022966	-6.225357	5.83E-09	4.61E-07	10.178475
cg02050917	chr1	2173571	+	SKI	Body	1.4261062	1.531361	6.2250776	5.84E-09	4.62E-07	10.17715
cg23980468	chr17	9479939	-	WDR16;WDR16;STX8	TSS200;TSS200;TSS1500	-2.286809	-4.020322	-6.225047	5.84E-09	4.62E-07	10.177006
cg11280964	chr17	1359946	-	CRK;CRK	TSS1500;TSS1500	-0.448616	-3.830237	-6.22493	5.84E-09	4.62E-07	10.17645
cg07700925	chr10	129540267	-			-1.419104	-0.521338	-6.224815	5.84E-09	4.62E-07	10.175902

cg01149264	chr16	644081	+	RAB40C	Body	-1.92179	1.3746896	-6.227786	5.85E-09	4.63E-07	10.176004
cg11063877	chr12	50793961	+	LARP4;LARP4;LARP4;LARP4;LARP4;LARP4;LARP4	TSS1500;TSS1500;TSS1500;TSS1500;TSS1500;TSS1500;TSS1500	-1.822367	0.8513947	-6.224534	5.85E-09	4.63E-07	10.174571
cg01257383	chr11	46408399	+	CHRM4	TSS1500	-0.957984	-1.022217	-6.22418	5.86E-09	4.64E-07	10.172889
cg10507988	chr2	46593271	-	EPAS1	Body	2.3521918	2.5109141	6.2240509	5.87E-09	4.64E-07	10.172275
cg03452625	chr3	11537620	+	ATG7;ATG7;ATG7	Body;Body;Body	1.9031089	1.1955243	6.2239186	5.87E-09	4.64E-07	10.171647
cg27008230	chr10	116286623	-	ABLIM1;ABLIM1;ABLIM1;ABLIM1;ABLIM1	Body;1stExon;Body;5'UTR;Body	-0.86641	-3.624624	-6.223411	5.89E-09	4.65E-07	10.169237
cg26407917	chr7	169721	+			-1.449738	0.7052756	-6.223254	5.89E-09	4.65E-07	10.168494
cg23908943	chr19	54213920	+	MIR524	TSS1500	-1.681181	0.5892332	-6.22259	5.91E-09	4.67E-07	10.165341
cg17886399	chr3	62362660	+			1.1445221	2.0856494	6.2224968	5.91E-09	4.67E-07	10.164898
cg22212237	chr12	49730055	-	C1QL4	1stExon	1.4732113	-0.90101	6.2219728	5.93E-09	4.68E-07	10.162411
cg22384356	chr8	124195192	-	FAM83A;FAM83A	1stExon;1stExon	-1.062063	-0.354968	-6.221983	5.93E-09	4.68E-07	10.162458
cg01272668	chr14	100091498	+			-1.384417	0.8745611	-6.221735	5.93E-09	4.68E-07	10.161281
cg13606889	chr11	121335457	-	SORL1	Body	-1.447098	-1.753647	-6.221399	5.94E-09	4.69E-07	10.159686
cg16312954	chr7	142632296	+	TRPV5	TSS1500	-1.637618	0.3428597	-6.221029	5.95E-09	4.70E-07	10.157929
cg17030173	chr5	16180062	-	11-Mar	TSS200	2.6309509	-0.526075	6.2209188	5.96E-09	4.70E-07	10.157408
cg14841601	chr4	124340166	+			-1.630838	-0.123635	-6.220458	5.97E-09	4.71E-07	10.155219
cg01656244	chr8	65284080	+			1.5641304	-0.902969	6.2200762	5.98E-09	4.72E-07	10.153409
cg00575153	chr8	145047469	-	PLEC1;PLEC1	Body;Body	-1.20466	-1.652327	-6.219695	5.99E-09	4.73E-07	10.151599
cg27645228	chr2	74663698	+	RTKN;RTKN;RTKN	Body;5'UTR;Body	0.8450804	1.1762343	6.2194341	6.00E-09	4.73E-07	10.150362
cg15887709	chr7	1270976	-			1.0980826	-1.214446	6.2191749	6.01E-09	4.74E-07	10.149132

cg17916432	chr2	208989209	-	CRYGD;CRYGD	1stExon;5'UTR	0.7985714	0.3166593	6.2223701	6.01E-09	4.74E-07	10.150335
cg04396112	chr7	2151007	-	MAD1L1;MAD1L1;MAD1L1	Body;Body;Body	1.366	1.7563134	6.2190054	6.01E-09	4.74E-07	10.148328
cg01013171	chr14	75716369	-			-1.517099	-0.9378	-6.218848	6.02E-09	4.74E-07	10.147579
cg02829743	chr6	105400884	+			1.7301038	-0.004866	6.2186702	6.02E-09	4.74E-07	10.146737
cg20345923	chr2	1983190	+	MYT1L	Body	-1.535412	0.2946005	-6.218696	6.02E-09	4.74E-07	10.14686
cg12338657	chr10	80384267	+			-1.316845	0.0719146	-6.218495	6.03E-09	4.75E-07	10.145907
cg04926881	chr7	29024378	+			-1.291932	-2.134307	-6.21767	6.05E-09	4.77E-07	10.141993
cg10340033	chr2	220071009	+	ZFAND2B	TSS1500	-0.922845	-2.952882	-6.217436	6.06E-09	4.77E-07	10.140879
cg09958065	chr10	12648526	-	CAMK1D;CAMK1D	Body;Body	-1.39429	-0.973062	-6.217277	6.07E-09	4.77E-07	10.140125
cg26920905	chr18	21718735	+	CABYR;CABYR;CABYR;CABYR;CABYR;CABYR	TSS1500;TSS1500;TSS1500;TSS1500;TSS1500;TSS1500	-1.154503	-3.930974	-6.220483	6.07E-09	4.77E-07	10.141393
cg11963912	chr17	72439044	+	GPRC5C;GPRC5C	Body;Body	-1.022849	-2.573508	-6.217213	6.07E-09	4.77E-07	10.139823
cg01954305	chr8	135817117	+			1.6462315	1.2533258	6.2165784	6.09E-09	4.79E-07	10.136812
cg03584506	chr11	39689828	+			-1.349325	-1.653753	-6.21637	6.09E-09	4.79E-07	10.135823
cg05057910	chr7	27260441	+			1.6070587	-0.391645	6.216276	6.10E-09	4.79E-07	10.135377
cg03690956	chr10	118900324	+			1.4244411	-0.370549	6.2161922	6.10E-09	4.79E-07	10.13498
cg22623133	chr7	27195960	+	HOXA7	1stExon	2.5586964	-1.585251	6.2227166	6.10E-09	4.79E-07	10.137582
cg23088126	chr1	207078912	-	FAIM3;FAIM3;FAIM3	Body;Body;Body	-1.476506	0.1848092	-6.215418	6.12E-09	4.81E-07	10.131305
cg20269185	chr3	70048926	-			-1.406485	0.9283416	-6.215328	6.12E-09	4.81E-07	10.130881
cg23696214	chr7	141136170	+			-1.236568	-0.810974	-6.215359	6.12E-09	4.81E-07	10.131027
cg15328703	chr12	126142896	+	TMEM132B	3'UTR	-1.600809	0.0715413	-6.215252	6.13E-09	4.81E-07	10.130519

cg22243000	chr17	37813369	-	STARD3;STARD3;STARD3	Body;Body;Body	0.7142179	1.6217663	6.2150799	6.13E-09	4.82E-07	10.129703
cg23897083	chr12	34755568	-			-1.074703	-0.404858	-6.214759	6.14E-09	4.82E-07	10.12818
cg19657174	chr22	19746268	-	TBX1;TBX1;TBX1	5'UTR;5'UTR;5'UTR	1.6436972	0.1814681	6.2144888	6.15E-09	4.83E-07	10.126899
cg19839325	chr19	18761132	-	KLHL26	Body	1.0865199	-0.253424	6.2140247	6.16E-09	4.84E-07	10.124698
cg00474840	chr19	18120819	+	ARRDC2;ARRDC2	Body;Body	1.7615945	4.5907536	6.2171031	6.17E-09	4.84E-07	10.125383
cg01352906	chr1	206679195	+			-1.078963	-0.672778	-6.213561	6.18E-09	4.85E-07	10.122498
cg22540040	chr12	68978235	-			-1.238585	-1.310678	-6.213146	6.19E-09	4.86E-07	10.12053
cg21072567	chr14	62035431	-			-1.402027	-1.269532	-6.216247	6.19E-09	4.86E-07	10.12108
cg05305099	chr16	57657514	-	GPR56;GPR56	5'UTR;5'UTR	-1.493834	0.3187096	-6.212951	6.20E-09	4.86E-07	10.119603
cg00664697	chr16	80061290	+			-1.352758	-2.412109	-6.212818	6.20E-09	4.86E-07	10.118974
cg07373723	chr7	110838461	-	IMMP2L	Body	-1.741083	0.1272219	-6.212651	6.21E-09	4.86E-07	10.118183
cg08946575	chr1	85931072	+	DDAH1;DDAH1	TSS200;5'UTR	-1.497343	-4.115376	-6.212462	6.21E-09	4.87E-07	10.117287
cg16326403	chr20	31123990	-			-0.828261	-3.552102	-6.21222	6.22E-09	4.87E-07	10.11614
cg02495395	chr3	50383368	-	ZMYND10	TSS1500	-0.844456	-1.820673	-6.212128	6.22E-09	4.87E-07	10.115701
cg11021940	chr5	115299475	-	LVRN	Body	0.9231912	-0.072754	6.2119615	6.23E-09	4.88E-07	10.114913
cg13305823	chr6	29454557	+	MAS1L	1stExon	-1.506016	-1.974074	-6.211173	6.25E-09	4.90E-07	10.111173
cg05910407	chr8	93584836	-			-1.658705	-0.025588	-6.210979	6.26E-09	4.90E-07	10.110253
cg07538946	chr5	131705188	-	SLC22A5	TSS1500	-1.085887	-4.687909	-6.210812	6.26E-09	4.90E-07	10.10946
cg03120185	chr6	100054118	+	PRDM13	TSS1500	1.5294721	-0.842677	6.2105873	6.27E-09	4.91E-07	10.108396
cg06588028	chr12	6745568	+	LPAR5	TSS1500	-1.120031	-0.897973	-6.210589	6.27E-09	4.91E-07	10.108404

cg04209913	chr5	37836246	-	GDNF;GDNF;GDNF	TSS1500;5'UTR;TSS1500	2.5863103	-0.177628	6.2105424	6.27E-09	4.91E-07	10.108183
cg07267845	chr7	157345566	-	PTPRN2;PTPRN2;PTPRN2	Body;Body;Body	-1.569411	-0.498351	-6.213657	6.27E-09	4.91E-07	10.109065
cg04281464	chr14	70014873	-			3.4595147	-1.6524	6.2104084	6.28E-09	4.91E-07	10.107548
cg00216961	chr12	6054731	+	ANO2	Body	-1.302489	-2.242518	-6.210026	6.29E-09	4.92E-07	10.105734
cg18916060	chr14	57266029	-			1.0596459	1.3945981	6.2097632	6.30E-09	4.92E-07	10.104488
cg23168531	chr7	1270601	+			1.7357503	-1.009123	6.2129714	6.30E-09	4.92E-07	10.105817
cg12417689	chr17	259426	+	C17orf97	TSS1500	-0.921591	0.2366382	-6.20939	6.31E-09	4.93E-07	10.10272
cg17480554	chr5	52334107	-	ITGA2	Body	-1.505314	0.3468243	-6.208983	6.32E-09	4.94E-07	10.100787
cg00589006	chr16	70415864	+	ST3GAL2	Body	-0.773899	-1.625204	-6.208936	6.32E-09	4.94E-07	10.100566
cg06275853	chr11	20617937	-			1.4692708	-0.18226	6.208636	6.33E-09	4.94E-07	10.099144
cg04731448	chr11	628628	-	SCT	TSS1500	-2.390732	0.3291589	-6.208335	6.34E-09	4.95E-07	10.097715
cg04286194	chr5	54519023	-			1.6419123	0.1001785	6.2114644	6.34E-09	4.95E-07	10.098433
cg05451842	chr14	93581139	+	ITPK1;ITPK1;ITPK1	Body;Body;Body	-0.59634	-3.686761	-6.208049	6.35E-09	4.96E-07	10.09636
cg01502872	chr6	33173119	+	HSD17B8	Body	-1.508759	-1.279966	-6.211121	6.35E-09	4.96E-07	10.096809
cg16908824	chr19	54260952	-	MIR1283-2	TSS1500	-1.646542	-0.163714	-6.207106	6.38E-09	4.98E-07	10.091892
cg22164891	chr20	52199729	+	ZNF217	TSS200	-2.15488	-2.775056	-6.207008	6.38E-09	4.98E-07	10.091425
cg20536041	chr2	45029004	-			2.0654653	-1.115876	6.206653	6.39E-09	4.99E-07	10.089743
cg25740640	chr13	45961680	+	LOC100190939	Body	1.0478623	1.5844923	6.2064346	6.40E-09	4.99E-07	10.088708
cg18297736	chr4	85416865	+	NKX6-1	Body	1.3572341	-1.523513	6.2063369	6.40E-09	4.99E-07	10.088245
cg06812574	chr10	18331664	+	SLC39A12;SLC39A12	Body;Body	-1.224311	-1.778309	-6.20566	6.42E-09	5.01E-07	10.085036

cg07160992	chr14	105244271	+	AKT1;AKT1;AKT1	Body;Body;Body	0.5883658	2.7827412	6.2053664	6.43E-09	5.02E-07	10.083645
cg11579116	chr19	6990919	-	EMR4P	TSS200	-0.96853	-0.75332	-6.20505	6.44E-09	5.02E-07	10.082145
cg20646120	chr20	60982720	-	CABLES2	TSS1500	-0.803668	-2.927603	-6.205018	6.44E-09	5.02E-07	10.081992
cg19597382	chr4	147559423	-	POU4F2	TSS1500	2.5719443	-1.418158	6.2045997	6.46E-09	5.03E-07	10.080011
cg07850274	chr4	110748770	+	RRH	TSS1500	0.795699	2.4019747	6.2045353	6.46E-09	5.03E-07	10.079706
cg18788940	chr11	20385085	-	HTATIP2;HTATIP2;HTATIP2;HTATIP2;HTATIP2	TSS1500;TSS200;TSS1500;TSS200;TSS1500	-1.098987	-1.171527	-6.204033	6.48E-09	5.04E-07	10.077326
cg02551743	chr2	66673428	-	MEIS1	Body	1.2209284	-0.322479	6.2039289	6.48E-09	5.05E-07	10.076832
cg21367256	chr20	62795658	+	MYT1	TSS200	-1.699015	-0.313904	-6.203808	6.48E-09	5.05E-07	10.076259
cg23903597	chr17	61704154	+	MAP3K3;MAP3K3	Body;Body	1.2115318	0.8831964	6.2036989	6.49E-09	5.05E-07	10.075742
cg01812047	chr1	44482771	+	SLC6A9;SLC6A9;SLC6A9	Body;1stExon;1stExon	1.2528076	5.4275456	6.2163331	6.50E-09	5.06E-07	10.078935
cg04801402	chr6	46138912	+	ENPP5	TSS200	-0.85605	-3.275663	-6.203067	6.51E-09	5.06E-07	10.07275
cg06672160	chr5	1699896	-			-1.503557	-0.351124	-6.202515	6.52E-09	5.08E-07	10.070131
cg11438039	chr3	62145697	+	PTPRG	Body	1.2193069	1.3300474	6.2023384	6.53E-09	5.08E-07	10.069295
cg09798801	chr4	137277541	-			-1.773459	-0.269053	-6.201949	6.54E-09	5.09E-07	10.067449
cg01054938	chr17	48712923	-	ABCC3;ABCC3	Body;Body	-1.467261	0.2740732	-6.201793	6.55E-09	5.09E-07	10.066709
cg03996195	chr18	55095229	+			1.5918194	-1.702898	6.2018023	6.55E-09	5.09E-07	10.066755
cg17282145	chr1	231005552	+	C1orf198;C1orf198	TSS1500;TSS1500	1.1850967	1.4817908	6.2016919	6.55E-09	5.09E-07	10.066232
cg19878838	chr4	77135506	-	SCARB2	TSS1500	-0.719527	-3.111375	-6.201493	6.56E-09	5.10E-07	10.065288
cg05908105	chr10	5489767	+	NET1;NET1	Body;Body	-1.418327	-2.906662	-6.204457	6.56E-09	5.10E-07	10.06527
cg11699517	chr17	79419796	+	BAHCC1	Body	-2.42189	2.9533096	-6.201091	6.57E-09	5.10E-07	10.063385

cg10384725	chr11	63035103	+			-1.842478	-0.483014	-6.200961	6.57E-09	5.11E-07	10.062768
cg16506566	chr5	10420680	+	6-Mar	Body	0.8639466	2.1013219	6.2005625	6.59E-09	5.12E-07	10.06088
cg19520234	chr4	141391053	-			1.1208946	0.4069275	6.2005549	6.59E-09	5.12E-07	10.060844
cg04317962	chr16	79623625	+			2.0639933	-0.053194	6.2004519	6.59E-09	5.12E-07	10.060357
cg05222982	chr13	28545214	+			1.658983	-0.464425	6.2003067	6.60E-09	5.12E-07	10.059669
cg03328485	chr16	62267020	+			-1.699389	0.8191053	-6.199785	6.61E-09	5.13E-07	10.057199
cg07706758	chr16	2133801	-	TSC2;TSC2;TSC2	Body;Body;Body	0.961578	4.3802514	6.1996265	6.62E-09	5.13E-07	10.056446
cg25863289	chr15	102193416	+	TM2D3;TM2D3	TSS1500;TSS1500	-2.568523	1.7745755	-6.202703	6.62E-09	5.14E-07	10.057219
cg26400113	chr6	138120219	+			-1.546382	0.6148776	-6.199189	6.63E-09	5.14E-07	10.054372
cg16065021	chr12	127940654	-			1.6357276	-0.379208	6.1991485	6.63E-09	5.14E-07	10.054182
cg07428782	chr17	31861540	-	ACCN1	Body	-1.322947	-1.180323	-6.198776	6.65E-09	5.15E-07	10.052419
cg19815017	chr10	44856047	+			-1.827217	0.9434834	-6.204817	6.66E-09	5.16E-07	10.053229
cg07528209	chr2	121549725	+			-1.771826	0.5036621	-6.198107	6.67E-09	5.17E-07	10.049248
cg05933997	chr16	86735122	+			-1.436638	1.164541	-6.197835	6.68E-09	5.17E-07	10.047958
cg01772854	chr5	1176225	-			-1.164453	0.0867893	-6.197391	6.69E-09	5.18E-07	10.045856
cg26128092	chr1	3567550	+	WDR8	TSS1500	1.551855	-0.919806	6.1973499	6.69E-09	5.18E-07	10.045662
cg11751344	chr8	92622592	+			-1.903136	-0.353073	-6.197057	6.70E-09	5.19E-07	10.044273
cg09427944	chr16	30579519	-			-1.730611	-1.948382	-6.195624	6.75E-09	5.23E-07	10.037487
cg14585371	chr16	51168816	-			0.8534546	-0.519575	6.1951883	6.76E-09	5.24E-07	10.035425
cg18330370	chr2	983636	+	SNTG2	Body	-1.607777	0.4714461	-6.194541	6.79E-09	5.25E-07	10.032361

cg08535978	chr3	152092595	+	MBNL1;MBNL1;MBNL1;MBNL1;MBNL1;MBNL1;MBNL1	Body;Body;Body;Body;Body;Body;Body	1.0956853	1.2635962	6.194496	6.79E-09	5.25E-07	10.032147
cg26741746	chr6	134871919	+			1.0295815	1.1659819	6.1944396	6.79E-09	5.25E-07	10.03188
cg20031845	chr7	50306663	+			-1.671908	-1.326297	-6.194337	6.79E-09	5.26E-07	10.031393
cg23089825	chr1	170630558	-			2.3950539	-0.753289	6.1972819	6.80E-09	5.26E-07	10.031583
cg26959384	chr19	57272621	-			-1.424168	-0.231577	-6.194072	6.80E-09	5.26E-07	10.030139
cg03441853	chr15	73075003	+	ADPGK;ADPGK;ADPGK	Body;Body;Body	-1.080101	-2.416296	-6.193846	6.81E-09	5.27E-07	10.029069
cg25591868	chr1	182573931	-	RGS16	TSS1500	-1.019774	-2.899205	-6.193644	6.82E-09	5.27E-07	10.028111
cg13534580	chr2	238662655	+	LRRFIP1;LRRFIP1;LRRFIP1;LRRFIP1;LRRFIP1	Body;Body;Body;Body;Body	0.8137583	2.7863933	6.1967668	6.82E-09	5.27E-07	10.028896
cg24233194	chr10	113526840	-			-1.178613	-0.541365	-6.193531	6.82E-09	5.27E-07	10.027577
cg02447239	chr2	235936137	+	SH3BP4	5'UTR	-1.134656	0.1871644	-6.193351	6.83E-09	5.27E-07	10.026726
cg19463518	chr19	634035	-	POLRMT	TSS1500	-0.906419	-2.742709	-6.199823	6.82E-09	5.27E-07	10.029389
cg26161878	chr3	8613388	+	LOH3CR2A	TSS200	0.8413461	2.3191348	6.193329	6.83E-09	5.27E-07	10.026621
cg24788028	chr19	30869034	-	ZNF536	5'UTR	-1.638954	0.0378845	-6.193165	6.83E-09	5.28E-07	10.025844
cg03564620	chr14	23878667	-	MYH6	TSS1500	-1.190226	-0.535671	-6.193019	6.84E-09	5.28E-07	10.025156
cg04389897	chr6	10398601	-	TFAP2A;TFAP2A;TFAP2A	3'UTR;3'UTR;3'UTR	2.0794496	-0.567221	6.1927663	6.85E-09	5.28E-07	10.023957
cg02240748	chr16	1057796	-			-0.771253	-0.859378	-6.192667	6.85E-09	5.29E-07	10.023485
cg00594408	chr6	29595349	+	GABBR1;GABBR1;GABBR1	Body;1stExon;Body	1.0675606	-1.086741	6.1922107	6.86E-09	5.29E-07	10.021327
cg00626856	chr5	1885950	+			1.6625335	-0.955584	6.192281	6.86E-09	5.29E-07	10.02166
cg10061927	chr14	88620324	-			-1.393085	0.5448596	-6.192253	6.86E-09	5.29E-07	10.021527
cg10512745	chr1	50884480	-	DMRTA2	Body	3.4421566	-0.839816	6.1922067	6.86E-09	5.29E-07	10.021308

cg15391794	chr7	141973451	-			-1.323218	-0.895292	-6.192257	6.86E-09	5.29E-07	10.021547
cg13400493	chr7	44826786	-			-1.400097	0.6830562	-6.192026	6.87E-09	5.30E-07	10.020454
cg01352175	chr18	44773985	+			1.3411609	-1.033153	6.191682	6.88E-09	5.31E-07	10.018824
cg14339750	chr3	52271331	-	TWF2	Body	-0.951618	-0.206023	-6.19153	6.89E-09	5.31E-07	10.018106
cg23348010	chr17	18908324	+	SLC5A10;SLC5A10;FAM83G	Body;Body;TSS1500	-0.885938	-1.155146	-6.191508	6.89E-09	5.31E-07	10.017999
cg03014326	chr18	14748285	-	ANKRD30B;ANKRD30B	1stExon;5'UTR	2.4346851	-0.464554	6.1978908	6.89E-09	5.31E-07	10.03136
cg07977202	chr11	66311348	-	ZDHHC24	Body	1.1171528	0.8918932	6.1913875	6.89E-09	5.31E-07	10.01743
cg25506432	chr7	27184065	-	HOXA5	TSS1500	1.4024342	0.5567616	6.1906199	6.92E-09	5.33E-07	10.013796
cg04746199	chr2	114034534	+	PAX8;PAX8;PAX8;PAX8;PAX8;LOC440839	Body;Body;Body;Body;Body;Body	1.1882489	-1.327341	6.1900795	6.94E-09	5.34E-07	10.011238
cg09102610	chr4	94427508	-	GRID2	Body	-1.519432	0.3425932	-6.190079	6.94E-09	5.34E-07	10.011234
cg22187125	chr19	58565865	+	ZSCAN1	3'UTR	-1.549222	0.1067884	-6.190119	6.94E-09	5.34E-07	10.011424
cg26361533	chr12	2445561	+	CACNA1C;CACNA1C;CACNA1C;CACNA1C;CACNA1C;CACNA1C;CACNA1C;CACNA1C;CACNA1C;CACNA1C;CACNA1C;CACNA1C;CACNA1C;CACNA1C;CACNA1C;CACNA1C;CACNA1C;CACNA	Body;Body;Body;Body;Body;Body;Body;Body;Body;Body;Body;Body;Body;Body;Body;Body;Body;Body;Body;Body;Body;Body;Body	-1.793386	-2.287363	-6.190062	6.94E-09	5.34E-07	10.011155
cg06002516	chr12	57666521	-	R3HDM2	Body	1.2820764	0.9711477	6.1897613	6.95E-09	5.35E-07	10.009732
cg06672560	chr20	54580175	-	CBLN4	TSS200	1.2855544	-1.448956	6.1897491	6.95E-09	5.35E-07	10.009674
cg23287510	chr8	144156462	+			-1.779803	-0.141141	-6.189214	6.97E-09	5.36E-07	10.007141
cg21743907	chr7	129423053	+			1.9806295	-0.058312	6.1891139	6.97E-09	5.36E-07	10.006668
cg12451099	chr20	37356466	+	SLC32A1	Body	1.4244783	-0.519577	6.1884312	6.99E-09	5.38E-07	10.003437
cg27480727	chr2	223164831	-	PAX3;PAX3;PAX3;PAX3;PAX3;CCDC140;PAX3;PAX3;PAX3	TSS1500;TSS1500;TSS1500;TSS1500;TSS1500;5'UTR;TSS1500;TSS1500;TSS1500	1.3865044	-0.391917	6.1880568	7.01E-09	5.39E-07	10.001665
cg07788092	chr19	9473691	+	ZNF177	TSS200	1.6948786	-0.660425	6.1877822	7.02E-09	5.39E-07	10.000365
cg04723343	chr2	238647913	+	LRRFIP1;LRRFIP1;LRRFIP1;LRRFIP1;LRRFIP1	Body;Body;Body;Body;Body	1.1283224	1.589456	6.1876413	7.02E-09	5.40E-07	9.9996984

cg12195273	chr17	20060038	-	CYTSB;CYTSB;CYTSB;CYTSB	Body;Body;Body;Body	-0.723557	-3.663597	-6.190345	7.04E-09	5.41E-07	9.9987956
cg15375488	chr2	4050845	+			-1.351363	0.3649552	-6.187053	7.04E-09	5.41E-07	9.9969134
cg11566154	chr6	80579376	-			-0.82302	-2.967723	-6.186251	7.07E-09	5.43E-07	9.9931212
cg13437554	chr19	2272716	+	OAZ1	Body	-1.341825	0.4762611	-6.185553	7.09E-09	5.45E-07	9.9898177
cg02164046	chr3	187388148	+	SST;SST	1stExon;5'UTR	2.0542607	-0.744656	6.1884103	7.10E-09	5.45E-07	9.989399
cg07933234	chr10	3441625	-			1.4824341	1.0579444	6.1852373	7.10E-09	5.45E-07	9.988323
cg18100753	chr8	1356513	+			-1.026223	-0.186844	-6.18522	7.10E-09	5.45E-07	9.9882435
cg12938556	chr13	114812918	-	RASA3	Body	-1.351787	0.666513	-6.185041	7.11E-09	5.46E-07	9.987393
cg18502477	chr11	57946199	+	OR9Q1	5'UTR	-1.75793	0.6699596	-6.185054	7.11E-09	5.46E-07	9.987454
cg10853063	chr12	120315422	+	CIT	TSS1500	-0.349462	-3.759562	-6.184797	7.12E-09	5.46E-07	9.9862388
cg00156001	chr22	50195025	+	BRD1	Body	0.9139824	2.3473492	6.1845324	7.13E-09	5.47E-07	9.984988
cg21621782	chr2	240168836	-	HDAC4	Body	-1.692475	0.5342065	-6.184417	7.13E-09	5.47E-07	9.984443
cg22444720	chr6	126240414	+	NCOA7;NCOA7	Body;Body	-0.605657	-4.049613	-6.184436	7.13E-09	5.47E-07	9.9845327
cg04466484	chr16	85497023	-			-0.776299	-1.468295	-6.184192	7.14E-09	5.47E-07	9.9833798
cg06800849	chr16	89180587	-	ACSF3;ACSF3;ACSF3	Body;Body;Body	-2.001109	2.4916975	-6.186338	7.18E-09	5.50E-07	9.9796082
cg11751806	chr12	43945680	+	ADAMTS20	1stExon	1.6877862	-1.524854	6.1828685	7.19E-09	5.51E-07	9.9771165
cg17342588	chr7	27737875	-			-1.482169	-0.709777	-6.182829	7.19E-09	5.51E-07	9.9769302
cg24381587	chr1	158736312	+	OR6N1	1stExon	-1.711586	-0.097807	-6.182737	7.19E-09	5.51E-07	9.9764961
cg01900006	chr16	88814613	+	FAM38A	Body	-1.877329	0.8962816	-6.18266	7.19E-09	5.51E-07	9.9761322
cg05165862	chr12	50030609	+	PRPF40B;PRPF40B	Body;Body	1.1898427	1.3606802	6.1825928	7.20E-09	5.51E-07	9.9758124

cg10966440	chr11	132745701	-	OPCML;OPCML	Body;Body	-1.696469	-0.441126	-6.182546	7.20E-09	5.51E-07	9.9755905
cg20087519	chr10	135379242	-	SYCE1;SYCE1;SYCE1	TSS200;TSS200;5'UTR	1.0708992	1.0429855	6.1825236	7.20E-09	5.51E-07	9.9754851
cg01567783	chr14	103686559	-			0.9663732	0.292337	6.1823465	7.21E-09	5.52E-07	9.9746475
cg14658493	chr7	27184077	-	HOXA5	TSS1500	1.6964682	0.5196878	6.1822895	7.21E-09	5.52E-07	9.9743778
cg16625119	chr16	86653176	-			-1.251101	0.0024673	-6.182114	7.21E-09	5.52E-07	9.9735462
cg11560197	chr5	140182956	-	PCDHA2;PCDHA1;PCDHA3;PCDHA3;PCDHA1	Body;Body;1stExon;1stExon;Body	0.9156856	0.2307658	6.1820206	7.22E-09	5.52E-07	9.9731059
cg13747435	chr9	130631782	+	AK1	Body	0.8586346	3.4159837	6.1819651	7.22E-09	5.52E-07	9.9728433
cg21307155	chr20	3216740	-	SLC4A11	Body	-1.119386	-0.089461	-6.181923	7.22E-09	5.52E-07	9.9726448
cg26130488	chr17	59530104	-			2.0086128	-0.182759	6.181754	7.23E-09	5.53E-07	9.9718451
cg26686150	chr12	93637466	-			-0.948993	-1.989374	-6.181693	7.23E-09	5.53E-07	9.9715573
cg26203839	chr8	142174631	-	DENND3	Body	1.1141416	0.4831335	6.1816066	7.23E-09	5.53E-07	9.9711477
cg20740711	chr2	113450279	+			-1.857051	-2.136762	-6.181261	7.24E-09	5.54E-07	9.9695132
cg27132732	chr15	32202752	-			-1.614658	-0.088062	-6.181213	7.25E-09	5.54E-07	9.9692868
cg09832443	chr7	28612680	+	CREB5;CREB5;CREB5	Body;Body;Body	-1.402584	-0.496919	-6.180887	7.26E-09	5.54E-07	9.9677444
cg16296417	chr8	126285443	+	NSMCE2	Body	-1.334971	-1.140847	-6.180607	7.27E-09	5.55E-07	9.9664188
cg03386765	chr11	118763674	+	CXCR5;CXCR5	Body;TSS1500	-0.990399	-2.81103	-6.180487	7.27E-09	5.55E-07	9.9658525
cg03005261	chr4	110480828	-	CCDC109B	TSS1500	-1.166979	-2.612073	-6.180288	7.28E-09	5.56E-07	9.9649124
cg20078624	chr15	22922301	+	CYFIP1	5'UTR	1.5814764	3.0814411	6.1802535	7.28E-09	5.56E-07	9.9647479
cg11273000	chr19	340378	-	MIER2	Body	0.847476	0.86806	6.180165	7.28E-09	5.56E-07	9.9643295
cg16001495	chr21	38080526	-	SIM2;SIM2	Body;Body	1.9975547	-1.250659	6.263912	7.28E-09	5.56E-07	9.9957364

cg02356566	chr10	2453060	+			-1.45899	-0.181615	-6.179996	7.29E-09	5.56E-07	9.9635281
cg04093645	chr5	127872037	-	FBN2	Body	1.1122907	1.6511589	6.1795376	7.31E-09	5.57E-07	9.9613628
cg15887459	chr11	18230903	-	LOC494141;LOC494141;LOC494141	Body;Body;Body	1.3317294	-0.305794	6.1795475	7.31E-09	5.57E-07	9.9614095
cg02725001	chr16	86711219	+			-1.966707	1.0003387	-6.17914	7.32E-09	5.58E-07	9.9594839
cg09119665	chr14	74181285	-	PNMA1	TSS200	-0.993419	-3.222407	-6.178743	7.33E-09	5.59E-07	9.9576036
cg14208234	chr9	97763410	+	C9orf3	Body	1.053884	1.1879588	6.1786259	7.34E-09	5.59E-07	9.9570512
cg21838979	chr2	106681945	+	C2orf40	TSS200	1.2417743	-0.326531	6.1773988	7.38E-09	5.63E-07	9.9512493
cg02700891	chr3	129693586	+	TRH;TRH	5'UTR;1stExon	2.5079079	-1.164752	6.177181	7.39E-09	5.63E-07	9.9502194
cg07355189	chr10	45923374	+	ALOX5	Body	1.7442005	0.7818965	6.177199	7.39E-09	5.63E-07	9.9503044
cg15267232	chr10	8097689	+	GATA3;GATA3	Body;Body	2.5269337	-0.716485	6.1758963	7.44E-09	5.67E-07	9.9441458
cg01433914	chr10	111792543	-	ADD3;ADD3;ADD3	5'UTR;5'UTR;5'UTR	0.9890903	2.0853256	6.1757521	7.44E-09	5.67E-07	9.9434639
cg00929376	chr1	25258236	+	RUNX3;RUNX3	TSS1500;Body	1.2827244	0.3976905	6.1756288	7.45E-09	5.67E-07	9.9428813
cg11872743	chr17	48858651	-			1.0651989	0.5337066	6.1746994	7.48E-09	5.70E-07	9.9384876
cg27158867	chr11	537638	-	LRRC56	5'UTR	-0.922752	-3.102901	-6.174383	7.49E-09	5.70E-07	9.9369901
cg02358761	chr2	197036780	+	STK17B	TSS1500	-0.568384	-4.263785	-6.174102	7.50E-09	5.71E-07	9.9356648
cg15834395	chr10	134261413	+	C10orf91	Body	-1.096597	-1.486678	-6.174046	7.51E-09	5.71E-07	9.9353969
cg05177629	chr20	43834580	-	SEMG1	TSS1500	-1.406025	-0.877256	-6.173934	7.51E-09	5.71E-07	9.9348679
cg16790682	chr12	57984821	+	PIP4K2C;PIP4K2C;PIP4K2C;PIP4K2C	TSS200;TSS200;TSS200;TSS200	-0.536169	-3.871882	-6.173885	7.51E-09	5.71E-07	9.9346359
cg21884231	chr3	170303721	-	SLC7A14;SLC7A14	5'UTR;1stExon	2.3022882	-0.975687	6.173874	7.51E-09	5.71E-07	9.9345865
cg20554353	chr7	72839549	-			-1.109809	-0.457686	-6.173452	7.53E-09	5.72E-07	9.93259

cg09328228	chr20	43538527	+	PABPC1L	TSS200	-0.919639	-3.856662	-6.173023	7.54E-09	5.74E-07	9.9305624
cg07568203	chr11	5365377	-	OR51B5	TSS1500	-1.680326	0.5027809	-6.172859	7.55E-09	5.74E-07	9.9297911
cg06086990	chr21	31970928	-			-1.562853	0.4607999	-6.17282	7.55E-09	5.74E-07	9.9296061
cg26980034	chr6	53530624	-	KLHL31	TSS200	1.4055606	1.4315392	6.1722165	7.57E-09	5.76E-07	9.9267522
cg05131483	chr16	23706242	-	ERN2	Body	1.6413238	-0.933298	6.1751351	7.58E-09	5.76E-07	9.9267135
cg09684112	chr10	13701447	-	FRMD4A	Body	1.2631433	0.9124058	6.1718961	7.59E-09	5.76E-07	9.9252384
cg22563907	chr6	35919050	+	SLC26A8;SLC26A8	Body;Body	0.8320969	2.0839796	6.1715332	7.60E-09	5.77E-07	9.9235233
cg02184008	chr11	44333072	+	ALX4	TSS1500	1.4375144	-0.86198	6.1710901	7.62E-09	5.78E-07	9.9214293
cg13169011	chr14	60973462	+			1.603968	-0.540286	6.1708795	7.62E-09	5.79E-07	9.9204344
cg09220838	chr3	121468945	+	GOLGB1	TSS1500	-0.637191	-5.539135	-6.170148	7.65E-09	5.81E-07	9.9169788
cg11680590	chr15	92701885	-	SLCO3A1;SLCO3A1	Body;Body	-1.524359	-0.289687	-6.170174	7.65E-09	5.81E-07	9.9171004
cg19733740	chr11	82609924	-	PRCP;PRCP	Body;Body	0.8128056	1.391419	6.1701725	7.65E-09	5.81E-07	9.9170934
cg01243371	chr16	86547386	-	FOXF1	3'UTR	1.5862403	0.0476393	6.1699617	7.66E-09	5.81E-07	9.9160976
cg05455971	chr8	1616422	-	DLGAP2	Body	-1.203322	-0.047882	-6.169679	7.67E-09	5.82E-07	9.9147641
cg11698888	chr7	156813023	+			1.6693747	-0.813261	6.1721665	7.69E-09	5.84E-07	9.9129623
cg26422656	chr2	238585054	-	LRRFIP1	Body	1.0262296	2.3117257	6.1687947	7.70E-09	5.84E-07	9.910584
cg11429271	chr15	45722346	-	C15orf48;C15orf48	TSS1500;TSS1500	-1.361597	-2.574499	-6.168397	7.72E-09	5.85E-07	9.9087042
cg25503903	chr3	138679512	-			1.9451084	-1.163285	6.1681977	7.73E-09	5.86E-07	9.9077631
cg02691192	chr17	59693501	+			-1.443177	-1.668511	-6.168037	7.73E-09	5.86E-07	9.9070019
cg02110836	chr17	71953083	-			-1.182044	-0.383833	-6.167544	7.75E-09	5.87E-07	9.9046773

cg02655630	chr20	44658941	-	SLC12A5;SLC12A5	Body;Body	1.0996549	-0.364584	6.1675412	7.75E-09	5.87E-07	9.9046618
cg09469079	chr2	240599954	-			-1.0293	-0.017515	-6.167605	7.75E-09	5.87E-07	9.9049653
cg21547628	chr11	364623	-			-1.092045	-0.160726	-6.167481	7.75E-09	5.87E-07	9.9043764
cg22669120	chr12	5024586	+	KCNA1	3'UTR	-1.408254	-0.913543	-6.167296	7.76E-09	5.88E-07	9.9035022
cg01425666	chr7	5468436	-			-1.39176	-3.401817	-6.16694	7.77E-09	5.89E-07	9.9018232
cg07381930	chr10	99608915	+	GOLGA7B	TSS1500	-0.792556	-1.455443	-6.169829	7.78E-09	5.89E-07	9.9019366
cg02652369	chr6	31111076	-	CCHCR1;CCHCR1;CCHCR1	Body;Body;Body	0.9899652	0.8301973	6.1661813	7.80E-09	5.91E-07	9.898238
cg09780241	chr4	111535597	-			2.3944154	-0.816141	6.1661394	7.80E-09	5.91E-07	9.8980401
cg16021428	chr5	7395395	+	ADCY2	TSS1500	2.2708808	-0.664239	6.1660664	7.81E-09	5.91E-07	9.8976955
cg01554149	chr16	86285923	-			-1.454655	0.1815329	-6.165738	7.82E-09	5.91E-07	9.8961445
cg08616061	chr5	140753570	-	PCDHGA4;PCDHGA1;PCDHGA6;PCDHGA5;PCDHGB1;PCDHGA3;PCDHGA2;PCDHGA6;PCDHGB2;PCDHGB3	Body;Body;TSS200;Body;Body;Body;Body;TSS200;Body;Body	1.0462573	0.1039122	6.1688858	7.82E-09	5.91E-07	9.8972298
cg02426464	chr17	1481305	-	SLC43A2	Body	-0.878406	-1.795324	-6.165287	7.84E-09	5.93E-07	9.8940155
cg12033781	chr1	154600788	-	ADAR	TSS1500	-1.062613	-3.567709	-6.164667	7.86E-09	5.94E-07	9.8910855
cg05980200	chr2	137181418	+			-1.003741	-2.784246	-6.164405	7.87E-09	5.95E-07	9.8898488
cg06475541	chr13	34731213	+			-0.984323	-2.429166	-6.16414	7.88E-09	5.96E-07	9.8885955
cg26751580	chr21	18885132	-	CXADR	TSS200	-1.058637	-5.729719	-6.167043	7.89E-09	5.96E-07	9.8887934
cg20465661	chr1	248551409	-	OR2T6	1stExon	-1.622983	-0.430685	-6.163797	7.89E-09	5.96E-07	9.8869753
cg27405731	chr7	101571203	+	CUX1;CUX1;CUX1	Body;Body;Body	1.3918106	1.2948867	6.1637474	7.90E-09	5.96E-07	9.8867432
cg11710211	chr19	56609064	-	ZNF787	Body	1.7255821	3.7728636	6.1699036	7.90E-09	5.97E-07	9.8883167
cg01458495	chr3	48310544	+	ZNF589	3'UTR	-1.275457	0.4470147	-6.163513	7.91E-09	5.97E-07	9.8856357

cg21950166	chr1	27189349	-	SFN	TSS1500	-1.225118	-0.859309	-6.163472	7.91E-09	5.97E-07	9.8854448
cg03290602	chr14	101514051	-	MIR889	TSS200	-1.774547	0.555653	-6.162884	7.93E-09	5.99E-07	9.8826656
cg05949889	chr11	106336898	+			-1.763657	-0.011165	-6.162821	7.93E-09	5.99E-07	9.8823699
cg25805393	chr18	74692214	-	MBP;MBP;MBP;MBP;MBP	3'UTR;3'UTR;3'UTR;3'UTR;3'UTR	0.8385038	2.2382405	6.1624077	7.95E-09	6.00E-07	9.8804168
cg06538684	chr12	12511223	+	LOH12CR2;LOH12CR1	TSS1500;Body	-1.330727	-1.340601	-6.162346	7.95E-09	6.00E-07	9.8801244
cg23068913	chr10	23463377	+			2.4671402	-0.950391	6.1620042	7.96E-09	6.01E-07	9.8785117
cg12715136	chr10	100004513	-	C10orf28	3'UTR	0.9530202	2.8032313	6.1619491	7.97E-09	6.01E-07	9.8782518
cg17265115	chr4	734692	-	PCGF3	Body	0.9047616	0.9293365	6.1615588	7.98E-09	6.02E-07	9.8764087
cg02117364	chr2	240972338	-			-0.677479	-1.597704	-6.161148	8.00E-09	6.03E-07	9.8744711
cg03990033	chr1	208084030	+	CD34;CD34	Body;Body	1.5611491	0.0154876	6.1611154	8.00E-09	6.03E-07	9.8743153
cg24065451	chr11	128419512	-	ETS1	Body	1.512053	2.6622944	6.1642103	8.00E-09	6.03E-07	9.8754392
cg00790098	chr2	223167400	-	CCDC140	5'UTR	2.3221132	-1.259795	6.1608124	8.01E-09	6.04E-07	9.8728849
cg20333001	chr2	242212896	-	HDLBP;HDLBP	5'UTR;TSS1500	1.2461442	2.2120182	6.1605496	8.02E-09	6.04E-07	9.8716442
cg01077058	chr2	241083864	+			-1.59975	-0.28036	-6.160166	8.04E-09	6.05E-07	9.8698331
cg14977066	chr1	50891195	-			1.961845	-1.221267	6.1601907	8.04E-09	6.05E-07	9.8699499
cg00988129	chr8	75232556	-	JPH1	Body	-0.77123	-3.542297	-6.159689	8.05E-09	6.07E-07	9.867583
cg05255275	chr14	60952097	+	C14orf39	5'UTR	1.882376	-1.188159	6.1589232	8.09E-09	6.09E-07	9.8639664
cg02092102	chr7	128555896	+			1.4123657	-0.658853	6.1582223	8.11E-09	6.11E-07	9.8606577
cg07222434	chr16	20395031	-	PDILT	Body	-1.196796	-2.79302	-6.158249	8.11E-09	6.11E-07	9.8607855
cg08352755	chr5	145758879	+			1.060804	0.109702	6.1579403	8.12E-09	6.11E-07	9.859327

cg02919422	chr8	55370544	-	SOX17;SOX17	5'UTR;1stExon	1.2834017	-0.499191	6.1609962	8.13E-09	6.12E-07	9.8602881
cg06351425	chr6	28482191	+	GPX6	Body	-1.7813	-0.306391	-6.157522	8.14E-09	6.12E-07	9.8573505
cg10770023	chr22	51016644	-	CPT1B;CPT1B;CPT1B;CHKB-CPT1B;CPT1B;CPT1B;CPT1B;CPT1B	5'UTR;5'UTR;TSS200;Body;5'UTR;5'UTR;5'UTR;5'UTR	1.242602	0.7143894	6.157404	8.15E-09	6.13E-07	9.8567958
cg04235416	chr14	101530343	+	MIR412;MIR541;MIR409	TSS1500;TSS1500;TSS1500	-1.468079	0.4141877	-6.157343	8.15E-09	6.13E-07	9.8565085
cg16130857	chr10	102900262	-			1.6143087	-1.256286	6.1570377	8.16E-09	6.14E-07	9.8550669
cg24066641	chr5	179554466	+	RASGEF1C	Body	-0.986101	0.3743059	-6.156558	8.18E-09	6.15E-07	9.8528045
cg02924487	chr1	3649344	+	TP73;TP73;TP73;TP73	Body;Body;3'UTR;3'UTR	1.0859521	1.2760224	6.1561324	8.20E-09	6.16E-07	9.8507944
cg21756208	chr3	134146405	+			-1.224631	-0.184137	-6.156076	8.20E-09	6.16E-07	9.8505299
cg04138181	chr16	23690918	-	PLK1	Body	-1.586767	-4.142331	-6.16232	8.20E-09	6.16E-07	9.8528844
cg04136036	chr16	87683014	+	JPH3	Body	-1.088594	-0.171991	-6.155912	8.21E-09	6.16E-07	9.8497545
cg25340966	chr1	119532195	-	TBX15	TSS200	1.997628	-0.669034	6.1558277	8.21E-09	6.17E-07	9.8493566
cg12782180	chr7	127880932	+	LEP	TSS1500	1.3908088	0.8113787	6.1552253	8.23E-09	6.18E-07	9.8465136
cg01796333	chr2	242034995	-	MTERFD2;MTERFD2;MTERFD2;MTERFD2	Body;3'UTR;Body;Body	0.88536	1.868905	6.1549612	8.24E-09	6.19E-07	9.8452675
cg22826333	chr4	48987791	-	CWH43	TSS1500	1.0884409	-0.320729	6.1547107	8.25E-09	6.20E-07	9.8440853
cg23697406	chr7	155744359	+			-1.605897	0.1831728	-6.154728	8.25E-09	6.20E-07	9.8441659
cg16929955	chr17	77375461	-	HRNBP3	5'UTR	-1.107963	-0.309235	-6.154626	8.26E-09	6.20E-07	9.843686
cg25436157	chr18	21018562	-	C18orf45	TSS1500	-0.966334	-1.387218	-6.154283	8.27E-09	6.21E-07	9.8420692
cg00629625	chr20	44663630	+	SLC12A5;SLC12A5	Body;Body	-1.268778	-0.350895	-6.153657	8.30E-09	6.22E-07	9.8391157
cg06088032	chr3	50383227	+	ZMYND10	TSS200	-1.801953	-3.146526	-6.153415	8.31E-09	6.23E-07	9.8379742
cg04257163	chr12	89620072	-			-1.158667	-1.086402	-6.153354	8.31E-09	6.23E-07	9.8376851

cg01284415	chr19	49457725	-	BAX;BAX;BAX;BAX;BAX	TSS1500;TSS1500;TSS1500;TSS1500;TSS1500	-1.03183	-1.41214	-6.152783	8.33E-09	6.25E-07	9.8349909
cg05173373	chr12	15942933	+	EPS8	TSS1500	-0.762018	-3.800807	-6.152818	8.33E-09	6.25E-07	9.835154
cg20701333	chr19	48965566	-	KCNJ14;KCNJ14	1stExon;Body	0.8799439	2.0411728	6.1527377	8.33E-09	6.25E-07	9.8347764
cg07338984	chr12	48194303	-	HDAC7;HDAC7	Body;Body	0.5505459	2.6469091	6.1524159	8.35E-09	6.26E-07	9.8332585
cg00228799	chr9	131580591	-	ENDOG	TSS200	-0.585257	-4.262631	-6.152292	8.35E-09	6.26E-07	9.8326738
cg01642550	chr16	89098327	-			-1.556519	-0.657333	-6.152307	8.35E-09	6.26E-07	9.8327452
cg07802909	chr5	169817797	+	KCNMB1;KCNIP1	TSS1500;Body	-1.426282	0.2304309	-6.152339	8.35E-09	6.26E-07	9.8328972
cg08206318	chr5	134363637	+	PITX1	3'UTR	1.3524447	-0.423246	6.1520172	8.36E-09	6.26E-07	9.8313774
cg26700320	chr22	48946066	-	FAM19A5	Body	-1.868329	0.7100164	-6.151236	8.40E-09	6.29E-07	9.8276921
cg06585203	chr6	159238500	+	EZR;EZR	Body;Body	-0.735628	-3.476569	-6.151134	8.40E-09	6.29E-07	9.82721
cg15527308	chr18	54813067	-			-1.368281	0.3652925	-6.15106	8.40E-09	6.29E-07	9.8268608
cg14031381	chr17	75181131	+	SEC14L1;SEC14L1;SEC14L1;SEC14L1;SEC14L1	Body;Body;TSS200;Body;Body	0.9981224	1.603714	6.1506779	8.42E-09	6.30E-07	9.8250597
cg04337653	chr6	100905587	+	SIM1	Body	1.5941948	-0.704151	6.1505902	8.42E-09	6.30E-07	9.824646
cg10363926	chr2	109921282	+	SH3RF3	Body	-0.951627	-1.108057	-6.150607	8.42E-09	6.30E-07	9.824723
cg12498580	chr12	7070732	+			-0.927295	-1.700835	-6.150571	8.42E-09	6.30E-07	9.8245533
cg05333146	chr16	67183725	+	B3GNT9	Body	0.9921159	0.9087423	6.1504042	8.43E-09	6.30E-07	9.8237689
cg19629631	chr7	2060116	-	MAD1L1;MAD1L1;MAD1L1	Body;Body;Body	0.9326238	1.5780934	6.1504391	8.43E-09	6.30E-07	9.8239332
cg04133658	chr3	129161504	-	IFT122;IFT122;IFT122;IFT122	Body;Body;Body;Body	1.0151257	1.1370802	6.1499606	8.45E-09	6.32E-07	9.8216764
cg11695831	chr17	31611767	-	ACCN1;ACCN1	Body;Body	-1.6961	0.6130342	-6.149887	8.45E-09	6.32E-07	9.8213287
cg20852851	chr2	240168848	-	HDAC4	Body	-3.560978	1.5400548	-6.152926	8.46E-09	6.32E-07	9.8222646

cg03460239	chr11	124311153	-	OR8B8	TSS200	-1.586759	0.9404155	-6.149665	8.46E-09	6.32E-07	9.8202812
cg09447450	chr6	40563644	+			-0.979378	0.3085751	-6.152508	8.47E-09	6.33E-07	9.8202983
cg13076829	chr18	45530698	-			-1.358937	0.6165841	-6.149085	8.49E-09	6.34E-07	9.8175486
cg14085715	chr7	20257162	+	MACC1	TSS200	-1.252438	-2.392963	-6.149049	8.49E-09	6.34E-07	9.8173774
cg16561172	chr8	128746032	+			-1.622373	-3.620813	-6.161634	8.50E-09	6.34E-07	9.8215717
cg11035122	chr14	101491994	-	MIR758;MIR329-2;MIR329-1;MIR323	TSS1500;TSS1500;TSS1500;TSS200	-1.265952	0.1360596	-6.14858	8.51E-09	6.35E-07	9.8151672
cg13860387	chr6	26456230	-			0.7823413	1.8617729	6.1479885	8.53E-09	6.37E-07	9.8123759
cg16829244	chr10	134491593	+	INPP5A	Body	0.875399	1.0858442	6.1478995	8.54E-09	6.37E-07	9.8119558
cg17972655	chr15	90709123	-			-1.094255	-2.103104	-6.147906	8.53E-09	6.37E-07	9.8119861
cg01923075	chr7	105223735	-			0.671407	1.9921903	6.147702	8.54E-09	6.37E-07	9.8110245
cg27071152	chr7	39649443	+	LOC646999	Body	2.5136037	0.1940965	6.1477286	8.54E-09	6.37E-07	9.8111499
cg06012215	chr10	111216867	-			0.9445253	-0.21784	6.1473464	8.56E-09	6.38E-07	9.8093478
cg19275220	chr7	108168721	-			-2.222459	0.8296253	-6.147253	8.56E-09	6.38E-07	9.8089058
cg02507181	chr12	10251536	-	CLEC1A;CLEC1A	5'UTR;1stExon	1.3074367	0.986935	6.1471197	8.57E-09	6.39E-07	9.8082788
cg02058408	chr17	20687681	+			1.8690551	-0.102119	6.1469619	8.57E-09	6.39E-07	9.8075346
cg23677657	chr7	96619076	+	DLX6AS	Body	1.0705047	-0.039559	6.1466209	8.59E-09	6.40E-07	9.8059269
cg18405544	chr5	67615770	-			-1.141545	0.4056613	-6.14647	8.60E-09	6.40E-07	9.8052151
cg08917117	chr10	115059021	-			-1.565587	0.1669488	-6.146344	8.60E-09	6.41E-07	9.8046217
cg01871127	chr2	177026301	+			1.5854778	-1.333287	6.1460106	8.61E-09	6.42E-07	9.8030492
cg01381829	chr22	40573054	-	TNRC6B;TNRC6B;TNRC6B	Body;TSS1500;TSS1500	-1.2472	-0.935631	-6.145773	8.62E-09	6.42E-07	9.8019269

cg20604028	chr8	29413587	+			-1.448985	0.0059788	-6.145762	8.63E-09	6.42E-07	9.8018786
cg02058924	chr12	133465592	+	CHFR;CHFR;CHFR;CHFR;CHFR	TSS1500;TSS1500;TSS1500;TSS1500;TSS1500	0.9413885	2.0302943	6.1454219	8.64E-09	6.43E-07	9.8002737
cg13136655	chr6	149806131	+	ZC3H12D;ZC3H12D	5'UTR;1stExon	-1.25862	-2.235248	-6.145359	8.64E-09	6.43E-07	9.799977
cg17419049	chr2	74097688	+			0.9969064	2.3266097	6.1449888	8.66E-09	6.44E-07	9.7982316
cg21119375	chr2	44393385	-			0.9336573	2.1021064	6.1449013	8.66E-09	6.45E-07	9.7978193
cg21594383	chr8	711581	-			-1.3486	-0.065419	-6.144329	8.69E-09	6.46E-07	9.7951198
cg27637363	chr17	27945387	+	CORO6	Body	-0.685629	-1.896146	-6.143943	8.70E-09	6.47E-07	9.7933
cg11849281	chr7	35494507	+			1.4217263	-1.221119	6.1438848	8.70E-09	6.47E-07	9.7930269
cg05107535	chr16	3242850	-			1.3631469	0.7362457	6.1435962	8.72E-09	6.48E-07	9.7916666
cg24373865	chr1	44716226	+	ERI3	Body	0.7339676	0.7238301	6.142676	8.76E-09	6.51E-07	9.7873291
cg03659519	chr18	74961966	-	GALR1	TSS200	2.5637626	-1.775885	6.1422903	8.77E-09	6.52E-07	9.7855113
cg00766482	chr19	6269688	+	MLLT1	Body	1.581653	2.8572645	6.1422445	8.78E-09	6.52E-07	9.7852953
cg01091565	chr15	90294762	+	MESP1	TSS1500	-1.521184	1.0548643	-6.142112	8.78E-09	6.52E-07	9.7846689
cg07731047	chr11	1994977	-			-1.447116	0.5714476	-6.142008	8.79E-09	6.53E-07	9.7841791
cg19113081	chr11	114548872	-	FAM55B	TSS1500	-1.396721	-1.504467	-6.141891	8.79E-09	6.53E-07	9.7836302
cg01496136	chr16	57375219	-			-1.170079	-0.625539	-6.141691	8.80E-09	6.53E-07	9.7826842
cg02484673	chr16	87674713	+	JPH3	Body	-1.171282	0.0563943	-6.14171	8.80E-09	6.53E-07	9.7827783
cg24722950	chr14	62035475	-			-1.691795	-1.523229	-6.141424	8.81E-09	6.54E-07	9.7814296
cg00347620	chr12	115109730	-	TBX3;TBX3	Body;Body	1.001094	0.0728823	6.144139	8.83E-09	6.55E-07	9.780896
cg09923978	chr11	58207761	-	OR5B12	TSS200	-1.665727	0.4433187	-6.141079	8.83E-09	6.55E-07	9.7797999

cg17953764	chr4	48492845	+	ZAR1	1stExon	0.8014591	1.0631657	6.1410884	8.83E-09	6.55E-07	9.7798463
cg23720064	chr6	100895679	+	SIM1	Body	1.7836928	-1.499791	6.1411252	8.82E-09	6.55E-07	9.7800199
cg25074066	chr11	62690043	-	CHRM1	TSS1500	-1.07141	-0.675584	-6.144186	8.83E-09	6.55E-07	9.780855
cg25642955	chr5	33050111	+			-1.500802	-0.265536	-6.141021	8.83E-09	6.55E-07	9.779527
cg13699355	chr2	468179	-			3.1178957	-1.123616	6.1407897	8.84E-09	6.55E-07	9.7784387
cg03758732	chr2	2030712	-	MYT1L	5'UTR	-1.490611	1.0082654	-6.140685	8.84E-09	6.56E-07	9.7779458
cg16358044	chr4	164245970	-	NPY1R	3'UTR	-1.699539	0.4581922	-6.140568	8.85E-09	6.56E-07	9.7773931
cg14557064	chr5	1752840	+			-2.260099	0.324518	-6.146757	8.85E-09	6.56E-07	9.7797006
cg17560832	chr10	130757336	+			-1.373707	0.0630368	-6.140483	8.85E-09	6.56E-07	9.7769917
cg20826151	chr7	100743732	+			-0.94447	-2.378828	-6.140246	8.86E-09	6.57E-07	9.7758758
cg11937768	chr10	65344030	+	REEP3	Body	0.9306189	2.583653	6.140035	8.87E-09	6.57E-07	9.774882
cg21491201	chr7	30545074	-	GGCT	TSS1500	-1.051047	-1.040275	-6.14001	8.87E-09	6.57E-07	9.7747639
cg08991210	chr12	133010785	-			-1.375824	0.9727444	-6.139725	8.88E-09	6.58E-07	9.77342
cg22557091	chr11	31827920	+	PAX6;PAX6;PAX6	Body;Body;Body	1.8655706	-1.444529	6.1395095	8.89E-09	6.58E-07	9.7724056
cg21106407	chr14	57262177	+			1.676201	-0.848452	6.1422637	8.91E-09	6.60E-07	9.7720713
cg05903736	chr2	240197131	-	HDAC4	Body	-1.403496	-1.504553	-6.139034	8.91E-09	6.60E-07	9.7701649
cg09424526	chr2	223163809	-	PAX3;PAX3;PAX3;CCDC140;PAX3;PAX3;PAX3;PAX3;PAX3	TSS200;TSS200;TSS200;5'UTR;TSS200;TSS200;TSS200;TSS200;TSS200	2.6226458	-1.333098	6.1390265	8.91E-09	6.60E-07	9.7701298
cg09671258	chr1	180202530	+	LHX4	Body	2.9262836	-0.909199	6.1390079	8.92E-09	6.60E-07	9.7700424
cg18845375	chr2	237173852	-	ASB18	TSS1500	-1.40834	1.0706274	-6.138884	8.92E-09	6.60E-07	9.7694575
cg19843540	chr4	183957914	-	FAM92A3	TSS1500	-1.447129	0.3107683	-6.138192	8.95E-09	6.62E-07	9.7661969

cg23018448	chr19	10811855	-	QTRT1	TSS1500	-1.123199	-1.394162	-6.13794	8.96E-09	6.63E-07	9.7650094
cg04923174	chr1	158549277	+	OR10X1	1stExon	-1.766302	-0.283259	-6.13772	8.97E-09	6.63E-07	9.7639758
cg15158045	chr13	95827029	-	ABCC4;ABCC4	Body;Body	0.8245027	2.5516365	6.1376747	8.97E-09	6.63E-07	9.7637604
cg07784084	chr3	26751141	+	LRRC3B	5'UTR	-1.479073	0.2533427	-6.136943	9.01E-09	6.66E-07	9.7603126
cg26469011	chr10	75886930	+	AP3M1;AP3M1	Body;Body	-0.89223	1.3745935	-6.136951	9.01E-09	6.66E-07	9.7603507
cg10689404	chr17	35300645	-			1.5194012	0.210469	6.1399422	9.01E-09	6.66E-07	9.7608869
cg22389137	chr4	113432227	-			1.364329	-0.982358	6.1367127	9.02E-09	6.66E-07	9.7592285
cg06764173	chr16	89632174	-			-1.369034	-2.908281	-6.136584	9.02E-09	6.66E-07	9.7586235
cg03582285	chr17	40700314	+			-1.156362	-1.11226	-6.13955	9.03E-09	6.67E-07	9.7593044
cg14942536	chr14	101520169	-	MIR134;MIR668;MIR382	TSS1500;TSS1500;TSS1500	-1.483266	0.3169742	-6.136404	9.03E-09	6.67E-07	9.7577738
cg17550009	chr20	61974165	-			-2.677743	2.2671406	-6.136318	9.03E-09	6.67E-07	9.7573678
cg13624163	chr11	442018	-	ANO9	TSS200	-0.894834	-5.112701	-6.136026	9.05E-09	6.68E-07	9.7559915
cg18449135	chr19	48833062	+	EMP3	Body	-1.786188	0.8055777	-6.135861	9.05E-09	6.68E-07	9.7552174
cg17470067	chr2	45156156	+			1.5844866	-1.460261	6.1357386	9.06E-09	6.68E-07	9.7546394
cg06720467	chr1	27189440	-	SFN	TSS200	-1.086718	-1.013981	-6.135543	9.07E-09	6.69E-07	9.7537165
cg22380693	chr19	54676948	-	TMC4;TMC4	TSS200;TSS200	-0.747079	-5.227088	-6.1355	9.07E-09	6.69E-07	9.7535138
cg16253580	chr7	143701215	+	OR6B1	1stExon	-1.573903	-0.348088	-6.135092	9.09E-09	6.70E-07	9.7515946
cg10604646	chr1	163172649	-	RGS5;RGS5	1stExon;5'UTR	1.3134796	1.7783239	6.1380279	9.10E-09	6.71E-07	9.7521435
cg07995570	chr1	91192025	+			1.6981407	-1.905867	6.1348405	9.10E-09	6.71E-07	9.7504089
cg24770292	chr9	136223781	+	SURF1;SURF2	TSS1500;Body	-0.316933	-4.204645	-6.134403	9.12E-09	6.72E-07	9.7483491

cg16664405	chr10	111216962	+			1.9886863	-0.414122	6.1334435	9.16E-09	6.75E-07	9.7438293
cg19583265	chr2	101086756	-	NMS	TSS200	-1.745648	0.539068	-6.133314	9.17E-09	6.76E-07	9.7432205
cg17592231	chr11	43604758	-			0.7715989	0.298957	6.132986	9.18E-09	6.77E-07	9.7416745
cg22550446	chr5	8059446	+			-2.000297	0.2115672	-6.132939	9.18E-09	6.77E-07	9.7414536
cg00456557	chr6	74008688	+	C6orf147	Body	0.8370003	1.5239791	6.1326175	9.20E-09	6.77E-07	9.7399395
cg18832152	chr1	155035183	+	ADAM15;EFNA4;ADAM15;ADAM15;EFNA4;ADAM15;ADAM15;EFNA4;ADAM15	3'UTR;TSS1500;3'UTR;3'UTR;TSS1500;3'UTR;3'UTR;TSS1500;3'UTR	-1.083397	-2.394851	-6.132642	9.20E-09	6.77E-07	9.7400546
cg23238119	chr6	74009041	+	C6orf147	Body	1.3929846	1.5738766	6.1356553	9.20E-09	6.77E-07	9.740985
cg05946353	chr17	79609272	-	TSPAN10	TSS200	-1.362508	0.1939081	-6.132412	9.21E-09	6.78E-07	9.7389726
cg17191462	chr6	50690975	-	TFAP2D	Body	2.2542224	-1.250296	6.1323165	9.21E-09	6.78E-07	9.7385219
cg14285788	chr2	128458556	-	SFT2D3	TSS200	-1.495176	-0.69898	-6.131468	9.25E-09	6.81E-07	9.7345257
cg20487548	chr20	37854852	+	LOC339568	TSS1500	-1.7012	0.527692	-6.131473	9.25E-09	6.81E-07	9.7345482
cg06712013	chr12	49759545	-	SPATS2	TSS1500	-1.625845	-0.411789	-6.131154	9.27E-09	6.82E-07	9.7330461
cg08300570	chr7	92353454	-	CDK6;CDK6	Body;Body	1.1293083	1.7534654	6.1341384	9.27E-09	6.82E-07	9.7338523
cg04995095	chr17	72619556	-	CD300E	Body	-1.647883	-0.348314	-6.13089	9.28E-09	6.82E-07	9.7318033
cg22830638	chr2	73209573	-	SFXN5	Body	-0.944358	0.5510029	-6.130771	9.28E-09	6.83E-07	9.7312447
cg13173392	chr11	69245569	-			-1.406766	0.1250194	-6.130645	9.29E-09	6.83E-07	9.7306523
cg16739441	chr12	21926524	+	KCNJ8	Body	1.6647453	-0.298018	6.1306288	9.29E-09	6.83E-07	9.7305749
cg17028595	chr10	80725361	-	LOC283050;LOC283050;LOC283050	Body;Body;Body	-1.435191	0.6272882	-6.13051	9.29E-09	6.83E-07	9.7300142
cg14086122	chr12	123921793	+	RILPL2	TSS1500	-0.842793	-1.772185	-6.130444	9.30E-09	6.83E-07	9.7297036
cg03962950	chr16	945795	+	LMF1	Body	-1.38735	0.5700713	-6.130261	9.31E-09	6.84E-07	9.7288425

cg20425384	chr12	132657819	+			-1.559214	-1.828693	-6.130284	9.31E-09	6.84E-07	9.728949
cg21648069	chr12	1157718	+	ERC1;ERC1;ERC1;ERC1;ERC1	Body;Body;Body;Body;Body	1.4623588	1.2194478	6.1301507	9.31E-09	6.84E-07	9.7283238
cg25751961	chr11	128711233	-	KCNJ1;KCNJ1;KCNJ1;KCNJ1;KCNJ1	5'UTR;5'UTR;5'UTR;Body;5'UTR	-1.649224	1.1973419	-6.130096	9.31E-09	6.84E-07	9.7280654
cg18724037	chr15	27127627	-	GABRA5;GABRA5	Body;Body	-0.924271	-0.741896	-6.129952	9.32E-09	6.84E-07	9.7273861
cg04137837	chr14	97647221	-			-1.227925	0.4450426	-6.129908	9.32E-09	6.84E-07	9.7271832
cg19254119	chr19	9609422	-	ZNF560	TSS200	2.1708271	-0.714566	6.129845	9.33E-09	6.84E-07	9.7268843
cg25458520	chr6	36099195	-	MAPK13	Body	-0.562512	-3.508756	-6.129787	9.33E-09	6.84E-07	9.7266112
cg21723559	chr20	44048174	-	PIGT	Body	-1.348174	-0.715671	-6.129725	9.33E-09	6.85E-07	9.726317
cg13916459	chr12	85673221	+	ALX1	TSS1500	2.058107	0.1601201	6.1327826	9.33E-09	6.85E-07	9.7272148
cg10595547	chr10	119310911	+			1.7524165	-0.337609	6.1296495	9.33E-09	6.85E-07	9.725964
cg24427895	chr11	20230313	-			1.3588717	-0.089104	6.1294355	9.34E-09	6.85E-07	9.7249565
cg11084334	chr3	9594264	-	LHFPL4	Body	1.0958205	1.2245329	6.1291728	9.36E-09	6.86E-07	9.7237196
cg26379822	chr6	134758490	+			-1.192998	-1.434648	-6.128998	9.36E-09	6.86E-07	9.7228948
cg01152019	chr2	177015044	+	HOXD4;MIR10B	TSS1500;Body	1.5949611	-1.226467	6.1287144	9.38E-09	6.87E-07	9.7215616
cg03207666	chr7	27183950	+	HOXA5	TSS1500	1.3298796	0.7692376	6.1287239	9.38E-09	6.87E-07	9.7216065
cg23064609	chr2	175208594	-			2.4232093	-0.121205	6.1286529	9.38E-09	6.87E-07	9.721272
cg24456654	chr4	41754125	-			1.5728607	-0.475934	6.1286054	9.38E-09	6.87E-07	9.7210483
cg26464796	chr5	54601060	+	DHX29	Body	1.238798	1.3305972	6.1284802	9.39E-09	6.88E-07	9.7204589
cg24630516	chr11	75852389	+	UVRAG	Body	1.8209207	4.0580133	6.1346476	9.39E-09	6.88E-07	9.734191
cg03280063	chr4	893186	-	GAK	Body	0.7287825	1.380226	6.1282867	9.40E-09	6.88E-07	9.7195483

cg14082127	chr6	101847349	+	GRIK2;GRIK2;GRIK2	Body;Body;Body	1.4907065	-0.863628	6.1281553	9.40E-09	6.88E-07	9.7189296
cg17552333	chr5	124821012	+			-1.162928	-2.822987	-6.127959	9.41E-09	6.89E-07	9.7180058
cg19998432	chr9	125391618	-	OR1B1	1stExon	-0.923749	-1.201082	-6.127442	9.44E-09	6.90E-07	9.7155704
cg21557724	chr2	3317928	-	TSSC1	Body	0.7857106	1.3496564	6.1273005	9.44E-09	6.91E-07	9.7149057
cg14118515	chr2	176948728	+	EVX2	TSS200	2.7625333	0.0673428	6.1270934	9.45E-09	6.91E-07	9.7139312
cg17163729	chr2	554372	+			-1.604075	-0.370217	-6.130056	9.46E-09	6.92E-07	9.7143947
cg23606421	chr9	140330621	-	ENTPD8;ENTPD8	Body;Body	-1.042412	-0.656134	-6.126995	9.46E-09	6.92E-07	9.7134668
cg26228531	chr3	135956534	+			0.9193327	1.5161697	6.1266685	9.47E-09	6.92E-07	9.7119311
cg03963963	chr10	44434410	-			-1.351273	0.3597107	-6.126157	9.50E-09	6.94E-07	9.709522
cg08534628	chr13	100650073	+			0.9056837	-0.116675	6.1259949	9.50E-09	6.95E-07	9.7087608
cg24576804	chr5	180056040	-	FLT4;FLT4	Body;Body	-1.176385	0.1676497	-6.125777	9.51E-09	6.95E-07	9.7077339
cg11625005	chr5	1295737	+	TERT;TERT	TSS1500;TSS1500	1.2137548	0.7691861	6.1255804	9.52E-09	6.96E-07	9.7068099
cg17242937	chr4	111561114	+			1.6745564	-0.3749	6.1256038	9.52E-09	6.96E-07	9.70692
cg18961681	chr7	32467657	-			1.9987645	-0.385553	6.1253714	9.53E-09	6.96E-07	9.7058261
cg16780688	chr8	120220073	+	MAL2	TSS1500	-1.198301	-0.692891	-6.125173	9.54E-09	6.97E-07	9.704894
cg00491064	chr11	19681786	-	NAV2	Body	-1.08049	-0.802195	-6.127605	9.57E-09	6.99E-07	9.7031395
cg05328547	chr7	19185139	-	FERD3L	TSS200	1.7525095	-1.578879	6.1243583	9.58E-09	6.99E-07	9.7010587
cg23880668	chr2	176946790	+	EVX2	Body	1.7173747	-1.481397	6.1240877	9.59E-09	7.00E-07	9.6997851
cg26789453	chr12	112452424	-	TMEM116;ERP29;ERP29	TSS1500;Body;Body	-1.697675	-0.824992	-6.123944	9.60E-09	7.00E-07	9.6991078
cg26328335	chr12	50354840	+	AQP5	TSS1500	-1.813066	-1.75217	-6.123728	9.61E-09	7.01E-07	9.6980941

cg27321079	chr7	100034454	-	C7orf47	TSS1500	-1.126488	-3.190565	-6.126337	9.63E-09	7.03E-07	9.6971809
cg01009664	chr3	129693613	+	TRH;TRH	5'UTR;1stExon	2.0622874	-0.92166	6.1230583	9.64E-09	7.03E-07	9.6949416
cg19748485	chr5	140743927	+	PCDHGA4;PCDHGA2;PCDHGA5;PCDHGB2;PCDHGA1;PCDHGB1;PCDHGA3;PCDHGA5	Body;Body;1stExon;Body;Body;Body;Body;1stExon	0.8551488	0.5033497	6.1261639	9.64E-09	7.03E-07	9.6963693
cg26607337	chr5	145758881	+			1.1364078	0.1980546	6.1229956	9.64E-09	7.03E-07	9.6946465
cg01635405	chr10	124896713	-	HMX3	Body	1.769459	-1.336834	6.1228776	9.65E-09	7.03E-07	9.6940914
cg02026535	chr19	7580217	+	ZNF358	TSS1500	1.1555139	1.3967215	6.1228752	9.65E-09	7.03E-07	9.6940799
cg03975694	chr19	38042472	-	ZNF540;ZNF540	5'UTR;1stExon	1.7054001	-0.576477	6.1229208	9.65E-09	7.03E-07	9.6942944
cg14162076	chr12	8665092	-	CLEC4D	TSS1500	-1.284985	0.1135624	-6.122799	9.65E-09	7.03E-07	9.6937215
cg19544065	chr2	2178847	+	MYT1L	5'UTR	-1.201686	-0.385196	-6.122416	9.67E-09	7.04E-07	9.6919213
cg19589396	chr8	103937374	+			-1.344499	-1.349133	-6.122397	9.67E-09	7.04E-07	9.6918278
cg26449951	chr2	219754840	+	WNT10A	Body	1.4067317	3.1940448	6.1224091	9.67E-09	7.04E-07	9.6918869
cg15248242	chr11	125603160	-			-1.690828	0.7592922	-6.122278	9.68E-09	7.05E-07	9.6912694
cg19489885	chr5	178684416	-	ADAMTS2;ADAMTS2	Body;Body	-1.191763	1.1331393	-6.121986	9.69E-09	7.06E-07	9.6898956
cg10798682	chr3	125820372	+	SLC41A3;SLC41A3	5'UTR;1stExon	0.7021265	1.3323093	6.1218974	9.70E-09	7.06E-07	9.6894793
cg18719170	chr2	240969975	+	OR6B2	TSS200	-0.73606	-2.357407	-6.121914	9.69E-09	7.06E-07	9.6895584
cg11540692	chr6	100912072	-	SIM1	TSS1500	1.1891154	-0.732359	6.121752	9.70E-09	7.06E-07	9.6887955
cg18202928	chr5	15692835	-	FBXL7	Body	-2.319469	0.5113822	-6.1246	9.71E-09	7.07E-07	9.6887583
cg02011392	chr6	101847541	-	GRIK2;GRIK2;GRIK2	Body;Body;Body	1.891474	-1.127935	6.1211761	9.73E-09	7.08E-07	9.686086
cg05640754	chr4	137732125	-			-1.715808	0.9205415	-6.121197	9.73E-09	7.08E-07	9.6861823
cg27172877	chr11	124806777	-	HEPACAM	TSS1500	-1.58602	0.6029657	-6.12043	9.77E-09	7.10E-07	9.6825761

cg24627789	chr8	1338463	-			-1.521279	0.4099805	-6.120316	9.77E-09	7.10E-07	9.68204
cg09950423	chr16	49856768	+	ZNF423	5'UTR	-1.790345	0.7440583	-6.119965	9.79E-09	7.11E-07	9.6803892
cg18609078	chr4	13538807	+			1.1513561	-0.150422	6.120014	9.79E-09	7.11E-07	9.680619
cg19653589	chr19	2614177	+	GNG7	5'UTR	-0.899156	-2.071928	-6.120015	9.79E-09	7.11E-07	9.6806255
cg26931050	chr6	130757762	-	TMEM200A	TSS1500	-2.17422	0.3398379	-6.123038	9.79E-09	7.11E-07	9.6814173
cg03254916	chr1	201978949	-	ELF3;ELF3	TSS1500;TSS1500	-1.012969	-3.716539	-6.119537	9.81E-09	7.13E-07	9.6783776
cg04147875	chr16	1587610	-	IFT140;TMEM204	Body;Body	0.7558176	1.5089567	6.1193993	9.81E-09	7.13E-07	9.6777277
cg10979436	chr7	27242005	+			2.3502993	-2.11767	6.1193208	9.82E-09	7.13E-07	9.6773585
cg17939805	chr18	44773988	+			1.4355739	-1.065197	6.1189074	9.84E-09	7.14E-07	9.6754138
cg26277754	chr5	50673033	-			1.0431792	-0.067257	6.1186007	9.85E-09	7.15E-07	9.6739716
cg11364256	chr7	42005232	-	GLI3	Body	-1.40934	0.310144	-6.118554	9.86E-09	7.15E-07	9.6737498
cg20902975	chr8	11138403	+			-0.883529	-1.003713	-6.118453	9.86E-09	7.15E-07	9.6732768
cg26202762	chr15	25432236	-	SNORD115-10	TSS1500	-1.463652	0.1503164	-6.12153	9.86E-09	7.15E-07	9.6745992
cg19029181	chr3	147130536	-	ZIC1	Body	1.5333009	0.8853853	6.1335023	9.90E-09	7.18E-07	9.6758835
cg05522011	chr4	81122726	-	PRDM8;PRDM8	Body;Body	1.6166157	1.9828169	6.1173352	9.91E-09	7.19E-07	9.6680197
cg01995289	chr8	145939351	+			1.152423	1.5107875	6.117192	9.92E-09	7.20E-07	9.6673464
cg18276135	chr11	194498	-	LOC653486;SCGB1C1	3'UTR;3'UTR	-0.913463	-0.264641	-6.11708	9.93E-09	7.20E-07	9.6668192
cg05756685	chr1	12571625	-	VPS13D;VPS13D	3'UTR;3'UTR	0.8886243	0.6440767	6.1169845	9.93E-09	7.20E-07	9.6663704
cg07677157	chr12	66050928	-			-2.391677	-1.77725	-6.116909	9.94E-09	7.20E-07	9.6660143
cg11324259	chr8	143999374	-	CYP11B2	TSS200	-1.354447	-0.061641	-6.116942	9.93E-09	7.20E-07	9.6661692

cg26763394	chr3	45957664	+			-1.166659	-0.735644	-6.116581	9.95E-09	7.21E-07	9.6644744
cg01524278	chr12	54355460	-			1.4970463	-0.623777	6.1160841	9.98E-09	7.23E-07	9.6621364
cg03403966	chr4	85504051	-	CDS1	TSS200	-1.027287	-5.395087	-6.115952	9.98E-09	7.23E-07	9.6615157
cg02646706	chr16	20053474	-	GPR139	Body	-1.698393	0.5246256	-6.115291	1.00E-08	7.25E-07	9.6584078
cg22618337	chr10	115510928	+	C10orf81	TSS1500	-1.915047	0.9027717	-6.115071	1.00E-08	7.26E-07	9.6573723
cg24830876	chr3	56836259	-	ARHGEF3;ARHGEF3	Body;TSS1500	-0.362497	-4.171968	-6.115056	1.00E-08	7.26E-07	9.6573026
cg14663264	chr1	91192798	+			1.5217424	-0.242341	6.114718	1.00E-08	7.27E-07	9.6557131
cg03922251	chr11	118842390	+	FOXR1	TSS200	0.8935219	0.5753827	6.1146646	1.00E-08	7.27E-07	9.6554617
cg16158484	chr10	1981672	-			-1.572964	0.5802226	-6.114496	1.01E-08	7.28E-07	9.6546684
cg16313343	chr14	105768109	+	BRF1	TSS1500	-1.335535	-2.131756	-6.114262	1.01E-08	7.28E-07	9.6535678
cg24599434	chr3	172166242	-	GHSR;GHSR	TSS200;TSS200	1.8118948	-1.561111	6.1171489	1.01E-08	7.29E-07	9.6540279
cg10698928	chr8	65290320	+	MIR124-2	TSS1500	2.9326486	-1.490342	6.1134305	1.01E-08	7.31E-07	9.6496598
cg02442509	chr2	45156207	-			1.63894	-1.828384	6.11312	1.01E-08	7.32E-07	9.6482
cg25690589	chr15	60285478	-			1.0381423	1.1643592	6.1128121	1.01E-08	7.33E-07	9.6467526
cg00791074	chr6	151186169	+	MTHFD1L	TSS1500	-1.453679	-0.773412	-6.112577	1.01E-08	7.34E-07	9.6456458
cg13555689	chr14	101348566	+	MIR127;RTL1	TSS1500;1stExon	-1.307845	1.5435979	-6.112419	1.02E-08	7.34E-07	9.6449068
cg20316883	chr8	138493473	+			-1.805923	-0.067321	-6.112419	1.02E-08	7.34E-07	9.6449025
cg05890855	chr2	216783091	-			-1.295322	-1.81151	-6.11232	1.02E-08	7.34E-07	9.6444384
cg11430718	chr3	57113702	-	ARHGEF3	TSS1500	-1.197566	-1.723987	-6.112228	1.02E-08	7.34E-07	9.6440078
cg26736232	chr11	628603	-	SCT	TSS1500	-2.082018	-0.406767	-6.11218	1.02E-08	7.34E-07	9.6437792

cg01680823	chr17	37822045	-	TCAP	Body	-1.350135	0.1007065	-6.111963	1.02E-08	7.35E-07	9.6427613
cg04960007	chr17	5820294	-			-1.434639	-0.477006	-6.111983	1.02E-08	7.35E-07	9.6428537
cg07611933	chr16	2773245	-			-1.744558	0.8887045	-6.111894	1.02E-08	7.35E-07	9.6424346
cg23498925	chr13	52952113	-	THSD1;THSD1	Body;Body	0.8503606	1.076565	6.1118711	1.02E-08	7.35E-07	9.642329
cg26348348	chr2	133428993	+	LYPD1;LYPD1;LYPD1	1stExon;5'UTR;TSS1500	-1.983065	-1.435366	-6.114862	1.02E-08	7.35E-07	9.6430246
cg21042456	chr7	1270558	+			2.5805273	-1.138919	6.1117266	1.02E-08	7.35E-07	9.64165
cg11343938	chr3	72978007	-	GXYLT2	Body	0.9022306	2.5005391	6.1115602	1.02E-08	7.36E-07	9.6408678
cg18229521	chr14	92790285	-	SLC24A4;SLC24A4;SLC24A4	1stExon;5'UTR;1stExon	2.4451142	-0.753508	6.1115081	1.02E-08	7.36E-07	9.6406228
cg12297292	chr13	114126424	-	DCUN1D2	Body	0.9744632	1.8213525	6.1114482	1.02E-08	7.36E-07	9.6403412
cg01791874	chr5	16180055	-	11-Mar	TSS200	3.0057823	-1.50777	6.1106915	1.02E-08	7.39E-07	9.6367848
cg01967642	chr1	38229476	+	EPHA10;EPHA10	Body;Body	-1.123405	-1.494794	-6.110649	1.02E-08	7.39E-07	9.6365828
cg13942103	chr1	111177829	-			-1.227329	-0.352401	-6.110432	1.03E-08	7.39E-07	9.6355661
cg15564098	chr7	27281346	-	EVX1	TSS1500	2.1686268	-0.132066	6.1100564	1.03E-08	7.40E-07	9.6338001
cg27110491	chr7	48132109	-	UPP1;UPP1	5'UTR;5'UTR	-1.474061	0.1473563	-6.110073	1.03E-08	7.40E-07	9.6338761
cg01271812	chr2	66671478	-	MEIS1	Body	1.2681085	0.1743408	6.109952	1.03E-08	7.41E-07	9.6333092
cg10394610	chr2	234215965	-	SAG	TSS1500	1.0522222	3.0056985	6.1095772	1.03E-08	7.42E-07	9.6315481
cg16662043	chr16	48846231	+			-1.434291	0.2326126	-6.109458	1.03E-08	7.42E-07	9.6309866
cg18677871	chr5	1103325	-	SLC12A7	Body	0.9364614	0.8028392	6.1092768	1.03E-08	7.43E-07	9.6301362
cg02174359	chr6	43654967	+	MRPS18A	Body	-1.004522	-2.930253	-6.109017	1.03E-08	7.44E-07	9.628917
cg00796728	chr11	94276898	-	FUT4	TSS200	-0.60196	-3.539861	-6.10881	1.03E-08	7.44E-07	9.6279406

cg02109484	chr2	74875253	+	C2orf65	TSS200	1.0642672	0.5009809	6.1117417	1.03E-08	7.45E-07	9.6283819
cg04228935	chr21	36399258	+	RUNX1	Body	2.280291	-1.766639	6.1086239	1.03E-08	7.45E-07	9.627068
cg22613968	chr1	23953556	+	MDS2	TSS1500	-1.289734	-0.861263	-6.107935	1.04E-08	7.47E-07	9.6238317
cg24082440	chr21	33672186	-	MRAP;MRAP	Body;Body	1.0738278	1.5824607	6.1078319	1.04E-08	7.47E-07	9.6233465
cg06434732	chr16	12322653	-	SNX29	Body	-0.794114	-1.877746	-6.107359	1.04E-08	7.49E-07	9.6211265
cg21251475	chr12	116970856	+	NCRNA00173;NCRNA00173	TSS1500;TSS1500	-0.864035	-2.139249	-6.107018	1.04E-08	7.50E-07	9.6195237
cg10648179	chr2	73986783	+			1.1875511	0.0833079	6.1069578	1.04E-08	7.50E-07	9.6192393
cg11706983	chr7	27233454	+			1.4877417	-0.491515	6.1067855	1.04E-08	7.51E-07	9.61843
cg26829395	chr15	40217855	+			-1.488449	-1.198313	-6.106485	1.05E-08	7.52E-07	9.6170162
cg07172334	chr21	31655261	-	KRTAP24-1;KRTAP24-1	1stExon;5'UTR	-1.616336	-0.618849	-6.105507	1.05E-08	7.55E-07	9.6124212
cg12911095	chr5	38468306	+			1.0781685	1.8496408	6.1054107	1.05E-08	7.55E-07	9.6119707
cg14807365	chr17	18908235	+	SLC5A10;SLC5A10;FAM83G	Body;Body;TSS200	-0.664973	-4.784197	-6.104659	1.05E-08	7.58E-07	9.6084384
cg12393318	chr5	87981068	+	LOC645323	TSS1500	1.8379878	-0.798821	6.1045961	1.06E-08	7.58E-07	9.6081441
cg13645732	chr19	45901649	+	PPP1R13L;PPP1R13L	5'UTR;5'UTR	3.1654997	-1.608142	6.1107377	1.06E-08	7.58E-07	9.6107072
cg20555674	chr17	37321631	-	ARL5C	Body	2.3227513	-0.770577	6.10437	1.06E-08	7.58E-07	9.6070824
cg23874437	chr19	38886110	-	SPRED3	Body	1.5059767	-0.204229	6.1043863	1.06E-08	7.58E-07	9.6071588
cg05221326	chr12	126172639	-			-1.272101	-0.469567	-6.104305	1.06E-08	7.59E-07	9.6067772
cg15412759	chr6	50683213	+	TFAP2D	Body	1.6721929	-0.880796	6.1042766	1.06E-08	7.59E-07	9.6066437
cg15481472	chr6	159225665	+	EZR;EZR	Body;Body	-1.387122	0.5717841	-6.103901	1.06E-08	7.60E-07	9.604878
cg27026045	chr8	2242024	-			-1.510203	1.255768	-6.103922	1.06E-08	7.60E-07	9.6049765

cg10022788	chr19	3136019	+	GNA15	TSS200	-1.072295	-1.447008	-6.103848	1.06E-08	7.60E-07	9.6046308
cg08237971	chr1	27290846	+			0.8856938	2.3248957	6.1037597	1.06E-08	7.60E-07	9.6042154
cg16746901	chr11	2428431	-	TRPM5	Body	-1.864948	0.8624943	-6.106495	1.06E-08	7.61E-07	9.6037681
cg18087266	chr1	25257629	+	RUNX3;RUNX3	TSS1500;Body	0.9808493	0.1222832	6.1032376	1.06E-08	7.62E-07	9.6017633
cg00043591	chr7	156874860	+			-1.256731	-0.341478	-6.10278	1.06E-08	7.63E-07	9.5996144
cg17065772	chr3	14991718	+	NR2C2	5'UTR	0.9528337	1.4191569	6.1020475	1.07E-08	7.66E-07	9.5961738
cg17551002	chr1	158517849	+	OR6Y1	1stExon	-1.790704	-0.12517	-6.102021	1.07E-08	7.66E-07	9.5960478
cg19271190	chr6	31554946	+	LST1;LST1;LST1;LST1;LST1;LST1;LST1;LST1	Body;TSS200;5'UTR;5'UTR;TSS200;TSS200;Body;5'UTR	-1.164156	-1.511791	-6.101871	1.07E-08	7.66E-07	9.5953462
cg11850659	chr6	164254856	-			-1.69865	1.204147	-6.101803	1.07E-08	7.66E-07	9.5950232
cg23254184	chr14	53620237	+	DDHD1;DDHD1;DDHD1	TSS200;TSS200;TSS200	-0.630422	-3.852703	-6.101807	1.07E-08	7.66E-07	9.5950455
cg23883696	chr18	70534298	-	NETO1;NETO1;NETO1	TSS1500;TSS1500;Body	1.6029859	-0.613253	6.1015596	1.07E-08	7.67E-07	9.5938825
cg26214026	chr12	111926384	-	ATXN2	Body	1.6277987	1.2004322	6.1014685	1.07E-08	7.67E-07	9.5934548
cg11946503	chr5	134870514	+	NEUROG1;NEUROG1	1stExon;3'UTR	1.4560809	-0.790967	6.1011322	1.07E-08	7.68E-07	9.5918753
cg04240373	chr1	109969611	+	PSMA5	TSS1500	-0.65092	-3.275897	-6.100792	1.08E-08	7.70E-07	9.5902785
cg14298777	chr19	28399736	-			-1.189452	-0.36972	-6.100717	1.08E-08	7.70E-07	9.5899265
cg17071446	chr4	85402497	+			1.516489	-0.61076	6.1037718	1.08E-08	7.70E-07	9.5909944
cg14284630	chr11	56018532	-	OR5T3	TSS1500	-1.511413	-0.863062	-6.100607	1.08E-08	7.70E-07	9.5894106
cg24693341	chr4	41746947	-	PHOX2B	3'UTR	1.849827	-1.145375	6.1004868	1.08E-08	7.70E-07	9.5888448
cg09597710	chr7	1121124	-	C7orf50;C7orf50;C7orf50	Body;Body;Body	2.2449513	3.9760791	6.1000333	1.08E-08	7.72E-07	9.5867153
cg20571967	chr10	73856465	+	ASCC1	3'UTR	0.9258051	2.2157767	6.0998619	1.08E-08	7.72E-07	9.5859104

cg06302118	chr12	51785656	+	GALNT6	TSS1500	-0.504554	-3.753292	-6.099814	1.08E-08	7.72E-07	9.5856845
cg24057775	chr1	2838930	-			-1.114778	-1.73175	-6.099297	1.08E-08	7.74E-07	9.5832567
cg07120889	chr1	150535935	-			-1.249126	-1.879474	-6.099119	1.08E-08	7.75E-07	9.582421
cg05300762	chr19	16990306	+	SIN3B	3'UTR	0.6974307	2.0416576	6.1020821	1.08E-08	7.75E-07	9.5830713
cg13590979	chr20	61803906	+			-1.138062	-0.02187	-6.098727	1.09E-08	7.76E-07	9.5805829
cg11700800	chr5	92906633	+	FLJ42709;FLJ42709;FLJ42709	Body;Body;TSS200	1.3275466	-0.500726	6.1013713	1.09E-08	7.77E-07	9.5800067
cg04974290	chr17	5001047	-			2.1089193	-0.98755	6.0978681	1.09E-08	7.79E-07	9.5765495
cg16641055	chr16	88846292	+	FAM38A	Body	0.9259678	1.4105232	6.0975333	1.09E-08	7.80E-07	9.5749778
cg08975917	chr10	134867969	+			-0.847633	-0.562179	-6.097373	1.09E-08	7.81E-07	9.5742244
cg02409722	chr4	140429978	-	SETD7	3'UTR	0.7695139	1.9302555	6.096842	1.10E-08	7.82E-07	9.5717328
cg25469406	chr11	134288122	+			-1.509596	0.2270256	-6.096834	1.10E-08	7.82E-07	9.5716955
cg08469255	chr6	30851069	+	DDR1;DDR1	TSS1500;TSS1500	-1.102702	-0.187883	-6.099688	1.10E-08	7.83E-07	9.5718476
cg09542210	chr3	157821536	+	SHOX2;SHOX2;SHOX2	Body;Body;Body	2.0945837	-1.274519	6.0961542	1.10E-08	7.85E-07	9.5685041
cg00711496	chr19	50191497	-	C19orf76;PRMT1;PRMT1;PRMT1	TSS1500;Body;Body;Body	1.1474451	1.5467557	6.0959567	1.10E-08	7.85E-07	9.5675773
cg16062877	chr17	25798415	+	KSR1	TSS1500	-1.115181	0.3262399	-6.095903	1.10E-08	7.85E-07	9.5673266
cg10490842	chr22	51016604	-	CPT1B;CPT1B;CPT1B;CHKB-CPT1B;CPT1B;CPT1B;CPT1B;CPT1B	5'UTR;5'UTR;TSS200;Body;5'UTR;5'UTR;5'UTR;5'UTR	0.9446606	0.2754448	6.0955907	1.10E-08	7.86E-07	9.5658594
cg03020635	chr14	82526476	-			-1.303213	-0.557336	-6.098507	1.10E-08	7.87E-07	9.5665792
cg04484550	chr1	2980163	-	FLJ42875	Body	1.3909064	1.9775383	6.0954652	1.10E-08	7.87E-07	9.5652703
cg21183329	chr9	124976219	+	LHX6;LHX6	Body;Body	-1.810506	-2.684325	-6.09853	1.10E-08	7.87E-07	9.5666868
cg15667493	chr5	55404179	+	ANKRD55;ANKRD55	Body;Body	1.2958273	1.2940864	6.095216	1.10E-08	7.87E-07	9.5641009

cg25307168	chr20	37353035	-	SLC32A1	TSS200	2.1109871	-1.081903	6.0951384	1.11E-08	7.88E-07	9.5637364
cg01310330	chr7	1787194	+	ELFN1	3'UTR	-0.968781	-0.189579	-6.095098	1.11E-08	7.88E-07	9.5635465
cg13992911	chr5	1003504	+			-0.922781	-2.380599	-6.094815	1.11E-08	7.88E-07	9.5622193
cg21349914	chr2	79411794	-			-1.586345	0.1758512	-6.094848	1.11E-08	7.88E-07	9.5623717
cg20248163	chr11	74957383	+			-1.170764	-0.291289	-6.094363	1.11E-08	7.90E-07	9.5600964
cg20947259	chr1	201572159	+			-1.893329	0.9092859	-6.094149	1.11E-08	7.91E-07	9.5590933
cg01591152	chr11	119994800	+	TRIM29	Body	-1.185909	-0.600952	-6.093809	1.11E-08	7.92E-07	9.5574966
cg15144153	chr2	74663598	-	RTKN;RTKN;RTKN	Body;5'UTR;Body	0.7200708	1.4988061	6.1091839	1.11E-08	7.93E-07	9.5618875
cg11969108	chr7	154582418	+	DPP6;DPP6;DPP6	Body;Body;Body	-1.274698	-1.130915	-6.092737	1.12E-08	7.96E-07	9.5524688
cg02047286	chr5	1744345	-			-1.513515	-0.117911	-6.092597	1.12E-08	7.96E-07	9.5518108
cg04687486	chr8	33330940	+	FUT10	TSS1500	-0.763487	-2.105008	-6.092511	1.12E-08	7.96E-07	9.5514063
cg20895669	chr13	113622539	-	MCF2L	TSS1500	-1.103488	-0.101986	-6.092539	1.12E-08	7.96E-07	9.5515401
cg23729777	chr15	81547011	+	IL16	Body	-1.127028	-1.287863	-6.092475	1.12E-08	7.96E-07	9.551237
cg10148473	chr12	75601824	+	KCNC2;KCNC2;KCNC2	5'UTR;5'UTR;5'UTR	2.0591206	-1.315061	6.0921003	1.12E-08	7.98E-07	9.5494792
cg26654851	chr18	21269104	+	LAMA3;LAMA3	TSS1500;TSS1500	-0.783866	-3.868297	-6.092098	1.12E-08	7.98E-07	9.549468
cg09594291	chr19	58879022	-	ZNF837;ZNF837	3'UTR;3'UTR	1.0221345	0.5385267	6.0918801	1.12E-08	7.98E-07	9.5484464
cg01850889	chr2	130636190	+			1.2341744	1.7367728	6.0917623	1.12E-08	7.99E-07	9.5478934
cg19244562	chr7	149528625	+	SSPO	Body	-1.49672	0.1932161	-6.091675	1.12E-08	7.99E-07	9.5474837
cg14167415	chr5	77146999	-			1.2178432	0.8536134	6.0914996	1.12E-08	7.99E-07	9.5466608
cg00947782	chr6	30039142	+	RNF39;RNF39	Body;Body	1.0896283	0.1395657	6.0911547	1.13E-08	8.00E-07	9.5450428

cg22070406	chr18	55019708	-	ST8SIA3	TSS200	1.4297611	-1.157139	6.0911308	1.13E-08	8.00E-07	9.5449305
cg22209573	chr17	79652244	-	ARL16;HGS	TSS1500;Body	-1.527407	0.7749944	-6.09118	1.13E-08	8.00E-07	9.5451636
cg24890104	chr6	27648895	-			1.5134277	-1.050208	6.0912137	1.13E-08	8.00E-07	9.5453196
cg24141911	chr5	168196172	+	SLIT3;MIR218-2	Body;TSS1500	-1.291155	0.4744331	-6.090948	1.13E-08	8.01E-07	9.5440724
cg02636004	chr3	119747339	-	GSK3B;GSK3B	Body;Body	1.2171528	1.4616079	6.0908748	1.13E-08	8.01E-07	9.5437294
cg25729445	chr6	29595347	+	GABBR1;GABBR1;GABBR1	Body;1stExon;Body	1.6609412	-0.993565	6.0905652	1.13E-08	8.02E-07	9.542277
cg02798576	chr10	101290168	-			1.8690552	-0.244741	6.0903912	1.13E-08	8.03E-07	9.5414606
cg25895695	chr22	48945618	-	FAM19A5	Body	-1.415352	0.1089688	-6.090195	1.13E-08	8.03E-07	9.5405426
cg11611448	chr2	127453782	+	GYPC;GYPC	3'UTR;3'UTR	-1.779656	0.2290708	-6.09013	1.13E-08	8.03E-07	9.5402349
cg00241941	chr7	56550751	+			-0.868993	-1.410325	-6.093046	1.13E-08	8.04E-07	9.5407229
cg16635314	chr16	31580299	+	CSDAP1	Body	2.4520796	-2.107654	6.0897755	1.13E-08	8.05E-07	9.5385724
cg20025658	chr2	219031640	+	CXCR1;CXCR1	1stExon;5'UTR	-1.321271	-1.221996	-6.089732	1.13E-08	8.05E-07	9.5383672
cg03602280	chr18	11148914	-	FAM38B	TSS200	2.2242957	-0.558569	6.0890655	1.14E-08	8.07E-07	9.5352419
cg27091787	chr3	50360690	-	HYAL2	TSS1500	1.140444	1.0133818	6.089069	1.14E-08	8.07E-07	9.5352582
cg22660933	chr17	46608355	+	HOXB1	TSS200	1.9435222	3.3396517	6.0920557	1.14E-08	8.07E-07	9.5363518
cg23423766	chr6	13614841	+	NOL7	TSS1500	-0.777103	-3.55011	-6.088652	1.14E-08	8.08E-07	9.5333003
:h.12.41606343I	chr12	43320076	+			-1.195459	-3.842581	-6.088566	1.14E-08	8.08E-07	9.5328965
cg15311814	chr7	35296213	+			1.6323484	-0.21895	6.0884198	1.14E-08	8.09E-07	9.532213
cg25053413	chr14	50467526	+	C14orf182	Body	-1.839124	-3.064942	-6.088148	1.14E-08	8.10E-07	9.5309364
cg04231085	chr16	85561302	-			1.2562323	2.1059472	6.0878769	1.15E-08	8.11E-07	9.5296667

cg09709142	chr13	97919888	+	MBNL2;MBNL2	5'UTR;5'UTR	1.4329238	2.825286	6.0908609	1.15E-08	8.11E-07	9.5304855
cg23523480	chr5	153204284	+			-1.509371	0.5497926	-6.087836	1.15E-08	8.11E-07	9.529475
cg00976797	chr16	1562314	-	IFT140	Body	1.526794	2.0428294	6.0874799	1.15E-08	8.12E-07	9.5278045
cg24671734	chr12	107909364	-	BTBD11	Body	-1.557616	-1.981916	-6.087154	1.15E-08	8.13E-07	9.526278
cg06805925	chr17	16233044	+			-1.247108	-1.430655	-6.086721	1.15E-08	8.15E-07	9.5242477
cg06410537	chr5	170736572	+	TLX3	1stExon	1.6986285	-0.718295	6.086613	1.15E-08	8.15E-07	9.5237389
cg10222925	chr8	88885186	-	DCAF4L2	1stExon	-1.896978	0.1256894	-6.086621	1.15E-08	8.15E-07	9.5237772
cg05922911	chr11	57795433	-	OR9Q1	5'UTR	-1.669498	0.3375964	-6.086493	1.15E-08	8.15E-07	9.5231772
cg25684999	chr10	23480803	-	PTF1A	TSS1500	1.7461142	-0.769722	6.0864488	1.15E-08	8.15E-07	9.5229686
cg14701867	chr10	64193068	-	ZNF365;ZNF365	Body;Body	-1.655957	-2.399406	-6.08605	1.16E-08	8.17E-07	9.5210963
cg20043679	chr2	84517321	+	LOC388965	TSS1500	-1.592726	0.108112	-6.085431	1.16E-08	8.19E-07	9.5181963
cg14596108	chr22	49146584	+	FAM19A5;FAM19A5	3'UTR;3'UTR	-1.713017	0.1241457	-6.085296	1.16E-08	8.19E-07	9.5175651
cg16621790	chr3	50383485	+	ZMYND10	TSS1500	-1.287048	-2.815711	-6.088281	1.16E-08	8.20E-07	9.5186726
cg10292376	chr12	52995070	+	KRT72;KRT72;KRT72	1stExon;1stExon;1stExon	0.8793338	-0.425442	6.0849705	1.16E-08	8.21E-07	9.5160364
cg00266983	chr7	1277521	-			2.6238761	-0.529561	6.0846736	1.16E-08	8.22E-07	9.5146446
cg18731327	chr3	147114352	+	ZIC4;ZIC4;ZIC4	Body;Body;Body	1.1568614	-1.050803	6.0846201	1.16E-08	8.22E-07	9.5143933
cg16280667	chr11	118754593	+	CXCR5;CXCR5	5'UTR;1stExon	-1.763415	0.8723452	-6.084378	1.16E-08	8.22E-07	9.5132603
cg21051964	chr12	58022620	-	B4GALNT1	Body	1.6211487	3.5205567	6.090193	1.17E-08	8.24E-07	9.5142826
cg17001194	chr11	116216209	-			-1.52403	0.0893139	-6.084	1.17E-08	8.24E-07	9.5114861
cg10422709	chr15	53083978	-			1.0670017	1.0329754	6.0839274	1.17E-08	8.24E-07	9.5111457

cg21305834	chr6	99291401	-			1.4939695	-1.335866	6.0868045	1.17E-08	8.24E-07	9.5114892
cg07917176	chr20	48522792	-	SPATA2;SPATA2	Body;Body	1.3647088	4.866556	6.0866654	1.17E-08	8.25E-07	9.5108378
cg26673195	chr17	41052729	-	G6PC	TSS200	-1.393408	-1.263552	-6.083646	1.17E-08	8.25E-07	9.5098246
cg15430661	chr12	52403817	-	GRASP	Body	1.1678281	1.6654652	6.0834258	1.17E-08	8.25E-07	9.508794
cg14131536	chr2	98340921	-	ZAP70	Body	1.4036015	1.8476406	6.0832932	1.17E-08	8.26E-07	9.5081722
cg03144922	chr4	111540992	-	PITX2;PITX2;PITX2	Body;Body;Body	1.8911339	-0.272659	6.0831194	1.17E-08	8.26E-07	9.5073574
cg21038785	chr15	53087068	-			1.6818076	-1.269451	6.0829325	1.17E-08	8.27E-07	9.5064813
cg06817840	chr4	122744213	-	CCNA2	Body	-0.88312	-4.26313	-6.082542	1.18E-08	8.28E-07	9.5046523
cg13625026	chr11	1146419	+			-1.399762	-1.175	-6.082437	1.18E-08	8.29E-07	9.5041577
cg15713389	chr5	5568588	+			-2.069879	0.3357257	-6.085464	1.18E-08	8.29E-07	9.5054834
cg17402103	chr10	2267250	+			-1.388888	0.0627891	-6.082405	1.18E-08	8.29E-07	9.5040105
cg01558785	chr6	29638162	-	MOG;MOG;MOG;MOG;MOG;MOG;MOG;MOG;MOG;MOG;<br>MOG	3'UTR;Body;3'UTR;Body;Body;Body;Body;Body;Body<br>;Body;Body	-1.362347	0.3619454	-6.082308	1.18E-08	8.29E-07	9.5035553
cg10202994	chr2	220071050	+	ZFAND2B	TSS1500	-0.449532	-2.922143	-6.082271	1.18E-08	8.29E-07	9.5033798
cg04583232	chr11	22362874	+	SLC17A6	Body	1.1392794	-1.167876	6.0821093	1.18E-08	8.29E-07	9.5026221
cg02164741	chr17	77537018	-			-1.233251	-0.589407	-6.081882	1.18E-08	8.30E-07	9.5015565
cg01248385	chr13	113636953	-	MCF2L;MCF2L	Body;Body	1.6177864	3.9329863	6.0842795	1.18E-08	8.33E-07	9.4999388
cg14487944	chr11	46261352	+			-0.988247	-1.270298	-6.087178	1.18E-08	8.33E-07	9.5001852
cg14214856	chr10	36965362	+			-1.238924	-1.607115	-6.08091	1.18E-08	8.34E-07	9.4969999
cg22280333	chr11	193016	-	SCGB1C1;LOC653486	TSS200;TSS200	-1.014162	0.1357626	-6.080525	1.19E-08	8.35E-07	9.4951976
cg13939859	chr1	91183519	+	BARHL2	TSS1500	2.8226889	-1.804421	6.0804659	1.19E-08	8.35E-07	9.4949184

cg03618016	chr1	247769804	+	OR2G3	1stExon	-1.87925	0.5178209	-6.080363	1.19E-08	8.35E-07	9.4944366
cg00074145	chr15	53075505	+	ONECUT1	Body	2.1728733	0.1747585	6.0802537	1.19E-08	8.36E-07	9.493924
cg05020604	chr19	37096329	-	ZNF529;ZNF382;ZNF529;ZNF529	TSS200;5'UTR;TSS200;TSS200	3.2550322	-1.173842	6.0802717	1.19E-08	8.36E-07	9.4940083
cg10029842	chr1	75595919	-	LHX8	5'UTR	1.7644582	-1.957511	6.0802972	1.19E-08	8.36E-07	9.4941281
cg09118558	chr11	20132641	-	NAV2;NAV2;NAV2;NAV2	Body;Body;Body;Body	-1.120138	-3.41575	-6.082924	1.19E-08	8.37E-07	9.4935963
cg18384960	chr14	101489528	+	MIR299;MIR411	TSS1500;TSS200	-1.581778	0.516026	-6.079862	1.19E-08	8.37E-07	9.4920885
cg09484032	chr12	54329854	-			1.3718003	-0.14418	6.0787841	1.20E-08	8.41E-07	9.4870363
cg00548098	chr16	11295667	-			-1.247893	-1.08192	-6.078689	1.20E-08	8.41E-07	9.4865897
cg11736940	chr1	155265026	+	PKLR;PKLR	Body;Body	0.9540659	-0.302436	6.0786274	1.20E-08	8.41E-07	9.4863022
cg19676181	chr4	56660398	+			1.1429938	1.3196642	6.0782852	1.20E-08	8.43E-07	9.4846985
cg04781796	chr1	1959747	-	GABRD	Body	-2.405956	1.6146991	-6.078122	1.20E-08	8.43E-07	9.4839329
cg13943025	chr11	2210306	+			-1.339326	-0.336782	-6.077762	1.20E-08	8.45E-07	9.4822475
cg25997796	chr10	53350074	+	PRKG1;PRKG1	Body;Body	-1.917968	0.6506297	-6.076651	1.21E-08	8.49E-07	9.4770428
cg02307239	chr4	174421558	+			1.1075206	0.3624109	6.0764331	1.21E-08	8.50E-07	9.4760196
cg08393041	chr3	147109426	+	ZIC4;ZIC4;ZIC4;ZIC4;ZIC4	Body;Body;Body;Body;Body	1.7184629	-0.415428	6.0763184	1.21E-08	8.50E-07	9.4754826
cg15552491	chr11	119037285	-			-1.414712	-0.154509	-6.076195	1.21E-08	8.51E-07	9.4749023
cg07428004	chr7	127912372	+			1.1177587	0.0618895	6.0757736	1.21E-08	8.52E-07	9.4729298
cg23099587	chr4	74570525	-			-1.218204	-2.881454	-6.075451	1.22E-08	8.53E-07	9.4714203
cg21363377	chr7	127651533	-	SND1	Body	1.174428	0.6730888	6.0753882	1.22E-08	8.54E-07	9.4711242
cg19038490	chr17	71733130	+			-1.392309	0.447177	-6.075242	1.22E-08	8.54E-07	9.4704396

cg20067612	chr14	101489415	-	MIR299;MIR411	TSS1500;TSS1500	-1.389071	0.4958935	-6.07509	1.22E-08	8.54E-07	9.4697279
cg22399790	chr20	21501378	+			1.6997558	-1.703908	6.0751038	1.22E-08	8.54E-07	9.4697919
cg19092396	chr1	1143751	+			-1.130402	-1.704283	-6.07489	1.22E-08	8.55E-07	9.4687882
cg03700024	chr2	74666700	+	RTKN;RTKN;RTKN	Body;5'UTR;Body	0.8449331	-0.162289	6.0745763	1.22E-08	8.56E-07	9.4673208
cg10816943	chr1	145382734	+			-0.474952	-3.321002	-6.074337	1.22E-08	8.57E-07	9.4661995
cg05289678	chr7	150549560	+	ABP1	TSS200	-1.010742	-1.103895	-6.07426	1.22E-08	8.57E-07	9.4658388
cg11192022	chr1	156571543	+	GPATCH4;GPATCH4	TSS1500;TSS1500	-0.437865	-4.212237	-6.073925	1.23E-08	8.58E-07	9.4642694
cg17929273	chr22	37967569	-	LGALS2	Body	-1.56034	0.4445958	-6.0739	1.23E-08	8.58E-07	9.4641519
cg26441627	chr11	2044066	+			-1.105974	0.0318596	-6.073927	1.23E-08	8.58E-07	9.4642774
cg02653559	chr3	147112081	+	ZIC4;ZIC4;ZIC4	Body;Body;Body	2.9271146	-1.388916	6.0734158	1.23E-08	8.60E-07	9.4618846
cg26692003	chr3	13063165	-	IQSEC1	Body	-1.700758	1.1419929	-6.073406	1.23E-08	8.60E-07	9.4618389
cg05324273	chr10	60086738	+			0.9019515	1.194503	6.0733689	1.23E-08	8.60E-07	9.4616649
cg03015368	chr16	10065222	-	GRIN2A;GRIN2A;GRIN2A	Body;Body;Body	-1.601459	-0.321713	-6.073155	1.23E-08	8.61E-07	9.4606634
cg09267427	chr12	124953924	+	NCOR2;NCOR2	Body;Body	1.241949	0.3740128	6.073188	1.23E-08	8.61E-07	9.4608175
cg05190002	chr2	219262192	+			-0.706978	-2.868463	-6.073073	1.23E-08	8.61E-07	9.4602787
cg01964152	chr6	30095546	-			1.6199478	-1.043858	6.072997	1.23E-08	8.61E-07	9.4599229
cg09929879	chr10	343794	-	DIP2C	Body	1.2917706	2.3835564	6.0729843	1.23E-08	8.61E-07	9.4598633
cg16797735	chr16	57653530	+	GPR56;GPR56	TSS1500;TSS1500	-1.953618	1.0446917	-6.072641	1.23E-08	8.62E-07	9.4582534
cg19808205	chr1	67217984	+	TCTEX1D1	TSS200	1.6707968	-0.433305	6.072441	1.23E-08	8.63E-07	9.4573187
cg23634413	chr3	116534169	-			-1.632842	0.1433346	-6.072211	1.24E-08	8.64E-07	9.4562425

cg18961589	chr10	133598669	+			-1.564836	1.1654193	-6.071741	1.24E-08	8.66E-07	9.4540402
cg01101020	chr12	132562425	-	EP400	3'UTR	0.8048107	1.7629641	6.0714603	1.24E-08	8.67E-07	9.4527256
cg15490335	chr1	10723174	+	CASZ1;CASZ1	Body;Body	0.7287926	0.6892136	6.0711835	1.24E-08	8.68E-07	9.4514293
cg10488012	chr5	3306368	-			-1.322511	-0.676989	-6.070575	1.25E-08	8.70E-07	9.4485784
cg01211627	chr3	55733455	-	ERC2	Body	0.8242394	1.985391	6.0704427	1.25E-08	8.71E-07	9.4479602
cg18519834	chr20	60303394	+	CDH4	Body	-1.271695	-0.66265	-6.07036	1.25E-08	8.71E-07	9.4475721
cg10266211	chr4	4860082	-	MSX1	TSS1500	0.8577632	0.0047694	6.0701218	1.25E-08	8.72E-07	9.4464575
cg04022112	chr6	33146323	-	COL11A2;COL11A2;COL11A2	Body;Body;Body	-1.128844	-0.05239	-6.069911	1.25E-08	8.73E-07	9.4454689
cg00723044	chr14	71276570	+	MAP3K9	TSS1500	-0.559728	-4.114886	-6.069292	1.25E-08	8.75E-07	9.4425722
cg00373436	chr3	111721343	-	TAGLN3;TAGLN3;TAGLN3	Body;Body;Body	-1.182925	-0.688651	-6.06921	1.25E-08	8.75E-07	9.4421893
cg10636246	chr1	159046973	+	AIM2	TSS1500	-1.488997	-1.304679	-6.068571	1.26E-08	8.78E-07	9.4391952
cg08123106	chr8	810937	+			-1.161528	0.0536573	-6.068414	1.26E-08	8.78E-07	9.4384624
cg25996077	chr2	228331319	-			-1.088173	-1.663044	-6.06843	1.26E-08	8.78E-07	9.4385354
cg04794690	chr1	17768059	-			-1.237291	-1.640213	-6.068065	1.26E-08	8.80E-07	9.4368259
cg23251248	chr14	55907374	-	TBPL2	TSS200	1.7093516	1.6112165	6.0706787	1.26E-08	8.81E-07	9.4363137
cg20638582	chr6	32151457	+	AGER;AGER	Body;Body	0.7532856	1.1122801	6.0675717	1.26E-08	8.81E-07	9.4345175
cg23909079	chr10	87403707	+	GRID1	Body	-1.50578	0.759892	-6.067398	1.27E-08	8.82E-07	9.4337044
cg24084564	chr19	39892799	+			-1.293895	-0.371515	-6.067358	1.27E-08	8.82E-07	9.4335164
cg10473367	chr16	1309550	-			-1.519317	-0.29553	-6.067092	1.27E-08	8.83E-07	9.4322724
cg11949262	chr7	20816184	+			1.3002355	-0.48	6.0671228	1.27E-08	8.83E-07	9.432416

cg15978561	chr2	240196996	+	HDAC4	Body	-1.936276	-2.339734	-6.066888	1.27E-08	8.84E-07	9.4313162
cg14096482	chr5	53625860	-			-1.420296	0.2395781	-6.066828	1.27E-08	8.84E-07	9.4310369
cg08797598	chr7	4751611	+	FOXK1	Body	-2.235898	1.113951	-6.069345	1.27E-08	8.86E-07	9.4298042
cg13241663	chr17	33125534	+			-1.204049	-0.780506	-6.065973	1.27E-08	8.87E-07	9.4270334
cg23940612	chr2	218898788	+			1.1685556	-0.170885	6.0655379	1.28E-08	8.89E-07	9.4249968
cg18975416	chr1	25349562	+			-1.900544	-1.648544	-6.065104	1.28E-08	8.91E-07	9.4229643
cg08271259	chr1	219504623	-			-1.76121	0.1765197	-6.064969	1.28E-08	8.91E-07	9.4223324
cg22890825	chr10	80725166	-	LOC283050;LOC283050;LOC283050	Body;Body;Body	-1.04515	-0.485633	-6.064927	1.28E-08	8.91E-07	9.4221387
cg00240178	chr6	74232108	-	EEF1A1	TSS1500	-1.441698	-0.477893	-6.064793	1.28E-08	8.92E-07	9.4215098
cg06228507	chr20	61637360	-	BHLHE23;BHLHE23	3'UTR;1stExon	1.3673418	-1.047733	6.0647161	1.28E-08	8.92E-07	9.4211505
cg15390649	chr12	114986632	+			-1.534192	0.2885327	-6.067417	1.28E-08	8.93E-07	9.421067
cg18114463	chr1	1476551	+	C1orf70	TSS1500	0.8455486	1.7539051	6.0643222	1.28E-08	8.93E-07	9.4193069
cg00966255	chr16	10479562	+			-1.303789	0.8309543	-6.064221	1.29E-08	8.93E-07	9.4188318
cg23063647	chr4	206339	-	ZNF876P	TSS200	1.5011414	0.2724962	6.064205	1.29E-08	8.93E-07	9.4187584
cg19755459	chr12	19284928	+	PLEKHA5;PLEKHA5	Body;Body	-1.696027	0.1121744	-6.06403	1.29E-08	8.94E-07	9.4179387
cg12475507	chr1	3567852	-	TP73;WDR8	TSS1500;TSS1500	1.1465692	0.2173599	6.0664464	1.29E-08	8.96E-07	9.4165309
cg14759482	chr13	114280854	+	TFDP1;TFDP1	Body;Body	0.7318946	1.1895257	6.0633921	1.29E-08	8.96E-07	9.4149539
cg13685015	chr3	39221003	+			1.2259289	2.0958166	6.0632921	1.29E-08	8.97E-07	9.4144861
cg20957553	chr1	87791719	+			-0.796041	-2.3704	-6.063253	1.29E-08	8.97E-07	9.414304
cg06849778	chr1	16847214	+			1.5206977	-1.085615	6.0631945	1.29E-08	8.97E-07	9.4140295

cg14206523	chr15	35017944	+			-1.380838	-0.413875	-6.063051	1.29E-08	8.97E-07	9.4133581
cg16580289	chr6	90025307	+	GABRR2	TSS1500	0.7012005	2.0961916	6.062641	1.30E-08	8.99E-07	9.411439
cg04556126	chr3	147106561	-	ZIC4;ZIC4;ZIC4;ZIC4;ZIC4	3'UTR;Body;3'UTR;Body;3'UTR	1.6849898	-0.214896	6.0623939	1.30E-08	9.00E-07	9.4102827
cg04810745	chr2	189064557	-			-1.750423	0.2708598	-6.062392	1.30E-08	9.00E-07	9.410273
cg00388812	chr8	99961376	-	OSR2;OSR2	Body;Body	1.0220486	-0.014554	6.0623049	1.30E-08	9.00E-07	9.4098664
cg14210790	chr15	70877277	-			-1.051631	0.1312072	-6.062299	1.30E-08	9.00E-07	9.4098371
cg03339704	chr16	89050731	-			-1.10534	-0.187121	-6.062085	1.30E-08	9.01E-07	9.4088354
cg07362328	chr1	50886393	+	DMRTA2	Body	1.4714854	-1.078072	6.0619942	1.30E-08	9.01E-07	9.4084127
cg25764899	chr1	47697948	+			3.1347719	-1.304713	6.0650072	1.30E-08	9.01E-07	9.4098052
cg08587845	chr5	134870506	+	NEUROG1;NEUROG1	1stExon;3'UTR	1.1606306	-0.800977	6.0619461	1.30E-08	9.01E-07	9.4081875
cg14742937	chr8	39172097	+	ADAM5P	TSS200	1.7028218	0.3920531	6.0649217	1.30E-08	9.01E-07	9.4091314
cg06740578	chr10	131142473	+			-1.338376	-0.109893	-6.061805	1.30E-08	9.01E-07	9.4075261
cg18022224	chr2	176964404	+	HOXD12	TSS200	1.5418681	-1.452459	6.0618139	1.30E-08	9.01E-07	9.4075688
cg13882284	chr4	4859996	-	MSX1	TSS1500	0.9658703	-0.155858	6.0616698	1.30E-08	9.02E-07	9.4068945
cg22210145	chr13	28545263	+			1.8867033	-0.501729	6.0615438	1.30E-08	9.02E-07	9.4063053
cg19535394	chr4	6577205	+	MAN2B2	Body	-0.446324	-4.116779	-6.06115	1.30E-08	9.04E-07	9.4044634
cg15693937	chr5	76429218	-	LOC728723	Body	-1.227033	-0.509253	-6.060945	1.31E-08	9.04E-07	9.4035017
cg02267488	chr4	71858644	-	DCK	TSS1500	-1.123024	-0.514125	-6.060865	1.31E-08	9.04E-07	9.4031309
cg24759919	chr19	7702494	-	STXBP2;STXBP2	Body;Body	-1.057645	-3.384191	-6.063872	1.31E-08	9.04E-07	9.4042276
cg19666687	chr14	98154028	+			-1.7691	0.8360552	-6.060761	1.31E-08	9.05E-07	9.4026443

cg03743720	chr2	241460002	-	ANKMY1;ANKMY1	Body;Body	0.9670895	0.1156781	6.0607164	1.31E-08	9.05E-07	9.4024337
cg00411411	chr10	103455165	+	FBXW4	TSS1500	-0.864109	-0.325093	-6.060538	1.31E-08	9.05E-07	9.401597
cg19809667	chr19	54401945	+	PRKCG	Body	1.1404299	-1.277814	6.0602634	1.31E-08	9.06E-07	9.4003144
cg01807407	chr6	6004686	+	NRN1	Body	1.7339278	-0.814407	6.065775	1.31E-08	9.09E-07	9.4124596
cg12193365	chr5	172945385	+			-1.199908	-0.487344	-6.059573	1.31E-08	9.09E-07	9.3970849
cg18693395	chr14	52735421	-	PTGDR	Body	2.4323932	-1.092678	6.0596033	1.31E-08	9.09E-07	9.3972263
cg17679619	chr4	74542972	-			-1.50672	-0.360067	-6.05916	1.32E-08	9.11E-07	9.3951524
cg00301372	chr2	139654347	-			-1.401218	-1.346475	-6.059123	1.32E-08	9.11E-07	9.3949818
cg07357987	chr2	223289306	+	SGPP2	TSS200	-0.655453	-3.175336	-6.059028	1.32E-08	9.11E-07	9.3945367
cg13910860	chr9	36986407	+	PAX5	Body	2.0882234	-2.371143	6.0589213	1.32E-08	9.11E-07	9.3940359
cg21300993	chr2	237123218	-	ASB18	Body	0.6451939	0.0532205	6.0587067	1.32E-08	9.12E-07	9.3930322
cg05350607	chr1	151777000	+	LINGO4	5'UTR	-1.349989	0.1470051	-6.058535	1.32E-08	9.13E-07	9.3922301
cg07580591	chr15	68851466	-			-2.16873	-2.795795	-6.064316	1.32E-08	9.14E-07	9.39343
cg08220696	chr17	61820713	-	STRADA;STRADA;STRADA;STRADA;STRADA;STRADA	TSS1500;TSS1500;TSS1500;TSS1500;TSS1500;TSS1500	-2.106755	1.0820802	-6.058272	1.32E-08	9.14E-07	9.391001
cg06634862	chr4	54457645	-	LNX1;LNX1	5'UTR;1stExon	-1.159775	-0.629654	-6.058148	1.32E-08	9.14E-07	9.3904172
cg22961278	chr4	81107087	-	PRDM8	5'UTR	1.6527689	-0.506428	6.0579112	1.33E-08	9.15E-07	9.389311
cg21165064	chr1	156402678	-			1.7494249	-1.699497	6.0577131	1.33E-08	9.16E-07	9.3883843
cg24342013	chr13	48891544	+	RB1	Body	1.0903103	3.1064452	6.0572257	1.33E-08	9.18E-07	9.3861047
cg00123055	chr8	55370951	+	SOX17	1stExon	1.4851279	-0.539107	6.0569877	1.33E-08	9.19E-07	9.3849918
cg09863149	chr8	87877382	-	CNBD1	TSS1500	-2.060425	0.8268519	-6.056776	1.33E-08	9.19E-07	9.3840026

cg02557922	chr2	595312	+			-1.1313	-0.582639	-6.056687	1.33E-08	9.20E-07	9.3835841
cg23097079	chr10	135054967	+	VENTX	3'UTR	-0.84146	-1.503546	-6.05934	1.34E-08	9.21E-07	9.3833289
cg16063656	chr2	499710	-			-1.641624	0.3849757	-6.056239	1.34E-08	9.21E-07	9.3814892
cg22624391	chr11	1937457	+			-1.219054	-0.262196	-6.058985	1.34E-08	9.22E-07	9.393957
cg11439695	chr12	2561024	-	CACNA1C;CACNA1C;CACNA1C;CACNA1C;CACNA1C;CACNA1C;CACNA1C;CACNA1C;CACNA1C;CACNA1C;CACNA1C;CACNA1C;CACNA1C;CACNA1C;CACNA1C;CACNA1C;CACNA1C;CACNA	Body;Body;Body;Body;Body;Body;Body;Body;Body;Body;Body;Body;Body;Body;Body;Body;Body;Body;Body;Body;Body	-1.089037	-1.237057	-6.055926	1.34E-08	9.23E-07	9.3800245
cg13605690	chr2	169746671	-	SPC25	5'UTR	-0.875315	-4.158648	-6.055895	1.34E-08	9.23E-07	9.3798824
cg21100077	chr13	100627366	-			1.410332	-0.458827	6.0580247	1.34E-08	9.26E-07	9.376913
cg18682226	chr7	71798428	-	CALN1;CALN1	5'UTR;Body	-1.313447	-0.143014	-6.054939	1.34E-08	9.27E-07	9.3754129
cg20418725	chr2	202122474	-	CASP8;CASP8;CASP8;CASP8;CASP8	5'UTR;5'UTR;5'UTR;TSS1500;5'UTR	-0.844763	-3.72333	-6.054912	1.34E-08	9.27E-07	9.3752821
cg15555712	chr5	149573484	-	SLC6A7	Body	-1.095522	-0.50299	-6.054783	1.35E-08	9.27E-07	9.3746831
cg09416154	chr6	109701201	-	CD164;CD164;CD164;CD164;CD164	Body;Body;Body;Body;Body	-1.513852	-1.975427	-6.054435	1.35E-08	9.28E-07	9.3730519
cg14215310	chr8	1414188	-			-2.153359	1.3983768	-6.054451	1.35E-08	9.28E-07	9.3731282
cg22586653	chr12	115136098	+			1.5896643	-1.468938	6.0540477	1.35E-08	9.30E-07	9.3712428
cg19229692	chr7	131830279	+	PLXNA4	Body	-1.890703	0.2875021	-6.053951	1.35E-08	9.30E-07	9.3707899
cg21163701	chr8	85165845	+	RALYL;RALYL;RALYL;RALYL	5'UTR;5'UTR;5'UTR;Body	-1.597039	-0.258933	-6.053895	1.35E-08	9.30E-07	9.3705272
cg11837293	chr20	52356911	-			-2.489154	-1.574436	-6.053278	1.36E-08	9.33E-07	9.3676451
cg12646227	chr1	46805334	+	NSUN4	TSS1500	-2.011676	0.4444939	-6.053161	1.36E-08	9.33E-07	9.3670986
cg08076830	chr18	44774923	-			1.3013395	-0.368986	6.0530163	1.36E-08	9.34E-07	9.3664204
cg00679556	chr6	30080782	+	TRIM31	5'UTR	-1.449393	0.5501594	-6.05295	1.36E-08	9.34E-07	9.3661097
cg24528164	chr6	30312580	-	RPP21	TSS1500	-0.711941	-1.811255	-6.052872	1.36E-08	9.34E-07	9.3657458

cg24766821	chr2	186603639	+			1.5054499	-0.379639	6.0528379	1.36E-08	9.34E-07	9.3655867
cg25825506	chr7	96626659	-	DLX6AS	Body	1.6631778	-0.966261	6.0528062	1.36E-08	9.34E-07	9.3654384
cg27234490	chr3	58174435	-			0.9440041	2.041303	6.0527343	1.36E-08	9.34E-07	9.365102
ch.14.1499103R	chr14	93412334	+	ITPK1;ITPK1;ITPK1	Body;Body;Body	-0.628584	-4.568188	-6.052287	1.36E-08	9.36E-07	9.3630087
cg03087607	chr6	134210667	-	TCF21;TCF21	1stExon;1stExon	1.4095852	-1.553787	6.0521522	1.36E-08	9.36E-07	9.362381
cg06622151	chr2	45168246	+	SIX3	TSS1500	1.856045	-0.872041	6.0551663	1.36E-08	9.36E-07	9.3638421
cg16021909	chr1	248021091	+	TRIM58	Body	2.5918824	-1.207925	6.0521984	1.36E-08	9.36E-07	9.3625969
cg24756865	chr14	103652182	-			-0.844314	-3.052441	-6.052098	1.36E-08	9.36E-07	9.3621268
cg03726236	chr16	28935666	+	RABEP2	Body	-0.978119	-0.718869	-6.054873	1.36E-08	9.37E-07	9.362195
cg12626882	chr17	11786412	+	DNAH9;DNAH9;DNAH9	5'UTR;Body;1stExon	-1.282182	-1.28229	-6.051825	1.37E-08	9.37E-07	9.3608522
cg06046216	chr1	247680201	+			-2.056153	0.959496	-6.051598	1.37E-08	9.38E-07	9.3597899
cg05722981	chr4	111533509	-			1.4051392	-0.774547	6.0511898	1.37E-08	9.40E-07	9.357882
cg22248052	chr6	50791419	+	TFAP2B	Body	1.8894386	-0.611722	6.0510529	1.37E-08	9.40E-07	9.3572421
cg24006721	chr1	25258225	+	RUNX3;RUNX3	TSS1500;Body	1.4523899	0.2213941	6.0508545	1.37E-08	9.41E-07	9.3563147
cg06626591	chr1	107810111	-	NTNG1;NTNG1;NTNG1	Body;Body;Body	-1.766558	0.7379819	-6.050519	1.37E-08	9.43E-07	9.3547442
cg03290827	chr17	75180760	+	SEC14L1;SEC14L1;SEC14L1;SEC14L1;SEC14L1	Body;Body;TSS1500;Body;Body	1.0713925	1.4557048	6.0504064	1.37E-08	9.43E-07	9.3542199
cg20935245	chr15	42234343	-	EHD4	Body	0.8445248	2.4698508	6.0502353	1.38E-08	9.44E-07	9.3534203
cg26820037	chr10	103534501	+	FGF8;FGF8;FGF8;FGF8	Body;Body;Body;Body	1.3581827	-0.562356	6.0499265	1.38E-08	9.45E-07	9.3519767
cg07010948	chr13	79181613	+			0.9159644	-0.602784	6.0496141	1.38E-08	9.46E-07	9.3505168
cg04752871	chr2	121412432	-			1.1770391	0.3011306	6.0495645	1.38E-08	9.46E-07	9.3502848

cg06708731	chr9	136501420	+	DBH	TSS200	-1.210806	0.0259603	-6.04939	1.38E-08	9.47E-07	9.3494699
cg24700929	chr12	114855417	+			-1.340199	-0.441496	-6.049331	1.38E-08	9.47E-07	9.3491923
cg16985708	chr7	73247674	+			-0.99787	-1.28661	-6.052262	1.38E-08	9.47E-07	9.3502847
cg13804316	chr1	28286884	-	XKR8	Body	-0.528505	-3.898353	-6.04916	1.38E-08	9.48E-07	9.3483954
cg00470636	chr8	26440349	-	DPYSL2	Body	1.4010718	1.1204763	6.0490535	1.38E-08	9.48E-07	9.3478968
cg02189843	chr19	10625775	+	S1PR5;S1PR5	5'UTR;5'UTR	0.9916337	-0.080355	6.0490299	1.38E-08	9.48E-07	9.3477862
cg27380904	chr5	178808910	+			-0.971042	-0.569241	-6.048912	1.38E-08	9.48E-07	9.3472337
cg04571509	chr15	51199642	+	AP4E1	TSS1500	-1.030848	-2.334212	-6.048797	1.39E-08	9.49E-07	9.3466975
cg01857186	chr11	123811110	-	OR4D5	1stExon	-1.973705	0.9073537	-6.04864	1.39E-08	9.49E-07	9.3459663
cg02936347	chr16	20338199	-	GP2;GP2;GP2;GP2	5'UTR;5'UTR;5'UTR;5'UTR	-1.430656	0.0261047	-6.048622	1.39E-08	9.49E-07	9.3458809
cg16472904	chr11	7597402	-	PPFIBP2	Body	-1.087417	-1.5666	-6.048466	1.39E-08	9.50E-07	9.3451523
cg05014721	chr11	118842387	+	FOXR1	TSS200	1.3899124	1.801864	6.0483537	1.39E-08	9.50E-07	9.344626
cg05033369	chr1	161676469	+	FCRLA	TSS1500	-1.669804	-1.592944	-6.048377	1.39E-08	9.50E-07	9.3447336
cg21186560	chr9	13284899	-			-1.060834	-0.483828	-6.048235	1.39E-08	9.50E-07	9.3440703
cg06471596	chr7	101579936	-	CUX1;CUX1;CUX1	Body;Body;Body	1.7983418	1.2369657	6.0572942	1.39E-08	9.50E-07	9.3599264
cg20109054	chr6	31804109	-	C6orf48;C6orf48;SNORD52	5'UTR;5'UTR;TSS1500	-0.568475	-4.251428	-6.047791	1.39E-08	9.52E-07	9.3419966
cg03361164	chr6	100915805	-			1.6502093	-0.769339	6.0507226	1.39E-08	9.52E-07	9.343101
cg25401594	chr19	57323858	+	PEG3;PEG3;PEG3;ZIM2;ZIM2;PEG3;ZIM2;PEG3;PEG3AS	3'UTR;3'UTR;3'UTR;Body;Body;3'UTR;Body;3'UTR;Body	-2.101663	1.0979107	-6.047571	1.39E-08	9.53E-07	9.3409701
cg19345602	chr11	125615388	-	PATE1	TSS1500	-1.218338	-0.747768	-6.04752	1.39E-08	9.53E-07	9.3407316
cg14034778	chr14	78640203	-			-1.354418	0.1868792	-6.04733	1.40E-08	9.54E-07	9.3398401

cg19192101	chr17	9129315	-	NTN1	Body	1.6788049	2.1070288	6.0496	1.40E-08	9.57E-07	9.3378624
cg24728105	chr11	2418157	+	CD81	3'UTR	1.386249	5.1293221	6.0494209	1.40E-08	9.57E-07	9.3367497
cg15776494	chr19	51327928	-	KLK1	TSS1500	-0.929948	-0.276628	-6.046301	1.40E-08	9.58E-07	9.3350357
cg09713225	chr20	400412	-	RBCK1;RBCK1	Body;Body	-1.714775	0.8603413	-6.046097	1.40E-08	9.59E-07	9.3340828
cg00160262	chr1	158533225	+	OR6P1	1stExon	-1.572243	-0.315318	-6.045958	1.40E-08	9.59E-07	9.3334336
cg12126990	chr2	100588044	-	AFF3;AFF3	Body;Body	-1.583604	0.0363003	-6.04597	1.40E-08	9.59E-07	9.3334871
cg14336501	chr17	35424059	+			0.8748081	1.9258731	6.0451182	1.41E-08	9.63E-07	9.329508
cg10132208	chr19	58545542	+	ZSCAN1	5'UTR	1.3963185	-0.112344	6.0449604	1.41E-08	9.63E-07	9.3287705
cg00626422	chr7	22894849	-	SNORD93	TSS1500	-1.597788	-3.160246	-6.044901	1.41E-08	9.64E-07	9.328491
cg02727104	chr13	36729247	+			1.635385	-1.509785	6.0445309	1.41E-08	9.65E-07	9.3267641
cg13858747	chr11	1331831	-	LOC255512;TOLLIP	Body;TSS1500	-1.575899	0.4773488	-6.044134	1.42E-08	9.67E-07	9.3249091
cg02551980	chr5	112628419	+	MCC;MCC	Body;Body	1.1881272	1.9623018	6.0438989	1.42E-08	9.68E-07	9.3238119
cg04880751	chr1	241695164	+	KMO	TSS1500	-1.076057	-3.545888	-6.043902	1.42E-08	9.68E-07	9.3238243
cg08842032	chr17	48612147	-	EPN3	5'UTR	-1.492597	0.2885044	-6.043919	1.42E-08	9.68E-07	9.3239064
cg22881914	chr14	52536147	-	NID2	TSS1500	2.4180623	-0.57729	6.043781	1.42E-08	9.68E-07	9.3232609
cg07757094	chr16	86260847	-			-1.212506	-0.027322	-6.043748	1.42E-08	9.68E-07	9.3231074
cg06123942	chr15	45722796	-	C15orf48;C15orf48;C15orf48;C15orf48	1stExon;5'UTR;5'UTR;1stExon	-1.350113	-3.300561	-6.049667	1.42E-08	9.68E-07	9.325141
cg25993718	chr20	54580196	+	CBLN4	TSS200	1.267429	-1.262313	6.0436694	1.42E-08	9.68E-07	9.3227398
cg13096701	chr4	160188727	-	RAPGEF2	TSS1500	1.214013	1.3148412	6.0434967	1.42E-08	9.69E-07	9.321933
cg13424923	chr2	65258738	+			-1.144719	-1.553049	-6.043415	1.42E-08	9.69E-07	9.3215529

cg14356608	chr7	19184257	+			2.2333383	-0.932651	6.0433739	1.42E-08	9.69E-07	9.3213592
cg23004409	chr17	43128367	+	DCAKD;DCAKD	5'UTR;5'UTR	-0.700593	-2.347235	-6.043392	1.42E-08	9.69E-07	9.3214419
cg18184910	chr6	47624247	-	GPR111	TSS200	-1.468415	0.0072402	-6.04288	1.43E-08	9.71E-07	9.3190535
cg11902748	chr20	36035466	-			-1.135946	0.536724	-6.042625	1.43E-08	9.72E-07	9.3178591
cg00965023	chr19	11785188	+	ZNF833	Body	1.3055819	-0.586954	6.0422209	1.43E-08	9.74E-07	9.3159736
cg16506815	chr2	162101123	-			-1.432065	-0.82592	-6.041918	1.43E-08	9.75E-07	9.3145602
cg04666975	chr14	60977353	-	SIX6	Body	1.5384666	-1.783054	6.0416419	1.43E-08	9.76E-07	9.3132695
cg00360267	chr20	3735052	+	C20orf27	Body	0.9858732	5.6017345	6.0601	1.44E-08	9.77E-07	9.3201464
cg11862144	chr18	61143961	-	SERPINB5	TSS200	-3.192963	1.2322784	-6.044263	1.44E-08	9.77E-07	9.3129654
cg21890551	chr14	62035585	-			-1.639119	-2.976353	-6.044245	1.44E-08	9.77E-07	9.3126051
cg27508551	chr7	27195918	-	HOXA7	1stExon	1.9318864	-1.229718	6.0409949	1.44E-08	9.79E-07	9.3102478
cg10386298	chr1	221050491	-			1.7404244	-0.584586	6.0409341	1.44E-08	9.79E-07	9.3099638
cg24641186	chr6	50804147	-	TFAP2B	Body	1.4210172	-0.109925	6.0408284	1.44E-08	9.79E-07	9.3094703
cg06890522	chr17	6917393	+	RNASEK;C17orf49;C17orf49;C17orf49	Body;TSS1500;TSS1500;TSS1500	-1.026573	-0.114388	-6.040661	1.44E-08	9.80E-07	9.3086871
cg09226786	chr7	96622204	-	DLX6AS	Body	1.6763366	-1.73378	6.0406297	1.44E-08	9.80E-07	9.3085424
cg18950778	chr11	32455362	-	WT1;WT1;WT1;WT1	Body;Body;Body;Body	1.1715886	-1.146021	6.0405889	1.44E-08	9.80E-07	9.3083521
cg04547244	chr7	25019044	-	OSBPL3;OSBPL3;OSBPL3;OSBPL3	5'UTR;5'UTR;5'UTR;5'UTR	-0.883715	-4.341817	-6.04054	1.44E-08	9.80E-07	9.3081247
cg08569678	chr8	143781397	-	LY6K;LY6K;LY6K	TSS200;TSS200;TSS200	-1.795336	-2.003748	-6.040441	1.44E-08	9.80E-07	9.3076626
cg25414639	chr15	88360194	-			-1.851083	0.6587261	-6.040173	1.44E-08	9.81E-07	9.3064097
cg11760857	chr17	18163729	+	SMCR7;SMCR7;SMCR7	TSS200;TSS1500;TSS200	-0.416385	-4.076567	-6.03988	1.45E-08	9.83E-07	9.3050392

cg05922610	chr6	108495985	+	NR2E1	Body	1.7655437	-0.743647	6.0397708	1.45E-08	9.83E-07	9.3045313
cg23617193	chr1	64971761	-	CACHD1	Body	-1.073399	-2.126675	-6.039797	1.45E-08	9.83E-07	9.304653
cg03799024	chr19	54187209	-	MIR515-2;MIR515-1	TSS1500;TSS1500	-1.49042	-0.562006	-6.039558	1.45E-08	9.84E-07	9.303537
cg12011522	chr2	179192421	+	OSBPL6;OSBPL6	Body;Body	-1.157074	-1.59987	-6.039196	1.45E-08	9.85E-07	9.3018465
cg17937101	chr8	1765066	+	MIR596	TSS1500	-2.090295	-2.657862	-6.039098	1.45E-08	9.86E-07	9.3013898
cg10547761	chr20	5829461	-	C20orf196	Body	-1.462807	-0.574779	-6.038996	1.45E-08	9.86E-07	9.3009114
cg16163826	chr11	128756444	+			-1.326993	-1.097556	-6.038917	1.45E-08	9.86E-07	9.3005444
cg21101465	chr13	28493404	+	PDX1	TSS1500	1.3744695	-0.089912	6.0418704	1.45E-08	9.86E-07	9.3015297
cg07055259	chr2	236300855	+			1.0547514	3.7766739	6.0505673	1.46E-08	9.88E-07	9.3030274
cg13445177	chr1	151967021	-	S100A10	TSS1500	-1.48291	-0.580869	-6.038372	1.46E-08	9.88E-07	9.2980004
cg13756014	chr2	204451053	+			-1.598699	1.4329711	-6.04131	1.46E-08	9.88E-07	9.2989155
cg01307130	chr10	71329040	-			2.3283336	-1.154004	6.0382784	1.46E-08	9.88E-07	9.2975631
cg13399544	chr8	144649678	-	C8orf73	Body	0.9690548	0.6926432	6.0408122	1.46E-08	9.90E-07	9.2965954
cg20528837	chr3	45052889	-	EXOSC7;EXOSC7	3'UTR;Body	1.072348	1.5194456	6.0377469	1.46E-08	9.91E-07	9.2950816
cg03873826	chr16	68407311	+	SMPD3	5'UTR	-1.002317	-0.18925	-6.037667	1.46E-08	9.91E-07	9.2947104
cg24059677	chr6	26572124	+			1.1129359	-2.098834	6.0375985	1.46E-08	9.91E-07	9.2943886
cg14279856	chr6	30851086	-	DDR1;DDR1	TSS1500;TSS1500	-1.533293	-0.053878	-6.037152	1.47E-08	9.93E-07	9.2923054
cg04677937	chr9	113016526	-	TXN	Body	-1.634712	1.1866317	-6.036984	1.47E-08	9.94E-07	9.29152
cg07834841	chr10	130185342	+			-1.949214	0.623969	-6.036859	1.47E-08	9.94E-07	9.2909359
cg14103446	chr5	33008910	+			-1.557719	-0.478631	-6.036698	1.47E-08	9.95E-07	9.2901841

cg26630231	chr13	112726482	+			1.8880109	-1.055946	6.0365198	1.47E-08	9.95E-07	9.2893532
cg00102920	chr6	18156401	+	TPMT;KDM1B	TSS1500;5'UTR	-0.384898	-4.437693	-6.03632	1.47E-08	9.96E-07	9.288422
cg24766553	chr11	118842444	-	FOXR1;FOXR1	5'UTR;1stExon	0.7772717	0.7644328	6.0361171	1.47E-08	9.97E-07	9.2874732
cg00572560	chr10	30093517	+			-1.206216	-0.546974	-6.036005	1.47E-08	9.97E-07	9.2869488
cg20212364	chr4	101745102	+			-1.403474	-0.101394	-6.035984	1.47E-08	9.97E-07	9.2868511
cg05267543	chr1	155036802	+	EFNA4;EFNA4;EFNA4	Body;Body;Body	-0.466577	-3.946957	-6.035861	1.48E-08	9.98E-07	9.2862799
cg23156916	chr8	688132	-			2.4830309	-2.139335	6.0387224	1.48E-08	9.98E-07	9.2871313
cg17594624	chr11	77813234	-	ALG8;ALG8	3'UTR;Body	1.0810505	2.3442239	6.0354962	1.48E-08	9.99E-07	9.284575
cg27369542	chr17	14207890	-	HS3ST3B1;MGC12916	Body;Body	1.1685789	1.1664068	6.0355185	1.48E-08	9.99E-07	9.2846788
cg13588599	chr9	131102140	+	SLC27A4	TSS1500	-1.205039	-0.599183	-6.035338	1.48E-08	1.00E-06	9.2838377
cg20970886	chr3	192568714	+	C3orf59	Body	-1.894685	-0.180421	-6.035244	1.48E-08	1.00E-06	9.2833967
cg08361180	chr10	103432427	+	FBXW4	Body	1.3577831	2.2079525	6.0350423	1.48E-08	1.00E-06	9.2824563
cg21751145	chr2	190744274	-			0.8924282	1.6069549	6.0348674	1.48E-08	1.00E-06	9.2816398
cg12928346	chr15	97321851	-			-1.501947	0.3786012	-6.034224	1.49E-08	1.00E-06	9.2786374
cg12990614	chr22	38379749	+	SOX10	Body	0.8659734	0.3505792	6.0342376	1.49E-08	1.00E-06	9.2787002
cg13754877	chr14	101943654	+			-1.028067	-1.120222	-6.034255	1.49E-08	1.00E-06	9.2787827
cg26031046	chr12	56754826	+	STAT2;APOF	TSS1500;3'UTR	-1.481335	0.569809	-6.034201	1.49E-08	1.00E-06	9.2785308
cg03420389	chr16	48720568	+			-1.30913	-0.191729	-6.034025	1.49E-08	1.00E-06	9.2777086
cg12993163	chr3	157821407	+	SHOX2;SHOX2;SHOX2	Body;Body;Body	2.0713334	-1.04924	6.0340407	1.49E-08	1.00E-06	9.2777816
cg08404354	chr10	115311837	+	HABP2	TSS1500	-1.192555	-0.201381	-6.033966	1.49E-08	1.00E-06	9.2774309

cg07169289	chr6	136534480	-			0.7731024	2.1306085	6.0336795	1.49E-08	1.01E-06	9.2760957
cg18155101	chr1	41345795	+			0.8937325	2.5947673	6.0332865	1.49E-08	1.01E-06	9.2742614
cg18640514	chr7	53664246	-			-1.311045	-0.552752	-6.03305	1.50E-08	1.01E-06	9.2731601
cg18921771	chr14	60952196	-	C14orf39	5'UTR	2.25942	-1.412376	6.0329893	1.50E-08	1.01E-06	9.2728747
cg14663607	chr2	219754816	-	WNT10A	Body	0.7996559	1.3977471	6.0327259	1.50E-08	1.01E-06	9.2716453
cg08152263	chr22	35795274	-	MCM5	TSS1500	-1.824914	1.0184406	-6.03264	1.50E-08	1.01E-06	9.2712459
cg08695113	chr10	74842425	-	P4HA1;P4HA1;P4HA1;P4HA1	5'UTR;5'UTR;5'UTR;5'UTR	1.048753	2.0053838	6.0326199	1.50E-08	1.01E-06	9.271151
cg07236958	chr8	140624727	+			-1.456138	-0.452165	-6.032414	1.50E-08	1.01E-06	9.2701904
cg20000464	chr11	76261997	+	C11orf30	3'UTR	1.157871	2.3858033	6.032317	1.50E-08	1.01E-06	9.2697374
cg00513208	chr10	12085641	-	UPF2;UPF2	TSS1500;TSS1500	-1.194255	-1.120998	-6.032016	1.50E-08	1.01E-06	9.2683347
cg15765502	chr7	20816521	-			1.2883828	-0.667772	6.0318146	1.50E-08	1.01E-06	9.2673929
cg11661236	chr17	74944775	+	MGAT5B;MGAT5B	Body;Body	-1.395632	0.2171007	-6.031632	1.51E-08	1.01E-06	9.2665388
cg24881420	chr5	170735754	+	TLX3	TSS1500	1.5675441	-0.414396	6.0316568	1.51E-08	1.01E-06	9.2666567
cg26410635	chr12	96350720	+	AMDHD1	Body	1.2172249	1.1110669	6.0316518	1.51E-08	1.01E-06	9.2666332
cg20995977	chr1	4856996	+			-1.533147	0.1947438	-6.03143	1.51E-08	1.01E-06	9.2655994
cg14384283	chr9	136869371	+			-1.106725	0.3782161	-6.03136	1.51E-08	1.02E-06	9.2652696
cg00321234	chr4	141217605	-	SCOC	5'UTR	-1.154654	-0.637699	-6.031177	1.51E-08	1.02E-06	9.2644196
cg14299508	chr8	105907690	+			-1.97227	0.5594284	-6.030829	1.51E-08	1.02E-06	9.2627965
cg09171112	chr5	166711594	+	ODZ2	TSS1500	-1.435624	-0.46047	-6.030728	1.51E-08	1.02E-06	9.2623244
cg14811102	chr15	53077010	+	ONECUT1	Body	1.0903755	-0.324044	6.0307477	1.51E-08	1.02E-06	9.2624148

cg14277848	chr1	156570825	+	GPATCH4;GPATCH4	Body;5'UTR	-0.86512	-4.451841	-6.030449	1.51E-08	1.02E-06	9.2610221
cg18956547	chr2	219031719	-	CXCR1	TSS200	-1.106012	-2.704419	-6.030247	1.52E-08	1.02E-06	9.2600778
cg19402483	chr22	36806001	+			-0.835561	-2.304488	-6.030306	1.52E-08	1.02E-06	9.2603532
cg26254045	chr19	38886658	+	SPRED3	Body	1.6606242	-0.109507	6.0302763	1.52E-08	1.02E-06	9.2602154
cg18813527	chr8	1408243	-			-1.40332	-0.279097	-6.030066	1.52E-08	1.02E-06	9.2592367
cg00025405	chr5	2135863	-			-1.510168	-0.472761	-6.029922	1.52E-08	1.02E-06	9.2585606
cg21149811	chr2	1151809	+	SNTG2	Body	-1.09125	-0.021973	-6.029903	1.52E-08	1.02E-06	9.258474
cg04832466	chr13	112760088	-			2.568114	-2.517293	6.0297871	1.52E-08	1.02E-06	9.2579333
cg15324651	chr6	137816620	+	OLIG3	TSS1500	1.1341046	-1.062872	6.0297784	1.52E-08	1.02E-06	9.2578929
cg25964007	chr17	9479910	-	WDR16;WDR16;STX8	TSS200;TSS200;TSS1500	-1.774726	-2.953127	-6.029465	1.52E-08	1.02E-06	9.2564321
cg08942939	chr1	119532542	+	TBX15	TSS1500	1.6345564	-0.925436	6.0293907	1.52E-08	1.02E-06	9.2560839
cg08089153	chr11	1956292	+	TNNT3;TNNT3;TNNT3;TNNT3	Body;Body;Body;Body	-0.996402	-0.992432	-6.029241	1.52E-08	1.02E-06	9.2553844
cg27304328	chr1	160519425	+	CD84	Body	-1.542631	0.5311986	-6.029127	1.52E-08	1.02E-06	9.2548543
cg07852825	chr3	172166182	-	GHSR;GHSR	1stExon;1stExon	1.0258268	-0.323822	6.0287411	1.53E-08	1.03E-06	9.2530537
cg18174834	chr1	212740460	-	ATF3	5'UTR	-0.810772	-3.378425	-6.028608	1.53E-08	1.03E-06	9.2524316
cg05700681	chr16	57392764	-	CCL22	1stExon	-1.190692	0.5289653	-6.028526	1.53E-08	1.03E-06	9.2520492
cg20271361	chr17	48666821	-	CACNA1G;CACNA1G;CACNA1G;CACNA1G;CACNA1G;CACNA1G;CACNA1G;CACNA1G;CACNA1G;CACNA1G;CACNA1G;CACNA1G;CACNA1G;CACNA1G;CACNA1G	Body;Body;Body;Body;Body;Body;Body;Body;Body;Body;Body;Body;Body;Body;Body	-0.965318	-2.642399	-6.028432	1.53E-08	1.03E-06	9.2516111
cg11491247	chr1	155035530	-	EFNA4;EFNA4;EFNA4	TSS1500;TSS1500;TSS1500	-1.093117	-2.338326	-6.027592	1.54E-08	1.03E-06	9.2476928
cg21148362	chr3	62353708	+			2.4490432	-2.377084	6.0394672	1.54E-08	1.03E-06	9.2515134
cg18099058	chr11	55737045	-	OR10AG1	TSS1500	-1.336872	-0.193221	-6.027251	1.54E-08	1.03E-06	9.2461047

cg04466022	chr6	37618123	+	MDGA1	Body	1.6876637	3.3120035	6.0421077	1.54E-08	1.03E-06	9.2505628
cg13470063	chr1	43766206	-	TIE1	TSS1500	1.1299429	1.1773878	6.0268404	1.54E-08	1.03E-06	9.244188
cg24724664	chr2	172945383	+	MAP1D	3'UTR	1.278454	0.0784534	6.0264378	1.54E-08	1.04E-06	9.2423103
cg20312687	chr20	29956585	+	DEFB118	Body	-1.701006	-0.109469	-6.025836	1.55E-08	1.04E-06	9.2395042
cg14717557	chr6	100895618	-	SIM1	Body	1.5769957	-0.265178	6.0257744	1.55E-08	1.04E-06	9.2392165
cg07122986	chr16	5452280	+			-1.178398	-0.529714	-6.025606	1.55E-08	1.04E-06	9.2384298
cg25843346	chr6	31762555	-	VARS	Body	-0.833258	-2.191097	-6.028465	1.55E-08	1.04E-06	9.2393381
cg19795722	chr11	132080630	+	NTM;NTM;NTM;NTM	Body;Body;Body;Body	-1.922128	0.6673619	-6.025478	1.55E-08	1.04E-06	9.2378345
cg12426748	chr11	22454152	+			1.3338859	-1.408325	6.0253316	1.55E-08	1.04E-06	9.2371515
cg17770910	chr8	143851427	-	LYNX1;LYNX1	TSS200;Body	-2.46761	0.9327868	-6.025412	1.55E-08	1.04E-06	9.2375287
cg18345386	chr3	62363466	-			1.1803642	-1.13561	6.0253859	1.55E-08	1.04E-06	9.2374046
cg22835157	chr15	41218352	-			-0.887353	-1.815787	-6.025325	1.55E-08	1.04E-06	9.2371229
cg14432910	chr15	68121931	-	LBXCOR1	Body	1.3741853	-1.010154	6.025244	1.55E-08	1.04E-06	9.2367432
cg03721976	chr17	36859825	-			-1.138847	-5.138562	-6.025118	1.55E-08	1.04E-06	9.2361576
cg10457539	chr4	85418584	-	NKX6-1	Body	1.2388338	-0.652644	6.0246854	1.56E-08	1.04E-06	9.2341382
cg09415754	chr6	10385392	+			1.3600522	-0.089328	6.0242781	1.56E-08	1.04E-06	9.2322391
cg14064488	chr7	26331273	+	SNX10	TSS1500	-0.885084	-4.441124	-6.024245	1.56E-08	1.04E-06	9.2320823
cg04651603	chr12	54321302	-			1.3471016	-1.524672	6.0235464	1.57E-08	1.05E-06	9.228827
cg13019143	chr7	96633060	-	DLX6AS	Body	1.260695	-0.871954	6.0232681	1.57E-08	1.05E-06	9.2275295
cg13495205	chr1	4714033	-	AJAP1;AJAP1	TSS1500;TSS1500	2.8324034	-1.107529	6.0231823	1.57E-08	1.05E-06	9.2271297

cg09338875	chr16	4818532	-	ZNF500	TSS1500	-0.767926	-4.194833	-6.022691	1.57E-08	1.05E-06	9.2248402
cg00346985	chr1	46940516	-			1.2748983	0.9309409	6.0283097	1.58E-08	1.05E-06	9.2260344
cg01199721	chr12	120632764	+	GCN1L1	TSS1500	-0.421784	-3.602967	-6.022288	1.58E-08	1.05E-06	9.2229598
cg00865849	chr5	54518208	+			1.1444578	-0.400921	6.0218468	1.58E-08	1.06E-06	9.2209033
cg19715771	chr17	77810912	+	CBX4	Body	-1.090113	-1.620403	-6.0218	1.58E-08	1.06E-06	9.2206847
cg00926215	chr2	223162049	-	PAX3;PAX3;PAX3;CCDC140;PAX3;PAX3;PAX3;PAX3;PAX3	Body;Body;Body;TSS1500;Body;Body;Body;Body;Body	1.7943144	-1.173646	6.0212968	1.58E-08	1.06E-06	9.2183397
cg02167020	chr10	102899949	-			1.934812	-1.925008	6.0212486	1.58E-08	1.06E-06	9.2181147
cg26286961	chr8	19460208	-	CSGALNACT1;CSGALNACT1;CSGALNACT1	TSS200;Body;5'UTR	1.3382494	0.7195412	6.0212767	1.58E-08	1.06E-06	9.2182458
cg19084726	chr1	145075538	+	PDE4DIP	Body	2.0451065	-1.161357	6.0211113	1.58E-08	1.06E-06	9.2174748
cg06496484	chr2	176946509	+	EVX2	Body	1.5644094	-0.136727	6.020878	1.59E-08	1.06E-06	9.2163871
cg18375236	chr19	30024706	-	VSTM2B	Body	-0.819059	-1.124077	-6.020656	1.59E-08	1.06E-06	9.2153523
cg04909432	chr17	72356237	-	BTBD17	Body	-1.098246	0.0263156	-6.020591	1.59E-08	1.06E-06	9.2150511
cg04637478	chr5	140753655	-	PCDHGA4;PCDHGA1;PCDHGA6;PCDHGA5;PCDHGB1;PCDHGA3;PCDHGA6;PCDHGA2;PCDHGB2;PCDHGB3	Body;Body;1stExon;Body;Body;Body;1stExon;Body;Body;Body	1.2642808	-0.684066	6.0259833	1.59E-08	1.06E-06	9.2152196
cg21059786	chr2	10361064	-			-1.646992	-0.150993	-6.019791	1.60E-08	1.06E-06	9.2113221
cg27315333	chr2	63285950	-			1.6463638	-0.49849	6.0194168	1.60E-08	1.07E-06	9.2095765
cg23463742	chr1	27287082	-	C1orf172	TSS200	-1.165995	-4.220749	-6.025212	1.60E-08	1.07E-06	9.2113463
cg07745344	chr8	514802	-			1.0320208	1.2939506	6.0191607	1.60E-08	1.07E-06	9.2083829
cg21353911	chr2	186603398	-			1.4168284	-0.719829	6.018811	1.60E-08	1.07E-06	9.2067531
cg12020396	chr19	55593580	+	EPS8L1;EPS8L1	Body;Body	1.8365467	-0.40099	6.0186196	1.60E-08	1.07E-06	9.2058609
cg04264560	chr16	27125068	-			-1.157786	-0.179348	-6.018514	1.61E-08	1.07E-06	9.2053706

cg02406469	chr15	83381070	+			-1.26311	-0.441853	-6.018325	1.61E-08	1.07E-06	9.2044885
cg16562724	chr16	68270631	-	ESRP2	TSS1500	-0.879573	-1.547878	-6.018326	1.61E-08	1.07E-06	9.2044937
cg10919344	chr11	59210634	+	OR5A1	TSS200	-1.412873	-0.619051	-6.017617	1.61E-08	1.07E-06	9.201187
cg06151243	chr17	5000872	-			1.5394687	-0.886393	6.0170352	1.62E-08	1.08E-06	9.1984775
cg12863443	chr10	99894013	+	C10orf28	TSS1500	-1.322236	0.1497289	-6.016982	1.62E-08	1.08E-06	9.1982319
cg15312943	chr11	17843855	-	SERGEF	Body	1.1858376	0.851196	6.0168807	1.62E-08	1.08E-06	9.1977578
cg14658067	chr7	1273394	+	UNCX	Body	2.0416184	-0.885303	6.0167698	1.62E-08	1.08E-06	9.1972408
cg13237945	chr13	43355739	+	C13orf30;C13orf30	1stExon;5'UTR	1.0460549	1.5464284	6.0165985	1.62E-08	1.08E-06	9.1964428
cg02046340	chr20	48552742	+	RNF114	TSS200	-0.68554	-3.587978	-6.016438	1.62E-08	1.08E-06	9.1956943
cg02161900	chr10	28035631	-	MKX	TSS1500	1.5029138	-1.12124	6.0164666	1.62E-08	1.08E-06	9.1958283
cg04953061	chr14	53620393	-	DDHD1;DDHD1;DDHD1	TSS1500;TSS1500;TSS1500	-0.862345	-1.492911	-6.016308	1.62E-08	1.08E-06	9.1950871
cg21487856	chr2	54828502	-	SPTBN1;SPTBN1	Body;Body	1.3233351	0.6476183	6.0162931	1.62E-08	1.08E-06	9.1950198
cg19107511	chr12	52476217	-			1.1368608	0.1918337	6.0160501	1.62E-08	1.08E-06	9.1938875
cg10945695	chr5	32871131	-			-1.759426	0.5881289	-6.015676	1.63E-08	1.08E-06	9.1921458
cg05103992	chr6	159290440	+			-1.382297	-4.617196	-6.018524	1.63E-08	1.08E-06	9.1930675
cg25742246	chr1	214153460	+			2.0263188	-1.1116	6.0148173	1.63E-08	1.09E-06	9.1881439
cg17827477	chr22	49487343	+			-1.239283	0.3467053	-6.014769	1.63E-08	1.09E-06	9.187918
cg19276014	chr10	133598638	+			-2.759079	2.5625846	-6.014732	1.63E-08	1.09E-06	9.1877485
cg19375196	chr17	45773559	-	TBKBP1	1stExon	0.8001922	0.5092342	6.0144807	1.64E-08	1.09E-06	9.1865759
cg01544903	chr16	56697229	-			2.3721215	-0.487461	6.0144312	1.64E-08	1.09E-06	9.1863452

cg26218269	chr4	151503227	+	LRBA;MAB21L2;MAB21L2	Body;1stExon;5'UTR	0.9272406	2.3231135	6.0142773	1.64E-08	1.09E-06	9.1856283
cg19755003	chr22	46489736	+	LOC400931	Body	0.8321476	0.5071109	6.0141123	1.64E-08	1.09E-06	9.1848596
cg20012537	chr10	134928794	+	GPR123	Body	-0.958025	-0.967288	-6.01411	1.64E-08	1.09E-06	9.1848504
cg10991475	chr11	134651602	+			-1.304154	-1.67387	-6.01405	1.64E-08	1.09E-06	9.1845686
cg21684179	chr1	110576933	-	FAM40A	TSS1500	-1.241542	-0.517234	-6.01397	1.64E-08	1.09E-06	9.1841984
cg03384552	chr11	36807594	-			-1.480702	0.0066176	-6.013922	1.64E-08	1.09E-06	9.1839726
cg16770238	chr18	14747661	-	ANKRD30B	TSS1500	1.1557224	0.7506545	6.0136632	1.64E-08	1.09E-06	9.1827677
cg16101077	chr10	129676142	+	CLRN3	3'UTR	-1.232243	-0.424961	-6.013474	1.64E-08	1.09E-06	9.1818857
cg04166042	chr1	50896246	+			1.8049802	-0.505893	6.0134061	1.65E-08	1.09E-06	9.1815698
cg17875429	chr12	52432565	+			0.9685468	0.754227	6.0125729	1.65E-08	1.10E-06	9.1776888
cg09273059	chr10	71077221	-	HK1;HK1;HK1;HK1;HK1	Body;TSS1500;Body;Body;Body	-0.934817	0.0572222	-6.012526	1.65E-08	1.10E-06	9.1774727
cg04902729	chr1	181452707	-	CACNA1E	TSS200	1.9017426	-1.24334	6.0124059	1.65E-08	1.10E-06	9.1769111
cg06241779	chr18	21718779	+	CABYR;CABYR;CABYR;CABYR;CABYR;CABYR	TSS200;TSS200;TSS200;TSS200;TSS1500;TSS200	-1.015731	-3.309626	-6.011802	1.66E-08	1.10E-06	9.1740989
cg12298745	chr18	76737545	+			1.5079697	-0.516831	6.0114326	1.66E-08	1.10E-06	9.1723778
cg22127773	chr17	7754785	+	KDM6B	Body	-2.189437	2.6846689	-6.011433	1.66E-08	1.10E-06	9.1723802
cg07234028	chr4	2043594	+	C4orf48;C4orf48	TSS200;TSS200	-0.673955	-3.46731	-6.011378	1.66E-08	1.10E-06	9.1721227
cg27177779	chr7	2753825	-	AMZ1	3'UTR	0.8236283	3.5216115	6.011212	1.66E-08	1.10E-06	9.1713504
cg03275459	chr15	95889928	+			-1.721156	-0.071887	-6.010976	1.66E-08	1.10E-06	9.1702523
cg27008316	chr12	34753697	+			-0.759928	-2.675543	-6.010533	1.67E-08	1.11E-06	9.1681865
cg23065934	chr5	16180266	-	11-Mar	TSS1500	2.3837755	-1.178854	6.013119	1.67E-08	1.11E-06	9.1676404

cg17696160	chr14	37136192	-	PAX9	Body	1.6823254	-1.252485	6.0095882	1.68E-08	1.11E-06	9.1637888
cg12018140	chr5	122434326	+	PRDM6	Body	1.0314262	0.7801289	6.0094293	1.68E-08	1.11E-06	9.1630487
cg17305368	chr7	76022370	-	SRCRB4D	Body	-0.696801	-5.485962	-6.009432	1.68E-08	1.11E-06	9.1630605
cg11078221	chr12	115135934	-			1.5600829	-1.511958	6.0092675	1.68E-08	1.11E-06	9.1622957
cg19942083	chr12	7070562	-			-1.222723	-1.709873	-6.009244	1.68E-08	1.11E-06	9.1621865
cg05563515	chr6	30039027	-	RNF39;RNF39	Body;Body	1.5799459	2.4654259	6.0090598	1.68E-08	1.11E-06	9.1613284
cg07052041	chr10	135092104	+			-1.206544	-1.622324	-6.009051	1.68E-08	1.11E-06	9.1612872
cg05363616	chr1	119529219	-	TBX15	5'UTR	1.2417305	-0.399889	6.0087171	1.68E-08	1.11E-06	9.1597329
cg08305436	chr19	22806184	+			1.5162849	0.2558495	6.0086834	1.68E-08	1.11E-06	9.1595758
cg08317412	chr11	124481193	-	PANX3	TSS1500	-1.076786	-0.208351	-6.008436	1.69E-08	1.11E-06	9.1584236
cg11906285	chr5	23315719	-			-2.006009	0.1364412	-6.011265	1.69E-08	1.12E-06	9.1590203
cg07109438	chr10	118561572	+			-1.375745	0.9927142	-6.008204	1.69E-08	1.12E-06	9.1573429
cg08087668	chr8	58116908	+			-1.138062	0.964108	-6.007905	1.69E-08	1.12E-06	9.1559505
cg08718490	chr4	52943099	+	SPATA18	Body	1.8642927	-0.285799	6.0108171	1.69E-08	1.12E-06	9.1699082
cg03886192	chr5	161495049	-	GABRG2;GABRG2;GABRG2	1stExon;1stExon;1stExon	1.698835	-0.943601	6.0077638	1.69E-08	1.12E-06	9.1552944
cg21883598	chr15	45404157	+	DUOX2	Body	2.0313823	-0.8704	6.0106368	1.69E-08	1.12E-06	9.1563819
cg17786697	chr5	122430509	-	PRDM6	Body	1.0106522	-0.354716	6.0076168	1.69E-08	1.12E-06	9.1546098
cg03276489	chr2	240449683	+			-0.898969	0.8985796	-6.007194	1.70E-08	1.12E-06	9.1526409
cg10727432	chr14	72083707	+	SIPA1L1	Body	-1.110492	0.6428137	-6.007083	1.70E-08	1.12E-06	9.1521265
cg01200264	chr14	101505523	+	MIR654;MIR376B;MIR376C;MIR376A2	TSS1500;TSS1500;TSS1500;TSS1500	-1.444523	-0.064794	-6.007035	1.70E-08	1.12E-06	9.1519035

cg18055067	chr5	148803404	-	LOC728264	Body	0.969368	1.1163974	6.0065769	1.70E-08	1.12E-06	9.1497686
cg16443812	chr22	50987294	-	KLHDC7B	1stExon	1.9376103	-1.329477	6.006513	1.70E-08	1.12E-06	9.1494715
cg14375890	chr6	13355672	+			-1.728904	-0.009512	-6.006272	1.70E-08	1.12E-06	9.1483501
cg04311653	chr10	133204241	-			-2.508776	0.8395326	-6.012015	1.70E-08	1.12E-06	9.1500429
cg05049825	chr2	124944568	+	CNTNAP5	Body	-1.946675	0.6905466	-6.006134	1.70E-08	1.12E-06	9.147706
cg16794506	chr2	119600002	+	EN1	3'UTR	2.236192	-0.925247	6.0061576	1.70E-08	1.12E-06	9.1478167
cg17180631	chr1	201708788	-	NAV1;NAV1	TSS200;Body	-1.745286	-3.163958	-6.005973	1.71E-08	1.13E-06	9.1469555
cg14909495	chr7	150549472	-	ABP1	TSS200	-0.892996	-1.596208	-6.005794	1.71E-08	1.13E-06	9.1461253
cg24376337	chr11	56430801	+	OR5AR1;OR8U8	TSS1500;Body	-1.676886	0.759094	-6.005751	1.71E-08	1.13E-06	9.1459263
cg14450238	chr2	219262134	-			-0.5563	-4.170379	-6.005709	1.71E-08	1.13E-06	9.1457305
cg18479593	chr14	57265560	+			2.182618	-0.602695	6.0085108	1.71E-08	1.13E-06	9.1592056
cg03599224	chr6	31541349	-	LTA;LTA	Body;Body	-1.048651	0.0306009	-6.005407	1.71E-08	1.13E-06	9.1443252
cg00971396	chr3	184081400	+	POLR2H	Body	-0.415658	-3.924935	-6.005271	1.71E-08	1.13E-06	9.14369
cg02207219	chr6	30080968	+	TRIM31	TSS200	-1.371795	-0.730348	-6.005115	1.71E-08	1.13E-06	9.1429626
cg05923226	chr19	15121516	+	CCDC105	TSS200	1.0334797	0.1723127	6.004986	1.71E-08	1.13E-06	9.1423636
cg12179658	chr3	11178652	-	HRH1	TSS200	-1.06414	-0.310577	-6.005015	1.71E-08	1.13E-06	9.1424979
cg20417953	chr17	72426797	-	GPRC5C	TSS1500	-1.296354	-2.914786	-6.007694	1.72E-08	1.13E-06	9.1426996
cg07697082	chr8	82753677	-	SNX16;SNX16;SNX16	5'UTR;5'UTR;5'UTR	-0.7668	-3.132161	-6.004112	1.72E-08	1.13E-06	9.1382944
cg20069718	chr12	50898249	+	DIP2B	TSS1500	-2.281829	1.8200683	-6.003985	1.72E-08	1.13E-06	9.1377029
cg05917988	chr6	44281197	+	AARS2	TSS200	-0.71708	-2.691087	-6.003904	1.72E-08	1.13E-06	9.1373279

cg00989365	chr14	70014548	+			1.5720996	0.080076	6.0038448	1.72E-08	1.13E-06	9.1370519
cg03050096	chr14	101530580	+	MIR412;MIR541;MIR409;MIR369	TSS1500;TSS1500;TSS1500;TSS1500	-0.968633	0.1479798	-6.003601	1.73E-08	1.14E-06	9.1359154
cg12485428	chr17	1549098	-	SCARF1;SCARF1;SCARF1;SCARF1;SCARF1	TSS200;TSS200;TSS200;TSS200;TSS200	0.9925849	0.2640708	6.0033604	1.73E-08	1.14E-06	9.1347978
cg01714553	chr17	60459065	+	EFCAB3;EFCAB3	Body;5'UTR	-1.04482	-0.276694	-6.003152	1.73E-08	1.14E-06	9.1338282
cg10608596	chr19	11785125	-	ZNF833	Body	1.7135813	0.5650979	6.0030371	1.73E-08	1.14E-06	9.1332932
cg02947450	chr10	22766861	-			0.9740197	1.3277608	6.0118875	1.73E-08	1.14E-06	9.1365378
cg05995576	chr15	35049543	+			-1.19365	-0.642252	-6.002964	1.73E-08	1.14E-06	9.1329547
cg09572195	chr20	55202447	+			1.7462362	-1.77931	6.0058394	1.73E-08	1.14E-06	9.134082
cg04655520	chr7	134576239	+	CALD1;CALD1;CALD1;CALD1;CALD1;CALD1;CALD1	Body;1stExon;5'UTR;1stExon;5'UTR;Body;Body	-1.293642	-2.931287	-6.002671	1.73E-08	1.14E-06	9.1315879
cg15152595	chr1	204972818	+	NFASC;NFASC;NFASC;NFASC	Body;Body;Body;Body	-1.615337	0.200787	-6.002677	1.73E-08	1.14E-06	9.1316178
cg02657401	chr19	22469223	+			1.8837116	-0.159268	6.0023859	1.74E-08	1.14E-06	9.1302629
cg07351059	chr16	29999179	+	TAOK2;TAOK2	Body;Body	1.4452361	5.0820498	6.017344	1.74E-08	1.14E-06	9.1359968
cg02716614	chr6	131101115	-			-1.830281	0.2543191	-6.002084	1.74E-08	1.14E-06	9.1288558
cg19810954	chr21	46496510	-	ADARB1;ADARB1;ADARB1;ADARB1;ADARB1;ADARB1;ADARB 1	5'UTR;5'UTR;5'UTR;Body;Body;5'UTR;Body	0.9916724	0.8039599	6.0019807	1.74E-08	1.14E-06	9.1283775
cg10006515	chr19	34895882	+	PDCD2L	Body	-0.755007	-2.018682	-6.001634	1.74E-08	1.14E-06	9.1267624
cg21842967	chr14	53508673	+	DDHD1;DDHD1;DDHD1	3'UTR;3'UTR;3'UTR	1.0411883	1.5990093	6.0009957	1.75E-08	1.15E-06	9.1237944
cg01518398	chr2	74406523	+	MOBKL1B	TSS1500	-0.897527	-0.693231	-6.000928	1.75E-08	1.15E-06	9.1234784
cg05085500	chr6	19692172	+			1.6904792	-0.845168	6.0005022	1.75E-08	1.15E-06	9.1214981
cg12183861	chr17	45773468	+	TBKBP1;TBKBP1	1stExon;5'UTR	0.7134989	0.9570429	6.000346	1.75E-08	1.15E-06	9.1207717
cg15634980	chr6	36253026	+	PNPLA1;PNPLA1;PNPLA1	5'UTR;Body;5'UTR	2.1040907	-1.206133	6.0001785	1.75E-08	1.15E-06	9.1199922

cg12554573	chr3	51976667	-	PARP3;RRP9;PARP3;PARP3;PARP3	5'UTR;TSS1500;1stExon;1stExon;5'UTR	-0.892	-0.629169	-5.999916	1.76E-08	1.15E-06	9.1187716
cg07126399	chr14	101530537	-	MIR412;MIR541;MIR409;MIR369	TSS1500;TSS1500;TSS1500;TSS1500	-1.331726	-0.225206	-5.999537	1.76E-08	1.15E-06	9.1170072
cg22559878	chr17	1038005	-	ABR;ABR	Body;5'UTR	0.8094116	1.4151231	5.9994671	1.76E-08	1.16E-06	9.1166827
cg19247475	chr5	140789745	+	PCDHGA4;PCDHGA9;PCDHGA1;PCDHGB1;PCDHGB6;PCDHGB6;PCDHGB3;PCDHGA6;PCDHGA8;PCDHGA5;PCDHGB4;PCDHGA3;PCDHGA2;PCDHGB2;PCDHGA7;PCDHGB5	Body;Body;Body;Body;1stExon;1stExon;Body;Body;Body;Body;Body;Body;Body;Body;Body;Body	0.9992662	0.4138405	5.9990772	1.76E-08	1.16E-06	9.1148689
cg16821385	chr11	46467232	-	AMBRA1	Body	0.676439	2.5846209	5.9990228	1.76E-08	1.16E-06	9.114616
cg20067471	chr6	32765281	-			-0.831139	-3.508223	-5.998957	1.76E-08	1.16E-06	9.1143078
cg26252167	chr6	110300573	-	GPR6	1stExon	1.725648	-1.047271	6.0108649	1.77E-08	1.16E-06	9.1193132
cg22502382	chr12	32701732	-	FGD4	5'UTR	1.4034928	-0.181497	5.9988579	1.77E-08	1.16E-06	9.1138486
cg03815116	chr4	44654206	-	YIPF7	TSS1500	-1.483711	0.1098682	-5.998752	1.77E-08	1.16E-06	9.1133576
cg01369750	chr8	688018	-			2.0829894	-1.73986	5.998612	1.77E-08	1.16E-06	9.1127049
cg10483261	chr7	1632661	+			-0.97337	-1.555932	-5.998612	1.77E-08	1.16E-06	9.1127035
cg05944137	chr7	155301734	-	CNPY1	5'UTR	1.2987488	-0.530714	5.9982224	1.77E-08	1.16E-06	9.1108924
cg14781521	chr3	129063090	-			-1.645902	-3.145106	-5.998192	1.77E-08	1.16E-06	9.1107503
cg24604013	chr13	112721325	+	SOX1	TSS1500	1.9911466	-0.761082	5.9978351	1.77E-08	1.16E-06	9.1090909
cg16836355	chr15	41218333	-			-1.16918	-2.063708	-6.000668	1.78E-08	1.16E-06	9.1097709
cg09173768	chr2	176989349	-	HOXD9	3'UTR	1.8341605	0.1447792	5.9977057	1.78E-08	1.16E-06	9.1084895
cg05406637	chr14	51332290	+			0.7892574	1.4729506	5.997173	1.78E-08	1.17E-06	9.1060116
cg13781149	chr6	27114655	+	HIST1H2BK;HIST1H2AH	TSS200;TSS1500	-0.663079	-5.218271	-5.997166	1.78E-08	1.17E-06	9.1059802
cg15839752	chr12	6933689	-	GPR162;GPR162	Body;Body	-1.196693	0.0896253	-5.997173	1.78E-08	1.17E-06	9.1060115
cg21865751	chr14	96283686	+			-1.550458	0.135302	-5.996959	1.78E-08	1.17E-06	9.1050161

cg03585053	chr2	240169028	-	HDAC4	Body	-1.992905	0.6331761	-5.996842	1.78E-08	1.17E-06	9.1044735
cg04928338	chr16	5286492	+			-1.551275	-0.050072	-5.996832	1.78E-08	1.17E-06	9.1044235
cg05955251	chr11	2447537	+			-1.825279	1.3269931	-5.996886	1.78E-08	1.17E-06	9.1046765
cg19712603	chr13	53422777	+	PCDH8;PCDH8	TSS200;TSS200	2.4511852	-1.190546	5.9967937	1.78E-08	1.17E-06	9.1042476
cg06275859	chr4	1407694	-			1.3035947	-0.794017	5.9961105	1.79E-08	1.17E-06	9.1010703
cg00792424	chr6	10386160	-			1.5769846	-0.814811	5.9960623	1.79E-08	1.17E-06	9.100846
cg10122050	chr10	106059062	-	GSTO2	3'UTR	1.0467146	1.1547436	5.9960434	1.79E-08	1.17E-06	9.1007579
cg23685969	chr10	73976821	+	ASCC1;C10orf104	TSS1500;5'UTR	-1.326	-0.541457	-5.99599	1.79E-08	1.17E-06	9.1005091
cg00246301	chr2	101086963	-	NMS	1stExon	-1.470644	0.2855213	-5.995832	1.79E-08	1.17E-06	9.0997771
cg12951849	chr18	11148932	-	FAM38B	TSS200	1.9672661	-1.480301	5.9957175	1.79E-08	1.17E-06	9.0992424
cg07333608	chr2	240445555	-			-1.217941	-0.25723	-5.995577	1.79E-08	1.17E-06	9.0985894
cg18577239	chr12	53955664	-	ATF7;ATF7;ATF7	Body;Body;Body	-1.454055	-0.494675	-5.995573	1.79E-08	1.17E-06	9.0985708
cg16510350	chr4	112477798	-			-1.366568	-0.1282	-5.995283	1.80E-08	1.17E-06	9.0972229
cg23878241	chr12	111019734	-	PPTC7	Body	-1.583112	-2.162608	-5.994964	1.80E-08	1.17E-06	9.0957366
cg02792792	chr5	115152420	-	CDO1	TSS200	1.7357437	-1.399587	5.994851	1.80E-08	1.18E-06	9.0952132
cg22024572	chr22	38866743	-	KDELR3;KDELR3	Body;Body	0.895124	2.2622345	5.994701	1.80E-08	1.18E-06	9.0945154
cg04598774	chr11	31821590	-	PAX6;PAX6;PAX6	Body;Body;Body	1.3147154	-1.208862	5.9945671	1.80E-08	1.18E-06	9.0938931
cg19537558	chr5	167550996	-	ODZ2	Body	-1.769145	0.5524057	-5.994527	1.80E-08	1.18E-06	9.0937069
cg21026566	chr14	101759654	+			-1.56513	0.5196928	-5.994197	1.81E-08	1.18E-06	9.0921727
cg03188495	chr19	3136515	+	GNA15	1stExon	-0.645689	-1.050415	-5.993777	1.81E-08	1.18E-06	9.0902191

cg26579578	chr11	69707303	-			-2.647278	2.4509265	-6.005693	1.81E-08	1.18E-06	9.0957041
cg20388165	chr15	101748973	+	CHSY1	Body	0.9044094	1.6186747	5.9935056	1.81E-08	1.18E-06	9.0889572
cg24359868	chr21	30261524	-			-1.397641	0.6437727	-5.9934	1.81E-08	1.18E-06	9.0884682
cg27166177	chr22	49498376	+			-1.214437	-0.836376	-5.993091	1.82E-08	1.18E-06	9.0870295
cg17298751	chr11	22363370	+	SLC17A6	Body	1.3271752	0.5487694	5.9928566	1.82E-08	1.19E-06	9.0859401
cg07502829	chr13	112708288	-			1.2336488	-0.57462	5.9924813	1.82E-08	1.19E-06	9.084195
cg07344468	chr11	127811816	-			-1.242401	-1.32139	-5.992177	1.82E-08	1.19E-06	9.0827805
cg08618451	chr5	96117388	-	ERAP1;ERAP1	Body;Body	1.4044327	1.9613813	5.9918497	1.83E-08	1.19E-06	9.0812588
cg14151372	chr10	108045557	+			-1.374672	-0.352776	-5.991665	1.83E-08	1.19E-06	9.0803985
cg23008177	chr1	155036577	+	EFNA4;EFNA4;EFNA4	Body;Body;Body	1.0238232	-1.845667	5.9916673	1.83E-08	1.19E-06	9.080411
cg22984992	chr19	13056423	+	RAD23A	TSS1500	-1.123523	-2.3137	-5.991326	1.83E-08	1.19E-06	9.078825
cg09664215	chr1	158325254	-	CD1E;CD1E;CD1E;CD1E;CD1E;CD1E	Body;Body;Body;Body;Body;Body	-1.704191	0.43306	-5.993915	1.83E-08	1.19E-06	9.0784105
cg22498784	chr19	35982399	+	KRTDAP	TSS1500	-1.41316	-0.115707	-5.990687	1.84E-08	1.20E-06	9.0758533
cg03760191	chr10	22623047	-			1.2536758	2.5677679	5.9905137	1.84E-08	1.20E-06	9.0750486
cg04057858	chr8	10383763	-	T-SP1	Body	-1.095702	-0.538011	-5.990428	1.84E-08	1.20E-06	9.0746515
cg24686357	chr4	48272266	-	TEC	TSS1500	-0.889681	-4.614719	-5.993216	1.84E-08	1.20E-06	9.0754529
cg04662056	chr17	73506017	-	CASKIN2;CASKIN2	Body;TSS200	1.0895906	1.4168886	5.9902259	1.84E-08	1.20E-06	9.0737108
cg08123277	chr3	24537469	+	THRB;THRB;THRB	TSS1500;TSS1500;TSS1500	-1.239427	-0.805127	-5.990153	1.84E-08	1.20E-06	9.0733715
cg21140843	chr17	1398405	-	INPP5K;INPP5K;INPP5K	3'UTR;3'UTR;3'UTR	0.7105043	1.7235607	5.9897254	1.85E-08	1.20E-06	9.0713848
cg22739858	chr5	142053365	-	FGF1;FGF1;FGF1;FGF1;FGF1;FGF1	5'UTR;5'UTR;5'UTR;5'UTR;Body;Body	-0.974179	-0.198981	-5.989708	1.85E-08	1.20E-06	9.0713031

cg06132727	chr4	174422191	-			1.5623695	0.1057706	5.9895635	1.85E-08	1.20E-06	9.0706324
cg26251325	chr5	54518745	+			1.8274675	-1.249833	5.989488	1.85E-08	1.20E-06	9.0702812
cg23015341	chr6	50813341	+	TFAP2B	3'UTR	1.0061041	-0.775103	5.9890863	1.85E-08	1.20E-06	9.0684143
cg16557204	chr11	27264214	-			-1.498146	-1.620425	-5.988616	1.86E-08	1.21E-06	9.0662305
cg09117114	chr16	67998030	-	SLC12A4;SLC12A4;SLC12A4;SLC12A4;SLC12A4	Body;Body;TSS200;Body;Body	1.0873252	0.7222438	5.9885095	1.86E-08	1.21E-06	9.0657338
cg19497031	chr13	79176272	-	POU4F1	Body	3.0022251	-1.661387	5.9884236	1.86E-08	1.21E-06	9.0653344
cg22541735	chr2	176988203	-	HOXD9	1stExon	2.5397194	-0.882206	5.9883753	1.86E-08	1.21E-06	9.0651101
cg04543289	chr16	24267559	-	CACNG3;CACNG3	1stExon;5'UTR	1.2791563	-0.809281	5.988314	1.86E-08	1.21E-06	9.0648253
cg14489474	chr18	905611	-	ADCYAP1;ADCYAP1	Body;Body	1.3220011	-0.257898	5.9879916	1.86E-08	1.21E-06	9.0633272
cg18282455	chr13	36729109	-			1.788934	-1.317843	5.9875458	1.87E-08	1.21E-06	9.0612553
cg15370180	chr10	23484326	-			1.0868524	-1.36447	5.9873804	1.87E-08	1.21E-06	9.0604868
cg22129180	chr8	49892980	-			-1.542377	-0.413668	-5.987331	1.87E-08	1.21E-06	9.0602558
cg14757492	chr19	19029194	-	COPE;DDX49;COPE;COPE	Body;TSS1500;Body;Body	-1.227394	0.4093025	-5.98714	1.87E-08	1.21E-06	9.0593699
cg18161904	chr22	42978394	-	RRP7B	TSS1500	-0.699073	-2.919326	-5.987145	1.87E-08	1.21E-06	9.059395
cg23462788	chr3	38941491	-	SCN11A	Body	-1.41166	1.0347205	-5.986829	1.87E-08	1.21E-06	9.0579231
cg02391509	chr3	27766382	+			1.2278497	-0.544188	5.9867089	1.87E-08	1.22E-06	9.0573668
cg19477793	chr17	52027331	+			-1.722765	0.6364612	-5.98664	1.87E-08	1.22E-06	9.0570451
cg15812020	chr13	25506235	-	LOC646405	Body	1.1034458	0.1484141	5.9865932	1.87E-08	1.22E-06	9.0568289
cg12373951	chr3	133503437	+			-1.19876	-0.761953	-5.986487	1.87E-08	1.22E-06	9.0563349
cg03830329	chr6	100897081	-	SIM1	Body	1.2792832	-0.882945	5.9864234	1.88E-08	1.22E-06	9.0560401

cg17284804	chr1	67218135	-	TCTEX1D1	TSS200	2.3775491	-1.367209	5.9893408	1.87E-08	1.22E-06	9.0574684
cg01308087	chr2	145137193	-			1.1921153	1.9681703	5.9861965	1.88E-08	1.22E-06	9.054986
cg17003736	chr3	147111308	+	ZIC4;ZIC4;ZIC4;ZIC4;ZIC4	Body;TSS1500;Body;Body;TSS1500	1.2827413	-1.121516	5.9862211	1.88E-08	1.22E-06	9.0551
cg25527882	chr6	41698885	+	TFEB;TFEB	5'UTR;5'UTR	-1.152076	0.6262164	-5.9862	1.88E-08	1.22E-06	9.0550042
cg05581394	chr12	40500619	-	SLC2A13	TSS1500	-1.550699	0.5271202	-5.986164	1.88E-08	1.22E-06	9.0548365
cg02400740	chr7	19157938	-	TWIST1	TSS1500	2.8836218	-2.401316	5.9888908	1.88E-08	1.22E-06	9.0553803
cg07945323	chr21	31972672	-	KRTAP6-2;KRTAP22-1	TSS1500;TSS1500	-1.595169	0.2533088	-5.985926	1.88E-08	1.22E-06	9.053729
cg01568244	chr16	67218584	+	KIAA0895L;EXOC3L	TSS1500;Body	-0.922994	-3.241729	-5.985867	1.88E-08	1.22E-06	9.053455
cg09043518	chr17	80290106	-	SECTM1	5'UTR	-1.547534	-0.343629	-5.98575	1.88E-08	1.22E-06	9.0529098
cg22655521	chr17	48194939	-	SAMD14	Body	-1.669453	-3.905191	-5.985631	1.88E-08	1.22E-06	9.0523563
cg06515111	chr10	118153009	+			-2.241063	2.3625399	-5.985256	1.89E-08	1.22E-06	9.0506174
cg24415208	chr19	54466441	-	CACNG8	1stExon	1.8495677	-0.482226	5.9852085	1.89E-08	1.22E-06	9.0503958
cg18699439	chr19	40928820	-	SERTAD1	Body	0.6700693	1.2302946	5.9849291	1.89E-08	1.22E-06	9.0490976
cg17871793	chr20	50416694	-	SALL4	Body	-0.54519	-3.327644	-5.98455	1.89E-08	1.22E-06	9.047337
cg26615224	chr19	1621124	+	TCF3;TCF3	Body;Body	-1.653968	2.8801676	-5.993402	1.89E-08	1.22E-06	9.0512301
cg05436845	chr11	65378622	-	MAP3K11	Body	1.0014599	0.9894355	5.9842946	1.89E-08	1.23E-06	9.0461503
cg24705717	chr2	155448944	+			-1.145132	0.0250717	-5.984051	1.90E-08	1.23E-06	9.0450174
cg12978253	chr10	134258602	+	C10orf91	TSS200	-0.686173	-1.336783	-5.98394	1.90E-08	1.23E-06	9.0445041
cg13563725	chr15	27212911	-			1.4169231	-0.745844	5.9839198	1.90E-08	1.23E-06	9.044409
cg19949932	chr11	30531731	-	MPPED2;MPPED2	Body;Body	-1.836235	0.5504227	-5.983443	1.90E-08	1.23E-06	9.0421943

cg24324628	chr6	138866882	+	NHSL1	Body	1.4366136	1.0343557	5.9834332	1.90E-08	1.23E-06	9.0421488
cg27457631	chr2	484237	+			-1.561305	0.3236286	-5.98342	1.90E-08	1.23E-06	9.042086
cg13023870	chr14	37124018	+			1.8659639	0.4330917	5.9833209	1.90E-08	1.23E-06	9.0416272
cg26874367	chr2	129381792	-			-1.856023	-1.632751	-5.983191	1.90E-08	1.23E-06	9.041025
cg07719492	chr8	70983348	+	PRDM14	5'UTR	2.3236742	-1.166174	5.9830714	1.91E-08	1.23E-06	9.0404682
cg02996583	chr8	142237188	-	SLC45A4	Body	1.2835428	1.9486957	5.9828665	1.91E-08	1.23E-06	9.0395164
cg11859607	chr18	909154	-	ADCYAP1;ADCYAP1	Body;Body	1.6917565	-1.042906	5.9824793	1.91E-08	1.23E-06	9.0377182
cg18583697	chr8	35576222	+	UNC5D	Body	-1.461411	0.2800342	-5.982371	1.91E-08	1.24E-06	9.0372134
cg02457319	chr9	138671108	+	KCNT1	Body	-1.152437	-0.999278	-5.982285	1.91E-08	1.24E-06	9.0368152
cg05511722	chr8	55379566	-			1.5135761	-1.50816	5.9822052	1.91E-08	1.24E-06	9.0364451
cg03680338	chr6	22043967	+	FLJ22536	Body	1.004376	0.6206526	5.9819732	1.92E-08	1.24E-06	9.0353675
cg01649611	chr2	43521066	-	THADA;THADA	Body;Body	-1.61152	-3.154017	-5.981737	1.92E-08	1.24E-06	9.0342724
cg04694492	chr16	4387567	+	GLIS2	3'UTR	1.3808196	4.2119973	5.9817477	1.92E-08	1.24E-06	9.03432
cg11246938	chr12	115112433	-	TBX3;TBX3	Body;Body	1.5534627	1.3096882	5.9817844	1.92E-08	1.24E-06	9.0344905
cg16486109	chr11	613632	-	IRF7;IRF7;IRF7	Body;Body;Body	-1.372158	-0.266422	-5.981764	1.92E-08	1.24E-06	9.0343981
cg16179952	chr2	947117	-	SNTG2	Body	1.1431694	-0.491412	5.9813767	1.92E-08	1.24E-06	9.0325974
cg12463722	chr17	56232499	+	OR4D1	TSS200	-1.322157	-0.2235	-5.987141	1.92E-08	1.24E-06	9.0349975
cg01198364	chr5	35946095	-			-1.428767	-0.111959	-5.980986	1.93E-08	1.24E-06	9.0307831
cg16085553	chr18	74958286	-			-1.601404	0.7061237	-5.980784	1.93E-08	1.24E-06	9.0298457
cg20823772	chr8	99962013	+	OSR2;OSR2	Body;Body	1.3748003	0.0930845	5.9807358	1.93E-08	1.24E-06	9.029621

cg17911539	chr3	126260814	+	CHST13	Body	1.0330045	0.7795877	5.9803932	1.93E-08	1.24E-06	9.0280301
cg16288089	chr7	97361408	-	TAC1;TAC1;TAC1;TAC1;TAC1;TAC1;TAC1;TAC1	5'UTR;1stExon;1stExon;1stExon;5'UTR;5'UTR;1stExon;5'UTR	2.6063602	-0.662078	5.9803348	1.93E-08	1.24E-06	9.0277588
cg00174508	chr12	107774298	+	BTBD11	Body	-1.344342	-0.673327	-5.979936	1.93E-08	1.25E-06	9.0259064
cg17386213	chr6	108488335	+	NR2E1	Body	2.3606722	-1.06813	5.9799372	1.93E-08	1.25E-06	9.0259127
cg17298305	chr1	153911925	+	DENND4B	Body	0.6505747	2.3243138	5.9798114	1.94E-08	1.25E-06	9.0253284
cg09539739	chr16	48277559	+	LONP2	TSS1500	-1.537568	-0.221207	-5.979487	1.94E-08	1.25E-06	9.0238219
cg24461524	chr19	4207451	-	ANKRD24	Body	-1.128488	-0.378894	-5.979291	1.94E-08	1.25E-06	9.0229111
cg05638689	chr11	65314454	+	LTBP3;LTBP3;LTBP3	Body;Body;Body	-1.423577	-2.278543	-5.987779	1.94E-08	1.25E-06	9.0252218
cg07736657	chr16	19777512	+	IQCK	Body	1.0498843	1.7590281	5.9789515	1.94E-08	1.25E-06	9.0213356
cg27038198	chr7	86686461	+	KIAA1324L	Body	1.1709662	1.9567864	5.9786858	1.95E-08	1.25E-06	9.0201018
cg21811143	chr2	119599748	+	EN1	3'UTR	1.8250641	-1.578692	5.9785065	1.95E-08	1.25E-06	9.0192694
cg17644208	chr2	25142391	-	ADCY3	TSS1500	-0.739746	-3.046709	-5.984183	1.95E-08	1.25E-06	9.0210026
cg22618924	chr5	126054151	-			0.9561174	1.034471	5.9813053	1.95E-08	1.25E-06	9.0199089
cg25246082	chr4	39034284	-	TMEM156	TSS1500	-1.542963	0.4005279	-5.978381	1.95E-08	1.25E-06	9.0186888
cg21584518	chr7	142033426	-			-1.600304	0.063302	-5.978293	1.95E-08	1.25E-06	9.018278
cg22146357	chr8	143781406	-	LY6K;LY6K;LY6K	TSS200;TSS200;TSS200	-1.661566	-1.863004	-5.978023	1.95E-08	1.26E-06	9.017026
cg17337672	chr10	123354171	-	FGFR2;FGFR2;FGFR2;FGFR2;FGFR2;FGFR2;FGFR2;FGFR2;FGFR2	5'UTR;5'UTR;TSS1500;5'UTR;TSS1500;5'UTR;5'UTR;TSS1500;5'UTR	1.3447397	2.5651561	5.9777244	1.96E-08	1.26E-06	9.0156384
cg15555527	chr4	80885981	-	ANTXR2	Body	1.2695445	1.6711687	5.9772729	1.96E-08	1.26E-06	9.0135423
cg25987102	chr11	116709735	-	APOA1	TSS1500	-1.248318	1.1869972	-5.976995	1.96E-08	1.26E-06	9.0122539
cg24527098	chr6	28697846	+			1.5097006	-0.952898	5.9769452	1.96E-08	1.26E-06	9.0120214

cg25260543	chr7	100946792	+			1.3195281	-0.760661	5.9768888	1.96E-08	1.26E-06	9.0117594
cg12094863	chr6	164092588	+			-1.226982	0.259147	-5.976702	1.97E-08	1.26E-06	9.0108939
cg01638741	chr16	54404381	-			0.9719497	2.3094075	5.9766428	1.97E-08	1.26E-06	9.0106176
cg18886062	chr19	49224454	+	MAMSTR;RASIP1	TSS1500;Body	-0.965767	-1.670261	-5.976574	1.97E-08	1.26E-06	9.0102975
cg08216021	chr8	110552896	-	EBAG9;EBAG9	5'UTR;5'UTR	-1.009822	-3.307722	-5.976089	1.97E-08	1.27E-06	9.0080482
cg22539420	chr2	200329680	+			1.481494	-0.867242	5.9759559	1.97E-08	1.27E-06	9.0074291
cg01610231	chr4	111555314	-	PITX2;PITX2	5'UTR;5'UTR	1.6587553	-1.438385	5.9757379	1.97E-08	1.27E-06	9.0064172
cg02241330	chr19	15122142	+	CCDC105	1stExon	1.3307215	-1.108327	5.9756911	1.98E-08	1.27E-06	9.0061999
cg07065111	chr2	5831213	-			2.0988125	-0.860773	5.9751891	1.98E-08	1.27E-06	9.00387
cg18520125	chr13	28903926	-	FLT1	Body	1.3498833	1.3849907	5.9751632	1.98E-08	1.27E-06	9.0037495
cg15607672	chr14	57277227	-	OTX2	TSS200	1.6780398	-0.167947	5.9746792	1.98E-08	1.27E-06	9.0015032
cg00253403	chr4	71553554	-	UTP3	TSS1500	-0.702227	-3.760473	-5.974497	1.99E-08	1.27E-06	9.0006591
cg26025557	chr3	42842344	+	HIGD1A;HIGD1A;HIGD1A	Body;5'UTR;5'UTR	1.2324261	1.692965	5.9743479	1.99E-08	1.28E-06	8.9999659
cg21317965	chr6	50803850	-	TFAP2B	Body	1.5384943	-0.264593	5.9742593	1.99E-08	1.28E-06	8.9995544
cg03529261	chr4	185209641	-			-1.247628	-0.586039	-5.976947	1.99E-08	1.28E-06	8.9999936
cg03606020	chr16	86868192	+			-0.873252	-0.976165	-5.973925	1.99E-08	1.28E-06	8.9980014
cg03109101	chr10	119305095	+	EMX2;EMX2;EMX2OS	Body;Body;TSS1500	1.1677703	-0.957082	5.9738017	1.99E-08	1.28E-06	8.9974307
cg15455864	chr19	46319398	-	SYMPK;RSPH6A	Body;TSS1500	1.1231686	0.4592113	5.9735416	2.00E-08	1.28E-06	8.9962239
cg11586034	chr1	150117110	-	VPS45	3'UTR	0.7332287	0.8294955	5.973264	2.00E-08	1.28E-06	8.9949358
cg26515755	chr16	15878612	+	MYH11;MYH11;MYH11;MYH11	Body;Body;Body;Body	-1.378136	0.1482041	-5.973066	2.00E-08	1.28E-06	8.9940163

cg20624923	chr10	120355427	-	PRLHR	TSS1500	1.3267466	-0.484811	5.9728234	2.00E-08	1.28E-06	8.992891
cg13448828	chr2	111826490	+	ACOXL;ACOXL;ACOXL	Body;Body;Body	-1.663362	0.7120611	-5.972774	2.00E-08	1.28E-06	8.9926605
cg15100945	chr1	90287507	-	LRRC8D;LRRC8D;LRRC8D	1stExon;5'UTR;5'UTR	-1.017863	-4.12163	-5.972218	2.01E-08	1.29E-06	8.9900812
cg10472919	chr5	135266805	-	FBXL21	5'UTR	1.3953039	0.5618107	5.9720882	2.01E-08	1.29E-06	8.9894797
cg02633723	chr12	133173041	-			-2.421526	1.7709545	-5.98067	2.01E-08	1.29E-06	9.0036594
cg09341730	chr19	40269534	-	LEUTX	5'UTR	-1.493378	0.2337399	-5.971892	2.01E-08	1.29E-06	8.9885691
cg26385126	chr12	124912021	+	NCOR2;NCOR2	Body;Body	1.0446402	1.0570005	5.9746357	2.01E-08	1.29E-06	8.9889936
cg07307484	chr17	4743324	+	MINK1;MINK1;MINK1;MINK1	Body;Body;Body;Body	0.9388279	1.9577631	5.9715918	2.01E-08	1.29E-06	8.9871764
cg17450733	chr12	45443875	+	DBX2	Body	1.5928755	-0.373199	5.971465	2.02E-08	1.29E-06	8.9865878
cg09378441	chr7	36429182	+	ANLN;KIAA0895	TSS1500;Body	-0.64797	-4.499854	-5.971214	2.02E-08	1.29E-06	8.9854222
cg23636802	chr6	32054441	+	TNXB	Body	-1.183225	-0.188058	-5.971236	2.02E-08	1.29E-06	8.9855254
cg22807449	chr17	46622522	-	HOXB2	TSS200	1.6268855	0.1261748	5.9709527	2.02E-08	1.29E-06	8.984211
cg12141218	chr1	209799248	+	LAMB3;LAMB3;LAMB3	Body;Body;Body	1.1275172	1.9991704	5.9766154	2.02E-08	1.29E-06	8.9856864
cg20896233	chr2	186603561	-			1.8530823	-1.52375	5.9705569	2.02E-08	1.29E-06	8.9823747
cg23230830	chr1	248903100	-	LOC646627	1stExon	-1.732094	-0.668602	-5.97025	2.03E-08	1.30E-06	8.9809516
cg23922739	chr12	54338743	-	HOXC13	Body	1.7880797	-1.362269	5.9730358	2.03E-08	1.30E-06	8.9818704
cg27616109	chr17	34093677	+	MMP28	Body	-1.819831	1.1543684	-5.973016	2.03E-08	1.30E-06	8.9817792
cg02567344	chr1	179010239	-	FAM20B	5'UTR	-1.287387	2.5730488	-5.970053	2.03E-08	1.30E-06	8.980038
cg13868393	chr17	75136249	-	SEC14L1;SEC14L1;SEC14L1;SEC14L1	TSS1500;TSS1500;TSS1500;TSS1500	-0.737987	-3.055026	-5.969978	2.03E-08	1.30E-06	8.9796909
cg19021221	chr1	52834398	+			-0.683474	-3.039336	-5.969927	2.03E-08	1.30E-06	8.9794538

cg15962969	chr6	138866887	+	NHSL1	Body	1.5766748	1.1640414	5.9697755	2.03E-08	1.30E-06	8.9787499
cg25729068	chr14	92303235	+	TC2N;TC2N;TC2N	TSS1500;5'UTR;TSS1500	-0.656963	-4.169423	-5.96978	2.03E-08	1.30E-06	8.9787728
cg19857461	chr17	78703048	+	RPTOR;RPTOR	Body;Body	1.337948	1.3166293	5.9696334	2.03E-08	1.30E-06	8.9780907
cg04938966	chr16	86530790	-			1.1951067	-0.93433	5.9695127	2.03E-08	1.30E-06	8.9775306
cg11582484	chr15	25491315	-	SNORD115-10;SNORD115-42	TSS1500;TSS1500	-1.29326	0.1941475	-5.969483	2.04E-08	1.30E-06	8.9773932
cg15708618	chr11	1994117	+			-1.164919	0.1471684	-5.96941	2.04E-08	1.30E-06	8.9770555
cg17322667	chr11	61159837	-	TMEM216	TSS200	-0.652913	-3.568862	-5.969435	2.04E-08	1.30E-06	8.9771688
cg21671052	chr12	119027591	+			-1.11576	-0.702195	-5.969141	2.04E-08	1.30E-06	8.9758072
cg14424579	chr2	27274309	-	AGBL5;AGBL5	TSS200;TSS200	-0.800457	-4.384465	-5.968999	2.04E-08	1.30E-06	8.9751472
cg11127249	chr10	6104630	+	IL2RA	TSS1500	-1.381209	-1.663768	-5.971762	2.04E-08	1.30E-06	8.975971
cg02606480	chr8	6366994	-	ANGPT2;ANGPT2;ANGPT2;MCPH1	Body;Body;Body;Body	0.915651	1.0331298	5.9683517	2.05E-08	1.31E-06	8.9721451
cg04383058	chr11	67273507	-	PITPNM1;PITPNM1	TSS1500;TSS1500	-1.212309	-2.367813	-5.96834	2.05E-08	1.31E-06	8.9720893
cg16721977	chr10	116286841	-	ABLIM1;ABLIM1;ABLIM1;ABLIM1	Body;Body;Body;TSS200	-0.939754	-4.112376	-5.968329	2.05E-08	1.31E-06	8.9720377
cg22873539	chr4	147554210	+			1.5176175	-1.092599	5.9680133	2.05E-08	1.31E-06	8.9705759
cg03059382	chr16	1602140	+	IFT140;TMEM204	Body;Body	0.8128152	1.1287035	5.9677475	2.05E-08	1.31E-06	8.9693427
cg07455504	chr16	1080144	-			-2.151041	3.3422293	-5.967483	2.06E-08	1.31E-06	8.9681167
cg13802013	chr20	37303351	+			1.6540324	-0.6741	5.9674609	2.06E-08	1.31E-06	8.9680134
cg22311458	chr11	65265909	-	MALAT1	Body	-0.714963	-4.474948	-5.967474	2.06E-08	1.31E-06	8.9680729
cg02894109	chr4	16359846	+			-1.352273	0.9012912	-5.967264	2.06E-08	1.31E-06	8.9671014
cg03319695	chr18	59156627	-	CDH20	TSS1500	-1.248386	-0.342206	-5.966995	2.06E-08	1.31E-06	8.9658515

cg20038219	chr7	73241729	+			-1.193114	-1.376664	-5.966968	2.06E-08	1.31E-06	8.9657265
cg11012061	chr5	145722468	-			1.4074389	-1.160515	5.9667195	2.06E-08	1.31E-06	8.9645752
cg19843451	chr1	45769454	-	LOC400752	TSS200	1.2748015	-4.841718	5.9695498	2.06E-08	1.31E-06	8.9657229
cg15666275	chr19	57274897	-			-1.625238	0.7919014	-5.966413	2.07E-08	1.32E-06	8.9631519
cg08260245	chr22	51016501	+	CPT1B;CPT1B;CHKB-CPT1B;CPT1B;CPT1B;CPT1B;CPT1B;CPT1B;CPT1B	5'UTR;5'UTR;Body;5'UTR;5'UTR;5'UTR;1stExon;5'UTR;5'UTR	1.2611571	1.6418155	5.9663189	2.07E-08	1.32E-06	8.9627173
cg08312626	chr6	27534137	-			1.7907476	-0.928175	5.9691092	2.07E-08	1.32E-06	8.9636819
cg24599107	chr1	161049443	+	PVRL4	Body	1.307306	2.7392198	5.9662894	2.07E-08	1.32E-06	8.9625803
cg26470926	chr11	106197651	+			-1.68454	0.8627152	-5.966251	2.07E-08	1.32E-06	8.9624004
cg24738311	chr15	89952632	+			2.2484054	-1.315851	5.9661242	2.07E-08	1.32E-06	8.9618147
cg18504190	chr11	1649947	-	HCCA2;KRTAP5-5	Body;TSS1500	-0.948721	-1.113066	-5.96608	2.07E-08	1.32E-06	8.9616078
cg26311734	chr4	94755520	+			1.5186624	-0.330531	5.9658854	2.07E-08	1.32E-06	8.960707
cg24144440	chr1	119526882	-	TBX15	5'UTR	1.2664743	-0.519593	5.9658151	2.07E-08	1.32E-06	8.9603809
cg14888916	chr8	99951420	-			1.661124	-0.24813	5.9657441	2.07E-08	1.32E-06	8.9600519
cg22855933	chr2	157179512	+			1.2382879	0.8391319	5.9655532	2.07E-08	1.32E-06	8.9591666
cg20348858	chr10	119305335	-	EMX2;EMX2;EMX2OS	Body;Body;TSS1500	1.1220511	-1.049255	5.9644638	2.09E-08	1.33E-06	8.9541152
cg18867912	chr19	33623072	+	WDR88;WDR88	1stExon;5'UTR	1.2526011	3.8705893	5.9643867	2.09E-08	1.33E-06	8.9537578
cg27574980	chr6	31744636	+	C6orf27	5'UTR	-0.942761	-2.176782	-5.964351	2.09E-08	1.33E-06	8.9535928
cg26687072	chr17	46608345	-	HOXB1	TSS200	1.4833949	1.1837179	5.9642064	2.09E-08	1.33E-06	8.9529219
cg15164103	chr7	130126871	+	MEST	5'UTR	-1.046597	-1.570947	-5.96403	2.09E-08	1.33E-06	8.9521045
cg19969606	chr12	113901665	+	LHX5	Body	1.6219435	-1.605188	5.9640483	2.09E-08	1.33E-06	8.9521887

cg05818824	chr17	62051709	+	SCN4A	TSS1500	-1.292958	-0.808726	-5.963945	2.09E-08	1.33E-06	8.951708
cg07301795	chr5	157001698	-	ADAM19	Body	1.2982622	-0.432904	5.9639263	2.09E-08	1.33E-06	8.9516229
cg04416111	chr16	87234895	+			-1.320195	-0.956892	-5.966221	2.10E-08	1.33E-06	8.9500151
cg01689404	chr17	35423942	+			1.207928	0.3621715	5.9632095	2.10E-08	1.33E-06	8.9482999
cg04860852	chr1	200993069	+	KIF21B	TSS1500	-1.076349	-2.628447	-5.963225	2.10E-08	1.33E-06	8.9483701
cg08744726	chr7	149323101	+	ZNF767;ZNF767	TSS1500;TSS1500	-0.97605	-1.398579	-5.963014	2.10E-08	1.33E-06	8.9473929
cg25423174	chr3	12236303	-			-1.319873	-0.274407	-5.962755	2.10E-08	1.33E-06	8.9461929
cg25654677	chr2	1926671	-	MYT1L	Body	-1.348398	0.1071504	-5.962702	2.10E-08	1.33E-06	8.9459476
cg01231009	chr8	130641185	-			-1.61233	0.6870196	-5.962461	2.11E-08	1.34E-06	8.9448318
cg12939270	chr6	28494445	-	GPX5;GPX5	Body;Body	-1.712714	0.305024	-5.962409	2.11E-08	1.34E-06	8.9445874
cg23663774	chr4	111555440	-	PITX2;PITX2	5'UTR;5'UTR	2.5843538	-1.438565	5.9651185	2.11E-08	1.34E-06	8.9449126
cg18369913	chr11	51515083	-	OR4C46	TSS200	-1.392055	-0.901785	-5.962128	2.11E-08	1.34E-06	8.9432852
cg17208360	chr15	25483088	-	SNORD115-37	TSS200	-1.215321	-0.058671	-5.962004	2.11E-08	1.34E-06	8.9427113
cg23348270	chr2	176969612	+			1.9506744	-0.526949	5.9619577	2.11E-08	1.34E-06	8.9424966
cg05036173	chr2	108994528	-	SULT1C4;SULT1C4	5'UTR;1stExon	1.135306	-0.216352	5.9705916	2.11E-08	1.34E-06	8.9461147
cg08365609	chr19	33726655	-			-1.363698	-1.108581	-5.961567	2.11E-08	1.34E-06	8.940686
cg13555116	chr13	25862332	-	MTMR6	TSS1500	-1.086613	0.336733	-5.961311	2.12E-08	1.34E-06	8.9394994
cg13692222	chr14	105683925	+	BRF1;BRF1	Body;Body	1.4513751	3.5882188	5.9640239	2.12E-08	1.34E-06	8.9401368
cg16178415	chr10	82265445	+	TSPAN14;TSPAN14	Body;Body	0.867594	-3.470327	5.9611115	2.12E-08	1.34E-06	8.9385741
cg06590170	chr6	33969828	-			1.553522	1.4713654	5.9606491	2.12E-08	1.35E-06	8.936431

cg24766041	chr19	17375445	-	USHBP1	5'UTR	1.3280961	2.1378617	5.9605599	2.12E-08	1.35E-06	8.9360176
cg11994630	chr2	74663482	+	RTKN;RTKN;RTKN	Body;5'UTR;Body	1.0824564	2.8154479	5.9602349	2.13E-08	1.35E-06	8.9345111
cg20694988	chr1	116694667	-			-0.924996	-3.479995	-5.959808	2.13E-08	1.35E-06	8.9325314
cg02145866	chr3	99353080	+	MIR548G	Body	-1.736236	0.5808097	-5.958891	2.14E-08	1.36E-06	8.9282807
cg23481873	chr20	464190	-	CSNK2A1;CSNK2A1;CSNK2A1	3'UTR;3'UTR;3'UTR	0.7242572	1.6058197	5.958325	2.15E-08	1.36E-06	8.92566
cg07630345	chr6	31164804	-	HCG27	TSS1500	-0.871092	-3.073334	-5.958273	2.15E-08	1.36E-06	8.9254173
cg17918002	chr10	28035719	-	MKX	TSS1500	1.6248504	-1.396093	5.958216	2.15E-08	1.36E-06	8.9251549
cg17367077	chr5	159850153	-	PTTG1	Body	-1.376265	0.0382864	-5.95809	2.15E-08	1.36E-06	8.9245726
cg20805961	chr1	91361641	-			1.1120235	1.9952338	5.9578947	2.15E-08	1.36E-06	8.9236662
cg04094178	chr1	4195569	-			-1.336	-0.782126	-5.957462	2.16E-08	1.36E-06	8.921659
cg03072569	chr16	76309968	-	CNTNAP4	TSS1500	-1.698151	0.4318189	-5.957206	2.16E-08	1.37E-06	8.9204759
cg08331163	chr11	59271519	-	OR4D11	1stExon	-1.264053	0.0076971	-5.957202	2.16E-08	1.37E-06	8.9204583
cg19923650	chr5	172659730	-	NKX2-5;NKX2-5;NKX2-5	3'UTR;Body;3'UTR	2.2762888	-1.151623	5.960078	2.16E-08	1.37E-06	8.9215829
cg16154524	chr17	26633895	-	FLJ40504	Body	0.8757025	2.1795885	5.956882	2.16E-08	1.37E-06	8.9189737
cg23158877	chr11	86012876	+	C11orf73;C11orf73;C11orf73	TSS1500;TSS1500;TSS1500	-0.974066	-2.572271	-5.956756	2.16E-08	1.37E-06	8.9183894
cg02906074	chr14	73102873	-			-1.784179	0.8283767	-5.956087	2.17E-08	1.37E-06	8.9152903
cg12020230	chr4	78432901	+	CXCL13	TSS200	0.8774323	1.8425455	5.9561567	2.17E-08	1.37E-06	8.9156133
cg22946562	chr2	1880012	-	MYT1L	Body	-1.884695	0.3969436	-5.956112	2.17E-08	1.37E-06	8.9154064
cg24593464	chr11	120008998	-	TRIM29	TSS200	-1.331466	-0.176026	-5.956087	2.17E-08	1.37E-06	8.9152915
cg07191524	chr16	1077624	-			-1.215256	0.0547318	-5.955949	2.17E-08	1.37E-06	8.9146509

cg20916523	chr3	10184584	-	VHL;VHL	Body;Body	-1.72448	-0.801928	-5.961251	2.18E-08	1.37E-06	8.9149321
cg09676013	chr6	33080500	-	HLA-DPB2	Body	-0.940616	-1.811693	-5.955407	2.18E-08	1.38E-06	8.9121423
cg17450930	chr7	150065170	-	REPIN1;REPIN1;REPIN1	TSS1500;TSS1500;TSS1500	-0.531257	-4.237338	-5.955377	2.18E-08	1.38E-06	8.9120018
cg25085755	chr5	139294323	-	NRG2;NRG2;NRG2;NRG2	Body;Body;Body;Body	-0.687395	-1.293646	-5.95528	2.18E-08	1.38E-06	8.9115507
cg01900836	chr7	38743230	+			-2.045319	0.8998907	-5.955218	2.18E-08	1.38E-06	8.911267
cg04837071	chr9	140318649	+	EXD3;NOXA1	TSS1500;Body	-0.801475	-1.057592	-5.954665	2.19E-08	1.38E-06	8.9087015
cg18501230	chr8	1338706	-			-1.0502	-0.554838	-5.954444	2.19E-08	1.38E-06	8.9076801
cg07799299	chr8	131344855	-	ASAP1	Body	1.1147749	1.5008863	5.954168	2.19E-08	1.38E-06	8.906401
cg17228821	chr11	62529407	-	POLR2G	Body	-0.477491	-4.518235	-5.954102	2.19E-08	1.38E-06	8.9060966
cg19249874	chr7	28895537	+			-1.442281	0.5143646	-5.95409	2.19E-08	1.38E-06	8.9060396
cg06973615	chr2	175208761	+			1.6174051	-0.203929	5.9538147	2.20E-08	1.38E-06	8.9047645
cg17144184	chr8	143221499	+			-1.761037	0.2326466	-5.953729	2.20E-08	1.38E-06	8.9043656
cg15415194	chr8	859154	+			-1.078239	-1.022169	-5.953522	2.20E-08	1.39E-06	8.9034087
cg10941916	chr8	19615741	+			-1.252168	-0.215111	-5.953215	2.20E-08	1.39E-06	8.9019858
cg14176752	chr6	100913250	-			1.0636964	-0.562189	5.9529408	2.20E-08	1.39E-06	8.9007165
cg15536830	chr18	43245137	-	SLC14A2	Body	-0.586041	-3.856345	-5.95565	2.21E-08	1.39E-06	8.9010984
cg03142554	chr6	82958230	+	IBTK	TSS1500	-1.266923	-1.722645	-5.952691	2.21E-08	1.39E-06	8.8995617
cg17495715	chr2	176950273	+			1.3970203	-0.296993	5.9523575	2.21E-08	1.39E-06	8.8980151
cg07336617	chr5	76935080	+	OTP	TSS1500	1.3481904	-0.812259	5.9523073	2.21E-08	1.39E-06	8.8977828
cg05380558	chr19	5905215	+	VMAC;NDUFA11	Body;TSS1500	-0.696942	-2.514712	-5.951612	2.22E-08	1.40E-06	8.8945605

cg10805039	chr17	750241	+	NXN	Body	1.6581215	-0.881668	5.9513999	2.22E-08	1.40E-06	8.8935806
cg14174928	chr1	245747991	+	KIF26B	Body	0.7125518	2.5192458	5.9514357	2.22E-08	1.40E-06	8.8937463
cg14339466	chr13	27927490	-			-1.323393	-1.514257	-5.951425	2.22E-08	1.40E-06	8.8936969
cg27116912	chr2	176947940	-	EVX2	Body	2.2566181	-1.57531	5.954119	2.22E-08	1.40E-06	8.8943088
cg04191306	chr14	104854974	+			-1.403908	0.7833142	-5.950923	2.23E-08	1.40E-06	8.8913717
cg19442900	chr11	17226015	-			0.8196893	1.2155465	5.9506953	2.23E-08	1.40E-06	8.8903178
cg14588399	chr8	87242630	+	SLC7A13	TSS200	-1.578778	-0.659956	-5.950568	2.23E-08	1.40E-06	8.8897283
cg12559031	chr17	48046365	-	DLX4	TSS200	-0.495551	-3.095209	-5.950201	2.23E-08	1.40E-06	8.8880272
cg16765088	chr15	99640471	-			-0.87021	-1.499939	-5.950202	2.23E-08	1.40E-06	8.8880323
cg18864804	chr10	117642441	+	ATRNL1	Body	-1.249974	0.1938338	-5.950264	2.23E-08	1.40E-06	8.8883194
cg22378919	chr1	119522188	-	TBX15	5'UTR	1.5753724	-0.646601	5.9502433	2.23E-08	1.40E-06	8.8882248
cg07917644	chr15	101766122	+	CHSY1	Body	1.2323213	1.632409	5.9498174	2.24E-08	1.41E-06	8.8862525
cg23270529	chr1	243584668	-	SDCCAG8	Body	-2.950193	0.1867724	-5.949715	2.24E-08	1.41E-06	8.8857776
cg05708550	chr5	137688227	-	KDM3B	TSS200	-0.597471	-2.697109	-5.949649	2.24E-08	1.41E-06	8.8854722
cg14507845	chr9	137418069	-			-0.823066	-1.580342	-5.949431	2.24E-08	1.41E-06	8.8844641
cg08548498	chr20	43883546	+	SLPI	TSS1500	-1.102917	-2.716913	-5.949346	2.24E-08	1.41E-06	8.8840688
cg04930596	chr12	124864528	+	NCOR2;NCOR2	Body;Body	0.9313869	0.1784426	5.9492884	2.24E-08	1.41E-06	8.8838035
cg14741870	chr6	30174091	-	TRIM26	5'UTR	-0.976315	0.9686944	-5.94919	2.24E-08	1.41E-06	8.8833464
cg13646020	chr4	41750390	-	PHOX2B	1stExon	2.0202095	-1.285696	5.9488782	2.25E-08	1.41E-06	8.8819043
cg01016169	chr12	51441443	+	LETMD1;LETMD1	TSS1500;TSS1500	-1.268763	-0.094662	-5.948372	2.25E-08	1.41E-06	8.879563

cg18164281	chr20	2797721	-	LOC100288797	3'UTR	-1.177381	-0.211659	-5.948111	2.26E-08	1.42E-06	8.8783513
cg18404706	chr19	18761564	-	KLHL26	Body	1.5121868	0.9837818	5.9477816	2.26E-08	1.42E-06	8.8768276
cg02604095	chr9	32955790	+			1.0092179	0.5604849	5.9476724	2.26E-08	1.42E-06	8.876322
cg16713687	chr8	55366538	-			1.5737198	-1.204004	5.9476888	2.26E-08	1.42E-06	8.876398
cg21812313	chr1	94158618	+			-1.182213	-0.858212	-5.947706	2.26E-08	1.42E-06	8.8764765
cg14592399	chr4	41881933	+			1.2434555	-0.216329	5.9475234	2.26E-08	1.42E-06	8.8756321
cg00526273	chr20	59864749	-	CDH4	Body	-1.663704	0.9936932	-5.946857	2.27E-08	1.42E-06	8.8725499
cg15197583	chr15	69320701	-	MIR548H4;NOX5	Body;Body	-1.050915	-0.230662	-5.946769	2.27E-08	1.42E-06	8.872142
cg13784855	chr8	128806938	+	MIR1204;PVT1	TSS1500;Body	-0.711135	-4.027643	-5.946635	2.27E-08	1.42E-06	8.8715208
cg16365424	chr1	234700636	+			-1.951428	0.1993459	-5.946636	2.27E-08	1.42E-06	8.8715224
cg23199517	chr3	71292739	-	FOXP1;FOXP1	5'UTR;5'UTR	0.7957498	2.4099213	5.9464999	2.27E-08	1.43E-06	8.8708948
cg27639662	chr4	81111393	+	PRDM8	5'UTR	0.9698321	-0.018758	5.9464364	2.27E-08	1.43E-06	8.8706006
cg16884902	chr11	20617974	-			1.8843423	-1.216746	5.9492425	2.28E-08	1.43E-06	8.883126
cg02508697	chr11	123809942	+	OR4D5	TSS1500	-1.856026	1.5507929	-5.945987	2.28E-08	1.43E-06	8.8685226
cg02367160	chr5	166614884	-			-1.382956	0.008748	-5.945921	2.28E-08	1.43E-06	8.8682154
cg18229196	chr10	134827324	-			-1.083571	-0.650382	-5.945823	2.28E-08	1.43E-06	8.8677594
cg18653372	chr6	112647973	-			-1.218058	0.1105605	-5.948367	2.28E-08	1.43E-06	8.8677163
cg25691167	chr7	19184961	+	FERD3L	1stExon	2.6479409	-1.033751	5.9453952	2.29E-08	1.43E-06	8.8657815
cg15415452	chr13	112759355	-			2.2735709	-0.950032	5.9452668	2.29E-08	1.43E-06	8.8651872
cg24110050	chr1	67217853	-	TCTEX1D1	TSS1500	2.0180802	-1.374593	5.9451741	2.29E-08	1.43E-06	8.8647582

cg07673979	chr17	45270216	+			-1.508242	-2.689	-5.945137	2.29E-08	1.43E-06	8.8645887
cg27177554	chr10	16562102	+	C1QL3	Body	1.6643689	-0.100937	5.944991	2.29E-08	1.43E-06	8.8639111
cg16576033	chr1	235268621	+			-1.633016	-0.997505	-5.944938	2.29E-08	1.43E-06	8.8636662
cg14636534	chr5	87968528	+	LOC645323;LOC645323;LOC645323	Body;Body;Body	1.4019828	0.0658145	5.944744	2.29E-08	1.43E-06	8.8627677
cg01866955	chr6	19692217	-			2.1724034	-1.672048	5.9438991	2.30E-08	1.44E-06	8.8588579
cg06142122	chr16	85695032	+	KIAA0182;KIAA0182	Body;Body	1.0085792	1.8933045	5.943639	2.31E-08	1.44E-06	8.8576542
cg24127869	chr21	37506839	-	CBR3	TSS1500	-0.602929	-3.272984	-5.943657	2.31E-08	1.44E-06	8.8577371
cg11993160	chr18	12404041	+			-1.514922	-2.73942	-5.943484	2.31E-08	1.44E-06	8.856939
cg05985821	chr8	328586	-			-1.278922	-1.333424	-5.946154	2.31E-08	1.44E-06	8.8574888
cg05652445	chr16	3345075	+			0.8651057	1.1580685	5.943164	2.31E-08	1.44E-06	8.8554563
cg09005679	chr20	57875326	-	EDN3;EDN3;EDN3;EDN3	TSS200;TSS200;TSS200;TSS200	1.3767101	-1.214534	5.9425882	2.32E-08	1.45E-06	8.8527922
cg26317237	chr14	104685248	+			-1.567027	1.2186515	-5.942457	2.32E-08	1.45E-06	8.8521843
cg13457496	chr7	2609648	-	IQCE;IQCE	Body;Body	1.3176543	3.2710453	5.9423459	2.32E-08	1.45E-06	8.851671
cg26844246	chr5	170736277	-	TLX3	TSS200	1.3906199	-0.128043	5.9419957	2.32E-08	1.45E-06	8.8500509
cg05095290	chr14	103995143	-	TRMT61A	TSS1500	-0.537406	-3.15823	-5.941696	2.33E-08	1.45E-06	8.8486649
cg19514928	chr1	95583636	-	TMEM56	5'UTR	-0.546116	-3.684844	-5.941378	2.33E-08	1.46E-06	8.8471911
cg23058652	chr19	11319463	-	DOCK6	Body	1.2007971	3.3577698	5.9412634	2.33E-08	1.46E-06	8.8466626
cg09122423	chr11	107540995	-			-1.228291	-1.447722	-5.9411	2.33E-08	1.46E-06	8.8459049
cg18530729	chr8	30514814	-	GTF2E2	5'UTR	-0.391838	-4.4015	-5.941101	2.33E-08	1.46E-06	8.8459093
cg07019857	chr5	106822849	-	EFNA5	Body	0.7921109	0.5196975	5.9406074	2.34E-08	1.46E-06	8.8436281

cg01446894	chr20	21688203	+	PAX1	Body	0.9417256	0.3874457	5.9402744	2.34E-08	1.46E-06	8.8420873
cg13195526	chr12	68647498	-	IL22	TSS1500	-1.272204	-0.883963	-5.940153	2.34E-08	1.46E-06	8.8415253
cg14897188	chr17	6917185	+	RNASEK;C17orf49;C17orf49;C17orf49	Body;TSS1500;TSS1500;TSS1500	-1.018761	-0.682193	-5.940045	2.35E-08	1.46E-06	8.8410277
cg14845812	chr2	45240373	-			1.373849	0.1559815	5.9398534	2.35E-08	1.46E-06	8.8401401
cg06876648	chr17	77181544	-	HRNBP3	5'UTR	-1.095674	-0.504344	-5.939679	2.35E-08	1.47E-06	8.8393335
cg17726575	chr2	11606945	-	E2F6;E2F6;E2F6;E2F6;E2F6	TSS1500;TSS1500;TSS1500;TSS1500;TSS1500	-1.316618	-2.036488	-5.939653	2.35E-08	1.47E-06	8.8392128
cg24715988	chr1	244237941	+			-0.85442	-2.878516	-5.939251	2.36E-08	1.47E-06	8.8373514
cg26279745	chr14	24801970	-	ADCY4	Body	-1.049944	-0.636461	-5.941971	2.36E-08	1.47E-06	8.8381623
cg04837832	chr10	101290958	+			1.3003873	-0.086007	5.9389261	2.36E-08	1.47E-06	8.8358509
cg12807237	chr10	134729045	-			-1.291344	0.1233511	-5.938632	2.36E-08	1.47E-06	8.8344897
cg25537125	chr2	108993602	+	SULT1C4	TSS1500	1.2604439	2.5659386	5.9410507	2.37E-08	1.47E-06	8.8339108
cg17161180	chr1	165086088	-			-1.752126	-0.676945	-5.938085	2.37E-08	1.48E-06	8.8319627
cg06234624	chr17	27181559	-	ERAL1	TSS1500	-0.439013	-3.26981	-5.937712	2.37E-08	1.48E-06	8.8302373
cg25165975	chr6	31855202	-	EHMT2;EHMT2	Body;Body	-0.852604	-0.473592	-5.937527	2.37E-08	1.48E-06	8.8293821
cg20754979	chr17	4764504	-	MINK1;MINK1;MINK1;MINK1	Body;Body;Body;Body	0.7864282	1.7936401	5.9371031	2.38E-08	1.48E-06	8.8274195
cg03567652	chr19	11242272	-	LDLR	3'UTR	0.8378866	0.3146012	5.9370462	2.38E-08	1.48E-06	8.8271564
cg11717507	chr12	114838825	-	TBX5;TBX5;TBX5;TBX5	Body;Body;Body;Body	1.6943206	-1.20885	5.9369784	2.38E-08	1.48E-06	8.826843
cg10296688	chr13	52598408	+	UTP14C;ALG11	TSS1500;Body	0.8002023	2.9340962	5.9365921	2.39E-08	1.49E-06	8.8250566
cg08893692	chr5	72595741	+			2.2810346	-0.690524	5.9365616	2.39E-08	1.49E-06	8.8249157
cg24619785	chr2	9416069	-	ASAP2;ASAP2	Body;Body	0.7826362	2.1389349	5.9364132	2.39E-08	1.49E-06	8.8242295

cg26530942	chr2	232110727	+	ARMC9	Body	-0.977115	-1.28811	-5.936121	2.39E-08	1.49E-06	8.8228765
cg05852824	chr15	26543817	-			-1.063898	-0.665512	-5.936038	2.39E-08	1.49E-06	8.8224921
cg16733705	chr12	85667616	-			1.616193	-0.383498	5.9359464	2.39E-08	1.49E-06	8.8220708
cg20014094	chr1	16053725	+	PLEKHM2	Body	0.7877269	3.3465665	5.9416637	2.39E-08	1.49E-06	8.824224
cg22749589	chr20	21501710	+			2.0220715	-1.118401	5.9359017	2.39E-08	1.49E-06	8.821864
cg03799957	chr8	42249026	+	VDAC3;VDAC3	TSS1500;TSS1500	-1.162938	-3.8155	-5.93575	2.40E-08	1.49E-06	8.8211626
cg10109500	chr3	172165884	-	GHSR;GHSR	1stExon;1stExon	2.514725	-1.667439	5.9351686	2.40E-08	1.49E-06	8.8184743
cg14557690	chr9	139978293	+	UAP1L1	3'UTR	-1.206459	0.1066222	-5.93481	2.41E-08	1.50E-06	8.8168166
cg21281463	chr19	38423760	-	SIPA1L3	5'UTR	-1.224369	-1.341682	-5.934792	2.41E-08	1.50E-06	8.8167326
cg26151206	chr1	158390649	+	OR10K2	1stExon	-2.231987	0.8238535	-5.934688	2.41E-08	1.50E-06	8.8162533
cg13549152	chr1	39456418	+	AKIRIN1;AKIRIN1	TSS1500;TSS1500	-0.895723	-2.973947	-5.934599	2.41E-08	1.50E-06	8.8158394
cg14184078	chr20	54579814	+	CBLN4;CBLN4	5'UTR;1stExon	1.5106972	-0.470181	5.934626	2.41E-08	1.50E-06	8.8159657
cg27093376	chr17	5185073	-	RABEP1;RABEP1	TSS1500;TSS1500	-1.13537	0.7046115	-5.934276	2.41E-08	1.50E-06	8.8143478
cg21807034	chr18	61144150	-	SERPINB5;SERPINB5	1stExon;5'UTR	-2.419027	1.0900686	-5.934233	2.41E-08	1.50E-06	8.8141504
cg15447913	chr1	23162550	+	EPHB2;EPHB2	Body;Body	1.6526338	3.5359796	5.934099	2.41E-08	1.50E-06	8.8135292
cg03547017	chr17	37031281	-	LASP1	Body	1.1239923	0.3289495	5.9340624	2.41E-08	1.50E-06	8.8133601
cg16646298	chr6	30698987	-	FLOT1	Body	-1.485445	-0.584187	-5.933718	2.42E-08	1.50E-06	8.8117694
cg00875805	chr4	140656749	+	MAML3	Body	1.8808994	-0.399189	5.933527	2.42E-08	1.50E-06	8.8108848
cg09833701	chr11	1959051	-	TNNT3;TNNT3;TNNT3;TNNT3	Body;Body;Body;Body	-1.184083	-0.368532	-5.933529	2.42E-08	1.50E-06	8.8108951
cg07993003	chr19	55454132	-	NLRP7;NLRP7;NLRP7	5'UTR;5'UTR;5'UTR	-1.101791	-0.037411	-5.933136	2.43E-08	1.51E-06	8.8090789

cg03574723	chr10	125425994	-	GPR26	1stExon	1.717275	-1.118745	5.9330791	2.43E-08	1.51E-06	8.8088142
cg09244100	chr2	219697159	-	PRKAG3	TSS1500	-1.260607	0.4645462	-5.932594	2.43E-08	1.51E-06	8.8065721
cg05163588	chr7	73498778	+	LIMK1	Body	-0.438201	-3.604951	-5.932539	2.43E-08	1.51E-06	8.8063154
cg13538006	chr9	95897313	-	NINJ1	TSS1500	-0.844496	-1.316906	-5.932475	2.43E-08	1.51E-06	8.8060198
cg15617545	chr11	57365018	-	SERPING1;SERPING1	TSS1500;TSS200	1.3157677	0.3381792	5.932395	2.43E-08	1.51E-06	8.8056519
cg05339415	chr14	105107459	-			-0.857813	-1.502502	-5.932164	2.44E-08	1.51E-06	8.8045826
cg05767489	chr1	10725218	+	CASZ1;CASZ1	Body;Body	0.9884237	2.7653647	5.9348149	2.44E-08	1.51E-06	8.8051173
cg09624551	chr3	72128386	-			-1.308063	-0.45353	-5.931805	2.44E-08	1.51E-06	8.8029255
cg27513573	chr10	131768098	-			2.633259	-1.795065	5.9316807	2.44E-08	1.51E-06	8.8023505
cg02805890	chr6	27778836	+			-1.600465	-2.412888	-5.931394	2.45E-08	1.52E-06	8.8010231
cg20694425	chr4	55235418	+			0.9276083	2.0353868	5.9309731	2.45E-08	1.52E-06	8.7990801
cg24184689	chr15	74723271	-	SEMA7A;SEMA7A;SEMA7A	Body;Body;5'UTR	-1.184787	-0.069752	-5.930694	2.45E-08	1.52E-06	8.7977912
cg21306240	chr1	38230845	+	EPHA10;EPHA10	TSS200;TSS200	2.2227041	-0.929105	5.9303419	2.46E-08	1.52E-06	8.7961627
cg15867307	chr2	238110829	+			-1.415112	-0.2802	-5.930306	2.46E-08	1.52E-06	8.7959982
cg07700393	chr5	33788976	-	ADAMTS12	Body	-1.543828	-0.498706	-5.9301	2.46E-08	1.52E-06	8.7950464
cg24474037	chr8	145865124	-			1.1210206	1.2885623	5.9297056	2.47E-08	1.53E-06	8.7932222
cg23575099	chr14	55603874	+	LGALS3;LGALS3	TSS200;5'UTR	1.1606599	0.7997361	5.9296511	2.47E-08	1.53E-06	8.7929703
cg18560915	chr4	54430218	+	LNX1	Body	-1.201949	-0.487032	-5.929184	2.47E-08	1.53E-06	8.7908133
cg03281194	chr1	2776972	+			-1.578019	0.1134155	-5.931899	2.47E-08	1.53E-06	8.7913623
cg24882551	chr4	85403032	+			1.6396588	-1.097132	5.9289426	2.47E-08	1.53E-06	8.7896965

cg09347582	chr1	233497916	+	KIAA1804	Body	1.3552051	1.9945465	5.9287795	2.48E-08	1.53E-06	8.7889429
cg07996594	chr1	25257505	-	RUNX3;RUNX3	TSS1500;Body	1.6768476	1.1595732	5.9285302	2.48E-08	1.53E-06	8.7877907
cg07164722	chr1	247587378	-	NLRP3;NLRP3;NLRP3;NLRP3;NLRP3	Body;Body;Body;Body;Body	-1.955691	0.8159554	-5.928425	2.48E-08	1.53E-06	8.7873049
cg07817024	chr1	119535500	-			1.6839335	-0.490282	5.9282129	2.48E-08	1.54E-06	8.7863248
cg16399049	chr16	22825843	+	HS3ST2	TSS200	1.462503	-0.911678	5.9282012	2.48E-08	1.54E-06	8.7862704
cg09578028	chr2	176987411	-	HOXD9	TSS200	1.8309201	-0.325157	5.9278658	2.49E-08	1.54E-06	8.7847207
cg20493497	chr17	75830718	+			-1.315548	-2.406573	-5.92779	2.49E-08	1.54E-06	8.7843702
cg16419441	chr8	105478683	-	DPYS	Body	2.0568689	-0.163458	5.9276341	2.49E-08	1.54E-06	8.7836503
cg19048532	chr7	27148002	+	HOXA3;HOXA3;HOXA3	Body;Body;Body	1.0386445	0.9232537	5.9275728	2.49E-08	1.54E-06	8.7833673
cg07820868	chr1	119528638	+	TBX15	5'UTR	1.4642244	-0.137073	5.9274463	2.49E-08	1.54E-06	8.7827825
cg25527090	chr10	118894180	+	VAX1;VAX1	Body;Body	1.8504649	-0.060364	5.9272534	2.50E-08	1.54E-06	8.7818917
cg02171905	chr15	67035337	-	SMAD6;SMAD6;SMAD6	Body;Body;Body	0.7773577	1.6688223	5.9272137	2.50E-08	1.54E-06	8.7817082
cg18515872	chr14	72943461	-	RGS6	Body	-1.438368	-2.744152	-5.92712	2.50E-08	1.54E-06	8.7812737
cg05898591	chr5	174805715	-			-0.836275	-3.101048	-5.927069	2.50E-08	1.54E-06	8.7810372
cg00400987	chr16	28915821	-	ATP2A1;ATP2A1;RABEP2	3'UTR;3'UTR;3'UTR	0.7702616	1.7642294	5.9269521	2.50E-08	1.54E-06	8.7804993
cg16592658	chr19	4229887	-	EBI3	Body	-1.186812	-0.868926	-5.926883	2.50E-08	1.54E-06	8.780181
cg19573564	chr2	74607697	-	DCTN1;DCTN1	TSS1500;TSS1500	0.475315	1.9133502	5.9268857	2.50E-08	1.54E-06	8.7801927
cg19576556	chr17	80024177	+	DUS1L	TSS1500	-1.246531	-0.461028	-5.926899	2.50E-08	1.54E-06	8.7802544
cg24480735	chr19	54219381	+	MIR521-2	TSS1500	-1.608103	0.282577	-5.926987	2.50E-08	1.54E-06	8.7806602
cg06717850	chr19	57617602	+			1.3113724	-0.250367	5.9267474	2.50E-08	1.54E-06	8.7795539

cg26703066	chr11	10813944	+			-0.829394	0.4290846	-5.926682	2.50E-08	1.54E-06	8.7792503
cg15786900	chr11	44333131	+	ALX4	TSS1500	1.2679102	-0.544517	5.9293796	2.50E-08	1.54E-06	8.779737
cg04543008	chr11	71955332	+	PHOX2A	TSS200	2.6858458	-1.621048	5.926426	2.51E-08	1.55E-06	8.7780689
cg15584790	chr6	29692475	+	HLA-F;HLA-F;HLA-F	Body;Body;Body	1.2848495	2.0041384	5.9263428	2.51E-08	1.55E-06	8.7776846
cg16605327	chr12	114846849	+	TBX5;TBX5	TSS1500;TSS1500	1.7198602	-1.492427	5.9263338	2.51E-08	1.55E-06	8.7776429
cg01663563	chr15	90775513	-	CIB1	Body	-1.159951	0.9952916	-5.92592	2.51E-08	1.55E-06	8.7757293
cg02325832	chr4	13542883	+	NKX3-2	3'UTR	1.2772519	-0.23953	5.9287142	2.51E-08	1.55E-06	8.7766672
cg07584855	chr1	221055545	+	HLX	Body	1.759643	2.1938263	5.9258848	2.51E-08	1.55E-06	8.7755689
cg01476739	chr2	241853884	-			-0.635353	-3.25711	-5.925497	2.52E-08	1.55E-06	8.7737774
cg19987768	chr17	750306	-	NXN	Body	2.0947217	-1.192237	5.9254832	2.52E-08	1.55E-06	8.7737139
cg10319905	chr14	98097986	+			-1.408722	-0.779406	-5.925356	2.52E-08	1.55E-06	8.7731283
cg23987336	chr17	7959907	+			-1.252646	-1.953444	-5.925326	2.52E-08	1.55E-06	8.7729871
cg05944840	chr16	89401621	-	ANKRD11	5'UTR	0.6585789	0.0303794	5.9249377	2.52E-08	1.55E-06	8.7711939
cg13541578	chr17	77419374	-	HRNBP3	5'UTR	-0.814072	0.1343733	-5.924943	2.52E-08	1.55E-06	8.77122
cg05358175	chr5	156693748	+	CYFIP2;CYFIP2	5'UTR;5'UTR	-0.536359	-3.246612	-5.924149	2.53E-08	1.56E-06	8.7675513
cg22465123	chr18	29598437	-	RNF125	TSS200	-0.552439	-4.037868	-5.924092	2.53E-08	1.56E-06	8.7672893
cg13736376	chr4	111539291	+	PITX2;PITX2;PITX2	Body;Body;Body	1.4334288	-1.003143	5.9238876	2.54E-08	1.56E-06	8.7663436
cg05388262	chr4	76995796	+	ART3;ART3;ART3	TSS200;5'UTR;TSS200	1.1441323	1.7576224	5.9265925	2.54E-08	1.56E-06	8.767177
cg19140429	chr17	48858746	+			0.9946784	0.7920502	5.9237124	2.54E-08	1.56E-06	8.7655344
cg09500738	chr15	87911464	-			-1.21245	-0.335106	-5.923562	2.54E-08	1.56E-06	8.7648394

cg16656895	chr10	119305317	-	EMX2;EMX2;EMX2OS	Body;Body;TSS1500	1.2540132	-0.439528	5.9232243	2.54E-08	1.57E-06	8.7632802
cg05284727	chr4	1874366	+	WHSC1	5'UTR	-1.041671	-2.120516	-5.923175	2.54E-08	1.57E-06	8.7630547
cg24337215	chr1	155881944	-	RIT1	TSS1500	-3.199311	1.5602361	-5.92302	2.55E-08	1.57E-06	8.7623367
cg08200419	chr10	134261480	-	C10orf91	Body	-0.956837	-1.064448	-5.922652	2.55E-08	1.57E-06	8.7606357
cg12332524	chr11	45733155	-			-1.520236	-0.267567	-5.922415	2.55E-08	1.57E-06	8.7595441
cg20015412	chr4	10147100	-			-1.490695	0.3314969	-5.922087	2.56E-08	1.57E-06	8.7580269
cg13401658	chr2	177311027	+			-1.628424	1.3580995	-5.921944	2.56E-08	1.57E-06	8.7573687
cg24310126	chr10	124514711	-	FLJ46361	TSS1500	-1.35142	0.0761013	-5.921692	2.56E-08	1.58E-06	8.7562057
cg23075337	chr7	8481460	-	NXPH1	Body	1.6962942	-0.177845	5.9216538	2.56E-08	1.58E-06	8.7560277
cg24057107	chr7	117970353	+			-1.241482	-1.129224	-5.921625	2.56E-08	1.58E-06	8.7558951
cg17754510	chr6	10391412	+			3.2856427	-1.038272	5.9239071	2.57E-08	1.58E-06	8.7544945
cg14355941	chr14	101526485	-	MIR496	TSS1500	-1.798428	0.5393519	-5.920867	2.57E-08	1.58E-06	8.7523945
cg08454125	chr3	46747930	-	TMIE	Body	-1.003266	-0.506532	-5.920759	2.57E-08	1.58E-06	8.7518974
cg06173758	chr1	5957461	-	NPHP4	Body	0.5112104	1.9433972	5.920718	2.57E-08	1.58E-06	8.7517067
cg20014398	chr11	31825792	-	PAX6;PAX6;PAX6	Body;Body;Body	2.224693	0.0337801	5.9203212	2.58E-08	1.58E-06	8.749875
cg12071888	chr1	91195159	-			2.012307	-0.517062	5.9230208	2.58E-08	1.58E-06	8.7507061
cg19554569	chr11	5364833	-	OR51B5	TSS200	-1.534308	-0.151794	-5.920201	2.58E-08	1.58E-06	8.7493212
cg20696451	chr10	14881628	+	HSPA14	Body	-1.111507	-0.680246	-5.919979	2.58E-08	1.59E-06	8.748297
cg02896970	chr16	87460610	-	ZCCHC14	Body	0.7132888	2.3750323	5.9199434	2.58E-08	1.59E-06	8.7481304
cg03978169	chr6	33091357	-	HLA-DPB2	Body	-1.396881	0.2217183	-5.919741	2.59E-08	1.59E-06	8.7471974

cg09382096	chr11	31827593	-	PAX6;PAX6;PAX6	Body;Body;Body	1.831736	-1.298646	5.9194032	2.59E-08	1.59E-06	8.7456366
cg20611680	chr1	16554171	-			1.8247676	-1.312279	5.919348	2.59E-08	1.59E-06	8.7453817
cg02783918	chr16	86547544	-	FOXF1	3'UTR	1.2195128	-0.245462	5.9190615	2.60E-08	1.59E-06	8.7440591
cg14354327	chr10	131763676	+			1.227998	-0.579911	5.9190604	2.60E-08	1.59E-06	8.7440541
cg09906857	chr16	56557397	-			-0.738041	-0.554762	-5.918833	2.60E-08	1.59E-06	8.7430035
cg16262756	chr1	154156180	-	TPM3;TPM3;TPM3;TPM3;TPM3	TSS1500;TSS1500;Body;TSS1500;TSS1500	-0.66183	-3.301873	-5.918809	2.60E-08	1.59E-06	8.7428929
cg12664119	chr1	119532352	+	TBX15	TSS200	0.9657394	0.1263914	5.9184796	2.60E-08	1.60E-06	8.7413731
cg24544803	chr6	29596840	+	GABBR1;GABBR1;GABBR1	TSS1500;Body;Body	-1.151721	-1.821034	-5.918459	2.60E-08	1.60E-06	8.7412796
cg23048334	chr4	99850664	+	EIF4E;EIF4E;EIF4E;EIF4E;EIF4E	5'UTR;1stExon;5'UTR;TSS1500;1stExon	-0.390792	-4.501444	-5.918335	2.60E-08	1.60E-06	8.7407034
cg07208057	chr2	84516404	+	LOC388965	TSS1500	-1.55509	0.3795242	-5.921054	2.61E-08	1.60E-06	8.7413386
cg21123417	chr5	1103453	+	SLC12A7	Body	1.2071136	0.3212745	5.9181916	2.61E-08	1.60E-06	8.7400437
cg12432980	chr20	5987794	-	CRLS1;CRLS1	Body;TSS200	-0.745641	-2.389508	-5.918107	2.61E-08	1.60E-06	8.7396551
cg02431866	chr8	14710899	+	SGCZ	Body	-1.489283	0.2805246	-5.917945	2.61E-08	1.60E-06	8.7389038
cg16909733	chr2	241459847	+	ANKMY1;ANKMY1	Body;Body	1.5955735	-1.418653	5.9177653	2.61E-08	1.60E-06	8.7380756
cg00211821	chr17	16978673	-	MPRIP;MPRIP	Body;Body	1.0845899	1.2717042	5.9176896	2.61E-08	1.60E-06	8.7377265
cg11502555	chr13	100627341	-			1.6567888	-0.792165	5.9175562	2.61E-08	1.60E-06	8.7371104
cg17094296	chr4	169931799	-	CBR4	TSS1500	-0.469672	-4.586492	-5.917569	2.61E-08	1.60E-06	8.7371709
cg11299964	chr9	128469783	+	MAPKAP1;MAPKAP1;MAPKAP1;MAPKAP1;MAPKAP1;MAPKAP1	TSS1500;TSS1500;TSS1500;TSS1500;TSS1500;TSS1500	-0.744076	-2.919691	-5.917511	2.61E-08	1.60E-06	8.7369013
cg27086014	chr19	52004992	-	SIGLEC12;SIGLEC12;SIGLEC12	1stExon;5'UTR;TSS1500	-1.121667	-1.318834	-5.917471	2.62E-08	1.60E-06	8.7367151
cg05709458	chr11	74948173	+			-1.317689	0.1315214	-5.917074	2.62E-08	1.60E-06	8.7348843

cg16208053	chr10	135379215	-	SYCE1;SYCE1;SYCE1	TSS200;TSS200;5'UTR	1.5716687	2.2258874	5.9170601	2.62E-08	1.60E-06	8.7348207
cg10917952	chr11	5989222	+	OR56A5	1stExon	-1.267075	-0.126253	-5.919598	2.62E-08	1.60E-06	8.7349246
cg21627409	chr5	140810106	+	PCDHGA4;PCDHGA11;PCDHGA11;PCDHGA9;PCDHGA1;PCDHGB1;PCDHGB6;PCDHGA12;PCDHGB3;PCDHGB7;PCDHGA6;PCDHGA8;PCDHGA10;PCDHGA5;PCDHGB4;PCDHGA3;PCDHGA2	Body;Body;Body;Body;Body;Body;Body;TSS200;Body;Body;Body;Body;Body;Body;Body;Body;TSS200;Body;Body;Body	1.8414257	-0.699611	5.9223488	2.63E-08	1.61E-06	8.7355234
cg04857257	chr8	90913896	+	OSGIN2;OSGIN2	TSS200;TSS1500	-1.250462	-0.875911	-5.91622	2.63E-08	1.61E-06	8.7309416
cg12180703	chr20	37353037	-	SLC32A1	TSS200	1.7780979	-0.898052	5.9158891	2.64E-08	1.61E-06	8.7294164
cg06982544	chr1	150254546	+	C1orf51	TSS1500	-1.712056	-4.623712	-5.921047	2.64E-08	1.61E-06	8.7298406
cg15309879	chr8	933232	-			-1.687181	0.6909525	-5.915401	2.64E-08	1.61E-06	8.727163
cg18263455	chr15	69374595	-	MIR548H4;TMEM84	Body;Body	1.1959125	0.9374001	5.9153703	2.64E-08	1.61E-06	8.7270221
cg23704627	chr22	39152758	+	UNC84B	TSS1500	-0.902504	-2.266865	-5.915131	2.65E-08	1.62E-06	8.7259186
cg15480897	chr4	151505467	-	LRBA;MAB21L2;MAB21L2	Body;3'UTR;1stExon	0.9234011	2.3210709	5.9149864	2.65E-08	1.62E-06	8.7252505
cg22661082	chr2	189558080	-			-1.743153	0.782913	-5.914923	2.65E-08	1.62E-06	8.7249567
cg23105419	chr2	43496213	+	THADA;THADA	Body;Body	1.1956485	0.4892436	5.9147264	2.65E-08	1.62E-06	8.7240505
cg08393317	chr19	54445463	+	CACNG7	Body	1.8337243	-0.5664	5.9145871	2.65E-08	1.62E-06	8.7234081
cg00698204	chr21	38080694	-	SIM2;SIM2	Body;Body	1.2665558	-0.775633	5.9144857	2.65E-08	1.62E-06	8.7229399
cg13933279	chr15	88418502	-			-1.076762	-1.957653	-5.91425	2.66E-08	1.62E-06	8.7218507
cg13572780	chr10	24792697	-	KIAA1217;KIAA1217;KIAA1217	Body;Body;Body	-1.142087	-2.668766	-5.91405	2.66E-08	1.62E-06	8.7209286
cg19442493	chr17	77375519	-	HRNBP3	5'UTR	-0.713004	-1.122996	-5.913669	2.66E-08	1.63E-06	8.719172
cg14223654	chr2	45397802	+			1.0338134	0.0251392	5.9164275	2.66E-08	1.63E-06	8.7203148
cg09159022	chr8	4849522	+	CSMD1	Body	1.5009852	-0.742244	5.9134487	2.67E-08	1.63E-06	8.7181551
cg05100070	chr6	100896760	-	SIM1	Body	1.0646233	-0.414208	5.913343	2.67E-08	1.63E-06	8.7176674

cg22897141	chr11	64577338	-	MEN1;MEN1;MEN1;MEN1;MEN1;MEN1;MEN1	Body;Body;Body;Body;Body;Body;Body	-1.744719	-3.299913	-5.921335	2.67E-08	1.63E-06	8.7192463
cg21009965	chr1	75593596	+	LHX8	TSS1500	1.374692	-1.273044	5.9126637	2.68E-08	1.63E-06	8.7145333
cg04907218	chr12	132826672	-	GALNT9	Body	-1.11215	-0.120724	-5.912455	2.68E-08	1.63E-06	8.7135689
cg18801579	chr5	1003897	-			-1.600791	-0.284898	-5.912236	2.68E-08	1.64E-06	8.7125597
cg07588253	chr1	115860698	-	NGF	5'UTR	-1.834309	0.4866316	-5.91214	2.68E-08	1.64E-06	8.712116
cg21917524	chr11	74200334	+			1.1091767	2.303851	5.9121473	2.68E-08	1.64E-06	8.7121508
cg10927449	chr2	63286621	-			1.2864175	-0.584456	5.9119422	2.69E-08	1.64E-06	8.7112047
cg09323727	chr2	176936436	-			2.2650187	-0.209212	5.911217	2.70E-08	1.64E-06	8.7078594
cg16247269	chr5	148513941	+			-0.791363	-0.872871	-5.911163	2.70E-08	1.64E-06	8.7076121
cg23111196	chr5	60603263	-			-1.467151	0.7445108	-5.911027	2.70E-08	1.64E-06	8.7069808
cg03947979	chr6	78176785	+			1.7298058	-1.092378	5.9109873	2.70E-08	1.64E-06	8.7067999
cg24029436	chr3	88199835	+	C3orf38	Body	-0.862258	-3.615473	-5.910875	2.70E-08	1.64E-06	8.706282
cg06702880	chr4	155663094	-			2.2671244	-1.691552	5.9104244	2.71E-08	1.65E-06	8.7042031
cg06760238	chr10	134400036	-	INPP5A	Body	1.1104426	0.5049178	5.9104401	2.71E-08	1.65E-06	8.7042758
cg17353337	chr6	138866898	+	NHSL1	Body	1.549605	0.7517067	5.910413	2.71E-08	1.65E-06	8.7041509
cg25212131	chr5	140683670	+	SLC25A2	TSS200	1.013589	1.7274693	5.9132532	2.71E-08	1.65E-06	8.7056904
cg24394172	chr4	46995203	+	GABRA4	Body	1.1881657	0.0819438	5.9103699	2.71E-08	1.65E-06	8.703952
cg11448366	chr15	101601096	-	LRRK1	Body	0.9734227	1.4767871	5.9102191	2.71E-08	1.65E-06	8.7032563
cg08564375	chr15	25436682	+			-0.984785	-0.094428	-5.910049	2.71E-08	1.65E-06	8.7024697
cg11720054	chr11	124481218	-	PANX3	TSS1500	-0.955137	-0.23906	-5.910072	2.71E-08	1.65E-06	8.7025772

cg13636887	chr14	105189936	-	ADSSL1	TSS1500	-0.594788	-1.257516	-5.909786	2.71E-08	1.65E-06	8.7012573
cg11742898	chr7	127636374	+	C7orf54;SND1	TSS1500;Body	0.7009825	1.9901748	5.9097167	2.71E-08	1.65E-06	8.7009392
cg23892568	chr12	10283049	-	CLEC7A;CLEC7A;CLEC7A;CLEC7A;CLEC7A;CLEC7A	TSS200;TSS200;TSS200;TSS200;TSS200;TSS200	-1.628066	0.7884457	-5.909683	2.72E-08	1.65E-06	8.7007858
cg11990559	chr2	240727278	-			-2.205193	0.9000335	-5.909444	2.72E-08	1.65E-06	8.6996806
cg25310700	chr10	5702083	-	ASB13;ASB13	Body;Body	-0.914685	-1.017126	-5.909447	2.72E-08	1.65E-06	8.6996963
cg16199460	chr5	5087391	-			-1.379912	-1.181886	-5.909328	2.72E-08	1.65E-06	8.699146
cg11645155	chr19	47288114	+	SLC1A5;SLC1A5;SLC1A5;SLC1A5	1stExon;5'UTR;Body;5'UTR	-0.680226	-4.15097	-5.909102	2.72E-08	1.65E-06	8.6981048
cg15325961	chr8	49183143	+			-1.055652	-1.104314	-5.90891	2.73E-08	1.66E-06	8.69722
cg13411286	chr2	191365138	+	MFSD6	3'UTR	1.6216942	1.5788197	5.9115212	2.73E-08	1.66E-06	8.6977125
cg04383383	chr19	310430	-	MIER2	Body	0.900103	1.4807522	5.9086231	2.73E-08	1.66E-06	8.6958955
cg16476940	chr7	101927973	+	SH2B2	TSS1500	-1.567905	0.9952833	-5.908557	2.73E-08	1.66E-06	8.6955892
cg23693487	chr8	30670746	+	PPP2CB;PPP2CB	TSS1500;TSS1500	-1.097039	-0.629207	-5.908502	2.73E-08	1.66E-06	8.6953362
cg26014538	chr3	147126763	-	ZIC1	TSS1500	2.4453832	0.4828053	5.9083383	2.73E-08	1.66E-06	8.6945821
cg07055769	chr14	52555443	+			-1.805157	0.5460207	-5.908214	2.73E-08	1.66E-06	8.6940079
cg03285468	chr18	61143933	+	SERPINB5	TSS1500	-1.379336	0.0485042	-5.908173	2.74E-08	1.66E-06	8.6938215
cg23366832	chr6	90712882	-	BACH2;BACH2	Body;Body	-1.420823	1.0048775	-5.908106	2.74E-08	1.66E-06	8.6935107
cg00618155	chr10	72459629	-	ADAMTS14;ADAMTS14	Body;Body	-1.578342	0.7196146	-5.908075	2.74E-08	1.66E-06	8.693367
cg20756891	chr5	170743951	+			1.2227356	-0.492228	5.9077504	2.74E-08	1.66E-06	8.6918711
cg06877024	chr6	28743586	-			0.9829695	-0.65423	5.9076121	2.74E-08	1.66E-06	8.6912333
cg08580187	chr16	84027871	+	NECAB2	Body	-1.208757	-0.862881	-5.907359	2.75E-08	1.67E-06	8.6900673

cg17046586	chr12	50665774	-	LIMA1;LIMA1	5'UTR;5'UTR	1.6384381	0.7607426	5.9072706	2.75E-08	1.67E-06	8.6896586
cg01190024	chr10	8078357	+			1.3879451	-0.905695	5.9066316	2.76E-08	1.67E-06	8.6867122
cg19628333	chr12	56914766	-	RBMS2	TSS1500	1.6183287	0.9819353	5.9063993	2.76E-08	1.67E-06	8.6856409
cg18336674	chr5	170735264	-	TLX3	TSS1500	1.2571267	0.1884023	5.905876	2.77E-08	1.68E-06	8.6832281
cg20777920	chr6	10404848	-	TFAP2A;TFAP2A;TFAP2A	Body;Body;Body	-1.036413	-4.452703	-5.905902	2.77E-08	1.68E-06	8.6833493
cg06768670	chr8	143182662	-			-1.400717	-0.199858	-5.908511	2.77E-08	1.68E-06	8.6838517
cg09889948	chr17	48858873	+			0.9716851	0.5761649	5.9055019	2.77E-08	1.68E-06	8.6815033
cg00621899	chr7	100486423	-	UFSP1;UFSP1	1stExon;3'UTR	-1.462276	0.7763627	-5.905057	2.78E-08	1.68E-06	8.6794538
cg08411049	chr18	61144250	+	SERPINB5;SERPINB5	1stExon;5'UTR	-1.549385	-0.141897	-5.904672	2.78E-08	1.69E-06	8.6776779
cg26291600	chr10	45923705	-	ALOX5	Body	1.1408134	0.9448294	5.9044447	2.78E-08	1.69E-06	8.6766297
cg27142536	chr11	20618198	-			1.7178429	-0.321371	5.9043908	2.79E-08	1.69E-06	8.6763813
cg04462561	chr13	24837937	-	SPATA13;SPATA13	Body;Body	1.2022897	-0.344342	5.9042421	2.79E-08	1.69E-06	8.6756959
cg03578464	chr3	8928666	+	RAD18	Body	0.6561819	2.2296911	5.9041581	2.79E-08	1.69E-06	8.6753085
cg04399632	chr16	11837547	-	TXNDC11	TSS1500	-1.226153	-1.852989	-5.904095	2.79E-08	1.69E-06	8.6750173
cg23816037	chr2	127455623	-			-1.808119	1.0223	-5.90406	2.79E-08	1.69E-06	8.6748541
cg07397033	chr10	98031201	+	BLNK;BLNK;BLNK;BLNK	5'UTR;5'UTR;1stExon;1stExon	-0.986597	-1.628927	-5.906815	2.79E-08	1.69E-06	8.6760412
cg17552471	chr7	4678661	-			-1.032841	-0.665967	-5.90387	2.79E-08	1.69E-06	8.6739801
cg03974889	chr1	91192162	+			1.4394612	-1.265545	5.9037995	2.79E-08	1.69E-06	8.6736555
cg00334691	chr15	25468075	+	SNORD115-11;SNORD115-15;SNORD115-21;SNORD115-43;SNORD115-36;SNORD115-29	TSS1500;Body;Body;TSS1500;TSS1500;TSS1500	-1.366726	0.5242351	-5.903681	2.79E-08	1.69E-06	8.6731113
cg25567594	chr20	21683478	-			1.6557285	-0.909005	5.9036962	2.79E-08	1.69E-06	8.6731793

cg01813877	chr14	103369717	-	TRAF3;TRAF3;TRAF3	Body;Body;Body	1.6253728	3.8978524	5.903615	2.80E-08	1.69E-06	8.6728051
cg18121003	chr11	86085757	+	CCDC81;CCDC81	TSS200;TSS200	2.1308609	-0.557171	5.9064071	2.80E-08	1.69E-06	8.6738637
cg21028562	chr3	50287909	-	GNAI2;GNAI2	Body;Body	0.6386259	0.7087553	5.9033404	2.80E-08	1.69E-06	8.6715393
cg27074174	chr6	101847318	-	GRIK2;GRIK2;GRIK2	Body;Body;Body	1.5813821	-1.156726	5.9033297	2.80E-08	1.69E-06	8.6714898
cg24822053	chr19	18979397	+	GDF1;LASS1	3'UTR;3'UTR	1.7468408	3.916813	5.9029783	2.80E-08	1.69E-06	8.6698703
cg25406518	chr11	61102165	+	DAK;DDB1	5'UTR;TSS1500	-0.994092	-0.468241	-5.902997	2.80E-08	1.69E-06	8.6699562
cg26904406	chr6	111927312	-	TRAF3IP2;TRAF3IP2;TRAF3IP2;TRAF3IP2;TRAF3IP2	1stExon;Body;5'UTR;1stExon;5'UTR	-0.59459	-4.119947	-5.90291	2.81E-08	1.70E-06	8.669554
cg19544372	chr19	9609405	-	ZNF560	TSS200	1.3938268	-0.788352	5.9055773	2.81E-08	1.70E-06	8.6703444
cg04456219	chr7	17274337	+			-2.138436	-1.334007	-5.902612	2.81E-08	1.70E-06	8.668184
cg05425602	chr8	135812896	+	MIR30B	TSS200	1.6193077	1.8170956	5.9024472	2.81E-08	1.70E-06	8.6674226
cg10477621	chr7	19184950	-	FERD3L	1stExon	1.6401788	-0.139295	5.9023201	2.81E-08	1.70E-06	8.6668366
cg20303399	chr8	26722965	-	ADRA1A;ADRA1A;ADRA1A;ADRA1A	TSS200;TSS200;TSS200;TSS200	1.1842181	-0.943571	5.9022935	2.81E-08	1.70E-06	8.6667138
cg20608990	chr2	202097607	+	CASP8;CASP8;CASP8;CASP8	TSS1500;TSS1500;TSS1500;TSS1500	-1.280352	-1.165286	-5.902297	2.81E-08	1.70E-06	8.666728
cg20885089	chr1	110162701	+	AMPD2;AMPD2;AMPD2	1stExon;TSS1500;5'UTR	-0.373502	-3.965223	-5.902207	2.81E-08	1.70E-06	8.6663167
cg00184376	chr13	28499045	+	PDX1	3'UTR	1.1216208	-1.149288	5.9019986	2.82E-08	1.70E-06	8.6653549
cg20058187	chr11	121665730	+			-1.341418	0.2828371	-5.901873	2.82E-08	1.70E-06	8.6647764
cg00824767	chr10	23488272	+			1.5378428	-1.269394	5.9017306	2.82E-08	1.70E-06	8.6641196
cg01637011	chr1	200992964	-	KIF21B	TSS200	-1.37948	-3.657265	-5.901628	2.82E-08	1.70E-06	8.6636474
cg15836733	chr15	42875249	+	STARD9	Body	0.7699968	2.4479842	5.9013291	2.83E-08	1.71E-06	8.6622692
cg04043571	chr9	127265989	+	NR5A1	5'UTR	1.2492201	-1.644521	5.903847	2.83E-08	1.71E-06	8.6620793

cg13997435	chr1	153538406	-	S100A2	TSS200	-1.015298	-2.095407	-5.90106	2.83E-08	1.71E-06	8.6610288
cg16692196	chr1	85040371	+	CTBS	TSS1500	-1.239799	-5.27808	-5.903784	2.83E-08	1.71E-06	8.662091
cg27642784	chr3	157813670	+			1.6175019	1.2518756	5.9034485	2.84E-08	1.71E-06	8.6605466
cg26818625	chr8	72471153	-			2.641579	-1.160408	5.9033635	2.84E-08	1.71E-06	8.673737
cg18884447	chr14	105018912	+			-1.299507	0.8570892	-5.900458	2.84E-08	1.71E-06	8.6582543
cg06773295	chr1	3028577	+	PRDM16;PRDM16	Body;Body	1.0764681	2.6086187	5.9003402	2.84E-08	1.71E-06	8.6577124
cg19248676	chr12	115135926	-			1.3198969	-1.069703	5.900339	2.84E-08	1.71E-06	8.6577069
cg16136079	chr13	113811690	+	PROZ	TSS1500	0.784249	2.2246955	5.9001043	2.84E-08	1.71E-06	8.6566251
cg24459147	chr21	38068581	-			1.1159894	-0.794055	5.900088	2.84E-08	1.71E-06	8.65655
cg17940268	chr1	166124106	-	MIR921;FAM78B	TSS200;Body	-1.826132	1.0666763	-5.902758	2.84E-08	1.71E-06	8.6573709
cg16145324	chr6	36020012	+	MAPK14;MAPK14;MAPK14;MAPK14	Body;Body;Body;Body	1.1734203	1.141728	5.8997475	2.85E-08	1.72E-06	8.654981
cg22603393	chr1	39878781	-	KIAA0754;MACF1;MACF1	1stExon;Body;Body	0.7903871	1.5942131	5.8997052	2.85E-08	1.72E-06	8.654786
cg17474524	chr20	62168831	+	PTK6	TSS200	-1.12532	-1.616935	-5.899574	2.85E-08	1.72E-06	8.6541795
cg16291276	chr1	208084071	+	CD34;CD34	Body;Body	1.8443609	-0.748843	5.8994056	2.85E-08	1.72E-06	8.6534057
cg16426293	chr17	40192112	+			0.946194	0.8043216	5.8991499	2.86E-08	1.72E-06	8.6522276
cg25018951	chr4	25235369	+	PI4K2B	TSS1500	-1.272988	-2.741911	-5.899032	2.86E-08	1.72E-06	8.6516863
cg10084644	chr7	99775521	+	STAG3;GPC2	TSS200;TSS1500	1.5940687	-0.462452	5.8989742	2.86E-08	1.72E-06	8.6514182
cg14682528	chr11	111798182	-			-0.524573	-4.644628	-5.898841	2.86E-08	1.72E-06	8.650805
cg03983645	chr11	20181887	-	DBX1	TSS200	1.8885921	-1.091619	5.8988026	2.86E-08	1.72E-06	8.6506276
cg01393985	chr6	89927651	+	GABRR1	TSS200	0.9531537	1.9042965	5.8984807	2.87E-08	1.72E-06	8.6491445

cg18839416	chr1	12805849	+	C1orf158	TSS1500	-1.136113	-0.573359	-5.898503	2.87E-08	1.72E-06	8.6492459
cg13803688	chr1	110610452	-	ALX3	Body	1.2191004	0.8047918	5.8984357	2.87E-08	1.72E-06	8.648937
cg12294118	chr2	202879585	-			1.1212958	0.9903985	5.8983531	2.87E-08	1.72E-06	8.6485567
cg22188918	chr7	100882175	+	CLDN15	TSS1500	0.8438883	1.6219148	5.8983335	2.87E-08	1.72E-06	8.6484663
cg19909036	chr15	90294776	+	MESP1	TSS1500	-0.965458	-1.949353	-5.898166	2.87E-08	1.73E-06	8.6476942
cg11225330	chr1	1196747	-	UBE2J2;UBE2J2;LOC100128842;UBE2J2;UBE2J2	Body;Body;Body;Body;Body	0.7925983	1.4954748	5.8979061	2.87E-08	1.73E-06	8.6464972
cg03273715	chr19	54415702	+	CACNG7	TSS1500	-0.890501	-0.284152	-5.897579	2.88E-08	1.73E-06	8.6449916
cg08909802	chr8	145049419	+	PLEC1	Body	-1.139641	-2.632321	-5.897017	2.89E-08	1.73E-06	8.6424003
cg25343246	chr12	34756291	-			-0.912038	-1.836949	-5.896919	2.89E-08	1.73E-06	8.6419499
cg00960906	chr5	31769846	+			-1.218919	-0.871678	-5.899362	2.89E-08	1.74E-06	8.6414434
cg11218176	chr1	16403434	+			-1.343566	0.1773646	-5.89638	2.89E-08	1.74E-06	8.6394676
cg18484189	chr11	7984973	-	NLRP10	1stExon	-1.003365	-0.277351	-5.89632	2.90E-08	1.74E-06	8.63919
cg04861230	chr1	159682497	-	CRP	3'UTR	-1.537373	0.1189663	-5.896202	2.90E-08	1.74E-06	8.6386476
cg05468171	chr16	84733172	+	USP10	TSS1500	-0.740407	-3.414947	-5.896215	2.90E-08	1.74E-06	8.6387087
cg22322465	chr4	174459201	-	NBLA00301	Body	1.0573526	-0.880501	5.8962179	2.90E-08	1.74E-06	8.6387205
cg20122518	chr7	810958	+	HEATR2	Body	0.9743274	-0.455448	5.896171	2.90E-08	1.74E-06	8.6385046
cg23726526	chr9	96711316	+			2.0100622	-1.644815	5.8959525	2.90E-08	1.74E-06	8.6374979
cg11264499	chr5	79286765	-	MTX3;MTX3	5'UTR;Body	-0.693712	-2.589302	-5.89581	2.90E-08	1.74E-06	8.6368399
cg24296397	chr3	49692537	+	BSN	Body	-1.270578	-0.098915	-5.898553	2.90E-08	1.74E-06	8.6377205
cg07643930	chr16	2060265	-	ZNF598	TSS1500	-0.924429	-3.123911	-5.895538	2.91E-08	1.74E-06	8.6355886

cg22485810	chr17	29248725	+	ADAP2	TSS200	-0.948709	-1.556275	-5.895509	2.91E-08	1.74E-06	8.6354544
cg00827893	chr13	33162946	+	PDS5B	5'UTR	1.3400303	0.1446701	5.8952057	2.91E-08	1.75E-06	8.6340585
cg04095339	chr5	1879917	+	IRX4	Body	0.9683266	0.4208311	5.8950841	2.91E-08	1.75E-06	8.6334984
cg07343027	chr17	80024200	+	DUS1L	TSS1500	-1.279107	-1.441737	-5.894824	2.92E-08	1.75E-06	8.6322986
cg20297976	chr11	193062	-	SCGB1C1;LOC653486	TSS200;TSS200	-1.241103	0.8664215	-5.894844	2.92E-08	1.75E-06	8.6323947
cg16205854	chr20	57875318	-	EDN3;EDN3;EDN3;EDN3	TSS200;TSS200;TSS200;TSS200	1.1783448	-1.347534	5.8941667	2.93E-08	1.75E-06	8.629273
cg18776616	chr10	72235645	-			-1.454838	-0.061441	-5.894145	2.93E-08	1.75E-06	8.6291721
cg27542422	chr12	122884354	-	CLIP1;CLIP1	5'UTR;5'UTR	-0.831479	-2.404838	-5.894133	2.93E-08	1.75E-06	8.62912
cg18924324	chr6	100057143	+	PRDM13	Body	2.6086106	-0.475098	5.8937771	2.93E-08	1.76E-06	8.6274792
cg23397023	chr7	149112887	-			0.8025417	0.3366584	5.893725	2.93E-08	1.76E-06	8.6272392
cg01114124	chr2	240171748	+	HDAC4	Body	1.350393	1.3702225	5.8964468	2.93E-08	1.76E-06	8.6283347
cg06637017	chr5	1092391	-	SLC12A7	Body	0.782118	1.5868559	5.8933737	2.94E-08	1.76E-06	8.6256213
cg01286685	chr6	30039025	-	RNF39;RNF39	Body;Body	1.1579909	1.4588097	5.8931522	2.94E-08	1.76E-06	8.6246015
cg07510303	chr6	143748526	-	ADAT2	3'UTR	-0.510829	-2.692681	-5.893131	2.94E-08	1.76E-06	8.6245016
cg26694290	chr15	93457211	+	CHD2;CHD2	Body;Body	-1.229123	0.7092109	-5.893066	2.94E-08	1.76E-06	8.6242059
cg17437419	chr15	95400067	+			-1.324488	0.5611229	-5.892888	2.94E-08	1.76E-06	8.6233864
cg10617784	chr2	71134586	-	VAX2	Body	1.7767353	-0.529672	5.8924517	2.95E-08	1.77E-06	8.6213761
cg19306866	chr21	31970997	+			-1.168737	0.5135978	-5.8924	2.95E-08	1.77E-06	8.6211395
cg24305156	chr11	45871936	+	CRY2;CRY2	Body;Body	1.0345301	-0.17777	5.8923742	2.95E-08	1.77E-06	8.6210189
cg25389145	chr8	141220490	-	TRAPPC9;TRAPPC9	Body;Body	0.903489	0.3825111	5.8923928	2.95E-08	1.77E-06	8.6211046

cg25820699	chr12	75601803	+	KCNC2;KCNC2;KCNC2	5'UTR;5'UTR;5'UTR	2.8834707	-2.23923	5.8948722	2.95E-08	1.77E-06	8.6210939
cg17019053	chr2	74875263	+	C2orf65	TSS200	1.229288	0.5814857	5.891999	2.96E-08	1.77E-06	8.6192914
cg03832522	chr2	218898668	+			1.3461617	0.4179385	5.8918519	2.96E-08	1.77E-06	8.6186141
cg22760287	chr11	107575901	+			-1.267389	-0.442948	-5.891881	2.96E-08	1.77E-06	8.6187466
cg16385583	chr1	247711595	+	C1orf150	TSS1500	-1.56639	-1.961113	-5.891578	2.96E-08	1.77E-06	8.6173554
cg26732155	chr15	37236977	+	MEIS2;MEIS2;MEIS2;MEIS2;MEIS2;MEIS2;MEIS2	Body;Body;Body;Body;Body;Body;Body	0.7169515	1.8207689	5.8915332	2.96E-08	1.77E-06	8.6171468
cg25144135	chr1	1144012	-			-1.181807	-1.325055	-5.891357	2.97E-08	1.77E-06	8.6163345
cg17710804	chr1	36771844	-	C1orf113;C1orf113	TSS1500;TSS200	-1.053336	-1.44427	-5.896826	2.97E-08	1.77E-06	8.6181795
cg27507473	chr16	86002958	-			-0.884281	-2.533724	-5.89118	2.97E-08	1.77E-06	8.6155212
cg17689601	chr10	83651137	+	NRG3;NRG3;NRG3	Body;Body;Body	-1.890184	0.7810088	-5.890716	2.97E-08	1.78E-06	8.6133857
cg01343344	chr1	116694665	-			-0.684368	-3.663411	-5.890566	2.98E-08	1.78E-06	8.6126938
cg00587009	chr6	106429129	-			2.1617755	-1.524253	5.8904632	2.98E-08	1.78E-06	8.6122207
cg07555084	chr6	27186199	-			1.1553201	1.8220675	5.890456	2.98E-08	1.78E-06	8.6121877
cg11632679	chr20	56759082	+			-1.283105	-0.708303	-5.890094	2.98E-08	1.78E-06	8.6105207
cg12217625	chr1	159825628	+	VSIG8;C1orf204	Body;TSS1500	0.7582768	1.8678849	5.8895256	2.99E-08	1.79E-06	8.6079045
cg20443357	chr10	134269240	+			-1.221993	0.9318016	-5.889409	2.99E-08	1.79E-06	8.6073688
cg23797471	chr6	135504410	+	MYB;MYB;MYB;MYB;MYB;MYB;MYB;MYB	Body;Body;Body;Body;Body;Body;Body;Body	-0.680476	-4.045911	-5.889345	2.99E-08	1.79E-06	8.6070742
cg21619594	chr7	31146128	-	ADCYAP1R1	Body	-1.414188	0.4732964	-5.889223	3.00E-08	1.79E-06	8.6065123
cg02596960	chr12	114985224	-			-1.332894	0.4538835	-5.889097	3.00E-08	1.79E-06	8.6059321
cg14534089	chr13	111061002	+	COL4A2	Body	1.54757	2.7946606	5.8888087	3.00E-08	1.79E-06	8.6046049

cg16023122	chr1	201708836	+	NAV1;NAV1	TSS200;Body	-1.768213	-2.746209	-5.888496	3.01E-08	1.79E-06	8.6031659
cg20982011	chr11	1950657	-	TNNT3;TNNT3;TNNT3;TNNT3	Body;Body;Body;Body	-1.204343	-0.095209	-5.88843	3.01E-08	1.79E-06	8.6028635
cg03439301	chr7	136190456	-			-1.455246	-1.208234	-5.888371	3.01E-08	1.79E-06	8.6025909
cg19865472	chr19	617133	+	HCN2	3'UTR	-2.12999	2.2311009	-5.899602	3.01E-08	1.79E-06	8.6314825
cg01712948	chr2	89165761	-			-1.61858	-1.239091	-5.888221	3.01E-08	1.79E-06	8.6018977
cg20506659	chr6	33132943	+	COL11A2;COL11A2;COL11A2	Body;Body;Body	-1.426394	-0.911104	-5.887902	3.01E-08	1.80E-06	8.6004335
cg24377694	chr20	1975372	-	PDYN	TSS1500	-2.671042	1.2558059	-5.887591	3.02E-08	1.80E-06	8.5989989
cg05583632	chr22	25081868	-			0.9752076	-1.794973	5.8874918	3.02E-08	1.80E-06	8.5985435
cg00543474	chr10	134260981	-	C10orf91	Body	-0.981371	-1.298557	-5.887411	3.02E-08	1.80E-06	8.5981723
cg17376299	chr17	74931583	+	MGAT5B;MGAT5B	Body;Body	-1.70794	0.1227065	-5.887259	3.02E-08	1.80E-06	8.5974743
cg01553004	chr14	77495672	-	C14orf4	TSS1500	-1.118309	-1.016038	-5.887204	3.02E-08	1.80E-06	8.5972211
cg08319999	chr5	124054948	-	ZNF608	Body	1.0898189	1.1808198	5.8866884	3.03E-08	1.81E-06	8.5948467
cg18531916	chr10	130124378	-			-1.344443	-0.274477	-5.886537	3.03E-08	1.81E-06	8.5941475
cg07118041	chr17	79213681	+	C17orf89;C17orf56	Body;TSS1500	-0.41998	-4.147249	-5.88575	3.05E-08	1.81E-06	8.5905261
cg08519008	chr11	123815202	-	OR6T1	TSS1500	-1.693172	0.295984	-5.885777	3.05E-08	1.81E-06	8.5906547
cg01530082	chr6	32784327	+	HLA-DOB	Body	-1.746836	1.0729412	-5.885191	3.05E-08	1.82E-06	8.587956
cg08636922	chr21	46420451	+	NCRNA00162	Body	-1.262276	0.2268605	-5.885149	3.05E-08	1.82E-06	8.5877628
cg14065153	chr17	38512388	+	RARA;RARA;RARA;RARA	Body;Body;Body;Body	0.8009159	1.347922	5.8849368	3.06E-08	1.82E-06	8.5867864
cg02430935	chr10	124909028	-	HMX2	Body	1.5449706	-0.813483	5.884411	3.07E-08	1.82E-06	8.5843676
cg17181043	chr1	64961535	-	CACHD1	Body	-1.571392	-0.163328	-5.884379	3.07E-08	1.82E-06	8.5842216

cg06204948	chr11	63656090	+	MARK2;MARK2;MARK2;MARK2;MARK2;MARK2	Body;1stExon;Body;5'UTR;Body;Body	-1.081787	-1.867291	-5.884199	3.07E-08	1.83E-06	8.5833916
cg19693031	chr1	145441552	+	TXNIP	3'UTR	1.5644004	0.9292363	5.8839781	3.07E-08	1.83E-06	8.5823759
cg22378237	chr15	94867583	-	MCTP2;MCTP2	Body;Body	-0.979976	-2.4083	-5.883676	3.08E-08	1.83E-06	8.5809865
cg01084740	chr14	61748243	+	TMEM30B;TMEM30B	1stExon;5'UTR	-0.971144	-1.356142	-5.883572	3.08E-08	1.83E-06	8.5805094
cg27303223	chr10	28035822	-	MKX	TSS1500	1.5064074	-0.970104	5.8835628	3.08E-08	1.83E-06	8.5804653
cg00867889	chr8	133056586	+	OC90	Body	-1.819565	0.56674	-5.883404	3.08E-08	1.83E-06	8.5797342
cg18880986	chr7	5534951	+	FBXL18	Body	-1.418532	1.0100782	-5.883399	3.08E-08	1.83E-06	8.5797103
cg08785215	chr3	57993979	-	FLNB;FLNB;FLNB;FLNB	TSS200;TSS200;TSS200;TSS200	-0.569077	-3.886351	-5.883151	3.08E-08	1.83E-06	8.578571
cg12810084	chr7	27225319	-	HOXA11AS;HOXA11	Body;TSS1500	1.1262801	-0.861103	5.8831783	3.08E-08	1.83E-06	8.5786961
cg03192874	chr2	187474432	+	ITGAV;ITGAV;ITGAV	Body;Body;Body	0.7943729	1.9714383	5.8830754	3.09E-08	1.83E-06	8.578223
cg11270070	chr3	50383118	-	ZMYND10;ZMYND10	1stExon;5'UTR	-2.210856	-3.333188	-5.885727	3.09E-08	1.83E-06	8.5787576
cg03402235	chr15	42749336	+	ZFP106	1stExon	1.1190716	0.5816924	5.8828375	3.09E-08	1.83E-06	8.5771287
cg25004981	chr15	101085028	+	LASS3	TSS200	1.5595175	3.2935346	5.8941137	3.09E-08	1.83E-06	8.5821288
cg08413646	chr5	149996740	-	SYNPO;SYNPO	5'UTR;TSS1500	-1.299148	0.9156981	-5.882603	3.09E-08	1.84E-06	8.5760496
cg06211255	chr11	65153842	-	FRMD8	TSS200	-0.829757	-1.90146	-5.882247	3.10E-08	1.84E-06	8.5744108
cg25121227	chr13	28000282	-	GTF3A	Body	-1.066095	-3.331627	-5.882046	3.10E-08	1.84E-06	8.5734866
cg12416080	chr3	26750855	-	LRRC3B	5'UTR	-1.45412	0.5100377	-5.882016	3.10E-08	1.84E-06	8.5733476
cg10007534	chr21	46378354	-	C21orf70	Body	1.5633599	3.9832731	5.8816424	3.11E-08	1.84E-06	8.5716313
cg10648542	chr5	178416050	-	GRM6	Body	-1.197702	0.6093129	-5.881548	3.11E-08	1.84E-06	8.5711968
cg01687040	chr12	55043559	-	DCD	TSS1500	-1.514972	-0.478676	-5.881519	3.11E-08	1.84E-06	8.5710638

cg13317079	chr2	197504192	-	CCDC150	TSS200	-0.567871	-2.648083	-5.881464	3.11E-08	1.84E-06	8.5708109
cg16427420	chr17	74526161	+	CYGB	Body	0.9493522	0.774871	5.8813019	3.11E-08	1.85E-06	8.5700649
cg23909343	chr1	212457915	+	PPP2R5A	TSS1500	-0.680335	-3.122865	-5.881324	3.11E-08	1.85E-06	8.5701683
cg22076757	chr1	109849705	+	MYBPHL	TSS200	-0.693478	-1.334577	-5.880865	3.12E-08	1.85E-06	8.5680552
cg00983705	chr6	16690986	+	ATXN1;ATXN1	5'UTR;5'UTR	-1.857123	-0.251798	-5.880738	3.12E-08	1.85E-06	8.5674723
cg08056629	chr1	154359587	-			-1.086516	-1.045292	-5.880679	3.12E-08	1.85E-06	8.5671999
cg04081402	chr22	31003587	-	TCN2	Body	0.9590127	1.3503326	5.880558	3.12E-08	1.85E-06	8.5666438
cg02008627	chr16	6318633	+	A2BP1;A2BP1	5'UTR;5'UTR	-1.582515	0.5439083	-5.880436	3.12E-08	1.85E-06	8.5660814
cg08817836	chr7	16955875	+			-1.134571	-0.389287	-5.880092	3.13E-08	1.85E-06	8.5645027
cg00032609	chr20	57606607	-	ATP5E;ATP5E	Body;Body	-0.879211	-2.251427	-5.880059	3.13E-08	1.85E-06	8.5643497
cg07851738	chr8	131896788	+	ADCY8	Body	-1.725694	0.3310619	-5.879876	3.13E-08	1.86E-06	8.563508
cg09732255	chr2	2190278	-	MYT1L	5'UTR	-1.780335	-0.09343	-5.879659	3.14E-08	1.86E-06	8.5625103
cg23288059	chr14	38724945	+	CLEC14A	1stExon	1.7444546	-1.333116	5.8796134	3.14E-08	1.86E-06	8.5622996
cg06099459	chr12	25150530	+	C12orf77	TSS200	-1.243501	1.8652757	-5.879327	3.14E-08	1.86E-06	8.5609839
cg02255732	chr11	31150189	+			-1.609491	0.4367622	-5.879257	3.14E-08	1.86E-06	8.5606606
cg24001710	chr2	119606695	-	EN1	TSS1500	1.498419	-1.285079	5.8792175	3.14E-08	1.86E-06	8.560479
cg05175318	chr14	79747774	+	NRXN3;NRXN3;NRXN3	Body;Body;Body	-0.926698	-1.702679	-5.879089	3.14E-08	1.86E-06	8.5598875
cg27183818	chr1	85764021	-			-1.146863	-1.680149	-5.878978	3.15E-08	1.86E-06	8.5593771
cg21054147	chr22	49495056	-			-2.054303	1.6658207	-5.878819	3.15E-08	1.86E-06	8.5586484
cg06242000	chr3	183966155	+	ALG3;ALG3;ALG3;ECE2;ECE2;ALG3	Body;Body;Body;TSS1500;TSS1500;Body	-0.683443	-4.305147	-5.878276	3.16E-08	1.87E-06	8.5561517

cg04875128	chr15	31775895	-	OTUD7A	Body	1.3295226	-0.415258	5.8781358	3.16E-08	1.87E-06	8.5555053
cg11141743	chr4	17188725	-			-1.221218	-0.346672	-5.877937	3.16E-08	1.87E-06	8.5545908
cg09276998	chr10	29991884	-	SVIL	5'UTR	1.8268084	-2.385001	5.8777278	3.17E-08	1.87E-06	8.5536292
cg10779656	chr6	46138787	-	ENPP5	TSS200	-0.553942	-3.682332	-5.877629	3.17E-08	1.87E-06	8.5531727
cg27405791	chr4	41882953	-			1.6115282	-1.023782	5.8776339	3.17E-08	1.87E-06	8.5531973
cg00157656	chr8	644663	+	ERICH1	Body	-1.971674	1.1982548	-5.877516	3.17E-08	1.87E-06	8.5526542
cg09600411	chr12	110885245	+	ARPC3	Body	1.0008655	1.2034686	5.8771257	3.17E-08	1.88E-06	8.550861
cg08298351	chr6	100916916	-			1.3955798	0.3349517	5.87694	3.18E-08	1.88E-06	8.5500071
cg12983089	chr11	74800716	+	OR2AT4	1stExon	-1.508246	0.3882261	-5.876953	3.18E-08	1.88E-06	8.5500669
cg00079598	chr11	123676903	+	OR6M1	1stExon	-1.314556	-0.079408	-5.876557	3.18E-08	1.88E-06	8.5482448
cg13651876	chr9	37434131	+	GRHPR	Body	0.5940565	1.9438896	5.8765839	3.18E-08	1.88E-06	8.5483701
cg15737123	chr6	33091567	-	HLA-DPB2	Body	-1.74595	-0.258384	-5.876583	3.18E-08	1.88E-06	8.5483672
cg17960080	chr17	26926868	-	SPAG5	TSS1500	-0.92572	-3.15856	-5.876518	3.18E-08	1.88E-06	8.548065
cg13316854	chr2	177027856	+	HOXD3	TSS1500	1.4853849	-0.76274	5.8792072	3.18E-08	1.88E-06	8.5488117
cg12885287	chr2	240259002	+	HDAC4	Body	1.2519577	1.8943339	5.8762555	3.19E-08	1.88E-06	8.5468602
cg11294364	chr19	56367316	+	NLRP4	Body	-1.854822	0.9441785	-5.876168	3.19E-08	1.88E-06	8.5464579
cg01492538	chr7	2774543	-	GNA12	Body	0.9536162	0.9078994	5.875955	3.19E-08	1.88E-06	8.545479
cg01933695	chr14	77495691	+	C14orf4	TSS1500	-1.095259	-3.077321	-5.875981	3.19E-08	1.88E-06	8.5455966
cg18311708	chr7	149322908	-	ZNF767;ZNF767	TSS1500;TSS1500	-1.295385	-2.134426	-5.875957	3.19E-08	1.88E-06	8.5454876
cg24931520	chr6	99291587	-			1.2727841	-1.159443	5.8758354	3.19E-08	1.88E-06	8.544929

cg09978583	chr6	112380564	-	WISP3;WISP3	Body;Body	-1.369196	0.6987098	-5.875621	3.20E-08	1.89E-06	8.5439446
cg03969079	chr10	63759909	-	ARID5B	Body	-2.030838	2.385111	-5.881071	3.20E-08	1.89E-06	8.5455858
cg13916877	chr17	55601697	+	MSI2;MSI2	Body;Body	-1.482555	1.0820141	-5.875136	3.21E-08	1.89E-06	8.5417128
cg06063171	chr11	190242	-			-1.072646	0.4668864	-5.877282	3.21E-08	1.90E-06	8.5399722
cg21902817	chr12	50794088	+	LARP4;LARP4;LARP4;LARP4;LARP4;LARP4;LARP4	TSS1500;TSS1500;TSS1500;TSS1500;TSS1500;TSS1500;TSS1500	-0.987267	-2.726359	-5.874346	3.22E-08	1.90E-06	8.538082
cg22086566	chr7	3989097	+	SDK1	Body	1.096813	1.687011	5.8767441	3.22E-08	1.90E-06	8.5378096
cg16205058	chr14	20924369	-	APEX1;APEX1;OSGEP;APEX1	Body;Body;TSS1500;Body	-1.252789	-2.838411	-5.873922	3.22E-08	1.90E-06	8.5361351
cg22285621	chr11	67071322	-	SSH3	Body	1.2622544	-1.771166	5.8738976	3.22E-08	1.90E-06	8.5360219
cg06407634	chr20	33460576	-	GGT7	1stExon	-0.874712	-3.872511	-5.873857	3.22E-08	1.90E-06	8.5358332
cg08498585	chr19	34266019	+			-1.044757	-0.378096	-5.873819	3.23E-08	1.90E-06	8.535661
cg17367472	chr21	32254161	-	KRTAP11-1	TSS1500	-1.325931	0.0324639	-5.873782	3.23E-08	1.90E-06	8.5354903
cg13524919	chr21	34396506	+			1.1059256	0.203863	5.8764029	3.23E-08	1.90E-06	8.5362436
cg06106653	chr10	84719878	-	NRG3;NRG3;NRG3	Body;Body;Body	-1.540022	-0.565842	-5.873232	3.23E-08	1.91E-06	8.5329631
cg26282283	chr7	2704368	+	TTYH3	3'UTR	-1.237911	-1.271122	-5.873011	3.24E-08	1.91E-06	8.5319492
cg05275832	chr2	27984686	-			-1.635271	0.8301975	-5.872832	3.24E-08	1.91E-06	8.5311256
cg10187894	chr10	38015525	+			1.5200068	2.375717	5.8756036	3.24E-08	1.91E-06	8.532575
cg10211891	chr6	33141687	+	COL11A2;COL11A2;COL11A2	Body;Body;Body	-1.311433	0.1483281	-5.872879	3.24E-08	1.91E-06	8.5313404
cg10262662	chr16	70721696	+	VAC14	3'UTR	0.6621403	0.6376646	5.8728532	3.24E-08	1.91E-06	8.5312219
cg18362003	chr11	122051630	+	LOC399959	Body	-2.088562	-0.067878	-5.872888	3.24E-08	1.91E-06	8.5313833
cg23527387	chr2	100056660	-	REV1;REV1	Body;Body	0.9553383	1.0680689	5.8728237	3.24E-08	1.91E-06	8.5310866

cg05798059	chr11	30444984	-	MPPED2;MPPED2	Body;Body	-1.502055	-0.445673	-5.87279	3.24E-08	1.91E-06	8.5309335
cg06550539	chr10	134698937	-			-2.238476	1.4711614	-5.872736	3.24E-08	1.91E-06	8.5306845
cg20084504	chr13	28369514	-			1.4392907	-1.124876	5.8725808	3.24E-08	1.91E-06	8.5299702
cg20057066	chr12	116971500	-	NCRNA00173;NCRNA00173	Body;Body	-0.6234	-3.851551	-5.872224	3.25E-08	1.91E-06	8.5283283
cg07302069	chr7	27196286	+	HOXA7;HOXA7	5'UTR;1stExon	3.9078216	-1.488551	5.8720309	3.25E-08	1.91E-06	8.5274432
cg05901613	chr19	30709527	-			-1.384623	-0.152521	-5.871983	3.25E-08	1.91E-06	8.5272251
cg02925601	chr6	27648605	-			1.5575345	-0.651172	5.8718674	3.26E-08	1.91E-06	8.526692
cg11382666	chr6	20032841	+			0.9286881	2.1790887	5.8717828	3.26E-08	1.91E-06	8.5263032
cg14213394	chr8	38508585	+			1.0235933	0.9326124	5.8716325	3.26E-08	1.92E-06	8.5256124
cg16327839	chr11	133715264	+	SPATA19	1stExon	-1.712488	0.4492343	-5.87157	3.26E-08	1.92E-06	8.5253235
cg03740025	chr7	127340829	+	SND1	Body	0.8171086	1.674382	5.8713359	3.26E-08	1.92E-06	8.5242495
cg14640525	chr3	38876856	-			-1.347475	0.6503239	-5.870995	3.27E-08	1.92E-06	8.5226827
cg15998961	chr12	133360718	+	GOLGA3	Body	0.8164614	4.5222363	5.8737249	3.27E-08	1.92E-06	8.5236471
cg09557462	chr8	26721736	-	ADRA1A;ADRA1A;ADRA1A;ADRA1A	1stExon;1stExon;1stExon;1stExon	1.5587322	-0.819709	5.8709184	3.27E-08	1.92E-06	8.5223315
cg13933043	chr7	130696871	-	FLJ43663;FLJ43663	Body;Body	-1.822505	-3.323988	-5.870801	3.27E-08	1.92E-06	8.5217931
cg17373345	chr8	66946188	-	DNAJC5B	5'UTR	-1.177637	-1.291171	-5.8706	3.28E-08	1.92E-06	8.5208666
cg21561712	chr11	118895504	+	SLC37A4;SLC37A4;SLC37A4;SLC37A4;SLC37A4	3'UTR;3'UTR;3'UTR;3'UTR;3'UTR	0.7385478	0.0962604	5.8704285	3.28E-08	1.92E-06	8.5200802
cg00715682	chr19	39881530	-	PAF1;PAF1;MED29	5'UTR;1stExon;TSS1500	1.2077199	-2.800629	5.8758458	3.28E-08	1.93E-06	8.5222534
cg16550264	chr12	115813214	-			-1.720834	-2.3866	-5.875708	3.28E-08	1.93E-06	8.5213062
cg05691158	chr12	77668964	-			-1.680917	0.2333942	-5.869949	3.29E-08	1.93E-06	8.5178772

cg04992950	chr7	1423072	-			-1.212026	0.1810098	-5.869857	3.29E-08	1.93E-06	8.5174555
cg23029159	chr14	101524635	-	MIR154	TSS1500	-1.317733	-0.055137	-5.86985	3.29E-08	1.93E-06	8.5174215
cg00291661	chr16	30581198	+	ZNF688;ZNF688	3'UTR;3'UTR	1.006173	4.6151723	5.8695709	3.29E-08	1.93E-06	8.5161405
cg14896157	chr3	5137549	-			1.261067	-1.206328	5.8695727	3.29E-08	1.93E-06	8.5161484
cg18838346	chr6	30615813	-	C6orf136;C6orf136;C6orf136	Body;Body;Body	-0.581095	-4.454699	-5.87235	3.29E-08	1.93E-06	8.5173394
cg24932585	chr19	12666317	-			1.2850264	0.3445569	5.869575	3.29E-08	1.93E-06	8.5161591
cg21498326	chr21	26735730	+			-1.666157	0.5418258	-5.869466	3.29E-08	1.93E-06	8.5156577
cg00248242	chr2	240867059	-			-1.572206	0.2034945	-5.869309	3.30E-08	1.93E-06	8.5149385
cg05307752	chr2	144462886	+	ARHGAP15	Body	-1.947593	-1.50367	-5.869329	3.30E-08	1.93E-06	8.515028
cg07321467	chr4	81960704	-	BMP3	Body	-1.025918	-2.138556	-5.869364	3.29E-08	1.93E-06	8.515189
cg12531611	chr6	11212619	-	NEDD9;NEDD9;NEDD9	Body;Body;Body	1.1061957	1.7427461	5.8693232	3.30E-08	1.93E-06	8.5150024
cg27350402	chr6	27550691	-			1.1963916	-0.298479	5.8692527	3.30E-08	1.93E-06	8.5146785
cg22121687	chr7	65037720	-			1.0684249	-0.813682	5.8691183	3.30E-08	1.93E-06	8.5140612
cg09158651	chr7	1793240	-			-1.305167	0.3802257	-5.86906	3.30E-08	1.93E-06	8.513794
cg13580649	chr4	177467794	+			-1.690841	1.2172627	-5.86901	3.30E-08	1.93E-06	8.5135645
cg03129645	chr7	141947898	+	MOXD2	TSS1500	-1.647389	0.2956318	-5.868824	3.30E-08	1.93E-06	8.5127084
cg06637517	chr3	52407344	-	DNAH1	Body	2.5756169	-0.402804	5.8682813	3.31E-08	1.94E-06	8.5102161
cg06896987	chr2	177003735	-			1.5002758	-0.520255	5.8709259	3.31E-08	1.94E-06	8.5230216
cg07363137	chr5	76923737	-			1.1767078	0.3188643	5.8764381	3.32E-08	1.94E-06	8.5265462
cg27120999	chr14	65008050	+	HSPA2	1stExon	1.0414117	-0.231919	5.8680902	3.32E-08	1.94E-06	8.5093381

cg03411938	chr11	2700003	+	KCNQ1;KCNQ1OT1;KCNQ1	Body;Body;Body	1.0788652	0.4044202	5.8680397	3.32E-08	1.94E-06	8.5091065
cg05983315	chr19	58545837	+	ZSCAN1	5'UTR	2.0122359	-0.489313	5.8680195	3.32E-08	1.94E-06	8.5090137
cg24784129	chr15	57215558	+	TCF12;TCF12;TCF12;TCF12	Body;Body;Body;Body	1.303977	2.1285661	5.8679575	3.32E-08	1.94E-06	8.5087289
cg25188760	chr20	5301547	+			-1.70644	-0.184753	-5.867755	3.32E-08	1.94E-06	8.5077975
cg01962471	chr6	157876936	+	ZDHHC14;ZDHHC14	Body;Body	1.0056356	1.0197011	5.8676286	3.32E-08	1.94E-06	8.5072179
cg22130834	chr1	55267046	+	TTC22;TTC22	TSS200;TSS200	-0.587781	-4.174522	-5.867639	3.32E-08	1.94E-06	8.5072671
cg18395354	chr7	3801491	+	SDK1	Body	-1.72419	-0.169518	-5.867547	3.32E-08	1.94E-06	8.5068433
cg02730990	chr6	168122688	-			-1.342644	-0.160099	-5.867281	3.33E-08	1.95E-06	8.5056227
cg03365733	chr6	100055662	-	PRDM13	Body	1.1911718	0.0568685	5.8672185	3.33E-08	1.95E-06	8.5053346
cg15157581	chr11	43966188	+			-0.746895	-3.180042	-5.875596	3.33E-08	1.95E-06	8.5088924
cg02284800	chr16	872520	+			-0.977622	-0.098296	-5.866947	3.33E-08	1.95E-06	8.5040853
cg21291134	chr1	159558512	+	APCS	3'UTR	-1.834988	0.0005636	-5.866911	3.33E-08	1.95E-06	8.5039198
cg13193239	chr4	111539479	+	PITX2;PITX2;PITX2	Body;Body;Body	1.0456073	-0.900931	5.8665934	3.34E-08	1.95E-06	8.5024633
cg15169077	chr6	46139019	+	ENPP5	TSS1500	-0.883678	-2.59502	-5.866583	3.34E-08	1.95E-06	8.5024156
cg13473383	chr11	57434378	+	ZDHHC5	TSS1500	-0.612702	-3.039265	-5.866202	3.35E-08	1.95E-06	8.500665
cg22024112	chr12	109245582	+	SSH1;SSH1	Body;Body	0.6262441	2.3308375	5.8661159	3.35E-08	1.95E-06	8.5002706
cg20201639	chr6	86388914	-	SNHG5	TSS1500	-0.455282	-4.47995	-5.86607	3.35E-08	1.95E-06	8.5000618
cg06249604	chr6	30039206	+	RNF39;RNF39	Body;Body	1.3960772	0.5935458	5.8659725	3.35E-08	1.96E-06	8.4996118
cg00175403	chr1	12223543	+			-0.715992	-1.386257	-5.865842	3.35E-08	1.96E-06	8.4990127
cg15127733	chr20	3713584	-	HSPA12B	5'UTR	0.8796669	0.0989694	5.8657641	3.35E-08	1.96E-06	8.4986548

cg25355291	chr8	29369401	+			-1.449806	0.5513052	-5.865303	3.36E-08	1.96E-06	8.4965378
cg00962793	chr14	104852641	+			-0.9766	-0.141968	-5.865224	3.36E-08	1.96E-06	8.4961726
cg06544111	chr7	27281344	-	EVX1	TSS1500	1.8203032	-0.528341	5.8650542	3.36E-08	1.96E-06	8.4953946
cg17141902	chr9	95897216	+	NINJ1	TSS1500	-0.83518	-0.458737	-5.865072	3.36E-08	1.96E-06	8.4954753
cg00529958	chr13	100624288	-	ZIC5	TSS200	1.6860984	-0.504295	5.864938	3.37E-08	1.96E-06	8.494861
cg14292522	chr12	100535489	+	UHRF1BP1L;UHRF1BP1L	Body;Body	-1.118784	-1.844336	-5.864984	3.36E-08	1.96E-06	8.4950745
cg23679471	chr10	102473810	-			1.1590951	-1.323647	5.8649294	3.37E-08	1.96E-06	8.4948218
cg15176213	chr19	36643572	+	COX7A1;COX7A1	5'UTR;1stExon	1.0254734	0.3426425	5.8648578	3.37E-08	1.96E-06	8.4944926
cg12853633	chr14	57264919	+			1.9925271	-0.93028	5.8673971	3.37E-08	1.96E-06	8.4949256
cg01176947	chr6	10385279	-			1.5180118	-1.770012	5.8645789	3.37E-08	1.97E-06	8.4932122
cg13416517	chr2	620106	-			-1.613661	0.6537399	-5.864273	3.38E-08	1.97E-06	8.4918054
cg09284397	chr7	128312406	-	FAM71F2;FAM71F2;FAM71F2;FAM71F2	5'UTR;1stExon;5'UTR;1stExon	0.6462407	1.0218893	5.8639412	3.38E-08	1.97E-06	8.4902838
cg07812953	chr11	32130759	-			-1.401197	0.3746161	-5.863832	3.38E-08	1.97E-06	8.4897838
cg22083866	chr15	70328989	-			-1.51555	0.2947496	-5.863518	3.39E-08	1.97E-06	8.4883401
cg11029358	chr21	46875433	+	COL18A1;COL18A1;COL18A1;COL18A1;COL18A1	5'UTR;1stExon;5'UTR;Body;1stExon	-0.877128	-1.601683	-5.863428	3.39E-08	1.97E-06	8.4879255
cg00187120	chr7	54731309	+			-1.157827	-0.553654	-5.863309	3.39E-08	1.98E-06	8.4873808
cg06375148	chr1	209958343	-	C1orf74	TSS1500	-0.98663	-3.326142	-5.863327	3.39E-08	1.98E-06	8.4874617
cg26975787	chr12	2613778	+	CACNA1C;CACNA1C;CACNA1C;CACNA1C;CACNA1C;CACNA1C<br>;CACNA1C;CACNA1C;CACNA1C;CACNA1C;CACNA1C;CACNA1<br>C;CACNA1C;CACNA1C;CACNA1C;CACNA1C;CACNA1C;CACNA	Body;Body;Body;Body;Body;Body;Body;Body;Body;<br>Body;Body;Body;Body;Body;Body;Body;Body;Body;<br>Body;Body;Body;Body;Body	-1.460292	0.5116393	-5.86327	3.39E-08	1.98E-06	8.4872013
cg15203028	chr11	1029188	-	MUC6	Body	-1.66343	1.2326064	-5.86316	3.39E-08	1.98E-06	8.4866958
cg08234372	chr16	86094189	+			-1.125068	0.2324728	-5.862772	3.40E-08	1.98E-06	8.4849156

cg12639192	chr10	1166782	+	WDR37	Body	0.5674085	1.5705306	5.8627165	3.40E-08	1.98E-06	8.4846611
cg02696327	chr16	49312543	+	CBLN1	3'UTR	1.6701695	-0.40676	5.8626418	3.40E-08	1.98E-06	8.4843183
cg11874760	chr6	14118415	-	CD83;CD83	Body;Body	-1.296575	-2.327451	-5.86508	3.41E-08	1.98E-06	8.4843021
cg14153740	chr7	141957702	+	TRYX3	5'UTR	-1.775538	0.4616758	-5.862287	3.41E-08	1.98E-06	8.4826871
cg12917072	chr5	33758285	+	ADAMTS12	Body	-1.799335	0.5428822	-5.862043	3.41E-08	1.99E-06	8.4815713
cg24537688	chr2	137250617	-			-1.259259	-0.007841	-5.861775	3.42E-08	1.99E-06	8.4803401
cg21774964	chr6	127687368	+			-1.530217	0.8331122	-5.861739	3.42E-08	1.99E-06	8.480174
cg07053841	chr6	29012588	+	OR2W1	1stExon	-1.427853	-1.368496	-5.861488	3.42E-08	1.99E-06	8.4790218
cg21758733	chr5	14359544	-	TRIO	Body	0.6057986	1.8328141	5.8612013	3.43E-08	1.99E-06	8.4777051
cg07890553	chr3	119182858	+	TMEM39A	TSS1500	-1.533473	-1.046238	-5.861027	3.43E-08	1.99E-06	8.4769041
cg14191493	chr11	62760308	+	SLC22A8	3'UTR	-1.382476	0.4084689	-5.860957	3.43E-08	1.99E-06	8.4765856
cg01300684	chr4	141230118	+	SCOC	5'UTR	-1.173857	-0.131333	-5.860896	3.43E-08	1.99E-06	8.4763041
cg12236880	chr15	68564635	+			-1.320345	-0.152201	-5.860609	3.44E-08	2.00E-06	8.4749858
cg06974428	chr10	22972299	-	PIP4K2A	Body	-1.668344	-0.31186	-5.860503	3.44E-08	2.00E-06	8.4744978
cg26407316	chr14	50801745	+	CDKL1	Body	0.6941129	1.8738025	5.8605158	3.44E-08	2.00E-06	8.474559
cg11660826	chr6	150286508	+	ULBP1	Body	2.3433732	-1.757713	5.8602891	3.44E-08	2.00E-06	8.473518
cg24813704	chr6	32800549	+	TAP2;TAP2	Body;Body	1.345034	2.9932388	5.8629703	3.44E-08	2.00E-06	8.4746288
cg02989940	chr16	31539234	+	AHSP;AHSP	5'UTR;1stExon	-1.459645	0.3143003	-5.859836	3.45E-08	2.00E-06	8.4714398
cg11392848	chr8	49987774	+	C8orf22	Body	-1.890663	-0.150512	-5.859847	3.45E-08	2.00E-06	8.4714912
cg07755735	chr2	20870362	-	GDF7	Body	0.7601282	0.1122177	5.8597009	3.45E-08	2.00E-06	8.4708185

cg07340145	chr16	86220038	+			1.278854	2.2485819	5.8595021	3.45E-08	2.00E-06	8.4699059
cg27621129	chr7	19152121	-			1.1982347	-1.126537	5.8594708	3.45E-08	2.01E-06	8.4697623
cg26683398	chr5	179220589	-	LTC4S	TSS1500	1.0810677	0.7410219	5.8593195	3.46E-08	2.01E-06	8.4690681
cg02107844	chr15	92612836	+	SLCO3A1;SLCO3A1	Body;Body	-1.404702	-1.134099	-5.85895	3.46E-08	2.01E-06	8.4673713
cg03857640	chr16	46607688	+			-1.129567	0.3417983	-5.858994	3.46E-08	2.01E-06	8.4675721
cg08364772	chr2	554341	+			-1.660378	0.5411755	-5.861772	3.46E-08	2.01E-06	8.4688264
cg21850691	chr1	205649807	+	SLC45A3	TSS200	-0.480308	-4.435075	-5.859056	3.46E-08	2.01E-06	8.4678577
cg23870168	chr2	240121206	+	HDAC4	Body	1.0001766	0.6299288	5.858975	3.46E-08	2.01E-06	8.4674869
cg25620430	chr15	53093405	-			1.5209269	-1.380793	5.8590316	3.46E-08	2.01E-06	8.467747
cg15020206	chr5	3142929	+			-1.702023	-0.345932	-5.85814	3.48E-08	2.02E-06	8.4636563
cg18156793	chr20	32031936	-	SNTA1	TSS1500	-0.590216	-3.779473	-5.857612	3.49E-08	2.02E-06	8.4612339
cg07734106	chr14	96117415	-	TCL6	TSS200	-1.187893	-0.391731	-5.857145	3.49E-08	2.02E-06	8.4590884
cg12579684	chr14	101391505	+			-1.507437	-0.276098	-5.856929	3.50E-08	2.03E-06	8.4580963
cg07705639	chr1	174992861	-	MRPS14	TSS1500	-1.339546	-2.495666	-5.856672	3.50E-08	2.03E-06	8.4569181
cg19590421	chr14	51327604	-			-1.053954	-2.050587	-5.856669	3.50E-08	2.03E-06	8.456905
cg19497444	chr11	2930794	+	SLC22A18;SLC22A18	Body;Body	1.000315	1.3391146	5.8565922	3.50E-08	2.03E-06	8.4565528
cg08565197	chr2	240196284	-	HDAC4	Body	-1.192268	-0.816061	-5.85654	3.50E-08	2.03E-06	8.4563114
cg04944090	chr16	85635486	-			1.6241611	3.0629088	5.856359	3.51E-08	2.03E-06	8.4554828
cg26143452	chr14	55907385	-	TBPL2	TSS200	1.4358101	0.218156	5.8563723	3.51E-08	2.03E-06	8.4555439
cg13708803	chr8	138898243	+			-1.223697	-1.847546	-5.856248	3.51E-08	2.03E-06	8.4549755

cg04751149	chr2	66673449	-	MEIS1	Body	1.2096376	-0.41879	5.8561541	3.51E-08	2.03E-06	8.4545428
cg25149238	chr2	45030384	-			1.3450907	-0.032567	5.8561432	3.51E-08	2.03E-06	8.4544928
cg05995496	chr6	30126535	-	TRIM10;TRIM10	Body;Body	-1.094908	-1.089169	-5.856092	3.51E-08	2.03E-06	8.4542577
cg09517450	chr5	1209057	+	SLC6A19	Body	-2.091232	1.421317	-5.856048	3.51E-08	2.03E-06	8.4540561
cg15842276	chr11	92702648	-	MTNR1B	TSS200	0.8632101	-0.373028	5.855924	3.51E-08	2.03E-06	8.4534874
cg06919159	chr5	80286535	-	RASGRF2	Body	-1.613027	1.1930626	-5.855852	3.52E-08	2.03E-06	8.4531574
cg16086730	chr5	9782142	+	LOC285692	Body	-1.610527	-0.135685	-5.855766	3.52E-08	2.03E-06	8.4527624
cg00463767	chr2	63282043	-	OTX1	Body	1.3269159	-0.441008	5.8557265	3.52E-08	2.03E-06	8.4525812
cg21678461	chr11	20180700	-	DBX1	Body	2.5439317	-2.187704	5.855643	3.52E-08	2.03E-06	8.4521979
cg06580745	chr17	4692790	-	GLTPD2	Body	-0.550769	-2.709145	-5.855472	3.52E-08	2.04E-06	8.4514113
cg08237401	chr1	94057789	-	BCAR3	Body	-1.413097	-0.506502	-5.855441	3.52E-08	2.04E-06	8.4512721
cg23046711	chr8	143662932	+			-2.305437	2.5188427	-5.855318	3.52E-08	2.04E-06	8.4507058
cg02784956	chr3	33619441	-	CLASP2	Body	1.007667	2.2831541	5.8552254	3.53E-08	2.04E-06	8.4502821
cg03818576	chr11	40312660	+	LRRC4C	5'UTR	-1.665143	-0.289915	-5.854909	3.53E-08	2.04E-06	8.4488285
cg19454081	chr7	152593563	-			-0.959912	-0.768156	-5.854798	3.53E-08	2.04E-06	8.4483219
cg05342240	chr16	9246537	-			0.7739055	1.2478126	5.8546693	3.53E-08	2.04E-06	8.447731
cg11116300	chr14	101515879	+	MIR655	TSS200	-0.990713	-0.451155	-5.854377	3.54E-08	2.04E-06	8.4463915
cg27211408	chr7	2060130	+	MAD1L1;MAD1L1;MAD1L1	Body;Body;Body	0.7769376	1.2619951	5.8543754	3.54E-08	2.04E-06	8.4463827
cg23413104	chr2	118982263	-			1.2046766	-0.786209	5.8542893	3.54E-08	2.05E-06	8.445988
cg26579145	chr16	22829691	-	HS3ST2	Body	-1.171047	-0.948433	-5.854161	3.54E-08	2.05E-06	8.4453985

cg19196335	chr7	27184094	+	HOXA5	TSS1500	1.308912	0.828212	5.8539404	3.55E-08	2.05E-06	8.4443874
cg12090942	chr14	77279508	-	ANGEL1	TSS1500	-0.92615	0.0669157	-5.853877	3.55E-08	2.05E-06	8.4440987
cg20354882	chr12	96350795	-	AMDHD1	Body	0.9313345	0.5300515	5.8538008	3.55E-08	2.05E-06	8.4437472
cg06360427	chr18	74962672	-	GALR1	1stExon	1.7977024	-0.735827	5.8536937	3.55E-08	2.05E-06	8.4432561
cg04907257	chr5	7395318	-	ADCY2	TSS1500	1.4784682	-0.824046	5.8526698	3.57E-08	2.06E-06	8.4385597
cg02222728	chr8	55371829	+	SOX17	Body	1.2040388	-1.10561	5.8525838	3.57E-08	2.06E-06	8.4381651
cg00184452	chr1	50887477	+	DMRTA2	5'UTR	0.9451169	-0.689077	5.8521953	3.58E-08	2.06E-06	8.4363834
cg14112356	chr21	46348443	-	ITGB2	5'UTR	-0.888767	-1.327902	-5.852196	3.58E-08	2.06E-06	8.4363851
cg09158821	chr17	1508609	+	SLC43A2	Body	0.8559785	1.1971856	5.8519352	3.58E-08	2.07E-06	8.4351907
cg13143529	chr13	28492971	-	PDX1	TSS1500	1.696109	-2.46646	5.8519628	3.58E-08	2.07E-06	8.4353171
cg21170029	chr1	209799330	-	LAMB3;LAMB3;LAMB3	Body;Body;Body	1.0353891	2.3063776	5.8519114	3.58E-08	2.07E-06	8.4350813
cg23706819	chr4	89152212	+			1.226388	1.035687	5.8511473	3.60E-08	2.07E-06	8.4315775
cg02643054	chr7	27206544	+	HOXA9	TSS1500	1.6020863	-0.8983	5.8509815	3.60E-08	2.07E-06	8.4308172
cg06942110	chr7	1545385	-	INTS1	TSS1500	-0.934818	-0.619053	-5.85097	3.60E-08	2.07E-06	8.4307632
cg08262002	chr4	16575323	+	LDB2;LDB2	Body;Body	-0.969102	-1.063239	-5.850918	3.60E-08	2.07E-06	8.4305251
cg04203950	chr17	4443191	-	SPNS2;MYBBP1A;MYBBP1A	3'UTR;Body;Body	0.9866041	3.7716818	5.8532281	3.61E-08	2.08E-06	8.4296834
cg02961101	chr16	48533155	+			-1.39486	-1.073655	-5.850458	3.61E-08	2.08E-06	8.4284156
cg03175417	chr22	20050298	+	C22orf25	Body	-1.529359	0.3114433	-5.850276	3.61E-08	2.08E-06	8.4275799
cg16694634	chr8	144952216	-			-0.821453	-1.486704	-5.850279	3.61E-08	2.08E-06	8.4275938
cg22699620	chr21	46348691	-	ITGB2;ITGB2	5'UTR;1stExon	-0.98798	-1.545565	-5.850083	3.61E-08	2.08E-06	8.4266972

cg27655168	chr17	39059398	+			-1.391721	-0.105052	-5.850028	3.61E-08	2.08E-06	8.4264464
cg12761728	chr6	32800523	+	TAP2;TAP2	Body;Body	1.8204679	2.8285895	5.858255	3.62E-08	2.08E-06	8.4415765
cg13692543	chr9	35510933	-	RUSC2	5'UTR	1.7260048	0.5913436	5.8498389	3.62E-08	2.08E-06	8.4255778
cg26357344	chr12	53300293	-	KRT8	TSS1500	-1.446537	0.2949717	-5.849845	3.62E-08	2.08E-06	8.4256053
cg18235026	chr7	129419139	-			-0.807431	-4.580683	-5.849616	3.62E-08	2.08E-06	8.4245549
cg04388863	chr16	48269349	-	ABCC11;ABCC11	TSS1500;TSS1500	-1.292717	0.9073016	-5.849527	3.62E-08	2.08E-06	8.4241484
cg21046148	chr5	72731905	-			1.9425519	-0.804105	5.8606201	3.62E-08	2.08E-06	8.4291193
cg11912272	chr2	201204807	-	SPATS2L;SPATS2L;SPATS2L;SPATS2L	5'UTR;5'UTR;5'UTR;5'UTR	0.8509344	0.9840436	5.8494396	3.62E-08	2.09E-06	8.4237471
cg11865119	chr7	130125932	-	MEST	TSS200	-1.128633	-2.563601	-5.849384	3.63E-08	2.09E-06	8.42349
cg12404831	chr2	192114017	+	MYO1B;MYO1B;MYO1B	5'UTR;5'UTR;5'UTR	1.3022367	-0.160755	5.8518249	3.63E-08	2.09E-06	8.4232578
cg18797590	chr4	15480643	-	CC2D2A;CC2D2A;CC2D2A	Body;Body;Body	-1.254746	-1.665098	-5.84913	3.63E-08	2.09E-06	8.4223275
cg13191508	chr8	144600686	+	ZC3H3	Body	1.1889181	2.5081213	5.8488632	3.63E-08	2.09E-06	8.4211042
cg03156170	chr17	55673870	+	MSI2;MSI2	Body;Body	1.7647965	2.7647514	5.851401	3.64E-08	2.09E-06	8.4216263
cg04379703	chr3	13053208	-	IQSEC1	Body	-1.29459	-1.429479	-5.848714	3.64E-08	2.09E-06	8.4204196
cg23003783	chr5	178422071	+	GRM6;GRM6	1stExon;5'UTR	1.3463242	-0.956124	5.8486732	3.64E-08	2.09E-06	8.420233
cg12710176	chr1	207226467	-	PFKFB2;PFKFB2	TSS200;TSS200	-0.387598	-4.01502	-5.851333	3.64E-08	2.09E-06	8.4210066
cg19256331	chr15	89915212	-			1.1215091	-0.767377	5.8485538	3.64E-08	2.09E-06	8.419686
cg02331025	chr5	37835965	-	GDNF;GDNF;GDNF	TSS1500;5'UTR;TSS1500	0.9193658	1.1178633	5.8483515	3.64E-08	2.09E-06	8.4187585
cg04413320	chr13	58208929	+	PCDH17	1stExon	1.5231928	-1.121381	5.8482713	3.64E-08	2.09E-06	8.4183904
cg17495087	chr8	96085689	+			1.7782784	-0.189425	5.8481831	3.65E-08	2.09E-06	8.4179864

cg17545399	chr10	49459800	+	FRMPD2;FRMPD2	Body;Body	-1.0202	-0.467473	-5.847958	3.65E-08	2.10E-06	8.4169562
cg21436572	chr8	70623422	+	SLCO5A1;SLCO5A1;SLCO5A1	Body;Body;Body	1.595882	0.6698797	5.8478683	3.65E-08	2.10E-06	8.4165432
cg00334063	chr3	147106010	-	ZIC4;ZIC4;ZIC4;ZIC4;ZIC4	3'UTR;Body;3'UTR;Body;3'UTR	2.0422471	-0.230103	5.8559097	3.66E-08	2.10E-06	8.419228
cg02736395	chr1	227748424	-			0.9261089	0.6238922	5.8476515	3.66E-08	2.10E-06	8.4155492
cg16428251	chr3	137483479	-	SOX14	TSS200	1.5604356	-1.302651	5.8476595	3.66E-08	2.10E-06	8.4155859
cg19190699	chr15	101514156	+	LRRK1	Body	0.9605235	0.1958741	5.847614	3.66E-08	2.10E-06	8.4153773
cg01145686	chr2	501496	-			-1.521462	0.3213241	-5.847534	3.66E-08	2.10E-06	8.4150123
cg13053992	chr21	45663171	+			-1.212737	0.2260444	-5.847482	3.66E-08	2.10E-06	8.4147744
cg08324862	chr12	77800655	-			-1.800071	-0.588597	-5.847192	3.66E-08	2.10E-06	8.4134448
cg09438605	chr15	80757169	-	ARNT2	Body	-1.361245	0.6724188	-5.847007	3.67E-08	2.10E-06	8.4125965
cg10131026	chr8	122823862	-			-1.795027	-0.406359	-5.847021	3.67E-08	2.10E-06	8.4126588
cg27056742	chr1	4231293	+			-1.341806	0.0626821	-5.846736	3.67E-08	2.11E-06	8.4113525
cg25538881	chr2	129294825	-			-1.352898	0.0015931	-5.846663	3.67E-08	2.11E-06	8.4110193
cg22105145	chr18	5196397	+	LOC642597	Body	1.226579	-1.551253	5.8466067	3.67E-08	2.11E-06	8.4107601
cg22372096	chr19	3810782	+	ZFR2	Body	-1.356817	1.2919199	-5.846624	3.67E-08	2.11E-06	8.4108372
cg23585168	chr6	32178215	+	NOTCH4	Body	-0.810566	-0.832038	-5.848975	3.68E-08	2.11E-06	8.4102103
cg00206356	chr4	56659922	-			1.1161109	-0.649765	5.8461674	3.68E-08	2.11E-06	8.4087466
cg06359056	chr16	1107364	-			-0.881116	-0.945667	-5.845854	3.69E-08	2.11E-06	8.407312
cg07855319	chr16	69123283	+			-0.913568	-1.757382	-5.845861	3.69E-08	2.11E-06	8.4073412
cg26449294	chr11	83169193	-	DLG2;DLG2;DLG2;DLG2	3'UTR;3'UTR;3'UTR;3'UTR	-1.650114	0.3604653	-5.845863	3.69E-08	2.11E-06	8.4073512

cg07055879	chr7	143747474	+	OR2A5	TSS200	-1.437677	-0.42726	-5.845824	3.69E-08	2.11E-06	8.407173
cg00647217	chr1	165086866	-			2.9172526	-2.616938	5.8457437	3.69E-08	2.11E-06	8.4068045
cg06950023	chr19	2041985	+	MKNK2;MKNK2	Body;Body	-2.20222	-3.956743	-5.851213	3.69E-08	2.11E-06	8.4088838
cg13816321	chr9	973198	+			1.7134264	-0.385237	5.8454605	3.69E-08	2.11E-06	8.4055068
cg02484343	chr16	2284734	-	E4F1	Body	-2.402496	2.9331113	-5.845196	3.70E-08	2.12E-06	8.4042924
cg10520226	chr1	2811554	-			-0.982653	-0.895558	-5.845156	3.70E-08	2.12E-06	8.4041101
cg01345315	chr12	43945998	+	ADAMTS20	TSS1500	1.4607748	-0.781409	5.8450662	3.70E-08	2.12E-06	8.4036995
cg07569562	chr2	130343885	-			-1.97411	0.9865168	-5.845056	3.70E-08	2.12E-06	8.4036516
cg24518098	chr1	2146288	-			0.9456943	-0.39101	5.8446452	3.71E-08	2.12E-06	8.4017701
cg00148114	chr7	5468197	-			-0.907584	-2.309424	-5.844038	3.72E-08	2.13E-06	8.3989855
cg00087417	chr6	35887678	+	SRPK1	Body	-0.783359	-4.377231	-5.84399	3.72E-08	2.13E-06	8.3987698
cg14991487	chr2	176987404	-	HOXD9	TSS200	1.6629579	-1.077153	5.8439206	3.72E-08	2.13E-06	8.3984497
cg10249570	chr19	2947905	+			0.9337964	1.6257625	5.843154	3.74E-08	2.14E-06	8.394937
cg16192707	chr6	99292347	+			2.1085485	-0.677909	5.8458304	3.74E-08	2.14E-06	8.3958169
cg18866015	chr18	49868552	+	DCC	Body	2.0551834	-0.90562	5.8431336	3.74E-08	2.14E-06	8.3948433
cg13430053	chr2	150932623	-			-1.485336	-0.008552	-5.842954	3.74E-08	2.14E-06	8.3940221
cg03323794	chr7	151215397	-	RHEB	Body	-1.036869	-4.434594	-5.842771	3.74E-08	2.14E-06	8.3931825
cg08423533	chr10	28030628	+	MKX	Body	1.3122139	-0.695993	5.8427873	3.74E-08	2.14E-06	8.3932565
cg22639293	chr3	141516638	+	GRK7	Body	2.1535351	-2.189396	5.8427062	3.74E-08	2.14E-06	8.392885
cg02838825	chr1	234613490	-	TARBP1	Body	-1.28564	-0.349154	-5.842344	3.75E-08	2.14E-06	8.3912236

cg02564291	chr5	122430235	+	PRDM6	Body	1.3866311	-1.064843	5.842259	3.75E-08	2.14E-06	8.3908362
cg21158815	chr14	75988735	-	BATF	TSS200	-1.752553	-1.929888	-5.842103	3.75E-08	2.14E-06	8.3901232
cg00273449	chr2	74875268	+	C2orf65	TSS200	0.9017262	-0.242755	5.8417139	3.76E-08	2.15E-06	8.3883384
cg10520543	chr12	125810688	+	TMEM132B	TSS1500	-1.294302	-0.777817	-5.841624	3.76E-08	2.15E-06	8.3879247
cg13486820	chr1	91182288	+	BARHL2	1stExon	1.2200087	-0.983251	5.8410585	3.77E-08	2.15E-06	8.3853362
cg10593165	chr4	5116565	-	STK32B	Body	-0.965179	-0.549291	-5.840892	3.78E-08	2.16E-06	8.3845737
cg24828167	chr4	5899040	+			-1.538072	0.1988475	-5.840908	3.78E-08	2.16E-06	8.3846448
cg24834659	chr19	22805803	-			1.5155902	-0.679116	5.8408151	3.78E-08	2.16E-06	8.3842209
cg18818866	chr12	50221061	+	NCKAP5L;LOC100286844;LOC100286844;LOC100286844	5'UTR;TSS1500;TSS1500;TSS1500	1.1955805	0.3365935	5.8407839	3.78E-08	2.16E-06	8.384078
cg24125282	chr17	11395707	+	SHISA6	Body	-1.700766	0.6647524	-5.840303	3.79E-08	2.16E-06	8.381875
cg18839637	chr5	140723684	+	PCDHGA2;PCDHGA3;PCDHGA3;PCDHGA1	Body;1stExon;1stExon;Body	1.1844075	0.5883465	5.8401101	3.79E-08	2.16E-06	8.3809915
cg02452586	chr1	233497903	+	KIAA1804	Body	1.0890836	1.3137384	5.839905	3.79E-08	2.16E-06	8.3800522
cg15946590	chr6	30421089	-			1.1252015	0.0221093	5.8399283	3.79E-08	2.16E-06	8.3801587
cg12884605	chr12	66123314	+			0.958917	-0.855406	5.8397417	3.80E-08	2.16E-06	8.379304
cg26329192	chr12	108687373	+	CMKLR1;CMKLR1;CMKLR1;CMKLR1	5'UTR;5'UTR;5'UTR;5'UTR	-1.516587	0.2130145	-5.839669	3.80E-08	2.17E-06	8.3789717
cg02774282	chr6	109615419	-	CCDC162	TSS200	-1.523399	0.1157644	-5.8396	3.80E-08	2.17E-06	8.378657
cg06803853	chr16	2234031	+	CASKIN1	Body	-2.144914	3.1364067	-5.839022	3.81E-08	2.17E-06	8.3760068
cg14570051	chr18	10955104	+	FAM38B	Body	-1.814664	0.8214441	-5.838813	3.81E-08	2.17E-06	8.3750501
cg01870330	chr4	1534685	+			-1.419513	0.9572471	-5.838424	3.82E-08	2.18E-06	8.3732695
cg21236500	chr9	139779897	-	TRAF2	TSS1500	-1.554177	0.627699	-5.838362	3.82E-08	2.18E-06	8.3729864

cg00780520	chr8	128808554	+	PVT1	Body	-1.701086	0.1569573	-5.838305	3.82E-08	2.18E-06	8.3727245
cg21573135	chr15	71595576	+	THSD4	Body	0.6809923	1.3705622	5.8382436	3.82E-08	2.18E-06	8.3724422
cg22905350	chr3	27401810	+	NEK10	5'UTR	-1.622396	0.9619959	-5.838154	3.83E-08	2.18E-06	8.3720344
cg17033359	chr11	57638157	+			-1.186754	-0.943913	-5.837905	3.83E-08	2.18E-06	8.3708916
cg15972544	chr2	20551587	+			-0.962461	-2.464464	-5.83785	3.83E-08	2.18E-06	8.3706382
cg00767581	chr2	177015125	+	HOXD4;MIR10B	TSS1500;Body	1.2722548	-0.398668	5.8377752	3.83E-08	2.18E-06	8.3702973
cg16367976	chr22	50969678	-	TYMP;ODF3B;TYMP;TYMP	TSS1500;Body;TSS1500;TSS1500	-1.175307	-4.719075	-5.839985	3.84E-08	2.19E-06	8.369073
cg25883762	chr8	1328682	-			-1.413799	0.5553553	-5.83705	3.85E-08	2.19E-06	8.3669767
cg22476027	chr6	108492642	+	NR2E1	Body	1.5954385	-0.19566	5.8394684	3.85E-08	2.19E-06	8.3667114
cg20074021	chr18	74695457	+	MBP;MBP;MBP;MBP;MBP	Body;Body;Body;Body;Body	-0.423629	-4.258697	-5.836665	3.85E-08	2.19E-06	8.3652152
cg11918450	chr2	33359198	-	LTBP1;LTBP1;LTBP1;LTBP1;LTBP1	Body;TSS1500;TSS1500;TSS1500;TSS1500	-1.248682	-0.662906	-5.836395	3.86E-08	2.19E-06	8.363975
cg02423618	chr15	97325725	-	SPATA8	TSS1500	-1.042304	-0.515434	-5.836199	3.86E-08	2.20E-06	8.3630809
cg19375492	chr19	22441140	-			-1.530846	-0.298753	-5.836138	3.86E-08	2.20E-06	8.3627984
cg15726557	chr19	617267	-	POLRMT	3'UTR	-1.183713	0.2053964	-5.836088	3.86E-08	2.20E-06	8.3625694
cg18597220	chr14	80010617	-	NRXN3;NRXN3;NRXN3	Body;Body;Body	-1.410261	1.1976771	-5.836037	3.86E-08	2.20E-06	8.3623392
cg22115994	chr21	44834701	+	SIK1	3'UTR	-1.016889	-1.857945	-5.836015	3.86E-08	2.20E-06	8.3622382
cg02875253	chr16	3314612	+			-0.84796	-3.869004	-5.835531	3.87E-08	2.20E-06	8.3600213
cg23042086	chr8	102236831	-			0.8722568	0.8716337	5.8355417	3.87E-08	2.20E-06	8.3600696
cg00871979	chr18	11982063	-	IMPA2	Body	-0.779829	-4.718147	-5.835126	3.88E-08	2.21E-06	8.3581649
cg16362949	chr17	1390671	-	MYO1C;MYO1C	5'UTR;Body	-0.932511	-1.843565	-5.834937	3.88E-08	2.21E-06	8.3573

cg05852625	chr12	128748582	-			-1.696957	0.3010933	-5.834802	3.89E-08	2.21E-06	8.3566819
cg00003187	chr1	8739981	+	RERE;RERE	5'UTR;5'UTR	1.0403468	2.5060399	5.8346907	3.89E-08	2.21E-06	8.3561735
cg11930477	chr11	31841980	+			1.3764452	-1.438314	5.8345733	3.89E-08	2.21E-06	8.3556363
cg26346796	chr6	32054867	-	TNXB	Body	-0.999335	-0.863014	-5.842002	3.91E-08	2.22E-06	8.3554443
cg14947846	chr2	129227604	+			-1.310494	0.0531035	-5.833466	3.91E-08	2.22E-06	8.350565
cg04892643	chr7	18906433	+	HDAC9;HDAC9;HDAC9	Body;Body;Body	-1.136369	-1.385518	-5.833132	3.92E-08	2.22E-06	8.3490403
cg12138818	chr13	95201227	+			-0.752176	-3.461331	-5.832876	3.92E-08	2.23E-06	8.3478642
cg03431727	chr2	217234404	+	4-Mar	Body	-1.386728	0.2205781	-5.832736	3.93E-08	2.23E-06	8.3472262
cg03039990	chr14	101317622	-	MIR770;MEG3;MEG3;MEG3	TSS1500;Body;Body;Body	-1.06999	-0.545423	-5.832596	3.93E-08	2.23E-06	8.346585
cg08453030	chr3	171177320	-	TNIK;TNIK;TNIK;TNIK;TNIK;TNIK;TNIK;TNIK;TNIK	Body;Body;Body;Body;Body;Body;Body;Body;Body	-0.640973	-4.386014	-5.83238	3.93E-08	2.23E-06	8.3455953
cg25713625	chr2	120022835	-	STEAP3;STEAP3;STEAP3	3'UTR;3'UTR;3'UTR	-0.65852	-1.32983	-5.832329	3.93E-08	2.23E-06	8.3453613
cg25361850	chr19	52391789	+	ZNF577;ZNF577;ZNF577	TSS1500;TSS1500;TSS1500	1.1996195	0.752844	5.8322295	3.94E-08	2.23E-06	8.344907
cg13246235	chr6	12749978	+	PHACTR1	Body	2.0515654	0.5618037	5.8321988	3.94E-08	2.23E-06	8.3447665
cg06641153	chr18	55021669	-	ST8SIA3	Body	1.0888617	-1.275114	5.8320162	3.94E-08	2.23E-06	8.343931
cg24561641	chr11	16533876	-			0.7970086	1.6975001	5.8319311	3.94E-08	2.24E-06	8.3435411
cg14009098	chr1	234041306	+	SLC35F3	Body	1.5254993	-1.206043	5.8317907	3.94E-08	2.24E-06	8.3428986
cg03976856	chr1	2764669	+			-1.328639	-1.390824	-5.831623	3.95E-08	2.24E-06	8.3421327
cg05802386	chr1	43422920	+	SLC2A1	Body	-1.727522	-0.232035	-5.831529	3.95E-08	2.24E-06	8.3417018
cg14732324	chr5	528621	+			3.7864094	-1.861185	5.8314931	3.95E-08	2.24E-06	8.3415365
cg17037491	chr6	25280491	-	LRRC16A	Body	-0.585584	-2.413875	-5.831318	3.95E-08	2.24E-06	8.3407347

cg20265951	chr8	134466907	-			-1.206321	0.2404342	-5.831311	3.95E-08	2.24E-06	8.3407024
cg16450249	chr13	98868494	-	FARP1;FARP1	Body;Body	-1.095204	-0.405345	-5.830889	3.96E-08	2.24E-06	8.3387732
cg01521917	chr17	56238140	-			-1.452211	-0.023242	-5.830771	3.96E-08	2.25E-06	8.3382301
cg25363564	chr10	111540348	-			-1.247537	-0.371584	-5.83074	3.96E-08	2.25E-06	8.3380898
cg16544169	chr8	23564717	+	NKX2-6	TSS1500	0.8690201	-0.282354	5.8306667	3.96E-08	2.25E-06	8.3377546
cg14021133	chr7	20834283	+			1.5535051	-0.068705	5.8304011	3.97E-08	2.25E-06	8.3365392
cg05572621	chr21	31972533	-	KRTAP6-2;KRTAP22-1	TSS1500;TSS1500	-1.367305	-0.123929	-5.830338	3.97E-08	2.25E-06	8.3362509
cg02530022	chr7	96642708	+	DLX6AS	Body	1.6511724	-0.498381	5.8302843	3.97E-08	2.25E-06	8.3360047
cg11190987	chr5	64334489	-			1.0660718	1.8583949	5.8302569	3.97E-08	2.25E-06	8.3358791
cg14327531	chr10	8097331	-	GATA3;GATA3	5'UTR;5'UTR	1.7782653	-0.813903	5.8301996	3.97E-08	2.25E-06	8.3356169
cg00582971	chr5	178422128	+	GRM6	TSS200	2.23908	-0.599328	5.8298366	3.98E-08	2.25E-06	8.333956
cg24923694	chr8	25905747	-			1.6875639	-0.663724	5.8298164	3.98E-08	2.25E-06	8.3338636
cg04074481	chr12	4917740	-	KCNA6	TSS1500	-1.381206	0.5136393	-5.829787	3.98E-08	2.25E-06	8.333727
cg08483912	chr10	132632041	-			-3.074721	2.5626198	-5.829571	3.99E-08	2.25E-06	8.3327401
cg09932758	chr12	58022542	-	B4GALNT1	Body	0.8873707	1.1881146	5.8294508	3.99E-08	2.26E-06	8.3321904
cg00177388	chr5	72677886	-			1.4632266	-0.483308	5.8289462	4.00E-08	2.26E-06	8.3298814
cg06833110	chr2	66673316	-	MEIS1	Body	2.380905	-0.142899	5.8284151	4.01E-08	2.27E-06	8.3274515
cg04527018	chr3	126261298	+	CHST13	Body	1.4649799	1.5545565	5.8283731	4.01E-08	2.27E-06	8.3272596
cg01764252	chr5	87991071	+			2.0875659	-0.535663	5.8336974	4.01E-08	2.27E-06	8.3291897
cg26035201	chr7	1607146	+	PSMG3;PSMG3	3'UTR;3'UTR	-1.49278	0.2741849	-5.828269	4.01E-08	2.27E-06	8.3267815

cg04436994	chr1	221055665	-	HLX	Body	1.3980658	1.7487912	5.8278321	4.02E-08	2.27E-06	8.3247841
cg22391205	chr21	47458587	-			-1.639025	0.3099695	-5.827847	4.02E-08	2.27E-06	8.3248502
cg03930153	chr3	176868896	-	TBL1XR1	5'UTR	-1.138832	-2.296436	-5.827612	4.02E-08	2.27E-06	8.3237794
cg08246090	chr14	22370776	-			-1.45265	-0.331768	-5.827622	4.02E-08	2.27E-06	8.3238208
cg01777586	chr17	80163174	-	CCDC57	5'UTR	-1.315015	0.0115209	-5.830161	4.03E-08	2.27E-06	8.3244795
cg10177827	chr14	101511425	+	MIR487B;MIR381	TSS1500;TSS1500	-1.220142	0.2335148	-5.827545	4.02E-08	2.27E-06	8.3234691
cg16695570	chr14	101532886	-	MIR656	TSS200	-1.318816	0.4351937	-5.827497	4.02E-08	2.27E-06	8.3232502
cg18947951	chr2	63285739	+			1.9343843	-0.969384	5.8275243	4.02E-08	2.27E-06	8.3233758
cg11195360	chr11	124311399	-	OR8B8	TSS1500	-1.747308	0.3034603	-5.827378	4.03E-08	2.27E-06	8.3227046
cg04589521	chr2	239890141	-			-1.372135	0.4818311	-5.827173	4.03E-08	2.28E-06	8.3217676
cg03146219	chr11	71189514	-	NADSYN1	Body	1.4719961	3.3587279	5.8296896	4.03E-08	2.28E-06	8.322324
cg15993786	chr19	54204773	-	MIR518B	TSS1500	-1.745765	0.8290407	-5.826976	4.03E-08	2.28E-06	8.3208668
cg06679720	chr13	51417686	+	DLEU7	1stExon	1.6038806	-1.154023	5.8269421	4.04E-08	2.28E-06	8.3207125
cg02006405	chr6	6055150	+			-1.661277	-0.357245	-5.826832	4.04E-08	2.28E-06	8.3202089
cg03601644	chr19	54498336	+	CACNG6;CACNG6;CACNG6	Body;Body;Body	-1.406809	0.7292745	-5.826587	4.04E-08	2.28E-06	8.3190886
cg16883726	chr8	50729333	+			-1.641705	0.1763864	-5.826581	4.04E-08	2.28E-06	8.3190586
cg23518037	chr15	79526621	-			-1.09727	-0.678385	-5.82924	4.04E-08	2.28E-06	8.3199569
cg15703773	chr7	156813342	-			1.5675322	-0.936197	5.8265218	4.04E-08	2.28E-06	8.3187899
cg12865818	chr8	96126599	+			-1.725024	0.3783667	-5.826439	4.05E-08	2.28E-06	8.3184125
cg08278108	chr17	48042917	+			1.7503693	-0.990455	5.8262947	4.05E-08	2.28E-06	8.317751

cg00869668	chr17	1549012	-	SCARF1;SCARF1;SCARF1;SCARF1;SCARF1;SCARF1;SCARF1;SCARF1	5'UTR;1stExon;5'UTR;Body;1stExon;Body;5'UTR;1stExon	1.1030585	0.8581258	5.834407	4.05E-08	2.28E-06	8.3214769
cg03833216	chr17	51603096	+			-1.548104	-0.514681	-5.826241	4.05E-08	2.28E-06	8.3175037
cg05812269	chr18	21033260	+	RIOK3;RIOK3	1stExon;5'UTR	0.8141019	-1.3584	5.8260244	4.05E-08	2.28E-06	8.3165146
cg01008602	chr8	27144085	+	TRIM35	3'UTR	1.0572011	1.1159362	5.8258507	4.06E-08	2.29E-06	8.3157201
cg23960736	chr1	4171594	-			-1.650437	-0.383107	-5.825665	4.06E-08	2.29E-06	8.3148692
cg04093499	chr15	73193992	+			-1.307751	0.2026853	-5.825528	4.06E-08	2.29E-06	8.3142445
cg20211134	chr8	138979023	-			-1.741989	-0.662022	-5.825566	4.06E-08	2.29E-06	8.3144191
cg20821276	chr17	65243119	+			-0.870826	-1.516919	-5.825512	4.06E-08	2.29E-06	8.3141722
cg03217729	chr16	11222890	-	CLEC16A	Body	1.1703113	-0.820141	5.8251354	4.07E-08	2.29E-06	8.3124483
cg04652522	chr5	179124978	+	CANX;CANX	TSS1500;TSS1500	-0.860297	-1.81931	-5.824953	4.07E-08	2.29E-06	8.3116144
cg17473656	chr4	190286944	-			-1.283095	0.0583201	-5.824924	4.07E-08	2.29E-06	8.3114813
cg18892132	chr2	85803209	+	VAMP8	TSS1500	1.0638748	2.3383894	5.8275256	4.08E-08	2.29E-06	8.3124382
cg04261138	chr5	87968636	-	LOC645323;LOC645323;LOC645323	Body;Body;Body	1.482146	-1.12692	5.8245235	4.08E-08	2.30E-06	8.3096496
cg13194045	chr1	165914462	-			-1.235116	-0.800512	-5.824162	4.09E-08	2.30E-06	8.3079963
cg03233940	chr19	37894644	-			1.022496	2.2072841	5.8238044	4.10E-08	2.31E-06	8.306361
cg01072821	chr8	10382165	-	T-SP1	TSS1500	-1.060584	-0.705824	-5.823665	4.10E-08	2.31E-06	8.3057253
cg24497955	chr6	3364503	-	SLC22A23;SLC22A23	Body;Body	-1.443557	0.5756587	-5.823589	4.10E-08	2.31E-06	8.3053759
cg10435838	chr5	5034303	-	LOC340094	TSS200	-1.638939	-0.37598	-5.822468	4.12E-08	2.32E-06	8.3002502
cg05238769	chr21	34399260	+	OLIG2	Body	2.4477388	-2.128106	5.8221947	4.13E-08	2.32E-06	8.2990002
cg18766475	chr1	179923022	-	CEP350	TSS1500	-1.088649	-1.701587	-5.822095	4.13E-08	2.32E-06	8.2985429

cg03589296	chr2	66666292	+	MEIS1	Body	1.1558482	-0.533205	5.8220256	4.13E-08	2.32E-06	8.298227
cg01202519	chr11	85645743	-			1.2787103	0.7963326	5.8219941	4.13E-08	2.32E-06	8.298083
cg08512345	chr5	112043275	-	APC;APC	5'UTR;1stExon	0.8524816	-4.792508	5.8219257	4.13E-08	2.32E-06	8.2977699
cg24363820	chr22	51016703	-	CPT1B;CPT1B;CPT1B;CHKB-CPT1B;CPT1B;CPT1B;CPT1B;CPT1B	5'UTR;5'UTR;TSS200;Body;5'UTR;5'UTR;5'UTR;5'UTR	1.2754675	0.6837585	5.8218875	4.13E-08	2.32E-06	8.2975953
cg19757573	chr14	101506246	-	MIR654;MIR376B;MIR376A2;MIR300;MIR376A1	TSS1500;TSS1500;TSS200;TSS1500;TSS1500	-1.901846	0.2631662	-5.824519	4.14E-08	2.32E-06	8.2987052
cg15396204	chr1	107348393	+			-1.249113	-0.119156	-5.821759	4.14E-08	2.32E-06	8.2970069
cg20163243	chr9	95571181	+	ANKRD19	TSS1500	-0.845422	-3.807884	-5.821611	4.14E-08	2.33E-06	8.2963333
cg04384626	chr4	41882580	+			1.5058804	-0.760079	5.8214561	4.14E-08	2.33E-06	8.295623
cg27475169	chr11	119190850	+			0.8078952	1.0357568	5.8212597	4.15E-08	2.33E-06	8.2947248
cg06892635	chr17	8649228	-	CCDC42;CCDC42	TSS1500;TSS1500	-0.723002	-5.131617	-5.821194	4.15E-08	2.33E-06	8.2944257
cg16538749	chr8	1054487	-			-1.603871	1.0162822	-5.821168	4.15E-08	2.33E-06	8.294307
cg00841725	chr13	113655538	+	MCF2L;MCF2L	Body;Body	-1.052674	-1.393778	-5.821048	4.15E-08	2.33E-06	8.2937576
cg04048259	chr20	57875346	-	EDN3;EDN3;EDN3;EDN3	TSS200;TSS200;TSS200;TSS200	2.2481903	-1.137656	5.8209492	4.15E-08	2.33E-06	8.2933054
cg09053536	chr12	115117551	+	TBX3;TBX3	Body;Body	1.3836437	1.0027765	5.8205482	4.16E-08	2.34E-06	8.2914721
cg23943272	chr17	45851891	+			-0.921784	-0.909283	-5.820485	4.16E-08	2.34E-06	8.2911837
cg02147240	chr14	73011054	+	RGS6	Body	-1.244675	0.3223723	-5.820384	4.16E-08	2.34E-06	8.2907235
cg18473090	chr19	49436786	+	DHDH	TSS200	-0.992368	-5.823417	-5.820405	4.16E-08	2.34E-06	8.2908197
cg17836014	chr2	201630341	-			-1.25519	0.1133447	-5.82027	4.17E-08	2.34E-06	8.2901991
cg16441900	chr17	750848	+	NXN	Body	1.0358178	0.6111078	5.8201794	4.17E-08	2.34E-06	8.2897863
cg16537676	chr6	30851624	-	DDR1;DDR1	TSS1500;TSS1500	-1.111719	-0.553275	-5.819929	4.17E-08	2.34E-06	8.2886407

cg19370054	chr2	98964186	+	CNGA3;CNGA3	5'UTR;5'UTR	1.9452732	-1.338032	5.8225376	4.17E-08	2.34E-06	8.2896593
cg22074858	chr1	89488430	-	GBP3;GBP3	1stExon;5'UTR	-0.913001	-3.686717	-5.819822	4.18E-08	2.34E-06	8.2881515
cg22501998	chr1	38155367	+	C1orf109	Body	-1.14176	-5.642504	-5.825235	4.17E-08	2.34E-06	8.2909104
cg26609631	chr13	28366814	-	GSX1;GSX1	5'UTR;1stExon	1.993309	-0.848364	5.8195735	4.18E-08	2.34E-06	8.287016
cg01881265	chr17	934534	-	ABR;ABR;ABR	Body;Body;Body	0.6586498	1.1259266	5.8194692	4.18E-08	2.34E-06	8.2865393
cg07519873	chr19	54483188	+	CACNG8	Body	2.0461067	-1.819318	5.8194424	4.18E-08	2.34E-06	8.2864172
cg01876130	chr2	97308580	+	FER1L5	1stExon	1.1783048	1.1875548	5.8187738	4.20E-08	2.35E-06	8.2833606
cg05582719	chr6	73043502	+	RIMS1;RIMS1;RIMS1;RIMS1;RIMS1	Body;Body;Body;Body;Body	-1.555358	-0.1512	-5.818722	4.20E-08	2.35E-06	8.2831232
cg18093372	chr2	119607603	-			1.8093438	-0.709019	5.818707	4.20E-08	2.35E-06	8.2830556
cg00537979	chr21	46876731	+	COL18A1;COL18A1;COL18A1	Body;Body;1stExon	-1.818563	1.9027234	-5.818614	4.20E-08	2.35E-06	8.2826281
cg06671450	chr9	36985987	-	PAX5	Body	1.2329143	-1.483567	5.8182882	4.21E-08	2.36E-06	8.2811413
cg05961294	chr2	45028264	-			1.1293568	-0.176675	5.8180347	4.21E-08	2.36E-06	8.2799824
cg15791438	chr22	31893238	-	SFI1;SFI1	5'UTR;5'UTR	-1.136174	-1.453196	-5.817994	4.21E-08	2.36E-06	8.2797961
cg09199250	chr11	2191147	+	TH;TH;TH	Body;Body;Body	-1.157576	0.4683725	-5.817546	4.22E-08	2.36E-06	8.2777472
cg13338798	chr6	28557846	+			1.5060286	-0.980294	5.8175689	4.22E-08	2.36E-06	8.2778537
cg13828600	chr7	23144799	-	KLHL7;KLHL7	TSS1500;TSS1500	-0.67726	-3.479247	-5.817508	4.22E-08	2.36E-06	8.2775741
cg02035043	chr4	74486457	-	RASSF6;RASSF6	TSS200;TSS1500	-0.810478	-3.123484	-5.817218	4.23E-08	2.37E-06	8.2762505
cg07257571	chr10	22979312	-	PIP4K2A	Body	-1.008598	1.8055566	-5.817145	4.23E-08	2.37E-06	8.2759152
cg13168221	chr1	22962032	-	C1QA	TSS1500	-1.379562	-0.143771	-5.817055	4.23E-08	2.37E-06	8.2755052
cg05035315	chr11	132691484	+	OPCML;OPCML	Body;Body	-1.457954	-0.54826	-5.819702	4.23E-08	2.37E-06	8.2764009

cg14239592	chr19	58715251	+	ZNF274;ZNF274;ZNF274	Body;Body;5'UTR	1.215995	2.0519643	5.8169957	4.23E-08	2.37E-06	8.2752338
cg25738529	chr12	49938018	+	KCNH3	Body	0.7019354	0.070108	5.8168998	4.23E-08	2.37E-06	8.2747957
cg03692563	chr11	31846414	+			1.3198487	-0.427225	5.816789	4.24E-08	2.37E-06	8.2742895
cg02102832	chr16	1581834	-	IFT140	Body	0.844926	1.7911088	5.8165004	4.24E-08	2.37E-06	8.2729703
cg07116919	chr7	136558341	+	CHRM2;CHRM2;CHRM2;CHRM2;CHRM2;CHRM2;CHRM2;CHRM2	5'UTR;5'UTR;5'UTR;5'UTR;5'UTR;5'UTR;5'UTR;5'UTR	-1.805555	0.4794535	-5.815892	4.25E-08	2.38E-06	8.2701898
cg08682607	chr14	97208170	-			0.9347037	0.4252422	5.8159096	4.25E-08	2.38E-06	8.2702705
cg13382840	chr15	88119539	+	NCRNA00052	TSS1500	-1.545261	1.1235776	-5.815557	4.26E-08	2.38E-06	8.2686599
cg10177394	chr6	10385126	+			1.3876589	-0.950085	5.8152076	4.27E-08	2.39E-06	8.2670631
cg23425762	chr18	45664130	+			-1.339324	-2.082302	-5.814984	4.27E-08	2.39E-06	8.2660415
cg12547939	chr13	104949913	+			-1.605683	0.426773	-5.814802	4.28E-08	2.39E-06	8.2652077
cg04348023	chr1	70384012	-	LRRC7;PIN1L	Body;TSS1500	-1.344365	-0.128516	-5.81439	4.28E-08	2.39E-06	8.263327
cg11705975	chr10	120354248	-	PRLHR	Body	0.9470614	0.9484189	5.8139639	4.29E-08	2.40E-06	8.2613803
cg20159095	chr7	157342543	+	PTPRN2;PTPRN2;PTPRN2	Body;Body;Body	-1.828447	0.5498945	-5.8139	4.29E-08	2.40E-06	8.2610907
cg14425564	chr9	126794772	-	LHX2	Body	1.7174418	-0.265974	5.8138295	4.30E-08	2.40E-06	8.2607662
cg01049782	chr7	30717954	+	CRHR2	Body	-1.25475	-0.325017	-5.818904	4.30E-08	2.40E-06	8.2620541
cg01746241	chr9	34370835	-	KIAA1161	Body	1.0113678	0.8352876	5.8135204	4.30E-08	2.40E-06	8.2593544
cg19893473	chr3	177778301	+			1.0086942	1.4739687	5.8134206	4.30E-08	2.40E-06	8.2588982
cg27054144	chr4	185092121	-	ENPP6	Body	-1.324253	0.2675625	-5.813032	4.31E-08	2.41E-06	8.2571229
cg10129884	chr16	65794720	+			-1.661746	0.540535	-5.812813	4.32E-08	2.41E-06	8.2561236
cg11800608	chr20	56724999	+	C20orf85	TSS1500	-1.286188	-0.515839	-5.812588	4.32E-08	2.41E-06	8.2550939

cg16264595	chr1	4619207	-			-1.437344	-0.029228	-5.812489	4.32E-08	2.41E-06	8.2546431
cg17437939	chr7	97361252	-	TAC1;TAC1;TAC1;TAC1	TSS200;TSS200;TSS200;TSS200	2.0478948	-0.664129	5.8122403	4.33E-08	2.41E-06	8.2535064
cg06348773	chr16	1087129	-			-0.78586	-1.287061	-5.812074	4.33E-08	2.42E-06	8.2527473
cg05422883	chr2	43072932	+			0.986858	0.8369928	5.8117945	4.34E-08	2.42E-06	8.2514702
cg27228433	chr16	71475716	+			1.1678352	1.1906358	5.8118173	4.34E-08	2.42E-06	8.2515745
cg14312063	chr3	85010606	-	CADM2;CADM2	Body;Body	1.1145103	0.7867944	5.8115617	4.34E-08	2.42E-06	8.2504068
cg06722606	chr13	107687552	+			-1.531337	0.8274451	-5.811426	4.35E-08	2.42E-06	8.2497867
cg18389931	chr10	72978724	-	UNC5B	Body	-1.098654	0.2121749	-5.811225	4.35E-08	2.42E-06	8.2488686
cg24085502	chr11	119066850	+	CCDC153	TSS1500	-1.199416	0.6432431	-5.810918	4.36E-08	2.43E-06	8.2474656
cg00765705	chr12	124865130	-	NCOR2;NCOR2	Body;Body	1.3289142	1.276662	5.8108021	4.36E-08	2.43E-06	8.2469376
cg06756919	chr14	37135898	-	PAX9	Body	1.4992258	-0.53951	5.8105489	4.36E-08	2.43E-06	8.2457809
cg20598190	chr2	240866924	+			-1.454814	-0.806162	-5.810455	4.37E-08	2.43E-06	8.2453515
cg14395731	chr7	158571518	-	ESYT2	Body	1.5525514	2.8666835	5.8100043	4.38E-08	2.44E-06	8.2432939
cg27175287	chr1	47883234	+	FOXE3;FOXE3	3'UTR;1stExon	1.035735	-0.243792	5.8099399	4.38E-08	2.44E-06	8.2429996
cg26119330	chr2	64677720	-			0.6440349	2.3314332	5.8099066	4.38E-08	2.44E-06	8.2428479
cg02662495	chr12	34759279	+			-0.914748	-1.022667	-5.809811	4.38E-08	2.44E-06	8.2424127
cg19477978	chr17	16998439	+	MPRIP;MPRIP	Body;Body	0.6384561	1.9180338	5.8097375	4.38E-08	2.44E-06	8.2420754
cg17431391	chr19	38042109	-	ZNF540	TSS200	1.193224	-0.077702	5.8096819	4.38E-08	2.44E-06	8.2418217
cg27254338	chr17	34192710	+	C17orf66	Body	-1.013928	-0.936406	-5.809661	4.38E-08	2.44E-06	8.2417275
cg19241468	chr3	122399344	+	PARP14	TSS1500	-0.521848	-3.732216	-5.809329	4.39E-08	2.44E-06	8.2402113

cg19080053	chr6	5132887	+	LYRM4;LYRM4	3'UTR;Body	1.3466418	-0.438307	5.8091486	4.39E-08	2.44E-06	8.2393863
cg18569335	chr17	40171970	+	NKIRAS2;NKIRAS2;NKIRAS2;NKIRAS2;NKIRAS2	TSS200;TSS200;TSS200;TSS200;5'UTR	-0.899278	-3.719099	-5.80898	4.40E-08	2.45E-06	8.2386168
cg00159764	chr20	35059674	-	DLGAP4	5'UTR	-1.241862	-0.339201	-5.808872	4.40E-08	2.45E-06	8.2381227
cg14604681	chr2	63283355	-	OTX1	Body	1.0515206	-0.302665	5.8088917	4.40E-08	2.45E-06	8.2382133
cg09195271	chr1	20142497	-	RNF186	TSS1500	-1.273841	-0.385779	-5.808782	4.40E-08	2.45E-06	8.2377122
cg21983531	chr7	74075317	-	GTF2I;GTF2I;GTF2I;GTF2I;GTF2I	5'UTR;5'UTR;5'UTR;5'UTR;5'UTR	-1.380932	-1.385847	-5.80879	4.40E-08	2.45E-06	8.2377478
cg00464927	chr11	68139121	+	LRP5	Body	-1.760732	2.6570338	-5.811328	4.40E-08	2.45E-06	8.2385108
cg06370771	chr1	18960940	+	PAX7;PAX7;PAX7	Body;Body;Body	1.9207285	-1.542691	5.8086024	4.40E-08	2.45E-06	8.2368923
cg15014975	chr1	25257547	-	RUNX3;RUNX3	TSS1500;Body	1.8587128	0.3916122	5.8193917	4.41E-08	2.45E-06	8.253552
cg10437900	chr7	23518135	+			-1.114338	-0.063774	-5.808307	4.41E-08	2.45E-06	8.2355454
cg14948822	chr2	27545006	+	MPV17	Body	-1.459678	0.7319858	-5.807904	4.42E-08	2.46E-06	8.233704
cg01884917	chr17	1038155	-	ABR;ABR	Body;5'UTR	1.5603103	1.8341677	5.8078694	4.42E-08	2.46E-06	8.2335451
cg25820728	chr7	1684706	+			-1.462935	-0.462445	-5.810363	4.42E-08	2.46E-06	8.2464371
cg03465652	chr2	121625735	+	GLI2	Body	1.119492	-0.668806	5.8073411	4.43E-08	2.46E-06	8.231133
cg02464487	chr6	149806235	+	ZC3H12D	TSS200	-1.114772	-1.764419	-5.807154	4.43E-08	2.46E-06	8.2302799
cg26361999	chr6	33969961	+			0.7958828	0.2860987	5.8068956	4.44E-08	2.47E-06	8.2290993
cg10993442	chr1	25257587	-	RUNX3;RUNX3	TSS1500;Body	0.8960417	0.0156855	5.8068351	4.44E-08	2.47E-06	8.2288228
cg13172153	chr1	201391747	+	TNNI1	TSS1500	-1.350896	0.0749826	-5.806842	4.44E-08	2.47E-06	8.2288528
cg23448390	chr7	34889338	-	NPSR1;NPSR1	3'UTR;Body	-1.040897	-0.45236	-5.806611	4.45E-08	2.47E-06	8.2278007
cg27363021	chr13	72202615	-	DACH1;DACH1;DACH1	Body;Body;Body	-1.521529	0.9538861	-5.806346	4.45E-08	2.47E-06	8.2265908

cg11873482	chr7	97361244	-	TAC1;TAC1;TAC1;TAC1	TSS200;TSS200;TSS200;TSS200	2.0158685	-1.12146	5.8087365	4.46E-08	2.47E-06	8.2263769
cg02103822	chr1	147246329	+	GJA5	TSS1500	-1.767301	0.8711033	-5.806039	4.46E-08	2.47E-06	8.2251869
cg02489377	chr9	125391390	-	OR1B1	1stExon	-1.034502	-0.431152	-5.806012	4.46E-08	2.47E-06	8.2250644
cg11181763	chr5	158379078	+	EBF1	Body	-1.669128	0.9004661	-5.805982	4.46E-08	2.47E-06	8.2249292
cg17044356	chr17	7758228	-	TMEM88	TSS200	1.5151709	0.6229706	5.8085999	4.46E-08	2.47E-06	8.225754
cg02535674	chr20	52226352	+			-1.455206	-2.552884	-5.808543	4.46E-08	2.48E-06	8.2258108
cg25143990	chr12	28127783	+			2.6623273	-1.645433	5.8058437	4.46E-08	2.48E-06	8.2242971
cg11562312	chr8	144406532	-	TOP1MT	Body	0.5955716	1.4288173	5.8057516	4.46E-08	2.48E-06	8.2238768
cg20779917	chr7	121946959	+			1.2914581	-0.694836	5.8057726	4.46E-08	2.48E-06	8.2239724
cg03650608	chr19	8647531	-	ADAMTS10	Body	-1.620902	0.6878619	-5.805551	4.47E-08	2.48E-06	8.2229589
cg21660861	chr7	16700959	+	BZW2;BZW2;BZW2	Body;5'UTR;5'UTR	-1.504369	0.3924812	-5.80552	4.47E-08	2.48E-06	8.2228182
cg09429111	chr9	96792562	+	PTPDC1	TSS1500	-0.551184	-3.577205	-5.805357	4.47E-08	2.48E-06	8.2220755
cg03294491	chr18	45458243	+	SMAD2;SMAD2;SMAD2	TSS1500;TSS1500;TSS1500	-1.19588	-1.270412	-5.805295	4.47E-08	2.48E-06	8.2217937
cg15580167	chr2	175629219	+	CHRNA1;CHRNA1	TSS200;TSS200	-1.753662	0.948267	-5.805189	4.48E-08	2.48E-06	8.2213071
cg19972648	chr10	79110632	-	KCNMA1;KCNMA1;KCNMA1;KCNMA1	Body;Body;Body;Body	-1.446465	-0.669977	-5.805069	4.48E-08	2.48E-06	8.2207607
cg12505170	chr5	72595685	-			2.2643014	-0.854519	5.8043761	4.49E-08	2.49E-06	8.217598
cg04902327	chr1	248019234	-	TRIM58	TSS1500	-1.676677	0.3254862	-5.804009	4.50E-08	2.49E-06	8.2159205
cg17365076	chr11	116274125	+			-1.84552	0.4927767	-5.803987	4.50E-08	2.49E-06	8.2158227
cg20874031	chr2	69206176	+	GKN1	Body	-1.346314	-0.536303	-5.803984	4.50E-08	2.49E-06	8.2158095
cg01362243	chr2	45160389	-			1.1749706	0.1364665	5.8064344	4.51E-08	2.50E-06	8.2158818

cg02329599	chr3	40547855	-	ZNF620;ZNF620	Body;Body	-0.455337	-4.418806	-5.803529	4.51E-08	2.50E-06	8.2137328
cg17381426	chr7	140374125	-	ADCK2	Body	-1.343121	-0.063114	-5.803542	4.51E-08	2.50E-06	8.2137901
cg19752861	chr2	74153213	-	DGUOK;DGUOK	TSS1500;TSS1500	-2.763708	0.9203757	-5.811433	4.52E-08	2.50E-06	8.2447306
cg19848529	chr6	170483067	+			1.2682406	2.4636513	5.8028854	4.53E-08	2.50E-06	8.2107947
cg08951271	chr6	30850543	-	DDR1	TSS1500	-1.133179	-1.047622	-5.805123	4.53E-08	2.51E-06	8.2102228
cg11203211	chr8	144301594	+			-1.599724	0.0897316	-5.80242	4.54E-08	2.51E-06	8.2086699
cg10255237	chr12	90150622	+			-2.016643	-1.795788	-5.80228	4.54E-08	2.51E-06	8.208034
cg12446088	chr1	245730665	-	KIF26B	Body	1.0398622	0.4625855	5.8021132	4.54E-08	2.51E-06	8.2072707
cg08172065	chr8	70981796	+	PRDM14	Body	1.2163325	-1.882211	5.8018898	4.55E-08	2.52E-06	8.2062515
cg01247127	chr20	1538366	-	SIRPD	TSS200	-1.364647	0.0191006	-5.801113	4.56E-08	2.52E-06	8.2027054
cg16641358	chr5	15930438	+	FBXL7	Body	-2.096712	0.3030011	-5.801074	4.57E-08	2.52E-06	8.202528
cg27621931	chr13	24368124	+	MIPEP	Body	0.8916093	1.9365335	5.8010109	4.57E-08	2.52E-06	8.2022411
cg00147248	chr7	136568546	+	CHRM2;CHRM2;CHRM2;CHRM2;CHRM2;CHRM2;CHRM2;CHRM2	5'UTR;5'UTR;5'UTR;5'UTR;5'UTR;5'UTR;5'UTR;5'UTR	-1.822296	1.0348905	-5.803492	4.57E-08	2.53E-06	8.2027869
cg13568258	chr19	48823427	+	CCDC114	TSS200	-0.762456	-3.23557	-5.800796	4.57E-08	2.53E-06	8.2012607
cg25186802	chr4	10149849	+			-0.863879	-0.395635	-5.800554	4.58E-08	2.53E-06	8.2001542
cg26917140	chr1	91184770	-			1.4962966	-0.729397	5.8004166	4.58E-08	2.53E-06	8.1995295
cg05238741	chr12	52214758	-	FIGNL2	1stExon	-1.550409	-1.840485	-5.800378	4.58E-08	2.53E-06	8.1993548
cg11344572	chr1	10702712	-	CASZ1	Body	1.3615965	2.3760699	5.8029429	4.58E-08	2.53E-06	8.1999693
cg19079351	chr10	61126168	-			0.9965549	2.1162748	5.8002664	4.58E-08	2.53E-06	8.1988441
cg10398455	chr2	72904298	-	EXOC6B	Body	0.8310603	1.7133482	5.8001904	4.58E-08	2.53E-06	8.1984975

cg12137450	chr8	22458046	+	C8orf58	Body	1.0577596	0.3970002	5.8002068	4.58E-08	2.53E-06	8.1985724
cg12534216	chr6	30095570	-			1.8950218	-1.353591	5.8000006	4.59E-08	2.53E-06	8.1976317
cg19716018	chr19	1438056	-	RPS15	TSS1500	-1.041581	-1.378351	-5.799913	4.59E-08	2.53E-06	8.1972328
cg13285077	chr11	134651819	+			-1.128095	-0.674141	-5.799858	4.59E-08	2.53E-06	8.1969815
cg09803959	chr17	78865514	+	RPTOR;RPTOR	Body;Body	-1.632749	-1.980709	-5.799723	4.59E-08	2.54E-06	8.1963629
cg12598338	chr13	28368172	+			1.1001873	-1.640106	5.7994231	4.60E-08	2.54E-06	8.1949967
cg13858975	chr15	52016644	-	LYSMD2;LYSMD2	Body;Body	0.8588279	2.6675369	5.7991435	4.61E-08	2.54E-06	8.1937215
cg18623980	chr2	45240563	-			2.7943394	-1.138881	5.7990772	4.61E-08	2.54E-06	8.1934187
cg14377171	chr9	138022131	+			-1.353575	0.2880835	-5.798743	4.62E-08	2.55E-06	8.1918931
cg26272623	chr1	119532320	+	TBX15	TSS200	0.9636386	-0.336926	5.7987284	4.62E-08	2.55E-06	8.1918279
:h.1.145732754I	chr1	147266130	+			-0.78944	-4.592724	-5.79874	4.62E-08	2.55E-06	8.1918808
cg18130044	chr20	21682362	+			2.6766152	-1.130819	5.7985962	4.62E-08	2.55E-06	8.1912246
cg16400631	chr1	91300215	-			2.1562389	-0.878716	5.7983976	4.62E-08	2.55E-06	8.1903187
cg01368780	chr6	169937196	-	WDR27	Body	-1.970862	1.1063326	-5.797895	4.63E-08	2.55E-06	8.1880244
cg14120984	chr14	104627874	+	KIF26A	Body	-0.949555	-0.186867	-5.797915	4.63E-08	2.55E-06	8.1881163
cg21253590	chr7	102073120	-	ORAI2;ORAI2	TSS1500;TSS1500	-1.306059	0.5076671	-5.797752	4.64E-08	2.56E-06	8.187376
cg01602666	chr12	83176430	+	TMTC2	Body	1.0663504	1.9500421	5.7977168	4.64E-08	2.56E-06	8.1872134
cg14945456	chr17	79791791	+	DYSFIP1;FAM195B;FAM195B	Body;TSS1500;TSS1500	-0.605635	-2.963628	-5.797528	4.64E-08	2.56E-06	8.1863507
cg09246103	chr1	8456466	-	RERE;RERE;RERE	5'UTR;Body;Body	1.1722197	0.398971	5.7974526	4.64E-08	2.56E-06	8.1860081
cg00339556	chr5	16180048	-	11-Mar	TSS200	3.1865557	-1.752566	5.7991646	4.67E-08	2.57E-06	8.1827556

cg25443851	chr5	87991388	+			1.7213995	-1.761713	5.804562	4.67E-08	2.57E-06	8.1850278
cg13941682	chr4	702545	-	PCGF3	5'UTR	1.2429967	1.2361626	5.795529	4.69E-08	2.58E-06	8.1772355
cg24756642	chr2	1597123	-			-1.536859	0.640319	-5.795529	4.69E-08	2.58E-06	8.1772369
cg03743678	chr16	47048004	-			0.9564691	2.3469893	5.7954451	4.69E-08	2.58E-06	8.1768531
cg01348293	chr7	27260113	-			1.5764608	-0.834149	5.7951525	4.70E-08	2.59E-06	8.1755187
cg14244493	chr9	137515845	-			-1.298733	0.2398747	-5.794983	4.70E-08	2.59E-06	8.1747452
cg20872937	chr18	74961968	-	GALR1	TSS200	2.9364078	-1.786713	5.7948675	4.70E-08	2.59E-06	8.1742192
cg00857435	chr7	90932279	-			-1.25046	-1.248865	-5.797434	4.70E-08	2.59E-06	8.1748729
cg23584735	chr8	10686021	+	PINX1	Body	0.777808	2.8030565	5.7948062	4.70E-08	2.59E-06	8.1739396
cg06958563	chr5	87980911	-	LOC645323	TSS1500	2.0365087	-0.742933	5.7999925	4.71E-08	2.59E-06	8.1880101
cg14598764	chr3	125820484	-	SLC41A3	TSS200	0.7596938	1.018846	5.7945779	4.71E-08	2.59E-06	8.1728986
cg21198300	chr4	140429869	-	SETD7	3'UTR	0.9388546	1.7128735	5.7942084	4.72E-08	2.59E-06	8.171214
cg00551679	chr16	86547530	-	FOXF1	3'UTR	1.1316735	-0.724863	5.7941418	4.72E-08	2.59E-06	8.17091
cg05907939	chr8	129055563	-	PVT1	Body	1.0088429	1.6283354	5.7941202	4.72E-08	2.59E-06	8.1708116
cg02525108	chr11	122847582	-			1.1276662	-0.934686	5.793929	4.72E-08	2.60E-06	8.1699399
cg03531247	chr2	50573485	-	NRXN1;NRXN1;NRXN1	Body;Body;Body	1.9735846	-0.956084	5.7939084	4.72E-08	2.60E-06	8.169846
cg03855388	chr11	68580018	+	CPT1A;CPT1A	Body;Body	1.4240808	4.675476	5.7936423	4.73E-08	2.60E-06	8.1686326
cg05611719	chr17	73902048	+	MRPL38	TSS1500	-1.225135	-1.867038	-5.793537	4.73E-08	2.60E-06	8.1681548
cg27475088	chr11	194509	-	SCGB1C1	3'UTR	-0.775933	-0.051053	-5.793361	4.74E-08	2.60E-06	8.16735
cg03715846	chr9	126761082	+			1.179436	-0.115391	5.7928699	4.75E-08	2.61E-06	8.1651114

cg06711656	chr3	140660335	-	SLC25A36;SLC25A36	TSS1500;TSS1500	-0.624272	-4.730968	-5.792823	4.75E-08	2.61E-06	8.1648983
cg07059009	chr2	64494859	+			0.9436027	1.3849391	5.7928281	4.75E-08	2.61E-06	8.1649208
cg16240162	chr3	147106890	+	ZIC4;ZIC4;ZIC4;ZIC4;ZIC4	Body;Body;Body;Body;Body	1.6642206	-0.463634	5.7927634	4.75E-08	2.61E-06	8.1646257
cg04782470	chr19	18208422	-	MAST3	TSS200	-0.991611	-3.434394	-5.792625	4.75E-08	2.61E-06	8.1639948
cg19625116	chr11	94276903	-	FUT4	TSS200	-0.503427	-3.345728	-5.792618	4.75E-08	2.61E-06	8.1639646
cg25547580	chr18	55103734	+	ONECUT2	1stExon	2.3655414	-0.705198	5.7949927	4.76E-08	2.61E-06	8.1640757
cg06379876	chr1	41220106	+	NFYC;NFYC;NFYC;NFYC;NFYC;MIR30E	Body;Body;Body;Body;Body;Body	2.3097216	3.2658803	5.7948791	4.76E-08	2.61E-06	8.1632398
cg20604286	chr2	58273438	+	VRK2;VRK2;VRK2;VRK2;VRK2;VRK2	TSS1500;TSS1500;TSS1500;TSS1500;TSS1500;TSS1500	-0.89003	-2.479529	-5.79758	4.76E-08	2.61E-06	8.1791035
cg05734973	chr3	15139826	-	ZFYVE20	5'UTR	-0.757216	-2.168306	-5.792172	4.76E-08	2.61E-06	8.1619306
cg00866399	chr2	73148052	+	EMX1	Body	1.3930625	-1.308937	5.791981	4.77E-08	2.62E-06	8.1610591
cg17201542	chr3	122818413	-	PDIA5;PDIA5	Body;Body	0.6786377	2.6923185	5.7919592	4.77E-08	2.62E-06	8.1609594
cg19257274	chr1	207990250	+	LOC148696	TSS1500	1.4047168	1.7951816	5.7946463	4.77E-08	2.62E-06	8.1624985
cg24681963	chr2	72012367	-			-1.281779	-0.520666	-5.791903	4.77E-08	2.62E-06	8.1607025
cg13998871	chr10	50902733	-	C10orf53;C10orf53	3'UTR;Body	-1.151745	-0.226699	-5.794506	4.77E-08	2.62E-06	8.1618592
cg14367020	chr6	99292286	+			2.1030708	-0.487226	5.791834	4.77E-08	2.62E-06	8.1603888
cg14784699	chr10	31607330	+	ZEB1;ZEB1;ZEB1;LOC220930;ZEB1	TSS1500;TSS200;TSS1500;Body;TSS1500	-0.736344	-5.199036	-5.794459	4.77E-08	2.62E-06	8.1616442
cg04276058	chr1	19254031	-	IFFO2	Body	-1.370495	-1.173189	-5.791747	4.77E-08	2.62E-06	8.1599909
cg24901091	chr5	164576826	+			-1.697354	1.2096387	-5.791541	4.78E-08	2.62E-06	8.1590523
cg15434211	chr17	4442193	-	SPNS2;MYBBP1A;MYBBP1A	3'UTR;3'UTR;3'UTR	0.6994342	1.2290177	5.7914825	4.78E-08	2.62E-06	8.1587868
cg25514273	chr13	70681804	-	KLHL1;ATXN8OS	1stExon;Body	1.7395213	-0.461575	5.7914813	4.78E-08	2.62E-06	8.1587812

cg05616355	chr11	124480990	-	PANX3	TSS1500	-1.15774	-0.669453	-5.791414	4.78E-08	2.62E-06	8.1584759
cg20969424	chr5	73690902	+			-1.092495	-1.08707	-5.791211	4.78E-08	2.62E-06	8.1575499
cg26836696	chr5	145722201	+			1.956075	-1.406594	5.7912068	4.78E-08	2.62E-06	8.1575298
cg17906267	chr1	95582329	-	TMEM56	TSS1500	-0.72262	-3.215515	-5.790959	4.79E-08	2.62E-06	8.1564025
cg19411738	chr10	13481909	-	BEND7	Body	-0.983833	-1.999591	-5.790963	4.79E-08	2.62E-06	8.156419
cg22192489	chr5	134521633	+			-1.013942	-1.509344	-5.790924	4.79E-08	2.62E-06	8.1562426
cg14405625	chr9	136855902	+	VAV2;VAV2	Body;Body	-0.992873	0.335614	-5.793421	4.79E-08	2.63E-06	8.1566005
cg00145253	chr1	75602857	+	LHX8	Body	1.0618974	-0.472627	5.7906393	4.80E-08	2.63E-06	8.1549433
cg04513214	chr5	17057446	+			-1.361281	-0.509488	-5.790238	4.81E-08	2.63E-06	8.1531162
cg06645921	chr12	8025394	+	SLC2A14	5'UTR	1.3256504	-0.240035	5.7902556	4.81E-08	2.63E-06	8.1531943
cg09959931	chr4	671550	-	MYL5	TSS200	-1.278666	0.1247682	-5.7929	4.81E-08	2.63E-06	8.1545505
cg17477162	chr1	91176464	-			1.8024847	-1.036825	5.7902971	4.81E-08	2.63E-06	8.1533835
cg19683073	chr5	79553267	+	SERINC5	TSS1500	-1.501595	0.1171354	-5.790328	4.80E-08	2.63E-06	8.153524
cg27639613	chr2	28408263	-	BRE;BRE;BRE;BRE;BRE	Body;Body;Body;Body;Body	-1.541103	-0.104014	-5.790277	4.81E-08	2.63E-06	8.1532935
cg25756406	chr10	134597884	-			1.1360414	0.5522994	5.7898951	4.81E-08	2.63E-06	8.1515515
cg24460544	chr1	3085446	-	PRDM16;PRDM16	Body;Body	-0.792039	-1.563875	-5.789715	4.82E-08	2.64E-06	8.1507283
cg18450930	chr11	5444223	-	OR51Q1	1stExon	-1.537803	0.6433855	-5.789687	4.82E-08	2.64E-06	8.1506024
cg15790820	chr11	2292510	+	ASCL2	TSS1500	2.8035228	-1.762674	5.7896238	4.82E-08	2.64E-06	8.1503151
cg27113419	chr16	58533979	-	NDRG4;NDRG4;NDRG4	Body;Body;Body	-1.147853	-1.351018	-5.789504	4.82E-08	2.64E-06	8.1497695
cg05047401	chr1	156131213	+	SEMA4A	Body	1.6806795	0.4062939	5.789353	4.83E-08	2.64E-06	8.1490807

cg09351315	chr17	77775823	-			1.09357	-0.403645	5.7893254	4.83E-08	2.64E-06	8.1489549
cg07811198	chr7	153585368	+	DPP6	Body	1.1939544	-0.461581	5.7891086	4.83E-08	2.64E-06	8.1479669
cg09105687	chr1	2536647	+	MMEL1	Body	-1.429177	0.5792575	-5.788893	4.84E-08	2.64E-06	8.1469828
ch.11.1416715R	chr11	68206429	+	LRP5	Body	-0.899121	-4.256141	-5.78878	4.84E-08	2.64E-06	8.1464714
cg12558519	chr19	18748017	-	KLHL26	Body	-0.936374	-4.146319	-5.78844	4.85E-08	2.65E-06	8.1449195
cg18993949	chr4	15481269	+	CC2D2A;CC2D2A;CC2D2A	Body;Body;Body	-0.827937	-1.620345	-5.788297	4.85E-08	2.65E-06	8.1442698
cg21503476	chr5	169531595	+	FOXI1;FOXI1	TSS1500;TSS1500	-1.058871	0.1497483	-5.787793	4.86E-08	2.66E-06	8.1419702
cg07928551	chr14	71275530	-	MAP3K9	1stExon	-0.838932	-5.283444	-5.790314	4.87E-08	2.66E-06	8.1424605
cg19350375	chr10	98624529	+	LCOR;LCOR;LCOR	5'UTR;5'UTR;5'UTR	-1.796112	1.2878316	-5.787605	4.87E-08	2.66E-06	8.1411143
cg01981760	chr16	53737576	+	FTO;RPGRIP1L;RPGRIP1L	TSS1500;5'UTR;5'UTR	-0.446499	-3.287288	-5.787272	4.87E-08	2.66E-06	8.1395987
cg14240790	chr11	6378691	+			-1.010296	-0.86186	-5.787196	4.88E-08	2.66E-06	8.1392511
cg20944283	chr1	159172620	+	CADM3;CADM3	3'UTR;3'UTR	-1.364917	-0.354398	-5.787206	4.88E-08	2.66E-06	8.1392972
cg04111071	chr7	19813299	-	TMEM196	TSS1500	1.9618497	-0.687919	5.7870745	4.88E-08	2.66E-06	8.1386981
cg02656891	chr1	34642519	+	C1orf94;C1orf94	5'UTR;TSS200	1.8583608	-0.922499	5.787045	4.88E-08	2.66E-06	8.1385635
cg08768395	chr5	33889461	+	ADAMTS12	Body	-1.526767	-1.292538	-5.786669	4.89E-08	2.67E-06	8.1368489
cg13313616	chr11	2127569	+			-1.610148	0.6355631	-5.786636	4.89E-08	2.67E-06	8.1366987
cg05413872	chr14	57261256	+			1.6924031	-1.524918	5.7866055	4.89E-08	2.67E-06	8.1365612
cg01311151	chr12	85671976	+			0.9367396	-0.108522	5.7863819	4.90E-08	2.67E-06	8.1355425
cg21448057	chr13	84453836	-	SLITRK1	1stExon	1.7239497	-0.940145	5.7863441	4.90E-08	2.67E-06	8.1353702
cg23508201	chr17	38677884	+			-1.244454	0.148981	-5.786331	4.90E-08	2.67E-06	8.1353123

cg26167841	chr10	102807218	-			1.1029443	-0.222877	5.7863156	4.90E-08	2.67E-06	8.1352404
cg16221927	chr14	101436540	+			-1.789591	0.8112759	-5.786189	4.90E-08	2.67E-06	8.1346637
cg02938414	chr4	174444166	-			1.0432501	-0.908039	5.7859689	4.91E-08	2.67E-06	8.1336611
cg06649410	chr6	154360483	+	OPRM1;OPRM1;OPRM1;OPRM1;OPRM1;OPRM1;OPRM1;OPRM1;OPRM1;OPRM1;OPRM1;OPRM1;OPRM1;OPRM1;OPRM1;OPRM1;OPRM1;OPRM1;OPRM1	1stExon;1stExon;1stExon;5'UTR;5'UTR;5'UTR;Body;5'UTR;1stExon;5'UTR;1stExon;5'UTR;1stExon;TSS200;5'UTR;5'UTR;5'UTR;Body;1stExon;1stExon	1.2815203	-1.015248	5.7859545	4.91E-08	2.67E-06	8.1335955
cg04044224	chr2	238499793	-	RAB17	TSS200	-1.018557	-0.931477	-5.785814	4.91E-08	2.68E-06	8.1329543
cg01774019	chr4	11637533	+			-1.468918	1.0062162	-5.78534	4.92E-08	2.68E-06	8.1307975
cg20651995	chr2	69525471	+			-1.133778	-0.867006	-5.785127	4.92E-08	2.68E-06	8.1298241
cg23255141	chr15	51199395	+			-1.253266	-0.38758	-5.785131	4.92E-08	2.68E-06	8.1298417
cg13420744	chr1	12679306	-	DHRS3	TSS1500	-0.845045	-3.437365	-5.785049	4.93E-08	2.68E-06	8.1294723
cg19469357	chr7	8474830	-	NXPH1	5'UTR	1.5021805	-1.946125	5.7850712	4.93E-08	2.68E-06	8.1295715
cg19396666	chr10	125866280	+			-0.987909	-1.14563	-5.784972	4.93E-08	2.68E-06	8.1291175
cg16891968	chr6	29698284	+	LOC285830;LOC285830	Body;Body	-1.652099	0.2910936	-5.784887	4.93E-08	2.68E-06	8.1287312
cg05324368	chr20	25370336	-	ABHD12;ABHD12	Body;Body	-0.830293	-3.311777	-5.784791	4.93E-08	2.69E-06	8.1282958
cg01323954	chr15	83654524	+	FAM103A1	TSS1500	-1.158213	-2.266708	-5.784737	4.93E-08	2.69E-06	8.1280504
cg06003388	chr16	59363443	+			-1.435207	-0.100658	-5.784669	4.94E-08	2.69E-06	8.1277377
cg05155605	chr16	878130	+			-1.206866	0.3102713	-5.784584	4.94E-08	2.69E-06	8.1273527
cg07598370	chr11	56409533	+	OR8U8;OR5AP2	Body;1stExon	-1.503873	0.6439978	-5.787114	4.94E-08	2.69E-06	8.1282197
cg08227526	chr4	155663529	-			2.2256403	-0.677535	5.7844406	4.94E-08	2.69E-06	8.126699
cg14010720	chr20	62168878	+	PTK6	TSS200	-1.089885	-0.998054	-5.784351	4.94E-08	2.69E-06	8.1262886
cg19598514	chr11	442149	-	ANO9	TSS200	-0.815773	-2.626073	-5.784317	4.94E-08	2.69E-06	8.1261342

cg08243465	chr20	56274036	+	PMEPA1;PMEPA1;PMEPA1;PMEPA1	5'UTR;5'UTR;Body;Body	-1.66676	-0.889669	-5.784105	4.95E-08	2.69E-06	8.1251685
cg06733602	chr16	86554372	+			-0.840456	-1.236109	-5.784043	4.95E-08	2.69E-06	8.1248893
cg08979895	chr4	111540126	+	PITX2;PITX2;PITX2	Body;Body;Body	1.2671129	-0.172039	5.7839311	4.95E-08	2.69E-06	8.124378
cg11597131	chr2	176988939	-	HOXD9	3'UTR	1.991514	-1.031133	5.7833751	4.97E-08	2.70E-06	8.1218456
cg19404723	chr8	1449313	+	DLGAP2	TSS1500	-1.497648	0.2781548	-5.783297	4.97E-08	2.70E-06	8.1214889
cg10167849	chr6	34206152	-	HMGA1;HMGA1;HMGA1;HMGA1;HMGA1;HMGA1	5'UTR;5'UTR;5'UTR;5'UTR;5'UTR;TSS1500	-1.441637	-2.219309	-5.782984	4.98E-08	2.70E-06	8.1200666
cg19440734	chr3	48264753	-	CAMP	TSS200	-1.06741	-0.927368	-5.782995	4.97E-08	2.70E-06	8.1201146
cg07071426	chr3	39470608	+			0.8675397	1.2888541	5.7829536	4.98E-08	2.70E-06	8.1199262
cg17299437	chr4	54974644	-			1.9574483	0.219649	5.7829157	4.98E-08	2.70E-06	8.1197534
ch.10.1515233F	chr10	73039321	+	UNC5B	Body	-0.683516	-4.025763	-5.782756	4.98E-08	2.71E-06	8.1190257
cg11273471	chr15	25438403	-	SNORD115-13	TSS200	-1.220171	0.2512637	-5.782656	4.98E-08	2.71E-06	8.1185698
cg09889350	chr16	56995813	-	CETP	TSS200	0.8376177	0.7334369	5.7825287	4.99E-08	2.71E-06	8.117991
cg10145246	chr1	119527884	+	TBX15	5'UTR	1.3408623	-0.146091	5.7821741	4.99E-08	2.71E-06	8.1163762
cg26155032	chr15	97319937	+			-1.508379	0.9412645	-5.782051	5.00E-08	2.71E-06	8.1158161
cg09234672	chr16	51187750	-			1.3028284	-0.979006	5.7845016	5.00E-08	2.72E-06	8.1160152
cg07479786	chr1	150967828	+	ANXA9	3'UTR	1.1880411	1.6204387	5.781797	5.00E-08	2.72E-06	8.114659
cg19055803	chr19	12947164	+	RTBDN	TSS1500	-0.614548	-3.358892	-5.781632	5.01E-08	2.72E-06	8.1139086
cg17310258	chr11	31847173	+			1.1008836	0.7621602	5.7815613	5.01E-08	2.72E-06	8.1135856
cg27649239	chr15	68120393	-	LBXCOR1	Body	1.9271135	-0.118959	5.7815576	5.01E-08	2.72E-06	8.1135685
cg07229767	chr4	3372206	-	RGS12;RGS12;RGS12	Body;Body;Body	0.9153475	1.5269642	5.7814612	5.01E-08	2.72E-06	8.1131298

cg00368577	chr12	52915064	-	KRT5	TSS1500	-1.055275	-0.422077	-5.784025	5.01E-08	2.72E-06	8.1141674
cg03151469	chr2	176936091	-			1.0312274	-0.285998	5.7813961	5.01E-08	2.72E-06	8.1128331
cg18424634	chr3	147127097	-	ZIC1	TSS200	2.4335798	-2.36149	5.7810516	5.02E-08	2.72E-06	8.1112644
cg19651694	chr11	71955164	-	PHOX2A;PHOX2A	1stExon;5'UTR	1.6918426	-1.054599	5.7810026	5.02E-08	2.72E-06	8.1110413
cg07582825	chr3	152769889	-			-1.503424	-0.093274	-5.780859	5.03E-08	2.73E-06	8.1103876
cg04239645	chr17	59564867	-			0.7557922	0.7352237	5.7803283	5.04E-08	2.73E-06	8.1079714
cg15689121	chr1	38618977	-			-1.673199	0.3463358	-5.780332	5.04E-08	2.73E-06	8.1079873
cg18630667	chr19	37096323	-	ZNF529;ZNF382;ZNF529;ZNF529	TSS200;5'UTR;TSS200;TSS200	2.8836548	-0.778	5.7802767	5.04E-08	2.73E-06	8.107736
cg10940093	chr6	47104079	-			-1.595452	0.6437018	-5.78003	5.05E-08	2.73E-06	8.1066134
cg08014964	chr17	10372972	+	MYH4	TSS200	-1.284173	-0.888385	-5.779737	5.05E-08	2.74E-06	8.1052772
cg01452847	chr17	46711341	+	MIR196A1	TSS1500	2.0582308	-0.324307	5.7792904	5.06E-08	2.74E-06	8.1032458
cg08604097	chr7	8480865	-	NXPH1	Body	2.2633729	-1.063211	5.7820086	5.06E-08	2.74E-06	8.104678
cg10624665	chr17	42636570	-	FZD2	1stExon	0.8172025	-0.647734	5.7793373	5.06E-08	2.74E-06	8.1034591
cg14462402	chr11	120678447	+	GRIK4	Body	-1.085538	-1.416089	-5.779322	5.06E-08	2.74E-06	8.1033896
cg15932610	chr12	108820116	+			-1.59314	0.7774176	-5.779292	5.06E-08	2.74E-06	8.1032527
cg14028577	chr9	89259694	-			-1.473687	0.5318205	-5.778905	5.07E-08	2.75E-06	8.1014914
cg02940147	chr11	124747263	+	ROBO3	Body	1.497707	0.9671352	5.7788228	5.07E-08	2.75E-06	8.1011172
cg03518417	chr1	6483657	-	ESPN	TSS1500	-0.463844	-3.861005	-5.778836	5.07E-08	2.75E-06	8.1011749
cg22507154	chr1	91185233	-			1.3495828	1.2737337	5.7788008	5.08E-08	2.75E-06	8.1010167
cg25517810	chr2	105474385	-			1.890507	-0.208462	5.7838779	5.08E-08	2.75E-06	8.1023978

cg16413327	chr16	31215451	-	PYCARD;PYCARD	TSS1500;TSS1500	-1.288049	0.5184069	-5.778286	5.09E-08	2.75E-06	8.0986714
cg15651267	chr5	167028535	+	ODZ2	Body	-1.607379	0.4263605	-5.77823	5.09E-08	2.75E-06	8.0984173
cg08276645	chr2	241459502	-	ANKMY1;ANKMY1	Body;Body	1.3441229	0.6392823	5.780733	5.09E-08	2.75E-06	8.0991994
cg12797746	chr8	1110520	+			-1.176284	-0.688576	-5.778105	5.09E-08	2.75E-06	8.0978483
cg13305657	chr1	25258181	-	RUNX3;RUNX3	TSS1500;Body	0.8019989	0.5697594	5.7834379	5.09E-08	2.75E-06	8.1003999
cg17284236	chr19	12708001	+	ZNF490	Body	1.2283483	1.7767446	5.7781534	5.09E-08	2.75E-06	8.0980698
cg22480875	chr12	77417567	+	E2F7	3'UTR	0.827643	1.6555215	5.7779824	5.09E-08	2.76E-06	8.0972912
cg20454888	chr18	44139159	+	LOXHD1;LOXHD1;LOXHD1	5'UTR;1stExon;Body	-1.453955	0.4955724	-5.777749	5.10E-08	2.76E-06	8.0962294
cg01650904	chr20	37354889	+	SLC32A1	Body	1.3087628	-1.413759	5.7776742	5.10E-08	2.76E-06	8.0958884
cg04921646	chr1	2851502	-			-0.890248	-0.677611	-5.777662	5.10E-08	2.76E-06	8.0958321
cg05723989	chr1	210407677	+	C1orf133;SERTAD4	TSS1500;5'UTR	-0.919884	-2.846947	-5.780278	5.10E-08	2.76E-06	8.0971311
cg02987523	chr13	103978230	-			-1.596566	0.504653	-5.777188	5.11E-08	2.76E-06	8.0936753
cg08697689	chr13	84457340	-	SLITRK1	TSS1500	1.2966322	0.281741	5.7771918	5.11E-08	2.76E-06	8.0936924
cg08091192	chr3	147114986	-	ZIC4;ZIC4;ZIC4	Body;Body;Body	1.5569695	-1.321725	5.7770429	5.12E-08	2.76E-06	8.0930147
cg00434573	chr19	2041960	+	MKNK2;MKNK2	Body;Body	-1.259843	-1.069722	-5.77696	5.12E-08	2.77E-06	8.0926376
cg09142313	chr5	72677859	-			2.024573	-0.580664	5.7767225	5.13E-08	2.77E-06	8.0915568
cg11162104	chr1	204677873	-			-1.224989	-0.838012	-5.776702	5.13E-08	2.77E-06	8.0914648
cg11728821	chr11	58034405	-	OR10W1;OR10W1	3'UTR;1stExon	-1.448412	-0.477355	-5.776741	5.12E-08	2.77E-06	8.0916388
cg13592064	chr6	100915634	+			1.3194065	0.0200972	5.7767363	5.13E-08	2.77E-06	8.0916193
cg23640411	chr10	102433458	-			-1.297059	0.180907	-5.776606	5.13E-08	2.77E-06	8.0910259

cg01611679	chr7	99051465	-	CPSF4;CPSF4	Body;Body	0.554195	0.9248033	5.7764139	5.13E-08	2.77E-06	8.0901521
cg02947418	chr16	59789071	+	LOC644649	Body	-1.561149	1.2169393	-5.776344	5.13E-08	2.77E-06	8.0898338
cg13240116	chr7	27147513	+	HOXA3;HOXA3;HOXA3	3'UTR;3'UTR;3'UTR	0.971988	0.6693681	5.7763632	5.13E-08	2.77E-06	8.0899214
cg14283447	chr21	43735412	+	TFF3	1stExon	-1.15538	-0.38504	-5.776284	5.14E-08	2.77E-06	8.089559
cg25102449	chr19	18761273	-	KLHL26	Body	0.9797912	-0.105069	5.7760049	5.14E-08	2.77E-06	8.0882906
cg03971454	chr1	43770557	+	TIE1	Body	1.5081321	1.8811596	5.7759707	5.14E-08	2.77E-06	8.0881347
cg12637676	chr8	11587161	+	GATA4	Body	-1.377508	-0.299178	-5.775832	5.15E-08	2.78E-06	8.087502
cg00412851	chr6	15463343	+	JARID2	Body	1.2057882	0.9993311	5.7782323	5.15E-08	2.78E-06	8.0878317
cg04161526	chr6	31696519	+	DDAH2	Body	0.9330516	-0.378984	5.7755645	5.15E-08	2.78E-06	8.0862862
cg08032924	chr16	66613096	-	CMTM2	TSS1500	1.7682214	-0.230153	5.7754308	5.16E-08	2.78E-06	8.0856777
cg15987088	chr3	172166234	-	GHSR;GHSR	TSS200;TSS200	1.9647506	-2.244095	5.7754315	5.16E-08	2.78E-06	8.0856812
cg04811540	chr7	1351903	+			-1.124657	-0.02392	-5.775401	5.16E-08	2.78E-06	8.08554
cg12719622	chr10	134694878	-			-1.307115	0.8218791	-5.774996	5.17E-08	2.79E-06	8.0837009
cg09848445	chr10	43601862	-	RET;RET	Body;Body	-2.138961	3.4037911	-5.785654	5.17E-08	2.79E-06	8.0881223
cg07072463	chr5	180047184	-	FLT4;FLT4	Body;Body	-1.463582	0.533514	-5.774698	5.17E-08	2.79E-06	8.0823447
cg22256354	chr8	131554148	-			-1.358772	-1.392085	-5.774454	5.18E-08	2.79E-06	8.0812345
cg26738437	chr17	34308465	+	CCL16;CCL16	1stExon;5'UTR	-1.106711	0.2361185	-5.774165	5.19E-08	2.79E-06	8.0799188
cg21235508	chr9	128067284	+	GAPVD1	Body	0.6433252	2.3458283	5.774101	5.19E-08	2.80E-06	8.0796265
cg02908900	chr7	15726821	+	MEOX2	TSS1500	2.1341765	-1.462674	5.7736689	5.20E-08	2.80E-06	8.0776603
cg10745499	chr6	100906685	+	SIM1	Body	1.289735	-0.110448	5.7735358	5.20E-08	2.80E-06	8.077055

cg02567289	chr1	247805255	+			-1.82818	0.4474775	-5.773504	5.20E-08	2.80E-06	8.0769097
cg25651783	chr1	155265033	+	PKLR;PKLR	Body;Body	1.5015663	-1.325638	5.773461	5.21E-08	2.80E-06	8.0767143
cg01166925	chr4	41880280	-			1.1067371	0.5844542	5.7732783	5.21E-08	2.80E-06	8.075883
cg05938001	chr2	172952948	+	DLX1;DLX1	Body;3'UTR	1.3571061	-0.446049	5.7731166	5.21E-08	2.81E-06	8.0751473
cg26271876	chr2	54754922	-	RPL23AP32;SPTBN1	TSS1500;Body	0.8932797	1.9633673	5.7731361	5.21E-08	2.81E-06	8.0752361
cg06585708	chr5	3602413	-			0.9873599	-0.466063	5.772819	5.22E-08	2.81E-06	8.0737936
cg04136490	chr14	21053613	-	RNASE11	5'UTR	-1.692668	0.2400639	-5.772755	5.22E-08	2.81E-06	8.0735016
cg00654888	chr2	119603133	+	EN1	Body	0.9212736	-0.456306	5.7723881	5.23E-08	2.81E-06	8.071833
cg17448336	chr3	147141588	-			1.1041104	0.3217403	5.7723824	5.23E-08	2.81E-06	8.0718074
cg02066813	chr7	57247707	+	LOC642006	Body	-2.551804	2.1093049	-5.772332	5.23E-08	2.81E-06	8.0715762
cg17288397	chr5	72715731	+			2.4764585	-1.854772	5.7722199	5.24E-08	2.82E-06	8.0710677
cg04608651	chr17	40557508	+	PTRF	Body	1.1178095	3.265691	5.7716426	5.25E-08	2.82E-06	8.0684416
cg22031964	chr11	62677251	-	CHRM1	Body	2.1220056	2.5564172	5.7715934	5.25E-08	2.82E-06	8.0682176
cg17454920	chr14	74185993	+	C14orf43;C14orf43	3'UTR;3'UTR	0.7548388	2.364859	5.7714705	5.25E-08	2.82E-06	8.0676586
cg26748578	chr2	65217211	+	SLC1A4;SLC1A4	1stExon;5'UTR	-1.474826	-1.718619	-5.771473	5.25E-08	2.82E-06	8.0676709
cg16594273	chr16	58131312	-			-1.566745	-2.014393	-5.771442	5.26E-08	2.82E-06	8.0675292
cg20448594	chr7	73940352	-	GTF2IRD1;GTF2IRD1	Body;Body	-1.468581	-0.555757	-5.771278	5.26E-08	2.83E-06	8.0667817
cg14269525	chr14	98214031	-			-1.47339	-0.103594	-5.771047	5.27E-08	2.83E-06	8.065734
cg25377796	chr4	8643957	-			-1.8316	0.8722094	-5.770997	5.27E-08	2.83E-06	8.0655069
cg03603062	chr7	95224434	-	PDK4	Body	1.1324718	2.008585	5.770848	5.27E-08	2.83E-06	8.0648272

cg06540636	chr19	44174071	+	PLAUR;PLAUR;PLAUR	Body;Body;Body	-0.840536	-3.324178	-5.770799	5.27E-08	2.83E-06	8.0646022
cg19659741	chr8	39172099	+	ADAM5P	TSS200	1.5628982	0.6564794	5.7759164	5.28E-08	2.83E-06	8.0659401
cg00860322	chr5	54528989	-	CCNO	1stExon	-0.396869	-3.622924	-5.770451	5.28E-08	2.84E-06	8.063022
cg03461430	chr8	143201610	-			-1.076588	-0.906351	-5.770339	5.28E-08	2.84E-06	8.0625122
cg08316234	chr17	51899386	+	KIF2B	TSS1500	-1.572446	0.7272692	-5.770047	5.29E-08	2.84E-06	8.0611851
cg27143703	chr17	77961719	+	TBC1D16	Body	-1.903295	1.0342393	-5.769959	5.29E-08	2.84E-06	8.0607851
cg11452087	chr1	2918752	+			-1.273482	0.1739498	-5.769625	5.30E-08	2.85E-06	8.0592665
cg21546821	chr2	10975271	-			-1.143437	0.0448518	-5.76929	5.31E-08	2.85E-06	8.0577398
cg05682581	chr8	929958	+			-1.0859	-1.632393	-5.769253	5.31E-08	2.85E-06	8.0575702
cg17021798	chr7	226444	-	FAM20C	Body	-1.234339	0.6852691	-5.77184	5.31E-08	2.85E-06	8.0587863
cg16266494	chr2	238499782	-	RAB17	TSS200	-1.04404	-1.013447	-5.769153	5.31E-08	2.85E-06	8.057117
cg03048654	chr10	118893138	-	VAX1;VAX1	3'UTR;Body	2.5676427	-0.899781	5.7689186	5.32E-08	2.85E-06	8.0560516
cg13204568	chr1	12679255	+	DHRS3	TSS1500	-1.185903	-4.774057	-5.774061	5.32E-08	2.85E-06	8.0578427
cg19868631	chr7	54609776	+	VSTM2A	TSS1500	3.0654316	-2.026662	5.7740602	5.32E-08	2.85E-06	8.0727303
cg23409075	chr8	91013007	+	DECR1	TSS1500	-0.578762	-3.715876	-5.771262	5.33E-08	2.86E-06	8.055839
cg07192594	chr16	71221950	-	HYDIN;HYDIN	5'UTR;5'UTR	-1.339693	0.248539	-5.768551	5.33E-08	2.86E-06	8.0543781
cg16401207	chr10	53182118	+	PRKG1;PRKG1	Body;Body	-1.153068	-0.575201	-5.768518	5.33E-08	2.86E-06	8.0542296
cg01759597	chr7	96626788	+	DLX6AS	Body	1.810706	-1.565824	5.7736478	5.33E-08	2.86E-06	8.0563008
cg05727149	chr16	75375801	-	CFDP1	Body	1.0646923	1.5358558	5.7682901	5.33E-08	2.86E-06	8.0531934
cg04564515	chr21	46445691	+			-1.34866	0.4813971	-5.768022	5.34E-08	2.86E-06	8.0519755

cg18154567	chr15	51464013	+			1.1765401	0.5094004	5.7679691	5.34E-08	2.86E-06	8.0517338
cg20663219	chr7	73130521	-	STX1A;STX1A	Body;Body	-1.340335	0.3380512	-5.767744	5.35E-08	2.87E-06	8.0507097
cg04370314	chr13	100643312	+			2.2871258	-1.698273	5.7755237	5.35E-08	2.87E-06	8.0532899
cg22441310	chr2	63277222	+	OTX1	TSS1500	-0.745202	-3.650973	-5.767416	5.36E-08	2.87E-06	8.0492173
cg25036357	chr8	84606468	+			-2.435624	2.2996864	-5.767357	5.36E-08	2.87E-06	8.04895
cg00135497	chr4	155661849	-			1.2714168	1.6257676	5.7699138	5.36E-08	2.87E-06	8.0497155
cg17165841	chr2	207507001	-	LOC200726	TSS200	1.474276	-1.156054	5.7669666	5.37E-08	2.87E-06	8.0471751
cg14630993	chr9	7274452	-			-1.0962	-0.333692	-5.766249	5.39E-08	2.88E-06	8.0439108
cg15414028	chr10	14644188	-	FAM107B	Body	-1.297505	-2.150087	-5.76607	5.39E-08	2.89E-06	8.0430994
cg16654801	chr10	119301763	-	EMX2;EMX2OS;EMX2	TSS200;Body;TSS200	2.005971	-1.484353	5.7661002	5.39E-08	2.89E-06	8.0432361
cg26378984	chr11	116374243	+			-1.559161	0.0586014	-5.766093	5.39E-08	2.89E-06	8.0432023
cg05043349	chr8	65290113	-			1.8978284	-1.48289	5.7659126	5.40E-08	2.89E-06	8.042383
cg12819548	chr11	120256570	+	ARHGEF12	Body	1.0120434	1.733373	5.7658242	5.40E-08	2.89E-06	8.0419811
cg05331807	chr7	157224035	+			-1.837632	1.7096657	-5.768286	5.40E-08	2.89E-06	8.0423222
cg17128799	chr1	2458466	-	PANK4	TSS1500	-1.178452	-0.773899	-5.76512	5.42E-08	2.90E-06	8.0387785
cg11052202	chr6	99290546	-			1.6809187	-0.771775	5.7676561	5.42E-08	2.90E-06	8.0397869
cg09175734	chr5	169510081	-	DOCK2	3'UTR	-1.720687	1.019315	-5.767623	5.42E-08	2.90E-06	8.0396349
cg18816826	chr1	237168540	+			-1.182888	-0.682429	-5.764588	5.43E-08	2.90E-06	8.0363603
cg27088150	chr3	62353894	-			1.528678	-1.551111	5.7645097	5.43E-08	2.90E-06	8.0360053
cg25382573	chr12	93096844	-	C12orf74;C12orf74	5'UTR;1stExon	-1.410968	-0.075668	-5.766841	5.44E-08	2.91E-06	8.0357633

cg20547131	chr14	101531510	+	MIR412;MIR409;MIR410;MIR369	TSS1500;TSS200;TSS1500;TSS1500	-0.943868	-0.872574	-5.764083	5.44E-08	2.91E-06	8.0340634
cg03964696	chr2	74734085	-	PCGF1	Body	-1.167983	-0.70727	-5.764052	5.44E-08	2.91E-06	8.0339242
cg27006721	chr10	49760566	+	ARHGAP22	Body	-0.888167	-1.155482	-5.763299	5.46E-08	2.92E-06	8.0305041
cg12129480	chr1	158549410	-	OR10X1	1stExon	-1.822745	0.0180656	-5.763271	5.46E-08	2.92E-06	8.0303746
cg01075271	chr14	38080314	-			1.6713895	-0.732376	5.7630534	5.47E-08	2.92E-06	8.0293859
cg11870309	chr3	12927951	-			-1.056119	0.7313089	-5.763047	5.47E-08	2.92E-06	8.0293549
cg03538383	chr12	110399167	+	GIT2;GIT2;GIT2;GIT2;GIT2;GIT2	Body;Body;Body;Body;Body;Body	1.1851561	1.4170921	5.7630168	5.47E-08	2.92E-06	8.0292196
cg05691168	chr2	216176809	+	ATIC;ATIC	1stExon;5'UTR	-0.725715	-4.959671	-5.762963	5.47E-08	2.92E-06	8.0289738
cg18168672	chr20	61905223	+	ARFGAP1;ARFGAP1	5'UTR;5'UTR	-0.704181	-1.340895	-5.762951	5.47E-08	2.92E-06	8.028919
cg22352078	chr8	41424982	-			1.4569881	-0.355099	5.7627952	5.48E-08	2.92E-06	8.0282121
cg02947214	chr14	105998115	-			-1.227526	0.9464548	-5.762748	5.48E-08	2.92E-06	8.0279973
cg00410719	chr16	56621065	+			-1.334409	0.6124217	-5.762583	5.48E-08	2.93E-06	8.0272471
cg01136167	chr7	65037704	-			1.2466903	-0.780754	5.7625788	5.48E-08	2.93E-06	8.0272289
cg05978707	chr2	1204376	-	SNTG2	Body	-1.485873	0.1839407	-5.762544	5.48E-08	2.93E-06	8.0270701
cg23295754	chr20	1639250	-	SIRPG;SIRPG;SIRPG	TSS1500;TSS1500;TSS1500	-1.558682	0.024504	-5.762527	5.48E-08	2.93E-06	8.0269927
cg15575017	chr2	237010430	+	AGAP1;AGAP1	Body;Body	0.8261735	2.2598713	5.762375	5.49E-08	2.93E-06	8.0263024
cg02497486	chr13	30085687	+	SLC7A1	3'UTR	0.6784256	2.2067819	5.7622828	5.49E-08	2.93E-06	8.0258837
cg13891220	chr4	111549953	+	PITX2;PITX2	Body;Body	1.2060635	-0.331731	5.7622854	5.49E-08	2.93E-06	8.0258951
cg09437994	chr16	89035147	+	CBFA2T3	Body	-1.936378	2.2375956	-5.761982	5.50E-08	2.93E-06	8.0245177
cg02869444	chr10	110998760	+			-1.592371	0.3991952	-5.761929	5.50E-08	2.93E-06	8.0242748

cg04948438	chr10	99301906	+	UBTD1	Body	-1.198991	0.1591982	-5.761948	5.50E-08	2.93E-06	8.0243621
cg04789818	chr16	49005567	-			-1.326367	0.2899739	-5.761616	5.51E-08	2.94E-06	8.0228533
cg18126060	chr12	4923430	-	KCNA6;KCNA6	3'UTR;1stExon	-1.558406	0.1819798	-5.761571	5.51E-08	2.94E-06	8.0226478
cg21332500	chr7	27233480	+			1.2684646	0.4836905	5.7615298	5.51E-08	2.94E-06	8.0224614
cg13215593	chr2	558823	-			-1.277046	0.089486	-5.761181	5.52E-08	2.94E-06	8.0208747
cg19640007	chr4	146600771	-	C4orf51	TSS1500	-1.2039	-1.372482	-5.761051	5.52E-08	2.94E-06	8.0202858
cg23125492	chr11	31821298	+	PAX6;PAX6;PAX6	Body;Body;Body	1.4025073	-0.309897	5.7609483	5.52E-08	2.94E-06	8.0198188
cg25647583	chr15	91427184	+	FES;FES;FES;FES	TSS1500;TSS1500;TSS1500;TSS1500	1.0030314	-0.28367	5.7605664	5.53E-08	2.95E-06	8.0180835
cg23098371	chr1	92012061	+			-1.469225	1.2420329	-5.760289	5.54E-08	2.95E-06	8.0168241
cg01215442	chr19	18980443	+	GDF1;LASS1	Body;3'UTR	0.9356417	-0.541942	5.7601297	5.55E-08	2.95E-06	8.0160992
cg10783469	chr19	52391234	+	ZNF577;ZNF577;ZNF577	TSS200;TSS200;TSS200	1.5967845	0.0973149	5.7653986	5.54E-08	2.95E-06	8.0185641
cg22830507	chr12	115095025	+			-1.181032	-0.398653	-5.759661	5.56E-08	2.96E-06	8.0139692
cg11081307	chr20	57770619	-	ZNF831	Body	-1.873626	0.7060392	-5.759633	5.56E-08	2.96E-06	8.013843
cg05070250	chr11	36620199	+	C11orf74;RAG2	5'UTR;TSS1500	-1.549062	0.5795647	-5.759205	5.57E-08	2.96E-06	8.0118999
cg23091159	chr4	174422043	+			0.7464933	0.9691228	5.7617296	5.57E-08	2.97E-06	8.0125626
cg13897222	chr1	221449786	-			-1.67741	0.5297719	-5.759063	5.57E-08	2.97E-06	8.0112542
cg02331198	chr6	106988121	+	AIM1	Body	0.965659	1.1084471	5.7589846	5.58E-08	2.97E-06	8.0108966
cg16052237	chr21	48015365	+			-1.383324	0.6745513	-5.758616	5.59E-08	2.97E-06	8.0092229
cg12488585	chr12	132434050	+	EP400	TSS1500	-1.154737	-0.167792	-5.758548	5.59E-08	2.97E-06	8.0089136
cg02623400	chr1	50513749	+	ELAVL4;ELAVL4	1stExon;5'UTR	1.4846577	-0.49363	5.7584077	5.59E-08	2.97E-06	8.0082757

cg16346321	chr1	10709185	+	CASZ1;CASZ1	Body;Body	0.6606345	1.761881	5.7584229	5.59E-08	2.97E-06	8.0083447
cg04132540	chr8	56851846	-	LYN;LYN	5'UTR;5'UTR	-1.118844	-1.840372	-5.75828	5.59E-08	2.97E-06	8.0076955
cg05999364	chr8	3269952	+	CSMD1	Body	-1.241243	-1.248183	-5.758145	5.60E-08	2.98E-06	8.0070817
cg02005873	chr6	10385839	-			1.5927394	-0.218119	5.7580333	5.60E-08	2.98E-06	8.0065748
cg03546235	chr10	102590290	+			1.1801809	-1.393947	5.7580221	5.60E-08	2.98E-06	8.006524
cg11819702	chr5	73529573	+			-1.313728	0.6060424	-5.7576	5.61E-08	2.98E-06	8.0046083
cg13249591	chr1	151966892	+	S100A10	TSS200	-1.06636	-3.15067	-5.757299	5.62E-08	2.99E-06	8.0032374
cg16844941	chr8	11555178	+			1.5782808	-1.627184	5.7568986	5.63E-08	2.99E-06	8.0014207
cg03102841	chr11	85646010	-			0.8539825	0.5608567	5.7565018	5.64E-08	3.00E-06	7.9996182
cg04044203	chr11	124631218	+	ESAM	Body	1.3969102	0.7321773	5.7564803	5.64E-08	3.00E-06	7.9995208
cg14861089	chr10	102895043	-	TLX1	Body	2.6476875	-0.866184	5.7563018	5.65E-08	3.00E-06	7.9987098
cg10085057	chr16	31235824	-	TRIM72	Body	0.8169935	0.0107756	5.7561784	5.65E-08	3.00E-06	7.9981493
cg03327403	chr12	53298911	+	KRT8	TSS200	-1.22822	-0.189726	-5.756011	5.65E-08	3.00E-06	7.9973877
cg09074113	chr2	20870087	+	GDF7	Body	1.0516815	1.2033188	5.7560136	5.65E-08	3.00E-06	7.9974008
cg11567288	chr6	26758166	-			1.2167687	-0.923065	5.7560948	5.65E-08	3.00E-06	7.9977696
cg17360781	chr2	200329486	-			1.1068201	-1.209297	5.7560535	5.65E-08	3.00E-06	7.997582
cg21699330	chr7	26193032	-	NFE2L3	Body	-1.538322	-1.791479	-5.756065	5.65E-08	3.00E-06	7.9976335
cg07978738	chr10	116298339	-	ABLIM1;ABLIM1;ABLIM1	Body;Body;Body	-1.131597	-0.772943	-5.755804	5.66E-08	3.00E-06	7.9964475
cg25756066	chr1	85931083	+	DDAH1;DDAH1	TSS200;5'UTR	-1.122719	-4.078772	-5.755782	5.66E-08	3.00E-06	7.9963509
cg07147166	chr4	147569185	+			2.0174352	-1.578565	5.7556166	5.67E-08	3.01E-06	7.9955979

cg10862620	chr17	37749866	-			-1.575233	-0.171124	-5.755262	5.67E-08	3.01E-06	7.9939869
cg22682646	chr12	114216448	+			-1.390695	0.1520891	-5.755264	5.67E-08	3.01E-06	7.9939976
cg03162314	chr19	51600935	-	CTU1	3'UTR	-0.660001	-1.506876	-5.755232	5.68E-08	3.01E-06	7.9938515
cg02809028	chr2	495143	-			-1.051434	-0.262084	-5.755139	5.68E-08	3.01E-06	7.9934302
cg09643136	chr2	223176683	-			1.2466549	0.1221453	5.755091	5.68E-08	3.01E-06	7.9932108
cg14002960	chr18	31739423	-	NOL4	Body	1.9816449	-0.855315	5.7550765	5.68E-08	3.01E-06	7.9931448
cg01973483	chr12	123591123	+	PITPNM2	5'UTR	0.8786201	1.4219999	5.7549595	5.68E-08	3.01E-06	7.9926136
cg04676482	chr4	111536216	+			1.0807064	-0.157979	5.7549338	5.68E-08	3.01E-06	7.9924972
cg10395685	chr11	86085715	-	CCDC81;CCDC81	TSS200;TSS200	1.7969442	-1.413936	5.7549413	5.68E-08	3.01E-06	7.992531
cg22876908	chr12	56325149	+	DGKA;DGKA;DGKA;DGKA	TSS1500;TSS1500;TSS1500;5'UTR	-0.728957	-1.598166	-5.76009	5.69E-08	3.01E-06	7.994512
cg26069973	chr7	80080213	+			-1.337446	-1.126258	-5.75483	5.69E-08	3.01E-06	7.9920272
cg08292467	chr5	140782510	-	PCDHGA4;PCDHGA6;PCDHGA9;PCDHGA1;PCDHGA8;PCDHGA5;PCDHGB1;PCDHGB4;PCDHGA3;PCDHGA9;PCDHGA2;PCDHGA7;PCDHGB2;PCDHGB5;PCDHGB3	Body;Body;TSS200;Body;Body;Body;Body;Body;Body;TSS200;Body;Body;Body;Body;Body	1.1261086	0.0813109	5.7547944	5.69E-08	3.01E-06	7.9918639
cg05016408	chr5	150326174	-	LOC134466	TSS200	1.9029686	-0.027264	5.7573619	5.69E-08	3.01E-06	7.993073
cg26991761	chr11	67071064	+	SSH3;SSH3	5'UTR;1stExon	1.1162641	-1.846691	5.7571876	5.69E-08	3.02E-06	7.9919576
cg26879813	chr5	180541897	+			1.0424818	-1.102762	5.7543123	5.70E-08	3.02E-06	7.9896748
cg00876272	chr14	103265614	-	TRAF3;TRAF3;TRAF3	5'UTR;5'UTR;5'UTR	-1.02018	1.9559112	-5.754144	5.70E-08	3.02E-06	7.9889098
cg05184519	chr11	132080258	+	NTM;NTM;NTM;NTM	Body;Body;Body;Body	-1.256274	-0.690644	-5.7541	5.71E-08	3.02E-06	7.9887118
cg06357807	chr6	135503203	+	MYB;MYB;MYB;MYB;MYB;MYB;MYB;MYB	Body;Body;Body;Body;Body;Body;Body;Body	-0.488314	-4.26167	-5.753871	5.71E-08	3.02E-06	7.9876693
cg10679156	chr6	27228101	-			1.2755798	-0.198566	5.7538589	5.71E-08	3.02E-06	7.9876159
cg12371587	chr22	42093157	+	C22orf46	3'UTR	-0.641531	-3.340181	-5.753904	5.71E-08	3.02E-06	7.9878194

cg02878382	chr14	104665702	-			-1.3725	0.2726442	-5.753717	5.72E-08	3.03E-06	7.986972
cg24038105	chr1	15679299	-	FHAD1	Body	-0.862201	-0.944244	-5.753618	5.72E-08	3.03E-06	7.9865209
cg10888244	chr1	161049543	+	PVRL4	Body	0.9299432	1.7034069	5.7535848	5.72E-08	3.03E-06	7.9863711
cg18674828	chr12	132839436	+	GALNT9	Body	-1.229742	0.1314437	-5.753131	5.73E-08	3.03E-06	7.9843125
cg10753636	chr19	54193013	+	MIR520A	TSS1500	-1.63265	0.377603	-5.753085	5.73E-08	3.03E-06	7.9841003
cg10502244	chr12	48399295	-	COL2A1;COL2A1	TSS1500;TSS1500	0.9572452	1.2723201	5.7527039	5.74E-08	3.04E-06	7.9823716
cg12364786	chr15	64056664	+	HERC1	Body	1.5782362	1.0874375	5.7523447	5.75E-08	3.04E-06	7.9807408
cg22016779	chr2	230452311	-	DNER	Body	-1.066737	-2.380996	-5.752052	5.76E-08	3.05E-06	7.979411
cg14940165	chr2	177021702	+			1.662419	-1.297482	5.7511826	5.79E-08	3.06E-06	7.9754648
cg06021880	chr12	50105418	+			-1.184235	-0.316956	-5.750998	5.79E-08	3.06E-06	7.974627
cg25355213	chr17	73780246	-	UNK	TSS1500	-1.222398	-1.654504	-5.750997	5.79E-08	3.06E-06	7.9746226
cg08115553	chr17	13215554	+			-1.495888	0.18437	-5.750897	5.79E-08	3.06E-06	7.9741697
cg00850552	chr21	46236351	-	SUMO3	Body	0.8924994	1.579392	5.7508395	5.79E-08	3.06E-06	7.9739076
cg06564232	chr6	50810993	+	TFAP2B	Body	1.8171885	-0.505428	5.7508007	5.80E-08	3.06E-06	7.9737315
cg24127989	chr7	128049508	-	IMPDH1;IMPDH1;IMPDH1;IMPDH1	Body;Body;Body;Body	-1.069557	-3.172143	-5.750432	5.81E-08	3.07E-06	7.9720573
cg26815032	chr6	55956315	-	COL21A1	Body	-1.337506	-0.831873	-5.750398	5.81E-08	3.07E-06	7.9719017
cg12308369	chr15	73193612	+			-1.10121	-0.034938	-5.750345	5.81E-08	3.07E-06	7.971664
cg10682566	chr5	127033878	-			-1.39523	0.4938186	-5.750294	5.81E-08	3.07E-06	7.9714324
cg12294678	chr7	150686728	+	NOS3	TSS1500	0.8339977	1.7879147	5.7501553	5.81E-08	3.07E-06	7.970802
cg08646805	chr7	40338163	+	C7orf10	Body	-1.912629	0.8847801	-5.749871	5.82E-08	3.07E-06	7.9695112

cg18422371	chr11	36593664	+	RAG1	5'UTR	-1.487403	-0.322033	-5.749825	5.82E-08	3.07E-06	7.9693043
cg21636683	chr5	140683699	+	SLC25A2	TSS200	0.8363472	1.0999399	5.7497998	5.82E-08	3.07E-06	7.9691882
cg17060964	chr11	55796572	+	OR5AS1	TSS1500	-1.26596	-1.035188	-5.749761	5.82E-08	3.07E-06	7.9690143
cg00623111	chr16	1559692	-	TELO2	Body	0.5477812	2.0368704	5.7495323	5.83E-08	3.08E-06	7.9679738
cg27235829	chr1	158390759	-	OR10K2	TSS200	-1.826037	0.8066485	-5.749452	5.83E-08	3.08E-06	7.9676096
cg16736889	chr16	20421627	+	ACSM5	5'UTR	-1.480356	-0.401009	-5.749397	5.83E-08	3.08E-06	7.9673615
cg07207982	chr6	34984930	-	ANKS1A	Body	1.6184099	0.7204456	5.749369	5.84E-08	3.08E-06	7.967233
cg07936456	chr16	7708060	+	A2BP1;A2BP1;A2BP1;A2BP1;A2BP1;A2BP1	Body;Body;Body;Body;Body;Body	-1.654051	0.9400594	-5.749216	5.84E-08	3.08E-06	7.9665387
cg17818471	chr2	177012640	-			1.3647653	-0.552451	5.7492257	5.84E-08	3.08E-06	7.9665827
cg20744163	chr10	80999841	+	ZMIZ1	Body	2.284904	2.1163714	5.7490202	5.84E-08	3.08E-06	7.96565
cg27382409	chr10	47088062	-	PPYR1	3'UTR	-0.897147	-0.889766	-5.749031	5.84E-08	3.08E-06	7.9656972
cg01843946	chr14	37124434	+			1.4478741	-0.126989	5.7489129	5.85E-08	3.08E-06	7.9651628
cg17074821	chr11	75046354	-	ARRB1;MIR326;ARRB1	Body;TSS200;Body	0.9353717	1.8803279	5.7489265	5.85E-08	3.08E-06	7.9652247
cg18543971	chr15	86686938	+	AGBL1	5'UTR	-1.647906	0.8759432	-5.748876	5.85E-08	3.08E-06	7.9649937
cg13581866	chr3	11138138	+			-0.783404	-2.503521	-5.748775	5.85E-08	3.08E-06	7.9645349
cg00157228	chr9	138682403	+	KCNT1	Body	-1.337311	0.5909098	-5.748663	5.85E-08	3.08E-06	7.9640274
cg14605874	chr6	100915395	+			1.450376	-0.563739	5.7512778	5.85E-08	3.08E-06	7.9654869
cg15399466	chr5	50696059	+			0.9634545	-0.67497	5.7620834	5.85E-08	3.08E-06	7.9850559
cg16616467	chr16	89642914	-	CPNE7;CPNE7	Body;Body	1.4246669	2.5919033	5.7486543	5.86E-08	3.08E-06	7.9639892
cg20282814	chr1	43423072	+	SLC2A1	Body	-1.854365	0.1499177	-5.748529	5.86E-08	3.09E-06	7.9634207

cg03900492	chr7	157345659	-	PTPRN2;PTPRN2;PTPRN2	Body;Body;Body	-1.517305	-0.569582	-5.748474	5.86E-08	3.09E-06	7.9631702
cg05717833	chr15	69706383	-	KIF23;KIF23	TSS1500;TSS1500	-0.389973	-3.96129	-5.74832	5.86E-08	3.09E-06	7.9624713
cg03443455	chr2	171671795	+	GAD1;GAD1	TSS1500;TSS1500	1.1643328	-0.337506	5.7482866	5.87E-08	3.09E-06	7.9623204
cg03422651	chr16	2542324	+	TBC1D24	5'UTR	0.6911032	2.0715417	5.7481388	5.87E-08	3.09E-06	7.9616499
cg08040836	chr12	53273301	+			-0.967394	-1.54265	-5.748146	5.87E-08	3.09E-06	7.9616833
cg14469928	chr2	125733618	+			-1.727164	0.3386037	-5.748086	5.87E-08	3.09E-06	7.9614078
cg09147985	chr11	40314618	-	LRRC4C;LRRC4C	5'UTR;1stExon	0.9826931	-0.381905	5.7506249	5.87E-08	3.09E-06	7.9622015
cg03843000	chr12	114846968	-	TBX5;TBX5	TSS1500;TSS1500	1.8248871	-1.611093	5.7475869	5.88E-08	3.10E-06	7.9591451
cg13409449	chr10	8097354	-	GATA3;GATA3	5'UTR;5'UTR	2.354774	-1.730342	5.7475736	5.89E-08	3.10E-06	7.9590847
cg09682848	chr12	132928795	+			-0.897173	-0.505063	-5.747229	5.89E-08	3.10E-06	7.957523
cg13611006	chr17	45983023	+	SP2	Body	1.0512735	0.2933024	5.7469994	5.90E-08	3.10E-06	7.9564795
cg21695661	chr1	63796263	+			1.5538745	-0.768103	5.7468572	5.91E-08	3.11E-06	7.9558342
cg00250391	chr11	93850000	-			-0.979665	0.0436359	-5.746744	5.91E-08	3.11E-06	7.9553207
cg09076431	chr1	197889163	+	LHX9;LHX9	Body;Body	1.3160402	-1.260172	5.7467476	5.91E-08	3.11E-06	7.9553366
cg25389328	chr17	38222062	+	THRA;THRA	5'UTR;5'UTR	0.9091851	-0.751995	5.7467172	5.91E-08	3.11E-06	7.9551986
cg18462653	chr20	29978176	+	DEFB119;DEFB119;DEFB119	Body;Body;Body	-1.183769	-0.443562	-5.746591	5.91E-08	3.11E-06	7.9546245
cg01900413	chr11	128419356	+	ETS1	Body	1.3801622	-0.461449	5.7465177	5.91E-08	3.11E-06	7.9542936
cg14263518	chr9	134249904	-			-1.720646	1.8113617	-5.748935	5.92E-08	3.11E-06	7.9697072
cg01054478	chr5	180100963	-			1.9920348	-1.459343	5.7462819	5.92E-08	3.11E-06	7.9532239
cg11391335	chr1	119521928	-	TBX15	5'UTR	1.3496347	0.1600772	5.7462715	5.92E-08	3.11E-06	7.9531767

cg03325522	chr8	30859349	+	PURG	Body	-1.467503	0.6814455	-5.746191	5.92E-08	3.11E-06	7.9528122
cg12280113	chr19	56250127	-	NLRP9	TSS1500	-1.224442	-0.348608	-5.746091	5.93E-08	3.11E-06	7.9523565
cg21195843	chr20	37077836	-	SNORA60;SNHG11	TSS200;Body	0.9110525	1.4781736	5.7460439	5.93E-08	3.11E-06	7.9521441
cg07153966	chr7	27147590	-	HOXA3;HOXA3;HOXA3	Body;Body;Body	1.5436016	0.6956412	5.745916	5.93E-08	3.12E-06	7.9515634
cg03975815	chr1	240270565	+	FMN2	Body	-1.169787	-1.132015	-5.745827	5.93E-08	3.12E-06	7.9511592
cg10965508	chr6	43231063	+	TTBK1	Body	-0.740901	-1.306775	-5.745837	5.93E-08	3.12E-06	7.9512045
cg26809868	chr16	2886233	+			-0.895361	-2.314478	-5.745812	5.93E-08	3.12E-06	7.9510897
cg20904010	chr22	33402393	-	SYN3;SYN3;SYN3	1stExon;Body;1stExon	-1.463061	0.7709611	-5.745725	5.94E-08	3.12E-06	7.950698
cg22387174	chr7	155302770	-	CNPY1	5'UTR	1.7301508	-1.948337	5.745728	5.94E-08	3.12E-06	7.9507105
cg02930348	chr20	59968939	-	CDH4	Body	-1.438274	-0.189751	-5.745617	5.94E-08	3.12E-06	7.9502056
cg15095917	chr17	7482694	+	CD68;CD68	TSS200;TSS200	-1.298938	-1.843436	-5.745589	5.94E-08	3.12E-06	7.9500819
cg00203347	chr12	108603943	-	WSCD2	Body	-2.884199	2.7879536	-5.745407	5.95E-08	3.12E-06	7.9492557
cg05329352	chr10	112838983	-	ADRA2A	1stExon	0.779425	-0.398101	5.7453187	5.95E-08	3.12E-06	7.9488535
cg08044097	chr8	55371013	-	SOX17	Body	1.5327764	-0.708038	5.7452708	5.95E-08	3.12E-06	7.9486362
cg23224042	chr8	145936442	+			-1.036803	-0.529669	-5.74515	5.95E-08	3.12E-06	7.9480895
cg14153494	chr17	56162028	-	DYNLL2	5'UTR	-0.658363	-3.57178	-5.745072	5.96E-08	3.12E-06	7.9477348
cg10314760	chr10	123353654	+	FGFR2;FGFR2;FGFR2;FGFR2;FGFR2;FGFR2;FGFR2;FGFR2;FGFR2;FGFR2	TSS200;TSS200;5'UTR;5'UTR;1stExon;5'UTR;5'UTR;5'UTR;5'UTR;5'UTR	1.2579202	2.2229879	5.7449018	5.96E-08	3.13E-06	7.9469626
cg25101380	chr2	2605594	+			-1.669098	0.8691472	-5.744494	5.97E-08	3.13E-06	7.9451112
cg02874667	chr19	31206903	-			-1.771394	1.5458493	-5.74418	5.98E-08	3.13E-06	7.9436865
cg25147632	chr10	84027418	-	NRG3;NRG3;NRG3	Body;Body;Body	-1.470453	0.6178292	-5.744175	5.98E-08	3.13E-06	7.9436637

cg23495279	chr8	4849827	+	CSMD1	Body	1.7338898	-0.807443	5.7441319	5.98E-08	3.14E-06	7.9434697
cg08320989	chr4	177575257	-			-1.410782	-1.180859	-5.744022	5.98E-08	3.14E-06	7.9429696
cg00808170	chr5	140807787	+	PCDHGA4;PCDHGA11;PCDHGA11;PCDHGA9;PCDHGA1;PCDHGB1;PCDHGB8P;PCDHGB6;PCDHGB3;PCDHGB7;PCDHGA6;PCDHGA8;PCDHGA10;PCDHGA5;PCDHGB4;PCDHGA3;PCDHGA2	Body;Body;Body;Body;Body;Body;Body;Body;Body;Body;Body;Body;Body;Body;Body;Body;Body;Body;Body	0.7834374	0.2757296	5.7438483	5.99E-08	3.14E-06	7.9421834
cg04256864	chr10	119292300	+	EMX2OS	Body	1.7126771	-2.392394	5.7435073	6.00E-08	3.14E-06	7.9406367
cg14788768	chr5	24170286	-			-2.226856	1.6051795	-5.74352	6.00E-08	3.14E-06	7.9406943
cg10546219	chr17	29336262	-			-1.416129	-2.659619	-5.743474	6.00E-08	3.14E-06	7.9404832
cg01218619	chr4	25090298	-			2.0794119	-0.42288	5.7430116	6.01E-08	3.15E-06	7.9383883
cg04960342	chr16	86870745	-			-1.631422	1.0946992	-5.743042	6.01E-08	3.15E-06	7.9385261
cg15475650	chr10	120938899	-	PRDX3;PRDX3	TSS1500;TSS1500	-0.752264	-3.100046	-5.743026	6.01E-08	3.15E-06	7.9384513
cg26269966	chr1	213646397	-			-1.354028	-0.335114	-5.742882	6.02E-08	3.15E-06	7.9377996
cg13658899	chr9	139094665	-	LHX3;LHX3	Body;Body	1.4876792	-0.82647	5.7428541	6.02E-08	3.15E-06	7.9376736
cg05965387	chr13	51418053	-	DLEU7	TSS200	1.1829589	0.646104	5.7428053	6.02E-08	3.15E-06	7.9374526
cg20671613	chr12	10753798	+	KLRA1	TSS1500	0.7180896	2.6514153	5.7425354	6.03E-08	3.15E-06	7.9362283
cg20332645	chr4	1400869	+			1.296386	-0.775963	5.7423365	6.03E-08	3.16E-06	7.9353262
cg01158680	chr7	79083997	-	MAGI2	TSS1500	1.6477027	-1.170561	5.744804	6.04E-08	3.16E-06	7.9361533
cg13696012	chr20	31595360	-	BPIL1	TSS200	-1.704465	1.0612181	-5.742161	6.04E-08	3.16E-06	7.9345306
cg26435149	chr3	55605611	-	ERC2	3'UTR	-0.86286	-1.141203	-5.742138	6.04E-08	3.16E-06	7.9344242
cg06807029	chr3	78928130	+	ROBO1;ROBO1;ROBO1;ROBO1	Body;Body;Body;Body	-0.92411	-0.609652	-5.741562	6.06E-08	3.17E-06	7.9318123
cg10211193	chr10	44782092	+			-1.255361	-0.825806	-5.741444	6.06E-08	3.17E-06	7.9312785
cg00944163	chr17	11390262	-	SHISA6	Body	-1.277614	0.2707404	-5.741285	6.06E-08	3.17E-06	7.9305576

cg21697512	chr1	208081541	-	CD34;CD34	Body;Body	1.2099782	1.1921728	5.7409038	6.07E-08	3.18E-06	7.9288286
cg17833265	chr17	48042135	+			1.5343222	-0.189513	5.7407091	6.08E-08	3.18E-06	7.9279458
cg18448949	chr2	176993089	-	HOXD8	TSS1500	2.3305498	-1.044652	5.7406033	6.08E-08	3.18E-06	7.9274662
cg25610200	chr7	142636371	+	C7orf34	TSS1500	-1.396823	0.1686517	-5.748455	6.08E-08	3.18E-06	7.9311696
cg10168330	chr8	27262164	+	PTK2B;PTK2B;PTK2B;PTK2B	Body;Body;Body;Body	-1.263378	1.2580254	-5.740541	6.08E-08	3.18E-06	7.9271838
cg26730416	chr11	4233502	+			2.1550753	-0.702295	5.7399643	6.10E-08	3.19E-06	7.9245686
cg07143331	chr22	31322680	-	MORC2	3'UTR	0.7382679	2.1146098	5.7395992	6.11E-08	3.19E-06	7.9229131
cg03183618	chr2	134964228	+			-1.359216	0.7860821	-5.73938	6.12E-08	3.20E-06	7.9219199
cg13784080	chr14	57275413	-	OTX2	5'UTR	1.8822463	-1.271074	5.7394007	6.12E-08	3.20E-06	7.922013
cg26572865	chr8	144213592	+			-1.388711	0.4458918	-5.739162	6.12E-08	3.20E-06	7.9209317
cg02856420	chr7	101571219	+	CUX1;CUX1;CUX1	Body;Body;Body	0.9918264	0.0193096	5.7390959	6.13E-08	3.20E-06	7.9206315
cg08989214	chr16	87492071	-	ZCCHC14	Body	1.1699293	2.7690452	5.7388803	6.13E-08	3.20E-06	7.919654
cg01794265	chr5	14582549	+	FAM105A	Body	-0.832844	-3.435578	-5.738613	6.14E-08	3.21E-06	7.9184422
cg03536885	chr7	27163820	-	HOXA3	5'UTR	1.2243729	0.4232034	5.7385295	6.14E-08	3.21E-06	7.9180633
cg14179581	chr1	50881502	+			1.7942931	-1.412394	5.7384685	6.14E-08	3.21E-06	7.9177867
cg03758150	chr19	30866192	+	ZNF536	5'UTR	2.7597218	-1.616087	5.7384421	6.15E-08	3.21E-06	7.917667
cg16618789	chr7	10116380	-			-1.186757	-1.20371	-5.738392	6.15E-08	3.21E-06	7.9174411
cg00054741	chr19	54201247	+	MIR523	TSS1500	-1.832542	1.0638271	-5.738326	6.15E-08	3.21E-06	7.9171397
cg24116870	chr12	43946284	+	ADAMTS20	TSS1500	1.5730266	-1.44763	5.7383447	6.15E-08	3.21E-06	7.9172255
cg13604337	chr12	54321653	-			2.5582591	-2.63165	5.7407663	6.15E-08	3.21E-06	7.9178675

cg22328644	chr12	109028385	-	SELPLG	TSS1500	-1.253932	-0.699261	-5.738046	6.16E-08	3.21E-06	7.9158706
cg10741153	chr19	29284353	+			1.7591959	-2.03716	5.7379914	6.16E-08	3.21E-06	7.9156241
cg12145085	chr14	73289895	-	DPF3	Body	-1.573687	0.4823225	-5.73791	6.16E-08	3.21E-06	7.9152565
cg11269599	chr19	52391304	+	ZNF577;ZNF577;ZNF577	TSS200;TSS200;TSS200	1.8774642	-0.719928	5.7377117	6.17E-08	3.21E-06	7.914356
cg12529273	chr4	54458246	-	LNX1	TSS1500	-1.300449	-0.049631	-5.73745	6.17E-08	3.22E-06	7.913168
cg19765377	chr10	82049858	+	MAT1A	TSS1500	-1.111755	-2.647372	-5.737379	6.18E-08	3.22E-06	7.9128485
cg08157459	chr13	112176430	-			-1.387592	0.2307826	-5.73727	6.18E-08	3.22E-06	7.9123516
cg16273943	chr8	38175684	-	WHSC1L1;WHSC1L1	Body;Body	0.8883789	1.1917576	5.7372434	6.18E-08	3.22E-06	7.9122331
cg23899424	chr20	60456296	-	CDH4	Body	-0.729579	-0.573889	-5.737231	6.18E-08	3.22E-06	7.9121784
cg13972124	chr9	132539744	+			-0.869152	-2.620291	-5.737153	6.18E-08	3.22E-06	7.9118218
cg05224741	chr6	10422322	+			3.2341902	-1.625177	5.7368873	6.19E-08	3.22E-06	7.910619
cg07805777	chr11	15962932	+			1.3605415	0.8729337	5.7368449	6.19E-08	3.23E-06	7.9104267
cg25388882	chr1	85098598	+	C1orf180	Body	-1.054357	-1.524695	-5.739149	6.20E-08	3.23E-06	7.9105449
cg04275692	chr7	45610627	+			-1.613476	-0.64066	-5.735704	6.23E-08	3.24E-06	7.9052549
cg01917716	chr11	59824541	+	MS4A3;MS4A3;MS4A3	5'UTR;5'UTR;5'UTR	-1.237909	-0.205971	-5.735551	6.23E-08	3.24E-06	7.9045612
cg01993576	chr6	44187674	+	SLC29A1;SLC29A1	5'UTR;5'UTR	-1.096694	-1.246445	-5.735567	6.23E-08	3.24E-06	7.9046341
cg10231746	chr17	3337719	-	OR1E2	TSS1500	-1.081932	-1.356424	-5.735597	6.23E-08	3.24E-06	7.9047713
cg26024530	chr10	130281732	+			-2.053629	1.8676012	-5.740394	6.24E-08	3.25E-06	7.9057154
cg18270113	chr7	5603125	-			-1.003943	-2.300821	-5.740368	6.24E-08	3.25E-06	7.9049328
cg11827910	chr11	31825756	-	PAX6;PAX6;PAX6	Body;Body;Body	2.0202826	-0.015763	5.7351578	6.24E-08	3.25E-06	7.9027803

cg09255851	chr16	24555610	+	RBBP6;RBBP6;RBBP6	Body;Body;Body	1.475389	-0.238735	5.7351067	6.24E-08	3.25E-06	7.9025484
cg00534274	chr4	139163341	-	SLC7A11;SLC7A11	1stExon;5'UTR	-0.988846	-2.207745	-5.735021	6.25E-08	3.25E-06	7.9021603
cg10502231	chr1	224363449	-			1.2334182	0.1740586	5.7397245	6.26E-08	3.26E-06	7.9175244
cg07931792	chr19	12707809	+	ZNF490	Body	1.5458599	3.8204034	5.7343867	6.26E-08	3.26E-06	7.8992856
cg24704177	chr2	177027710	-	HOXD3	TSS1500	1.4402087	-1.06968	5.734035	6.27E-08	3.26E-06	7.8976918
cg03796321	chr15	86162442	+	AKAP13;AKAP13	Body;Body	2.5131719	-2.048215	5.7338932	6.28E-08	3.26E-06	7.8970494
cg23395341	chr7	141175075	-			-1.877779	0.4406323	-5.733702	6.28E-08	3.27E-06	7.8961844
cg12711760	chr11	20132923	+	NAV2;NAV2;NAV2;NAV2	Body;Body;Body;Body	-1.222348	-1.817978	-5.738812	6.29E-08	3.27E-06	7.898566
cg06572465	chr5	140800929	-	PCDHGA4;PCDHGA9;PCDHGA1;PCDHGB1;PCDHGB6;PCDHGB3;PCDHGA11;PCDHGB7;PCDHGA6;PCDHGA8;PCDHGA11;PCDHGA10;PCDHGA5;PCDHGB4;PCDHGA3;PCDHGA11;PCDHGA	Body;Body;Body;Body;Body;Body;1stExon;Body;Body;Body;1stExon;Body;Body;Body;Body;1stExon;Body;Body;Body;Body	1.7074865	-1.219422	5.7335698	6.29E-08	3.27E-06	7.8955838
cg15215348	chr19	18344520	-	PDE4C	5'UTR	1.619616	1.5697819	5.7333348	6.30E-08	3.27E-06	7.8945189
cg06641537	chr7	142020422	+			-1.242372	-0.11229	-5.733263	6.30E-08	3.27E-06	7.8941928
cg21836370	chr12	85667451	+			1.2548383	-0.128883	5.7332722	6.30E-08	3.27E-06	7.8942353
cg02249292	chr10	28035655	+	MKX	TSS1500	1.3377808	-0.844671	5.7332049	6.30E-08	3.27E-06	7.8939306
cg05713242	chr8	23145039	-	R3HCC1	TSS1500	-1.168477	-1.229494	-5.732922	6.31E-08	3.28E-06	7.8926487
cg07951949	chr16	64985514	-	CDH11	Body	-1.263205	-0.545058	-5.732958	6.31E-08	3.28E-06	7.8928104
cg17109725	chr1	18707479	-			-0.976617	-1.468285	-5.732941	6.31E-08	3.28E-06	7.8927364
cg06567855	chr2	85977911	-			-1.167898	-2.416575	-5.732405	6.32E-08	3.28E-06	7.8903076
cg08640923	chr2	220117945	+	TUBA4B;TUBA4A	TSS200;Body	-1.714103	-3.560003	-5.732237	6.33E-08	3.29E-06	7.8895472
cg14353137	chr8	67873799	-			2.6820198	-1.500506	5.7321702	6.33E-08	3.29E-06	7.8892424
cg26657240	chr15	81475061	+			-1.420817	-2.28026	-5.731674	6.34E-08	3.29E-06	7.8869941

cg05560435	chr5	172671526	+			2.405379	-1.587591	5.731562	6.35E-08	3.30E-06	7.8864869
cg12250807	chr2	136598602	-	MCM6	Body	1.2142007	0.636386	5.7315172	6.35E-08	3.30E-06	7.886284
h.12.105153690	chr12	106629560	+			-0.724641	-4.053661	-5.731541	6.35E-08	3.30E-06	7.8863928
cg16559630	chr7	56301065	+			-1.471885	0.2373505	-5.731253	6.36E-08	3.30E-06	7.885086
cg05364567	chr17	5019761	-	ZNF232	5'UTR	1.2683607	-0.48591	5.7309361	6.37E-08	3.30E-06	7.8836515
cg17861424	chr4	17318093	-			-1.638215	1.1046313	-5.730701	6.37E-08	3.31E-06	7.8825885
cg05129325	chr1	215255161	-	KCNK2;KCNK2;KCNK2	TSS1500;TSS1500;Body	1.7724256	-0.624224	5.7305823	6.38E-08	3.31E-06	7.8820486
cg02131862	chr21	31654844	+	KRTAP24-1	1stExon	-0.863448	-0.297348	-5.730397	6.38E-08	3.31E-06	7.8812091
cg09555118	chr6	6004337	-	NRN1	Body	1.4024808	-1.312793	5.7329869	6.38E-08	3.31E-06	7.8826586
cg24587268	chr20	60813121	+	OSBPL2;OSBPL2	TSS1500;TSS1500	-0.707595	-3.882927	-5.735339	6.39E-08	3.31E-06	7.88253
cg19272720	chr1	204289444	-	PLEKHA6	5'UTR	-1.220277	0.0395775	-5.732626	6.39E-08	3.31E-06	7.8810262
cg07998554	chr19	14785776	-	EMR3	TSS200	-1.103967	-1.656538	-5.729924	6.40E-08	3.32E-06	7.8790648
cg20010506	chr12	62585467	+	FAM19A2;FAM19A2	5'UTR;1stExon	1.5932537	-0.295355	5.7298794	6.40E-08	3.32E-06	7.8788651
cg16361253	chr6	47624276	-	GPR111	TSS200	-1.105809	-0.325364	-5.729813	6.40E-08	3.32E-06	7.8785653
cg10839475	chr12	113959012	-			-1.14963	-0.835581	-5.729679	6.41E-08	3.32E-06	7.8779589
cg04815334	chr11	71210295	-	NADSYN1	Body	0.840784	1.8602662	5.7296494	6.41E-08	3.32E-06	7.8778229
cg01565703	chr14	103245090	+	TRAF3;TRAF3;TRAF3	5'UTR;5'UTR;5'UTR	-0.715746	-3.289835	-5.732168	6.41E-08	3.32E-06	7.8789539
cg16988810	chr6	31762650	+	VARS	Body	-1.120211	-2.426894	-5.729587	6.41E-08	3.32E-06	7.8775394
cg03030267	chr20	57394000	-	GNASAS;MIR298;MIR296	Body;TSS1500;TSS1500	-1.325046	-1.006936	-5.729491	6.41E-08	3.32E-06	7.8771067
cg11295724	chr20	1538353	-	SIRPD	TSS200	-1.32434	-0.079982	-5.729397	6.41E-08	3.32E-06	7.8766817

cg04772947	chr11	57363540	-	SERPING1	TSS1500	-1.192989	-0.181277	-5.729303	6.42E-08	3.32E-06	7.8762562
cg09059319	chr8	99986175	+			1.2427635	-0.650552	5.729247	6.42E-08	3.32E-06	7.8760005
cg02978297	chr1	94057587	-	BCAR3	Body	-1.580455	-1.491636	-5.728908	6.43E-08	3.33E-06	7.8744632
cg13314863	chr10	94594490	+	EXOC6;EXOC6	5'UTR;1stExon	0.6892725	1.417807	5.7287462	6.43E-08	3.33E-06	7.8737325
cg15162922	chr5	132162758	-	SHROOM1	TSS1500	0.6035274	2.004591	5.7287331	6.43E-08	3.33E-06	7.8736729
cg13654588	chr10	120354081	-	PRLHR	Body	1.4017095	-0.56389	5.7286711	6.44E-08	3.33E-06	7.8733922
cg01899676	chr12	106988413	-	RFX4	Body	1.2268697	0.2459055	5.7337861	6.44E-08	3.33E-06	7.8904735
cg05381729	chr20	61278414	+	SLCO4A1	5'UTR	-1.060645	-0.349694	-5.728604	6.44E-08	3.33E-06	7.8730899
cg00459078	chr17	43024659	-	KIF18B	5'UTR	-0.335312	-3.898504	-5.728384	6.44E-08	3.33E-06	7.8720924
cg13982366	chr9	138018566	-			-1.366538	0.4752534	-5.728361	6.45E-08	3.33E-06	7.8719888
cg09871315	chr7	27142682	+	HOXA2	TSS1500	1.6722264	-0.147508	5.7281698	6.45E-08	3.34E-06	7.8711221
cg10608166	chr12	115887445	-			-1.423754	0.3732813	-5.730638	6.45E-08	3.34E-06	7.8720339
cg07464571	chr7	127881001	+	LEP	TSS1500	0.8997722	-0.124003	5.7277412	6.46E-08	3.34E-06	7.8691811
cg10831246	chr17	18553663	+			1.3746705	0.8133788	5.7277649	6.46E-08	3.34E-06	7.8692884
cg10511357	chr1	7196075	-	CAMTA1	Body	-1.179219	-0.416583	-5.727235	6.48E-08	3.35E-06	7.8668907
cg18186343	chr14	101317620	-	MIR770;MEG3;MEG3;MEG3	TSS1500;Body;Body;Body	-1.1724	-0.734422	-5.727211	6.48E-08	3.35E-06	7.8667791
cg08519134	chr8	1137026	+			-1.250128	0.3395761	-5.727043	6.49E-08	3.35E-06	7.8660212
cg09376835	chr8	131347294	-	ASAP1	Body	0.8990946	0.7324487	5.7270431	6.49E-08	3.35E-06	7.8660198
cg07256668	chr11	64398137	+	NRXN2;NRXN2;NRXN2	Body;Body;Body	1.005102	3.1897448	5.7268326	6.49E-08	3.35E-06	7.8650663
cg08152065	chr15	27092400	+			-1.283726	-0.22541	-5.726451	6.50E-08	3.36E-06	7.8633404

cg00839132	chr18	74692235	-	MBP;MBP;MBP;MBP;MBP	3'UTR;3'UTR;3'UTR;3'UTR;3'UTR	1.01821	2.0439472	5.7263689	6.51E-08	3.36E-06	7.862967
cg15723222	chr17	59222476	-	BCAS3;BCAS3	Body;Body	1.3003892	1.3784706	5.7263419	6.51E-08	3.36E-06	7.8628448
cg13329248	chr1	34642745	-	C1orf94;C1orf94;C1orf94	5'UTR;1stExon;5'UTR	1.1418409	-1.480973	5.7262617	6.51E-08	3.36E-06	7.8624815
cg26689077	chr12	53599806	-	ITGB7	5'UTR	-1.209725	-1.188377	-5.726185	6.51E-08	3.36E-06	7.8621353
cg01964337	chr12	105104782	-	CHST11	Body	1.3443192	0.1766455	5.7259685	6.52E-08	3.37E-06	7.8611538
cg12306333	chr15	97312064	-			-0.961238	-0.840213	-5.725866	6.52E-08	3.37E-06	7.8606914
cg26748297	chr6	40346044	+	TDRG1	TSS200	-1.335904	0.6128981	-5.725813	6.52E-08	3.37E-06	7.8604478
cg24364398	chr2	86565216	-	REEP1;REEP1;REEP1;REEP1	TSS200;TSS1500;TSS1500;TSS1500	-0.899212	-2.215888	-5.728172	6.53E-08	3.37E-06	7.8608803
cg18217235	chr6	50817157	-			1.5628612	-0.769489	5.72492	6.55E-08	3.38E-06	7.8564069
cg25408237	chr1	9656984	+	TMEM201;TMEM201	Body;Body	1.1320133	2.1434989	5.727341	6.56E-08	3.38E-06	7.8567932
cg02759725	chr8	133072665	+	OC90	TSS1500	-1.281208	-0.715737	-5.72448	6.56E-08	3.39E-06	7.8544147
cg05036153	chr9	140128254	+	SLC34A3	Body	-1.008264	-1.313035	-5.724519	6.56E-08	3.39E-06	7.8545923
cg13974773	chr6	27463217	+			2.3772155	-1.443302	5.7244729	6.56E-08	3.39E-06	7.8543827
cg22853797	chr8	19172126	-	SH2D4A	5'UTR	-0.459958	-3.091271	-5.724473	6.56E-08	3.39E-06	7.8543816
cg14589553	chr9	140275263	+	EXD3	Body	-1.488138	1.0958936	-5.724288	6.57E-08	3.39E-06	7.8535456
cg02712949	chr8	87570714	-	CPNE3	3'UTR	0.7309845	2.0910037	5.7241558	6.57E-08	3.39E-06	7.8529473
cg10580056	chr15	35086928	+	ACTC1	Body	0.845157	-0.384805	5.7236362	6.59E-08	3.40E-06	7.8505953
cg18911395	chr12	113907122	+	LHX5	Body	1.3040306	-0.63916	5.7234602	6.60E-08	3.40E-06	7.8497988
cg07699978	chr12	63194001	-	PPM1H	Body	-1.62591	0.2715653	-5.723011	6.61E-08	3.41E-06	7.847766
cg13853761	chr11	64863232	+	C11orf2	TSS1500	0.956936	1.6477089	5.7229712	6.61E-08	3.41E-06	7.8475855

cg19956147	chr17	800431	-	NXN	Body	0.9135584	0.2140385	5.7228317	6.62E-08	3.41E-06	7.846954
cg22460123	chr12	52638294	-	KRT7	Body	-0.899684	-1.65949	-5.722551	6.62E-08	3.41E-06	7.845682
cg07908160	chr17	56296982	+	MKS1;MKS1	TSS200;TSS1500	-0.932707	-2.469191	-5.72221	6.64E-08	3.42E-06	7.8441417
cg13888983	chr9	132981932	-	FREQ;FREQ	Body;Body	0.9664912	3.5644689	5.7220505	6.64E-08	3.42E-06	7.8434184
cg09735390	chr5	164658718	+			-1.813785	1.1193559	-5.72197	6.64E-08	3.42E-06	7.843052
cg16869108	chr3	10184319	+	VHL;VHL	Body;Body	-1.144022	0.2856264	-5.721967	6.64E-08	3.42E-06	7.8430419
cg05149537	chr22	45709699	-	FAM118A;FAM118A	5'UTR;5'UTR	0.8774579	2.2839395	5.7218796	6.65E-08	3.42E-06	7.8426445
cg26860855	chr7	152618527	+			-1.505302	0.3654161	-5.721863	6.65E-08	3.42E-06	7.8425698
cg27179533	chr7	101006052	+	EMID2	TSS200	2.4210588	-1.964304	5.7218555	6.65E-08	3.42E-06	7.8425355
cg19800670	chr1	86081701	-			0.8520805	1.8557418	5.7217777	6.65E-08	3.42E-06	7.8421833
cg21853021	chr11	2920146	+	SLC22A18AS;SLC22A18	Body;TSS1500	-1.293103	-3.536178	-5.72174	6.65E-08	3.42E-06	7.8420123
cg10995381	chr5	7877198	-	MTRR;MTRR	Body;Body	-1.298775	-0.791835	-5.72168	6.65E-08	3.42E-06	7.8417416
cg04073934	chr4	3372272	+	RGS12;RGS12;RGS12	Body;Body;Body	0.7318604	1.6839337	5.7214849	6.66E-08	3.43E-06	7.8408586
cg24896649	chr4	188916726	-	ZFP42	TSS200	1.9471215	-1.097061	5.7239852	6.66E-08	3.43E-06	7.8416219
cg03394188	chr16	4863878	+	GLYR1	Body	0.7228571	2.5191556	5.7210701	6.67E-08	3.43E-06	7.8389815
cg20104341	chr12	54338937	+	HOXC13	Body	2.5840926	-2.388836	5.723201	6.68E-08	3.44E-06	7.8380773
cg24740818	chr15	76440405	-	C15orf27	Body	-1.288852	-2.869335	-5.725407	6.70E-08	3.44E-06	7.8505607
cg16271437	chr20	23123952	+			-1.385566	-2.716248	-5.720106	6.70E-08	3.45E-06	7.8346207
cg19415202	chr11	133706356	+			-1.318981	-0.602851	-5.71971	6.71E-08	3.45E-06	7.8328283
cg07870999	chr7	155260895	-			1.8683274	-0.65005	5.7195149	6.72E-08	3.45E-06	7.8319439

cg20954987	chr12	48578223	+	C12orf68	1stExon	1.5701461	-0.983411	5.7195255	6.72E-08	3.45E-06	7.831992
cg22576976	chr19	56852136	+			-1.458702	-0.783239	-5.719517	6.72E-08	3.45E-06	7.8319544
cg00143249	chr7	156798810	-	MNX1;MNX1	Body;Body	1.4880414	-0.642522	5.71941	6.72E-08	3.46E-06	7.8314692
cg02129814	chr6	84799458	+	MRAP2	3'UTR	-1.149136	0.1249483	-5.719311	6.73E-08	3.46E-06	7.8310202
cg10543582	chr2	199202432	+			-1.719733	0.8816222	-5.719309	6.73E-08	3.46E-06	7.8310124
cg14665523	chr10	89421684	+	PAPSS2;PAPSS2	Body;Body	1.2169435	1.5954783	5.7192447	6.73E-08	3.46E-06	7.8307214
cg01914621	chr7	155260474	-			1.5689976	-0.963514	5.7191022	6.73E-08	3.46E-06	7.8300769
cg15610488	chr13	113492103	+	ATP11A;ATP11A	Body;Body	0.9864091	2.0027926	5.7191088	6.73E-08	3.46E-06	7.8301064
cg19792599	chr11	125774406	-	DDX25;PUS3;DDX25	5'UTR;TSS1500;1stExon	1.8876642	-1.078705	5.7190421	6.74E-08	3.46E-06	7.829805
cg23154270	chr2	137527566	+			-1.438277	-0.638582	-5.719032	6.74E-08	3.46E-06	7.8297582
cg00609834	chr11	13984596	-	SPON1	1stExon	1.3095062	-1.259358	5.7188323	6.74E-08	3.46E-06	7.8288554
cg06878548	chr8	514821	-			1.4086047	1.6011199	5.7188143	6.74E-08	3.46E-06	7.8287743
cg13313226	chr11	124665869	-	C11orf61	Body	0.8868194	1.7676345	5.7188661	6.74E-08	3.46E-06	7.8290084
cg16105569	chr12	27627468	+	C12orf70	Body	-1.695263	0.3266843	-5.718875	6.74E-08	3.46E-06	7.8290471
cg21547371	chr3	52869521	-	MUSTN1	TSS1500	0.967606	1.0431804	5.7188282	6.74E-08	3.46E-06	7.8288372
cg06608359	chr6	28831300	+			-0.915814	-2.557674	-5.71875	6.74E-08	3.46E-06	7.8284834
cg13816423	chr6	41513166	-	FOXP4;FOXP4;FOXP4	TSS1500;TSS1500;TSS1500	-1.075517	-1.104074	-5.71875	6.74E-08	3.46E-06	7.828481
cg16419354	chr1	179713349	+	FAM163A	5'UTR	1.5261041	-0.698415	5.7185587	6.75E-08	3.46E-06	7.8276177
cg23508701	chr12	76331877	+			0.8741117	1.6308428	5.7185714	6.75E-08	3.46E-06	7.8276753
cg19570924	chr10	3457015	+			1.0945083	1.4578184	5.7183165	6.76E-08	3.47E-06	7.8265218

cg07696099	chr8	133573809	-	HPYR1	TSS200	-1.491111	-1.051679	-5.71815	6.76E-08	3.47E-06	7.8257665
cg23644854	chr9	132500429	+			-0.879724	-3.231512	-5.717835	6.77E-08	3.47E-06	7.8243456
cg05070372	chr16	87027489	-			-1.20805	1.1352843	-5.717805	6.77E-08	3.47E-06	7.8242076
cg21495622	chr22	24199125	+	SLC2A11;SLC2A11;SLC2A11;SLC2A11;SLC2A11	1stExon;5'UTR;TSS1500;1stExon;5'UTR	-1.229092	-0.999782	-5.722781	6.78E-08	3.48E-06	7.8254984
cg02506008	chr12	115861520	+			-1.071282	-2.184765	-5.717567	6.78E-08	3.48E-06	7.8231307
cg19339848	chr6	74170937	+	MTO1;MTO1;MTO1	TSS1500;TSS1500;TSS1500	-1.041571	-2.926371	-5.717467	6.79E-08	3.48E-06	7.8226782
cg09036188	chr17	4802888	-	CHRNE;C17orf107	Body;TSS200	-1.614866	-1.994167	-5.71738	6.79E-08	3.48E-06	7.8222865
cg07526763	chr9	140485720	+	ZMYND19	TSS1500	-0.480608	-3.166571	-5.717094	6.80E-08	3.48E-06	7.8209921
cg24454829	chr11	22363053	-	SLC17A6	Body	1.1421698	-1.139727	5.7170801	6.80E-08	3.48E-06	7.8209289
cg06120000	chr16	67480229	+	ATP6V0D1	Body	1.008695	0.9020299	5.7166547	6.81E-08	3.49E-06	7.8190046
cg16873130	chr7	136586950	-	CHRM2;CHRM2;CHRM2;CHRM2;CHRM2;CHRM2;CHRM2;MIR490;CHRM2	5'UTR;5'UTR;5'UTR;5'UTR;5'UTR;5'UTR;5'UTR;TSS1500;5'UTR	-1.724463	0.7516117	-5.716372	6.82E-08	3.49E-06	7.8177257
cg22875024	chr20	31995970	-	SNTA1	3'UTR	0.8105142	1.0158952	5.7163577	6.82E-08	3.49E-06	7.8176612
cg25862975	chr6	108454984	-			-0.906854	-3.333477	-5.716313	6.82E-08	3.49E-06	7.8174607
cg05091238	chr3	157813609	+			2.2305192	-0.634074	5.7213453	6.83E-08	3.50E-06	7.8196883
cg06082906	chr20	25277813	-	ABHD12;PYGB	Body;3'UTR	-0.85961	0.4209493	-5.716224	6.83E-08	3.50E-06	7.8170567
cg02878244	chr11	20181911	-	DBX1	TSS200	1.9737771	-0.541827	5.7160781	6.83E-08	3.50E-06	7.8163966
cg05548130	chr10	130820766	+			-1.383249	-0.456373	-5.716025	6.83E-08	3.50E-06	7.8161565
cg09942424	chr17	74913419	+	MGAT5B;MGAT5B	Body;Body	-1.732129	0.1602072	-5.715925	6.84E-08	3.50E-06	7.8157019
cg23158811	chr19	19729732	-	PBX4	TSS1500	-0.864913	-3.535025	-5.715742	6.84E-08	3.50E-06	7.8148769
cg14852227	chr4	86705640	-	ARHGAP24;ARHGAP24	Body;5'UTR	0.7710499	2.2503112	5.7156698	6.84E-08	3.50E-06	7.8145497

cg01103601	chr2	239288872	-	TRAF3IP1;TRAF3IP1	Body;Body	-1.907988	1.3388507	-5.715583	6.85E-08	3.50E-06	7.8141553
cg22209441	chr7	65579017	-	CRCP;CRCP;CRCP;CRCP;CRCP	TSS1500;TSS1500;TSS1500;TSS1500;TSS1500	-0.848031	0.2767476	-5.715533	6.85E-08	3.50E-06	7.813933
cg11195707	chr14	104852706	-			-1.24034	1.5164448	-5.715397	6.85E-08	3.51E-06	7.8133144
cg16565154	chr1	151129062	-	TNFAIP8L2	TSS200	-1.625879	-1.081606	-5.717824	6.86E-08	3.51E-06	7.8141125
cg22643751	chr7	855365	+	UNC84A;UNC84A	TSS1500;TSS1500	-1.817402	-2.102243	-5.731003	6.86E-08	3.51E-06	7.8199476
cg02280859	chr1	1711185	-	NADK	TSS1500	-1.169332	0.2073983	-5.714495	6.88E-08	3.52E-06	7.8092362
cg22846876	chr19	38959746	-	RYR1;RYR1	Body;Body	-1.271826	0.3864391	-5.714515	6.88E-08	3.52E-06	7.8093277
cg08798307	chr7	141190187	+			-1.414915	-0.035964	-5.714425	6.88E-08	3.52E-06	7.8089209
cg15927357	chr18	47013900	+	C18orf32	TSS1500	-1.131625	-2.964429	-5.714353	6.89E-08	3.52E-06	7.8085947
cg08553524	chr5	164884295	+			-1.725847	0.7630169	-5.714314	6.89E-08	3.52E-06	7.8084185
cg18836689	chr14	69708392	-	EXD2	3'UTR	1.0296217	0.6712607	5.7141303	6.89E-08	3.52E-06	7.8075875
cg04108360	chr11	67771791	-	UNC93B1	TSS200	-0.575944	-2.042186	-5.714084	6.89E-08	3.52E-06	7.8073797
cg24694502	chr1	4736187	+	AJAP1;AJAP1	Body;Body	-1.741645	0.9455823	-5.714077	6.89E-08	3.52E-06	7.8073481
cg13117164	chr4	54960232	-			1.852576	-1.364369	5.7140471	6.90E-08	3.52E-06	7.8072113
cg23662675	chr20	45985596	+	ZMYND8;ZMYND8;ZMYND8	TSS200;TSS200;TSS200	-0.950039	-4.708472	-5.713949	6.90E-08	3.52E-06	7.8067686
cg02507296	chr10	104211328	-	C10orf95	TSS200	-0.659207	-1.19719	-5.713894	6.90E-08	3.52E-06	7.8065192
cg02155398	chr2	45160490	+			1.8353699	-0.457846	5.7138112	6.90E-08	3.53E-06	7.8061445
cg26575914	chr1	228397977	+	OBSCN;OBSCN	5'UTR;5'UTR	-0.821627	-0.793494	-5.713774	6.90E-08	3.53E-06	7.8059762
cg24857294	chr2	121010915	+	RALB	5'UTR	0.80598	-2.241611	5.7136169	6.91E-08	3.53E-06	7.8052658
cg06282802	chr2	127529446	+			-1.012149	-0.542882	-5.715928	6.92E-08	3.53E-06	7.8055517

cg07603449	chr1	11986842	-	KIAA2013	TSS1500	-0.655399	-0.469285	-5.713391	6.92E-08	3.53E-06	7.8042439
cg06657050	chr15	53083587	-	ONECUT1	TSS1500	1.4320857	-1.424943	5.7132814	6.92E-08	3.53E-06	7.8037486
cg15547143	chr10	89417335	-			1.2411921	0.7914519	5.7130687	6.93E-08	3.54E-06	7.8027872
cg01213519	chr5	134386369	+			1.191468	0.0874951	5.7129022	6.93E-08	3.54E-06	7.8020343
cg07924387	chr1	31885880	-	SERINC2	TSS200	-0.530964	-2.716943	-5.712661	6.94E-08	3.54E-06	7.8009416
cg20497968	chr11	127145287	-			-1.642241	-0.159113	-5.71521	6.94E-08	3.54E-06	7.8019764
cg27050153	chr14	102030110	+			1.2025969	-0.569213	5.7126465	6.94E-08	3.54E-06	7.800878
cg12934788	chr18	44099028	+	LOXHD1;LOXHD1;LOXHD1;LOXHD1	1stExon;Body;5'UTR;Body	-1.165558	-0.550827	-5.712295	6.95E-08	3.55E-06	7.7992904
cg05100432	chr14	104889005	-			-1.445761	0.6910788	-5.712228	6.96E-08	3.55E-06	7.7989881
cg05938149	chr16	88498622	-	ZNF469	Body	-1.04411	-0.497532	-5.711899	6.97E-08	3.55E-06	7.7974999
cg20727434	chr5	170740547	+			2.24727	-1.112517	5.7115055	6.98E-08	3.56E-06	7.7957197
cg03572260	chr17	37808339	-	STARD3;STARD3;STARD3	5'UTR;5'UTR;5'UTR	1.0194315	1.2341102	5.7114617	6.98E-08	3.56E-06	7.7955215
cg08326879	chr10	133143121	+			-1.184548	-0.139567	-5.711391	6.98E-08	3.56E-06	7.7952016
cg00187129	chr16	66580422	-	TK2	Body	0.9773611	1.4418276	5.7112061	6.99E-08	3.56E-06	7.794366
cg02065293	chr2	1928480	-	MYT1L	Body	-1.940556	0.7825116	-5.711057	6.99E-08	3.56E-06	7.7936916
cg12806121	chr1	10601389	+	PEX14	Body	0.9693243	0.8804191	5.7109336	7.00E-08	3.57E-06	7.7931342
cg13740135	chr19	44006432	-	PHLDB3	Body	1.477572	2.8414858	5.7109563	7.00E-08	3.57E-06	7.7932367
cg21623566	chr18	49868513	-	DCC	Body	1.4074866	-0.901427	5.7109707	7.00E-08	3.57E-06	7.7933017
cg02667102	chr5	3277120	-			-1.426615	-0.535051	-5.710066	7.03E-08	3.58E-06	7.7892105
cg14314029	chr7	121956643	-			1.7802955	-0.798486	5.7100411	7.03E-08	3.58E-06	7.7890995

cg23105471	chr12	58241215	-	CTDSP2	TSS1500	-1.294344	-1.668052	-5.710063	7.03E-08	3.58E-06	7.7891975
cg08379987	chr13	31507139	+	C13orf26	Body	1.2886387	0.5772237	5.7099499	7.03E-08	3.58E-06	7.7886875
cg24921140	chr3	156260776	-	SSR3	3'UTR	0.9424537	0.7467525	5.7098752	7.03E-08	3.58E-06	7.7883495
cg06020207	chr10	131766412	+			0.9653016	-1.218023	5.7122987	7.04E-08	3.58E-06	7.7891622
cg10894072	chr1	51985215	+	EPS15	TSS1500	-0.578448	-3.438639	-5.709706	7.04E-08	3.58E-06	7.7875846
cg09858237	chr17	9479929	+	WDR16;WDR16;STX8	TSS200;TSS200;TSS1500	-1.891212	-4.478666	-5.714723	7.04E-08	3.58E-06	7.7894782
cg17030233	chr2	119607573	-			1.1384401	-1.000648	5.7095909	7.04E-08	3.58E-06	7.7870649
cg17272642	chr11	124747244	-	ROBO3	Body	0.7571377	-0.760437	5.7093049	7.05E-08	3.59E-06	7.7857719
cg10982443	chr14	20903611	+	KLHL33	5'UTR	1.6551695	1.8910461	5.7092485	7.05E-08	3.59E-06	7.785517
cg25321114	chr11	67926276	+	SUV420H1	Body	0.8650639	1.8842142	5.7091648	7.06E-08	3.59E-06	7.785139
cg20885782	chr4	41875606	+			1.5623184	-0.986512	5.709032	7.06E-08	3.59E-06	7.7845388
cg07568015	chr11	13367266	+	ARNTL;ARNTL;ARNTL	5'UTR;5'UTR;5'UTR	0.89032	1.8100903	5.7086416	7.07E-08	3.60E-06	7.782774
cg20158671	chr19	56007515	+	SSC5D	Body	-1.651503	0.7159001	-5.708479	7.08E-08	3.60E-06	7.7820385
cg26597242	chr5	161274540	+	GABRA1;GABRA1;GABRA1;GABRA1;GABRA1	TSS1500;TSS1500;TSS1500;5'UTR;TSS200	1.7817229	-0.664316	5.7108817	7.08E-08	3.60E-06	7.7827658
cg18528367	chr6	100912822	-	SIM1	TSS1500	1.3915121	-1.014416	5.7081951	7.09E-08	3.60E-06	7.7807561
cg17737681	chr2	172952136	-	DLX1;DLX1	Body;Body	1.5385038	-0.296824	5.7130113	7.10E-08	3.61E-06	7.781761
cg21170796	chr1	119528164	+	TBX15	5'UTR	1.3259969	-0.069588	5.707534	7.11E-08	3.61E-06	7.7777685
cg14344102	chr6	100054817	+	PRDM13;PRDM13	1stExon;5'UTR	1.6185356	-1.925874	5.7074848	7.11E-08	3.62E-06	7.7775464
cg02148612	chr1	248018816	-			-1.146469	-0.985241	-5.707247	7.12E-08	3.62E-06	7.7764739
cg02456087	chr7	27291541	-			1.2593212	-1.580446	5.7071183	7.13E-08	3.62E-06	7.77589

cg14852603	chr6	164585948	-			-1.363098	-0.273524	-5.70714	7.12E-08	3.62E-06	7.7759863
cg17302155	chr6	100061111	+	PRDM13	Body	2.2910836	-0.868535	5.707126	7.12E-08	3.62E-06	7.775925
cg16861076	chr8	11421594	-	BLK	Body	1.8197312	-1.698051	5.7070073	7.13E-08	3.62E-06	7.7753885
cg17099568	chr8	65284438	+			1.4825413	-1.118796	5.7068228	7.14E-08	3.62E-06	7.7745547
cg09060914	chr1	169599558	+	SELP	TSS200	1.0092647	1.453134	5.7067364	7.14E-08	3.62E-06	7.7741643
cg12869229	chr12	56618373	+	OBFC2B	5'UTR	-0.634037	-4.320784	-5.706054	7.16E-08	3.64E-06	7.7710812
cg18846954	chr12	133028690	+			-1.135777	0.2202672	-5.70603	7.16E-08	3.64E-06	7.7709748
cg20090283	chr17	36718549	-	SRCIN1	Body	1.5043151	0.1192415	5.7058208	7.17E-08	3.64E-06	7.7700274
cg08375222	chr13	31311963	+	ALOX5AP	Body	-1.344224	-0.582742	-5.705687	7.17E-08	3.64E-06	7.7694221
cg18301410	chr7	132192892	+	PLXNA4;PLXNA4;PLXNA4	Body;Body;Body	-1.418663	0.2409271	-5.705434	7.18E-08	3.65E-06	7.7682782
cg10828316	chr14	78869352	-	NRXN3	TSS1500	-1.531042	0.1520288	-5.705365	7.18E-08	3.65E-06	7.7679697
cg09692368	chr8	1307280	-			-1.182327	0.2413735	-5.705313	7.19E-08	3.65E-06	7.7677352
cg10447615	chr1	109506805	+	CLCC1;CLCC1	TSS1500;TSS1500	-1.099004	-1.679271	-5.705228	7.19E-08	3.65E-06	7.7673491
cg10099827	chr16	85551514	-			1.5189384	4.1990879	5.7072438	7.21E-08	3.66E-06	7.7660155
cg27476623	chr11	40139315	-	LRRC4C	5'UTR	-1.998426	0.6164144	-5.704696	7.21E-08	3.66E-06	7.7649458
cg11816838	chr3	150484093	-			-1.329429	1.0855983	-5.704503	7.21E-08	3.66E-06	7.7640737
cg19212949	chr19	57323840	+	PEG3;PEG3;PEG3;ZIM2;ZIM2;PEG3;ZIM2;PEG3;PEG3AS	3'UTR;3'UTR;3'UTR;Body;Body;3'UTR;Body;3'UTR;TSS200	-2.161886	-0.040802	-5.706996	7.21E-08	3.66E-06	7.7782632
cg23680900	chr15	75017924	-	CYP1A1	TSS200	-0.641946	-3.09741	-5.704461	7.21E-08	3.66E-06	7.7638845
cg19815327	chr3	32023353	-	ZNF860;ZNF860;OSBPL10	5'UTR;1stExon;TSS200	-1.014118	-3.431453	-5.704411	7.22E-08	3.66E-06	7.7636562
cg06386307	chr6	100895211	+	SIM1	Body	1.0939251	-0.176094	5.7041888	7.22E-08	3.66E-06	7.7626546

cg23149825	chr7	145395722	+			-1.762525	1.0625308	-5.70406	7.23E-08	3.66E-06	7.762072
cg24988255	chr7	27225396	+	HOXA11AS;HOXA11	Body;TSS1500	1.3942238	-0.635255	5.7040284	7.23E-08	3.66E-06	7.76193
cg00744739	chr2	240983326	-	PRR21	TSS1500	-1.429048	-0.063717	-5.703914	7.23E-08	3.67E-06	7.7614121
cg18652346	chr6	28733673	-			1.3557785	-0.54002	5.7039227	7.23E-08	3.67E-06	7.7614526
cg04362790	chr14	100223811	-			-2.140797	0.9567439	-5.703632	7.24E-08	3.67E-06	7.7601385
cg12308407	chr6	32081491	+			0.9527269	0.7447997	5.7034248	7.25E-08	3.67E-06	7.7592033
cg08656837	chr7	155744544	-			-1.822606	0.7399167	-5.703368	7.25E-08	3.67E-06	7.7589484
cg05380019	chr7	19146555	-			1.4677339	-0.479549	5.7032993	7.25E-08	3.67E-06	7.7586364
cg13830791	chr10	132936706	+	TCERG1L	Body	-1.694652	0.8782285	-5.703171	7.26E-08	3.68E-06	7.7580571
cg26185061	chr10	81744786	+			-0.838736	-0.52853	-5.702619	7.28E-08	3.69E-06	7.7555645
cg25137030	chr15	45722589	-	C15orf48;C15orf48	TSS200;TSS200	-1.482081	-4.868062	-5.702472	7.28E-08	3.69E-06	7.7548992
cg04870732	chr11	123754732	+	TMEM225	Body	-1.328772	-0.109014	-5.702315	7.29E-08	3.69E-06	7.7541894
cg18623836	chr2	10261907	+	RRM2;RRM2	TSS1500;TSS1500	-1.333395	-0.221869	-5.702198	7.29E-08	3.69E-06	7.7536639
cg27195914	chr16	66907460	-			-1.33504	-2.593473	-5.704589	7.30E-08	3.69E-06	7.7543717
cg00754989	chr15	72530044	+			-1.186908	-1.05553	-5.701992	7.30E-08	3.69E-06	7.7527308
cg08417730	chr16	50189512	-	PAPD5;PAPD5	Body;Body	-0.965055	-2.783738	-5.70197	7.30E-08	3.69E-06	7.7526336
cg04585937	chr14	101506532	-	MIR376B;MIR654;MIR300;MIR376A1	TSS1500;TSS200;TSS1500;TSS1500	-1.457177	0.2383586	-5.701876	7.30E-08	3.69E-06	7.7522096
cg09451574	chr4	113069076	+	C4orf32	Body	1.0888118	0.9634184	5.7018852	7.30E-08	3.69E-06	7.7522493
cg22234080	chr22	29876942	-	NEFH	1stExon	1.1344915	-0.630054	5.7018581	7.30E-08	3.69E-06	7.752127
cg02666638	chr19	40732804	+	CNTD2	TSS1500	-0.737416	-1.978514	-5.701607	7.31E-08	3.70E-06	7.7509908

cg13457462	chr8	49801972	+			-1.311853	-0.577403	-5.701583	7.31E-08	3.70E-06	7.7508841
cg20737500	chr4	5865654	-	CRMP1;CRMP1	Body;Body	-1.01168	-0.309486	-5.70161	7.31E-08	3.70E-06	7.7510066
cg08384322	chr16	81094757	-	C16orf46;C16orf46	3'UTR;Body	0.9271723	2.0393512	5.7014711	7.32E-08	3.70E-06	7.7503794
cg12151656	chr3	9850558	+	TTLL3	TSS1500	-0.769304	-1.290925	-5.701489	7.32E-08	3.70E-06	7.7504611
cg14440640	chr14	61578115	-			1.2350268	0.8095193	5.7015142	7.32E-08	3.70E-06	7.750574
ch.20.1295406F	chr20	56228606	+	PMEPA1;PMEPA1;PMEPA1;PMEPA1	Body;Body;Body;Body	-0.880313	-4.17719	-5.701431	7.32E-08	3.70E-06	7.7501961
cg09739536	chr5	178339445	-	ZFP2	5'UTR	-1.661605	0.0978073	-5.70102	7.33E-08	3.71E-06	7.7483405
cg13048903	chr13	21755056	+			-1.054373	-1.173933	-5.703513	7.33E-08	3.71E-06	7.7491846
cg10596006	chr3	49711089	+	APEH	TSS1500	-1.194828	-3.864343	-5.700937	7.34E-08	3.71E-06	7.7479661
cg06588201	chr4	174430487	+			1.1151519	-0.075595	5.700655	7.35E-08	3.71E-06	7.746694
cg01793704	chr7	130126024	-	MEST	TSS200	-1.138573	-3.008606	-5.700499	7.35E-08	3.71E-06	7.7459881
cg09450260	chr7	155193250	+			-0.935588	-0.688543	-5.700355	7.36E-08	3.71E-06	7.7453396
cg21789085	chr8	2024594	-	MYOM2	Body	-1.573685	0.1899659	-5.700344	7.36E-08	3.71E-06	7.7452903
cg01399981	chr16	87812658	+			-1.112303	-2.906293	-5.700237	7.36E-08	3.72E-06	7.7448053
cg26048630	chr1	170640675	+	PRRX1;PRRX1	Body;Body	2.9882531	-2.034316	5.7002338	7.36E-08	3.72E-06	7.7447918
cg02471153	chr8	99440331	+	KCNS2	Body	1.1213868	-0.029898	5.7001499	7.36E-08	3.72E-06	7.7444131
cg12299478	chr10	111935236	+			-1.204683	0.4230855	-5.699959	7.37E-08	3.72E-06	7.7435517
cg21929183	chr5	140807225	+	PCDHGA4;PCDHGA11;PCDHGA11;PCDHGA9;PCDHGA1;PCDHGB1;PCDHGB8P;PCDHGB6;PCDHGB3;PCDHGB7;PCDHGA6;PCDHGA8;PCDHGA10;PCDHGA5;PCDHGB4;PCDHGA3;PCDHGA2	Body;Body;Body;Body;Body;Body;Body;Body;Body;Body;Body;Body;Body;Body;Body;Body;Body;Body	1.5560065	1.0578939	5.7024538	7.37E-08	3.72E-06	7.744407
cg26406441	chr1	33427141	-	RNF19B;RNF19B	Body;Body	-1.633763	1.537829	-5.699745	7.38E-08	3.72E-06	7.7425838
cg09448088	chr13	113635690	-	MCF2L;MCF2L	Body;Body	0.6834288	2.0344221	5.6989579	7.40E-08	3.74E-06	7.7390311

cg16484526	chr12	25150608	+	C12orf77	TSS1500	-1.336193	1.4790173	-5.698974	7.40E-08	3.74E-06	7.7391049
cg01514538	chr1	2980380	+	FLJ42875	Body	1.0162648	1.6117689	5.6988527	7.41E-08	3.74E-06	7.7385562
cg26775087	chr3	15382808	-	SH3BP5;SH3BP5	1stExon;5'UTR	0.7795867	1.0412975	5.6988165	7.41E-08	3.74E-06	7.7383924
cg16764177	chr6	164252644	+			-1.582249	0.6691068	-5.698608	7.42E-08	3.74E-06	7.7374489
cg08716684	chr12	133337116	+	ANKLE2	Body	-0.954889	-0.544879	-5.698482	7.42E-08	3.74E-06	7.7368837
cg27317694	chr13	49105050	-	RCBTB2	5'UTR	-0.997076	2.0771671	-5.698491	7.42E-08	3.74E-06	7.7369242
cg03190891	chr10	97201172	-	SORBS1;SORBS1;SORBS1;SORBS1;SORBS1;SORBS1;SORBS1	5'UTR;TSS1500;TSS1500;TSS1500;TSS1500;5'UTR;TSS1500	-1.430638	-0.292326	-5.698367	7.43E-08	3.74E-06	7.7363639
cg21319323	chr2	177001263	-			2.2617848	-1.11959	5.6979925	7.44E-08	3.75E-06	7.7346724
cg04287574	chr1	201619622	-	NAV1	Body	-1.349482	-1.678689	-5.697963	7.44E-08	3.75E-06	7.7345385
cg15570148	chr3	61235985	+	FHIT;FHIT	5'UTR;5'UTR	-1.175413	-1.700834	-5.697916	7.44E-08	3.75E-06	7.7343261
cg27123859	chr6	107230619	-			1.1271493	-0.818216	5.6976396	7.45E-08	3.75E-06	7.7330795
cg14237025	chr17	34309207	-	CCL16	TSS1500	-1.200639	-0.019904	-5.697519	7.46E-08	3.76E-06	7.7325337
cg05246283	chr7	73270172	-			-0.430805	-3.813051	-5.697132	7.47E-08	3.76E-06	7.7307882
cg23104436	chr11	15838747	+			-1.333319	1.4832424	-5.696909	7.48E-08	3.77E-06	7.7297814
cg05207834	chr17	49199381	-	SPAG9;SPAG9	TSS1500;TSS1500	-1.143533	-4.171689	-5.701408	7.50E-08	3.78E-06	7.7294822
cg06912936	chr5	169935197	-	KCNIP1;KCNIP1;KCNIP1	Body;Body;Body	-1.404852	0.1644094	-5.695734	7.52E-08	3.79E-06	7.724479
cg01603862	chr8	1041336	-			-1.363191	0.051891	-5.695697	7.52E-08	3.79E-06	7.724313
cg01934781	chr12	12223947	+	BCL2L14;BCL2L14;BCL2L14;BCL2L14	5'UTR;TSS1500;1stExon;TSS1500	-1.224902	-0.988284	-5.695268	7.53E-08	3.79E-06	7.7223742
cg02384911	chr17	907886	-	ABR;ABR;ABR	3'UTR;3'UTR;3'UTR	1.034901	0.9465891	5.6952427	7.54E-08	3.79E-06	7.7222604
cg23154781	chr15	80634195	-			-1.337815	0.2986731	-5.695166	7.54E-08	3.79E-06	7.721914

cg27423027	chr6	131147800	+	LOC285733	TSS1500	-1.788668	0.9224359	-5.697267	7.55E-08	3.80E-06	7.736608
cg21439129	chr7	72844212	+			-1.096788	0.1653737	-5.694711	7.55E-08	3.80E-06	7.7198602
cg24629972	chr8	43147439	-	POTEA;POTEA	TSS200;TSS200	-1.477651	0.1784841	-5.694489	7.56E-08	3.81E-06	7.7188571
cg12438155	chr1	4223893	-			-1.949631	-0.258032	-5.694423	7.56E-08	3.81E-06	7.7185616
cg13689591	chr3	124239629	+	KALRN	Body	-1.621644	-0.137029	-5.696761	7.57E-08	3.81E-06	7.7187389
cg26925231	chr8	15094380	-	SGCZ	Body	1.5465028	-1.018619	5.6942276	7.57E-08	3.81E-06	7.7176794
cg02710296	chr1	182921851	+	C1orf14	Body	1.8080177	0.9376656	5.6967013	7.57E-08	3.81E-06	7.7188065
cg01292846	chr17	33672543	-			0.6888297	0.9169589	5.6941279	7.58E-08	3.81E-06	7.7172296
cg06434454	chr5	5146320	-	ADAMTS16	Body	-2.859898	1.9611897	-5.694104	7.58E-08	3.81E-06	7.7171235
cg22774211	chr1	120439014	-	ADAM30;ADAM30	1stExon;5'UTR	0.8389201	0.3631098	5.6938694	7.58E-08	3.81E-06	7.7160631
cg08717880	chr2	177014849	+	HOXD4;MIR10B	TSS1500;TSS200	0.9902183	-0.133494	5.6937603	7.59E-08	3.81E-06	7.7155709
cg06793062	chr16	76343868	+	CNTNAP4;CNTNAP4	Body;Body	-1.279506	0.0820978	-5.693534	7.60E-08	3.82E-06	7.7145508
cg23173688	chr1	86081894	-			0.8622034	1.1777253	5.6934635	7.60E-08	3.82E-06	7.7142313
cg26913833	chr2	232388288	-	NMUR1	3'UTR	0.9438013	0.8040499	5.6956141	7.61E-08	3.83E-06	7.7139063
cg10155304	chr12	109107559	-	CORO1C	5'UTR	0.7917309	1.1442176	5.6929989	7.62E-08	3.83E-06	7.712135
cg18235662	chr6	164975487	-			-1.59547	-0.248506	-5.692957	7.62E-08	3.83E-06	7.7119443
cg03740167	chr10	23463746	-			1.4387384	-1.249092	5.6927651	7.62E-08	3.83E-06	7.71108
cg09473282	chr6	35115955	+			1.1874221	1.1722238	5.6926865	7.63E-08	3.83E-06	7.7107257
cg07292251	chr5	176829198	-	F12	3'UTR	1.1101617	3.1562151	5.6925319	7.63E-08	3.83E-06	7.7100279
cg19954017	chr17	80333257	+	UTS2R	1stExon	1.6137791	-0.159223	5.6950693	7.63E-08	3.83E-06	7.7111164

cg21618620	chr12	54408427	-			1.2933702	-1.137687	5.6925899	7.63E-08	3.83E-06	7.7102896
cg26751356	chr13	112712635	-			1.666916	-0.841874	5.6925652	7.63E-08	3.83E-06	7.7101783
cg09260773	chr1	119529266	+	TBX15	5'UTR	1.3771586	-1.204125	5.6924816	7.63E-08	3.83E-06	7.7098009
cg08844390	chr1	16298537	-	ZBTB17	5'UTR	0.6033585	2.7088124	5.6923822	7.64E-08	3.83E-06	7.7093527
cg14407667	chr1	51985209	+	EPS15	TSS1500	-0.474917	-3.577778	-5.69242	7.64E-08	3.83E-06	7.7095234
cg16926809	chr1	10732464	-	CASZ1;CASZ1	Body;Body	0.947696	0.8514191	5.6923503	7.64E-08	3.83E-06	7.7092086
cg19457909	chr17	59206070	-	BCAS3;BCAS3	Body;Body	0.9156544	1.3008079	5.6923557	7.64E-08	3.83E-06	7.7092329
cg04956471	chr2	234328457	+	DGKD;DGKD	Body;Body	-0.937389	-1.561374	-5.692118	7.65E-08	3.84E-06	7.7081626
cg05803237	chr11	92702628	-	MTNR1B	TSS200	0.9308426	-0.190269	5.6920691	7.65E-08	3.84E-06	7.7079398
cg08288818	chr15	55920079	+	PRTG	Body	-1.119944	-0.725334	-5.692082	7.65E-08	3.84E-06	7.707997
cg11917750	chr5	123979287	-	ZNF608	Body	0.8166374	3.0855595	5.6920412	7.65E-08	3.84E-06	7.7078141
cg08575883	chr10	3291355	+			-1.486871	-0.746131	-5.69196	7.65E-08	3.84E-06	7.7074483
cg01644731	chr12	58236712	+	CTDSP2	Body	1.1707457	0.9774422	5.691673	7.66E-08	3.84E-06	7.7061529
cg01053241	chr8	17352834	-			-1.825411	0.8829748	-5.691503	7.67E-08	3.85E-06	7.7053852
cg21884064	chr5	2177644	-			-1.737093	0.0345428	-5.69114	7.68E-08	3.85E-06	7.703748
cg06449084	chr8	1498266	-	DLGAP2	Body	-1.319478	0.5933155	-5.690972	7.69E-08	3.85E-06	7.7029924
cg26522684	chr7	56563018	-	DKFZp434L192	TSS1500	-1.264419	-0.135711	-5.690519	7.70E-08	3.86E-06	7.7009488
cg26060255	chr20	3025968	+	MRPS26;GNRH2;GNRH2;GNRH2	TSS1500;Body;Body;Body	-0.894707	-1.548048	-5.69041	7.71E-08	3.86E-06	7.7004569
cg27641628	chr7	134833031	-	TMEM140	5'UTR	-0.862585	-3.840693	-5.689743	7.73E-08	3.88E-06	7.697445
cg04809380	chr16	1244525	-	CACNA1H;CACNA1H	Body;Body	-1.309949	0.7650244	-5.689261	7.75E-08	3.88E-06	7.6952742

cg21043424	chr7	73300908	-			1.0281058	-0.356331	5.6891544	7.75E-08	3.88E-06	7.694792
cg20206377	chr11	2323272	+	TSPAN32;C11orf21;TSPAN32;C11orf21	1stExon;TSS200;5'UTR;TSS200	-1.011989	-0.760324	-5.688903	7.76E-08	3.89E-06	7.6936564
cg09427644	chr11	125657662	+	PATE3	TSS1500	-1.180189	-0.538596	-5.688657	7.77E-08	3.89E-06	7.6925496
cg13462129	chr7	96651281	+	DLX5	Body	1.3186754	-0.097212	5.6886379	7.77E-08	3.89E-06	7.6924622
cg16431978	chr21	31797932	+	KRTAP13-3	1stExon	-1.237443	-0.830726	-5.688615	7.77E-08	3.89E-06	7.6923593
cg19720302	chr17	65990670	+	C17orf58;C17orf58	TSS1500;TSS1500	-1.30196	-0.641923	-5.688673	7.77E-08	3.89E-06	7.6926199
cg09255521	chr11	125312257	+			-1.510942	0.6185695	-5.688557	7.78E-08	3.89E-06	7.6920954
cg14517004	chr22	48946660	-	FAM19A5	Body	-1.915198	1.1829164	-5.687877	7.80E-08	3.91E-06	7.6890289
cg06869505	chr1	15527517	+	TMEM51;TMEM51;TMEM51;TMEM51	5'UTR;5'UTR;5'UTR;5'UTR	-1.290287	-0.646432	-5.687654	7.81E-08	3.91E-06	7.6880274
cg14275842	chr17	4802813	+	CHRNE;C17orf107	Body;TSS200	-2.341952	-3.54987	-5.687653	7.81E-08	3.91E-06	7.6880215
cg05110828	chr3	61570099	-	PTPRG	Body	1.1877816	2.0532695	5.6875545	7.81E-08	3.91E-06	7.6875766
cg14587524	chr19	38183262	-	ZNF781	TSS200	2.9731461	-1.116703	5.6873335	7.82E-08	3.91E-06	7.68658
cg06346993	chr6	44093974	+	TMEM63B;MRPL14	TSS1500;5'UTR	-1.1161	-1.391562	-5.687109	7.83E-08	3.92E-06	7.6855688
cg03711129	chr2	71124762	-			1.1637897	-0.49348	5.6870497	7.83E-08	3.92E-06	7.6853003
cg26140120	chr8	124219575	+	FAM83A;FAM83A	Body;Body	0.8728773	0.8488051	5.6868235	7.84E-08	3.92E-06	7.6842804
cg13157483	chr11	125145672	+	PKNOX2	5'UTR	-1.262161	0.443317	-5.68921	7.84E-08	3.92E-06	7.6850565
cg26935168	chr1	6260346	-	RPL22	TSS1500	0.8815767	-0.36925	5.6866208	7.85E-08	3.92E-06	7.6833662
cg06182099	chr8	9789639	-			-1.119717	0.3058169	-5.686337	7.86E-08	3.93E-06	7.6820875
cg27474957	chr11	1567578	-	HCCA2	Body	-0.765525	-2.487401	-5.686188	7.86E-08	3.93E-06	7.6814145
cg20879468	chr5	133983708	-	SEC24A	TSS1500	-0.680905	-4.275246	-5.686112	7.87E-08	3.93E-06	7.6810715

cg18325622	chr5	16180259	-	11-Mar	TSS1500	1.8616655	-0.889802	5.6883756	7.88E-08	3.94E-06	7.680962
cg10269365	chr2	223166989	+	CCDC140	5'UTR	1.4687367	-0.374471	5.6858403	7.88E-08	3.94E-06	7.679847
cg15256305	chr17	36734942	+	SRCIN1	Body	1.4803887	-0.316803	5.6856371	7.88E-08	3.94E-06	7.6789311
cg22558685	chr1	212894643	-			-1.2904	-0.438242	-5.685313	7.90E-08	3.95E-06	7.6774699
cg02390725	chr11	2925768	+	SLC22A18;SLC22A18;SLC22A18AS	Body;Body;TSS1500	-1.397114	0.3383739	-5.685288	7.90E-08	3.95E-06	7.6773587
cg11328677	chr7	154540294	-	DPP6;DPP6;DPP6	Body;Body;Body	-1.156483	0.3266737	-5.684929	7.91E-08	3.95E-06	7.6757382
cg18722124	chr19	30862050	+	ZNF536	TSS1500	-1.694456	0.3494703	-5.684891	7.91E-08	3.95E-06	7.6755659
cg19206146	chr6	28661759	+			1.7175063	0.4565116	5.6847248	7.92E-08	3.95E-06	7.674818
cg00860712	chr1	203274134	+	BTG2	TSS1500	-1.020688	-1.667559	-5.684525	7.93E-08	3.96E-06	7.6739161
cg26610808	chr10	102046685	+	BLOC1S2;BLOC1S2	TSS1500;TSS1500	-0.750872	-3.711762	-5.68427	7.93E-08	3.96E-06	7.6727686
cg21343777	chr4	3341177	-	RGS12;RGS12	Body;Body	0.8237215	4.0252547	5.6841467	7.94E-08	3.96E-06	7.6722118
cg22016731	chr1	40311142	+	TRIT1	Body	-1.346964	0.060507	-5.684009	7.94E-08	3.97E-06	7.6715929
cg08550101	chr7	20823981	-	SP8;SP8	Body;Body	1.3379448	-0.981545	5.683932	7.95E-08	3.97E-06	7.6712443
cg22730004	chr1	158656718	-	SPTA1	TSS1500	-1.814935	-1.366776	-5.683789	7.95E-08	3.97E-06	7.6705975
cg07937271	chr4	13542719	-	NKX3-2	3'UTR	1.2417899	-0.641123	5.6836301	7.96E-08	3.97E-06	7.669883
cg17070005	chr8	13395492	-			-1.618848	1.1293411	-5.683381	7.97E-08	3.98E-06	7.6687617
cg18787437	chr1	16499673	-			-1.074548	-0.516223	-5.685766	7.97E-08	3.98E-06	7.6695496
cg15874390	chr14	96752011	+	ATG2B	3'UTR	0.9673747	0.3901129	5.6855695	7.98E-08	3.98E-06	7.6683271
cg03723716	chr11	111609239	+	PPP2R1B;PPP2R1B	Body;3'UTR	1.112934	1.7163575	5.6830023	7.98E-08	3.98E-06	7.6670535
cg26308012	chr8	141359592	+	TRAPPC9;TRAPPC9	Body;Body	0.6877653	-0.069177	5.6829081	7.99E-08	3.98E-06	7.6666289

cg14640592	chr1	203044726	-	PPFIA4	Body	1.3940685	-0.032608	5.6828713	7.99E-08	3.98E-06	7.6664628
cg20996561	chr22	40082316	+	CACNA1I;CACNA1I	Body;Body	1.3022996	0.9365426	5.6852951	7.99E-08	3.98E-06	7.6674282
cg18606630	chr14	75400792	+			-0.944287	-2.003393	-5.682389	8.01E-08	3.99E-06	7.6642911
cg03676298	chr3	48078103	-	MAP4;MAP4;MAP4	5'UTR;5'UTR;5'UTR	1.0597678	1.5353644	5.6823214	8.01E-08	3.99E-06	7.6639846
cg17067638	chr15	25432270	-	SNORD115-10	TSS1500	-1.139297	0.079492	-5.68203	8.02E-08	4.00E-06	7.6626713
cg21241823	chr21	43300024	+	PRDM15;PRDM15	TSS1500;TSS1500	-0.945238	-1.887624	-5.682012	8.02E-08	4.00E-06	7.6625892
cg00938395	chr1	26633206	+	UBXN11;UBXN11;UBXN11	5'UTR;TSS200;TSS200	-0.514671	-4.301352	-5.681721	8.03E-08	4.00E-06	7.6612786
cg05940984	chr11	32448678	+	WT1;WT1;WT1;WT1	Body;Body;Body;Body	2.3689915	-1.850749	5.6841901	8.03E-08	4.00E-06	7.6624546
cg00086710	chr4	89204074	+	PPM1K	5'UTR	-1.341881	-2.51058	-5.681624	8.03E-08	4.00E-06	7.6608423
cg00423704	chr20	21077665	-			-1.320058	0.1670509	-5.681545	8.04E-08	4.00E-06	7.6604868
cg12210286	chr19	40732316	+	CNTD2	1stExon	-1.685707	-3.199736	-5.681379	8.04E-08	4.01E-06	7.6597384
cg26378179	chr14	87477349	-			-1.61684	-0.26893	-5.681347	8.04E-08	4.01E-06	7.6595938
cg01881287	chr7	105223849	+			0.7627534	1.6117102	5.6810782	8.06E-08	4.01E-06	7.6583819
cg03530754	chr12	128751460	-	TMEM132C	TSS1500	2.0452373	-1.056547	5.6836046	8.05E-08	4.01E-06	7.6594823
cg08185255	chr10	80063568	-			-1.031279	0.0182921	-5.68116	8.05E-08	4.01E-06	7.6587522
cg11056055	chr7	116356738	+	MET;MET	Body;Body	-1.349023	1.0107269	-5.681147	8.05E-08	4.01E-06	7.6586911
cg12324970	chr10	104000446	+	PITX3	5'UTR	0.931859	0.1461803	5.6835965	8.05E-08	4.01E-06	7.6597826
cg05576992	chr8	969029	+			-1.237088	0.1573929	-5.681005	8.06E-08	4.01E-06	7.6580502
cg26506790	chr16	85873323	+			-0.752274	-0.299252	-5.68102	8.06E-08	4.01E-06	7.6581205
cg17362434	chr11	69832232	+			1.4658311	-0.488785	5.6808586	8.06E-08	4.01E-06	7.657392

cg19544919	chr19	18451228	+	PGPEP1	TSS200	-0.661016	-3.668474	-5.680626	8.07E-08	4.02E-06	7.6563449
cg05461386	chr5	178371587	+	ZNF454	Body	-1.641198	1.1526025	-5.680587	8.07E-08	4.02E-06	7.6561675
cg26552650	chr6	113682475	+			-1.646579	0.9308544	-5.680555	8.07E-08	4.02E-06	7.6560238
cg23817893	chr11	86085932	-	CCDC81;CCDC81;CCDC81;CCDC81	1stExon;5'UTR;1stExon;5'UTR	1.8222467	-0.546269	5.6804442	8.08E-08	4.02E-06	7.6555247
cg09884341	chr5	87974464	+	LOC645323;LOC645323	Body;TSS1500	1.6659835	-1.099493	5.6799402	8.10E-08	4.03E-06	7.6532538
cg04874562	chr11	5462540	+	OR51I1	1stExon	-1.034828	-0.423059	-5.679674	8.11E-08	4.03E-06	7.6520553
cg09175338	chr17	10372273	-	MYH4	5'UTR	-1.30764	-0.194154	-5.679717	8.11E-08	4.03E-06	7.6522495
cg16468773	chr8	82644668	+	CHMP4C	TSS200	-0.414243	-3.073158	-5.682176	8.11E-08	4.03E-06	7.6533918
cg13450944	chr3	66533056	-	LRIG1	Body	1.0497823	2.3861907	5.6794606	8.12E-08	4.03E-06	7.6510928
cg07680129	chr4	1808029	+	FGFR3;FGFR3;FGFR3	Body;Body;Body	-0.745118	-0.027893	-5.679287	8.12E-08	4.04E-06	7.6503117
cg01218180	chr6	168079427	+			-1.492438	-1.238433	-5.679209	8.13E-08	4.04E-06	7.6499597
cg00333679	chr6	33943783	+			1.166207	-0.612616	5.6791502	8.13E-08	4.04E-06	7.6496943
cg06847624	chr5	176827671	-	PFN3	TSS200	1.8502989	-0.652272	5.6784621	8.15E-08	4.05E-06	7.6465945
cg14468932	chr13	37961201	+			-1.616228	0.9123879	-5.678205	8.16E-08	4.06E-06	7.6454346
cg23452969	chr11	62691182	+			-1.216953	-1.718832	-5.678105	8.17E-08	4.06E-06	7.644987
cg24392479	chr17	79934870	-	ASPSCR1	TSS1500	-0.63472	-4.164431	-5.677971	8.17E-08	4.06E-06	7.64438
cg09614415	chr10	129535509	-	FOXI2	TSS200	1.4692425	-0.660365	5.6778677	8.18E-08	4.06E-06	7.6439167
cg23695133	chr20	21087075	+			1.9742544	-0.931037	5.6777048	8.18E-08	4.06E-06	7.6431827
cg13462219	chr8	144649510	-	C8orf73	Body	1.174402	0.8685199	5.677498	8.19E-08	4.07E-06	7.6422509
cg27001894	chr10	13022624	-	CCDC3	Body	1.130036	0.0295256	5.6772729	8.20E-08	4.07E-06	7.6412372

cg23038813	chr10	103599520	+	KCNIP2;KCNIP2;KCNIP2;KCNIP2;KCNIP2;KCNIP2;KCNIP2	Body;Body;Body;1stExon;Body;Body;Body	-1.673318	-3.388729	-5.677234	8.20E-08	4.07E-06	7.6410611
cg04436634	chr1	158575773	+	OR10Z1	TSS1500	-1.72716	-0.584254	-5.677147	8.21E-08	4.07E-06	7.6406719
cg04761722	chr16	10274315	+	GRIN2A;GRIN2A;GRIN2A	5'UTR;5'UTR;5'UTR	1.3312177	-1.48297	5.6771622	8.20E-08	4.07E-06	7.6407382
cg17473896	chr11	67272980	+	PITPNM1;PITPNM1	TSS200;TSS200	-0.463026	-3.734963	-5.676977	8.21E-08	4.07E-06	7.6399046
cg01109169	chr2	71134630	+	VAX2	Body	1.3548746	-0.718129	5.6768859	8.22E-08	4.08E-06	7.6394939
cg15648389	chr12	54448769	+	HOXC4;HOXC4	Body;Body	1.3901042	0.0599305	5.6765194	8.23E-08	4.08E-06	7.6378428
cg07797853	chr6	41472032	-			-0.721349	-3.532479	-5.676176	8.24E-08	4.09E-06	7.6362951
cg08258066	chr20	30605656	+	C20orf160	Body	-1.502541	-2.8326	-5.678622	8.24E-08	4.09E-06	7.6370626
cg08529882	chr10	124902456	+			1.3158909	-0.917057	5.6760992	8.25E-08	4.09E-06	7.6359505
cg22374057	chr18	11148682	-	FAM38B;FAM38B	5'UTR;1stExon	1.1842811	-0.61892	5.6759045	8.25E-08	4.09E-06	7.6350733
cg17782848	chr5	170006832	-	KCNIP1;KCNIP1;KCNIP1	Body;Body;Body	-1.358595	0.7229574	-5.675742	8.26E-08	4.10E-06	7.6343421
cg00160667	chr1	29587357	+	PTPRU;PTPRU;PTPRU	Body;Body;Body	1.1365468	1.9944185	5.6756709	8.26E-08	4.10E-06	7.6340213
cg20094831	chr10	44699412	-			-1.638641	0.6468855	-5.675604	8.27E-08	4.10E-06	7.6337179
cg22763649	chr20	21694307	+	PAX1	Body	0.9785867	-0.333328	5.675571	8.27E-08	4.10E-06	7.6335714
cg02156692	chr7	2749751	-	AMZ1	Body	-1.131654	-0.647603	-5.675384	8.27E-08	4.10E-06	7.6327305
cg11835657	chr14	95967697	-			-1.430686	0.4074542	-5.675143	8.28E-08	4.10E-06	7.6316432
cg10824107	chr6	10382069	-			0.889913	0.0217112	5.6750773	8.29E-08	4.10E-06	7.631348
cg27514017	chr10	130684739	+			-1.524279	0.808234	-5.67507	8.29E-08	4.10E-06	7.6313168
cg00654657	chr1	99728691	+	LPPR4;LPPR4	TSS1500;TSS1500	-1.259376	0.1727844	-5.674977	8.29E-08	4.11E-06	7.6308984
cg08641514	chr7	25987606	-			-1.650577	1.3154548	-5.674696	8.30E-08	4.11E-06	7.62963

cg14409029	chr11	19138243	+	ZDHHC13;ZDHHC13	TSS1500;TSS1500	-1.747578	-1.763779	-5.674621	8.30E-08	4.11E-06	7.6292944
cg16596440	chr4	140684596	+	MAML3	Body	1.3879546	-0.133004	5.6745454	8.31E-08	4.11E-06	7.6289525
cg27296341	chr6	154485839	+	IPCEF1;OPRM1;IPCEF1;IPCEF1	Body;Body;Body;Body	1.4984588	0.1936979	5.6745187	8.31E-08	4.11E-06	7.6288325
cg07561747	chr1	200983313	-	KIF21B	Body	1.1690943	0.3787194	5.6769522	8.31E-08	4.11E-06	7.6295501
cg02201132	chr2	242212884	-	HDLBP;HDLBP	5'UTR;TSS1500	1.0772099	1.9059856	5.6742656	8.32E-08	4.12E-06	7.6276925
cg24342696	chr1	39617089	+	MACF1	Body	1.0845359	1.6181274	5.6741617	8.32E-08	4.12E-06	7.6272246
cg26160564	chr1	3826643	-	LOC100133612	Body	-2.111553	-0.129417	-5.674167	8.32E-08	4.12E-06	7.6272488
cg14833896	chr13	53869898	-			-1.106719	-1.430145	-5.674128	8.32E-08	4.12E-06	7.6270745
cg03146625	chr12	54448729	+	HOXC4;HOXC4	Body;Body	1.9239355	0.7718931	5.6791009	8.32E-08	4.12E-06	7.6291609
cg09470142	chr2	190744385	-			0.8812971	3.2675599	5.6740817	8.32E-08	4.12E-06	7.6268645
cg05944004	chr20	59546092	-			-1.381296	-0.371142	-5.67377	8.34E-08	4.12E-06	7.6254624
cg24122498	chr17	4648682	-	ZMYND15;ZMYND15	Body;Body	1.3342634	0.080471	5.6762405	8.34E-08	4.12E-06	7.6263491
cg04995717	chr9	27109293	+	TEK;TEK	5'UTR;1stExon	1.2209967	0.2784189	5.6735875	8.34E-08	4.13E-06	7.624639
cg16266809	chr20	30943371	-			-1.362868	0.1275779	-5.673576	8.34E-08	4.13E-06	7.6245896
cg00910518	chr1	91195213	+			1.8477035	-1.012213	5.6733317	8.35E-08	4.13E-06	7.6234874
cg06059360	chr3	42657618	-	NKTR	Body	1.0450399	2.1048887	5.6732896	8.36E-08	4.13E-06	7.6232979
cg00181834	chr10	23463015	-			1.1081219	-0.86866	5.6731364	8.36E-08	4.13E-06	7.6226077
cg25231776	chr8	10480438	-	RP1L1	Body	-1.217158	-0.023205	-5.673013	8.37E-08	4.14E-06	7.6220502
cg10908953	chr11	121322456	+	SORL1	TSS1500	-0.956672	-1.474096	-5.672949	8.37E-08	4.14E-06	7.6217634
cg04934246	chr11	131410981	-	NTM	Body	-1.942418	0.9704649	-5.672813	8.37E-08	4.14E-06	7.6211524

cg00252418	chr7	154540325	+	DPP6;DPP6;DPP6	Body;Body;Body	-1.122307	-0.879194	-5.672217	8.40E-08	4.15E-06	7.6184699
cg10654803	chr5	41809936	-	OXCT1	Body	1.3555443	1.6924426	5.6720843	8.40E-08	4.15E-06	7.6178709
cg11274148	chr17	78876656	+	RPTOR;RPTOR	Body;Body	0.9082047	4.5868274	5.6823125	8.40E-08	4.15E-06	7.6385493
cg03524083	chr18	70534316	+	NETO1;NETO1;NETO1	TSS1500;TSS1500;Body	1.7559822	-1.251093	5.6720172	8.41E-08	4.15E-06	7.6175691
cg17097782	chr2	19551860	-	OSR1	3'UTR	1.8493261	-0.992951	5.6738938	8.43E-08	4.16E-06	7.6293903
cg10877084	chr5	87967728	-	LOC645323;LOC645323;LOC645323	Body;Body;Body	2.0941234	-1.460057	5.6713491	8.43E-08	4.16E-06	7.6145612
cg13784312	chr9	134609065	-	RAPGEF1	Body	-1.981416	-0.779407	-5.671347	8.43E-08	4.16E-06	7.6145518
cg17179923	chr10	28956088	+			-1.622925	0.351734	-5.671305	8.43E-08	4.16E-06	7.614361
cg22734676	chr4	185139478	-	ENPP6	TSS1500	1.0201409	1.7210026	5.673788	8.43E-08	4.16E-06	7.6310961
cg12792526	chr21	43098958	-	NCRNA00111	TSS1500	-0.782537	-1.504309	-5.671182	8.44E-08	4.17E-06	7.6138104
cg14416311	chr9	89560881	+	GAS1	1stExon	2.6973096	-0.868854	5.6711668	8.44E-08	4.17E-06	7.6137405
cg25037384	chr2	63277127	+	OTX1	TSS1500	-1.102891	-3.183178	-5.671126	8.44E-08	4.17E-06	7.6135571
cg18657700	chr2	241807708	-	AGXT	TSS1500	-1.347546	-0.462335	-5.670936	8.45E-08	4.17E-06	7.6127024
cg15834355	chr12	54442075	-	HOXC4	5'UTR	0.7406186	2.0572427	5.6732536	8.46E-08	4.17E-06	7.6129175
cg18961101	chr12	4923116	-	KCNA6;KCNA6	3'UTR;1stExon	-1.561149	0.9822205	-5.670729	8.46E-08	4.17E-06	7.6117702
cg24813736	chr5	63255045	-			1.4337645	-0.93506	5.670641	8.46E-08	4.17E-06	7.6113736
cg25593309	chr7	155754630	-			-0.866935	-0.777833	-5.670425	8.47E-08	4.18E-06	7.6104036
cg10362335	chr19	6670109	+	TNFSF14;TNFSF14	5'UTR;5'UTR	-1.063865	-1.77863	-5.669827	8.49E-08	4.19E-06	7.607709
cg24008577	chr3	62353504	-			1.5548686	-1.640894	5.6698244	8.49E-08	4.19E-06	7.6076978
cg23337845	chr4	170641374	+	CLCN3;CLCN3	3'UTR;3'UTR	0.9348817	1.8858559	5.6697054	8.50E-08	4.19E-06	7.6071625

cg17694130	chr4	14858290	+			-1.753561	-3.363774	-5.67211	8.50E-08	4.19E-06	7.6081167
cg02066518	chr2	114463932	+			0.6614822	1.262808	5.6695027	8.51E-08	4.19E-06	7.60625
cg00843416	chr17	40914631	+	LOC100190938;RAMP2;LOC100190938	TSS1500;Body;TSS1500	1.057816	1.6079933	5.669414	8.51E-08	4.19E-06	7.6058508
cg00345744	chr7	156929167	+			-1.003539	-2.4249	-5.669352	8.51E-08	4.19E-06	7.6055721
cg17174372	chr11	126522485	+	KIRREL3;KIRREL3	Body;Body	-1.49729	0.5140109	-5.669254	8.52E-08	4.20E-06	7.6051307
cg12767196	chr1	175525986	+	TNR	5'UTR	-1.575885	0.1181472	-5.669206	8.52E-08	4.20E-06	7.6049153
cg18454510	chr6	40363184	+	LRFN2	Body	-1.601689	-0.12677	-5.669173	8.52E-08	4.20E-06	7.6047664
cg06163371	chr1	38230887	+	EPHA10;EPHA10	TSS200;TSS200	1.6246358	-1.3311	5.6690952	8.52E-08	4.20E-06	7.604416
cg06539006	chr2	207814687	+	CPO	Body	-1.044731	-1.060446	-5.668946	8.53E-08	4.20E-06	7.6037439
cg17486097	chr8	35093411	-	UNC5D	Body	1.6257202	-0.843749	5.6689552	8.53E-08	4.20E-06	7.603786
cg25800765	chr12	85673347	+	ALX1	TSS1500	1.5066942	-0.578092	5.6688397	8.53E-08	4.20E-06	7.603266
cg26639039	chr20	44657812	-	SLC12A5;SLC12A5	TSS200;Body	1.5695885	-1.86513	5.6688258	8.53E-08	4.20E-06	7.6032034
cg08845685	chr2	8167037	-			-1.572208	0.562413	-5.668759	8.54E-08	4.20E-06	7.6029021
cg24859617	chr11	125656966	-	PATE3	TSS1500	-1.343535	-0.104529	-5.668773	8.53E-08	4.20E-06	7.6029647
cg17725037	chr8	15653664	-			-1.681907	0.7613934	-5.668678	8.54E-08	4.20E-06	7.6025395
cg22827864	chr6	72298424	+			1.3509614	-0.296663	5.6685068	8.55E-08	4.21E-06	7.6017678
cg03199745	chr12	54454445	+	FLJ12825	Body	0.9756867	1.0431303	5.6684571	8.55E-08	4.21E-06	7.6015441
cg01578632	chr16	69958298	-	WWP2;WWP2	TSS1500;Body	0.6925558	1.9750094	5.6683136	8.55E-08	4.21E-06	7.6008984
cg07672076	chr10	76789460	+	MYST4	Body	0.7293302	2.7606201	5.6681755	8.56E-08	4.21E-06	7.6002767
cg08766211	chr15	79118175	+			1.0367139	0.8536512	5.6681478	8.56E-08	4.21E-06	7.6001522

cg00834922	chr5	178545514	-	ADAMTS2	Body	-0.861669	-1.203763	-5.668047	8.56E-08	4.21E-06	7.5996973
cg05683292	chr5	155133584	-			-1.485902	0.8573188	-5.667908	8.57E-08	4.21E-06	7.5990753
cg09313705	chr17	46622491	-	HOXB2	TSS200	1.4358348	-0.319825	5.6679009	8.57E-08	4.21E-06	7.599041
cg22877504	chr2	216844602	+	MREG	Body	-1.026176	0.0440546	-5.667928	8.57E-08	4.21E-06	7.5991637
cg02553039	chr22	49563521	-			-1.350158	-0.164758	-5.667498	8.59E-08	4.22E-06	7.5972259
cg22337744	chr6	159463893	+	TAGAP;TAGAP;TAGAP	5'UTR;Body;Body	-0.761386	-3.940703	-5.667115	8.60E-08	4.23E-06	7.5955068
cg07722722	chr1	45187551	+	C1orf228	Body	-1.052731	-2.568724	-5.66703	8.61E-08	4.23E-06	7.5951232
cg16229671	chr2	75937132	-	C2orf3	Body	-1.145187	-1.094198	-5.66704	8.60E-08	4.23E-06	7.5951659
cg08696165	chr2	45172169	+	SIX3	3'UTR	2.1056692	-0.807845	5.6669254	8.61E-08	4.23E-06	7.5946517
cg20996682	chr7	79084048	+	MAGI2	TSS1500	1.4652139	-1.118008	5.6668575	8.61E-08	4.23E-06	7.5943462
cg26517714	chr5	170736245	-	TLX3	TSS200	1.482905	-0.919325	5.6667466	8.62E-08	4.23E-06	7.5938471
cg14664303	chr11	2445288	+	TRPM5	TSS1500	-0.995573	0.1018376	-5.666701	8.62E-08	4.23E-06	7.5936425
cg10625365	chr1	168196658	+	SFT2D2	Body	-1.113907	-3.096383	-5.66907	8.62E-08	4.24E-06	7.5941116
cg22914729	chr4	111541339	-	PITX2;PITX2;PITX2	Body;Body;Body	1.6135846	-1.16149	5.6664897	8.63E-08	4.24E-06	7.5926909
cg17827020	chr1	180202784	+	LHX4	Body	1.6056559	-1.821913	5.6664443	8.63E-08	4.24E-06	7.592487
cg03296810	chr15	88826454	+			-1.011919	-1.113434	-5.668681	8.64E-08	4.24E-06	7.5923643
cg13702629	chr15	66546648	-	MEGF11	TSS1500	1.4396478	-1.060313	5.6662035	8.64E-08	4.24E-06	7.5914033
cg00688447	chr8	55379910	-			2.2019397	-1.15398	5.6659701	8.65E-08	4.25E-06	7.5903532
cg15456082	chr14	59444979	+			-1.436062	-0.534414	-5.664854	8.69E-08	4.27E-06	7.5853328
cg01757116	chr13	34118017	+			-1.060004	-0.796514	-5.664829	8.69E-08	4.27E-06	7.585219

cg00478884	chr14	20978888	-	RNASE10	1stExon	-1.410828	-0.197928	-5.664401	8.71E-08	4.28E-06	7.583295
cg22540135	chr19	51898904	-			-1.043573	-1.669952	-5.66429	8.72E-08	4.28E-06	7.5827936
cg03672274	chr4	4860468	-	MSX1	TSS1500	1.3395464	-1.18814	5.6640753	8.73E-08	4.28E-06	7.5818288
cg02757432	chr10	125426048	+	GPR26	1stExon	1.1022216	-1.059623	5.6638919	8.73E-08	4.28E-06	7.5810042
cg11206634	chr2	128458399	-	SFT2D3	TSS200	-1.420338	-0.43484	-5.663802	8.74E-08	4.29E-06	7.5805988
cg08134091	chr6	11213750	+	NEDD9;NEDD9;NEDD9	Body;Body;Body	0.9744557	0.5110051	5.6636452	8.74E-08	4.29E-06	7.5798942
cg23785114	chr8	1153402	+			-1.397186	0.7107087	-5.663405	8.75E-08	4.29E-06	7.5788125
cg06265809	chr14	101525784	+	MIR496;MIR154	TSS1500;TSS1500	-1.3277	0.1687043	-5.663211	8.76E-08	4.30E-06	7.5779429
cg11093142	chr13	49075016	+	RCBTB2	Body	-1.144727	-1.465669	-5.663102	8.77E-08	4.30E-06	7.5774524
cg14544225	chr17	11139650	+			-1.512717	0.1429729	-5.663108	8.77E-08	4.30E-06	7.5774798
cg24716343	chr1	233431953	-	PCNXL2	TSS1500	-0.360147	-3.421397	-5.663064	8.77E-08	4.30E-06	7.5772804
cg01994902	chr16	1576069	-	IFT140	Body	-0.795554	-0.393411	-5.662986	8.77E-08	4.30E-06	7.5769285
cg05788138	chr11	55577775	+	OR5L1	TSS1500	-1.228332	-0.8613	-5.662941	8.77E-08	4.30E-06	7.5767277
cg17434154	chr1	217306590	-	ESRRG	5'UTR	1.0408868	1.0828838	5.6627442	8.78E-08	4.30E-06	7.5758419
cg16540391	chr6	151042035	+	PLEKHG1	5'UTR	-0.888002	-2.674994	-5.662545	8.79E-08	4.31E-06	7.5749439
cg18930354	chr3	147109629	+	ZIC4;ZIC4;ZIC4;ZIC4;ZIC4	Body;Body;Body;Body;Body	1.2571375	-1.632538	5.6625568	8.79E-08	4.31E-06	7.574999
cg14456683	chr3	147127010	-	ZIC1	TSS200	1.2352261	0.0052736	5.6624503	8.79E-08	4.31E-06	7.5745199
cg14232851	chr9	80111634	+	GNA14	Body	-0.949751	-0.917027	-5.661948	8.81E-08	4.32E-06	7.5722617
cg04204669	chr11	2193742	-	TH;TH;TH	TSS1500;TSS1500;TSS1500	-0.94984	-0.680033	-5.661741	8.82E-08	4.32E-06	7.5713321
cg25054821	chr12	119216236	+			-1.305779	-0.092643	-5.661707	8.82E-08	4.32E-06	7.5711793

cg10187167	chr2	3990508	-			-1.152904	0.5002221	-5.661617	8.83E-08	4.32E-06	7.5707715
cg00987918	chr6	30115829	-	TRIM40	3'UTR	-1.031588	-0.879922	-5.6614	8.84E-08	4.33E-06	7.5697953
cg17753169	chr8	41523177	+	ANK1;ANK1;ANK1;ANK1;ANK1;ANK1;ANK1;ANK1	TSS1500;TSS1500;Body;TSS1500;Body;Body;Body;Body	-1.563177	0.5491591	-5.663858	8.84E-08	4.33E-06	7.5710332
cg25210835	chr1	110254828	-	GSTM5	TSS200	1.4462485	0.4676158	5.6612442	8.84E-08	4.33E-06	7.5690961
cg04324666	chr19	23386784	+			1.1487519	-0.935725	5.6609823	8.85E-08	4.33E-06	7.567918
cg26256263	chr1	25258146	+	RUNX3;RUNX3	TSS1500;Body	1.7720201	0.4553066	5.6609517	8.85E-08	4.33E-06	7.5677805
cg02285791	chr6	27550747	+			1.7762034	1.3064997	5.6633503	8.86E-08	4.33E-06	7.5684142
cg05743885	chr7	27196365	+	HOXA7	TSS200	2.0383503	-1.348767	5.6608313	8.86E-08	4.33E-06	7.5672394
cg12189551	chr14	60952945	+	C14orf39	TSS200	1.9288512	-1.314162	5.6608275	8.86E-08	4.33E-06	7.567222
cg15316843	chr8	65282946	+			2.305317	-0.90987	5.6607413	8.86E-08	4.34E-06	7.5668344
cg00625776	chr12	53297384	+	KRT8	Body	-0.673779	-2.816853	-5.660576	8.87E-08	4.34E-06	7.5660925
cg14258078	chr17	62051773	+	SCN4A	TSS1500	-1.553565	0.5468599	-5.660533	8.87E-08	4.34E-06	7.5658974
cg00466249	chr17	79269884	-	SLC38A10;SLC38A10	TSS1500;TSS1500	-1.010687	-0.865413	-5.660329	8.88E-08	4.34E-06	7.5649786
cg16148891	chr15	102347868	-			-1.256139	-0.581693	-5.660348	8.88E-08	4.34E-06	7.5650639
cg21185289	chr2	74743437	-	TLX2	3'UTR	3.3161293	-1.663522	5.6603004	8.88E-08	4.34E-06	7.5648519
cg04123776	chr1	170630602	+			3.4014846	-2.016257	5.6648944	8.90E-08	4.35E-06	7.581172
cg16310491	chr5	87973597	-	LOC645323;LOC645323	Body;Body	1.3105971	-0.613033	5.6599257	8.90E-08	4.35E-06	7.5631671
cg16453617	chr16	1257884	-	CACNA1H;CACNA1H	Body;Body	-1.268474	0.2447934	-5.659908	8.90E-08	4.35E-06	7.5630883
cg17911318	chr18	74962000	-	GALR1	TSS200	2.4323428	-1.529046	5.6599679	8.90E-08	4.35E-06	7.5633567
cg11385906	chr8	28745680	-	INTS9;INTS9;INTS9	Body;Body;Body	1.0381013	0.8300573	5.6598152	8.90E-08	4.35E-06	7.5626702

cg06776976	chr1	212588580	+	TMEM206	TSS1500	-0.942124	-1.623312	-5.659751	8.90E-08	4.35E-06	7.5623802
cg08977702	chr2	168407472	-			-1.792286	0.655777	-5.659613	8.91E-08	4.35E-06	7.5617592
cg02851873	chr10	80907003	-	ZMIZ1	5'UTR	0.8953936	0.5479871	5.6592473	8.93E-08	4.36E-06	7.5601171
cg15402156	chr19	57669719	-	DUXA	Body	-0.917534	-0.667637	-5.659006	8.94E-08	4.36E-06	7.5590311
cg19907968	chr7	1560598	-			-2.108067	1.2484205	-5.659027	8.93E-08	4.36E-06	7.5591262
cg03052128	chr12	85671811	+			1.3268249	-0.842902	5.6587802	8.95E-08	4.37E-06	7.558017
cg21057473	chr11	5346163	-	OR51B2	TSS1500	-1.377384	-0.30296	-5.658735	8.95E-08	4.37E-06	7.5578146
cg13192013	chr4	160188736	-	RAPGEF2	TSS1500	1.1463494	0.7635718	5.666212	8.95E-08	4.37E-06	7.576354
cg00853940	chr2	234847683	+	TRPM8	Body	1.5148887	-1.09208	5.6585566	8.95E-08	4.37E-06	7.5570118
cg00021242	chr15	29773425	+	FAM189A1	Body	-1.470515	0.5692053	-5.658409	8.96E-08	4.37E-06	7.5563479
cg16582419	chr6	30038998	-	RNF39;RNF39	Body;Body	1.1641618	1.5720655	5.6583666	8.96E-08	4.37E-06	7.5561575
cg09971653	chr16	48559770	-			-0.944011	0.1155475	-5.658314	8.96E-08	4.37E-06	7.5559232
cg14035553	chr1	21029443	-	KIF17;KIF17	Body;Body	-1.046327	-0.590634	-5.658196	8.97E-08	4.38E-06	7.5553926
cg26616780	chr20	1546019	-	SIRPB1;SIRPB1	3'UTR;3'UTR	-1.595627	0.2309408	-5.658064	8.98E-08	4.38E-06	7.5547992
cg09545109	chr3	50388910	+	TUSC4;CYB561D2	TSS1500;Body	-0.724	0.0123778	-5.65799	8.98E-08	4.38E-06	7.5544643
cg22635088	chr5	2096922	+			-1.117639	-0.879971	-5.657964	8.98E-08	4.38E-06	7.5543477
cg10283844	chr2	10487314	+	HPCAL1;HPCAL1	5'UTR;5'UTR	1.7820874	2.267464	5.6579287	8.98E-08	4.38E-06	7.5541891
cg13066016	chr1	63786083	+			1.3723952	-1.279945	5.6577457	8.99E-08	4.38E-06	7.5533663
cg01004363	chr10	88729464	-	AGAP11;C10orf116	TSS1500;Body	1.1381776	0.3468039	5.6576547	8.99E-08	4.38E-06	7.5529575
cg04324521	chr16	1248501	-	CACNA1H;CACNA1H	Body;Body	-1.392999	1.0742618	-5.657606	8.99E-08	4.38E-06	7.552737

cg05353872	chr1	248366399	-	OR2M3	1stExon	-1.87697	0.3008304	-5.657524	9.00E-08	4.39E-06	7.5523719
cg00963169	chr1	50513927	+	ELAVL4	Body	2.3230866	-1.314064	5.657446	9.00E-08	4.39E-06	7.552019
cg20289911	chr20	816362	-	FAM110A;FAM110A	TSS1500;5'UTR	0.8953921	-1.023202	5.6574436	9.00E-08	4.39E-06	7.5520086
cg26384036	chr13	111059652	+	COL4A2	Body	0.8957324	1.3207293	5.6594688	9.02E-08	4.40E-06	7.5513249
cg03451574	chr1	61542546	-	NFIA	TSS1500	-0.386492	-4.171058	-5.656933	9.02E-08	4.40E-06	7.5497111
cg03526920	chr14	77291706	-	C14orf166B	TSS1500	-0.971139	-0.336892	-5.656797	9.03E-08	4.40E-06	7.5491027
cg13678973	chr1	50513870	+	ELAVL4	Body	2.4120254	-0.951499	5.6564554	9.04E-08	4.40E-06	7.5475664
cg05474726	chr8	65291523	+	MIR124-2	TSS200	1.6348831	-1.190545	5.658397	9.07E-08	4.42E-06	7.5465134
cg10838757	chr5	130507273	-	LYRM7	Body	-0.607409	-3.36819	-5.655904	9.07E-08	4.42E-06	7.5450867
cg13689563	chr19	12277357	+	ZNF136	Body	0.9294687	1.6127547	5.6557868	9.07E-08	4.42E-06	7.5445616
cg19136783	chr4	16598466	-	LDB2;LDB2	Body;Body	-1.626541	1.1515898	-5.655672	9.08E-08	4.42E-06	7.5440466
cg05050341	chr9	130617534	+	ENG;ENG	TSS1500;TSS1500	0.9205142	1.3684917	5.6555162	9.08E-08	4.42E-06	7.5433454
cg26812411	chr11	129150282	+			-1.280823	-2.743923	-5.655536	9.08E-08	4.42E-06	7.5434329
cg09796501	chr1	26233435	+	STMN1;STMN1;STMN1;STMN1	TSS1500;TSS1500;TSS1500;TSS200	-1.277882	-2.987422	-5.660419	9.09E-08	4.42E-06	7.5456746
cg10818219	chr1	43124459	+	PPIH	Body	-0.37333	-4.796405	-5.65518	9.10E-08	4.43E-06	7.5418347
cg00630991	chr12	12252249	+	BCL2L14;BCL2L14;BCL2L14	3'UTR;3'UTR;3'UTR	1.1097626	1.5556998	5.6551486	9.10E-08	4.43E-06	7.5416933
cg00714874	chr1	161679461	+	FCRLA	Body	-0.827072	-0.977548	-5.655051	9.10E-08	4.43E-06	7.541255
cg04313338	chr16	55735794	+	SLC6A2	Body	-0.727124	-2.01782	-5.654976	9.11E-08	4.43E-06	7.5409181
cg06842954	chr4	111559066	-	PITX2;PITX2	TSS1500;TSS1500	1.8920836	-0.457856	5.6573309	9.11E-08	4.43E-06	7.5417281
cg16993794	chr22	24199328	-	SLC2A11;SLC2A11;SLC2A11	5'UTR;Body;TSS1500	-0.89656	-3.639345	-5.6548	9.11E-08	4.43E-06	7.5401256

cg17095652	chr15	40161645	+	GPR176	Body	-1.082333	-1.523724	-5.65469	9.12E-08	4.43E-06	7.5396335
cg02550308	chr16	69310261	+	SNTB2	Body	1.0124663	1.8634722	5.6543291	9.13E-08	4.44E-06	7.5380106
cg02768875	chr6	43603375	+	MAD2L1BP;MAD2L1BP	Body;TSS1500	-0.588415	-2.304209	-5.654139	9.14E-08	4.44E-06	7.5371572
cg08995609	chr11	66104115	+	RIN1	TSS200	-1.383028	-3.323011	-5.654094	9.14E-08	4.45E-06	7.536956
cg19022006	chr18	61143945	-	SERPINB5	TSS200	-2.840602	1.0659078	-5.653984	9.15E-08	4.45E-06	7.5364598
cg02320543	chr14	61109545	+			1.5218373	-0.115481	5.6538497	9.15E-08	4.45E-06	7.5358566
cg17827767	chr10	86001154	+	LRIT1	1stExon	-1.245582	0.0324651	-5.653665	9.16E-08	4.45E-06	7.5350249
cg07109804	chr15	83654517	+	FAM103A1	TSS1500	-1.047798	-2.275102	-5.653566	9.17E-08	4.45E-06	7.5345838
cg12415917	chr11	6047917	-	OR56A1;OR56A1	3'UTR;1stExon	-1.510104	0.3782986	-5.65348	9.17E-08	4.46E-06	7.5341941
cg00552318	chr22	41633632	-	CHADL	Body	-1.067075	-1.926616	-5.653277	9.18E-08	4.46E-06	7.5332827
cg01423251	chr6	32765322	+			-1.119223	-2.281596	-5.653143	9.18E-08	4.46E-06	7.5326803
cg25127692	chr3	185406536	-	IGF2BP2;IGF2BP2	Body;Body	-1.768624	0.5640444	-5.653136	9.19E-08	4.46E-06	7.5326487
cg04420907	chr17	9480215	+	WDR16;WDR16;STX8	Body;Body;TSS1500	-1.09087	-3.034765	-5.655405	9.19E-08	4.47E-06	7.532742
cg12921481	chr1	158747277	+	OR6N2	1stExon	-1.78479	0.6398272	-5.652863	9.20E-08	4.47E-06	7.5314232
cg18984452	chr9	1046103	-			1.1534267	-0.422246	5.6527239	9.20E-08	4.47E-06	7.5307982
cg07625194	chr6	50683782	+	TFAP2D	Body	1.7767417	-1.93649	5.6524712	9.21E-08	4.47E-06	7.5296629
cg24146331	chr10	102590225	+			1.4417533	-1.169873	5.6523551	9.22E-08	4.48E-06	7.5291414
cg11014073	chr12	64460951	-	SRGAP1	Body	0.6599095	2.7277003	5.6523028	9.22E-08	4.48E-06	7.5289065
cg11062417	chr5	76578232	-	PDE8B;PDE8B;PDE8B;PDE8B;PDE8B	Body;Body;Body;Body;Body	0.9197617	0.6754621	5.6521552	9.23E-08	4.48E-06	7.5282432
cg14254748	chr1	62660421	-	L1TD1;L1TD1	TSS200;TSS200	1.3459543	0.5065002	5.6520151	9.23E-08	4.48E-06	7.5276139

cg27521476	chr10	102896376	-	TLX1	Body	0.9777151	-0.920038	5.6520209	9.23E-08	4.48E-06	7.52764
cg17007164	chr2	6778799	+			-1.623169	-0.005957	-5.651941	9.24E-08	4.48E-06	7.5272799
cg14069619	chr17	8275273	-	KRBA2	TSS1500	0.919227	2.025338	5.6519142	9.24E-08	4.48E-06	7.5271605
cg21975390	chr11	8703324	+	RPL27A	TSS1500	-0.858784	-0.976232	-5.65187	9.24E-08	4.48E-06	7.5269626
cg01691827	chr16	86599189	-			0.8485017	0.1057509	5.6518151	9.24E-08	4.48E-06	7.5267156
cg01613010	chr7	20835627	-			1.5703996	0.0743513	5.6517652	9.24E-08	4.48E-06	7.5264911
cg09444685	chr5	159355481	+	ADRA1B	Body	-0.963554	-1.562568	-5.651774	9.24E-08	4.48E-06	7.52653
cg15495091	chr13	113491819	+	ATP11A;ATP11A	Body;Body	1.8491803	3.6467392	5.651593	9.25E-08	4.49E-06	7.5257175
cg08343600	chr10	43894095	+	HNRNPF;HNRNPF;HNRNPF;HNRNPF;HNRNPF	5'UTR;TSS1500;5'UTR;5'UTR;5'UTR	-1.344442	-0.371388	-5.65151	9.26E-08	4.49E-06	7.5253433
cg17679470	chr4	189602161	+			-1.254419	0.6288446	-5.651139	9.27E-08	4.49E-06	7.5236792
cg24783510	chr19	17862150	-	FCHO1;FCHO1;FCHO1;FCHO1	5'UTR;TSS200;TSS200;5'UTR	-1.268546	-1.115204	-5.65087	9.28E-08	4.50E-06	7.5224721
cg20776977	chr1	2564584	+	MMEL1	TSS200	-0.709388	-1.731261	-5.650803	9.29E-08	4.50E-06	7.5221711
cg26798039	chr6	47120567	+			0.8292951	1.505678	5.6508262	9.29E-08	4.50E-06	7.522273
cg10085326	chr11	102826680	+	MMP13	TSS1500	-1.518761	0.4685443	-5.650774	9.29E-08	4.50E-06	7.5220396
cg07631533	chr16	13135565	-	SHISA9	Body	-1.18105	-0.897265	-5.650507	9.30E-08	4.51E-06	7.5208413
cg03526459	chr1	146549940	-			1.3644254	0.7964358	5.6504524	9.30E-08	4.51E-06	7.5205942
cg04374654	chr11	75479408	-	DGAT2	TSS1500	-0.848484	-3.483792	-5.650275	9.31E-08	4.51E-06	7.5197968
cg14344261	chr9	36986380	+	PAX5	Body	2.5162517	-1.871428	5.652667	9.31E-08	4.51E-06	7.5204581
cg09098707	chr12	108525359	+	WSCD2	5'UTR	-1.050502	-0.947354	-5.649979	9.32E-08	4.51E-06	7.5184656
cg13096208	chr18	55019843	+	ST8SIA3;ST8SIA3	1stExon;5'UTR	2.4073658	-0.938835	5.6522184	9.33E-08	4.52E-06	7.5184458

cg23345292	chr18	44099184	+	LOXHD1;LOXHD1;LOXHD1	TSS200;Body;Body	-1.118385	-0.694977	-5.649637	9.34E-08	4.52E-06	7.516932
cg14197071	chr4	81110459	+	PRDM8	5'UTR	1.257969	0.3906155	5.6494017	9.35E-08	4.52E-06	7.515875
cg07536847	chr1	18957232	-	PAX7;PAX7;PAX7	TSS1500;TSS1500;TSS1500	2.0205805	-1.037642	5.6516567	9.36E-08	4.53E-06	7.516268
cg23801338	chr1	985239	-	AGRN	Body	-1.944607	2.8897171	-5.654125	9.36E-08	4.53E-06	7.5174663
cg26966083	chr11	2185376	+	TH;TH;TH	3'UTR;3'UTR;3'UTR	-1.089703	0.1089679	-5.64918	9.36E-08	4.53E-06	7.5148775
cg00057663	chr2	105945629	-	TGFBRAP1;TGFBRAP1	5'UTR;5'UTR	-0.547657	-2.702479	-5.648975	9.37E-08	4.53E-06	7.5139591
cg00709966	chr7	27253143	-			0.8679813	0.2703406	5.6490404	9.36E-08	4.53E-06	7.5142521
cg15088574	chr1	4848991	+			-1.283169	-0.734655	-5.649069	9.36E-08	4.53E-06	7.5143789
cg18174678	chr1	42801761	+	FOXJ3	TSS1500	-1.003337	-1.934503	-5.649028	9.36E-08	4.53E-06	7.5141951
cg20802826	chr6	30782080	-			-1.198805	-0.054464	-5.653949	9.37E-08	4.53E-06	7.5163231
cg24186774	chr14	63757917	+	RHOJ	3'UTR	-1.475513	0.4434994	-5.648993	9.37E-08	4.53E-06	7.5140381
cg18687314	chr20	43733672	-			-1.014485	-0.352858	-5.648738	9.38E-08	4.53E-06	7.5128938
cg25248045	chr2	102381786	+	MAP4K4;MAP4K4;MAP4K4	Body;Body;Body	0.7945757	1.3556802	5.6486949	9.38E-08	4.53E-06	7.5127005
cg18273840	chr5	45695643	-	HCN1	Body	1.7973787	-0.210289	5.6485778	9.38E-08	4.54E-06	7.5121749
cg03205252	chr16	51187536	-			1.4989087	-1.614508	5.6485515	9.38E-08	4.54E-06	7.5120566
cg17563034	chr4	5021268	-	CYTL1	TSS200	1.5385787	-0.822327	5.6483347	9.39E-08	4.54E-06	7.5110829
cg04744713	chr8	142517440	-	FLJ43860	TSS200	-1.448935	-0.908371	-5.64814	9.40E-08	4.54E-06	7.5102089
cg04877287	chr11	132278166	+			-1.611426	0.3598853	-5.648124	9.40E-08	4.54E-06	7.5101352
cg23208152	chr3	50383134	-	ZMYND10;ZMYND10	1stExon;5'UTR	-2.303679	-4.239622	-5.660827	9.40E-08	4.54E-06	7.515861
cg25214093	chr10	126135810	-			1.4890033	-1.181585	5.6480326	9.41E-08	4.54E-06	7.5097265

cg13123578	chr7	154683249	-	DPP6;DPP6;DPP6	Body;Body;Body	-1.327138	0.559879	-5.648005	9.41E-08	4.55E-06	7.5096044
cg05254221	chr10	61050062	-	FAM13C;FAM13C;FAM13C;FAM13C	Body;Body;Body;Body	-1.489671	-0.366016	-5.647857	9.42E-08	4.55E-06	7.5089392
cg15974196	chr5	11791728	+	CTNND2	Body	-1.75317	-0.00241	-5.654757	9.44E-08	4.56E-06	7.5099287
cg00374101	chr8	87878605	+	CNBD1	TSS200	-1.829338	0.4630894	-5.64718	9.45E-08	4.56E-06	7.5058994
cg12559228	chr19	50191882	+	C19orf76	TSS200	0.6840637	1.2236478	5.647045	9.45E-08	4.56E-06	7.5052918
cg16734549	chr17	43488551	+	ARHGAP27	5'UTR	0.7817151	1.4867471	5.6470157	9.45E-08	4.56E-06	7.5051603
cg01758167	chr12	131357429	+	RAN	Body	-0.46126	-4.431983	-5.646932	9.46E-08	4.56E-06	7.5047866
cg17656763	chr12	65927079	-			-1.423446	-0.756743	-5.646913	9.46E-08	4.56E-06	7.5046992
cg02341815	chr5	33936752	-	RXFP3;RXFP3	1stExon;5'UTR	1.1089327	-1.444625	5.6468646	9.46E-08	4.57E-06	7.5044818
cg05573844	chr12	54354590	-			1.499709	-0.164983	5.6467479	9.46E-08	4.57E-06	7.503958
cg13878456	chr17	38020344	-	IKZF3;IKZF3;IKZF3;IKZF3;IKZF3;IKZF3	Body;Body;Body;Body;Body;Body	-0.654656	-2.656159	-5.65153	9.47E-08	4.57E-06	7.5058421
cg21877907	chr11	131506338	+	NTM	Body	-1.510693	0.4234173	-5.649038	9.47E-08	4.57E-06	7.5041788
cg07147449	chr12	115136072	+			2.5105064	-1.513567	5.6489145	9.48E-08	4.57E-06	7.5036269
cg04578317	chr14	105189737	-	ADSSL1	TSS1500	-1.008339	0.543979	-5.645899	9.50E-08	4.58E-06	7.5001445
cg22914987	chr12	4054555	+			-1.594684	0.7214581	-5.648396	9.50E-08	4.58E-06	7.501646
cg23746577	chr18	45555438	+	ZBTB7C	3'UTR	-1.420902	0.8332364	-5.645909	9.50E-08	4.58E-06	7.5001908
cg22509504	chr17	19322114	-			-1.309956	-0.148292	-5.645803	9.51E-08	4.58E-06	7.4997135
cg09122414	chr1	38391771	+	INPP5B	Body	-1.322365	-1.797046	-5.645684	9.51E-08	4.59E-06	7.4991804
cg03614193	chr18	55021542	+	ST8SIA3	Body	1.4235253	0.2472694	5.6456278	9.51E-08	4.59E-06	7.4989289
cg27399031	chr4	37224078	-			-1.431362	0.8315609	-5.645503	9.52E-08	4.59E-06	7.4983703

cg11345693	chr17	79170810	+	AZI1;AZI1	Body;Body	0.6730049	0.8715023	5.6452853	9.53E-08	4.59E-06	7.4973911
cg21234123	chr9	139435339	+	NOTCH1	Body	1.0125629	1.6818067	5.6452675	9.53E-08	4.59E-06	7.4973113
cg05927017	chr18	60988155	+			-0.755441	-3.910095	-5.645164	9.54E-08	4.59E-06	7.4968489
cg09841150	chr6	30106949	+	TRIM40	Body	-1.647127	0.1319713	-5.644881	9.55E-08	4.60E-06	7.4955749
cg24479137	chr1	239699220	-			-2.000637	1.0263562	-5.644739	9.55E-08	4.60E-06	7.4949396
cg08761208	chr15	65693289	+	IGDCC4	Body	-0.935362	-1.697257	-5.644681	9.56E-08	4.60E-06	7.4946794
cg27367721	chr16	20917257	-	LYRM1;LYRM1;LYRM1	5'UTR;5'UTR;5'UTR	1.112773	0.9840121	5.6443341	9.57E-08	4.61E-06	7.4931215
cg01023759	chr16	89983766	+	MC1R	TSS1500	-1.187831	0.2964897	-5.644185	9.58E-08	4.61E-06	7.4924507
cg03270167	chr2	238768916	-	RAMP1	Body	-1.08889	-3.309857	-5.643956	9.59E-08	4.62E-06	7.4914251
cg16065473	chr15	88576884	-	NTRK3;NTRK3;NTRK3	Body;Body;Body	-1.732497	0.7545775	-5.643947	9.59E-08	4.62E-06	7.4913845
cg07074042	chr22	30783944	-	RNF215	TSS1500	-1.292499	0.0623155	-5.643866	9.59E-08	4.62E-06	7.4910185
cg24550149	chr1	55246954	+	TTC22	Body	1.2013254	0.7042683	5.6461149	9.60E-08	4.62E-06	7.4910737
cg20903926	chr1	55271677	-	C1orf177;C1orf177	TSS200;TSS200	0.9811259	1.5753855	5.6435373	9.61E-08	4.62E-06	7.4895449
cg00733786	chr14	54795898	-			-1.377165	-3.068387	-5.643513	9.61E-08	4.62E-06	7.4894369
cg09634202	chr10	15387710	-	FAM171A1	Body	-1.300361	0.5330112	-5.643372	9.62E-08	4.63E-06	7.4888019
cg04645914	chr19	52452317	-			1.5514316	-0.09593	5.6432926	9.62E-08	4.63E-06	7.4884465
cg21526778	chr1	243584861	-	SDCCAG8	Body	-2.247222	1.00817	-5.642652	9.65E-08	4.64E-06	7.4855694
cg07311759	chr14	21623804	-	OR5AU1	1stExon	-1.374917	0.3576519	-5.644984	9.65E-08	4.64E-06	7.4860056
cg25481636	chr2	983326	+	SNTG2	Body	-1.571646	0.9170527	-5.642302	9.66E-08	4.65E-06	7.4840008
cg05431947	chr11	12621248	+			-1.092685	-1.551704	-5.642189	9.67E-08	4.65E-06	7.4834932

cg07486821	chr16	60405704	+			-1.17808	-0.304795	-5.642035	9.68E-08	4.65E-06	7.4828043
cg16285941	chr8	144212930	-			-0.646785	-1.217803	-5.642021	9.68E-08	4.65E-06	7.4827381
cg16153042	chr6	3282307	-	SLC22A23;SLC22A23	Body;Body	0.8793797	0.5253985	5.6418598	9.68E-08	4.66E-06	7.482016
cg16520796	chr16	75964393	+			-1.52289	0.2988718	-5.641873	9.68E-08	4.66E-06	7.4820732
cg04661674	chr6	29596874	-	GABBR1;GABBR1;GABBR1	TSS1500;Body;Body	-0.993161	-3.909017	-5.641733	9.69E-08	4.66E-06	7.4814484
cg17036007	chr1	156460747	-	MEF2D	5'UTR	0.7707416	-0.677234	5.6416088	9.70E-08	4.66E-06	7.4808897
cg18235690	chr19	46386962	+	IRF2BP1;IRF2BP1	3'UTR;1stExon	-1.162103	-1.23641	-5.64157	9.70E-08	4.66E-06	7.4807166
cg16791528	chr2	79347532	-	REG1A	TSS200	-1.641549	1.142782	-5.641422	9.70E-08	4.66E-06	7.4800514
cg12629008	chr1	58625006	+	DAB1	5'UTR	-1.594013	-0.19431	-5.640933	9.73E-08	4.67E-06	7.4778554
cg22683277	chr2	105362983	+			-1.563461	0.4243207	-5.640919	9.73E-08	4.67E-06	7.4777967
cg13563074	chr11	7728814	+	OVCH2	TSS1500	-1.217421	-2.018673	-5.640696	9.74E-08	4.68E-06	7.4767956
cg11031737	chr11	27255755	+			-0.965547	0.7120393	-5.640623	9.74E-08	4.68E-06	7.4764667
cg12308746	chr2	172952415	-	DLX1;DLX1	Body;Body	1.5705048	-0.496633	5.6405397	9.74E-08	4.68E-06	7.4760927
cg15696462	chr5	162995646	-			-1.627599	1.4082056	-5.640506	9.75E-08	4.68E-06	7.4759427
cg21301805	chr1	119534644	+			1.5510019	-0.751101	5.6404772	9.75E-08	4.68E-06	7.4758122
cg12513880	chr12	115136298	-			2.0283658	-1.27747	5.6428923	9.75E-08	4.68E-06	7.4766287
cg22928599	chr19	46999403	-	PNMAL2	TSS1500	-0.813691	-1.129691	-5.640238	9.76E-08	4.68E-06	7.4747371
cg10842141	chr10	23489232	-			1.5871742	-2.31702	5.6401887	9.76E-08	4.68E-06	7.4745177
cg21282997	chr2	103035835	+	IL18RAP	5'UTR	-1.537237	-0.749023	-5.639952	9.77E-08	4.69E-06	7.4734567
cg20636352	chr2	200468832	-			1.3268248	0.0933402	5.6398065	9.78E-08	4.69E-06	7.4728028

cg03432085	chr11	126521914	+	KIRREL3;KIRREL3	Body;Body	-1.644796	0.9370597	-5.639684	9.78E-08	4.69E-06	7.4722539
cg03440799	chr1	179334176	-	C1orf125;C1orf125	TSS1500;TSS1500	-1.883685	0.3953963	-5.639699	9.78E-08	4.69E-06	7.4723227
cg11721554	chr14	101528156	-	MIR377	TSS1500	-1.112596	-0.112888	-5.639757	9.78E-08	4.69E-06	7.4725826
cg20364632	chr6	49636226	-			-1.087733	-1.747166	-5.639672	9.78E-08	4.69E-06	7.4721988
cg27153655	chr14	94760403	-	SERPINA10;SERPINA10	TSS1500;TSS1500	-1.556353	0.6597366	-5.639703	9.78E-08	4.69E-06	7.4723394
cg09635874	chr13	98952518	+	FARP1	Body	1.0373033	1.1752625	5.6396121	9.79E-08	4.69E-06	7.4719308
cg10192198	chr9	973184	-			1.5550051	-0.560526	5.6395801	9.79E-08	4.69E-06	7.4717873
cg00923934	chr8	55795838	-			-1.324039	-0.474533	-5.641921	9.79E-08	4.69E-06	7.4722768
cg12109291	chr2	45232117	+			1.8418851	-1.633317	5.6394712	9.79E-08	4.69E-06	7.4712983
cg15425827	chr8	124194965	-	FAM83A;FAM83A;FAM83A;FAM83A	1stExon;1stExon;5'UTR;5'UTR	-0.832413	-1.253681	-5.639331	9.80E-08	4.70E-06	7.4706713
cg22790986	chr11	123757153	-	TMEM225	TSS1500	-1.89384	1.1208986	-5.639233	9.80E-08	4.70E-06	7.4702308
cg21737226	chr8	53326668	+			1.2187737	0.2104929	5.6391695	9.81E-08	4.70E-06	7.4699451
cg02855363	chr17	11457882	-	SHISA6	Body	-0.869337	-0.331743	-5.638901	9.82E-08	4.70E-06	7.4687413
cg04363282	chr2	233877969	+	NGEF	TSS200	-1.06059	-0.735761	-5.638888	9.82E-08	4.70E-06	7.4686825
cg06365303	chr20	2801844	-			1.2202249	-0.398821	5.6388747	9.82E-08	4.70E-06	7.4686223
cg25596297	chr2	223162875	+	PAX3;PAX3;PAX3;CCDC140;CCDC140;PAX3;PAX3;PAX3;PAX3;PAX3	Body;Body;Body;5'UTR;1stExon;Body;Body;Body;Body;Body	2.0480413	-1.437796	5.6389266	9.82E-08	4.70E-06	7.4688553
ch.17.1646506R	chr17	62164903	+	ERN1	Body	-0.694431	-4.160076	-5.638648	9.83E-08	4.71E-06	7.4676042
cg01792749	chr3	38835179	-	SCN10A	Body	-1.155943	-0.963357	-5.641012	9.83E-08	4.71E-06	7.4685466
cg18098722	chr11	556889	-	C11orf35	Body	0.8857525	2.7187226	5.6385533	9.84E-08	4.71E-06	7.4671806
cg21206652	chr8	144790187	-	LOC100130274	1stExon	0.9514814	0.1581238	5.6384954	9.84E-08	4.71E-06	7.4669207

cg11462533	chr1	40156840	-	HPCAL4	5'UTR	-0.783857	-2.704245	-5.638373	9.84E-08	4.71E-06	7.4663718
cg22688241	chr12	55369150	-	KIAA0748;KIAA0748	5'UTR;5'UTR	-1.81075	1.2041722	-5.638342	9.84E-08	4.71E-06	7.4662344
cg20699079	chr2	137199850	-			-1.340209	-0.600598	-5.64833	9.85E-08	4.71E-06	7.4862816
cg16905506	chr1	155035748	-	EFNA4;EFNA4;EFNA4	TSS1500;TSS1500;TSS1500	-1.263642	-1.954719	-5.637989	9.86E-08	4.72E-06	7.4646509
cg01802545	chr14	93196432	+	LGMN;LGMN	Body;Body	-1.454544	-3.308244	-5.63792	9.86E-08	4.72E-06	7.4643386
cg06277849	chr4	186316674	-	ANKRD37	TSS1500	-0.980417	-3.338231	-5.637923	9.86E-08	4.72E-06	7.4643519
cg01399485	chr1	10733030	+	CASZ1;CASZ1	Body;Body	0.7067615	1.6514806	5.6378187	9.87E-08	4.72E-06	7.4638852
cg16982077	chr5	138842197	+	ECSCR	1stExon	0.6949977	0.8390643	5.6374916	9.88E-08	4.73E-06	7.462418
cg19390271	chr12	70645360	+	CNOT2	5'UTR	1.2123557	0.1049368	5.6374594	9.89E-08	4.73E-06	7.4622739
cg08977536	chr2	66808851	-			1.4768889	-1.63137	5.637416	9.89E-08	4.73E-06	7.4620789
cg16251016	chr19	11643100	-			-0.975915	-1.052559	-5.637324	9.89E-08	4.73E-06	7.4616679
cg13498800	chr1	182031601	+	ZNF648	TSS1500	-1.311028	0.0080394	-5.637196	9.90E-08	4.73E-06	7.4610915
cg14497054	chr12	34261116	-			1.1972211	0.5580605	5.6371477	9.90E-08	4.73E-06	7.4608754
cg26777456	chr12	54454250	-	FLJ12825	Body	1.2119094	-0.286055	5.6369688	9.91E-08	4.74E-06	7.460073
cg03813377	chr6	30737455	-			-0.95336	-1.596075	-5.636835	9.91E-08	4.74E-06	7.4594732
cg06638795	chr2	42719933	-	KCNG3;KCNG3	Body;Body	1.0878282	0.7972989	5.6367581	9.92E-08	4.74E-06	7.4591282
cg18810022	chr2	134989811	-			-1.150809	0.7229043	-5.636766	9.92E-08	4.74E-06	7.4591642
cg14662181	chr1	192408849	+			-1.749087	0.1652292	-5.636691	9.92E-08	4.74E-06	7.4588269
cg15345206	chr10	23463706	+			1.3239667	-1.419044	5.6366734	9.92E-08	4.74E-06	7.4587481
cg22157099	chr2	15830172	+			-1.266395	-0.534532	-5.636566	9.93E-08	4.74E-06	7.4582667

cg07178066	chr16	87440505	-	ZCCHC14	3'UTR	-1.326088	1.4161935	-5.636399	9.93E-08	4.75E-06	7.457517
cg10271112	chr10	57394513	-			-1.373416	0.5847437	-5.636057	9.95E-08	4.75E-06	7.4559831
cg20784768	chr1	234041456	+	SLC35F3	Body	1.050276	0.0183475	5.6359951	9.95E-08	4.75E-06	7.4557061
cg26043322	chr1	159507162	-	OR10J5	TSS1500	-1.735733	0.2578073	-5.638366	9.96E-08	4.76E-06	7.456692
cg02434059	chr17	29248604	-	ADAP2	TSS200	-1.316124	-1.563616	-5.635292	9.99E-08	4.77E-06	7.4525548
cg00355286	chr16	55525729	+	MMP2;MMP2	Body;Body	-1.157048	-0.382215	-5.635222	9.99E-08	4.77E-06	7.4522387
cg02077256	chr17	15133981	-	PMP22;PMP22;PMP22	3'UTR;3'UTR;3'UTR	-1.542414	-0.199146	-5.635246	9.99E-08	4.77E-06	7.4523454
cg14950344	chr17	44810162	+	NSF	Body	1.0090917	1.4282584	5.635211	9.99E-08	4.77E-06	7.4521898
cg17825194	chr12	124864681	+	NCOR2;NCOR2	Body;Body	0.7671394	-0.698923	5.6351566	9.99E-08	4.77E-06	7.451946
cg18246640	chr10	112136000	+			0.8825638	1.8057668	5.6351394	9.99E-08	4.77E-06	7.4518685
cg00903099	chr7	154862441	+	HTR5A	TSS200	1.2825731	-1.293738	5.6348702	1.00E-07	4.77E-06	7.4506614
cg13804738	chr10	133000233	-	TCERG1L	Body	-1.512905	0.4313454	-5.634812	1.00E-07	4.78E-06	7.4503999
cg08901339	chr10	98031261	+	BLNK;BLNK;BLNK;BLNK	5'UTR;5'UTR;1stExon;1stExon	-1.059793	-1.418802	-5.634658	1.00E-07	4.78E-06	7.4497105
cg01054755	chr5	138728685	-	LOC389333	1stExon	-2.025135	-3.623971	-5.634602	1.00E-07	4.78E-06	7.4494608
cg12041340	chr6	27125500	+			-0.816987	-4.689419	-5.634459	1.00E-07	4.78E-06	7.4488158
cg21545720	chr1	32405083	+	PTP4A2;PTP4A2	TSS1500;TSS1500	-1.363701	-0.115286	-5.634422	1.00E-07	4.78E-06	7.4486539
cg25221239	chr10	131764419	+			1.3320191	-0.493894	5.6341064	1.00E-07	4.79E-06	7.4472365
cg26834627	chr16	58686097	+			-1.364328	0.419496	-5.634097	1.00E-07	4.79E-06	7.4471928
cg09867953	chr16	23706666	+	ERN2	Body	0.9090636	0.6038842	5.6340275	1.00E-07	4.79E-06	7.4468829
cg18912728	chr6	45631106	+			1.1913052	-0.128019	5.636313	1.01E-07	4.79E-06	7.4474971

cg24465329	chr2	172452502	-			-1.455032	0.5614197	-5.633876	1.01E-07	4.79E-06	7.4462042
cg01081096	chr7	150721518	+	ATG9B;ATG9B	1stExon;5'UTR	-1.159515	-0.288667	-5.633726	1.01E-07	4.79E-06	7.445532
cg23956238	chr19	55672036	-	C19orf51	Body	-0.645906	-2.499406	-5.635582	1.01E-07	4.81E-06	7.4442225
cg14476745	chr9	124979356	-	LHX6;LHX6	Body;Body	1.3645397	2.3511851	5.6354996	1.01E-07	4.81E-06	7.4438549
cg15110296	chr12	12509705	-	LOH12CR1;LOH12CR2	TSS1500;Body	-0.912348	-2.420208	-5.632949	1.01E-07	4.81E-06	7.4420453
cg24427504	chr12	133481520	+			2.9005544	-2.453531	5.6354	1.01E-07	4.81E-06	7.4430645
cg00141925	chr12	9231867	-	A2M	Body	-1.510011	0.9731226	-5.632844	1.01E-07	4.81E-06	7.4415781
cg12351587	chr4	85405251	+			1.0492004	-0.570227	5.6326875	1.01E-07	4.81E-06	7.440875
cg22490454	chr2	468934	+			1.2258741	-0.389293	5.6326003	1.01E-07	4.82E-06	7.4404838
cg07485198	chr8	95246699	-			1.8014958	-1.364232	5.6324439	1.01E-07	4.82E-06	7.4397826
cg06166371	chr8	872030	-			-1.507743	0.6142517	-5.632281	1.01E-07	4.82E-06	7.4390524
cg10126205	chr7	19158664	+	TWIST1	TSS1500	2.165812	-0.9858	5.6322228	1.01E-07	4.82E-06	7.4387919
cg04787728	chr16	3598600	-	NLRC3	Body	-1.049833	-0.711076	-5.632153	1.01E-07	4.82E-06	7.4384775
cg11872970	chr4	125571757	-			-0.926044	3.8921761	-5.634459	1.01E-07	4.83E-06	7.4388526
cg00875989	chr18	44774054	-			1.1690962	-0.00112	5.6319875	1.01E-07	4.83E-06	7.4377368
cg16626067	chr16	66613266	-	CMTM2	TSS200	2.0857978	-0.666044	5.6318934	1.01E-07	4.83E-06	7.4373152
cg17421805	chr1	159781662	-	FCRL6	Body	-1.181695	-0.131126	-5.631616	1.02E-07	4.83E-06	7.4360735
cg24743122	chr2	16692578	-			-1.100986	-0.02077	-5.631617	1.02E-07	4.83E-06	7.436074
cg22643110	chr10	72319345	+	KIAA1274	Body	-0.685316	-1.556074	-5.634032	1.02E-07	4.83E-06	7.4369377
cg12608565	chr17	4802692	-	CHRNE;C17orf107	Body;TSS1500	-1.849736	-2.041411	-5.63126	1.02E-07	4.84E-06	7.4344766

cg04744409	chr6	105388191	-			1.093466	0.5731013	5.6311986	1.02E-07	4.84E-06	7.4342005
cg06714981	chr7	127716013	+	SND1	Body	0.7205983	1.8160225	5.6310076	1.02E-07	4.85E-06	7.4333444
cg03514660	chr12	131418057	-			-1.088427	-1.560007	-5.630925	1.02E-07	4.85E-06	7.4329719
cg07197785	chr1	111813690	-			1.6281628	0.281351	5.6308284	1.02E-07	4.85E-06	7.4325409
cg08579420	chr17	31594469	+	ACCN1;ACCN1	Body;Body	-1.616967	0.4347754	-5.630715	1.02E-07	4.85E-06	7.4320344
cg00091960	chr7	126829514	-	GRM8;GRM8;GRM8	Body;Body;Body	-1.440602	0.2887361	-5.63306	1.02E-07	4.85E-06	7.4329317
cg20054385	chr12	133060217	+			-1.115409	0.1935111	-5.630514	1.02E-07	4.85E-06	7.4311313
cg07434271	chr11	31840698	-	PAX6	TSS1500	1.8579375	-0.985486	5.6304517	1.02E-07	4.85E-06	7.4308528
cg14884828	chr10	14603607	+	FAM107B	Body	-1.467791	-0.738943	-5.630388	1.02E-07	4.86E-06	7.4305661
cg26946259	chr2	119599545	+			1.4030295	-0.930528	5.630148	1.02E-07	4.86E-06	7.4294917
cg10601940	chr2	223159305	-	PAX3;PAX3;PAX3;PAX3;PAX3;PAX3;PAX3;PAX3	Body;Body;Body;Body;Body;Body;Body;Body	1.2506722	-0.381166	5.6299771	1.02E-07	4.86E-06	7.4287257
cg15702277	chr1	8477935	-	RERE;RERE;RERE	5'UTR;Body;Body	0.8975794	0.0156199	5.6299294	1.02E-07	4.86E-06	7.4285116
cg09659227	chr17	46797291	+			1.4297679	-1.225808	5.629608	1.03E-07	4.87E-06	7.4270711
cg02081266	chr17	59529618	-			2.529943	-1.521567	5.629522	1.03E-07	4.87E-06	7.426686
cg26059468	chr8	55370579	+	SOX17;SOX17	5'UTR;1stExon	1.8405989	-0.853764	5.631904	1.03E-07	4.87E-06	7.4277572
cg20682639	chr5	31799285	-	PDZD2;PDZD2	5'UTR;1stExon	-1.318976	-1.299724	-5.629361	1.03E-07	4.88E-06	7.4259659
cg09144520	chr10	134842984	-			-0.720057	-1.202152	-5.629221	1.03E-07	4.88E-06	7.4253385
cg12173150	chr6	170338591	+			0.9043686	0.211461	5.6292052	1.03E-07	4.88E-06	7.425266
cg05262436	chr6	137815569	+	OLIG3	TSS200	1.9090689	-1.352899	5.6339987	1.03E-07	4.88E-06	7.4270384
cg16219583	chr5	135702333	+	TRPC7;TRPC7;TRPC7	TSS1500;TSS1500;TSS1500	-1.229299	-0.336526	-5.628966	1.03E-07	4.88E-06	7.4241955

cg24185447	chr5	15935879	-	FBXL7	Body	-2.263595	0.4741074	-5.628779	1.03E-07	4.89E-06	7.4233552
cg02048645	chr17	49199316	-	SPAG9;SPAG9	TSS1500;TSS1500	-0.539624	-4.002214	-5.630982	1.03E-07	4.89E-06	7.4232862
cg17588293	chr1	154986238	+	ZBTB7B	5'UTR	1.3856031	0.1470722	5.6283227	1.03E-07	4.90E-06	7.4213115
cg20967028	chr12	14996272	-	ART4;ART4	5'UTR;1stExon	0.7185787	2.4998525	5.6282284	1.03E-07	4.90E-06	7.4208889
cg24831711	chr6	16337921	-	ATXN1;ATXN1	5'UTR;5'UTR	1.2692335	0.6866791	5.6281368	1.03E-07	4.90E-06	7.4204783
cg03452174	chr17	27045113	+	RAB34;RAB34;RAB34;RAB34;RAB34;RAB34;RAB34	Body;Body;TSS1500;Body;TSS1500;Body;Body	-1.276087	-1.877428	-5.627986	1.03E-07	4.90E-06	7.4198014
cg18049571	chr8	54791151	-	RGS20	Body	1.4706853	-0.570708	5.6279691	1.03E-07	4.90E-06	7.4197267
cg19383075	chr1	220866716	+	C1orf115	Body	0.8966442	2.2226788	5.6279558	1.03E-07	4.90E-06	7.4196671
cg27205928	chr7	1062652	+	C7orf50;C7orf50;C7orf50;MIR339	Body;Body;Body;Body	-1.688316	2.6287382	-5.628023	1.03E-07	4.90E-06	7.4199694
cg27420135	chr11	425289	+	ANO9	Body	0.9403201	1.2078325	5.6278676	1.03E-07	4.90E-06	7.4192722
cg02313118	chr14	104852104	-			-1.348773	0.2779273	-5.627634	1.04E-07	4.91E-06	7.4182251
cg20499861	chr10	31607228	-	ZEB1;ZEB1;ZEB1;LOC220930;ZEB1	TSS1500;TSS200;TSS1500;Body;TSS1500	-0.486585	-4.229169	-5.627496	1.04E-07	4.91E-06	7.4176092
cg12897164	chr8	124529273	-	FBXO32;FBXO32	5'UTR;Body	-1.378825	-2.224315	-5.627346	1.04E-07	4.91E-06	7.4169355
cg10249542	chr7	70359655	-			-1.282451	0.3065594	-5.627279	1.04E-07	4.91E-06	7.4166347
cg00658743	chr2	620044	+			-1.276606	-1.793075	-5.627249	1.04E-07	4.91E-06	7.4164981
cg13626830	chr5	80460495	+	RASGRF2	Body	-1.213694	1.4944622	-5.627193	1.04E-07	4.91E-06	7.4162513
cg07957035	chr16	49623763	-	ZNF423	Body	-1.296978	0.2170915	-5.627146	1.04E-07	4.91E-06	7.4160373
cg19857227	chr14	95240340	+			0.9171812	0.3556377	5.6294267	1.04E-07	4.92E-06	7.4163242
cg23889236	chr11	706100	+	EPS8L2	TSS200	-0.879787	-5.32571	-5.626889	1.04E-07	4.92E-06	7.4148888
cg15362155	chr11	62066533	+	SCGB1D4;SCGB1D4	5'UTR;1stExon	-1.048851	-0.656396	-5.62678	1.04E-07	4.92E-06	7.4143995

cg23404737	chr11	123892725	+	OR10G9	TSS1500	-1.566756	0.3378797	-5.626752	1.04E-07	4.92E-06	7.4142758
cg21171610	chr4	179086552	+			-1.199579	0.383279	-5.626672	1.04E-07	4.92E-06	7.4139174
cg06834698	chr11	7961985	-	OR10A3	TSS1500	-1.407362	0.6426713	-5.626633	1.04E-07	4.92E-06	7.413741
cg15891991	chr1	117396695	-			-1.004105	-1.442384	-5.626499	1.04E-07	4.93E-06	7.4131423
cg06940505	chr1	156877122	-	PEAR1	Body	-1.290282	-0.264399	-5.626377	1.04E-07	4.93E-06	7.4125931
cg04257238	chr7	15972810	+			-1.517008	0.2251856	-5.626348	1.04E-07	4.93E-06	7.4124638
cg15359390	chr2	79365892	+	REG1P	TSS1500	-1.161943	-0.719394	-5.626317	1.04E-07	4.93E-06	7.412324
cg18301891	chr14	102027172	-	DIO3;MIR1247	TSS1500;TSS1500	2.2090523	-1.218279	5.6311376	1.04E-07	4.93E-06	7.4142492
cg00294267	chr16	49572227	-	ZNF423	Body	-0.932831	-0.278502	-5.626147	1.04E-07	4.93E-06	7.411562
cg04731038	chr17	48858636	-			0.9835806	0.3723965	5.6259468	1.04E-07	4.94E-06	7.4106664
cg13635710	chr13	56216872	+			-1.34571	0.5106818	-5.625794	1.04E-07	4.94E-06	7.4099827
cg01291856	chr11	1706894	-	HCCA2;FAM99B	Body;TSS200	-0.680091	-0.82181	-5.625641	1.04E-07	4.94E-06	7.409295
cg06657888	chr7	62531318	+			-0.852142	-1.628363	-5.625569	1.05E-07	4.94E-06	7.4089723
cg13453424	chr9	98260248	+	PTCH1;PTCH1;PTCH1;PTCH1;PTCH1;PTCH1;PTCH1	5'UTR;5'UTR;Body;Body;5'UTR;5'UTR;Body	1.314604	0.1009976	5.6279684	1.05E-07	4.94E-06	7.4101443
cg00287773	chr5	139594619	-	C5orf32	Body	-1.376653	-1.069689	-5.625453	1.05E-07	4.94E-06	7.4084534
cg19601636	chr22	20001066	-	ARVCF	5'UTR	1.0971106	-1.001331	5.6254592	1.05E-07	4.94E-06	7.4084818
cg01316476	chr7	25899194	-			1.0076173	1.3470008	5.6252443	1.05E-07	4.95E-06	7.407519
cg11236311	chr7	105172168	-	RINT1	TSS1500	-1.508322	-2.354423	-5.625197	1.05E-07	4.95E-06	7.4073053
cg27218212	chr2	136743579	+	DARS	TSS1500	-0.71571	-3.762935	-5.627629	1.05E-07	4.95E-06	7.4244292
cg08012275	chr11	1464300	+	BRSK2	Body	-1.673717	1.2883738	-5.63005	1.05E-07	4.95E-06	7.4097426

cg21205320	chr2	3648280	+	COLEC11;COLEC11	5'UTR;5'UTR	-0.984871	0.7285935	-5.624928	1.05E-07	4.95E-06	7.4061034
cg21876964	chr22	36851623	+			-1.056902	-1.81523	-5.624932	1.05E-07	4.95E-06	7.4061204
cg03069242	chr12	4555720	+	FGF6	TSS1500	-1.560123	0.4516568	-5.624864	1.05E-07	4.95E-06	7.4058162
cg09250199	chr1	92415145	-	BRDT;BRDT	5'UTR;1stExon	-2.587533	1.3278379	-5.624713	1.05E-07	4.96E-06	7.4051381
cg12091498	chr12	47226255	-			1.1519479	-0.280779	5.6246934	1.05E-07	4.96E-06	7.4050514
cg04967578	chr18	13641894	-	C18orf1;C18orf1;C18orf1;C18orf1;C18orf1;C18orf1	Body;Body;Body;Body;Body;Body	1.5175562	0.1847015	5.6243979	1.05E-07	4.96E-06	7.4037279
cg24755177	chr19	54850645	-	LILRA4	TSS1500	-2.358628	1.5779422	-5.626791	1.05E-07	4.96E-06	7.404531
cg13433881	chr2	96804750	+	ASTL	TSS1500	-0.999813	-2.532565	-5.623867	1.05E-07	4.97E-06	7.4013488
cg24847685	chr3	147087653	-			2.1220638	-0.908624	5.6237926	1.05E-07	4.98E-06	7.4010168
cg24249450	chr7	33894296	-			-1.631889	0.5455608	-5.623763	1.05E-07	4.98E-06	7.4008822
cg12334488	chr2	20871173	-	GDF7	Body	0.9148554	1.1649285	5.6236128	1.05E-07	4.98E-06	7.4002115
cg17974053	chr10	130010309	-			-0.83456	-3.258576	-5.625825	1.06E-07	4.98E-06	7.400211
cg26157385	chr1	248020641	+	TRIM58	1stExon	1.6096565	-0.524214	5.6333367	1.06E-07	4.98E-06	7.4195226
cg20830514	chr7	158936538	-	VIPR2	Body	1.7395011	-1.234587	5.6233008	1.06E-07	4.98E-06	7.3988142
cg24257495	chr8	1601314	-	DLGAP2	Body	-1.801617	1.5705955	-5.623273	1.06E-07	4.98E-06	7.3986898
cg17334937	chr10	54538305	-			1.1769267	1.7505267	5.6354432	1.06E-07	4.99E-06	7.4027195
cg18935453	chr11	68167030	+	LRP5	Body	1.2811178	0.0571363	5.6227566	1.06E-07	4.99E-06	7.3963767
cg22370716	chr6	33133538	+	COL11A2;COL11A2;COL11A2	Body;Body;Body	-0.970119	-0.055166	-5.622757	1.06E-07	4.99E-06	7.3963795
cg25743345	chr17	73506230	-	CASKIN2;CASKIN2	TSS1500;Body	0.8793485	1.5174585	5.6227305	1.06E-07	4.99E-06	7.39626
cg12622597	chr5	134363053	+			1.1102325	-1.365615	5.6224932	1.06E-07	5.00E-06	7.3951972

cg21653462	chr6	42021921	-	TAF8	Body	0.7265939	2.3862461	5.6224281	1.06E-07	5.00E-06	7.3949057
cg06123346	chr19	36054519	+	ATP4A	1stExon	-0.904006	-1.15306	-5.622347	1.06E-07	5.00E-06	7.3945412
cg22953017	chr2	201451303	-	AOX1	Body	0.9205827	-0.290121	5.6222318	1.06E-07	5.00E-06	7.3940269
cg25471956	chr8	101859439	+			-0.468204	-3.685202	-5.622013	1.06E-07	5.01E-06	7.3930454
cg12444328	chr1	197887955	+	LHX9;LHX9	Body;Body	1.9497023	-1.139672	5.6217471	1.06E-07	5.01E-06	7.3918562
cg05492170	chr4	111536341	+			1.2667045	-0.497937	5.6216584	1.06E-07	5.02E-06	7.3914591
cg20450058	chr13	111152708	-	COL4A2	Body	-1.499114	0.5033601	-5.621583	1.06E-07	5.02E-06	7.3911202
cg05678658	chr2	1500117	-	TPO;TPO;TPO;TPO	Body;Body;Body;Body	-1.14354	-0.512539	-5.621516	1.07E-07	5.02E-06	7.3908232
cg23110019	chr17	77181644	-	HRNBP3	5'UTR	-1.011107	-0.380071	-5.621512	1.07E-07	5.02E-06	7.3908034
cg07473138	chr10	122213951	+			-1.38419	-0.150096	-5.621435	1.07E-07	5.02E-06	7.3904601
cg01828733	chr1	201708932	+	NAV1;NAV1	TSS200;Body	-1.496589	-1.577918	-5.621377	1.07E-07	5.02E-06	7.3901972
cg13835852	chr9	120660089	+			-1.453892	0.5452281	-5.621297	1.07E-07	5.02E-06	7.38984
cg16146353	chr2	115199477	+	DPP10	TSS1500	-1.340991	-0.874371	-5.628698	1.07E-07	5.02E-06	7.3931546
cg12205669	chr14	82086140	+			-1.398198	0.3798537	-5.62106	1.07E-07	5.02E-06	7.3887784
cg26784891	chr6	30884124	+	VARS2;VARS2;VARS2	Body;Body;Body	0.8622338	0.883883	5.6210731	1.07E-07	5.02E-06	7.3888383
cg22108469	chr5	174158984	+			1.4266805	-1.471016	5.6210252	1.07E-07	5.03E-06	7.3886238
cg23892439	chr7	39332884	-	POU6F2;POU6F2	Body;Body	0.9994705	1.0669237	5.6208748	1.07E-07	5.03E-06	7.3879504
cg09379485	chr19	53758297	+	ZNF677	TSS200	2.3979421	-1.954057	5.6208396	1.07E-07	5.03E-06	7.3877929
cg10137253	chr14	101507590	+	MIR300	TSS200	-1.415245	0.3497498	-5.620744	1.07E-07	5.03E-06	7.3873626
cg12377113	chr1	12775955	-	AADACL3;AADACL3	TSS200;TSS200	-1.551609	0.5130885	-5.620714	1.07E-07	5.03E-06	7.3872299

cg06994420	chr2	66672553	-	MEIS1	Body	1.1489133	0.0141719	5.6439594	1.07E-07	5.03E-06	7.3980019
cg25017369	chr4	83486706	-			-1.017787	0.3162636	-5.620557	1.07E-07	5.03E-06	7.3865268
cg04526480	chr1	20838582	+			-1.002965	-1.120947	-5.620317	1.07E-07	5.04E-06	7.3854506
cg08114696	chr3	146964409	-			-1.02925	-1.253636	-5.620339	1.07E-07	5.04E-06	7.3855526
cg15491432	chr4	26585218	+	TBC1D19	TSS1500	-0.764772	-4.107448	-5.627531	1.07E-07	5.04E-06	7.3879461
cg16142906	chr22	32750777	+	RFPL3	TSS200	1.7484185	4.4647012	5.6200232	1.07E-07	5.04E-06	7.3841374
cg18261246	chr11	20230330	+			0.9860127	-0.699309	5.6199713	1.07E-07	5.04E-06	7.3839053
cg15847600	chr8	143995000	-	CYP11B2	Body	-1.298253	-0.441168	-5.619794	1.07E-07	5.05E-06	7.3831123
cg18974111	chr1	5732348	+			-0.865928	-1.174944	-5.619443	1.08E-07	5.06E-06	7.3815408
cg07757887	chr8	104382523	-	CTHRC1	TSS1500	-0.981119	-0.351834	-5.619371	1.08E-07	5.06E-06	7.3812196
cg13672200	chr12	54387530	-			1.2909436	0.4274774	5.6192692	1.08E-07	5.06E-06	7.3807618
cg26380116	chr12	125927452	+	TMEM132B	Body	-1.256443	-0.108773	-5.619271	1.08E-07	5.06E-06	7.380769
cg13840239	chr9	89218392	+			-1.451432	0.0098898	-5.618944	1.08E-07	5.06E-06	7.3793066
cg14793287	chr12	48578573	+	C12orf68;C12orf68	3'UTR;1stExon	1.0673481	-0.528628	5.6188897	1.08E-07	5.07E-06	7.379063
cg25612057	chr4	5368330	+	STK32B	Body	-1.122588	0.2121334	-5.618773	1.08E-07	5.07E-06	7.378541
cg08459783	chr4	2259249	+	MXD4	Body	0.715395	1.0379886	5.6186504	1.08E-07	5.07E-06	7.377992
cg03190219	chr7	25898688	-			1.484151	1.810815	5.6185647	1.08E-07	5.07E-06	7.3776083
cg00782323	chr12	30351139	+			-1.484366	0.2432247	-5.620834	1.08E-07	5.07E-06	7.377888
cg16002305	chr14	97879656	-			2.2133821	-0.938027	5.6207116	1.08E-07	5.08E-06	7.3776875
cg18900271	chr2	110863886	-	MALL	Body	-1.021579	-1.954426	-5.618264	1.08E-07	5.08E-06	7.3762609

cg26875655	chr12	111746046	-	CUX2	Body	-1.0631	-0.460443	-5.617856	1.08E-07	5.09E-06	7.3744365
cg22300320	chr17	79027117	-	BAIAP2;BAIAP2;BAIAP2;BAIAP2	Body;Body;Body;Body	1.3011689	2.059557	5.6178307	1.08E-07	5.09E-06	7.3743228
cg21942490	chr7	27206237	+	HOXA9	TSS1500	1.1454893	-1.221909	5.617653	1.08E-07	5.09E-06	7.3735272
cg26517074	chr16	3998975	+			0.9799507	1.1087207	5.617579	1.08E-07	5.09E-06	7.373196
cg20556503	chr21	46349095	+	ITGB2	TSS1500	-1.12118	1.3165937	-5.617511	1.09E-07	5.09E-06	7.3728916
cg15379170	chr2	233476236	+	EFHD1	Body	-0.901439	-0.339821	-5.619822	1.09E-07	5.09E-06	7.3733627
cg04672506	chr6	33117384	-			-1.190263	-0.090063	-5.61732	1.09E-07	5.09E-06	7.372039
cg13601650	chr14	105865464	+			-1.044027	0.5079876	-5.617323	1.09E-07	5.09E-06	7.3720492
cg25577821	chr15	53098475	+			1.503944	-0.477979	5.6173452	1.09E-07	5.09E-06	7.3721498
cg10809783	chr6	148784879	-	SASH1	Body	0.944118	2.1495532	5.6170791	1.09E-07	5.10E-06	7.3709585
cg16182932	chr11	113863433	-			-0.808706	-3.007989	-5.617082	1.09E-07	5.10E-06	7.3709732
cg20928950	chr13	113976443	+	LAMP1	Body	0.5728867	1.6902803	5.6170883	1.09E-07	5.10E-06	7.3709999
cg26094789	chr14	37135586	-	PAX9	Body	1.3767761	0.4630211	5.6170518	1.09E-07	5.10E-06	7.3708364
cg06570224	chr3	157812475	+			1.04274	0.1368269	5.6192512	1.09E-07	5.10E-06	7.371159
cg20991483	chr7	1215631	+			-0.696705	-0.696071	-5.616742	1.09E-07	5.11E-06	7.369451
cg11213520	chr12	113901529	-	LHX5	Body	2.9483861	-2.017846	5.6166635	1.09E-07	5.11E-06	7.3690988
cg15965134	chr3	137480671	+			1.3879326	-1.020953	5.6166251	1.09E-07	5.11E-06	7.3689267
cg05766802	chr2	118982056	-			2.1140517	-1.418559	5.6165686	1.09E-07	5.11E-06	7.368674
cg26479667	chr5	3339581	-			-2.724094	0.416922	-5.621404	1.09E-07	5.11E-06	7.3704236
cg14568384	chr10	104220744	-	TMEM180	TSS1500	-0.87058	-2.504095	-5.616483	1.09E-07	5.11E-06	7.368293

cg20946805	chr3	138821772	+	BPESC1	TSS1500	-1.390736	0.6454828	-5.616394	1.09E-07	5.11E-06	7.3678932
cg10747185	chr3	176862267	-	TBL1XR1	5'UTR	-1.695886	0.5778535	-5.616311	1.09E-07	5.11E-06	7.36752
cg15241316	chr2	60680761	-	BCL11A;BCL11A	Body;Body	0.7164041	2.2920911	5.6162794	1.09E-07	5.11E-06	7.3673797
cg27389185	chr19	38042123	-	ZNF540	TSS200	1.2606981	-0.991495	5.6161647	1.09E-07	5.11E-06	7.3668665
cg00316875	chr18	12912338	+			0.8305122	0.2868528	5.6209841	1.09E-07	5.11E-06	7.3692517
cg06698795	chr3	126260615	+	CHST13	Body	1.2083255	0.6611449	5.6160964	1.09E-07	5.11E-06	7.3665606
cg13411229	chr8	143823898	-	SLURP1	TSS200	-1.12991	-0.006049	-5.616029	1.09E-07	5.12E-06	7.3662613
cg03665605	chr7	99064212	+	ATP5J2;ATP5J2;ATP5J2;ATP5J2	TSS1500;TSS1500;TSS1500;TSS1500	-1.182204	0.9059042	-5.618427	1.09E-07	5.12E-06	7.3674731
cg02680487	chr6	30851529	+	DDR1;DDR1	TSS1500;TSS1500	-0.851886	-0.866482	-5.618243	1.09E-07	5.12E-06	7.3663049
cg13288164	chr5	140501380	-	PCDHB4	TSS200	1.2794788	1.0735459	5.6158041	1.09E-07	5.12E-06	7.3652528
cg01336781	chr8	23563182	+	NKX2-6	Body	1.5181185	-2.092196	5.615157	1.10E-07	5.13E-06	7.3623568
cg00698908	chr12	128750971	-	TMEM132C	TSS1500	1.1896192	-0.945783	5.6151054	1.10E-07	5.13E-06	7.3621259
cg04746197	chr11	2951300	+	PHLDA2	TSS1500	-0.772391	-2.833049	-5.617397	1.10E-07	5.14E-06	7.3628697
cg11199639	chr8	39172111	+	ADAM5P	TSS200	1.4651362	0.6154985	5.6149424	1.10E-07	5.14E-06	7.3613967
cg19611822	chr15	96101553	-			-1.487697	0.7923498	-5.614938	1.10E-07	5.14E-06	7.361375
cg23051970	chr17	75284193	-	SEPT9;SEPT9;SEPT9	1stExon;5'UTR;Body	-1.702783	1.3820192	-5.614868	1.10E-07	5.14E-06	7.3610626
cg24804195	chr5	87968844	+	LOC645323;LOC645323;LOC645323	Body;Body;Body	1.7491371	-0.153459	5.6146775	1.10E-07	5.14E-06	7.3602113
cg07999668	chr12	8765336	+	AICDA	Body	-1.414271	0.6443226	-5.614598	1.10E-07	5.14E-06	7.3598575
cg04422802	chr19	22446026	-			-1.062841	-0.254984	-5.614479	1.10E-07	5.15E-06	7.3593246
cg16656493	chr6	42893167	+	PTCRA	Body	1.2224683	1.9855015	5.6144648	1.10E-07	5.15E-06	7.3592597

cg11407681	chr1	166039918	-	FAM78B	Body	-1.779697	0.7885546	-5.614393	1.10E-07	5.15E-06	7.3589384
cg03286031	chr10	102592366	+			-1.056949	-1.643382	-5.614365	1.10E-07	5.15E-06	7.3588125
cg05915139	chr12	123582277	-	PITPNM2	5'UTR	0.9016338	0.6451458	5.61421	1.10E-07	5.15E-06	7.3581198
cg23146663	chr5	151066662	+	SPARC	TSS200	1.6914028	-1.226644	5.6142064	1.10E-07	5.15E-06	7.3581037
cg02764897	chr21	31768311	+	KRTAP13-1	TSS200	-1.276578	-0.230041	-5.614166	1.10E-07	5.15E-06	7.3579211
cg07717903	chr5	140753536	+	PCDHGA4;PCDHGA1;PCDHGA6;PCDHGA5;PCDHGB1;PCDHGA3;PCDHGA2;PCDHGA6;PCDHGB2;PCDHGB3	Body;Body;TSS200;Body;Body;Body;Body;TSS200;Body;Body	1.0026807	0.4905574	5.614132	1.10E-07	5.15E-06	7.3577705
cg07713192	chr7	100757628	-			-1.00214	-0.497144	-5.614043	1.10E-07	5.15E-06	7.3573742
cg14274989	chr2	119606302	-	EN1	TSS1500	1.2959675	-1.2876	5.6160211	1.11E-07	5.16E-06	7.3567226
cg21560697	chr2	10054883	-	TAF1B	Body	-1.572464	0.137337	-5.613524	1.11E-07	5.16E-06	7.3550495
cg25951075	chr5	179528386	+	RASGEF1C	3'UTR	-0.888944	-0.371595	-5.613517	1.11E-07	5.16E-06	7.3550194
cg21944234	chr3	177234095	-			1.0638924	1.5300138	5.6158941	1.11E-07	5.16E-06	7.3558074
cg06696815	chr4	1284574	+	MAEA;MAEA	Body;Body	-0.602116	-2.643381	-5.613399	1.11E-07	5.16E-06	7.3544933
cg23202139	chr13	38155024	-	POSTN;POSTN;POSTN;POSTN	Body;Body;Body;Body	-1.412692	0.5699937	-5.61335	1.11E-07	5.17E-06	7.3542723
cg09595050	chr4	81110205	+	PRDM8	5'UTR	1.0872359	0.9197977	5.6132975	1.11E-07	5.17E-06	7.3540371
cg14514991	chr1	158737820	-	OR6N1	TSS1500	-1.559323	-0.269346	-5.613256	1.11E-07	5.17E-06	7.3538511
cg06666025	chr16	66613278	-	CMTM2	TSS200	1.9716582	-0.809783	5.6130979	1.11E-07	5.17E-06	7.3531443
cg11710595	chr2	15260455	-			-1.45178	0.4941861	-5.613036	1.11E-07	5.17E-06	7.3528673
cg17320707	chr10	119307931	-	EMX2;EMX2	3'UTR;3'UTR	1.8698336	-0.971924	5.6129485	1.11E-07	5.17E-06	7.3524758
cg02306156	chr4	188952355	+			-1.397345	0.8625986	-5.612857	1.11E-07	5.17E-06	7.3520648
cg24791025	chr8	65285897	+			1.4435324	-1.617606	5.6128428	1.11E-07	5.17E-06	7.352003

cg03308038	chr10	132866576	+			-1.542515	0.0989279	-5.612714	1.11E-07	5.18E-06	7.3514246
cg18834058	chr7	4832174	-			0.8664093	0.9184044	5.6126998	1.11E-07	5.18E-06	7.3513631
cg16636104	chr7	104004514	-	LHFPL3	Body	-1.571996	0.0953599	-5.612473	1.11E-07	5.18E-06	7.3503491
cg13109911	chr1	160178210	+	PEA15	5'UTR	0.6173115	0.3831246	5.6122249	1.11E-07	5.19E-06	7.3492389
cg20887073	chr6	10381764	-			1.4476214	-1.181047	5.6122078	1.11E-07	5.19E-06	7.3491625
cg23919865	chr19	587177	-			-0.966233	-0.924747	-5.612247	1.11E-07	5.19E-06	7.3493389
cg02078710	chr14	59066094	-			-0.999999	-0.646704	-5.612084	1.11E-07	5.19E-06	7.3486078
cg18655633	chr19	48107418	+			-1.131755	-1.404516	-5.611728	1.12E-07	5.20E-06	7.3470161
cg23670794	chr3	111313065	+	ZBED2;CD96;CD96	5'UTR;Body;Body	-1.324056	-0.235601	-5.611746	1.11E-07	5.20E-06	7.3470958
cg23886141	chr19	52297519	+	FPR3	TSS1500	-1.553921	0.5250709	-5.614145	1.12E-07	5.20E-06	7.3483392
cg13939546	chr7	142428231	-			-1.756244	0.5678247	-5.61408	1.12E-07	5.20E-06	7.3480482
cg03752977	chr8	144330404	+	ZFP41	5'UTR	-1.360893	-0.559085	-5.611575	1.12E-07	5.20E-06	7.3463326
cg04010817	chr16	62273362	-			-1.599114	0.7854691	-5.613958	1.12E-07	5.20E-06	7.3471549
cg16927040	chr3	187388128	-	SST;SST	1stExon;5'UTR	1.9024041	-1.196452	5.6115572	1.12E-07	5.20E-06	7.3462521
cg23203997	chr15	73231661	-			-1.449637	0.4795535	-5.611527	1.12E-07	5.20E-06	7.3461154
cg27603069	chr11	65123374	-	TIGD3	Body	0.8763773	-0.503652	5.6114301	1.12E-07	5.20E-06	7.3456836
cg26007849	chr14	90874616	-	CALM1;CALM1	3'UTR;3'UTR	1.1751773	1.831991	5.611355	1.12E-07	5.20E-06	7.3453476
cg12048965	chr10	3848384	+			-1.49225	-1.466572	-5.611008	1.12E-07	5.21E-06	7.3437949
cg21872908	chr1	55271673	-	C1orf177;C1orf177	TSS200;TSS200	0.9547579	1.0179472	5.6107922	1.12E-07	5.21E-06	7.3428307
cg10543947	chr22	36635882	+	APOL2;APOL2;APOL2	TSS200;1stExon;5'UTR	-1.658834	-3.362672	-5.618096	1.12E-07	5.21E-06	7.3462341

cg05929574	chr20	62611299	+	SAMD10;PRPF6	TSS1500;TSS1500	-0.636201	-4.50894	-5.610565	1.12E-07	5.22E-06	7.3418126
cg16167639	chr10	103889899	-			-1.148839	1.7894801	-5.610566	1.12E-07	5.22E-06	7.3418172
cg27074221	chr14	101500080	+	MIR495	TSS200	-1.651924	0.0572037	-5.610598	1.12E-07	5.22E-06	7.3419637
cg14240536	chr11	65783221	-			0.7246612	0.9364584	5.6104115	1.12E-07	5.22E-06	7.3411277
cg21480725	chr15	30212981	+			-1.214618	-1.376129	-5.61012	1.12E-07	5.23E-06	7.3398238
cg08779982	chr16	2131825	+	TSC2;TSC2;TSC2	Body;Body;Body	1.1428175	2.1752135	5.610051	1.12E-07	5.23E-06	7.3395157
cg22466653	chr17	56232357	-	OR4D1	TSS200	-1.679825	0.5146792	-5.612454	1.12E-07	5.23E-06	7.3404369
cg15856055	chr10	135121295	+	TUBGCP2;ZNF511	5'UTR;TSS1500	-0.968014	-1.720252	-5.609958	1.12E-07	5.23E-06	7.3391006
cg13570972	chr11	31839632	-	PAX6	TSS200	1.2541942	-0.184624	5.6097897	1.13E-07	5.23E-06	7.3383471
cg22353551	chr1	156357839	-			1.3894636	-0.390539	5.6096492	1.13E-07	5.23E-06	7.3377189
cg14780282	chr4	177922514	-			-1.635032	0.5367257	-5.609624	1.13E-07	5.23E-06	7.3376066
cg20286882	chr10	33432514	+			-1.188062	-0.229969	-5.609549	1.13E-07	5.24E-06	7.3372707
cg18315834	chr14	64909119	-	MTHFD1	Body	1.2392496	2.3628469	5.6094763	1.13E-07	5.24E-06	7.3369454
cg17679427	chr10	35480749	+	CREM;CREM;CREM;CREM;CREM;CREM;CREM;CREM;CREM;CREM;CREM	Body;Body;5'UTR;Body;Body;Body;Body;Body;5'UTR;Body;Body	1.0525459	0.8453244	5.6091639	1.13E-07	5.24E-06	7.3355484
cg08580057	chr6	118238090	-	SLC35F1	Body	-1.59209	0.3104077	-5.609028	1.13E-07	5.25E-06	7.3349397
cg16519487	chr13	20876063	-			0.6227378	-0.138713	5.6084605	1.13E-07	5.26E-06	7.3324033
cg12590494	chr1	151008411	-	BNIPL;BNIPL	TSS1500;TSS1500	1.0648381	1.6076663	5.6084355	1.13E-07	5.26E-06	7.3322912
cg22949578	chr1	85667951	-	SYDE2	TSS1500	-0.846838	-2.93011	-5.608401	1.13E-07	5.26E-06	7.3321391
cg09474331	chr19	54926805	+	TTYH1;TTYH1	1stExon;1stExon	3.0013014	-1.692848	5.6079829	1.13E-07	5.27E-06	7.3302677
cg11068352	chr7	142761497	-	OR6W1P	TSS1500	-1.786407	0.7856183	-5.607893	1.14E-07	5.27E-06	7.3298665

cg16804284	chr14	29237480	+	FOXG1	1stExon	1.6891425	-0.848343	5.6077265	1.14E-07	5.28E-06	7.3291213
cg08892455	chr17	1231715	-			-0.929315	-1.38407	-5.607671	1.14E-07	5.28E-06	7.3288715
cg05618934	chr4	1407592	-			1.430449	0.7641262	5.6075747	1.14E-07	5.28E-06	7.3284426
cg13292042	chr4	111535334	+			1.8012606	-1.19219	5.6074864	1.14E-07	5.28E-06	7.3280478
cg14959729	chr6	166580693	+	T	Body	1.1510316	-1.08026	5.6075119	1.14E-07	5.28E-06	7.3281619
cg16980245	chr15	25492469	+	SNORD115-42;SNORD115-10	TSS200;TSS200	-0.836511	-0.153454	-5.607482	1.14E-07	5.28E-06	7.3280296
cg06714480	chr2	182544307	-	NEUROD1	5'UTR	1.5706548	-0.986888	5.6074303	1.14E-07	5.28E-06	7.3277967
cg24803719	chr17	45855879	-			1.551315	-0.331494	5.6074194	1.14E-07	5.28E-06	7.3277481
cg04385523	chr3	44381576	+	C3orf23;C3orf23;C3orf23	5'UTR;5'UTR;5'UTR	1.0179793	2.0284294	5.6072544	1.14E-07	5.28E-06	7.3270106
cg07010146	chr10	105239205	-	CALHM3	TSS1500	-1.22849	0.0338761	-5.607054	1.14E-07	5.29E-06	7.3261125
cg02755847	chr6	29232651	-			-1.002784	-1.028466	-5.607007	1.14E-07	5.29E-06	7.3259027
cg21001184	chr7	27205262	+	HOXA9	TSS200	1.9405632	-0.258765	5.6069391	1.14E-07	5.29E-06	7.3256011
cg00699219	chr4	174422442	+			1.0812511	-0.575806	5.6068224	1.14E-07	5.29E-06	7.3250793
cg22995449	chr14	75988765	+	BATF	TSS200	-1.120711	-2.071669	-5.606845	1.14E-07	5.29E-06	7.3251795
cg08271909	chr4	155661691	-			0.9601054	1.7512942	5.6067819	1.14E-07	5.29E-06	7.3248982
cg03828328	chr5	140744556	-	PCDHGA4;PCDHGA2;PCDHGA5;PCDHGB2;PCDHGA1;PCDHGB1;PCDHGA3;PCDHGA5	Body;Body;1stExon;Body;Body;Body;Body;1stExon	0.9545024	0.0201739	5.6091683	1.14E-07	5.29E-06	7.3261119
cg08525845	chr11	20181456	+	DBX1	Body	1.0453017	-0.779362	5.6067294	1.14E-07	5.29E-06	7.3246634
cg27625491	chr7	80266930	+	CD36;CD36;CD36	TSS1500;TSS1500;5'UTR	1.3604498	0.6132745	5.6067031	1.14E-07	5.29E-06	7.3245456
cg23036136	chr6	30955979	+	MUC21	3'UTR	0.6463765	1.9435282	5.6137298	1.14E-07	5.30E-06	7.3267807
cg00823843	chr1	244094885	+			0.9909131	0.311124	5.6059273	1.15E-07	5.31E-06	7.3210779

cg09626867	chr3	45053351	+	EXOSC7	Body	1.427404	-1.066612	5.6058168	1.15E-07	5.31E-06	7.3205839
cg12338097	chr18	22929407	-	ZNF521	Body	1.6240205	-1.776196	5.6057888	1.15E-07	5.31E-06	7.3204588
cg16886882	chr18	47014260	-	C18orf32	TSS1500	-1.256784	-0.273883	-5.605649	1.15E-07	5.31E-06	7.3198347
cg02079420	chr8	82753780	-	SNX16;SNX16;SNX16	5'UTR;5'UTR;5'UTR	-0.635185	-3.68737	-5.605615	1.15E-07	5.31E-06	7.3196838
cg00691240	chr1	52498502	-	TXNDC12;KTI12	Body;1stExon	-1.26536	-3.565185	-5.605421	1.15E-07	5.32E-06	7.3188157
cg26422784	chr11	62063821	+	SCGB1D4	3'UTR	-1.38497	0.4878423	-5.605246	1.15E-07	5.32E-06	7.3180325
cg15304679	chr7	53104001	-	POM121L12	1stExon	-1.190965	0.6096195	-5.607593	1.15E-07	5.32E-06	7.3187296
cg05344949	chr3	62364077	+			1.1902631	-1.164071	5.6051153	1.15E-07	5.32E-06	7.317448
cg26457248	chr18	74840216	-	MBP;MBP	5'UTR;5'UTR	-1.228986	-1.41498	-5.60491	1.15E-07	5.33E-06	7.316529
cg15724256	chr2	119602857	+	EN1	Body	1.7599114	-0.775562	5.6045845	1.15E-07	5.34E-06	7.3150754
cg07812589	chr16	49195595	-			-1.276006	-0.517664	-5.604469	1.15E-07	5.34E-06	7.3145579
cg03774463	chr7	96636616	-	DLX6AS;DLX6	Body;Body	1.7005705	-0.440953	5.6043808	1.15E-07	5.34E-06	7.3141651
cg01964039	chr11	131239703	+	NTM	TSS1500	-1.643503	0.5352728	-5.604263	1.15E-07	5.34E-06	7.3136395
cg26886976	chr17	72588412	-	CD300LD;C17orf77	TSS200;Body	-1.313654	-1.415008	-5.604183	1.16E-07	5.34E-06	7.3132809
cg20413454	chr1	53163625	+	C1orf163	Body	-0.911478	-3.447269	-5.604051	1.16E-07	5.35E-06	7.3126926
cg00707406	chr5	140167620	-	PCDHA1;PCDHA1;PCDHA1	Body;1stExon;1stExon	1.0384989	0.6110975	5.6040265	1.16E-07	5.35E-06	7.3125817
cg02017282	chr17	44896923	-	WNT3	TSS1500	1.3574459	-1.359181	5.6038506	1.16E-07	5.35E-06	7.3117955
cg26035118	chr8	97171126	-	GDF6	Body	1.2410185	-1.654467	5.603858	1.16E-07	5.35E-06	7.3118285
cg11375072	chr20	35885301	-	GHRH	TSS200	-1.094771	-0.009294	-5.603671	1.16E-07	5.35E-06	7.3109912
cg23750445	chr12	54347938	-	HOXC12	TSS1500	0.8217549	-1.115336	5.6059058	1.16E-07	5.36E-06	7.3115462

cg18677812	chr3	38936985	-	SCN11A	Body	-1.560734	0.959578	-5.603457	1.16E-07	5.36E-06	7.3100342
cg05490712	chr11	31838674	+	PAX6	5'UTR	1.9603661	-1.3511	5.6033249	1.16E-07	5.36E-06	7.3094462
cg17642201	chr6	159549216	-			1.1129196	2.1298617	5.6033444	1.16E-07	5.36E-06	7.3095334
cg05711710	chr10	93793140	-			1.0420367	1.1139848	5.603192	1.16E-07	5.36E-06	7.3088523
cg04095257	chr7	1062669	+	C7orf50;MIR339;C7orf50;C7orf50	Body;TSS200;Body;Body	-0.93019	1.5231778	-5.60297	1.16E-07	5.37E-06	7.3078587
cg27612447	chr16	31229759	-	PYDC1;TRIM72	TSS1500;Body	-1.207443	0.4534271	-5.602971	1.16E-07	5.37E-06	7.3078633
cg22117968	chr21	46768124	+			1.5022288	2.8414018	5.6029278	1.16E-07	5.37E-06	7.3076717
cg24136205	chr13	100624293	-	ZIC5	TSS200	1.3044927	-0.418468	5.6026667	1.16E-07	5.37E-06	7.3065045
cg23189410	chr3	147125712	+	ZIC4;ZIC1	TSS1500;TSS1500	1.8745965	0.5511881	5.6022438	1.17E-07	5.38E-06	7.3046149
cg25247859	chr6	30095174	-			1.1901419	-0.070296	5.6021713	1.17E-07	5.39E-06	7.3042912
cg03053579	chr10	102987483	-	LBX1	Body	1.2002362	-0.805426	5.6018377	1.17E-07	5.39E-06	7.3028006
cg04697262	chr10	130338886	+			1.4427979	-1.085134	5.6018091	1.17E-07	5.39E-06	7.3026727
cg24075113	chr7	100465289	-	TRIP6	Body	-0.957943	-1.366649	-5.601528	1.17E-07	5.40E-06	7.3014152
cg04787888	chr16	86547322	+	FOXF1	3'UTR	1.3546637	-0.588056	5.6012212	1.17E-07	5.41E-06	7.3000457
cg06966245	chr1	30043002	-			-1.124119	-0.381566	-5.601075	1.17E-07	5.41E-06	7.2993932
cg22904737	chr17	36735245	+	SRCIN1	Body	1.488753	-0.553041	5.6034986	1.17E-07	5.41E-06	7.3008028
cg02306139	chr5	148638033	+	ABLIM3	3'UTR	-1.40582	0.5033208	-5.600892	1.17E-07	5.41E-06	7.2985771
cg15444735	chr13	56140478	+			-1.481343	0.461047	-5.600881	1.17E-07	5.41E-06	7.2985247
cg00405078	chr6	34504890	-			-0.707386	-0.912016	-5.60081	1.17E-07	5.41E-06	7.2982093
cg15511120	chr11	6598119	+			0.8756173	0.332594	5.6008294	1.17E-07	5.41E-06	7.2982954

cg14294859	chr9	130862556	-	SLC25A25;SLC25A25;SLC25A25;SLC25A25	Body;Body;Body;5'UTR	2.1622033	0.305074	5.6007656	1.17E-07	5.41E-06	7.2980105
cg18359618	chr14	104648508	-			-0.893491	-0.117358	-5.600699	1.17E-07	5.42E-06	7.2977116
ch.19.714487R	chr19	16978952	+	SIN3B	Body	-0.678869	-5.017896	-5.600642	1.17E-07	5.42E-06	7.2974603
cg10815253	chr2	116925524	+			-1.612617	0.4725631	-5.600605	1.17E-07	5.42E-06	7.297293
cg16402415	chr15	57021851	+	ZNF280D;ZNF280D;ZNF280D	5'UTR;5'UTR;5'UTR	0.9738291	1.1478917	5.600524	1.17E-07	5.42E-06	7.2969309
cg07720856	chr2	232572668	+	PTMA;PTMA	TSS1500;TSS1500	-0.810816	-3.424629	-5.600347	1.18E-07	5.42E-06	7.2961415
cg20768638	chr3	36950004	+	TRANK1	Body	-0.671211	-3.350984	-5.600267	1.18E-07	5.42E-06	7.2957808
cg01845764	chr11	127112911	-			-1.712165	0.5847103	-5.600131	1.18E-07	5.43E-06	7.2951761
cg26678012	chr15	78857577	+	CHRNA5	TSS1500	-0.733388	-4.128207	-5.600148	1.18E-07	5.43E-06	7.2952523
cg04838847	chr8	110587155	-	GOLSYN;GOLSYN;GOLSYN;GOLSYN;GOLSYN;GOLSYN;GOLSYN;GOLSYN;GOLSYN;GOLSYN;GOLSYN;GOLSYN;GOLSYN;GOLSYN:GOLSYN	Body;Body;Body;Body;Body;Body;Body;Body;Body;Body;Body;Body;Body;Body;Body	-1.936736	3.383603	-5.602441	1.18E-07	5.43E-06	7.2957335
cg03267466	chr17	55752319	-	MSI2	Body	1.2069397	3.5475931	5.6048462	1.18E-07	5.43E-06	7.2972698
cg03475285	chr8	54789207	-	RGS20	Body	1.7862877	-1.572967	5.5999694	1.18E-07	5.43E-06	7.2944532
cg18418928	chr1	23953778	+	MDS2	TSS200	-1.272355	-0.589605	-5.599935	1.18E-07	5.43E-06	7.2942978
cg19389293	chr18	13641872	-	C18orf1;C18orf1;C18orf1;C18orf1;C18orf1;C18orf1	Body;Body;Body;Body;Body;Body	1.4370719	-0.587271	5.5998672	1.18E-07	5.43E-06	7.2939969
cg22498453	chr7	98805084	+	KPNA7	1stExon	-1.62936	0.7563998	-5.599745	1.18E-07	5.43E-06	7.2934495
cg05000039	chr8	94754024	-	RBM12B	TSS1500	-1.317333	-0.490128	-5.599691	1.18E-07	5.43E-06	7.2932117
cg11267630	chr17	46811527	-			2.2150489	-1.502911	5.6021009	1.18E-07	5.43E-06	7.2945661
cg01605366	chr11	106255565	-			-1.740372	-0.621586	-5.599597	1.18E-07	5.43E-06	7.29279
cg04943241	chr4	5898879	+			-1.051616	-0.390326	-5.599557	1.18E-07	5.44E-06	7.2926096
cg04910228	chr1	231739450	+	TSNAX-DISC1;TSNAX-DISC1;TSNAX-DISC1;TSNAX-DISC1;TSNAX-DISC1;TSNAX-DISC1;TSNAX-DISC1;TSNAX-DISC1	Body;Body;Body;Body;Body;Body;Body;Body	-1.095871	-0.889389	-5.599458	1.18E-07	5.44E-06	7.2921694

cg24299136	chr19	2511707	-	GNG7	3'UTR	-2.403726	2.9872676	-5.622616	1.18E-07	5.44E-06	7.3326595
cg24553417	chr13	50244794	-	EBPL	Body	1.0812676	0.3425839	5.5994528	1.18E-07	5.44E-06	7.2921455
cg25967897	chr7	100865997	-	ZNHIT1	Body	0.6235191	1.8164346	5.5994785	1.18E-07	5.44E-06	7.2922602
cg13614286	chr9	131038327	-	C9orf119;GOLGA2	TSS200;TSS200	1.3733019	-2.49757	5.5992105	1.18E-07	5.44E-06	7.2910631
cg25019989	chr4	54961835	-			1.3295983	-1.301421	5.5990692	1.18E-07	5.44E-06	7.2904321
cg22264813	chr7	158710861	-	WDR60	Body	0.4633718	0.9547195	5.5988368	1.18E-07	5.45E-06	7.2893939
cg26149682	chr6	10417663	+	TFAP2A	Body	1.1855541	-0.095494	5.5983864	1.19E-07	5.46E-06	7.287382
cg20164479	chr17	10325617	-	MYH8	TSS1500	-1.495139	0.4228536	-5.600758	1.19E-07	5.46E-06	7.2885759
cg14671011	chr5	134544423	+			-1.066994	-0.130845	-5.598182	1.19E-07	5.46E-06	7.2864683
cg22309923	chr7	35297281	+			1.0318459	-1.478978	5.5979533	1.19E-07	5.47E-06	7.2854478
cg24918790	chr22	20785148	+	SCARF2;SCARF2	Body;Body	-0.746245	-0.863437	-5.597279	1.19E-07	5.49E-06	7.2824373
cg13398027	chr18	46447840	+	SMAD7	Body	1.671556	2.2477204	5.5971734	1.19E-07	5.49E-06	7.2819648
cg04612488	chr17	8113907	+	AURKB	TSS200	-1.187131	-5.593005	-5.601958	1.19E-07	5.49E-06	7.2836926
cg17127702	chr1	226594323	-	PARP1	Body	-0.952741	-2.616255	-5.597022	1.19E-07	5.49E-06	7.2812901
cg05878390	chr4	151502935	-	MAB21L2;LRBA	TSS200;Body	0.8271576	2.3027565	5.5969868	1.19E-07	5.49E-06	7.2811313
cg13846270	chr13	51417929	+	DLEU7	TSS200	1.2598327	0.2545542	5.5968434	1.20E-07	5.49E-06	7.2804907
cg22373770	chr1	9224198	+			-0.926332	-2.597591	-5.596855	1.20E-07	5.49E-06	7.2805417
cg25528806	chr11	124190035	+	OR8D2	1stExon	-1.366914	-0.538431	-5.596867	1.20E-07	5.49E-06	7.2805971
cg03240301	chr7	23387383	+	IGF2BP3	Body	-1.269224	-1.368252	-5.596596	1.20E-07	5.50E-06	7.2793843
cg22702857	chr12	4269985	-			-1.476991	0.0318485	-5.598706	1.20E-07	5.51E-06	7.2790695

cg07969622	chr22	22901569	-	LOC648691;PRAME;PRAME;PRAME;PRAME;PRAME	TSS200;TSS200;TSS200;5'UTR;5'UTR;5'UTR	-0.745396	-0.945524	-5.596277	1.20E-07	5.51E-06	7.2779628
cg11996390	chr2	227654510	+	IRS1	3'UTR	0.6298153	2.2912064	5.5962026	1.20E-07	5.51E-06	7.2776294
cg19448144	chr6	32169707	-	NOTCH4	Body	-1.148047	0.709021	-5.596193	1.20E-07	5.51E-06	7.2775848
cg05104995	chr10	49460249	+	FRMPD2;FRMPD2	Body;Body	-1.520141	0.4651308	-5.596102	1.20E-07	5.51E-06	7.2771809
cg09466667	chr3	42140775	+	TRAK1	Body	0.7196591	1.1613302	5.5961004	1.20E-07	5.51E-06	7.2771728
cg06043315	chr8	144640306	+	GSDMD;GSDMD	TSS200;5'UTR	-0.784696	-3.163256	-5.595937	1.20E-07	5.51E-06	7.2764446
cg11694519	chr11	111783563	+	CRYAB;HSPB2	TSS1500;1stExon	1.9299797	-0.118383	5.5959329	1.20E-07	5.51E-06	7.2764249
cg14412490	chr11	77880406	-			1.4511825	0.1816021	5.5952499	1.20E-07	5.53E-06	7.2733755
cg15133201	chr1	248852044	+			-1.950712	1.0464895	-5.595167	1.20E-07	5.53E-06	7.273007
cg21877855	chr1	63989125	+	ITGB3BP;EFCAB7;EFCAB7	TSS1500;1stExon;5'UTR	-0.480438	-4.620496	-5.595057	1.21E-07	5.53E-06	7.2725127
cg11247103	chr1	201915768	+	LMOD1	TSS200	-0.70771	-3.50898	-5.594635	1.21E-07	5.54E-06	7.2706279
cg21215550	chr9	969544	-			2.1107977	0.4516588	5.5969072	1.21E-07	5.54E-06	7.2710484
cg21402364	chr15	74257642	+			-0.962707	-1.159445	-5.594506	1.21E-07	5.54E-06	7.2700517
cg01142668	chr16	2086846	-	SLC9A3R2;SLC9A3R2	Body;Body	1.7144098	3.8786058	5.5968095	1.21E-07	5.55E-06	7.270613
cg05372679	chr15	48064730	+	SEMA6D;SEMA6D;SEMA6D;SEMA6D;SEMA6D	3'UTR;3'UTR;3'UTR;3'UTR;3'UTR	-1.369423	0.0446831	-5.594363	1.21E-07	5.55E-06	7.2694174
cg04932056	chr17	49199280	-	SPAG9;SPAG9	TSS1500;TSS1500	-0.502822	-4.429532	-5.59434	1.21E-07	5.55E-06	7.2693141
cg24366168	chr19	3097712	-	GNA11	Body	1.8335234	2.2961059	5.5943031	1.21E-07	5.55E-06	7.2691481
cg08107735	chr1	6260360	+	RPL22	TSS1500	0.9646628	1.6337339	5.594003	1.21E-07	5.55E-06	7.2678082
cg09774787	chr16	55690381	-	SLC6A2	TSS200	2.5168375	-1.744562	5.5940209	1.21E-07	5.55E-06	7.2678883
cg20311730	chr11	7985183	+	NLRP10	TSS200	-1.353482	0.2864072	-5.593955	1.21E-07	5.55E-06	7.2675955

cg03671802	chr18	56941473	-	RAX	TSS1500	1.285949	-0.695312	5.5939092	1.21E-07	5.56E-06	7.2673896
cg20073553	chr4	13545291	+	NKX3-2	Body	1.3656348	-1.064483	5.5952805	1.22E-07	5.58E-06	7.263795
cg19757261	chr10	44222463	+			-1.382259	-0.600034	-5.592529	1.22E-07	5.59E-06	7.2612272
cg00222472	chr1	164545783	+	PBX1	Body	0.9630366	1.0105419	5.5921417	1.22E-07	5.60E-06	7.2594994
cg03804732	chr12	14136365	-			-1.829328	0.7087963	-5.592065	1.22E-07	5.60E-06	7.2591583
cg18500830	chr10	12395689	-	CAMK1D;CAMK1D	Body;Body	-1.139432	2.0070704	-5.592083	1.22E-07	5.60E-06	7.2592373
cg18184092	chr1	8412759	+	RERE;RERE;RERE	3'UTR;3'UTR;3'UTR	0.9662106	0.1278092	5.5918367	1.22E-07	5.61E-06	7.2581383
cg26508928	chr18	14145866	-			-0.914447	-0.193134	-5.591719	1.22E-07	5.61E-06	7.2576121
cg21892967	chr19	10306043	-	DNMT1;DNMT1	TSS1500;TSS1500	-0.63137	-3.435381	-5.591457	1.23E-07	5.61E-06	7.2564449
cg22693055	chr6	107897986	+	SOBP	Body	-1.122971	0.1912079	-5.591452	1.23E-07	5.61E-06	7.2564205
cg05168229	chr13	45390049	-			-1.039165	-2.136954	-5.591346	1.23E-07	5.62E-06	7.2559497
cg10536101	chr1	16400330	+	FAM131C	TSS1500	-0.576694	-3.173637	-5.595946	1.23E-07	5.62E-06	7.2576257
cg19674065	chr4	26858928	+			-0.610734	-4.772864	-5.591116	1.23E-07	5.62E-06	7.2549227
cg13794404	chr9	126816959	-			-0.99665	-1.687687	-5.59106	1.23E-07	5.62E-06	7.2546695
cg21230745	chr5	87974414	+	LOC645323;LOC645323	Body;TSS200	1.7418909	-0.957032	5.5909958	1.23E-07	5.62E-06	7.2543853
cg03489382	chr7	128432457	+	CCDC136;CCDC136	1stExon;5'UTR	0.8513675	0.7330091	5.5909453	1.23E-07	5.62E-06	7.2541597
cg18703515	chr10	44830587	+			-1.705645	0.2991374	-5.590962	1.23E-07	5.62E-06	7.254235
cg20584157	chr3	64149428	-	PRICKLE2	Body	-1.242077	-0.143999	-5.590896	1.23E-07	5.62E-06	7.2539394
cg02859319	chr3	50674664	+	MAPKAPK3	Body	0.6719537	1.5469385	5.5908017	1.23E-07	5.63E-06	7.2535189
cg20918463	chr13	99638055	-	DOCK9;DOCK9	Body;Body	0.6399741	2.666934	5.5907048	1.23E-07	5.63E-06	7.2530863

cg20175390	chr2	119599459	-			1.1326427	-0.21347	5.5928679	1.23E-07	5.63E-06	7.2533907
cg09230250	chr11	112676851	+			-1.296903	0.3196746	-5.590428	1.23E-07	5.63E-06	7.251851
cg04810997	chr7	143140674	-	TAS2R60	1stExon	-1.753315	0.4555161	-5.59038	1.23E-07	5.63E-06	7.2516354
cg23592947	chr1	63786306	+			1.1377025	0.0820167	5.5903935	1.23E-07	5.63E-06	7.2516973
cg09732036	chr20	9511102	+	C20orf103	3'UTR	-1.380808	-0.246553	-5.590266	1.23E-07	5.64E-06	7.2511278
cg14209730	chr2	64632636	-			1.0281656	0.9534231	5.5902554	1.23E-07	5.64E-06	7.251081
cg16657724	chr10	50916667	+	C10orf53	3'UTR	-1.51082	0.5435098	-5.590156	1.23E-07	5.64E-06	7.2506378
cg04206219	chr6	1602137	+			0.7112216	1.5469172	5.5925201	1.23E-07	5.64E-06	7.2518403
cg09233659	chr4	88832540	+			-1.030462	-2.385567	-5.590063	1.23E-07	5.64E-06	7.2502239
cg12077754	chr2	75089669	+	HK2	Body	1.1377758	2.006201	5.5900565	1.23E-07	5.64E-06	7.2501933
cg16387141	chr6	26757842	-			1.2300954	-0.777444	5.5900845	1.23E-07	5.64E-06	7.2503183
cg08756614	chr16	5263828	+			-1.41942	0.325713	-5.589592	1.24E-07	5.65E-06	7.2481218
cg23202253	chr12	26902211	-	ITPR2	Body	-1.648971	-0.728162	-5.589455	1.24E-07	5.65E-06	7.2475093
cg00078513	chr15	25488114	+	SNORD115-40	TSS1500	-0.72838	-0.93207	-5.589358	1.24E-07	5.65E-06	7.2470748
cg09387528	chr21	47876058	-			1.0512885	0.6215055	5.5891254	1.24E-07	5.66E-06	7.2460384
cg05000772	chr17	48194500	+	SAMD14	Body	-1.278406	0.0555401	-5.588932	1.24E-07	5.66E-06	7.2451765
cg19458529	chr5	66347164	+	MAST4;MAST4	Body;Body	-0.789975	-0.627687	-5.588772	1.24E-07	5.67E-06	7.2444616
cg17157798	chr12	129595394	+	TMEM132D	Body	-1.450529	0.1854225	-5.588727	1.24E-07	5.67E-06	7.2442607
cg10633609	chr4	190705649	-			-1.023331	-0.334631	-5.588494	1.24E-07	5.67E-06	7.2432211
cg03367622	chr7	144967385	+			-1.580683	0.3051585	-5.588358	1.24E-07	5.68E-06	7.2426161

cg24128979	chr19	29222213	+			-1.88428	0.1420085	-5.590714	1.24E-07	5.68E-06	7.2437898
cg15401968	chr17	1179554	-			-0.493887	-3.438427	-5.588267	1.24E-07	5.68E-06	7.2422088
cg04453552	chr16	57394387	+	CCL22	Body	-2.076631	3.3850326	-5.588153	1.24E-07	5.68E-06	7.2417018
cg02936740	chr4	68995995	+	TMPRSS11F	TSS1500	-1.593686	1.3185335	-5.588067	1.25E-07	5.68E-06	7.2413141
cg08062023	chr3	111393015	+	PLCXD2;PLCXD2	TSS1500;TSS1500	-0.804272	-2.690928	-5.588076	1.25E-07	5.68E-06	7.2413571
cg02123262	chr6	20879651	-	CDKAL1	Body	-0.979954	2.1894895	-5.587892	1.25E-07	5.69E-06	7.2405359
cg21115004	chr1	67520352	-	SLC35D1	TSS1500	-0.674248	-3.730276	-5.587854	1.25E-07	5.69E-06	7.2403676
cg19987665	chr2	223159250	+	PAX3;PAX3;PAX3;PAX3;PAX3;PAX3;PAX3;PAX3	Body;Body;Body;Body;Body;Body;Body;Body	1.5459079	-1.153275	5.5876963	1.25E-07	5.69E-06	7.2396622
cg17927926	chr2	39103310	+	MORN2;DHX57	5'UTR;TSS1500	-0.34771	-4.150117	-5.587561	1.25E-07	5.69E-06	7.23906
cg14077219	chr6	111303792	+	RPF2	Body	-0.614839	-3.670093	-5.587382	1.25E-07	5.70E-06	7.2382581
cg14535884	chr9	117349621	+	ATP6V1G1	TSS1500	-0.76289	-2.806802	-5.587319	1.25E-07	5.70E-06	7.2379803
cg20386316	chr8	72470982	+			2.2077795	-0.75791	5.5871777	1.25E-07	5.70E-06	7.2373486
cg00468320	chr8	109800379	-	TMEM74	TSS1500	-1.630715	1.0165442	-5.58708	1.25E-07	5.70E-06	7.2369125
cg24446242	chr6	32185340	+	NOTCH4	Body	-1.114205	-0.056845	-5.586941	1.25E-07	5.71E-06	7.2362916
cg17187764	chr12	7818654	+	APOBEC1	TSS200	-1.429974	-0.036464	-5.586835	1.25E-07	5.71E-06	7.2358185
cg01187920	chr5	71015162	+	CARTPT	1stExon	1.2303414	-0.623207	5.5866897	1.25E-07	5.71E-06	7.235172
cg04863713	chr1	150521201	-	ADAMTSL4;ADAMTSL4	TSS1500;TSS1500	-0.833825	-3.134242	-5.586687	1.25E-07	5.71E-06	7.2351591
cg06792538	chr1	2954250	+			-1.014474	-0.307234	-5.586729	1.25E-07	5.71E-06	7.2353475
cg11151402	chr5	159820339	-	C5orf54	3'UTR	0.6175235	1.9941462	5.586635	1.25E-07	5.71E-06	7.2349277
cg14262681	chr20	21084420	+			3.3886035	-1.135579	5.5865996	1.25E-07	5.71E-06	7.2347699

cg23030339	chr3	31703127	-	OSBPL10	3'UTR	1.4932505	1.8398785	5.586445	1.25E-07	5.72E-06	7.2340802
cg00682263	chr15	66188803	-	MEGF11	3'UTR	1.6356882	0.4819774	5.5863746	1.26E-07	5.72E-06	7.2337662
cg19301017	chr6	30884713	-	VARS2;VARS2;VARS2	Body;Body;Body	1.0307885	2.02451	5.5863919	1.26E-07	5.72E-06	7.2338432
cg18571451	chr4	16507312	+	LDB2;LDB2	Body;3'UTR	-1.595925	1.3920945	-5.586292	1.26E-07	5.72E-06	7.2333967
cg09516476	chr6	10881925	+	GCM2	Body	1.4723717	-1.536705	5.5861841	1.26E-07	5.72E-06	7.2329165
cg12765028	chr4	13526659	-			0.9756849	-0.03883	5.5859841	1.26E-07	5.72E-06	7.2320244
cg01715455	chr20	25062827	-	VSX1;VSX1	TSS200;TSS200	2.160418	-1.623707	5.5882205	1.26E-07	5.73E-06	7.2326805
cg06002616	chr8	101225028	+	SPAG1;SPAG1	Body;Body	-0.770525	-3.665358	-5.585812	1.26E-07	5.73E-06	7.2312575
cg12184660	chr19	2885997	-			-1.000604	0.6258775	-5.585836	1.26E-07	5.73E-06	7.2313618
cg00889398	chr13	28502089	+			1.4195166	-0.941093	5.5857614	1.26E-07	5.73E-06	7.2310313
cg15618336	chr12	52182759	-	SCN8A	Body	-1.682163	1.045784	-5.585665	1.26E-07	5.73E-06	7.2306006
cg09263904	chr12	58219817	+	CTDSP2;MIR26A2	Body;TSS1500	0.7405974	1.0634037	5.585501	1.26E-07	5.73E-06	7.2298697
cg06851000	chr1	33627109	-	TRIM62	Body	1.0987804	-0.231456	5.5854412	1.26E-07	5.74E-06	7.229603
cg11376227	chr4	113333069	+	ALPK1;ALPK1	Body;Body	0.7956807	1.8188484	5.5853493	1.26E-07	5.74E-06	7.229193
cg25747670	chr1	115722330	-			-0.988923	0.1923619	-5.585348	1.26E-07	5.74E-06	7.2291886
cg02022572	chr15	75932759	+	IMP3	TSS200	1.3453752	-4.721968	5.587632	1.26E-07	5.74E-06	7.2297071
cg12930930	chr11	5372503	-	OR51B6	TSS1500	-1.471803	-0.469073	-5.585242	1.26E-07	5.74E-06	7.2287151
cg21534423	chr19	24154607	+			2.3365304	-1.711087	5.5851107	1.26E-07	5.74E-06	7.228129
cg15102821	chr1	1844801	+	CALML6	TSS1500	-0.91353	-1.003376	-5.585023	1.26E-07	5.74E-06	7.2277372
cg01616446	chr6	43196715	+	C6orf108;C6orf108	Body;Body	-0.632257	-4.860175	-5.58495	1.26E-07	5.74E-06	7.2274118

cg25049698	chr6	50692605	+	TFAP2D	Body	1.6794701	-1.103198	5.5849708	1.26E-07	5.74E-06	7.2275048
cg04995300	chr3	66848608	+			1.1868584	-0.896229	5.5848423	1.26E-07	5.75E-06	7.2269318
cg23620236	chr14	45368282	+	C14orf28	5'UTR	1.0292205	1.4335199	5.5848181	1.26E-07	5.75E-06	7.2268238
cg02271895	chr5	127872958	-	FBN2	Body	1.2404443	-1.470513	5.584575	1.27E-07	5.75E-06	7.2257399
cg05627696	chr4	16933239	+			-1.587856	0.6339751	-5.584472	1.27E-07	5.75E-06	7.2252819
cg21813377	chr1	4192817	-			-1.434063	0.746641	-5.589213	1.27E-07	5.76E-06	7.2272947
cg18781988	chr5	140723549	+	PCDHGA2;PCDHGA3;PCDHGA1;PCDHGA3	Body;TSS200;Body;TSS200	1.684967	-0.062588	5.5843579	1.27E-07	5.76E-06	7.2247715
cg14482741	chr17	4802906	-	CHRNE;C17orf107	Body;TSS200	-1.843311	-2.987446	-5.584288	1.27E-07	5.76E-06	7.2244618
cg11280525	chr5	87955695	-	LOC645323	Body	1.2275181	-1.063221	5.5840356	1.27E-07	5.76E-06	7.223334
cg03733219	chr19	38886319	+	SPRED3	Body	2.1120452	-1.003272	5.583964	1.27E-07	5.76E-06	7.2230149
cg06797880	chr14	100112250	-	HHIPL1;HHIPL1	Body;Body	-1.357315	-0.938837	-5.591162	1.27E-07	5.77E-06	7.2263799
cg14460403	chr10	119301766	-	EMX2;EMX2OS;EMX2	TSS200;Body;TSS200	1.960412	-1.807211	5.583912	1.27E-07	5.77E-06	7.2227827
cg06872331	chr2	119981960	+	STEAP3;STEAP3;STEAP3	5'UTR;5'UTR;5'UTR	-0.640976	-3.29386	-5.58381	1.27E-07	5.77E-06	7.2223276
cg14829066	chr15	88559141	+	NTRK3;NTRK3;NTRK3	Body;Body;Body	-1.179488	-0.425622	-5.58375	1.27E-07	5.77E-06	7.2220623
cg15411840	chr6	109615455	-	CCDC162	TSS200	-1.512551	0.8079428	-5.583417	1.27E-07	5.78E-06	7.2205752
cg02202502	chr16	86371442	+	LOC732275	Body	-1.374823	0.5931718	-5.583375	1.27E-07	5.78E-06	7.2203878
cg05452406	chr6	10881891	+	GCM2	Body	1.95454	-0.439368	5.5832665	1.27E-07	5.78E-06	7.2199045
cg23967544	chr5	172672684	+			1.5601901	-0.61958	5.5828719	1.28E-07	5.79E-06	7.2181448
cg09105442	chr10	1228439	+	ADARB2	3'UTR	-1.194456	0.5693593	-5.582843	1.28E-07	5.79E-06	7.218014
cg10728351	chr4	122628020	+			-1.818464	-1.009422	-5.582689	1.28E-07	5.79E-06	7.2173306

cg17840707	chr15	86951099	-	AGBL1	Body	-1.597994	0.6295638	-5.582506	1.28E-07	5.80E-06	7.2165115
cg08417719	chr11	123814620	-	OR6T1	TSS200	-1.769339	1.1165616	-5.582293	1.28E-07	5.80E-06	7.2155638
cg21011616	chr2	177015992	-	HOXD4	TSS200	1.3767715	-1.032268	5.5821343	1.28E-07	5.81E-06	7.2148561
cg16606320	chr4	56685972	-	LOC644145	TSS1500	1.4095451	-0.015692	5.5820334	1.28E-07	5.81E-06	7.2144064
cg21197871	chr8	96036803	-	C8orf38	TSS1500	-1.043706	-2.013244	-5.582034	1.28E-07	5.81E-06	7.2144076
cg24686610	chr3	147125782	-	ZIC4;ZIC1	TSS1500;TSS1500	1.9699893	-1.103097	5.5819106	1.28E-07	5.81E-06	7.2138587
cg04377609	chr3	9358914	-			1.1077006	0.0267628	5.5818543	1.28E-07	5.81E-06	7.2136076
cg17475172	chr8	87878728	-	CNBD1	1stExon	-1.722885	0.2063787	-5.581677	1.28E-07	5.82E-06	7.2128186
cg22494858	chr1	110611712	+	ALX3	Body	1.084047	-1.103046	5.581197	1.29E-07	5.83E-06	7.2106774
cg24428232	chr1	166124419	-	MIR921;FAM78B	TSS1500;Body	-0.992463	-1.304561	-5.581081	1.29E-07	5.83E-06	7.2101608
cg26810767	chr16	79303841	-			-1.472821	-0.550823	-5.581034	1.29E-07	5.83E-06	7.2099513
cg05007035	chr12	104234495	-	NT5DC3	Body	-0.352511	-4.134297	-5.580801	1.29E-07	5.84E-06	7.2089114
cg05866854	chr2	171784945	+	GORASP2	TSS1500	-1.933342	0.6817957	-5.580671	1.29E-07	5.84E-06	7.2083329
cg26292442	chr19	10693912	+	AP1M2	Body	0.74185	0.7864617	5.5806747	1.29E-07	5.84E-06	7.2083489
cg03192598	chr19	12305854	+			2.3774394	-0.462826	5.5828875	1.29E-07	5.84E-06	7.2085757
cg19581424	chr2	42396302	-	EML4;EML4	TSS200;TSS200	-0.762885	-3.19381	-5.582874	1.29E-07	5.84E-06	7.2088702
cg25843426	chr7	1497307	+	MICALL2	Body	-1.075626	-0.675133	-5.580519	1.29E-07	5.84E-06	7.2076548
cg11100309	chr12	57637268	-	STAC3	3'UTR	-0.934774	-3.405905	-5.580317	1.29E-07	5.85E-06	7.206753
cg17283453	chr19	9473880	+	ZNF177	5'UTR	1.6938296	-0.844761	5.580282	1.29E-07	5.85E-06	7.2065982
cg09584358	chr17	14201680	-			1.098786	0.3566424	5.5801328	1.29E-07	5.85E-06	7.2059332

cg22365132	chr6	5205542	+	LYRM4;LYRM4;LYRM4	Body;Body;Body	0.8955531	1.0144739	5.5801324	1.29E-07	5.85E-06	7.2059312
cg27248029	chr17	71039644	+	SLC39A11;SLC39A11	Body;Body	0.8156943	0.4363722	5.5799887	1.29E-07	5.85E-06	7.2052907
cg02088996	chr7	41817771	-	LOC285954	Body	-1.32759	-1.17101	-5.579892	1.29E-07	5.85E-06	7.2048591
cg09527769	chr4	190582456	-			-1.374375	0.4097972	-5.579899	1.29E-07	5.85E-06	7.2048931
cg20666585	chr15	31523186	+			-1.548061	0.9598192	-5.579905	1.29E-07	5.85E-06	7.2049161
cg25070639	chr17	41170693	+	VAT1	Body	0.7714361	0.2860804	5.5798454	1.29E-07	5.85E-06	7.2046522
cg06704773	chr1	173836824	-	GAS5;SNORD76;SNORD75;SNORD77;ZBTB37;ZBTB37;SNORD74	Body;TSS1500;TSS1500;TSS1500;TSS1500;TSS1500;Body	-0.671718	-4.566375	-5.579597	1.30E-07	5.86E-06	7.2035442
cg16678925	chr17	3100972	-	OR1A2	1stExon	-1.216075	-0.118385	-5.579617	1.30E-07	5.86E-06	7.2036337
cg27185978	chr8	25095288	-	DOCK5	Body	1.2564062	0.0771602	5.5795194	1.30E-07	5.86E-06	7.2031989
cg19785773	chr8	142398412	+			-0.890641	-1.016995	-5.579344	1.30E-07	5.87E-06	7.2024174
cg05728596	chr2	219128475	+	GPBAR1;GPBAR1;GPBAR1	3'UTR;3'UTR;3'UTR	1.0684794	2.0067093	5.5791576	1.30E-07	5.87E-06	7.2015861
cg23340017	chr10	102895133	+	TLX1	Body	1.1953499	-0.881133	5.5790843	1.30E-07	5.87E-06	7.2012597
cg07015195	chr8	109093450	-	RSPO2	Body	1.3986585	-0.194376	5.5789929	1.30E-07	5.87E-06	7.2008522
cg15898113	chr8	142532182	+			-1.242572	-0.208007	-5.57897	1.30E-07	5.87E-06	7.2007501
cg07665115	chr8	133062851	-	OC90	Body	-1.253452	0.3263613	-5.578933	1.30E-07	5.87E-06	7.2005839
cg10107473	chr2	46566094	+	EPAS1	Body	1.1497567	-0.185629	5.578908	1.30E-07	5.87E-06	7.200474
cg13026772	chr11	125098532	-	PKNOX2	5'UTR	-1.378013	-0.158134	-5.578816	1.30E-07	5.88E-06	7.2000657
cg21138550	chr17	1966796	-	SMG6;SMG6	Body;Body	0.8363003	1.2665377	5.5788032	1.30E-07	5.88E-06	7.2000067
cg01933073	chr4	186732942	+	SORBS2;SORBS2;SORBS2;SORBS2;SORBS2;SORBS2;SORBS2	5'UTR;5'UTR;5'UTR;1stExon;TSS1500;1stExon;TSS1500	-1.138777	-1.174602	-5.578674	1.30E-07	5.88E-06	7.1994294
cg15163151	chr8	100906373	+	COX6C	TSS1500	-1.525571	0.4878758	-5.581012	1.30E-07	5.88E-06	7.2002239

cg01008263	chr16	70605696	-	SF3B3	Body	0.8629433	3.7515408	5.5833803	1.30E-07	5.88E-06	7.2017164
cg00701514	chr16	1183462	+			-2.35233	2.1385284	-5.578484	1.30E-07	5.88E-06	7.1985822
cg23330788	chr4	21699988	-	KCNIP4;KCNIP4	5'UTR;TSS1500	-1.478377	0.4396359	-5.578376	1.30E-07	5.88E-06	7.1981018
cg09363733	chr2	85767277	-	MAT2A	Body	-1.020945	-3.011877	-5.578286	1.30E-07	5.89E-06	7.1977012
cg01258751	chr6	100911949	+	SIM1	TSS1500	1.5068763	-1.163793	5.5780728	1.30E-07	5.89E-06	7.1967514
cg06513470	chr16	90066769	+	AFG3L1	Body	-1.510864	0.6230587	-5.578022	1.30E-07	5.89E-06	7.1965233
cg14289469	chr14	103010887	+			-0.901804	-2.11493	-5.578007	1.30E-07	5.89E-06	7.1964605
cg14343017	chr7	1632867	+			-1.890275	0.8560024	-5.577919	1.31E-07	5.89E-06	7.1960669
cg27574071	chr4	1219498	+	CTBP1;CTBP1	Body;Body	0.9927629	4.9253731	5.5779301	1.31E-07	5.89E-06	7.1961156
cg26495839	chr19	11484842	+	C19orf39	TSS1500	-1.068077	-2.457656	-5.577837	1.31E-07	5.89E-06	7.1956994
cg03344433	chr5	84126213	-			-2.227043	1.7488976	-5.577789	1.31E-07	5.89E-06	7.1954889
cg15000279	chr19	29285009	+			1.4462917	-1.211949	5.577755	1.31E-07	5.90E-06	7.1953354
cg11735242	chr1	4644030	+			-1.743238	0.3145816	-5.577408	1.31E-07	5.90E-06	7.1937889
cg02264079	chr16	31053489	+			0.988524	3.1368459	5.5770295	1.31E-07	5.91E-06	7.1921024
cg05710479	chr1	7120104	+	CAMTA1	Body	-1.537245	0.8159993	-5.576659	1.31E-07	5.92E-06	7.1904519
cg20060080	chr15	44063940	+	PDIA3	3'UTR	0.9812079	2.5074997	5.5766062	1.31E-07	5.92E-06	7.1902159
cg27109129	chr4	74864165	-	CXCL5	Body	2.3715128	-0.251931	5.5765636	1.31E-07	5.92E-06	7.1900264
cg04722901	chr5	16812392	+	MYO10	Body	0.9343284	1.4340268	5.5788897	1.31E-07	5.92E-06	7.1907787
cg19821682	chr1	4849063	-			-1.166453	-0.413057	-5.576532	1.31E-07	5.92E-06	7.1898858
cg11950860	chr1	152730231	+	KPRP	TSS1500	-1.364815	-0.959118	-5.576354	1.32E-07	5.93E-06	7.1890912

cg05788638	chr14	94759020	-	SERPINA10;SERPINA10;SERPINA10	1stExon;5'UTR;5'UTR	-1.558638	1.0598808	-5.576255	1.32E-07	5.93E-06	7.1886493
cg06729642	chr17	43324793	-	LOC100133991	TSS1500	-0.43883	-3.955636	-5.575965	1.32E-07	5.94E-06	7.1873575
cg14874750	chr7	101006063	+	EMID2	TSS200	2.4706838	-2.024856	5.5755161	1.32E-07	5.95E-06	7.1853588
cg25119415	chr1	158799935	-	MNDA	TSS1500	-1.547867	-0.433182	-5.57539	1.32E-07	5.95E-06	7.1847974
cg13522776	chr9	71785587	-	TJP2	5'UTR	0.8632773	1.4268556	5.5751909	1.32E-07	5.96E-06	7.1839099
cg20073686	chr11	105481863	+	GRIA4;GRIA4;GRIA4;GRIA4	Body;Body;Body;Body	1.5665656	-1.230884	5.5750887	1.32E-07	5.96E-06	7.1834548
cg11467638	chr4	154707153	+	SFRP2	Body	-0.845586	-3.713529	-5.574658	1.33E-07	5.97E-06	7.1815351
cg07768103	chr5	175956344	+	RNF44	Body	-1.818348	3.6722768	-5.58429	1.33E-07	5.97E-06	7.185817
cg04793268	chr11	7728370	-	OVCH2	TSS1500	-1.041226	-2.246591	-5.574518	1.33E-07	5.97E-06	7.1809138
cg10632395	chr6	137815648	-	OLIG3	TSS200	1.2535775	-0.702906	5.5744072	1.33E-07	5.98E-06	7.1804188
cg02336104	chr3	71481486	-	FOXP1;FOXP1	5'UTR;5'UTR	0.8272077	1.3386807	5.5742481	1.33E-07	5.98E-06	7.1797101
cg00565679	chr12	115371893	-			-1.799819	0.2499905	-5.573961	1.33E-07	5.99E-06	7.1784293
cg03524243	chr7	22396146	-	RAPGEF5	5'UTR	-0.492443	-3.455007	-5.573942	1.33E-07	5.99E-06	7.1783465
cg23542902	chr3	193807749	-			-1.324059	-0.925561	-5.573839	1.33E-07	5.99E-06	7.1778874
cg05157140	chr7	1272545	-	UNCX	TSS200	2.5803974	-1.456435	5.573804	1.33E-07	5.99E-06	7.1777317
cg23358612	chr7	1275258	-	UNCX	Body	1.7702991	-0.966487	5.5760061	1.33E-07	5.99E-06	7.1783005
cg16840358	chr3	27765484	-			1.0780391	-0.618881	5.5735099	1.33E-07	6.00E-06	7.1764216
cg01449136	chr14	61109317	-			1.1800638	-0.248745	5.5733916	1.33E-07	6.00E-06	7.1758944
cg09387382	chr17	78418426	-			-1.072537	-1.951351	-5.573335	1.33E-07	6.00E-06	7.1756432
cg19267861	chr20	61809724	-	MIR124-3	TSS200	1.8502989	-1.024624	5.573333	1.33E-07	6.00E-06	7.1756336

cg00323305	chr3	24537182	-	THRB;THRB;THRB	TSS1500;TSS1500;TSS1500	-1.776377	-3.923099	-5.573202	1.33E-07	6.00E-06	7.1750522
cg21512644	chr4	156130016	-	NPY2R;NPY2R	5'UTR;1stExon	1.7723896	-0.644698	5.5731877	1.33E-07	6.00E-06	7.1749864
cg11545923	chr15	27272330	-	GABRG3	Body	-1.346168	-0.245318	-5.572995	1.34E-07	6.01E-06	7.1741291
cg12441928	chr3	46249985	+	CCR1	TSS200	-1.314696	-3.654718	-5.572962	1.34E-07	6.01E-06	7.1739801
cg03217253	chr19	53758055	+	ZNF677	5'UTR	2.138402	-1.529588	5.5728153	1.34E-07	6.01E-06	7.1733277
cg13930766	chr9	122577345	+			-1.405061	-0.258936	-5.572749	1.34E-07	6.01E-06	7.1730302
cg22078710	chr15	53087599	-			1.9042045	-2.318313	5.5775215	1.34E-07	6.01E-06	7.1749591
cg24074477	chr22	17956455	+	CECR2	TSS200	1.2672663	-0.13535	5.5727623	1.34E-07	6.01E-06	7.1730916
cg26123647	chr2	2195399	+	MYT1L	5'UTR	-1.183677	-1.396037	-5.572695	1.34E-07	6.01E-06	7.1727913
cg00859478	chr14	60978094	-	SIX6	3'UTR	1.4939316	-1.621191	5.5726652	1.34E-07	6.01E-06	7.1726589
cg18738581	chr3	9944183	-	IL17RE;IL17RE;IL17RE	TSS200;TSS1500;TSS1500	-0.948231	-1.15977	-5.572545	1.34E-07	6.02E-06	7.1721243
cg03731797	chr19	42900540	-			-0.924463	-0.905281	-5.572455	1.34E-07	6.02E-06	7.171724
cg08720250	chr12	107637226	+			-0.947952	-0.598185	-5.572449	1.34E-07	6.02E-06	7.1716977
cg00534479	chr5	168196464	+	SLIT3;MIR218-2	Body;TSS1500	-1.122725	0.138192	-5.572346	1.34E-07	6.02E-06	7.1712376
cg13047869	chr3	10149882	-	C3orf24;C3orf24;C3orf24;C3orf24	1stExon;1stExon;5'UTR;5'UTR	1.3755294	3.6521144	5.5820096	1.34E-07	6.02E-06	7.1760798
cg17963840	chr8	26722861	-	ADRA1A;ADRA1A;ADRA1A;ADRA1A;ADRA1A;ADRA1A;ADRA1A;ADRA1A	1stExon;5'UTR;1stExon;1stExon;5'UTR;5'UTR;5'UTR;1stExon	0.9587683	-0.549784	5.57197	1.34E-07	6.03E-06	7.169563
cg13577628	chr1	119534899	-			1.2085622	-0.10194	5.5718817	1.34E-07	6.03E-06	7.1691698
cg07569216	chr15	53075533	+	ONECUT1	Body	1.358255	-0.451949	5.5717267	1.34E-07	6.03E-06	7.1684792
cg07912689	chr20	56195458	+	ZBP1;ZBP1;ZBP1;ZBP1;ZBP1;ZBP1;ZBP1;ZBP1	1stExon;1stExon;1stExon;5'UTR;5'UTR;5'UTR;1stExon;5'UTR	-1.325436	-1.800665	-5.571507	1.35E-07	6.04E-06	7.1675008
cg03502791	chr14	54413931	+			-1.320166	-1.197798	-5.571412	1.35E-07	6.04E-06	7.1670793

cg02401454	chr16	230343	-	HBQ1;HBQ1	1stExon;5'UTR	2.6535338	-1.731769	5.5737332	1.35E-07	6.04E-06	7.1681893
cg01504656	chr11	16627903	-			-0.619248	-3.874616	-5.571188	1.35E-07	6.05E-06	7.1660815
cg10100887	chr20	56273259	+	PMEPA1;PMEPA1;PMEPA1;PMEPA1	5'UTR;5'UTR;Body;Body	-1.42461	-0.401305	-5.571098	1.35E-07	6.05E-06	7.1656789
cg27307183	chr6	4890095	-	CDYL;CDYL;CDYL;CDYL	5'UTR;Body;Body;TSS200	0.9464081	1.0936944	5.5710842	1.35E-07	6.05E-06	7.1656182
cg20322837	chr11	57005876	+	APLNR;APLNR	TSS1500;TSS1500	-1.373205	0.1455723	-5.57106	1.35E-07	6.05E-06	7.1655106
cg18617005	chr5	140787504	-	PCDHGA4;PCDHGA6;PCDHGA9;PCDHGA1;PCDHGA8;PCDHGA5;PCDHGB1;PCDHGB6;PCDHGB4;PCDHGA3;PCDHGA2;PCDHGA7;PCDHGB2;PCDHGB6;PCDHGB5;PCDHGB3	Body;Body;Body;Body;Body;Body;Body;TSS1500;Body;Body;Body;Body;Body;TSS1500;Body;Body	2.6607255	-1.390752	5.5732677	1.35E-07	6.05E-06	7.1823462
cg16433357	chr6	80340561	-	SH3BGRL2	TSS1500	-0.682804	-2.829122	-5.570849	1.35E-07	6.05E-06	7.1645712
cg16018124	chr8	94627566	-			-1.234415	-0.634563	-5.570646	1.35E-07	6.06E-06	7.1636651
cg09430586	chr19	54242124	+	MIR518A2	TSS1500	-1.636582	0.7920338	-5.570566	1.35E-07	6.06E-06	7.1633093
cg02887114	chr19	54568236	-	VSTM1	TSS1500	-1.033552	0.2375297	-5.570392	1.35E-07	6.06E-06	7.1625349
cg21281009	chr18	14748298	-	ANKRD30B;ANKRD30B	1stExon;5'UTR	2.1091968	-0.92109	5.5776333	1.35E-07	6.06E-06	7.1824224
cg22958248	chr12	56545428	-	MYL6B	TSS1500	-0.773082	-1.064898	-5.570408	1.35E-07	6.06E-06	7.1626061
cg06932535	chr14	32350324	-			-1.434971	-0.168733	-5.570295	1.35E-07	6.06E-06	7.1621017
cg18003659	chr2	175629267	-	CHRNA1;CHRNA1	TSS200;TSS200	-1.704076	0.7127524	-5.57023	1.35E-07	6.07E-06	7.1618144
cg21793948	chr18	60988096	+	BCL2;BCL2	TSS1500;TSS1500	-1.368072	-5.044338	-5.570233	1.35E-07	6.07E-06	7.1618287
cg24627900	chr8	133573739	+	HPYR1	TSS200	-1.393792	-0.749333	-5.570099	1.35E-07	6.07E-06	7.1612297
cg12440258	chr5	50265272	+			1.4097079	-0.747063	5.5747287	1.35E-07	6.07E-06	7.1771055
cg22478310	chr6	100911744	-	SIM1	TSS200	1.2571239	-0.841602	5.5699676	1.35E-07	6.07E-06	7.160646
cg02077084	chr1	248549941	-	OR2T6	TSS1500	-1.449031	0.1113776	-5.569786	1.36E-07	6.07E-06	7.1598385
cg18405691	chr11	55322753	-	OR4C15	1stExon	-1.785117	0.8808189	-5.569778	1.36E-07	6.07E-06	7.1598028

cg20504856	chr6	19692011	+			1.6333173	-1.786602	5.5697894	1.36E-07	6.07E-06	7.1598524
cg02079645	chr2	167744466	-			-1.822969	1.4288671	-5.569697	1.36E-07	6.08E-06	7.1594405
cg20949198	chr6	134317925	-	SLC2A12	Body	-1.898587	1.3254123	-5.569561	1.36E-07	6.08E-06	7.1588354
cg03878722	chr16	76666860	+			-1.463007	0.8667765	-5.569453	1.36E-07	6.08E-06	7.1583543
cg10447095	chr16	60678710	+			-1.443738	0.5412207	-5.568997	1.36E-07	6.09E-06	7.1563226
cg26990102	chr2	119606746	-	EN1	TSS1500	2.3293412	-1.618985	5.5688678	1.36E-07	6.10E-06	7.1557492
cg15835664	chr1	62787266	+			-1.501533	0.7642137	-5.568569	1.36E-07	6.10E-06	7.1544189
cg01025233	chr5	72732060	+			0.9350096	0.1417292	5.5683492	1.37E-07	6.11E-06	7.1534406
cg11204152	chr14	72638695	+	RGS6	Body	-1.352093	0.3082123	-5.56829	1.37E-07	6.11E-06	7.1531767
cg06853455	chr12	113787113	+			1.0818286	1.5618912	5.5680439	1.37E-07	6.12E-06	7.1520814
cg08338216	chr10	121577530	-	INPP5F;INPP5F	Body;TSS1500	0.7465536	1.9488164	5.5679646	1.37E-07	6.12E-06	7.1517282
cg10592926	chr6	157876961	-	ZDHHC14;ZDHHC14	Body;Body	1.3903347	2.1779271	5.5677993	1.37E-07	6.12E-06	7.1509925
cg13429423	chr22	44568775	+	PARVG;PARVG	TSS200;TSS200	-1.334599	0.0461257	-5.567734	1.37E-07	6.12E-06	7.1506999
cg03577157	chr5	80716263	+	RNU5E;SSBP2;RNU5D	Body;3'UTR;Body	-1.415276	-1.261038	-5.567537	1.37E-07	6.13E-06	7.1498247
cg18541087	chr19	3293448	+	BRUNOL5	3'UTR	-1.097614	0.3940047	-5.567223	1.37E-07	6.14E-06	7.1484263
cg23490887	chr6	117584596	+			1.4499999	-1.361873	5.5670978	1.37E-07	6.14E-06	7.1478696
cg23507945	chr6	137477184	+	IL22RA2;IL22RA2;IL22RA2	Body;Body;Body	-1.992199	-1.484299	-5.566928	1.37E-07	6.15E-06	7.1471126
cg26159860	chr11	133469193	+			-1.570728	-0.232696	-5.566819	1.37E-07	6.15E-06	7.1466278
cg21463368	chr6	167263025	+	RPS6KA2	Body	-2.121755	1.7979318	-5.566608	1.38E-07	6.15E-06	7.145688
cg11078674	chr14	73030458	-	RGS6	3'UTR	-1.202014	0.2565541	-5.566276	1.38E-07	6.16E-06	7.1442115

cg13597013	chr17	78922527	-	RPTOR;RPTOR	Body;Body	0.7062991	1.543698	5.5661435	1.38E-07	6.17E-06	7.1436223
cg26137103	chr4	40193114	+			-1.781586	0.5705756	-5.566085	1.38E-07	6.17E-06	7.1433626
cg00884334	chr15	34787920	-			1.1206823	-1.176832	5.5659615	1.38E-07	6.17E-06	7.1428123
cg16668520	chr8	55387024	+			-1.684333	0.2750279	-5.565968	1.38E-07	6.17E-06	7.1428421
cg21918595	chr1	44784097	+	ERI3	Body	-1.015456	-1.835152	-5.568228	1.38E-07	6.17E-06	7.1437073
cg04889069	chr8	57030408	-			0.8670897	0.7052399	5.5657613	1.38E-07	6.17E-06	7.141921
cg03496533	chr17	46035183	+	PRR15L	TSS200	-0.92139	-1.017658	-5.565667	1.38E-07	6.17E-06	7.1415001
cg10805082	chr1	179199199	+	ABL2;ABL2;ABL2;ABL2;ABL2	TSS1500;TSS1500;TSS1500;TSS1500;TSS1500	-0.495783	-3.763541	-5.565531	1.38E-07	6.18E-06	7.140897
cg20593059	chr12	115286012	+			-1.457071	0.5826687	-5.56555	1.38E-07	6.18E-06	7.1409805
cg27223272	chr7	152618877	+			-1.811394	0.9986189	-5.565542	1.38E-07	6.18E-06	7.1409472
cg20828084	chr15	81070851	-	KIAA1199	TSS1500	-1.409824	0.9859153	-5.564834	1.39E-07	6.20E-06	7.1377956
cg10542584	chr3	62356223	+	FEZF2	Body	1.1966866	-1.192854	5.5647797	1.39E-07	6.20E-06	7.1375528
cg17217059	chr4	152329364	+	FAM160A1	TSS1500	-0.780936	-2.568318	-5.564783	1.39E-07	6.20E-06	7.1375657
cg20167074	chr1	151967023	-	S100A10	TSS1500	-1.674427	-1.115064	-5.564734	1.39E-07	6.20E-06	7.1373473
cg07888234	chr18	59992252	-	TNFRSF11A	TSS1500	-0.594744	-3.111586	-5.564686	1.39E-07	6.20E-06	7.137134
cg19772011	chr6	39015339	+	GLP1R	TSS1500	-0.979717	-1.971293	-5.564642	1.39E-07	6.20E-06	7.136938
cg25115993	chr6	150285475	+	ULBP1	Body	1.9167073	-2.881977	5.5645331	1.39E-07	6.20E-06	7.1364554
cg09661370	chr7	27225299	+	HOXA11AS;HOXA11	Body;TSS1500	1.2226118	-0.000382	5.564448	1.39E-07	6.20E-06	7.1360766
cg18299068	chr4	1305425	-	MAEA;MAEA	Body;Body	0.8281754	1.9332867	5.5643953	1.39E-07	6.20E-06	7.1358421
cg21121082	chr11	128318549	-			-1.505017	0.9120803	-5.564438	1.39E-07	6.20E-06	7.1360313

cg22772747	chr7	27164285	-	HOXA3	5'UTR	1.341147	0.5831394	5.564412	1.39E-07	6.20E-06	7.1359163
cg23856293	chr20	62795341	+	MYT1	TSS1500	-1.937382	0.844959	-5.564438	1.39E-07	6.20E-06	7.1360319
cg11394278	chr22	49566994	+			-1.496433	-0.09066	-5.564368	1.39E-07	6.20E-06	7.1357195
cg23229770	chr2	129491004	-			-1.388299	-1.221746	-5.564237	1.39E-07	6.21E-06	7.1351375
cg09792881	chr1	50884544	-	DMRTA2	Body	2.1978621	-0.989496	5.5639663	1.39E-07	6.21E-06	7.1339332
cg15298486	chr10	102046690	+	BLOC1S2;BLOC1S2	TSS1500;TSS1500	-0.723855	-3.468334	-5.563918	1.39E-07	6.21E-06	7.133719
cg07396047	chr16	87735077	+	LOC100129637	Body	-1.19731	1.8108896	-5.563769	1.39E-07	6.22E-06	7.1330558
cg08733172	chr4	174431860	-			0.8390474	0.5862237	5.5659648	1.40E-07	6.22E-06	7.1336499
cg23478293	chr14	57279275	-	OTX2OS1	TSS1500	1.8178234	-1.254411	5.5635908	1.40E-07	6.22E-06	7.1322621
cg02165692	chr2	30142586	+	ALK	Body	0.7317539	1.7205472	5.5634886	1.40E-07	6.22E-06	7.1318076
cg05187003	chr21	34641507	-	IL10RB	Body	-0.781458	-0.940668	-5.563345	1.40E-07	6.23E-06	7.1311708
cg13072214	chr8	145060799	+	PARP10	TSS200	-0.94609	-1.943053	-5.563311	1.40E-07	6.23E-06	7.1310193
cg08199953	chr16	51183473	-	SALL1;SALL1	Body;5'UTR	1.8163479	-0.928888	5.5632413	1.40E-07	6.23E-06	7.1307072
cg12142424	chr14	94759873	+	SERPINA10;SERPINA10	TSS1500;TSS1500	-0.968003	-1.509214	-5.563194	1.40E-07	6.23E-06	7.1304964
cg23239109	chr3	43732098	+	ABHD5	TSS1500	-1.082129	-4.498339	-5.563203	1.40E-07	6.23E-06	7.1305351
cg14386668	chr17	3337334	-	OR1E2	TSS200	-1.436409	0.2209692	-5.563117	1.40E-07	6.23E-06	7.1301524
cg06197769	chr12	12868983	-	CDKN1B	TSS1500	-1.51918	-2.82191	-5.565275	1.40E-07	6.23E-06	7.1302283
cg20041088	chr1	91192803	+			1.1712025	-0.683567	5.562929	1.40E-07	6.23E-06	7.1293178
cg09024228	chr1	116707400	+			0.9411097	0.5598269	5.5628152	1.40E-07	6.24E-06	7.1288114
cg10047173	chr12	14996587	+	ART4	TSS200	1.163911	1.5701746	5.5628161	1.40E-07	6.24E-06	7.1288156

cg06498267	chr5	45695922	-	HCN1	1stExon	1.3295132	-0.91426	5.5627275	1.40E-07	6.24E-06	7.1284212
cg16081096	chr3	12980202	+	IQSEC1;IQSEC1	Body;Body	0.6812276	1.30606	5.5627256	1.40E-07	6.24E-06	7.128413
cg04452092	chr5	153438080	-			-1.113868	-0.079457	-5.562698	1.40E-07	6.24E-06	7.1282915
cg06646708	chr6	18123224	-	NHLRC1	TSS1500	-1.335792	0.0627861	-5.562633	1.40E-07	6.24E-06	7.1280029
cg14706200	chr19	2472062	-			-0.905638	0.8654987	-5.562641	1.40E-07	6.24E-06	7.1280352
cg21063296	chr19	10304422	+	DNMT1;DNMT1	Body;Body	-0.734598	-1.947716	-5.562606	1.40E-07	6.24E-06	7.1278827
cg01564671	chr17	56296913	-	MKS1;MKS1;MKS1	1stExon;5'UTR;TSS1500	-0.599399	-3.905793	-5.562528	1.40E-07	6.24E-06	7.1275337
cg06002471	chr21	32014696	+	KRTAP20-3	TSS1500	-1.48238	1.1936245	-5.562443	1.40E-07	6.24E-06	7.1271561
cg07510346	chr8	2035751	+	MYOM2	Body	-1.022049	-0.385463	-5.562354	1.40E-07	6.24E-06	7.1267604
cg02136690	chr13	114905809	+			-1.105025	-1.59087	-5.562194	1.40E-07	6.25E-06	7.1260459
cg14066757	chr19	44285568	-	KCNN4	TSS200	-1.04907	-1.551665	-5.562212	1.40E-07	6.25E-06	7.12613
cg18708986	chr12	52652590	+			1.2496395	0.4325744	5.5618143	1.41E-07	6.26E-06	7.1243586
cg25402228	chr15	47474134	-			-1.494135	0.8281397	-5.561749	1.41E-07	6.26E-06	7.1240697
cg16960046	chr7	55053391	+			-1.29273	0.016747	-5.561705	1.41E-07	6.26E-06	7.123872
cg01182683	chr12	52586635	-	KRT80;KRT80	TSS1500;TSS1500	-1.264437	0.8468222	-5.561599	1.41E-07	6.26E-06	7.1234019
cg22360016	chr2	105276153	-			-1.113402	-0.942554	-5.561186	1.41E-07	6.27E-06	7.1215633
cg00778928	chr2	128380062	-	MYO7B	Body	-0.793885	-0.807202	-5.561132	1.41E-07	6.27E-06	7.1213248
cg14860680	chr15	74528243	-	CCDC33	TSS1500	-1.088521	0.0968271	-5.560796	1.41E-07	6.28E-06	7.119828
cg01434649	chr1	3567870	-	TP73;WDR8	TSS1500;TSS1500	1.3453606	0.5310365	5.5729429	1.41E-07	6.28E-06	7.1258123
cg26610167	chr19	55720817	+	PTPRH;PTPRH;PTPRH;PTPRH	1stExon;5'UTR;5'UTR;1stExon	-1.367932	0.0875211	-5.560605	1.42E-07	6.29E-06	7.1189808

cg03670393	chr4	81128690	+			1.2001118	0.0508242	5.5604923	1.42E-07	6.29E-06	7.1184781
cg17349632	chr7	131830544	-	PLXNA4	Body	-1.76151	0.6077484	-5.560276	1.42E-07	6.30E-06	7.1175155
cg00817377	chr7	73441057	+	ELN;ELN;ELN;ELN;ELN	TSS1500;TSS1500;TSS1500;TSS1500;TSS1500	-0.756794	-3.00129	-5.560047	1.42E-07	6.30E-06	7.1164957
cg15919833	chr5	96079175	+	CAST;CAST;CAST;CAST;CAST;CAST;CAST;CAST;CAST;CAST;CAST	Body;Body;Body;Body;Body;Body;TSS200;Body;Body;Body;Body	0.6470264	2.1915106	5.5598965	1.42E-07	6.31E-06	7.1158278
cg11347582	chr6	168502341	+			2.3531147	-0.445673	5.5598036	1.42E-07	6.31E-06	7.1154147
cg03989260	chr1	119529360	-	TBX15	5'UTR	1.0881399	-0.518539	5.5597705	1.42E-07	6.31E-06	7.1152675
cg09781307	chr11	27648324	-	BDNFOS	Body	-1.153562	-0.771173	-5.559682	1.42E-07	6.31E-06	7.1148732
cg25665573	chr5	137369222	-	FAM13B;FAM13B;FAM13B	TSS1500;TSS1500;TSS1500	-0.367931	-3.229176	-5.559679	1.42E-07	6.31E-06	7.1148627
cg15193614	chr5	2239477	-			-1.670508	0.2137538	-5.559547	1.42E-07	6.31E-06	7.1142718
cg23243400	chr7	20818844	+			1.4978446	-0.073791	5.5593282	1.42E-07	6.32E-06	7.1133003
cg25060890	chr1	213224164	+	RPS6KC1;RPS6KC1	TSS1500;TSS1500	-1.388771	0.2312594	-5.559245	1.42E-07	6.32E-06	7.1129297
cg00368604	chr10	131126358	-			-1.510264	0.295988	-5.55907	1.43E-07	6.32E-06	7.1121529
cg04662532	chr12	54330125	+			1.3365693	0.2529533	5.5590337	1.43E-07	6.32E-06	7.1119906
cg14629571	chr9	120470582	+	TLR4;TLR4;TLR4	Body;Body;Body	-1.801192	1.2737964	-5.559033	1.43E-07	6.32E-06	7.1119884
cg20597842	chr12	7818585	-	APOBEC1	TSS200	-1.705477	0.4569178	-5.559056	1.43E-07	6.32E-06	7.112088
cg23045972	chr3	118230228	-			-1.346949	-0.414177	-5.558807	1.43E-07	6.33E-06	7.1109809
cg09258804	chr17	59532564	-	TBX4	TSS1500	1.4641375	-0.753613	5.561053	1.43E-07	6.33E-06	7.1114702
cg16731157	chr1	24830054	+	RCAN3	5'UTR	-0.723989	-3.284572	-5.560888	1.43E-07	6.34E-06	7.1110954
cg18555117	chr14	53620401	+	DDHD1;DDHD1;DDHD1	TSS1500;TSS1500;TSS1500	-1.146714	-3.107704	-5.558235	1.43E-07	6.34E-06	7.108438
cg00282510	chr9	100611516	+			1.9044549	-1.20809	5.5580301	1.43E-07	6.35E-06	7.1075275

cg15711902	chr1	170630457	-			1.8415273	-1.382827	5.558026	1.43E-07	6.35E-06	7.1075095
cg09476347	chr16	613343	+	C16orf11	Body	-1.438059	1.1564364	-5.557923	1.43E-07	6.35E-06	7.1070534
cg04793090	chr3	128903277	+	CNBP;CNBP;CNBP;CNBP;CNBP;CNBP	TSS1500;TSS1500;TSS1500;TSS1500;TSS1500;TSS1500	-0.514179	-3.13229	-5.557863	1.43E-07	6.35E-06	7.1067825
cg09745307	chr5	35048945	-	AGXT2	TSS1500	-1.777619	0.0051688	-5.557775	1.43E-07	6.36E-06	7.1063915
cg01174771	chr14	32670315	+			-0.631684	-4.025588	-5.557458	1.44E-07	6.36E-06	7.1049845
cg19721065	chr12	113549986	+	RASAL1	Body	1.1795985	1.3273922	5.5574335	1.44E-07	6.36E-06	7.1048749
cg17165266	chr12	53342635	+	KRT18;KRT18	TSS200;TSS1500	-0.525043	-5.096156	-5.557399	1.44E-07	6.36E-06	7.1047194
cg18011916	chr7	35295120	-	TBX20;TBX20	TSS1500;TSS1500	1.5298738	-0.317387	5.5573263	1.44E-07	6.37E-06	7.104398
cg15855970	chr6	99273338	-			1.4104165	-1.408941	5.557269	1.44E-07	6.37E-06	7.1041432
cg19177125	chr18	19752414	-	GATA6	Body	1.4143798	0.6677135	5.5571411	1.44E-07	6.37E-06	7.1035747
cg15075226	chr3	128734262	-	CCDC48	Body	0.7535348	0.3441928	5.5569856	1.44E-07	6.37E-06	7.1028833
cg10214581	chr14	94811949	-			-1.078218	-0.911431	-5.556875	1.44E-07	6.38E-06	7.1023905
cg26173946	chr7	48126643	-			-1.197195	0.7844227	-5.556879	1.44E-07	6.38E-06	7.1024084
cg09189497	chr10	2147517	-			-1.371523	0.7156527	-5.556671	1.44E-07	6.38E-06	7.1014839
cg02861380	chr1	75596758	-	LHX8	Body	2.291771	-1.857226	5.5587445	1.44E-07	6.39E-06	7.1015737
cg10879722	chr2	91816195	-			-0.559065	-0.270334	-5.55638	1.44E-07	6.39E-06	7.1001889
cg05311909	chr10	23484025	-			1.3461387	-2.116692	5.5561727	1.44E-07	6.39E-06	7.099269
cg26151467	chr13	112724221	-	SOX1;SOX1	1stExon;3'UTR	1.4965304	-0.349528	5.556175	1.44E-07	6.39E-06	7.0992793
cg11305134	chr3	65950683	-	MAGI1;MAGI1;MAGI1	Body;Body;Body	-1.054345	1.7123665	-5.556028	1.45E-07	6.40E-06	7.0986237
cg26098117	chr6	125803002	-			-1.472962	0.1778832	-5.556016	1.45E-07	6.40E-06	7.0985723

cg05091519	chr11	31839552	-	PAX6	TSS200	2.519263	-2.190528	5.5583442	1.45E-07	6.40E-06	7.0997958
cg13560452	chr6	137813584	+	OLIG3;OLIG3	1stExon;3'UTR	1.2392465	0.2453195	5.5558913	1.45E-07	6.40E-06	7.0980179
cg10023530	chr18	11148783	+	FAM38B	TSS200	1.3461778	-1.301922	5.5558369	1.45E-07	6.40E-06	7.0977762
cg20902104	chr14	106307623	+			-1.259719	0.1422366	-5.555834	1.45E-07	6.40E-06	7.0977611
cg02095401	chr5	167545264	+	ODZ2	Body	-1.255162	0.1653653	-5.555669	1.45E-07	6.40E-06	7.0970289
cg17700835	chr6	27390890	+			1.0422877	1.4952629	5.5556487	1.45E-07	6.40E-06	7.0969396
cg01200640	chr8	687384	-			2.6709476	-1.930947	5.5603637	1.45E-07	6.40E-06	7.0991406
cg12017745	chr11	65352327	-	EHBP1L1	Body	1.5043261	2.1846186	5.5577848	1.45E-07	6.41E-06	7.0969555
cg12800047	chr11	106566411	+	GUCY1A2	Body	-1.521902	0.0751604	-5.555355	1.45E-07	6.41E-06	7.0956326
cg11953088	chr2	217240840	+			-1.195281	-1.022065	-5.555253	1.45E-07	6.41E-06	7.0951796
cg04973183	chr4	160118923	-			0.8140091	1.6833762	5.5551137	1.45E-07	6.42E-06	7.0945611
cg04398526	chr16	1035197	+	SOX8	Body	-1.327138	0.8322853	-5.555075	1.45E-07	6.42E-06	7.0943874
cg16058493	chr1	91189167	-			1.9725772	-0.180446	5.5621802	1.45E-07	6.42E-06	7.0978005
cg22953960	chr10	22766081	+			1.1446601	1.1004794	5.5548635	1.45E-07	6.42E-06	7.0934491
cg02698668	chr19	44261472	+			-1.028325	-1.129287	-5.5548	1.45E-07	6.42E-06	7.0931651
cg19071452	chr2	152146944	+	NMI	TSS1500	-1.79372	-1.951035	-5.554766	1.45E-07	6.42E-06	7.0930169
cg26751645	chr20	43945364	+	RBPJL	Body	1.0692444	0.0766861	5.5547304	1.45E-07	6.42E-06	7.0928573
cg06586046	chr17	78038103	-	CCDC40	Body	-0.861466	-0.726073	-5.554618	1.45E-07	6.43E-06	7.0923588
cg19917412	chr11	64815189	-	NAALADL1	Body	1.3091835	-0.377548	5.5546202	1.45E-07	6.43E-06	7.0923674
cg22112832	chr2	71098542	-			-0.549186	-1.985288	-5.554462	1.46E-07	6.43E-06	7.0916639

cg05938409	chr16	85290060	-			-1.468386	0.029239	-5.554418	1.46E-07	6.43E-06	7.0914677
cg26251098	chr11	131319616	-	NTM	Body	-1.682642	0.2928012	-5.554419	1.46E-07	6.43E-06	7.091471
cg20328399	chr5	15928089	-	FBXL7	Body	-1.498316	0.4864416	-5.554009	1.46E-07	6.44E-06	7.0896494
cg24354933	chr19	14049294	+	PODNL1;PODNL1;PODNL1	Body;TSS200;Body	-0.657632	-2.282745	-5.556301	1.46E-07	6.44E-06	7.0907236
cg01912955	chr6	100906383	-	SIM1	Body	1.1108824	-0.544594	5.5538701	1.46E-07	6.44E-06	7.0890331
cg07083862	chr2	1417248	+	TPO;TPO;TPO;TPO;TPO;TPO	1stExon;1stExon;5'UTR;TSS1500;5'UTR;TSS1500	-1.948396	1.0571267	-5.55373	1.46E-07	6.45E-06	7.0884085
cg14255644	chr1	228289747	+	C1orf35	Body	-0.769699	-1.241889	-5.553664	1.46E-07	6.45E-06	7.0881192
cg04011469	chr2	82864753	+			-1.672024	0.1276965	-5.555878	1.46E-07	6.45E-06	7.088847
cg06389950	chr16	75240536	-	CTRB2	Body	-0.572634	-0.615423	-5.553513	1.46E-07	6.45E-06	7.0874469
cg07487731	chr5	140616495	+	PCDHB18	Body	1.1436137	-0.748204	5.5535436	1.46E-07	6.45E-06	7.0875819
cg12506165	chr12	116997072	-	MAP1LC3B2	TSS200	-1.14931	-0.909518	-5.555904	1.46E-07	6.45E-06	7.0889596
cg24603439	chr7	15436238	-	TMEM195	Body	-2.120879	1.6964785	-5.553546	1.46E-07	6.45E-06	7.0875915
cg23170495	chr10	130519824	+			-1.509909	0.4696867	-5.553445	1.46E-07	6.45E-06	7.0871453
cg07025650	chr2	54710513	+	SPTBN1	5'UTR	1.1943537	0.8361003	5.5532628	1.46E-07	6.46E-06	7.0863339
cg04871460	chr1	1991521	-	PRKCZ	Body	0.6058358	0.7999243	5.5532262	1.46E-07	6.46E-06	7.0861716
cg16458596	chr14	99584812	+			0.9054812	-0.702954	5.5528745	1.47E-07	6.47E-06	7.0846086
cg23654401	chr7	55606442	+	VOPP1	Body	-2.027651	-1.197937	-5.552591	1.47E-07	6.47E-06	7.0833484
cg10746542	chr14	38678924	-	SSTR1	Body	1.0807456	-1.498624	5.5525384	1.47E-07	6.48E-06	7.0831146
cg07474269	chr5	166722513	+	ODZ2	Body	-1.236394	-0.261366	-5.552495	1.47E-07	6.48E-06	7.0829199
cg08070327	chr7	27184059	-	HOXA5	TSS1500	1.536367	0.7094949	5.5524704	1.47E-07	6.48E-06	7.0828126

cg18328265	chr19	418899	+	SHC2	3'UTR	-2.050391	2.2207908	-5.55456	1.47E-07	6.48E-06	7.0829947
cg20233073	chr4	54960226	-			1.2881546	-1.082595	5.5521279	1.47E-07	6.49E-06	7.0812902
cg00941900	chr14	101357601	-			-1.118991	-0.048193	-5.552067	1.47E-07	6.49E-06	7.0810196
cg23095584	chr2	237077704	-	GBX2	TSS1500	1.194556	-0.770936	5.5520078	1.47E-07	6.49E-06	7.0807569
cg23725321	chr10	102279690	+	SEC31B	TSS200	0.8752128	-0.060586	5.55196	1.47E-07	6.49E-06	7.0805442
cg17004906	chr1	11727668	+	FBXO6	5'UTR	1.0369801	1.0107056	5.5519171	1.47E-07	6.49E-06	7.0803535
cg17466857	chr7	27225528	+	HOXA11AS;HOXA11	Body;TSS1500	2.5152997	-1.47307	5.5562726	1.48E-07	6.50E-06	7.0809997
cg13545297	chr12	54404315	+	HOXC8	Body	1.4618062	1.1160012	5.5609541	1.48E-07	6.51E-06	7.0828599
cg09523275	chr6	28227093	+	NKAPL	TSS200	1.2545798	-0.023465	5.5513005	1.48E-07	6.51E-06	7.0776138
cg06713116	chr7	154720452	-	LOC100132707;LOC100132707	Body;Body	-0.797771	-3.801981	-5.551143	1.48E-07	6.51E-06	7.0769133
cg09165441	chr10	103044194	-			2.2215822	-0.927691	5.5510708	1.48E-07	6.51E-06	7.0765933
cg24896460	chr5	73188814	-	RGNEF	Body	-1.029975	-0.106347	-5.551091	1.48E-07	6.51E-06	7.076681
cg02919564	chr6	47210376	+	TNFRSF21	Body	-1.535357	-4.035367	-5.550955	1.48E-07	6.51E-06	7.0760778
cg02620403	chr5	110404837	-			-1.494071	1.0881907	-5.550917	1.48E-07	6.51E-06	7.0759086
cg22033759	chr17	1182267	+	TUSC5	TSS1500	-1.289577	0.1868821	-5.550886	1.48E-07	6.51E-06	7.0757709
cg15826426	chr15	97325761	+	SPATA8	TSS1500	-1.627907	0.5116863	-5.550588	1.48E-07	6.52E-06	7.0744484
cg17090237	chr11	8060223	+	TUB;TUB	5'UTR;1stExon	-0.892294	-0.403457	-5.550425	1.48E-07	6.53E-06	7.0737236
cg27642416	chr8	86554026	-			-1.710228	-0.185301	-5.550274	1.48E-07	6.53E-06	7.0730538
cg09649486	chr10	131641336	-	EBF3	Body	-1.273789	0.3867363	-5.550088	1.49E-07	6.54E-06	7.0722252
cg05218696	chr14	101402494	+	SNORD113-4	TSS1500	-1.497939	0.4370566	-5.550042	1.49E-07	6.54E-06	7.0720216

cg20975733	chr15	87155056	+	AGBL1	Body	-1.12629	-0.605876	-5.550042	1.49E-07	6.54E-06	7.0720217
cg14095959	chr4	77723415	+			1.1660456	-0.077348	5.55202	1.49E-07	6.55E-06	7.071723
cg04771285	chr12	117557630	-			-0.875785	-0.957883	-5.5496	1.49E-07	6.55E-06	7.0700592
cg23341182	chr10	102046768	+	BLOC1S2;BLOC1S2	TSS1500;TSS1500	-0.853377	-2.49486	-5.549582	1.49E-07	6.55E-06	7.0699792
cg06749213	chr14	20387292	+	OR4K5	TSS1500	-1.69095	0.5274022	-5.549345	1.49E-07	6.55E-06	7.0689252
cg05884214	chr7	1712054	+			-1.339971	0.7019605	-5.549306	1.49E-07	6.55E-06	7.0687514
cg08721187	chr7	131626708	+			-1.393925	0.5390704	-5.549168	1.49E-07	6.56E-06	7.068139
cg07475000	chr7	22162928	-	RAPGEF5	Body	1.4120674	0.1586961	5.5661149	1.49E-07	6.57E-06	7.1053495
cg24507955	chr15	49339070	+	SECISBP2L	TSS1500	-1.079499	-1.663606	-5.548902	1.49E-07	6.57E-06	7.0669578
cg09810089	chr1	211652688	+	RD3;RD3	Body;Body	1.2437143	1.3819077	5.5560077	1.49E-07	6.57E-06	7.0708478
cg04157138	chr14	58331898	-			1.1059688	-0.305859	5.5485491	1.50E-07	6.57E-06	7.06539
cg25398727	chr8	10756220	+	XKR6	Body	-1.309531	0.37021	-5.548492	1.50E-07	6.58E-06	7.0651355
cg00351537	chr19	7580275	+	ZNF358	TSS1500	1.0424309	0.8933945	5.5484361	1.50E-07	6.58E-06	7.0648882
cg08198071	chr2	109742356	-			-1.660016	0.2918357	-5.547991	1.50E-07	6.59E-06	7.0629109
cg02433979	chr7	128824829	-			-1.400295	0.0402603	-5.547963	1.50E-07	6.59E-06	7.0627844
cg05671644	chr7	102632303	+	FBXL13;FBXL13	Body;Body	-1.216444	-0.496315	-5.547597	1.50E-07	6.60E-06	7.0611622
cg02849693	chr19	54402455	-	PRKCG	Body	1.3021068	-0.150315	5.5471868	1.51E-07	6.61E-06	7.0593391
cg13022977	chr12	6420252	-	PLEKHG6;PLEKHG6;PLEKHG6	1stExon;5'UTR;5'UTR	-1.18665	-1.939926	-5.551691	1.51E-07	6.62E-06	7.0606935
cg12039490	chr8	99258458	-	NIPAL2	Body	0.6162414	1.869538	5.5469257	1.51E-07	6.62E-06	7.0581793
cg07664000	chr17	28659293	-	TMIGD1	5'UTR	-1.581473	-1.844922	-5.546596	1.51E-07	6.63E-06	7.0567136

cg11663667	chr4	155662515	-			1.1429111	-0.276423	5.5463734	1.51E-07	6.63E-06	7.0557266
ch.7.1905850F	chr7	90038364	+	CLDN12	5'UTR	-0.877435	-3.139042	-5.546378	1.51E-07	6.63E-06	7.0557459
cg02407048	chr7	77046134	-	PION	TSS1500	-1.008367	-1.075493	-5.546075	1.51E-07	6.64E-06	7.0544006
cg13436155	chr13	100644217	+			1.3032379	-0.344333	5.5460319	1.51E-07	6.64E-06	7.0542096
cg21544416	chr15	71125838	-	LARP6	Body	-1.305669	0.6951639	-5.54832	1.51E-07	6.64E-06	7.0549476
cg12062198	chr8	22419460	-	SORBS3	Body	-0.774845	-4.283748	-5.54591	1.52E-07	6.65E-06	7.0536695
cg15241920	chr19	54926514	+	TTYH1;TTYH1	TSS200;TSS200	3.2406723	-2.674269	5.5458988	1.52E-07	6.65E-06	7.0536189
cg10502121	chr7	53286906	-			1.148662	-0.056609	5.5458224	1.52E-07	6.65E-06	7.0532797
cg02764346	chr2	166651375	-	GALNT3	TSS1500	-0.593496	-3.84271	-5.545767	1.52E-07	6.65E-06	7.0530337
cg25748108	chr20	39968608	-	LPIN3	TSS1500	-0.914258	-1.404372	-5.545786	1.52E-07	6.65E-06	7.0531195
cg20256245	chr22	29877285	+	NEFH	Body	1.3862426	-1.436941	5.5456846	1.52E-07	6.65E-06	7.0526677
cg06934265	chr14	101521477	+	MIR453;MIR485;MIR668	TSS1500;TSS1500;TSS200	-1.262436	1.0087361	-5.545429	1.52E-07	6.66E-06	7.0515308
cg14353278	chr2	21229231	+	APOB	Body	-1.732611	0.9991247	-5.545437	1.52E-07	6.66E-06	7.0515671
cg27361370	chr3	157813327	+			1.4988297	-0.639597	5.5452876	1.52E-07	6.66E-06	7.0509047
cg02344820	chr7	155167511	-			2.2788094	-2.866403	5.5475214	1.52E-07	6.66E-06	7.0517658
cg24964130	chr2	133197577	-	GPR39	Body	-1.676541	0.7065966	-5.545188	1.52E-07	6.66E-06	7.050461
cg02519751	chr3	147126753	-	ZIC1	TSS1500	1.8299745	0.6468947	5.5450382	1.52E-07	6.67E-06	7.049797
cg17066275	chr10	93044533	-			0.7535109	2.3282889	5.5448791	1.52E-07	6.67E-06	7.0490908
cg05997362	chr8	56791748	+	LYN;LYN	TSS1500;TSS1500	-0.924744	-2.461627	-5.544787	1.52E-07	6.67E-06	7.0486808
cg03065175	chr4	155661758	-			1.4862578	2.5696037	5.567092	1.52E-07	6.68E-06	7.0906221

cg12475759	chr1	4714672	-	AJAP1;AJAP1	TSS1500;TSS1500	2.0195036	-1.178331	5.5445373	1.52E-07	6.68E-06	7.0475733
cg00291292	chr20	61702795	-			-1.314828	-0.198846	-5.54443	1.53E-07	6.68E-06	7.0470966
cg18473196	chr19	49222487	+	MAMSTR	5'UTR	1.050329	1.920361	5.5444328	1.53E-07	6.68E-06	7.0471091
cg07291563	chr19	48949441	-	GRWD1	1stExon	-0.497609	-4.195755	-5.544408	1.53E-07	6.68E-06	7.0469981
cg22187630	chr19	13616871	+	CACNA1A;CACNA1A	1stExon;1stExon	1.5045154	-1.010613	5.5443141	1.53E-07	6.68E-06	7.0465819
cg02820040	chr2	241836501	-	C2orf54	TSS1500	-0.823569	-1.018447	-5.544185	1.53E-07	6.68E-06	7.0460074
cg14502847	chr9	18490009	+	ADAMTSL1;ADAMTSL1	Body;Body	-1.5166	-0.23663	-5.544196	1.53E-07	6.68E-06	7.0460557
cg18702197	chr2	177027641	+	HOXD3	TSS1500	1.460169	-1.447925	5.5437888	1.53E-07	6.70E-06	7.0442499
cg10846615	chr2	119599189	+			1.8984743	-0.741254	5.5483911	1.53E-07	6.70E-06	7.0460746
cg11275487	chr5	37836119	+	GDNF;GDNF;GDNF	TSS1500;5'UTR;TSS1500	0.9952451	1.1670309	5.5436785	1.53E-07	6.70E-06	7.0437599
cg08037115	chr14	104837541	-			-1.370802	0.4068703	-5.543181	1.53E-07	6.71E-06	7.0415505
cg10191581	chr21	32255112	-	KRTAP11-1	TSS1500	-1.737972	1.1640307	-5.543053	1.54E-07	6.72E-06	7.040985
cg07957294	chr1	170638917	+	PRRX1;PRRX1	Body;Body	1.2575845	0.1611961	5.5430162	1.54E-07	6.72E-06	7.0408198
cg19112359	chr2	169346613	-	LASS6	Body	-1.536098	-1.248586	-5.542924	1.54E-07	6.72E-06	7.0404088
cg12522833	chr18	74981730	+	GALR1	3'UTR	-1.791543	1.3239576	-5.54288	1.54E-07	6.72E-06	7.0402132
cg11015348	chr11	25070714	-	LUZP2	Body	-1.656718	0.1241207	-5.542696	1.54E-07	6.72E-06	7.0393961
cg02338965	chr1	11476001	-			-1.337465	-0.327612	-5.542635	1.54E-07	6.73E-06	7.0391286
cg04574507	chr1	158301038	+	CD1B	Body	-1.850033	0.5877617	-5.542628	1.54E-07	6.73E-06	7.0390964
cg11357542	chr2	66669737	+	MEIS1	Body	1.0904718	1.1950173	5.5425102	1.54E-07	6.73E-06	7.0385733
cg19038230	chr13	47126445	-	LRCH1;LRCH1;LRCH1	TSS1500;TSS1500;TSS1500	-0.973176	-2.128999	-5.542499	1.54E-07	6.73E-06	7.0385259

cg21803927	chr8	14412919	-	SGCZ	Body	-1.383764	-0.073764	-5.54254	1.54E-07	6.73E-06	7.0387037
cg21435684	chr17	80255457	-			-1.717171	-2.580305	-5.551979	1.54E-07	6.73E-06	7.0597329
cg08726248	chr11	637032	+	DRD4	TSS1500	1.668536	-1.474736	5.5423301	1.54E-07	6.73E-06	7.037774
cg04408897	chr6	29596901	+	GABBR1;GABBR1;GABBR1	TSS1500;Body;Body	-0.614609	-3.327423	-5.541733	1.54E-07	6.75E-06	7.0351249
cg12284854	chr1	40939078	-			-1.023875	-0.625752	-5.541546	1.55E-07	6.75E-06	7.034295
cg21448788	chr12	10826971	+	STYK1	TSS200	-0.502242	-4.375011	-5.541437	1.55E-07	6.76E-06	7.0338109
cg09539720	chr7	150885458	-	ASB10;ASB10;ASB10	TSS1500;TSS1500;TSS1500	-0.83666	-1.639719	-5.541165	1.55E-07	6.77E-06	7.0326014
cg23501406	chr11	637035	+	DRD4	TSS1500	1.7145868	-1.001542	5.5408586	1.55E-07	6.77E-06	7.0312427
cg20170533	chr13	51417846	+	DLEU7;DLEU7	1stExon;5'UTR	1.8800766	-0.631709	5.5407893	1.55E-07	6.78E-06	7.0309351
cg19350197	chr6	32782988	-	HLA-DOB	Body	-1.295728	1.0103004	-5.544288	1.56E-07	6.81E-06	7.0279033
cg14909902	chr4	9884128	-	SLC2A9;SLC2A9	Body;Body	-0.561356	1.1459451	-5.539375	1.56E-07	6.82E-06	7.0246599
cg06565975	chr8	143823917	-	SLURP1	TSS200	-1.79037	0.106589	-5.539199	1.56E-07	6.82E-06	7.0238762
cg15874877	chr5	140799572	+	PCDHGA4;PCDHGA9;PCDHGA1;PCDHGB1;PCDHGB7;PCDHGB 6;PCDHGA11;PCDHGB3;PCDHGA6;PCDHGA8;PCDHGA10;PC DHGA5;PCDHGB7;PCDHGB4;PCDHGA3;PCDHGA2;PCDHGA11	Body;Body;Body;Body;1stExon;Body;TSS1500;Body; Body;Body;Body;Body;1stExon;Body;Body;Body;TSS 1500;TSS1500;Body;Body;Body	0.9579732	0.8165725	5.5391617	1.56E-07	6.82E-06	7.023712
cg03369289	chr7	152619168	+			-1.064801	-0.130869	-5.539137	1.56E-07	6.82E-06	7.0236019
cg01886524	chr3	128225824	+	LOC90246	TSS1500	-1.232404	-0.60433	-5.550904	1.57E-07	6.83E-06	7.0450349
cg02708506	chr10	131238682	-			-1.19772	0.1652796	-5.538774	1.57E-07	6.83E-06	7.0219919
cg03110703	chr3	108249446	+	MYH15	TSS1500	-1.304383	0.8395307	-5.538762	1.57E-07	6.83E-06	7.0219386
cg10880006	chr4	81122687	-	PRDM8;PRDM8	Body;Body	1.2065101	1.0500519	5.5387798	1.57E-07	6.83E-06	7.0220174
cg22940031	chr7	127841735	-			1.3658841	-1.623562	5.5386685	1.57E-07	6.84E-06	7.0215236
cg19509393	chr2	177016490	+	HOXD4	1stExon	1.7608812	-0.758729	5.538573	1.57E-07	6.84E-06	7.0210995

cg09457527	chr15	25487893	-	SNORD115-40	TSS1500	-1.094781	-0.147617	-5.538258	1.57E-07	6.85E-06	7.0197003
cg07908874	chr10	135123006	-	ZNF511;TUBGCP2	Body;TSS1500	-1.387359	0.6663691	-5.540498	1.57E-07	6.85E-06	7.0202666
cg16643706	chr5	111289503	+	C5orf13;C5orf13	Body;Body	-1.58575	0.6615214	-5.53818	1.57E-07	6.85E-06	7.0193554
cg09677252	chr12	110283596	+			-0.972529	-2.266438	-5.538086	1.57E-07	6.85E-06	7.0189397
cg22495460	chr2	25135724	-	ADCY3	Body	1.0306263	1.8473043	5.5378981	1.57E-07	6.86E-06	7.0181051
cg00045227	chr11	56467944	-	OR8U8;OR9G9;OR9G1	Body;1stExon;1stExon	-0.996309	-1.172577	-5.53783	1.57E-07	6.86E-06	7.0178009
cg04776040	chr1	210607796	+	HHAT;HHAT;HHAT;HHAT;HHAT;HHAT	Body;Body;Body;Body;Body;Body	0.7470511	1.927163	5.537781	1.57E-07	6.86E-06	7.0175856
cg04902968	chr1	5771047	-			-1.334552	-0.279288	-5.537711	1.57E-07	6.86E-06	7.0172749
cg27328211	chr15	25464436	+	SNORD115-15;SNORD115-21;HBII-52-27	Body;Body;TSS1500	-1.022787	0.2107047	-5.537712	1.57E-07	6.86E-06	7.0172775
cg05061769	chr15	59700360	+			0.9061923	0.9103868	5.5376397	1.57E-07	6.86E-06	7.0169588
cg26612418	chr1	5539958	+			-1.156995	-0.154949	-5.537444	1.58E-07	6.87E-06	7.0160908
cg00981928	chr7	56563152	-	DKFZp434L192	TSS1500	-0.934887	-1.104934	-5.537315	1.58E-07	6.87E-06	7.0155196
cg06803821	chr16	50533237	-			-1.565633	1.1834228	-5.537223	1.58E-07	6.87E-06	7.0151084
cg22119665	chr22	23745131	-	ZDHHC8P	TSS1500	-0.859636	-1.261674	-5.537179	1.58E-07	6.87E-06	7.0149139
cg06952236	chr15	38856341	-	RASGRP1;RASGRP1	Body;Body	-0.404022	-3.340763	-5.537034	1.58E-07	6.88E-06	7.0142716
cg14413466	chr17	79170920	+	AZI1;AZI1	Body;Body	0.8273714	2.3309347	5.5390436	1.58E-07	6.89E-06	7.0138231
cg20738406	chr15	90233884	-	WDR93;PEX11A;PEX11A	TSS200;5'UTR;1stExon	-0.393493	-4.559656	-5.536695	1.58E-07	6.89E-06	7.0127658
cg06732684	chr11	50257752	+	LOC441601	TSS200	0.8353323	0.1166043	5.5364663	1.58E-07	6.89E-06	7.011753
cg04035553	chr8	143757498	+			-0.945686	-1.083969	-5.538406	1.59E-07	6.90E-06	7.0113571
cg10640072	chr8	99985888	-			1.1469804	-0.184898	5.5360615	1.59E-07	6.90E-06	7.0099571

cg27127492	chr8	96036905	+	C8orf38	TSS1500	-0.89123	-1.933357	-5.535906	1.59E-07	6.91E-06	7.0092655
cg23305899	chr14	101539489	+			-1.699967	-0.784204	-5.538143	1.59E-07	6.91E-06	7.0098312
cg23235622	chr20	34039349	-			-1.745308	0.6389751	-5.535611	1.59E-07	6.92E-06	7.0079607
cg25395812	chr17	43306344	+	FMNL1	Body	-1.043865	-1.633811	-5.535543	1.59E-07	6.92E-06	7.0076587
cg21498471	chr19	33183713	+	NUDT19	Body	-0.813404	-2.058443	-5.535429	1.59E-07	6.92E-06	7.007153
cg11304383	chr12	7818635	-	APOBEC1	TSS200	-1.427818	-0.161626	-5.535124	1.59E-07	6.93E-06	7.0058009
cg23205629	chr8	33421410	-	RNF122	Body	1.0183022	1.0465793	5.5351412	1.59E-07	6.93E-06	7.0058752
cg04127587	chr5	176829112	-	PFN3	TSS1500	0.9923597	0.9546349	5.5350059	1.59E-07	6.93E-06	7.0052748
cg15731655	chr12	54357604	+	HOTAIR	Body	1.9684713	-1.06957	5.5373631	1.59E-07	6.93E-06	7.0067365
cg20974826	chr6	33141040	-	COL11A2;COL11A2;COL11A2	Body;Body;Body	-1.002061	-0.665036	-5.534997	1.59E-07	6.93E-06	7.005237
cg25388861	chr4	139644828	-			-1.41568	-0.466512	-5.535024	1.59E-07	6.93E-06	7.0053563
cg00221494	chr13	98794593	-	FARP1;FARP1	TSS1500;TSS1500	-1.0069	-2.124329	-5.534851	1.59E-07	6.93E-06	7.0045869
cg25225607	chr3	13925627	-			-1.290624	0.1796179	-5.534865	1.59E-07	6.93E-06	7.0046513
cg10333520	chr3	36987040	+	TRANK1	TSS1500	0.6233345	1.2055674	5.5345492	1.60E-07	6.94E-06	7.0032494
cg00569447	chr5	50695224	-			1.0285774	-0.962363	5.5343092	1.60E-07	6.95E-06	7.0021851
cg06499647	chr2	106681983	-	C2orf40	TSS200	2.0099314	-1.868666	5.5340755	1.60E-07	6.96E-06	7.0011485
cg19270409	chr8	1430753	-			-1.126316	-0.028646	-5.533911	1.60E-07	6.96E-06	7.0004172
cg02485729	chr12	128994427	+	TMEM132C	Body	-1.482335	-0.0654	-5.533855	1.60E-07	6.96E-06	7.000169
cg03239552	chr12	54339653	-	HOXC13	3'UTR	2.463174	-1.5641	5.5361883	1.60E-07	6.96E-06	7.0015325
cg11453079	chr20	43058872	+	HNF4A;HNF4A	3'UTR;3'UTR	-1.290552	-1.100945	-5.533715	1.60E-07	6.97E-06	6.999549

cg23935006	chr15	32215789	-			-1.934249	1.8681583	-5.533695	1.60E-07	6.97E-06	6.9994625
cg04354077	chr20	5295137	-	PROKR2	TSS200	-1.180802	-0.141681	-5.533467	1.61E-07	6.97E-06	6.9984519
cg23400451	chr17	10373728	+	MYH4	TSS1500	-1.46725	0.1008509	-5.533464	1.61E-07	6.97E-06	6.9984387
cg03148124	chr2	174890849	-			0.7930129	2.2860096	5.5332839	1.61E-07	6.98E-06	6.9976378
h.13.106104868	chr13	107306867	+			-0.658796	-4.168648	-5.533203	1.61E-07	6.98E-06	6.9972788
cg20214328	chr19	39378302	+	SIRT2;SIRT2	Body;Body	-1.033044	-2.007639	-5.533158	1.61E-07	6.98E-06	6.9970791
cg01367627	chr7	98477438	+	TRRAP	5'UTR	-0.93248	-2.435345	-5.533105	1.61E-07	6.98E-06	6.9968463
cg09405758	chr16	49670437	+	ZNF423	Body	-1.231829	0.63952	-5.532934	1.61E-07	6.99E-06	6.9960862
cg26718561	chr6	125470950	-			-1.184007	0.2316578	-5.532924	1.61E-07	6.99E-06	6.9960436
cg15493035	chr10	116659330	-	FAM160B1	3'UTR	-1.478957	0.1221282	-5.532724	1.61E-07	6.99E-06	6.9951573
cg05095591	chr3	147129184	-	ZIC1	Body	1.647236	-0.991356	5.5325819	1.61E-07	6.99E-06	6.994525
cg14072120	chr22	37640402	+	RAC2	TSS200	-0.838816	-3.10476	-5.532409	1.61E-07	7.00E-06	6.9937566
cg21532760	chr2	99120630	+	INPP4A;INPP4A;INPP4A;INPP4A	5'UTR;5'UTR;5'UTR;5'UTR	0.8634151	1.8514917	5.5419231	1.61E-07	7.00E-06	6.9984062
cg12852187	chr2	231903372	-	C2orf72	Body	1.2529994	-0.306257	5.5344865	1.61E-07	7.01E-06	6.9939946
cg21587238	chr5	42953590	-			1.9337601	1.0408958	5.5343326	1.62E-07	7.01E-06	6.9929529
cg26906217	chr11	17251956	-			-1.506121	-2.852296	-5.531965	1.62E-07	7.01E-06	6.9917893
cg04437966	chr5	60954562	-	FLJ37543	Body	-0.717938	-4.325562	-5.539008	1.62E-07	7.01E-06	6.9952904
cg07254706	chr5	30870768	+			-1.697846	0.2063366	-5.531696	1.62E-07	7.02E-06	6.9905969
cg00025357	chr1	17577455	-	PADI3	Body	-1.167876	-1.958898	-5.531609	1.62E-07	7.02E-06	6.9902094
cg07512361	chr7	101944430	-	SH2B2	Body	1.1726035	0.4449036	5.5315844	1.62E-07	7.02E-06	6.990102

cg03744332	chr2	223173061	-			1.4592198	-0.855991	5.5313747	1.62E-07	7.03E-06	6.9891723
cg18049638	chr6	28733695	+			1.1843532	-0.674861	5.5308022	1.62E-07	7.04E-06	6.9866345
cg00280812	chr12	52585844	-	KRT80;KRT80	TSS200;TSS200	-1.172872	-0.642296	-5.530711	1.63E-07	7.05E-06	6.9862309
cg01609559	chr6	46138957	-	ENPP5	TSS1500	-0.824563	-3.462307	-5.530676	1.63E-07	7.05E-06	6.9860755
cg18737081	chr10	80999807	+	ZMIZ1	Body	1.5620617	1.8198431	5.5305608	1.63E-07	7.05E-06	6.985564
cg23933216	chr1	145059467	+	PDE4DIP	Body	-0.66423	-1.669627	-5.530254	1.63E-07	7.06E-06	6.9842027
cg14659008	chr8	102090315	+			-2.051609	1.017231	-5.530195	1.63E-07	7.06E-06	6.9839408
cg20515679	chr21	39056255	+	KCNJ6	Body	-1.068156	-0.542418	-5.530181	1.63E-07	7.06E-06	6.9838822
cg02169391	chr7	130126176	-	MEST;MEST	1stExon;5'UTR	-0.807247	-3.310575	-5.530147	1.63E-07	7.06E-06	6.983728
cg07056079	chr6	32765314	+			-1.146747	-2.636872	-5.53241	1.63E-07	7.06E-06	6.9844393
cg01868729	chr12	83251284	+	TMTC2	Body	0.9227416	1.9074387	5.5298317	1.63E-07	7.07E-06	6.9823319
cg06560169	chr5	172129028	-			-0.868067	-1.73982	-5.529767	1.63E-07	7.07E-06	6.9820439
cg26519930	chr16	76665665	+			-1.416547	1.5036637	-5.529633	1.63E-07	7.08E-06	6.9814524
cg12587954	chr1	200993200	+	KIF21B	TSS1500	-1.462944	-1.726965	-5.531809	1.63E-07	7.08E-06	6.9821385
cg25273520	chr15	59713427	-			0.9169128	1.2502119	5.5293532	1.64E-07	7.08E-06	6.9802109
cg22399984	chr6	1601802	-			1.1340419	-0.536018	5.5291794	1.64E-07	7.09E-06	6.9794404
cg08106190	chr14	67937737	-	TMEM229B	3'UTR	-1.146829	0.7827765	-5.528994	1.64E-07	7.09E-06	6.9786187
cg19170009	chr10	17277756	+	VIM	Body	0.6867634	1.4253658	5.5289547	1.64E-07	7.09E-06	6.9784446
cg16845394	chr8	109096007	+	RSPO2	TSS200	1.6206456	-0.70246	5.5289072	1.64E-07	7.09E-06	6.9782339
cg17266431	chr1	1711069	-	NADK	TSS1500	-1.216363	0.3522823	-5.52889	1.64E-07	7.09E-06	6.9781562

cg20728490	chr10	98064175	-	DNTT;DNTT;DNTT;DNTT	5'UTR;1stExon;1stExon;5'UTR	-1.023672	-1.141627	-5.528806	1.64E-07	7.10E-06	6.977787
cg02297838	chr13	92002454	+	MIR19A;MIR17HG;MIR92A1;MIR17HG;MIR20A;MIR17;MIR19B1;MIR18A	TSS1500;Body;TSS1500;Body;TSS1500;TSS1500;TSS1500;TSS1500	-1.12161	-1.566082	-5.528336	1.64E-07	7.11E-06	6.9757027
cg02474858	chr22	30233609	-	ASCC2	5'UTR	-0.947166	-0.964374	-5.528308	1.64E-07	7.11E-06	6.9755803
cg09416096	chr2	120488830	-			-1.225167	1.1430206	-5.535331	1.64E-07	7.11E-06	6.97943
cg11709405	chr1	24649398	-	GRHL3;GRHL3;GRHL3	TSS200;Body;TSS200	-1.298568	-0.763147	-5.528282	1.64E-07	7.11E-06	6.9754614
cg25123670	chr14	94588068	+			-0.723364	-1.793069	-5.5283	1.64E-07	7.11E-06	6.9755413
cg14524936	chr11	20627597	+	SLC6A5	Body	1.3164852	-0.090655	5.5280678	1.65E-07	7.12E-06	6.9745135
cg20542190	chr19	54850619	-	LILRA4	TSS200	-0.915405	-1.13727	-5.527803	1.65E-07	7.12E-06	6.9733399
cg16933229	chr4	111561352	+			1.6369518	-0.804082	5.5277636	1.65E-07	7.13E-06	6.9731654
cg23044087	chr15	97328778	-	SPATA8	3'UTR	-1.690334	0.451727	-5.534585	1.65E-07	7.13E-06	6.9925009
cg06166915	chr16	57415838	+	CX3CL1	Body	-0.798062	0.382426	-5.527446	1.65E-07	7.13E-06	6.9717592
cg17022548	chr5	139294892	-	NRG2;NRG2;NRG2;NRG2	Body;Body;Body;Body	-1.603731	-0.559845	-5.527381	1.65E-07	7.13E-06	6.9714703
cg19027507	chr1	27320128	-	TRNP1	TSS200	-0.408445	-3.625319	-5.527386	1.65E-07	7.13E-06	6.9714925
cg20180370	chr6	50691003	-	TFAP2D	Body	2.2701665	-1.549694	5.5273902	1.65E-07	7.13E-06	6.9715106
cg06845853	chr11	32461240	+	WIT1	Body	1.6007096	-0.691148	5.5317173	1.65E-07	7.15E-06	6.9867803
cg14298457	chr2	177012117	-			1.2732745	-0.643669	5.526801	1.66E-07	7.15E-06	6.9688997
cg19153300	chr11	134794980	+			-1.418671	-0.425789	-5.52681	1.66E-07	7.15E-06	6.9689412
cg02782840	chr13	25290701	+			1.3227118	1.5439307	5.526728	1.66E-07	7.15E-06	6.968576
cg22253945	chr2	105858489	-	GPR45	1stExon	-0.846454	-0.020001	-5.526458	1.66E-07	7.16E-06	6.9673776
cg09912552	chr8	134250937	+	NDRG1;NDRG1	3'UTR;3'UTR	0.6775563	1.748781	5.5262846	1.66E-07	7.17E-06	6.9666113

cg12750431	chr10	44873037	+	CXCL12;CXCL12;CXCL12	3'UTR;Body;Body	-1.629708	0.6788929	-5.526129	1.66E-07	7.17E-06	6.9659232
cg21996227	chr1	156130826	-	SEMA4A	Body	2.1635148	-0.803562	5.525648	1.66E-07	7.19E-06	6.9637907
cg05057932	chr20	62006836	-			-1.167117	-0.525268	-5.525609	1.66E-07	7.19E-06	6.9636167
cg19360930	chr2	20111778	-	WDR35;WDR35	3'UTR;3'UTR	-1.835801	1.427693	-5.525421	1.67E-07	7.19E-06	6.9627834
cg16478977	chr14	77689345	-	TMEM63C	Body	0.9373088	0.9902485	5.5246521	1.67E-07	7.22E-06	6.9593782
cg14943722	chr3	50341009	+	HYAL1;HYAL1;HYAL1;HYAL1;HYAL1;HYAL1	TSS200;TSS200;5'UTR;TSS200;TSS200;TSS200	1.3420581	0.7265584	5.5246109	1.67E-07	7.22E-06	6.9591959
cg20755170	chr5	145720024	-	POU4F3	3'UTR	1.9491698	-0.978124	5.5246031	1.67E-07	7.22E-06	6.9591614
cg27481559	chr2	87037038	-			1.2112694	-0.25574	5.5244451	1.67E-07	7.22E-06	6.9584611
cg26171523	chr19	13858605	+	CCDC130	TSS200	-1.100245	-5.257666	-5.524232	1.68E-07	7.23E-06	6.9575171
cg12544557	chr17	39019545	-	KRT12	Body	0.82684	0.5943801	5.5241348	1.68E-07	7.23E-06	6.9570864
cg07431199	chr15	41218265	+			-1.14766	-2.292941	-5.524029	1.68E-07	7.23E-06	6.9566173
cg14590098	chr8	65290848	+	MIR124-2	TSS1500	1.5661355	-2.000822	5.5238646	1.68E-07	7.24E-06	6.9558896
cg12656020	chr1	91185028	-			1.5002093	-0.926417	5.5284652	1.68E-07	7.24E-06	6.9582852
cg20911718	chr14	24867491	-	NYNRIN	TSS1500	1.0582124	0.7722036	5.5234913	1.68E-07	7.25E-06	6.9542359
cg07143815	chr8	66474918	+			-1.098443	-1.797689	-5.523181	1.68E-07	7.26E-06	6.9528628
cg08070771	chr3	147125758	-	ZIC4;ZIC1	TSS1500;TSS1500	1.8892854	0.1165726	5.5350063	1.68E-07	7.26E-06	6.9720649
cg22797461	chr19	40943746	+			-1.311312	0.471151	-5.522924	1.69E-07	7.27E-06	6.9517246
cg06429138	chr11	72860376	+			1.1253911	1.2233476	5.5228697	1.69E-07	7.27E-06	6.9514824
cg20914879	chr14	56566808	+			1.1964055	1.6301047	5.5228862	1.69E-07	7.27E-06	6.9515556
cg27394794	chr10	52600370	-	A1CF;A1CF;A1CF	Body;Body;Body	-1.520245	-0.306983	-5.522848	1.69E-07	7.27E-06	6.9513875

cg27560687	chr7	62555691	+			-1.650661	0.479105	-5.522794	1.69E-07	7.27E-06	6.9511472
cg06386478	chr8	19460136	+	CSGALNACT1;CSGALNACT1;CSGALNACT1	TSS200;Body;5'UTR	1.3168489	-0.498171	5.5225595	1.69E-07	7.27E-06	6.9501086
cg07318128	chr17	46683806	+	HOXB6	TSS1500	1.1753521	1.6510682	5.5225689	1.69E-07	7.27E-06	6.9501504
cg10020787	chr10	50626838	-			-0.972204	-0.791772	-5.522528	1.69E-07	7.27E-06	6.9499682
cg19853936	chr6	19692304	+			1.2910365	-1.007193	5.5224465	1.69E-07	7.28E-06	6.9496083
cg25360518	chr12	4554623	+	FGF6	1stExon	-1.258137	0.4246577	-5.522429	1.69E-07	7.28E-06	6.9495308
cg08119325	chr3	195613212	-	TNK2;TNK2	Body;Body	0.7248138	0.4724144	5.5223046	1.69E-07	7.28E-06	6.9489795
cg01621390	chr12	5195150	+			-1.186264	-0.601427	-5.522223	1.69E-07	7.28E-06	6.9486195
cg01578585	chr16	19721878	+	C16orf88	Body	1.0680943	2.7402272	5.5221579	1.69E-07	7.28E-06	6.94833
cg27385773	chr3	50201416	+	SEMA3F	Body	0.7740532	0.3771975	5.5221278	1.69E-07	7.28E-06	6.9481967
cg00033909	chr7	9674902	+	PER4	Body	-1.356758	-0.860952	-5.522092	1.69E-07	7.28E-06	6.948038
cg09957391	chr12	57984777	-	PIP4K2C;PIP4K2C;PIP4K2C;PIP4K2C	TSS200;TSS200;TSS200;TSS200	-0.498132	-4.177184	-5.521921	1.69E-07	7.29E-06	6.9472815
cg22286936	chr6	50683593	+	TFAP2D	Body	1.3599286	-0.559861	5.5219128	1.69E-07	7.29E-06	6.9472444
cg10982851	chr7	4747166	-	FOXK1	Body	1.2822846	2.4799693	5.5218706	1.69E-07	7.29E-06	6.9470576
cg02814416	chr7	141947168	-	MOXD2	TSS1500	-1.379892	0.3542299	-5.521396	1.70E-07	7.31E-06	6.9449544
cg02084669	chr14	74707360	-	VSX2	Body	1.3144636	-1.770213	5.5213648	1.70E-07	7.31E-06	6.9448173
cg22228439	chr1	46766431	-	LRRC41	Body	0.7607502	0.8386372	5.5213232	1.70E-07	7.31E-06	6.9446331
cg24746315	chr11	125660444	-	PATE3	Body	-1.526314	0.4585808	-5.520796	1.70E-07	7.32E-06	6.9423006
cg10083417	chr16	17818508	+			-1.334338	-0.416933	-5.523028	1.70E-07	7.33E-06	6.9429175
cg04187039	chr16	89048704	-			-0.74526	-0.456051	-5.520656	1.70E-07	7.33E-06	6.9416781

cg15415136	chr19	38042418	+	ZNF540;ZNF540	5'UTR;1stExon	1.4870586	-0.371924	5.5206456	1.70E-07	7.33E-06	6.9416326
cg24154937	chr12	5153711	+	KCNA5	1stExon	0.956336	-0.415168	5.5206646	1.70E-07	7.33E-06	6.9417165
cg18326021	chr10	106401479	-	SORCS3	1stExon	2.1193053	-1.575593	5.5204926	1.70E-07	7.33E-06	6.9409547
cg02499308	chr2	135594780	+	ACMSD	TSS1500	-1.388072	-0.291277	-5.520402	1.71E-07	7.33E-06	6.940555
cg11807188	chr1	65924844	+	LEPR;LEPR;LEPR	5'UTR;5'UTR;5'UTR	-0.994742	0.4289905	-5.520361	1.71E-07	7.33E-06	6.9403732
cg26724070	chr16	50707467	+	SNX20;SNX20;SNX20	Body;Body;Body	0.8292194	2.333806	5.520204	1.71E-07	7.34E-06	6.9396769
cg06235145	chr4	17034835	-			-1.139568	0.4453279	-5.52009	1.71E-07	7.34E-06	6.9391712
cg24643381	chr12	43941378	+	ADAMTS20	Body	-1.499298	-0.035219	-5.519995	1.71E-07	7.34E-06	6.938752
cg26405695	chr12	39836148	+	KIF21A	Body	-0.466261	-3.708036	-5.519858	1.71E-07	7.35E-06	6.9381451
cg13022679	chr5	140207498	-	PCDHA2;PCDHA1;PCDHA1;PCDHA6;PCDHA5;PCDHA6;PCDHA3;PCDHA4;PCDHA6	Body;Body;Body;TSS200;Body;TSS200;Body;Body;TSS200	1.4240729	0.1610444	5.5196399	1.71E-07	7.35E-06	6.9371793
cg26386189	chr11	1706902	-	HCCA2;FAM99B	Body;TSS200	-0.532046	-1.010973	-5.519643	1.71E-07	7.35E-06	6.9371936
cg13620034	chr8	72755052	+	MSC	Body	2.3161556	-1.492942	5.5195347	1.71E-07	7.36E-06	6.9367136
cg03900808	chr5	149871453	+			0.5542564	0.9055849	5.5194002	1.71E-07	7.36E-06	6.9361179
cg05217213	chr6	88412782	+	AKIRIN2	TSS1500	-1.516715	0.2534996	-5.51944	1.71E-07	7.36E-06	6.9362923
cg22762388	chr7	141176413	-			-1.820512	0.9879923	-5.519416	1.71E-07	7.36E-06	6.936187
cg00400532	chr15	53093509	-			1.1312554	-0.997903	5.5193224	1.71E-07	7.36E-06	6.9357734
cg12317601	chr10	119301760	-	EMX2;EMX2OS;EMX2	TSS200;Body;TSS200	1.9613413	-0.928495	5.5189451	1.72E-07	7.37E-06	6.9341029
cg03201337	chr10	69609995	-			-1.512922	-1.983318	-5.518881	1.72E-07	7.37E-06	6.9338188
cg07415085	chr11	48977834	-			-1.030945	2.9147665	-5.518811	1.72E-07	7.38E-06	6.9335077
cg12076463	chr4	85418265	-	NKX6-1	Body	1.3024695	0.5012523	5.5187206	1.72E-07	7.38E-06	6.933109

cg09262882	chr19	34899586	+	PDCD2L	Body	0.7366655	1.4551826	5.5184834	1.72E-07	7.39E-06	6.932059
cg07279557	chr21	46875417	+	COL18A1;COL18A1;COL18A1	TSS200;Body;TSS200	-1.283937	-1.669229	-5.518199	1.72E-07	7.39E-06	6.9307976
cg10316270	chr4	155702266	+	RBM46	TSS200	-1.229792	0.1232286	-5.518159	1.72E-07	7.39E-06	6.9306208
cg14451926	chr8	23562918	-	NKX2-6	Body	1.2313553	-1.007926	5.5179223	1.72E-07	7.40E-06	6.9295748
cg02642585	chr21	32202284	-	KRTAP7-1	TSS1500	-1.358622	0.5899559	-5.517868	1.73E-07	7.40E-06	6.9293349
cg06836656	chr1	2876347	-			-1.726169	0.3958112	-5.517721	1.73E-07	7.41E-06	6.9286856
cg15095427	chr1	234651180	-			-1.095361	0.3273509	-5.517716	1.73E-07	7.41E-06	6.9286595
cg19057459	chr19	19551640	-	GATAD2A	5'UTR	0.8746874	1.7141258	5.5177474	1.73E-07	7.41E-06	6.9288006
cg07376374	chr20	37854477	-	LOC339568	TSS1500	-1.058404	-0.42989	-5.517622	1.73E-07	7.41E-06	6.9282434
cg26347851	chr20	61754869	-			-1.114444	-0.272846	-5.519782	1.73E-07	7.41E-06	6.9285609
cg11938455	chr6	15622032	-	DTNBP1;DTNBP1;DTNBP1	Body;Body;Body	1.4269857	-1.189213	5.5173917	1.73E-07	7.42E-06	6.9272259
cg16616521	chr11	31821388	+	PAX6;PAX6;PAX6	Body;Body;Body	2.4545438	-1.389368	5.5173561	1.73E-07	7.42E-06	6.9270682
cg27063969	chr5	169133966	+	DOCK2	Body	-0.773845	-0.99375	-5.517323	1.73E-07	7.42E-06	6.9269238
cg02577745	chr14	101334582	+	MIR493	TSS1500	-1.377732	-0.077158	-5.517196	1.73E-07	7.42E-06	6.926361
cg26431018	chr14	102054550	-			-1.223617	-0.221576	-5.517136	1.73E-07	7.42E-06	6.926092
cg24902038	chr1	2428882	+	PLCH2	Body	-1.138089	-0.101911	-5.51709	1.73E-07	7.42E-06	6.9258924
cg22960971	chr20	5099001	+	PCNA;PCNA	Body;Body	-1.076644	-0.921387	-5.516897	1.73E-07	7.43E-06	6.9250368
cg12574576	chr10	44363776	+			-1.103756	-0.517784	-5.516849	1.73E-07	7.43E-06	6.9248255
cg01950077	chr6	109610932	+			-0.98797	-0.45772	-5.516801	1.73E-07	7.43E-06	6.9246107
cg02381539	chr8	65290109	-			1.6459646	-1.181712	5.51667	1.73E-07	7.43E-06	6.9240312

cg19255241	chr1	248058104	+	OR2W3	TSS1500	-1.49785	0.3488848	-5.51654	1.74E-07	7.44E-06	6.9234575
cg01207916	chr15	79500886	+	MIR184	TSS1500	-1.188757	-0.330935	-5.516348	1.74E-07	7.44E-06	6.9226071
cg23319460	chr11	65123510	+	TIGD3	Body	0.9842484	0.2705263	5.5162597	1.74E-07	7.45E-06	6.9222151
cg00320597	chr8	1339461	-			-1.259981	0.4429286	-5.516134	1.74E-07	7.45E-06	6.9216568
cg06621027	chr2	80179759	+	CTNNA2;CTNNA2	Body;Body	-1.467725	-1.60398	-5.515978	1.74E-07	7.45E-06	6.9209669
cg06600287	chr1	53387719	-	ECHDC2	TSS1500	-0.968539	-0.60027	-5.515902	1.74E-07	7.46E-06	6.9206338
cg05581451	chr4	111553999	-	PITX2;PITX2	Body;Body	1.2837003	-0.584561	5.5156179	1.74E-07	7.46E-06	6.9193747
cg08141873	chr11	534632	+	HRAS;HRAS;HRAS	5'UTR;5'UTR;5'UTR	-0.982696	-2.093918	-5.515571	1.74E-07	7.47E-06	6.9191686
cg10513302	chr6	30698905	+	FLOT1	Body	-1.013687	-1.51953	-5.515268	1.75E-07	7.48E-06	6.9178265
cg12447911	chr15	95515060	+			-1.361991	0.2112267	-5.515241	1.75E-07	7.48E-06	6.9177066
cg01108370	chr7	151330008	+	PRKAG2;PRKAG2;PRKAG2	Body;TSS1500;Body	-1.144604	-2.360977	-5.51751	1.75E-07	7.48E-06	6.9188811
cg19355929	chr11	20618001	-			1.5246326	-1.47617	5.5151976	1.75E-07	7.48E-06	6.9175145
cg01882871	chr17	32575548	-			-1.280295	-0.680147	-5.514904	1.75E-07	7.48E-06	6.9162137
cg06648156	chr6	28481660	-	GPX6	Body	-1.260054	0.0022403	-5.514591	1.75E-07	7.49E-06	6.9148306
cg07079663	chr8	21581910	+	GFRA2;GFRA2;GFRA2	Body;Body;Body	-1.417068	0.4759945	-5.514611	1.75E-07	7.49E-06	6.9149193
cg17899889	chr1	4191180	+			-1.329657	0.2414736	-5.514546	1.75E-07	7.49E-06	6.9146296
cg06629775	chr2	55459073	-	RPS27A;C2orf63;RPS27A;RPS27A;C2orf63	5'UTR;5'UTR;1stExon;TSS1500;5'UTR	-0.550539	-5.239316	-5.514482	1.75E-07	7.50E-06	6.9143469
cg04741366	chr2	4022242	+			-1.533348	0.3703165	-5.514341	1.75E-07	7.50E-06	6.9137216
cg09337375	chr10	131812798	+			-1.328255	0.7298596	-5.514159	1.76E-07	7.51E-06	6.9129168
cg03318924	chr12	49728150	-	C1QL4	Body	0.9197568	1.2257856	5.5141347	1.76E-07	7.51E-06	6.9128107

cg25060172	chr4	76555980	+	CDKL2	TSS1500	-1.63147	-3.243742	-5.525652	1.76E-07	7.52E-06	6.9176481
cg17526483	chr7	27281404	-	EVX1	TSS1500	0.9292087	-0.086361	5.5136825	1.76E-07	7.52E-06	6.9108098
cg27528510	chr7	101006058	+	EMID2	TSS200	2.3272437	-1.553227	5.5134109	1.76E-07	7.53E-06	6.909608
cg03981954	chr7	121942010	-	FEZF1;FEZF1	3'UTR;3'UTR	1.9502124	-0.954287	5.5130968	1.76E-07	7.54E-06	6.9082182
cg17503085	chr20	62095546	+	KCNQ2;KCNQ2;KCNQ2;KCNQ2;KCNQ2	Body;Body;Body;Body;Body	-1.161905	-0.405693	-5.513089	1.76E-07	7.54E-06	6.9081847
cg27316036	chr6	27228188	-			0.9981024	0.5255364	5.5129191	1.77E-07	7.54E-06	6.907432
cg14882265	chr7	27184375	+	HOXA5	TSS1500	1.1154033	0.0214617	5.5128859	1.77E-07	7.54E-06	6.9072852
cg11876705	chr19	38918253	-	RASGRP4;RASGRP4;RASGRP4;RASGRP4;RASGRP4;RASGRP4;RASGRP4	TSS1500;TSS1500;TSS1500;TSS1500;TSS1500;TSS1500;TSS1500	-1.251322	-0.780801	-5.512817	1.77E-07	7.55E-06	6.9069811
cg04321303	chr7	44107504	+			-1.404381	0.1359091	-5.512749	1.77E-07	7.55E-06	6.9066781
cg24153813	chr14	26731696	+			-1.366535	0.0042974	-5.512575	1.77E-07	7.55E-06	6.9059081
cg27281285	chr12	120315368	+	CIT	TSS1500	-0.382786	-3.879519	-5.512346	1.77E-07	7.56E-06	6.904895
cg06752054	chr21	42733747	+	MX2	TSS1500	-2.021052	-3.007116	-5.5122	1.77E-07	7.56E-06	6.9042518
cg16197857	chr18	75402282	+			-1.686218	1.8314343	-5.512125	1.77E-07	7.57E-06	6.9039179
cg04561937	chr22	17443619	-	GAB4	3'UTR	-1.283698	0.5505416	-5.511985	1.77E-07	7.57E-06	6.9032977
cg12609530	chr7	62541571	-			-0.930724	-0.226452	-5.512004	1.77E-07	7.57E-06	6.9033845
cg18295183	chr5	161494711	-	GABRG2;GABRG2;GABRG2;GABRG2;GABRG2;GABRG2	1stExon;1stExon;5'UTR;5'UTR;5'UTR;1stExon	1.2644083	-0.958814	5.5120117	1.77E-07	7.57E-06	6.9034177
cg03059876	chr20	61905353	+	ARFGAP1;ARFGAP1	5'UTR;5'UTR	-0.84038	-0.399508	-5.511949	1.77E-07	7.57E-06	6.9031404
cg14780990	chr15	93181182	-	FAM174B	Body	-1.170423	0.4523904	-5.511864	1.77E-07	7.57E-06	6.9027626
cg01869288	chr15	31523257	-			-1.41951	0.6646932	-5.513777	1.78E-07	7.58E-06	6.9023836
cg17942121	chr6	100911964	+	SIM1	TSS1500	1.5525699	-0.925177	5.5113336	1.78E-07	7.59E-06	6.9004179

cg22763718	chr13	53422780	+	PCDH8;PCDH8	TSS200;TSS200	2.4347439	-1.589905	5.5110963	1.78E-07	7.60E-06	6.8993681
cg08263708	chr6	85476373	+			1.3847411	-1.371943	5.5107276	1.78E-07	7.61E-06	6.8977371
cg23676151	chr6	108491372	+	NR2E1	Body	1.4519621	-0.450845	5.5106773	1.78E-07	7.61E-06	6.8975146
cg01004063	chr14	88606476	-			-0.867891	-1.823562	-5.510624	1.78E-07	7.61E-06	6.8972808
cg00041014	chr1	52498044	+	KTI12;TXNDC12;KTI12	3'UTR;Body;1stExon	-1.375727	0.8589545	-5.510469	1.79E-07	7.61E-06	6.8965941
cg18532070	chr7	41940672	-			-1.687896	0.3696996	-5.510486	1.79E-07	7.61E-06	6.8966703
cg27531490	chr6	31542459	+	TNF	TSS1500	-0.96105	-0.240879	-5.510485	1.79E-07	7.61E-06	6.8966651
cg05928342	chr19	9473598	+	ZNF177	TSS200	1.9844466	-1.365827	5.5126797	1.79E-07	7.62E-06	6.8975338
cg10977352	chr2	220862582	+			-1.179322	0.0696653	-5.510182	1.79E-07	7.62E-06	6.895322
cg14371636	chr2	63277208	+	OTX1	TSS1500	-0.521432	-4.495431	-5.512411	1.79E-07	7.62E-06	6.8963486
cg20445197	chr2	72057817	-			-1.074637	-0.193139	-5.509996	1.79E-07	7.63E-06	6.8945032
cg20764887	chr5	178004012	+	COL23A1	Body	2.0609938	-1.303486	5.5099816	1.79E-07	7.63E-06	6.8944377
cg03214622	chr17	77115633	+	HRNBP3	5'UTR	-1.543686	0.7240907	-5.509935	1.79E-07	7.63E-06	6.8942299
cg18659248	chr10	3679062	-			-1.925055	0.3227047	-5.509873	1.79E-07	7.63E-06	6.8939577
cg22822219	chr14	24105017	+	DHRS2;DHRS2	TSS1500;TSS1500	1.3577738	2.5959698	5.5120774	1.79E-07	7.63E-06	6.8945089
cg05350879	chr4	88451223	+	SPARCL1;SPARCL1	TSS1500;TSS1500	0.849159	1.3506094	5.509715	1.79E-07	7.63E-06	6.8932585
cg04701505	chr5	145719413	+	POU4F3	Body	1.0009922	-1.061043	5.5096427	1.79E-07	7.64E-06	6.8929384
cg05854698	chr10	134910850	+	GPR123	Body	-1.067516	-0.015847	-5.509341	1.79E-07	7.65E-06	6.8916037
cg00792107	chr12	133181600	-			-0.9797	-1.28447	-5.509231	1.80E-07	7.65E-06	6.8911181
cg01575414	chr13	112161295	-			-1.280334	1.0300267	-5.509286	1.80E-07	7.65E-06	6.8913605

cg06857094	chr6	31762442	+	VARS	Body	-1.121043	-1.509002	-5.509255	1.80E-07	7.65E-06	6.8912244
cg10860802	chr7	154632602	-	DPP6;DPP6;DPP6	Body;Body;Body	-2.29674	1.6885005	-5.509252	1.80E-07	7.65E-06	6.8912118
cg17753347	chr1	197883190	-	LHX9	Body	1.036985	0.1932926	5.509226	1.80E-07	7.65E-06	6.8910955
cg13651137	chr9	136522662	+	DBH	Body	-0.681166	-0.988991	-5.509202	1.80E-07	7.65E-06	6.8909903
cg24638828	chr7	72974572	-			-1.367607	-2.041131	-5.508893	1.80E-07	7.66E-06	6.8896217
cg17146570	chr20	57875362	-	EDN3;EDN3;EDN3;EDN3	TSS200;TSS200;TSS200;TSS200	2.3954893	-2.194986	5.5085478	1.80E-07	7.67E-06	6.8880962
cg00019495	chr4	57547525	-	HOPX;HOPX;HOPX;HOPX;HOPX;HOPX	5'UTR;1stExon;1stExon;5'UTR;5'UTR;1stExon	-1.008819	-2.551738	-5.508392	1.80E-07	7.67E-06	6.8874083
cg17480076	chr1	91190251	+			1.0627237	-0.74844	5.5106896	1.80E-07	7.67E-06	6.8887422
cg26794477	chr3	50357592	+	HYAL2;HYAL2	Body;Body	1.1489165	1.2726733	5.5083044	1.80E-07	7.67E-06	6.8870202
cg20935862	chr9	77643594	+	C9orf41	TSS1500	-0.398518	-4.867806	-5.508223	1.80E-07	7.68E-06	6.8866578
cg07994696	chr16	11680075	-	LITAF;LITAF;LITAF;LITAF;LITAF	5'UTR;1stExon;5'UTR;Body;5'UTR	-0.798544	-1.553035	-5.508145	1.80E-07	7.68E-06	6.8863166
cg09351881	chr4	188190184	-			-1.399616	-0.553709	-5.508115	1.80E-07	7.68E-06	6.8861809
cg18811269	chr3	70048838	+			-1.471677	0.9741711	-5.508141	1.80E-07	7.68E-06	6.8862982
cg25106913	chr6	27648354	+			1.4611555	-0.882893	5.5081186	1.80E-07	7.68E-06	6.8861983
cg09348580	chr19	14373659	+			-1.412647	0.4128841	-5.508023	1.81E-07	7.68E-06	6.8857776
cg00221654	chr11	44333030	+	ALX4	TSS1500	1.3317164	-1.124147	5.510152	1.81E-07	7.68E-06	6.8863677
cg12800734	chr17	27889135	+	ABHD15	3'UTR	1.0385992	0.2741894	5.5077458	1.81E-07	7.69E-06	6.88455
cg00136400	chr8	144000430	+	CYP11B2	TSS1500	-1.491875	0.4798871	-5.507715	1.81E-07	7.69E-06	6.8844152
cg01693650	chr3	45883855	+	LZTFL1	TSS1500	-0.984965	-3.50529	-5.507441	1.81E-07	7.70E-06	6.8832041
cg24519867	chr2	168103543	-	XIRP2;XIRP2	Body;Body	-1.626216	0.5720446	-5.507062	1.81E-07	7.71E-06	6.8815266

cg20067765	chr7	16524513	-			-1.740037	1.2748308	-5.506664	1.82E-07	7.72E-06	6.8797657
cg24980653	chr4	1399235	+			2.3548129	-0.430819	5.5089583	1.82E-07	7.72E-06	6.8810957
cg06095695	chr6	28831653	+			-0.337083	-3.908808	-5.506577	1.82E-07	7.73E-06	6.8793801
cg08640475	chr16	85551478	-			1.4267622	4.3414095	5.5088003	1.82E-07	7.73E-06	6.8800339
cg21652122	chr22	28837586	+	TTC28	Body	0.9267101	1.2505403	5.5064634	1.82E-07	7.73E-06	6.8788796
cg01637404	chr16	86322358	-			-1.100963	-0.996412	-5.506329	1.82E-07	7.73E-06	6.8782844
cg00205786	chr16	49669842	+	ZNF423	Body	-1.068162	0.8829941	-5.506288	1.82E-07	7.73E-06	6.8781064
cg16086559	chr15	96911531	-			1.3795821	-0.166869	5.5059386	1.82E-07	7.74E-06	6.8765597
cg17131579	chr6	33256216	-	PFDN6;WDR46;WDR46	TSS1500;Body;Body	-0.745263	-2.118049	-5.505903	1.82E-07	7.74E-06	6.876403
cg20270932	chr13	110790353	+			-0.557249	-4.181503	-5.505921	1.82E-07	7.74E-06	6.8764822
cg16408555	chr2	8787097	+			-1.107663	0.6510093	-5.505571	1.83E-07	7.75E-06	6.8749336
cg16463460	chr11	32454718	-	WT1;WT1;WT1;WT1	Body;Body;Body;Body	1.0529419	-0.167976	5.5052708	1.83E-07	7.76E-06	6.8736074
cg00675372	chr10	130201069	+			-1.624058	0.6093475	-5.505212	1.83E-07	7.77E-06	6.8733459
cg08305378	chr8	118953376	-	EXT1	Body	0.9306769	0.298063	5.5051284	1.83E-07	7.77E-06	6.8729777
cg01109135	chr14	105532252	+	GPR132	TSS1500	-0.9359	-1.489238	-5.505055	1.83E-07	7.77E-06	6.8726517
cg09618933	chr8	23571724	-			1.3908115	-0.288932	5.5050517	1.83E-07	7.77E-06	6.8726389
cg11192416	chr1	231296968	-			1.5329875	-1.55718	5.5048729	1.83E-07	7.78E-06	6.8718482
cg27648056	chr19	18085190	-	KCNN1	Body	-1.468814	0.4755819	-5.504619	1.83E-07	7.78E-06	6.8707259
cg10582795	chr2	161172036	-	RBMS1;RBMS1	Body;Body	0.8857456	1.4449982	5.504519	1.84E-07	7.79E-06	6.870284
cg02664479	chr20	62690218	-	TCEA2	5'UTR	-1.070951	0.1230263	-5.504427	1.84E-07	7.79E-06	6.8698781

cg20507963	chr4	13541863	-			1.5259611	-0.491287	5.5044482	1.84E-07	7.79E-06	6.8699711
cg17976390	chr10	6130479	-	RBM17;RBM17	TSS1500;TSS1500	-0.918329	-2.319393	-5.504148	1.84E-07	7.80E-06	6.8686439
cg09307788	chr10	119311459	-			1.1342098	-0.542002	5.5040627	1.84E-07	7.80E-06	6.8682671
cg07000643	chr12	133183142	+			-1.048867	-0.322731	-5.504036	1.84E-07	7.80E-06	6.8681488
cg04556349	chr16	76349097	+	CNTNAP4;CNTNAP4	Body;Body	-1.412624	0.0528738	-5.503907	1.84E-07	7.80E-06	6.8675769
cg09019936	chr2	162283434	-			1.8564567	-1.422136	5.5038074	1.84E-07	7.81E-06	6.8671387
cg08252579	chr3	62359773	+	FEZF2	TSS1500	1.5207988	-1.388044	5.5037076	1.84E-07	7.81E-06	6.8666976
cg08103568	chr4	100737138	+	DAPP1	TSS1500	-0.936413	0.0244589	-5.503552	1.84E-07	7.81E-06	6.86601
cg06511917	chr10	23482255	-	PTF1A	Body	1.0850308	-1.065987	5.5035253	1.84E-07	7.81E-06	6.865892
cg17421991	chr7	156795356	-			1.7318816	-1.054416	5.5057839	1.84E-07	7.82E-06	6.8667146
cg08555866	chr16	3345604	-			0.8610371	1.9147644	5.5033031	1.85E-07	7.82E-06	6.8649096
cg09629193	chr16	691570	+	FAM195A	TSS1500	-0.879536	-3.25239	-5.503221	1.85E-07	7.82E-06	6.8645468
cg01838497	chr8	10512769	+	RP1L1	TSS200	-0.881571	-1.281085	-5.505482	1.85E-07	7.82E-06	6.8657461
cg20653032	chr11	7259254	+			-1.503641	-0.285624	-5.502955	1.85E-07	7.83E-06	6.8633696
cg19175193	chr6	41007450	-	UNC5CL	TSS1500	-1.623027	0.7549316	-5.502719	1.85E-07	7.84E-06	6.8623287
cg13139843	chr21	31852793	-	KRTAP19-1	TSS200	-1.336773	-0.273335	-5.502544	1.85E-07	7.84E-06	6.861553
cg13007843	chr14	61120662	-			1.4617374	-0.919967	5.5021831	1.86E-07	7.86E-06	6.8599601
cg24832498	chr11	5528669	-	UBQLN3	3'UTR	-1.173927	-0.155356	-5.501929	1.86E-07	7.87E-06	6.8588389
cg02595760	chr6	100066673	-			1.2228227	-1.064711	5.5018707	1.86E-07	7.87E-06	6.8585796
cg22851691	chr17	46711446	+			1.8919778	-0.437263	5.5017039	1.86E-07	7.87E-06	6.8578427

cg02600258	chr5	75013682	-	C5orf37	TSS1500	-0.87438	-0.281662	-5.501585	1.86E-07	7.88E-06	6.8573178
cg01131100	chr14	101495047	+	MIR494	TSS1500	-1.386284	0.4798914	-5.501529	1.86E-07	7.88E-06	6.8570699
cg26756625	chr9	126779875	+	LHX2	Body	1.4552281	-1.625457	5.5014959	1.86E-07	7.88E-06	6.8569236
cg14470895	chr5	115152431	-	CDO1	TSS200	2.3505975	-1.719046	5.5036797	1.86E-07	7.88E-06	6.8577914
cg02431562	chr2	96809970	-	DUSP2	Body	-1.199653	-0.093598	-5.501214	1.86E-07	7.89E-06	6.8556765
cg05378861	chr20	61803065	+			-1.282495	-0.341586	-5.501147	1.86E-07	7.89E-06	6.8553815
cg11375053	chr11	6702453	-	MRPL17	3'UTR	1.0257598	1.543518	5.5011777	1.86E-07	7.89E-06	6.8555175
cg19328294	chr16	2908554	+	PRSS22	TSS1500	-0.872334	-2.571073	-5.501151	1.86E-07	7.89E-06	6.8553976
cg24168221	chr20	23587099	-	CST9	TSS1500	-1.069742	-1.404623	-5.500984	1.87E-07	7.89E-06	6.8546616
cg26700716	chr2	183730940	-	FRZB	1stExon	1.3953385	-0.559332	5.5009794	1.87E-07	7.89E-06	6.8546411
cg05619598	chr19	33569228	-			-0.972091	-2.295477	-5.503188	1.87E-07	7.89E-06	6.8552548
cg27137084	chr12	129035348	-	TMEM132C	Body	-1.315181	0.1173763	-5.503179	1.87E-07	7.89E-06	6.8552169
cg25840173	chr7	45914379	-			-1.311255	-0.072348	-5.500769	1.87E-07	7.90E-06	6.85371
cg00507135	chr10	27528611	+	ACBD5;ACBD5;ACBD5	Body;Body;Body	-1.275159	0.1012894	-5.500579	1.87E-07	7.90E-06	6.8528726
cg03270204	chr6	30851638	-	DDR1;DDR1	TSS1500;TSS1500	-1.153219	0.2720598	-5.502506	1.87E-07	7.91E-06	6.8522468
cg19588154	chr2	60154029	+			1.0187248	4.1522028	5.5002097	1.87E-07	7.91E-06	6.8512406
cg14959260	chr4	9917839	+	SLC2A9;SLC2A9	Body;Body	-1.19548	-0.672406	-5.500104	1.87E-07	7.92E-06	6.8507725
cg23621013	chr7	135433353	+	FAM180A;FAM180A	1stExon;5'UTR	-1.31551	-0.902769	-5.500077	1.87E-07	7.92E-06	6.8506551
cg00396687	chr15	44069062	-	ELL3	1stExon	-0.46275	-4.243195	-5.499717	1.88E-07	7.93E-06	6.849064
cg10594543	chr5	33649717	-	ADAMTS12	Body	-1.822237	0.9307433	-5.499634	1.88E-07	7.93E-06	6.8486975

cg10166609	chr2	71383612	+			-1.061271	-0.283538	-5.499607	1.88E-07	7.93E-06	6.8485764
cg08629884	chr4	1719983	-	TMEM129;TMEM129	Body;Body	-2.260237	2.6274466	-5.499332	1.88E-07	7.94E-06	6.8473647
cg20766179	chr3	134361914	+	KY	Body	-1.19999	0.0945601	-5.499326	1.88E-07	7.94E-06	6.8473379
cg23330281	chr11	19372234	-	NAV2	TSS200	-1.648826	0.2720763	-5.499034	1.88E-07	7.95E-06	6.846047
cg18500300	chr7	45128199	+	NACAD	Body	0.8494	0.2359768	5.4988099	1.88E-07	7.96E-06	6.8450564
cg11854154	chr11	10472133	-	AMPD3	TSS200	-1.163492	-4.623001	-5.498585	1.89E-07	7.97E-06	6.844063
cg00692151	chr3	132386753	+	UBA5;UBA5	Body;Body	0.92153	2.3293745	5.4983762	1.89E-07	7.97E-06	6.8431405
cg07116631	chr16	3345463	-			0.9813662	1.4327705	5.4983813	1.89E-07	7.97E-06	6.8431635
cg08943045	chr1	9043919	-			-0.872318	-1.854674	-5.498277	1.89E-07	7.98E-06	6.8427039
cg08981537	chr2	54858444	+	SPTBN1;SPTBN1	Body;Body	0.8098858	0.9129398	5.4980943	1.89E-07	7.98E-06	6.8418955
cg07291439	chr3	147125926	-	ZIC1	TSS1500	1.1906053	-0.552177	5.4979514	1.89E-07	7.99E-06	6.8412642
cg04838988	chr8	70984437	+	PRDM14	TSS1500	1.4190743	-1.309923	5.4977239	1.89E-07	7.99E-06	6.8402597
cg01319892	chr10	130268832	+			-0.912661	-0.07369	-5.497495	1.90E-07	8.00E-06	6.8392466
cg08701543	chr5	169928875	+	KCNIP1	Body	-1.091637	0.0336951	-5.499778	1.90E-07	8.00E-06	6.8402069
cg10209391	chr1	26633129	+	UBXN11;UBXN11;UBXN11;UBXN11;UBXN11	5'UTR;1stExon;5'UTR;1stExon;5'UTR	-0.471051	-4.505234	-5.497398	1.90E-07	8.00E-06	6.8388205
cg19702785	chr20	43727089	-	KCNS1	Body	0.9513012	-0.831745	5.497394	1.90E-07	8.00E-06	6.8388025
cg06541857	chr3	134203789	-	ANAPC13;ANAPC13;CEP63;CEP63;CEP63;ANAPC13;CEP63	5'UTR;Body;TSS1500;TSS1500;TSS1500;Body;TSS1500	-0.959759	-2.67456	-5.497371	1.90E-07	8.00E-06	6.8387024
cg09881503	chr22	19506979	-	CDC45L	3'UTR	0.5785367	1.6373205	5.4973208	1.90E-07	8.00E-06	6.8384791
cg13515842	chr9	127215440	-	GPR144	Body	0.8043944	0.6460154	5.4971091	1.90E-07	8.01E-06	6.8375439
cg21847393	chr3	50383483	+	ZMYND10	TSS1500	-1.475129	-3.338588	-5.497088	1.90E-07	8.01E-06	6.8374505

cg15089806	chr1	43418199	-	SLC2A1	Body	-1.077029	0.0761632	-5.496948	1.90E-07	8.01E-06	6.8368318
cg25042258	chr7	148427662	+	CUL1	Body	0.9743044	0.2807634	5.4968584	1.90E-07	8.02E-06	6.8364368
cg12610887	chr17	9479912	-	WDR16;WDR16;STX8	TSS200;TSS200;TSS1500	-1.730661	-2.3251	-5.498833	1.90E-07	8.03E-06	6.8532074
cg21945930	chr7	96631580	+	DLX6AS	Body	1.1958355	-0.603883	5.4963801	1.91E-07	8.03E-06	6.8343246
cg02962903	chr2	239887658	-			-1.510914	0.5748868	-5.498618	1.91E-07	8.03E-06	6.8350936
cg21069005	chr8	11537430	-			1.1781617	-0.230899	5.4963276	1.91E-07	8.03E-06	6.8340926
cg01798261	chr20	21378495	-	NKX2-4	TSS1500	0.9704004	-0.018082	5.4962319	1.91E-07	8.04E-06	6.8336702
cg05589845	chr7	27282444	-	EVX1;EVX1	1stExon;5'UTR	1.4542996	-1.458861	5.4962067	1.91E-07	8.04E-06	6.8335588
cg14058027	chr12	132839125	+	GALNT9	Body	-1.951313	1.8387125	-5.496181	1.91E-07	8.04E-06	6.8334467
cg16746576	chr8	99962118	-	OSR2;OSR2	Body;Body	1.5589056	-0.72886	5.4961886	1.91E-07	8.04E-06	6.8334787
cg26841048	chr17	46622454	+	HOXB2	TSS200	1.3469467	0.2302733	5.4962051	1.91E-07	8.04E-06	6.8335516
cg27507569	chr3	134045136	-			0.8933166	1.0628568	5.4961142	1.91E-07	8.04E-06	6.8331502
cg10195962	chr10	47656369	+			-0.832509	-3.387578	-5.495997	1.91E-07	8.04E-06	6.8326324
cg13842240	chr4	13539213	-			0.8377596	-0.903636	5.4960074	1.91E-07	8.04E-06	6.8326788
cg02397368	chr5	2564206	-			-1.682574	-0.513797	-5.498196	1.91E-07	8.04E-06	6.8332313
cg11068289	chr17	78955884	+			-1.546648	-0.648421	-5.498207	1.91E-07	8.04E-06	6.8332792
cg08654915	chr3	157813417	-			1.015317	-0.457847	5.4958927	1.91E-07	8.04E-06	6.8321721
cg06624951	chr12	133055588	-			-0.969966	-0.755465	-5.49568	1.91E-07	8.05E-06	6.8312336
cg21644009	chr6	28494863	+	GPX5;GPX5	Body;Body	-1.702474	0.7864549	-5.495574	1.91E-07	8.05E-06	6.8307636
cg22462235	chr1	226129481	+	LEFTY2	TSS1500	0.9403771	0.6083693	5.4954595	1.91E-07	8.06E-06	6.8302592

cg23346516	chr17	76421646	+	DNAH17	Body	1.2174391	1.4346147	5.495381	1.91E-07	8.06E-06	6.8299124
cg08577743	chr9	38621011	-	C9orf122;ANKRD18A	TSS200;TSS1500	-0.519736	-2.900208	-5.495349	1.91E-07	8.06E-06	6.8297728
cg16019434	chr18	76736785	+			1.3401457	-0.543187	5.4953332	1.91E-07	8.06E-06	6.8297015
cg24633312	chr7	43300742	+	HECW1	Body	-0.897801	-0.720407	-5.495261	1.92E-07	8.06E-06	6.8293809
cg11835068	chr1	4714974	+	AJAP1;AJAP1	TSS200;TSS200	1.9362317	-1.491876	5.4951616	1.92E-07	8.06E-06	6.8289438
cg22793130	chr8	144163464	-			-1.071128	-0.900613	-5.495153	1.92E-07	8.06E-06	6.8289075
cg13803727	chr9	89445247	-			-1.208835	-0.179401	-5.495057	1.92E-07	8.06E-06	6.8284818
cg14207326	chr4	100025015	-			0.814529	1.9283637	5.4948865	1.92E-07	8.07E-06	6.8277292
cg24575275	chr7	1094737	+	C7orf50;C7orf50;C7orf50	Body;Body;Body	1.0830886	-0.566234	5.4948323	1.92E-07	8.07E-06	6.8274896
cg04951293	chr2	231688289	-			-0.902251	-0.064477	-5.494789	1.92E-07	8.07E-06	6.8272965
cg14412111	chr7	91522266	-			-1.26114	-0.927143	-5.49475	1.92E-07	8.07E-06	6.8271245
cg17341370	chr6	150068017	-	NUP43	TSS1500	-0.931561	-1.735443	-5.494768	1.92E-07	8.07E-06	6.8272046
cg00064020	chr3	183894761	+	AP2M1;AP2M1	5'UTR;5'UTR	0.9950194	0.7316601	5.4944487	1.92E-07	8.08E-06	6.8257962
cg12065616	chr12	94954828	+			-1.145549	-1.142063	-5.494454	1.92E-07	8.08E-06	6.8258209
cg22782268	chr13	92050791	-	GPC5	TSS200	1.2982855	-4.256008	5.4943716	1.92E-07	8.08E-06	6.8254556
cg26560699	chr5	171918659	-			-1.260741	0.4889793	-5.496324	1.93E-07	8.10E-06	6.8249757
cg13219080	chr1	942161	-			-1.568661	0.4259273	-5.493912	1.93E-07	8.10E-06	6.8234247
cg16081499	chr12	129000862	+	TMEM132C	Body	-1.422341	0.7980048	-5.493766	1.93E-07	8.10E-06	6.8227822
cg25645462	chr16	57653702	+	GPR56;GPR56	TSS1500;TSS1500	-0.953917	0.0470452	-5.49377	1.93E-07	8.10E-06	6.8228003
cg14898177	chr12	2486634	+	CACNA1C;CACNA1C;CACNA1C;CACNA1C;CACNA1C;CACNA1C ;CACNA1C;CACNA1C;CACNA1C;CACNA1C;CACNA1C;CACNA1 C;CACNA1C;CACNA1C;CACNA1C;CACNA1C;CACNA1C;CACNA	Body;Body;Body;Body;Body;Body;Body;Body; Body;Body;Body;Body;Body;Body;Body;Body; Body;Body;Body;Body;Body	-1.391759	0.5502165	-5.493733	1.93E-07	8.10E-06	6.8226351

cg14174583	chr2	1051469	-	SNTG2	Body	-1.653745	0.799682	-5.493686	1.93E-07	8.10E-06	6.8224308
cg05938889	chr8	10583827	-	SOX7	Body	0.9746745	1.2103663	5.493574	1.93E-07	8.11E-06	6.8219343
cg13306815	chr6	140364611	+			-1.315037	-0.057644	-5.493415	1.93E-07	8.11E-06	6.8212318
cg15069995	chr1	150692971	-	HORMAD1	5'UTR	0.7996741	3.7396526	5.4933507	1.93E-07	8.12E-06	6.8209486
cg01397223	chr5	85756331	-			-1.020218	0.929045	-5.493264	1.93E-07	8.12E-06	6.8205672
cg12598025	chr5	172091816	-	NEURL1B	Body	0.9594638	1.8084089	5.4932521	1.93E-07	8.12E-06	6.820513
cg06639923	chr7	5534978	-	FBXL18	Body	-1.218463	0.8745007	-5.493164	1.93E-07	8.12E-06	6.8201232
cg11486694	chr10	53367157	+	PRKG1;PRKG1	Body;Body	-1.499696	-0.536412	-5.493075	1.93E-07	8.12E-06	6.8197315
cg19591908	chr14	101459254	+	SNORD114-31	TSS1500	-1.530627	0.3064823	-5.493076	1.93E-07	8.12E-06	6.8197355
cg17294725	chr7	1272559	-	UNCX	TSS200	1.6351253	-1.057313	5.497442	1.94E-07	8.13E-06	6.8214158
cg07803420	chr6	30851517	+	DDR1;DDR1	TSS1500;TSS1500	-0.901013	-0.60771	-5.492726	1.94E-07	8.13E-06	6.8181928
cg00991875	chr6	161100376	+			1.7825172	-0.018201	5.4925387	1.94E-07	8.14E-06	6.8173641
cg24047124	chr14	52031748	-	FRMD6	5'UTR	-1.529251	0.4466734	-5.492518	1.94E-07	8.14E-06	6.817274
cg06512128	chr1	113245695	+	RHOC;RHOC;RHOC	Body;Body;Body	0.8188551	1.32317	5.4924252	1.94E-07	8.14E-06	6.8168628
cg10553204	chr2	20871195	-	GDF7	3'UTR	1.4076993	1.8775483	5.4923376	1.94E-07	8.14E-06	6.8164763
cg00915830	chr12	34448653	+			-1.109513	0.3858416	-5.492115	1.94E-07	8.15E-06	6.8154953
cg04255230	chr2	74727010	+	LBX2	Body	1.3143411	0.9442353	5.4920218	1.94E-07	8.15E-06	6.8150821
cg12229082	chr7	150721650	-	ATG9B	TSS200	-1.077273	-0.47566	-5.49187	1.95E-07	8.16E-06	6.8144107
cg26568226	chr15	22913326	+	CYFIP1	5'UTR	-1.456438	2.6726016	-5.49166	1.95E-07	8.17E-06	6.8134855
cg12434982	chr1	197890479	-	LHX9;LHX9	Body;Body	0.8024195	0.515005	5.4915275	1.95E-07	8.17E-06	6.8129005

cg01460202	chr16	23706506	+	ERN2	Body	1.2002989	-1.03643	5.4914568	1.95E-07	8.17E-06	6.8125886
cg08313939	chr20	37353117	+	SLC32A1;SLC32A1	5'UTR;1stExon	2.2356203	-1.776822	5.4914153	1.95E-07	8.17E-06	6.8124051
cg13692446	chr13	112759719	-			2.3335678	-0.979558	5.4914161	1.95E-07	8.17E-06	6.8124088
cg15664905	chr19	44009429	+	PHLDB3	TSS1500	0.8035759	1.2840809	5.4914202	1.95E-07	8.17E-06	6.8124269
cg01569083	chr6	31543289	-	TNF	TSS200	-1.231604	-0.747888	-5.491267	1.95E-07	8.18E-06	6.8117515
cg24704759	chr10	133446251	+			-1.295177	-0.243594	-5.49127	1.95E-07	8.18E-06	6.8117655
cg23230564	chr5	31470890	+	RNASEN;RNASEN	Body;Body	1.2902434	0.8288044	5.4912047	1.95E-07	8.18E-06	6.8114757
cg05599030	chr17	37356047	-	RPL19	TSS1500	-0.761779	-3.246105	-5.491007	1.95E-07	8.18E-06	6.8106038
cg22798977	chr1	13909743	-	PDPN;PDPN	TSS1500;TSS1500	0.9132694	-1.204457	5.4909643	1.95E-07	8.19E-06	6.8104148
cg00050618	chr16	27414418	-	IL21R;IL21R	TSS200;5'UTR	-1.356962	0.2871954	-5.490903	1.95E-07	8.19E-06	6.8101433
cg24463066	chr4	48377724	+	SLAIN2	Body	-1.615427	1.1458208	-5.490801	1.96E-07	8.19E-06	6.8096934
cg26079428	chr11	106513618	+			-1.49936	0.1309536	-5.490819	1.96E-07	8.19E-06	6.8097752
cg20842915	chr7	39665132	-	RALA	5'UTR	1.3024099	0.7475027	5.4906545	1.96E-07	8.19E-06	6.8090475
cg18426908	chr10	130087149	+			-1.242822	0.41653	-5.490392	1.96E-07	8.20E-06	6.8078888
cg03401963	chr2	125755746	-			-1.47422	0.4484496	-5.490282	1.96E-07	8.21E-06	6.8074052
cg26315862	chr13	21520519	-			1.9996887	-0.531053	5.4902901	1.96E-07	8.21E-06	6.8074392
cg02198144	chr16	85462102	-			-1.654485	1.2626294	-5.48974	1.96E-07	8.22E-06	6.8050102
cg25103417	chr3	157812018	-			1.2916695	0.3367813	5.4897314	1.96E-07	8.22E-06	6.8049738
cg23331421	chr19	30866202	+	ZNF536	5'UTR	1.9162145	-0.883175	5.4896981	1.97E-07	8.23E-06	6.8048267
cg06547034	chr10	87403729	-	GRID1	Body	-1.568003	1.4797893	-5.489523	1.97E-07	8.23E-06	6.804056

cg06294416	chr12	66628232	+	IRAK3;IRAK3	Body;Body	-1.610989	0.4758845	-5.489382	1.97E-07	8.23E-06	6.803431
cg08112616	chr14	101360188	+	MEG8	TSS1500	-1.427988	0.7430473	-5.489396	1.97E-07	8.23E-06	6.8034955
cg22117172	chr7	91764530	+	CYP51A1;CYP51A1	TSS1500;TSS1500	-0.986717	-2.704414	-5.489284	1.97E-07	8.24E-06	6.8029973
cg02453208	chr1	156897930	+	C1orf92	Body	-0.788689	-3.705978	-5.489088	1.97E-07	8.24E-06	6.8021365
cg06384463	chr1	91182708	-	BARHL2	1stExon	1.40179	-2.34277	5.4890782	1.97E-07	8.24E-06	6.8020912
cg23882545	chr2	202122653	-	CASP8;CASP8;CASP8;CASP8;CASP8	5'UTR;5'UTR;5'UTR;5'UTR;TSS200	-0.670296	-3.76374	-5.488933	1.97E-07	8.25E-06	6.8014485
cg19691260	chr15	42186945	-	SPTBN5	TSS1500	-0.9144	-1.336242	-5.488705	1.97E-07	8.26E-06	6.8004454
cg11014124	chr5	140782297	-	PCDHGA4;PCDHGA6;PCDHGA1;PCDHGA8;PCDHGA5;PCDHGB1;PCDHGB4;PCDHGA3;PCDHGA2;PCDHGA7;PCDHGB2;PCDHGA9;PCDHGB5;PCDHGA9;PCDHGB3	Body;Body;Body;Body;Body;Body;Body;Body;Body;Body;Body;TSS1500;Body;TSS1500;Body	1.6281097	-0.574857	5.4885664	1.98E-07	8.26E-06	6.7998333
cg15700197	chr1	159409207	+	OR10J1	TSS1500	-1.70891	-0.031619	-5.488513	1.98E-07	8.26E-06	6.7995956
cg15902864	chr5	33504903	+			-1.292941	-0.513158	-5.488401	1.98E-07	8.27E-06	6.7991045
cg09495482	chr6	29408232	-	OR10C1	1stExon	-0.802629	-0.095428	-5.488378	1.98E-07	8.27E-06	6.7990014
cg19405484	chr8	29513210	-			-1.705189	-1.351995	-5.488008	1.98E-07	8.28E-06	6.7973685
cg26672573	chr16	19991691	+			-1.259625	0.1305772	-5.487843	1.98E-07	8.28E-06	6.7966414
cg22821300	chr15	79296585	-	RASGRF1;RASGRF1;RASGRF1	Body;Body;5'UTR	-1.126786	0.6176161	-5.487711	1.98E-07	8.29E-06	6.7960611
cg03265268	chr8	11839764	-	DEFB135	TSS200	-1.289777	-0.017194	-5.48745	1.99E-07	8.30E-06	6.7949073
cg10152447	chr4	16324929	-			-1.593513	1.233264	-5.487386	1.99E-07	8.30E-06	6.7946252
cg16684817	chr1	28202173	-	C1orf38;C1orf38;C1orf38	Body;Body;Body	-1.446426	-0.17041	-5.48737	1.99E-07	8.30E-06	6.7945538
cg07925587	chr12	52583324	-	KRT80;KRT80	Body;Body	-1.41427	-1.825397	-5.487293	1.99E-07	8.30E-06	6.7942161
cg14847917	chr12	78635247	-			-1.211326	-0.082221	-5.487041	1.99E-07	8.31E-06	6.7931045
cg09868780	chr5	115299071	+	LVRN	Body	1.2662331	-0.726857	5.4869957	1.99E-07	8.31E-06	6.7929034

cg05557266	chr3	49057259	-	DALRD3;DALRD3;NDUFAF3;NDUFAF3	5'UTR;TSS1500;TSS1500;TSS1500	-1.229424	-1.192521	-5.486944	1.99E-07	8.31E-06	6.7926769
cg08195983	chr3	61235829	-	FHIT;FHIT	5'UTR;5'UTR	-1.002022	-1.060238	-5.486731	1.99E-07	8.32E-06	6.7917338
cg14721506	chr20	61758358	-			-1.342412	-0.021916	-5.486672	1.99E-07	8.32E-06	6.7914738
cg15606663	chr21	31812603	-	KRTAP15-1	TSS200	-1.404882	0.1822479	-5.486675	1.99E-07	8.32E-06	6.7914891
cg24401219	chr6	72892597	-	RIMS1	Body	-0.979808	-2.503181	-5.486636	1.99E-07	8.32E-06	6.7913162
cg03738936	chr16	76670197	-			-1.62018	0.9030166	-5.486468	1.99E-07	8.33E-06	6.7905757
cg12743248	chr8	49636840	-	EFCAB1;EFCAB1;EFCAB1	3'UTR;3'UTR;Body	-1.558666	-0.60742	-5.486397	2.00E-07	8.33E-06	6.7902631
cg19959677	chr14	61120783	+			1.1944133	0.1554263	5.4863822	2.00E-07	8.33E-06	6.7901969
cg00126638	chr6	29795583	-	HLA-G	5'UTR	1.0598609	0.1360126	5.4862461	2.00E-07	8.33E-06	6.7895967
cg20981086	chr14	101923256	+			1.7878107	-0.307985	5.4859214	2.00E-07	8.34E-06	6.7881642
cg23552192	chr8	59323544	+	UBXN2B	TSS1500	-0.968988	-3.069183	-5.485532	2.00E-07	8.36E-06	6.7864453
cg16709353	chr11	68856817	+	TPCN2	3'UTR	-1.410331	-1.747274	-5.485454	2.00E-07	8.36E-06	6.7861013
cg15466176	chr12	114781410	-			-1.623981	0.6720329	-5.485143	2.01E-07	8.37E-06	6.7847313
cg16105687	chr19	22805801	-			1.3568614	-0.261123	5.485159	2.01E-07	8.37E-06	6.7848014
cg17357062	chr9	137810348	-	FCN1	TSS1500	-1.300888	-0.032408	-5.485139	2.01E-07	8.37E-06	6.784715
cg07442409	chr14	60973773	-			1.3218268	-1.63497	5.484987	2.01E-07	8.37E-06	6.7840428
cg10433327	chr15	56209506	-	NEDD4;NEDD4	TSS200;Body	-1.291934	-0.235747	-5.485002	2.01E-07	8.37E-06	6.7841109
cg02150820	chr17	77390045	+	HRNBP3	5'UTR	-0.994587	-0.510695	-5.48492	2.01E-07	8.38E-06	6.7837472
cg10333802	chr14	71373888	-	PCNX	TSS1500	-0.785984	-4.055387	-5.484931	2.01E-07	8.38E-06	6.7837978
cg03221073	chr1	186157555	-	HMCN1;MIR548F1	Body;Body	-1.570012	0.0341967	-5.484735	2.01E-07	8.38E-06	6.7829304

cg24011351	chr11	62782446	-	SLC22A8	5'UTR	-1.438466	0.71838	-5.484599	2.01E-07	8.39E-06	6.7823327
cg20479063	chr4	10016908	-	SLC2A9;SLC2A9	Body;Body	-1.014246	-0.900073	-5.4843	2.01E-07	8.40E-06	6.7810145
cg25948831	chr17	78038002	-	CCDC40	Body	-1.597096	0.9868026	-5.484292	2.01E-07	8.40E-06	6.7809761
cg20133923	chr20	3027467	+	MRPS26	Body	-0.417553	-2.908604	-5.484162	2.02E-07	8.40E-06	6.7804054
cg13314145	chr7	98246001	-	NPTX2	TSS1500	2.5369609	-1.56618	5.4839212	2.02E-07	8.41E-06	6.7793426
cg03013118	chr2	218375971	-	DIRC3	Body	-0.908536	-1.262157	-5.483719	2.02E-07	8.41E-06	6.7784509
cg05785155	chr2	182547401	+			1.3023653	-1.30249	5.4837365	2.02E-07	8.41E-06	6.7785282
cg23648516	chr13	78467552	+			-1.680075	0.9847717	-5.483752	2.02E-07	8.41E-06	6.7785968
cg26827533	chr8	22458122	-	C8orf58	Body	1.2051342	0.654929	5.4837182	2.02E-07	8.41E-06	6.7784472
cg07802350	chr2	176956573	-	HOXD13	TSS1500	2.5224071	-1.552336	5.4858712	2.02E-07	8.42E-06	6.7961738
cg26333450	chr5	141139633	+			-0.992634	-0.262957	-5.483473	2.02E-07	8.42E-06	6.7773656
cg14789175	chr1	11534690	-			-1.089169	-0.400715	-5.483341	2.02E-07	8.43E-06	6.776786
cg26492446	chr20	61638574	+	BHLHE23	TSS200	3.1770341	-1.626346	5.4832615	2.02E-07	8.43E-06	6.7764333
cg15448372	chr2	129226666	+			-1.669306	1.0157084	-5.483231	2.02E-07	8.43E-06	6.7762975
cg03631131	chr3	147115506	+	ZIC4;ZIC4;ZIC4	Body;Body;Body	1.0516548	-1.070968	5.4831388	2.03E-07	8.43E-06	6.7758925
cg00485849	chr4	74486170	-	RASSF6;RASSF6	1stExon;TSS200	-0.781121	-4.255756	-5.482856	2.03E-07	8.44E-06	6.7746472
cg10826175	chr10	55016939	-			-1.229227	0.0701298	-5.48256	2.03E-07	8.45E-06	6.7733403
cg12524698	chr11	808556	-	RPLP2	TSS1500	-1.056157	-0.005284	-5.48249	2.03E-07	8.45E-06	6.7730326
cg27417606	chr14	21503144	-	RNASE13	TSS1500	-1.210948	-0.217645	-5.482368	2.03E-07	8.46E-06	6.772492
cg24541426	chr2	177029459	-	HOXD3	5'UTR	1.3398699	-0.207709	5.4845741	2.03E-07	8.46E-06	6.7731899

cg07349217	chr8	133878997	-	TG	TSS1500	-1.51531	0.7644766	-5.48449	2.03E-07	8.46E-06	6.773189
cg10888461	chr4	113300821	-	ALPK1;ALPK1	Body;Body	0.6800153	2.3159805	5.4822365	2.03E-07	8.46E-06	6.7719139
cg17355329	chr5	76937980	-			0.9429879	0.1610693	5.4820586	2.04E-07	8.47E-06	6.7711296
cg01489419	chr16	26293057	+			-1.441255	0.5175559	-5.482034	2.04E-07	8.47E-06	6.7710216
cg02834280	chr2	9253005	-			-1.256301	0.7362827	-5.481947	2.04E-07	8.47E-06	6.770639
cg20597215	chr11	59224664	-	OR4D6	1stExon	-1.539645	0.2914162	-5.481954	2.04E-07	8.47E-06	6.7706691
cg02712464	chr13	30951156	-			-0.933823	-1.573826	-5.481709	2.04E-07	8.48E-06	6.7695892
cg26521448	chr16	11876426	-	ZC3H7A	TSS200	-0.849137	1.3168325	-5.481549	2.04E-07	8.48E-06	6.7688838
cg05730368	chr6	42146052	+	GUCA1A	Body	1.0945986	-0.477373	5.4814379	2.04E-07	8.49E-06	6.768393
cg04262140	chr17	46827626	+			2.2106183	-1.722804	5.4836422	2.04E-07	8.49E-06	6.7694538
cg04694812	chr5	63257554	-	HTR1A	TSS200	1.3554771	-1.328123	5.4813033	2.04E-07	8.49E-06	6.7677993
cg24838764	chr3	122720612	+	SEMA5B	5'UTR	-1.367323	0.9892242	-5.481276	2.04E-07	8.49E-06	6.767678
cg00914781	chr1	47902275	-	FOXD2;FOXD2	1stExon;5'UTR	-0.423052	-3.794941	-5.48105	2.05E-07	8.50E-06	6.7666811
cg06463800	chr13	25754183	+			-1.413165	-3.35593	-5.480872	2.05E-07	8.50E-06	6.7658977
cg22730412	chr1	51700609	-	RNF11	TSS1500	-1.433543	-1.194911	-5.480887	2.05E-07	8.50E-06	6.7659633
cg12087123	chr17	40700043	-			-1.48974	0.5944237	-5.48078	2.05E-07	8.51E-06	6.7654908
cg26048448	chr1	197879380	-			1.3004924	-1.327918	5.4807269	2.05E-07	8.51E-06	6.7652586
cg20033619	chr15	39523161	-			0.8361713	1.367386	5.4806126	2.05E-07	8.51E-06	6.7647546
cg18135379	chr1	161981673	+	OLFML2B	Body	1.1570228	2.5584574	5.4805543	2.05E-07	8.51E-06	6.7644977
cg05968822	chr1	226071684	+	TMEM63A	TSS1500	-1.179496	0.1535729	-5.480529	2.05E-07	8.51E-06	6.7643872

cg08506924	chr16	3931286	-	CREBBP;CREBBP	TSS1500;TSS1500	-0.495659	-3.0117	-5.480309	2.05E-07	8.52E-06	6.7634143
cg13806095	chr20	13620048	+	TASP1	TSS1500	-1.214794	-2.768291	-5.480325	2.05E-07	8.52E-06	6.7634889
cg07881405	chr5	3597487	+	IRX1	Body	2.6994915	-1.591439	5.4801181	2.05E-07	8.53E-06	6.7625746
cg24468199	chr11	65410028	+	SIPA1;SIPA1	Body;Body	0.916805	1.4225829	5.4801093	2.05E-07	8.53E-06	6.7625362
cg16284238	chr6	87196595	-			-1.422546	0.0346297	-5.480066	2.05E-07	8.53E-06	6.7623469
cg09471343	chr2	102884397	-			-1.362931	0.84009	-5.479931	2.06E-07	8.53E-06	6.7617479
cg26682580	chr12	128752058	-	TMEM132C	Body	2.3606453	-2.11362	5.4796699	2.06E-07	8.54E-06	6.7605991
cg26857670	chr7	27225448	+	HOXA11AS;HOXA11	Body;TSS1500	1.6351683	-1.234939	5.479605	2.06E-07	8.54E-06	6.7603129
cg24471894	chr9	2838508	-	KIAA0020	5'UTR	1.1155752	-1.870313	5.4793808	2.06E-07	8.55E-06	6.759325
cg01466491	chr19	10523363	-			-1.380208	-0.19159	-5.479134	2.06E-07	8.56E-06	6.7582381
cg06689045	chr3	120025420	-			0.8139632	1.7292534	5.4791441	2.06E-07	8.56E-06	6.7582815
cg04287330	chr1	43423596	+	SLC2A1	Body	-0.88905	-4.28741	-5.478945	2.07E-07	8.57E-06	6.7574029
cg09141965	chr7	1269385	-			1.5063592	-0.474734	5.478834	2.07E-07	8.57E-06	6.7569149
cg11183632	chr20	21503152	+			2.4800696	-1.876991	5.478791	2.07E-07	8.57E-06	6.7567254
cg25808809	chr13	114917199	-			-0.879902	-1.292183	-5.478707	2.07E-07	8.57E-06	6.7563533
cg15319470	chr12	62546183	-	FAM19A2	5'UTR	-1.26406	-1.180039	-5.478174	2.07E-07	8.59E-06	6.7540075
cg22021794	chr5	54177306	-			-1.215485	0.3055698	-5.478103	2.07E-07	8.60E-06	6.7536939
cg05180522	chr14	98101740	+			-1.261536	0.8404638	-5.477947	2.07E-07	8.60E-06	6.753006
cg18082979	chr1	32085118	-	HCRTR1	Body	-0.94852	-0.402288	-5.477926	2.07E-07	8.60E-06	6.7529147
cg12066473	chr4	186733331	+	SORBS2;SORBS2;SORBS2;SORBS2;SORBS2;SORBS2;SORBS2	5'UTR;5'UTR;5'UTR;1stExon;TSS1500;1stExon;TSS1500	-1.157166	-0.781367	-5.4778	2.08E-07	8.60E-06	6.7523566

cg24345551	chr6	33360652	-	KIFC1	Body	-0.761763	-0.604087	-5.477795	2.08E-07	8.60E-06	6.7523355
cg12227172	chr19	52391411	-	ZNF577;ZNF577;ZNF577	TSS1500;TSS200;TSS1500	2.3007782	-1.062969	5.4776687	2.08E-07	8.61E-06	6.751779
cg16202734	chr17	37321890	+	ARL5C	Body	0.9534058	-0.158314	5.4776337	2.08E-07	8.61E-06	6.7516251
cg00599770	chr7	27196825	-	HOXA7	TSS1500	0.9924726	-0.74589	5.4775965	2.08E-07	8.61E-06	6.7514609
cg13437581	chr18	44777799	-			2.0667021	-1.666127	5.4798497	2.08E-07	8.61E-06	6.7523881
cg07586760	chr22	49487396	+			-0.995727	0.0371084	-5.477468	2.08E-07	8.61E-06	6.7508968
cg27409401	chr16	614852	-	C16orf11	Body	-1.350526	1.5447424	-5.477446	2.08E-07	8.61E-06	6.7507976
cg02611848	chr2	74875387	+	C2orf65	TSS1500	1.1079623	0.7189226	5.477332	2.08E-07	8.62E-06	6.7502954
cg02715629	chr8	124193817	-	FAM83A;FAM83A	TSS1500;TSS1500	-1.419046	0.0097257	-5.477339	2.08E-07	8.62E-06	6.7503265
cg09331106	chr2	10260230	+			-0.832615	-0.448736	-5.477313	2.08E-07	8.62E-06	6.7502096
cg27404824	chr10	1454848	+	ADARB2	Body	-1.263344	-0.453035	-5.477247	2.08E-07	8.62E-06	6.7499192
cg10888348	chr7	4763137	-	FOXK1	Body	0.8212534	0.7212722	5.476903	2.08E-07	8.63E-06	6.7484049
cg23845250	chr12	132944590	+			-1.040015	-1.390209	-5.476828	2.09E-07	8.63E-06	6.7480725
cg16793394	chr14	57278084	-	OTX2	TSS1500	2.0339649	-0.568447	5.4790146	2.09E-07	8.63E-06	6.7490809
cg26613361	chr16	31830668	-			0.883481	0.7911761	5.4789512	2.09E-07	8.64E-06	6.7484331
cg05973555	chr2	84114418	-			-1.753976	0.1664399	-5.476611	2.09E-07	8.64E-06	6.7471187
cg06476685	chr2	241459638	+	ANKMY1;ANKMY1	Body;Body	1.1681164	0.1450591	5.4765288	2.09E-07	8.64E-06	6.7467561
cg09287629	chr2	203036208	+			0.8258678	0.1738348	5.4763559	2.09E-07	8.65E-06	6.7459942
cg00663077	chr4	188916709	-	ZFP42	TSS1500	1.8496932	-0.559362	5.4760673	2.09E-07	8.66E-06	6.7447228
cg16370491	chr12	75601928	-	KCNC2;KCNC2;KCNC2	5'UTR;5'UTR;5'UTR	1.6724172	-1.42027	5.4759927	2.09E-07	8.66E-06	6.744394

cg27054847	chr6	131386869	-			-1.192846	0.0840206	-5.475769	2.10E-07	8.67E-06	6.7434076
cg10956857	chr1	3308549	+	PRDM16;PRDM16	Body;Body	1.2285489	1.666448	5.4756845	2.10E-07	8.67E-06	6.7430363
cg01497613	chr17	48041301	+			1.4861909	-1.154846	5.4754615	2.10E-07	8.68E-06	6.7420535
cg03289416	chr15	75166202	+	SCAMP2	TSS1500	-1.118587	-3.361282	-5.477749	2.10E-07	8.68E-06	6.7431421
cg24074594	chr1	161676581	+	FCRLA	TSS200	-1.666033	0.3024492	-5.475472	2.10E-07	8.68E-06	6.7420997
cg07196505	chr16	7565837	-	A2BP1;A2BP1;A2BP1;A2BP1;A2BP1;A2BP1	Body;Body;Body;Body;Body;Body	-1.319029	-1.068806	-5.475117	2.10E-07	8.69E-06	6.7405358
cg18758976	chr6	158430311	-	SYNJ2	Body	0.7823233	0.0937596	5.4751168	2.10E-07	8.69E-06	6.7405352
cg24818200	chr5	140501365	-	PCDHB4	TSS1500	1.1626985	0.6780669	5.4750501	2.10E-07	8.69E-06	6.7402415
cg02320740	chr10	106400259	+	SORCS3	TSS1500	2.703878	-2.434991	5.4749986	2.10E-07	8.69E-06	6.7400144
cg13487918	chr3	38067350	+	PLCD1;PLCD1;PLCD1	TSS1500;TSS1500;Body	-1.286655	-0.186752	-5.474926	2.10E-07	8.69E-06	6.7396961
cg11049075	chr17	42199063	+	HDAC5;HDAC5	5'UTR;5'UTR	1.0751546	0.1132128	5.474733	2.11E-07	8.70E-06	6.7388441
cg26874872	chr16	26156362	+			-1.181439	-1.64585	-5.474702	2.11E-07	8.70E-06	6.7387088
cg06195280	chr17	6658706	+	XAF1;XAF1	TSS1500;TSS1500	0.9238633	1.1619637	5.4745428	2.11E-07	8.71E-06	6.7380064
cg22794244	chr11	123892873	+	OR10G9	TSS1500	-1.390043	-0.13377	-5.474542	2.11E-07	8.71E-06	6.7380044
cg01634458	chr16	51168553	-			1.4994626	-0.81556	5.4744093	2.11E-07	8.71E-06	6.7374181
cg13595212	chr13	28369574	+			1.3493782	-1.910067	5.4740931	2.11E-07	8.72E-06	6.7360254
cg13798780	chr7	105162888	+	PUS7	TSS1500	-0.660577	-4.730692	-5.474068	2.11E-07	8.72E-06	6.7359145
cg08693172	chr19	46526333	+	PGLYRP1	TSS200	1.4500902	-0.402474	5.4739767	2.11E-07	8.73E-06	6.7355126
cg11840804	chr12	57388184	+	GPR182	TSS200	0.6788756	2.0811992	5.4739468	2.11E-07	8.73E-06	6.735381
cg03812546	chr12	118728016	-	TAOK3	5'UTR	1.0029453	1.807118	5.4738645	2.11E-07	8.73E-06	6.7350183

cg07683388	chr2	198029332	+	ANKRD44	Body	0.8635491	1.3631964	5.4737109	2.12E-07	8.73E-06	6.7343417
cg21706229	chr17	70112877	+			1.348815	-0.340936	5.4736643	2.12E-07	8.74E-06	6.7341367
cg09936839	chr19	4181854	+	SIRT6;ANKRD24	Body;TSS1500	-0.91818	-0.097831	-5.473589	2.12E-07	8.74E-06	6.7338059
cg10503208	chr11	5545506	-			-1.414174	-0.238045	-5.473116	2.12E-07	8.76E-06	6.7317214
cg09968702	chr19	36160759	+	UPK1A	Body	-0.866535	-0.942416	-5.472921	2.12E-07	8.76E-06	6.7308635
cg11907797	chr5	140763125	+	PCDHGA4;PCDHGA7;PCDHGA6;PCDHGA1;PCDHGA5;PCDHGB1;PCDHGA3;PCDHGA2;PCDHGB2;PCDHGA7;PCDHGB3	Body;1stExon;Body;Body;Body;Body;Body;Body;Body;1stExon;Body	1.1073665	-0.357838	5.4729404	2.12E-07	8.76E-06	6.730948
cg21184518	chr9	124777955	+	TTLL11;TTLL11	Body;Body	0.6262375	1.8146313	5.4729279	2.12E-07	8.76E-06	6.7308932
cg26792755	chr7	140714919	-	MRPS33;MRPS33	TSS200;TSS1500	-1.707865	-3.931093	-5.472954	2.12E-07	8.76E-06	6.7310093
cg27315579	chr16	22956167	+			-1.855845	1.0412592	-5.472958	2.12E-07	8.76E-06	6.7310237
cg02414555	chr2	79216742	-			-1.390319	0.8889314	-5.472607	2.13E-07	8.77E-06	6.7294798
cg13986762	chr1	220960934	+	MOSC1	Body	-1.086924	-1.803157	-5.472582	2.13E-07	8.77E-06	6.7293679
cg00261162	chr1	28907478	-	SNORA16A;SNHG12;SNORA44;SNORA61	Body;Body;TSS1500;TSS1500	-0.590785	-4.678267	-5.472412	2.13E-07	8.78E-06	6.7286194
cg01759112	chr17	5980904	+	WSCD1	5'UTR	-1.594825	0.5704214	-5.472288	2.13E-07	8.78E-06	6.7280755
cg05537653	chr11	47612070	+	C1QTNF4	Body	1.090316	1.290956	5.4766949	2.13E-07	8.79E-06	6.7301489
cg12886406	chr3	13579728	-			-1.164772	0.1739542	-5.472112	2.13E-07	8.79E-06	6.7272976
cg05546264	chr4	187507476	+			1.0141214	2.1411565	5.4720531	2.13E-07	8.79E-06	6.7270403
cg14244636	chr10	100055918	+			-1.006454	-0.885273	-5.471534	2.14E-07	8.81E-06	6.7247558
cg19676502	chr14	50438151	+			-1.570147	-1.461098	-5.473772	2.14E-07	8.81E-06	6.7256449
cg01923516	chr7	3989167	+	SDK1	Body	0.852146	2.058688	5.4713387	2.14E-07	8.81E-06	6.7238942
cg19673440	chr2	2402353	-			-1.56431	0.6243484	-5.471295	2.14E-07	8.82E-06	6.7237022

cg10702818	chr3	32023473	+	ZNF860;OSBPL10	5'UTR;TSS1500	-0.869593	-1.025337	-5.471257	2.14E-07	8.82E-06	6.7235339
cg06804433	chr6	50682485	+	TFAP2D	Body	1.7248273	-2.267815	5.4711662	2.14E-07	8.82E-06	6.7231346
cg04624228	chr8	38411598	+			-1.279061	-1.8006	-5.47306	2.14E-07	8.83E-06	6.7225107
cg26033293	chr10	49864600	+			-0.482679	-3.829241	-5.470797	2.14E-07	8.83E-06	6.7215096
cg13353015	chr1	34547254	+	CSMD2	Body	-0.759023	1.9424923	-5.472992	2.14E-07	8.83E-06	6.7222148
cg08453036	chr1	215255572	+	KCNK2;KCNK2;KCNK2	TSS1500;TSS1500;Body	1.5728855	-1.593234	5.4706876	2.15E-07	8.84E-06	6.7210271
cg20731469	chr6	108495678	-	NR2E1	Body	1.5344775	-1.298937	5.4728938	2.15E-07	8.84E-06	6.7391534
cg24996930	chr14	77229233	+	VASH1	1stExon	0.8778624	1.1268444	5.4704804	2.15E-07	8.84E-06	6.7201145
cg09062961	chr2	1204735	-	SNTG2	Body	-1.756444	0.7730186	-5.470273	2.15E-07	8.85E-06	6.719201
cg21552014	chr3	146026497	-			1.107525	1.0323147	5.4698906	2.15E-07	8.86E-06	6.7175178
cg06607679	chr17	47551100	-			-0.955668	-0.695067	-5.469865	2.15E-07	8.86E-06	6.7174034
cg20634226	chr7	25264629	+	NPVF	3'UTR	-1.326414	0.7143533	-5.469772	2.15E-07	8.87E-06	6.7169938
cg17621718	chr3	42306737	+	CCK	TSS1500	1.8889708	-1.313135	5.4697402	2.15E-07	8.87E-06	6.7168554
cg05384724	chr12	124155451	-	TCTN2;TCTN2	TSS1500;TSS1500	-0.959636	-1.614523	-5.469642	2.16E-07	8.87E-06	6.716421
cg01079651	chr21	46847648	-	COL18A1	Body	-1.047438	-0.247938	-5.46942	2.16E-07	8.88E-06	6.7154449
cg20948486	chr1	156863129	-	PEAR1	TSS1500	1.3586148	-0.875835	5.4692801	2.16E-07	8.88E-06	6.7148301
cg15142042	chr5	3963277	-			-1.912002	0.1709049	-5.469214	2.16E-07	8.89E-06	6.7145406
cg00599463	chr20	30609716	-	C20orf160	Body	-1.34857	0.1560032	-5.468969	2.16E-07	8.90E-06	6.7134603
cg09480162	chr13	73301047	-	C13orf37;C13orf34	Body;TSS1500	-0.344016	-4.153319	-5.468931	2.16E-07	8.90E-06	6.7132911
cg24009250	chr14	98017621	-			-1.350349	0.6145559	-5.468476	2.17E-07	8.91E-06	6.7112895

cg10329468	chr20	44802548	+	CDH22	3'UTR	-0.997977	-0.358409	-5.468342	2.17E-07	8.92E-06	6.7106982
cg19412743	chr4	190747919	+			-1.092903	-0.733827	-5.468351	2.17E-07	8.92E-06	6.710739
cg22732253	chr2	63280164	-	OTX1	Body	0.8940237	0.0915053	5.4682095	2.17E-07	8.92E-06	6.7101168
cg23294090	chr8	139509672	+	FAM135B	TSS1500	1.8079385	-1.06548	5.4678768	2.17E-07	8.94E-06	6.7086523
cg09577469	chr7	1457117	+			-1.36607	-1.600597	-5.467813	2.17E-07	8.94E-06	6.7083702
cg04887082	chr10	95094925	+	MYOF;MYOF	Body;Body	-0.825622	-0.772397	-5.467477	2.18E-07	8.95E-06	6.7068923
cg21255356	chr6	169446912	+			-1.03765	-0.71366	-5.467429	2.18E-07	8.95E-06	6.7066833
cg07565042	chr11	10543456	+	RNF141	Body	1.1253067	0.9982282	5.4672983	2.18E-07	8.96E-06	6.7061059
cg27181349	chr1	36574854	-			-1.399241	-0.992077	-5.467193	2.18E-07	8.96E-06	6.7056443
cg14412857	chr14	64675538	+	SYNE2;SYNE2	Body;Body	1.0228322	2.3337939	5.4671318	2.18E-07	8.96E-06	6.7053729
cg00820056	chr8	144265337	+			-0.78802	-0.879287	-5.466813	2.18E-07	8.97E-06	6.7039701
cg09349723	chr2	199013270	+	PLCL1	3'UTR	-1.699549	0.6667227	-5.466741	2.18E-07	8.98E-06	6.7036524
cg24674368	chr14	97879637	-			1.2644953	0.481945	5.4689674	2.18E-07	8.98E-06	6.704515
cg26210364	chr11	26800135	-			-1.42106	0.1315687	-5.466681	2.19E-07	8.98E-06	6.70339
cg20879085	chr7	115850441	-	TES	TSS200	-1.062705	-4.73252	-5.466605	2.19E-07	8.98E-06	6.7030525
cg14452619	chr9	8627950	-	PTPRD;PTPRD;PTPRD;PTPRD;PTPRD;PTPRD	Body;Body;Body;Body;Body;Body	-1.856061	0.9991147	-5.466572	2.19E-07	8.98E-06	6.702907
cg00808563	chr5	5493320	-			-1.201262	0.8006603	-5.466353	2.19E-07	8.99E-06	6.7019446
cg08327428	chr10	109641026	-			-1.065066	-0.807985	-5.466141	2.19E-07	8.99E-06	6.7010104
cg05461143	chr7	150232206	-			-1.514883	0.8831176	-5.466118	2.19E-07	9.00E-06	6.7009093
cg13432339	chr4	58211039	-			-1.392545	-2.079173	-5.466052	2.19E-07	9.00E-06	6.7006192

cg11085294	chr1	177057631	-	ASTN1;ASTN1	Body;Body	-1.765846	0.6275569	-5.465991	2.19E-07	9.00E-06	6.7003508
cg26275799	chr6	150255758	+			-1.179951	-0.734507	-5.465943	2.19E-07	9.00E-06	6.7001419
cg19671648	chr8	79027162	-			-1.642883	0.7894991	-5.465823	2.19E-07	9.00E-06	6.699611
cg00364325	chr15	89342191	-			-1.320077	0.4225809	-5.465709	2.19E-07	9.01E-06	6.6991124
cg08339189	chr9	124260498	+	GGTA1	Body	-0.655076	-3.021506	-5.465615	2.20E-07	9.01E-06	6.6986981
cg04243368	chr22	32366128	-			-0.935783	-2.805775	-5.465448	2.20E-07	9.02E-06	6.6979635
cg18161327	chr13	100627703	-			1.7149048	0.2917792	5.4677006	2.20E-07	9.02E-06	6.6993166
cg15410236	chr19	948452	+	ARID3A	Body	-1.461744	2.2399049	-5.465418	2.20E-07	9.02E-06	6.6978322
cg02873204	chr6	164757735	-			-1.528784	0.5987328	-5.465309	2.20E-07	9.02E-06	6.6973486
cg03827689	chr3	32501676	+			-0.99413	-1.649172	-5.465152	2.20E-07	9.03E-06	6.6966601
cg19238840	chr16	20339175	+	GP2;GP2;GP2;GP2	TSS1500;TSS1500;TSS1500;TSS1500	-0.957996	-1.442266	-5.465091	2.20E-07	9.03E-06	6.6963895
cg09059945	chr7	130126018	-	MEST	TSS200	-0.935375	-2.915166	-5.46492	2.20E-07	9.03E-06	6.6956404
cg14279528	chr9	36572411	-	MELK	TSS1500	-1.416376	0.7135707	-5.464869	2.20E-07	9.03E-06	6.695414
cg20821800	chr13	54422912	-			-1.128447	-0.751725	-5.464756	2.20E-07	9.04E-06	6.6949154
cg07257768	chr6	30684736	+	MDC1	5'UTR	-0.931744	-3.177697	-5.466773	2.21E-07	9.05E-06	6.6948709
cg02205391	chr6	31762409	-	VARS	Body	-1.039657	-2.451073	-5.46443	2.21E-07	9.05E-06	6.6934844
cg08877521	chr6	170453256	+			1.189771	1.8946368	5.4643507	2.21E-07	9.05E-06	6.693134
cg26628811	chr8	1132142	+			-1.500365	1.3146081	-5.464334	2.21E-07	9.05E-06	6.6930586
cg04179952	chr1	201619295	-	NAV1	Body	-1.231303	-1.952614	-5.464176	2.21E-07	9.06E-06	6.6923666
cg05304729	chr1	158800024	-	MNDA	TSS1500	-1.319715	-1.443186	-5.464078	2.21E-07	9.06E-06	6.6919334

cg21296230	chr15	33010536	-	GREM1	5'UTR	1.4617066	-0.741771	5.4662839	2.21E-07	9.06E-06	6.6927194
cg03330642	chr5	178411124	-	GRM6	Body	-0.415999	-0.259742	-5.46379	2.21E-07	9.07E-06	6.6906674
cg03758367	chr12	54867542	-	GTSF1	TSS200	-1.336694	0.5894219	-5.463732	2.22E-07	9.07E-06	6.6904115
cg10781870	chr17	28648320	-	TMIGD1	Body	-1.293767	0.0545392	-5.463714	2.22E-07	9.07E-06	6.6903306
cg15015920	chr5	16179873	-	MARCH11;MARCH11	1stExon;5'UTR	2.0154349	-1.377254	5.4636106	2.22E-07	9.08E-06	6.6898776
cg23259394	chr1	24634964	-			-1.352067	0.0904314	-5.463628	2.22E-07	9.08E-06	6.6899532
cg10255761	chr3	49210029	-	KLHDC8B	5'UTR	0.9460545	0.248527	5.463572	2.22E-07	9.08E-06	6.6897077
cg09015246	chr16	10971105	-	CIITA;CIITA	1stExon;5'UTR	-1.576133	-1.515862	-5.463486	2.22E-07	9.08E-06	6.6893288
cg20710709	chr14	37135671	+	PAX9	Body	1.2773698	-0.253212	5.4634521	2.22E-07	9.08E-06	6.6891801
cg26942121	chr11	8832203	+	ST5;ST5;ST5;ST5	5'UTR;5'UTR;1stExon;5'UTR	-1.65628	-2.386385	-5.465701	2.22E-07	9.08E-06	6.6905285
cg11668844	chr13	113655622	+	MCF2L;MCF2L	Body;Body	-0.923791	-1.692165	-5.463355	2.22E-07	9.08E-06	6.6887549
cg11872672	chr7	157514730	+	PTPRN2;PTPRN2;PTPRN2	Body;Body;Body	-1.153529	-0.143778	-5.463365	2.22E-07	9.08E-06	6.6887989
cg07114810	chr12	7070679	-			-0.915602	-2.559174	-5.463292	2.22E-07	9.08E-06	6.6884772
cg22675604	chr15	97320595	-			-0.973489	0.3431468	-5.463262	2.22E-07	9.08E-06	6.6883442
cg19589277	chr12	119313382	+			-0.991211	-0.670697	-5.463097	2.22E-07	9.09E-06	6.6876171
cg00998387	chr8	65281572	-			1.5438852	-1.71812	5.4629466	2.22E-07	9.10E-06	6.6869561
cg08548396	chr6	30095549	+			2.4009561	-0.904677	5.4628982	2.22E-07	9.10E-06	6.6867431
cg21008145	chr8	49090318	+			-1.151953	0.9101561	-5.462877	2.22E-07	9.10E-06	6.6866483
cg22645961	chr8	2669372	-			-1.079809	0.071485	-5.462876	2.22E-07	9.10E-06	6.6866461
cg23010048	chr19	52391257	+	ZNF577;ZNF577;ZNF577	TSS200;TSS200;TSS200	1.599972	-0.112512	5.4625051	2.23E-07	9.11E-06	6.6850141

cg01715572	chr2	232093057	-	ARMC9	Body	-1.182652	0.307183	-5.462473	2.23E-07	9.11E-06	6.6848728
cg00167670	chr4	13922944	-			-1.160634	-1.028481	-5.462376	2.23E-07	9.11E-06	6.6844469
cg15979173	chr1	91182128	+	BARHL2	1stExon	1.3804378	0.1896461	5.4642734	2.23E-07	9.13E-06	6.701323
cg04177826	chr22	50985681	+	KLHDC7B	TSS1500	0.8698245	-0.487283	5.4617601	2.24E-07	9.14E-06	6.6817365
cg21528204	chr6	100050506	-			1.7325675	-1.400872	5.4708876	2.24E-07	9.14E-06	6.6865263
cg15448726	chr12	53273497	+			-0.728251	-2.810947	-5.461673	2.24E-07	9.14E-06	6.6813525
cg08311885	chr10	16032954	-			-1.168549	-0.275416	-5.461599	2.24E-07	9.14E-06	6.6810285
cg11666040	chr10	135012454	+	KNDC1	Body	-2.366325	1.528209	-5.463683	2.24E-07	9.15E-06	6.6987328
cg20530855	chr10	125755151	+			-1.109414	-3.116523	-5.463604	2.24E-07	9.15E-06	6.6809406
cg13902101	chr15	101738453	+	CHSY1	Body	0.7137216	2.3562098	5.4612175	2.24E-07	9.16E-06	6.67935
cg25855592	chr4	1496293	+			-1.542701	0.2095434	-5.463371	2.24E-07	9.16E-06	6.6799186
cg10724054	chr17	3904396	+			-1.1104	0.0130362	-5.463345	2.24E-07	9.16E-06	6.679803
cg24281267	chr6	35479648	+	TULP1	Body	1.6849149	0.2198005	5.4609976	2.24E-07	9.16E-06	6.6783826
cg06083483	chr17	32646006	-	CCL8	TSS200	-1.298102	-1.306662	-5.460919	2.24E-07	9.16E-06	6.6780372
cg16822191	chr20	62046387	-	KCNQ2;KCNQ2;KCNQ2;KCNQ2	Body;Body;Body;Body	-1.777034	1.6928146	-5.460924	2.24E-07	9.16E-06	6.6780583
cg13379558	chr19	14797552	+			-1.159866	-0.54874	-5.460634	2.25E-07	9.18E-06	6.6767819
cg25936482	chr5	15928686	-	FBXL7	Body	-1.807055	0.6417951	-5.46041	2.25E-07	9.18E-06	6.6757989
cg19375537	chr1	46951589	-			1.889468	-1.134158	5.4603469	2.25E-07	9.19E-06	6.6755208
cg01946364	chr5	175296158	-	CPLX2	5'UTR	-1.201624	0.4922056	-5.460302	2.25E-07	9.19E-06	6.6753242
cg24365766	chr4	82367672	-	RASGEF1B	Body	1.0591309	1.2218206	5.4601956	2.25E-07	9.19E-06	6.6748554

cg15422685	chr3	62356774	-	FEZF2	Body	1.0773531	-0.101129	5.4624012	2.25E-07	9.19E-06	6.6760294
cg20467957	chr11	31845529	-			1.0711576	-0.43146	5.4600994	2.25E-07	9.19E-06	6.6744322
cg24416513	chr2	176994764	-	HOXD8;HOXD8	5'UTR;1stExon	2.8587385	-0.790837	5.4644637	2.25E-07	9.20E-06	6.6764435
cg04544908	chr5	178833490	+			-1.102635	-0.025702	-5.459905	2.25E-07	9.20E-06	6.6735772
cg00732878	chr2	2654357	+			-1.16081	-0.066853	-5.459696	2.26E-07	9.21E-06	6.6726582
cg00361898	chr15	25488753	-	SNORD115-40	TSS200	-1.268206	0.4251449	-5.459459	2.26E-07	9.22E-06	6.671614
cg06379721	chr10	102590401	+			1.1271041	-1.111158	5.4593368	2.26E-07	9.22E-06	6.6710785
cg14994183	chr3	12066807	+	SYN2;SYN2	Body;Body	-1.076372	-1.663522	-5.459329	2.26E-07	9.22E-06	6.6710452
cg12582728	chr17	36162433	+			-1.582655	0.015917	-5.459111	2.26E-07	9.23E-06	6.670084
cg04447362	chr2	237654635	+			-1.193161	-0.926159	-5.458999	2.26E-07	9.23E-06	6.6695911
cg14006181	chr14	29254942	+	C14orf23	Body	1.5091245	-0.40785	5.4609053	2.27E-07	9.25E-06	6.6690869
cg19738404	chr2	127644612	+			-0.870155	-0.451964	-5.458583	2.27E-07	9.25E-06	6.6677633
cg21069434	chr2	2192072	+	MYT1L	5'UTR	-1.303546	-0.365469	-5.45858	2.27E-07	9.25E-06	6.6677527
cg19403534	chr10	120355419	-	PRLHR	TSS1500	1.2220626	-0.260423	5.4584638	2.27E-07	9.25E-06	6.6672397
cg19813025	chr5	5139643	+	ADAMTS16	TSS1500	0.9942454	-0.775821	5.4583085	2.27E-07	9.26E-06	6.6665568
cg16711450	chr11	2183505	-	INS;INS-IGF2;INS-IGF2	TSS1500;TSS1500;TSS1500	-1.029226	-0.144517	-5.458262	2.27E-07	9.26E-06	6.6663518
cg06143897	chr10	112404754	-	RBM20	Body	1.2412916	-0.693959	5.4579814	2.27E-07	9.27E-06	6.6651187
cg16122269	chr8	49170175	-			-1.706122	0.4851775	-5.457752	2.28E-07	9.28E-06	6.6641093
cg01836861	chr6	46094735	+			0.8224639	2.6859671	5.4576217	2.28E-07	9.28E-06	6.6635371
cg13120756	chr17	42386950	+	RUNDC3A;RUNDC3A;RUNDC3A	Body;Body;Body	-1.162868	0.1787476	-5.4576	2.28E-07	9.28E-06	6.6634434

cg13458609	chr9	130608923	-	ENG;ENG	Body;Body	1.3121218	0.5946233	5.4575832	2.28E-07	9.28E-06	6.6633677
cg21214467	chr19	55894758	-			-0.802423	-2.119787	-5.457354	2.28E-07	9.29E-06	6.6623595
cg05940231	chr1	119532189	-	TBX15	TSS200	1.3979283	-0.560382	5.4571417	2.28E-07	9.30E-06	6.6614266
cg03962359	chr6	154166263	-			-1.36585	0.0431043	-5.457117	2.28E-07	9.30E-06	6.6613195
cg06475633	chr17	3805065	+	P2RX1	Body	-2.051233	2.3084996	-5.456964	2.29E-07	9.31E-06	6.660645
cg24020826	chr8	140630180	-	KCNK9	3'UTR	-1.465192	-0.292498	-5.456948	2.29E-07	9.31E-06	6.6605767
cg22717014	chr4	111542473	-	PITX2;PITX2;PITX2	Body;Body;Body	2.1450011	-1.122018	5.456809	2.29E-07	9.31E-06	6.6599641
cg10371050	chr10	133055449	-	TCERG1L	Body	-1.354918	0.9390138	-5.456786	2.29E-07	9.31E-06	6.6598639
cg04701034	chr14	52536175	-	NID2	TSS1500	2.3719614	-0.630736	5.4588642	2.29E-07	9.32E-06	6.6601229
cg10998242	chr3	53530847	+	CACNA1D;CACNA1D;CACNA1D	Body;Body;Body	0.7265952	2.3866975	5.4565118	2.29E-07	9.32E-06	6.6586572
cg26990587	chr7	100253901	-	ACTL6B	Body	1.9569307	-1.120866	5.4564402	2.29E-07	9.32E-06	6.6583424
cg21120678	chr7	140103973	+	RAB19;RAB19	1stExon;5'UTR	-0.983218	-3.232891	-5.456294	2.29E-07	9.33E-06	6.6577001
cg14082075	chr9	135644622	+	C9orf98	Body	-1.043482	-3.441962	-5.456167	2.29E-07	9.33E-06	6.65714
cg07846297	chr4	174439990	-			1.1681156	-1.292088	5.4561406	2.29E-07	9.33E-06	6.6570254
cg04284535	chr17	41624145	-	ETV4;ETV4	TSS1500;TSS1500	-0.932996	-2.4989	-5.456025	2.29E-07	9.34E-06	6.6565155
cg01120173	chr17	5019669	+	ZNF232	5'UTR	1.3381803	-0.387578	5.4558535	2.30E-07	9.34E-06	6.6557636
cg14910227	chr14	101500125	-	MIR495	Body	-1.419066	-0.02929	-5.455834	2.30E-07	9.34E-06	6.655676
cg23037411	chr4	178808604	+	LOC285501	Body	-1.450219	0.4462179	-5.45583	2.30E-07	9.34E-06	6.6556599
cg00798876	chr7	2670978	-	TTYH3	TSS1500	-0.727528	-1.650906	-5.455786	2.30E-07	9.34E-06	6.6554676
cg01806921	chr22	28011160	+			-0.912486	-0.919301	-5.455778	2.30E-07	9.34E-06	6.6554325

cg16342842	chr10	18270704	-	SLC39A12;SLC39A12	Body;Body	-1.368216	0.7084708	-5.455699	2.30E-07	9.35E-06	6.6550856
cg20127859	chr8	144656271	+	C8orf73	TSS1500	-1.053815	-0.612849	-5.455523	2.30E-07	9.35E-06	6.6543097
cg01302656	chr14	29254853	+	C14orf23	Body	1.220494	-0.536512	5.4554963	2.30E-07	9.35E-06	6.6541932
cg13319042	chr5	151067848	+	SPARC	TSS1500	-1.05451	-0.707764	-5.455419	2.30E-07	9.36E-06	6.6538547
cg12564097	chr19	40732762	+	CNTD2	TSS200	-1.12547	-2.401243	-5.45535	2.30E-07	9.36E-06	6.6535509
cg15588266	chr20	43538539	+	PABPC1L	TSS200	-0.525757	-3.40254	-5.455079	2.30E-07	9.37E-06	6.6523608
cg23508908	chr6	84771783	+	MRAP2	Body	-1.260859	0.5498989	-5.455016	2.31E-07	9.37E-06	6.6520816
cg09560465	chr7	33923628	+			-1.735992	0.9386885	-5.454868	2.31E-07	9.38E-06	6.6514299
cg04681600	chr14	38725536	+	CLEC14A;CLEC14A	1stExon;5'UTR	1.1876978	-0.934117	5.4547139	2.31E-07	9.38E-06	6.6507543
cg21420622	chr7	105162941	-	PUS7	TSS1500	-0.395985	-3.71054	-5.454671	2.31E-07	9.38E-06	6.6505678
cg11868461	chr17	75830800	+			-1.675089	-2.15566	-5.454593	2.31E-07	9.38E-06	6.6502209
cg23023970	chr10	134385698	-	INPP5A	Body	-1.160962	-2.164741	-5.454604	2.31E-07	9.38E-06	6.6502716
cg04552921	chr10	80262264	-			-1.31121	0.7634087	-5.45456	2.31E-07	9.38E-06	6.6500793
cg10566776	chr20	37074185	+	SNHG11	TSS1500	-1.697011	-0.015149	-5.454503	2.31E-07	9.39E-06	6.6498293
cg03208936	chr1	98515087	-			2.1222726	-2.386917	5.4566386	2.31E-07	9.39E-06	6.6507226
cg13663303	chr5	35046992	+	AGXT2	Body	-1.732144	-0.174768	-5.4544	2.31E-07	9.39E-06	6.6493726
cg09419102	chr11	65550444	-			-0.620453	-0.815466	-5.454323	2.31E-07	9.39E-06	6.649037
cg14604066	chr9	139590572	+			-1.14494	0.1920213	-5.454328	2.31E-07	9.39E-06	6.6490582
cg08195247	chr2	176933286	-			1.6487896	-1.098249	5.4542847	2.31E-07	9.39E-06	6.6488679
cg00305193	chr1	26158583	-	FAM54B;FAM54B;FAM54B;FAM54B	3'UTR;3'UTR;3'UTR;3'UTR	0.9359543	0.2986938	5.4542544	2.31E-07	9.39E-06	6.6487348

cg22665383	chr11	55339961	-	OR4C16	1stExon	-1.557505	0.1480954	-5.456477	2.31E-07	9.39E-06	6.6646996
cg17319956	chr12	115671392	-			-1.54806	0.6881629	-5.454097	2.32E-07	9.40E-06	6.6480427
cg14582763	chr19	52956683	-	ZNF578	TSS200	1.5194651	-0.628876	5.4540086	2.32E-07	9.40E-06	6.6476544
cg15199678	chr7	96642510	-	DLX6AS	Body	1.3468749	-0.926031	5.456249	2.32E-07	9.40E-06	6.6490123
cg12453631	chr3	157812226	+			1.075817	-0.18308	5.4537906	2.32E-07	9.41E-06	6.6466966
cg03599357	chr18	76737392	-			1.1729672	-2.013701	5.4535652	2.32E-07	9.41E-06	6.6457059
cg05846851	chr16	10172054	+	GRIN2A;GRIN2A;GRIN2A	Body;Body;Body	-1.552453	-0.572117	-5.453555	2.32E-07	9.41E-06	6.6456631
cg12936335	chr6	150286212	-	ULBP1	Body	1.404387	-1.715649	5.4535513	2.32E-07	9.41E-06	6.6456449
cg02696067	chr6	30850977	-	DDR1;DDR1	TSS1500;TSS1500	-1.313075	-1.217355	-5.453482	2.32E-07	9.42E-06	6.6453411
cg18699337	chr17	44159144	+	KIAA1267	Body	0.8244503	1.3360602	5.4534688	2.32E-07	9.42E-06	6.6452821
cg07235577	chr8	51039471	+	SNTG1	5'UTR	-1.565124	-0.874241	-5.453427	2.32E-07	9.42E-06	6.6450973
cg09469566	chr13	112717244	-			1.4173133	-0.474005	5.4555963	2.32E-07	9.42E-06	6.6461473
cg23432345	chr7	27196153	+	HOXA7	1stExon	2.3479658	-1.884968	5.4533909	2.32E-07	9.42E-06	6.6449398
cg24161652	chr6	50813859	-	TFAP2B	3'UTR	1.4185161	-1.426367	5.4533645	2.32E-07	9.42E-06	6.6448239
cg07482372	chr11	2951201	+	PHLDA2	TSS1500	-0.970411	-2.570893	-5.453261	2.32E-07	9.42E-06	6.6443695
cg06639053	chr12	111837470	-			-1.571436	-0.725844	-5.453209	2.32E-07	9.42E-06	6.6441394
cg16693870	chr14	96752231	-	ATG2B	Body	0.8561646	2.7315198	5.4576233	2.33E-07	9.43E-06	6.6464408
cg04853995	chr12	112550460	+			-1.051063	-0.221277	-5.453069	2.33E-07	9.43E-06	6.6435253
cg22026610	chr12	54694925	+	NFE2	TSS200	-0.583459	-4.16202	-5.452769	2.33E-07	9.44E-06	6.6422068
cg06798887	chr16	17898759	-			-1.440847	0.5233449	-5.452493	2.33E-07	9.45E-06	6.6409954

cg17152757	chr3	172166258	-	GHSR;GHSR	TSS200;TSS200	1.4458389	-1.401179	5.4524048	2.33E-07	9.45E-06	6.6406065
cg18867480	chr11	27744816	+	BDNF	TSS1500	1.6509319	-1.482521	5.4546214	2.33E-07	9.45E-06	6.6418681
cg10643691	chr8	95246514	+			2.2746752	-0.587917	5.4546007	2.33E-07	9.45E-06	6.6417771
cg14934413	chr11	20184162	+			1.100592	-0.829335	5.4523414	2.33E-07	9.45E-06	6.6403282
cg02665650	chr6	34984838	-	ANKS1A	Body	1.5666215	0.5428257	5.452297	2.33E-07	9.45E-06	6.6401331
cg12990639	chr2	119471314	-			-1.739353	1.0438164	-5.452264	2.34E-07	9.45E-06	6.6399873
cg24148361	chr17	12388639	-			-0.977788	-0.836824	-5.452281	2.33E-07	9.45E-06	6.6400641
cg14532497	chr4	174439756	-			0.7458553	-0.311835	5.4522295	2.34E-07	9.45E-06	6.6398363
cg01022219	chr18	13641735	-	C18orf1;C18orf1;C18orf1;C18orf1;C18orf1;C18orf1	Body;Body;Body;Body;Body;Body	1.5990685	0.4663627	5.4544169	2.34E-07	9.46E-06	6.6409704
cg00009553	chr16	61854084	+	CDH8	Body	-1.519137	0.470769	-5.452035	2.34E-07	9.46E-06	6.63898
cg00541544	chr22	39150255	+	UNC84B	5'UTR	-1.052778	-2.611073	-5.452025	2.34E-07	9.46E-06	6.6389388
cg01263222	chr16	83932332	-	MLYCD	TSS1500	-0.578366	-3.614015	-5.452058	2.34E-07	9.46E-06	6.6390827
cg07563944	chr16	89038263	+	CBFA2T3	Body	-0.858667	-0.67011	-5.451998	2.34E-07	9.46E-06	6.6388176
cg07716249	chr2	133428422	-	LYPD1;LYPD1;LYPD1	1stExon;5'UTR;5'UTR	-0.588472	-2.209708	-5.454101	2.34E-07	9.46E-06	6.6395837
cg12127811	chr10	92079600	+			-1.175282	-0.305267	-5.451577	2.34E-07	9.48E-06	6.636969
cg02571055	chr6	158896790	+	TULP4;TULP4	Body;Body	-1.710399	0.7291864	-5.451416	2.34E-07	9.48E-06	6.6362628
cg24584161	chr15	40452931	-	BUB1B	TSS1500	-1.192571	0.62861	-5.451216	2.35E-07	9.49E-06	6.6353852
cg02543636	chr7	30810183	+	FAM188B	TSS1500	-1.702584	0.5897141	-5.451176	2.35E-07	9.49E-06	6.6352055
cg23867673	chr10	73404170	+	CDH23;CDH23	Body;Body	-1.159261	-1.001643	-5.45117	2.35E-07	9.49E-06	6.6351809
cg00610000	chr2	58272914	+	VRK2;VRK2;VRK2;VRK2;VRK2;VRK2	TSS1500;TSS1500;TSS1500;TSS1500;TSS1500;TSS1500	-1.570476	1.1797023	-5.451006	2.35E-07	9.50E-06	6.6344597

cg11452391	chr5	16180315	-	11-Mar	TSS1500	1.9164272	-1.768284	5.4509894	2.35E-07	9.50E-06	6.6343877
cg27005118	chr17	13972210	-	COX10	TSS1500	-1.03137	-1.050391	-5.45102	2.35E-07	9.50E-06	6.6345228
cg00592781	chr12	66122874	+			1.5225694	-0.368929	5.453158	2.35E-07	9.50E-06	6.6354458
cg03330223	chr16	86371363	-	LOC732275	Body	-1.264218	0.4719544	-5.450882	2.35E-07	9.50E-06	6.6339164
cg04483701	chr16	86253703	-			-1.025324	-0.582012	-5.450872	2.35E-07	9.50E-06	6.6338717
cg00779206	chr12	2914519	-	FKBP4	3'UTR	0.8895807	0.7942955	5.4507077	2.35E-07	9.50E-06	6.6331503
cg08640634	chr6	108486450	-	NR2E1	TSS1500	2.1657064	-0.770081	5.4506621	2.35E-07	9.51E-06	6.6329499
cg20713968	chr8	1737564	-			1.3134086	0.6979046	5.4506818	2.35E-07	9.51E-06	6.6330363
cg21774338	chr1	50884242	-	DMRTA2	3'UTR	1.4838501	-0.423107	5.4528151	2.35E-07	9.51E-06	6.633941
cg14561217	chr1	210425254	-			-1.449875	-5.901851	-5.457205	2.35E-07	9.51E-06	6.6525405
cg01159576	chr9	129386206	+	LMX1B	Body	1.2426931	-1.192783	5.4503265	2.36E-07	9.52E-06	6.6314754
cg13200838	chr1	91194708	+			1.5259866	-1.472932	5.4502121	2.36E-07	9.52E-06	6.6309731
cg21814290	chr8	10466918	-	RP1L1	Body	-0.921566	0.2934641	-5.450065	2.36E-07	9.53E-06	6.6303248
cg11926444	chr3	119137289	+	CDGAP	3'UTR	1.2705635	3.2151919	5.4499374	2.36E-07	9.53E-06	6.6297661
cg00011861	chr14	103607265	+			-0.713671	-2.400745	-5.449835	2.36E-07	9.54E-06	6.6293151
cg07395354	chr7	15727127	+	MEOX2	TSS1500	1.7476483	-0.495084	5.4497895	2.36E-07	9.54E-06	6.6291165
cg02580969	chr6	28483341	+	GPX6	Body	-1.159777	-0.257716	-5.449652	2.36E-07	9.54E-06	6.6285136
cg02743222	chr5	170736335	+	TLX3;TLX3	5'UTR;1stExon	1.6570116	-0.918804	5.4496505	2.36E-07	9.54E-06	6.6285058
cg02788731	chr1	247751498	+	OR2G2	TSS200	-1.554296	0.4708209	-5.449577	2.36E-07	9.54E-06	6.6281816
cg08474786	chr17	17491465	+	PEMT	Body	0.8170915	1.5221726	5.4494366	2.37E-07	9.55E-06	6.6275662

cg06931523	chr8	854757	-			-1.11247	0.2500092	-5.44923	2.37E-07	9.56E-06	6.6266578
cg00575674	chr1	61314297	-			-0.974627	-2.563503	-5.449158	2.37E-07	9.56E-06	6.6263429
cg09312809	chr2	233878004	+	NGEF	TSS200	-1.009893	-0.147728	-5.449049	2.37E-07	9.56E-06	6.6258618
cg04959480	chr4	154711675	-	SFRP2	TSS1500	1.0335963	0.6755251	5.4489341	2.37E-07	9.57E-06	6.6253593
cg20844619	chr4	152875860	-			-1.372298	0.3461378	-5.451131	2.37E-07	9.57E-06	6.6265509
cg02631468	chr20	25062777	-	VSX1;VSX1	TSS200;TSS200	1.9922448	-1.470216	5.4488413	2.37E-07	9.57E-06	6.6249516
cg13823791	chr17	17184990	-	COPS3	TSS1500	-1.108444	-3.424847	-5.448675	2.37E-07	9.58E-06	6.6242202
cg00436476	chr6	26240505	+	HIST1H4F	TSS200	2.3754746	-2.157172	5.4485663	2.38E-07	9.58E-06	6.6237437
cg02773403	chr10	62053458	+	ANK3	Body	0.9234081	1.5568827	5.4485832	2.37E-07	9.58E-06	6.623818
cg03164800	chr4	80836552	-	ANTXR2	Body	0.9410095	1.6346793	5.4484849	2.38E-07	9.58E-06	6.623386
cg10257302	chr14	55907427	+	TBPL2	TSS200	1.4632846	0.7651651	5.4484412	2.38E-07	9.58E-06	6.6231944
cg11355029	chr19	6276660	-	MLLT1	Body	0.7853899	0.4399081	5.4483532	2.38E-07	9.58E-06	6.6228079
cg05481257	chr2	20870211	-	GDF7	Body	0.9858426	0.1006059	5.4483255	2.38E-07	9.59E-06	6.622686
cg21669326	chr7	47267691	-			-1.401889	-1.508573	-5.448262	2.38E-07	9.59E-06	6.6224082
cg11740103	chr7	150020945	+	ACTR3C;LRRC61;LRRC61	TSS200;5'UTR;5'UTR	-0.97708	-2.48802	-5.44818	2.38E-07	9.59E-06	6.6220453
cg15978017	chr13	29042758	+	FLT1;FLT1;FLT1;FLT1	Body;Body;Body;Body	-1.070139	-0.120136	-5.448196	2.38E-07	9.59E-06	6.6221178
cg23672425	chr1	11249037	-	MTOR;ANGPTL7	Body;TSS1500	0.6842008	2.8894822	5.4482017	2.38E-07	9.59E-06	6.6221424
cg14258250	chr9	139255778	-			0.7925471	1.3695303	5.4481018	2.38E-07	9.59E-06	6.6217034
cg20288916	chr11	56545039	-			-1.251562	-0.098143	-5.447941	2.38E-07	9.60E-06	6.6209978
cg13826890	chr14	57275282	-	OTX2	5'UTR	1.9364589	-0.987054	5.450125	2.38E-07	9.60E-06	6.6368672

cg27599015	chr20	50175489	+	NFATC2	Body	-1.729138	0.5521928	-5.447914	2.38E-07	9.60E-06	6.6208804
cg07241457	chr20	61787786	-			-1.530228	0.2228612	-5.447808	2.38E-07	9.60E-06	6.6204148
cg08378505	chr17	73127297	+	NT5C	Body	-0.758817	-4.518869	-5.45008	2.38E-07	9.60E-06	6.6219399
cg13832669	chr2	119609429	-			2.2934907	-1.55055	5.4691762	2.38E-07	9.60E-06	6.6327163
cg14720274	chr15	61014131	-	RORA	Body	-0.980149	-0.432814	-5.447761	2.38E-07	9.60E-06	6.6202047
cg26024424	chr12	34552411	+			-1.569282	-0.945794	-5.447778	2.38E-07	9.60E-06	6.6202817
cg27245185	chr12	99257038	+	ANKS1B;ANKS1B;ANKS1B	Body;Body;Body	-1.58383	0.8832463	-5.450027	2.38E-07	9.60E-06	6.6217069
cg00996827	chr1	2838805	+			-1.211101	-0.160594	-5.449926	2.38E-07	9.60E-06	6.6359935
cg04052038	chr21	31588123	+	CLDN8	1stExon	-1.993275	0.9936879	-5.449943	2.38E-07	9.60E-06	6.6360683
cg19794939	chr11	27494405	-	LGR4	TSS200	-0.452989	-3.753513	-5.447629	2.39E-07	9.60E-06	6.6196272
cg24453118	chr13	47229927	+	LRCH1;LRCH1;LRCH1	Body;Body;Body	1.3977867	-0.405402	5.4470966	2.39E-07	9.62E-06	6.6172889
cg00303378	chr1	159825552	-	VSIG8;C1orf204	Body;TSS1500	0.6170631	1.5778741	5.4492463	2.39E-07	9.63E-06	6.6179103
cg07142723	chr8	105431269	-	DPYS	Body	-0.922183	-1.496022	-5.446957	2.39E-07	9.63E-06	6.6166759
cg17282466	chr4	69046856	-	LOC100130017	TSS1500	-1.335706	0.4386871	-5.446933	2.39E-07	9.63E-06	6.6165706
cg08226379	chr4	111560807	+			0.9090757	-1.029411	5.4467418	2.40E-07	9.64E-06	6.6157309
cg01209909	chr20	55500189	+			1.0314219	-1.645399	5.4467144	2.40E-07	9.64E-06	6.6156108
cg06226384	chr17	64873351	-	CACNG5;CACNG5	TSS200;TSS200	-1.112774	-0.871154	-5.446648	2.40E-07	9.64E-06	6.6153205
cg23493167	chr2	219920195	+	IHH	Body	-1.082761	0.9548396	-5.446546	2.40E-07	9.64E-06	6.6148698
cg12620005	chr1	51779655	+	TTC39A;TTC39A	Body;Body	-1.223561	0.3387137	-5.446488	2.40E-07	9.64E-06	6.6146152
cg14210971	chr11	3014102	+	NAP1L4	TSS1500	-0.444026	-4.176427	-5.446363	2.40E-07	9.65E-06	6.614068

cg07721777	chr10	130201251	-			-1.497283	0.6004449	-5.446288	2.40E-07	9.65E-06	6.6137372
cg04519775	chr2	231849693	-			-1.032488	-0.394687	-5.446184	2.40E-07	9.65E-06	6.6132828
cg26712428	chr10	105218771	+	CALHM1	TSS200	-0.980972	-0.7308	-5.446161	2.40E-07	9.65E-06	6.613182
cg07844518	chr16	85387556	+			-0.823453	-0.332928	-5.446047	2.40E-07	9.66E-06	6.6126789
cg18956562	chr7	144149304	-	TPK1;TPK1	3'UTR;3'UTR	-1.247517	0.0110968	-5.44601	2.40E-07	9.66E-06	6.6125193
cg25331127	chr10	133608464	+			-1.369258	1.1746816	-5.445895	2.40E-07	9.66E-06	6.6120125
cg01417062	chr10	63246532	+			-1.505	0.5126943	-5.445822	2.41E-07	9.66E-06	6.6116914
cg08123701	chr1	57955106	-	DAB1	5'UTR	-1.508715	-0.627244	-5.445836	2.40E-07	9.66E-06	6.6117556
cg21033632	chr5	64486421	+	ADAMTS6	Body	-1.441552	0.6111062	-5.445808	2.41E-07	9.66E-06	6.6116309
cg06121469	chr15	44956098	-	SPG11;SPG11	TSS1500;TSS1500	-0.759702	-4.14797	-5.445697	2.41E-07	9.67E-06	6.6111454
cg17050984	chr5	14397222	+	TRIO	Body	0.6519115	2.4459314	5.4456941	2.41E-07	9.67E-06	6.6111308
cg14539856	chr10	118187002	-	PNLIPRP3	TSS1500	-1.18345	-0.483279	-5.445662	2.41E-07	9.67E-06	6.61099
cg20366906	chr13	53422381	+	PCDH8;PCDH8	1stExon;1stExon	0.9675284	-0.418692	5.4456485	2.41E-07	9.67E-06	6.6109305
cg12881901	chr8	2130812	+			-1.106575	0.1375922	-5.445536	2.41E-07	9.67E-06	6.6104356
cg08555653	chr3	138659021	-			1.5846743	-0.498353	5.4452999	2.41E-07	9.68E-06	6.6094
cg18093866	chr6	30851753	-	DDR1;DDR1	TSS200;TSS1500	-0.797682	-1.750825	-5.445219	2.41E-07	9.68E-06	6.6090429
cg11159417	chr5	148822491	+			0.8288034	1.5603432	5.4451269	2.41E-07	9.69E-06	6.6086402
cg17546817	chr5	57881537	+	RAB3C	Body	-1.172608	0.8157315	-5.445063	2.41E-07	9.69E-06	6.608359
cg14793844	chr2	100516880	-	AFF3;AFF3	Body;Body	-1.551034	0.1399415	-5.444896	2.42E-07	9.70E-06	6.6076253
cg16123202	chr7	1287145	+			1.5807599	-0.541401	5.4448429	2.42E-07	9.70E-06	6.6073933

cg22851944	chr6	101847388	-	GRIK2;GRIK2;GRIK2	Body;Body;Body	1.5444514	-1.776043	5.444711	2.42E-07	9.70E-06	6.6068143
cg09319072	chr18	44775413	+			0.8447996	-0.364281	5.4446661	2.42E-07	9.70E-06	6.6066173
cg08486875	chr19	23386892	-			0.943176	-0.579698	5.4446386	2.42E-07	9.70E-06	6.6064963
cg00975971	chr5	14744206	-	ANKH	Body	-1.413138	0.2447067	-5.444513	2.42E-07	9.71E-06	6.6059431
cg27219221	chr20	30585327	-	XKR7	3'UTR	-1.267535	1.7208851	-5.444394	2.42E-07	9.71E-06	6.6054213
cg12180551	chr1	56975583	+	PPAP2B;PPAP2B	Body;Body	1.0810371	1.1424468	5.444322	2.42E-07	9.72E-06	6.6051068
cg06506966	chr19	53758289	+	ZNF677	TSS200	2.1761716	-1.313316	5.4440729	2.42E-07	9.73E-06	6.6040133
cg27280332	chr12	77378563	+			-1.293153	0.5109324	-5.444004	2.43E-07	9.73E-06	6.6037099
cg03186999	chr17	7165420	+	CLDN7;CLDN7	5'UTR;1stExon	-0.633417	-2.046991	-5.443554	2.43E-07	9.75E-06	6.6017359
cg04235146	chr12	110718311	+	ATP2A2;ATP2A2;ATP2A2	TSS1500;TSS1500;TSS1500	-0.506357	-3.93101	-5.443563	2.43E-07	9.75E-06	6.6017766
cg19092981	chr22	19751654	+	TBX1;TBX1;TBX1	Body;Body;Body	1.5426398	1.4789183	5.4501682	2.43E-07	9.75E-06	6.6047043
cg19901523	chr14	107253273	+			-1.895975	-0.892769	-5.443253	2.43E-07	9.76E-06	6.6004148
cg22195627	chr19	12305869	+			1.926283	-0.248716	5.4431961	2.43E-07	9.76E-06	6.6001641
cg26507629	chr16	49632903	+	ZNF423	Body	-1.759621	0.7208	-5.443135	2.44E-07	9.76E-06	6.5998963
cg14865516	chr5	43557751	-	PAIP1;PAIP1;PAIP1	TSS1500;TSS1500;TSS1500	-0.664822	-4.204042	-5.442909	2.44E-07	9.77E-06	6.5989031
cg08539991	chr19	36203832	-	ZBTB32;ZBTB32	5'UTR;1stExon	-1.112213	-0.107045	-5.442821	2.44E-07	9.77E-06	6.5985182
cg22546168	chr10	135050326	+	VENTX	TSS1500	1.570505	-0.943685	5.4449932	2.44E-07	9.77E-06	6.5992583
cg16209310	chr14	96117286	-	TCL6	TSS1500	-1.579208	0.8221475	-5.442727	2.44E-07	9.78E-06	6.5981033
cg07694404	chr10	133572173	-			-1.145838	0.3361922	-5.442624	2.44E-07	9.78E-06	6.5976536
cg19453059	chr20	29955259	-	DEFB118	TSS1500	-1.319458	0.1299462	-5.442638	2.44E-07	9.78E-06	6.5977139

cg06084534	chr1	158725300	-	OR6K6	1stExon	-1.633025	0.4273543	-5.442278	2.44E-07	9.79E-06	6.5961324
cg09243507	chr16	6640880	+	A2BP1;A2BP1	5'UTR;5'UTR	-1.095803	-0.189526	-5.442261	2.44E-07	9.79E-06	6.5960616
cg01106926	chr4	13539255	+			1.3353334	-0.954041	5.4421717	2.45E-07	9.80E-06	6.5956682
cg06817669	chr15	77518882	-	C15orf5;SGK269	TSS1500;5'UTR	1.2019677	1.2970491	5.4442439	2.45E-07	9.80E-06	6.5959735
cg19924352	chr8	124195402	-	FAM83A;FAM83A	1stExon;1stExon	-1.336912	0.257982	-5.44193	2.45E-07	9.81E-06	6.5946076
cg06155641	chr9	138531512	+	GLT6D1	TSS200	-0.871236	-0.745138	-5.446354	2.45E-07	9.81E-06	6.6141913
cg13349346	chr13	28550189	+			1.0915515	-0.626279	5.441813	2.45E-07	9.81E-06	6.5940939
cg18948794	chr10	134894048	-			-0.820714	-0.619578	-5.441794	2.45E-07	9.81E-06	6.5940108
cg04952324	chr7	73727108	-	CLIP2;CLIP2	5'UTR;5'UTR	-1.200396	-0.074355	-5.441739	2.45E-07	9.81E-06	6.593768
cg24943405	chr6	105936926	-			1.1554209	1.5894827	5.4415753	2.45E-07	9.82E-06	6.5930504
cg16456576	chr16	86409251	-			-1.689992	0.7087959	-5.441547	2.45E-07	9.82E-06	6.5929256
cg22327427	chr8	95653287	-	ESRP1;ESRP1;ESRP1;ESRP1;ESRP1	TSS200;TSS200;TSS200;TSS200;TSS200	-0.379914	-3.68499	-5.441472	2.45E-07	9.82E-06	6.5925951
cg05670004	chr10	135259565	-			0.867664	0.5492735	5.4458398	2.45E-07	9.82E-06	6.5944267
cg13453847	chr5	5034274	-	LOC340094	TSS200	-1.475468	-1.020897	-5.441364	2.45E-07	9.82E-06	6.592125
cg20905680	chr6	32151723	-	AGER;AGER	Body;Body	0.6635413	1.8897262	5.4413079	2.46E-07	9.82E-06	6.591877
cg02304033	chr10	129346680	-	NPS	TSS1500	-1.367831	-0.239334	-5.441237	2.46E-07	9.83E-06	6.591564
cg26345619	chr12	2602405	-	CACNA1C;CACNA1C;CACNA1C;CACNA1C;CACNA1C;CACNA1C ;CACNA1C;CACNA1C;CACNA1C;CACNA1C;CACNA1C;CACNA1 C;CACNA1C;CACNA1C;CACNA1C;CACNA1C;CACNA1C;CACNA	Body;Body;Body;Body;Body;Body;Body;Body;Body; Body;Body;Body;Body;Body;Body;Body;Body;Body; Body;Body;Body;Body;Body	-1.972314	1.5800222	-5.441242	2.46E-07	9.83E-06	6.5915875
cg21096345	chr3	32432942	-	CMTM7;CMTM7	TSS1500;TSS1500	-0.885482	-2.564037	-5.441166	2.46E-07	9.83E-06	6.5912543
cg05516272	chr11	66311334	-	ZDHHC24	Body	1.0843709	0.5563595	5.4433204	2.46E-07	9.83E-06	6.5922992
cg06236559	chr18	45458303	+	SMAD2;SMAD2;SMAD2	TSS1500;TSS1500;TSS1500	-1.205765	-1.114833	-5.441024	2.46E-07	9.83E-06	6.590632

cg17627328	chr5	5146343	+	ADAMTS16	Body	-1.787054	0.3703784	-5.441018	2.46E-07	9.83E-06	6.5906028
cg10641258	chr21	43484279	-			-1.159794	-1.012954	-5.440971	2.46E-07	9.83E-06	6.5904006
cg01236166	chr16	59390195	+			-1.362047	-0.629027	-5.440857	2.46E-07	9.84E-06	6.5898968
cg19024599	chr2	109886730	-	SH3RF3	Body	-1.361502	-0.205434	-5.442688	2.46E-07	9.85E-06	6.5895261
cg12865837	chr6	100911526	+	SIM1;SIM1	1stExon;5'UTR	1.2654936	-0.071966	5.4403282	2.47E-07	9.86E-06	6.5875777
cg11081752	chr5	117380176	-			-1.374044	0.4901169	-5.440011	2.47E-07	9.87E-06	6.5861867
cg21682780	chr1	200211351	+			1.1143959	2.6994659	5.439671	2.47E-07	9.89E-06	6.5846944
cg06031011	chr6	35152001	+			-1.193392	0.7028891	-5.439546	2.48E-07	9.89E-06	6.584146
cg12975230	chr18	73167671	-			2.778329	-1.912484	5.4395566	2.48E-07	9.89E-06	6.5841922
cg14504259	chr9	970882	+			1.2941778	-0.17921	5.4393971	2.48E-07	9.90E-06	6.5834923
cg01203136	chr2	241922076	+			-1.011527	-0.700658	-5.439325	2.48E-07	9.90E-06	6.583175
cg03716984	chr10	54538505	-			1.469246	1.9220269	5.4393295	2.48E-07	9.90E-06	6.5831957
cg11707031	chr8	22602401	+	PEBP4	Body	-1.128405	0.584626	-5.439238	2.48E-07	9.90E-06	6.5827961
cg26261502	chr11	31846533	-			1.4380405	-1.16096	5.4386532	2.49E-07	9.93E-06	6.5802289
cg20821187	chr1	200452917	-			-1.405121	-0.219789	-5.438398	2.49E-07	9.94E-06	6.579111
cg21880020	chr6	168453194	+			-1.054874	0.8265707	-5.43834	2.49E-07	9.94E-06	6.5788545
cg09798888	chr12	108239646	+			2.0008768	0.0180204	5.43825	2.49E-07	9.94E-06	6.57846
cg14651616	chr11	64563992	-	MAP4K2	Body	0.7040601	1.2949775	5.438198	2.49E-07	9.94E-06	6.5782317
cg17207736	chr8	142237307	-	SLC45A4	Body	1.1478186	1.3796068	5.4381034	2.49E-07	9.95E-06	6.5778168
cg09667650	chr11	56956373	-	LRRC55	3'UTR	-1.503115	0.575819	-5.43801	2.49E-07	9.95E-06	6.5774052

cg22777724	chr17	46622516	-	HOXB2	TSS200	1.5737036	-0.167833	5.4379287	2.49E-07	9.95E-06	6.5770507
cg04461228	chr5	92906678	-	FLJ42709;FLJ42709;FLJ42709	Body;Body;TSS200	1.1093192	-0.708194	5.4397078	2.50E-07	9.97E-06	6.5937227
cg25825488	chr10	15761876	-	ITGA8	TSS200	2.1938055	-1.73925	5.4394969	2.50E-07	9.98E-06	6.5751684
cg17657898	chr3	31317297	-			-1.073431	-0.229932	-5.43726	2.50E-07	9.98E-06	6.5741191
cg10054857	chr18	61816543	+	C18orf20	TSS1500	-1.484094	0.8996688	-5.437205	2.50E-07	9.98E-06	6.5738766
cg00786305	chr2	176977220	-			1.3000361	-0.545326	5.436931	2.51E-07	9.99E-06	6.5726739
cg10166314	chr10	2006842	+			-1.083499	0.2485771	-5.436895	2.51E-07	1.00E-05	6.572517
cg03478122	chr2	66809241	-			1.2364445	-1.537325	5.4367915	2.51E-07	1.00E-05	6.5720622
cg04626878	chr20	62168809	+	PTK6	TSS200	-1.015665	-1.586433	-5.436739	2.51E-07	1.00E-05	6.5718336
cg04083776	chr20	56778717	+			-1.370795	0.2557731	-5.438894	2.51E-07	1.00E-05	6.5729031
cg19473529	chr20	48726783	+	UBE2V1;UBE2V1;UBE2V1;TMEM189-UBE2V1;UBE2V1	Body;Body;Body;Body;Body	1.5416227	-1.024146	5.4364785	2.51E-07	1.00E-05	6.5706891
cg27009439	chr10	131142880	+			-1.380199	0.0528735	-5.436312	2.51E-07	1.00E-05	6.5699605
cg07817608	chr12	76219528	-			-1.29581	0.1114187	-5.436269	2.51E-07	1.00E-05	6.5697686
cg23304647	chr7	2778058	-	GNA12	Body	0.8118723	0.7728103	5.4362772	2.51E-07	1.00E-05	6.5698064
cg15355015	chr7	101943990	-	SH2B2	Body	0.9066173	1.5005179	5.4358185	2.52E-07	1.00E-05	6.5677946
cg14826417	chr7	73244073	-	CLDN4	TSS1500	-0.862401	-1.270404	-5.435632	2.52E-07	1.00E-05	6.5669776
cg05967403	chr13	28367245	-	GSX1	Body	1.2232835	-0.206178	5.4398443	2.52E-07	1.01E-05	6.5681833
cg24618354	chr12	88535429	+	TMTC3;CEP290	TSS1500;5'UTR	-0.710587	-3.37698	-5.435386	2.52E-07	1.01E-05	6.5658973
cg04276938	chr5	40802085	-			-1.336681	1.2454307	-5.435352	2.52E-07	1.01E-05	6.5657477
cg14013695	chr7	27184176	-	HOXA5	TSS1500	1.7029445	0.2799127	5.4351688	2.53E-07	1.01E-05	6.5649454

cg10186456	chr16	2892912	+	TMPRSS8	TSS200	0.8446853	0.3313576	5.4351045	2.53E-07	1.01E-05	6.5646634
cg23533267	chr11	120942758	+	TBCEL;TBCEL	Body;Body	1.2528637	0.5156644	5.4350073	2.53E-07	1.01E-05	6.5642371
cg23250700	chr19	13056068	-	RAD23A	TSS1500	-0.94067	-0.174277	-5.434929	2.53E-07	1.01E-05	6.5638926
cg05917419	chr5	124013536	-	ZNF608	Body	1.1721447	-0.341517	5.4347532	2.53E-07	1.01E-05	6.563123
cg07696033	chr11	44326410	-	ALX4	Body	2.2545001	-2.800231	5.4346172	2.53E-07	1.01E-05	6.5625266
cg05127369	chr15	96876470	-	NR2F2;NR2F2;NR2F2;NR2F2;MIR1469	TSS200;Body;Body;5'UTR;TSS200	0.8184262	-2.881705	5.4367409	2.53E-07	1.01E-05	6.5634697
cg25984671	chr20	591083	+	TCF15	TSS200	2.3349154	-0.853847	5.4367058	2.53E-07	1.01E-05	6.5633157
cg01363324	chr9	124979353	-	LHX6;LHX6	Body;Body	0.9247538	1.1667053	5.4366605	2.53E-07	1.01E-05	6.5627422
cg18693704	chr16	66731313	+	CMTM4;CMTM4	TSS1500;TSS1500	-0.779405	-2.46233	-5.434451	2.53E-07	1.01E-05	6.5617965
cg05054531	chr12	132929737	+			-1.533421	-0.327958	-5.436541	2.54E-07	1.01E-05	6.5622169
cg09149672	chr1	158261151	+	CD1C	Body	-2.132728	0.9528282	-5.4343	2.54E-07	1.01E-05	6.5611375
cg06074123	chr4	56660460	+			0.9817132	2.2827812	5.4341635	2.54E-07	1.01E-05	6.5605371
cg19940644	chr1	12112386	+			-1.155799	-2.87714	-5.434085	2.54E-07	1.01E-05	6.5601939
cg13651690	chr14	106320748	+			-2.316819	2.215293	-5.433951	2.54E-07	1.01E-05	6.5596059
cg21346043	chr8	86351067	-	CA3;CA3	1stExon;5'UTR	1.6860787	-1.013116	5.4339663	2.54E-07	1.01E-05	6.5596722
cg13153698	chr8	860815	-			-1.56352	0.9459187	-5.433931	2.54E-07	1.01E-05	6.5595155
cg23095451	chr4	83437412	-	TMEM150C	5'UTR	-1.002422	-0.086466	-5.433842	2.54E-07	1.01E-05	6.5591275
cg06400301	chr1	248438569	-	OR2T33	TSS1500	-1.133447	-1.100263	-5.433521	2.54E-07	1.01E-05	6.5577201
cg11053663	chr11	58034457	-	OR10W1	1stExon	-1.403949	0.2589646	-5.4335	2.55E-07	1.01E-05	6.5576294
cg11416433	chr15	25429261	+	SNORD115-8	TSS200	-0.621518	-1.160562	-5.433548	2.54E-07	1.01E-05	6.5578383

cg15174117	chr15	68913662	-	CORO2B	Body	1.0906803	1.2371651	5.4335557	2.54E-07	1.01E-05	6.557872
cg19537070	chr5	161112334	+	GABRA6	TSS1500	-1.197292	-0.059405	-5.433492	2.55E-07	1.01E-05	6.5575922
cg09766710	chr1	247767976	+	OR2G3	TSS1500	-1.331111	-0.38647	-5.433461	2.55E-07	1.01E-05	6.5574557
cg15400655	chr4	107995	-	ZNF718	Body	1.1045302	-0.369571	5.4356054	2.55E-07	1.01E-05	6.5584963
cg18257955	chr15	25454202	-	SNORD115-15;SNORD115-21;SNORD115-22	Body;Body;TSS1500	-1.27643	0.3968579	-5.433263	2.55E-07	1.01E-05	6.5565868
cg05162202	chr1	2939117	+	ACTRT2	1stExon	-1.550993	1.5068941	-5.433128	2.55E-07	1.01E-05	6.5559984
cg06715628	chr12	52482292	-			-0.952416	-0.417338	-5.43307	2.55E-07	1.01E-05	6.5557437
cg00556419	chr1	210580909	+	HHAT;HHAT;HHAT;HHAT;HHAT;HHAT	Body;Body;Body;Body;Body;Body	-0.871329	-2.840996	-5.433027	2.55E-07	1.01E-05	6.5555557
cg25696972	chr6	1594827	+			-1.306853	0.4692581	-5.432959	2.55E-07	1.01E-05	6.5552567
cg24084836	chr14	77767735	-	POMT2	Body	-1.184283	-0.36596	-5.432654	2.56E-07	1.02E-05	6.5539194
cg24663419	chr2	58862141	-			-1.313387	-0.03079	-5.432616	2.56E-07	1.02E-05	6.5537535
cg04415176	chr2	176957842	+	HOXD13	1stExon	1.8528776	-0.553312	5.432225	2.56E-07	1.02E-05	6.5520382
cg11282657	chr17	79615101	-	TSPAN10	Body	1.1849918	-0.069182	5.4321078	2.56E-07	1.02E-05	6.5515241
cg11624345	chr19	44278551	+	KCNN4	Body	0.8528942	0.0545828	5.4320516	2.56E-07	1.02E-05	6.5512777
cg07457626	chr6	155347581	-			-1.577715	1.1677203	-5.432031	2.56E-07	1.02E-05	6.5511867
cg02226645	chr8	65291287	+	MIR124-2	TSS1500	1.5354487	-1.434486	5.4319607	2.56E-07	1.02E-05	6.5508795
cg16720405	chr3	122790178	-	PDIA5;PDIA5	Body;Body	0.8525997	1.5811676	5.4363058	2.56E-07	1.02E-05	6.5527035
cg21806580	chr5	18746010	-			-1.206301	-1.438001	-5.431735	2.57E-07	1.02E-05	6.5498912
cg07160014	chr11	47959926	+			1.068964	-0.07496	5.4315216	2.57E-07	1.02E-05	6.5489544
cg04041679	chr12	118537229	+	VSIG10	Body	-1.522284	0.3014461	-5.43145	2.57E-07	1.02E-05	6.5486414

cg11640773	chr16	58535416	-	NDRG4;NDRG4;NDRG4	Body;Body;Body	-1.157445	-1.871052	-5.431465	2.57E-07	1.02E-05	6.5487065
cg21865150	chr17	46804239	-	HOXB13	Body	1.971231	-0.644763	5.431364	2.57E-07	1.02E-05	6.5482636
cg04961235	chr7	4901628	+	PAPOLB;RADIL	TSS200;Body	1.0433413	0.4044698	5.4334895	2.57E-07	1.02E-05	6.5492307
cg05671386	chr16	3550239	+	CLUAP1	TSS1500	1.1835903	-0.705534	5.433481	2.57E-07	1.02E-05	6.5488178
cg01287975	chr7	97361241	-	TAC1;TAC1;TAC1;TAC1	TSS200;TSS200;TSS200;TSS200	1.7604855	-0.485527	5.4311833	2.57E-07	1.02E-05	6.5474717
cg18083704	chr3	9851847	+	TTLL3	TSS200	1.2736699	-5.110897	5.4311312	2.57E-07	1.02E-05	6.547243
cg01588826	chr19	36352459	+	KIRREL2;KIRREL2;KIRREL2	Body;Body;Body	1.045303	-0.385509	5.4309507	2.58E-07	1.02E-05	6.5464521
cg14012310	chr13	79181947	+			0.9479403	-0.309921	5.4331474	2.58E-07	1.02E-05	6.547733
cg20955507	chr6	30853258	+	DDR1;DDR1	5'UTR;5'UTR	-0.779559	-1.569482	-5.430869	2.58E-07	1.02E-05	6.546095
cg20168806	chr6	10382828	-			2.5543735	-1.408487	5.4330535	2.58E-07	1.02E-05	6.5473218
cg05297854	chr22	50623687	+	TRABD	TSS1500	3.0439925	-0.718318	5.4328026	2.58E-07	1.02E-05	6.5462235
cg19358493	chr10	119300942	-	EMX2;EMX2OS;EMX2	TSS1500;Body;TSS1500	1.4109165	-0.494949	5.4304373	2.58E-07	1.02E-05	6.5442019
cg06101576	chr7	150072968	+			-1.024804	1.4778503	-5.430211	2.58E-07	1.02E-05	6.5432106
cg01921126	chr11	2171026	-	IGF2;INS-IGF2;INS-IGF2	TSS200;Body;Body	-1.020024	-1.245488	-5.430139	2.58E-07	1.02E-05	6.5428926
cg23287992	chr6	31734390	+	C6orf27	Body	1.0116934	0.6713016	5.4299262	2.59E-07	1.03E-05	6.5419615
cg13362028	chr2	5689033	+			-1.188685	-0.95384	-5.429858	2.59E-07	1.03E-05	6.5416621
cg08499046	chr11	31824327	+	PAX6;PAX6;PAX6	Body;Body;Body	1.8605519	-0.424059	5.4297333	2.59E-07	1.03E-05	6.5411164
cg06516124	chr11	32450591	+	WT1;WT1;WT1;WT1	Body;Body;Body;Body	1.3456579	-1.382549	5.4295969	2.59E-07	1.03E-05	6.5405186
cg03011928	chr16	83023240	+	CDH13	Body	-1.276942	-0.295508	-5.429389	2.59E-07	1.03E-05	6.5396091
cg00702547	chr16	34214362	-			0.9065792	-0.187476	5.4292905	2.59E-07	1.03E-05	6.5391756

cg24765360	chr2	119611101	-			1.1667426	-0.934404	5.4291195	2.60E-07	1.03E-05	6.5384265
cg00786657	chr15	88515810	-	NTRK3;NTRK3	Body;Body	-1.609255	1.1292089	-5.428886	2.60E-07	1.03E-05	6.5374046
cg10991081	chr1	201960135	+	RNPEP	Body	0.5808203	2.4140712	5.4288816	2.60E-07	1.03E-05	6.537384
cg12157941	chr7	155567841	+	RBM33	3'UTR	1.1586049	3.8710932	5.4309364	2.60E-07	1.03E-05	6.5553761
cg03143118	chr6	42893093	-	PTCRA	Body	0.7623514	-0.190885	5.428523	2.60E-07	1.03E-05	6.5358126
cg08821747	chr4	39641752	+	C4orf34	TSS1500	-1.073049	-0.720009	-5.428416	2.61E-07	1.03E-05	6.5353438
cg01340991	chr6	33397233	-	SYNGAP1	Body	1.0208149	0.9555861	5.4282421	2.61E-07	1.03E-05	6.5345814
cg04047221	chr13	112723477	+	SOX1;SOX1	1stExon;3'UTR	1.6214225	-0.962224	5.4282651	2.61E-07	1.03E-05	6.5346825
cg06929724	chr13	97991122	+	MBNL2;MBNL2	Body;Body	0.7976307	1.2490289	5.4282446	2.61E-07	1.03E-05	6.5345927
cg12973118	chr2	223168941	+	CCDC140	Body	1.7333006	-1.84272	5.4370659	2.61E-07	1.03E-05	6.5383254
cg22689249	chr6	10555262	+	GCNT2;GCNT2	TSS1500;Body	0.8795346	1.4277333	5.4280554	2.61E-07	1.03E-05	6.5337633
cg02778609	chr16	23568302	+	UBFD1;EARS2;EARS2	TSS1500;Body;Body	-1.091851	-1.187106	-5.427919	2.61E-07	1.03E-05	6.5331665
cg06968788	chr22	30877907	+	SDC4P	TSS200	-1.128212	-0.12381	-5.427279	2.62E-07	1.04E-05	6.5303628
cg11932205	chr7	37888257	+	TXNDC3;TXNDC3	1stExon;5'UTR	-1.180112	0.4745356	-5.429495	2.62E-07	1.04E-05	6.5317465
cg14760020	chr3	170529777	-			-1.228982	-0.580035	-5.427297	2.62E-07	1.04E-05	6.5304422
cg14781394	chr19	16441457	+			-0.649167	-2.312792	-5.427318	2.62E-07	1.04E-05	6.5305342
cg22098115	chr2	131721768	-	ARHGEF4;ARHGEF4	Body;Body	2.3581747	-1.800785	5.4295212	2.62E-07	1.04E-05	6.5318597
cg26451345	chr6	47666248	+	GPR115	TSS200	-1.629868	0.341946	-5.427231	2.62E-07	1.04E-05	6.5301511
cg11697169	chr8	27457461	-	CLU;CLU;CLU	Body;Body;Body	0.9269049	1.3796229	5.4271502	2.62E-07	1.04E-05	6.5297971
cg25032793	chr18	76125627	+			-1.200638	0.2202242	-5.427136	2.62E-07	1.04E-05	6.5297346

cg20641545	chr12	89984390	-	ATP2B1;ATP2B1	3'UTR;3'UTR	0.8680125	2.1511748	5.4266987	2.63E-07	1.04E-05	6.5278194
cg05916059	chr16	26146070	-	HS3ST4	Body	-1.505092	0.0866776	-5.42659	2.63E-07	1.04E-05	6.5273422
cg25172145	chr22	22296625	-	PPM1F	Body	0.762495	2.4685785	5.4265703	2.63E-07	1.04E-05	6.5272566
cg26889659	chr6	684090	+	EXOC2	5'UTR	1.6823197	2.4262177	5.4285644	2.63E-07	1.04E-05	6.5272964
cg18220087	chr11	134783448	+			-0.751169	-0.434432	-5.426036	2.63E-07	1.04E-05	6.5249153
cg24624901	chr7	19184273	+			1.6598113	-1.281399	5.4260111	2.63E-07	1.04E-05	6.5248068
cg01994190	chr2	119768574	-			-0.926345	-1.283673	-5.425931	2.64E-07	1.04E-05	6.5244579
cg15769756	chr13	107489008	+			-1.305807	0.2014947	-5.432633	2.64E-07	1.04E-05	6.5280742
cg00196414	chr2	176947764	-	EVX2	Body	1.4864772	-1.811105	5.4258936	2.64E-07	1.04E-05	6.5242922
cg15652933	chr6	33141807	+	COL11A2;COL11A2;COL11A2	Body;Body;Body	-0.688014	-1.383267	-5.425812	2.64E-07	1.04E-05	6.5239327
cg08370178	chr5	43034043	-			0.9281898	0.6787059	5.4255766	2.64E-07	1.04E-05	6.5229034
cg03222834	chr16	13177060	+	SHISA9	Body	-0.945652	-1.027178	-5.425347	2.64E-07	1.04E-05	6.5218994
cg10103000	chr1	187609278	+			-1.644847	0.0593114	-5.425115	2.64E-07	1.04E-05	6.5208825
cg27343917	chr2	75751211	-	FAM176A;FAM176A	5'UTR;5'UTR	-1.032324	-0.507434	-5.425111	2.64E-07	1.04E-05	6.520866
cg10537708	chr10	134843768	+			-1.260081	-0.899596	-5.425074	2.65E-07	1.04E-05	6.5207014
cg01203286	chr2	46873926	-			1.0734004	0.442463	5.4250214	2.65E-07	1.04E-05	6.5204718
cg15567237	chr7	157277418	-			-1.051584	-0.151867	-5.427177	2.65E-07	1.05E-05	6.5212263
cg22022716	chr8	26276541	+			1.0671946	0.9223303	5.4249628	2.65E-07	1.05E-05	6.5202151
cg21225548	chr9	100864335	+	TRIM14;TRIM14;TRIM14;TRIM14	Body;Body;Body;Body	-1.32653	-1.611338	-5.424721	2.65E-07	1.05E-05	6.5191549
cg25656762	chr5	60927728	+			1.0797452	0.1906654	5.4245642	2.65E-07	1.05E-05	6.5184688

cg08151809	chr7	151480352	+	PRKAG2;PRKAG2	Body;Body	-0.566522	-2.782077	-5.426629	2.65E-07	1.05E-05	6.5192058
cg26401551	chr1	8889780	-			-0.637482	-2.841331	-5.424325	2.65E-07	1.05E-05	6.517421
cg19918057	chr4	1508098	+			-0.961806	-0.175842	-5.424214	2.66E-07	1.05E-05	6.5169341
cg00937742	chr2	133429299	+	LYPD1;LYPD1	TSS1500;TSS1500	-1.089774	-1.707093	-5.424059	2.66E-07	1.05E-05	6.5162581
cg01473038	chr2	218873170	-			-0.804425	0.8203677	-5.423951	2.66E-07	1.05E-05	6.5157823
cg02018408	chr16	25269986	+	ZKSCAN2	TSS1500	-0.879352	-2.185608	-5.4239	2.66E-07	1.05E-05	6.5155617
cg17034079	chr3	16652470	-			-1.241301	0.8389619	-5.423875	2.66E-07	1.05E-05	6.5154519
cg20747380	chr7	27142275	+	HOXA2;HOXA2	1stExon;5'UTR	1.1189499	-0.001776	5.4236993	2.66E-07	1.05E-05	6.5146811
cg11334771	chr11	71955337	+	PHOX2A	TSS200	2.0720118	-1.437194	5.423341	2.67E-07	1.05E-05	6.5131117
cg26980954	chr19	51955472	-	SIGLEC8	3'UTR	-1.344827	-0.41872	-5.423264	2.67E-07	1.05E-05	6.5127739
cg23626762	chr8	1283442	+			-1.504236	0.802801	-5.423092	2.67E-07	1.05E-05	6.5120216
cg08077673	chr7	130126368	-	MEST	5'UTR	-0.91728	-2.203392	-5.422866	2.67E-07	1.05E-05	6.51103
cg05754861	chr8	99961768	-	OSR2;OSR2	Body;Body	0.9903375	0.4397012	5.4228103	2.67E-07	1.05E-05	6.5107879
cg20334738	chr4	151502939	-	MAB21L2;LRBA	TSS200;Body	0.5921119	2.8582167	5.4228154	2.67E-07	1.05E-05	6.5108101
cg10453343	chr1	161049655	+	PVRL4	Body	0.6288762	0.4857163	5.4227328	2.67E-07	1.05E-05	6.5104485
cg19404692	chr2	129663717	+			-1.28923	0.9127632	-5.422702	2.67E-07	1.05E-05	6.5103118
cg12676322	chr8	145936124	-			-1.225802	-0.431674	-5.422515	2.68E-07	1.06E-05	6.5094941
cg00073460	chr6	149806502	+	ZC3H12D	TSS1500	-1.255304	-1.395928	-5.422339	2.68E-07	1.06E-05	6.5087237
cg00766188	chr15	89953370	+			1.5190478	-1.370004	5.422275	2.68E-07	1.06E-05	6.5084439
cg06466839	chr11	129062277	-	RICS	TSS200	-1.154288	-0.334399	-5.422224	2.68E-07	1.06E-05	6.5082183

cg15797110	chr7	27196314	+	HOXA7	TSS200	2.0760371	-1.679959	5.4220128	2.68E-07	1.06E-05	6.5072958
cg00355019	chr11	119994877	+	TRIM29	Body	-1.62938	0.8759369	-5.421785	2.69E-07	1.06E-05	6.5062985
cg20995928	chr1	120229863	-			-1.298684	-0.368787	-5.421517	2.69E-07	1.06E-05	6.5051238
cg26328757	chr1	201979764	-	ELF3;ELF3;ELF3;ELF3	5'UTR;5'UTR;1stExon;1stExon	-0.841954	0.2499739	-5.421488	2.69E-07	1.06E-05	6.5049967
cg20818806	chr13	110885926	+	COL4A1	Body	0.9950278	0.8648059	5.4214427	2.69E-07	1.06E-05	6.5047995
cg17769229	chr10	83639690	+	NRG3;NRG3;NRG3	Body;Body;Body	-1.53502	0.6264654	-5.421405	2.69E-07	1.06E-05	6.504635
cg01260146	chr1	48175984	+			-0.487142	-3.480206	-5.42136	2.69E-07	1.06E-05	6.504439
cg12560128	chr12	113416061	-	OAS2;OAS2;OAS2	TSS1500;TSS1500;TSS1500	-1.20569	-2.696166	-5.421322	2.69E-07	1.06E-05	6.5042709
cg18740359	chr19	56393163	-	NLRP4	3'UTR	-1.382297	-0.196418	-5.421284	2.69E-07	1.06E-05	6.5041031
cg24483785	chr6	33133329	-	COL11A2;COL11A2;COL11A2	Body;Body;Body	-1.128647	0.1106294	-5.421254	2.69E-07	1.06E-05	6.5039746
cg07338464	chr5	148244123	-			1.1917729	0.5048994	5.4211188	2.69E-07	1.06E-05	6.5033815
cg15032941	chr11	11778177	+			-1.258489	0.0523532	-5.420988	2.70E-07	1.06E-05	6.502809
cg20236424	chr13	101910811	-	NALCN	Body	-1.362253	0.4573589	-5.420969	2.70E-07	1.06E-05	6.5027274
cg02757516	chr10	134921759	-	GPR123	Body	-1.221665	-0.411431	-5.420924	2.70E-07	1.06E-05	6.502529
cg10035294	chr2	223164925	-	PAX3;PAX3;PAX3;PAX3;PAX3;CCDC140;PAX3;PAX3;PAX3	TSS1500;TSS1500;TSS1500;TSS1500;TSS1500;5'UTR;TSS1500;TSS1500;TSS1500	1.6590902	-2.304193	5.4209312	2.70E-07	1.06E-05	6.5025602
cg26098816	chr10	2210963	+			-1.558011	0.0671349	-5.420754	2.70E-07	1.06E-05	6.5017846
cg19072291	chr7	65617362	+	CRCP;CRCP;CRCP;CRCP;CRCP	3'UTR;3'UTR;Body;3'UTR;3'UTR	1.3213599	0.6862288	5.4207228	2.70E-07	1.06E-05	6.5016478
cg22495211	chr7	56514965	-	LOC650226	Body	-1.864979	0.7802006	-5.420539	2.70E-07	1.06E-05	6.5008423
cg08756898	chr2	129228081	+			-1.279029	-0.12972	-5.420444	2.70E-07	1.06E-05	6.5004274
cg09249494	chr6	6588846	+	LY86;LOC285780	TSS200;Body	-1.359086	-1.563389	-5.420439	2.70E-07	1.06E-05	6.5004065

cg27416957	chr22	38890913	+	DDX17;DDX17	Body;Body	0.6847807	2.190912	5.4204638	2.70E-07	1.06E-05	6.5005138
cg13202816	chr11	32605193	-	EIF3M	TSS200	-0.487921	-4.062287	-5.420385	2.70E-07	1.06E-05	6.5001708
cg01416712	chr4	41750864	-	PHOX2B;PHOX2B	1stExon;5'UTR	1.2903001	-0.785129	5.4196481	2.71E-07	1.07E-05	6.496943
cg11283429	chr13	28549574	-			1.1384586	-0.846941	5.4196444	2.71E-07	1.07E-05	6.4969268
cg22491927	chr19	13617091	-	CACNA1A;CACNA1A;CACNA1A;CACNA1A	5'UTR;1stExon;1stExon;5'UTR	2.8149854	-1.358087	5.4196593	2.71E-07	1.07E-05	6.496992
cg23549179	chr12	113228545	+	RPH3A;RPH3A	TSS1500;TSS1500	-1.566615	0.8616697	-5.419281	2.72E-07	1.07E-05	6.4953372
cg08018572	chr16	1014592	-	LMF1	Body	-1.293812	-0.625815	-5.419256	2.72E-07	1.07E-05	6.4952251
cg12357366	chr3	177158101	+			0.6635138	2.2513329	5.419219	2.72E-07	1.07E-05	6.4950644
cg25912717	chr1	155176951	+	THBS3	Body	-0.67376	-3.750076	-5.418934	2.72E-07	1.07E-05	6.4938192
cg23016129	chr10	22634224	-	SPAG6;SPAG6	TSS200;TSS200	1.2513002	-0.632857	5.4188934	2.72E-07	1.07E-05	6.4936392
cg26537274	chr19	57379813	+			-1.683308	0.8345145	-5.418804	2.72E-07	1.07E-05	6.4932461
cg23196549	chr4	110480700	-	CCDC109B	TSS1500	-1.368939	0.23106	-5.418589	2.73E-07	1.07E-05	6.4923082
cg22569973	chr10	127408789	+	C10orf137	Body	-0.3956	-4.518852	-5.418549	2.73E-07	1.07E-05	6.4921331
cg25336874	chr11	16632311	-			1.1218717	-0.328467	5.4183567	2.73E-07	1.07E-05	6.4912904
cg11804538	chr19	57336314	+	PEG3;PEG3;ZIM2;PEG3;PEG3;PEG3;PEG3;ZIM2;ZIM2	5'UTR;5'UTR;5'UTR;1stExon;5'UTR;5'UTR;5'UTR;5'UTR;5'UTR	-1.717668	0.8727051	-5.418093	2.73E-07	1.07E-05	6.4901343
cg21391373	chr6	112306329	+			-1.223831	0.062395	-5.418106	2.73E-07	1.07E-05	6.4901933
cg25320749	chr6	28494732	+	GPX5;GPX5	Body;Body	-1.626475	0.0536147	-5.418111	2.73E-07	1.07E-05	6.4902156
cg09017434	chr5	16179660	+	11-Mar	1stExon	1.1666123	0.1284574	5.4180002	2.73E-07	1.07E-05	6.4897298
cg12491169	chr11	40137955	+	LRRC4C	5'UTR	-1.727815	0.7042209	-5.417899	2.73E-07	1.07E-05	6.4892881
cg12485926	chr11	58170303	+	OR5B3	1stExon	-1.770694	0.632901	-5.417676	2.74E-07	1.07E-05	6.4883101

cg18410680	chr17	7758397	-	TMEM88	1stExon	1.5040512	2.7495247	5.4176765	2.74E-07	1.07E-05	6.4883132
cg23955593	chr1	163393693	+			-1.461974	1.1573261	-5.417676	2.74E-07	1.07E-05	6.488313
cg19865375	chr7	86629801	-	KIAA1324L;KIAA1324L	Body;Body	0.8443056	0.2363549	5.4198247	2.74E-07	1.07E-05	6.4894476
cg04967039	chr4	8292202	+	HTRA3	Body	-1.17546	0.3980883	-5.417582	2.74E-07	1.07E-05	6.4879016
cg13537416	chr9	124506555	-	DAB2IP;DAB2IP	Body;5'UTR	1.3027427	0.3495029	5.41753	2.74E-07	1.07E-05	6.4876721
cg04110105	chr3	46395356	+	CCR2;CCR2;CCR2;CCR2	5'UTR;1stExon;1stExon;5'UTR	-1.432543	-1.319038	-5.417375	2.74E-07	1.08E-05	6.4869945
cg24139093	chr15	97491245	+			1.4726034	-0.444886	5.417331	2.74E-07	1.08E-05	6.4868012
cg00435173	chr17	40284046	+	RAB5C;RAB5C	5'UTR;5'UTR	0.9042646	0.9790365	5.4173104	2.74E-07	1.08E-05	6.4867109
cg27425146	chr1	25062925	+			0.9825154	0.4264804	5.4172198	2.74E-07	1.08E-05	6.4863147
cg08291024	chr1	165324411	+	LMX1A	Body	1.5839365	-1.476727	5.4170964	2.74E-07	1.08E-05	6.4857747
cg26501046	chr2	218466307	-	DIRC3	Body	-1.126016	0.1034493	-5.416879	2.75E-07	1.08E-05	6.4848241
cg24524694	chr10	52829575	+	PRKG1	Body	-1.496205	0.8413085	-5.416858	2.75E-07	1.08E-05	6.4847324
cg09225922	chr20	42286289	+			-0.573891	-4.360964	-5.41678	2.75E-07	1.08E-05	6.4843893
cg06285293	chr15	25447222	+	SNORD115-19;SNORD115-17;SNORD115-18	TSS1500;TSS1500;TSS1500	-1.15343	0.6500136	-5.416626	2.75E-07	1.08E-05	6.4837167
cg12260798	chr6	26240519	-	HIST1H4F	TSS200	2.2570598	-1.179825	5.4166342	2.75E-07	1.08E-05	6.4837518
cg18542688	chr2	241290875	-			0.9979717	1.4062769	5.4256361	2.75E-07	1.08E-05	6.4885029
cg20798152	chr5	71014900	-	CARTPT	TSS200	0.8391025	-0.644235	5.4166315	2.75E-07	1.08E-05	6.4837401
cg20044918	chr10	134908622	-	GPR123	Body	-1.453792	1.0899877	-5.416592	2.75E-07	1.08E-05	6.4835665
cg25083091	chr8	10646690	+	PINX1	Body	1.1221994	1.6525063	5.4165362	2.75E-07	1.08E-05	6.483323
cg26328687	chr11	96076794	-	MAML2	TSS1500	-0.937961	-2.298424	-5.416378	2.75E-07	1.08E-05	6.4826303

cg25061701	chr17	62608856	-	SMURF2	Body	1.0241138	1.4329952	5.4162767	2.75E-07	1.08E-05	6.4821877
cg16452248	chr16	613526	+	C16orf11	Body	-2.404856	1.8288815	-5.41612	2.76E-07	1.08E-05	6.4815038
cg14148016	chr9	138874126	+			-1.298015	0.920949	-5.416027	2.76E-07	1.08E-05	6.4810935
cg14060944	chr9	125124080	+			-1.027176	-0.350942	-5.415873	2.76E-07	1.08E-05	6.4804196
cg27501878	chr20	61638588	+	BHLHE23	TSS1500	2.92673	-2.018479	5.4156551	2.76E-07	1.08E-05	6.4794677
cg01757168	chr3	128215565	-			1.3008209	-0.065089	5.4155821	2.76E-07	1.08E-05	6.4791483
cg19720260	chr11	114549291	+	FAM55B;FAM55B	5'UTR;1stExon	-1.185758	-1.47813	-5.415471	2.76E-07	1.08E-05	6.4786631
cg04930211	chr22	49069896	+	FAM19A5;FAM19A5	Body;Body	-1.439328	0.8361091	-5.41536	2.77E-07	1.08E-05	6.478176
cg26090020	chr17	32903717	-	C17orf102	3'UTR	-1.550542	0.3989971	-5.415284	2.77E-07	1.08E-05	6.4778453
cg27015332	chr12	89479076	-			0.6236027	1.2504006	5.4152551	2.77E-07	1.08E-05	6.4777176
cg23274660	chr20	44655227	-	SLC12A5	Body	-1.263706	-1.953295	-5.4174	2.77E-07	1.08E-05	6.4788507
cg05673966	chr16	85431095	-			-1.209488	0.0630369	-5.415092	2.77E-07	1.08E-05	6.4770031
cg05287437	chr3	16215248	-	GALNTL2	TSS1500	-0.957607	-0.258901	-5.415023	2.77E-07	1.08E-05	6.4767008
cg13018903	chr11	124806901	+	HEPACAM	TSS1500	-1.58289	1.2937386	-5.414828	2.77E-07	1.09E-05	6.4758501
cg25362709	chr11	124413728	-	OR8B12	TSS200	-1.46219	0.1639699	-5.414472	2.78E-07	1.09E-05	6.4742926
cg06829391	chr16	9857151	-	GRIN2A;GRIN2A;GRIN2A	Body;Body;3'UTR	-1.479947	1.1662696	-5.41436	2.78E-07	1.09E-05	6.4737996
cg05006211	chr22	48896486	+	FAM19A5	Body	-0.943315	0.1479283	-5.414199	2.78E-07	1.09E-05	6.4730967
cg19823649	chr11	44951252	+	TSPAN18;TSPAN18	Body;3'UTR	-1.014512	0.4404007	-5.414139	2.78E-07	1.09E-05	6.4728349
cg00371453	chr19	7854482	-	CLEC4GP1	Body	-1.65695	-2.158856	-5.414009	2.78E-07	1.09E-05	6.4722666
cg19062099	chr5	113744106	+	KCNN2	Body	-1.194172	-0.73918	-5.413815	2.79E-07	1.09E-05	6.4714191

cg04243401	chr14	37128378	+	PAX9	5'UTR	1.5684061	-1.307492	5.4159689	2.79E-07	1.09E-05	6.4725956
cg14757107	chr6	16277615	-	GMPR	Body	-1.351917	-0.011123	-5.413732	2.79E-07	1.09E-05	6.4710541
cg12049093	chr1	175804286	-			-1.67898	0.9507432	-5.413624	2.79E-07	1.09E-05	6.4705797
cg07424400	chr19	39786778	+	IL29	TSS200	-1.090112	-0.654836	-5.415782	2.79E-07	1.09E-05	6.4714014
cg00451054	chr8	77913321	+	PXMP3;PXMP3	TSS1500;TSS1500	-1.319052	0.1423337	-5.413104	2.79E-07	1.09E-05	6.4683054
cg25076197	chr7	104681440	+	MLL5;MLL5	Body;Body	0.7963491	1.9192969	5.4130909	2.79E-07	1.09E-05	6.4682498
cg08863830	chr1	2202028	-	SKI	Body	1.3110521	1.2719524	5.4217826	2.80E-07	1.09E-05	6.47172
cg02721902	chr17	46825371	+			1.0582521	0.1905135	5.4127595	2.80E-07	1.09E-05	6.4668004
cg22284058	chr8	142237359	+	SLC45A4	Body	0.8889469	1.0215461	5.412742	2.80E-07	1.09E-05	6.466724
cg27539234	chr17	7218945	+	GPS2	TSS1500	-0.460392	-3.199611	-5.412739	2.80E-07	1.09E-05	6.4667091
cg22548438	chr2	119607636	+			1.4397705	-1.3775	5.4127064	2.80E-07	1.09E-05	6.466568
cg11538998	chr17	21371533	-			-1.463132	1.0361185	-5.412668	2.80E-07	1.09E-05	6.4663997
cg16035633	chr7	150700728	-	NOS3;NOS3;NOS3;NOS3	3'UTR;3'UTR;Body;Body	-0.985624	-1.197357	-5.412578	2.80E-07	1.09E-05	6.4660086
cg24091438	chr7	150019955	-	LRRC61;ACTR3C;LRRC61	TSS1500;5'UTR;TSS1500	-0.898909	-1.136554	-5.412568	2.80E-07	1.09E-05	6.4659631
cg18863922	chr1	156889882	-	C1orf92	TSS1500	-1.253961	-0.598593	-5.412443	2.80E-07	1.10E-05	6.4654183
cg01882150	chr11	63933998	+	MACROD1	TSS1500	-0.335162	-3.171664	-5.412344	2.80E-07	1.10E-05	6.4649828
cg23516310	chr7	73417197	+			-1.268467	-1.517502	-5.412321	2.80E-07	1.10E-05	6.464883
cg19451848	chr5	42176806	-			-1.587311	-0.352115	-5.414386	2.81E-07	1.10E-05	6.4656805
cg09631909	chr22	35389016	+			-1.183721	-0.997741	-5.411832	2.81E-07	1.10E-05	6.4627436
cg02249074	chr9	99639692	-			1.3137122	-1.27728	5.4116966	2.81E-07	1.10E-05	6.4621516

cg23415434	chr2	131798869	+	ARHGEF4;ARHGEF4	Body;Body	1.0424014	1.2645442	5.411629	2.81E-07	1.10E-05	6.461856
cg25287708	chr1	11596434	+	PTCHD2	Body	-1.261216	0.6518224	-5.41152	2.81E-07	1.10E-05	6.4613801
cg02014853	chr6	29595335	+	GABBR1;GABBR1;GABBR1	Body;1stExon;Body	1.0182954	0.1097124	5.4112827	2.82E-07	1.10E-05	6.4603415
cg17095179	chr1	9969424	+	CTNNBIP1;CTNNBIP1	5'UTR;5'UTR	-0.623473	-3.711073	-5.411093	2.82E-07	1.10E-05	6.4595126
cg15937784	chr19	54230170	+	MIR526A2	TSS200	-1.125698	-0.842839	-5.411029	2.82E-07	1.10E-05	6.4592307
cg10767141	chr7	27225143	+	HOXA11AS;HOXA11	Body;TSS1500	1.4890143	-1.220327	5.4108883	2.82E-07	1.10E-05	6.4586167
cg14859460	chr5	178422244	+	GRM6	TSS200	1.2782282	-1.000434	5.4108247	2.82E-07	1.10E-05	6.4583385
cg18020955	chr1	147790806	+			1.2532248	-0.572706	5.4106741	2.83E-07	1.10E-05	6.4576799
cg12661262	chr6	21691612	-	FLJ22536	Body	0.7907875	2.1099992	5.4104049	2.83E-07	1.10E-05	6.4565027
cg15562346	chr2	239403295	-			-1.486189	1.837366	-5.410395	2.83E-07	1.10E-05	6.4564615
cg14850784	chr16	1430103	+	UNKL	TSS1500	-1.56584	0.2619705	-5.410271	2.83E-07	1.10E-05	6.4559173
cg02019774	chr3	138658470	+			1.7381721	-1.841468	5.410224	2.83E-07	1.10E-05	6.4557119
cg23314055	chr12	51455536	-	CSRNP2	3'UTR	0.8892322	1.8331741	5.4101193	2.83E-07	1.10E-05	6.4552542
cg10092198	chr11	6129548	-	OR56B4	1stExon	-1.283885	-0.726433	-5.409952	2.84E-07	1.11E-05	6.4545216
cg21141234	chr6	100067169	+			1.0258294	-1.268413	5.4099737	2.83E-07	1.11E-05	6.4546173
cg25640519	chr3	141516705	+	GRK7	Body	1.9570673	-0.381175	5.4099591	2.83E-07	1.11E-05	6.4545535
cg10824915	chr1	916863	-	C1orf170	Body	-0.685972	-0.719556	-5.409614	2.84E-07	1.11E-05	6.4530439
cg07021275	chr8	1325845	-			-1.132574	-0.177561	-5.409437	2.84E-07	1.11E-05	6.4522726
cg26092367	chr6	145431290	+			-1.503817	0.9521298	-5.409142	2.85E-07	1.11E-05	6.4509816
cg16134191	chr11	31847509	-			1.0597727	-1.145905	5.4090769	2.85E-07	1.11E-05	6.4506965

cg00955544	chr21	31767222	+	KRTAP13-1	TSS1500	-1.360357	0.6840572	-5.408972	2.85E-07	1.11E-05	6.4502397
cg10305789	chr20	37434167	+	PPP1R16B	TSS200	1.8629644	-0.892632	5.4088247	2.85E-07	1.11E-05	6.4495938
cg20424973	chr2	3045241	-			-1.289936	0.1749271	-5.408814	2.85E-07	1.11E-05	6.4495449
cg05959932	chr6	2382734	+			1.2627997	1.9800101	5.4085695	2.85E-07	1.11E-05	6.448478
cg10682155	chr21	38077473	-	SIM2;SIM2	Body;Body	2.372473	-2.696035	5.4129758	2.85E-07	1.11E-05	6.4507983
cg16330965	chr15	66790390	+	SNAPC5	TSS1500	-0.897634	-0.529548	-5.408341	2.86E-07	1.11E-05	6.4474801
cg18358845	chr5	178381935	-	ZNF454	Body	-1.588179	0.5908775	-5.40835	2.86E-07	1.11E-05	6.4475187
cg14834881	chr4	92034737	-	FAM190A	Body	-0.938117	0.0451057	-5.408313	2.86E-07	1.11E-05	6.4473587
cg04895616	chr19	44331009	-	ZNF283	TSS1500	0.9106592	0.0502228	5.4082882	2.86E-07	1.11E-05	6.4472481
cg20293594	chr7	27196296	+	HOXA7	TSS200	2.3022433	-1.939339	5.4078292	2.86E-07	1.11E-05	6.4452415
cg00665650	chr5	138676787	+	PAIP2;PAIP2	TSS1500;TSS1500	-1.00234	-2.136052	-5.407664	2.86E-07	1.12E-05	6.44452
cg26363579	chr8	1107269	+			-0.884468	-0.164941	-5.407599	2.87E-07	1.12E-05	6.4442346
cg27497038	chr10	125401743	+			-1.211079	0.381898	-5.407444	2.87E-07	1.12E-05	6.4435578
cg13364075	chr7	157320369	+			-1.414575	0.3065243	-5.407362	2.87E-07	1.12E-05	6.4431976
cg06507590	chr5	166745042	+	ODZ2	Body	-1.428685	0.6086591	-5.407319	2.87E-07	1.12E-05	6.4430095
cg11243283	chr10	109910629	+			-1.489491	0.2015127	-5.407285	2.87E-07	1.12E-05	6.4428643
cg25921502	chr10	50363896	-	C10orf128	3'UTR	-1.232922	-0.012284	-5.407246	2.87E-07	1.12E-05	6.442692
cg23891273	chr12	102036400	+	MYBPC1;MYBPC1;MYBPC1;MYBPC1	Body;Body;Body;Body	2.5066335	-0.997435	5.4071691	2.87E-07	1.12E-05	6.4423563
cg07835051	chr18	56935446	-	RAX	3'UTR	1.5964847	-1.358526	5.4071468	2.87E-07	1.12E-05	6.4422586
cg00574412	chr17	27892866	+	ABHD15	Body	-0.888172	-0.521289	-5.407126	2.87E-07	1.12E-05	6.4421678

cg20050826	chr12	52995295	+	KRT72;KRT72;KRT72;KRT72;KRT72;KRT72	1stExon;5'UTR;1stExon;5'UTR;1stExon;5'UTR	1.03915	-0.279712	5.4070935	2.87E-07	1.12E-05	6.4420259
cg26327666	chr5	76937963	+			1.7807073	-0.204127	5.4114548	2.87E-07	1.12E-05	6.444164
cg00795248	chr1	197888305	-	LHX9;LHX9	Body;Body	1.5617831	-1.236262	5.406845	2.88E-07	1.12E-05	6.4409398
cg09968709	chr20	29845320	+	DEFB115	TSS200	-1.541252	0.3356955	-5.409033	2.88E-07	1.12E-05	6.4423007
cg20495738	chr12	2338399	+	CACNA1C;CACNA1C;CACNA1C;CACNA1C;CACNA1C;CACNA1C<br>;CACNA1C;CACNA1C;CACNA1C;CACNA1C;CACNA1C;CACNA1<br>C;CACNA1C;CACNA1C;CACNA1C;CACNA1C;CACNA1C;CACNA	Body;Body;Body;Body;Body;Body;Body;Body;Body;<br>Body;Body;Body;Body;Body;Body;Body;Body;Body;<br>Body;Body;Body;Body;Body	-1.399966	-0.918737	-5.406714	2.88E-07	1.12E-05	6.4403672
cg05091115	chr10	65280959	-	REEP3	TSS200	-0.927939	-5.006879	-5.406481	2.88E-07	1.12E-05	6.4393467
cg22190437	chr2	229045417	+	SPHKAP;SPHKAP	Body;Body	1.4159616	-0.837833	5.406069	2.89E-07	1.12E-05	6.4375478
cg07834574	chr17	4802847	-	CHRNE;C17orf107	Body;TSS200	-1.926977	-2.376198	-5.405664	2.89E-07	1.12E-05	6.4357798
cg06362313	chr12	6645287	-	GAPDH	Body	-1.234152	-1.363616	-5.405471	2.89E-07	1.13E-05	6.4349347
cg22320365	chr17	36718198	+	SRCIN1	Body	1.2902102	-0.055063	5.4075752	2.89E-07	1.13E-05	6.4359369
cg03059921	chr20	61451730	-	COL9A3	Body	-1.336438	0.3770294	-5.4053	2.90E-07	1.13E-05	6.434189
cg08506654	chr5	172673098	-			1.0675562	-0.237099	5.4052382	2.90E-07	1.13E-05	6.4339171
cg03480068	chr10	1749547	+	ADARB2	Body	-1.606767	0.70254	-5.405166	2.90E-07	1.13E-05	6.4336012
cg08291907	chr10	53011793	+	PRKG1;PRKG1	Body;Body	-1.673181	0.191567	-5.405138	2.90E-07	1.13E-05	6.4334802
cg15477680	chr7	150889435	+			-1.568123	1.7859727	-5.405102	2.90E-07	1.13E-05	6.4333198
cg21572897	chr3	119277821	-	CD80	5'UTR	-1.209146	0.6086566	-5.405096	2.90E-07	1.13E-05	6.4332949
cg04419012	chr16	66835618	-	CCDC79	TSS200	0.9505269	4.3098524	5.4139364	2.90E-07	1.13E-05	6.437972
cg04663916	chr13	50265991	+	EBPL	TSS1500	-1.114375	-2.198119	-5.404966	2.90E-07	1.13E-05	6.4327294
cg17283039	chr22	29787625	+			0.7671991	1.4658379	5.4049867	2.90E-07	1.13E-05	6.4328179
cg25006511	chr6	105726047	-	PREP	Body	0.9074404	3.7268998	5.4116015	2.90E-07	1.13E-05	6.4359751

cg07503203	chr8	2005940	+	MYOM2	Body	-1.328866	1.329579	-5.406869	2.90E-07	1.13E-05	6.4324757
cg01484487	chr2	74726892	-	LBX2	Body	1.1332654	-0.577306	5.4046703	2.90E-07	1.13E-05	6.4314353
cg14036402	chr1	15743671	-	EFHD2	Body	-1.273859	-1.341705	-5.404579	2.91E-07	1.13E-05	6.431035
cg15977719	chr7	25021503	+			-0.446511	-4.328867	-5.404495	2.91E-07	1.13E-05	6.4306708
cg11886405	chr11	133699751	-			-1.309263	0.1703966	-5.40416	2.91E-07	1.13E-05	6.4292052
cg10032387	chr4	38988166	-	TMEM156	Body	-0.869916	0.8777612	-5.408452	2.91E-07	1.13E-05	6.4310688
cg07333191	chr4	13526769	+			1.3420557	0.1070489	5.4039608	2.91E-07	1.13E-05	6.428335
cg11089489	chr14	37124313	-			1.2809712	-0.329972	5.4039711	2.91E-07	1.13E-05	6.4283803
cg10156274	chr12	12247855	+	BCL2L14;BCL2L14;BCL2L14	Body;Body;3'UTR	1.1056759	1.3394238	5.4038879	2.91E-07	1.13E-05	6.4280166
cg24391471	chr3	44974926	-	ZDHHC3;ZDHHC3;ZDHHC3	Body;Body;Body	0.751538	2.3413184	5.4059351	2.92E-07	1.13E-05	6.4437909
cg25536171	chr13	108535144	+			-1.484708	-0.225792	-5.405868	2.92E-07	1.13E-05	6.4281066
cg27435498	chr7	150655387	+	KCNH2;KCNH2	Body;Body	1.0162688	0.09989	5.4036151	2.92E-07	1.13E-05	6.4268246
cg09579869	chr1	10738769	-	CASZ1;CASZ1	Body;Body	0.7841646	1.3446249	5.4035844	2.92E-07	1.13E-05	6.4266905
cg24109894	chr1	36108212	-	PSMB2	TSS1500	-1.159099	-0.376622	-5.403443	2.92E-07	1.13E-05	6.426074
cg13414654	chr2	10224232	+			-1.433999	-0.630663	-5.40339	2.92E-07	1.13E-05	6.4258405
cg21934588	chr1	10532847	-	DFFA;DFFA	TSS1500;TSS1500	-0.513212	-4.054342	-5.403233	2.92E-07	1.13E-05	6.4251532
cg26733975	chr7	56551640	-			-1.124531	-0.327219	-5.403076	2.93E-07	1.13E-05	6.4244707
cg07730329	chr5	140810137	+	PCDHGA4;PCDHGA11;PCDHGA11;PCDHGA9;PCDHGA1;PCDHGB1;PCDHGB6;PCDHGA12;PCDHGB3;PCDHGB7;PCDHGA6;PCDHGA8;PCDHGA10;PCDHGA5;PCDHGB4;PCDHGA3;PCDHGA2	Body;Body;Body;Body;Body;Body;Body;TSS200;Body;Body;Body;Body;Body;Body;Body;Body;TSS200;Body;Body;Body	1.2462606	0.1919958	5.4025338	2.93E-07	1.14E-05	6.4221006
cg14314674	chr9	970654	-			1.2171442	-1.35813	5.4025388	2.93E-07	1.14E-05	6.4221223
cg19495428	chr10	135123067	+	ZNF511;TUBGCP2	Body;TSS1500	-1.958285	1.1294244	-5.404653	2.93E-07	1.14E-05	6.440703

cg18866586	chr5	178803207	-			-1.30252	0.6737984	-5.402459	2.93E-07	1.14E-05	6.4217718
cg05242592	chr16	20120165	+			-1.425666	0.8460681	-5.402436	2.93E-07	1.14E-05	6.421672
cg22970357	chr2	119600408	-	EN1	3'UTR	1.2027248	-0.526247	5.4024024	2.93E-07	1.14E-05	6.4215264
cg03408904	chr22	17956462	+	CECR2	TSS200	1.253581	-0.271733	5.4021704	2.94E-07	1.14E-05	6.4205128
cg03110787	chr19	6217641	+	MLLT1	Body	0.7797596	1.4085913	5.4020971	2.94E-07	1.14E-05	6.4201927
cg16852704	chr2	74743243	+	TLX2	Body	1.1615007	-1.310196	5.4019252	2.94E-07	1.14E-05	6.4194417
cg16937268	chr20	21684417	+			1.784609	-0.330377	5.4016295	2.95E-07	1.14E-05	6.4181502
cg24810473	chr5	139090314	-			-1.435978	-3.926291	-5.401625	2.95E-07	1.14E-05	6.4181284
cg02994956	chr22	29876534	-	NEFH	1stExon	0.9006536	-0.201628	5.4080762	2.95E-07	1.14E-05	6.4359072
cg09340639	chr1	157789662	-	FCRL1;FCRL1;FCRL1	Body;Body;Body	-1.391789	-2.253986	-5.401375	2.95E-07	1.14E-05	6.4170399
cg25354466	chr8	65281397	+			1.3851106	-1.536274	5.4013877	2.95E-07	1.14E-05	6.4170937
cg14555736	chr12	128852982	-	TMEM132C	Body	-1.369666	-0.064515	-5.401346	2.95E-07	1.14E-05	6.4169103
cg25104511	chr17	38333503	-	RAPGEFL1	TSS1500	-0.735541	-1.594703	-5.401034	2.95E-07	1.14E-05	6.4155486
cg04495670	chr11	69634296	+	FGF3	TSS200	1.870176	-1.04927	5.4009886	2.95E-07	1.14E-05	6.4153505
cg08441806	chr10	134599149	-	NKX6-2	1stExon	1.3306246	-0.766579	5.4008996	2.95E-07	1.14E-05	6.4149618
cg19086905	chr15	102342791	-			-1.244566	-0.293884	-5.40089	2.96E-07	1.14E-05	6.414918
cg25271672	chr1	156462624	+	MEF2D	5'UTR	1.1188075	-2.067202	5.4009076	2.95E-07	1.14E-05	6.4149968
cg17054386	chr2	219855054	-	CRYBA2;CRYBA2;CRYBA2	Body;Body;Body	0.8370717	0.0428705	5.400721	2.96E-07	1.14E-05	6.4141815
cg05768419	chr1	174417967	+	GPR52;RABGAP1L	1stExon;Body	1.2750599	1.8069172	5.4006949	2.96E-07	1.14E-05	6.4140675
cg04666911	chr11	1872756	+	LSP1	TSS1500	-0.901749	-0.208309	-5.400601	2.96E-07	1.15E-05	6.4136578

cg19128082	chr3	38537279	-	EXOG;EXOG	TSS1500;TSS1500	-1.001516	0.0645525	-5.400471	2.96E-07	1.15E-05	6.4130906
cg14391419	chr7	19158647	+	TWIST1	TSS1500	2.2424953	-1.493664	5.4004289	2.96E-07	1.15E-05	6.4129059
cg23883632	chr14	71174292	+			-1.222371	-0.837008	-5.400304	2.96E-07	1.15E-05	6.4123608
cg08190545	chr19	28403514	+			-1.58432	0.2566053	-5.400269	2.96E-07	1.15E-05	6.4122067
cg26240231	chr7	1148101	+	C7orf50;C7orf50;C7orf50	Body;Body;Body	1.6729889	3.1552819	5.4002067	2.96E-07	1.15E-05	6.4119355
cg00347746	chr19	48970082	+			-0.923191	-0.23849	-5.400158	2.96E-07	1.15E-05	6.4117211
cg13973849	chr9	138657014	+	KCNT1	Body	-1.027338	0.209953	-5.400003	2.97E-07	1.15E-05	6.4110463
cg12091642	chr14	61104566	-			2.3352545	-0.735592	5.4065625	2.97E-07	1.15E-05	6.4318514
cg23399286	chr8	133072873	-	OC90	TSS1500	-1.25588	-0.727596	-5.399903	2.97E-07	1.15E-05	6.4106102
cg09638704	chr1	247654260	-	OR2W5	TSS200	-1.046741	-0.633586	-5.399717	2.97E-07	1.15E-05	6.4097967
cg02514022	chr4	4391898	-	D4S234E;D4S234E	Body;Body	-1.211068	0.2248111	-5.399658	2.97E-07	1.15E-05	6.4095398
cg08677655	chr17	1549108	-	SCARF1;SCARF1;SCARF1;SCARF1;SCARF1	TSS200;TSS200;TSS200;TSS200;TSS200	1.1964997	-0.086125	5.3995007	2.97E-07	1.15E-05	6.4088518
cg19880947	chr17	1510000	-	SLC43A2	Body	-0.951235	1.2180007	-5.399487	2.97E-07	1.15E-05	6.4087927
cg24411972	chr10	134754396	+	C10orf93	Body	-1.144356	-0.651649	-5.3993	2.98E-07	1.15E-05	6.4079736
cg14331623	chr9	134155084	-			-0.904995	0.072954	-5.399219	2.98E-07	1.15E-05	6.4076228
cg14930633	chr22	46519133	+			-0.762327	-1.829092	-5.399157	2.98E-07	1.15E-05	6.407353
cg19404979	chr19	48498031	+	ELSPBP1;ELSPBP1	1stExon;5'UTR	-0.995965	-1.133652	-5.4011	2.98E-07	1.15E-05	6.4073027
cg15087860	chr7	20816253	-			1.6903569	-0.78324	5.3987512	2.98E-07	1.15E-05	6.405579
cg20644952	chr4	6717431	-	CNO	TSS1500	-0.910309	-1.039879	-5.398674	2.99E-07	1.15E-05	6.4052424
cg01994994	chr16	48999049	+			-1.50697	1.0186949	-5.398609	2.99E-07	1.15E-05	6.4049583

cg21041579	chr14	95907997	+	C14orf49	Body	-1.454062	0.5356437	-5.398602	2.99E-07	1.15E-05	6.4049257
cg08975834	chr10	133036065	+	TCERG1L	Body	-1.415391	0.5510105	-5.398459	2.99E-07	1.15E-05	6.4043029
cg16330786	chr2	75326898	-	TACR1;TACR1	Body;Body	-1.103126	-0.088094	-5.398416	2.99E-07	1.15E-05	6.4041156
cg05738403	chr6	33142936	-	COL11A2;COL11A2;COL11A2	Body;Body;Body	-1.177014	0.4663068	-5.400452	2.99E-07	1.15E-05	6.404472
cg25121621	chr15	45926780	+	SQRDL	TSS1500	-0.587346	-2.368465	-5.397913	3.00E-07	1.16E-05	6.4019179
cg21215767	chr2	119599819	+	EN1	3'UTR	1.3841143	-0.825784	5.397623	3.00E-07	1.16E-05	6.4006526
cg15896978	chr11	116709264	-	APOA1	TSS1500	-1.172932	0.1435576	-5.397464	3.00E-07	1.16E-05	6.3999605
cg10469791	chr4	42895240	+	GRXCR1	TSS200	-1.135668	-1.06883	-5.397339	3.00E-07	1.16E-05	6.3994109
cg14028671	chr9	139937558	+	NPDC1	Body	1.4567671	1.7035119	5.397219	3.01E-07	1.16E-05	6.3988887
cg14180030	chr9	123475675	-	MEGF9	Body	-0.787725	-2.731951	-5.397138	3.01E-07	1.16E-05	6.3985355
cg09759994	chr2	113238376	-	TTL	TSS1500	-0.985944	-1.96307	-5.397118	3.01E-07	1.16E-05	6.3984494
cg06390077	chr7	141385142	+	KIAA1147	Body	0.8586772	1.7821539	5.3965777	3.01E-07	1.16E-05	6.3960888
cg15196820	chr2	131486606	-	GPR148	TSS200	-0.999066	-0.573882	-5.396575	3.01E-07	1.16E-05	6.3960791
cg10682299	chr4	111550154	-	PITX2;PITX2	Body;Body	1.6318556	-0.937624	5.3964604	3.02E-07	1.16E-05	6.3955771
cg20830867	chr3	182402080	-			-1.321559	-0.595101	-5.396445	3.02E-07	1.16E-05	6.3955091
cg18857588	chr20	61916279	-	ARFGAP1;ARFGAP1	Body;Body	1.0275185	2.4754155	5.3963388	3.02E-07	1.16E-05	6.395046
cg10621809	chr12	95840313	-			0.8826391	0.9699825	5.3961101	3.02E-07	1.16E-05	6.3940479
cg10975001	chr1	33627155	-	TRIM62	Body	0.99261	-0.098544	5.3959321	3.02E-07	1.17E-05	6.3932705
cg01689438	chr3	147140930	-			1.6268191	-1.297836	5.3955215	3.03E-07	1.17E-05	6.3914784
cg11126767	chr14	60978275	-	SIX6	3'UTR	1.1136417	-0.15999	5.3954972	3.03E-07	1.17E-05	6.3913724

cg22514674	chr12	56473743	-	ERBB3;ERBB3	TSS200;TSS200	-0.358745	-4.012261	-5.395513	3.03E-07	1.17E-05	6.3914425
cg03544320	chr4	5894691	+	CRMP1	1stExon	1.8417155	-0.160911	5.395464	3.03E-07	1.17E-05	6.3912274
cg11027221	chr11	67770665	-	UNC93B1	Body	-1.139938	-0.539877	-5.39538	3.03E-07	1.17E-05	6.3908608
cg06211724	chr1	183155437	-	LAMC2;LAMC2;LAMC2;LAMC2	1stExon;5'UTR;5'UTR;1stExon	-1.025267	-2.035802	-5.394947	3.04E-07	1.17E-05	6.3889684
cg05538118	chr16	32493059	+			-1.01042	-1.273208	-5.394925	3.04E-07	1.17E-05	6.3888736
cg21374754	chr3	42452862	+	LYZL4	TSS1500	-1.021086	-0.836184	-5.394828	3.04E-07	1.17E-05	6.3884513
cg11728809	chr12	34554731	-			-0.983685	-0.591002	-5.394754	3.04E-07	1.17E-05	6.3881298
cg18986967	chr7	141176264	+			-1.438559	-1.280347	-5.394713	3.04E-07	1.17E-05	6.3879481
cg23654431	chr12	48298967	-	VDR;VDR	TSS200;TSS200	-0.426249	-4.104312	-5.394673	3.04E-07	1.17E-05	6.387774
cg02744705	chr4	151178090	-	DCLK2;DCLK2	3'UTR;3'UTR	-1.428221	0.1594808	-5.39462	3.04E-07	1.17E-05	6.3875423
cg04870460	chr1	146644887	+	PRKAB2	TSS1500	-0.958314	-3.14005	-5.39457	3.04E-07	1.17E-05	6.3873261
cg20014974	chr1	8271918	-			-0.903991	-1.242806	-5.394581	3.04E-07	1.17E-05	6.3873719
cg24314434	chr19	5851762	-	FUT3;FUT3;FUT3;FUT3	TSS1500;TSS1500;TSS1500;TSS1500	-1.073192	0.0743431	-5.394514	3.04E-07	1.17E-05	6.3870806
cg26650638	chr16	86320857	+			2.0534157	-1.106074	5.3944518	3.04E-07	1.17E-05	6.3868093
cg13016237	chr7	154681513	+	DPP6;DPP6;DPP6	Body;Body;Body	-1.433964	0.4283922	-5.394387	3.04E-07	1.17E-05	6.3865284
cg13386351	chr17	9479935	+	WDR16;WDR16;STX8	TSS200;TSS200;TSS1500	-1.653009	-3.443974	-5.394381	3.04E-07	1.17E-05	6.3864994
cg25290307	chr7	101005910	-	EMID2	TSS1500	1.2732086	0.1777734	5.394402	3.04E-07	1.17E-05	6.3865916
cg13579752	chr5	153577937	+	GALNT10	Body	-0.752228	-0.528331	-5.394065	3.05E-07	1.17E-05	6.3851214
cg19956605	chr22	21921881	+	UBE2L3;UBE2L3;UBE2L3	TSS200;TSS200;TSS200	-0.393866	-4.241713	-5.394077	3.05E-07	1.17E-05	6.3851745
cg06106063	chr13	78550939	-	EDNRB	TSS1500	-1.262543	0.5344761	-5.393886	3.05E-07	1.17E-05	6.3843388

cg02469871	chr12	103218581	+			1.4665237	-1.372363	5.3936539	3.05E-07	1.18E-05	6.3833269
cg19153763	chr7	27196371	+	HOXA7	TSS200	1.5668087	-1.842819	5.3936283	3.05E-07	1.18E-05	6.3832151
cg18587476	chr2	24397810	-	C2orf84	TSS200	1.1680568	1.0961008	5.3932894	3.06E-07	1.18E-05	6.3817361
cg03020554	chr8	99951730	+			1.4730843	-1.357919	5.3952893	3.06E-07	1.18E-05	6.3819621
cg00364016	chr6	10113648	-			1.9292764	-1.038222	5.3930625	3.06E-07	1.18E-05	6.3807458
cg07976787	chr16	25598190	+			-1.076686	-1.498692	-5.393063	3.06E-07	1.18E-05	6.3807496
cg17389956	chr12	126167667	+			-1.141368	-1.356638	-5.393025	3.06E-07	1.18E-05	6.3805804
cg09553242	chr5	72597015	-			1.0781293	-0.229328	5.3929473	3.06E-07	1.18E-05	6.3802432
cg14696458	chr1	91185157	-			1.1505045	-0.575282	5.3928621	3.07E-07	1.18E-05	6.3798713
cg20234976	chr6	100895907	+	SIM1	Body	1.5856364	-1.593181	5.3928198	3.07E-07	1.18E-05	6.3796868
cg19602728	chr4	1496334	+			-1.771715	-0.114229	-5.392521	3.07E-07	1.18E-05	6.3783845
cg11536851	chr7	4832359	+			0.7463543	0.845093	5.3924754	3.07E-07	1.18E-05	6.378184
cg03561338	chr14	88601933	-			-1.23906	0.0311961	-5.392376	3.07E-07	1.18E-05	6.3777512
cg05256269	chr2	176956841	+	HOXD13	TSS1500	1.1256382	-1.084309	5.3923071	3.07E-07	1.18E-05	6.3774497
cg00085448	chr12	113416442	+	OAS2;OAS2;OAS2	1stExon;1stExon;1stExon	-0.912407	-3.3757	-5.394311	3.08E-07	1.18E-05	6.3780782
cg10019018	chr12	119214021	-			-1.403279	0.173383	-5.392142	3.08E-07	1.18E-05	6.3767302
cg14627921	chr17	76593346	-			-1.244703	1.5160013	-5.392114	3.08E-07	1.18E-05	6.3766063
cg13376438	chr7	108168711	-			-1.721323	0.3880883	-5.39201	3.08E-07	1.18E-05	6.3761513
cg16214492	chr17	76128676	-	TMC8;TMC6	Body;TSS200	-0.947979	-1.342722	-5.391827	3.08E-07	1.18E-05	6.3753556
cg08628911	chr16	1199592	-			-1.435742	0.8151993	-5.391754	3.08E-07	1.18E-05	6.3750379

cg16808587	chr4	174432425	+			0.8604032	0.0695148	5.3917261	3.08E-07	1.18E-05	6.3749143
cg17422774	chr10	124901928	+			1.8797762	-1.985373	5.3938581	3.08E-07	1.18E-05	6.3761055
cg23092086	chr16	55909573	+	CES7;CES7	TSS1500;TSS1500	-1.454002	0.6695729	-5.391481	3.08E-07	1.18E-05	6.3738466
cg13647536	chr7	27260466	+			1.3842179	-0.962481	5.391313	3.09E-07	1.19E-05	6.3731123
cg02161292	chr5	140821687	+	PCDHGA4;PCDHGA12;PCDHGA11;PCDHGA11;PCDHGA9;PCDHGA1;PCDHGB1;PCDHGB6;PCDHGB3;PCDHGB7;PCDHGA6;PCDHGA8;PCDHGA10;PCDHGA5;PCDHGB4;PCDHGA3;PCDHG	Body;Body;Body;Body;Body;Body;Body;Body;Body;Body;Body;Body;Body;Body;Body;Body;Body;Body;Body;Body	0.9470975	0.9980201	5.3912135	3.09E-07	1.19E-05	6.3726779
cg25123866	chr11	64528328	-	PYGM;PYGM	TSS200;TSS200	-0.771148	-1.325603	-5.391163	3.09E-07	1.19E-05	6.3724596
cg01828111	chr16	15802082	+	MYH11;NDE1;NDE1;MYH11;MYH11;MYH11	Body;Body;Body;Body;3'UTR;3'UTR	0.6915438	1.8433452	5.3931879	3.09E-07	1.19E-05	6.3731847
cg04402891	chr20	61668469	+	NCRNA00029	TSS200	-0.948095	-0.088114	-5.390794	3.09E-07	1.19E-05	6.3708489
cg06361127	chr1	182024689	+	ZNF648	3'UTR	-1.347673	0.3690512	-5.390674	3.10E-07	1.19E-05	6.3703263
cg17297305	chr1	185287148	+	IVNS1ABP	TSS1500	-0.499556	-3.559369	-5.390602	3.10E-07	1.19E-05	6.370009
cg21424940	chr7	19158666	+	TWIST1	TSS1500	1.2806404	-1.236113	5.3905991	3.10E-07	1.19E-05	6.3699976
cg07094075	chr17	1182236	+	TUSC5	TSS1500	-1.27808	0.7243153	-5.390293	3.10E-07	1.19E-05	6.368663
cg17254386	chr5	70897396	+	MCCC2	Body	-1.381347	0.4116996	-5.390101	3.10E-07	1.19E-05	6.3678257
cg24298684	chr20	54823878	+	MC3R;MC3R	5'UTR;1stExon	-0.97856	-1.229564	-5.389837	3.11E-07	1.19E-05	6.3666733
cg16493071	chr16	1225126	+	CACNA1H;CACNA1H	Body;Body	-2.900214	1.813507	-5.389662	3.11E-07	1.19E-05	6.3659105
cg19489494	chr6	42146143	-	GUCA1A	Body	0.8426385	0.0389341	5.3896845	3.11E-07	1.19E-05	6.3660078
cg26287741	chr6	138867125	-	NHSL1	Body	1.0076994	0.1033059	5.3896754	3.11E-07	1.19E-05	6.3659679
cg07609206	chr2	1270354	-	SNTG2	Body	-1.152633	0.3128644	-5.38961	3.11E-07	1.19E-05	6.365682
cg14614689	chr10	133019987	-	TCERG1L	Body	-1.135432	-0.366122	-5.388969	3.12E-07	1.20E-05	6.3628851
cg21551253	chr12	131418217	+			-1.216075	-0.581598	-5.388849	3.12E-07	1.20E-05	6.3623653

cg22340508	chr19	22891978	+			1.4589426	0.3255508	5.3888545	3.12E-07	1.20E-05	6.3623874
cg11329129	chr11	705692	+	EPS8L2	TSS1500	-0.744977	-0.905237	-5.38883	3.12E-07	1.20E-05	6.3622799
cg02776313	chr22	50965782	+	TYMP;SCO2;TYMP;TYMP;SCO2	Body;TSS1500;Body;Body;TSS1500	-0.903475	0.1778544	-5.388808	3.12E-07	1.20E-05	6.3621853
cg12682972	chr2	102444908	+	MAP4K4;MAP4K4;MAP4K4	Body;Body;Body	1.0650105	1.3304585	5.3885736	3.13E-07	1.20E-05	6.3611621
cg02124758	chr10	124910217	-			1.2977682	-0.356346	5.3884371	3.13E-07	1.20E-05	6.360567
cg11165158	chr16	86613094	+	FOXL1	1stExon	1.3923285	-1.516645	5.3884525	3.13E-07	1.20E-05	6.3606338
cg15584221	chr1	210546902	+	HHAT;HHAT;HHAT;HHAT;HHAT;HHAT	Body;Body;Body;Body;Body;Body	-1.701106	0.9208049	-5.388494	3.13E-07	1.20E-05	6.360815
cg22614891	chr1	3567163	+	WDR8	TSS1500	0.8908013	-1.449361	5.3884452	3.13E-07	1.20E-05	6.3606021
cg26843711	chr5	140753449	+	PCDHGA4;PCDHGA6;PCDHGA1;PCDHGA5;PCDHGB1;PCDHGA6;PCDHGA3;PCDHGA2;PCDHGB2;PCDHGB3	Body;TSS1500;Body;Body;Body;TSS1500;Body;Body;Body;Body	1.1312842	-0.854616	5.3884644	3.13E-07	1.20E-05	6.3606857
cg13100449	chr1	184836205	+	FAM129A	Body	-2.225033	-1.415381	-5.388291	3.13E-07	1.20E-05	6.3599304
cg15584070	chr2	129439854	+			-1.312799	0.2482356	-5.388118	3.13E-07	1.20E-05	6.359176
cg16196812	chr10	120355139	+	PRLHR;PRLHR	5'UTR;1stExon	0.8703942	-0.86081	5.3880322	3.13E-07	1.20E-05	6.358801
cg07527126	chr17	11165833	+	SHISA6	Body	-1.437242	-0.168476	-5.387863	3.14E-07	1.20E-05	6.3580609
cg16225405	chr11	67141422	-	LOC100130987;CLCF1;CLCF1	Body;TSS1500;5'UTR	-0.699172	-3.948593	-5.3878	3.14E-07	1.20E-05	6.3577898
cg25792351	chr2	43414162	+			0.8914869	1.3352105	5.3877297	3.14E-07	1.20E-05	6.3574816
cg22335340	chr12	7055153	+	PTPN6;C12orf57	TSS1500;3'UTR	-0.780574	-0.086535	-5.387671	3.14E-07	1.20E-05	6.3572276
cg15961219	chr14	53960406	-			-1.423972	-0.691923	-5.387114	3.15E-07	1.21E-05	6.3547949
:h.8.103893680I	chr8	103824504	+			-0.714437	-4.24233	-5.386759	3.15E-07	1.21E-05	6.3532465
cg18196453	chr6	125334555	+	RNF217	Body	-1.084258	-0.169808	-5.386675	3.15E-07	1.21E-05	6.3528808
cg10708898	chr1	35309303	-			0.8206885	-0.357546	5.3865876	3.15E-07	1.21E-05	6.3525009

cg07681935	chr4	174452461	-	NBLA00301;HAND2	Body;TSS1500	2.138205	-0.583353	5.3886994	3.16E-07	1.21E-05	6.3712707
cg13858909	chr15	29489350	+	FAM189A1	Body	-1.210926	0.3707047	-5.386546	3.16E-07	1.21E-05	6.3523206
cg14530143	chr11	122179528	-			-1.760485	0.3361018	-5.386475	3.16E-07	1.21E-05	6.3520084
cg16158220	chr2	79383891	-			-1.334575	0.1108372	-5.386109	3.16E-07	1.21E-05	6.3504148
cg22101174	chr2	240230452	-	HDAC4	Body	1.0243623	1.5203263	5.3881396	3.16E-07	1.21E-05	6.3511907
cg05121006	chr4	85402397	-			1.3698106	-0.819995	5.3857537	3.17E-07	1.21E-05	6.3488649
cg00431198	chr16	76266534	+			-1.701238	0.9086999	-5.385682	3.17E-07	1.21E-05	6.3485511
cg24169669	chr14	38092080	+			1.4906455	1.6675969	5.3897333	3.17E-07	1.21E-05	6.3499428
cg02998394	chr4	99849011	-	EIF4E;EIF4E;EIF4E	Body;Body;Body	-0.66844	-3.425178	-5.385158	3.18E-07	1.21E-05	6.3462673
cg04849985	chr1	68215783	+	GNG12	5'UTR	-1.119892	-1.992626	-5.38516	3.18E-07	1.21E-05	6.3462757
cg04025150	chr1	34642862	-	C1orf94;C1orf94;C1orf94	5'UTR;1stExon;5'UTR	1.7635769	-1.400209	5.3872026	3.18E-07	1.22E-05	6.3471102
cg15952370	chr11	124190997	-	OR8D2	TSS1500	-1.363876	-0.678278	-5.385022	3.18E-07	1.22E-05	6.3456768
cg01033419	chr6	56387378	-	DST;DST;DST;DST;DST	Body;Body;Body;Body;Body	0.8433486	1.2510352	5.3848464	3.18E-07	1.22E-05	6.3449091
cg13863764	chr1	11561908	+	PTCHD2	Body	1.0147214	1.653602	5.3869028	3.18E-07	1.22E-05	6.3454219
cg25975689	chr12	54330354	-			1.63231	-2.093453	5.3847536	3.18E-07	1.22E-05	6.3445046
cg02119840	chr7	150020900	-	ACTR3C;LRRC61;LRRC61	TSS200;5'UTR;5'UTR	-0.925478	-3.065306	-5.384574	3.18E-07	1.22E-05	6.3437233
cg07136029	chr4	24690304	-			-1.301661	1.3861551	-5.384503	3.18E-07	1.22E-05	6.343413
cg10293403	chr6	10421670	-			1.9023976	-1.597586	5.3843595	3.19E-07	1.22E-05	6.3427866
cg05687083	chr7	5371160	+	TNRC18	Body	-1.873516	-0.76763	-5.384337	3.19E-07	1.22E-05	6.3426879
cg13414270	chr2	45465395	-			-1.441722	-1.496408	-5.384294	3.19E-07	1.22E-05	6.3425019

cg13784017	chr1	68166689	-			-1.245387	0.0477103	-5.384001	3.19E-07	1.22E-05	6.3412233
cg21129006	chr12	4023078	+			-1.531206	-0.145632	-5.383985	3.19E-07	1.22E-05	6.3411557
cg23302945	chr2	594950	-			-1.262913	0.2333643	-5.383984	3.19E-07	1.22E-05	6.3411505
cg22001211	chr2	61766532	-	XPO1	TSS1500	-1.037796	-2.952588	-5.383939	3.19E-07	1.22E-05	6.3409554
cg17327828	chr14	101435254	+	SNORD114-12;SNORD114-13	TSS200;TSS1500	-1.297406	0.063921	-5.3836	3.20E-07	1.22E-05	6.3394737
cg03072621	chr16	6069929	-	A2BP1;A2BP1;A2BP1;A2BP1	5'UTR;1stExon;1stExon;5'UTR	1.5688757	-1.306019	5.38336	3.20E-07	1.22E-05	6.3384296
cg04063045	chr2	71124531	-			1.0638421	-1.146366	5.3833004	3.20E-07	1.22E-05	6.3381697
cg02346706	chr15	101645225	-			-1.039294	-0.179607	-5.382885	3.21E-07	1.23E-05	6.3363602
cg13782213	chr9	3183159	-			-1.376724	0.5811058	-5.384998	3.21E-07	1.23E-05	6.3371254
cg15880113	chr14	101592480	+			-1.038008	0.5788071	-5.382758	3.21E-07	1.23E-05	6.3358064
cg27198040	chr12	28912345	-			-1.338485	-0.20034	-5.382556	3.21E-07	1.23E-05	6.3349248
cg11041817	chr17	46685327	-	HOXB7	Body	0.8806181	0.9062996	5.3824808	3.21E-07	1.23E-05	6.3345976
cg10746936	chr14	95240143	+			1.9433101	-0.800533	5.3824094	3.22E-07	1.23E-05	6.3342862
cg25130710	chr12	133159752	+	FBRSL1	Body	1.3418861	2.7304137	5.3823629	3.22E-07	1.23E-05	6.3340838
cg04566694	chr1	9312021	+	H6PD	Body	0.7348937	1.7511954	5.3823247	3.22E-07	1.23E-05	6.3339171
cg08599909	chr7	1299363	-			-0.947177	-0.208028	-5.382307	3.22E-07	1.23E-05	6.3338416
cg14677048	chr6	4282075	+			-1.122241	-2.779759	-5.382285	3.22E-07	1.23E-05	6.3337457
cg13824156	chr22	24199059	-	SLC2A11;SLC2A11;SLC2A11;SLC2A11;SLC2A11	1stExon;5'UTR;TSS1500;1stExon;5'UTR	-0.856323	-1.754751	-5.38221	3.22E-07	1.23E-05	6.3334178
cg04265797	chr14	38679952	+	SSTR1	3'UTR	1.235363	-1.170758	5.3821567	3.22E-07	1.23E-05	6.3331849
cg07903472	chr2	168846657	+	STK39	Body	1.000036	1.4608956	5.3819808	3.22E-07	1.23E-05	6.3324182

cg07138024	chr7	25608323	+			-1.68777	0.2397849	-5.381558	3.23E-07	1.23E-05	6.3305749
cg27102864	chr16	58535418	-	NDRG4;NDRG4;NDRG4	Body;Body;Body	-1.112945	-1.994245	-5.381381	3.23E-07	1.23E-05	6.329804
cg13382769	chr7	79084078	+	MAGI2	TSS1500	1.5247496	-1.067007	5.3834732	3.23E-07	1.23E-05	6.3304892
cg18799217	chr5	87985589	-			1.2308007	-1.200167	5.381271	3.23E-07	1.23E-05	6.329325
cg08318726	chr12	114844265	-	TBX5;TBX5;TBX5	TSS1500;5'UTR;5'UTR	1.9964679	-1.1258	5.3832753	3.23E-07	1.23E-05	6.330011
cg20026737	chr12	88389682	-	C12orf50	Body	-0.765924	-3.273433	-5.381143	3.23E-07	1.23E-05	6.3287687
cg16686960	chr22	31022898	-	TCN2	3'UTR	-0.820975	-0.104099	-5.383063	3.24E-07	1.23E-05	6.328703
cg01272790	chr2	79255434	+	REG3G;REG3G	3'UTR;3'UTR	-1.472768	0.4271846	-5.380889	3.24E-07	1.23E-05	6.3276609
cg22789714	chr11	46261208	-			-0.786391	-1.761511	-5.396642	3.24E-07	1.23E-05	6.3363453
cg25195899	chr17	25640203	+	WSB1;WSB1	3'UTR;3'UTR	1.0952332	1.6226203	5.3808063	3.24E-07	1.23E-05	6.3273002
cg05237374	chr14	66980127	+	GPHN;GPHN	Body;Body	1.2696433	0.4361214	5.3807204	3.24E-07	1.23E-05	6.3269258
cg02582191	chr2	240970850	-	OR6B2	TSS1500	-1.039855	-0.234578	-5.380674	3.24E-07	1.24E-05	6.3267215
cg13813458	chr17	35085134	-			1.4855141	0.2590841	5.3804882	3.24E-07	1.24E-05	6.325914
cg05697849	chr1	50513766	+	ELAVL4;ELAVL4	1stExon;5'UTR	1.5222934	-0.012109	5.3804625	3.24E-07	1.24E-05	6.325802
cg04832767	chr7	96632841	+	DLX6AS	Body	1.7416117	-1.82245	5.3803354	3.25E-07	1.24E-05	6.325248
cg24555906	chr10	134882170	+			-0.905361	-0.287637	-5.380241	3.25E-07	1.24E-05	6.324838
cg18907180	chr14	97499987	+			1.4190279	-1.528729	5.3801761	3.25E-07	1.24E-05	6.3245541
cg01670677	chr11	71955322	+	PHOX2A	TSS200	2.7854426	-2.745977	5.3799832	3.25E-07	1.24E-05	6.3237133
cg23340214	chr12	106974225	-			1.0457056	0.1286711	5.3799315	3.25E-07	1.24E-05	6.323488
cg01127423	chr11	45742908	-			-0.874642	-0.776817	-5.381945	3.25E-07	1.24E-05	6.32422

cg01616956	chr2	232393196	+	NMUR1	Body	1.0338346	2.1670102	5.3797802	3.25E-07	1.24E-05	6.3228291
cg25371634	chr2	176981422	+	HOXD10	TSS200	1.7357748	-1.300919	5.3797789	3.25E-07	1.24E-05	6.3228234
cg13804476	chr13	113976694	+	LAMP1	Body	0.9952093	2.4465039	5.3796518	3.26E-07	1.24E-05	6.3222697
cg09390241	chr4	8174148	-			-1.887714	0.216718	-5.379534	3.26E-07	1.24E-05	6.3217567
cg00158122	chr10	101290029	+			0.7132799	0.3378181	5.3816544	3.26E-07	1.24E-05	6.3225726
cg10000052	chr19	5048426	-	KDM4B	Body	-0.526731	-3.206538	-5.379369	3.26E-07	1.24E-05	6.3210384
cg12656085	chr2	85173360	+			-1.399719	-0.662927	-5.381408	3.26E-07	1.24E-05	6.3215012
cg03764062	chr11	64008174	-	FKBP2;FKBP2;FKBP2	TSS1500;TSS1500;TSS1500	-0.361258	-4.059612	-5.379078	3.26E-07	1.24E-05	6.3197682
cg14699728	chr11	66188522	-	NPAS4;NPAS4	5'UTR;1stExon	1.9634769	-0.257217	5.3789882	3.27E-07	1.24E-05	6.3193783
cg03272310	chr17	39704454	+			-1.266515	-2.012505	-5.378967	3.27E-07	1.24E-05	6.3192879
cg01826682	chr2	105459097	-			1.3415553	-1.479532	5.3788626	3.27E-07	1.24E-05	6.3188313
cg05848884	chr8	74848047	+			-1.265749	-0.601347	-5.378813	3.27E-07	1.24E-05	6.3186162
cg10233463	chr17	56246650	-	OR4D2	TSS1500	-1.601793	0.4240133	-5.378474	3.27E-07	1.25E-05	6.3171366
cg18372620	chr11	102963125	-	DCUN1D5	TSS200	-0.515636	-4.10662	-5.377991	3.28E-07	1.25E-05	6.3150327
cg06460076	chr11	45711402	-			-1.428425	0.7999696	-5.377944	3.28E-07	1.25E-05	6.3148285
cg12679230	chr3	155463133	-			-1.188996	-2.400779	-5.377954	3.28E-07	1.25E-05	6.3148749
cg08466740	chr16	6534829	+	A2BP1;A2BP1	5'UTR;5'UTR	-1.096653	-0.579019	-5.377836	3.28E-07	1.25E-05	6.3143597
cg03422944	chr10	105344723	+	NEURL	Body	0.7767118	0.2170768	5.3778068	3.28E-07	1.25E-05	6.314232
cg25555059	chr5	128301488	-	SLC27A6;SLC27A6;SLC27A6	5'UTR;1stExon;5'UTR	1.0768885	-0.315476	5.3775135	3.29E-07	1.25E-05	6.3129541
cg05775862	chr10	31026101	+			-1.781546	-2.090953	-5.377358	3.29E-07	1.25E-05	6.3122776

cg19378216	chr20	31870775	+	C20orf114	TSS200	-1.261251	-0.355823	-5.377249	3.29E-07	1.25E-05	6.3118036
cg03660331	chr7	56564743	-	DKFZp434L192	Body	-1.049211	-1.071352	-5.377128	3.29E-07	1.25E-05	6.311274
cg12457270	chr10	116678438	-			-1.247155	0.1904707	-5.377034	3.30E-07	1.25E-05	6.3108671
cg03160217	chr2	117814299	+			-1.47335	0.6292686	-5.37698	3.30E-07	1.25E-05	6.3106292
cg11015549	chr19	1026207	+	CNN2;CNN2	TSS200;TSS200	-0.79238	-3.161364	-5.376938	3.30E-07	1.25E-05	6.3104489
cg12080717	chr1	248004886	+	OR11L1	1stExon	-1.104104	-0.268336	-5.376952	3.30E-07	1.25E-05	6.3105072
cg17325792	chr1	77042560	-	ST6GALNAC3;ST6GALNAC3	3'UTR;Body	-1.637373	0.9813636	-5.376971	3.30E-07	1.25E-05	6.3105914
cg18538751	chr2	233879149	-	NGEF	TSS1500	-1.222791	-0.261433	-5.376798	3.30E-07	1.25E-05	6.3098374
cg06732666	chr2	208890496	+	PLEKHM3	TSS1500	-1.073951	-2.520731	-5.376627	3.30E-07	1.25E-05	6.3090922
cg09232478	chr1	119548852	+			1.4638687	-0.52926	5.3766122	3.30E-07	1.25E-05	6.3090286
cg08639586	chr19	34111483	-	CHST8;CHST8	TSS1500;TSS1500	-1.205835	0.1718756	-5.376508	3.30E-07	1.25E-05	6.3085745
cg15333426	chr4	10463620	-			1.2429815	-1.021724	5.3786536	3.30E-07	1.25E-05	6.3098987
cg03817667	chr7	54609953	+	VSTM2A	TSS200	1.776759	-0.291761	5.376425	3.30E-07	1.25E-05	6.3082132
cg00087728	chr6	33385262	+	CUTA;CUTA;CUTA;CUTA;CUTA	Body;Body;Body;1stExon;Body	-0.461584	-4.631959	-5.376368	3.31E-07	1.25E-05	6.307967
cg23684318	chr10	133061213	-	TCERG1L	Body	-1.47332	0.9959005	-5.376234	3.31E-07	1.26E-05	6.3073809
cg23934404	chr13	112758491	-			2.2438232	-1.944868	5.3762209	3.31E-07	1.26E-05	6.3073241
cg08708790	chr16	22176873	+			-0.973845	-0.520021	-5.376169	3.31E-07	1.26E-05	6.3070968
cg15087179	chr6	50676699	+			1.6150685	-0.971313	5.3760173	3.31E-07	1.26E-05	6.3064373
cg10038618	chr1	3567719	+	TP73;WDR8	TSS1500;TSS1500	1.1025279	-0.29624	5.3758968	3.31E-07	1.26E-05	6.3059128
cg23577033	chr10	75636861	+			-1.324942	0.3431922	-5.375525	3.32E-07	1.26E-05	6.3042923

cg23642130	chr19	30866365	+	ZNF536	5'UTR	2.3253568	-1.312293	5.377611	3.32E-07	1.26E-05	6.3049787
cg11792470	chr14	104630673	-	KIF26A	Body	-2.596559	2.0571944	-5.375421	3.32E-07	1.26E-05	6.3038393
cg22402753	chr11	56512503	-	OR9G4	TSS1500	-1.43276	0.1558688	-5.375389	3.32E-07	1.26E-05	6.3036997
cg19871361	chr10	11505280	+	USP6NL;USP6NL	Body;Body	0.9884503	3.4254565	5.3753148	3.32E-07	1.26E-05	6.3033781
cg12399700	chr12	113902262	+	LHX5	Body	2.2546468	-1.398995	5.3752607	3.32E-07	1.26E-05	6.3031425
cg03579107	chr2	867791	+			-0.762599	-0.34251	-5.375009	3.33E-07	1.26E-05	6.3020456
cg07463848	chr8	49985379	+	C8orf22	5'UTR	-1.518075	0.6086308	-5.374978	3.33E-07	1.26E-05	6.3019122
cg08552214	chr2	176931864	-			1.5631849	-0.834391	5.3749641	3.33E-07	1.26E-05	6.301851
cg12271797	chr8	50891864	+	SNTG1	5'UTR	-1.423962	0.652397	-5.374986	3.33E-07	1.26E-05	6.3019475
cg24011260	chr7	153584748	-	DPP6;DPP6	1stExon;5'UTR	1.7899533	-2.170413	5.3749311	3.33E-07	1.26E-05	6.3017075
cg06298530	chr15	25481210	-	SNORD115-11;SNORD115-29;SNORD115-36;SNORD115-43	TSS200;TSS200;TSS200;TSS200	-1.235181	-0.33285	-5.37461	3.33E-07	1.26E-05	6.3003092
cg22853542	chr8	128812713	-	PVT1	Body	-1.373793	0.8304789	-5.374592	3.33E-07	1.26E-05	6.3002302
cg12623852	chr1	228075452	-			1.3728373	-0.311933	5.3745616	3.33E-07	1.26E-05	6.3000981
cg17529832	chr2	105461096	-			2.8291548	-2.429707	5.3743923	3.34E-07	1.26E-05	6.299361
cg06895005	chr16	86168540	+			-1.280187	0.818226	-5.374294	3.34E-07	1.26E-05	6.2989348
cg08403064	chr11	44068709	-	ACCSL	TSS1500	-1.260261	-0.371913	-5.374165	3.34E-07	1.27E-05	6.2983703
cg00066942	chr2	149401448	-	EPC2	TSS1500	-0.928989	-3.508086	-5.374102	3.34E-07	1.27E-05	6.2980978
cg25882256	chr1	25257759	-	RUNX3;RUNX3	TSS1500;Body	1.0211051	0.2980052	5.3741106	3.34E-07	1.27E-05	6.2981345
cg17048700	chr15	95985725	-	LOC145820;LOC145820	Body;Body	-1.205371	0.1709557	-5.373876	3.34E-07	1.27E-05	6.2971119
cg03037030	chr6	31543300	+	TNF	TSS200	-1.510108	-1.678763	-5.373782	3.34E-07	1.27E-05	6.2967048

cg17239057	chr1	224363575	+			1.2961181	1.5537222	5.3737774	3.34E-07	1.27E-05	6.2966838
cg24488469	chr1	25426427	+			-0.889349	-1.958141	-5.373811	3.34E-07	1.27E-05	6.296832
cg23598604	chr1	99732472	-	LPPR4;LPPR4	Body;Body	-1.431258	-0.231478	-5.373693	3.35E-07	1.27E-05	6.2963178
cg06288900	chr16	25868139	-	HS3ST4	Body	-1.271885	-0.32795	-5.373506	3.35E-07	1.27E-05	6.2955013
cg00098144	chr4	13525235	-			1.5526816	-0.964938	5.3733456	3.35E-07	1.27E-05	6.2948037
cg06710735	chr3	48264567	+	CAMP	TSS1500	-1.416293	0.4358106	-5.375353	3.35E-07	1.27E-05	6.2955409
cg07197493	chr11	9884649	-	SBF2	Body	1.1823851	0.6360149	5.3753537	3.35E-07	1.27E-05	6.2951605
cg08094614	chr12	53319217	-			-1.354572	1.1342826	-5.373214	3.35E-07	1.27E-05	6.2942299
cg24147840	chr8	117682246	+	EIF3H	Body	0.8910141	1.0824035	5.3732342	3.35E-07	1.27E-05	6.2943187
cg07325893	chr11	46293567	-			-0.820134	0.1551277	-5.373115	3.35E-07	1.27E-05	6.2938002
cg26356694	chr11	72948278	-			-0.849196	-0.110659	-5.373051	3.36E-07	1.27E-05	6.2935227
cg21714266	chr6	146350748	-	GRM1;GRM1	Body;Body	1.3499703	-0.397572	5.372914	3.36E-07	1.27E-05	6.292925
cg00178551	chr8	1164044	-			-0.985532	0.0932199	-5.372812	3.36E-07	1.27E-05	6.2924817
cg02516845	chr16	84076320	+	SLC38A8	TSS1500	-0.964881	-0.33537	-5.372809	3.36E-07	1.27E-05	6.2924693
cg24693551	chr6	28697819	+			1.5315154	-1.501972	5.3728233	3.36E-07	1.27E-05	6.29253
cg02963613	chr2	1425319	+	TPO;TPO;TPO;TPO	Body;Body;Body;Body	-1.184343	0.2394579	-5.372692	3.36E-07	1.27E-05	6.2919573
cg20427318	chr6	134757763	+			-1.539762	-0.988577	-5.372602	3.36E-07	1.27E-05	6.2915644
cg14537800	chr4	15964950	-	FGFBP2	TSS200	-0.682647	-2.552392	-5.372426	3.37E-07	1.27E-05	6.2907998
cg12329275	chr10	11912597	+	C10orf47	3'UTR	0.8114485	2.6003925	5.3722967	3.37E-07	1.27E-05	6.2902375
cg00991179	chr16	56000067	+			-0.866265	-1.634008	-5.372217	3.37E-07	1.27E-05	6.2898898

cg15723195	chr11	58911845	-	FAM111A;FAM111A;FAM111A;FAM111A;FAM111A	5'UTR;5'UTR;5'UTR;TSS1500;TSS1500	-1.300047	0.2509307	-5.371967	3.37E-07	1.28E-05	6.2888022
cg10063845	chr2	80363347	+	CTNNA2;CTNNA2	Body;Body	-1.634939	0.8013005	-5.374006	3.37E-07	1.28E-05	6.2893015
cg07387286	chr8	39172120	+	ADAM5P	TSS200	1.4166722	0.5470163	5.3739515	3.37E-07	1.28E-05	6.2890629
cg03228766	chr7	57878945	+			-1.006281	-0.433614	-5.371629	3.38E-07	1.28E-05	6.2873322
cg23727983	chr11	125774082	-	DDX25;PUS3	TSS200;TSS1500	2.7785804	-1.561054	5.3734688	3.38E-07	1.28E-05	6.2869641
cg11389960	chr15	31277661	-	MTMR10	Body	0.8761564	0.9626055	5.3711733	3.38E-07	1.28E-05	6.2853475
cg14566901	chr11	57948705	-	OR9Q1	3'UTR	-1.626329	0.3714819	-5.371033	3.39E-07	1.28E-05	6.2847385
cg00997079	chr2	80529990	+	CTNNA2;LRRTM1;CTNNA2	Body;Body;Body	1.0322442	-1.0612	5.3731162	3.39E-07	1.28E-05	6.2858157
cg12110537	chr3	122721909	+	SEMA5B	5'UTR	-1.534145	1.3983977	-5.370919	3.39E-07	1.28E-05	6.2842416
cg08755026	chr2	85819614	-	VAMP5	Body	-1.300615	0.4386769	-5.370859	3.39E-07	1.28E-05	6.2839779
cg20668321	chr5	153192843	+	GRIA1;GRIA1	3'UTR;3'UTR	-1.375712	0.4883897	-5.370861	3.39E-07	1.28E-05	6.2839891
cg04303500	chr20	59602012	-			-1.41523	0.7711261	-5.370837	3.39E-07	1.28E-05	6.2838827
cg20154287	chr19	1267350	-			-0.738809	-2.257724	-5.370818	3.39E-07	1.28E-05	6.2838021
cg12445786	chr15	89533560	-			-1.072647	-0.236932	-5.370785	3.39E-07	1.28E-05	6.283657
cg18159860	chr5	72678420	+			0.9898968	-0.64779	5.370755	3.39E-07	1.28E-05	6.2835269
cg18800143	chr1	16393791	-	FAM131C	Body	-1.01142	-0.246796	-5.370704	3.39E-07	1.28E-05	6.2833026
cg26818489	chr11	119380820	-			-0.988676	-1.612864	-5.37067	3.39E-07	1.28E-05	6.2831583
cg11682350	chr6	33240284	+	VPS52;RPS18	TSS1500;Body	-0.537739	-3.594346	-5.372761	3.39E-07	1.28E-05	6.2842709
cg05194362	chr2	114036444	-	PAX8;PAX8;PAX8;PAX8;PAX8;PAX8;PAX8;LOC440839;PAX8;PAX8;PAX8	1stExon;5'UTR;5'UTR;1stExon;5'UTR;1stExon;1stExon;Body;5'UTR;1stExon;5'UTR	0.5232633	1.5516933	5.3705792	3.39E-07	1.28E-05	6.2827617
cg18391899	chr10	23462616	-			1.4694336	-1.354839	5.3705758	3.39E-07	1.28E-05	6.2827469

cg24653728	chr11	124294778	-	OR8B4	TSS200	-1.339159	0.4667585	-5.370516	3.39E-07	1.28E-05	6.2824878
cg13628577	chr5	122431121	-	PRDM6	Body	1.9435868	-1.086505	5.3704798	3.40E-07	1.28E-05	6.282329
cg11874882	chr17	31910113	-	ACCN1	Body	-1.425917	0.3714834	-5.370444	3.40E-07	1.28E-05	6.2821747
cg10576132	chr7	37899045	-	TXNDC3	Body	-1.51129	-0.112239	-5.370355	3.40E-07	1.28E-05	6.2817858
cg20618433	chr19	11266919	+	SPC24	TSS1500	-0.943195	-0.802964	-5.370266	3.40E-07	1.28E-05	6.281399
cg19291576	chr9	969530	-			1.5990253	-0.511609	5.3702328	3.40E-07	1.28E-05	6.2812539
cg20080282	chr11	107896210	+	CUL5	Body	0.6367948	1.596172	5.3701518	3.40E-07	1.28E-05	6.2809014
cg11358741	chr12	125093204	+			-0.935215	-1.147833	-5.370069	3.40E-07	1.28E-05	6.2805422
cg19402335	chr1	159410002	-	OR10J1	1stExon	-1.770671	0.9478626	-5.370025	3.40E-07	1.28E-05	6.2803503
cg26147480	chr10	126490099	+	FAM175B	TSS1500	-1.105019	1.4041011	-5.370005	3.40E-07	1.28E-05	6.2802626
cg06430061	chr5	75013592	-	C5orf37	TSS1500	-0.890556	-1.424556	-5.369914	3.40E-07	1.28E-05	6.2798678
cg26263263	chr10	118893430	+	VAX1;VAX1	3'UTR;Body	1.0759591	-0.603551	5.3698258	3.41E-07	1.28E-05	6.2794828
cg26680097	chr3	70048832	+			-2.507912	2.6368813	-5.371875	3.41E-07	1.28E-05	6.2804198
cg08269733	chr16	62057179	-	CDH8	5'UTR	-1.261297	0.4057079	-5.369566	3.41E-07	1.29E-05	6.278354
cg04330449	chr5	134871166	+	NEUROG1	1stExon	1.8345986	-1.036909	5.3694725	3.41E-07	1.29E-05	6.2779453
cg13611822	chr7	8483710	+	NXPH1	Body	1.5804939	-1.3538	5.3691616	3.42E-07	1.29E-05	6.2765921
cg24771804	chr18	55107755	+	ONECUT2	Body	1.5387347	-1.874186	5.3690957	3.42E-07	1.29E-05	6.2763054
cg17930201	chr1	155828174	-	SYT11;GON4L;GON4L	TSS1500;TSS1500;TSS1500	-1.438128	-0.597996	-5.368934	3.42E-07	1.29E-05	6.2755998
cg26382586	chr2	131797606	-	ARHGEF4;ARHGEF4	Body;Body	0.9634209	-0.506299	5.368948	3.42E-07	1.29E-05	6.2756627
cg08812189	chr3	147110367	-	ZIC4;ZIC4;ZIC4;ZIC4;ZIC4	Body;TSS200;TSS200;Body;Body	1.6938277	-1.013466	5.3688454	3.42E-07	1.29E-05	6.2752165

cg19734896	chr1	166888684	+	ILDR2	Body	-1.9819	0.9957648	-5.368855	3.42E-07	1.29E-05	6.2752589
cg20122943	chr19	54483532	-	CACNG8	Body	2.427264	-1.747594	5.368746	3.42E-07	1.29E-05	6.2747839
cg25125340	chr12	131172703	+			-1.284075	0.1491704	-5.368705	3.42E-07	1.29E-05	6.2746035
cg21492137	chr14	101525939	-	MIR496;MIR154	TSS1500;TSS200	-1.51469	0.7126151	-5.368606	3.42E-07	1.29E-05	6.2741736
cg26247168	chr11	119994722	+	TRIM29	Body	-1.986211	1.6469333	-5.368439	3.43E-07	1.29E-05	6.2734491
cg02519286	chr12	6642354	-	GAPDH	TSS1500	-0.699453	-3.113857	-5.368361	3.43E-07	1.29E-05	6.2731086
cg03991953	chr2	241764454	+			-1.164277	-0.004191	-5.368337	3.43E-07	1.29E-05	6.2730044
cg02613295	chr7	2564256	-	LFNG;LFNG;LFNG;LFNG	Body;Body;Body;Body	-0.907466	0.0615895	-5.370379	3.43E-07	1.29E-05	6.2735314
cg04011062	chr7	142724374	-	OR9A2	TSS200	-1.605225	0.8868904	-5.368201	3.43E-07	1.29E-05	6.2724121
cg12798338	chr17	76128683	-	TMC8;TMC6	Body;TSS200	-1.005519	-0.865305	-5.370193	3.43E-07	1.29E-05	6.2731089
cg12555334	chr7	56120290	+	CCT6A;PSPH;CCT6A	Body;TSS1500;Body	-0.938215	-1.464295	-5.367985	3.43E-07	1.29E-05	6.2714717
cg02852670	chr14	20903410	+	KLHL33	5'UTR	1.3690394	0.4291191	5.3678201	3.44E-07	1.29E-05	6.2707551
cg12673103	chr5	134364717	-	PITX1	Body	1.1860289	-0.646382	5.367805	3.44E-07	1.29E-05	6.2706894
cg11108060	chr2	111879843	-	BCL2L11;BCL2L11;BCL2L11	5'UTR;5'UTR;5'UTR	-0.454651	-3.917802	-5.367551	3.44E-07	1.29E-05	6.2695827
cg19545560	chr19	51688987	-			-0.80157	-0.674252	-5.369698	3.44E-07	1.29E-05	6.2709555
cg15980797	chr6	137813648	-	OLIG3;OLIG3	1stExon;3'UTR	1.3346168	-0.581965	5.3694868	3.44E-07	1.30E-05	6.2700394
cg05635600	chr10	2519129	-			-1.545929	1.1099357	-5.367231	3.45E-07	1.30E-05	6.2681921
cg02726924	chr21	43299868	-	PRDM15;PRDM15	TSS1500;TSS1500	-0.901202	-5.641105	-5.367176	3.45E-07	1.30E-05	6.2679529
cg20380768	chr7	130125511	+	MEST	TSS1500	-1.455657	-0.014558	-5.366935	3.45E-07	1.30E-05	6.2669055
cg26445794	chr16	10837274	-	NUBP1	TSS1500	-0.828523	-1.72806	-5.366904	3.45E-07	1.30E-05	6.2667712

cg24921858	chr12	12223989	-	BCL2L14;BCL2L14;BCL2L14;BCL2L14	5'UTR;TSS1500;1stExon;TSS1500	-1.217543	-1.370995	-5.36664	3.45E-07	1.30E-05	6.2656222
cg10646254	chr19	51326980	+	KLK1	1stExon	-0.645477	-1.167716	-5.366531	3.46E-07	1.30E-05	6.2651457
cg05423768	chr10	134692616	-			-1.21944	-0.094021	-5.366301	3.46E-07	1.30E-05	6.2641464
cg21045575	chr13	59169584	+			-1.317935	-0.426239	-5.366204	3.46E-07	1.30E-05	6.263723
cg27018424	chr6	33173425	-	HSD17B8	Body	-0.920293	-2.470342	-5.366134	3.46E-07	1.30E-05	6.2634219
cg02378847	chr15	74422750	+	ISLR2;ISLR2;LOC283731;ISLR2;ISLR2;ISLR2	5'UTR;5'UTR;TSS1500;1stExon;5'UTR;5'UTR	1.6809164	-1.540352	5.3660893	3.46E-07	1.30E-05	6.2632255
cg03521696	chr6	29795595	+	HLA-G	5'UTR	1.8475954	-0.832819	5.3660306	3.46E-07	1.30E-05	6.2629701
cg18118198	chr14	25100740	+	GZMB	Body	-1.029869	-0.265091	-5.36603	3.46E-07	1.30E-05	6.2629691
cg06020182	chr2	365232	+			-1.530751	0.6457019	-5.365983	3.47E-07	1.30E-05	6.2627641
cg08649201	chr6	6004464	-	NRN1	Body	1.1908455	-1.339055	5.3659775	3.47E-07	1.30E-05	6.2627392
cg03726785	chr20	1532543	+	SIRPD	Body	-1.450643	0.0789385	-5.36595	3.47E-07	1.30E-05	6.26262
cg09256941	chr16	20086891	+			-1.174846	-0.473281	-5.365695	3.47E-07	1.30E-05	6.2615098
cg08146945	chr16	86538307	-			0.852484	0.0741915	5.3655462	3.47E-07	1.30E-05	6.2608631
cg12305265	chr6	162065571	-	PARK2;PARK2;PARK2	Body;Body;Body	-1.648818	-0.168081	-5.365355	3.48E-07	1.31E-05	6.2600331
cg06307046	chr7	16685776	-	BZW2;BZW2;BZW2;ANKMY2;BZW2;BZW2	Body;1stExon;5'UTR;TSS1500;5'UTR;1stExon	1.0982494	-3.750931	5.3674661	3.48E-07	1.31E-05	6.261259
cg03330762	chr19	49686116	-	TRPM4	Body	0.6062378	2.6165564	5.3651763	3.48E-07	1.31E-05	6.2592541
cg08361126	chr10	15762091	+	ITGA8	TSS1500	1.44155	-1.278058	5.3650837	3.48E-07	1.31E-05	6.2588512
cg25711246	chr12	4917426	-	KCNA6	TSS1500	-1.111078	-0.284649	-5.364934	3.48E-07	1.31E-05	6.2582011
cg08173709	chr3	71103443	-	FOXP1	Body	0.8747932	2.0320417	5.3648966	3.48E-07	1.31E-05	6.2580376
cg00574563	chr17	35299270	+	LHX1	Body	1.4593798	-1.240463	5.366932	3.48E-07	1.31E-05	6.2589384

cg00046899	chr13	97777690	-			-0.996902	-1.954183	-5.364772	3.48E-07	1.31E-05	6.2574954
cg26629715	chr8	89379430	+			-1.822268	0.2451044	-5.364736	3.48E-07	1.31E-05	6.2573394
cg27438128	chr10	43951555	-	ZNF487	Body	-0.785226	0.0709875	-5.364587	3.49E-07	1.31E-05	6.25669
cg02725334	chr22	32146705	+	C22orf30	TSS1500	1.0600434	1.6004719	5.3642194	3.49E-07	1.31E-05	6.2550924
cg05736768	chr8	105479248	+	DPYS;DPYS	1stExon;5'UTR	1.6960712	-0.728281	5.3642514	3.49E-07	1.31E-05	6.2552313
cg22736872	chr5	601900	-			-2.185035	0.6037843	-5.364215	3.49E-07	1.31E-05	6.2550734
cg16234978	chr7	1704095	-			1.7411887	0.1456944	5.3684326	3.49E-07	1.31E-05	6.2722252
cg09848114	chr6	30130698	-	TRIM15	TSS1500	-1.5805	0.8420574	-5.364032	3.50E-07	1.31E-05	6.2542764
cg11484872	chr6	31543169	-	TNF	TSS200	-1.270938	0.0774855	-5.364034	3.50E-07	1.31E-05	6.2542845
cg21987785	chr19	36019198	-	SBSN;SBSN;SBSN;SBSN;SBSN;SBSN	5'UTR;1stExon;5'UTR;1stExon;5'UTR;1stExon	-0.997097	-0.459034	-5.363868	3.50E-07	1.31E-05	6.2535628
cg24693287	chr1	31885850	-	SERINC2	TSS200	-1.018563	-5.128546	-5.363798	3.50E-07	1.31E-05	6.2532616
cg16768850	chr11	76397220	+	GUCY2E	Body	-1.137774	0.0853361	-5.363777	3.50E-07	1.31E-05	6.2531697
cg24954895	chr5	140800761	-	PCDHGA4;PCDHGA9;PCDHGA1;PCDHGB1;PCDHGB6;PCDHGA11;PCDHGA11;PCDHGA11;PCDHGB3;PCDHGA11;PCDHGB7;PCDHGA6;PCDHGA8;PCDHGA11;PCDHGA10;PCDHGA5;PCDHG	Body;Body;Body;Body;Body;5'UTR;5'UTR;5'UTR;Body;1stExon;Body;Body;Body;1stExon;Body;Body;Body;Body;1stExon;Body;Body;Body;Body	1.5075936	-0.71465	5.363586	3.50E-07	1.31E-05	6.2523375
cg21437561	chr4	187026872	+			-0.630377	-3.111925	-5.36356	3.50E-07	1.31E-05	6.2522239
cg02995013	chr10	43365572	+			-0.833014	0.5623629	-5.3634	3.51E-07	1.31E-05	6.2515302
cg19344013	chr18	47721741	+	MYO5B	TSS1500	-0.351036	-3.890884	-5.363402	3.51E-07	1.31E-05	6.2515388
cg26185071	chr5	67506875	+			-1.219735	1.3304648	-5.363215	3.51E-07	1.32E-05	6.2507232
cg05090319	chr22	49297925	-			-2.63803	2.8537896	-5.363116	3.51E-07	1.32E-05	6.250292
cg24874191	chr5	43280334	-	MGC42105	Body	-1.704357	0.6931898	-5.363115	3.51E-07	1.32E-05	6.2502874
cg11940526	chr10	80311367	-			-1.399348	0.3804313	-5.363009	3.51E-07	1.32E-05	6.2498285

cg08267250	chr10	134872255	-			-1.389966	-2.442664	-5.362947	3.51E-07	1.32E-05	6.249558
cg03008387	chr20	62089152	+	KCNQ2;KCNQ2;KCNQ2;KCNQ2;KCNQ2	Body;Body;Body;Body;Body	-1.472406	-1.188217	-5.371654	3.51E-07	1.32E-05	6.2536841
cg24422198	chr18	59992387	+	TNFRSF11A	TSS200	-0.513157	-3.095438	-5.362889	3.51E-07	1.32E-05	6.2493073
cg11048797	chr17	80450903	-			-1.811082	0.988281	-5.36278	3.52E-07	1.32E-05	6.2488307
ch.19.239668R	chr19	4434073	+	CHAF1A	Body	-0.466595	-4.195097	-5.362772	3.52E-07	1.32E-05	6.248796
cg08495027	chr22	31364830	-	MORC2;TUG1	TSS1500;TSS1500	-0.336027	-3.245583	-5.362733	3.52E-07	1.32E-05	6.248629
cg00036585	chr6	164441131	+			-1.330684	0.6129627	-5.362483	3.52E-07	1.32E-05	6.2475427
cg21971285	chr11	31841818	-			1.0298659	-0.645975	5.362467	3.52E-07	1.32E-05	6.247472
cg01533082	chr14	58567637	+	C14orf37	Body	-1.511197	0.5841771	-5.362219	3.53E-07	1.32E-05	6.2463944
cg08151623	chr16	3242737	-			1.2689109	0.7278	5.3642916	3.53E-07	1.32E-05	6.247469
cg22658979	chr11	102826469	-	MMP13	TSS200	-1.647736	1.4209746	-5.36431	3.53E-07	1.32E-05	6.247164
cg00951213	chr8	1585929	+	DLGAP2	Body	-1.279632	-0.342132	-5.362073	3.53E-07	1.32E-05	6.2457569
cg13527233	chr19	3097570	-	GNA11	Body	1.2609123	1.6486628	5.3620794	3.53E-07	1.32E-05	6.2457863
cg24859937	chr8	38411708	+			-1.42317	-3.204455	-5.361751	3.53E-07	1.32E-05	6.2443587
cg12800270	chr6	32085328	-	ATF6B;ATF6B	Body;Body	0.4936948	1.6229861	5.3615992	3.54E-07	1.32E-05	6.2436985
cg24792113	chr4	69313197	-	TMPRSS11E;TMPRSS11E	1stExon;5'UTR	-1.019181	-0.867278	-5.361614	3.53E-07	1.32E-05	6.243765
cg19642505	chr6	30224156	-			-1.339304	-0.513165	-5.361547	3.54E-07	1.32E-05	6.2434729
cg25931491	chr11	1422468	+	BRSK2	Body	-2.233705	2.0777383	-5.361494	3.54E-07	1.32E-05	6.2432399
cg05471296	chr7	27196302	+	HOXA7	TSS200	2.2048606	-1.53014	5.3614125	3.54E-07	1.32E-05	6.2428869
cg16543094	chr11	20229517	-			2.5319658	-1.878694	5.3634235	3.54E-07	1.32E-05	6.2436989

cg18717146	chr19	1478715	-	C19orf25	Body	-1.096499	-2.226916	-5.361208	3.54E-07	1.33E-05	6.2419964
cg26203328	chr15	45018905	+			-1.6683	-3.173844	-5.361043	3.54E-07	1.33E-05	6.2412787
cg08381046	chr5	112294315	+			-1.006017	-1.067044	-5.360896	3.55E-07	1.33E-05	6.2406394
cg14128315	chr4	1227091	-	CTBP1;CTBP1	Body;Body	0.7569813	0.0042082	5.3608804	3.55E-07	1.33E-05	6.2405738
cg11031372	chr17	79173614	-	AZI1;AZI1	Body;Body	0.4474613	2.0400326	5.3606901	3.55E-07	1.33E-05	6.2397465
cg25537993	chr19	58545182	+	ZSCAN1	TSS1500	2.1636392	-0.253711	5.3606333	3.55E-07	1.33E-05	6.2394993
cg01184591	chr7	57249065	-	LOC642006	TSS1500	-1.000186	-0.819331	-5.360433	3.55E-07	1.33E-05	6.2386281
cg09852918	chr20	24929822	+	CST7	TSS200	-1.357672	-1.848137	-5.362509	3.55E-07	1.33E-05	6.2575665
cg14462779	chr10	76803669	-	DUPD1	Body	0.6820611	0.4722192	5.360353	3.56E-07	1.33E-05	6.2382809
cg00445405	chr2	63281844	-	OTX1	Body	1.2416114	0.0637958	5.3603132	3.56E-07	1.33E-05	6.2381082
cg03169508	chr6	28557901	+			1.2809605	-0.55908	5.360311	3.56E-07	1.33E-05	6.2380983
cg00498211	chr2	65217568	-	SLC1A4;SLC1A4	Body;5'UTR	-1.213153	-1.366267	-5.360242	3.56E-07	1.33E-05	6.2377973
cg14789645	chr13	98794353	+	FARP1;FARP1	TSS1500;TSS1500	-0.93248	-2.024648	-5.360187	3.56E-07	1.33E-05	6.2375598
cg15773794	chr2	172957583	-			1.1319782	-0.414026	5.360134	3.56E-07	1.33E-05	6.2373291
cg13982098	chr5	87955859	+	LOC645323	Body	1.1380342	-0.73598	5.3601135	3.56E-07	1.33E-05	6.2372397
cg15431544	chr7	27225476	-	HOXA11AS;HOXA11	Body;TSS1500	1.213391	-1.301478	5.3600886	3.56E-07	1.33E-05	6.2371318
cg08883223	chr1	197888880	-	LHX9;LHX9	Body;Body	2.1343966	-0.773087	5.3643529	3.56E-07	1.33E-05	6.2396978
cg08166214	chr16	1027357	-			-0.783067	-0.154194	-5.359867	3.56E-07	1.33E-05	6.2361676
cg14058329	chr7	27183946	+	HOXA5	TSS1500	1.1259944	0.8730045	5.3618868	3.56E-07	1.33E-05	6.2366395
cg09057033	chr8	144313828	-			-1.020477	-1.561723	-5.359499	3.57E-07	1.33E-05	6.2345679

cg07121693	chr10	44148437	-			-0.829753	-1.519448	-5.359408	3.57E-07	1.33E-05	6.2341738
cg11238564	chr16	67218787	+	KIAA0895L;EXOC3L	TSS1500;Body	-0.802766	-1.882187	-5.359361	3.57E-07	1.33E-05	6.233971
cg25388800	chr5	137990243	+			-1.818414	-1.8745	-5.359099	3.58E-07	1.34E-05	6.2328294
cg09038267	chr10	104524861	+	C10orf26	Body	1.2130335	0.9295066	5.3589857	3.58E-07	1.34E-05	6.2323378
cg26759179	chr10	121125566	+	GRK5	Body	1.0513602	0.4070382	5.3589497	3.58E-07	1.34E-05	6.2321813
cg23696949	chr1	183155644	-	LAMC2;LAMC2	Body;Body	-0.90917	-1.253593	-5.358664	3.58E-07	1.34E-05	6.2309379
cg10523860	chr14	103875565	-	MARK3;MARK3;MARK3;MARK3;MARK3	Body;Body;Body;Body;Body	0.7357106	1.9128012	5.358621	3.58E-07	1.34E-05	6.2307529
cg05376362	chr6	74826447	-			-1.629975	0.5287165	-5.358494	3.59E-07	1.34E-05	6.2302027
cg04571833	chr2	173836756	+	RAPGEF4;RAPGEF4	Body;Body	-1.265655	0.5423855	-5.358427	3.59E-07	1.34E-05	6.2299108
cg16076997	chr1	47905067	-	FOXD2	1stExon	0.9278071	-1.199732	5.3584393	3.59E-07	1.34E-05	6.2299632
cg25332717	chr2	106020669	-	FHL2	5'UTR	-1.066299	-0.89778	-5.358372	3.59E-07	1.34E-05	6.2296727
cg24692170	chr1	205327054	+	KLHDC8A	TSS1500	-1.54186	0.7445085	-5.358169	3.59E-07	1.34E-05	6.228788
cg11618057	chr20	60469115	-	CDH4	Body	-1.329956	-0.143032	-5.357905	3.60E-07	1.34E-05	6.2276423
cg21972567	chr5	158350708	+	EBF1	Body	-1.499461	0.330513	-5.357753	3.60E-07	1.34E-05	6.2269826
cg03927304	chr16	3017593	-	KREMEN2;KREMEN2	Body;Body	0.702851	0.1394977	5.3576082	3.60E-07	1.34E-05	6.2263511
cg15990724	chr12	100866802	+	NR1H4	TSS1500	-1.315465	0.4684542	-5.357323	3.60E-07	1.34E-05	6.2251137
cg00003298	chr12	113901210	-	LHX5	Body	2.8240813	-1.783676	5.3568792	3.61E-07	1.35E-05	6.2231834
cg02538637	chr17	79520379	-	C17orf70;C17orf70	TSS1500;TSS1500	-0.344954	-3.718274	-5.356822	3.61E-07	1.35E-05	6.222934
cg05379350	chr17	27917157	-	GIT1;GIT1	TSS1500;TSS1500	-1.146154	-1.871837	-5.356607	3.62E-07	1.35E-05	6.2219991
cg25159927	chr3	49947934	-	MON1A;MON1A	Body;Body	0.9743475	3.613035	5.3583808	3.62E-07	1.35E-05	6.2214192

cg02295509	chr17	48256402	+			-0.933617	0.1468632	-5.356204	3.62E-07	1.35E-05	6.2202494
cg24384519	chr7	31125198	+	ADCYAP1R1	Body	-1.473913	0.493662	-5.356156	3.62E-07	1.35E-05	6.2200409
cg04475868	chr16	86380594	+	LOC732275	TSS1500	-1.311362	0.1622175	-5.356136	3.62E-07	1.35E-05	6.2199538
cg23371877	chr7	152067904	-	MLL3	Body	0.9715934	1.731567	5.3582002	3.63E-07	1.35E-05	6.2210226
cg10429608	chr2	238864632	+			2.0523912	-2.053573	5.3560529	3.63E-07	1.35E-05	6.219593
cg17256609	chr8	41643375	+	ANK1;ANK1;ANK1;ANK1;ANK1	Body;Body;Body;Body;Body	-1.334945	0.7177066	-5.356007	3.63E-07	1.35E-05	6.2193916
cg23289079	chr5	122430476	+	PRDM6	Body	1.6487274	-0.149216	5.3811586	3.63E-07	1.35E-05	6.2471039
cg06419850	chr17	4688683	+	VMO1;VMO1;VMO1;VMO1	3'UTR;3'UTR;3'UTR;Body	1.7161179	-0.741341	5.3556229	3.63E-07	1.35E-05	6.2177248
cg14239983	chr11	121337384	-	SORL1	Body	-0.869199	-1.107406	-5.355556	3.63E-07	1.35E-05	6.2174325
cg07859439	chr2	197792757	+	PGAP1	TSS1500	-1.272887	-2.958554	-5.355514	3.63E-07	1.35E-05	6.2172533
cg17542751	chr22	44568725	-	PARVG;PARVG	TSS200;TSS200	-0.932225	-0.989339	-5.355478	3.63E-07	1.35E-05	6.2170953
cg10933691	chr10	28436499	+	MPP7	Body	1.1000223	2.0687545	5.3554395	3.64E-07	1.35E-05	6.2169284
cg12403329	chr17	8371203	+	NDEL1;NDEL1	3'UTR;3'UTR	0.7336305	2.5325243	5.3575154	3.64E-07	1.35E-05	6.2359265
cg02105666	chr1	999724	+			-0.990909	-5.342804	-5.355264	3.64E-07	1.36E-05	6.2161639
cg12140851	chr7	26897612	-	SKAP2	Body	1.5875604	-0.353947	5.3552584	3.64E-07	1.36E-05	6.2161412
cg08210399	chr3	180784531	-			-1.299669	-1.193321	-5.355226	3.64E-07	1.36E-05	6.2160015
cg25044651	chr5	115298720	+	LVRN	1stExon	1.1463267	-0.844344	5.3551757	3.64E-07	1.36E-05	6.2157821
cg22782492	chr3	10184007	-	VHL;VHL	Body;Body	-0.413605	-4.158705	-5.35509	3.64E-07	1.36E-05	6.2154091
cg06388730	chr7	35297699	-			1.0568186	-0.424406	5.3550595	3.64E-07	1.36E-05	6.2152773
cg16047279	chr17	38717242	+	CCR7	Body	-2.01294	-1.219845	-5.355027	3.64E-07	1.36E-05	6.215135

cg21579399	chr2	11890700	+	LPIN1	5'UTR	-1.585135	-1.371496	-5.355029	3.64E-07	1.36E-05	6.2151463
cg23306526	chr2	45171583	+	SIX3	Body	1.293276	-1.854383	5.3550067	3.64E-07	1.36E-05	6.215048
cg01213381	chr14	57277761	-	OTX2	TSS1500	1.6038319	-1.067322	5.3549105	3.64E-07	1.36E-05	6.2146298
cg07303577	chr12	3280014	-	TSPAN9;TSPAN9	5'UTR;5'UTR	-1.594708	1.0248051	-5.354806	3.65E-07	1.36E-05	6.2141741
cg01577187	chr2	106362832	+	NCK2;NCK2	5'UTR;5'UTR	-1.149623	-2.297562	-5.354786	3.65E-07	1.36E-05	6.2140877
cg26981170	chr4	2662888	+	FAM193A	Body	0.7421439	2.2283818	5.3545479	3.65E-07	1.36E-05	6.2130549
cg14526039	chr3	49210221	-	KLHDC8B	Body	0.7924973	0.3479597	5.3564454	3.65E-07	1.36E-05	6.2134073
cg02367133	chr2	241612327	-			1.8082928	-1.48506	5.3542198	3.66E-07	1.36E-05	6.2116296
cg13300580	chr1	27440539	+	SLC9A1	Body	0.7563329	0.7379344	5.3541821	3.66E-07	1.36E-05	6.2114657
cg11944284	chr11	2904663	+	CDKN1C;CDKN1C;CDKN1C	3'UTR;3'UTR;3'UTR	0.9368076	1.6448453	5.3541339	3.66E-07	1.36E-05	6.2112563
cg15516314	chr14	101347290	-	RTL1;MIR431;MIR433	1stExon;TSS200;TSS1500	-1.494007	0.9688549	-5.356182	3.66E-07	1.36E-05	6.2122661
cg20449685	chr19	58545728	-	ZSCAN1	5'UTR	1.7484303	-1.038587	5.3540335	3.66E-07	1.36E-05	6.2108204
cg09640621	chr10	129346391	-	NPS	TSS1500	-1.418859	-0.011791	-5.353969	3.66E-07	1.36E-05	6.2105394
cg16841748	chr8	142710298	+			-1.056047	-0.359859	-5.356038	3.66E-07	1.36E-05	6.2112539
cg15114651	chr19	47289410	+	SLC1A5;SLC1A5;SLC1A5	TSS1500;Body;5'UTR	-0.749682	-0.834579	-5.353885	3.66E-07	1.36E-05	6.2101773
cg00176949	chr16	86160795	+			-1.037553	0.3446788	-5.353799	3.66E-07	1.36E-05	6.2098019
cg17654747	chr2	125279777	-	CNTNAP5	Body	-1.184815	0.1396444	-5.35372	3.66E-07	1.36E-05	6.209457
cg01462537	chr10	2992189	+			-1.472988	-0.426802	-5.353649	3.67E-07	1.36E-05	6.2091482
cg03362215	chr6	78176806	+			1.3902772	-0.891113	5.353658	3.67E-07	1.36E-05	6.2091891
cg20218614	chr8	124194935	-	FAM83A;FAM83A;FAM83A;FAM83A	1stExon;1stExon;5'UTR;5'UTR	-1.025598	-1.083086	-5.353662	3.67E-07	1.36E-05	6.2092081

cg06375062	chr2	40149258	-			-1.627552	0.7659566	-5.353614	3.67E-07	1.36E-05	6.2089982
cg06150195	chr16	11275874	+	CLEC16A	3'UTR	-1.563003	-1.833881	-5.355632	3.67E-07	1.36E-05	6.2098774
cg18081393	chr17	733750	-	NXN	Body	0.834202	1.7492143	5.3534118	3.67E-07	1.36E-05	6.2081197
cg25525825	chr6	28889919	+	TRIM27	Body	-1.197679	-0.232841	-5.353295	3.67E-07	1.36E-05	6.2076115
cg05840707	chr5	173639013	+			-1.303217	0.9061217	-5.35318	3.67E-07	1.36E-05	6.2071141
cg10313368	chr1	8935228	-	ENO1	5'UTR	-1.227495	0.5320257	-5.35305	3.68E-07	1.37E-05	6.206549
cg05582340	chr12	113630080	+	C12orf52	3'UTR	0.8688961	1.6615296	5.3527448	3.68E-07	1.37E-05	6.2052228
cg15473084	chr5	50265600	-			1.2417502	-1.226385	5.3527179	3.68E-07	1.37E-05	6.2051059
cg16531209	chr8	99951696	-			1.7482057	-0.856008	5.3526006	3.68E-07	1.37E-05	6.2045966
cg05116777	chr9	125377176	-	OR1Q1	1stExon	-0.949929	-0.654133	-5.352488	3.68E-07	1.37E-05	6.2041079
cg12062545	chr12	104532339	+	NFYB	TSS1500	-0.634796	-4.034299	-5.354596	3.68E-07	1.37E-05	6.204997
cg11181587	chr10	121772185	+			-1.092083	0.0157361	-5.352169	3.69E-07	1.37E-05	6.2027239
cg05485137	chr10	28035864	-	MKX	TSS1500	1.6990283	-0.983706	5.3520839	3.69E-07	1.37E-05	6.2023525
cg15055149	chr16	34208209	-			0.7691153	1.7531426	5.3519883	3.69E-07	1.37E-05	6.2019376
cg02795546	chr1	247697875	-	OR2C3	TSS1500	-1.831188	0.9172984	-5.35194	3.69E-07	1.37E-05	6.2017263
cg13877801	chr7	138666834	-	KIAA1549;KIAA1549	TSS1500;TSS1500	-0.823798	-2.47944	-5.351912	3.69E-07	1.37E-05	6.2016083
cg12377139	chr10	22634218	-	SPAG6;SPAG6	TSS200;TSS200	1.3934877	-0.285532	5.351887	3.69E-07	1.37E-05	6.2014978
cg00458351	chr10	133609529	-			-1.50423	0.7776182	-5.351857	3.70E-07	1.37E-05	6.2013688
cg02809611	chr16	3253368	-	OR1F1	TSS1500	-0.855637	-0.727516	-5.351748	3.70E-07	1.37E-05	6.2008943
cg12393668	chr17	42768503	+	CCDC43;CCDC43	TSS1500;TSS1500	-1.137717	-0.776693	-5.351524	3.70E-07	1.37E-05	6.1999215

cg26657086	chr6	32373089	+	BTNL2	Body	-1.329321	0.0606024	-5.351526	3.70E-07	1.37E-05	6.1999281
cg09032137	chr11	19362896	-			-1.559279	0.4530991	-5.351484	3.70E-07	1.37E-05	6.1997455
cg05582286	chr11	69218633	+			-1.306578	0.2195495	-5.351451	3.70E-07	1.37E-05	6.1996031
cg04037905	chr10	97047067	-	PDLIM1	Body	1.2457983	0.7630197	5.3514211	3.70E-07	1.37E-05	6.1994745
cg20052760	chr6	10510789	+			-0.857319	-2.048027	-5.351317	3.70E-07	1.37E-05	6.1990218
cg26708874	chr8	8580598	-			1.160285	0.0063238	5.3511885	3.71E-07	1.37E-05	6.1984643
cg09683350	chr15	89914466	-			2.0210674	-1.472654	5.3510811	3.71E-07	1.38E-05	6.1979982
cg26477856	chr12	56324915	+	DGKA;DGKA;DGKA;DGKA	TSS1500;TSS1500;TSS1500;TSS200	-1.237807	-0.338109	-5.350926	3.71E-07	1.38E-05	6.1973238
cg11791577	chr12	4958491	-	KCNA6	3'UTR	-0.96367	-2.986738	-5.350907	3.71E-07	1.38E-05	6.1972408
cg09532095	chr2	39963854	-	THUMPD2;THUMPD2	Body;3'UTR	0.8718547	1.5431724	5.3508127	3.71E-07	1.38E-05	6.1968325
cg11371231	chr11	55927697	+	OR8K5	1stExon	-1.263933	-0.300501	-5.350801	3.71E-07	1.38E-05	6.1967819
cg26254792	chr1	60280088	-	HOOK1	TSS1500	-0.76012	-2.873204	-5.350608	3.72E-07	1.38E-05	6.1959452
cg04373948	chr12	90806795	+			-1.56101	-0.024726	-5.350522	3.72E-07	1.38E-05	6.1955685
cg10696677	chr6	41681327	-	TFEB;TFEB	5'UTR;5'UTR	-1.05631	-0.794869	-5.35048	3.72E-07	1.38E-05	6.1953899
cg24004532	chr6	53517075	+	KLHL31	Body	-1.084659	-2.846771	-5.350426	3.72E-07	1.38E-05	6.1951542
cg16764236	chr17	32611290	-	CCL11	TSS1500	-1.555133	0.3531418	-5.350406	3.72E-07	1.38E-05	6.1950682
cg03142256	chr3	75958299	+			-1.251961	0.73806	-5.350383	3.72E-07	1.38E-05	6.1949664
cg24938743	chr13	98960620	-	FARP1	Body	0.8104309	1.9849913	5.3503437	3.72E-07	1.38E-05	6.1947963
cg23072711	chr5	167474670	+	ODZ2	Body	-0.764492	-2.58091	-5.350312	3.72E-07	1.38E-05	6.1946565
cg02866628	chr7	54612475	-	VSTM2A	Body	1.977075	-2.110832	5.3502489	3.72E-07	1.38E-05	6.1943844

cg26270899	chr9	139590760	+			-1.222977	-0.710572	-5.350179	3.72E-07	1.38E-05	6.1940794
cg21641573	chr4	1210478	+	CTBP1;CTBP1	Body;Body	0.6353446	2.1102988	5.3501342	3.72E-07	1.38E-05	6.1938868
cg21071097	chr17	4803053	+	CHRNE;C17orf107;C17orf107	Body;5'UTR;1stExon	-1.247796	-2.19699	-5.350107	3.72E-07	1.38E-05	6.193769
cg03053290	chr1	95698378	+	RWDD3;RWDD3	TSS1500;TSS1500	0.9942146	1.4216722	5.349721	3.73E-07	1.38E-05	6.1920925
cg02584377	chr12	122489545	+	BCL7A;BCL7A	Body;Body	1.1552806	1.1084303	5.3494558	3.74E-07	1.38E-05	6.1909411
cg05208878	chr22	22901596	-	PRAME;PRAME;PRAME;PRAME;LOC648691;PRAME;PRAME;PRAME;PRAME	TSS200;5'UTR;1stExon;1stExon;TSS200;TSS200;5'UTR;5'UTR;1stExon	-0.706656	-1.484289	-5.349363	3.74E-07	1.38E-05	6.1905377
cg19778455	chr3	42924913	+			-0.751087	-0.567293	-5.349352	3.74E-07	1.38E-05	6.1904928
cg05899471	chr14	101456433	+	SNORD114-29	Body	-1.495933	0.6463791	-5.34926	3.74E-07	1.38E-05	6.1900927
cg17865955	chr6	33601177	+	ITPR3	Body	-0.789249	-0.663592	-5.351345	3.74E-07	1.38E-05	6.1908925
cg12287358	chr6	29796032	+	HLA-G	Body	1.4238204	-1.681745	5.3491324	3.74E-07	1.38E-05	6.1895374
cg16243079	chr7	142428294	-			-1.54799	0.3895717	-5.348938	3.74E-07	1.39E-05	6.1886935
cg05331791	chr20	62383275	+	ZBTB46	Body	-2.336782	1.3727199	-5.348817	3.75E-07	1.39E-05	6.1881702
cg19927816	chr19	46319153	-	SYMPK;RSPH6A	Body;TSS1500	0.7743184	1.0050295	5.3487851	3.75E-07	1.39E-05	6.1880295
cg17755730	chr3	50285392	-	GNAI2;GNAI2	Body;Body	0.5942785	2.2992479	5.3487314	3.75E-07	1.39E-05	6.1877967
cg04093159	chr5	166211825	+			-2.633006	3.1690276	-5.348312	3.76E-07	1.39E-05	6.1859759
cg07959771	chr7	121956740	-			1.8789288	-1.236729	5.348194	3.76E-07	1.39E-05	6.1854636
cg01464104	chr11	56043040	-	OR5T1	TSS200	-1.381759	-0.497554	-5.347904	3.76E-07	1.39E-05	6.1842029
cg14793406	chr12	115135906	-			1.230289	-1.453015	5.3476164	3.77E-07	1.39E-05	6.1829568
cg17061340	chr18	74692145	+	MBP;MBP;MBP;MBP;MBP	3'UTR;3'UTR;3'UTR;3'UTR;3'UTR	0.856672	1.0505057	5.3474379	3.77E-07	1.39E-05	6.1821818
cg20018806	chr11	59633874	-	TCN1	1stExon	-1.408267	-0.021892	-5.347454	3.77E-07	1.39E-05	6.1822538

cg20817131	chr7	27184167	-	HOXA5	TSS1500	1.4532059	0.0437239	5.3474441	3.77E-07	1.39E-05	6.1822087
cg03498081	chr16	80061357	+			-1.217402	-2.249971	-5.347407	3.77E-07	1.39E-05	6.1820459
cg25136495	chr12	6729718	-	LPAR5;LPAR5	Body;Body	1.6848956	-0.109438	5.3473966	3.77E-07	1.39E-05	6.1820025
cg14838566	chr1	37791043	-			-1.404878	0.4949083	-5.347373	3.77E-07	1.39E-05	6.1818983
cg05020625	chr6	43252858	+	TTBK1	Body	2.4735501	-2.713565	5.3472546	3.77E-07	1.39E-05	6.1813865
cg23205886	chr5	138611766	+	SNHG4;MATR3	Body;5'UTR	-0.977058	-1.041134	-5.347014	3.78E-07	1.40E-05	6.180343
cg07033372	chr20	37353032	-	SLC32A1	TSS200	2.3639497	-1.961495	5.3468664	3.78E-07	1.40E-05	6.1797013
cg01734062	chr17	48351821	+	TMEM92;TMEM92;TMEM92	1stExon;5'UTR;5'UTR	-1.014315	-1.983749	-5.346633	3.78E-07	1.40E-05	6.1786885
cg12408990	chr5	179634671	+	RASGEF1C	5'UTR	1.5811127	0.6759239	5.3466347	3.78E-07	1.40E-05	6.1786957
cg07411620	chr2	176948759	-	EVX2	TSS200	1.9256258	-0.121094	5.3465025	3.79E-07	1.40E-05	6.1781222
cg09494418	chr11	60131271	-			-0.6672	-3.070309	-5.346334	3.79E-07	1.40E-05	6.1773927
cg27496116	chr13	43660964	+	DNAJC15	Body	0.8384156	1.6731763	5.3483641	3.79E-07	1.40E-05	6.1779696
cg02449336	chr7	143805356	-	OR2A2	TSS1500	-1.199245	-0.792116	-5.346018	3.79E-07	1.40E-05	6.1760214
cg08271031	chr22	44568812	-	PARVG;PARVG	TSS200;TSS200	-1.08482	-0.904145	-5.345968	3.80E-07	1.40E-05	6.1758021
cg03038245	chr10	630310	-	DIP2C	Body	0.6337852	1.8796617	5.3459457	3.80E-07	1.40E-05	6.1757057
cg15778437	chr11	31839521	+	PAX6	TSS200	2.6120667	-1.607417	5.3523371	3.80E-07	1.40E-05	6.1791396
cg04508649	chr1	11249046	-	MTOR;ANGPTL7	Body;TSS1500	0.6180671	3.4634801	5.3457976	3.80E-07	1.40E-05	6.1750631
cg13839957	chr1	6165483	-	CHD5	3'UTR	-0.979914	-0.854622	-5.345759	3.80E-07	1.40E-05	6.1748956
cg00300672	chr2	125077436	-	CNTNAP5	Body	-1.340571	-1.146848	-5.345739	3.80E-07	1.40E-05	6.174808
cg13672736	chr9	135114066	+	NTNG2	Body	-2.332289	1.0747831	-5.3457	3.80E-07	1.40E-05	6.1746379

cg17489748	chr10	117887197	-	GFRA1;GFRA1;GFRA1	Body;Body;Body	-1.531937	0.5733349	-5.347797	3.80E-07	1.40E-05	6.1758983
cg08911391	chr1	20988590	+	DDOST	TSS1500	-0.895851	-1.971709	-5.345598	3.80E-07	1.40E-05	6.1741953
cg16993015	chr11	8006143	-			-1.125978	-0.253578	-5.345492	3.80E-07	1.40E-05	6.1737372
cg26675654	chr7	1269693	+			1.2762882	-0.809862	5.3454022	3.81E-07	1.40E-05	6.1733471
cg15658676	chr6	53530538	+	KLHL31	TSS200	1.1992574	1.0913151	5.3453248	3.81E-07	1.40E-05	6.1730115
cg23206851	chr7	27143046	-	HOXA2	TSS1500	1.1513408	-1.062115	5.3452859	3.81E-07	1.40E-05	6.1728428
cg13140267	chr2	96971704	-	SNRNP200	TSS1500	-0.898726	-3.34307	-5.345243	3.81E-07	1.40E-05	6.1726584
cg22537597	chr7	149530984	-	SSPO	3'UTR	-0.88993	-0.613035	-5.345097	3.81E-07	1.41E-05	6.1720243
cg00019759	chr1	212687842	-			1.0605392	-0.498224	5.3447904	3.82E-07	1.41E-05	6.1706926
cg26683361	chr22	31460131	-			-1.432112	0.2943846	-5.344789	3.82E-07	1.41E-05	6.1706846
cg13441058	chr2	45028269	-			0.9523291	0.9217228	5.346666	3.82E-07	1.41E-05	6.1706092
cg16328369	chr1	248058642	-	OR2W3	TSS1500	-1.716454	0.5447852	-5.344563	3.82E-07	1.41E-05	6.169707
cg10803767	chr2	231688923	+			-1.191384	0.2843107	-5.346502	3.82E-07	1.41E-05	6.1698973
cg11650648	chr17	9741522	+	GLP2R	Body	-1.015652	-1.457181	-5.34426	3.83E-07	1.41E-05	6.1683928
cg19204131	chr6	10385320	-			1.3686628	-0.792391	5.3438075	3.83E-07	1.41E-05	6.1664281
cg19620758	chr11	102826565	-	MMP13	TSS200	-1.66014	0.6021246	-5.343666	3.84E-07	1.41E-05	6.1658161
cg23207990	chr4	154710535	-	SFRP2	TSS1500	1.8409917	-1.025177	5.3436124	3.84E-07	1.41E-05	6.1655817
cg03512426	chr10	131763683	+			0.9358773	-0.955619	5.343509	3.84E-07	1.41E-05	6.1651333
cg27622506	chr3	24537160	+	THRB;THRB;THRB	TSS1500;TSS1500;TSS1500	-1.588304	-4.12794	-5.345618	3.84E-07	1.41E-05	6.1664581
cg09165170	chr1	10601579	-	PEX14	Body	1.0346559	1.6608487	5.3434439	3.84E-07	1.41E-05	6.1648508

cg10399824	chr10	121048354	+	GRK5	Body	0.9589024	1.6448398	5.3434124	3.84E-07	1.41E-05	6.164714
cg16927372	chr17	32367929	+	ACCN1	Body	-1.238255	-0.455131	-5.343375	3.84E-07	1.41E-05	6.1645533
cg05525649	chr6	32156269	+	PBX2	Body	1.057134	0.5684892	5.3453528	3.84E-07	1.42E-05	6.1653068
cg09559882	chr19	46142291	+	EML2;MIR330	Body;Body	-0.614235	-3.989985	-5.343255	3.84E-07	1.42E-05	6.1640321
cg12304520	chr5	140810123	-	PCDHGA4;PCDHGA11;PCDHGA11;PCDHGA9;PCDHGA1;PCDHGB1;PCDHGB6;PCDHGA12;PCDHGB3;PCDHGB7;PCDHGA6;PCDHGA8;PCDHGA10;PCDHGA5;PCDHGB4;PCDHGA3;PCDHGA2	Body;Body;Body;Body;Body;Body;Body;TSS200;Body;Body;Body;Body;Body;Body;Body;Body;TSS200;Body;Body;Body	1.5251292	0.000155	5.3432344	3.84E-07	1.42E-05	6.1639419
cg18521771	chr9	117373239	-	C9orf91	TSS1500	-0.577737	-0.597615	-5.343242	3.84E-07	1.42E-05	6.1639728
cg08942191	chr15	80729093	-	ARNT2	Body	-1.381381	-0.39417	-5.343086	3.85E-07	1.42E-05	6.1632995
cg15808943	chr2	176994665	-	HOXD8;HOXD8	5'UTR;1stExon	2.5357814	-1.566678	5.3471687	3.85E-07	1.42E-05	6.1652412
cg06000994	chr19	30866194	+	ZNF536	5'UTR	1.3451048	-1.286589	5.3428964	3.85E-07	1.42E-05	6.1624756
cg22791045	chr11	133403793	-	OPCML	TSS1500	-1.518999	0.3197604	-5.342871	3.85E-07	1.42E-05	6.1623636
cg03832010	chr5	175031838	+			-1.220821	-0.00601	-5.342749	3.85E-07	1.42E-05	6.1618358
cg00590761	chr1	55008319	-			-1.030128	-3.889476	-5.344829	3.85E-07	1.42E-05	6.1630358
cg10236239	chr2	108994514	-	SULT1C4;SULT1C4	5'UTR;1stExon	1.0575943	0.1402896	5.3426602	3.85E-07	1.42E-05	6.1614508
cg14531530	chr4	41362253	-	LIMCH1;LIMCH1;LIMCH1	TSS1500;TSS1500;TSS1500	-0.668679	-3.382506	-5.342643	3.85E-07	1.42E-05	6.1613758
cg19708337	chr1	93544307	-	MTF2;MTF2;MTF2;MTF2	TSS1500;TSS1500;TSS1500;TSS1500	-0.902371	-2.279762	-5.342677	3.85E-07	1.42E-05	6.1615258
cg23335976	chr10	18240598	+	SLC39A12;SLC39A12	TSS200;TSS200	-1.128171	0.652517	-5.342554	3.85E-07	1.42E-05	6.1609896
cg01122241	chr4	41882748	+			0.952834	-0.429655	5.3424927	3.86E-07	1.42E-05	6.1607242
cg10778841	chr10	106401319	-	SORCS3	1stExon	2.2845383	-1.331538	5.3445182	3.86E-07	1.42E-05	6.1613011
cg16567330	chr6	106577558	+			-1.415409	-1.445632	-5.344516	3.86E-07	1.42E-05	6.1616815
cg02914112	chr6	99096390	-			-1.570613	0.1409909	-5.342378	3.86E-07	1.42E-05	6.1602257

cg23553368	chr11	5950798	-			-1.007844	0.0404806	-5.342363	3.86E-07	1.42E-05	6.1601622
cg16670155	chr19	18557220	+	ELL	Body	1.6952889	4.1757666	5.3423018	3.86E-07	1.42E-05	6.1598965
cg17320698	chr11	30510489	-	MPPED2;MPPED2	Body;Body	-1.032636	-1.394163	-5.344237	3.86E-07	1.42E-05	6.1604701
cg00938540	chr20	61166085	+	C20orf166	Body	-1.490517	-0.002378	-5.341843	3.87E-07	1.42E-05	6.1579059
cg11606215	chr4	84035922	+	PLAC8;PLAC8	TSS200;TSS200	-1.093664	-1.007873	-5.341763	3.87E-07	1.42E-05	6.1575607
cg17018767	chr18	44099221	-	LOXHD1;LOXHD1;LOXHD1	TSS200;Body;Body	-1.038306	-0.307534	-5.341755	3.87E-07	1.42E-05	6.1575254
cg26218411	chr15	59226409	-	SLTM;SLTM	TSS1500;TSS1500	-0.587909	-2.972913	-5.341776	3.87E-07	1.42E-05	6.1576168
cg22388232	chr8	861072	+			-1.171457	0.7255952	-5.341705	3.87E-07	1.42E-05	6.1573062
cg19019849	chr4	13533145	+			1.8669333	-2.180647	5.3416205	3.87E-07	1.42E-05	6.1569414
cg23225103	chr1	205040765	+	CNTN2	Body	-1.064932	-0.453216	-5.341542	3.87E-07	1.42E-05	6.1566003
cg13128443	chr14	78669728	-			-1.591399	0.1268175	-5.341492	3.87E-07	1.42E-05	6.156384
cg26636590	chr4	46911374	-	COX7B2	TSS200	-1.507602	0.792678	-5.341451	3.87E-07	1.42E-05	6.1562079
cg05545891	chr11	56113768	-	OR8K1	1stExon	-1.672597	0.711583	-5.341407	3.88E-07	1.42E-05	6.1560167
cg14431443	chr12	114846431	+	TBX5;TBX5	TSS200;TSS200	0.790415	-0.480657	5.3412994	3.88E-07	1.42E-05	6.1555487
cg07143863	chr12	64954729	-			-1.29141	-0.141601	-5.340827	3.89E-07	1.43E-05	6.1534994
cg11152825	chr20	44881449	-	CDH22	TSS1500	-1.515166	0.0795942	-5.340784	3.89E-07	1.43E-05	6.1533143
cg08476244	chr2	120867222	-	EPB41L5	Body	-1.053747	-0.899283	-5.340679	3.89E-07	1.43E-05	6.152859
cg23209990	chr4	55097576	+	PDGFRA	5'UTR	2.7049399	-2.266942	5.3406008	3.89E-07	1.43E-05	6.1525188
cg16541026	chr3	49026934	-	P4HTM;P4HTM	TSS1500;TSS1500	-0.855495	-1.391436	-5.340571	3.89E-07	1.43E-05	6.1523873
cg17914107	chr4	169481028	+	PALLD;PALLD	Body;Body	-1.007057	0.3030477	-5.340351	3.89E-07	1.43E-05	6.1514351

cg20296493	chr8	124219725	+	FAM83A;FAM83A	Body;Body	0.8229989	0.6498851	5.3445953	3.89E-07	1.43E-05	6.171997
cg03308092	chr22	17488275	-	GAB4	Body	1.3471808	0.1390505	5.3399624	3.90E-07	1.43E-05	6.1497502
cg02587606	chr10	77352231	-			1.1783186	1.4028521	5.339795	3.90E-07	1.43E-05	6.1490245
cg09681167	chr2	237078026	+	GBX2	TSS1500	2.0128745	-2.203202	5.3418679	3.90E-07	1.43E-05	6.1502082
cg24067911	chr6	16729610	+	ATXN1;ATXN1	5'UTR;5'UTR	-1.384221	-0.713174	-5.339706	3.91E-07	1.43E-05	6.1486397
cg13366863	chr5	177505451	+			-1.113855	-0.639746	-5.339612	3.91E-07	1.43E-05	6.1482307
cg27095592	chr8	8580746	-			1.3047198	-1.057672	5.3394834	3.91E-07	1.43E-05	6.1476733
cg13961576	chr10	1228316	-	ADARB2	3'UTR	-0.938347	-0.312936	-5.339254	3.91E-07	1.44E-05	6.1466775
cg15815435	chr4	11605526	-			-1.215758	-0.043251	-5.339254	3.91E-07	1.44E-05	6.1466783
cg03613942	chr5	86416413	+			-1.162638	-0.836241	-5.339213	3.91E-07	1.44E-05	6.1465001
cg02168303	chr7	27265309	+			1.9365553	-0.912134	5.3388954	3.92E-07	1.44E-05	6.1451235
cg06802812	chr5	40679165	-	PTGER4	TSS1500	-1.206014	-4.980158	-5.33864	3.92E-07	1.44E-05	6.1440181
cg24042854	chr3	194930170	+	C3orf21	Body	0.6798787	2.5250608	5.3386552	3.92E-07	1.44E-05	6.1440818
cg18634506	chr7	99007560	+	BUD31;PDAP1	5'UTR;TSS1500	-0.958021	-1.861038	-5.3385	3.93E-07	1.44E-05	6.143407
cg20291222	chr12	75784541	-	GLIPR1L2	TSS1500	-0.872125	-3.487463	-5.338417	3.93E-07	1.44E-05	6.1430489
cg23424003	chr7	101006035	+	EMID2	TSS200	2.2178221	-0.930788	5.3405124	3.93E-07	1.44E-05	6.1439478
cg11033588	chr6	140495807	+			-1.494894	0.5186553	-5.338259	3.93E-07	1.44E-05	6.1423651
cg17504999	chr4	134072723	-	PCDH10;PCDH10	1stExon;1stExon	2.4020781	-2.162656	5.3382429	3.93E-07	1.44E-05	6.1422945
cg00160981	chr1	161691911	+	FCRLB	TSS1500	-1.3131	-0.983956	-5.338215	3.93E-07	1.44E-05	6.1421726
cg07715909	chr11	1750738	-	HCCA2	Body	-0.612451	-1.465829	-5.338162	3.93E-07	1.44E-05	6.141942

cg03129884	chr2	95692727	+	MAL;MAL;MAL;MAL	Body;Body;Body;Body	1.215185	-0.416465	5.342372	3.93E-07	1.44E-05	6.1444851
cg08454485	chr3	105086213	+	ALCAM;ALCAM	5'UTR;1stExon	-0.333435	-3.963013	-5.337999	3.94E-07	1.44E-05	6.141236
:h.2.128981206l	chr2	129264738	+			-0.511357	-4.620933	-5.342178	3.94E-07	1.44E-05	6.1432426
cg01190484	chr6	30104593	-	TRIM40;TRIM40	1stExon;5'UTR	-1.298816	-1.756608	-5.337887	3.94E-07	1.44E-05	6.1407525
cg01917657	chr22	50617092	+	PANX2;PANX2;PANX2	Body;Body;Body	0.9576901	1.4886974	5.3377826	3.94E-07	1.44E-05	6.1402986
cg23319289	chr14	82292196	+			-1.61695	0.5940556	-5.33777	3.94E-07	1.44E-05	6.1402444
cg21752270	chr11	2813528	+	KCNQ1;KCNQ1	Body;Body	-1.024931	0.7511941	-5.337607	3.94E-07	1.44E-05	6.1395369
cg10687131	chr2	20871002	+	GDF7	Body	1.2180751	-0.280031	5.3373805	3.95E-07	1.45E-05	6.1385556
cg10476585	chr13	94499653	-	GPC6	Body	-1.417768	0.3222465	-5.337301	3.95E-07	1.45E-05	6.1382127
cg11061975	chr20	1639325	+	SIRPG;SIRPG;SIRPG	TSS1500;TSS1500;TSS1500	-1.422546	0.4095679	-5.337208	3.95E-07	1.45E-05	6.1378097
cg16607685	chr5	140181889	-	PCDHA2;PCDHA1;PCDHA3;PCDHA3;PCDHA1	Body;Body;1stExon;1stExon;Body	1.0306793	0.3661313	5.3371605	3.95E-07	1.45E-05	6.1376018
cg19604060	chr5	22928822	+			-1.743237	0.2992239	-5.33712	3.95E-07	1.45E-05	6.1374265
cg18930910	chr6	30039151	+	RNF39;RNF39	Body;Body	0.7895532	-0.527214	5.3389933	3.96E-07	1.45E-05	6.137369
cg27610921	chr17	6026638	-	WSCD1	3'UTR	-1.584215	0.9764582	-5.336704	3.96E-07	1.45E-05	6.1356243
cg12593541	chr1	181110845	+			-1.097043	-2.166601	-5.336537	3.96E-07	1.45E-05	6.1348993
cg12308216	chr6	30853255	+	DDR1;DDR1	5'UTR;5'UTR	-0.622087	-1.392731	-5.336479	3.96E-07	1.45E-05	6.1346464
cg09793121	chr21	34398263	-	OLIG2;OLIG2	1stExon;5'UTR	1.8001556	-0.665155	5.3518501	3.96E-07	1.45E-05	6.1427912
cg14293300	chr21	34399361	+	OLIG2	Body	1.1715437	-1.510255	5.3362054	3.97E-07	1.45E-05	6.1334617
cg18539122	chr13	79169872	+			1.2825188	-0.709446	5.3359575	3.97E-07	1.45E-05	6.1323872
cg17023149	chr4	13536504	+			1.3356359	-1.918184	5.3358214	3.97E-07	1.45E-05	6.1317974

cg03253616	chr15	71372288	-			-0.75567	-0.740418	-5.33575	3.98E-07	1.46E-05	6.1314894
cg13937208	chr13	78551078	-	EDNRB	TSS1500	-1.540226	0.6760433	-5.335688	3.98E-07	1.46E-05	6.1312193
cg01817009	chr16	67978182	-	SLC12A4;SLC12A4;SLC12A4;LCAT;SLC12A4;SLC12A4	3'UTR;3'UTR;3'UTR;TSS200;3'UTR;3'UTR	0.6710706	1.9033629	5.3355884	3.98E-07	1.46E-05	6.1307876
cg11195363	chr12	61915276	-			-1.38765	-0.267433	-5.335527	3.98E-07	1.46E-05	6.1305232
cg27156584	chr11	58207784	-	OR5B12	TSS200	-1.6231	1.2514163	-5.33541	3.98E-07	1.46E-05	6.1300154
cg10589281	chr11	924500	+	AP2A2	TSS1500	0.9512098	1.7946715	5.3353912	3.98E-07	1.46E-05	6.1299327
cg27190014	chr8	128971471	-	MIR1205;PVT1	TSS1500;Body	0.762918	1.2125485	5.3353197	3.98E-07	1.46E-05	6.1296228
cg09071889	chr8	80523413	+	STMN2;STMN2	5'UTR;1stExon	1.4489506	-1.080321	5.3352998	3.98E-07	1.46E-05	6.1295368
cg13604462	chr10	88163667	+			-1.480562	1.2734957	-5.335273	3.98E-07	1.46E-05	6.1294224
cg14041603	chr6	168394198	-			1.1205832	1.6375832	5.3352179	3.99E-07	1.46E-05	6.1291817
cg02261780	chr19	3097728	+	GNA11	Body	1.2802459	1.512937	5.3351237	3.99E-07	1.46E-05	6.1287733
cg03243965	chr19	16254516	+	HSH2D	5'UTR	-1.076686	-3.433747	-5.337156	3.99E-07	1.46E-05	6.1298059
cg23916646	chr6	31744545	-	C6orf27	Body	-0.88109	-1.667123	-5.335019	3.99E-07	1.46E-05	6.1283212
cg05080651	chr16	66985153	+			-1.150884	0.693281	-5.334731	3.99E-07	1.46E-05	6.1270714
cg08404427	chr6	164527247	+			-1.168203	0.6176778	-5.334741	3.99E-07	1.46E-05	6.1271147
cg11946618	chr2	240727230	+			-1.351753	0.4421626	-5.334626	4.00E-07	1.46E-05	6.1266146
cg20963227	chr4	41868203	+			1.1384692	-0.837136	5.3346352	4.00E-07	1.46E-05	6.1266563
cg03620964	chr16	76268785	-			-1.491473	0.9165428	-5.334493	4.00E-07	1.46E-05	6.1260397
cg15260552	chr12	64338402	+	SRGAP1	Body	0.9447183	1.5909721	5.3344547	4.00E-07	1.46E-05	6.1258742
cg05166473	chr16	88103629	-	BANP;BANP	Body;Body	-1.323502	2.0935996	-5.3344	4.00E-07	1.46E-05	6.1256357

cg02251393	chr2	159651559	-	DAPL1	TSS1500	-1.101257	-0.916911	-5.33438	4.00E-07	1.46E-05	6.1255493
cg23047137	chr3	24089778	-			-0.958974	-1.156271	-5.333944	4.01E-07	1.46E-05	6.1236598
cg26456223	chr4	13540430	-			0.8696825	0.0806431	5.3337227	4.01E-07	1.47E-05	6.122702
cg02887623	chr14	101531755	-	MIR369;MIR410;MIR412;MIR656	TSS200;TSS1500;TSS200;TSS1500	-1.574875	1.5715705	-5.333664	4.01E-07	1.47E-05	6.1224477
cg15145341	chr13	25506340	+	LOC646405	Body	1.4406463	0.0110224	5.3336147	4.01E-07	1.47E-05	6.1222341
cg04366628	chr4	26290463	+			-1.431233	-0.582842	-5.333522	4.02E-07	1.47E-05	6.1218343
cg00898102	chr6	32029152	+	TNXB	Body	-1.340311	0.9808093	-5.333483	4.02E-07	1.47E-05	6.1216633
cg05144620	chr17	5969641	+			-0.847735	-1.22937	-5.333397	4.02E-07	1.47E-05	6.121292
cg09275956	chr1	50896251	+			2.0026649	-0.876372	5.3353563	4.02E-07	1.47E-05	6.1220136
cg02491912	chr16	74809091	+	FA2H	TSS1500	-0.385717	-2.950396	-5.33317	4.02E-07	1.47E-05	6.1203092
cg24154024	chr1	228550401	+	OBSCN	Body	-1.97691	2.2284479	-5.333166	4.02E-07	1.47E-05	6.1202879
cg26343889	chr15	68848244	-			-0.925758	0.1315321	-5.333146	4.02E-07	1.47E-05	6.1202019
cg27362525	chr17	5019638	+	ZNF232	5'UTR	1.6999105	-0.589331	5.3329895	4.03E-07	1.47E-05	6.1195252
cg03789934	chr19	56342665	-	NLRP11	5'UTR	-0.904446	-0.640997	-5.33288	4.03E-07	1.47E-05	6.1190522
cg14817655	chr4	188916724	-	ZFP42	TSS200	1.7712634	-0.857857	5.3328574	4.03E-07	1.47E-05	6.1189528
cg09934892	chr16	3559551	+	CLUAP1;CLUAP1	Body;TSS1500	-1.10106	0.1947971	-5.332766	4.03E-07	1.47E-05	6.1185547
cg13320538	chr8	2419822	-			-1.275374	0.4274474	-5.332608	4.03E-07	1.47E-05	6.1178706
cg10395806	chr11	70280601	-	CTTN;CTTN	Body;Body	-1.387382	2.7021838	-5.332536	4.03E-07	1.47E-05	6.1175591
cg24432073	chr4	76555634	+	CDKL2;CDKL2	5'UTR;1stExon	-0.80619	-4.344231	-5.332342	4.04E-07	1.47E-05	6.116718
cg14717069	chr13	103945151	-			-1.167493	-0.574082	-5.332071	4.04E-07	1.47E-05	6.1155446

cg24523685	chr21	36062813	-	CLIC6	Body	-1.669019	0.2599751	-5.332012	4.04E-07	1.47E-05	6.1152905
cg23521140	chr2	37617601	+			-2.334998	-3.235234	-5.33194	4.05E-07	1.48E-05	6.1149775
cg23247185	chr3	14530356	+	SLC6A6;SLC6A6	3'UTR;3'UTR	1.0028845	1.1609853	5.331656	4.05E-07	1.48E-05	6.1137479
cg16591502	chr13	76210838	+	LMO7	Body	-0.326947	-3.324241	-5.331608	4.05E-07	1.48E-05	6.1135396
cg20502080	chr14	73137874	-	DPF3	Body	-1.614633	1.4072949	-5.331568	4.05E-07	1.48E-05	6.1133662
cg05726701	chr1	248005057	+	OR11L1	1stExon	-1.385586	0.4686029	-5.331481	4.05E-07	1.48E-05	6.1129889
cg14239307	chr10	135379213	-	SYCE1;SYCE1;SYCE1	TSS200;TSS200;5'UTR	1.1457159	1.3816168	5.3314782	4.05E-07	1.48E-05	6.1129774
cg19366147	chr8	1717660	-	CLN8	5'UTR	1.1371679	2.4792299	5.3314663	4.05E-07	1.48E-05	6.1129258
cg01684562	chr6	80713632	+	TTK;TTK	TSS1500;TSS1500	-0.899702	-3.044103	-5.331411	4.05E-07	1.48E-05	6.1126873
cg04819096	chr12	54339167	+	HOXC13	3'UTR	1.4709852	-1.301881	5.3333746	4.06E-07	1.48E-05	6.1130463
cg00812438	chr12	127940086	-			1.8961134	-1.09433	5.3312011	4.06E-07	1.48E-05	6.111777
cg11073558	chr8	97171901	-	GDF6	Body	1.9743363	-1.225959	5.3311671	4.06E-07	1.48E-05	6.1116298
cg02097792	chr11	2058046	-			-0.687764	-2.610466	-5.331063	4.06E-07	1.48E-05	6.1111795
cg09509503	chr1	58353581	+	DAB1	5'UTR	-1.617944	1.4053872	-5.330818	4.07E-07	1.48E-05	6.1101194
cg13389211	chr17	78027740	-	CCDC40	Body	0.8259715	1.2228429	5.3308198	4.07E-07	1.48E-05	6.1101253
cg19935850	chr4	41753439	+			1.2642843	-0.840564	5.3306873	4.07E-07	1.48E-05	6.1095515
cg06179765	chr7	23287352	+	GPNMB;GPNMB	Body;Body	2.1668708	-1.914862	5.3305539	4.07E-07	1.48E-05	6.1089735
cg14260889	chr10	17275599	-	VIM	Body	1.2236068	2.016284	5.3305465	4.07E-07	1.48E-05	6.1089416
cg18361098	chr14	37136391	+	PAX9	Body	1.7517768	-1.098846	5.3303219	4.08E-07	1.48E-05	6.1079688
cg08318212	chr20	57875364	-	EDN3;EDN3;EDN3;EDN3	TSS200;TSS200;TSS200;TSS200	2.326534	-2.026016	5.3323723	4.08E-07	1.48E-05	6.1091003

cg16122716	chr1	206679959	+	RASSF5;RASSF5	TSS1500;TSS1500	-0.998686	0.2382379	-5.330231	4.08E-07	1.48E-05	6.1075758
cg04075546	chr7	54143022	-			-1.508432	0.5621819	-5.330112	4.08E-07	1.49E-05	6.1070586
cg03316587	chr8	142245164	+			-0.938362	-1.034977	-5.330073	4.08E-07	1.49E-05	6.10689
cg00562107	chr11	37532293	+			-1.51429	0.1977826	-5.330052	4.08E-07	1.49E-05	6.1067979
cg06390651	chr2	177023667	+			1.7958751	-0.802434	5.3341822	4.08E-07	1.49E-05	6.1267208
cg15541611	chr10	104211319	-	C10orf95	TSS200	-0.71838	-1.267479	-5.336281	4.08E-07	1.49E-05	6.1277137
cg11717597	chr1	156046546	+	MEX3A	Body	1.1087872	1.2743353	5.3297016	4.09E-07	1.49E-05	6.1052819
cg06857674	chr5	175110641	-	HRH2;HRH2	Body;1stExon	-1.357924	1.3543732	-5.331717	4.09E-07	1.49E-05	6.1062646
cg00105628	chr20	21087165	-			1.0534517	-0.476794	5.3294233	4.09E-07	1.49E-05	6.1040766
cg00525823	chr18	11148510	+	FAM38B	Body	1.1364082	-0.840887	5.3294044	4.09E-07	1.49E-05	6.1039946
cg10767350	chr8	50822476	-			1.4350689	-1.615866	5.3290602	4.10E-07	1.49E-05	6.1025041
cg14332049	chr6	153241931	-			-1.429969	0.0139	-5.329025	4.10E-07	1.49E-05	6.1023503
cg23649708	chr8	85097049	-	RALYL;RALYL;RALYL;RALYL	TSS200;5'UTR;5'UTR;5'UTR	1.9079789	-1.197749	5.3290152	4.10E-07	1.49E-05	6.1023093
cg05106892	chr15	99670731	+	SYNM;SYNM	Body;Body	1.1738908	2.7270663	5.3287369	4.10E-07	1.49E-05	6.1011041
cg22916646	chr5	162672583	+			-1.19595	0.2687665	-5.328668	4.11E-07	1.49E-05	6.1008035
cg08071609	chr6	117086040	-	FAM162B	Body	1.4293518	-1.56893	5.3305882	4.11E-07	1.49E-05	6.1013817
cg27079680	chr5	150326190	+	LOC134466	TSS200	1.0145587	-0.941276	5.32834	4.11E-07	1.50E-05	6.099385
cg01749904	chr16	89687052	-	DPEP1;DPEP1;DPEP1	5'UTR;1stExon;5'UTR	-0.965724	-0.767394	-5.328174	4.11E-07	1.50E-05	6.0986677
cg16010178	chr17	48611054	+	EPN3	5'UTR	-0.926144	-2.163457	-5.328177	4.11E-07	1.50E-05	6.0986791
cg09528449	chr8	144241120	+	LY6H;LY6H;LY6H	Body;5'UTR;Body	1.0812844	-0.848695	5.3280985	4.12E-07	1.50E-05	6.0983392

cg00607194	chr12	47479795	+			-1.28626	-1.231597	-5.330143	4.12E-07	1.50E-05	6.0994544
cg08823287	chr3	149049903	-	TM4SF18	Body	1.0411291	1.9015549	5.3280561	4.12E-07	1.50E-05	6.0981557
cg22602513	chr1	160429714	-			-1.603058	0.11476	-5.328037	4.12E-07	1.50E-05	6.0980718
cg20902783	chr14	21494071	-	NDRG2;NDRG2;NDRG2;NDRG2;NDRG2;NDRG2;NDRG2;NDRG2	TSS1500;TSS200;TSS200;TSS1500;TSS1500;TSS1500;TSS200;TSS200	0.9355135	-0.402934	5.3279549	4.12E-07	1.50E-05	6.0977177
cg22220806	chr19	57337469	+	PEG3;PEG3;PEG3;ZIM2;PEG3;PEG3;ZIM2;ZIM2	TSS1500;5'UTR;5'UTR;5'UTR;5'UTR;5'UTR;5'UTR;5'UTR	-1.357834	-0.181145	-5.327874	4.12E-07	1.50E-05	6.0973671
cg24058604	chr7	27142437	+	HOXA2	TSS200	2.2281164	-1.710822	5.3278265	4.12E-07	1.50E-05	6.0971615
cg12912022	chr7	62554288	-			-1.355871	-0.267362	-5.327763	4.12E-07	1.50E-05	6.0968886
cg07236424	chr22	39715474	-	SNORD43;RPL3;RPL3	TSS1500;Body;Body	-0.340024	-3.948724	-5.327514	4.13E-07	1.50E-05	6.0958066
cg03000596	chr7	73624373	-	LAT2;LAT2;LAT2;LAT2;LAT2;LAT2	5'UTR;5'UTR;5'UTR;1stExon;1stExon;1stExon	-1.055989	-2.223558	-5.327367	4.13E-07	1.50E-05	6.0951719
cg20229534	chr6	10882250	-	GCM2	TSS200	2.5316421	-2.423657	5.3273184	4.13E-07	1.50E-05	6.0949613
cg26149244	chr17	80329964	-			2.2917485	-1.622771	5.3273356	4.13E-07	1.50E-05	6.0950359
cg04001520	chr5	166988043	-	ODZ2	Body	-1.373338	0.1062225	-5.32718	4.13E-07	1.50E-05	6.0943601
cg24761867	chr5	138728704	-	LOC389333	1stExon	-1.520882	-4.070336	-5.327187	4.13E-07	1.50E-05	6.0943945
cg08566882	chr11	131348843	-	NTM	Body	-1.537002	0.1713768	-5.327111	4.13E-07	1.50E-05	6.094064
cg02140020	chr12	2613914	-	CACNA1C;CACNA1C;CACNA1C;CACNA1C;CACNA1C;CACNA1C;CACNA1C;CACNA1C;CACNA1C;CACNA1C;CACNA1C;CACNA1C;CACNA1C;CACNA1C;CACNA1C;CACNA1C;CACNA1C;CACNA	Body;Body;Body;Body;Body;Body;Body;Body;Body;Body;Body;Body;Body;Body;Body;Body;Body;Body;Body;Body;Body;Body;Body	-1.801934	1.0574833	-5.326994	4.14E-07	1.50E-05	6.0935578
cg22786667	chr7	47641179	-			-1.440776	-0.809267	-5.32687	4.14E-07	1.50E-05	6.093019
cg25732462	chr19	44324898	-	LYPD5	TSS200	2.202477	-1.229964	5.3267956	4.14E-07	1.50E-05	6.092698
cg01089319	chr2	171676809	+	GAD1;GAD1	Body;Body	1.2995423	-0.516302	5.3267576	4.14E-07	1.50E-05	6.0925334
cg06621060	chr10	102413151	+			-1.168997	-2.78334	-5.326483	4.15E-07	1.50E-05	6.0913448
cg20384702	chr10	2359772	+			-1.426337	0.352163	-5.326486	4.15E-07	1.50E-05	6.0913564

cg06272038	chr11	2292512	+	ASCL2	TSS1500	2.4998744	-1.431375	5.3264569	4.15E-07	1.50E-05	6.0912316
cg07795402	chr1	2938954	-	ACTRT2	1stExon	-1.686083	1.8928758	-5.326442	4.15E-07	1.50E-05	6.0911685
cg07981869	chr19	34174665	+	CHST8;CHST8;CHST8	5'UTR;5'UTR;TSS1500	-1.921443	1.4134199	-5.326279	4.15E-07	1.51E-05	6.0904615
cg14485901	chr9	139913752	-	ABCA2;ABCA2	Body;Body	-1.293762	0.4441027	-5.326091	4.15E-07	1.51E-05	6.089648
cg16170767	chr6	99290737	-			1.3018374	-0.865598	5.326076	4.15E-07	1.51E-05	6.0895826
cg05869699	chr10	82258734	-	TSPAN14;TSPAN14	Body;Body	1.5222177	-2.687851	5.3260528	4.15E-07	1.51E-05	6.0894822
cg12252069	chr7	26897597	-	SKAP2	Body	1.5315948	-0.317554	5.3257704	4.16E-07	1.51E-05	6.0882595
cg14207728	chr1	67217886	+	TCTEX1D1	TSS1500	2.1326777	-1.922837	5.3257452	4.16E-07	1.51E-05	6.0881505
cg10969919	chr5	135694224	+	TRPC7;TRPC7;TRPC7	Body;Body;Body	-1.3285	0.2080845	-5.325648	4.16E-07	1.51E-05	6.087728
cg12800803	chr20	60717299	-	PSMA7	Body	-1.062742	-4.225254	-5.325541	4.16E-07	1.51E-05	6.0872672
cg07115767	chr7	19146255	+			1.1122917	-0.74905	5.3253866	4.17E-07	1.51E-05	6.0865979
cg14616234	chr9	84305683	-			-0.915175	-0.59916	-5.325194	4.17E-07	1.51E-05	6.0857655
cg04914562	chr1	62207459	+	INADL	TSS1500	-0.923867	-1.33811	-5.32511	4.17E-07	1.51E-05	6.0853994
cg14367229	chr13	28367741	-	GSX1	Body	1.1977719	-0.548249	5.325128	4.17E-07	1.51E-05	6.0854788
cg01425409	chr3	187413913	+			-1.182568	-0.294516	-5.324978	4.17E-07	1.51E-05	6.084831
cg08293075	chr6	32156294	+	PBX2	Body	0.9172772	-0.151383	5.32494	4.18E-07	1.51E-05	6.0846646
cg20569855	chr1	109824440	-	PSRC1;PSRC1;PSRC1;PSRC1	Body;Body;Body;Body	0.8177278	1.6900081	5.3312006	4.18E-07	1.51E-05	6.0878335
cg23527468	chr3	133418844	+			-1.211656	-0.069369	-5.324805	4.18E-07	1.51E-05	6.0840826
cg06790305	chr21	32412191	+	KRTAP19-8	TSS1500	-1.480226	0.3360004	-5.324565	4.18E-07	1.52E-05	6.0830401
cg26279783	chr6	33143356	+	COL11A2;COL11A2;COL11A2	Body;Body;Body	-1.595609	0.7266363	-5.326498	4.19E-07	1.52E-05	6.0836917

cg00822495	chr14	57276490	+	OTX2	5'UTR	1.7155206	-1.845073	5.3243501	4.19E-07	1.52E-05	6.0821113
cg00913879	chr17	76421538	-	DNAH17	Body	0.8670246	0.9883402	5.3240134	4.19E-07	1.52E-05	6.0806543
cg20027419	chr6	35734095	-			0.8094158	2.0685212	5.3239342	4.19E-07	1.52E-05	6.0803112
cg14055835	chr19	55384515	-	FCAR;FCAR;FCAR;FCAR;FCAR;FCAR;FCAR;FCAR;FCAR	TSS1500;TSS1500;TSS1500;TSS1500;TSS1500;TSS1500;TSS1500;TSS1500;TSS1500	-1.307217	-1.60681	-5.323824	4.20E-07	1.52E-05	6.0798355
cg11341142	chr12	72152267	-	RAB21	Body	1.0468415	1.7215443	5.3236104	4.20E-07	1.52E-05	6.0789099
cg14595462	chr9	138453863	-	PAEP;PAEP	Body;Body	-0.83436	0.2978668	-5.323491	4.20E-07	1.52E-05	6.078393
cg13546514	chr3	9712113	+	MTMR14;MTMR14;MTMR14	Body;Body;Body	1.2117899	1.7830951	5.3233657	4.21E-07	1.52E-05	6.0778508
cg22856279	chr21	44830477	+			-1.196459	-0.98635	-5.323376	4.21E-07	1.52E-05	6.0778949
cg03938015	chr1	161953806	-	OLFML2B	Body	-1.016889	0.4486479	-5.323283	4.21E-07	1.52E-05	6.077494
cg00399374	chr8	82644943	+	CHMP4C	1stExon	-0.813389	-4.02965	-5.323252	4.21E-07	1.52E-05	6.0773584
cg01528028	chr13	100649800	+			1.1019172	-1.363861	5.3231551	4.21E-07	1.52E-05	6.0769392
cg01695643	chr14	95237330	-	GSC	TSS1500	1.012262	1.049703	5.3231168	4.21E-07	1.52E-05	6.0767738
cg15407213	chr6	33145550	-	COL11A2;COL11A2;COL11A2	Body;Body;Body	-1.451005	0.8210948	-5.323053	4.21E-07	1.52E-05	6.0764986
cg00750606	chr1	20899121	+			-0.797173	-0.815288	-5.322506	4.22E-07	1.53E-05	6.0741323
cg11935147	chr1	145075831	+	PDE4DIP	1stExon	1.1802474	-0.915106	5.3245395	4.22E-07	1.53E-05	6.0748338
cg12808596	chr11	60640645	+	ZP1	Body	1.0154446	1.118019	5.3223718	4.22E-07	1.53E-05	6.0735497
cg02675308	chr4	76556042	-	CDKL2	TSS1500	-1.39872	-4.788003	-5.322303	4.23E-07	1.53E-05	6.0732508
cg02313492	chr20	60467407	-	CDH4	Body	-0.897923	-1.748682	-5.322224	4.23E-07	1.53E-05	6.0729108
cg04222933	chr1	160783181	-	LY9	Body	-1.093134	-1.352096	-5.322174	4.23E-07	1.53E-05	6.0726917
cg25934680	chr17	31203833	+	MYO1D	1stExon	-0.402402	-4.165948	-5.322182	4.23E-07	1.53E-05	6.0727284

cg16433258	chr18	7114654	-	LAMA1	Body	-1.248572	-0.249867	-5.321929	4.23E-07	1.53E-05	6.0716336
cg10083898	chr12	66123380	+			1.594673	-0.753736	5.3218844	4.23E-07	1.53E-05	6.0714405
cg00548911	chr17	33788026	-			-1.124904	-0.539904	-5.321606	4.24E-07	1.53E-05	6.0702363
cg01619929	chr22	36806443	+			-0.872169	-1.550251	-5.321614	4.24E-07	1.53E-05	6.0702702
cg07714276	chr6	7169632	+	RREB1;RREB1;RREB1;RREB1	5'UTR;5'UTR;5'UTR;5'UTR	0.9318602	0.2479621	5.3216351	4.24E-07	1.53E-05	6.0703618
cg22438763	chr1	47900256	-	FOXD2;MGC12982	TSS1500;Body	1.6825422	0.1302595	5.321569	4.24E-07	1.53E-05	6.0700759
cg24340655	chr1	162955625	-			-1.643657	0.8944922	-5.321565	4.24E-07	1.53E-05	6.0700582
cg26709285	chr20	48099146	-	KCNB1;KCNB1	1stExon;5'UTR	1.4276003	-0.45481	5.3215013	4.24E-07	1.53E-05	6.0697829
cg14215702	chr7	155252317	+	EN2	Body	1.4883284	-0.91965	5.3213693	4.24E-07	1.53E-05	6.0692119
cg26019295	chr12	54409212	-	HOXC4;HOXC6;HOXC5	TSS1500;TSS1500;TSS1500	1.5653549	-0.727318	5.321346	4.24E-07	1.53E-05	6.0691112
cg15455084	chr19	29281888	-			-1.651435	0.1758602	-5.321144	4.25E-07	1.54E-05	6.0682357
cg01492523	chr4	155413789	-	DCHS2;DCHS2	TSS1500;TSS1500	1.0667628	0.0201856	5.3209997	4.25E-07	1.54E-05	6.0676129
cg17108958	chr1	221129545	-			-1.399627	-0.017219	-5.323006	4.25E-07	1.54E-05	6.0685982
cg23154497	chr10	134919573	+	GPR123	Body	-1.108084	0.1822061	-5.320889	4.25E-07	1.54E-05	6.067133
cg26922697	chr6	33284542	+	ZBTB22;ZBTB22	Body;Body	0.5585382	0.3147055	5.3207701	4.26E-07	1.54E-05	6.0666195
cg02738298	chr3	51749852	-	GRM2;GRM2	Body;Body	-0.57851	-0.820506	-5.320727	4.26E-07	1.54E-05	6.0664349
cg09767822	chr11	20178040	+	DBX1	Body	1.0529851	-0.115508	5.3207343	4.26E-07	1.54E-05	6.0664645
cg02874376	chr14	101193397	+	DLK1;DLK1	1stExon;5'UTR	2.3302574	-1.203701	5.3206907	4.26E-07	1.54E-05	6.0662759
cg15013801	chr10	73976790	+	ASCC1;C10orf104	TSS1500;5'UTR	-1.470154	-0.430721	-5.32488	4.26E-07	1.54E-05	6.0869339
cg17241289	chr7	71747632	+	CALN1;CALN1	5'UTR;Body	-1.544554	0.3347889	-5.320529	4.26E-07	1.54E-05	6.0655746

cg16110032	chr11	76030131	+			-0.670394	-4.103676	-5.320502	4.26E-07	1.54E-05	6.0654609
cg07679679	chr22	37717098	+			-1.09582	-0.25063	-5.320443	4.26E-07	1.54E-05	6.0652043
cg13570284	chr15	87807847	-			-1.06449	-0.836496	-5.320244	4.27E-07	1.54E-05	6.0643433
cg12073319	chr4	15965025	+	FGFBP2	TSS200	-0.83973	-0.882718	-5.320129	4.27E-07	1.54E-05	6.0638469
cg03969902	chr1	2980546	+	FLJ42875	Body	0.7253601	0.6004078	5.3199466	4.27E-07	1.54E-05	6.0630568
cg07648740	chr7	154864630	+	HTR5A	Body	-1.127052	-1.265463	-5.319951	4.27E-07	1.54E-05	6.0630737
cg07834069	chr17	72524918	-	CD300LB	Body	-1.551516	0.2075731	-5.319906	4.27E-07	1.54E-05	6.0628799
cg12836959	chr3	147098568	-			1.9068646	-1.01245	5.3220031	4.27E-07	1.54E-05	6.0638712
cg15772157	chr10	22766320	-			0.8271677	0.2086597	5.3199261	4.27E-07	1.54E-05	6.0629678
cg18845377	chr7	156795892	-			2.2937995	-2.105493	5.3218639	4.27E-07	1.54E-05	6.0636621
cg08775137	chr4	41880319	-			1.3700676	0.6161011	5.3280999	4.28E-07	1.54E-05	6.0652232
cg10763247	chr3	61066614	+	FHIT;FHIT	5'UTR;5'UTR	0.7562994	2.578396	5.3195604	4.28E-07	1.54E-05	6.0613859
cg25025879	chr12	53359317	-			1.537058	-1.50449	5.3194757	4.28E-07	1.54E-05	6.0610197
cg24547910	chr4	100632951	+			-0.865318	-0.549684	-5.319355	4.28E-07	1.54E-05	6.0604979
cg02491905	chr2	10975305	+			-1.046775	0.106731	-5.319332	4.28E-07	1.54E-05	6.0603981
cg07960360	chr14	96128403	-	TCL6	Body	-2.547221	1.7910393	-5.321211	4.29E-07	1.55E-05	6.0608389
cg02106941	chr8	24772137	+	NEFM;NEFM	TSS1500;1stExon	1.3085091	-1.700702	5.319062	4.29E-07	1.55E-05	6.0592302
cg22240704	chr3	20202403	+	SGOL1;SGOL1;SGOL1	3'UTR;3'UTR;3'UTR	0.6019206	2.6026839	5.3190702	4.29E-07	1.55E-05	6.0592653
cg11132336	chr7	54705907	+			-1.395354	0.0594023	-5.318934	4.29E-07	1.55E-05	6.0586742
cg14089503	chr8	37755555	+	RAB11FIP1;RAB11FIP1	Body;Body	-1.150883	-1.71451	-5.318801	4.29E-07	1.55E-05	6.0581004

cg01515155	chr11	55579363	-	OR5L1	1stExon	-1.712437	0.3783461	-5.318682	4.30E-07	1.55E-05	6.0575863
cg09508434	chr5	110919885	-			1.0472246	1.7853068	5.3186197	4.30E-07	1.55E-05	6.057317
cg18758281	chr2	161808953	+			0.9972046	0.9981743	5.3186062	4.30E-07	1.55E-05	6.0572587
cg00809969	chr7	27181067	-	HOXA5	3'UTR	1.2190732	0.7541345	5.318511	4.30E-07	1.55E-05	6.0568466
cg22593432	chr13	32575905	-			-1.134622	-0.108202	-5.318244	4.30E-07	1.55E-05	6.0556934
cg03188976	chr7	134443864	-			-0.997711	-1.106386	-5.318076	4.31E-07	1.55E-05	6.0549644
cg06127313	chr17	74945219	-	MGAT5B;MGAT5B	3'UTR;3'UTR	-1.476186	0.7670487	-5.318024	4.31E-07	1.55E-05	6.05474
cg06991300	chr4	122301724	+	QRFPR	1stExon	1.4054795	-0.30754	5.3180174	4.31E-07	1.55E-05	6.0547119
cg08038219	chr20	62031924	+			-1.637108	0.1994508	-5.318018	4.31E-07	1.55E-05	6.0547138
cg09231675	chr17	79523642	-			-1.727082	1.0121768	-5.318015	4.31E-07	1.55E-05	6.0547006
cg23217025	chr2	128173482	-			-0.849681	-1.836681	-5.318084	4.31E-07	1.55E-05	6.0549987
cg13434411	chr9	970297	+			1.8751011	-0.57144	5.3176152	4.32E-07	1.55E-05	6.0529722
cg23437479	chr3	49940353	-	MST1R	1stExon	-1.691438	0.688671	-5.317561	4.32E-07	1.55E-05	6.0527365
cg05988358	chr10	74082489	-			-0.93566	0.0317231	-5.31749	4.32E-07	1.56E-05	6.0524308
cg19984146	chr10	90968091	-	CH25H	TSS1500	-1.030941	-2.816643	-5.317442	4.32E-07	1.56E-05	6.0522243
cg04860664	chr1	43136315	+	PPIH	3'UTR	0.6351012	2.6295871	5.3174086	4.32E-07	1.56E-05	6.0520788
cg20814095	chr17	4802981	+	CHRNE;C17orf107;C17orf107	Body;5'UTR;1stExon	-2.097343	-3.207616	-5.321561	4.32E-07	1.56E-05	6.0545708
cg26146988	chr14	102034601	-			-1.192841	0.540413	-5.319433	4.32E-07	1.56E-05	6.0531584
cg12411994	chr7	150017070	-	ACTR3C	5'UTR	0.6545739	2.4128573	5.3172363	4.32E-07	1.56E-05	6.0513339
cg00119186	chr1	158670124	-	OR6K2	1stExon	-1.763785	0.8999999	-5.317198	4.32E-07	1.56E-05	6.0511682

cg24863335	chr18	70535406	-	NETO1	TSS1500	1.594479	-1.144445	5.3171696	4.33E-07	1.56E-05	6.0510452
cg07524398	chr22	39261722	+	CBX6	3'UTR	0.809632	0.6990238	5.3171114	4.33E-07	1.56E-05	6.0507937
cg20747577	chr7	26897253	-	SKAP2	Body	1.7164697	-0.971927	5.3171251	4.33E-07	1.56E-05	6.0508527
cg02006426	chr2	72012453	+			-1.12936	0.2786946	-5.317031	4.33E-07	1.56E-05	6.0504464
cg05068206	chr6	30095195	-			1.2064306	-0.118972	5.3170242	4.33E-07	1.56E-05	6.0504165
cg26253500	chr7	136641740	-	CHRM2;CHRM2;CHRM2;CHRM2;CHRM2;CHRM2;CHRM2;CHRM2	5'UTR;5'UTR;5'UTR;5'UTR;5'UTR;5'UTR;5'UTR;5'UTR	-1.620394	0.3065154	-5.317003	4.33E-07	1.56E-05	6.0503244
cg01626493	chr15	93115885	+	LOC100144604	TSS1500	-1.303223	0.2040456	-5.316971	4.33E-07	1.56E-05	6.0501869
cg13951797	chr16	2204381	-	TRAF7	TSS1500	-1.188603	-1.357811	-5.316847	4.33E-07	1.56E-05	6.0496481
cg17174023	chr22	50987453	+	KLHDC7B	1stExon	1.7228487	-0.239246	5.3166621	4.34E-07	1.56E-05	6.0488506
cg05268769	chr14	75733932	+			1.0785098	0.6753376	5.316609	4.34E-07	1.56E-05	6.0486208
cg04250451	chr1	25257784	+	RUNX3;RUNX3	TSS1500;Body	1.0645973	0.937515	5.3186148	4.34E-07	1.56E-05	6.0492319
cg13691192	chr17	2624945	+			-0.935626	-0.037893	-5.316518	4.34E-07	1.56E-05	6.0482255
cg15122966	chr2	102135168	+			-1.134906	0.0475367	-5.316513	4.34E-07	1.56E-05	6.0482078
cg00688840	chr14	106363051	-			-1.209622	-2.497049	-5.316401	4.34E-07	1.56E-05	6.0477204
cg08638320	chr1	47900265	-	FOXD2;MGC12982	TSS1500;Body	1.5439695	0.5839201	5.3164031	4.34E-07	1.56E-05	6.0477304
cg09166091	chr2	216484055	-			-1.526455	-1.216635	-5.316264	4.34E-07	1.56E-05	6.0471304
cg20430077	chr10	102983513	-			1.0056733	-1.256542	5.3162561	4.34E-07	1.56E-05	6.047095
cg12090740	chr18	60988058	-	BCL2;BCL2	TSS1500;TSS1500	-0.865193	-2.861358	-5.316175	4.34E-07	1.56E-05	6.0467439
cg00692462	chr1	4773089	+	AJAP1;AJAP1	Body;Body	-1.221039	0.1162344	-5.316085	4.35E-07	1.56E-05	6.0463538
cg00552235	chr7	100943163	+			2.1646241	-0.672982	5.3160203	4.35E-07	1.56E-05	6.0460753

cg02303068	chr12	131831940	-			-2.225555	0.8576337	-5.317875	4.35E-07	1.56E-05	6.0464309
cg01236299	chr17	9819556	-	GAS7;GAS7;GAS7;GAS7	3'UTR;3'UTR;3'UTR;3'UTR	-1.104027	0.3669872	-5.315737	4.35E-07	1.56E-05	6.0448501
cg12920361	chr7	150445474	-	LOC100128542	TSS1500	-1.421018	0.4118053	-5.31576	4.35E-07	1.56E-05	6.0449503
cg15246895	chr17	17672475	+	RAI1	5'UTR	0.4778224	0.175382	5.3157487	4.35E-07	1.56E-05	6.044901
cg16290689	chr13	31506685	-	C13orf26	TSS200	0.8462285	0.25932	5.3156266	4.36E-07	1.56E-05	6.0443731
cg07736627	chr11	44322491	-	ALX4	Body	-0.931898	0.1712839	-5.315565	4.36E-07	1.56E-05	6.0441075
cg05580415	chr17	80553970	-	FOXK2	Body	-0.940656	0.4956456	-5.315499	4.36E-07	1.56E-05	6.0438215
cg05028750	chr4	1544598	-			-0.857493	-0.224241	-5.315411	4.36E-07	1.57E-05	6.0434415
cg11380108	chr2	75835911	+			-0.87195	-1.01141	-5.315398	4.36E-07	1.57E-05	6.0433841
cg10541517	chr12	128753103	-	TMEM132C	Body	2.2924221	-2.198646	5.3260123	4.36E-07	1.57E-05	6.0488786
cg16734913	chr11	55681277	+	OR5W2	1stExon	-1.543897	0.6860826	-5.315175	4.36E-07	1.57E-05	6.0424217
cg01461840	chr10	130085088	+			1.4194639	-1.834373	5.3150991	4.37E-07	1.57E-05	6.0420922
cg02527056	chr4	180649176	-			-1.190178	0.173652	-5.31513	4.37E-07	1.57E-05	6.0422256
cg20617977	chr4	38053457	-	TBC1D1	Body	1.1946164	1.3414649	5.3151038	4.37E-07	1.57E-05	6.0421127
cg14385280	chr18	30051183	-	FAM59A	TSS1500	-1.160874	-6.180834	-5.316961	4.37E-07	1.57E-05	6.042481
cg14834653	chr10	123353791	-	FGFR2;FGFR2;FGFR2;FGFR2;FGFR2;FGFR2;FGFR2;FGFR2;FGFR2	TSS200;5'UTR;5'UTR;TSS1500;5'UTR;TSS1500;5'UTR;5'UTR;5'UTR	1.0452694	1.8975119	5.3147178	4.37E-07	1.57E-05	6.0404436
cg19754520	chr10	101825118	-	CPN1	Body	1.2161323	1.1432808	5.3146568	4.37E-07	1.57E-05	6.0401802
cg14092259	chr1	41220011	-	MIR30E;NFYC;NFYC;NFYC;NFYC;NFYC	TSS200;Body;Body;Body;Body;Body	1.4448537	1.8909479	5.3145445	4.38E-07	1.57E-05	6.0396944
cg23464619	chr13	95513506	+			-1.222593	-2.751281	-5.314554	4.38E-07	1.57E-05	6.039737
cg02396865	chr8	120867706	+	DSCC1	Body	-0.389704	-4.382662	-5.318654	4.38E-07	1.57E-05	6.0416204

cg05241461	chr19	22816980	-	ZNF492	TSS200	2.1095953	-1.335384	5.3144517	4.38E-07	1.57E-05	6.0392935
cg05945230	chr17	12425767	-			-1.376937	0.1996849	-5.314467	4.38E-07	1.57E-05	6.0393591
cg08196032	chr14	95239751	+			1.558218	-1.818986	5.3165355	4.38E-07	1.57E-05	6.0402508
cg01349621	chr20	61165941	+	C20orf166	Body	-1.145875	-0.789929	-5.314337	4.38E-07	1.57E-05	6.038796
cg22052659	chr8	114444989	+	CSMD3;CSMD3	Body;Body	2.2958983	-1.387621	5.3143531	4.38E-07	1.57E-05	6.0388673
cg12687617	chr15	89948930	-			1.3376895	-0.195796	5.3142743	4.38E-07	1.57E-05	6.0385265
cg24414344	chr17	39019556	-	KRT12	Body	0.8015624	1.0161839	5.3140658	4.39E-07	1.57E-05	6.037625
cg19371339	chr1	152657198	+	LCE2B	TSS1500	-1.382045	-0.533964	-5.31398	4.39E-07	1.57E-05	6.0372545
cg21599081	chr4	1511568	+			-1.068223	-0.087141	-5.31372	4.39E-07	1.57E-05	6.0361304
cg18203974	chr13	75039802	+			-1.010443	-1.050152	-5.3137	4.39E-07	1.57E-05	6.0360427
cg04573550	chr14	38680313	+	SSTR1	3'UTR	1.462216	-1.215375	5.3132918	4.40E-07	1.58E-05	6.0342791
cg01277983	chr22	43835600	+	MPPED1	Body	-1.277038	-0.046632	-5.313224	4.40E-07	1.58E-05	6.0339874
cg25276223	chr1	179334251	-	C1orf125;C1orf125	TSS1500;TSS1500	-1.204516	0.8918493	-5.313231	4.40E-07	1.58E-05	6.0340161
cg26441291	chr16	85289802	-			-1.542975	1.7132174	-5.313047	4.41E-07	1.58E-05	6.0332193
cg04622802	chr11	27015872	-	FIBIN;FIBIN	5'UTR;1stExon	1.0922736	-0.434465	5.3130009	4.41E-07	1.58E-05	6.0330219
cg07582829	chr19	54408440	-	PRKCG	Body	-0.731447	-1.658829	-5.312991	4.41E-07	1.58E-05	6.032981
cg08316699	chr6	150357289	+			-0.936414	-1.202999	-5.312763	4.41E-07	1.58E-05	6.0319941
cg09294937	chr2	219028997	-	CXCR1	Body	-1.271572	0.5126824	-5.312742	4.41E-07	1.58E-05	6.0319048
cg09911083	chr17	7754956	+	KDM6B	Body	-0.896763	-0.163687	-5.312751	4.41E-07	1.58E-05	6.0319415
cg25598840	chr7	27142618	+	HOXA2	TSS1500	1.7609442	-0.865253	5.3127775	4.41E-07	1.58E-05	6.0320561

cg00219321	chr5	153318647	+			-1.527883	1.0488343	-5.312643	4.41E-07	1.58E-05	6.0314732
cg15415259	chr6	134158982	+	MGC34034;MGC34034	Body;Body	-1.023659	-1.542869	-5.312183	4.42E-07	1.58E-05	6.0294882
cg22681721	chr6	35477358	-	TULP1	Body	-1.010747	0.532291	-5.312141	4.43E-07	1.58E-05	6.0293048
cg01380361	chr1	207225647	-	PFKFB2;PFKFB2;YOD1	TSS1500;TSS1500;TSS1500	-1.795146	0.4306707	-5.312077	4.43E-07	1.58E-05	6.029029
cg03975306	chr8	11519192	+			0.9380495	2.0509461	5.3120769	4.43E-07	1.58E-05	6.0290282
cg03165383	chr18	74981431	+	GALR1	3'UTR	-1.7096	1.2692182	-5.314064	4.43E-07	1.58E-05	6.0295796
cg08096367	chr15	95384471	+			-1.525805	1.2087196	-5.31189	4.43E-07	1.59E-05	6.0282216
cg21806015	chr7	8483249	+	NXPH1	Body	1.4316091	-1.434571	5.3118428	4.43E-07	1.59E-05	6.0280163
cg13241144	chr12	34463891	+			-1.113462	-0.432488	-5.311774	4.43E-07	1.59E-05	6.027719
cg00199549	chr22	35795374	+	MCM5	TSS1500	-1.780634	1.2931454	-5.311713	4.43E-07	1.59E-05	6.0274546
cg01572696	chr4	995849	+	IDUA	Body	1.0914327	-0.237196	5.3116693	4.43E-07	1.59E-05	6.0272665
cg01641371	chr17	26712092	-	SARM1	Body	-0.716104	-1.058065	-5.31376	4.43E-07	1.59E-05	6.0286598
cg22911200	chr8	139124425	-			-1.23371	-0.388017	-5.311728	4.43E-07	1.59E-05	6.0275182
cg27360326	chr13	53422691	-	PCDH8;PCDH8;PCDH8;PCDH8	1stExon;5'UTR;1stExon;5'UTR	2.0980616	-0.999532	5.3116837	4.43E-07	1.59E-05	6.0273287
cg03805566	chr3	183245952	+	KLHL6	Body	-0.971771	2.1253824	-5.311599	4.44E-07	1.59E-05	6.0269637
cg07431286	chr5	72526496	+			2.0350591	-1.44964	5.311187	4.44E-07	1.59E-05	6.0251823
cg27158462	chr15	26873818	-	GABRB3;GABRB3	Body;Body	-1.234919	0.5283903	-5.311166	4.44E-07	1.59E-05	6.0250902
cg07917031	chr1	232571335	+	SIPA1L2	Body	1.0334871	0.771566	5.3111224	4.45E-07	1.59E-05	6.024903
cg23167287	chr2	230139541	+			-1.390412	0.4890665	-5.311029	4.45E-07	1.59E-05	6.0244975
cg14203721	chr19	52266642	+	FPR2;FPR2;FPR2	5'UTR;1stExon;5'UTR	-1.423716	0.3426972	-5.310994	4.45E-07	1.59E-05	6.0243471

cg22300957	chr4	41748857	+	PHOX2B	Body	1.1530793	-0.958597	5.3108298	4.45E-07	1.59E-05	6.0236385
cg12498094	chr2	40356782	-	SLC8A1;SLC8A1;SLC8A1;SLC8A1	Body;Body;Body;Body	-1.226404	0.168058	-5.310797	4.45E-07	1.59E-05	6.0234971
cg01947130	chr15	60287196	+			2.2723524	-1.560577	5.3127289	4.45E-07	1.59E-05	6.0238155
cg23201695	chr8	1272959	+			-1.725637	1.3638554	-5.310631	4.46E-07	1.59E-05	6.0227818
cg03134809	chr11	1365011	-			-1.27336	0.0130018	-5.310526	4.46E-07	1.59E-05	6.0223249
cg18748085	chr5	22372872	+	CDH12	5'UTR	-1.616788	0.3686341	-5.310539	4.46E-07	1.59E-05	6.022381
cg22575043	chr7	1664952	+			-1.220024	-0.14074	-5.312622	4.46E-07	1.59E-05	6.0233549
cg16368763	chr1	231296640	-			0.8114928	-0.514048	5.3104599	4.46E-07	1.59E-05	6.0220402
cg16032050	chr10	50605158	-			1.3252218	-1.900869	5.3104129	4.46E-07	1.59E-05	6.0218373
cg02988755	chr13	110918331	-	COL4A1	Body	0.7623962	0.9653511	5.3098619	4.47E-07	1.60E-05	6.0194565
cg14006798	chr20	30222136	+			-1.068192	-1.12473	-5.311859	4.47E-07	1.60E-05	6.0200616
cg01477473	chr17	73128192	-	NT5C	TSS1500	-0.6372	-4.022728	-5.31178	4.47E-07	1.60E-05	6.0201131
cg07781678	chr5	143267380	+			-1.193919	1.849895	-5.309597	4.48E-07	1.60E-05	6.0183139
cg16573755	chr2	172958337	+			1.5484913	0.2113116	5.3095944	4.48E-07	1.60E-05	6.0183007
cg16847760	chr8	28912915	+			0.9395462	0.0621683	5.3094638	4.48E-07	1.60E-05	6.0177365
cg21056723	chr4	56686117	+	LOC644145	TSS200	1.8875877	-3.238904	5.3092332	4.48E-07	1.60E-05	6.0167401
cg10837429	chr5	177411733	-			1.3910231	-1.835991	5.3111823	4.49E-07	1.60E-05	6.0175338
cg11009001	chr6	165715240	-	C6orf118	Body	-1.277281	0.2231802	-5.309082	4.49E-07	1.60E-05	6.0160876
cg22587479	chr2	219738226	+	WNT6	Body	2.0369557	-2.312523	5.3087759	4.49E-07	1.60E-05	6.0147643
cg00162064	chr1	44998151	+	RNF220	Body	-1.100721	-1.981196	-5.30862	4.50E-07	1.60E-05	6.0140922

cg07896832	chr8	67975874	-	CSPP1;CSPP1;COPS5	TSS1500;TSS1500;TSS1500	-1.636115	-0.108133	-5.308617	4.50E-07	1.60E-05	6.0140789
cg13088556	chr6	117583893	+			1.0692873	0.4551777	5.3086045	4.50E-07	1.60E-05	6.014024
cg13879937	chr15	42265413	-	EHD4	TSS1500	-1.272856	-0.562903	-5.3086	4.50E-07	1.60E-05	6.0140033
cg20377376	chr3	48031771	-	MAP4;MAP4;MAP4	Body;Body;Body	-0.86888	-2.659198	-5.30852	4.50E-07	1.60E-05	6.0136585
cg04505972	chr2	173508040	-			-1.182016	-0.381864	-5.30841	4.50E-07	1.61E-05	6.0131818
cg10094691	chr21	42210827	-	DSCAM	Body	-1.333142	-0.023696	-5.308357	4.50E-07	1.61E-05	6.012954
cg08509270	chr14	101513595	+	MIR889;MIR539	TSS1500;TSS200	-1.068987	0.1212536	-5.308277	4.50E-07	1.61E-05	6.0126093
cg13374432	chr15	86192745	-	AKAP13;AKAP13	Body;Body	1.3793298	-0.452972	5.3082502	4.50E-07	1.61E-05	6.0124933
cg09142858	chr16	85146088	-	FAM92B;FAM92B	5'UTR;1stExon	-0.794155	-0.116087	-5.308172	4.51E-07	1.61E-05	6.0121563
cg10522607	chr2	1595933	-			1.4589533	-1.563171	5.3081532	4.51E-07	1.61E-05	6.0120742
cg16201210	chr20	30607220	-	C20orf160	Body	-0.453383	-3.462031	-5.308105	4.51E-07	1.61E-05	6.0118655
cg01949002	chr1	2203589	-	SKI	Body	1.0804708	-0.803752	5.3080719	4.51E-07	1.61E-05	6.011723
cg15128898	chr5	35618029	-	SPEF2;SPEF2;SPEF2;SPEF2	5'UTR;5'UTR;1stExon;1stExon	-1.422799	-3.257677	-5.309918	4.51E-07	1.61E-05	6.0116833
cg19547694	chr22	50174005	+	BRD1;LOC90834	Body;TSS200	0.6159841	1.9033657	5.3098998	4.51E-07	1.61E-05	6.0116053
cg00017842	chr2	119528421	-			-1.209039	-0.901227	-5.307697	4.51E-07	1.61E-05	6.0101058
cg20018563	chr15	92690264	+	SLCO3A1;SLCO3A1	Body;Body	0.9546443	0.6183607	5.3076744	4.52E-07	1.61E-05	6.0100061
cg25449821	chr17	6004572	+	WSCD1	Body	-1.205272	0.3569277	-5.307486	4.52E-07	1.61E-05	6.0091916
cg05855917	chr3	147113700	-	ZIC4;ZIC4;ZIC4	Body;Body;Body	1.789469	-1.325849	5.3095361	4.52E-07	1.61E-05	6.0104301
cg06235973	chr19	14183619	+	LOC113230	TSS1500	-0.571007	-3.208615	-5.307443	4.52E-07	1.61E-05	6.0090074
cg27549878	chr19	38886554	+	SPRED3	Body	2.1887755	-0.941883	5.3073681	4.52E-07	1.61E-05	6.0086829

cg00166722	chr3	10149974	-	C3orf24;C3orf24	TSS200;TSS200	1.1493726	2.9721562	5.309333	4.52E-07	1.61E-05	6.0091594
cg08868915	chr1	248097906	-			-1.266863	-0.293753	-5.307131	4.53E-07	1.61E-05	6.0076596
cg16707952	chr6	30851632	-	DDR1;DDR1	TSS1500;TSS1500	-1.067001	-0.191462	-5.307142	4.53E-07	1.61E-05	6.0077083
cg12976793	chr12	96584672	+			-1.442972	1.6763986	-5.30914	4.53E-07	1.61E-05	6.0083261
cg17971015	chr19	12306198	+			1.5357915	-0.861462	5.3069771	4.53E-07	1.61E-05	6.0069941
cg26663590	chr16	28959310	-			-1.340237	-0.068481	-5.306918	4.53E-07	1.61E-05	6.0067408
cg02421734	chr19	55172736	+	LILRB4;LILRB4	TSS1500;TSS1500	-1.250059	-0.572858	-5.306767	4.53E-07	1.61E-05	6.0060878
cg00446472	chr16	89042082	-	CBFA2T3	Body	-1.032964	-0.75977	-5.306727	4.53E-07	1.61E-05	6.0059149
cg19564450	chr2	23023791	+			-1.305951	0.1928081	-5.306404	4.54E-07	1.62E-05	6.0045182
cg07385423	chr1	161697574	+	FCRLB	3'UTR	-1.630907	-2.190867	-5.306319	4.54E-07	1.62E-05	6.0041533
cg08806779	chr16	86965605	-			1.2161437	1.3033578	5.3062757	4.54E-07	1.62E-05	6.0039648
cg08151514	chr7	57247748	+	LOC642006	Body	-1.460482	1.053894	-5.306251	4.54E-07	1.62E-05	6.0038574
cg09169953	chr4	111555102	+	PITX2;PITX2	5'UTR;5'UTR	1.270087	-1.70592	5.3061583	4.55E-07	1.62E-05	6.0034577
cg22869726	chr21	34398265	-	OLIG2;OLIG2	1stExon;5'UTR	2.1991567	-0.707493	5.3061502	4.55E-07	1.62E-05	6.0034227
cg19730699	chr10	44231015	+			-0.945438	-0.815701	-5.306126	4.55E-07	1.62E-05	6.0033163
cg17033166	chr12	52221959	-			-0.712463	-1.90502	-5.306052	4.55E-07	1.62E-05	6.0029975
cg20894686	chr6	50803903	+	TFAP2B	Body	1.4899726	-1.419175	5.3059472	4.55E-07	1.62E-05	6.0025459
cg10078750	chr4	2421179	-	ZFYVE28	TSS1500	-0.420886	-3.564058	-5.305661	4.56E-07	1.62E-05	6.0013117
cg22976218	chr2	182544321	-	NEUROD1	5'UTR	1.5811419	-0.489419	5.3056229	4.56E-07	1.62E-05	6.0011453
cg02057782	chr13	26042598	+	ATP8A2	Body	0.866774	-0.192801	5.3055907	4.56E-07	1.62E-05	6.0010065

cg13742513	chr19	6433825	+	SLC25A41	TSS200	-1.032163	-0.228921	-5.305546	4.56E-07	1.62E-05	6.0008116
cg01494399	chr16	81709890	+	CMIP;CMIP	Body;Body	0.7493636	1.3463566	5.3055074	4.56E-07	1.62E-05	6.0006464
cg02386354	chr6	112042446	+	FYN;FYN;FYN	5'UTR;5'UTR;TSS1500	0.9396864	2.3746257	5.3049367	4.57E-07	1.63E-05	5.9981821
cg06916239	chr2	223162881	+	PAX3;PAX3;PAX3;CCDC140;CCDC140;PAX3;PAX3;PAX3;PAX3;PAX3	Body;Body;Body;5'UTR;1stExon;Body;Body;Body;Body;Body	1.9500762	-0.941713	5.3048897	4.57E-07	1.63E-05	5.9979793
cg15017067	chr4	17643749	+	FAM184B	Body	0.9646992	0.9185633	5.3048427	4.57E-07	1.63E-05	5.9977763
cg12750151	chr11	55586264	+	OR5D18	TSS1500	-0.953332	-1.31877	-5.304778	4.57E-07	1.63E-05	5.9974977
cg04223832	chr14	101515855	+	MIR655	TSS200	-1.025938	0.2131298	-5.304565	4.58E-07	1.63E-05	5.9965768
cg13394491	chr5	15737401	-	FBXL7	Body	-1.649528	-0.460481	-5.304488	4.58E-07	1.63E-05	5.9962425
cg06283052	chr10	44774998	+			-1.203437	-0.112923	-5.304438	4.58E-07	1.63E-05	5.9960272
cg09212803	chr10	62899394	-			0.9048517	1.6978076	5.3044121	4.58E-07	1.63E-05	5.9959167
cg16108887	chr7	25608305	+			-1.443306	-0.185059	-5.304231	4.59E-07	1.63E-05	5.9951329
cg20596629	chr8	15096774	-	SGCZ	TSS1500	-1.339803	0.088971	-5.304115	4.59E-07	1.63E-05	5.9946349
cg05270344	chr7	79083635	+	MAGI2	TSS1500	1.6394514	-1.887522	5.3040355	4.59E-07	1.63E-05	5.9942907
cg26019112	chr11	31821313	+	PAX6;PAX6;PAX6	Body;Body;Body	1.6123449	-0.939754	5.304007	4.59E-07	1.63E-05	5.9941678
cg23332813	chr6	33133370	-	COL11A2;COL11A2;COL11A2	Body;Body;Body	-1.20046	0.4128909	-5.30601	4.59E-07	1.63E-05	5.994824
cg04058752	chr11	76751369	+	B3GNT6	Body	0.9063468	1.4165041	5.3080833	4.59E-07	1.63E-05	5.996456
cg02697519	chr1	10769910	-	CASZ1;CASZ1	5'UTR;5'UTR	0.8812272	2.0921023	5.3038147	4.59E-07	1.63E-05	5.9933376
cg03706086	chr8	71381216	-			-1.314196	0.5448809	-5.303757	4.60E-07	1.63E-05	5.9930884
cg15117754	chr3	10150083	+	C3orf24;C3orf24	TSS200;TSS200	0.8667538	1.2341752	5.3058544	4.60E-07	1.63E-05	5.9945475
cg18721397	chr5	32584912	-	SUB1	TSS1500	-1.551238	-0.443261	-5.305821	4.60E-07	1.63E-05	5.9940083

cg03476007	chr4	143768727	+	INPP4B;INPP4B	TSS1500;TSS1500	-1.037083	-1.837795	-5.303538	4.60E-07	1.63E-05	5.9921407
cg00158333	chr17	47075855	-	IGF2BP1;IGF2BP1	Body;Body	1.5810229	-1.310381	5.3034922	4.60E-07	1.63E-05	5.9919451
cg03299042	chr2	63283806	-	OTX1	3'UTR	1.0614244	0.3027104	5.3034964	4.60E-07	1.63E-05	5.991963
cg09757087	chr16	1585644	+	IFT140;TMEM204	Body;Body	0.9574533	0.9613621	5.3034269	4.60E-07	1.63E-05	5.9916629
cg09468231	chr7	129709893	-	KLHDC10	TSS1500	-0.686143	-3.137899	-5.303222	4.61E-07	1.64E-05	5.9907771
cg21214566	chr9	138549731	-			-1.322758	0.1405953	-5.303043	4.61E-07	1.64E-05	5.9900043
cg23474173	chr14	23888492	-	MIR208B;MYH7	TSS1500;Body	-1.234178	0.1688992	-5.303037	4.61E-07	1.64E-05	5.9899789
cg15916646	chr7	27225127	+	HOXA11AS;HOXA11	Body;TSS1500	0.9703866	-0.233693	5.3029073	4.61E-07	1.64E-05	5.9894197
cg19234591	chr1	26632948	+	UBXN11;UBXN11;UBXN11	5'UTR;5'UTR;5'UTR	-1.087259	-5.099153	-5.304978	4.61E-07	1.64E-05	5.9903746
cg01972751	chr6	137814728	-	OLIG3	1stExon	2.1639979	-0.671988	5.3048763	4.62E-07	1.64E-05	5.9903293
cg04093349	chr15	66998947	-	SMAD6;SMAD6	Body;Body	1.7909788	2.0525216	5.3027963	4.62E-07	1.64E-05	5.9889407
cg09873544	chr10	132882986	+			-0.999169	-0.01789	-5.302756	4.62E-07	1.64E-05	5.9887684
cg11497372	chr6	27513092	-			2.0093701	-1.727587	5.3047604	4.62E-07	1.64E-05	5.9894345
cg19159759	chr13	20989818	-	CRYL1	Body	0.9613663	1.1775254	5.3025846	4.62E-07	1.64E-05	5.9880268
cg21186708	chr13	112716728	+			1.2713474	-1.299571	5.3025201	4.62E-07	1.64E-05	5.9877483
cg03051449	chr6	26501812	+	BTN1A1	Body	1.2818191	-1.25023	5.3023603	4.63E-07	1.64E-05	5.9870585
cg22747076	chr12	54447873	+	HOXC4;HOXC4	1stExon;Body	2.9180427	-1.75563	5.3022875	4.63E-07	1.64E-05	5.9867444
cg04014685	chr3	115509674	-			-1.573357	0.6639598	-5.302231	4.63E-07	1.64E-05	5.9864989
cg24567694	chr7	154719693	+	LOC100132707;LOC100132707	TSS1500;TSS1500	-0.745361	-4.235005	-5.304283	4.63E-07	1.64E-05	5.9877715
cg25499650	chr11	7728057	+	OVCH2	TSS200	-1.022342	-0.743941	-5.302249	4.63E-07	1.64E-05	5.9865768

cg26968498	chr4	26174157	-			0.8500086	1.423122	5.3022004	4.63E-07	1.64E-05	5.9863683
cg16043357	chr10	118897904	+	VAX1;VAX1	TSS200;TSS200	1.5750931	-0.67668	5.3017782	4.64E-07	1.64E-05	5.9845458
cg19921377	chr19	7580209	+	ZNF358	TSS1500	1.2916241	1.6095262	5.3017578	4.64E-07	1.64E-05	5.9844576
cg07143083	chr7	70597921	+	WBSCR17	1stExon	1.3488216	-0.59577	5.3015243	4.64E-07	1.65E-05	5.9834499
cg26026598	chr1	181142292	+			-1.416535	0.0954194	-5.301513	4.64E-07	1.65E-05	5.9834011
cg26394308	chr17	74067861	-	SRP68	Body	-0.843866	-3.030794	-5.301479	4.64E-07	1.65E-05	5.9832531
cg16396980	chr16	6633344	+	A2BP1;A2BP1	5'UTR;5'UTR	-1.372201	0.7856702	-5.301371	4.65E-07	1.65E-05	5.9827875
cg04388548	chr13	114905640	+			-1.008182	-0.492949	-5.301288	4.65E-07	1.65E-05	5.9824298
cg19466563	chr4	88450506	+	SPARCL1;SPARCL1;SPARCL1;SPARCL1	1stExon;1stExon;5'UTR;5'UTR	1.0573245	0.4000341	5.3012425	4.65E-07	1.65E-05	5.9822337
cg01177854	chr16	68270387	+	ESRP2	TSS1500	-0.565502	-2.081588	-5.301104	4.65E-07	1.65E-05	5.9816364
cg04158698	chr1	201523041	+			0.8629327	1.1791571	5.301113	4.65E-07	1.65E-05	5.9816743
cg24865210	chr5	158689727	+	UBLCP1	TSS1500	-1.002518	-2.016665	-5.301082	4.65E-07	1.65E-05	5.9815414
cg06217245	chr20	33103252	-	DYNLRB1	TSS1500	-0.860061	-3.449324	-5.301052	4.65E-07	1.65E-05	5.9814114
cg00526455	chr19	19285099	-	LOC729991-MEF2B;LOC729991-MEF2B;LOC729991-MEF2B	Body;5'UTR;Body	-1.42492	0.9160167	-5.300742	4.66E-07	1.65E-05	5.9800716
cg15697852	chr5	140098576	-	VTRNA1-2	Body	-1.604165	0.3878825	-5.300657	4.66E-07	1.65E-05	5.979708
cg08832234	chr8	138493933	+			-1.181203	-0.906126	-5.3006	4.66E-07	1.65E-05	5.9794594
cg25045972	chr14	101405842	+	SNORD113-6	TSS200	-1.495218	0.4448874	-5.300513	4.66E-07	1.65E-05	5.979086
cg25368586	chr13	111561018	+	ANKRD10	Body	-1.550881	0.5513524	-5.300527	4.66E-07	1.65E-05	5.9791432
cg21328389	chr8	109145172	-			-1.259386	0.2913885	-5.300405	4.67E-07	1.65E-05	5.978617
cg05221057	chr13	112711587	-			1.8421378	-1.287772	5.2999069	4.68E-07	1.66E-05	5.9764691

cg12835181	chr18	56931575	-			1.3306034	-1.185032	5.2998027	4.68E-07	1.66E-05	5.9760194
cg26280578	chr20	61916036	-	ARFGAP1;ARFGAP1	Body;Body	1.2365211	3.0794316	5.2998103	4.68E-07	1.66E-05	5.9760524
cg02072834	chr14	76589894	-			-1.023181	-1.473938	-5.299631	4.68E-07	1.66E-05	5.9752788
cg21609640	chr1	166890380	+	ILDR2	Body	1.1619715	-0.533461	5.299603	4.68E-07	1.66E-05	5.9751578
cg15450445	chr3	14365151	+			0.8523846	0.8524936	5.299549	4.68E-07	1.66E-05	5.9749245
cg18327049	chr14	103470338	+	CDC42BPB	Body	0.8343138	4.4149359	5.3014911	4.69E-07	1.66E-05	5.975734
cg05477165	chr2	129074982	-	HS6ST1	Body	-1.063415	-2.599933	-5.299404	4.69E-07	1.66E-05	5.974301
cg23877608	chr19	48823413	+	CCDC114	TSS200	-0.927886	-2.781103	-5.299319	4.69E-07	1.66E-05	5.9739337
cg06070324	chr15	75322058	-	PPCDC	Body	0.8430816	0.266641	5.2991482	4.69E-07	1.66E-05	5.9731951
cg16624013	chr6	35437101	+	RPL10A	Body	-0.565803	-3.042959	-5.298716	4.70E-07	1.66E-05	5.9713296
cg10526277	chr3	157813363	+			1.5608276	-0.776626	5.2981967	4.71E-07	1.67E-05	5.9690893
cg01464835	chr19	30016147	-	VSTM2B	TSS1500	2.8016682	-2.114987	5.2981391	4.71E-07	1.67E-05	5.9688411
cg05493344	chr9	136567064	-	SARDH;SARDH	Body;Body	-0.999545	0.8107164	-5.298133	4.71E-07	1.67E-05	5.9688137
cg21997401	chr4	12225077	+			-1.433536	0.8909601	-5.297913	4.72E-07	1.67E-05	5.9678656
cg13981026	chr9	101726137	+	COL15A1	Body	-1.332627	1.0808896	-5.297836	4.72E-07	1.67E-05	5.9675346
cg03722525	chr11	67381554	+			-1.032085	-2.971342	-5.297794	4.72E-07	1.67E-05	5.9673518
cg13035910	chr6	100052728	-			1.0818221	-0.668128	5.2978001	4.72E-07	1.67E-05	5.9673781
cg24793022	chr6	160296499	+			-1.615557	0.5970783	-5.297635	4.73E-07	1.67E-05	5.9666662
cg08686462	chr11	2171184	+	INS-IGF2;IGF2;INS-IGF2	Body;TSS1500;Body	-0.803258	-1.891027	-5.297542	4.73E-07	1.67E-05	5.9662643
cg24593372	chr1	54562121	+	C1orf83	Body	0.7323574	0.2141999	5.2975254	4.73E-07	1.67E-05	5.9661931

cg14447710	chr9	137458222	+			-2.373477	2.915735	-5.296921	4.74E-07	1.68E-05	5.9635871
cg01368075	chr3	128523902	-	RAB7A	Body	0.8551173	1.5735035	5.2967867	4.74E-07	1.68E-05	5.9630063
cg17561365	chr7	157335526	-	PTPRN2;PTPRN2;PTPRN2	Body;Body;Body	-1.541742	0.5538716	-5.296779	4.74E-07	1.68E-05	5.9629717
cg12383352	chr6	31744612	+	C6orf27	5'UTR	-0.681218	-2.3571	-5.296221	4.76E-07	1.68E-05	5.960566
cg22434835	chr12	108603985	-	WSCD2	Body	-2.435961	2.4918607	-5.296149	4.76E-07	1.68E-05	5.9602569
cg10767223	chr5	1295799	+	TERT;TERT	TSS1500;TSS1500	1.0939115	0.1341909	5.2960852	4.76E-07	1.68E-05	5.9599801
cg00791406	chr12	14118880	-	GRIN2B	5'UTR	-1.412023	0.4038686	-5.30017	4.76E-07	1.68E-05	5.9615761
cg22723502	chr6	26240528	-	HIST1H4F	TSS200	2.7410735	-1.229726	5.3044658	4.76E-07	1.68E-05	5.9643596
cg10858215	chr2	177004604	-			2.2961887	-2.38256	5.2959974	4.76E-07	1.68E-05	5.9596014
cg03563308	chr18	56887279	-	GRP;GRP;GRP	TSS200;TSS200;TSS200	2.2938598	-1.978824	5.3000568	4.76E-07	1.68E-05	5.9801167
cg15003158	chr5	68628431	-			-0.96627	-3.201592	-5.295696	4.77E-07	1.68E-05	5.9583024
cg15112369	chr11	34195914	+	ABTB2	Body	-0.585818	-4.140908	-5.295699	4.77E-07	1.68E-05	5.9583149
cg02722637	chr12	4922070	-	KCNA6;KCNA6	3'UTR;1stExon	-1.423937	-0.038649	-5.295666	4.77E-07	1.68E-05	5.9581704
cg17473782	chr13	114780772	-	RASA3	Body	-2.025689	1.0222245	-5.295582	4.77E-07	1.68E-05	5.9578092
cg22353755	chr19	28397061	+			-1.440083	0.4387654	-5.295589	4.77E-07	1.68E-05	5.9578406
cg13833923	chr14	79746627	-	NRXN3;NRXN3;NRXN3;NRXN3;NRXN3	Body;1stExon;1stExon;5'UTR;5'UTR	-1.135481	-2.261402	-5.295396	4.77E-07	1.68E-05	5.9570053
cg23068476	chr12	2613703	-	CACNA1C;CACNA1C;CACNA1C;CACNA1C;CACNA1C;CACNA1C;CACNA1C;CACNA1C;CACNA1C;CACNA1C;CACNA1C;CACNA1C;CACNA1C;CACNA1C;CACNA1C;CACNA1C;CACNA1C;CACNA	Body;Body;Body;Body;Body;Body;Body;Body;Body;Body;Body;Body;Body;Body;Body;Body;Body;Body;Body;Body;Body;Body;Body	-1.697259	1.2677487	-5.295034	4.78E-07	1.69E-05	5.9554462
cg04207084	chr7	157477846	-	PTPRN2;PTPRN2;PTPRN2	Body;Body;Body	1.8423348	-1.223255	5.2949419	4.78E-07	1.69E-05	5.9550489
cg05600421	chr11	30597981	+	MPPED2;MPPED2	Body;Body	-1.677511	0.0686558	-5.294884	4.78E-07	1.69E-05	5.9547982
cg17403817	chr6	118480809	+	SLC35F1	Body	-1.631343	1.0068876	-5.294867	4.78E-07	1.69E-05	5.9547243

cg25092681	chr5	16180033	-	11-Mar	TSS200	2.6656044	-2.043199	5.2969083	4.78E-07	1.69E-05	5.9743121
cg07451946	chr10	2337473	-			-1.600296	0.9341688	-5.294712	4.79E-07	1.69E-05	5.9540571
cg20732478	chr2	176993643	-	HOXD8	TSS1500	1.4315858	-0.886674	5.2947279	4.79E-07	1.69E-05	5.954126
cg08883438	chr19	31163217	-			-1.491965	-0.053023	-5.294636	4.79E-07	1.69E-05	5.9537281
cg22304239	chr19	3968427	-	DAPK3	Body	0.96129	1.3400868	5.2946533	4.79E-07	1.69E-05	5.9538043
cg14139311	chr10	81208625	+			-1.144498	1.2276687	-5.294559	4.79E-07	1.69E-05	5.9533961
cg01315916	chr5	3591609	-			1.1166027	-0.591198	5.2944179	4.79E-07	1.69E-05	5.9527888
cg18530299	chr8	50818283	+			-1.2745	-1.878428	-5.296304	4.80E-07	1.69E-05	5.9533818
cg21961487	chr12	106979430	-	RFX4	Body	1.3200798	-1.305766	5.2939807	4.80E-07	1.69E-05	5.9509037
cg11474811	chr11	2443869	+	TRPM5	Body	-1.21009	0.6592398	-5.293882	4.81E-07	1.69E-05	5.9504761
cg06934867	chr1	225651959	-			-1.213914	0.0460127	-5.293798	4.81E-07	1.69E-05	5.9501136
cg12277278	chr20	61636767	-			1.0227137	-1.320363	5.2978688	4.81E-07	1.70E-05	5.9520677
cg13457172	chr10	118897847	+	VAX1;VAX1	TSS200;TSS200	2.5243147	-2.082162	5.2936559	4.81E-07	1.70E-05	5.9495029
cg01350686	chr1	24439075	+	MYOM3	TSS1500	-0.809785	-2.403304	-5.293612	4.81E-07	1.70E-05	5.949314
cg18997129	chr7	143105850	+	EPHA1	1stExon	-0.768842	-1.683511	-5.293557	4.81E-07	1.70E-05	5.9490743
cg21024112	chr6	41376993	+			0.4306056	0.8070631	5.2934994	4.81E-07	1.70E-05	5.948828
cg05292376	chr17	79095236	-	AATK	Body	-1.262721	0.3125815	-5.293351	4.82E-07	1.70E-05	5.9481876
cg16955074	chr12	121671600	-	P2RX4	3'UTR	0.7825755	2.069879	5.2932887	4.82E-07	1.70E-05	5.9479196
cg18627816	chr19	39686695	+	NCCRP1	TSS1500	-1.083362	-1.352702	-5.293183	4.82E-07	1.70E-05	5.9474639
cg27456885	chr21	31972913	+	KRTAP22-1	TSS1500	-1.20507	0.5785942	-5.293187	4.82E-07	1.70E-05	5.947482

cg04095776	chr6	31106941	-	PSORS1C1;PSORS1C2;PSORS1C2	Body;1stExon;5'UTR	-0.917422	-0.617826	-5.293122	4.82E-07	1.70E-05	5.9472004
cg17975258	chr6	159639330	-	FNDC1	Body	-1.802756	1.0590296	-5.292885	4.83E-07	1.70E-05	5.9461803
cg04017533	chr18	55020099	-	ST8SIA3	1stExon	1.500198	-1.382396	5.2928639	4.83E-07	1.70E-05	5.9460878
cg26527487	chr1	99469819	+	LPPR5;LPPR5	Body;Body	1.3122186	-1.210306	5.2926508	4.83E-07	1.70E-05	5.9451689
cg21977507	chr17	12145856	-			1.0893556	0.8728814	5.2925884	4.83E-07	1.70E-05	5.9448999
cg00486280	chr15	34988869	-			-0.855468	-1.190271	-5.292524	4.84E-07	1.70E-05	5.9446232
cg14928510	chr19	55449439	+	NLRP7;NLRP7;NLRP7	Body;Body;Body	-1.462924	-0.765358	-5.2925	4.84E-07	1.70E-05	5.944517
cg19623418	chr20	55499548	+			1.760123	-0.969631	5.2924458	4.84E-07	1.70E-05	5.9442849
cg07706695	chr1	111179008	-			-1.39943	-0.046033	-5.292392	4.84E-07	1.70E-05	5.9440538
cg16201038	chr13	28544760	-	CDX2	TSS1500	2.145669	-1.82241	5.2944366	4.84E-07	1.70E-05	5.9453376
cg17781313	chr6	50817374	-			1.4570916	-1.241466	5.2922976	4.84E-07	1.70E-05	5.9436459
cg02840025	chr9	1045050	+			1.1810826	-0.700973	5.2920872	4.85E-07	1.71E-05	5.9427388
cg00006081	chr10	124908907	-	HMX2	Body	0.8882079	-0.799201	5.2918844	4.85E-07	1.71E-05	5.9418647
cg01485009	chr20	61311408	+			-1.817982	0.7472524	-5.293804	4.85E-07	1.71E-05	5.9422177
cg04381888	chr12	48195549	-	HDAC7;HDAC7	Body;Body	0.7948799	-0.122265	5.2918517	4.85E-07	1.71E-05	5.9417235
cg06546469	chr2	2736144	+			-1.033882	-1.178709	-5.293811	4.85E-07	1.71E-05	5.9426414
cg08233833	chr1	10725237	+	CASZ1;CASZ1	Body;Body	0.5443108	2.6717088	5.2917498	4.85E-07	1.71E-05	5.941284
cg19907796	chr2	71134694	-	VAX2	Body	1.2153287	-0.277471	5.2917735	4.85E-07	1.71E-05	5.9413865
cg24467349	chr1	110254835	-	GSTM5	TSS200	1.4968333	0.6233569	5.2918185	4.85E-07	1.71E-05	5.9415804
cg25744579	chr2	11724901	-	GREB1;GREB1;GREB1	Body;Body;Body	-1.302428	0.8816206	-5.291758	4.85E-07	1.71E-05	5.9413212

cg01584103	chr7	71578347	+	CALN1;CALN1	Body;Body	-1.189764	-0.757107	-5.291601	4.86E-07	1.71E-05	5.9406446
cg20801711	chr8	55278980	-			-1.439296	-0.841769	-5.291615	4.86E-07	1.71E-05	5.9407018
cg07501070	chr10	92613939	-	HTR7;HTR7;HTR7	Body;Body;Body	-1.257668	0.8552188	-5.291298	4.86E-07	1.71E-05	5.939335
cg24540114	chr7	156812941	-			1.4090085	-0.934165	5.2912856	4.86E-07	1.71E-05	5.939283
cg05612973	chr8	787052	+			-2.22337	2.2906238	-5.291146	4.87E-07	1.71E-05	5.9386792
cg11129772	chr7	53879728	+			-1.52411	-0.071636	-5.292867	4.87E-07	1.71E-05	5.9385776
cg07184423	chr6	139294818	-	REPS1;REPS1	Body;Body	0.9224357	0.8664297	5.2928387	4.87E-07	1.71E-05	5.9384562
cg25200152	chr1	147790458	-			2.1150195	0.1247607	5.2906226	4.88E-07	1.71E-05	5.9364251
cg05519187	chr17	35303387	+			1.7535882	-1.356015	5.290135	4.89E-07	1.72E-05	5.9343232
cg22852840	chr6	47749714	+	OPN5	TSS200	-1.166901	-0.386505	-5.290137	4.89E-07	1.72E-05	5.9343307
cg02560364	chr16	63219709	+			-1.303642	0.3897901	-5.28993	4.89E-07	1.72E-05	5.9334394
cg00442646	chr6	159278649	-	OSTCL	Body	-0.944011	0.6385003	-5.289885	4.89E-07	1.72E-05	5.9332476
cg04842146	chr8	85097056	-	RALYL;RALYL;RALYL;RALYL	TSS200;5'UTR;5'UTR;5'UTR	2.751818	-1.723464	5.2898631	4.89E-07	1.72E-05	5.9331512
cg00704780	chr2	242207920	-	HDLBP;HDLBP	5'UTR;5'UTR	0.7577823	0.9730311	5.291785	4.90E-07	1.72E-05	5.9335219
cg22149137	chr17	46804414	+	HOXB13	Body	1.2747773	-0.990969	5.2896929	4.90E-07	1.72E-05	5.9324177
cg16430687	chr20	33762474	+	PROCR	Body	1.0507477	1.1801374	5.289649	4.90E-07	1.72E-05	5.9322285
cg21237481	chr5	172227661	+			0.9826879	2.5105269	5.2894911	4.90E-07	1.72E-05	5.9315477
cg07361056	chr10	29769470	-	SVIL;SVIL	Body;Body	-0.702128	0.3835659	-5.28947	4.90E-07	1.72E-05	5.9314549
cg03881775	chr3	147111135	-	ZIC4;ZIC4;ZIC4;ZIC4;ZIC4	Body;TSS1500;Body;Body;TSS1500	1.3540405	-0.90362	5.2893693	4.90E-07	1.72E-05	5.9310228
cg25737664	chr20	1538671	-	SIRPD	TSS1500	-1.583465	1.0450425	-5.28928	4.91E-07	1.72E-05	5.930638

cg06535308	chr11	17497693	+	ABCC8	Body	1.2592402	-1.632155	5.2892395	4.91E-07	1.72E-05	5.9304635
cg14874541	chr3	124304033	-	KALRN;KALRN	Body;Body	-1.020199	-2.996867	-5.289225	4.91E-07	1.72E-05	5.930402
cg06424168	chr16	18994622	+	TMC7;TMC7	TSS1500;TSS1500	-0.975022	0.3909589	-5.289132	4.91E-07	1.72E-05	5.9300012
cg13155535	chr3	15917288	-			0.9830768	1.8358818	5.289077	4.91E-07	1.72E-05	5.929763
cg18324824	chr10	117884227	+	GFRA1;GFRA1;GFRA1	Body;Body;Body	-1.438631	0.5221677	-5.289078	4.91E-07	1.72E-05	5.9297674
cg27047671	chr14	69006140	+	RAD51L1	Body	0.5142417	2.3941537	5.2889355	4.91E-07	1.72E-05	5.929153
cg25724515	chr11	131926464	+	NTM;NTM;NTM;NTM	Body;Body;Body;Body	-1.664589	0.9888899	-5.288624	4.92E-07	1.73E-05	5.9278092
cg01318557	chr7	73624319	-	LAT2;LAT2;LAT2;LAT2;LAT2;LAT2	5'UTR;5'UTR;5'UTR;1stExon;1stExon;1stExon	-1.207739	-2.768786	-5.290459	4.93E-07	1.73E-05	5.9282118
cg06124793	chr11	1939725	+	TNNT3;TNNT3;TNNT3;TNNT3	TSS1500;TSS1500;TSS1500;TSS1500	-1.220296	0.2632952	-5.288279	4.93E-07	1.73E-05	5.9263219
cg21587469	chr12	56323816	+	DGKA	TSS1500	-1.483117	-0.645399	-5.288263	4.93E-07	1.73E-05	5.9262553
cg22547540	chr6	32055007	-	TNXB	Body	-1.169158	-0.461668	-5.288267	4.93E-07	1.73E-05	5.9262706
cg02105481	chr2	2853515	+			-0.827141	-0.260702	-5.288226	4.93E-07	1.73E-05	5.9260955
cg02212339	chr17	3493666	-	TRPV1;TRPV1;TRPV1;TRPV1	Body;Body;Body;Body	-1.757787	2.9898707	-5.288169	4.93E-07	1.73E-05	5.9258479
cg21197796	chr9	111313996	+			-1.199094	-0.031512	-5.288145	4.93E-07	1.73E-05	5.9257471
cg08315202	chr7	98246006	-	NPTX2	TSS1500	2.3880269	-0.988066	5.288056	4.93E-07	1.73E-05	5.9253629
cg04548815	chr1	116184387	-	VANGL1	TSS200	-0.818268	-2.370698	-5.287803	4.94E-07	1.73E-05	5.9242724
cg06959142	chr1	91182731	-	BARHL2	1stExon	1.3205516	-1.487802	5.2878012	4.94E-07	1.73E-05	5.9242647
cg08776619	chr3	42743921	-	HHATL;HHATL	5'UTR;TSS1500	-0.957029	-0.178784	-5.287802	4.94E-07	1.73E-05	5.9242682
cg07687548	chr2	172965280	-	DLX2	Body	1.9144713	-2.223202	5.2877742	4.94E-07	1.73E-05	5.9241484
cg03437157	chr6	100052633	-			0.9312836	-0.953224	5.2877389	4.94E-07	1.73E-05	5.9239966

cg08125539	chr17	47079959	+	IGF2BP1;IGF2BP1	Body;Body	-1.434752	0.4038794	-5.287693	4.94E-07	1.73E-05	5.923799
cg19350020	chr11	118489198	-	PHLDB1;PHLDB1;PHLDB1	Body;Body;Body	1.1471915	1.4422933	5.2876724	4.94E-07	1.73E-05	5.9237098
cg15300436	chr2	2321788	+	LOC730811;MYT1L	TSS1500;5'UTR	-2.013445	-0.045773	-5.298223	4.94E-07	1.73E-05	5.9304709
cg04080041	chr5	140811253	+	PCDHGA4;PCDHGA11;PCDHGA11;PCDHGA9;PCDHGA1;PCDHGB1;PCDHGB6;PCDHGA12;PCDHGB3;PCDHGB7;PCDHGA6;PCDHGA8;PCDHGA10;PCDHGA12;PCDHGA5;PCDHGB4;PCDHGA	Body;Body;Body;Body;Body;Body;Body;1stExon;Body;Body;Body;Body;1stExon;Body;Body;Body;Body;Body;Body;Body	0.9423417	-0.734509	5.2875318	4.95E-07	1.73E-05	5.9231038
cg08135904	chr6	32181025	-	NOTCH4	Body	-1.189541	0.1525083	-5.287532	4.95E-07	1.73E-05	5.9231029
cg08755224	chr20	21001116	-			1.0766549	-0.802419	5.2875397	4.95E-07	1.73E-05	5.9231379
cg16248937	chr2	76925398	-			-1.292807	1.1492431	-5.28951	4.95E-07	1.73E-05	5.9241231
cg13645481	chr19	56369522	+	NLRP4	Body	-1.146793	-0.013355	-5.287419	4.95E-07	1.73E-05	5.9226196
cg04781095	chr2	184123747	+			-1.220691	0.5216562	-5.287393	4.95E-07	1.73E-05	5.922505
cg16280141	chr5	63257753	+	HTR1A	TSS1500	1.3533541	-1.766324	5.2873731	4.95E-07	1.73E-05	5.9224202
cg13729210	chr5	3606561	-			1.2027994	-0.748571	5.2873402	4.95E-07	1.73E-05	5.9222784
cg21610556	chr4	181452599	-			-1.432045	0.7351611	-5.289344	4.95E-07	1.73E-05	5.9234126
cg06785999	chr14	60975964	+	SIX6;SIX6	1stExon;5'UTR	2.5539941	-1.625443	5.2872394	4.95E-07	1.73E-05	5.921844
cg13084731	chr7	20834411	-			1.6085014	-1.778078	5.2872321	4.95E-07	1.73E-05	5.9218128
cg08215169	chr13	112144901	-			-1.295738	-0.120755	-5.287129	4.95E-07	1.73E-05	5.9213676
cg13540981	chr6	32011506	+	TNXB;TNXB	Body;Body	-1.091427	-0.206029	-5.287047	4.96E-07	1.73E-05	5.9210167
cg15808795	chr2	240582510	-			-1.687807	0.8629701	-5.287072	4.96E-07	1.73E-05	5.921121
cg26501154	chr6	113966694	+			-1.63975	1.0191993	-5.28705	4.96E-07	1.73E-05	5.921029
cg04234971	chr7	157316329	+			-1.174678	0.7000739	-5.28701	4.96E-07	1.74E-05	5.9208557
cg00026553	chr15	90562839	-	ZNF710	5'UTR	0.918791	1.3662242	5.2869211	4.96E-07	1.74E-05	5.9204727

cg01224715	chr5	140811520	+	PCDHGA4;PCDHGA11;PCDHGA11;PCDHGA9;PCDHGA1;PCDHGB1;PCDHGB6;PCDHGA12;PCDHGB3;PCDHGB7;PCDHGA6;PCDHGA8;PCDHGA10;PCDHGA12;PCDHGA5;PCDHGB4;PCDHGA	Body;Body;Body;Body;Body;Body;Body;1stExon;Body;Body;Body;Body;1stExon;Body;Body;Body;Body;Body;Body;Body	1.033561	0.5128862	5.2868919	4.96E-07	1.74E-05	5.9203466
cg26123605	chr3	147141614	+			1.5722917	-1.410705	5.2868716	4.96E-07	1.74E-05	5.9202591
cg15368905	chr6	78172337	-	HTR1B	1stExon	2.4583319	-2.486143	5.2888192	4.96E-07	1.74E-05	5.9207543
cg09666573	chr3	11267627	+	HRH1;HRH1;HRH1	5'UTR;TSS200;5'UTR	-0.949293	-1.802596	-5.286678	4.96E-07	1.74E-05	5.919425
cg13280041	chr6	138866865	+	NHSL1	Body	1.4763393	0.7827056	5.2885905	4.97E-07	1.74E-05	5.9197702
cg03552937	chr7	44795713	+	ZMIZ2;ZMIZ2	TSS200;5'UTR	0.8055571	0.3125627	5.2864652	4.97E-07	1.74E-05	5.9185082
cg13957558	chr19	11435095	-	TSPAN16;RAB3D	Body;3'UTR	-1.143242	-2.767316	-5.286205	4.98E-07	1.74E-05	5.9173889
cg01723606	chr7	101944275	-	SH2B2	Body	1.466213	2.6586573	5.2860847	4.98E-07	1.74E-05	5.9168687
cg20993909	chr8	916135	-			-0.664956	-1.249757	-5.286083	4.98E-07	1.74E-05	5.9168606
cg06354392	chr2	176970064	-			1.6641514	-1.115181	5.2859961	4.98E-07	1.74E-05	5.9164874
cg11605835	chr5	3597695	-	IRX1	Body	1.5245999	-1.285379	5.2859852	4.98E-07	1.74E-05	5.9164403
cg19255307	chr11	69593809	+			-0.848566	-1.216844	-5.286017	4.98E-07	1.74E-05	5.9165771
cg05469819	chr6	122115942	-			-1.294166	0.0826139	-5.285935	4.98E-07	1.74E-05	5.9162248
cg02120584	chr2	45171340	-	SIX3	Body	0.986425	0.0345829	5.2857393	4.99E-07	1.74E-05	5.915381
cg16622061	chr16	86888736	+			-0.90376	-1.993385	-5.285708	4.99E-07	1.74E-05	5.9152458
cg18789918	chr13	84452994	-	SLITRK1;SLITRK1	3'UTR;1stExon	2.1907454	-0.691692	5.2876952	4.99E-07	1.74E-05	5.9163143
cg13726218	chr13	72438249	-	DACH1;DACH1;DACH1	Body;Body;Body	1.4263301	-0.635117	5.2855731	4.99E-07	1.74E-05	5.914665
cg26107754	chr3	156848845	-			-1.21117	0.4814036	-5.285382	4.99E-07	1.75E-05	5.9138432
cg09939462	chr17	25708131	-			-0.78224	-0.912197	-5.285362	4.99E-07	1.75E-05	5.9137544
cg11712990	chr5	87967845	+	LOC645323;LOC645323;LOC645323	Body;Body;Body	2.0935068	-1.402557	5.2851498	5.00E-07	1.75E-05	5.9128412

cg12707135	chr6	28945182	+			1.3384668	-0.428262	5.2851356	5.00E-07	1.75E-05	5.9127803
cg06440960	chr1	155828926	+	SYT11	TSS1500	-1.453511	-2.845745	-5.285011	5.00E-07	1.75E-05	5.9122447
cg23665802	chr13	92002338	+	MIR19A;MIR17HG;MIR92A1;MIR17HG;MIR20A;MIR17;MIR19B1;MIR18A	TSS1500;Body;TSS1500;Body;TSS1500;TSS1500;TSS1500;TSS1500	-1.207738	-1.07876	-5.284996	5.00E-07	1.75E-05	5.9121774
cg01097408	chr1	228073635	+			-1.055992	-1.187158	-5.284937	5.00E-07	1.75E-05	5.9119238
cg00585714	chr8	97156859	+	GDF6	Body	0.9101475	-0.756188	5.2849125	5.00E-07	1.75E-05	5.9118193
cg25153233	chr20	37854269	-	LOC339568	TSS1500	-1.102919	-1.598104	-5.284891	5.01E-07	1.75E-05	5.9117253
cg12997720	chr7	27225058	+	HOXA11AS;HOXA11	Body;TSS1500	2.1110477	-0.43201	5.2869037	5.01E-07	1.75E-05	5.9125107
cg10634728	chr19	418906	+	SHC2	3'UTR	-2.231505	2.3074475	-5.284736	5.01E-07	1.75E-05	5.9110581
cg00768993	chr2	200335874	-	FLJ32063	Body	1.7040983	-1.178042	5.2846722	5.01E-07	1.75E-05	5.9107841
cg21006600	chr12	2342206	-	CACNA1C;CACNA1C;CACNA1C;CACNA1C;CACNA1C;CACNA1C;CACNA1C;CACNA1C;CACNA1C;CACNA1C;CACNA1C;CACNA1C:CACNA1C:CACNA1C:CACNA1C:CACNA1C:CACNA1C:CACNA	Body;Body;Body;Body;Body;Body;Body;Body;Body;Body;Body;Body;Body;Body;Body;Body;Body;Body;Body:Body:Body:Body:Body	-1.399826	0.5149936	-5.284666	5.01E-07	1.75E-05	5.9107575
cg09049451	chr11	64774295	-			-1.368606	1.1911137	-5.284551	5.01E-07	1.75E-05	5.9102619
cg17910564	chr8	42248702	+	VDAC3;VDAC3	TSS1500;TSS1500	-1.101915	-0.452362	-5.284485	5.01E-07	1.75E-05	5.9099786
cg08080660	chr8	56773721	-			-0.771287	-2.746172	-5.284398	5.02E-07	1.75E-05	5.9096043
cg14423617	chr14	95058208	+	SERPINA5	Body	-1.512008	0.8220653	-5.284329	5.02E-07	1.75E-05	5.9093051
cg24900654	chr19	44285594	-	KCNN4	TSS200	-0.939116	-0.211121	-5.284331	5.02E-07	1.75E-05	5.9093151
cg19954978	chr5	176233844	+			-1.231467	0.5167082	-5.284258	5.02E-07	1.75E-05	5.9089981
cg19928493	chr15	87667390	+			-1.691871	0.3691843	-5.286254	5.02E-07	1.75E-05	5.9101121
cg10521852	chr19	19739820	+	LPAR2	TSS1500	-0.895847	-1.496488	-5.284151	5.02E-07	1.75E-05	5.9085397
cg14249155	chr9	124976040	+	LHX6;LHX6	Body;Body	-0.924586	-1.831652	-5.284067	5.02E-07	1.75E-05	5.9081768
cg04758329	chr16	58132534	+			-1.097952	0.1450282	-5.284004	5.03E-07	1.75E-05	5.9079037

cg17139666	chr4	174421440	-			1.1245172	-0.371669	5.2839145	5.03E-07	1.75E-05	5.9075203
cg01347682	chr4	140656794	+	MAML3	Body	1.3527863	-1.271657	5.2838591	5.03E-07	1.75E-05	5.9072818
cg07159802	chr1	247921568	+	OR1C1	1stExon	-1.69009	0.6170636	-5.283872	5.03E-07	1.75E-05	5.9073379
cg19995130	chr2	130000478	-			-1.045229	-0.195824	-5.283867	5.03E-07	1.75E-05	5.9073161
cg04438558	chr5	140723899	+	PCDHGA2;PCDHGA3;PCDHGA3;PCDHGA1	Body;1stExon;1stExon;Body	1.0295289	0.2037945	5.2834965	5.04E-07	1.76E-05	5.9057199
cg00691081	chr16	6379449	-	A2BP1;A2BP1	5'UTR;5'UTR	-1.541107	0.7506316	-5.283377	5.04E-07	1.76E-05	5.9052047
cg07481031	chr2	192890299	+	TMEFF2	Body	0.9859475	2.0943581	5.2853845	5.04E-07	1.76E-05	5.9063717
cg07403258	chr13	112200864	+			-2.553956	2.2573558	-5.283316	5.04E-07	1.76E-05	5.9049432
cg26332310	chr1	91182305	-	BARHL2	1stExon	1.4717193	-0.904165	5.2832381	5.04E-07	1.76E-05	5.9046069
cg10334489	chr17	25798878	+	KSR1	TSS200	-1.024204	-0.000936	-5.283193	5.04E-07	1.76E-05	5.9044117
cg16949584	chr10	135337429	-			-1.033239	-0.199827	-5.283146	5.04E-07	1.76E-05	5.9042104
cg01309395	chr6	33091795	-	HLA-DPB2	Body	-1.433475	0.3766482	-5.283031	5.05E-07	1.76E-05	5.903717
cg07727170	chr15	70458214	+			1.0615607	0.6627125	5.2829569	5.05E-07	1.76E-05	5.9033959
cg14270434	chr9	100613647	-			1.3390985	-0.170411	5.2828669	5.05E-07	1.76E-05	5.9030084
cg06581175	chr7	96632635	-	DLX6AS	Body	1.5098164	-1.055233	5.2827975	5.05E-07	1.76E-05	5.9027095
cg22538757	chr12	34755126	-			-1.308059	0.0914723	-5.282806	5.05E-07	1.76E-05	5.9027468
cg24499517	chr6	111408763	-	SLC16A10	TSS200	-0.689554	-5.787953	-5.284752	5.05E-07	1.76E-05	5.922067
cg25315312	chr7	2020528	-	MAD1L1;MAD1L1;MAD1L1	Body;Body;Body	-2.012321	2.7197966	-5.282661	5.06E-07	1.76E-05	5.9021199
cg13547767	chr17	16205608	+	PIGL	Body	0.6539478	1.9802873	5.2825157	5.06E-07	1.76E-05	5.901496
cg17679987	chr8	106535882	+	ZFPM2	Body	-1.399962	-0.585248	-5.282528	5.06E-07	1.76E-05	5.9015497

cg06151165	chr20	25062254	-	VSX1;VSX1	Body;Body	1.7466603	-1.451067	5.2844467	5.06E-07	1.76E-05	5.9019394
cg21748751	chr6	35309867	+	PPARD;PPARD	TSS1500;TSS1500	-0.946964	-1.781551	-5.28441	5.06E-07	1.76E-05	5.901782
cg09269866	chr1	47900197	+	FOXD2;MGC12982	TSS1500;Body	1.289616	-1.024001	5.2843388	5.06E-07	1.76E-05	5.9018733
cg26595278	chr11	32009163	+			1.954091	-1.373537	5.2841868	5.07E-07	1.76E-05	5.9008215
cg05769349	chr12	114847043	+	TBX5;TBX5	TSS1500;TSS1500	1.7400472	-1.475942	5.2820203	5.07E-07	1.77E-05	5.8993627
cg10624729	chr1	78298207	+	FAM73A	Body	1.0642737	0.9921461	5.2819607	5.07E-07	1.77E-05	5.8991059
cg10848367	chr11	62009065	-	SCGB1D2	TSS1500	-1.076125	-0.919557	-5.284	5.07E-07	1.77E-05	5.9004164
cg20595271	chr12	111889200	+	SH2B3	3'UTR	0.9120149	1.5871891	5.2819918	5.07E-07	1.77E-05	5.8992398
cg00733567	chr10	133663272	+			-0.896778	-0.447506	-5.281861	5.07E-07	1.77E-05	5.8986787
cg11120865	chr14	21853037	-	SUPT16H	TSS1500	-0.987841	-3.263636	-5.281835	5.07E-07	1.77E-05	5.8985658
cg20489228	chr12	115125549	+			-0.930165	-1.526086	-5.283869	5.07E-07	1.77E-05	5.899455
cg01251150	chr5	67535553	+	PIK3R1	Body	1.2594568	-1.307547	5.2817936	5.08E-07	1.77E-05	5.8983865
cg06544316	chr11	93394605	+	KIAA1731	TSS1500	-0.856961	-3.011539	-5.281685	5.08E-07	1.77E-05	5.8979182
cg24395452	chr4	52943197	-	SPATA18	Body	1.0129461	-0.092355	5.2816939	5.08E-07	1.77E-05	5.8979573
cg11811559	chr14	96178999	-	TCL1A;TCL1A	Body;Body	-1.448942	0.4425479	-5.281551	5.08E-07	1.77E-05	5.8973424
cg00169412	chr6	27463347	-			1.6021159	-1.640887	5.2834885	5.08E-07	1.77E-05	5.8978178
cg19796981	chr22	50438501	-	IL17REL	Body	1.7536742	-0.986855	5.2814515	5.08E-07	1.77E-05	5.8969136
cg12616923	chr8	128228107	-			-1.326997	-0.878327	-5.28336	5.09E-07	1.77E-05	5.8972659
cg11796827	chr7	6435978	-	RAC1;RAC1	Body;Body	0.7602021	0.9154148	5.2833187	5.09E-07	1.77E-05	5.8970873
cg12805865	chr11	27061899	+	BBOX1	TSS1500	-1.177757	-0.341518	-5.281212	5.09E-07	1.77E-05	5.8958827

cg10065823	chr9	96108467	-	C9orf129;C9orf129	1stExon;5'UTR	1.4530641	-0.999201	5.2811915	5.09E-07	1.77E-05	5.8957939
cg06487191	chr17	8090696	+			-0.546762	-3.725397	-5.281169	5.09E-07	1.77E-05	5.8956982
cg01796223	chr7	129933014	-	CPA4;CPA4;CPA4;CPA4	1stExon;1stExon;5'UTR;5'UTR	-1.210527	-0.437479	-5.281134	5.09E-07	1.77E-05	5.895547
cg10211626	chr22	42085361	-	C22orf46;NHP2L1	TSS1500;TSS1500	-0.502159	-0.793732	-5.28089	5.10E-07	1.77E-05	5.894495
cg25282354	chr4	177314845	-			-1.200903	-0.808559	-5.280888	5.10E-07	1.77E-05	5.8944865
cg06459066	chr14	64855287	+	MTHFD1	Body	-0.358293	-4.27154	-5.280794	5.10E-07	1.77E-05	5.8940835
cg21801176	chr6	167544964	+	CCR6;CCR6	5'UTR;5'UTR	-1.199547	0.5149184	-5.280804	5.10E-07	1.77E-05	5.8941263
cg00088797	chr3	12976288	+	IQSEC1;IQSEC1	Body;Body	0.8692702	0.5089241	5.2806847	5.10E-07	1.77E-05	5.8936119
cg23091122	chr1	110024289	-	SYPL2	3'UTR	-1.011534	-0.362682	-5.280684	5.10E-07	1.77E-05	5.8936067
cg20788021	chr3	58164168	-			-1.085583	0.262747	-5.280566	5.10E-07	1.77E-05	5.8931005
cg25918827	chr2	62533140	+			-1.166103	-0.333911	-5.280539	5.10E-07	1.77E-05	5.8929841
cg19619416	chr1	1992232	-	PRKCZ	Body	0.75782	2.1361253	5.2804793	5.11E-07	1.77E-05	5.8927276
cg01935400	chr17	80393124	+	HEXDC	Body	-1.426863	3.3390837	-5.280265	5.11E-07	1.78E-05	5.8918058
cg14037837	chr11	31839314	-	PAX6	5'UTR	1.5847795	-0.928262	5.2802134	5.11E-07	1.78E-05	5.8915829
cg00830086	chr8	979504	-			-1.068198	-0.143949	-5.280191	5.11E-07	1.78E-05	5.8914878
cg18780257	chr3	192126023	+	FGF12;FGF12;FGF12	5'UTR;1stExon;Body	1.7437988	-1.005096	5.2801706	5.11E-07	1.78E-05	5.8913985
cg23815702	chr3	52869738	+	MUSTN1	TSS1500	0.6180197	-0.060458	5.2801199	5.11E-07	1.78E-05	5.8911802
cg27341860	chr1	248099880	+	OR2L13	TSS1500	-1.363116	0.6024616	-5.280125	5.11E-07	1.78E-05	5.8912003
cg02287710	chr14	102027660	+	DIO3;MIR1247	TSS200;TSS1500	1.5166724	0.4697804	5.2800562	5.12E-07	1.78E-05	5.8909061
cg24074153	chr14	47572921	+	MDGA2;MDGA2	Body;Body	-1.747692	0.8168834	-5.279901	5.12E-07	1.78E-05	5.8902367

cg10578996	chr19	29455873	+	LOC148145	TSS200	-1.354111	-0.539873	-5.279864	5.12E-07	1.78E-05	5.8900804
cg03734750	chr4	17040881	-			-1.217937	-0.111153	-5.279787	5.12E-07	1.78E-05	5.8897466
cg00134210	chr10	14644132	-	FAM107B	Body	-1.413951	-2.812938	-5.279703	5.12E-07	1.78E-05	5.8893856
cg23931381	chr19	18119847	-	ARRDC2;ARRDC2	Body;Body	1.1876912	2.849011	5.2796548	5.12E-07	1.78E-05	5.8891781
cg08762008	chr11	13191022	+			-0.981311	1.9288235	-5.279499	5.13E-07	1.78E-05	5.888506
cg23306063	chr19	10463879	-	TYK2	Body	0.8906214	2.2307138	5.2794041	5.13E-07	1.78E-05	5.8880986
cg21787848	chr12	13463480	-			-1.293559	0.4407172	-5.279341	5.13E-07	1.78E-05	5.8878255
cg14421582	chr1	3481668	-	MEGF6	Body	-1.129393	-0.173231	-5.279308	5.13E-07	1.78E-05	5.8876863
cg09896867	chr12	113734442	-	TPCN1;TPCN1	3'UTR;3'UTR	0.4380069	2.2451187	5.2792662	5.13E-07	1.78E-05	5.8875053
cg00758881	chr16	58534681	-	NDRG4;NDRG4;NDRG4	Body;Body;Body	-1.670526	-1.845219	-5.279173	5.14E-07	1.78E-05	5.8871038
cg10776493	chr7	16684321	-	BZW2;BZW2;ANKMY2;BZW2	TSS1500;TSS1500;Body;TSS1500	-0.459322	-4.452418	-5.279033	5.14E-07	1.78E-05	5.8864995
cg17314160	chr12	110399253	+	GIT2;GIT2;GIT2;GIT2;GIT2;GIT2	Body;Body;Body;Body;Body;Body	0.9652345	2.2455849	5.2790167	5.14E-07	1.78E-05	5.886431
cg25007722	chr1	247803033	+			0.8695228	0.5600399	5.2789479	5.14E-07	1.78E-05	5.8861351
cg19373760	chr1	247679988	-			-1.126751	-0.952022	-5.278599	5.15E-07	1.79E-05	5.8846345
cg18443253	chr14	38678420	-	SSTR1	5'UTR	1.683967	-1.307542	5.2785529	5.15E-07	1.79E-05	5.8844349
cg19288904	chr15	98836248	+			2.397531	-1.764578	5.2784729	5.15E-07	1.79E-05	5.8840902
cg20329405	chr1	197888123	-	LHX9;LHX9	Body;Body	1.5491964	-1.230693	5.2784798	5.15E-07	1.79E-05	5.8841201
cg01951963	chr16	85942346	-	IRF8	Body	-1.375135	-0.339804	-5.278143	5.16E-07	1.79E-05	5.8826718
cg03011535	chr20	42544794	-	TOX2;TOX2;TOX2;TOX2;TOX2	5'UTR;Body;1stExon;1stExon;5'UTR	2.8615833	-2.022681	5.2780097	5.16E-07	1.79E-05	5.8820967
cg15844441	chr17	51902426	+	KIF2B;KIF2B	1stExon;3'UTR	-1.543951	0.9390846	-5.277879	5.17E-07	1.79E-05	5.8815341

cg14721139	chr21	43772498	+	TFF2	TSS1500	-0.944435	0.7719498	-5.277796	5.17E-07	1.79E-05	5.8811765
cg00074184	chr1	116519323	-	SLC22A15	1stExon	-0.427808	-4.169129	-5.277616	5.17E-07	1.79E-05	5.8804018
cg07223180	chr13	20989142	-	CRYL1	Body	0.9586079	0.4821235	5.2775872	5.17E-07	1.79E-05	5.8802784
cg08155741	chr11	63994308	+	TRPT1;NUDT22;TRPT1;TRPT1;TRPT1;TRPT1;NUDT22;TRPT1;NUDT22	TSS1500;Body;TSS1500;TSS1500;TSS1500;TSS1500;Body;TSS1500;Body	-1.10284	-2.687161	-5.277549	5.17E-07	1.79E-05	5.8801128
cg14288639	chr9	122351409	+			-1.41827	0.5579633	-5.277544	5.17E-07	1.79E-05	5.8800926
cg12506597	chr6	31699709	-	CLIC1	Body	1.0464834	1.4097866	5.2774601	5.18E-07	1.79E-05	5.8797312
cg01392180	chr6	5133475	-	LYRM4;LYRM4	3'UTR;Body	0.9497022	2.1904886	5.2774089	5.18E-07	1.79E-05	5.8795109
cg07236001	chr11	1392360	-			-1.12647	-0.143995	-5.277334	5.18E-07	1.79E-05	5.8791872
cg19587904	chr13	28492425	-			2.0929203	-1.78917	5.2771951	5.18E-07	1.79E-05	5.8785906
cg27183456	chr4	113333163	+	ALPK1;ALPK1	Body;Body	1.1302386	4.1245092	5.2770543	5.19E-07	1.80E-05	5.8779847
cg23896695	chr8	128931535	-	PVT1	Body	-1.346504	-0.740075	-5.277021	5.19E-07	1.80E-05	5.8778427
cg03715413	chr5	180053982	-	FLT4;FLT4	Body;Body	-0.817188	0.4504413	-5.276923	5.19E-07	1.80E-05	5.8774212
cg12046677	chr12	2360663	+	CACNA1C;CACNA1C;CACNA1C;CACNA1C;CACNA1C;CACNA1C;CACNA1C;CACNA1C;CACNA1C;CACNA1C;CACNA1C;CACNA1C;CACNA1C;CACNA1C;CACNA1C;CACNA1C;CACNA1C;CACNA	Body;Body;Body;Body;Body;Body;Body;Body;Body;Body;Body;Body;Body;Body;Body;Body;Body;Body;Body;Body;Body;Body;Body	-1.424555	0.3566448	-5.276905	5.19E-07	1.80E-05	5.8773403
cg16096308	chr5	137072504	-	KLHL3	TSS1500	0.9039096	1.0415258	5.2767872	5.19E-07	1.80E-05	5.8768352
cg17463145	chr7	79084011	+	MAGI2	TSS1500	1.0312283	-0.350869	5.2767197	5.19E-07	1.80E-05	5.8765447
cg06148330	chr8	1778042	+	ARHGEF10	5'UTR	-0.820943	-0.308544	-5.276661	5.19E-07	1.80E-05	5.8762911
cg06691616	chr4	139531098	+			-1.414734	-0.004022	-5.27664	5.19E-07	1.80E-05	5.8762009
cg10782923	chr12	56329731	-	DGKA;DGKA;DGKA;DGKA	5'UTR;5'UTR;5'UTR;5'UTR	0.8748482	0.9224311	5.276673	5.19E-07	1.80E-05	5.876344
cg20539752	chr2	176994305	+	HOXD8	TSS200	2.1934813	-1.912977	5.278673	5.19E-07	1.80E-05	5.8771105
cg23586423	chr1	228547510	-	OBSCN;OBSCN	Body;Body	-0.890548	1.4802357	-5.276615	5.20E-07	1.80E-05	5.8760937

cg13126652	chr8	105368434	-	TM7SF4	3'UTR	-1.63297	1.1557743	-5.276472	5.20E-07	1.80E-05	5.8754809
cg16726869	chr4	127454704	-			-0.839891	-1.123288	-5.276376	5.20E-07	1.80E-05	5.8750677
cg15358855	chr11	129830237	+	PRDM10;PRDM10	Body;Body	-1.786696	0.3144753	-5.276332	5.20E-07	1.80E-05	5.8748765
cg01984304	chr1	91191813	+			1.3592862	-1.36783	5.2762736	5.20E-07	1.80E-05	5.8746253
cg12610471	chr10	22634199	-	SPAG6;SPAG6	TSS200;TSS200	1.387026	-0.37622	5.276206	5.21E-07	1.80E-05	5.8743341
cg16463700	chr2	108993737	+	SULT1C4	TSS1500	0.9220194	1.1459033	5.2758275	5.21E-07	1.80E-05	5.8727058
cg19673176	chr17	77116058	+	HRNBP3	5'UTR	-1.092364	-0.442758	-5.275724	5.22E-07	1.80E-05	5.8722592
cg07716052	chr11	32454748	-	WT1;WT1;WT1;WT1	Body;Body;Body;Body	0.9778669	-0.797622	5.2756668	5.22E-07	1.80E-05	5.8720143
cg03031672	chr20	60450178	-	CDH4	Body	-1.23593	-0.131711	-5.275481	5.22E-07	1.81E-05	5.8712162
cg16345720	chr11	64727184	-	C11orf85	5'UTR	-1.502658	0.7049381	-5.279558	5.22E-07	1.81E-05	5.872964
cg27598583	chr16	86337662	+			0.8384592	1.0901784	5.2754648	5.22E-07	1.81E-05	5.8711452
cg10454127	chr16	86386091	-			-0.692541	-0.881943	-5.275293	5.23E-07	1.81E-05	5.8704056
cg01240795	chr5	72528314	-			1.1545107	-0.330983	5.2751729	5.23E-07	1.81E-05	5.8698891
cg01425762	chr16	81666633	+	CMIP;CMIP	Body;Body	-0.970223	-1.08745	-5.275058	5.23E-07	1.81E-05	5.8693952
cg01301339	chr5	180673096	-			-0.820559	-2.454703	-5.274903	5.24E-07	1.81E-05	5.8687273
cg20711812	chr19	1775422	+	ONECUT3	Body	1.5129993	-0.4444	5.2749165	5.24E-07	1.81E-05	5.868786
cg07126525	chr6	166218519	-			2.2036956	-1.027787	5.2786802	5.24E-07	1.81E-05	5.8700043
cg19907293	chr2	217448253	-			-1.672256	1.3941966	-5.274549	5.24E-07	1.81E-05	5.8672051
cg25305993	chr1	181382934	+			-1.227399	-0.082659	-5.274395	5.25E-07	1.81E-05	5.8665435
cg02398629	chr15	70283067	+			0.6705764	0.5376175	5.2743352	5.25E-07	1.81E-05	5.8662852

cg02478708	chr19	55401611	+	FCAR;FCAR;FCAR;FCAR;FCAR;FCAR;FCAR;FCAR	3'UTR;3'UTR;3'UTR;3'UTR;3'UTR;3'UTR;3'UTR;3'UTR	-1.487219	0.3056292	-5.274351	5.25E-07	1.81E-05	5.8663546
cg21032945	chr5	5652728	+			-1.679663	0.8194399	-5.274228	5.25E-07	1.81E-05	5.8658237
cg16615154	chr6	40567553	+			1.1638334	-0.340811	5.2739161	5.26E-07	1.82E-05	5.8644821
cg19145082	chr3	142854187	+			-1.260375	-1.075874	-5.27389	5.26E-07	1.82E-05	5.8643685
cg00812557	chr4	38073835	+	TBC1D1	Body	0.9167525	-0.616781	5.2737614	5.26E-07	1.82E-05	5.8638167
cg24390965	chr12	57637169	-			-1.089186	-4.863484	-5.273423	5.27E-07	1.82E-05	5.8623592
cg15460584	chr4	8540687	+			-0.725693	-1.28883	-5.273393	5.27E-07	1.82E-05	5.8622336
cg04123507	chr12	52695270	+	KRT86	TSS1500	1.1345692	1.5535141	5.2733303	5.27E-07	1.82E-05	5.8619622
cg19220282	chr2	65217528	+	SLC1A4;SLC1A4	Body;5'UTR	-1.358436	-1.136572	-5.273186	5.28E-07	1.82E-05	5.8613394
cg05230642	chr15	25439252	+	SNORD115-14	TSS1500	-0.998482	0.055922	-5.272922	5.28E-07	1.82E-05	5.8602063
cg03945902	chr10	111216805	-			1.2731173	-1.442778	5.2746907	5.29E-07	1.83E-05	5.8599952
cg08238215	chr2	66673985	-	MEIS1	Body	1.0921254	-0.1405	5.272648	5.29E-07	1.83E-05	5.8590273
cg17146110	chr11	67751163	+			-1.195172	-0.220185	-5.274683	5.29E-07	1.83E-05	5.8599611
cg21170007	chr9	138465739	+			-1.711793	1.7935988	-5.272657	5.29E-07	1.83E-05	5.8590658
cg23656380	chr20	60877581	+	ADRM1;ADRM1	TSS1500;TSS1500	-0.430277	-4.449421	-5.272563	5.29E-07	1.83E-05	5.8586632
cg00030420	chr5	140683772	+	SLC25A2	TSS200	0.6706124	1.9183405	5.2724616	5.29E-07	1.83E-05	5.8582253
cg17150789	chr3	43097994	+	FAM198A	3'UTR	-1.26682	1.3029559	-5.272374	5.30E-07	1.83E-05	5.8578497
cg09937438	chr7	1095005	-	C7orf50;C7orf50;C7orf50	Body;Body;Body	1.3275814	0.3482234	5.27223	5.30E-07	1.83E-05	5.8572294
cg04367164	chr11	76902992	-	MYO7A;MYO7A	Body;Body	-0.983726	-0.342965	-5.272183	5.30E-07	1.83E-05	5.8570268
cg09043962	chr17	79694615	-			-1.850527	0.708282	-5.272173	5.30E-07	1.83E-05	5.8569851

cg22740492	chr11	31820658	+	PAX6;PAX6;PAX6	Body;Body;Body	1.3343164	-1.450303	5.2721597	5.30E-07	1.83E-05	5.8569273
cg23413527	chr15	95145293	-			-1.688656	1.1005463	-5.272108	5.30E-07	1.83E-05	5.8567033
cg24599329	chr11	23811425	+			-1.46101	0.1689602	-5.271999	5.30E-07	1.83E-05	5.8562348
cg26548883	chr17	34329899	+	CCL14-CCL15;CCL15;CCL14-CCL15	TSS1500;TSS1500;TSS1500	-1.411275	0.2548928	-5.271886	5.31E-07	1.83E-05	5.8557498
cg09420907	chr12	4273763	-			1.8521236	-1.132783	5.2738634	5.31E-07	1.83E-05	5.8568401
cg10944063	chr2	120233706	-	SCTR	Body	-1.097608	-0.71049	-5.271738	5.31E-07	1.83E-05	5.8551151
cg17327268	chr18	70502513	+	NETO1;NETO1;NETO1	Body;3'UTR;Body	-1.782721	0.3225825	-5.271734	5.31E-07	1.83E-05	5.8550974
cg24899545	chr6	7112846	+	RREB1;RREB1;RREB1;RREB1	5'UTR;5'UTR;5'UTR;5'UTR	-0.829663	-1.219577	-5.271587	5.31E-07	1.83E-05	5.8544658
cg10601582	chr19	22605234	+	ZNF98	TSS200	1.5548072	-0.919053	5.2712837	5.32E-07	1.83E-05	5.8531597
cg25956089	chr3	168822459	-	MECOM;MECOM;MECOM;MECOM;MECOM;MECOM	Body;Body;Body;Body;Body;Body	-1.374039	-2.63888	-5.271258	5.32E-07	1.83E-05	5.8530484
cg13393830	chr19	52391605	+	ZNF577;ZNF577;ZNF577	TSS1500;TSS1500;TSS1500	1.2159245	0.7489762	5.2712347	5.32E-07	1.83E-05	5.8529491
cg21699044	chr8	65281689	+			1.6967037	-0.833595	5.2711835	5.32E-07	1.83E-05	5.8527287
cg03241971	chr7	2638278	-	IQCE;IQCE	Body;Body	0.5719141	1.7130372	5.2711552	5.32E-07	1.83E-05	5.852607
cg24874998	chr3	163021378	+			-1.193626	-0.585709	-5.271118	5.33E-07	1.84E-05	5.8524486
cg04477404	chr4	161403211	+			-2.376463	2.2356023	-5.270998	5.33E-07	1.84E-05	5.8519329
cg24951886	chr10	13526236	-	BEND7;BEND7	Body;Body	-1.614502	0.2323678	-5.270884	5.33E-07	1.84E-05	5.851441
cg15902297	chr7	55614512	+	VOPP1	Body	-1.047434	0.3887089	-5.270775	5.33E-07	1.84E-05	5.8509734
cg16549085	chr11	2951981	+	PHLDA2	TSS1500	-1.099612	-3.809947	-5.272765	5.34E-07	1.84E-05	5.8706254
cg00456672	chr15	62358751	+	C2CD4A	TSS1500	-0.948414	-0.321983	-5.27061	5.34E-07	1.84E-05	5.8502614
cg06779449	chr20	42544663	-	TOX2;TOX2;TOX2	TSS200;Body;TSS200	2.4634919	-1.746818	5.2706233	5.34E-07	1.84E-05	5.8503197

cg11644424	chr6	157258856	+	ARID1B;ARID1B;ARID1B	Body;Body;Body	0.9394362	1.8691561	5.2705532	5.34E-07	1.84E-05	5.8500181
cg12044000	chr2	1605890	+			1.0211579	1.6790695	5.2705126	5.34E-07	1.84E-05	5.8498435
cg14399060	chr2	177015070	+	HOXD4;MIR10B	TSS1500;Body	1.3342636	-1.608095	5.2702578	5.35E-07	1.84E-05	5.8487481
cg13381984	chr7	127881344	-	LEP;LEP	1stExon;5'UTR	1.1455965	0.2363204	5.2701597	5.35E-07	1.84E-05	5.8483261
cg16304215	chr5	76928810	+	OTP	Body	2.2993856	-1.743812	5.2701887	5.35E-07	1.84E-05	5.8484508
cg25972591	chr19	58627987	+	ZSCAN18;ZSCAN18	Body;Body	-1.732609	0.8489862	-5.270172	5.35E-07	1.84E-05	5.8483779
cg25923609	chr5	3607092	-			1.6158283	0.6731714	5.2700764	5.35E-07	1.84E-05	5.8479682
cg08074534	chr6	100895143	-	SIM1	Body	1.9036282	-1.401776	5.2700363	5.35E-07	1.84E-05	5.8477956
cg10202788	chr5	175900443	-	FAF2	Body	0.8707445	2.5836571	5.2699744	5.35E-07	1.84E-05	5.8475293
cg24814117	chr5	77591744	+	AP3B1	TSS1500	-0.757423	-3.042365	-5.269836	5.36E-07	1.84E-05	5.8469355
cg01895124	chr19	3584954	+	GIPC3	TSS1500	-1.096215	1.0103755	-5.26973	5.36E-07	1.84E-05	5.8464805
cg03532483	chr16	22860396	+	HS3ST2	Body	-1.632371	1.2000262	-5.269623	5.36E-07	1.84E-05	5.8460192
cg10791580	chr6	131147870	+	LOC285733	TSS1500	-1.347754	0.3568431	-5.269589	5.36E-07	1.84E-05	5.845871
cg12820006	chr14	101491497	+	MIR758;MIR1197;MIR323	TSS1500;TSS1500;TSS1500	-1.419019	1.1336678	-5.269597	5.36E-07	1.84E-05	5.8459055
cg06103624	chr15	66998926	-	SMAD6;SMAD6	Body;Body	1.6470278	2.2219769	5.2695052	5.36E-07	1.85E-05	5.8455121
cg08811309	chr5	87968749	+	LOC645323;LOC645323;LOC645323	Body;Body;Body	1.4125513	-1.043205	5.2695219	5.36E-07	1.85E-05	5.8455841
cg10821813	chr10	129562528	-			-1.669151	1.1451946	-5.269464	5.37E-07	1.85E-05	5.8453336
cg26778630	chr16	86331580	+			-1.018536	-1.355507	-5.26923	5.37E-07	1.85E-05	5.8443297
cg27626318	chr16	70835285	-	VAC14	TSS1500	-0.446566	-4.927199	-5.269233	5.37E-07	1.85E-05	5.8443405
cg26630171	chr19	3459270	+	NFIC;NFIC	Body;Body	1.4132427	2.1121645	5.2691212	5.37E-07	1.85E-05	5.8438609

cg16594007	chr19	57641963	+	USP29	Body	-1.267459	-0.009095	-5.269065	5.38E-07	1.85E-05	5.8436187
cg04062391	chr19	9609204	+	ZNF560	5'UTR	1.4511396	-1.821252	5.2689421	5.38E-07	1.85E-05	5.8430912
cg00564524	chr8	142629256	-			-1.399447	0.1740069	-5.268823	5.38E-07	1.85E-05	5.8425811
cg13794530	chr7	158937494	+	VIPR2;VIPR2	1stExon;5'UTR	2.3174799	-1.571231	5.2687577	5.38E-07	1.85E-05	5.8422983
cg21592803	chr19	54199923	+	MIR525	TSS1500	-1.701349	1.2378457	-5.268759	5.38E-07	1.85E-05	5.8423059
cg07377422	chr19	46318633	+	RSPH6A	TSS200	1.1561836	1.1677828	5.2685137	5.39E-07	1.85E-05	5.8412495
cg26209676	chr19	56154382	+	ZNF581;ZNF580;ZNF580;ZNF580	TSS1500;1stExon;Body;Body	-1.107713	-2.305056	-5.268479	5.39E-07	1.85E-05	5.8410987
cg18358723	chr5	87955618	-	LOC645323	Body	1.2305068	-0.533124	5.2683997	5.39E-07	1.85E-05	5.8407592
cg10604040	chr11	65559109	+	OVOL1	Body	-0.942069	0.2815096	-5.268179	5.40E-07	1.85E-05	5.8398102
cg20072442	chr2	80530255	-	CTNNA2;LRRTM1;CTNNA2	Body;Body;Body	2.2096493	-1.732601	5.2677297	5.41E-07	1.86E-05	5.8378792
cg20246277	chr12	5867313	-	ANO2	Body	-1.334939	0.987407	-5.267461	5.41E-07	1.86E-05	5.8367239
cg03078400	chr6	31762644	+	VARS	Body	-1.531473	-3.63186	-5.267243	5.42E-07	1.86E-05	5.835787
cg25598488	chr12	34258505	+			-1.546266	0.7940512	-5.26715	5.42E-07	1.86E-05	5.8353879
cg03706175	chr2	47597331	-	EPCAM	Body	-1.463438	-0.526295	-5.267078	5.42E-07	1.86E-05	5.8350761
cg16393182	chr1	18472902	-	IGSF21	Body	-1.287264	0.5148428	-5.267085	5.42E-07	1.86E-05	5.8351064
cg16728114	chr1	156697793	-	C1orf66;ISG20L2;C1orf66	TSS1500;TSS200;TSS1500	-0.540363	-3.643621	-5.266828	5.43E-07	1.86E-05	5.8340041
cg00440032	chr12	127257679	+			-1.949854	1.3353719	-5.268731	5.43E-07	1.87E-05	5.8343963
cg06551168	chr19	308524	+	MIER2	Body	0.850022	1.0384446	5.2666443	5.43E-07	1.87E-05	5.8332135
cg16638920	chr5	87974012	+	LOC645323;LOC645323	Body;Body	1.1371593	-0.015952	5.2664428	5.44E-07	1.87E-05	5.8323475
cg08620044	chr5	87980976	+	LOC645323	TSS1500	2.120288	-1.38739	5.2812029	5.44E-07	1.87E-05	5.8738701

cg21541638	chr3	61236016	-	FHIT;FHIT	5'UTR;5'UTR	-0.997677	-1.632081	-5.266068	5.45E-07	1.87E-05	5.8307351
cg15995592	chr2	160614976	-	7-Mar	Body	1.3453296	0.3942742	5.2657459	5.46E-07	1.87E-05	5.8293525
cg12612445	chr21	46353573	+	C21orf67	Body	-1.042551	0.7865516	-5.265694	5.46E-07	1.87E-05	5.8291293
cg20167366	chr6	30094980	-			1.1809837	0.1572233	5.2676791	5.46E-07	1.87E-05	5.8302815
cg13732865	chr4	154711512	+	SFRP2	TSS1500	0.9903749	0.8663868	5.2676494	5.46E-07	1.87E-05	5.8297536
cg03233332	chr7	66118400	-			-1.411103	0.9370662	-5.265358	5.47E-07	1.88E-05	5.8276862
cg00366934	chr6	135503837	-	MYB;MYB;MYB;MYB;MYB;MYB;MYB;MYB	Body;Body;Body;Body;Body;Body;Body;Body	-0.527405	-4.728755	-5.267239	5.47E-07	1.88E-05	5.8279919
cg08109815	chr6	142409831	-	NMBR;NMBR	5'UTR;1stExon	1.1408161	-0.797457	5.2649184	5.48E-07	1.88E-05	5.8257966
cg03982609	chr22	43740798	-	SCUBE1	TSS1500	-1.67092	1.6092546	-5.264875	5.48E-07	1.88E-05	5.8256103
cg18391323	chr13	20716432	+	GJA3	Body	1.0007142	-0.982349	5.26471	5.48E-07	1.88E-05	5.8249008
cg11152302	chr14	24867472	-	NYNRIN	TSS1500	1.0344158	0.4902874	5.2646512	5.48E-07	1.88E-05	5.8246481
cg20225745	chr12	52585849	+	KRT80;KRT80	TSS200;TSS200	-0.995477	-0.682784	-5.264521	5.49E-07	1.88E-05	5.8240874
cg27420819	chr16	874894	+			-1.127272	0.4588234	-5.264502	5.49E-07	1.88E-05	5.8240058
cg14305278	chr9	971046	+			1.7995768	-1.029532	5.2644597	5.49E-07	1.88E-05	5.8238255
cg02597923	chr5	75374482	+			-1.353401	1.1033058	-5.264433	5.49E-07	1.88E-05	5.8237104
cg08133486	chr6	100911746	+	SIM1	TSS200	1.6317802	-0.600863	5.2643548	5.49E-07	1.88E-05	5.8233746
cg09486778	chr2	223169609	+	CCDC140	3'UTR	1.5707023	-0.54393	5.2643494	5.49E-07	1.88E-05	5.8233513
cg04319617	chr3	51749897	-	GRM2;GRM2	Body;Body	-1.607385	2.1912129	-5.264151	5.50E-07	1.88E-05	5.8224985
cg16309319	chr11	6191393	+	OR52B2	1stExon	-1.607882	0.7562061	-5.264121	5.50E-07	1.88E-05	5.8223693
cg24921221	chr8	12584192	-	LONRF1	Body	-1.164029	-2.349295	-5.264106	5.50E-07	1.88E-05	5.8223077

cg02574464	chr16	57940653	+	CNGB1	Body	1.0474186	0.3153969	5.2639615	5.50E-07	1.89E-05	5.8216846
cg22546130	chr22	19950026	-	COMT;COMT;COMT;COMT	5'UTR;5'UTR;5'UTR;TSS200	-0.831308	-1.420961	-5.263944	5.50E-07	1.89E-05	5.821609
cg21299491	chr1	3057366	+	PRDM16;PRDM16	Body;Body	1.5541728	3.9390905	5.2638117	5.50E-07	1.89E-05	5.821041
cg02716218	chr7	17813208	-			-1.708039	1.2037084	-5.265741	5.51E-07	1.89E-05	5.8219619
cg18282099	chr11	131965129	-	NTM;NTM;NTM;NTM	Body;Body;Body;Body	-1.262981	0.1086677	-5.2634	5.51E-07	1.89E-05	5.8192734
cg16692227	chr8	119567541	-	SAMD12;SAMD12	Body;Body	1.2737047	1.4073521	5.2633705	5.52E-07	1.89E-05	5.8191456
cg14051535	chr17	33641253	-			1.222719	-1.740606	5.2632044	5.52E-07	1.89E-05	5.818432
cg07033624	chr13	112716623	-			1.1555939	-1.354638	5.2631022	5.52E-07	1.89E-05	5.8179931
cg21578195	chr20	62168727	-	PTK6	TSS200	-0.813964	-1.23724	-5.263111	5.52E-07	1.89E-05	5.818031
cg04557452	chr11	62691251	-			-2.145551	-3.337426	-5.26509	5.52E-07	1.89E-05	5.8377293
cg21188533	chr3	53700263	-	CACNA1D;CACNA1D;CACNA1D	Body;Body;Body	0.8631671	1.7199702	5.2630281	5.52E-07	1.89E-05	5.8176746
cg06970472	chr4	40910981	-	APBB2;APBB2;APBB2	Body;Body;Body	-0.971529	-2.416889	-5.262986	5.52E-07	1.89E-05	5.817492
cg22660196	chr6	143932803	+	PHACTR2;PHACTR2	Body;Body	-0.946067	1.1595998	-5.262994	5.52E-07	1.89E-05	5.817526
cg21488538	chr2	98964436	-	CNGA3;CNGA3	5'UTR;5'UTR	2.0948579	-0.939059	5.2629661	5.53E-07	1.89E-05	5.8174082
cg21614638	chr4	100737821	-	DAPP1	TSS200	-1.44562	-1.177159	-5.262943	5.53E-07	1.89E-05	5.8173079
cg08661470	chr4	1556833	-			-1.016353	0.3101242	-5.262814	5.53E-07	1.89E-05	5.8167545
cg03605761	chr19	663973	+	RNF126	TSS1500	-1.132131	0.3189039	-5.262719	5.53E-07	1.89E-05	5.8163463
cg06029905	chr11	2155521	-	INS-IGF2;IGF2;IGF2;MIR483;IGF2	Body;Body;Body;TSS200;Body	-1.122091	-0.12741	-5.262662	5.53E-07	1.89E-05	5.8161005
cg23954787	chr8	72470700	-			1.4372598	-1.073772	5.2626702	5.53E-07	1.89E-05	5.8161369
cg00069417	chr16	87874279	+	SLC7A5	Body	0.5341928	2.306382	5.2626402	5.53E-07	1.89E-05	5.8160082

cg18990042	chr12	48399169	-	COL2A1;COL2A1	TSS1500;TSS1500	1.0021722	-0.320539	5.2626189	5.53E-07	1.89E-05	5.8159167
cg21713522	chr7	27957308	+	JAZF1	Body	-1.520511	0.4465095	-5.262566	5.54E-07	1.89E-05	5.8156903
cg14079243	chr9	88358062	-	AGTPBP1	TSS1500	-0.706702	-0.689295	-5.262521	5.54E-07	1.89E-05	5.8154966
cg04250681	chr14	71949117	+			1.1932364	2.7357823	5.262497	5.54E-07	1.89E-05	5.815393
cg11441533	chr12	64295363	-	SRGAP1	Body	-1.321542	0.8247165	-5.262456	5.54E-07	1.89E-05	5.8152155
cg21368143	chr12	25150626	+	C12orf77	TSS1500	-1.339979	1.8005598	-5.262204	5.54E-07	1.90E-05	5.8141359
cg22871559	chr1	206730254	-	RASSF5;RASSF5;RASSF5	Body;TSS1500;Body	-0.647149	-3.402602	-5.262098	5.55E-07	1.90E-05	5.8136786
cg14276133	chr9	89061100	+			-1.303968	0.5105493	-5.261822	5.55E-07	1.90E-05	5.8124948
cg25753024	chr11	67070913	-	SSH3	TSS200	1.4227221	-2.773215	5.2617353	5.56E-07	1.90E-05	5.8121211
cg01363284	chr16	85695124	+	KIAA0182;KIAA0182	Body;Body	0.7688023	1.3241885	5.2617021	5.56E-07	1.90E-05	5.8119786
cg23128495	chr12	106254483	-			-1.117543	-1.166108	-5.261563	5.56E-07	1.90E-05	5.8113816
cg24392577	chr11	65314556	-	LTBP3;LTBP3;LTBP3	Body;Body;Body	-1.037769	-0.38499	-5.261518	5.56E-07	1.90E-05	5.8111889
cg19439651	chr10	130299087	-			-1.340321	0.3358021	-5.261451	5.56E-07	1.90E-05	5.8109002
cg06674117	chr17	30488883	-	RHOT1;RHOT1;RHOT1	Body;Body;Body	-1.271759	0.7997956	-5.261294	5.57E-07	1.90E-05	5.8102271
cg13486532	chr6	10421460	+			2.2174889	-1.110884	5.2612839	5.57E-07	1.90E-05	5.810182
cg27093637	chr11	5410452	+	OR51M1	TSS200	-1.627517	0.4986253	-5.261214	5.57E-07	1.90E-05	5.8098815
cg15565065	chr17	46655476	-	HOXB4	1stExon	2.7384553	-1.480146	5.2650435	5.57E-07	1.91E-05	5.8110976
cg03653880	chr2	26254790	+			-1.27679	0.498122	-5.26085	5.58E-07	1.91E-05	5.8083179
cg03231647	chr2	237081650	-			1.1516665	-0.79179	5.2606821	5.58E-07	1.91E-05	5.8075975
cg13267619	chr3	36812001	-			-1.368247	1.4070958	-5.260619	5.58E-07	1.91E-05	5.8073257

cg19978416	chr7	73699563	-			-1.398218	0.1498676	-5.260524	5.59E-07	1.91E-05	5.8069177
cg08386867	chr19	55148602	+	LILRB1;LILRB1;LILRB1;LILRB1	3'UTR;3'UTR;3'UTR;3'UTR	-1.576265	1.2593666	-5.260413	5.59E-07	1.91E-05	5.8064399
cg19399370	chr6	74268449	-			-1.260252	1.1483602	-5.260325	5.59E-07	1.91E-05	5.8060647
cg05444514	chr6	466935	+			-0.954696	-0.045689	-5.260293	5.59E-07	1.91E-05	5.8059277
cg13099139	chr1	43901596	+	KIAA0467	Body	0.8140419	1.0462209	5.2602681	5.59E-07	1.91E-05	5.8058195
cg10315366	chr10	134911047	-	GPR123	Body	-1.037606	0.3871293	-5.260234	5.59E-07	1.91E-05	5.8056721
cg21962423	chr12	113913887	+			2.4665136	-2.681896	5.2601115	5.60E-07	1.91E-05	5.8051467
cg27421298	chr17	35303285	+			1.9592617	-2.541339	5.2600295	5.60E-07	1.91E-05	5.8047946
cg22666373	chr2	63284768	-			1.1368434	-0.360691	5.2597861	5.60E-07	1.91E-05	5.8037494
cg26589669	chr10	106028206	-	GSTO2	TSS1500	-0.962614	-1.886009	-5.261603	5.61E-07	1.92E-05	5.8042071
cg05345160	chr6	144555498	+			-1.24833	0.8285414	-5.259563	5.61E-07	1.92E-05	5.8027922
cg14405528	chr22	47372700	+	TBC1D22A	Body	1.3220942	2.8995828	5.2614879	5.61E-07	1.92E-05	5.8033114
cg18581950	chr2	219124271	+	GPBAR1;GPBAR1;GPBAR1	TSS1500;TSS1500;TSS1500	1.1802829	0.8711903	5.2594466	5.61E-07	1.92E-05	5.8022913
cg03560973	chr2	25390400	+	POMC;POMC	5'UTR;5'UTR	1.0129575	0.0609474	5.2594242	5.61E-07	1.92E-05	5.8021954
cg11566267	chr12	132895346	+	GALNT9	Body	-0.983082	0.0431495	-5.259409	5.61E-07	1.92E-05	5.8021283
cg23871640	chr1	110611807	-	ALX3	Body	1.1458249	-1.42366	5.2593364	5.62E-07	1.92E-05	5.801818
cg24252910	chr15	90234070	+	PEX11A;WDR93;WDR93	TSS200;1stExon;5'UTR	-0.360417	-4.071803	-5.25933	5.62E-07	1.92E-05	5.8017917
cg19772092	chr2	45177632	-			0.6361794	0.87495	5.2592722	5.62E-07	1.92E-05	5.8015425
cg10179251	chr2	30142742	+	ALK	Body	0.8233081	0.6141237	5.2592489	5.62E-07	1.92E-05	5.8014424
cg11061422	chr10	73136913	-			-0.963752	-1.430439	-5.259025	5.62E-07	1.92E-05	5.8004789

cg23178186	chr11	1321213	+	TOLLIP	Body	1.1617035	1.8896713	5.2590402	5.62E-07	1.92E-05	5.8005463
cg09257824	chr11	32455642	+	WT1;WT1;WT1;WT1	Body;Body;Body;Body	1.3133068	-1.609272	5.2588192	5.63E-07	1.92E-05	5.7995973
cg14431547	chr9	36572421	-	MELK	TSS1500	-1.82826	1.2488536	-5.258617	5.63E-07	1.92E-05	5.7987281
cg07725167	chr11	66185078	+			-1.222647	-0.194295	-5.258458	5.64E-07	1.92E-05	5.7980468
cg27160726	chr2	128587544	+			-1.426561	-1.50584	-5.258416	5.64E-07	1.92E-05	5.7978652
cg14506696	chr4	100737830	-	DAPP1	TSS200	-1.312147	-1.383513	-5.258373	5.64E-07	1.92E-05	5.7976812
cg16049367	chr22	41701763	-	ZC3H7B	5'UTR	0.8844084	1.820211	5.258321	5.64E-07	1.92E-05	5.7974583
cg01454519	chr20	23860242	-	CST5	1stExon	-1.507775	0.4120315	-5.258184	5.65E-07	1.92E-05	5.7968683
cg11299264	chr19	18920396	-			-0.759333	-2.094322	-5.258181	5.65E-07	1.92E-05	5.796859
cg14786116	chr1	235268232	+			-1.151068	-1.184265	-5.258159	5.65E-07	1.92E-05	5.7967644
cg01034884	chr16	86408564	-			-1.650463	1.6934916	-5.258134	5.65E-07	1.92E-05	5.7966557
cg25367999	chr11	114248617	+			-1.391654	0.7378966	-5.25809	5.65E-07	1.92E-05	5.796467
cg01924561	chr1	43416103	+	SLC2A1	Body	-1.538702	0.6008449	-5.258063	5.65E-07	1.92E-05	5.7963519
cg27076223	chr4	40632858	-	RBM47	TSS1500	-0.907548	-0.707576	-5.258025	5.65E-07	1.93E-05	5.7961853
cg03002848	chr10	135050343	+	VENTX	TSS1500	1.8747675	-1.445403	5.2577884	5.66E-07	1.93E-05	5.7951713
cg03263123	chr2	236209563	-			-1.15458	-0.202167	-5.25774	5.66E-07	1.93E-05	5.7949628
cg16491739	chr14	105031052	+			-0.870278	-0.872884	-5.259742	5.66E-07	1.93E-05	5.7958231
cg26402630	chr15	28053930	+	OCA2	Body	-1.40996	0.7291282	-5.257477	5.66E-07	1.93E-05	5.7938351
cg11778001	chr2	191512851	-	NAB1	TSS1500	-0.972151	-2.299678	-5.257443	5.66E-07	1.93E-05	5.7936887
cg18161211	chr4	177716461	-			-1.334207	0.7736502	-5.25737	5.67E-07	1.93E-05	5.7933761

cg13185413	chr6	30039202	+	RNF39;RNF39	Body;Body	1.1047584	0.9592432	5.261285	5.67E-07	1.93E-05	5.7954065
cg09396895	chr7	25021067	+	OSBPL3;OSBPL3;OSBPL3;OSBPL3	TSS1500;TSS1500;TSS1500;TSS1500	-0.798582	-3.569907	-5.257061	5.67E-07	1.93E-05	5.7920495
cg16964025	chr7	157482215	+	PTPRN2;PTPRN2;PTPRN2	Body;Body;Body	1.3394233	-1.328651	5.2569828	5.68E-07	1.93E-05	5.7917129
cg23424933	chr2	45157613	-			1.3291139	-0.415489	5.2569829	5.68E-07	1.93E-05	5.7917134
cg03950614	chr6	108486517	-	NR2E1	TSS1500	1.1945817	-0.548175	5.256908	5.68E-07	1.93E-05	5.7913919
cg17969662	chr1	221101412	-			-1.590885	-0.080272	-5.256823	5.68E-07	1.93E-05	5.791027
cg01528052	chr6	30095728	-			2.090896	-1.099886	5.2586028	5.69E-07	1.94E-05	5.7909374
cg25526001	chr10	85939451	+	C10orf99	Body	-0.902004	-1.23376	-5.256536	5.69E-07	1.94E-05	5.7897938
cg05048523	chr1	228502681	-	OBSCN;OBSCN	Body;Body	-1.367003	0.6283734	-5.256483	5.69E-07	1.94E-05	5.7895691
cg11917595	chr2	10534602	-	HPCAL1;HPCAL1	5'UTR;5'UTR	0.9583849	5.1311727	5.266692	5.69E-07	1.94E-05	5.7947115
cg02654940	chr6	34206400	+	HMGA1;HMGA1;HMGA1;HMGA1;HMGA1;HMGA1	5'UTR;5'UTR;5'UTR;5'UTR;5'UTR;TSS200	-1.270528	-0.361769	-5.256257	5.69E-07	1.94E-05	5.7885967
cg25645310	chr10	116581305	-	FAM160B1;FAM160B1	TSS200;TSS200	-0.710911	-4.764915	-5.256108	5.70E-07	1.94E-05	5.787956
cg07054292	chr2	127963330	+	CYP27C1;CYP27C1	1stExon;5'UTR	0.7818271	1.2732839	5.2579638	5.70E-07	1.94E-05	5.788599
cg04784475	chr19	38183130	+	ZNF781;ZNF781	5'UTR;1stExon	2.3269479	-2.003249	5.2577411	5.71E-07	1.94E-05	5.7872421
cg14036226	chr9	131464506	+	PKN3	TSS1500	-0.528724	-0.743436	-5.255703	5.71E-07	1.94E-05	5.786221
cg05093169	chr11	71955401	+	PHOX2A	TSS200	1.3895205	-1.079069	5.255514	5.71E-07	1.94E-05	5.785408
cg22545356	chr10	88716659	-	MMRN2	Body	0.7688872	1.0919325	5.2554686	5.71E-07	1.94E-05	5.7852132
cg10481065	chr20	37357371	+	SLC32A1	3'UTR	1.333241	-1.296067	5.2553545	5.72E-07	1.94E-05	5.7847235
cg24632241	chr2	80530431	-	CTNNA2;LRRTM1;CTNNA2	Body;Body;Body	1.9544125	-1.695406	5.2552696	5.72E-07	1.95E-05	5.784359
cg00530870	chr5	34864283	-	TTC23L	Body	0.8614563	2.3820497	5.255222	5.72E-07	1.95E-05	5.7841546

cg05854601	chr2	40351367	-	SLC8A1;SLC8A1;SLC8A1;SLC8A1	Body;Body;Body;Body	-1.522365	0.3016679	-5.255209	5.72E-07	1.95E-05	5.7840977
cg09533145	chr5	178563145	-	ADAMTS2	Body	-0.698707	-0.928579	-5.25506	5.73E-07	1.95E-05	5.78346
cg08632095	chr5	172639423	+			-1.187965	0.6116275	-5.254894	5.73E-07	1.95E-05	5.7827466
cg27534912	chr1	158258802	-	CD1C	TSS1500	-1.481576	-0.296239	-5.254886	5.73E-07	1.95E-05	5.7827136
cg23217622	chr1	156405438	+			2.3953577	-0.701622	5.2548519	5.73E-07	1.95E-05	5.7825665
cg08347500	chr16	54316049	-			2.6325172	-1.525889	5.2630108	5.73E-07	1.95E-05	5.8029092
cg25736251	chr3	61235869	+	FHIT;FHIT	5'UTR;5'UTR	-1.301422	-0.886837	-5.254607	5.74E-07	1.95E-05	5.7815141
cg00933411	chr8	13373090	-	DLC1;DLC1	TSS1500;TSS1500	-1.413385	-0.806915	-5.254497	5.74E-07	1.95E-05	5.7810421
cg01515508	chr1	10728270	-	CASZ1;CASZ1	Body;Body	1.4956686	2.8023914	5.2544912	5.74E-07	1.95E-05	5.7810183
cg08572611	chr7	100253914	-	ACTL6B	Body	1.9056209	-1.010405	5.2604101	5.75E-07	1.95E-05	5.7837061
cg07676145	chr10	73506646	+	CDH23	Body	1.3238301	0.03608	5.2541386	5.75E-07	1.95E-05	5.7795052
cg02671826	chr6	157191263	-	ARID1B;ARID1B;ARID1B	Body;Body;Body	0.8953001	2.2069512	5.2541122	5.75E-07	1.95E-05	5.7793918
cg10356204	chr19	2255744	-	JSRP1	TSS1500	-0.862358	0.2467201	-5.254001	5.75E-07	1.95E-05	5.7789148
cg05384868	chr18	20714428	+	CABLES1;CABLES1	TSS1500;TSS200	-0.814392	-2.581919	-5.253953	5.75E-07	1.95E-05	5.7787086
cg06094523	chr1	43398103	-	SLC2A1	Body	-0.598195	-1.134834	-5.253935	5.75E-07	1.95E-05	5.7786313
cg20241375	chr4	295862	+			-1.538075	1.2607832	-5.253902	5.76E-07	1.95E-05	5.7784906
cg23792314	chr12	25056243	+	BCAT1	Body	1.7217719	-1.425245	5.2539002	5.76E-07	1.95E-05	5.7784822
cg00201870	chr7	53103789	+	POM121L12	1stExon	-1.049081	0.1063356	-5.253873	5.76E-07	1.95E-05	5.7783642
cg16699818	chr16	48479333	-			1.006806	1.6820723	5.2556779	5.76E-07	1.96E-05	5.778799
cg19350115	chr6	136043961	+			-1.237647	-1.052158	-5.253654	5.76E-07	1.96E-05	5.7774251

cg02547394	chr13	112721789	-	SOX1	TSS200	1.2836354	-0.496161	5.2534646	5.77E-07	1.96E-05	5.7766126
cg25670567	chr12	132694774	+	GALNT9	Body	-1.657817	0.8399838	-5.253464	5.77E-07	1.96E-05	5.7766094
cg27072996	chr19	822635	-	LPPR3	TSS1500	1.6335188	-0.402972	5.2534954	5.77E-07	1.96E-05	5.7767448
cg27090678	chr16	85514649	-			-1.235185	0.8174891	-5.253283	5.77E-07	1.96E-05	5.7758332
cg08160970	chr19	54180782	+	MIR515-2;MIR515-1	TSS1500;TSS1500	-1.131964	0.0474253	-5.253256	5.77E-07	1.96E-05	5.7757163
cg05961809	chr4	154710523	-	SFRP2	TSS1500	2.0549452	-1.394878	5.2550706	5.78E-07	1.96E-05	5.7757935
cg11043993	chr10	50536966	-			-1.141722	-0.283319	-5.253025	5.78E-07	1.96E-05	5.7747277
cg14431528	chr14	101521542	+	MIR453;MIR485;MIR668	TSS1500;TSS1500;TSS200	-1.037459	0.2709819	-5.25294	5.78E-07	1.96E-05	5.77436
cg03947749	chr20	62330072	+	ARFRP1;ARFRP1	3'UTR;3'UTR	-1.211559	0.8933751	-5.252552	5.79E-07	1.96E-05	5.7726976
cg27531587	chr20	30619137	+	C20orf160	3'UTR	-0.918891	-1.251675	-5.252475	5.79E-07	1.97E-05	5.7723651
cg03686593	chr18	11149470	+	FAM38B	TSS1500	2.5428322	-1.000098	5.2523849	5.79E-07	1.97E-05	5.7719799
cg04100397	chr2	110875551	+	MALL	TSS1500	0.8207128	1.4941088	5.2522417	5.80E-07	1.97E-05	5.7713657
cg12127159	chr7	33809972	+			-1.217745	-0.051627	-5.252155	5.80E-07	1.97E-05	5.7709954
cg15694809	chr11	101786117	+	ANGPTL5;KIAA1377	5'UTR;1stExon	-0.48588	-3.746573	-5.252162	5.80E-07	1.97E-05	5.7710258
cg22515303	chr2	145037160	-	GTDC1;GTDC1	5'UTR;5'UTR	0.9286613	2.1300084	5.2521403	5.80E-07	1.97E-05	5.7709307
cg25462858	chr10	105032988	+			-0.798392	-2.209929	-5.252106	5.80E-07	1.97E-05	5.7707838
cg17107893	chr19	36912880	+			1.2713955	-0.391326	5.2520209	5.80E-07	1.97E-05	5.7704182
cg26426437	chr12	121686502	+	CAMKK2;CAMKK2;CAMKK2;CAMKK2;CAMKK2;CAMKK2;CAMKK2	Body;Body;Body;Body;Body;Body;Body	-1.642182	2.0552229	-5.25203	5.80E-07	1.97E-05	5.7704587
cg11307417	chr3	48310616	+	ZNF589	3'UTR	-1.493481	1.2011615	-5.251758	5.81E-07	1.97E-05	5.7692884
cg10762199	chr14	61934948	+	PRKCH	Body	1.0513026	1.7315009	5.2514707	5.82E-07	1.97E-05	5.7680579

cg19377250	chr7	100463206	+	SLC12A9	Body	-1.45184	0.0640942	-5.251229	5.82E-07	1.97E-05	5.7670229
cg11039361	chr11	63655748	-	MARK2;MARK2;MARK2;MARK2;MARK2	TSS1500;Body;Body;Body;Body	-0.925055	-3.421424	-5.251209	5.83E-07	1.97E-05	5.7669373
cg00827895	chr6	36805384	-	CPNE5	Body	-1.120413	-0.017425	-5.261445	5.83E-07	1.98E-05	5.7880242
cg20574436	chr14	99701416	+	BCL11B;BCL11B	Body;Body	1.7248545	-1.009571	5.253102	5.83E-07	1.98E-05	5.7673561
cg06081750	chr12	1018307	+	WNK1	3'UTR	0.7879227	1.2554134	5.2509825	5.83E-07	1.98E-05	5.7659635
cg13987700	chr9	137657798	-	COL5A1	Body	-1.234182	-0.129382	-5.252866	5.83E-07	1.98E-05	5.7667459
cg19978312	chr5	179634688	+	RASGEF1C	5'UTR	1.4173565	0.8279821	5.250793	5.84E-07	1.98E-05	5.7651508
cg21071075	chr11	56099798	-			-1.054009	0.5778537	-5.250802	5.84E-07	1.98E-05	5.7651911
cg10791343	chr7	53103287	+	POM121L12	TSS200	-2.520897	2.6428026	-5.250774	5.84E-07	1.98E-05	5.7650712
cg02311725	chr20	30639816	+	HCK	TSS1500	0.8643942	-0.465616	5.2506867	5.84E-07	1.98E-05	5.7646949
cg00256046	chr1	217306763	-	ESRRG	5'UTR	0.9476388	1.7861727	5.2505415	5.84E-07	1.98E-05	5.764072
cg04267467	chr7	139762256	-	PARP12	Body	-0.723566	-3.477261	-5.250412	5.85E-07	1.98E-05	5.7635183
cg00733728	chr13	79171230	+			1.5228361	-1.133674	5.2503189	5.85E-07	1.98E-05	5.7631171
cg19635897	chr15	33360195	+	FMN1	TSS200	-0.90992	-1.885924	-5.250253	5.85E-07	1.98E-05	5.7628348
cg19503963	chr17	2085005	+	SMG6;SMG6	Body;Body	0.9166464	0.5112664	5.2502023	5.85E-07	1.98E-05	5.762617
cg10061384	chr1	2466275	+			-0.910736	-0.737691	-5.252166	5.85E-07	1.98E-05	5.7637479
cg17071868	chr6	33047056	-	HLA-DPB1	Body	-1.352692	0.798753	-5.250176	5.85E-07	1.98E-05	5.7625041
cg06537019	chr1	178420683	-	RASAL2;RASAL2	Body;Body	1.0584136	1.7544148	5.2501232	5.85E-07	1.98E-05	5.7622777
cg14269191	chr11	5346249	-	OR51B2	TSS1500	-1.520443	0.0645542	-5.250023	5.86E-07	1.98E-05	5.7618493
cg24249695	chr6	37896180	+	ZFAND3	Body	0.5924371	1.5855146	5.2499465	5.86E-07	1.98E-05	5.7615199

cg06067593	chr4	176918809	-	GPM6A;GPM6A	5'UTR;Body	-1.206447	-0.246145	-5.24981	5.86E-07	1.98E-05	5.7609342
cg11027879	chr5	118794305	+	HSD17B4	Body	1.0413647	1.6842381	5.2497688	5.86E-07	1.98E-05	5.7607575
cg13050981	chr17	9479965	+	WDR16;WDR16;WDR16;WDR16;STX8	5'UTR;1stExon;5'UTR;1stExon;TSS1500	-1.194629	-3.71891	-5.249666	5.87E-07	1.98E-05	5.7603148
cg00202829	chr15	74609868	+	CCDC33;CCDC33	Body;TSS1500	-0.575743	-1.745704	-5.24961	5.87E-07	1.99E-05	5.7600776
cg14056644	chr4	111559105	+	PITX2;PITX2	TSS1500;TSS1500	1.0420666	-1.283357	5.2494149	5.87E-07	1.99E-05	5.7592398
cg01581360	chr12	122986464	-	ZCCHC8	TSS1500	-1.400657	-0.052636	-5.249345	5.87E-07	1.99E-05	5.7589378
cg17298489	chr10	3109555	+	PFKP	TSS200	-0.442219	-3.912498	-5.249343	5.87E-07	1.99E-05	5.758931
cg06477444	chr19	50411521	-	NUP62;NUP62;IL4I1;NUP62;NUP62	Body;Body;5'UTR;Body;Body	-1.410266	2.831531	-5.24915	5.88E-07	1.99E-05	5.7581047
cg15333932	chr5	16735498	+	MYO10	Body	-1.307989	-0.072047	-5.249143	5.88E-07	1.99E-05	5.7580734
cg04245540	chr5	55148534	+	IL31RA	Body	-1.101689	-1.542593	-5.249045	5.88E-07	1.99E-05	5.7576513
cg19445097	chr13	114075643	+			-1.218855	-0.06456	-5.248717	5.89E-07	1.99E-05	5.7562447
cg02929780	chr11	59282555	-	OR4D9	1stExon	-1.077626	0.4879301	-5.248384	5.90E-07	1.99E-05	5.7548204
cg05482942	chr10	102899285	+			2.1937366	-1.627607	5.2482307	5.90E-07	2.00E-05	5.7541613
cg15822328	chr1	1072197	-			-0.822062	-0.868775	-5.252225	5.90E-07	2.00E-05	5.7913967
cg02384543	chr19	58879857	-	ZNF837;ZNF837	Body;Body	1.2008581	4.9444903	5.2480839	5.91E-07	2.00E-05	5.7535318
cg05115375	chr15	33360485	+	FMN1	TSS1500	-0.886587	-2.725723	-5.247992	5.91E-07	2.00E-05	5.7531381
cg13720108	chr3	62364032	-			1.5518778	-1.474113	5.2479636	5.91E-07	2.00E-05	5.7530156
cg21412973	chr4	1408557	+			1.077403	-0.401972	5.2478959	5.91E-07	2.00E-05	5.7527256
cg23097006	chr20	25063817	-	VSX1;VSX1	TSS1500;TSS1500	1.5275018	-1.924216	5.2476716	5.92E-07	2.00E-05	5.7517636
cg07870307	chr5	140743780	+	PCDHGA4;PCDHGA2;PCDHGB2;PCDHGA5;PCDHGA1;PCDHGB1;PCDHGA5;PCDHGA3	Body;Body;Body;TSS200;Body;Body;TSS200;Body	1.7526533	-1.374051	5.2601649	5.92E-07	2.00E-05	5.7766751

cg15003208	chr3	52189217	+	WDR51A;WDR51A;WDR51A	TSS1500;TSS1500;TSS1500	-0.673279	-3.397939	-5.247359	5.93E-07	2.00E-05	5.7504229
cg01152025	chr9	15305873	-	TTC39B;TTC39B;TTC39B;TTC39B	Body;Body;Body;Body	-1.177932	-0.30505	-5.24693	5.94E-07	2.01E-05	5.7485842
cg09609598	chr7	49709938	+			-1.61727	0.7115733	-5.246856	5.94E-07	2.01E-05	5.7482656
cg12936469	chr6	161583516	-	C6orf59;AGPAT4	TSS1500;Body	-1.086518	1.4020905	-5.246688	5.94E-07	2.01E-05	5.7475471
cg22707397	chr19	49020528	-			-1.904385	1.1299338	-5.246698	5.94E-07	2.01E-05	5.74759
cg04843067	chr2	177016037	+	HOXD4	TSS200	0.8649264	-0.701673	5.2466046	5.95E-07	2.01E-05	5.7471886
cg11770920	chr2	218465695	+	DIRC3	Body	-1.339868	1.4983971	-5.246621	5.95E-07	2.01E-05	5.7472572
cg14079463	chr6	127796989	-	C6orf174	Body	1.1822947	1.1402296	5.2465011	5.95E-07	2.01E-05	5.7467451
cg25367332	chr11	125135875	-	PKNOX2	5'UTR	-1.366411	1.3541554	-5.246492	5.95E-07	2.01E-05	5.7467046
cg22844623	chr1	228336130	-	GJC2;GUK1;GUK1;GUK1	TSS1500;Body;Body;Body	0.734663	1.0738811	5.2464091	5.95E-07	2.01E-05	5.7463507
cg03318573	chr14	57856465	+	NAA30	TSS1500	-1.028204	-0.329554	-5.246167	5.96E-07	2.01E-05	5.7453111
cg04958794	chr14	97685274	-			1.6389195	-1.549486	5.2461358	5.96E-07	2.01E-05	5.745179
cg00530925	chr10	23463094	+			2.4941135	-2.287758	5.2460409	5.96E-07	2.01E-05	5.7447719
cg16325174	chr13	108365238	+	FAM155A	Body	-1.609776	0.4223996	-5.24584	5.97E-07	2.01E-05	5.7439088
cg21784917	chr7	79084038	-	MAGI2	TSS1500	1.697755	-1.298464	5.2457902	5.97E-07	2.01E-05	5.7436973
cg22679120	chr7	2353402	+	SNX8	Body	-0.806131	-0.839913	-5.245725	5.97E-07	2.02E-05	5.7434177
cg26070020	chr1	165201385	+	LMX1A	Body	-1.234256	0.08525	-5.245516	5.98E-07	2.02E-05	5.7425201
cg27133034	chr11	12235611	-	MICAL2	Body	-1.215329	0.3399982	-5.245508	5.98E-07	2.02E-05	5.7424889
cg19285800	chr13	21520379	-			1.7810963	-1.038348	5.2454057	5.98E-07	2.02E-05	5.7420488
cg09950681	chr11	393392	-	PKP3	TSS1500	-1.214743	0.709117	-5.245183	5.99E-07	2.02E-05	5.7410924

cg00552087	chr8	3316893	-	CSMD1	Body	-2.5916	2.6578406	-5.251243	5.99E-07	2.02E-05	5.7600339
cg03032677	chr6	36648794	+	CDKN1A;CDKN1A	5'UTR;5'UTR	-1.217063	0.5933279	-5.245037	5.99E-07	2.02E-05	5.7404682
cg03187073	chr6	4890079	-	CDYL;CDYL;CDYL;CDYL	5'UTR;Body;Body;TSS200	0.9639488	0.9825667	5.2449341	5.99E-07	2.02E-05	5.7400272
cg23012855	chr1	235111328	+			-1.53992	0.4460246	-5.244876	5.99E-07	2.02E-05	5.7397777
cg23522915	chr12	48199277	-	HDAC7;HDAC7	Body;Body	0.5595376	0.1706511	5.2448878	5.99E-07	2.02E-05	5.7398289
cg21053474	chr4	46737408	-	COX7B2	5'UTR	-1.246002	0.1945881	-5.244828	5.99E-07	2.02E-05	5.7395746
cg25338036	chr8	3047536	-	CSMD1	Body	-2.392836	2.2834906	-5.244676	6.00E-07	2.02E-05	5.7389191
cg16817826	chr3	62356323	+	FEZF2	Body	1.9727897	-2.114361	5.2443602	6.01E-07	2.03E-05	5.7375674
cg05628567	chr1	156783944	-	SH2D2A;SH2D2A;SH2D2A;SH2D2A;SH2D2A	Body;Body;Body;Body;Body	-0.759869	-4.944813	-5.244244	6.01E-07	2.03E-05	5.7370711
cg16721427	chr2	4050797	-			-1.200958	0.260147	-5.2442	6.01E-07	2.03E-05	5.7368801
cg10735319	chr14	65121324	-			-1.219152	-3.754024	-5.244163	6.01E-07	2.03E-05	5.7367219
cg00917373	chr14	61764607	-			0.6736083	3.0680625	5.2441312	6.01E-07	2.03E-05	5.736586
cg13361185	chr1	91185749	-			0.9984196	-1.073692	5.2437618	6.02E-07	2.03E-05	5.7350027
cg17304457	chr12	115775889	+			-1.270544	0.3022783	-5.243759	6.02E-07	2.03E-05	5.7349908
cg24402880	chr4	84035837	+	PLAC8;PLAC8;PLAC8;PLAC8	1stExon;5'UTR;5'UTR;1stExon	-1.010567	-1.107134	-5.243734	6.02E-07	2.03E-05	5.7348842
cg24834823	chr8	38386476	-	C8orf86	TSS1500	0.7050544	1.3450533	5.2437425	6.02E-07	2.03E-05	5.7349199
cg01807825	chr4	80329368	-	GK2;GK2	1stExon;5'UTR	-0.989695	-0.340695	-5.243669	6.03E-07	2.03E-05	5.7346068
cg25761955	chr20	61679122	-			-1.398288	0.7259843	-5.243644	6.03E-07	2.03E-05	5.7344958
cg25384000	chr11	55605161	+	OR5D16	TSS1500	-1.141965	-0.91775	-5.243603	6.03E-07	2.03E-05	5.7343215
cg26556336	chr15	87197932	-	AGBL1	Body	-1.565452	0.6794471	-5.243454	6.03E-07	2.03E-05	5.733684

cg10729496	chr3	10149963	-	C3orf24;C3orf24	TSS200;TSS200	0.8096588	2.5955385	5.251576	6.03E-07	2.03E-05	5.7375159
cg25260137	chr8	65282185	+			2.0804526	-1.379191	5.2453908	6.03E-07	2.03E-05	5.7534371
cg04330389	chr19	18084986	+	KCNN1	Body	-2.465892	3.4459568	-5.243219	6.04E-07	2.03E-05	5.7326746
cg04956701	chr11	131903650	-	NTM;NTM;NTM;NTM	Body;Body;Body;Body	-1.583678	0.2721697	-5.243205	6.04E-07	2.03E-05	5.7326162
cg14168530	chr2	45155991	-			2.7445133	-1.86498	5.2431938	6.04E-07	2.03E-05	5.7325682
cg15904283	chr10	23484867	-			1.3264701	-0.974521	5.2432158	6.04E-07	2.03E-05	5.7326624
cg00397673	chr18	56940418	-	RAX	1stExon	1.366186	-0.672904	5.2431551	6.04E-07	2.03E-05	5.7324025
cg14067101	chr9	975825	-	DMRT3	TSS1500	1.1287835	-0.386751	5.2429722	6.04E-07	2.04E-05	5.7316186
cg10514097	chr16	86544787	+	FOXF1	1stExon	1.8630054	-1.504314	5.2429265	6.05E-07	2.04E-05	5.7314228
cg24633978	chr2	176972581	+	HOXD11	1stExon	1.4611824	-1.085192	5.242782	6.05E-07	2.04E-05	5.7308034
cg05143012	chr7	10974404	-	NDUFA4	Body	0.9769785	1.8161537	5.2426507	6.05E-07	2.04E-05	5.7302407
cg02488338	chr5	63798214	-			1.5300347	-0.600694	5.2426093	6.05E-07	2.04E-05	5.7300633
cg07440398	chr14	99712966	+	BCL11B;BCL11B	Body;Body	1.1685547	-0.837357	5.2426109	6.05E-07	2.04E-05	5.7300705
cg14515453	chr7	19158041	+	TWIST1	TSS1500	1.3389624	-1.312868	5.2423914	6.06E-07	2.04E-05	5.7291298
cg19613432	chr2	119606151	+	EN1	TSS1500	1.1202588	-1.489862	5.2423922	6.06E-07	2.04E-05	5.7291332
cg22234712	chr8	96085385	-			1.2472927	0.3801576	5.2421916	6.07E-07	2.04E-05	5.7282737
cg03731828	chr6	33129106	-			-0.728682	-2.683417	-5.242152	6.07E-07	2.04E-05	5.7281039
cg06219125	chr16	24373675	+	CACNG3	3'UTR	-1.481517	0.7931459	-5.242096	6.07E-07	2.04E-05	5.7278626
cg16723982	chr15	40225618	+	EIF2AK4	TSS1500	-1.02287	-0.450123	-5.24213	6.07E-07	2.04E-05	5.7280088
cg18186672	chr2	71679841	+	DYSF;DYSF;DYSF;DYSF;DYSF;DYSF;DYSF	TSS1500;TSS1500;TSS1500;TSS1500;TSS1500;TSS1500;TSS1500	-0.933341	-1.306949	-5.242097	6.07E-07	2.04E-05	5.7278687

cg17682535	chr11	56237629	-	OR8U8;OR5M3	Body;1stExon	-1.565167	0.9339656	-5.242078	6.07E-07	2.04E-05	5.7277869
cg06418867	chr10	128153186	+	C10orf90	Body	-1.277598	0.6084174	-5.241982	6.07E-07	2.04E-05	5.7273735
cg12416304	chr2	134884764	+			0.7255819	2.4807573	5.2420053	6.07E-07	2.04E-05	5.7274753
cg16071219	chr13	48986124	-	LPAR6;LPAR6;RB1;LPAR6	Body;Body;Body;1stExon	1.7579553	-0.493531	5.2419743	6.07E-07	2.04E-05	5.7273424
cg01144019	chr15	63188741	-			-1.665351	1.580645	-5.241951	6.07E-07	2.04E-05	5.727242
cg25531987	chr17	34346381	-	CCL23;CCL23	TSS1500;TSS1500	-1.237427	0.1223398	-5.241859	6.08E-07	2.04E-05	5.726848
cg03333634	chr8	11555251	+			1.5589575	-1.00745	5.2417774	6.08E-07	2.04E-05	5.7264986
cg03739177	chr17	27889826	-	ABHD15	Body	0.8640293	2.2432685	5.2417576	6.08E-07	2.04E-05	5.7264137
cg14269477	chr7	142630597	+	TRPV5;TRPV5	5'UTR;1stExon	-1.291731	0.413231	-5.24168	6.08E-07	2.04E-05	5.7260828
cg03043406	chr1	45242356	-	RPS8;SNORD38A	Body;TSS1500	-1.306557	-0.350713	-5.241608	6.08E-07	2.04E-05	5.7257748
cg16139380	chr6	33142968	-	COL11A2;COL11A2;COL11A2	Body;Body;Body	-1.206379	0.4622298	-5.241615	6.08E-07	2.04E-05	5.7258048
cg22688043	chr11	55944198	+	OR5J2	1stExon	-1.638095	0.8767904	-5.241592	6.08E-07	2.04E-05	5.7257054
cg12710899	chr14	57261702	+			1.8272696	-1.517245	5.2435405	6.08E-07	2.04E-05	5.7268094
cg05883269	chr5	109333225	-			-1.44258	1.1939543	-5.241467	6.09E-07	2.04E-05	5.7251675
cg04732303	chr14	76848081	-	ESRRB	5'UTR	-1.373949	0.660097	-5.24141	6.09E-07	2.05E-05	5.7249232
cg17884327	chr3	197840760	+			1.8947125	0.4483765	5.241295	6.09E-07	2.05E-05	5.7244315
cg20145598	chr19	2775950	+	SGTA	5'UTR	0.8892275	1.1035713	5.240886	6.10E-07	2.05E-05	5.7226793
cg14047361	chr17	80015160	+	GPS1;GPS1	3'UTR;3'UTR	-0.570679	1.1505408	-5.240858	6.10E-07	2.05E-05	5.7225583
cg26163578	chr19	57154589	-			1.4061661	-0.552267	5.2405467	6.11E-07	2.05E-05	5.7212258
cg01040960	chr3	196505017	-	PAK2	5'UTR	0.5694161	2.519547	5.2405094	6.11E-07	2.05E-05	5.7210658

cg21900624	chr12	113013363	+			1.5236829	-0.534347	5.2425011	6.11E-07	2.05E-05	5.722361
cg21173150	chr12	53321588	-			-1.317278	0.9714493	-5.240383	6.12E-07	2.05E-05	5.7205255
cg11163179	chr8	110587335	-	GOLSYN;GOLSYN;GOLSYN;GOLSYN;GOLSYN;GOLSYN;GOLSYN;GOLSYN;GOLSYN;GOLSYN;GOLSYN;GOLSYN;GOLSYN;GOLSYN;GOLSYN	Body;Body;Body;Body;Body;Body;Body;Body;Body;Body;Body;Body;Body;Body;Body	-0.720371	1.2896298	-5.240297	6.12E-07	2.05E-05	5.7201567
cg13485564	chr2	223167617	-	CCDC140	5'UTR	1.4504888	-1.419998	5.2401022	6.12E-07	2.06E-05	5.7193217
cg16418183	chr16	51360402	+			-1.107285	-0.264226	-5.242114	6.12E-07	2.06E-05	5.7207048
cg24771877	chr12	114234123	+			1.0831193	1.2150509	5.2420852	6.12E-07	2.06E-05	5.7201769
cg23434592	chr8	139489757	-	FAM135B	5'UTR	-1.164604	-0.855404	-5.239953	6.13E-07	2.06E-05	5.7186805
cg00286621	chr1	27709881	-	CD164L2	TSS200	-0.978123	-3.029876	-5.239875	6.13E-07	2.06E-05	5.7183492
cg14045814	chr2	219267341	-	CTDSP1;CTDSP1;MIR26B	Body;Body;TSS200	0.9834385	0.5984398	5.2397137	6.13E-07	2.06E-05	5.7176573
cg18485530	chr13	59547037	-			-1.274258	-0.180895	-5.239722	6.13E-07	2.06E-05	5.7176938
cg04013994	chr2	817595	-			-1.399516	0.1617359	-5.239572	6.14E-07	2.06E-05	5.7170484
cg15388210	chr1	36005826	-	KIAA0319L	Body	0.8592477	0.0460195	5.2395362	6.14E-07	2.06E-05	5.716897
cg05798501	chr7	30492171	-	NOD1	Body	1.4269188	2.5346793	5.2394244	6.14E-07	2.06E-05	5.7164178
cg09364122	chr1	942198	-			-2.31538	2.1729223	-5.241433	6.14E-07	2.06E-05	5.7173873
cg03807298	chr5	174159039	+			1.8727061	-1.740349	5.2393188	6.14E-07	2.06E-05	5.7159657
cg01928411	chr12	50793981	+	LARP4;LARP4;LARP4;LARP4;LARP4;LARP4;LARP4	TSS1500;TSS1500;TSS1500;TSS1500;TSS1500;TSS1500;TSS1500	-1.689166	0.7964453	-5.239247	6.15E-07	2.06E-05	5.7156597
cg05929192	chr2	97192266	+			-1.274503	1.3701208	-5.239272	6.15E-07	2.06E-05	5.7157671
cg17329292	chr1	33895890	-			-0.640598	-2.455417	-5.23924	6.15E-07	2.06E-05	5.7156296
cg10083071	chr6	151347666	+	MTHFD1L	Body	0.8909907	2.119047	5.2391703	6.15E-07	2.06E-05	5.7153294
cg05191879	chr8	144650730	-	C8orf73	Body	2.2926876	-1.232922	5.239015	6.15E-07	2.06E-05	5.7146644

cg03117976	chr18	11149435	+	FAM38B	TSS1500	2.7632225	-1.757295	5.2409841	6.15E-07	2.06E-05	5.7158694
cg16479539	chr12	54345212	+			2.1521213	-1.927698	5.2388315	6.16E-07	2.06E-05	5.7138783
cg16808373	chr11	122850140	+	BSX	Body	1.1294664	-0.653054	5.2388396	6.16E-07	2.06E-05	5.7139128
cg03727202	chr15	53075718	-	ONECUT1	Body	1.3671706	-1.115201	5.2386337	6.16E-07	2.07E-05	5.7130309
cg15404785	chr14	61727792	-			-1.085593	-0.904763	-5.238649	6.16E-07	2.07E-05	5.7130984
cg18698431	chr12	105956397	-			-1.479534	-0.695922	-5.238657	6.16E-07	2.07E-05	5.7131318
cg07753598	chr2	219267634	-	CTDSP1;CTDSP1	Body;Body	0.8434218	2.5115357	5.2385941	6.16E-07	2.07E-05	5.7128616
cg09695592	chr10	134787953	-			-1.721039	1.2013439	-5.238558	6.17E-07	2.07E-05	5.7127077
cg07893921	chr6	137809124	+			2.0306792	-1.926864	5.2425552	6.17E-07	2.07E-05	5.7144436
cg13826452	chr6	26758395	+			1.2787685	0.2983816	5.2383121	6.17E-07	2.07E-05	5.7116537
cg18462521	chr2	64879911	-	SERTAD2	5'UTR	-0.905566	-5.298585	-5.238282	6.17E-07	2.07E-05	5.7115235
cg04299274	chr17	25798741	+	KSR1	TSS1500	-0.991726	-0.394655	-5.237935	6.18E-07	2.07E-05	5.710037
cg07609862	chr11	92702912	-	MTNR1B	1stExon	0.985916	-0.586038	5.2379465	6.18E-07	2.07E-05	5.7100878
cg00162231	chr3	11035357	+	SLC6A1	5'UTR	1.7279084	-0.907099	5.2378121	6.19E-07	2.07E-05	5.709512
cg26816421	chr22	32022314	-	PISD	5'UTR	0.8884465	0.4770753	5.2376867	6.19E-07	2.07E-05	5.7089753
cg00381541	chr17	40192279	-			0.8877175	0.7292124	5.2375727	6.19E-07	2.07E-05	5.708487
cg09371112	chr11	12222570	-	MICAL2	Body	0.9165042	0.5717791	5.237515	6.19E-07	2.07E-05	5.7082399
cg12200716	chr11	15581327	-			-0.98608	-0.254558	-5.237473	6.20E-07	2.07E-05	5.708058
cg01062443	chr19	1609369	-	TCF3;TCF3	3'UTR;3'UTR	-1.039707	-1.773891	-5.237392	6.20E-07	2.08E-05	5.7077139
cg24573261	chr21	38350069	-	HLCS	5'UTR	-1.040081	-1.120598	-5.237364	6.20E-07	2.08E-05	5.7075935

cg12649374	chr2	911318	+			-1.796838	1.6997347	-5.237301	6.20E-07	2.08E-05	5.7073216
cg15606654	chr18	75690241	-			-1.29502	0.7599429	-5.237271	6.20E-07	2.08E-05	5.7071953
cg17077610	chr10	22766143	-			0.7795916	-0.322155	5.2372324	6.20E-07	2.08E-05	5.7070297
cg12312338	chr6	33145571	-	COL11A2;COL11A2;COL11A2	Body;Body;Body	-2.189422	2.5001303	-5.236975	6.21E-07	2.08E-05	5.7059283
cg18910298	chr14	101471487	-			-1.219379	-2.366812	-5.236901	6.21E-07	2.08E-05	5.7056094
cg27186851	chr7	2566750	-	LFNG;LFNG;LFNG;LFNG	Body;Body;Body;Body	-1.246301	0.3825622	-5.23683	6.21E-07	2.08E-05	5.7053081
cg04472725	chr3	141516232	+	GRK7	Body	1.7139863	-1.632395	5.2367579	6.22E-07	2.08E-05	5.7049974
cg01400884	chr6	28583971	+			0.719969	0.2342206	5.236687	6.22E-07	2.08E-05	5.7046939
cg27093949	chr7	107771400	+	LAMB4	TSS1500	-1.407084	0.7916794	-5.236696	6.22E-07	2.08E-05	5.7047309
cg26014409	chr1	158324471	-	CD1E;CD1E;CD1E;CD1E;CD1E;CD1E	Body;Body;Body;Body;Body;Body	-1.561646	-0.666443	-5.236633	6.22E-07	2.08E-05	5.7044647
cg00009293	chr7	44151172	+	AEBP1	Body	-1.024234	0.708616	-5.236475	6.22E-07	2.08E-05	5.7037854
cg08929327	chr11	45938923	-	PEX16;PEX16	Body;Body	-0.599553	-2.957331	-5.236243	6.23E-07	2.08E-05	5.7027931
cg15193133	chr17	19883474	+			1.1165038	1.5678315	5.2361926	6.23E-07	2.08E-05	5.7025771
cg16638248	chr11	10596059	+	MRVI1;MRVI1;MRVI1;MRVI1	3'UTR;3'UTR;3'UTR;3'UTR	-0.808265	-0.711513	-5.236198	6.23E-07	2.08E-05	5.7026009
cg22861116	chr5	140762582	-	PCDHGA4;PCDHGA7;PCDHGA6;PCDHGA1;PCDHGA5;PCDHGB1;PCDHGA3;PCDHGA2;PCDHGB2;PCDHGA7;PCDHGB3	Body;1stExon;Body;Body;Body;Body;Body;Body;Body;1stExon;Body	1.7174566	-0.410322	5.2402031	6.23E-07	2.08E-05	5.7211875
cg15676015	chr11	67272636	+	PITPNM1;PITPNM1	5'UTR;5'UTR	-1.216715	-5.171278	-5.236055	6.24E-07	2.08E-05	5.7019891
cg04653364	chr16	89058690	-			-1.161799	0.3599365	-5.235932	6.24E-07	2.09E-05	5.7014595
cg24579604	chr10	1827839	-			-1.093568	-0.888855	-5.235931	6.24E-07	2.09E-05	5.7014577
cg10792987	chr19	18575406	+	ELL	Body	1.2410245	-0.721251	5.2377151	6.24E-07	2.09E-05	5.7014795
cg23619399	chr13	79169753	-			1.4987969	-0.366381	5.2355413	6.25E-07	2.09E-05	5.6997884

cg00516092	chr20	31177275	-			-0.922107	-0.909391	-5.235447	6.25E-07	2.09E-05	5.6993863
cg03023900	chr17	62085236	+	ICAM2;ICAM2;ICAM2;ICAM2;ICAM2	TSS1500;5'UTR;5'UTR;5'UTR;5'UTR	0.8826085	1.9174235	5.2353819	6.25E-07	2.09E-05	5.699106
cg03554221	chr8	142450231	-	FLJ43860	Body	-0.951634	-0.426876	-5.235392	6.25E-07	2.09E-05	5.6991492
cg07872854	chr19	6203421	+			-1.429338	1.1573374	-5.235209	6.26E-07	2.09E-05	5.698367
cg23947828	chr14	101992137	-			-0.751271	-1.420123	-5.237048	6.26E-07	2.09E-05	5.6990333
cg07892167	chr8	23144983	+	R3HCC1	TSS1500	-1.231591	-0.319982	-5.234985	6.27E-07	2.09E-05	5.6974065
cg03971808	chr4	3322833	+	RGS12;RGS12	Body;Body	1.026377	1.4624874	5.2368879	6.27E-07	2.09E-05	5.6979416
cg07371504	chr17	3433451	+	TRPV3	Body	1.2205134	0.9273396	5.2348789	6.27E-07	2.09E-05	5.6969524
cg12608433	chr2	172945144	+	MAP1D	3'UTR	0.8725285	-0.271464	5.234849	6.27E-07	2.09E-05	5.6968245
cg14712186	chr1	91184413	+			2.2215792	-2.092829	5.2347553	6.27E-07	2.09E-05	5.6964232
cg20666307	chr5	144490992	+			-1.367232	-0.116266	-5.234717	6.27E-07	2.10E-05	5.6962583
cg00667343	chr1	244624249	+	C1orf101;C1orf101	TSS1500;TSS1500	-1.192218	-0.851827	-5.234401	6.28E-07	2.10E-05	5.6949052
cg00971508	chr15	98291886	+	LOC91948;LOC91948	Body;Body	-1.279209	0.7266753	-5.234406	6.28E-07	2.10E-05	5.6949295
cg14678084	chr7	127627251	+	SND1	Body	0.8337973	2.1115812	5.2343851	6.28E-07	2.10E-05	5.6948384
cg13512859	chr9	140127379	-	SLC34A3	Body	-0.72824	-0.748701	-5.234352	6.28E-07	2.10E-05	5.6946975
cg07661480	chr18	46447737	-	SMAD7	3'UTR	0.9733834	1.1291891	5.2343069	6.28E-07	2.10E-05	5.6945038
cg13153009	chr8	144561626	+	ZC3H3	Body	1.1324661	3.3195997	5.2343129	6.28E-07	2.10E-05	5.6945293
cg24453699	chr1	91190891	-			1.6335587	-0.610516	5.2342553	6.29E-07	2.10E-05	5.6942827
cg03265969	chr7	54617631	+	VSTM2A	Body	-1.763289	0.3838947	-5.234211	6.29E-07	2.10E-05	5.6940922
cg26466587	chr6	100442120	-	MCHR2;MCHR2	TSS200;TSS200	1.9487396	-0.592152	5.2342065	6.29E-07	2.10E-05	5.6940738

cg05241830	chr5	17128822	+			-1.371751	0.4209721	-5.234171	6.29E-07	2.10E-05	5.6939222
cg03481488	chr10	81091172	+			-1.057972	1.0299685	-5.234099	6.29E-07	2.10E-05	5.6936135
cg20154206	chr2	128458240	-	SFT2D3	TSS1500	-0.975081	-0.900174	-5.234042	6.29E-07	2.10E-05	5.6933716
cg16112248	chr7	54164018	-			-1.507154	0.5613918	-5.233995	6.29E-07	2.10E-05	5.6931705
cg09373727	chr1	32405360	-	PTP4A2;PTP4A2	TSS1500;TSS1500	-1.677454	0.3603626	-5.23362	6.30E-07	2.10E-05	5.6915622
cg23602752	chr1	165089433	-			-1.365391	-0.152698	-5.233604	6.30E-07	2.10E-05	5.6914952
cg17078629	chr3	187419942	-	RTP2;RTP2	5'UTR;1stExon	-1.215884	-0.138756	-5.23354	6.31E-07	2.10E-05	5.6912223
cg25924694	chr12	102872603	+	IGF1;IGF1;IGF1;IGF1	Body;Body;TSS200;Body	-0.872084	-2.887731	-5.233542	6.31E-07	2.10E-05	5.6912305
cg10929628	chr13	112169571	-			-0.965574	0.1879801	-5.233384	6.31E-07	2.10E-05	5.6905527
cg26155802	chr15	102344469	-	OR4F6	TSS1500	-1.31047	0.2608522	-5.233201	6.32E-07	2.11E-05	5.6897697
cg19513004	chr6	34206683	+	HMGA1;HMGA1;HMGA1;HMGA1;HMGA1;HMGA1;HMGA1	5'UTR;1stExon;5'UTR;5'UTR;5'UTR;5'UTR;5'UTR	-1.083883	1.3156127	-5.233053	6.32E-07	2.11E-05	5.6891368
cg02330818	chr20	62001057	-			-1.060402	-0.800853	-5.233002	6.32E-07	2.11E-05	5.6889191
cg07166266	chr17	74067713	-	SRP68	Body	-1.059326	-2.423841	-5.232968	6.32E-07	2.11E-05	5.6887745
cg09250367	chr14	101492132	+	MIR758;MIR323;MIR329-2;MIR329-1	TSS1500;Body;TSS1500;TSS1500	-0.988281	0.4271665	-5.232949	6.32E-07	2.11E-05	5.6886921
cg13471712	chr11	45952237	-	PHF21A;PHF21A	3'UTR;3'UTR	-1.26786	0.4773039	-5.232939	6.32E-07	2.11E-05	5.6886504
cg17145370	chr2	63285097	+			1.161477	-0.447918	5.2326825	6.33E-07	2.11E-05	5.6875503
cg06871344	chr17	452707	-	VPS53;VPS53	Body;Body	1.3013786	1.2945208	5.2325903	6.33E-07	2.11E-05	5.6871559
cg11223361	chr6	32023102	-	TNXB	Body	-1.229868	-0.201532	-5.232512	6.34E-07	2.11E-05	5.6868221
cg12311002	chr17	34391735	+	CCL18	1stExon	-1.351891	0.0197907	-5.23237	6.34E-07	2.11E-05	5.6862122
cg02412803	chr16	1099138	+			-0.868526	-0.043022	-5.232287	6.34E-07	2.11E-05	5.6858569

cg03515829	chr7	134233829	+			-1.245998	-0.547586	-5.232278	6.34E-07	2.11E-05	5.6858194
cg24362812	chr18	11148769	+	FAM38B	TSS200	2.072494	-1.480544	5.2321569	6.35E-07	2.11E-05	5.6853009
cg25731211	chr17	79694636	-			-2.388179	0.4989205	-5.232078	6.35E-07	2.11E-05	5.6849626
cg23530553	chr17	46622536	+	HOXB2	TSS200	1.2451252	0.3996539	5.2320393	6.35E-07	2.12E-05	5.6847975
cg20277905	chr20	61809716	-	MIR124-3	TSS200	1.2980542	-1.402912	5.2319502	6.35E-07	2.12E-05	5.6844165
cg04029933	chr20	31068427	+	C20orf112	5'UTR	0.9956542	2.6081696	5.2315972	6.36E-07	2.12E-05	5.6829058
cg21515956	chr14	104608332	-	KIF26A	Body	-1.193501	-0.067954	-5.23161	6.36E-07	2.12E-05	5.6829599
cg21571658	chr15	70785399	+			-1.723259	-2.467391	-5.231527	6.36E-07	2.12E-05	5.6826052
cg00184457	chr8	8946301	-			-1.45324	-1.361876	-5.23151	6.36E-07	2.12E-05	5.6825309
cg00867406	chr16	89997889	+	TUBB3	Body	-1.885278	2.6643973	-5.231491	6.36E-07	2.12E-05	5.6824505
cg00146027	chr10	33299147	+			-0.939267	-1.639976	-5.231406	6.37E-07	2.12E-05	5.6820886
cg18593945	chr21	32015231	+	KRTAP20-3	1stExon	-1.412767	0.6779477	-5.231255	6.37E-07	2.12E-05	5.6814399
cg18513023	chr6	44009266	+			-1.236851	0.3303104	-5.23103	6.38E-07	2.12E-05	5.6804768
cg26181334	chr7	4832535	+			0.7518468	0.8059295	5.2309941	6.38E-07	2.12E-05	5.6803247
cg23595927	chr4	671851	-	MYL5	Body	-0.9147	-0.326428	-5.230949	6.38E-07	2.12E-05	5.6801297
cg00589737	chr5	77252276	-			-1.21057	0.356536	-5.230711	6.39E-07	2.13E-05	5.6791134
cg07028396	chr10	93805682	+			1.0284451	0.5996964	5.2304797	6.39E-07	2.13E-05	5.6781237
cg24158157	chr10	124581726	-			-1.010323	-0.166619	-5.230467	6.39E-07	2.13E-05	5.6780685
cg26901111	chr19	12251917	+	ZNF20	TSS1500	-0.759906	-2.039903	-5.230467	6.39E-07	2.13E-05	5.6780714
cg01079599	chr14	59295181	+			-1.417534	1.2027151	-5.230262	6.40E-07	2.13E-05	5.677194

cg10440578	chr1	115397857	-	SYCP1	5'UTR	2.0609113	-1.029199	5.2322036	6.40E-07	2.13E-05	5.6783193
cg13197457	chr14	74059282	-	ACOT4	Body	-1.16624	0.4016802	-5.230158	6.40E-07	2.13E-05	5.6767475
cg13459498	chr20	42544792	-	TOX2;TOX2;TOX2;TOX2;TOX2	5'UTR;Body;1stExon;1stExon;5'UTR	2.7330427	-1.996862	5.23013	6.40E-07	2.13E-05	5.6766275
cg20316538	chr2	218937982	+	RUFY4	Body	-1.04664	0.0346222	-5.23013	6.40E-07	2.13E-05	5.6766266
cg05712938	chr5	3187456	+			-1.5283	0.0349622	-5.230084	6.40E-07	2.13E-05	5.6764291
cg25023666	chr21	41585021	+	DSCAM	Body	-1.334296	0.2149995	-5.229962	6.41E-07	2.13E-05	5.675908
cg01796166	chr14	95239586	-			2.2026335	-1.487956	5.2298211	6.41E-07	2.13E-05	5.6753056
cg09728607	chr16	51190065	-			2.5614129	-3.27963	5.2318069	6.41E-07	2.13E-05	5.6927335
cg20260607	chr12	49388987	-	DDN	3'UTR	0.947472	-0.283456	5.2297781	6.41E-07	2.13E-05	5.6751217
cg16971668	chr3	27764810	+	EOMES	TSS1500	1.6151046	-1.004675	5.2297428	6.41E-07	2.13E-05	5.6749708
cg01624197	chr14	92252480	+	TC2N;TC2N;TC2N	Body;Body;Body	0.7455026	2.750085	5.2297226	6.42E-07	2.13E-05	5.6748843
cg07552803	chr8	24771326	-	NEFM;NEFM	TSS1500;1stExon	1.4406808	-0.769211	5.2296552	6.42E-07	2.13E-05	5.6745959
cg14763548	chr20	25062447	-	VSX1;VSX1	1stExon;1stExon	3.0597876	-2.119222	5.229532	6.42E-07	2.13E-05	5.674069
cg26176665	chr17	16994978	+	MPRIP;MPRIP	Body;Body	0.7329873	2.8442899	5.2295308	6.42E-07	2.13E-05	5.6740637
cg15422147	chr18	61144339	+	SERPINB5	5'UTR	-1.371233	-0.028257	-5.229502	6.42E-07	2.13E-05	5.6739401
cg19759051	chr1	2030817	-	PRKCZ;PRKCZ	Body;5'UTR	0.7777623	1.6561002	5.2293259	6.43E-07	2.14E-05	5.6731871
cg00152340	chr8	144297019	-	GPIHBP1	Body	-1.308043	0.7244235	-5.229271	6.43E-07	2.14E-05	5.672951
cg19435284	chr4	13532671	-			1.0772074	-1.386239	5.2292042	6.43E-07	2.14E-05	5.6726666
cg16557178	chr17	46804309	-	HOXB13	Body	1.2444613	-0.921845	5.2291119	6.43E-07	2.14E-05	5.6722716
cg25368103	chr8	93516589	+			-1.393951	-0.244389	-5.229123	6.43E-07	2.14E-05	5.6723199

cg02266732	chr5	63257710	-	HTR1A	TSS200	1.2967528	-1.793904	5.2289376	6.44E-07	2.14E-05	5.671526
cg21784383	chr1	217311172	+	ESRRG	TSS200	1.4234133	-0.572449	5.2287563	6.44E-07	2.14E-05	5.6707505
cg03015610	chr16	58163699	+	C16orf80	TSS1500	-0.441335	-4.10456	-5.228665	6.45E-07	2.14E-05	5.6703614
cg13834623	chr6	25652407	-	SCGN	TSS200	1.6329651	-0.801397	5.2283891	6.45E-07	2.14E-05	5.6691796
cg07039362	chr16	55908870	-	CES7;CES7	Body;Body	-1.298339	0.6098376	-5.228264	6.46E-07	2.14E-05	5.6686439
cg23214464	chr7	140373596	+	ADCK2	1stExon	-0.966335	-2.398708	-5.228212	6.46E-07	2.14E-05	5.6684237
cg26025505	chr11	35138084	+			-0.772178	-0.070945	-5.228218	6.46E-07	2.14E-05	5.6684475
cg20152088	chr1	181616694	+	CACNA1E	Body	-1.031621	-1.030183	-5.228157	6.46E-07	2.14E-05	5.6681883
cg25380622	chr5	150695684	+	SLC36A2	3'UTR	-0.446084	-3.665382	-5.228163	6.46E-07	2.14E-05	5.6682109
cg14114377	chr19	45756845	-	MARK4	Body	-0.903179	-1.381406	-5.232015	6.46E-07	2.15E-05	5.670243
cg17982478	chr11	17036403	+	PLEKHA7	TSS1500	-0.566842	-3.710546	-5.227935	6.47E-07	2.15E-05	5.667239
cg04720330	chr11	2949813	-	PHLDA2	3'UTR	-1.127147	-2.363847	-5.227897	6.47E-07	2.15E-05	5.6670728
cg14890224	chr11	2156694	+	MIR483;INS-IGF2;IGF2;IGF2;IGF2	TSS1500;Body;Body;Body;Body	-0.825305	-0.200513	-5.227732	6.47E-07	2.15E-05	5.6663671
cg27015161	chr2	24816070	+	NCOA1;NCOA1;NCOA1	5'UTR;5'UTR;5'UTR	1.387775	1.8804302	5.2276422	6.48E-07	2.15E-05	5.6659849
cg21836358	chr1	63790202	+	FOXD3;FOXD3	3'UTR;1stExon	1.4327177	-0.202918	5.2275207	6.48E-07	2.15E-05	5.665465
cg06735127	chr12	124805929	-			-1.201302	0.5992241	-5.227486	6.48E-07	2.15E-05	5.6653166
cg23722274	chr10	98063967	+	DNTT;DNTT	TSS200;TSS200	-1.330875	0.9132729	-5.227118	6.49E-07	2.15E-05	5.6637435
cg14964285	chr12	4435533	-	C12orf5	Body	0.6848865	1.5083874	5.2269858	6.49E-07	2.15E-05	5.6631774
cg10300684	chr14	29236323	-	FOXG1;FOXG1	1stExon;5'UTR	1.7287749	-1.101358	5.2269337	6.50E-07	2.15E-05	5.6629542
cg07759206	chr19	14185448	+	LOC113230	Body	-0.677568	-2.513903	-5.226893	6.50E-07	2.16E-05	5.6627812

cg11277090	chr17	72848048	-	GRIN2C	Body	-0.907532	-0.864048	-5.226864	6.50E-07	2.16E-05	5.6626569
cg16798252	chr2	200334402	+	FLJ32063	Body	1.1936493	-0.532895	5.2267105	6.50E-07	2.16E-05	5.6619996
cg02990598	chr19	1955419	-	C19orf34;CSNK1G2	TSS1500;5'UTR	-0.781896	0.0637507	-5.226621	6.51E-07	2.16E-05	5.6616182
cg24506767	chr6	52264762	-	PAQR8	5'UTR	-0.607741	-3.374112	-5.226634	6.50E-07	2.16E-05	5.6616742
cg09858281	chr8	142456070	+	FLJ43860	Body	-1.076744	-0.236753	-5.226505	6.51E-07	2.16E-05	5.6611194
cg00095930	chr12	109569116	+			-0.854277	-1.170431	-5.22627	6.52E-07	2.16E-05	5.6601146
cg25406374	chr10	3442760	-			1.2135646	1.6540554	5.2260871	6.52E-07	2.16E-05	5.6593338
cg03880642	chr10	105560568	+	SH3PXD2A	Body	-1.195358	-0.85405	-5.22577	6.53E-07	2.16E-05	5.6579776
cg11539857	chr6	436594	+			-1.765189	0.9959094	-5.225674	6.53E-07	2.17E-05	5.6575656
cg09868640	chr15	27539735	+	GABRG3	Body	-1.372093	0.4660058	-5.225452	6.54E-07	2.17E-05	5.6566173
cg05736424	chr2	127646592	+			-1.605532	0.495773	-5.225405	6.54E-07	2.17E-05	5.6564181
cg09552641	chr19	55173510	+	LILRB4;LILRB4	TSS1500;TSS1500	-1.171963	0.2546031	-5.225342	6.54E-07	2.17E-05	5.6561492
cg27219182	chr17	70680294	-	SLC39A11;SLC39A11	Body;Body	-1.095413	-0.631462	-5.225254	6.55E-07	2.17E-05	5.655771
cg17199483	chr16	16042767	-	ABCC1;ABCC1;ABCC1;ABCC1;ABCC1	TSS1500;TSS1500;TSS1500;TSS1500;TSS1500	-0.78664	-1.497231	-5.225226	6.55E-07	2.17E-05	5.6556523
cg15727708	chr11	125061462	+	PKNOX2	5'UTR	-1.004771	0.2041572	-5.227179	6.55E-07	2.17E-05	5.6568488
cg26060535	chr6	29948514	-			0.6648614	2.1661654	5.2251795	6.55E-07	2.17E-05	5.6554526
cg10959353	chr2	229046515	+	SPHKAP;SPHKAP	TSS200;TSS200	1.6481701	-0.923985	5.2250734	6.55E-07	2.17E-05	5.654999
cg07352859	chr16	89050672	+			-0.897521	-1.124215	-5.22505	6.55E-07	2.17E-05	5.6548992
cg11299537	chr11	60531387	+	MS4A15;MS4A15	5'UTR;Body	-1.320812	0.5471432	-5.22495	6.55E-07	2.17E-05	5.6544714
cg16324018	chr2	85580627	-	ELMOD3;ELMOD3;ELMOD3;RETSAT;ELMOD3	TSS1500;TSS1500;TSS1500;Body;TSS1500	-1.509391	-0.083222	-5.224806	6.56E-07	2.17E-05	5.6538569

cg25612428	chr5	10649867	-	ANKRD33B	Body	0.9466667	2.0929286	5.2246772	6.56E-07	2.17E-05	5.6533049
cg17208528	chr6	134211114	-	TCF21;TCF21	Body;Body	2.5236127	-1.992352	5.2243393	6.57E-07	2.18E-05	5.6518599
cg04552206	chr10	102896546	-	TLX1	Body	0.9376101	-1.119411	5.2239563	6.58E-07	2.18E-05	5.6502226
cg09353931	chr2	242762571	-			-1.417817	0.3077622	-5.223894	6.59E-07	2.18E-05	5.6499553
cg22984898	chr5	162617091	+			-1.282208	0.50043	-5.223806	6.59E-07	2.18E-05	5.6495797
cg13870866	chr7	35293130	-	TBX20;TBX20	1stExon;1stExon	1.0065482	-0.477915	5.2237528	6.59E-07	2.18E-05	5.6493526
cg23854087	chr2	121615574	+	GLI2	Body	-0.898156	0.1429959	-5.223621	6.59E-07	2.18E-05	5.6487887
cg21929564	chr5	42757171	-	CCDC152	5'UTR	1.5521852	0.7399933	5.2235651	6.59E-07	2.18E-05	5.64855
cg19625689	chr8	1051510	-			-0.960866	-0.026876	-5.223499	6.60E-07	2.18E-05	5.6482672
cg20308663	chr13	100523121	+	CLYBL	Body	-2.69616	1.9227339	-5.225471	6.60E-07	2.18E-05	5.6495566
cg16298813	chr1	2878455	+			-1.369407	0.9732957	-5.223378	6.60E-07	2.18E-05	5.6477508
cg24091954	chr7	132333580	+	PLXNA4	TSS200	-1.212802	1.1829263	-5.223368	6.60E-07	2.18E-05	5.6477082
cg05933789	chr2	97190408	+			-1.207591	0.4160386	-5.223338	6.60E-07	2.18E-05	5.6475807
cg22682373	chr12	22697774	+	KIAA0528	TSS1500	-0.903332	-3.085019	-5.22482	6.62E-07	2.19E-05	5.646776
cg14236662	chr2	176988284	+	HOXD9	1stExon	1.7245068	-0.865599	5.2227978	6.62E-07	2.19E-05	5.6452699
cg19830983	chr17	75125896	-			1.0953865	1.5699944	5.2226675	6.62E-07	2.19E-05	5.6447129
cg03678729	chr2	144658851	-			-1.589631	1.2567947	-5.222634	6.62E-07	2.19E-05	5.6445717
cg17526441	chr5	4856273	+			-1.954234	0.7153228	-5.22247	6.63E-07	2.19E-05	5.6438694
cg25505610	chr11	32605184	-	EIF3M	TSS1500	-0.506278	-4.577877	-5.222115	6.64E-07	2.20E-05	5.6423522
cg06453088	chr4	5887725	+	CRMP1;CRMP1	Body;Body	-1.195844	-0.48629	-5.222089	6.64E-07	2.20E-05	5.6422406

cg15460294	chr4	5896960	-			-1.321829	0.8772585	-5.221921	6.64E-07	2.20E-05	5.6415236
cg23930313	chr12	25056083	+	BCAT1	Body	2.642445	-2.400884	5.2219023	6.64E-07	2.20E-05	5.6414421
cg08515811	chr17	77921489	-	TBC1D16	Body	-1.753528	2.185368	-5.22381	6.65E-07	2.20E-05	5.6424632
cg07851948	chr11	56085745	+	OR8K3	TSS200	-1.749821	1.5150376	-5.221736	6.65E-07	2.20E-05	5.6407314
cg24021956	chr7	79083509	-	MAGI2	TSS1500	1.8746567	-1.245056	5.221702	6.65E-07	2.20E-05	5.6405858
cg23365739	chr14	57277178	+	OTX2;OTX2	1stExon;5'UTR	1.0528548	-0.002357	5.2216582	6.65E-07	2.20E-05	5.6403988
cg17881513	chr8	10717687	+			-1.222485	0.4276514	-5.221481	6.66E-07	2.20E-05	5.6396401
cg25336902	chr8	1327904	-			-1.049257	0.4584093	-5.22126	6.66E-07	2.20E-05	5.6386956
cg20480740	chr11	123899875	+	OR10G8	TSS1500	-1.379259	0.5846258	-5.223047	6.67E-07	2.20E-05	5.6387991
cg25117362	chr10	104221598	-	TMEM180	5'UTR	-0.822636	-2.572453	-5.221012	6.67E-07	2.20E-05	5.6376368
cg05543520	chr3	66500703	-	LRIG1	Body	1.2223649	0.5100833	5.2209314	6.67E-07	2.20E-05	5.6372925
cg24667437	chr5	30608171	+			-1.498872	-0.359013	-5.2207	6.68E-07	2.21E-05	5.6363015
cg20682146	chr1	164545843	-	PBX1	Body	0.8170958	0.2173342	5.222593	6.68E-07	2.21E-05	5.6372662
cg25734913	chr5	172672390	+			1.1931952	-1.09734	5.220527	6.69E-07	2.21E-05	5.6355641
cg04157272	chr2	68017838	-			-1.531088	1.1797483	-5.220479	6.69E-07	2.21E-05	5.6353605
cg14716990	chr10	129533731	+			1.8718976	-0.617153	5.2204945	6.69E-07	2.21E-05	5.6354255
cg00529742	chr1	209607166	+			-1.233341	-0.723861	-5.222436	6.69E-07	2.21E-05	5.6361881
cg09466312	chr4	580474	+			-1.073807	0.2950104	-5.22037	6.69E-07	2.21E-05	5.6348926
cg06948658	chr12	2552733	+	CACNA1C;CACNA1C;CACNA1C;CACNA1C;CACNA1C;CACNA1C ;CACNA1C;CACNA1C;CACNA1C;CACNA1C;CACNA1C;CACNA1 C;CACNA1C;CACNA1C;CACNA1C;CACNA1C;CACNA1C;CACNA	Body;Body;Body;Body;Body;Body;Body;Body;Body; Body;Body;Body;Body;Body;Body;Body;Body;Body; Body;Body;Body;Body;Body	-1.66345	0.688471	-5.220164	6.70E-07	2.21E-05	5.6340114
cg13528289	chr4	1559561	-			-1.002774	-0.040033	-5.222082	6.70E-07	2.21E-05	5.6350838

cg18318649	chr5	87974369	+	LOC645323;LOC645323	Body;TSS200	2.0937177	-0.941063	5.2220609	6.70E-07	2.21E-05	5.6345876
cg15769920	chr12	56545649	+	MYL6B	TSS1500	-0.559953	-3.52971	-5.220034	6.70E-07	2.21E-05	5.6334568
cg19102271	chr9	77567974	+	C9orf40	TSS200	-0.469848	-3.634089	-5.220008	6.70E-07	2.21E-05	5.6333473
cg19802639	chr22	39263202	-	CBX6	Body	0.715154	1.5301588	5.2199781	6.70E-07	2.21E-05	5.6332187
cg00715811	chr1	247654140	-	OR2W5	TSS1500	-1.569829	0.4208089	-5.219909	6.70E-07	2.21E-05	5.6329251
cg01534390	chr14	23265401	+	SLC7A7;SLC7A7;SLC7A7	Body;Body;Body	-1.473155	0.144702	-5.219912	6.70E-07	2.21E-05	5.6329354
cg27151303	chr7	27184821	-			1.2498028	-0.160339	5.2198116	6.71E-07	2.21E-05	5.6325072
cg07030287	chr2	242174797	+	HDLBP;HDLBP	Body;Body	0.5419302	-0.181128	5.2196079	6.71E-07	2.22E-05	5.6316365
cg05210558	chr11	131410879	-	NTM	Body	-1.538142	0.2058282	-5.219486	6.72E-07	2.22E-05	5.6311137
cg27203560	chr3	193629645	-			0.9767951	-0.354335	5.2194553	6.72E-07	2.22E-05	5.6309848
cg00354641	chr6	30071503	-	TRIM31	Body	-0.926422	-2.647327	-5.219378	6.72E-07	2.22E-05	5.6306535
cg09533709	chr5	156900363	-	NIPAL4	3'UTR	0.92023	1.9487513	5.2193884	6.72E-07	2.22E-05	5.6306989
cg08172927	chr14	101927997	-			-1.198041	0.1780731	-5.219133	6.73E-07	2.22E-05	5.629606
cg00321614	chr5	172856932	-			-1.189906	-0.938352	-5.220811	6.74E-07	2.22E-05	5.6292513
cg02647520	chr6	33173218	-	HSD17B8	Body	-0.806524	-0.667946	-5.218821	6.74E-07	2.22E-05	5.6282741
cg25423563	chr13	95248771	-	TGDS	TSS1500	-0.82797	-2.884698	-5.218732	6.74E-07	2.22E-05	5.6278961
cg14634534	chr2	119616825	+			1.4440979	-1.403963	5.2187024	6.74E-07	2.22E-05	5.6277677
cg24654028	chr11	8854482	-	ST5	5'UTR	-0.828905	-1.427712	-5.218566	6.74E-07	2.22E-05	5.6271832
cg02704158	chr3	138656042	+			1.5848997	-1.859105	5.2182898	6.75E-07	2.23E-05	5.6260049
cg18386618	chr1	1268144	-	TAS1R3	Body	-1.147878	-0.067603	-5.222265	6.75E-07	2.23E-05	5.6278265

cg04490714	chr16	55690564	+	SLC6A2;SLC6A2	1stExon;5'UTR	1.5211277	-1.813475	5.2181921	6.76E-07	2.23E-05	5.6255873
cg04715245	chr1	154377091	-	IL6R;IL6R	TSS1500;TSS1500	-1.020148	-1.989401	-5.220079	6.76E-07	2.23E-05	5.626127
cg04050824	chr8	11538245	+			0.9629034	-0.294803	5.2180335	6.76E-07	2.23E-05	5.6249099
cg25648436	chr19	58554527	-	ZSCAN1	Body	1.4102552	0.4057586	5.2179743	6.76E-07	2.23E-05	5.6246568
cg03121281	chr7	96627253	-	DLX6AS	Body	1.3539777	-1.374797	5.2176635	6.77E-07	2.23E-05	5.6233292
cg26664528	chr15	69327013	+	MIR548H4;NOX5	Body;Body	-1.435845	0.5743336	-5.219502	6.78E-07	2.23E-05	5.6240738
cg16961648	chr6	28743427	-			0.958135	-0.9973	5.2173008	6.78E-07	2.23E-05	5.62178
cg08739738	chr12	1963310	+	CACNA2D4	Body	-1.336736	-0.182817	-5.219035	6.79E-07	2.24E-05	5.6216737
cg07488872	chr2	16955374	+			-1.450719	-0.559054	-5.216902	6.79E-07	2.24E-05	5.6200769
cg21196839	chr9	116358024	-	RGS3;RGS3;RGS3;RGS3;RGS3	Body;Body;Body;Body;Body	0.8969236	1.3813601	5.216907	6.79E-07	2.24E-05	5.6200975
cg03074925	chr6	10425625	-			0.8835322	-0.819355	5.2167974	6.80E-07	2.24E-05	5.6196294
cg19407943	chr2	130031761	-			-1.28489	0.4046169	-5.21669	6.80E-07	2.24E-05	5.6191689
cg22852353	chr1	165171672	+	LMX1A;LMX1A;LMX1A	3'UTR;3'UTR;3'UTR	-1.717936	0.8744856	-5.216671	6.80E-07	2.24E-05	5.6190895
cg09228833	chr20	52199778	+	ZNF217	TSS200	-1.624776	-1.746749	-5.216606	6.80E-07	2.24E-05	5.618811
cg10367023	chr2	223177287	+			1.3662304	-0.915301	5.2165955	6.80E-07	2.24E-05	5.6187669
cg08177968	chr16	59788728	+	LOC644649	Body	-1.595714	1.2337618	-5.216515	6.81E-07	2.24E-05	5.6184217
cg05943574	chr1	248685382	-	OR2G6	1stExon	-1.995354	1.2681729	-5.216441	6.81E-07	2.24E-05	5.6181085
cg13208088	chr6	10392519	-			1.4547567	0.2241397	5.2163286	6.81E-07	2.24E-05	5.6176269
cg12334247	chr2	169312418	-	LASS6	TSS1500	-0.550858	-3.232057	-5.216242	6.81E-07	2.24E-05	5.6172589
cg04475027	chr12	128752040	-	TMEM132C	Body	2.0947366	-1.267003	5.2162188	6.82E-07	2.24E-05	5.6171579

cg21544402	chr1	151129160	-	TNFAIP8L2;TNFAIP8L2	1stExon;5'UTR	-0.937879	-2.607767	-5.216203	6.82E-07	2.24E-05	5.6170895
cg17199800	chr7	96632777	-	DLX6AS	Body	1.4797746	-1.152793	5.2179965	6.82E-07	2.25E-05	5.6176488
cg22809871	chr13	102572229	+	FGF14	Body	1.9761277	-1.184171	5.2160148	6.82E-07	2.25E-05	5.6162869
cg20012024	chr1	160991549	-	F11R	TSS1500	-0.893458	-2.321463	-5.215884	6.83E-07	2.25E-05	5.6157275
cg03080142	chr8	50822801	+			1.400894	-1.262917	5.2157437	6.83E-07	2.25E-05	5.6151287
cg24749089	chr2	153322823	-	FMNL2	Body	-1.436923	-0.324607	-5.215703	6.83E-07	2.25E-05	5.6149547
cg26691305	chr10	112896689	-			-1.129924	0.0280155	-5.215709	6.83E-07	2.25E-05	5.6149788
cg03766998	chr18	12378044	+	AFG3L2	TSS1500	-0.752668	-2.995742	-5.215341	6.84E-07	2.25E-05	5.6134073
cg20331980	chr6	149554503	-			-1.013262	-0.320208	-5.215262	6.84E-07	2.25E-05	5.6130721
cg11983942	chr11	132946934	+	OPCML	Body	-0.929303	-0.926655	-5.215244	6.85E-07	2.25E-05	5.6129931
cg22697574	chr1	34642605	-	C1orf94;C1orf94;C1orf94	5'UTR;1stExon;5'UTR	1.4513504	-1.0766	5.2171783	6.85E-07	2.25E-05	5.614158
cg03506028	chr7	35494689	+			0.9872042	1.0288444	5.2170894	6.85E-07	2.25E-05	5.6137787
cg02911509	chr22	44568770	+	PARVG;PARVG	TSS200;TSS200	-1.077835	-0.519857	-5.21493	6.86E-07	2.25E-05	5.6116534
cg27317647	chr8	145562760	-			1.0619311	-0.252422	5.2148802	6.86E-07	2.25E-05	5.6114409
cg16174681	chr5	108220453	-	FER	Body	1.1560464	-0.25997	5.2148349	6.86E-07	2.25E-05	5.6112476
cg11358114	chr2	45233390	-	SIX2	Body	0.991742	1.3326731	5.2146274	6.86E-07	2.26E-05	5.6103616
cg18401367	chr5	176107201	+			2.4899716	-1.382592	5.2145326	6.87E-07	2.26E-05	5.6099566
cg22164298	chr4	114276834	+	ANK2;ANK2;ANK2	Body;Body;Body	-1.879749	-1.402932	-5.214502	6.87E-07	2.26E-05	5.6098243
cg20467147	chr1	248005038	+	OR11L1	1stExon	-1.437733	0.9290037	-5.214275	6.88E-07	2.26E-05	5.6088554
cg10019467	chr4	40632788	+	RBM47	TSS1500	-1.181612	-0.874925	-5.214178	6.88E-07	2.26E-05	5.6084417

cg04236178	chr1	217310572	-	ESRRG	5'UTR	1.085005	-0.107666	5.2139589	6.88E-07	2.26E-05	5.607507
cg01955203	chr8	124515502	-	FBXO32;FBXO32	3'UTR;3'UTR	1.1194042	1.3844652	5.2137576	6.89E-07	2.26E-05	5.6066472
cg00046156	chr5	2109785	+			-1.343538	-0.547033	-5.213639	6.89E-07	2.27E-05	5.6061395
cg10964330	chr1	11565584	+	PTCHD2	Body	-1.56987	0.6859813	-5.213612	6.90E-07	2.27E-05	5.6060266
cg13136556	chr21	46424880	-	NCRNA00162	TSS1500	-1.065665	-0.072211	-5.213394	6.90E-07	2.27E-05	5.6050958
cg08857221	chr1	37941361	-	ZC3H12A	Body	-1.018878	-0.581926	-5.213357	6.90E-07	2.27E-05	5.6049365
cg08825075	chr16	3934709	-			-0.958093	-0.599737	-5.213141	6.91E-07	2.27E-05	5.6040145
cg09159795	chr7	151127123	-	CRYGN	3'UTR	-0.8982	-2.179926	-5.213133	6.91E-07	2.27E-05	5.6039826
cg19682786	chr7	88424985	-	ZNF804B;MGC26647;MGC26647	Body;1stExon;5'UTR	-1.550837	0.5565319	-5.213111	6.91E-07	2.27E-05	5.6038869
cg26149275	chr2	176950007	+	EVX2	TSS1500	1.7499893	-0.751159	5.2130566	6.91E-07	2.27E-05	5.6036544
cg06820621	chr5	92938232	-			1.7922588	-1.292599	5.212892	6.92E-07	2.27E-05	5.6029514
cg27658330	chr13	68682777	+			-1.403608	0.7692486	-5.212886	6.92E-07	2.27E-05	5.602924
cg15557758	chr1	13828828	-			-0.953211	-0.562585	-5.214747	6.92E-07	2.27E-05	5.6037857
cg27613455	chr17	38020644	+	IKZF3;IKZF3;IKZF3;IKZF3;IKZF3;IKZF3	TSS1500;TSS1500;TSS1500;TSS1500;TSS1500;TSS1500	-0.469636	-3.699332	-5.212762	6.92E-07	2.27E-05	5.6023972
cg03266453	chr2	119606055	-	EN1	TSS1500	1.3561335	-1.424239	5.2124956	6.93E-07	2.28E-05	5.6012594
cg18156192	chr1	112320833	+	KCND3;KCND3	Body;Body	-1.17611	-0.616084	-5.212189	6.94E-07	2.28E-05	5.5999518
cg13734860	chr8	109206912	+			1.2493262	2.0491479	5.2121256	6.94E-07	2.28E-05	5.5996798
cg02213103	chr3	181421548	-	SOX2OT	Body	1.8642614	-0.130549	5.2118733	6.95E-07	2.28E-05	5.5986025
cg01446287	chr5	10649381	-	ANKRD33B	Body	0.7177233	1.8772689	5.2118354	6.95E-07	2.28E-05	5.5984407
cg19464524	chr10	93870321	-	CPEB3	Body	-0.914602	-1.041486	-5.211759	6.95E-07	2.28E-05	5.598113

cg19303187	chr2	172968881	-	DLX2	TSS1500	-0.775448	-2.53403	-5.211615	6.96E-07	2.28E-05	5.5975016
cg01654824	chr4	76943946	+	ART3;CXCL10	5'UTR;Body	-0.995929	2.2422565	-5.211571	6.96E-07	2.28E-05	5.5973101
cg00299737	chr16	49671991	-	ZNF423	Body	-2.124085	1.922861	-5.211548	6.96E-07	2.28E-05	5.5972151
cg14825736	chr5	142077949	-	FGF1;FGF1;FGF1;FGF1	TSS1500;TSS1500;TSS1500;TSS1500	-1.095833	-0.109369	-5.211535	6.96E-07	2.28E-05	5.5971578
cg11796258	chr1	175355362	+	TNR	Body	-1.329363	0.3356136	-5.211512	6.96E-07	2.28E-05	5.5970589
cg02225346	chr2	8833307	-			-1.525069	0.8542634	-5.211492	6.96E-07	2.28E-05	5.596973
cg07291100	chr3	139812907	-	CLSTN2	Body	-1.177295	-1.363692	-5.211354	6.97E-07	2.28E-05	5.5963878
cg00257455	chr1	65530538	-			1.0891346	-2.471745	5.2111543	6.97E-07	2.29E-05	5.5955336
cg26752613	chr7	30492160	-	NOD1	Body	1.2069244	1.8395307	5.2110595	6.97E-07	2.29E-05	5.5951287
cg01009920	chr8	8153650	-			-1.632401	1.092505	-5.212957	6.98E-07	2.29E-05	5.5961557
cg08097290	chr7	22472508	-	MGC87042	Body	-1.464753	0.9265416	-5.21083	6.98E-07	2.29E-05	5.5941512
cg12196573	chr7	27195602	-	HOXA7	Body	1.6552452	-1.376943	5.2106976	6.99E-07	2.29E-05	5.593584
cg00095099	chr16	20377894	-	PDILT	Body	-1.47452	1.2521122	-5.210616	6.99E-07	2.29E-05	5.5932354
cg03880683	chr6	36561606	-	SFRS3	TSS1500	-0.776548	-4.781766	-5.216607	6.99E-07	2.29E-05	5.5964111
cg17504164	chr1	79653656	-			-1.406443	0.4414434	-5.210576	6.99E-07	2.29E-05	5.593066
cg26434983	chr15	89952904	+			1.3768742	-0.651455	5.2125152	6.99E-07	2.29E-05	5.5938617
cg01826354	chr17	81006497	-	B3GNTL1	Body	-1.376386	-2.537991	-5.210498	6.99E-07	2.29E-05	5.59273
cg01637233	chr5	76933224	-	OTP	Body	1.2649466	-1.824347	5.2103329	7.00E-07	2.29E-05	5.5920273
cg08425203	chr3	128764940	+			1.3533161	-0.627723	5.2103121	7.00E-07	2.29E-05	5.5919386
cg05288172	chr8	103751006	-			-1.01297	-1.755376	-5.210252	7.00E-07	2.29E-05	5.5916838

cg05367173	chr17	62045564	+	SCN4A	Body	-1.505828	1.3087985	-5.210123	7.00E-07	2.29E-05	5.5911335
cg26808436	chr6	108492696	-	NR2E1	Body	0.977333	0.2759474	5.2119587	7.01E-07	2.30E-05	5.5914887
cg26892635	chr16	3253043	+	OR1F1	TSS1500	-0.949298	-2.805617	-5.209928	7.01E-07	2.30E-05	5.5902988
cg14407697	chr10	18424093	-			-1.437806	0.6468579	-5.209802	7.01E-07	2.30E-05	5.589761
cg12280317	chr1	152008083	+	S100A11	Body	-1.005316	-2.925048	-5.209722	7.02E-07	2.30E-05	5.5894182
cg15531386	chr11	116334079	-			-1.595368	-0.480304	-5.211682	7.02E-07	2.30E-05	5.5907173
cg14659956	chr5	17189703	-	LOC285696	Body	-0.387242	-4.221491	-5.2097	7.02E-07	2.30E-05	5.5893269
cg13582457	chr5	172324773	-	ERGIC1	Body	1.0199554	1.3291104	5.2094663	7.02E-07	2.30E-05	5.5883288
cg18744668	chr8	99962347	-	OSR2;OSR2	Body;Body	0.9039996	1.8591785	5.2094571	7.03E-07	2.30E-05	5.5882896
cg00940261	chr14	95786011	+	CLMN	Body	-0.515754	-5.250314	-5.20924	7.03E-07	2.30E-05	5.5873612
cg03190130	chr11	43543270	-			-1.157949	0.6223146	-5.211153	7.03E-07	2.30E-05	5.5884626
cg16654432	chr3	105167621	+	ALCAM	Body	0.9102621	0.6939347	5.2091392	7.04E-07	2.30E-05	5.5869331
cg10923855	chr12	34488830	-			-1.240549	0.3873859	-5.209088	7.04E-07	2.30E-05	5.5867128
cg13502128	chr6	32039874	-	TNXB	Body	-1.250788	1.4715473	-5.209087	7.04E-07	2.30E-05	5.5867091
cg15084499	chr1	227505916	-	CDC42BPA;CDC42BPA	TSS200;TSS200	1.036929	-2.076476	5.2089692	7.04E-07	2.30E-05	5.5862077
cg01074356	chr1	57884286	-	DAB1	5'UTR	-1.573935	1.0516563	-5.210853	7.04E-07	2.30E-05	5.5867751
cg22570757	chr2	238253330	+	COL6A3;COL6A3;COL6A3	Body;Body;Body	-1.130242	-0.405906	-5.208583	7.05E-07	2.31E-05	5.5845582
cg22303045	chr6	100917022	+			1.3352059	-1.626286	5.2085566	7.05E-07	2.31E-05	5.5844467
cg03081675	chr6	154540097	+	IPCEF1;OPRM1;IPCEF1;IPCEF1	Body;Body;Body;Body	0.8738714	1.2622176	5.2084657	7.06E-07	2.31E-05	5.5840591
cg08985530	chr12	77694045	+			-1.689672	0.5201704	-5.208482	7.06E-07	2.31E-05	5.584129

cg20998200	chr11	22370099	+	SLC17A6	Body	-1.190263	-0.787465	-5.208475	7.06E-07	2.31E-05	5.5840975
cg21665774	chr19	34790692	-	KIAA0355	5'UTR	0.9174514	1.9701148	5.2084735	7.06E-07	2.31E-05	5.5840924
cg27157482	chr7	27215606	-	HOXA10	Body	1.1585075	0.7340678	5.2085134	7.05E-07	2.31E-05	5.5842625
cg19514854	chr17	39678394	-			-0.980471	-0.571437	-5.208444	7.06E-07	2.31E-05	5.5839667
cg17578548	chr6	33132727	-	COL11A2;COL11A2;COL11A2	Body;Body;Body	-0.689407	-1.208823	-5.208348	7.06E-07	2.31E-05	5.5835572
cg02390801	chr15	74528565	+	CCDC33	TSS200	-0.922745	-1.256367	-5.208286	7.06E-07	2.31E-05	5.5832926
cg15898536	chr5	1753301	-			-1.136476	-0.414975	-5.208134	7.07E-07	2.31E-05	5.5826456
cg11815914	chr18	59992350	+	TNFRSF11A	TSS200	-0.796711	-4.330308	-5.208059	7.07E-07	2.31E-05	5.5823243
cg03023089	chr22	36806580	+			-0.825704	-1.25719	-5.207701	7.08E-07	2.31E-05	5.5807951
cg26432539	chr15	97326893	+	SPATA8	1stExon	-1.245407	0.1401516	-5.207674	7.08E-07	2.32E-05	5.5806816
cg01427630	chr16	13270646	+	SHISA9	Body	-1.240654	0.2969441	-5.207643	7.08E-07	2.32E-05	5.5805495
cg19309079	chr2	133175311	+	GPR39	1stExon	-1.23125	0.140944	-5.207406	7.09E-07	2.32E-05	5.5795395
cg05662655	chr18	28682828	+	DSC2;DSC2	TSS1500;TSS1500	-1.165865	0.6161889	-5.207389	7.09E-07	2.32E-05	5.5794644
cg27135989	chr8	61088342	-			-1.598765	0.2418268	-5.207173	7.10E-07	2.32E-05	5.5785433
cg00178728	chr4	22516158	-	GPR125	Body	-1.104869	-0.419566	-5.207131	7.10E-07	2.32E-05	5.5783639
cg04546999	chr1	152956429	-	SPRR1A	TSS200	-1.806648	0.0005452	-5.207038	7.10E-07	2.32E-05	5.5779672
cg16495212	chr15	83317306	+	CPEB1	TSS1500	0.8456168	-0.246156	5.2070137	7.10E-07	2.32E-05	5.577864
cg07804076	chr5	140741730	+	PCDHGA4;PCDHGA2;PCDHGB2;PCDHGA1;PCDHGB1;PCDHGA3;PCDHGB2	Body;Body;1stExon;Body;Body;Body;1stExon	0.8758461	-0.534249	5.206877	7.11E-07	2.32E-05	5.5772806
cg07203024	chr1	228560604	-	OBSCN	Body	-0.782979	-0.450039	-5.206816	7.11E-07	2.32E-05	5.5770189
cg21832150	chr1	902025	+	PLEKHN1;PLEKHN1	Body;Body	-0.421615	-3.481928	-5.206678	7.11E-07	2.32E-05	5.5764321

cg20584905	chr2	223163784	-	PAX3;PAX3;PAX3;CCDC140;PAX3;PAX3;PAX3;PAX3;PAX3	TSS200;TSS200;TSS200;5'UTR;TSS200;TSS200;TSS200;TSS200;TSS200	1.2926215	-1.012414	5.2066191	7.11E-07	2.32E-05	5.5761802
cg14936188	chr18	40647794	+	RIT2	Body	-1.467355	0.8395894	-5.206486	7.12E-07	2.33E-05	5.5756145
cg01518591	chr6	29408229	-	OR10C1	1stExon	-1.158439	0.1559023	-5.206431	7.12E-07	2.33E-05	5.5753796
cg12001304	chr2	229045020	+	SPHKAP;SPHKAP	Body;Body	1.61238	-0.768904	5.2063938	7.12E-07	2.33E-05	5.5752191
cg13931250	chr1	179777372	-	FAM163A	5'UTR	-1.339399	1.6531052	-5.206334	7.12E-07	2.33E-05	5.574965
cg07805542	chr1	9779309	-	PIK3CD	Body	-1.715221	-0.777105	-5.206311	7.12E-07	2.33E-05	5.5748646
cg04085571	chr11	1872753	+	LSP1	TSS1500	-1.222334	-0.068469	-5.206267	7.13E-07	2.33E-05	5.5746782
cg09709600	chr11	65359521	-	EHBP1L1	Body	1.3276504	1.9570409	5.2062508	7.13E-07	2.33E-05	5.5746094
cg09100343	chr16	57147202	-	CPNE2	Body	-0.741547	0.3635206	-5.206205	7.13E-07	2.33E-05	5.5744133
cg12456714	chr5	16180393	+	11-Mar	TSS1500	1.9549335	-0.788507	5.2060883	7.13E-07	2.33E-05	5.5739159
cg00032366	chr19	5692159	-	LONP1	Body	-0.833216	-0.186293	-5.206012	7.13E-07	2.33E-05	5.5735889
cg00396783	chr8	22864006	-	RHOBTB2;RHOBTB2;RHOBTB2	Body;Body;Body	0.5307252	2.7479776	5.2060274	7.13E-07	2.33E-05	5.5736561
cg04037228	chr3	45636386	-	LIMD1	1stExon	-0.782598	-1.429609	-5.205994	7.13E-07	2.33E-05	5.5735135
cg24215776	chr11	65152051	-			1.0177908	0.2130061	5.2058989	7.14E-07	2.33E-05	5.5731079
cg12447154	chr13	107002422	-			-1.31995	-1.230235	-5.205853	7.14E-07	2.33E-05	5.5729124
cg11399254	chr1	47694517	-	TAL1	5'UTR	0.7495876	0.9752836	5.2058269	7.14E-07	2.33E-05	5.5728011
cg01138078	chr7	1220000	-			-1.346513	1.3684062	-5.205804	7.14E-07	2.33E-05	5.5727037
cg16293631	chr17	38020606	-	IKZF3;IKZF3;IKZF3;IKZF3;IKZF3;IKZF3	TSS200;TSS200;TSS200;TSS200;TSS200;TSS200	-1.199456	-6.675663	-5.207704	7.14E-07	2.33E-05	5.5733527
cg06575035	chr5	140744449	-	PCDHGA4;PCDHGA2;PCDHGA5;PCDHGB2;PCDHGA1;PCDHGB1;PCDHGA3;PCDHGA5	Body;Body;1stExon;Body;Body;Body;Body;1stExon	0.841748	0.4094425	5.205712	7.14E-07	2.33E-05	5.5723108
cg08082810	chr14	61108391	-			1.1409766	0.0409644	5.2056788	7.14E-07	2.33E-05	5.5721693

cg19611809	chr15	53080209	+	ONECUT1	Body	1.4077943	-1.603299	5.2055964	7.15E-07	2.33E-05	5.5718179
cg02168291	chr16	82671520	+	CDH13	Body	-1.286042	0.2871664	-5.205568	7.15E-07	2.33E-05	5.5716971
cg12187394	chr13	112761328	-			0.9420498	-0.26649	5.2055409	7.15E-07	2.33E-05	5.5715812
cg09988837	chr1	224811939	-	CNIH3	Body	-1.108836	-1.911437	-5.205493	7.15E-07	2.33E-05	5.5713788
cg15272362	chr20	25062823	-	VSX1;VSX1	TSS200;TSS200	1.845176	-1.436656	5.2052898	7.16E-07	2.33E-05	5.57051
cg00793591	chr14	70014715	-			1.2249201	-1.822535	5.2049907	7.17E-07	2.34E-05	5.5692344
cg22643811	chr22	48885446	+	FAM19A5	1stExon	1.4783585	-1.415374	5.2049399	7.17E-07	2.34E-05	5.5690175
cg01839464	chr18	49868477	-	DCC	Body	1.5541943	-1.342154	5.2047696	7.17E-07	2.34E-05	5.5682912
cg03224418	chr20	62611858	+	SAMD10;PRPF6	TSS1500;TSS1500	-0.96813	-1.443981	-5.204574	7.18E-07	2.34E-05	5.5674563
cg14593033	chr17	56296980	+	MKS1;MKS1	TSS200;TSS1500	-0.812204	-2.353811	-5.204575	7.18E-07	2.34E-05	5.5674618
cg13657884	chr5	52046775	+			-1.080359	1.8750312	-5.204542	7.18E-07	2.34E-05	5.5673219
cg16409261	chr20	61703735	+			1.8723887	-1.810114	5.2043489	7.19E-07	2.34E-05	5.5664971
cg12074585	chr12	7280958	-	RBP5	Body	1.3542779	4.2516104	5.2043058	7.19E-07	2.34E-05	5.566313
cg20596162	chr15	45408861	+	DUOXA2	Body	1.0723576	-1.225786	5.2043168	7.19E-07	2.34E-05	5.56636
cg26702670	chr15	90456619	+	C15orf38	TSS1500	-1.461704	0.6909142	-5.204071	7.20E-07	2.34E-05	5.5653128
cg13478928	chr1	75596724	-	LHX8	Body	1.3697005	-0.946528	5.2040368	7.20E-07	2.34E-05	5.5651658
cg16015593	chr14	51794932	-			-1.300795	-0.791779	-5.203978	7.20E-07	2.35E-05	5.5649172
cg25778479	chr7	16686771	+	BZW2;BZW2;ANKMY2;BZW2	Body;5'UTR;TSS1500;5'UTR	-1.157093	-1.723304	-5.203879	7.20E-07	2.35E-05	5.5644935
cg03717367	chr9	139980219	+	LOC100289341;MAN1B1	Body;TSS1500	-0.995634	-0.824744	-5.203589	7.21E-07	2.35E-05	5.5632572
cg23884241	chr7	27169957	-	HOXA4	1stExon	0.5969218	0.1925091	5.2036102	7.21E-07	2.35E-05	5.5633465

cg25255082	chr12	115272264	+			-1.399781	-0.224877	-5.203589	7.21E-07	2.35E-05	5.5632561
cg17507671	chr4	111542063	-	PITX2;PITX2;PITX2	Body;Body;Body	1.4713718	-1.556409	5.2033592	7.22E-07	2.35E-05	5.5622765
cg21282549	chr19	51925128	-			2.6352435	-1.519084	5.2031941	7.22E-07	2.35E-05	5.5615723
cg18202449	chr1	50513654	-	ELAVL4	TSS200	1.730765	-1.619883	5.2030848	7.23E-07	2.35E-05	5.5611063
cg20728514	chr22	44568423	-	PARVG;PARVG	TSS1500;TSS1500	-0.985339	-0.195095	-5.202996	7.23E-07	2.35E-05	5.5607256
cg24997562	chr12	121476720	+	OASL;OASL	1stExon;1stExon	-0.759754	-3.276278	-5.202954	7.23E-07	2.35E-05	5.5605472
cg16870215	chr4	76481269	+	C4orf26;C4orf26	5'UTR;1stExon	-1.221181	-0.42095	-5.202898	7.23E-07	2.35E-05	5.5603076
cg04044720	chr3	22413680	-			1.1373901	-0.74115	5.2028682	7.24E-07	2.36E-05	5.5601827
cg06611922	chr10	118893174	-	VAX1;VAX1	3'UTR;Body	2.097548	-2.058331	5.2027174	7.24E-07	2.36E-05	5.5595397
cg10568624	chr9	100619991	-			1.6598554	-1.051986	5.202635	7.24E-07	2.36E-05	5.5591882
cg08994082	chr19	2525459	+	GNG7	5'UTR	-1.184077	-0.260117	-5.202552	7.25E-07	2.36E-05	5.5588329
cg12108974	chr8	95653291	-	ESRP1;ESRP1;ESRP1;ESRP1;ESRP1	TSS200;TSS200;TSS200;TSS200;TSS200	-0.635961	-4.281066	-5.204542	7.24E-07	2.36E-05	5.560291
cg15977904	chr22	48888940	+	FAM19A5	Body	-1.232236	0.4680343	-5.202572	7.24E-07	2.36E-05	5.5589184
cg18305853	chr2	2713323	+			-2.013612	1.6893324	-5.202548	7.25E-07	2.36E-05	5.5588161
cg14995062	chr11	102826570	-	MMP13	TSS200	-1.586703	1.0614752	-5.202397	7.25E-07	2.36E-05	5.5581752
cg00676536	chr2	41924253	-			-1.355093	0.0575244	-5.202133	7.26E-07	2.36E-05	5.5570476
cg00645755	chr7	1514031	+	INTS1	Body	-0.973695	-0.546319	-5.202114	7.26E-07	2.36E-05	5.5569685
cg04086012	chr4	189060900	-	TRIML1	1stExon	-1.155026	0.4685475	-5.202095	7.26E-07	2.36E-05	5.5568837
cg18055610	chr6	30095419	-			1.682868	-1.423482	5.2019028	7.27E-07	2.36E-05	5.5560662
cg21308760	chr7	141629801	+	CLEC5A	3'UTR	-1.611419	1.4735799	-5.201847	7.27E-07	2.36E-05	5.5558304

cg22767348	chr4	4669484	-			0.7117636	1.070387	5.2017247	7.27E-07	2.36E-05	5.5553071
cg10497023	chr5	180057034	-	FLT4;FLT4	Body;Body	-1.10051	1.1387451	-5.201629	7.28E-07	2.37E-05	5.5548987
cg24539937	chr7	136633191	+	CHRM2;CHRM2;CHRM2;CHRM2;CHRM2;CHRM2;CHRM2;CHRM2	5'UTR;5'UTR;5'UTR;5'UTR;5'UTR;5'UTR;5'UTR;5'UTR	-1.679932	1.0570471	-5.2015	7.28E-07	2.37E-05	5.5543481
cg08575330	chr2	175199828	+	SP9	1stExon	2.9958655	-2.116947	5.2014615	7.28E-07	2.37E-05	5.5541846
cg16301768	chr3	183894727	-	AP2M1;AP2M1	5'UTR;5'UTR	1.1297602	1.6817524	5.2094802	7.28E-07	2.37E-05	5.5587455
cg02119091	chr8	11553749	-			-0.93229	-2.13558	-5.201389	7.28E-07	2.37E-05	5.5538742
cg02721693	chr1	182922705	+	C1orf14	TSS200	-1.248526	-0.708704	-5.20133	7.29E-07	2.37E-05	5.553626
cg21561953	chr7	144967622	+			-1.039326	-0.706922	-5.201297	7.29E-07	2.37E-05	5.5534823
cg20597619	chr8	1636504	-	DLGAP2	Body	-0.901762	0.1599031	-5.201264	7.29E-07	2.37E-05	5.5533416
cg08844913	chr1	177724656	+			-1.757219	0.4891305	-5.201244	7.29E-07	2.37E-05	5.5532559
cg20382626	chr1	85156797	+	SSX2IP;SSX2IP;SSX2IP;SSX2IP;SSX2IP	TSS1500;TSS1500;TSS1500;TSS1500;TSS1500	-0.381815	-3.656401	-5.201137	7.29E-07	2.37E-05	5.5528035
cg22916722	chr14	76605056	+			2.5200038	-2.040657	5.2028652	7.30E-07	2.37E-05	5.5531501
cg04962428	chr17	1957748	-	HIC1	TSS1500	-1.505239	-3.764569	-5.200788	7.30E-07	2.37E-05	5.5513139
cg26306437	chr20	24930041	+	CST7;CST7	1stExon;5'UTR	-1.114366	-0.704149	-5.200748	7.30E-07	2.37E-05	5.551143
cg06578276	chr17	79068856	+	BAIAP2;BAIAP2;BAIAP2;BAIAP2	Body;Body;Body;Body	-1.239453	-1.352942	-5.200549	7.31E-07	2.37E-05	5.5502937
cg05369766	chr8	50629982	-			-1.823721	0.9836127	-5.200187	7.32E-07	2.38E-05	5.548751
cg24516259	chr1	2428864	-	PLCH2	Body	-1.830759	0.7484452	-5.200185	7.32E-07	2.38E-05	5.5487422
cg23390871	chr11	2183708	-	INS;INS-IGF2;INS-IGF2	TSS1500;TSS1500;TSS1500	-1.065693	0.257788	-5.200129	7.32E-07	2.38E-05	5.5485032
cg07436074	chr6	162071140	-	PARK2;PARK2;PARK2	Body;Body;Body	-1.014811	-0.943943	-5.200106	7.33E-07	2.38E-05	5.5484051
cg17993900	chr8	55380008	-			1.3619984	-0.594077	5.2019467	7.33E-07	2.38E-05	5.5488288

cg20585530	chr14	60975912	+	SIX6	TSS200	1.972987	-0.92542	5.1997781	7.34E-07	2.38E-05	5.5470086
cg08454015	chr1	240078452	+			-1.431491	0.0715877	-5.199688	7.34E-07	2.38E-05	5.5466235
cg18459449	chr14	68976569	-	RAD51L1	Body	0.9636331	1.7593656	5.1996694	7.34E-07	2.38E-05	5.5465456
cg10409560	chr4	76481299	-	C4orf26	1stExon	-1.548715	0.8083522	-5.199416	7.35E-07	2.39E-05	5.5454658
cg15985470	chr12	4554756	-	FGF6;FGF6	5'UTR;1stExon	-0.644816	-1.132505	-5.199357	7.35E-07	2.39E-05	5.5452125
cg10821845	chr8	144241104	-	LY6H;LY6H;LY6H	Body;5'UTR;Body	2.0685165	-2.023219	5.1987976	7.37E-07	2.39E-05	5.5428295
cg04275695	chr6	149806273	+	ZC3H12D	TSS200	-1.270977	-2.579656	-5.198581	7.38E-07	2.39E-05	5.5419076
cg15985775	chr11	20183267	-	DBX1	TSS1500	1.083985	-0.934374	5.1985332	7.38E-07	2.39E-05	5.541703
cg00887902	chr8	56436393	+	XKR4	Body	-1.184019	-0.238943	-5.198502	7.38E-07	2.39E-05	5.5415708
cg25889711	chr10	102473609	-			2.1481017	-2.485832	5.1984754	7.38E-07	2.39E-05	5.5414564
cg05504354	chr12	88974893	+	KITLG;KITLG	TSS1500;TSS1500	-1.485671	1.096039	-5.198444	7.38E-07	2.39E-05	5.5413245
cg27264322	chr1	1992046	+	PRKCZ	Body	1.8871715	2.486025	5.2003793	7.38E-07	2.39E-05	5.542155
cg04539172	chr6	32036611	+	TNXB	Body	-1.096932	0.1414104	-5.198408	7.38E-07	2.39E-05	5.5411684
cg23302570	chr2	139538601	-	NXPH2	TSS1500	1.5985821	-0.568074	5.2002902	7.38E-07	2.39E-05	5.542186
cg21104449	chr3	196485278	+	PAK2	5'UTR	-2.051568	2.1159769	-5.200028	7.39E-07	2.40E-05	5.5406615
cg24175649	chr3	124304161	+	KALRN;KALRN	Body;Body	-0.951867	-2.469831	-5.198089	7.39E-07	2.40E-05	5.5398079
cg25750004	chr17	77962682	-	TBC1D16	Body	-1.096694	0.0141043	-5.198051	7.39E-07	2.40E-05	5.5396481
cg08132940	chr7	1081526	+	C7orf50;C7orf50;C7orf50	Body;Body;Body	0.8917513	1.6158818	5.1980011	7.39E-07	2.40E-05	5.5394354
cg23684410	chr11	116897558	-	SIK3	Body	-1.302007	-1.530549	-5.19792	7.40E-07	2.40E-05	5.5390905
cg03278271	chr12	14436768	-			-1.548519	0.2926097	-5.197828	7.40E-07	2.40E-05	5.5386975

cg12565335	chr2	1990242	-	MYT1L	5'UTR	-1.584559	0.5302092	-5.197794	7.40E-07	2.40E-05	5.5385511
cg14508508	chr15	27113322	-	GABRA5;GABRA5	5'UTR;5'UTR	1.4708064	-1.378485	5.1996934	7.40E-07	2.40E-05	5.5396454
cg19585597	chr5	150326193	+	LOC134466	TSS200	1.3142153	-1.027315	5.1977227	7.40E-07	2.40E-05	5.5382489
cg02590088	chr2	47761452	-	KCNK12	Body	-1.253401	-0.371292	-5.197631	7.41E-07	2.40E-05	5.5378595
cg05802545	chr1	107921855	+	NTNG1;NTNG1;NTNG1	Body;Body;Body	-1.062943	0.0093868	-5.197612	7.41E-07	2.40E-05	5.537778
cg01181150	chr7	154407878	-	DPP6;DPP6;DPP6	Body;Body;Body	-1.296392	0.1904895	-5.197523	7.41E-07	2.40E-05	5.5373989
cg16827475	chr10	131767526	+			2.1674518	-1.057504	5.2014513	7.41E-07	2.40E-05	5.5396066
cg26164488	chr2	64440295	+			-1.377602	-0.92289	-5.197407	7.41E-07	2.40E-05	5.5369026
cg13104274	chr1	169078316	+	ATP1B1;ATP1B1	Body;Body	-1.312507	-0.395673	-5.197284	7.42E-07	2.40E-05	5.5363802
cg01152072	chr4	27097275	-			-1.133547	1.3095909	-5.19723	7.42E-07	2.40E-05	5.5361496
cg03660603	chr3	86998418	-	VGLL3	Body	-1.372775	0.1285629	-5.197178	7.42E-07	2.40E-05	5.5359263
cg09136129	chr11	116692084	-	APOA4	Body	-0.866566	0.906638	-5.197173	7.42E-07	2.40E-05	5.5359086
cg24545728	chr17	79102937	+	AATK	Body	-0.989331	1.0033444	-5.197155	7.42E-07	2.40E-05	5.5358286
cg16320249	chr17	46719761	+			1.2639813	-1.086878	5.1971	7.42E-07	2.40E-05	5.5355955
cg00616129	chr12	10827014	+	STYK1	TSS200	-0.651682	-3.633864	-5.197056	7.43E-07	2.40E-05	5.5354072
cg19877103	chr8	143851371	-	LYNX1;LYNX1	TSS200;Body	-0.956738	-0.218893	-5.196914	7.43E-07	2.41E-05	5.5348042
cg20656587	chr17	44895317	-	WNT3	Body	1.2563165	-0.561126	5.1967885	7.43E-07	2.41E-05	5.5342681
cg05143420	chr14	64926333	+	MTHFD1	3'UTR	0.8064433	1.7952112	5.1965937	7.44E-07	2.41E-05	5.5334381
cg23015917	chr1	67485337	+	SLC35D1	Body	1.0149146	0.0138461	5.1965876	7.44E-07	2.41E-05	5.5334124
cg17349199	chr10	118429452	+	C10orf82;C10orf82	1stExon;5'UTR	0.7729688	0.6253869	5.1963613	7.45E-07	2.41E-05	5.5324482

cg11804928	chr17	38220298	+	THRA;THRA	5'UTR;5'UTR	1.3869874	-0.99046	5.1961357	7.46E-07	2.41E-05	5.5314868
cg06155341	chr11	66048759	-	CNIH2	Body	-0.963308	0.0331729	-5.196015	7.46E-07	2.41E-05	5.5309729
cg27508769	chr19	7701802	-	STXBP2;STXBP2	TSS200;TSS200	-0.474971	-2.980787	-5.196031	7.46E-07	2.41E-05	5.5310417
cg17865114	chr16	3067759	-	CLDN6	5'UTR	0.9422544	0.8434448	5.1979467	7.46E-07	2.41E-05	5.5318001
cg23609982	chr1	154377403	+	IL6R;IL6R	TSS1500;TSS1500	-0.955055	-1.372545	-5.195859	7.47E-07	2.42E-05	5.530308
cg24698371	chr19	6110817	-	RFX2;RFX2	TSS200;TSS200	0.7161988	-4.714489	5.1957389	7.47E-07	2.42E-05	5.5297965
cg23041822	chr15	24917974	-			-1.280102	-0.588644	-5.195576	7.47E-07	2.42E-05	5.5291008
cg22255095	chr20	60472331	+	CDH4	Body	-1.295066	-0.432291	-5.195533	7.48E-07	2.42E-05	5.5289187
cg02773433	chr11	94133505	-	GPR83	Body	0.9411251	-0.437696	5.1953577	7.48E-07	2.42E-05	5.5281725
cg13428477	chr3	122748086	+			-1.215163	-0.146731	-5.195374	7.48E-07	2.42E-05	5.5282414
cg17012665	chr8	1974195	-			-0.848089	-3.115381	-5.195369	7.48E-07	2.42E-05	5.5282214
cg22840780	chr13	58207650	+	PCDH17	1stExon	1.5510888	-2.106219	5.195376	7.48E-07	2.42E-05	5.5282506
cg18615133	chr12	116997095	+	MAP1LC3B2	TSS200	-1.214614	-0.396847	-5.195167	7.49E-07	2.42E-05	5.5273602
cg17934948	chr1	215255716	+	KCNK2;KCNK2;KCNK2	TSS1500;TSS1500;Body	1.5248706	-1.664991	5.1951199	7.49E-07	2.42E-05	5.5271596
cg15247069	chr2	204822542	+	ICOS	Body	-1.796258	1.4401243	-5.194826	7.50E-07	2.43E-05	5.5259073
cg08610968	chr10	133797327	+			-1.450175	0.9117427	-5.194736	7.50E-07	2.43E-05	5.525524
cg13391638	chr7	112430772	+	TMEM168	TSS1500	-0.79342	-3.370947	-5.194579	7.51E-07	2.43E-05	5.5248547
cg01780754	chr8	77913262	-	PXMP3;PXMP3	TSS1500;TSS1500	-1.147582	-3.62484	-5.194288	7.52E-07	2.43E-05	5.5236167
cg25273160	chr11	56955011	+	LRRC55	3'UTR	-1.262363	0.1201995	-5.196097	7.52E-07	2.43E-05	5.5243412
cg12988424	chr3	62355884	-	FEZF2	Body	1.4218649	-1.122302	5.194012	7.53E-07	2.43E-05	5.5224402

cg20792062	chr12	5153288	+	KCNA5;KCNA5	5'UTR;1stExon	1.001567	-1.108696	5.194007	7.53E-07	2.43E-05	5.5224191
cg01270424	chr2	237078509	+			1.5113363	-1.214773	5.1957977	7.53E-07	2.43E-05	5.5226549
cg22344727	chr12	81102035	-	MYF6	Body	1.1569818	-0.762194	5.1937554	7.54E-07	2.44E-05	5.5213472
cg01777643	chr1	224805287	+	CNIH3	Body	2.1402317	-1.390159	5.1956679	7.54E-07	2.44E-05	5.5225138
cg25678587	chr11	6129408	-	OR56B4	1stExon	-1.261651	-0.196135	-5.193617	7.54E-07	2.44E-05	5.5207566
cg01295203	chr8	70984199	+	PRDM14	TSS1500	1.2982299	-0.708729	5.1935354	7.54E-07	2.44E-05	5.5204104
cg09502809	chr1	32248172	+			0.8582308	0.8906065	5.1935187	7.54E-07	2.44E-05	5.5203393
cg26174533	chr4	2011724	-	WHSC2	TSS1500	-0.419368	-4.191576	-5.193347	7.55E-07	2.44E-05	5.5196099
cg11800832	chr19	40732305	+	CNTD2	1stExon	-1.764295	-2.93581	-5.19329	7.55E-07	2.44E-05	5.5193639
cg14940871	chr18	77386828	+			-1.834645	-0.108012	-5.193294	7.55E-07	2.44E-05	5.5193827
cg06896909	chr7	19813297	-	TMEM196	TSS1500	1.1649236	-0.283107	5.1931662	7.56E-07	2.44E-05	5.5188378
cg12280471	chr1	247587794	+	NLRP3;NLRP3;NLRP3;NLRP3;NLRP3	Body;Body;Body;Body;Body	-1.133438	-0.677305	-5.193097	7.56E-07	2.44E-05	5.5185424
cg04470557	chr5	131630995	-	SLC22A4	Body	-1.086903	-2.325267	-5.193055	7.56E-07	2.44E-05	5.5183635
cg01134139	chr6	168435636	+	KIF25;KIF25	Body;Body	-1.184471	0.4609958	-5.192951	7.56E-07	2.44E-05	5.5179205
cg12591668	chr1	192520335	+			-1.849947	-1.437687	-5.192809	7.57E-07	2.44E-05	5.5173172
cg19344315	chr6	16669649	+	ATXN1;ATXN1	5'UTR;5'UTR	-1.220419	-0.960195	-5.192812	7.57E-07	2.44E-05	5.5173299
cg25279294	chr17	56232260	+	OR4D1	TSS1500	-1.56392	0.9488528	-5.192735	7.57E-07	2.44E-05	5.517002
cg11736104	chr1	176430983	+	PAPPA2;PAPPA2	TSS1500;TSS1500	-1.517016	-0.37324	-5.192674	7.57E-07	2.44E-05	5.5167433
cg17491560	chr5	6338529	+	FLJ33360	TSS1500	-1.846214	1.0017527	-5.192459	7.58E-07	2.45E-05	5.5158273
cg11689753	chr10	6821048	+			-0.985479	-0.994495	-5.192109	7.59E-07	2.45E-05	5.5143353

cg20223392	chr8	328499	+			-2.438906	1.0532035	-5.191919	7.60E-07	2.45E-05	5.5135249
cg01758512	chr6	96463902	-	FUT9;FUT9	5'UTR;1stExon	1.3573678	-0.878263	5.1918852	7.60E-07	2.45E-05	5.5133829
cg08147050	chr11	93394607	+	KIAA1731	TSS1500	-0.767464	-3.347455	-5.191832	7.60E-07	2.45E-05	5.5131563
cg08249698	chr16	6610161	-	A2BP1;A2BP1	5'UTR;5'UTR	-1.162075	-0.509459	-5.191652	7.61E-07	2.45E-05	5.5123895
cg07429284	chr5	132107420	-	SEPT8;SEPT8;SEPT8;SEPT8	Body;5'UTR;Body;Body	0.7105856	-0.339901	5.1916107	7.61E-07	2.45E-05	5.512214
cg02139586	chr16	59792836	+			-1.015796	-0.892769	-5.191465	7.61E-07	2.46E-05	5.5115938
cg06067393	chr3	86780723	-			-1.43477	2.3275951	-5.191476	7.61E-07	2.46E-05	5.5116424
cg19384289	chr2	176994365	-	HOXD8	TSS200	2.0589893	-0.975692	5.191464	7.61E-07	2.46E-05	5.5115895
cg01178854	chr1	154913744	+			-1.085129	1.2273069	-5.191444	7.61E-07	2.46E-05	5.5115035
cg22820316	chr1	119532736	-	TBX15	TSS1500	1.3452307	-0.224544	5.1914098	7.62E-07	2.46E-05	5.5113586
cg07510423	chr2	106681998	-	C2orf40	TSS200	1.5790014	-1.699234	5.1932348	7.62E-07	2.46E-05	5.5121631
cg02081733	chr15	68120412	-	LBXCOR1	Body	1.203514	-0.915551	5.1911949	7.62E-07	2.46E-05	5.5104434
cg19050940	chr13	76445108	+			-0.768604	-1.315014	-5.191214	7.62E-07	2.46E-05	5.5105245
cg22052948	chr5	140778342	+	PCDHGA4;PCDHGB5;PCDHGA6;PCDHGA1;PCDHGA8;PCDHGA5;PCDHGB1;PCDHGB5;PCDHGB4;PCDHGA3;PCDHGA2;PCDHGA7;PCDHGB2;PCDHGB3	Body;1stExon;Body;Body;Body;Body;Body;1stExon;Body;Body;Body;Body;Body;Body	1.2911134	-0.424987	5.1912096	7.62E-07	2.46E-05	5.5105063
cg16336556	chr2	33295138	-	LTBP1	Body	-1.420505	-0.899611	-5.191063	7.63E-07	2.46E-05	5.5098821
cg22812280	chr11	5173605	+	OR52A1	TSS200	-1.443976	0.3039788	-5.191033	7.63E-07	2.46E-05	5.5097525
cg00501006	chr15	25480975	-	SNORD115-29;SNORD115-11;SNORD115-43;SNORD115-36	TSS1500;TSS1500;TSS1500;TSS1500	-1.355857	1.1334634	-5.191012	7.63E-07	2.46E-05	5.5096654
cg17998530	chr22	35388865	-			-0.905121	-1.918256	-5.190918	7.63E-07	2.46E-05	5.5092645
cg06203976	chr1	159140445	+	CADM3;CADM3	TSS1500;TSS1500	-1.399211	0.215705	-5.190793	7.64E-07	2.46E-05	5.5087325
cg20008734	chr11	321324	+	IFITM3	TSS1500	0.9726258	0.4839416	5.1905563	7.64E-07	2.46E-05	5.5077245

cg18804963	chr11	132456073	-	OPCML;OPCML	Body;Body	-1.275357	0.0179343	-5.190435	7.65E-07	2.46E-05	5.5072103
cg12446045	chr1	25857419	+			-1.303009	0.5080317	-5.190285	7.65E-07	2.47E-05	5.5065707
cg27589654	chr16	27060571	-			-0.986802	-0.526921	-5.190112	7.66E-07	2.47E-05	5.5058321
cg02730156	chr21	38069950	+			1.6311892	-1.87851	5.1900829	7.66E-07	2.47E-05	5.5057094
cg22524061	chr8	99959430	+	OSR2;OSR2	5'UTR;5'UTR	1.7410577	1.4141834	5.1900283	7.66E-07	2.47E-05	5.505477
cg04459499	chr6	39772468	+	DAAM2	5'UTR	-1.111168	-0.290204	-5.189924	7.67E-07	2.47E-05	5.505033
cg25285904	chr18	73628634	+			1.5778469	-1.342067	5.1899335	7.67E-07	2.47E-05	5.5050732
cg04389398	chr7	103767075	-	ORC5L	3'UTR	1.3358952	1.8811892	5.189773	7.67E-07	2.47E-05	5.5043901
cg24869056	chr10	100205558	+	HPS1;HPS1	5'UTR;5'UTR	0.7143797	0.884096	5.1897193	7.67E-07	2.47E-05	5.5041612
cg17516247	chr1	174933574	-			-1.378377	-1.740627	-5.189688	7.67E-07	2.47E-05	5.5040285
cg20201270	chr2	45397874	+			1.3518091	0.5708342	5.191535	7.68E-07	2.47E-05	5.5049341
cg21930712	chr19	51761116	+	C19orf75;C19orf75	1stExon;5'UTR	-1.063999	-0.33427	-5.189465	7.68E-07	2.47E-05	5.5030801
cg08271804	chr19	22816896	-	ZNF492	TSS1500	1.67469	-1.205812	5.1893989	7.68E-07	2.47E-05	5.5027973
cg20248654	chr13	91825341	-			-1.271983	0.7220218	-5.18928	7.69E-07	2.48E-05	5.5022914
cg23913995	chr20	21490665	-			1.6943457	-0.683788	5.1892869	7.69E-07	2.48E-05	5.5023209
cg07113642	chr20	2781262	-	CPXM1;CPXM1	5'UTR;1stExon	3.4359315	-1.87071	5.1891532	7.69E-07	2.48E-05	5.5017516
cg13638420	chr11	32448293	+	WT1;WT1;WT1;WT1	Body;Body;Body;Body	1.0704962	-0.672837	5.1890335	7.70E-07	2.48E-05	5.501242
cg06092815	chr2	229046406	-	SPHKAP;SPHKAP	TSS200;TSS200	1.2101204	-0.925927	5.1888028	7.70E-07	2.48E-05	5.5002601
cg19180555	chr12	5982295	+	ANO2	Body	-1.304774	0.2199921	-5.188764	7.71E-07	2.48E-05	5.5000935
cg09655253	chr6	39895417	-	MOCS1;MOCS1;MOCS1;MOCS1;MOCS1;MOCS1;MOCS1;MOCS1	5'UTR;1stExon;1stExon;1stExon;Body;Body;5'UTR;5'UTR	-1.575814	0.2381897	-5.188526	7.71E-07	2.48E-05	5.499083

cg01017926	chr16	30581228	-	ZNF688;ZNF688	3'UTR;3'UTR	0.6130777	3.3975369	5.1884919	7.72E-07	2.48E-05	5.4989367
cg20131968	chr17	61850012	-	CCDC47	5'UTR	-0.968128	-1.561258	-5.188401	7.72E-07	2.48E-05	5.4985491
cg19743881	chr7	115850438	-	TES	TSS200	-0.614726	-3.722903	-5.188178	7.73E-07	2.49E-05	5.4976018
cg05295038	chr20	61340107	-	NTSR1	TSS200	2.7271685	-2.633999	5.1900121	7.73E-07	2.49E-05	5.4984583
cg04302194	chr5	3599012	+	IRX1	Body	1.3646646	-1.283989	5.1879877	7.73E-07	2.49E-05	5.4967909
cg21271420	chr6	164527436	-			-1.244397	0.331913	-5.187759	7.74E-07	2.49E-05	5.4958171
cg25797887	chr7	96627821	+	DLX6AS	Body	1.919354	-1.519456	5.1936249	7.74E-07	2.49E-05	5.4996216
cg03204678	chr6	26614535	-			2.2364561	-2.416602	5.187642	7.74E-07	2.49E-05	5.4953195
cg04819775	chr5	87974436	-	LOC645323;LOC645323	Body;TSS200	1.2195783	-0.83422	5.1876303	7.74E-07	2.49E-05	5.4952697
cg23241448	chr17	750379	+	NXN	Body	2.8781009	-2.604077	5.1876242	7.75E-07	2.49E-05	5.4952437
cg16654610	chr4	471390	+	ZNF721	5'UTR	-1.727282	1.1306547	-5.187556	7.75E-07	2.49E-05	5.4949515
cg25023596	chr12	118627943	-	TAOK3	Body	0.8902946	1.231415	5.1874863	7.75E-07	2.49E-05	5.494657
cg00253681	chr12	14996583	+	ART4	TSS200	0.9331591	1.1779659	5.1874427	7.75E-07	2.49E-05	5.4944711
cg25798782	chr2	242795282	-	PDCD1	Body	-1.020788	-0.145511	-5.187302	7.76E-07	2.49E-05	5.4938706
cg02177231	chr1	119529930	+	TBX15	5'UTR	1.6588026	-0.847743	5.1892173	7.76E-07	2.49E-05	5.4950792
cg02805814	chr19	53088482	+			0.7413411	1.8794725	5.187249	7.76E-07	2.49E-05	5.4936469
cg14638209	chr2	10260280	-			-0.487653	-3.442035	-5.187193	7.76E-07	2.49E-05	5.4934107
cg13275538	chr10	132913255	-	TCERG1L	Body	-1.197192	0.8357487	-5.187154	7.76E-07	2.49E-05	5.4932418
cg01868128	chr1	152483675	+	LCE5A	5'UTR	-0.98558	-0.649337	-5.187064	7.76E-07	2.50E-05	5.4928585
cg07850987	chr17	46645972	-	HOXB3	5'UTR	1.1696314	-0.36004	5.1868729	7.77E-07	2.50E-05	5.4920463

cg25585185	chr11	133231644	+	OPCML	Body	-1.162835	0.5545004	-5.186749	7.78E-07	2.50E-05	5.491518
cg04092699	chr2	76975220	-	LRRTM4	3'UTR	-1.336946	1.1130756	-5.186553	7.78E-07	2.50E-05	5.4906871
cg09010107	chr1	170638807	+	PRRX1;PRRX1	Body;Body	1.2281353	-0.091078	5.1863952	7.79E-07	2.50E-05	5.4900136
cg14851701	chr12	131831956	+			-1.11206	-0.458337	-5.186404	7.79E-07	2.50E-05	5.490053
cg24892966	chr6	27526023	+			2.0417313	-0.27436	5.18636	7.79E-07	2.50E-05	5.489864
cg08645889	chr2	20425310	-	SDC1;SDC1	TSS1500;TSS200	-0.356614	-4.403614	-5.186336	7.79E-07	2.50E-05	5.4897626
cg03860037	chr13	28497359	+	PDX1	Body	1.2056515	-1.271207	5.1861513	7.80E-07	2.50E-05	5.488976
cg02085660	chr8	145751749	-	LRRC24;MGC70857	5'UTR;3'UTR	-1.231	0.6175265	-5.186088	7.80E-07	2.50E-05	5.4887055
cg11494699	chr11	36588217	-	RAG1	TSS1500	-1.30856	1.014897	-5.18593	7.80E-07	2.51E-05	5.4880354
cg22142968	chr12	52238436	+			-1.262095	0.1664776	-5.187797	7.81E-07	2.51E-05	5.4886298
cg07739205	chr8	50823541	+	SNTG1	TSS1500	2.2781107	-1.81126	5.1874995	7.82E-07	2.51E-05	5.4877772
cg27090087	chr6	54172378	-	TINAG	TSS1500	-1.120839	-1.014647	-5.18554	7.82E-07	2.51E-05	5.4863762
cg01234044	chr7	1277585	-			1.3817983	-1.833346	5.1854739	7.82E-07	2.51E-05	5.4860935
cg12369353	chr13	36728963	-			1.3334119	-0.708834	5.1854819	7.82E-07	2.51E-05	5.4861274
cg13609167	chr4	2827061	-	SH3BP2;SH3BP2;SH3BP2;SH3BP2	Body;Body;Body;Body	0.551567	1.0316607	5.1853218	7.83E-07	2.51E-05	5.4854464
cg24843380	chr5	178367827	+	ZNF454	TSS1500	2.1328345	-1.148674	5.1851309	7.83E-07	2.51E-05	5.4846341
cg26716753	chr12	126105989	+	TMEM132B	Body	-1.623238	0.6425628	-5.185127	7.83E-07	2.51E-05	5.4846163
cg03354707	chr19	41115390	+	LTBP4;LTBP4;LTBP4	Body;Body;Body	-0.782104	-1.76626	-5.185048	7.83E-07	2.51E-05	5.4842798
cg13468767	chr17	9672024	-			-1.184752	-1.574186	-5.184995	7.84E-07	2.51E-05	5.4840553
cg24900666	chr17	46608329	-	HOXB1	TSS200	1.4495357	2.5059658	5.1847699	7.84E-07	2.52E-05	5.4830985

cg07774193	chr5	30863935	-			-1.361003	-1.100948	-5.184733	7.85E-07	2.52E-05	5.4829393
cg02063817	chr7	27253521	-			0.800043	0.2294721	5.1846816	7.85E-07	2.52E-05	5.4827227
cg01925738	chr5	140480770	+	PCDHB3	1stExon	1.7620564	1.2147684	5.184623	7.85E-07	2.52E-05	5.4824733
cg02537108	chr11	77907224	-	USP35	5'UTR	0.7762618	0.7766647	5.1845949	7.85E-07	2.52E-05	5.4823538
cg25951210	chr2	177016619	+	HOXD4	1stExon	1.3029909	-0.447846	5.1845603	7.85E-07	2.52E-05	5.4822066
cg26995656	chr14	88121644	+			-1.515354	0.337004	-5.184427	7.86E-07	2.52E-05	5.4816377
cg03596178	chr17	76138514	-	TMC8	3'UTR	-1.17492	-0.159464	-5.184312	7.86E-07	2.52E-05	5.4811504
cg15511801	chr10	88296171	-			1.0119921	0.0342144	5.1843224	7.86E-07	2.52E-05	5.4811948
cg01447854	chr1	228556332	+	OBSCN	Body	-1.345509	0.4517592	-5.184157	7.87E-07	2.52E-05	5.4804916
cg09579953	chr2	101925467	+	RNF149	TSS1500	-0.483044	-3.281426	-5.18417	7.87E-07	2.52E-05	5.4805451
cg04617948	chr10	129535138	-	FOXI2	TSS1500	1.381545	-1.563396	5.1841035	7.87E-07	2.52E-05	5.4802635
cg06829584	chr10	75659483	-			-1.34479	0.2227361	-5.184081	7.87E-07	2.52E-05	5.4801669
cg09675824	chr20	61695785	-			-1.227978	0.2209086	-5.183885	7.88E-07	2.52E-05	5.4793333
cg18453551	chr3	128226565	+	LOC90246	TSS200	-1.133232	-0.833562	-5.183767	7.88E-07	2.53E-05	5.4788313
cg09821185	chr14	101923366	-			1.7304788	-0.991991	5.1836806	7.88E-07	2.53E-05	5.4784644
cg08950105	chr9	96622335	+			-1.179785	0.52411	-5.183641	7.88E-07	2.53E-05	5.4782968
cg21631443	chr15	100049225	-			-1.099441	0.4208306	-5.183652	7.88E-07	2.53E-05	5.4783444
cg18175808	chr1	63786271	-			1.1334095	-0.678138	5.1853474	7.89E-07	2.53E-05	5.4782191
cg01574663	chr16	22825769	+	HS3ST2	TSS200	1.2755282	-1.090631	5.1833265	7.90E-07	2.53E-05	5.4769581
cg17546247	chr3	147125714	+	ZIC4;ZIC1	TSS1500;TSS1500	1.1375182	0.0510962	5.1833237	7.90E-07	2.53E-05	5.4769463

cg05229802	chr16	75282362	+	BCAR1;BCAR1;BCAR1;BCAR1;BCAR1;BCAR1;BCAR1;BCAR1	Body;Body;Body;Body;Body;Body;TSS200;Body	0.8798752	1.5187261	5.1832931	7.90E-07	2.53E-05	5.4768164
cg26263675	chr17	72743191	-	RAB37;RAB37;RAB37;RAB37	3'UTR;3'UTR;3'UTR;3'UTR	1.0985269	-0.925258	5.1832278	7.90E-07	2.53E-05	5.4765384
cg09178678	chr5	153853798	-			0.7381715	-0.630829	5.183043	7.91E-07	2.53E-05	5.4757524
cg12152002	chr14	57279496	-	OTX2OS1	TSS1500	1.4684781	-0.941555	5.1830323	7.91E-07	2.53E-05	5.4757068
cg08660562	chr12	66627900	+	IRAK3;IRAK3	Body;Body	-1.601418	0.6535804	-5.183014	7.91E-07	2.53E-05	5.4756299
cg11982293	chr2	175205387	+			1.0860504	-1.066277	5.1829609	7.91E-07	2.53E-05	5.4754034
cg26922483	chr15	97322838	-			-1.066251	0.1179955	-5.182939	7.91E-07	2.53E-05	5.4753115
cg21177502	chr8	50968578	-	SNTG1	5'UTR	-1.319277	-0.471421	-5.182837	7.91E-07	2.53E-05	5.4748752
cg08726863	chr2	128458401	-	SFT2D3	TSS200	-1.329222	-0.040788	-5.182752	7.92E-07	2.53E-05	5.4745131
cg05826245	chr12	10827400	+	STYK1	TSS1500	-1.21118	-2.447524	-5.182642	7.92E-07	2.53E-05	5.474048
cg09876519	chr6	50675610	+			1.1780723	-0.692436	5.182634	7.92E-07	2.53E-05	5.474013
cg11992940	chr17	34094454	-	MMP28	Body	-0.740015	-0.32083	-5.182637	7.92E-07	2.53E-05	5.4740247
cg21013206	chr11	113347208	+	DRD2;DRD2	TSS1500;TSS1500	-1.045605	-0.383511	-5.182633	7.92E-07	2.53E-05	5.4740085
cg15825731	chr1	224805753	-	CNIH3	Body	2.2179641	-2.53689	5.18242	7.93E-07	2.54E-05	5.4731028
cg23581088	chr6	3912160	-			-1.166627	-0.199925	-5.182417	7.93E-07	2.54E-05	5.4730901
cg20213166	chr11	6191635	+	OR52B2	TSS200	-1.567408	0.6508211	-5.182276	7.93E-07	2.54E-05	5.4724896
cg02173977	chr14	74532360	-	C14orf45;ALDH6A1	3'UTR;Body	0.8472652	1.5299234	5.1822255	7.93E-07	2.54E-05	5.4722755
cg04594598	chr8	145105319	+			1.3902901	-1.557404	5.18214	7.94E-07	2.54E-05	5.4719119
cg03112433	chr7	90338555	+	CDK14	TSS200	-1.522354	-0.244487	-5.182073	7.94E-07	2.54E-05	5.4716253
cg26532664	chr1	2739523	+			-1.166165	0.7702413	-5.182086	7.94E-07	2.54E-05	5.4716808

cg07706111	chr5	90459841	+	GPR98;GPR98	3'UTR;Body	-1.231238	1.3180616	-5.182037	7.94E-07	2.54E-05	5.4714757
cg27116186	chr19	5480734	+			-0.777668	-1.617498	-5.181732	7.95E-07	2.54E-05	5.4701756
cg06005396	chr19	590541	+	HCN2	1stExon	1.2900572	-1.314877	5.1816162	7.96E-07	2.54E-05	5.4696842
cg01851208	chr10	82168043	-	C10orf58	TSS200	-0.525501	-3.951004	-5.183527	7.96E-07	2.54E-05	5.4704846
cg05260129	chr3	50388622	-	TUSC4;CYB561D2	TSS200;5'UTR	-0.525064	-4.885143	-5.183377	7.96E-07	2.55E-05	5.4702617
cg05467160	chr2	176945526	-	EVX2	Body	1.5245615	-0.805167	5.1814134	7.96E-07	2.55E-05	5.4688219
cg01806238	chr17	56597682	-	SEPT4;SEPT4;SEPT4	3'UTR;3'UTR;3'UTR	0.9929645	1.2552946	5.1813585	7.96E-07	2.55E-05	5.4685883
cg21699506	chr4	80786707	+			-1.122711	-1.385987	-5.181268	7.97E-07	2.55E-05	5.4682029
cg05404091	chr12	133182886	-			-0.946032	0.1408693	-5.181175	7.97E-07	2.55E-05	5.4678087
cg24183678	chr4	16919935	+			-1.318724	0.5563293	-5.183083	7.97E-07	2.55E-05	5.468597
cg18131548	chr1	47696212	+	TAL1	TSS1500	1.1117979	-0.385416	5.1810963	7.97E-07	2.55E-05	5.4674732
cg09825979	chr7	27179432	+			1.1641011	0.9106914	5.1810339	7.98E-07	2.55E-05	5.4672081
cg24697925	chr19	1782411	+	ATP8B3	3'UTR	-1.432531	2.7989838	-5.180997	7.98E-07	2.55E-05	5.4670519
cg00028056	chr2	220407171	+	CHPF	Body	-1.269932	0.3123684	-5.180967	7.98E-07	2.55E-05	5.466922
cg04297406	chr4	16609513	-	LDB2;LDB2	Body;Body	-1.282659	1.1110217	-5.180926	7.98E-07	2.55E-05	5.4667507
cg26279116	chr12	25429198	+			-1.497298	1.1027865	-5.180878	7.98E-07	2.55E-05	5.4665434
cg01657995	chr6	31804883	+	C6orf48;C6orf48;SNORD52	5'UTR;5'UTR;Body	-1.35539	-1.882623	-5.180649	7.99E-07	2.55E-05	5.465572
cg14774165	chr3	11812037	+			-0.602069	-4.555138	-5.180452	8.00E-07	2.55E-05	5.4647339
cg26389913	chr12	97631769	-			-1.59105	0.1175086	-5.180373	8.00E-07	2.56E-05	5.4643978
cg09142144	chr5	35939847	-	CAPSL;CAPSL	TSS1500;TSS1500	-1.657359	0.2434254	-5.180237	8.00E-07	2.56E-05	5.4638207

cg23792592	chr20	61150637	+	MIR1-1;C20orf166	TSS1500;5'UTR	-0.957272	0.1415382	-5.179863	8.02E-07	2.56E-05	5.4622275
cg11646259	chr7	34026657	+	BMPER	Body	-1.645102	0.6806063	-5.179769	8.02E-07	2.56E-05	5.4618308
cg02857898	chr19	46582429	+			0.9101651	-0.171802	5.1797526	8.02E-07	2.56E-05	5.46176
cg21462844	chr8	67628165	-	SGK3	5'UTR	-1.301054	-1.14226	-5.179692	8.02E-07	2.56E-05	5.4615016
cg01049870	chr14	95601239	-	DICER1;DICER1	5'UTR;5'UTR	0.8564804	1.8202887	5.1795932	8.03E-07	2.56E-05	5.4610825
cg18661205	chr5	140613884	-	PCDHB18	TSS200	1.7373621	-0.710233	5.1796061	8.03E-07	2.56E-05	5.4611372
cg09638248	chr10	15577075	-	ITGA8	Body	-1.362562	1.1902653	-5.179485	8.03E-07	2.56E-05	5.4606244
cg00792184	chr14	73355148	-	DPF3	Body	-1.152661	0.2686767	-5.179441	8.03E-07	2.56E-05	5.4604348
cg01618697	chr7	2913864	-			-1.50328	-0.063839	-5.179326	8.04E-07	2.57E-05	5.4599468
cg03215422	chr5	135266509	+	FBXL21	5'UTR	1.1701614	-0.502934	5.1793273	8.04E-07	2.57E-05	5.4599519
cg06635722	chr4	13532622	+			1.3340379	-1.262779	5.1792843	8.04E-07	2.57E-05	5.4597689
cg05062178	chr3	124605247	-	ITGB5	Body	-0.841366	-2.440856	-5.17923	8.04E-07	2.57E-05	5.4595386
cg10155147	chr2	106500829	-	NCK2;NCK2;NCK2	Body;Body;Body	0.7395845	2.1596116	5.1792182	8.04E-07	2.57E-05	5.4594878
cg14490025	chr6	41703872	+	TFEB;TFEB	5'UTR;TSS1500	-1.712109	1.2174385	-5.179201	8.04E-07	2.57E-05	5.4594137
cg10279148	chr16	8960126	-	CARHSP1;CARHSP1	5'UTR;5'UTR	0.9943444	1.3015048	5.1791058	8.05E-07	2.57E-05	5.4590102
cg21759048	chr2	1500255	-	TPO;TPO;TPO;TPO	Body;Body;Body;Body	-1.529242	0.6341763	-5.178963	8.05E-07	2.57E-05	5.4584023
cg08507393	chr17	11553929	-	DNAH9	Body	-1.493853	0.7867959	-5.178795	8.06E-07	2.57E-05	5.4576898
cg19527959	chr6	168394191	-			1.198447	1.9686352	5.1787526	8.06E-07	2.57E-05	5.4575086
cg13813355	chr2	175210231	+			-1.564671	0.127963	-5.182612	8.06E-07	2.57E-05	5.4595871
cg12387464	chr11	123775935	-	OR8D4	TSS1500	-1.435257	0.0882159	-5.178594	8.06E-07	2.57E-05	5.4568356

cg05893353	chr3	115335126	+			-1.290482	0.139612	-5.178502	8.07E-07	2.57E-05	5.4564431
cg11543944	chr12	52644410	+			1.0785014	0.6135496	5.1783777	8.07E-07	2.57E-05	5.455915
cg26533107	chr16	86614804	-	FOXL1;FOXL1	1stExon;3'UTR	-1.186413	0.7086268	-5.178266	8.08E-07	2.58E-05	5.4554381
cg03717570	chr17	73316496	-	GRB2;GRB2	Body;Body	-1.755687	3.8294158	-5.178227	8.08E-07	2.58E-05	5.4552758
cg05537796	chr14	101531806	+	MIR369;MIR410;MIR412;MIR656	TSS200;TSS1500;Body;TSS1500	-1.123279	0.8659651	-5.17822	8.08E-07	2.58E-05	5.4552426
cg14781262	chr5	169398708	+	FAM196B;DOCK2	5'UTR;Body	-1.648257	1.2536821	-5.178163	8.08E-07	2.58E-05	5.4550015
cg03165356	chr2	216882347	+			-1.378719	-0.199166	-5.178096	8.08E-07	2.58E-05	5.4547157
cg00444390	chr11	7950225	-	OR10A6	TSS200	-1.617506	0.3022965	-5.178014	8.08E-07	2.58E-05	5.4543706
cg01454540	chr10	134782773	-			-1.536102	0.627431	-5.177952	8.09E-07	2.58E-05	5.4541037
cg05377120	chr12	10456885	-	KLRD1	TSS200	-1.489926	-0.250252	-5.1779	8.09E-07	2.58E-05	5.4538824
cg01978105	chr16	67678756	-	RLTPR	TSS1500	1.0048546	-2.222426	5.1778366	8.09E-07	2.58E-05	5.453615
cg25101764	chr2	227291401	-			-1.554072	0.5825002	-5.177598	8.10E-07	2.58E-05	5.4526024
cg25407736	chr1	68296179	-	GNG12	5'UTR	1.2128194	0.8993684	5.1773616	8.11E-07	2.58E-05	5.451596
cg14582400	chr13	112726118	-			1.3112917	-0.448921	5.1770959	8.12E-07	2.59E-05	5.4504668
cg17396638	chr15	91423337	-	FURIN	Body	-0.874012	0.2601837	-5.177104	8.12E-07	2.59E-05	5.450502
cg24135645	chr15	95403291	+			-1.2768	0.73728	-5.177105	8.12E-07	2.59E-05	5.4505055
cg10471825	chr16	73557323	-			-1.56441	-0.127288	-5.17695	8.12E-07	2.59E-05	5.4498461
cg10669129	chr15	25444152	-	SNORD115-16	TSS1500	-1.107064	-0.189779	-5.176874	8.13E-07	2.59E-05	5.4495221
cg06555227	chr7	157366979	+	PTPRN2;PTPRN2;PTPRN2	Body;Body;Body	-1.360705	-0.275931	-5.176575	8.14E-07	2.59E-05	5.4482545
cg14498492	chr10	76995113	-	COMTD1	Body	-0.355095	-3.240301	-5.176422	8.14E-07	2.59E-05	5.4476024

cg24221738	chr4	166125552	+			-1.383105	0.2767409	-5.176378	8.14E-07	2.59E-05	5.4474147
cg02979850	chr11	55682603	+	OR5W2	TSS1500	-1.476966	0.5575535	-5.176247	8.15E-07	2.59E-05	5.4468597
cg00992039	chr19	45903658	+	PPP1R13L;PPP1R13L	5'UTR;5'UTR	-1.321998	0.2700638	-5.17617	8.15E-07	2.60E-05	5.4465318
cg01281157	chr5	178422260	+	GRM6	TSS200	1.2497002	-1.140541	5.1760931	8.15E-07	2.60E-05	5.4462047
cg14093792	chr12	322888	-	SLC6A12;SLC6A12;SLC6A12	TSS200;TSS1500;5'UTR	-0.835423	-0.573097	-5.176122	8.15E-07	2.60E-05	5.4463292
cg17220055	chr1	42248998	-	HIVEP3;HIVEP3	5'UTR;5'UTR	-1.21154	-0.670364	-5.176106	8.15E-07	2.60E-05	5.4462577
cg23068499	chr2	176986026	+	HOXD9	TSS1500	1.2757653	-0.596213	5.1761055	8.15E-07	2.60E-05	5.4462573
cg09469986	chr13	31017306	+			-1.248635	1.329667	-5.176009	8.16E-07	2.60E-05	5.4458489
cg24312063	chr19	22444509	-			1.5603979	-1.657567	5.1759791	8.16E-07	2.60E-05	5.4457204
cg10210076	chr8	143852085	+	LYNX1;LYNX1	Body;TSS1500	-1.14228	0.4387431	-5.175893	8.16E-07	2.60E-05	5.4453556
cg16041686	chr4	111560462	+			1.4670422	-0.970172	5.1758303	8.16E-07	2.60E-05	5.4450879
cg15373239	chr10	119303225	+	EMX2OS;EMX2;EMX2	Body;Body;Body	1.3313161	-0.974438	5.1758135	8.16E-07	2.60E-05	5.4450168
cg25124943	chr10	4117248	+			-1.017603	0.3487763	-5.17578	8.17E-07	2.60E-05	5.4448752
cg05446322	chr13	36053408	+	MIR548F5;NBEA	Body;Body	1.0827604	0.8556264	5.1756458	8.17E-07	2.60E-05	5.4443042
cg14741855	chr7	141974226	+			-1.515698	0.7353056	-5.175684	8.17E-07	2.60E-05	5.4444652
cg16202331	chr4	13532655	+			2.1862603	-1.517224	5.1795211	8.17E-07	2.60E-05	5.446905
cg17725019	chr22	31677931	+	PIK3IP1;PIK3IP1	3'UTR;3'UTR	0.6609197	2.0900198	5.1756586	8.17E-07	2.60E-05	5.4443585
cg07312240	chr16	11406995	+			-1.305169	0.8359591	-5.175604	8.17E-07	2.60E-05	5.4441275
cg01571001	chr3	128215433	-			1.6403422	0.561626	5.177397	8.18E-07	2.60E-05	5.4444519
cg04844495	chr1	179050404	+	TOR3A	TSS1500	-1.127574	0.9081214	-5.179347	8.18E-07	2.60E-05	5.4461667

cg08736072	chr16	1471543	-	C16orf91	Body	-0.98253	-0.72559	-5.175317	8.18E-07	2.60E-05	5.4429074
cg15023513	chr15	87667171	+			-1.273721	-0.052932	-5.175052	8.19E-07	2.60E-05	5.441779
cg25743482	chr11	19775505	+	NAV2;NAV2;NAV2	Body;Body;Body	-1.234036	-0.382701	-5.174991	8.19E-07	2.61E-05	5.4415195
cg01976810	chr17	751709	+	NXN	Body	0.9805459	1.8600133	5.1749596	8.20E-07	2.61E-05	5.4413881
cg09586743	chr5	155134244	+			-1.140342	-0.741844	-5.174934	8.20E-07	2.61E-05	5.4412812
cg10424264	chr5	180056301	+	FLT4;FLT4	Body;Body	-1.622986	1.3701719	-5.174877	8.20E-07	2.61E-05	5.4410361
cg06667761	chr4	134073627	+	PCDH10;PCDH10	1stExon;1stExon	1.1333013	-0.959104	5.1747395	8.20E-07	2.61E-05	5.4404529
cg12551957	chr7	5535006	-	FBXL18	Body	-0.891753	0.7210608	-5.174752	8.20E-07	2.61E-05	5.4405064
cg13636952	chr7	142447956	+			-1.079216	-2.522579	-5.174555	8.21E-07	2.61E-05	5.4396707
cg12033779	chr8	810796	-			-1.184237	1.7636183	-5.174515	8.21E-07	2.61E-05	5.4394988
cg01441699	chr1	2848427	+			-0.998107	-0.202271	-5.17443	8.21E-07	2.61E-05	5.4391385
cg15576517	chr19	7711153	+	STXBP2;STXBP2	Body;Body	0.9878795	1.8211968	5.1744453	8.21E-07	2.61E-05	5.4392028
cg08357120	chr2	105413372	+			-1.592511	1.7111651	-5.174353	8.22E-07	2.61E-05	5.4388089
cg17947175	chr5	1003134	-			-3.157157	1.3605489	-5.174207	8.22E-07	2.61E-05	5.4381925
cg18966401	chr1	201979478	+	ELF3;ELF3	TSS1500;TSS1500	-0.8299	-1.470599	-5.174221	8.22E-07	2.61E-05	5.4382484
cg12367543	chr22	48602194	-			-1.358097	0.8173585	-5.174157	8.22E-07	2.61E-05	5.4379786
cg01966974	chr1	212830395	-			-0.981404	-1.851001	-5.174021	8.23E-07	2.61E-05	5.4374019
cg14715696	chr2	176945122	-	EVX2	Body	2.6740419	-2.538425	5.1739729	8.23E-07	2.61E-05	5.437196
cg06777750	chr5	1666551	+			-0.85922	-0.60818	-5.173826	8.24E-07	2.62E-05	5.4365714
cg08104310	chr1	176831160	+	ASTN1	3'UTR	-1.110734	-0.758819	-5.173653	8.24E-07	2.62E-05	5.4358366

cg12345982	chr6	160389645	+	IGF2R	TSS1500	-0.352791	-3.975793	-5.173282	8.26E-07	2.62E-05	5.4342627
cg05329893	chr4	71384359	-	AMTN;AMTN	1stExon;5'UTR	-1.058936	-0.273102	-5.173192	8.26E-07	2.62E-05	5.4338787
cg00245850	chr8	143925513	+	GML	Body	-0.899003	-1.978818	-5.173141	8.26E-07	2.62E-05	5.4336598
cg01069159	chr5	161275865	+	GABRA1;GABRA1;GABRA1;GABRA1;GABRA1	5'UTR;5'UTR;5'UTR;TSS200;5'UTR	1.0451584	-0.78448	5.1730698	8.26E-07	2.62E-05	5.4333594
cg01665118	chr13	77502805	+	BTF3L1	Body	-1.127344	-0.194229	-5.17302	8.27E-07	2.62E-05	5.4331465
cg00007644	chr11	20623259	-	SLC6A5	Body	1.1157043	-1.951636	5.172802	8.27E-07	2.63E-05	5.4322217
cg00662556	chr18	74963364	-	GALR1	Body	1.0137278	-1.084209	5.1727902	8.27E-07	2.63E-05	5.4321717
cg03987648	chr4	140958771	-	MAML3	Body	-0.941265	-1.037483	-5.172746	8.28E-07	2.63E-05	5.431985
cg04161859	chr10	73404657	+	CDH23;CDH23	Body;Body	-1.057326	-0.519031	-5.172695	8.28E-07	2.63E-05	5.4317672
cg27573344	chr17	9808600	-	RCVRN;RCVRN	1stExon;5'UTR	-1.068867	0.1001962	-5.172694	8.28E-07	2.63E-05	5.4317634
cg10457446	chr17	4401043	-	SPNS2	TSS1500	-1.442042	0.3503112	-5.172565	8.28E-07	2.63E-05	5.4312145
cg21934189	chr7	68252808	-			-1.333103	-0.162377	-5.172556	8.28E-07	2.63E-05	5.4311778
cg17192377	chr18	44677553	-	HDHD2	TSS1500	-0.919163	-2.238002	-5.172452	8.29E-07	2.63E-05	5.4307359
cg21536074	chr7	42105481	-	GLI3	Body	-1.572104	-0.079332	-5.172456	8.29E-07	2.63E-05	5.4307507
cg10832108	chr5	134870517	+	NEUROG1;NEUROG1	1stExon;3'UTR	1.0874366	-1.071336	5.1723886	8.29E-07	2.63E-05	5.4304656
cg08795964	chr8	72468987	+			1.7087064	-0.792221	5.1722362	8.30E-07	2.63E-05	5.4298184
cg18360873	chr10	133051669	+	TCERG1L	Body	-1.206303	0.0566399	-5.172225	8.30E-07	2.63E-05	5.42977
cg18714204	chr5	134093972	-	DDX46	TSS1500	-0.998993	-0.735913	-5.172224	8.30E-07	2.63E-05	5.4297682
cg05177932	chr16	89631655	-			-1.499694	1.0835343	-5.171973	8.31E-07	2.63E-05	5.4287011
cg15873327	chr19	8398677	+	KANK3	Body	-0.620221	-0.866903	-5.171972	8.31E-07	2.63E-05	5.4286948

cg15580355	chr3	183003859	-	MCF2L2	Body	-0.489817	-3.721507	-5.171742	8.31E-07	2.64E-05	5.4277185
cg14970186	chr8	57701690	+			-1.411365	0.0491162	-5.171676	8.32E-07	2.64E-05	5.4274374
cg18697636	chr5	524503	-	SLC9A3;SLC9A3	5'UTR;1stExon	1.6272548	-0.881668	5.1716533	8.32E-07	2.64E-05	5.4273425
cg20698421	chr2	65217623	+	SLC1A4;SLC1A4	Body;5'UTR	-1.246007	-0.459709	-5.171515	8.32E-07	2.64E-05	5.4267536
cg26876101	chr1	2321877	+	RER1;MORN1	TSS1500;Body	-0.997154	-0.28571	-5.171428	8.33E-07	2.64E-05	5.4263866
cg08475827	chr2	152266230	-	RIF1	TSS200	-0.787668	-3.118995	-5.171194	8.33E-07	2.64E-05	5.4253939
cg26250989	chr1	6333502	-	ACOT7;ACOT7;ACOT7;ACOT7	Body;Body;Body;Body	-1.294881	0.746137	-5.171175	8.33E-07	2.64E-05	5.4253124
cg09586034	chr1	175355293	-	TNR	Body	-1.627244	0.9833827	-5.170971	8.34E-07	2.64E-05	5.4244445
cg01401344	chr4	85422998	+			0.9380282	-1.426904	5.1707449	8.35E-07	2.65E-05	5.4234848
cg13267264	chr8	70983600	+	PRDM14	TSS200	2.5093566	-0.949207	5.1707537	8.35E-07	2.65E-05	5.423522
cg05573306	chr3	183894763	+	AP2M1;AP2M1	5'UTR;5'UTR	1.1178203	1.0070068	5.1726384	8.35E-07	2.65E-05	5.4246718
cg01089498	chr4	11428985	-	HS3ST1	5'UTR	2.5017617	-1.247295	5.1706284	8.35E-07	2.65E-05	5.4229898
cg07128890	chr11	59807164	-	PLAC1L	TSS1500	-1.725229	1.1661881	-5.170583	8.36E-07	2.65E-05	5.4227954
cg12473697	chr1	2205770	-	SKI	Body	1.1009026	1.819809	5.1725047	8.36E-07	2.65E-05	5.4241047
cg02052265	chr1	247712110	+	C1orf150	TSS1500	-1.457331	-0.837702	-5.170518	8.36E-07	2.65E-05	5.4225202
cg20399616	chr12	25055967	-	BCAT1	Body	3.4378852	-0.972625	5.1704754	8.36E-07	2.65E-05	5.4223401
cg24948406	chr17	46608268	-	HOXB1;HOXB1	5'UTR;1stExon	1.4968534	1.5948273	5.1704639	8.36E-07	2.65E-05	5.4222913
cg22919908	chr4	1573613	-			-0.989653	0.2738232	-5.17028	8.37E-07	2.65E-05	5.4215116
cg20680284	chr7	35497958	-			-0.793486	-0.305189	-5.171955	8.38E-07	2.65E-05	5.4217726
cg02598079	chr2	64834106	-			-1.588965	-1.148498	-5.169927	8.38E-07	2.65E-05	5.4200111

cg17754945	chr1	85514684	-	MCOLN3	TSS1500	-0.679777	-2.906253	-5.169795	8.39E-07	2.65E-05	5.419449
cg27102629	chr10	76573155	-			0.7683821	0.5710938	5.1697401	8.39E-07	2.66E-05	5.4192179
cg14563921	chr15	87455477	-	AGBL1	Body	-1.450314	0.7669038	-5.169687	8.39E-07	2.66E-05	5.4189907
cg18523666	chr7	150129578	+			-1.446615	0.9940979	-5.169663	8.39E-07	2.66E-05	5.4188903
cg18741044	chr3	117507563	+			-1.221569	0.0001844	-5.169648	8.39E-07	2.66E-05	5.4188267
cg16301890	chr10	119301464	+	EMX2;EMX2OS;EMX2	TSS1500;Body;TSS1500	1.4460927	-0.956442	5.1696063	8.39E-07	2.66E-05	5.4186498
cg09735723	chr10	106402042	-	SORCS3	Body	1.5640906	-1.996884	5.1694945	8.40E-07	2.66E-05	5.4181748
cg12668399	chr3	141516458	-	GRK7	Body	1.7792573	-1.896613	5.1694425	8.40E-07	2.66E-05	5.4179541
cg16021896	chr11	134147525	-	GLB1L3	Body	0.9349269	-0.610475	5.1694441	8.40E-07	2.66E-05	5.417961
cg16255914	chr19	53832438	+			1.3674223	-1.077382	5.1694521	8.40E-07	2.66E-05	5.4179949
cg08049717	chr3	134091412	+	AMOTL2	5'UTR	1.4277331	0.4266402	5.1694228	8.40E-07	2.66E-05	5.4178703
cg00206542	chr11	59210965	-	OR5A1	1stExon	-1.418467	0.5574359	-5.169345	8.40E-07	2.66E-05	5.417541
cg21072457	chr5	151192481	-			0.7562459	2.0185477	5.1692176	8.41E-07	2.66E-05	5.4169992
cg04622795	chr2	73158609	+	EMX1	Body	-1.024931	0.2134732	-5.169056	8.41E-07	2.66E-05	5.4163111
cg22891862	chr10	101279697	+			1.0768817	-1.136473	5.1690171	8.42E-07	2.66E-05	5.4161479
cg20677171	chr19	56608297	-	ZNF787	Body	0.8851162	1.2371828	5.1688767	8.42E-07	2.66E-05	5.415552
cg17708240	chr19	54134782	-	DPRX	TSS1500	-1.257521	0.4318406	-5.168599	8.43E-07	2.67E-05	5.4143745
cg14993283	chr6	100906182	+	SIM1	Body	0.5805386	-0.393241	5.1684495	8.44E-07	2.67E-05	5.4137381
cg19670290	chr15	91477210	-	HDDC3;UNC45A;UNC45A	TSS1500;5'UTR;TSS1500	-0.683625	-1.83393	-5.168435	8.44E-07	2.67E-05	5.4136772
cg03408135	chr1	207842833	-	CR1L	Body	1.4818332	-0.746765	5.1682304	8.44E-07	2.67E-05	5.412808

cg15045356	chr11	11969590	+	USP47	Body	1.1205288	2.8425466	5.1680847	8.45E-07	2.67E-05	5.4121894
cg26300517	chr4	38732661	+			-1.301975	-0.427819	-5.168084	8.45E-07	2.67E-05	5.4121869
cg27517681	chr1	119528848	+	TBX15	5'UTR	1.3139109	-0.065315	5.1697751	8.46E-07	2.67E-05	5.4125268
cg21724796	chr17	8113904	+	AURKB	TSS200	-1.079475	-5.356841	-5.167828	8.46E-07	2.67E-05	5.4111016
cg20656868	chr19	3177310	+	S1PR4	TSS1500	-0.80197	-2.023293	-5.167796	8.46E-07	2.67E-05	5.410964
cg08116462	chr11	31823141	+	PAX6;PAX6;PAX6	Body;Body;Body	1.3739353	-1.448697	5.16776	8.46E-07	2.67E-05	5.410811
cg19127613	chr6	74403105	-			-1.472764	1.275374	-5.167629	8.47E-07	2.68E-05	5.4102567
cg20951255	chr7	131291256	-			-0.910003	-1.851952	-5.167491	8.47E-07	2.68E-05	5.4096686
cg22084410	chr3	51987688	+			-0.853367	-0.572057	-5.167495	8.47E-07	2.68E-05	5.409685
cg14091631	chr1	225117076	-	DNAH14;DNAH14;DNAH14	TSS1500;TSS1500;TSS1500	-1.035279	-1.029472	-5.169346	8.47E-07	2.68E-05	5.4107085
cg12305431	chr7	27157855	-	HOXA3;HOXA3	5'UTR;5'UTR	1.4753075	1.1673431	5.1673969	8.48E-07	2.68E-05	5.4092696
cg22207732	chr2	44065791	-	ABCG8;ABCG5	TSS1500;1stExon	-0.920695	0.1892618	-5.167332	8.48E-07	2.68E-05	5.4089957
cg21331947	chr6	36720936	+	CPNE5	Body	-1.176787	0.0160117	-5.167283	8.48E-07	2.68E-05	5.4087851
cg01688536	chr7	35296968	-			2.3665476	-1.094186	5.1690488	8.49E-07	2.68E-05	5.4090322
cg05393023	chr1	168194826	+	SFT2D2	TSS1500	-1.235725	-1.803734	-5.16713	8.49E-07	2.68E-05	5.408137
cg00001854	chr16	47008491	+	DNAJA2	TSS1500	-1.117455	1.7881856	-5.166944	8.49E-07	2.68E-05	5.4073483
cg04684114	chr1	17476074	+			0.8844701	-0.536724	5.1667629	8.50E-07	2.68E-05	5.4065783
cg07131210	chr7	1025825	+	CYP2W1	Body	-0.996553	0.5671952	-5.168655	8.50E-07	2.68E-05	5.4073604
cg08451870	chr12	57398787	+	ZBTB39	5'UTR	0.6624938	0.3935083	5.1667407	8.50E-07	2.68E-05	5.4064842
cg26402660	chr7	27179268	+			1.4207453	0.5494632	5.1667277	8.50E-07	2.68E-05	5.4064291

cg05144814	chr11	126944154	+			-0.926686	-0.725168	-5.166677	8.50E-07	2.68E-05	5.4062129
cg13179074	chr1	167064091	-	DUSP27	1stExon	-1.310224	0.3713024	-5.168422	8.51E-07	2.69E-05	5.4063727
cg06329574	chr11	69634334	+	FGF3	TSS200	1.4446915	-0.563904	5.1662998	8.52E-07	2.69E-05	5.4046127
cg04912984	chr1	116184219	+	VANGL1	TSS1500	-0.866222	0.0008348	-5.166125	8.52E-07	2.69E-05	5.4038701
cg02324227	chr5	180591594	-			0.7002639	-0.212239	5.1661058	8.52E-07	2.69E-05	5.4037894
cg24535956	chr8	791770	-			-1.444174	1.7543621	-5.165999	8.53E-07	2.69E-05	5.4033368
cg24728099	chr8	80706829	-			-0.932019	1.6064946	-5.165979	8.53E-07	2.69E-05	5.4032505
cg09453636	chr2	208990238	+	CRYGD	TSS1500	-0.969671	-0.296355	-5.165853	8.53E-07	2.69E-05	5.4027183
cg01117697	chr12	49730211	+	C1QL4	1stExon	1.2009956	-1.931567	5.165805	8.54E-07	2.69E-05	5.4025128
cg15696460	chr12	131159658	-			-1.163507	0.2020281	-5.165819	8.54E-07	2.69E-05	5.40257
cg24996161	chr5	140735397	+	PCDHGA2;PCDHGA4;PCDHGA1;PCDHGB1;PCDHGA3;PCDHGA4	Body;1stExon;Body;Body;Body;1stExon	1.3184011	1.2236656	5.1676311	8.54E-07	2.69E-05	5.4034358
cg11674664	chr1	152158135	-			-0.808768	-3.535871	-5.165677	8.54E-07	2.69E-05	5.4019693
cg22800273	chr3	50655332	-	MAPKAPK3	Body	-0.868043	-2.262484	-5.165658	8.54E-07	2.69E-05	5.40189
cg19883813	chr10	8098005	-	GATA3;GATA3	Body;Body	1.5244293	-1.303017	5.1654992	8.55E-07	2.70E-05	5.401215
cg04006516	chr21	32007639	-	KRTAP20-2	1stExon	-1.416319	0.8998065	-5.165415	8.55E-07	2.70E-05	5.4008566
cg02613300	chr11	123776728	-	OR8D4	TSS1500	-1.355265	-0.295402	-5.165346	8.55E-07	2.70E-05	5.4005635
cg10243004	chr2	128616167	-	POLR2D	TSS1500	-1.809404	0.5190276	-5.167218	8.56E-07	2.70E-05	5.4016839
cg02314201	chr10	134843775	+			-1.141442	-0.439665	-5.165154	8.56E-07	2.70E-05	5.3997486
cg09945147	chr14	88625382	-			-1.27075	0.5475221	-5.165145	8.56E-07	2.70E-05	5.3997108
cg19737225	chr19	17392320	+	ANKLE1	TSS200	-0.889362	-1.492729	-5.165097	8.56E-07	2.70E-05	5.3995081

cg18501555	chr4	111554966	-	PITX2;PITX2	5'UTR;5'UTR	1.331011	-1.275701	5.1650694	8.56E-07	2.70E-05	5.3993907
cg20240931	chr19	2613894	+	GNG7	5'UTR	-0.543619	-3.43482	-5.164822	8.57E-07	2.70E-05	5.3983416
cg03874653	chr3	123380206	+	MYLK;MYLK;MYLK;MYLK	Body;Body;Body;Body	1.0129647	-0.438639	5.1647216	8.58E-07	2.70E-05	5.3979152
cg01157046	chr2	223419421	-	SGPP2	Body	0.9958519	0.7826743	5.164606	8.58E-07	2.70E-05	5.3974246
cg03373781	chr20	30582113	+	XKR7	Body	-0.888424	-0.739966	-5.164553	8.58E-07	2.70E-05	5.3971979
cg01810763	chr12	104683571	-	TXNRD1;TXNRD1;TXNRD1;TXNRD1;TXNRD1	5'UTR;5'UTR;Body;Body;5'UTR	-0.836069	-0.268189	-5.164481	8.59E-07	2.71E-05	5.3968943
cg14517119	chr5	45678610	-	HCN1	Body	-1.815517	0.3467044	-5.16448	8.59E-07	2.71E-05	5.3968911
cg06926146	chr1	170629502	-			1.1278017	-0.645946	5.1644572	8.59E-07	2.71E-05	5.3967929
cg09511477	chr7	99964511	-	PILRB;PILRB;PILRB	Body;Body;Body	-1.723087	1.0638668	-5.164109	8.60E-07	2.71E-05	5.3953168
cg05279172	chr17	7113697	+	DLG4;DLG4	Body;Body	-1.076711	0.1757378	-5.164074	8.60E-07	2.71E-05	5.3951666
cg25579729	chr5	15993627	-			-1.770634	0.856269	-5.163999	8.61E-07	2.71E-05	5.394847
cg07252363	chr6	33360697	+	KIFC1	Body	-0.799218	-1.117259	-5.16391	8.61E-07	2.71E-05	5.394471
cg21175685	chr3	99378032	+	COL8A1;MIR548G;COL8A1	5'UTR;Body;5'UTR	-1.475367	-0.112562	-5.163725	8.62E-07	2.71E-05	5.3936856
cg03820608	chr11	131547319	+	NTM	Body	-1.422225	-0.09358	-5.163653	8.62E-07	2.71E-05	5.3933802
cg01372939	chr16	5283418	-			-1.263257	-0.225551	-5.16356	8.62E-07	2.71E-05	5.3929874
cg23248452	chr5	9631117	+	TAS2R1	TSS1500	-1.209457	-0.67789	-5.163456	8.63E-07	2.72E-05	5.3925465
cg19411025	chr5	87968118	-	LOC645323;LOC645323;LOC645323	Body;Body;Body	1.2089432	-1.099648	5.1651747	8.63E-07	2.72E-05	5.3930229
cg10660965	chr19	53762839	+	VN1R2;VN1R2	3'UTR;1stExon	-1.510304	0.9774845	-5.163118	8.64E-07	2.72E-05	5.3911118
cg06996389	chr10	132546174	+			-1.816107	1.0317911	-5.162828	8.65E-07	2.72E-05	5.3898789
cg23353046	chr8	931125	+			-1.414866	0.7964928	-5.162812	8.65E-07	2.72E-05	5.3898146

cg25406138	chr4	147560770	-	POU4F2	Body	1.4874727	-1.589446	5.1627852	8.65E-07	2.72E-05	5.3896989
cg06607384	chr14	91579859	+	C14orf159;C14orf159;C14orf159;C14orf159;C14orf159	TSS1500;TSS1500;TSS1500;TSS1500;TSS1500	1.1593538	-0.820267	5.1627622	8.65E-07	2.72E-05	5.3896014
cg27022558	chr11	64141041	-			-1.381212	-0.175106	-5.166607	8.65E-07	2.72E-05	5.3921847
cg14377596	chr1	201709135	-	NAV1;NAV1;NAV1	1stExon;Body;5'UTR	-2.028615	-2.579016	-5.164642	8.65E-07	2.72E-05	5.3903519
cg14382719	chr9	124291856	+			1.0352332	0.81103	5.1625526	8.66E-07	2.73E-05	5.388712
cg07477282	chr15	44956107	-	SPG11;SPG11	TSS1500;TSS1500	-0.515616	-3.684631	-5.162507	8.66E-07	2.73E-05	5.3885182
:h.22.26442001I	chr22	28112001	+			-0.497481	-4.984108	-5.162163	8.68E-07	2.73E-05	5.3870598
cg25097946	chr10	97200985	-	SORBS1;SORBS1;SORBS1;SORBS1;SORBS1;SORBS1;SORBS1	5'UTR;TSS200;TSS200;TSS200;5'UTR;TSS200;TSS200	-1.516523	0.8543308	-5.162136	8.68E-07	2.73E-05	5.3869441
cg06602478	chr3	195159296	-	ACAP2	Body	-1.729477	1.4107121	-5.162083	8.68E-07	2.73E-05	5.3867199
cg08767044	chr16	11678785	-	LITAF;LITAF;LITAF;LITAF	5'UTR;5'UTR;Body;5'UTR	-1.675586	-1.084499	-5.162074	8.68E-07	2.73E-05	5.3866804
cg09975620	chr13	28498384	-	PDX1	Body	2.1931721	-1.669808	5.1640169	8.68E-07	2.73E-05	5.3881162
cg06815826	chr21	43725202	-			-0.856374	0.1268779	-5.162004	8.68E-07	2.73E-05	5.3863864
cg20808234	chr6	19692082	-			1.5848005	-1.918536	5.1619391	8.68E-07	2.73E-05	5.3861095
cg10180281	chr7	26897714	-	SKAP2	Body	1.1529795	-0.382375	5.1637491	8.69E-07	2.73E-05	5.3865659
cg11546137	chr2	176972563	+	HOXD11	1stExon	1.8483835	-0.717936	5.1617549	8.69E-07	2.73E-05	5.3853283
cg14215689	chr9	139334807	-	INPP5E;SEC16A	TSS1500;3'UTR	0.8187082	0.3643066	5.1614018	8.71E-07	2.74E-05	5.3838307
cg18073788	chr6	130992677	+			-1.322276	-0.21164	-5.16124	8.71E-07	2.74E-05	5.3831446
cg10134910	chr4	1076041	+	RNF212;RNF212	Body;Body	-0.707082	-1.434637	-5.161147	8.72E-07	2.74E-05	5.3827479
cg16300300	chr13	28501366	+			1.9522338	-1.577097	5.1649629	8.72E-07	2.74E-05	5.3843849
cg16099404	chr2	202336282	-	STRADB	Body	-1.155527	0.7675713	-5.161005	8.72E-07	2.74E-05	5.382149

cg05583676	chr10	132461246	-			-3.053759	2.2662442	-5.162866	8.72E-07	2.74E-05	5.382823
cg27576271	chr12	93096811	-	C12orf74	TSS200	-1.179758	0.1536925	-5.160786	8.73E-07	2.74E-05	5.3812181
cg17926940	chr14	97685060	-			1.9540495	-2.436647	5.1606135	8.74E-07	2.75E-05	5.3804868
cg25425089	chr10	131214209	-			-1.432127	0.5205254	-5.160584	8.74E-07	2.75E-05	5.3803608
cg24694665	chr15	37782428	+			-1.21655	-0.607807	-5.160475	8.74E-07	2.75E-05	5.3799015
cg27420946	chr15	32404311	+	CHRNA7	Body	-1.503154	1.230801	-5.160443	8.74E-07	2.75E-05	5.379765
cg27508281	chr16	87027322	+			-1.758792	1.592195	-5.160442	8.74E-07	2.75E-05	5.3797584
cg02505216	chr12	1943889	-	LRTM2;LRTM2;CACNA2D4;LRTM2	3'UTR;3'UTR;Body;3'UTR	-1.00865	0.477068	-5.1604	8.74E-07	2.75E-05	5.3795823
cg09160776	chr4	56659924	-			0.9503826	-0.286815	5.1602885	8.75E-07	2.75E-05	5.3791083
cg02341759	chr15	86685279	+	AGBL1;AGBL1	1stExon;5'UTR	-1.198542	-0.028463	-5.160205	8.75E-07	2.75E-05	5.3787532
cg09828575	chr12	132663675	-			-1.159611	-0.402586	-5.160032	8.76E-07	2.75E-05	5.3780187
cg07800544	chr5	35129387	+	PRLR	5'UTR	-1.707396	0.2075125	-5.159897	8.76E-07	2.75E-05	5.3774468
cg09562675	chr5	1245669	+	SLC6A18	Body	-1.107264	-0.545432	-5.159913	8.76E-07	2.75E-05	5.3775154
cg10906826	chr2	240985618	+	OR6B3	TSS200	-0.898393	-1.371993	-5.159894	8.76E-07	2.75E-05	5.3774342
cg03509079	chr16	26031138	-	HS3ST4	Body	-1.126622	-3.587198	-5.159824	8.77E-07	2.75E-05	5.3771402
cg13085627	chr15	101597169	-	LRRK1	Body	0.9675807	1.7019502	5.1597409	8.77E-07	2.75E-05	5.3767861
cg22131234	chr7	54609968	+	VSTM2A	TSS200	1.4513608	-0.980739	5.1597436	8.77E-07	2.75E-05	5.3767976
cg19562321	chr5	150727045	-	SLC36A2;SLC36A2	1stExon;5'UTR	-1.189062	-0.767916	-5.159608	8.78E-07	2.75E-05	5.3762221
cg11752894	chr1	205196012	+	TMCC2	TSS1500	-1.163394	-0.441146	-5.159567	8.78E-07	2.75E-05	5.3760485
cg13819319	chr14	104917395	+			-1.828831	2.0697001	-5.159465	8.78E-07	2.76E-05	5.3756161

cg08193369	chr13	32889315	-	BRCA2	TSS1500	-0.448117	-4.414795	-5.159303	8.79E-07	2.76E-05	5.3749274
cg05685892	chr6	30421091	-			1.1677047	1.21766	5.1592098	8.79E-07	2.76E-05	5.3745338
cg16004593	chr6	32369487	-	BTNL2	Body	-1.060057	-2.301002	-5.159054	8.80E-07	2.76E-05	5.3738718
cg07218880	chr13	115046279	-	UPF3A;UPF3A	TSS1500;TSS1500	-1.001365	-1.907467	-5.159015	8.80E-07	2.76E-05	5.3737095
cg13749670	chr9	137611646	+	COL5A1	Body	-0.853225	-0.218773	-5.159004	8.80E-07	2.76E-05	5.3736611
cg10136560	chr4	140737518	+	MAML3	Body	1.0682083	0.4848229	5.158953	8.80E-07	2.76E-05	5.3734451
cg06181508	chr3	147102840	-			0.8054108	-1.321722	5.158931	8.80E-07	2.76E-05	5.3733516
cg03280026	chr7	112430707	-	TMEM168	TSS1500	-0.666342	-2.29486	-5.15881	8.81E-07	2.76E-05	5.3728382
cg18321051	chr19	12251893	+	ZNF20	TSS1500	-0.942291	-2.25893	-5.158803	8.81E-07	2.76E-05	5.3728093
cg00533390	chr1	245028657	+	HNRNPU;HNRNPU	TSS1500;TSS1500	-1.183449	-0.732659	-5.158735	8.81E-07	2.76E-05	5.3725224
cg17604312	chr6	30850829	+	DDR1;DDR1	TSS1500;TSS1500	-0.64724	-0.149114	-5.160505	8.81E-07	2.76E-05	5.3732371
cg25490477	chr6	133078831	+	VNN2;VNN2	1stExon;Body	-1.359216	0.9953341	-5.158562	8.82E-07	2.76E-05	5.3717877
cg20968678	chr2	216948982	-	TMEM169;TMEM169;TMEM169;TMEM169	5'UTR;5'UTR;5'UTR;5'UTR	-1.063179	-0.369944	-5.158537	8.82E-07	2.76E-05	5.3716815
cg25257905	chr19	57335807	+	PEG3;ZIM2;PEG3;ZIM2;PEG3;PEG3;PEG3;ZIM2	5'UTR;5'UTR;5'UTR;5'UTR;1stExon;Body;Body;5'UTR	-1.95635	1.7125635	-5.158487	8.82E-07	2.77E-05	5.3714676
cg26403843	chr5	158634085	-	RNF145	Body	-1.427717	-2.202878	-5.158268	8.83E-07	2.77E-05	5.3705401
cg21669966	chr6	42224838	-	TRERF1	Body	0.8125472	2.0718358	5.158079	8.83E-07	2.77E-05	5.3697391
cg06792186	chr7	127637778	+	C7orf54;SND1	Body;Body	1.2134713	1.6853635	5.1579223	8.84E-07	2.77E-05	5.3690746
cg01372811	chr16	85970529	+			-0.946661	-0.096484	-5.157887	8.84E-07	2.77E-05	5.3689232
cg18430555	chr5	170740937	-			1.7039569	-0.891251	5.157879	8.84E-07	2.77E-05	5.3688912
cg10352418	chr16	66876938	-	CA7	TSS1500	-1.133446	0.8611255	-5.157762	8.85E-07	2.77E-05	5.3683935

cg16274890	chr3	127299325	-	TPRA1;TPRA1;TPRA1	5'UTR;5'UTR;TSS200	0.7423448	0.4674343	5.1576888	8.85E-07	2.77E-05	5.3680848
cg03164969	chr11	1855884	+	SYT8	Body	-0.724544	-0.291249	-5.157531	8.86E-07	2.78E-05	5.3674141
cg02843158	chr16	66575847	+	TK2	Body	0.8804001	2.3033071	5.1574559	8.86E-07	2.78E-05	5.3670975
cg08498647	chr11	118550644	+	TREH	TSS1500	-0.789296	-0.114319	-5.157434	8.86E-07	2.78E-05	5.3670063
cg11968804	chr1	212688447	+			0.9788737	0.061901	5.1574429	8.86E-07	2.78E-05	5.3670424
cg12852210	chr5	138898624	-			-0.702912	1.0648746	-5.157428	8.86E-07	2.78E-05	5.3669771
cg13644528	chr9	36163544	-	GLIPR2	3'UTR	0.9616509	0.1576269	5.1571273	8.87E-07	2.78E-05	5.3657042
cg15687300	chr8	62084457	+			-1.39901	0.1378975	-5.157082	8.87E-07	2.78E-05	5.3655128
cg25223771	chr8	145105503	-			2.0702321	-0.927345	5.156997	8.88E-07	2.78E-05	5.365152
cg14813485	chr2	196460621	+			-1.199199	0.8403368	-5.156896	8.88E-07	2.78E-05	5.364723
cg06565368	chr11	131907820	-	NTM;NTM;NTM;NTM	Body;Body;Body;Body	-1.414039	0.4980898	-5.158732	8.88E-07	2.78E-05	5.3653131
cg17732044	chr15	90234207	+	PEX11A;WDR93	TSS1500;5'UTR	-0.448743	-4.255535	-5.156787	8.89E-07	2.78E-05	5.3642603
cg25604731	chr2	595071	+			-1.140136	0.0583863	-5.158658	8.89E-07	2.78E-05	5.365415
cg03007522	chr8	11562685	-	GATA4	5'UTR	2.0029988	-0.981119	5.1566415	8.89E-07	2.78E-05	5.3636449
cg15797102	chr4	17158272	+			-1.602846	1.5126513	-5.156614	8.89E-07	2.78E-05	5.3635263
cg04525189	chr7	158936508	-	VIPR2	Body	1.4563917	-1.688966	5.1584045	8.90E-07	2.78E-05	5.3639246
cg18221168	chr10	50971607	+	OGDHL;OGDHL;OGDHL	TSS1500;TSS1500;TSS1500	-1.11857	0.2245169	-5.156511	8.90E-07	2.78E-05	5.363091
cg23405212	chr2	208989382	-	CRYGD	TSS200	1.7733128	-1.595868	5.1564487	8.90E-07	2.79E-05	5.3628275
cg07805981	chr12	56075321	+	METTL7B	TSS200	-0.844009	-2.45839	-5.156393	8.90E-07	2.79E-05	5.3625897
cg10472263	chr14	101452368	+	SNORD114-25;SNORD114-26	TSS200;TSS1500	-1.304344	0.2228832	-5.156362	8.90E-07	2.79E-05	5.3624588

cg17185622	chr8	98103016	-	PGCP	Body	-1.753652	1.0569958	-5.156278	8.91E-07	2.79E-05	5.3621039
cg20951444	chr1	33337112	-	FNDC5	TSS1500	-1.143189	0.1946648	-5.156154	8.91E-07	2.79E-05	5.361577
cg13696135	chr12	49396782	-	PRKAG1;PRKAG1	Body;Body	-0.797003	0.5124814	-5.156113	8.91E-07	2.79E-05	5.3614062
cg24928333	chr18	44790141	-			1.4417677	-1.326029	5.1560542	8.91E-07	2.79E-05	5.3611552
cg09669874	chr2	24836601	-	NCOA1;NCOA1;NCOA1	5'UTR;5'UTR;5'UTR	-1.035754	1.1358019	-5.155935	8.92E-07	2.79E-05	5.3606508
cg19122389	chr20	21687378	-	PAX1	Body	1.137148	-1.516093	5.1559244	8.92E-07	2.79E-05	5.3606052
cg02357046	chr4	1686926	+	FAM53A	TSS1500	-1.143633	0.5196708	-5.155871	8.92E-07	2.79E-05	5.3603797
cg14691921	chr3	12055127	-	SYN2;SYN2	Body;Body	-1.36588	0.7203035	-5.155326	8.94E-07	2.80E-05	5.3580683
cg03493668	chr11	77776607	-	THRSP	3'UTR	-0.752946	1.1629082	-5.155295	8.95E-07	2.80E-05	5.3579355
cg21137236	chr4	113333072	+	ALPK1;ALPK1	Body;Body	0.7784274	1.87148	5.1551762	8.95E-07	2.80E-05	5.3574338
cg06599543	chr6	165749446	+	PDE10A;PDE10A	Body;Body	-1.38021	1.2602683	-5.15506	8.95E-07	2.80E-05	5.3569402
cg05508585	chr10	118900001	-			1.3735981	-2.057109	5.155035	8.96E-07	2.80E-05	5.3568354
cg22115805	chr15	88804912	-			-1.320322	0.8438006	-5.155001	8.96E-07	2.80E-05	5.35669
cg15945235	chr10	90611604	+	ANKRD22;ANKRD22	1stExon;5'UTR	-0.9644	-2.042694	-5.154909	8.96E-07	2.80E-05	5.3562998
cg05482722	chr13	52598175	+	UTP14C;ALG11	TSS1500;Body	0.5652605	3.31086	5.1548799	8.96E-07	2.80E-05	5.3561783
cg09936218	chr7	38331871	+			-1.489439	0.3626759	-5.154869	8.96E-07	2.80E-05	5.3561316
cg11290225	chr8	145692591	+	KIFC2	Body	-0.733655	-3.153478	-5.154772	8.97E-07	2.80E-05	5.3557194
cg16655905	chr19	38755330	-	SPINT2;SPINT2;SPINT2;SPINT2	5'UTR;1stExon;5'UTR;1stExon	-0.818803	-6.067034	-5.156686	8.97E-07	2.80E-05	5.357063
cg18132256	chr22	24190340	+			-1.051174	1.9461693	-5.154717	8.97E-07	2.80E-05	5.3554887
cg24008234	chr15	40531443	-	PAK6;PAK6;PAK6	5'UTR;5'UTR;TSS1500	-0.693335	-5.424091	-5.154697	8.97E-07	2.80E-05	5.3554012

cg25691763	chr6	13874001	-			-0.658263	-3.315032	-5.15458	8.97E-07	2.80E-05	5.3549087
cg03120289	chr1	99470550	+	LPPR5;LPPR5	TSS200;TSS200	2.1763241	-1.844363	5.1545358	8.98E-07	2.80E-05	5.3547199
cg07986943	chr2	176932579	-			0.9881367	-1.223937	5.154367	8.98E-07	2.81E-05	5.3540044
cg00741609	chr10	102893925	+	TLX1	Body	1.2871198	-1.324597	5.1542151	8.99E-07	2.81E-05	5.3533608
cg00640053	chr7	155305911	+	CNPY1	5'UTR	-1.369328	-0.074024	-5.154196	8.99E-07	2.81E-05	5.3532791
cg00295794	chr13	100641409	+			1.9855206	-0.632964	5.1540301	9.00E-07	2.81E-05	5.3525766
cg09516210	chr1	2825189	+			-0.606862	-0.924874	-5.154062	8.99E-07	2.81E-05	5.3527117
cg23810463	chr11	127208447	-			-1.490751	-0.165604	-5.154044	8.99E-07	2.81E-05	5.3526356
cg26608174	chr17	46711035	-	MIR196A1	TSS1500	1.7336968	-0.788294	5.1537015	9.01E-07	2.81E-05	5.3511843
cg08314660	chr11	394199	-	PKP3	TSS200	-0.744385	0.0330062	-5.153553	9.01E-07	2.81E-05	5.3505537
cg02267483	chr5	140810404	-	PCDHGA4;PCDHGA11;PCDHGA11;PCDHGA9;PCDHGA1;PCDHGB1;PCDHGB6;PCDHGA12;PCDHGB3;PCDHGB7;PCDHGA6;PCDHGA8;PCDHGA10;PCDHGA12;PCDHGA5;PCDHGB4;PCDHGA	Body;Body;Body;Body;Body;Body;Body;1stExon;Body;Body;Body;Body;Body;1stExon;Body;Body;Body;Body;Body;Body;Body	1.0457742	-0.511707	5.1553676	9.02E-07	2.82E-05	5.3510663
cg19561972	chr12	130711426	+			-1.554951	0.5521313	-5.153396	9.02E-07	2.82E-05	5.3498886
cg25371919	chr2	63284481	-			1.7604986	-0.039107	5.1534077	9.02E-07	2.82E-05	5.3499394
cg27478707	chr6	32909781	+	HLA-DMB	TSS1500	-0.918363	1.6529769	-5.153377	9.02E-07	2.82E-05	5.3498091
cg14162629	chr7	2720394	+	AMZ1	5'UTR	-0.979416	0.5170343	-5.155153	9.03E-07	2.82E-05	5.3501583
cg01248426	chr8	87111155	-	ATP6V0D2;ATP6V0D2	5'UTR;1stExon	-1.214924	-0.322708	-5.153078	9.03E-07	2.82E-05	5.3485411
cg00470341	chr2	183237787	+	PDE1A;PDE1A	Body;Body	-1.609803	-0.300283	-5.153061	9.03E-07	2.82E-05	5.3484694
cg06127719	chr18	11150784	+			-1.334025	1.1083709	-5.152898	9.04E-07	2.82E-05	5.3477797
cg23583920	chr17	41923945	-	CD300LG;CD300LG;CD300LG;CD300LG	TSS1500;TSS1500;TSS1500;TSS1500	-0.787164	-3.089485	-5.152908	9.04E-07	2.82E-05	5.3478211
cg10326182	chr8	1340874	+			-1.255494	0.6557523	-5.152834	9.04E-07	2.82E-05	5.3475085

cg19494811	chr6	34356253	+	NUDT3	Body	0.9397826	1.6021479	5.1528357	9.04E-07	2.82E-05	5.3475155
cg20143982	chr12	7341252	+	PEX5;PEX5;PEX5;PEX5	TSS1500;TSS1500;TSS1500;TSS1500	-0.611221	-3.062233	-5.152672	9.05E-07	2.82E-05	5.3468213
cg11380072	chr3	81540527	-	GBE1	Body	0.9402629	0.8193415	5.1525905	9.05E-07	2.82E-05	5.3464768
cg23019889	chr1	146544044	+			1.1908356	0.5682502	5.1525889	9.05E-07	2.82E-05	5.3464699
cg12172441	chr6	28176163	-			1.8384665	-0.240688	5.1525628	9.05E-07	2.82E-05	5.3463592
cg08897422	chr7	8666902	+	NXPH1	Body	-1.490048	0.7154954	-5.1525	9.06E-07	2.82E-05	5.3460916
cg00001269	chr20	48959004	-			-1.75879	0.7392154	-5.152449	9.06E-07	2.83E-05	5.3458781
cg06774893	chr16	68011109	-	DPEP3;DPEP3	Body;Body	-0.677433	-1.055417	-5.152293	9.07E-07	2.83E-05	5.3452158
cg24696967	chr6	165747029	-	PDE10A;PDE10A	Body;Body	-1.548312	1.173842	-5.152271	9.07E-07	2.83E-05	5.3451238
cg17457014	chr1	55254401	+	TTC22;TTC22	Body;Body	0.8632466	0.9190667	5.154158	9.07E-07	2.83E-05	5.3459462
cg17471836	chr5	118691007	-	TNFAIP8;TNFAIP8	Body;TSS1500	-1.224907	-0.169019	-5.152163	9.07E-07	2.83E-05	5.3446652
cg23502089	chr6	1286762	-			-1.397119	1.2186012	-5.151933	9.08E-07	2.83E-05	5.3436895
cg06150772	chr17	77300674	-	HRNBP3	5'UTR	-1.334936	-0.523679	-5.151916	9.08E-07	2.83E-05	5.3436171
cg09188099	chr7	27245747	-			1.0869174	-1.028087	5.1516793	9.09E-07	2.83E-05	5.3426162
cg06135689	chr17	35085060	-			1.2699964	-0.554021	5.1516588	9.09E-07	2.83E-05	5.3425292
cg02627966	chr7	54899537	-			-1.382584	-0.377753	-5.151484	9.10E-07	2.84E-05	5.3417885
cg02675047	chr14	101393226	+	SNORD113-2	TSS1500	-1.445629	0.8648563	-5.151473	9.10E-07	2.84E-05	5.3417433
cg26800551	chr15	25459423	-	SNORD115-15;SNORD115-21;SNORD115-25	Body;Body;TSS1500	-1.40498	0.460827	-5.151337	9.10E-07	2.84E-05	5.3411661
cg16027403	chr1	247978791	+	OR14A16	1stExon	-1.930934	1.2676099	-5.151282	9.11E-07	2.84E-05	5.3409351
cg00508851	chr16	49783173	+	ZNF423	Body	-1.48001	1.2550909	-5.151235	9.11E-07	2.84E-05	5.3407328

cg18685594	chr6	134211217	+	TCF21;TCF21	Body;Body	1.1782895	-1.358895	5.1530932	9.11E-07	2.84E-05	5.341857
cg04307853	chr17	6021418	+	WSCD1	Body	-1.161709	0.287319	-5.151092	9.11E-07	2.84E-05	5.3401269
cg17343563	chr6	50803945	+	TFAP2B	Body	1.6069385	-0.793557	5.1510441	9.12E-07	2.84E-05	5.3399253
cg11583041	chr6	169740781	-			-1.367657	0.8258249	-5.151011	9.12E-07	2.84E-05	5.3397873
cg03804083	chr3	168891416	-	MECOM	Body	-1.519851	-0.253412	-5.15095	9.12E-07	2.84E-05	5.3395278
cg16459281	chr3	72621000	-			1.1262534	1.0665611	5.1508251	9.12E-07	2.84E-05	5.3389977
cg11421182	chr20	36153479	-	BLCAP;BLCAP;BLCAP;BLCAP;BLCAP	5'UTR;TSS1500;5'UTR;5'UTR;5'UTR	-1.04441	-1.207851	-5.150769	9.13E-07	2.84E-05	5.3387587
cg17940587	chr10	89292231	+	MINPP1	Body	1.4804788	-0.714867	5.1545925	9.13E-07	2.84E-05	5.3409299
cg21960265	chr6	32015635	+	TNXB	Body	-1.736113	1.4506622	-5.150751	9.13E-07	2.84E-05	5.3386822
cg23935420	chr11	65123609	-	TIGD3	Body	0.8185421	0.7511488	5.1506234	9.13E-07	2.84E-05	5.3381435
cg16975959	chr2	223184510	+			-0.259123	-3.184667	-5.150575	9.13E-07	2.84E-05	5.3379403
cg20910807	chr20	57875384	+	EDN3;EDN3;EDN3;EDN3	TSS200;TSS200;TSS200;TSS200	1.1214333	-1.119106	5.1505595	9.14E-07	2.84E-05	5.3378725
cg22225479	chr17	25681463	+			-0.87347	-1.991191	-5.150584	9.13E-07	2.84E-05	5.3379764
cg12640930	chr1	175051172	+	TNN	Body	-1.206921	0.8355877	-5.15025	9.15E-07	2.85E-05	5.3365604
cg17201760	chr4	206562	-	ZNF876P	Body	1.3738372	-0.953569	5.1501108	9.15E-07	2.85E-05	5.3359721
cg19874938	chr12	121131378	+	MLEC	Body	1.0176466	1.6384277	5.1500983	9.15E-07	2.85E-05	5.3359192
cg25319067	chr6	10383084	-			1.7832374	-0.25425	5.1500084	9.16E-07	2.85E-05	5.3355386
cg00061635	chr17	8311836	+			-0.904178	-0.671944	-5.149963	9.16E-07	2.85E-05	5.3353453
cg20736321	chr11	1029162	-	MUC6	Body	-1.435692	0.7297091	-5.149962	9.16E-07	2.85E-05	5.3353406
cg11848788	chr1	39041399	+			-1.740402	-0.19722	-5.149776	9.17E-07	2.85E-05	5.3345552

cg02526439	chr8	89053427	+	MMP16	3'UTR	-1.734115	0.5633008	-5.149712	9.17E-07	2.85E-05	5.3342811
cg11548303	chr8	23567350	+			1.3114465	-1.098435	5.1516283	9.17E-07	2.85E-05	5.335658
cg15159131	chr11	132107699	+	NTM;NTM;NTM;NTM	Body;Body;Body;Body	-1.529478	1.5058894	-5.149742	9.17E-07	2.85E-05	5.3344119
cg12532546	chr7	2062523	+	MAD1L1;MAD1L1;MAD1L1	Body;Body;Body	0.9039129	2.3023553	5.1495954	9.17E-07	2.85E-05	5.3337892
cg14116799	chr10	43800154	-			-0.747507	-3.076379	-5.149566	9.18E-07	2.85E-05	5.3336662
cg21442730	chr13	25754851	-			-1.439476	0.8317185	-5.14949	9.18E-07	2.85E-05	5.3333431
cg01371799	chr7	1264049	+			1.4161253	-1.229079	5.149439	9.18E-07	2.85E-05	5.3331266
cg12423553	chr21	47541643	-	COL6A2;COL6A2;COL6A2	Body;Body;Body	-1.511429	0.9593558	-5.151337	9.18E-07	2.85E-05	5.3344277
cg25484904	chr4	48988015	-	CWH43	TSS1500	1.0035008	-0.569041	5.1492395	9.19E-07	2.86E-05	5.3322818
cg19657945	chr8	144367392	+			-1.720316	0.7540121	-5.152887	9.20E-07	2.86E-05	5.3337224
cg09230996	chr10	102498419	-			1.3490769	-1.438538	5.1489533	9.20E-07	2.86E-05	5.3310698
cg24360034	chr11	124414205	-	OR8B12	TSS1500	-1.251326	-0.693931	-5.148865	9.20E-07	2.86E-05	5.3306959
cg05743346	chr7	71800987	+	CALN1;CALN1	5'UTR;Body	1.8896948	-1.938501	5.1487329	9.21E-07	2.86E-05	5.3301365
cg19378892	chr1	159283536	-	OR10J3	1stExon	-1.750801	1.0036334	-5.148632	9.21E-07	2.86E-05	5.3297089
cg14128411	chr2	1926907	+	MYT1L	Body	-1.562768	0.2646254	-5.148134	9.23E-07	2.87E-05	5.3276015
cg18335991	chr15	74724562	+	SEMA7A;SEMA7A;SEMA7A	Body;Body;5'UTR	-0.847606	-0.228052	-5.148084	9.24E-07	2.87E-05	5.3273895
cg05641656	chr19	58728610	-			0.7808144	-0.214468	5.1479897	9.24E-07	2.87E-05	5.3269892
cg03336334	chr15	29407987	-	APBA2;APBA2	Body;Body	1.6998901	-0.709648	5.147945	9.24E-07	2.87E-05	5.3268002
cg18625538	chr6	87832609	+			1.1897997	0.4862787	5.1479066	9.24E-07	2.87E-05	5.3266375
cg07266404	chr12	54447584	-	HOXC4;HOXC4	5'UTR;TSS200	1.9370684	-1.417532	5.1478852	9.24E-07	2.87E-05	5.326547

cg13858407	chr2	235935606	+	SH3BP4	5'UTR	-0.718061	0.0765757	-5.147849	9.25E-07	2.87E-05	5.3263922
cg07144328	chr5	1854953	-			-1.392514	-0.427603	-5.147831	9.25E-07	2.87E-05	5.3263152
cg25166437	chr17	1549284	+	SCARF1;SCARF1;SCARF1;SCARF1;SCARF1	TSS1500;TSS1500;TSS1500;TSS1500;TSS1500	0.9261563	-0.288629	5.1477623	9.25E-07	2.87E-05	5.3260264
cg06878361	chr19	52390810	-	ZNF577;ZNF577;ZNF577	Body;5'UTR;5'UTR	1.3267212	-0.303353	5.147739	9.25E-07	2.87E-05	5.3259276
cg09619146	chr10	125651034	+	CPXM2	1stExon	1.0075396	-0.118121	5.1476862	9.25E-07	2.87E-05	5.3257041
cg13125224	chr6	6587445	+	LY86;LOC285780	TSS1500;Body	-1.384364	0.8540776	-5.147691	9.25E-07	2.87E-05	5.3257259
cg08662757	chr11	67070967	-	SSH3;SSH3	5'UTR;1stExon	1.6254223	-2.810227	5.1475577	9.26E-07	2.87E-05	5.3251603
cg08877269	chr8	1370236	+			-1.425465	0.9171442	-5.149423	9.26E-07	2.88E-05	5.3263276
cg17076890	chr10	23481421	-	PTF1A	TSS200	1.166378	-0.894593	5.1474917	9.26E-07	2.88E-05	5.3248806
cg08825084	chr2	119602862	+	EN1	Body	1.0963298	-1.512473	5.1474556	9.26E-07	2.88E-05	5.3247279
cg12280901	chr6	28501816	-	GPX5;GPX5	Body;3'UTR	-1.099132	-0.632957	-5.149262	9.27E-07	2.88E-05	5.3252301
cg23647410	chr1	221051066	-			0.9525784	1.290425	5.1511946	9.27E-07	2.88E-05	5.326569
cg15605011	chr1	110162477	-	AMPD2;AMPD2;AMPD2	1stExon;TSS1500;5'UTR	-0.86666	-4.282696	-5.147196	9.27E-07	2.88E-05	5.323628
cg00254017	chr5	56119093	-	MAP3K1	Body	0.8388213	0.667137	5.1471034	9.28E-07	2.88E-05	5.3232366
cg07010373	chr14	101390989	+	SNORD113-1	TSS200	-1.20146	-0.083469	-5.147086	9.28E-07	2.88E-05	5.3231617
cg11416095	chr1	31627721	+			-0.800311	-5.448928	-5.148967	9.28E-07	2.88E-05	5.3243982
cg13135437	chr12	127209458	+			-1.572847	1.3537589	-5.147067	9.28E-07	2.88E-05	5.3230844
cg13731609	chr8	82753739	+	SNX16;SNX16;SNX16	5'UTR;5'UTR;5'UTR	-0.563933	-2.338102	-5.147039	9.28E-07	2.88E-05	5.3229633
cg00477017	chr3	196731150	-	MFI2	Body	-1.507128	0.2263688	-5.146911	9.28E-07	2.88E-05	5.322422
cg02304465	chr2	176931543	-			1.13044	0.4354887	5.1468974	9.29E-07	2.88E-05	5.3223645

cg17287235	chr10	131768149	+			1.4635762	-1.275802	5.1468879	9.29E-07	2.88E-05	5.3223241
cg21154627	chr3	170303190	-	SLC7A14	5'UTR	1.4039637	-1.633174	5.1468739	9.29E-07	2.88E-05	5.3222647
cg13483447	chr1	41849431	-			-1.529194	-0.105202	-5.146822	9.29E-07	2.88E-05	5.3220438
cg24183098	chr14	101296297	+	MEG3;MEG3;MEG3	Body;Body;Body	-1.071792	-0.051035	-5.146828	9.29E-07	2.88E-05	5.3220689
cg10587082	chr1	208293478	-	PLXNA2	Body	-0.906472	-1.18551	-5.146684	9.29E-07	2.88E-05	5.3214607
cg06176750	chr11	7112356	+	RBMXL2;RBMXL2	3'UTR;1stExon	-1.477816	-0.187297	-5.146599	9.30E-07	2.88E-05	5.3210997
cg07354728	chr11	537717	+	LRRC56	5'UTR	-0.587416	-2.399483	-5.148405	9.30E-07	2.88E-05	5.321605
cg14327393	chr9	75141633	+	TMC1	5'UTR	0.9475715	0.1693841	5.1464641	9.30E-07	2.88E-05	5.32053
cg10724576	chr8	144561662	+	ZC3H3	Body	0.6337898	2.226829	5.1463263	9.31E-07	2.89E-05	5.3199467
cg15823461	chr11	60699566	+	TMEM132A;TMEM132A	Body;Body	-1.100058	1.968084	-5.146247	9.31E-07	2.89E-05	5.3196096
cg07382435	chr1	50880864	-			1.4001861	-0.482845	5.1461625	9.32E-07	2.89E-05	5.3192531
cg26557693	chr7	27265159	-			1.070109	-0.500743	5.1479319	9.32E-07	2.89E-05	5.3200223
cg24454144	chr20	37353126	+	SLC32A1;SLC32A1	5'UTR;1stExon	2.8356396	-1.924763	5.1458145	9.33E-07	2.89E-05	5.3177798
cg05442408	chr1	2706173	+			1.3934978	-1.888985	5.145713	9.33E-07	2.89E-05	5.3173503
cg17892178	chr3	6902824	-	GRM7;GRM7;GRM7;GRM7	1stExon;5'UTR;5'UTR;1stExon	1.4159393	-1.100998	5.1455997	9.34E-07	2.89E-05	5.3168708
cg11691504	chr8	1448750	+	DLGAP2	TSS1500	-1.048918	0.3339204	-5.145568	9.34E-07	2.89E-05	5.3167364
cg10852684	chr10	43903588	-	HNRNPF;HNRNPF;HNRNPF;HNRNPF	5'UTR;TSS1500;5'UTR;5'UTR	-0.377909	-3.892581	-5.145364	9.35E-07	2.90E-05	5.3158725
cg16366073	chr21	32185576	+	KRTAP8-1	TSS200	-1.533025	0.9557925	-5.145302	9.35E-07	2.90E-05	5.3156097
cg17184165	chr3	53855929	+	CHDH	Body	-0.953743	-0.535846	-5.145269	9.35E-07	2.90E-05	5.3154698
cg17932055	chr8	144237110	-			-1.514661	0.862997	-5.145213	9.36E-07	2.90E-05	5.3152319

cg08604987	chr7	57269554	+			-1.14357	0.4469303	-5.145124	9.36E-07	2.90E-05	5.3148579
cg10245988	chr3	12046420	-	SYN2;SYN2	Body;Body	1.7420684	-0.97795	5.1449418	9.37E-07	2.90E-05	5.3140857
cg10451116	chr15	37350381	+	MEIS2;MEIS2;MEIS2;MEIS2;MEIS2;MEIS2;MEIS2	Body;Body;Body;Body;Body;Body;Body	-1.232505	0.2136216	-5.14495	9.37E-07	2.90E-05	5.3141217
cg12440549	chr13	28541477	+	CDX2	Body	1.6470802	-1.858854	5.1449126	9.37E-07	2.90E-05	5.313962
cg04938317	chr6	32015737	+	TNXB	Body	-1.705344	1.3825039	-5.144828	9.37E-07	2.90E-05	5.3136045
cg22193385	chr12	52638005	-	KRT7	Body	-1.055206	-2.778148	-5.14671	9.37E-07	2.90E-05	5.3144354
cg25504884	chr11	114842544	-			-1.549166	-0.097928	-5.144683	9.38E-07	2.90E-05	5.3129918
cg04317399	chr7	27170313	+	HOXA4	1stExon	1.0652403	-0.690382	5.1446329	9.38E-07	2.90E-05	5.3127785
cg17988187	chr2	74612222	-	LOC100189589	TSS1500	0.7698215	1.198803	5.1446008	9.38E-07	2.90E-05	5.3126422
cg02721182	chr6	28805254	+			-0.691776	-2.892607	-5.144341	9.39E-07	2.91E-05	5.311543
cg09964012	chr14	105036453	+			-1.15457	0.4947995	-5.144281	9.39E-07	2.91E-05	5.311288
cg18786171	chr10	75935758	+	ADK;ADK	Body;TSS1500	-0.645239	-1.01808	-5.14427	9.39E-07	2.91E-05	5.3112442
cg11089364	chr15	25480987	-	SNORD115-29;SNORD115-11;SNORD115-43;SNORD115-36	TSS1500;TSS1500;TSS1500;TSS1500	-1.760686	1.1596168	-5.146125	9.40E-07	2.91E-05	5.3119637
cg00697992	chr2	139537131	+	NXPH2	Body	1.7912283	-0.956994	5.1440852	9.40E-07	2.91E-05	5.3104603
cg10416294	chr15	28049119	-	OCA2	Body	-1.521713	0.8929047	-5.144086	9.40E-07	2.91E-05	5.3104635
cg16210461	chr19	22994561	-			-1.366474	0.053104	-5.143904	9.41E-07	2.91E-05	5.3096937
cg00855458	chr16	9845943	+			-1.168509	-0.352575	-5.143825	9.41E-07	2.91E-05	5.3093587
cg25751787	chr11	31847880	-			1.0743451	-1.478268	5.1437053	9.42E-07	2.91E-05	5.3088522
cg24612707	chr7	128094774	-	C7orf68;C7orf68	TSS1500;TSS1500	0.7831392	-0.338362	5.143682	9.42E-07	2.91E-05	5.308754
cg26210445	chr2	162273326	+	TBR1	1stExon	2.321149	-0.684409	5.1454383	9.42E-07	2.92E-05	5.3090603

cg07028950	chr8	144302512	+			-0.976006	-0.386947	-5.14351	9.43E-07	2.92E-05	5.3080277
cg03554962	chr16	86404423	-			-0.990469	0.0204067	-5.147297	9.43E-07	2.92E-05	5.3101036
cg13764516	chr9	139648911	+	LCN8	3'UTR	-0.952165	-0.282852	-5.143437	9.43E-07	2.92E-05	5.3077173
cg07221438	chr22	48607198	-			-1.210745	-0.10451	-5.143203	9.44E-07	2.92E-05	5.3067288
cg16429725	chr16	57798273	+	KIFC3;KIFC3;KIFC3	Body;Body;Body	-1.120576	-0.695122	-5.145063	9.44E-07	2.92E-05	5.3074728
cg18257574	chr2	43358343	-			-1.233747	-1.974986	-5.143025	9.45E-07	2.92E-05	5.3059722
cg07148968	chr10	26072483	-			-1.396955	0.0444954	-5.142993	9.45E-07	2.92E-05	5.3058384
cg14655316	chr5	138861855	+	TMEM173	5'UTR	1.1985006	-2.86001	5.1429427	9.45E-07	2.92E-05	5.3056251
cg20457051	chr6	47668044	-	GPR115	5'UTR	-1.523659	0.3170019	-5.144716	9.45E-07	2.92E-05	5.3064264
cg15287850	chr3	186739599	+	ST6GAL1;ST6GAL1;ST6GAL1	5'UTR;5'UTR;TSS200	-1.352087	0.9151187	-5.142793	9.46E-07	2.92E-05	5.304991
cg22061523	chr17	76183551	+	AFMID;TK1;AFMID;AFMID	Body;TSS1500;Body;Body	-0.766439	-2.716991	-5.142607	9.46E-07	2.93E-05	5.3042063
cg18217048	chr8	9742024	-			-0.833052	-0.639519	-5.142463	9.47E-07	2.93E-05	5.3035969
cg15583029	chr10	134446066	-	INPP5A	Body	0.6987689	0.4645576	5.1423672	9.47E-07	2.93E-05	5.3031901
cg25574024	chr11	2161894	-	IGF2AS;INS-IGF2;IGF2;IGF2;IGF2AS	Body;Body;5'UTR;5'UTR;Body	1.7625312	-1.294299	5.1423506	9.47E-07	2.93E-05	5.3031198
cg11685134	chr19	30011945	-			-1.11161	-0.53324	-5.142246	9.48E-07	2.93E-05	5.3026758
cg05172122	chr1	119526783	-	TBX15	5'UTR	0.9263615	0.0234344	5.1421132	9.48E-07	2.93E-05	5.3021151
cg10640333	chr2	63280625	-	OTX1	Body	1.4230091	0.7180743	5.1420626	9.49E-07	2.93E-05	5.301901
cg23479586	chr15	52860292	-	ARPP19	Body	-0.511352	-3.794043	-5.142073	9.49E-07	2.93E-05	5.3019452
cg05200057	chr5	139172852	-			-1.398818	0.9490435	-5.142014	9.49E-07	2.93E-05	5.3016976
cg26963271	chr1	66259081	+	PDE4B;PDE4B	5'UTR;5'UTR	2.3267028	-2.222169	5.141911	9.49E-07	2.93E-05	5.3012595

cg05297677	chr10	2359853	+			-1.007437	-0.495727	-5.141822	9.50E-07	2.93E-05	5.3008849
cg26251101	chr2	46768944	-	RHOQ	TSS1500	-0.529574	-3.665616	-5.141765	9.50E-07	2.93E-05	5.3006432
cg14146966	chr2	61757674	-	XPO1	Body	0.9525263	0.6790677	5.1417097	9.50E-07	2.93E-05	5.3004078
cg21545390	chr7	96627190	+	DLX6AS	Body	1.5260715	-0.984934	5.1415318	9.51E-07	2.94E-05	5.2996553
cg10344081	chr4	76555777	+	CDKL2	TSS200	-1.238755	-4.966573	-5.14143	9.51E-07	2.94E-05	5.2992233
cg11515873	chr6	32117401	-	PRRT1	Body	1.6109436	5.3296352	5.1511403	9.52E-07	2.94E-05	5.3244514
cg04098866	chr16	51190201	-			2.1148259	-2.261428	5.1432472	9.52E-07	2.94E-05	5.3002163
cg10828284	chr22	50528333	+	MOV10L1;MOV10L1;MOV10L1	TSS200;TSS200;TSS1500	1.3740252	1.2305537	5.1412845	9.52E-07	2.94E-05	5.2986092
cg26371512	chr7	112430779	-	TMEM168	TSS1500	-0.946792	-3.667036	-5.141264	9.52E-07	2.94E-05	5.2985243
cg18271789	chr7	4832112	-			0.8334012	0.6109858	5.141192	9.52E-07	2.94E-05	5.2982176
cg01749491	chr2	176948693	+	EVX2	TSS200	2.2211671	-0.437979	5.1411452	9.53E-07	2.94E-05	5.2980197
cg26452056	chr10	131770909	+			1.3064254	-1.561779	5.1410783	9.53E-07	2.94E-05	5.2977367
cg15636792	chr21	47552628	+	COL6A2	3'UTR	-1.124518	0.8711427	-5.141019	9.53E-07	2.94E-05	5.2974845
cg18164611	chr1	46958628	-			1.3421252	-0.488504	5.1410005	9.53E-07	2.94E-05	5.2974075
cg04567731	chr17	59532519	-	TBX4	TSS1500	1.384408	-0.929723	5.1408304	9.54E-07	2.94E-05	5.2966878
cg10229453	chr12	68142120	+			-1.511348	0.8974008	-5.140731	9.54E-07	2.94E-05	5.2962686
cg00877938	chr4	8692828	-			-0.895918	-0.50882	-5.140687	9.54E-07	2.94E-05	5.2960805
cg13946792	chr19	57337648	+	PEG3;PEG3;PEG3;ZIM2;PEG3;PEG3;ZIM2;ZIM2	TSS1500;5'UTR;5'UTR;5'UTR;5'UTR;5'UTR;5'UTR;5'UTR	-1.784356	0.823629	-5.142579	9.54E-07	2.94E-05	5.2969729
cg15460816	chr12	25150402	-	C12orf77	TSS200	-1.360206	0.9979239	-5.140583	9.55E-07	2.95E-05	5.2956417
cg15929797	chr5	172660796	+	NKX2-5;NKX2-5;NKX2-5	Body;Body;Body	1.9654911	0.3209497	5.1405992	9.55E-07	2.95E-05	5.2957096

cg21595175	chr5	180670701	-	SNORD95;GNB2L1	TSS1500;1stExon	-0.829494	-5.8711	-5.140462	9.55E-07	2.95E-05	5.29513
cg17454086	chr1	155903354	+	KIAA0907	Body	-0.950534	1.275831	-5.144243	9.56E-07	2.95E-05	5.2972075
cg03178489	chr11	56511248	+	OR9G4	1stExon	-1.435989	0.4334532	-5.140304	9.56E-07	2.95E-05	5.2944605
cg03155345	chr4	11642410	+			-0.802072	-0.064213	-5.140226	9.56E-07	2.95E-05	5.2941316
cg17555806	chr10	74448117	+			-1.164932	-0.68123	-5.142052	9.57E-07	2.95E-05	5.295165
cg04513411	chr14	95046934	+	SERPINA5	TSS1500	-1.461349	0.8801238	-5.140143	9.57E-07	2.95E-05	5.2937783
cg05043461	chr5	140735027	+	PCDHGA2;PCDHGA4;PCDHGA1;PCDHGB1;PCDHGA3;PCDHGA4	Body;1stExon;Body;Body;Body;1stExon	0.9527591	-1.227576	5.1400622	9.57E-07	2.95E-05	5.2934381
cg18021161	chr15	76440448	-	C15orf27	Body	-1.270632	-4.297121	-5.14179	9.58E-07	2.95E-05	5.294057
cg19487738	chr5	87785433	-			1.2095496	0.9618495	5.1397866	9.58E-07	2.95E-05	5.2922723
cg18964082	chr17	32895602	+			-1.096741	-0.436033	-5.139523	9.59E-07	2.96E-05	5.291157
cg01104549	chr1	201479959	-			-1.020739	2.4454409	-5.139468	9.60E-07	2.96E-05	5.2909237
cg04003871	chr2	241290895	-			1.1450086	1.0375711	5.1413037	9.60E-07	2.96E-05	5.2920034
cg17463745	chr1	16554402	+			1.5413047	-0.049032	5.1393792	9.60E-07	2.96E-05	5.290549
ch.17.1457304R	chr17	55195361	+	AKAP1	Body	-0.4759	-4.609096	-5.139233	9.61E-07	2.96E-05	5.2899301
cg07495574	chr22	49564611	+			-1.234669	0.3505968	-5.139213	9.61E-07	2.96E-05	5.2898454
cg20919922	chr2	75185588	-	POLE4	TSS200	-0.886724	-5.021493	-5.140992	9.61E-07	2.96E-05	5.3101503
cg13726814	chr2	120230682	-	SCTR	Body	-1.573966	1.2124546	-5.138964	9.62E-07	2.96E-05	5.2887934
cg03103944	chr7	1601985	-			0.7633465	0.6521101	5.1405869	9.63E-07	2.97E-05	5.2885557
cg11630148	chr10	134405380	+	INPP5A	Body	-1.136651	0.7243895	-5.138565	9.64E-07	2.97E-05	5.2871053
cg10343364	chr16	3559568	+	CLUAP1;CLUAP1	Body;TSS1500	-0.989551	0.0710415	-5.138404	9.64E-07	2.97E-05	5.2864229

cg03107310	chr10	86094757	+	FAM190B	5'UTR	0.8820989	1.1324942	5.1383671	9.64E-07	2.97E-05	5.2862684
cg00030588	chr16	87100811	+			-1.003746	-0.383711	-5.138258	9.65E-07	2.97E-05	5.2858055
cg05196835	chr4	38005666	+	TBC1D1	Body	-1.108206	1.365048	-5.138215	9.65E-07	2.97E-05	5.2856253
cg06353345	chr11	5322976	-	OR51B4	1stExon	-1.400166	0.3129458	-5.138222	9.65E-07	2.97E-05	5.285655
cg26051165	chr7	120742600	+	C7orf58;C7orf58	Body;Body	-1.179447	0.5786804	-5.138104	9.65E-07	2.97E-05	5.2851542
cg05630943	chr16	76521159	-	CNTNAP4;CNTNAP4	Body;Body	-1.100913	0.2537273	-5.13806	9.66E-07	2.97E-05	5.2849696
cg22576950	chr15	68522787	+	CLN6	TSS1500	-0.957455	0.073823	-5.137934	9.66E-07	2.97E-05	5.2844382
cg02524983	chr3	188012918	+	LPP;LPP;LPP	5'UTR;5'UTR;5'UTR	-1.638311	-2.040626	-5.139552	9.67E-07	2.98E-05	5.2841854
cg24147428	chr11	65409760	-	SIPA1;SIPA1	Body;Body	1.3285936	0.8947019	5.1376463	9.67E-07	2.98E-05	5.2832201
cg05138681	chr9	98782756	-	NCRNA00092	Body	0.7131605	1.0065852	5.1375144	9.68E-07	2.98E-05	5.2826623
cg19445758	chr6	167632873	-			-1.012713	-0.339658	-5.137471	9.68E-07	2.98E-05	5.2824798
cg27653595	chr8	1010174	-			-1.250681	0.6586181	-5.137395	9.69E-07	2.98E-05	5.2821572
cg11382394	chr1	2564504	-	MMEL1	TSS200	-1.357683	0.5867297	-5.137354	9.69E-07	2.98E-05	5.2819848
cg19015654	chr11	62008949	+	SCGB1D2	TSS1500	-1.065231	0.1160639	-5.137315	9.69E-07	2.98E-05	5.28182
cg13792444	chr9	119245443	+	ASTN2;ASTN2;ASTN2;ASTN2	Body;Body;Body;Body	0.8942356	0.3070689	5.1371683	9.70E-07	2.98E-05	5.2811988
cg03702686	chr6	33244172	-	B3GALT4;RPS18	TSS1500;Body	-1.067687	0.0387354	-5.137144	9.70E-07	2.98E-05	5.2810976
cg18264210	chr2	465195	-			-1.288841	1.1487448	-5.137098	9.70E-07	2.98E-05	5.2809037
cg20203768	chr8	142631398	+			-0.829576	-0.993102	-5.137039	9.70E-07	2.98E-05	5.2806507
cg19131272	chr13	106457767	-			-1.36828	0.1400892	-5.13694	9.70E-07	2.99E-05	5.2802324
cg20537629	chr7	79083944	+	MAGI2	TSS1500	0.9514519	-0.870479	5.1368919	9.71E-07	2.99E-05	5.2800301

cg10899768	chr8	102506635	-	GRHL2	Body	-1.160111	-1.56973	-5.136827	9.71E-07	2.99E-05	5.2797565
cg13767245	chr7	75831678	-	SRRM3	5'UTR	-0.672065	-1.683066	-5.138707	9.71E-07	2.99E-05	5.2810318
cg08520451	chr19	996720	-			-0.66083	-3.382636	-5.136717	9.71E-07	2.99E-05	5.2792927
cg04086239	chr16	24067174	-	PRKCB;PRKCB	Body;Body	-1.373161	0.0836363	-5.136671	9.72E-07	2.99E-05	5.2790951
cg15432711	chr11	63952213	+	STIP1	TSS1500	-0.863854	-0.927968	-5.136623	9.72E-07	2.99E-05	5.278895
cg00040103	chr2	46040236	+	PRKCE	Body	0.9636399	1.5318737	5.1363121	9.73E-07	2.99E-05	5.2775788
cg06478823	chr16	20774873	-	ACSM3;ACSM3	TSS1500;TSS1500	-1.059328	-2.187982	-5.136147	9.74E-07	2.99E-05	5.2768815
cg24746938	chr2	225543274	+			-1.173111	-0.246504	-5.135977	9.75E-07	3.00E-05	5.276161
cg10143067	chr10	118892423	-	VAX1	Body	1.9251938	-2.299051	5.1355851	9.76E-07	3.00E-05	5.274505
cg11518938	chr1	159138217	+			-1.427243	-0.436435	-5.135553	9.76E-07	3.00E-05	5.2743699
cg23588121	chr6	100905038	-	SIM1	Body	1.0065646	0.3865259	5.1355408	9.77E-07	3.00E-05	5.2743178
cg05673079	chr10	110084660	+			-1.225006	-0.266039	-5.135503	9.77E-07	3.00E-05	5.2741595
cg12844277	chr14	47544244	-	MDGA2;MDGA2	Body;Body	-1.638418	0.569518	-5.135436	9.77E-07	3.00E-05	5.2738729
cg21163617	chr7	157384658	-	PTPRN2;PTPRN2;PTPRN2	Body;Body;Body	-1.643814	0.1851288	-5.135447	9.77E-07	3.00E-05	5.2739221
cg09487575	chr16	47882395	-			-1.522955	1.0613099	-5.135364	9.77E-07	3.00E-05	5.2735697
cg06624731	chr7	1889793	-	MAD1L1;MAD1L1;MAD1L1	Body;Body;Body	1.189395	4.2519151	5.1353251	9.77E-07	3.00E-05	5.2734061
cg23667749	chr1	110631389	+			-0.856587	-1.873108	-5.137113	9.78E-07	3.00E-05	5.2742985
cg04071689	chr5	166354050	-			-2.146457	3.4001051	-5.135199	9.78E-07	3.00E-05	5.2728732
cg17200768	chr13	28503373	-			2.4603007	-2.371747	5.1351559	9.78E-07	3.00E-05	5.2726905
cg09941712	chr15	81068966	+			-1.37085	-3.720774	-5.135093	9.78E-07	3.01E-05	5.272424

cg18275316	chr5	3607063	+			0.6904154	-0.078667	5.134995	9.79E-07	3.01E-05	5.2720105
cg22778788	chr10	101294300	-	NKX2-3	Body	0.8783231	-0.025159	5.1349906	9.79E-07	3.01E-05	5.2719919
cg04897713	chr8	11832762	+	DEFB136	TSS1500	-0.614308	-1.304031	-5.134774	9.80E-07	3.01E-05	5.2710773
cg12880658	chr5	115152386	-	CDO1;CDO1	1stExon;5'UTR	1.6410753	-1.74127	5.1345958	9.81E-07	3.01E-05	5.270323
cg07228702	chr10	18385632	+			-1.032696	-0.205647	-5.134495	9.81E-07	3.01E-05	5.2698969
cg16651768	chr1	222340714	+			-1.581169	-0.477691	-5.134389	9.82E-07	3.01E-05	5.2694498
cg20547407	chr7	155255369	-	EN2	Body	1.061547	-0.417415	5.1343765	9.82E-07	3.01E-05	5.2693958
cg11578532	chr1	161008127	+	TSTD1;TSTD1;TSTD1	Body;Body;Body	-1.095864	-1.446932	-5.136172	9.82E-07	3.01E-05	5.2898276
cg04498110	chr3	39306129	-	CX3CR1;CX3CR1;CX3CR1;CX3CR1	3'UTR;3'UTR;3'UTR;3'UTR	-1.249236	0.7187575	-5.134266	9.82E-07	3.01E-05	5.2689277
cg17209188	chr7	23387396	+	IGF2BP3	Body	-1.335336	-0.733137	-5.134225	9.82E-07	3.01E-05	5.2687568
cg27258272	chr11	57424773	-	CLP1;CLP1	TSS1500;TSS1500	-0.820311	-3.079897	-5.134159	9.83E-07	3.02E-05	5.2684752
cg03138124	chr2	68290812	+	C1D;C1D	TSS1500;TSS1500	-1.713643	0.6576997	-5.134069	9.83E-07	3.02E-05	5.2680953
cg07163547	chr1	165413323	-	RXRG;RXRG	Body;Body	1.4682127	-1.108381	5.1338919	9.84E-07	3.02E-05	5.2673477
cg23493412	chr17	63491688	+			-1.146552	-1.511527	-5.137697	9.84E-07	3.02E-05	5.2695838
cg05774978	chr1	146649842	+	PDIA3P	Body	1.2118097	-0.09091	5.133782	9.84E-07	3.02E-05	5.2668832
cg17118948	chr17	37350252	-	CACNB1;CACNB1;CACNB1	Body;Body;Body	0.5826863	1.6109356	5.1336833	9.85E-07	3.02E-05	5.2664657
cg17833169	chr1	67600547	-			1.5918798	-0.789394	5.1355817	9.85E-07	3.02E-05	5.2674148
cg24432464	chr11	58085823	-			-1.232967	-0.361286	-5.135598	9.84E-07	3.02E-05	5.2674852
cg05603527	chr2	71131629	-	VAX2	Body	1.1283791	0.2649921	5.1336066	9.85E-07	3.02E-05	5.2661415
cg17619823	chr8	37823475	+	ADRB3	1stExon	2.0453646	-2.103407	5.1392199	9.86E-07	3.02E-05	5.2687202

cg20321319	chr8	61088307	-			-1.229424	-1.321836	-5.133402	9.86E-07	3.02E-05	5.2652787
cg12752325	chr11	2137875	+			-0.87953	-0.341909	-5.133367	9.86E-07	3.02E-05	5.2651302
cg01337931	chr22	32651693	+	SLC5A4	TSS1500	-1.003546	0.7162225	-5.133186	9.87E-07	3.03E-05	5.2643626
cg09621472	chr3	113439314	-	NAA50	3'UTR	0.8174366	2.3697335	5.1331881	9.87E-07	3.03E-05	5.2643728
cg11051429	chr11	58207266	+	OR5B12	1stExon	-1.53029	0.9141642	-5.1332	9.87E-07	3.03E-05	5.2644223
cg21687740	chr1	157164556	-			1.3769946	1.8739382	5.1350611	9.87E-07	3.03E-05	5.2656361
cg22025210	chr5	116356275	+			-1.407506	1.1093285	-5.133081	9.87E-07	3.03E-05	5.2639187
cg02024466	chr11	6277275	-			-1.417386	0.7434304	-5.133047	9.87E-07	3.03E-05	5.2637767
cg25007422	chr14	97211894	-			-1.103468	-0.175887	-5.133023	9.88E-07	3.03E-05	5.263676
cg23939875	chr11	8832272	-	ST5;ST5;ST5	5'UTR;5'UTR;TSS200	-1.266979	-1.690062	-5.132915	9.88E-07	3.03E-05	5.2632168
cg03132806	chr5	178414179	-	GRM6	Body	-1.153649	0.8575993	-5.132836	9.88E-07	3.03E-05	5.2628831
cg04591032	chr17	46827458	+			1.9431024	-1.406938	5.1327369	9.89E-07	3.03E-05	5.2624661
cg15381162	chr14	23517736	+	CDH24;CDH24	Body;Body	0.5433254	2.4823816	5.1323822	9.90E-07	3.03E-05	5.2609673
cg15451729	chr11	364805	+			-1.783367	1.7050831	-5.132387	9.90E-07	3.03E-05	5.2609871
cg13863617	chr12	8186708	+	FOXJ2	5'UTR	0.9071182	1.2037337	5.1322539	9.91E-07	3.04E-05	5.260425
cg23925201	chr2	130001008	+			-1.01357	-0.521473	-5.132236	9.91E-07	3.04E-05	5.2603512
cg11545457	chr4	53724684	+			-0.930442	0.6055571	-5.132166	9.91E-07	3.04E-05	5.2600515
cg07872300	chr8	1553395	-	DLGAP2	Body	-1.057198	0.3940812	-5.132046	9.92E-07	3.04E-05	5.2595454
cg14042653	chr9	8454918	-	PTPRD;PTPRD;PTPRD;PTPRD;PTPRD;PTPRD	Body;Body;Body;Body;Body;Body	-1.575148	1.6201292	-5.133837	9.92E-07	3.04E-05	5.2604677
cg15131808	chr5	528580	+			3.6577912	-2.348098	5.1337768	9.92E-07	3.04E-05	5.279732

cg11005831	chr5	15928441	+	FBXL7	Body	-2.360746	1.7673015	-5.131857	9.93E-07	3.04E-05	5.2587461
cg10070788	chr17	76220955	+	BIRC5;EPR1;BIRC5;BIRC5	3'UTR;TSS200;3'UTR;3'UTR	1.1417993	1.8829026	5.1317413	9.93E-07	3.04E-05	5.2582589
cg20512711	chr11	31841524	+			2.0463182	-1.250645	5.1373179	9.94E-07	3.04E-05	5.2781077
cg04627863	chr2	234741670	+			-1.229977	-0.369069	-5.131391	9.95E-07	3.05E-05	5.2567776
cg02132714	chr17	46656690	+	HOXB4	TSS1500	1.7215454	0.0353397	5.1313118	9.95E-07	3.05E-05	5.2564438
cg04778012	chr7	99775508	+	STAG3;GPC2	TSS200;TSS1500	1.34548	0.2165661	5.1313122	9.95E-07	3.05E-05	5.2564458
cg10343742	chr3	137483506	-	SOX14	TSS200	0.8290645	-1.036094	5.1329244	9.96E-07	3.05E-05	5.2566163
cg02352370	chr6	32054845	-	TNXB	Body	-0.915192	-1.303265	-5.130979	9.97E-07	3.05E-05	5.2550379
cg04455639	chr17	74944779	+	MGAT5B;MGAT5B	Body;Body	-1.567508	0.4502455	-5.130977	9.97E-07	3.05E-05	5.2550299
cg13870520	chr4	77131705	-	SCARB2	Body	1.0231884	0.23902	5.1309138	9.97E-07	3.05E-05	5.2547622
cg01483824	chr19	48945886	+	GRIN2D	Body	1.5509432	3.6667429	5.1308188	9.97E-07	3.05E-05	5.2543611
cg07203767	chr2	242973721	-			-1.204719	0.318841	-5.13067	9.98E-07	3.05E-05	5.2537339
cg13387826	chr1	2005260	+	PRKCZ;PRKCZ;PRKCZ	1stExon;Body;5'UTR	0.9664674	1.807129	5.132499	9.98E-07	3.05E-05	5.2548207
cg09607276	chr11	637491	+	DRD4	1stExon	1.9524637	-1.133346	5.1305043	9.99E-07	3.06E-05	5.2530323
cg03943826	chr15	72612556	+	BRUNOL6	TSS200	-0.477023	-3.622715	-5.130398	9.99E-07	3.06E-05	5.2525838
cg13629783	chr17	5689617	-			-0.893122	-0.35818	-5.130279	1.00E-06	3.06E-05	5.2520809
cg10065209	chr1	1267559	-	TAS1R3	Body	-2.259707	1.59409	-5.13019	1.00E-06	3.06E-05	5.2517036
cg12149795	chr21	47882121	-	DIP2A;DIP2A;DIP2A;DIP2A;DIP2A;DIP2A;DIP2A	Body;Body;Body;Body;Body;Body;Body	1.200669	0.4319349	5.130187	1.00E-06	3.06E-05	5.2516919
cg21462732	chr6	136871881	-	MAP7	TSS200	-0.524338	-3.345326	-5.130214	1.00E-06	3.06E-05	5.2518073
cg05729745	chr22	49937644	-			-1.038003	-0.487514	-5.130039	1.00E-06	3.06E-05	5.2510657

cg05383657	chr12	34757822	-			-0.875538	-0.347658	-5.130013	1.00E-06	3.06E-05	5.250958
cg09775263	chr6	10884094	-			1.138238	-0.395514	5.129976	1.00E-06	3.06E-05	5.2508001
cg03730389	chr18	55315731	-	ATP8B1	Body	0.6552207	2.6038284	5.1298464	1.00E-06	3.06E-05	5.2502527
cg15089077	chr1	55246867	-	TTC22	3'UTR	0.7224561	0.0173886	5.1297131	1.00E-06	3.06E-05	5.2496898
cg05244952	chr10	108927230	-			-1.444836	0.9206879	-5.129691	1.00E-06	3.06E-05	5.2495971
cg00107488	chr22	19930437	-	COMT;TXNRD2	5'UTR;TSS1500	-1.067428	-2.353449	-5.129618	1.00E-06	3.06E-05	5.2492896
cg27088787	chr15	101839929	+			0.6839682	2.4689063	5.129555	1.00E-06	3.07E-05	5.2490217
cg21127068	chr3	147124417	-	ZIC4;ZIC4	TSS1500;TSS200	1.85957	-1.635845	5.1295278	1.00E-06	3.07E-05	5.2489068
cg03938558	chr1	181381335	+			-1.046101	-0.887574	-5.129405	1.00E-06	3.07E-05	5.248387
cg16265906	chr5	115152326	+	CDO1;CDO1	1stExon;5'UTR	1.4455973	-1.810765	5.1293461	1.00E-06	3.07E-05	5.2481393
cg24929264	chr17	34325375	-	CCL15;CCL14-CCL15;CCL14-CCL15	Body;Body;Body	-0.924027	-0.631801	-5.129183	1.00E-06	3.07E-05	5.2474495
cg14416371	chr11	43602847	-	MIR129-2	TSS200	3.1047706	-1.555114	5.1310007	1.00E-06	3.07E-05	5.2484975
cg03105556	chr2	80329553	-	CTNNA2;CTNNA2	Body;Body	-1.327875	-0.445441	-5.129104	1.00E-06	3.07E-05	5.2471163
cg14958635	chr5	134870977	-	NEUROG1	1stExon	1.3110558	-0.976675	5.1290044	1.01E-06	3.07E-05	5.246696
cg00188704	chr7	27140797	-	HOXA2	Body	1.2234961	0.615381	5.1288991	1.01E-06	3.07E-05	5.2462512
cg25223055	chr19	40928736	-	SERTAD1	3'UTR	0.5698862	0.1443418	5.1288564	1.01E-06	3.07E-05	5.2460709
cg07854132	chr12	29650795	-	OVCH1	TSS200	-1.696672	1.2390283	-5.130692	1.01E-06	3.07E-05	5.2467739
cg13919285	chr20	57876125	+	EDN3;EDN3;EDN3;EDN3	Body;Body;Body;Body	0.9999557	-0.708455	5.1287979	1.01E-06	3.07E-05	5.2458235
cg04724177	chr16	12554036	+	SNX29	Body	-1.119008	3.9999484	-5.128601	1.01E-06	3.08E-05	5.2449929
cg05891292	chr7	53103310	-	POM121L12	TSS200	-2.583188	3.0791322	-5.130481	1.01E-06	3.08E-05	5.2458843

cg22660578	chr17	35294029	-	LHX1	TSS1500	2.1052636	-1.027515	5.1286111	1.01E-06	3.08E-05	5.2450345
cg06883126	chr5	140457585	-			1.3018915	-0.091732	5.1285354	1.01E-06	3.08E-05	5.2447148
cg19159365	chr1	67218505	-	TCTEX1D1	5'UTR	1.2063578	-0.722087	5.1285346	1.01E-06	3.08E-05	5.2447116
cg05863502	chr9	140771990	-	CACNA1B	TSS1500	1.5481584	-0.406426	5.1284289	1.01E-06	3.08E-05	5.2442651
cg16518348	chr8	1281696	+			-1.171606	0.0662754	-5.130166	1.01E-06	3.08E-05	5.2445546
cg04961101	chr20	37303484	+			1.3027058	-0.716206	5.1281221	1.01E-06	3.08E-05	5.2429692
cg12115882	chr11	133449267	-			-1.069497	0.5016316	-5.128129	1.01E-06	3.08E-05	5.2429997
cg01421252	chr16	5590677	+			-1.258969	-0.790024	-5.127926	1.01E-06	3.08E-05	5.2421396
cg20159490	chr6	66493769	-			-1.360125	0.0862398	-5.127925	1.01E-06	3.08E-05	5.2421357
cg05218035	chr15	25449617	+			-0.893436	-0.10466	-5.127858	1.01E-06	3.08E-05	5.2418528
cg06236573	chr10	100007832	+	LOXL4	3'UTR	0.5207612	1.8851944	5.1278519	1.01E-06	3.08E-05	5.2418279
cg05235171	chr11	117958104	-	TMPRSS4;TMPRSS4	Body;Body	0.6155276	0.3414912	5.1277757	1.01E-06	3.08E-05	5.2415063
cg26998537	chr14	57277366	+	OTX2	TSS200	1.3380503	-0.226835	5.1277534	1.01E-06	3.08E-05	5.2414122
cg12669271	chr8	118987299	+	EXT1	Body	1.0429825	1.5335481	5.1277172	1.01E-06	3.08E-05	5.241259
cg20099806	chr17	61849988	-	CCDC47	5'UTR	-0.928837	-1.978529	-5.127663	1.01E-06	3.09E-05	5.2410309
cg08459169	chr2	130760544	-			-0.911043	0.0862693	-5.127543	1.01E-06	3.09E-05	5.2405252
cg19516647	chr1	2458256	+	PANK4	TSS1500	-1.441748	-4.704139	-5.127513	1.01E-06	3.09E-05	5.240398
cg22943986	chr7	27142700	+	HOXA2	TSS1500	2.2111784	-1.149485	5.1275048	1.01E-06	3.09E-05	5.2403621
cg00416130	chr1	35257924	-	GJA4	TSS1500	-0.983397	0.0191248	-5.127232	1.01E-06	3.09E-05	5.2392116
cg09370016	chr1	100193837	-	FRRS1	Body	0.7217318	2.5233637	5.1271042	1.01E-06	3.09E-05	5.2386705

cg17650274	chr20	25064127	+	VSX1;VSX1	TSS1500;TSS1500	1.2419552	-0.581355	5.127104	1.01E-06	3.09E-05	5.2386697
cg21473782	chr17	79099819	+	MIR657;AATK;MIR338	TSS1500;Body;TSS200	-1.37368	0.6065002	-5.127111	1.01E-06	3.09E-05	5.2387011
cg25025966	chr2	159581121	+			-0.786876	-0.78838	-5.128942	1.01E-06	3.09E-05	5.2398095
cg04176246	chr17	55434640	+	MSI2;MSI2	Body;Body	-0.914687	-0.745698	-5.126924	1.01E-06	3.09E-05	5.2379084
cg06164660	chr12	128751582	+	TMEM132C	TSS1500	2.4453579	-1.984466	5.1325968	1.01E-06	3.09E-05	5.2412552
cg02185007	chr8	117587078	-			-1.504213	-0.677133	-5.126829	1.02E-06	3.09E-05	5.2375084
cg24336547	chr20	1539702	+	SIRPD	TSS1500	-1.638864	0.7118432	-5.126803	1.02E-06	3.09E-05	5.2373997
cg00722081	chr10	61147668	-			-1.221077	-0.339056	-5.126771	1.02E-06	3.09E-05	5.2372626
cg11234328	chr10	135043586	-	UTF1	TSS200	0.884937	-0.938031	5.1267434	1.02E-06	3.09E-05	5.2371467
cg19224201	chr2	119615015	-			1.5403576	-1.057276	5.1285954	1.02E-06	3.09E-05	5.2379284
cg01232121	chr8	58177661	+			-1.597763	-0.429194	-5.126546	1.02E-06	3.10E-05	5.2363133
cg14091978	chr1	34652610	-	C1orf94;C1orf94	Body;5'UTR	-1.194887	-0.260196	-5.126471	1.02E-06	3.10E-05	5.235996
cg07367144	chr7	151833529	-	MLL3	3'UTR	0.559829	3.2233425	5.1264191	1.02E-06	3.10E-05	5.2357774
cg08807414	chr16	85608505	-			0.8559099	0.7736956	5.1263639	1.02E-06	3.10E-05	5.2355443
cg23189692	chr3	184050393	-	EIF4G1;EIF4G1;EIF4G1;EIF4G1;EIF4G1	Body;Body;Body;Body;Body	1.291263	1.5641776	5.1263007	1.02E-06	3.10E-05	5.2352772
cg05168580	chr14	103572194	+	C14orf73	Body	-1.157402	-1.383306	-5.126274	1.02E-06	3.10E-05	5.2351647
cg10865119	chr6	170190112	-	C6orf122;C6orf208	Body;TSS200	-0.413462	-3.465502	-5.126258	1.02E-06	3.10E-05	5.2350974
cg23058673	chr7	5422234	-	TNRC18	Body	0.492625	1.5793745	5.1261063	1.02E-06	3.10E-05	5.2344566
cg24693704	chr1	16068067	-	TMEM82	TSS1500	-0.444019	-3.350484	-5.12605	1.02E-06	3.10E-05	5.2342183
cg04498349	chr16	10274317	+	GRIN2A;GRIN2A;GRIN2A	5'UTR;5'UTR;5'UTR	0.9449204	-0.647001	5.1278574	1.02E-06	3.10E-05	5.234815

cg16548961	chr5	176928448	+			-0.781158	-1.064134	-5.125933	1.02E-06	3.10E-05	5.2337257
cg04436207	chr3	147136463	-			0.9999271	-0.312793	5.1258462	1.02E-06	3.10E-05	5.2333583
cg10966830	chr4	115280987	+			-0.825828	-0.506029	-5.125842	1.02E-06	3.10E-05	5.2333398
cg24371425	chr1	11561497	+	PTCHD2	Body	0.9905421	0.6012412	5.1315878	1.02E-06	3.10E-05	5.2561114
cg26972272	chr14	57283582	+	OTX2OS1	Body	1.2959139	-1.631956	5.1258718	1.02E-06	3.10E-05	5.2334664
cg05240350	chr10	72221844	+			-0.970983	-1.318653	-5.125803	1.02E-06	3.10E-05	5.2331746
cg22405653	chr21	32128800	-	KRTAP21-1	TSS1500	-1.151629	-0.500999	-5.125798	1.02E-06	3.10E-05	5.2331552
cg14563954	chr3	147141286	+			1.1186852	-0.515108	5.1255953	1.02E-06	3.11E-05	5.2322987
cg03413097	chr7	156811422	+			1.8073858	-1.294783	5.1255461	1.02E-06	3.11E-05	5.232091
cg13406339	chr8	811003	+			-0.635378	-0.841599	-5.129335	1.02E-06	3.11E-05	5.2339129
cg08762472	chr5	1891789	-			-1.030673	-0.573352	-5.125436	1.02E-06	3.11E-05	5.2316261
cg12277416	chr19	2859153	+			0.6445289	0.6855145	5.1254141	1.02E-06	3.11E-05	5.2315338
cg00134787	chr17	10419499	-	MYH1	Body	-1.498783	0.5293621	-5.125267	1.02E-06	3.11E-05	5.2309147
cg10994564	chr3	119306022	-	ADPRH	Body	-1.24472	-0.099523	-5.125281	1.02E-06	3.11E-05	5.2309711
cg07934434	chr10	87898692	-	GRID1	Body	-1.214217	0.4121247	-5.125108	1.02E-06	3.11E-05	5.2302428
cg04724319	chr17	1009219	-	ABR;ABR;ABR	Body;Body;Body	0.9749227	0.6175288	5.1250523	1.02E-06	3.11E-05	5.2300061
cg17079556	chr7	57252146	+			-1.32369	0.0575891	-5.125009	1.02E-06	3.11E-05	5.2298241
cg23208513	chr8	101118377	-	RGS22	TSS200	1.6727779	-1.478873	5.1249657	1.02E-06	3.11E-05	5.2296408
cg22708902	chr8	23100981	-	CHMP7	TSS200	-1.231049	-1.491446	-5.12491	1.02E-06	3.11E-05	5.2294047
cg02922689	chr3	67047651	-	KBTBD8	TSS1500	-1.27774	1.2810788	-5.124833	1.02E-06	3.11E-05	5.2290786

cg03760919	chr8	93103361	-	RUNX1T1	5'UTR	-1.145855	-0.599124	-5.124726	1.02E-06	3.12E-05	5.228629
cg07379055	chr19	12305553	-			1.4681178	0.0359387	5.1247295	1.02E-06	3.12E-05	5.2286435
cg21821214	chr2	176969223	-			1.5383196	-1.040321	5.1246876	1.02E-06	3.12E-05	5.2284667
cg05767404	chr1	247712591	+	C1orf150	Body	-1.451319	-0.583221	-5.124423	1.03E-06	3.12E-05	5.2273511
cg10988673	chr15	74423898	-	ISLR2;ISLR2;ISLR2;ISLR2	5'UTR;5'UTR;5'UTR;5'UTR	-1.165253	-2.632307	-5.124389	1.03E-06	3.12E-05	5.227207
cg26256104	chr20	57765815	+	ZNF831	TSS1500	-1.508933	0.3350912	-5.124378	1.03E-06	3.12E-05	5.2271616
cg13551505	chr12	2014983	-	CACNA2D4	Body	-1.00074	-0.680716	-5.124331	1.03E-06	3.12E-05	5.2269597
cg21548021	chr8	15873055	+			-1.2997	0.3797217	-5.124301	1.03E-06	3.12E-05	5.2268358
cg26799209	chr8	72754953	-	MSC	Body	1.84906	-1.403693	5.1243106	1.03E-06	3.12E-05	5.2268752
cg22969079	chr11	75046350	-	ARRB1;MIR326;ARRB1	Body;TSS200;Body	0.7872047	1.8047015	5.1242725	1.03E-06	3.12E-05	5.2267142
cg23455517	chr19	49315679	+	BCAT2;BCAT2;BCAT2	TSS1500;TSS1500;TSS1500	-0.951682	-4.203241	-5.124193	1.03E-06	3.12E-05	5.2263783
cg02262553	chr4	87849250	-			-1.086379	-1.006941	-5.126051	1.03E-06	3.12E-05	5.2276169
cg26446499	chr5	77253555	+			1.0678224	0.1791279	5.1240992	1.03E-06	3.12E-05	5.2259824
cg08377819	chr6	28956268	+			2.2041342	-1.046499	5.1276615	1.03E-06	3.12E-05	5.2268599
cg21342910	chr17	48042779	+			2.0971706	-1.567678	5.1237641	1.03E-06	3.13E-05	5.2245682
cg11523799	chr6	43479817	-	C6orf154;YIPF3	TSS1500;3'UTR	0.7515254	1.3937198	5.1236208	1.03E-06	3.13E-05	5.2239634
cg20469625	chr8	106582085	-	ZFPM2	Body	-1.695174	0.7442442	-5.123605	1.03E-06	3.13E-05	5.223897
cg03537591	chr10	72727859	+			-1.165147	0.4274046	-5.1235	1.03E-06	3.13E-05	5.2234541
cg05579037	chr7	27184853	-			1.1330597	0.7546897	5.1234979	1.03E-06	3.13E-05	5.2234444
cg10831324	chr15	79386035	+			-1.179811	0.2263684	-5.123527	1.03E-06	3.13E-05	5.2235679

cg18004701	chr7	157481628	-	PTPRN2;PTPRN2;PTPRN2	Body;Body;Body	1.7807973	-1.382604	5.1234504	1.03E-06	3.13E-05	5.2232439
cg07149785	chr11	69455028	-	CCND1	TSS1500	-0.596656	-3.925208	-5.123267	1.03E-06	3.13E-05	5.2224691
cg14744050	chr19	2280748	-	C19orf35	Body	-0.840898	-0.193483	-5.12314	1.03E-06	3.13E-05	5.2219333
cg01571928	chr22	49639725	+			-1.104324	-0.513645	-5.122989	1.03E-06	3.13E-05	5.2212966
cg01964852	chr7	27146262	-	HOXA3;HOXA3;HOXA3	3'UTR;3'UTR;3'UTR	1.1946998	0.1348377	5.1267087	1.03E-06	3.14E-05	5.240372
cg13476854	chr1	119549263	+			2.0274107	-1.956241	5.1227236	1.03E-06	3.14E-05	5.2201763
cg02646225	chr8	86020307	-	LRRCC1;LRRCC1	Body;Body	-1.006254	-1.157047	-5.122469	1.03E-06	3.14E-05	5.2191009
cg01256139	chr2	161228737	+	RBMS1;RBMS1	Body;Body	1.060563	0.4817289	5.1223518	1.04E-06	3.14E-05	5.2186071
cg13925692	chr3	350789	+	CHL1	5'UTR	1.2269814	-0.144017	5.124102	1.04E-06	3.14E-05	5.2189776
cg03605164	chr4	44447076	-	KCTD8	Body	-1.566481	1.3066684	-5.122145	1.04E-06	3.15E-05	5.2177333
cg04363624	chr1	47697965	+			2.0660115	-1.296627	5.1258495	1.04E-06	3.15E-05	5.2196457
cg17985646	chr7	35293759	+	TBX20;TBX20	TSS200;TSS200	2.6366342	-0.743137	5.1220654	1.04E-06	3.15E-05	5.2173981
cg08284310	chr12	2864481	-			-1.629935	1.1569014	-5.122001	1.04E-06	3.15E-05	5.2171265
cg19926444	chr19	12624742	+	ZNF709	TSS200	1.5729431	0.1205971	5.121993	1.04E-06	3.15E-05	5.2170929
cg03213396	chr19	53760449	-	VN1R2	TSS1500	-1.321188	0.2731223	-5.121953	1.04E-06	3.15E-05	5.2169235
cg06676637	chr18	56940333	-	RAX	1stExon	1.0773746	-0.91407	5.1219623	1.04E-06	3.15E-05	5.216963
cg09281805	chr7	4751840	+	FOXK1	Body	-2.414947	-0.783776	-5.121827	1.04E-06	3.15E-05	5.216394
cg01470356	chr4	41746614	+	PHOX2B	3'UTR	1.0221686	0.6332064	5.1216969	1.04E-06	3.15E-05	5.215843
cg14273116	chr4	107897	+	ZNF718	Body	1.0897598	-1.178528	5.1216959	1.04E-06	3.15E-05	5.2158388
cg06099971	chr15	42386816	-	PLA2G4D	TSS200	-0.865583	-0.759829	-5.121635	1.04E-06	3.15E-05	5.2155817

cg25950278	chr12	49388412	+			1.2138671	-0.316869	5.1235118	1.04E-06	3.15E-05	5.2164894
cg02583589	chr3	19791456	+			-0.945835	-0.555899	-5.121573	1.04E-06	3.15E-05	5.2153182
cg07048608	chr1	158581040	+	SPTA1	3'UTR	-1.731788	0.6051431	-5.121525	1.04E-06	3.15E-05	5.2151177
cg05725404	chr16	58534157	-	NDRG4;NDRG4;NDRG4	Body;Body;Body	-0.988021	-0.881173	-5.121427	1.04E-06	3.15E-05	5.214706
cg13711885	chr3	113821393	-			-1.35181	0.8310769	-5.121326	1.04E-06	3.15E-05	5.2142773
cg22010052	chr5	115298704	+	LVRN	1stExon	1.2003339	-0.452369	5.1213167	1.04E-06	3.15E-05	5.2142389
cg18512262	chr11	6049729	-	OR56A1	TSS1500	-1.470527	-0.214073	-5.12127	1.04E-06	3.15E-05	5.2140425
cg15645674	chr8	143333674	+	TSNARE1	Body	-1.180415	0.3010808	-5.121229	1.04E-06	3.15E-05	5.2138696
cg26727372	chr18	48404491	+	ME2;ME2	TSS1500;TSS1500	-0.902887	-2.170149	-5.121239	1.04E-06	3.15E-05	5.2139109
cg04523868	chr11	64563983	+	MAP4K2	Body	0.7445376	0.8669422	5.1210995	1.04E-06	3.16E-05	5.213322
cg14164044	chr15	89952135	-			1.8903221	-1.902365	5.1229419	1.04E-06	3.16E-05	5.2341038
cg17726105	chr11	122023188	-	MIR100;LOC399959	TSS200;Body	-1.827844	0.5606615	-5.121035	1.04E-06	3.16E-05	5.2130484
cg23087108	chr1	1120617	-	TTLL10;TTLL10	3'UTR;Body	-2.09877	3.5274682	-5.12673	1.04E-06	3.16E-05	5.233211
cg14166284	chr1	221055719	-	HLX	Body	0.913911	1.0891634	5.1209525	1.04E-06	3.16E-05	5.2127018
cg11701312	chr19	58897497	+	RPS5	TSS1500	-0.704672	-0.963738	-5.120862	1.04E-06	3.16E-05	5.212319
cg16214267	chr5	66299592	+	MAST4;MAST4	Body;Body	-0.595561	-3.331509	-5.120353	1.04E-06	3.16E-05	5.2101725
cg23667759	chr11	134632869	-			-0.807782	-0.217046	-5.12018	1.05E-06	3.17E-05	5.2094416
cg14772301	chr3	193589937	+			-1.751372	0.938533	-5.120138	1.05E-06	3.17E-05	5.209264
cg05741102	chr8	143636744	+			-0.915337	-0.868139	-5.11988	1.05E-06	3.17E-05	5.2081749
cg11269295	chr15	29489548	-	FAM189A1	Body	-1.31518	1.3340988	-5.119653	1.05E-06	3.17E-05	5.2072206

cg16959747	chr12	7276714	-	RBP5	3'UTR	0.7527165	0.8403629	5.119664	1.05E-06	3.17E-05	5.2072652
cg10005224	chr12	54424964	+	HOXC4;HOXC5	5'UTR;Body	1.3633688	-1.02076	5.1196177	1.05E-06	3.17E-05	5.2070701
cg27559747	chr1	27916108	+	AHDC1	5'UTR	0.4410153	0.9194543	5.1195575	1.05E-06	3.17E-05	5.2068159
cg10406538	chr17	6024454	-	WSCD1	3'UTR	-1.495345	1.0386837	-5.119531	1.05E-06	3.17E-05	5.206705
cg05794927	chr1	248059252	+	OR2W3	1stExon	-1.389102	0.676046	-5.119388	1.05E-06	3.18E-05	5.2061007
cg04634493	chr10	80828642	+	LOC283050;LOC283050;LOC283050;ZMIZ1	TSS1500;TSS1500;TSS1500;TSS200	0.9178442	-2.523116	5.1192774	1.05E-06	3.18E-05	5.2056345
cg18223625	chr20	61955800	-	COL20A1	Body	-1.417632	1.5057205	-5.121097	1.05E-06	3.18E-05	5.2067301
cg07811255	chr10	7149062	+			-1.309547	0.2421959	-5.119111	1.05E-06	3.18E-05	5.2049336
cg24432768	chr1	158149974	-	CD1D	5'UTR	-1.527418	-0.658612	-5.119117	1.05E-06	3.18E-05	5.2049571
cg26461267	chr4	155662795	+			1.2501841	-0.328084	5.1191138	1.05E-06	3.18E-05	5.2049441
cg09580755	chr1	76761200	+	ST6GALNAC3;ST6GALNAC3	Body;Body	-1.192955	-0.142181	-5.120864	1.05E-06	3.18E-05	5.2057493
cg24504927	chr12	85667353	+			2.9501509	-1.621613	5.1227747	1.05E-06	3.18E-05	5.2266486
cg02485642	chr16	832958	+	MSLNL	TSS200	-0.991347	0.5257776	-5.118872	1.05E-06	3.18E-05	5.2039234
cg14011368	chr14	96341538	+			-1.287657	0.5320031	-5.118771	1.05E-06	3.18E-05	5.2034977
cg09602288	chr6	10382800	-			1.7829112	-0.556847	5.1186208	1.05E-06	3.18E-05	5.2028644
cg16873414	chr22	44568699	-	PARVG;PARVG	TSS200;TSS200	-1.150035	-0.32424	-5.118584	1.05E-06	3.18E-05	5.2027094
cg25913233	chr5	151066683	-	SPARC	TSS200	1.1881745	-1.868834	5.1204345	1.05E-06	3.18E-05	5.2039402
cg21997672	chr7	123221351	-			-0.971075	-1.585874	-5.118457	1.05E-06	3.19E-05	5.2021716
cg02903275	chr14	67653661	+			-0.962829	-1.480765	-5.118144	1.05E-06	3.19E-05	5.2008539
cg07793807	chr1	226823363	+	ITPKB	Body	-1.181525	-0.277213	-5.117905	1.06E-06	3.19E-05	5.1998435

cg10704680	chr11	2064747	-			-0.936137	-0.400005	-5.117883	1.06E-06	3.19E-05	5.1997503
cg10180052	chr16	2018558	+	RNF151	Body	0.6597903	1.1490459	5.1196523	1.06E-06	3.19E-05	5.2002223
cg09977703	chr19	46854563	+	PPP5C	Body	1.1475762	-0.581564	5.1176572	1.06E-06	3.20E-05	5.1987999
cg26423322	chr2	33905480	-			-1.400755	-0.288169	-5.117641	1.06E-06	3.20E-05	5.1987307
cg02658730	chr1	98510182	+			0.8910109	0.0909697	5.1175975	1.06E-06	3.20E-05	5.1985482
cg24847625	chr15	40660063	+	DISP2	Body	-0.7251	-1.029506	-5.117609	1.06E-06	3.20E-05	5.1985969
cg00579805	chr1	185822829	+	HMCN1	Body	-1.596645	0.7378041	-5.117478	1.06E-06	3.20E-05	5.1980443
cg04180086	chr5	1878499	-	IRX4	Body	1.5275503	-1.217721	5.1174948	1.06E-06	3.20E-05	5.198115
cg09395969	chr16	67918001	-	EDC4;NRN1L	Body;TSS1500	0.4183393	2.3889795	5.1174823	1.06E-06	3.20E-05	5.1980619
cg20406033	chr20	48103474	+			-0.94923	-1.435284	-5.117411	1.06E-06	3.20E-05	5.1977624
cg05513274	chr12	114212759	-			-1.406575	0.0311192	-5.117383	1.06E-06	3.20E-05	5.1976422
cg12492380	chr7	98872169	+	MYH16	Body	-1.465454	0.2048278	-5.117355	1.06E-06	3.20E-05	5.1975247
cg11479165	chr15	65068594	-	RBPMS2	TSS1500	0.8719527	-0.996193	5.1172339	1.06E-06	3.20E-05	5.1970144
cg05770240	chr11	124502244	+	TBRG1;TBRG1	3'UTR;Body	0.5586586	2.7102779	5.1172158	1.06E-06	3.20E-05	5.1969379
cg24456130	chr10	131768029	+			1.9233193	-2.650918	5.1170989	1.06E-06	3.20E-05	5.1964451
cg26332903	chr8	1132189	+			-0.939124	0.2824754	-5.117083	1.06E-06	3.20E-05	5.1963793
cg17400812	chr7	41854825	-			-1.610261	0.5415692	-5.117067	1.06E-06	3.20E-05	5.1963096
cg04714110	chr10	21799143	-			0.7987204	0.2662644	5.1168795	1.06E-06	3.20E-05	5.1955199
cg10580513	chr1	248111283	+	OR2L8;OR2L13	TSS1500;5'UTR	-1.220647	-0.265727	-5.118746	1.06E-06	3.20E-05	5.2164521
cg12095594	chr1	223306857	+	TLR5	5'UTR	-0.742584	-2.436402	-5.116829	1.06E-06	3.20E-05	5.1953077

cg02291365	chr13	25317267	+			-1.370159	-0.743161	-5.116745	1.06E-06	3.21E-05	5.194951
cg05075545	chr19	4183450	-	ANKRD24;ANKRD24;SIRT6	1stExon;5'UTR;TSS1500	-1.321811	0.3010278	-5.116698	1.06E-06	3.21E-05	5.194756
cg17812123	chr15	101696922	+			-1.851609	1.55188	-5.11662	1.06E-06	3.21E-05	5.1944242
cg21454362	chr7	2609875	-	IQCE;IQCE	Body;Body	1.0546108	0.8527341	5.116489	1.06E-06	3.21E-05	5.193873
cg08480826	chr5	87981253	+	LOC645323	TSS1500	2.1438593	-1.202944	5.1180584	1.06E-06	3.21E-05	5.1939286
cg26391674	chr20	3148934	+	ProSAPiP1;ProSAPiP1	1stExon;5'UTR	-0.79216	-1.831587	-5.116136	1.06E-06	3.21E-05	5.1923862
cg25369015	chr1	9599256	+	SLC25A33	TSS1500	-0.852043	-5.132727	-5.116073	1.06E-06	3.21E-05	5.1921198
cg05162306	chr14	95826734	-			1.3806393	0.0536338	5.1160531	1.06E-06	3.21E-05	5.1920348
cg11416384	chr4	37246827	+	KIAA1239	1stExon	2.6427716	-1.928563	5.1197889	1.06E-06	3.21E-05	5.2141021
cg15417641	chr3	53700141	-	CACNA1D;CACNA1D;CACNA1D	Body;Body;Body	0.87102	1.724443	5.1159846	1.06E-06	3.21E-05	5.191746
cg21549497	chr21	44592641	-	CRYAA	3'UTR	-0.883511	0.786311	-5.115957	1.07E-06	3.21E-05	5.191629
cg15852891	chr5	76934946	-	OTP	TSS1500	1.5908851	-2.302169	5.1158687	1.07E-06	3.22E-05	5.1912569
cg05529816	chr17	4802735	-	CHRNE;C17orf107	Body;TSS1500	-1.915048	-1.985057	-5.119606	1.07E-06	3.22E-05	5.1933579
cg18729001	chr12	45443808	+	DBX2	Body	1.3876093	0.1958564	5.1158071	1.07E-06	3.22E-05	5.1909971
cg14020914	chr19	53798641	+			-1.666148	0.8226816	-5.115737	1.07E-06	3.22E-05	5.1907018
cg14538785	chr9	112112130	+			-1.106232	-0.479436	-5.11574	1.07E-06	3.22E-05	5.1907164
cg26385993	chr17	80788710	+	TBCD;ZNF750	Body;Body	0.7691957	3.8551206	5.1156072	1.07E-06	3.22E-05	5.1901546
cg20939830	chr1	2814879	-			-1.25237	0.6735447	-5.115484	1.07E-06	3.22E-05	5.1896343
cg27051315	chr2	238864959	+			1.3992782	-0.743237	5.1153224	1.07E-06	3.22E-05	5.1889536
cg03957547	chr16	9524433	-			-1.564991	1.5558419	-5.117102	1.07E-06	3.22E-05	5.1894766

cg03619332	chr16	585859	-	SOLH	5'UTR	-0.966593	0.3177188	-5.115142	1.07E-06	3.22E-05	5.188191
cg18468394	chr5	170736021	-	TLX3	TSS1500	2.0251768	-2.620441	5.1150563	1.07E-06	3.22E-05	5.1878316
cg06819298	chr11	2445216	-	TRPM5	TSS1500	-0.945982	0.2257507	-5.114999	1.07E-06	3.23E-05	5.1875915
cg12486498	chr1	230975106	-	C1orf198;C1orf198;C1orf198	3'UTR;3'UTR;3'UTR	1.0363763	2.5771406	5.1149618	1.07E-06	3.23E-05	5.1874331
cg00848461	chr5	176732093	+	PRELID1;RAB24;RAB24	Body;TSS1500;TSS1500	-0.930239	0.0288517	-5.114806	1.07E-06	3.23E-05	5.1867746
cg05634376	chr11	66659993	+	PC;PC;PC	5'UTR;5'UTR;5'UTR	-1.471638	0.6889769	-5.116657	1.07E-06	3.23E-05	5.1876014
cg14317639	chr14	92721505	-			1.2311438	-0.693965	5.1146323	1.07E-06	3.23E-05	5.1860436
cg10145533	chr8	20350779	+			-1.392629	0.3673941	-5.114603	1.07E-06	3.23E-05	5.1859217
cg06546806	chr2	237078704	+			2.361008	-1.581922	5.1143986	1.07E-06	3.23E-05	5.1850582
cg07456815	chr8	56106825	-	XKR4	Body	-1.086151	-0.242832	-5.114393	1.07E-06	3.23E-05	5.1850342
cg07189394	chr3	131802515	+			-1.603075	0.6221699	-5.114355	1.07E-06	3.23E-05	5.1848758
cg15982130	chr2	236300720	+			0.686521	1.8193346	5.1143573	1.07E-06	3.23E-05	5.1848844
cg08944961	chr12	111537195	-	CUX2	Body	-1.392317	-0.541125	-5.114171	1.07E-06	3.23E-05	5.1840983
cg03462380	chr15	27112902	-	GABRA5;GABRA5	5'UTR;5'UTR	1.2438011	-0.844456	5.1141434	1.07E-06	3.23E-05	5.1839825
cg09426383	chr7	26897182	+	SKAP2	Body	1.6969731	-0.354558	5.1140898	1.07E-06	3.24E-05	5.1837564
cg25125060	chr6	108488915	-	NR2E1	Body	1.1778421	-0.74932	5.1140969	1.07E-06	3.24E-05	5.1837864
cg08875705	chr12	6665330	-	IFFO1;IFFO1	TSS200;TSS200	1.6923027	0.1666318	5.1158732	1.07E-06	3.24E-05	5.1847241
cg11199827	chr10	49881056	+			-1.55824	1.7246317	-5.12164	1.07E-06	3.24E-05	5.187866
cg24708694	chr4	135248946	-			-1.400767	0.9755562	-5.113993	1.07E-06	3.24E-05	5.1833501
cg26980111	chr8	132321917	+			-1.815386	-0.234311	-5.113919	1.07E-06	3.24E-05	5.1830368

cg00936309	chr6	33141824	+	COL11A2;COL11A2;COL11A2	Body;Body;Body	-1.174279	0.1848384	-5.115634	1.08E-06	3.24E-05	5.1837157
cg07197230	chr22	17956641	+	CECR2	1stExon	1.6115097	-0.768649	5.11363	1.08E-06	3.24E-05	5.181818
cg22039458	chr2	74756240	+	HTRA2;HTRA2;AUP1	TSS1500;TSS1500;Body	-0.421397	-3.903367	-5.113628	1.08E-06	3.24E-05	5.1818106
cg14819820	chr17	56246435	+	OR4D2	TSS1500	-1.37796	0.5719648	-5.11321	1.08E-06	3.25E-05	5.1800462
cg02682520	chr5	159895160	+			-0.849619	-2.890981	-5.113119	1.08E-06	3.25E-05	5.1796646
cg02509086	chr8	70945457	+			1.4750815	-1.766758	5.1130447	1.08E-06	3.25E-05	5.1793506
cg21338225	chr7	30179748	+	C7orf41	Body	1.227288	-0.051897	5.1130062	1.08E-06	3.25E-05	5.1791884
cg22082350	chr4	4385026	-			-1.015142	-0.161435	-5.112838	1.08E-06	3.25E-05	5.1784786
cg02178846	chr11	7988687	+			-0.882441	-0.639308	-5.112583	1.08E-06	3.25E-05	5.1774028
cg23180780	chr2	12260486	-			-1.249315	-0.72119	-5.11249	1.08E-06	3.25E-05	5.1770143
cg04274236	chr19	54171275	-	MIR512-1;MIR512-2	TSS1500;TSS1500	-1.466539	0.4607468	-5.112299	1.08E-06	3.26E-05	5.1762092
cg25865120	chr15	48625058	-	DUT;DUT;DUT	Body;Body;Body	-1.38223	-1.1047	-5.112184	1.08E-06	3.26E-05	5.1757239
cg09205595	chr7	150782539	+	AGAP3;AGAP3	TSS1500;TSS1500	0.9561562	-0.08876	5.1117919	1.08E-06	3.26E-05	5.1740704
cg25750363	chr12	133156565	-	FBRSL1	Body	0.6389428	3.0407505	5.1117615	1.09E-06	3.26E-05	5.1739419
cg21012788	chr4	8642306	+			-2.131195	1.9803922	-5.115316	1.09E-06	3.27E-05	5.1753085
cg21949305	chr22	24828655	-	C22orf45;ADORA2A	Body;5'UTR	1.3246449	0.8198625	5.1115078	1.09E-06	3.27E-05	5.172873
cg21191241	chr8	63385433	-	NKAIN3	Body	-1.387477	0.5485007	-5.113195	1.09E-06	3.27E-05	5.1730223
cg24475782	chr1	119530932	-	TBX15	5'UTR	1.8861047	-1.114459	5.1112474	1.09E-06	3.27E-05	5.1717755
cg03739470	chr8	144746992	-			-0.663513	-0.927697	-5.111182	1.09E-06	3.27E-05	5.1714993
cg15658784	chr11	2951243	+	PHLDA2	TSS1500	-1.052046	-3.550614	-5.114939	1.09E-06	3.27E-05	5.173723

cg16562275	chr12	133481390	-			1.9166443	-1.184786	5.1111372	1.09E-06	3.27E-05	5.1713112
cg09143605	chr2	137184365	-			-1.356126	1.0403245	-5.110979	1.09E-06	3.27E-05	5.1706426
cg06800578	chr1	44683416	-	DMAP1;DMAP1;DMAP1	Body;Body;Body	-0.850199	-2.854805	-5.110908	1.09E-06	3.27E-05	5.1703458
cg07684063	chr7	152683010	+			-1.279052	0.6762629	-5.110745	1.09E-06	3.28E-05	5.1696584
cg25039158	chr7	127365616	+	SND1	Body	0.4543362	2.6657402	5.1106655	1.09E-06	3.28E-05	5.1693231
cg26974383	chr2	79347666	+	REG1A	5'UTR	-1.361048	0.1191168	-5.110653	1.09E-06	3.28E-05	5.1692719
cg12693436	chr10	47130971	-			-0.910025	0.5345703	-5.110615	1.09E-06	3.28E-05	5.16911
cg21669226	chr8	39917609	-			-1.490113	0.9321392	-5.110281	1.09E-06	3.28E-05	5.1677042
cg23791011	chr10	124219564	-	HTRA1	TSS1500	-1.459517	-0.465758	-5.1102	1.09E-06	3.28E-05	5.1673625
cg08701183	chr7	21209781	+			1.3003067	0.0130588	5.1101175	1.09E-06	3.28E-05	5.167014
cg01926906	chr8	65499701	+			1.2485599	-1.504084	5.1119494	1.09E-06	3.28E-05	5.1677797
cg07300060	chr16	54323335	-			0.5856014	-0.488034	5.1099908	1.09E-06	3.29E-05	5.1664802
cg27073337	chr11	133998086	-	JAM3	Body	-1.398585	0.1835821	-5.109794	1.09E-06	3.29E-05	5.1656512
cg02335185	chr20	60491905	-	CDH4	Body	-1.183727	-0.085653	-5.109674	1.10E-06	3.29E-05	5.1651457
cg05998301	chr2	224562787	-			-1.17496	1.8192195	-5.109631	1.10E-06	3.29E-05	5.1649661
cg27397757	chr1	54199825	-	GLIS1;GLIS1	5'UTR;1stExon	-1.482599	1.1999479	-5.109396	1.10E-06	3.29E-05	5.1639758
cg05202985	chr12	114926138	+			-1.189725	0.3669525	-5.109366	1.10E-06	3.29E-05	5.1638472
cg03527086	chr11	64074137	-	ESRRA	5'UTR	-0.893968	-2.276433	-5.109308	1.10E-06	3.29E-05	5.1636011
cg07428136	chr17	46811492	-			1.5775212	-0.79643	5.109166	1.10E-06	3.30E-05	5.1630047
cg13985817	chr4	56685845	+	LOC644145	TSS1500	1.0731386	-0.586516	5.1090646	1.10E-06	3.30E-05	5.1625775

cg20814774	chr4	10467319	-			-1.183008	-0.129481	-5.108974	1.10E-06	3.30E-05	5.1621962
cg27519828	chr16	85394177	-			-0.54255	-0.924307	-5.108803	1.10E-06	3.30E-05	5.1614751
cg06277472	chr19	30936625	+	ZNF536	Body	-1.303319	0.7404245	-5.108692	1.10E-06	3.30E-05	5.1610083
cg10310168	chr1	4376380	+			-1.25565	-0.486267	-5.108672	1.10E-06	3.30E-05	5.160925
cg01638770	chr8	87080395	-	PSKH2	Body	-1.588221	1.0486458	-5.108634	1.10E-06	3.30E-05	5.1607634
cg04098263	chr6	2382649	+			1.2246018	1.708726	5.1086092	1.10E-06	3.30E-05	5.160659
cg14093715	chr9	126776180	-	LHX2	Body	2.3678158	-1.809241	5.1086152	1.10E-06	3.30E-05	5.1606844
cg21978924	chr11	44332756	-	ALX4	TSS1500	1.9032166	-2.26416	5.1122779	1.10E-06	3.30E-05	5.1822074
cg17485838	chr7	27138712	-			1.7125884	0.3033458	5.1083862	1.10E-06	3.31E-05	5.1597196
cg22399458	chr11	112038039	-	TEX12	TSS200	0.6854307	2.8395796	5.1083545	1.10E-06	3.31E-05	5.159586
cg24302683	chr2	218142840	+			-0.954239	-0.446169	-5.108286	1.10E-06	3.31E-05	5.1592964
cg06898923	chr10	27792905	-	RAB18	TSS1500	-0.598631	-4.242834	-5.108231	1.10E-06	3.31E-05	5.1590664
cg04027548	chr12	21926447	-	KCNJ8	Body	1.2874593	0.8696296	5.1081894	1.10E-06	3.31E-05	5.1588903
cg24412204	chr1	240934971	-			-1.752852	0.4178516	-5.108109	1.10E-06	3.31E-05	5.1585513
cg24917359	chr17	77044255	+	C1QTNF1;C1QTNF1;C1QTNF1	3'UTR;3'UTR;3'UTR	-1.592629	0.2292766	-5.108068	1.10E-06	3.31E-05	5.1583806
cg19304150	chr1	114696350	+	SYT6;SYT6	5'UTR;1stExon	2.1613706	-1.775201	5.1079804	1.10E-06	3.31E-05	5.1580102
cg07459248	chr17	77194008	+	HRNBP3	5'UTR	-1.360624	0.4605485	-5.107922	1.10E-06	3.31E-05	5.1577633
cg03730533	chr7	26897685	+	SKAP2	Body	2.0534839	-0.515476	5.1134227	1.10E-06	3.31E-05	5.1769748
cg13702185	chr4	3438579	-	RGS12;RGS12	Body;Body	-1.137882	0.91036	-5.107662	1.10E-06	3.31E-05	5.1566686
cg03072786	chr13	59707460	-			-1.27695	1.2582023	-5.107537	1.11E-06	3.31E-05	5.1561427

cg06278714	chr7	43287968	-	HECW1	Body	-1.012578	-0.686318	-5.107538	1.11E-06	3.31E-05	5.1561464
cg12255501	chr8	2421548	-			-1.306641	0.1267307	-5.10939	1.11E-06	3.31E-05	5.1574308
cg13952688	chr17	21305114	+	KCNJ12	5'UTR	-0.863258	-0.190546	-5.107549	1.11E-06	3.31E-05	5.1561932
cg23087707	chr12	132863869	+	GALNT9	Body	-1.998912	0.99971	-5.107447	1.11E-06	3.32E-05	5.1557631
cg02589576	chr1	201344793	+	TNNT2;TNNT2;TNNT2;TNNT2	5'UTR;5'UTR;5'UTR;5'UTR	-0.907	-0.602687	-5.107361	1.11E-06	3.32E-05	5.1554001
cg15739437	chr5	110407538	-	TSLP;TSLP;TSLP	1stExon;TSS1500;5'UTR	-0.911362	-1.288704	-5.107347	1.11E-06	3.32E-05	5.1553412
cg07759833	chr13	44619208	-			-1.186107	0.3139908	-5.107065	1.11E-06	3.32E-05	5.1541527
cg25633678	chr8	65284473	+			1.8984681	-0.874104	5.1070557	1.11E-06	3.32E-05	5.1541151
cg11869773	chr14	101489781	+	MIR299	TSS1500	-2.096462	1.7603146	-5.106894	1.11E-06	3.32E-05	5.153434
cg26339193	chr5	168731918	+			-1.348427	1.5420453	-5.106848	1.11E-06	3.32E-05	5.15324
cg02532700	chr22	37257404	+	NCF4;NCF4	Body;Body	-1.429314	-1.848435	-5.106741	1.11E-06	3.32E-05	5.1527875
cg18821418	chr11	75046267	-	ARRB1;MIR326;ARRB1	Body;TSS200;Body	1.0404242	1.4778403	5.1066882	1.11E-06	3.33E-05	5.1525671
cg06598634	chr4	63416083	+			-1.565542	1.3241031	-5.106639	1.11E-06	3.33E-05	5.1523592
cg15653173	chr13	112723034	-	SOX1	1stExon	1.7185822	-0.37956	5.1064186	1.11E-06	3.33E-05	5.1514316
cg06891458	chr15	41951943	-	MGA;MGA	TSS1500;TSS1500	1.1292612	-0.067491	5.1063494	1.11E-06	3.33E-05	5.1511403
cg10180058	chr5	1765590	+			-1.381574	0.2913187	-5.106206	1.11E-06	3.33E-05	5.1505355
cg14112635	chr17	76250769	+			-0.459529	-4.209951	-5.105951	1.11E-06	3.33E-05	5.1494624
cg22614239	chr3	147106208	+	ZIC4;ZIC4;ZIC4;ZIC4;ZIC4	3'UTR;Body;3'UTR;Body;3'UTR	1.5562773	-1.062075	5.1097022	1.11E-06	3.33E-05	5.1689192
cg11352083	chr17	46674514	-	LOC404266;LOC404266;LOC404266;LOC404266;HOXB6;LOC 404266	Body;Body;Body;Body;Body;Body	1.2019528	-1.52188	5.1057566	1.11E-06	3.34E-05	5.1486436
cg03562414	chr19	52391078	+	ZNF577;ZNF577;ZNF577	Body;5'UTR;5'UTR	1.6974801	-0.249461	5.1055742	1.12E-06	3.34E-05	5.1478753

cg18096939	chr14	101513572	+	MIR889;MIR539	TSS1500;TSS200	-1.15117	0.6188032	-5.105575	1.12E-06	3.34E-05	5.1478796
cg13357922	chr10	135251512	-			-0.915025	-1.783539	-5.105553	1.12E-06	3.34E-05	5.1477843
cg17755321	chr6	31546085	-	TNF	3'UTR	-0.98808	1.7851142	-5.10543	1.12E-06	3.34E-05	5.14727
cg14944647	chr11	43602857	-	MIR129-2	TSS200	2.4903077	-1.968801	5.1053528	1.12E-06	3.34E-05	5.1469432
cg06826870	chr12	125811164	+	TMEM132B;TMEM132B	1stExon;5'UTR	-1.187624	0.1997389	-5.105327	1.12E-06	3.34E-05	5.146833
cg15294851	chr2	71679959	+	DYSF;DYSF;DYSF;DYSF;DYSF;DYSF;DYSF	TSS1500;TSS1500;TSS1500;TSS1500;TSS1500;TSS1500;TSS1500	-0.579864	-3.346298	-5.105289	1.12E-06	3.34E-05	5.1466732
cg09449947	chr3	38694294	+			-0.951586	-0.821774	-5.105152	1.12E-06	3.34E-05	5.1460963
cg19223299	chr6	100057160	+	PRDM13	Body	1.8672841	-1.413821	5.1051039	1.12E-06	3.34E-05	5.1458949
cg07465344	chr17	4849412	+	PFN1	Body	-1.297442	-0.111048	-5.105054	1.12E-06	3.35E-05	5.1456868
cg00336700	chr2	176943704	+			1.1935826	-0.259152	5.1049901	1.12E-06	3.35E-05	5.1454156
cg00001809	chr14	101154025	+			-0.92291	0.2705508	-5.104896	1.12E-06	3.35E-05	5.1450196
cg13917589	chr4	732749	+	PCGF3	Body	-1.162665	1.552838	-5.104506	1.12E-06	3.35E-05	5.1433777
cg03610527	chr2	242174889	+	HDLBP;HDLBP	Body;Body	0.8949323	1.9548119	5.1042391	1.12E-06	3.36E-05	5.1422536
cg14026459	chr9	23820979	+	ELAVL2;ELAVL2;ELAVL2	5'UTR;5'UTR;5'UTR	2.2801803	-2.933896	5.1060042	1.12E-06	3.36E-05	5.1431863
cg00731785	chr4	15704680	-	BST1;BST1	1stExon;5'UTR	3.2238327	-2.791321	5.1040434	1.12E-06	3.36E-05	5.1414296
cg00652908	chr16	46878164	+			0.886147	-0.581215	5.1039068	1.12E-06	3.36E-05	5.1408544
cg14879760	chr5	50673965	-			1.4161456	-0.491514	5.1039119	1.12E-06	3.36E-05	5.1408759
cg22699727	chr1	247698341	-	OR2C3	TSS1500	-1.403563	0.5127019	-5.103794	1.12E-06	3.36E-05	5.1403777
cg02489552	chr19	15121531	+	CCDC105	TSS200	0.9017444	-0.550802	5.103773	1.12E-06	3.36E-05	5.1402911
cg12614105	chr7	24324435	+	NPY	5'UTR	1.5744217	-1.254528	5.1036878	1.12E-06	3.36E-05	5.1399323

cg01161877	chr5	158690922	-	UBLCP1	5'UTR	-1.041394	-0.106019	-5.103664	1.12E-06	3.36E-05	5.1398319
cg14977433	chr4	134941108	-			-0.975059	-0.4525	-5.103621	1.12E-06	3.36E-05	5.1396519
cg07030704	chr11	112792759	+			-1.153656	-0.955722	-5.103448	1.13E-06	3.37E-05	5.138921
cg08284371	chr4	36245424	+	ARAP2	5'UTR	-0.474441	-2.656831	-5.103467	1.13E-06	3.37E-05	5.139001
cg12476188	chr15	66999484	+	SMAD6;SMAD6;SMAD6	TSS1500;Body;Body	1.0786542	1.0420242	5.1034592	1.13E-06	3.37E-05	5.13897
cg24360137	chr6	90598826	-			-1.157498	0.0049088	-5.103487	1.13E-06	3.37E-05	5.139087
cg01771096	chr6	161638100	-	AGPAT4	Body	-1.564795	0.8852792	-5.103329	1.13E-06	3.37E-05	5.1384229
cg01279418	chr16	89623374	-	SPG7	Body	0.7496644	3.3633542	5.1089527	1.13E-06	3.37E-05	5.1586155
cg02251243	chr2	74210857	-			-0.821552	-1.071841	-5.103233	1.13E-06	3.37E-05	5.1380163
cg01947943	chr6	29012859	+	OR2W1	1stExon	-1.650402	0.2405222	-5.103209	1.13E-06	3.37E-05	5.1379175
cg13534095	chr4	12926002	+			-1.325131	0.5905789	-5.103103	1.13E-06	3.37E-05	5.1374705
cg08781738	chr8	117876328	-	RAD21	Body	1.1099574	1.8646601	5.1030189	1.13E-06	3.37E-05	5.1371162
cg21486233	chr12	29375141	-	FAR2	TSS1500	-1.821412	1.2260087	-5.108602	1.13E-06	3.37E-05	5.1399585
cg07309576	chr11	5463768	-	OR51I1	TSS1500	-1.688849	0.9970876	-5.102918	1.13E-06	3.37E-05	5.136691
cg20154938	chr8	37793789	+	GOT1L1	Body	-1.024936	0.179058	-5.102872	1.13E-06	3.37E-05	5.1364981
cg03993839	chr16	55851398	-	CES1;CES1;CES1	Body;Body;Body	-1.106678	0.4493797	-5.102847	1.13E-06	3.37E-05	5.1363932
cg18369325	chr14	68736214	+	RAD51L1;RAD51L1;RAD51L1	Body;Body;Body	0.6298178	1.9405633	5.1028226	1.13E-06	3.37E-05	5.1362897
cg07368661	chr17	73506203	+	CASKIN2;CASKIN2	TSS1500;Body	0.9890873	1.4253421	5.1027161	1.13E-06	3.37E-05	5.1358415
cg12407791	chr17	73824354	+	UNC13D	Body	0.7941849	0.6936018	5.1026961	1.13E-06	3.37E-05	5.1357574
cg23248887	chr14	38679643	+	SSTR1	Body	1.9450745	-0.153373	5.1045638	1.13E-06	3.37E-05	5.136704

cg20871721	chr11	30488071	+	MPPED2;MPPED2	Body;Body	-1.270131	0.7949424	-5.102678	1.13E-06	3.37E-05	5.1356817
cg26095658	chr2	71017887	+	FIGLA	TSS200	1.1104924	-0.233368	5.1026539	1.13E-06	3.37E-05	5.1355797
cg26677584	chr13	28541468	+	CDX2	Body	1.508274	-1.730696	5.1026328	1.13E-06	3.37E-05	5.1354909
cg17746316	chr2	9401362	-	ASAP2;ASAP2	Body;Body	1.1811575	1.490862	5.1026075	1.13E-06	3.37E-05	5.1353843
cg10196163	chr14	105560628	-			-0.65501	-1.879045	-5.102455	1.13E-06	3.38E-05	5.1347439
cg22345214	chr4	46995509	-	GABRA4;GABRA4	5'UTR;1stExon	1.3402697	-1.972407	5.1024313	1.13E-06	3.38E-05	5.1346429
cg24412662	chr1	2866667	-			-1.95923	0.8008131	-5.102333	1.13E-06	3.38E-05	5.1342285
cg07280182	chr22	49810275	-			-1.462138	0.2851674	-5.102247	1.13E-06	3.38E-05	5.1338687
cg15975217	chr4	140656926	+	MAML3	Body	1.2758274	-1.030984	5.102228	1.13E-06	3.38E-05	5.133787
cg17387577	chr12	124864657	+	NCOR2;NCOR2	Body;Body	0.9473964	-0.243548	5.1039518	1.13E-06	3.38E-05	5.1345543
cg00832923	chr7	150270224	-	GIMAP4	3'UTR	-1.770244	1.2929626	-5.102013	1.13E-06	3.38E-05	5.1328813
cg01041256	chr15	27125580	+	GABRA5;GABRA5	Body;Body	-1.381801	0.8187208	-5.101864	1.13E-06	3.38E-05	5.1322549
cg01212562	chr21	46868092	-	COL18A1	Body	-1.13103	0.4504167	-5.101785	1.13E-06	3.38E-05	5.1319233
cg26814075	chr7	127881298	-	LEP	TSS200	0.9823816	0.2619864	5.1016551	1.13E-06	3.39E-05	5.1313754
cg01598642	chr11	5272039	+	HBG1	TSS1500	-1.388584	1.1065793	-5.101564	1.14E-06	3.39E-05	5.1309899
cg02858594	chr21	34400211	-	OLIG2	3'UTR	1.4173249	-1.26846	5.1033618	1.14E-06	3.39E-05	5.1320736
cg14287565	chr9	127630689	-	ARPC5L	TSS1500	-1.179553	-3.114447	-5.101476	1.14E-06	3.39E-05	5.1306217
cg08393937	chr5	78277492	-	ARSB;ARSB	Body;Body	-1.288668	-0.253671	-5.101447	1.14E-06	3.39E-05	5.1304996
cg10454258	chr16	27443933	+	IL21R;IL21R;IL21R	Body;Body;Body	-1.048413	-0.822344	-5.101417	1.14E-06	3.39E-05	5.1303714
cg04661810	chr4	154231075	+	TRIM2;ANXA2P1;TRIM2	Body;TSS1500;Body	-0.572475	2.2449787	-5.101387	1.14E-06	3.39E-05	5.1302477

cg02246426	chr8	97162431	+	GDF6	Body	1.296905	0.6267228	5.1011852	1.14E-06	3.39E-05	5.1293976
cg00844298	chr7	9826908	-			-1.313676	-0.026018	-5.101153	1.14E-06	3.39E-05	5.1292623
cg05971966	chr5	40681137	+	PTGER4	Body	2.4714949	-0.68522	5.1030016	1.14E-06	3.39E-05	5.130559
cg12815355	chr2	75894077	+	C2orf3	Body	1.1825458	1.185834	5.1011013	1.14E-06	3.39E-05	5.1290445
cg02007288	chr8	30513708	+	GTF2E2	5'UTR	-1.619955	0.039844	-5.101078	1.14E-06	3.39E-05	5.1289462
cg05289892	chr11	67070816	+	SSH3	TSS200	1.6898057	-2.497544	5.1010406	1.14E-06	3.39E-05	5.128789
cg06999856	chr10	135260170	+			0.7641553	1.4066377	5.1010549	1.14E-06	3.39E-05	5.1288491
cg00840332	chr7	127881269	-	LEP	TSS200	1.6318253	-0.395063	5.100769	1.14E-06	3.40E-05	5.1276459
cg10139717	chr8	2363092	-			-1.284286	1.6075068	-5.100776	1.14E-06	3.40E-05	5.1276758
cg25077137	chr7	16566884	-			-1.346146	1.3238075	-5.100679	1.14E-06	3.40E-05	5.1272661
cg25875702	chr17	76501582	+	DNAH17	Body	-0.841157	0.2961922	-5.100676	1.14E-06	3.40E-05	5.1272535
cg02770983	chr11	31822341	+	PAX6;PAX6;PAX6	Body;Body;Body	1.2736533	-0.218822	5.1005118	1.14E-06	3.40E-05	5.1265635
cg17685019	chr11	30251556	-	FSHB;FSHB	TSS1500;TSS1500	-1.015492	-0.841081	-5.100524	1.14E-06	3.40E-05	5.1266136
cg27454064	chr12	64215611	+			1.3206374	0.4512355	5.1042202	1.14E-06	3.40E-05	5.1459202
cg09789636	chr1	248021163	-	TRIM58	Body	1.4830674	-1.468677	5.1004277	1.14E-06	3.40E-05	5.1262094
cg17942750	chr9	140683527	-	EHMT1	Body	1.2359297	1.4882917	5.1003326	1.14E-06	3.40E-05	5.1258095
cg22944499	chr2	207507163	-	LOC200726;LOC200726	5'UTR;1stExon	1.1848587	-0.943478	5.1000673	1.14E-06	3.40E-05	5.1246929
cg02203420	chr9	19786951	+	SLC24A2	TSS200	-1.517727	0.9631233	-5.099979	1.14E-06	3.40E-05	5.1243214
cg11884933	chr7	2774414	-	GNA12	Body	0.8682744	1.1628102	5.0999686	1.14E-06	3.40E-05	5.1242775
cg18541254	chr13	112709550	+			0.982664	-0.493544	5.1000049	1.14E-06	3.40E-05	5.1244305

cg22644321	chr8	126446923	-	TRIB1	Body	-1.186359	-1.712573	-5.099961	1.14E-06	3.40E-05	5.1242445
cg10954182	chr12	104532377	+	NFYB	TSS1500	-0.78599	-2.277037	-5.099921	1.14E-06	3.40E-05	5.1240774
cg25007250	chr7	150687712	+	NOS3	TSS1500	-1.603892	1.729856	-5.099821	1.14E-06	3.41E-05	5.1236554
cg22830663	chr2	131975750	-	POTEE	TSS200	-1.425647	1.2702831	-5.099747	1.14E-06	3.41E-05	5.1233461
cg06798106	chr8	55380185	+			1.3127296	-0.307218	5.0997083	1.14E-06	3.41E-05	5.1231824
cg04056774	chr16	46672846	-			-1.005507	0.2621832	-5.09967	1.14E-06	3.41E-05	5.1230195
cg08496123	chr19	52225777	+	HAS1	Body	-1.249636	-0.042239	-5.099396	1.15E-06	3.41E-05	5.1218671
cg17218466	chr1	202928564	+	ADIPOR1;ADIPOR1	TSS1500;TSS1500	-1.326926	-0.418619	-5.101202	1.15E-06	3.41E-05	5.1399009
cg15508280	chr14	101526089	+	MIR496;MIR154	TSS1500;TSS200	-1.40051	1.0122957	-5.099241	1.15E-06	3.41E-05	5.1212159
cg02571560	chr6	32037587	+	TNXB	Body	-1.087821	0.5530909	-5.099127	1.15E-06	3.41E-05	5.1207345
cg26882224	chr15	89386800	+	ACAN;ACAN	Body;Body	-2.060294	1.7121144	-5.10841	1.15E-06	3.42E-05	5.1241967
cg01355932	chr8	982926	+			-1.051137	-0.16565	-5.098805	1.15E-06	3.42E-05	5.1193811
cg17658568	chr11	122033987	+	LOC399959	Body	-1.61855	0.1514027	-5.09882	1.15E-06	3.42E-05	5.1194463
cg07419021	chr2	162274436	-	TBR1	Body	1.7665172	-1.478585	5.1082297	1.15E-06	3.42E-05	5.1238554
cg07512206	chr19	15725989	+	CYP4F8	TSS200	-0.806466	-0.587649	-5.098696	1.15E-06	3.42E-05	5.1189223
cg21577889	chr1	60281077	+	HOOK1	Body	-0.714363	-2.284452	-5.098591	1.15E-06	3.42E-05	5.1184812
cg20739013	chr3	27761677	+	EOMES	Body	1.0066587	0.453695	5.0985155	1.15E-06	3.42E-05	5.1181634
cg09351082	chr10	97319133	+	SORBS1;SORBS1	5'UTR;5'UTR	1.0420133	1.9592797	5.0984154	1.15E-06	3.42E-05	5.1177422
cg08645400	chr16	49307062	+			-1.398737	0.4899496	-5.098306	1.15E-06	3.43E-05	5.1172822
cg04142323	chr5	87981449	+	LOC645323	TSS1500	1.3969595	-1.027777	5.0981736	1.15E-06	3.43E-05	5.1167247

cg13212362	chr1	21648026	-	ECE1	Body	0.9405424	1.5863715	5.0980529	1.15E-06	3.43E-05	5.1162171
cg21868063	chr17	74869001	-	MGAT5B;MGAT5B	Body;1stExon	-1.259697	-0.607793	-5.098	1.15E-06	3.43E-05	5.1159953
cg27616666	chr2	168193427	-			-1.582578	-0.302182	-5.097945	1.15E-06	3.43E-05	5.1157647
cg18081258	chr14	21494161	-	NDRG2;NDRG2;NDRG2;NDRG2;NDRG2;NDRG2;NDRG2;NDRG2	TSS1500;TSS1500;TSS1500;TSS1500;TSS1500;TSS1500;TSS1500;TSS1500	0.8979342	0.0446403	5.0978527	1.15E-06	3.43E-05	5.1153749
cg02251742	chr3	156009319	+	KCNAB1;KCNAB1;KCNAB1;KCNAB1	Body;1stExon;5'UTR;Body	1.3470597	-1.797631	5.0976599	1.15E-06	3.43E-05	5.1145636
cg18388910	chr15	101085061	+	LASS3	TSS200	0.8540436	1.4841469	5.0993911	1.16E-06	3.44E-05	5.1153818
cg16353624	chr6	74160974	+	C6orf150	Body	-1.262219	-3.512741	-5.097444	1.16E-06	3.44E-05	5.1136561
cg25883348	chr2	234883862	+	TRPM8	Body	-0.922686	-0.399205	-5.097416	1.16E-06	3.44E-05	5.1135389
cg01520454	chr1	2304375	+	MORN1	Body	-0.900963	0.1147664	-5.097375	1.16E-06	3.44E-05	5.1133658
cg17120792	chr11	55705284	+	OR5I1	TSS1500	-1.334513	-0.300485	-5.097264	1.16E-06	3.44E-05	5.1128973
cg18592365	chr1	79472536	-	ELTD1	TSS200	0.7637489	-0.878531	5.0972106	1.16E-06	3.44E-05	5.1126734
cg25278353	chr10	91717815	-			-1.245763	0.2427683	-5.097078	1.16E-06	3.44E-05	5.1121151
cg00469269	chr2	241534583	-	CAPN10;CAPN10;CAPN10;CAPN10	Body;Body;Body;Body	0.4556581	1.9388508	5.0970364	1.16E-06	3.44E-05	5.1119406
cg24534583	chr7	5709854	+	RNF216;RNF216	Body;Body	0.8784966	1.3736751	5.0970284	1.16E-06	3.44E-05	5.1119071
cg03012452	chr6	12754496	+	PHACTR1	Body	-1.603645	1.1124485	-5.096985	1.16E-06	3.44E-05	5.1117258
cg24080247	chr21	38082613	+	SIM2;SIM2	Body;Body	1.9523815	-0.874128	5.0966602	1.16E-06	3.45E-05	5.1103583
cg00707134	chr16	66906974	+			-0.857266	-1.191106	-5.096631	1.16E-06	3.45E-05	5.1102348
cg08305879	chr8	2127340	-			-1.438262	0.9353067	-5.096511	1.16E-06	3.45E-05	5.1097298
cg09537551	chr8	99950917	-			1.6529957	-0.879175	5.0965022	1.16E-06	3.45E-05	5.1096938
cg05835105	chr4	111543401	-	PITX2;PITX2;PITX2	Body;Body;Body	2.2176619	-0.205763	5.0963183	1.16E-06	3.45E-05	5.1089203

cg16901302	chr2	2692889	-			-1.42174	0.4217269	-5.096262	1.16E-06	3.45E-05	5.1086848
cg03802191	chr1	234615169	+	TARBP1	TSS1500	-1.056964	-0.496613	-5.096223	1.16E-06	3.45E-05	5.1085187
cg00069003	chr5	140164106	-			-1.174052	-0.388428	-5.096121	1.16E-06	3.45E-05	5.1080909
cg24000937	chr19	475139	-	ODF3L2	TSS200	-1.056902	-0.036605	-5.096054	1.16E-06	3.45E-05	5.1078086
cg11558875	chr12	97647115	+			-1.56737	1.0443436	-5.096037	1.16E-06	3.45E-05	5.1077352
cg22051586	chr4	881820	+	GAK	Body	0.6145066	2.6332581	5.0959249	1.16E-06	3.45E-05	5.1072656
cg19526568	chr13	100643087	-			0.8861777	-0.449786	5.0958781	1.16E-06	3.46E-05	5.1070689
cg01511557	chr1	50514319	+	ELAVL4	Body	1.0471669	0.1458834	5.0957546	1.16E-06	3.46E-05	5.1065495
cg09682796	chr3	50383167	-	ZMYND10	TSS200	-1.868728	-3.25363	-5.103303	1.16E-06	3.46E-05	5.1114125
cg07022289	chr1	238226289	-			-1.60493	1.0028495	-5.095596	1.17E-06	3.46E-05	5.1058805
cg15489799	chr1	1098380	-			-0.638812	-0.927894	-5.095587	1.17E-06	3.46E-05	5.1058456
cg04739648	chr5	25907524	+			-1.505689	-0.328437	-5.095492	1.17E-06	3.46E-05	5.1054454
cg15159060	chr11	30251197	-	FSHB;FSHB	TSS1500;TSS1500	-0.872007	-1.066528	-5.095465	1.17E-06	3.46E-05	5.1053325
cg16736018	chr1	156661648	-			-0.882216	-2.351876	-5.095441	1.17E-06	3.46E-05	5.1052306
cg13759645	chr17	31671421	-	ACCN1	Body	-1.403107	0.9997469	-5.095223	1.17E-06	3.46E-05	5.1043148
cg25751484	chr19	13915817	+	ZSWIM4	Body	1.2191515	2.3095469	5.0950814	1.17E-06	3.47E-05	5.103718
cg15722952	chr3	194924602	-	C3orf21	Body	0.676938	0.7683364	5.0948934	1.17E-06	3.47E-05	5.1029276
cg04736751	chr16	7807001	+			-1.552316	1.2283734	-5.09482	1.17E-06	3.47E-05	5.1026204
cg07162608	chr1	239828159	-	CHRM3	5'UTR	-1.186311	0.7102336	-5.094622	1.17E-06	3.47E-05	5.1017862
cg01856892	chr16	1388728	+	BAIAP3	Body	-1.033637	0.3917731	-5.094573	1.17E-06	3.47E-05	5.1015802

cg10475970	chr5	140772512	-	PCDHGA4;PCDHGA6;PCDHGA1;PCDHGA5;PCDHGB1;PCDHGB4;PCDHGA3;PCDHGA2;PCDHGA8;PCDHGA7;PCDHGB2;PCDHGA8;PCDHGB3	Body;Body;Body;Body;Body;Body;Body;Body;1stExon;Body;Body;1stExon;Body	0.9911114	-0.64586	5.0944186	1.17E-06	3.47E-05	5.1009307
cg14794936	chr10	88471856	-	LDB3;LDB3;LDB3	Body;Body;Body	-0.650393	-3.920044	-5.09799	1.17E-06	3.48E-05	5.1029575
cg08019151	chr3	38836991	+	SCN10A	TSS1500	-1.353409	0.9099668	-5.094133	1.17E-06	3.48E-05	5.0997309
cg20274553	chr6	169939263	-	WDR27	Body	0.6642957	2.9224822	5.0978128	1.17E-06	3.48E-05	5.1022121
cg06621744	chr14	37052470	+	NKX2-8	TSS1500	1.1518833	-1.194316	5.0957847	1.17E-06	3.48E-05	5.1002293
cg25406011	chr11	1254421	+	MUC5B	Body	-0.727068	0.0921362	-5.093942	1.17E-06	3.48E-05	5.098928
cg02077688	chr12	127257703	-			-1.576224	1.0947833	-5.09392	1.17E-06	3.48E-05	5.0988324
cg01301382	chr18	15198067	-			1.6367239	-0.111525	5.0937677	1.17E-06	3.48E-05	5.098194
cg12011136	chr7	4901750	-	PAPOLB;RADIL	TSS200;Body	0.8357044	-0.481061	5.0936734	1.18E-06	3.48E-05	5.0977974
cg12028548	chr20	590799	+	TCF15	1stExon	2.2310559	-2.156571	5.093672	1.18E-06	3.48E-05	5.0977916
cg02812763	chr15	35010826	-			-1.34588	1.5918833	-5.093648	1.18E-06	3.48E-05	5.0976901
cg21696987	chr6	33140459	+	COL11A2;COL11A2;COL11A2	Body;Body;Body	-0.670286	-0.963219	-5.093639	1.18E-06	3.48E-05	5.0976521
cg06865612	chr2	946999	-	SNTG2	Body	1.0313431	-1.457997	5.0936043	1.18E-06	3.48E-05	5.0975068
cg13616190	chr8	88041484	+	CNBD1	Body	-1.759903	0.6389427	-5.093506	1.18E-06	3.49E-05	5.0970941
cg14216068	chr7	27146445	+	HOXA3;HOXA3;HOXA3	3'UTR;3'UTR;3'UTR	1.6953516	1.344937	5.0930886	1.18E-06	3.49E-05	5.0953385
cg18875012	chr3	172165517	+	GHSR;GHSR	1stExon;1stExon	1.1372768	-2.008042	5.0930543	1.18E-06	3.49E-05	5.0951943
cg25022866	chr7	54609819	+	VSTM2A	TSS200	1.2073884	-1.652192	5.0929853	1.18E-06	3.49E-05	5.0949042
cg11650926	chr5	5568539	-			-1.616652	0.8544536	-5.09278	1.18E-06	3.50E-05	5.0940412
cg13897914	chr15	41695294	+	NDUFAF1	TSS1500	-1.252975	-0.737704	-5.092635	1.18E-06	3.50E-05	5.0934326
cg13193591	chr11	120377997	-			-1.470511	0.5017879	-5.092589	1.18E-06	3.50E-05	5.0932366

cg08787039	chr1	182921857	+	C1orf14	Body	1.2120625	1.331301	5.0925212	1.18E-06	3.50E-05	5.0929532
cg09920043	chr12	50617546	-	LIMA1;LIMA1;LIMA1	Body;TSS1500;Body	-1.914633	3.5136257	-5.092484	1.18E-06	3.50E-05	5.0927946
cg24422202	chr21	31744647	-	KRTAP13-2	TSS200	-1.201426	0.3445567	-5.092455	1.18E-06	3.50E-05	5.0926754
cg01139861	chr7	50343298	+	IKZF1	TSS1500	1.0892052	-0.551371	5.0923503	1.18E-06	3.50E-05	5.0922345
cg04340918	chr8	19577028	-			-1.154417	0.2465571	-5.092341	1.18E-06	3.50E-05	5.0921969
cg16284507	chr10	18334434	-			-0.93716	-0.718316	-5.09232	1.18E-06	3.50E-05	5.0921073
cg07134252	chr6	168581459	-			-1.501552	0.5383924	-5.092265	1.18E-06	3.50E-05	5.0918754
cg19200333	chr10	108918391	-	SORCS1;SORCS1	Body;Body	-1.47535	0.815114	-5.092221	1.18E-06	3.50E-05	5.0916923
cg02959108	chr9	1042202	-			1.2512367	-1.205478	5.0919497	1.18E-06	3.51E-05	5.0905503
cg17192862	chr12	130921663	-	RIMBP2	Body	-1.408666	0.2754451	-5.093776	1.18E-06	3.51E-05	5.0913653
cg01288598	chr6	14118227	+	CD83;CD83	Body;Body	-0.40349	-3.614727	-5.091915	1.18E-06	3.51E-05	5.0904026
cg04835431	chr14	105684005	+	BRF1;BRF1	Body;Body	1.4190102	3.899916	5.0919015	1.18E-06	3.51E-05	5.0903481
cg18845797	chr5	7794275	+	ADCY2	Body	-1.313832	-0.175286	-5.093661	1.18E-06	3.51E-05	5.0908823
cg09628216	chr15	25465328	+	SNORD115-15;SNORD115-21;HBII-52-27	Body;Body;TSS1500	-0.796055	-0.006652	-5.091357	1.19E-06	3.51E-05	5.0880586
cg16854826	chr10	80889523	+	ZMIZ1	5'UTR	0.6661242	0.6927657	5.0913607	1.19E-06	3.51E-05	5.0880747
cg17304713	chr12	19383622	+	PLEKHA5;PLEKHA5	5'UTR;Body	-0.85947	4.7702529	-5.093079	1.19E-06	3.52E-05	5.0884396
cg18224977	chr1	5398602	+			-1.328675	0.2746045	-5.091164	1.19E-06	3.52E-05	5.087247
cg04835091	chr17	35406	+			-1.307318	0.8664522	-5.091139	1.19E-06	3.52E-05	5.0871432
cg03631457	chr10	20230737	+	PLXDC2	Body	-1.337385	-0.115445	-5.090951	1.19E-06	3.52E-05	5.0863537
cg02087075	chr6	44226395	-	SLC35B2;NFKBIE	TSS1500;3'UTR	-0.751869	1.177647	-5.09093	1.19E-06	3.52E-05	5.0862657

cg00045275	chr1	43389643	+			-0.780767	-4.28535	-5.092717	1.19E-06	3.52E-05	5.0873435
cg08904058	chr15	29859427	-	FAM189A1	Body	-0.974671	-0.079722	-5.090886	1.19E-06	3.52E-05	5.0860782
cg01785490	chr1	4659688	-			-1.199589	-0.152098	-5.090737	1.19E-06	3.52E-05	5.085455
cg04032066	chr11	20622721	-	SLC6A5	Body	1.3457463	-1.460878	5.0906158	1.19E-06	3.52E-05	5.0849437
cg04240548	chr22	50469202	-	TTLL8	Body	-0.623073	-2.30573	-5.090654	1.19E-06	3.52E-05	5.0851029
cg07596645	chr1	201347917	-	TNNT2;TNNT2;TNNT2;TNNT2	TSS1500;TSS1500;TSS1500;TSS1500	-1.070597	0.0100356	-5.090623	1.19E-06	3.52E-05	5.0849741
cg21027535	chr15	80634507	-			-1.041669	-0.238112	-5.09062	1.19E-06	3.52E-05	5.0849631
cg09411682	chr12	26145384	+	RASSF8;RASSF8;RASSF8	5'UTR;5'UTR;5'UTR	-1.092122	-0.446085	-5.090571	1.19E-06	3.52E-05	5.0847567
cg22866754	chr8	788100	+			-1.261531	0.6742203	-5.090503	1.19E-06	3.52E-05	5.0844701
cg23366979	chr5	6411510	-			-3.194124	3.611918	-5.094135	1.19E-06	3.52E-05	5.0867829
cg06882571	chr16	27801626	+	GSG1L;GSG1L	3'UTR;3'UTR	-0.713333	-1.666398	-5.090316	1.19E-06	3.53E-05	5.0836846
cg16033151	chr17	27966740	-	SSH2	Body	-0.956731	-1.749636	-5.090311	1.19E-06	3.53E-05	5.0836618
cg17840719	chr5	178368253	-	ZNF454;ZNF454	1stExon;5'UTR	1.9279812	-0.113375	5.0902533	1.19E-06	3.53E-05	5.0834202
cg22060367	chr2	161957720	-			0.7741726	2.3660412	5.0901603	1.19E-06	3.53E-05	5.0830294
cg05210266	chr8	65282507	-			1.6343149	-1.250731	5.0900812	1.19E-06	3.53E-05	5.0826967
cg25046772	chr15	75527224	-			-0.804532	-0.682578	-5.090083	1.19E-06	3.53E-05	5.0827062
cg03387497	chr20	17680945	+	BANF2;BANF2;BANF2	5'UTR;5'UTR;Body	-1.483647	1.0179323	-5.089991	1.19E-06	3.53E-05	5.0823156
cg21031917	chr6	62596341	-	KHDRBS2	Body	-1.571958	-0.412074	-5.089972	1.19E-06	3.53E-05	5.082239
cg08829841	chr10	129535348	+	FOXI2	TSS200	1.1020358	-0.635369	5.0917731	1.19E-06	3.53E-05	5.0833823
cg16747828	chr12	12938425	+	APOLD1;APOLD1	Body;TSS200	0.7084389	1.6761431	5.0898302	1.20E-06	3.53E-05	5.0816418

cg24710886	chr1	31538937	+	PUM1;PUM1	TSS1500;TSS1500	-0.465131	-3.640833	-5.089692	1.20E-06	3.53E-05	5.0810616
cg06233018	chr21	41552656	+	DSCAM	Body	-1.173364	0.3514784	-5.089649	1.20E-06	3.53E-05	5.0808816
cg19216792	chr7	26897202	+	SKAP2	Body	1.2729518	-0.569915	5.0895815	1.20E-06	3.53E-05	5.080597
cg24360617	chr22	39353495	-	APOBEC3A	TSS200	-0.712888	-1.773056	-5.089483	1.20E-06	3.54E-05	5.0801844
cg06218344	chr8	142394747	-			-1.101628	-1.098629	-5.089346	1.20E-06	3.54E-05	5.0796058
cg10484085	chr6	32041689	-	TNXB	Body	-1.321809	0.5981079	-5.091171	1.20E-06	3.54E-05	5.080856
cg03343128	chr1	247712140	+	C1orf150	TSS1500	-1.289374	-0.734487	-5.089238	1.20E-06	3.54E-05	5.0791539
cg16008054	chr8	1441386	+			-0.906362	1.4507848	-5.089246	1.20E-06	3.54E-05	5.0791851
cg03832839	chr19	54401967	-	PRKCG	Body	1.4172744	-2.223552	5.0889931	1.20E-06	3.54E-05	5.0781241
cg05081497	chr20	4804710	-	RASSF2	TSS1500	0.8258987	-0.126304	5.0888684	1.20E-06	3.54E-05	5.0776001
cg24364282	chr6	114126759	-			-1.387525	1.0773546	-5.088783	1.20E-06	3.54E-05	5.0772406
cg01916697	chr5	148541626	-	ABLIM3	Body	-1.086823	1.5482222	-5.088689	1.20E-06	3.55E-05	5.0768444
cg27222147	chr12	2224755	-	CACNA1C;CACNA1C;CACNA1C;CACNA1C;CACNA1C;CACNA1C<br>;CACNA1C;CACNA1C;CACNA1C;CACNA1C;CACNA1C;CACNA1<br>C;CACNA1C;CACNA1C;CACNA1C;CACNA1C;CACNA1C;CACNA	Body;Body;Body;Body;Body;Body;Body;Body;Body;<br>Body;Body;Body;Body;Body;Body;Body;Body;Body;<br>Body;Body;Body;Body;Body	-1.062416	0.690762	-5.088466	1.20E-06	3.55E-05	5.075909
cg09521575	chr8	19041618	-			-1.185773	1.7121959	-5.088425	1.20E-06	3.55E-05	5.0757377
cg10635122	chr19	52391090	+	ZNF577;ZNF577;ZNF577;ZNF577	Body;5'UTR;5'UTR;1stExon	1.6977426	-0.505246	5.088304	1.20E-06	3.55E-05	5.0752288
cg17013303	chr11	58189993	+	OR5B2	1stExon	-1.255346	0.0927159	-5.088236	1.20E-06	3.55E-05	5.0749432
cg05586667	chr16	19971547	+			-1.184341	0.072466	-5.088105	1.20E-06	3.55E-05	5.0743926
cg18513344	chr3	195531298	+	MUC4;MUC4;MUC4	Body;Body;Body	-0.686992	-1.768792	-5.088107	1.20E-06	3.55E-05	5.0744025
cg22602592	chr20	61956679	-	COL20A1	Body	-1.11721	0.0944803	-5.088034	1.20E-06	3.55E-05	5.0740957
cg19955928	chr2	240152257	-	HDAC4	Body	0.8317152	0.6895515	5.0880014	1.21E-06	3.55E-05	5.0739573

cg11605903	chr6	78176622	+			1.5863303	-1.429668	5.0878525	1.21E-06	3.56E-05	5.0733315
cg20844427	chr13	106470099	+			-1.197933	-0.533614	-5.087867	1.21E-06	3.56E-05	5.0733928
cg09080522	chr22	44568387	+	PARVG;PARVG	TSS1500;TSS1500	-1.300761	-0.21621	-5.08782	1.21E-06	3.56E-05	5.0731934
cg01185626	chr1	75594016	+	LHX8	TSS200	1.4260898	-1.583967	5.0876486	1.21E-06	3.56E-05	5.0724749
cg08292485	chr20	29993663	+	DEFB121	Body	-1.20818	-1.691946	-5.087412	1.21E-06	3.56E-05	5.0714791
cg19594218	chr1	62660556	-	L1TD1;L1TD1;L1TD1;L1TD1	1stExon;5'UTR;5'UTR;1stExon	1.2312964	-0.500077	5.0892551	1.21E-06	3.56E-05	5.0728123
cg12413242	chr12	2292890	-	CACNA1C;CACNA1C;CACNA1C;CACNA1C;CACNA1C;CACNA1C ;CACNA1C;CACNA1C;CACNA1C;CACNA1C;CACNA1C;CACNA1 C;CACNA1C;CACNA1C;CACNA1C;CACNA1C;CACNA1C;CACNA	Body;Body;Body;Body;Body;Body;Body;Body; Body;Body;Body;Body;Body;Body;Body;Body; Body;Body;Body;Body;Body	-1.078296	-0.123366	-5.08725	1.21E-06	3.57E-05	5.070802
cg04103198	chr20	1601704	+	SIRPB1;SIRPB1;SIRPB1	TSS1500;TSS1500;TSS1500	-1.613338	0.9076845	-5.087138	1.21E-06	3.57E-05	5.0703314
cg08158518	chr3	140866589	+	SPSB4	3'UTR	-1.364155	0.2083016	-5.086973	1.21E-06	3.57E-05	5.0696384
cg03935116	chr12	31476565	-	FAM60A;FAM60A;FLJ13224;FAM60A	5'UTR;5'UTR;TSS1500;5'UTR	-0.895095	-1.91941	-5.086891	1.21E-06	3.57E-05	5.0692941
cg23384185	chr18	657227	+	C18orf56;TYMS	3'UTR;TSS1500	-0.576379	-4.125887	-5.086886	1.21E-06	3.57E-05	5.0692718
cg17299899	chr18	76738478	-			1.4664486	-0.791252	5.0867834	1.21E-06	3.57E-05	5.0688403
cg05668422	chr13	114026346	-			-1.157427	0.0496961	-5.086633	1.21E-06	3.57E-05	5.0682093
cg26383838	chr12	93096819	-	C12orf74	TSS200	-1.136058	-0.029885	-5.0884	1.21E-06	3.57E-05	5.0862108
cg26948823	chr16	1255116	+	CACNA1H;CACNA1H	Body;Body	-0.866643	-0.496107	-5.088223	1.21E-06	3.58E-05	5.0684815
cg02603518	chr20	61717939	+			-1.621063	0.8944845	-5.086277	1.21E-06	3.58E-05	5.0667109
cg22077222	chr5	166681042	+			-1.375474	1.2062099	-5.091867	1.21E-06	3.58E-05	5.0693714
cg02006324	chr8	2997052	+	CSMD1	Body	-1.450232	1.3486772	-5.086241	1.21E-06	3.58E-05	5.0665627
cg16243197	chr1	182807962	+	DHX9	TSS1500	-1.688857	0.5689548	-5.086212	1.21E-06	3.58E-05	5.0664396
cg01746044	chr22	30234775	+	ASCC2	TSS1500	-0.99106	-2.530406	-5.086173	1.21E-06	3.58E-05	5.0662752

cg04929703	chr1	91300390	-			1.3566568	-0.702462	5.0861199	1.22E-06	3.58E-05	5.066053
cg02073680	chr16	87633963	+			-1.040242	-0.030803	-5.086088	1.22E-06	3.58E-05	5.0659206
cg18164784	chr5	140762229	+	PCDHGA4;PCDHGA6;PCDHGA1;PCDHGA5;PCDHGB1;PCDHGA3;PCDHGA2;PCDHGB2;PCDHGA7;PCDHGA7;PCDHGB3	Body;Body;Body;Body;Body;Body;Body;Body;TSS1500;TSS1500;Body	1.3831896	-1.002218	5.0860027	1.22E-06	3.58E-05	5.0655608
cg08928145	chr19	19625364	+	TSSK6;TSSK6	3'UTR;1stExon	0.7711537	0.4619236	5.0859612	1.22E-06	3.58E-05	5.0653864
cg19029904	chr13	79161619	-			1.3242216	-0.950144	5.0859324	1.22E-06	3.58E-05	5.0652653
cg14866339	chr14	101487756	-	MIR379	TSS1500	-1.428751	0.764543	-5.085797	1.22E-06	3.58E-05	5.0646948
cg01502353	chr6	56733843	+	DST	Body	-1.323859	0.5429834	-5.085777	1.22E-06	3.58E-05	5.0646112
cg07045857	chr9	13323113	-			-0.932301	0.0494324	-5.085583	1.22E-06	3.59E-05	5.0637989
cg01252713	chr2	235549589	+			-1.500981	0.1745369	-5.085384	1.22E-06	3.59E-05	5.062962
cg10111328	chr12	130683535	-			-1.250354	-0.544107	-5.08535	1.22E-06	3.59E-05	5.0628199
cg25952192	chr18	21656374	+	TTC39C;TTC39C	Body;Body	-1.06288	-2.127371	-5.085019	1.22E-06	3.59E-05	5.0614269
cg06159896	chr12	95840443	+			0.9674056	0.3867206	5.0849904	1.22E-06	3.59E-05	5.0613089
cg08453928	chr16	80333922	+			-1.547741	0.2082192	-5.084929	1.22E-06	3.60E-05	5.0610524
cg21145245	chr5	150593684	-	CCDC69	Body	1.4267143	-0.973124	5.0845544	1.22E-06	3.60E-05	5.0594776
cg20775254	chr2	95940705	-	PROM2;PROM2;PROM2	Body;Body;Body	-1.103413	-0.332088	-5.084501	1.22E-06	3.60E-05	5.059254
cg12810096	chr17	32484112	-	ACCN1	TSS1500	2.0205129	-2.541878	5.0863066	1.22E-06	3.60E-05	5.0600132
cg00962781	chr4	159131393	+	TMEM144	TSS200	-0.821807	-2.877931	-5.08438	1.22E-06	3.60E-05	5.0587472
cg18526607	chr5	54519307	+			1.210526	-0.489754	5.0842029	1.23E-06	3.61E-05	5.0580017
cg22630169	chr19	13108217	-	NFIX	Body	1.1108731	0.3386068	5.0839707	1.23E-06	3.61E-05	5.0570265
cg17046776	chr11	8832283	-	ST5;ST5;ST5	5'UTR;5'UTR;TSS200	-1.400873	-2.038374	-5.083858	1.23E-06	3.61E-05	5.0565547

cg00394823	chr16	20775166	-	ACSM3;ACSM3	TSS200;TSS200	-1.142663	-2.183864	-5.083761	1.23E-06	3.61E-05	5.0561447
cg15707455	chr1	28157011	+	PPP1R8;PPP1R8;PPP1R8	TSS1500;TSS1500;TSS1500	-0.541265	-3.679017	-5.083664	1.23E-06	3.61E-05	5.0557383
cg09770570	chr17	65646042	+	PITPNC1;PITPNC1	Body;Body	-0.974912	0.7122418	-5.083614	1.23E-06	3.61E-05	5.0555269
cg24592906	chr6	100911550	+	SIM1;SIM1	1stExon;5'UTR	1.5444968	-1.344664	5.0835608	1.23E-06	3.61E-05	5.0553053
cg11241278	chr17	74023465	+	EVPL;EVPL	5'UTR;1stExon	-0.619383	0.0339919	-5.083465	1.23E-06	3.62E-05	5.0549019
cg23581186	chr21	32119309	+	KRTAP21-2	1stExon	-1.299174	0.3105527	-5.083475	1.23E-06	3.62E-05	5.0549449
cg18338293	chr11	66277283	-	BBS1	TSS1500	0.5179322	2.0752939	5.083437	1.23E-06	3.62E-05	5.0547854
cg25038773	chr8	6638180	+			-0.795069	-0.460726	-5.083297	1.23E-06	3.62E-05	5.0541989
cg18669209	chr18	44777736	-			2.2623979	-1.480507	5.0832652	1.23E-06	3.62E-05	5.054064
cg27616541	chr17	40837288	+	CNTNAP1	Body	1.0358711	2.7340076	5.088718	1.23E-06	3.62E-05	5.0570438
cg13586160	chr15	25445634	+	SNORD115-19;SNORD115-17;SNORD115-18	TSS1500;TSS1500;TSS1500	-0.883666	0.2230226	-5.082919	1.23E-06	3.62E-05	5.0526104
cg24641276	chr5	170743715	-			1.299396	-1.694625	5.082911	1.23E-06	3.62E-05	5.0525765
cg07147846	chr3	10801997	-	LOC285370	Body	-1.109692	-0.329317	-5.082892	1.23E-06	3.62E-05	5.0524958
cg05601974	chr7	150941389	+	SMARCD3;SMARCD3;SMARCD3	Body;Body;Body	-1.015883	-0.105132	-5.082824	1.23E-06	3.62E-05	5.0522118
cg05030450	chr5	167480853	+	ODZ2	Body	-1.39251	-0.435093	-5.08274	1.23E-06	3.62E-05	5.0518578
cg14289246	chr4	154710475	-	SFRP2	TSS1500	1.9122715	-2.165671	5.082726	1.23E-06	3.62E-05	5.0518001
cg21216477	chr12	54440773	-	HOXC4	5'UTR	1.0354201	-0.402457	5.0827196	1.23E-06	3.62E-05	5.0517729
cg05093535	chr2	100466754	+	AFF3;AFF3	Body;Body	-1.211643	0.0982087	-5.082656	1.23E-06	3.63E-05	5.0515051
cg08905654	chr12	14996743	+	ART4	TSS1500	0.6895631	2.497034	5.0826594	1.23E-06	3.63E-05	5.0515202
cg19772066	chr19	44953982	-	ZNF229	TSS1500	-1.244039	-0.296823	-5.082602	1.23E-06	3.63E-05	5.05128

cg27022827	chr19	3728751	+	TJP3	Body	-0.638513	-0.796763	-5.082585	1.23E-06	3.63E-05	5.0512072
cg03963250	chr10	44198369	-			-2.527	2.6041315	-5.084375	1.23E-06	3.63E-05	5.0523373
cg25875213	chr19	38183055	+	ZNF781;ZNF781	5'UTR;1stExon	2.9612379	-2.179383	5.0823492	1.24E-06	3.63E-05	5.050218
cg02164574	chr2	176985592	+			1.901919	-1.471458	5.0822938	1.24E-06	3.63E-05	5.0499852
cg20555040	chr5	110404682	-			-1.394351	1.5842416	-5.08229	1.24E-06	3.63E-05	5.049969
cg04382374	chr22	49080302	-	FAM19A5;FAM19A5	Body;Body	-1.502253	0.7790989	-5.082142	1.24E-06	3.63E-05	5.0493501
cg26927516	chr7	97735499	+	LMTK2	TSS1500	-1.213485	1.5779964	-5.082073	1.24E-06	3.63E-05	5.0490575
cg21509040	chr20	61814830	+			-0.380255	-3.095089	-5.082048	1.24E-06	3.63E-05	5.0489527
cg02322373	chr2	71134524	+	VAX2	Body	1.0399596	-1.100139	5.0820104	1.24E-06	3.63E-05	5.0487954
cg10517919	chr1	226707583	+			-1.18179	-0.560258	-5.081888	1.24E-06	3.63E-05	5.0482814
cg00920572	chr12	54349349	+	HOXC12	Body	0.9519808	0.3172862	5.0818644	1.24E-06	3.63E-05	5.0481826
cg25977206	chr14	97499484	+			1.5225571	-1.360865	5.0818206	1.24E-06	3.64E-05	5.0479986
cg09349604	chr4	41880747	+			1.7159412	-1.555978	5.0817896	1.24E-06	3.64E-05	5.0478685
cg05562851	chr12	121151805	-	UNC119B	Body	1.1891	-2.201298	5.0816344	1.24E-06	3.64E-05	5.0472169
cg08998657	chr16	46893929	-			-1.195341	0.0405779	-5.08155	1.24E-06	3.64E-05	5.0468626
cg22700220	chr1	237247258	-	RYR2	Body	-1.161539	-0.607776	-5.081511	1.24E-06	3.64E-05	5.0466969
cg14036128	chr11	134296018	-			-1.120579	-0.399027	-5.081434	1.24E-06	3.64E-05	5.0463746
cg12464561	chr4	1401729	+			0.9965771	-1.522433	5.0813554	1.24E-06	3.64E-05	5.0460456
cg14993912	chr5	83683585	+			-1.43487	1.6272164	-5.08135	1.24E-06	3.64E-05	5.0460237
cg01042641	chr16	1575979	+	IFT140	Body	-1.367784	0.4108105	-5.081075	1.24E-06	3.65E-05	5.0448687

cg20427378	chr6	33143129	-	COL11A2;COL11A2;COL11A2	Body;Body;Body	-1.011659	0.0872285	-5.081047	1.24E-06	3.65E-05	5.0447519
cg04835284	chr10	102279758	-	SEC31B	TSS200	0.8741623	-0.508276	5.0810186	1.24E-06	3.65E-05	5.0446321
cg13877974	chr1	201708888	-	NAV1;NAV1	TSS200;Body	-1.383429	-2.086058	-5.08095	1.24E-06	3.65E-05	5.0443438
cg24331722	chr8	62633628	-			-1.606795	0.8607342	-5.080952	1.24E-06	3.65E-05	5.0443516
cg10184983	chr17	36734911	+	SRCIN1	Body	3.0326944	-1.385842	5.08083	1.24E-06	3.65E-05	5.0438403
cg14696926	chr8	2093220	-	MYOM2	3'UTR	-1.215733	-0.175676	-5.080795	1.24E-06	3.65E-05	5.0436934
cg14726117	chr17	77761133	+	CBX2	3'UTR	-1.437928	0.9464447	-5.080753	1.24E-06	3.65E-05	5.0435166
cg26372498	chr2	168614674	+			-1.373603	0.7877628	-5.080765	1.24E-06	3.65E-05	5.0435667
cg09385371	chr6	142959676	-	LOC153910;LOC153910	TSS1500;TSS1500	-1.237612	-0.079771	-5.080645	1.24E-06	3.65E-05	5.0430635
cg19871940	chr14	57264287	-			2.4462474	-1.402387	5.0843388	1.24E-06	3.65E-05	5.0452782
cg23561531	chr11	127328847	-			-1.110473	-0.744454	-5.080544	1.25E-06	3.65E-05	5.0426407
cg07682547	chr5	153863417	-			1.9924243	-2.055052	5.084147	1.25E-06	3.65E-05	5.0449151
cg20841047	chr2	207506675	-	LOC200726	TSS1500	1.0498537	-1.367091	5.0804363	1.25E-06	3.65E-05	5.0421876
cg20688651	chr12	66220754	+	HMGA2;RPSAP52;HMGA2	Body;TSS200;Body	-1.614216	-0.419515	-5.080409	1.25E-06	3.65E-05	5.0420717
cg19614454	chr6	139265575	+	REPS1;REPS1	Body;Body	-1.320865	1.1005841	-5.080216	1.25E-06	3.66E-05	5.0412623
cg17248730	chr3	27772201	+			1.4291552	-1.566233	5.0801644	1.25E-06	3.66E-05	5.0410464
cg14105536	chr6	47666329	+	GPR115;GPR115	5'UTR;1stExon	-1.242906	0.5490232	-5.080145	1.25E-06	3.66E-05	5.0409659
cg02550537	chr15	25450131	-	SNORD115-21;SNORD115-20;SNORD115-15	TSS1500;TSS1500;TSS1500	-1.596449	0.8114178	-5.080118	1.25E-06	3.66E-05	5.0408505
cg20230508	chr11	69500644	-			-0.614064	-4.048457	-5.080079	1.25E-06	3.66E-05	5.0406861
cg08515427	chr16	848997	-	GNG13	Body	-1.167724	0.5801065	-5.079988	1.25E-06	3.66E-05	5.0403068

cg24511738	chr8	9762716	+			2.1578273	-1.78427	5.0799376	1.25E-06	3.66E-05	5.0400946
cg05891572	chr7	33462512	+	BBS9;BBS9;BBS9;BBS9	Body;Body;Body;Body	0.7723155	1.9590458	5.0797626	1.25E-06	3.66E-05	5.0393602
cg14994060	chr5	134376489	-			1.3643504	-1.327799	5.0797318	1.25E-06	3.66E-05	5.039231
cg06487334	chr6	155759026	+	NOX3	Body	1.0425871	1.7006721	5.0833948	1.25E-06	3.66E-05	5.0417645
cg22941315	chr1	175511259	+	TNR	5'UTR	-1.629293	0.2060546	-5.079695	1.25E-06	3.66E-05	5.0390783
cg03197830	chr15	73371746	+	NEO1	Body	0.8271572	2.6465386	5.0795728	1.25E-06	3.66E-05	5.0385634
cg04581004	chr12	7281333	-	RBP5	1stExon	0.680777	2.0108438	5.0794365	1.25E-06	3.66E-05	5.0379914
cg06630988	chr12	126135376	+	TMEM132B	Body	-1.153087	-0.633365	-5.081256	1.25E-06	3.66E-05	5.038829
cg02891945	chr16	85863447	-			-0.956647	-0.842646	-5.081185	1.25E-06	3.67E-05	5.0389605
cg06531870	chr13	89037260	+			-1.154003	-0.783026	-5.079202	1.25E-06	3.67E-05	5.0370072
cg26128079	chr12	127212416	-			1.607536	-2.029313	5.0810058	1.25E-06	3.67E-05	5.0382085
cg08431567	chr19	57175043	-	ZNF835	Body	-1.462601	0.5851841	-5.079053	1.25E-06	3.67E-05	5.0363841
cg02168592	chr1	9416127	-	SPSB1	Body	0.6559704	1.1966647	5.0790137	1.25E-06	3.67E-05	5.0362171
cg02041108	chr5	35946185	-			-2.079673	1.7379562	-5.078951	1.25E-06	3.67E-05	5.0359544
cg13336690	chr6	32976484	+	HLA-DOA	Body	-1.310065	0.446561	-5.08833	1.25E-06	3.67E-05	5.0410869
cg00721170	chr4	74864239	-	CXCL5	1stExon	1.6854861	-0.792545	5.0788136	1.25E-06	3.67E-05	5.0353775
cg15047223	chr12	8687513	+	CLEC4E	Body	-1.733927	1.2297615	-5.078805	1.25E-06	3.67E-05	5.035342
cg06010667	chr6	169309818	-			-1.116459	1.8181468	-5.078709	1.26E-06	3.67E-05	5.0349365
cg11837391	chr3	128199781	+	GATA2;GATA2;GATA2	3'UTR;3'UTR;3'UTR	1.0445488	1.5582881	5.0786927	1.26E-06	3.67E-05	5.0348699
cg25372195	chr12	55042293	-	DCD	TSS200	-1.279492	0.0537352	-5.078622	1.26E-06	3.67E-05	5.034573

cg18609037	chr17	79099246	+	AATK;MIR657	Body;TSS200	-0.911606	0.6732373	-5.078563	1.26E-06	3.68E-05	5.0343278
cg05243629	chr7	143190369	+			-1.529142	0.4381802	-5.078507	1.26E-06	3.68E-05	5.0340909
cg06878311	chr6	32043191	-	TNXB	Body	-0.907138	0.5966817	-5.078527	1.26E-06	3.68E-05	5.0341748
cg27635714	chr6	21748765	-	FLJ22536	Body	-1.244929	0.2239367	-5.078509	1.26E-06	3.68E-05	5.0341004
cg21303011	chr3	24537177	-	THRB;THRB;THRB	TSS1500;TSS1500;TSS1500	-1.061721	-3.139032	-5.078488	1.26E-06	3.68E-05	5.0340098
cg24114813	chr5	172128837	-			-1.289207	-0.700274	-5.07819	1.26E-06	3.68E-05	5.0327604
cg01550348	chr1	85809159	+	DDAH1;DDAH1	Body;Body	1.1020861	1.3384113	5.0780374	1.26E-06	3.68E-05	5.0321202
cg06624020	chr19	58632805	+			-1.513429	0.6011938	-5.078024	1.26E-06	3.68E-05	5.032066
cg02601140	chr7	63250517	-			1.0295967	0.2487907	5.0778611	1.26E-06	3.69E-05	5.0313804
cg02331883	chr5	140798095	+	PCDHGA4;PCDHGA9;PCDHGA1;PCDHGB1;PCDHGB7;PCDHGB6;PCDHGB3;PCDHGA6;PCDHGA8;PCDHGA10;PCDHGA5;PCDHGB4;PCDHGB7;PCDHGA3;PCDHGA2;PCDHGB2;PCDHGA7;P	Body;Body;Body;Body;1stExon;Body;Body;Body;Body;Body;Body;1stExon;Body;Body;Body;Body;Body	1.0202066	-0.010083	5.0778074	1.26E-06	3.69E-05	5.031155
cg00162344	chr4	8583937	-	GPR78	Body	-0.640275	-1.977079	-5.077696	1.26E-06	3.69E-05	5.0306886
cg23674011	chr8	29706233	+			-1.415266	1.6713753	-5.077573	1.26E-06	3.69E-05	5.0301712
cg02517632	chr3	8769188	+			-1.320516	1.3217488	-5.077508	1.26E-06	3.69E-05	5.0298976
cg14322224	chr1	85931226	-	DDAH1;DDAH1	TSS1500;5'UTR	-0.826428	-1.898791	-5.077286	1.26E-06	3.69E-05	5.0289667
cg04149039	chr7	155249019	+			1.309634	-0.70405	5.0771791	1.26E-06	3.69E-05	5.0285189
cg11072637	chr7	65773051	-	TPST1	Body	-1.653852	-0.732485	-5.076744	1.27E-06	3.70E-05	5.0266936
cg16353006	chr1	197882469	-	LHX9	Body	1.6668714	-0.924429	5.0767034	1.27E-06	3.70E-05	5.0265231
cg06244417	chr9	137809628	-	FCN1	1stExon	-1.159537	0.4858758	-5.076679	1.27E-06	3.70E-05	5.0264222
cg15126544	chr2	171678954	+	GAD1;GAD1	Body;Body	2.8368307	-1.797758	5.0765411	1.27E-06	3.70E-05	5.0258424
cg02908282	chr8	145502297	+	BOP1	Body	0.7086391	2.0629057	5.0764587	1.27E-06	3.71E-05	5.0254966

cg12731773	chr11	846055	-	TSPAN4;TSPAN4;TSPAN4;TSPAN4;TSPAN4;TSPAN4;TSPAN4	5'UTR;5'UTR;5'UTR;5'UTR;5'UTR;5'UTR;5'UTR	0.929307	0.323835	5.076423	1.27E-06	3.71E-05	5.0253469
cg09469812	chr1	2899497	-			-0.805766	-0.4633	-5.076387	1.27E-06	3.71E-05	5.025194
cg08453903	chr17	71688187	-			-1.480654	-0.167718	-5.076317	1.27E-06	3.71E-05	5.0249023
cg05056825	chr2	110457974	+			1.0096515	0.9370042	5.0762792	1.27E-06	3.71E-05	5.0247436
cg07454154	chr10	106176905	-	CCDC147	Body	-1.728581	1.039691	-5.078109	1.27E-06	3.71E-05	5.026064
cg21188858	chr5	158303922	+	EBF1	Body	-1.381906	1.2925592	-5.076255	1.27E-06	3.71E-05	5.0246431
cg09589443	chr20	35489221	+	C20orf117;C20orf117	Body;TSS1500	0.908219	1.4440045	5.0761381	1.27E-06	3.71E-05	5.0241514
cg24637364	chr1	66259084	+	PDE4B;PDE4B	5'UTR;5'UTR	1.9295497	-1.517756	5.0760647	1.27E-06	3.71E-05	5.0238436
cg23350273	chr3	97982397	-	OR5H6	TSS1500	-1.194114	-0.903003	-5.07602	1.27E-06	3.71E-05	5.0236543
cg11002923	chr3	49237327	+	CCDC36;CCDC36	5'UTR;5'UTR	0.7890227	1.5809947	5.0759167	1.27E-06	3.71E-05	5.0232227
cg10101521	chr2	176933132	-			1.0427742	-0.91349	5.0758499	1.27E-06	3.71E-05	5.0229425
cg01285926	chr7	56242554	-			1.1349046	1.1265792	5.0756846	1.27E-06	3.71E-05	5.0222492
cg00590320	chr13	114287374	-	TFDP1;TFDP1	Body;Body	0.6331447	2.5078286	5.0754944	1.27E-06	3.72E-05	5.0214513
cg26273886	chr20	17950694	-	SNX5;C20orf72	TSS1500;Body	-0.617559	-4.230582	-5.075452	1.27E-06	3.72E-05	5.0212734
cg20676788	chr13	49379791	-			-1.523071	-0.184905	-5.075415	1.27E-06	3.72E-05	5.0211174
cg04041283	chr8	1414952	+			-0.885979	-2.368497	-5.075391	1.27E-06	3.72E-05	5.0210174
cg26774043	chr11	1647150	+	HCCA2	Body	-1.258166	0.7035905	-5.075353	1.27E-06	3.72E-05	5.0208582
cg20303331	chr17	36945575	-	PIP4K2B	Body	0.8481663	-1.140711	5.0752768	1.27E-06	3.72E-05	5.0205386
cg21383592	chr4	190569145	-			-1.055923	0.4909785	-5.075199	1.28E-06	3.72E-05	5.0202121
cg02952295	chr2	234913188	+	TRPM8	Body	-1.427359	-0.246462	-5.074991	1.28E-06	3.72E-05	5.0193383

cg13223721	chr7	131720532	-			-1.568188	0.4283272	-5.074995	1.28E-06	3.72E-05	5.0193566
cg26566927	chr16	1148030	-			-0.651462	-0.343419	-5.075017	1.28E-06	3.72E-05	5.0194505
cg06575723	chr22	42948520	+	SERHL2	TSS1500	-1.355713	1.689716	-5.07488	1.28E-06	3.73E-05	5.0188742
cg10570993	chr5	177755504	-	COL23A1	Body	-0.985578	0.0282107	-5.074868	1.28E-06	3.73E-05	5.0188232
cg06660332	chr1	47009707	-			1.4871251	-0.834355	5.0747997	1.28E-06	3.73E-05	5.0185375
cg19764143	chr14	60974499	+	SIX6	TSS1500	1.4785779	-1.256769	5.0748093	1.28E-06	3.73E-05	5.0185776
cg20971054	chr14	104797641	+			-1.495865	1.5440856	-5.074744	1.28E-06	3.73E-05	5.0183048
cg02750098	chr5	172656021	+			1.1605498	-1.538905	5.0745469	1.28E-06	3.73E-05	5.017477
cg14829409	chr4	113568820	-	LARP7;LARP7	Body;Body	0.6368201	2.0848879	5.0744885	1.28E-06	3.73E-05	5.0172322
cg00411945	chr10	45324999	+			-1.438659	0.7259052	-5.076196	1.28E-06	3.73E-05	5.0176233
cg12053147	chr4	2221831	-	POLN	Body	-0.552613	-3.303551	-5.074224	1.28E-06	3.73E-05	5.0161238
cg02704181	chr5	15572568	-	FBXL7	Body	-2.214645	0.7549443	-5.074166	1.28E-06	3.73E-05	5.0158804
cg17018096	chr19	8657692	+	ADAMTS10	Body	1.0553161	-1.177931	5.0739407	1.28E-06	3.74E-05	5.0149346
cg14126408	chr10	44705342	-			-1.142765	-0.967578	-5.073799	1.28E-06	3.74E-05	5.0143398
cg05196969	chr15	76627594	+			2.224906	-1.721057	5.0737731	1.28E-06	3.74E-05	5.0142318
cg07156115	chr7	17379753	-	AHR	Body	0.923936	1.5921049	5.0735726	1.28E-06	3.74E-05	5.013391
cg14460768	chr2	161283478	+	RBMS1;RBMS1	Body;Body	0.5728793	2.7441312	5.0735411	1.28E-06	3.74E-05	5.0132592
cg01874697	chr10	106401517	+	SORCS3	1stExon	1.2729541	-1.762404	5.0735123	1.28E-06	3.74E-05	5.0131381
cg12816961	chr10	134598927	-	NKX6-2	Body	2.1086346	-1.739422	5.0734709	1.28E-06	3.74E-05	5.0129647
cg11406667	chr6	11272495	-	NEDD9	Body	-1.250879	1.0717326	-5.073402	1.29E-06	3.75E-05	5.0126742

cg01311313	chr8	35252359	+	UNC5D	Body	-1.035903	-0.895357	-5.07322	1.29E-06	3.75E-05	5.0119109
cg19856263	chr11	127012786	+			-1.368732	0.6035002	-5.073173	1.29E-06	3.75E-05	5.0117148
cg17092340	chr13	112110810	+			-1.164303	0.343501	-5.07485	1.29E-06	3.75E-05	5.032348
cg17345620	chr13	97640663	-	OXGR1	5'UTR	-1.255721	0.3127789	-5.073016	1.29E-06	3.75E-05	5.0110559
cg20285470	chr14	92786551	-			-1.384377	1.1908804	-5.074819	1.29E-06	3.75E-05	5.0118529
cg22495968	chr2	5622155	-			-1.699694	0.2233756	-5.07298	1.29E-06	3.75E-05	5.0109066
cg01670876	chr14	74891361	-	TMEM90A	5'UTR	-1.33462	-0.065162	-5.072861	1.29E-06	3.75E-05	5.0104073
cg24384882	chr13	110961824	-	COL4A2	Body	0.761118	2.187947	5.0728187	1.29E-06	3.75E-05	5.0102299
cg00493422	chr4	57547872	+	HOPX;HOPX;HOPX	TSS200;TSS200;TSS200	-0.936161	-2.147588	-5.07278	1.29E-06	3.75E-05	5.010069
cg09196959	chr6	30104440	-	TRIM40	TSS200	-1.394536	-0.919364	-5.072775	1.29E-06	3.75E-05	5.0100467
cg01585096	chr19	35981517	+	KRTDAP	TSS200	-1.020531	-0.529998	-5.072501	1.29E-06	3.76E-05	5.0088987
cg11377054	chr13	112189885	-			-1.165119	0.2279356	-5.072515	1.29E-06	3.76E-05	5.0089548
cg11773243	chr7	136613718	+	CHRM2;CHRM2;CHRM2;CHRM2;CHRM2;CHRM2;CHRM2;CHRM2	5'UTR;5'UTR;5'UTR;5'UTR;5'UTR;5'UTR;5'UTR;5'UTR	-1.766414	1.1956294	-5.072416	1.29E-06	3.76E-05	5.0085419
cg17252884	chr3	15369681	+	SH3BP5;SH3BP5	Body;5'UTR	-0.84279	-2.302625	-5.074186	1.29E-06	3.76E-05	5.0091995
cg14016363	chr7	100486297	-			-0.846988	-1.072959	-5.072251	1.29E-06	3.76E-05	5.0078492
cg26458072	chr10	102279694	+	SEC31B	TSS200	0.9481634	-0.240502	5.0740499	1.29E-06	3.76E-05	5.0086306
cg13750949	chr14	105293957	+			-0.708228	-3.127617	-5.072149	1.29E-06	3.76E-05	5.0074207
cg27092704	chr3	153066830	-			-1.252149	1.3186123	-5.072163	1.29E-06	3.76E-05	5.0074819
cg16720947	chr8	145048137	+	PLEC1;PLEC1	Body;TSS1500	-1.158304	-2.253241	-5.072064	1.29E-06	3.76E-05	5.0070661
cg23839680	chr7	56118238	+	CCT6A;PSPH;CCT6A	TSS1500;5'UTR;TSS1500	-1.352429	-0.910484	-5.072056	1.29E-06	3.76E-05	5.0070314

cg08597802	chr1	19657280	-			-0.917153	0.7433713	-5.071982	1.29E-06	3.76E-05	5.0067198
cg05891899	chr11	1326879	-	TOLLIP	Body	0.9530198	0.9037903	5.071732	1.29E-06	3.77E-05	5.0056734
cg12413579	chr1	36108206	-	PSMB2	TSS1500	-0.951189	-1.14333	-5.071665	1.30E-06	3.77E-05	5.0053935
cg20200460	chr10	130165501	-			-1.297264	-0.317426	-5.071612	1.30E-06	3.77E-05	5.0051708
cg20713092	chr7	100946111	-			1.4016022	-0.720124	5.0715513	1.30E-06	3.77E-05	5.0049156
cg01405242	chr8	131705773	-			-1.254979	0.1701151	-5.073249	1.30E-06	3.77E-05	5.0052766
cg02174748	chr14	101507570	+	MIR300	TSS200	-0.993172	-0.208928	-5.071407	1.30E-06	3.77E-05	5.0043104
cg08555552	chr4	13536409	+			1.4840971	-2.419633	5.0714	1.30E-06	3.77E-05	5.0042813
cg23141355	chr3	44063593	-			2.0936628	-0.692713	5.0711967	1.30E-06	3.77E-05	5.0034291
cg04239863	chr16	87093670	+			-1.09937	0.6407198	-5.071105	1.30E-06	3.78E-05	5.003043
cg14175690	chr1	119527638	-	TBX15	5'UTR	1.6286833	0.183934	5.0710611	1.30E-06	3.78E-05	5.0028605
cg06446824	chr1	2535440	+	MMEL1	Body	-0.988728	0.137153	-5.070994	1.30E-06	3.78E-05	5.0025793
cg15266705	chr12	115136096	+			2.3425331	-2.022263	5.0709584	1.30E-06	3.78E-05	5.0024303
cg21067465	chr11	86304288	-	ME3;ME3;ME3	Body;Body;Body	-1.00419	-2.105719	-5.070942	1.30E-06	3.78E-05	5.0023616
cg08150914	chr3	36778755	-	DCLK3	Body	-1.056632	1.5600046	-5.070925	1.30E-06	3.78E-05	5.0022892
cg18229092	chr2	196228235	+			-1.41051	0.6658151	-5.070906	1.30E-06	3.78E-05	5.0022121
cg13521941	chr9	140330627	-	ENTPD8;ENTPD8	Body;Body	-0.839891	-0.769015	-5.070882	1.30E-06	3.78E-05	5.0021106
cg19599407	chr15	33360214	-	FMN1	TSS200	-0.849056	-1.157663	-5.070863	1.30E-06	3.78E-05	5.0020285
cg07635132	chr16	30441833	+	DCTPP1	TSS1500	-1.183033	-0.352217	-5.070658	1.30E-06	3.78E-05	5.0011692
cg01348055	chr16	10272788	-	GRIN2A;GRIN2A;GRIN2A	Body;Body;Body	-0.960827	-0.437372	-5.070639	1.30E-06	3.78E-05	5.0010917

cg15642666	chr19	34174388	-	CHST8;CHST8;CHST8	5'UTR;5'UTR;TSS1500	-1.217893	-0.561404	-5.07054	1.30E-06	3.78E-05	5.0006758
cg07679492	chr16	85233194	-			1.2335535	2.4573406	5.0704591	1.30E-06	3.78E-05	5.000337
cg23280506	chr17	14201938	+			1.4686134	-0.457768	5.0943738	1.30E-06	3.78E-05	5.0145366
cg04997124	chr14	101393397	+	SNORD113-2	TSS1500	-1.330894	0.2828242	-5.070402	1.30E-06	3.78E-05	5.0000991
cg06368760	chr2	237018205	-	AGAP1;AGAP1	Body;Body	0.6835304	2.6640977	5.070425	1.30E-06	3.78E-05	5.0001943
cg22449745	chr1	38156939	-	CDCA8;C1orf109	TSS1500;TSS1500	-0.816381	-1.034127	-5.070408	1.30E-06	3.78E-05	5.0001215
cg16131748	chr13	99959606	+	GPR183;GPR183;UBAC2;UBAC2;UBAC2	5'UTR;1stExon;Body;Body;Body	-1.200936	-1.301579	-5.070374	1.30E-06	3.78E-05	4.9999798
cg00849365	chr16	5074999	+	NAGPA	3'UTR	-0.991162	-1.024913	-5.070318	1.30E-06	3.78E-05	4.9997471
cg01160347	chr14	55908301	+	TBPL2	TSS1500	-1.30521	1.210381	-5.072136	1.30E-06	3.78E-05	5.0006135
cg02459416	chr17	7758256	-	TMEM88	TSS200	1.3762649	1.9514439	5.0718882	1.30E-06	3.79E-05	5.0000056
cg19331215	chr20	23545539	-	CST9L	3'UTR	-1.182585	0.0680924	-5.070044	1.30E-06	3.79E-05	4.9985982
cg24264042	chr3	16738417	-			-1.207084	0.6385599	-5.07183	1.30E-06	3.79E-05	4.9993315
cg24707416	chr7	1277703	+			1.4398432	-2.241152	5.0699661	1.30E-06	3.79E-05	4.9982706
cg02827132	chr16	1052529	+			-2.449841	1.6462207	-5.069896	1.31E-06	3.79E-05	4.997978
cg06976260	chr11	116703588	-	APOC3	Body	-0.99339	1.2871526	-5.069904	1.31E-06	3.79E-05	4.9980082
cg01145333	chr7	42954591	-			0.9094943	2.6894843	5.0698174	1.31E-06	3.79E-05	4.9976473
cg08735716	chr2	129126375	+			-0.959382	0.0300392	-5.069769	1.31E-06	3.79E-05	4.9974438
cg21335942	chr16	58549945	-	SETD6;SETD6	Body;Body	-1.267987	-5.088271	-5.071583	1.31E-06	3.79E-05	4.9987264
cg22129276	chr2	169926103	-	DHRS9;DHRS9	5'UTR;5'UTR	-0.875445	-1.301419	-5.071585	1.31E-06	3.79E-05	4.9987368
cg24525151	chr7	63648835	-			-1.244652	-0.147991	-5.069769	1.31E-06	3.79E-05	4.9974429

cg09341796	chr22	48730484	-			-0.981563	-0.288775	-5.069735	1.31E-06	3.79E-05	4.9972998
cg18732651	chr11	41557290	-			-1.45304	0.5313973	-5.069715	1.31E-06	3.79E-05	4.997218
cg12208782	chr2	84541650	-			-1.201102	0.70325	-5.069353	1.31E-06	3.80E-05	4.9957019
cg16042652	chr13	40396777	+			-1.349391	0.7942726	-5.069316	1.31E-06	3.80E-05	4.9955442
cg02329029	chr22	18559261	+	PEX26;PEX26	TSS1500;TSS1500	-1.280275	1.8650729	-5.06918	1.31E-06	3.80E-05	4.9949741
cg08507422	chr7	1272512	-	UNCX	TSS200	2.5095792	-2.341336	5.0747087	1.31E-06	3.80E-05	5.0154084
cg19953452	chr17	254590	-			-1.126056	0.3055403	-5.069154	1.31E-06	3.80E-05	4.9948661
cg11113536	chr11	115790529	-			-1.265266	-0.393858	-5.069136	1.31E-06	3.80E-05	4.9947926
cg14994944	chr4	110084489	+	COL25A1;COL25A1	Body;Body	-1.356613	0.8899216	-5.06911	1.31E-06	3.80E-05	4.9946822
cg14055212	chr7	104445585	-	LHFPL3	Body	-0.776503	-0.810033	-5.070715	1.31E-06	3.80E-05	4.9950946
cg16008138	chr17	60885892	+	10-Mar	TSS200	-0.940481	-0.249886	-5.068855	1.31E-06	3.80E-05	4.9936144
cg07483650	chr1	248525007	-	OR2T4	1stExon	-1.049855	-1.228862	-5.068813	1.31E-06	3.80E-05	4.9934383
cg09384400	chr3	42546922	-	VIPR1	Body	0.720241	0.9975628	5.068761	1.31E-06	3.80E-05	4.9932198
cg10480735	chr2	61371057	-	C2orf74	TSS1500	0.4599531	2.9089573	5.0687685	1.31E-06	3.80E-05	4.9932509
cg20141733	chr1	73425038	+			-1.476797	0.6392322	-5.068753	1.31E-06	3.80E-05	4.9931879
cg05441133	chr10	48416891	+	GDF2	TSS200	1.0483017	1.0809755	5.0687011	1.31E-06	3.80E-05	4.9929687
cg07163640	chr2	366012	-			-1.315852	0.6930911	-5.068707	1.31E-06	3.80E-05	4.9929935
cg25012143	chr2	117814536	+			-1.578371	1.3518564	-5.068659	1.31E-06	3.80E-05	4.9927909
cg01785706	chr12	118570077	+			-0.973004	-1.218014	-5.068621	1.31E-06	3.80E-05	4.9926347
cg25474312	chr21	31914550	-	KRTAP19-6	TSS1500	-1.394054	0.9031223	-5.068493	1.31E-06	3.81E-05	4.9920974

cg24628816	chr1	6849430	+	CAMTA1	Body	1.0437199	1.6910913	5.0684735	1.31E-06	3.81E-05	4.9920147
cg11325267	chr3	137480853	+			1.1207178	-1.002867	5.0684325	1.31E-06	3.81E-05	4.991843
cg12769732	chr4	170913525	-	MFAP3L;MFAP3L	Body;5'UTR	-1.181443	0.5350552	-5.068446	1.31E-06	3.81E-05	4.9919015
cg21899473	chr14	104980775	+			-1.020535	0.3476174	-5.068392	1.31E-06	3.81E-05	4.991674
cg00250414	chr7	157522373	-	PTPRN2;PTPRN2;PTPRN2	Body;Body;Body	-1.138002	0.1992609	-5.068358	1.31E-06	3.81E-05	4.9915301
cg17805202	chr1	239937440	-	CHRM3	5'UTR	-1.840586	1.3464514	-5.068367	1.31E-06	3.81E-05	4.9915689
cg00809888	chr4	76862425	-	NAAA;NAAA	TSS1500;TSS1500	-1.153436	-4.213477	-5.068208	1.31E-06	3.81E-05	4.9909033
cg10679688	chr13	100624965	-	ZIC5	TSS1500	1.2685717	-0.944663	5.0682053	1.31E-06	3.81E-05	4.9908908
cg19118533	chr6	111927323	-	TRAF3IP2;TRAF3IP2;TRAF3IP2;TRAF3IP2;TRAF3IP2	1stExon;Body;5'UTR;1stExon;5'UTR	-0.460882	-3.812647	-5.070006	1.32E-06	3.81E-05	4.9916978
cg23429042	chr3	32023402	+	ZNF860;OSBPL10	5'UTR;TSS200	-0.780222	-2.250113	-5.068202	1.31E-06	3.81E-05	4.9908777
cg06651311	chr4	37246230	+	KIAA1239	TSS1500	2.1554401	-1.316088	5.0680569	1.32E-06	3.81E-05	4.9902689
cg24696500	chr5	191492	+	LRRC14B	TSS200	-0.713491	-2.166015	-5.068062	1.32E-06	3.81E-05	4.99029
cg02952078	chr1	205419204	-			-0.604925	-1.983879	-5.06801	1.32E-06	3.81E-05	4.9900707
cg24334045	chr22	49376354	-			-0.959014	-0.444947	-5.067966	1.32E-06	3.81E-05	4.9898859
cg21721022	chr6	29407834	-	OR10C1	1stExon	-1.066487	-0.276487	-5.067824	1.32E-06	3.81E-05	4.9892929
cg02579389	chr21	46407004	-			-1.312486	0.0905014	-5.06778	1.32E-06	3.81E-05	4.9891085
cg10819981	chr6	167756099	+	TTLL2	3'UTR	-1.557472	0.9903643	-5.069465	1.32E-06	3.82E-05	4.98986
cg10431265	chr5	159626030	+	FABP6;FABP6	5'UTR;TSS200	-0.79043	-1.586046	-5.067553	1.32E-06	3.82E-05	4.9881586
cg20795401	chr2	173291908	-	ITGA6;ITGA6	TSS1500;TSS1500	-0.967356	-2.130421	-5.0675	1.32E-06	3.82E-05	4.9879365
cg01195545	chr7	150253908	-			-1.32236	-2.056194	-5.067376	1.32E-06	3.82E-05	4.9874141

cg04022561	chr5	50673245	+			1.1685518	-0.940066	5.0674003	1.32E-06	3.82E-05	4.9875177
cg21831463	chr7	132372840	-			-1.09855	-1.232173	-5.067377	1.32E-06	3.82E-05	4.9874189
cg22628926	chr17	4844700	+	SLC25A11;RNF167;SLC25A11;SLC25A11	TSS1500;Body;TSS1500;TSS1500	-0.84275	-1.59669	-5.067154	1.32E-06	3.82E-05	4.9864845
cg19756025	chr6	67658563	-			-1.593249	1.4312111	-5.066917	1.32E-06	3.83E-05	4.985492
cg25767345	chr18	54788969	-			1.2748446	-0.064032	5.0669102	1.32E-06	3.83E-05	4.9854638
cg10775000	chr2	119910430	+			-1.292197	0.0519776	-5.066863	1.32E-06	3.83E-05	4.9852651
cg05574272	chr5	33938213	+	RXFP3	1stExon	0.9056769	-0.002454	5.0667709	1.32E-06	3.83E-05	4.9848801
cg02408311	chr11	5364676	+	OR51B5	1stExon	-1.646092	0.9035059	-5.066748	1.32E-06	3.83E-05	4.9847833
cg25657713	chr6	100038708	+			1.2926426	-0.311529	5.0685526	1.32E-06	3.83E-05	4.9856115
cg04611717	chr12	132897840	+	GALNT9	Body	-1.018038	-0.269896	-5.06666	1.32E-06	3.83E-05	4.9844155
cg15638709	chr10	110226387	+			1.5185437	-0.657863	5.0665196	1.32E-06	3.83E-05	4.9838275
cg02468320	chr12	2404134	-	CACNA1C;CACNA1C;CACNA1C;CACNA1C;CACNA1C;CACNA1C ;CACNA1C;CACNA1C;CACNA1C;CACNA1C;CACNA1C;CACNA1 C;CACNA1C;CACNA1C;CACNA1C;CACNA1C;CACNA1C;CACNA	Body;Body;Body;Body;Body;Body;Body;Body;Body; Body;Body;Body;Body;Body;Body;Body;Body;Body; Body;Body;Body;Body;Body	-1.273188	-1.444362	-5.066482	1.32E-06	3.83E-05	4.9836709
cg21531300	chr14	75988800	+	BATF;BATF	5'UTR;1stExon	-1.250437	-0.903058	-5.066402	1.33E-06	3.83E-05	4.9833345
cg00130693	chr1	226110727	+	PYCR2	Body	-1.302131	0.1089125	-5.068143	1.33E-06	3.83E-05	4.9843265
cg26647303	chr19	2041955	-	MKNK2;MKNK2	Body;Body	-0.915883	-1.502549	-5.066267	1.33E-06	3.83E-05	4.982769
cg07822469	chr1	182585393	+			-1.058872	-3.845649	-5.066126	1.33E-06	3.84E-05	4.9821778
cg15146859	chr18	74961737	+	GALR1	TSS1500	2.6503852	-2.315271	5.067917	1.33E-06	3.84E-05	4.9829511
cg01461862	chr10	27548591	+			0.797804	0.2555282	5.0660715	1.33E-06	3.84E-05	4.9819502
cg13884617	chr7	47987703	-	PKD1L1	Body	0.69939	1.30658	5.0659746	1.33E-06	3.84E-05	4.9815441
cg16287451	chr8	145936947	-			-1.115159	-0.576603	-5.065979	1.33E-06	3.84E-05	4.9815619

cg25274263	chr1	214613732	+	PTPN14	Body	-1.588051	0.9470878	-5.065968	1.33E-06	3.84E-05	4.9815154
cg11744006	chr22	49786128	+			-1.324392	0.7272542	-5.06591	1.33E-06	3.84E-05	4.9812747
cg03271520	chr17	3100171	+	OR1A2	TSS1500	-1.271978	0.1532873	-5.067638	1.33E-06	3.84E-05	4.9822123
cg11150901	chr1	241694605	+	KMO	TSS1500	-0.744856	-4.357698	-5.065745	1.33E-06	3.84E-05	4.9805831
cg10235817	chr4	3768555	-	ADRA2C	1stExon	0.834024	-0.627615	5.0656423	1.33E-06	3.84E-05	4.980152
cg14847243	chr3	184104362	+	CHRD	Body	1.0763364	1.8984853	5.0656573	1.33E-06	3.84E-05	4.980215
cg00539976	chr5	1878508	-	IRX4	Body	1.6531342	-0.383108	5.0655078	1.33E-06	3.84E-05	4.9795886
cg16438688	chr1	156815529	-	NTRK1;INSRR	Body;Body	1.0409237	-0.741729	5.0655133	1.33E-06	3.84E-05	4.9796116
cg09290600	chr4	41753032	-			1.6122317	-1.152624	5.0653304	1.33E-06	3.85E-05	4.9788457
cg11321190	chr3	37239890	+			-0.873884	-1.419007	-5.065294	1.33E-06	3.85E-05	4.9786918
cg18369680	chr20	41984014	-			-1.246396	-0.433847	-5.065304	1.33E-06	3.85E-05	4.9787348
cg03604424	chr5	14171590	+	TRIO	Body	-1.1365	0.5828848	-5.067048	1.33E-06	3.85E-05	4.9797419
cg00097228	chr1	63783167	-			-0.623212	-4.881158	-5.065211	1.33E-06	3.85E-05	4.9783467
cg14445389	chr2	100426973	+	AFF3;AFF3	Body;Body	-1.392741	-0.057779	-5.065154	1.33E-06	3.85E-05	4.9781047
cg19956866	chr7	100610552	-			-0.666366	-0.691478	-5.06516	1.33E-06	3.85E-05	4.9781312
cg02185402	chr20	400361	+	RBCK1;RBCK1	Body;Body	-1.613234	1.565191	-5.065089	1.33E-06	3.85E-05	4.9778343
cg09276486	chr1	6999547	+	CAMTA1	Body	-1.36203	0.4561629	-5.065061	1.33E-06	3.85E-05	4.9777162
cg15357821	chr5	176316988	+	HK3	Body	-1.136527	-0.179593	-5.064988	1.33E-06	3.85E-05	4.9774115
cg14059959	chr13	111144447	-	COL4A2	Body	-0.897781	0.3464158	-5.064934	1.33E-06	3.85E-05	4.9771852
cg21475610	chr4	78077151	-	CCNG2	TSS1500	-1.025759	-0.675998	-5.06485	1.33E-06	3.85E-05	4.9768313

cg22322679	chr6	33244178	-	B3GALT4;RPS18	TSS1500;Body	-0.886571	-0.02302	-5.064646	1.34E-06	3.86E-05	4.9759789
cg12011267	chr14	104314583	+	PPP1R13B	TSS1500	-0.708289	-3.150169	-5.066436	1.34E-06	3.86E-05	4.9771834
cg23328050	chr15	90234224	+	PEX11A;WDR93	TSS1500;5'UTR	-0.572736	-3.966087	-5.064431	1.34E-06	3.86E-05	4.9750791
cg01447112	chr7	6703803	-			2.324264	-1.331585	5.0643978	1.34E-06	3.86E-05	4.9749391
cg10709593	chr2	172952883	+	DLX1;DLX1	Body;3'UTR	1.4404657	-0.646906	5.0643671	1.34E-06	3.86E-05	4.9748105
cg00997130	chr3	10854266	-			-0.976656	-0.780287	-5.064213	1.34E-06	3.86E-05	4.974164
cg02339465	chr15	48665853	-			-1.289998	1.4361151	-5.064167	1.34E-06	3.86E-05	4.9739737
cg01277162	chr13	54604033	-			-1.119655	-0.097183	-5.064102	1.34E-06	3.86E-05	4.9737021
cg08290756	chr17	80882768	-	TBCD	Body	-1.127494	1.0567045	-5.064025	1.34E-06	3.86E-05	4.9733765
cg25034941	chr13	47326127	-	LRCH1	3'UTR	-1.531458	-1.309362	-5.064039	1.34E-06	3.86E-05	4.9734359
cg20256649	chr2	1941523	-	MYT1L	Body	-1.175468	0.7944639	-5.063938	1.34E-06	3.87E-05	4.9730115
cg02279147	chr8	87243677	-	SLC7A13	TSS1500	-1.614737	0.8495004	-5.063883	1.34E-06	3.87E-05	4.9727809
cg00983482	chr10	130083707	+			-1.10303	0.1134349	-5.06379	1.34E-06	3.87E-05	4.9723919
cg16511229	chr7	130126153	+	MEST;MEST	1stExon;5'UTR	-1.187249	-2.273018	-5.063776	1.34E-06	3.87E-05	4.9723368
cg25793571	chr3	128597852	-	ACAD9	TSS1500	-0.590067	-3.997843	-5.063559	1.34E-06	3.87E-05	4.9714265
cg20570464	chr1	42144398	+	HIVEP3;HIVEP3	5'UTR;5'UTR	-1.291973	-0.193188	-5.063465	1.34E-06	3.87E-05	4.9710341
cg13912849	chr3	56762402	-	ARHGEF3;ARHGEF3;ARHGEF3	3'UTR;3'UTR;3'UTR	0.93057	1.909526	5.0633966	1.34E-06	3.87E-05	4.9707456
cg10790429	chr12	106974623	+			1.3672681	-1.747269	5.062924	1.35E-06	3.88E-05	4.9687667
cg02412169	chr5	1732744	-			-1.269429	-0.357332	-5.062741	1.35E-06	3.88E-05	4.9679986
cg09569097	chr7	129419030	-			-0.619973	-4.159572	-5.062753	1.35E-06	3.88E-05	4.9680487

cg01487981	chr6	133120407	+	C6orf192	TSS1500	-0.623995	-3.898322	-5.062557	1.35E-06	3.89E-05	4.9672297
cg08573038	chr12	110399302	+	GIT2;GIT2;GIT2;GIT2;GIT2;GIT2	Body;Body;Body;Body;Body;Body	0.8979943	1.5319886	5.0625345	1.35E-06	3.89E-05	4.9671355
cg27305357	chr5	140773864	-	PCDHGA4;PCDHGA6;PCDHGA1;PCDHGA5;PCDHGB1;PCDHGB4;PCDHGA3;PCDHGA2;PCDHGA8;PCDHGA7;PCDHGB2;PCDHGA8;PCDHGB3	Body;Body;Body;Body;Body;Body;Body;Body;1stExon;Body;Body;1stExon;Body	0.6767704	-0.617046	5.0623857	1.35E-06	3.89E-05	4.9665126
cg24794228	chr19	52391166	+	ZNF577;ZNF577;ZNF577;ZNF577;ZNF577	Body;5'UTR;5'UTR;1stExon;1stExon	1.8556646	-0.512163	5.0658628	1.35E-06	3.89E-05	4.9675436
cg07007382	chr6	32578070	+			-0.964519	-2.473372	-5.061824	1.35E-06	3.90E-05	4.9641617
cg04681472	chr5	149227220	+	PPARGC1B	3'UTR	0.7262719	2.1648586	5.0617979	1.35E-06	3.90E-05	4.9640511
cg24358599	chr8	126250639	-	NSMCE2	Body	1.1102535	1.8359491	5.0617363	1.35E-06	3.90E-05	4.9637932
cg09827071	chr12	57569262	+	LRP1	Body	-0.777433	-1.064045	-5.061637	1.35E-06	3.90E-05	4.963376
cg10692140	chr6	30496072	-			-1.070059	-2.721644	-5.061441	1.35E-06	3.90E-05	4.962558
cg09744840	chr10	74469027	-	CCDC109A	Body	1.3849164	0.1610362	5.0613682	1.36E-06	3.90E-05	4.9622518
cg11613415	chr14	21510384	-	RNASE7;RNASE7	1stExon;5'UTR	-1.317604	0.6836496	-5.061354	1.36E-06	3.90E-05	4.9621941
cg08022436	chr2	155555414	-	KCNJ3	1stExon	1.2376772	-1.25226	5.0611884	1.36E-06	3.91E-05	4.9614992
cg09321403	chr6	13555624	+			1.0266523	0.7024916	5.0610188	1.36E-06	3.91E-05	4.9607892
cg22782986	chr11	79152821	-	ODZ4	TSS1500	1.5520724	-1.524715	5.060979	1.36E-06	3.91E-05	4.9606223
cg22092292	chr17	3462255	+	TRPV3	TSS1500	-1.094358	0.2775306	-5.060838	1.36E-06	3.91E-05	4.9600306
cg07244202	chr10	127750970	-	ADAM12;ADAM12	Body;Body	-1.195633	-0.923814	-5.060814	1.36E-06	3.91E-05	4.9599305
cg21549299	chr21	40984760	+	C21orf88;C21orf88	TSS200;TSS200	1.8510522	-1.535697	5.0607668	1.36E-06	3.91E-05	4.9597342
cg22060735	chr4	56213336	+	SRD5A3	Body	-1.45963	-0.295532	-5.060742	1.36E-06	3.91E-05	4.9596289
cg12608507	chr2	207025477	+	EEF1B2;EEF1B2;SNORD51;NDUFS1;SNORA41;EEF1B2	Body;Body;TSS1500;TSS1500;TSS1500;Body	-1.079304	-1.627509	-5.060673	1.36E-06	3.91E-05	4.9593401
cg16326674	chr4	122301816	-	QRFPR;QRFPR	1stExon;5'UTR	2.1241594	-1.360943	5.0606472	1.36E-06	3.91E-05	4.9592335

cg04519462	chr17	77387741	-	HRNBP3	5'UTR	-0.816942	-2.295275	-5.060566	1.36E-06	3.92E-05	4.9588937
cg15010372	chr1	175832735	-			-1.382935	0.4210056	-5.060526	1.36E-06	3.92E-05	4.9587277
cg09042277	chr12	114840854	-	TBX5;TBX5;TBX5;TBX5	Body;Body;5'UTR;Body	1.3657146	-1.237542	5.0604106	1.36E-06	3.92E-05	4.9582429
cg00831710	chr8	144240945	+	LY6H;LY6H;LY6H	Body;Body;Body	1.0324746	-0.429469	5.0603122	1.36E-06	3.92E-05	4.957831
cg01862363	chr17	8928028	-	NTN1	Body	1.1386403	0.9946656	5.0603156	1.36E-06	3.92E-05	4.9578452
cg21442881	chr8	142160081	-	DENND3	Body	0.7829502	1.5116369	5.0603554	1.36E-06	3.92E-05	4.9580119
cg21704448	chr6	132410188	-			-1.395848	0.5844811	-5.060334	1.36E-06	3.92E-05	4.9579239
cg23789148	chr15	97321146	-			-0.76378	-0.978422	-5.060359	1.36E-06	3.92E-05	4.9580285
cg24233679	chr19	18761792	+	KLHL26	Body	0.6105132	0.4173418	5.0621373	1.36E-06	3.92E-05	4.9591999
cg06014958	chr6	10425553	-			1.4506758	-1.22595	5.0602144	1.36E-06	3.92E-05	4.9574214
cg13499324	chr9	3441530	+	RFX3;RFX3	5'UTR;5'UTR	-1.332051	0.4744379	-5.060229	1.36E-06	3.92E-05	4.957483
cg25940557	chr1	158575177	+	OR10Z1	TSS1500	-1.367312	-0.086826	-5.060228	1.36E-06	3.92E-05	4.9574786
cg13375295	chr19	45344725	+			-1.006722	-0.85521	-5.060183	1.36E-06	3.92E-05	4.9572917
cg12073594	chr20	61903495	-	ARFGAP1;ARFGAP1	TSS1500;TSS1500	-0.605835	-2.635142	-5.060033	1.36E-06	3.92E-05	4.9566614
cg12550241	chr1	248859041	+			-1.227863	-0.362436	-5.060002	1.36E-06	3.92E-05	4.9565311
cg06822689	chr3	141516291	-	GRK7	Body	1.86932	-1.48141	5.0598441	1.36E-06	3.92E-05	4.9558716
cg12838303	chr6	154360670	+	OPRM1;OPRM1;OPRM1;OPRM1;OPRM1;OPRM1;OPRM1;OPRM1;OPRM1;OPRM1;OPRM1;OPRM1;OPRM1;OPRM1;OPRM1;OPRM1;OPRM1;OPRM1;OPRM1;OPRM1	1stExon;1stExon;1stExon;5'UTR;5'UTR;1stExon;5'UTR;5'UTR;Body;5'UTR;1stExon;5'UTR;1stExon;5'UTR;1stExon;5'UTR;5'UTR;5'UTR;Body;1stExon;1stExon	1.2969925	-1.187054	5.059793	1.36E-06	3.92E-05	4.9556577
cg15235798	chr13	112720244	+			1.4728979	-0.508144	5.0653147	1.36E-06	3.92E-05	4.9791462
cg14318679	chr17	700464	+			1.0344634	1.9405503	5.0632244	1.37E-06	3.93E-05	4.9573906
cg21207450	chr15	29570795	-	FAM189A1	Body	-0.977986	-0.266662	-5.059587	1.37E-06	3.93E-05	4.9547933

cg05915362	chr6	159274480	-	OSTCL	Body	-0.662594	-3.270029	-5.05953	1.37E-06	3.93E-05	4.9545553
cg05042706	chr1	48703955	+	SLC5A9;SLC5A9	Body;Body	-0.874494	-0.506257	-5.059181	1.37E-06	3.93E-05	4.9530951
cg00691451	chr2	168609834	+			-1.198511	1.9946143	-5.059077	1.37E-06	3.93E-05	4.9526585
cg04750099	chr11	57958248	-	OR9Q2	1stExon	-1.260441	0.1808879	-5.059007	1.37E-06	3.94E-05	4.952367
cg04780434	chr10	134402787	-	INPP5A	Body	0.7774398	0.5661392	5.0589942	1.37E-06	3.94E-05	4.9523142
cg11118422	chr6	28557883	+			1.3257678	-0.176686	5.0590016	1.37E-06	3.94E-05	4.9523448
cg19610529	chr14	61108807	-			1.4297535	0.1657438	5.0589829	1.37E-06	3.94E-05	4.9522668
cg09224821	chr17	80329944	-			1.2478933	-1.166026	5.0589525	1.37E-06	3.94E-05	4.9521396
cg10541864	chr4	174450722	+	HAND2;NBLA00301;HAND2	5'UTR;TSS1500;1stExon	1.7813014	-0.440575	5.0589312	1.37E-06	3.94E-05	4.9520504
cg13986969	chr9	73984868	-			-1.360843	0.4940984	-5.058942	1.37E-06	3.94E-05	4.9520936
cg02008511	chr5	54737053	+	PPAP2A;PPAP2A	Body;Body	-0.791779	1.9694351	-5.05891	1.37E-06	3.94E-05	4.9519607
cg04251307	chr12	10827012	-	STYK1	TSS200	-0.653722	-2.976638	-5.058864	1.37E-06	3.94E-05	4.9517673
cg01236063	chr11	16632088	+			1.0517333	0.4406873	5.0587094	1.37E-06	3.94E-05	4.9511218
cg15104644	chr1	231297103	+			2.2889723	-1.876828	5.0586782	1.37E-06	3.94E-05	4.9509912
cg00333870	chr7	51870809	-			-0.869872	-0.373434	-5.05857	1.37E-06	3.94E-05	4.9505406
cg00432233	chr6	6292977	+	F13A1	Body	-1.517362	1.0699103	-5.058546	1.37E-06	3.94E-05	4.9504399
cg02344833	chr19	58545285	-	ZSCAN1	TSS200	2.0253559	-1.26654	5.0585555	1.37E-06	3.94E-05	4.9504779
cg15594444	chr1	59893192	-	FGGY;FGGY	Body;Body	-1.707227	0.8298638	-5.058068	1.37E-06	3.95E-05	4.9484379
cg18023666	chr5	173148154	+			0.8064732	0.9633878	5.0580663	1.37E-06	3.95E-05	4.9484307
cg09293559	chr16	72911729	-	ZFHX3;ZFHX3	Body;Body	-1.352381	1.5126303	-5.057939	1.38E-06	3.95E-05	4.947897

cg05899507	chr14	101349120	+	MIR127;RTL1	TSS200;1stExon	-1.152445	0.9024726	-5.059561	1.38E-06	3.95E-05	4.9479954
cg11142705	chr3	42306974	-	CCK	TSS1500	1.4867924	-1.89598	5.0577637	1.38E-06	3.95E-05	4.9471641
cg25284213	chr6	52009154	-	MIR206	Body	-1.036201	-0.568102	-5.057739	1.38E-06	3.95E-05	4.9470615
cg04638205	chr5	3002493	-			-1.782811	0.4092286	-5.057667	1.38E-06	3.95E-05	4.9467592
cg24086536	chr19	56444551	+	NLRP13	TSS1500	-1.55194	0.9082543	-5.057618	1.38E-06	3.95E-05	4.9465542
cg22705388	chr7	19443825	-			-1.313106	1.2611612	-5.057434	1.38E-06	3.96E-05	4.9457832
cg26753302	chr18	61143993	+	SERPINB5	TSS200	-1.441747	-0.097395	-5.057398	1.38E-06	3.96E-05	4.9456349
cg23580945	chr17	37186341	-			-1.065233	0.5876656	-5.057258	1.38E-06	3.96E-05	4.945049
cg07441143	chr8	143824001	+	SLURP1	TSS200	-0.869683	-0.388021	-5.057119	1.38E-06	3.96E-05	4.9444651
cg07924653	chr4	16883525	-	LDB2;LDB2	Body;Body	-1.729444	1.6969159	-5.057131	1.38E-06	3.96E-05	4.9445185
cg02172150	chr7	70597774	+	WBSCR17	TSS200	1.6645092	-1.571345	5.0571021	1.38E-06	3.96E-05	4.9443957
cg05330708	chr16	1077597	-			-1.17116	0.3515117	-5.056861	1.38E-06	3.97E-05	4.9433856
cg06956444	chr1	8001309	-	TNFRSF9	TSS1500	-1.253109	0.6851307	-5.056825	1.38E-06	3.97E-05	4.9432347
cg20770516	chr7	104681396	-	MLL5;MLL5	5'UTR;5'UTR	0.7641634	1.1028832	5.0568206	1.38E-06	3.97E-05	4.9432175
cg16512667	chr1	5809346	+			-1.255566	0.9107675	-5.056801	1.38E-06	3.97E-05	4.9431355
cg08440077	chr2	241854588	-			-1.166003	0.0014492	-5.056745	1.38E-06	3.97E-05	4.9429011
cg25561762	chr13	20876028	+			1.3260856	-0.755984	5.0567521	1.38E-06	3.97E-05	4.9429311
cg04651781	chr4	1398798	+			1.5296675	-1.057263	5.0566679	1.38E-06	3.97E-05	4.9425788
cg13246744	chr13	91826938	-			-1.315943	1.7358864	-5.056673	1.38E-06	3.97E-05	4.9425988
cg01709766	chr11	5640318	+	TRIM6-TRIM34;TRIM34	Body;TSS1500	-0.989466	-0.023075	-5.056612	1.38E-06	3.97E-05	4.9423437

cg06530338	chr2	19561612	+			2.6197315	-1.55309	5.0565766	1.38E-06	3.97E-05	4.9421968
cg24513522	chr17	77393929	-	HRNBP3	5'UTR	-0.856663	-0.280444	-5.056575	1.38E-06	3.97E-05	4.9421893
cg15209440	chr1	179696814	+			-1.188458	0.0691464	-5.056456	1.38E-06	3.97E-05	4.9416937
cg18118147	chr19	51128640	+	SYT3;SYT3;SYT3	Body;Body;Body	-2.317082	1.2669992	-5.058167	1.39E-06	3.97E-05	4.942168
cg07885629	chr7	129422915	-			1.4041331	-1.261961	5.0562705	1.39E-06	3.97E-05	4.9409159
cg10596768	chr17	41053112	-	G6PC	1stExon	-1.253915	-0.368807	-5.065615	1.39E-06	3.97E-05	4.9463807
cg22535084	chr7	1426162	-			-1.223067	0.8966712	-5.056284	1.39E-06	3.97E-05	4.9409708
cg18172877	chr5	1878672	+	IRX4	Body	2.0212364	-1.328524	5.0562381	1.39E-06	3.97E-05	4.9407804
cg24592790	chr8	27450047	+			-0.916885	-4.277031	-5.05624	1.39E-06	3.97E-05	4.9407877
cg08431709	chr10	85602560	+			-1.391199	0.9918493	-5.056161	1.39E-06	3.97E-05	4.9404573
cg24750674	chr17	79097243	+	AATK	Body	-1.410002	1.0667301	-5.057963	1.39E-06	3.97E-05	4.9417454
cg11812928	chr1	227164459	+	CABC1	Body	0.485399	0.6828925	5.0561022	1.39E-06	3.97E-05	4.9402117
cg26294716	chr21	31972923	+	KRTAP22-1	TSS1500	-1.426095	1.3746763	-5.056058	1.39E-06	3.97E-05	4.9400272
cg07403610	chr3	152438075	+			-1.58811	0.3351434	-5.056001	1.39E-06	3.97E-05	4.9397895
cg10132917	chr7	30951801	-	AQP1	1stExon	1.1246574	0.4890755	5.0559889	1.39E-06	3.97E-05	4.9397377
cg15645309	chr14	75988747	-	BATF	TSS200	-1.58799	-0.942313	-5.055652	1.39E-06	3.98E-05	4.9383284
cg04068564	chr20	57795246	+	ZNF831	Body	-1.81528	0.8057716	-5.055417	1.39E-06	3.98E-05	4.9373447
cg25494730	chr8	6664028	-			-1.087863	0.3975494	-5.055354	1.39E-06	3.99E-05	4.9370808
cg20842326	chr7	157476887	-	PTPRN2;PTPRN2;PTPRN2	Body;Body;Body	1.3044438	-1.450249	5.055286	1.39E-06	3.99E-05	4.936797
cg14016057	chr4	54975250	+			0.9636191	-0.594339	5.0551427	1.39E-06	3.99E-05	4.9361978

cg20410114	chr6	3160502	-			-1.469577	-0.172182	-5.0551	1.39E-06	3.99E-05	4.9360172
cg01635742	chr3	64561075	-	ADAMTS9	Body	-1.098856	1.3135917	-5.054955	1.39E-06	3.99E-05	4.9354131
cg18308516	chr11	393648	-	PKP3	TSS1500	-0.601273	-1.128877	-5.054909	1.39E-06	3.99E-05	4.9352204
cg07834418	chr10	107409739	-			-1.421142	0.019885	-5.054795	1.39E-06	3.99E-05	4.9347442
cg15292275	chr13	109149011	+			1.9729635	-2.434744	5.0547829	1.39E-06	3.99E-05	4.9346924
cg19686152	chr15	52121542	-	TMOD3	TSS1500	-0.701905	-0.765083	-5.054803	1.39E-06	3.99E-05	4.9347761
cg15861585	chr11	637038	+	DRD4	TSS1500	1.7006611	-0.771832	5.0547388	1.40E-06	3.99E-05	4.9345078
cg08358332	chr2	30789342	-	LCLAT1;LCLAT1	Body;Body	0.9193383	1.6734116	5.0545092	1.40E-06	4.00E-05	4.9335477
cg19360330	chr13	95355347	-			1.6284344	-1.436322	5.0543032	1.40E-06	4.00E-05	4.9326858
cg11432962	chr13	95354741	+			1.4816853	-1.554203	5.054266	1.40E-06	4.00E-05	4.9325301
cg04342924	chr8	142185183	-	DENND3	Body	-0.549577	-3.544999	-5.054206	1.40E-06	4.00E-05	4.9322774
cg26186727	chr18	70534534	+	NETO1;NETO1	1stExon;5'UTR	1.3652839	-1.316095	5.0540837	1.40E-06	4.00E-05	4.9317677
cg20122727	chr4	75719957	-	BTC	TSS200	-0.70004	-2.474751	-5.05769	1.40E-06	4.00E-05	4.934277
cg09026722	chr1	156867080	+	PEAR1	5'UTR	-1.395333	0.686632	-5.054	1.40E-06	4.00E-05	4.9314156
cg02768671	chr6	169470822	+			-0.836654	-0.716531	-5.053895	1.40E-06	4.01E-05	4.9309791
cg06222885	chr4	182889207	-			-1.424514	0.7193277	-5.053881	1.40E-06	4.01E-05	4.9309187
cg17073306	chr4	671791	-	MYL5;MYL5	1stExon;5'UTR	-0.904505	-0.65027	-5.053803	1.40E-06	4.01E-05	4.9305933
cg00126148	chr4	111533517	+			1.2595304	-1.376897	5.0536931	1.40E-06	4.01E-05	4.9301339
cg16474701	chr8	27321661	-	CHRNA2	Body	-1.353616	1.4626455	-5.053543	1.40E-06	4.01E-05	4.9295062
cg12543918	chr5	38403957	+	EGFLAM;EGFLAM;EGFLAM;EGFLAM;EGFLAM	Body;5'UTR;1stExon;1stExon;5'UTR	-1.246418	-0.103527	-5.053372	1.40E-06	4.01E-05	4.92879

cg16651895	chr16	77910291	+	VAT1L	Body	-1.25501	-0.438526	-5.053299	1.40E-06	4.02E-05	4.928485
cg23656213	chr20	25177389	-	ENTPD6;ENTPD6	5'UTR;Body	-0.962657	-1.203803	-5.053309	1.40E-06	4.02E-05	4.9285274
cg14479709	chr8	1249007	-			-1.142437	-0.526191	-5.053162	1.40E-06	4.02E-05	4.9279127
cg15707110	chr8	144311102	+			-1.227922	-0.001443	-5.053137	1.41E-06	4.02E-05	4.9278102
cg03918288	chr8	145101324	+	SPATC1;SPATC1	Body;Body	-0.872648	-1.027861	-5.053074	1.41E-06	4.02E-05	4.9275463
cg10649130	chr11	9682641	-			-1.208556	-0.433153	-5.052911	1.41E-06	4.02E-05	4.9268649
cg11493661	chr6	32016239	-	TNXB	Body	-1.041684	-1.425819	-5.052854	1.41E-06	4.02E-05	4.9266243
cg25321854	chr18	6916835	-			-1.299772	0.2867283	-5.05283	1.41E-06	4.02E-05	4.9265222
cg01220257	chr19	45260935	+	BCL3	Body	0.817153	0.20895	5.0542449	1.41E-06	4.03E-05	4.9262101
cg01152727	chr12	29940622	-			-1.146192	0.5392338	-5.0523	1.41E-06	4.03E-05	4.924308
cg10024505	chr14	76818832	+			-0.854681	0.0164519	-5.052287	1.41E-06	4.03E-05	4.9242538
cg05408203	chr16	1258830	+	CACNA1H;CACNA1H	Body;Body	-1.282151	1.3016365	-5.054064	1.41E-06	4.03E-05	4.9254526
cg01983682	chr2	162283979	+			0.8998472	-0.30537	5.0521514	1.41E-06	4.03E-05	4.9236866
cg13510050	chr14	24550395	+	NRL	3'UTR	-0.476883	-4.18427	-5.052037	1.41E-06	4.03E-05	4.9232093
cg06554192	chr18	75691425	-			-1.141698	0.3287236	-5.051877	1.41E-06	4.04E-05	4.9225398
cg09244312	chr13	42120657	-			-1.0779	0.6933018	-5.051865	1.41E-06	4.04E-05	4.9224899
cg02714994	chr19	15870836	+			-0.897922	-0.683038	-5.051813	1.41E-06	4.04E-05	4.9222698
cg14265075	chr1	157016521	-	ARHGEF11;ARHGEF11	TSS1500;TSS1500	-0.892868	-2.431881	-5.053628	1.41E-06	4.04E-05	4.9232019
cg23985388	chr17	31350176	+	ACCN1;ACCN1	Body;Body	-1.340135	0.6293881	-5.05183	1.41E-06	4.04E-05	4.9223408
cg15246805	chr6	133584189	-	EYA4;EYA4;EYA4	5'UTR;5'UTR;5'UTR	-1.404783	0.4207218	-5.051634	1.41E-06	4.04E-05	4.9215228

cg18112117	chr8	101965903	-	YWHAZ;YWHAZ	TSS1500;TSS1500	-0.478013	-3.961232	-5.051613	1.41E-06	4.04E-05	4.9214361
cg21963436	chr13	28541142	+	CDX2	Body	1.6328174	-2.093105	5.0516255	1.41E-06	4.04E-05	4.9214872
cg22679725	chr1	27702662	-	FCN3;FCN3	TSS1500;TSS1500	0.7469051	2.3585313	5.0515759	1.41E-06	4.04E-05	4.92128
cg13767755	chr2	223162800	-	PAX3;PAX3;PAX3;PAX3;PAX3;PAX3;PAX3;PAX3;CCDC140	Body;Body;Body;Body;Body;Body;Body;Body;TSS200	1.2884799	-0.930459	5.0515278	1.42E-06	4.04E-05	4.9210788
cg01774894	chr17	46674395	+	LOC404266;LOC404266;LOC404266;LOC404266;HOXB6;LOC404266	Body;Body;Body;Body;Body;Body	2.4961291	-0.634551	5.056934	1.42E-06	4.04E-05	4.9242621
cg13217260	chr2	176981469	-	HOXD10	TSS200	1.5160836	-1.72256	5.0514249	1.42E-06	4.04E-05	4.9206485
cg16887183	chr15	80658049	+			-1.349223	0.2424647	-5.051395	1.42E-06	4.04E-05	4.9205241
cg02849956	chr19	4634827	-			-1.145431	-1.185538	-5.051253	1.42E-06	4.04E-05	4.919928
cg16239184	chr6	135079577	+			-1.169509	-1.400451	-5.051238	1.42E-06	4.04E-05	4.9198683
cg15556502	chr11	43602845	-	MIR129-2	TSS200	2.7539324	-1.986487	5.0527073	1.42E-06	4.05E-05	4.9193555
cg14797295	chr7	93641451	+			-1.22412	0.4840265	-5.050819	1.42E-06	4.05E-05	4.9181158
cg00055679	chr13	79170430	-			0.9008284	-0.657477	5.0507652	1.42E-06	4.05E-05	4.9178904
cg08203192	chr2	45880618	+	PRKCE	Body	0.8539597	1.9726604	5.0507719	1.42E-06	4.05E-05	4.9179183
cg26657101	chr15	97322869	+			-1.044142	0.7956392	-5.050793	1.42E-06	4.05E-05	4.9180064
cg03128163	chr10	131686154	-	EBF3	Body	-1.181226	0.7920798	-5.050639	1.42E-06	4.05E-05	4.9173608
cg03109827	chr10	133110349	-	TCERG1L	TSS1500	2.245375	-1.648751	5.0506121	1.42E-06	4.05E-05	4.91725
cg20011459	chr5	15936642	-	FBXL7	Body	-1.399102	0.9377346	-5.050561	1.42E-06	4.05E-05	4.9170345
cg20707023	chr5	141133392	-			-1.15846	-2.405677	-5.050554	1.42E-06	4.05E-05	4.9170068
cg09299053	chr7	57509762	-	ZNF716	TSS200	-1.665639	0.6332702	-5.050398	1.42E-06	4.06E-05	4.9163554
cg18933494	chr11	8616016	-	STK33	TSS1500	-0.578329	-3.284652	-5.050353	1.42E-06	4.06E-05	4.9161682

cg13355424	chr1	157165134	+			0.8670415	-0.031247	5.0502847	1.42E-06	4.06E-05	4.9158813
cg07877559	chr5	11903299	+	CTNND2	Body	1.3918242	-1.198508	5.0502439	1.42E-06	4.06E-05	4.9157106
cg08530879	chr12	51071743	-	DIP2B	Body	1.1106615	0.672915	5.0519567	1.42E-06	4.06E-05	4.9162205
cg25928474	chr17	48716301	+	ABCC3;ABCC3	Body;Body	-1.317727	0.5140408	-5.05008	1.42E-06	4.06E-05	4.9150269
cg01547344	chr5	153282014	+			-1.545962	1.1792728	-5.049973	1.42E-06	4.06E-05	4.9145768
cg18158670	chr15	99433225	+	IGF1R	Body	-1.210443	1.0293345	-5.04992	1.43E-06	4.06E-05	4.9143548
cg00741900	chr14	102027797	+	DIO3;DIO3;MIR1247	5'UTR;1stExon;TSS1500	1.166498	-0.721758	5.0498571	1.43E-06	4.06E-05	4.9140938
cg21797500	chr13	84406289	-			-1.548385	0.9052547	-5.049788	1.43E-06	4.07E-05	4.9138059
cg01963002	chr7	97735575	-	LMTK2	TSS1500	-0.517951	-2.046106	-5.049641	1.43E-06	4.07E-05	4.9131913
cg16764362	chr16	87682674	-	JPH3	Body	-0.864392	-0.701181	-5.049657	1.43E-06	4.07E-05	4.9132571
cg17167253	chr2	119603383	+	EN1	Body	1.3972971	-1.12924	5.0496375	1.43E-06	4.07E-05	4.9131755
cg02587316	chr19	37096321	-	ZNF529;ZNF382;ZNF529;ZNF529	TSS200;5'UTR;TSS200;TSS200	2.2244849	-1.189816	5.0494367	1.43E-06	4.07E-05	4.9123361
cg21806750	chr1	86673476	+			-1.532796	1.1784024	-5.049444	1.43E-06	4.07E-05	4.912366
cg07092157	chr12	89353329	-			-1.500229	0.5221077	-5.049361	1.43E-06	4.07E-05	4.9120203
cg11983576	chr15	53090807	-			1.5798031	-1.470795	5.0493071	1.43E-06	4.07E-05	4.9117944
cg23262218	chr10	101290270	-			1.0706179	-0.50367	5.0492338	1.43E-06	4.07E-05	4.9114883
cg11020821	chr15	79128547	-			-1.363904	0.6308377	-5.049186	1.43E-06	4.07E-05	4.911287
cg25121437	chr11	125322169	-	FEZ1	Body	0.8443355	1.69937	5.0491435	1.43E-06	4.07E-05	4.9111106
cg21399832	chr4	117847410	-			1.5632174	-0.296106	5.0490969	1.43E-06	4.07E-05	4.9109157
cg00794881	chr1	19043573	+	PAX7;PAX7;PAX7	Body;Body;Body	1.2297322	-1.690325	5.0508794	1.43E-06	4.07E-05	4.9293654

cg23367107	chr12	115136287	-			2.5424834	-2.990256	5.0489406	1.43E-06	4.08E-05	4.9102624
cg01827999	chr17	17791914	-	TOM1L2;TOM1L2	Body;Body	0.6156209	-0.144775	5.0488167	1.43E-06	4.08E-05	4.9097446
cg02202933	chr14	105101693	+			-1.071986	0.2717832	-5.048798	1.43E-06	4.08E-05	4.9096672
cg04642300	chr6	109288592	-	ARMC2	Body	-1.180282	-0.425214	-5.048769	1.43E-06	4.08E-05	4.9095452
cg17657179	chr19	52227309	-	HAS1	TSS200	1.3363576	-1.065833	5.0486802	1.43E-06	4.08E-05	4.9091742
cg19303305	chr4	8277560	+	HTRA3	Body	-0.856048	0.1306523	-5.048642	1.43E-06	4.08E-05	4.9090136
cg02846841	chr6	28945341	-			1.1145841	0.4120989	5.0485856	1.43E-06	4.08E-05	4.9087786
cg13439414	chr8	93385654	-			-1.341134	0.5888069	-5.050365	1.43E-06	4.08E-05	4.9100028
cg21513316	chr14	101532296	+	MIR410;MIR656	Body;TSS1500	-1.024532	0.4160313	-5.048504	1.43E-06	4.08E-05	4.9084366
cg23012591	chr3	73923792	-			-1.16894	-0.288698	-5.048418	1.43E-06	4.08E-05	4.9080796
cg22845912	chr8	134059874	-	SLA;TG;SLA;SLA	Body;Body;Body;Body	-1.308339	0.2432497	-5.048378	1.43E-06	4.08E-05	4.9079109
cg19680583	chr5	83409549	-	EDIL3	Body	-1.502789	1.6242177	-5.05015	1.43E-06	4.08E-05	4.9086757
cg04293602	chr11	65553660	+	OVOL1	TSS1500	0.8717333	0.6222275	5.0483218	1.44E-06	4.08E-05	4.9076759
cg16779976	chr10	98031125	+	BLNK;BLNK	1stExon;1stExon	-1.021835	-0.236608	-5.048027	1.44E-06	4.09E-05	4.9064425
cg22282365	chr18	76727282	+			-1.406452	1.1090717	-5.047962	1.44E-06	4.09E-05	4.9061723
cg07152755	chr2	105474472	+			1.1538451	-0.799174	5.0477737	1.44E-06	4.09E-05	4.9053854
cg03682112	chr18	77623598	+	KCNG2	TSS200	1.1232648	2.5371624	5.0511367	1.44E-06	4.10E-05	4.906934
cg07865418	chr5	6411408	+			-1.359214	-0.427764	-5.047548	1.44E-06	4.10E-05	4.9044435
cg10297191	chr6	46620661	+	CYP39A1;SLC25A27	TSS200;TSS200	1.511312	-4.126827	5.0475395	1.44E-06	4.10E-05	4.9044065
cg12641739	chr1	235062535	-			-1.077375	0.3346588	-5.047512	1.44E-06	4.10E-05	4.9042906

cg05281206	chr17	32682770	+	CCL13	TSS1500	-1.62689	0.1552362	-5.047478	1.44E-06	4.10E-05	4.9041494
cg22593953	chr3	186781783	+	ST6GAL1;ST6GAL1;ST6GAL1	Body;Body;Body	-1.113486	-0.238419	-5.047416	1.44E-06	4.10E-05	4.9038905
cg23003832	chr9	131486918	+	ZDHHC12	TSS1500	-0.609852	-2.592861	-5.04742	1.44E-06	4.10E-05	4.9039078
cg27662750	chr16	65606213	+	LOC283867	Body	-1.546421	1.2140388	-5.047441	1.44E-06	4.10E-05	4.9039968
cg06135139	chr20	61637856	+	BHLHE23	1stExon	1.8673291	-0.840172	5.0473308	1.44E-06	4.10E-05	4.9035345
cg26314330	chr3	27772633	+			1.4598959	-1.372966	5.047304	1.44E-06	4.10E-05	4.9034226
cg27308982	chr18	77196450	-	NFATC1;NFATC1;NFATC1;NFATC1;NFATC1	Body;5'UTR;Body;Body;Body	-1.310206	1.4979887	-5.047241	1.44E-06	4.10E-05	4.9031611
cg01236132	chr13	112723581	+	SOX1;SOX1	1stExon;3'UTR	1.2368427	-0.532943	5.0471107	1.44E-06	4.10E-05	4.9026146
cg01360115	chr22	50747878	-			0.9062944	0.0251412	5.047044	1.44E-06	4.10E-05	4.9023361
cg19925558	chr1	1072370	-			-0.686196	-2.601678	-5.04885	1.44E-06	4.10E-05	4.9032489
cg02523844	chr7	79083507	-	MAGI2	TSS1500	1.7889242	-1.354282	5.0468608	1.44E-06	4.11E-05	4.9015702
cg02298116	chr14	57264579	+			1.3679049	-1.271588	5.0468288	1.44E-06	4.11E-05	4.9014366
cg15067713	chr3	137433605	+			-1.78335	1.9527782	-5.046779	1.44E-06	4.11E-05	4.9012269
cg11557618	chr2	172974138	+			0.8392613	-0.303836	5.0467509	1.45E-06	4.11E-05	4.9011109
cg13844500	chr11	123495493	+			-1.438291	0.7493223	-5.046738	1.45E-06	4.11E-05	4.9010553
cg15839431	chr19	19639596	-	YJEFN3	TSS200	1.2455762	2.4522568	5.046701	1.45E-06	4.11E-05	4.9009025
cg21763211	chr19	12932850	-			-1.088723	0.5589183	-5.046709	1.45E-06	4.11E-05	4.900936
cg18770739	chr12	56031142	-	OR10P1	1stExon	-0.784601	-0.236725	-5.046676	1.45E-06	4.11E-05	4.9007995
cg07450086	chr3	150320765	-	SELT	TSS1500	-0.819453	-2.188416	-5.046593	1.45E-06	4.11E-05	4.9004514
cg04424621	chr6	27101941	+	HIST1H2BJ	TSS1500	-1.647297	-2.079902	-5.046515	1.45E-06	4.11E-05	4.9001245

cg15208197	chr1	156561146	+	APOA1BP	TSS1500	-1.264956	-1.622143	-5.046529	1.45E-06	4.11E-05	4.9001833
cg03312805	chr22	48889627	+	FAM19A5	Body	-1.4237	0.5820962	-5.046375	1.45E-06	4.11E-05	4.8995423
cg27054610	chr6	32186164	+	NOTCH4	Body	-1.269762	0.6597639	-5.04637	1.45E-06	4.11E-05	4.8995199
cg18831371	chr5	176037325	+	GPRIN1	TSS200	-0.419801	-4.15403	-5.048005	1.45E-06	4.11E-05	4.8997204
cg26153182	chr7	154703374	+			-1.611833	0.8666783	-5.045876	1.45E-06	4.12E-05	4.8974572
cg08130572	chr6	112081102	+	FYN;FYN	5'UTR;TSS1500	1.2646395	-0.406059	5.0457124	1.45E-06	4.12E-05	4.8967719
cg02516330	chr8	20457026	+			-1.095171	0.1973145	-5.045671	1.45E-06	4.12E-05	4.8965981
cg06679538	chr15	101458783	-	LRRK1	TSS1500	-1.090458	-3.382276	-5.045602	1.45E-06	4.12E-05	4.8963102
cg16670307	chr11	131720640	-	NTM	Body	1.1299404	1.6611841	5.0456025	1.45E-06	4.12E-05	4.8963126
cg25079743	chr16	30441674	+	DCTPP1	TSS1500	-0.461476	-4.087256	-5.04557	1.45E-06	4.12E-05	4.8961749
cg14302471	chr7	158799777	-	LOC154822	TSS1500	-2.288263	1.4309466	-5.045491	1.45E-06	4.13E-05	4.8958462
cg00584450	chr16	86612312	+	FOXL1;FOXL1	1stExon;5'UTR	1.2291059	-0.69957	5.0454688	1.45E-06	4.13E-05	4.8957539
cg16251205	chr7	153586592	-	DPP6	Body	-1.520735	0.8461096	-5.045378	1.45E-06	4.13E-05	4.8953762
cg24330456	chr6	30038955	-	RNF39;RNF39	Body;Body	1.1552608	2.5714744	5.0471472	1.45E-06	4.13E-05	4.8965716
cg07027430	chr16	7354685	+	A2BP1;A2BP1;A2BP1	Body;Body;Body	1.2210919	-0.678809	5.0450689	1.46E-06	4.13E-05	4.8940832
cg10904109	chr6	146755494	-	GRM1;GRM1	3'UTR;Body	1.0721031	-0.785567	5.0450353	1.46E-06	4.13E-05	4.8939429
cg25952581	chr7	27170961	-	HOXA4	TSS1500	1.0480239	-0.653425	5.0448328	1.46E-06	4.14E-05	4.8930968
cg22151446	chr5	140810805	+	PCDHGA4;PCDHGA11;PCDHGA11;PCDHGA9;PCDHGA1;PCDH GB1;PCDHGB6;PCDHGA12;PCDHGB3;PCDHGB7;PCDHGA6;PC DHGA8;PCDHGA10;PCDHGA12;PCDHGA5;PCDHGB4;PCDHGA	Body;Body;Body;Body;Body;Body;Body;1stExon;Bod y;Body;Body;Body;Body;1stExon;Body;Body;Body;B ody;Body;Body;Body	1.1817059	-0.536983	5.0443858	1.46E-06	4.14E-05	4.8912297
cg12036544	chr10	22013777	-	MLLT10;MLLT10	Body;Body	1.2345107	-3.188385	5.0443504	1.46E-06	4.14E-05	4.8910816
cg27504369	chr22	39353550	+	APOBEC3A;APOBEC3A	1stExon;5'UTR	-1.074301	-0.954043	-5.044356	1.46E-06	4.14E-05	4.8911044

cg26545519	chr2	418075	+			-1.403145	0.0679219	-5.044335	1.46E-06	4.14E-05	4.8910163
cg20810396	chr13	53779590	-			-1.215159	0.6787324	-5.044155	1.46E-06	4.15E-05	4.8902661
cg12193327	chr2	31454631	+			1.0464351	1.0167272	5.0440257	1.46E-06	4.15E-05	4.8897253
cg08832018	chr3	156543647	-	LEKR1	TSS1500	-1.027199	-1.002046	-5.043969	1.46E-06	4.15E-05	4.8894893
cg26992245	chr8	29848579	-			-1.566087	-0.243254	-5.043903	1.46E-06	4.15E-05	4.8892127
cg03545404	chr5	149556350	-	CDX1	Body	-0.967284	-1.510253	-5.043774	1.46E-06	4.15E-05	4.888672
cg09341793	chr14	94405681	-	ASB2	Body	2.0046028	-0.179456	5.0455814	1.46E-06	4.15E-05	4.8900367
cg14741939	chr3	147127137	+	ZIC1	TSS200	1.3552816	-1.167264	5.0437376	1.46E-06	4.15E-05	4.8885218
cg19310458	chr2	80361612	-	CTNNA2;CTNNA2	Body;Body	-1.498989	0.3937407	-5.043716	1.46E-06	4.15E-05	4.8884302
cg01032675	chr19	3136430	+	GNA15;GNA15	1stExon;5'UTR	-0.573403	0.2628806	-5.043671	1.46E-06	4.15E-05	4.8882439
cg06632549	chr6	31529993	+			-1.123045	-0.352757	-5.043629	1.46E-06	4.15E-05	4.8880702
cg11375458	chr2	177003996	-			1.1039919	0.2718835	5.0436132	1.47E-06	4.15E-05	4.8880024
cg02076869	chr7	155301917	+	CNPY1	5'UTR	1.5414487	-1.516916	5.0435391	1.47E-06	4.15E-05	4.887693
cg14987564	chr14	60557946	+	C14orf135	TSS1500	-0.538618	-3.135622	-5.043505	1.47E-06	4.16E-05	4.8875501
cg18236250	chr4	16791106	+	LDB2;LDB2	Body;Body	-1.64387	1.3640093	-5.043425	1.47E-06	4.16E-05	4.8872151
cg01084270	chr5	35904569	+	CAPSL;CAPSL	3'UTR;3'UTR	-1.759811	0.448989	-5.043379	1.47E-06	4.16E-05	4.887025
cg24055703	chr20	46417680	-			-1.225908	-0.041069	-5.043265	1.47E-06	4.16E-05	4.8865476
cg14104036	chr4	154266957	-	MND1	Body	-0.639841	-3.319629	-5.043199	1.47E-06	4.16E-05	4.8862716
cg07561162	chr16	10174417	-	GRIN2A;GRIN2A;GRIN2A	Body;Body;Body	-1.236414	-2.629258	-5.043184	1.47E-06	4.16E-05	4.886208
cg09026448	chr5	168852416	+			-0.992264	0.4629218	-5.043166	1.47E-06	4.16E-05	4.8861332

cg05854217	chr2	1595819	+			1.1871697	-1.118629	5.0431078	1.47E-06	4.16E-05	4.8858913
cg05090966	chr7	157278058	+			-1.120805	-0.02186	-5.04301	1.47E-06	4.16E-05	4.8854827
cg00312921	chr4	113441703	+			1.4042196	-1.284436	5.0429475	1.47E-06	4.16E-05	4.8852218
cg06118609	chr11	7982706	-	NLRP10	Body	-1.147542	-0.097982	-5.04296	1.47E-06	4.16E-05	4.885273
cg08832227	chr12	5020693	+	KCNA1	Body	1.2030546	-0.358175	5.0429731	1.47E-06	4.16E-05	4.8853288
cg07733920	chr10	116636356	-	FAM160B1	Body	-0.735648	0.623417	-5.042894	1.47E-06	4.16E-05	4.8849992
cg22606224	chr19	12266489	-	ZNF625	5'UTR	1.8631605	-0.787047	5.0428505	1.47E-06	4.16E-05	4.8848168
cg07969668	chr8	142841913	-			-1.118804	-0.242039	-5.042775	1.47E-06	4.16E-05	4.8844999
cg27182510	chr6	128830356	+	PTPRK;PTPRK	Body;Body	-1.152251	1.3298648	-5.042731	1.47E-06	4.17E-05	4.8843173
cg16057598	chr2	172967819	-	DLX2	TSS1500	-0.879083	-3.716215	-5.042617	1.47E-06	4.17E-05	4.8838416
cg05666607	chr5	145718405	-	POU4F3	TSS200	1.8884454	-2.127279	5.0425737	1.47E-06	4.17E-05	4.8836605
cg12033297	chr1	176998801	+	MIR488;ASTN1;ASTN1	TSS1500;Body;Body	-1.084827	-0.344765	-5.042523	1.47E-06	4.17E-05	4.8834483
cg21591938	chr12	29195033	+			-1.394187	0.4365613	-5.042484	1.47E-06	4.17E-05	4.8832852
cg11251234	chr10	101825055	+	CPN1	Body	0.6921011	-0.899283	5.0424299	1.47E-06	4.17E-05	4.8830603
cg21246665	chr14	94759743	+	SERPINA10;SERPINA10	TSS1500;TSS200	-1.045575	-1.143719	-5.042362	1.47E-06	4.17E-05	4.8827785
cg19146001	chr13	22245089	-	FGF9	TSS200	-0.379411	-3.831513	-5.042313	1.47E-06	4.17E-05	4.8825708
cg22714668	chr5	145453107	-			0.7428568	0.3989599	5.0421671	1.47E-06	4.17E-05	4.8819626
cg19304088	chr4	111547790	+	PITX2;PITX2	Body;Body	1.5990655	-1.786373	5.0421336	1.47E-06	4.17E-05	4.8818227
cg17809181	chr7	157674091	+	PTPRN2;PTPRN2;PTPRN2	Body;Body;Body	-1.160571	0.5575714	-5.04208	1.47E-06	4.17E-05	4.8815991
cg13130031	chr14	99584939	+			0.7704997	-0.745286	5.0420164	1.48E-06	4.18E-05	4.881333

cg15162000	chr6	77265160	-			-1.332211	0.2029443	-5.041999	1.48E-06	4.18E-05	4.8812603
cg16621833	chr6	117587588	-	VGLL2;VGLL2	Body;Body	1.2936148	-0.500361	5.0419405	1.48E-06	4.18E-05	4.8810163
cg22027471	chr22	32651776	-	SLC5A4	TSS1500	-1.326348	-0.527533	-5.041932	1.48E-06	4.18E-05	4.8809796
cg26386449	chr7	112430654	-	TMEM168	TSS200	-0.534048	-3.495525	-5.041834	1.48E-06	4.18E-05	4.8805727
cg04917446	chr17	46699073	-	HOXB9	3'UTR	1.2253109	-0.177435	5.0414358	1.48E-06	4.18E-05	4.8789086
cg00020348	chr7	142749542	-	OR6V1	1stExon	-1.402077	0.6143247	-5.041333	1.48E-06	4.19E-05	4.8784801
cg01720455	chr2	176948343	-	EVX2	1stExon	1.2989795	-1.122031	5.0412895	1.48E-06	4.19E-05	4.8782976
cg18592195	chr1	227963171	-	SNAP47	Body	1.1139422	5.3569018	5.0486248	1.48E-06	4.19E-05	4.8827296
cg09183146	chr16	1429863	+	UNKL	TSS200	-0.949383	-1.226157	-5.040955	1.48E-06	4.19E-05	4.8769025
cg00914963	chr17	77966262	-	TBC1D16	Body	-0.6268	-1.469488	-5.040785	1.48E-06	4.20E-05	4.8761903
cg04697148	chr7	24325079	+	NPY	Body	1.3942801	-0.996179	5.0407306	1.48E-06	4.20E-05	4.8759641
cg22491320	chr2	133998732	-	NCKAP5;NCKAP5	Body;Body	1.034123	0.8703958	5.0406034	1.48E-06	4.20E-05	4.875433
cg15734764	chr10	134266555	-			-0.503226	-2.245179	-5.040442	1.49E-06	4.20E-05	4.8747588
cg12819144	chr4	165953018	-	TRIM60	TSS200	-1.030499	0.6941145	-5.040426	1.49E-06	4.20E-05	4.874691
cg01920398	chr16	89703023	+	DPEP1;DPEP1	Body;Body	-0.809475	-0.092647	-5.040396	1.49E-06	4.20E-05	4.8745662
cg02332982	chr12	45444420	-	DBX2	1stExon	2.0643545	-1.385209	5.0403136	1.49E-06	4.20E-05	4.8742232
cg07134608	chr19	49436579	+	DHDH	TSS1500	-0.703698	-2.621615	-5.0403	1.49E-06	4.20E-05	4.8741654
cg08421271	chr6	33284646	-	ZBTB22;ZBTB22	Body;Body	0.9176482	0.9130299	5.0439352	1.49E-06	4.20E-05	4.8760352
cg08328134	chr19	57279184	+			-1.79913	1.7435171	-5.040097	1.49E-06	4.20E-05	4.8733188
cg12642246	chr17	46059753	+			0.615908	1.6426174	5.0400938	1.49E-06	4.20E-05	4.8733053

cg24867501	chr22	50925337	+	MIOX	1stExon	-0.880387	-0.139989	-5.040098	1.49E-06	4.20E-05	4.8733219
cg25253677	chr19	54485730	+	CACNG8	Body	2.4617315	-1.883505	5.0397044	1.49E-06	4.21E-05	4.8716796
cg26958654	chr15	25437871	-	SNORD115-13	TSS1500	-1.340536	1.1151174	-5.039569	1.49E-06	4.21E-05	4.8711145
cg27646205	chr19	11545491	+	CCDC151;PRKCSH;PRKCSH	Body;TSS1500;TSS1500	-0.814615	-1.32568	-5.039569	1.49E-06	4.21E-05	4.8711142
cg01346748	chr16	4321202	-	TFAP4	Body	-0.381692	-4.001739	-5.041339	1.49E-06	4.21E-05	4.8719061
cg02836046	chr21	38120824	+	SIM2	3'UTR	-1.366607	1.054203	-5.039492	1.49E-06	4.21E-05	4.8707939
cg06400119	chr7	158046222	+	PTPRN2;PTPRN2;PTPRN2	Body;Body;Body	-1.801497	0.3500443	-5.039343	1.49E-06	4.22E-05	4.8701694
cg08887114	chr1	185229087	+	C1orf26;C1orf26	Body;Body	0.931986	1.6226663	5.0392515	1.49E-06	4.22E-05	4.8697888
cg10541534	chr4	140429995	-	SETD7	3'UTR	0.6265711	2.295371	5.0391554	1.49E-06	4.22E-05	4.8693876
cg00035630	chr11	33036664	-	DEPDC7;DEPDC7	TSS1500;TSS1500	-1.530057	0.1612681	-5.039109	1.49E-06	4.22E-05	4.8691946
cg18428006	chr5	177633449	+	HNRNPAB;HNRNPAB	Body;Body	-1.097994	0.2544198	-5.039023	1.49E-06	4.22E-05	4.8688334
cg20532925	chr3	17186314	+			-1.144564	0.5297042	-5.038994	1.50E-06	4.22E-05	4.8687134
cg07845352	chr6	118260820	+	SLC35F1	Body	-1.77798	0.596339	-5.038931	1.50E-06	4.22E-05	4.8684511
cg17924283	chr10	134298762	+			-1.138452	1.283474	-5.038926	1.50E-06	4.22E-05	4.8684288
cg21779805	chr3	187982495	-	LPP;LPP;LPP	5'UTR;5'UTR;5'UTR	1.1731807	0.1824725	5.0386079	1.50E-06	4.23E-05	4.8671022
cg12251895	chr2	207980866	+	KLF7	Body	1.2044764	0.9577895	5.0385262	1.50E-06	4.23E-05	4.8667613
cg17834755	chr7	129423218	-			1.1326497	-1.077465	5.0384949	1.50E-06	4.23E-05	4.8666304
cg03534410	chr3	12801112	+	TMEM40	TSS1500	-1.040821	-0.111657	-5.040263	1.50E-06	4.23E-05	4.8880356
cg04166335	chr16	29996068	+	TAOK2;TAOK2	Body;Body	1.0053543	1.8479745	5.0384133	1.50E-06	4.23E-05	4.8662899
cg00690392	chr1	225697019	+	ENAH;ENAH	Body;Body	-1.573528	0.8957469	-5.038325	1.50E-06	4.23E-05	4.8659214

cg10583311	chr12	133082545	-	FBRSL1	Body	0.773722	0.1707619	5.0382552	1.50E-06	4.23E-05	4.8656299
cg00043788	chr20	25062754	+	VSX1;VSX1;VSX1;VSX1	5'UTR;1stExon;5'UTR;1stExon	1.3152708	-1.60158	5.0400213	1.50E-06	4.23E-05	4.8664101
cg03369382	chr7	139427635	-	HIPK2;HIPK2	Body;Body	0.9766994	1.9064028	5.0381905	1.50E-06	4.23E-05	4.8653602
cg24099605	chr14	95035773	-	SERPINA4	Body	-1.209638	0.8523904	-5.038107	1.50E-06	4.24E-05	4.8650128
cg03574652	chr3	187100815	-			0.9611781	1.1068676	5.0380575	1.50E-06	4.24E-05	4.8648048
cg26160492	chr3	38009911	-	CTDSPL;CTDSPL;MIR26A1	Body;Body;TSS1500	0.8310624	4.007181	5.0380545	1.50E-06	4.24E-05	4.8647923
cg06679270	chr15	101084980	+	LASS3	TSS200	1.3516199	2.0220806	5.0379652	1.50E-06	4.24E-05	4.8644198
cg09258315	chr20	62053155	-	KCNQ2;KCNQ2;KCNQ2;KCNQ2	Body;Body;Body;Body	-1.116889	0.3831521	-5.037917	1.50E-06	4.24E-05	4.8642202
cg04948284	chr11	65313608	+	LTBP3;LTBP3;LTBP3	Body;Body;Body	-0.974981	-0.936295	-5.037884	1.50E-06	4.24E-05	4.8640796
[truncated: 403,357 more chars]
