# Supplementary material for: A Sight of the Diagnostic Value of Aberrant Cell-Free DNA Methylation in Lung Cancer
Source: Dis Markers. 2022 Jan 27;2022:9619357. doi: 10.1155/2022/9619357 (PMC8814721; doi:10.1155/2022/9619357)
Supplement: Supplementary 6 — Table S3: GO analysis of DMGs in cfDNA samples. [file 9619357.f6.pdf]

Table S3. GO analysis of DMGs in cfDNA samples

ONTOLOGY	ID	Description	GeneRatio	BgRatio	pvalue	p.adjust	qvalue	Count	geneID
Biological Process	GO:0002283	neutrophil activation involved in immune response	127/2279	490/18866	1.61E-17	1.19E-13	1.08E-13	127	MS4A3/DEFA4/AZU1/ELANE/LRG1/S100P/SLPI/TRPM2/CTSZ/PRTN3/S100A8/CTSG/VAT1/CDA/TIMP2/ABR/MPO/AMPD3/SERPINA1/PLAU/SLC2A5/KCNAB2/OLR1/GSN/C3AR1/LCN2/TOLLIP/RNASE3/UBR4/HEXB/CRISPLD2/CHI3L1/LPCAT1/GALNS/SIRPB1/CD55/TMEM30A/CD177/SLC11A1/CLEC4C/TNFRSF1B/DOK3/RAP1B/CEACAM6/RAB5C/RHOG/RAB5B/TSPAN14/PLD1/RAB31/FGR/COTL1/ORMDL3/FOLR3/DYNC1H1/FPR2/FPR1/ATP8A1/MNDA/MGAM/CLEC5A/PADI2/CTSD/HLA-C/JUP/CHIT1/BRI3/PKP1/SLC15A4/RHOF/ATP11A/CTSH/TUBB4B/RAB27A/PTPN6/TNFAIP6/CPNE1/PGAM1/PTPRN2/PTPRJ/HBB/FCN1/CD59/PGLYRP1/CREG1/OLFM4/PTPRC/CAB39/CXCR1/GRN/BCR/LAIR1/CTSB/CAT/PNP/ITGB2/PDXK/ATP8B4/ARSB/DNASE1L3/PKM/PSMB7/P2RX1/PRAM1/LTF/CD68/ANO6/SPTAN1/DOCK2/CYFIP1/CD33/GMFG/PSMD13/CDK13/IMPDH1/DBNL/SVIP/CEACAM3/ADGRG3/ARHGAP45/STING1/IRAG2/ATP6V0C/ACP3/VPS35L/ADGRE3/CRACR2A
Biological Process	GO:0043312	neutrophil degranulation	126/2279	487/18866	2.51E-17	1.19E-13	1.08E-13	126	MS4A3/DEFA4/AZU1/ELANE/LRG1/S100P/SLPI/TRPM2/CTSZ/PRTN3/S100A8/CTSG/VAT1/CDA/TIMP2/ABR/MPO/AMPD3/SERPINA1/PLAU/SLC2A5/KCNAB2/OLR1/GSN/C3AR1/LCN2/TOLLIP/RNASE3/UBR4/HEXB/CRISPLD2/CHI3L1/LPCAT1/GALNS/SIRPB1/CD55/TMEM30A/CD177/SLC11A1/CLEC4C/TNFRSF1B/DOK3/RAP1B/CEACAM6/RAB5C/RHOG/RAB5B/TSPAN14/PLD1/RAB31/FGR/COTL1/ORMDL3/FOLR3/DYNC1H1/FPR2/FPR1/ATP8A1/MNDA/MGAM/CLEC5A/PADI2/CTSD/HLA-C/JUP/CHIT1/BRI3/PKP1/SLC15A4/RHOF/ATP11A/CTSH/TUBB4B/RAB27A/PTPN6/TNFAIP6/CPNE1/PGAM1/PTPRN2/PTPRJ/HBB/FCN1/CD59/PGLYRP1/CREG1/OLFM4/PTPRC/CAB39/CXCR1/GRN/BCR/LAIR1/CTSB/CAT/PNP/ITGB2/PDXK/ATP8B4/ARSB/PKM/PSMB7/P2RX1/PRAM1/LTF/CD68/ANO6/SPTAN1/DOCK2/CYFIP1/CD33/GMFG/PSMD13/CDK13/IMPDH1/DBNL/SVIP/CEACAM3/ADGRG3/ARHGAP45/STING1/IRAG2/ATP6V0C/ACP3/VPS35L/ADGRE3/CRACR2A
Biological Process	GO:0007015	actin filament organization	98/2279	434/18866	4.31E-10	1.36E-06	1.23E-06	98	VILL/ARHGEF10/TRIM27/TACSTD2/GAS7/SSH1/GSN/TTN/KANK1/ELMO1/RHOH/SHROOM3/PSTPIP1/ITGB1BP1/MSRB1/PIK3R1/RHOG/SMAD3/ARRB1/WIPF1/CTTN/COTL1/CAPG/CYFIP2/MICAL1/NCK2/MSRB2/SH3PXD2B/MICAL2/WHAMM/MICAL3/ARAP1/BAIAP2L1/CUL3/CDC42EP1/BIN1/RHOF/BAIAP2/SYNPO2L/ACTN1/CORO7/ACTN4/SSH3/PREX1/PXN/WNT11/ITGB5/PPFIA1/WASL/FER/RHOBTB2/TMOD3/SVIL/MYO5A/PFN1/ESPN/CSF3/GMFB/GHRL/ACTG1/WDR1/SPTA1/ARHGAP25/PRKCI/CASS4/MIR138-2/NCKAP1/ADD1/SAMD14/ARHGEF10L/BCL2/ARHGEF18/CORO1C/AVIL/PRKCE/FLII/ARHGAP17/ARHGEF2/MYO1C/FSCN3/ARHGEF15/SPTAN1/SHROOM1/TMOD1/ARHGAP12/CYFIP1/GMFG/EZR/BAIAP2L2/MYADM/SPTBN4/SYNPO/DBNL/PLEKHG2/RFLNA/CATIP/PRKN/CYRIB
Biological Process	GO:0010256	endomembrane system organization	102/2279	468/18866	1.50E-09	3.56E-06	3.22E-06	102	DCTN1/SH3TC2/STX5/GSN/ACRBP/VTI1A/TANGO2/CSNK1D/CREB1/RAB2A/SYNE1/LYST/CRB1/NDRG1/RAB5B/PRKCA/ARHGEF7/DNM2/ANXA6/MYO18A/LMAN2/WHAMM/RTN4/ANO7/PACSIN2/BAIAP2L1/BIN1/MYH9/FAM174B/BAIAP2/RAB27A/SNX3/ATP8B1/RTN3/ZNF385A/CLU/MIA3/ASAP1/WASL/COG1/PRKCB/MYO5A/NUP93/EPB41L3/RAB43/VPS18/PDE4DIP/MTSS1/HTT/PTPRC/ABCA1/VCPIP1/HOOK2/NUMA1/USP6NL/PPP2R1A/SPTA1/GORASP2/DNM1/ATP8B4/MIR138-2/NEK6/PI4K2A/YWHAZ/MICALL1/CORO1C/COL5A1/VMP1/FA2H/UBXN2A/F2R/RAB30/FER1L6/TOR1B/TMED2/ANO6/DYSF/ATL1/ANK2/AP3D1/ESYT1/NDEL1/FER1L4/ANKLE2/KIFC3/LPIN1/BAIAP2L2/FLOT1/CASQ1/BAG5/BANF1/DTNBP1/SYPL2/MVB12A/VPS51/SEC31A/PHETA1/GRAMD2A/PRKN/MFSD14A/ARMH3/DOP1B
Biological Process	GO:0030099	myeloid cell differentiation	95/2279	431/18866	2.99E-09	5.69E-06	5.15E-06	95	MAEA/CEBPE/PRTN3/KLF1/ZFPM1/IRF8/FOXP1/SBNO2/PPARG/PIK3CD/CREB1/MIR145/RUNX1/CSF3R/INPP5D/TRIM10/FLI1/ASH2L/PIK3R1/ITPKB/TNRC6B/GAB2/PABPC4/PRKCA/TNF/UBASH3B/CSF1R/AGO1/HIPK2/CLEC5A/CBFA2T3/ZBTB16/SLC11A2/TGFBR2/TESC/THBS1/MYH9/BRD1/DLL1/PIP4K2A/RIPK1/ACTN1/APP/RBPJ/ITGA2B/PTPN6/ZNF385A/HDAC1/MTURN/TGFB1/CD101/TMOD3/PRKCB/KMT2D/RARA/TMEM178A/LILRB4/NF1/LRRK1/SP3/CSF3/RARG/ALAS1/TMEM64/SFXN1/SETD1A/MEIS2/WDR1/CREBBP/LRRC17/TET2/ETS1/HOXA9/ADD1/RB1/MEIS1/MEF2C/EPAS1/TSPAN2/OSTM1/LTF/CEBPB/TRIB1/FAM20C/EPB42/KAT7/INHBA/ERCC2/LIF/HOXB7/FADD/MB/TMEM14C/H3C1/CCN4
Biological Process	GO:0043547	positive regulation of GTPase activity	88/2279	406/18866	2.61E-08	4.14E-05	3.74E-05	88	PRTN3/ARHGEF10/TBCD/ABR/SMAP2/RIN2/DENND1A/MYO9B/ARHGDIB/DOCK1/ADAP1/TBC1D16/ARHGAP26/RHOG/ACAP2/TBC1D2/ARRB1/ARHGEF7/SEC23B/CCL5/TBC1D10A/ARHGAP31/RASAL2/RIN3/AGAP1/ARAP1/GIT1/RGS6/RASA3/PREX1/VPS9D1/WNT11/DOCK8/ASAP1/RALBP1/SYNGAP1/RGS12/RABGAP1L/SEMA4D/RAPGEF2/IQSEC1/TBC1D22B/NF1/RGS17/ASAP2/CCL20/SRGAP1/USP6NL/BCR/SIPA1L1/PLCB1/SNX18/ARHGAP25/TBC1D14/DOCK10/SIPA1L2/NTRK1/RGS3/ARHGEF10L/GARNL3/TBC1D2B/SGSM2/CORO1C/DNM1L/TBC1D1/ARHGAP17/F2R/ARHGAP15/RGS10/BCAR3/TSC2/RSU1/ARHGEF15/CHN2/ARHGAP1/ARHGAP8/DOCK2/RGL3/ARHGAP12/ARHGAP10/NDEL1/RAP1GAP2/CCL22/TBC1D22A/VAV3/ARHGAP23/NPRL2/ARHGAP45
Biological Process	GO:0043087	regulation of GTPase activity	99/2279	481/18866	5.55E-08	7.53E-05	6.81E-05	99	PRTN3/ARHGEF10/TBCD/PLXNC1/ABR/SMAP2/FGD4/RHOH/RIN2/DENND1A/MYO9B/ARHGDIB/DOCK1/ADAP1/ITGB1BP1/TBC1D16/ARHGAP26/RHOG/ACAP2/TBC1D2/ARRB1/ARHGEF7/SEC23B/CCL5/TBC1D10A/EPHB3/FGD5/ARHGAP31/RASAL2/RIN3/AGAP1/ARAP1/GIT1/RGS6/RASA3/PREX1/VPS9D1/WNT11/PTPRN2/DOCK8/ASAP1/RALBP1/SYNGAP1/RGS12/RABGAP1L/SEMA4D/RAPGEF2/IQSEC1/TBC1D22B/NF1/RGS17/ASAP2/CCL20/VAV2/SH3BP4/SRGAP1/USP6NL/BCR/SIPA1L1/PLCB1/SNX18/ARHGAP25/TBC1D14/ARRB2/DOCK10/SIPA1L2/NTRK1/RGS3/ARHGEF10L/GARNL3/TBC1D2B/SGSM2/CORO1C/DNM1L/TBC1D1/ARHGAP17/F2R/ARHGAP15/RGS10/BCAR3/TMED2/TSC2/RSU1/ARHGEF15/CHN2/ARHGAP1/ARHGAP8/DOCK2/RGL3/ARHGAP12/ARHGAP10/NDEL1/RAP1GAP2/CCL22/TBC1D22A/VAV3/ARHGAP23/NPRL2/ARHGAP45
Biological Process	GO:0051056	regulation of small GTPase mediated signal transduction	73/2279	323/18866	6.92E-08	8.22E-05	7.44E-05	73	ARHGEF10/TIMP2/ABR/TRIO/FGD4/KANK1/RHOH/DENND1A/AKAP13/MYO9B/ARHGDIB/ITPKB/ARHGAP26/RHOG/ARRB1/ARHGEF7/DNM2/ARHGAP31/ITSN1/DENND3/ARAP1/CUL3/LRRC59/RHOF/SYNPO2L/RASA3/PREX1/RALBP1/PLEKHG5/SYNGAP1/RHOBTB2/EPS8L3/IQSEC1/EPS8L1/NF1/MCF2L/VAV2/ABCA1/SRGAP1/BCR/ARHGEF25/SIPA1L1/ARHGAP25/SIPA1L2/NTRK1/GARNL3/ARHGEF18/NOTCH1/CYTH4/ARHGAP17/F2R/ARHGEF28/ARHGAP15/FOXM1/ARHGEF2/GPR4/TSC2/PDGFRB/CHN2/ARHGAP1/ARHGAP8/RALGPS1/ARHGAP12/ARHGAP10/FLOT1/RAP1GAP2/FBXO8/VAV3/ARHGAP23/PLEKHG2/PRAG1/ARHGAP45/CYRIB
Biological Process	GO:0045785	positive regulation of cell adhesion	90/2279	428/18866	8.03E-08	8.48E-05	7.67E-05	90	AZU1/ELANE/FUT4/HLA-DMB/PRKCZ/P4HB/RREB1/CALR/FUT7/SPOCK2/IL10/VIT/RHOH/LCK/MYB/RIN2/RUNX1/SIRPB1/CD55/ANGPT1/DOCK1/ITGB1BP1/NLRP3/PIK3R1/ITPKB/CEACAM6/CCR2/PIK3R6/SMAD3/PRKCA/ARHGEF7/DNM2/TNF/CCL5/GRAP2/NCK2/TNFSF13B/ZFHX3/ZBTB16/JUP/TGFBR2/DISC1/NINJ1/RNASE10/PTPN6/EGFLAM/ILK/PREX1/SMAD7/ZMIZ1/DOCK8/JAK1/ZBTB7B/PTPRJ/RARA/LILRB4/CCDC88B/OLFM4/PTPRC/CSK/PLEKHA2/BCL10/SPTA1/ETS1/PNP/CASS4/ITGB2/NOD2/GCNT2/PRKCE/SIRPG/KIFAP3/HLX/RUNX3/RSU1/DYSF/CYLD/AP3D1/RELA/PTPRU/FLOT1/SLC7A1/MYADM/NODAL/LIF/FADD/VAV3/CYRIB/VSIR/PLPP3

Biological Process	GO:0022407	regulation of cell-cell adhesion	90/2279	439/18866	2.66E-07	0.0002529	0.0002288	90	ELANE/FUT4/HLA-DMB/PRKCZ/FUT7/IL10/CD9/MAD1L1/RHOH/LCK/MYB/RUNX1/SIRPB1/CD55/NLRP3/PIK3R1/ITPKB/CEACAM6/CCR2/PIK3R6/PRKCA/SPINT2/LAX1/TNF/UBASH3B/CCL5/GRAP2/NCK2/LOXL3/SFTPD/EPHB3/TNFSF13B/DUSP22/ZBTB16/CCM2L/TGFBR2/ZDHHC2/RNASE10/MBP/PTPN6/MIR27A/SMAD7/TNFRSF21/MIA3/ZMIZ1/DOCK8/JAK1/ZBTB7B/TGFB1/IL1RN/TIGIT/RARA/LILRB4/ZC3H8/BLK/CCDC88B/PTPRC/CSK/BCL10/SPTA1/ETS1/PNP/ITGB2/CDH1/NOD2/NOTCH1/GCNT2/DUSP3/NOTCH4/SIRPG/KIFAP3/HLX/CEBPB/RUNX3/CYLD/AP3D1/ADTRP/RELA/PTPRU/FLOT1/SLC7A1/MYADM/NODAL/FADD/PELI1/TMEM131L/CYRIB/VSIR/MARCHF7/PLPP3
Biological Process	GO:0008154	actin polymerization or depolymerization	53/2279	221/18866	6.13E-07	0.0005292	0.0004788	53	VILL/TRIM27/GAS7/SSH1/GSN/KANK1/PSTPIP1/MSRB1/WIPF1/CTTN/COTL1/CAPG/CYFIP2/MICAL1/NCK2/MSRB2/MICAL2/WHAMM/MICAL3/BAIAP2L1/CDC42EP1/BIN1/BAIAP2/CORO7/SSH3/PREX1/WASL/FER/TMOD3/SVIL/PFN1/CSF3/GMFB/GHRL/WDR1/SPTA1/NCKAP1/ADD1/AVIL/PRKCE/FLII/MYO1C/SPTAN1/TMOD1/CYFIP1/GMFG/BAIAP2L2/MYADM/SPTBN4/DBNL/PLEKHG2/CATIP/CYRIB
Biological Process	GO:0032970	regulation of actin filament-based process	83/2279	405/18866	7.81E-07	0.0006182	0.0005594	83	TRPM2/VILL/ARHGEF10/PDE4D/TRIM27/TACSTD2/SSH1/GSN/KANK1/RHOH/CACNA1C/AKAP13/ARHGDIB/ITGB1BP1/PIK3R1/RHOG/SMAD3/CTTN/COTL1/CAPG/CYFIP2/CSF1R/NCK2/GJA5/SH3PXD2B/WHAMM/JUP/ARAP1/BAIAP2L1/CDC42EP1/BIN1/MYH9/RHOF/BAIAP2/SYNPO2L/NOS1AP/SSH3/ILK/PREX1/PXN/WNT11/PPFIA1/WASL/FER/RHOBTB2/TMOD3/SVIL/PFN1/JAM3/CSF3/GMFB/ACTG1/WDR1/SPTA1/MIR138-2/NCKAP1/ADD1/ARHGEF10L/ARHGEF18/AVIL/MEF2C/PRKCE/FLII/ARHGAP17/MYO1C/PDGFRB/ARHGEF15/SPTAN1/ANK2/FRMD6/TMOD1/CYFIP1/GMFG/BAIAP2L2/MYADM/CCL27/SPTBN4/SYNPO/DAPK3/DBNL/PLEKHG2/PRKN/CYRIB
Biological Process	GO:2001238	positive regulation of extrinsic apoptotic signaling pathway	20/2279	52/18866	1.08E-06	0.0007326	0.0006629	20	TNF/TRAF2/DEDD2/THBS1/RIPK1/NF1/PTPRC/ITM2C/BMPR1B/STK3/BCL10/PPP2R1A/TRPS1/PPP2R1B/TNFSF12/CYLD/G0S2/INHBA/FADD/DAPK3
Biological Process	GO:0018209	peptidyl-serine modification	71/2279	333/18866	1.08E-06	0.0007326	0.0006629	71	PRKCZ/PDE4D/RPTOR/LMTK2/MARK3/GALNT2/VRK2/MAST4/DYRK4/SPOCK2/CSNK1D/TNKS/ULK1/HIPK3/ANGPT1/ERN1/SRPK2/ARRB1/PRKCA/TNF/STK38L/SGK1/NCK2/HIPK2/TGFBR2/SMYD3/RIPK1/AKT3/APP/EGFLAM/RPS6KA4/ILK/SMAD7/LATS2/TGFB1/PRKCH/DGKQ/PRKCB/AXIN1/DYRK2/CAMK1D/CSF3/SH2D3C/CAB39/CSNK1E/PDGFB/MKNK1/PRKCI/ARRB2/FNIP2/NEK6/PLCL1/MORC3/CLSPN/BCL2/PRKCE/GGNBP2/CHEK2/BCAR3/MAPKAPK3/STK32C/MARK2/SGK2/INPP5F/NTMT1/SPTBN4/NTF4/LIF/EIF4G1/PRKAA1/GRK2
Biological Process	GO:0018105	peptidyl-serine phosphorylation	67/2279	310/18866	1.30E-06	0.0008173	0.0007396	67	PRKCZ/PDE4D/RPTOR/LMTK2/MARK3/VRK2/MAST4/DYRK4/CSNK1D/TNKS/ULK1/HIPK3/ANGPT1/ERN1/SRPK2/ARRB1/PRKCA/TNF/STK38L/SGK1/NCK2/HIPK2/TGFBR2/SMYD3/RIPK1/AKT3/APP/RPS6KA4/ILK/SMAD7/LATS2/TGFB1/PRKCH/DGKQ/PRKCB/AXIN1/DYRK2/CAMK1D/CSF3/SH2D3C/CAB39/CSNK1E/PDGFB/MKNK1/PRKCI/ARRB2/FNIP2/NEK6/PLCL1/MORC3/CLSPN/BCL2/PRKCE/GGNBP2/CHEK2/BCAR3/MAPKAPK3/STK32C/MARK2/SGK2/INPP5F/SPTBN4/NTF4/LIF/EIF4G1/PRKAA1/GRK2
Biological Process	GO:0032956	regulation of actin cytoskeleton organization	75/2279	360/18866	1.38E-06	0.0008173	0.0007396	75	TRPM2/VILL/ARHGEF10/TRIM27/TACSTD2/SSH1/GSN/KANK1/RHOH/AKAP13/ARHGDIB/ITGB1BP1/PIK3R1/RHOG/SMAD3/CTTN/COTL1/CAPG/CYFIP2/CSF1R/NCK2/SH3PXD2B/WHAMM/ARAP1/BAIAP2L1/CDC42EP1/BIN1/RHOF/BAIAP2/SYNPO2L/SSH3/ILK/PREX1/PXN/WNT11/PPFIA1/WASL/FER/RHOBTB2/TMOD3/SVIL/PFN1/JAM3/CSF3/GMFB/ACTG1/WDR1/SPTA1/MIR138-2/NCKAP1/ADD1/ARHGEF10L/ARHGEF18/AVIL/MEF2C/PRKCE/FLII/ARHGAP17/MYO1C/PDGFRB/ARHGEF15/SPTAN1/TMOD1/CYFIP1/GMFG/BAIAP2L2/MYADM/CCL27/SPTBN4/SYNPO/DAPK3/DBNL/PLEKHG2/PRKN/CYRIB
Biological Process	GO:0030041	actin filament polymerization	47/2279	193/18866	1.64E-06	0.0009178	0.0008305	47	VILL/TRIM27/GAS7/SSH1/GSN/KANK1/PSTPIP1/MSRB1/CTTN/COTL1/CAPG/CYFIP2/NCK2/MSRB2/WHAMM/BAIAP2L1/CDC42EP1/BIN1/BAIAP2/CORO7/SSH3/PREX1/WASL/FER/TMOD3/SVIL/PFN1/CSF3/GMFB/SPTA1/NCKAP1/ADD1/AVIL/PRKCE/FLII/MYO1C/SPTAN1/TMOD1/CYFIP1/GMFG/BAIAP2L2/MYADM/SPTBN4/DBNL/PLEKHG2/CATIP/CYRIB
Biological Process	GO:0051091	positive regulation of DNA-binding transcription factor activity	60/2279	270/18866	1.78E-06	0.0009392	0.0008498	60	HDAC4/ARID5B/PRKCZ/NLRC4/S100A8/TRIM27/IL10/AIM2/TRIM38/PPARG/TFDP1/TRIM8/NLRP3/SMAD3/TNF/TRAF2/TRAF1/HIPK2/TLR9/JUP/ERC1/TRAPPC9/RIPK1/APP/RPS6KA4/CLU/CRTC3/PRKCH/FER/PRKCB/RIPK3/COPS5/TRIM5/SLCO3A1/UBE2V1/CAMK1D/CSF3/STK3/BCL10/CAT/PRKCI/ITGB2/IRAK2/NTRK1/IL18RAP/NOD2/TCF3/STK36/RNF25/TP53BP1/IL18R1/ARHGEF2/LTF/RELA/FLOT1/NODAL/CX3CR1/TRIM26/STING1/PLPP3

Biological Process	GO:0006644	phospholipid metabolic process	89/2279	455/18866	2.48E-06	0.0011778	0.0010657	89	CPNE6/OSBPL5/PLA2G15/SYNJ2/PLBD2/PCYT1A/PIK3CD/SPTLC2/MFSD2A/IDI1/HEXB/INPP5A/VAC14/LPCAT1/PIP5K1B/INPP5D/LIPC/ABHD16A/PIK3R1/ITPKB/PIGL/PIK3R6/PIGU/PLD1/GPLD1/FGR/SMPDL3B/CSF1R/FPR2/TTC7A/PIGN/PGP/ACP6/PITPNM2/SLC44A4/INPP4A/PIK3R5/PIP4K2A/PITPNM1/CPNE1/FLT1/SPHK2/TGFB1/SGMS1/TTC7B/PGS1/DGKQ/SH3YL1/CHPT1/NR1H2/VAV2/ATG14/PCTP/GNB3/PLEKHA2/PDGFB/ABHD16B/DGKA/DGKD/INPP5E/PLCB1/OSBPL10/IMPA2/SGMS2/MIR138-2/PI4K2A/SGPP1/NOD2/PI4KA/PIGQ/PLA1A/ABHD5/PDGFRB/RUFY1/WDR81/CHKB/HMGCS1/PLCG1/PTDSS2/INPP5F/LPIN1/CDS2/DPAGT1/VAV3/AGPAT5/ETNK2/IP6K1/SLA2/PLPP3
Biological Process	GO:0007009	plasma membrane organization	32/2279	113/18866	2.52E-06	0.0011778	0.0010657	32	SH3TC2/GSN/CRB1/NDRG1/DNM2/ANXA6/WHAMM/ANO7/PACSIN2/BAIAP2L1/BIN1/MYH9/BAIAP2/CLU/ASAP1/WASL/EPB41L3/MTSS1/PTPRC/SPTA1/MIR138-2/MICALL1/COL5A1/FA2H/FER1L6/ANO6/DYSF/ANK2/FER1L4/BAIAP2L2/FLOT1/SYPL2
Biological Process	GO:0051014	actin filament severing	10/2279	16/18866	2.60E-06	0.0011778	0.0010657	10	VILL/GSN/CAPG/MYH9/FMNL1/SVIL/GMFB/AVIL/FLII/GMFG
Biological Process	GO:0051258	protein polymerization	64/2279	300/18866	3.53E-06	0.0015224	0.0013775	64	VILL/TRIM27/TBCD/DCTN1/GAS7/SSH1/GSN/CASQ2/KANK1/CSNK1D/NDE1/PSTPIP1/MSRB1/CTTN/DNM2/COTL1/CAPG/CYFIP2/NCK2/MSRB2/WHAMM/BAIAP2L1/NAV3/CDC42EP1/BIN1/BAIAP2/CORO7/SSH3/PREX1/WASL/FER/TMOD3/SVIL/PFN1/CSF3/GMFB/NUMA1/SPTA1/DNM1/NCKAP1/ADD1/DNM3/DNM1L/AVIL/TPPP3/PRKCE/FLII/MYO1C/SPTAN1/MX1/TMOD1/CYFIP1/TUBGCP2/NDEL1/GMFG/BAIAP2L2/CASQ1/MYADM/SPTBN4/DBNL/PLEKHG2/CATIP/PRKN/CYRIB
Biological Process	GO:0051092	positive regulation of NF-kappaB transcription factor activity	40/2279	159/18866	4.07E-06	0.0016798	0.00152	40	PRKCZ/NLRC4/S100A8/TRIM27/AIM2/TRIM38/TRIM8/NLRP3/TNF/TRAF2/TRAF1/TLR9/ERC1/TRAPPC9/RIPK1/APP/RPS6KA4/CLU/PRKCH/FER/PRKCB/RIPK3/TRIM5/SLCO3A1/UBE2V1/BCL10/CAT/PRKCI/ITGB2/IRAK2/NTRK1/IL18RAP/NOD2/RNF25/IL18R1/ARHGEF2/LTF/RELA/FLOT1/CX3CR1
Biological Process	GO:0110053	regulation of actin filament organization	60/2279	278/18866	4.78E-06	0.0018935	0.0017133	60	VILL/ARHGEF10/TRIM27/TACSTD2/SSH1/GSN/KANK1/ITGB1BP1/PIK3R1/SMAD3/CTTN/COTL1/CAPG/CYFIP2/NCK2/SH3PXD2B/WHAMM/ARAP1/BAIAP2L1/CDC42EP1/BIN1/BAIAP2/SYNPO2L/SSH3/PREX1/PXN/WNT11/PPFIA1/WASL/FER/TMOD3/SVIL/PFN1/CSF3/GMFB/ACTG1/WDR1/SPTA1/MIR138-2/NCKAP1/ADD1/ARHGEF10L/ARHGEF18/AVIL/PRKCE/FLII/MYO1C/ARHGEF15/SPTAN1/TMOD1/CYFIP1/GMFG/BAIAP2L2/MYADM/SPTBN4/SYNPO/DBNL/PLEKHG2/PRKN/CYRIB
Biological Process	GO:0048193	Golgi vesicle transport	75/2279	374/18866	5.98E-06	0.0022346	0.002022	75	CTSZ/OSBPL5/DCTN1/SORL1/SERPINA1/AP2A1/TGFA/STX5/VTI1A/ANKFY1/CSNK1D/CD55/KIF13A/DNM2/TRAPPC10/RAB31/SEC23B/MYO18A/DYNC1H1/LMAN2/WHAMM/YIF1A/CUL3/CUX1/TRAPPC9/SNX3/CORO7/ERGIC1/UVRAG/MIA3/COG1/DCTN2/BICD2/VAMP5/CNIH2/SEC22C/MYO5A/CD59/TRAPPC3L/COG8/HTT/SEC13/CSK/NUMA1/CREB3L2/SPTA1/TBC1D14/ANKRD28/PRKCI/SORT1/SEC24C/NBAS/AP1G2/SGSM2/CNIH4/COG5/KIFAP3/EPS15/LMF1/TMED2/ARF5/SPTAN1/ANK2/AP3D1/SPTBN4/VPS51/CUX2/SEC31A/TEX261/TRAPPC2B/MIA2/VPS35L/TRAPPC2/PLPP3/DOP1B
Biological Process	GO:0048017	inositol lipid-mediated signaling	46/2279	196/18866	6.12E-06	0.0022346	0.002022	46	GSN/PIK3CD/PRR5L/PIP5K1B/ANGPT1/PIK3AP1/PIK3R1/GAB2/PLD1/FGR/TNF/CCL5/CSF1R/UNC5B/PITPNM2/PIK3R5/PIP4K2A/PTPN6/IGF1R/FLT1/FGFR1/SEMA4D/PPP2R5C/NF1/PDGFD/PRR5/CSF3/PDGFB/INPP5E/PLCB1/CAT/PLCL1/NTRK1/PI4KA/F2R/UBE3A/PDGFC/NEDD4/TSC2/PDGFRB/PPP1R16B/TYRO3/KBTBD2/INPP5F/PLCD3/EZR
Biological Process	GO:0007159	leukocyte cell-cell adhesion	73/2279	364/18866	7.90E-06	0.0026478	0.0023958	73	ELANE/FUT4/HLA-DMB/PRKCZ/S100A8/FUT7/IL10/OLR1/MAD1L1/RHOH/LCK/MYB/RUNX1/SIRPB1/CD55/CD177/NLRP3/PIK3R1/ITPKB/CCR2/PIK3R6/LAX1/TNF/CCL5/GRAP2/NCK2/LOXL3/SFTPD/TNFSF13B/DUSP22/ZBTB16/TGFBR2/PTPN6/MIR27A/SMAD7/TNFRSF21/MIA3/ZMIZ1/DOCK8/ZBTB7B/TIGIT/SEMA4D/RARA/LILRB4/ZC3H8/CCDC88B/TNIP1/PTPRC/CSK/BCL10/SPTA1/ETS1/PNP/ITGB2/NOD2/DUSP3/SIRPG/HLX/CEBPB/RUNX3/CYLD/AP3D1/ADTRP/RELA/EZR/SLC7A1/CX3CR1/FADD/PELI1/TMEM131L/CYRIB/VSIR/MARCHF7

Biological Process	GO:0048015	phosphatidylinositol-mediated signaling	45/2279	192/18866	7.97E-06	0.0026478	0.0023958	45	GSN/PIK3CD/PRR5L/PIP5K1B/ANGPT1/PIK3AP1/PIK3R1/GAB2/FGR/TNF/CCL5/CSF1R/UNC5B/PITPNM2/PIK3R5/PIP4K2A/PTPN6/IGF1R/FLT1/FGFR1/SEMA4D/PPP2R5C/NF1/PDGFD/PRR5/CSF3/PDGFB/INPP5E/PLCB1/CAT/PLCL1/NTRK1/PI4KA/F2R/UBE3A/PDGFC/NEDD4/TSC2/PDGFRB/PPP1R16B/TYRO3/KBTBD2/INPP5F/PLCD3/EZR
Biological Process	GO:0051090	regulation of DNA-binding transcription factor activity	87/2279	455/18866	8.16E-06	0.0026478	0.0023958	87	HDAC4/ARID5B/PRKCZ/NLRC4/S100A8/TRIM27/GFI1/IL10/AIM2/TRIM38/PPARG/TFDP1/TRIM8/WWP2/PRMT2/NLRP3/SMAD3/ARRB1/SUFU/TNF/TRAF2/TNFAIP3/SGK1/TRAF1/MIR101-2/HIPK2/TLR9/TRAIP/JUP/ERC1/TRAPPC9/RIPK1/APP/MIR27A/TCF7L2/RPS6KA4/CLU/SMAD7/MTURN/CRTC3/PRKCH/FER/FANCA/PRKCB/RIPK3/COPS5/TRIM5/SLCO3A1/UBE2V1/MED13/CAMK1D/CSF3/STK3/BCL10/NLRC5/CAT/PRKCI/ARRB2/ITGB2/MIR138-2/IRAK2/NTRK1/IL18RAP/NOD2/RB1/TCF3/STK36/RNF25/TP53BP1/IL18R1/SP100/ARHGEF2/LTF/TRIB1/CYLD/RELA/FLOT1/PARP10/NODAL/CX3CR1/PELI1/COMMD6/TRIM26/PAXIP1/ADGRG3/STING1/PLPP3
Biological Process	GO:0045216	cell-cell junction organization	48/2279	210/18866	8.36E-06	0.0026478	0.0023958	48	PRTN3/TBCD/CD9/CADM3/TLN2/HDAC7/TJP2/RUNX1/CD177/SMAD3/PRKCA/TNF/CSF1R/GJA5/CCM2/JUP/PKP1/ACTN4/SMAD7/WNT11/CDH3/TGFB1/PRKCH/PLEKHA7/PERP/EPB41L3/JAM3/NUMB/ACTG1/CSK/GRHL2/CDH4/WDR1/PRKCI/CDH1/ADD1/MPP7/F2R/SDK2/CNTNAP1/MYO1C/ANK2/CADM1/CDH11/KIFC3/NPHP4/NECTIN1/NECTIN4
Biological Process	GO:0014068	positive regulation of phosphatidylinositol 3-kinase signaling	26/2279	88/18866	9.27E-06	0.0028423	0.0025718	26	PIK3CD/PRR5L/ANGPT1/PIK3AP1/PIK3R1/FGR/TNF/CCL5/UNC5B/PIK3R5/PTPN6/IGF1R/FLT1/FGFR1/SEMA4D/PDGFD/PRR5/CSF3/PDGFB/CAT/NTRK1/F2R/UBE3A/PDGFC/NEDD4/PDGFRB
Biological Process	GO:0001933	negative regulation of protein phosphorylation	85/2279	444/18866	9.84E-06	0.0028647	0.0025921	85	PRKCZ/PDE4D/TRIM27/RPTOR/SPRED2/PRKAG2/SORL1/SH3BP5L/PRR5L/ITPRIP/PPP1R1B/HIPK3/ANGPT1/PRKAR1B/ITGB1BP1/DUSP14/SLC8A1/ARRB1/LDLRAD4/LAX1/UBASH3B/TNFAIP3/MICAL1/NCK2/FBXO7/SH3BP5/DUSP22/TESC/CTDSPL/SMYD3/PTPN6/MIR24-2/ILK/PRKRIP1/SMAD7/IGF1R/LATS2/TGFB1/PHB/PTPRJ/SEMA4D/DGKQ/NF1/LRRK1/MLLT1/GMFB/WWTR1/TNIP1/ATG14/PTPRC/CSK/PPP2R1A/ENG/ARRB2/TAOK3/PPP2R5D/MIR138-2/RGS3/RB1/PER1/CORO1C/DUSP3/GGNBP2/ZBED3/FOXM1/DUS2/TSC2/FOXO1/HHEX/TRIB1/CYLD/HEXIM1/INPP5F/GMFG/EZR/MYADM/DTNBP1/MECOM/LIF/EIF4G1/NDRG2/SIRT3/NIBAN1/PRKN/PLPP3
Biological Process	GO:1902903	regulation of supramolecular fiber organization	74/2279	373/18866	1.02E-05	0.0028647	0.0025921	74	VILL/ARHGEF10/TRIM27/TBCD/TACSTD2/DCTN1/SSH1/GSN/KANK1/AKAP13/ITGB1BP1/PIK3R1/SMAD3/CTTN/COTL1/CAPG/CYFIP2/NCK2/SH3PXD2B/WHAMM/ARAP1/BAIAP2L1/NAV3/CDC42EP1/BIN1/BAIAP2/SYNPO2L/APP/SSH3/PREX1/CLU/PXN/WNT11/PPFIA1/WASL/FER/TMOD3/SVIL/PFN1/CSF3/GMFB/ACTG1/NUMA1/WDR1/SPTA1/MIR138-2/NCKAP1/ADD1/ARHGEF10L/AEBP1/RB1/ARHGEF18/MAP1A/AVIL/MEF2C/PRKCE/FLII/ARHGEF2/MYO1C/ARHGEF15/SPTAN1/TMOD1/CYFIP1/GMFG/BAIAP2L2/MYADM/SPTBN4/SPEF1/SYNPO/DAPK3/DBNL/PLEKHG2/PRKN/CYRIB
Biological Process	GO:0042326	negative regulation of phosphorylation	91/2279	484/18866	1.03E-05	0.0028647	0.0025921	91	HDAC4/PRKCZ/PDE4D/TRIM27/RPTOR/SPRED2/PRKAG2/SORL1/SH3BP5L/PRR5L/ITPRIP/RHOH/PPP1R1B/HIPK3/ANGPT1/PRKAR1B/ITGB1BP1/DUSP14/SLC8A1/ARRB1/LDLRAD4/LAX1/UBASH3B/TNFAIP3/MICAL1/NCK2/FBXO7/SH3BP5/DUSP22/CBFA2T3/TESC/CTDSPL/SMYD3/PTPN6/MIR24-2/ILK/PRKRIP1/SMAD7/IGF1R/LATS2/TGFB1/PHB/PTPRJ/SEMA4D/DGKQ/NF1/LRRK1/MLLT1/GMFB/WWTR1/TNIP1/ATG14/PTPRC/CSK/PPP2R1A/ENG/ARRB2/TAOK3/PPP2R5D/MIR138-2/RGS3/RB1/PER1/CORO1C/DUSP3/GGNBP2/ZBED3/FOXM1/DUS2/TSC2/FOXO1/HHEX/TRIB1/CYLD/HEXIM1/ANKLE2/INPP5F/GMFG/EZR/MYADM/DTNBP1/INHBA/MECOM/LIF/EIF4G1/NDRG2/SIRT3/NPRL2/NIBAN1/PRKN/PLPP3
Biological Process	GO:0007599	hemostasis	70/2279	348/18866	1.08E-05	0.0029152	0.0026378	70	PRTN3/ZFPM1/EHD1/GP9/SERPINA1/PLAU/CD9/ENTPD1/ITPR2/LCK/DOCK6/CD177/FLI1/PRKAR1B/DOCK1/PIK3R1/PIK3R6/PABPC4/ARRB1/PRKCA/UBASH3B/MICAL1/MYL12A/SERPINB2/ITPR1/THBS1/MYH9/RAB27A/PIK3R5/ITGA2B/PTPN6/ZNF385A/ILK/HDAC1/DOCK8/F12/PRKCH/DGKQ/PRKCB/HBB/ADAMTS13/BLK/CD59/VAV2/GNA11/ITGB3/ACTG1/PDGFB/DGKA/DGKD/NFE2L2/ARRB2/PAPSS2/YWHAZ/GNG2/P2RX1/PRKCE/GNA12/CPB2/F2R/ANO6/HPS6/TYRO3/IRF2/ADTRP/P2RX5/DTNBP1/ANXA7/VAV3/H3C1
Biological Process	GO:0022409	positive regulation of cell-cell adhesion	59/2279	279/18866	1.10E-05	0.0029152	0.0026378	59	ELANE/FUT4/HLA-DMB/PRKCZ/FUT7/IL10/RHOH/LCK/MYB/RUNX1/SIRPB1/CD55/NLRP3/PIK3R1/ITPKB/CEACAM6/CCR2/PIK3R6/TNF/CCL5/GRAP2/NCK2/TNFSF13B/ZBTB16/TGFBR2/RNASE10/PTPN6/SMAD7/ZMIZ1/DOCK8/JAK1/ZBTB7B/RARA/LILRB4/CCDC88B/PTPRC/CSK/BCL10/SPTA1/ETS1/PNP/ITGB2/NOD2/GCNT2/SIRPG/KIFAP3/HLX/RUNX3/CYLD/AP3D1/RELA/PTPRU/FLOT1/SLC7A1/NODAL/FADD/CYRIB/VSIR/PLPP3

Biological Process	GO:2001235	positive regulation of apoptotic signaling pathway	43/2279	183/18866	1.17E-05	0.0030121	0.0027254	43	TRIM39/S100A8/TFDP1/GSN/LCK/YWHAH/PLAGL2/BMF/SMAD3/TNF/TRAF2/NCK2/DEDD2/THBS1/CTSH/RIPK1/NACC2/CIDEB/RIPK3/NF1/PTPRC/ITM2C/BMPR1B/STK3/BCL10/PPP2R1A/TRPS1/YWHAZ/FIS1/BCL2/DNM1L/PPP2R1B/TP53BP2/BCL2L1/TNFSF12/CYLD/YWHAQ/G0S2/ING5/INHBA/FADD/DAPK3/FBH1
Biological Process	GO:0061448	connective tissue development	56/2279	262/18866	1.32E-05	0.0032978	0.002984	56	CSGALNACT1/ARID5B/ZNF516/SORL1/SLC25A25/SPTLC2/MEF2D/CHI3L1/RUNX1/LRP5L/CHSY1/SMAD3/TGFBI/GPLD1/ANXA6/SH3PXD2B/CHST11/ZBTB16/TGFBR2/SMAD7/POC1A/WNT11/FGFR1/PAX7/TGFB1/RARA/POR/PDGFD/GHRL/RARG/BMPR1B/WNT5B/RUNX2/IL6R/PDGFB/FTO/CREB3L2/KLF7/TRPS1/NOTCH1/SLC39A13/COL5A1/MEF2C/NPPC/GPR4/RUNX3/PDGFRB/CRIP1/ACAT1/GDF6/RELA/ZNF219/PAXIP1/RFLNA/SPART/CCN4
Biological Process	GO:0002683	negative regulation of immune system process	87/2279	463/18866	1.63E-05	0.0039707	0.0035928	87	TRIM27/ZFPM1/ABR/IL10/TRIM38/PPARG/MAD1L1/RUNX1/CD55/INPP5D/PIK3R1/ITPKB/CCR2/FGR/LAX1/TNF/UBASH3B/TNFAIP3/LOXL3/SFTPD/CD84/FBXO7/MNDA/DUSP22/PADI2/ZBTB16/THBS1/DLL1/C5AR2/HLA-F/PTPN6/MIR24-2/MIR27A/SMAD7/TNFRSF21/MIA3/WASL/ZBTB7B/TGFB1/FER/PTPRJ/TIGIT/RARA/TMEM178A/LILRB4/NF1/ZC3H8/BLK/CD59/NR1H2/PGLYRP1/SEC14L1/PTPRC/BCL10/GRN/NLRC5/BCR/MEIS2/CNR2/LRRC17/PLCB1/NFE2L2/ARRB2/HOXA9/MEIS1/NOTCH1/DUSP3/ELF1/BANK1/LTF/HLX/CD68/CEBPB/RUNX3/TRIB1/TYRO3/ADTRP/CD96/EZR/INHBA/FADD/PELI1/TMEM131L/VSIR/ILRUN/MARCHF7/SLA2
Biological Process	GO:0030833	regulation of actin filament polymerization	41/2279	174/18866	1.73E-05	0.0040998	0.0037096	41	VILL/TRIM27/SSH1/GSN/KANK1/CTTN/COTL1/CAPG/CYFIP2/NCK2/WHAMM/BAIAP2L1/CDC42EP1/BIN1/BAIAP2/SSH3/PREX1/WASL/FER/TMOD3/SVIL/PFN1/CSF3/GMFB/SPTA1/NCKAP1/ADD1/AVIL/PRKCE/FLII/MYO1C/SPTAN1/TMOD1/CYFIP1/GMFG/BAIAP2L2/MYADM/SPTBN4/DBNL/PLEKHG2/CYRIB
Biological Process	GO:0002573	myeloid leukocyte differentiation	47/2279	210/18866	1.85E-05	0.0042876	0.0038796	47	CEBPE/PRTN3/ZFPM1/FOXP1/SBNO2/PPARG/PIK3CD/CREB1/MIR145/RUNX1/INPP5D/PIK3R1/GAB2/PRKCA/TNF/UBASH3B/CSF1R/CBFA2T3/TGFBR2/TESC/MYH9/RIPK1/APP/RBPJ/TGFB1/CD101/RARA/TMEM178A/LILRB4/NF1/LRRK1/SP3/CSF3/TMEM64/LRRC17/RB1/MEF2C/TSPAN2/OSTM1/LTF/CEBPB/TRIB1/FAM20C/INHBA/LIF/FADD/CCN4
Biological Process	GO:0022604	regulation of cell morphogenesis	92/2279	499/18866	2.04E-05	0.004575	0.0041396	92	SEMA6B/CPNE6/P4HB/PLXNC1/RREB1/TACSTD2/PARVB/CALR/FMNL3/KIF13B/FGD4/KANK1/RHOH/MFSD2A/HEXB/SHROOM3/YWHAH/DOCK1/ITGB1BP1/RHOG/ARHGEF7/CTTN/DNM2/FGR/CPNE5/ZEB2/CSF1R/EPHB3/BCL11A/FGD5/SEMA4A/RTN4/ARAP1/CDC42EP1/TNIK/DISC1/MYH9/RHOF/CUX1/BAIAP2/SEMA4B/FMNL1/ACTN4/IFRD1/ILK/LIMD1/PREX1/SDC2/SYNGAP1/RHOBTB2/SEMA4D/RAPGEF2/SMURF1/ZSWIM5/SS18L2/EPB41L3/OLFM4/CDH4/WDR1/PALM/SIPA1L1/SPTA1/CASS4/ITGB2/ARHGEF18/DNM3/CORO1C/CDKL3/STRIP1/DNM1L/SYT17/GNA12/UBE3A/PDZD8/ARHGAP15/NEDD4/HECW2/EPB42/MARK2/CYFIP1/TRPV2/NDEL1/EZR/MYADM/ANXA7/CUX2/DAPK3/DBNL/PRAG1/SPART/PALM2AKAP2/PRKN
Biological Process	GO:1903037	regulation of leukocyte cell-cell adhesion	66/2279	329/18866	2.09E-05	0.004575	0.0041396	66	ELANE/FUT4/HLA-DMB/PRKCZ/FUT7/IL10/MAD1L1/RHOH/LCK/MYB/RUNX1/SIRPB1/CD55/NLRP3/PIK3R1/ITPKB/CCR2/PIK3R6/LAX1/TNF/CCL5/GRAP2/NCK2/LOXL3/SFTPD/TNFSF13B/DUSP22/ZBTB16/TGFBR2/PTPN6/MIR27A/SMAD7/TNFRSF21/MIA3/ZMIZ1/DOCK8/ZBTB7B/TIGIT/RARA/LILRB4/ZC3H8/CCDC88B/PTPRC/CSK/BCL10/SPTA1/ETS1/PNP/ITGB2/NOD2/DUSP3/SIRPG/HLX/CEBPB/RUNX3/CYLD/AP3D1/ADTRP/RELA/SLC7A1/FADD/PELI1/TMEM131L/CYRIB/VSIR/MARCHF7
Biological Process	GO:0030168	platelet activation	38/2279	158/18866	2.12E-05	0.004575	0.0041396	38	GP9/CD9/ITPR2/LCK/PIK3R1/PIK3R6/ARRB1/PRKCA/UBASH3B/MYL12A/ITPR1/MYH9/PIK3R5/ITGA2B/PTPN6/ILK/PRKCH/DGKQ/PRKCB/HBB/ADAMTS13/BLK/VAV2/GNA11/ITGB3/ACTG1/PDGFB/DGKA/DGKD/ARRB2/YWHAZ/GNG2/P2RX1/PRKCE/GNA12/F2R/TYRO3/VAV3
Biological Process	GO:0002062	chondrocyte differentiation	29/2279	108/18866	2.23E-05	0.0047166	0.0042678	29	MEF2D/RUNX1/CHSY1/SMAD3/TGFBI/GPLD1/ANXA6/CHST11/ZBTB16/TGFBR2/SMAD7/POC1A/FGFR1/TGFB1/POR/RARG/BMPR1B/WNT5B/RUNX2/CREB3L2/TRPS1/MEF2C/NPPC/RUNX3/GDF6/RELA/ZNF219/RFLNA/CCN4

Biological Process	GO:0030111	regulation of Wnt signaling pathway	73/2279	375/18866	2.29E-05	0.0047215	0.0042722	73	RNF220/KANK1/CSNK1D/TNKS/CCDC88C/MIR145/RUNX1/SKI/SMAD3/AMFR/ZEB2/TNFAIP3/VGLL4/FAM53B/UBAC2/NFATC1/JUP/CUL3/DISC1/GRB10/SNX3/APP/RBPJ/TCF7L2/CDK14/ILK/LIMD1/HDAC1/LATS2/WNT11/CDH3/TGFB1/KREMEN1/AXIN1/TLE1/LRRK1/WWTR1/TMEM64/WNT5B/STK3/CSNK1E/PSMF1/RNF213/PPP2R1A/KREMEN2/ARNTL/NXN/PSMB7/TTC21B/NOTCH1/ZBED3/TSC2/FOXO1/CHD8/HHEX/CYLD/ANKRD6/RUVBL1/ZBED2/PPM1N/PTPRU/CCNY/GID8/DAPK3/PSMD13/TSKU/NPHP4/TMEM131L/ADGRA2/PRKN/ATP6V0C/PLPP3/CCN4
Biological Process	GO:0050766	positive regulation of phagocytosis	21/2279	68/18866	3.15E-05	0.0063694	0.0057633	21	AZU1/ABR/CALR/PPARG/SIRPB1/SLC11A1/DNM2/RAB31/TNF/MYO18A/SFTPD/FPR2/LMAN2/RAB27A/PTPRJ/CAMK1D/PTPRC/BCR/SIRPG/ANO6/DOCK2
Biological Process	GO:1990845	adaptive thermogenesis	37/2279	155/18866	3.22E-05	0.006373	0.0057665	37	ZNF516/SORL1/THADA/LCN2/ACSL1/MAP2K6/LNPEP/ACOT13/CCR2/RHEB/DYNC1H1/GRB10/RBPJ/PRKAB1/IGF1R/ZBTB7B/NR1H2/GHRL/PCTP/ARNTL/PRDM16/PLCL1/PER2/RB1/NOTCH1/EPAS1/IL18R1/PDGFC/CEBPB/GADD45G/SCD/TRPV2/ZNF423/LPIN1/G0S2/BSCL2/IP6K1
Biological Process	GO:0014066	regulation of phosphatidylinositol 3-kinase signaling	32/2279	127/18866	3.48E-05	0.0064343	0.005822	32	PIK3CD/PRR5L/PIP5K1B/ANGPT1/PIK3AP1/PIK3R1/FGR/TNF/CCL5/UNC5B/PIK3R5/PIP4K2A/PTPN6/IGF1R/FLT1/FGFR1/SEMA4D/PPP2R5C/PDGFD/PRR5/CSF3/PDGFB/INPP5E/CAT/NTRK1/F2R/UBE3A/PDGFC/NEDD4/TSC2/PDGFRB/PPP1R16B
Biological Process	GO:0106106	cold-induced thermogenesis	35/2279	144/18866	3.48E-05	0.0064343	0.005822	35	ZNF516/LCN2/ACSL1/MAP2K6/LNPEP/ACOT13/CCR2/RHEB/DYNC1H1/GRB10/RBPJ/PRKAB1/IGF1R/ZBTB7B/NR1H2/GHRL/PCTP/ARNTL/PRDM16/PLCL1/PER2/RB1/NOTCH1/EPAS1/IL18R1/PDGFC/CEBPB/GADD45G/SCD/TRPV2/ZNF423/LPIN1/G0S2/BSCL2/IP6K1
Biological Process	GO:0120161	regulation of cold-induced thermogenesis	35/2279	144/18866	3.48E-05	0.0064343	0.005822	35	ZNF516/LCN2/ACSL1/MAP2K6/LNPEP/ACOT13/CCR2/RHEB/DYNC1H1/GRB10/RBPJ/PRKAB1/IGF1R/ZBTB7B/NR1H2/GHRL/PCTP/ARNTL/PRDM16/PLCL1/PER2/RB1/NOTCH1/EPAS1/IL18R1/PDGFC/CEBPB/GADD45G/SCD/TRPV2/ZNF423/LPIN1/G0S2/BSCL2/IP6K1
Biological Process	GO:0031532	actin cytoskeleton reorganization	28/2279	105/18866	3.52E-05	0.0064343	0.005822	28	TRPM2/PARVB/GSN/ARHGDIB/CTTN/CSF1R/WHAMM/ARAP1/BAIAP2L1/TNIK/MYH9/BAIAP2/FER/CSF3/SIPA1L1/CASS4/RALA/INSRR/S1PR2/PARVG/PDLIM4/CDC42BPB/GMFG/EZR/BAIAP2L2/CCL27/DTNBP1/DAPK3
Biological Process	GO:0008360	regulation of cell shape	37/2279	156/18866	3.74E-05	0.0066988	0.0060613	37	PLXNC1/PARVB/FMNL3/FGD4/RHOH/HEXB/SHROOM3/RHOG/FGR/CSF1R/FGD5/SEMA4A/ARAP1/CDC42EP1/MYH9/RHOF/BAIAP2/FMNL1/LIMD1/RHOBTB2/SEMA4D/EPB41L3/WDR1/PALM/SPTA1/ITGB2/ARHGEF18/GNA12/ARHGAP15/EPB42/MARK2/CYFIP1/EZR/ANXA7/DAPK3/PRAG1/PALM2AKAP2
Biological Process	GO:0001659	temperature homeostasis	40/2279	174/18866	3.97E-05	0.0069869	0.006322	40	TRPM2/ZNF516/LCN2/ACSL1/MAP2K6/LNPEP/ACOT13/CCR2/RHEB/TNF/DYNC1H1/GRB10/RBPJ/PRKAB1/IGF1R/ZBTB7B/NR1H2/GHRL/PCTP/FTO/ARNTL/PRDM16/PLCL1/PER2/RB1/NOTCH1/EPAS1/IL18R1/PDGFC/CEBPB/FOXO1/GADD45G/SCD/TRPV2/ZNF423/LPIN1/G0S2/ACADVL/BSCL2/IP6K1

Biological Process	GO:1904018	positive regulation of vasculature development	50/2279	235/18866	4.23E-05	0.0073144	0.0066183	50	LRG1/IL10/C3AR1/MIR140/MAP3K3/MIR143/MIR145/RIN2/CHI3L1/HDAC7/RUNX1/PIK3R6/PRKCA/MIR199A1/MIR101-2/HIPK2/JUP/TGFBR2/THBS1/CTSH/DLL1/AKT3/MIR27A/MIR23A/FLT1/JAK1/AGO2/RAPGEF2/PRKCB/AQP1/PDGFD/BMPER/HK2/GHRL/IL6R/GRN/PDGFB/ETS1/NFE2L2/ENG/ITGB2/PKM/MIR199A2/NOTCH1/AGTR1/PPP1R16B/TNFSF12/PLCG1/NODAL/CX3CR1
Biological Process	GO:0007596	blood coagulation	67/2279	343/18866	4.36E-05	0.0073951	0.0066913	67	PRTN3/ZFPM1/EHD1/GP9/SERPINA1/PLAU/CD9/ENTPD1/ITPR2/LCK/DOCK6/CD177/PRKAR1B/DOCK1/PIK3R1/PIK3R6/PABPC4/ARRB1/PRKCA/UBASH3B/MICAL1/MYL12A/SERPINB2/ITPR1/THBS1/MYH9/RAB27A/PIK3R5/ITGA2B/PTPN6/ILK/HDAC1/DOCK8/F12/PRKCH/DGKQ/PRKCB/HBB/ADAMTS13/BLK/CD59/VAV2/GNA11/ITGB3/ACTG1/PDGFB/DGKA/DGKD/NFE2L2/ARRB2/PAPSS2/YWHAZ/GNG2/P2RX1/PRKCE/GNA12/CPB2/F2R/ANO6/HPS6/TYRO3/IRF2/ADTRP/P2RX5/DTNBP1/VAV3/H3C1
Biological Process	GO:0032535	regulation of cellular component size	73/2279	383/18866	4.72E-05	0.0078549	0.0071074	73	SEMA6B/VILL/TRIM27/RPTOR/SSH1/CLCN6/GSN/KANK1/CREB1/CTTN/DNM2/COTL1/CAPG/CYFIP2/NCK2/WHAMM/SEMA4A/RTN4/BAIAP2L1/CDC42EP1/BIN1/DISC1/BAIAP2/SEMA4B/SSH3/IFRD1/ILK/PREX1/WASL/FER/SEMA4D/TMOD3/AQP1/SVIL/PFN1/CDHR2/CSF3/GMFB/VAV2/RARG/GNB3/CDH4/WDR1/SPTA1/NCKAP1/ADD1/CDKL3/AVIL/PRKCE/FLII/PEX11B/MYO1C/ANO6/SPTAN1/TMOD1/CYFIP1/TRPV2/WDTC1/NDEL1/GMFG/SLC12A1/EZR/BAIAP2L2/RAP1GAP2/MYADM/SPTBN4/SLC12A7/ANXA7/VAV3/DBNL/PLEKHG2/SPART/CYRIB
Biological Process	GO:0042110	T cell activation	88/2279	483/18866	4.80E-05	0.0078549	0.0071074	88	HLA-DMB/IL27/PRKCZ/ZFPM1/LAT/FOXP1/FUT7/IL10/KIF13B/GSN/MAD1L1/PIK3CD/RHOH/LCK/MYB/RUNX1/SIRPB1/CD55/SLC11A1/TNFRSF1B/NLRP3/PIK3R1/ITPKB/CCR2/PIK3R6/SMAD3/LAX1/CCL5/GRAP2/NCK2/LOXL3/SFTPD/NFATC2/HSH2D/TNFSF13B/DUSP22/SEMA4A/ZBTB16/TGFBR2/MYH9/RAB27A/PTPN6/MIR27A/PREX1/SMAD7/TNFRSF21/ZMIZ1/DOCK8/LY9/CCR9/ZBTB7B/TIGIT/FANCA/RARA/RIPK3/LILRB4/ZC3H8/SP3/CCDC88B/PTPRC/CSK/RUNX2/BCL10/CLPTM1/SPTA1/PNP/NOD2/BCL2/DUSP3/IL18R1/SIRPG/NEDD4/HLX/CEBPB/RUNX3/CYLD/DOCK2/AP3D1/BCL3/SLC7A1/FADD/PELI1/JAML/TMEM131L/CYRIB/VSIR/MARCHF7/SLA2
Biological Process	GO:0050863	regulation of T cell activation	65/2279	332/18866	5.24E-05	0.0084323	0.0076298	65	HLA-DMB/IL27/PRKCZ/LAT/IL10/GSN/MAD1L1/RHOH/LCK/MYB/RUNX1/SIRPB1/CD55/TNFRSF1B/NLRP3/PIK3R1/ITPKB/CCR2/PIK3R6/LAX1/CCL5/GRAP2/NCK2/LOXL3/SFTPD/NFATC2/TNFSF13B/DUSP22/ZBTB16/TGFBR2/PTPN6/MIR27A/SMAD7/TNFRSF21/ZMIZ1/DOCK8/ZBTB7B/TIGIT/FANCA/RARA/RIPK3/LILRB4/ZC3H8/CCDC88B/PTPRC/CSK/BCL10/CLPTM1/SPTA1/PNP/NOD2/DUSP3/SIRPG/HLX/CEBPB/RUNX3/CYLD/AP3D1/SLC7A1/FADD/PELI1/TMEM131L/CYRIB/VSIR/MARCHF7
Biological Process	GO:1903706	regulation of hemopoiesis	90/2279	498/18866	5.38E-05	0.0085216	0.0077107	90	IL27/PRKCZ/ZFPM1/FOXP1/RHOH/CREB1/MIR145/MYB/RUNX1/CSF3R/INPP5D/ASH2L/NLRP3/PIK3R1/ITPKB/CCR2/PIK3R6/TNRC6B/PRKCA/TNF/UBASH3B/LOXL3/NFATC2/FBXO7/AGO1/TLR9/ZBTB16/TGFBR2/TESC/THBS1/BRD1/DLL1/RIPK1/ITGA2B/PTPN6/TCF12/SMAD7/HDAC1/ZMIZ1/MTURN/ZBTB7B/TGFB1/CD101/FANCA/PRKCB/KMT2D/RARA/TMEM178A/LILRB4/NF1/ZC3H8/PGLYRP1/CSF3/PTPRC/RARG/TMEM64/SETD1A/CLPTM1/PSMF1/MEIS2/GFI1B/CREBBP/LRRC17/ETS1/PNP/NFE2L2/PUS7/HOXA9/RB1/MEIS1/TCF3/PSMB7/NOTCH1/MEF2C/LTF/HLX/CEBPB/RUNX3/TRIB1/CYLD/AP3D1/KAT7/INHBA/LIF/FADD/PSMD13/PCID2/TMEM131L/VSIR/H3C1
Biological Process	GO:0007265	Ras protein signal transduction	67/2279	346/18866	5.76E-05	0.0089716	0.0081179	67	KSR1/ARHGEF10/RREB1/TIMP2/LAT/KANK1/ELMO1/RHOH/DENND1A/AKAP13/MYO9B/ARHGDIB/ITPKB/DOK3/RAP1B/RHOG/ARRB1/PLD1/DNM2/GRAP2/DOK1/RCC1/DENND3/CUL3/CDC42EP1/LRRC59/DOK2/SYNPO2L/RASA3/PLEKHG5/SYNGAP1/NISCH/RERG/RAPGEF2/EPS8L3/IQSEC1/EPS8L1/NF1/ABCA1/BCR/ARHGEF25/NCKAP1/RALA/NTRK1/RB1/ARHGEF18/NOTCH1/DNMT1/GNA12/CYTH4/ARHGAP17/F2R/ARHGEF28/RAB30/FOXM1/ARHGEF2/GPR4/AGTR1/PDGFRB/ARHGAP1/RALGPS1/CYFIP1/FLOT1/FBXO8/DBNL/PRAG1/RIPOR1
Biological Process	GO:0050764	regulation of phagocytosis	26/2279	97/18866	6.01E-05	0.0092067	0.0083306	26	AZU1/PRTN3/ABR/CALR/PPARG/SIRPB1/SLC11A1/DNM2/RAB31/FGR/TNF/MYO18A/SFTPD/FPR2/LMAN2/RAB27A/SNX3/PTPRJ/CAMK1D/PTPRC/CSK/BCR/SIRPG/ANO6/DYSF/DOCK2
Biological Process	GO:2001233	regulation of apoptotic signaling pathway	77/2279	413/18866	6.40E-05	0.0096481	0.0087299	77	TRIM39/S100A8/P4HB/ENO1/TFDP1/GSN/ITPRIP/LCK/URI1/YWHAH/PAM16/PLAGL2/BMF/SMAD3/CTTN/TNF/TRAF2/TNFAIP3/NCK2/TRAF1/DEDD2/UNC5B/THBS1/RRM2B/CTSH/RIPK1/GNAI2/NACC2/ZNF385A/TCF7L2/CLU/HDAC1/CIDEB/FGFR1/SGMS1/RIPK3/NF1/HTT/PTPRC/ITM2C/BMPR1B/STK3/BCL10/PPP2R1A/NOL3/MAPK8IP2/NFE2L2/TRPS1/ARRB2/ANKRD2/YWHAZ/FIS1/DAPK2/RB1/BCL2/PIH1D1/DNM1L/PPP2R1B/SP100/ARHGEF2/TP53BP2/BCL2L1/TNFSF12/CYLD/YWHAQ/RELA/G0S2/ING5/BAG5/GRINA/INHBA/CX3CR1/FADD/DAPK3/PRKN/MARCHF7/FBH1

Biological Process	GO:0071222	cellular response to lipopolysaccharide	45/2279	208/18866	6.59E-05	0.0096638	0.0087442	45	DEFA4/CEBPE/PDE4D/CTSG/IRF8/GFI1/SBNO2/IL10/LCN2/MIR140/CD55/TNFRSF1B/NLRP3/PRKCA/TNF/TNFAIP3/CCL5/ABCC2/SPON2/SCIMP/TGFB1/PABPN1/LITAF/SGMS1/LY86/RARA/TRIM5/ADAMTS13/PRPF8/CSF3/ABCA1/BCL10/BCR/IRAK2/NOD2/MEF2C/PRKCE/LTF/CASP1/CD68/CEBPB/TRIB1/ANKRD1/RELA/CX3CR1
Biological Process	GO:0001819	positive regulation of cytokine production	82/2279	447/18866	6.73E-05	0.0096638	0.0087442	82	AZU1/ELANE/IL27/PRKCZ/NLRC4/PDE4D/ZFPM1/IRF8/SORL1/IL10/AIM2/IRF5/C3AR1/IL16/C1QTNF3/CREB1/CLEC9A/MIR145/MYB/RUNX1/SLC11A1/NLRP3/IL1R1/PIK3R1/SETD2/CCR2/SMAD3/FGR/TNF/TRAF2/POLR2F/TLR9/MNDA/CLEC5A/SPON2/CCM2L/THBS1/CRCP/RIPK1/CD226/APP/HLA-F/MBP/CLU/WNT11/DEFB124/SCIMP/LY9/TGFB1/PHB/ZNF580/PTPRJ/TIGIT/FCN1/RARA/CXCL17/CCDC88B/SEC14L1/SLC7A5/PTPRC/BCL10/IL6R/CREBBP/PLCB1/NOD2/POU2F2/IL18R1/DDX41/F2R/ARHGEF2/CASP1/CEBPB/CADM1/RELA/BCL3/FLOT1/NODAL/FADD/PELI1/STING1/CYRIB/STMP1
Biological Process	GO:0008064	regulation of actin polymerization or depolymerization	42/2279	190/18866	6.79E-05	0.0096638	0.0087442	42	VILL/TRIM27/SSH1/GSN/KANK1/CTTN/COTL1/CAPG/CYFIP2/NCK2/WHAMM/BAIAP2L1/CDC42EP1/BIN1/BAIAP2/SSH3/PREX1/WASL/FER/TMOD3/SVIL/PFN1/CSF3/GMFB/WDR1/SPTA1/NCKAP1/ADD1/AVIL/PRKCE/FLII/MYO1C/SPTAN1/TMOD1/CYFIP1/GMFG/BAIAP2L2/MYADM/SPTBN4/DBNL/PLEKHG2/CYRIB
Biological Process	GO:0002695	negative regulation of leukocyte activation	41/2279	184/18866	6.81E-05	0.0096638	0.0087442	41	ABR/IL10/MAD1L1/RUNX1/INPP5D/CCR2/FGR/LAX1/TNFAIP3/LOXL3/SFTPD/CD84/FBXO7/MNDA/DUSP22/HLA-F/PTPN6/MIR27A/SMAD7/TNFRSF21/ZBTB7B/FER/TIGIT/ZC3H8/BLK/PGLYRP1/PTPRC/GRN/BCR/CNR2/DUSP3/BANK1/HLX/CEBPB/RUNX3/TYRO3/INHBA/PELI1/TMEM131L/VSIR/MARCHF7
Biological Process	GO:0046486	glycerolipid metabolic process	80/2279	434/18866	6.94E-05	0.0096694	0.0087492	80	PNPLA2/CPNE6/OSBPL5/PLA2G15/SYNJ2/SORL1/MGLL/PCYT1A/PIK3CD/ACSL1/MFSD2A/INPP5A/VAC14/LPCAT1/PIP5K1B/INPP5D/LIPC/ABHD16A/PIK3R1/ITPKB/PIGL/PIK3R6/PIGU/PLD1/GPLD1/CSF1R/FPR2/TTC7A/PIGN/PGP/ACP6/PITPNM2/SLC44A4/INPP4A/PIK3R5/PIP4K2A/PITPNM1/CPNE1/TTC7B/PGS1/DGKQ/SH3YL1/CHPT1/NR1H2/ATG14/PCTP/GNB3/PLEKHA2/PDGFB/ABHD16B/DGKA/DGKD/INPP5E/FABP6/PLCB1/CAT/OSBPL10/IMPA2/PI4K2A/PI4KA/PIGQ/PLA1A/AVIL/LMF1/ABHD5/PCK2/PDGFRB/RUFY1/CHKB/SREBF1/PTDSS2/CPT1A/INPP5F/LPIN1/CDS2/LYPLA2/AGPAT5/ETNK2/IP6K1/SLA2
Biological Process	GO:1902105	regulation of leukocyte differentiation	58/2279	290/18866	7.02E-05	0.0096694	0.0087492	58	IL27/PRKCZ/ZFPM1/FOXP1/RHOH/CREB1/MIR145/MYB/RUNX1/INPP5D/NLRP3/PIK3R1/ITPKB/CCR2/PIK3R6/PRKCA/TNF/UBASH3B/LOXL3/NFATC2/FBXO7/TLR9/ZBTB16/TGFBR2/TESC/RIPK1/PTPN6/SMAD7/ZMIZ1/ZBTB7B/TGFB1/CD101/FANCA/RARA/TMEM178A/LILRB4/NF1/ZC3H8/PGLYRP1/PTPRC/TMEM64/CLPTM1/LRRC17/PNP/RB1/LTF/HLX/CEBPB/RUNX3/TRIB1/CYLD/AP3D1/INHBA/LIF/FADD/PCID2/TMEM131L/VSIR
Biological Process	GO:0030098	lymphocyte differentiation	70/2279	368/18866	7.13E-05	0.0096773	0.0087564	70	HDAC4/IL27/PRKCZ/ZFPM1/FOXP1/FUT7/IL10/PIK3CD/RHOH/LCK/MYB/RUNX1/INPP5D/NLRP3/PIK3R1/ITPKB/CCR2/PIK3R6/LOXL3/LYL1/NFATC2/FBXO7/TLR9/SEMA4A/ZBTB16/TGFBR2/DLL1/RBPJ/PTPN6/PREX1/SMAD7/ZMIZ1/LY9/CCR9/ZBTB7B/PTPRJ/FANCA/RARA/RIPK3/LILRB4/CD79A/ZC3H8/SP3/PGLYRP1/CMTM7/PTPRC/RUNX2/CLPTM1/PNP/DOCK10/NTRK1/POU2F2/BCL2/TCF3/IL18R1/HLX/RUNX3/HHEX/CYLD/DOCK2/AP3D1/TYRO3/BCL3/KAT7/INHBA/FADD/PCID2/ADGRG3/TMEM131L/VSIR
Biological Process	GO:0050817	coagulation	67/2279	349/18866	7.57E-05	0.0099225	0.0089783	67	PRTN3/ZFPM1/EHD1/GP9/SERPINA1/PLAU/CD9/ENTPD1/ITPR2/LCK/DOCK6/CD177/PRKAR1B/DOCK1/PIK3R1/PIK3R6/PABPC4/ARRB1/PRKCA/UBASH3B/MICAL1/MYL12A/SERPINB2/ITPR1/THBS1/MYH9/RAB27A/PIK3R5/ITGA2B/PTPN6/ILK/HDAC1/DOCK8/F12/PRKCH/DGKQ/PRKCB/HBB/ADAMTS13/BLK/CD59/VAV2/GNA11/ITGB3/ACTG1/PDGFB/DGKA/DGKD/NFE2L2/ARRB2/PAPSS2/YWHAZ/GNG2/P2RX1/PRKCE/GNA12/CPB2/F2R/ANO6/HPS6/TYRO3/IRF2/ADTRP/P2RX5/DTNBP1/VAV3/H3C1
Biological Process	GO:0030832	regulation of actin filament length	42/2279	191/18866	7.70E-05	0.0099225	0.0089783	42	VILL/TRIM27/SSH1/GSN/KANK1/CTTN/COTL1/CAPG/CYFIP2/NCK2/WHAMM/BAIAP2L1/CDC42EP1/BIN1/BAIAP2/SSH3/PREX1/WASL/FER/TMOD3/SVIL/PFN1/CSF3/GMFB/WDR1/SPTA1/NCKAP1/ADD1/AVIL/PRKCE/FLII/MYO1C/SPTAN1/TMOD1/CYFIP1/GMFG/BAIAP2L2/MYADM/SPTBN4/DBNL/PLEKHG2/CYRIB

Biological Process	GO:0043254	regulation of protein-containing complex assembly	82/2279	449/18866	7.87E-05	0.0099225	0.0089783	82	VILL/PRKCZ/TRIM27/TBCD/DCTN1/SORL1/SPIDR/SSH1/GSN/KANK1/CREB1/ULK1/RAP1B/BMF/CTTN/LDLRAD4/MEFV/TNF/COTL1/TRAF2/CAPG/CYFIP2/NCK2/WHAMM/BAIAP2L1/NAV3/CDC42EP1/BIN1/BAIAP2/SSH3/PREX1/CLU/ASAP1/WASL/TGFB1/FER/TMOD3/SVIL/PFN1/VPS18/JAM3/NR1H2/CSF3/GMFB/ATF7IP/ABCA1/NUMA1/GFAP/ZDHHC1/SPTA1/FNIP2/NCKAP1/ADD1/RB1/MPP7/PIH1D1/MED25/AVIL/TPPP3/PRKCE/FLII/ARHGEF2/MYO1C/ERCC1/SPTAN1/TMOD1/CYFIP1/GMFG/BAIAP2L2/LMO4/XPA/MYADM/SPTBN4/ERCC2/EIF4G1/DBNL/PLEKHG2/SVIP/TFIP11/CYRIB/STMP1/SLF1
Biological Process	GO:0002237	response to molecule of bacterial origin	68/2279	356/18866	7.88E-05	0.0099225	0.0089783	68	DEFA4/ELANE/CEBPE/SLPI/PDE4D/S100A8/CTSG/IRF8/GFI1/ABR/MPO/FOXP1/SBNO2/IL10/IRF5/CSF2RB/LCN2/MIR140/CD55/SLC11A1/TNFRSF1B/NLRP3/GGT1/PRKCA/TNF/TNFAIP3/CCL5/TLR9/ABCC2/SPON2/COMT/ALPL/DEFB124/SCIMP/TGFB1/PABPN1/FER/LITAF/SGMS1/LY86/RARA/TRIM5/ADAMTS13/PRPF8/CSF3/ABCA1/BCL10/MGST2/BCR/CNR2/IRAK2/NOD2/NOTCH1/MEF2C/PRKCE/F2R/LTF/CASP1/CD68/CEBPB/MAPKAPK3/TRIB1/ANKRD1/CD96/RELA/CX3CR1/PELI1/CYRIB
Biological Process	GO:0006650	glycerophospholipid metabolic process	66/2279	343/18866	7.94E-05	0.0099225	0.0089783	66	CPNE6/OSBPL5/PLA2G15/SYNJ2/PCYT1A/PIK3CD/MFSD2A/INPP5A/VAC14/LPCAT1/PIP5K1B/INPP5D/LIPC/ABHD16A/PIK3R1/ITPKB/PIGL/PIK3R6/PIGU/PLD1/GPLD1/CSF1R/FPR2/TTC7A/PIGN/PGP/ACP6/PITPNM2/SLC44A4/INPP4A/PIK3R5/PIP4K2A/PITPNM1/CPNE1/TTC7B/PGS1/DGKQ/SH3YL1/CHPT1/NR1H2/PCTP/PLEKHA2/PDGFB/ABHD16B/DGKA/DGKD/INPP5E/PLCB1/OSBPL10/IMPA2/PI4K2A/PI4KA/PIGQ/PLA1A/ABHD5/PDGFRB/RUFY1/CHKB/PTDSS2/INPP5F/LPIN1/CDS2/AGPAT5/ETNK2/IP6K1/SLA2
Biological Process	GO:0019932	second-messenger-mediated signaling	83/2279	456/18866	8.04E-05	0.0099225	0.0089783	83	AZU1/HDAC4/TRPM2/KSR1/PDE4D/LAT/MGRN1/ADCY2/CASQ2/ITPR2/INPP5A/CACNA1C/AKAP13/PRKAR1B/ADCY9/CCR2/SLC8A1/PRKCA/TNF/FPR2/FPR1/NFATC2/ITPR1/CAMKK2/NFATC1/ACKR2/THBS1/JSRP1/GNAI2/NOS1AP/CAMTA1/MRAP/RCAN1/CRTC3/P2RY6/CCR9/PTPRJ/RAPGEF2/HRH1/DGKQ/AQP1/MCTP2/DEFB1/MYO5A/DYRK2/GIPR/HTT/CCL20/PTPRC/CXCR1/ABCA1/PDGFB/PPP2R1A/DGKD/CNR2/TPCN2/PDE9A/ADCY4/SAMD14/FIS1/S1PR2/GSTO1/GNG2/LPAR2/FHL2/NPPC/S1PR4/GPR4/AGTR1/ANK2/CHERP/PDE3B/PLCG1/P2RX5/NR5A2/CASQ1/CX3CR1/PRKAA1/IRAG1/ADGRG3/STIMATE/SLA2/ADGRE3
Biological Process	GO:0071219	cellular response to molecule of bacterial origin	47/2279	222/18866	8.04E-05	0.0099225	0.0089783	47	DEFA4/CEBPE/PDE4D/CTSG/IRF8/GFI1/SBNO2/IL10/LCN2/MIR140/CD55/TNFRSF1B/NLRP3/PRKCA/TNF/TNFAIP3/CCL5/ABCC2/SPON2/DEFB124/SCIMP/TGFB1/PABPN1/LITAF/SGMS1/LY86/RARA/TRIM5/ADAMTS13/PRPF8/CSF3/ABCA1/BCL10/BCR/IRAK2/NOD2/MEF2C/PRKCE/LTF/CASP1/CD68/CEBPB/TRIB1/ANKRD1/RELA/CX3CR1/CYRIB
Biological Process	GO:0045123	cellular extravasation	20/2279	67/18866	8.25E-05	0.0100463	0.0090902	20	AZU1/ELANE/FUT4/PRTN3/ABR/FUT7/PIK3CD/CD177/IL1R1/CCR2/TNF/FER/RIPK3/PDGFD/BCR/PLCB1/ITGB2/CX3CR1/FADD/JAML
Biological Process	GO:0060348	bone development	44/2279	204/18866	8.51E-05	0.0102336	0.0092598	44	CSGALNACT1/ZFPM1/FOXP1/SLC38A10/FOXN3/MEF2D/LRP5L/FLI1/AKAP13/SKI/CHSY1/ANKRD11/ANXA6/SH3PXD2B/TGFBR2/ALPL/PIP4K2A/PTPN6/ZNF385A/POC1A/XYLT1/SEMA4D/RARA/POR/LRRK1/GHRL/PTPRC/RARG/BMPR1B/RUNX2/LRRC17/ENG/PAPSS2/MEIS1/MEF2C/PDGFC/NPPC/LTF/ANO6/FAM20C/RFLNA/P3H1/NSD2/CCN4
Biological Process	GO:0006900	vesicle budding from membrane	28/2279	110/18866	8.64E-05	0.0102553	0.0092794	28	CTSZ/SERPINA1/TGFA/STX5/CSNK1D/DNM2/TRAPPC10/SEC23B/MYO18A/CUL3/TRAPPC9/SNX3/MIA3/WASL/CNIH2/CD59/SEC13/ANKRD28/PRKCI/DNM1/SEC24C/DNM3/TMED2/AP3D1/MX1/SEC31A/PRKN/TRAPPC2
Biological Process	GO:1900027	regulation of ruffle assembly	12/2279	30/18866	9.94E-05	0.0116567	0.0105474	12	TACSTD2/DEF8/KANK1/RHOG/SH3YL1/EPS8L3/EPS8L1/PFN1/PLEKHM1/CORO1C/CYFIP1/NDEL1

Biological Process	GO:0032496	response to lipopolysaccharide	64/2279	334/18866	0.0001146	0.0132739	0.0120107	64	DEFA4/ELANE/CEBPE/SLPI/PDE4D/S100A8/CTSG/IRF8/GFI1/ABR/MPO/FOXP1/SBNO2/IL10/CSF2RB/LCN2/MIR140/CD55/SLC11A1/TNFRSF1B/NLRP3/GGT1/PRKCA/TNF/TNFAIP3/CCL5/ABCC2/SPON2/COMT/ALPL/SCIMP/TGFB1/PABPN1/FER/LITAF/SGMS1/LY86/RARA/TRIM5/ADAMTS13/PRPF8/CSF3/ABCA1/BCL10/MGST2/BCR/CNR2/IRAK2/NOD2/NOTCH1/MEF2C/PRKCE/F2R/LTF/CASP1/CD68/CEBPB/MAPKAPK3/TRIB1/ANKRD1/CD96/RELA/CX3CR1/PELI1
Biological Process	GO:0050866	negative regulation of cell activation	44/2279	207/18866	0.0001213	0.0138652	0.0125457	44	ABR/IL10/CD9/MAD1L1/RUNX1/INPP5D/CCR2/FGR/LAX1/UBASH3B/TNFAIP3/LOXL3/SFTPD/CD84/FBXO7/MNDA/DUSP22/HLA-F/PTPN6/MIR27A/SMAD7/TNFRSF21/ZBTB7B/FER/TIGIT/ZC3H8/BLK/PGLYRP1/PTPRC/GRN/PDGFB/BCR/CNR2/DUSP3/BANK1/HLX/CEBPB/RUNX3/TYRO3/INHBA/PELI1/TMEM131L/VSIR/MARCHF7
Biological Process	GO:0045017	glycerolipid biosynthetic process	54/2279	270/18866	0.0001226	0.0138652	0.0125457	54	PNPLA2/CPNE6/PLA2G15/SYNJ2/PCYT1A/PIK3CD/ACSL1/MFSD2A/VAC14/LPCAT1/PIP5K1B/INPP5D/PIK3R1/PIGL/PIK3R6/PIGU/PLD1/GPLD1/FPR2/PIGN/ACP6/PITPNM2/SLC44A4/INPP4A/PIK3R5/PIP4K2A/PITPNM1/CPNE1/PGS1/DGKQ/SH3YL1/CHPT1/NR1H2/PCTP/PLEKHA2/PDGFB/DGKA/DGKD/INPP5E/PI4K2A/PI4KA/PIGQ/AVIL/ABHD5/PCK2/RUFY1/CHKB/SREBF1/PTDSS2/INPP5F/LPIN1/CDS2/AGPAT5/ETNK2
Biological Process	GO:0045651	positive regulation of macrophage differentiation	8/2279	15/18866	0.0001312	0.0146609	0.0132657	8	MIR145/PRKCA/RIPK1/TGFB1/RB1/TRIB1/LIF/FADD
Biological Process	GO:0008654	phospholipid biosynthetic process	54/2279	271/18866	0.0001354	0.0149627	0.0135388	54	CPNE6/SYNJ2/PCYT1A/PIK3CD/SPTLC2/MFSD2A/IDI1/HEXB/VAC14/LPCAT1/PIP5K1B/INPP5D/PIK3R1/PIGL/PIK3R6/PIGU/PLD1/FPR2/PIGN/ACP6/PITPNM2/SLC44A4/INPP4A/PIK3R5/PIP4K2A/PITPNM1/CPNE1/SPHK2/SGMS1/PGS1/DGKQ/SH3YL1/CHPT1/PCTP/PLEKHA2/PDGFB/DGKA/DGKD/INPP5E/SGMS2/PI4K2A/PI4KA/PIGQ/ABHD5/RUFY1/CHKB/HMGCS1/PTDSS2/INPP5F/LPIN1/CDS2/DPAGT1/AGPAT5/ETNK2
Biological Process	GO:0071216	cellular response to biotic stimulus	50/2279	246/18866	0.0001422	0.0155333	0.0140551	50	DEFA4/CEBPE/PDE4D/CTSG/IRF8/GFI1/SBNO2/IL10/LCN2/MIR140/CD55/TNFRSF1B/NLRP3/PRKCA/TNF/TNFAIP3/CCL5/ABCC2/SPON2/EME1/DEFB124/SCIMP/TGFB1/PABPN1/LITAF/SGMS1/LY86/RARA/TRIM5/ADAMTS13/PRPF8/CSF3/ABCA1/BCL10/BCR/IRAK2/NOD2/MEF2C/PRKCE/LTF/CASP1/CD68/CEBPB/TRIB1/ANKRD1/SLX4/RELA/CX3CR1/CYRIB/FBH1
Biological Process	GO:0014065	phosphatidylinositol 3-kinase signaling	35/2279	154/18866	0.0001489	0.0158958	0.0143831	35	PIK3CD/PRR5L/PIP5K1B/ANGPT1/PIK3AP1/PIK3R1/FGR/TNF/CCL5/UNC5B/PIK3R5/PIP4K2A/PTPN6/IGF1R/FLT1/FGFR1/SEMA4D/PPP2R5C/NF1/PDGFD/PRR5/CSF3/PDGFB/INPP5E/CAT/NTRK1/F2R/UBE3A/PDGFC/NEDD4/TSC2/PDGFRB/PPP1R16B/TYRO3/KBTBD2
Biological Process	GO:0006022	aminoglycan metabolic process	38/2279	172/18866	0.0001489	0.0158958	0.0143831	38	CSGALNACT1/AP2A1/SPOCK2/HS3ST3B1/DSE/HEXB/CHI3L1/GALNS/ANGPT1/CHSY1/CHST11/LYG2/CHIT1/CHST15/NDST1/CHST2/EGFLAM/SDC2/XYLT1/TGFB1/STAB2/B4GALT7/PGLYRP1/PDGFB/ST3GAL4/B4GALT3/LYVE1/ARSB/GCNT2/PDGFRB/CHI3L2/PGLYRP4/HAS3/ST3GAL2/B3GNT2/B4GAT1/CEMIP2/PXYLP1
Biological Process	GO:0019722	calcium-mediated signaling	46/2279	222/18866	0.0001618	0.0170813	0.0154558	46	AZU1/HDAC4/TRPM2/PDE4D/LAT/CASQ2/CACNA1C/CCR2/SLC8A1/TNF/FPR2/NFATC2/ITPR1/CAMKK2/NFATC1/ACKR2/JSRP1/CAMTA1/RCAN1/P2RY6/CCR9/PTPRJ/MCTP2/DEFB1/MYO5A/DYRK2/HTT/CCL20/PTPRC/CXCR1/TPCN2/SAMD14/FIS1/GSTO1/FHL2/AGTR1/ANK2/CHERP/PLCG1/P2RX5/NR5A2/CASQ1/CX3CR1/PRKAA1/STIMATE/SLA2

Biological Process	GO:0045444	fat cell differentiation	48/2279	235/18866	0.0001692	0.0174728	0.01581	48	LRG1/ARID5B/ZFPM1/RREB1/ZNF516/CEBPD/TRIO/PPARG/C1QTNF3/CREB1/SMAD3/TNF/SH3PXD2B/ZBTB16/ZNF385A/TCF7L2/LRRC8C/ZBTB7B/TGFB1/GRK5/CTBP1/TGFB1I1/WWTR1/CTBP2/TMEM64/WNT5B/STK3/GNB3/PLCB1/FTO/AAMDC/SORT1/ARNTL/PRDM16/PER2/CEBPB/FOXO1/DYSF/GDF6/SREBF1/LAMB3/NOC3L/ZADH2/MB/BSCL2/ANGPTL8/TLCD3B/CCN4
Biological Process	GO:1903039	positive regulation of leukocyte cell-cell adhesion	48/2279	235/18866	0.0001692	0.0174728	0.01581	48	ELANE/FUT4/HLA-DMB/PRKCZ/FUT7/RHOH/LCK/MYB/RUNX1/SIRPB1/CD55/NLRP3/PIK3R1/ITPKB/CCR2/PIK3R6/TNF/CCL5/GRAP2/NCK2/TNFSF13B/ZBTB16/TGFBR2/PTPN6/ZMIZ1/DOCK8/ZBTB7B/RARA/LILRB4/CCDC88B/PTPRC/CSK/BCL10/SPTA1/ETS1/PNP/ITGB2/NOD2/SIRPG/HLX/RUNX3/CYLD/AP3D1/RELA/SLC7A1/FADD/CYRIB/VSIR
Biological Process	GO:0031589	cell-substrate adhesion	67/2279	359/18866	0.0001798	0.0183723	0.0166239	67	PRKCZ/TBCD/P4HB/RREB1/TACSTD2/PARVB/CALR/PLAU/SPOCK2/COL17A1/VIT/KANK1/RIN2/ANGPT1/DOCK1/ITGB1BP1/PIK3R1/CEACAM6/SMAD3/ARHGEF7/CTTN/DNM2/EPHB3/DUSP22/WHAMM/JUP/THBS1/DISC1/NINJ1/ACTN1/ITGA2B/EGFLAM/ACTN4/ILK/PREX1/PXN/ITGB5/PPFIA1/FER/PTPRJ/NF1/ADAMTS13/JAM3/TESK2/OLFM4/ITGB3/ACTG1/PLEKHA2/BCR/CASS4/ITGB2/LYVE1/BCL2/LYPD5/NOTCH1/CORO1C/PARVG/GCNT2/DUSP3/PRKCE/RSU1/TYRO3/CD96/LAMB3/ITGB6/MYADM/DAPK3
Biological Process	GO:0002703	regulation of leukocyte mediated immunity	44/2279	211/18866	0.0001912	0.0193256	0.0174865	44	PRKCZ/ABR/FUT7/IL10/CD177/TNFRSF1B/NLRP3/IL1R1/CCR2/PIK3R6/GAB2/FGR/TNF/TRAF2/CD84/DUSP22/CD226/HLA-F/PTPN6/SMAD7/SCIMP/SPHK2/TGFB1/FER/NCR1/RIPK3/BLK/PTPRC/BCR/ARRB2/ITGB2/IL18RAP/DNASE1L3/NOD2/TP53BP1/PRAM1/IL18R1/TNFSF13/CADM1/CD96/FADD/PAXIP1/CYRIB/NSD2
Biological Process	GO:0046777	protein autophosphorylation	48/2279	237/18866	0.0002086	0.0207058	0.0187354	48	AATK/LMTK2/MARK3/VRK2/MAP3K3/LCK/ULK1/ERN1/AAK1/FGR/CSF1R/EPHB3/CAMKK2/TNIK/SLA/RIPK1/IGF1R/FLT1/FGFR1/FER/CLK3/PPP2R5C/GRK5/RIPK3/STK24/PDGFD/BLK/PTPRC/CSK/PDGFB/BCR/MKNK1/ENG/TAOK3/PPP2R5E/PPP2R5D/NEK6/NTRK1/INSRR/DAPK2/GRK7/PDGFC/CHEK2/MAPKAPK3/PDGFRB/TYRO3/MARK2/DAPK3
Biological Process	GO:0003332	negative regulation of extracellular matrix constituent secretion	4/2279	4/18866	0.0002124	0.0207058	0.0187354	4	TNFRSF1B/TNFRSF1A/NOTCH1/ADTRP
Biological Process	GO:0010761	fibroblast migration	15/2279	46/18866	0.0002126	0.0207058	0.0187354	15	ARID5B/PRR5L/MIR145/ITGB1BP1/SLC8A1/ARHGEF7/THBS1/TNS1/ILK/TGFB1/FER/MTA2/CORO1C/PRKCE/GNA12
Biological Process	GO:0002685	regulation of leukocyte migration	44/2279	212/18866	0.0002136	0.0207058	0.0187354	44	ELANE/FUT4/ABR/CALR/FUT7/CD9/C3AR1/RHOH/IL1R1/CCR2/RHOG/TNF/CCL5/CSF1R/FPR2/PADI2/THBS1/C5AR2/APP/ITGA2B/MIR24-2/MIA3/DEFB124/DOCK8/WASL/ZNF580/RIPK3/PDGFD/JAM3/CAMK1D/CXCL17/CCL20/IL6R/C10orf99/BCR/PLCB1/DAPK2/DNM1L/ANO6/DYSF/ADTRP/CCL27/CX3CR1/FADD
Biological Process	GO:0045637	regulation of myeloid cell differentiation	52/2279	263/18866	0.000218	0.0208659	0.0188802	52	ZFPM1/FOXP1/CREB1/MIR145/RUNX1/CSF3R/INPP5D/ASH2L/PIK3R1/ITPKB/TNRC6B/PRKCA/TNF/UBASH3B/AGO1/ZBTB16/TESC/THBS1/BRD1/DLL1/RIPK1/ITGA2B/HDAC1/MTURN/TGFB1/CD101/PRKCB/KMT2D/RARA/TMEM178A/LILRB4/NF1/CSF3/RARG/TMEM64/SETD1A/MEIS2/CREBBP/LRRC17/ETS1/HOXA9/RB1/MEIS1/MEF2C/LTF/CEBPB/TRIB1/KAT7/INHBA/LIF/FADD/H3C1

Biological Process	GO:0006888	endoplasmic reticulum to Golgi vesicle-mediated transport	43/2279	206/18866	0.0002196	0.0208659	0.0188802	43	CTSZ/DCTN1/SORL1/SERPINA1/TGFA/STX5/VTI1A/CSNK1D/CD55/TRAPPC10/SEC23B/DYNC1H1/LMAN2/WHAMM/YIF1A/CUL3/TRAPPC9/ERGIC1/MIA3/COG1/DCTN2/CNIH2/SEC22C/CD59/TRAPPC3L/COG8/SEC13/CREB3L2/SPTA1/ANKRD28/SEC24C/CNIH4/COG5/LMF1/TMED2/SPTAN1/ANK2/SPTBN4/SEC31A/TEX261/TRAPPC2B/MIA2/TRAPPC2
Biological Process	GO:0006909	phagocytosis	70/2279	382/18866	0.0002291	0.0215484	0.0194977	70	AZU1/ELANE/CEBPE/PRTN3/IRF8/ABR/CALR/PPARG/GSN/ELMO1/TM9SF4/RHOH/SIRPB1/LYST/SLC11A1/DOCK1/PIK3R1/NCF4/CCR2/RHOG/WIPF1/DNM2/RAB31/FGR/TNF/CYFIP2/MYO18A/SFTPD/FPR2/LMAN2/IGHV6-1/SPON2/THBS1/MYH9/BAIAP2/RAB27A/SNX3/COLEC12/NCF2/P2RY6/WASL/MARCO/PTPRJ/RHOBTB2/FCN1/RARA/CAMK1D/VAV2/ITGB3/PTPRC/ACTG1/CSK/ABCA1/BCR/ARHGAP25/ITGB2/NCKAP1/CORO1C/PRKCE/SIRPG/MYO1C/ANO6/DYSF/ICAM5/DOCK2/TYRO3/ARHGAP12/PLCG1/CYFIP1/VAV3
Biological Process	GO:0010634	positive regulation of epithelial cell migration	38/2279	176/18866	0.0002446	0.0225824	0.0204333	38	RREB1/FOXP1/CALR/NRP2/MAP3K3/MIR143/RIN2/HDAC7/ANGPT1/DOCK1/ITGB1BP1/PRKCA/MIR199A1/GPLD1/MIR101-2/STAT5A/RTN4/TGFBR2/THBS1/CTSH/AKT3/MIR27A/MIR23A/FGFR1/TGFB1/ZNF580/IQSEC1/PFN1/ITGB3/GRN/PDGFB/ETS1/NFE2L2/MIR199A2/PRKCE/PLCG1/ADGRA2/PLPP3
Biological Process	GO:0061515	myeloid cell development	20/2279	72/18866	0.0002448	0.0225824	0.0204333	20	MAEA/ZFPM1/FOXP1/FLI1/PABPC4/SLC11A2/BRD1/PIP4K2A/APP/PTPN6/ZNF385A/TMOD3/LRRK1/ALAS1/MEIS1/TSPAN2/LTF/FAM20C/EPB42/ERCC2
Biological Process	GO:0046718	viral entry into host cell	31/2279	134/18866	0.0002479	0.0226464	0.0204913	31	TRIM27/P4HB/TRIM38/GSN/TRIM8/WWP2/SLC10A1/CD55/TRIM10/SLC1A5/CLEC5A/SNX3/UVRAG/ITGB5/PHB/FCN1/TRIM5/VPS18/ITGB3/CTSB/TPCN2/NPC1/SLC52A2/EPS15/TYRO3/ITGB6/SIVA1/TRIM26/GRK2/NECTIN1/NECTIN4
Biological Process	GO:0030203	glycosaminoglycan metabolic process	35/2279	158/18866	0.0002522	0.0228249	0.0206528	35	CSGALNACT1/AP2A1/SPOCK2/HS3ST3B1/DSE/HEXB/GALNS/ANGPT1/CHSY1/CHST11/LYG2/CHST15/NDST1/CHST2/EGFLAM/SDC2/XYLT1/TGFB1/STAB2/B4GALT7/PGLYRP1/PDGFB/ST3GAL4/B4GALT3/LYVE1/ARSB/GCNT2/PDGFRB/PGLYRP4/HAS3/ST3GAL2/B3GNT2/B4GAT1/CEMIP2/PXYLP1
Biological Process	GO:0032332	positive regulation of chondrocyte differentiation	9/2279	20/18866	0.0002592	0.0231832	0.020977	9	SMAD3/ZBTB16/SMAD7/POR/BMPR1B/RUNX2/GDF6/RELA/ZNF219
Biological Process	GO:0019216	regulation of lipid metabolic process	77/2279	431/18866	0.0002611	0.0231832	0.020977	77	PNPLA2/PRKAG2/GFI1/RXRA/SORL1/PPARG/NCOR2/ACSL1/CREB1/MFSD2A/IDI1/LPCAT1/SIN3B/OPA3/VDR/PIK3R1/PIK3R6/MBTPS1/GPLD1/FGR/TNF/ORMDL3/ABCG1/FPR2/PIK3R5/MIR27A/TNFRSF1A/TNFRSF21/CRTC3/FLT1/SPHK2/TGFB1/ACADM/RDH10/DGKQ/SF1/POR/NR1H2/VAV2/ATG14/ALAS1/ABCA1/NCOA1/GNB3/ACACB/PDGFB/CREBBP/MIR138-2/NOD2/CHD9/RB1/ACACA/AVIL/FHL2/PRKCE/LMF1/ABHD5/AGTR1/PDGFRB/WDR81/NCOA2/ANKRD1/HMGCS1/SCD/SREBF1/PDE3B/WDTC1/CPT1A/G0S2/SIRT3/VAV3/ACADVL/BSCL2/PRKAA1/ANGPTL8/RUBCNL/SLA2
Biological Process	GO:0018107	peptidyl-threonine phosphorylation	28/2279	117/18866	0.0002673	0.0235117	0.0212742	28	AZU1/RPTOR/SPRED2/PRKAG2/LMTK2/DYRK4/TNKS/ULK1/CHI3L1/HIPK3/PRKCA/HIPK2/TGFBR2/APP/SMAD7/TGFB1/DGKQ/PRKCB/AXIN1/DYRK2/CAB39/PPP2R5D/BCL2/S1PR2/CDC42BPB/MARK2/EIF4G1/GRK2

Biological Process	GO:0070534	protein K63-linked ubiquitination	17/2279	57/18866	0.000279	0.024321	0.0220065	17	TRIM27/WWP2/TRAF2/TRAF1/UBE2O/OTUB2/TRIM5/UBE2V1/MIR138-2/OTUB1/NOD2/TRAF3IP2/NEDD4/TRIP12/PARP10/PELI1/PRKN
Biological Process	GO:1902107	positive regulation of leukocyte differentiation	35/2279	159/18866	0.0002865	0.0247474	0.0223923	35	PRKCZ/RHOH/CREB1/MIR145/MYB/RUNX1/INPP5D/NLRP3/ITPKB/PIK3R6/PRKCA/TNF/ZBTB16/TGFBR2/TESC/RIPK1/ZMIZ1/ZBTB7B/TGFB1/CD101/RARA/LILRB4/PTPRC/TMEM64/PNP/RB1/HLX/RUNX3/TRIB1/CYLD/AP3D1/LIF/FADD/PCID2/VSIR
Biological Process	GO:0031638	zymogen activation	17/2279	58/18866	0.0003508	0.0297638	0.0269314	17	CTSZ/NLRC4/ENO1/PLAU/GGT1/CYFIP2/TBC1D10A/THBS1/CTSH/CIDEB/F12/PERP/CPB2/ASPH/FADD/C1RL/VSIR
Biological Process	GO:0033673	negative regulation of kinase activity	52/2279	268/18866	0.0003509	0.0297638	0.0269314	52	TRIM27/RPTOR/SPRED2/PRKAG2/SORL1/SH3BP5L/ITPRIP/RHOH/PPP1R1B/HIPK3/PRKAR1B/ITGB1BP1/DUSP14/SLC8A1/LAX1/UBASH3B/TNFAIP3/FBXO7/SH3BP5/DUSP22/TESC/SMYD3/PTPN6/MIR24-2/ILK/PRKRIP1/IGF1R/LATS2/PTPRJ/NF1/MLLT1/GMFB/WWTR1/PTPRC/CSK/PPP2R1A/TAOK3/MIR138-2/RGS3/RB1/CORO1C/DUSP3/GGNBP2/DUS2/TSC2/HHEX/TRIB1/HEXIM1/GMFG/DTNBP1/NPRL2/PRKN
Biological Process	GO:0046474	glycerophospholipid biosynthetic process	46/2279	230/18866	0.0003748	0.0315146	0.0285156	46	CPNE6/SYNJ2/PCYT1A/PIK3CD/MFSD2A/VAC14/LPCAT1/PIP5K1B/INPP5D/PIK3R1/PIGL/PIK3R6/PIGU/PLD1/FPR2/PIGN/ACP6/PITPNM2/SLC44A4/INPP4A/PIK3R5/PIP4K2A/PITPNM1/CPNE1/PGS1/DGKQ/SH3YL1/CHPT1/PCTP/PLEKHA2/PDGFB/DGKA/DGKD/INPP5E/PI4K2A/PI4KA/PIGQ/ABHD5/RUFY1/CHKB/PTDSS2/INPP5F/LPIN1/CDS2/AGPAT5/ETNK2
Biological Process	GO:0050854	regulation of antigen receptor-mediated signaling pathway	19/2279	69/18866	0.0003915	0.0324895	0.0293976	19	FOXP1/LCK/RUNX1/DUSP22/CD226/PTPN6/PRKCH/PTPRJ/PRKCB/LILRB4/BLK/PTPRC/BCL10/DUSP3/ELF1/CYLD/RELA/EZR/SLA2
Biological Process	GO:0072511	divalent inorganic cation transport	83/2279	478/18866	0.0003948	0.0324895	0.0293976	83	TRPM2/PDE4D/TRIM27/RCVRN/SLC25A25/SLC39A11/THADA/IL16/CASQ2/ITPR2/LCK/CACNA1C/SLC11A1/CACNA2D4/SLC24A4/VDR/MYLK/SLC8A1/PKD1L1/ANXA6/UBASH3B/CDH23/CCL5/SPG7/CD84/TLR9/ABCC2/ATP2B2/ITPR1/SLC11A2/BIN1/TRPM6/JSRP1/AHNAK/GNAI2/NOS1AP/PTPN6/RASA3/NIPAL3/TMEM163/P2RY6/SPHK2/PHB/PRKCB/MYO5A/HTT/PTPRC/ATP2A3/PDGFB/ZDHHC17/NOL3/TPCN2/ARRB2/CNNM2/TRPM3/BCL2/GSTO1/SLC39A13/P2RX1/DNM1L/PRKCE/TMEM37/F2R/TMC1/PDGFRB/ANO6/DYSF/CACNA1B/ANK2/CHERP/PLCG1/ATP7B/P2RX5/TRPV2/CD33/CASQ1/FGF14/SLC41A1/GRAMD2A/CBARP/STIMATE/SARAF/CRACR2A
Biological Process	GO:0010762	regulation of fibroblast migration	12/2279	34/18866	0.0003967	0.0324895	0.0293976	12	PRR5L/MIR145/ITGB1BP1/SLC8A1/ARHGEF7/THBS1/TGFB1/FER/MTA2/CORO1C/PRKCE/GNA12
Biological Process	GO:0032271	regulation of protein polymerization	46/2279	231/18866	0.0004143	0.0336422	0.0304406	46	VILL/TRIM27/TBCD/DCTN1/SSH1/GSN/KANK1/CTTN/COTL1/CAPG/CYFIP2/NCK2/WHAMM/BAIAP2L1/NAV3/CDC42EP1/BIN1/BAIAP2/SSH3/PREX1/WASL/FER/TMOD3/SVIL/PFN1/CSF3/GMFB/NUMA1/SPTA1/NCKAP1/ADD1/AVIL/TPPP3/PRKCE/FLII/MYO1C/SPTAN1/TMOD1/CYFIP1/GMFG/BAIAP2L2/MYADM/SPTBN4/DBNL/PLEKHG2/CYRIB

Biological Process	GO:0018210	peptidyl-threonine modification	29/2279	126/18866	0.0004231	0.0340694	0.0308272	29	AZU1/RPTOR/SPRED2/PRKAG2/LMTK2/GALNT2/DYRK4/TNKS/ULK1/CHI3L1/HIPK3/PRKCA/HIPK2/TGFBR2/APP/SMAD7/TGFB1/DGKQ/PRKCB/AXIN1/DYRK2/CAB39/PPP2R5D/BCL2/S1PR2/CDC42BPB/MARK2/EIF4G1/GRK2
Biological Process	GO:0010595	positive regulation of endothelial cell migration	30/2279	132/18866	0.0004271	0.0340968	0.030852	30	FOXP1/CALR/NRP2/MAP3K3/MIR143/RIN2/HDAC7/ANGPT1/ITGB1BP1/PRKCA/MIR199A1/GPLD1/MIR101-2/STAT5A/THBS1/AKT3/MIR27A/MIR23A/FGFR1/TGFB1/ZNF580/ITGB3/GRN/PDGFB/ETS1/NFE2L2/MIR199A2/PLCG1/ADGRA2/PLPP3
Biological Process	GO:0097193	intrinsic apoptotic signaling pathway	55/2279	290/18866	0.0004348	0.0344244	0.0311484	55	S100A8/P4HB/ENO1/LCK/BRCA2/URI1/ERN1/TNFRSF1B/PIK3R1/PLAGL2/BMF/ZNF622/TNF/TRAF2/NCK2/HIPK2/ITPR1/CUL3/RRM2B/NACC2/ZNF385A/TNFRSF1A/CLU/HDAC1/CIDEB/SGMS1/RIPK3/PERP/STK24/CDIP1/DYRK2/ATP2A3/NOL3/NFE2L2/FNIP2/ANKRD2/SGPP1/FIS1/DAPK2/BCL2/DNM1L/CHEK2/ARHGEF2/TP53BP2/CEBPB/CRIP1/BCL2L1/CYLD/BCL3/BAG5/GRINA/XPA/PRKN/MARCHF7/FBH1
Biological Process	GO:0097178	ruffle assembly	14/2279	44/18866	0.0004558	0.0357906	0.0323846	14	TACSTD2/DEF8/KANK1/RHOG/SH3YL1/EPS8L3/EPS8L1/PFN1/PLEKHM1/CORO1C/PDGFRB/CYFIP1/NDEL1/DBNL
Biological Process	GO:0006790	sulfur compound metabolic process	67/2279	371/18866	0.0004681	0.0361636	0.0327221	67	CSGALNACT1/SPOCK2/HS3ST3B1/ACSL1/DSE/HEXB/FAR2/GADL1/GALNS/ANGPT1/CHSY1/GGT1/ACOT13/SULT6B1/ARSG/LPO/CBS/MICAL1/MICAL2/CHST11/ABCC2/STAT5A/MSRA/ABHD14B/COMT/CHST15/CNDP2/NDST1/CHST2/HSCB/OGDH/EGFLAM/ACOT7/ACSS1/XYLT1/PC/SULT1A2/SLC26A1/GSTM5/AGXT/ACACB/MGST2/ST3GAL4/ACSS2/B4GALT3/NFE2L2/MTHFD1/ARSB/PAPSS2/ADO/GSTO1/ACACA/ACSF3/MAT1A/HMGCL/GLRX2/ADI1/ACAT1/HMGCS1/SCD/TPST1/GSTO2/SLC25A1/ST3GAL2/B3GNT2/B4GAT1/ACP3
Biological Process	GO:0051235	maintenance of location	60/2279	324/18866	0.0004682	0.0361636	0.0327221	60	TRPM2/LTBP1/PNPLA2/PDE4D/S100A8/EHD1/SORL1/CALR/IL10/HK1/PPARG/GSN/LCN2/TMEM159/CASQ2/ITPR2/LCK/HEXB/SYNE1/CACNA1C/VPS13D/SLC8A1/SUFU/TNF/ANXA6/UBASH3B/ABCG1/ITPR1/JSRP1/PTPN6/RASA3/UVRAG/P2RY6/MYO5A/NR1H2/HTT/HK2/ITGB3/PTPRC/ABCA1/ACACB/FTO/NOL3/TPCN2/MORC3/GSTO1/PRKCE/SP100/F2R/ABHD5/DYSF/ANK2/AP3D1/CHERP/PLCG1/ATP7B/BCL3/CASQ1/CDS2/BSCL2
Biological Process	GO:0001667	ameboidal-type cell migration	83/2279	481/18866	0.0004823	0.0369542	0.0334374	83	SEMA6B/S100P/ARID5B/RREB1/TACSTD2/FOXP1/CALR/NRP2/PPARG/PRR5L/KANK1/MAP3K3/MIR143/MIR145/RIN2/HDAC7/ANGPT1/ARHGDIB/DOCK1/ITGB1BP1/SLC8A1/PRKCA/ARHGEF7/MIR199A1/GPLD1/TNF/ANXA6/ZEB2/MIR101-2/SEMA4A/STAT5A/RTN4/JUP/TGFBR2/THBS1/TNS1/MYH9/CTSH/SEMA4B/AKT3/MIR24-2/MIR27A/MIR23A/ILK/MIA3/PXN/WNT11/PLEKHG5/LTB4R2/FGFR1/AGO2/TGFB1/ZNF580/FER/SEMA4D/IQSEC1/NF1/PFN1/BMPER/ITGB3/GRN/PDGFB/MTA2/ETS1/NFE2L2/ITGB2/VASH1/ARSB/MIR199A2/NOTCH1/CORO1C/MEF2C/PRKCE/GNA12/SP100/TNFSF12/PLCG1/ADTRP/PDXDC1/NODAL/PAXIP1/ADGRA2/PLPP3
Biological Process	GO:0030837	negative regulation of actin filament polymerization	18/2279	65/18866	0.000512	0.0389136	0.0352104	18	VILL/SSH1/GSN/KANK1/CAPG/SSH3/TMOD3/SVIL/PFN1/SPTA1/ADD1/AVIL/FLII/SPTAN1/TMOD1/MYADM/SPTBN4/CYRIB
Biological Process	GO:0007041	lysosomal transport	26/2279	110/18866	0.0005334	0.0400737	0.0362601	26	SORL1/MGRN1/ANKFY1/VPS53/LYST/TGFBRAP1/KIF13A/SNX27/DENND3/BIN1/CLU/VPS18/CLEC16A/ATG14/HOOK2/NUMA1/GRN/SORT1/NPC1/ARSB/NEDD4/AP3D1/NCOA4/MVB12A/VPS51/PRKN

Biological Process	GO:0045766	positive regulation of angiogenesis	42/2279	208/18866	0.0005357	0.0400737	0.0362601	42	LRG1/IL10/C3AR1/MAP3K3/MIR143/CHI3L1/HDAC7/RUNX1/PIK3R6/PRKCA/MIR199A1/MIR101-2/HIPK2/JUP/TGFBR2/THBS1/CTSH/DLL1/AKT3/MIR27A/MIR23A/FLT1/JAK1/AGO2/PRKCB/AQP1/BMPER/HK2/GHRL/GRN/ETS1/NFE2L2/ENG/ITGB2/PKM/MIR199A2/AGTR1/PPP1R16B/TNFSF12/PLCG1/NODAL/CX3CR1
Biological Process	GO:1902914	regulation of protein polyubiquitination	10/2279	26/18866	0.0005497	0.0407998	0.0369171	10	OTUB2/MIR138-2/OTUB1/NOD2/TRIP12/DYSF/RNF40/PARP10/PRKN/MARCHF7
Biological Process	GO:0097529	myeloid leukocyte migration	44/2279	222/18866	0.0006056	0.0446042	0.0403595	44	AZU1/PRTN3/PDE4D/S100A8/FUT7/CD9/C3AR1/PIK3CD/RHOH/CSF3R/CD177/IL1R1/CCR2/RHOG/CCL5/CSF1R/SFTPD/FPR2/THBS1/C5AR2/MIR24-2/PREX1/DEFB124/FLT1/HRH1/PDGFD/JAM3/CAMK1D/CXCL17/CCL20/CXCR1/IL6R/PDGFB/WDR1/PLCB1/ITGB2/DAPK2/DNM1L/ANO6/DYSF/CCL22/CX3CR1/VAV3/JAML
Biological Process	GO:0070838	divalent metal ion transport	81/2279	471/18866	0.0006222	0.0453854	0.0410663	81	TRPM2/PDE4D/TRIM27/RCVRN/SLC25A25/SLC39A11/THADA/IL16/CASQ2/ITPR2/LCK/CACNA1C/SLC11A1/CACNA2D4/SLC24A4/VDR/MYLK/SLC8A1/PKD1L1/ANXA6/UBASH3B/CDH23/CCL5/SPG7/CD84/TLR9/ATP2B2/ITPR1/SLC11A2/BIN1/TRPM6/JSRP1/AHNAK/GNAI2/NOS1AP/PTPN6/RASA3/NIPAL3/TMEM163/P2RY6/SPHK2/PHB/PRKCB/MYO5A/HTT/PTPRC/ATP2A3/PDGFB/ZDHHC17/NOL3/TPCN2/ARRB2/CNNM2/TRPM3/BCL2/GSTO1/SLC39A13/P2RX1/DNM1L/PRKCE/TMEM37/F2R/TMC1/PDGFRB/ANO6/DYSF/CACNA1B/ANK2/CHERP/PLCG1/P2RX5/TRPV2/CD33/CASQ1/FGF14/SLC41A1/GRAMD2A/CBARP/STIMATE/SARAF/CRACR2A
Biological Process	GO:0044409	entry into host	32/2279	147/18866	0.0006258	0.0453854	0.0410663	32	TRIM27/P4HB/TRIM38/GSN/TRIM8/WWP2/SLC10A1/CD55/TRIM10/SLC1A5/CLEC5A/SNX3/UVRAG/ITGB5/PHB/FCN1/TRIM5/VPS18/ITGB3/CTSB/TPCN2/NPC1/CDH1/SLC52A2/EPS15/TYRO3/ITGB6/SIVA1/TRIM26/GRK2/NECTIN1/NECTIN4
Biological Process	GO:1903708	positive regulation of hemopoiesis	41/2279	204/18866	0.0006832	0.0491761	0.0444963	41	PRKCZ/RHOH/CREB1/MIR145/MYB/RUNX1/INPP5D/NLRP3/ITPKB/PIK3R6/PRKCA/TNF/ZBTB16/TGFBR2/TESC/BRD1/RIPK1/ZMIZ1/MTURN/ZBTB7B/TGFB1/CD101/RARA/LILRB4/CSF3/PTPRC/TMEM64/ETS1/PNP/RB1/HLX/RUNX3/TRIB1/CYLD/AP3D1/KAT7/INHBA/LIF/FADD/PCID2/VSIR
Biological Process	GO:0051250	negative regulation of lymphocyte activation	33/2279	154/18866	0.0006958	0.0495934	0.0448738	33	IL10/MAD1L1/RUNX1/INPP5D/FGR/LAX1/TNFAIP3/LOXL3/SFTPD/FBXO7/MNDA/DUSP22/HLA-F/PTPN6/MIR27A/SMAD7/TNFRSF21/ZBTB7B/TIGIT/ZC3H8/BLK/PGLYRP1/DUSP3/BANK1/HLX/CEBPB/RUNX3/TYRO3/INHBA/PELI1/TMEM131L/VSIR/MARCHF7
Biological Process	GO:0034446	substrate adhesion-dependent cell spreading	25/2279	106/18866	0.0006995	0.0495934	0.0448738	25	P4HB/RREB1/TACSTD2/PARVB/CALR/KANK1/DOCK1/ITGB1BP1/PIK3R1/ARHGEF7/DNM2/EPHB3/ACTN4/ILK/PREX1/PXN/FER/OLFM4/ITGB3/CASS4/CORO1C/PARVG/TYRO3/LAMB3/MYADM
Biological Process	GO:0051348	negative regulation of transferase activity	55/2279	296/18866	0.000725	0.0508037	0.045969	55	TRIM27/RPTOR/SPRED2/PRKAG2/SORL1/SH3BP5L/PPARG/ITPRIP/RHOH/PPP1R1B/HIPK3/PRKAR1B/ITGB1BP1/DUSP14/SLC8A1/LAX1/UBASH3B/TNFAIP3/FBXO7/SH3BP5/DUSP22/TESC/SMYD3/PTPN6/MIR24-2/ILK/PRKRIP1/SMAD7/IGF1R/LATS2/PTPRJ/NF1/MLLT1/GMFB/WWTR1/PTPRC/CSK/PPP2R1A/TAOK3/MIR138-2/RGS3/RB1/CORO1C/DUSP3/GGNBP2/DUS2/TSC2/HHEX/TRIB1/HEXIM1/GMFG/BAG5/DTNBP1/NPRL2/PRKN

Biological Process	GO:0051208	sequestering of calcium ion	28/2279	124/18866	0.0007272	0.0508037	0.045969	28	TRPM2/PDE4D/CALR/CASQ2/ITPR2/LCK/CACNA1C/SLC8A1/ANXA6/UBASH3B/ITPR1/JSRP1/PTPN6/RASA3/P2RY6/MYO5A/HTT/PTPRC/NOL3/TPCN2/GSTO1/PRKCE/F2R/ANK2/CHERP/PLCG1/ATP7B/CASQ1
Biological Process	GO:0006040	amino sugar metabolic process	13/2279	41/18866	0.0007496	0.0519863	0.0470391	13	CSGALNACT1/NAGK/AMDHD2/CHI3L1/NPL/MGAT3/CHIT1/CHST2/ST6GAL1/FN3K/GFPT2/CHI3L2/DPAGT1
Biological Process	GO:0071375	cellular response to peptide hormone stimulus	60/2279	330/18866	0.0007561	0.0520566	0.0471027	60	PRKCZ/SORL1/FUT7/PPARG/ADCY2/KANK1/CREB1/PTPRE/MIR143/MIR145/PRKAR1B/ADCY9/PIK3R1/RAP1B/GPLD1/RAB31/STAT5A/BAIAP2L1/BAIAP2/GRB10/GNAI2/ATP6V1B2/ZDHHC7/PXN/IGF1R/ZBTB7B/CRHBP/FER/PRKCB/POR/MYO5A/ARID1B/GHRL/CSK/AGTRAP/PRKCI/NFE2L2/ADCY4/INSRR/PKM/ATP6V0B/BCAR3/PCK2/AGTR1/MYO1C/TSC2/FOXO1/MBD5/SOGA1/SREBF1/PDE3B/CYFIP1/RELA/WDTC1/NDEL1/LPIN1/PRKAA1/MARS1/ATP6V0C/ATP6V1C1
Biological Process	GO:0061572	actin filament bundle organization	34/2279	161/18866	0.0007662	0.0523749	0.0473907	34	ARHGEF10/TACSTD2/ITGB1BP1/PIK3R1/SMAD3/ARRB1/MICAL1/SH3PXD2B/ARAP1/BAIAP2L1/CUL3/BAIAP2/SYNPO2L/ACTN1/ACTN4/PXN/WNT11/ITGB5/PPFIA1/PFN1/ESPN/ACTG1/MIR138-2/ADD1/ARHGEF10L/ARHGEF18/FSCN3/ARHGEF15/SHROOM1/EZR/BAIAP2L2/SYNPO/RFLNA/PRKN
Biological Process	GO:0016197	endosomal transport	47/2279	244/18866	0.0007733	0.0524806	0.0474863	47	TRIM27/EHD1/LMTK2/DCTN1/SORL1/STX5/VTI1A/ANKFY1/VPS53/LYST/DENND1A/ACAP2/HEATR5A/TRAPPC10/SNX27/TBC1D10A/ITSN1/ERC1/SNX3/ZDHHC2/VPS26B/UBE2O/AKAP5/EPS15L1/AP5S1/CLEC16A/ABCA1/NUMA1/SNX18/TBC1D14/SORT1/MICALL1/SGSM2/CORO1C/DNM1L/EPS15/ARHGAP1/AP3D1/CLN5/INPP5F/MVB12A/VPS51/CLTCL1/ZFYVE9/PHETA1/PRKN/VPS35L
Biological Process	GO:0051650	establishment of vesicle localization	42/2279	212/18866	0.0008022	0.0536288	0.0485253	42	CTSZ/PRKCZ/SERPINA1/TGFA/STX5/CSNK1D/NDE1/KIF13A/DNM2/TRAPPC10/DYNC1H1/C17orf75/CUL3/TRAPPC9/RAB27A/ACTN4/CDH3/WASL/DCTN2/CNIH2/MYO5A/CD59/HTT/SEC13/KIF5C/ANKRD28/DNM1/SEC24C/KIF1B/DNM3/KIFAP3/TMED2/MYO1C/AP3D1/MX1/FBXW11/NDEL1/DTNBP1/SEC31A/IRAG2/PRKN/TRAPPC2
Biological Process	GO:0016570	histone modification	80/2279	468/18866	0.0008041	0.0536288	0.0485253	80	HDAC4/GFI1/BRD4/BAZ1B/KDM4B/SRCAP/MYB/HDAC7/SIN3B/BRCA2/PRMT2/SKI/ASH2L/SETD2/KANSL1/ARRB1/PRKCA/KDM2A/C6orf89/PADI2/USP3/SMYD3/UHRF1/BRD1/EHMT2/USP36/EPC1/OTUB2/RPS6KA4/TAF10/HDAC1/WDR82/KANSL2/SPHK2/ZBTB7B/PAX7/PHB/ENY2/PRKCB/KMT2D/MTA1/CTBP1/KDM1B/KDM4C/SETD1B/NCOA1/SETD1A/CREBBP/RSF1/TET2/MTA2/ZNF335/PRDM16/PHF19/PER2/OTUB1/USP49/PER1/KDM7A/DNMT1/PIH1D1/PCGF3/TRIP12/SREBF1/RUVBL1/RNF40/KAT7/DYDC2/DYDC1/NTMT1/ING5/FBL/MECOM/LIF/ELK4/SIRT3/PAXIP1/PRKAA1/NSD3/NSD2
Biological Process	GO:0032147	activation of protein kinase activity	60/2279	331/18866	0.0008171	0.0536288	0.0485253	60	PRKCZ/KSR1/RPTOR/PRKAG2/PILRB/TGFA/ADCY2/MAP3K3/MIR143/CHI3L1/MAP2K6/SLC11A1/ANGPT1/PRKAR1B/ERN1/ITGB1BP1/ADCY9/ARRB1/TNF/TRAF2/CCL5/FPR1/TGFBR2/TNIK/THBS1/RIPK1/WNT11/PHB/DGKQ/AXIN1/RIPK3/UBE2V1/MUC20/CXCL17/PRR5/ITGB3/GHRL/PTPRC/CAB39/STK3/PDE6H/IL6R/PDGFB/MAP3K14/TAOK3/ADCY4/IRAK2/NTRK1/NOD2/CLSPN/S1PR2/F2R/PDGFC/PRRC1/MAPKAPK3/GADD45G/MARK2/DBNL/PRKAA1/MAP3K20
Biological Process	GO:0034113	heterotypic cell-cell adhesion	17/2279	62/18866	0.0008201	0.0536288	0.0485253	17	IL10/LCK/CEACAM6/TNF/JUP/RNASE10/MBP/IL1RN/PERP/JAM3/ITGB3/PTPRC/ITGB2/GCNT2/FLOT1/MYADM/GLDN

Biological Process	GO:1903510	mucopolysaccharide metabolic process	26/2279	113/18866	0.0008225	0.0536288	0.0485253	26	CSGALNACT1/AP2A1/SPOCK2/DSE/HEXB/GALNS/ANGPT1/CHSY1/CHST11/CHST15/NDST1/CHST2/EGFLAM/XYLT1/TGFB1/STAB2/PDGFB/ST3GAL4/B4GALT3/LYVE1/ARSB/HAS3/ST3GAL2/B3GNT2/B4GAT1/CEMIP2
Biological Process	GO:0048703	embryonic viscerocranium morphogenesis	6/2279	11/18866	0.0008297	0.0536288	0.0485253	6	MTHFD1L/CHST11/NDST1/RDH10/MTHFD1/MEF2C
Biological Process	GO:1902915	negative regulation of protein polyubiquitination	6/2279	11/18866	0.0008297	0.0536288	0.0485253	6	OTUB2/MIR138-2/OTUB1/TRIP12/DYSF/PARP10
Biological Process	GO:2001236	regulation of extrinsic apoptotic signaling pathway	34/2279	162/18866	0.0008594	0.0551695	0.0499193	34	ITPRIP/CTTN/TNF/TRAF2/TNFAIP3/TRAF1/DEDD2/UNC5B/THBS1/RIPK1/TCF7L2/FGFR1/NF1/HTT/PTPRC/ITM2C/BMPR1B/STK3/BCL10/PPP2R1A/NOL3/TRPS1/BCL2/PPP2R1B/SP100/ARHGEF2/BCL2L1/TNFSF12/CYLD/RELA/G0S2/INHBA/FADD/DAPK3
Biological Process	GO:0045747	positive regulation of Notch signaling pathway	16/2279	57/18866	0.0008757	0.055836	0.0505224	16	MAML3/SLC35C2/MAML1/ITGB1BP1/AAK1/TSPAN14/MAML2/DLL1/RBPJ/ZMIZ1/EPN2/CREBBP/PRKCI/NOD2/NOTCH1/NOTCH4
Biological Process	GO:0045649	regulation of macrophage differentiation	9/2279	23/18866	0.000899	0.0566283	0.0512393	9	MIR145/PRKCA/RIPK1/TGFB1/RB1/TRIB1/INHBA/LIF/FADD
Biological Process	GO:0035264	multicellular organism growth	32/2279	150/18866	0.0009	0.0566283	0.0512393	32	ARID5B/PDE4D/SLC25A25/CREB1/MFSD2A/ANKRD11/CCM2/SH3PXD2B/STAT5A/APP/TAF10/APBA2/RARA/SLC1A2/WWTR1/GHRL/RARG/GRHL2/FTO/ADD1/BCL2/RAI1/NPPC/TMED2/MBD5/ERCC1/WDTC1/EZR/XPA/SPTBN4/ERCC2/ETNK2
Biological Process	GO:0006469	negative regulation of protein kinase activity	47/2279	246/18866	0.0009271	0.0576447	0.0521589	47	TRIM27/RPTOR/SPRED2/PRKAG2/SORL1/SH3BP5L/ITPRIP/PPP1R1B/HIPK3/PRKAR1B/ITGB1BP1/DUSP14/SLC8A1/LAX1/UBASH3B/TNFAIP3/FBXO7/SH3BP5/DUSP22/TESC/SMYD3/PTPN6/MIR24-2/ILK/PRKRIP1/IGF1R/LATS2/PTPRJ/NF1/MLLT1/GMFB/WWTR1/PTPRC/PPP2R1A/TAOK3/RGS3/RB1/CORO1C/DUSP3/GGNBP2/DUS2/TSC2/HHEX/TRIB1/HEXIM1/GMFG/DTNBP1
Biological Process	GO:0018022	peptidyl-lysine methylation	29/2279	132/18866	0.0009413	0.0576447	0.0521589	29	GFI1/BRD4/METTL21A/MYB/ASH2L/SETD2/SMYD3/EHMT2/WDR82/KMT2D/KDM4C/SETD1B/SETD1A/TET2/ZNF335/METTL22/PRDM16/PHF19/DNMT1/PIH1D1/DYDC2/DYDC1/MECOM/PAXIP1/EEF1AKMT3/EEF1AKMT2/NSD3/NSD2/CSKMT

Biological Process	GO:0120163	negative regulation of cold-induced thermogenesis	14/2279	47/18866	0.0009554	0.0576447	0.0521589	14	MAP2K6/LNPEP/ACOT13/RHEB/RBPJ/NR1H2/PCTP/ARNTL/PLCL1/RB1/NOTCH1/IL18R1/ZNF423/IP6K1
Biological Process	GO:0098713	leucine import across plasma membrane	4/2279	5/18866	0.0009597	0.0576447	0.0521589	4	SLC43A2/SLC7A5/SLC43A1/SLC7A8
Biological Process	GO:1901563	response to camptothecin	4/2279	5/18866	0.0009597	0.0576447	0.0521589	4	BLM/SPIDR/RECQL5/PRKAA1
Biological Process	GO:1902339	positive regulation of apoptotic process involved in morphogenesis	4/2279	5/18866	0.0009597	0.0576447	0.0521589	4	TNFRSF1B/VDR/TNFRSF1A/NOTCH1
Biological Process	GO:1903801	L-leucine import across plasma membrane	4/2279	5/18866	0.0009597	0.0576447	0.0521589	4	SLC43A2/SLC7A5/SLC43A1/SLC7A8
Biological Process	GO:0016050	vesicle organization	61/2279	340/18866	0.0009687	0.0576447	0.0521589	61	CTSZ/CALR/SERPINA1/TGFA/STX5/ACRBP/VTI1A/ANKFY1/CSNK1D/CREB1/LYST/TGFBRAP1/RAB5B/KIF13A/DNM2/TRAPPC10/SEC23B/ZEB2/MYO18A/CUL3/TRAPPC9/RAB27A/SNX3/ZDHHC2/ZNF385A/UVRAG/MIA3/SNX11/WASL/CNIH2/VPS18/CD59/SEC13/ABCA1/HOOK2/ANKRD28/PRKCI/SORT1/DNM1/SEC24C/PI4K2A/BCL2/DNM3/CORO1C/F2R/EPS15/TMED2/DYSF/AP3D1/MX1/DTNBP1/MVB12A/TSNARE1/SEC31A/VAV3/PHETA1/IRAG2/PRKN/TRAPPC2/MFSD14A/RUBCNL
Biological Process	GO:0051017	actin filament bundle assembly	33/2279	157/18866	0.0009883	0.0576447	0.0521589	33	ARHGEF10/TACSTD2/ITGB1BP1/PIK3R1/SMAD3/ARRB1/MICAL1/SH3PXD2B/ARAP1/BAIAP2L1/CUL3/BAIAP2/SYNPO2L/ACTN1/ACTN4/PXN/WNT11/ITGB5/PPFIA1/PFN1/ESPN/ACTG1/MIR138-2/ADD1/ARHGEF10L/ARHGEF18/FSCN3/ARHGEF15/SHROOM1/EZR/BAIAP2L2/SYNPO/PRKN
Biological Process	GO:0034349	glial cell apoptotic process	7/2279	15/18866	0.000989	0.0576447	0.0521589	7	PRKCA/TRAF2/DLL1/TNFRSF21/PRKCH/PRKCI/RB1
Biological Process	GO:0097202	activation of cysteine-type endopeptidase activity	7/2279	15/18866	0.000989	0.0576447	0.0521589	7	NLRC4/CYFIP2/TBC1D10A/CIDEB/PERP/ASPH/FADD

Biological Process	GO:1900029	positive regulation of ruffle assembly	7/2279	15/18866	0.000989	0.0576447	0.0521589	7	DEF8/EPS8L3/EPS8L1/PFN1/PLEKHM1/CYFIP1/NDEL1
Biological Process	GO:0097306	cellular response to alcohol	22/2279	91/18866	0.0010051	0.0577537	0.0522576	22	BLM/SPIDR/PPARG/ADCY2/ITPR2/GPLD1/LARP1/JUP/RECQL5/P2RY6/SPHK2/FDX1/MLC1/ABCA1/CDH1/GNG2/ACACA/PRKCE/GRAMD1A/HMGCS1/INHBA/PRKAA1
Biological Process	GO:0090132	epithelium migration	65/2279	368/18866	0.0010236	0.0577537	0.0522576	65	S100P/RREB1/TACSTD2/FOXP1/CALR/NRP2/PPARG/KANK1/MAP3K3/MIR143/RIN2/HDAC7/ANGPT1/DOCK1/ITGB1BP1/PRKCA/MIR199A1/GPLD1/TNF/ZEB2/MIR101-2/SEMA4A/STAT5A/RTN4/JUP/TGFBR2/THBS1/MYH9/CTSH/AKT3/MIR24-2/MIR27A/MIR23A/MIA3/PXN/PLEKHG5/LTB4R2/FGFR1/TGFB1/ZNF580/IQSEC1/NF1/PFN1/BMPER/ITGB3/GRHL2/GRN/PDGFB/ETS1/NFE2L2/ITGB2/VASH1/ARSB/MIR199A2/NOTCH1/CORO1C/MEF2C/PRKCE/SP100/TNFSF12/PLCG1/ADTRP/PAXIP1/ADGRA2/PLPP3
Biological Process	GO:0019058	viral life cycle	61/2279	341/18866	0.0010438	0.0577537	0.0522576	61	SLPI/TRIM27/P4HB/TRIM38/GSN/TRIM8/WWP2/SLC10A1/CD55/TRIM10/RAE1/SLC1A5/SRPK2/TNF/CCL5/CLEC5A/LARP1/SNX3/UVRAG/ITGB5/PC/PHB/FCN1/CTBP1/NUP93/TRIM5/RAB43/VPS18/CTBP2/TNIP1/ITGB3/SEC13/USP6NL/CTSB/TPCN2/NPC1/BCL2/PI4KA/NOTCH1/SLC52A2/EPS15/LTF/NEDD4/ILF3/MX1/TYRO3/ITGB6/NR5A2/PARP10/BANF1/SIVA1/MVB12A/ISG20/TRIM26/NFIA/SPCS1/GRK2/PRKN/SHFL/NECTIN1/NECTIN4
Biological Process	GO:0003330	regulation of extracellular matrix constituent secretion	5/2279	8/18866	0.0010455	0.0577537	0.0522576	5	TNFRSF1B/TNFRSF1A/NOTCH1/CPB2/ADTRP
Biological Process	GO:0003373	dynamin family protein polymerization involved in membrane fission	5/2279	8/18866	0.0010455	0.0577537	0.0522576	5	DNM2/DNM1/DNM3/DNM1L/MX1
Biological Process	GO:0003374	dynamin family protein polymerization involved in mitochondrial fission	5/2279	8/18866	0.0010455	0.0577537	0.0522576	5	DNM2/DNM1/DNM3/DNM1L/MX1
Biological Process	GO:0034351	negative regulation of glial cell apoptotic process	5/2279	8/18866	0.0010455	0.0577537	0.0522576	5	PRKCA/TRAF2/DLL1/PRKCH/PRKCI
Biological Process	GO:0043313	regulation of neutrophil degranulation	5/2279	8/18866	0.0010455	0.0577537	0.0522576	5	ABR/CD177/BCR/ITGB2/PRAM1

Biological Process	GO:1990961	xenobiotic detoxification by transmembrane export across the plasma membrane	5/2279	8/18866	0.0010455	0.0577537	0.0522576	5	ABCC2/OSCP1/RALBP1/SLC22A5/PDZK1
Biological Process	GO:0030042	actin filament depolymerization	16/2279	58/18866	0.0010756	0.0587311	0.053142	16	VILL/GSN/CAPG/MICAL1/MICAL2/MICAL3/TMOD3/SVIL/WDR1/SPTA1/ADD1/AVIL/FLII/SPTAN1/TMOD1/SPTBN4
Biological Process	GO:0043551	regulation of phosphatidylinositol 3-kinase activity	16/2279	58/18866	0.0010756	0.0587311	0.053142	16	PIK3R1/PIK3R6/FGR/FPR2/PIK3R5/FLT1/TGFB1/VAV2/ATG14/PDGFB/MIR138-2/NOD2/PDGFRB/WDR81/VAV3/SLA2
Biological Process	GO:0007162	negative regulation of cell adhesion	55/2279	301/18866	0.0010873	0.0590308	0.0534132	55	TBCD/PLXNC1/TACSTD2/IL10/CD9/MAD1L1/KANK1/RUNX1/ANGPT1/ARHGDIB/ITGB1BP1/PIK3R1/TGFBI/SPINT2/LAX1/UBASH3B/LOXL3/SFTPD/DUSP22/CCM2L/THBS1/MBP/PTPN6/ACTN4/MIR27A/SMAD7/TNFRSF21/MIA3/ZBTB7B/TGFB1/IL1RN/TIGIT/SEMA4D/NF1/ZC3H8/JAM3/PTPRC/MIR138-2/CDH1/NOTCH1/CORO1C/GCNT2/DUSP3/NOTCH4/HLX/CEBPB/RUNX3/PDE3B/ADTRP/ADAMDEC1/MYADM/PELI1/TMEM131L/VSIR/MARCHF7
Biological Process	GO:0034109	homotypic cell-cell adhesion	21/2279	86/18866	0.0011297	0.0602981	0.0545599	21	CD9/TJP2/PRKCA/UBASH3B/CCL5/MYL12A/CCM2L/JUP/MYH9/ITGA2B/PTPN6/ILK/JAK1/HBB/BLK/ITGB3/ACTG1/TYRO3/PTPRU/CEACAM5/PLPP3
Biological Process	GO:0035023	regulation of Rho protein signal transduction	21/2279	86/18866	0.0011297	0.0602981	0.0545599	21	ARHGEF10/KANK1/AKAP13/MYO9B/ARHGDIB/ARRB1/CUL3/SYNPO2L/EPS8L3/EPS8L1/ABCA1/BCR/ARHGEF25/ARHGEF18/F2R/ARHGEF28/ARHGEF2/GPR4/PDGFRB/FLOT1/PRAG1
Biological Process	GO:1903901	negative regulation of viral life cycle	21/2279	86/18866	0.0011297	0.0602981	0.0545599	21	SLPI/TRIM27/GSN/TRIM8/TRIM10/SRPK2/TNF/CCL5/SNX3/FCN1/TRIM5/TNIP1/LTF/ILF3/MX1/PARP10/BANF1/ISG20/TRIM26/PRKN/SHFL
Biological Process	GO:1900024	regulation of substrate adhesion-dependent cell spreading	15/2279	53/18866	0.0011451	0.0607333	0.0549537	15	P4HB/RREB1/TACSTD2/CALR/KANK1/DOCK1/ITGB1BP1/ARHGEF7/DNM2/ACTN4/PREX1/OLFM4/CASS4/CORO1C/MYADM
Biological Process	GO:0060828	regulation of canonical Wnt signaling pathway	54/2279	295/18866	0.0011506	0.0607333	0.0549537	54	RNF220/KANK1/CSNK1D/TNKS/CCDC88C/MIR145/SMAD3/AMFR/FAM53B/UBAC2/JUP/CUL3/RBPJ/TCF7L2/CDK14/ILK/LIMD1/HDAC1/LATS2/WNT11/CDH3/TGFB1/KREMEN1/AXIN1/TLE1/LRRK1/WWTR1/TMEM64/WNT5B/STK3/CSNK1E/PSMF1/KREMEN2/ARNTL/PSMB7/TTC21B/NOTCH1/ZBED3/FOXO1/CHD8/CYLD/ANKRD6/RUVBL1/ZBED2/PPM1N/PTPRU/CCNY/GID8/DAPK3/PSMD13/NPHP4/TMEM131L/ADGRA2/PRKN

Biological Process	GO:0016569	covalent chromatin modification	81/2279	481/18866	0.0011725	0.0611906	0.0553674	81	HDAC4/GFI1/BRD4/BAZ1B/KDM4B/SRCAP/MYB/HDAC7/SIN3B/BRCA2/PRMT2/SKI/ASH2L/SETD2/KANSL1/ARRB1/PRKCA/KDM2A/C6orf89/PADI2/USP3/SMYD3/UHRF1/BRD1/EHMT2/USP36/EPC1/OTUB2/RPS6KA4/TAF10/HDAC1/WDR82/KANSL2/SPHK2/ZBTB7B/PAX7/PHB/ENY2/PRKCB/KMT2D/MTA1/CTBP1/KDM1B/ATF7IP/KDM4C/SETD1B/NCOA1/SETD1A/CREBBP/RSF1/TET2/MTA2/ZNF335/PRDM16/PHF19/PER2/OTUB1/USP49/PER1/KDM7A/DNMT1/PIH1D1/PCGF3/TRIP12/SREBF1/RUVBL1/RNF40/KAT7/DYDC2/DYDC1/NTMT1/ING5/FBL/MECOM/LIF/ELK4/SIRT3/PAXIP1/PRKAA1/NSD3/NSD2
Biological Process	GO:0051648	vesicle localization	44/2279	229/18866	0.0011768	0.0611906	0.0553674	44	CTSZ/PRKCZ/SERPINA1/TGFA/STX5/CSNK1D/NDE1/KIF13A/DNM2/TRAPPC10/DYNC1H1/C17orf75/CUL3/TRAPPC9/RAB27A/SYN3/ACTN4/CDH3/WASL/DCTN2/CNIH2/MYO5A/SYN2/CD59/HTT/SEC13/KIF5C/ANKRD28/DNM1/SEC24C/KIF1B/DNM3/KIFAP3/TMED2/MYO1C/AP3D1/MX1/FBXW11/NDEL1/DTNBP1/SEC31A/IRAG2/PRKN/TRAPPC2
Biological Process	GO:0002691	regulation of cellular extravasation	11/2279	33/18866	0.0011897	0.0611906	0.0553674	11	ELANE/FUT4/ABR/FUT7/IL1R1/CCR2/RIPK3/PDGFD/BCR/PLCB1/FADD
Biological Process	GO:0018212	peptidyl-tyrosine modification	66/2279	377/18866	0.0011907	0.0611906	0.0553674	66	PRKCZ/AATK/LMTK2/DYRK4/TGFA/SH3BP5L/BAZ1B/TTN/LCK/HIPK3/MAP2K6/ANGPT1/FGR/TNF/CCL5/CSF1R/NCK2/EPHB3/SH3BP5/HIPK2/DUSP22/CAMKK2/SLA/APP/PTPN6/TNFRSF1A/IGF1R/FLT1/JAK1/FGFR1/TGFB1/FER/PTPRJ/CLK3/SEMA4D/DGKQ/PDGFD/LRRK1/DYRK2/BLK/CSF3/TESK2/ITGB3/GHRL/PTPRC/CSK/IL6R/PDGFB/PPP2R1A/CASS4/ARRB2/ITGB2/NTRK1/INSRR/PRKCE/GGNBP2/BANK1/PDGFC/ARHGEF2/PDGFRB/TYRO3/TPST1/INPP5F/CRLF1/PARP9/LIF
Biological Process	GO:0010799	regulation of peptidyl-threonine phosphorylation	14/2279	48/18866	0.0012009	0.0611906	0.0553674	14	AZU1/RPTOR/SPRED2/PRKAG2/CHI3L1/APP/SMAD7/TGFB1/DGKQ/AXIN1/CAB39/PPP2R5D/S1PR2/EIF4G1
Biological Process	GO:0048701	embryonic cranial skeleton morphogenesis	14/2279	48/18866	0.0012009	0.0611906	0.0553674	14	SETD2/SMAD3/MTHFD1L/CHST11/TGFBR2/NDST1/RDH10/GRHL2/RUNX2/IFT140/MTHFD1/ALX3/MEF2C/NODAL
Biological Process	GO:0048010	vascular endothelial growth factor receptor signaling pathway	23/2279	98/18866	0.0012044	0.0611906	0.0553674	23	NRP2/ELMO1/DOCK1/PIK3R1/NCF4/CYFIP2/NCK2/SHB/BAIAP2/GRB10/NCF2/PXN/FLT1/EPN2/PRKCB/VAV2/ITGB3/NCKAP1/NEDD4/MAPKAPK3/HHEX/CYFIP1/VAV3
Biological Process	GO:0050856	regulation of T cell receptor signaling pathway	13/2279	43/18866	0.0012347	0.0619594	0.0560631	13	LCK/DUSP22/CD226/PTPN6/PTPRJ/LILRB4/BCL10/DUSP3/ELF1/CYLD/RELA/EZR/SLA2
Biological Process	GO:1903146	regulation of autophagy of mitochondrion	13/2279	43/18866	0.0012347	0.0619594	0.0560631	13	VPS13D/CTTN/FBXO7/CAMKK2/USP36/SMURF1/CLEC16A/HTT/HK2/DNM1L/TSC2/SREBF1/PRKN

Biological Process	GO:0032640	tumor necrosis factor production	26/2279	116/18866	0.0012391	0.0619594	0.0560631	26	TRIM27/FOXP1/IL10/MIR140/ANGPT1/PIK3R1/TNFAIP3/TLR9/SPON2/RIPK1/C5AR2/APP/PTPN6/CLU/SPHK2/PTPRJ/RARA/PTPRC/ARRB2/NOD2/ARHGEF2/LTF/CD33/FADD/VSIR/ILRUN
Biological Process	GO:0048194	Golgi vesicle budding	20/2279	81/18866	0.0012663	0.062785	0.0568101	20	CTSZ/SERPINA1/TGFA/STX5/CSNK1D/TRAPPC10/SEC23B/MYO18A/CUL3/TRAPPC9/MIA3/CNIH2/CD59/SEC13/ANKRD28/PRKCI/SEC24C/TMED2/SEC31A/TRAPPC2
Biological Process	GO:0002822	regulation of adaptive immune response based on somatic recombination of immune receptors built from immunoglobulin superfamily domains	32/2279	153/18866	0.0012741	0.062785	0.0568101	32	IL27/PRKCZ/FUT7/IL10/SLC11A1/TNFRSF1B/NLRP3/IL1R1/CCR2/TNF/TRAF2/TNFAIP3/LOXL3/TNFSF13B/DUSP22/CD226/HLA-F/PTPN6/SMAD7/ZBTB7B/TGFB1/RIPK3/PTPRC/TP53BP1/MEF2C/IL18R1/HLX/TNFSF13/FADD/PAXIP1/CYRIB/NSD2
Biological Process	GO:0050995	negative regulation of lipid catabolic process	9/2279	24/18866	0.0012854	0.062785	0.0568101	9	SORL1/MFSD2A/GPLD1/TNF/CRTC3/ACACB/PDE3B/BSCL2/PRKAA1
Biological Process	GO:1903306	negative regulation of regulated secretory pathway	9/2279	24/18866	0.0012854	0.062785	0.0568101	9	ABR/RAP1B/CCR2/CD84/GNAI2/HLA-F/BCR/NOTCH1/CBARP
Biological Process	GO:0097191	extrinsic apoptotic signaling pathway	44/2279	230/18866	0.0012886	0.062785	0.0568101	44	LCN2/ITPRIP/TNFRSF1B/PIK3R1/SMAD3/CTTN/TNF/TRAF2/TNFAIP3/TRAF1/DEDD2/UNC5B/THBS1/RIPK1/TCF7L2/TNFRSF1A/FGFR1/TGFB1/NF1/HTT/PTPRC/ITM2C/BMPR1B/STK3/BCL10/IL6R/PPP2R1A/NOL3/TRPS1/SORT1/SGPP1/BCL2/PPP2R1B/SP100/ARHGEF2/BCL2L1/TNFSF12/CYLD/RELA/G0S2/SIVA1/INHBA/FADD/DAPK3
Biological Process	GO:0010810	regulation of cell-substrate adhesion	42/2279	217/18866	0.0012958	0.0628129	0.0568354	42	PRKCZ/TBCD/P4HB/RREB1/TACSTD2/CALR/PLAU/SPOCK2/VIT/KANK1/RIN2/DOCK1/ITGB1BP1/PIK3R1/CEACAM6/SMAD3/ARHGEF7/DNM2/DUSP22/JUP/THBS1/DISC1/NINJ1/EGFLAM/ACTN4/ILK/PREX1/PTPRJ/NF1/OLFM4/ACTG1/PLEKHA2/CASS4/BCL2/NOTCH1/CORO1C/GCNT2/DUSP3/PRKCE/RSU1/MYADM/DAPK3
Biological Process	GO:0010631	epithelial cell migration	64/2279	365/18866	0.0013445	0.0648262	0.058657	64	S100P/RREB1/TACSTD2/FOXP1/CALR/NRP2/PPARG/KANK1/MAP3K3/MIR143/RIN2/HDAC7/ANGPT1/DOCK1/ITGB1BP1/PRKCA/MIR199A1/GPLD1/TNF/ZEB2/MIR101-2/SEMA4A/STAT5A/RTN4/JUP/TGFBR2/THBS1/MYH9/CTSH/AKT3/MIR24-2/MIR27A/MIR23A/MIA3/PXN/PLEKHG5/LTB4R2/FGFR1/TGFB1/ZNF580/IQSEC1/NF1/PFN1/BMPER/ITGB3/GRN/PDGFB/ETS1/NFE2L2/ITGB2/VASH1/ARSB/MIR199A2/NOTCH1/CORO1C/MEF2C/PRKCE/SP100/TNFSF12/PLCG1/ADTRP/PAXIP1/ADGRA2/PLPP3
Biological Process	GO:0043112	receptor metabolic process	39/2279	198/18866	0.001351	0.0648262	0.058657	39	LMTK2/SORL1/AP2A1/IL10/CD9/PPARG/ANGPT1/TBC1D16/ARRB1/DNM2/MIR199A1/RAB31/TNF/ARAP1/ANKRD13B/ANKRD13D/UVRAG/HDAC1/TGFB1/SMURF1/NUMB/ITGB3/ANKRD13A/CXCR1/GSG1L/ARRB2/ITGB2/DNM1/DNM3/MIR199A2/EPS15/NEDD4/MX1/INPP5F/EZR/FLOT1/MVB12A/PHETA1/GRK2

Biological Process	GO:0002761	regulation of myeloid leukocyte differentiation	27/2279	123/18866	0.0014033	0.0669997	0.0606237	27	ZFPM1/FOXP1/CREB1/MIR145/RUNX1/INPP5D/PIK3R1/PRKCA/TNF/UBASH3B/TESC/RIPK1/TGFB1/CD101/RARA/TMEM178A/LILRB4/NF1/TMEM64/LRRC17/RB1/LTF/CEBPB/TRIB1/INHBA/LIF/FADD
Biological Process	GO:0048660	regulation of smooth muscle cell proliferation	35/2279	173/18866	0.0014367	0.0682526	0.0617574	35	ELANE/HDAC4/FOXP1/IL10/PPARG/MIR140/MEF2D/MIR143/MIR145/MYB/ERN1/TNF/TNFAIP3/CCL5/TGFBR2/THBS1/COMT/GNAI2/MIR27A/TCF7L2/ILK/P2RY6/PHB/SF1/PDGFD/IL6R/PDGFB/DNMT1/MEF2C/NPPC/PDGFRB/VIPR2/TRIB1/NDRG2/CCN4
Biological Process	GO:0034138	toll-like receptor 3 signaling pathway	8/2279	20/18866	0.0014702	0.0688074	0.0622594	8	TNFAIP3/RIPK1/COLEC12/SCIMP/WDFY1/FLOT1/FADD/PELI1
Biological Process	GO:0098884	postsynaptic neurotransmitter receptor internalization	8/2279	20/18866	0.0014702	0.0688074	0.0622594	8	DNM2/NUMB/ITGB3/GSG1L/DNM1/DNM3/EPS15/MX1
Biological Process	GO:0140239	postsynaptic endocytosis	8/2279	20/18866	0.0014702	0.0688074	0.0622594	8	DNM2/NUMB/ITGB3/GSG1L/DNM1/DNM3/EPS15/MX1
Biological Process	GO:0016311	dephosphorylation	82/2279	492/18866	0.0014886	0.0690339	0.0624644	82	LMTK2/SYNJ2/SSH1/CTDP1/PTPRE/LCK/INPP5A/MGAT5/PPP1R1B/INPP5D/URI1/PFKFB4/DUSP14/SMAD3/CABIN1/GPLD1/TNF/UBASH3B/PHLPP1/DUSP22/PGP/ACP6/CTDSPL/CCDC8/ALPL/INPP4A/GNAI2/PTPN6/CAMTA1/SSH3/ELFN2/RCAN1/SMG7/PHACTR2/DUSP28/PTPRN2/CDC25A/TGFB1/FARP1/PTPRJ/SEMA4D/PPP2R5C/RIPK3/LRRK1/HTT/PTPRC/SMG6/PPP2R1A/INPP5E/PALD1/PPP1CB/IMPA2/PPP2R5E/PPP2R5D/DAPP1/SGPP1/CA3/SGPP2/BCL2/MEF2C/DUSP3/PPP2R1B/PDGFRB/WDR81/PPP1R16B/RPRD1B/CDC14A/NT5DC3/FBXW11/CD33/SPOCD1/ANKLE2/LHPP/INPP5F/PPM1N/PTPRU/LPIN1/PFKFB3/ACP3/PTPA/PLPP3/PXYLP1
Biological Process	GO:0070278	extracellular matrix constituent secretion	6/2279	12/18866	0.0014902	0.0690339	0.0624644	6	TNFRSF1B/TNFRSF1A/ENG/NOTCH1/CPB2/ADTRP
Biological Process	GO:0002687	positive regulation of leukocyte migration	30/2279	142/18866	0.0015041	0.0690339	0.0624644	30	ELANE/FUT4/CALR/FUT7/C3AR1/IL1R1/CCR2/TNF/CCL5/CSF1R/FPR2/THBS1/APP/ITGA2B/MIA3/DEFB124/DOCK8/ZNF580/PDGFD/CAMK1D/CXCL17/CCL20/IL6R/DAPK2/DNM1L/ANO6/DYSF/CCL27/CX3CR1/FADD
Biological Process	GO:0031333	negative regulation of protein-containing complex assembly	30/2279	142/18866	0.0015041	0.0690339	0.0624644	30	VILL/PRKCZ/TBCD/SORL1/SSH1/GSN/KANK1/ULK1/LDLRAD4/MEFV/CAPG/SSH3/CLU/TMOD3/SVIL/PFN1/JAM3/SPTA1/ADD1/AVIL/FLII/ARHGEF2/SPTAN1/TMOD1/LMO4/MYADM/SPTBN4/SVIP/TFIP11/CYRIB

Biological Process	GO:0018108	peptidyl-tyrosine phosphorylation	65/2279	374/18866	0.0015563	0.0706411	0.0639186	65	PRKCZ/AATK/LMTK2/DYRK4/TGFA/SH3BP5L/BAZ1B/TTN/LCK/HIPK3/MAP2K6/ANGPT1/FGR/TNF/CCL5/CSF1R/NCK2/EPHB3/SH3BP5/HIPK2/DUSP22/CAMKK2/SLA/APP/PTPN6/TNFRSF1A/IGF1R/FLT1/JAK1/FGFR1/TGFB1/FER/PTPRJ/CLK3/SEMA4D/DGKQ/PDGFD/LRRK1/DYRK2/BLK/CSF3/TESK2/ITGB3/GHRL/PTPRC/CSK/IL6R/PDGFB/PPP2R1A/CASS4/ARRB2/ITGB2/NTRK1/INSRR/PRKCE/GGNBP2/BANK1/PDGFC/ARHGEF2/PDGFRB/TYRO3/INPP5F/CRLF1/PARP9/LIF
Biological Process	GO:0090130	tissue migration	65/2279	374/18866	0.0015563	0.0706411	0.0639186	65	S100P/RREB1/TACSTD2/FOXP1/CALR/NRP2/PPARG/KANK1/MAP3K3/MIR143/RIN2/HDAC7/ANGPT1/DOCK1/ITGB1BP1/PRKCA/MIR199A1/GPLD1/TNF/ZEB2/MIR101-2/SEMA4A/STAT5A/RTN4/JUP/TGFBR2/THBS1/MYH9/CTSH/AKT3/MIR24-2/MIR27A/MIR23A/MIA3/PXN/PLEKHG5/LTB4R2/FGFR1/TGFB1/ZNF580/IQSEC1/NF1/PFN1/BMPER/ITGB3/GRHL2/GRN/PDGFB/ETS1/NFE2L2/ITGB2/VASH1/ARSB/MIR199A2/NOTCH1/CORO1C/MEF2C/PRKCE/SP100/TNFSF12/PLCG1/ADTRP/PAXIP1/ADGRA2/PLPP3
Biological Process	GO:0060612	adipose tissue development	13/2279	44/18866	0.0015614	0.0706411	0.0639186	13	ARID5B/ZNF516/SORL1/SLC25A25/SPTLC2/LRP5L/SH3PXD2B/GHRL/FTO/KLF7/ACAT1/PAXIP1/SPART
Biological Process	GO:0042908	xenobiotic transport	11/2279	34/18866	0.001569	0.0706492	0.0639259	11	NR1I2/ABCC1/ABCC2/OSCP1/LRRC8C/RALBP1/SLC7A5/SLC22A5/ABCC3/SLC2A1/PDZK1
Biological Process	GO:0006672	ceramide metabolic process	23/2279	100/18866	0.0016059	0.071806	0.0649727	23	PLA2G15/FUT7/SPTLC2/HEXB/GALC/TNF/ORMDL3/CERS6/TNFRSF1A/SPHK2/SGMS1/ST8SIA6/PPP2R1A/SGMS2/B4GALT3/P2RX1/FA2H/CERS2/CERK/SIRT3/PRKAA1/TLCD3B/PLPP3
Biological Process	GO:0001841	neural tube formation	24/2279	106/18866	0.0016174	0.071806	0.0649727	24	SHROOM3/SKI/SETD2/SPINT2/MTHFD1L/SUFU/ZEB2/MIB1/PAX2/TGFB1/RARA/PFN1/RARG/STK3/GRHL2/BCL10/MTHFD1/RALA/TMED2/TSC2/TEAD2/LMO4/NODAL/BRD2
Biological Process	GO:0046632	alpha-beta T cell differentiation	24/2279	106/18866	0.0016174	0.071806	0.0649727	24	IL27/PRKCZ/ZFPM1/FOXP1/FUT7/MYB/RUNX1/NLRP3/ITPKB/LOXL3/SEMA4A/ZBTB16/TGFBR2/SMAD7/LY9/ZBTB7B/RARA/PNP/BCL2/IL18R1/HLX/RUNX3/AP3D1/BCL3
Biological Process	GO:0031032	actomyosin structure organization	39/2279	200/18866	0.0016382	0.0723948	0.0655054	39	ARHGEF10/TACSTD2/TTN/CASQ2/MYH11/AKAP13/ITGB1BP1/PIK3R1/SMAD3/ARRB1/MYO18A/SH3PXD2B/TNNT3/ARAP1/CUL3/MYH9/SYNPO2L/PXN/WNT11/ITGB5/PPFIA1/TMOD3/FRMD3/PFN1/EPB41L3/ACTG1/WDR1/MIR138-2/ARHGEF10L/ARHGEF18/MEF2C/FLII/PDGFRB/ARHGEF15/CDC42BPB/ANKRD1/FRMD6/TMOD1/CASQ1
Biological Process	GO:0045619	regulation of lymphocyte differentiation	36/2279	181/18866	0.0016951	0.0745605	0.067465	36	IL27/PRKCZ/RHOH/MYB/RUNX1/INPP5D/NLRP3/ITPKB/CCR2/PIK3R6/LOXL3/NFATC2/FBXO7/TLR9/ZBTB16/TGFBR2/PTPN6/SMAD7/ZMIZ1/ZBTB7B/FANCA/RARA/LILRB4/ZC3H8/PGLYRP1/PTPRC/CLPTM1/PNP/HLX/RUNX3/CYLD/AP3D1/INHBA/PCID2/TMEM131L/VSIR

Biological Process	GO:0046578	regulation of Ras protein signal transduction	38/2279	194/18866	0.0017147	0.0750732	0.0679289	38	ARHGEF10/TIMP2/KANK1/DENND1A/AKAP13/MYO9B/ARHGDIB/ITPKB/ARRB1/DNM2/DENND3/CUL3/LRRC59/SYNPO2L/RASA3/SYNGAP1/EPS8L3/IQSEC1/EPS8L1/NF1/ABCA1/BCR/ARHGEF25/NTRK1/ARHGEF18/NOTCH1/CYTH4/ARHGAP17/F2R/ARHGEF28/FOXM1/ARHGEF2/GPR4/PDGFRB/RALGPS1/FLOT1/FBXO8/PRAG1
Biological Process	GO:0035697	CD8-positive, alpha-beta T cell extravasation	3/2279	3/18866	0.0017607	0.075142	0.0679912	3	CCR2/RIPK3/FADD
Biological Process	GO:1904578	response to thapsigargin	3/2279	3/18866	0.0017607	0.075142	0.0679912	3	CLU/FIS1/DNM1L
Biological Process	GO:1904579	cellular response to thapsigargin	3/2279	3/18866	0.0017607	0.075142	0.0679912	3	CLU/FIS1/DNM1L
Biological Process	GO:2000449	regulation of CD8-positive, alpha-beta T cell extravasation	3/2279	3/18866	0.0017607	0.075142	0.0679912	3	CCR2/RIPK3/FADD
Biological Process	GO:0048659	smooth muscle cell proliferation	35/2279	175/18866	0.0017659	0.075142	0.0679912	35	ELANE/HDAC4/FOXP1/IL10/PPARG/MIR140/MEF2D/MIR143/MIR145/MYB/ERN1/TNF/TNFAIP3/CCL5/TGFBR2/THBS1/COMT/GNAI2/MIR27A/TCF7L2/ILK/P2RY6/PHB/SF1/PDGFD/IL6R/PDGFB/DNMT1/MEF2C/NPPC/PDGFRB/VIPR2/TRIB1/NDRG2/CCN4
Biological Process	GO:0001890	placenta development	32/2279	156/18866	0.0017766	0.075142	0.0679912	32	BSG/RXRA/IL10/PPARG/VDR/SETD2/SPINT2/E2F7/HSD17B2/RBPJ/SP3/DNAJB6/DAZAP1/GHRL/STK3/GRHL2/NCOA1/PDGFB/CTSB/GJB3/VASH1/TPPP3/EPAS1/GGNBP2/STOX2/TMED2/CEBPB/PCDH12/PLCD3/NODAL/LIF/ETNK2
Biological Process	GO:0050870	positive regulation of T cell activation	41/2279	214/18866	0.0017928	0.075142	0.0679912	41	HLA-DMB/PRKCZ/RHOH/LCK/MYB/RUNX1/SIRPB1/CD55/NLRP3/PIK3R1/ITPKB/CCR2/PIK3R6/CCL5/GRAP2/NCK2/TNFSF13B/ZBTB16/TGFBR2/PTPN6/ZMIZ1/DOCK8/ZBTB7B/RARA/LILRB4/CCDC88B/PTPRC/CSK/BCL10/SPTA1/PNP/NOD2/SIRPG/HLX/RUNX3/CYLD/AP3D1/SLC7A1/FADD/CYRIB/VSIR
Biological Process	GO:0016358	dendrite development	46/2279	247/18866	0.0017943	0.075142	0.0679912	46	MFSD2A/YWHAH/ZMYND8/BTBD3/NCK2/EPHB3/BCL11A/ITSN1/SLC11A2/TNIK/DISC1/CUX1/BAIAP2/APP/ILK/PREX1/SDC2/ASAP1/WASL/SYNGAP1/FARP1/SEMA4D/RAPGEF2/SS18L2/IQSEC1/ARID1B/CPEB3/CAMK1D/GHRL/SIPA1L1/MAPK8IP2/KLF7/DOCK10/DNM3/MAP1A/CDKL3/DNM1L/MEF2C/SHANK2/UBE3A/NEDD4/HECW2/CYFIP1/DTNBP1/CUX2/DBNL

Biological Process	GO:0036010	protein localization to endosome	9/2279	25/18866	0.0017953	0.075142	0.0679912	9	SORL1/TOLLIP/TMEM30A/PACSIN2/MGAT3/ANKRD13A/ZDHHC1/MICALL1/EZR
Biological Process	GO:0071677	positive regulation of mononuclear cell migration	9/2279	25/18866	0.0017953	0.075142	0.0679912	9	CCR2/TNF/CCL5/FPR2/DEFB124/PDGFD/CXCL17/ANO6/CCL27
Biological Process	GO:0051283	negative regulation of sequestering of calcium ion	26/2279	119/18866	0.0018265	0.0757813	0.0685696	26	TRPM2/PDE4D/CASQ2/ITPR2/LCK/CACNA1C/SLC8A1/ANXA6/UBASH3B/ITPR1/JSRP1/PTPN6/RASA3/P2RY6/MYO5A/HTT/PTPRC/NOL3/TPCN2/GSTO1/PRKCE/F2R/ANK2/CHERP/PLCG1/CASQ1
Biological Process	GO:0071706	tumor necrosis factor superfamily cytokine production	26/2279	119/18866	0.0018265	0.0757813	0.0685696	26	TRIM27/FOXP1/IL10/MIR140/ANGPT1/PIK3R1/TNFAIP3/TLR9/SPON2/RIPK1/C5AR2/APP/PTPN6/CLU/SPHK2/PTPRJ/RARA/PTPRC/ARRB2/NOD2/ARHGEF2/LTF/CD33/FADD/VSIR/ILRUN
Biological Process	GO:0046834	lipid phosphorylation	18/2279	72/18866	0.0018667	0.0771107	0.0697725	18	PIK3CD/PIP5K1B/PIK3R1/ITPKB/PIK3R6/TTC7A/PIP4K2A/SPHK2/TTC7B/DGKQ/DGKA/DGKD/IMPA2/PI4K2A/PI4KA/CERK/IP6K1/SLA2
Biological Process	GO:0050804	modulation of chemical synaptic transmission	76/2279	454/18866	0.0019233	0.0788411	0.0713382	76	PRKCZ/ABR/SSH1/RNF19A/SORCS2/CREB1/YWHAH/PRKAR1B/ZMYND8/RAP1B/CCR2/TNF/PACSIN2/DISC1/GIT1/BAIAP2/ZDHHC2/GNAI2/APP/SYN3/AKAP5/GRIK4/APBA2/PLEKHG5/SYNGAP1/NISCH/DLGAP4/RAPGEF2/HRH1/PRKCB/CNIH2/RARA/MCTP2/NF1/NRXN1/VPS18/CPEB3/GHRL/CHRM2/SH3GL1/BCR/CNR2/SIPA1L1/PLCB1/BEGAIN/MAPK8IP2/ARRB2/DNM1/PLCL1/NTRK1/CDH1/SLC1A3/S1PR2/MAP1A/P2RX1/DNM1L/MEF2C/SHANK2/PRKCE/F2R/CACNA1B/PLCG1/CYFIP1/CDH11/NPTX1/GRID1/FLOT1/DTNBP1/AKAP7/NTF4/CX3CR1/CUX2/SYNPO/FGF14/LRFN2/PRKN
Biological Process	GO:1901653	cellular response to peptide	68/2279	398/18866	0.0019252	0.0788411	0.0713382	68	PRKCZ/KLF1/SORL1/FUT7/PPARG/ADCY2/MIR140/KANK1/ABCC1/CREB1/PTPRE/MIR143/MIR145/PRKAR1B/ADCY9/PIK3R1/RAP1B/GPLD1/RAB31/FPR2/STAT5A/BAIAP2L1/BAIAP2/GRB10/GNAI2/APP/ATP6V1B2/ZDHHC7/PXN/IGF1R/ZBTB7B/CRHBP/FER/PRKCB/POR/MYO5A/ARID1B/GHRL/CSK/AGTRAP/PRKCI/NFE2L2/ADCY4/INSRR/NOD2/FIS1/PKM/ATP6V0B/ARHGEF2/BCAR3/PCK2/AGTR1/MYO1C/TSC2/FOXO1/MBD5/SOGA1/SREBF1/PDE3B/CYFIP1/RELA/WDTC1/NDEL1/LPIN1/PRKAA1/MARS1/ATP6V0C/ATP6V1C1
Biological Process	GO:0006816	calcium ion transport	72/2279	426/18866	0.0019335	0.0788411	0.0713382	72	TRPM2/PDE4D/TRIM27/RCVRN/SLC25A25/THADA/IL16/CASQ2/ITPR2/LCK/CACNA1C/CACNA2D4/SLC24A4/VDR/MYLK/SLC8A1/PKD1L1/ANXA6/UBASH3B/CDH23/CCL5/SPG7/CD84/TLR9/ATP2B2/ITPR1/BIN1/TRPM6/JSRP1/AHNAK/GNAI2/NOS1AP/PTPN6/RASA3/P2RY6/SPHK2/PHB/PRKCB/MYO5A/HTT/PTPRC/ATP2A3/PDGFB/NOL3/TPCN2/ARRB2/TRPM3/BCL2/GSTO1/P2RX1/DNM1L/PRKCE/TMEM37/F2R/TMC1/PDGFRB/ANO6/DYSF/CACNA1B/ANK2/CHERP/PLCG1/P2RX5/TRPV2/CD33/CASQ1/FGF14/GRAMD2A/CBARP/STIMATE/SARAF/CRACR2A
Biological Process	GO:0016049	cell growth	81/2279	490/18866	0.0020022	0.0812964	0.0735599	81	SEMA6B/PRKCZ/S100A8/RPTOR/CPNE6/CDA/ENO1/NRP2/PPARG/CTDP1/RPS6KA1/SERTAD2/MFSD2A/ULK1/APBB2/EXOSC2/URI1/AKAP13/PRMT2/SMAD3/CTTN/DNM2/MIR199A1/CPNE5/ZEB2/CYFIP2/SGK1/BCL11A/VGLL4/SEMA4A/RTN4/KRT17/TGFBR2/MEG3/DISC1/SLC44A4/SEMA4B/APP/MIR24-2/MIR23A/DERL2/SMARCA2/IFRD1/MUC12/ILK/SMAD7/CPNE1/WNT11/SPHK2/TGFB1/PHB/PTPRJ/SEMA4D/RERG/SMURF1/CHPT1/KMT2D/EPB41L3/CDHR2/RARG/CDH4/SH3BP4/PPP2R1A/RB1/BCL2/MIR199A2/CDKL3/SYT17/SLIT3/AGTR1/CYFIP1/TRPV2/SGK2/NDEL1/SLIT1/SCGB3A1/INHBA/EIF4G1/DBNL/SPART/PRKN

Biological Process	GO:0030279	negative regulation of ossification	20/2279	84/18866	0.0020312	0.0814848	0.0737304	20	HDAC4/HDAC7/SKI/CHSY1/SMAD3/SUFU/TNF/RBPJ/LIMD1/SMAD7/KREMEN1/SEMA4D/SMURF1/TMEM64/KREMEN2/MIR138-2/BCL2/NOTCH1/MEF2C/RFLNA
Biological Process	GO:0099177	regulation of trans-synaptic signaling	76/2279	455/18866	0.0020424	0.0814848	0.0737304	76	PRKCZ/ABR/SSH1/RNF19A/SORCS2/CREB1/YWHAH/PRKAR1B/ZMYND8/RAP1B/CCR2/TNF/PACSIN2/DISC1/GIT1/BAIAP2/ZDHHC2/GNAI2/APP/SYN3/AKAP5/GRIK4/APBA2/PLEKHG5/SYNGAP1/NISCH/DLGAP4/RAPGEF2/HRH1/PRKCB/CNIH2/RARA/MCTP2/NF1/NRXN1/VPS18/CPEB3/GHRL/CHRM2/SH3GL1/BCR/CNR2/SIPA1L1/PLCB1/BEGAIN/MAPK8IP2/ARRB2/DNM1/PLCL1/NTRK1/CDH1/SLC1A3/S1PR2/MAP1A/P2RX1/DNM1L/MEF2C/SHANK2/PRKCE/F2R/CACNA1B/PLCG1/CYFIP1/CDH11/NPTX1/GRID1/FLOT1/DTNBP1/AKAP7/NTF4/CX3CR1/CUX2/SYNPO/FGF14/LRFN2/PRKN
Biological Process	GO:0007030	Golgi organization	31/2279	151/18866	0.0020475	0.0814848	0.0737304	31	STX5/VTI1A/TANGO2/CSNK1D/RAB2A/SYNE1/ARHGEF7/DNM2/MYO18A/LMAN2/FAM174B/ATP8B1/COG1/RAB43/PDE4DIP/HTT/VCPIP1/NUMA1/USP6NL/GORASP2/ATP8B4/PI4K2A/YWHAZ/VMP1/UBXN2A/RAB30/TMED2/KIFC3/BAG5/VPS51/ARMH3
Biological Process	GO:0055001	muscle cell development	36/2279	183/18866	0.0020661	0.0814848	0.0737304	36	HDAC4/CTDP1/MYO18B/TTN/CASQ2/MYH11/AKAP13/SKI/MAML1/SLC8A1/MIR199A1/NFATC2/TNNT3/BIN1/SMYD3/SYNPO2L/MIR24-2/MIR23A/TMOD3/ACTG1/WDR1/ENG/BCL2/MIR199A2/NOTCH1/MEF2C/FHL2/FLII/PDGFRB/DYSF/ANK2/ANKRD1/TMOD1/CASQ1/SYPL2/MYORG
Biological Process	GO:0120032	regulation of plasma membrane bounded cell projection assembly	36/2279	183/18866	0.0020661	0.0814848	0.0737304	36	HDAC4/TRPM2/TACSTD2/DEF8/CDKL1/KANK1/ZMYND8/RHOG/PLD1/DNM2/ARAP1/CDC42EP1/ATP8B1/CEP135/WASL/FER/SH3YL1/EPS8L3/EPS8L1/PFN1/ESPN/HTT/IFT140/PALM/PLEKHM1/SDCCAG8/NCKAP1/RALA/DNM3/CORO1C/AVIL/PPP1R16B/CYLD/CYFIP1/NDEL1/SEPTIN9
Biological Process	GO:0034332	adherens junction organization	17/2279	67/18866	0.002079	0.0814848	0.0737304	17	TBCD/CADM3/JUP/SMAD7/CDH3/PLEKHA7/JAM3/NUMB/CSK/CDH4/CDH1/ADD1/CADM1/CDH11/KIFC3/NECTIN1/NECTIN4
Biological Process	GO:0043550	regulation of lipid kinase activity	17/2279	67/18866	0.002079	0.0814848	0.0737304	17	PIK3R1/PIK3R6/FGR/FPR2/PIK3R5/FLT1/TGFB1/VAV2/ATG14/PDGFB/MIR138-2/NOD2/RB1/PDGFRB/WDR81/VAV3/SLA2
Biological Process	GO:0031529	ruffle organization	15/2279	56/18866	0.0020996	0.0814848	0.0737304	15	TACSTD2/DEF8/KANK1/RHOG/CSF1R/SH3YL1/EPS8L3/EPS8L1/PFN1/PLEKHM1/CORO1C/PDGFRB/CYFIP1/NDEL1/DBNL
Biological Process	GO:0006024	glycosaminoglycan biosynthetic process	24/2279	108/18866	0.0021126	0.0814848	0.0737304	24	CSGALNACT1/AP2A1/HS3ST3B1/DSE/ANGPT1/CHSY1/CHST11/CHST15/NDST1/CHST2/SDC2/XYLT1/TGFB1/B4GALT7/PDGFB/ST3GAL4/B4GALT3/GCNT2/PDGFRB/HAS3/ST3GAL2/B3GNT2/B4GAT1/PXYLP1

Biological Process	GO:0003418	growth plate cartilage chondrocyte differentiation	5/2279	9/18866	0.0021184	0.0814848	0.0737304	5	ANXA6/TGFBR2/POC1A/RARG/NPPC
Biological Process	GO:0010692	regulation of alkaline phosphatase activity	5/2279	9/18866	0.0021184	0.0814848	0.0737304	5	SMAD3/GPLD1/TNF/SEMA4D/MEF2C
Biological Process	GO:0071550	death-inducing signaling complex assembly	5/2279	9/18866	0.0021184	0.0814848	0.0737304	5	TNF/TRAF2/RIPK1/TNFRSF1A/FADD
Biological Process	GO:1900045	negative regulation of protein K63-linked ubiquitination	5/2279	9/18866	0.0021184	0.0814848	0.0737304	5	OTUB2/MIR138-2/OTUB1/TRIP12/PARP10
Biological Process	GO:0051651	maintenance of location in cell	41/2279	216/18866	0.0021457	0.0820971	0.0742844	41	TRPM2/PDE4D/S100A8/SORL1/CALR/HK1/GSN/LCN2/CASQ2/ITPR2/LCK/SYNE1/CACNA1C/VPS13D/SLC8A1/ANXA6/UBASH3B/ITPR1/JSRP1/PTPN6/RASA3/UVRAG/P2RY6/MYO5A/HTT/HK2/PTPRC/NOL3/TPCN2/MORC3/GSTO1/PRKCE/SP100/F2R/ANK2/AP3D1/CHERP/PLCG1/ATP7B/BCL3/CASQ1
Biological Process	GO:0002697	regulation of immune effector process	78/2279	470/18866	0.0021516	0.0820971	0.0742844	78	HLA-DMB/IL27/PRKCZ/ABR/FUT7/IL10/AIM2/TRIM38/C3AR1/MYB/CD55/CD177/TNFRSF1B/NLRP3/IL1R1/CCR2/PIK3R6/GAB2/FGR/TNF/TRAF2/TNFAIP3/MYO18A/LOXL3/CD84/TLR9/DUSP22/SPON2/C5AR2/CD226/HLA-F/PTPN6/CLU/SMAD7/SCIMP/SPHK2/ZBTB7B/TGFB1/PHB/FER/PTPRJ/NCR1/RARA/RIPK3/BLK/CD59/C1S/PGLYRP1/SEC14L1/SLC7A5/PTPRC/BCL10/GRN/BCR/ZDHHC1/ARRB2/ITGB2/IL18RAP/DNASE1L3/NOD2/TP53BP1/PRAM1/IL18R1/TRAF3IP2/CPB2/HLX/TNFSF13/C1QB/CADM1/CD96/HERC5/PARP9/FADD/PAXIP1/STING1/CYRIB/ILRUN/NSD2
Biological Process	GO:0090114	COPII-coated vesicle budding	18/2279	73/18866	0.0022029	0.0837209	0.0757536	18	CTSZ/SERPINA1/TGFA/STX5/CSNK1D/TRAPPC10/SEC23B/CUL3/TRAPPC9/MIA3/CNIH2/CD59/SEC13/ANKRD28/SEC24C/TMED2/SEC31A/TRAPPC2
Biological Process	GO:0030100	regulation of endocytosis	40/2279	210/18866	0.0022584	0.0854863	0.077351	40	AP2A1/PPARG/ANKFY1/CD177/ANGPT1/AAK1/RAB5C/RAB5B/ARRB1/DNM2/MIR199A1/RAB31/ITSN1/PACSIN2/STON1/BIN1/ANKRD13B/DLL1/MIB1/ACTN4/MIR27A/ANKRD13D/CLU/WASL/DGKQ/NR1H2/NUMB/ITGB3/ANKRD13A/SH3GL1/DGKD/GSG1L/ARRB2/DNM1/MIR199A2/DNM1L/TSC2/ANO6/RUFY1/FLOT1
Biological Process	GO:0032272	negative regulation of protein polymerization	19/2279	79/18866	0.0022957	0.0865524	0.0783156	19	VILL/TBCD/SSH1/GSN/KANK1/CAPG/SSH3/TMOD3/SVIL/PFN1/SPTA1/ADD1/AVIL/FLII/SPTAN1/TMOD1/MYADM/SPTBN4/CYRIB

Biological Process	GO:0051282	regulation of sequestering of calcium ion	26/2279	121/18866	0.0023389	0.0878338	0.0794752	26	TRPM2/PDE4D/CASQ2/ITPR2/LCK/CACNA1C/SLC8A1/ANXA6/UBASH3B/ITPR1/JSRP1/PTPN6/RASA3/P2RY6/MYO5A/HTT/PTPRC/NOL3/TPCN2/GSTO1/PRKCE/F2R/ANK2/CHERP/PLCG1/CASQ1
Biological Process	GO:0032609	interferon-gamma production	25/2279	115/18866	0.0023773	0.0887297	0.0802857	25	IL27/PDE4D/TRIM27/ZFPM1/IRF8/IL10/SLC11A1/IL1R1/CCR2/TNF/TLR9/CD226/MIR24-2/RARA/RIPK3/PGLYRP1/SLC7A5/IL18RAP/IL18R1/CD96/BCL3/INHBA/FADD/CYRIB/VSIR
Biological Process	GO:0051897	positive regulation of protein kinase B signaling	35/2279	178/18866	0.0023814	0.0887297	0.0802857	35	TGFA/PIK3CD/LCK/MIR143/CHI3L1/ANGPT1/PIK3AP1/ITGB1BP1/PIK3R1/RHOG/MIR199A1/TNF/ITSN1/RTN4/THBS1/PIK3R5/TCF7L2/ILK/CPNE1/IGF1R/FGFR1/TGFB1/PHB/PTPRJ/CSF3/STK3/PDGFB/ENG/ARRB2/MIR138-2/MIR199A2/GCNT2/PDGFRB/CX3CR1/MYORG
Biological Process	GO:0001843	neural tube closure	21/2279	91/18866	0.0024006	0.0890158	0.0805446	21	SHROOM3/SKI/SETD2/SPINT2/MTHFD1L/SUFU/ZEB2/PAX2/TGFB1/RARA/PFN1/RARG/GRHL2/BCL10/MTHFD1/RALA/TMED2/TSC2/TEAD2/LMO4/BRD2
Biological Process	GO:0033157	regulation of intracellular protein transport	48/2279	264/18866	0.0024079	0.0890158	0.0805446	48	SORL1/KCNE1/SH3TC2/TFDP1/PRR5L/TM9SF4/TMEM30A/YWHAH/ANGPT1/ITGB1BP1/PIK3R1/SETD2/SMAD3/SUFU/UBAC2/JUP/EDEM1/USP36/SNX3/ZDHHC2/TCF7L2/DERL2/ABLIM3/AKAP5/UBE2J2/TGFB1/NF1/EDEM2/NUMA1/ITGB2/YWHAZ/CDH1/FIS1/BCL2/TTC21B/CABP1/SP100/TP53BP2/MYO1C/MIEF1/SREBF1/YWHAQ/NDEL1/PDZK1/SVIP/PRKAA1/PRKN/RIPOR1
Biological Process	GO:0033002	muscle cell proliferation	45/2279	244/18866	0.0024324	0.0893592	0.0808554	45	ELANE/HDAC4/RXRA/FOXP1/IL10/PPARG/MIR140/MEF2D/MIR143/MIR145/MYB/ANGPT1/ERN1/MIR199A1/TNF/TNFAIP3/CCL5/VGLL4/TGFBR2/THBS1/COMT/RIPK1/GNAI2/RBPJ/MIR27A/TCF7L2/ILK/P2RY6/FGFR1/PHB/SF1/PDGFD/IL6R/PDGFB/MEIS1/MIR199A2/NOTCH1/DNMT1/MEF2C/NPPC/PDGFRB/VIPR2/TRIB1/NDRG2/CCN4
Biological Process	GO:0031579	membrane raft organization	9/2279	26/18866	0.0024548	0.0893592	0.0808554	9	GSN/PACSIN2/PTPRC/NPC1/MIR138-2/FA2H/DOCK2/FLOT1/MYADM
Biological Process	GO:0034114	regulation of heterotypic cell-cell adhesion	9/2279	26/18866	0.0024548	0.0893592	0.0808554	9	IL10/LCK/CEACAM6/TNF/MBP/IL1RN/GCNT2/FLOT1/MYADM
Biological Process	GO:0070633	transepithelial transport	9/2279	26/18866	0.0024548	0.0893592	0.0808554	9	ABCC1/GPLD1/ABCC2/ITPR1/P2RY6/AQP1/SLC1A2/SLC1A3/SLC23A1

Biological Process	GO:0042921	glucocorticoid receptor signaling pathway	6/2279	13/18866	0.0024858	0.0894619	0.0809483	6	CALR/YWHAH/PHB/ARNTL/PER1/NEDD4
Biological Process	GO:0046348	amino sugar catabolic process	6/2279	13/18866	0.0024858	0.0894619	0.0809483	6	NAGK/AMDHD2/CHI3L1/NPL/CHIT1/CHI3L2
Biological Process	GO:1902563	regulation of neutrophil activation	6/2279	13/18866	0.0024858	0.0894619	0.0809483	6	ABR/CD177/GRN/BCR/ITGB2/PRAM1
Biological Process	GO:0046488	phosphatidylinositol metabolic process	36/2279	185/18866	0.0025052	0.0894804	0.080965	36	SYNJ2/PIK3CD/INPP5A/VAC14/PIP5K1B/INPP5D/PIK3R1/ITPKB/PIGL/PIK3R6/PIGU/GPLD1/CSF1R/FPR2/TTC7A/PIGN/PITPNM2/INPP4A/PIK3R5/PIP4K2A/PITPNM1/TTC7B/SH3YL1/PLEKHA2/PDGFB/INPP5E/PLCB1/IMPA2/PI4K2A/PI4KA/PIGQ/PDGFRB/RUFY1/INPP5F/IP6K1/SLA2
Biological Process	GO:0060491	regulation of cell projection assembly	36/2279	185/18866	0.0025052	0.0894804	0.080965	36	HDAC4/TRPM2/TACSTD2/DEF8/CDKL1/KANK1/ZMYND8/RHOG/PLD1/DNM2/ARAP1/CDC42EP1/ATP8B1/CEP135/WASL/FER/SH3YL1/EPS8L3/EPS8L1/PFN1/ESPN/HTT/IFT140/PALM/PLEKHM1/SDCCAG8/NCKAP1/RALA/DNM3/CORO1C/AVIL/PPP1R16B/CYLD/CYFIP1/NDEL1/SEPTIN9
Biological Process	GO:0051693	actin filament capping	12/2279	41/18866	0.0025549	0.0909127	0.0822611	12	VILL/GSN/CAPG/TMOD3/SVIL/SPTA1/ADD1/AVIL/FLII/SPTAN1/TMOD1/SPTBN4
Biological Process	GO:0071224	cellular response to peptidoglycan	4/2279	6/18866	0.0026026	0.0919243	0.0831764	4	NLRP3/DEFB124/NOD2/RELA
Biological Process	GO:1903613	regulation of protein tyrosine phosphatase activity	4/2279	6/18866	0.0026026	0.0919243	0.0831764	4	MGAT5/GNAI2/PTPRC/CD33
Biological Process	GO:0001893	maternal placenta development	11/2279	36/18866	0.0026235	0.092145	0.0833761	11	BSG/RXRA/VDR/DAZAP1/GHRL/CTSB/TPPP3/STOX2/TMED2/NODAL/LIF

Biological Process	GO:0010811	positive regulation of cell-substrate adhesion	26/2279	122/18866	0.002638	0.092145	0.0833761	26	PRKCZ/P4HB/RREB1/CALR/SPOCK2/VIT/RIN2/DOCK1/ITGB1BP1/CEACAM6/SMAD3/ARHGEF7/DNM2/JUP/DISC1/NINJ1/EGFLAM/ILK/PREX1/PTPRJ/OLFM4/PLEKHA2/CASS4/PRKCE/RSU1/MYADM
Biological Process	GO:1990266	neutrophil migration	26/2279	122/18866	0.002638	0.092145	0.0833761	26	PRTN3/PDE4D/S100A8/FUT7/C3AR1/PIK3CD/RHOH/CSF3R/CD177/IL1R1/RHOG/CCL5/C5AR2/PREX1/JAM3/CAMK1D/CCL20/CXCR1/WDR1/ITGB2/DAPK2/DNM1L/DYSF/CCL22/VAV3/JAML
Biological Process	GO:0051701	interaction with host	40/2279	212/18866	0.0026963	0.0938359	0.0849061	40	TRIM27/P4HB/RXRA/TRIM38/GSN/TRIM8/WWP2/SLC10A1/ULK1/CD55/TRIM10/SLC1A5/HIPK2/CLEC5A/SNX3/UVRAG/ITGB5/PC/PABPN1/PHB/FCN1/TRIM5/VPS18/TNIP1/ITGB3/CTSB/TPCN2/NPC1/CDH1/SLC52A2/EPS15/BCL2L1/TYRO3/CPSF4/ITGB6/SIVA1/TRIM26/GRK2/NECTIN1/NECTIN4
Biological Process	GO:0032680	regulation of tumor necrosis factor production	24/2279	110/18866	0.0027313	0.094029	0.0850808	24	TRIM27/FOXP1/IL10/MIR140/ANGPT1/PIK3R1/TNFAIP3/TLR9/SPON2/RIPK1/C5AR2/PTPN6/CLU/PTPRJ/RARA/PTPRC/ARRB2/NOD2/ARHGEF2/LTF/CD33/FADD/VSIR/ILRUN
Biological Process	GO:0046635	positive regulation of alpha-beta T cell activation	16/2279	63/18866	0.002748	0.094029	0.0850808	16	PRKCZ/MYB/RUNX1/CD55/NLRP3/ITPKB/CCR2/ZBTB16/TGFBR2/ZBTB7B/RARA/PTPRC/PNP/HLX/RUNX3/AP3D1
Biological Process	GO:0002367	cytokine production involved in immune response	23/2279	104/18866	0.0027592	0.094029	0.0850808	23	PRKCZ/IL10/CD55/SLC11A1/TNFRSF1B/NLRP3/IL1R1/CCR2/TNF/TRAF2/SPON2/CD226/HLA-F/SMAD7/SCIMP/SPHK2/TGFB1/SLC7A5/BCL10/IL18RAP/NOD2/IL18R1/CD96
Biological Process	GO:0032649	regulation of interferon-gamma production	23/2279	104/18866	0.0027592	0.094029	0.0850808	23	IL27/PDE4D/ZFPM1/IRF8/IL10/SLC11A1/IL1R1/CCR2/TNF/TLR9/CD226/MIR24-2/RARA/RIPK3/PGLYRP1/SLC7A5/IL18R1/CD96/BCL3/INHBA/FADD/CYRIB/VSIR
Biological Process	GO:0042147	retrograde transport, endosome to Golgi	21/2279	92/18866	0.0027636	0.094029	0.0850808	21	TRIM27/DCTN1/STX5/VTI1A/ANKFY1/VPS53/HEATR5A/TRAPPC10/TBC1D10A/ERC1/SNX3/VPS26B/UBE2O/NUMA1/TBC1D14/SGSM2/CLN5/VPS51/CLTCL1/PHETA1/PRKN
Biological Process	GO:0060606	tube closure	21/2279	92/18866	0.0027636	0.094029	0.0850808	21	SHROOM3/SKI/SETD2/SPINT2/MTHFD1L/SUFU/ZEB2/PAX2/TGFB1/RARA/PFN1/RARG/GRHL2/BCL10/MTHFD1/RALA/TMED2/TSC2/TEAD2/LMO4/BRD2

Biological Process	GO:0014020	primary neural tube formation	22/2279	98/18866	0.0027711	0.094029	0.0850808	22	SHROOM3/SKI/SETD2/SPINT2/MTHFD1L/SUFU/ZEB2/PAX2/TGFB1/RARA/PFN1/RARG/GRHL2/BCL10/MTHFD1/RALA/TMED2/TSC2/TEAD2/LMO4/NODAL/BRD2
Biological Process	GO:0042493	response to drug	67/2279	397/18866	0.0028137	0.0951336	0.0860802	67	HDAC4/TIMP2/CALR/IL10/AIM2/PPARG/NR1I2/LCN2/ACSL1/ABCC1/CREB1/LCK/MAP2K6/TMEM30A/RAP1B/SLC8A1/ARRB1/GPLD1/NFATC2/ABCC2/TGFBR2/RECQL5/THBS1/COMT/DDC/ATP8B1/FECH/CDH3/CRHBP/SLC19A1/AQP1/POR/ENO3/SLC1A2/PARP4/ABCA1/NCOA1/ACACB/CAT/KCNQ1/PNP/PPOX/NFE2L2/ENG/ABCC3/NPC1/NTRK1/CDH1/SLC1A3/BCL2/MEF2C/GNA12/CPB2/CHEK2/NPPC/BCAR3/ANKRD1/HMGCS1/SREBF1/RELA/CPT1A/RPH3AL/DHODH/HSD11B2/INHBA/SST/VAV3
Biological Process	GO:0030217	T cell differentiation	46/2279	253/18866	0.0029243	0.0981765	0.0888336	46	IL27/PRKCZ/ZFPM1/FOXP1/FUT7/PIK3CD/RHOH/LCK/MYB/RUNX1/NLRP3/ITPKB/CCR2/PIK3R6/LOXL3/NFATC2/SEMA4A/ZBTB16/TGFBR2/PREX1/SMAD7/ZMIZ1/LY9/CCR9/ZBTB7B/FANCA/RARA/RIPK3/LILRB4/ZC3H8/SP3/PTPRC/RUNX2/CLPTM1/PNP/BCL2/IL18R1/HLX/RUNX3/CYLD/DOCK2/AP3D1/BCL3/FADD/TMEM131L/VSIR
Biological Process	GO:0051924	regulation of calcium ion transport	46/2279	253/18866	0.0029243	0.0981765	0.0888336	46	PDE4D/TRIM27/RCVRN/THADA/IL16/CASQ2/CACNA1C/MYLK/SLC8A1/UBASH3B/CCL5/CD84/TLR9/BIN1/JSRP1/AHNAK/GNAI2/NOS1AP/PTPN6/P2RY6/SPHK2/MYO5A/HTT/PDGFB/ARRB2/BCL2/GSTO1/P2RX1/PRKCE/F2R/TMC1/PDGFRB/DYSF/CACNA1B/ANK2/PLCG1/P2RX5/TRPV2/CD33/CASQ1/FGF14/GRAMD2A/CBARP/STIMATE/SARAF/CRACR2A
Biological Process	GO:0043405	regulation of MAP kinase activity	59/2279	342/18866	0.0029524	0.0984362	0.0890686	59	ELANE/KSR1/SPRED2/SORL1/TGFA/MAP3K3/HIPK3/MAP2K6/AKAP13/ERN1/DUSP14/PIK3R6/ARRB1/LAX1/TNF/ZEB2/TRAF2/FPR1/TLR9/TNIK/THBS1/PIK3R5/RIPK1/PTPN6/ILK/IGF1R/FLT1/FGFR1/TGFB1/PTPRJ/NF1/PDGFD/UBE2V1/MUC20/CXCL17/GHRL/PTPRC/CSK/PDE6H/PDGFB/PPP2R1A/MAP3K14/TAOK3/IRAK2/NTRK1/RGS3/NOD2/S1PR2/DUSP3/F2R/PDGFC/MAPKAPK3/PDGFRB/GADD45G/TRIB1/DTNBP1/DBNL/PRKAA1/MAP3K20
Biological Process	GO:0010888	negative regulation of lipid storage	8/2279	22/18866	0.0029902	0.0984362	0.0890686	8	PNPLA2/PPARG/TNF/ABCG1/NR1H2/ITGB3/ABCA1/ABHD5
Biological Process	GO:0035584	calcium-mediated signaling using intracellular calcium source	8/2279	22/18866	0.0029902	0.0984362	0.0890686	8	AZU1/TRPM2/PTPRJ/DEFB1/CCL20/PTPRC/FIS1/STIMATE
Biological Process	GO:0030225	macrophage differentiation	13/2279	47/18866	0.0029942	0.0984362	0.0890686	13	CEBPE/MIR145/PRKCA/CSF1R/RIPK1/APP/TGFB1/RB1/TSPAN2/TRIB1/INHBA/LIF/FADD
Biological Process	GO:0043300	regulation of leukocyte degranulation	13/2279	47/18866	0.0029942	0.0984362	0.0890686	13	ABR/CD177/CCR2/GAB2/FGR/CD84/HLA-F/SPHK2/FER/BLK/BCR/ITGB2/PRAM1

Biological Process	GO:0046638	positive regulation of alpha-beta T cell differentiation	13/2279	47/18866	0.0029942	0.0984362	0.0890686	13	PRKCZ/MYB/RUNX1/NLRP3/ITPKB/ZBTB16/TGFBR2/ZBTB7B/RARA/PNP/HLX/RUNX3/AP3D1
Biological Process	GO:0072659	protein localization to plasma membrane	50/2279	281/18866	0.0031329	0.1026399	0.0928722	50	PRKCZ/EHD1/BSG/ITGB1BP1/PIK3R1/MAP7/RHOG/TSPAN14/KIF13A/RAB31/TNF/PACS2/TTC7A/JUP/TESC/TNIK/ZDHHC2/TNFRSF1A/MRAP/AKAP5/SLC9A3R2/ZDHHC7/PPFIA1/TGFB1/PRKCH/RAB40C/RAPGEF2/TTC7B/SMURF1/VAMP5/MYO5A/EPB41L3/NUMB/CSK/PALM/GORASP2/PRKCI/RILPL1/CDH1/PRAM1/PRKCE/TMED2/BCL2L1/ANK2/EZR/PDZK1/FLOT1/MYADM/SPTBN4/FYB1
Biological Process	GO:0002819	regulation of adaptive immune response	33/2279	168/18866	0.0031665	0.1033847	0.0935462	33	IL27/PRKCZ/TRIM27/FUT7/IL10/SLC11A1/TNFRSF1B/NLRP3/IL1R1/CCR2/TNF/TRAF2/TNFAIP3/LOXL3/TNFSF13B/DUSP22/CD226/HLA-F/PTPN6/SMAD7/ZBTB7B/TGFB1/RIPK3/PTPRC/TP53BP1/MEF2C/IL18R1/HLX/TNFSF13/FADD/PAXIP1/CYRIB/NSD2
Biological Process	GO:0001894	tissue homeostasis	47/2279	261/18866	0.0032223	0.1048451	0.0948676	47	PIWIL4/DEF8/MFSD2A/TJP2/LPCAT1/INPP5D/ANGPT1/CRB1/CCR2/ANKRD11/PRKCA/UBASH3B/TNFAIP3/CDH23/CSF1R/SFTPD/TLR9/CTSH/MBP/CCDC66/MTF1/CDH3/NF1/LRRK1/JAM3/WWTR1/ITGB3/ACTG1/TMEM64/CSK/SLC22A5/PLEKHM1/ESRRB/ADD1/NOD2/RB1/BCL2/NOTCH1/ACACA/GCNT2/CDHR1/EPAS1/F2R/LTF/PDGFRB/SLC2A1/NPHP4
Biological Process	GO:0046637	regulation of alpha-beta T cell differentiation	16/2279	64/18866	0.003261	0.1057424	0.0956795	16	IL27/PRKCZ/MYB/RUNX1/NLRP3/ITPKB/LOXL3/ZBTB16/TGFBR2/SMAD7/ZBTB7B/RARA/PNP/HLX/RUNX3/AP3D1
Biological Process	GO:0016571	histone methylation	29/2279	143/18866	0.0033763	0.108982	0.0986108	29	GFI1/BRD4/MYB/PRMT2/ASH2L/SETD2/SMYD3/EHMT2/WDR82/PAX7/KMT2D/KDM4C/SETD1B/SETD1A/CREBBP/TET2/ZNF335/PRDM16/PHF19/DNMT1/PIH1D1/DYDC2/DYDC1/NTMT1/FBL/MECOM/PAXIP1/NSD3/NSD2
Biological Process	GO:0070528	protein kinase C signaling	10/2279	32/18866	0.0033838	0.108982	0.0986108	10	AZU1/PRKCZ/WNT11/SPHK2/DGKQ/PDGFB/DGKD/ANKRD1/FLOT1/MYADM
Biological Process	GO:0006661	phosphatidylinositol biosynthetic process	25/2279	118/18866	0.0034209	0.1094339	0.0990197	25	SYNJ2/PIK3CD/VAC14/PIP5K1B/INPP5D/PIK3R1/PIGL/PIK3R6/PIGU/FPR2/PIGN/PITPNM2/INPP4A/PIK3R5/PIP4K2A/PITPNM1/SH3YL1/PLEKHA2/PDGFB/INPP5E/PI4K2A/PI4KA/PIGQ/RUFY1/INPP5F
Biological Process	GO:0051209	release of sequestered calcium ion into cytosol	25/2279	118/18866	0.0034209	0.1094339	0.0990197	25	TRPM2/PDE4D/CASQ2/ITPR2/LCK/CACNA1C/SLC8A1/UBASH3B/ITPR1/JSRP1/PTPN6/RASA3/P2RY6/MYO5A/HTT/PTPRC/NOL3/TPCN2/GSTO1/PRKCE/F2R/ANK2/CHERP/PLCG1/CASQ1

Biological Process	GO:1902904	negative regulation of supramolecular fiber organization	31/2279	156/18866	0.0034563	0.1101958	0.0997091	31	VILL/TBCD/TACSTD2/SSH1/GSN/KANK1/CAPG/ARAP1/NAV3/SSH3/CLU/PPFIA1/TMOD3/SVIL/PFN1/GMFB/SPTA1/MIR138-2/ADD1/ARHGEF18/AVIL/FLII/ARHGEF2/SPTAN1/TMOD1/GMFG/MYADM/SPTBN4/SPEF1/PRKN/CYRIB
Biological Process	GO:0052126	movement in host environment	33/2279	169/18866	0.0034891	0.1103755	0.0998716	33	TRIM27/P4HB/TRIM38/GSN/TRIM8/WWP2/SLC10A1/CD55/TRIM10/SLC1A5/CLEC5A/SNX3/UVRAG/ITGB5/PC/PHB/FCN1/TRIM5/VPS18/ITGB3/CTSB/TPCN2/NPC1/CDH1/SLC52A2/EPS15/TYRO3/ITGB6/SIVA1/TRIM26/GRK2/NECTIN1/NECTIN4
Biological Process	GO:0070374	positive regulation of ERK1 and ERK2 cascade	40/2279	215/18866	0.0034895	0.1103755	0.0998716	40	PRKCZ/CHI3L1/ANGPT1/RAP1B/ARRB1/PRKCA/TNF/CCL5/CSF1R/FPR2/C5AR2/GNAI2/APP/MIR24-2/MIR27A/MIR23A/MTURN/SCIMP/P2RY6/TGFB1/PHB/MARCO/RAPGEF2/PDGFD/BMPER/CCL20/PTPRC/PDGFB/ARRB2/NTRK1/NOD2/NOTCH1/GCNT2/F2R/PDGFC/PDGFRB/ARHGAP8/NODAL/CCL22/DENND2B
Biological Process	GO:0010927	cellular component assembly involved in morphogenesis	24/2279	112/18866	0.0034968	0.1103755	0.0998716	24	CD9/ACRBP/TTN/CASQ2/MYH11/AKAP13/TNNT3/PMP22/SYNPO2L/ILK/TMOD3/EPB41L3/ACTG1/WDR1/MEF2C/FLII/CNTNAP1/PDGFRB/ANK2/ANKRD1/TMOD1/CASQ1/ERCC2/MFSD14A
Biological Process	GO:0007221	positive regulation of transcription of Notch receptor target	7/2279	18/18866	0.0035236	0.1104876	0.0999731	7	MAML3/MAML1/MAML2/RBPJ/CREBBP/NOTCH1/NOTCH4
Biological Process	GO:0046597	negative regulation of viral entry into host cell	7/2279	18/18866	0.0035236	0.1104876	0.0999731	7	GSN/TRIM8/TRIM10/SNX3/FCN1/TRIM5/TRIM26
Biological Process	GO:2000379	positive regulation of reactive oxygen species metabolic process	23/2279	106/18866	0.0035592	0.1112355	0.1006498	23	HDAC4/CD177/SMAD3/DNM2/TNF/FPR2/TGFBR2/THBS1/RAB27A/RIPK1/GNAI2/NOS1AP/MIR24-2/CLU/TGFB1/HBB/RIPK3/PDGFB/NFE2L2/ITGB2/AGTR1/PDGFRB/SIRT3
Biological Process	GO:0007034	vacuolar transport	30/2279	150/18866	0.0036035	0.1118844	0.101237	30	SORL1/MGRN1/VTI1A/ANKFY1/VPS53/LYST/TGFBRAP1/VPS13D/KIF13A/SNX27/DENND3/BIN1/CLU/SMURF1/VPS18/CLEC16A/ATG14/HOOK2/NUMA1/GRN/SORT1/NPC1/ARSB/AP1G2/NEDD4/AP3D1/NCOA4/MVB12A/VPS51/PRKN
Biological Process	GO:0097530	granulocyte migration	30/2279	150/18866	0.0036035	0.1118844	0.101237	30	PRTN3/PDE4D/S100A8/FUT7/C3AR1/PIK3CD/RHOH/CSF3R/CD177/IL1R1/RHOG/CCL5/CSF1R/THBS1/C5AR2/PREX1/HRH1/JAM3/CAMK1D/CXCL17/CCL20/CXCR1/WDR1/ITGB2/DAPK2/DNM1L/DYSF/CCL22/VAV3/JAML

Biological Process	GO:1901342	regulation of vasculature development	73/2279	444/18866	0.0037106	0.114835	0.1039068	73	LRG1/RNH1/IL10/PPARG/C3AR1/MIR140/MAP3K3/MIR143/MIR145/RIN2/CHI3L1/HDAC7/RUNX1/ITGB1BP1/CCR2/PIK3R6/PRKCA/COL4A2/MIR199A1/TNF/TNFAIP3/NFATC2/MIR101-2/AGO1/HIPK2/SEMA4A/NFATC1/JUP/TGFBR2/THBS1/CTSH/DLL1/AKT3/MIR24-2/MIR27A/MIR23A/FLT1/JAK1/AGO2/RAPGEF2/EPN2/PRKCB/AQP1/NF1/PDGFD/BMPER/HK2/GHRL/IL6R/GRN/PDGFB/ETS1/NFE2L2/ENG/ITGB2/VASH1/MIR138-2/PKM/MIR199A2/NOTCH1/DNMT1/SP100/GPR4/AGTR1/PPP1R16B/HHEX/TNFSF12/PDE3B/PLCG1/NODAL/CX3CR1/CEMIP2/ADGRA2
Biological Process	GO:0060326	cell chemotaxis	54/2279	311/18866	0.0037609	0.115691	0.1046813	54	AZU1/TRPM2/PDE4D/S100A8/CALR/IL10/C3AR1/IL16/PIK3CD/ABCC1/CSF3R/LYST/CCR2/RHOG/CCL5/CSF1R/SFTPD/FPR2/PADI2/ACKR2/THBS1/C5AR2/PREX1/DEFB124/FLT1/PLEKHG5/CCR9/FGFR1/ZNF580/HRH1/PDGFD/JAM3/CAMK1D/CXCL17/CCL20/CXCR1/IL6R/C10orf99/PDGFB/CNR2/ARRB2/ITGB2/DAPK2/NOTCH1/DNM1L/AGTR1/PDGFRB/ANO6/DYSF/CCL27/CCL22/CX3CR1/VAV3/JAML
Biological Process	GO:0010944	negative regulation of transcription by competitive promoter binding	5/2279	10/18866	0.0038166	0.115691	0.1046813	5	CREB1/KDM2A/SMAD7/PHB/HHEX
Biological Process	GO:0015893	drug transport	5/2279	10/18866	0.0038166	0.115691	0.1046813	5	TMEM30A/ABCC2/ATP8B1/SLC19A1/ABCC3
Biological Process	GO:0034350	regulation of glial cell apoptotic process	5/2279	10/18866	0.0038166	0.115691	0.1046813	5	PRKCA/TRAF2/DLL1/PRKCH/PRKCI
Biological Process	GO:0030177	positive regulation of Wnt signaling pathway	35/2279	183/18866	0.0038182	0.115691	0.1046813	35	RNF220/KANK1/CSNK1D/TNKS/MIR145/SKI/SMAD3/ZEB2/TNFAIP3/FAM53B/JUP/DISC1/RBPJ/ILK/CDH3/TGFB1/AXIN1/LRRK1/CSNK1E/PSMF1/ARNTL/PSMB7/TTC21B/ZBED3/HHEX/ANKRD6/RUVBL1/ZBED2/PPM1N/GID8/DAPK3/PSMD13/ADGRA2/ATP6V0C/CCN4
Biological Process	GO:0060070	canonical Wnt signaling pathway	58/2279	339/18866	0.0038321	0.115691	0.1046813	58	RNF220/KANK1/CSNK1D/TNKS/CCDC88C/MIR145/SMAD3/AMFR/FAM53B/UBAC2/JUP/CUL3/DISC1/RBPJ/TCF7L2/CDK14/ILK/LIMD1/HDAC1/LATS2/WNT11/CDH3/TGFB1/KREMEN1/AXIN1/TLE1/LRRK1/WWTR1/RARG/TMEM64/WNT5B/STK3/CSNK1E/PSMF1/KREMEN2/ARNTL/FRAT2/PSMB7/TTC21B/NOTCH1/ZBED3/FOXO1/CHD8/CYLD/ANKRD6/RUVBL1/ZBED2/PPM1N/PTPRU/CCNY/GID8/DAPK3/PSMD13/NPHP4/TMEM131L/ADGRA2/PRKN/PLPP3
Biological Process	GO:0051098	regulation of binding	62/2279	367/18866	0.0038512	0.115691	0.1046813	62	HDAC4/SLPI/CTSZ/ZFPM1/MARK3/SORL1/IL10/PPARG/TNKS/ANGPT1/SKI/HMBOX1/ITGB1BP1/SMAD3/AMFR/ARRB1/ARHGEF7/UBASH3B/HIPK2/CCM2L/DISC1/APP/MIR27A/TCF7L2/TAF10/CPNE1/PAX7/TGFB1/RAPGEF2/RARA/CSF3/CTBP2/STK3/ATP2A3/CSNK1E/PDGFB/RSF1/SPTA1/ZNF462/ARRB2/PLCL1/PER2/ADD1/RB1/MED25/CDCA5/SP100/CYLD/MARK2/BCL3/CLN5/FLOT1/DTNBP1/ERCC2/PARP9/LIF/EIF4G1/TFIP11/STING1/PRKN/ILRUN/FBH1
Biological Process	GO:0006638	neutral lipid metabolic process	28/2279	138/18866	0.0038971	0.115691	0.1046813	28	PNPLA2/PLA2G15/SORL1/MGLL/ACSL1/MFSD2A/LIPC/ABHD16A/GPLD1/PGS1/DGKQ/NR1H2/ATG14/GNB3/ABHD16B/DGKA/DGKD/FABP6/CAT/AVIL/LMF1/ABHD5/PCK2/SREBF1/CPT1A/LPIN1/LYPLA2/AGPAT5

Biological Process	GO:0006639	acylglycerol metabolic process	28/2279	138/18866	0.0038971	0.115691	0.1046813	28	PNPLA2/PLA2G15/SORL1/MGLL/ACSL1/MFSD2A/LIPC/ABHD16A/GPLD1/PGS1/DGKQ/NR1H2/ATG14/GNB3/ABHD16B/DGKA/DGKD/FABP6/CAT/AVIL/LMF1/ABHD5/PCK2/SREBF1/CPT1A/LPIN1/LYPLA2/AGPAT5
Biological Process	GO:0031958	corticosteroid receptor signaling pathway	6/2279	14/18866	0.0039087	0.115691	0.1046813	6	CALR/YWHAH/PHB/ARNTL/PER1/NEDD4
Biological Process	GO:0045217	cell-cell junction maintenance	6/2279	14/18866	0.0039087	0.115691	0.1046813	6	PRTN3/CD177/CSF1R/PLEKHA7/F2R/KIFC3
Biological Process	GO:0072672	neutrophil extravasation	6/2279	14/18866	0.0039087	0.115691	0.1046813	6	PRTN3/FUT7/PIK3CD/CD177/IL1R1/JAML
Biological Process	GO:1900044	regulation of protein K63-linked ubiquitination	6/2279	14/18866	0.0039087	0.115691	0.1046813	6	OTUB2/MIR138-2/OTUB1/NOD2/TRIP12/PARP10
Biological Process	GO:1901569	fatty acid derivative catabolic process	6/2279	14/18866	0.0039087	0.115691	0.1046813	6	BDH1/ABHD16A/ACOT7/CYP4F3/ACAT1/LYPLA2
Biological Process	GO:1903555	regulation of tumor necrosis factor superfamily cytokine production	24/2279	113/18866	0.0039426	0.1160967	0.1050484	24	TRIM27/FOXP1/IL10/MIR140/ANGPT1/PIK3R1/TNFAIP3/TLR9/SPON2/RIPK1/C5AR2/PTPN6/CLU/PTPRJ/RARA/PTPRC/ARRB2/NOD2/ARHGEF2/LTF/CD33/FADD/VSIR/ILRUN
Biological Process	GO:0002369	T cell cytokine production	12/2279	43/18866	0.0039469	0.1160967	0.1050484	12	PRKCZ/CD55/SLC11A1/TNFRSF1B/NLRP3/IL1R1/CCR2/TRAF2/HLA-F/SMAD7/IL18RAP/IL18R1
Biological Process	GO:2001056	positive regulation of cysteine-type endopeptidase activity	30/2279	151/18866	0.0039893	0.1169816	0.1058491	30	NLRC4/S100A8/GRAMD4/AIM2/PPARG/GSN/LCK/NLRP3/SMAD3/ARRB1/MEFV/TNF/TRAF2/CYFIP2/TBC1D10A/CTSD/CTSH/RIPK1/CIDEB/PERP/BCL10/ATP2A3/FIS1/P2RX1/F2R/CASP1/BCL2L13/ASPH/NODAL/FADD

Biological Process	GO:0009416	response to light stimulus	55/2279	319/18866	0.0040481	0.1174915	0.1063104	55	ELANE/RCVRN/PDE6A/MFAP4/CREB1/PPP1R1B/BRCA2/CACNA2D4/CRB1/PIK3R1/BMF/ARRB1/DNM2/HUS1/TANC1/APP/PITPNM1/FECH/CCDC66/GNAT2/SYNGAP1/CDC25A/HRH1/AQP1/MTA1/NF1/SLC1A2/GNA11/MEIS2/CREBBP/CAT/PPP1CB/COPS3/PER2/SLC1A3/BCL2/PER1/FBXL6/GRK7/RHNO1/NEDD4/ERCC1/CRIP1/USP2/HMGCS1/RELA/BCL3/EYS/POLD3/XPA/CABP4/ERCC2/SYNPO/NPHP4/PRKAA1
Biological Process	GO:0048588	developmental cell growth	43/2279	237/18866	0.0040752	0.1174915	0.1063104	43	SEMA6B/PRKCZ/CPNE6/NRP2/CTDP1/ULK1/AKAP13/PRMT2/CTTN/DNM2/MIR199A1/CPNE5/ZEB2/CYFIP2/BCL11A/SEMA4A/RTN4/TGFBR2/DISC1/SEMA4B/APP/MIR24-2/MIR23A/IFRD1/ILK/SMAD7/CPNE1/SEMA4D/SMURF1/KMT2D/RARG/CDH4/MIR199A2/CDKL3/SYT17/SLIT3/CYFIP1/TRPV2/NDEL1/SLIT1/DBNL/SPART/PRKN
Biological Process	GO:0000209	protein polyubiquitination	58/2279	340/18866	0.0040898	0.1174915	0.1063104	58	TRIM27/RNF144A/FZR1/MGRN1/RNF19A/TRIM38/WWP2/TNKS/LNPEP/AMFR/TRAF2/TNFAIP3/TRAF1/FBXO7/RUSC1/CUL3/DCAF15/UBE2O/OTUB2/CDC16/FBXW2/UBE2J2/FBXO40/SMURF1/LMO7/TRIM5/UBE2V1/UBE2Q2/FBXL19/PSMF1/RNF213/FBXL13/MIR138-2/FBXL18/RNF144B/OTUB1/NOD2/BCL2/PSMB7/TRAF3IP2/UBE3A/NEDD4/ANAPC7/TRIP12/RNF14/DYSF/HECW2/FBXW11/RNF40/PARP10/HERC5/UBE2D2/PELI1/PSMD13/MARCHF6/MARCHF8/PRKN/MARCHF7
Biological Process	GO:0010506	regulation of autophagy	59/2279	347/18866	0.0040968	0.1174915	0.1063104	59	FOXK1/TRIM27/RPTOR/PRKAG2/IL10/TRIM38/TRIM8/SPTLC2/ULK1/ERN1/VPS13D/BMF/CTTN/MIR199A1/MEFV/RHEB/FBXO7/LARP1/ITPR1/CAMKK2/USP36/PIP4K2A/VPS26B/ATP6V1B2/FEZ2/PRKAB1/UVRAG/TMEM150B/SMURF1/RUFY4/TRIM5/CLEC16A/HTT/HK2/SH3BP4/GFAP/TPCN2/TBC1D14/NPC1/DAPK2/BCL2/MIR199A2/DNM1L/ATP6V0B/NEDD4/TSC2/FOXO1/SOGA1/SREBF1/XPA/EIF4G1/DAPK3/SVIP/NPRL2/PRKAA1/STING1/PRKN/ATP6V0C/ATP6V1C1
Biological Process	GO:0051222	positive regulation of protein transport	60/2279	354/18866	0.0041005	0.1174915	0.1063104	60	PRKCZ/SORL1/NADK/IL10/TFDP1/TTN/C1QTNF3/PRR5L/TM9SF4/MYRIP/CLEC9A/TMEM30A/YWHAH/ITGB1BP1/PIK3R1/SMAD3/FRMD4A/ARRB1/GPLD1/FGR/TNF/MYO18A/ABCG1/CLEC5A/JUP/EDEM1/USP36/ZDHHC2/TCF7L2/ABLIM3/AKAP5/UBE2J2/TGFB1/GIPR/BLK/NR1H2/GHRL/EDEM2/ITGB2/MYOM1/YWHAZ/CDH1/FIS1/BCL2/DNM1L/PRKCE/TP53BP2/MYO1C/MIEF1/ANKRD1/CADM1/YWHAQ/CD33/RPH3AL/EZR/PDZK1/C2CD2L/SIRT3/PRKAA1/RIPOR1
Biological Process	GO:0018027	peptidyl-lysine dimethylation	8/2279	23/18866	0.0041056	0.1174915	0.1063104	8	SETD2/EHMT2/KMT2D/SETD1B/SETD1A/PIH1D1/EEF1AKMT2/CSKMT
Biological Process	GO:0050860	negative regulation of T cell receptor signaling pathway	8/2279	23/18866	0.0041056	0.1174915	0.1063104	8	DUSP22/PTPN6/PTPRJ/LILRB4/DUSP3/ELF1/EZR/SLA2
Biological Process	GO:1904996	positive regulation of leukocyte adhesion to vascular endothelial cell	8/2279	23/18866	0.0041056	0.1174915	0.1063104	8	ELANE/FUT4/FUT7/CCR2/TNF/ETS1/ITGB2/RELA
Biological Process	GO:0002718	regulation of cytokine production involved in immune response	19/2279	83/18866	0.0041481	0.1180299	0.1067977	19	PRKCZ/IL10/TNFRSF1B/NLRP3/IL1R1/CCR2/TNF/TRAF2/SPON2/CD226/HLA-F/SMAD7/SCIMP/TGFB1/SLC7A5/BCL10/NOD2/IL18R1/CD96

Biological Process	GO:0001892	embryonic placenta development	20/2279	89/18866	0.0041603	0.1180299	0.1067977	20	IL10/SETD2/SPINT2/E2F7/RBPJ/SP3/DNAJB6/STK3/GRHL2/NCOA1/PDGFB/VASH1/EPAS1/GGNBP2/TMED2/CEBPB/PCDH12/PLCD3/NODAL/LIF
Biological Process	GO:0006479	protein methylation	35/2279	184/18866	0.0041804	0.1180299	0.1067977	35	GFI1/BRD4/METTL21A/MYB/PRMT2/ASH2L/SNRPD3/SETD2/SMYD3/EHMT2/WDR82/PAX7/KMT2D/KDM4C/SETD1B/SETD1A/CREBBP/TET2/ZNF335/METTL22/PRDM16/PHF19/DNMT1/PIH1D1/DYDC2/DYDC1/NTMT1/FBL/MECOM/PAXIP1/EEF1AKMT3/EEF1AKMT2/NSD3/NSD2/CSKMT
Biological Process	GO:0008213	protein alkylation	35/2279	184/18866	0.0041804	0.1180299	0.1067977	35	GFI1/BRD4/METTL21A/MYB/PRMT2/ASH2L/SNRPD3/SETD2/SMYD3/EHMT2/WDR82/PAX7/KMT2D/KDM4C/SETD1B/SETD1A/CREBBP/TET2/ZNF335/METTL22/PRDM16/PHF19/DNMT1/PIH1D1/DYDC2/DYDC1/NTMT1/FBL/MECOM/PAXIP1/EEF1AKMT3/EEF1AKMT2/NSD3/NSD2/CSKMT
Biological Process	GO:0007223	Wnt signaling pathway, calcium modulating pathway	11/2279	38/18866	0.0041865	0.1180299	0.1067977	11	PDE6A/TNRC6B/AGO1/NFATC1/TCF7L2/GNAT2/WNT11/AGO2/PLCB1/GNG2/GNAO1
Biological Process	GO:0042093	T-helper cell differentiation	15/2279	60/18866	0.004319	0.1198569	0.1084508	15	IL27/PRKCZ/ZFPM1/FOXP1/MYB/NLRP3/LOXL3/SEMA4A/SMAD7/LY9/ZBTB7B/RARA/IL18R1/HLX/BCL3
Biological Process	GO:0042692	muscle cell differentiation	65/2279	390/18866	0.0043296	0.1198569	0.1084508	65	HDAC4/EHD1/RXRA/CALR/CD9/CTDP1/MYO18B/TTN/CASQ2/MIR140/MIR145/MYH11/SYNE1/AKAP13/SKI/MAML1/SLC8A1/MIR199A1/TANC1/NFATC2/TNNT3/NFATC1/BIN1/SMYD3/MYH9/EPC1/SYNPO2L/DLL1/RBPJ/MIR24-2/MIR23A/IFRD1/TGFB1/FBXO40/TMOD3/RARA/ACTG1/PDGFB/WDR1/ENG/ARRB2/SORT1/ANKRD2/RB1/BCL2/TCF3/MIR199A2/NOTCH1/DNMT1/MEF2C/FHL2/FLII/PLD3/PDGFRB/DYSF/ANK2/ANKRD1/TMOD1/FLOT1/SCGB3A1/CASQ1/SYPL2/CEACAM5/MYORG/MRTFA
Biological Process	GO:0010821	regulation of mitochondrion organization	36/2279	191/18866	0.0043351	0.1198569	0.1084508	36	VAT1/TFDP1/YWHAH/INF2/VPS13D/PAM16/BMF/CTTN/FBXO7/CAMKK2/USP36/CLU/ABLIM3/UBE2J2/CIDEB/SMURF1/CLEC16A/HTT/HK2/NOL3/ARRB2/FNIP2/YWHAZ/FIS1/BCL2/DNM1L/TP53BP2/MIEF1/TSC2/BCL2L1/SREBF1/YWHAQ/DHODH/PRKAA1/PRKN/CYRIB
Biological Process	GO:0043552	positive regulation of phosphatidylinositol 3-kinase activity	10/2279	33/18866	0.0043378	0.1198569	0.1084508	10	FGR/FPR2/FLT1/TGFB1/VAV2/ATG14/PDGFB/NOD2/PDGFRB/VAV3
Biological Process	GO:0045920	negative regulation of exocytosis	10/2279	33/18866	0.0043378	0.1198569	0.1084508	10	ABR/RAP1B/CCR2/CD84/GNAI2/HLA-F/BCR/NOTCH1/CBARP/PRKN

Biological Process	GO:0022011	myelination in peripheral nervous system	9/2279	28/18866	0.0043396	0.1198569	0.1084508	9	ARHGEF10/SH3TC2/SKI/LGI4/NDRG1/ILK/NF1/FA2H/CNTNAP1
Biological Process	GO:0032292	peripheral nervous system axon ensheathment	9/2279	28/18866	0.0043396	0.1198569	0.1084508	9	ARHGEF10/SH3TC2/SKI/LGI4/NDRG1/ILK/NF1/FA2H/CNTNAP1
Biological Process	GO:0016482	cytosolic transport	32/2279	165/18866	0.0044289	0.1219674	0.1103604	32	TRIM27/LMTK2/DCTN1/SORL1/AP2A1/STX5/VTI1A/ANKFY1/VPS53/HEATR5A/TRAPPC10/TBC1D10A/ERC1/SNX3/VPS26B/CORO7/UBE2O/HOOK2/NUMA1/TBC1D14/SORT1/KIF1B/SGSM2/EPS15/WDR81/CLN5/EZR/VPS51/CLTCL1/PHETA1/PRKN/DOP1B
Biological Process	GO:0007249	I-kappaB kinase/NF-kappaB signaling	50/2279	286/18866	0.0044923	0.1230021	0.1112967	50	TRIM39/TRIM27/BRD4/TRIM38/TRIM8/MAP3K3/RHOH/AKAP13/ANGPT1/TNF/TRAF2/TNFAIP3/CXXC5/TRAF1/TLR9/ERC1/RIPK1/TNFRSF1A/HDAC1/CPNE1/DEFB124/PLEKHG5/SPHK2/LITAF/PRKCB/TLE1/RIPK3/TRIM5/UBE2V1/TNIP1/BCL10/ZDHHC17/MAP3K14/NEK6/IRAK2/NOD2/PER1/PRKCE/TRAF3IP2/F2R/LTF/CASP1/RELA/BCL3/PPM1N/CX3CR1/FADD/PELI1/EEF1D/PRKN
Biological Process	GO:0043542	endothelial cell migration	50/2279	286/18866	0.0044923	0.1230021	0.1112967	50	S100P/FOXP1/CALR/NRP2/PPARG/MAP3K3/MIR143/RIN2/HDAC7/ANGPT1/ITGB1BP1/PRKCA/MIR199A1/GPLD1/TNF/MIR101-2/SEMA4A/STAT5A/JUP/THBS1/MYH9/AKT3/MIR24-2/MIR27A/MIR23A/MIA3/PXN/PLEKHG5/FGFR1/TGFB1/ZNF580/NF1/BMPER/ITGB3/GRN/PDGFB/ETS1/NFE2L2/ITGB2/VASH1/MIR199A2/NOTCH1/MEF2C/SP100/TNFSF12/PLCG1/ADTRP/PAXIP1/ADGRA2/PLPP3
Biological Process	GO:0048207	vesicle targeting, rough ER to cis-Golgi	16/2279	66/18866	0.0045242	0.1231645	0.1114436	16	CTSZ/SERPINA1/TGFA/STX5/CSNK1D/TRAPPC10/CUL3/TRAPPC9/CNIH2/CD59/SEC13/ANKRD28/SEC24C/TMED2/SEC31A/TRAPPC2
Biological Process	GO:0048208	COPII vesicle coating	16/2279	66/18866	0.0045242	0.1231645	0.1114436	16	CTSZ/SERPINA1/TGFA/STX5/CSNK1D/TRAPPC10/CUL3/TRAPPC9/CNIH2/CD59/SEC13/ANKRD28/SEC24C/TMED2/SEC31A/TRAPPC2
Biological Process	GO:0043122	regulation of I-kappaB kinase/NF-kappaB signaling	45/2279	252/18866	0.0045578	0.1237237	0.1119496	45	TRIM39/TRIM27/BRD4/TRIM38/TRIM8/MAP3K3/RHOH/AKAP13/ANGPT1/TNF/TRAF2/TNFAIP3/CXXC5/TRAF1/TLR9/RIPK1/TNFRSF1A/HDAC1/CPNE1/PLEKHG5/SPHK2/LITAF/PRKCB/TLE1/TRIM5/UBE2V1/TNIP1/BCL10/ZDHHC17/MAP3K14/NEK6/NOD2/PER1/PRKCE/TRAF3IP2/F2R/LTF/CASP1/RELA/PPM1N/CX3CR1/FADD/PELI1/EEF1D/PRKN
Biological Process	GO:0046631	alpha-beta T cell activation	29/2279	146/18866	0.0046061	0.1246791	0.1128141	29	IL27/PRKCZ/ZFPM1/FOXP1/FUT7/MYB/RUNX1/CD55/NLRP3/ITPKB/CCR2/LOXL3/SEMA4A/ZBTB16/TGFBR2/SMAD7/LY9/ZBTB7B/RARA/PTPRC/PNP/BCL2/IL18R1/HLX/RUNX3/DOCK2/AP3D1/BCL3/VSIR

Biological Process	GO:0045862	positive regulation of proteolysis	62/2279	370/18866	0.0046364	0.1247895	0.112914	62	NLRC4/S100A8/RNF144A/ENO1/FZR1/GRAMD4/RNF19A/AIM2/PPARG/GSN/CSNK1D/LCK/TNFRSF1B/NLRP3/SMAD3/ARRB1/MEFV/GPLD1/TNF/TRAF2/CYFIP2/TBC1D10A/CBFA2T3/CTSD/DISC1/EDEM1/MYH9/CTSH/RIPK1/APP/MBP/CLU/SMAD7/CIDEB/F12/SMURF1/AXIN1/PERP/TNIP1/EDEM2/BCL10/ATP2A3/CSNK1E/GRN/NFE2L2/RNF144B/FIS1/P2RX1/F2R/CASP1/BCL2L13/RNF14/TRIB1/ASPH/FBXW11/AURKAIP1/RNF40/NODAL/FADD/PRKN/ANGPTL8/VSIR
Biological Process	GO:1904951	positive regulation of establishment of protein localization	62/2279	370/18866	0.0046364	0.1247895	0.112914	62	PRKCZ/SORL1/NADK/IL10/TFDP1/TTN/C1QTNF3/PRR5L/TM9SF4/MYRIP/CLEC9A/TMEM30A/YWHAH/ITGB1BP1/PIK3R1/SMAD3/FRMD4A/ARRB1/GPLD1/FGR/TNF/MYO18A/ABCG1/CLEC5A/JUP/EDEM1/USP36/ZDHHC2/CEP135/TCF7L2/ABLIM3/AKAP5/UBE2J2/TGFB1/GIPR/BLK/NR1H2/GHRL/EDEM2/ITGB2/MYOM1/YWHAZ/CDH1/FIS1/BCL2/DNM1L/PRKCE/TP53BP2/MYO1C/MIEF1/ANKRD1/CADM1/YWHAQ/CD33/RPH3AL/EZR/PDZK1/CCT6A/C2CD2L/SIRT3/PRKAA1/RIPOR1
Biological Process	GO:0030595	leukocyte chemotaxis	42/2279	232/18866	0.0046739	0.1254426	0.113505	42	AZU1/TRPM2/PDE4D/S100A8/CALR/IL10/C3AR1/IL16/PIK3CD/CSF3R/LYST/CCR2/CCL5/CSF1R/SFTPD/FPR2/PADI2/THBS1/C5AR2/PREX1/DEFB124/FLT1/ZNF580/HRH1/JAM3/CAMK1D/CXCL17/CCL20/CXCR1/IL6R/C10orf99/PDGFB/CNR2/ITGB2/DAPK2/DNM1L/ANO6/DYSF/CCL27/CCL22/VAV3/JAML
Biological Process	GO:0034329	cell junction assembly	71/2279	434/18866	0.0047117	0.1261005	0.1141002	71	TBCD/SPOCK2/CD9/COL17A1/TLN2/HDAC7/RUNX1/ITGB1BP1/RAP1B/SMAD3/PRKCA/ARHGEF7/CTTN/COLQ/TNF/GJA5/EPHB3/DUSP22/WHAMM/SEMA4A/JUP/PKP1/THBS1/TNS1/ACTN1/APP/ACTN4/ILK/SMAD7/WNT11/FARP1/PRKCH/PTPRJ/SEMA4D/RAPGEF2/EPB41L3/NRXN1/KIRREL3/JAM3/TESK2/GHRL/ACTG1/GRHL2/BCR/WDR1/NRXN2/PRKCI/NTRK1/CDH1/MPP7/BCL2/DNM3/CORO1C/VMP1/MEF2C/DUSP3/SHANK2/SDK2/CNTNAP1/MYO1C/ANK2/LAMB3/CDH11/FLOT1/SLIT1/CUX2/EIF4G1/DAPK3/NPHP4/DBNL/NECTIN1
Biological Process	GO:0044272	sulfur compound biosynthetic process	36/2279	192/18866	0.0047302	0.1261005	0.1141002	36	CSGALNACT1/ACSL1/DSE/ANGPT1/CHSY1/GGT1/CBS/CHST11/CHST15/CNDP2/NDST1/CHST2/ACSS1/XYLT1/SLC26A1/GSTM5/ACACB/MGST2/ST3GAL4/ACSS2/B4GALT3/NFE2L2/MTHFD1/PAPSS2/GSTO1/ACACA/ACSF3/MAT1A/ADI1/ACAT1/SCD/GSTO2/SLC25A1/ST3GAL2/B3GNT2/B4GAT1
Biological Process	GO:0043367	CD4-positive, alpha-beta T cell differentiation	18/2279	78/18866	0.0047382	0.1261005	0.1141002	18	IL27/PRKCZ/ZFPM1/FOXP1/FUT7/MYB/RUNX1/NLRP3/LOXL3/SEMA4A/SMAD7/LY9/ZBTB7B/RARA/IL18R1/HLX/RUNX3/BCL3
Biological Process	GO:0007266	Rho protein signal transduction	28/2279	140/18866	0.0048042	0.1275003	0.1153668	28	ARHGEF10/KANK1/AKAP13/MYO9B/ARHGDIB/RHOG/ARRB1/CUL3/CDC42EP1/SYNPO2L/PLEKHG5/EPS8L3/EPS8L1/ABCA1/BCR/ARHGEF25/ARHGEF18/GNA12/F2R/ARHGEF28/ARHGEF2/GPR4/AGTR1/PDGFRB/ARHGAP1/FLOT1/PRAG1/RIPOR1
Biological Process	GO:0002696	positive regulation of leukocyte activation	67/2279	406/18866	0.0048289	0.1277735	0.115614	67	HLA-DMB/PRKCZ/IL10/RHOH/LCK/MIR145/MYB/RUNX1/SIRPB1/CD55/INPP5D/CD177/NLRP3/PIK3R1/ITPKB/CCR2/PIK3R6/GAB2/FGR/CCL5/GRAP2/NCK2/NFATC2/TNFSF13B/TLR9/IGHV6-1/ZBTB16/TGFBR2/THBS1/CD226/HLA-F/PTPN6/ZMIZ1/DOCK8/SPHK2/ZBTB7B/TGFB1/RARA/LILRB4/CCDC88B/PTPRC/CD320/CSK/BCL10/SPTA1/ZNF335/PNP/ITGB2/NOD2/BCL2/TP53BP1/MEF2C/SIRPG/HLX/RUNX3/TNFSF13/CYLD/AP3D1/SLC7A1/FADD/PELI1/PCID2/PAXIP1/VAV3/CYRIB/VSIR/NSD2
Biological Process	GO:0030835	negative regulation of actin filament depolymerization	12/2279	44/18866	0.0048414	0.1277735	0.115614	12	VILL/GSN/CAPG/TMOD3/SVIL/SPTA1/ADD1/AVIL/FLII/SPTAN1/TMOD1/SPTBN4

Biological Process	GO:0006874	cellular calcium ion homeostasis	74/2279	456/18866	0.0048655	0.1280527	0.1158666	74	ELANE/TRPM2/PDE4D/CALR/C3AR1/THADA/CASQ2/ITPR2/LCK/HEXB/CD55/CACNA1C/SLC24A4/VDR/CCR2/SMAD3/SLC8A1/MIR199A1/ANXA6/UBASH3B/CDH23/CCL5/PACS2/FPR2/FPR1/ATP2B2/ITPR1/ACKR2/DISC1/JSRP1/C5AR2/APP/PTPN6/RASA3/GNAT2/P2RY6/CCR9/PRKCB/TMEM178A/MYO5A/GIPR/HTT/GHRL/PTPRC/TMEM64/CXCR1/ATP2A3/NOL3/TPCN2/IMMT/FIS1/BCL2/MIR199A2/GSTO1/P2RX1/LPAR2/PRKCE/F2R/PDZD8/S1PR4/GPR4/AGTR1/ANK2/CHERP/PLCG1/ATP7B/P2RX5/TRPV2/CASQ1/GRINA/SYPL2/ANXA7/CX3CR1/SYNPO
Biological Process	GO:0010632	regulation of epithelial cell migration	52/2279	301/18866	0.0048804	0.1280908	0.1159011	52	RREB1/TACSTD2/FOXP1/CALR/NRP2/PPARG/MAP3K3/MIR143/RIN2/HDAC7/ANGPT1/DOCK1/ITGB1BP1/PRKCA/MIR199A1/GPLD1/TNF/MIR101-2/SEMA4A/STAT5A/RTN4/JUP/TGFBR2/THBS1/CTSH/AKT3/MIR24-2/MIR27A/MIR23A/FGFR1/TGFB1/ZNF580/IQSEC1/NF1/PFN1/BMPER/ITGB3/GRN/PDGFB/ETS1/NFE2L2/VASH1/ARSB/MIR199A2/NOTCH1/CORO1C/MEF2C/PRKCE/SP100/PLCG1/ADGRA2/PLPP3
Biological Process	GO:0072503	cellular divalent inorganic cation homeostasis	79/2279	492/18866	0.0048979	0.1281962	0.1159965	79	ELANE/TRPM2/PDE4D/S100A8/CALR/C3AR1/THADA/CASQ2/ITPR2/LCK/HEXB/CD55/CACNA1C/SLC11A1/SLC24A4/VDR/CCR2/SMAD3/SLC8A1/MIR199A1/ANXA6/UBASH3B/CDH23/CCL5/PACS2/FPR2/FPR1/ATP2B2/ITPR1/ACKR2/DISC1/JSRP1/C5AR2/APP/PTPN6/RASA3/GNAT2/P2RY6/CCR9/PRKCB/TMEM178A/MYO5A/GIPR/HTT/GHRL/PTPRC/TMEM64/CXCR1/ATP2A3/NOL3/TPCN2/IMMT/FIS1/BCL2/MIR199A2/GSTO1/SLC39A13/P2RX1/LPAR2/PRKCE/F2R/PDZD8/S1PR4/GPR4/AGTR1/ANK2/AP3D1/CHERP/PLCG1/ATP7B/P2RX5/TRPV2/CASQ1/GRINA/SYPL2/ANXA7/CX3CR1/SYNPO/SLC41A1
Biological Process	GO:0032869	cellular response to insulin stimulus	41/2279	226/18866	0.0049565	0.1292147	0.1169181	41	PRKCZ/SORL1/FUT7/PPARG/KANK1/PTPRE/PIK3R1/GPLD1/RAB31/BAIAP2L1/BAIAP2/GRB10/GNAI2/ATP6V1B2/ZDHHC7/IGF1R/ZBTB7B/FER/PRKCB/MYO5A/PRKCI/INSRR/PKM/ATP6V0B/BCAR3/PCK2/MYO1C/TSC2/FOXO1/SOGA1/SREBF1/PDE3B/CYFIP1/RELA/WDTC1/NDEL1/LPIN1/PRKAA1/MARS1/ATP6V0C/ATP6V1C1
Biological Process	GO:0006023	aminoglycan biosynthetic process	24/2279	115/18866	0.0049776	0.1292147	0.1169181	24	CSGALNACT1/AP2A1/HS3ST3B1/DSE/ANGPT1/CHSY1/CHST11/CHST15/NDST1/CHST2/SDC2/XYLT1/TGFB1/B4GALT7/PDGFB/ST3GAL4/B4GALT3/GCNT2/PDGFRB/HAS3/ST3GAL2/B3GNT2/B4GAT1/PXYLP1
Biological Process	GO:0031623	receptor internalization	24/2279	115/18866	0.0049776	0.1292147	0.1169181	24	CD9/ANGPT1/ARRB1/DNM2/MIR199A1/RAB31/ANKRD13B/ANKRD13D/NUMB/ITGB3/ANKRD13A/CXCR1/GSG1L/ARRB2/ITGB2/DNM1/DNM3/MIR199A2/EPS15/NEDD4/MX1/EZR/FLOT1/GRK2
Biological Process	GO:0003417	growth plate cartilage development	7/2279	19/18866	0.005004	0.1295453	0.1172172	7	ANXA6/TGFBR2/POC1A/RARA/POR/RARG/NPPC
Biological Process	GO:0050867	positive regulation of cell activation	69/2279	421/18866	0.0050525	0.130444	0.1180304	69	HLA-DMB/PRKCZ/IL10/RPS6KA1/RHOH/LCK/MIR145/MYB/RUNX1/SIRPB1/CD55/INPP5D/CD177/NLRP3/PIK3R1/ITPKB/CCR2/PIK3R6/GAB2/FGR/CCL5/GRAP2/NCK2/NFATC2/TNFSF13B/TLR9/IGHV6-1/ZBTB16/TGFBR2/THBS1/CD226/HLA-F/PTPN6/ZMIZ1/DOCK8/SPHK2/ZBTB7B/TGFB1/RARA/LILRB4/CCDC88B/PTPRC/CD320/CSK/BCL10/SPTA1/ZNF335/PNP/ITGB2/NOD2/BCL2/TP53BP1/MEF2C/SIRPG/HLX/RUNX3/PDGFRB/TNFSF13/CYLD/AP3D1/SLC7A1/FADD/PELI1/PCID2/PAXIP1/VAV3/CYRIB/VSIR/NSD2
Biological Process	GO:0007163	establishment or maintenance of cell polarity	40/2279	220/18866	0.0052554	0.1353151	0.1224379	40	PRKCZ/PARVB/DCTN1/GSN/KANK1/RHOH/NDE1/CRB1/RAP1B/MAP7/RHOG/FRMD4A/SPINT2/SNX27/MYO18A/MYH9/RHOF/ILK/WNT11/DOCK8/FRMD4B/RHOBTB2/AQP1/JAM3/HTT/NUMA1/WDR1/PRKCI/SDCCAG8/MPP7/PARVG/ARHGEF2/FSCN3/NEK3/CDC42BPB/DOCK2/MARK2/FBXW11/NDEL1/CYRIB

Biological Process	GO:0046513	ceramide biosynthetic process	16/2279	67/18866	0.0052913	0.1358728	0.1229426	16	SPTLC2/TNF/ORMDL3/CERS6/TNFRSF1A/SPHK2/SGMS1/ST8SIA6/SGMS2/B4GALT3/P2RX1/FA2H/CERS2/SIRT3/PRKAA1/TLCD3B
Biological Process	GO:0035710	CD4-positive, alpha-beta T cell activation	21/2279	97/18866	0.0053429	0.1362154	0.1232526	21	IL27/PRKCZ/ZFPM1/FOXP1/FUT7/MYB/RUNX1/CD55/NLRP3/LOXL3/SEMA4A/TGFBR2/SMAD7/LY9/ZBTB7B/RARA/IL18R1/HLX/RUNX3/BCL3/VSIR
Biological Process	GO:0120162	positive regulation of cold-induced thermogenesis	21/2279	97/18866	0.0053429	0.1362154	0.1232526	21	ZNF516/LCN2/ACSL1/CCR2/DYNC1H1/GRB10/PRKAB1/IGF1R/ZBTB7B/GHRL/PRDM16/PER2/EPAS1/PDGFC/CEBPB/GADD45G/SCD/TRPV2/LPIN1/G0S2/BSCL2
Biological Process	GO:0032456	endocytic recycling	13/2279	50/18866	0.0053514	0.1362154	0.1232526	13	EHD1/LMTK2/SORL1/VPS53/DENND1A/ACAP2/SNX3/MICALL1/EPS15/ARHGAP1/INPP5F/VPS51/VPS35L
Biological Process	GO:0002706	regulation of lymphocyte mediated immunity	30/2279	154/18866	0.005362	0.1362154	0.1232526	30	PRKCZ/FUT7/IL10/TNFRSF1B/NLRP3/IL1R1/CCR2/PIK3R6/TNF/TRAF2/DUSP22/CD226/HLA-F/PTPN6/SMAD7/TGFB1/NCR1/RIPK3/PTPRC/ARRB2/IL18RAP/TP53BP1/IL18R1/TNFSF13/CADM1/CD96/FADD/PAXIP1/CYRIB/NSD2
Biological Process	GO:0015914	phospholipid transport	20/2279	91/18866	0.0054183	0.1369558	0.1239225	20	OSBPL5/ABCC1/MFSD2A/TMEM30A/ABCG1/ATP8A1/ANO7/PLTP/PITPNM2/ATP11A/ATP8B1/PITPNM1/PITPNA/PCTP/ABCA1/OSBPL10/ATP8B4/PITPNC1/ANO6/C2CD2L
Biological Process	GO:0030851	granulocyte differentiation	10/2279	34/18866	0.0054914	0.1369558	0.1239225	10	CEBPE/ZFPM1/RUNX1/INPP5D/CBFA2T3/TESC/RARA/SP3/CSF3/TRIB1
Biological Process	GO:0043276	anoikis	10/2279	34/18866	0.0054914	0.1369558	0.1239225	10	TFDP1/CEACAM6/BMF/TLE1/DAPK2/BCL2/NOTCH1/CHEK2/TSC2/CEACAM5
Biological Process	GO:0030091	protein repair	4/2279	7/18866	0.0054921	0.1369558	0.1239225	4	MSRB1/MSRB2/MSRA/FN3K

Biological Process	GO:0030913	paranodal junction assembly	4/2279	7/18866	0.0054921	0.1369558	0.1239225	4	CD9/EPB41L3/CNTNAP1/ANK2
Biological Process	GO:0050882	voluntary musculoskeletal movement	4/2279	7/18866	0.0054921	0.1369558	0.1239225	4	VTI1A/HIPK2/ITPR1/MAP1A
Biological Process	GO:1904747	positive regulation of apoptotic process involved in development	4/2279	7/18866	0.0054921	0.1369558	0.1239225	4	TNFRSF1B/VDR/TNFRSF1A/NOTCH1
Biological Process	GO:0034968	histone lysine methylation	24/2279	116/18866	0.0055744	0.1386451	0.125451	24	GFI1/BRD4/MYB/ASH2L/SETD2/SMYD3/EHMT2/WDR82/KMT2D/KDM4C/SETD1B/SETD1A/TET2/ZNF335/PRDM16/PHF19/DNMT1/PIH1D1/DYDC2/DYDC1/MECOM/PAXIP1/NSD3/NSD2
Biological Process	GO:0008277	regulation of G protein-coupled receptor signaling pathway	29/2279	148/18866	0.0056195	0.1389092	0.12569	29	PDE6A/MGRN1/MGLL/ARRB1/PRKCA/DNM2/CCL5/GIT1/GNAI2/RGS6/MRAP/ZDHHC7/GRK6/RGS12/PHB/DGKQ/GRK5/GNG7/GIPR/PDE6H/PLCB1/ARRB2/DNM1/RGS3/GRK7/RGS10/RPH3AL/GRK2/ACP3
Biological Process	GO:0015721	bile acid and bile salt transport	9/2279	29/18866	0.0056289	0.1389092	0.12569	9	RXRA/SLC51A/SLC10A1/ABCC2/ATP8B1/NCOA1/ABCC3/NCOA2/AQP9
Biological Process	GO:0070861	regulation of protein exit from endoplasmic reticulum	9/2279	29/18866	0.0056289	0.1389092	0.12569	9	SORL1/TM9SF4/TMEM30A/UBAC2/EDEM1/DERL2/EDEM2/NUMA1/SVIP
Biological Process	GO:0002221	pattern recognition receptor signaling pathway	37/2279	201/18866	0.0057649	0.1418964	0.1283929	37	S100A8/GFI1/GRAMD4/MIR140/MAP2K6/PIK3AP1/SMPDL3B/TNFAIP3/SFTPD/TLR9/RTN4/RIPK1/COLEC12/SCIMP/PHB/FCN1/UBE2V1/TREML4/SEC14L1/TNIP1/BCL10/CTSB/ARRB2/ITGB2/IRAK2/NOD2/PRKCE/WDFY1/LTF/MAPKAPK3/CYLD/TYRO3/RELA/FLOT1/UBE2D2/FADD/PELI1
Biological Process	GO:0010763	positive regulation of fibroblast migration	6/2279	15/18866	0.0058552	0.1430094	0.1293999	6	MIR145/SLC8A1/ARHGEF7/THBS1/TGFB1/PRKCE

Biological Process	GO:0090148	membrane fission	6/2279	15/18866	0.0058552	0.1430094	0.1293999	6	DNM2/DNM1/DNM3/CORO1C/DNM1L/MX1
Biological Process	GO:1905820	positive regulation of chromosome separation	6/2279	15/18866	0.0058552	0.1430094	0.1293999	6	NSMCE2/CUL3/CDC16/NUMA1/RB1/ANAPC7
Biological Process	GO:0050773	regulation of dendrite development	30/2279	155/18866	0.0058995	0.1431525	0.1295295	30	MFSD2A/YWHAH/ZMYND8/BCL11A/ITSN1/TNIK/DISC1/CUX1/BAIAP2/ILK/PREX1/SDC2/ASAP1/SEMA4D/RAPGEF2/SS18L2/CPEB3/CAMK1D/SIPA1L1/DNM3/CDKL3/DNM1L/MEF2C/SHANK2/UBE3A/NEDD4/HECW2/CYFIP1/CUX2/DBNL
Biological Process	GO:0048525	negative regulation of viral process	22/2279	104/18866	0.0059063	0.1431525	0.1295295	22	SLPI/TRIM27/GSN/TRIM8/TRIM10/SRPK2/TNF/CCL5/SNX3/HDAC1/FCN1/TRIM5/TNIP1/LTF/ILF3/MX1/PARP10/BANF1/ISG20/TRIM26/PRKN/SHFL
Biological Process	GO:0120034	positive regulation of plasma membrane bounded cell projection assembly	22/2279	104/18866	0.0059063	0.1431525	0.1295295	22	HDAC4/DEF8/ZMYND8/DNM2/ARAP1/CDC42EP1/CEP135/WASL/EPS8L3/EPS8L1/PFN1/ESPN/HTT/PALM/PLEKHM1/NCKAP1/RALA/DNM3/AVIL/CYFIP1/NDEL1/SEPTIN9
Biological Process	GO:0002294	CD4-positive, alpha-beta T cell differentiation involved in immune response	15/2279	62/18866	0.0059938	0.1449032	0.1311136	15	IL27/PRKCZ/ZFPM1/FOXP1/MYB/NLRP3/LOXL3/SEMA4A/SMAD7/LY9/ZBTB7B/RARA/IL18R1/HLX/BCL3
Biological Process	GO:0007409	axonogenesis	77/2279	482/18866	0.0061551	0.1470026	0.1330131	77	SEMA6B/PLXNC1/LMTK2/BSG/NRP2/TRIO/KIF13B/PIK3CD/RHOH/CREB1/ULK1/APBB2/PIK3R1/RHOG/GAB2/PRKCA/DRGX/CTTN/DNM2/ZEB2/CSF1R/DOK1/EPHB3/SEMA4A/RTN4/UNC5B/DISC1/DOK2/BAIAP2/GRB10/SEMA4B/APP/IFRD1/FEZ2/ILK/PAX2/PITPNA/SYNGAP1/SEMA4D/ZSWIM5/NRXN1/NUMB/DSCAML1/BMPR1B/KIF5C/CDH4/SIPA1L1/SPTA1/KLF7/NTRK1/BCL2/NOTCH1/MYOT/MAP1A/CDKL3/SLIT3/ATL1/SPTAN1/PLCG1/MARK2/CYFIP1/TRPV2/CDH11/NDEL1/NPTX1/EZR/FLOT1/SLIT1/SPTBN4/OTX2/LYPLA2/TSKU/DBNL/UNC5D/B3GNT2/SPART/NECTIN1
Biological Process	GO:0045582	positive regulation of T cell differentiation	20/2279	92/18866	0.0061557	0.1470026	0.1330131	20	PRKCZ/RHOH/MYB/RUNX1/NLRP3/ITPKB/PIK3R6/ZBTB16/TGFBR2/ZMIZ1/ZBTB7B/RARA/LILRB4/PTPRC/PNP/HLX/RUNX3/CYLD/AP3D1/VSIR
Biological Process	GO:1904888	cranial skeletal system development	16/2279	68/18866	0.0061608	0.1470026	0.1330131	16	FOXN3/SETD2/SMAD3/MTHFD1L/SH3PXD2B/CHST11/TGFBR2/NDST1/RDH10/GRHL2/RUNX2/IFT140/MTHFD1/ALX3/MEF2C/NODAL

Biological Process	GO:0070142	synaptic vesicle budding	5/2279	11/18866	0.0063057	0.1470026	0.1330131	5	DNM2/DNM1/DNM3/AP3D1/MX1
Biological Process	GO:1903265	positive regulation of tumor necrosis factor-mediated signaling pathway	5/2279	11/18866	0.0063057	0.1470026	0.1330131	5	TRAF2/RIPK1/CPNE1/CASP1/PRKN
Biological Process	GO:0071675	regulation of mononuclear cell migration	13/2279	51/18866	0.0064045	0.1470026	0.1330131	13	C3AR1/CCR2/TNF/CCL5/CSF1R/FPR2/THBS1/DEFB124/PDGFD/CXCL17/PLCB1/ANO6/CCL27
Biological Process	GO:0000416	positive regulation of histone H3-K36 methylation	3/2279	4/18866	0.0064055	0.1470026	0.1330131	3	BRD4/PAXIP1/NSD3
Biological Process	GO:0002374	cytokine secretion involved in immune response	3/2279	4/18866	0.0064055	0.1470026	0.1330131	3	NLRP3/SPHK2/NOD2
Biological Process	GO:0008594	photoreceptor cell morphogenesis	3/2279	4/18866	0.0064055	0.1470026	0.1330131	3	MFSD2A/CDHR1/CABP4
Biological Process	GO:0015722	canalicular bile acid transport	3/2279	4/18866	0.0064055	0.1470026	0.1330131	3	ABCC2/ABCC3/AQP9
Biological Process	GO:0019541	propionate metabolic process	3/2279	4/18866	0.0064055	0.1470026	0.1330131	3	ACSS1/ACSS2/PCK2
Biological Process	GO:0032474	otolith morphogenesis	3/2279	4/18866	0.0064055	0.1470026	0.1330131	3	SLC44A4/LRIG1/TTC39C

Biological Process	GO:0033183	negative regulation of histone ubiquitination	3/2279	4/18866	0.0064055	0.1470026	0.1330131	3	OTUB2/OTUB1/TRIP12
Biological Process	GO:0051138	positive regulation of NK T cell differentiation	3/2279	4/18866	0.0064055	0.1470026	0.1330131	3	ZBTB16/TGFBR2/AP3D1
Biological Process	GO:0072429	response to intra-S DNA damage checkpoint signaling	3/2279	4/18866	0.0064055	0.1470026	0.1330131	3	EME1/SLX4/FBH1
Biological Process	GO:0072757	cellular response to camptothecin	3/2279	4/18866	0.0064055	0.1470026	0.1330131	3	BLM/SPIDR/RECQL5
Biological Process	GO:1901314	regulation of histone H2A K63-linked ubiquitination	3/2279	4/18866	0.0064055	0.1470026	0.1330131	3	OTUB2/OTUB1/TRIP12
Biological Process	GO:1901315	negative regulation of histone H2A K63-linked ubiquitination	3/2279	4/18866	0.0064055	0.1470026	0.1330131	3	OTUB2/OTUB1/TRIP12
Biological Process	GO:1904016	response to Thyroglobulin triiodothyronine	3/2279	4/18866	0.0064055	0.1470026	0.1330131	3	NCOA1/SLC2A1/NCOA2
Biological Process	GO:2000437	regulation of monocyte extravasation	3/2279	4/18866	0.0064055	0.1470026	0.1330131	3	CCR2/PDGFD/PLCB1
Biological Process	GO:2000491	positive regulation of hepatic stellate cell activation	3/2279	4/18866	0.0064055	0.1470026	0.1330131	3	RPS6KA1/MYB/PDGFRB

Biological Process	GO:0098732	macromolecule deacylation	23/2279	111/18866	0.0064415	0.1474724	0.1334382	23	HDAC4/SPRED2/HDAC7/SIN3B/SKI/ABHD16A/C6orf89/HDAC1/SPHK2/ZBTB7B/PHB/MTA1/CTBP1/ABHD16B/MTA2/PER2/PER1/NEK3/SREBF1/LYPLA2/ELK4/SIRT3/PRKAA1
Biological Process	GO:0008286	insulin receptor signaling pathway	28/2279	143/18866	0.006492	0.147915	0.1338387	28	PRKCZ/SORL1/FUT7/KANK1/PTPRE/PIK3R1/GPLD1/BAIAP2L1/BAIAP2/GRB10/GNAI2/ATP6V1B2/IGF1R/ZBTB7B/FER/PRKCB/INSRR/ATP6V0B/BCAR3/TSC2/FOXO1/SOGA1/SREBF1/RELA/NDEL1/PRKAA1/ATP6V0C/ATP6V1C1
Biological Process	GO:0045598	regulation of fat cell differentiation	28/2279	143/18866	0.006492	0.147915	0.1338387	28	ZFPM1/RREB1/TRIO/PPARG/CREB1/SMAD3/TNF/SH3PXD2B/ZBTB16/ZNF385A/ZBTB7B/TGFB1/TGFB1I1/WWTR1/TMEM64/WNT5B/STK3/GNB3/FTO/AAMDC/SORT1/ARNTL/PRDM16/CEBPB/FOXO1/ZADH2/TLCD3B/CCN4
Biological Process	GO:0010876	lipid localization	71/2279	440/18866	0.0065442	0.1487466	0.1345912	71	PNPLA2/EHD1/PRKAG2/OSBPL5/RXRA/PPARG/SLC51A/TMEM159/ACSL1/ABCC1/SLC10A1/MFSD2A/HEXB/MYB/MAP2K6/TMEM30A/LIPC/TNF/ABCG1/ATP8A1/ABCC2/HDLBP/ANO7/PLTP/THBS1/PITPNM2/STARD6/ATP11A/ATP8B1/PITPNM1/MIR27A/OSBPL6/CLU/PITPNA/SLCO3A1/NR1H2/LRP10/ITGB3/GHRL/PCTP/ABCA1/NCOA1/ACACB/FABP6/FTO/OSBPL10/TEX2/ABCC3/ATP8B4/NPC1/SGPP1/SPNS3/ACACA/PDZD8/PITPNC1/ABHD5/AGTR1/SLC2A1/ANO6/DYSF/NCOA2/GRAMD1A/ESYT1/CPT1A/INHBA/VPS51/CDS2/C2CD2L/AQP9/BSCL2/PRKN
Biological Process	GO:0031334	positive regulation of protein-containing complex assembly	45/2279	257/18866	0.0065671	0.1489119	0.1347407	45	TRIM27/DCTN1/SPIDR/GSN/CREB1/RAP1B/BMF/CTTN/TNF/NCK2/WHAMM/BAIAP2L1/NAV3/CDC42EP1/BIN1/BAIAP2/CLU/ASAP1/WASL/TGFB1/FER/PFN1/NR1H2/CSF3/GMFB/ABCA1/NUMA1/ZDHHC1/FNIP2/NCKAP1/MPP7/PIH1D1/MED25/TPPP3/PRKCE/MYO1C/ERCC1/CYFIP1/GMFG/BAIAP2L2/XPA/ERCC2/EIF4G1/STMP1/SLF1
Biological Process	GO:0045621	positive regulation of lymphocyte differentiation	22/2279	105/18866	0.0066401	0.1495093	0.1352813	22	PRKCZ/RHOH/MYB/RUNX1/INPP5D/NLRP3/ITPKB/PIK3R6/ZBTB16/TGFBR2/ZMIZ1/ZBTB7B/RARA/LILRB4/PTPRC/PNP/HLX/RUNX3/CYLD/AP3D1/PCID2/VSIR
Biological Process	GO:1902106	negative regulation of leukocyte differentiation	22/2279	105/18866	0.0066401	0.1495093	0.1352813	22	ZFPM1/RUNX1/INPP5D/PIK3R1/UBASH3B/LOXL3/FBXO7/SMAD7/ZBTB7B/RARA/TMEM178A/LILRB4/NF1/ZC3H8/PGLYRP1/LRRC17/LTF/HLX/RUNX3/TRIB1/INHBA/TMEM131L
Biological Process	GO:0002699	positive regulation of immune effector process	40/2279	223/18866	0.0066407	0.1495093	0.1352813	40	HLA-DMB/PRKCZ/MYB/CD177/NLRP3/IL1R1/CCR2/GAB2/FGR/TNF/TRAF2/MYO18A/CD84/TLR9/SPON2/CD226/HLA-F/SCIMP/SPHK2/TGFB1/PHB/FER/PTPRJ/RARA/BLK/SLC7A5/PTPRC/BCL10/ITGB2/IL18RAP/NOD2/TP53BP1/IL18R1/HLX/TNFSF13/CADM1/FADD/PAXIP1/CYRIB/NSD2
Biological Process	GO:0002698	negative regulation of immune effector process	25/2279	124/18866	0.0066828	0.1501012	0.1358169	25	ABR/IL10/TRIM38/CD55/CCR2/TNF/LOXL3/CD84/DUSP22/HLA-F/PTPN6/SMAD7/ZBTB7B/TGFB1/FER/CD59/PGLYRP1/SEC14L1/PTPRC/GRN/BCR/ARRB2/HLX/CD96/ILRUN

Biological Process	GO:0021915	neural tube development	31/2279	163/18866	0.0067494	0.150177	0.1358855	31	SHROOM3/SKI/SETD2/SPINT2/MTHFD1L/SUFU/ZEB2/MIB1/PAX2/PAX7/TGFB1/RARA/NF1/PFN1/RARG/STK3/GRHL2/BCL10/IFT140/MTHFD1/RALA/NOTCH1/TMED2/TSC2/TEAD2/SSBP3/ALDH1A2/LMO4/NODAL/HES3/BRD2
Biological Process	GO:0051494	negative regulation of cytoskeleton organization	31/2279	163/18866	0.0067494	0.150177	0.1358855	31	VILL/TBCD/TACSTD2/SSH1/GSN/KANK1/CAPG/ARAP1/NAV3/SSH3/PPFIA1/TMOD3/SVIL/PFN1/ESPN/GMFB/SPTA1/MIR138-2/ADD1/ARHGEF18/AVIL/FLII/ARHGEF2/SPTAN1/TMOD1/GMFG/MYADM/SPTBN4/SPEF1/PRKN/CYRIB
Biological Process	GO:0048016	inositol phosphate-mediated signaling	14/2279	57/18866	0.0067694	0.150177	0.1358855	14	ITPR2/INPP5A/TNF/NFATC2/ITPR1/NFATC1/CAMTA1/RCAN1/HRH1/DYRK2/PDGFB/FHL2/CHERP/STIMATE
Biological Process	GO:0051568	histone H3-K4 methylation	14/2279	57/18866	0.0067694	0.150177	0.1358855	14	GFI1/MYB/ASH2L/WDR82/KMT2D/SETD1B/SETD1A/TET2/ZNF335/DNMT1/PIH1D1/DYDC2/DYDC1/PAXIP1
Biological Process	GO:0070372	regulation of ERK1 and ERK2 cascade	52/2279	306/18866	0.0067909	0.150177	0.1358855	52	PRKCZ/SPRED2/MIR145/CHI3L1/ANGPT1/ITGB1BP1/RAP1B/ARRB1/PRKCA/TNF/CCL5/CSF1R/FPR2/C5AR2/GNAI2/APP/PTPN6/MIR24-2/MIR27A/MIR23A/MTURN/SCIMP/P2RY6/TGFB1/PHB/MARCO/RAPGEF2/PDGFD/BMPER/CCL20/TNIP1/PTPRC/CSK/PDGFB/ARRB2/YWHAZ/NTRK1/NOD2/NOTCH1/GCNT2/DUSP3/F2R/PDGFC/PDGFRB/ARHGAP8/EZR/NODAL/CCL22/LIF/NDRG2/SIRT3/DENND2B
Biological Process	GO:0042552	myelination	27/2279	137/18866	0.0067977	0.150177	0.1358855	27	ARHGEF10/SH3TC2/CD9/HEXB/SKI/LGI4/TNFRSF1B/NDRG1/GALC/EIF2B5/TG/PMP22/MBP/ILK/CLU/TNFRSF21/RARA/MYO5A/NF1/EPB41L3/JAM3/RARG/TSPAN2/FA2H/CNTNAP1/ANK2/ERCC2
Biological Process	GO:0045580	regulation of T cell differentiation	29/2279	150/18866	0.0068126	0.150177	0.1358855	29	IL27/PRKCZ/RHOH/MYB/RUNX1/NLRP3/ITPKB/CCR2/PIK3R6/LOXL3/NFATC2/ZBTB16/TGFBR2/SMAD7/ZMIZ1/ZBTB7B/FANCA/RARA/LILRB4/ZC3H8/PTPRC/CLPTM1/PNP/HLX/RUNX3/CYLD/AP3D1/TMEM131L/VSIR
Biological Process	GO:1905039	carboxylic acid transmembrane transport	29/2279	150/18866	0.0068126	0.150177	0.1358855	29	SLC38A4/PRKAG2/SLC38A10/SLC16A3/SLC1A5/SLC15A4/THBS1/LRRC8C/SLC43A2/SLC19A1/SLC1A2/SLC7A7/SLC7A5/ACACB/SLC1A6/PER2/SLC16A1/SLC36A3/SLC1A3/ACACA/SLC43A1/SLC2A1/SLC7A8/SLC13A5/CPT1A/SLC7A1/SLC25A1/SLC23A1/AQP9
Biological Process	GO:0000083	regulation of transcription involved in G1/S transition of mitotic cell cycle	10/2279	35/18866	0.0068714	0.1504263	0.1361111	10	GFI1/BRD4/TFDP1/E2F7/CDK14/ESRRB/CCNA1/RB1/E2F6/BACH1

Biological Process	GO:0033144	negative regulation of intracellular steroid hormone receptor signaling pathway	10/2279	35/18866	0.0068714	0.1504263	0.1361111	10	FOXP1/CALR/NCOR2/LBH/ZNF366/HDAC1/PHB/ARNTL/PER1/NODAL
Biological Process	GO:0098751	bone cell development	10/2279	35/18866	0.0068714	0.1504263	0.1361111	10	ZFPM1/FOXP1/FLI1/PIP4K2A/PTPN6/ZNF385A/LRRK1/MEIS1/LTF/FAM20C
Biological Process	GO:0032088	negative regulation of NF-kappaB transcription factor activity	20/2279	93/18866	0.0069732	0.1522848	0.1377927	20	GFI1/IL10/AIM2/PRMT2/NLRP3/ARRB1/TNFAIP3/TLR9/TRAIP/MIR27A/MTURN/NLRC5/CAT/ARRB2/MIR138-2/IRAK2/CYLD/PARP10/PELI1/COMMD6
Biological Process	GO:0002287	alpha-beta T cell activation involved in immune response	15/2279	63/18866	0.0070081	0.1522848	0.1377927	15	IL27/PRKCZ/ZFPM1/FOXP1/MYB/NLRP3/LOXL3/SEMA4A/SMAD7/LY9/ZBTB7B/RARA/IL18R1/HLX/BCL3
Biological Process	GO:0002293	alpha-beta T cell differentiation involved in immune response	15/2279	63/18866	0.0070081	0.1522848	0.1377927	15	IL27/PRKCZ/ZFPM1/FOXP1/MYB/NLRP3/LOXL3/SEMA4A/SMAD7/LY9/ZBTB7B/RARA/IL18R1/HLX/BCL3
Biological Process	GO:0030308	negative regulation of cell growth	35/2279	190/18866	0.0070204	0.1522848	0.1377927	35	SEMA6B/CDA/ENO1/PPARG/CTDP1/SERTAD2/APBB2/SMAD3/MIR199A1/BCL11A/VGLL4/SEMA4A/RTN4/MEG3/SEMA4B/SMARCA2/IFRD1/WNT11/SPHK2/TGFB1/PHB/PTPRJ/SEMA4D/RERG/CDHR2/SH3BP4/PPP2R1A/RB1/BCL2/MIR199A2/CDKL3/SLIT3/SCGB3A1/INHBA/SPART
Biological Process	GO:0097581	lamellipodium organization	19/2279	87/18866	0.007093	0.1532176	0.1386367	19	HDAC4/RREB1/PARVB/KANK1/ARHGEF7/CTTN/DNM2/NCK2/WHAMM/SPATA13/ABLIM3/FER/VAV2/NCKAP1/CORO1C/AVIL/CYFIP1/ABLIM1/VAV3
Biological Process	GO:0010508	positive regulation of autophagy	26/2279	131/18866	0.0071075	0.1532176	0.1386367	26	TRIM27/TRIM38/TRIM8/SPTLC2/ULK1/VPS13D/MEFV/FBXO7/LARP1/CAMKK2/PIP4K2A/UVRAG/SMURF1/RUFY4/TRIM5/CLEC16A/HTT/HK2/SH3BP4/TSC2/FOXO1/SVIP/NPRL2/PRKAA1/STING1/PRKN
Biological Process	GO:0061028	establishment of endothelial barrier	12/2279	46/18866	0.0071118	0.1532176	0.1386367	12	PDE4D/TJP2/RAP1B/TNF/TNFRSF1A/RAPGEF2/ENG/ADD1/S1PR2/PPP1R16B/EZR/MYADM

Biological Process	GO:0043242	negative regulation of protein-containing complex disassembly	18/2279	81/18866	0.0071692	0.1541055	0.1394401	18	VILL/GSN/TNF/CAPG/NAV3/TMOD3/SVIL/CLEC16A/SPTA1/ADD1/AVIL/SCAF8/FLII/ARHGEF2/SPTAN1/TMOD1/SPTBN4/SPEF1
Biological Process	GO:0010800	positive regulation of peptidyl-threonine phosphorylation	9/2279	30/18866	0.0071947	0.1543054	0.139621	9	AZU1/RPTOR/PRKAG2/CHI3L1/APP/TGFB1/AXIN1/CAB39/S1PR2
Biological Process	GO:0032528	microvillus organization	8/2279	25/18866	0.0072664	0.1551418	0.1403778	8	PLD1/TNIK/ATP8B1/RAPGEF2/CDHR2/ESPN/EZR/GLDN
Biological Process	GO:0050857	positive regulation of antigen receptor-mediated signaling pathway	8/2279	25/18866	0.0072664	0.1551418	0.1403778	8	FOXP1/LCK/CD226/PRKCH/PRKCB/PTPRC/CYLD/RELA
Biological Process	GO:0046328	regulation of JNK cascade	34/2279	184/18866	0.0074331	0.1583442	0.1432755	34	HIPK3/ERN1/ZNF622/TNF/ZEB2/TRAF2/TRAF1/PHLPP1/HIPK2/TLR9/DUSP22/TNIK/RIPK1/APP/IGF1R/MTURN/AXIN1/COPS5/SH3RF3/STK3/PLCB1/MAPK8IP2/TAOK3/NOD2/PER1/DUSP3/GADD45G/CYLD/ANKRD6/DTNBP1/MECOM/DBNL/PRKN/MAP3K20
Biological Process	GO:0008643	carbohydrate transport	29/2279	151/18866	0.0074837	0.158711	0.1436074	29	PRKAG2/SLC2A5/MFSD2A/SLC45A4/MIR143/PIK3R1/SLC2A14/TNF/GRB10/SLC2A9/ZDHHC7/HNF1A/PRKCB/AQP1/SLC1A2/HK2/PRKCI/SLC37A1/NFE2L2/SORT1/AQP3/SLC5A10/SLC2A1/RTN2/EZR/ASPSCR1/CLTCL1/SLC23A1/AQP9
Biological Process	GO:1903825	organic acid transmembrane transport	29/2279	151/18866	0.0074837	0.158711	0.1436074	29	SLC38A4/PRKAG2/SLC38A10/SLC16A3/SLC1A5/SLC15A4/THBS1/LRRC8C/SLC43A2/SLC19A1/SLC1A2/SLC7A7/SLC7A5/ACACB/SLC1A6/PER2/SLC16A1/SLC36A3/SLC1A3/ACACA/SLC43A1/SLC2A1/SLC7A8/SLC13A5/CPT1A/SLC7A1/SLC25A1/SLC23A1/AQP9
Biological Process	GO:0032330	regulation of chondrocyte differentiation	13/2279	52/18866	0.0076157	0.160944	0.1456278	13	SMAD3/ZBTB16/SMAD7/POR/RARG/BMPR1B/RUNX2/TRPS1/GDF6/RELA/ZNF219/RFLNA/CCN4
Biological Process	GO:0022408	negative regulation of cell-cell adhesion	35/2279	191/18866	0.0076229	0.160944	0.1456278	35	IL10/CD9/MAD1L1/RUNX1/SPINT2/LAX1/UBASH3B/LOXL3/SFTPD/DUSP22/CCM2L/MBP/PTPN6/MIR27A/SMAD7/TNFRSF21/MIA3/ZBTB7B/TGFB1/IL1RN/TIGIT/ZC3H8/CDH1/NOTCH1/DUSP3/NOTCH4/HLX/CEBPB/RUNX3/ADTRP/MYADM/PELI1/TMEM131L/VSIR/MARCHF7

Biological Process	GO:0021700	developmental maturation	49/2279	287/18866	0.0076943	0.1620915	0.1466662	49	MAEA/PPARG/BRCA2/LGI4/SLFN14/LYL1/BCL11A/RFX3/DISC1/BRD1/ZDHHC2/APP/RBPJ/PAX2/CDH3/FGFR1/XYLT1/SEMA4D/DEFB1/MYO5A/NF1/PFN1/NRXN1/RUNX2/PPP2R1A/PALM/FEV/YWHAZ/RB1/POU2F2/BCL2/DMC1/EPAS1/NPPC/LTF/ANO6/ARHGEF15/FAM20C/EPB42/AP3D1/CLN5/ALDH1A2/SPTBN4/ERCC2/GLDN/CX3CR1/NFIA/RFLNA/ANGPTL8
Biological Process	GO:1903707	negative regulation of hemopoiesis	30/2279	158/18866	0.0077883	0.1637087	0.1481294	30	ZFPM1/RUNX1/INPP5D/PIK3R1/ITPKB/UBASH3B/LOXL3/FBXO7/ZBTB16/DLL1/SMAD7/ZBTB7B/RARA/TMEM178A/LILRB4/NF1/ZC3H8/PGLYRP1/MEIS2/LRRC17/NFE2L2/HOXA9/MEIS1/NOTCH1/LTF/HLX/RUNX3/TRIB1/INHBA/TMEM131L
Biological Process	GO:2000116	regulation of cysteine-type endopeptidase activity	42/2279	239/18866	0.0079094	0.165887	0.1501004	42	NLRC4/S100A8/GRAMD4/AIM2/PPARG/GSN/RPS6KA1/LCK/NLRP3/SMAD3/ARRB1/MEFV/TNF/TRAF2/CYFIP2/MICAL1/TBC1D10A/CTSD/THBS1/CTSH/RIPK1/PAX2/CIDEB/AQP1/PERP/POR/DNAJB6/BCL10/ATP2A3/NOL3/ARRB2/FIS1/PIH1D1/P2RX1/F2R/LTF/CASP1/BCL2L13/ASPH/NODAL/FADD/PCID2
Biological Process	GO:0002763	positive regulation of myeloid leukocyte differentiation	14/2279	58/18866	0.007954	0.1664555	0.1506148	14	CREB1/MIR145/RUNX1/PRKCA/TNF/TESC/RIPK1/TGFB1/CD101/TMEM64/RB1/TRIB1/LIF/FADD
Biological Process	GO:0006643	membrane lipid metabolic process	38/2279	212/18866	0.0080885	0.1688986	0.1528254	38	PLA2G15/FUT7/SPTLC2/HEXB/PIGL/PIGU/GPLD1/GALC/TNF/SMPDL3B/ORMDL3/CERS6/PIGN/TNFRSF1A/SPHK2/SGMS1/B3GNT5/MGST2/ST8SIA6/ST3GAL4/PPP2R1A/SGMS2/B4GALT3/TEX2/SGPP1/SGPP2/PIGQ/P2RX1/FA2H/ESYT1/CERS2/PDXDC1/CERK/SIRT3/ST3GAL2/PRKAA1/TLCD3B/PLPP3
Biological Process	GO:0031346	positive regulation of cell projection organization	64/2279	394/18866	0.0081922	0.1704012	0.154185	64	HDAC4/CPNE6/PLXNC1/RREB1/DEF8/AP2A1/LCN2/TMEM30A/ZMYND8/ARHGEF7/DNM2/CPNE5/ZEB2/ITSN1/ARAP1/NAV3/CDC42EP1/DISC1/CUX1/BAIAP2/SNX3/CEP135/ILK/WASL/FGFR1/SEMA4D/RAPGEF2/SMURF1/EPS8L3/SS18L2/EPS8L1/PFN1/CPEB3/CAMK1D/ESPN/HTT/CDH4/GRN/PALM/CREB3L2/PLEKHM1/PRKCI/NFE2L2/NCKAP1/RALA/ARSB/NTRK1/DNM3/CORO1C/CDKL3/DNM1L/AVIL/SHANK2/SYT17/ANKRD1/MARK2/CYFIP1/TRPV2/NDEL1/CUX2/DBNL/SEPTIN9/PRKN/P3H1
Biological Process	GO:0002224	toll-like receptor signaling pathway	29/2279	152/18866	0.0082085	0.1704012	0.154185	29	S100A8/GFI1/GRAMD4/MIR140/PIK3AP1/SMPDL3B/TNFAIP3/SFTPD/TLR9/RTN4/RIPK1/COLEC12/SCIMP/TREML4/TNIP1/BCL10/CTSB/ARRB2/ITGB2/IRAK2/PRKCE/WDFY1/LTF/MAPKAPK3/TYRO3/FLOT1/UBE2D2/FADD/PELI1
Biological Process	GO:0051146	striated muscle cell differentiation	50/2279	295/18866	0.0082212	0.1704012	0.154185	50	HDAC4/EHD1/RXRA/CALR/CD9/CTDP1/MYO18B/TTN/CASQ2/MYH11/AKAP13/SKI/MAML1/SLC8A1/MIR199A1/TANC1/NFATC2/TNNT3/SMYD3/MYH9/SYNPO2L/DLL1/RBPJ/MIR24-2/MIR23A/TGFB1/TMOD3/RARA/ACTG1/WDR1/ARRB2/SORT1/ANKRD2/RB1/BCL2/MIR199A2/NOTCH1/MEF2C/FHL2/FLII/PLD3/PDGFRB/DYSF/ANKRD1/TMOD1/FLOT1/SCGB3A1/CASQ1/CEACAM5/MYORG
Biological Process	GO:0006901	vesicle coating	16/2279	70/18866	0.0082448	0.1704012	0.154185	16	CTSZ/SERPINA1/TGFA/STX5/CSNK1D/TRAPPC10/CUL3/TRAPPC9/CNIH2/CD59/SEC13/ANKRD28/SEC24C/TMED2/SEC31A/TRAPPC2

Biological Process	GO:0007272	ensheathment of neurons	27/2279	139/18866	0.0082681	0.1704012	0.154185	27	ARHGEF10/SH3TC2/CD9/HEXB/SKI/LGI4/TNFRSF1B/NDRG1/GALC/EIF2B5/TG/PMP22/MBP/ILK/CLU/TNFRSF21/RARA/MYO5A/NF1/EPB41L3/JAM3/RARG/TSPAN2/FA2H/CNTNAP1/ANK2/ERCC2
Biological Process	GO:0008366	axon ensheathment	27/2279	139/18866	0.0082681	0.1704012	0.154185	27	ARHGEF10/SH3TC2/CD9/HEXB/SKI/LGI4/TNFRSF1B/NDRG1/GALC/EIF2B5/TG/PMP22/MBP/ILK/CLU/TNFRSF21/RARA/MYO5A/NF1/EPB41L3/JAM3/RARG/TSPAN2/FA2H/CNTNAP1/ANK2/ERCC2
Biological Process	GO:0030397	membrane disassembly	6/2279	16/18866	0.008423	0.1706321	0.154394	6	DCTN1/PRKCA/PRKCB/NEK6/NDEL1/LPIN1
Biological Process	GO:0033033	negative regulation of myeloid cell apoptotic process	6/2279	16/18866	0.008423	0.1706321	0.154394	6	MAEA/ITPKB/CCL5/CLEC5A/NOD2/BCL2
Biological Process	GO:0034116	positive regulation of heterotypic cell-cell adhesion	6/2279	16/18866	0.008423	0.1706321	0.154394	6	IL10/LCK/CEACAM6/TNF/GCNT2/FLOT1
Biological Process	GO:0051081	nuclear envelope disassembly	6/2279	16/18866	0.008423	0.1706321	0.154394	6	DCTN1/PRKCA/PRKCB/NEK6/NDEL1/LPIN1
Biological Process	GO:0051127	positive regulation of actin nucleation	6/2279	16/18866	0.008423	0.1706321	0.154394	6	TRIM27/GSN/WHAMM/WASL/NCKAP1/CYFIP1
Biological Process	GO:0071380	cellular response to prostaglandin E stimulus	6/2279	16/18866	0.008423	0.1706321	0.154394	6	PPARG/P2RY6/GNG2/ACACA/PRKCE/PRKAA1
Biological Process	GO:0072109	glomerular mesangium development	6/2279	16/18866	0.008423	0.1706321	0.154394	6	PDGFD/IL6R/PDGFB/NOTCH1/GPR4/PDGFRB

Biological Process	GO:0099170	postsynaptic modulation of chemical synaptic transmission	6/2279	16/18866	0.008423	0.1706321	0.154394	6	RNF19A/GHRL/PLCB1/CDH1/CYFIP1/SYNPO
Biological Process	GO:0071402	cellular response to lipoprotein particle stimulus	10/2279	36/18866	0.0085052	0.1719311	0.1555693	10	CD9/PPARG/ABCG1/MIA3/ABCA1/ITGB2/NPC1/CD68/HMGCS1/ADTRP
Biological Process	GO:0050708	regulation of protein secretion	58/2279	352/18866	0.0085437	0.1723437	0.1559427	58	SORL1/NADK/IL10/TTN/C1QTNF3/ITPR2/MYRIP/CLEC9A/CACNA1C/LRP5L/RAB11FIP1/FRMD4A/ARRB1/PRKCA/MIR199A1/GPLD1/FGR/TNF/TRAF2/MYO18A/CCL5/ABCG1/GJA5/RHBDF2/TLR9/CLEC5A/ITPR1/RFX3/TCF7L2/TGFB1/ENY2/GIPR/BLK/NR1H2/GHRL/KLF7/ARNTL/MYOM1/PER2/SLC16A1/MIR199A2/DNM1L/PRKCE/F2R/SLC2A1/ANKRD1/SREBF1/ADTRP/CADM1/CPT1A/CD33/RPH3AL/EZR/C2CD2L/SIRT3/PRKN/P3H1/CCN4
Biological Process	GO:0045639	positive regulation of myeloid cell differentiation	21/2279	101/18866	0.0086138	0.1733902	0.1568896	21	CREB1/MIR145/RUNX1/INPP5D/PRKCA/TNF/TESC/BRD1/RIPK1/MTURN/TGFB1/CD101/CSF3/TMEM64/ETS1/RB1/TRIB1/KAT7/INHBA/LIF/FADD
Biological Process	GO:0048813	dendrite morphogenesis	28/2279	146/18866	0.0086446	0.1736417	0.1571172	28	YWHAH/BTBD3/EPHB3/SLC11A2/TNIK/CUX1/BAIAP2/ILK/SDC2/WASL/FARP1/SEMA4D/RAPGEF2/SS18L2/SIPA1L1/MAPK8IP2/KLF7/DOCK10/DNM3/CDKL3/DNM1L/SHANK2/UBE3A/NEDD4/HECW2/DTNBP1/CUX2/DBNL
Biological Process	GO:0007254	JNK cascade	38/2279	213/18866	0.0087287	0.1748865	0.1582435	38	HIPK3/ERN1/ZNF622/TNF/ZEB2/TRAF2/TRAF1/PHLPP1/HIPK2/TLR9/DUSP22/TNIK/RIPK1/APP/IGF1R/MTURN/AXIN1/COPS5/UBE2V1/SH3RF3/SH2D3C/STK3/PLCB1/MAPK8IP2/TAOK3/IRAK2/NOD2/PER1/DUSP3/GADD45G/TRIB1/CYLD/ANKRD6/DTNBP1/MECOM/DBNL/PRKN/MAP3K20
Biological Process	GO:0010769	regulation of cell morphogenesis involved in differentiation	52/2279	310/18866	0.0087434	0.1748865	0.1582435	52	SEMA6B/P4HB/PLXNC1/RREB1/TACSTD2/CALR/KIF13B/KANK1/YWHAH/DOCK1/ITGB1BP1/ARHGEF7/CTTN/DNM2/ZEB2/EPHB3/SEMA4A/RTN4/TNIK/DISC1/CUX1/BAIAP2/SEMA4B/ACTN4/IFRD1/ILK/PREX1/SDC2/SYNGAP1/SEMA4D/RAPGEF2/ZSWIM5/SS18L2/OLFM4/CDH4/SIPA1L1/CASS4/DNM3/CORO1C/CDKL3/DNM1L/UBE3A/NEDD4/HECW2/MARK2/CYFIP1/TRPV2/NDEL1/MYADM/CUX2/DBNL/SPART
Biological Process	GO:0045834	positive regulation of lipid metabolic process	29/2279	153/18866	0.0089902	0.1789036	0.1618783	29	PNPLA2/PPARG/CREB1/MFSD2A/GPLD1/FGR/TNF/ABCG1/FPR2/TNFRSF1A/FLT1/SPHK2/TGFB1/RDH10/POR/NR1H2/VAV2/ATG14/PDGFB/NOD2/PRKCE/ABHD5/AGTR1/PDGFRB/SREBF1/CPT1A/SIRT3/VAV3/PRKAA1
Biological Process	GO:0008333	endosome to lysosome transport	13/2279	53/18866	0.0090007	0.1789036	0.1618783	13	MGRN1/LYST/TGFBRAP1/KIF13A/SNX27/DENND3/BIN1/VPS18/CLEC16A/ATG14/HOOK2/SORT1/MVB12A

Biological Process	GO:0045599	negative regulation of fat cell differentiation	13/2279	53/18866	0.0090007	0.1789036	0.1618783	13	ZFPM1/TRIO/SMAD3/TNF/TGFB1/TGFB1I1/WWTR1/SORT1/ARNTL/FOXO1/ZADH2/TLCD3B/CCN4
Biological Process	GO:0014044	Schwann cell development	9/2279	31/18866	0.0090727	0.1793754	0.1623052	9	ARHGEF10/SH3TC2/SKI/LGI4/NDRG1/ILK/NF1/FA2H/CNTNAP1
Biological Process	GO:1905476	negative regulation of protein localization to membrane	9/2279	31/18866	0.0090727	0.1793754	0.1623052	9	KCNE1/ITGB1BP1/MRAP/PPFIA1/LZTFL1/TGFB1/NUMB/CSK/BCL2L1
Biological Process	GO:0055074	calcium ion homeostasis	74/2279	468/18866	0.0090811	0.1793754	0.1623052	74	ELANE/TRPM2/PDE4D/CALR/C3AR1/THADA/CASQ2/ITPR2/LCK/HEXB/CD55/CACNA1C/SLC24A4/VDR/CCR2/SMAD3/SLC8A1/MIR199A1/ANXA6/UBASH3B/CDH23/CCL5/PACS2/FPR2/FPR1/ATP2B2/ITPR1/ACKR2/DISC1/JSRP1/C5AR2/APP/PTPN6/RASA3/GNAT2/P2RY6/CCR9/PRKCB/TMEM178A/MYO5A/GIPR/HTT/GHRL/PTPRC/TMEM64/CXCR1/ATP2A3/NOL3/TPCN2/IMMT/FIS1/BCL2/MIR199A2/GSTO1/P2RX1/LPAR2/PRKCE/F2R/PDZD8/S1PR4/GPR4/AGTR1/ANK2/CHERP/PLCG1/ATP7B/P2RX5/TRPV2/CASQ1/GRINA/SYPL2/ANXA7/CX3CR1/SYNPO
Biological Process	GO:0008625	extrinsic apoptotic signaling pathway via death domain receptors	19/2279	89/18866	0.0091019	0.1794135	0.1623397	19	ITPRIP/PIK3R1/TNF/TRAF2/TNFAIP3/DEDD2/THBS1/RIPK1/TNFRSF1A/NF1/BMPR1B/STK3/TRPS1/SORT1/BCL2/SP100/ARHGEF2/BCL2L1/FADD
Biological Process	GO:0010959	regulation of metal ion transport	64/2279	396/18866	0.0091372	0.179736	0.1626315	64	PDE4D/TRIM27/RCVRN/KCNE1/KCNAB2/THADA/IL16/CASQ2/WWP2/FXYD2/CACNA1C/YWHAH/FXYD1/MYLK/SLC8A1/DNM2/UBASH3B/CCL5/CD84/TLR9/TESC/BIN1/JSRP1/AHNAK/GNAI2/NOS1AP/PTPN6/ACTN4/KCNIP1/P2RY6/SPHK2/MYO5A/HTT/CAB39/PDGFB/SNTA1/KCNQ1/ARRB2/BCL2/PER1/GSTO1/P2RX1/PRKCE/F2R/TMC1/NEDD4/PDGFRB/ANO6/DYSF/CACNA1B/ANK2/HECW2/PLCG1/P2RX5/TRPV2/CD33/CASQ1/SPTBN4/FGF14/GRAMD2A/CBARP/STIMATE/SARAF/CRACR2A
Biological Process	GO:0031663	lipopolysaccharide-mediated signaling pathway	14/2279	59/18866	0.0092969	0.1804668	0.1632928	14	CD55/PRKCA/TNF/TNFAIP3/CCL5/SCIMP/TGFB1/LY86/TRIM5/BCL10/IRAK2/PRKCE/LTF/TRIB1
Biological Process	GO:0002693	positive regulation of cellular extravasation	7/2279	21/18866	0.0092975	0.1804668	0.1632928	7	ELANE/FUT4/FUT7/IL1R1/CCR2/PDGFD/FADD
Biological Process	GO:0030220	platelet formation	7/2279	21/18866	0.0092975	0.1804668	0.1632928	7	ZFPM1/MYH9/ACTN1/PTPN6/ZNF385A/WDR1/MEF2C

Biological Process	GO:0032288	myelin assembly	7/2279	21/18866	0.0092975	0.1804668	0.1632928	7	CD9/PMP22/ILK/EPB41L3/CNTNAP1/ANK2/ERCC2
Biological Process	GO:1903599	positive regulation of autophagy of mitochondrion	7/2279	21/18866	0.0092975	0.1804668	0.1632928	7	VPS13D/FBXO7/CAMKK2/SMURF1/HTT/HK2/PRKN
Biological Process	GO:0008593	regulation of Notch signaling pathway	22/2279	108/18866	0.0093011	0.1804668	0.1632928	22	MAML3/WWP2/SLC35C2/MAML1/ITGB1BP1/AAK1/TSPAN14/ARRB1/MAML2/EGFL7/DLL1/RBPJ/ZMIZ1/EPN2/CREBBP/PRKCI/TSPEAR/NOD2/NOTCH1/NOTCH4/DLK1/PRAG1
Biological Process	GO:0009896	positive regulation of catabolic process	72/2279	454/18866	0.0093073	0.1804668	0.1632928	72	PNPLA2/TRIM27/RNF144A/SORL1/FZR1/RNF19A/STX5/TRIM38/TRIM8/WWP2/PRR5L/SPTLC2/CSNK1D/ULK1/LPCAT1/TNFRSF1B/PFKFB4/VPS13D/TNRC6B/MEFV/GPLD1/TNF/TNFAIP3/FBXO7/VGLL4/CBFA2T3/LARP1/CAMKK2/ZC3H12D/DISC1/EDEM1/PIP4K2A/APP/CLU/SMAD7/UVRAG/AGO2/SMURF1/AXIN1/RUFY4/TRIM5/CLEC16A/CPEB3/HTT/HK2/EDEM2/SH3BP4/CSNK1E/FTO/NFE2L2/ESRRB/RNF144B/PRKCE/NEDD4/ABHD5/TSC2/RNF14/FOXO1/TRIB1/TNFSF12/HECW2/CPT1A/EZR/RNF40/PFKFB3/SVIP/NPRL2/PRKAA1/GRSF1/STING1/PRKN/VSIR
Biological Process	GO:0031664	regulation of lipopolysaccharide-mediated signaling pathway	8/2279	26/18866	0.0094049	0.1810721	0.1638405	8	CD55/PRKCA/TNFAIP3/SCIMP/LY86/TRIM5/LTF/TRIB1
Biological Process	GO:0060074	synapse maturation	8/2279	26/18866	0.0094049	0.1810721	0.1638405	8	DISC1/PFN1/NRXN1/PALM/YWHAZ/ARHGEF15/CX3CR1/NFIA
Biological Process	GO:0060765	regulation of androgen receptor signaling pathway	8/2279	26/18866	0.0094049	0.1810721	0.1638405	8	FOXP1/NCOR2/PRMT2/HDAC1/PHB/ARRB2/RNF14/NODAL
Biological Process	GO:0043434	response to peptide hormone	71/2279	447/18866	0.0094195	0.1810721	0.1638405	71	PRKCZ/BSG/SORL1/FUT7/IL10/PPARG/ADCY2/KANK1/CREB1/PTPRE/MIR143/MIR145/PRKAR1B/ADCY9/PIK3R1/RAP1B/GPLD1/RAB31/EIF2B5/ABCC2/STAT5A/BAIAP2L1/BAIAP2/GRB10/GNAI2/ATP6V1B2/ZDHHC7/PXN/IGF1R/ZBTB7B/CRHBP/FER/PRKCB/POR/MYO5A/ARID1B/GHRL/CSK/AGTRAP/CAT/PRKCI/NFE2L2/SORT1/ADCY4/INSRR/PKM/ATP6V0B/BCAR3/PCK2/AGTR1/MYO1C/TSC2/SLC2A1/FOXO1/MBD5/HHEX/SOGA1/SREBF1/KBTBD2/PDE3B/CYFIP1/RELA/WDTC1/NDEL1/LPIN1/RNF40/HSD11B2/PRKAA1/MARS1/ATP6V0C/ATP6V1C1
Biological Process	GO:0032729	positive regulation of interferon-gamma production	15/2279	65/18866	0.0094466	0.1810721	0.1638405	15	IL27/PDE4D/ZFPM1/IRF8/SLC11A1/IL1R1/CCR2/TNF/TLR9/CD226/SLC7A5/IL18R1/BCL3/FADD/CYRIB

Biological Process	GO:0000266	mitochondrial fission	11/2279	42/18866	0.0094719	0.1810721	0.1638405	11	INF2/DNM2/DNM1/FIS1/DNM3/DNM1L/MIEF1/MX1/DHODH/PRKN/CYRIB
Biological Process	GO:1902622	regulation of neutrophil migration	11/2279	42/18866	0.0094719	0.1810721	0.1638405	11	FUT7/C3AR1/RHOH/IL1R1/RHOG/C5AR2/JAM3/CAMK1D/DAPK2/DNM1L/DYSF
Biological Process	GO:0009306	protein secretion	73/2279	462/18866	0.0096636	0.1822909	0.1649433	73	SORL1/NADK/IL10/TTN/C1QTNF3/ITPR2/TANGO2/MYRIP/CLEC9A/CACNA1C/LRP5L/NLRP3/RAB11FIP1/FRMD4A/ARRB1/PRKCA/MIR199A1/GPLD1/FGR/TNF/TRAF2/MYO18A/CCL5/ABCG1/GJA5/RHBDF2/TNFSF13B/TLR9/CLEC5A/ITPR1/RFX3/TCF7L2/MIA3/HNF1A/PTPRN2/SPHK2/TGFB1/IL1RN/ENY2/MYO5A/GIPR/BLK/NR1H2/GHRL/KLF7/ARNTL/MYOM1/PER2/SLC16A1/NOD2/POU2F2/MIR199A2/DNM1L/PRKCE/TRAF3IP2/F2R/LMF1/SLC2A1/DYSF/ANKRD1/SREBF1/ADTRP/CADM1/CPT1A/CD33/RPH3AL/EZR/C2CD2L/SIRT3/MIA2/PRKN/P3H1/CCN4
Biological Process	GO:0031331	positive regulation of cellular catabolic process	63/2279	390/18866	0.0097342	0.1822909	0.1649433	63	PNPLA2/TRIM27/RNF144A/FZR1/RNF19A/TRIM38/TRIM8/PRR5L/SPTLC2/CSNK1D/ULK1/TNFRSF1B/PFKFB4/VPS13D/TNRC6B/MEFV/GPLD1/TNF/TNFAIP3/FBXO7/VGLL4/CBFA2T3/LARP1/CAMKK2/ZC3H12D/DISC1/EDEM1/PIP4K2A/APP/CLU/SMAD7/UVRAG/AGO2/SMURF1/AXIN1/RUFY4/TRIM5/CLEC16A/CPEB3/HTT/HK2/EDEM2/SH3BP4/CSNK1E/FTO/NFE2L2/ESRRB/RNF144B/ABHD5/TSC2/RNF14/FOXO1/TRIB1/CPT1A/EZR/RNF40/PFKFB3/SVIP/NPRL2/PRKAA1/GRSF1/STING1/PRKN
Biological Process	GO:0010755	regulation of plasminogen activation	5/2279	12/18866	0.0097454	0.1822909	0.1649433	5	CTSZ/ENO1/THBS1/F12/CPB2
Biological Process	GO:0032494	response to peptidoglycan	5/2279	12/18866	0.0097454	0.1822909	0.1649433	5	IRF5/NLRP3/DEFB124/NOD2/RELA
Biological Process	GO:0035845	photoreceptor cell outer segment organization	5/2279	12/18866	0.0097454	0.1822909	0.1649433	5	MFSD2A/CRB1/IFT140/CDHR1/NPHP4
Biological Process	GO:0043301	negative regulation of leukocyte degranulation	5/2279	12/18866	0.0097454	0.1822909	0.1649433	5	ABR/CCR2/CD84/HLA-F/BCR
Biological Process	GO:1902337	regulation of apoptotic process involved in morphogenesis	5/2279	12/18866	0.0097454	0.1822909	0.1649433	5	TNFRSF1B/VDR/TNFRSF1A/PAX2/NOTCH1

Biological Process	GO:2001241	positive regulation of extrinsic apoptotic signaling pathway in absence of ligand	5/2279	12/18866	0.0097454	0.1822909	0.1649433	5	NF1/PPP2R1A/PPP2R1B/INHBA/DAPK3
Biological Process	GO:0001701	in utero embryonic development	62/2279	383/18866	0.0098227	0.1822909	0.1649433	62	RXRA/IL10/MYO18B/BRCA2/ANGPT1/ADCY9/SETD2/ANKRD11/SMAD3/SPINT2/CMIP/CCM2/CHST11/E2F7/RTN4/HSD17B2/TGFBR2/CUL3/MYH9/HM13/ATP11A/RBPJ/MIB1/ZMIZ1/APBA2/FGFR1/RDH10/SP3/DNAJB6/KDM4C/STK3/GRHL2/NCOA1/ST8SIA6/PDGFB/ZNF335/GJB3/VASH1/COPS3/ADD1/NXN/NOTCH1/EPAS1/GGNBP2/GNA12/TMED2/CEBPB/PCDH12/PDGFRB/CHD8/BCL2L1/NSRP1/PLCG1/WDTC1/NDEL1/PLCD3/NODAL/HES3/ERCC2/LIF/ETNK2/TENT5C
Biological Process	GO:0006855	drug transmembrane transport	4/2279	8/18866	0.0099386	0.1822909	0.1649433	4	TMEM30A/ABCC2/ATP8B1/ABCC3
Biological Process	GO:0015820	leucine transport	4/2279	8/18866	0.0099386	0.1822909	0.1649433	4	SLC43A2/SLC7A5/SLC43A1/SLC7A8
Biological Process	GO:0016185	synaptic vesicle budding from presynaptic endocytic zone membrane	4/2279	8/18866	0.0099386	0.1822909	0.1649433	4	DNM2/DNM1/DNM3/MX1
Biological Process	GO:0035696	monocyte extravasation	4/2279	8/18866	0.0099386	0.1822909	0.1649433	4	CCR2/PDGFD/PLCB1/JAML
Biological Process	GO:0035701	hematopoietic stem cell migration	4/2279	8/18866	0.0099386	0.1822909	0.1649433	4	CCR2/GPLD1/JAM3/PTPRC
Biological Process	GO:0043353	enucleate erythrocyte differentiation	4/2279	8/18866	0.0099386	0.1822909	0.1649433	4	MAEA/SP3/RB1/MB
Biological Process	GO:0048227	plasma membrane to endosome transport	4/2279	8/18866	0.0099386	0.1822909	0.1649433	4	RAB5C/RAB5B/USP6NL/SORT1

Biological Process	GO:0051665	membrane raft localization	4/2279	8/18866	0.0099386	0.1822909	0.1649433	4	GSN/PTPRC/RALA/DOCK2
Biological Process	GO:0090235	regulation of metaphase plate congression	4/2279	8/18866	0.0099386	0.1822909	0.1649433	4	MAD1L1/DYNC1H1/NUMA1/SPAG5
Biological Process	GO:0097527	necroptotic signaling pathway	4/2279	8/18866	0.0099386	0.1822909	0.1649433	4	TNF/RIPK1/RIPK3/FADD
Biological Process	GO:0098597	observational learning	4/2279	8/18866	0.0099386	0.1822909	0.1649433	4	NF1/NRXN1/HTT/NRXN2
Biological Process	GO:1903238	positive regulation of leukocyte tethering or rolling	4/2279	8/18866	0.0099386	0.1822909	0.1649433	4	ELANE/FUT4/FUT7/CCR2
Biological Process	GO:1902905	positive regulation of supramolecular fiber organization	37/2279	208/18866	0.0100075	0.183104	0.165679	37	ARHGEF10/TRIM27/DCTN1/GSN/ITGB1BP1/SMAD3/CTTN/NCK2/SH3PXD2B/WHAMM/BAIAP2L1/NAV3/CDC42EP1/BIN1/BAIAP2/SYNPO2L/APP/CLU/PXN/WNT11/WASL/FER/PFN1/CSF3/GMFB/NUMA1/WDR1/NCKAP1/ARHGEF10L/RB1/PRKCE/MYO1C/ARHGEF15/CYFIP1/GMFG/BAIAP2L2/SYNPO
Biological Process	GO:0071692	protein localization to extracellular region	74/2279	470/18866	0.0100215	0.183104	0.165679	74	LTBP1/SORL1/NADK/IL10/TTN/C1QTNF3/ITPR2/TANGO2/MYRIP/CLEC9A/CACNA1C/LRP5L/NLRP3/RAB11FIP1/FRMD4A/ARRB1/PRKCA/MIR199A1/GPLD1/FGR/TNF/TRAF2/MYO18A/CCL5/ABCG1/GJA5/RHBDF2/TNFSF13B/TLR9/CLEC5A/ITPR1/RFX3/TCF7L2/MIA3/HNF1A/PTPRN2/SPHK2/TGFB1/IL1RN/ENY2/MYO5A/GIPR/BLK/NR1H2/GHRL/KLF7/ARNTL/MYOM1/PER2/SLC16A1/NOD2/POU2F2/MIR199A2/DNM1L/PRKCE/TRAF3IP2/F2R/LMF1/SLC2A1/DYSF/ANKRD1/SREBF1/ADTRP/CADM1/CPT1A/CD33/RPH3AL/EZR/C2CD2L/SIRT3/MIA2/PRKN/P3H1/CCN4
Biological Process	GO:0035592	establishment of protein localization to extracellular region	73/2279	463/18866	0.0101527	0.1851451	0.1675259	73	SORL1/NADK/IL10/TTN/C1QTNF3/ITPR2/TANGO2/MYRIP/CLEC9A/CACNA1C/LRP5L/NLRP3/RAB11FIP1/FRMD4A/ARRB1/PRKCA/MIR199A1/GPLD1/FGR/TNF/TRAF2/MYO18A/CCL5/ABCG1/GJA5/RHBDF2/TNFSF13B/TLR9/CLEC5A/ITPR1/RFX3/TCF7L2/MIA3/HNF1A/PTPRN2/SPHK2/TGFB1/IL1RN/ENY2/MYO5A/GIPR/BLK/NR1H2/GHRL/KLF7/ARNTL/MYOM1/PER2/SLC16A1/NOD2/POU2F2/MIR199A2/DNM1L/PRKCE/TRAF3IP2/F2R/LMF1/SLC2A1/DYSF/ANKRD1/SREBF1/ADTRP/CADM1/CPT1A/CD33/RPH3AL/EZR/C2CD2L/SIRT3/MIA2/PRKN/P3H1/CCN4
Biological Process	GO:0032388	positive regulation of intracellular transport	39/2279	222/18866	0.0102462	0.1864934	0.1687458	39	EHD1/SORL1/TFDP1/PRR5L/TM9SF4/TMEM30A/YWHAH/ITGB1BP1/PIK3R1/SMAD3/DYNC1H1/JUP/EDEM1/USP36/ZDHHC2/TCF7L2/ABLIM3/AKAP5/UBE2J2/TGFB1/MLC1/EDEM2/NUMA1/ITGB2/YWHAZ/CDH1/FIS1/BCL2/NEDD4/TP53BP2/MYO1C/MIEF1/SPAG5/YWHAQ/EZR/PDZK1/PRKAA1/PRKN/RIPOR1

Biological Process	GO:0045730	respiratory burst	10/2279	37/18866	0.0104211	0.1889512	0.1709698	10	MPO/PIK3CD/CD55/SLC11A1/NCF4/NCF2/PGAM1/CAMK1D/GRN/BCR
Biological Process	GO:0090218	positive regulation of lipid kinase activity	10/2279	37/18866	0.0104211	0.1889512	0.1709698	10	FGR/FPR2/FLT1/TGFB1/VAV2/ATG14/PDGFB/NOD2/PDGFRB/VAV3
Biological Process	GO:1905475	regulation of protein localization to membrane	35/2279	195/18866	0.010479	0.1896406	0.1715935	35	PRKCZ/SSH1/KCNE1/TFDP1/GSN/YWHAH/ZMYND8/ITGB1BP1/PIK3R1/RHOG/TNF/ZDHHC2/MRAP/AKAP5/ZDHHC7/PPFIA1/LZTFL1/TGFB1/PRKCH/NUMB/CSK/PRKCI/ITGB2/YWHAZ/FIS1/BCL2/PRKCE/TP53BP2/MYO1C/MIEF1/BCL2L1/YWHAQ/EZR/PDZK1/PRKN
Biological Process	GO:0015748	organophosphate ester transport	26/2279	135/18866	0.0105171	0.1897301	0.1716745	26	OSBPL5/SLC25A25/ABCC1/MFSD2A/TMEM30A/ABCG1/ATP8A1/ANO7/PLTP/PITPNM2/ATP11A/SLC44A4/ATP8B1/PITPNM1/PITPNA/PCTP/ABCA1/OSBPL10/SLC37A1/ATP8B4/SLC33A1/PITPNC1/ABCC11/ANO6/SLC25A1/C2CD2L
Biological Process	GO:0043244	regulation of protein-containing complex disassembly	24/2279	122/18866	0.0105239	0.1897301	0.1716745	24	VILL/GSN/TNF/CAPG/NAV3/UVRAG/IGF1R/TMOD3/SVIL/CLEC16A/WDR1/SPTA1/ADD1/MAP1A/AVIL/SCAF8/FLII/ARHGEF2/SPTAN1/ASPH/TMOD1/SPTBN4/SPEF1/SHFL
Biological Process	GO:0030834	regulation of actin filament depolymerization	13/2279	54/18866	0.0105754	0.1899277	0.1718533	13	VILL/GSN/CAPG/TMOD3/SVIL/WDR1/SPTA1/ADD1/AVIL/FLII/SPTAN1/TMOD1/SPTBN4
Biological Process	GO:0032623	interleukin-2 production	13/2279	54/18866	0.0105754	0.1899277	0.1718533	13	PDE4D/TRIM27/HDAC7/RUNX1/SLC11A1/CCR2/TRAF2/TNFAIP3/SFTPD/NAV3/PTPRC/PNP/EZR
Biological Process	GO:0043406	positive regulation of MAP kinase activity	45/2279	264/18866	0.0105949	0.1899277	0.1718533	45	ELANE/KSR1/TGFA/MAP3K3/MAP2K6/AKAP13/ERN1/PIK3R6/ARRB1/TNF/ZEB2/TRAF2/FPR1/TLR9/TNIK/THBS1/PIK3R5/RIPK1/ILK/FLT1/FGFR1/TGFB1/PDGFD/UBE2V1/MUC20/CXCL17/GHRL/PTPRC/CSK/PDE6H/PDGFB/MAP3K14/TAOK3/IRAK2/NTRK1/NOD2/S1PR2/F2R/PDGFC/MAPKAPK3/PDGFRB/GADD45G/DBNL/PRKAA1/MAP3K20
Biological Process	GO:0008630	intrinsic apoptotic signaling pathway in response to DNA damage	21/2279	103/18866	0.0107725	0.1923854	0.1740772	21	BRCA2/TNFRSF1B/PIK3R1/TNF/HIPK2/NACC2/ZNF385A/TNFRSF1A/CLU/CIDEB/CDIP1/DYRK2/FNIP2/BCL2/CHEK2/CRIP1/BCL2L1/BCL3/XPA/MARCHF7/FBH1

Biological Process	GO:0048661	positive regulation of smooth muscle cell proliferation	21/2279	103/18866	0.0107725	0.1923854	0.1740772	21	ELANE/HDAC4/FOXP1/IL10/MEF2D/MYB/ERN1/TNF/CCL5/TGFBR2/THBS1/GNAI2/MIR27A/P2RY6/PHB/PDGFD/IL6R/PDGFB/DNMT1/PDGFRB/CCN4
Biological Process	GO:1903828	negative regulation of cellular protein localization	23/2279	116/18866	0.0110168	0.1963803	0.1776919	23	KCNE1/ANGPT1/ITGB1BP1/SUFU/UBAC2/DERL2/MRAP/PPFIA1/LZTFL1/TGFB1/LILRB4/NF1/NUMB/ANKRD13A/CSK/INPP5E/ASTN2/MAP1A/CABP1/SP100/BCL2L1/SVIP/ILRUN
Biological Process	GO:0007160	cell-matrix adhesion	40/2279	230/18866	0.0110973	0.1969042	0.1781659	40	PRKCZ/PLAU/COL17A1/RIN2/ITGB1BP1/PIK3R1/CEACAM6/SMAD3/ARHGEF7/CTTN/DUSP22/WHAMM/JUP/THBS1/DISC1/NINJ1/ACTN1/ITGA2B/ILK/ITGB5/PPFIA1/PTPRJ/NF1/ADAMTS13/JAM3/TESK2/ITGB3/ACTG1/PLEKHA2/BCR/ITGB2/LYVE1/BCL2/LYPD5/CORO1C/PARVG/DUSP3/CD96/ITGB6/DAPK3
Biological Process	GO:0006367	transcription initiation from RNA polymerase II promoter	34/2279	189/18866	0.0111354	0.1969042	0.1781659	34	RXRA/MAML3/PPARG/NR1I2/CREB1/MAML1/VDR/E2F3/MAML2/POLR2F/RBPJ/TAF10/HNF1A/RARA/MED13/NR1H2/WWTR1/ATF7IP/RARG/RUNX2/CREBBP/CCNH/ESRRB/MED8/NOTCH1/MED25/SUB1/NOTCH4/ERCC1/TEAD2/NR5A2/XPA/ERCC2/PAXIP1
Biological Process	GO:0032872	regulation of stress-activated MAPK cascade	41/2279	237/18866	0.011168	0.1969042	0.1781659	41	MAP3K3/HIPK3/ERN1/ZNF622/TNF/ZEB2/TRAF2/TRAF1/PHLPP1/HIPK2/TLR9/DUSP22/TNIK/RIPK1/APP/IGF1R/MTURN/SCIMP/AXIN1/COPS5/SH3RF3/STK3/PLCB1/MAPK8IP2/TAOK3/MIR138-2/NOD2/PER1/DUSP3/FOXM1/FOXO1/GADD45G/GDF6/CYLD/ANKRD6/EZR/DTNBP1/MECOM/DBNL/PRKN/MAP3K20
Biological Process	GO:0006476	protein deacetylation	20/2279	97/18866	0.0111705	0.1969042	0.1781659	20	HDAC4/SPRED2/HDAC7/SIN3B/SKI/C6orf89/HDAC1/SPHK2/ZBTB7B/PHB/MTA1/CTBP1/MTA2/PER2/PER1/NEK3/SREBF1/ELK4/SIRT3/PRKAA1
Biological Process	GO:0032651	regulation of interleukin-1 beta production	20/2279	97/18866	0.0111705	0.1969042	0.1781659	20	AZU1/NLRC4/FOXP1/AIM2/NLRP3/SMAD3/MEFV/TNF/TNFAIP3/MIR101-2/MNDA/APP/GHRL/ARRB2/NOD2/F2R/CASP1/CD33/CX3CR1/STMP1
Biological Process	GO:0046634	regulation of alpha-beta T cell activation	20/2279	97/18866	0.0111705	0.1969042	0.1781659	20	IL27/PRKCZ/MYB/RUNX1/CD55/NLRP3/ITPKB/CCR2/LOXL3/ZBTB16/TGFBR2/SMAD7/ZBTB7B/RARA/PTPRC/PNP/HLX/RUNX3/AP3D1/VSIR
Biological Process	GO:0002063	chondrocyte development	9/2279	32/18866	0.0112988	0.1975092	0.1787133	9	CHSY1/CHST11/TGFBR2/SMAD7/POC1A/RARG/BMPR1B/RUNX2/RFLNA

Biological Process	GO:0002724	regulation of T cell cytokine production	9/2279	32/18866	0.0112988	0.1975092	0.1787133	9	PRKCZ/TNFRSF1B/NLRP3/IL1R1/CCR2/TRAF2/HLA-F/SMAD7/IL18R1
Biological Process	GO:0045761	regulation of adenylate cyclase activity	9/2279	32/18866	0.0112988	0.1975092	0.1787133	9	TIMP2/CACNA1C/CCR2/GNAI2/AKAP5/LTB4R2/NF1/PALM/GABBR2
Biological Process	GO:0061036	positive regulation of cartilage development	9/2279	32/18866	0.0112988	0.1975092	0.1787133	9	SMAD3/ZBTB16/SMAD7/POR/BMPR1B/RUNX2/GDF6/RELA/ZNF219
Biological Process	GO:0055002	striated muscle cell development	31/2279	169/18866	0.0113463	0.1975092	0.1787133	31	HDAC4/CTDP1/MYO18B/TTN/CASQ2/MYH11/AKAP13/SKI/MAML1/SLC8A1/MIR199A1/NFATC2/TNNT3/SMYD3/SYNPO2L/MIR24-2/MIR23A/TMOD3/ACTG1/WDR1/BCL2/MIR199A2/MEF2C/FHL2/FLII/PDGFRB/DYSF/ANKRD1/TMOD1/CASQ1/MYORG
Biological Process	GO:0051251	positive regulation of lymphocyte activation	58/2279	357/18866	0.0113476	0.1975092	0.1787133	58	HLA-DMB/PRKCZ/RHOH/LCK/MYB/RUNX1/SIRPB1/CD55/INPP5D/NLRP3/PIK3R1/ITPKB/CCR2/PIK3R6/CCL5/GRAP2/NCK2/NFATC2/TNFSF13B/TLR9/IGHV6-1/ZBTB16/TGFBR2/HLA-F/PTPN6/ZMIZ1/DOCK8/ZBTB7B/TGFB1/RARA/LILRB4/CCDC88B/PTPRC/CD320/CSK/BCL10/SPTA1/ZNF335/PNP/NOD2/BCL2/TP53BP1/MEF2C/SIRPG/HLX/RUNX3/TNFSF13/CYLD/AP3D1/SLC7A1/FADD/PELI1/PCID2/PAXIP1/VAV3/CYRIB/VSIR/NSD2
Biological Process	GO:0014074	response to purine-containing compound	28/2279	149/18866	0.011351	0.1975092	0.1787133	28	TRPM2/BSG/SSH1/KCNE1/PPARG/CASQ2/ITPR2/RAP1B/SLC8A1/APP/P2RY6/FDX1/CRHBP/RAPGEF2/DGKQ/AQP1/AGXT/KCNQ1/PER1/P2RX1/HMGCS1/SREBF1/P2RX5/RELA/EZR/DHODH/AQP9/PRKAA1
Biological Process	GO:0045124	regulation of bone resorption	11/2279	43/18866	0.0113712	0.1975092	0.1787133	11	DEF8/INPP5D/PRKCA/UBASH3B/TNFAIP3/CSF1R/NF1/ITGB3/TMEM64/CSK/PLEKHM1
Biological Process	GO:0009135	purine nucleoside diphosphate metabolic process	26/2279	136/18866	0.0115498	0.1998808	0.1808592	26	FOXK1/HDAC4/PRKAG2/AMPD3/ENO1/HK1/TJP2/PFKFB4/RAE1/CBFA2T3/DHTKD1/APP/OGDH/ADPGK/PGAM1/NUP93/ENO3/HK2/SEC13/GUK1/ESRRB/PKM/AK2/AK5/PFKFB3/PRKAA1
Biological Process	GO:0009179	purine ribonucleoside diphosphate metabolic process	26/2279	136/18866	0.0115498	0.1998808	0.1808592	26	FOXK1/HDAC4/PRKAG2/AMPD3/ENO1/HK1/TJP2/PFKFB4/RAE1/CBFA2T3/DHTKD1/APP/OGDH/ADPGK/PGAM1/NUP93/ENO3/HK2/SEC13/GUK1/ESRRB/PKM/AK2/AK5/PFKFB3/PRKAA1

Biological Process	GO:0006978	DNA damage response, signal transduction by p53 class mediator resulting in transcription of p21 class mediator	6/2279	17/18866	0.0117074	0.2011437	0.1820019	6	BRCA2/HIPK2/ZNF385A/SP100/FOXM1/CHEK2
Biological Process	GO:0030033	microvillus assembly	6/2279	17/18866	0.0117074	0.2011437	0.1820019	6	PLD1/TNIK/ATP8B1/RAPGEF2/ESPN/EZR
Biological Process	GO:0035855	megakaryocyte development	6/2279	17/18866	0.0117074	0.2011437	0.1820019	6	ZFPM1/FLI1/PIP4K2A/PTPN6/ZNF385A/MEIS1
Biological Process	GO:0070293	renal absorption	6/2279	17/18866	0.0117074	0.2011437	0.1820019	6	GSN/SGK1/HNF1A/HBB/KCNQ1/AQP3
Biological Process	GO:0010594	regulation of endothelial cell migration	41/2279	238/18866	0.0119614	0.2034952	0.1841296	41	FOXP1/CALR/NRP2/PPARG/MAP3K3/MIR143/RIN2/HDAC7/ANGPT1/ITGB1BP1/PRKCA/MIR199A1/GPLD1/TNF/MIR101-2/SEMA4A/STAT5A/JUP/THBS1/AKT3/MIR24-2/MIR27A/MIR23A/FGFR1/TGFB1/ZNF580/NF1/BMPER/ITGB3/GRN/PDGFB/ETS1/NFE2L2/VASH1/MIR199A2/NOTCH1/MEF2C/SP100/PLCG1/ADGRA2/PLPP3
Biological Process	GO:0006735	NADH regeneration	8/2279	27/18866	0.0119785	0.2034952	0.1841296	8	FOXK1/ENO1/HK1/ADPGK/PGAM1/ENO3/HK2/PKM
Biological Process	GO:0035459	vesicle cargo loading	8/2279	27/18866	0.0119785	0.2034952	0.1841296	8	KIF13A/SEC23B/MIA3/SEC13/SEC24C/TMED2/SEC31A/MIA2
Biological Process	GO:0061621	canonical glycolysis	8/2279	27/18866	0.0119785	0.2034952	0.1841296	8	FOXK1/ENO1/HK1/ADPGK/PGAM1/ENO3/HK2/PKM
Biological Process	GO:0061718	glucose catabolic process to pyruvate	8/2279	27/18866	0.0119785	0.2034952	0.1841296	8	FOXK1/ENO1/HK1/ADPGK/PGAM1/ENO3/HK2/PKM

Biological Process	GO:0099590	neurotransmitter receptor internalization	8/2279	27/18866	0.0119785	0.2034952	0.1841296	8	DNM2/NUMB/ITGB3/GSG1L/DNM1/DNM3/EPS15/MX1
Biological Process	GO:0047496	vesicle transport along microtubule	12/2279	49/18866	0.0119942	0.2034952	0.1841296	12	PRKCZ/NDE1/KIF13A/DYNC1H1/CNIH2/HTT/KIF1B/KIFAP3/AP3D1/FBXW11/NDEL1/DTNBP1
Biological Process	GO:0000422	autophagy of mitochondrion	17/2279	79/18866	0.0121686	0.2049888	0.1854811	17	VPS13D/CTTN/FBXO7/WIPI2/CAMKK2/USP36/SMURF1/CLEC16A/HTT/ATG14/HK2/FIS1/DNM1L/TSC2/SREBF1/MARK2/PRKN
Biological Process	GO:0016575	histone deacetylation	17/2279	79/18866	0.0121686	0.2049888	0.1854811	17	HDAC4/HDAC7/SIN3B/SKI/C6orf89/HDAC1/SPHK2/ZBTB7B/PHB/MTA1/CTBP1/MTA2/PER2/PER1/SREBF1/ELK4/SIRT3
Biological Process	GO:0043536	positive regulation of blood vessel endothelial cell migration	17/2279	79/18866	0.0121686	0.2049888	0.1854811	17	MAP3K3/MIR143/HDAC7/ANGPT1/PRKCA/MIR101-2/STAT5A/THBS1/AKT3/MIR27A/MIR23A/FGFR1/TGFB1/PDGFB/ETS1/NFE2L2/PLCG1
Biological Process	GO:0061726	mitochondrion disassembly	17/2279	79/18866	0.0121686	0.2049888	0.1854811	17	VPS13D/CTTN/FBXO7/WIPI2/CAMKK2/USP36/SMURF1/CLEC16A/HTT/ATG14/HK2/FIS1/DNM1L/TSC2/SREBF1/MARK2/PRKN
Biological Process	GO:0051216	cartilage development	35/2279	197/18866	0.0122071	0.2051159	0.1855961	35	CSGALNACT1/MEF2D/CHI3L1/RUNX1/CHSY1/SMAD3/TGFBI/GPLD1/ANXA6/CHST11/ZBTB16/TGFBR2/SMAD7/POC1A/WNT11/FGFR1/PAX7/TGFB1/RARA/POR/GHRL/RARG/BMPR1B/WNT5B/RUNX2/CREB3L2/TRPS1/MEF2C/NPPC/RUNX3/GDF6/RELA/ZNF219/RFLNA/CCN4
Biological Process	GO:0036344	platelet morphogenesis	7/2279	22/18866	0.0122409	0.2051159	0.1855961	7	ZFPM1/MYH9/ACTN1/PTPN6/ZNF385A/WDR1/MEF2C
Biological Process	GO:0062033	positive regulation of mitotic sister chromatid segregation	7/2279	22/18866	0.0122409	0.2051159	0.1855961	7	NSMCE2/CUL3/CDC16/NUMA1/RB1/ANAPC7/SLF1

Biological Process	GO:0060350	endochondral bone morphogenesis	13/2279	55/18866	0.0123562	0.2059281	0.1863311	13	CSGALNACT1/MEF2D/ANXA6/TGFBR2/ALPL/POC1A/RARA/POR/RARG/BMPR1B/RUNX2/MEF2C/NPPC
Biological Process	GO:0035148	tube formation	28/2279	150/18866	0.0123926	0.2059281	0.1863311	28	SHROOM3/SKI/ITGB1BP1/SETD2/SPINT2/MTHFD1L/SUFU/ZEB2/MIB1/PAX2/TGFB1/RARA/PFN1/RARG/STK3/GRHL2/BCL10/SDCCAG8/MTHFD1/RALA/YWHAZ/NOTCH1/TMED2/TSC2/TEAD2/LMO4/NODAL/BRD2
Biological Process	GO:0009895	negative regulation of catabolic process	53/2279	323/18866	0.0124613	0.2059281	0.1863311	53	TRIM39/FOXK1/HDAC4/TIMP2/SORL1/IL10/MFSD2A/SLC11A1/PAM16/SF3B3/BMF/SMAD3/MIR199A1/GPLD1/SUFU/TNF/HIPK2/CBFA2T3/UBAC2/LARP1/MGAT3/PKP1/USP36/SNX3/DERL2/FEZ2/ZDHHC7/CRTC3/PHB/CLEC16A/ACACB/PSMF1/TBC1D14/NPC1/NBAS/BCL2/MIR199A2/MAP1A/PAIP1/TSC2/DYSF/PDE3B/SECISBP2/RELA/SYNCRIP/BAG5/EIF4G1/SVIP/BSCL2/PRKAA1/TENT5C/TENT5A/MARCHF7
Biological Process	GO:0030316	osteoclast differentiation	20/2279	98/18866	0.012485	0.2059281	0.1863311	20	FOXP1/SBNO2/CREB1/INPP5D/PIK3R1/GAB2/TNF/UBASH3B/CSF1R/TMEM178A/LILRB4/NF1/LRRK1/TMEM64/LRRC17/OSTM1/LTF/CEBPB/FAM20C/CCN4
Biological Process	GO:0030239	myofibril assembly	15/2279	67/18866	0.0125106	0.2059281	0.1863311	15	TTN/CASQ2/MYH11/AKAP13/TNNT3/SYNPO2L/TMOD3/ACTG1/WDR1/MEF2C/FLII/PDGFRB/ANKRD1/TMOD1/CASQ1
Biological Process	GO:0046847	filopodium assembly	14/2279	61/18866	0.012513	0.2059281	0.1863311	14	TRPM2/FGD4/ZMYND8/FGD5/SPATA13/ARAP1/WASL/ESPN/PALM/RALA/DNM3/S1PR2/PPP1R16B/EZR
Biological Process	GO:0046596	regulation of viral entry into host cell	10/2279	38/18866	0.0126477	0.2059281	0.1863311	10	TRIM27/P4HB/TRIM38/GSN/TRIM8/TRIM10/SNX3/FCN1/TRIM5/TRIM26
Biological Process	GO:1900026	positive regulation of substrate adhesion-dependent cell spreading	10/2279	38/18866	0.0126477	0.2059281	0.1863311	10	P4HB/RREB1/CALR/DOCK1/ARHGEF7/DNM2/PREX1/OLFM4/CASS4/MYADM
Biological Process	GO:1903900	regulation of viral life cycle	29/2279	157/18866	0.0127508	0.2059281	0.1863311	29	SLPI/TRIM27/P4HB/TRIM38/GSN/TRIM8/TRIM10/SRPK2/TNF/CCL5/LARP1/SNX3/PC/PHB/FCN1/TRIM5/TNIP1/BCL2/NOTCH1/LTF/ILF3/MX1/NR5A2/PARP10/BANF1/ISG20/TRIM26/PRKN/SHFL

Biological Process	GO:0032652	regulation of interleukin-1 production	22/2279	111/18866	0.0127652	0.2059281	0.1863311	22	AZU1/NLRC4/FOXP1/IL10/AIM2/IL16/NLRP3/SMAD3/MEFV/TNF/TNFAIP3/MIR101-2/MNDA/APP/GHRL/ARRB2/NOD2/F2R/CASP1/CD33/CX3CR1/STMP1
Biological Process	GO:0046031	ADP metabolic process	24/2279	124/18866	0.0128034	0.2059281	0.1863311	24	FOXK1/HDAC4/PRKAG2/AMPD3/ENO1/HK1/PFKFB4/RAE1/CBFA2T3/DHTKD1/APP/OGDH/ADPGK/PGAM1/NUP93/ENO3/HK2/SEC13/ESRRB/PKM/AK2/AK5/PFKFB3/PRKAA1
Biological Process	GO:0071902	positive regulation of protein serine/threonine kinase activity	56/2279	345/18866	0.0128923	0.2059281	0.1863311	56	ELANE/KSR1/RPTOR/TGFA/ACSL1/MAP3K3/MAP2K6/AKAP13/ERN1/PIK3R6/ARRB1/TNF/ZEB2/TRAF2/CSF1R/FPR1/TLR9/TNIK/THBS1/PIK3R5/RIPK1/ILK/FLT1/FGFR1/TGFB1/RAPGEF2/PDGFD/UBE2V1/MUC20/CXCL17/GHRL/PTPRC/CAB39/CSK/STK3/PDE6H/PDGFB/MAP3K14/TAOK3/IRAK2/NTRK1/NOD2/S1PR2/PIH1D1/F2R/PDGFC/LTF/MAPKAPK3/PDGFRB/GADD45G/CCNY/CCNYL1/DBNL/DAZAP2/PRKAA1/MAP3K20
Biological Process	GO:0002791	regulation of peptide secretion	61/2279	381/18866	0.0129657	0.2059281	0.1863311	61	S100A8/SORL1/NADK/IL10/TTN/C1QTNF3/ITPR2/MYRIP/CLEC9A/CACNA1C/LRP5L/RAB11FIP1/FRMD4A/ARRB1/PRKCA/MIR199A1/GPLD1/FGR/TNF/TRAF2/MYO18A/CCL5/ABCG1/GJA5/RHBDF2/TLR9/CLEC5A/ITSN1/ITPR1/RFX3/TCF7L2/TGFB1/CRHBP/ENY2/GIPR/BLK/NR1H2/GHRL/KLF7/ARNTL/MYOM1/PER2/SLC16A1/MIR199A2/DNM1L/PRKCE/F2R/SLC2A1/ANKRD1/SREBF1/ADTRP/CADM1/CPT1A/CD33/RPH3AL/EZR/C2CD2L/SIRT3/PRKN/P3H1/CCN4
Biological Process	GO:0045765	regulation of angiogenesis	64/2279	403/18866	0.0131965	0.2059281	0.1863311	64	LRG1/RNH1/IL10/PPARG/C3AR1/MAP3K3/MIR143/MIR145/CHI3L1/HDAC7/RUNX1/ITGB1BP1/CCR2/PIK3R6/PRKCA/COL4A2/MIR199A1/TNF/TNFAIP3/MIR101-2/AGO1/HIPK2/SEMA4A/JUP/TGFBR2/THBS1/CTSH/DLL1/AKT3/MIR24-2/MIR27A/MIR23A/FLT1/JAK1/AGO2/EPN2/PRKCB/AQP1/NF1/BMPER/HK2/GHRL/GRN/ETS1/NFE2L2/ENG/ITGB2/VASH1/MIR138-2/PKM/MIR199A2/NOTCH1/SP100/GPR4/AGTR1/PPP1R16B/HHEX/TNFSF12/PDE3B/PLCG1/NODAL/CX3CR1/CEMIP2/ADGRA2
Biological Process	GO:0016331	morphogenesis of embryonic epithelium	28/2279	151/18866	0.01351	0.2059281	0.1863311	28	SHROOM3/SKI/SETD2/SPINT2/MTHFD1L/SUFU/ZEB2/MIB1/PAX2/TGFB1/RDH10/RARA/PFN1/TGFB1I1/RARG/STK3/GRHL2/BCL10/MTHFD1/RALA/TMED2/TSC2/TEAD2/ALDH1A2/LMO4/NODAL/GRSF1/BRD2
Biological Process	GO:0060249	anatomical structure homeostasis	73/2279	469/18866	0.0135441	0.2059281	0.1863311	73	PIWIL4/BLM/DCTN1/DEF8/MFSD2A/TNKS/POLE/TJP2/LPCAT1/BRCA2/INPP5D/ANGPT1/HMBOX1/CRB1/CCR2/ANKRD11/PRKCA/RTEL1/HUS1/UBASH3B/TNFAIP3/NSMCE2/CDH23/CSF1R/SFTPD/TLR9/YLPM1/ATP2B2/POLA2/CTSH/MBP/CCDC66/MTF1/CDH3/NF1/LRRK1/JAM3/WWTR1/ITGB3/ACTG1/SMARCAL1/TMEM64/CSK/SMG6/SLC22A5/PLEKHM1/ESRRB/ADD1/NOD2/RB1/BCL2/NOTCH1/ACACA/MAP1A/GCNT2/P2RX1/CDHR1/EPAS1/SP100/F2R/LTF/PDGFRB/SLC2A1/ERCC1/TYRO3/SLX4/POLD3/CCT6A/POLE4/NPHP4/PRKAA1/TFIP11/PRKN
Biological Process	GO:0050654	chondroitin sulfate proteoglycan metabolic process	11/2279	44/18866	0.0135478	0.2059281	0.1863311	11	CSGALNACT1/SPOCK2/DSE/HEXB/CHSY1/CHST11/CHST15/EGFLAM/XYLT1/ARSB/PXYLP1
Biological Process	GO:0070302	regulation of stress-activated protein kinase signaling cascade	41/2279	240/18866	0.013687	0.2059281	0.1863311	41	MAP3K3/HIPK3/ERN1/ZNF622/TNF/ZEB2/TRAF2/TRAF1/PHLPP1/HIPK2/TLR9/DUSP22/TNIK/RIPK1/APP/IGF1R/MTURN/SCIMP/AXIN1/COPS5/SH3RF3/STK3/PLCB1/MAPK8IP2/TAOK3/MIR138-2/NOD2/PER1/DUSP3/FOXM1/FOXO1/GADD45G/GDF6/CYLD/ANKRD6/EZR/DTNBP1/MECOM/DBNL/PRKN/MAP3K20

Biological Process	GO:0019318	hexose metabolic process	43/2279	254/18866	0.0137201	0.2059281	0.1863311	43	MAEA/FUT4/FOXK1/ENO1/FUT7/HK1/C1QTNF3/PFKFB4/GPLD1/TNF/PGP/CHST15/ADPGK/PGAM1/GLYCTK/PC/NISCH/DGKQ/ENO3/DYRK2/HK2/GHRL/GNB3/ACACB/PPP1CB/PMM2/ESRRB/PER2/PKM/PCK2/FOXO1/NCOA2/MAN2C1/SOGA1/KBTBD2/WDTC1/CPT1A/PFKFB3/SLC25A1/PRKAA1/PRKN/SDHAF3/RUBCNL
Biological Process	GO:0034331	cell junction maintenance	9/2279	33/18866	0.0139091	0.2059281	0.1863311	9	PRTN3/DCTN1/CD177/CSF1R/SYNGAP1/PLEKHA7/CTBP2/F2R/KIFC3
Biological Process	GO:0048384	retinoic acid receptor signaling pathway	9/2279	33/18866	0.0139091	0.2059281	0.1863311	9	RXRA/CALR/PPARG/ACTN4/RARA/NR1H2/CTBP2/RARG/ALDH1A2
Biological Process	GO:0070371	ERK1 and ERK2 cascade	53/2279	325/18866	0.0139687	0.2059281	0.1863311	53	PRKCZ/SPRED2/MIR145/CHI3L1/ANGPT1/ITGB1BP1/RAP1B/ARRB1/PRKCA/TNF/CCL5/CSF1R/FPR2/CTSH/C5AR2/GNAI2/APP/PTPN6/MIR24-2/MIR27A/MIR23A/MTURN/SCIMP/P2RY6/TGFB1/PHB/MARCO/RAPGEF2/PDGFD/BMPER/CCL20/TNIP1/PTPRC/CSK/PDGFB/ARRB2/YWHAZ/NTRK1/NOD2/NOTCH1/GCNT2/DUSP3/F2R/PDGFC/PDGFRB/ARHGAP8/EZR/NODAL/CCL22/LIF/NDRG2/SIRT3/DENND2B
Biological Process	GO:0042176	regulation of protein catabolic process	63/2279	397/18866	0.0140534	0.2059281	0.1863311	63	TRIM39/RNF144A/TIMP2/SORL1/FZR1/IL10/RNF19A/STX5/WWP2/CSNK1D/LPCAT1/TNFRSF1B/SF3B3/SMAD3/GPLD1/SUFU/TNF/TNFAIP3/HIPK2/VGLL4/CBFA2T3/UBAC2/MGAT3/ODC1/DISC1/EDEM1/SNX3/ZDHHC2/DERL2/CLU/SMAD7/PLEKHG5/PHB/SMURF1/AXIN1/CLEC16A/EDEM2/CSNK1E/PSMF1/GFAP/NFE2L2/ARNTL/CDH1/RNF144B/MAP1A/GNA12/UBXN2A/UBE3A/CHEK2/NEDD4/RNF14/FOXO1/DYSF/TRIB1/TNFSF12/HECW2/RELA/EZR/RNF40/BAG5/SVIP/PRKN/MARCHF7
Biological Process	GO:0048199	vesicle targeting, to, from or within Golgi	16/2279	74/18866	0.0140795	0.2059281	0.1863311	16	CTSZ/SERPINA1/TGFA/STX5/CSNK1D/TRAPPC10/CUL3/TRAPPC9/CNIH2/CD59/SEC13/ANKRD28/SEC24C/TMED2/SEC31A/TRAPPC2
Biological Process	GO:0001838	embryonic epithelial tube formation	24/2279	125/18866	0.0140828	0.2059281	0.1863311	24	SHROOM3/SKI/SETD2/SPINT2/MTHFD1L/SUFU/ZEB2/MIB1/PAX2/TGFB1/RARA/PFN1/RARG/STK3/GRHL2/BCL10/MTHFD1/RALA/TMED2/TSC2/TEAD2/LMO4/NODAL/BRD2
Biological Process	GO:0001767	establishment of lymphocyte polarity	5/2279	13/18866	0.0142826	0.2059281	0.1863311	5	GSN/SNX27/MYH9/DOCK8/DOCK2
Biological Process	GO:0002430	complement receptor mediated signaling pathway	5/2279	13/18866	0.0142826	0.2059281	0.1863311	5	C3AR1/GPLD1/FPR2/FPR1/C5AR2

Biological Process	GO:0009650	UV protection	5/2279	13/18866	0.0142826	0.2059281	0.1863311	5	MFAP4/CAT/ERCC1/XPA/ERCC2
Biological Process	GO:0010745	negative regulation of macrophage derived foam cell differentiation	5/2279	13/18866	0.0142826	0.2059281	0.1863311	5	PPARG/ABCG1/NR1H2/ITGB3/ABCA1
Biological Process	GO:0030656	regulation of vitamin metabolic process	5/2279	13/18866	0.0142826	0.2059281	0.1863311	5	GFI1/VDR/TNF/RDH10/CD320
Biological Process	GO:0045842	positive regulation of mitotic metaphase/anaphase transition	5/2279	13/18866	0.0142826	0.2059281	0.1863311	5	NSMCE2/CUL3/CDC16/RB1/ANAPC7
Biological Process	GO:0048096	chromatin-mediated maintenance of transcription	5/2279	13/18866	0.0142826	0.2059281	0.1863311	5	SERTAD2/PADI2/ZMIZ1/ARID1B/SMARCD1
Biological Process	GO:0048505	regulation of timing of cell differentiation	5/2279	13/18866	0.0142826	0.2059281	0.1863311	5	DLL1/RBPJ/NOTCH1/NODAL/HES3
Biological Process	GO:0099566	regulation of postsynaptic cytosolic calcium ion concentration	5/2279	13/18866	0.0142826	0.2059281	0.1863311	5	SLC8A1/ATP2B2/ITPR1/MYO5A/SYNPO
Biological Process	GO:1900025	negative regulation of substrate adhesion-dependent cell spreading	5/2279	13/18866	0.0142826	0.2059281	0.1863311	5	TACSTD2/KANK1/ITGB1BP1/ACTN4/CORO1C
Biological Process	GO:1901970	positive regulation of mitotic sister chromatid separation	5/2279	13/18866	0.0142826	0.2059281	0.1863311	5	NSMCE2/CUL3/CDC16/RB1/ANAPC7

Biological Process	GO:1905666	regulation of protein localization to endosome	5/2279	13/18866	0.0142826	0.2059281	0.1863311	5	SORL1/MGAT3/ANKRD13A/ZDHHC1/EZR
Biological Process	GO:0030888	regulation of B cell proliferation	14/2279	62/18866	0.0144145	0.2059281	0.1863311	14	IL10/INPP5D/NFATC2/TNFSF13B/TLR9/MNDA/TNFRSF21/BLK/PTPRC/CD320/BCL2/MEF2C/PELI1/VAV3
Biological Process	GO:0050714	positive regulation of protein secretion	31/2279	172/18866	0.0144581	0.2059281	0.1863311	31	SORL1/NADK/IL10/TTN/C1QTNF3/MYRIP/CLEC9A/FRMD4A/ARRB1/GPLD1/FGR/TNF/MYO18A/ABCG1/CLEC5A/TCF7L2/TGFB1/GIPR/BLK/NR1H2/GHRL/MYOM1/DNM1L/PRKCE/ANKRD1/CADM1/CD33/RPH3AL/EZR/C2CD2L/SIRT3
Biological Process	GO:0032868	response to insulin	47/2279	283/18866	0.0144814	0.2059281	0.1863311	47	PRKCZ/SORL1/FUT7/IL10/PPARG/KANK1/PTPRE/PIK3R1/GPLD1/RAB31/BAIAP2L1/BAIAP2/GRB10/GNAI2/ATP6V1B2/ZDHHC7/IGF1R/ZBTB7B/FER/PRKCB/MYO5A/CAT/PRKCI/SORT1/INSRR/PKM/ATP6V0B/BCAR3/PCK2/MYO1C/TSC2/SLC2A1/FOXO1/SOGA1/SREBF1/KBTBD2/PDE3B/CYFIP1/RELA/WDTC1/NDEL1/LPIN1/HSD11B2/PRKAA1/MARS1/ATP6V0C/ATP6V1C1
Biological Process	GO:0002085	inhibition of neuroepithelial cell differentiation	3/2279	5/18866	0.0145742	0.2059281	0.1863311	3	DLL1/NOTCH1/NODAL
Biological Process	GO:0003415	chondrocyte hypertrophy	3/2279	5/18866	0.0145742	0.2059281	0.1863311	3	TGFBR2/SMAD7/RARG
Biological Process	GO:0003431	growth plate cartilage chondrocyte development	3/2279	5/18866	0.0145742	0.2059281	0.1863311	3	TGFBR2/POC1A/RARG
Biological Process	GO:0010891	negative regulation of sequestering of triglyceride	3/2279	5/18866	0.0145742	0.2059281	0.1863311	3	PNPLA2/PPARG/ABHD5
Biological Process	GO:0014004	microglia differentiation	3/2279	5/18866	0.0145742	0.2059281	0.1863311	3	APP/TGFB1/TSPAN2

Biological Process	GO:0031033	myosin filament organization	3/2279	5/18866	0.0145742	0.2059281	0.1863311	3	TTN/MYH11/DAPK3
Biological Process	GO:0048298	positive regulation of isotype switching to IgA isotypes	3/2279	5/18866	0.0145742	0.2059281	0.1863311	3	TGFB1/TNFSF13/NSD2
Biological Process	GO:0048625	myoblast fate commitment	3/2279	5/18866	0.0145742	0.2059281	0.1863311	3	TCF7L2/IFRD1/EPAS1
Biological Process	GO:0060414	aorta smooth muscle tissue morphogenesis	3/2279	5/18866	0.0145742	0.2059281	0.1863311	3	MIR143/MIR145/MYLK
Biological Process	GO:0070345	negative regulation of fat cell proliferation	3/2279	5/18866	0.0145742	0.2059281	0.1863311	3	TFDP1/E2F3/PER2
Biological Process	GO:0072144	glomerular mesangial cell development	3/2279	5/18866	0.0145742	0.2059281	0.1863311	3	PDGFB/NOTCH1/GPR4
Biological Process	GO:1905832	positive regulation of spindle assembly	3/2279	5/18866	0.0145742	0.2059281	0.1863311	3	DYNC1H1/NUMA1/SPAG5
Biological Process	GO:2000370	positive regulation of clathrin-dependent endocytosis	3/2279	5/18866	0.0145742	0.2059281	0.1863311	3	DNM2/WASL/DGKD
Biological Process	GO:2000407	regulation of T cell extravasation	3/2279	5/18866	0.0145742	0.2059281	0.1863311	3	CCR2/RIPK3/FADD

Biological Process	GO:0000117	regulation of transcription involved in G2/M transition of mitotic cell cycle	2/2279	2/18866	0.0145868	0.2059281	0.1863311	2	ESRRB/BACH1
Biological Process	GO:0001831	trophectodermal cellular morphogenesis	2/2279	2/18866	0.0145868	0.2059281	0.1863311	2	CUL3/NODAL
Biological Process	GO:0002728	negative regulation of natural killer cell cytokine production	2/2279	2/18866	0.0145868	0.2059281	0.1863311	2	HLA-F/CD96
Biological Process	GO:0006083	acetate metabolic process	2/2279	2/18866	0.0145868	0.2059281	0.1863311	2	ACSS1/ACSS2
Biological Process	GO:0006668	sphinganine-1-phosphate metabolic process	2/2279	2/18866	0.0145868	0.2059281	0.1863311	2	SPHK2/SGPP1
Biological Process	GO:0009183	purine deoxyribonucleoside diphosphate biosynthetic process	2/2279	2/18866	0.0145868	0.2059281	0.1863311	2	GUK1/AK5
Biological Process	GO:0009257	10-formyltetrahydrofolate biosynthetic process	2/2279	2/18866	0.0145868	0.2059281	0.1863311	2	MTHFD1L/MTHFD1
Biological Process	GO:0019413	acetate biosynthetic process	2/2279	2/18866	0.0145868	0.2059281	0.1863311	2	ACSS1/ACSS2
Biological Process	GO:0019417	sulfur oxidation	2/2279	2/18866	0.0145868	0.2059281	0.1863311	2	MICAL1/MICAL2

Biological Process	GO:0019427	acetyl-CoA biosynthetic process from acetate	2/2279	2/18866	0.0145868	0.2059281	0.1863311	2	ACSS1/ACSS2
Biological Process	GO:0019542	propionate biosynthetic process	2/2279	2/18866	0.0145868	0.2059281	0.1863311	2	ACSS1/ACSS2
Biological Process	GO:0032603	fractalkine production	2/2279	2/18866	0.0145868	0.2059281	0.1863311	2	AZU1/TNF
Biological Process	GO:0035408	histone H3-T6 phosphorylation	2/2279	2/18866	0.0145868	0.2059281	0.1863311	2	PRKCA/PRKCB
Biological Process	GO:0035526	retrograde transport, plasma membrane to Golgi	2/2279	2/18866	0.0145868	0.2059281	0.1863311	2	RAB43/USP6NL
Biological Process	GO:0035674	tricarboxylic acid transmembrane transport	2/2279	2/18866	0.0145868	0.2059281	0.1863311	2	SLC13A5/SLC25A1
Biological Process	GO:0035698	CD8-positive, alpha-beta cytotoxic T cell extravasation	2/2279	2/18866	0.0145868	0.2059281	0.1863311	2	RIPK3/FADD
Biological Process	GO:0043314	negative regulation of neutrophil degranulation	2/2279	2/18866	0.0145868	0.2059281	0.1863311	2	ABR/BCR
Biological Process	GO:0044663	establishment or maintenance of cell type involved in phenotypic switching	2/2279	2/18866	0.0145868	0.2059281	0.1863311	2	MIR143/MIR145

Biological Process	GO:0044855	plasma membrane raft distribution	2/2279	2/18866	0.0145868	0.2059281	0.1863311	2	GSN/PTPRC
Biological Process	GO:0044856	plasma membrane raft localization	2/2279	2/18866	0.0145868	0.2059281	0.1863311	2	GSN/PTPRC
Biological Process	GO:0045659	negative regulation of neutrophil differentiation	2/2279	2/18866	0.0145868	0.2059281	0.1863311	2	INPP5D/TRIB1
Biological Process	GO:0045994	positive regulation of translational initiation by iron	2/2279	2/18866	0.0145868	0.2059281	0.1863311	2	RXRA/TNF
Biological Process	GO:0046778	modification by virus of host mRNA processing	2/2279	2/18866	0.0145868	0.2059281	0.1863311	2	PABPN1/CPSF4
Biological Process	GO:0060357	regulation of leucine import	2/2279	2/18866	0.0145868	0.2059281	0.1863311	2	SLC43A2/SLC43A1
Biological Process	GO:0060358	negative regulation of leucine import	2/2279	2/18866	0.0145868	0.2059281	0.1863311	2	SLC43A2/SLC43A1
Biological Process	GO:0060844	arterial endothelial cell fate commitment	2/2279	2/18866	0.0145868	0.2059281	0.1863311	2	DLL1/RBPJ
Biological Process	GO:0061766	positive regulation of lung blood pressure	2/2279	2/18866	0.0145868	0.2059281	0.1863311	2	MIR199A1/MIR199A2

Biological Process	GO:0071716	leukotriene transport	2/2279	2/18866	0.0145868	0.2059281	0.1863311	2	ABCC1/ABCC2
Biological Process	GO:0071846	actin filament debranching	2/2279	2/18866	0.0145868	0.2059281	0.1863311	2	GMFB/GMFG
Biological Process	GO:0071963	establishment or maintenance of cell polarity regulating cell shape	2/2279	2/18866	0.0145868	0.2059281	0.1863311	2	PARVB/MARK2
Biological Process	GO:0090108	positive regulation of high-density lipoprotein particle assembly	2/2279	2/18866	0.0145868	0.2059281	0.1863311	2	NR1H2/ABCA1
Biological Process	GO:1900756	protein processing in phagocytic vesicle	2/2279	2/18866	0.0145868	0.2059281	0.1863311	2	GSN/MYH9
Biological Process	GO:1902960	negative regulation of aspartic-type endopeptidase activity involved in amyloid precursor protein catabolic process	2/2279	2/18866	0.0145868	0.2059281	0.1863311	2	SORL1/BIN1
Biological Process	GO:1902996	regulation of neurofibrillary tangle assembly	2/2279	2/18866	0.0145868	0.2059281	0.1863311	2	SORL1/CLU
Biological Process	GO:1903526	negative regulation of membrane tubulation	2/2279	2/18866	0.0145868	0.2059281	0.1863311	2	DNM2/WASL
Biological Process	GO:1903565	negative regulation of protein localization to cilium	2/2279	2/18866	0.0145868	0.2059281	0.1863311	2	LZTFL1/INPP5E

Biological Process	GO:1903921	regulation of protein processing in phagocytic vesicle	2/2279	2/18866	0.0145868	0.2059281	0.1863311	2	GSN/MYH9
Biological Process	GO:1903923	positive regulation of protein processing in phagocytic vesicle	2/2279	2/18866	0.0145868	0.2059281	0.1863311	2	GSN/MYH9
Biological Process	GO:1904017	cellular response to Thyroglobulin triiodothyronine	2/2279	2/18866	0.0145868	0.2059281	0.1863311	2	NCOA1/NCOA2
Biological Process	GO:1904675	regulation of somatic stem cell division	2/2279	2/18866	0.0145868	0.2059281	0.1863311	2	MIR145/LBH
Biological Process	GO:1904719	positive regulation of AMPA glutamate receptor clustering	2/2279	2/18866	0.0145868	0.2059281	0.1863311	2	SSH1/ZDHHC2
Biological Process	GO:1905424	regulation of Wnt-mediated midbrain dopaminergic neuron differentiation	2/2279	2/18866	0.0145868	0.2059281	0.1863311	2	CSNK1D/CSNK1E
Biological Process	GO:1905426	positive regulation of Wnt-mediated midbrain dopaminergic neuron differentiation	2/2279	2/18866	0.0145868	0.2059281	0.1863311	2	CSNK1D/CSNK1E
Biological Process	GO:1905532	regulation of leucine import across plasma membrane	2/2279	2/18866	0.0145868	0.2059281	0.1863311	2	SLC43A2/SLC43A1
Biological Process	GO:1905533	negative regulation of leucine import across plasma membrane	2/2279	2/18866	0.0145868	0.2059281	0.1863311	2	SLC43A2/SLC43A1

Biological Process	GO:1905704	positive regulation of inhibitory synapse assembly	2/2279	2/18866	0.0145868	0.2059281	0.1863311	2	SEMA4A/SEMA4D
Biological Process	GO:1905751	positive regulation of endosome to plasma membrane protein transport	2/2279	2/18866	0.0145868	0.2059281	0.1863311	2	ZDHHC2/AKAP5
Biological Process	GO:2000451	positive regulation of CD8-positive, alpha-beta T cell extravasation	2/2279	2/18866	0.0145868	0.2059281	0.1863311	2	CCR2/FADD
Biological Process	GO:2000452	regulation of CD8-positive, alpha-beta cytotoxic T cell extravasation	2/2279	2/18866	0.0145868	0.2059281	0.1863311	2	RIPK3/FADD
Biological Process	GO:2000471	regulation of hematopoietic stem cell migration	2/2279	2/18866	0.0145868	0.2059281	0.1863311	2	CCR2/PTPRC
Biological Process	GO:2000473	positive regulation of hematopoietic stem cell migration	2/2279	2/18866	0.0145868	0.2059281	0.1863311	2	CCR2/PTPRC
Biological Process	GO:2001295	malonyl-CoA biosynthetic process	2/2279	2/18866	0.0145868	0.2059281	0.1863311	2	ACACB/ACACA
Biological Process	GO:0006090	pyruvate metabolic process	28/2279	152/18866	0.0147072	0.2073185	0.1875892	28	FOXK1/HDAC4/PRKAG2/BSG/ENO1/HK1/SLC16A3/PFKFB4/RAE1/CBFA2T3/DHTKD1/APP/OGDH/ADPGK/PGAM1/PC/NUP93/ENO3/AGXT/HK2/SEC13/ESRRB/HOGA1/SLC16A1/PKM/PCK2/PFKFB3/PRKAA1
Biological Process	GO:0032611	interleukin-1 beta production	21/2279	106/18866	0.0148016	0.2083401	0.1885135	21	AZU1/NLRC4/FOXP1/AIM2/NLRP3/SMAD3/MEFV/TNF/TNFAIP3/MIR101-2/MNDA/APP/GHRL/ABCA1/ARRB2/NOD2/F2R/CASP1/CD33/CX3CR1/STMP1

Biological Process	GO:0001558	regulation of cell growth	66/2279	420/18866	0.0149435	0.2100266	0.1900395	66	SEMA6B/S100A8/RPTOR/CPNE6/CDA/ENO1/PPARG/CTDP1/RPS6KA1/SERTAD2/MFSD2A/APBB2/EXOSC2/URI1/SMAD3/CTTN/DNM2/MIR199A1/CPNE5/SGK1/BCL11A/VGLL4/SEMA4A/RTN4/KRT17/MEG3/DISC1/SLC44A4/SEMA4B/DERL2/SMARCA2/IFRD1/MUC12/ILK/SMAD7/WNT11/SPHK2/TGFB1/PHB/PTPRJ/SEMA4D/RERG/SMURF1/CHPT1/EPB41L3/CDHR2/CDH4/SH3BP4/PPP2R1A/RB1/BCL2/MIR199A2/CDKL3/SYT17/SLIT3/AGTR1/CYFIP1/TRPV2/SGK2/NDEL1/SCGB3A1/INHBA/EIF4G1/DBNL/SPART/PRKN
Biological Process	GO:0070588	calcium ion transmembrane transport	51/2279	312/18866	0.0150075	0.210615	0.190572	51	TRPM2/PDE4D/SLC25A25/THADA/CASQ2/ITPR2/LCK/CACNA1C/CACNA2D4/SLC24A4/SLC8A1/PKD1L1/ANXA6/UBASH3B/SPG7/TLR9/ATP2B2/ITPR1/BIN1/TRPM6/JSRP1/AHNAK/NOS1AP/PTPN6/RASA3/P2RY6/PHB/MYO5A/HTT/PTPRC/ATP2A3/NOL3/TPCN2/TRPM3/GSTO1/PRKCE/TMEM37/F2R/TMC1/ANO6/DYSF/CACNA1B/ANK2/CHERP/PLCG1/TRPV2/CASQ1/FGF14/CBARP/STIMATE/CRACR2A
Biological Process	GO:0061620	glycolytic process through glucose-6-phosphate	8/2279	28/18866	0.0150341	0.2106769	0.190628	8	FOXK1/ENO1/HK1/ADPGK/PGAM1/ENO3/HK2/PKM
Biological Process	GO:0050777	negative regulation of immune response	29/2279	159/18866	0.0150592	0.2107181	0.1906652	29	TRIM27/ABR/IL10/PPARG/CD55/INPP5D/CCR2/TNF/TNFAIP3/LOXL3/CD84/DUSP22/HLA-F/PTPN6/SMAD7/ZBTB7B/TGFB1/FER/CD59/NR1H2/PGLYRP1/PTPRC/GRN/NLRC5/BCR/ARRB2/HLX/TYRO3/CD96
Biological Process	GO:0009185	ribonucleoside diphosphate metabolic process	26/2279	139/18866	0.0151474	0.2109142	0.1908427	26	FOXK1/HDAC4/PRKAG2/AMPD3/ENO1/HK1/TJP2/PFKFB4/RAE1/CBFA2T3/DHTKD1/APP/OGDH/ADPGK/PGAM1/NUP93/ENO3/HK2/SEC13/GUK1/ESRRB/PKM/AK2/AK5/PFKFB3/PRKAA1
Biological Process	GO:0014037	Schwann cell differentiation	10/2279	39/18866	0.0152137	0.2109142	0.1908427	10	ARHGEF10/SH3TC2/SKI/LGI4/NDRG1/ILK/NF1/FA2H/CNTNAP1/RELA
Biological Process	GO:0016601	Rac protein signal transduction	10/2279	39/18866	0.0152137	0.2109142	0.1908427	10	ELMO1/RHOH/RHOG/DNM2/NISCH/NF1/NCKAP1/ARHGAP17/CYFIP1/DBNL
Biological Process	GO:0030204	chondroitin sulfate metabolic process	10/2279	39/18866	0.0152137	0.2109142	0.1908427	10	CSGALNACT1/SPOCK2/DSE/HEXB/CHSY1/CHST11/CHST15/EGFLAM/XYLT1/ARSB
Biological Process	GO:2000249	regulation of actin cytoskeleton reorganization	10/2279	39/18866	0.0152137	0.2109142	0.1908427	10	TRPM2/ARHGDIB/CSF1R/BAIAP2L1/BAIAP2/CSF3/GMFG/BAIAP2L2/CCL27/DAPK3

Biological Process	GO:0009755	hormone-mediated signaling pathway	35/2279	200/18866	0.0152286	0.2109142	0.1908427	35	RXRA/FOXP1/CALR/PPARG/NCOR2/ACSL1/RUNX1/AKAP13/YWHAH/PRMT2/LBH/PADI2/ZNF366/HDAC1/LATS2/ZMIZ1/PHB/CRHBP/AXIN1/KMT2D/RARA/GHRL/RARG/NCOA1/ARRB2/ARNTL/ESRRB/PER1/UBE3A/NEDD4/RNF14/NCOA4/NR5A2/NODAL/SST
Biological Process	GO:2000377	regulation of reactive oxygen species metabolic process	35/2279	200/18866	0.0152286	0.2109142	0.1908427	35	HDAC4/IL10/CD177/SMAD3/DNM2/MIR199A1/TNF/FPR2/TGFBR2/THBS1/RAB27A/RIPK1/GNAI2/NOS1AP/MIR24-2/CLU/PAX2/SPHK2/TGFB1/HBB/RIPK3/HK2/PDGFB/BCR/NFE2L2/ITGB2/BCL2/MIR199A2/FOXM1/AGTR1/PDGFRB/FOXO1/CX3CR1/SIRT3/PRKN
Biological Process	GO:0032409	regulation of transporter activity	48/2279	291/18866	0.0152992	0.2115836	0.1914484	48	PDE4D/KCNE1/PPARG/THADA/CASQ2/WWP2/FXYD2/YWHAH/FXYD1/CCR2/DNM2/SGK1/TLR9/KCTD7/TESC/JSRP1/AHNAK/RIPK1/APP/NOS1AP/ACTN4/P2RY6/SPHK2/CRHBP/CNIH2/HTR3A/MYO5A/HTT/CAB39/MAPK8IP2/GSG1L/SYNGR3/BCL2/GSTO1/MEF2C/SHANK2/PRKCE/NEDD4/DYSF/ANK2/HECW2/SGK2/PDZK1/CASQ1/FGF14/CBARP/STIMATE/CRACR2A
Biological Process	GO:2001242	regulation of intrinsic apoptotic signaling pathway	30/2279	166/18866	0.0153679	0.2120696	0.1918881	30	S100A8/P4HB/ENO1/LCK/URI1/PLAGL2/BMF/NCK2/RRM2B/NACC2/ZNF385A/CLU/HDAC1/SGMS1/RIPK3/NOL3/NFE2L2/ANKRD2/FIS1/DAPK2/BCL2/DNM1L/ARHGEF2/BCL2L1/CYLD/BAG5/GRINA/PRKN/MARCHF7/FBH1
Biological Process	GO:0007411	axon guidance	47/2279	284/18866	0.015379	0.2120696	0.1918881	47	SEMA6B/PLXNC1/BSG/NRP2/TRIO/PIK3CD/RHOH/APBB2/PIK3R1/RHOG/GAB2/PRKCA/DRGX/CSF1R/DOK1/EPHB3/SEMA4A/UNC5B/DOK2/GRB10/SEMA4B/APP/FEZ2/SEMA4D/ZSWIM5/NRXN1/DSCAML1/BMPR1B/KIF5C/CDH4/SPTA1/KLF7/NTRK1/NOTCH1/MYOT/SLIT3/SPTAN1/PLCG1/CYFIP1/EZR/SLIT1/SPTBN4/OTX2/LYPLA2/UNC5D/B3GNT2/NECTIN1
Biological Process	GO:0030219	megakaryocyte differentiation	20/2279	100/18866	0.0154805	0.2128518	0.1925959	20	ZFPM1/RUNX1/FLI1/ASH2L/TNRC6B/AGO1/TESC/THBS1/PIP4K2A/ITGA2B/PTPN6/ZNF385A/HDAC1/MTURN/KMT2D/SP3/SETD1A/MEIS1/MEF2C/H3C1
Biological Process	GO:0045807	positive regulation of endocytosis	20/2279	100/18866	0.0154805	0.2128518	0.1925959	20	AP2A1/PPARG/ANKFY1/ANGPT1/ARRB1/DNM2/RAB31/ITSN1/DLL1/MIB1/ACTN4/CLU/WASL/SH3GL1/DGKD/ARRB2/DNM1/DNM1L/ANO6/FLOT1
Biological Process	GO:0048705	skeletal system morphogenesis	39/2279	228/18866	0.0155667	0.2132297	0.1929378	39	CSGALNACT1/ARID5B/FOXN3/MEF2D/LRP5L/SKI/CHSY1/SETD2/ANKRD11/SMAD3/MTHFD1L/FGR/ANXA6/SH3PXD2B/CHST11/TGFBR2/ALPL/NDST1/POC1A/FGFR1/RDH10/RARA/POR/DSCAML1/RARG/BMPR1B/GRHL2/RUNX2/IFT140/MTHFD1/HOXA9/ALX3/MEF2C/NPPC/LTF/PDGFRB/NODAL/HOXB7/RFLNA
Biological Process	GO:0048872	homeostasis of number of cells	43/2279	256/18866	0.0155875	0.2132297	0.1929378	43	MAEA/KLF1/ZFPM1/LAT/AMPD3/PIK3CD/INPP5D/TRIM10/ITPKB/CCR2/TNFAIP3/TNFSF13B/HIPK2/SLC11A2/BRD1/TMOD3/RIPK3/ZC3H8/JAM3/SP3/ALAS1/SFXN1/BCL10/BCR/SPTA1/ETS1/DOCK10/ADD1/RB1/BCL2/NOTCH1/MEF2C/EPAS1/UBAP2L/F2R/CYLD/EPB42/KAT7/INHBA/ERCC2/FADD/MB/TMEM14C

Biological Process	GO:0001938	positive regulation of endothelial cell proliferation	22/2279	113/18866	0.0155972	0.2132297	0.1929378	22	LRG1/RPTOR/NRP2/IL10/PRKCA/EGFL7/MIR101-2/STAT5A/AKT3/MIR27A/MIR23A/FGFR1/ZNF580/NF1/ITGB3/GHRL/PDGFB/AGTR1/DYSF/PPP1R16B/TNFSF12/PLCG1
Biological Process	GO:0071695	anatomical structure maturation	40/2279	235/18866	0.0155978	0.2132297	0.1929378	40	MAEA/PPARG/BRCA2/LGI4/SLFN14/LYL1/BCL11A/RFX3/BRD1/APP/RBPJ/PAX2/CDH3/FGFR1/XYLT1/SEMA4D/DEFB1/MYO5A/NF1/RUNX2/PPP2R1A/FEV/RB1/POU2F2/BCL2/DMC1/EPAS1/NPPC/LTF/ANO6/FAM20C/EPB42/CLN5/ALDH1A2/SPTBN4/ERCC2/GLDN/CX3CR1/RFLNA/ANGPTL8
Biological Process	GO:0042772	DNA damage response, signal transduction resulting in transcription	6/2279	18/18866	0.0157994	0.2150668	0.1946001	6	BRCA2/HIPK2/ZNF385A/SP100/FOXM1/CHEK2
Biological Process	GO:2000303	regulation of ceramide biosynthetic process	6/2279	18/18866	0.0157994	0.2150668	0.1946001	6	TNF/ORMDL3/TNFRSF1A/SPHK2/SIRT3/PRKAA1
Biological Process	GO:0032703	negative regulation of interleukin-2 production	7/2279	23/18866	0.0158001	0.2150668	0.1946001	7	TRIM27/HDAC7/TNFAIP3/SFTPD/NAV3/PTPRC/EZR
Biological Process	GO:0033143	regulation of intracellular steroid hormone receptor signaling pathway	16/2279	75/18866	0.0159478	0.2151981	0.1947189	16	FOXP1/CALR/NCOR2/RUNX1/PRMT2/LBH/ZNF366/HDAC1/PHB/AXIN1/KMT2D/ARRB2/ARNTL/PER1/RNF14/NODAL
Biological Process	GO:0046323	glucose import	16/2279	75/18866	0.0159478	0.2151981	0.1947189	16	PRKAG2/MIR143/PIK3R1/TNF/GRB10/ZDHHC7/HNF1A/SLC1A2/HK2/PRKCI/NFE2L2/SORT1/SLC2A1/RTN2/ASPSCR1/CLTCL1
Biological Process	GO:0099518	vesicle cytoskeletal trafficking	16/2279	75/18866	0.0159478	0.2151981	0.1947189	16	PRKCZ/NDE1/KIF13A/DYNC1H1/ACTN4/WASL/CNIH2/MYO5A/HTT/KIF1B/KIFAP3/MYO1C/AP3D1/FBXW11/NDEL1/DTNBP1
Biological Process	GO:1900076	regulation of cellular response to insulin stimulus	16/2279	75/18866	0.0159478	0.2151981	0.1947189	16	PRKCZ/SORL1/FUT7/KANK1/PTPRE/PIK3R1/GPLD1/BAIAP2L1/GRB10/GNAI2/ZBTB7B/PRKCB/MYO1C/TSC2/RELA/PRKAA1

Biological Process	GO:0033292	T-tubule organization	4/2279	9/18866	0.0161948	0.2151981	0.1947189	4	BIN1/DYSF/ANK2/SYPL2
Biological Process	GO:0034139	regulation of toll-like receptor 3 signaling pathway	4/2279	9/18866	0.0161948	0.2151981	0.1947189	4	TNFAIP3/WDFY1/FLOT1/PELI1
Biological Process	GO:0043129	surfactant homeostasis	4/2279	9/18866	0.0161948	0.2151981	0.1947189	4	LPCAT1/SFTPD/CTSH/EPAS1
Biological Process	GO:0045542	positive regulation of cholesterol biosynthetic process	4/2279	9/18866	0.0161948	0.2151981	0.1947189	4	ABCG1/POR/SREBF1/PRKAA1
Biological Process	GO:0045762	positive regulation of adenylate cyclase activity	4/2279	9/18866	0.0161948	0.2151981	0.1947189	4	TIMP2/CACNA1C/AKAP5/NF1
Biological Process	GO:0045955	negative regulation of calcium ion-dependent exocytosis	4/2279	9/18866	0.0161948	0.2151981	0.1947189	4	RAP1B/GNAI2/NOTCH1/CBARP
Biological Process	GO:0046951	ketone body biosynthetic process	4/2279	9/18866	0.0161948	0.2151981	0.1947189	4	BDH1/HMGCL/ACAT1/ACSS3
Biological Process	GO:0048478	replication fork protection	4/2279	9/18866	0.0161948	0.2151981	0.1947189	4	BLM/BRCA2/SMARCAL1/FBH1
Biological Process	GO:0072102	glomerulus morphogenesis	4/2279	9/18866	0.0161948	0.2151981	0.1947189	4	KIRREL3/MTSS1/MEF2C/PDGFRB

Biological Process	GO:0106120	positive regulation of sterol biosynthetic process	4/2279	9/18866	0.0161948	0.2151981	0.1947189	4	ABCG1/POR/SREBF1/PRKAA1
Biological Process	GO:1901072	glucosamine-containing compound catabolic process	4/2279	9/18866	0.0161948	0.2151981	0.1947189	4	AMDHD2/CHI3L1/CHIT1/CHI3L2
Biological Process	GO:1903423	positive regulation of synaptic vesicle recycling	4/2279	9/18866	0.0161948	0.2151981	0.1947189	4	SH3GL1/DNM1/DNM3/DNM1L
Biological Process	GO:1904177	regulation of adipose tissue development	4/2279	9/18866	0.0161948	0.2151981	0.1947189	4	SORL1/SH3PXD2B/GHRL/KLF7
Biological Process	GO:0030032	lamellipodium assembly	15/2279	69/18866	0.0162983	0.216271	0.1956896	15	HDAC4/PARVB/ARHGEF7/DNM2/NCK2/WHAMM/SPATA13/ABLIM3/FER/VAV2/NCKAP1/AVIL/CYFIP1/ABLIM1/VAV3
Biological Process	GO:0097485	neuron projection guidance	47/2279	285/18866	0.0163214	0.2162759	0.1956941	47	SEMA6B/PLXNC1/BSG/NRP2/TRIO/PIK3CD/RHOH/APBB2/PIK3R1/RHOG/GAB2/PRKCA/DRGX/CSF1R/DOK1/EPHB3/SEMA4A/UNC5B/DOK2/GRB10/SEMA4B/APP/FEZ2/SEMA4D/ZSWIM5/NRXN1/DSCAML1/BMPR1B/KIF5C/CDH4/SPTA1/KLF7/NTRK1/NOTCH1/MYOT/SLIT3/SPTAN1/PLCG1/CYFIP1/EZR/SLIT1/SPTBN4/OTX2/LYPLA2/UNC5D/B3GNT2/NECTIN1
Biological Process	GO:0035601	protein deacylation	21/2279	107/18866	0.0163813	0.2167667	0.1961382	21	HDAC4/SPRED2/HDAC7/SIN3B/SKI/C6orf89/HDAC1/SPHK2/ZBTB7B/PHB/MTA1/CTBP1/MTA2/PER2/PER1/NEK3/SREBF1/LYPLA2/ELK4/SIRT3/PRKAA1
Biological Process	GO:0043491	protein kinase B signaling	46/2279	278/18866	0.0164075	0.2168123	0.1961795	46	TGFA/PIK3CD/LCK/MIR143/MIR145/CHI3L1/ANGPT1/PIK3AP1/ITGB1BP1/PIK3R1/RHOG/MIR199A1/TNF/CCL5/PHLPP1/ITSN1/RTN4/THBS1/PIK3R5/TCF7L2/ILK/CPNE1/IGF1R/PAX2/FGFR1/TGFB1/PHB/PTPRJ/PPP2R5C/CSF3/STK3/PDGFB/ENG/ARRB2/MIR138-2/NTRK1/MIR199A2/GCNT2/BANK1/TSC2/PDGFRB/TYRO3/ADTRP/INPP5F/CX3CR1/MYORG
Biological Process	GO:0002762	negative regulation of myeloid leukocyte differentiation	12/2279	51/18866	0.0164574	0.2171688	0.196502	12	ZFPM1/INPP5D/PIK3R1/UBASH3B/RARA/TMEM178A/LILRB4/NF1/LRRC17/LTF/TRIB1/INHBA

Biological Process	GO:0030258	lipid modification	45/2279	271/18866	0.0164827	0.2172009	0.1965311	45	PRKAG2/SYNJ2/PPARG/PIK3CD/MFSD2A/INPP5A/PIP5K1B/INPP5D/PIK3R1/ITPKB/PIK3R6/AMACR/ABCG1/TTC7A/ACOXL/PIP4K2A/MIR27A/SPHK2/ACADM/TTC7B/DGKQ/POR/ACACB/ST3GAL4/DGKA/DGKD/INPP5E/IMPA2/ACOX2/PI4K2A/SGPP1/SGPP2/PI4KA/FA2H/AGTR1/ACAT1/CPT1A/INPP5F/CERK/ST3GAL2/ACADVL/PRKAA1/IP6K1/SLA2/PLPP3
Biological Process	GO:0097553	calcium ion transmembrane import into cytosol	26/2279	140/18866	0.0165277	0.2172295	0.196557	26	TRPM2/PDE4D/CASQ2/ITPR2/LCK/CACNA1C/SLC8A1/UBASH3B/ITPR1/JSRP1/PTPN6/RASA3/P2RY6/MYO5A/HTT/PTPRC/NOL3/TPCN2/GSTO1/PRKCE/F2R/ANK2/CHERP/PLCG1/TRPV2/CASQ1
Biological Process	GO:2000514	regulation of CD4-positive, alpha-beta T cell activation	14/2279	63/18866	0.0165306	0.2172295	0.196557	14	IL27/PRKCZ/MYB/RUNX1/CD55/NLRP3/LOXL3/TGFBR2/SMAD7/ZBTB7B/RARA/HLX/RUNX3/VSIR
Biological Process	GO:0046854	phosphatidylinositol phosphorylation	13/2279	57/18866	0.016602	0.2178673	0.1971341	13	PIK3CD/PIP5K1B/PIK3R1/ITPKB/PIK3R6/TTC7A/PIP4K2A/TTC7B/IMPA2/PI4K2A/PI4KA/IP6K1/SLA2
Biological Process	GO:0009100	glycoprotein metabolic process	65/2279	415/18866	0.0166982	0.2188272	0.1980026	65	CSGALNACT1/FUT4/GALNT2/FUT7/SPOCK2/RPN1/XXYLT1/HS3ST3B1/DSE/SLC35C2/HEXB/MGAT5/ST6GALNAC1/GALNT12/CHSY1/AMFR/MGAT4A/MIR101-2/CHST11/MGAT3/EDEM1/CHST15/NDST1/EGFLAM/TCF7L2/DERL2/MUC12/PCSK6/ASGR2/AGO2/XYLT1/GXYLT2/B4GALT7/POMT2/ADAMTS13/MUC20/B3GNT5/ST6GAL1/TNIP1/ITM2C/BMPR1B/EDEM2/ST8SIA6/ST3GAL4/PLCB1/TET2/B4GALT3/PMM2/NPC1/ARSB/BCL2/GALNT9/FUT11/GCNT2/GFPT2/LMF1/ALG11/PHLDA1/MAN1C1/DPAGT1/ST3GAL2/MARCHF6/B3GNT2/B4GAT1/PXYLP1
Biological Process	GO:1901879	regulation of protein depolymerization	18/2279	88/18866	0.0168086	0.2199702	0.1990368	18	VILL/GSN/CAPG/NAV3/TMOD3/SVIL/WDR1/SPTA1/ADD1/MAP1A/AVIL/FLII/ARHGEF2/SPTAN1/ASPH/TMOD1/SPTBN4/SPEF1
Biological Process	GO:0043094	cellular metabolic compound salvage	9/2279	34/18866	0.0169395	0.2205574	0.1995682	9	CDA/AMPD3/TK2/UCK2/PNP/PDXK/GMPR2/ADI1/TK1
Biological Process	GO:0055094	response to lipoprotein particle	9/2279	34/18866	0.0169395	0.2205574	0.1995682	9	CD9/PPARG/MIA3/ABCA1/ITGB2/NPC1/CD68/HMGCS1/ADTRP
Biological Process	GO:2000780	negative regulation of double-strand break repair	9/2279	34/18866	0.0169395	0.2205574	0.1995682	9	RMI2/RECQL5/OTUB2/OTUB1/TP53BP1/TRIP12/TFIP11/CYREN/FBH1

Biological Process	GO:0019079	viral genome replication	24/2279	127/18866	0.0169463	0.2205574	0.1995682	24	SLPI/TRIM38/SRPK2/TNF/CCL5/LARP1/PHB/CTBP1/CTBP2/TNIP1/BCL2/PI4KA/NOTCH1/LTF/ILF3/MX1/NR5A2/PARP10/BANF1/ISG20/NFIA/GRK2/PRKN/SHFL
Biological Process	GO:0031098	stress-activated protein kinase signaling cascade	49/2279	300/18866	0.0170735	0.22176	0.2006563	49	MAP3K3/CCDC88C/HIPK3/ERN1/ZNF622/TNF/ZEB2/TRAF2/TRAF1/CCM2/PHLPP1/HIPK2/TLR9/DUSP22/TNIK/RIPK1/APP/IGF1R/MTURN/SCIMP/AXIN1/COPS5/UBE2V1/SH3RF3/SH2D3C/STK3/PLCB1/MAPK8IP2/TAOK3/MIR138-2/IRAK2/NOD2/PER1/DUSP3/FOXM1/FOXO1/GADD45G/GDF6/TRIB1/CYLD/ANKRD6/FBXW11/EZR/KAT7/DTNBP1/MECOM/DBNL/PRKN/MAP3K20
Biological Process	GO:0008361	regulation of cell size	32/2279	181/18866	0.0171087	0.22176	0.2006563	32	SEMA6B/RPTOR/CLCN6/CREB1/CTTN/DNM2/SEMA4A/RTN4/DISC1/SEMA4B/IFRD1/ILK/SEMA4D/AQP1/VAV2/RARG/GNB3/CDH4/ADD1/CDKL3/ANO6/CYFIP1/TRPV2/WDTC1/NDEL1/SLC12A1/RAP1GAP2/SLC12A7/ANXA7/VAV3/DBNL/SPART
Biological Process	GO:0010950	positive regulation of endopeptidase activity	32/2279	181/18866	0.0171087	0.22176	0.2006563	32	NLRC4/S100A8/GRAMD4/AIM2/PPARG/GSN/LCK/NLRP3/SMAD3/ARRB1/MEFV/TNF/TRAF2/CYFIP2/TBC1D10A/CTSD/CTSH/RIPK1/MBP/CIDEB/PERP/BCL10/ATP2A3/FIS1/P2RX1/F2R/CASP1/BCL2L13/ASPH/NODAL/FADD/VSIR
Biological Process	GO:0030838	positive regulation of actin filament polymerization	20/2279	101/18866	0.017176	0.2220263	0.2008973	20	TRIM27/GSN/CTTN/NCK2/WHAMM/BAIAP2L1/CDC42EP1/BIN1/BAIAP2/WASL/FER/PFN1/CSF3/GMFB/NCKAP1/PRKCE/MYO1C/CYFIP1/GMFG/BAIAP2L2
Biological Process	GO:0060191	regulation of lipase activity	20/2279	101/18866	0.017176	0.2220263	0.2008973	20	PRKCZ/LIPC/CCL5/PCSK6/FLT1/P2RY6/FGFR1/PHB/POR/NR1H2/SORT1/LPAR2/S1PR4/LMF1/ABHD5/AGTR1/PDGFRB/PLCG1/NTF4/ANGPTL8
Biological Process	GO:0043123	positive regulation of I-kappaB kinase/NF-kappaB signaling	33/2279	188/18866	0.0172913	0.2231538	0.2019175	33	TRIM27/BRD4/TRIM38/TRIM8/MAP3K3/AKAP13/TNF/TRAF2/CXXC5/TLR9/RIPK1/TNFRSF1A/PLEKHG5/LITAF/PRKCB/TRIM5/UBE2V1/BCL10/ZDHHC17/MAP3K14/NEK6/NOD2/PRKCE/TRAF3IP2/F2R/LTF/CASP1/RELA/CX3CR1/FADD/PELI1/EEF1D/PRKN
Biological Process	GO:0051403	stress-activated MAPK cascade	47/2279	286/18866	0.0173102	0.2231538	0.2019175	47	MAP3K3/HIPK3/ERN1/ZNF622/TNF/ZEB2/TRAF2/TRAF1/CCM2/PHLPP1/HIPK2/TLR9/DUSP22/TNIK/RIPK1/APP/IGF1R/MTURN/SCIMP/AXIN1/COPS5/UBE2V1/SH3RF3/SH2D3C/STK3/PLCB1/MAPK8IP2/TAOK3/MIR138-2/IRAK2/NOD2/PER1/DUSP3/FOXM1/FOXO1/GADD45G/GDF6/TRIB1/CYLD/ANKRD6/FBXW11/EZR/DTNBP1/MECOM/DBNL/PRKN/MAP3K20
Biological Process	GO:0032386	regulation of intracellular transport	57/2279	358/18866	0.0176055	0.2266534	0.2050841	57	EHD1/SORL1/KCNE1/SH3TC2/TFDP1/PRR5L/TM9SF4/TMEM30A/YWHAH/ANGPT1/ITGB1BP1/PIK3R1/SETD2/SMAD3/SUFU/DYNC1H1/UBAC2/JUP/EDEM1/USP36/SNX3/ZDHHC2/TCF7L2/DERL2/ABLIM3/AKAP5/UBE2J2/TGFB1/MLC1/CNIH2/NF1/EDEM2/NUMA1/ITGB2/YWHAZ/CDH1/FIS1/BCL2/TTC21B/CABP1/SP100/NEDD4/TP53BP2/MYO1C/MIEF1/ARHGAP1/SPAG5/SREBF1/YWHAQ/NDEL1/INPP5F/EZR/PDZK1/SVIP/PRKAA1/PRKN/RIPOR1

Biological Process	GO:0051495	positive regulation of cytoskeleton organization	39/2279	230/18866	0.0177712	0.2284763	0.2067334	39	ARHGEF10/TRIM27/DCTN1/GSN/ITGB1BP1/SMAD3/CTTN/NCK2/DYNC1H1/SH3PXD2B/WHAMM/BAIAP2L1/NAV3/CDC42EP1/BIN1/BAIAP2/SYNPO2L/POC1A/PXN/WNT11/WASL/FER/PFN1/CSF3/GMFB/NUMA1/WDR1/NCKAP1/ARHGEF10L/PRKCE/MYO1C/ARHGEF15/SPAG5/CYFIP1/GMFG/BAIAP2L2/CCL27/PFDN2/SYNPO
Biological Process	GO:0032612	interleukin-1 production	23/2279	121/18866	0.0179055	0.2298927	0.2080151	23	AZU1/NLRC4/FOXP1/IL10/AIM2/IL16/NLRP3/SMAD3/MEFV/TNF/TNFAIP3/MIR101-2/MNDA/APP/GHRL/ABCA1/ARRB2/NOD2/F2R/CASP1/CD33/CX3CR1/STMP1
Biological Process	GO:0051668	localization within membrane	19/2279	95/18866	0.0179733	0.2304516	0.2085208	19	SSH1/GSN/ITGB1BP1/COLQ/SEC23B/ZDHHC2/MIA3/SYNGAP1/CNIH2/NRXN1/SEC13/PTPRC/NRXN2/ITGB2/SEC24C/RALA/RILPL1/DOCK2/SEC31A
Biological Process	GO:0030010	establishment of cell polarity	26/2279	141/18866	0.0180058	0.2305564	0.2086156	26	PRKCZ/DCTN1/GSN/KANK1/NDE1/CRB1/RAP1B/FRMD4A/SNX27/MYO18A/MYH9/DOCK8/FRMD4B/JAM3/HTT/NUMA1/PRKCI/SDCCAG8/MPP7/ARHGEF2/NEK3/DOCK2/MARK2/FBXW11/NDEL1/CYRIB
Biological Process	GO:0001909	leukocyte mediated cytotoxicity	21/2279	108/18866	0.0180903	0.2307057	0.2087507	21	AZU1/ELANE/CTSG/LYST/PIK3R6/SLAMF7/CTSH/TUBB4B/RAB27A/CD226/HLA-F/PTPN6/NCR1/RIPK3/PTPRC/ARRB2/IL18RAP/DNASE1L3/CADM1/FADD/CYRIB
Biological Process	GO:0007229	integrin-mediated signaling pathway	21/2279	108/18866	0.0180903	0.2307057	0.2087507	21	ITGAE/LAT/CD177/DOCK1/ITGB1BP1/FGR/LOXL3/CCM2/CUL3/MYH9/ITGA2B/ILK/ITGB5/ADAMTS13/ITGB3/ITGB2/PRAM1/ITGB6/VAV3/FYB1/PLPP3
Biological Process	GO:0032526	response to retinoic acid	21/2279	108/18866	0.0180903	0.2307057	0.2087507	21	RXRA/PPARG/CREB1/MYB/HSD17B2/TESC/CTSH/WNT11/PAX2/AQP1/RARA/RARG/WNT5B/ABCA1/NCOA1/GJB3/AQP3/PDGFRB/TEAD2/SREBF1/ALDH1A2
Biological Process	GO:0000096	sulfur amino acid metabolic process	10/2279	40/18866	0.0181475	0.230816	0.2088505	10	GADL1/GGT1/CBS/MSRA/COMT/AGXT/MTHFD1/ADO/MAT1A/ADI1
Biological Process	GO:0006623	protein targeting to vacuole	10/2279	40/18866	0.0181475	0.230816	0.2088505	10	SORL1/VTI1A/VPS13D/CLU/SMURF1/NUMA1/SORT1/NEDD4/AP3D1/NCOA4

Biological Process	GO:0010921	regulation of phosphatase activity	31/2279	175/18866	0.018223	0.2314667	0.2094392	31	LMTK2/MGAT5/PPP1R1B/URI1/SMAD3/CABIN1/GPLD1/TNF/CCDC8/GNAI2/ELFN2/RCAN1/PHACTR2/FARP1/SEMA4D/PPP2R5C/RIPK3/HTT/PTPRC/PPP2R1A/PPP2R5E/PPP2R5D/MEF2C/PPP2R1B/PDGFRB/WDR81/PPP1R16B/CD33/SPOCD1/ANKLE2/PTPA
Biological Process	GO:2001252	positive regulation of chromosome organization	32/2279	182/18866	0.0184268	0.2337429	0.2114989	32	BRD4/TNKS/MYB/HMBOX1/ARRB1/RTEL1/NSMCE2/C6orf89/CUL3/CDC16/RPS6KA4/SPHK2/ZBTB7B/PAX7/CTBP1/ATF7IP/NUMA1/PHF19/RB1/DNMT1/PIH1D1/ANAPC7/ERCC1/SREBF1/SLX4/RNF40/KAT7/CCT6A/LIF/PAXIP1/NSD3/SLF1
Biological Process	GO:0044839	cell cycle G2/M phase transition	45/2279	273/18866	0.0185825	0.2342787	0.2119837	45	TRIM39/BLM/BRD4/DCTN1/FZR1/CSNK1D/FOXN3/NDE1/DNM2/HUS1/DYNC1H1/TUBB4B/CEP135/APP/CDK14/CDC25A/CEP164/DCTN2/CEP78/CSNK1E/PSMF1/PPP2R1A/PLCB1/CCNH/PPP1CB/TUBA4A/TAOK3/SDCCAG8/ZFYVE19/ESRRB/CLSPN/PSMB7/FBXL6/FOXM1/CHEK2/BACH1/FBXW11/PHLDA1/CEP72/CCNY/NAE1/PSMD13/PAXIP1/ABRAXAS1/BABAM2
Biological Process	GO:0006470	protein dephosphorylation	52/2279	323/18866	0.0185891	0.2342787	0.2119837	52	LMTK2/SSH1/CTDP1/PTPRE/LCK/MGAT5/PPP1R1B/URI1/DUSP14/CABIN1/TNF/UBASH3B/PHLPP1/DUSP22/PGP/CTDSPL/GNAI2/PTPN6/CAMTA1/SSH3/ELFN2/RCAN1/PHACTR2/DUSP28/PTPRN2/CDC25A/TGFB1/PTPRJ/PPP2R5C/LRRK1/HTT/PTPRC/PPP2R1A/PALD1/PPP1CB/PPP2R5E/PPP2R5D/DAPP1/BCL2/DUSP3/PPP2R1B/PDGFRB/PPP1R16B/RPRD1B/CDC14A/FBXW11/CD33/ANKLE2/LHPP/PPM1N/PTPRU/PTPA
Biological Process	GO:0033032	regulation of myeloid cell apoptotic process	8/2279	29/18866	0.018617	0.2342787	0.2119837	8	MAEA/PIK3CD/ITPKB/CCL5/CLEC5A/NOD2/BCL2/MEF2C
Biological Process	GO:0042634	regulation of hair cycle	8/2279	29/18866	0.018617	0.2342787	0.2119837	8	TNF/KRT17/CDH3/EPS8L3/NUMA1/ARNTL/PER1/FA2H
Biological Process	GO:0045671	negative regulation of osteoclast differentiation	8/2279	29/18866	0.018617	0.2342787	0.2119837	8	INPP5D/PIK3R1/UBASH3B/TMEM178A/LILRB4/NF1/LRRC17/LTF
Biological Process	GO:0061615	glycolytic process through fructose-6-phosphate	8/2279	29/18866	0.018617	0.2342787	0.2119837	8	FOXK1/ENO1/HK1/ADPGK/PGAM1/ENO3/HK2/PKM
Biological Process	GO:0007565	female pregnancy	34/2279	196/18866	0.0187258	0.2353364	0.2129407	34	KLF1/BSG/RXRA/CALR/FUT7/LNPEP/ARHGDIB/VDR/ABCC2/PNOC/TGFBR2/COMT/AGO2/SPHK2/CRHBP/SLC19A1/RARA/SP3/DAZAP1/GHRL/CTSB/ETS1/BCL2/VMP1/TPPP3/STOX2/TMED2/SLC2A1/DHODH/HSD11B2/NODAL/LIF/PRLHR/PSG2

Biological Process	GO:1901019	regulation of calcium ion transmembrane transporter activity	18/2279	89/18866	0.0187624	0.2354841	0.2130744	18	PDE4D/THADA/CASQ2/TLR9/JSRP1/AHNAK/NOS1AP/P2RY6/MYO5A/HTT/GSTO1/DYSF/ANK2/CASQ1/FGF14/CBARP/STIMATE/CRACR2A
Biological Process	GO:0001885	endothelial cell development	14/2279	64/18866	0.0188757	0.2365935	0.2140782	14	PDE4D/TJP2/RAP1B/TNF/CCM2/TNFRSF1A/RAPGEF2/ENG/ADD1/S1PR2/NOTCH4/PPP1R16B/EZR/MYADM
Biological Process	GO:0051261	protein depolymerization	22/2279	115/18866	0.0189028	0.2366209	0.214103	22	VILL/GSN/CAPG/MICAL1/MICAL2/MICAL3/NAV3/TMOD3/SVIL/SH3GL1/WDR1/SPTA1/ADD1/MAP1A/AVIL/FLII/ARHGEF2/SPTAN1/ASPH/TMOD1/SPTBN4/SPEF1
Biological Process	GO:0060996	dendritic spine development	20/2279	102/18866	0.0190129	0.2373745	0.2147849	20	ZMYND8/NCK2/EPHB3/ITSN1/DISC1/BAIAP2/ASAP1/WASL/IQSEC1/ARID1B/CPEB3/SIPA1L1/DOCK10/DNM3/DNM1L/MEF2C/SHANK2/UBE3A/CUX2/DBNL
Biological Process	GO:0150115	cell-substrate junction organization	20/2279	102/18866	0.0190129	0.2373745	0.2147849	20	COL17A1/ITGB1BP1/SMAD3/ARHGEF7/CTTN/DUSP22/WHAMM/THBS1/TNS1/ACTN1/PTPRJ/IQSEC1/TESK2/ACTG1/BCR/BCL2/CORO1C/DUSP3/LAMB3/DAPK3
Biological Process	GO:0002886	regulation of myeloid leukocyte mediated immunity	13/2279	58/18866	0.0191004	0.2376477	0.215032	13	ABR/CD177/CCR2/GAB2/FGR/CD84/SPHK2/FER/BLK/BCR/ITGB2/DNASE1L3/PRAM1
Biological Process	GO:0035306	positive regulation of dephosphorylation	13/2279	58/18866	0.0191004	0.2376477	0.215032	13	SMAD3/GPLD1/CAMTA1/TGFB1/RIPK3/PTPRC/PPP2R5D/MEF2C/PDGFRB/PPP1R16B/CD33/ANKLE2/PTPA
Biological Process	GO:0006998	nuclear envelope organization	12/2279	52/18866	0.0191099	0.2376477	0.215032	12	DCTN1/PRKCA/PRKCB/NUP93/PPP2R1A/NEK6/UBXN2A/TOR1B/NDEL1/ANKLE2/LPIN1/BANF1
Biological Process	GO:1903305	regulation of regulated secretory pathway	29/2279	162/18866	0.0191372	0.237677	0.2150586	29	ABR/CD177/RAP1B/CCR2/GAB2/FGR/MICAL1/CD84/GIT1/RAB27A/GNAI2/HLA-F/APBA2/SPHK2/FER/PRKCB/VPS18/BLK/CHRM2/BCR/ITGB2/NOTCH1/P2RX1/DNM1L/PRAM1/SYT17/RPH3AL/DTNBP1/CBARP

Biological Process	GO:2001021	negative regulation of response to DNA damage stimulus	17/2279	83/18866	0.0195287	0.2422221	0.2191712	17	RMI2/RTEL1/RECQL5/OTUB2/ZNF385A/CLU/OTUB1/BCL2/TP53BP1/CHEK2/TRIP12/ERCC1/BCL2L1/TFIP11/MARCHF7/CYREN/FBH1
Biological Process	GO:0048638	regulation of developmental growth	56/2279	353/18866	0.0197532	0.2426178	0.2195292	56	SEMA6B/CPNE6/CTDP1/CREB1/MFSD2A/CTTN/DNM2/MIR199A1/COLQ/CPNE5/SH3PXD2B/BCL11A/VGLL4/SEMA4A/STAT5A/RTN4/TGFBR2/DISC1/SEMA4B/DLL1/RIPK1/APP/RBPJ/IFRD1/ILK/SMAD7/LATS2/FGFR1/SEMA4D/SMURF1/POR/GHRL/STK3/ACACB/CDH4/PLCB1/FTO/MEIS1/BCL2/MIR199A2/NOTCH1/RAI1/CDKL3/MEF2C/SYT17/NPPC/HLX/MBD5/CYFIP1/TRPV2/NDEL1/EZR/SPTBN4/DBNL/SPART/PRKN
Biological Process	GO:0001959	regulation of cytokine-mediated signaling pathway	32/2279	183/18866	0.0198245	0.2426178	0.2195292	32	GFI1/VRK2/PPARG/RUNX1/ANGPT1/IL1R1/TNF/TRAF2/TNFAIP3/CCL5/TRAF1/TRAIP/PADI2/RIPK1/PTPN6/MIR24-2/MIR27A/TNFRSF1A/CPNE1/JAK1/IL1RN/NR1H2/PTPRC/NLRC5/IRAK2/SLIT3/ELF1/CASP1/CYLD/PARP9/FADD/PRKN
Biological Process	GO:0043433	negative regulation of DNA-binding transcription factor activity	32/2279	183/18866	0.0198245	0.2426178	0.2195292	32	HDAC4/GFI1/IL10/AIM2/WWP2/PRMT2/NLRP3/ARRB1/SUFU/TNFAIP3/MIR101-2/TLR9/TRAIP/MIR27A/TCF7L2/SMAD7/MTURN/MED13/NLRC5/CAT/ARRB2/MIR138-2/IRAK2/RB1/SP100/TRIB1/CYLD/PARP10/PELI1/COMMD6/PAXIP1/ADGRG3
Biological Process	GO:1903034	regulation of response to wounding	32/2279	183/18866	0.0198245	0.2426178	0.2195292	32	RREB1/PLAU/IL10/CD9/KANK1/MYLK/SMAD3/PRKCA/TNF/UBASH3B/TNFAIP3/SERPINB2/THBS1/MYH9/F12/KREMEN1/STK24/BLK/ACTG1/GRN/PDGFB/NFE2L2/PRKCE/CPB2/F2R/ANO6/ADTRP/CERS2/NDEL1/INPP5F/PLPP3/CCN4
Biological Process	GO:0090630	activation of GTPase activity	21/2279	109/18866	0.0199352	0.2426178	0.2195292	21	ARHGEF10/ABR/TBC1D16/RHOG/TBC1D2/TBC1D10A/RABGAP1L/TBC1D22B/USP6NL/BCR/SIPA1L1/ARHGAP25/TBC1D14/TBC1D2B/SGSM2/CORO1C/TBC1D1/ARHGEF15/NDEL1/TBC1D22A/ARHGAP45
Biological Process	GO:0032469	endoplasmic reticulum calcium ion homeostasis	7/2279	24/18866	0.0200349	0.2426178	0.2195292	7	THADA/PACS2/ITPR1/APP/FIS1/BCL2/GRINA
Biological Process	GO:0034123	positive regulation of toll-like receptor signaling pathway	7/2279	24/18866	0.0200349	0.2426178	0.2195292	7	TLR9/RTN4/TREML4/WDFY1/LTF/FLOT1/PELI1
Biological Process	GO:0051004	regulation of lipoprotein lipase activity	7/2279	24/18866	0.0200349	0.2426178	0.2195292	7	LIPC/PCSK6/NR1H2/SORT1/LMF1/ABHD5/ANGPTL8

Biological Process	GO:0051896	regulation of protein kinase B signaling	42/2279	253/18866	0.0200356	0.2426178	0.2195292	42	TGFA/PIK3CD/LCK/MIR143/MIR145/CHI3L1/ANGPT1/PIK3AP1/ITGB1BP1/PIK3R1/RHOG/MIR199A1/TNF/PHLPP1/ITSN1/RTN4/THBS1/PIK3R5/TCF7L2/ILK/CPNE1/IGF1R/FGFR1/TGFB1/PHB/PTPRJ/PPP2R5C/CSF3/STK3/PDGFB/ENG/ARRB2/MIR138-2/NTRK1/MIR199A2/GCNT2/BANK1/TSC2/PDGFRB/INPP5F/CX3CR1/MYORG
Biological Process	GO:0003413	chondrocyte differentiation involved in endochondral bone morphogenesis	5/2279	14/18866	0.0200458	0.2426178	0.2195292	5	ANXA6/TGFBR2/POC1A/RARG/NPPC
Biological Process	GO:0014854	response to inactivity	5/2279	14/18866	0.0200458	0.2426178	0.2195292	5	HDAC4/IL10/CAT/PKM/CASQ1
Biological Process	GO:0032933	SREBP signaling pathway	5/2279	14/18866	0.0200458	0.2426178	0.2195292	5	AMFR/ZBTB7B/ARHGEF10L/SREBF1/SPRING1
Biological Process	GO:0040034	regulation of development, heterochronic	5/2279	14/18866	0.0200458	0.2426178	0.2195292	5	DLL1/RBPJ/NOTCH1/NODAL/HES3
Biological Process	GO:0050872	white fat cell differentiation	5/2279	14/18866	0.0200458	0.2426178	0.2195292	5	PPARG/CTBP1/CTBP2/PRDM16/PER2
Biological Process	GO:1900239	regulation of phenotypic switching	5/2279	14/18866	0.0200458	0.2426178	0.2195292	5	MIR140/MIR143/MIR145/PDGFB/DNMT1
Biological Process	GO:1902101	positive regulation of metaphase/anaphase transition of cell cycle	5/2279	14/18866	0.0200458	0.2426178	0.2195292	5	NSMCE2/CUL3/CDC16/RB1/ANAPC7
Biological Process	GO:1903054	negative regulation of extracellular matrix organization	5/2279	14/18866	0.0200458	0.2426178	0.2195292	5	TNFRSF1B/TNFRSF1A/TGFB1/NOTCH1/ADTRP

Biological Process	GO:1904152	regulation of retrograde protein transport, ER to cytosol	5/2279	14/18866	0.0200458	0.2426178	0.2195292	5	UBAC2/EDEM1/DERL2/EDEM2/SVIP
Biological Process	GO:1904748	regulation of apoptotic process involved in development	5/2279	14/18866	0.0200458	0.2426178	0.2195292	5	TNFRSF1B/VDR/TNFRSF1A/PAX2/NOTCH1
Biological Process	GO:0050679	positive regulation of epithelial cell proliferation	36/2279	211/18866	0.020218	0.2443906	0.2211333	36	LRG1/RPTOR/RREB1/NRP2/TGFA/IL10/PRKCA/TNFAIP3/EGFL7/CCL5/MIR101-2/STAT5A/RTN4/C5AR2/AKT3/MIR27A/MIR23A/TCF7L2/PAX2/CDH3/FGFR1/ZNF580/NF1/ITGB3/GHRL/GRN/PDGFB/NOD2/NOTCH1/AGTR1/DYSF/PPP1R16B/TNFSF12/PLCG1/NODAL/JAML
Biological Process	GO:0005996	monosaccharide metabolic process	48/2279	296/18866	0.0203955	0.2459494	0.2225437	48	MAEA/FUT4/FOXK1/ENO1/FUT7/HK1/C1QTNF3/PFKFB4/GPLD1/TNF/PGP/CHST15/ADPGK/PGAM1/GLYCTK/PC/NISCH/DGKQ/ENO3/DYRK2/HK2/GHRL/GNB3/ACACB/PPP1CB/PMM2/ESRRB/PER2/PKM/GSTO1/PRKCE/PCK2/SLC2A1/FOXO1/NCOA2/MAN2C1/SOGA1/KBTBD2/WDTC1/CPT1A/PFKFB3/GSTO2/SLC25A1/SLC23A1/PRKAA1/PRKN/SDHAF3/RUBCNL
Biological Process	GO:0071312	cellular response to alkaloid	9/2279	35/18866	0.0204246	0.2459494	0.2225437	9	BLM/SPIDR/CASQ2/SLC8A1/RECQL5/DDC/CRHBP/CASP7/BCL2L1
Biological Process	GO:1901223	negative regulation of NIK/NF-kappaB signaling	9/2279	35/18866	0.0204246	0.2459494	0.2225437	9	C1QTNF3/HDAC7/NLRP3/CPNE1/LITAF/CYLD/RELA/FBXW11/ADGRG3
Biological Process	GO:0006665	sphingolipid metabolic process	29/2279	163/18866	0.0206754	0.2465022	0.2230439	29	PLA2G15/FUT7/SPTLC2/HEXB/GALC/TNF/SMPDL3B/ORMDL3/CERS6/TNFRSF1A/SPHK2/SGMS1/ST8SIA6/PPP2R1A/SGMS2/B4GALT3/TEX2/SGPP1/SGPP2/P2RX1/FA2H/ESYT1/CERS2/PDXDC1/CERK/SIRT3/PRKAA1/TLCD3B/PLPP3
Biological Process	GO:0015749	monosaccharide transmembrane transport	22/2279	116/18866	0.0207484	0.2465022	0.2230439	22	PRKAG2/SLC2A5/MIR143/PIK3R1/SLC2A14/TNF/GRB10/SLC2A9/ZDHHC7/HNF1A/PRKCB/SLC1A2/HK2/PRKCI/NFE2L2/SORT1/SLC5A10/SLC2A1/RTN2/ASPSCR1/CLTCL1/SLC23A1
Biological Process	GO:0030518	intracellular steroid hormone receptor signaling pathway	22/2279	116/18866	0.0207484	0.2465022	0.2230439	22	FOXP1/CALR/NCOR2/RUNX1/YWHAH/PRMT2/LBH/PADI2/ZNF366/HDAC1/ZMIZ1/PHB/AXIN1/KMT2D/ARRB2/ARNTL/PER1/UBE3A/NEDD4/RNF14/NCOA4/NODAL

Biological Process	GO:0002076	osteoblast development	6/2279	19/18866	0.0207818	0.2465022	0.2230439	6	HDAC4/LRP5L/SMAD3/CLEC5A/LIMD1/RUNX2
Biological Process	GO:0007213	G protein-coupled acetylcholine receptor signaling pathway	6/2279	19/18866	0.0207818	0.2465022	0.2230439	6	GNAI2/GNA11/CHRM2/PLCB1/RGS10/GRK2
Biological Process	GO:0071800	podosome assembly	6/2279	19/18866	0.0207818	0.2465022	0.2230439	6	GSN/TNF/SH3PXD2B/ASAP1/ARHGEF2/DBNL
Biological Process	GO:0090026	positive regulation of monocyte chemotaxis	6/2279	19/18866	0.0207818	0.2465022	0.2230439	6	CCR2/CCL5/FPR2/DEFB124/CXCL17/ANO6
Biological Process	GO:0090153	regulation of sphingolipid biosynthetic process	6/2279	19/18866	0.0207818	0.2465022	0.2230439	6	TNF/ORMDL3/TNFRSF1A/SPHK2/SIRT3/PRKAA1
Biological Process	GO:0097320	plasma membrane tubulation	6/2279	19/18866	0.0207818	0.2465022	0.2230439	6	DNM2/WHAMM/PACSIN2/ASAP1/WASL/MICALL1
Biological Process	GO:1905038	regulation of membrane lipid metabolic process	6/2279	19/18866	0.0207818	0.2465022	0.2230439	6	TNF/ORMDL3/TNFRSF1A/SPHK2/SIRT3/PRKAA1
Biological Process	GO:1905288	vascular associated smooth muscle cell apoptotic process	6/2279	19/18866	0.0207818	0.2465022	0.2230439	6	PPARG/MIR140/E2F3/MIR24-2/MIR138-2/DNMT1
Biological Process	GO:1905459	regulation of vascular associated smooth muscle cell apoptotic process	6/2279	19/18866	0.0207818	0.2465022	0.2230439	6	PPARG/MIR140/E2F3/MIR24-2/MIR138-2/DNMT1

Biological Process	GO:0018205	peptidyl-lysine modification	63/2279	405/18866	0.0208122	0.246555	0.2230917	63	HDAC4/GFI1/BRD4/SRCAP/TOLLIP/METTL21A/MYB/BRCA2/ASH2L/SETD2/RAE1/KANSL1/ARRB1/NSMCE2/LOXL3/BCL11A/SMYD3/BRD1/EHMT2/EPC1/RPS6KA4/TAF10/ZMIZ1/WDR82/KANSL2/SPHK2/KMT2D/CTBP1/POR/RNF212/NUP93/KDM4C/SETD1B/NCOA1/SETD1A/CREBBP/RSF1/TET2/ZNF335/METTL22/PRDM16/PHF19/PER1/DNMT1/PIH1D1/RUVBL1/RELA/KAT7/DYDC2/DYDC1/LOXL4/ING5/MECOM/LIF/SIRT3/PAXIP1/PRKAA1/P3H3/EEF1AKMT3/EEF1AKMT2/NSD3/NSD2/CSKMT
Biological Process	GO:0002292	T cell differentiation involved in immune response	15/2279	71/18866	0.0209095	0.2470909	0.2235766	15	IL27/PRKCZ/ZFPM1/FOXP1/MYB/NLRP3/LOXL3/SEMA4A/SMAD7/LY9/ZBTB7B/RARA/IL18R1/HLX/BCL3
Biological Process	GO:1901880	negative regulation of protein depolymerization	15/2279	71/18866	0.0209095	0.2470909	0.2235766	15	VILL/GSN/CAPG/NAV3/TMOD3/SVIL/SPTA1/ADD1/AVIL/FLII/ARHGEF2/SPTAN1/TMOD1/SPTBN4/SPEF1
Biological Process	GO:0045926	negative regulation of growth	42/2279	254/18866	0.0212867	0.2512365	0.2273277	42	SEMA6B/CDA/ENO1/PPARG/CTDP1/SERTAD2/APBB2/SMAD3/MIR199A1/BCL11A/VGLL4/SEMA4A/RTN4/TGFBR2/MEG3/SEMA4B/RIPK1/SMARCA2/IFRD1/WNT11/SPHK2/TGFB1/PHB/PTPRJ/SEMA4D/RERG/CDHR2/STK3/SH3BP4/PPP2R1A/RB1/MEIS1/BCL2/MIR199A2/NOTCH1/RAI1/CDKL3/SLIT3/SCGB3A1/ING5/INHBA/SPART
Biological Process	GO:0090181	regulation of cholesterol metabolic process	14/2279	65/18866	0.0214641	0.2525387	0.228506	14	IDI1/MBTPS1/ABCG1/DGKQ/POR/GNB3/ACACB/ACACA/LMF1/HMGCS1/SCD/SREBF1/ACADVL/PRKAA1
Biological Process	GO:0070663	regulation of leukocyte proliferation	40/2279	240/18866	0.0214745	0.2525387	0.228506	40	HLA-DMB/IL27/IL10/MAD1L1/CD55/INPP5D/TNFRSF1B/CCR2/TNFAIP3/CCL5/CSF1R/NCK2/SFTPD/NFATC2/TNFSF13B/TLR9/MNDA/TGFBR2/PTPN6/TNFRSF21/ZBTB7B/RIPK3/BLK/CCDC88B/PTPRC/CD320/SPTA1/ZNF335/PNP/BCL2/MEF2C/CEBPB/HHEX/SLC7A1/FADD/PELI1/VAV3/TMEM131L/VSIR/MARCHF7
Biological Process	GO:0089718	amino acid import across plasma membrane	10/2279	41/18866	0.0214768	0.2525387	0.228506	10	SLC1A5/SLC43A2/SLC1A2/SLC7A5/SLC1A6/PER2/SLC1A3/SLC43A1/SLC7A8/SLC7A1
Biological Process	GO:0044706	multi-multicellular organism process	38/2279	226/18866	0.0215873	0.253486	0.2293631	38	KLF1/BSG/RXRA/CALR/FUT7/HEXB/LNPEP/ARHGDIB/VDR/SH3PXD2B/ABCC2/PNOC/TGFBR2/COMT/AGO2/SPHK2/CRHBP/SLC19A1/RARA/SP3/DAZAP1/GHRL/CTSB/ETS1/ARNTL/BCL2/P2RX1/VMP1/TPPP3/STOX2/TMED2/SLC2A1/DHODH/HSD11B2/NODAL/LIF/PRLHR/PSG2
Biological Process	GO:0050670	regulation of lymphocyte proliferation	37/2279	219/18866	0.0216107	0.253486	0.2293631	37	HLA-DMB/IL27/IL10/MAD1L1/CD55/INPP5D/TNFRSF1B/CCR2/CCL5/NCK2/SFTPD/NFATC2/TNFSF13B/TLR9/MNDA/TGFBR2/PTPN6/TNFRSF21/ZBTB7B/RIPK3/BLK/CCDC88B/PTPRC/CD320/SPTA1/ZNF335/PNP/BCL2/MEF2C/CEBPB/SLC7A1/FADD/PELI1/VAV3/TMEM131L/VSIR/MARCHF7

Biological Process	GO:0090263	positive regulation of canonical Wnt signaling pathway	27/2279	150/18866	0.0216797	0.253981	0.229811	27	RNF220/KANK1/CSNK1D/TNKS/MIR145/SMAD3/FAM53B/JUP/RBPJ/ILK/CDH3/TGFB1/AXIN1/LRRK1/CSNK1E/PSMF1/ARNTL/PSMB7/TTC21B/ZBED3/RUVBL1/ZBED2/PPM1N/GID8/DAPK3/PSMD13/ADGRA2
Biological Process	GO:0032387	negative regulation of intracellular transport	13/2279	59/18866	0.0218709	0.2559057	0.2315526	13	KCNE1/ANGPT1/ITGB1BP1/SUFU/UBAC2/SNX3/DERL2/CNIH2/NF1/CABP1/SP100/ARHGAP1/SVIP
Biological Process	GO:1904659	glucose transmembrane transport	21/2279	110/18866	0.0219228	0.2561969	0.231816	21	PRKAG2/SLC2A5/MIR143/PIK3R1/SLC2A14/TNF/GRB10/SLC2A9/ZDHHC7/HNF1A/PRKCB/SLC1A2/HK2/PRKCI/NFE2L2/SORT1/SLC5A10/SLC2A1/RTN2/ASPSCR1/CLTCL1
Biological Process	GO:0035722	interleukin-12-mediated signaling pathway	11/2279	47/18866	0.0219745	0.2564866	0.2320782	11	P4HB/IL10/RAP1B/SERPINB2/AIP/JAK1/PITPNA/PLCB1/RALA/CA1/GSTO1
Biological Process	GO:0018023	peptidyl-lysine trimethylation	12/2279	53/18866	0.0220686	0.2568484	0.2324056	12	BRD4/SETD2/WDR82/KMT2D/KDM4C/SETD1B/SETD1A/TET2/ZNF335/PIH1D1/NSD3/CSKMT
Biological Process	GO:0071320	cellular response to cAMP	12/2279	53/18866	0.0220686	0.2568484	0.2324056	12	KCNE1/ITPR2/RAP1B/SLC8A1/APP/FDX1/CRHBP/RAPGEF2/AQP1/KCNQ1/EZR/AQP9
Biological Process	GO:0071674	mononuclear cell migration	19/2279	97/18866	0.0220866	0.2568484	0.2324056	19	C3AR1/CCR2/TNF/CCL5/CSF1R/FPR2/THBS1/DEFB124/FLT1/PDGFD/CXCL17/CCL20/IL6R/PDGFB/PLCB1/ANO6/CCL27/CCL22/JAML
Biological Process	GO:1901293	nucleoside phosphate biosynthetic process	44/2279	269/18866	0.0223	0.2590126	0.2343638	44	PRKAG2/RCVRN/AMPD3/ENO1/NADK/ADCY2/ACSL1/ADCY9/NMNAT3/RRM2B/ACOT7/VPS9D1/TK2/ACSS1/SPHK2/TGFB1/SLC26A1/UCK2/ACACB/ACSS2/PNP/GUK1/ADCY4/MTHFD1/PPCDC/PAPSS2/PKM/ACACA/ACSF3/AK2/AK5/NPPC/ACAT1/SCD/LHPP/DCTD/PARP10/DHODH/PARP9/SLC25A1/TK1/PANK4/IMPDH1/ATP5PD
Biological Process	GO:0030183	B cell differentiation	25/2279	137/18866	0.0226154	0.2623553	0.2373884	25	HDAC4/IL10/INPP5D/PIK3R1/LYL1/TLR9/DLL1/RBPJ/PTPN6/PTPRJ/CD79A/SP3/CMTM7/PTPRC/DOCK10/NTRK1/POU2F2/BCL2/TCF3/HHEX/CYLD/BCL3/INHBA/PCID2/ADGRG3

Biological Process	GO:0043666	regulation of phosphoprotein phosphatase activity	22/2279	117/18866	0.0227308	0.2628717	0.2378556	22	LMTK2/MGAT5/PPP1R1B/URI1/CABIN1/TNF/GNAI2/ELFN2/RCAN1/PHACTR2/PPP2R5C/HTT/PTPRC/PPP2R1A/PPP2R5E/PPP2R5D/PPP2R1B/PDGFRB/PPP1R16B/CD33/ANKLE2/PTPA
Biological Process	GO:0006884	cell volume homeostasis	8/2279	30/18866	0.0227706	0.2628717	0.2378556	8	CLCN6/AQP1/GNB3/ADD1/ANO6/SLC12A1/SLC12A7/ANXA7
Biological Process	GO:0051125	regulation of actin nucleation	8/2279	30/18866	0.0227706	0.2628717	0.2378556	8	TRIM27/GSN/WHAMM/WASL/GMFB/NCKAP1/CYFIP1/GMFG
Biological Process	GO:0070723	response to cholesterol	8/2279	30/18866	0.0227706	0.2628717	0.2378556	8	GPLD1/TGFBR2/TGFB1/MLC1/ABCA1/GRAMD1A/HMGCS1/INHBA
Biological Process	GO:0002456	T cell mediated immunity	20/2279	104/18866	0.0231403	0.2664918	0.2411312	20	PRKCZ/FUT7/CD55/SLC11A1/TNFRSF1B/NLRP3/IL1R1/CCR2/TRAF2/DUSP22/CTSH/RAB27A/HLA-F/SMAD7/RIPK3/PTPRC/IL18RAP/IL18R1/FADD/CYRIB
Biological Process	GO:0043279	response to alkaloid	20/2279	104/18866	0.0231403	0.2664918	0.2411312	20	BLM/SPIDR/PPARG/CASQ2/PPP1R1B/SLC8A1/DNM2/RECQL5/DDC/CRHBP/HTR3A/CASP7/PRKCE/UBE3A/BCL2L1/RELA/DHODH/HNMT/FADD/PRKAA1
Biological Process	GO:0034103	regulation of tissue remodeling	18/2279	91/18866	0.0231903	0.2667448	0.2413602	18	ABR/DEF8/MIR143/INPP5D/VDR/PRKCA/MIR199A1/UBASH3B/TNFAIP3/CSF1R/TGFB1/NF1/ITGB3/TMEM64/CSK/BCR/PLEKHM1/MIR199A2
Biological Process	GO:1904062	regulation of cation transmembrane transport	55/2279	349/18866	0.023296	0.2676368	0.2421673	55	PDE4D/KCNE1/KCNAB2/THADA/CASQ2/WWP2/FXYD2/CACNA1C/YWHAH/FXYD1/CCR2/SLC8A1/DNM2/UBASH3B/TLR9/TESC/BIN1/JSRP1/AHNAK/APP/NOS1AP/PTPN6/ACTN4/KCNIP1/P2RY6/SPHK2/CRHBP/SLC43A2/CNIH2/MYO5A/HTT/CAB39/SNTA1/MAPK8IP2/KCNQ1/GSG1L/GSTO1/MEF2C/SHANK2/PRKCE/F2R/TMC1/SLC43A1/NEDD4/ANO6/DYSF/ANK2/HECW2/PLCG1/PDZK1/CASQ1/FGF14/CBARP/STIMATE/CRACR2A
Biological Process	GO:0061035	regulation of cartilage development	15/2279	72/18866	0.0235551	0.270116	0.2444106	15	SMAD3/ZBTB16/SMAD7/WNT11/RARA/POR/RARG/BMPR1B/RUNX2/TRPS1/GDF6/RELA/ZNF219/RFLNA/CCN4

Biological Process	GO:0006869	lipid transport	61/2279	393/18866	0.0235687	0.270116	0.2444106	61	PRKAG2/OSBPL5/RXRA/PPARG/SLC51A/ACSL1/ABCC1/SLC10A1/MFSD2A/MYB/MAP2K6/TMEM30A/LIPC/ABCG1/ATP8A1/ABCC2/HDLBP/ANO7/PLTP/THBS1/PITPNM2/STARD6/ATP11A/ATP8B1/PITPNM1/MIR27A/OSBPL6/CLU/PITPNA/SLCO3A1/NR1H2/LRP10/ITGB3/GHRL/PCTP/ABCA1/NCOA1/ACACB/FABP6/OSBPL10/TEX2/ABCC3/ATP8B4/NPC1/SGPP1/SPNS3/ACACA/PDZD8/PITPNC1/AGTR1/SLC2A1/ANO6/NCOA2/GRAMD1A/ESYT1/CPT1A/INHBA/VPS51/C2CD2L/AQP9/PRKN
Biological Process	GO:0009101	glycoprotein biosynthetic process	54/2279	342/18866	0.023604	0.2701946	0.2444816	54	CSGALNACT1/FUT4/GALNT2/FUT7/RPN1/XXYLT1/HS3ST3B1/DSE/SLC35C2/MGAT5/ST6GALNAC1/GALNT12/CHSY1/MGAT4A/MIR101-2/CHST11/MGAT3/CHST15/NDST1/TCF7L2/MUC12/ASGR2/AGO2/XYLT1/GXYLT2/B4GALT7/POMT2/MUC20/B3GNT5/ST6GAL1/TNIP1/ITM2C/BMPR1B/ST8SIA6/ST3GAL4/PLCB1/TET2/B4GALT3/PMM2/NPC1/BCL2/GALNT9/FUT11/GCNT2/GFPT2/LMF1/ALG11/PHLDA1/MAN1C1/DPAGT1/ST3GAL2/B3GNT2/B4GAT1/PXYLP1
Biological Process	GO:0044282	small molecule catabolic process	69/2279	452/18866	0.0237506	0.2715451	0.2457037	69	FUT4/FOXK1/CDA/ACAD8/DPYS/PLA2G15/SYNJ2/ENO1/HAL/FUT7/NAGK/HK1/ENTPD1/MFSD2A/HEXB/INPP5A/GADL1/AMDHD2/BDH1/HGD/ABHD16A/AMACR/NPL/ALDH2/CBS/ACOXL/AKR1D1/MTHFS/OGDH/NUDT3/ADPGK/ALDH4A1/ENOSF1/PGAM1/GLYCTK/FAH/TGFB1/CYP4F3/STAB2/ACADM/SULT1A2/HIBADH/ENO3/AGXT/HK2/ACACB/INPP5E/UPB1/IMPA2/PNP/ACOX2/LYVE1/HOGA1/ADO/BCKDHA/PKM/ACSF3/MAT1A/HMGCL/PCK2/CRYM/ACAT1/ADTRP/CPT1A/LPIN1/HNMT/LYPLA2/ACADVL/CEMIP2
Biological Process	GO:0060402	calcium ion transport into cytosol	28/2279	158/18866	0.0237799	0.2715536	0.2457114	28	TRPM2/PDE4D/CASQ2/ITPR2/LCK/CACNA1C/SLC8A1/UBASH3B/ITPR1/JSRP1/PTPN6/RASA3/P2RY6/MYO5A/HTT/PTPRC/NOL3/TPCN2/BCL2/GSTO1/PRKCE/F2R/ANK2/CHERP/PLCG1/P2RX5/TRPV2/CASQ1
Biological Process	GO:0006352	DNA-templated transcription, initiation	41/2279	249/18866	0.0241306	0.2722976	0.2463846	41	RXRA/TAF1C/MAML3/PPARG/NR1I2/CREB1/MAML1/VDR/E2F3/MAML2/POLR2F/CRCP/RBPJ/TAF10/HNF1A/RARA/MED13/NR1H2/WWTR1/ATF7IP/RARG/RUNX2/BRF1/CREBBP/RSF1/CCNH/ESRRB/MED8/NOTCH1/PIH1D1/MED25/SUB1/NOTCH4/TAF1D/MORC1/ERCC1/TEAD2/NR5A2/XPA/ERCC2/PAXIP1
Biological Process	GO:0034394	protein localization to cell surface	14/2279	66/18866	0.0243097	0.2722976	0.2463846	14	KCNAB2/TM9SF4/CD177/ANGPT1/PIGU/TNF/WNT11/SMURF1/FCN1/JAM3/ASTN2/MAP1A/ANK2/PTPRU
Biological Process	GO:0046626	regulation of insulin receptor signaling pathway	14/2279	66/18866	0.0243097	0.2722976	0.2463846	14	PRKCZ/SORL1/FUT7/KANK1/PTPRE/PIK3R1/BAIAP2L1/GRB10/GNAI2/ZBTB7B/PRKCB/TSC2/RELA/PRKAA1
Biological Process	GO:0045622	regulation of T-helper cell differentiation	9/2279	36/18866	0.0243977	0.2722976	0.2463846	9	IL27/PRKCZ/MYB/NLRP3/LOXL3/SMAD7/ZBTB7B/RARA/HLX
Biological Process	GO:0045738	negative regulation of DNA repair	9/2279	36/18866	0.0243977	0.2722976	0.2463846	9	RMI2/RECQL5/OTUB2/OTUB1/TP53BP1/TRIP12/TFIP11/CYREN/FBH1

Biological Process	GO:0097484	dendrite extension	9/2279	36/18866	0.0243977	0.2722976	0.2463846	9	CPNE6/CPNE5/CYFIP2/BCL11A/SMURF1/CDKL3/SYT17/CYFIP1/PRKN
Biological Process	GO:0001865	NK T cell differentiation	4/2279	10/18866	0.0244469	0.2722976	0.2463846	4	ZBTB16/TGFBR2/ZBTB7B/AP3D1
Biological Process	GO:0002676	regulation of chronic inflammatory response	4/2279	10/18866	0.0244469	0.2722976	0.2463846	4	IL10/TNF/TNFAIP3/CCL5
Biological Process	GO:0002887	negative regulation of myeloid leukocyte mediated immunity	4/2279	10/18866	0.0244469	0.2722976	0.2463846	4	ABR/CCR2/CD84/BCR
Biological Process	GO:0002934	desmosome organization	4/2279	10/18866	0.0244469	0.2722976	0.2463846	4	PRKCA/JUP/PERP/NECTIN1
Biological Process	GO:0003376	sphingosine-1-phosphate receptor signaling pathway	4/2279	10/18866	0.0244469	0.2722976	0.2463846	4	SPHK2/S1PR2/S1PR4/EZR
Biological Process	GO:0010887	negative regulation of cholesterol storage	4/2279	10/18866	0.0244469	0.2722976	0.2463846	4	PPARG/ABCG1/NR1H2/ABCA1
Biological Process	GO:0015937	coenzyme A biosynthetic process	4/2279	10/18866	0.0244469	0.2722976	0.2463846	4	ACOT7/PPCDC/ACAT1/PANK4
Biological Process	GO:0044857	plasma membrane raft organization	4/2279	10/18866	0.0244469	0.2722976	0.2463846	4	PACSIN2/MIR138-2/FA2H/FLOT1

Biological Process	GO:0070327	thyroid hormone transport	4/2279	10/18866	0.0244469	0.2722976	0.2463846	4	ABCC2/SLC7A5/CRYM/SLC7A8
Biological Process	GO:0070341	fat cell proliferation	4/2279	10/18866	0.0244469	0.2722976	0.2463846	4	TFDP1/E2F3/FTO/PER2
Biological Process	GO:0070344	regulation of fat cell proliferation	4/2279	10/18866	0.0244469	0.2722976	0.2463846	4	TFDP1/E2F3/FTO/PER2
Biological Process	GO:0070544	histone H3-K36 demethylation	4/2279	10/18866	0.0244469	0.2722976	0.2463846	4	KDM4B/KDM2A/KDM4C/KDM7A
Biological Process	GO:0070942	neutrophil mediated cytotoxicity	4/2279	10/18866	0.0244469	0.2722976	0.2463846	4	AZU1/ELANE/CTSG/DNASE1L3
Biological Process	GO:0090527	actin filament reorganization	4/2279	10/18866	0.0244469	0.2722976	0.2463846	4	GSN/WHAMM/ARAP1/CASS4
Biological Process	GO:2000659	regulation of interleukin-1-mediated signaling pathway	4/2279	10/18866	0.0244469	0.2722976	0.2463846	4	VRK2/IL1R1/MIR27A/IL1RN
Biological Process	GO:0009260	ribonucleotide biosynthetic process	32/2279	186/18866	0.024526	0.272299	0.2463858	32	PRKAG2/AMPD3/ENO1/ADCY2/ACSL1/ADCY9/ACOT7/VPS9D1/ACSS1/SPHK2/TGFB1/SLC26A1/UCK2/ACACB/ACSS2/GUK1/ADCY4/PPCDC/PAPSS2/PKM/ACACA/ACSF3/AK2/AK5/NPPC/ACAT1/SCD/DHODH/SLC25A1/PANK4/IMPDH1/ATP5PD
Biological Process	GO:0002705	positive regulation of leukocyte mediated immunity	25/2279	138/18866	0.0245523	0.272299	0.2463858	25	PRKCZ/CD177/NLRP3/IL1R1/GAB2/FGR/TNF/TRAF2/CD226/HLA-F/SCIMP/SPHK2/TGFB1/PTPRC/ITGB2/IL18RAP/NOD2/TP53BP1/IL18R1/TNFSF13/CADM1/FADD/PAXIP1/CYRIB/NSD2

Biological Process	GO:0032944	regulation of mononuclear cell proliferation	37/2279	221/18866	0.0245665	0.272299	0.2463858	37	HLA-DMB/IL27/IL10/MAD1L1/CD55/INPP5D/TNFRSF1B/CCR2/CCL5/NCK2/SFTPD/NFATC2/TNFSF13B/TLR9/MNDA/TGFBR2/PTPN6/TNFRSF21/ZBTB7B/RIPK3/BLK/CCDC88B/PTPRC/CD320/SPTA1/ZNF335/PNP/BCL2/MEF2C/CEBPB/SLC7A1/FADD/PELI1/VAV3/TMEM131L/VSIR/MARCHF7
Biological Process	GO:0002793	positive regulation of peptide secretion	33/2279	193/18866	0.024601	0.272299	0.2463858	33	S100A8/SORL1/NADK/IL10/TTN/C1QTNF3/MYRIP/CLEC9A/FRMD4A/ARRB1/GPLD1/FGR/TNF/MYO18A/ABCG1/CLEC5A/ITSN1/TCF7L2/TGFB1/GIPR/BLK/NR1H2/GHRL/MYOM1/DNM1L/PRKCE/ANKRD1/CADM1/CD33/RPH3AL/EZR/C2CD2L/SIRT3
Biological Process	GO:0034219	carbohydrate transmembrane transport	22/2279	118/18866	0.0248559	0.272299	0.2463858	22	PRKAG2/SLC2A5/MIR143/PIK3R1/SLC2A14/TNF/GRB10/SLC2A9/ZDHHC7/HNF1A/PRKCB/SLC1A2/HK2/PRKCI/NFE2L2/SORT1/SLC5A10/SLC2A1/RTN2/ASPSCR1/CLTCL1/SLC23A1
Biological Process	GO:0050868	negative regulation of T cell activation	22/2279	118/18866	0.0248559	0.272299	0.2463858	22	IL10/MAD1L1/RUNX1/LAX1/LOXL3/SFTPD/DUSP22/PTPN6/MIR27A/SMAD7/TNFRSF21/ZBTB7B/TIGIT/ZC3H8/DUSP3/HLX/CEBPB/RUNX3/PELI1/TMEM131L/VSIR/MARCHF7
Biological Process	GO:0030865	cortical cytoskeleton organization	13/2279	60/18866	0.0249298	0.272299	0.2463858	13	CALR/FMNL3/RHOH/RHOG/TNF/RHOF/FMNL1/RHOBTB2/EPB41L3/WDR1/NCKAP1/STRIP1/EZR
Biological Process	GO:0050994	regulation of lipid catabolic process	13/2279	60/18866	0.0249298	0.272299	0.2463858	13	PNPLA2/SORL1/MFSD2A/GPLD1/TNF/CRTC3/ACACB/PRKCE/ABHD5/PDE3B/CPT1A/BSCL2/PRKAA1
Biological Process	GO:0046339	diacylglycerol metabolic process	7/2279	25/18866	0.0250004	0.272299	0.2463858	7	PNPLA2/PLA2G15/PGS1/DGKQ/DGKA/DGKD/AVIL
Biological Process	GO:1903421	regulation of synaptic vesicle recycling	7/2279	25/18866	0.0250004	0.272299	0.2463858	7	DGKQ/SH3GL1/DNM1/DNM3/DNM1L/CYFIP1/FGF14
Biological Process	GO:1904376	negative regulation of protein localization to cell periphery	7/2279	25/18866	0.0250004	0.272299	0.2463858	7	MRAP/PPFIA1/LZTFL1/TGFB1/NUMB/CSK/BCL2L1

Biological Process	GO:1904385	cellular response to angiotensin	7/2279	25/18866	0.0250004	0.272299	0.2463858	7	MIR143/MIR145/ARID1B/AGTRAP/NFE2L2/AGTR1/RELA
Biological Process	GO:0042063	gliogenesis	49/2279	307/18866	0.0251002	0.272299	0.2463858	49	AZU1/ARHGEF10/S100A8/SH3TC2/CD9/PPARG/GSN/CREB1/HEXB/MYB/SKI/LGI4/TNFRSF1B/CRB1/NDRG1/CCR2/RHEB/TNF/CSF1R/FPR2/EIF2B5/RTN4/BIN1/DISC1/DLL1/APP/ILK/CLU/TNFRSF21/HDAC1/ZMIZ1/PAX2/TGFB1/PRKCH/NF1/CSK/GRN/WDR1/PRKCI/TTC21B/NOTCH1/TSPAN2/FA2H/CNTNAP1/RELA/CERS2/ERCC2/LIF/CX3CR1
Biological Process	GO:1903169	regulation of calcium ion transmembrane transport	27/2279	152/18866	0.0253488	0.272299	0.2463858	27	PDE4D/THADA/CASQ2/CACNA1C/SLC8A1/UBASH3B/TLR9/BIN1/JSRP1/AHNAK/NOS1AP/PTPN6/P2RY6/MYO5A/HTT/GSTO1/PRKCE/F2R/TMC1/DYSF/ANK2/PLCG1/CASQ1/FGF14/CBARP/STIMATE/CRACR2A
Biological Process	GO:0002824	positive regulation of adaptive immune response based on somatic recombination of immune receptors built from immunoglobulin superfamily domains	20/2279	105/18866	0.0254452	0.272299	0.2463858	20	PRKCZ/SLC11A1/NLRP3/IL1R1/CCR2/TNF/TRAF2/TNFSF13B/CD226/HLA-F/TGFB1/PTPRC/TP53BP1/IL18R1/HLX/TNFSF13/FADD/PAXIP1/CYRIB/NSD2
Biological Process	GO:0097006	regulation of plasma lipoprotein particle levels	20/2279	105/18866	0.0254452	0.272299	0.2463858	20	P4HB/EHD1/MPO/AP2A1/MFSD2A/LIPC/PLAGL2/MIR199A1/GPLD1/ABCG1/HDLBP/PLTP/MIR27A/NR1H2/CSK/ABCA1/NPC1/MIR199A2/LMF1/AGTR1
Biological Process	GO:0043370	regulation of CD4-positive, alpha-beta T cell differentiation	11/2279	48/18866	0.0254916	0.272299	0.2463858	11	IL27/PRKCZ/MYB/RUNX1/NLRP3/LOXL3/SMAD7/ZBTB7B/RARA/HLX/RUNX3
Biological Process	GO:2001239	regulation of extrinsic apoptotic signaling pathway in absence of ligand	11/2279	48/18866	0.0254916	0.272299	0.2463858	11	TNF/UNC5B/RIPK1/FGFR1/NF1/PPP2R1A/BCL2/PPP2R1B/BCL2L1/INHBA/DAPK3
Biological Process	GO:0031058	positive regulation of histone modification	18/2279	92/18866	0.025682	0.272299	0.2463858	18	BRD4/MYB/ARRB1/C6orf89/RPS6KA4/SPHK2/ZBTB7B/PAX7/CTBP1/PHF19/DNMT1/PIH1D1/SREBF1/RNF40/KAT7/LIF/PAXIP1/NSD3
Biological Process	GO:0106027	neuron projection organization	18/2279	92/18866	0.025682	0.272299	0.2463858	18	DCTN1/ZMYND8/CTTN/TANC1/EPHB3/BAIAP2/APP/IGF1R/WASL/SIPA1L1/DOCK10/DNM3/MAP1A/DNM1L/SHANK2/UBE3A/CUX2/DBNL

Biological Process	GO:0050920	regulation of chemotaxis	38/2279	229/18866	0.0260508	0.272299	0.2463858	38	AZU1/ELANE/SEMA6B/CALR/C3AR1/IL16/CCR2/SMAD3/CCL5/CSF1R/FPR2/SEMA4A/PADI2/THBS1/SEMA4B/C5AR2/DEFB124/FGFR1/TGFB1/ZNF580/SEMA4D/ZSWIM5/PDGFD/JAM3/CAMK1D/CXCL17/IL6R/PDGFB/DAPK2/NOTCH1/DNM1L/DUSP3/PDGFRB/ANO6/DYSF/CCL27/ADGRA2/CYRIB
Biological Process	GO:0009152	purine ribonucleotide biosynthetic process	30/2279	173/18866	0.0260581	0.272299	0.2463858	30	PRKAG2/AMPD3/ENO1/ADCY2/ACSL1/ADCY9/ACOT7/VPS9D1/ACSS1/SPHK2/TGFB1/SLC26A1/ACACB/ACSS2/GUK1/ADCY4/PPCDC/PAPSS2/PKM/ACACA/ACSF3/AK2/AK5/NPPC/ACAT1/SCD/SLC25A1/PANK4/IMPDH1/ATP5PD
Biological Process	GO:0051656	establishment of organelle localization	66/2279	432/18866	0.0261005	0.272299	0.2463858	66	CTSZ/PRKCZ/LAT/DCTN1/SERPINA1/TGFA/STX5/MAD1L1/PIK3CD/CSNK1D/NDE1/GAB2/KIF13A/DNM2/TRAPPC10/FGR/DYNC1H1/CD84/CHAMP1/C17orf75/CUL3/MYH9/TRAPPC9/RAB27A/ACTN4/MIS12/CDH3/WASL/SPHK2/FER/DCTN2/BICD2/CNIH2/MYO5A/BLK/CD59/HTT/SEC13/KIF5C/NUMA1/ANKRD28/DNM1/SEC24C/YWHAZ/RAD21L1/KIF1B/DNM3/CDCA5/ARHGEF2/KIFAP3/TMED2/MYO1C/SPAG5/AP3D1/MX1/FBXW11/NDEL1/EZR/SLIT1/DTNBP1/SEC31A/TERB2/IRAG2/PRKN/TRAPPC2/RIPOR1
Biological Process	GO:0050821	protein stabilization	32/2279	187/18866	0.0262732	0.272299	0.2463858	32	TRIM39/CALR/CREB1/AAK1/PIK3R1/SMAD3/MIR101-2/RTN4/TESC/USP36/CLU/SMAD7/PHB/PFN1/ATF7IP/STK3/GRN/CCNH/MORC3/ZBED3/CHEK2/NAA16/USP2/WDR81/ANK2/FLOT1/BAG5/CCT6A/PRKN/P3H1/MARCHF7/PLPP3
Biological Process	GO:0010952	positive regulation of peptidase activity	34/2279	201/18866	0.0263248	0.272299	0.2463858	34	NLRC4/S100A8/GRAMD4/AIM2/PPARG/GSN/LCK/NLRP3/SMAD3/ARRB1/MEFV/TNF/TRAF2/CYFIP2/TBC1D10A/CTSD/CTSH/RIPK1/APP/MBP/CIDEB/PERP/BCL10/ATP2A3/GRN/FIS1/P2RX1/F2R/CASP1/BCL2L13/ASPH/NODAL/FADD/VSIR
Biological Process	GO:0009314	response to radiation	68/2279	447/18866	0.0263533	0.272299	0.2463858	68	ELANE/BLM/RCVRN/PDE6A/SPIDR/MFAP4/CREB1/PPP1R1B/BRCA2/CACNA2D4/CRB1/PIK3R1/BMF/ARRB1/DNM2/HUS1/TANC1/APP/PITPNM1/FECH/CCDC66/GNAT2/SYNGAP1/CDC25A/HRH1/AQP1/MTA1/NF1/SLC1A2/GNA11/PTPRC/MEIS2/CREBBP/CAT/PPP1CB/COPS3/PER2/NTRK1/SLC1A3/BCL2/DMC1/PER1/TP53BP1/FBXL6/GRK7/RHNO1/CHEK2/NEDD4/ERCC1/CRIP1/USP2/BCL2L1/HMGCS1/RELA/BCL3/EYS/POLD3/XPA/CABP4/ERCC2/SYNPO/NPHP4/PAXIP1/EEF1D/PRKAA1/MAP3K20/ABRAXAS1/BABAM2
Biological Process	GO:0002709	regulation of T cell mediated immunity	15/2279	73/18866	0.0264436	0.272299	0.2463858	15	PRKCZ/FUT7/TNFRSF1B/NLRP3/IL1R1/CCR2/TRAF2/DUSP22/HLA-F/SMAD7/RIPK3/PTPRC/IL18R1/FADD/CYRIB
Biological Process	GO:0015807	L-amino acid transport	15/2279	73/18866	0.0264436	0.272299	0.2463858	15	SLC11A1/SLC1A5/SLC15A4/SLC43A2/SLC1A2/SLC7A7/SLC7A5/SFXN1/SLC1A6/PER2/SLC36A3/SLC1A3/SLC43A1/SLC7A8/SLC7A1
Biological Process	GO:0070227	lymphocyte apoptotic process	15/2279	73/18866	0.0264436	0.272299	0.2463858	15	FOXP1/IL10/CCL5/HSH2D/RIPK1/PTCRA/TNFRSF21/DOCK8/RIPK3/ZC3H8/BLK/BCL10/TRAF3IP2/FADD/GIMAP8

Biological Process	GO:0000270	peptidoglycan metabolic process	3/2279	6/18866	0.0265458	0.272299	0.2463858	3	LYG2/PGLYRP1/PGLYRP4
Biological Process	GO:0006030	chitin metabolic process	3/2279	6/18866	0.0265458	0.272299	0.2463858	3	CHI3L1/CHIT1/CHI3L2
Biological Process	GO:0006032	chitin catabolic process	3/2279	6/18866	0.0265458	0.272299	0.2463858	3	CHI3L1/CHIT1/CHI3L2
Biological Process	GO:0006686	sphingomyelin biosynthetic process	3/2279	6/18866	0.0265458	0.272299	0.2463858	3	SPTLC2/SGMS1/SGMS2
Biological Process	GO:0009136	purine nucleoside diphosphate biosynthetic process	3/2279	6/18866	0.0265458	0.272299	0.2463858	3	GUK1/AK2/AK5
Biological Process	GO:0009180	purine ribonucleoside diphosphate biosynthetic process	3/2279	6/18866	0.0265458	0.272299	0.2463858	3	GUK1/AK2/AK5
Biological Process	GO:0009253	peptidoglycan catabolic process	3/2279	6/18866	0.0265458	0.272299	0.2463858	3	LYG2/PGLYRP1/PGLYRP4
Biological Process	GO:0010694	positive regulation of alkaline phosphatase activity	3/2279	6/18866	0.0265458	0.272299	0.2463858	3	SMAD3/GPLD1/MEF2C
Biological Process	GO:0010757	negative regulation of plasminogen activation	3/2279	6/18866	0.0265458	0.272299	0.2463858	3	CTSZ/THBS1/CPB2

Biological Process	GO:0014916	regulation of lung blood pressure	3/2279	6/18866	0.0265458	0.272299	0.2463858	3	SMAD3/MIR199A1/MIR199A2
Biological Process	GO:0015942	formate metabolic process	3/2279	6/18866	0.0265458	0.272299	0.2463858	3	HAL/MTHFD1L/MTHFS
Biological Process	GO:0019262	N-acetylneuraminate catabolic process	3/2279	6/18866	0.0265458	0.272299	0.2463858	3	NAGK/AMDHD2/NPL
Biological Process	GO:0034499	late endosome to Golgi transport	3/2279	6/18866	0.0265458	0.272299	0.2463858	3	SNX3/NUMA1/SGSM2
Biological Process	GO:0035434	copper ion transmembrane transport	3/2279	6/18866	0.0265458	0.272299	0.2463858	3	SLC11A2/ATOX1/ATP7B
Biological Process	GO:0042866	pyruvate biosynthetic process	3/2279	6/18866	0.0265458	0.272299	0.2463858	3	AGXT/HOGA1/PKM
Biological Process	GO:0043152	induction of bacterial agglutination	3/2279	6/18866	0.0265458	0.272299	0.2463858	3	RNASE3/SFTPD/SPON2
Biological Process	GO:0043376	regulation of CD8-positive, alpha-beta T cell differentiation	3/2279	6/18866	0.0265458	0.272299	0.2463858	3	RUNX1/ZBTB7B/RUNX3
Biological Process	GO:0044648	histone H3-K4 dimethylation	3/2279	6/18866	0.0265458	0.272299	0.2463858	3	KMT2D/SETD1B/SETD1A

Biological Process	GO:0045634	regulation of melanocyte differentiation	3/2279	6/18866	0.0265458	0.272299	0.2463858	3	ZEB2/GNA11/BCL2
Biological Process	GO:0048296	regulation of isotype switching to IgA isotypes	3/2279	6/18866	0.0265458	0.272299	0.2463858	3	TGFB1/TNFSF13/NSD2
Biological Process	GO:0051136	regulation of NK T cell differentiation	3/2279	6/18866	0.0265458	0.272299	0.2463858	3	ZBTB16/TGFBR2/AP3D1
Biological Process	GO:0060842	arterial endothelial cell differentiation	3/2279	6/18866	0.0265458	0.272299	0.2463858	3	DLL1/RBPJ/NOTCH1
Biological Process	GO:0061419	positive regulation of transcription from RNA polymerase II promoter in response to hypoxia	3/2279	6/18866	0.0265458	0.272299	0.2463858	3	RBPJ/NFE2L2/NOTCH1
Biological Process	GO:0070535	histone H2A K63-linked ubiquitination	3/2279	6/18866	0.0265458	0.272299	0.2463858	3	OTUB2/OTUB1/TRIP12
Biological Process	GO:0072008	glomerular mesangial cell differentiation	3/2279	6/18866	0.0265458	0.272299	0.2463858	3	PDGFB/NOTCH1/GPR4
Biological Process	GO:0072126	positive regulation of glomerular mesangial cell proliferation	3/2279	6/18866	0.0265458	0.272299	0.2463858	3	PDGFD/IL6R/PDGFB
Biological Process	GO:0072143	mesangial cell development	3/2279	6/18866	0.0265458	0.272299	0.2463858	3	PDGFB/NOTCH1/GPR4

Biological Process	GO:0097198	histone H3-K36 trimethylation	3/2279	6/18866	0.0265458	0.272299	0.2463858	3	BRD4/SETD2/NSD3
Biological Process	GO:1901341	positive regulation of store-operated calcium channel activity	3/2279	6/18866	0.0265458	0.272299	0.2463858	3	CASQ1/STIMATE/CRACR2A
Biological Process	GO:1902564	negative regulation of neutrophil activation	3/2279	6/18866	0.0265458	0.272299	0.2463858	3	ABR/GRN/BCR
Biological Process	GO:1903525	regulation of membrane tubulation	3/2279	6/18866	0.0265458	0.272299	0.2463858	3	DNM2/ASAP1/WASL
Biological Process	GO:1903721	positive regulation of I-kappaB phosphorylation	3/2279	6/18866	0.0265458	0.272299	0.2463858	3	TNF/TRAF2/CX3CR1
Biological Process	GO:1904179	positive regulation of adipose tissue development	3/2279	6/18866	0.0265458	0.272299	0.2463858	3	SORL1/SH3PXD2B/GHRL
Biological Process	GO:1905274	regulation of modification of postsynaptic actin cytoskeleton	3/2279	6/18866	0.0265458	0.272299	0.2463858	3	ITSN1/BAIAP2/CYFIP1
Biological Process	GO:1905634	regulation of protein localization to chromatin	3/2279	6/18866	0.0265458	0.272299	0.2463858	3	SETD2/RB1/CDCA5
Biological Process	GO:2000323	negative regulation of glucocorticoid receptor signaling pathway	3/2279	6/18866	0.0265458	0.272299	0.2463858	3	PHB/ARNTL/PER1

Biological Process	GO:2000489	regulation of hepatic stellate cell activation	3/2279	6/18866	0.0265458	0.272299	0.2463858	3	RPS6KA1/MYB/PDGFRB
Biological Process	GO:0043401	steroid hormone mediated signaling pathway	25/2279	139/18866	0.0266148	0.272299	0.2463858	25	RXRA/FOXP1/CALR/NCOR2/RUNX1/AKAP13/YWHAH/PRMT2/LBH/PADI2/ZNF366/HDAC1/ZMIZ1/PHB/AXIN1/KMT2D/ARRB2/ARNTL/ESRRB/PER1/UBE3A/NEDD4/RNF14/NCOA4/NODAL
Biological Process	GO:0030522	intracellular receptor signaling pathway	43/2279	265/18866	0.0266962	0.272299	0.2463858	43	RXRA/FOXP1/CALR/PPARG/NR1I2/NCOR2/RUNX1/MAP2K6/YWHAH/PRMT2/VDR/TNFAIP3/LBH/PADI2/ZNF366/ACTN4/HDAC1/ZMIZ1/PHB/AXIN1/KMT2D/RARA/UBE2V1/NR1H2/SEC14L1/CTBP2/RARG/NCOA1/ARRB2/ARNTL/IRAK2/NOD2/PER1/UBE3A/NEDD4/RNF14/CYLD/SREBF1/RELA/NCOA4/ALDH1A2/NR5A2/NODAL
Biological Process	GO:0002544	chronic inflammatory response	6/2279	20/18866	0.0267283	0.272299	0.2463858	6	S100A8/IL10/TNF/TNFAIP3/CCL5/THBS1
Biological Process	GO:0007194	negative regulation of adenylate cyclase activity	6/2279	20/18866	0.0267283	0.272299	0.2463858	6	CCR2/GNAI2/AKAP5/LTB4R2/PALM/GABBR2
Biological Process	GO:0032495	response to muramyl dipeptide	6/2279	20/18866	0.0267283	0.272299	0.2463858	6	IRF5/TNFAIP3/NOD2/NOTCH1/ARHGEF2/RELA
Biological Process	GO:0045618	positive regulation of keratinocyte differentiation	6/2279	20/18866	0.0267283	0.272299	0.2463858	6	VDR/PRKCH/NUMA1/NOTCH1/ZBED2/MACROH2A2
Biological Process	GO:0060353	regulation of cell adhesion molecule production	6/2279	20/18866	0.0267283	0.272299	0.2463858	6	FUT7/MIR101-2/COLEC12/NOTCH1/NOTCH4/FLOT1
Biological Process	GO:0070932	histone H3 deacetylation	6/2279	20/18866	0.0267283	0.272299	0.2463858	6	HDAC4/HDAC7/HDAC1/PER2/PER1/ELK4

Biological Process	GO:0007044	cell-substrate junction assembly	19/2279	99/18866	0.0268841	0.272299	0.2463858	19	COL17A1/ITGB1BP1/SMAD3/ARHGEF7/CTTN/DUSP22/WHAMM/THBS1/TNS1/ACTN1/PTPRJ/TESK2/ACTG1/BCR/BCL2/CORO1C/DUSP3/LAMB3/DAPK3
Biological Process	GO:0014910	regulation of smooth muscle cell migration	17/2279	86/18866	0.0269875	0.272299	0.2463858	17	HDAC4/SORL1/PLAU/SSH1/MIR140/MIR143/CCL5/ILK/P2RY6/PDGFD/PDGFB/NFE2L2/BCL2/MEF2C/PDGFRB/TRIB1/CCN4
Biological Process	GO:2000106	regulation of leukocyte apoptotic process	17/2279	86/18866	0.0269875	0.272299	0.2463858	17	FOXP1/IL10/PIK3CD/ITPKB/CCL5/HSH2D/PTCRA/DOCK8/RAPGEF2/RIPK3/ZC3H8/BLK/BCL10/NOD2/MEF2C/FADD/GIMAP8
Biological Process	GO:0042542	response to hydrogen peroxide	26/2279	146/18866	0.0270225	0.272299	0.2463858	26	TRPM2/IL10/LCN2/MYB/SLC8A1/TNFAIP3/RIPK1/PAX2/ZNF580/HBB/AQP1/RIPK3/STK24/PDGFD/CAT/ETS1/NFE2L2/IL18RAP/BCL2/UBE3A/GLRX2/PDGFRB/FOXO1/RELA/MB/PRKAA1
Biological Process	GO:0034976	response to endoplasmic reticulum stress	47/2279	294/18866	0.0270828	0.272299	0.2463858	47	P4HB/DCTN1/CALR/ERN1/PIK3R1/AMFR/MBTPS1/RHBDD2/TRAF2/NCK2/EIF2B5/UBAC2/ITPR1/YIF1A/THBS1/EDEM1/HM13/DERL2/ZBTB17/PDIA5/CLU/UBE2J2/CLGN/TMX1/POMT2/COPS5/NR1H2/EDEM2/ATP2A3/CREB3L2/GORASP2/NFE2L2/ADD1/BCL2/CEBPB/BCL2L1/FLOT1/GRINA/KLHDC3/EIF4G1/UBXN8/SEC31A/SVIP/ACADVL/MARCHF6/NIBAN1/PRKN
Biological Process	GO:0010822	positive regulation of mitochondrion organization	22/2279	119/18866	0.02713	0.272299	0.2463858	22	TFDP1/YWHAH/VPS13D/BMF/FBXO7/CAMKK2/USP36/ABLIM3/UBE2J2/CIDEB/SMURF1/HTT/HK2/YWHAZ/FIS1/BCL2/DNM1L/TP53BP2/MIEF1/YWHAQ/PRKAA1/PRKN
Biological Process	GO:1901222	regulation of NIK/NF-kappaB signaling	22/2279	119/18866	0.02713	0.272299	0.2463858	22	CALR/C1QTNF3/CHI3L1/HDAC7/NLRP3/TNF/TRAF2/AGO1/TLR9/APP/ACTN4/ILK/CPNE1/PHB/LITAF/NOD2/IL18R1/CYLD/RELA/FBXW11/BCL3/ADGRG3
Biological Process	GO:0000098	sulfur amino acid catabolic process	5/2279	15/18866	0.0271411	0.272299	0.2463858	5	GADL1/CBS/AGXT/ADO/MAT1A
Biological Process	GO:0001325	formation of extrachromosomal circular DNA	5/2279	15/18866	0.0271411	0.272299	0.2463858	5	BLM/RTEL1/SMARCAL1/ERCC1/SLX4

Biological Process	GO:0002091	negative regulation of receptor internalization	5/2279	15/18866	0.0271411	0.272299	0.2463858	5	MIR199A1/ANKRD13B/ANKRD13D/ANKRD13A/MIR199A2
Biological Process	GO:0002467	germinal center formation	5/2279	15/18866	0.0271411	0.272299	0.2463858	5	TNFAIP3/TNFSF13B/KLHL6/MEF2C/BCL3
Biological Process	GO:0020027	hemoglobin metabolic process	5/2279	15/18866	0.0271411	0.272299	0.2463858	5	ALAS1/CAT/ADD1/EPB42/INHBA
Biological Process	GO:0030852	regulation of granulocyte differentiation	5/2279	15/18866	0.0271411	0.272299	0.2463858	5	RUNX1/INPP5D/TESC/RARA/TRIB1
Biological Process	GO:0045064	T-helper 2 cell differentiation	5/2279	15/18866	0.0271411	0.272299	0.2463858	5	PRKCZ/NLRP3/RARA/HLX/BCL3
Biological Process	GO:0060766	negative regulation of androgen receptor signaling pathway	5/2279	15/18866	0.0271411	0.272299	0.2463858	5	FOXP1/NCOR2/HDAC1/PHB/NODAL
Biological Process	GO:0071501	cellular response to sterol depletion	5/2279	15/18866	0.0271411	0.272299	0.2463858	5	AMFR/ZBTB7B/ARHGEF10L/SREBF1/SPRING1
Biological Process	GO:0072160	nephron tubule epithelial cell differentiation	5/2279	15/18866	0.0271411	0.272299	0.2463858	5	PAX2/MTSS1/WWTR1/MEF2C/LIF
Biological Process	GO:0090110	COPII-coated vesicle cargo loading	5/2279	15/18866	0.0271411	0.272299	0.2463858	5	SEC23B/MIA3/SEC13/SEC24C/SEC31A

Biological Process	GO:0090656	t-circle formation	5/2279	15/18866	0.0271411	0.272299	0.2463858	5	BLM/RTEL1/SMARCAL1/ERCC1/SLX4
Biological Process	GO:0090737	telomere maintenance via telomere trimming	5/2279	15/18866	0.0271411	0.272299	0.2463858	5	BLM/RTEL1/SMARCAL1/ERCC1/SLX4
Biological Process	GO:0006026	aminoglycan catabolic process	14/2279	67/18866	0.0274265	0.2736515	0.2476096	14	HEXB/CHI3L1/GALNS/LYG2/CHIT1/SDC2/TGFB1/STAB2/PGLYRP1/LYVE1/ARSB/CHI3L2/PGLYRP4/CEMIP2
Biological Process	GO:0033866	nucleoside bisphosphate biosynthetic process	14/2279	67/18866	0.0274265	0.2736515	0.2476096	14	ACSL1/ACOT7/ACSS1/SLC26A1/ACACB/ACSS2/PPCDC/PAPSS2/ACACA/ACSF3/ACAT1/SCD/SLC25A1/PANK4
Biological Process	GO:0034030	ribonucleoside bisphosphate biosynthetic process	14/2279	67/18866	0.0274265	0.2736515	0.2476096	14	ACSL1/ACOT7/ACSS1/SLC26A1/ACACB/ACSS2/PPCDC/PAPSS2/ACACA/ACSF3/ACAT1/SCD/SLC25A1/PANK4
Biological Process	GO:0034033	purine nucleoside bisphosphate biosynthetic process	14/2279	67/18866	0.0274265	0.2736515	0.2476096	14	ACSL1/ACOT7/ACSS1/SLC26A1/ACACB/ACSS2/PPCDC/PAPSS2/ACACA/ACSF3/ACAT1/SCD/SLC25A1/PANK4
Biological Process	GO:1903672	positive regulation of sprouting angiogenesis	14/2279	67/18866	0.0274265	0.2736515	0.2476096	14	IL10/MAP3K3/HDAC7/MIR101-2/DLL1/AKT3/MIR27A/MIR23A/JAK1/BMPER/GHRL/PKM/AGTR1/PPP1R16B
Biological Process	GO:0003299	muscle hypertrophy in response to stress	8/2279	31/18866	0.0275351	0.2736515	0.2476096	8	HDAC4/SMAD3/MIR199A1/CAMTA2/MIR199A2/MEF2C/FOXO1/INPP5F
Biological Process	GO:0014887	cardiac muscle adaptation	8/2279	31/18866	0.0275351	0.2736515	0.2476096	8	HDAC4/SMAD3/MIR199A1/CAMTA2/MIR199A2/MEF2C/FOXO1/INPP5F

Biological Process	GO:0014898	cardiac muscle hypertrophy in response to stress	8/2279	31/18866	0.0275351	0.2736515	0.2476096	8	HDAC4/SMAD3/MIR199A1/CAMTA2/MIR199A2/MEF2C/FOXO1/INPP5F
Biological Process	GO:0050858	negative regulation of antigen receptor-mediated signaling pathway	8/2279	31/18866	0.0275351	0.2736515	0.2476096	8	DUSP22/PTPN6/PTPRJ/LILRB4/DUSP3/ELF1/EZR/SLA2
Biological Process	GO:0030038	contractile actin filament bundle assembly	20/2279	106/18866	0.0279204	0.2768296	0.2504853	20	ARHGEF10/TACSTD2/ITGB1BP1/PIK3R1/SMAD3/ARRB1/SH3PXD2B/ARAP1/CUL3/SYNPO2L/PXN/WNT11/ITGB5/PPFIA1/PFN1/ACTG1/MIR138-2/ARHGEF10L/ARHGEF18/ARHGEF15
Biological Process	GO:0043149	stress fiber assembly	20/2279	106/18866	0.0279204	0.2768296	0.2504853	20	ARHGEF10/TACSTD2/ITGB1BP1/PIK3R1/SMAD3/ARRB1/SH3PXD2B/ARAP1/CUL3/SYNPO2L/PXN/WNT11/ITGB5/PPFIA1/PFN1/ACTG1/MIR138-2/ARHGEF10L/ARHGEF18/ARHGEF15
Biological Process	GO:0001818	negative regulation of cytokine production	56/2279	360/18866	0.0279423	0.2768296	0.2504853	56	ELANE/TRIM27/ZFPM1/IL10/C1QTNF3/MIR140/MIR145/HDAC7/SLC11A1/NLRP3/ARRB1/MIR199A1/MEFV/TNF/TNFAIP3/SFTPD/CD84/MIR101-2/TLR9/TRAIP/NAV3/THBS1/DLL1/C5AR2/HLA-F/PTPN6/MIR24-2/RPS6KA4/SMAD7/TNFRSF21/WNT11/CDH3/FGFR1/TGFB1/TIGIT/RARA/PGLYRP1/GHRL/PTPRC/CSK/ASB1/NLRC5/ARRB2/MIR199A2/LTF/CYLD/CD96/CD33/BCL3/EZR/HERC5/INHBA/CX3CR1/NDRG2/VSIR/ILRUN
Biological Process	GO:0035303	regulation of dephosphorylation	35/2279	209/18866	0.0280386	0.2774948	0.2510871	35	LMTK2/MGAT5/PPP1R1B/URI1/SMAD3/CABIN1/GPLD1/TNF/CCDC8/GNAI2/CAMTA1/ELFN2/RCAN1/SMG7/PHACTR2/TGFB1/FARP1/SEMA4D/PPP2R5C/RIPK3/HTT/PTPRC/SMG6/PPP2R1A/PPP2R5E/PPP2R5D/MEF2C/PPP2R1B/PDGFRB/WDR81/PPP1R16B/CD33/SPOCD1/ANKLE2/PTPA
Biological Process	GO:1901136	carbohydrate derivative catabolic process	33/2279	195/18866	0.0281257	0.2780674	0.2516052	33	PDE4D/CDA/DPYS/AMPD3/NAGK/HEXB/AMDHD2/CHI3L1/GALNS/NPL/GALC/AOAH/LYG2/CHIT1/EDEM1/NUDT3/ACOT7/SDC2/UNG/TGFB1/STAB2/NTHL1/PGLYRP1/EDEM2/UPB1/PDE9A/PNP/LYVE1/ARSB/ACAT1/CHI3L2/PGLYRP4/CEMIP2
Biological Process	GO:0043954	cellular component maintenance	13/2279	61/18866	0.0282926	0.278597	0.2520844	13	PRTN3/DCTN1/CD177/ZMYND8/CTTN/CSF1R/TANC1/IGF1R/SYNGAP1/PLEKHA7/CTBP2/F2R/KIFC3
Biological Process	GO:0045071	negative regulation of viral genome replication	13/2279	61/18866	0.0282926	0.278597	0.2520844	13	SLPI/SRPK2/TNF/CCL5/TNIP1/LTF/ILF3/MX1/PARP10/BANF1/ISG20/PRKN/SHFL

Biological Process	GO:0090303	positive regulation of wound healing	13/2279	61/18866	0.0282926	0.278597	0.2520844	13	RREB1/KANK1/MYLK/THBS1/F12/ACTG1/NFE2L2/PRKCE/CPB2/F2R/ANO6/PLPP3/CCN4
Biological Process	GO:0031397	negative regulation of protein ubiquitination	16/2279	80/18866	0.0282966	0.278597	0.2520844	16	ARRB1/TNFAIP3/MIR101-2/OTUB2/SMAD7/ARRB2/MIR138-2/PER2/OTUB1/NXN/PRKCE/TRIP12/DYSF/BAG5/PARP10/MARCHF7
Biological Process	GO:0072175	epithelial tube formation	24/2279	133/18866	0.0283469	0.2787457	0.252219	24	SHROOM3/SKI/SETD2/SPINT2/MTHFD1L/SUFU/ZEB2/MIB1/PAX2/TGFB1/RARA/PFN1/RARG/STK3/GRHL2/BCL10/MTHFD1/RALA/TMED2/TSC2/TEAD2/LMO4/NODAL/BRD2
Biological Process	GO:0014909	smooth muscle cell migration	18/2279	93/18866	0.0283704	0.2787457	0.252219	18	HDAC4/SORL1/PLAU/SSH1/MIR140/MIR143/CCL5/ILK/P2RY6/PDGFD/ITGB3/PDGFB/NFE2L2/BCL2/MEF2C/PDGFRB/TRIB1/CCN4
Biological Process	GO:0048545	response to steroid hormone	54/2279	346/18866	0.0287823	0.2825009	0.2556169	54	RXRA/FOXP1/CALR/IL10/NCOR2/RUNX1/URI1/AKAP13/YWHAH/PRMT2/TNF/LBH/ABCC2/PADI2/TGFBR2/SMYD3/THBS1/ALPL/ZNF366/FECH/HDAC1/ZMIZ1/TGFB1/PHB/IL1RN/AXIN1/AQP1/KMT2D/AGXT/NCOA1/ENG/ARRB2/ARNTL/NPC1/ESRRB/BCL2/PER1/NOTCH1/SLIT3/UBE3A/NEDD4/PCK2/RNF14/FOXO1/NCOA2/SREBF1/ADTRP/RELA/NCOA4/PTPRU/HSD11B2/NODAL/HNMT/SST
Biological Process	GO:0010614	negative regulation of cardiac muscle hypertrophy	9/2279	37/18866	0.0288902	0.2829754	0.2560462	9	CTDP1/MIR145/TNFRSF1B/SMAD3/MIR199A1/TNFRSF1A/MIR199A2/NOTCH1/FOXO1
Biological Process	GO:1905314	semi-lunar valve development	9/2279	37/18866	0.0288902	0.2829754	0.2560462	9	TNFRSF1B/GJA5/NFATC1/RBPJ/TNFRSF1A/TGFB1/RB1/NOTCH1/SLIT3
Biological Process	GO:0002720	positive regulation of cytokine production involved in immune response	12/2279	55/18866	0.0289793	0.2832633	0.2563067	12	PRKCZ/NLRP3/IL1R1/TRAF2/SPON2/CD226/HLA-F/SCIMP/SLC7A5/BCL10/NOD2/IL18R1
Biological Process	GO:0032655	regulation of interleukin-12 production	12/2279	55/18866	0.0289793	0.2832633	0.2563067	12	IRF8/FOXP1/IL10/IRF5/IL16/MEFV/TLR9/THBS1/SCIMP/TIGIT/PLCB1/ARRB2

Biological Process	GO:0007043	cell-cell junction assembly	26/2279	147/18866	0.0291827	0.2841368	0.257097	26	TBCD/CD9/TLN2/HDAC7/RUNX1/PRKCA/TNF/GJA5/JUP/PKP1/ACTN4/SMAD7/WNT11/PRKCH/EPB41L3/JAM3/ACTG1/GRHL2/WDR1/PRKCI/MPP7/CNTNAP1/MYO1C/ANK2/CDH11/NPHP4
Biological Process	GO:0046467	membrane lipid biosynthetic process	26/2279	147/18866	0.0291827	0.2841368	0.257097	26	SPTLC2/PIGL/PIGU/TNF/ORMDL3/CERS6/PIGN/TNFRSF1A/SPHK2/SGMS1/B3GNT5/ST8SIA6/ST3GAL4/SGMS2/B4GALT3/SGPP1/SGPP2/PIGQ/P2RX1/FA2H/CERS2/SIRT3/ST3GAL2/PRKAA1/TLCD3B/PLPP3
Biological Process	GO:0007595	lactation	11/2279	49/18866	0.0293987	0.2841368	0.257097	11	NCOR2/CREB1/VDR/STAT5A/ZBTB7B/HK2/NCOA1/USF2/SLC29A1/ATP7B/DHODH
Biological Process	GO:0010712	regulation of collagen metabolic process	11/2279	49/18866	0.0293987	0.2841368	0.257097	11	MFAP4/PPARG/MIR145/MYB/TGFB1/ENG/ARRB2/F2R/NPPC/PDGFRB/VSIR
Biological Process	GO:0032527	protein exit from endoplasmic reticulum	11/2279	49/18866	0.0293987	0.2841368	0.257097	11	SORL1/TM9SF4/TMEM30A/UBAC2/EDEM1/HM13/DERL2/SEC13/EDEM2/NUMA1/SVIP
Biological Process	GO:0032663	regulation of interleukin-2 production	11/2279	49/18866	0.0293987	0.2841368	0.257097	11	PDE4D/TRIM27/HDAC7/RUNX1/CCR2/TRAF2/TNFAIP3/SFTPD/NAV3/PTPRC/EZR
Biological Process	GO:0060711	labyrinthine layer development	11/2279	49/18866	0.0293987	0.2841368	0.257097	11	IL10/SPINT2/RBPJ/DNAJB6/GRHL2/NCOA1/VASH1/GGNBP2/TMED2/PCDH12/PLCD3
Biological Process	GO:0071349	cellular response to interleukin-12	11/2279	49/18866	0.0293987	0.2841368	0.257097	11	P4HB/IL10/RAP1B/SERPINB2/AIP/JAK1/PITPNA/PLCB1/RALA/CA1/GSTO1
Biological Process	GO:1990090	cellular response to nerve growth factor stimulus	11/2279	49/18866	0.0293987	0.2841368	0.257097	11	EHD1/CREB1/ACAP2/APP/RAPGEF2/SORT1/NTRK1/MICALL1/KIF1B/UBE3A/NTF4

Biological Process	GO:0030521	androgen receptor signaling pathway	10/2279	43/18866	0.0294275	0.2841368	0.257097	10	FOXP1/NCOR2/PRMT2/HDAC1/ZMIZ1/PHB/ARRB2/UBE3A/RNF14/NODAL
Biological Process	GO:0090207	regulation of triglyceride metabolic process	10/2279	43/18866	0.0294275	0.2841368	0.257097	10	PNPLA2/SORL1/MFSD2A/GPLD1/NR1H2/ATG14/GNB3/LMF1/ABHD5/SREBF1
Biological Process	GO:0090317	negative regulation of intracellular protein transport	10/2279	43/18866	0.0294275	0.2841368	0.257097	10	KCNE1/ANGPT1/ITGB1BP1/SUFU/UBAC2/DERL2/NF1/CABP1/SP100/SVIP
Biological Process	GO:0002262	myeloid cell homeostasis	27/2279	154/18866	0.0294806	0.2842331	0.2571842	27	MAEA/KLF1/ZFPM1/AMPD3/PIK3CD/INPP5D/TRIM10/ITPKB/CCR2/HIPK2/SLC11A2/BRD1/TMOD3/JAM3/SP3/ALAS1/SFXN1/ETS1/ADD1/RB1/EPAS1/EPB42/KAT7/INHBA/ERCC2/MB/ TMEM14C
Biological Process	GO:0002702	positive regulation of production of molecular mediator of immune response	19/2279	100/18866	0.029559	0.2842331	0.2571842	19	PRKCZ/NLRP3/IL1R1/TRAF2/TLR9/SPON2/CD226/HLA-F/SCIMP/TGFB1/SLC7A5/PTPRC/BCL10/NOD2/TP53BP1/IL18R1/TNFSF13/PAXIP1/NSD2
Biological Process	GO:0045069	regulation of viral genome replication	19/2279	100/18866	0.029559	0.2842331	0.2571842	19	SLPI/TRIM38/SRPK2/TNF/CCL5/LARP1/PHB/TNIP1/BCL2/NOTCH1/LTF/ILF3/MX1/NR5A2/PARP10/BANF1/ISG20/PRKN/SHFL
Biological Process	GO:0036465	synaptic vesicle recycling	15/2279	74/18866	0.029587	0.2842331	0.2571842	15	SYNJ2/DENND1A/DNM2/ITSN1/STON1/DGKQ/ACTG1/SH3GL1/DNM1/DNM3/DNM1L/AP3D1/MX1/CYFIP1/FGF14
Biological Process	GO:1903036	positive regulation of response to wounding	15/2279	74/18866	0.029587	0.2842331	0.2571842	15	RREB1/KANK1/MYLK/THBS1/F12/ACTG1/GRN/NFE2L2/PRKCE/CPB2/F2R/ANO6/NDEL1/PLPP3/CCN4
Biological Process	GO:0060759	regulation of response to cytokine stimulus	33/2279	196/18866	0.0300288	0.2878698	0.2604748	33	GFI1/VRK2/PPARG/RUNX1/ANGPT1/IL1R1/TNF/TRAF2/TNFAIP3/CCL5/TRAF1/TRAIP/PADI2/RIPK1/PTPN6/MIR24-2/MIR27A/TNFRSF1A/CPNE1/JAK1/IL1RN/NR1H2/PTPRC/NLRC5/MIR138-2/IRAK2/SLIT3/ELF1/CASP1/CYLD/PARP9/FADD/PRKN

Biological Process	GO:0090316	positive regulation of intracellular protein transport	31/2279	182/18866	0.0300432	0.2878698	0.2604748	31	SORL1/TFDP1/PRR5L/TM9SF4/TMEM30A/YWHAH/ITGB1BP1/PIK3R1/SMAD3/JUP/EDEM1/USP36/ZDHHC2/TCF7L2/ABLIM3/AKAP5/UBE2J2/TGFB1/EDEM2/ITGB2/YWHAZ/CDH1/FIS1/BCL2/TP53BP2/MYO1C/MIEF1/YWHAQ/PDZK1/PRKAA1/RIPOR1
Biological Process	GO:0043534	blood vessel endothelial cell migration	32/2279	189/18866	0.0300565	0.2878698	0.2604748	32	PPARG/MAP3K3/MIR143/HDAC7/ANGPT1/ITGB1BP1/PRKCA/MIR199A1/GPLD1/TNF/MIR101-2/STAT5A/JUP/THBS1/MYH9/AKT3/MIR24-2/MIR27A/MIR23A/MIA3/FGFR1/TGFB1/NF1/PDGFB/ETS1/NFE2L2/VASH1/MIR199A2/NOTCH1/MEF2C/PLCG1/ADTRP
Biological Process	GO:0071621	granulocyte chemotaxis	23/2279	127/18866	0.0301895	0.2885619	0.261101	23	PDE4D/S100A8/C3AR1/PIK3CD/CSF3R/CCL5/CSF1R/THBS1/C5AR2/PREX1/HRH1/JAM3/CAMK1D/CXCL17/CCL20/CXCR1/ITGB2/DAPK2/DNM1L/DYSF/CCL22/VAV3/JAML
Biological Process	GO:1905477	positive regulation of protein localization to membrane	23/2279	127/18866	0.0301895	0.2885619	0.261101	23	SSH1/TFDP1/YWHAH/ITGB1BP1/PIK3R1/RHOG/TNF/ZDHHC2/AKAP5/PRKCH/PRKCI/ITGB2/YWHAZ/FIS1/BCL2/PRKCE/TP53BP2/MYO1C/MIEF1/YWHAQ/EZR/PDZK1/PRKN
Biological Process	GO:1990778	protein localization to cell periphery	53/2279	340/18866	0.0306612	0.2915353	0.2637915	53	PRKCZ/EHD1/BSG/ITGB1BP1/PIK3R1/MAP7/RHOG/TSPAN14/KIF13A/RAB31/TNF/PACS2/TTC7A/JUP/TESC/TNIK/ZDHHC2/TNFRSF1A/MRAP/AKAP5/SLC9A3R2/ZDHHC7/PPFIA1/LZTFL1/TGFB1/PRKCH/RAB40C/RAPGEF2/TTC7B/SMURF1/VAMP5/MYO5A/EPB41L3/NUMB/CSK/NUMA1/PALM/GORASP2/PRKCI/RILPL1/CDH1/KIF1B/PRAM1/PRKCE/TMED2/BCL2L1/ANK2/EZR/PDZK1/FLOT1/MYADM/SPTBN4/FYB1
Biological Process	GO:0001783	B cell apoptotic process	7/2279	26/18866	0.0307461	0.2915353	0.2637915	7	FOXP1/IL10/HSH2D/TNFRSF21/BLK/BCL10/TRAF3IP2
Biological Process	GO:0015012	heparan sulfate proteoglycan biosynthetic process	7/2279	26/18866	0.0307461	0.2915353	0.2637915	7	CSGALNACT1/HS3ST3B1/DSE/NDST1/TCF7L2/XYLT1/PXYLP1
Biological Process	GO:0030318	melanocyte differentiation	7/2279	26/18866	0.0307461	0.2915353	0.2637915	7	ZEB2/RAB27A/MYO5A/GNA11/BCL2/MEF2C/HPS6
Biological Process	GO:0051016	barbed-end actin filament capping	7/2279	26/18866	0.0307461	0.2915353	0.2637915	7	VILL/GSN/CAPG/SVIL/ADD1/AVIL/FLII

Biological Process	GO:0060307	regulation of ventricular cardiac muscle cell membrane repolarization	7/2279	26/18866	0.0307461	0.2915353	0.2637915	7	KCNE1/GJA5/NOS1AP/WDR1/SNTA1/KCNQ1/ANK2
Biological Process	GO:0072012	glomerulus vasculature development	7/2279	26/18866	0.0307461	0.2915353	0.2637915	7	ANGPT1/PDGFD/IL6R/PDGFB/NOTCH1/GPR4/PDGFRB
Biological Process	GO:1901071	glucosamine-containing compound metabolic process	7/2279	26/18866	0.0307461	0.2915353	0.2637915	7	NAGK/AMDHD2/CHI3L1/MGAT3/CHIT1/CHST2/CHI3L2
Biological Process	GO:0016054	organic acid catabolic process	45/2279	282/18866	0.0308371	0.2918156	0.2640451	45	ACAD8/PLA2G15/HAL/NAGK/MFSD2A/HEXB/GADL1/AMDHD2/HGD/ABHD16A/AMACR/NPL/CBS/ACOXL/AKR1D1/MTHFS/OGDH/ALDH4A1/ENOSF1/FAH/TGFB1/CYP4F3/STAB2/ACADM/HIBADH/AGXT/ACACB/ACOX2/LYVE1/HOGA1/ADO/BCKDHA/ACSF3/MAT1A/HMGCL/PCK2/CRYM/ACAT1/ADTRP/CPT1A/LPIN1/HNMT/LYPLA2/ACADVL/CEMIP2
Biological Process	GO:0046395	carboxylic acid catabolic process	45/2279	282/18866	0.0308371	0.2918156	0.2640451	45	ACAD8/PLA2G15/HAL/NAGK/MFSD2A/HEXB/GADL1/AMDHD2/HGD/ABHD16A/AMACR/NPL/CBS/ACOXL/AKR1D1/MTHFS/OGDH/ALDH4A1/ENOSF1/FAH/TGFB1/CYP4F3/STAB2/ACADM/HIBADH/AGXT/ACACB/ACOX2/LYVE1/HOGA1/ADO/BCKDHA/ACSF3/MAT1A/HMGCL/PCK2/CRYM/ACAT1/ADTRP/CPT1A/LPIN1/HNMT/LYPLA2/ACADVL/CEMIP2
Biological Process	GO:0051591	response to cAMP	18/2279	94/18866	0.0312639	0.2952667	0.2671678	18	BSG/KCNE1/ITPR2/RAP1B/SLC8A1/APP/FDX1/CRHBP/RAPGEF2/DGKQ/AQP1/AGXT/KCNQ1/PER1/SREBF1/RELA/EZR/AQP9
Biological Process	GO:0060349	bone morphogenesis	18/2279	94/18866	0.0312639	0.2952667	0.2671678	18	CSGALNACT1/FOXN3/MEF2D/LRP5L/SKI/CHSY1/ANXA6/TGFBR2/ALPL/POC1A/RARA/POR/RARG/BMPR1B/RUNX2/MEF2C/NPPC/LTF
Biological Process	GO:0008645	hexose transmembrane transport	21/2279	114/18866	0.0314307	0.2960806	0.2679043	21	PRKAG2/SLC2A5/MIR143/PIK3R1/SLC2A14/TNF/GRB10/SLC2A9/ZDHHC7/HNF1A/PRKCB/SLC1A2/HK2/PRKCI/NFE2L2/SORT1/SLC5A10/SLC2A1/RTN2/ASPSCR1/CLTCL1
Biological Process	GO:0019915	lipid storage	16/2279	81/18866	0.0314436	0.2960806	0.2679043	16	PNPLA2/EHD1/PPARG/TMEM159/HEXB/TNF/ABCG1/NR1H2/ITGB3/ABCA1/ACACB/FTO/ABHD5/DYSF/CDS2/BSCL2

Biological Process	GO:0051279	regulation of release of sequestered calcium ion into cytosol	16/2279	81/18866	0.0314436	0.2960806	0.2679043	16	PDE4D/CASQ2/CACNA1C/SLC8A1/UBASH3B/JSRP1/PTPN6/P2RY6/MYO5A/HTT/GSTO1/PRKCE/F2R/ANK2/PLCG1/CASQ1
Biological Process	GO:0070527	platelet aggregation	13/2279	62/18866	0.0319746	0.3004854	0.2718899	13	CD9/PRKCA/UBASH3B/MYL12A/MYH9/ITGA2B/PTPN6/ILK/HBB/BLK/ITGB3/ACTG1/TYRO3
Biological Process	GO:0140115	export across plasma membrane	13/2279	62/18866	0.0319746	0.3004854	0.2718899	13	KCNE1/ABCC1/FXYD2/FXYD1/SLC8A1/ABCC2/OSCP1/RALBP1/ABCA1/SLC22A5/KCNQ1/ANO6/PDZK1
Biological Process	GO:0006066	alcohol metabolic process	59/2279	385/18866	0.0320789	0.3011646	0.2725044	59	PNPLA2/GFI1/OSBPL5/SYNJ2/RXRA/SPTLC2/IDI1/INPP5A/INPP5D/DHRS9/LIPC/VDR/ITPKB/MBTPS1/TNF/ALDH2/ABCG1/HDLBP/PGP/AKR1D1/INPP4A/APP/NUDT3/ACSS1/P2RY6/FGFR1/SPHK2/FDX1/RDH10/HRH1/SULT1A2/DGKQ/POR/ABCA1/GNB3/ACACB/INPP5E/PLCB1/CAT/ACSS2/IMPA2/NPC1/SGPP1/SGPP2/ACACA/LMF1/PCK2/DYSF/HMGCS1/SCD/SREBF1/PLCG1/PLCD3/ALDH1A2/DPAGT1/ACADVL/PRKAA1/IP6K1/PLPP3
Biological Process	GO:0016241	regulation of macroautophagy	30/2279	176/18866	0.0321103	0.3011646	0.2725044	30	RPTOR/PRKAG2/SPTLC2/ULK1/ERN1/VPS13D/RHEB/LARP1/USP36/PIP4K2A/VPS26B/ATP6V1B2/FEZ2/PRKAB1/UVRAG/SMURF1/RUFY4/CLEC16A/HTT/TBC1D14/NPC1/ATP6V0B/NEDD4/TSC2/NPRL2/PRKAA1/STING1/PRKN/ATP6V0C/ATP6V1C1
Biological Process	GO:0030218	erythrocyte differentiation	22/2279	121/18866	0.0321485	0.3012254	0.2725594	22	MAEA/KLF1/ZFPM1/INPP5D/TRIM10/HIPK2/SLC11A2/BRD1/TMOD3/SP3/ALAS1/SFXN1/ETS1/ADD1/RB1/EPAS1/EPB42/KAT7/INHBA/ERCC2/MB/TMEM14C
Biological Process	GO:0000086	G2/M transition of mitotic cell cycle	41/2279	254/18866	0.0322035	0.3014438	0.2727571	41	TRIM39/BLM/BRD4/DCTN1/CSNK1D/FOXN3/NDE1/DNM2/HUS1/DYNC1H1/TUBB4B/CEP135/APP/CDK14/CDC25A/CEP164/DCTN2/CEP78/CSNK1E/PSMF1/PPP2R1A/PLCB1/CCNH/PPP1CB/TUBA4A/TAOK3/SDCCAG8/ZFYVE19/ESRRB/CLSPN/PSMB7/FBXL6/FOXM1/CHEK2/BACH1/FBXW11/PHLDA1/CEP72/CCNY/NAE1/PSMD13
Biological Process	GO:0051271	negative regulation of cellular component movement	61/2279	400/18866	0.0324262	0.3032292	0.2743725	61	SEMA6B/TACSTD2/ABR/CALR/CD9/PPARG/MIR140/KANK1/ARHGDIB/ZMYND8/ITGB1BP1/SPINT2/LDLRAD4/MIR199A1/TNF/MIR101-2/DUSP22/SEMA4A/PADI2/JUP/NAV3/BIN1/THBS1/SEMA4B/C5AR2/ACTN1/ACTN4/MIR24-2/ILK/SMAD7/MIA3/WNT11/WASL/NISCH/TGFB1/PTPRJ/SEMA4D/CNIH2/NF1/STK24/SRGAP1/BCR/PLCB1/NFE2L2/ENG/VASH1/MIR138-2/CDH1/BCL2/MIR199A2/NOTCH1/CORO1C/MEF2C/DUSP3/SP100/TRIB1/ADTRP/CERS2/PTPRU/NODAL/CX3CR1
Biological Process	GO:0003012	muscle system process	70/2279	467/18866	0.0327007	0.3054959	0.2764236	70	HDAC4/PDE4D/ENO1/KCNE1/CTDP1/GSN/TTN/CASQ2/MIR143/MIR145/MYH11/MAP2K6/CACNA1C/AKAP13/FXYD1/TNFRSF1B/MYLK/SMAD3/SLC8A1/PRKCA/CTTN/MIR199A1/ANXA6/GJA5/MYL12A/TNNT3/JUP/BIN1/JSRP1/NOS1AP/MIR24-2/MIR23A/TNFRSF1A/SMAD7/PXN/ITGB5/SCN1A/PABPN1/TMOD3/CAMTA2/GHRL/CHRM2/SNTA1/TPCN2/KCNQ1/PDE9A/SNTB1/MYOM1/ANKRD2/MIR199A2/NOTCH1/MYOT/GSTO1/GNAO1/P2RX1/MEF2C/F2R/FOXO1/DYSF/ANK2/ASPH/TMOD1/INPP5F/MYL7/CASQ1/SMTN/LTB4R/DAPK3/MB/GRK2

Biological Process	GO:0003176	aortic valve development	8/2279	32/18866	0.0329472	0.307073	0.2778505	8	TNFRSF1B/NFATC1/RBPJ/TNFRSF1A/TGFB1/RB1/NOTCH1/SLIT3
Biological Process	GO:0050869	negative regulation of B cell activation	8/2279	32/18866	0.0329472	0.307073	0.2778505	8	IL10/INPP5D/TNFAIP3/MNDA/TNFRSF21/BLK/BANK1/INHBA
Biological Process	GO:0070228	regulation of lymphocyte apoptotic process	12/2279	56/18866	0.0329665	0.307073	0.2778505	12	FOXP1/IL10/CCL5/HSH2D/PTCRA/DOCK8/RIPK3/ZC3H8/BLK/BCL10/FADD/GIMAP8
Biological Process	GO:0071887	leukocyte apoptotic process	20/2279	108/18866	0.0334101	0.3082117	0.2788809	20	FOXP1/IL10/PIK3CD/ITPKB/CCL5/HSH2D/RIPK1/PTCRA/TNFRSF21/DOCK8/RAPGEF2/RIPK3/ZC3H8/BLK/BCL10/NOD2/MEF2C/TRAF3IP2/FADD/GIMAP8
Biological Process	GO:0030178	negative regulation of Wnt signaling pathway	36/2279	219/18866	0.0335863	0.3082117	0.2788809	36	CCDC88C/AMFR/VGLL4/UBAC2/NFATC1/CUL3/GRB10/TCF7L2/LIMD1/HDAC1/LATS2/WNT11/KREMEN1/AXIN1/TLE1/WWTR1/TMEM64/WNT5B/STK3/PSMF1/RNF213/KREMEN2/NXN/PSMB7/NOTCH1/TSC2/FOXO1/CHD8/CYLD/ANKRD6/PTPRU/PSMD13/TSKU/NPHP4/TMEM131L/PRKN
Biological Process	GO:0002029	desensitization of G protein-coupled receptor signaling pathway	6/2279	21/18866	0.0337006	0.3082117	0.2788809	6	ARRB1/DNM2/GIPR/ARRB2/DNM1/GRK2
Biological Process	GO:0003177	pulmonary valve development	6/2279	21/18866	0.0337006	0.3082117	0.2788809	6	TNFRSF1B/GJA5/NFATC1/RBPJ/TNFRSF1A/NOTCH1
Biological Process	GO:0016973	poly(A)+ mRNA export from nucleus	6/2279	21/18866	0.0337006	0.3082117	0.2788809	6	NXF1/PABPN1/ENY2/NUP93/HHEX/PCID2
Biological Process	GO:0022401	negative adaptation of signaling pathway	6/2279	21/18866	0.0337006	0.3082117	0.2788809	6	ARRB1/DNM2/GIPR/ARRB2/DNM1/GRK2

Biological Process	GO:0036507	protein demannosylation	6/2279	21/18866	0.0337006	0.3082117	0.2788809	6	AMFR/EDEM1/DERL2/EDEM2/MAN1C1/MARCHF6
Biological Process	GO:0036508	protein alpha-1,2-demannosylation	6/2279	21/18866	0.0337006	0.3082117	0.2788809	6	AMFR/EDEM1/DERL2/EDEM2/MAN1C1/MARCHF6
Biological Process	GO:0043371	negative regulation of CD4-positive, alpha-beta T cell differentiation	6/2279	21/18866	0.0337006	0.3082117	0.2788809	6	RUNX1/LOXL3/SMAD7/ZBTB7B/HLX/RUNX3
Biological Process	GO:0060216	definitive hemopoiesis	6/2279	21/18866	0.0337006	0.3082117	0.2788809	6	ZFPM1/LYL1/SP3/BCR/HOXA9/MEIS1
Biological Process	GO:0071397	cellular response to cholesterol	6/2279	21/18866	0.0337006	0.3082117	0.2788809	6	GPLD1/MLC1/ABCA1/GRAMD1A/HMGCS1/INHBA
Biological Process	GO:0080182	histone H3-K4 trimethylation	6/2279	21/18866	0.0337006	0.3082117	0.2788809	6	WDR82/KMT2D/SETD1B/SETD1A/TET2/ZNF335
Biological Process	GO:0090201	negative regulation of release of cytochrome c from mitochondria	6/2279	21/18866	0.0337006	0.3082117	0.2788809	6	PAM16/CLU/NOL3/ARRB2/BCL2L1/PRKN
Biological Process	GO:0014009	glial cell proliferation	11/2279	50/18866	0.0337159	0.3082117	0.2788809	11	CREB1/MYB/SKI/LGI4/TNF/CSF1R/CLU/PRKCH/PRKCI/NOTCH1/CERS2
Biological Process	GO:0046850	regulation of bone remodeling	11/2279	50/18866	0.0337159	0.3082117	0.2788809	11	DEF8/INPP5D/PRKCA/UBASH3B/TNFAIP3/CSF1R/NF1/ITGB3/TMEM64/CSK/PLEKHM1

Biological Process	GO:0070671	response to interleukin-12	11/2279	50/18866	0.0337159	0.3082117	0.2788809	11	P4HB/IL10/RAP1B/SERPINB2/AIP/JAK1/PITPNA/PLCB1/RALA/CA1/GSTO1
Biological Process	GO:0030278	regulation of ossification	35/2279	212/18866	0.0337976	0.3082117	0.2788809	35	HDAC4/CEBPD/HDAC7/SKI/CHSY1/SMAD3/SLC8A1/SUFU/TNF/ZBTB16/RBPJ/ILK/LIMD1/SMAD7/TGFB1/KREMEN1/SEMA4D/SMURF1/BMPR1B/TMEM64/RUNX2/IL6R/KREMEN2/SGMS2/MIR138-2/BCL2/NOTCH1/MEF2C/NPPC/LTF/CEBPB/ANO6/FAM20C/RFLNA/CCN4
Biological Process	GO:0009150	purine ribonucleotide metabolic process	61/2279	401/18866	0.0338805	0.3082117	0.2788809	61	FOXK1/HDAC4/PDE4D/PRKAG2/AMPD3/ENO1/HK1/ADCY2/ACSL1/FAR2/TJP2/ADCY9/PFKFB4/ACOT13/SULT6B1/RAE1/CBFA2T3/ABHD14B/DHTKD1/APP/OGDH/NUDT3/ADPGK/ACOT7/VPS9D1/PGAM1/ACSS1/SPHK2/TGFB1/SULT1A2/SLC26A1/NUP93/ENO3/HK2/SEC13/ACACB/ACSS2/PDE9A/GUK1/ADCY4/ESRRB/PPCDC/PAPSS2/PKM/ACACA/ACSF3/AK2/GMPR2/AK5/HMGCL/NPPC/ACAT1/HMGCS1/SCD/TPST1/PFKFB3/SLC25A1/PANK4/IMPDH1/PRKAA1/ATP5PD
Biological Process	GO:0086005	ventricular cardiac muscle cell action potential	9/2279	38/18866	0.033931	0.3082117	0.2788809	9	KCNE1/CACNA1C/GJA5/JUP/BIN1/NOS1AP/SNTA1/KCNQ1/ANK2
Biological Process	GO:0017145	stem cell division	10/2279	44/18866	0.034098	0.3082117	0.2788809	10	MIR145/LBH/ZBTB16/CUL3/FGFR1/WWTR1/NUMB/ESRRB/NOTCH1/ARHGEF2
Biological Process	GO:0048489	synaptic vesicle transport	10/2279	44/18866	0.034098	0.3082117	0.2788809	10	DNM2/RAB27A/CNIH2/KIF5C/DNM1/DNM3/AP3D1/MX1/DTNBP1/PRKN
Biological Process	GO:0120009	intermembrane lipid transfer	10/2279	44/18866	0.034098	0.3082117	0.2788809	10	OSBPL5/ABCG1/PLTP/PITPNM2/PITPNM1/PITPNA/ABCA1/PITPNC1/GRAMD1A/C2CD2L
Biological Process	GO:1903053	regulation of extracellular matrix organization	10/2279	44/18866	0.034098	0.3082117	0.2788809	10	TNFRSF1B/SMAD3/TNFRSF1A/TGFB1/ETS1/AEBP1/RB1/NOTCH1/CPB2/ADTRP
Biological Process	GO:0051051	negative regulation of transport	72/2279	483/18866	0.0341367	0.3082117	0.2788809	72	PRTN3/TRIM27/ABR/KCNE1/THADA/CASQ2/WWP2/MIR143/ANGPT1/TNFRSF1B/ITGB1BP1/RAP1B/CCR2/RAB11FIP1/FRMD4A/MIR199A1/SUFU/TNF/CD84/RHBDF2/TLR9/UBAC2/PACSIN2/BIN1/THBS1/COMT/ANKRD13B/GRB10/SNX3/GNAI2/HLA-F/MIR27A/TNFRSF1A/ANKRD13D/DERL2/CRHBP/ENY2/SLC43A2/PRKCB/CNIH2/NF1/NR1H2/ITGB3/ANKRD13A/GHRL/CAB39/CSK/BCR/KLF7/BCL2/MIR199A2/NOTCH1/GSTO1/CABP1/PRKCE/SP100/F2R/SLC43A1/NEDD4/DYSF/ARHGAP1/HECW2/SREBF1/ADTRP/CYFIP1/YWHAQ/CD33/INHBA/LIF/SVIP/CBARP/PRKN

Biological Process	GO:0009259	ribonucleotide metabolic process	63/2279	416/18866	0.0341557	0.3082117	0.2788809	63	FOXK1/HDAC4/PDE4D/PRKAG2/AMPD3/ENO1/HK1/ADCY2/ACSL1/FAR2/TJP2/ADCY9/PFKFB4/ACOT13/SULT6B1/RAE1/CBFA2T3/ABHD14B/DHTKD1/APP/OGDH/NUDT3/ADPGK/ACOT7/VPS9D1/PGAM1/ACSS1/SPHK2/TGFB1/SULT1A2/SLC26A1/UCK2/NUP93/ENO3/HK2/SEC13/ACACB/ACSS2/PDE9A/GUK1/ADCY4/ESRRB/PPCDC/PAPSS2/PKM/ACACA/ACSF3/AK2/GMPR2/AK5/HMGCL/NPPC/ACAT1/HMGCS1/SCD/TPST1/PFKFB3/DHODH/SLC25A1/PANK4/IMPDH1/PRKAA1/ATP5PD
Biological Process	GO:0006096	glycolytic process	21/2279	115/18866	0.0342295	0.3082117	0.2788809	21	FOXK1/HDAC4/PRKAG2/ENO1/HK1/PFKFB4/RAE1/CBFA2T3/DHTKD1/APP/OGDH/ADPGK/PGAM1/NUP93/ENO3/HK2/SEC13/ESRRB/PKM/PFKFB3/PRKAA1
Biological Process	GO:0051480	regulation of cytosolic calcium ion concentration	55/2279	357/18866	0.0342596	0.3082117	0.2788809	55	TRPM2/PDE4D/C3AR1/CASQ2/ITPR2/LCK/CD55/CACNA1C/CCR2/SMAD3/SLC8A1/MIR199A1/UBASH3B/CDH23/FPR2/FPR1/ATP2B2/ITPR1/ACKR2/JSRP1/C5AR2/PTPN6/RASA3/GNAT2/P2RY6/CCR9/TMEM178A/MYO5A/GIPR/HTT/GHRL/PTPRC/TMEM64/CXCR1/NOL3/TPCN2/FIS1/BCL2/MIR199A2/GSTO1/P2RX1/LPAR2/PRKCE/F2R/S1PR4/GPR4/AGTR1/ANK2/CHERP/PLCG1/P2RX5/TRPV2/CASQ1/CX3CR1/SYNPO
Biological Process	GO:0006903	vesicle targeting	18/2279	95/18866	0.0343708	0.3082117	0.2788809	18	CTSZ/SERPINA1/TGFA/STX5/CSNK1D/TRAPPC10/C17orf75/CUL3/TRAPPC9/CNIH2/CD59/SEC13/ANKRD28/SEC24C/TMED2/SEC31A/IRAG2/TRAPPC2
Biological Process	GO:0045638	negative regulation of myeloid cell differentiation	18/2279	95/18866	0.0343708	0.3082117	0.2788809	18	ZFPM1/INPP5D/PIK3R1/ITPKB/UBASH3B/ZBTB16/DLL1/RARA/TMEM178A/LILRB4/NF1/MEIS2/LRRC17/HOXA9/MEIS1/LTF/TRIB1/INHBA
Biological Process	GO:0048814	regulation of dendrite morphogenesis	18/2279	95/18866	0.0343708	0.3082117	0.2788809	18	YWHAH/TNIK/CUX1/BAIAP2/ILK/SDC2/SEMA4D/RAPGEF2/SS18L2/SIPA1L1/DNM3/CDKL3/DNM1L/UBE3A/NEDD4/HECW2/CUX2/DBNL
Biological Process	GO:1903725	regulation of phospholipid metabolic process	18/2279	95/18866	0.0343708	0.3082117	0.2788809	18	LPCAT1/PIK3R1/PIK3R6/FGR/FPR2/PIK3R5/FLT1/TGFB1/VAV2/ATG14/GNB3/PDGFB/MIR138-2/NOD2/PDGFRB/WDR81/VAV3/SLA2
Biological Process	GO:0048285	organelle fission	71/2279	476/18866	0.0347852	0.3082117	0.2788809	71	ARHGEF10/CALR/FZR1/TGFA/CTDP1/MAD1L1/TTN/TNKS/MOV10L1/NDE1/BRCA2/INF2/PRKCA/DNM2/NSMCE2/RCC1/CHAMP1/CUL3/EME1/CCDC8/MEIOB/CDC16/MIS12/DCTN2/FANCA/PRKCB/RNF212/TACC3/STAG1/NUMA1/PDGFB/PPP2R1A/PLCB1/MSTO1/DNM1/NEK6/CCNA1/FIS1/RB1/RAD21L1/DMC1/DNM3/DNM1L/CDCA5/DIS3L2/PEX11B/CHEK2/ANAPC7/MIEF1/PDGFRB/SPAG5/HECW2/CDC14A/MX1/SLX4/TEX12/AURKAIP1/NDEL1/LPIN1/DHODH/LIF/KLHDC3/DAPK3/PSMD13/CDK13/PCID2/TERB2/PRKN/CYRIB/ABRAXAS1/SLF1
Biological Process	GO:0002604	regulation of dendritic cell antigen processing and presentation	4/2279	11/18866	0.0348133	0.3082117	0.2788809	4	SLC11A1/THBS1/NOD2/CD68

Biological Process	GO:0006054	N-acetylneuraminate metabolic process	4/2279	11/18866	0.0348133	0.3082117	0.2788809	4	NAGK/AMDHD2/NPL/ST6GAL1
Biological Process	GO:0006853	carnitine shuttle	4/2279	11/18866	0.0348133	0.3082117	0.2788809	4	PRKAG2/ACACB/ACACA/CPT1A
Biological Process	GO:0008655	pyrimidine-containing compound salvage	4/2279	11/18866	0.0348133	0.3082117	0.2788809	4	CDA/TK2/UCK2/TK1
Biological Process	GO:0019852	L-ascorbic acid metabolic process	4/2279	11/18866	0.0348133	0.3082117	0.2788809	4	GSTO1/SLC2A1/GSTO2/SLC23A1
Biological Process	GO:0032754	positive regulation of interleukin-5 production	4/2279	11/18866	0.0348133	0.3082117	0.2788809	4	PRKCZ/PDE4D/NLRP3/RARA
Biological Process	GO:0034115	negative regulation of heterotypic cell-cell adhesion	4/2279	11/18866	0.0348133	0.3082117	0.2788809	4	IL10/MBP/IL1RN/MYADM
Biological Process	GO:0043097	pyrimidine nucleoside salvage	4/2279	11/18866	0.0348133	0.3082117	0.2788809	4	CDA/TK2/UCK2/TK1
Biological Process	GO:0045628	regulation of T-helper 2 cell differentiation	4/2279	11/18866	0.0348133	0.3082117	0.2788809	4	PRKCZ/NLRP3/RARA/HLX
Biological Process	GO:0045945	positive regulation of transcription by RNA polymerase III	4/2279	11/18866	0.0348133	0.3082117	0.2788809	4	RPTOR/ZC3H8/BRF1/CHD8

Biological Process	GO:0046950	cellular ketone body metabolic process	4/2279	11/18866	0.0348133	0.3082117	0.2788809	4	BDH1/HMGCL/ACAT1/ACSS3
Biological Process	GO:0048875	chemical homeostasis within a tissue	4/2279	11/18866	0.0348133	0.3082117	0.2788809	4	LPCAT1/SFTPD/CTSH/EPAS1
Biological Process	GO:0060368	regulation of Fc receptor mediated stimulatory signaling pathway	4/2279	11/18866	0.0348133	0.3082117	0.2788809	4	CD226/PTPRJ/PTPRC/CSK
Biological Process	GO:0060837	blood vessel endothelial cell differentiation	4/2279	11/18866	0.0348133	0.3082117	0.2788809	4	CCM2/DLL1/RBPJ/NOTCH1
Biological Process	GO:0070587	regulation of cell-cell adhesion involved in gastrulation	4/2279	11/18866	0.0348133	0.3082117	0.2788809	4	IL10/MBP/IL1RN/MYADM
Biological Process	GO:0070778	L-aspartate transmembrane transport	4/2279	11/18866	0.0348133	0.3082117	0.2788809	4	SLC1A5/SLC1A2/SLC1A6/SLC1A3
Biological Process	GO:0071372	cellular response to follicle-stimulating hormone stimulus	4/2279	11/18866	0.0348133	0.3082117	0.2788809	4	POR/NOTCH1/HMGCS1/INHBA
Biological Process	GO:0072110	glomerular mesangial cell proliferation	4/2279	11/18866	0.0348133	0.3082117	0.2788809	4	PDGFD/IL6R/PDGFB/PDGFRB
Biological Process	GO:0090193	positive regulation of glomerulus development	4/2279	11/18866	0.0348133	0.3082117	0.2788809	4	PAX2/PDGFD/IL6R/PDGFB

Biological Process	GO:0090205	positive regulation of cholesterol metabolic process	4/2279	11/18866	0.0348133	0.3082117	0.2788809	4	ABCG1/POR/SREBF1/PRKAA1
Biological Process	GO:2000392	regulation of lamellipodium morphogenesis	4/2279	11/18866	0.0348133	0.3082117	0.2788809	4	RREB1/KANK1/ARHGEF7/CORO1C
Biological Process	GO:1902930	regulation of alcohol biosynthetic process	16/2279	82/18866	0.0348405	0.3082117	0.2788809	16	GFI1/IDI1/VDR/MBTPS1/TNF/ABCG1/P2RY6/HRH1/DGKQ/POR/ACACB/ACACA/HMGCS1/SCD/SREBF1/PRKAA1
Biological Process	GO:0034101	erythrocyte homeostasis	23/2279	129/18866	0.0354514	0.3124574	0.2827226	23	MAEA/KLF1/ZFPM1/AMPD3/INPP5D/TRIM10/HIPK2/SLC11A2/BRD1/TMOD3/SP3/ALAS1/SFXN1/ETS1/ADD1/RB1/EPAS1/EPB42/KAT7/INHBA/ERCC2/MB/TMEM14C
Biological Process	GO:0110020	regulation of actomyosin structure organization	19/2279	102/18866	0.0354992	0.3124574	0.2827226	19	ARHGEF10/TACSTD2/AKAP13/ITGB1BP1/PIK3R1/SMAD3/SH3PXD2B/ARAP1/SYNPO2L/PXN/WNT11/PPFIA1/PFN1/ACTG1/MIR138-2/ARHGEF10L/ARHGEF18/MEF2C/ARHGEF15
Biological Process	GO:0002523	leukocyte migration involved in inflammatory response	5/2279	16/18866	0.0356493	0.3124574	0.2827226	5	ELANE/S100A8/FUT7/JAM3/ITGB2
Biological Process	GO:0010452	histone H3-K36 methylation	5/2279	16/18866	0.0356493	0.3124574	0.2827226	5	BRD4/SETD2/PAXIP1/NSD3/NSD2
Biological Process	GO:0030214	hyaluronan catabolic process	5/2279	16/18866	0.0356493	0.3124574	0.2827226	5	HEXB/TGFB1/STAB2/LYVE1/CEMIP2
Biological Process	GO:0046134	pyrimidine nucleoside biosynthetic process	5/2279	16/18866	0.0356493	0.3124574	0.2827226	5	CDA/TK2/UCK2/DHODH/TK1

Biological Process	GO:0048820	hair follicle maturation	5/2279	16/18866	0.0356493	0.3124574	0.2827226	5	RBPJ/CDH3/MYO5A/NF1/ERCC2
Biological Process	GO:0070863	positive regulation of protein exit from endoplasmic reticulum	5/2279	16/18866	0.0356493	0.3124574	0.2827226	5	SORL1/TM9SF4/TMEM30A/EDEM1/EDEM2
Biological Process	GO:1903358	regulation of Golgi organization	5/2279	16/18866	0.0356493	0.3124574	0.2827226	5	STX5/DNM2/PDE4DIP/USP6NL/ARMH3
Biological Process	GO:1903729	regulation of plasma membrane organization	5/2279	16/18866	0.0356493	0.3124574	0.2827226	5	GSN/DNM2/MYH9/ASAP1/WASL
Biological Process	GO:0030198	extracellular matrix organization	60/2279	395/18866	0.0360132	0.315356	0.2853453	60	ITGAE/CSGALNACT1/ELANE/CTSG/TIMP2/BSG/COL23A1/MFAP4/SPOCK2/COL17A1/VIT/APBB2/CRISPLD2/MYH11/TNFRSF1B/SMAD3/TGFBI/SPINT2/COL4A2/COLQ/TNF/LOXL3/SH3PXD2B/THBS1/APP/ITGA2B/EGFLAM/TNFRSF1A/TNXB/COL12A1/ITGB5/TGFB1/NF1/ADAMTS13/JAM3/ADAMTSL5/DNAJB6/ITGB3/PDGFB/COL14A1/ETS1/ENG/ITGB2/CDH1/AEBP1/RB1/NOTCH1/COL5A1/ST7/CPB2/COL28A1/ICAM5/ADTRP/LAMB3/BCL3/FLOT1/ITGB6/LOXL4/ERCC2/HAS3
Biological Process	GO:0071383	cellular response to steroid hormone stimulus	34/2279	206/18866	0.0361365	0.3161441	0.2860584	34	RXRA/FOXP1/CALR/NCOR2/RUNX1/URI1/AKAP13/YWHAH/PRMT2/LBH/ABCC2/PADI2/SMYD3/ZNF366/FECH/HDAC1/ZMIZ1/PHB/AXIN1/AQP1/KMT2D/ARRB2/ARNTL/NPC1/ESRRB/PER1/UBE3A/NEDD4/PCK2/RNF14/FOXO1/ADTRP/NCOA4/NODAL
Biological Process	GO:0001935	endothelial cell proliferation	33/2279	199/18866	0.0363359	0.3175965	0.2873726	33	LRG1/RPTOR/NRP2/IL10/PPARG/ERN1/ITGB1BP1/PRKCA/TNF/EGFL7/MIR101-2/STAT5A/THBS1/AKT3/MIR24-2/MIR27A/MIR23A/FLT1/FGFR1/ZNF580/NF1/BMPER/ITGB3/GHRL/PDGFB/VASH1/MEF2C/AGTR1/DYSF/PPP1R16B/TNFSF12/PLCG1/ALDH1A2
Biological Process	GO:0060193	positive regulation of lipase activity	15/2279	76/18866	0.0366841	0.318593	0.2882743	15	PRKCZ/CCL5/FLT1/P2RY6/FGFR1/PHB/NR1H2/LPAR2/S1PR4/LMF1/ABHD5/AGTR1/PDGFRB/PLCG1/NTF4
Biological Process	GO:0043535	regulation of blood vessel endothelial cell migration	28/2279	164/18866	0.0367082	0.318593	0.2882743	28	PPARG/MAP3K3/MIR143/HDAC7/ANGPT1/ITGB1BP1/PRKCA/MIR199A1/TNF/MIR101-2/STAT5A/JUP/THBS1/AKT3/MIR24-2/MIR27A/MIR23A/FGFR1/TGFB1/NF1/PDGFB/ETS1/NFE2L2/VASH1/MIR199A2/NOTCH1/MEF2C/PLCG1

Biological Process	GO:0050769	positive regulation of neurogenesis	72/2279	485/18866	0.0369279	0.318593	0.2882743	72	CPNE6/PLXNC1/TIMP2/AP2A1/PPARG/CYB5D2/CSNK1D/MYB/TMEM30A/ZMYND8/TNFRSF1B/CCR2/RHEB/TNF/CPNE5/ZEB2/ITSN1/BIN1/DISC1/CUX1/BAIAP2/SNX3/TCF12/ILK/HDAC1/CPNE1/FGFR1/TGFB1/PRKCH/SEMA4D/RAPGEF2/SMURF1/SS18L2/RARA/CPEB3/CAMK1D/NUMB/KDM4C/NCOA1/CDH4/CSNK1E/GRN/CREB3L2/ZNF335/PRKCI/NFE2L2/ARSB/NTRK1/BCL2/TCF3/NOTCH1/CDKL3/DNM1L/AVIL/MEF2C/SHANK2/SYT17/ARHGEF2/GDF6/ANKRD1/MARK2/CYFIP1/TRPV2/RELA/NDEL1/LIF/CX3CR1/CUX2/EIF4G1/DBNL/PRKN/P3H1
Biological Process	GO:0006757	ATP generation from ADP	21/2279	116/18866	0.0372096	0.318593	0.2882743	21	FOXK1/HDAC4/PRKAG2/ENO1/HK1/PFKFB4/RAE1/CBFA2T3/DHTKD1/APP/OGDH/ADPGK/PGAM1/NUP93/ENO3/HK2/SEC13/ESRRB/PKM/PFKFB3/PRKAA1
Biological Process	GO:0003416	endochondral bone growth	7/2279	27/18866	0.0373147	0.318593	0.2882743	7	ANXA6/TGFBR2/POC1A/RARA/POR/RARG/NPPC
Biological Process	GO:0010714	positive regulation of collagen metabolic process	7/2279	27/18866	0.0373147	0.318593	0.2882743	7	MYB/TGFB1/ENG/ARRB2/F2R/PDGFRB/VSIR
Biological Process	GO:0048799	animal organ maturation	7/2279	27/18866	0.0373147	0.318593	0.2882743	7	PAX2/XYLT1/SEMA4D/LTF/ANO6/ALDH1A2/RFLNA
Biological Process	GO:0060544	regulation of necroptotic process	7/2279	27/18866	0.0373147	0.318593	0.2882743	7	MIR101-2/RIPK1/RIPK3/ARHGEF2/CYLD/FADD/PELI1
Biological Process	GO:0062098	regulation of programmed necrotic cell death	7/2279	27/18866	0.0373147	0.318593	0.2882743	7	MIR101-2/RIPK1/RIPK3/ARHGEF2/CYLD/FADD/PELI1
Biological Process	GO:0090140	regulation of mitochondrial fission	7/2279	27/18866	0.0373147	0.318593	0.2882743	7	INF2/FIS1/DNM1L/MIEF1/DHODH/PRKN/CYRIB
Biological Process	GO:0002090	regulation of receptor internalization	12/2279	57/18866	0.0373303	0.318593	0.2882743	12	ANGPT1/ARRB1/MIR199A1/ANKRD13B/ANKRD13D/NUMB/ITGB3/ANKRD13A/GSG1L/ARRB2/MIR199A2/FLOT1

Biological Process	GO:0032615	interleukin-12 production	12/2279	57/18866	0.0373303	0.318593	0.2882743	12	IRF8/FOXP1/IL10/IRF5/IL16/MEFV/TLR9/THBS1/SCIMP/TIGIT/PLCB1/ARRB2
Biological Process	GO:0097479	synaptic vesicle localization	12/2279	57/18866	0.0373303	0.318593	0.2882743	12	DNM2/RAB27A/SYN3/CNIH2/SYN2/KIF5C/DNM1/DNM3/AP3D1/MX1/DTNBP1/PRKN
Biological Process	GO:1903556	negative regulation of tumor necrosis factor superfamily cytokine production	12/2279	57/18866	0.0373303	0.318593	0.2882743	12	TRIM27/IL10/MIR140/TNFAIP3/C5AR2/PTPN6/RARA/ARRB2/LTF/CD33/VSIR/ILRUN
Biological Process	GO:0043062	extracellular structure organization	60/2279	396/18866	0.0376063	0.318593	0.2882743	60	ITGAE/CSGALNACT1/ELANE/CTSG/TIMP2/BSG/COL23A1/MFAP4/SPOCK2/COL17A1/VIT/APBB2/CRISPLD2/MYH11/TNFRSF1B/SMAD3/TGFBI/SPINT2/COL4A2/COLQ/TNF/LOXL3/SH3PXD2B/THBS1/APP/ITGA2B/EGFLAM/TNFRSF1A/TNXB/COL12A1/ITGB5/TGFB1/NF1/ADAMTS13/JAM3/ADAMTSL5/DNAJB6/ITGB3/PDGFB/COL14A1/ETS1/ENG/ITGB2/CDH1/AEBP1/RB1/NOTCH1/COL5A1/ST7/CPB2/COL28A1/ICAM5/ADTRP/LAMB3/BCL3/FLOT1/ITGB6/LOXL4/ERCC2/HAS3
Biological Process	GO:0006006	glucose metabolic process	35/2279	214/18866	0.0381127	0.318593	0.2882743	35	MAEA/FOXK1/ENO1/HK1/C1QTNF3/GPLD1/TNF/PGP/ADPGK/PGAM1/PC/NISCH/DGKQ/ENO3/DYRK2/HK2/GHRL/GNB3/ACACB/PPP1CB/ESRRB/PER2/PKM/PCK2/FOXO1/NCOA2/SOGA1/KBTBD2/WDTC1/CPT1A/SLC25A1/PRKAA1/PRKN/SDHAF3/RUBCNL
Biological Process	GO:0032873	negative regulation of stress-activated MAPK cascade	11/2279	51/18866	0.0384621	0.318593	0.2882743	11	HIPK3/TAOK3/MIR138-2/PER1/DUSP3/FOXM1/FOXO1/CYLD/EZR/MECOM/PRKN
Biological Process	GO:0070303	negative regulation of stress-activated protein kinase signaling cascade	11/2279	51/18866	0.0384621	0.318593	0.2882743	11	HIPK3/TAOK3/MIR138-2/PER1/DUSP3/FOXM1/FOXO1/CYLD/EZR/MECOM/PRKN
Biological Process	GO:1902893	regulation of pri-miRNA transcription by RNA polymerase II	11/2279	51/18866	0.0384621	0.318593	0.2882743	11	HDAC4/IL10/PPARG/SMAD3/TNF/TGFB1/LILRB4/PDGFB/ETS1/SREBF1/RELA
Biological Process	GO:0001570	vasculogenesis	16/2279	83/18866	0.038497	0.318593	0.2882743	16	MYO18B/RIN2/HDAC7/SETD2/EGFL7/CCM2/TGFBR2/ZMIZ1/TGFB1/RAPGEF2/ENG/NOTCH1/FOXM1/PDGFRB/TEAD2/PAXIP1

Biological Process	GO:0048041	focal adhesion assembly	16/2279	83/18866	0.038497	0.318593	0.2882743	16	ITGB1BP1/SMAD3/ARHGEF7/CTTN/DUSP22/WHAMM/THBS1/ACTN1/PTPRJ/TESK2/ACTG1/BCR/BCL2/CORO1C/DUSP3/DAPK3
Biological Process	GO:0048644	muscle organ morphogenesis	16/2279	83/18866	0.038497	0.318593	0.2882743	16	ARID5B/ZFPM1/RXRA/TTN/MIR143/MIR145/MYLK/CCM2L/RBPJ/SMAD7/PAX7/TGFB1/ENG/NOTCH1/ANKRD1/LIF
Biological Process	GO:0032273	positive regulation of protein polymerization	24/2279	137/18866	0.0387024	0.318593	0.2882743	24	TRIM27/DCTN1/GSN/CTTN/NCK2/WHAMM/BAIAP2L1/NAV3/CDC42EP1/BIN1/BAIAP2/WASL/FER/PFN1/CSF3/GMFB/NUMA1/NCKAP1/TPPP3/PRKCE/MYO1C/CYFIP1/GMFG/BAIAP2L2
Biological Process	GO:1903038	negative regulation of leukocyte cell-cell adhesion	24/2279	137/18866	0.0387024	0.318593	0.2882743	24	IL10/MAD1L1/RUNX1/LAX1/LOXL3/SFTPD/DUSP22/PTPN6/MIR27A/SMAD7/TNFRSF21/MIA3/ZBTB7B/TIGIT/ZC3H8/DUSP3/HLX/CEBPB/RUNX3/ADTRP/PELI1/TMEM131L/VSIR/MARCHF7
Biological Process	GO:0030593	neutrophil chemotaxis	19/2279	103/18866	0.0387791	0.318593	0.2882743	19	PDE4D/S100A8/C3AR1/PIK3CD/CSF3R/CCL5/C5AR2/PREX1/JAM3/CAMK1D/CCL20/CXCR1/ITGB2/DAPK2/DNM1L/DYSF/CCL22/VAV3/JAML
Biological Process	GO:0002700	regulation of production of molecular mediator of immune response	25/2279	144/18866	0.0389768	0.318593	0.2882743	25	PRKCZ/IL10/TNFRSF1B/NLRP3/IL1R1/CCR2/TNF/TRAF2/TLR9/SPON2/CD226/HLA-F/SMAD7/SCIMP/TGFB1/SLC7A5/PTPRC/BCL10/NOD2/TP53BP1/IL18R1/TNFSF13/CD96/PAXIP1/NSD2
Biological Process	GO:0050921	positive regulation of chemotaxis	25/2279	144/18866	0.0389768	0.318593	0.2882743	25	AZU1/CALR/C3AR1/IL16/CCR2/SMAD3/CCL5/CSF1R/FPR2/THBS1/DEFB124/FGFR1/TGFB1/ZNF580/PDGFD/CAMK1D/CXCL17/IL6R/PDGFB/DAPK2/DNM1L/PDGFRB/ANO6/DYSF/CCL27
Biological Process	GO:0033028	myeloid cell apoptotic process	8/2279	33/18866	0.0390394	0.318593	0.2882743	8	MAEA/PIK3CD/ITPKB/CCL5/CLEC5A/NOD2/BCL2/MEF2C
Biological Process	GO:0042339	keratan sulfate metabolic process	8/2279	33/18866	0.0390394	0.318593	0.2882743	8	HEXB/GALNS/CHST2/ST3GAL4/B4GALT3/ST3GAL2/B3GNT2/B4GAT1

Biological Process	GO:0046627	negative regulation of insulin receptor signaling pathway	8/2279	33/18866	0.0390394	0.318593	0.2882743	8	PRKCZ/KANK1/PTPRE/GRB10/PRKCB/TSC2/RELA/PRKAA1
Biological Process	GO:2000758	positive regulation of peptidyl-lysine acetylation	8/2279	33/18866	0.0390394	0.318593	0.2882743	8	ARRB1/RPS6KA4/SPHK2/PIH1D1/KAT7/LIF/PAXIP1/PRKAA1
Biological Process	GO:0035567	non-canonical Wnt signaling pathway	26/2279	151/18866	0.0391607	0.318593	0.2882743	26	PDE6A/AP2A1/CSNK1D/CCDC88C/TNRC6B/VANGL1/AGO1/NFATC1/PRICKLE2/TCF7L2/GNAT2/WNT11/AGO2/SMURF1/PFN1/CSNK1E/PSMF1/RNF213/PLCB1/ARRB2/DAAM1/PSMB7/GNG2/GNAO1/ANKRD6/PSMD13
Biological Process	GO:0001974	blood vessel remodeling	10/2279	45/18866	0.0392617	0.318593	0.2882743	10	ABR/MIR143/CRB1/CCR2/RBPJ/TGFB1/BCR/MEF2C/EPAS1/LIF
Biological Process	GO:0043090	amino acid import	10/2279	45/18866	0.0392617	0.318593	0.2882743	10	SLC1A5/SLC43A2/SLC1A2/SLC7A5/SLC1A6/PER2/SLC1A3/SLC43A1/SLC7A8/SLC7A1
Biological Process	GO:0043114	regulation of vascular permeability	10/2279	45/18866	0.0392617	0.318593	0.2882743	10	AZU1/ABR/TJP2/ANGPT1/MIR23A/TGFB1/PTPRJ/HRH1/BCR/GPR4
Biological Process	GO:0045214	sarcomere organization	10/2279	45/18866	0.0392617	0.318593	0.2882743	10	TTN/CASQ2/AKAP13/TNNT3/SYNPO2L/ACTG1/WDR1/MEF2C/ANKRD1/CASQ1
Biological Process	GO:0045616	regulation of keratinocyte differentiation	10/2279	45/18866	0.0392617	0.318593	0.2882743	10	RUNX1/VDR/PRKCH/GRHL2/NUMA1/SGPP1/NOTCH1/AQP3/ZBED2/MACROH2A2
Biological Process	GO:0052372	modulation by symbiont of entry into host	10/2279	45/18866	0.0392617	0.318593	0.2882743	10	TRIM27/P4HB/TRIM38/GSN/TRIM8/TRIM10/SNX3/FCN1/TRIM5/TRIM26

Biological Process	GO:0098815	modulation of excitatory postsynaptic potential	10/2279	45/18866	0.0392617	0.318593	0.2882743	10	PRKCZ/SSH1/ZMYND8/BAIAP2/APP/NRXN1/SH3GL1/S1PR2/SHANK2/CUX2
Biological Process	GO:1990138	neuron projection extension	29/2279	172/18866	0.0392664	0.318593	0.2882743	29	SEMA6B/PRKCZ/CPNE6/NRP2/ULK1/CTTN/DNM2/CPNE5/CYFIP2/BCL11A/SEMA4A/RTN4/DISC1/SEMA4B/IFRD1/ILK/CPNE1/SEMA4D/SMURF1/CDH4/CDKL3/SYT17/SLIT3/CYFIP1/TRPV2/NDEL1/SLIT1/DBNL/PRKN
Biological Process	GO:0062197	cellular response to chemical stress	55/2279	360/18866	0.0392776	0.318593	0.2882743	55	TRPM2/P4HB/MPO/VRK2/IL10/PPARG/LCN2/PRR5L/MYB/RAD52/NCF4/MYLK/ZNF622/DNM2/TNF/TRAF2/TNFAIP3/FBXO7/CAMKK2/MGAT3/MSRA/RIPK1/LRRC8C/NCF2/PXN/PAX2/ZNF580/FER/AQP1/RIPK3/AIFM2/STK24/PDGFD/CAB39/NOL3/CAT/ETS1/NFE2L2/ARNTL/ANKRD2/IL18RAP/BCL2/EPAS1/GLRX2/ARHGEF2/PDGFRB/SLC2A1/FOXO1/DYSF/RELA/BAG5/TXNRD2/SIRT3/PRKAA1/PRKN
Biological Process	GO:0001503	ossification	62/2279	412/18866	0.0394828	0.318593	0.2882743	62	CSGALNACT1/HDAC4/SBNO2/CEBPD/MEF2D/HDAC7/RUNX1/LRP5L/SKI/CHSY1/PAM16/SMAD3/SLC8A1/GPLD1/FGR/SUFU/TNF/SH3PXD2B/CLEC5A/ZBTB16/ALPL/RBPJ/ILK/LIMD1/SMAD7/ASGR2/WNT11/XYLT1/TGFB1/PHB/KREMEN1/SEMA4D/SMURF1/NF1/SP3/WWTR1/BMPR1B/TMEM64/RUNX2/IL6R/CAT/KREMEN2/SGMS2/SORT1/MIR138-2/BCL2/NOTCH1/MEF2C/FHL2/NPPC/LTF/CEBPB/RUNX3/ANO6/SND1/FAM20C/CDH11/SYNCRIP/FBL/ERCC2/RFLNA/CCN4
Biological Process	GO:0032412	regulation of ion transmembrane transporter activity	42/2279	265/18866	0.0395441	0.318593	0.2882743	42	PDE4D/KCNE1/THADA/CASQ2/WWP2/FXYD2/YWHAH/FXYD1/CCR2/DNM2/TLR9/TESC/JSRP1/AHNAK/RIPK1/APP/NOS1AP/ACTN4/P2RY6/SPHK2/CRHBP/CNIH2/HTR3A/MYO5A/HTT/CAB39/MAPK8IP2/GSG1L/GSTO1/MEF2C/SHANK2/PRKCE/NEDD4/DYSF/ANK2/HECW2/PDZK1/CASQ1/FGF14/CBARP/STIMATE/CRACR2A
Biological Process	GO:0014741	negative regulation of muscle hypertrophy	9/2279	39/18866	0.0395462	0.318593	0.2882743	9	CTDP1/MIR145/TNFRSF1B/SMAD3/MIR199A1/TNFRSF1A/MIR199A2/NOTCH1/FOXO1
Biological Process	GO:0035886	vascular associated smooth muscle cell differentiation	9/2279	39/18866	0.0395462	0.318593	0.2882743	9	MIR140/MIR145/NFATC2/NFATC1/EPC1/PDGFB/ENG/NOTCH1/DNMT1
Biological Process	GO:0042092	type 2 immune response	9/2279	39/18866	0.0395462	0.318593	0.2882743	9	PRKCZ/IL10/NLRP3/CCR2/RARA/NOD2/BCL6B/HLX/BCL3
Biological Process	GO:0002821	positive regulation of adaptive immune response	20/2279	110/18866	0.0396638	0.318593	0.2882743	20	PRKCZ/SLC11A1/NLRP3/IL1R1/CCR2/TNF/TRAF2/TNFSF13B/CD226/HLA-F/TGFB1/PTPRC/TP53BP1/IL18R1/HLX/TNFSF13/FADD/PAXIP1/CYRIB/NSD2

Biological Process	GO:0090100	positive regulation of transmembrane receptor protein serine/threonine kinase signaling pathway	20/2279	110/18866	0.0396638	0.318593	0.2882743	20	LRG1/MIR140/HIPK2/THBS1/RBPJ/UBE2O/ILK/TGFB1/NUP93/TGFB1I1/NUMA1/CREBBP/ENG/NOTCH1/RBPMS/GDF6/ZC3H3/ZNF423/INHBA/NODAL
Biological Process	GO:0002874	regulation of chronic inflammatory response to antigenic stimulus	2/2279	3/18866	0.0402391	0.318593	0.2882743	2	IL10/TNF
Biological Process	GO:0003138	primary heart field specification	2/2279	3/18866	0.0402391	0.318593	0.2882743	2	WNT11/MEF2C
Biological Process	GO:0003162	atrioventricular node development	2/2279	3/18866	0.0402391	0.318593	0.2882743	2	MAML1/NOTCH1
Biological Process	GO:0003192	mitral valve formation	2/2279	3/18866	0.0402391	0.318593	0.2882743	2	ZFPM1/NOTCH1
Biological Process	GO:0003430	growth plate cartilage chondrocyte growth	2/2279	3/18866	0.0402391	0.318593	0.2882743	2	TGFBR2/RARG
Biological Process	GO:0006447	regulation of translational initiation by iron	2/2279	3/18866	0.0402391	0.318593	0.2882743	2	RXRA/TNF
Biological Process	GO:0006679	glucosylceramide biosynthetic process	2/2279	3/18866	0.0402391	0.318593	0.2882743	2	FA2H/PRKAA1
Biological Process	GO:0009436	glyoxylate catabolic process	2/2279	3/18866	0.0402391	0.318593	0.2882743	2	AGXT/HOGA1

Biological Process	GO:0010693	negative regulation of alkaline phosphatase activity	2/2279	3/18866	0.0402391	0.318593	0.2882743	2	TNF/SEMA4D
Biological Process	GO:0010908	regulation of heparan sulfate proteoglycan biosynthetic process	2/2279	3/18866	0.0402391	0.318593	0.2882743	2	TCF7L2/PXYLP1
Biological Process	GO:0010909	positive regulation of heparan sulfate proteoglycan biosynthetic process	2/2279	3/18866	0.0402391	0.318593	0.2882743	2	TCF7L2/PXYLP1
Biological Process	GO:0021558	trochlear nerve development	2/2279	3/18866	0.0402391	0.318593	0.2882743	2	PHOX2A/HES3
Biological Process	GO:0021852	pyramidal neuron migration	2/2279	3/18866	0.0402391	0.318593	0.2882743	2	DISC1/ZMIZ1
Biological Process	GO:0030185	nitric oxide transport	2/2279	3/18866	0.0402391	0.318593	0.2882743	2	HBB/AQP1
Biological Process	GO:0030241	skeletal muscle myosin thick filament assembly	2/2279	3/18866	0.0402391	0.318593	0.2882743	2	TTN/MYH11
Biological Process	GO:0031134	sister chromatid biorientation	2/2279	3/18866	0.0402391	0.318593	0.2882743	2	CHAMP1/RB1
Biological Process	GO:0032916	positive regulation of transforming growth factor beta3 production	2/2279	3/18866	0.0402391	0.318593	0.2882743	2	CREB1/SMAD3

Biological Process	GO:0035793	positive regulation of metanephric mesenchymal cell migration by platelet-derived growth factor receptor-beta signaling pathway	2/2279	3/18866	0.0402391	0.318593	0.2882743	2	PDGFB/PDGFRB
Biological Process	GO:0035879	plasma membrane lactate transport	2/2279	3/18866	0.0402391	0.318593	0.2882743	2	SLC16A3/SLC16A1
Biological Process	GO:0039656	modulation by virus of host gene expression	2/2279	3/18866	0.0402391	0.318593	0.2882743	2	PABPN1/CPSF4
Biological Process	GO:0045218	zonula adherens maintenance	2/2279	3/18866	0.0402391	0.318593	0.2882743	2	PLEKHA7/KIFC3
Biological Process	GO:0045658	regulation of neutrophil differentiation	2/2279	3/18866	0.0402391	0.318593	0.2882743	2	INPP5D/TRIB1
Biological Process	GO:0045872	positive regulation of rhodopsin gene expression	2/2279	3/18866	0.0402391	0.318593	0.2882743	2	MFSD2A/NRL
Biological Process	GO:0045914	negative regulation of catecholamine metabolic process	2/2279	3/18866	0.0402391	0.318593	0.2882743	2	COMT/ITGB2
Biological Process	GO:0045963	negative regulation of dopamine metabolic process	2/2279	3/18866	0.0402391	0.318593	0.2882743	2	COMT/ITGB2
Biological Process	GO:0051176	positive regulation of sulfur metabolic process	2/2279	3/18866	0.0402391	0.318593	0.2882743	2	COMT/NFE2L2

Biological Process	GO:0060003	copper ion export	2/2279	3/18866	0.0402391	0.318593	0.2882743	2	ATOX1/ATP7B
Biological Process	GO:0060313	negative regulation of blood vessel remodeling	2/2279	3/18866	0.0402391	0.318593	0.2882743	2	ABR/BCR
Biological Process	GO:0060399	positive regulation of growth hormone receptor signaling pathway	2/2279	3/18866	0.0402391	0.318593	0.2882743	2	GHRL/MBD5
Biological Process	GO:0060671	epithelial cell differentiation involved in embryonic placenta development	2/2279	3/18866	0.0402391	0.318593	0.2882743	2	SPINT2/GRHL2
Biological Process	GO:0060672	epithelial cell morphogenesis involved in placental branching	2/2279	3/18866	0.0402391	0.318593	0.2882743	2	SPINT2/GRHL2
Biological Process	GO:0060846	blood vessel endothelial cell fate commitment	2/2279	3/18866	0.0402391	0.318593	0.2882743	2	DLL1/RBPJ
Biological Process	GO:0060847	endothelial cell fate specification	2/2279	3/18866	0.0402391	0.318593	0.2882743	2	DLL1/RBPJ
Biological Process	GO:0062232	prostanoid catabolic process	2/2279	3/18866	0.0402391	0.318593	0.2882743	2	ABHD16A/LYPLA2
Biological Process	GO:0070105	positive regulation of interleukin-6-mediated signaling pathway	2/2279	3/18866	0.0402391	0.318593	0.2882743	2	GFI1/RIPK1

Biological Process	GO:0071688	striated muscle myosin thick filament assembly	2/2279	3/18866	0.0402391	0.318593	0.2882743	2	TTN/MYH11
Biological Process	GO:0071930	negative regulation of transcription involved in G1/S transition of mitotic cell cycle	2/2279	3/18866	0.0402391	0.318593	0.2882743	2	E2F7/RB1
Biological Process	GO:0071955	recycling endosome to Golgi transport	2/2279	3/18866	0.0402391	0.318593	0.2882743	2	NUMA1/TBC1D14
Biological Process	GO:0097101	blood vessel endothelial cell fate specification	2/2279	3/18866	0.0402391	0.318593	0.2882743	2	DLL1/RBPJ
Biological Process	GO:0097116	gephyrin clustering involved in postsynaptic density assembly	2/2279	3/18866	0.0402391	0.318593	0.2882743	2	NRXN1/NRXN2
Biological Process	GO:0097343	ripoptosome assembly	2/2279	3/18866	0.0402391	0.318593	0.2882743	2	RIPK1/CYLD
Biological Process	GO:1900238	regulation of metanephric mesenchymal cell migration by platelet-derived growth factor receptor-beta signaling pathway	2/2279	3/18866	0.0402391	0.318593	0.2882743	2	PDGFB/PDGFRB
Biological Process	GO:1901026	ripoptosome assembly involved in necroptotic process	2/2279	3/18866	0.0402391	0.318593	0.2882743	2	RIPK1/CYLD
Biological Process	GO:1901895	negative regulation of ATPase-coupled calcium transmembrane transporter activity	2/2279	3/18866	0.0402391	0.318593	0.2882743	2	THADA/TLR9

Biological Process	GO:1902044	regulation of Fas signaling pathway	2/2279	3/18866	0.0402391	0.318593	0.2882743	2	ZDHHC7/SP100
Biological Process	GO:1902948	negative regulation of tau-protein kinase activity	2/2279	3/18866	0.0402391	0.318593	0.2882743	2	SORL1/RB1
Biological Process	GO:1902988	neurofibrillary tangle assembly	2/2279	3/18866	0.0402391	0.318593	0.2882743	2	SORL1/CLU
Biological Process	GO:1903614	negative regulation of protein tyrosine phosphatase activity	2/2279	3/18866	0.0402391	0.318593	0.2882743	2	MGAT5/GNAI2
Biological Process	GO:1903615	positive regulation of protein tyrosine phosphatase activity	2/2279	3/18866	0.0402391	0.318593	0.2882743	2	PTPRC/CD33
Biological Process	GO:1904958	positive regulation of midbrain dopaminergic neuron differentiation	2/2279	3/18866	0.0402391	0.318593	0.2882743	2	CSNK1D/CSNK1E
Biological Process	GO:1905246	negative regulation of aspartic-type peptidase activity	2/2279	3/18866	0.0402391	0.318593	0.2882743	2	SORL1/BIN1
Biological Process	GO:1905344	prostaglandin catabolic process	2/2279	3/18866	0.0402391	0.318593	0.2882743	2	ABHD16A/LYPLA2
Biological Process	GO:1905395	response to flavonoid	2/2279	3/18866	0.0402391	0.318593	0.2882743	2	FIS1/DNM1L

Biological Process	GO:1905616	regulation of miRNA mediated inhibition of translation	2/2279	3/18866	0.0402391	0.318593	0.2882743	2	AGO2/EIF4G1
Biological Process	GO:1905618	positive regulation of miRNA mediated inhibition of translation	2/2279	3/18866	0.0402391	0.318593	0.2882743	2	AGO2/EIF4G1
Biological Process	GO:1990456	mitochondrion-endoplasmic reticulum membrane tethering	2/2279	3/18866	0.0402391	0.318593	0.2882743	2	PACS2/PDZD8
Biological Process	GO:2000361	regulation of prostaglandin-E synthase activity	2/2279	3/18866	0.0402391	0.318593	0.2882743	2	ITGB2/NOD2
Biological Process	GO:2000363	positive regulation of prostaglandin-E synthase activity	2/2279	3/18866	0.0402391	0.318593	0.2882743	2	ITGB2/NOD2
Biological Process	GO:2000409	positive regulation of T cell extravasation	2/2279	3/18866	0.0402391	0.318593	0.2882743	2	CCR2/FADD
Biological Process	GO:2000439	positive regulation of monocyte extravasation	2/2279	3/18866	0.0402391	0.318593	0.2882743	2	CCR2/PDGFD
Biological Process	GO:2000591	positive regulation of metanephric mesenchymal cell migration	2/2279	3/18866	0.0402391	0.318593	0.2882743	2	PDGFB/PDGFRB
Biological Process	GO:2000640	positive regulation of SREBP signaling pathway	2/2279	3/18866	0.0402391	0.318593	0.2882743	2	ZBTB7B/SPRING1

Biological Process	GO:2000819	regulation of nucleotide-excision repair	2/2279	3/18866	0.0402391	0.318593	0.2882743	2	RAD52/KAT7
Biological Process	GO:2001255	positive regulation of histone H3-K36 trimethylation	2/2279	3/18866	0.0402391	0.318593	0.2882743	2	BRD4/NSD3
Biological Process	GO:2001293	malonyl-CoA metabolic process	2/2279	3/18866	0.0402391	0.318593	0.2882743	2	ACACB/ACACA
Biological Process	GO:0032835	glomerulus development	13/2279	64/18866	0.0403525	0.3189597	0.2886061	13	ANGPT1/PAX2/PDGFD/KIRREL3/MTSS1/WWTR1/IL6R/PDGFB/BCL2/NOTCH1/MEF2C/GPR4/PDGFRB
Biological Process	GO:2000401	regulation of lymphocyte migration	13/2279	64/18866	0.0403525	0.3189597	0.2886061	13	CCR2/CCL5/PADI2/APP/MIA3/DOCK8/WASL/RIPK3/CCL20/C10orf99/ADTRP/CCL27/FADD
Biological Process	GO:0014015	positive regulation of gliogenesis	15/2279	77/18866	0.0406594	0.3197425	0.2893144	15	PPARG/MYB/TNFRSF1B/CCR2/RHEB/TNF/BIN1/HDAC1/TGFB1/PRKCH/PRKCI/NOTCH1/RELA/LIF/CX3CR1
Biological Process	GO:0072522	purine-containing compound biosynthetic process	34/2279	208/18866	0.0407587	0.3197425	0.2893144	34	PRKAG2/RCVRN/AMPD3/ENO1/ADCY2/ACSL1/ADCY9/ACOT7/VPS9D1/ACSS1/SPHK2/TGFB1/SLC26A1/ACACB/ACSS2/PNP/GUK1/ADCY4/MTHFD1/PPCDC/PAPSS2/PKM/ACACA/ACSF3/AK2/GMPR2/AK5/NPPC/ACAT1/SCD/SLC25A1/PANK4/IMPDH1/ATP5PD
Biological Process	GO:1901184	regulation of ERBB signaling pathway	18/2279	97/18866	0.0412563	0.3197425	0.2893144	18	TGFA/SH3TC2/ARHGEF7/NCK2/DOK1/RHBDF2/RTN4/ARAP1/APP/RBPJ/EPS15L1/FER/PTPRJ/PDE6H/DGKD/DUSP3/EPS15/MVB12A
Biological Process	GO:0046390	ribose phosphate biosynthetic process	32/2279	194/18866	0.0413438	0.3197425	0.2893144	32	PRKAG2/AMPD3/ENO1/ADCY2/ACSL1/ADCY9/ACOT7/VPS9D1/ACSS1/SPHK2/TGFB1/SLC26A1/UCK2/ACACB/ACSS2/GUK1/ADCY4/PPCDC/PAPSS2/PKM/ACACA/ACSF3/AK2/AK5/NPPC/ACAT1/SCD/DHODH/SLC25A1/PANK4/IMPDH1/ATP5PD

Biological Process	GO:0006766	vitamin metabolic process	23/2279	131/18866	0.0413685	0.3197425	0.2893144	23	GFI1/ABCC1/VDR/MTHFD1L/TNF/PLTP/MTHFS/FGFR1/PC/CYP4F3/RDH10/SLC19A1/CD320/PDXK/MTHFD1/GSTO1/SLC52A2/SLC2A1/ALDH1A2/GSTO2/SLC23A1/AOX1/ACP3
Biological Process	GO:0006979	response to oxidative stress	68/2279	458/18866	0.0414612	0.3197425	0.2893144	68	TRPM2/P4HB/MPO/VRK2/IL10/LCN2/PRR5L/MYB/RAD52/NCF4/SLC8A1/ZNF622/LPO/DNM2/TNF/TRAF2/TNFAIP3/FBXO7/MSRB2/ABCC2/CAMKK2/MGAT3/MSRA/RRM2B/RIPK1/APP/NDUFS2/NCF2/PXN/PAX2/MTF1/ZNF580/FER/HBB/AQP1/NDUFS8/RIPK3/AIFM2/STK24/PDGFD/ATOX1/NOL3/CAT/ETS1/NFE2L2/ACOX2/ARNTL/ANKRD2/IL18RAP/CA3/BCL2/RBPMS/EPAS1/UBE3A/GLRX2/PDGFRB/FOXO1/ERCC1/RELA/SGK2/BAG5/XPA/TXNRD2/ERCC2/SIRT3/MB/PRKAA1/PRKN
Biological Process	GO:0002285	lymphocyte activation involved in immune response	31/2279	187/18866	0.0415825	0.3197425	0.2893144	31	HLA-DMB/IL27/PRKCZ/ZFPM1/FOXP1/IL10/MYB/SLC11A1/NLRP3/LOXL3/SEMA4A/RAB27A/DLL1/HLA-F/SMAD7/UNG/LY9/ZBTB7B/TGFB1/RARA/PGLYRP1/PTPRC/DOCK10/TP53BP1/IL18R1/HLX/ERCC1/TNFSF13/BCL3/PAXIP1/NSD2
Biological Process	GO:0048639	positive regulation of developmental growth	31/2279	187/18866	0.0415825	0.3197425	0.2893144	31	CPNE6/CREB1/MIR199A1/CPNE5/SH3PXD2B/TGFBR2/DISC1/DLL1/RBPJ/ILK/SMAD7/FGFR1/SEMA4D/SMURF1/GHRL/ACACB/CDH4/PLCB1/BCL2/MIR199A2/NOTCH1/MEF2C/SYT17/HLX/CYFIP1/TRPV2/NDEL1/EZR/SPTBN4/DBNL/PRKN
Biological Process	GO:0009165	nucleotide biosynthetic process	42/2279	266/18866	0.0416634	0.3197425	0.2893144	42	PRKAG2/RCVRN/AMPD3/ENO1/NADK/ADCY2/ACSL1/ADCY9/NMNAT3/RRM2B/ACOT7/VPS9D1/TK2/ACSS1/SPHK2/TGFB1/SLC26A1/UCK2/ACACB/ACSS2/PNP/GUK1/ADCY4/MTHFD1/PPCDC/PAPSS2/PKM/ACACA/ACSF3/AK2/AK5/NPPC/ACAT1/SCD/DCTD/PARP10/DHODH/PARP9/SLC25A1/PANK4/IMPDH1/ATP5PD
Biological Process	GO:0001779	natural killer cell differentiation	6/2279	22/18866	0.0417479	0.3197425	0.2893144	6	PIK3CD/SP3/PGLYRP1/PTPRC/TYRO3/KAT7
Biological Process	GO:0006312	mitotic recombination	6/2279	22/18866	0.0417479	0.3197425	0.2893144	6	BRCA2/RAD52/NSMCE2/DMC1/ERCC1/ERCC2
Biological Process	GO:0006379	mRNA cleavage	6/2279	22/18866	0.0417479	0.3197425	0.2893144	6	FIP1L1/ERN1/CPSF3/AGO2/CPSF4/TUT1
Biological Process	GO:0006622	protein targeting to lysosome	6/2279	22/18866	0.0417479	0.3197425	0.2893144	6	SORL1/CLU/NUMA1/SORT1/NEDD4/NCOA4

Biological Process	GO:0023058	adaptation of signaling pathway	6/2279	22/18866	0.0417479	0.3197425	0.2893144	6	ARRB1/DNM2/GIPR/ARRB2/DNM1/GRK2
Biological Process	GO:0034104	negative regulation of tissue remodeling	6/2279	22/18866	0.0417479	0.3197425	0.2893144	6	ABR/INPP5D/UBASH3B/TNFAIP3/CSK/BCR
Biological Process	GO:0060352	cell adhesion molecule production	6/2279	22/18866	0.0417479	0.3197425	0.2893144	6	FUT7/MIR101-2/COLEC12/NOTCH1/NOTCH4/FLOT1
Biological Process	GO:0071379	cellular response to prostaglandin stimulus	6/2279	22/18866	0.0417479	0.3197425	0.2893144	6	PPARG/P2RY6/GNG2/ACACA/PRKCE/PRKAA1
Biological Process	GO:0033135	regulation of peptidyl-serine phosphorylation	25/2279	145/18866	0.0418913	0.3197425	0.2893144	25	PDE4D/RPTOR/ANGPT1/ARRB1/TNF/NCK2/SMYD3/APP/SMAD7/TGFB1/AXIN1/CSF3/SH2D3C/ARRB2/FNIP2/PLCL1/BCL2/GGNBP2/BCAR3/INPP5F/SPTBN4/NTF4/LIF/EIF4G1/PRKAA1
Biological Process	GO:0034599	cellular response to oxidative stress	48/2279	310/18866	0.0421121	0.3197425	0.2893144	48	TRPM2/P4HB/MPO/VRK2/IL10/LCN2/PRR5L/MYB/RAD52/NCF4/ZNF622/DNM2/TNF/TRAF2/TNFAIP3/FBXO7/CAMKK2/MGAT3/MSRA/RIPK1/NCF2/PXN/PAX2/ZNF580/FER/AQP1/RIPK3/AIFM2/STK24/PDGFD/NOL3/CAT/ETS1/NFE2L2/ARNTL/ANKRD2/IL18RAP/BCL2/EPAS1/GLRX2/PDGFRB/FOXO1/RELA/BAG5/TXNRD2/SIRT3/PRKAA1/PRKN
Biological Process	GO:0090287	regulation of cellular response to growth factor stimulus	48/2279	310/18866	0.0421121	0.3197425	0.2893144	48	LRG1/LTBP1/SPRED2/SORL1/MIR140/SKI/SMAD3/LDLRAD4/DNM2/MIR199A1/CYFIP2/HIPK2/CHST11/TGFBR2/THBS1/GRB10/DLL1/RBPJ/UBE2O/ILK/PCSK6/SMAD7/FGFR1/TGFB1/EPN2/PRKCB/SMURF1/TGFB1I1/BMPER/ITGB3/RUNX2/HTRA4/NUMA1/CREBBP/ENG/PRDM16/MIR199A2/NOTCH1/NEDD4/HTRA3/MYO1C/HHEX/FAM20C/CYFIP1/ZNF423/OTX2/SPART/ADGRA2
Biological Process	GO:0003300	cardiac muscle hypertrophy	19/2279	104/18866	0.0422749	0.3197425	0.2893144	19	HDAC4/CTDP1/TTN/MIR145/AKAP13/TNFRSF1B/SMAD3/PRKCA/MIR199A1/MIR24-2/MIR23A/TNFRSF1A/CAMTA2/PDE9A/MIR199A2/NOTCH1/MEF2C/FOXO1/INPP5F
Biological Process	GO:0032231	regulation of actin filament bundle assembly	19/2279	104/18866	0.0422749	0.3197425	0.2893144	19	ARHGEF10/TACSTD2/ITGB1BP1/PIK3R1/SMAD3/SH3PXD2B/ARAP1/SYNPO2L/PXN/WNT11/PPFIA1/PFN1/ACTG1/MIR138-2/ARHGEF10L/ARHGEF18/ARHGEF15/SYNPO/PRKN

Biological Process	GO:0000414	regulation of histone H3-K36 methylation	3/2279	7/18866	0.0423362	0.3197425	0.2893144	3	BRD4/PAXIP1/NSD3
Biological Process	GO:0006657	CDP-choline pathway	3/2279	7/18866	0.0423362	0.3197425	0.2893144	3	PCYT1A/CHPT1/CHKB
Biological Process	GO:0009188	ribonucleoside diphosphate biosynthetic process	3/2279	7/18866	0.0423362	0.3197425	0.2893144	3	GUK1/AK2/AK5
Biological Process	GO:0015793	glycerol transport	3/2279	7/18866	0.0423362	0.3197425	0.2893144	3	AQP1/AQP3/AQP9
Biological Process	GO:0021648	vestibulocochlear nerve morphogenesis	3/2279	7/18866	0.0423362	0.3197425	0.2893144	3	NRP2/ATP8B1/PAX2
Biological Process	GO:0031580	membrane raft distribution	3/2279	7/18866	0.0423362	0.3197425	0.2893144	3	GSN/PTPRC/DOCK2
Biological Process	GO:0031585	regulation of inositol 1,4,5-trisphosphate-sensitive calcium-release channel activity	3/2279	7/18866	0.0423362	0.3197425	0.2893144	3	P2RY6/MYO5A/HTT
Biological Process	GO:0034141	positive regulation of toll-like receptor 3 signaling pathway	3/2279	7/18866	0.0423362	0.3197425	0.2893144	3	WDFY1/FLOT1/PELI1
Biological Process	GO:0036466	synaptic vesicle recycling via endosome	3/2279	7/18866	0.0423362	0.3197425	0.2893144	3	ITSN1/DNM1L/AP3D1

Biological Process	GO:0042297	vocal learning	3/2279	7/18866	0.0423362	0.3197425	0.2893144	3	NRXN1/HTT/NRXN2
Biological Process	GO:0042940	D-amino acid transport	3/2279	7/18866	0.0423362	0.3197425	0.2893144	3	SLC1A2/SFXN1/SLC1A3
Biological Process	GO:0044854	plasma membrane raft assembly	3/2279	7/18866	0.0423362	0.3197425	0.2893144	3	PACSIN2/MIR138-2/FLOT1
Biological Process	GO:0045630	positive regulation of T-helper 2 cell differentiation	3/2279	7/18866	0.0423362	0.3197425	0.2893144	3	PRKCZ/NLRP3/RARA
Biological Process	GO:0048290	isotype switching to IgA isotypes	3/2279	7/18866	0.0423362	0.3197425	0.2893144	3	TGFB1/TNFSF13/NSD2
Biological Process	GO:0051597	response to methylmercury	3/2279	7/18866	0.0423362	0.3197425	0.2893144	3	FECH/ARSB/ANK2
Biological Process	GO:0052651	monoacylglycerol catabolic process	3/2279	7/18866	0.0423362	0.3197425	0.2893144	3	MGLL/ABHD16A/ABHD16B
Biological Process	GO:0060369	positive regulation of Fc receptor mediated stimulatory signaling pathway	3/2279	7/18866	0.0423362	0.3197425	0.2893144	3	CD226/PTPRJ/PTPRC
Biological Process	GO:0060462	lung lobe development	3/2279	7/18866	0.0423362	0.3197425	0.2893144	3	TGFBR2/GRHL2/LIF

Biological Process	GO:0060463	lung lobe morphogenesis	3/2279	7/18866	0.0423362	0.3197425	0.2893144	3	TGFBR2/GRHL2/LIF
Biological Process	GO:0060558	regulation of calcidiol 1-monooxygenase activity	3/2279	7/18866	0.0423362	0.3197425	0.2893144	3	GFI1/VDR/TNF
Biological Process	GO:0060717	chorion development	3/2279	7/18866	0.0423362	0.3197425	0.2893144	3	E2F7/DNAJB6/PAXIP1
Biological Process	GO:0061762	CAMKK-AMPK signaling cascade	3/2279	7/18866	0.0423362	0.3197425	0.2893144	3	CAMKK2/HTT/PRKAA1
Biological Process	GO:0070100	negative regulation of chemokine-mediated signaling pathway	3/2279	7/18866	0.0423362	0.3197425	0.2893144	3	CCL5/PADI2/SLIT3
Biological Process	GO:0070837	dehydroascorbic acid transport	3/2279	7/18866	0.0423362	0.3197425	0.2893144	3	SLC2A14/SLC2A1/SLC23A1
Biological Process	GO:0070944	neutrophil-mediated killing of bacterium	3/2279	7/18866	0.0423362	0.3197425	0.2893144	3	AZU1/ELANE/CTSG
Biological Process	GO:0072007	mesangial cell differentiation	3/2279	7/18866	0.0423362	0.3197425	0.2893144	3	PDGFB/NOTCH1/GPR4
Biological Process	GO:0072257	metanephric nephron tubule epithelial cell differentiation	3/2279	7/18866	0.0423362	0.3197425	0.2893144	3	PAX2/WWTR1/LIF

Biological Process	GO:0072307	regulation of metanephric nephron tubule epithelial cell differentiation	3/2279	7/18866	0.0423362	0.3197425	0.2893144	3	PAX2/WWTR1/LIF
Biological Process	GO:0090240	positive regulation of histone H4 acetylation	3/2279	7/18866	0.0423362	0.3197425	0.2893144	3	ARRB1/PIH1D1/KAT7
Biological Process	GO:0098596	imitative learning	3/2279	7/18866	0.0423362	0.3197425	0.2893144	3	NRXN1/HTT/NRXN2
Biological Process	GO:1900244	positive regulation of synaptic vesicle endocytosis	3/2279	7/18866	0.0423362	0.3197425	0.2893144	3	SH3GL1/DNM1/DNM1L
Biological Process	GO:1904429	regulation of t-circle formation	3/2279	7/18866	0.0423362	0.3197425	0.2893144	3	RTEL1/ERCC1/SLX4
Biological Process	GO:2000394	positive regulation of lamellipodium morphogenesis	3/2279	7/18866	0.0423362	0.3197425	0.2893144	3	RREB1/ARHGEF7/CORO1C
Biological Process	GO:2000638	regulation of SREBP signaling pathway	3/2279	7/18866	0.0423362	0.3197425	0.2893144	3	AMFR/ZBTB7B/SPRING1
Biological Process	GO:0002704	negative regulation of leukocyte mediated immunity	11/2279	52/18866	0.0436541	0.328131	0.2969046	11	ABR/CCR2/CD84/DUSP22/HLA-F/PTPN6/SMAD7/PTPRC/BCR/ARRB2/CD96
Biological Process	GO:0010883	regulation of lipid storage	11/2279	52/18866	0.0436541	0.328131	0.2969046	11	PNPLA2/EHD1/PPARG/TNF/ABCG1/NR1H2/ITGB3/ABCA1/ACACB/FTO/ABHD5

Biological Process	GO:0033059	cellular pigmentation	11/2279	52/18866	0.0436541	0.328131	0.2969046	11	SHROOM3/LYST/KIF13A/ZEB2/RAB27A/CDH3/DCTN2/MYO5A/BCL2/AP3D1/DTNBP1
Biological Process	GO:0071622	regulation of granulocyte chemotaxis	11/2279	52/18866	0.0436541	0.328131	0.2969046	11	C3AR1/CCL5/CSF1R/THBS1/C5AR2/JAM3/CAMK1D/CXCL17/DAPK2/DNM1L/DYSF
Biological Process	GO:0072666	establishment of protein localization to vacuole	11/2279	52/18866	0.0436541	0.328131	0.2969046	11	SORL1/VTI1A/VPS13D/TNFAIP3/CLU/SMURF1/NUMA1/SORT1/NEDD4/AP3D1/NCOA4
Biological Process	GO:1990089	response to nerve growth factor	11/2279	52/18866	0.0436541	0.328131	0.2969046	11	EHD1/CREB1/ACAP2/APP/RAPGEF2/SORT1/NTRK1/MICALL1/KIF1B/UBE3A/NTF4
Biological Process	GO:0051492	regulation of stress fiber assembly	17/2279	91/18866	0.0438718	0.3295067	0.2981494	17	ARHGEF10/TACSTD2/ITGB1BP1/PIK3R1/SMAD3/SH3PXD2B/ARAP1/SYNPO2L/PXN/WNT11/PPFIA1/PFN1/ACTG1/MIR138-2/ARHGEF10L/ARHGEF18/ARHGEF15
Biological Process	GO:0003014	renal system process	22/2279	125/18866	0.0442279	0.3319192	0.3003323	22	GSN/ADCY2/PRKAR1B/ADCY9/SGK1/GJA5/COMT/RRM2B/GNAI2/PRKRIP1/HNF1A/HBB/AQP1/PDGFB/BCR/KCNQ1/ADCY4/BCL2/AQP3/F2R/AGTR1/HSD11B2
Biological Process	GO:0006282	regulation of DNA repair	23/2279	132/18866	0.0445853	0.3336653	0.3019122	23	FAM168A/SPIDR/RMI2/RAD52/SETD2/RTEL1/WRNIP1/RECQL5/OTUB2/UBE2V1/OTUB1/TP53BP1/FOXM1/TRIP12/KAT7/PARP9/TFIP11/NSD2/CYREN/ABRAXAS1/SLF1/FBH1/BABAM2
Biological Process	GO:0044331	cell-cell adhesion mediated by cadherin	7/2279	28/18866	0.0447416	0.3336653	0.3019122	7	FER/PLEKHA7/CDHR2/NOTCH1/NOTCH4/PTPRU/FLOT1
Biological Process	GO:0061437	renal system vasculature development	7/2279	28/18866	0.0447416	0.3336653	0.3019122	7	ANGPT1/PDGFD/IL6R/PDGFB/NOTCH1/GPR4/PDGFRB

Biological Process	GO:0061440	kidney vasculature development	7/2279	28/18866	0.0447416	0.3336653	0.3019122	7	ANGPT1/PDGFD/IL6R/PDGFB/NOTCH1/GPR4/PDGFRB
Biological Process	GO:0095500	acetylcholine receptor signaling pathway	7/2279	28/18866	0.0447416	0.3336653	0.3019122	7	LY6G6D/GNAI2/GNA11/CHRM2/PLCB1/RGS10/GRK2
Biological Process	GO:1902932	positive regulation of alcohol biosynthetic process	7/2279	28/18866	0.0447416	0.3336653	0.3019122	7	TNF/ABCG1/P2RY6/HRH1/POR/SREBF1/PRKAA1
Biological Process	GO:1905063	regulation of vascular associated smooth muscle cell differentiation	7/2279	28/18866	0.0447416	0.3336653	0.3019122	7	MIR140/MIR145/NFATC2/NFATC1/PDGFB/ENG/DNMT1
Biological Process	GO:1990776	response to angiotensin	7/2279	28/18866	0.0447416	0.3336653	0.3019122	7	MIR143/MIR145/ARID1B/AGTRAP/NFE2L2/AGTR1/RELA
Biological Process	GO:0035304	regulation of protein dephosphorylation	24/2279	139/18866	0.0448256	0.3340302	0.3022424	24	LMTK2/MGAT5/PPP1R1B/URI1/CABIN1/TNF/GNAI2/CAMTA1/ELFN2/RCAN1/PHACTR2/TGFB1/PPP2R5C/HTT/PTPRC/PPP2R1A/PPP2R5E/PPP2R5D/PPP2R1B/PDGFRB/PPP1R16B/CD33/ANKLE2/PTPA
Biological Process	GO:0060395	SMAD protein signal transduction	15/2279	78/18866	0.0449326	0.3345649	0.3027262	15	LNPEP/SKI/SMAD3/HIPK2/SMAD7/ZMIZ1/TGFB1/NUP93/WWTR1/RBPMS/SUB1/SLC33A1/GDF6/INHBA/NODAL
Biological Process	GO:0010771	negative regulation of cell morphogenesis involved in differentiation	18/2279	98/18866	0.0450499	0.3351752	0.3032784	18	SEMA6B/TACSTD2/KANK1/YWHAH/ITGB1BP1/SEMA4A/RTN4/SEMA4B/ACTN4/IFRD1/SYNGAP1/SEMA4D/RAPGEF2/DNM3/CORO1C/CDKL3/UBE3A/SPART
Biological Process	GO:0006895	Golgi to endosome transport	5/2279	17/18866	0.0456251	0.337079	0.3050011	5	AP2A1/CORO7/SORT1/EPS15/DOP1B

Biological Process	GO:0006991	response to sterol depletion	5/2279	17/18866	0.0456251	0.337079	0.3050011	5	AMFR/ZBTB7B/ARHGEF10L/SREBF1/SPRING1
Biological Process	GO:0007158	neuron cell-cell adhesion	5/2279	17/18866	0.0456251	0.337079	0.3050011	5	NINJ2/NCAM2/NRXN1/NRXN2/ASTN2
Biological Process	GO:0030889	negative regulation of B cell proliferation	5/2279	17/18866	0.0456251	0.337079	0.3050011	5	IL10/INPP5D/MNDA/TNFRSF21/BLK
Biological Process	GO:0031000	response to caffeine	5/2279	17/18866	0.0456251	0.337079	0.3050011	5	PPARG/CASQ2/SLC8A1/DHODH/PRKAA1
Biological Process	GO:0032695	negative regulation of interleukin-12 production	5/2279	17/18866	0.0456251	0.337079	0.3050011	5	IL10/MEFV/THBS1/TIGIT/ARRB2
Biological Process	GO:0036166	phenotypic switching	5/2279	17/18866	0.0456251	0.337079	0.3050011	5	MIR140/MIR143/MIR145/PDGFB/DNMT1
Biological Process	GO:1904380	endoplasmic reticulum mannose trimming	5/2279	17/18866	0.0456251	0.337079	0.3050011	5	AMFR/EDEM1/DERL2/EDEM2/MARCHF6
Biological Process	GO:2000811	negative regulation of anoikis	5/2279	17/18866	0.0456251	0.337079	0.3050011	5	CEACAM6/TLE1/BCL2/NOTCH1/CEACAM5
Biological Process	GO:0034142	toll-like receptor 4 signaling pathway	9/2279	40/18866	0.0457588	0.3370895	0.3050105	9	MIR140/PIK3AP1/TNFAIP3/SCIMP/ITGB2/PRKCE/WDFY1/LTF/PELI1

Biological Process	GO:1902895	positive regulation of pri-miRNA transcription by RNA polymerase II	9/2279	40/18866	0.0457588	0.3370895	0.3050105	9	IL10/PPARG/SMAD3/TNF/TGFB1/PDGFB/ETS1/SREBF1/RELA
Biological Process	GO:0006109	regulation of carbohydrate metabolic process	34/2279	210/18866	0.0458116	0.3370895	0.3050105	34	MAEA/FOXK1/HDAC4/PRKAG2/AP2A1/C1QTNF3/PFKFB4/RAE1/GPLD1/CBFA2T3/PGP/APP/PGAM1/P2RY6/TGFB1/HRH1/DGKQ/NUP93/DYRK2/SEC13/GNB3/ACACB/PDGFB/PPP1CB/ESRRB/PRKCE/FOXO1/NCOA2/SOGA1/PFKFB3/PRKAA1/PRKN/SDHAF3/RUBCNL
Biological Process	GO:0009649	entrainment of circadian clock	8/2279	34/18866	0.0458393	0.3370895	0.3050105	8	PHLPP1/MTA1/GNA11/PPP1CB/PER2/PER1/FBXL6/USP2
Biological Process	GO:0010922	positive regulation of phosphatase activity	8/2279	34/18866	0.0458393	0.3370895	0.3050105	8	SMAD3/GPLD1/RIPK3/PTPRC/MEF2C/PDGFRB/CD33/PTPA
Biological Process	GO:0050715	positive regulation of cytokine secretion	8/2279	34/18866	0.0458393	0.3370895	0.3050105	8	SORL1/IL10/C1QTNF3/CLEC9A/FGR/TNF/CLEC5A/CADM1
Biological Process	GO:0010639	negative regulation of organelle organization	62/2279	416/18866	0.0465214	0.3418405	0.3093094	62	VILL/TBCD/VAT1/TACSTD2/SSH1/GSN/MAD1L1/KANK1/TNKS/SKI/PAM16/RTEL1/DNM2/CAPG/ARAP1/NAV3/OTUB2/SSH3/FEZ2/CLU/PPFIA1/SPHK2/TMOD3/SVIL/CTBP1/PFN1/CLEC16A/ESPN/GMFB/KDM4C/MCM2/SMG6/NOL3/SPTA1/ARRB2/FNIP2/MIR138-2/ADD1/OTUB1/ARHGEF18/DNMT1/PIH1D1/DNM1L/AVIL/FLII/ARHGEF2/TRIP12/TSC2/ERCC1/BCL2L1/SPTAN1/TMOD1/SLX4/AURKAIP1/GMFG/MYADM/SPTBN4/SPEF1/LIF/PCID2/PRKN/CYRIB
Biological Process	GO:2000779	regulation of double-strand break repair	16/2279	85/18866	0.0466254	0.3420884	0.3095337	16	SPIDR/RMI2/SETD2/RTEL1/RECQL5/OTUB2/OTUB1/TP53BP1/FOXM1/TRIP12/PARP9/TFIP11/NSD2/CYREN/SLF1/FBH1
Biological Process	GO:0001768	establishment of T cell polarity	4/2279	12/18866	0.0473472	0.3420884	0.3095337	4	GSN/MYH9/DOCK8/DOCK2
Biological Process	GO:0002468	dendritic cell antigen processing and presentation	4/2279	12/18866	0.0473472	0.3420884	0.3095337	4	SLC11A1/THBS1/NOD2/CD68

Biological Process	GO:0007077	mitotic nuclear envelope disassembly	4/2279	12/18866	0.0473472	0.3420884	0.3095337	4	PRKCA/PRKCB/NEK6/LPIN1
Biological Process	GO:0009162	deoxyribonucleoside monophosphate metabolic process	4/2279	12/18866	0.0473472	0.3420884	0.3095337	4	TK2/GUK1/DCTD/TK1
Biological Process	GO:0015803	branched-chain amino acid transport	4/2279	12/18866	0.0473472	0.3420884	0.3095337	4	SLC43A2/SLC7A5/SLC43A1/SLC7A8
Biological Process	GO:0032621	interleukin-18 production	4/2279	12/18866	0.0473472	0.3420884	0.3095337	4	IL10/NLRP3/CD84/TLR9
Biological Process	GO:0033182	regulation of histone ubiquitination	4/2279	12/18866	0.0473472	0.3420884	0.3095337	4	OTUB2/OTUB1/TRIP12/RNF40
Biological Process	GO:0034086	maintenance of sister chromatid cohesion	4/2279	12/18866	0.0473472	0.3420884	0.3095337	4	TNKS/NSMCE2/RB1/SLF1
Biological Process	GO:0034088	maintenance of mitotic sister chromatid cohesion	4/2279	12/18866	0.0473472	0.3420884	0.3095337	4	TNKS/NSMCE2/RB1/SLF1
Biological Process	GO:0038180	nerve growth factor signaling pathway	4/2279	12/18866	0.0473472	0.3420884	0.3095337	4	RAPGEF2/SORT1/NTRK1/NTF4
Biological Process	GO:0046689	response to mercury ion	4/2279	12/18866	0.0473472	0.3420884	0.3095337	4	BSG/ABCC2/AQP1/AQP9

Biological Process	GO:0070586	cell-cell adhesion involved in gastrulation	4/2279	12/18866	0.0473472	0.3420884	0.3095337	4	IL10/MBP/IL1RN/MYADM
Biological Process	GO:0090154	positive regulation of sphingolipid biosynthetic process	4/2279	12/18866	0.0473472	0.3420884	0.3095337	4	TNF/TNFRSF1A/SPHK2/SIRT3
Biological Process	GO:0090520	sphingolipid mediated signaling pathway	4/2279	12/18866	0.0473472	0.3420884	0.3095337	4	SPHK2/S1PR2/S1PR4/EZR
Biological Process	GO:0097284	hepatocyte apoptotic process	4/2279	12/18866	0.0473472	0.3420884	0.3095337	4	GSN/STK3/RB1/BCL2L1
Biological Process	GO:0098885	modification of postsynaptic actin cytoskeleton	4/2279	12/18866	0.0473472	0.3420884	0.3095337	4	ITSN1/BAIAP2/PFN1/CYFIP1
Biological Process	GO:1902224	ketone body metabolic process	4/2279	12/18866	0.0473472	0.3420884	0.3095337	4	BDH1/HMGCL/ACAT1/ACSS3
Biological Process	GO:1905461	positive regulation of vascular associated smooth muscle cell apoptotic process	4/2279	12/18866	0.0473472	0.3420884	0.3095337	4	PPARG/MIR140/E2F3/MIR24-2
Biological Process	GO:1905668	positive regulation of protein localization to endosome	4/2279	12/18866	0.0473472	0.3420884	0.3095337	4	SORL1/MGAT3/ZDHHC1/EZR
Biological Process	GO:2000304	positive regulation of ceramide biosynthetic process	4/2279	12/18866	0.0473472	0.3420884	0.3095337	4	TNF/TNFRSF1A/SPHK2/SIRT3

Biological Process	GO:2001135	regulation of endocytic recycling	4/2279	12/18866	0.0473472	0.3420884	0.3095337	4	EHD1/SORL1/ARHGAP1/INPP5F
Biological Process	GO:0043647	inositol phosphate metabolic process	14/2279	72/18866	0.047523	0.3428371	0.3102112	14	SYNJ2/INPP5A/INPP5D/ITPKB/INPP4A/NUDT3/P2RY6/HRH1/INPP5E/PLCB1/IMPA2/PLCG1/PLCD3/IP6K1
Biological Process	GO:0050663	cytokine secretion	14/2279	72/18866	0.047523	0.3428371	0.3102112	14	SORL1/IL10/C1QTNF3/CLEC9A/NLRP3/FGR/TNF/TLR9/CLEC5A/SPHK2/NOD2/DYSF/CADM1/CCN4
Biological Process	GO:0022898	regulation of transmembrane transporter activity	43/2279	276/18866	0.0478034	0.344598	0.3118045	43	PDE4D/KCNE1/THADA/CASQ2/WWP2/FXYD2/YWHAH/FXYD1/CCR2/DNM2/TLR9/TESC/JSRP1/AHNAK/RIPK1/APP/NOS1AP/ACTN4/P2RY6/SPHK2/CRHBP/CNIH2/HTR3A/MYO5A/HTT/CAB39/MAPK8IP2/GSG1L/BCL2/GSTO1/MEF2C/SHANK2/PRKCE/NEDD4/DYSF/ANK2/HECW2/PDZK1/CASQ1/FGF14/CBARP/STIMATE/CRACR2A
Biological Process	GO:0006997	nucleus organization	23/2279	133/18866	0.0479806	0.3454536	0.3125787	23	DCTN1/SYNE1/SRPK2/PRKCA/HIPK2/RTN4/BIN1/USP36/PRKCB/SF1/NUP93/NUMA1/PPP2R1A/ETS1/NEK6/UBXN2A/TOR1B/NDEL1/ANKLE2/LPIN1/BANF1/IRAG2/MFSD14A
Biological Process	GO:1903321	negative regulation of protein modification by small protein conjugation or removal	17/2279	92/18866	0.0479948	0.3454536	0.3125787	17	ARRB1/TNFAIP3/MIR101-2/OTUB2/SMAD7/ARRB2/MIR138-2/PER2/OTUB1/NXN/PRKCE/TRIP12/DYSF/RELA/BAG5/PARP10/MARCHF7
Biological Process	GO:0010977	negative regulation of neuron projection development	26/2279	154/18866	0.0481659	0.3464222	0.3134551	26	SEMA6B/CTSZ/GFI1/KANK1/YWHAH/BCL11A/SEMA4A/RTN4/PMP22/SEMA4B/IFRD1/ASAP1/SYNGAP1/KREMEN1/SEMA4D/RAPGEF2/ITM2C/DNM3/CDKL3/UBE3A/CERS2/INPP5F/BAG5/RAP1GAP2/PRAG1/SPART
Biological Process	GO:0043393	regulation of protein binding	34/2279	211/18866	0.0485058	0.3486037	0.315429	34	HDAC4/SLPI/CTSZ/ZFPM1/MARK3/SORL1/IL10/ANGPT1/ITGB1BP1/AMFR/ARRB1/UBASH3B/HIPK2/CCM2L/DISC1/APP/TCF7L2/RAPGEF2/CSF3/STK3/ATP2A3/CSNK1E/PDGFB/SPTA1/ARRB2/PLCL1/ADD1/CYLD/MARK2/FLOT1/DTNBP1/TFIP11/STING1/PRKN
Biological Process	GO:0006302	double-strand break repair	41/2279	262/18866	0.049271	0.3524981	0.3189528	41	BLM/SPIDR/BRCA2/RMI2/RAD52/SETD2/TDP1/RTEL1/HUS1/NSMCE2/KDM2A/MCM5/RECQL5/EME1/MEIOB/OTUB2/UVRAG/MTA1/AP5S1/MCM2/SMARCAL1/OTUB1/RAD21L1/DMC1/TP53BP1/CDCA5/FOXM1/CHEK2/TRIP12/ERCC1/SLX4/PARP9/PAXIP1/TFIP11/PAXX/NSD2/CYREN/ABRAXAS1/SLF1/FBH1/BABAM2

Biological Process	GO:0007632	visual behavior	11/2279	53/18866	0.0493074	0.3524981	0.3189528	11	CREB1/PPP1R1B/TANC1/APP/SYNGAP1/HRH1/NF1/SLC1A2/MEIS2/SYNPO/NPHP4
Biological Process	GO:0032731	positive regulation of interleukin-1 beta production	11/2279	53/18866	0.0493074	0.3524981	0.3189528	11	AZU1/NLRC4/AIM2/NLRP3/SMAD3/TNF/MNDA/APP/NOD2/CASP1/STMP1
Biological Process	GO:0032964	collagen biosynthetic process	11/2279	53/18866	0.0493074	0.3524981	0.3189528	11	PPARG/MIR145/MYB/TGFB1/ENG/ARRB2/COL5A1/F2R/NPPC/PDGFRB/P3H3
Biological Process	GO:0045668	negative regulation of osteoblast differentiation	11/2279	53/18866	0.0493074	0.3524981	0.3189528	11	HDAC4/HDAC7/SKI/SMAD3/SUFU/TNF/LIMD1/SEMA4D/TMEM64/MIR138-2/NOTCH1
Biological Process	GO:0045744	negative regulation of G protein-coupled receptor signaling pathway	11/2279	53/18866	0.0493074	0.3524981	0.3189528	11	MGRN1/ARRB1/DNM2/CCL5/GNAI2/MRAP/GIPR/ARRB2/DNM1/RPH3AL/GRK2
Biological Process	GO:0061614	pri-miRNA transcription by RNA polymerase II	11/2279	53/18866	0.0493074	0.3524981	0.3189528	11	HDAC4/IL10/PPARG/SMAD3/TNF/TGFB1/LILRB4/PDGFB/ETS1/SREBF1/RELA
Biological Process	GO:0010721	negative regulation of cell development	52/2279	343/18866	0.0494465	0.3531707	0.3195614	52	SEMA6B/CTSZ/GFI1/TACSTD2/SORL1/CALR/CTDP1/KANK1/LSM1/YWHAH/SKI/ITGB1BP1/MIR199A1/TNF/BCL11A/SEMA4A/RTN4/PMP22/SEMA4B/DLL1/APP/MIB1/ACTN4/IFRD1/ASAP1/SYNGAP1/KREMEN1/SEMA4D/RAPGEF2/NF1/ITM2C/MEIS1/BCL2/DNM3/MIR199A2/NOTCH1/CORO1C/ZHX2/CDKL3/UBE3A/ARHGEF2/NPPC/LTF/CERS2/INPP5F/BAG5/RAP1GAP2/NODAL/HES3/RFLNA/PRAG1/SPART
Biological Process	GO:0006164	purine nucleotide biosynthetic process	32/2279	197/18866	0.0494889	0.3531707	0.3195614	32	PRKAG2/RCVRN/AMPD3/ENO1/ADCY2/ACSL1/ADCY9/ACOT7/VPS9D1/ACSS1/SPHK2/TGFB1/SLC26A1/ACACB/ACSS2/GUK1/ADCY4/MTHFD1/PPCDC/PAPSS2/PKM/ACACA/ACSF3/AK2/AK5/NPPC/ACAT1/SCD/SLC25A1/PANK4/IMPDH1/ATP5PD
Biological Process	GO:0042246	tissue regeneration	15/2279	79/18866	0.049513	0.3531707	0.3195614	15	CD9/GSN/NINJ2/MIR199A1/TGFBR2/NINJ1/IFRD1/PAX7/ENO3/CPQ/PKM/MIR199A2/NOTCH1/DYSF/EYS

Biological Process	GO:0014812	muscle cell migration	19/2279	106/18866	0.04994	0.3556818	0.3218335	19	HDAC4/SORL1/PLAU/SSH1/MIR140/MIR143/ITGB1BP1/CCL5/ILK/P2RY6/PDGFD/ITGB3/PDGFB/NFE2L2/BCL2/MEF2C/PDGFRB/TRIB1/CCN4
Biological Process	GO:1905269	positive regulation of chromatin organization	19/2279	106/18866	0.04994	0.3556818	0.3218335	19	BRD4/MYB/ARRB1/C6orf89/RPS6KA4/SPHK2/ZBTB7B/PAX7/CTBP1/ATF7IP/PHF19/DNMT1/PIH1D1/SREBF1/RNF40/KAT7/LIF/PAXIP1/NSD3
Biological Process	GO:0002228	natural killer cell mediated immunity	13/2279	66/18866	0.050168	0.3565039	0.3225774	13	LYST/PIK3R6/SLAMF7/TUBB4B/RAB27A/CD226/HLA-F/PTPN6/NCR1/ARRB2/IL18RAP/CADM1/CD96
Biological Process	GO:0032715	negative regulation of interleukin-6 production	13/2279	66/18866	0.050168	0.3565039	0.3225774	13	IL10/C1QTNF3/MIR140/ARRB1/TNF/TNFAIP3/MIR101-2/TLR9/C5AR2/PTPN6/GHRL/CSK/ARRB2
Biological Process	GO:0032922	circadian regulation of gene expression	13/2279	66/18866	0.050168	0.3565039	0.3225774	13	CSNK1D/KDM2A/ZFHX3/HDAC1/MTA1/CSNK1E/PPP1CB/ARNTL/PER2/PER1/RAI1/USP2/NCOA2
Biological Process	GO:0035821	modulation of process of other organism	20/2279	113/18866	0.0505877	0.359063	0.324893	20	DEFA4/SLPI/RXRA/CTDP1/ULK1/CCL5/CSF1R/SFTPD/HIPK2/HDAC1/PC/PABPN1/PHB/AQP1/TNIP1/MPEG1/LTF/BCL2L1/CPSF4/PRKN
Biological Process	GO:0006907	pinocytosis	6/2279	23/18866	0.050906	0.359063	0.324893	6	ANKFY1/DNM2/ACTN4/NR1H2/MAPKAPK3/DOCK2
Biological Process	GO:0007617	mating behavior	6/2279	23/18866	0.050906	0.359063	0.324893	6	HDAC4/HEXB/PPP1R1B/APP/NCOA1/MAPK8IP2
Biological Process	GO:0031280	negative regulation of cyclase activity	6/2279	23/18866	0.050906	0.359063	0.324893	6	CCR2/GNAI2/AKAP5/LTB4R2/PALM/GABBR2

Biological Process	GO:0031639	plasminogen activation	6/2279	23/18866	0.050906	0.359063	0.324893	6	CTSZ/ENO1/PLAU/THBS1/F12/CPB2
Biological Process	GO:0035743	CD4-positive, alpha-beta T cell cytokine production	6/2279	23/18866	0.050906	0.359063	0.324893	6	PRKCZ/CD55/NLRP3/IL1R1/IL18RAP/IL18R1
Biological Process	GO:0060044	negative regulation of cardiac muscle cell proliferation	6/2279	23/18866	0.050906	0.359063	0.324893	6	MIR199A1/VGLL4/TGFBR2/RIPK1/MEIS1/MIR199A2
Biological Process	GO:0071404	cellular response to low-density lipoprotein particle stimulus	6/2279	23/18866	0.050906	0.359063	0.324893	6	CD9/PPARG/ABCA1/ITGB2/NPC1/HMGCS1
Biological Process	GO:1902001	fatty acid transmembrane transport	6/2279	23/18866	0.050906	0.359063	0.324893	6	PRKAG2/THBS1/ACACB/ACACA/SLC2A1/CPT1A
Biological Process	GO:1903077	negative regulation of protein localization to plasma membrane	6/2279	23/18866	0.050906	0.359063	0.324893	6	MRAP/PPFIA1/TGFB1/NUMB/CSK/BCL2L1
Biological Process	GO:0045445	myoblast differentiation	16/2279	86/18866	0.0511141	0.3599439	0.32569	16	MAML1/TNF/DLL1/RBPJ/TCF7L2/IFRD1/PLEKHM3/ILK/TGFB1/PLCB1/ANKRD2/RB1/NOTCH1/CMTM5/MEF2C/EPAS1
Biological Process	GO:0006378	mRNA polyadenylation	10/2279	47/18866	0.0511445	0.3599439	0.32569	10	FIP1L1/CPSF3/APP/CPEB3/SCAF8/ZC3H3/RNF40/TUT1/PAPOLG/GRSF1
Biological Process	GO:0034204	lipid translocation	10/2279	47/18866	0.0511445	0.3599439	0.32569	10	ABCC1/TMEM30A/ABCG1/ATP8A1/ANO7/ATP11A/ATP8B1/ABCA1/ATP8B4/ANO6

Biological Process	GO:0009132	nucleoside diphosphate metabolic process	26/2279	155/18866	0.0514784	0.3620256	0.3275736	26	FOXK1/HDAC4/PRKAG2/AMPD3/ENO1/HK1/TJP2/PFKFB4/RAE1/CBFA2T3/DHTKD1/APP/OGDH/ADPGK/PGAM1/NUP93/ENO3/HK2/SEC13/GUK1/ESRRB/PKM/AK2/AK5/PFKFB3/PRKAA1
Biological Process	GO:0043280	positive regulation of cysteine-type endopeptidase activity involved in apoptotic process	23/2279	134/18866	0.0515589	0.3623234	0.3278431	23	NLRC4/S100A8/GRAMD4/PPARG/GSN/LCK/NLRP3/SMAD3/ARRB1/TNF/TRAF2/CTSD/CTSH/RIPK1/BCL10/ATP2A3/FIS1/P2RX1/F2R/CASP1/BCL2L13/NODAL/FADD
Biological Process	GO:0001889	liver development	24/2279	141/18866	0.0516277	0.3625389	0.3280381	24	ARID5B/IL10/RPS6KA1/SMAD3/TNFAIP3/E2F7/CUL3/TAF10/HNF1A/RARA/NF1/SP3/PKM/NOTCH1/CPB2/HMGCL/HLX/PCK2/CEBPB/ACAT1/HMGCS1/RELA/ALDH1A2/NODAL
Biological Process	GO:0007178	transmembrane receptor protein serine/threonine kinase signaling pathway	54/2279	359/18866	0.0519504	0.364535	0.3298443	54	LRG1/PRKCZ/LTBP1/SPRED2/SORL1/MIR140/CREB1/LNPEP/SKI/TGFBRAP1/SMAD3/LDLRAD4/DNM2/HIPK2/DUSP22/CHST11/TGFBR2/THBS1/RBPJ/UBE2O/ILK/PCSK6/SMAD7/PXN/ZMIZ1/ITGB5/TGFB1/SMURF1/NUP93/TGFB1I1/BMPER/WWTR1/BMPR1B/RUNX2/HTRA4/NUMA1/CREBBP/ENG/ARRB2/PRDM16/ARHGEF18/NOTCH1/GCNT2/RBPMS/SUB1/SLC33A1/HTRA3/GDF6/ZC3H3/ZNF423/INHBA/NODAL/ZFYVE9/SPART
Biological Process	GO:0007204	positive regulation of cytosolic calcium ion concentration	49/2279	322/18866	0.052183	0.3658975	0.3310771	49	TRPM2/PDE4D/C3AR1/CASQ2/ITPR2/LCK/CD55/CACNA1C/CCR2/SLC8A1/MIR199A1/UBASH3B/FPR2/FPR1/ITPR1/ACKR2/JSRP1/C5AR2/PTPN6/RASA3/GNAT2/P2RY6/CCR9/MYO5A/GIPR/HTT/GHRL/PTPRC/CXCR1/NOL3/TPCN2/FIS1/BCL2/MIR199A2/GSTO1/LPAR2/PRKCE/F2R/S1PR4/GPR4/AGTR1/ANK2/CHERP/PLCG1/P2RX5/TRPV2/CASQ1/CX3CR1/SYNPO
Biological Process	GO:0030510	regulation of BMP signaling pathway	17/2279	93/18866	0.0523845	0.3667687	0.3318653	17	SORL1/MIR140/SKI/HIPK2/RBPJ/UBE2O/ILK/PCSK6/SMAD7/SMURF1/BMPER/NUMA1/ENG/NOTCH1/HTRA3/ZNF423/SPART
Biological Process	GO:0032677	regulation of interleukin-8 production	17/2279	93/18866	0.0523845	0.3667687	0.3318653	17	ELANE/IL10/ARRB1/TNF/TLR9/RIPK1/C5AR2/ZNF580/FCN1/PTPRC/BCL10/NOD2/F2R/RELA/CD33/BCL3/FADD
Biological Process	GO:1903779	regulation of cardiac conduction	14/2279	73/18866	0.0525249	0.3673817	0.33242	14	PDE4D/CASQ2/ITPR2/FXYD2/FXYD1/SLC8A1/GJA5/ATP2B2/ITPR1/ATP2A3/NPPC/ANK2/ASPH/CASQ1
Biological Process	GO:0030890	positive regulation of B cell proliferation	9/2279	41/18866	0.0525881	0.3673817	0.33242	9	NFATC2/TNFSF13B/TLR9/PTPRC/CD320/BCL2/MEF2C/PELI1/VAV3

Biological Process	GO:0050832	defense response to fungus	9/2279	41/18866	0.0525881	0.3673817	0.33242	9	DEFA4/ELANE/S100A8/CTSG/MPO/COTL1/SPON2/C10orf99/LTF
Biological Process	GO:0032732	positive regulation of interleukin-1 production	12/2279	60/18866	0.0528266	0.3687771	0.3336827	12	AZU1/NLRC4/AIM2/IL16/NLRP3/SMAD3/TNF/MNDA/APP/NOD2/CASP1/STMP1
Biological Process	GO:0072521	purine-containing compound metabolic process	69/2279	472/18866	0.0530126	0.3690156	0.3338984	69	FOXK1/HDAC4/PDE4D/PRKAG2/RCVRN/AMPD3/ENO1/HK1/ADCY2/ACSL1/FAR2/TJP2/ADCY9/PFKFB4/ACOT13/SULT6B1/MACROD1/RAE1/CBFA2T3/ABHD14B/DHTKD1/SLC2A9/APP/OGDH/NUDT3/ADPGK/ACOT7/VPS9D1/PGAM1/ACSS1/SPHK2/TGFB1/SULT1A2/SLC26A1/SLC22A11/NUP93/ENO3/HK2/SEC13/ACACB/ACSS2/PDE9A/PNP/GUK1/ADCY4/ESRRB/MTHFD1/PPCDC/PAPSS2/PKM/ACACA/ACSF3/AK2/GMPR2/AK5/HMGCL/NPPC/ACAT1/HMGCS1/SCD/TPST1/PFKFB3/SLC25A1/PANK4/IMPDH1/PRKAA1/AOX1/ATP5PD/ACP3
Biological Process	GO:0036336	dendritic cell migration	7/2279	29/18866	0.0530539	0.3690156	0.3338984	7	TRPM2/CALR/CCR2/CCL5/DOCK8/CXCR1/ANO6
Biological Process	GO:0060351	cartilage development involved in endochondral bone morphogenesis	7/2279	29/18866	0.0530539	0.3690156	0.3338984	7	ANXA6/TGFBR2/POC1A/RARA/POR/RARG/NPPC
Biological Process	GO:2000108	positive regulation of leukocyte apoptotic process	7/2279	29/18866	0.0530539	0.3690156	0.3338984	7	IL10/PIK3CD/CCL5/RAPGEF2/ZC3H8/BLK/MEF2C
Biological Process	GO:0098656	anion transmembrane transport	48/2279	315/18866	0.0531198	0.3690156	0.3338984	48	SLC38A4/PRKAG2/CLCN6/SLC38A10/ABCC1/SLC16A3/SLC35C2/SLC24A4/FXYD1/SLC1A5/ANO7/SLC15A4/THBS1/SLC44A4/RIPK1/LRRC8C/SLC13A4/SLC43A2/SLC19A1/SLC26A1/SLC1A2/SLC7A7/SLC7A5/ACACB/SLC1A6/SLC37A1/MFSD10/PER2/SLC16A1/SLC36A3/SLC1A3/LRRC8B/ACACA/SLC43A1/ANO10/SLC2A1/ANO6/SLC7A8/SLC13A5/CPT1A/SLC12A1/ANO8/SLC7A1/SLC12A7/SLC25A1/SLC23A1/AQP9/PACC1
Biological Process	GO:0030513	positive regulation of BMP signaling pathway	8/2279	35/18866	0.0533696	0.3690156	0.3338984	8	MIR140/RBPJ/UBE2O/ILK/NUMA1/ENG/NOTCH1/ZNF423
Biological Process	GO:0034390	smooth muscle cell apoptotic process	8/2279	35/18866	0.0533696	0.3690156	0.3338984	8	PPARG/MIR140/ARRB1/E2F3/MIR24-2/ARRB2/MIR138-2/DNMT1

Biological Process	GO:0034391	regulation of smooth muscle cell apoptotic process	8/2279	35/18866	0.0533696	0.3690156	0.3338984	8	PPARG/MIR140/ARRB1/E2F3/MIR24-2/ARRB2/MIR138-2/DNMT1
Biological Process	GO:0036314	response to sterol	8/2279	35/18866	0.0533696	0.3690156	0.3338984	8	GPLD1/TGFBR2/TGFB1/MLC1/ABCA1/GRAMD1A/HMGCS1/INHBA
Biological Process	GO:0060251	regulation of glial cell proliferation	8/2279	35/18866	0.0533696	0.3690156	0.3338984	8	CREB1/MYB/SKI/TNF/PRKCH/PRKCI/NOTCH1/CERS2
Biological Process	GO:0097009	energy homeostasis	8/2279	35/18866	0.0533696	0.3690156	0.3338984	8	AMPD3/MFSD2A/DLL1/CRTC3/ACACB/FOXO1/EIF4G1/PRKAA1
Biological Process	GO:1900077	negative regulation of cellular response to insulin stimulus	8/2279	35/18866	0.0533696	0.3690156	0.3338984	8	PRKCZ/KANK1/PTPRE/GRB10/PRKCB/TSC2/RELA/PRKAA1
Biological Process	GO:0070301	cellular response to hydrogen peroxide	18/2279	100/18866	0.0533733	0.3690156	0.3338984	18	TRPM2/IL10/LCN2/MYB/TNFAIP3/RIPK1/PAX2/ZNF580/AQP1/RIPK3/PDGFD/ETS1/NFE2L2/IL18RAP/PDGFRB/FOXO1/RELA/PRKAA1
Biological Process	GO:0019693	ribose phosphate metabolic process	63/2279	427/18866	0.0534045	0.3690156	0.3338984	63	FOXK1/HDAC4/PDE4D/PRKAG2/AMPD3/ENO1/HK1/ADCY2/ACSL1/FAR2/TJP2/ADCY9/PFKFB4/ACOT13/SULT6B1/RAE1/CBFA2T3/ABHD14B/DHTKD1/APP/OGDH/NUDT3/ADPGK/ACOT7/VPS9D1/PGAM1/ACSS1/SPHK2/TGFB1/SULT1A2/SLC26A1/UCK2/NUP93/ENO3/HK2/SEC13/ACACB/ACSS2/PDE9A/GUK1/ADCY4/ESRRB/PPCDC/PAPSS2/PKM/ACACA/ACSF3/AK2/GMPR2/AK5/HMGCL/NPPC/ACAT1/HMGCS1/SCD/TPST1/PFKFB3/DHODH/SLC25A1/PANK4/IMPDH1/PRKAA1/ATP5PD
Biological Process	GO:0001936	regulation of endothelial cell proliferation	30/2279	184/18866	0.0534648	0.3691637	0.3340324	30	LRG1/RPTOR/NRP2/IL10/PPARG/PRKCA/TNF/EGFL7/MIR101-2/STAT5A/THBS1/AKT3/MIR24-2/MIR27A/MIR23A/FLT1/FGFR1/ZNF580/NF1/ITGB3/GHRL/PDGFB/VASH1/MEF2C/AGTR1/DYSF/PPP1R16B/TNFSF12/PLCG1/ALDH1A2
Biological Process	GO:0099003	vesicle-mediated transport in synapse	35/2279	220/18866	0.0535649	0.3695861	0.3344146	35	SYNJ2/DENND1A/PRKAR1B/RAP1B/PLD1/DNM2/ITSN1/STON1/GIT1/DDC/SYN3/APBA2/DGKQ/PRKCB/NRXN1/VPS18/SYN2/CTBP2/NUMB/ITGB3/ACTG1/CHRM2/SH3GL1/GSG1L/DNM1/DNM3/P2RX1/DNM1L/SYT17/EPS15/AP3D1/MX1/CYFIP1/DTNBP1/FGF14

Biological Process	GO:0014897	striated muscle hypertrophy	19/2279	107/18866	0.0541214	0.3728845	0.3373991	19	HDAC4/CTDP1/TTN/MIR145/AKAP13/TNFRSF1B/SMAD3/PRKCA/MIR199A1/MIR24-2/MIR23A/TNFRSF1A/CAMTA2/PDE9A/MIR199A2/NOTCH1/MEF2C/FOXO1/INPP5F
Biological Process	GO:0030148	sphingolipid biosynthetic process	19/2279	107/18866	0.0541214	0.3728845	0.3373991	19	SPTLC2/TNF/ORMDL3/CERS6/TNFRSF1A/SPHK2/SGMS1/ST8SIA6/SGMS2/B4GALT3/SGPP1/SGPP2/P2RX1/FA2H/CERS2/SIRT3/PRKAA1/TLCD3B/PLPP3
Biological Process	GO:0016051	carbohydrate biosynthetic process	34/2279	213/18866	0.0542422	0.373446	0.3379072	34	CSGALNACT1/MAEA/ENO1/AP2A1/C1QTNF3/ST6GALNAC1/CHST11/PGP/CHST15/NDST1/PGAM1/P2RY6/PC/TGFB1/HRH1/DGKQ/ENO3/DYRK2/ST8SIA6/PDGFB/ST3GAL4/PPP1CB/IMPA2/ESRRB/PER2/PCK2/SLC2A1/FOXO1/SOGA1/SLC25A1/HAS3/ST3GAL2/B3GNT2/SDHAF3
Biological Process	GO:0003279	cardiac septum development	20/2279	114/18866	0.0546636	0.3760745	0.3402856	20	LTBP1/ZFPM1/NRP2/MAML1/DNM2/SUFU/GJA5/TGFBR2/NDST1/RBPJ/SMAD7/WNT11/RARA/ENG/NOTCH1/SLIT3/ANK2/LMO4/CPLANE1/NSD2
Biological Process	GO:0031056	regulation of histone modification	25/2279	149/18866	0.0551653	0.3792513	0.34316	25	GFI1/BRD4/MYB/SKI/ARRB1/C6orf89/OTUB2/RPS6KA4/SPHK2/ZBTB7B/PAX7/CTBP1/KDM4C/ZNF335/PHF19/OTUB1/DNMT1/PIH1D1/TRIP12/SREBF1/RNF40/KAT7/LIF/PAXIP1/NSD3
Biological Process	GO:0046939	nucleotide phosphorylation	23/2279	135/18866	0.0553245	0.3800709	0.3439017	23	FOXK1/HDAC4/PRKAG2/ENO1/HK1/PFKFB4/RAE1/CBFA2T3/DHTKD1/APP/OGDH/ADPGK/PGAM1/NUP93/ENO3/HK2/SEC13/GUK1/ESRRB/PKM/AK5/PFKFB3/PRKAA1
Biological Process	GO:0009620	response to fungus	11/2279	54/18866	0.0554355	0.3802833	0.3440938	11	DEFA4/ELANE/S100A8/CTSG/MPO/COTL1/SPON2/SCIMP/BCL10/C10orf99/LTF
Biological Process	GO:0032757	positive regulation of interleukin-8 production	11/2279	54/18866	0.0554355	0.3802833	0.3440938	11	ELANE/TNF/TLR9/RIPK1/ZNF580/FCN1/BCL10/NOD2/F2R/RELA/FADD
Biological Process	GO:0071300	cellular response to retinoic acid	13/2279	67/18866	0.0556428	0.3814299	0.3451313	13	PPARG/CREB1/MYB/TESC/WNT11/PAX2/AQP1/RARG/WNT5B/ABCA1/GJB3/TEAD2/ALDH1A2

Biological Process	GO:0048013	ephrin receptor signaling pathway	16/2279	87/18866	0.0558959	0.3828888	0.3464514	16	AP2A1/ARHGEF7/NCK2/EPHB3/ITSN1/GIT1/RBPJ/SDC2/WASL/VAV2/ACTG1/SIPA1L1/DNM1/NTRK1/ARHGEF28/VAV3
Biological Process	GO:0044262	cellular carbohydrate metabolic process	45/2279	294/18866	0.0559996	0.3833228	0.3468441	45	CSGALNACT1/MAEA/FOXK1/HDAC4/PRKAG2/SYNJ2/NAGK/HK1/C1QTNF3/INPP5A/PFKFB4/GPLD1/AOAH/MGAM/CBFA2T3/PGP/NDST1/ENOSF1/PGAM1/P2RY6/HRH1/DGKQ/DYRK2/HK2/GNB3/ACACB/INPP5E/PPP1CB/IMPA2/ESRRB/PER2/PRKCE/PCK2/PCDH12/SLC2A1/FOXO1/DYSF/NCOA2/SOGA1/PFKFB3/HAS3/B3GNT2/PRKN/SDHAF3/RUBCNL
Biological Process	GO:0051304	chromosome separation	17/2279	94/18866	0.0570475	0.3877581	0.3508573	17	MAD1L1/NSMCE2/RECQL5/CUL3/EME1/MEIOB/CDC16/TACC3/NUMA1/PPP2R1A/NEK6/RB1/DIS3L2/ANAPC7/HECW2/SLX4/PCID2
Biological Process	GO:0010885	regulation of cholesterol storage	5/2279	18/18866	0.0570965	0.3877581	0.3508573	5	EHD1/PPARG/ABCG1/NR1H2/ABCA1
Biological Process	GO:0034199	activation of protein kinase A activity	5/2279	18/18866	0.0570965	0.3877581	0.3508573	5	ADCY2/PRKAR1B/ADCY9/ADCY4/PRRC1
Biological Process	GO:0043217	myelin maintenance	5/2279	18/18866	0.0570965	0.3877581	0.3508573	5	SH3TC2/NDRG1/CLU/EPB41L3/FA2H
Biological Process	GO:0051782	negative regulation of cell division	5/2279	18/18866	0.0570965	0.3877581	0.3508573	5	BLM/MIR145/E2F7/C10orf99/ZFYVE19
Biological Process	GO:0051923	sulfation	5/2279	18/18866	0.0570965	0.3877581	0.3508573	5	HS3ST3B1/CHSY1/NDST1/SULT1A2/TPST1
Biological Process	GO:0071545	inositol phosphate catabolic process	5/2279	18/18866	0.0570965	0.3877581	0.3508573	5	SYNJ2/INPP5A/NUDT3/INPP5E/IMPA2

Biological Process	GO:0099010	modification of postsynaptic structure	5/2279	18/18866	0.0570965	0.3877581	0.3508573	5	ITSN1/BAIAP2/PFN1/CYFIP1/SYNPO
Biological Process	GO:1901524	regulation of mitophagy	5/2279	18/18866	0.0570965	0.3877581	0.3508573	5	VPS13D/SMURF1/CLEC16A/TSC2/PRKN
Biological Process	GO:1905049	negative regulation of metallopeptidase activity	5/2279	18/18866	0.0570965	0.3877581	0.3508573	5	TIMP2/SORL1/MIR199A1/MIR24-2/MIR199A2
Biological Process	GO:1990000	amyloid fibril formation	5/2279	18/18866	0.0570965	0.3877581	0.3508573	5	GSN/RIPK1/APP/CLU/RIPK3
Biological Process	GO:0060401	cytosolic calcium ion transport	29/2279	178/18866	0.0572645	0.3884654	0.3514972	29	TRPM2/PDE4D/CASQ2/ITPR2/LCK/CACNA1C/SLC8A1/UBASH3B/ITPR1/JSRP1/PTPN6/RASA3/P2RY6/MYO5A/HTT/PTPRC/ATP2A3/NOL3/TPCN2/BCL2/GSTO1/PRKCE/F2R/ANK2/CHERP/PLCG1/P2RX5/TRPV2/CASQ1
Biological Process	GO:0034121	regulation of toll-like receptor signaling pathway	14/2279	74/18866	0.0578771	0.3884654	0.3514972	14	GFI1/GRAMD4/MIR140/SMPDL3B/TNFAIP3/TLR9/RTN4/TREML4/ARRB2/WDFY1/LTF/TYRO3/FLOT1/PELI1
Biological Process	GO:0051865	protein autoubiquitination	14/2279	74/18866	0.0578771	0.3884654	0.3514972	14	RNF220/WWP2/AMFR/TRAF2/CUL3/UHRF1/MTA1/SH3RF3/RNF213/UBE3A/RNF11/UBE2D2/PRKN/MARCHF7
Biological Process	GO:0014911	positive regulation of smooth muscle cell migration	10/2279	48/18866	0.0578953	0.3884654	0.3514972	10	HDAC4/SSH1/MIR143/CCL5/P2RY6/PDGFD/PDGFB/BCL2/PDGFRB/CCN4
Biological Process	GO:0042461	photoreceptor cell development	10/2279	48/18866	0.0578953	0.3884654	0.3514972	10	MFSD2A/CRB1/GNAT2/IFT140/PRKCI/RPGRIP1/CDHR1/NRL/CABP4/NPHP4

Biological Process	GO:0043631	RNA polyadenylation	10/2279	48/18866	0.0578953	0.3884654	0.3514972	10	FIP1L1/CPSF3/APP/CPEB3/SCAF8/ZC3H3/RNF40/TUT1/PAPOLG/GRSF1
Biological Process	GO:0046461	neutral lipid catabolic process	10/2279	48/18866	0.0578953	0.3884654	0.3514972	10	PNPLA2/SORL1/MGLL/LIPC/ABHD16A/GPLD1/ABHD16B/FABP6/ABHD5/LYPLA2
Biological Process	GO:0046464	acylglycerol catabolic process	10/2279	48/18866	0.0578953	0.3884654	0.3514972	10	PNPLA2/SORL1/MGLL/LIPC/ABHD16A/GPLD1/ABHD16B/FABP6/ABHD5/LYPLA2
Biological Process	GO:0099637	neurotransmitter receptor transport	10/2279	48/18866	0.0578953	0.3884654	0.3514972	10	DNM2/NUMB/ITGB3/KIF5C/GSG1L/DNM1/DNM3/EPS15/AP3D1/MX1
Biological Process	GO:0060560	developmental growth involved in morphogenesis	37/2279	236/18866	0.0579003	0.3884654	0.3514972	37	SEMA6B/PRKCZ/CPNE6/NRP2/ULK1/CTTN/DNM2/CPNE5/ZEB2/CYFIP2/BCL11A/SEMA4A/RTN4/TGFBR2/DISC1/SEMA4B/APP/IFRD1/ILK/CPNE1/WNT11/SEMA4D/RDH10/SMURF1/RARG/CDH4/NOTCH1/CDKL3/SYT17/SLIT3/CYFIP1/TRPV2/NDEL1/SLIT1/DBNL/SPART/PRKN
Biological Process	GO:0010522	regulation of calcium ion transport into cytosol	18/2279	101/18866	0.0579155	0.3884654	0.3514972	18	PDE4D/CASQ2/CACNA1C/SLC8A1/UBASH3B/JSRP1/PTPN6/P2RY6/MYO5A/HTT/BCL2/GSTO1/PRKCE/F2R/ANK2/PLCG1/P2RX5/CASQ1
Biological Process	GO:0032637	interleukin-8 production	18/2279	101/18866	0.0579155	0.3884654	0.3514972	18	ELANE/IL10/CHI3L1/ARRB1/TNF/TLR9/RIPK1/C5AR2/ZNF580/FCN1/PTPRC/BCL10/NOD2/F2R/RELA/CD33/BCL3/FADD
Biological Process	GO:0042116	macrophage activation	18/2279	101/18866	0.0579155	0.3884654	0.3514972	18	AZU1/FOXP1/SBNO2/IL10/MIR145/SLC11A1/TNF/MYO18A/FPR2/THBS1/APP/CLU/CRTC3/PTPRC/GRN/ITGB2/PRKCE/DYSF
Biological Process	GO:0071559	response to transforming growth factor beta	40/2279	258/18866	0.0580982	0.3884654	0.3514972	40	LRG1/PRKCZ/LTBP1/SPRED2/CREB1/SKI/TGFBRAP1/SMAD3/COL4A2/LDLRAD4/DNM2/HIPK2/DUSP22/CHST11/ZFHX3/TGFBR2/THBS1/MIR27A/SMAD7/PXN/ZMIZ1/ITGB5/TGFB1/SMURF1/PDGFD/TGFB1I1/HTRA4/CREBBP/ENG/ARRB2/PRDM16/ARHGEF18/GCNT2/MEF2C/HTRA3/RUNX3/ANKRD1/NODAL/ZFYVE9/CX3CR1

Biological Process	GO:0010770	positive regulation of cell morphogenesis involved in differentiation	26/2279	157/18866	0.0585913	0.3884654	0.3514972	26	P4HB/PLXNC1/RREB1/CALR/DOCK1/ARHGEF7/DNM2/ZEB2/DISC1/CUX1/BAIAP2/ILK/PREX1/SEMA4D/SS18L2/OLFM4/CDH4/CASS4/CDKL3/DNM1L/CYFIP1/TRPV2/NDEL1/MYADM/CUX2/DBNL
Biological Process	GO:0003170	heart valve development	12/2279	61/18866	0.0588349	0.3884654	0.3514972	12	ZFPM1/TNFRSF1B/GJA5/NFATC1/TGFBR2/RBPJ/TNFRSF1A/TGFB1/RB1/NOTCH1/MEF2C/SLIT3
Biological Process	GO:0016266	O-glycan processing	12/2279	61/18866	0.0588349	0.3884654	0.3514972	12	GALNT2/XXYLT1/GALNT12/MUC12/GXYLT2/MUC20/B3GNT5/ST6GAL1/ST3GAL4/GALNT9/ST3GAL2/B3GNT2
Biological Process	GO:0033619	membrane protein proteolysis	12/2279	61/18866	0.0588349	0.3884654	0.3514972	12	PRTN3/TIMP2/IL10/TNFRSF1B/MBTPS1/GPLD1/TNF/MYH9/HM13/CTSH/TGFB1/RELA
Biological Process	GO:0042267	natural killer cell mediated cytotoxicity	12/2279	61/18866	0.0588349	0.3884654	0.3514972	12	LYST/PIK3R6/SLAMF7/TUBB4B/RAB27A/CD226/HLA-F/PTPN6/NCR1/ARRB2/IL18RAP/CADM1
Biological Process	GO:0045576	mast cell activation	12/2279	61/18866	0.0588349	0.3884654	0.3514972	12	LAT/PIK3CD/RHOH/NDRG1/GAB2/FGR/CD84/CD226/SPHK2/FER/BLK/CNR2
Biological Process	GO:0046324	regulation of glucose import	12/2279	61/18866	0.0588349	0.3884654	0.3514972	12	PRKAG2/MIR143/PIK3R1/TNF/GRB10/SLC1A2/HK2/PRKCI/NFE2L2/RTN2/ASPSCR1/CLTCL1
Biological Process	GO:1902041	regulation of extrinsic apoptotic signaling pathway via death domain receptors	12/2279	61/18866	0.0588349	0.3884654	0.3514972	12	ITPRIP/TRAF2/TNFAIP3/THBS1/RIPK1/BMPR1B/STK3/TRPS1/SP100/ARHGEF2/BCL2L1/FADD
Biological Process	GO:0021782	glial cell development	21/2279	122/18866	0.0592132	0.3884654	0.3514972	21	ARHGEF10/S100A8/SH3TC2/CD9/GSN/SKI/LGI4/NDRG1/TNF/FPR2/EIF2B5/DLL1/APP/ILK/CLU/NF1/GRN/TSPAN2/FA2H/CNTNAP1/ERCC2

Biological Process	GO:0072593	reactive oxygen species metabolic process	44/2279	288/18866	0.0596819	0.3884654	0.3514972	44	HDAC4/MPO/IL10/CD177/NCF4/SMAD3/LPO/DNM2/MIR199A1/TNF/SFTPD/FPR2/SH3PXD2B/TGFBR2/THBS1/RAB27A/RIPK1/GNAI2/NOS1AP/MIR24-2/PREX1/CLU/NCF2/PAX2/SPHK2/TGFB1/HBB/RIPK3/POR/HK2/PDGFB/BCR/CAT/NFE2L2/ITGB2/BCL2/MIR199A2/FOXM1/AGTR1/PDGFRB/FOXO1/CX3CR1/SIRT3/PRKN
Biological Process	GO:0001504	neurotransmitter uptake	9/2279	42/18866	0.0600498	0.3884654	0.3514972	9	SLC1A2/ITGB3/SLC1A6/PER2/SYNGR3/SLC1A3/SLC29A1/FLOT1/PRKN
Biological Process	GO:1904037	positive regulation of epithelial cell apoptotic process	9/2279	42/18866	0.0600498	0.3884654	0.3514972	9	GSN/MIR101-2/THBS1/MIR24-2/PRKCI/ARRB2/SORT1/ANO6/PLCG1
Biological Process	GO:0002407	dendritic cell chemotaxis	6/2279	24/18866	0.0611964	0.3884654	0.3514972	6	TRPM2/CALR/CCR2/CCL5/CXCR1/ANO6
Biological Process	GO:0002719	negative regulation of cytokine production involved in immune response	6/2279	24/18866	0.0611964	0.3884654	0.3514972	6	IL10/TNF/HLA-F/SMAD7/TGFB1/CD96
Biological Process	GO:0034035	purine ribonucleoside bisphosphate metabolic process	6/2279	24/18866	0.0611964	0.3884654	0.3514972	6	SULT6B1/ABHD14B/SULT1A2/SLC26A1/PAPSS2/TPST1
Biological Process	GO:0034629	cellular protein-containing complex localization	6/2279	24/18866	0.0611964	0.3884654	0.3514972	6	NACC2/SMAD7/WASL/KLHL21/CEP72/EZR
Biological Process	GO:0034695	response to prostaglandin E	6/2279	24/18866	0.0611964	0.3884654	0.3514972	6	PPARG/P2RY6/GNG2/ACACA/PRKCE/PRKAA1
Biological Process	GO:0048745	smooth muscle tissue development	6/2279	24/18866	0.0611964	0.3884654	0.3514972	6	MIR143/MIR145/MYLK/NF1/ENG/PDGFRB

Biological Process	GO:0050427	3'-phosphoadenosine 5'-phosphosulfate metabolic process	6/2279	24/18866	0.0611964	0.3884654	0.3514972	6	SULT6B1/ABHD14B/SULT1A2/SLC26A1/PAPSS2/TPST1
Biological Process	GO:0090208	positive regulation of triglyceride metabolic process	6/2279	24/18866	0.0611964	0.3884654	0.3514972	6	PNPLA2/MFSD2A/GPLD1/NR1H2/ABHD5/SREBF1
Biological Process	GO:0097186	amelogenesis	6/2279	24/18866	0.0611964	0.3884654	0.3514972	6	CSF3R/SLC24A4/PERP/FOXO1/FAM20C/NECTIN1
Biological Process	GO:2000209	regulation of anoikis	6/2279	24/18866	0.0611964	0.3884654	0.3514972	6	CEACAM6/TLE1/BCL2/NOTCH1/CHEK2/CEACAM5
Biological Process	GO:0031647	regulation of protein stability	45/2279	296/18866	0.0613516	0.3884654	0.3514972	45	TRIM39/CALR/GSN/CREB1/CCDC88C/AAK1/PIK3R1/SMAD3/FBXO7/MIR101-2/RTN4/USP3/CUL3/TESC/CTSH/USP36/CLU/SMAD7/ASGR2/PHB/PFN1/HTT/ATF7IP/STK3/GRN/CREBBP/CCNH/MORC3/BCL2/ZBED3/CHEK2/NAA16/USP2/WDR81/ANK2/ASPH/SREBF1/FBXW11/FLOT1/BAG5/CCT6A/PRKN/P3H1/MARCHF7/PLPP3
Biological Process	GO:0002244	hematopoietic progenitor cell differentiation	28/2279	172/18866	0.061357	0.3884654	0.3514972	28	CEBPD/KCNAB2/MYB/RUNX1/CSF1R/RBM47/ACP6/TMEM91/DHTKD1/PTPN6/TCF12/FLT1/TGFB1/JAM3/PTPRC/SETD1A/PSMF1/NFE2L2/PUS7/BCL2/TCF3/PSMB7/NOTCH1/SSBP3/INHBA/ERCC2/PSMD13/AGPAT5
Biological Process	GO:0016239	positive regulation of macroautophagy	13/2279	68/18866	0.0615082	0.3884654	0.3514972	13	SPTLC2/ULK1/VPS13D/LARP1/PIP4K2A/UVRAG/SMURF1/RUFY4/CLEC16A/HTT/TSC2/STING1/PRKN
Biological Process	GO:0040014	regulation of multicellular organism growth	13/2279	68/18866	0.0615082	0.3884654	0.3514972	13	CREB1/MFSD2A/SH3PXD2B/STAT5A/APP/GHRL/FTO/BCL2/RAI1/NPPC/MBD5/EZR/SPTBN4
Biological Process	GO:0072665	protein localization to vacuole	13/2279	68/18866	0.0615082	0.3884654	0.3514972	13	SORL1/VTI1A/VPS13D/TNFAIP3/RTN4/CLU/SMURF1/SH3BP4/NUMA1/SORT1/NEDD4/AP3D1/NCOA4

Biological Process	GO:0003230	cardiac atrium development	8/2279	36/18866	0.0616473	0.3884654	0.3514972	8	ZFPM1/GJA5/CCM2L/WNT11/ENG/NOTCH1/ANK2/NSD2
Biological Process	GO:0006007	glucose catabolic process	8/2279	36/18866	0.0616473	0.3884654	0.3514972	8	FOXK1/ENO1/HK1/ADPGK/PGAM1/ENO3/HK2/PKM
Biological Process	GO:0014808	release of sequestered calcium ion into cytosol by sarcoplasmic reticulum	8/2279	36/18866	0.0616473	0.3884654	0.3514972	8	PDE4D/CASQ2/CACNA1C/SLC8A1/NOL3/GSTO1/ANK2/CASQ1
Biological Process	GO:0031572	G2 DNA damage checkpoint	8/2279	36/18866	0.0616473	0.3884654	0.3514972	8	TRIM39/BLM/FZR1/FOXN3/TAOK3/CLSPN/ABRAXAS1/BABAM2
Biological Process	GO:0043368	positive T cell selection	8/2279	36/18866	0.0616473	0.3884654	0.3514972	8	ZFPM1/ITPKB/LOXL3/LY9/PTPRC/BCL2/CYLD/DOCK2
Biological Process	GO:0050931	pigment cell differentiation	8/2279	36/18866	0.0616473	0.3884654	0.3514972	8	ZEB2/RAB27A/MYO5A/GNA11/BCL2/MEF2C/HPS6/AP3D1
Biological Process	GO:0071634	regulation of transforming growth factor beta production	8/2279	36/18866	0.0616473	0.3884654	0.3514972	8	LTBP1/CREB1/MYB/SMAD3/THBS1/WNT11/CDH3/ITGB6
Biological Process	GO:2000516	positive regulation of CD4-positive, alpha-beta T cell activation	8/2279	36/18866	0.0616473	0.3884654	0.3514972	8	PRKCZ/MYB/CD55/NLRP3/TGFBR2/ZBTB7B/RARA/HLX
Biological Process	GO:0002138	retinoic acid biosynthetic process	3/2279	8/18866	0.0617747	0.3884654	0.3514972	3	DHRS9/RDH10/ALDH1A2

Biological Process	GO:0003062	regulation of heart rate by chemical signal	3/2279	8/18866	0.0617747	0.3884654	0.3514972	3	PDE4D/NOS1AP/SREBF1
Biological Process	GO:0003433	chondrocyte development involved in endochondral bone morphogenesis	3/2279	8/18866	0.0617747	0.3884654	0.3514972	3	TGFBR2/POC1A/RARG
Biological Process	GO:0006477	protein sulfation	3/2279	8/18866	0.0617747	0.3884654	0.3514972	3	HS3ST3B1/NDST1/TPST1
Biological Process	GO:0007172	signal complex assembly	3/2279	8/18866	0.0617747	0.3884654	0.3514972	3	NCK2/PXN/MAPK8IP2
Biological Process	GO:0030853	negative regulation of granulocyte differentiation	3/2279	8/18866	0.0617747	0.3884654	0.3514972	3	INPP5D/RARA/TRIB1
Biological Process	GO:0030854	positive regulation of granulocyte differentiation	3/2279	8/18866	0.0617747	0.3884654	0.3514972	3	RUNX1/TESC/TRIB1
Biological Process	GO:0032287	peripheral nervous system myelin maintenance	3/2279	8/18866	0.0617747	0.3884654	0.3514972	3	SH3TC2/NDRG1/FA2H
Biological Process	GO:0032906	transforming growth factor beta2 production	3/2279	8/18866	0.0617747	0.3884654	0.3514972	3	SMAD3/WNT11/CDH3
Biological Process	GO:0032909	regulation of transforming growth factor beta2 production	3/2279	8/18866	0.0617747	0.3884654	0.3514972	3	SMAD3/WNT11/CDH3

Biological Process	GO:0034091	regulation of maintenance of sister chromatid cohesion	3/2279	8/18866	0.0617747	0.3884654	0.3514972	3	TNKS/NSMCE2/SLF1
Biological Process	GO:0034182	regulation of maintenance of mitotic sister chromatid cohesion	3/2279	8/18866	0.0617747	0.3884654	0.3514972	3	TNKS/NSMCE2/SLF1
Biological Process	GO:0035425	autocrine signaling	3/2279	8/18866	0.0617747	0.3884654	0.3514972	3	S100A8/CD68/CX3CR1
Biological Process	GO:0035655	interleukin-18-mediated signaling pathway	3/2279	8/18866	0.0617747	0.3884654	0.3514972	3	PDGFB/IL18RAP/IL18R1
Biological Process	GO:0035733	hepatic stellate cell activation	3/2279	8/18866	0.0617747	0.3884654	0.3514972	3	RPS6KA1/MYB/PDGFRB
Biological Process	GO:0035744	T-helper 1 cell cytokine production	3/2279	8/18866	0.0617747	0.3884654	0.3514972	3	IL1R1/IL18RAP/IL18R1
Biological Process	GO:0036289	peptidyl-serine autophosphorylation	3/2279	8/18866	0.0617747	0.3884654	0.3514972	3	MARK3/ERN1/RIPK1
Biological Process	GO:0038203	TORC2 signaling	3/2279	8/18866	0.0617747	0.3884654	0.3514972	3	PRR5L/SIK3/PRR5
Biological Process	GO:0046476	glycosylceramide biosynthetic process	3/2279	8/18866	0.0617747	0.3884654	0.3514972	3	B4GALT3/FA2H/PRKAA1

Biological Process	GO:0048840	otolith development	3/2279	8/18866	0.0617747	0.3884654	0.3514972	3	SLC44A4/LRIG1/TTC39C
Biological Process	GO:0050932	regulation of pigment cell differentiation	3/2279	8/18866	0.0617747	0.3884654	0.3514972	3	ZEB2/GNA11/BCL2
Biological Process	GO:0062043	positive regulation of cardiac epithelial to mesenchymal transition	3/2279	8/18866	0.0617747	0.3884654	0.3514972	3	TGFBR2/ENG/NOTCH1
Biological Process	GO:0070943	neutrophil-mediated killing of symbiont cell	3/2279	8/18866	0.0617747	0.3884654	0.3514972	3	AZU1/ELANE/CTSG
Biological Process	GO:0071351	cellular response to interleukin-18	3/2279	8/18866	0.0617747	0.3884654	0.3514972	3	PDGFB/IL18RAP/IL18R1
Biological Process	GO:0072396	response to cell cycle checkpoint signaling	3/2279	8/18866	0.0617747	0.3884654	0.3514972	3	EME1/SLX4/FBH1
Biological Process	GO:0072402	response to DNA integrity checkpoint signaling	3/2279	8/18866	0.0617747	0.3884654	0.3514972	3	EME1/SLX4/FBH1
Biological Process	GO:0072423	response to DNA damage checkpoint signaling	3/2279	8/18866	0.0617747	0.3884654	0.3514972	3	EME1/SLX4/FBH1
Biological Process	GO:0097692	histone H3-K4 monomethylation	3/2279	8/18866	0.0617747	0.3884654	0.3514972	3	KMT2D/SETD1B/SETD1A

Biological Process	GO:0099004	calmodulin dependent kinase signaling pathway	3/2279	8/18866	0.0617747	0.3884654	0.3514972	3	CAMKK2/HTT/PRKAA1
Biological Process	GO:0099541	trans-synaptic signaling by lipid	3/2279	8/18866	0.0617747	0.3884654	0.3514972	3	NRXN1/PLCB1/F2R
Biological Process	GO:0099542	trans-synaptic signaling by endocannabinoid	3/2279	8/18866	0.0617747	0.3884654	0.3514972	3	NRXN1/PLCB1/F2R
Biological Process	GO:1900125	regulation of hyaluronan biosynthetic process	3/2279	8/18866	0.0617747	0.3884654	0.3514972	3	AP2A1/TGFB1/PDGFB
Biological Process	GO:1901339	regulation of store-operated calcium channel activity	3/2279	8/18866	0.0617747	0.3884654	0.3514972	3	CASQ1/STIMATE/CRACR2A
Biological Process	GO:1901523	icosanoid catabolic process	3/2279	8/18866	0.0617747	0.3884654	0.3514972	3	ABHD16A/CYP4F3/LYPLA2
Biological Process	GO:1901724	positive regulation of cell proliferation involved in kidney development	3/2279	8/18866	0.0617747	0.3884654	0.3514972	3	PDGFD/IL6R/PDGFB
Biological Process	GO:1902455	negative regulation of stem cell population maintenance	3/2279	8/18866	0.0617747	0.3884654	0.3514972	3	MIR145/PAX2/ZNF706
Biological Process	GO:1902965	regulation of protein localization to early endosome	3/2279	8/18866	0.0617747	0.3884654	0.3514972	3	SORL1/MGAT3/EZR

Biological Process	GO:1902966	positive regulation of protein localization to early endosome	3/2279	8/18866	0.0617747	0.3884654	0.3514972	3	SORL1/MGAT3/EZR
Biological Process	GO:1903564	regulation of protein localization to cilium	3/2279	8/18866	0.0617747	0.3884654	0.3514972	3	CCDC66/LZTFL1/INPP5E
Biological Process	GO:1903719	regulation of I-kappaB phosphorylation	3/2279	8/18866	0.0617747	0.3884654	0.3514972	3	TNF/TRAF2/CX3CR1
Biological Process	GO:1904238	pericyte cell differentiation	3/2279	8/18866	0.0617747	0.3884654	0.3514972	3	PDGFB/NOTCH1/GPR4
Biological Process	GO:2000074	regulation of type B pancreatic cell development	3/2279	8/18866	0.0617747	0.3884654	0.3514972	3	RHEB/RFX3/ARNTL
Biological Process	GO:2000322	regulation of glucocorticoid receptor signaling pathway	3/2279	8/18866	0.0617747	0.3884654	0.3514972	3	PHB/ARNTL/PER1
Biological Process	GO:0010001	glial cell differentiation	36/2279	230/18866	0.0618764	0.3884654	0.3514972	36	ARHGEF10/S100A8/SH3TC2/CD9/PPARG/GSN/SKI/LGI4/TNFRSF1B/CRB1/NDRG1/RHEB/TNF/FPR2/EIF2B5/BIN1/DLL1/APP/ILK/CLU/TNFRSF21/HDAC1/PAX2/TGFB1/NF1/CSK/GRN/WDR1/TTC21B/NOTCH1/TSPAN2/FA2H/CNTNAP1/RELA/ERCC2/LIF
Biological Process	GO:0098657	import into cell	36/2279	230/18866	0.0618764	0.3884654	0.3514972	36	TRPM2/KCNJ15/SLC2A5/ACSL1/FXYD2/SLC1A5/SLC8A1/DNM2/SLC11A2/THBS1/SLC43A2/SLC19A1/SLC1A2/SLC7A5/NUMB/ITGB3/SLC1A6/GSG1L/DNM1/PER2/SYNGR3/SLC1A3/DNM3/EPS15/LTF/SLC43A1/SLC2A1/SLC7A8/MX1/SLC29A1/TRPV2/SLC12A1/FLOT1/SLC7A1/SLC12A7/PRKN
Biological Process	GO:0006684	sphingomyelin metabolic process	4/2279	13/18866	0.0620427	0.3884654	0.3514972	4	SPTLC2/SMPDL3B/SGMS1/SGMS2

Biological Process	GO:0007171	activation of transmembrane receptor protein tyrosine kinase activity	4/2279	13/18866	0.0620427	0.3884654	0.3514972	4	PILRB/ANGPT1/DGKQ/PDGFC
Biological Process	GO:0015801	aromatic amino acid transport	4/2279	13/18866	0.0620427	0.3884654	0.3514972	4	SLC15A4/SLC7A5/SLC7A8/SLC7A1
Biological Process	GO:0031223	auditory behavior	4/2279	13/18866	0.0620427	0.3884654	0.3514972	4	NRXN1/HTT/NRXN2/SLC1A3
Biological Process	GO:0032530	regulation of microvillus organization	4/2279	13/18866	0.0620427	0.3884654	0.3514972	4	PLD1/ATP8B1/CDHR2/EZR
Biological Process	GO:0035563	positive regulation of chromatin binding	4/2279	13/18866	0.0620427	0.3884654	0.3514972	4	HMBOX1/CTBP2/MED25/PARP9
Biological Process	GO:0042308	negative regulation of protein import into nucleus	4/2279	13/18866	0.0620427	0.3884654	0.3514972	4	ANGPT1/SUFU/NF1/CABP1
Biological Process	GO:0051764	actin crosslink formation	4/2279	13/18866	0.0620427	0.3884654	0.3514972	4	BAIAP2L1/BAIAP2/ACTN1/BAIAP2L2
Biological Process	GO:0060312	regulation of blood vessel remodeling	4/2279	13/18866	0.0620427	0.3884654	0.3514972	4	ABR/MIR143/TGFB1/BCR
Biological Process	GO:0060456	positive regulation of digestive system process	4/2279	13/18866	0.0620427	0.3884654	0.3514972	4	AQP1/NR1H2/GHRL/SLC22A5

Biological Process	GO:0071415	cellular response to purine-containing compound	4/2279	13/18866	0.0620427	0.3884654	0.3514972	4	TRPM2/CASQ2/SLC8A1/P2RY6
Biological Process	GO:0090677	reversible differentiation	4/2279	13/18866	0.0620427	0.3884654	0.3514972	4	MIR140/MIR145/PDGFB/DNMT1
Biological Process	GO:0098712	L-glutamate import across plasma membrane	4/2279	13/18866	0.0620427	0.3884654	0.3514972	4	SLC1A2/SLC1A6/PER2/SLC1A3
Biological Process	GO:1902916	positive regulation of protein polyubiquitination	4/2279	13/18866	0.0620427	0.3884654	0.3514972	4	NOD2/RNF40/PRKN/MARCHF7
Biological Process	GO:1903236	regulation of leukocyte tethering or rolling	4/2279	13/18866	0.0620427	0.3884654	0.3514972	4	ELANE/FUT4/FUT7/CCR2
Biological Process	GO:1904590	negative regulation of protein import	4/2279	13/18866	0.0620427	0.3884654	0.3514972	4	ANGPT1/SUFU/NF1/CABP1
Biological Process	GO:0001954	positive regulation of cell-matrix adhesion	11/2279	55/18866	0.0620495	0.3884654	0.3514972	11	PRKCZ/RIN2/ITGB1BP1/CEACAM6/SMAD3/JUP/DISC1/NINJ1/ILK/PTPRJ/PLEKHA2
Biological Process	GO:0032720	negative regulation of tumor necrosis factor production	11/2279	55/18866	0.0620495	0.3884654	0.3514972	11	TRIM27/IL10/MIR140/TNFAIP3/C5AR2/PTPN6/RARA/ARRB2/CD33/VSIR/ILRUN
Biological Process	GO:0045620	negative regulation of lymphocyte differentiation	11/2279	55/18866	0.0620495	0.3884654	0.3514972	11	RUNX1/LOXL3/FBXO7/SMAD7/ZBTB7B/ZC3H8/PGLYRP1/HLX/RUNX3/INHBA/TMEM131L

Biological Process	GO:0071825	protein-lipid complex subunit organization	11/2279	55/18866	0.0620495	0.3884654	0.3514972	11	P4HB/MPO/MFSD2A/LIPC/PLAGL2/ABCG1/PLTP/BIN1/NR1H2/ABCA1/AGTR1
Biological Process	GO:0000132	establishment of mitotic spindle orientation	7/2279	30/18866	0.0622706	0.3884654	0.3514972	7	DCTN1/NDE1/HTT/NUMA1/ARHGEF2/FBXW11/NDEL1
Biological Process	GO:0001844	protein insertion into mitochondrial membrane involved in apoptotic signaling pathway	7/2279	30/18866	0.0622706	0.3884654	0.3514972	7	TFDP1/YWHAH/BMF/YWHAZ/BCL2/TP53BP2/YWHAQ
Biological Process	GO:0034110	regulation of homotypic cell-cell adhesion	7/2279	30/18866	0.0622706	0.3884654	0.3514972	7	CD9/PRKCA/UBASH3B/CCL5/CCM2L/JAK1/BLK
Biological Process	GO:0035066	positive regulation of histone acetylation	7/2279	30/18866	0.0622706	0.3884654	0.3514972	7	ARRB1/RPS6KA4/SPHK2/PIH1D1/KAT7/LIF/PAXIP1
Biological Process	GO:0048147	negative regulation of fibroblast proliferation	7/2279	30/18866	0.0622706	0.3884654	0.3514972	7	PPARG/SKI/B4GALT7/NF1/MORC3/MED25/PARP10
Biological Process	GO:0051491	positive regulation of filopodium assembly	7/2279	30/18866	0.0622706	0.3884654	0.3514972	7	ZMYND8/ARAP1/WASL/ESPN/PALM/RALA/DNM3
Biological Process	GO:0099625	ventricular cardiac muscle cell membrane repolarization	7/2279	30/18866	0.0622706	0.3884654	0.3514972	7	KCNE1/GJA5/NOS1AP/WDR1/SNTA1/KCNQ1/ANK2
Biological Process	GO:1904837	beta-catenin-TCF complex assembly	7/2279	30/18866	0.0622706	0.3884654	0.3514972	7	ASH2L/TCF7L2/HDAC1/TLE1/KMT2D/CREBBP/RUVBL1

Biological Process	GO:1905144	response to acetylcholine	7/2279	30/18866	0.0622706	0.3884654	0.3514972	7	LY6G6D/GNAI2/GNA11/CHRM2/PLCB1/RGS10/GRK2
Biological Process	GO:1905145	cellular response to acetylcholine	7/2279	30/18866	0.0622706	0.3884654	0.3514972	7	LY6G6D/GNAI2/GNA11/CHRM2/PLCB1/RGS10/GRK2
Biological Process	GO:0051224	negative regulation of protein transport	25/2279	151/18866	0.0628248	0.391409	0.3541608	25	KCNE1/WWP2/ANGPT1/ITGB1BP1/RAB11FIP1/FRMD4A/MIR199A1/SUFU/RHBDF2/UBAC2/SNX3/DERL2/ENY2/NF1/GHRL/CSK/KLF7/MIR199A2/CABP1/SP100/F2R/SREBF1/ADTRP/SVIP/PRKN
Biological Process	GO:0061041	regulation of wound healing	25/2279	151/18866	0.0628248	0.391409	0.3541608	25	RREB1/PLAU/CD9/KANK1/MYLK/SMAD3/PRKCA/TNF/UBASH3B/TNFAIP3/SERPINB2/THBS1/MYH9/F12/BLK/ACTG1/PDGFB/NFE2L2/PRKCE/CPB2/F2R/ANO6/ADTRP/PLPP3/CCN4
Biological Process	GO:0061008	hepaticobiliary system development	24/2279	144/18866	0.0631743	0.39329	0.3558627	24	ARID5B/IL10/RPS6KA1/SMAD3/TNFAIP3/E2F7/CUL3/TAF10/HNF1A/RARA/NF1/SP3/PKM/NOTCH1/CPB2/HMGCL/HLX/PCK2/CEBPB/ACAT1/HMGCS1/RELA/ALDH1A2/NODAL
Biological Process	GO:0014896	muscle hypertrophy	19/2279	109/18866	0.0632095	0.39329	0.3558627	19	HDAC4/CTDP1/TTN/MIR145/AKAP13/TNFRSF1B/SMAD3/PRKCA/MIR199A1/MIR24-2/MIR23A/TNFRSF1A/CAMTA2/PDE9A/MIR199A2/NOTCH1/MEF2C/FOXO1/INPP5F
Biological Process	GO:0030534	adult behavior	23/2279	137/18866	0.0634333	0.3944238	0.3568887	23	PPP1R1B/LGI4/HIPK2/APP/SCN1A/CRHBP/NRXN1/SLC1A2/WDR47/GHRL/NRXN2/ARRB2/NPC1/SHANK2/TMOD1/NTAN1/INPP5F/SPTBN4/NPY/NTF4/PRLHR/FADD/PRKN
Biological Process	GO:0032418	lysosome localization	14/2279	75/18866	0.0635873	0.394865	0.3572878	14	LAT/DEF8/PIK3CD/GAB2/FGR/CD84/MYH9/SPHK2/FER/BLK/PLEKHM1/KIF1B/HPS6/NDEL1
Biological Process	GO:0035924	cellular response to vascular endothelial growth factor stimulus	14/2279	75/18866	0.0635873	0.394865	0.3572878	14	NRP2/ERN1/ITGB1BP1/MIR199A1/DLL1/MIR27A/MIR23A/FLT1/MIR199A2/NOTCH1/MYO1C/PDGFRB/RELA/ADGRA2

Biological Process	GO:0043409	negative regulation of MAPK cascade	30/2279	187/18866	0.0636671	0.3951019	0.3575022	30	SPRED2/SORL1/HIPK3/ITGB1BP1/DUSP14/ARRB1/LAX1/PTPN6/IGF1R/PHB/PTPRJ/NF1/TNIP1/PTPRC/CSK/PPP2R1A/TAOK3/MIR138-2/RGS3/PER1/DUSP3/FOXM1/FOXO1/CYLD/EZR/MECOM/LIF/NDRG2/SIRT3/PRKN
Biological Process	GO:0009612	response to mechanical stimulus	33/2279	209/18866	0.0646744	0.4010912	0.3629216	33	HDAC4/MPO/CLCN6/PPARG/TTN/CHI3L1/SLC8A1/PKD1L1/JUP/TGFBR2/THBS1/TNFRSF1A/WNT11/SCN1A/AQP1/NRXN1/HTT/BCL10/MEIS2/NRXN2/MAP3K14/ETS1/ENG/NTRK1/SLC1A3/TMC1/CASP1/SLC2A1/ANKRD1/RELA/XPA/FADD/PPL
Biological Process	GO:0001649	osteoblast differentiation	36/2279	231/18866	0.0651091	0.4022301	0.363952	36	HDAC4/CEBPD/MEF2D/HDAC7/LRP5L/SKI/SMAD3/SUFU/TNF/SH3PXD2B/CLEC5A/ALPL/ILK/LIMD1/WNT11/PHB/SEMA4D/NF1/WWTR1/BMPR1B/TMEM64/RUNX2/IL6R/CAT/MIR138-2/NOTCH1/MEF2C/FHL2/NPPC/LTF/CEBPB/SND1/FAM20C/SYNCRIP/FBL/CCN4
Biological Process	GO:0099173	postsynapse organization	28/2279	173/18866	0.0651091	0.4022301	0.363952	28	NRP2/SSH1/ZMYND8/NDRG1/CTTN/COLQ/TANC1/EPHB3/BAIAP2/ZDHHC2/ACTN1/NOS1AP/IGF1R/WASL/SYNGAP1/NRXN1/GHRL/ACTG1/NRXN2/SIPA1L1/DOCK10/DNM3/DNM1L/SHANK2/UBE3A/CUX2/DBNL/LRFN2
Biological Process	GO:0031279	regulation of cyclase activity	10/2279	49/18866	0.0652021	0.4022301	0.363952	10	RCVRN/TIMP2/CACNA1C/CCR2/GNAI2/AKAP5/LTB4R2/NF1/PALM/GABBR2
Biological Process	GO:0097720	calcineurin-mediated signaling	10/2279	49/18866	0.0652021	0.4022301	0.363952	10	TNF/NFATC2/NFATC1/CAMTA1/RCAN1/DYRK2/FHL2/CHERP/NR5A2/STIMATE
Biological Process	GO:0010518	positive regulation of phospholipase activity	12/2279	62/18866	0.0652814	0.4022301	0.363952	12	PRKCZ/CCL5/FLT1/P2RY6/FGFR1/PHB/LPAR2/S1PR4/AGTR1/PDGFRB/PLCG1/NTF4
Biological Process	GO:0010803	regulation of tumor necrosis factor-mediated signaling pathway	12/2279	62/18866	0.0652814	0.4022301	0.363952	12	TNF/TRAF2/TNFAIP3/TRAF1/TRAIP/RIPK1/MIR24-2/TNFRSF1A/CPNE1/CASP1/CYLD/PRKN
Biological Process	GO:0048488	synaptic vesicle endocytosis	12/2279	62/18866	0.0652814	0.4022301	0.363952	12	SYNJ2/DENND1A/DNM2/ITSN1/STON1/DGKQ/ACTG1/SH3GL1/DNM1/DNM3/DNM1L/MX1

Biological Process	GO:0140238	presynaptic endocytosis	12/2279	62/18866	0.0652814	0.4022301	0.363952	12	SYNJ2/DENND1A/DNM2/ITSN1/STON1/DGKQ/ACTG1/SH3GL1/DNM1/DNM3/DNM1L/MX1
Biological Process	GO:1902475	L-alpha-amino acid transmembrane transport	12/2279	62/18866	0.0652814	0.4022301	0.363952	12	SLC1A5/SLC15A4/SLC43A2/SLC1A2/SLC7A7/SLC7A5/SLC1A6/PER2/SLC1A3/SLC43A1/SLC7A8/SLC7A1
Biological Process	GO:2001244	positive regulation of intrinsic apoptotic signaling pathway	12/2279	62/18866	0.0652814	0.4022301	0.363952	12	S100A8/LCK/PLAGL2/BMF/NCK2/NACC2/RIPK3/FIS1/BCL2/DNM1L/BCL2L1/FBH1
Biological Process	GO:0007179	transforming growth factor beta receptor signaling pathway	32/2279	202/18866	0.0655861	0.4037661	0.3653419	32	LRG1/PRKCZ/LTBP1/SPRED2/CREB1/SKI/TGFBRAP1/SMAD3/LDLRAD4/DNM2/HIPK2/DUSP22/CHST11/TGFBR2/THBS1/SMAD7/PXN/ZMIZ1/ITGB5/TGFB1/SMURF1/TGFB1I1/HTRA4/CREBBP/ENG/ARRB2/PRDM16/ARHGEF18/GCNT2/HTRA3/NODAL/ZFYVE9
Biological Process	GO:1903531	negative regulation of secretion by cell	27/2279	166/18866	0.0657684	0.4037661	0.3653419	27	ABR/TNFRSF1B/RAP1B/CCR2/RAB11FIP1/FRMD4A/MIR199A1/CD84/RHBDF2/GNAI2/HLA-F/TNFRSF1A/CRHBP/ENY2/NF1/GHRL/BCR/KLF7/MIR199A2/NOTCH1/F2R/SREBF1/ADTRP/INHBA/LIF/CBARP/PRKN
Biological Process	GO:0045666	positive regulation of neuron differentiation	56/2279	380/18866	0.0663852	0.4037661	0.3653419	56	CPNE6/PLXNC1/TIMP2/AP2A1/CYB5D2/CSNK1D/TMEM30A/ZMYND8/CPNE5/ZEB2/ITSN1/DISC1/CUX1/BAIAP2/SNX3/TCF12/ILK/CPNE1/FGFR1/SEMA4D/RAPGEF2/SMURF1/SS18L2/RARA/CPEB3/CAMK1D/KDM4C/NCOA1/CDH4/CSNK1E/GRN/CREB3L2/PRKCI/NFE2L2/ARSB/NTRK1/BCL2/TCF3/CDKL3/DNM1L/AVIL/MEF2C/SHANK2/SYT17/ARHGEF2/GDF6/ANKRD1/MARK2/CYFIP1/TRPV2/NDEL1/CUX2/EIF4G1/DBNL/PRKN/P3H1
Biological Process	GO:0050808	synapse organization	63/2279	433/18866	0.0668505	0.4037661	0.3653419	63	DCTN1/NRP2/SSH1/SPOCK2/IL10/ZMYND8/NDRG1/CTTN/COLQ/TNF/TANC1/EPHB3/SEMA4A/ITSN1/DISC1/BAIAP2/ZDHHC2/ACTN1/APP/NOS1AP/IGF1R/PPFIA1/WASL/SYNGAP1/SPARCL1/FARP1/SEMA4D/MYO5A/PFN1/NRXN1/KIRREL3/CTBP2/GHRL/ACTG1/PPFIBP2/NRXN2/PALM/SIPA1L1/DOCK10/YWHAZ/NTRK1/CDH1/DNM3/MYOT/DNM1L/MEF2C/SHANK2/F2R/UBE3A/SDK2/NEDD4/ARHGEF15/C1QB/CYFIP1/SLIT1/CX3CR1/CUX2/EIF4G1/SYNPO/DBNL/LRFN2/NFIA/NECTIN1
Biological Process	GO:0050792	regulation of viral process	34/2279	217/18866	0.0671686	0.4037661	0.3653419	34	SLPI/TRIM27/P4HB/TRIM38/CTDP1/GSN/TRIM8/TRIM10/SRPK2/TNF/POLR2F/CCL5/CSF1R/LARP1/SNX3/HDAC1/PC/PHB/FCN1/TRIM5/TNIP1/RSF1/BCL2/NOTCH1/LTF/ILF3/MX1/NR5A2/PARP10/BANF1/ISG20/TRIM26/PRKN/SHFL
Biological Process	GO:0002690	positive regulation of leukocyte chemotaxis	17/2279	96/18866	0.067217	0.4037661	0.3653419	17	CALR/C3AR1/CCR2/CCL5/CSF1R/FPR2/THBS1/DEFB124/ZNF580/CAMK1D/CXCL17/IL6R/DAPK2/DNM1L/ANO6/DYSF/CCL27

Biological Process	GO:0006898	receptor-mediated endocytosis	49/2279	328/18866	0.0676479	0.4037661	0.3653419	49	SORL1/CALR/AP2A1/CD9/CLEC9A/ANGPT1/AAK1/ARRB1/CTTN/DNM2/MIR199A1/RAB31/SFTPD/FPR2/ITSN1/TGFBR2/ANKRD13B/DLL1/MASP1/COLEC12/ANKRD13D/CLU/WASL/MARCO/STAB2/HBB/NUMB/ITGB3/ANKRD13A/CXCR1/DGKD/TPCN2/GSG1L/ARRB2/ITGB2/DNM1/AP1G2/MICALL1/DNM3/MIR199A2/EPS15/NEDD4/MX1/INPP5F/EZR/FLOT1/CLTCL1/DBNL/GRK2
Biological Process	GO:0042113	B cell activation	49/2279	328/18866	0.0676479	0.4037661	0.3653419	49	HDAC4/IL10/PIK3CD/INPP5D/PIK3R1/LAX1/TNFAIP3/LYL1/NFATC2/TNFSF13B/TLR9/MNDA/IGHV6-1/DLL1/RBPJ/PTPN6/TNFRSF21/UNG/TGFB1/PHB/PTPRJ/PRKCB/CD79A/BLK/SP3/CMTM7/PTPRC/CD320/DOCK10/NTRK1/NOD2/POU2F2/BCL2/TCF3/TP53BP1/MEF2C/BANK1/ERCC1/HHEX/TNFSF13/CYLD/BCL3/INHBA/PELI1/PCID2/PAXIP1/VAV3/ADGRG3/NSD2
Biological Process	GO:0048662	negative regulation of smooth muscle cell proliferation	13/2279	69/18866	0.0677719	0.4037661	0.3653419	13	IL10/PPARG/MIR140/MIR143/MIR145/TNFAIP3/COMT/ILK/SF1/MEF2C/VIPR2/TRIB1/NDRG2
Biological Process	GO:0033865	nucleoside bisphosphate metabolic process	23/2279	138/18866	0.0677837	0.4037661	0.3653419	23	ACSL1/FAR2/ACOT13/SULT6B1/ABHD14B/OGDH/ACOT7/ACSS1/SULT1A2/SLC26A1/ACACB/ACSS2/PPCDC/PAPSS2/ACACA/ACSF3/HMGCL/ACAT1/HMGCS1/SCD/TPST1/SLC25A1/PANK4
Biological Process	GO:0033875	ribonucleoside bisphosphate metabolic process	23/2279	138/18866	0.0677837	0.4037661	0.3653419	23	ACSL1/FAR2/ACOT13/SULT6B1/ABHD14B/OGDH/ACOT7/ACSS1/SULT1A2/SLC26A1/ACACB/ACSS2/PPCDC/PAPSS2/ACACA/ACSF3/HMGCL/ACAT1/HMGCS1/SCD/TPST1/SLC25A1/PANK4
Biological Process	GO:0034032	purine nucleoside bisphosphate metabolic process	23/2279	138/18866	0.0677837	0.4037661	0.3653419	23	ACSL1/FAR2/ACOT13/SULT6B1/ABHD14B/OGDH/ACOT7/ACSS1/SULT1A2/SLC26A1/ACACB/ACSS2/PPCDC/PAPSS2/ACACA/ACSF3/HMGCL/ACAT1/HMGCS1/SCD/TPST1/SLC25A1/PANK4
Biological Process	GO:0046330	positive regulation of JNK cascade	23/2279	138/18866	0.0677837	0.4037661	0.3653419	23	ERN1/ZNF622/TNF/ZEB2/TRAF2/TRAF1/HIPK2/TLR9/DUSP22/TNIK/RIPK1/APP/MTURN/AXIN1/SH3RF3/STK3/PLCB1/TAOK3/NOD2/GADD45G/ANKRD6/DBNL/MAP3K20
Biological Process	GO:0045667	regulation of osteoblast differentiation	22/2279	131/18866	0.0680706	0.4037661	0.3653419	22	HDAC4/CEBPD/HDAC7/SKI/SMAD3/SUFU/TNF/ILK/LIMD1/SEMA4D/BMPR1B/TMEM64/RUNX2/IL6R/MIR138-2/NOTCH1/MEF2C/NPPC/LTF/CEBPB/FAM20C/CCN4
Biological Process	GO:0061462	protein localization to lysosome	9/2279	43/18866	0.0681557	0.4037661	0.3653419	9	SORL1/RTN4/CLU/SH3BP4/NUMA1/SORT1/NEDD4/AP3D1/NCOA4

Biological Process	GO:0050796	regulation of insulin secretion	29/2279	181/18866	0.0682036	0.4037661	0.3653419	29	NADK/ITPR2/MYRIP/CACNA1C/LRP5L/ARRB1/PRKCA/GPLD1/TNF/CCL5/ITPR1/RFX3/TCF7L2/ENY2/GIPR/BLK/GHRL/KLF7/ARNTL/PER2/SLC16A1/PRKCE/SLC2A1/SREBF1/CPT1A/RPH3AL/C2CD2L/SIRT3/PRKN
Biological Process	GO:0031929	TOR signaling	21/2279	124/18866	0.0682383	0.4037661	0.3653419	21	RPTOR/PRR5L/MIR199A1/RHEB/LARP1/DISC1/DGKQ/CLEC16A/SIK3/PRR5/SEC13/SH3BP4/ARNTL/MIR199A2/PIH1D1/GNA12/TSC2/TNFAIP8L1/TTI1/NPRL2/PRKAA1
Biological Process	GO:0051099	positive regulation of binding	28/2279	174/18866	0.0690198	0.4037661	0.3653419	28	MARK3/PPARG/SKI/HMBOX1/AMFR/ARRB1/HIPK2/APP/TCF7L2/TGFB1/RAPGEF2/RARA/CSF3/CTBP2/STK3/SPTA1/PLCL1/ADD1/RB1/MED25/CDCA5/CLN5/FLOT1/ERCC2/PARP9/EIF4G1/STING1/PRKN
Biological Process	GO:0019320	hexose catabolic process	11/2279	56/18866	0.0691587	0.4037661	0.3653419	11	FUT4/FOXK1/ENO1/FUT7/HK1/ADPGK/PGAM1/GLYCTK/ENO3/HK2/PKM
Biological Process	GO:0044273	sulfur compound catabolic process	11/2279	56/18866	0.0691587	0.4037661	0.3653419	11	HEXB/GADL1/GALNS/GGT1/CBS/ACOT7/AGXT/ARSB/ADO/MAT1A/ACAT1
Biological Process	GO:0050732	negative regulation of peptidyl-tyrosine phosphorylation	11/2279	56/18866	0.0691587	0.4037661	0.3653419	11	PRKCZ/SH3BP5L/SH3BP5/DUSP22/PTPN6/SEMA4D/LRRK1/PTPRC/PPP2R1A/GGNBP2/INPP5F
Biological Process	GO:0045665	negative regulation of neuron differentiation	35/2279	225/18866	0.0695873	0.4037661	0.3653419	35	SEMA6B/CTSZ/GFI1/CALR/KANK1/LSM1/YWHAH/BCL11A/SEMA4A/RTN4/PMP22/SEMA4B/DLL1/APP/MIB1/IFRD1/ASAP1/SYNGAP1/KREMEN1/SEMA4D/RAPGEF2/ITM2C/MEIS1/DNM3/NOTCH1/ZHX2/CDKL3/UBE3A/CERS2/INPP5F/BAG5/RAP1GAP2/HES3/PRAG1/SPART
Biological Process	GO:0003281	ventricular septum development	14/2279	76/18866	0.0696623	0.4037661	0.3653419	14	LTBP1/ZFPM1/DNM2/SUFU/GJA5/TGFBR2/RBPJ/SMAD7/WNT11/NOTCH1/SLIT3/LMO4/CPLANE1/NSD2
Biological Process	GO:0060415	muscle tissue morphogenesis	14/2279	76/18866	0.0696623	0.4037661	0.3653419	14	ZFPM1/RXRA/TTN/MIR143/MIR145/MYLK/CCM2L/RBPJ/SMAD7/PAX7/TGFB1/ENG/NOTCH1/ANKRD1

Biological Process	GO:0042129	regulation of T cell proliferation	27/2279	167/18866	0.069797	0.4037661	0.3653419	27	HLA-DMB/IL27/IL10/MAD1L1/CD55/TNFRSF1B/CCR2/CCL5/NCK2/SFTPD/TNFSF13B/TGFBR2/PTPN6/TNFRSF21/ZBTB7B/RIPK3/CCDC88B/PTPRC/SPTA1/PNP/CEBPB/SLC7A1/FADD/PELI1/TMEM131L/VSIR/MARCHF7
Biological Process	GO:0001991	regulation of systemic arterial blood pressure by circulatory renin-angiotensin	5/2279	19/18866	0.070066	0.4037661	0.3653419	5	CTSZ/CTSG/GJA5/F2R/AOPEP
Biological Process	GO:0006670	sphingosine metabolic process	5/2279	19/18866	0.070066	0.4037661	0.3653419	5	SPTLC2/SPHK2/SGPP1/SGPP2/PLPP3
Biological Process	GO:0010759	positive regulation of macrophage chemotaxis	5/2279	19/18866	0.070066	0.4037661	0.3653419	5	C3AR1/CCL5/CSF1R/THBS1/CXCL17
Biological Process	GO:0032674	regulation of interleukin-5 production	5/2279	19/18866	0.070066	0.4037661	0.3653419	5	PRKCZ/PDE4D/NLRP3/TNFRSF21/RARA
Biological Process	GO:0032930	positive regulation of superoxide anion generation	5/2279	19/18866	0.070066	0.4037661	0.3653419	5	CD177/FPR2/GNAI2/TGFB1/ITGB2
Biological Process	GO:0034393	positive regulation of smooth muscle cell apoptotic process	5/2279	19/18866	0.070066	0.4037661	0.3653419	5	PPARG/MIR140/ARRB1/E2F3/MIR24-2
Biological Process	GO:0050908	detection of light stimulus involved in visual perception	5/2279	19/18866	0.070066	0.4037661	0.3653419	5	CACNA2D4/CRB1/CCDC66/GNAT2/EYS
Biological Process	GO:0050962	detection of light stimulus involved in sensory perception	5/2279	19/18866	0.070066	0.4037661	0.3653419	5	CACNA2D4/CRB1/CCDC66/GNAT2/EYS

Biological Process	GO:2000104	negative regulation of DNA-dependent DNA replication	5/2279	19/18866	0.070066	0.4037661	0.3653419	5	BLM/BRCA2/SMARCAL1/CHEK2/FBH1
Biological Process	GO:2000251	positive regulation of actin cytoskeleton reorganization	5/2279	19/18866	0.070066	0.4037661	0.3653419	5	BAIAP2L1/BAIAP2/CSF3/BAIAP2L2/CCL27
Biological Process	GO:0002040	sprouting angiogenesis	31/2279	196/18866	0.0702066	0.4037661	0.3653419	31	IL10/MAP3K3/HDAC7/ANGPT1/ITGB1BP1/MIR199A1/GPLD1/MIR101-2/E2F7/THBS1/DLL1/AKT3/MIR24-2/MIR27A/MIR23A/ESM1/MIA3/JAK1/EPN2/BMPER/GHRL/RNF213/MIR138-2/PKM/MIR199A2/NOTCH1/AGTR1/PPP1R16B/ADTRP/CEMIP2/ADGRA2
Biological Process	GO:0006163	purine nucleotide metabolic process	64/2279	442/18866	0.0703551	0.4037661	0.3653419	64	FOXK1/HDAC4/PDE4D/PRKAG2/RCVRN/AMPD3/ENO1/HK1/ADCY2/ACSL1/FAR2/TJP2/ADCY9/PFKFB4/ACOT13/SULT6B1/RAE1/CBFA2T3/ABHD14B/DHTKD1/APP/OGDH/NUDT3/ADPGK/ACOT7/VPS9D1/PGAM1/ACSS1/SPHK2/TGFB1/SULT1A2/SLC26A1/NUP93/ENO3/HK2/SEC13/ACACB/ACSS2/PDE9A/PNP/GUK1/ADCY4/ESRRB/MTHFD1/PPCDC/PAPSS2/PKM/ACACA/ACSF3/AK2/GMPR2/AK5/HMGCL/NPPC/ACAT1/HMGCS1/SCD/TPST1/PFKFB3/SLC25A1/PANK4/IMPDH1/PRKAA1/ATP5PD
Biological Process	GO:0030212	hyaluronan metabolic process	8/2279	37/18866	0.0706838	0.4037661	0.3653419	8	AP2A1/HEXB/TGFB1/STAB2/PDGFB/LYVE1/HAS3/CEMIP2
Biological Process	GO:0033363	secretory granule organization	8/2279	37/18866	0.0706838	0.4037661	0.3653419	8	ACRBP/CREB1/LYST/ZNF385A/ABCA1/F2R/DTNBP1/MFSD14A
Biological Process	GO:0060260	regulation of transcription initiation from RNA polymerase II promoter	8/2279	37/18866	0.0706838	0.4037661	0.3653419	8	CREB1/HNF1A/MED13/ATF7IP/SUB1/ERCC1/XPA/PAXIP1
Biological Process	GO:0090322	regulation of superoxide metabolic process	8/2279	37/18866	0.0706838	0.4037661	0.3653419	8	CD177/TNF/FPR2/GNAI2/TGFB1/NFE2L2/ITGB2/SIRT3
Biological Process	GO:0098926	postsynaptic signal transduction	8/2279	37/18866	0.0706838	0.4037661	0.3653419	8	LY6G6D/GNAI2/GNA11/CHRM2/PLCB1/RGS10/RELA/GRK2

Biological Process	GO:1903514	release of sequestered calcium ion into cytosol by endoplasmic reticulum	8/2279	37/18866	0.0706838	0.4037661	0.3653419	8	PDE4D/CASQ2/CACNA1C/SLC8A1/NOL3/GSTO1/ANK2/CASQ1
Biological Process	GO:1904994	regulation of leukocyte adhesion to vascular endothelial cell	8/2279	37/18866	0.0706838	0.4037661	0.3653419	8	ELANE/FUT4/FUT7/CCR2/TNF/ETS1/ITGB2/RELA
Biological Process	GO:0050803	regulation of synapse structure or activity	37/2279	240/18866	0.0707123	0.4037661	0.3653419	37	DCTN1/NRP2/SSH1/IL10/ZMYND8/DNM2/COLQ/TNF/EPHB3/SEMA4A/ITSN1/DISC1/BAIAP2/ZDHHC2/APP/SYNGAP1/SEMA4D/NRXN1/GHRL/SIPA1L1/DNM1/YWHAZ/NTRK1/DNM3/DNM1L/MEF2C/UBE3A/NEDD4/ARHGEF15/MX1/CYFIP1/SLIT1/CUX2/EIF4G1/DBNL/LRFN2/NECTIN1
Biological Process	GO:0048145	regulation of fibroblast proliferation	15/2279	83/18866	0.0710637	0.4037661	0.3653419	15	PPARG/CREB1/MYB/SKI/ZMIZ1/B4GALT7/AQP1/NF1/PDGFD/PDGFB/MORC3/MED25/PDGFC/PDGFRB/PARP10
Biological Process	GO:0040013	negative regulation of locomotion	58/2279	397/18866	0.0714455	0.4037661	0.3653419	58	ELANE/SEMA6B/TACSTD2/ABR/CALR/PPARG/MIR140/KANK1/ARHGDIB/ZMYND8/ITGB1BP1/SPINT2/LDLRAD4/MIR199A1/TNF/MIR101-2/DUSP22/SEMA4A/PADI2/JUP/NAV3/THBS1/SEMA4B/C5AR2/MIR24-2/ILK/SMAD7/MIA3/WNT11/WASL/NISCH/TGFB1/PTPRJ/SEMA4D/NF1/STK24/GHRL/SRGAP1/BCR/PLCB1/NFE2L2/ENG/VASH1/MIR138-2/CDH1/BCL2/MIR199A2/NOTCH1/CORO1C/MEF2C/DUSP3/SP100/TRIB1/ADTRP/CERS2/PTPRU/NODAL/CX3CR1
Biological Process	GO:0031667	response to nutrient levels	68/2279	473/18866	0.0718326	0.4037661	0.3653419	68	FOXK1/RPTOR/PRKAG2/MPO/SORL1/SLC25A25/PPARG/GSN/LCN2/ACSL1/MFSD2A/TNKS/ULK1/CHSY1/VDR/SLC8A1/WIPI2/LARP1/TGFBR2/COMT/ALPL/EHMT2/GNAI2/WNT11/PAX2/RARA/POR/STK24/CLEC16A/GIPR/ATG14/GHRL/ABCA1/NCOA1/ACACB/BCL10/CAT/NFE2L2/ARSB/NTRK1/SLC16A1/NOD2/FIS1/PKM/BCL2/AQP3/DNM1L/HMGCL/USF2/CD68/PCK2/SLC2A1/FOXO1/ERCC1/ACAT1/HMGCS1/SREBF1/RELA/ALDH1A2/DHODH/NPY/HSD11B2/EIF4G1/SST/NPRL2/PRKAA1/MARS1/RIPOR1
Biological Process	GO:0070555	response to interleukin-1	33/2279	211/18866	0.0718408	0.4037661	0.3653419	33	HDAC4/VRK2/LCN2/TOLLIP/MAP3K3/CHI3L1/IL1R1/PRKCA/CCL5/MIR101-2/ABCC2/APP/MIR27A/RPS6KA4/IL1RN/UBE2V1/CCL20/PSMF1/PLCB1/ETS1/PRKCI/IRAK2/OTUB1/NOD2/PSMB7/CEBPB/ANKRD1/RELA/FBXW11/HNMT/CCL22/PELI1/PSMD13
Biological Process	GO:0006029	proteoglycan metabolic process	16/2279	90/18866	0.0720641	0.4037661	0.3653419	16	CSGALNACT1/SPOCK2/HS3ST3B1/DSE/HEXB/CHSY1/CHST11/CHST15/NDST1/EGFLAM/TCF7L2/XYLT1/B4GALT7/BMPR1B/ARSB/PXYLP1
Biological Process	GO:0070542	response to fatty acid	16/2279	90/18866	0.0720641	0.4037661	0.3653419	16	PPARG/ACSL1/CREB1/P2RY6/GIPR/CAT/SMARCD1/GNG2/ACACA/PRKCE/HMGCL/FOXO1/SCD/SREBF1/CPT1A/PRKAA1

Biological Process	GO:0046530	photoreceptor cell differentiation	12/2279	63/18866	0.0721735	0.4037661	0.3653419	12	MFSD2A/CRB1/GNAT2/IFT140/PRKCI/NOTCH1/RPGRIP1/CDHR1/SDK2/NRL/CABP4/NPHP4
Biological Process	GO:0050650	chondroitin sulfate proteoglycan biosynthetic process	7/2279	31/18866	0.0724018	0.4037661	0.3653419	7	CSGALNACT1/DSE/CHSY1/CHST11/CHST15/XYLT1/PXYLP1
Biological Process	GO:0060261	positive regulation of transcription initiation from RNA polymerase II promoter	7/2279	31/18866	0.0724018	0.4037661	0.3653419	7	CREB1/HNF1A/MED13/SUB1/ERCC1/XPA/PAXIP1
Biological Process	GO:0070229	negative regulation of lymphocyte apoptotic process	7/2279	31/18866	0.0724018	0.4037661	0.3653419	7	FOXP1/CCL5/HSH2D/PTCRA/DOCK8/BCL10/FADD
Biological Process	GO:0098868	bone growth	7/2279	31/18866	0.0724018	0.4037661	0.3653419	7	ANXA6/TGFBR2/POC1A/RARA/POR/RARG/NPPC
Biological Process	GO:0099623	regulation of cardiac muscle cell membrane repolarization	7/2279	31/18866	0.0724018	0.4037661	0.3653419	7	KCNE1/GJA5/NOS1AP/WDR1/SNTA1/KCNQ1/ANK2
Biological Process	GO:2000406	positive regulation of T cell migration	7/2279	31/18866	0.0724018	0.4037661	0.3653419	7	CCR2/CCL5/APP/DOCK8/CCL20/CCL27/FADD
Biological Process	GO:2000515	negative regulation of CD4-positive, alpha-beta T cell activation	7/2279	31/18866	0.0724018	0.4037661	0.3653419	7	RUNX1/LOXL3/SMAD7/ZBTB7B/HLX/RUNX3/VSIR
Biological Process	GO:0000423	mitophagy	6/2279	25/18866	0.0726267	0.4037661	0.3653419	6	VPS13D/SMURF1/CLEC16A/ATG14/TSC2/PRKN

Biological Process	GO:0010954	positive regulation of protein processing	6/2279	25/18866	0.0726267	0.4037661	0.3653419	6	NLRC4/ENO1/GSN/MYH9/F12/ANGPTL8
Biological Process	GO:0015740	C4-dicarboxylate transport	6/2279	25/18866	0.0726267	0.4037661	0.3653419	6	SLC1A5/LRRC8C/SLC1A2/SLC1A6/SLC1A3/SLC13A5
Biological Process	GO:0043302	positive regulation of leukocyte degranulation	6/2279	25/18866	0.0726267	0.4037661	0.3653419	6	CD177/GAB2/FGR/HLA-F/SPHK2/ITGB2
Biological Process	GO:0045662	negative regulation of myoblast differentiation	6/2279	25/18866	0.0726267	0.4037661	0.3653419	6	TNF/DLL1/TGFB1/ANKRD2/NOTCH1/CMTM5
Biological Process	GO:0046639	negative regulation of alpha-beta T cell differentiation	6/2279	25/18866	0.0726267	0.4037661	0.3653419	6	RUNX1/LOXL3/SMAD7/ZBTB7B/HLX/RUNX3
Biological Process	GO:0046697	decidualization	6/2279	25/18866	0.0726267	0.4037661	0.3653419	6	BSG/VDR/GHRL/CTSB/TPPP3/LIF
Biological Process	GO:0060669	embryonic placenta morphogenesis	6/2279	25/18866	0.0726267	0.4037661	0.3653419	6	IL10/SETD2/SPINT2/DNAJB6/GRHL2/NCOA1
Biological Process	GO:1903859	regulation of dendrite extension	6/2279	25/18866	0.0726267	0.4037661	0.3653419	6	CPNE6/CPNE5/BCL11A/SMURF1/SYT17/PRKN
Biological Process	GO:0048704	embryonic skeletal system morphogenesis	17/2279	97/18866	0.0727336	0.4037661	0.3653419	17	SETD2/SMAD3/MTHFD1L/CHST11/TGFBR2/NDST1/RDH10/DSCAML1/GRHL2/RUNX2/IFT140/MTHFD1/HOXA9/ALX3/MEF2C/NODAL/HOXB7

Biological Process	GO:1901655	cellular response to ketone	17/2279	97/18866	0.0727336	0.4037661	0.3653419	17	PPARG/ADCY2/ABCC2/LARP1/SMYD3/FECH/P2RY6/SPHK2/FDX1/AQP1/GNG2/ACACA/PRKCE/PCK2/FOXO1/NCOA4/PRKAA1
Biological Process	GO:0019751	polyol metabolic process	22/2279	132/18866	0.0727677	0.4037661	0.3653419	22	SYNJ2/SPTLC2/INPP5A/INPP5D/ITPKB/PGP/INPP4A/NUDT3/P2RY6/SPHK2/HRH1/INPP5E/PLCB1/IMPA2/SGPP1/SGPP2/PCK2/DYSF/PLCG1/PLCD3/IP6K1/PLPP3
Biological Process	GO:0048732	gland development	64/2279	443/18866	0.072886	0.4037661	0.3653419	64	ARID5B/RREB1/RXRA/IL10/RPS6KA1/NCOR2/MAD1L1/CREB1/BRCA2/LRP5L/VDR/SMAD3/TNF/TNFAIP3/CSF1R/LBH/EPHB3/E2F7/STAT5A/RTN4/TGFBR2/CUL3/TG/RBPJ/TAF10/HNF1A/WNT11/FGFR1/ZBTB7B/TGFB1/RARA/RIPK3/NF1/SP3/HK2/RARG/NCOA1/ETS1/CDH1/HOXA9/PKM/BCL2/NOTCH1/NOTCH4/FA2H/CPB2/HMGCL/UBE3A/USF2/HLX/PCK2/CEBPB/PDGFRB/CRIP1/ACAT1/HMGCS1/SLC29A1/ATP7B/RELA/ALDH1A2/LMO4/DHODH/NODAL/FADD
Biological Process	GO:0001961	positive regulation of cytokine-mediated signaling pathway	10/2279	50/18866	0.0730737	0.4037661	0.3653419	10	GFI1/IL1R1/TRAF2/RIPK1/CPNE1/NLRC5/CASP1/PARP9/FADD/PRKN
Biological Process	GO:0008038	neuron recognition	10/2279	50/18866	0.0730737	0.4037661	0.3653419	10	BSG/NCAM2/EPHB3/RTN4/APP/TNFRSF21/DSCAML1/YWHAZ/PCDH12/OPCML
Biological Process	GO:0019674	NAD metabolic process	10/2279	50/18866	0.0730737	0.4037661	0.3653419	10	FOXK1/ENO1/NADK/HK1/NMNAT3/ADPGK/PGAM1/ENO3/HK2/PKM
Biological Process	GO:0032760	positive regulation of tumor necrosis factor production	10/2279	50/18866	0.0730737	0.4037661	0.3653419	10	PIK3R1/TLR9/SPON2/RIPK1/CLU/PTPRJ/PTPRC/NOD2/ARHGEF2/FADD
Biological Process	GO:0045540	regulation of cholesterol biosynthetic process	10/2279	50/18866	0.0730737	0.4037661	0.3653419	10	IDI1/MBTPS1/ABCG1/POR/ACACB/ACACA/HMGCS1/SCD/SREBF1/PRKAA1
Biological Process	GO:0051653	spindle localization	10/2279	50/18866	0.0730737	0.4037661	0.3653419	10	DCTN1/NDE1/DYNC1H1/MYH9/WASL/HTT/NUMA1/ARHGEF2/FBXW11/NDEL1

Biological Process	GO:0097300	programmed necrotic cell death	10/2279	50/18866	0.0730737	0.4037661	0.3653419	TNF/TRAF2/MIR101-2/RIPK1/RIPK3/DNM1L/ARHGEF2/CYLD/FADD/PELI1	10
Biological Process	GO:0106118	regulation of sterol biosynthetic process	10/2279	50/18866	0.0730737	0.4037661	0.3653419	IDI1/MBTPS1/ABCG1/POR/ACACB/ACACA/HMGCS1/SCD/SREBF1/PRKAA1	10
Biological Process	GO:1903727	positive regulation of phospholipid metabolic process	10/2279	50/18866	0.0730737	0.4037661	0.3653419	FGR/FPR2/FLT1/TGFB1/VAV2/ATG14/PDGFB/NOD2/PDGFRB/VAV3	10
Biological Process	GO:0001952	regulation of cell-matrix adhesion	21/2279	125/18866	0.0730866	0.4037661	0.3653419	PRKCZ/PLAU/RIN2/ITGB1BP1/PIK3R1/CEACAM6/SMAD3/DUSP22/JUP/THBS1/DISC1/NINJ1/ILK/PTPRJ/NF1/ACTG1/PLEKHA2/BCL2/CORO1C/DUSP3/DAPK3	21
Biological Process	GO:0030968	endoplasmic reticulum unfolded protein response	21/2279	125/18866	0.0730866	0.4037661	0.3653419	DCTN1/CALR/ERN1/PIK3R1/AMFR/MBTPS1/RHBDD2/NCK2/YIF1A/EDEM1/DERL2/ZBTB17/PDIA5/CLGN/COPS5/CREB3L2/NFE2L2/ADD1/KLHDC3/SEC31A/ACADVL	21
Biological Process	GO:0006892	post-Golgi vesicle-mediated transport	18/2279	104/18866	0.0731288	0.4037661	0.3653419	OSBPL5/SORL1/AP2A1/VTI1A/ANKFY1/KIF13A/DNM2/RAB31/CORO7/VAMP5/MYO5A/CSK/SORT1/AP1G2/EPS15/AP3D1/VPS35L/DOP1B	18
Biological Process	GO:0042303	molting cycle	19/2279	111/18866	0.0732954	0.4037661	0.3653419	TNF/KRT17/RBPJ/LRIG1/HDAC1/CDH3/EPS8L3/MYO5A/NF1/NUMA1/ARNTL/BCL2/PER1/NOTCH1/FA2H/TGM3/RELA/INHBA/ERCC2	19
Biological Process	GO:0042633	hair cycle	19/2279	111/18866	0.0732954	0.4037661	0.3653419	TNF/KRT17/RBPJ/LRIG1/HDAC1/CDH3/EPS8L3/MYO5A/NF1/NUMA1/ARNTL/BCL2/PER1/NOTCH1/FA2H/TGM3/RELA/INHBA/ERCC2	19
Biological Process	GO:1903008	organelle disassembly	19/2279	111/18866	0.0732954	0.4037661	0.3653419	STX5/VPS13D/CTTN/FBXO7/WIPI2/CAMKK2/USP36/SMURF1/CLEC16A/HTT/ATG14/HK2/FIS1/DNM1L/TSC2/SREBF1/MARK2/STING1/PRKN	19

Biological Process	GO:0034765	regulation of ion transmembrane transport	70/2279	489/18866	0.0736862	0.4037661	0.3653419	70	PDE4D/KCNJ15/KCNE1/KCNAB2/THADA/CASQ2/WWP2/FXYD2/CACNA1C/YWHAH/CACNA2D4/FXYD1/CCR2/SLC8A1/DNM2/UBASH3B/GJA5/TLR9/KCNQ2/TESC/BIN1/THBS1/JSRP1/AHNAK/RIPK1/APP/NOS1AP/PTPN6/ACTN4/KCNIP1/SCN1A/P2RY6/SPHK2/KCNK6/CRHBP/SLC43A2/CNIH2/HTR3A/MYO5A/HTT/CAB39/WDR1/SNTA1/TPCN2/MAPK8IP2/KCNQ1/GSG1L/PER2/GSTO1/MEF2C/SHANK2/PRKCE/TMEM37/F2R/TMC1/SLC43A1/NEDD4/ANO6/DYSF/CACNA1B/ANK2/HECW2/PLCG1/YWHAQ/PDZK1/CASQ1/FGF14/CBARP/STIMATE/CRACR2A
Biological Process	GO:0045931	positive regulation of mitotic cell cycle	27/2279	168/18866	0.0739938	0.4037661	0.3653419	27	RPTOR/BRD4/TGFA/TFDP1/BRCA2/PRKCA/NSMCE2/CUL3/APP/CDC16/FGFR1/CDC25A/TMOD3/NUMA1/PDGFB/MEIS2/PLCB1/RB1/DUSP3/CDCA5/ANAPC7/PDGFRB/USP2/PLRG1/EIF4G1/PCID2/SLF1
Biological Process	GO:0000429	carbon catabolite regulation of transcription from RNA polymerase II promoter	2/2279	4/18866	0.0740726	0.4037661	0.3653419	2	NCOA1/USF2
Biological Process	GO:0000436	carbon catabolite activation of transcription from RNA polymerase II promoter	2/2279	4/18866	0.0740726	0.4037661	0.3653419	2	NCOA1/USF2
Biological Process	GO:0001766	membrane raft polarization	2/2279	4/18866	0.0740726	0.4037661	0.3653419	2	GSN/DOCK2
Biological Process	GO:0001878	response to yeast	2/2279	4/18866	0.0740726	0.4037661	0.3653419	2	ELANE/MPO
Biological Process	GO:0002051	osteoblast fate commitment	2/2279	4/18866	0.0740726	0.4037661	0.3653419	2	SH3PXD2B/RUNX2
Biological Process	GO:0002074	extraocular skeletal muscle development	2/2279	4/18866	0.0740726	0.4037661	0.3653419	2	MYH15/MYOM1
Biological Process	GO:0002159	desmosome assembly	2/2279	4/18866	0.0740726	0.4037661	0.3653419	2	PRKCA/JUP

Biological Process	GO:0002265	astrocyte activation involved in immune response	2/2279	4/18866	0.0740726	0.4037661	0.3653419	2	APP/GRN
Biological Process	GO:0002378	immunoglobulin biosynthetic process	2/2279	4/18866	0.0740726	0.4037661	0.3653419	2	GALNT2/PTPRC
Biological Process	GO:0002434	immune complex clearance	2/2279	4/18866	0.0740726	0.4037661	0.3653419	2	CCR2/CLU
Biological Process	GO:0002439	chronic inflammatory response to antigenic stimulus	2/2279	4/18866	0.0740726	0.4037661	0.3653419	2	IL10/TNF
Biological Process	GO:0002605	negative regulation of dendritic cell antigen processing and presentation	2/2279	4/18866	0.0740726	0.4037661	0.3653419	2	THBS1/CD68
Biological Process	GO:0003105	negative regulation of glomerular filtration	2/2279	4/18866	0.0740726	0.4037661	0.3653419	2	GJA5/F2R
Biological Process	GO:0003273	cell migration involved in endocardial cushion formation	2/2279	4/18866	0.0740726	0.4037661	0.3653419	2	ENG/NOTCH1
Biological Process	GO:0003383	apical constriction	2/2279	4/18866	0.0740726	0.4037661	0.3653419	2	CCDC88C/FRMD6
Biological Process	GO:0003419	growth plate cartilage chondrocyte proliferation	2/2279	4/18866	0.0740726	0.4037661	0.3653419	2	POR/NPPC

Biological Process	GO:0006574	valine catabolic process	2/2279	4/18866	0.0740726	0.4037661	0.3653419	2	ACAD8/HIBADH
Biological Process	GO:0006660	phosphatidylserine catabolic process	2/2279	4/18866	0.0740726	0.4037661	0.3653419	2	ABHD16A/ABHD16B
Biological Process	GO:0006842	tricarboxylic acid transport	2/2279	4/18866	0.0740726	0.4037661	0.3653419	2	SLC13A5/SLC25A1
Biological Process	GO:0006933	negative regulation of cell adhesion involved in substrate-bound cell migration	2/2279	4/18866	0.0740726	0.4037661	0.3653419	2	ITGB1BP1/PTPRC
Biological Process	GO:0007296	vitellogenesis	2/2279	4/18866	0.0740726	0.4037661	0.3653419	2	ZMIZ1/ETV6
Biological Process	GO:0007468	regulation of rhodopsin gene expression	2/2279	4/18866	0.0740726	0.4037661	0.3653419	2	MFSD2A/NRL
Biological Process	GO:0009093	cysteine catabolic process	2/2279	4/18866	0.0740726	0.4037661	0.3653419	2	CBS/AGXT
Biological Process	GO:0009182	purine deoxyribonucleoside diphosphate metabolic process	2/2279	4/18866	0.0740726	0.4037661	0.3653419	2	GUK1/AK5
Biological Process	GO:0009189	deoxyribonucleoside diphosphate biosynthetic process	2/2279	4/18866	0.0740726	0.4037661	0.3653419	2	GUK1/AK5

Biological Process	GO:0010735	positive regulation of transcription via serum response element binding	2/2279	4/18866	0.0740726	0.4037661	0.3653419	2	MICAL2/MRTFA
Biological Process	GO:0014005	microglia development	2/2279	4/18866	0.0740726	0.4037661	0.3653419	2	APP/TSPAN2
Biological Process	GO:0014809	regulation of skeletal muscle contraction by regulation of release of sequestered calcium ion	2/2279	4/18866	0.0740726	0.4037661	0.3653419	2	GSTO1/CASQ1
Biological Process	GO:0015746	citrate transport	2/2279	4/18866	0.0740726	0.4037661	0.3653419	2	SLC13A5/SLC25A1
Biological Process	GO:0015879	carnitine transport	2/2279	4/18866	0.0740726	0.4037661	0.3653419	2	SLC22A5/PDZK1
Biological Process	GO:0018120	peptidyl-arginine ADP-ribosylation	2/2279	4/18866	0.0740726	0.4037661	0.3653419	2	ART5/ART4
Biological Process	GO:0019344	cysteine biosynthetic process	2/2279	4/18866	0.0740726	0.4037661	0.3653419	2	GGT1/CBS
Biological Process	GO:0019448	L-cysteine catabolic process	2/2279	4/18866	0.0740726	0.4037661	0.3653419	2	CBS/AGXT
Biological Process	GO:0019470	4-hydroxyproline catabolic process	2/2279	4/18866	0.0740726	0.4037661	0.3653419	2	ALDH4A1/HOGA1

Biological Process	GO:0019471	4-hydroxyproline metabolic process	2/2279	4/18866	0.0740726	0.4037661	0.3653419	2	ALDH4A1/HOGA1
Biological Process	GO:0019482	beta-alanine metabolic process	2/2279	4/18866	0.0740726	0.4037661	0.3653419	2	DPYS/UPB1
Biological Process	GO:0021557	oculomotor nerve development	2/2279	4/18866	0.0740726	0.4037661	0.3653419	2	PHOX2A/HES3
Biological Process	GO:0031034	myosin filament assembly	2/2279	4/18866	0.0740726	0.4037661	0.3653419	2	TTN/MYH11
Biological Process	GO:0031296	B cell costimulation	2/2279	4/18866	0.0740726	0.4037661	0.3653419	2	TNFSF13B/CD320
Biological Process	GO:0031587	positive regulation of inositol 1,4,5-trisphosphate-sensitive calcium-release channel activity	2/2279	4/18866	0.0740726	0.4037661	0.3653419	2	P2RY6/HTT
Biological Process	GO:0032097	positive regulation of response to food	2/2279	4/18866	0.0740726	0.4037661	0.3653419	2	GHRL/NPY
Biological Process	GO:0032100	positive regulation of appetite	2/2279	4/18866	0.0740726	0.4037661	0.3653419	2	GHRL/NPY
Biological Process	GO:0032417	positive regulation of sodium:proton antiporter activity	2/2279	4/18866	0.0740726	0.4037661	0.3653419	2	TESC/ACTN4

Biological Process	GO:0032605	hepatocyte growth factor production	2/2279	4/18866	0.0740726	0.4037661	0.3653419	2	MIR199A1/MIR199A2
Biological Process	GO:0032646	regulation of hepatocyte growth factor production	2/2279	4/18866	0.0740726	0.4037661	0.3653419	2	MIR199A1/MIR199A2
Biological Process	GO:0032686	negative regulation of hepatocyte growth factor production	2/2279	4/18866	0.0740726	0.4037661	0.3653419	2	MIR199A1/MIR199A2
Biological Process	GO:0032701	negative regulation of interleukin-18 production	2/2279	4/18866	0.0740726	0.4037661	0.3653419	2	IL10/CD84
Biological Process	GO:0034436	glycoprotein transport	2/2279	4/18866	0.0740726	0.4037661	0.3653419	2	ABCG1/GUK1
Biological Process	GO:0034465	response to carbon monoxide	2/2279	4/18866	0.0740726	0.4037661	0.3653419	2	IL10/DNM2
Biological Process	GO:0034552	respiratory chain complex II assembly	2/2279	4/18866	0.0740726	0.4037661	0.3653419	2	SDHAF1/SDHAF3
Biological Process	GO:0034553	mitochondrial respiratory chain complex II assembly	2/2279	4/18866	0.0740726	0.4037661	0.3653419	2	SDHAF1/SDHAF3
Biological Process	GO:0035788	cell migration involved in metanephros development	2/2279	4/18866	0.0740726	0.4037661	0.3653419	2	PDGFB/PDGFRB

Biological Process	GO:0035789	metanephric mesenchymal cell migration	2/2279	4/18866	0.0740726	0.4037661	0.3653419	2	PDGFB/PDGFRB
Biological Process	GO:0035799	ureter maturation	2/2279	4/18866	0.0740726	0.4037661	0.3653419	2	PAX2/ALDH1A2
Biological Process	GO:0036510	trimming of terminal mannose on C branch	2/2279	4/18866	0.0740726	0.4037661	0.3653419	2	EDEM1/EDEM2
Biological Process	GO:0042126	nitrate metabolic process	2/2279	4/18866	0.0740726	0.4037661	0.3653419	2	POR/MTARC1
Biological Process	GO:0042699	follicle-stimulating hormone signaling pathway	2/2279	4/18866	0.0740726	0.4037661	0.3653419	2	ARRB1/ARRB2
Biological Process	GO:0042904	9-cis-retinoic acid biosynthetic process	2/2279	4/18866	0.0740726	0.4037661	0.3653419	2	DHRS9/ALDH1A2
Biological Process	GO:0042905	9-cis-retinoic acid metabolic process	2/2279	4/18866	0.0740726	0.4037661	0.3653419	2	DHRS9/ALDH1A2
Biological Process	GO:0043315	positive regulation of neutrophil degranulation	2/2279	4/18866	0.0740726	0.4037661	0.3653419	2	CD177/ITGB2
Biological Process	GO:0043378	positive regulation of CD8-positive, alpha-beta T cell differentiation	2/2279	4/18866	0.0740726	0.4037661	0.3653419	2	RUNX1/RUNX3

Biological Process	GO:0045608	negative regulation of inner ear auditory receptor cell differentiation	2/2279	4/18866	0.0740726	0.4037661	0.3653419	2	DLL1/NOTCH1
Biological Process	GO:0045632	negative regulation of mechanoreceptor differentiation	2/2279	4/18866	0.0740726	0.4037661	0.3653419	2	DLL1/NOTCH1
Biological Process	GO:0046439	L-cysteine metabolic process	2/2279	4/18866	0.0740726	0.4037661	0.3653419	2	CBS/AGXT
Biological Process	GO:0046952	ketone body catabolic process	2/2279	4/18866	0.0740726	0.4037661	0.3653419	2	BDH1/ACAT1
Biological Process	GO:0051790	short-chain fatty acid biosynthetic process	2/2279	4/18866	0.0740726	0.4037661	0.3653419	2	ACSS1/ACSS2
Biological Process	GO:0060528	secretory columnal luminar epithelial cell differentiation involved in prostate glandular acinus development	2/2279	4/18866	0.0740726	0.4037661	0.3653419	2	RXRA/NOTCH1
Biological Process	GO:0061589	calcium activated phosphatidylserine scrambling	2/2279	4/18866	0.0740726	0.4037661	0.3653419	2	ANO7/ANO6
Biological Process	GO:0070235	regulation of activation-induced cell death of T cells	2/2279	4/18866	0.0740726	0.4037661	0.3653419	2	RIPK3/FADD
Biological Process	GO:0070676	intralumenal vesicle formation	2/2279	4/18866	0.0740726	0.4037661	0.3653419	2	SNX3/PRKN

Biological Process	GO:0070777	D-aspartate transport	2/2279	4/18866	0.0740726	0.4037661	0.3653419	2	SLC1A2/SLC1A3
Biological Process	GO:0070779	D-aspartate import across plasma membrane	2/2279	4/18866	0.0740726	0.4037661	0.3653419	2	SLC1A2/SLC1A3
Biological Process	GO:0071211	protein targeting to vacuole involved in autophagy	2/2279	4/18866	0.0740726	0.4037661	0.3653419	2	CLU/SMURF1
Biological Process	GO:0071787	endoplasmic reticulum tubular network formation	2/2279	4/18866	0.0740726	0.4037661	0.3653419	2	RTN4/RTN3
Biological Process	GO:0072108	positive regulation of mesenchymal to epithelial transition involved in metanephros morphogenesis	2/2279	4/18866	0.0740726	0.4037661	0.3653419	2	PAX2/LIF
Biological Process	GO:0090038	negative regulation of protein kinase C signaling	2/2279	4/18866	0.0740726	0.4037661	0.3653419	2	DGKD/MYADM
Biological Process	GO:0090107	regulation of high-density lipoprotein particle assembly	2/2279	4/18866	0.0740726	0.4037661	0.3653419	2	NR1H2/ABCA1
Biological Process	GO:0090168	Golgi reassembly	2/2279	4/18866	0.0740726	0.4037661	0.3653419	2	VCPIP1/YWHAZ
Biological Process	GO:0097118	neuroligin clustering involved in postsynaptic membrane assembly	2/2279	4/18866	0.0740726	0.4037661	0.3653419	2	NRXN1/NRXN2

Biological Process	GO:0097167	circadian regulation of translation	2/2279	4/18866	0.0740726	0.4037661	0.3653419	2	PER2/PER1
Biological Process	GO:0097241	hematopoietic stem cell migration to bone marrow	2/2279	4/18866	0.0740726	0.4037661	0.3653419	2	GPLD1/JAM3
Biological Process	GO:0099039	sphingolipid translocation	2/2279	4/18866	0.0740726	0.4037661	0.3653419	2	ABCC1/ABCA1
Biological Process	GO:0099576	regulation of protein catabolic process at postsynapse, modulating synaptic transmission	2/2279	4/18866	0.0740726	0.4037661	0.3653419	2	RNF19A/CDH1
Biological Process	GO:0140009	L-aspartate import across plasma membrane	2/2279	4/18866	0.0740726	0.4037661	0.3653419	2	SLC1A5/SLC1A3
Biological Process	GO:1900126	negative regulation of hyaluronan biosynthetic process	2/2279	4/18866	0.0740726	0.4037661	0.3653419	2	AP2A1/TGFB1
Biological Process	GO:1900133	regulation of renin secretion into blood stream	2/2279	4/18866	0.0740726	0.4037661	0.3653419	2	GJA5/F2R
Biological Process	GO:1900240	negative regulation of phenotypic switching	2/2279	4/18866	0.0740726	0.4037661	0.3653419	2	MIR145/DNMT1
Biological Process	GO:1901671	positive regulation of superoxide dismutase activity	2/2279	4/18866	0.0740726	0.4037661	0.3653419	2	TNF/SIRT3

Biological Process	GO:1902565	positive regulation of neutrophil activation	2/2279	4/18866	0.0740726	0.4037661	0.3653419	2	CD177/ITGB2
Biological Process	GO:1902730	positive regulation of proteoglycan biosynthetic process	2/2279	4/18866	0.0740726	0.4037661	0.3653419	2	TCF7L2/PXYLP1
Biological Process	GO:1903463	regulation of mitotic cell cycle DNA replication	2/2279	4/18866	0.0740726	0.4037661	0.3653419	2	FGFR1/CHEK2
Biological Process	GO:1903903	regulation of establishment of T cell polarity	2/2279	4/18866	0.0740726	0.4037661	0.3653419	2	GSN/DOCK8
Biological Process	GO:1904154	positive regulation of retrograde protein transport, ER to cytosol	2/2279	4/18866	0.0740726	0.4037661	0.3653419	2	EDEM1/EDEM2
Biological Process	GO:1904340	positive regulation of dopaminergic neuron differentiation	2/2279	4/18866	0.0740726	0.4037661	0.3653419	2	CSNK1D/CSNK1E
Biological Process	GO:1904431	positive regulation of t-circle formation	2/2279	4/18866	0.0740726	0.4037661	0.3653419	2	ERCC1/SLX4
Biological Process	GO:1904505	regulation of telomere maintenance in response to DNA damage	2/2279	4/18866	0.0740726	0.4037661	0.3653419	2	RTEL1/ERCC1
Biological Process	GO:1904506	negative regulation of telomere maintenance in response to DNA damage	2/2279	4/18866	0.0740726	0.4037661	0.3653419	2	RTEL1/ERCC1

Biological Process	GO:1904833	positive regulation of removal of superoxide radicals	2/2279	4/18866	0.0740726	0.4037661	0.3653419	2	TNF/SIRT3
Biological Process	GO:1905180	positive regulation of cardiac muscle tissue regeneration	2/2279	4/18866	0.0740726	0.4037661	0.3653419	2	MIR199A1/MIR199A2
Biological Process	GO:1905702	regulation of inhibitory synapse assembly	2/2279	4/18866	0.0740726	0.4037661	0.3653419	2	SEMA4A/SEMA4D
Biological Process	GO:1905908	positive regulation of amyloid fibril formation	2/2279	4/18866	0.0740726	0.4037661	0.3653419	2	APP/CLU
Biological Process	GO:1990258	histone glutamine methylation	2/2279	4/18866	0.0740726	0.4037661	0.3653419	2	CREBBP/FBL
Biological Process	GO:1990743	protein sialylation	2/2279	4/18866	0.0740726	0.4037661	0.3653419	2	ST3GAL4/ST3GAL2
Biological Process	GO:1990809	endoplasmic reticulum tubular network membrane organization	2/2279	4/18866	0.0740726	0.4037661	0.3653419	2	RTN4/ATL1
Biological Process	GO:1990910	response to hypobaric hypoxia	2/2279	4/18866	0.0740726	0.4037661	0.3653419	2	FIS1/DNM1L
Biological Process	GO:2000589	regulation of metanephric mesenchymal cell migration	2/2279	4/18866	0.0740726	0.4037661	0.3653419	2	PDGFB/PDGFRB

Biological Process	GO:2000622	regulation of nuclear-transcribed mRNA catabolic process, nonsense-mediated decay	2/2279	4/18866	0.0740726	0.4037661	0.3653419	2	NBAS/SECISBP2
Biological Process	GO:2000623	negative regulation of nuclear-transcribed mRNA catabolic process, nonsense-mediated decay	2/2279	4/18866	0.0740726	0.4037661	0.3653419	2	NBAS/SECISBP2
Biological Process	GO:2000670	positive regulation of dendritic cell apoptotic process	2/2279	4/18866	0.0740726	0.4037661	0.3653419	2	RAPGEF2/BLK
Biological Process	GO:2000981	negative regulation of inner ear receptor cell differentiation	2/2279	4/18866	0.0740726	0.4037661	0.3653419	2	DLL1/NOTCH1
Biological Process	GO:2001013	epithelial cell proliferation involved in renal tubule morphogenesis	2/2279	4/18866	0.0740726	0.4037661	0.3653419	2	MTSS1/MEF2C
Biological Process	GO:2001253	regulation of histone H3-K36 trimethylation	2/2279	4/18866	0.0740726	0.4037661	0.3653419	2	BRD4/NSD3
Biological Process	GO:0051604	protein maturation	44/2279	293/18866	0.074593	0.4063691	0.3676972	44	CTSZ/NLRC4/CTSG/SORL1/ENO1/CALR/PLAU/GSN/ASPRV1/GGT1/TSPAN14/CYFIP2/SPG7/TBC1D10A/MIPEP/AIP/TESC/CPD/THBS1/MYH9/HM13/CTSH/HSCB/PCSK6/CIDEB/F12/PERP/ADAMTS13/CREBBP/NOL3/AEBP1/CPB2/NAA16/CASP1/LMF1/ASPH/ATP7B/CLN5/FADD/C1RL/SPCS1/ANGPTL8/VSIR/AOPEP
Biological Process	GO:1903829	positive regulation of cellular protein localization	50/2279	338/18866	0.0749721	0.4082006	0.3693544	50	SORL1/SSH1/TFDP1/TRIM8/PRR5L/TM9SF4/TMEM30A/YWHAH/ITGB1BP1/PIK3R1/RHOG/SMAD3/TNF/RTN4/MGAT3/JUP/EDEM1/USP36/ZDHHC2/TCF7L2/ABLIM3/AKAP5/UBE2J2/TGFB1/PRKCH/EDEM2/NUMA1/ZDHHC1/PRKCI/ITGB2/YWHAZ/CDH1/FIS1/BCL2/MAP1A/PRKCE/TP53BP2/MYO1C/MIEF1/CYLD/YWHAQ/EZR/PDZK1/KAT7/CCT6A/PARP9/LIF/PRKAA1/PRKN/RIPOR1
Biological Process	GO:0097305	response to alcohol	36/2279	234/18866	0.0755259	0.4109803	0.3718696	36	S100A8/BLM/SPIDR/PPARG/GSN/ADCY2/ITPR2/GPLD1/LARP1/JUP/TGFBR2/RECQL5/FECH/P2RY6/SPHK2/TGFB1/FDX1/CRHBP/MLC1/RARA/HTR3A/CSF3/ABCA1/CAT/CDH1/CA3/GNG2/ACACA/SLIT3/PRKCE/NPPC/BCL2L1/GRAMD1A/HMGCS1/INHBA/PRKAA1

Biological Process	GO:0007422	peripheral nervous system development	14/2279	77/18866	0.0761074	0.4139073	0.374518	14	ARHGEF10/SH3TC2/RUNX1/SKI/LGI4/NDRG1/PMP22/ILK/NF1/FA2H/CNTNAP1/RUNX3/RELA/NTF4
Biological Process	GO:0045840	positive regulation of mitotic nuclear division	11/2279	57/18866	0.0767698	0.4154372	0.3759023	11	TGFA/NSMCE2/CUL3/CDC16/NUMA1/PDGFB/RB1/CDCA5/ANAPC7/PDGFRB/SLF1
Biological Process	GO:1905517	macrophage migration	11/2279	57/18866	0.0767698	0.4154372	0.3759023	11	AZU1/CD9/C3AR1/CCR2/CCL5/CSF1R/SFTPD/THBS1/MIR24-2/CXCL17/CX3CR1
Biological Process	GO:0032965	regulation of collagen biosynthetic process	9/2279	44/18866	0.0769134	0.4154372	0.3759023	9	PPARG/MIR145/MYB/TGFB1/ENG/ARRB2/F2R/NPPC/PDGFRB
Biological Process	GO:0033173	calcineurin-NFAT signaling cascade	9/2279	44/18866	0.0769134	0.4154372	0.3759023	9	TNF/NFATC2/NFATC1/CAMTA1/RCAN1/DYRK2/FHL2/CHERP/STIMATE
Biological Process	GO:0035307	positive regulation of protein dephosphorylation	9/2279	44/18866	0.0769134	0.4154372	0.3759023	9	CAMTA1/TGFB1/PTPRC/PPP2R5D/PDGFRB/PPP1R16B/CD33/ANKLE2/PTPA
Biological Process	GO:0035329	hippo signaling	9/2279	44/18866	0.0769134	0.4154372	0.3759023	9	MARK3/TJP2/VGLL4/LIMD1/LATS2/WWTR1/STK3/TEAD2/NPHP4
Biological Process	GO:0042088	T-helper 1 type immune response	9/2279	44/18866	0.0769134	0.4154372	0.3759023	9	IL27/SLC11A1/IL1R1/CCR2/SEMA4A/IL18RAP/IL18R1/HLX/BCL3
Biological Process	GO:0044060	regulation of endocrine process	9/2279	44/18866	0.0769134	0.4154372	0.3759023	9	C1QTNF3/RAB11FIP1/GJA5/FGFR1/CRHBP/GHRL/F2R/AGTR1/INHBA

Biological Process	GO:0045332	phospholipid translocation	9/2279	44/18866	0.0769134	0.4154372	0.3759023	9	ABCC1/TMEM30A/ATP8A1/ANO7/ATP11A/ATP8B1/ABCA1/ATP8B4/ANO6
Biological Process	GO:0045687	positive regulation of glial cell differentiation	9/2279	44/18866	0.0769134	0.4154372	0.3759023	9	PPARG/TNFRSF1B/RHEB/BIN1/HDAC1/TGFB1/NOTCH1/RELA/LIF
Biological Process	GO:1901985	positive regulation of protein acetylation	9/2279	44/18866	0.0769134	0.4154372	0.3759023	9	ARRB1/RPS6KA4/SPHK2/ARNTL/PIH1D1/KAT7/LIF/PAXIP1/PRKAA1
Biological Process	GO:2000404	regulation of T cell migration	9/2279	44/18866	0.0769134	0.4154372	0.3759023	9	CCR2/CCL5/APP/DOCK8/RIPK3/CCL20/C10orf99/CCL27/FADD
Biological Process	GO:0033045	regulation of sister chromatid segregation	15/2279	84/18866	0.0772901	0.4167611	0.3771002	15	MAD1L1/TNKS/RMI2/NSMCE2/CUL3/CDC16/TACC3/NUMA1/NEK6/RB1/CDCA5/ANAPC7/HECW2/PCID2/SLF1
Biological Process	GO:0048144	fibroblast proliferation	15/2279	84/18866	0.0772901	0.4167611	0.3771002	15	PPARG/CREB1/MYB/SKI/ZMIZ1/B4GALT7/AQP1/NF1/PDGFD/PDGFB/MORC3/MED25/PDGFC/PDGFRB/PARP10
Biological Process	GO:0097061	dendritic spine organization	15/2279	84/18866	0.0772901	0.4167611	0.3771002	15	ZMYND8/CTTN/TANC1/EPHB3/BAIAP2/IGF1R/WASL/SIPA1L1/DOCK10/DNM3/DNM1L/SHANK2/UBE3A/CUX2/DBNL
Biological Process	GO:0006165	nucleoside diphosphate phosphorylation	22/2279	133/18866	0.0776804	0.4186283	0.3787897	22	FOXK1/HDAC4/PRKAG2/ENO1/HK1/PFKFB4/RAE1/CBFA2T3/DHTKD1/APP/OGDH/ADPGK/PGAM1/NUP93/ENO3/HK2/SEC13/ESRRB/PKM/AK5/PFKFB3/PRKAA1
Biological Process	GO:0055007	cardiac muscle cell differentiation	21/2279	126/18866	0.0781629	0.4205909	0.3805656	21	RXRA/CALR/CTDP1/MYO18B/TTN/MYH11/AKAP13/MAML1/SLC8A1/MIR199A1/DLL1/RBPJ/MIR24-2/MIR23A/TGFB1/RARA/ARRB2/MIR199A2/MEF2C/FHL2/PDGFRB

Biological Process	GO:0048259	regulation of receptor-mediated endocytosis	18/2279	105/18866	0.0787431	0.4205909	0.3805656	18	AP2A1/ANGPT1/AAK1/ARRB1/DNM2/MIR199A1/ANKRD13B/ANKRD13D/CLU/WASL/NUMB/ITGB3/ANKRD13A/DGKD/GSG1L/ARRB2/MIR199A2/FLOT1
Biological Process	GO:0006878	cellular copper ion homeostasis	4/2279	14/18866	0.0788414	0.4205909	0.3805656	4	COX19/APP/ATOX1/ATP7B
Biological Process	GO:0010649	regulation of cell communication by electrical coupling	4/2279	14/18866	0.0788414	0.4205909	0.3805656	4	PDE4D/CASQ2/SLC8A1/GJA5
Biological Process	GO:0010896	regulation of triglyceride catabolic process	4/2279	14/18866	0.0788414	0.4205909	0.3805656	4	PNPLA2/SORL1/GPLD1/ABHD5
Biological Process	GO:0010989	negative regulation of low-density lipoprotein particle clearance	4/2279	14/18866	0.0788414	0.4205909	0.3805656	4	MIR199A1/MIR27A/CSK/MIR199A2
Biological Process	GO:0030213	hyaluronan biosynthetic process	4/2279	14/18866	0.0788414	0.4205909	0.3805656	4	AP2A1/TGFB1/PDGFB/HAS3
Biological Process	GO:0034616	response to laminar fluid shear stress	4/2279	14/18866	0.0788414	0.4205909	0.3805656	4	SMAD7/ABCA1/ETS1/NFE2L2
Biological Process	GO:0038166	angiotensin-activated signaling pathway	4/2279	14/18866	0.0788414	0.4205909	0.3805656	4	MIR143/MIR145/AGTRAP/AGTR1
Biological Process	GO:0046037	GMP metabolic process	4/2279	14/18866	0.0788414	0.4205909	0.3805656	4	TJP2/GUK1/GMPR2/IMPDH1

Biological Process	GO:0051938	L-glutamate import	4/2279	14/18866	0.0788414	0.4205909	0.3805656	4	SLC1A2/SLC1A6/PER2/SLC1A3
Biological Process	GO:0055064	chloride ion homeostasis	4/2279	14/18866	0.0788414	0.4205909	0.3805656	4	ABCC2/SLC12A1/CA12/SLC12A7
Biological Process	GO:0072216	positive regulation of metanephros development	4/2279	14/18866	0.0788414	0.4205909	0.3805656	4	PAX2/PDGFB/PDGFRB/LIF
Biological Process	GO:0090128	regulation of synapse maturation	4/2279	14/18866	0.0788414	0.4205909	0.3805656	4	DISC1/NRXN1/YWHAZ/ARHGEF15
Biological Process	GO:0090239	regulation of histone H4 acetylation	4/2279	14/18866	0.0788414	0.4205909	0.3805656	4	ARRB1/CTBP1/PIH1D1/KAT7
Biological Process	GO:0098787	mRNA cleavage involved in mRNA processing	4/2279	14/18866	0.0788414	0.4205909	0.3805656	4	FIP1L1/ERN1/CPSF4/TUT1
Biological Process	GO:1902894	negative regulation of pri-miRNA transcription by RNA polymerase II	4/2279	14/18866	0.0788414	0.4205909	0.3805656	4	HDAC4/LILRB4/PDGFB/RELA
Biological Process	GO:1905064	negative regulation of vascular associated smooth muscle cell differentiation	4/2279	14/18866	0.0788414	0.4205909	0.3805656	4	NFATC2/NFATC1/PDGFB/DNMT1
Biological Process	GO:0001505	regulation of neurotransmitter levels	36/2279	235/18866	0.0792429	0.4223235	0.3821333	36	RAP1B/COLQ/COMT/GIT1/SLC44A4/SYN3/PTPRN2/PPFIA1/APBA2/PRKCB/MCTP2/NF1/NRXN1/VPS18/SYN2/SLC1A2/CTBP2/ITGB3/CHRM2/SLC1A6/NRXN2/PER2/SYNGR3/SLC1A3/RPH3A/P2RX1/DNM1L/MEF2C/SYT17/CACNA1B/SLC29A1/FLOT1/DTNBP1/ZNF219/HNMT/PRKN

Biological Process	GO:0006936	muscle contraction	53/2279	362/18866	0.0792551	0.4223235	0.3821333	53	HDAC4/PDE4D/ENO1/KCNE1/TTN/CASQ2/MIR143/MIR145/MYH11/MAP2K6/CACNA1C/FXYD1/MYLK/SLC8A1/CTTN/ANXA6/GJA5/MYL12A/TNNT3/JUP/BIN1/JSRP1/NOS1AP/SMAD7/PXN/ITGB5/SCN1A/PABPN1/TMOD3/GHRL/CHRM2/SNTA1/TPCN2/KCNQ1/SNTB1/MYOM1/ANKRD2/MYOT/GSTO1/GNAO1/P2RX1/F2R/DYSF/ANK2/ASPH/TMOD1/MYL7/CASQ1/SMTN/LTB4R/DAPK3/MB/GRK2
Biological Process	GO:0035265	organ growth	30/2279	191/18866	0.0793143	0.422402	0.3822043	30	RXRA/CTDP1/AKAP13/MIR199A1/ANXA6/VGLL4/CCM2L/TGFBR2/DLL1/RIPK1/RBPJ/MIR24-2/MIR23A/POC1A/LATS2/FGFR1/RARA/POR/RARG/STK3/ACACB/MEIS1/BCL2/MIR199A2/NOTCH1/MEF2C/UBE3A/NPPC/HLX/PDGFRB
Biological Process	GO:0045453	bone resorption	12/2279	64/18866	0.0795168	0.4226826	0.3824582	12	DEF8/INPP5D/PRKCA/UBASH3B/TNFAIP3/CSF1R/NF1/LRRK1/ITGB3/TMEM64/CSK/PLEKHM1
Biological Process	GO:0051306	mitotic sister chromatid separation	12/2279	64/18866	0.0795168	0.4226826	0.3824582	12	MAD1L1/NSMCE2/CUL3/CDC16/TACC3/PPP2R1A/NEK6/RB1/DIS3L2/ANAPC7/HECW2/PCID2
Biological Process	GO:0060135	maternal process involved in female pregnancy	12/2279	64/18866	0.0795168	0.4226826	0.3824582	12	KLF1/BSG/RXRA/VDR/DAZAP1/GHRL/CTSB/TPPP3/STOX2/TMED2/NODAL/LIF
Biological Process	GO:0030073	insulin secretion	33/2279	213/18866	0.0795449	0.4226826	0.3824582	33	NADK/ITPR2/MYRIP/CACNA1C/LRP5L/ARRB1/PRKCA/GPLD1/TNF/CCL5/ITPR1/RFX3/TCF7L2/HNF1A/PTPRN2/IL1RN/ENY2/MYO5A/GIPR/BLK/GHRL/KLF7/ARNTL/PER2/SLC16A1/PRKCE/SLC2A1/SREBF1/CPT1A/RPH3AL/C2CD2L/SIRT3/PRKN
Biological Process	GO:0043010	camera-type eye development	49/2279	332/18866	0.0796402	0.4229523	0.3827022	49	SPRED2/RXRA/PDE6A/FZR1/SLC25A25/LIMK2/MFSD2A/LPCAT1/CACNA1C/LRP5L/SKI/CRB1/SMAD3/ZEB2/HIPK2/TGFBR2/MYH15/NINJ1/DLL1/HDAC1/GNAT2/PAX2/FLT1/RDH10/RARA/NF1/CRYBB1/SP3/RARG/BMPR1B/WNT5B/GRHL2/IFT140/MYOM1/MEIS1/RPGRIP1/ZHX2/SDK2/BCAR3/NRL/PDGFRB/ARHGEF15/TMOD1/ALDH1A2/INHBA/CABP4/TSKU/NPHP4/NECTIN1
Biological Process	GO:1904950	negative regulation of establishment of protein localization	25/2279	155/18866	0.0803173	0.4257612	0.3852439	25	KCNE1/WWP2/ANGPT1/ITGB1BP1/RAB11FIP1/FRMD4A/MIR199A1/SUFU/RHBDF2/UBAC2/SNX3/DERL2/ENY2/NF1/GHRL/CSK/KLF7/MIR199A2/CABP1/SP100/F2R/SREBF1/ADTRP/SVIP/PRKN
Biological Process	GO:0009225	nucleotide-sugar metabolic process	8/2279	38/18866	0.0804845	0.4257612	0.3852439	8	CSGALNACT1/NAGK/AMDHD2/UXS1/GUK1/PMM2/GFPT2/DPAGT1

Biological Process	GO:0010742	macrophage derived foam cell differentiation	8/2279	38/18866	0.0804845	0.4257612	0.3852439	8	PPARG/ABCG1/TGFB1/PRKCH/NR1H2/ITGB3/ABCA1/AGTR1
Biological Process	GO:0032094	response to food	8/2279	38/18866	0.0804845	0.4257612	0.3852439	8	MPO/SLC25A25/GHRL/BCL10/SLC16A1/SREBF1/NPY/HSD11B2
Biological Process	GO:0060306	regulation of membrane repolarization	8/2279	38/18866	0.0804845	0.4257612	0.3852439	8	KCNE1/CASQ2/GJA5/NOS1AP/WDR1/SNTA1/KCNQ1/ANK2
Biological Process	GO:0071604	transforming growth factor beta production	8/2279	38/18866	0.0804845	0.4257612	0.3852439	8	LTBP1/CREB1/MYB/SMAD3/THBS1/WNT11/CDH3/ITGB6
Biological Process	GO:0090077	foam cell differentiation	8/2279	38/18866	0.0804845	0.4257612	0.3852439	8	PPARG/ABCG1/TGFB1/PRKCH/NR1H2/ITGB3/ABCA1/AGTR1
Biological Process	GO:0090090	negative regulation of canonical Wnt signaling pathway	29/2279	184/18866	0.0805276	0.4257612	0.3852439	29	CCDC88C/AMFR/UBAC2/CUL3/TCF7L2/LIMD1/HDAC1/LATS2/WNT11/KREMEN1/AXIN1/TLE1/WWTR1/TMEM64/WNT5B/STK3/PSMF1/KREMEN2/PSMB7/NOTCH1/FOXO1/CHD8/CYLD/ANKRD6/PTPRU/PSMD13/NPHP4/TMEM131L/PRKN
Biological Process	GO:0043277	apoptotic cell clearance	10/2279	51/18866	0.0815156	0.4275745	0.3868846	10	RHOH/CCR2/RHOG/THBS1/MARCO/RHOBTB2/FCN1/RARA/ITGB3/TYRO3
Biological Process	GO:0046460	neutral lipid biosynthetic process	10/2279	51/18866	0.0815156	0.4275745	0.3868846	10	PNPLA2/PLA2G15/ACSL1/MFSD2A/GPLD1/NR1H2/AVIL/PCK2/SREBF1/LPIN1
Biological Process	GO:0046463	acylglycerol biosynthetic process	10/2279	51/18866	0.0815156	0.4275745	0.3868846	10	PNPLA2/PLA2G15/ACSL1/MFSD2A/GPLD1/NR1H2/AVIL/PCK2/SREBF1/LPIN1

Biological Process	GO:0071827	plasma lipoprotein particle organization	10/2279	51/18866	0.0815156	0.4275745	0.3868846	10	P4HB/MPO/MFSD2A/LIPC/PLAGL2/ABCG1/PLTP/NR1H2/ABCA1/AGTR1
Biological Process	GO:0007029	endoplasmic reticulum organization	13/2279	71/18866	0.0815179	0.4275745	0.3868846	13	LMAN2/RTN4/RTN3/MIA3/MYO5A/VCPIP1/TOR1B/ATL1/ESYT1/CASQ1/SEC31A/GRAMD2A/DOP1B
Biological Process	GO:0033627	cell adhesion mediated by integrin	13/2279	71/18866	0.0815179	0.4275745	0.3868846	13	PLAU/ITGB1BP1/CCL5/TESC/PTPN6/ITGB5/JAM3/ITGB3/ITGB2/PDE3B/ITGB6/LIF/PLPP3
Biological Process	GO:0002064	epithelial cell development	34/2279	221/18866	0.0821369	0.4275745	0.3868846	34	PDE4D/CCDC88C/TJP2/SHROOM3/RAP1B/SPINT2/RHEB/TNF/CCM2/RFX3/DLL1/TNFRSF1A/FGFR1/RAPGEF2/RARA/RARG/WNT5B/GRHL2/PDGFB/ENG/ARNTL/RILPL1/NTRK1/ADD1/NOTCH1/S1PR2/NOTCH4/TMC1/GPR4/PPP1R16B/FRMD6/TMOD1/EZR/MYADM
Biological Process	GO:0099504	synaptic vesicle cycle	31/2279	199/18866	0.0822516	0.4275745	0.3868846	31	SYNJ2/DENND1A/PRKAR1B/RAP1B/PLD1/DNM2/ITSN1/STON1/GIT1/DDC/SYN3/APBA2/DGKQ/PRKCB/NRXN1/VPS18/SYN2/CTBP2/ACTG1/CHRM2/SH3GL1/DNM1/DNM3/P2RX1/DNM1L/SYT17/AP3D1/MX1/CYFIP1/DTNBP1/FGF14
Biological Process	GO:0046683	response to organophosphorus	22/2279	134/18866	0.0828114	0.4275745	0.3868846	22	BSG/SSH1/KCNE1/ITPR2/RAP1B/SLC8A1/APP/P2RY6/FDX1/CRHBP/RAPGEF2/DGKQ/AQP1/AGXT/KCNQ1/PER1/P2RX1/SREBF1/P2RX5/RELA/EZR/AQP9
Biological Process	GO:0032874	positive regulation of stress-activated MAPK cascade	27/2279	170/18866	0.0829006	0.4275745	0.3868846	27	MAP3K3/ERN1/ZNF622/TNF/ZEB2/TRAF2/TRAF1/HIPK2/TLR9/DUSP22/TNIK/RIPK1/APP/MTURN/SCIMP/AXIN1/SH3RF3/STK3/PLCB1/MAPK8IP2/TAOK3/NOD2/GADD45G/GDF6/ANKRD6/DBNL/MAP3K20
Biological Process	GO:0034614	cellular response to reactive oxygen species	27/2279	170/18866	0.0829006	0.4275745	0.3868846	27	TRPM2/MPO/IL10/LCN2/MYB/DNM2/TNF/TRAF2/TNFAIP3/CAMKK2/RIPK1/PXN/PAX2/ZNF580/FER/AQP1/RIPK3/PDGFD/ETS1/NFE2L2/IL18RAP/GLRX2/PDGFRB/FOXO1/RELA/SIRT3/PRKAA1
Biological Process	GO:0060998	regulation of dendritic spine development	14/2279	78/18866	0.0829268	0.4275745	0.3868846	14	ZMYND8/ITSN1/DISC1/BAIAP2/ASAP1/CPEB3/SIPA1L1/DNM3/DNM1L/MEF2C/SHANK2/UBE3A/CUX2/DBNL

Biological Process	GO:0072332	intrinsic apoptotic signaling pathway by p53 class mediator	14/2279	78/18866	0.0829268	0.4275745	0.3868846	14	BRCA2/HIPK2/RRM2B/ZNF385A/PERP/CDIP1/DYRK2/ANKRD2/BCL2/CHEK2/TP53BP2/BCL3/PRKN/MARCHF7
Biological Process	GO:0043414	macromolecule methylation	47/2279	318/18866	0.0829855	0.4275745	0.3868846	47	PIWIL4/GFI1/BRD4/THADA/METTL21A/MOV10L1/MYB/PRMT2/ASH2L/SNRPD3/SETD2/GATAD2A/SMYD3/EHMT2/WDR82/PAX7/METTL6/KMT2D/KDM1B/ATF7IP/KDM4C/SETD1B/GRHL2/SETD1A/CREBBP/TET2/MTA2/ZNF335/METTL22/PRDM16/PHF19/DNMT1/PIH1D1/MORC1/RBM15B/DYDC2/DYDC1/NTMT1/FBL/MECOM/METTL8/PAXIP1/EEF1AKMT3/EEF1AKMT2/NSD3/NSD2/CSKMT
Biological Process	GO:0010743	regulation of macrophage derived foam cell differentiation	7/2279	32/18866	0.083449	0.4275745	0.3868846	7	PPARG/ABCG1/PRKCH/NR1H2/ITGB3/ABCA1/AGTR1
Biological Process	GO:0010880	regulation of release of sequestered calcium ion into cytosol by sarcoplasmic reticulum	7/2279	32/18866	0.083449	0.4275745	0.3868846	7	PDE4D/CASQ2/CACNA1C/SLC8A1/GSTO1/ANK2/CASQ1
Biological Process	GO:0030201	heparan sulfate proteoglycan metabolic process	7/2279	32/18866	0.083449	0.4275745	0.3868846	7	CSGALNACT1/HS3ST3B1/DSE/NDST1/TCF7L2/XYLT1/PXYLP1
Biological Process	GO:0044818	mitotic G2/M transition checkpoint	7/2279	32/18866	0.083449	0.4275745	0.3868846	7	TRIM39/BLM/FOXN3/HUS1/TAOK3/CLSPN/NAE1
Biological Process	GO:0050849	negative regulation of calcium-mediated signaling	7/2279	32/18866	0.083449	0.4275745	0.3868846	7	CASQ2/ITPR1/RCAN1/DYRK2/GSTO1/FHL2/SLA2
Biological Process	GO:0032259	methylation	54/2279	371/18866	0.0836808	0.4275745	0.3868846	54	PIWIL4/GFI1/BRD4/THADA/METTL21A/MOV10L1/MYB/PRMT2/ASH2L/SNRPD3/SETD2/GATAD2A/SMYD3/COMT/EHMT2/WDR82/PAX7/METTL6/KMT2D/KDM1B/TPMT/ATF7IP/KDM4C/SETD1B/PRDM2/GRHL2/SETD1A/CREBBP/TET2/MTA2/ZNF335/METTL22/PRDM16/PHF19/DNMT1/GSTO1/PIH1D1/MAT1A/SMYD4/MORC1/RBM15B/DYDC2/DYDC1/NTMT1/FBL/HNMT/MECOM/METTL8/PAXIP1/EEF1AKMT3/EEF1AKMT2/NSD3/NSD2/CSKMT
Biological Process	GO:0006767	water-soluble vitamin metabolic process	15/2279	85/18866	0.0838611	0.4275745	0.3868846	15	ABCC1/MTHFD1L/MTHFS/PC/SLC19A1/CD320/PDXK/MTHFD1/GSTO1/SLC52A2/SLC2A1/GSTO2/SLC23A1/AOX1/ACP3

Biological Process	GO:0030101	natural killer cell activation	15/2279	85/18866	0.0838611	0.4275745	0.3868846	15	PIK3CD/FGR/SNX27/SLAMF7/DCAF15/RAB27A/HLA-F/NCR1/SP3/PGLYRP1/PTPRC/ITGB2/IL18R1/TYRO3/KAT7
Biological Process	GO:0032092	positive regulation of protein binding	15/2279	85/18866	0.0838611	0.4275745	0.3868846	15	MARK3/AMFR/ARRB1/HIPK2/APP/TCF7L2/RAPGEF2/CSF3/STK3/SPTA1/PLCL1/ADD1/FLOT1/STING1/PRKN
Biological Process	GO:0060537	muscle tissue development	59/2279	409/18866	0.083902	0.4275745	0.3868846	59	HDAC4/ZFPM1/RXRA/CALR/CTDP1/MYO18B/TTN/CREB1/MEF2D/MIR143/MIR145/MYH11/AKAP13/SKI/MAML1/MYLK/SMAD3/SLC8A1/MIR199A1/GJA5/VGLL4/CCM2L/TGFBR2/MYH15/DLL1/RIPK1/RBPJ/MIR24-2/MIR23A/IFRD1/SMAD7/FGFR1/PAX7/TGFB1/IGSF8/VAMP5/SVIL/RARA/NF1/ENG/ARRB2/ARNTL/MYOM1/ANKRD2/RB1/MEIS1/BCL2/MIR199A2/NOTCH1/MEF2C/FHL2/HLX/PDGFRB/ANKRD1/FLOT1/ALDH1A2/CASQ1/PRKAA1/MYORG
Biological Process	GO:0055088	lipid homeostasis	26/2279	163/18866	0.0840476	0.4275745	0.3868846	26	PNPLA2/EHD1/PPARG/NR1I2/THADA/C1QTNF3/LRP5L/LIPC/VDR/ORMDL3/ABCG1/ACOXL/RTN4/ASGR2/MED13/NR1H2/ABCA1/ACOX2/NPC1/ACACA/USF2/ABHD5/NR5A2/RALY/PRKAA1/ANGPTL8
Biological Process	GO:0033044	regulation of chromosome organization	52/2279	356/18866	0.084069	0.4275745	0.3868846	52	GFI1/BRD4/MAD1L1/TNKS/MYB/SKI/HMBOX1/RMI2/ARRB1/RTEL1/NSMCE2/C6orf89/YLPM1/PADI2/CUL3/OTUB2/CDC16/RPS6KA4/SPHK2/ZBTB7B/PAX7/CTBP1/TACC3/ATF7IP/KDM4C/MCM2/SETD1A/SMG6/NUMA1/ZNF335/NEK6/PHF19/OTUB1/RB1/DNMT1/PIH1D1/CDCA5/ANAPC7/TRIP12/ERCC1/HECW2/SREBF1/SLX4/RNF40/KAT7/PARP10/CCT6A/LIF/PCID2/PAXIP1/NSD3/SLF1
Biological Process	GO:0003333	amino acid transmembrane transport	16/2279	92/18866	0.0844119	0.4275745	0.3868846	16	SLC38A4/SLC38A10/SLC1A5/SLC15A4/LRRC8C/SLC43A2/SLC1A2/SLC7A7/SLC7A5/SLC1A6/PER2/SLC36A3/SLC1A3/SLC43A1/SLC7A8/SLC7A1
Biological Process	GO:0002726	positive regulation of T cell cytokine production	5/2279	20/18866	0.0845119	0.4275745	0.3868846	5	PRKCZ/NLRP3/IL1R1/TRAF2/IL18R1
Biological Process	GO:0002827	positive regulation of T-helper 1 type immune response	5/2279	20/18866	0.0845119	0.4275745	0.3868846	5	SLC11A1/IL1R1/CCR2/IL18R1/HLX
Biological Process	GO:0002902	regulation of B cell apoptotic process	5/2279	20/18866	0.0845119	0.4275745	0.3868846	5	FOXP1/IL10/HSH2D/BLK/BCL10

Biological Process	GO:0007252	I-kappaB phosphorylation	5/2279	20/18866	0.0845119	0.4275745	0.3868846	5	TNF/TRAF2/TLR9/ERC1/CX3CR1
Biological Process	GO:0008090	retrograde axonal transport	5/2279	20/18866	0.0845119	0.4275745	0.3868846	5	DYNC1H1/KIF1B/MAP1A/FBXW11/NDEL1
Biological Process	GO:0010878	cholesterol storage	5/2279	20/18866	0.0845119	0.4275745	0.3868846	5	EHD1/PPARG/ABCG1/NR1H2/ABCA1
Biological Process	GO:0032634	interleukin-5 production	5/2279	20/18866	0.0845119	0.4275745	0.3868846	5	PRKCZ/PDE4D/NLRP3/TNFRSF21/RARA
Biological Process	GO:0034315	regulation of Arp2/3 complex-mediated actin nucleation	5/2279	20/18866	0.0845119	0.4275745	0.3868846	5	WASL/GMFB/NCKAP1/CYFIP1/GMFG
Biological Process	GO:0043931	ossification involved in bone maturation	5/2279	20/18866	0.0845119	0.4275745	0.3868846	5	XYLT1/SEMA4D/LTF/ANO6/RFLNA
Biological Process	GO:0060546	negative regulation of necroptotic process	5/2279	20/18866	0.0845119	0.4275745	0.3868846	5	MIR101-2/RIPK1/ARHGEF2/FADD/PELI1
Biological Process	GO:0062099	negative regulation of programmed necrotic cell death	5/2279	20/18866	0.0845119	0.4275745	0.3868846	5	MIR101-2/RIPK1/ARHGEF2/FADD/PELI1
Biological Process	GO:0071636	positive regulation of transforming growth factor beta production	5/2279	20/18866	0.0845119	0.4275745	0.3868846	5	CREB1/MYB/SMAD3/THBS1/WNT11

Biological Process	GO:0097503	sialylation	5/2279	20/18866	0.0845119	0.4275745	0.3868846	5	ST6GALNAC1/ST6GAL1/ST8SIA6/ST3GAL4/ST3GAL2
Biological Process	GO:1902004	positive regulation of amyloid-beta formation	5/2279	20/18866	0.0845119	0.4275745	0.3868846	5	TNF/ABCG1/APP/CLU/RELA
Biological Process	GO:0002315	marginal zone B cell differentiation	3/2279	9/18866	0.0845647	0.4275745	0.3868846	3	DLL1/DOCK10/BCL3
Biological Process	GO:0002370	natural killer cell cytokine production	3/2279	9/18866	0.0845647	0.4275745	0.3868846	3	CD226/HLA-F/CD96
Biological Process	GO:0002480	antigen processing and presentation of exogenous peptide antigen via MHC class I, TAP-independent	3/2279	9/18866	0.0845647	0.4275745	0.3868846	3	LNPEP/HLA-C/HLA-F
Biological Process	GO:0002634	regulation of germinal center formation	3/2279	9/18866	0.0845647	0.4275745	0.3868846	3	TNFAIP3/TNFSF13B/MEF2C
Biological Process	GO:0002692	negative regulation of cellular extravasation	3/2279	9/18866	0.0845647	0.4275745	0.3868846	3	ABR/BCR/PLCB1
Biological Process	GO:0002727	regulation of natural killer cell cytokine production	3/2279	9/18866	0.0845647	0.4275745	0.3868846	3	CD226/HLA-F/CD96
Biological Process	GO:0006013	mannose metabolic process	3/2279	9/18866	0.0845647	0.4275745	0.3868846	3	HK1/PMM2/MAN2C1

Biological Process	GO:0006651	diacylglycerol biosynthetic process	3/2279	9/18866	0.0845647	0.4275745	0.3868846	3	PNPLA2/PLA2G15/AVIL
Biological Process	GO:0006681	galactosylceramide metabolic process	3/2279	9/18866	0.0845647	0.4275745	0.3868846	3	GALC/B4GALT3/FA2H
Biological Process	GO:0009157	deoxyribonucleoside monophosphate biosynthetic process	3/2279	9/18866	0.0845647	0.4275745	0.3868846	3	TK2/DCTD/TK1
Biological Process	GO:0010637	negative regulation of mitochondrial fusion	3/2279	9/18866	0.0845647	0.4275745	0.3868846	3	VAT1/DNM1L/PRKN
Biological Process	GO:0010911	regulation of isomerase activity	3/2279	9/18866	0.0845647	0.4275745	0.3868846	3	UHRF1/ITGB2/NOD2
Biological Process	GO:0010912	positive regulation of isomerase activity	3/2279	9/18866	0.0845647	0.4275745	0.3868846	3	UHRF1/ITGB2/NOD2
Biological Process	GO:0016102	diterpenoid biosynthetic process	3/2279	9/18866	0.0845647	0.4275745	0.3868846	3	DHRS9/RDH10/ALDH1A2
Biological Process	GO:0021603	cranial nerve formation	3/2279	9/18866	0.0845647	0.4275745	0.3868846	3	PHOX2A/ATP8B1/PAX2
Biological Process	GO:0030210	heparin biosynthetic process	3/2279	9/18866	0.0845647	0.4275745	0.3868846	3	CSGALNACT1/ANGPT1/NDST1

Biological Process	GO:0031666	positive regulation of lipopolysaccharide-mediated signaling pathway	3/2279	9/18866	0.0845647	0.4275745	0.3868846	3	PRKCA/SCIMP/LY86
Biological Process	GO:0034145	positive regulation of toll-like receptor 4 signaling pathway	3/2279	9/18866	0.0845647	0.4275745	0.3868846	3	WDFY1/LTF/PELI1
Biological Process	GO:0048539	bone marrow development	3/2279	9/18866	0.0845647	0.4275745	0.3868846	3	LRP5L/PTPRC/LRRC17
Biological Process	GO:0048702	embryonic neurocranium morphogenesis	3/2279	9/18866	0.0845647	0.4275745	0.3868846	3	MTHFD1L/NDST1/MTHFD1
Biological Process	GO:0048845	venous blood vessel morphogenesis	3/2279	9/18866	0.0845647	0.4275745	0.3868846	3	CCM2/ENG/NOTCH1
Biological Process	GO:0050847	progesterone receptor signaling pathway	3/2279	9/18866	0.0845647	0.4275745	0.3868846	3	PHB/UBE3A/NEDD4
Biological Process	GO:0051643	endoplasmic reticulum localization	3/2279	9/18866	0.0845647	0.4275745	0.3868846	3	MYO5A/ESYT1/GRAMD2A
Biological Process	GO:0060155	platelet dense granule organization	3/2279	9/18866	0.0845647	0.4275745	0.3868846	3	ABCA1/F2R/DTNBP1
Biological Process	GO:0060179	male mating behavior	3/2279	9/18866	0.0845647	0.4275745	0.3868846	3	HDAC4/HEXB/NCOA1

Biological Process	GO:0060556	regulation of vitamin D biosynthetic process	3/2279	9/18866	0.0845647	0.4275745	0.3868846	3	GFI1/VDR/TNF
Biological Process	GO:0061744	motor behavior	3/2279	9/18866	0.0845647	0.4275745	0.3868846	3	DCTN1/MFSD2A/PRKAA1
Biological Process	GO:0062042	regulation of cardiac epithelial to mesenchymal transition	3/2279	9/18866	0.0845647	0.4275745	0.3868846	3	TGFBR2/ENG/NOTCH1
Biological Process	GO:0071316	cellular response to nicotine	3/2279	9/18866	0.0845647	0.4275745	0.3868846	3	TNF/NTRK1/RELA
Biological Process	GO:0097119	postsynaptic density protein 95 clustering	3/2279	9/18866	0.0845647	0.4275745	0.3868846	3	ZMYND8/NRXN1/NRXN2
Biological Process	GO:0099159	regulation of modification of postsynaptic structure	3/2279	9/18866	0.0845647	0.4275745	0.3868846	3	ITSN1/BAIAP2/CYFIP1
Biological Process	GO:1901164	negative regulation of trophoblast cell migration	3/2279	9/18866	0.0845647	0.4275745	0.3868846	3	CALR/ARHGDIB/NODAL
Biological Process	GO:1903753	negative regulation of p38MAPK cascade	3/2279	9/18866	0.0845647	0.4275745	0.3868846	3	MIR138-2/CYLD/EZR
Biological Process	GO:1903867	extraembryonic membrane development	3/2279	9/18866	0.0845647	0.4275745	0.3868846	3	E2F7/DNAJB6/PAXIP1

Biological Process	GO:1904153	negative regulation of retrograde protein transport, ER to cytosol	3/2279	9/18866	0.0845647	0.4275745	0.3868846	3	UBAC2/DERL2/SVIP
Biological Process	GO:0002708	positive regulation of lymphocyte mediated immunity	18/2279	106/18866	0.0846346	0.4275745	0.3868846	18	PRKCZ/NLRP3/IL1R1/TNF/TRAF2/CD226/HLA-F/TGFB1/PTPRC/IL18RAP/TP53BP1/IL18R1/TNFSF13/CADM1/FADD/PAXIP1/CYRIB/NSD2
Biological Process	GO:0051817	modulation of process of other organism involved in symbiotic interaction	17/2279	99/18866	0.0846508	0.4275745	0.3868846	17	RXRA/CTDP1/ULK1/CCL5/CSF1R/SFTPD/HIPK2/HDAC1/PC/PABPN1/PHB/AQP1/TNIP1/LTF/BCL2L1/CPSF4/PRKN
Biological Process	GO:0007566	embryo implantation	11/2279	58/18866	0.0848874	0.4275745	0.3868846	11	BSG/RXRA/CALR/FUT7/ARHGDIB/TGFBR2/AGO2/VMP1/TPPP3/NODAL/LIF
Biological Process	GO:0000188	inactivation of MAPK activity	6/2279	26/18866	0.0851909	0.4275745	0.3868846	6	SPRED2/DUSP14/LAX1/PPP2R1A/RGS3/DUSP3
Biological Process	GO:0002068	glandular epithelial cell development	6/2279	26/18866	0.0851909	0.4275745	0.3868846	6	RHEB/RFX3/DLL1/RARA/RARG/ARNTL
Biological Process	GO:0003081	regulation of systemic arterial blood pressure by renin-angiotensin	6/2279	26/18866	0.0851909	0.4275745	0.3868846	6	CTSZ/CTSG/GJA5/F2R/AGTR1/AOPEP
Biological Process	GO:0007064	mitotic sister chromatid cohesion	6/2279	26/18866	0.0851909	0.4275745	0.3868846	6	TNKS/NSMCE2/RB1/RAD21L1/CDCA5/SLF1
Biological Process	GO:0030206	chondroitin sulfate biosynthetic process	6/2279	26/18866	0.0851909	0.4275745	0.3868846	6	CSGALNACT1/DSE/CHSY1/CHST11/CHST15/XYLT1

Biological Process	GO:0032967	positive regulation of collagen biosynthetic process	6/2279	26/18866	0.0851909	0.4275745	0.3868846	6	MYB/TGFB1/ENG/ARRB2/F2R/PDGFRB
Biological Process	GO:0036315	cellular response to sterol	6/2279	26/18866	0.0851909	0.4275745	0.3868846	6	GPLD1/MLC1/ABCA1/GRAMD1A/HMGCS1/INHBA
Biological Process	GO:0042832	defense response to protozoan	6/2279	26/18866	0.0851909	0.4275745	0.3868846	6	IRF8/IL10/LYST/SLC11A1/CCDC88B/BCL3
Biological Process	GO:0043951	negative regulation of cAMP-mediated signaling	6/2279	26/18866	0.0851909	0.4275745	0.3868846	6	PDE4D/MGRN1/GNAI2/MRAP/CRTC3/PDE3B
Biological Process	GO:0048643	positive regulation of skeletal muscle tissue development	6/2279	26/18866	0.0851909	0.4275745	0.3868846	6	DLL1/ARNTL/BCL2/MEF2C/FLOT1/PRKAA1
Biological Process	GO:0051350	negative regulation of lyase activity	6/2279	26/18866	0.0851909	0.4275745	0.3868846	6	CCR2/GNAI2/AKAP5/LTB4R2/PALM/GABBR2
Biological Process	GO:0060561	apoptotic process involved in morphogenesis	6/2279	26/18866	0.0851909	0.4275745	0.3868846	6	TNFRSF1B/VDR/TNFRSF1A/PAX2/NOTCH1/PPP2R1B
Biological Process	GO:0060706	cell differentiation involved in embryonic placenta development	6/2279	26/18866	0.0851909	0.4275745	0.3868846	6	SPINT2/E2F7/DNAJB6/STK3/GRHL2/LIF
Biological Process	GO:0090025	regulation of monocyte chemotaxis	6/2279	26/18866	0.0851909	0.4275745	0.3868846	6	CCR2/CCL5/FPR2/DEFB124/CXCL17/ANO6

Biological Process	GO:1900739	regulation of protein insertion into mitochondrial membrane involved in apoptotic signaling pathway	6/2279	26/18866	0.0851909	0.4275745	0.3868846	6	TFDP1/YWHAH/YWHAZ/BCL2/TP53BP2/YWHAQ
Biological Process	GO:1900740	positive regulation of protein insertion into mitochondrial membrane involved in apoptotic signaling pathway	6/2279	26/18866	0.0851909	0.4275745	0.3868846	6	TFDP1/YWHAH/YWHAZ/BCL2/TP53BP2/YWHAQ
Biological Process	GO:1905523	positive regulation of macrophage migration	6/2279	26/18866	0.0851909	0.4275745	0.3868846	6	C3AR1/CCL5/CSF1R/THBS1/CXCL17/CX3CR1
Biological Process	GO:0009615	response to virus	51/2279	349/18866	0.0858581	0.4298823	0.3889727	51	AZU1/IL27/ENO1/AIM2/TRIM38/HNRNPUL1/IRF5/LCN2/LYST/URI1/NLRP3/SETD2/FGR/TNF/TNFAIP3/CCL5/TLR9/SPON2/ODC1/CRCP/CLU/PHB/RIPK3/TRIM5/AGBL5/SEC14L1/PTPRC/CHRM2/NLRC5/ZDHHC1/MAP3K14/POU2F2/BCL2/TRAF3IP2/DDX41/BCL2L1/ILF3/MX1/IRF2/RELA/BCL3/BANF1/HERC5/PARP9/CCL22/ISG20/FADD/STING1/SHFL/NCBP3/ILRUN
Biological Process	GO:0050727	regulation of inflammatory response	61/2279	425/18866	0.0862051	0.4298823	0.3889727	61	ELANE/S100A8/BRD4/ABR/FOXP1/SBNO2/FUT7/MGLL/IL10/PPARG/IL16/C1QTNF3/MIR140/ABCC1/MIR145/PIK3AP1/TNFRSF1B/NLRP3/GGT1/IL1R1/CCR2/SMAD3/MEFV/TNF/SMPDL3B/TNFAIP3/CCL5/AOAH/FPR2/TLR9/RIPK1/TNFAIP6/TNFRSF1A/F12/FANCA/CXCL17/PGLYRP1/TNIP1/GHRL/PTPRC/MGST2/GRN/BCR/CNR2/ETS1/MIR138-2/DNASE1L3/NOD2/RB1/PER1/BCL6B/PLD3/CASP1/GPR4/CEBPB/AGTR1/CYLD/TYRO3/RELA/STING1/CCN4
Biological Process	GO:0048771	tissue remodeling	28/2279	178/18866	0.086291	0.4298823	0.3889727	28	ABR/DEF8/MIR143/INPP5D/LRP5L/CRB1/VDR/CCR2/PRKCA/MIR199A1/UBASH3B/TNFAIP3/CSF1R/GJA5/RBPJ/TGFB1/NF1/LRRK1/ITGB3/TMEM64/CSK/BCR/PLEKHM1/MIR199A2/MEF2C/EPAS1/F2R/LIF
Biological Process	GO:0014002	astrocyte development	9/2279	45/18866	0.0863263	0.4298823	0.3889727	9	S100A8/TNF/FPR2/EIF2B5/DLL1/APP/NF1/GRN/TSPAN2
Biological Process	GO:0031057	negative regulation of histone modification	9/2279	45/18866	0.0863263	0.4298823	0.3889727	9	SKI/OTUB2/SPHK2/CTBP1/KDM4C/OTUB1/DNMT1/PIH1D1/TRIP12
Biological Process	GO:0042771	intrinsic apoptotic signaling pathway in response to DNA damage by p53 class mediator	9/2279	45/18866	0.0863263	0.4298823	0.3889727	9	BRCA2/HIPK2/ZNF385A/CDIP1/DYRK2/BCL2/CHEK2/BCL3/MARCHF7

Biological Process	GO:0051293	establishment of spindle localization	9/2279	45/18866	0.0863263	0.4298823	0.3889727	9	DCTN1/NDE1/DYNC1H1/MYH9/HTT/NUMA1/ARHGEF2/FBXW11/NDEL1
Biological Process	GO:0060412	ventricular septum morphogenesis	9/2279	45/18866	0.0863263	0.4298823	0.3889727	9	ZFPM1/GJA5/TGFBR2/RBPJ/SMAD7/WNT11/NOTCH1/SLIT3/NSD2
Biological Process	GO:0070266	necroptotic process	9/2279	45/18866	0.0863263	0.4298823	0.3889727	9	TNF/MIR101-2/RIPK1/RIPK3/DNM1L/ARHGEF2/CYLD/FADD/PELI1
Biological Process	GO:0090311	regulation of protein deacetylation	9/2279	45/18866	0.0863263	0.4298823	0.3889727	9	SPRED2/SKI/C6orf89/SPHK2/ZBTB7B/CTBP1/NEK3/SREBF1/PRKAA1
Biological Process	GO:0007568	aging	47/2279	319/18866	0.0863767	0.4298823	0.3889727	47	PDE4D/TIMP2/MPO/CALR/FZR1/IL10/GSN/MAP3K3/CREB1/BRCA2/INPP5D/TNFRSF1B/AMFR/COL4A2/NSMCE2/EIF2B5/TGFBR2/DDC/AKT3/APP/ILK/ZMIZ1/PAX2/LITAF/ENO3/SLC1A2/PRDM2/CAT/NFE2L2/ENG/ITGB2/VASH1/ARNTL/NEK6/NTRK1/MORC3/BCL2/FOXM1/GLRX2/CHEK2/CD68/PDGFRB/ERCC1/SREBF1/RELA/ERCC2/SIRT3
Biological Process	GO:0016052	carbohydrate catabolic process	31/2279	200/18866	0.0865617	0.4298823	0.3889727	31	FUT4/FOXK1/HDAC4/PRKAG2/ENO1/FUT7/HK1/HEXB/PFKFB4/RAE1/AOAH/MGAM/CBFA2T3/CHIT1/DHTKD1/APP/OGDH/ADPGK/ENOSF1/PGAM1/GLYCTK/NUP93/ENO3/HK2/SEC13/PPP1CB/ESRRB/PKM/MAN2C1/PFKFB3/PRKAA1
Biological Process	GO:0045604	regulation of epidermal cell differentiation	12/2279	65/18866	0.087315	0.4298823	0.3889727	12	RUNX1/VDR/DLL1/PRKCH/GRHL2/NUMA1/KLF7/SGPP1/NOTCH1/AQP3/ZBED2/MACROH2A2
Biological Process	GO:0062012	regulation of small molecule metabolic process	65/2279	456/18866	0.0875591	0.4298823	0.3889727	65	MAEA/FOXK1/HDAC4/PRKAG2/GFI1/CDA/RCVRN/ENO1/AP2A1/PPARG/NCOR2/C1QTNF3/MFSD2A/IDI1/PFKFB4/VDR/RAE1/MBTPS1/GPLD1/TNF/ABCG1/CBFA2T3/PGP/ODC1/COMT/APP/PGAM1/P2RY6/SPHK2/TGFB1/RDH10/HRH1/DGKQ/POR/NUP93/DYRK2/NR1H2/SLC7A7/SEC13/CD320/GNB3/ACACB/PSMF1/PDGFB/PPP1CB/ESRRB/PSMB7/ACACA/PRKCE/LMF1/FOXO1/NCOA2/HMGCS1/SCD/SOGA1/SREBF1/WDTC1/CPT1A/PFKFB3/PSMD13/ACADVL/PRKAA1/PRKN/SDHAF3/RUBCNL
Biological Process	GO:0007584	response to nutrient	27/2279	171/18866	0.0876143	0.4298823	0.3889727	27	PPARG/GSN/ACSL1/TNKS/VDR/SLC8A1/TGFBR2/ALPL/GNAI2/RARA/POR/GIPR/ABCA1/NCOA1/CAT/ARSB/SLC16A1/NOD2/PKM/AQP3/HMGCL/USF2/ERCC1/HMGCS1/RELA/ALDH1A2/SST

Biological Process	GO:0030856	regulation of epithelial cell differentiation	27/2279	171/18866	0.0876143	0.4298823	0.3889727	27	SPRED2/RUNX1/VDR/RHEB/TNF/ZEB2/RFX3/DLL1/TNFRSF1A/PAX2/PRKCH/WWTR1/GRHL2/NUMA1/KLF7/ARNTL/SGPP1/ADD1/NOTCH1/AQP3/S1PR2/NOTCH4/CEBPB/ZBED2/NODAL/LIF/MACROH2A2
Biological Process	GO:0051302	regulation of cell division	27/2279	171/18866	0.0876143	0.4298823	0.3889727	27	BLM/TGFA/MIR145/BRCA2/ITGB1BP1/SETD2/KIF13A/LBH/E2F7/CUL3/GIT1/DLL1/UVRAG/TGFB1/SVIL/CSPP1/PDGFD/C10orf99/PDGFB/CAT/ZFYVE19/ESRRB/KLHL21/PRKCE/PDGFC/BCL2L1/CDC14A
Biological Process	GO:0071560	cellular response to transforming growth factor beta stimulus	38/2279	252/18866	0.0876473	0.4298823	0.3889727	38	LRG1/PRKCZ/LTBP1/SPRED2/CREB1/SKI/TGFBRAP1/SMAD3/COL4A2/LDLRAD4/DNM2/HIPK2/DUSP22/CHST11/TGFBR2/THBS1/MIR27A/SMAD7/PXN/ZMIZ1/ITGB5/TGFB1/SMURF1/PDGFD/TGFB1I1/HTRA4/CREBBP/ENG/ARRB2/PRDM16/ARHGEF18/GCNT2/MEF2C/HTRA3/ANKRD1/NODAL/ZFYVE9/CX3CR1
Biological Process	GO:0043281	regulation of cysteine-type endopeptidase activity involved in apoptotic process	33/2279	215/18866	0.0877993	0.4298823	0.3889727	33	NLRC4/S100A8/GRAMD4/PPARG/GSN/RPS6KA1/LCK/NLRP3/SMAD3/ARRB1/TNF/TRAF2/MICAL1/CTSD/THBS1/CTSH/RIPK1/PAX2/AQP1/POR/DNAJB6/BCL10/ATP2A3/NOL3/ARRB2/FIS1/PIH1D1/P2RX1/F2R/CASP1/BCL2L13/NODAL/FADD
Biological Process	GO:0009123	nucleoside monophosphate metabolic process	13/2279	72/18866	0.0890083	0.4298823	0.3889727	13	AMPD3/TJP2/TK2/UCK2/GUK1/AK2/GMPR2/AK5/LHPP/DCTD/DHODH/TK1/IMPDH1
Biological Process	GO:0033047	regulation of mitotic sister chromatid segregation	13/2279	72/18866	0.0890083	0.4298823	0.3889727	13	MAD1L1/TNKS/NSMCE2/CUL3/CDC16/TACC3/NUMA1/NEK6/RB1/ANAPC7/HECW2/PCID2/SLF1
Biological Process	GO:0045600	positive regulation of fat cell differentiation	13/2279	72/18866	0.0890083	0.4298823	0.3889727	13	RREB1/PPARG/CREB1/SH3PXD2B/ZBTB16/ZNF385A/ZBTB7B/TMEM64/WNT5B/STK3/AAMDC/PRDM16/CEBPB
Biological Process	GO:0014013	regulation of gliogenesis	21/2279	128/18866	0.08901	0.4298823	0.3889727	21	PPARG/CREB1/MYB/SKI/TNFRSF1B/CCR2/RHEB/TNF/BIN1/TNFRSF21/HDAC1/TGFB1/PRKCH/NF1/WDR1/PRKCI/NOTCH1/RELA/CERS2/LIF/CX3CR1
Biological Process	GO:0030336	negative regulation of cell migration	51/2279	350/18866	0.0891705	0.4298823	0.3889727	51	TACSTD2/ABR/CALR/PPARG/MIR140/KANK1/ARHGDIB/ZMYND8/ITGB1BP1/LDLRAD4/MIR199A1/TNF/MIR101-2/DUSP22/PADI2/JUP/NAV3/THBS1/C5AR2/MIR24-2/ILK/SMAD7/MIA3/WNT11/WASL/NISCH/TGFB1/PTPRJ/NF1/STK24/SRGAP1/BCR/PLCB1/NFE2L2/ENG/VASH1/MIR138-2/CDH1/BCL2/MIR199A2/NOTCH1/CORO1C/MEF2C/DUSP3/SP100/TRIB1/ADTRP/CERS2/PTPRU/NODAL/CX3CR1

Biological Process	GO:0046942	carboxylic acid transport	51/2279	350/18866	0.0891705	0.4298823	0.3889727	51	SLC38A4/PRKAG2/RXRA/PPARG/SLC38A10/SLC51A/ACSL1/ABCC1/SLC16A3/SLC10A1/MFSD2A/MAP2K6/SLC11A1/SLC1A5/FOLR3/ABCC2/SLC15A4/THBS1/ATP8B1/LRRC8C/PPFIA1/SLC43A2/SLC19A1/SLC26A1/NF1/SLCO3A1/SLC1A2/SLC7A7/SLC7A5/AGXT/SFXN1/NCOA1/ACACB/SLC1A6/SLC16A5/ABCC3/PER2/SLC16A1/SLC36A3/SLC1A3/ACACA/SLC43A1/SLC2A1/SLC7A8/NCOA2/SLC13A5/CPT1A/SLC7A1/SLC25A1/SLC23A1/AQP9
Biological Process	GO:0006446	regulation of translational initiation	14/2279	79/18866	0.0901236	0.4298823	0.3889727	14	RXRA/KLHL25/TNF/CCL5/NCK2/EIF2B5/LARP1/CTIF/AGO2/MIF4GD/PAIP1/BANK1/EIF3H/EIF4G1
Biological Process	GO:0006826	iron ion transport	14/2279	79/18866	0.0901236	0.4298823	0.3889727	14	LMTK2/SLC25A37/LCN2/SLC11A1/DNM2/SLC11A2/ATP6V1B2/SFXN1/ATP6V0B/LTF/ARHGAP1/ATP6V0C/ATP6V1C1/NECTIN1
Biological Process	GO:0010827	regulation of glucose transmembrane transport	14/2279	79/18866	0.0901236	0.4298823	0.3889727	14	PRKAG2/MIR143/PIK3R1/TNF/GRB10/PRKCB/SLC1A2/HK2/PRKCI/NFE2L2/SLC2A1/RTN2/ASPSCR1/CLTCL1
Biological Process	GO:0050672	negative regulation of lymphocyte proliferation	14/2279	79/18866	0.0901236	0.4298823	0.3889727	14	IL10/MAD1L1/INPP5D/SFTPD/MNDA/PTPN6/TNFRSF21/ZBTB7B/BLK/CEBPB/PELI1/TMEM131L/VSIR/MARCHF7
Biological Process	GO:0052548	regulation of endopeptidase activity	62/2279	434/18866	0.0903156	0.4298823	0.3889727	62	SLPI/NLRC4/S100A8/CSTA/TIMP2/SORL1/SERPINA1/GRAMD4/SPOCK2/AIM2/PPARG/GSN/RPS6KA1/APLP2/LCK/SERPINB11/NLRP3/SMAD3/ARRB1/SPINT2/MEFV/TNF/TRAF2/CYFIP2/MICAL1/TBC1D10A/SERPINB2/CTSD/BIN1/THBS1/CTSH/RIPK1/APP/UBE2O/MBP/MIR24-2/HDAC1/PAX2/CIDEB/AQP1/PERP/POR/DNAJB6/BCL10/ATP2A3/PSMF1/CRIM1/NOL3/ARRB2/FIS1/PIH1D1/P2RX1/F2R/LTF/CASP1/BCL2L13/COL28A1/ASPH/NODAL/FADD/PCID2/VSIR
Biological Process	GO:0032411	positive regulation of transporter activity	19/2279	114/18866	0.090358	0.4298823	0.3889727	19	CCR2/DNM2/SGK1/KCTD7/TESC/NOS1AP/ACTN4/P2RY6/HTR3A/HTT/SYNGR3/GSTO1/ANK2/SGK2/PDZK1/CASQ1/FGF14/STIMATE/CRACR2A
Biological Process	GO:0016485	protein processing	34/2279	223/18866	0.0904186	0.4298823	0.3889727	34	CTSZ/NLRC4/CTSG/ENO1/PLAU/GSN/ASPRV1/GGT1/CYFIP2/SPG7/TBC1D10A/MIPEP/CPD/THBS1/MYH9/HM13/CTSH/PCSK6/CIDEB/F12/PERP/ADAMTS13/NOL3/AEBP1/CPB2/CASP1/ASPH/CLN5/FADD/C1RL/SPCS1/ANGPTL8/VSIR/AOPEP
Biological Process	GO:0051339	regulation of lyase activity	10/2279	52/18866	0.0905302	0.4298823	0.3889727	10	RCVRN/TIMP2/CACNA1C/CCR2/GNAI2/AKAP5/LTB4R2/NF1/PALM/GABBR2

Biological Process	GO:1903557	positive regulation of tumor necrosis factor superfamily cytokine production	10/2279	52/18866	0.0905302	0.4298823	0.3889727	10	PIK3R1/TLR9/SPON2/RIPK1/CLU/PTPRJ/PTPRC/NOD2/ARHGEF2/FADD
Biological Process	GO:0001776	leukocyte homeostasis	15/2279	86/18866	0.0907795	0.4298823	0.3889727	15	LAT/PIK3CD/ITPKB/CCR2/TNFAIP3/TNFSF13B/RIPK3/ZC3H8/JAM3/BCL10/SPTA1/DOCK10/BCL2/MEF2C/FADD
Biological Process	GO:0006919	activation of cysteine-type endopeptidase activity involved in apoptotic process	15/2279	86/18866	0.0907795	0.4298823	0.3889727	15	NLRC4/S100A8/PPARG/LCK/NLRP3/SMAD3/TNF/TRAF2/CTSH/RIPK1/ATP2A3/P2RX1/F2R/BCL2L13/FADD
Biological Process	GO:0070664	negative regulation of leukocyte proliferation	15/2279	86/18866	0.0907795	0.4298823	0.3889727	15	IL10/MAD1L1/INPP5D/TNFAIP3/SFTPD/MNDA/PTPN6/TNFRSF21/ZBTB7B/BLK/CEBPB/PELI1/TMEM131L/VSIR/MARCHF7
Biological Process	GO:0006656	phosphatidylcholine biosynthetic process	8/2279	39/18866	0.0910492	0.4298823	0.3889727	8	PCYT1A/MFSD2A/LPCAT1/SLC44A4/CHPT1/PCTP/CHKB/LPIN1
Biological Process	GO:0030866	cortical actin cytoskeleton organization	8/2279	39/18866	0.0910492	0.4298823	0.3889727	8	CALR/FMNL3/TNF/FMNL1/EPB41L3/NCKAP1/STRIP1/EZR
Biological Process	GO:0031076	embryonic camera-type eye development	8/2279	39/18866	0.0910492	0.4298823	0.3889727	8	HIPK2/PAX2/RDH10/RARA/SP3/RARG/IFT140/ALDH1A2
Biological Process	GO:0032691	negative regulation of interleukin-1 beta production	8/2279	39/18866	0.0910492	0.4298823	0.3889727	8	NLRP3/MEFV/TNFAIP3/MIR101-2/GHRL/ARRB2/CD33/CX3CR1
Biological Process	GO:0050775	positive regulation of dendrite morphogenesis	8/2279	39/18866	0.0910492	0.4298823	0.3889727	8	CUX1/BAIAP2/ILK/SS18L2/CDKL3/DNM1L/CUX2/DBNL

Biological Process	GO:1901021	positive regulation of calcium ion transmembrane transporter activity	8/2279	39/18866	0.0910492	0.4298823	0.3889727	8	P2RY6/HTT/GSTO1/ANK2/CASQ1/FGF14/STIMATE/CRACR2A
Biological Process	GO:0050848	regulation of calcium-mediated signaling	17/2279	100/18866	0.091057	0.4298823	0.3889727	17	PDE4D/CASQ2/TNF/ITPR1/JSRP1/CAMTA1/RCAN1/P2RY6/MYO5A/DYRK2/HTT/GSTO1/FHL2/CHERP/P2RX5/STIMATE/SLA2
Biological Process	GO:0035107	appendage morphogenesis	24/2279	150/18866	0.0914247	0.4298823	0.3889727	24	IQCE/CACNA1C/SKI/GJA5/CHST11/ZBTB16/HDAC1/FGFR1/RDH10/RARG/BMPR1B/GRHL2/RUNX2/IFT140/CREBBP/HOXA9/NOTCH1/ALX3/GNA12/ASPH/ALDH1A2/ZNF219/MAP3K20/CPLANE1
Biological Process	GO:0035108	limb morphogenesis	24/2279	150/18866	0.0914247	0.4298823	0.3889727	24	IQCE/CACNA1C/SKI/GJA5/CHST11/ZBTB16/HDAC1/FGFR1/RDH10/RARG/BMPR1B/GRHL2/RUNX2/IFT140/CREBBP/HOXA9/NOTCH1/ALX3/GNA12/ASPH/ALDH1A2/ZNF219/MAP3K20/CPLANE1
Biological Process	GO:0070661	leukocyte proliferation	46/2279	313/18866	0.0917755	0.4298823	0.3889727	46	HLA-DMB/IL27/IL10/MAD1L1/CD55/INPP5D/SLC11A1/TNFRSF1B/CCR2/TNFAIP3/CCL5/CSF1R/NCK2/SFTPD/NFATC2/TNFSF13B/TLR9/MNDA/TGFBR2/PTPN6/CLU/TNFRSF21/DOCK8/ZBTB7B/RIPK3/CD79A/BLK/CCDC88B/PTPRC/CD320/SPTA1/ZNF335/PNP/BCL2/MEF2C/CEBPB/HHEX/DOCK2/SLC7A1/FADD/PELI1/VAV3/IMPDH1/TMEM131L/VSIR/MARCHF7
Biological Process	GO:1903532	positive regulation of secretion by cell	46/2279	313/18866	0.0917755	0.4298823	0.3889727	46	SORL1/NADK/IL10/TTN/C1QTNF3/MYRIP/CREB1/CLEC9A/MYB/MAP2K6/CD177/GAB2/FRMD4A/ARRB1/GPLD1/FGR/TNF/MYO18A/ABCG1/CLEC5A/ITSN1/RAB27A/HLA-F/TCF7L2/FGFR1/SPHK2/TGFB1/GIPR/BLK/NR1H2/GHRL/ITGB2/MYOM1/DNM1L/PRKCE/CPB2/ANKRD1/CADM1/CD33/RPH3AL/EZR/DTNBP1/INHBA/C2CD2L/SIRT3/GRK2
Biological Process	GO:0046651	lymphocyte proliferation	42/2279	283/18866	0.0920095	0.4298823	0.3889727	42	HLA-DMB/IL27/IL10/MAD1L1/CD55/INPP5D/SLC11A1/TNFRSF1B/CCR2/CCL5/NCK2/SFTPD/NFATC2/TNFSF13B/TLR9/MNDA/TGFBR2/PTPN6/TNFRSF21/DOCK8/ZBTB7B/RIPK3/CD79A/BLK/CCDC88B/PTPRC/CD320/SPTA1/ZNF335/PNP/BCL2/MEF2C/CEBPB/DOCK2/SLC7A1/FADD/PELI1/VAV3/IMPDH1/TMEM131L/VSIR/MARCHF7
Biological Process	GO:0001906	cell killing	27/2279	172/18866	0.0925036	0.4298823	0.3889727	27	DEFA4/AZU1/ELANE/CTSG/LYST/CD55/PIK3R6/SLAMF7/CTSH/TUBB4B/RAB27A/CD226/HLA-F/PTPN6/NCR1/RIPK3/CD59/PGLYRP1/PTPRC/ARRB2/IL18RAP/DNASE1L3/LTF/PGLYRP4/CADM1/FADD/CYRIB
Biological Process	GO:0070304	positive regulation of stress-activated protein kinase signaling cascade	27/2279	172/18866	0.0925036	0.4298823	0.3889727	27	MAP3K3/ERN1/ZNF622/TNF/ZEB2/TRAF2/TRAF1/HIPK2/TLR9/DUSP22/TNIK/RIPK1/APP/MTURN/SCIMP/AXIN1/SH3RF3/STK3/PLCB1/MAPK8IP2/TAOK3/NOD2/GADD45G/GDF6/ANKRD6/DBNL/MAP3K20

Biological Process	GO:0014706	striated muscle tissue development	56/2279	389/18866	0.0926803	0.4298823	0.3889727	56	HDAC4/ZFPM1/RXRA/CALR/CTDP1/MYO18B/TTN/CREB1/MEF2D/MYH11/AKAP13/SKI/MAML1/SMAD3/SLC8A1/MIR199A1/GJA5/VGLL4/CCM2L/TGFBR2/MYH15/DLL1/RIPK1/RBPJ/MIR24-2/MIR23A/IFRD1/SMAD7/FGFR1/PAX7/TGFB1/IGSF8/VAMP5/SVIL/RARA/NF1/ENG/ARRB2/ARNTL/MYOM1/ANKRD2/RB1/MEIS1/BCL2/MIR199A2/NOTCH1/MEF2C/FHL2/HLX/PDGFRB/ANKRD1/FLOT1/ALDH1A2/CASQ1/PRKAA1/MYORG
Biological Process	GO:0043903	regulation of symbiotic process	35/2279	231/18866	0.0929404	0.4298823	0.3889727	35	SLPI/TRIM27/P4HB/TRIM38/CTDP1/GSN/TRIM8/TRIM10/SRPK2/TNF/POLR2F/CCL5/CSF1R/SFTPD/LARP1/SNX3/HDAC1/PC/PHB/FCN1/TRIM5/TNIP1/RSF1/BCL2/NOTCH1/LTF/ILF3/MX1/NR5A2/PARP10/BANF1/ISG20/TRIM26/PRKN/SHFL
Biological Process	GO:0048738	cardiac muscle tissue development	35/2279	231/18866	0.0929404	0.4298823	0.3889727	35	ZFPM1/RXRA/CALR/CTDP1/MYO18B/TTN/CREB1/MYH11/AKAP13/MAML1/SLC8A1/MIR199A1/GJA5/VGLL4/CCM2L/TGFBR2/DLL1/RIPK1/RBPJ/MIR24-2/MIR23A/SMAD7/FGFR1/TGFB1/RARA/ENG/ARRB2/MEIS1/MIR199A2/NOTCH1/MEF2C/FHL2/PDGFRB/ANKRD1/ALDH1A2
Biological Process	GO:0002429	immune response-activating cell surface receptor signaling pathway	68/2279	481/18866	0.0934365	0.4298823	0.3889727	68	PDE4D/LAT/FOXP1/C3AR1/PIK3CD/ELMO1/LCK/RUNX1/INPP5D/CLEC4C/DOCK1/PIK3R1/WIPF1/GPLD1/FGR/LAX1/CYFIP2/GRAP2/FPR2/FPR1/NFATC2/EIF2B5/MNDA/DUSP22/IGHV6-1/BAIAP2/C5AR2/CD226/PTPN6/MUC12/TNFRSF21/KLHL6/WASL/PRKCH/PTPRJ/PRKCB/FCN1/LILRB4/CD79A/MUC20/BLK/VAV2/PTPRC/ACTG1/CSK/BCL10/PSMF1/CREBBP/THEMIS2/NCKAP1/BCL2/PSMB7/MEF2C/DUSP3/PRAM1/PRKCE/ELF1/MYO1C/CYLD/PLCG1/CYFIP1/RELA/FBXW11/EZR/PSMD13/VAV3/FYB1/SLA2
Biological Process	GO:0002757	immune response-activating signal transduction	68/2279	481/18866	0.0934365	0.4298823	0.3889727	68	PDE4D/LAT/FOXP1/C3AR1/PIK3CD/ELMO1/LCK/RUNX1/INPP5D/CLEC4C/DOCK1/PIK3R1/WIPF1/GPLD1/FGR/LAX1/CYFIP2/GRAP2/FPR2/FPR1/NFATC2/EIF2B5/MNDA/DUSP22/IGHV6-1/BAIAP2/C5AR2/CD226/PTPN6/MUC12/TNFRSF21/KLHL6/WASL/PRKCH/PTPRJ/PRKCB/FCN1/LILRB4/CD79A/MUC20/BLK/VAV2/PTPRC/ACTG1/CSK/BCL10/PSMF1/CREBBP/THEMIS2/NCKAP1/BCL2/PSMB7/MEF2C/DUSP3/PRAM1/PRKCE/ELF1/MYO1C/CYLD/PLCG1/CYFIP1/RELA/FBXW11/EZR/PSMD13/VAV3/FYB1/SLA2
Biological Process	GO:0045454	cell redox homeostasis	11/2279	59/18866	0.0935132	0.4298823	0.3889727	11	P4HB/MPO/SLC11A1/NCF4/LPO/PDIA5/NCF2/TMX1/NFE2L2/GLRX2/TXNRD2
Biological Process	GO:0045815	positive regulation of gene expression, epigenetic	11/2279	59/18866	0.0935132	0.4298823	0.3889727	11	TAF1C/BAZ1B/SERTAD2/POLR2F/PADI2/ZMIZ1/SPHK2/ARID1B/SMARCD1/TAF1D/MYO1C
Biological Process	GO:0000041	transition metal ion transport	21/2279	129/18866	0.0947847	0.4298823	0.3889727	21	TRPM2/LMTK2/SLC25A37/SLC39A11/LCN2/SLC11A1/DNM2/ABCC2/SLC11A2/ATP6V1B2/TMEM163/SFXN1/ATOX1/SLC39A13/ATP6V0B/LTF/ARHGAP1/ATP7B/ATP6V0C/ATP6V1C1/NECTIN1
Biological Process	GO:0002576	platelet degranulation	21/2279	129/18866	0.0947847	0.4298823	0.3889727	21	SERPINA1/CD9/TTN/APLP2/LY6G6F/THBS1/ACTN1/APP/ITGA2B/ACTN4/CLU/PHACTR2/TGFB1/BLK/ITGB3/LHFPL2/PDGFB/WDR1/TUBA4A/P2RX1/CYRIB

Biological Process	GO:0035025	positive regulation of Rho protein signal transduction	7/2279	33/18866	0.0954055	0.4298823	0.3889727	7	AKAP13/ARRB1/SYNPO2L/F2R/GPR4/PDGFRB/PRAG1
Biological Process	GO:0051984	positive regulation of chromosome segregation	7/2279	33/18866	0.0954055	0.4298823	0.3889727	7	NSMCE2/CUL3/CDC16/NUMA1/RB1/ANAPC7/SLF1
Biological Process	GO:0090022	regulation of neutrophil chemotaxis	7/2279	33/18866	0.0954055	0.4298823	0.3889727	7	C3AR1/C5AR2/JAM3/CAMK1D/DAPK2/DNM1L/DYSF
Biological Process	GO:1902003	regulation of amyloid-beta formation	7/2279	33/18866	0.0954055	0.4298823	0.3889727	7	SORL1/TNF/ABCG1/BIN1/APP/CLU/RELA
Biological Process	GO:1902745	positive regulation of lamellipodium organization	7/2279	33/18866	0.0954055	0.4298823	0.3889727	7	HDAC4/RREB1/ARHGEF7/DNM2/NCKAP1/CORO1C/AVIL
Biological Process	GO:0048568	embryonic organ development	64/2279	451/18866	0.0955191	0.4298823	0.3889727	64	ZFPM1/ABR/IL10/POLE/SETD2/SMAD3/SPINT2/MTHFD1L/SUFU/TNF/GJA5/HIPK2/MICAL2/CHST11/E2F7/TGFBR2/SLC44A4/NDST1/DLL1/RBPJ/MIB1/LRIG1/WNT11/PAX2/FGFR1/TTC39C/TGFB1/RDH10/RARA/SP3/DNAJB6/DSCAML1/RARG/STK3/GRHL2/RUNX2/NCOA1/IFT140/PDGFB/BCR/ENG/VASH1/MTHFD1/HOXA9/NOTCH1/PBX4/ALX3/MEF2C/EPAS1/GGNBP2/PDGFC/HLX/TMED2/CEBPB/PCDH12/PDGFRB/ERCC1/TEAD2/PLCD3/ALDH1A2/NODAL/ERCC2/LIF/HOXB7
Biological Process	GO:0032768	regulation of monooxygenase activity	12/2279	66/18866	0.0955699	0.4298823	0.3889727	12	GFI1/VDR/DNM2/TNF/NOS1AP/CDH3/WASL/NOSTRIN/POR/CNR2/MIR138-2/NOSIP
Biological Process	GO:0090092	regulation of transmembrane receptor protein serine/threonine kinase signaling pathway	38/2279	254/18866	0.0957362	0.4298823	0.3889727	38	LRG1/LTBP1/SPRED2/SORL1/MIR140/SKI/SMAD3/LDLRAD4/DNM2/HIPK2/CHST11/TGFBR2/THBS1/RBPJ/UBE2O/ILK/PCSK6/SMAD7/TGFB1/SMURF1/NUP93/TGFB1I1/BMPER/WWTR1/HTRA4/NUMA1/CREBBP/ENG/PRDM16/NOTCH1/RBPMS/HTRA3/GDF6/ZC3H3/ZNF423/INHBA/NODAL/SPART
Biological Process	GO:0010863	positive regulation of phospholipase C activity	9/2279	46/18866	0.0963937	0.4298823	0.3889727	9	FLT1/P2RY6/FGFR1/PHB/LPAR2/S1PR4/PDGFRB/PLCG1/NTF4

Biological Process	GO:0032692	negative regulation of interleukin-1 production	9/2279	46/18866	0.0963937	0.4298823	0.3889727	9	IL10/NLRP3/MEFV/TNFAIP3/MIR101-2/GHRL/ARRB2/CD33/CX3CR1
Biological Process	GO:0035987	endodermal cell differentiation	9/2279	46/18866	0.0963937	0.4298823	0.3889727	9	SETD2/COL4A2/COL12A1/ITGB5/ITGB2/COL5A1/LAMB3/INHBA/NODAL
Biological Process	GO:0051489	regulation of filopodium assembly	9/2279	46/18866	0.0963937	0.4298823	0.3889727	9	TRPM2/ZMYND8/ARAP1/WASL/ESPN/PALM/RALA/DNM3/PPP1R16B
Biological Process	GO:2000142	regulation of DNA-templated transcription, initiation	9/2279	46/18866	0.0963937	0.4298823	0.3889727	9	CREB1/HNF1A/MED13/ATF7IP/SUB1/MORC1/ERCC1/XPA/PAXIP1
Biological Process	GO:0042098	T cell proliferation	30/2279	195/18866	0.0974087	0.4298823	0.3889727	30	HLA-DMB/IL27/IL10/MAD1L1/CD55/SLC11A1/TNFRSF1B/CCR2/CCL5/NCK2/SFTPD/TNFSF13B/TGFBR2/PTPN6/TNFRSF21/DOCK8/ZBTB7B/RIPK3/CCDC88B/PTPRC/SPTA1/PNP/CEBPB/DOCK2/SLC7A1/FADD/PELI1/TMEM131L/VSIR/MARCHF7
Biological Process	GO:0002281	macrophage activation involved in immune response	4/2279	15/18866	0.0976416	0.4298823	0.3889727	4	SBNO2/GRN/PRKCE/DYSF
Biological Process	GO:0007638	mechanosensory behavior	4/2279	15/18866	0.0976416	0.4298823	0.3889727	4	NRXN1/HTT/NRXN2/SLC1A3
Biological Process	GO:0010867	positive regulation of triglyceride biosynthetic process	4/2279	15/18866	0.0976416	0.4298823	0.3889727	4	MFSD2A/GPLD1/NR1H2/SREBF1
Biological Process	GO:0015936	coenzyme A metabolic process	4/2279	15/18866	0.0976416	0.4298823	0.3889727	4	ACOT7/PPCDC/ACAT1/PANK4

Biological Process	GO:0016114	terpenoid biosynthetic process	4/2279	15/18866	0.0976416	0.4298823	0.3889727	4	DHRS9/RDH10/HMGCS1/ALDH1A2
Biological Process	GO:0042362	fat-soluble vitamin biosynthetic process	4/2279	15/18866	0.0976416	0.4298823	0.3889727	4	GFI1/VDR/TNF/PLTP
Biological Process	GO:0043374	CD8-positive, alpha-beta T cell differentiation	4/2279	15/18866	0.0976416	0.4298823	0.3889727	4	RUNX1/ZBTB7B/BCL2/RUNX3
Biological Process	GO:0045475	locomotor rhythm	4/2279	15/18866	0.0976416	0.4298823	0.3889727	4	ZFHX3/MTA1/USP2/NCOA2
Biological Process	GO:0045779	negative regulation of bone resorption	4/2279	15/18866	0.0976416	0.4298823	0.3889727	4	INPP5D/UBASH3B/TNFAIP3/CSK
Biological Process	GO:0050862	positive regulation of T cell receptor signaling pathway	4/2279	15/18866	0.0976416	0.4298823	0.3889727	4	LCK/CD226/CYLD/RELA
Biological Process	GO:0060391	positive regulation of SMAD protein signal transduction	4/2279	15/18866	0.0976416	0.4298823	0.3889727	4	TGFB1/NUP93/RBPMS/NODAL
Biological Process	GO:0071801	regulation of podosome assembly	4/2279	15/18866	0.0976416	0.4298823	0.3889727	4	GSN/TNF/ASAP1/ARHGEF2
Biological Process	GO:0072148	epithelial cell fate commitment	4/2279	15/18866	0.0976416	0.4298823	0.3889727	4	DLL1/RBPJ/RARA/NOTCH1

Biological Process	GO:0090192	regulation of glomerulus development	4/2279	15/18866	0.0976416	0.4298823	0.3889727	4	PAX2/PDGFD/IL6R/PDGFB
Biological Process	GO:0090594	inflammatory response to wounding	4/2279	15/18866	0.0976416	0.4298823	0.3889727	4	CCR2/TGFB1/GRN/F2R
Biological Process	GO:1901550	regulation of endothelial cell development	4/2279	15/18866	0.0976416	0.4298823	0.3889727	4	TNF/TNFRSF1A/ADD1/S1PR2
Biological Process	GO:1902043	positive regulation of extrinsic apoptotic signaling pathway via death domain receptors	4/2279	15/18866	0.0976416	0.4298823	0.3889727	4	THBS1/BMPR1B/STK3/TRPS1
Biological Process	GO:1902187	negative regulation of viral release from host cell	4/2279	15/18866	0.0976416	0.4298823	0.3889727	4	TRIM27/TRIM8/TRIM5/TRIM26
Biological Process	GO:1903140	regulation of establishment of endothelial barrier	4/2279	15/18866	0.0976416	0.4298823	0.3889727	4	TNF/TNFRSF1A/ADD1/S1PR2
Biological Process	GO:2000402	negative regulation of lymphocyte migration	4/2279	15/18866	0.0976416	0.4298823	0.3889727	4	PADI2/MIA3/WASL/ADTRP
Biological Process	GO:2001256	regulation of store-operated calcium entry	4/2279	15/18866	0.0976416	0.4298823	0.3889727	4	CD84/CASQ1/GRAMD2A/SARAF
Biological Process	GO:0032945	negative regulation of mononuclear cell proliferation	14/2279	80/18866	0.0976994	0.4298823	0.3889727	14	IL10/MAD1L1/INPP5D/SFTPD/MNDA/PTPN6/TNFRSF21/ZBTB7B/BLK/CEBPB/PELI1/TMEM131L/VSIR/MARCHF7

Biological Process	GO:0045778	positive regulation of ossification	16/2279	94/18866	0.0980452	0.4298823	0.3889727	16	CEBPD/SMAD3/SLC8A1/ZBTB16/ILK/TGFB1/BMPR1B/RUNX2/IL6R/MEF2C/NPPC/LTF/CEBPB/ANO6/FAM20C/CCN4
Biological Process	GO:0030512	negative regulation of transforming growth factor beta receptor signaling pathway	15/2279	87/18866	0.0980469	0.4298823	0.3889727	15	LTBP1/SPRED2/SKI/SMAD3/LDLRAD4/DNM2/CHST11/TGFBR2/SMAD7/TGFB1/SMURF1/TGFB1I1/HTRA4/PRDM16/HTRA3
Biological Process	GO:0001562	response to protozoan	6/2279	27/18866	0.0988698	0.4298823	0.3889727	6	IRF8/IL10/LYST/SLC11A1/CCDC88B/BCL3
Biological Process	GO:0002825	regulation of T-helper 1 type immune response	6/2279	27/18866	0.0988698	0.4298823	0.3889727	6	IL27/SLC11A1/IL1R1/CCR2/IL18R1/HLX
Biological Process	GO:0006779	porphyrin-containing compound biosynthetic process	6/2279	27/18866	0.0988698	0.4298823	0.3889727	6	SLC11A2/FECH/ALAS1/SPTA1/PPOX/TMEM14C
Biological Process	GO:0033014	tetrapyrrole biosynthetic process	6/2279	27/18866	0.0988698	0.4298823	0.3889727	6	SLC11A2/FECH/ALAS1/SPTA1/PPOX/TMEM14C
Biological Process	GO:1903319	positive regulation of protein maturation	6/2279	27/18866	0.0988698	0.4298823	0.3889727	6	NLRC4/ENO1/GSN/MYH9/F12/ANGPTL8
Biological Process	GO:2000353	positive regulation of endothelial cell apoptotic process	6/2279	27/18866	0.0988698	0.4298823	0.3889727	6	MIR101-2/THBS1/MIR24-2/PRKCI/ANO6/PLCG1
Biological Process	GO:0015849	organic acid transport	51/2279	353/18866	0.0996326	0.4298823	0.3889727	51	SLC38A4/PRKAG2/RXRA/PPARG/SLC38A10/SLC51A/ACSL1/ABCC1/SLC16A3/SLC10A1/MFSD2A/MAP2K6/SLC11A1/SLC1A5/FOLR3/ABCC2/SLC15A4/THBS1/ATP8B1/LRRC8C/PPFIA1/SLC43A2/SLC19A1/SLC26A1/NF1/SLCO3A1/SLC1A2/SLC7A7/SLC7A5/AGXT/SFXN1/NCOA1/ACACB/SLC1A6/SLC16A5/ABCC3/PER2/SLC16A1/SLC36A3/SLC1A3/ACACA/SLC43A1/SLC2A1/SLC7A8/NCOA2/SLC13A5/CPT1A/SLC7A1/SLC25A1/SLC23A1/AQP9

Biological Process	GO:0043949	regulation of cAMP-mediated signaling	10/2279	53/18866	0.1001169	0.4298823	0.3889727	10	PDE4D/MGRN1/PRKAR1B/PRKCA/GNAI2/MRAP/CRTC3/RAPGEF2/GIPR/PDE3B
Biological Process	GO:0097035	regulation of membrane lipid distribution	10/2279	53/18866	0.1001169	0.4298823	0.3889727	10	ABCC1/TMEM30A/ABCG1/ATP8A1/ANO7/ATP11A/ATP8B1/ABCA1/ATP8B4/ANO6
Biological Process	GO:0002115	store-operated calcium entry	5/2279	21/18866	0.1003904	0.4298823	0.3889727	5	CD84/CASQ1/GRAMD2A/SARAF/CRACR2A
Biological Process	GO:0009110	vitamin biosynthetic process	5/2279	21/18866	0.1003904	0.4298823	0.3889727	5	GFI1/VDR/TNF/PLTP/PDXK
Biological Process	GO:0010801	negative regulation of peptidyl-threonine phosphorylation	5/2279	21/18866	0.1003904	0.4298823	0.3889727	5	SPRED2/SMAD7/DGKQ/PPP2R5D/EIF4G1
Biological Process	GO:0010893	positive regulation of steroid biosynthetic process	5/2279	21/18866	0.1003904	0.4298823	0.3889727	5	TNF/ABCG1/POR/SREBF1/PRKAA1
Biological Process	GO:0032616	interleukin-13 production	5/2279	21/18866	0.1003904	0.4298823	0.3889727	5	PRKCZ/NLRP3/TNFRSF21/SPHK2/RARA
Biological Process	GO:0046519	sphingoid metabolic process	5/2279	21/18866	0.1003904	0.4298823	0.3889727	5	SPTLC2/SPHK2/SGPP1/SGPP2/PLPP3
Biological Process	GO:0046823	negative regulation of nucleocytoplasmic transport	5/2279	21/18866	0.1003904	0.4298823	0.3889727	5	ANGPT1/SUFU/NF1/CABP1/SP100

Biological Process	GO:0048305	immunoglobulin secretion	5/2279	21/18866	0.1003904	0.4298823	0.3889727	5	TNF/TRAF2/TNFSF13B/POU2F2/TRAF3IP2
Biological Process	GO:0060713	labyrinthine layer morphogenesis	5/2279	21/18866	0.1003904	0.4298823	0.3889727	5	IL10/SPINT2/DNAJB6/GRHL2/NCOA1
Biological Process	GO:0060716	labyrinthine layer blood vessel development	5/2279	21/18866	0.1003904	0.4298823	0.3889727	5	RBPJ/VASH1/GGNBP2/TMED2/PLCD3
Biological Process	GO:2000737	negative regulation of stem cell differentiation	5/2279	21/18866	0.1003904	0.4298823	0.3889727	5	LBH/NFE2L2/ESRRB/NOTCH1/CDK13
Biological Process	GO:0048706	embryonic skeletal system development	21/2279	130/18866	0.1007954	0.4298823	0.3889727	21	SETD2/SMAD3/MTHFD1L/CHST11/TGFBR2/NDST1/WNT11/XYLT1/PAX7/RDH10/SP3/DSCAML1/GRHL2/RUNX2/IFT140/MTHFD1/HOXA9/ALX3/MEF2C/NODAL/HOXB7
Biological Process	GO:2000058	regulation of ubiquitin-dependent protein catabolic process	25/2279	159/18866	0.1008308	0.4298823	0.3889727	25	TRIM39/RNF144A/FZR1/RNF19A/CSNK1D/SUFU/HIPK2/CBFA2T3/DISC1/CLU/SMAD7/SMURF1/AXIN1/CLEC16A/CSNK1E/NFE2L2/RNF144B/MAP1A/GNA12/UBE3A/RNF14/TRIB1/BAG5/SVIP/PRKN
Biological Process	GO:0009914	hormone transport	47/2279	323/18866	0.1008937	0.4298823	0.3889727	47	NADK/C1QTNF3/ITPR2/MYRIP/CREB1/MYB/CACNA1C/LRP5L/RAB11FIP1/ARRB1/PRKCA/GPLD1/TNF/CCL5/ABCC2/ITSN1/ITPR1/RFX3/TCF7L2/HNF1A/PTPRN2/FGFR1/IL1RN/CRHBP/ENY2/MYO5A/GIPR/BLK/SLC7A5/GHRL/KLF7/ARNTL/PER2/SLC16A1/PRKCE/AGTR1/CRYM/SLC2A1/SLC7A8/SREBF1/CPT1A/RPH3AL/INHBA/LIF/C2CD2L/SIRT3/PRKN
Biological Process	GO:0048736	appendage development	28/2279	181/18866	0.1010023	0.4298823	0.3889727	28	IQCE/CACNA1C/SKI/GJA5/CHST11/ZBTB16/PRKAB1/HDAC1/FGFR1/KREMEN1/RDH10/RARA/RARG/BMPR1B/GRHL2/RUNX2/IFT140/CREBBP/KREMEN2/HOXA9/NOTCH1/ALX3/GNA12/ASPH/ALDH1A2/ZNF219/MAP3K20/CPLANE1
Biological Process	GO:0060173	limb development	28/2279	181/18866	0.1010023	0.4298823	0.3889727	28	IQCE/CACNA1C/SKI/GJA5/CHST11/ZBTB16/PRKAB1/HDAC1/FGFR1/KREMEN1/RDH10/RARA/RARG/BMPR1B/GRHL2/RUNX2/IFT140/CREBBP/KREMEN2/HOXA9/NOTCH1/ALX3/GNA12/ASPH/ALDH1A2/ZNF219/MAP3K20/CPLANE1

Biological Process	GO:0001654	eye development	55/2279	384/18866	0.1015686	0.4298823	0.3889727	55	SPRED2/RXRA/PDE6A/FZR1/SLC25A25/LIMK2/MFSD2A/LPCAT1/CACNA1C/LRP5L/SKI/CRB1/SMAD3/ZEB2/SH3PXD2B/HIPK2/TGFBR2/MYH15/NINJ1/DLL1/HDAC1/GNAT2/PAX2/FLT1/RDH10/RARA/NF1/CRYBB1/SP3/RARG/BMPR1B/WNT5B/GRHL2/IFT140/MEIS2/PRKCI/MYOM1/MEIS1/BCL2/RPGRIP1/ZHX2/PBX4/COL5A1/SDK2/BCAR3/NRL/PDGFRB/ARHGEF15/TMOD1/ALDH1A2/INHBA/CABP4/TSKU/NPHP4/NECTIN1
Biological Process	GO:0048675	axon extension	20/2279	123/18866	0.1019746	0.4298823	0.3889727	20	SEMA6B/NRP2/ULK1/CTTN/DNM2/SEMA4A/RTN4/DISC1/SEMA4B/IFRD1/ILK/SEMA4D/CDH4/CDKL3/SLIT3/CYFIP1/TRPV2/NDEL1/SLIT1/DBNL
Biological Process	GO:0050730	regulation of peptidyl-tyrosine phosphorylation	39/2279	263/18866	0.1021345	0.4298823	0.3889727	39	PRKCZ/TGFA/SH3BP5L/ANGPT1/TNF/CCL5/CSF1R/NCK2/SH3BP5/DUSP22/APP/PTPN6/TNFRSF1A/TGFB1/PTPRJ/SEMA4D/DGKQ/PDGFD/LRRK1/CSF3/ITGB3/GHRL/PTPRC/IL6R/PDGFB/PPP2R1A/CASS4/ARRB2/ITGB2/PRKCE/GGNBP2/BANK1/PDGFC/ARHGEF2/PDGFRB/INPP5F/CRLF1/PARP9/LIF
Biological Process	GO:0002714	positive regulation of B cell mediated immunity	8/2279	40/18866	0.1023718	0.4298823	0.3889727	8	TNF/CD226/TGFB1/PTPRC/TP53BP1/TNFSF13/PAXIP1/NSD2
Biological Process	GO:0002891	positive regulation of immunoglobulin mediated immune response	8/2279	40/18866	0.1023718	0.4298823	0.3889727	8	TNF/CD226/TGFB1/PTPRC/TP53BP1/TNFSF13/PAXIP1/NSD2
Biological Process	GO:0006730	one-carbon metabolic process	8/2279	40/18866	0.1023718	0.4298823	0.3889727	8	MTHFD1L/MTHFS/SFXN1/MTHFD1/CA3/CA1/MAT1A/CA12
Biological Process	GO:0030501	positive regulation of bone mineralization	8/2279	40/18866	0.1023718	0.4298823	0.3889727	8	SMAD3/SLC8A1/TGFB1/BMPR1B/MEF2C/LTF/ANO6/FAM20C
Biological Process	GO:0043403	skeletal muscle tissue regeneration	8/2279	40/18866	0.1023718	0.4298823	0.3889727	8	CD9/TGFBR2/IFRD1/PAX7/ENO3/PKM/DYSF/EYS
Biological Process	GO:0048713	regulation of oligodendrocyte differentiation	8/2279	40/18866	0.1023718	0.4298823	0.3889727	8	PPARG/TNFRSF1B/RHEB/TNFRSF21/HDAC1/NF1/WDR1/NOTCH1

Biological Process	GO:0060562	epithelial tube morphogenesis	48/2279	331/18866	0.1025062	0.4298823	0.3889727	48	CTSZ/TACSTD2/FOXP1/SHROOM3/LRP5L/SKI/VDR/SETD2/SMAD3/SPINT2/MTHFD1L/SUFU/TNF/ZEB2/CSF1R/CCM2/MICAL2/TGFBR2/CTSH/DLL1/RBPJ/MIB1/ILK/WNT11/PAX2/TGFB1/RDH10/RARA/PFN1/MTSS1/RARG/STK3/GRHL2/BCL10/ENG/MTHFD1/RALA/BCL2/NOTCH1/MEF2C/NOTCH4/TMED2/TSC2/TEAD2/LMO4/NODAL/HOXB7/BRD2
Biological Process	GO:0001836	release of cytochrome c from mitochondria	11/2279	60/18866	0.1026469	0.4298823	0.3889727	11	PAM16/BMF/CLU/CIDEB/NOL3/ARRB2/FIS1/BCL2/DNM1L/BCL2L1/PRKN
Biological Process	GO:0010823	negative regulation of mitochondrion organization	11/2279	60/18866	0.1026469	0.4298823	0.3889727	11	VAT1/PAM16/CLU/CLEC16A/NOL3/ARRB2/FNIP2/DNM1L/TSC2/BCL2L1/PRKN
Biological Process	GO:0035904	aorta development	11/2279	60/18866	0.1026469	0.4298823	0.3889727	11	LTBP1/MIR143/MIR145/MYLK/DNM2/SUFU/NDST1/RBPJ/ENG/NOTCH1/PDGFRB
Biological Process	GO:0007033	vacuole organization	27/2279	174/18866	0.1028136	0.4298823	0.3889727	27	ENO1/ULK1/HEXB/LYST/MBTPS1/PACS2/WIPI2/PIP4K2A/FEZ2/SMURF1/RUFY4/ENO3/RAB43/VPS18/ATG14/ABCA1/HOOK2/GRN/TPCN2/TBC1D14/ARSB/VMP1/UBXN2A/CLN5/NPRL2/STING1/ACP3
Biological Process	GO:2001020	regulation of response to DNA damage stimulus	34/2279	226/18866	0.103864	0.4298823	0.3889727	34	FAM168A/SPRED2/SPIDR/RMI2/RAD52/SETD2/RTEL1/WRNIP1/RECQL5/NACC2/OTUB2/ZNF385A/CLU/UBE2V1/OTUB1/BCL2/TP53BP1/FOXM1/CHEK2/TRIP12/ERCC1/BCL2L1/ANKRD1/KAT7/PARP9/PAXIP1/TFIP11/MARCHF7/NSD2/CYREN/ABRAXAS1/SLF1/FBH1/BABAM2
Biological Process	GO:0032943	mononuclear cell proliferation	42/2279	286/18866	0.1039846	0.4298823	0.3889727	42	HLA-DMB/IL27/IL10/MAD1L1/CD55/INPP5D/SLC11A1/TNFRSF1B/CCR2/CCL5/NCK2/SFTPD/NFATC2/TNFSF13B/TLR9/MNDA/TGFBR2/PTPN6/TNFRSF21/DOCK8/ZBTB7B/RIPK3/CD79A/BLK/CCDC88B/PTPRC/CD320/SPTA1/ZNF335/PNP/BCL2/MEF2C/CEBPB/DOCK2/SLC7A1/FADD/PELI1/VAV3/IMPDH1/TMEM131L/VSIR/MARCHF7
Biological Process	GO:0140014	mitotic nuclear division	42/2279	286/18866	0.1039846	0.4298823	0.3889727	42	ARHGEF10/TGFA/CTDP1/MAD1L1/TTN/TNKS/NDE1/PRKCA/NSMCE2/RCC1/CHAMP1/CUL3/CCDC8/CDC16/MIS12/DCTN2/PRKCB/TACC3/STAG1/NUMA1/PDGFB/PPP2R1A/MSTO1/NEK6/RB1/RAD21L1/CDCA5/DIS3L2/CHEK2/ANAPC7/PDGFRB/SPAG5/HECW2/CDC14A/AURKAIP1/NDEL1/LPIN1/DAPK3/CDK13/PCID2/ABRAXAS1/SLF1
Biological Process	GO:0002286	T cell activation involved in immune response	18/2279	109/18866	0.1039933	0.4298823	0.3889727	18	HLA-DMB/IL27/PRKCZ/ZFPM1/FOXP1/MYB/SLC11A1/NLRP3/LOXL3/SEMA4A/RAB27A/SMAD7/LY9/ZBTB7B/RARA/IL18R1/HLX/BCL3

Biological Process	GO:0038127	ERBB signaling pathway	23/2279	145/18866	0.1040154	0.4298823	0.3889727	23	TGFA/SH3TC2/PIK3R1/PRKCA/ARHGEF7/NCK2/DOK1/RHBDF2/RTN4/ARAP1/APP/RBPJ/EPS15L1/TGFB1/FER/PTPRJ/FAM83A/PDE6H/DGKD/DUSP3/EPS15/PLCG1/MVB12A
Biological Process	GO:0071322	cellular response to carbohydrate stimulus	23/2279	145/18866	0.1040154	0.4298823	0.3889727	23	NADK/SLC2A5/MAP2K6/LRP5L/ERN1/ARRB1/GPLD1/COLEC12/IGF1R/PTPRN2/PAX2/ENY2/PRKCB/GHRL/KLF7/FIS1/PIH1D1/PRKCE/CPB2/PCK2/SLC29A1/C2CD2L/PRKAA1
Biological Process	GO:0045670	regulation of osteoclast differentiation	12/2279	67/18866	0.1042813	0.4298823	0.3889727	12	CREB1/INPP5D/PIK3R1/TNF/UBASH3B/TMEM178A/LILRB4/NF1/TMEM64/LRRC17/LTF/CEBPB
Biological Process	GO:0034250	positive regulation of cellular amide metabolic process	26/2279	167/18866	0.1046288	0.4298823	0.3889727	26	RXRA/PLD1/TNF/CCL5/NCK2/ABCG1/EIF2B5/LARP1/KRT17/THBS1/APP/SAMD4A/TNFRSF1A/CLU/CTIF/SPHK2/MIF4GD/CPEB3/NFE2L2/PKM/PAIP1/RELA/BCL3/EIF4G1/SIRT3/NIBAN1
Biological Process	GO:0032006	regulation of TOR signaling	17/2279	102/18866	0.1047732	0.4298823	0.3889727	17	RPTOR/MIR199A1/RHEB/DGKQ/CLEC16A/SIK3/SEC13/SH3BP4/ARNTL/MIR199A2/PIH1D1/GNA12/TSC2/TNFAIP8L1/TTI1/NPRL2/PRKAA1
Biological Process	GO:0006801	superoxide metabolic process	13/2279	74/18866	0.1052322	0.4298823	0.3889727	13	MPO/CD177/NCF4/TNF/FPR2/SH3PXD2B/GNAI2/PREX1/NCF2/TGFB1/NFE2L2/ITGB2/SIRT3
Biological Process	GO:0002460	adaptive immune response based on somatic recombination of immune receptors built from immunoglobulin superfamily domains	53/2279	370/18866	0.106113	0.4298823	0.3889727	53	IL27/PRKCZ/FUT7/IL10/CD55/INPP5D/SLC11A1/TNFRSF1B/NLRP3/IL1R1/CCR2/TNF/TRAF2/TNFAIP3/LOXL3/TNFSF13B/DUSP22/IGHV6-1/SEMA4A/CTSH/RAB27A/CD226/HLA-F/PTPN6/CLU/SMAD7/UNG/KLHL6/LY9/ZBTB7B/TGFB1/PHB/RIPK3/BACH2/C1S/PTPRC/BCL10/IL18RAP/POU2F2/TP53BP1/MEF2C/IL18R1/HLX/ERCC1/TNFSF13/C1QB/BCL3/FADD/PAXIP1/C1RL/CYRIB/NSD2/SLA2
Biological Process	GO:0007050	cell cycle arrest	35/2279	234/18866	0.1063882	0.4298823	0.3889727	35	RPTOR/PRKAG2/CALR/TFDP1/APBB2/MAP2K6/ERN1/SMAD3/RHEB/E2F7/WHAMM/ZFHX3/BIN1/THBS1/PCBP4/ZNF385A/TCF7L2/ZBTB17/PRKAB1/ILK/TGFB1/ARID3A/CAB39/VASH1/RB1/NOTCH1/MED25/FOXM1/CHEK2/ZBTB49/CDC14A/INHBA/TBRG4/PRKAA1/MAP3K20
Biological Process	GO:0007157	heterophilic cell-cell adhesion via plasma membrane cell adhesion molecules	9/2279	47/18866	0.1071106	0.4298823	0.3889727	9	CADM3/CRB1/CEACAM6/CDH4/CADM1/CEACAM5/JAML/NECTIN1/NECTIN4

Biological Process	GO:0008542	visual learning	9/2279	47/18866	0.1071106	0.4298823	0.3889727	9	CREB1/PPP1R1B/TANC1/APP/SYNGAP1/HRH1/NF1/MEIS2/SYNPO
Biological Process	GO:0042551	neuron maturation	9/2279	47/18866	0.1071106	0.4298823	0.3889727	9	LGI4/BCL11A/APP/FEV/RB1/BCL2/CLN5/SPTBN4/GLDN
Biological Process	GO:0051047	positive regulation of secretion	49/2279	340/18866	0.1079265	0.4298823	0.3889727	49	S100A8/SORL1/NADK/IL10/TTN/C1QTNF3/MYRIP/CREB1/CLEC9A/MYB/MAP2K6/CD177/GAB2/FRMD4A/ARRB1/GPLD1/FGR/TNF/MYO18A/ABCG1/CLEC5A/ITSN1/RAB27A/GNAI2/HLA-F/TCF7L2/FGFR1/SPHK2/TGFB1/AQP1/GIPR/BLK/NR1H2/GHRL/ITGB2/MYOM1/DNM1L/PRKCE/CPB2/ANKRD1/CADM1/CD33/RPH3AL/EZR/DTNBP1/INHBA/C2CD2L/SIRT3/GRK2
Biological Process	GO:0001678	cellular glucose homeostasis	24/2279	153/18866	0.1082482	0.4298823	0.3889727	24	FOXK1/NADK/HK1/LRP5L/ERN1/PIK3R1/ARRB1/GPLD1/IGF1R/PTPRN2/PAX2/ENY2/HK2/GHRL/KLF7/FIS1/PIH1D1/PRKCE/CPB2/PCK2/FOXO1/SLC29A1/C2CD2L/PRKAA1
Biological Process	GO:0008203	cholesterol metabolic process	24/2279	153/18866	0.1082482	0.4298823	0.3889727	24	OSBPL5/RXRA/IDI1/LIPC/MBTPS1/ABCG1/HDLBP/AKR1D1/APP/FDX1/DGKQ/POR/ABCA1/GNB3/ACACB/CAT/NPC1/ACACA/LMF1/HMGCS1/SCD/SREBF1/ACADVL/PRKAA1
Biological Process	GO:0001881	receptor recycling	7/2279	34/18866	0.108256	0.4298823	0.3889727	7	LMTK2/SORL1/TBC1D16/ARAP1/EPS15/INPP5F/PHETA1
Biological Process	GO:0006891	intra-Golgi vesicle-mediated transport	7/2279	34/18866	0.108256	0.4298823	0.3889727	7	VTI1A/TRAPPC10/CUX1/COG1/TRAPPC3L/COG8/COG5
Biological Process	GO:0018149	peptide cross-linking	7/2279	34/18866	0.108256	0.4298823	0.3889727	7	CSTA/SPOCK2/TGM5/THBS1/EGFLAM/TGM3/EPB42
Biological Process	GO:0032232	negative regulation of actin filament bundle assembly	7/2279	34/18866	0.108256	0.4298823	0.3889727	7	TACSTD2/ARAP1/PPFIA1/PFN1/MIR138-2/ARHGEF18/PRKN

Biological Process	GO:0055022	negative regulation of cardiac muscle tissue growth	7/2279	34/18866	0.108256	0.4298823	0.3889727	7	CTDP1/MIR199A1/VGLL4/TGFBR2/RIPK1/MEIS1/MIR199A2
Biological Process	GO:0061117	negative regulation of heart growth	7/2279	34/18866	0.108256	0.4298823	0.3889727	7	CTDP1/MIR199A1/VGLL4/TGFBR2/RIPK1/MEIS1/MIR199A2
Biological Process	GO:0070050	neuron cellular homeostasis	7/2279	34/18866	0.108256	0.4298823	0.3889727	7	DCTN1/ATP2B2/MAP1A/P2RX1/TYRO3/PRKAA1/PRKN
Biological Process	GO:0070232	regulation of T cell apoptotic process	7/2279	34/18866	0.108256	0.4298823	0.3889727	7	CCL5/PTCRA/DOCK8/RIPK3/ZC3H8/FADD/GIMAP8
Biological Process	GO:2000036	regulation of stem cell population maintenance	7/2279	34/18866	0.108256	0.4298823	0.3889727	7	MIR145/WDR43/LBH/PAX2/ESRRB/NODAL/ZNF706
Biological Process	GO:0002688	regulation of leukocyte chemotaxis	20/2279	124/18866	0.1084623	0.4298823	0.3889727	20	CALR/C3AR1/CCR2/CCL5/CSF1R/FPR2/PADI2/THBS1/C5AR2/DEFB124/ZNF580/JAM3/CAMK1D/CXCL17/IL6R/DAPK2/DNM1L/ANO6/DYSF/CCL27
Biological Process	GO:0014902	myotube differentiation	19/2279	117/18866	0.1097933	0.4298823	0.3889727	19	HDAC4/EHD1/CD9/SKI/MAML1/TANC1/NFATC2/SMYD3/MYH9/SORT1/ANKRD2/BCL2/NOTCH1/MEF2C/PLD3/FLOT1/SCGB3A1/CEACAM5/MYORG
Biological Process	GO:0051341	regulation of oxidoreductase activity	19/2279	117/18866	0.1097933	0.4298823	0.3889727	19	GFI1/VDR/DNM2/TNF/GNAI2/NOS1AP/CDH3/WASL/NOSTRIN/SPHK2/RIPK3/POR/CNR2/MIR138-2/NOD2/AGTR1/NOSIP/SIRT3/PRKN
Biological Process	GO:1904019	epithelial cell apoptotic process	19/2279	117/18866	0.1097933	0.4298823	0.3889727	19	IL10/GSN/ANGPT1/TNF/TNFAIP3/MIR101-2/TGFBR2/THBS1/MIR24-2/TCF7L2/STK3/PRKCI/NFE2L2/ARRB2/SORT1/RB1/ANO6/BCL2L1/PLCG1

Biological Process	GO:0048880	sensory system development	56/2279	394/18866	0.1100322	0.4298823	0.3889727	56	SPRED2/RXRA/PDE6A/FZR1/SLC25A25/LIMK2/MFSD2A/LPCAT1/CACNA1C/LRP5L/SKI/CRB1/SMAD3/ZEB2/SH3PXD2B/HIPK2/TGFBR2/MYH15/SLC44A4/NINJ1/DLL1/HDAC1/GNAT2/PAX2/FLT1/RDH10/RARA/NF1/CRYBB1/SP3/RARG/BMPR1B/WNT5B/GRHL2/IFT140/MEIS2/PRKCI/MYOM1/MEIS1/BCL2/RPGRIP1/ZHX2/PBX4/COL5A1/SDK2/BCAR3/NRL/PDGFRB/ARHGEF15/TMOD1/ALDH1A2/INHBA/CABP4/TSKU/NPHP4/NECTIN1
Biological Process	GO:0006171	cAMP biosynthetic process	3/2279	10/18866	0.1103295	0.4298823	0.3889727	3	ADCY2/ADCY9/ADCY4
Biological Process	GO:0006285	base-excision repair, AP site formation	3/2279	10/18866	0.1103295	0.4298823	0.3889727	3	UNG/NTHL1/MUTYH
Biological Process	GO:0006547	histidine metabolic process	3/2279	10/18866	0.1103295	0.4298823	0.3889727	3	HAL/MTHFD1/HNMT
Biological Process	GO:0007084	mitotic nuclear envelope reassembly	3/2279	10/18866	0.1103295	0.4298823	0.3889727	3	PPP2R1A/ANKLE2/BANF1
Biological Process	GO:0009133	nucleoside diphosphate biosynthetic process	3/2279	10/18866	0.1103295	0.4298823	0.3889727	3	GUK1/AK2/AK5
Biological Process	GO:0009396	folic acid-containing compound biosynthetic process	3/2279	10/18866	0.1103295	0.4298823	0.3889727	3	MTHFD1L/MTHFS/MTHFD1
Biological Process	GO:0016559	peroxisome fission	3/2279	10/18866	0.1103295	0.4298823	0.3889727	3	FIS1/DNM1L/PEX11B
Biological Process	GO:0018026	peptidyl-lysine monomethylation	3/2279	10/18866	0.1103295	0.4298823	0.3889727	3	SETD2/EEF1AKMT2/CSKMT

Biological Process	GO:0019374	galactolipid metabolic process	3/2279	10/18866	0.1103295	0.4298823	0.3889727	3	GALC/B4GALT3/FA2H
Biological Process	GO:0021562	vestibulocochlear nerve development	3/2279	10/18866	0.1103295	0.4298823	0.3889727	3	NRP2/ATP8B1/PAX2
Biological Process	GO:0021859	pyramidal neuron differentiation	3/2279	10/18866	0.1103295	0.4298823	0.3889727	3	DISC1/ZMIZ1/UNC5D
Biological Process	GO:0030202	heparin metabolic process	3/2279	10/18866	0.1103295	0.4298823	0.3889727	3	CSGALNACT1/ANGPT1/NDST1
Biological Process	GO:0030953	astral microtubule organization	3/2279	10/18866	0.1103295	0.4298823	0.3889727	3	LIMK2/NUMA1/EZR
Biological Process	GO:0032661	regulation of interleukin-18 production	3/2279	10/18866	0.1103295	0.4298823	0.3889727	3	IL10/CD84/TLR9
Biological Process	GO:0032815	negative regulation of natural killer cell activation	3/2279	10/18866	0.1103295	0.4298823	0.3889727	3	FGR/HLA-F/PGLYRP1
Biological Process	GO:0033314	mitotic DNA replication checkpoint	3/2279	10/18866	0.1103295	0.4298823	0.3889727	3	HUS1/CLSPN/NAE1
Biological Process	GO:0034154	toll-like receptor 7 signaling pathway	3/2279	10/18866	0.1103295	0.4298823	0.3889727	3	PIK3AP1/SCIMP/TREML4

Biological Process	GO:0034163	regulation of toll-like receptor 9 signaling pathway	3/2279	10/18866	0.1103295	0.4298823	0.3889727	3	GRAMD4/TLR9/RTN4
Biological Process	GO:0035871	protein K11-linked deubiquitination	3/2279	10/18866	0.1103295	0.4298823	0.3889727	3	TNFAIP3/OTUB2/VCPIP1
Biological Process	GO:0035999	tetrahydrofolate interconversion	3/2279	10/18866	0.1103295	0.4298823	0.3889727	3	MTHFD1L/MTHFS/MTHFD1
Biological Process	GO:0042117	monocyte activation	3/2279	10/18866	0.1103295	0.4298823	0.3889727	3	AZU1/FOXP1/DYSF
Biological Process	GO:0044351	macropinocytosis	3/2279	10/18866	0.1103295	0.4298823	0.3889727	3	DNM2/MAPKAPK3/DOCK2
Biological Process	GO:0046487	glyoxylate metabolic process	3/2279	10/18866	0.1103295	0.4298823	0.3889727	3	ALDH4A1/AGXT/HOGA1
Biological Process	GO:0048251	elastic fiber assembly	3/2279	10/18866	0.1103295	0.4298823	0.3889727	3	MFAP4/MYH11/TNXB
Biological Process	GO:0048739	cardiac muscle fiber development	3/2279	10/18866	0.1103295	0.4298823	0.3889727	3	MYO18B/TTN/MYH11
Biological Process	GO:0050861	positive regulation of B cell receptor signaling pathway	3/2279	10/18866	0.1103295	0.4298823	0.3889727	3	FOXP1/PRKCH/PRKCB

Biological Process	GO:0051124	synaptic growth at neuromuscular junction	3/2279	10/18866	0.1103295	0.4298823	0.3889727	3	COLQ/APP/SHANK2
Biological Process	GO:0070099	regulation of chemokine-mediated signaling pathway	3/2279	10/18866	0.1103295	0.4298823	0.3889727	3	CCL5/PADI2/SLIT3
Biological Process	GO:0070862	negative regulation of protein exit from endoplasmic reticulum	3/2279	10/18866	0.1103295	0.4298823	0.3889727	3	UBAC2/DERL2/SVIP
Biological Process	GO:0072124	regulation of glomerular mesangial cell proliferation	3/2279	10/18866	0.1103295	0.4298823	0.3889727	3	PDGFD/IL6R/PDGFB
Biological Process	GO:0097278	complement-dependent cytotoxicity	3/2279	10/18866	0.1103295	0.4298823	0.3889727	3	CD55/RAB27A/CD59
Biological Process	GO:0098598	learned vocalization behavior or vocal learning	3/2279	10/18866	0.1103295	0.4298823	0.3889727	3	NRXN1/HTT/NRXN2
Biological Process	GO:0099532	synaptic vesicle endosomal processing	3/2279	10/18866	0.1103295	0.4298823	0.3889727	3	ITSN1/DNM1L/AP3D1
Biological Process	GO:0140042	lipid droplet formation	3/2279	10/18866	0.1103295	0.4298823	0.3889727	3	TMEM159/CDS2/BSCL2
Biological Process	GO:1902946	protein localization to early endosome	3/2279	10/18866	0.1103295	0.4298823	0.3889727	3	SORL1/MGAT3/EZR

Biological Process	GO:1904672	regulation of somatic stem cell population maintenance	3/2279	10/18866	0.1103295	0.4298823	0.3889727	3	MIR145/LBH/PAX2
Biological Process	GO:1990535	neuron projection maintenance	3/2279	10/18866	0.1103295	0.4298823	0.3889727	3	DCTN1/APP/MAP1A
Biological Process	GO:2000109	regulation of macrophage apoptotic process	3/2279	10/18866	0.1103295	0.4298823	0.3889727	3	CCL5/NOD2/MEF2C
Biological Process	GO:2000320	negative regulation of T-helper 17 cell differentiation	3/2279	10/18866	0.1103295	0.4298823	0.3889727	3	LOXL3/SMAD7/ZBTB7B
Biological Process	GO:2000601	positive regulation of Arp2/3 complex-mediated actin nucleation	3/2279	10/18866	0.1103295	0.4298823	0.3889727	3	WASL/NCKAP1/CYFIP1
Biological Process	GO:0035966	response to topologically incorrect protein	31/2279	205/18866	0.1103812	0.4298823	0.3889727	31	DCTN1/CALR/ERN1/PIK3R1/AMFR/MBTPS1/RHBDD2/NCK2/YIF1A/CUL3/THBS1/EDEM1/DERL2/ZBTB17/PDIA5/HSPA4L/CLU/UBE2J2/CLGN/F12/POMT2/COPS5/EDEM2/CREB3L2/NFE2L2/ADD1/TOR1B/KLHDC3/SEC31A/ACADVL/PRKN
Biological Process	GO:0031330	negative regulation of cellular catabolic process	39/2279	265/18866	0.1109413	0.4298823	0.3889727	39	TRIM39/FOXK1/TIMP2/SORL1/IL10/MFSD2A/SLC11A1/PAM16/BMF/MIR199A1/GPLD1/SUFU/HIPK2/UBAC2/LARP1/MGAT3/PKP1/USP36/DERL2/FEZ2/CLEC16A/ACACB/PSMF1/TBC1D14/NPC1/NBAS/BCL2/MIR199A2/MAP1A/PAIP1/TSC2/SECISBP2/SYNCRIP/BAG5/EIF4G1/SVIP/TENT5C/TENT5A/MARCHF7
Biological Process	GO:0006641	triglyceride metabolic process	18/2279	110/18866	0.1110109	0.4298823	0.3889727	18	PNPLA2/SORL1/MGLL/ACSL1/MFSD2A/LIPC/GPLD1/NR1H2/ATG14/GNB3/FABP6/CAT/LMF1/ABHD5/PCK2/SREBF1/CPT1A/LPIN1
Biological Process	GO:0000302	response to reactive oxygen species	35/2279	235/18866	0.1111386	0.4298823	0.3889727	35	TRPM2/MPO/IL10/LCN2/MYB/SLC8A1/DNM2/TNF/TRAF2/TNFAIP3/CAMKK2/RIPK1/PXN/PAX2/ZNF580/FER/HBB/AQP1/RIPK3/STK24/PDGFD/CAT/ETS1/NFE2L2/IL18RAP/BCL2/UBE3A/GLRX2/PDGFRB/FOXO1/RELA/TXNRD2/SIRT3/MB/PRKAA1

Biological Process	GO:0035335	peptidyl-tyrosine dephosphorylation	17/2279	103/18866	0.1120841	0.4298823	0.3889727	17	SSH1/PTPRE/DUSP14/UBASH3B/DUSP22/PGP/PTPN6/SSH3/DUSP28/PTPRN2/CDC25A/PTPRJ/PTPRC/PALD1/DUSP3/CDC14A/PTPRU
Biological Process	GO:0048709	oligodendrocyte differentiation	17/2279	103/18866	0.1120841	0.4298823	0.3889727	17	CD9/PPARG/GSN/TNFRSF1B/RHEB/EIF2B5/CLU/TNFRSF21/HDAC1/NF1/CSK/WDR1/NOTCH1/TSPAN2/FA2H/CNTNAP1/ERCC2
Biological Process	GO:0060079	excitatory postsynaptic potential	17/2279	103/18866	0.1120841	0.4298823	0.3889727	17	PRKCZ/SSH1/ZMYND8/BAIAP2/APP/NRXN1/GHRL/SH3GL1/MAPK8IP2/ARRB2/S1PR2/P2RX1/MEF2C/SHANK2/SLC29A1/P2RX5/CUX2
Biological Process	GO:0070498	interleukin-1-mediated signaling pathway	17/2279	103/18866	0.1120841	0.4298823	0.3889727	17	VRK2/TOLLIP/MAP3K3/IL1R1/MIR27A/RPS6KA4/IL1RN/UBE2V1/PSMF1/PLCB1/IRAK2/NOD2/PSMB7/RELA/FBXW11/PELI1/PSMD13
Biological Process	GO:0032102	negative regulation of response to external stimulus	61/2279	433/18866	0.1122515	0.4298823	0.3889727	61	ELANE/SEMA6B/ABR/PLAU/IL10/CD9/TRIM38/PPARG/C1QTNF3/MIR140/MIR145/TNFRSF1B/NLRP3/SMAD3/MEFV/TNF/UBASH3B/SMPDL3B/TNFAIP3/AOAH/FPR2/SERPINB2/SEMA4A/PADI2/THBS1/SEMA4B/C5AR2/HLA-F/TNFAIP6/TNFRSF1A/F12/KREMEN1/SEMA4D/NRXN1/CXCL17/NR1H2/PGLYRP1/SEC14L1/GHRL/PTPRC/GRN/PDGFB/NLRC5/BCR/CNR2/ETS1/ARRB2/MIR138-2/RB1/NOTCH1/DUSP3/CPB2/LTF/CHD8/TRIB1/TYRO3/ADTRP/CD96/CERS2/INPP5F/ILRUN
Biological Process	GO:0006027	glycosaminoglycan catabolic process	11/2279	61/18866	0.1122856	0.4298823	0.3889727	11	HEXB/GALNS/LYG2/SDC2/TGFB1/STAB2/PGLYRP1/LYVE1/ARSB/PGLYRP4/CEMIP2
Biological Process	GO:0061512	protein localization to cilium	11/2279	61/18866	0.1122856	0.4298823	0.3889727	11	EHD1/CSNK1D/TULP2/CCDC66/LZTFL1/IFT140/INPP5E/TTC21B/ZNF423/NPHP4/CPLANE1
Biological Process	GO:0061912	selective autophagy	11/2279	61/18866	0.1122856	0.4298823	0.3889727	11	SPTLC2/VPS13D/WIPI2/SMURF1/CLEC16A/HTT/ATG14/TSC2/WDR81/STING1/PRKN
Biological Process	GO:0010812	negative regulation of cell-substrate adhesion	12/2279	68/18866	0.113447	0.4298823	0.3889727	12	TBCD/TACSTD2/KANK1/ITGB1BP1/PIK3R1/DUSP22/THBS1/ACTN4/NF1/NOTCH1/CORO1C/GCNT2

Biological Process	GO:0051148	negative regulation of muscle cell differentiation	12/2279	68/18866	0.113447	0.4298823	0.3889727	12	HDAC4/CTDP1/MIR199A1/NFATC2/NFATC1/DLL1/PDGFB/ANKRD2/MIR199A2/NOTCH1/DNMT1/CEACAM5
Biological Process	GO:0022404	molting cycle process	15/2279	89/18866	0.1136298	0.4298823	0.3889727	15	TNF/KRT17/RBPJ/LRIG1/HDAC1/CDH3/MYO5A/NF1/NUMA1/BCL2/NOTCH1/TGM3/RELA/INHBA/ERCC2
Biological Process	GO:0022405	hair cycle process	15/2279	89/18866	0.1136298	0.4298823	0.3889727	15	TNF/KRT17/RBPJ/LRIG1/HDAC1/CDH3/MYO5A/NF1/NUMA1/BCL2/NOTCH1/TGM3/RELA/INHBA/ERCC2
Biological Process	GO:1903845	negative regulation of cellular response to transforming growth factor beta stimulus	15/2279	89/18866	0.1136298	0.4298823	0.3889727	15	LTBP1/SPRED2/SKI/SMAD3/LDLRAD4/DNM2/CHST11/TGFBR2/SMAD7/TGFB1/SMURF1/TGFB1I1/HTRA4/PRDM16/HTRA3
Biological Process	GO:0018146	keratan sulfate biosynthetic process	6/2279	28/18866	0.113632	0.4298823	0.3889727	6	CHST2/ST3GAL4/B4GALT3/ST3GAL2/B3GNT2/B4GAT1
Biological Process	GO:0030194	positive regulation of blood coagulation	6/2279	28/18866	0.113632	0.4298823	0.3889727	6	THBS1/F12/NFE2L2/CPB2/F2R/ANO6
Biological Process	GO:0034067	protein localization to Golgi apparatus	6/2279	28/18866	0.113632	0.4298823	0.3889727	6	SORL1/CSNK1D/VPS13D/BICD2/NUMA1/RIPOR1
Biological Process	GO:0045606	positive regulation of epidermal cell differentiation	6/2279	28/18866	0.113632	0.4298823	0.3889727	6	VDR/PRKCH/NUMA1/NOTCH1/ZBED2/MACROH2A2
Biological Process	GO:0045830	positive regulation of isotype switching	6/2279	28/18866	0.113632	0.4298823	0.3889727	6	TGFB1/PTPRC/TP53BP1/TNFSF13/PAXIP1/NSD2

Biological Process	GO:0048753	pigment granule organization	6/2279	28/18866	0.113632	0.4298823	0.3889727	6	LYST/KIF13A/ZEB2/BCL2/AP3D1/DTNBP1
Biological Process	GO:1900048	positive regulation of hemostasis	6/2279	28/18866	0.113632	0.4298823	0.3889727	6	THBS1/F12/NFE2L2/CPB2/F2R/ANO6
Biological Process	GO:2000171	negative regulation of dendrite development	6/2279	28/18866	0.113632	0.4298823	0.3889727	6	YWHAH/BCL11A/ASAP1/RAPGEF2/DNM3/UBE3A
Biological Process	GO:2000463	positive regulation of excitatory postsynaptic potential	6/2279	28/18866	0.113632	0.4298823	0.3889727	6	PRKCZ/SSH1/BAIAP2/NRXN1/SHANK2/CUX2
Biological Process	GO:0000320	re-entry into mitotic cell cycle	2/2279	5/18866	0.1137383	0.4298823	0.3889727	2	MIR199A1/MIR199A2
Biological Process	GO:0001887	selenium compound metabolic process	2/2279	5/18866	0.1137383	0.4298823	0.3889727	2	SCLY/MAT1A
Biological Process	GO:0002175	protein localization to paranode region of axon	2/2279	5/18866	0.1137383	0.4298823	0.3889727	2	EPB41L3/CNTNAP1
Biological Process	GO:0002513	tolerance induction to self antigen	2/2279	5/18866	0.1137383	0.4298823	0.3889727	2	TGFBR2/BLK
Biological Process	GO:0002677	negative regulation of chronic inflammatory response	2/2279	5/18866	0.1137383	0.4298823	0.3889727	2	IL10/TNFAIP3

Biological Process	GO:0002925	positive regulation of humoral immune response mediated by circulating immunoglobulin	2/2279	5/18866	0.1137383	0.4298823	0.3889727	2	TNF/PTPRC
Biological Process	GO:0003186	tricuspid valve morphogenesis	2/2279	5/18866	0.1137383	0.4298823	0.3889727	2	ZFPM1/TGFBR2
Biological Process	GO:0003406	retinal pigment epithelium development	2/2279	5/18866	0.1137383	0.4298823	0.3889727	2	MFSD2A/PAX2
Biological Process	GO:0006003	fructose 2,6-bisphosphate metabolic process	2/2279	5/18866	0.1137383	0.4298823	0.3889727	2	PFKFB4/PFKFB3
Biological Process	GO:0006172	ADP biosynthetic process	2/2279	5/18866	0.1137383	0.4298823	0.3889727	2	AK2/AK5
Biological Process	GO:0006572	tyrosine catabolic process	2/2279	5/18866	0.1137383	0.4298823	0.3889727	2	HGD/FAH
Biological Process	GO:0007386	compartment pattern specification	2/2279	5/18866	0.1137383	0.4298823	0.3889727	2	DLL1/NOTCH1
Biological Process	GO:0008291	acetylcholine metabolic process	2/2279	5/18866	0.1137383	0.4298823	0.3889727	2	COLQ/SLC44A4
Biological Process	GO:0009153	purine deoxyribonucleotide biosynthetic process	2/2279	5/18866	0.1137383	0.4298823	0.3889727	2	GUK1/AK5

Biological Process	GO:0009256	10-formyltetrahydrofolate metabolic process	2/2279	5/18866	0.1137383	0.4298823	0.3889727	2	MTHFD1L/MTHFD1
Biological Process	GO:0009912	auditory receptor cell fate commitment	2/2279	5/18866	0.1137383	0.4298823	0.3889727	2	RBPJ/NOTCH1
Biological Process	GO:0010815	bradykinin catabolic process	2/2279	5/18866	0.1137383	0.4298823	0.3889727	2	CTSH/XPNPEP1
Biological Process	GO:0010897	negative regulation of triglyceride catabolic process	2/2279	5/18866	0.1137383	0.4298823	0.3889727	2	SORL1/GPLD1
Biological Process	GO:0014722	regulation of skeletal muscle contraction by calcium ion signaling	2/2279	5/18866	0.1137383	0.4298823	0.3889727	2	GSTO1/CASQ1
Biological Process	GO:0015691	cadmium ion transport	2/2279	5/18866	0.1137383	0.4298823	0.3889727	2	SLC11A1/SLC11A2
Biological Process	GO:0015827	tryptophan transport	2/2279	5/18866	0.1137383	0.4298823	0.3889727	2	SLC7A5/SLC7A8
Biological Process	GO:0018057	peptidyl-lysine oxidation	2/2279	5/18866	0.1137383	0.4298823	0.3889727	2	LOXL3/LOXL4
Biological Process	GO:0019732	antifungal humoral response	2/2279	5/18866	0.1137383	0.4298823	0.3889727	2	DEFA4/LTF

Biological Process	GO:0021650	vestibulocochlear nerve formation	2/2279	5/18866	0.1137383	0.4298823	0.3889727	2	ATP8B1/PAX2
Biological Process	GO:0032237	activation of store-operated calcium channel activity	2/2279	5/18866	0.1137383	0.4298823	0.3889727	2	STIMATE/CRACR2A
Biological Process	GO:0032889	regulation of vacuole fusion, non-autophagic	2/2279	5/18866	0.1137383	0.4298823	0.3889727	2	ENO1/ENO3
Biological Process	GO:0032907	transforming growth factor beta3 production	2/2279	5/18866	0.1137383	0.4298823	0.3889727	2	CREB1/SMAD3
Biological Process	GO:0032910	regulation of transforming growth factor beta3 production	2/2279	5/18866	0.1137383	0.4298823	0.3889727	2	CREB1/SMAD3
Biological Process	GO:0033869	nucleoside bisphosphate catabolic process	2/2279	5/18866	0.1137383	0.4298823	0.3889727	2	ACOT7/ACAT1
Biological Process	GO:0034031	ribonucleoside bisphosphate catabolic process	2/2279	5/18866	0.1137383	0.4298823	0.3889727	2	ACOT7/ACAT1
Biological Process	GO:0034034	purine nucleoside bisphosphate catabolic process	2/2279	5/18866	0.1137383	0.4298823	0.3889727	2	ACOT7/ACAT1
Biological Process	GO:0034093	positive regulation of maintenance of sister chromatid cohesion	2/2279	5/18866	0.1137383	0.4298823	0.3889727	2	NSMCE2/SLF1

Biological Process	GO:0034184	positive regulation of maintenance of mitotic sister chromatid cohesion	2/2279	5/18866	0.1137383	0.4298823	0.3889727	2	NSMCE2/SLF1
Biological Process	GO:0034398	telomere tethering at nuclear periphery	2/2279	5/18866	0.1137383	0.4298823	0.3889727	2	RAD21L1/TERB2
Biological Process	GO:0035385	Roundabout signaling pathway	2/2279	5/18866	0.1137383	0.4298823	0.3889727	2	MYO9B/SLIT3
Biological Process	GO:0035441	cell migration involved in vasculogenesis	2/2279	5/18866	0.1137383	0.4298823	0.3889727	2	SETD2/PDGFRB
Biological Process	GO:0035787	cell migration involved in kidney development	2/2279	5/18866	0.1137383	0.4298823	0.3889727	2	PDGFB/PDGFRB
Biological Process	GO:0035977	protein deglycosylation involved in glycoprotein catabolic process	2/2279	5/18866	0.1137383	0.4298823	0.3889727	2	EDEM1/EDEM2
Biological Process	GO:0036337	Fas signaling pathway	2/2279	5/18866	0.1137383	0.4298823	0.3889727	2	ZDHHC7/SP100
Biological Process	GO:0036493	positive regulation of translation in response to endoplasmic reticulum stress	2/2279	5/18866	0.1137383	0.4298823	0.3889727	2	NCK2/EIF4G1
Biological Process	GO:0040016	embryonic cleavage	2/2279	5/18866	0.1137383	0.4298823	0.3889727	2	CUL3/ERCC2

Biological Process	GO:0042144	vacuole fusion, non-autophagic	2/2279	5/18866	0.1137383	0.4298823	0.3889727	2	ENO1/ENO3
Biological Process	GO:0042262	DNA protection	2/2279	5/18866	0.1137383	0.4298823	0.3889727	2	CBS/GLRX2
Biological Process	GO:0042816	vitamin B6 metabolic process	2/2279	5/18866	0.1137383	0.4298823	0.3889727	2	PDXK/AOX1
Biological Process	GO:0044821	meiotic telomere tethering at nuclear periphery	2/2279	5/18866	0.1137383	0.4298823	0.3889727	2	RAD21L1/TERB2
Biological Process	GO:0045053	protein retention in Golgi apparatus	2/2279	5/18866	0.1137383	0.4298823	0.3889727	2	SORL1/VPS13D
Biological Process	GO:0045636	positive regulation of melanocyte differentiation	2/2279	5/18866	0.1137383	0.4298823	0.3889727	2	ZEB2/BCL2
Biological Process	GO:0045900	negative regulation of translational elongation	2/2279	5/18866	0.1137383	0.4298823	0.3889727	2	CPEB3/SHFL
Biological Process	GO:0045990	carbon catabolite regulation of transcription	2/2279	5/18866	0.1137383	0.4298823	0.3889727	2	NCOA1/USF2
Biological Process	GO:0045991	carbon catabolite activation of transcription	2/2279	5/18866	0.1137383	0.4298823	0.3889727	2	NCOA1/USF2

Biological Process	GO:0046104	thymidine metabolic process	2/2279	5/18866	0.1137383	0.4298823	0.3889727	2	TK2/TK1
Biological Process	GO:0046125	pyrimidine deoxyribonucleoside metabolic process	2/2279	5/18866	0.1137383	0.4298823	0.3889727	2	TK2/TK1
Biological Process	GO:0046136	positive regulation of vitamin metabolic process	2/2279	5/18866	0.1137383	0.4298823	0.3889727	2	TNF/RDH10
Biological Process	GO:0048254	snoRNA localization	2/2279	5/18866	0.1137383	0.4298823	0.3889727	2	PIH1D1/FBL
Biological Process	GO:0048669	collateral sprouting in absence of injury	2/2279	5/18866	0.1137383	0.4298823	0.3889727	2	APP/SPART
Biological Process	GO:0048859	formation of anatomical boundary	2/2279	5/18866	0.1137383	0.4298823	0.3889727	2	NODAL/NTF4
Biological Process	GO:0060040	retinal bipolar neuron differentiation	2/2279	5/18866	0.1137383	0.4298823	0.3889727	2	ZHX2/CABP4
Biological Process	GO:0060120	inner ear receptor cell fate commitment	2/2279	5/18866	0.1137383	0.4298823	0.3889727	2	RBPJ/NOTCH1
Biological Process	GO:0060374	mast cell differentiation	2/2279	5/18866	0.1137383	0.4298823	0.3889727	2	ZFPM1/PIK3CD

Biological Process	GO:0060398	regulation of growth hormone receptor signaling pathway	2/2279	5/18866	0.1137383	0.4298823	0.3889727	2	GHRL/MBD5
Biological Process	GO:0061588	calcium activated phospholipid scrambling	2/2279	5/18866	0.1137383	0.4298823	0.3889727	2	ANO7/ANO6
Biological Process	GO:0061590	calcium activated phosphatidylcholine scrambling	2/2279	5/18866	0.1137383	0.4298823	0.3889727	2	ANO7/ANO6
Biological Process	GO:0061591	calcium activated galactosylceramide scrambling	2/2279	5/18866	0.1137383	0.4298823	0.3889727	2	ANO7/ANO6
Biological Process	GO:0061737	leukotriene signaling pathway	2/2279	5/18866	0.1137383	0.4298823	0.3889727	2	LTB4R2/LTB4R
Biological Process	GO:0070197	meiotic attachment of telomere to nuclear envelope	2/2279	5/18866	0.1137383	0.4298823	0.3889727	2	RAD21L1/TERB2
Biological Process	GO:0070574	cadmium ion transmembrane transport	2/2279	5/18866	0.1137383	0.4298823	0.3889727	2	SLC11A1/SLC11A2
Biological Process	GO:0070940	dephosphorylation of RNA polymerase II C-terminal domain	2/2279	5/18866	0.1137383	0.4298823	0.3889727	2	CTDP1/RPRD1B
Biological Process	GO:0071033	nuclear retention of pre-mRNA at the site of transcription	2/2279	5/18866	0.1137383	0.4298823	0.3889727	2	EXOSC2/PCID2

Biological Process	GO:0071205	protein localization to juxtaparanode region of axon	2/2279	5/18866	0.1137383	0.4298823	0.3889727	2	EPB41L3/CNTNAP1
Biological Process	GO:0071680	response to indole-3-methanol	2/2279	5/18866	0.1137383	0.4298823	0.3889727	2	JUP/CDH1
Biological Process	GO:0071681	cellular response to indole-3-methanol	2/2279	5/18866	0.1137383	0.4298823	0.3889727	2	JUP/CDH1
Biological Process	GO:0071895	odontoblast differentiation	2/2279	5/18866	0.1137383	0.4298823	0.3889727	2	CEBPB/FAM20C
Biological Process	GO:0071922	regulation of cohesin loading	2/2279	5/18866	0.1137383	0.4298823	0.3889727	2	RB1/CDCA5
Biological Process	GO:0072223	metanephric glomerular mesangium development	2/2279	5/18866	0.1137383	0.4298823	0.3889727	2	PDGFB/PDGFRB
Biological Process	GO:0072385	minus-end-directed organelle transport along microtubule	2/2279	5/18866	0.1137383	0.4298823	0.3889727	2	DYNC1H1/BICD2
Biological Process	GO:0072656	maintenance of protein location in mitochondrion	2/2279	5/18866	0.1137383	0.4298823	0.3889727	2	HK1/HK2
Biological Process	GO:0072708	response to sorbitol	2/2279	5/18866	0.1137383	0.4298823	0.3889727	2	MAP2K6/KAT7

Biological Process	GO:0090156	cellular sphingolipid homeostasis	2/2279	5/18866	0.1137383	0.4298823	0.3889727	2	ORMDL3/RTN4
Biological Process	GO:0090679	cell differentiation involved in phenotypic switching	2/2279	5/18866	0.1137383	0.4298823	0.3889727	2	MIR140/DNMT1
Biological Process	GO:0097240	chromosome attachment to the nuclear envelope	2/2279	5/18866	0.1137383	0.4298823	0.3889727	2	RAD21L1/TERB2
Biological Process	GO:0097466	ubiquitin-dependent glycoprotein ERAD pathway	2/2279	5/18866	0.1137383	0.4298823	0.3889727	2	EDEM1/EDEM2
Biological Process	GO:0097576	vacuole fusion	2/2279	5/18866	0.1137383	0.4298823	0.3889727	2	ENO1/ENO3
Biological Process	GO:0106071	positive regulation of adenylate cyclase-activating G protein-coupled receptor signaling pathway	2/2279	5/18866	0.1137383	0.4298823	0.3889727	2	PRKCA/MRAP
Biological Process	GO:0106104	regulation of glutamate receptor clustering	2/2279	5/18866	0.1137383	0.4298823	0.3889727	2	SSH1/ZDHHC2
Biological Process	GO:0120180	cell-substrate junction disassembly	2/2279	5/18866	0.1137383	0.4298823	0.3889727	2	IQSEC1/DUSP3
Biological Process	GO:0120181	focal adhesion disassembly	2/2279	5/18866	0.1137383	0.4298823	0.3889727	2	IQSEC1/DUSP3

Biological Process	GO:0120182	regulation of focal adhesion disassembly	2/2279	5/18866	0.1137383	0.4298823	0.3889727	2	IQSEC1/DUSP3
Biological Process	GO:0120183	positive regulation of focal adhesion disassembly	2/2279	5/18866	0.1137383	0.4298823	0.3889727	2	IQSEC1/DUSP3
Biological Process	GO:1900060	negative regulation of ceramide biosynthetic process	2/2279	5/18866	0.1137383	0.4298823	0.3889727	2	ORMDL3/PRKAA1
Biological Process	GO:1900113	negative regulation of histone H3-K9 trimethylation	2/2279	5/18866	0.1137383	0.4298823	0.3889727	2	KDM4C/PIH1D1
Biological Process	GO:1900619	acetate ester metabolic process	2/2279	5/18866	0.1137383	0.4298823	0.3889727	2	COLQ/SLC44A4
Biological Process	GO:1901628	positive regulation of postsynaptic membrane organization	2/2279	5/18866	0.1137383	0.4298823	0.3889727	2	SSH1/ZDHHC2
Biological Process	GO:1902617	response to fluoride	2/2279	5/18866	0.1137383	0.4298823	0.3889727	2	FIS1/FOXO1
Biological Process	GO:1902731	negative regulation of chondrocyte proliferation	2/2279	5/18866	0.1137383	0.4298823	0.3889727	2	SMAD7/BMPR1B
Biological Process	GO:1903297	regulation of hypoxia-induced intrinsic apoptotic signaling pathway	2/2279	5/18866	0.1137383	0.4298823	0.3889727	2	ENO1/NOL3

Biological Process	GO:1903298	negative regulation of hypoxia-induced intrinsic apoptotic signaling pathway	2/2279	5/18866	0.1137383	0.4298823	0.3889727	2	ENO1/NOL3
Biological Process	GO:1904382	mannose trimming involved in glycoprotein ERAD pathway	2/2279	5/18866	0.1137383	0.4298823	0.3889727	2	EDEM1/EDEM2
Biological Process	GO:1904502	regulation of lipophagy	2/2279	5/18866	0.1137383	0.4298823	0.3889727	2	SPTLC2/HTT
Biological Process	GO:1904504	positive regulation of lipophagy	2/2279	5/18866	0.1137383	0.4298823	0.3889727	2	SPTLC2/HTT
Biological Process	GO:1904673	negative regulation of somatic stem cell population maintenance	2/2279	5/18866	0.1137383	0.4298823	0.3889727	2	MIR145/PAX2
Biological Process	GO:1904717	regulation of AMPA glutamate receptor clustering	2/2279	5/18866	0.1137383	0.4298823	0.3889727	2	SSH1/ZDHHC2
Biological Process	GO:1904906	positive regulation of endothelial cell-matrix adhesion via fibronectin	2/2279	5/18866	0.1137383	0.4298823	0.3889727	2	RIN2/CEACAM6
Biological Process	GO:1904956	regulation of midbrain dopaminergic neuron differentiation	2/2279	5/18866	0.1137383	0.4298823	0.3889727	2	CSNK1D/CSNK1E
Biological Process	GO:1905007	positive regulation of epithelial to mesenchymal transition involved in endocardial cushion formation	2/2279	5/18866	0.1137383	0.4298823	0.3889727	2	TGFBR2/ENG

Biological Process	GO:1905232	cellular response to L-glutamate	2/2279	5/18866	0.1137383	0.4298823	0.3889727	2	BCL11A/BAIAP2
Biological Process	GO:1905420	vascular associated smooth muscle cell differentiation involved in phenotypic switching	2/2279	5/18866	0.1137383	0.4298823	0.3889727	2	MIR140/DNMT1
Biological Process	GO:1905451	positive regulation of Fc-gamma receptor signaling pathway involved in phagocytosis	2/2279	5/18866	0.1137383	0.4298823	0.3889727	2	PTPRJ/PTPRC
Biological Process	GO:1905605	positive regulation of blood-brain barrier permeability	2/2279	5/18866	0.1137383	0.4298823	0.3889727	2	TJP2/ANGPT1
Biological Process	GO:1905749	regulation of endosome to plasma membrane protein transport	2/2279	5/18866	0.1137383	0.4298823	0.3889727	2	ZDHHC2/AKAP5
Biological Process	GO:1905915	regulation of cell differentiation involved in phenotypic switching	2/2279	5/18866	0.1137383	0.4298823	0.3889727	2	MIR140/DNMT1
Biological Process	GO:1905930	regulation of vascular associated smooth muscle cell differentiation involved in phenotypic switching	2/2279	5/18866	0.1137383	0.4298823	0.3889727	2	MIR140/DNMT1
Biological Process	GO:1905962	glutamatergic neuron differentiation	2/2279	5/18866	0.1137383	0.4298823	0.3889727	2	ZHX2/CABP4
Biological Process	GO:2000172	regulation of branching morphogenesis of a nerve	2/2279	5/18866	0.1137383	0.4298823	0.3889727	2	BCL11A/RTN4

Biological Process	GO:2000554	regulation of T-helper 1 cell cytokine production	2/2279	5/18866	0.1137383	0.4298823	0.3889727	2	IL1R1/IL18R1
Biological Process	GO:2000556	positive regulation of T-helper 1 cell cytokine production	2/2279	5/18866	0.1137383	0.4298823	0.3889727	2	IL1R1/IL18R1
Biological Process	GO:2000563	positive regulation of CD4-positive, alpha-beta T cell proliferation	2/2279	5/18866	0.1137383	0.4298823	0.3889727	2	CD55/TGFBR2
Biological Process	GO:0008344	adult locomotory behavior	13/2279	75/18866	0.1139642	0.4298823	0.3889727	13	LGI4/HIPK2/APP/SCN1A/WDR47/ARRB2/NPC1/TMOD1/NTAN1/INPP5F/SPTBN4/NTF4/PRKN
Biological Process	GO:0032602	chemokine production	13/2279	75/18866	0.1139642	0.4298823	0.3889727	13	AZU1/S100A8/ZFPM1/FOXP1/IL10/C1QTNF3/MEFV/TNF/TLR9/MBP/DEFB124/IL6R/ACKR1
Biological Process	GO:0038034	signal transduction in absence of ligand	13/2279	75/18866	0.1139642	0.4298823	0.3889727	13	LCN2/TNF/UNC5B/RIPK1/FGFR1/NF1/PPP2R1A/BCL2/PPP2R1B/BCL2L1/INHBA/FADD/DAPK3
Biological Process	GO:0043627	response to estrogen	13/2279	75/18866	0.1139642	0.4298823	0.3889727	13	PPARG/ASH2L/ABCC2/TGFBR2/COMT/ZNF366/CRHBP/KMT2D/RARA/GHRL/ARSB/PDGFRB/NCOA4
Biological Process	GO:0097192	extrinsic apoptotic signaling pathway in absence of ligand	13/2279	75/18866	0.1139642	0.4298823	0.3889727	13	LCN2/TNF/UNC5B/RIPK1/FGFR1/NF1/PPP2R1A/BCL2/PPP2R1B/BCL2L1/INHBA/FADD/DAPK3
Biological Process	GO:1900006	positive regulation of dendrite development	13/2279	75/18866	0.1139642	0.4298823	0.3889727	13	ZMYND8/ITSN1/CUX1/BAIAP2/ILK/SS18L2/CPEB3/CDKL3/DNM1L/SHANK2/CYFIP1/CUX2/DBNL

Biological Process	GO:0030193	regulation of blood coagulation	14/2279	82/18866	0.1139879	0.4298823	0.3889727	14	PLAU/CD9/PRKCA/UBASH3B/SERPINB2/THBS1/F12/BLK/PDGFB/NFE2L2/CPB2/F2R/ANO6/ADTRP
Biological Process	GO:0045652	regulation of megakaryocyte differentiation	14/2279	82/18866	0.1139879	0.4298823	0.3889727	14	ZFPM1/RUNX1/ASH2L/TNRC6B/AGO1/TESC/THBS1/ITGA2B/HDAC1/MTURN/KMT2D/SETD1A/MEF2C/H3C1
Biological Process	GO:0006734	NADH metabolic process	8/2279	41/18866	0.1144406	0.4298823	0.3889727	8	FOXK1/ENO1/HK1/ADPGK/PGAM1/ENO3/HK2/PKM
Biological Process	GO:0035337	fatty-acyl-CoA metabolic process	8/2279	41/18866	0.1144406	0.4298823	0.3889727	8	ACSL1/FAR2/ACOT7/ACACA/ACSF3/ACAT1/SCD/SLC25A1
Biological Process	GO:0038083	peptidyl-tyrosine autophosphorylation	8/2279	41/18866	0.1144406	0.4298823	0.3889727	8	AATK/LCK/FGR/SLA/IGF1R/FER/BLK/NTRK1
Biological Process	GO:0051180	vitamin transport	8/2279	41/18866	0.1144406	0.4298823	0.3889727	8	ABCC1/SLC2A14/FOLR3/SLC19A1/CD320/SLC52A2/SLC2A1/SLC23A1
Biological Process	GO:0050852	T cell receptor signaling pathway	31/2279	206/18866	0.1156041	0.4298823	0.3889727	31	PDE4D/LAT/PIK3CD/LCK/INPP5D/PIK3R1/GRAP2/EIF2B5/DUSP22/CD226/PTPN6/TNFRSF21/PTPRJ/LILRB4/PTPRC/CSK/BCL10/PSMF1/THEMIS2/PSMB7/DUSP3/PRAM1/ELF1/CYLD/PLCG1/RELA/FBXW11/EZR/PSMD13/FYB1/SLA2
Biological Process	GO:2000146	negative regulation of cell motility	52/2279	365/18866	0.1162285	0.4298823	0.3889727	52	TACSTD2/ABR/CALR/PPARG/MIR140/KANK1/ARHGDIB/ZMYND8/ITGB1BP1/SPINT2/LDLRAD4/MIR199A1/TNF/MIR101-2/DUSP22/PADI2/JUP/NAV3/THBS1/C5AR2/MIR24-2/ILK/SMAD7/MIA3/WNT11/WASL/NISCH/TGFB1/PTPRJ/NF1/STK24/SRGAP1/BCR/PLCB1/NFE2L2/ENG/VASH1/MIR138-2/CDH1/BCL2/MIR199A2/NOTCH1/CORO1C/MEF2C/DUSP3/SP100/TRIB1/ADTRP/CERS2/PTPRU/NODAL/CX3CR1
Biological Process	GO:0150063	visual system development	55/2279	388/18866	0.1162914	0.4298823	0.3889727	55	SPRED2/RXRA/PDE6A/FZR1/SLC25A25/LIMK2/MFSD2A/LPCAT1/CACNA1C/LRP5L/SKI/CRB1/SMAD3/ZEB2/SH3PXD2B/HIPK2/TGFBR2/MYH15/NINJ1/DLL1/HDAC1/GNAT2/PAX2/FLT1/RDH10/RARA/NF1/CRYBB1/SP3/RARG/BMPR1B/WNT5B/GRHL2/IFT140/MEIS2/PRKCI/MYOM1/MEIS1/BCL2/RPGRIP1/ZHX2/PBX4/COL5A1/SDK2/BCAR3/NRL/PDGFRB/ARHGEF15/TMOD1/ALDH1A2/INHBA/CABP4/TSKU/NPHP4/NECTIN1

Biological Process	GO:0038061	NIK/NF-kappaB signaling	28/2279	184/18866	0.1172526	0.4298823	0.3889727	28	CALR/C1QTNF3/CHI3L1/HDAC7/NLRP3/TNF/TRAF2/AGO1/TLR9/APP/ACTN4/MIR27A/ILK/CPNE1/PHB/LITAF/RIPK3/PSMF1/MAP3K14/NOD2/PSMB7/IL18R1/CYLD/RELA/FBXW11/BCL3/PSMD13/ADGRG3
Biological Process	GO:0045927	positive regulation of growth	40/2279	274/18866	0.1175905	0.4298823	0.3889727	40	S100A8/RPTOR/CPNE6/RPS6KA1/CREB1/MFSD2A/EXOSC2/MIR199A1/CPNE5/SH3PXD2B/KRT17/TGFBR2/DISC1/SLC44A4/DLL1/RBPJ/DERL2/ILK/SMAD7/FGFR1/SEMA4D/SMURF1/GHRL/ACACB/CDH4/PLCB1/BCL2/MIR199A2/NOTCH1/MEF2C/SYT17/HLX/CYFIP1/TRPV2/NDEL1/EZR/SPTBN4/EIF4G1/DBNL/PRKN
Biological Process	GO:0002089	lens morphogenesis in camera-type eye	5/2279	22/18866	0.1176382	0.4298823	0.3889727	5	SKI/HIPK2/MEIS1/BCAR3/NECTIN1
Biological Process	GO:0006925	inflammatory cell apoptotic process	5/2279	22/18866	0.1176382	0.4298823	0.3889727	5	PIK3CD/ITPKB/CCL5/NOD2/MEF2C
Biological Process	GO:0007063	regulation of sister chromatid cohesion	5/2279	22/18866	0.1176382	0.4298823	0.3889727	5	TNKS/NSMCE2/RB1/CDCA5/SLF1
Biological Process	GO:0009083	branched-chain amino acid catabolic process	5/2279	22/18866	0.1176382	0.4298823	0.3889727	5	ACAD8/HIBADH/BCKDHA/HMGCL/ACAT1
Biological Process	GO:0032928	regulation of superoxide anion generation	5/2279	22/18866	0.1176382	0.4298823	0.3889727	5	CD177/FPR2/GNAI2/TGFB1/ITGB2
Biological Process	GO:0043369	CD4-positive or CD8-positive, alpha-beta T cell lineage commitment	5/2279	22/18866	0.1176382	0.4298823	0.3889727	5	ZFPM1/LOXL3/LY9/BCL2/CYLD
Biological Process	GO:0045624	positive regulation of T-helper cell differentiation	5/2279	22/18866	0.1176382	0.4298823	0.3889727	5	PRKCZ/MYB/NLRP3/RARA/HLX

Biological Process	GO:0072576	liver morphogenesis	5/2279	22/18866	0.1176382	0.4298823	0.3889727	5	RPS6KA1/TNFAIP3/CUL3/CPB2/CEBPB
Biological Process	GO:2000114	regulation of establishment of cell polarity	5/2279	22/18866	0.1176382	0.4298823	0.3889727	5	GSN/KANK1/RAP1B/DOCK8/CYRIB
Biological Process	GO:1902652	secondary alcohol metabolic process	25/2279	162/18866	0.1182505	0.4298823	0.3889727	25	OSBPL5/RXRA/IDI1/LIPC/MBTPS1/ABCG1/HDLBP/AKR1D1/APP/FGFR1/FDX1/DGKQ/POR/ABCA1/GNB3/ACACB/CAT/NPC1/ACACA/LMF1/HMGCS1/SCD/SREBF1/ACADVL/PRKAA1
Biological Process	GO:0001780	neutrophil homeostasis	4/2279	16/18866	0.1183056	0.4298823	0.3889727	4	PIK3CD/ITPKB/CCR2/JAM3
Biological Process	GO:0001977	renal system process involved in regulation of blood volume	4/2279	16/18866	0.1183056	0.4298823	0.3889727	4	GJA5/PDGFB/F2R/HSD11B2
Biological Process	GO:0002830	positive regulation of type 2 immune response	4/2279	16/18866	0.1183056	0.4298823	0.3889727	4	PRKCZ/NLRP3/RARA/NOD2
Biological Process	GO:0003222	ventricular trabecula myocardium morphogenesis	4/2279	16/18866	0.1183056	0.4298823	0.3889727	4	CCM2L/RBPJ/ENG/NOTCH1
Biological Process	GO:0006047	UDP-N-acetylglucosamine metabolic process	4/2279	16/18866	0.1183056	0.4298823	0.3889727	4	NAGK/AMDHD2/GFPT2/DPAGT1
Biological Process	GO:0006555	methionine metabolic process	4/2279	16/18866	0.1183056	0.4298823	0.3889727	4	MSRA/MTHFD1/MAT1A/ADI1

Biological Process	GO:0009263	deoxyribonucleotide biosynthetic process	4/2279	16/18866	0.1183056	0.4298823	0.3889727	4	RRM2B/GUK1/AK5/DCTD
Biological Process	GO:0019511	peptidyl-proline hydroxylation	4/2279	16/18866	0.1183056	0.4298823	0.3889727	4	P4HB/P4HA2/P3H3/P3H1
Biological Process	GO:0032354	response to follicle-stimulating hormone	4/2279	16/18866	0.1183056	0.4298823	0.3889727	4	POR/NOTCH1/HMGCS1/INHBA
Biological Process	GO:0034374	low-density lipoprotein particle remodeling	4/2279	16/18866	0.1183056	0.4298823	0.3889727	4	MPO/LIPC/ABCG1/AGTR1
Biological Process	GO:0043174	nucleoside salvage	4/2279	16/18866	0.1183056	0.4298823	0.3889727	4	CDA/TK2/UCK2/TK1
Biological Process	GO:0043249	erythrocyte maturation	4/2279	16/18866	0.1183056	0.4298823	0.3889727	4	MAEA/BRD1/EPB42/ERCC2
Biological Process	GO:0046855	inositol phosphate dephosphorylation	4/2279	16/18866	0.1183056	0.4298823	0.3889727	4	SYNJ2/INPP5A/INPP5E/IMPA2
Biological Process	GO:0050774	negative regulation of dendrite morphogenesis	4/2279	16/18866	0.1183056	0.4298823	0.3889727	4	YWHAH/RAPGEF2/DNM3/UBE3A
Biological Process	GO:0061450	trophoblast cell migration	4/2279	16/18866	0.1183056	0.4298823	0.3889727	4	CALR/ARHGDIB/AGO2/NODAL

Biological Process	GO:0070886	positive regulation of calcineurin-NFAT signaling cascade	4/2279	16/18866	0.1183056	0.4298823	0.3889727	4	TNF/CAMTA1/CHERP/STIMATE
Biological Process	GO:0071732	cellular response to nitric oxide	4/2279	16/18866	0.1183056	0.4298823	0.3889727	4	DNM2/TRAF2/AQP1/FOXO1
Biological Process	GO:0090083	regulation of inclusion body assembly	4/2279	16/18866	0.1183056	0.4298823	0.3889727	4	SORL1/CLU/DNAJB6/BAG5
Biological Process	GO:0090136	epithelial cell-cell adhesion	4/2279	16/18866	0.1183056	0.4298823	0.3889727	4	JUP/ITGB5/PLEKHA7/KIFC3
Biological Process	GO:0106058	positive regulation of calcineurin-mediated signaling	4/2279	16/18866	0.1183056	0.4298823	0.3889727	4	TNF/CAMTA1/CHERP/STIMATE
Biological Process	GO:1901163	regulation of trophoblast cell migration	4/2279	16/18866	0.1183056	0.4298823	0.3889727	4	CALR/ARHGDIB/AGO2/NODAL
Biological Process	GO:1902969	mitotic DNA replication	4/2279	16/18866	0.1183056	0.4298823	0.3889727	4	BRCA2/FGFR1/MCM2/CHEK2
Biological Process	GO:1903830	magnesium ion transmembrane transport	4/2279	16/18866	0.1183056	0.4298823	0.3889727	4	NIPAL3/ZDHHC17/CNNM2/SLC41A1
Biological Process	GO:0060324	face development	9/2279	48/18866	0.118468	0.4298823	0.3889727	9	ARID5B/CRISPLD2/SKI/ANKRD11/RARA/RARG/GRHL2/ASPH/ALDH1A2

Biological Process	GO:0070169	positive regulation of biomineral tissue development	9/2279	48/18866	0.118468	0.4298823	0.3889727	9	SMAD3/SLC8A1/TGFB1/BMPR1B/MEF2C/LTF/CEBPB/ANO6/FAM20C
Biological Process	GO:0110151	positive regulation of biomineralization	9/2279	48/18866	0.118468	0.4298823	0.3889727	9	SMAD3/SLC8A1/TGFB1/BMPR1B/MEF2C/LTF/CEBPB/ANO6/FAM20C
Biological Process	GO:1900274	regulation of phospholipase C activity	9/2279	48/18866	0.118468	0.4298823	0.3889727	9	FLT1/P2RY6/FGFR1/PHB/LPAR2/S1PR4/PDGFRB/PLCG1/NTF4
Biological Process	GO:0052547	regulation of peptidase activity	65/2279	466/18866	0.119914	0.4298823	0.3889727	65	SLPI/NLRC4/S100A8/CSTA/TIMP2/SORL1/SERPINA1/GRAMD4/SPOCK2/AIM2/PPARG/GSN/RPS6KA1/APLP2/LCK/SERPINB11/NLRP3/SMAD3/ARRB1/SPINT2/MIR199A1/MEFV/TNF/TRAF2/CYFIP2/MICAL1/TBC1D10A/SERPINB2/CTSD/BIN1/THBS1/CTSH/RIPK1/APP/UBE2O/MBP/MIR24-2/HDAC1/PAX2/CIDEB/AQP1/PERP/POR/DNAJB6/BCL10/ATP2A3/PSMF1/GRN/CRIM1/NOL3/ARRB2/FIS1/MIR199A2/PIH1D1/P2RX1/F2R/LTF/CASP1/BCL2L13/COL28A1/ASPH/NODAL/FADD/PCID2/VSIR
Biological Process	GO:0046883	regulation of hormone secretion	39/2279	267/18866	0.1202267	0.4298823	0.3889727	39	NADK/C1QTNF3/ITPR2/MYRIP/CREB1/MYB/CACNA1C/LRP5L/RAB11FIP1/ARRB1/PRKCA/GPLD1/TNF/CCL5/ITSN1/ITPR1/RFX3/TCF7L2/FGFR1/CRHBP/ENY2/GIPR/BLK/GHRL/KLF7/ARNTL/PER2/SLC16A1/PRKCE/AGTR1/SLC2A1/SREBF1/CPT1A/RPH3AL/INHBA/LIF/C2CD2L/SIRT3/PRKN
Biological Process	GO:0030509	BMP signaling pathway	24/2279	155/18866	0.1204825	0.4298823	0.3889727	24	SORL1/MIR140/SKI/SMAD3/HIPK2/RBPJ/UBE2O/ILK/PCSK6/SMAD7/TGFB1/SMURF1/BMPER/BMPR1B/RUNX2/NUMA1/ENG/NOTCH1/SLC33A1/HTRA3/GDF6/ZNF423/NODAL/SPART
Biological Process	GO:0048608	reproductive structure development	62/2279	443/18866	0.1205529	0.4298823	0.3889727	62	ARID5B/BSG/RXRA/IL10/PPARG/BRCA2/VDR/SETD2/ARRB1/SPINT2/EIF2B5/E2F7/CSDE1/HSD17B2/RBPJ/PTPN6/FANCA/RDH10/RARA/SF1/SP3/DNAJB6/DAZAP1/GHRL/RARG/BMPR1B/STK3/GRHL2/ASB1/NCOA1/PDGFB/CTSB/ARRB2/GJB3/VASH1/NTRK1/HOXA9/RAD21L1/BCL2/DMC1/NOTCH1/TPPP3/EPAS1/SLIT3/GGNBP2/STOX2/UBE3A/TMED2/CEBPB/PCDH12/PDGFRB/ERCC1/CRIP1/BCL2L1/HMGCS1/TYRO3/NCOA4/PLCD3/INHBA/NODAL/LIF/ETNK2
Biological Process	GO:0000409	regulation of transcription by galactose	1/2279	1/18866	0.1207993	0.4298823	0.3889727	1	NCOA1
Biological Process	GO:0000411	positive regulation of transcription by galactose	1/2279	1/18866	0.1207993	0.4298823	0.3889727	1	NCOA1

Biological Process	GO:0000431	regulation of transcription from RNA polymerase II promoter by galactose	1/2279	1/18866	0.1207993	0.4298823	0.3889727	1	NCOA1
Biological Process	GO:0000435	positive regulation of transcription from RNA polymerase II promoter by galactose	1/2279	1/18866	0.1207993	0.4298823	0.3889727	1	NCOA1
Biological Process	GO:0000732	strand displacement	1/2279	1/18866	0.1207993	0.4298823	0.3889727	1	RTEL1
Biological Process	GO:0001695	histamine catabolic process	1/2279	1/18866	0.1207993	0.4298823	0.3889727	1	HNMT
Biological Process	GO:0001770	establishment of natural killer cell polarity	1/2279	1/18866	0.1207993	0.4298823	0.3889727	1	SNX27
Biological Process	GO:0001949	sebaceous gland cell differentiation	1/2279	1/18866	0.1207993	0.4298823	0.3889727	1	FA2H
Biological Process	GO:0001971	negative regulation of activation of membrane attack complex	1/2279	1/18866	0.1207993	0.4298823	0.3889727	1	CD59
Biological Process	GO:0002148	hypochlorous acid metabolic process	1/2279	1/18866	0.1207993	0.4298823	0.3889727	1	MPO
Biological Process	GO:0002149	hypochlorous acid biosynthetic process	1/2279	1/18866	0.1207993	0.4298823	0.3889727	1	MPO

Biological Process	GO:0002174	mammary stem cell proliferation	1/2279	1/18866	0.1207993	0.4298823	0.3889727	1	LBH
Biological Process	GO:0002380	immunoglobulin secretion involved in immune response	1/2279	1/18866	0.1207993	0.4298823	0.3889727	1	POU2F2
Biological Process	GO:0002560	basophil mediated immunity	1/2279	1/18866	0.1207993	0.4298823	0.3889727	1	PI4K2A
Biological Process	GO:0002561	basophil degranulation	1/2279	1/18866	0.1207993	0.4298823	0.3889727	1	PI4K2A
Biological Process	GO:0002649	regulation of tolerance induction to self antigen	1/2279	1/18866	0.1207993	0.4298823	0.3889727	1	TGFBR2
Biological Process	GO:0002651	positive regulation of tolerance induction to self antigen	1/2279	1/18866	0.1207993	0.4298823	0.3889727	1	TGFBR2
Biological Process	GO:0002777	antimicrobial peptide biosynthetic process	1/2279	1/18866	0.1207993	0.4298823	0.3889727	1	ELANE
Biological Process	GO:0002780	antibacterial peptide biosynthetic process	1/2279	1/18866	0.1207993	0.4298823	0.3889727	1	ELANE
Biological Process	GO:0002812	biosynthetic process of antibacterial peptides active against Gram-negative bacteria	1/2279	1/18866	0.1207993	0.4298823	0.3889727	1	ELANE

Biological Process	GO:0002875	negative regulation of chronic inflammatory response to antigenic stimulus	1/2279	1/18866	0.1207993	0.4298823	0.3889727	1	IL10
Biological Process	GO:0003108	negative regulation of the force of heart contraction by chemical signal	1/2279	1/18866	0.1207993	0.4298823	0.3889727	1	GRK2
Biological Process	GO:0003172	sinoatrial valve development	1/2279	1/18866	0.1207993	0.4298823	0.3889727	1	MEF2C
Biological Process	GO:0003178	coronary sinus valve development	1/2279	1/18866	0.1207993	0.4298823	0.3889727	1	NOTCH1
Biological Process	GO:0003182	coronary sinus valve morphogenesis	1/2279	1/18866	0.1207993	0.4298823	0.3889727	1	NOTCH1
Biological Process	GO:0003185	sinoatrial valve morphogenesis	1/2279	1/18866	0.1207993	0.4298823	0.3889727	1	MEF2C
Biological Process	GO:0003270	Notch signaling pathway involved in regulation of secondary heart field cardioblast proliferation	1/2279	1/18866	0.1207993	0.4298823	0.3889727	1	NOTCH1
Biological Process	GO:0003294	atrial ventricular junction remodeling	1/2279	1/18866	0.1207993	0.4298823	0.3889727	1	GJA5
Biological Process	GO:0005982	starch metabolic process	1/2279	1/18866	0.1207993	0.4298823	0.3889727	1	MGAM

Biological Process	GO:0005983	starch catabolic process	1/2279	1/18866	0.1207993	0.4298823	0.3889727	1	MGAM
Biological Process	GO:0006117	acetaldehyde metabolic process	1/2279	1/18866	0.1207993	0.4298823	0.3889727	1	RELA
Biological Process	GO:0006148	inosine catabolic process	1/2279	1/18866	0.1207993	0.4298823	0.3889727	1	PNP
Biological Process	GO:0006173	dADP biosynthetic process	1/2279	1/18866	0.1207993	0.4298823	0.3889727	1	AK5
Biological Process	GO:0006185	dGDP biosynthetic process	1/2279	1/18866	0.1207993	0.4298823	0.3889727	1	GUK1
Biological Process	GO:0006452	translational frameshifting	1/2279	1/18866	0.1207993	0.4298823	0.3889727	1	SHFL
Biological Process	GO:0006507	GPI anchor release	1/2279	1/18866	0.1207993	0.4298823	0.3889727	1	GPLD1
Biological Process	GO:0006669	sphinganine-1-phosphate biosynthetic process	1/2279	1/18866	0.1207993	0.4298823	0.3889727	1	SPHK2
Biological Process	GO:0006713	glucocorticoid catabolic process	1/2279	1/18866	0.1207993	0.4298823	0.3889727	1	YWHAH

Biological Process	GO:0006738	nicotinamide riboside catabolic process	1/2279	1/18866	0.1207993	0.4298823	0.3889727	1	PNP
Biological Process	GO:0006843	mitochondrial citrate transmembrane transport	1/2279	1/18866	0.1207993	0.4298823	0.3889727	1	SLC25A1
Biological Process	GO:0008039	synaptic target recognition	1/2279	1/18866	0.1207993	0.4298823	0.3889727	1	YWHAZ
Biological Process	GO:0008049	male courtship behavior	1/2279	1/18866	0.1207993	0.4298823	0.3889727	1	HEXB
Biological Process	GO:0008052	sensory organ boundary specification	1/2279	1/18866	0.1207993	0.4298823	0.3889727	1	NTF4
Biological Process	GO:0008653	lipopolysaccharide metabolic process	1/2279	1/18866	0.1207993	0.4298823	0.3889727	1	AOAH
Biological Process	GO:0009087	methionine catabolic process	1/2279	1/18866	0.1207993	0.4298823	0.3889727	1	MAT1A
Biological Process	GO:0009104	lipopolysaccharide catabolic process	1/2279	1/18866	0.1207993	0.4298823	0.3889727	1	AOAH
Biological Process	GO:0009443	pyridoxal 5'-phosphate salvage	1/2279	1/18866	0.1207993	0.4298823	0.3889727	1	PDXK

Biological Process	GO:0009645	response to low light intensity stimulus	1/2279	1/18866	0.1207993	0.4298823	0.3889727	1	HMGCS1
Biological Process	GO:0010085	polarity specification of proximal/distal axis	1/2279	1/18866	0.1207993	0.4298823	0.3889727	1	NODAL
Biological Process	GO:0010160	formation of animal organ boundary	1/2279	1/18866	0.1207993	0.4298823	0.3889727	1	NTF4
Biological Process	GO:0010189	vitamin E biosynthetic process	1/2279	1/18866	0.1207993	0.4298823	0.3889727	1	PLTP
Biological Process	GO:0010585	glutamine secretion	1/2279	1/18866	0.1207993	0.4298823	0.3889727	1	SLC1A5
Biological Process	GO:0010768	negative regulation of transcription from RNA polymerase II promoter in response to UV-induced DNA damage	1/2279	1/18866	0.1207993	0.4298823	0.3889727	1	NEDD4
Biological Process	GO:0014006	regulation of microglia differentiation	1/2279	1/18866	0.1207993	0.4298823	0.3889727	1	TGFB1
Biological Process	GO:0014008	positive regulation of microglia differentiation	1/2279	1/18866	0.1207993	0.4298823	0.3889727	1	TGFB1
Biological Process	GO:0014810	positive regulation of skeletal muscle contraction by regulation of release of sequestered calcium ion	1/2279	1/18866	0.1207993	0.4298823	0.3889727	1	GSTO1

Biological Process	GO:0014813	skeletal muscle satellite cell commitment	1/2279	1/18866	0.1207993	0.4298823	0.3889727	1	PAX7
Biological Process	GO:0015675	nickel cation transport	1/2279	1/18866	0.1207993	0.4298823	0.3889727	1	SLC11A2
Biological Process	GO:0015692	lead ion transport	1/2279	1/18866	0.1207993	0.4298823	0.3889727	1	SLC11A2
Biological Process	GO:0015694	mercury ion transport	1/2279	1/18866	0.1207993	0.4298823	0.3889727	1	ABCC2
Biological Process	GO:0015707	nitrite transport	1/2279	1/18866	0.1207993	0.4298823	0.3889727	1	SLC11A1
Biological Process	GO:0015723	bilirubin transport	1/2279	1/18866	0.1207993	0.4298823	0.3889727	1	ABCC2
Biological Process	GO:0015728	mevalonate transport	1/2279	1/18866	0.1207993	0.4298823	0.3889727	1	SLC16A1
Biological Process	GO:0015829	valine transport	1/2279	1/18866	0.1207993	0.4298823	0.3889727	1	SLC7A8
Biological Process	GO:0015876	acetyl-CoA transport	1/2279	1/18866	0.1207993	0.4298823	0.3889727	1	SLC33A1

Biological Process	GO:0015904	tetracycline transmembrane transport	1/2279	1/18866	0.1207993	0.4298823	0.3889727	1	MFSD10
Biological Process	GO:0016333	morphogenesis of follicular epithelium	1/2279	1/18866	0.1207993	0.4298823	0.3889727	1	WDR1
Biological Process	GO:0016334	establishment or maintenance of polarity of follicular epithelium	1/2279	1/18866	0.1207993	0.4298823	0.3889727	1	WDR1
Biological Process	GO:0018011	N-terminal peptidyl-alanine methylation	1/2279	1/18866	0.1207993	0.4298823	0.3889727	1	NTMT1
Biological Process	GO:0018012	N-terminal peptidyl-alanine trimethylation	1/2279	1/18866	0.1207993	0.4298823	0.3889727	1	NTMT1
Biological Process	GO:0018013	N-terminal peptidyl-glycine methylation	1/2279	1/18866	0.1207993	0.4298823	0.3889727	1	NTMT1
Biological Process	GO:0018016	N-terminal peptidyl-proline dimethylation	1/2279	1/18866	0.1207993	0.4298823	0.3889727	1	NTMT1
Biological Process	GO:0018194	peptidyl-alanine modification	1/2279	1/18866	0.1207993	0.4298823	0.3889727	1	NTMT1
Biological Process	GO:0018969	thiocyanate metabolic process	1/2279	1/18866	0.1207993	0.4298823	0.3889727	1	LPO

Biological Process	GO:0021501	prechordal plate formation	1/2279	1/18866	0.1207993	0.4298823	0.3889727	1	SSBP3
Biological Process	GO:0021547	midbrain-hindbrain boundary initiation	1/2279	1/18866	0.1207993	0.4298823	0.3889727	1	SSBP3
Biological Process	GO:0021634	optic nerve formation	1/2279	1/18866	0.1207993	0.4298823	0.3889727	1	PAX2
Biological Process	GO:0021639	trochlear nerve morphogenesis	1/2279	1/18866	0.1207993	0.4298823	0.3889727	1	PHOX2A
Biological Process	GO:0021642	trochlear nerve formation	1/2279	1/18866	0.1207993	0.4298823	0.3889727	1	PHOX2A
Biological Process	GO:0021688	cerebellar molecular layer formation	1/2279	1/18866	0.1207993	0.4298823	0.3889727	1	DLL1
Biological Process	GO:0021703	locus ceruleus development	1/2279	1/18866	0.1207993	0.4298823	0.3889727	1	PHOX2A
Biological Process	GO:0030034	microvillar actin bundle assembly	1/2279	1/18866	0.1207993	0.4298823	0.3889727	1	ESPN
Biological Process	GO:0030037	actin filament reorganization involved in cell cycle	1/2279	1/18866	0.1207993	0.4298823	0.3889727	1	ARAP1

Biological Process	GO:0030393	fructoselysine metabolic process	1/2279	1/18866	0.1207993	0.4298823	0.3889727	1	FN3K
Biological Process	GO:0030653	beta-lactam antibiotic metabolic process	1/2279	1/18866	0.1207993	0.4298823	0.3889727	1	ABCC2
Biological Process	GO:0031038	myosin II filament organization	1/2279	1/18866	0.1207993	0.4298823	0.3889727	1	DAPK3
Biological Process	GO:0031161	phosphatidylinositol catabolic process	1/2279	1/18866	0.1207993	0.4298823	0.3889727	1	INPP5F
Biological Process	GO:0031337	positive regulation of sulfur amino acid metabolic process	1/2279	1/18866	0.1207993	0.4298823	0.3889727	1	COMT
Biological Process	GO:0032445	fructose import	1/2279	1/18866	0.1207993	0.4298823	0.3889727	1	SLC2A5
Biological Process	GO:0032458	slow endocytic recycling	1/2279	1/18866	0.1207993	0.4298823	0.3889727	1	MICALL1
Biological Process	GO:0032461	positive regulation of protein oligomerization	1/2279	1/18866	0.1207993	0.4298823	0.3889727	1	ZDHHC1
Biological Process	GO:0032475	otolith formation	1/2279	1/18866	0.1207993	0.4298823	0.3889727	1	SLC44A4

Biological Process	GO:0032498	detection of muramyl dipeptide	1/2279	1/18866	0.1207993	0.4298823	0.3889727	1	NOD2
Biological Process	GO:0032685	negative regulation of granulocyte macrophage colony-stimulating factor production	1/2279	1/18866	0.1207993	0.4298823	0.3889727	1	CD84
Biological Process	GO:0032771	regulation of monophenol monooxygenase activity	1/2279	1/18866	0.1207993	0.4298823	0.3889727	1	CDH3
Biological Process	GO:0032773	positive regulation of monophenol monooxygenase activity	1/2279	1/18866	0.1207993	0.4298823	0.3889727	1	CDH3
Biological Process	GO:0032848	negative regulation of cellular pH reduction	1/2279	1/18866	0.1207993	0.4298823	0.3889727	1	BCL2
Biological Process	GO:0032887	regulation of spindle elongation	1/2279	1/18866	0.1207993	0.4298823	0.3889727	1	NUMA1
Biological Process	GO:0032888	regulation of mitotic spindle elongation	1/2279	1/18866	0.1207993	0.4298823	0.3889727	1	NUMA1
Biological Process	GO:0032919	spermine acetylation	1/2279	1/18866	0.1207993	0.4298823	0.3889727	1	SAT2
Biological Process	GO:0032920	putrescine acetylation	1/2279	1/18866	0.1207993	0.4298823	0.3889727	1	SAT2

Biological Process	GO:0033214	siderophore-dependent iron import into cell	1/2279	1/18866	0.1207993	0.4298823	0.3889727	1	LTF
Biological Process	GO:0033319	UDP-D-xylose metabolic process	1/2279	1/18866	0.1207993	0.4298823	0.3889727	1	UXS1
Biological Process	GO:0033320	UDP-D-xylose biosynthetic process	1/2279	1/18866	0.1207993	0.4298823	0.3889727	1	UXS1
Biological Process	GO:0033396	beta-alanine biosynthetic process via 3-ureidopropionate	1/2279	1/18866	0.1207993	0.4298823	0.3889727	1	UPB1
Biological Process	GO:0033609	oxalate metabolic process	1/2279	1/18866	0.1207993	0.4298823	0.3889727	1	HOGA1
Biological Process	GO:0034014	response to triglyceride	1/2279	1/18866	0.1207993	0.4298823	0.3889727	1	GPLD1
Biological Process	GO:0034147	regulation of toll-like receptor 5 signaling pathway	1/2279	1/18866	0.1207993	0.4298823	0.3889727	1	TNFAIP3
Biological Process	GO:0034148	negative regulation of toll-like receptor 5 signaling pathway	1/2279	1/18866	0.1207993	0.4298823	0.3889727	1	TNFAIP3
Biological Process	GO:0034373	intermediate-density lipoprotein particle remodeling	1/2279	1/18866	0.1207993	0.4298823	0.3889727	1	LIPC

Biological Process	GO:0034670	chemotaxis to arachidonic acid	1/2279	1/18866	0.1207993	0.4298823	0.3889727	1	CREB1
Biological Process	GO:0035037	sperm entry	1/2279	1/18866	0.1207993	0.4298823	0.3889727	1	UBE3A
Biological Process	GO:0035444	nickel cation transmembrane transport	1/2279	1/18866	0.1207993	0.4298823	0.3889727	1	SLC11A2
Biological Process	GO:0035568	N-terminal peptidyl-proline methylation	1/2279	1/18866	0.1207993	0.4298823	0.3889727	1	NTMT1
Biological Process	GO:0035570	N-terminal peptidyl-serine methylation	1/2279	1/18866	0.1207993	0.4298823	0.3889727	1	NTMT1
Biological Process	GO:0035572	N-terminal peptidyl-serine dimethylation	1/2279	1/18866	0.1207993	0.4298823	0.3889727	1	NTMT1
Biological Process	GO:0035573	N-terminal peptidyl-serine trimethylation	1/2279	1/18866	0.1207993	0.4298823	0.3889727	1	NTMT1
Biological Process	GO:0035611	protein branching point deglutamylation	1/2279	1/18866	0.1207993	0.4298823	0.3889727	1	AGBL5
Biological Process	GO:0035623	renal glucose absorption	1/2279	1/18866	0.1207993	0.4298823	0.3889727	1	HNF1A

Biological Process	GO:0035704	helper T cell chemotaxis	1/2279	1/18866	0.1207993	0.4298823	0.3889727	1	CCR2
Biological Process	GO:0035705	T-helper 17 cell chemotaxis	1/2279	1/18866	0.1207993	0.4298823	0.3889727	1	CCR2
Biological Process	GO:0035724	CD24 biosynthetic process	1/2279	1/18866	0.1207993	0.4298823	0.3889727	1	PLCB1
Biological Process	GO:0035853	chromosome passenger complex localization to spindle midzone	1/2279	1/18866	0.1207993	0.4298823	0.3889727	1	KLHL21
Biological Process	GO:0036034	mediator complex assembly	1/2279	1/18866	0.1207993	0.4298823	0.3889727	1	MED25
Biological Process	GO:0036114	medium-chain fatty-acyl-CoA catabolic process	1/2279	1/18866	0.1207993	0.4298823	0.3889727	1	ACOT7
Biological Process	GO:0036116	long-chain fatty-acyl-CoA catabolic process	1/2279	1/18866	0.1207993	0.4298823	0.3889727	1	ACOT7
Biological Process	GO:0036179	osteoclast maturation	1/2279	1/18866	0.1207993	0.4298823	0.3889727	1	FAM20C
Biological Process	GO:0036290	protein trans-autophosphorylation	1/2279	1/18866	0.1207993	0.4298823	0.3889727	1	ERN1

Biological Process	GO:0036323	vascular endothelial growth factor receptor-1 signaling pathway	1/2279	1/18866	0.1207993	0.4298823	0.3889727	1	FLT1
Biological Process	GO:0038020	insulin receptor recycling	1/2279	1/18866	0.1207993	0.4298823	0.3889727	1	SORL1
Biological Process	GO:0038192	gastric inhibitory peptide signaling pathway	1/2279	1/18866	0.1207993	0.4298823	0.3889727	1	GIPR
Biological Process	GO:0042247	establishment of planar polarity of follicular epithelium	1/2279	1/18866	0.1207993	0.4298823	0.3889727	1	WDR1
Biological Process	GO:0042316	penicillin metabolic process	1/2279	1/18866	0.1207993	0.4298823	0.3889727	1	ABCC2
Biological Process	GO:0043310	negative regulation of eosinophil degranulation	1/2279	1/18866	0.1207993	0.4298823	0.3889727	1	CCR2
Biological Process	GO:0043322	negative regulation of natural killer cell degranulation	1/2279	1/18866	0.1207993	0.4298823	0.3889727	1	HLA-F
Biological Process	GO:0043519	regulation of myosin II filament organization	1/2279	1/18866	0.1207993	0.4298823	0.3889727	1	DAPK3
Biological Process	GO:0043602	nitrate catabolic process	1/2279	1/18866	0.1207993	0.4298823	0.3889727	1	POR

Biological Process	GO:0043977	histone H2A-K5 acetylation	1/2279	1/18866	0.1207993	0.4298823	0.3889727	1	SPHK2
Biological Process	GO:0043980	histone H2B-K12 acetylation	1/2279	1/18866	0.1207993	0.4298823	0.3889727	1	SPHK2
Biological Process	GO:0044328	canonical Wnt signaling pathway involved in positive regulation of endothelial cell migration	1/2279	1/18866	0.1207993	0.4298823	0.3889727	1	PLPP3
Biological Process	GO:0044329	canonical Wnt signaling pathway involved in positive regulation of cell-cell adhesion	1/2279	1/18866	0.1207993	0.4298823	0.3889727	1	PLPP3
Biological Process	GO:0044330	canonical Wnt signaling pathway involved in positive regulation of wound healing	1/2279	1/18866	0.1207993	0.4298823	0.3889727	1	PLPP3
Biological Process	GO:0044651	adhesion of symbiont to host epithelial cell	1/2279	1/18866	0.1207993	0.4298823	0.3889727	1	SFTPD
Biological Process	GO:0044858	plasma membrane raft polarization	1/2279	1/18866	0.1207993	0.4298823	0.3889727	1	GSN
Biological Process	GO:0045828	positive regulation of isoprenoid metabolic process	1/2279	1/18866	0.1207993	0.4298823	0.3889727	1	RDH10
Biological Process	GO:0045951	positive regulation of mitotic recombination	1/2279	1/18866	0.1207993	0.4298823	0.3889727	1	ERCC2

Biological Process	GO:0046054	dGMP metabolic process	1/2279	1/18866	0.1207993	0.4298823	0.3889727	1	GUK1
Biological Process	GO:0046092	deoxycytidine metabolic process	1/2279	1/18866	0.1207993	0.4298823	0.3889727	1	TK2
Biological Process	GO:0046204	nor-spermidine metabolic process	1/2279	1/18866	0.1207993	0.4298823	0.3889727	1	SAT2
Biological Process	GO:0046210	nitric oxide catabolic process	1/2279	1/18866	0.1207993	0.4298823	0.3889727	1	POR
Biological Process	GO:0046317	regulation of glucosylceramide biosynthetic process	1/2279	1/18866	0.1207993	0.4298823	0.3889727	1	PRKAA1
Biological Process	GO:0046318	negative regulation of glucosylceramide biosynthetic process	1/2279	1/18866	0.1207993	0.4298823	0.3889727	1	PRKAA1
Biological Process	GO:0046495	nicotinamide riboside metabolic process	1/2279	1/18866	0.1207993	0.4298823	0.3889727	1	PNP
Biological Process	GO:0046587	positive regulation of calcium-dependent cell-cell adhesion	1/2279	1/18866	0.1207993	0.4298823	0.3889727	1	KIFAP3
Biological Process	GO:0046657	folic acid catabolic process	1/2279	1/18866	0.1207993	0.4298823	0.3889727	1	MTHFS

Biological Process	GO:0046671	negative regulation of retinal cell programmed cell death	1/2279	1/18866	0.1207993	0.4298823	0.3889727	1	BCL2
Biological Process	GO:0046690	response to tellurium ion	1/2279	1/18866	0.1207993	0.4298823	0.3889727	1	HMGCS1
Biological Process	GO:0046711	GDP biosynthetic process	1/2279	1/18866	0.1207993	0.4298823	0.3889727	1	GUK1
Biological Process	GO:0046786	viral replication complex formation and maintenance	1/2279	1/18866	0.1207993	0.4298823	0.3889727	1	PI4KA
Biological Process	GO:0048219	inter-Golgi cisterna vesicle-mediated transport	1/2279	1/18866	0.1207993	0.4298823	0.3889727	1	COG5
Biological Process	GO:0048321	axial mesodermal cell differentiation	1/2279	1/18866	0.1207993	0.4298823	0.3889727	1	NODAL
Biological Process	GO:0048322	axial mesodermal cell fate commitment	1/2279	1/18866	0.1207993	0.4298823	0.3889727	1	NODAL
Biological Process	GO:0048327	axial mesodermal cell fate specification	1/2279	1/18866	0.1207993	0.4298823	0.3889727	1	NODAL
Biological Process	GO:0048698	negative regulation of collateral sprouting in absence of injury	1/2279	1/18866	0.1207993	0.4298823	0.3889727	1	SPART

Biological Process	GO:0050668	positive regulation of homocysteine metabolic process	1/2279	1/18866	0.1207993	0.4298823	0.3889727	1	COMT
Biological Process	GO:0051107	negative regulation of DNA ligation	1/2279	1/18866	0.1207993	0.4298823	0.3889727	1	TFIP11
Biological Process	GO:0051141	negative regulation of NK T cell proliferation	1/2279	1/18866	0.1207993	0.4298823	0.3889727	1	ZBTB7B
Biological Process	GO:0051232	meiotic spindle elongation	1/2279	1/18866	0.1207993	0.4298823	0.3889727	1	PPP2R1A
Biological Process	GO:0051460	negative regulation of corticotropin secretion	1/2279	1/18866	0.1207993	0.4298823	0.3889727	1	CRHBP
Biological Process	GO:0051686	establishment of ER localization	1/2279	1/18866	0.1207993	0.4298823	0.3889727	1	MYO5A
Biological Process	GO:0051780	behavioral response to nutrient	1/2279	1/18866	0.1207993	0.4298823	0.3889727	1	SLC16A1
Biological Process	GO:0051796	negative regulation of timing of catagen	1/2279	1/18866	0.1207993	0.4298823	0.3889727	1	CDH3
Biological Process	GO:0055048	anastral spindle assembly	1/2279	1/18866	0.1207993	0.4298823	0.3889727	1	NUMA1

Biological Process	GO:0060010	Sertoli cell fate commitment	1/2279	1/18866	0.1207993	0.4298823	0.3889727	1	RARA
Biological Process	GO:0060243	negative regulation of cell growth involved in contact inhibition	1/2279	1/18866	0.1207993	0.4298823	0.3889727	1	CDHR2
Biological Process	GO:0060377	negative regulation of mast cell differentiation	1/2279	1/18866	0.1207993	0.4298823	0.3889727	1	ZFPM1
Biological Process	GO:0060460	left lung morphogenesis	1/2279	1/18866	0.1207993	0.4298823	0.3889727	1	NODAL
Biological Process	GO:0060466	activation of meiosis involved in egg activation	1/2279	1/18866	0.1207993	0.4298823	0.3889727	1	PLCB1
Biological Process	GO:0060731	positive regulation of intestinal epithelial structure maintenance	1/2279	1/18866	0.1207993	0.4298823	0.3889727	1	SLC22A5
Biological Process	GO:0060802	epiblast cell-extraembryonic ectoderm cell signaling involved in anterior/posterior axis specification	1/2279	1/18866	0.1207993	0.4298823	0.3889727	1	NODAL
Biological Process	GO:0060843	venous endothelial cell differentiation	1/2279	1/18866	0.1207993	0.4298823	0.3889727	1	NOTCH1
Biological Process	GO:0060853	Notch signaling pathway involved in arterial endothelial cell fate commitment	1/2279	1/18866	0.1207993	0.4298823	0.3889727	1	DLL1

Biological Process	GO:0060901	regulation of hair cycle by canonical Wnt signaling pathway	1/2279	1/18866	0.1207993	0.4298823	0.3889727	1	CDH3
Biological Process	GO:0060981	cell migration involved in coronary angiogenesis	1/2279	1/18866	0.1207993	0.4298823	0.3889727	1	PDGFRB
Biological Process	GO:0061048	negative regulation of branching involved in lung morphogenesis	1/2279	1/18866	0.1207993	0.4298823	0.3889727	1	TNF
Biological Process	GO:0061159	establishment of bipolar cell polarity involved in cell morphogenesis	1/2279	1/18866	0.1207993	0.4298823	0.3889727	1	CRB1
Biological Process	GO:0061171	establishment of bipolar cell polarity	1/2279	1/18866	0.1207993	0.4298823	0.3889727	1	CRB1
Biological Process	GO:0061360	optic chiasma development	1/2279	1/18866	0.1207993	0.4298823	0.3889727	1	PAX2
Biological Process	GO:0061485	memory T cell proliferation	1/2279	1/18866	0.1207993	0.4298823	0.3889727	1	DOCK8
Biological Process	GO:0061534	gamma-aminobutyric acid secretion, neurotransmission	1/2279	1/18866	0.1207993	0.4298823	0.3889727	1	NF1
Biological Process	GO:0061580	colon epithelial cell migration	1/2279	1/18866	0.1207993	0.4298823	0.3889727	1	ARSB

Biological Process	GO:0061736	engulfment of target by autophagosome	1/2279	1/18866	0.1207993	0.4298823	0.3889727	1	SMURF1
Biological Process	GO:0061753	substrate localization to autophagosome	1/2279	1/18866	0.1207993	0.4298823	0.3889727	1	SMURF1
Biological Process	GO:0061767	negative regulation of lung blood pressure	1/2279	1/18866	0.1207993	0.4298823	0.3889727	1	SMAD3
Biological Process	GO:0061910	autophagosome-endosome fusion	1/2279	1/18866	0.1207993	0.4298823	0.3889727	1	RUBCNL
Biological Process	GO:0062036	sensory perception of hot stimulus	1/2279	1/18866	0.1207993	0.4298823	0.3889727	1	WDR47
Biological Process	GO:0070054	mRNA splicing, via endonucleolytic cleavage and ligation	1/2279	1/18866	0.1207993	0.4298823	0.3889727	1	ERN1
Biological Process	GO:0070429	negative regulation of nucleotide-binding oligomerization domain containing 1 signaling pathway	1/2279	1/18866	0.1207993	0.4298823	0.3889727	1	TNFAIP3
Biological Process	GO:0070637	pyridine nucleoside metabolic process	1/2279	1/18866	0.1207993	0.4298823	0.3889727	1	PNP
Biological Process	GO:0070638	pyridine nucleoside catabolic process	1/2279	1/18866	0.1207993	0.4298823	0.3889727	1	PNP

Biological Process	GO:0070715	sodium-dependent organic cation transport	1/2279	1/18866	0.1207993	0.4298823	0.3889727	1	SLC22A5
Biological Process	GO:0070926	regulation of ATP:ADP antiporter activity	1/2279	1/18866	0.1207993	0.4298823	0.3889727	1	RIPK1
Biological Process	GO:0070946	neutrophil-mediated killing of gram-positive bacterium	1/2279	1/18866	0.1207993	0.4298823	0.3889727	1	CTSG
Biological Process	GO:0071245	cellular response to carbon monoxide	1/2279	1/18866	0.1207993	0.4298823	0.3889727	1	DNM2
Biological Process	GO:0071348	cellular response to interleukin-11	1/2279	1/18866	0.1207993	0.4298823	0.3889727	1	SBNO2
Biological Process	GO:0071401	cellular response to triglyceride	1/2279	1/18866	0.1207993	0.4298823	0.3889727	1	GPLD1
Biological Process	GO:0071529	cementum mineralization	1/2279	1/18866	0.1207993	0.4298823	0.3889727	1	ALPL
Biological Process	GO:0071544	diphosphoinositol polyphosphate catabolic process	1/2279	1/18866	0.1207993	0.4298823	0.3889727	1	NUDT3
Biological Process	GO:0071802	negative regulation of podosome assembly	1/2279	1/18866	0.1207993	0.4298823	0.3889727	1	ARHGEF2

Biological Process	GO:0072019	proximal convoluted tubule development	1/2279	1/18866	0.1207993	0.4298823	0.3889727	1	ACAT1
Biological Process	GO:0072229	metanephric proximal convoluted tubule development	1/2279	1/18866	0.1207993	0.4298823	0.3889727	1	ACAT1
Biological Process	GO:0072255	metanephric glomerular mesangial cell development	1/2279	1/18866	0.1207993	0.4298823	0.3889727	1	PDGFB
Biological Process	GO:0072382	minus-end-directed vesicle transport along microtubule	1/2279	1/18866	0.1207993	0.4298823	0.3889727	1	DYNC1H1
Biological Process	GO:0072428	signal transduction involved in intra-S DNA damage checkpoint	1/2279	1/18866	0.1207993	0.4298823	0.3889727	1	CHEK2
Biological Process	GO:0075523	viral translational frameshifting	1/2279	1/18866	0.1207993	0.4298823	0.3889727	1	SHFL
Biological Process	GO:0085018	maintenance of symbiont-containing vacuole by host	1/2279	1/18866	0.1207993	0.4298823	0.3889727	1	AQP1
Biological Process	GO:0085032	modulation by symbiont of host I-kappaB kinase/NF-kappaB cascade	1/2279	1/18866	0.1207993	0.4298823	0.3889727	1	TNIP1
Biological Process	GO:0086024	adenylate cyclase-activating adrenergic receptor signaling pathway involved in positive regulation of heart rate	1/2279	1/18866	0.1207993	0.4298823	0.3889727	1	PDE4D

Biological Process	GO:0086044	atrial cardiac muscle cell to AV node cell communication by electrical coupling	1/2279	1/18866	0.1207993	0.4298823	0.3889727	1	GJA5
Biological Process	GO:0086054	bundle of His cell to Purkinje myocyte communication by electrical coupling	1/2279	1/18866	0.1207993	0.4298823	0.3889727	1	GJA5
Biological Process	GO:0086055	Purkinje myocyte to ventricular cardiac muscle cell communication by electrical coupling	1/2279	1/18866	0.1207993	0.4298823	0.3889727	1	GJA5
Biological Process	GO:0090010	transforming growth factor beta receptor signaling pathway involved in primitive streak formation	1/2279	1/18866	0.1207993	0.4298823	0.3889727	1	NODAL
Biological Process	GO:0090138	regulation of actin cytoskeleton organization by cell-cell adhesion	1/2279	1/18866	0.1207993	0.4298823	0.3889727	1	JAM3
Biological Process	GO:0090242	retinoic acid receptor signaling pathway involved in somitogenesis	1/2279	1/18866	0.1207993	0.4298823	0.3889727	1	ALDH1A2
Biological Process	GO:0090282	positive regulation of transcription involved in G2/M transition of mitotic cell cycle	1/2279	1/18866	0.1207993	0.4298823	0.3889727	1	ESRRB
Biological Process	GO:0090340	positive regulation of secretion of lysosomal enzymes	1/2279	1/18866	0.1207993	0.4298823	0.3889727	1	NR1H2
Biological Process	GO:0090410	malonate catabolic process	1/2279	1/18866	0.1207993	0.4298823	0.3889727	1	ACSF3

Biological Process	GO:0090625	mRNA cleavage involved in gene silencing by siRNA	1/2279	1/18866	0.1207993	0.4298823	0.3889727	1	AGO2
Biological Process	GO:0090720	primary adaptive immune response	1/2279	1/18866	0.1207993	0.4298823	0.3889727	1	BACH2
Biological Process	GO:0090721	primary adaptive immune response involving T cells and B cells	1/2279	1/18866	0.1207993	0.4298823	0.3889727	1	BACH2
Biological Process	GO:0097045	phosphatidylserine exposure on blood platelet	1/2279	1/18866	0.1207993	0.4298823	0.3889727	1	ANO6
Biological Process	GO:0097510	base-excision repair, AP site formation via deaminated base removal	1/2279	1/18866	0.1207993	0.4298823	0.3889727	1	UNG
Biological Process	GO:0098906	regulation of Purkinje myocyte action potential	1/2279	1/18866	0.1207993	0.4298823	0.3889727	1	GJA5
Biological Process	GO:0099089	establishment of endoplasmic reticulum localization to postsynapse	1/2279	1/18866	0.1207993	0.4298823	0.3889727	1	MYO5A
Biological Process	GO:0099564	modification of synaptic structure, modulating synaptic transmission	1/2279	1/18866	0.1207993	0.4298823	0.3889727	1	BAIAP2
Biological Process	GO:0099588	positive regulation of postsynaptic cytosolic calcium concentration	1/2279	1/18866	0.1207993	0.4298823	0.3889727	1	SYNPO

Biological Process	GO:0106096	response to ceramide	1/2279	1/18866	0.1207993	0.4298823	0.3889727	1	PDGFRB
Biological Process	GO:0110028	positive regulation of mitotic spindle organization	1/2279	1/18866	0.1207993	0.4298823	0.3889727	1	NUMA1
Biological Process	GO:0120093	regulation of peptidyl-lysine crotonylation	1/2279	1/18866	0.1207993	0.4298823	0.3889727	1	CDYL
Biological Process	GO:0120094	negative regulation of peptidyl-lysine crotonylation	1/2279	1/18866	0.1207993	0.4298823	0.3889727	1	CDYL
Biological Process	GO:0120158	positive regulation of collagen catabolic process	1/2279	1/18866	0.1207993	0.4298823	0.3889727	1	VSIR
Biological Process	GO:0120168	detection of hot stimulus involved in thermoception	1/2279	1/18866	0.1207993	0.4298823	0.3889727	1	WDR47
Biological Process	GO:0150089	multiple spine synapse organization	1/2279	1/18866	0.1207993	0.4298823	0.3889727	1	CX3CR1
Biological Process	GO:0150090	multiple spine synapse organization, single dendrite	1/2279	1/18866	0.1207993	0.4298823	0.3889727	1	CX3CR1
Biological Process	GO:1900010	regulation of corticotropin-releasing hormone receptor activity	1/2279	1/18866	0.1207993	0.4298823	0.3889727	1	CRHBP

Biological Process	GO:1900011	negative regulation of corticotropin-releasing hormone receptor activity	1/2279	1/18866	0.1207993	0.4298823	0.3889727	1	CRHBP
Biological Process	GO:1900054	positive regulation of retinoic acid biosynthetic process	1/2279	1/18866	0.1207993	0.4298823	0.3889727	1	RDH10
Biological Process	GO:1900134	negative regulation of renin secretion into blood stream	1/2279	1/18866	0.1207993	0.4298823	0.3889727	1	F2R
Biological Process	GO:1900148	negative regulation of Schwann cell migration	1/2279	1/18866	0.1207993	0.4298823	0.3889727	1	CERS2
Biological Process	GO:1900159	positive regulation of bone mineralization involved in bone maturation	1/2279	1/18866	0.1207993	0.4298823	0.3889727	1	LTF
Biological Process	GO:1900477	negative regulation of G1/S transition of mitotic cell cycle by negative regulation of transcription from RNA polymerase II promoter	1/2279	1/18866	0.1207993	0.4298823	0.3889727	1	NACC2
Biological Process	GO:1900480	regulation of diacylglycerol biosynthetic process	1/2279	1/18866	0.1207993	0.4298823	0.3889727	1	AVIL
Biological Process	GO:1900749	(R)-carnitine transport	1/2279	1/18866	0.1207993	0.4298823	0.3889727	1	SLC22A5
Biological Process	GO:1900753	doxorubicin transport	1/2279	1/18866	0.1207993	0.4298823	0.3889727	1	RALBP1

Biological Process	GO:1901086	benzylpenicillin metabolic process	1/2279	1/18866	0.1207993	0.4298823	0.3889727	1	ABCC2
Biological Process	GO:1901189	positive regulation of ephrin receptor signaling pathway	1/2279	1/18866	0.1207993	0.4298823	0.3889727	1	RBPJ
Biological Process	GO:1901297	positive regulation of canonical Wnt signaling pathway involved in cardiac muscle cell fate commitment	1/2279	1/18866	0.1207993	0.4298823	0.3889727	1	RBPJ
Biological Process	GO:1901609	negative regulation of vesicle transport along microtubule	1/2279	1/18866	0.1207993	0.4298823	0.3889727	1	CNIH2
Biological Process	GO:1901676	positive regulation of histone H3-K27 acetylation	1/2279	1/18866	0.1207993	0.4298823	0.3889727	1	LIF
Biological Process	GO:1901999	homogentisate metabolic process	1/2279	1/18866	0.1207993	0.4298823	0.3889727	1	FAH
Biological Process	GO:1902000	homogentisate catabolic process	1/2279	1/18866	0.1207993	0.4298823	0.3889727	1	FAH
Biological Process	GO:1902204	positive regulation of hepatocyte growth factor receptor signaling pathway	1/2279	1/18866	0.1207993	0.4298823	0.3889727	1	ESM1
Biological Process	GO:1902270	(R)-carnitine transmembrane transport	1/2279	1/18866	0.1207993	0.4298823	0.3889727	1	SLC22A5

Biological Process	GO:1902363	regulation of protein localization to spindle pole body	1/2279	1/18866	0.1207993	0.4298823	0.3889727	1	NUMA1
Biological Process	GO:1902365	positive regulation of protein localization to spindle pole body	1/2279	1/18866	0.1207993	0.4298823	0.3889727	1	NUMA1
Biological Process	GO:1902551	regulation of catalase activity	1/2279	1/18866	0.1207993	0.4298823	0.3889727	1	SIRT3
Biological Process	GO:1902553	positive regulation of catalase activity	1/2279	1/18866	0.1207993	0.4298823	0.3889727	1	SIRT3
Biological Process	GO:1902684	negative regulation of receptor localization to synapse	1/2279	1/18866	0.1207993	0.4298823	0.3889727	1	CNIH2
Biological Process	GO:1902761	positive regulation of chondrocyte development	1/2279	1/18866	0.1207993	0.4298823	0.3889727	1	SMAD7
Biological Process	GO:1902769	regulation of choline O-acetyltransferase activity	1/2279	1/18866	0.1207993	0.4298823	0.3889727	1	SORL1
Biological Process	GO:1902771	positive regulation of choline O-acetyltransferase activity	1/2279	1/18866	0.1207993	0.4298823	0.3889727	1	SORL1
Biological Process	GO:1902804	negative regulation of synaptic vesicle transport	1/2279	1/18866	0.1207993	0.4298823	0.3889727	1	CNIH2

Biological Process	GO:1902846	positive regulation of mitotic spindle elongation	1/2279	1/18866	0.1207993	0.4298823	0.3889727	1	NUMA1
Biological Process	GO:1902847	regulation of neuronal signal transduction	1/2279	1/18866	0.1207993	0.4298823	0.3889727	1	CLU
Biological Process	GO:1902858	propionyl-CoA metabolic process	1/2279	1/18866	0.1207993	0.4298823	0.3889727	1	ACAT1
Biological Process	GO:1902860	propionyl-CoA biosynthetic process	1/2279	1/18866	0.1207993	0.4298823	0.3889727	1	ACAT1
Biological Process	GO:1902910	positive regulation of melanosome transport	1/2279	1/18866	0.1207993	0.4298823	0.3889727	1	CDH3
Biological Process	GO:1902955	positive regulation of early endosome to recycling endosome transport	1/2279	1/18866	0.1207993	0.4298823	0.3889727	1	SORL1
Biological Process	GO:1902997	negative regulation of neurofibrillary tangle assembly	1/2279	1/18866	0.1207993	0.4298823	0.3889727	1	SORL1
Biological Process	GO:1902998	positive regulation of neurofibrillary tangle assembly	1/2279	1/18866	0.1207993	0.4298823	0.3889727	1	CLU
Biological Process	GO:1903041	regulation of chondrocyte hypertrophy	1/2279	1/18866	0.1207993	0.4298823	0.3889727	1	SMAD7

Biological Process	GO:1903043	positive regulation of chondrocyte hypertrophy	1/2279	1/18866	0.1207993	0.4298823	0.3889727	1	SMAD7
Biological Process	GO:1903406	regulation of sodium:potassium-exchanging ATPase activity	1/2279	1/18866	0.1207993	0.4298823	0.3889727	1	DNM2
Biological Process	GO:1903408	positive regulation of sodium:potassium-exchanging ATPase activity	1/2279	1/18866	0.1207993	0.4298823	0.3889727	1	DNM2
Biological Process	GO:1903435	positive regulation of constitutive secretory pathway	1/2279	1/18866	0.1207993	0.4298823	0.3889727	1	RAB27A
Biological Process	GO:1903465	positive regulation of mitotic cell cycle DNA replication	1/2279	1/18866	0.1207993	0.4298823	0.3889727	1	FGFR1
Biological Process	GO:1903568	negative regulation of protein localization to ciliary membrane	1/2279	1/18866	0.1207993	0.4298823	0.3889727	1	LZTFL1
Biological Process	GO:1903743	negative regulation of anterograde synaptic vesicle transport	1/2279	1/18866	0.1207993	0.4298823	0.3889727	1	CNIH2
Biological Process	GO:1903788	positive regulation of glutathione biosynthetic process	1/2279	1/18866	0.1207993	0.4298823	0.3889727	1	NFE2L2
Biological Process	GO:1903803	L-glutamine import across plasma membrane	1/2279	1/18866	0.1207993	0.4298823	0.3889727	1	SLC1A5

Biological Process	GO:1903810	L-histidine import across plasma membrane	1/2279	1/18866	0.1207993	0.4298823	0.3889727	1	SLC7A1
Biological Process	GO:1903906	regulation of plasma membrane raft polarization	1/2279	1/18866	0.1207993	0.4298823	0.3889727	1	GSN
Biological Process	GO:1903918	regulation of actin filament severing	1/2279	1/18866	0.1207993	0.4298823	0.3889727	1	MYH9
Biological Process	GO:1903919	negative regulation of actin filament severing	1/2279	1/18866	0.1207993	0.4298823	0.3889727	1	MYH9
Biological Process	GO:1903925	response to bisphenol A	1/2279	1/18866	0.1207993	0.4298823	0.3889727	1	CHEK2
Biological Process	GO:1903926	cellular response to bisphenol A	1/2279	1/18866	0.1207993	0.4298823	0.3889727	1	CHEK2
Biological Process	GO:1903996	negative regulation of non-membrane spanning protein tyrosine kinase activity	1/2279	1/18866	0.1207993	0.4298823	0.3889727	1	DUSP22
Biological Process	GO:1904210	VCP-NPL4-UFD1 AAA ATPase complex assembly	1/2279	1/18866	0.1207993	0.4298823	0.3889727	1	SVIP
Biological Process	GO:1904239	regulation of VCP-NPL4-UFD1 AAA ATPase complex assembly	1/2279	1/18866	0.1207993	0.4298823	0.3889727	1	SVIP

Biological Process	GO:1904240	negative regulation of VCP-NPL4-UFD1 AAA ATPase complex assembly	1/2279	1/18866	0.1207993	0.4298823	0.3889727	1	SVIP
Biological Process	GO:1904247	positive regulation of polynucleotide adenylyltransferase activity	1/2279	1/18866	0.1207993	0.4298823	0.3889727	1	PABPN1
Biological Process	GO:1904270	pyroptosome complex assembly	1/2279	1/18866	0.1207993	0.4298823	0.3889727	1	AIM2
Biological Process	GO:1904535	positive regulation of telomeric loop disassembly	1/2279	1/18866	0.1207993	0.4298823	0.3889727	1	RTEL1
Biological Process	GO:1904647	response to rotenone	1/2279	1/18866	0.1207993	0.4298823	0.3889727	1	KIF1B
Biological Process	GO:1904676	negative regulation of somatic stem cell division	1/2279	1/18866	0.1207993	0.4298823	0.3889727	1	MIR145
Biological Process	GO:1904677	positive regulation of somatic stem cell division	1/2279	1/18866	0.1207993	0.4298823	0.3889727	1	LBH
Biological Process	GO:1904766	negative regulation of macroautophagy by TORC1 signaling	1/2279	1/18866	0.1207993	0.4298823	0.3889727	1	CLEC16A
Biological Process	GO:1904799	regulation of neuron remodeling	1/2279	1/18866	0.1207993	0.4298823	0.3889727	1	BCL11A

Biological Process	GO:1904800	negative regulation of neuron remodeling	1/2279	1/18866	0.1207993	0.4298823	0.3889727	1	BCL11A
Biological Process	GO:1904876	negative regulation of DNA ligase activity	1/2279	1/18866	0.1207993	0.4298823	0.3889727	1	TFIP11
Biological Process	GO:1904897	regulation of hepatic stellate cell proliferation	1/2279	1/18866	0.1207993	0.4298823	0.3889727	1	MYB
Biological Process	GO:1904899	positive regulation of hepatic stellate cell proliferation	1/2279	1/18866	0.1207993	0.4298823	0.3889727	1	MYB
Biological Process	GO:1905045	negative regulation of Schwann cell proliferation involved in axon regeneration	1/2279	1/18866	0.1207993	0.4298823	0.3889727	1	CERS2
Biological Process	GO:1905068	positive regulation of canonical Wnt signaling pathway involved in heart development	1/2279	1/18866	0.1207993	0.4298823	0.3889727	1	RBPJ
Biological Process	GO:1905222	atrioventricular canal morphogenesis	1/2279	1/18866	0.1207993	0.4298823	0.3889727	1	ENG
Biological Process	GO:1905226	regulation of adhesion of symbiont to host epithelial cell	1/2279	1/18866	0.1207993	0.4298823	0.3889727	1	SFTPD
Biological Process	GO:1905313	transforming growth factor beta receptor signaling pathway involved in heart development	1/2279	1/18866	0.1207993	0.4298823	0.3889727	1	TGFB1

Biological Process	GO:1905317	inferior endocardial cushion morphogenesis	1/2279	1/18866	0.1207993	0.4298823	0.3889727	1	TGFBR2
Biological Process	GO:1905355	spine apparatus assembly	1/2279	1/18866	0.1207993	0.4298823	0.3889727	1	SYNPO
Biological Process	GO:1905528	positive regulation of Golgi lumen acidification	1/2279	1/18866	0.1207993	0.4298823	0.3889727	1	UBE3A
Biological Process	GO:1905535	regulation of eukaryotic translation initiation factor 4F complex assembly	1/2279	1/18866	0.1207993	0.4298823	0.3889727	1	EIF4G1
Biological Process	GO:1905537	positive regulation of eukaryotic translation initiation factor 4F complex assembly	1/2279	1/18866	0.1207993	0.4298823	0.3889727	1	EIF4G1
Biological Process	GO:1905578	regulation of ERBB3 signaling pathway	1/2279	1/18866	0.1207993	0.4298823	0.3889727	1	RTN4
Biological Process	GO:1905580	positive regulation of ERBB3 signaling pathway	1/2279	1/18866	0.1207993	0.4298823	0.3889727	1	RTN4
Biological Process	GO:1905590	fibronectin fibril organization	1/2279	1/18866	0.1207993	0.4298823	0.3889727	1	LOXL3
Biological Process	GO:1905610	regulation of mRNA cap binding	1/2279	1/18866	0.1207993	0.4298823	0.3889727	1	EIF4G1

Biological Process	GO:1905612	positive regulation of mRNA cap binding	1/2279	1/18866	0.1207993	0.4298823	0.3889727	1	EIF4G1
Biological Process	GO:1905667	negative regulation of protein localization to endosome	1/2279	1/18866	0.1207993	0.4298823	0.3889727	1	ANKRD13A
Biological Process	GO:1905673	positive regulation of lysosome organization	1/2279	1/18866	0.1207993	0.4298823	0.3889727	1	GRN
Biological Process	GO:1905834	response to pyrimidine ribonucleotide	1/2279	1/18866	0.1207993	0.4298823	0.3889727	1	P2RY6
Biological Process	GO:1905835	cellular response to pyrimidine ribonucleotide	1/2279	1/18866	0.1207993	0.4298823	0.3889727	1	P2RY6
Biological Process	GO:1905878	invadopodium disassembly	1/2279	1/18866	0.1207993	0.4298823	0.3889727	1	NAV3
Biological Process	GO:1905891	regulation of cellular response to thapsigargin	1/2279	1/18866	0.1207993	0.4298823	0.3889727	1	CLU
Biological Process	GO:1905892	negative regulation of cellular response to thapsigargin	1/2279	1/18866	0.1207993	0.4298823	0.3889727	1	CLU
Biological Process	GO:1905894	regulation of cellular response to tunicamycin	1/2279	1/18866	0.1207993	0.4298823	0.3889727	1	CLU

Biological Process	GO:1905895	negative regulation of cellular response to tunicamycin	1/2279	1/18866	0.1207993	0.4298823	0.3889727	1	CLU
Biological Process	GO:1905927	regulation of invadopodium disassembly	1/2279	1/18866	0.1207993	0.4298823	0.3889727	1	NAV3
Biological Process	GO:1905929	positive regulation of invadopodium disassembly	1/2279	1/18866	0.1207993	0.4298823	0.3889727	1	NAV3
Biological Process	GO:1990029	vasomotion	1/2279	1/18866	0.1207993	0.4298823	0.3889727	1	GJA5
Biological Process	GO:1990268	response to gold nanoparticle	1/2279	1/18866	0.1207993	0.4298823	0.3889727	1	MPO
Biological Process	GO:1990539	fructose import across plasma membrane	1/2279	1/18866	0.1207993	0.4298823	0.3889727	1	SLC2A5
Biological Process	GO:1990546	mitochondrial tricarboxylic acid transmembrane transport	1/2279	1/18866	0.1207993	0.4298823	0.3889727	1	SLC25A1
Biological Process	GO:1990579	peptidyl-serine trans-autophosphorylation	1/2279	1/18866	0.1207993	0.4298823	0.3889727	1	ERN1
Biological Process	GO:1990922	hepatic stellate cell proliferation	1/2279	1/18866	0.1207993	0.4298823	0.3889727	1	MYB

Biological Process	GO:2000101	regulation of mammary stem cell proliferation	1/2279	1/18866	0.1207993	0.4298823	0.3889727	1	LBH
Biological Process	GO:2000103	positive regulation of mammary stem cell proliferation	1/2279	1/18866	0.1207993	0.4298823	0.3889727	1	LBH
Biological Process	GO:2000173	negative regulation of branching morphogenesis of a nerve	1/2279	1/18866	0.1207993	0.4298823	0.3889727	1	BCL11A
Biological Process	GO:2000230	negative regulation of pancreatic stellate cell proliferation	1/2279	1/18866	0.1207993	0.4298823	0.3889727	1	PPARG
Biological Process	GO:2000308	negative regulation of tumor necrosis factor (ligand) superfamily member 11 production	1/2279	1/18866	0.1207993	0.4298823	0.3889727	1	LTF
Biological Process	GO:2000438	negative regulation of monocyte extravasation	1/2279	1/18866	0.1207993	0.4298823	0.3889727	1	PLCB1
Biological Process	GO:2000454	positive regulation of CD8-positive, alpha-beta cytotoxic T cell extravasation	1/2279	1/18866	0.1207993	0.4298823	0.3889727	1	FADD
Biological Process	GO:2000464	positive regulation of astrocyte chemotaxis	1/2279	1/18866	0.1207993	0.4298823	0.3889727	1	CCR2
Biological Process	GO:2000559	regulation of CD24 production	1/2279	1/18866	0.1207993	0.4298823	0.3889727	1	PLCB1

Biological Process	GO:2000560	positive regulation of CD24 production	1/2279	1/18866	0.1207993	0.4298823	0.3889727	1	PLCB1
Biological Process	GO:2000565	negative regulation of CD8-positive, alpha-beta T cell proliferation	1/2279	1/18866	0.1207993	0.4298823	0.3889727	1	VSIR
Biological Process	GO:2000595	regulation of optic nerve formation	1/2279	1/18866	0.1207993	0.4298823	0.3889727	1	PAX2
Biological Process	GO:2000597	positive regulation of optic nerve formation	1/2279	1/18866	0.1207993	0.4298823	0.3889727	1	PAX2
Biological Process	GO:2000661	positive regulation of interleukin-1-mediated signaling pathway	1/2279	1/18866	0.1207993	0.4298823	0.3889727	1	IL1R1
Biological Process	GO:2000830	positive regulation of parathyroid hormone secretion	1/2279	1/18866	0.1207993	0.4298823	0.3889727	1	FGFR1
Biological Process	GO:2001029	regulation of cellular glucuronidation	1/2279	1/18866	0.1207993	0.4298823	0.3889727	1	PRKCE
Biological Process	GO:2001031	positive regulation of cellular glucuronidation	1/2279	1/18866	0.1207993	0.4298823	0.3889727	1	PRKCE
Biological Process	GO:2001124	regulation of translational frameshifting	1/2279	1/18866	0.1207993	0.4298823	0.3889727	1	SHFL

Biological Process	GO:2001125	negative regulation of translational frameshifting	1/2279	1/18866	0.1207993	0.4298823	0.3889727	1	SHFL
Biological Process	GO:2001136	negative regulation of endocytic recycling	1/2279	1/18866	0.1207993	0.4298823	0.3889727	1	ARHGAP1
Biological Process	GO:2001176	regulation of mediator complex assembly	1/2279	1/18866	0.1207993	0.4298823	0.3889727	1	MED25
Biological Process	GO:2001178	positive regulation of mediator complex assembly	1/2279	1/18866	0.1207993	0.4298823	0.3889727	1	MED25
Biological Process	GO:2001246	negative regulation of phosphatidylcholine biosynthetic process	1/2279	1/18866	0.1207993	0.4298823	0.3889727	1	LPCAT1
Biological Process	GO:0030516	regulation of axon extension	16/2279	97/18866	0.1209224	0.4298823	0.3889727	16	SEMA6B/CTTN/DNM2/SEMA4A/RTN4/DISC1/SEMA4B/IFRD1/ILK/SEMA4D/CDH4/CDKL3/CYFIP1/TRPV2/NDEL1/DBNL
Biological Process	GO:0046890	regulation of lipid biosynthetic process	31/2279	207/18866	0.1209808	0.4298823	0.3889727	31	PRKAG2/GFI1/CREB1/MFSD2A/IDI1/LPCAT1/VDR/MBTPS1/GPLD1/TNF/ORMDL3/ABCG1/FPR2/TNFRSF1A/SPHK2/RDH10/DGKQ/SF1/POR/NR1H2/ACACB/PDGFB/ACACA/AVIL/HMGCS1/SCD/SREBF1/WDTC1/SIRT3/ACADVL/PRKAA1
Biological Process	GO:0051353	positive regulation of oxidoreductase activity	10/2279	55/18866	0.1209878	0.4298823	0.3889727	10	VDR/TNF/GNAI2/NOS1AP/CDH3/RIPK3/POR/NOD2/AGTR1/SIRT3
Biological Process	GO:0070741	response to interleukin-6	10/2279	55/18866	0.1209878	0.4298823	0.3889727	10	GFI1/SBNO2/CHI3L1/ABCC2/RIPK1/JAK1/PHB/FER/IL6R/RELA

Biological Process	GO:2000649	regulation of sodium ion transmembrane transporter activity	10/2279	55/18866	0.1209878	0.4298823	0.3889727	10	FXYD2/YWHAH/FXYD1/DNM2/TESC/ACTN4/PRKCE/NEDD4/HECW2/FGF14
Biological Process	GO:0042058	regulation of epidermal growth factor receptor signaling pathway	15/2279	90/18866	0.1219429	0.4322431	0.3911089	15	TGFA/ARHGEF7/NCK2/DOK1/RHBDF2/ARAP1/APP/EPS15L1/FER/PTPRJ/PDE6H/DGKD/DUSP3/EPS15/MVB12A
Biological Process	GO:0098773	skin epidermis development	15/2279	90/18866	0.1219429	0.4322431	0.3911089	15	TNF/KRT17/DLL1/RBPJ/HDAC1/CDH3/MYO5A/NF1/NUMA1/BCL2/NOTCH1/TGM3/RELA/INHBA/ERCC2
Biological Process	GO:0006471	protein ADP-ribosylation	7/2279	35/18866	0.1219776	0.4322431	0.3911089	7	TNKS/PARP4/ART5/ART4/PARP10/PARP9/SIRT3
Biological Process	GO:0035909	aorta morphogenesis	7/2279	35/18866	0.1219776	0.4322431	0.3911089	7	MIR143/MIR145/MYLK/RBPJ/ENG/NOTCH1/PDGFRB
Biological Process	GO:0040001	establishment of mitotic spindle localization	7/2279	35/18866	0.1219776	0.4322431	0.3911089	7	DCTN1/NDE1/HTT/NUMA1/ARHGEF2/FBXW11/NDEL1
Biological Process	GO:0048821	erythrocyte development	7/2279	35/18866	0.1219776	0.4322431	0.3911089	7	MAEA/SLC11A2/BRD1/TMOD3/ALAS1/EPB42/ERCC2
Biological Process	GO:0070884	regulation of calcineurin-NFAT signaling cascade	7/2279	35/18866	0.1219776	0.4322431	0.3911089	7	TNF/CAMTA1/RCAN1/DYRK2/FHL2/CHERP/STIMATE
Biological Process	GO:0032635	interleukin-6 production	26/2279	170/18866	0.1220162	0.4322431	0.3911089	26	IL10/IL16/C1QTNF3/MIR140/ARRB1/TNF/TNFAIP3/MIR101-2/TLR9/SPON2/C5AR2/APP/MBP/PTPN6/SCIMP/SPHK2/GHRL/CSK/BCL10/IL6R/ARRB2/IL18RAP/NOD2/POU2F2/F2R/ARHGEF2

Biological Process	GO:0002260	lymphocyte homeostasis	11/2279	62/18866	0.1224237	0.4332022	0.3919767	11	LAT/TNFAIP3/TNFSF13B/RIPK3/ZC3H8/BCL10/SPTA1/DOCK10/BCL2/MEF2C/FADD
Biological Process	GO:0032233	positive regulation of actin filament bundle assembly	11/2279	62/18866	0.1224237	0.4332022	0.3919767	11	ARHGEF10/ITGB1BP1/SMAD3/SH3PXD2B/SYNPO2L/PXN/WNT11/PFN1/ARHGEF10L/ARHGEF15/SYNPO
Biological Process	GO:0060997	dendritic spine morphogenesis	11/2279	62/18866	0.1224237	0.4332022	0.3919767	11	EPHB3/BAIAP2/WASL/SIPA1L1/DOCK10/DNM3/DNM1L/SHANK2/UBE3A/CUX2/DBNL
Biological Process	GO:1900046	regulation of hemostasis	14/2279	83/18866	0.1226972	0.4334584	0.3922085	14	PLAU/CD9/PRKCA/UBASH3B/SERPINB2/THBS1/F12/BLK/PDGFB/NFE2L2/CPB2/F2R/ANO6/ADTRP
Biological Process	GO:0010675	regulation of cellular carbohydrate metabolic process	23/2279	148/18866	0.1227242	0.4334584	0.3922085	23	MAEA/FOXK1/HDAC4/C1QTNF3/GPLD1/CBFA2T3/PGP/PGAM1/P2RY6/HRH1/DGKQ/DYRK2/GNB3/ACACB/PPP1CB/ESRRB/PRKCE/FOXO1/NCOA2/SOGA1/PRKN/SDHAF3/RUBCNL
Biological Process	GO:0061337	cardiac conduction	23/2279	148/18866	0.1227242	0.4334584	0.3922085	23	PDE4D/KCNE1/CASQ2/ITPR2/FXYD2/CACNA1C/CACNA2D4/FXYD1/SLC8A1/GJA5/ATP2B2/ITPR1/JUP/BIN1/KCNIP1/KCNK6/ATP2A3/KCNQ1/NPPC/ANK2/ASPH/CASQ1/SPTBN4
Biological Process	GO:0071901	negative regulation of protein serine/threonine kinase activity	23/2279	148/18866	0.1227242	0.4334584	0.3922085	23	RPTOR/SPRED2/PRKAG2/SORL1/HIPK3/PRKAR1B/DUSP14/SLC8A1/LAX1/TNFAIP3/FBXO7/PTPN6/MIR24-2/LATS2/PTPRJ/NF1/PPP2R1A/RGS3/RB1/DUSP3/HHEX/HEXIM1/DTNBP1
Biological Process	GO:1903670	regulation of sprouting angiogenesis	23/2279	148/18866	0.1227242	0.4334584	0.3922085	23	IL10/MAP3K3/HDAC7/ITGB1BP1/MIR199A1/MIR101-2/THBS1/DLL1/AKT3/MIR24-2/MIR27A/MIR23A/JAK1/EPN2/BMPER/GHRL/MIR138-2/PKM/MIR199A2/NOTCH1/AGTR1/PPP1R16B/CEMIP2
Biological Process	GO:1901888	regulation of cell junction assembly	30/2279	200/18866	0.123579	0.4363153	0.3947936	30	RUNX1/ITGB1BP1/RAP1B/SMAD3/COLQ/TNF/EPHB3/DUSP22/SEMA4A/THBS1/APP/PRKCH/PTPRJ/SEMA4D/RAPGEF2/NRXN1/GHRL/ACTG1/NTRK1/CORO1C/MEF2C/DUSP3/MYO1C/FLOT1/SLIT1/CUX2/EIF4G1/DAPK3/NPHP4/NECTIN1

Biological Process	GO:0051153	regulation of striated muscle cell differentiation	19/2279	119/18866	0.1240765	0.437909	0.3962356	19	HDAC4/EHD1/CTDP1/AKAP13/MAML1/MIR199A1/NFATC2/DLL1/RBPJ/TGFB1/ARRB2/ANKRD2/BCL2/MIR199A2/NOTCH1/MEF2C/FLOT1/SCGB3A1/CEACAM5
Biological Process	GO:0009581	detection of external stimulus	22/2279	141/18866	0.1249684	0.440893	0.3989357	22	RCVRN/PDE6A/TTN/CACNA2D4/CRB1/ARRB1/DRGX/PKD1L1/JUP/PITPNM1/CCDC66/GNAT2/SCN1A/WDR47/GNA11/ARRB2/NTRK1/TRPM3/GRK7/TMC1/EYS/CABP4
Biological Process	GO:0070997	neuron death	51/2279	360/18866	0.127163	0.4484691	0.4057908	51	HDAC4/CTSZ/SORL1/SSH1/IL10/CREB1/MYB/ANGPT1/TNFRSF1B/SRPK2/ARRB1/TNF/TRAF2/CCL5/FBXO7/HIPK2/ITSN1/UNC5B/APP/HLA-F/ILK/CLU/TNFRSF21/SYNGAP1/ZNF746/NF1/CSF3/GRN/PRKCI/ARRB2/ITGB2/RILPL1/NTRK1/FIS1/RB1/BCL2/MEF2C/F2R/ATN1/TP53BP2/CEBPB/BCL2L1/TYRO3/CRLF1/BAG5/NTF4/CX3CR1/NAE1/EIF4G1/FADD/PRKN
Biological Process	GO:0046621	negative regulation of organ growth	8/2279	42/18866	0.1272385	0.4485688	0.4058809	8	CTDP1/MIR199A1/VGLL4/TGFBR2/RIPK1/STK3/MEIS1/MIR199A2
Biological Process	GO:0030307	positive regulation of cell growth	26/2279	171/18866	0.1281848	0.4517374	0.408748	26	S100A8/RPTOR/CPNE6/RPS6KA1/MFSD2A/EXOSC2/MIR199A1/CPNE5/KRT17/DISC1/SLC44A4/DERL2/ILK/SMAD7/SEMA4D/SMURF1/CDH4/BCL2/MIR199A2/SYT17/CYFIP1/TRPV2/NDEL1/EIF4G1/DBNL/PRKN
Biological Process	GO:0006942	regulation of striated muscle contraction	16/2279	98/18866	0.1291914	0.4524511	0.4093939	16	HDAC4/PDE4D/CASQ2/CACNA1C/SLC8A1/GJA5/TNNT3/JUP/BIN1/NOS1AP/SMAD7/KCNQ1/GSTO1/ANK2/CASQ1/GRK2
Biological Process	GO:0045833	negative regulation of lipid metabolic process	16/2279	98/18866	0.1291914	0.4524511	0.4093939	16	GFI1/SORL1/MFSD2A/LPCAT1/GPLD1/TNF/ORMDL3/CRTC3/ACACB/PDGFB/MIR138-2/PDE3B/WDTC1/ACADVL/BSCL2/PRKAA1
Biological Process	GO:0031099	regeneration	30/2279	201/18866	0.1292908	0.4524511	0.4093939	30	IL10/CD9/PPARG/GSN/NINJ2/ULK1/MIR199A1/TGFBR2/NINJ1/IFRD1/PAX7/KREMEN1/STK24/ENO3/CPQ/JAM3/GRN/PKM/BCL2/MIR199A2/NOTCH1/CPB2/CEBPB/DYSF/CERS2/NDEL1/INPP5F/EYS/PTPRU/LPIN1
Biological Process	GO:0017015	regulation of transforming growth factor beta receptor signaling pathway	20/2279	127/18866	0.1294259	0.4524511	0.4093939	20	LRG1/LTBP1/SPRED2/SKI/SMAD3/LDLRAD4/DNM2/HIPK2/CHST11/TGFBR2/THBS1/SMAD7/TGFB1/SMURF1/TGFB1I1/HTRA4/CREBBP/ENG/PRDM16/HTRA3

Biological Process	GO:0030326	embryonic limb morphogenesis	20/2279	127/18866	0.1294259	0.4524511	0.4093939	20	CACNA1C/SKI/GJA5/CHST11/ZBTB16/HDAC1/FGFR1/RDH10/RARG/GRHL2/RUNX2/IFT140/CREBBP/HOXA9/NOTCH1/ALX3/GNA12/ALDH1A2/MAP3K20/CPLANE1
Biological Process	GO:0035113	embryonic appendage morphogenesis	20/2279	127/18866	0.1294259	0.4524511	0.4093939	20	CACNA1C/SKI/GJA5/CHST11/ZBTB16/HDAC1/FGFR1/RDH10/RARG/GRHL2/RUNX2/IFT140/CREBBP/HOXA9/NOTCH1/ALX3/GNA12/ALDH1A2/MAP3K20/CPLANE1
Biological Process	GO:0051053	negative regulation of DNA metabolic process	20/2279	127/18866	0.1294259	0.4524511	0.4093939	20	BLM/PPARG/TNKS/RMI2/PAM16/RTEL1/RECQL5/OTUB2/SMG6/OTUB1/TP53BP1/SUB1/NPPC/TRIP12/ERCC1/ANKRD1/SLX4/TFIP11/CYREN/FBH1
Biological Process	GO:0001782	B cell homeostasis	6/2279	29/18866	0.129435	0.4524511	0.4093939	6	TNFAIP3/TNFSF13B/BCL10/DOCK10/BCL2/MEF2C
Biological Process	GO:0002230	positive regulation of defense response to virus by host	6/2279	29/18866	0.129435	0.4524511	0.4093939	6	IL27/AIM2/ZDHHC1/TRAF3IP2/PARP9/STING1
Biological Process	GO:0006517	protein deglycosylation	6/2279	29/18866	0.129435	0.4524511	0.4093939	6	AMFR/EDEM1/DERL2/EDEM2/MAN1C1/MARCHF6
Biological Process	GO:0010882	regulation of cardiac muscle contraction by calcium ion signaling	6/2279	29/18866	0.129435	0.4524511	0.4093939	6	HDAC4/CASQ2/CACNA1C/SLC8A1/GSTO1/ANK2
Biological Process	GO:0030970	retrograde protein transport, ER to cytosol	6/2279	29/18866	0.129435	0.4524511	0.4093939	6	UBAC2/EDEM1/HM13/DERL2/EDEM2/SVIP
Biological Process	GO:0031063	regulation of histone deacetylation	6/2279	29/18866	0.129435	0.4524511	0.4093939	6	SKI/C6orf89/SPHK2/ZBTB7B/CTBP1/SREBF1

Biological Process	GO:0043153	entrainment of circadian clock by photoperiod	6/2279	29/18866	0.129435	0.4524511	0.4093939	6	MTA1/PPP1CB/PER2/PER1/FBXL6/USP2
Biological Process	GO:0045577	regulation of B cell differentiation	6/2279	29/18866	0.129435	0.4524511	0.4093939	6	INPP5D/TLR9/PTPN6/CYLD/INHBA/PCID2
Biological Process	GO:0048679	regulation of axon regeneration	6/2279	29/18866	0.129435	0.4524511	0.4093939	6	KREMEN1/STK24/GRN/CERS2/NDEL1/INPP5F
Biological Process	GO:0050820	positive regulation of coagulation	6/2279	29/18866	0.129435	0.4524511	0.4093939	6	THBS1/F12/NFE2L2/CPB2/F2R/ANO6
Biological Process	GO:0050855	regulation of B cell receptor signaling pathway	6/2279	29/18866	0.129435	0.4524511	0.4093939	6	FOXP1/RUNX1/PRKCH/PRKCB/BLK/ELF1
Biological Process	GO:0050901	leukocyte tethering or rolling	6/2279	29/18866	0.129435	0.4524511	0.4093939	6	ELANE/FUT4/FUT7/CCR2/TNF/CX3CR1
Biological Process	GO:0051569	regulation of histone H3-K4 methylation	6/2279	29/18866	0.129435	0.4524511	0.4093939	6	GFI1/MYB/ZNF335/DNMT1/PIH1D1/PAXIP1
Biological Process	GO:1903513	endoplasmic reticulum to cytosol transport	6/2279	29/18866	0.129435	0.4524511	0.4093939	6	UBAC2/EDEM1/HM13/DERL2/EDEM2/SVIP
Biological Process	GO:2000727	positive regulation of cardiac muscle cell differentiation	6/2279	29/18866	0.129435	0.4524511	0.4093939	6	MIR199A1/RBPJ/TGFB1/ARRB2/MIR199A2/MEF2C

Biological Process	GO:0002448	mast cell mediated immunity	9/2279	49/18866	0.1304528	0.4550044	0.4117041	9	LAT/PIK3CD/GAB2/FGR/CD84/SPON2/SPHK2/FER/BLK
Biological Process	GO:0002686	negative regulation of leukocyte migration	9/2279	49/18866	0.1304528	0.4550044	0.4117041	9	ABR/PADI2/C5AR2/MIR24-2/MIA3/WASL/BCR/PLCB1/ADTRP
Biological Process	GO:0042398	cellular modified amino acid biosynthetic process	9/2279	49/18866	0.1304528	0.4550044	0.4117041	9	GGT1/MTHFD1L/CNDP2/MTHFS/ACADM/MGST2/NFE2L2/MTHFD1/PTDSS2
Biological Process	GO:0070231	T cell apoptotic process	9/2279	49/18866	0.1304528	0.4550044	0.4117041	9	CCL5/RIPK1/PTCRA/DOCK8/RIPK3/ZC3H8/BCL10/FADD/GIMAP8
Biological Process	GO:0090199	regulation of release of cytochrome c from mitochondria	9/2279	49/18866	0.1304528	0.4550044	0.4117041	9	PAM16/BMF/CLU/CIDEB/NOL3/ARRB2/DNM1L/BCL2L1/PRKN
Biological Process	GO:2000107	negative regulation of leukocyte apoptotic process	9/2279	49/18866	0.1304528	0.4550044	0.4117041	9	FOXP1/ITPKB/CCL5/HSH2D/PTCRA/DOCK8/BCL10/NOD2/FADD
Biological Process	GO:0030901	midbrain development	15/2279	91/18866	0.1306003	0.4550179	0.4117164	15	CSNK1D/YWHAH/PHOX2A/PADI2/NDST1/MBP/FGFR1/CSNK1E/SYNGR3/KDM7A/YWHAQ/SYPL2/HES3/OTX2/NDRG2
Biological Process	GO:0045682	regulation of epidermis development	15/2279	91/18866	0.1306003	0.4550179	0.4117164	15	RUNX1/VDR/TNF/KRT17/DLL1/CDH3/PRKCH/GRHL2/NUMA1/KLF7/SGPP1/NOTCH1/AQP3/ZBED2/MACROH2A2
Biological Process	GO:0060333	interferon-gamma-mediated signaling pathway	15/2279	91/18866	0.1306003	0.4550179	0.4117164	15	IRF8/TRIM38/PPARG/IRF5/TRIM8/HLA-C/HLA-F/JAK1/TRIM5/NR1H2/NLRC5/SP100/IRF2/PARP9/TRIM26

Biological Process	GO:0048469	cell maturation	27/2279	179/18866	0.1317137	0.4587287	0.415074	27	MAEA/PPARG/BRCA2/LGI4/SLFN14/BCL11A/RFX3/BRD1/APP/FGFR1/DEFB1/RUNX2/PPP2R1A/FEV/RB1/POU2F2/BCL2/DMC1/EPAS1/NPPC/FAM20C/EPB42/CLN5/SPTBN4/ERCC2/GLDN/ANGPTL8
Biological Process	GO:0002712	regulation of B cell mediated immunity	10/2279	56/18866	0.132255	0.4599395	0.4161696	10	IL10/TNF/CD226/PTPN6/TGFB1/PTPRC/TP53BP1/TNFSF13/PAXIP1/NSD2
Biological Process	GO:0002889	regulation of immunoglobulin mediated immune response	10/2279	56/18866	0.132255	0.4599395	0.4161696	10	IL10/TNF/CD226/PTPN6/TGFB1/PTPRC/TP53BP1/TNFSF13/PAXIP1/NSD2
Biological Process	GO:0016447	somatic recombination of immunoglobulin gene segments	10/2279	56/18866	0.132255	0.4599395	0.4161696	10	IL10/UNG/TGFB1/PTPRC/TCF3/TP53BP1/ERCC1/TNFSF13/PAXIP1/NSD2
Biological Process	GO:0086002	cardiac muscle cell action potential involved in contraction	10/2279	56/18866	0.132255	0.4599395	0.4161696	10	KCNE1/CACNA1C/GJA5/JUP/BIN1/NOS1AP/SCN1A/SNTA1/KCNQ1/ANK2
Biological Process	GO:0003143	embryonic heart tube morphogenesis	12/2279	70/18866	0.1331226	0.4626182	0.4185934	12	SMAD3/SUFU/MICAL2/TGFBR2/DLL1/MIB1/ENG/NOTCH1/MEF2C/TMED2/TEAD2/NODAL
Biological Process	GO:0043967	histone H4 acetylation	12/2279	70/18866	0.1331226	0.4626182	0.4185934	12	BRCA2/KANSL1/ARRB1/EPC1/KANSL2/CTBP1/NCOA1/PER1/PIH1D1/RUVBL1/KAT7/ING5
Biological Process	GO:0032963	collagen metabolic process	18/2279	113/18866	0.1337513	0.4646329	0.4204164	18	PRTN3/MFAP4/PPARG/MIR145/MYB/CTSD/TNXB/TGFB1/CTSB/ENG/ARRB2/COL5A1/F2R/NPPC/PDGFRB/P3H3/VSIR/P3H1
Biological Process	GO:0060271	cilium assembly	53/2279	377/18866	0.1340984	0.4656685	0.4213534	53	EHD1/DCTN1/GSN/CDKL1/CSNK1D/NDE1/KIAA0586/TNPO1/DNM2/DYNC1H1/DNAJB13/RFX3/DISC1/TUBB4B/CEP135/CCDC66/ABLIM3/POC1A/ASAP1/TTC39C/CEP164/DCTN2/CLUAP1/CEP78/HTT/WWTR1/IQCG/CSNK1E/IFT140/PPP2R1A/TUBA4A/SDCCAG8/RILPL1/TTC21B/NOTCH1/STK36/RPGRIP1/AVIL/ARMC2/KIFAP3/CYLD/CDC14A/DNAI2/HYLS1/CEP72/ABLIM1/TTLL1/NPHP4/TXNDC15/SEPTIN9/CFAP221/CFAP46/CPLANE1

Biological Process	GO:1902749	regulation of cell cycle G2/M phase transition	32/2279	217/18866	0.1349563	0.4678207	0.4233007	32	TRIM39/BLM/BRD4/DCTN1/FZR1/CSNK1D/FOXN3/NDE1/HUS1/DYNC1H1/TUBB4B/CEP135/APP/CDC25A/CEP164/DCTN2/CEP78/CSNK1E/PSMF1/PPP2R1A/TUBA4A/TAOK3/SDCCAG8/ZFYVE19/CLSPN/PSMB7/CEP72/NAE1/PSMD13/PAXIP1/ABRAXAS1/BABAM2
Biological Process	GO:0034341	response to interferon-gamma	30/2279	202/18866	0.1351614	0.4678207	0.4233007	30	IRF8/TRIM38/PPARG/GSN/IRF5/TRIM8/SLC11A1/MEFV/CCL5/HLA-C/VPS26B/HLA-F/JAK1/TRIM5/RAB43/ADAMTS13/NR1H2/CCL20/ACTG1/NLRC5/SP100/CASP1/MYO1C/IRF2/SYNCRIP/PARP9/CCL22/DAPK3/TRIM26/SHFL
Biological Process	GO:0006493	protein O-linked glycosylation	17/2279	106/18866	0.1358158	0.4678207	0.4233007	17	GALNT2/XXYLT1/SLC35C2/GALNT12/MUC12/GXYLT2/POMT2/MUC20/B3GNT5/ST6GAL1/ST8SIA6/ST3GAL4/TET2/GALNT9/ST3GAL2/B3GNT2/B4GAT1
Biological Process	GO:0061458	reproductive system development	62/2279	447/18866	0.1358921	0.4678207	0.4233007	62	ARID5B/BSG/RXRA/IL10/PPARG/BRCA2/VDR/SETD2/ARRB1/SPINT2/EIF2B5/E2F7/CSDE1/HSD17B2/RBPJ/PTPN6/FANCA/RDH10/RARA/SF1/SP3/DNAJB6/DAZAP1/GHRL/RARG/BMPR1B/STK3/GRHL2/ASB1/NCOA1/PDGFB/CTSB/ARRB2/GJB3/VASH1/NTRK1/HOXA9/RAD21L1/BCL2/DMC1/NOTCH1/TPPP3/EPAS1/SLIT3/GGNBP2/STOX2/UBE3A/TMED2/CEBPB/PCDH12/PDGFRB/ERCC1/CRIP1/BCL2L1/HMGCS1/TYRO3/NCOA4/PLCD3/INHBA/NODAL/LIF/ETNK2
Biological Process	GO:0003283	atrial septum development	5/2279	23/18866	0.136175	0.4678207	0.4233007	5	ZFPM1/GJA5/WNT11/ANK2/NSD2
Biological Process	GO:0006359	regulation of transcription by RNA polymerase III	5/2279	23/18866	0.136175	0.4678207	0.4233007	5	RPTOR/ZC3H8/BRF1/CHD8/ZNF76
Biological Process	GO:0006582	melanin metabolic process	5/2279	23/18866	0.136175	0.4678207	0.4233007	5	ZEB2/CDH3/RAPGEF2/MYO5A/BCL2
Biological Process	GO:0006783	heme biosynthetic process	5/2279	23/18866	0.136175	0.4678207	0.4233007	5	SLC11A2/FECH/ALAS1/PPOX/TMEM14C
Biological Process	GO:0009226	nucleotide-sugar biosynthetic process	5/2279	23/18866	0.136175	0.4678207	0.4233007	5	NAGK/AMDHD2/UXS1/PMM2/GFPT2

Biological Process	GO:0016048	detection of temperature stimulus	5/2279	23/18866	0.136175	0.4678207	0.4233007	5	DRGX/WDR47/ARRB2/NTRK1/TRPM3
Biological Process	GO:0016578	histone deubiquitination	5/2279	23/18866	0.136175	0.4678207	0.4233007	5	USP3/USP36/TAF10/ENY2/USP49
Biological Process	GO:0018230	peptidyl-L-cysteine S-palmitoylation	5/2279	23/18866	0.136175	0.4678207	0.4233007	5	ZDHHC18/ZDHHC14/ZDHHC2/ZDHHC7/ZDHHC1
Biological Process	GO:0018231	peptidyl-S-diacylglycerol-L-cysteine biosynthetic process from peptidyl-cysteine	5/2279	23/18866	0.136175	0.4678207	0.4233007	5	ZDHHC18/ZDHHC14/ZDHHC2/ZDHHC7/ZDHHC1
Biological Process	GO:0031281	positive regulation of cyclase activity	5/2279	23/18866	0.136175	0.4678207	0.4233007	5	RCVRN/TIMP2/CACNA1C/AKAP5/NF1
Biological Process	GO:0035561	regulation of chromatin binding	5/2279	23/18866	0.136175	0.4678207	0.4233007	5	HMBOX1/CTBP2/MED25/PARP9/FBH1
Biological Process	GO:0036119	response to platelet-derived growth factor	5/2279	23/18866	0.136175	0.4678207	0.4233007	5	CREB1/FER/PDGFD/PDGFRB/MARS1
Biological Process	GO:0051043	regulation of membrane protein ectodomain proteolysis	5/2279	23/18866	0.136175	0.4678207	0.4233007	5	TIMP2/IL10/TNFRSF1B/GPLD1/TNF
Biological Process	GO:0060149	negative regulation of posttranscriptional gene silencing	5/2279	23/18866	0.136175	0.4678207	0.4233007	5	PPARG/NCOR2/TNF/TGFB1/RMRP

Biological Process	GO:0060547	negative regulation of necrotic cell death	5/2279	23/18866	0.136175	0.4678207	0.4233007	5	MIR101-2/RIPK1/ARHGEF2/FADD/PELI1
Biological Process	GO:0060967	negative regulation of gene silencing by RNA	5/2279	23/18866	0.136175	0.4678207	0.4233007	5	PPARG/NCOR2/TNF/TGFB1/RMRP
Biological Process	GO:0070584	mitochondrion morphogenesis	5/2279	23/18866	0.136175	0.4678207	0.4233007	5	OPA3/IMMT/FIS1/DNM1L/BCL2L1
Biological Process	GO:0070977	bone maturation	5/2279	23/18866	0.136175	0.4678207	0.4233007	5	XYLT1/SEMA4D/LTF/ANO6/RFLNA
Biological Process	GO:0072215	regulation of metanephros development	5/2279	23/18866	0.136175	0.4678207	0.4233007	5	PAX2/WWTR1/PDGFB/PDGFRB/LIF
Biological Process	GO:1901522	positive regulation of transcription from RNA polymerase II promoter involved in cellular response to chemical stimulus	5/2279	23/18866	0.136175	0.4678207	0.4233007	5	RBPJ/RUNX2/NFE2L2/NOTCH1/RELA
Biological Process	GO:1902254	negative regulation of intrinsic apoptotic signaling pathway by p53 class mediator	5/2279	23/18866	0.136175	0.4678207	0.4233007	5	RRM2B/ZNF385A/BCL2/PRKN/MARCHF7
Biological Process	GO:1903589	positive regulation of blood vessel endothelial cell proliferation involved in sprouting angiogenesis	5/2279	23/18866	0.136175	0.4678207	0.4233007	5	MIR101-2/MIR27A/MIR23A/AGTR1/PPP1R16B
Biological Process	GO:1903861	positive regulation of dendrite extension	5/2279	23/18866	0.136175	0.4678207	0.4233007	5	CPNE6/CPNE5/SMURF1/SYT17/PRKN

Biological Process	GO:2000810	regulation of bicellular tight junction assembly	5/2279	23/18866	0.136175	0.4678207	0.4233007	5	RUNX1/TNF/PRKCH/MYO1C/NPHP4
Biological Process	GO:0006213	pyrimidine nucleoside metabolic process	7/2279	36/18866	0.13654	0.4678207	0.4233007	7	CDA/DPYS/TK2/UCK2/UPB1/DHODH/TK1
Biological Process	GO:0009595	detection of biotic stimulus	7/2279	36/18866	0.13654	0.4678207	0.4233007	7	NLRC4/NLRP3/PTPRJ/CDHR2/PGLYRP1/NOD2/PGLYRP4
Biological Process	GO:0033572	transferrin transport	7/2279	36/18866	0.13654	0.4678207	0.4233007	7	LMTK2/DNM2/ATP6V1B2/ATP6V0B/ARHGAP1/ATP6V0C/ATP6V1C1
Biological Process	GO:0033762	response to glucagon	7/2279	36/18866	0.13654	0.4678207	0.4233007	7	ADCY2/CREB1/PRKAR1B/ADCY9/ABCC2/ADCY4/SREBF1
Biological Process	GO:0034405	response to fluid shear stress	7/2279	36/18866	0.13654	0.4678207	0.4233007	7	SMAD7/MTSS1/ABCA1/ETS1/NFE2L2/MEF2C/PDGFRB
Biological Process	GO:0046633	alpha-beta T cell proliferation	7/2279	36/18866	0.13654	0.4678207	0.4233007	7	CD55/CCR2/TGFBR2/ZBTB7B/PTPRC/DOCK2/VSIR
Biological Process	GO:0051294	establishment of spindle orientation	7/2279	36/18866	0.13654	0.4678207	0.4233007	7	DCTN1/NDE1/HTT/NUMA1/ARHGEF2/FBXW11/NDEL1
Biological Process	GO:0090313	regulation of protein targeting to membrane	7/2279	36/18866	0.13654	0.4678207	0.4233007	7	KCNE1/ITGB1BP1/ITGB2/FIS1/MYO1C/MIEF1/PDZK1

Biological Process	GO:0106056	regulation of calcineurin-mediated signaling	7/2279	36/18866	0.13654	0.4678207	0.4233007	7	TNF/CAMTA1/RCAN1/DYRK2/FHL2/CHERP/STIMATE
Biological Process	GO:0034763	negative regulation of transmembrane transport	20/2279	128/18866	0.1369103	0.4682883	0.4237239	20	KCNE1/THADA/CASQ2/MIR143/TNF/TLR9/BIN1/THBS1/GRB10/SLC43A2/PRKCB/CAB39/GSTO1/PRKCE/SLC43A1/NEDD4/DYSF/HECW2/YWHAQ/CBARP
Biological Process	GO:0045732	positive regulation of protein catabolic process	33/2279	225/18866	0.1375835	0.4682883	0.4237239	33	RNF144A/SORL1/FZR1/RNF19A/STX5/WWP2/CSNK1D/LPCAT1/TNFRSF1B/GPLD1/TNF/TNFAIP3/VGLL4/CBFA2T3/DISC1/EDEM1/CLU/SMAD7/SMURF1/AXIN1/EDEM2/CSNK1E/NFE2L2/RNF144B/NEDD4/RNF14/FOXO1/TRIB1/TNFSF12/HECW2/EZR/RNF40/PRKN
Biological Process	GO:0043473	pigmentation	16/2279	99/18866	0.1377775	0.4682883	0.4237239	16	SHROOM3/LYST/VANGL1/KIF13A/ZEB2/RAB27A/CDH3/DCTN2/MYO5A/NF1/GNA11/BCL2/MEF2C/HPS6/AP3D1/DTNBP1
Biological Process	GO:0007219	Notch signaling pathway	29/2279	195/18866	0.1382031	0.4682883	0.4237239	29	MAML3/WWP2/SLC35C2/MAML1/ITGB1BP1/AAK1/TSPAN14/ARRB1/MAML2/EGFL7/TGFBR2/DLL1/APP/RBPJ/MIB1/ZMIZ1/EPN2/PERP/AGXT/CREBBP/PRKCI/TSPEAR/NOD2/NOTCH1/NOTCH4/DLK1/ZNF423/HES3/PRAG1
Biological Process	GO:1902275	regulation of chromatin organization	29/2279	195/18866	0.1382031	0.4682883	0.4237239	29	GFI1/BRD4/MYB/SKI/ARRB1/C6orf89/PADI2/OTUB2/RPS6KA4/SPHK2/ZBTB7B/PAX7/CTBP1/ATF7IP/KDM4C/SETD1A/ZNF335/PHF19/OTUB1/DNMT1/PIH1D1/TRIP12/SREBF1/RNF40/KAT7/PARP10/LIF/PAXIP1/NSD3
Biological Process	GO:0003093	regulation of glomerular filtration	3/2279	11/18866	0.138648	0.4682883	0.4237239	3	GJA5/PDGFB/F2R
Biological Process	GO:0003139	secondary heart field specification	3/2279	11/18866	0.138648	0.4682883	0.4237239	3	RBPJ/WNT11/MEF2C
Biological Process	GO:0003157	endocardium development	3/2279	11/18866	0.138648	0.4682883	0.4237239	3	RBPJ/STK3/NOTCH1

Biological Process	GO:0003174	mitral valve development	3/2279	11/18866	0.138648	0.4682883	0.4237239	3	ZFPM1/GJA5/NOTCH1
Biological Process	GO:0006048	UDP-N-acetylglucosamine biosynthetic process	3/2279	11/18866	0.138648	0.4682883	0.4237239	3	NAGK/AMDHD2/GFPT2
Biological Process	GO:0014870	response to muscle inactivity	3/2279	11/18866	0.138648	0.4682883	0.4237239	3	HDAC4/PKM/CASQ1
Biological Process	GO:0032060	bleb assembly	3/2279	11/18866	0.138648	0.4682883	0.4237239	3	MYLK/PMP22/ANO6
Biological Process	GO:0033197	response to vitamin E	3/2279	11/18866	0.138648	0.4682883	0.4237239	3	PPARG/CAT/HMGCS1
Biological Process	GO:0035331	negative regulation of hippo signaling	3/2279	11/18866	0.138648	0.4682883	0.4237239	3	MARK3/VGLL4/LIMD1
Biological Process	GO:0042135	neurotransmitter catabolic process	3/2279	11/18866	0.138648	0.4682883	0.4237239	3	COLQ/COMT/HNMT
Biological Process	GO:0042985	negative regulation of amyloid precursor protein biosynthetic process	3/2279	11/18866	0.138648	0.4682883	0.4237239	3	MIR101-2/AGO2/ITM2C
Biological Process	GO:0043455	regulation of secondary metabolic process	3/2279	11/18866	0.138648	0.4682883	0.4237239	3	ZEB2/CDH3/RAPGEF2

Biological Process	GO:0043619	regulation of transcription from RNA polymerase II promoter in response to oxidative stress	3/2279	11/18866	0.138648	0.4682883	0.4237239	3	NFE2L2/ANKRD2/EPAS1
Biological Process	GO:0046501	protoporphyrinogen IX metabolic process	3/2279	11/18866	0.138648	0.4682883	0.4237239	3	FECH/ALAS1/PPOX
Biological Process	GO:0048021	regulation of melanin biosynthetic process	3/2279	11/18866	0.138648	0.4682883	0.4237239	3	ZEB2/CDH3/RAPGEF2
Biological Process	GO:0048103	somatic stem cell division	3/2279	11/18866	0.138648	0.4682883	0.4237239	3	MIR145/LBH/NOTCH1
Biological Process	GO:0051006	positive regulation of lipoprotein lipase activity	3/2279	11/18866	0.138648	0.4682883	0.4237239	3	NR1H2/LMF1/ABHD5
Biological Process	GO:0051573	negative regulation of histone H3-K9 methylation	3/2279	11/18866	0.138648	0.4682883	0.4237239	3	KDM4C/DNMT1/PIH1D1
Biological Process	GO:0051673	membrane disruption in other organism	3/2279	11/18866	0.138648	0.4682883	0.4237239	3	DEFA4/MPEG1/LTF
Biological Process	GO:0060354	negative regulation of cell adhesion molecule production	3/2279	11/18866	0.138648	0.4682883	0.4237239	3	MIR101-2/NOTCH1/NOTCH4
Biological Process	GO:0070106	interleukin-27-mediated signaling pathway	3/2279	11/18866	0.138648	0.4682883	0.4237239	3	IL27/JAK1/CRLF1

Biological Process	GO:0070673	response to interleukin-18	3/2279	11/18866	0.138648	0.4682883	0.4237239	3	PDGFB/IL18RAP/IL18R1
Biological Process	GO:0086023	adenylate cyclase-activating adrenergic receptor signaling pathway involved in heart process	3/2279	11/18866	0.138648	0.4682883	0.4237239	3	PDE4D/AKAP13/GNAI2
Biological Process	GO:0090269	fibroblast growth factor production	3/2279	11/18866	0.138648	0.4682883	0.4237239	3	CCM2L/WNT11/FGFR1
Biological Process	GO:0090270	regulation of fibroblast growth factor production	3/2279	11/18866	0.138648	0.4682883	0.4237239	3	CCM2L/WNT11/FGFR1
Biological Process	GO:0090657	telomeric loop disassembly	3/2279	11/18866	0.138648	0.4682883	0.4237239	3	BLM/RTEL1/SLX4
Biological Process	GO:0140052	cellular response to oxidised low-density lipoprotein particle stimulus	3/2279	11/18866	0.138648	0.4682883	0.4237239	3	MIA3/CD68/ADTRP
Biological Process	GO:1900376	regulation of secondary metabolite biosynthetic process	3/2279	11/18866	0.138648	0.4682883	0.4237239	3	ZEB2/CDH3/RAPGEF2
Biological Process	GO:1901033	positive regulation of response to reactive oxygen species	3/2279	11/18866	0.138648	0.4682883	0.4237239	3	TNF/RIPK1/SIRT3
Biological Process	GO:1901526	positive regulation of mitophagy	3/2279	11/18866	0.138648	0.4682883	0.4237239	3	VPS13D/SMURF1/PRKN

Biological Process	GO:1902065	response to L-glutamate	3/2279	11/18866	0.138648	0.4682883	0.4237239	3	CREB1/BCL11A/BAIAP2
Biological Process	GO:1905245	regulation of aspartic-type peptidase activity	3/2279	11/18866	0.138648	0.4682883	0.4237239	3	SORL1/BIN1/GRN
Biological Process	GO:2000035	regulation of stem cell division	3/2279	11/18866	0.138648	0.4682883	0.4237239	3	MIR145/LBH/ESRRB
Biological Process	GO:2000317	negative regulation of T-helper 17 type immune response	3/2279	11/18866	0.138648	0.4682883	0.4237239	3	LOXL3/SMAD7/ZBTB7B
Biological Process	GO:2000574	regulation of microtubule motor activity	3/2279	11/18866	0.138648	0.4682883	0.4237239	3	NDE1/FBXW11/NDEL1
Biological Process	GO:1905897	regulation of response to endoplasmic reticulum stress	15/2279	92/18866	0.1395981	0.4691931	0.4245426	15	ERN1/PIK3R1/NCK2/UBAC2/EDEM1/DERL2/CLU/COPS5/NR1H2/EDEM2/NFE2L2/BCL2L1/GRINA/SVIP/PRKN
Biological Process	GO:0009743	response to carbohydrate	34/2279	233/18866	0.140085	0.4691931	0.4245426	34	NADK/SLC2A5/MAP2K6/LRP5L/ERN1/RAP1B/SLC8A1/ARRB1/GPLD1/EIF2B5/TGFBR2/THBS1/COLEC12/TCF7L2/IGF1R/PTPRN2/PAX2/ENY2/PRKCB/GIPR/GHRL/CAT/KLF7/FIS1/PIH1D1/PRKCE/CPB2/PCK2/ERCC1/SREBF1/SLC29A1/KAT7/C2CD2L/PRKAA1
Biological Process	GO:2001234	negative regulation of apoptotic signaling pathway	34/2279	233/18866	0.140085	0.4691931	0.4245426	34	ENO1/ITPRIP/URI1/PAM16/CTTN/TNF/TRAF2/TNFAIP3/UNC5B/THBS1/RRM2B/RIPK1/GNAI2/ZNF385A/TCF7L2/CLU/HDAC1/HTT/NOL3/MAPK8IP2/NFE2L2/ARRB2/RB1/BCL2/PIH1D1/ARHGEF2/BCL2L1/RELA/BAG5/GRINA/CX3CR1/FADD/PRKN/MARCHF7
Biological Process	GO:0007519	skeletal muscle tissue development	24/2279	158/18866	0.1403576	0.4691931	0.4245426	24	HDAC4/MEF2D/SKI/MYH15/DLL1/PAX7/TGFB1/IGSF8/VAMP5/SVIL/NF1/ARNTL/MYOM1/ANKRD2/RB1/BCL2/NOTCH1/MEF2C/HLX/ANKRD1/FLOT1/CASQ1/PRKAA1/MYORG

Biological Process	GO:0000097	sulfur amino acid biosynthetic process	4/2279	17/18866	0.1406689	0.4691931	0.4245426	4	GGT1/CBS/MTHFD1/ADI1
Biological Process	GO:0002031	G protein-coupled receptor internalization	4/2279	17/18866	0.1406689	0.4691931	0.4245426	4	ARRB1/DNM2/ARRB2/DNM1
Biological Process	GO:0003198	epithelial to mesenchymal transition involved in endocardial cushion formation	4/2279	17/18866	0.1406689	0.4691931	0.4245426	4	TGFBR2/RBPJ/ENG/NOTCH1
Biological Process	GO:0006750	glutathione biosynthetic process	4/2279	17/18866	0.1406689	0.4691931	0.4245426	4	GGT1/CNDP2/MGST2/NFE2L2
Biological Process	GO:0006896	Golgi to vacuole transport	4/2279	17/18866	0.1406689	0.4691931	0.4245426	4	VTI1A/ANKFY1/AP1G2/AP3D1
Biological Process	GO:0009312	oligosaccharide biosynthetic process	4/2279	17/18866	0.1406689	0.4691931	0.4245426	4	ST6GALNAC1/ST3GAL4/SLC2A1/ST3GAL2
Biological Process	GO:0010224	response to UV-B	4/2279	17/18866	0.1406689	0.4691931	0.4245426	4	MFAP4/BCL2/CRIP1/RELA
Biological Process	GO:0015693	magnesium ion transport	4/2279	17/18866	0.1406689	0.4691931	0.4245426	4	NIPAL3/ZDHHC17/CNNM2/SLC41A1
Biological Process	GO:0016045	detection of bacterium	4/2279	17/18866	0.1406689	0.4691931	0.4245426	4	NLRC4/PGLYRP1/NOD2/PGLYRP4

Biological Process	GO:0021692	cerebellar Purkinje cell layer morphogenesis	4/2279	17/18866	0.1406689	0.4691931	0.4245426	4	DLL1/TTC21B/TTLL1/COQ8B
Biological Process	GO:0030575	nuclear body organization	4/2279	17/18866	0.1406689	0.4691931	0.4245426	4	SRPK2/HIPK2/SF1/ETS1
Biological Process	GO:0030730	sequestering of triglyceride	4/2279	17/18866	0.1406689	0.4691931	0.4245426	4	PNPLA2/PPARG/TNF/ABHD5
Biological Process	GO:0031065	positive regulation of histone deacetylation	4/2279	17/18866	0.1406689	0.4691931	0.4245426	4	C6orf89/ZBTB7B/CTBP1/SREBF1
Biological Process	GO:0036065	fucosylation	4/2279	17/18866	0.1406689	0.4691931	0.4245426	4	FUT4/FUT7/SLC35C2/FUT11
Biological Process	GO:0042953	lipoprotein transport	4/2279	17/18866	0.1406689	0.4691931	0.4245426	4	PPARG/MIA3/PRKCB/ZDHHC17
Biological Process	GO:0044546	NLRP3 inflammasome complex assembly	4/2279	17/18866	0.1406689	0.4691931	0.4245426	4	AIM2/NLRP3/MEFV/STMP1
Biological Process	GO:0045623	negative regulation of T-helper cell differentiation	4/2279	17/18866	0.1406689	0.4691931	0.4245426	4	LOXL3/SMAD7/ZBTB7B/HLX
Biological Process	GO:0046838	phosphorylated carbohydrate dephosphorylation	4/2279	17/18866	0.1406689	0.4691931	0.4245426	4	SYNJ2/INPP5A/INPP5E/IMPA2

Biological Process	GO:0046851	negative regulation of bone remodeling	4/2279	17/18866	0.1406689	0.4691931	0.4245426	4	INPP5D/UBASH3B/TNFAIP3/CSK
Biological Process	GO:0051580	regulation of neurotransmitter uptake	4/2279	17/18866	0.1406689	0.4691931	0.4245426	4	ITGB3/PER2/FLOT1/PRKN
Biological Process	GO:0055012	ventricular cardiac muscle cell differentiation	4/2279	17/18866	0.1406689	0.4691931	0.4245426	4	RXRA/RARA/MEF2C/FHL2
Biological Process	GO:0055070	copper ion homeostasis	4/2279	17/18866	0.1406689	0.4691931	0.4245426	4	COX19/APP/ATOX1/ATP7B
Biological Process	GO:0072673	lamellipodium morphogenesis	4/2279	17/18866	0.1406689	0.4691931	0.4245426	4	RREB1/KANK1/ARHGEF7/CORO1C
Biological Process	GO:0090141	positive regulation of mitochondrial fission	4/2279	17/18866	0.1406689	0.4691931	0.4245426	4	FIS1/DNM1L/MIEF1/PRKN
Biological Process	GO:0098962	regulation of postsynaptic neurotransmitter receptor activity	4/2279	17/18866	0.1406689	0.4691931	0.4245426	4	DLGAP4/CNIH2/BEGAIN/NPTX1
Biological Process	GO:1900242	regulation of synaptic vesicle endocytosis	4/2279	17/18866	0.1406689	0.4691931	0.4245426	4	DGKQ/SH3GL1/DNM1/DNM1L
Biological Process	GO:1904263	positive regulation of TORC1 signaling	4/2279	17/18866	0.1406689	0.4691931	0.4245426	4	CLEC16A/SIK3/SEC13/PIH1D1

Biological Process	GO:0008631	intrinsic apoptotic signaling pathway in response to oxidative stress	8/2279	43/18866	0.1407431	0.4691931	0.4245426	8	P4HB/ZNF622/STK24/NOL3/NFE2L2/BCL2/BAG5/PRKN
Biological Process	GO:0010470	regulation of gastrulation	8/2279	43/18866	0.1407431	0.4691931	0.4245426	8	IL10/MBP/FGFR1/IL1RN/COL5A1/MYADM/NODAL/OTX2
Biological Process	GO:0032008	positive regulation of TOR signaling	8/2279	43/18866	0.1407431	0.4691931	0.4245426	8	RPTOR/MIR199A1/RHEB/CLEC16A/SIK3/SEC13/MIR199A2/PIH1D1
Biological Process	GO:0050850	positive regulation of calcium-mediated signaling	8/2279	43/18866	0.1407431	0.4691931	0.4245426	8	TNF/CAMTA1/P2RY6/HTT/GSTO1/CHERP/P2RX5/STIMATE
Biological Process	GO:0051154	negative regulation of striated muscle cell differentiation	8/2279	43/18866	0.1407431	0.4691931	0.4245426	8	HDAC4/CTDP1/MIR199A1/DLL1/ANKRD2/MIR199A2/NOTCH1/CEACAM5
Biological Process	GO:0070296	sarcoplasmic reticulum calcium ion transport	8/2279	43/18866	0.1407431	0.4691931	0.4245426	8	PDE4D/CASQ2/CACNA1C/SLC8A1/NOL3/GSTO1/ANK2/CASQ1
Biological Process	GO:0006937	regulation of muscle contraction	26/2279	173/18866	0.1410778	0.4701439	0.4254029	26	HDAC4/PDE4D/ENO1/CASQ2/MIR143/MIR145/CACNA1C/SLC8A1/CTTN/ANXA6/GJA5/TNNT3/JUP/BIN1/NOS1AP/SMAD7/GHRL/CHRM2/KCNQ1/GSTO1/P2RX1/F2R/ANK2/CASQ1/DAPK3/GRK2
Biological Process	GO:0006970	response to osmotic stress	14/2279	85/18866	0.1412271	0.4701468	0.4254055	14	MYLK/MAP7/TNF/LRRC8C/MLC1/AQP1/CAB39/ARHGEF2/SLC2A1/DYSF/HNMT/ANXA7/SST/AQP9
Biological Process	GO:0045844	positive regulation of striated muscle tissue development	14/2279	85/18866	0.1412271	0.4701468	0.4254055	14	CREB1/MIR199A1/DLL1/RBPJ/FGFR1/TGFB1/ARRB2/ARNTL/BCL2/MIR199A2/NOTCH1/MEF2C/FLOT1/PRKAA1

Biological Process	GO:0048636	positive regulation of muscle organ development	14/2279	85/18866	0.1412271	0.4701468	0.4254055	14	CREB1/MIR199A1/DLL1/RBPJ/FGFR1/TGFB1/ARRB2/ARNTL/BCL2/MIR199A2/NOTCH1/MEF2C/FLOT1/PRKAA1
Biological Process	GO:0031345	negative regulation of cell projection organization	28/2279	188/18866	0.1413221	0.470298	0.4255424	28	SEMA6B/CTSZ/GFI1/TACSTD2/KANK1/YWHAH/DNM2/BCL11A/SEMA4A/RTN4/PMP22/SEMA4B/IFRD1/ASAP1/SYNGAP1/KREMEN1/SEMA4D/RAPGEF2/ITM2C/DNM3/CDKL3/UBE3A/CERS2/INPP5F/BAG5/RAP1GAP2/PRAG1/SPART
Biological Process	GO:0050671	positive regulation of lymphocyte proliferation	21/2279	136/18866	0.1418234	0.4718013	0.4269025	21	HLA-DMB/CD55/CCR2/CCL5/NCK2/NFATC2/TNFSF13B/TLR9/TGFBR2/CCDC88B/PTPRC/CD320/SPTA1/ZNF335/PNP/BCL2/MEF2C/SLC7A1/FADD/PELI1/VAV3
Biological Process	GO:0098693	regulation of synaptic vesicle cycle	18/2279	114/18866	0.1418875	0.4718493	0.426946	18	PRKAR1B/RAP1B/PLD1/GIT1/APBA2/DGKQ/PRKCB/NRXN1/VPS18/CHRM2/SH3GL1/DNM1/DNM3/P2RX1/DNM1L/CYFIP1/DTNBP1/FGF14
Biological Process	GO:0002711	positive regulation of T cell mediated immunity	9/2279	50/18866	0.1430482	0.4742153	0.4290868	9	PRKCZ/NLRP3/IL1R1/TRAF2/HLA-F/PTPRC/IL18R1/FADD/CYRIB
Biological Process	GO:0003044	regulation of systemic arterial blood pressure mediated by a chemical signal	9/2279	50/18866	0.1430482	0.4742153	0.4290868	9	CTSZ/PDE4D/CTSG/SLC2A5/GJA5/F2R/AGTR1/HSD11B2/AOPEP
Biological Process	GO:0010718	positive regulation of epithelial to mesenchymal transition	9/2279	50/18866	0.1430482	0.4742153	0.4290868	9	SMAD3/TGFBR2/TCF7L2/TGFB1/TGFB1I1/WWTR1/ENG/NOTCH1/GCNT2
Biological Process	GO:0030195	negative regulation of blood coagulation	9/2279	50/18866	0.1430482	0.4742153	0.4290868	9	PLAU/CD9/UBASH3B/SERPINB2/THBS1/F12/PDGFB/CPB2/ADTRP
Biological Process	GO:0050873	brown fat cell differentiation	9/2279	50/18866	0.1430482	0.4742153	0.4290868	9	LRG1/RREB1/ZNF516/ZBTB7B/FTO/PRDM16/CEBPB/LAMB3/MB

Biological Process	GO:0101023	vascular endothelial cell proliferation	9/2279	50/18866	0.1430482	0.4742153	0.4290868	9	PPARG/AKT3/MIR24-2/MIR27A/FLT1/FGFR1/GHRL/MEF2C/PLCG1
Biological Process	GO:1902743	regulation of lamellipodium organization	9/2279	50/18866	0.1430482	0.4742153	0.4290868	9	HDAC4/RREB1/KANK1/ARHGEF7/DNM2/FER/NCKAP1/CORO1C/AVIL
Biological Process	GO:1904707	positive regulation of vascular associated smooth muscle cell proliferation	9/2279	50/18866	0.1430482	0.4742153	0.4290868	9	IL10/MEF2D/ERN1/TNF/GNAI2/MIR27A/P2RY6/PDGFB/DNMT1
Biological Process	GO:1905562	regulation of vascular endothelial cell proliferation	9/2279	50/18866	0.1430482	0.4742153	0.4290868	9	PPARG/AKT3/MIR24-2/MIR27A/FLT1/FGFR1/GHRL/MEF2C/PLCG1
Biological Process	GO:0035690	cellular response to drug	12/2279	71/18866	0.1436185	0.4757738	0.4304971	12	AIM2/RAP1B/GPLD1/RECQL5/DDC/CRHBP/NCOA1/KCNQ1/NFE2L2/MEF2C/CHEK2/ANKRD1
Biological Process	GO:0051785	positive regulation of nuclear division	12/2279	71/18866	0.1436185	0.4757738	0.4304971	12	TGFA/NSMCE2/CUL3/CDC16/NUMA1/PDGFB/PLCB1/RB1/CDCA5/ANAPC7/PDGFRB/SLF1
Biological Process	GO:0001541	ovarian follicle development	10/2279	57/18866	0.1440604	0.4764214	0.431083	10	ARRB1/EIF2B5/BMPR1B/ARRB2/BCL2/DMC1/UBE3A/CEBPB/BCL2L1/INHBA
Biological Process	GO:0060760	positive regulation of response to cytokine stimulus	10/2279	57/18866	0.1440604	0.4764214	0.431083	10	GFI1/IL1R1/TRAF2/RIPK1/CPNE1/NLRC5/CASP1/PARP9/FADD/PRKN
Biological Process	GO:0071398	cellular response to fatty acid	10/2279	57/18866	0.1440604	0.4764214	0.431083	10	PPARG/CREB1/P2RY6/SMARCD1/GNG2/ACACA/PRKCE/SREBF1/CPT1A/PRKAA1

Biological Process	GO:0000082	G1/S transition of mitotic cell cycle	41/2279	287/18866	0.1440675	0.4764214	0.431083	41	RPTOR/GFI1/BRD4/TFDP1/POLE/PRMT2/E2F3/RCC1/FBXO7/E2F7/MCM5/CUL3/POLA2/CTDSPL/PCBP4/RIPK1/PTPN6/NACC2/ZNF385A/CDK14/TAF10/LATS2/CDC25A/ARID3A/MCM2/GFI1B/PLCB1/CCNH/ESRRB/MIR138-2/CCNA1/RB1/BCL2/E2F6/CHEK2/BACH1/PLRG1/INHBA/POLE4/EIF4G1/ZNF324
Biological Process	GO:0001947	heart looping	11/2279	64/18866	0.144165	0.4764214	0.431083	11	SMAD3/SUFU/MICAL2/TGFBR2/DLL1/MIB1/ENG/NOTCH1/MEF2C/TMED2/NODAL
Biological Process	GO:0030166	proteoglycan biosynthetic process	11/2279	64/18866	0.144165	0.4764214	0.431083	11	CSGALNACT1/HS3ST3B1/DSE/CHSY1/CHST11/CHST15/NDST1/TCF7L2/XYLT1/BMPR1B/PXYLP1
Biological Process	GO:0042130	negative regulation of T cell proliferation	11/2279	64/18866	0.144165	0.4764214	0.431083	11	IL10/MAD1L1/SFTPD/PTPN6/TNFRSF21/ZBTB7B/CEBPB/PELI1/TMEM131L/VSIR/MARCHF7
Biological Process	GO:0051983	regulation of chromosome segregation	17/2279	107/18866	0.1443177	0.4767601	0.4313895	17	MAD1L1/TNKS/RMI2/NSMCE2/DYNC1H1/CUL3/CDC16/TACC3/NUMA1/NEK6/RB1/CDCA5/ANAPC7/SPAG5/HECW2/PCID2/SLF1
Biological Process	GO:1903844	regulation of cellular response to transforming growth factor beta stimulus	20/2279	129/18866	0.1446398	0.4776582	0.4322021	20	LRG1/LTBP1/SPRED2/SKI/SMAD3/LDLRAD4/DNM2/HIPK2/CHST11/TGFBR2/THBS1/SMAD7/TGFB1/SMURF1/TGFB1I1/HTRA4/CREBBP/ENG/PRDM16/HTRA3
Biological Process	GO:0050678	regulation of epithelial cell proliferation	55/2279	395/18866	0.1452579	0.4795329	0.4338983	55	LRG1/RPTOR/RREB1/TACSTD2/NRP2/TGFA/IL10/PPARG/BRCA2/VDR/SMAD3/PRKCA/TNF/TNFAIP3/EGFL7/CCL5/MIR101-2/STAT5A/RTN4/THBS1/UHRF1/C5AR2/AKT3/MIR24-2/MIR27A/MIR23A/TCF7L2/PAX2/FLT1/CDH3/FGFR1/TGFB1/ZNF580/NF1/MTSS1/ITGB3/GHRL/GRN/PDGFB/VASH1/SGPP2/NOD2/RB1/NOTCH1/MEF2C/CPB2/AGTR1/RUNX3/DYSF/PPP1R16B/TNFSF12/PLCG1/ALDH1A2/NODAL/JAML
Biological Process	GO:0003209	cardiac atrium morphogenesis	6/2279	30/18866	0.1462261	0.4820592	0.4361843	6	ZFPM1/GJA5/CCM2L/ENG/NOTCH1/NSD2
Biological Process	GO:0019098	reproductive behavior	6/2279	30/18866	0.1462261	0.4820592	0.4361843	6	HDAC4/HEXB/PPP1R1B/APP/NCOA1/MAPK8IP2

Biological Process	GO:0043372	positive regulation of CD4-positive, alpha-beta T cell differentiation	6/2279	30/18866	0.1462261	0.4820592	0.4361843	6	PRKCZ/MYB/NLRP3/ZBTB7B/RARA/HLX
Biological Process	GO:0090314	positive regulation of protein targeting to membrane	6/2279	30/18866	0.1462261	0.4820592	0.4361843	6	ITGB1BP1/ITGB2/FIS1/MYO1C/MIEF1/PDZK1
Biological Process	GO:0009582	detection of abiotic stimulus	22/2279	144/18866	0.1463899	0.4822645	0.43637	22	RCVRN/PDE6A/TTN/CACNA2D4/CRB1/ARRB1/DRGX/PKD1L1/JUP/PITPNM1/CCDC66/GNAT2/SCN1A/WDR47/GNA11/ARRB2/NTRK1/TRPM3/GRK7/TMC1/EYS/CABP4
Biological Process	GO:0070585	protein localization to mitochondrion	22/2279	144/18866	0.1463899	0.4822645	0.43637	22	HK1/TFDP1/YWHAH/PAM16/BMF/FBXO7/MIPEP/AIP/USP36/ABLIM3/UBE2J2/HK2/YWHAZ/FIS1/BCL2/DNM1L/TIMM44/TP53BP2/SREBF1/YWHAQ/PRKAA1/PRKN
Biological Process	GO:0042752	regulation of circadian rhythm	19/2279	122/18866	0.1474683	0.4856487	0.4394322	19	PPARG/CSNK1D/CREB1/KDM2A/PHLPP1/ZFHX3/MTA1/GNA11/GHRL/CSNK1E/PPP1CB/ARNTL/PER2/PER1/FBXL6/UBE3A/USP2/FBXW11/PRKAA1
Biological Process	GO:0003205	cardiac chamber development	26/2279	174/18866	0.1477999	0.486572	0.4402676	26	LTBP1/ZFPM1/RXRA/NRP2/MAML1/DNM2/SUFU/GJA5/CCM2L/TGFBR2/NDST1/RBPJ/SMAD7/WNT11/TGFB1/RARA/GRHL2/ENG/NOTCH1/MEF2C/FHL2/SLIT3/ANK2/LMO4/CPLANE1/NSD2
Biological Process	GO:1903320	regulation of protein modification by small protein conjugation or removal	35/2279	242/18866	0.1481594	0.4875866	0.4411856	35	HDAC4/FZR1/TOLLIP/ANGPT1/ARRB1/TNFAIP3/MIR101-2/CUL3/OTUB2/SMAD7/ZMIZ1/AXIN1/MTA1/TNIP1/BCL10/ARRB2/MIR138-2/PER2/OTUB1/NOD2/NXN/DNM1L/PRKCE/UBXN2A/UBE3A/TRIP12/DYSF/RELA/RNF40/BAG5/PARP10/PELI1/PAXIP1/PRKN/MARCHF7
Biological Process	GO:0032946	positive regulation of mononuclear cell proliferation	21/2279	137/18866	0.1494793	0.4913795	0.4446176	21	HLA-DMB/CD55/CCR2/CCL5/NCK2/NFATC2/TNFSF13B/TLR9/TGFBR2/CCDC88B/PTPRC/CD320/SPTA1/ZNF335/PNP/BCL2/MEF2C/SLC7A1/FADD/PELI1/VAV3
Biological Process	GO:0051052	regulation of DNA metabolic process	51/2279	365/18866	0.1495294	0.4913795	0.4446176	51	FAM168A/BLM/SPIDR/IL10/PPARG/TFDP1/TNKS/HMBOX1/RMI2/PAM16/RAD52/SETD2/RTEL1/YLPM1/WRNIP1/RECQL5/OTUB2/TGFB1/UBE2V1/KDM1B/PARP4/PTPRC/GRHL2/SMG6/PDGFB/ARRB2/OTUB1/TP53BP1/SUB1/FOXM1/MORC1/NPPC/TRIP12/PDGFRB/ERCC1/TNFSF13/ANKRD1/SLX4/PARM1/KAT7/CCT6A/ERCC2/PARP9/PAXIP1/TFIP11/NSD2/CYREN/ABRAXAS1/SLF1/FBH1/BABAM2

Biological Process	GO:0048640	negative regulation of developmental growth	18/2279	115/18866	0.1502967	0.4913795	0.4446176	18	SEMA6B/CTDP1/MIR199A1/BCL11A/VGLL4/SEMA4A/RTN4/TGFBR2/SEMA4B/RIPK1/IFRD1/SEMA4D/STK3/MEIS1/MIR199A2/RAI1/CDKL3/SPART
Biological Process	GO:0009636	response to toxic substance	36/2279	250/18866	0.150394	0.4913795	0.4446176	36	MPO/LCN2/LPO/TNF/CCL5/ABCC2/TRPM6/OSCP1/DDC/MBP/FECH/RALBP1/HBB/ADAMTS13/MGST2/SLC22A5/ABTB2/CAT/NFE2L2/CDH1/FIS1/BCL2/NXN/GSTO1/EPHX1/PDGFRB/SLC7A8/PDZK1/XPA/TXNRD2/GSTO2/SLC23A1/SIRT3/PRXL2B/PRKN/MTARC1
Biological Process	GO:0008589	regulation of smoothened signaling pathway	14/2279	86/18866	0.1510364	0.4913795	0.4446176	14	MGRN1/CHSY1/SUFU/NDST1/POR/RUNX2/IFT140/CREBBP/RB1/TTC21B/STK36/OTX2/KCTD21/TXNDC15
Biological Process	GO:1901863	positive regulation of muscle tissue development	14/2279	86/18866	0.1510364	0.4913795	0.4446176	14	CREB1/MIR199A1/DLL1/RBPJ/FGFR1/TGFB1/ARRB2/ARNTL/BCL2/MIR199A2/NOTCH1/MEF2C/FLOT1/PRKAA1
Biological Process	GO:0071346	cellular response to interferon-gamma	27/2279	182/18866	0.1511873	0.4913795	0.4446176	27	IRF8/TRIM38/PPARG/GSN/IRF5/TRIM8/CCL5/HLA-C/VPS26B/HLA-F/JAK1/TRIM5/RAB43/ADAMTS13/NR1H2/CCL20/ACTG1/NLRC5/SP100/CASP1/MYO1C/IRF2/SYNCRIP/PARP9/CCL22/DAPK3/TRIM26
Biological Process	GO:0090288	negative regulation of cellular response to growth factor stimulus	27/2279	182/18866	0.1511873	0.4913795	0.4446176	27	LTBP1/SPRED2/SORL1/SKI/SMAD3/LDLRAD4/DNM2/MIR199A1/HIPK2/CHST11/TGFBR2/THBS1/SMAD7/TGFB1/EPN2/SMURF1/TGFB1I1/BMPER/HTRA4/PRDM16/MIR199A2/NOTCH1/NEDD4/HTRA3/HHEX/SPART/ADGRA2
Biological Process	GO:0009167	purine ribonucleoside monophosphate metabolic process	7/2279	37/18866	0.1519065	0.4913795	0.4446176	7	AMPD3/TJP2/GUK1/AK2/GMPR2/LHPP/IMPDH1
Biological Process	GO:0045191	regulation of isotype switching	7/2279	37/18866	0.1519065	0.4913795	0.4446176	7	IL10/TGFB1/PTPRC/TP53BP1/TNFSF13/PAXIP1/NSD2
Biological Process	GO:0048846	axon extension involved in axon guidance	7/2279	37/18866	0.1519065	0.4913795	0.4446176	7	SEMA6B/NRP2/SEMA4A/SEMA4B/SEMA4D/SLIT3/SLIT1

Biological Process	GO:0062208	positive regulation of pattern recognition receptor signaling pathway	7/2279	37/18866	0.1519065	0.4913795	0.4446176	7	TLR9/RTN4/TREML4/WDFY1/LTF/FLOT1/PELI1
Biological Process	GO:0070423	nucleotide-binding oligomerization domain containing signaling pathway	7/2279	37/18866	0.1519065	0.4913795	0.4446176	7	MAP2K6/TNFAIP3/UBE2V1/IRAK2/NOD2/CYLD/RELA
Biological Process	GO:1902284	neuron projection extension involved in neuron projection guidance	7/2279	37/18866	0.1519065	0.4913795	0.4446176	7	SEMA6B/NRP2/SEMA4A/SEMA4B/SEMA4D/SLIT3/SLIT1
Biological Process	GO:0051607	defense response to virus	37/2279	258/18866	0.1525239	0.4913795	0.4446176	37	AZU1/IL27/AIM2/TRIM38/IRF5/LYST/NLRP3/SETD2/TNFAIP3/TLR9/SPON2/CRCP/PHB/RIPK3/TRIM5/AGBL5/SEC14L1/PTPRC/NLRC5/ZDHHC1/MAP3K14/BCL2/TRAF3IP2/DDX41/BCL2L1/ILF3/MX1/IRF2/RELA/HERC5/PARP9/ISG20/FADD/STING1/SHFL/NCBP3/ILRUN
Biological Process	GO:0007492	endoderm development	13/2279	79/18866	0.1529333	0.4913795	0.4446176	13	SETD2/SMAD3/COL4A2/COL12A1/HDAC1/ITGB5/ITGB2/NOTCH1/COL5A1/LAMB3/SSBP3/INHBA/NODAL
Biological Process	GO:0050000	chromosome localization	13/2279	79/18866	0.1529333	0.4913795	0.4446176	13	MAD1L1/NDE1/DYNC1H1/CHAMP1/CUL3/MIS12/DCTN2/NUMA1/RAD21L1/CDCA5/SPAG5/NDEL1/TERB2
Biological Process	GO:0051303	establishment of chromosome localization	13/2279	79/18866	0.1529333	0.4913795	0.4446176	13	MAD1L1/NDE1/DYNC1H1/CHAMP1/CUL3/MIS12/DCTN2/NUMA1/RAD21L1/CDCA5/SPAG5/NDEL1/TERB2
Biological Process	GO:0000280	nuclear division	59/2279	428/18866	0.1539324	0.4913795	0.4446176	59	ARHGEF10/CALR/FZR1/TGFA/CTDP1/MAD1L1/TTN/TNKS/MOV10L1/NDE1/BRCA2/PRKCA/NSMCE2/RCC1/CHAMP1/CUL3/EME1/CCDC8/MEIOB/CDC16/MIS12/DCTN2/FANCA/PRKCB/RNF212/TACC3/STAG1/NUMA1/PDGFB/PPP2R1A/PLCB1/MSTO1/NEK6/CCNA1/RB1/RAD21L1/DMC1/CDCA5/DIS3L2/CHEK2/ANAPC7/PDGFRB/SPAG5/HECW2/CDC14A/SLX4/TEX12/AURKAIP1/NDEL1/LPIN1/LIF/KLHDC3/DAPK3/PSMD13/CDK13/PCID2/TERB2/ABRAXAS1/SLF1
Biological Process	GO:0044782	cilium organization	55/2279	397/18866	0.154282	0.4913795	0.4446176	55	EHD1/DCTN1/GSN/CDKL1/CSNK1D/NDE1/KIAA0586/TNPO1/DNM2/DYNC1H1/DNAJB13/RFX3/DISC1/TUBB4B/CEP135/CCDC66/ABLIM3/POC1A/ASAP1/TTC39C/CEP164/DCTN2/CLUAP1/CEP78/HTT/WWTR1/IQCG/CSNK1E/IFT140/PPP2R1A/TUBA4A/SDCCAG8/RILPL1/TTC21B/NOTCH1/STK36/RPGRIP1/AVIL/ARMC2/KIFAP3/CYLD/CDC14A/DNAI2/HYLS1/CEP72/ABLIM1/TTLL1/NPHP4/TXNDC15/SEPTIN9/CFAP221/CFAP61/CATIP/CFAP46/CPLANE1

Biological Process	GO:0051048	negative regulation of secretion	28/2279	190/18866	0.1543755	0.4913795	0.4446176	28	ABR/TNFRSF1B/RAP1B/CCR2/RAB11FIP1/FRMD4A/MIR199A1/CD84/RHBDF2/COMT/GNAI2/HLA-F/TNFRSF1A/CRHBP/ENY2/NF1/GHRL/BCR/KLF7/MIR199A2/NOTCH1/F2R/SREBF1/ADTRP/INHBA/LIF/CBARP/PRKN
Biological Process	GO:0009124	nucleoside monophosphate biosynthetic process	8/2279	44/18866	0.1549275	0.4913795	0.4446176	8	AMPD3/TK2/UCK2/LHPP/DCTD/DHODH/TK1/IMPDH1
Biological Process	GO:0033003	regulation of mast cell activation	8/2279	44/18866	0.1549275	0.4913795	0.4446176	8	GAB2/FGR/CD84/CD226/SPHK2/FER/BLK/CNR2
Biological Process	GO:0045684	positive regulation of epidermis development	8/2279	44/18866	0.1549275	0.4913795	0.4446176	8	VDR/TNF/KRT17/PRKCH/NUMA1/NOTCH1/ZBED2/MACROH2A2
Biological Process	GO:0060999	positive regulation of dendritic spine development	8/2279	44/18866	0.1549275	0.4913795	0.4446176	8	ZMYND8/ITSN1/BAIAP2/CPEB3/DNM1L/SHANK2/CUX2/DBNL
Biological Process	GO:0085029	extracellular matrix assembly	8/2279	44/18866	0.1549275	0.4913795	0.4446176	8	MFAP4/MYH11/SMAD3/TNXB/TGFB1/NOTCH1/LAMB3/HAS3
Biological Process	GO:1905521	regulation of macrophage migration	8/2279	44/18866	0.1549275	0.4913795	0.4446176	8	CD9/C3AR1/CCL5/CSF1R/THBS1/MIR24-2/CXCL17/CX3CR1
Biological Process	GO:0010573	vascular endothelial growth factor production	11/2279	65/18866	0.1557455	0.4913795	0.4446176	11	C3AR1/MIR140/CCR2/MIR199A1/TNF/TGFB1/CXCL17/IL6R/MIR199A2/NODAL/NDRG2
Biological Process	GO:0035308	negative regulation of protein dephosphorylation	11/2279	65/18866	0.1557455	0.4913795	0.4446176	11	LMTK2/MGAT5/PPP1R1B/URI1/CABIN1/TNF/GNAI2/ELFN2/PHACTR2/PPP1R16B/PTPA

Biological Process	GO:0044786	cell cycle DNA replication	11/2279	65/18866	0.1557455	0.4913795	0.4446176	11	POLE/BRCA2/RTEL1/E2F7/MCM5/POLA2/FGFR1/MCM2/CHEK2/POLD3/POLE4
Biological Process	GO:1902305	regulation of sodium ion transmembrane transport	11/2279	65/18866	0.1557455	0.4913795	0.4446176	11	FXYD2/YWHAH/FXYD1/DNM2/TESC/ACTN4/SNTA1/PRKCE/NEDD4/HECW2/FGF14
Biological Process	GO:0007173	epidermal growth factor receptor signaling pathway	19/2279	123/18866	0.1557797	0.4913795	0.4446176	19	TGFA/PIK3R1/ARHGEF7/NCK2/DOK1/RHBDF2/ARAP1/APP/EPS15L1/TGFB1/FER/PTPRJ/FAM83A/PDE6H/DGKD/DUSP3/EPS15/PLCG1/MVB12A
Biological Process	GO:1904375	regulation of protein localization to cell periphery	19/2279	123/18866	0.1557797	0.4913795	0.4446176	19	PRKCZ/PIK3R1/RHOG/TNF/ZDHHC2/MRAP/AKAP5/ZDHHC7/PPFIA1/LZTFL1/TGFB1/PRKCH/NUMB/CSK/NUMA1/PRKCI/PRKCE/BCL2L1/EZR
Biological Process	GO:0007095	mitotic G2 DNA damage checkpoint	5/2279	24/18866	0.1559062	0.4913795	0.4446176	5	TRIM39/BLM/FOXN3/TAOK3/CLSPN
Biological Process	GO:0007141	male meiosis I	5/2279	24/18866	0.1559062	0.4913795	0.4446176	5	MOV10L1/BRCA2/MEIOB/CCNA1/DMC1
Biological Process	GO:0010881	regulation of cardiac muscle contraction by regulation of the release of sequestered calcium ion	5/2279	24/18866	0.1559062	0.4913795	0.4446176	5	CASQ2/CACNA1C/SLC8A1/GSTO1/ANK2
Biological Process	GO:0030325	adrenal gland development	5/2279	24/18866	0.1559062	0.4913795	0.4446176	5	ARID5B/SMAD3/WNT11/NF1/PDGFRB
Biological Process	GO:0032753	positive regulation of interleukin-4 production	5/2279	24/18866	0.1559062	0.4913795	0.4446176	5	PRKCZ/NLRP3/RARA/SLC7A5/CEBPB

Biological Process	GO:0034143	regulation of toll-like receptor 4 signaling pathway	5/2279	24/18866	0.1559062	0.4913795	0.4446176	5	MIR140/TNFAIP3/WDFY1/LTF/PELI1
Biological Process	GO:0034505	tooth mineralization	5/2279	24/18866	0.1559062	0.4913795	0.4446176	5	ALPL/TSPEAR/FOXO1/FAM20C/NECTIN1
Biological Process	GO:0045821	positive regulation of glycolytic process	5/2279	24/18866	0.1559062	0.4913795	0.4446176	5	PFKFB4/APP/ESRRB/PFKFB3/PRKAA1
Biological Process	GO:0046835	carbohydrate phosphorylation	5/2279	24/18866	0.1559062	0.4913795	0.4446176	5	NAGK/HK1/PFKFB4/HK2/PFKFB3
Biological Process	GO:0051349	positive regulation of lyase activity	5/2279	24/18866	0.1559062	0.4913795	0.4446176	5	RCVRN/TIMP2/CACNA1C/AKAP5/NF1
Biological Process	GO:0060396	growth hormone receptor signaling pathway	5/2279	24/18866	0.1559062	0.4913795	0.4446176	5	PIK3R1/STAT5A/PXN/GHRL/MBD5
Biological Process	GO:0070841	inclusion body assembly	5/2279	24/18866	0.1559062	0.4913795	0.4446176	5	SORL1/CLU/DNAJB6/BAG5/PRKN
Biological Process	GO:0099563	modification of synaptic structure	5/2279	24/18866	0.1559062	0.4913795	0.4446176	5	ITSN1/BAIAP2/PFN1/CYFIP1/SYNPO
Biological Process	GO:1902993	positive regulation of amyloid precursor protein catabolic process	5/2279	24/18866	0.1559062	0.4913795	0.4446176	5	TNF/ABCG1/APP/CLU/RELA

Biological Process	GO:2000637	positive regulation of gene silencing by miRNA	5/2279	24/18866	0.1559062	0.4913795	0.4446176	5	RIPK1/LIMD1/AGO2/TGFB1/EIF4G1
Biological Process	GO:0002204	somatic recombination of immunoglobulin genes involved in immune response	9/2279	51/18866	0.1562338	0.4913795	0.4446176	9	IL10/UNG/TGFB1/PTPRC/TP53BP1/ERCC1/TNFSF13/PAXIP1/NSD2
Biological Process	GO:0002208	somatic diversification of immunoglobulins involved in immune response	9/2279	51/18866	0.1562338	0.4913795	0.4446176	9	IL10/UNG/TGFB1/PTPRC/TP53BP1/ERCC1/TNFSF13/PAXIP1/NSD2
Biological Process	GO:0006984	ER-nucleus signaling pathway	9/2279	51/18866	0.1562338	0.4913795	0.4446176	9	CALR/AMFR/MBTPS1/NCK2/ZBTB7B/NFE2L2/ARHGEF10L/SREBF1/SPRING1
Biological Process	GO:0009161	ribonucleoside monophosphate metabolic process	9/2279	51/18866	0.1562338	0.4913795	0.4446176	9	AMPD3/TJP2/UCK2/GUK1/AK2/GMPR2/LHPP/DHODH/IMPDH1
Biological Process	GO:0045190	isotype switching	9/2279	51/18866	0.1562338	0.4913795	0.4446176	9	IL10/UNG/TGFB1/PTPRC/TP53BP1/ERCC1/TNFSF13/PAXIP1/NSD2
Biological Process	GO:0045912	negative regulation of carbohydrate metabolic process	9/2279	51/18866	0.1562338	0.4913795	0.4446176	9	MAEA/HDAC4/AP2A1/C1QTNF3/CBFA2T3/PGP/TGFB1/SOGA1/PRKN
Biological Process	GO:0050885	neuromuscular process controlling balance	9/2279	51/18866	0.1562338	0.4913795	0.4446176	9	ABR/HEXB/CDH23/APP/CAMTA1/NRXN1/BCR/SLC1A3/CNTNAP1
Biological Process	GO:0060976	coronary vasculature development	9/2279	51/18866	0.1562338	0.4913795	0.4446176	9	LTBP1/MIR145/SETD2/DNM2/SUFU/NDST1/NOTCH1/PDGFRB/CPLANE1

Biological Process	GO:0071354	cellular response to interleukin-6	9/2279	51/18866	0.1562338	0.4913795	0.4446176	9	GFI1/SBNO2/ABCC2/RIPK1/JAK1/PHB/FER/IL6R/RELA
Biological Process	GO:1900047	negative regulation of hemostasis	9/2279	51/18866	0.1562338	0.4913795	0.4446176	9	PLAU/CD9/UBASH3B/SERPINB2/THBS1/F12/PDGFB/CPB2/ADTRP
Biological Process	GO:1903573	negative regulation of response to endoplasmic reticulum stress	9/2279	51/18866	0.1562338	0.4913795	0.4446176	9	NCK2/UBAC2/DERL2/CLU/NR1H2/BCL2L1/GRINA/SVIP/PRKN
Biological Process	GO:0003015	heart process	42/2279	297/18866	0.1563831	0.4913795	0.4446176	42	HDAC4/PDE4D/KCNE1/TTN/CASQ2/ITPR2/FXYD2/MAP2K6/CACNA1C/AKAP13/CACNA2D4/FXYD1/SLC8A1/GJA5/ATP2B2/ITPR1/JUP/BIN1/GNAI2/NOS1AP/SMAD7/KCNIP1/SCN1A/KCNK6/WWTR1/CHRM2/ATP2A3/CELF2/SNTA1/KCNQ1/GSTO1/GNAO1/DNM1L/EPAS1/NPPC/CACNA1B/ANK2/ASPH/SREBF1/CASQ1/SPTBN4/GRK2
Biological Process	GO:0007091	metaphase/anaphase transition of mitotic cell cycle	10/2279	58/18866	0.1563881	0.4913795	0.4446176	10	MAD1L1/NSMCE2/CUL3/CDC16/TACC3/NEK6/RB1/ANAPC7/HECW2/PCID2
Biological Process	GO:1903749	positive regulation of establishment of protein localization to mitochondrion	10/2279	58/18866	0.1563881	0.4913795	0.4446176	10	TFDP1/YWHAH/USP36/ABLIM3/UBE2J2/YWHAZ/BCL2/TP53BP2/YWHAQ/PRKAA1
Biological Process	GO:0090276	regulation of peptide hormone secretion	31/2279	213/18866	0.1564522	0.4913795	0.4446176	31	NADK/ITPR2/MYRIP/CACNA1C/LRP5L/ARRB1/PRKCA/GPLD1/TNF/CCL5/ITSN1/ITPR1/RFX3/TCF7L2/CRHBP/ENY2/GIPR/BLK/GHRL/KLF7/ARNTL/PER2/SLC16A1/PRKCE/SLC2A1/SREBF1/CPT1A/RPH3AL/C2CD2L/SIRT3/PRKN
Biological Process	GO:0000730	DNA recombinase assembly	2/2279	6/18866	0.1573345	0.4913795	0.4446176	2	RAD52/DMC1
Biological Process	GO:0000972	transcription-dependent tethering of RNA polymerase II gene DNA at nuclear periphery	2/2279	6/18866	0.1573345	0.4913795	0.4446176	2	RAE1/PCID2

Biological Process	GO:0001302	replicative cell aging	2/2279	6/18866	0.1573345	0.4913795	0.4446176	2	CHEK2/ERCC1
Biological Process	GO:0001999	renal response to blood flow involved in circulatory renin-angiotensin regulation of systemic arterial blood pressure	2/2279	6/18866	0.1573345	0.4913795	0.4446176	2	GJA5/F2R
Biological Process	GO:0002001	renin secretion into blood stream	2/2279	6/18866	0.1573345	0.4913795	0.4446176	2	GJA5/F2R
Biological Process	GO:0002326	B cell lineage commitment	2/2279	6/18866	0.1573345	0.4913795	0.4446176	2	BCL2/TCF3
Biological Process	GO:0002501	peptide antigen assembly with MHC protein complex	2/2279	6/18866	0.1573345	0.4913795	0.4446176	2	HLA-DMB/CALR
Biological Process	GO:0003097	renal water transport	2/2279	6/18866	0.1573345	0.4913795	0.4446176	2	AQP1/AQP3
Biological Process	GO:0003160	endocardium morphogenesis	2/2279	6/18866	0.1573345	0.4913795	0.4446176	2	RBPJ/NOTCH1
Biological Process	GO:0003175	tricuspid valve development	2/2279	6/18866	0.1573345	0.4913795	0.4446176	2	ZFPM1/TGFBR2
Biological Process	GO:0003284	septum primum development	2/2279	6/18866	0.1573345	0.4913795	0.4446176	2	GJA5/NSD2

Biological Process	GO:0006114	glycerol biosynthetic process	2/2279	6/18866	0.1573345	0.4913795	0.4446176	2	PGP/PCK2
Biological Process	GO:0006501	C-terminal protein lipidation	2/2279	6/18866	0.1573345	0.4913795	0.4446176	2	GPLD1/WIPI2
Biological Process	GO:0006682	galactosylceramide biosynthetic process	2/2279	6/18866	0.1573345	0.4913795	0.4446176	2	B4GALT3/FA2H
Biological Process	GO:0007182	common-partner SMAD protein phosphorylation	2/2279	6/18866	0.1573345	0.4913795	0.4446176	2	TGFBR2/TGFB1
Biological Process	GO:0007185	transmembrane receptor protein tyrosine phosphatase signaling pathway	2/2279	6/18866	0.1573345	0.4913795	0.4446176	2	TRIO/PTPRU
Biological Process	GO:0009186	deoxyribonucleoside diphosphate metabolic process	2/2279	6/18866	0.1573345	0.4913795	0.4446176	2	GUK1/AK5
Biological Process	GO:0009838	abscission	2/2279	6/18866	0.1573345	0.4913795	0.4446176	2	ZFYVE19/SPART
Biological Process	GO:0010626	negative regulation of Schwann cell proliferation	2/2279	6/18866	0.1573345	0.4913795	0.4446176	2	SKI/CERS2
Biological Process	GO:0010756	positive regulation of plasminogen activation	2/2279	6/18866	0.1573345	0.4913795	0.4446176	2	ENO1/F12

Biological Process	GO:0010792	DNA double-strand break processing involved in repair via single-strand annealing	2/2279	6/18866	0.1573345	0.4913795	0.4446176	2	RAD52/SLX4
Biological Process	GO:0015838	amino-acid betaine transport	2/2279	6/18866	0.1573345	0.4913795	0.4446176	2	SLC22A5/PDZK1
Biological Process	GO:0018364	peptidyl-glutamine methylation	2/2279	6/18866	0.1573345	0.4913795	0.4446176	2	CREBBP/FBL
Biological Process	GO:0019375	galactolipid biosynthetic process	2/2279	6/18866	0.1573345	0.4913795	0.4446176	2	B4GALT3/FA2H
Biological Process	GO:0019800	peptide cross-linking via chondroitin 4-sulfate glycosaminoglycan	2/2279	6/18866	0.1573345	0.4913795	0.4446176	2	SPOCK2/EGFLAM
Biological Process	GO:0021540	corpus callosum morphogenesis	2/2279	6/18866	0.1573345	0.4913795	0.4446176	2	ZEB2/TSKU
Biological Process	GO:0030579	ubiquitin-dependent SMAD protein catabolic process	2/2279	6/18866	0.1573345	0.4913795	0.4446176	2	SMURF1/TGFB1I1
Biological Process	GO:0031584	activation of phospholipase D activity	2/2279	6/18866	0.1573345	0.4913795	0.4446176	2	PRKCZ/CCL5
Biological Process	GO:0031999	negative regulation of fatty acid beta-oxidation	2/2279	6/18866	0.1573345	0.4913795	0.4446176	2	MFSD2A/ACACB

Biological Process	GO:0032106	positive regulation of response to extracellular stimulus	2/2279	6/18866	0.1573345	0.4913795	0.4446176	2	GHRL/NPY
Biological Process	GO:0032109	positive regulation of response to nutrient levels	2/2279	6/18866	0.1573345	0.4913795	0.4446176	2	GHRL/NPY
Biological Process	GO:0032286	central nervous system myelin maintenance	2/2279	6/18866	0.1573345	0.4913795	0.4446176	2	CLU/FA2H
Biological Process	GO:0032415	regulation of sodium:proton antiporter activity	2/2279	6/18866	0.1573345	0.4913795	0.4446176	2	TESC/ACTN4
Biological Process	GO:0032534	regulation of microvillus assembly	2/2279	6/18866	0.1573345	0.4913795	0.4446176	2	PLD1/ATP8B1
Biological Process	GO:0032962	positive regulation of inositol trisphosphate biosynthetic process	2/2279	6/18866	0.1573345	0.4913795	0.4446176	2	P2RY6/HRH1
Biological Process	GO:0033239	negative regulation of cellular amine metabolic process	2/2279	6/18866	0.1573345	0.4913795	0.4446176	2	COMT/ITGB2
Biological Process	GO:0034036	purine ribonucleoside bisphosphate biosynthetic process	2/2279	6/18866	0.1573345	0.4913795	0.4446176	2	SLC26A1/PAPSS2
Biological Process	GO:0034316	negative regulation of Arp2/3 complex-mediated actin nucleation	2/2279	6/18866	0.1573345	0.4913795	0.4446176	2	GMFB/GMFG

Biological Process	GO:0034334	adherens junction maintenance	2/2279	6/18866	0.1573345	0.4913795	0.4446176	2	PLEKHA7/KIFC3
Biological Process	GO:0036446	myofibroblast differentiation	2/2279	6/18866	0.1573345	0.4913795	0.4446176	2	MIR145/RB1
Biological Process	GO:0038145	macrophage colony-stimulating factor signaling pathway	2/2279	6/18866	0.1573345	0.4913795	0.4446176	2	CSF1R/DOK1
Biological Process	GO:0043570	maintenance of DNA repeat elements	2/2279	6/18866	0.1573345	0.4913795	0.4446176	2	RMI2/TCF7L2
Biological Process	GO:0044245	polysaccharide digestion	2/2279	6/18866	0.1573345	0.4913795	0.4446176	2	MGAM/CHIT1
Biological Process	GO:0046113	nucleobase catabolic process	2/2279	6/18866	0.1573345	0.4913795	0.4446176	2	DPYS/AOX1
Biological Process	GO:0048023	positive regulation of melanin biosynthetic process	2/2279	6/18866	0.1573345	0.4913795	0.4446176	2	ZEB2/CDH3
Biological Process	GO:0050428	3'-phosphoadenosine 5'-phosphosulfate biosynthetic process	2/2279	6/18866	0.1573345	0.4913795	0.4446176	2	SLC26A1/PAPSS2
Biological Process	GO:0051459	regulation of corticotropin secretion	2/2279	6/18866	0.1573345	0.4913795	0.4446176	2	CRHBP/GHRL

Biological Process	GO:0060136	embryonic process involved in female pregnancy	2/2279	6/18866	0.1573345	0.4913795	0.4446176	2	SP3/NODAL
Biological Process	GO:0060268	negative regulation of respiratory burst	2/2279	6/18866	0.1573345	0.4913795	0.4446176	2	GRN/BCR
Biological Process	GO:0060332	positive regulation of response to interferon-gamma	2/2279	6/18866	0.1573345	0.4913795	0.4446176	2	NLRC5/PARP9
Biological Process	GO:0060335	positive regulation of interferon-gamma-mediated signaling pathway	2/2279	6/18866	0.1573345	0.4913795	0.4446176	2	NLRC5/PARP9
Biological Process	GO:0060385	axonogenesis involved in innervation	2/2279	6/18866	0.1573345	0.4913795	0.4446176	2	NTRK1/NPTX1
Biological Process	GO:0060545	positive regulation of necroptotic process	2/2279	6/18866	0.1573345	0.4913795	0.4446176	2	RIPK1/RIPK3
Biological Process	GO:0061181	regulation of chondrocyte development	2/2279	6/18866	0.1573345	0.4913795	0.4446176	2	SMAD7/RFLNA
Biological Process	GO:0061734	parkin-mediated stimulation of mitophagy in response to mitochondrial depolarization	2/2279	6/18866	0.1573345	0.4913795	0.4446176	2	SMURF1/PRKN
Biological Process	GO:0061817	endoplasmic reticulum-plasma membrane tethering	2/2279	6/18866	0.1573345	0.4913795	0.4446176	2	ESYT1/GRAMD2A

Biological Process	GO:0062100	positive regulation of programmed necrotic cell death	2/2279	6/18866	0.1573345	0.4913795	0.4446176	2	RIPK1/RIPK3
Biological Process	GO:0070162	adiponectin secretion	2/2279	6/18866	0.1573345	0.4913795	0.4446176	2	C1QTNF3/RAB11FIP1
Biological Process	GO:0070163	regulation of adiponectin secretion	2/2279	6/18866	0.1573345	0.4913795	0.4446176	2	C1QTNF3/RAB11FIP1
Biological Process	GO:0071921	cohesin loading	2/2279	6/18866	0.1573345	0.4913795	0.4446176	2	RB1/CDCA5
Biological Process	GO:0072014	proximal tubule development	2/2279	6/18866	0.1573345	0.4913795	0.4446176	2	DLL1/ACAT1
Biological Process	GO:0072679	thymocyte migration	2/2279	6/18866	0.1573345	0.4913795	0.4446176	2	CCR2/CCL20
Biological Process	GO:0086029	Purkinje myocyte to ventricular cardiac muscle cell signaling	2/2279	6/18866	0.1573345	0.4913795	0.4446176	2	CASQ2/GJA5
Biological Process	GO:0086068	Purkinje myocyte to ventricular cardiac muscle cell communication	2/2279	6/18866	0.1573345	0.4913795	0.4446176	2	CASQ2/GJA5
Biological Process	GO:0089709	L-histidine transmembrane transport	2/2279	6/18866	0.1573345	0.4913795	0.4446176	2	SLC15A4/SLC7A1

Biological Process	GO:0090155	negative regulation of sphingolipid biosynthetic process	2/2279	6/18866	0.1573345	0.4913795	0.4446176	2	ORMDL3/PRKAA1
Biological Process	GO:0090241	negative regulation of histone H4 acetylation	2/2279	6/18866	0.1573345	0.4913795	0.4446176	2	CTBP1/PIH1D1
Biological Process	GO:0090272	negative regulation of fibroblast growth factor production	2/2279	6/18866	0.1573345	0.4913795	0.4446176	2	WNT11/FGFR1
Biological Process	GO:0090315	negative regulation of protein targeting to membrane	2/2279	6/18866	0.1573345	0.4913795	0.4446176	2	KCNE1/ITGB1BP1
Biological Process	GO:0090678	cell dedifferentiation involved in phenotypic switching	2/2279	6/18866	0.1573345	0.4913795	0.4446176	2	MIR145/PDGFB
Biological Process	GO:0090735	DNA repair complex assembly	2/2279	6/18866	0.1573345	0.4913795	0.4446176	2	RAD52/DMC1
Biological Process	GO:0098704	carbohydrate import across plasma membrane	2/2279	6/18866	0.1573345	0.4913795	0.4446176	2	SLC2A5/SLC2A1
Biological Process	GO:0106001	intestinal hexose absorption	2/2279	6/18866	0.1573345	0.4913795	0.4446176	2	SLC2A5/EZR
Biological Process	GO:0140271	hexose import across plasma membrane	2/2279	6/18866	0.1573345	0.4913795	0.4446176	2	SLC2A5/SLC2A1

Biological Process	GO:1900028	negative regulation of ruffle assembly	2/2279	6/18866	0.1573345	0.4913795	0.4446176	2	TACSTD2/KANK1
Biological Process	GO:1900157	regulation of bone mineralization involved in bone maturation	2/2279	6/18866	0.1573345	0.4913795	0.4446176	2	LTF/RFLNA
Biological Process	GO:1900241	positive regulation of phenotypic switching	2/2279	6/18866	0.1573345	0.4913795	0.4446176	2	MIR140/PDGFB
Biological Process	GO:1900378	positive regulation of secondary metabolite biosynthetic process	2/2279	6/18866	0.1573345	0.4913795	0.4446176	2	ZEB2/CDH3
Biological Process	GO:1901552	positive regulation of endothelial cell development	2/2279	6/18866	0.1573345	0.4913795	0.4446176	2	ADD1/S1PR2
Biological Process	GO:1901668	regulation of superoxide dismutase activity	2/2279	6/18866	0.1573345	0.4913795	0.4446176	2	TNF/SIRT3
Biological Process	GO:1902396	protein localization to bicellular tight junction	2/2279	6/18866	0.1573345	0.4913795	0.4446176	2	ACTN4/ACTG1
Biological Process	GO:1902498	regulation of protein autoubiquitination	2/2279	6/18866	0.1573345	0.4913795	0.4446176	2	MTA1/MARCHF7
Biological Process	GO:1903142	positive regulation of establishment of endothelial barrier	2/2279	6/18866	0.1573345	0.4913795	0.4446176	2	ADD1/S1PR2

Biological Process	GO:1903911	positive regulation of receptor clustering	2/2279	6/18866	0.1573345	0.4913795	0.4446176	2	SSH1/ZDHHC2
Biological Process	GO:1905005	regulation of epithelial to mesenchymal transition involved in endocardial cushion formation	2/2279	6/18866	0.1573345	0.4913795	0.4446176	2	TGFBR2/ENG
Biological Process	GO:1905174	regulation of vascular associated smooth muscle cell dedifferentiation	2/2279	6/18866	0.1573345	0.4913795	0.4446176	2	MIR145/PDGFB
Biological Process	GO:1905178	regulation of cardiac muscle tissue regeneration	2/2279	6/18866	0.1573345	0.4913795	0.4446176	2	MIR199A1/MIR199A2
Biological Process	GO:1990936	vascular associated smooth muscle cell dedifferentiation	2/2279	6/18866	0.1573345	0.4913795	0.4446176	2	MIR145/PDGFB
Biological Process	GO:2000110	negative regulation of macrophage apoptotic process	2/2279	6/18866	0.1573345	0.4913795	0.4446176	2	CCL5/NOD2
Biological Process	GO:2000348	regulation of CD40 signaling pathway	2/2279	6/18866	0.1573345	0.4913795	0.4446176	2	TNFAIP3/FANCA
Biological Process	GO:2001137	positive regulation of endocytic recycling	2/2279	6/18866	0.1573345	0.4913795	0.4446176	2	EHD1/SORL1
Biological Process	GO:2001186	negative regulation of CD8-positive, alpha-beta T cell activation	2/2279	6/18866	0.1573345	0.4913795	0.4446176	2	ZBTB7B/VSIR

Biological Process	GO:0007626	locomotory behavior	29/2279	198/18866	0.15738	0.4913795	0.4446176	29	HEXB/PPP1R1B/LGI4/CDH23/HIPK2/ZFHX3/APP/ZNF385A/APBA2/SCN1A/MTA1/MYO5A/ESPN/WDR47/GMFB/ARRB2/NPC1/MEIS1/GNAO1/PRKCE/CACNA1B/USP2/NCOA2/TMOD1/NTAN1/INPP5F/SPTBN4/NTF4/PRKN
Biological Process	GO:0001666	response to hypoxia	50/2279	359/18866	0.157856	0.4927037	0.4458158	50	P4HB/ENO1/PLAU/ITPR2/MIR140/TM9SF4/CREB1/MYB/NDRG1/SMAD3/SLC8A1/HIPK2/CBFA2T3/ITPR1/SLC11A2/TGFBR2/THBS1/RBPJ/ACTN4/LIMD1/AQP1/NF1/HK2/ALAS1/AGTRAP/PSMF1/CREBBP/NOL3/CAT/ETS1/NFE2L2/FIS1/PKM/BCL2/PSMB7/NOTCH1/AQP3/DNM1L/EPAS1/PRKCE/NPPC/SLC2A1/ANKRD1/SLC29A1/BACH1/HSD11B2/ERCC2/PSMD13/MB/PRKAA1
Biological Process	GO:0006986	response to unfolded protein	27/2279	183/18866	0.1580278	0.493078	0.4461545	27	DCTN1/CALR/ERN1/PIK3R1/AMFR/MBTPS1/RHBDD2/NCK2/YIF1A/THBS1/EDEM1/DERL2/ZBTB17/PDIA5/HSPA4L/UBE2J2/CLGN/COPS5/EDEM2/CREB3L2/NFE2L2/ADD1/TOR1B/KLHDC3/SEC31A/ACADVL/PRKN
Biological Process	GO:0030048	actin filament-based movement	23/2279	153/18866	0.1581429	0.4932751	0.4463328	23	PDE4D/KCNE1/TTN/CCDC88C/CACNA1C/MYO9B/WIPF1/GJA5/TNNT3/JUP/BIN1/MYH9/NOS1AP/ACTN4/SCN1A/WASL/MYO5A/SNTA1/KCNQ1/MYO1C/ANK2/FRMD6/TMOD1
Biological Process	GO:0071772	response to BMP	25/2279	168/18866	0.1583171	0.4934942	0.4465311	25	SORL1/MIR140/SKI/SMAD3/SPINT2/HIPK2/RBPJ/UBE2O/ILK/PCSK6/SMAD7/TGFB1/SMURF1/BMPER/BMPR1B/RUNX2/NUMA1/ENG/NOTCH1/SLC33A1/HTRA3/GDF6/ZNF423/NODAL/SPART
Biological Process	GO:0071773	cellular response to BMP stimulus	25/2279	168/18866	0.1583171	0.4934942	0.4465311	25	SORL1/MIR140/SKI/SMAD3/SPINT2/HIPK2/RBPJ/UBE2O/ILK/PCSK6/SMAD7/TGFB1/SMURF1/BMPER/BMPR1B/RUNX2/NUMA1/ENG/NOTCH1/SLC33A1/HTRA3/GDF6/ZNF423/NODAL/SPART
Biological Process	GO:0019319	hexose biosynthetic process	15/2279	94/18866	0.1585929	0.4940299	0.4470158	15	MAEA/ENO1/C1QTNF3/PGP/CHST15/PGAM1/PC/DGKQ/ENO3/PER2/PCK2/FOXO1/SOGA1/SLC25A1/SDHAF3
Biological Process	GO:1901992	positive regulation of mitotic cell cycle phase transition	15/2279	94/18866	0.1585929	0.4940299	0.4470158	15	RPTOR/BRD4/TFDP1/NSMCE2/CUL3/APP/CDC16/CDC25A/TMOD3/PLCB1/RB1/CDCA5/ANAPC7/PLRG1/EIF4G1
Biological Process	GO:0010976	positive regulation of neuron projection development	41/2279	290/18866	0.1601146	0.4986067	0.4511571	41	CPNE6/PLXNC1/AP2A1/TMEM30A/ZMYND8/CPNE5/ZEB2/ITSN1/DISC1/CUX1/BAIAP2/SNX3/ILK/FGFR1/SEMA4D/RAPGEF2/SMURF1/SS18L2/CPEB3/CAMK1D/CDH4/GRN/CREB3L2/PRKCI/NFE2L2/ARSB/NTRK1/CDKL3/DNM1L/AVIL/SHANK2/SYT17/ANKRD1/MARK2/CYFIP1/TRPV2/NDEL1/CUX2/DBNL/PRKN/P3H1

Biological Process	GO:0003231	cardiac ventricle development	20/2279	131/18866	0.1608221	0.5004816	0.4528535	LTBP1/ZFPM1/RXRA/DNM2/SUFU/GJA5/CCM2L/TGFBR2/RBPJ/SMAD7/WNT11/TGFB1/GRHL2/ENG/NOTCH1/MEF2C/SLIT3/LMO4/CPLANE1/NSD2	20
Biological Process	GO:0019218	regulation of steroid metabolic process	20/2279	131/18866	0.1608221	0.5004816	0.4528535	GFI1/IDI1/VDR/MBTPS1/TNF/ABCG1/MIR27A/DGKQ/SF1/POR/GNB3/ACACB/ACACA/LMF1/AGTR1/HMGCS1/SCD/SREBF1/ACADVL/PRKAA1	20
Biological Process	GO:0050673	epithelial cell proliferation	62/2279	453/18866	0.1611029	0.5011473	0.4534559	LRG1/RPTOR/RREB1/TACSTD2/NRP2/TGFA/IL10/PPARG/RPS6KA1/BRCA2/ERN1/ITGB1BP1/VDR/SMAD3/PRKCA/TNF/TNFAIP3/EGFL7/CCL5/C6orf89/MIR101-2/STAT5A/RTN4/THBS1/UHRF1/C5AR2/AKT3/MIR24-2/MIR27A/MIR23A/TCF7L2/PAX2/FLT1/CDH3/FGFR1/TGFB1/ZNF580/NF1/MTSS1/BMPER/ITGB3/GHRL/GRN/PDGFB/EHF/VASH1/SGPP2/NOD2/RB1/NOTCH1/MEF2C/CPB2/CEBPB/AGTR1/RUNX3/DYSF/PPP1R16B/TNFSF12/PLCG1/ALDH1A2/NODAL/JAML	62
Biological Process	GO:1901214	regulation of neuron death	45/2279	321/18866	0.1611415	0.5011473	0.4534559	HDAC4/CTSZ/SORL1/SSH1/IL10/CREB1/MYB/ANGPT1/TNFRSF1B/SRPK2/ARRB1/TNF/TRAF2/CCL5/FBXO7/HIPK2/ITSN1/UNC5B/HLA-F/ILK/CLU/SYNGAP1/ZNF746/NF1/CSF3/GRN/PRKCI/ARRB2/ITGB2/RILPL1/NTRK1/FIS1/BCL2/MEF2C/F2R/TP53BP2/CEBPB/BCL2L1/TYRO3/CRLF1/NTF4/CX3CR1/NAE1/EIF4G1/PRKN	45
Biological Process	GO:0001942	hair follicle development	14/2279	87/18866	0.1611988	0.5011615	0.4534687	TNF/KRT17/RBPJ/HDAC1/CDH3/MYO5A/NF1/NUMA1/BCL2/NOTCH1/TGM3/RELA/INHBA/ERCC2	14
Biological Process	GO:0006836	neurotransmitter transport	33/2279	229/18866	0.1614918	0.5017442	0.453996	SV2C/RAP1B/GIT1/DDC/SYN3/PTPRN2/PPFIA1/APBA2/PRKCB/MCTP2/NF1/NRXN1/VPS18/SYN2/SLC1A2/CTBP2/ITGB3/CHRM2/SLC1A6/NRXN2/PER2/SYNGR3/SLC1A3/RPH3A/P2RX1/DNM1L/MEF2C/SYT17/CACNA1B/SLC29A1/FLOT1/DTNBP1/PRKN	33
Biological Process	GO:0050807	regulation of synapse organization	33/2279	229/18866	0.1614918	0.5017442	0.453996	DCTN1/NRP2/SSH1/IL10/ZMYND8/COLQ/TNF/EPHB3/SEMA4A/ITSN1/DISC1/BAIAP2/ZDHHC2/APP/SEMA4D/NRXN1/GHRL/SIPA1L1/YWHAZ/NTRK1/DNM3/DNM1L/MEF2C/UBE3A/NEDD4/ARHGEF15/CYFIP1/SLIT1/CUX2/EIF4G1/DBNL/LRFN2/NECTIN1	33
Biological Process	GO:0034620	cellular response to unfolded protein	22/2279	146/18866	0.1617634	0.5024236	0.4546107	DCTN1/CALR/ERN1/PIK3R1/AMFR/MBTPS1/RHBDD2/NCK2/YIF1A/EDEM1/DERL2/ZBTB17/PDIA5/CLGN/COPS5/CREB3L2/NFE2L2/ADD1/KLHDC3/SEC31A/ACADVL/PRKN	22
Biological Process	GO:0006486	protein glycosylation	36/2279	252/18866	0.1620832	0.5030882	0.455212	FUT4/GALNT2/FUT7/RPN1/XXYLT1/SLC35C2/MGAT5/ST6GALNAC1/GALNT12/MGAT4A/MGAT3/MUC12/ASGR2/GXYLT2/B4GALT7/POMT2/MUC20/B3GNT5/ST6GAL1/ST8SIA6/ST3GAL4/TET2/B4GALT3/PMM2/NPC1/GALNT9/FUT11/GCNT2/GFPT2/LMF1/ALG11/MAN1C1/DPAGT1/ST3GAL2/B3GNT2/B4GAT1	36

Biological Process	GO:0043413	macromolecule glycosylation	36/2279	252/18866	0.1620832	0.5030882	0.455212	36	FUT4/GALNT2/FUT7/RPN1/XXYLT1/SLC35C2/MGAT5/ST6GALNAC1/GALNT12/MGAT4A/MGAT3/MUC12/ASGR2/GXYLT2/B4GALT7/POMT2/MUC20/B3GNT5/ST6GAL1/ST8SIA6/ST3GAL4/TET2/B4GALT3/PMM2/NPC1/GALNT9/FUT11/GCNT2/GFPT2/LMF1/ALG11/MAN1C1/DPAGT1/ST3GAL2/B3GNT2/B4GAT1
Biological Process	GO:0033138	positive regulation of peptidyl-serine phosphorylation	17/2279	109/18866	0.1621847	0.5032386	0.4553481	17	RPTOR/ANGPT1/ARRB1/TNF/SMYD3/APP/TGFB1/AXIN1/CSF3/SH2D3C/ARRB2/FNIP2/BCL2/BCAR3/NTF4/LIF/EIF4G1
Biological Process	GO:0035050	embryonic heart tube development	13/2279	80/18866	0.1636503	0.5041466	0.4561697	13	SMAD3/SUFU/GJA5/MICAL2/TGFBR2/DLL1/MIB1/ENG/NOTCH1/MEF2C/TMED2/TEAD2/NODAL
Biological Process	GO:1901224	positive regulation of NIK/NF-kappaB signaling	13/2279	80/18866	0.1636503	0.5041466	0.4561697	13	CALR/CHI3L1/TNF/TRAF2/AGO1/TLR9/APP/ACTN4/ILK/PHB/NOD2/IL18R1/RELA
Biological Process	GO:0000305	response to oxygen radical	6/2279	31/18866	0.163944	0.5041466	0.4561697	6	MPO/TNF/NFE2L2/GLRX2/TXNRD2/SIRT3
Biological Process	GO:0002828	regulation of type 2 immune response	6/2279	31/18866	0.163944	0.5041466	0.4561697	6	PRKCZ/NLRP3/CCR2/RARA/NOD2/HLX
Biological Process	GO:0009154	purine ribonucleotide catabolic process	6/2279	31/18866	0.163944	0.5041466	0.4561697	6	PDE4D/AMPD3/NUDT3/ACOT7/PDE9A/ACAT1
Biological Process	GO:0043171	peptide catabolic process	6/2279	31/18866	0.163944	0.5041466	0.4561697	6	LNPEP/GGT1/CTSH/ADAMTS13/CPQ/XPNPEP1
Biological Process	GO:0045940	positive regulation of steroid metabolic process	6/2279	31/18866	0.163944	0.5041466	0.4561697	6	TNF/ABCG1/POR/AGTR1/SREBF1/PRKAA1

Biological Process	GO:0051497	negative regulation of stress fiber assembly	6/2279	31/18866	0.163944	0.5041466	0.4561697	6	TACSTD2/ARAP1/PPFIA1/PFN1/MIR138-2/ARHGEF18
Biological Process	GO:0071549	cellular response to dexamethasone stimulus	6/2279	31/18866	0.163944	0.5041466	0.4561697	6	ABCC2/SMYD3/FECH/AQP1/PCK2/FOXO1
Biological Process	GO:0072538	T-helper 17 type immune response	6/2279	31/18866	0.163944	0.5041466	0.4561697	6	NLRP3/LOXL3/SMAD7/LY9/ZBTB7B/PHB
Biological Process	GO:1900181	negative regulation of protein localization to nucleus	6/2279	31/18866	0.163944	0.5041466	0.4561697	6	ANGPT1/SUFU/LILRB4/NF1/CABP1/ILRUN
Biological Process	GO:1902253	regulation of intrinsic apoptotic signaling pathway by p53 class mediator	6/2279	31/18866	0.163944	0.5041466	0.4561697	6	RRM2B/ZNF385A/ANKRD2/BCL2/PRKN/MARCHF7
Biological Process	GO:1902624	positive regulation of neutrophil migration	6/2279	31/18866	0.163944	0.5041466	0.4561697	6	C3AR1/IL1R1/CAMK1D/DAPK2/DNM1L/DYSF
Biological Process	GO:1904292	regulation of ERAD pathway	6/2279	31/18866	0.163944	0.5041466	0.4561697	6	UBAC2/EDEM1/DERL2/EDEM2/NFE2L2/SVIP
Biological Process	GO:0009063	cellular amino acid catabolic process	19/2279	124/18866	0.1643419	0.5041466	0.4561697	19	ACAD8/HAL/GADL1/HGD/CBS/OGDH/ALDH4A1/ENOSF1/FAH/HIBADH/AGXT/HOGA1/ADO/BCKDHA/MAT1A/HMGCL/CRYM/ACAT1/HNMT
Biological Process	GO:0051101	regulation of DNA binding	19/2279	124/18866	0.1643419	0.5041466	0.4561697	19	PPARG/TNKS/SKI/HIPK2/TAF10/CPNE1/PAX7/TGFB1/RSF1/ZNF462/PER2/RB1/CDCA5/SP100/BCL3/ERCC2/LIF/PRKN/ILRUN

Biological Process	GO:0006677	glycosylceramide metabolic process	4/2279	18/18866	0.1645467	0.5041466	0.4561697	4	GALC/B4GALT3/FA2H/PRKAA1
Biological Process	GO:0009070	serine family amino acid biosynthetic process	4/2279	18/18866	0.1645467	0.5041466	0.4561697	4	GGT1/CBS/AGXT/MTHFD1
Biological Process	GO:0010612	regulation of cardiac muscle adaptation	4/2279	18/18866	0.1645467	0.5041466	0.4561697	4	SMAD3/MIR199A1/MIR199A2/FOXO1
Biological Process	GO:0010832	negative regulation of myotube differentiation	4/2279	18/18866	0.1645467	0.5041466	0.4561697	4	HDAC4/ANKRD2/NOTCH1/CEACAM5
Biological Process	GO:0031468	nuclear envelope reassembly	4/2279	18/18866	0.1645467	0.5041466	0.4561697	4	PPP2R1A/UBXN2A/ANKLE2/BANF1
Biological Process	GO:0032656	regulation of interleukin-13 production	4/2279	18/18866	0.1645467	0.5041466	0.4561697	4	PRKCZ/NLRP3/TNFRSF21/RARA
Biological Process	GO:0032740	positive regulation of interleukin-17 production	4/2279	18/18866	0.1645467	0.5041466	0.4561697	4	LY9/TGFB1/SLC7A5/NOD2
Biological Process	GO:0034433	steroid esterification	4/2279	18/18866	0.1645467	0.5041466	0.4561697	4	ABCG1/MIR27A/AGTR1/ACAT1
Biological Process	GO:0034434	sterol esterification	4/2279	18/18866	0.1645467	0.5041466	0.4561697	4	ABCG1/MIR27A/AGTR1/ACAT1

Biological Process	GO:0034435	cholesterol esterification	4/2279	18/18866	0.1645467	0.5041466	0.4561697	4	ABCG1/MIR27A/AGTR1/ACAT1
Biological Process	GO:0035994	response to muscle stretch	4/2279	18/18866	0.1645467	0.5041466	0.4561697	4	TTN/SLC8A1/ANKRD1/RELA
Biological Process	GO:0036035	osteoclast development	4/2279	18/18866	0.1645467	0.5041466	0.4561697	4	FOXP1/LRRK1/LTF/FAM20C
Biological Process	GO:0043652	engulfment of apoptotic cell	4/2279	18/18866	0.1645467	0.5041466	0.4561697	4	RHOH/RHOG/THBS1/RHOBTB2
Biological Process	GO:0044872	lipoprotein localization	4/2279	18/18866	0.1645467	0.5041466	0.4561697	4	PPARG/MIA3/PRKCB/ZDHHC17
Biological Process	GO:0045721	negative regulation of gluconeogenesis	4/2279	18/18866	0.1645467	0.5041466	0.4561697	4	MAEA/C1QTNF3/PGP/SOGA1
Biological Process	GO:0048339	paraxial mesoderm development	4/2279	18/18866	0.1645467	0.5041466	0.4561697	4	SMAD3/WNT11/FGFR1/TEAD2
Biological Process	GO:0051895	negative regulation of focal adhesion assembly	4/2279	18/18866	0.1645467	0.5041466	0.4561697	4	ITGB1BP1/DUSP22/THBS1/CORO1C
Biological Process	GO:0071371	cellular response to gonadotropin stimulus	4/2279	18/18866	0.1645467	0.5041466	0.4561697	4	POR/NOTCH1/HMGCS1/INHBA

Biological Process	GO:0090036	regulation of protein kinase C signaling	4/2279	18/18866	0.1645467	0.5041466	0.4561697	4	WNT11/SPHK2/DGKD/MYADM
Biological Process	GO:0150118	negative regulation of cell-substrate junction organization	4/2279	18/18866	0.1645467	0.5041466	0.4561697	4	ITGB1BP1/DUSP22/THBS1/CORO1C
Biological Process	GO:1903242	regulation of cardiac muscle hypertrophy in response to stress	4/2279	18/18866	0.1645467	0.5041466	0.4561697	4	SMAD3/MIR199A1/MIR199A2/FOXO1
Biological Process	GO:2000369	regulation of clathrin-dependent endocytosis	4/2279	18/18866	0.1645467	0.5041466	0.4561697	4	AAK1/DNM2/WASL/DGKD
Biological Process	GO:2001185	regulation of CD8-positive, alpha-beta T cell activation	4/2279	18/18866	0.1645467	0.5041466	0.4561697	4	RUNX1/ZBTB7B/RUNX3/VSIR
Biological Process	GO:0060840	artery development	16/2279	102/18866	0.1653926	0.5054247	0.4573262	16	LTBP1/MIR143/MIR145/MYLK/DNM2/SUFU/GJA5/NDST1/RBPJ/SMAD7/WNT11/ZMIZ1/NF1/ENG/NOTCH1/PDGFRB
Biological Process	GO:1903076	regulation of protein localization to plasma membrane	16/2279	102/18866	0.1653926	0.5054247	0.4573262	16	PIK3R1/RHOG/TNF/ZDHHC2/MRAP/AKAP5/ZDHHC7/PPFIA1/TGFB1/PRKCH/NUMB/CSK/PRKCI/PRKCE/BCL2L1/EZR
Biological Process	GO:2000060	positive regulation of ubiquitin-dependent protein catabolic process	16/2279	102/18866	0.1653926	0.5054247	0.4573262	16	RNF144A/FZR1/RNF19A/CSNK1D/CBFA2T3/DISC1/CLU/SMAD7/SMURF1/AXIN1/CSNK1E/NFE2L2/RNF144B/RNF14/TRIB1/PRKN
Biological Process	GO:0016125	sterol metabolic process	25/2279	169/18866	0.1656579	0.5054247	0.4573262	25	PRKAG2/OSBPL5/RXRA/IDI1/LIPC/MBTPS1/ABCG1/HDLBP/AKR1D1/APP/FDX1/DGKQ/POR/ABCA1/GNB3/ACACB/CAT/NPC1/ACACA/LMF1/HMGCS1/SCD/SREBF1/ACADVL/PRKAA1

Biological Process	GO:0062013	positive regulation of small molecule metabolic process	23/2279	154/18866	0.165847	0.5054247	0.4573262	23	RCVRN/ENO1/PPARG/PFKFB4/GPLD1/TNF/ABCG1/COMT/APP/P2RY6/RDH10/HRH1/POR/DYRK2/NR1H2/PDGFB/ESRRB/PRKCE/FOXO1/SREBF1/CPT1A/PFKFB3/PRKAA1
Biological Process	GO:0006487	protein N-linked glycosylation	12/2279	73/18866	0.1658771	0.5054247	0.4573262	12	RPN1/MGAT5/MGAT4A/MGAT3/ASGR2/B4GALT7/ST6GAL1/PMM2/GFPT2/ALG11/MAN1C1/DPAGT1
Biological Process	GO:0010517	regulation of phospholipase activity	12/2279	73/18866	0.1658771	0.5054247	0.4573262	12	PRKCZ/CCL5/FLT1/P2RY6/FGFR1/PHB/LPAR2/S1PR4/AGTR1/PDGFRB/PLCG1/NTF4
Biological Process	GO:0051155	positive regulation of striated muscle cell differentiation	12/2279	73/18866	0.1658771	0.5054247	0.4573262	12	EHD1/MAML1/MIR199A1/NFATC2/RBPJ/TGFB1/ARRB2/BCL2/MIR199A2/MEF2C/FLOT1/SCGB3A1
Biological Process	GO:1903747	regulation of establishment of protein localization to mitochondrion	12/2279	73/18866	0.1658771	0.5054247	0.4573262	12	TFDP1/YWHAH/USP36/ABLIM3/UBE2J2/YWHAZ/BCL2/TP53BP2/SREBF1/YWHAQ/PRKAA1/PRKN
Biological Process	GO:0051402	neuron apoptotic process	35/2279	245/18866	0.1660226	0.5054247	0.4573262	35	HDAC4/CTSZ/IL10/MYB/ANGPT1/SRPK2/ARRB1/TNF/HIPK2/ITSN1/UNC5B/APP/ILK/TNFRSF21/SYNGAP1/NF1/GRN/PRKCI/ARRB2/NTRK1/FIS1/RB1/BCL2/MEF2C/F2R/ATN1/CEBPB/BCL2L1/TYRO3/CRLF1/NTF4/CX3CR1/NAE1/FADD/PRKN
Biological Process	GO:0015850	organic hydroxy compound transport	38/2279	268/18866	0.166068	0.5054247	0.4573262	38	OSBPL5/RXRA/PPARG/SLC51A/SLC16A3/SLC10A1/MYB/LIPC/ABCG1/ABCC2/PLTP/ATP8B1/MIR27A/OSBPL6/CLU/RALBP1/NISCH/AQP1/NR1H2/ITGB3/GHRL/ABCA1/NCOA1/OSBPL10/ABCC3/NPC1/MFSD10/SLC16A1/AQP3/P2RX1/SYT17/AGTR1/NCOA2/GRAMD1A/DTNBP1/AQP9/GRK2/PRKN
Biological Process	GO:0009247	glycolipid biosynthetic process	11/2279	66/18866	0.1677807	0.5054247	0.4573262	11	PIGL/PIGU/PIGN/B3GNT5/ST8SIA6/ST3GAL4/B4GALT3/PIGQ/FA2H/ST3GAL2/PRKAA1
Biological Process	GO:0016445	somatic diversification of immunoglobulins	11/2279	66/18866	0.1677807	0.5054247	0.4573262	11	IL10/UNG/TGFB1/PTPRC/TCF3/TP53BP1/CTNNBL1/ERCC1/TNFSF13/PAXIP1/NSD2

Biological Process	GO:0045669	positive regulation of osteoblast differentiation	11/2279	66/18866	0.1677807	0.5054247	0.4573262	11	CEBPD/ILK/BMPR1B/RUNX2/IL6R/MEF2C/NPPC/LTF/CEBPB/FAM20C/CCN4
Biological Process	GO:0046365	monosaccharide catabolic process	11/2279	66/18866	0.1677807	0.5054247	0.4573262	11	FUT4/FOXK1/ENO1/FUT7/HK1/ADPGK/PGAM1/GLYCTK/ENO3/HK2/PKM
Biological Process	GO:1905818	regulation of chromosome separation	11/2279	66/18866	0.1677807	0.5054247	0.4573262	11	MAD1L1/NSMCE2/CUL3/CDC16/TACC3/NUMA1/NEK6/RB1/ANAPC7/HECW2/PCID2
Biological Process	GO:0019433	triglyceride catabolic process	7/2279	38/18866	0.168034	0.5054247	0.4573262	7	PNPLA2/SORL1/MGLL/LIPC/GPLD1/FABP6/ABHD5
Biological Process	GO:0030224	monocyte differentiation	7/2279	38/18866	0.168034	0.5054247	0.4573262	7	FOXP1/PPARG/INPP5D/CSF1R/MYH9/SP3/MEF2C
Biological Process	GO:0032689	negative regulation of interferon-gamma production	7/2279	38/18866	0.168034	0.5054247	0.4573262	7	IL10/MIR24-2/RARA/PGLYRP1/CD96/INHBA/VSIR
Biological Process	GO:0035872	nucleotide-binding domain, leucine rich repeat containing receptor signaling pathway	7/2279	38/18866	0.168034	0.5054247	0.4573262	7	MAP2K6/TNFAIP3/UBE2V1/IRAK2/NOD2/CYLD/RELA
Biological Process	GO:0044003	modulation by symbiont of host process	7/2279	38/18866	0.168034	0.5054247	0.4573262	7	RXRA/ULK1/HIPK2/PABPN1/TNIP1/BCL2L1/CPSF4
Biological Process	GO:0048009	insulin-like growth factor receptor signaling pathway	7/2279	38/18866	0.168034	0.5054247	0.4573262	7	PIK3R1/IGF1R/CDH3/CRIM1/PLCB1/TSC2/MYORG

Biological Process	GO:0051567	histone H3-K9 methylation	7/2279	38/18866	0.168034	0.5054247	0.4573262	7	MYB/EHMT2/KDM4C/PRDM16/DNMT1/PIH1D1/MECOM
Biological Process	GO:0090224	regulation of spindle organization	7/2279	38/18866	0.168034	0.5054247	0.4573262	7	DCTN1/RAE1/RCC1/DYNC1H1/TACC3/NUMA1/SPAG5
Biological Process	GO:1901186	positive regulation of ERBB signaling pathway	7/2279	38/18866	0.168034	0.5054247	0.4573262	7	TGFA/DOK1/RTN4/ARAP1/RBPJ/PDE6H/DGKD
Biological Process	GO:1903131	mononuclear cell differentiation	7/2279	38/18866	0.168034	0.5054247	0.4573262	7	FOXP1/PPARG/INPP5D/CSF1R/MYH9/SP3/MEF2C
Biological Process	GO:2000144	positive regulation of DNA-templated transcription, initiation	7/2279	38/18866	0.168034	0.5054247	0.4573262	7	CREB1/HNF1A/MED13/SUB1/ERCC1/XPA/PAXIP1
Biological Process	GO:2000403	positive regulation of lymphocyte migration	7/2279	38/18866	0.168034	0.5054247	0.4573262	7	CCR2/CCL5/APP/DOCK8/CCL20/CCL27/FADD
Biological Process	GO:0097711	ciliary basal body-plasma membrane docking	15/2279	95/18866	0.1685764	0.5054247	0.4573262	15	DCTN1/CSNK1D/NDE1/DYNC1H1/TUBB4B/CEP135/CEP164/DCTN2/CEP78/CSNK1E/PPP2R1A/TUBA4A/SDCCAG8/CEP72/NPHP4
Biological Process	GO:1901987	regulation of cell cycle phase transition	66/2279	486/18866	0.1686614	0.5054247	0.4573262	66	TRIM39/RPTOR/BLM/BRD4/DCTN1/FZR1/TFDP1/MAD1L1/CSNK1D/FOXN3/NDE1/PRMT2/HUS1/NSMCE2/DYNC1H1/FBXO7/E2F7/CUL3/CTDSPL/PCBP4/TUBB4B/CEP135/RIPK1/APP/PTPN6/NACC2/ZNF385A/CDC16/CDC25A/ARID3A/CEP164/OVOL1/DCTN2/TMOD3/CEP78/CSNK1E/C10orf99/PSMF1/PPP2R1A/GFI1B/PLCB1/TUBA4A/TAOK3/SDCCAG8/ZFYVE19/MIR138-2/NEK6/CLSPN/RB1/BCL2/PSMB7/CDCA5/CHEK2/ANAPC7/HECW2/CDC14A/PLRG1/CEP72/ERCC2/NAE1/EIF4G1/PSMD13/PCID2/PAXIP1/ABRAXAS1/BABAM2
Biological Process	GO:0000185	activation of MAPKKK activity	3/2279	12/18866	0.1690816	0.5054247	0.4573262	3	KSR1/TNF/GADD45G

Biological Process	GO:0001765	membrane raft assembly	3/2279	12/18866	0.1690816	0.5054247	0.4573262	3	PACSIN2/MIR138-2/FLOT1
Biological Process	GO:0001778	plasma membrane repair	3/2279	12/18866	0.1690816	0.5054247	0.4573262	3	ANXA6/MYH9/DYSF
Biological Process	GO:0001867	complement activation, lectin pathway	3/2279	12/18866	0.1690816	0.5054247	0.4573262	3	MFAP4/MASP1/FCN1
Biological Process	GO:0002002	regulation of angiotensin levels in blood	3/2279	12/18866	0.1690816	0.5054247	0.4573262	3	CTSZ/CTSG/AOPEP
Biological Process	GO:0002003	angiotensin maturation	3/2279	12/18866	0.1690816	0.5054247	0.4573262	3	CTSZ/CTSG/AOPEP
Biological Process	GO:0002923	regulation of humoral immune response mediated by circulating immunoglobulin	3/2279	12/18866	0.1690816	0.5054247	0.4573262	3	TNF/PTPN6/PTPRC
Biological Process	GO:0006002	fructose 6-phosphate metabolic process	3/2279	12/18866	0.1690816	0.5054247	0.4573262	3	HK1/HK2/GFPT2
Biological Process	GO:0006069	ethanol oxidation	3/2279	12/18866	0.1690816	0.5054247	0.4573262	3	ALDH2/ACSS1/ACSS2
Biological Process	GO:0006465	signal peptide processing	3/2279	12/18866	0.1690816	0.5054247	0.4573262	3	HM13/CLN5/SPCS1

Biological Process	GO:0006534	cysteine metabolic process	3/2279	12/18866	0.1690816	0.5054247	0.4573262	3	GGT1/CBS/AGXT
Biological Process	GO:0008298	intracellular mRNA localization	3/2279	12/18866	0.1690816	0.5054247	0.4573262	3	EXOSC2/ZNF385A/PCID2
Biological Process	GO:0008343	adult feeding behavior	3/2279	12/18866	0.1690816	0.5054247	0.4573262	3	GHRL/NPY/PRLHR
Biological Process	GO:0009642	response to light intensity	3/2279	12/18866	0.1690816	0.5054247	0.4573262	3	GNAT2/CAT/HMGCS1
Biological Process	GO:0021670	lateral ventricle development	3/2279	12/18866	0.1690816	0.5054247	0.4573262	3	AQP1/NUMB/TSKU
Biological Process	GO:0032736	positive regulation of interleukin-13 production	3/2279	12/18866	0.1690816	0.5054247	0.4573262	3	PRKCZ/NLRP3/RARA
Biological Process	GO:0032957	inositol trisphosphate metabolic process	3/2279	12/18866	0.1690816	0.5054247	0.4573262	3	ITPKB/P2RY6/HRH1
Biological Process	GO:0033004	negative regulation of mast cell activation	3/2279	12/18866	0.1690816	0.5054247	0.4573262	3	CD84/FER/CNR2
Biological Process	GO:0034333	adherens junction assembly	3/2279	12/18866	0.1690816	0.5054247	0.4573262	3	TBCD/SMAD7/JAM3

Biological Process	GO:0035630	bone mineralization involved in bone maturation	3/2279	12/18866	0.1690816	0.5054247	0.4573262	3	LTF/ANO6/RFLNA
Biological Process	GO:0035739	CD4-positive, alpha-beta T cell proliferation	3/2279	12/18866	0.1690816	0.5054247	0.4573262	3	CD55/TGFBR2/VSIR
Biological Process	GO:0036005	response to macrophage colony-stimulating factor	3/2279	12/18866	0.1690816	0.5054247	0.4573262	3	CSF1R/DOK1/FER
Biological Process	GO:0036006	cellular response to macrophage colony-stimulating factor stimulus	3/2279	12/18866	0.1690816	0.5054247	0.4573262	3	CSF1R/DOK1/FER
Biological Process	GO:0042118	endothelial cell activation	3/2279	12/18866	0.1690816	0.5054247	0.4573262	3	FOXP1/BMPER/HOXA9
Biological Process	GO:0042178	xenobiotic catabolic process	3/2279	12/18866	0.1690816	0.5054247	0.4573262	3	ACSL1/ABCC2/GSTO1
Biological Process	GO:0042368	vitamin D biosynthetic process	3/2279	12/18866	0.1690816	0.5054247	0.4573262	3	GFI1/VDR/TNF
Biological Process	GO:0043696	dedifferentiation	3/2279	12/18866	0.1690816	0.5054247	0.4573262	3	MIR145/PDGFB/ESRRB
Biological Process	GO:0043697	cell dedifferentiation	3/2279	12/18866	0.1690816	0.5054247	0.4573262	3	MIR145/PDGFB/ESRRB

Biological Process	GO:0044090	positive regulation of vacuole organization	3/2279	12/18866	0.1690816	0.5054247	0.4573262	3	ULK1/PIP4K2A/GRN
Biological Process	GO:0045117	azole transmembrane transport	3/2279	12/18866	0.1690816	0.5054247	0.4573262	3	SLC15A4/SLC44A4/SLC7A1
Biological Process	GO:0046322	negative regulation of fatty acid oxidation	3/2279	12/18866	0.1690816	0.5054247	0.4573262	3	MFSD2A/ACACB/ACADVL
Biological Process	GO:0046929	negative regulation of neurotransmitter secretion	3/2279	12/18866	0.1690816	0.5054247	0.4573262	3	RAP1B/NF1/PRKN
Biological Process	GO:0051798	positive regulation of hair follicle development	3/2279	12/18866	0.1690816	0.5054247	0.4573262	3	TNF/KRT17/NUMA1
Biological Process	GO:0060213	positive regulation of nuclear-transcribed mRNA poly(A) tail shortening	3/2279	12/18866	0.1690816	0.5054247	0.4573262	3	TNRC6B/AGO2/CPEB3
Biological Process	GO:0060670	branching involved in labyrinthine layer morphogenesis	3/2279	12/18866	0.1690816	0.5054247	0.4573262	3	IL10/SPINT2/GRHL2
Biological Process	GO:0060856	establishment of blood-brain barrier	3/2279	12/18866	0.1690816	0.5054247	0.4573262	3	MFSD2A/ENG/ADGRA2
Biological Process	GO:0061365	positive regulation of triglyceride lipase activity	3/2279	12/18866	0.1690816	0.5054247	0.4573262	3	NR1H2/LMF1/ABHD5

Biological Process	GO:0070816	phosphorylation of RNA polymerase II C-terminal domain	3/2279	12/18866	0.1690816	0.5054247	0.4573262	3	BRD4/CCNH/CDK13
Biological Process	GO:0071888	macrophage apoptotic process	3/2279	12/18866	0.1690816	0.5054247	0.4573262	3	CCL5/NOD2/MEF2C
Biological Process	GO:0072531	pyrimidine-containing compound transmembrane transport	3/2279	12/18866	0.1690816	0.5054247	0.4573262	3	SLC44A4/SLC29A1/AQP9
Biological Process	GO:0072537	fibroblast activation	3/2279	12/18866	0.1690816	0.5054247	0.4573262	3	RPS6KA1/MYB/PDGFRB
Biological Process	GO:0072584	caveolin-mediated endocytosis	3/2279	12/18866	0.1690816	0.5054247	0.4573262	3	ITSN1/PACSIN2/MLC1
Biological Process	GO:0099149	regulation of postsynaptic neurotransmitter receptor internalization	3/2279	12/18866	0.1690816	0.5054247	0.4573262	3	NUMB/ITGB3/GSG1L
Biological Process	GO:1902855	regulation of non-motile cilium assembly	3/2279	12/18866	0.1690816	0.5054247	0.4573262	3	DNM2/CEP135/SEPTIN9
Biological Process	GO:1903909	regulation of receptor clustering	3/2279	12/18866	0.1690816	0.5054247	0.4573262	3	SSH1/GSN/ZDHHC2
Biological Process	GO:2000561	regulation of CD4-positive, alpha-beta T cell proliferation	3/2279	12/18866	0.1690816	0.5054247	0.4573262	3	CD55/TGFBR2/VSIR

Biological Process	GO:2000650	negative regulation of sodium ion transmembrane transporter activity	3/2279	12/18866	0.1690816	0.5054247	0.4573262	3	PRKCE/NEDD4/HECW2
Biological Process	GO:0050707	regulation of cytokine secretion	10/2279	59/18866	0.1692197	0.5054247	0.4573262	10	SORL1/IL10/C1QTNF3/CLEC9A/FGR/TNF/TLR9/CLEC5A/CADM1/CCN4
Biological Process	GO:0060043	regulation of cardiac muscle cell proliferation	10/2279	59/18866	0.1692197	0.5054247	0.4573262	10	MIR199A1/VGLL4/TGFBR2/RIPK1/RBPJ/FGFR1/MEIS1/MIR199A2/NOTCH1/MEF2C
Biological Process	GO:0098930	axonal transport	10/2279	59/18866	0.1692197	0.5054247	0.4573262	10	SPG7/DYNC1H1/CNIH2/KIF5C/KIF1B/MAP1A/AP3D1/FBXW11/NDEL1/DTNBP1
Biological Process	GO:0002715	regulation of natural killer cell mediated immunity	8/2279	45/18866	0.1697602	0.5059258	0.4577797	8	PIK3R6/CD226/HLA-F/NCR1/ARRB2/IL18RAP/CADM1/CD96
Biological Process	GO:0032365	intracellular lipid transport	8/2279	45/18866	0.1697602	0.5059258	0.4577797	8	PRKAG2/ABCG1/ABCA1/ACACB/NPC1/SGPP1/ACACA/CPT1A
Biological Process	GO:0032924	activin receptor signaling pathway	8/2279	45/18866	0.1697602	0.5059258	0.4577797	8	SKI/SMAD3/TGFBR2/SMAD7/GDF6/ZC3H3/INHBA/NODAL
Biological Process	GO:0034105	positive regulation of tissue remodeling	8/2279	45/18866	0.1697602	0.5059258	0.4577797	8	DEF8/MIR143/VDR/PRKCA/MIR199A1/TMEM64/PLEKHM1/MIR199A2
Biological Process	GO:0046839	phospholipid dephosphorylation	8/2279	45/18866	0.1697602	0.5059258	0.4577797	8	SYNJ2/INPP5A/INPP5D/INPP5E/SGPP1/SGPP2/INPP5F/PLPP3

Biological Process	GO:0055026	negative regulation of cardiac muscle tissue development	8/2279	45/18866	0.1697602	0.5059258	0.4577797	8	CTDP1/MIR199A1/VGLL4/TGFBR2/DLL1/RIPK1/MEIS1/MIR199A2
Biological Process	GO:0072583	clathrin-dependent endocytosis	8/2279	45/18866	0.1697602	0.5059258	0.4577797	8	AP2A1/AAK1/DNM2/ITSN1/DLL1/WASL/DGKD/INPP5F
Biological Process	GO:0003179	heart valve morphogenesis	9/2279	52/18866	0.1699857	0.505963	0.4578133	9	ZFPM1/GJA5/NFATC1/TGFBR2/TGFB1/RB1/NOTCH1/MEF2C/SLIT3
Biological Process	GO:0030514	negative regulation of BMP signaling pathway	9/2279	52/18866	0.1699857	0.505963	0.4578133	9	SORL1/SKI/HIPK2/SMAD7/SMURF1/BMPER/NOTCH1/HTRA3/SPART
Biological Process	GO:0051496	positive regulation of stress fiber assembly	9/2279	52/18866	0.1699857	0.505963	0.4578133	9	ARHGEF10/ITGB1BP1/SMAD3/SH3PXD2B/SYNPO2L/PXN/WNT11/ARHGEF10L/ARHGEF15
Biological Process	GO:0090329	regulation of DNA-dependent DNA replication	9/2279	52/18866	0.1699857	0.505963	0.4578133	9	BLM/BRCA2/E2F7/WRNIP1/FGFR1/SMARCAL1/CHEK2/KAT7/FBH1
Biological Process	GO:0002028	regulation of sodium ion transport	14/2279	88/18866	0.1717054	0.5106019	0.4620107	14	FXYD2/YWHAH/FXYD1/SLC8A1/DNM2/TESC/ACTN4/SNTA1/PER1/PRKCE/NEDD4/HECW2/SPTBN4/FGF14
Biological Process	GO:0046889	positive regulation of lipid biosynthetic process	14/2279	88/18866	0.1717054	0.5106019	0.4620107	14	CREB1/MFSD2A/GPLD1/TNF/ABCG1/FPR2/TNFRSF1A/SPHK2/RDH10/POR/NR1H2/SREBF1/SIRT3/PRKAA1
Biological Process	GO:0050818	regulation of coagulation	14/2279	88/18866	0.1717054	0.5106019	0.4620107	14	PLAU/CD9/PRKCA/UBASH3B/SERPINB2/THBS1/F12/BLK/PDGFB/NFE2L2/CPB2/F2R/ANO6/ADTRP

Biological Process	GO:0061013	regulation of mRNA catabolic process	30/2279	208/18866	0.1736655	0.5162689	0.4671384	30	PRR5L/EXOSC2/SLC11A1/TNRC6B/PABPC4/TNPO1/PRKCA/LARP1/ZC3H12D/PKP1/PCBP4/SAMD4A/AGO2/CPEB3/IGF2BP3/PSMF1/FTO/NBAS/YWHAZ/PSMB7/PAIP1/TNFSF13/SECISBP2/SYNCRIP/EIF4G1/PSMD13/PCID2/TBRG4/TENT5C/TENT5A
Biological Process	GO:0009411	response to UV	21/2279	140/18866	0.1738008	0.5163482	0.4672101	21	ELANE/MFAP4/BRCA2/PIK3R1/BMF/HUS1/CDC25A/AQP1/CREBBP/CAT/BCL2/RHNO1/NEDD4/ERCC1/CRIP1/RELA/BCL3/POLD3/XPA/ERCC2/PRKAA1
Biological Process	GO:0072655	establishment of protein localization to mitochondrion	21/2279	140/18866	0.1738008	0.5163482	0.4672101	21	HK1/TFDP1/YWHAH/PAM16/BMF/FBXO7/MIPEP/AIP/USP36/ABLIM3/UBE2J2/HK2/YWHAZ/FIS1/BCL2/TIMM44/TP53BP2/SREBF1/YWHAQ/PRKAA1/PRKN
Biological Process	GO:1901990	regulation of mitotic cell cycle phase transition	61/2279	448/18866	0.1738924	0.5164587	0.4673102	61	TRIM39/RPTOR/BLM/BRD4/DCTN1/FZR1/TFDP1/MAD1L1/CSNK1D/FOXN3/NDE1/PRMT2/HUS1/NSMCE2/DYNC1H1/FBXO7/E2F7/CUL3/CTDSPL/PCBP4/TUBB4B/CEP135/RIPK1/APP/PTPN6/NACC2/ZNF385A/CDC16/CDC25A/ARID3A/CEP164/DCTN2/TMOD3/CEP78/CSNK1E/PSMF1/PPP2R1A/GFI1B/PLCB1/TUBA4A/TAOK3/SDCCAG8/ZFYVE19/MIR138-2/NEK6/CLSPN/RB1/BCL2/PSMB7/CDCA5/CHEK2/ANAPC7/HECW2/CDC14A/PLRG1/CEP72/ERCC2/NAE1/EIF4G1/PSMD13/PCID2
Biological Process	GO:0034766	negative regulation of ion transmembrane transport	16/2279	103/18866	0.1751965	0.5201694	0.4706677	16	KCNE1/THADA/CASQ2/TLR9/BIN1/THBS1/SLC43A2/CAB39/GSTO1/PRKCE/SLC43A1/NEDD4/DYSF/HECW2/YWHAQ/CBARP
Biological Process	GO:0003071	renal system process involved in regulation of systemic arterial blood pressure	5/2279	25/18866	0.176726	0.5227503	0.4730031	5	GJA5/PDGFB/F2R/AGTR1/HSD11B2
Biological Process	GO:0009081	branched-chain amino acid metabolic process	5/2279	25/18866	0.176726	0.5227503	0.4730031	5	ACAD8/HIBADH/BCKDHA/HMGCL/ACAT1
Biological Process	GO:0032878	regulation of establishment or maintenance of cell polarity	5/2279	25/18866	0.176726	0.5227503	0.4730031	5	GSN/KANK1/RAP1B/DOCK8/CYRIB
Biological Process	GO:0034162	toll-like receptor 9 signaling pathway	5/2279	25/18866	0.176726	0.5227503	0.4730031	5	GRAMD4/PIK3AP1/TLR9/RTN4/IRAK2

Biological Process	GO:0045109	intermediate filament organization	5/2279	25/18866	0.176726	0.5227503	0.4730031	5	KRT17/PKP1/NEFM/DNAJB6/GFAP
Biological Process	GO:0060148	positive regulation of posttranscriptional gene silencing	5/2279	25/18866	0.176726	0.5227503	0.4730031	5	RIPK1/LIMD1/AGO2/TGFB1/EIF4G1
Biological Process	GO:0060330	regulation of response to interferon-gamma	5/2279	25/18866	0.176726	0.5227503	0.4730031	5	PPARG/JAK1/NR1H2/NLRC5/PARP9
Biological Process	GO:0060334	regulation of interferon-gamma-mediated signaling pathway	5/2279	25/18866	0.176726	0.5227503	0.4730031	5	PPARG/JAK1/NR1H2/NLRC5/PARP9
Biological Process	GO:0070920	regulation of production of small RNA involved in gene silencing by RNA	5/2279	25/18866	0.176726	0.5227503	0.4730031	5	NCOR2/TNF/RIPK1/TGFB1/RMRP
Biological Process	GO:0071378	cellular response to growth hormone stimulus	5/2279	25/18866	0.176726	0.5227503	0.4730031	5	PIK3R1/STAT5A/PXN/GHRL/MBD5
Biological Process	GO:1905564	positive regulation of vascular endothelial cell proliferation	5/2279	25/18866	0.176726	0.5227503	0.4730031	5	AKT3/MIR27A/FGFR1/GHRL/PLCG1
Biological Process	GO:2000311	regulation of AMPA receptor activity	5/2279	25/18866	0.176726	0.5227503	0.4730031	5	CNIH2/MAPK8IP2/GSG1L/MEF2C/SHANK2
Biological Process	GO:0032024	positive regulation of insulin secretion	12/2279	74/18866	0.1776148	0.5250524	0.475086	12	NADK/MYRIP/ARRB1/GPLD1/TCF7L2/GIPR/BLK/GHRL/PRKCE/RPH3AL/C2CD2L/SIRT3

Biological Process	GO:0046503	glycerolipid catabolic process	12/2279	74/18866	0.1776148	0.5250524	0.475086	12	PNPLA2/PLA2G15/SORL1/MGLL/LIPC/ABHD16A/GPLD1/ABHD16B/FABP6/ABHD5/INPP5F/LYPLA2
Biological Process	GO:0042177	negative regulation of protein catabolic process	20/2279	133/18866	0.1779434	0.5256523	0.4756289	20	TRIM39/TIMP2/IL10/SF3B3/SMAD3/SUFU/HIPK2/UBAC2/MGAT3/SNX3/DERL2/PHB/CLEC16A/PSMF1/MAP1A/DYSF/RELA/BAG5/SVIP/MARCHF7
Biological Process	GO:0090101	negative regulation of transmembrane receptor protein serine/threonine kinase signaling pathway	20/2279	133/18866	0.1779434	0.5256523	0.4756289	20	LTBP1/SPRED2/SORL1/SKI/SMAD3/LDLRAD4/DNM2/HIPK2/CHST11/TGFBR2/SMAD7/TGFB1/SMURF1/TGFB1I1/BMPER/HTRA4/PRDM16/NOTCH1/HTRA3/SPART
Biological Process	GO:0031644	regulation of nervous system process	22/2279	148/18866	0.1779837	0.5256523	0.4756289	22	PRKCZ/MGLL/SSH1/IL10/ZMYND8/TNFRSF1B/COMT/BAIAP2/APP/TNFRSF21/DLGAP4/CNIH2/NRXN1/GHRL/SH3GL1/BEGAIN/S1PR2/SHANK2/F2R/NPTX1/CUX2/ACP3
Biological Process	GO:0008202	steroid metabolic process	46/2279	332/18866	0.1787058	0.5276209	0.4774102	46	PRKAG2/GFI1/OSBPL5/RXRA/NR1I2/IDI1/DHRS9/LIPC/YWHAH/VDR/AMACR/MBTPS1/TNF/ABCG1/HDLBP/AKR1D1/HSD17B2/COMT/ATP8B1/APP/MIR27A/OSBPL6/FGFR1/FDX1/SULT1A2/DGKQ/SF1/POR/ABCA1/GNB3/ACACB/CAT/ACBD3/ACOX2/NPC1/ACACA/LMF1/AGTR1/ACAT1/HMGCS1/SCD/SREBF1/NR5A2/HSD11B2/ACADVL/PRKAA1
Biological Process	GO:0002548	monocyte chemotaxis	11/2279	67/18866	0.1802537	0.5313654	0.4807983	11	CCR2/CCL5/FPR2/DEFB124/FLT1/CXCL17/CCL20/IL6R/PDGFB/ANO6/CCL22
Biological Process	GO:0007040	lysosome organization	11/2279	67/18866	0.1802537	0.5313654	0.4807983	11	HEXB/LYST/MBTPS1/VPS18/ABCA1/HOOK2/GRN/TPCN2/ARSB/CLN5/ACP3
Biological Process	GO:0031060	regulation of histone methylation	11/2279	67/18866	0.1802537	0.5313654	0.4807983	11	GFI1/BRD4/MYB/PAX7/KDM4C/ZNF335/PHF19/DNMT1/PIH1D1/PAXIP1/NSD3
Biological Process	GO:0036498	IRE1-mediated unfolded protein response	11/2279	67/18866	0.1802537	0.5313654	0.4807983	11	DCTN1/ERN1/YIF1A/EDEM1/ZBTB17/PDIA5/COPS5/ADD1/KLHDC3/SEC31A/ACADVL

Biological Process	GO:0080171	lytic vacuole organization	11/2279	67/18866	0.1802537	0.5313654	0.4807983	11	HEXB/LYST/MBTPS1/VPS18/ABCA1/HOOK2/GRN/TPCN2/ARSB/CLN5/ACP3
Biological Process	GO:0040029	regulation of gene expression, epigenetic	30/2279	209/18866	0.1806158	0.5322676	0.4816146	30	HDAC4/TRIM27/TAF1C/BAZ1B/SERTAD2/POLR2F/PADI2/CDYL/EPC1/ZMIZ1/SPHK2/KMT2D/MTA1/CTBP1/ARID1B/KDM1B/KCNQ1/SMARCD1/ZNF335/PHF19/DNMT1/PCGF3/TAF1D/MORC1/MYO1C/RBM15B/DYDC2/DYDC1/MACROH2A2/H3C1
Biological Process	GO:0061387	regulation of extent of cell growth	17/2279	111/18866	0.1811654	0.5335563	0.4827806	17	SEMA6B/CTTN/DNM2/SEMA4A/RTN4/DISC1/SEMA4B/IFRD1/ILK/SEMA4D/CDH4/CDKL3/CYFIP1/TRPV2/NDEL1/DBNL/SPART
Biological Process	GO:1903509	liposaccharide metabolic process	17/2279	111/18866	0.1811654	0.5335563	0.4827806	17	HEXB/PIGL/PIGU/GPLD1/GALC/AOAH/PIGN/B3GNT5/ST8SIA6/ST3GAL4/B4GALT3/PIGQ/FA2H/ESYT1/CERK/ST3GAL2/PRKAA1
Biological Process	GO:0009261	ribonucleotide catabolic process	6/2279	32/18866	0.1825198	0.5338711	0.4830655	6	PDE4D/AMPD3/NUDT3/ACOT7/PDE9A/ACAT1
Biological Process	GO:0021591	ventricular system development	6/2279	32/18866	0.1825198	0.5338711	0.4830655	6	RAPGEF2/AQP1/NUMB/TTC21B/CORO1C/TSKU
Biological Process	GO:0032673	regulation of interleukin-4 production	6/2279	32/18866	0.1825198	0.5338711	0.4830655	6	PRKCZ/ZFPM1/NLRP3/RARA/SLC7A5/CEBPB
Biological Process	GO:0034694	response to prostaglandin	6/2279	32/18866	0.1825198	0.5338711	0.4830655	6	PPARG/P2RY6/GNG2/ACACA/PRKCE/PRKAA1
Biological Process	GO:0042219	cellular modified amino acid catabolic process	6/2279	32/18866	0.1825198	0.5338711	0.4830655	6	GGT1/ABHD16A/MTHFS/ALDH4A1/ABHD16B/HOGA1

Biological Process	GO:0043304	regulation of mast cell degranulation	6/2279	32/18866	0.1825198	0.5338711	0.4830655	6	GAB2/FGR/CD84/SPHK2/FER/BLK
Biological Process	GO:0046320	regulation of fatty acid oxidation	6/2279	32/18866	0.1825198	0.5338711	0.4830655	6	PRKAG2/PPARG/MFSD2A/ACACB/CPT1A/ACADVL
Biological Process	GO:0046949	fatty-acyl-CoA biosynthetic process	6/2279	32/18866	0.1825198	0.5338711	0.4830655	6	ACSL1/ACACA/ACSF3/ACAT1/SCD/SLC25A1
Biological Process	GO:0070570	regulation of neuron projection regeneration	6/2279	32/18866	0.1825198	0.5338711	0.4830655	6	KREMEN1/STK24/GRN/CERS2/NDEL1/INPP5F
Biological Process	GO:1901889	negative regulation of cell junction assembly	6/2279	32/18866	0.1825198	0.5338711	0.4830655	6	ITGB1BP1/TNF/DUSP22/THBS1/CORO1C/SLIT1
Biological Process	GO:0032515	negative regulation of phosphoprotein phosphatase activity	10/2279	60/18866	0.1825342	0.5338711	0.4830655	10	LMTK2/MGAT5/PPP1R1B/URI1/CABIN1/TNF/GNAI2/ELFN2/PHACTR2/PTPA
Biological Process	GO:0033260	nuclear DNA replication	10/2279	60/18866	0.1825342	0.5338711	0.4830655	10	POLE/BRCA2/RTEL1/MCM5/POLA2/FGFR1/MCM2/CHEK2/POLD3/POLE4
Biological Process	GO:0044784	metaphase/anaphase transition of cell cycle	10/2279	60/18866	0.1825342	0.5338711	0.4830655	10	MAD1L1/NSMCE2/CUL3/CDC16/TACC3/NEK6/RB1/ANAPC7/HECW2/PCID2
Biological Process	GO:0060042	retina morphogenesis in camera-type eye	10/2279	60/18866	0.1825342	0.5338711	0.4830655	10	MFSD2A/CRB1/HIPK2/DLL1/GNAT2/RPGRIP1/ZHX2/SDK2/NRL/CABP4

Biological Process	GO:1903428	positive regulation of reactive oxygen species biosynthetic process	10/2279	60/18866	0.1825342	0.5338711	0.4830655	10	HDAC4/SMAD3/DNM2/TNF/RAB27A/NOS1AP/MIR24-2/CLU/HBB/ITGB2
Biological Process	GO:0055006	cardiac cell development	14/2279	89/18866	0.1825462	0.5338711	0.4830655	14	CTDP1/MYO18B/TTN/MYH11/AKAP13/MAML1/SLC8A1/MIR199A1/MIR24-2/MIR23A/MIR199A2/NOTCH1/FHL2/PDGFRB
Biological Process	GO:0050851	antigen receptor-mediated signaling pathway	45/2279	325/18866	0.182974	0.5338711	0.4830655	45	PDE4D/LAT/FOXP1/PIK3CD/LCK/RUNX1/INPP5D/PIK3R1/LAX1/GRAP2/NFATC2/EIF2B5/MNDA/DUSP22/IGHV6-1/CD226/PTPN6/TNFRSF21/KLHL6/PRKCH/PTPRJ/PRKCB/LILRB4/CD79A/BLK/PTPRC/CSK/BCL10/PSMF1/THEMIS2/BCL2/PSMB7/MEF2C/DUSP3/PRAM1/ELF1/CYLD/PLCG1/RELA/FBXW11/EZR/PSMD13/VAV3/FYB1/SLA2
Biological Process	GO:0009311	oligosaccharide metabolic process	9/2279	53/18866	0.184277	0.5338711	0.4830655	9	FUT4/HEXB/ST6GALNAC1/MGAM/ST8SIA6/ST3GAL4/SLC2A1/MAN2C1/ST3GAL2
Biological Process	GO:0035384	thioester biosynthetic process	9/2279	53/18866	0.184277	0.5338711	0.4830655	9	ACSL1/ACSS1/ACACB/ACSS2/ACACA/ACSF3/ACAT1/SCD/SLC25A1
Biological Process	GO:0043124	negative regulation of I-kappaB kinase/NF-kappaB signaling	9/2279	53/18866	0.184277	0.5338711	0.4830655	9	TRIM39/RHOH/TNFAIP3/RIPK1/HDAC1/TLE1/TNIP1/PER1/PPM1N
Biological Process	GO:0045661	regulation of myoblast differentiation	9/2279	53/18866	0.184277	0.5338711	0.4830655	9	TNF/DLL1/ILK/TGFB1/PLCB1/ANKRD2/NOTCH1/CMTM5/MEF2C
Biological Process	GO:0051703	intraspecies interaction between organisms	9/2279	53/18866	0.184277	0.5338711	0.4830655	9	ATXN1/NRXN1/NRXN2/MAPK8IP2/SHANK2/LTF/CHD8/GRID1/CX3CR1
Biological Process	GO:0061756	leukocyte adhesion to vascular endothelial cell	9/2279	53/18866	0.184277	0.5338711	0.4830655	9	ELANE/FUT4/FUT7/CCR2/TNF/ETS1/ITGB2/RELA/CX3CR1

Biological Process	GO:0071616	acyl-CoA biosynthetic process	9/2279	53/18866	0.184277	0.5338711	0.4830655	9	ACSL1/ACSS1/ACACB/ACSS2/ACACA/ACSF3/ACAT1/SCD/SLC25A1
Biological Process	GO:0050890	cognition	42/2279	302/18866	0.1845616	0.5338711	0.4830655	42	PRKCZ/CREB1/MFSD2A/PPP1R1B/PRKAR1B/AMFR/TNF/SGK1/TANC1/ATP8A1/ATXN1/SLC11A2/MGAT3/COMT/APP/ZNF385A/SYNGAP1/CRHBP/HRH1/NF1/NRXN1/CPEB3/HTT/GMFB/ST3GAL4/MEIS2/NRXN2/PLCB1/MAPK8IP2/NTRK1/MAP1A/MEF2C/SHANK2/CEBPB/CYFIP1/NTAN1/NTF4/CX3CR1/CUX2/SYNPO/PRKN/DOP1B
Biological Process	GO:0006270	DNA replication initiation	7/2279	39/18866	0.1848744	0.5338711	0.4830655	7	POLE/MCM5/WRNIP1/POLA2/MCM2/KAT7/POLE4
Biological Process	GO:0009163	nucleoside biosynthetic process	7/2279	39/18866	0.1848744	0.5338711	0.4830655	7	CDA/TK2/UCK2/GUK1/DHODH/TK1/IMPDH1
Biological Process	GO:0010737	protein kinase A signaling	7/2279	39/18866	0.1848744	0.5338711	0.4830655	7	TTN/PRKAR1B/AIP/AKAP5/MYOM1/EZR/AKAP7
Biological Process	GO:0034205	amyloid-beta formation	7/2279	39/18866	0.1848744	0.5338711	0.4830655	7	SORL1/TNF/ABCG1/BIN1/APP/CLU/RELA
Biological Process	GO:0046329	negative regulation of JNK cascade	7/2279	39/18866	0.1848744	0.5338711	0.4830655	7	HIPK3/TAOK3/PER1/DUSP3/CYLD/MECOM/PRKN
Biological Process	GO:0086091	regulation of heart rate by cardiac conduction	7/2279	39/18866	0.1848744	0.5338711	0.4830655	7	KCNE1/CACNA1C/GJA5/JUP/BIN1/KCNQ1/ANK2
Biological Process	GO:1902229	regulation of intrinsic apoptotic signaling pathway in response to DNA damage	7/2279	39/18866	0.1848744	0.5338711	0.4830655	7	NACC2/ZNF385A/CLU/BCL2/BCL2L1/MARCHF7/FBH1

Biological Process	GO:0045581	negative regulation of T cell differentiation	8/2279	46/18866	0.1852058	0.5338711	0.4830655	8	RUNX1/LOXL3/SMAD7/ZBTB7B/ZC3H8/HLX/RUNX3/TMEM131L
Biological Process	GO:0048066	developmental pigmentation	8/2279	46/18866	0.1852058	0.5338711	0.4830655	8	ZEB2/RAB27A/MYO5A/GNA11/BCL2/MEF2C/HPS6/AP3D1
Biological Process	GO:1900744	regulation of p38MAPK cascade	8/2279	46/18866	0.1852058	0.5338711	0.4830655	8	MAP3K3/PHLPP1/MIR138-2/PER1/GADD45G/GDF6/CYLD/EZR
Biological Process	GO:1903050	regulation of proteolysis involved in cellular protein catabolic process	32/2279	225/18866	0.1854139	0.5338711	0.4830655	32	TRIM39/RNF144A/FZR1/RNF19A/CSNK1D/SUFU/HIPK2/CBFA2T3/UBAC2/DISC1/EDEM1/DERL2/CLU/SMAD7/SMURF1/AXIN1/CLEC16A/EDEM2/CSNK1E/PSMF1/NFE2L2/RNF144B/MAP1A/GNA12/UBE3A/RNF14/TRIB1/RNF40/BAG5/SVIP/PRKN/MARCHF7
Biological Process	GO:0044843	cell cycle G1/S phase transition	43/2279	310/18866	0.1859991	0.5338711	0.4830655	43	TRIM39/RPTOR/GFI1/BRD4/TFDP1/POLE/PRMT2/E2F3/RCC1/FBXO7/E2F7/MCM5/CUL3/POLA2/CTDSPL/PCBP4/RIPK1/PTPN6/NACC2/ZNF385A/CDK14/TAF10/LATS2/CDC25A/ARID3A/MCM2/C10orf99/GFI1B/PLCB1/CCNH/ESRRB/MIR138-2/CCNA1/RB1/BCL2/E2F6/CHEK2/BACH1/PLRG1/INHBA/POLE4/EIF4G1/ZNF324
Biological Process	GO:1903533	regulation of protein targeting	13/2279	82/18866	0.1861833	0.5338711	0.4830655	13	KCNE1/ITGB1BP1/USP36/ABLIM3/UBE2J2/ITGB2/FIS1/MYO1C/MIEF1/SREBF1/PDZK1/PRKAA1/PRKN
Biological Process	GO:0043524	negative regulation of neuron apoptotic process	22/2279	149/18866	0.186402	0.5338711	0.4830655	22	IL10/ANGPT1/ARRB1/HIPK2/ITSN1/UNC5B/ILK/SYNGAP1/GRN/PRKCI/ARRB2/NTRK1/BCL2/MEF2C/F2R/CEBPB/BCL2L1/TYRO3/CRLF1/NTF4/CX3CR1/PRKN
Biological Process	GO:1903364	positive regulation of cellular protein catabolic process	22/2279	149/18866	0.186402	0.5338711	0.4830655	22	RNF144A/FZR1/RNF19A/CSNK1D/TNFAIP3/VGLL4/CBFA2T3/DISC1/EDEM1/CLU/SMAD7/SMURF1/AXIN1/EDEM2/CSNK1E/NFE2L2/RNF144B/RNF14/TRIB1/EZR/RNF40/PRKN
Biological Process	GO:0090257	regulation of muscle system process	36/2279	256/18866	0.1869645	0.5338711	0.4830655	36	HDAC4/PDE4D/ENO1/CTDP1/CASQ2/MIR143/MIR145/CACNA1C/TNFRSF1B/SMAD3/SLC8A1/PRKCA/CTTN/MIR199A1/ANXA6/GJA5/TNNT3/JUP/BIN1/NOS1AP/TNFRSF1A/SMAD7/GHRL/CHRM2/KCNQ1/PDE9A/MIR199A2/NOTCH1/GSTO1/P2RX1/F2R/FOXO1/ANK2/CASQ1/DAPK3/GRK2

Biological Process	GO:0007188	adenylate cyclase-modulating G protein-coupled receptor signaling pathway	33/2279	233/18866	0.1876089	0.5338711	0.4830655	33	PDE4D/ADCY2/AKAP13/ADCY9/PRKCA/FPR2/FPR1/GNAZ/GNAI2/MRAP/AKAP5/GNAT2/MARCO/RAPGEF2/GRK5/GIPR/GNA11/CHRM2/ABCA1/PALM/CNR2/ADCY4/S1PR2/GNG2/GABBR1/GNAO1/LPAR2/GNA12/S1PR4/GPR4/VIPR2/ADGRG3/ADGRE3
Biological Process	GO:0042100	B cell proliferation	15/2279	97/18866	0.1894739	0.5338711	0.4830655	15	IL10/INPP5D/NFATC2/TNFSF13B/TLR9/MNDA/TNFRSF21/CD79A/BLK/PTPRC/CD320/BCL2/MEF2C/PELI1/VAV3
Biological Process	GO:0001910	regulation of leukocyte mediated cytotoxicity	12/2279	75/18866	0.1897387	0.5338711	0.4830655	12	PIK3R6/CD226/HLA-F/NCR1/RIPK3/PTPRC/ARRB2/IL18RAP/DNASE1L3/CADM1/FADD/CYRIB
Biological Process	GO:0030858	positive regulation of epithelial cell differentiation	12/2279	75/18866	0.1897387	0.5338711	0.4830655	12	VDR/ZEB2/RFX3/PAX2/PRKCH/NUMA1/ADD1/NOTCH1/S1PR2/ZBED2/LIF/MACROH2A2
Biological Process	GO:0045685	regulation of glial cell differentiation	12/2279	75/18866	0.1897387	0.5338711	0.4830655	12	PPARG/TNFRSF1B/RHEB/BIN1/TNFRSF21/HDAC1/TGFB1/NF1/WDR1/NOTCH1/RELA/LIF
Biological Process	GO:0048844	artery morphogenesis	12/2279	75/18866	0.1897387	0.5338711	0.4830655	12	MIR143/MIR145/MYLK/GJA5/RBPJ/SMAD7/WNT11/ZMIZ1/NF1/ENG/NOTCH1/PDGFRB
Biological Process	GO:0002710	negative regulation of T cell mediated immunity	4/2279	19/18866	0.1897416	0.5338711	0.4830655	4	DUSP22/HLA-F/SMAD7/PTPRC
Biological Process	GO:0006044	N-acetylglucosamine metabolic process	4/2279	19/18866	0.1897416	0.5338711	0.4830655	4	NAGK/AMDHD2/MGAT3/CHST2
Biological Process	GO:0007549	dosage compensation	4/2279	19/18866	0.1897416	0.5338711	0.4830655	4	CDYL/PCGF3/RBM15B/MACROH2A2

Biological Process	GO:0010985	negative regulation of lipoprotein particle clearance	4/2279	19/18866	0.1897416	0.5338711	0.4830655	4	MIR199A1/MIR27A/CSK/MIR199A2
Biological Process	GO:0015802	basic amino acid transport	4/2279	19/18866	0.1897416	0.5338711	0.4830655	4	SLC11A1/SLC15A4/SLC7A7/SLC7A1
Biological Process	GO:0019184	nonribosomal peptide biosynthetic process	4/2279	19/18866	0.1897416	0.5338711	0.4830655	4	GGT1/CNDP2/MGST2/NFE2L2
Biological Process	GO:0030050	vesicle transport along actin filament	4/2279	19/18866	0.1897416	0.5338711	0.4830655	4	ACTN4/WASL/MYO5A/MYO1C
Biological Process	GO:0033189	response to vitamin A	4/2279	19/18866	0.1897416	0.5338711	0.4830655	4	PPARG/RARA/CAT/ALDH1A2
Biological Process	GO:0045063	T-helper 1 cell differentiation	4/2279	19/18866	0.1897416	0.5338711	0.4830655	4	IL27/SEMA4A/IL18R1/HLX
Biological Process	GO:0045683	negative regulation of epidermis development	4/2279	19/18866	0.1897416	0.5338711	0.4830655	4	DLL1/CDH3/GRHL2/NOTCH1
Biological Process	GO:0045780	positive regulation of bone resorption	4/2279	19/18866	0.1897416	0.5338711	0.4830655	4	DEF8/PRKCA/TMEM64/PLEKHM1
Biological Process	GO:0046629	gamma-delta T cell activation	4/2279	19/18866	0.1897416	0.5338711	0.4830655	4	CCR9/PTPRC/NOD2/JAML

Biological Process	GO:0046852	positive regulation of bone remodeling	4/2279	19/18866	0.1897416	0.5338711	0.4830655	4	DEF8/PRKCA/TMEM64/PLEKHM1
Biological Process	GO:0060252	positive regulation of glial cell proliferation	4/2279	19/18866	0.1897416	0.5338711	0.4830655	4	MYB/TNF/PRKCH/PRKCI
Biological Process	GO:0070233	negative regulation of T cell apoptotic process	4/2279	19/18866	0.1897416	0.5338711	0.4830655	4	CCL5/PTCRA/DOCK8/FADD
Biological Process	GO:0098543	detection of other organism	4/2279	19/18866	0.1897416	0.5338711	0.4830655	4	NLRC4/PGLYRP1/NOD2/PGLYRP4
Biological Process	GO:0098911	regulation of ventricular cardiac muscle cell action potential	4/2279	19/18866	0.1897416	0.5338711	0.4830655	4	CACNA1C/JUP/BIN1/NOS1AP
Biological Process	GO:0150146	cell junction disassembly	4/2279	19/18866	0.1897416	0.5338711	0.4830655	4	IQSEC1/DUSP3/C1QB/CX3CR1
Biological Process	GO:1902170	cellular response to reactive nitrogen species	4/2279	19/18866	0.1897416	0.5338711	0.4830655	4	DNM2/TRAF2/AQP1/FOXO1
Biological Process	GO:1902176	negative regulation of oxidative stress-induced intrinsic apoptotic signaling pathway	4/2279	19/18866	0.1897416	0.5338711	0.4830655	4	NOL3/NFE2L2/BAG5/PRKN
Biological Process	GO:2000042	negative regulation of double-strand break repair via homologous recombination	4/2279	19/18866	0.1897416	0.5338711	0.4830655	4	RMI2/RECQL5/TP53BP1/FBH1

Biological Process	GO:2000319	regulation of T-helper 17 cell differentiation	4/2279	19/18866	0.1897416	0.5338711	0.4830655	4	NLRP3/LOXL3/SMAD7/ZBTB7B
Biological Process	GO:0005976	polysaccharide metabolic process	17/2279	112/18866	0.1910565	0.5338711	0.4830655	17	CSGALNACT1/PRKAG2/AP2A1/AOAH/MGAM/CHIT1/NDST1/TGFB1/DYRK2/PDGFB/PPP1CB/ESRRB/PER2/PCDH12/HAS3/B3GNT2/RUBCNL
Biological Process	GO:0099565	chemical synaptic transmission, postsynaptic	17/2279	112/18866	0.1910565	0.5338711	0.4830655	17	PRKCZ/SSH1/ZMYND8/BAIAP2/APP/NRXN1/GHRL/SH3GL1/MAPK8IP2/ARRB2/S1PR2/P2RX1/MEF2C/SHANK2/SLC29A1/P2RX5/CUX2
Biological Process	GO:0030879	mammary gland development	21/2279	142/18866	0.1911038	0.5338711	0.4830655	21	RREB1/NCOR2/CREB1/BRCA2/LRP5L/VDR/CSF1R/LBH/STAT5A/RTN4/TGFBR2/ZBTB7B/HK2/NCOA1/HOXA9/NOTCH4/USF2/CEBPB/SLC29A1/ATP7B/DHODH
Biological Process	GO:1903052	positive regulation of proteolysis involved in cellular protein catabolic process	19/2279	127/18866	0.1914837	0.5338711	0.4830655	19	RNF144A/FZR1/RNF19A/CSNK1D/CBFA2T3/DISC1/EDEM1/CLU/SMAD7/SMURF1/AXIN1/EDEM2/CSNK1E/NFE2L2/RNF144B/RNF14/TRIB1/RNF40/PRKN
Biological Process	GO:0022406	membrane docking	26/2279	180/18866	0.1918693	0.5338711	0.4830655	26	DCTN1/STX5/CSNK1D/NDE1/PACS2/DYNC1H1/TUBB4B/SNX3/CEP135/CEP164/DCTN2/CEP78/VPS18/CTBP2/ATG14/CSNK1E/PPP2R1A/TUBA4A/SDCCAG8/PDZD8/ESYT1/CEP72/EZR/TSNARE1/NPHP4/GRAMD2A
Biological Process	GO:0032642	regulation of chemokine production	11/2279	68/18866	0.1931463	0.5338711	0.4830655	11	ZFPM1/FOXP1/IL10/C1QTNF3/MEFV/TNF/TLR9/MBP/DEFB124/IL6R/ACKR1
Biological Process	GO:0034381	plasma lipoprotein particle clearance	11/2279	68/18866	0.1931463	0.5338711	0.4830655	11	EHD1/AP2A1/LIPC/MIR199A1/GPLD1/HDLBP/MIR27A/CSK/NPC1/MIR199A2/LMF1
Biological Process	GO:0045428	regulation of nitric oxide biosynthetic process	11/2279	68/18866	0.1931463	0.5338711	0.4830655	11	IL10/SMAD3/DNM2/MIR199A1/TNF/NOS1AP/CLU/HBB/ITGB2/MIR199A2/CX3CR1

Biological Process	GO:0072577	endothelial cell apoptotic process	11/2279	68/18866	0.1931463	0.5338711	0.4830655	11	IL10/ANGPT1/TNF/TNFAIP3/MIR101-2/THBS1/MIR24-2/PRKCI/NFE2L2/ANO6/PLCG1
Biological Process	GO:0051961	negative regulation of nervous system development	44/2279	319/18866	0.1932168	0.5338711	0.4830655	44	SEMA6B/CTSZ/GFI1/SORL1/CALR/KANK1/LSM1/YWHAH/SKI/SUFU/TNF/BCL11A/SEMA4A/RTN4/PMP22/SEMA4B/DLL1/APP/MIB1/IFRD1/TNFRSF21/ASAP1/SYNGAP1/KREMEN1/SEMA4D/RAPGEF2/NF1/ITM2C/MEIS1/DNM3/NOTCH1/ZHX2/CDKL3/UBE3A/ARHGEF2/ARHGEF15/CERS2/INPP5F/SLIT1/BAG5/RAP1GAP2/HES3/PRAG1/SPART
Biological Process	GO:0090501	RNA phosphodiester bond hydrolysis	24/2279	165/18866	0.1936121	0.5338711	0.4830655	24	PIWIL4/RNASE3/FIP1L1/EXOSC2/ERN1/CPSF3/SLFN14/RCL1/AGO1/ZC3H12D/RNASE10/AGO2/EDC3/NOB1/ERI3/SMG6/RMRP/NOP14/DIS3L2/SND1/TOE1/CPSF4/TUT1/ISG20
Biological Process	GO:0032465	regulation of cytokinesis	14/2279	90/18866	0.1937099	0.5338711	0.4830655	14	BRCA2/SETD2/KIF13A/E2F7/CUL3/GIT1/UVRAG/SVIL/CSPP1/ZFYVE19/KLHL21/PRKCE/BCL2L1/CDC14A
Biological Process	GO:0051783	regulation of nuclear division	27/2279	188/18866	0.1947584	0.5338711	0.4830655	27	CALR/FZR1/TGFA/MAD1L1/TNKS/NSMCE2/RCC1/CUL3/CCDC8/CDC16/TACC3/NUMA1/PDGFB/PLCB1/NEK6/RB1/CDCA5/ANAPC7/PDGFRB/HECW2/CDC14A/AURKAIP1/LIF/DAPK3/CDK13/PCID2/SLF1
Biological Process	GO:0031396	regulation of protein ubiquitination	30/2279	211/18866	0.1949549	0.5338711	0.4830655	30	FZR1/ANGPT1/ARRB1/TNFAIP3/MIR101-2/CUL3/OTUB2/SMAD7/AXIN1/MTA1/BCL10/ARRB2/MIR138-2/PER2/OTUB1/NOD2/NXN/DNM1L/PRKCE/UBXN2A/UBE3A/TRIP12/DYSF/RNF40/BAG5/PARP10/PELI1/PAXIP1/PRKN/MARCHF7
Biological Process	GO:0070665	positive regulation of leukocyte proliferation	22/2279	150/18866	0.1950197	0.5338711	0.4830655	22	HLA-DMB/CD55/CCR2/CCL5/CSF1R/NCK2/NFATC2/TNFSF13B/TLR9/TGFBR2/CCDC88B/PTPRC/CD320/SPTA1/ZNF335/PNP/BCL2/MEF2C/SLC7A1/FADD/PELI1/VAV3
Biological Process	GO:0070085	glycosylation	37/2279	265/18866	0.1952408	0.5338711	0.4830655	37	FUT4/GALNT2/FUT7/RPN1/XXYLT1/SLC35C2/MGAT5/ST6GALNAC1/GALNT12/MGAT4A/MGAT3/MUC12/ASGR2/GXYLT2/B4GALT7/POMT2/MUC20/B3GNT5/ST6GAL1/ST8SIA6/ST3GAL4/TET2/B4GALT3/PMM2/NPC1/GALNT9/FUT11/GCNT2/GFPT2/LMF1/ALG11/CLN5/MAN1C1/DPAGT1/ST3GAL2/B3GNT2/B4GAT1
Biological Process	GO:0002381	immunoglobulin production involved in immunoglobulin mediated immune response	10/2279	61/18866	0.1963085	0.5338711	0.4830655	10	IL10/UNG/TGFB1/PTPRC/POU2F2/TP53BP1/ERCC1/TNFSF13/PAXIP1/NSD2

Biological Process	GO:0010965	regulation of mitotic sister chromatid separation	10/2279	61/18866	0.1963085	0.5338711	0.4830655	10	MAD1L1/NSMCE2/CUL3/CDC16/TACC3/NEK6/RB1/ANAPC7/HECW2/PCID2
Biological Process	GO:0042733	embryonic digit morphogenesis	10/2279	61/18866	0.1963085	0.5338711	0.4830655	10	CHST11/ZBTB16/HDAC1/GRHL2/IFT140/CREBBP/NOTCH1/GNA12/MAP3K20/CPLANE1
Biological Process	GO:0033209	tumor necrosis factor-mediated signaling pathway	25/2279	173/18866	0.1968258	0.5338711	0.4830655	25	AIM2/TNFRSF1B/TNF/TRAF2/TNFAIP3/TRAF1/TNFSF13B/TRAIP/RIPK1/ACTN4/MIR24-2/TNFRSF1A/ILK/CPNE1/CDIP1/PSMF1/MAP3K14/PSMB7/CASP1/TNFSF12/TNFSF13/CYLD/RELA/PSMD13/PRKN
Biological Process	GO:0006260	DNA replication	38/2279	273/18866	0.1968993	0.5338711	0.4830655	38	BLM/POLE/BRCA2/RMI2/TOP1MT/RTEL1/HUS1/NFIC/NFIX/E2F7/MCM5/WRNIP1/ZBTB38/RECQL5/POLA2/EME1/RRM2B/EHMT2/IGHMBP2/FGFR1/MCM2/SMARCAL1/PPP2R1A/GINS3/CLSPN/PTMS/RHNO1/CHEK2/BCAR3/SLX4/CHAF1A/KAT7/POLD3/ING5/POLE4/POLI/NFIA/FBH1
Biological Process	GO:0050728	negative regulation of inflammatory response	28/2279	196/18866	0.1974557	0.5338711	0.4830655	28	ELANE/ABR/IL10/PPARG/C1QTNF3/MIR140/MIR145/TNFRSF1B/NLRP3/SMAD3/MEFV/SMPDL3B/TNFAIP3/AOAH/FPR2/TNFAIP6/TNFRSF1A/CXCL17/PGLYRP1/GHRL/PTPRC/GRN/BCR/CNR2/ETS1/MIR138-2/RB1/TYRO3
Biological Process	GO:0002088	lens development in camera-type eye	13/2279	83/18866	0.1979736	0.5338711	0.4830655	13	SPRED2/FZR1/SKI/SMAD3/ZEB2/HIPK2/TGFBR2/CRYBB1/WNT5B/MEIS1/BCAR3/TMOD1/NECTIN1
Biological Process	GO:0048708	astrocyte differentiation	13/2279	83/18866	0.1979736	0.5338711	0.4830655	13	S100A8/TNF/FPR2/EIF2B5/BIN1/DLL1/APP/NF1/GRN/TTC21B/NOTCH1/TSPAN2/LIF
Biological Process	GO:0055013	cardiac muscle cell development	13/2279	83/18866	0.1979736	0.5338711	0.4830655	13	CTDP1/MYO18B/TTN/MYH11/AKAP13/MAML1/SLC8A1/MIR199A1/MIR24-2/MIR23A/MIR199A2/FHL2/PDGFRB
Biological Process	GO:0055117	regulation of cardiac muscle contraction	13/2279	83/18866	0.1979736	0.5338711	0.4830655	13	HDAC4/PDE4D/CASQ2/CACNA1C/SLC8A1/GJA5/JUP/BIN1/NOS1AP/SMAD7/KCNQ1/GSTO1/ANK2

Biological Process	GO:0002507	tolerance induction	5/2279	26/18866	0.1985201	0.5338711	0.4830655	5	TNFAIP3/PHLPP1/TGFBR2/BLK/MARCHF7
Biological Process	GO:0003171	atrioventricular valve development	5/2279	26/18866	0.1985201	0.5338711	0.4830655	5	ZFPM1/GJA5/TGFBR2/NOTCH1/SLIT3
Biological Process	GO:0021952	central nervous system projection neuron axonogenesis	5/2279	26/18866	0.1985201	0.5338711	0.4830655	5	ZEB2/EPHB3/CDH11/SPTBN4/TSKU
Biological Process	GO:0032743	positive regulation of interleukin-2 production	5/2279	26/18866	0.1985201	0.5338711	0.4830655	5	PDE4D/RUNX1/CCR2/TRAF2/PTPRC
Biological Process	GO:0035902	response to immobilization stress	5/2279	26/18866	0.1985201	0.5338711	0.4830655	5	PPARG/SLC8A1/BRD1/ERCC1/HNMT
Biological Process	GO:0042730	fibrinolysis	5/2279	26/18866	0.1985201	0.5338711	0.4830655	5	PLAU/SERPINB2/THBS1/F12/CPB2
Biological Process	GO:0044068	modulation by symbiont of host cellular process	5/2279	26/18866	0.1985201	0.5338711	0.4830655	5	ULK1/PABPN1/TNIP1/BCL2L1/CPSF4
Biological Process	GO:0046174	polyol catabolic process	5/2279	26/18866	0.1985201	0.5338711	0.4830655	5	SYNJ2/INPP5A/NUDT3/INPP5E/IMPA2
Biological Process	GO:0051560	mitochondrial calcium ion homeostasis	5/2279	26/18866	0.1985201	0.5338711	0.4830655	5	ANXA6/DISC1/IMMT/FIS1/PDZD8

Biological Process	GO:0060219	camera-type eye photoreceptor cell differentiation	5/2279	26/18866	0.1985201	0.5338711	0.4830655	5	GNAT2/RPGRIP1/SDK2/NRL/CABP4
Biological Process	GO:0060390	regulation of SMAD protein signal transduction	5/2279	26/18866	0.1985201	0.5338711	0.4830655	5	TGFB1/NUP93/WWTR1/RBPMS/NODAL
Biological Process	GO:0090023	positive regulation of neutrophil chemotaxis	5/2279	26/18866	0.1985201	0.5338711	0.4830655	5	C3AR1/CAMK1D/DAPK2/DNM1L/DYSF
Biological Process	GO:0035051	cardiocyte differentiation	23/2279	158/18866	0.1986293	0.5338711	0.4830655	23	RXRA/CALR/CTDP1/MYO18B/TTN/MIR145/MYH11/AKAP13/MAML1/SLC8A1/MIR199A1/DLL1/RBPJ/MIR24-2/MIR23A/TGFB1/RARA/ARRB2/MIR199A2/NOTCH1/MEF2C/FHL2/PDGFRB
Biological Process	GO:0006968	cellular defense response	9/2279	54/18866	0.1990781	0.5338711	0.4830655	9	CCR2/MNDA/CLEC5A/NCF2/CCR9/NCR1/BCL10/RELA/CX3CR1
Biological Process	GO:0031050	dsRNA processing	9/2279	54/18866	0.1990781	0.5338711	0.4830655	9	NCOR2/ZC3H7A/SMAD3/TNF/AGO1/RIPK1/AGO2/TGFB1/RMRP
Biological Process	GO:0070918	production of small RNA involved in gene silencing by RNA	9/2279	54/18866	0.1990781	0.5338711	0.4830655	9	NCOR2/ZC3H7A/SMAD3/TNF/AGO1/RIPK1/AGO2/TGFB1/RMRP
Biological Process	GO:1901617	organic hydroxy compound biosynthetic process	36/2279	258/18866	0.2001323	0.5338711	0.4830655	36	PRKAG2/GFI1/SPTLC2/IDI1/VDR/ITPKB/AMACR/MBTPS1/TNF/ZEB2/ABCG1/PGP/PLTP/AKR1D1/DDC/OSBPL6/CDH3/P2RY6/SPHK2/RAPGEF2/HRH1/DGKQ/POR/MYO5A/ACACB/IMPA2/PDXK/ACOX2/PER2/ACACA/PCK2/HMGCS1/SCD/SREBF1/PRKAA1/IP6K1
Biological Process	GO:0050810	regulation of steroid biosynthetic process	15/2279	98/18866	0.2003684	0.5338711	0.4830655	15	GFI1/IDI1/VDR/MBTPS1/TNF/ABCG1/DGKQ/SF1/POR/ACACB/ACACA/HMGCS1/SCD/SREBF1/PRKAA1

Biological Process	GO:2001243	negative regulation of intrinsic apoptotic signaling pathway	15/2279	98/18866	0.2003684	0.5338711	0.4830655	15	ENO1/URI1/RRM2B/ZNF385A/CLU/HDAC1/NOL3/NFE2L2/BCL2/ARHGEF2/BCL2L1/BAG5/GRINA/PRKN/MARCHF7
Biological Process	GO:0000722	telomere maintenance via recombination	3/2279	13/18866	0.2011933	0.5338711	0.4830655	3	BRCA2/NSMCE2/ERCC1
Biological Process	GO:0002679	respiratory burst involved in defense response	3/2279	13/18866	0.2011933	0.5338711	0.4830655	3	MPO/PIK3CD/GRN
Biological Process	GO:0002903	negative regulation of B cell apoptotic process	3/2279	13/18866	0.2011933	0.5338711	0.4830655	3	FOXP1/HSH2D/BCL10
Biological Process	GO:0002921	negative regulation of humoral immune response	3/2279	13/18866	0.2011933	0.5338711	0.4830655	3	CD55/PTPN6/CD59
Biological Process	GO:0003161	cardiac conduction system development	3/2279	13/18866	0.2011933	0.5338711	0.4830655	3	MAML1/GJA5/NOTCH1
Biological Process	GO:0007183	SMAD protein complex assembly	3/2279	13/18866	0.2011933	0.5338711	0.4830655	3	SMAD3/LDLRAD4/TGFB1
Biological Process	GO:0007501	mesodermal cell fate specification	3/2279	13/18866	0.2011933	0.5338711	0.4830655	3	PAX2/FGFR1/NODAL
Biological Process	GO:0009265	2'-deoxyribonucleotide biosynthetic process	3/2279	13/18866	0.2011933	0.5338711	0.4830655	3	GUK1/AK5/DCTD

Biological Process	GO:0009437	carnitine metabolic process	3/2279	13/18866	0.2011933	0.5338711	0.4830655	3	ACADM/POR/CPT1A
Biological Process	GO:0010623	programmed cell death involved in cell development	3/2279	13/18866	0.2011933	0.5338711	0.4830655	3	NTRK1/DNASE1L3/BCL2
Biological Process	GO:0010635	regulation of mitochondrial fusion	3/2279	13/18866	0.2011933	0.5338711	0.4830655	3	VAT1/DNM1L/PRKN
Biological Process	GO:0010958	regulation of amino acid import across plasma membrane	3/2279	13/18866	0.2011933	0.5338711	0.4830655	3	SLC43A2/PER2/SLC43A1
Biological Process	GO:0015791	polyol transport	3/2279	13/18866	0.2011933	0.5338711	0.4830655	3	AQP1/AQP3/AQP9
Biological Process	GO:0018095	protein polyglutamylation	3/2279	13/18866	0.2011933	0.5338711	0.4830655	3	TTLL4/TTLL11/TTLL1
Biological Process	GO:0019755	one-carbon compound transport	3/2279	13/18866	0.2011933	0.5338711	0.4830655	3	AQP1/AQP3/AQP9
Biological Process	GO:0031665	negative regulation of lipopolysaccharide-mediated signaling pathway	3/2279	13/18866	0.2011933	0.5338711	0.4830655	3	TNFAIP3/LTF/TRIB1
Biological Process	GO:0035723	interleukin-15-mediated signaling pathway	3/2279	13/18866	0.2011933	0.5338711	0.4830655	3	STAT5A/JAK1/PLCB1

Biological Process	GO:0042635	positive regulation of hair cycle	3/2279	13/18866	0.2011933	0.5338711	0.4830655	3	TNF/KRT17/NUMA1
Biological Process	GO:0043247	telomere maintenance in response to DNA damage	3/2279	13/18866	0.2011933	0.5338711	0.4830655	3	RTEL1/ERCC1/TFIP11
Biological Process	GO:0046349	amino sugar biosynthetic process	3/2279	13/18866	0.2011933	0.5338711	0.4830655	3	NAGK/AMDHD2/GFPT2
Biological Process	GO:0046385	deoxyribose phosphate biosynthetic process	3/2279	13/18866	0.2011933	0.5338711	0.4830655	3	GUK1/AK5/DCTD
Biological Process	GO:0046415	urate metabolic process	3/2279	13/18866	0.2011933	0.5338711	0.4830655	3	SLC2A9/SLC22A11/PNP
Biological Process	GO:0048548	regulation of pinocytosis	3/2279	13/18866	0.2011933	0.5338711	0.4830655	3	ANKFY1/ACTN4/NR1H2
Biological Process	GO:0048711	positive regulation of astrocyte differentiation	3/2279	13/18866	0.2011933	0.5338711	0.4830655	3	BIN1/NOTCH1/LIF
Biological Process	GO:0051001	negative regulation of nitric-oxide synthase activity	3/2279	13/18866	0.2011933	0.5338711	0.4830655	3	CNR2/MIR138-2/NOSIP
Biological Process	GO:0060100	positive regulation of phagocytosis, engulfment	3/2279	13/18866	0.2011933	0.5338711	0.4830655	3	PPARG/RAB31/ANO6

Biological Process	GO:0061000	negative regulation of dendritic spine development	3/2279	13/18866	0.2011933	0.5338711	0.4830655	3	ASAP1/DNM3/UBE3A
Biological Process	GO:0061052	negative regulation of cell growth involved in cardiac muscle cell development	3/2279	13/18866	0.2011933	0.5338711	0.4830655	3	CTDP1/MIR199A1/MIR199A2
Biological Process	GO:0070914	UV-damage excision repair	3/2279	13/18866	0.2011933	0.5338711	0.4830655	3	ERCC1/POLD3/XPA
Biological Process	GO:0071287	cellular response to manganese ion	3/2279	13/18866	0.2011933	0.5338711	0.4830655	3	SLFN14/APP/PRKN
Biological Process	GO:0071350	cellular response to interleukin-15	3/2279	13/18866	0.2011933	0.5338711	0.4830655	3	STAT5A/JAK1/PLCB1
Biological Process	GO:0072182	regulation of nephron tubule epithelial cell differentiation	3/2279	13/18866	0.2011933	0.5338711	0.4830655	3	PAX2/WWTR1/LIF
Biological Process	GO:0072683	T cell extravasation	3/2279	13/18866	0.2011933	0.5338711	0.4830655	3	CCR2/RIPK3/FADD
Biological Process	GO:0072710	response to hydroxyurea	3/2279	13/18866	0.2011933	0.5338711	0.4830655	3	BLM/SPIDR/KAT7
Biological Process	GO:0098734	macromolecule depalmitoylation	3/2279	13/18866	0.2011933	0.5338711	0.4830655	3	ABHD16A/ABHD16B/LYPLA2

Biological Process	GO:0098789	pre-mRNA cleavage required for polyadenylation	3/2279	13/18866	0.2011933	0.5338711	0.4830655	3	FIP1L1/CPSF4/TUT1
Biological Process	GO:0098974	postsynaptic actin cytoskeleton organization	3/2279	13/18866	0.2011933	0.5338711	0.4830655	3	NOS1AP/ACTG1/DBNL
Biological Process	GO:0150105	protein localization to cell-cell junction	3/2279	13/18866	0.2011933	0.5338711	0.4830655	3	ACTN4/ACTG1/MPP7
Biological Process	GO:1902306	negative regulation of sodium ion transmembrane transport	3/2279	13/18866	0.2011933	0.5338711	0.4830655	3	PRKCE/NEDD4/HECW2
Biological Process	GO:1902947	regulation of tau-protein kinase activity	3/2279	13/18866	0.2011933	0.5338711	0.4830655	3	SORL1/CLU/RB1
Biological Process	GO:1903651	positive regulation of cytoplasmic transport	3/2279	13/18866	0.2011933	0.5338711	0.4830655	3	SORL1/EZR/PRKN
Biological Process	GO:1903789	regulation of amino acid transmembrane transport	3/2279	13/18866	0.2011933	0.5338711	0.4830655	3	SLC43A2/PER2/SLC43A1
Biological Process	GO:1905155	positive regulation of membrane invagination	3/2279	13/18866	0.2011933	0.5338711	0.4830655	3	PPARG/RAB31/ANO6
Biological Process	GO:1905244	regulation of modification of synaptic structure	3/2279	13/18866	0.2011933	0.5338711	0.4830655	3	ITSN1/BAIAP2/CYFIP1

Biological Process	GO:2000052	positive regulation of non-canonical Wnt signaling pathway	3/2279	13/18866	0.2011933	0.5338711	0.4830655	3	CSNK1D/CSNK1E/ANKRD6
Biological Process	GO:2000121	regulation of removal of superoxide radicals	3/2279	13/18866	0.2011933	0.5338711	0.4830655	3	TNF/NFE2L2/SIRT3
Biological Process	GO:0010559	regulation of glycoprotein biosynthetic process	8/2279	47/18866	0.2012253	0.5338711	0.4830655	8	MIR101-2/TCF7L2/AGO2/POMT2/ITM2C/PLCB1/BCL2/PXYLP1
Biological Process	GO:0035850	epithelial cell differentiation involved in kidney development	8/2279	47/18866	0.2012253	0.5338711	0.4830655	8	PAX2/MTSS1/WWTR1/PDGFB/NOTCH1/MEF2C/GPR4/LIF
Biological Process	GO:0043303	mast cell degranulation	8/2279	47/18866	0.2012253	0.5338711	0.4830655	8	LAT/PIK3CD/GAB2/FGR/CD84/SPHK2/FER/BLK
Biological Process	GO:0044088	regulation of vacuole organization	8/2279	47/18866	0.2012253	0.5338711	0.4830655	8	ENO1/ULK1/PIP4K2A/FEZ2/ENO3/GRN/TBC1D14/NPRL2
Biological Process	GO:0050919	negative chemotaxis	8/2279	47/18866	0.2012253	0.5338711	0.4830655	8	SEMA6B/NRP2/SEMA4A/SEMA4B/SEMA4D/ITGB3/SLIT3/SLIT1
Biological Process	GO:0071356	cellular response to tumor necrosis factor	41/2279	297/18866	0.2013744	0.5338711	0.4830655	41	HDAC4/AIM2/LCN2/CHI3L1/TNFRSF1B/TNF/TRAF2/TNFAIP3/CCL5/TRAF1/TNFSF13B/ABCC2/TRAIP/THBS1/RIPK1/ACTN4/MIR24-2/TNFRSF1A/ILK/TNFRSF21/CPNE1/CRHBP/SGMS1/ADAMTS13/CDIP1/PRPF8/CCL20/PSMF1/MAP3K14/NFE2L2/PSMB7/ARHGEF2/CASP1/TNFSF12/TNFSF13/CYLD/ANKRD1/RELA/CCL22/PSMD13/PRKN
Biological Process	GO:0009648	photoperiodism	6/2279	33/18866	0.2018785	0.5338711	0.4830655	6	MTA1/PPP1CB/PER2/PER1/FBXL6/USP2

Biological Process	GO:0033006	regulation of mast cell activation involved in immune response	6/2279	33/18866	0.2018785	0.5338711	0.4830655	6	GAB2/FGR/CD84/SPHK2/FER/BLK
Biological Process	GO:0035115	embryonic forelimb morphogenesis	6/2279	33/18866	0.2018785	0.5338711	0.4830655	6	CACNA1C/RDH10/RUNX2/HOXA9/ALX3/ALDH1A2
Biological Process	GO:0035590	purinergic nucleotide receptor signaling pathway	6/2279	33/18866	0.2018785	0.5338711	0.4830655	6	GNAI2/P2RY6/P2RX1/ANO6/P2RX5/ACP3
Biological Process	GO:0040018	positive regulation of multicellular organism growth	6/2279	33/18866	0.2018785	0.5338711	0.4830655	6	CREB1/SH3PXD2B/GHRL/BCL2/EZR/SPTBN4
Biological Process	GO:0046640	regulation of alpha-beta T cell proliferation	6/2279	33/18866	0.2018785	0.5338711	0.4830655	6	CD55/CCR2/TGFBR2/ZBTB7B/PTPRC/VSIR
Biological Process	GO:1905048	regulation of metallopeptidase activity	6/2279	33/18866	0.2018785	0.5338711	0.4830655	6	TIMP2/SORL1/MIR199A1/MBP/MIR24-2/MIR199A2
Biological Process	GO:1905508	protein localization to microtubule organizing center	6/2279	33/18866	0.2018785	0.5338711	0.4830655	6	CSNK1D/DISC1/DCTN2/NUMA1/SPAG5/CEP72
Biological Process	GO:0035967	cellular response to topologically incorrect protein	24/2279	166/18866	0.2019641	0.5338711	0.4830655	24	DCTN1/CALR/ERN1/PIK3R1/AMFR/MBTPS1/RHBDD2/NCK2/YIF1A/CUL3/EDEM1/DERL2/ZBTB17/PDIA5/CLGN/POMT2/COPS5/CREB3L2/NFE2L2/ADD1/KLHDC3/SEC31A/ACADVL/PRKN
Biological Process	GO:0009583	detection of light stimulus	12/2279	76/18866	0.2022324	0.5338711	0.4830655	12	RCVRN/PDE6A/CACNA2D4/CRB1/ARRB1/PITPNM1/CCDC66/GNAT2/GNA11/GRK7/EYS/CABP4

Biological Process	GO:1901215	negative regulation of neuron death	30/2279	212/18866	0.2023384	0.5338711	0.4830655	30	SORL1/IL10/CREB1/ANGPT1/TNFRSF1B/ARRB1/TRAF2/FBXO7/HIPK2/ITSN1/UNC5B/HLA-F/ILK/SYNGAP1/CSF3/GRN/PRKCI/ARRB2/NTRK1/BCL2/MEF2C/F2R/CEBPB/BCL2L1/TYRO3/CRLF1/NTF4/CX3CR1/EIF4G1/PRKN
Biological Process	GO:0001990	regulation of systemic arterial blood pressure by hormone	7/2279	40/18866	0.202375	0.5338711	0.4830655	7	CTSZ/CTSG/GJA5/F2R/AGTR1/HSD11B2/AOPEP
Biological Process	GO:0006284	base-excision repair	7/2279	40/18866	0.202375	0.5338711	0.4830655	7	FAM168A/POLE/NEIL3/UNG/NTHL1/MUTYH/XPA
Biological Process	GO:0009126	purine nucleoside monophosphate metabolic process	7/2279	40/18866	0.202375	0.5338711	0.4830655	7	AMPD3/TJP2/GUK1/AK2/GMPR2/LHPP/IMPDH1
Biological Process	GO:0010939	regulation of necrotic cell death	7/2279	40/18866	0.202375	0.5338711	0.4830655	7	MIR101-2/RIPK1/RIPK3/ARHGEF2/CYLD/FADD/PELI1
Biological Process	GO:0034314	Arp2/3 complex-mediated actin nucleation	7/2279	40/18866	0.202375	0.5338711	0.4830655	7	TRIM27/WHAMM/WASL/GMFB/NCKAP1/CYFIP1/GMFG
Biological Process	GO:0046636	negative regulation of alpha-beta T cell activation	7/2279	40/18866	0.202375	0.5338711	0.4830655	7	RUNX1/LOXL3/SMAD7/ZBTB7B/HLX/RUNX3/VSIR
Biological Process	GO:0050691	regulation of defense response to virus by host	7/2279	40/18866	0.202375	0.5338711	0.4830655	7	IL27/AIM2/TNFAIP3/ZDHHC1/TRAF3IP2/PARP9/STING1
Biological Process	GO:0090184	positive regulation of kidney development	7/2279	40/18866	0.202375	0.5338711	0.4830655	7	PAX2/PDGFD/IL6R/PDGFB/PDGFRB/LIF/HOXB7

Biological Process	GO:1902742	apoptotic process involved in development	7/2279	40/18866	0.202375	0.5338711	0.4830655	7	TNFRSF1B/VDR/TNFRSF1A/PAX2/NOTCH1/PPP2R1B/SLIT3
Biological Process	GO:1902991	regulation of amyloid precursor protein catabolic process	7/2279	40/18866	0.202375	0.5338711	0.4830655	7	SORL1/TNF/ABCG1/BIN1/APP/CLU/RELA
Biological Process	GO:0002248	connective tissue replacement involved in inflammatory response wound healing	2/2279	7/18866	0.2033338	0.5338711	0.4830655	2	TGFB1/F2R
Biological Process	GO:0002396	MHC protein complex assembly	2/2279	7/18866	0.2033338	0.5338711	0.4830655	2	HLA-DMB/CALR
Biological Process	GO:0002606	positive regulation of dendritic cell antigen processing and presentation	2/2279	7/18866	0.2033338	0.5338711	0.4830655	2	SLC11A1/NOD2
Biological Process	GO:0003256	regulation of transcription from RNA polymerase II promoter involved in myocardial precursor cell differentiation	2/2279	7/18866	0.2033338	0.5338711	0.4830655	2	RBPJ/NOTCH1
Biological Process	GO:0006573	valine metabolic process	2/2279	7/18866	0.2033338	0.5338711	0.4830655	2	ACAD8/HIBADH
Biological Process	GO:0009445	putrescine metabolic process	2/2279	7/18866	0.2033338	0.5338711	0.4830655	2	ODC1/SAT2
Biological Process	GO:0009992	cellular water homeostasis	2/2279	7/18866	0.2033338	0.5338711	0.4830655	2	AQP1/ANXA7

Biological Process	GO:0010216	maintenance of DNA methylation	2/2279	7/18866	0.2033338	0.5338711	0.4830655	2	UHRF1/DNMT1
Biological Process	GO:0010536	positive regulation of activation of Janus kinase activity	2/2279	7/18866	0.2033338	0.5338711	0.4830655	2	CCL5/IL6R
Biological Process	GO:0015727	lactate transport	2/2279	7/18866	0.2033338	0.5338711	0.4830655	2	SLC16A3/SLC16A1
Biological Process	GO:0015825	L-serine transport	2/2279	7/18866	0.2033338	0.5338711	0.4830655	2	SLC1A5/SFXN1
Biological Process	GO:0015871	choline transport	2/2279	7/18866	0.2033338	0.5338711	0.4830655	2	SLC44A4/SEC14L1
Biological Process	GO:0019065	receptor-mediated endocytosis of virus by host cell	2/2279	7/18866	0.2033338	0.5338711	0.4830655	2	TPCN2/EPS15
Biological Process	GO:0019401	alditol biosynthetic process	2/2279	7/18866	0.2033338	0.5338711	0.4830655	2	PGP/PCK2
Biological Process	GO:0019673	GDP-mannose metabolic process	2/2279	7/18866	0.2033338	0.5338711	0.4830655	2	GUK1/PMM2
Biological Process	GO:0021957	corticospinal tract morphogenesis	2/2279	7/18866	0.2033338	0.5338711	0.4830655	2	ZEB2/CDH11

Biological Process	GO:0030174	regulation of DNA-dependent DNA replication initiation	2/2279	7/18866	0.2033338	0.5338711	0.4830655	2	WRNIP1/KAT7
Biological Process	GO:0030578	PML body organization	2/2279	7/18866	0.2033338	0.5338711	0.4830655	2	HIPK2/ETS1
Biological Process	GO:0032226	positive regulation of synaptic transmission, dopaminergic	2/2279	7/18866	0.2033338	0.5338711	0.4830655	2	ARRB2/FLOT1
Biological Process	GO:0032466	negative regulation of cytokinesis	2/2279	7/18866	0.2033338	0.5338711	0.4830655	2	E2F7/ZFYVE19
Biological Process	GO:0032483	regulation of Rab protein signal transduction	2/2279	7/18866	0.2033338	0.5338711	0.4830655	2	DENND1A/DENND3
Biological Process	GO:0032510	endosome to lysosome transport via multivesicular body sorting pathway	2/2279	7/18866	0.2033338	0.5338711	0.4830655	2	LYST/MVB12A
Biological Process	GO:0032532	regulation of microvillus length	2/2279	7/18866	0.2033338	0.5338711	0.4830655	2	CDHR2/EZR
Biological Process	GO:0032847	regulation of cellular pH reduction	2/2279	7/18866	0.2033338	0.5338711	0.4830655	2	BCL2/UBE3A
Biological Process	GO:0032960	regulation of inositol trisphosphate biosynthetic process	2/2279	7/18866	0.2033338	0.5338711	0.4830655	2	P2RY6/HRH1

Biological Process	GO:0033029	regulation of neutrophil apoptotic process	2/2279	7/18866	0.2033338	0.5338711	0.4830655	2	PIK3CD/ITPKB
Biological Process	GO:0033864	positive regulation of NAD(P)H oxidase activity	2/2279	7/18866	0.2033338	0.5338711	0.4830655	2	GNAI2/AGTR1
Biological Process	GO:0035280	miRNA loading onto RISC involved in gene silencing by miRNA	2/2279	7/18866	0.2033338	0.5338711	0.4830655	2	AGO1/AGO2
Biological Process	GO:0035405	histone-threonine phosphorylation	2/2279	7/18866	0.2033338	0.5338711	0.4830655	2	PRKCA/PRKCB
Biological Process	GO:0035873	lactate transmembrane transport	2/2279	7/18866	0.2033338	0.5338711	0.4830655	2	SLC16A3/SLC16A1
Biological Process	GO:0040009	regulation of growth rate	2/2279	7/18866	0.2033338	0.5338711	0.4830655	2	GHRL/NOTCH1
Biological Process	GO:0045002	double-strand break repair via single-strand annealing	2/2279	7/18866	0.2033338	0.5338711	0.4830655	2	RAD52/SLX4
Biological Process	GO:0045110	intermediate filament bundle assembly	2/2279	7/18866	0.2033338	0.5338711	0.4830655	2	PKP1/NEFM
Biological Process	GO:0045162	clustering of voltage-gated sodium channels	2/2279	7/18866	0.2033338	0.5338711	0.4830655	2	SPTBN4/GLDN

Biological Process	GO:0050942	positive regulation of pigment cell differentiation	2/2279	7/18866	0.2033338	0.5338711	0.4830655	2	ZEB2/BCL2
Biological Process	GO:0051388	positive regulation of neurotrophin TRK receptor signaling pathway	2/2279	7/18866	0.2033338	0.5338711	0.4830655	2	CYFIP2/CYFIP1
Biological Process	GO:0051458	corticotropin secretion	2/2279	7/18866	0.2033338	0.5338711	0.4830655	2	CRHBP/GHRL
Biological Process	GO:0051694	pointed-end actin filament capping	2/2279	7/18866	0.2033338	0.5338711	0.4830655	2	TMOD3/TMOD1
Biological Process	GO:0051902	negative regulation of mitochondrial depolarization	2/2279	7/18866	0.2033338	0.5338711	0.4830655	2	HSH2D/BCL2
Biological Process	GO:0060331	negative regulation of response to interferon-gamma	2/2279	7/18866	0.2033338	0.5338711	0.4830655	2	PPARG/NR1H2
Biological Process	GO:0060336	negative regulation of interferon-gamma-mediated signaling pathway	2/2279	7/18866	0.2033338	0.5338711	0.4830655	2	PPARG/NR1H2
Biological Process	GO:0060839	endothelial cell fate commitment	2/2279	7/18866	0.2033338	0.5338711	0.4830655	2	DLL1/RBPJ
Biological Process	GO:0061002	negative regulation of dendritic spine morphogenesis	2/2279	7/18866	0.2033338	0.5338711	0.4830655	2	DNM3/UBE3A

Biological Process	GO:0061518	microglial cell proliferation	2/2279	7/18866	0.2033338	0.5338711	0.4830655	2	CSF1R/CLU
Biological Process	GO:0061724	lipophagy	2/2279	7/18866	0.2033338	0.5338711	0.4830655	2	SPTLC2/HTT
Biological Process	GO:0071072	negative regulation of phospholipid biosynthetic process	2/2279	7/18866	0.2033338	0.5338711	0.4830655	2	LPCAT1/PDGFB
Biological Process	GO:0071286	cellular response to magnesium ion	2/2279	7/18866	0.2033338	0.5338711	0.4830655	2	SLFN14/SLC41A1
Biological Process	GO:0071455	cellular response to hyperoxia	2/2279	7/18866	0.2033338	0.5338711	0.4830655	2	PPARG/FOXO1
Biological Process	GO:0071918	urea transmembrane transport	2/2279	7/18866	0.2033338	0.5338711	0.4830655	2	AQP3/AQP9
Biological Process	GO:0072383	plus-end-directed vesicle transport along microtubule	2/2279	7/18866	0.2033338	0.5338711	0.4830655	2	KIF13A/KIFAP3
Biological Process	GO:0075509	endocytosis involved in viral entry into host cell	2/2279	7/18866	0.2033338	0.5338711	0.4830655	2	TPCN2/EPS15
Biological Process	GO:0097211	cellular response to gonadotropin-releasing hormone	2/2279	7/18866	0.2033338	0.5338711	0.4830655	2	RAP1B/CRHBP

Biological Process	GO:0098910	regulation of atrial cardiac muscle cell action potential	2/2279	7/18866	0.2033338	0.5338711	0.4830655	2	GJA5/ANK2
Biological Process	GO:0150003	regulation of spontaneous synaptic transmission	2/2279	7/18866	0.2033338	0.5338711	0.4830655	2	APP/PRKN
Biological Process	GO:1900038	negative regulation of cellular response to hypoxia	2/2279	7/18866	0.2033338	0.5338711	0.4830655	2	ENO1/NOL3
Biological Process	GO:1901098	positive regulation of autophagosome maturation	2/2279	7/18866	0.2033338	0.5338711	0.4830655	2	UVRAG/CLEC16A
Biological Process	GO:1901388	regulation of transforming growth factor beta activation	2/2279	7/18866	0.2033338	0.5338711	0.4830655	2	LTBP1/ITGB6
Biological Process	GO:1901525	negative regulation of mitophagy	2/2279	7/18866	0.2033338	0.5338711	0.4830655	2	CLEC16A/TSC2
Biological Process	GO:1901642	nucleoside transmembrane transport	2/2279	7/18866	0.2033338	0.5338711	0.4830655	2	SLC29A3/SLC29A1
Biological Process	GO:1902231	positive regulation of intrinsic apoptotic signaling pathway in response to DNA damage	2/2279	7/18866	0.2033338	0.5338711	0.4830655	2	NACC2/FBH1
Biological Process	GO:1902803	regulation of synaptic vesicle transport	2/2279	7/18866	0.2033338	0.5338711	0.4830655	2	CNIH2/PRKN

Biological Process	GO:1903430	negative regulation of cell maturation	2/2279	7/18866	0.2033338	0.5338711	0.4830655	2	BCL11A/NPPC
Biological Process	GO:1903998	regulation of eating behavior	2/2279	7/18866	0.2033338	0.5338711	0.4830655	2	GHRL/TTC21B
Biological Process	GO:1904026	regulation of collagen fibril organization	2/2279	7/18866	0.2033338	0.5338711	0.4830655	2	AEBP1/RB1
Biological Process	GO:1904587	response to glycoprotein	2/2279	7/18866	0.2033338	0.5338711	0.4830655	2	EDEM1/EDEM2
Biological Process	GO:1905449	regulation of Fc-gamma receptor signaling pathway involved in phagocytosis	2/2279	7/18866	0.2033338	0.5338711	0.4830655	2	PTPRJ/PTPRC
Biological Process	GO:1905460	negative regulation of vascular associated smooth muscle cell apoptotic process	2/2279	7/18866	0.2033338	0.5338711	0.4830655	2	MIR138-2/DNMT1
Biological Process	GO:1905603	regulation of blood-brain barrier permeability	2/2279	7/18866	0.2033338	0.5338711	0.4830655	2	TJP2/ANGPT1
Biological Process	GO:1990144	intrinsic apoptotic signaling pathway in response to hypoxia	2/2279	7/18866	0.2033338	0.5338711	0.4830655	2	ENO1/NOL3
Biological Process	GO:2000553	positive regulation of T-helper 2 cell cytokine production	2/2279	7/18866	0.2033338	0.5338711	0.4830655	2	PRKCZ/NLRP3

Biological Process	GO:2000660	negative regulation of interleukin-1-mediated signaling pathway	2/2279	7/18866	0.2033338	0.5338711	0.4830655	2	MIR27A/IL1RN
Biological Process	GO:2001033	negative regulation of double-strand break repair via nonhomologous end joining	2/2279	7/18866	0.2033338	0.5338711	0.4830655	2	TFIP11/CYREN
Biological Process	GO:0006094	gluconeogenesis	14/2279	91/18866	0.2051844	0.5338711	0.4830655	14	MAEA/ENO1/C1QTNF3/PGP/PGAM1/PC/DGKQ/ENO3/PER2/PCK2/FOXO1/SOGA1/SLC25A1/SDHAF3
Biological Process	GO:0046849	bone remodeling	14/2279	91/18866	0.2051844	0.5338711	0.4830655	14	DEF8/INPP5D/LRP5L/PRKCA/UBASH3B/TNFAIP3/CSF1R/TGFB1/NF1/LRRK1/ITGB3/TMEM64/CSK/PLEKHM1
Biological Process	GO:1904063	negative regulation of cation transmembrane transport	14/2279	91/18866	0.2051844	0.5338711	0.4830655	14	KCNE1/THADA/CASQ2/TLR9/BIN1/SLC43A2/CAB39/GSTO1/PRKCE/SLC43A1/NEDD4/DYSF/HECW2/CBARP
Biological Process	GO:0071331	cellular response to hexose stimulus	20/2279	136/18866	0.2053008	0.5338711	0.4830655	20	NADK/SLC2A5/LRP5L/ERN1/ARRB1/GPLD1/IGF1R/PTPRN2/PAX2/ENY2/GHRL/KLF7/FIS1/PIH1D1/PRKCE/CPB2/PCK2/SLC29A1/C2CD2L/PRKAA1
Biological Process	GO:0060038	cardiac muscle cell proliferation	11/2279	69/18866	0.206438	0.5338711	0.4830655	11	RXRA/MIR199A1/VGLL4/TGFBR2/RIPK1/RBPJ/FGFR1/MEIS1/MIR199A2/NOTCH1/MEF2C
Biological Process	GO:0061371	determination of heart left/right asymmetry	11/2279	69/18866	0.206438	0.5338711	0.4830655	11	SMAD3/SUFU/MICAL2/TGFBR2/DLL1/MIB1/ENG/NOTCH1/MEF2C/TMED2/NODAL
Biological Process	GO:0071230	cellular response to amino acid stimulus	11/2279	69/18866	0.206438	0.5338711	0.4830655	11	RPTOR/TNF/BCL11A/BAIAP2/PDGFD/CPEB3/SH3BP4/DNMT1/PDGFC/CEBPB/BCL2L1

Biological Process	GO:0032675	regulation of interleukin-6 production	23/2279	159/18866	0.2072956	0.5338711	0.4830655	23	IL10/IL16/C1QTNF3/MIR140/ARRB1/TNF/TNFAIP3/MIR101-2/TLR9/SPON2/C5AR2/APP/MBP/PTPN6/SCIMP/GHRL/CSK/IL6R/ARRB2/NOD2/POU2F2/F2R/ARHGEF2
Biological Process	GO:0072331	signal transduction by p53 class mediator	37/2279	267/18866	0.2085005	0.5338711	0.4830655	37	SPRED2/BLM/PRKAG2/TFDP1/BRCA2/RMI2/NDRG1/HUS1/HIPK2/E2F7/PCBP4/RRM2B/EHMT2/ZNF385A/PRKAB1/TAF10/HDAC1/ARID3A/PERP/CDIP1/DYRK2/MTA2/ANKRD2/BCL2/SP100/RHNO1/FOXM1/CHEK2/TP53BP2/ANKRD1/BCL3/HEXIM1/ING5/PAXIP1/PRKAA1/PRKN/MARCHF7
Biological Process	GO:0006403	RNA localization	33/2279	236/18866	0.2085787	0.5338711	0.4830655	33	NXF1/FIP1L1/TNKS/EXOSC2/CPSF3/SETD2/RAE1/TGFBR2/ZNF385A/SMG7/PABPN1/ENY2/BICD2/NUP93/IGF2BP3/SEC13/KIF5C/SMG6/SNUPN/PIH1D1/MYO1C/PRPF6/HHEX/ZC3H3/RBM15B/RUVBL1/NOL6/CPSF4/FLOT1/CCT6A/FBL/PCID2/NCBP3
Biological Process	GO:0044242	cellular lipid catabolic process	33/2279	236/18866	0.2085787	0.5338711	0.4830655	33	PNPLA2/PLA2G15/SORL1/MGLL/PLBD2/MFSD2A/HEXB/LIPC/ABHD16A/AMACR/GPLD1/GALC/SMPDL3B/AOAH/ACOXL/CYP4F3/ACADM/ACACB/MGST2/ABHD16B/FABP6/ACOX2/ABHD5/PCK2/ACAT1/PLCG1/ADTRP/CPT1A/PDXDC1/INPP5F/LPIN1/LYPLA2/ACADVL
Biological Process	GO:0046434	organophosphate catabolic process	21/2279	144/18866	0.209232	0.5338711	0.4830655	21	PDE4D/PLA2G15/SYNJ2/AMPD3/PLBD2/INPP5A/LIPC/ABHD16A/SMPDL3B/NUDT3/ACOT7/UNG/NTHL1/ABHD16B/INPP5E/IMPA2/PDE9A/PNP/ACAT1/PLCG1/INPP5F
Biological Process	GO:0060078	regulation of postsynaptic membrane potential	21/2279	144/18866	0.209232	0.5338711	0.4830655	21	PRKCZ/SSH1/ZMYND8/BAIAP2/APP/GRIK4/NRXN1/GHRL/SH3GL1/MAPK8IP2/ARRB2/S1PR2/GABBR1/P2RX1/MEF2C/SHANK2/SLC29A1/P2RX5/GRID1/CUX2/FGF14
Biological Process	GO:1902850	microtubule cytoskeleton organization involved in mitosis	21/2279	144/18866	0.209232	0.5338711	0.4830655	21	ARHGEF10/DCTN1/TNKS/NDE1/SETD2/RAE1/RCC1/DYNC1H1/POC1A/DCTN2/TACC3/STAG1/HTT/NUMA1/MSTO1/CHEK2/ARHGEF2/FBXW11/NDEL1/PTPA/ABRAXAS1
Biological Process	GO:0050886	endocrine process	13/2279	84/18866	0.2100938	0.5338711	0.4830655	13	CTSZ/CTSG/C1QTNF3/RAB11FIP1/GJA5/FGFR1/CRHBP/GHRL/F2R/AGTR1/HSD11B2/INHBA/AOPEP
Biological Process	GO:0046879	hormone secretion	43/2279	314/18866	0.2101811	0.5338711	0.4830655	43	NADK/C1QTNF3/ITPR2/MYRIP/CREB1/MYB/CACNA1C/LRP5L/RAB11FIP1/ARRB1/PRKCA/GPLD1/TNF/CCL5/ITSN1/ITPR1/RFX3/TCF7L2/HNF1A/PTPRN2/FGFR1/IL1RN/CRHBP/ENY2/MYO5A/GIPR/BLK/GHRL/KLF7/ARNTL/PER2/SLC16A1/PRKCE/AGTR1/SLC2A1/SREBF1/CPT1A/RPH3AL/INHBA/LIF/C2CD2L/SIRT3/PRKN

Biological Process	GO:0048857	neural nucleus development	10/2279	62/18866	0.2105171	0.5338711	0.4830655	10	YWHAH/PHOX2A/PADI2/MBP/KIRREL3/SYNGR3/BCL2/YWHAQ/SYPL2/NDRG2
Biological Process	GO:0051851	modulation by host of symbiont process	10/2279	62/18866	0.2105171	0.5338711	0.4830655	10	CTDP1/CCL5/CSF1R/SFTPD/HDAC1/PC/PHB/AQP1/LTF/PRKN
Biological Process	GO:2000351	regulation of endothelial cell apoptotic process	10/2279	62/18866	0.2105171	0.5338711	0.4830655	10	ANGPT1/TNF/TNFAIP3/MIR101-2/THBS1/MIR24-2/PRKCI/NFE2L2/ANO6/PLCG1
Biological Process	GO:0001822	kidney development	39/2279	283/18866	0.2114564	0.5338711	0.4830655	39	ARID5B/TACSTD2/ANGPT1/SMAD3/ZBTB16/ODC1/RRM2B/CTSH/DLL1/ILK/SMAD7/WNT11/PAX2/FGFR1/OVOL1/RDH10/RARA/NF1/PDGFD/KIRREL3/MTSS1/BMPER/WWTR1/IL6R/PDGFB/CAT/BCL2/NOTCH1/MEF2C/GPR4/AGTR1/PDGFRB/ACAT1/CRLF1/ALDH1A2/LIF/HOXB7/FADD/CPLANE1
Biological Process	GO:0016579	protein deubiquitination	39/2279	283/18866	0.2114564	0.5338711	0.4830655	39	FOXK1/WDR20/NLRP3/SMAD3/TRAF2/TNFAIP3/USP3/USP36/SNX3/RIPK1/OTUB2/TAF10/SMAD7/CDC25A/MBD6/ENY2/COPS5/KDM1B/TNIP1/VCPIP1/PSMF1/ARRB2/CCNA1/OTUB1/CLSPN/USP49/PSMB7/USP54/MBD5/USP2/CYLD/RUVBL1/UCHL3/PSMD13/USP35/PRKN/MINDY1/ABRAXAS1/BABAM2
Biological Process	GO:0043255	regulation of carbohydrate biosynthetic process	15/2279	99/18866	0.2115453	0.5338711	0.4830655	15	MAEA/AP2A1/C1QTNF3/PGP/P2RY6/TGFB1/HRH1/DGKQ/DYRK2/PDGFB/PPP1CB/ESRRB/FOXO1/SOGA1/SDHAF3
Biological Process	GO:0062207	regulation of pattern recognition receptor signaling pathway	15/2279	99/18866	0.2115453	0.5338711	0.4830655	15	GFI1/GRAMD4/MIR140/SMPDL3B/TNFAIP3/TLR9/RTN4/TREML4/SEC14L1/ARRB2/WDFY1/LTF/TYRO3/FLOT1/PELI1
Biological Process	GO:0001655	urogenital system development	45/2279	330/18866	0.2124256	0.5338711	0.4830655	45	ARID5B/TACSTD2/RXRA/ANGPT1/SMAD3/EPHB3/ZBTB16/ODC1/RRM2B/CTSH/DLL1/ILK/SMAD7/WNT11/PAX2/FGFR1/OVOL1/RDH10/RARA/NF1/PDGFD/KIRREL3/MTSS1/BMPER/WWTR1/RARG/IL6R/PDGFB/CAT/BCL2/NOTCH1/MEF2C/UBE3A/GPR4/AGTR1/PDGFRB/CRIP1/ACAT1/CRLF1/ALDH1A2/LIF/HOXB7/FADD/NFIA/CPLANE1
Biological Process	GO:0051028	mRNA transport	22/2279	152/18866	0.2128332	0.5338711	0.4830655	22	NXF1/FIP1L1/TNKS/CPSF3/SETD2/RAE1/SMG7/PABPN1/ENY2/BICD2/NUP93/IGF2BP3/SEC13/KIF5C/SMG6/MYO1C/HHEX/ZC3H3/RBM15B/CPSF4/PCID2/NCBP3

Biological Process	GO:0007611	learning or memory	36/2279	260/18866	0.2137648	0.5338711	0.4830655	36	PRKCZ/CREB1/PPP1R1B/PRKAR1B/AMFR/SGK1/TANC1/ATP8A1/ATXN1/SLC11A2/COMT/APP/ZNF385A/SYNGAP1/CRHBP/HRH1/NF1/NRXN1/CPEB3/HTT/GMFB/MEIS2/NRXN2/PLCB1/MAPK8IP2/NTRK1/MAP1A/MEF2C/SHANK2/CEBPB/NTAN1/NTF4/CX3CR1/CUX2/SYNPO/PRKN
Biological Process	GO:1903362	regulation of cellular protein catabolic process	36/2279	260/18866	0.2137648	0.5338711	0.4830655	36	TRIM39/RNF144A/FZR1/RNF19A/CSNK1D/SUFU/TNFAIP3/HIPK2/VGLL4/CBFA2T3/UBAC2/MGAT3/DISC1/EDEM1/DERL2/CLU/SMAD7/SMURF1/AXIN1/CLEC16A/EDEM2/CSNK1E/PSMF1/NFE2L2/RNF144B/MAP1A/GNA12/UBE3A/RNF14/TRIB1/EZR/RNF40/BAG5/SVIP/PRKN/MARCHF7
Biological Process	GO:0001706	endoderm formation	9/2279	55/18866	0.2143566	0.5338711	0.4830655	9	SETD2/COL4A2/COL12A1/ITGB5/ITGB2/COL5A1/LAMB3/INHBA/NODAL
Biological Process	GO:0008347	glial cell migration	9/2279	55/18866	0.2143566	0.5338711	0.4830655	9	AZU1/CD9/HEXB/CCR2/RTN4/DISC1/ZMIZ1/CERS2/CX3CR1
Biological Process	GO:0014888	striated muscle adaptation	9/2279	55/18866	0.2143566	0.5338711	0.4830655	9	HDAC4/GSN/SMAD3/MIR199A1/CAMTA2/MIR199A2/MEF2C/FOXO1/INPP5F
Biological Process	GO:0030199	collagen fibril organization	9/2279	55/18866	0.2143566	0.5338711	0.4830655	9	LOXL3/TNXB/COL12A1/NF1/COL14A1/AEBP1/RB1/COL5A1/LOXL4
Biological Process	GO:0050819	negative regulation of coagulation	9/2279	55/18866	0.2143566	0.5338711	0.4830655	9	PLAU/CD9/UBASH3B/SERPINB2/THBS1/F12/PDGFB/CPB2/ADTRP
Biological Process	GO:0050879	multicellular organismal movement	9/2279	55/18866	0.2143566	0.5338711	0.4830655	9	VTI1A/HIPK2/TNNT3/ITPR1/JSRP1/GSTO1/MAP1A/CASQ1/MB
Biological Process	GO:0050881	musculoskeletal movement	9/2279	55/18866	0.2143566	0.5338711	0.4830655	9	VTI1A/HIPK2/TNNT3/ITPR1/JSRP1/GSTO1/MAP1A/CASQ1/MB

Biological Process	GO:0051452	intracellular pH reduction	9/2279	55/18866	0.2143566	0.5338711	0.4830655	9	SLC11A1/ATP6V1B2/GRN/BCL2/ATP6V0B/UBE3A/CLN5/ATP6V0C/ATP6V1C1
Biological Process	GO:0071326	cellular response to monosaccharide stimulus	20/2279	137/18866	0.2148429	0.5338711	0.4830655	20	NADK/SLC2A5/LRP5L/ERN1/ARRB1/GPLD1/IGF1R/PTPRN2/PAX2/ENY2/GHRL/KLF7/FIS1/PIH1D1/PRKCE/CPB2/PCK2/SLC29A1/C2CD2L/PRKAA1
Biological Process	GO:0060411	cardiac septum morphogenesis	12/2279	77/18866	0.215078	0.5338711	0.4830655	12	ZFPM1/NRP2/GJA5/TGFBR2/RBPJ/SMAD7/WNT11/RARA/ENG/NOTCH1/SLIT3/NSD2
Biological Process	GO:0002577	regulation of antigen processing and presentation	4/2279	20/18866	0.216049	0.5338711	0.4830655	4	SLC11A1/THBS1/NOD2/CD68
Biological Process	GO:0003323	type B pancreatic cell development	4/2279	20/18866	0.216049	0.5338711	0.4830655	4	RHEB/RFX3/DLL1/ARNTL
Biological Process	GO:0010042	response to manganese ion	4/2279	20/18866	0.216049	0.5338711	0.4830655	4	D2HGDH/SLFN14/APP/PRKN
Biological Process	GO:0010875	positive regulation of cholesterol efflux	4/2279	20/18866	0.216049	0.5338711	0.4830655	4	ABCG1/PLTP/NR1H2/ABCA1
Biological Process	GO:0010988	regulation of low-density lipoprotein particle clearance	4/2279	20/18866	0.216049	0.5338711	0.4830655	4	MIR199A1/MIR27A/CSK/MIR199A2
Biological Process	GO:0022038	corpus callosum development	4/2279	20/18866	0.216049	0.5338711	0.4830655	4	ZEB2/EPHB3/CORO1C/TSKU

Biological Process	GO:0032769	negative regulation of monooxygenase activity	4/2279	20/18866	0.216049	0.5338711	0.4830655	4	GFI1/CNR2/MIR138-2/NOSIP
Biological Process	GO:0043046	DNA methylation involved in gamete generation	4/2279	20/18866	0.216049	0.5338711	0.4830655	4	PIWIL4/MOV10L1/KDM1B/MORC1
Biological Process	GO:0043950	positive regulation of cAMP-mediated signaling	4/2279	20/18866	0.216049	0.5338711	0.4830655	4	PRKCA/MRAP/RAPGEF2/GIPR
Biological Process	GO:0046655	folic acid metabolic process	4/2279	20/18866	0.216049	0.5338711	0.4830655	4	MTHFD1L/MTHFS/SLC19A1/MTHFD1
Biological Process	GO:0046931	pore complex assembly	4/2279	20/18866	0.216049	0.5338711	0.4830655	4	RTN4/PLEKHA7/NUP93/ANO6
Biological Process	GO:0060039	pericardium development	4/2279	20/18866	0.216049	0.5338711	0.4830655	4	SETD2/SMAD3/CCM2/NOTCH1
Biological Process	GO:0060965	negative regulation of gene silencing by miRNA	4/2279	20/18866	0.216049	0.5338711	0.4830655	4	PPARG/NCOR2/TNF/TGFB1
Biological Process	GO:0071731	response to nitric oxide	4/2279	20/18866	0.216049	0.5338711	0.4830655	4	DNM2/TRAF2/AQP1/FOXO1
Biological Process	GO:0072234	metanephric nephron tubule development	4/2279	20/18866	0.216049	0.5338711	0.4830655	4	PAX2/WWTR1/ACAT1/LIF

Biological Process	GO:0086014	atrial cardiac muscle cell action potential	4/2279	20/18866	0.216049	0.5338711	0.4830655	4	CACNA1C/GJA5/KCNQ1/ANK2
Biological Process	GO:0086026	atrial cardiac muscle cell to AV node cell signaling	4/2279	20/18866	0.216049	0.5338711	0.4830655	4	CACNA1C/GJA5/KCNQ1/ANK2
Biological Process	GO:0086066	atrial cardiac muscle cell to AV node cell communication	4/2279	20/18866	0.216049	0.5338711	0.4830655	4	CACNA1C/GJA5/KCNQ1/ANK2
Biological Process	GO:0097062	dendritic spine maintenance	4/2279	20/18866	0.216049	0.5338711	0.4830655	4	ZMYND8/CTTN/TANC1/IGF1R
Biological Process	GO:0097709	connective tissue replacement	4/2279	20/18866	0.216049	0.5338711	0.4830655	4	MIR199A1/TGFB1/MIR199A2/F2R
Biological Process	GO:1901741	positive regulation of myoblast fusion	4/2279	20/18866	0.216049	0.5338711	0.4830655	4	EHD1/NFATC2/FLOT1/SCGB3A1
Biological Process	GO:0007051	spindle organization	26/2279	183/18866	0.2161774	0.5338711	0.4830655	26	ARHGEF10/DCTN1/LIMK2/CSNK1D/TNKS/RAE1/RCC1/DYNC1H1/MYH9/UVRAG/POC1A/DCTN2/TACC3/STAG1/NUMA1/PPP2R1A/MSTO1/NEK6/CHEK2/SPAG5/TUBGCP2/CEP72/EZR/NTMT1/PTPA/ABRAXAS1
Biological Process	GO:0071347	cellular response to interleukin-1	26/2279	183/18866	0.2161774	0.5338711	0.4830655	26	VRK2/LCN2/TOLLIP/MAP3K3/CHI3L1/IL1R1/CCL5/ABCC2/MIR27A/RPS6KA4/IL1RN/UBE2V1/CCL20/PSMF1/PLCB1/IRAK2/OTUB1/NOD2/PSMB7/CEBPB/ANKRD1/RELA/FBXW11/CCL22/PELI1/PSMD13
Biological Process	GO:0051928	positive regulation of calcium ion transport	18/2279	122/18866	0.2163707	0.5338711	0.4830655	18	MYLK/CCL5/P2RY6/SPHK2/HTT/PDGFB/ARRB2/GSTO1/F2R/PDGFRB/ANK2/PLCG1/P2RX5/TRPV2/CASQ1/FGF14/STIMATE/CRACR2A

Biological Process	GO:0031341	regulation of cell killing	14/2279	92/18866	0.2169563	0.5338711	0.4830655	14	CD55/PIK3R6/CD226/HLA-F/NCR1/RIPK3/CD59/PTPRC/ARRB2/IL18RAP/DNASE1L3/CADM1/FADD/CYRIB
Biological Process	GO:0030330	DNA damage response, signal transduction by p53 class mediator	16/2279	107/18866	0.2171925	0.5338711	0.4830655	16	SPRED2/TFDP1/BRCA2/NDRG1/HIPK2/E2F7/PCBP4/ZNF385A/ARID3A/SP100/FOXM1/CHEK2/ANKRD1/BCL3/PAXIP1/MARCHF7
Biological Process	GO:0034404	nucleobase-containing small molecule biosynthetic process	16/2279	107/18866	0.2171925	0.5338711	0.4830655	16	PDE4D/CDA/AMPD3/NUDT3/ACOT7/TK2/UNG/UCK2/NTHL1/PDE9A/PNP/GUK1/ACAT1/DHODH/TK1/IMPDH1
Biological Process	GO:0050905	neuromuscular process	16/2279	107/18866	0.2171925	0.5338711	0.4830655	16	ABR/DCTN1/KCNAB2/HEXB/OPA3/CDH23/APP/CAMTA1/SCN1A/NRXN1/BCR/SLC1A3/CNTNAP1/CHD8/SPART/PRKN
Biological Process	GO:0055017	cardiac muscle tissue growth	16/2279	107/18866	0.2171925	0.5338711	0.4830655	16	RXRA/CTDP1/AKAP13/MIR199A1/VGLL4/CCM2L/TGFBR2/RIPK1/RBPJ/MIR24-2/MIR23A/FGFR1/MEIS1/MIR199A2/NOTCH1/MEF2C
Biological Process	GO:2001237	negative regulation of extrinsic apoptotic signaling pathway	16/2279	107/18866	0.2171925	0.5338711	0.4830655	16	ITPRIP/CTTN/TNF/TRAF2/TNFAIP3/UNC5B/THBS1/RIPK1/TCF7L2/HTT/NOL3/BCL2/ARHGEF2/BCL2L1/RELA/FADD
Biological Process	GO:0043523	regulation of neuron apoptotic process	30/2279	214/18866	0.2175187	0.5338711	0.4830655	30	HDAC4/CTSZ/IL10/MYB/ANGPT1/SRPK2/ARRB1/TNF/HIPK2/ITSN1/UNC5B/ILK/SYNGAP1/NF1/GRN/PRKCI/ARRB2/NTRK1/FIS1/BCL2/MEF2C/F2R/CEBPB/BCL2L1/TYRO3/CRLF1/NTF4/CX3CR1/NAE1/PRKN
Biological Process	GO:0002279	mast cell activation involved in immune response	8/2279	48/18866	0.2177768	0.5338711	0.4830655	8	LAT/PIK3CD/GAB2/FGR/CD84/SPHK2/FER/BLK
Biological Process	GO:0002820	negative regulation of adaptive immune response	8/2279	48/18866	0.2177768	0.5338711	0.4830655	8	TRIM27/LOXL3/DUSP22/HLA-F/PTPN6/SMAD7/ZBTB7B/PTPRC

Biological Process	GO:0031952	regulation of protein autophosphorylation	8/2279	48/18866	0.2177768	0.5338711	0.4830655	8	PPP2R5C/PDGFD/PTPRC/PDGFB/ENG/PPP2R5E/PPP2R5D/PDGFC
Biological Process	GO:0033628	regulation of cell adhesion mediated by integrin	8/2279	48/18866	0.2177768	0.5338711	0.4830655	8	PLAU/ITGB1BP1/CCL5/TESC/PTPN6/JAM3/PDE3B/LIF
Biological Process	GO:0002431	Fc receptor mediated stimulatory signaling pathway	21/2279	145/18866	0.2185911	0.5338711	0.4830655	21	ELMO1/DOCK1/PIK3R1/WIPF1/FGR/CYFIP2/BAIAP2/CD226/WASL/PTPRJ/BLK/VAV2/PTPRC/ACTG1/CSK/NCKAP1/PRKCE/MYO1C/PLCG1/CYFIP1/VAV3
Biological Process	GO:0060538	skeletal muscle organ development	24/2279	168/18866	0.2191858	0.5338711	0.4830655	24	HDAC4/MEF2D/SKI/MYH15/DLL1/PAX7/TGFB1/IGSF8/VAMP5/SVIL/NF1/ARNTL/MYOM1/ANKRD2/RB1/BCL2/NOTCH1/MEF2C/HLX/ANKRD1/FLOT1/CASQ1/PRKAA1/MYORG
Biological Process	GO:0072001	renal system development	40/2279	292/18866	0.2193935	0.5338711	0.4830655	40	ARID5B/TACSTD2/ANGPT1/SMAD3/ZBTB16/ODC1/RRM2B/CTSH/DLL1/ILK/SMAD7/WNT11/PAX2/FGFR1/OVOL1/RDH10/RARA/NF1/PDGFD/KIRREL3/MTSS1/BMPER/WWTR1/IL6R/PDGFB/CAT/BCL2/NOTCH1/MEF2C/GPR4/AGTR1/PDGFRB/ACAT1/CRLF1/ALDH1A2/LIF/HOXB7/FADD/NFIA/CPLANE1
Biological Process	GO:0017157	regulation of exocytosis	31/2279	222/18866	0.2195328	0.5338711	0.4830655	31	ABR/CD177/RAP1B/CCR2/GAB2/FGR/MICAL1/CD84/GIT1/RAB27A/GNAI2/HLA-F/APBA2/SPHK2/FER/PRKCB/VPS18/BLK/CHRM2/BCR/ITGB2/RALA/NOTCH1/P2RX1/DNM1L/PRAM1/SYT17/RPH3AL/DTNBP1/CBARP/PRKN
Biological Process	GO:0008088	axo-dendritic transport	11/2279	70/18866	0.2201069	0.5338711	0.4830655	11	SPG7/DYNC1H1/APP/CNIH2/KIF5C/KIF1B/MAP1A/AP3D1/FBXW11/NDEL1/DTNBP1
Biological Process	GO:0007212	dopamine receptor signaling pathway	7/2279	41/18866	0.2204791	0.5338711	0.4830655	7	KLF16/GNA11/PALM/ARRB2/GNG2/GNAO1/DTNBP1
Biological Process	GO:0007618	mating	7/2279	41/18866	0.2204791	0.5338711	0.4830655	7	HDAC4/HEXB/PPP1R1B/APP/NCOA1/MAPK8IP2/P2RX1

Biological Process	GO:0031062	positive regulation of histone methylation	7/2279	41/18866	0.2204791	0.5338711	0.4830655	7	BRD4/MYB/PAX7/PHF19/DNMT1/PAXIP1/NSD3
Biological Process	GO:0035136	forelimb morphogenesis	7/2279	41/18866	0.2204791	0.5338711	0.4830655	7	CACNA1C/ZBTB16/RDH10/RUNX2/HOXA9/ALX3/ALDH1A2
Biological Process	GO:0042269	regulation of natural killer cell mediated cytotoxicity	7/2279	41/18866	0.2204791	0.5338711	0.4830655	7	PIK3R6/CD226/HLA-F/NCR1/ARRB2/IL18RAP/CADM1
Biological Process	GO:0072524	pyridine-containing compound metabolic process	7/2279	41/18866	0.2204791	0.5338711	0.4830655	7	NADK/NMNAT3/PNP/PDXK/PARP10/PARP9/AOX1
Biological Process	GO:0072528	pyrimidine-containing compound biosynthetic process	7/2279	41/18866	0.2204791	0.5338711	0.4830655	7	CDA/TK2/UCK2/AK5/DCTD/DHODH/TK1
Biological Process	GO:0099622	cardiac muscle cell membrane repolarization	7/2279	41/18866	0.2204791	0.5338711	0.4830655	7	KCNE1/GJA5/NOS1AP/WDR1/SNTA1/KCNQ1/ANK2
Biological Process	GO:1902042	negative regulation of extrinsic apoptotic signaling pathway via death domain receptors	7/2279	41/18866	0.2204791	0.5338711	0.4830655	7	ITPRIP/TRAF2/TNFAIP3/RIPK1/ARHGEF2/BCL2L1/FADD
Biological Process	GO:1903170	negative regulation of calcium ion transmembrane transport	7/2279	41/18866	0.2204791	0.5338711	0.4830655	7	THADA/CASQ2/TLR9/BIN1/GSTO1/DYSF/CBARP
Biological Process	GO:1905209	positive regulation of cardiocyte differentiation	7/2279	41/18866	0.2204791	0.5338711	0.4830655	7	MIR145/MIR199A1/RBPJ/TGFB1/ARRB2/MIR199A2/MEF2C

Biological Process	GO:0007628	adult walking behavior	5/2279	27/18866	0.2211679	0.5338711	0.4830655	5	HIPK2/SCN1A/ARRB2/NPC1/SPTBN4
Biological Process	GO:0008053	mitochondrial fusion	5/2279	27/18866	0.2211679	0.5338711	0.4830655	5	VAT1/SPG7/FIS1/DNM1L/PRKN
Biological Process	GO:0010996	response to auditory stimulus	5/2279	27/18866	0.2211679	0.5338711	0.4830655	5	NRXN1/HTT/NRXN2/SLC1A3/XPA
Biological Process	GO:0015949	nucleobase-containing small molecule interconversion	5/2279	27/18866	0.2211679	0.5338711	0.4830655	5	RRM2B/GUK1/AK2/AK5/DCTD
Biological Process	GO:0018126	protein hydroxylation	5/2279	27/18866	0.2211679	0.5338711	0.4830655	5	P4HB/P4HA2/ASPH/P3H3/P3H1
Biological Process	GO:0032201	telomere maintenance via semi-conservative replication	5/2279	27/18866	0.2211679	0.5338711	0.4830655	5	POLE/RTEL1/POLA2/POLD3/POLE4
Biological Process	GO:0032438	melanosome organization	5/2279	27/18866	0.2211679	0.5338711	0.4830655	5	LYST/KIF13A/ZEB2/AP3D1/DTNBP1
Biological Process	GO:0034389	lipid droplet organization	5/2279	27/18866	0.2211679	0.5338711	0.4830655	5	PNPLA2/TMEM159/CDS2/BSCL2/SPART
Biological Process	GO:0036037	CD8-positive, alpha-beta T cell activation	5/2279	27/18866	0.2211679	0.5338711	0.4830655	5	RUNX1/ZBTB7B/BCL2/RUNX3/VSIR

Biological Process	GO:0048668	collateral sprouting	5/2279	27/18866	0.2211679	0.5338711	0.4830655	5	ZEB2/APP/IFRD1/SEMA4D/SPART
Biological Process	GO:0070199	establishment of protein localization to chromosome	5/2279	27/18866	0.2211679	0.5338711	0.4830655	5	SPIDR/BRCA2/PIH1D1/CCT6A/MACROH2A2
Biological Process	GO:0072337	modified amino acid transport	5/2279	27/18866	0.2211679	0.5338711	0.4830655	5	ABCC1/FOLR3/SLC19A1/SLC22A5/PDZK1
Biological Process	GO:0072539	T-helper 17 cell differentiation	5/2279	27/18866	0.2211679	0.5338711	0.4830655	5	NLRP3/LOXL3/SMAD7/LY9/ZBTB7B
Biological Process	GO:1900078	positive regulation of cellular response to insulin stimulus	5/2279	27/18866	0.2211679	0.5338711	0.4830655	5	PRKCZ/SORL1/GNAI2/ZBTB7B/MYO1C
Biological Process	GO:1902175	regulation of oxidative stress-induced intrinsic apoptotic signaling pathway	5/2279	27/18866	0.2211679	0.5338711	0.4830655	5	P4HB/NOL3/NFE2L2/BAG5/PRKN
Biological Process	GO:0006658	phosphatidylserine metabolic process	6/2279	34/18866	0.2219404	0.5338711	0.4830655	6	OSBPL5/ABHD16A/ABHD16B/OSBPL10/PLA1A/PTDSS2
Biological Process	GO:0007202	activation of phospholipase C activity	6/2279	34/18866	0.2219404	0.5338711	0.4830655	6	P2RY6/PHB/LPAR2/S1PR4/PLCG1/NTF4
Biological Process	GO:0008156	negative regulation of DNA replication	6/2279	34/18866	0.2219404	0.5338711	0.4830655	6	BLM/BRCA2/HUS1/SMARCAL1/CHEK2/FBH1

Biological Process	GO:0008608	attachment of spindle microtubules to kinetochore	6/2279	34/18866	0.2219404	0.5338711	0.4830655	6	MAD1L1/CHAMP1/MIS12/RB1/SPAG5/ABRAXAS1
Biological Process	GO:0035633	maintenance of blood-brain barrier	6/2279	34/18866	0.2219404	0.5338711	0.4830655	6	MFSD2A/TJP2/ANGPT1/MBP/JAM3/ACTG1
Biological Process	GO:0048536	spleen development	6/2279	34/18866	0.2219404	0.5338711	0.4830655	6	RIPK3/BCL2/EPB42/BCL3/FADD/PCID2
Biological Process	GO:0048854	brain morphogenesis	6/2279	34/18866	0.2219404	0.5338711	0.4830655	6	PAX2/NF1/ZNF335/SHANK2/FBXW11/SLIT1
Biological Process	GO:0060236	regulation of mitotic spindle organization	6/2279	34/18866	0.2219404	0.5338711	0.4830655	6	DCTN1/RAE1/RCC1/DYNC1H1/TACC3/NUMA1
Biological Process	GO:0098901	regulation of cardiac muscle cell action potential	6/2279	34/18866	0.2219404	0.5338711	0.4830655	6	CACNA1C/GJA5/JUP/BIN1/NOS1AP/ANK2
Biological Process	GO:0060419	heart growth	17/2279	115/18866	0.2222343	0.5338711	0.4830655	17	RXRA/CTDP1/AKAP13/MIR199A1/VGLL4/CCM2L/TGFBR2/RIPK1/RBPJ/MIR24-2/MIR23A/FGFR1/ACACB/MEIS1/MIR199A2/NOTCH1/MEF2C
Biological Process	GO:0010507	negative regulation of autophagy	13/2279	85/18866	0.2225281	0.5338711	0.4830655	13	FOXK1/IL10/BMF/MIR199A1/USP36/FEZ2/CLEC16A/TBC1D14/NPC1/BCL2/MIR199A2/TSC2/EIF4G1
Biological Process	GO:0072384	organelle transport along microtubule	13/2279	85/18866	0.2225281	0.5338711	0.4830655	13	PRKCZ/NDE1/KIF13A/DYNC1H1/BICD2/CNIH2/HTT/KIF1B/KIFAP3/AP3D1/FBXW11/NDEL1/DTNBP1

Biological Process	GO:0072527	pyrimidine-containing compound metabolic process	13/2279	85/18866	0.2225281	0.5338711	0.4830655	13	CDA/DPYS/TK2/UNG/UCK2/NTHL1/UPB1/TET2/AK5/DCTD/DHODH/TK1/ACP3
Biological Process	GO:0046364	monosaccharide biosynthetic process	15/2279	100/18866	0.2229927	0.5338711	0.4830655	15	MAEA/ENO1/C1QTNF3/PGP/CHST15/PGAM1/PC/DGKQ/ENO3/PER2/PCK2/FOXO1/SOGA1/SLC25A1/SDHAF3
Biological Process	GO:0036293	response to decreased oxygen levels	50/2279	371/18866	0.223005	0.5338711	0.4830655	50	P4HB/ENO1/PLAU/ITPR2/MIR140/TM9SF4/CREB1/MYB/NDRG1/SMAD3/SLC8A1/HIPK2/CBFA2T3/ITPR1/SLC11A2/TGFBR2/THBS1/RBPJ/ACTN4/LIMD1/AQP1/NF1/HK2/ALAS1/AGTRAP/PSMF1/CREBBP/NOL3/CAT/ETS1/NFE2L2/FIS1/PKM/BCL2/PSMB7/NOTCH1/AQP3/DNM1L/EPAS1/PRKCE/NPPC/SLC2A1/ANKRD1/SLC29A1/BACH1/HSD11B2/ERCC2/PSMD13/MB/PRKAA1
Biological Process	GO:0072329	monocarboxylic acid catabolic process	20/2279	138/18866	0.2245852	0.5338711	0.4830655	20	PLA2G15/MFSD2A/ABHD16A/AMACR/ACOXL/AKR1D1/FAH/CYP4F3/ACADM/AGXT/ACACB/ACOX2/HOGA1/PCK2/ACAT1/ADTRP/CPT1A/LPIN1/LYPLA2/ACADVL
Biological Process	GO:0010830	regulation of myotube differentiation	10/2279	63/18866	0.2251328	0.5338711	0.4830655	10	HDAC4/EHD1/MAML1/NFATC2/ANKRD2/BCL2/NOTCH1/FLOT1/SCGB3A1/CEACAM5
Biological Process	GO:0055008	cardiac muscle tissue morphogenesis	10/2279	63/18866	0.2251328	0.5338711	0.4830655	10	ZFPM1/RXRA/TTN/CCM2L/RBPJ/SMAD7/TGFB1/ENG/NOTCH1/ANKRD1
Biological Process	GO:0070059	intrinsic apoptotic signaling pathway in response to endoplasmic reticulum stress	10/2279	63/18866	0.2251328	0.5338711	0.4830655	10	ERN1/TRAF2/NCK2/ITPR1/ATP2A3/BCL2/CEBPB/BCL2L1/GRINA/PRKN
Biological Process	GO:0000070	mitotic sister chromatid segregation	23/2279	161/18866	0.2251591	0.5338711	0.4830655	23	MAD1L1/TTN/TNKS/NSMCE2/CHAMP1/CUL3/CDC16/MIS12/DCTN2/TACC3/NUMA1/PPP2R1A/MSTO1/NEK6/RB1/RAD21L1/CDCA5/DIS3L2/ANAPC7/SPAG5/HECW2/PCID2/SLF1
Biological Process	GO:0000105	histidine biosynthetic process	1/2279	2/18866	0.2270118	0.5338711	0.4830655	1	MTHFD1

Biological Process	GO:0000301	retrograde transport, vesicle recycling within Golgi	1/2279	2/18866	0.2270118	0.5338711	0.4830655	1	CUX1
Biological Process	GO:0000390	spliceosomal complex disassembly	1/2279	2/18866	0.2270118	0.5338711	0.4830655	1	TFIP11
Biological Process	GO:0000973	posttranscriptional tethering of RNA polymerase II gene DNA at nuclear periphery	1/2279	2/18866	0.2270118	0.5338711	0.4830655	1	PCID2
Biological Process	GO:0001300	chronological cell aging	1/2279	2/18866	0.2270118	0.5338711	0.4830655	1	ENG
Biological Process	GO:0001507	acetylcholine catabolic process in synaptic cleft	1/2279	2/18866	0.2270118	0.5338711	0.4830655	1	COLQ
Biological Process	GO:0002017	regulation of blood volume by renal aldosterone	1/2279	2/18866	0.2270118	0.5338711	0.4830655	1	HSD11B2
Biological Process	GO:0002032	desensitization of G protein-coupled receptor signaling pathway by arrestin	1/2279	2/18866	0.2270118	0.5338711	0.4830655	1	ARRB2
Biological Process	GO:0002121	inter-male aggressive behavior	1/2279	2/18866	0.2270118	0.5338711	0.4830655	1	KIRREL3
Biological Process	GO:0002268	follicular dendritic cell differentiation	1/2279	2/18866	0.2270118	0.5338711	0.4830655	1	BCL3

Biological Process	GO:0002280	monocyte activation involved in immune response	1/2279	2/18866	0.2270118	0.5338711	0.4830655	1	DYSF
Biological Process	GO:0002304	gamma-delta intraepithelial T cell differentiation	1/2279	2/18866	0.2270118	0.5338711	0.4830655	1	CCR9
Biological Process	GO:0002305	CD8-positive, gamma-delta intraepithelial T cell differentiation	1/2279	2/18866	0.2270118	0.5338711	0.4830655	1	CCR9
Biological Process	GO:0002337	B-1a B cell differentiation	1/2279	2/18866	0.2270118	0.5338711	0.4830655	1	CMTM7
Biological Process	GO:0002384	hepatic immune response	1/2279	2/18866	0.2270118	0.5338711	0.4830655	1	IL6R
Biological Process	GO:0002397	MHC class I protein complex assembly	1/2279	2/18866	0.2270118	0.5338711	0.4830655	1	CALR
Biological Process	GO:0002502	peptide antigen assembly with MHC class I protein complex	1/2279	2/18866	0.2270118	0.5338711	0.4830655	1	CALR
Biological Process	GO:0002522	leukocyte migration involved in immune response	1/2279	2/18866	0.2270118	0.5338711	0.4830655	1	FUT7
Biological Process	GO:0002542	Factor XII activation	1/2279	2/18866	0.2270118	0.5338711	0.4830655	1	F12

Biological Process	GO:0002543	activation of blood coagulation via clotting cascade	1/2279	2/18866	0.2270118	0.5338711	0.4830655	1	ANO6
Biological Process	GO:0002661	regulation of B cell tolerance induction	1/2279	2/18866	0.2270118	0.5338711	0.4830655	1	TGFBR2
Biological Process	GO:0002663	positive regulation of B cell tolerance induction	1/2279	2/18866	0.2270118	0.5338711	0.4830655	1	TGFBR2
Biological Process	GO:0002752	cell surface pattern recognition receptor signaling pathway	1/2279	2/18866	0.2270118	0.5338711	0.4830655	1	FCN1
Biological Process	GO:0002876	positive regulation of chronic inflammatory response to antigenic stimulus	1/2279	2/18866	0.2270118	0.5338711	0.4830655	1	TNF
Biological Process	GO:0003127	detection of nodal flow	1/2279	2/18866	0.2270118	0.5338711	0.4830655	1	PKD1L1
Biological Process	GO:0003169	coronary vein morphogenesis	1/2279	2/18866	0.2270118	0.5338711	0.4830655	1	NOTCH1
Biological Process	GO:0003193	pulmonary valve formation	1/2279	2/18866	0.2270118	0.5338711	0.4830655	1	GJA5
Biological Process	GO:0003195	tricuspid valve formation	1/2279	2/18866	0.2270118	0.5338711	0.4830655	1	ZFPM1

Biological Process	GO:0003213	cardiac right atrium morphogenesis	1/2279	2/18866	0.2270118	0.5338711	0.4830655	1	NOTCH1
Biological Process	GO:0003252	negative regulation of cell proliferation involved in heart valve morphogenesis	1/2279	2/18866	0.2270118	0.5338711	0.4830655	1	NOTCH1
Biological Process	GO:0003290	atrial septum secundum morphogenesis	1/2279	2/18866	0.2270118	0.5338711	0.4830655	1	NSD2
Biological Process	GO:0003292	cardiac septum cell differentiation	1/2279	2/18866	0.2270118	0.5338711	0.4830655	1	MAML1
Biological Process	GO:0003420	regulation of growth plate cartilage chondrocyte proliferation	1/2279	2/18866	0.2270118	0.5338711	0.4830655	1	POR
Biological Process	GO:0006050	mannosamine metabolic process	1/2279	2/18866	0.2270118	0.5338711	0.4830655	1	NAGK
Biological Process	GO:0006051	N-acetylmannosamine metabolic process	1/2279	2/18866	0.2270118	0.5338711	0.4830655	1	NAGK
Biological Process	GO:0006196	AMP catabolic process	1/2279	2/18866	0.2270118	0.5338711	0.4830655	1	AMPD3
Biological Process	GO:0006212	uracil catabolic process	1/2279	2/18866	0.2270118	0.5338711	0.4830655	1	DPYS

Biological Process	GO:0006423	cysteinyl-tRNA aminoacylation	1/2279	2/18866	0.2270118	0.5338711	0.4830655	1	CARS2
Biological Process	GO:0006424	glutamyl-tRNA aminoacylation	1/2279	2/18866	0.2270118	0.5338711	0.4830655	1	EARS2
Biological Process	GO:0006431	methionyl-tRNA aminoacylation	1/2279	2/18866	0.2270118	0.5338711	0.4830655	1	MARS1
Biological Process	GO:0006437	tyrosyl-tRNA aminoacylation	1/2279	2/18866	0.2270118	0.5338711	0.4830655	1	YARS1
Biological Process	GO:0006478	peptidyl-tyrosine sulfation	1/2279	2/18866	0.2270118	0.5338711	0.4830655	1	TPST1
Biological Process	GO:0006480	N-terminal protein amino acid methylation	1/2279	2/18866	0.2270118	0.5338711	0.4830655	1	NTMT1
Biological Process	GO:0006535	cysteine biosynthetic process from serine	1/2279	2/18866	0.2270118	0.5338711	0.4830655	1	CBS
Biological Process	GO:0006550	isoleucine catabolic process	1/2279	2/18866	0.2270118	0.5338711	0.4830655	1	ACAT1
Biological Process	GO:0006581	acetylcholine catabolic process	1/2279	2/18866	0.2270118	0.5338711	0.4830655	1	COLQ

Biological Process	GO:0006876	cellular cadmium ion homeostasis	1/2279	2/18866	0.2270118	0.5338711	0.4830655	1	SLC11A1
Biological Process	GO:0007402	ganglion mother cell fate determination	1/2279	2/18866	0.2270118	0.5338711	0.4830655	1	NTF4
Biological Process	GO:0007518	myoblast fate determination	1/2279	2/18866	0.2270118	0.5338711	0.4830655	1	IFRD1
Biological Process	GO:0007521	muscle cell fate determination	1/2279	2/18866	0.2270118	0.5338711	0.4830655	1	MEF2C
Biological Process	GO:0007529	establishment of synaptic specificity at neuromuscular junction	1/2279	2/18866	0.2270118	0.5338711	0.4830655	1	F2R
Biological Process	GO:0009115	xanthine catabolic process	1/2279	2/18866	0.2270118	0.5338711	0.4830655	1	AOX1
Biological Process	GO:0009407	toxin catabolic process	1/2279	2/18866	0.2270118	0.5338711	0.4830655	1	NFE2L2
Biological Process	GO:0010142	farnesyl diphosphate biosynthetic process, mevalonate pathway	1/2279	2/18866	0.2270118	0.5338711	0.4830655	1	HMGCS1
Biological Process	GO:0010512	negative regulation of phosphatidylinositol biosynthetic process	1/2279	2/18866	0.2270118	0.5338711	0.4830655	1	PDGFB

Biological Process	GO:0010609	mRNA localization resulting in posttranscriptional regulation of gene expression	1/2279	2/18866	0.2270118	0.5338711	0.4830655	1	ZNF385A
Biological Process	GO:0010645	regulation of cell communication by chemical coupling	1/2279	2/18866	0.2270118	0.5338711	0.4830655	1	GJA5
Biological Process	GO:0010652	positive regulation of cell communication by chemical coupling	1/2279	2/18866	0.2270118	0.5338711	0.4830655	1	GJA5
Biological Process	GO:0010731	protein glutathionylation	1/2279	2/18866	0.2270118	0.5338711	0.4830655	1	FXYD1
Biological Process	GO:0010732	regulation of protein glutathionylation	1/2279	2/18866	0.2270118	0.5338711	0.4830655	1	FXYD1
Biological Process	GO:0010734	negative regulation of protein glutathionylation	1/2279	2/18866	0.2270118	0.5338711	0.4830655	1	FXYD1
Biological Process	GO:0010767	regulation of transcription from RNA polymerase II promoter in response to UV-induced DNA damage	1/2279	2/18866	0.2270118	0.5338711	0.4830655	1	NEDD4
Biological Process	GO:0010813	neuropeptide catabolic process	1/2279	2/18866	0.2270118	0.5338711	0.4830655	1	CTSH
Biological Process	GO:0010848	regulation of chromatin disassembly	1/2279	2/18866	0.2270118	0.5338711	0.4830655	1	PADI2

Biological Process	GO:0015680	protein maturation by copper ion transfer	1/2279	2/18866	0.2270118	0.5338711	0.4830655	1	ATP7B
Biological Process	GO:0015786	UDP-glucose transmembrane transport	1/2279	2/18866	0.2270118	0.5338711	0.4830655	1	SLC35C2
Biological Process	GO:0015817	histidine transport	1/2279	2/18866	0.2270118	0.5338711	0.4830655	1	SLC15A4
Biological Process	GO:0015819	lysine transport	1/2279	2/18866	0.2270118	0.5338711	0.4830655	1	SLC7A1
Biological Process	GO:0015823	phenylalanine transport	1/2279	2/18866	0.2270118	0.5338711	0.4830655	1	SLC7A5
Biological Process	GO:0015882	L-ascorbic acid transmembrane transport	1/2279	2/18866	0.2270118	0.5338711	0.4830655	1	SLC23A1
Biological Process	GO:0015916	fatty-acyl-CoA transport	1/2279	2/18866	0.2270118	0.5338711	0.4830655	1	SLC33A1
Biological Process	GO:0016036	cellular response to phosphate starvation	1/2279	2/18866	0.2270118	0.5338711	0.4830655	1	COMT
Biological Process	GO:0016182	synaptic vesicle budding from endosome	1/2279	2/18866	0.2270118	0.5338711	0.4830655	1	AP3D1

Biological Process	GO:0016320	endoplasmic reticulum membrane fusion	1/2279	2/18866	0.2270118	0.5338711	0.4830655	1	VCPIP1
Biological Process	GO:0016998	cell wall macromolecule catabolic process	1/2279	2/18866	0.2270118	0.5338711	0.4830655	1	LYG2
Biological Process	GO:0016999	antibiotic metabolic process	1/2279	2/18866	0.2270118	0.5338711	0.4830655	1	ABCC2
Biological Process	GO:0019072	viral genome packaging	1/2279	2/18866	0.2270118	0.5338711	0.4830655	1	PC
Biological Process	GO:0019074	viral RNA genome packaging	1/2279	2/18866	0.2270118	0.5338711	0.4830655	1	PC
Biological Process	GO:0019075	virus maturation	1/2279	2/18866	0.2270118	0.5338711	0.4830655	1	MVB12A
Biological Process	GO:0019265	glycine biosynthetic process, by transamination of glyoxylate	1/2279	2/18866	0.2270118	0.5338711	0.4830655	1	AGXT
Biological Process	GO:0019483	beta-alanine biosynthetic process	1/2279	2/18866	0.2270118	0.5338711	0.4830655	1	UPB1
Biological Process	GO:0019543	propionate catabolic process	1/2279	2/18866	0.2270118	0.5338711	0.4830655	1	PCK2

Biological Process	GO:0019858	cytosine metabolic process	1/2279	2/18866	0.2270118	0.5338711	0.4830655	1	CDA
Biological Process	GO:0021622	oculomotor nerve morphogenesis	1/2279	2/18866	0.2270118	0.5338711	0.4830655	1	PHOX2A
Biological Process	GO:0021623	oculomotor nerve formation	1/2279	2/18866	0.2270118	0.5338711	0.4830655	1	PHOX2A
Biological Process	GO:0021633	optic nerve structural organization	1/2279	2/18866	0.2270118	0.5338711	0.4830655	1	PAX2
Biological Process	GO:0021649	vestibulocochlear nerve structural organization	1/2279	2/18866	0.2270118	0.5338711	0.4830655	1	NRP2
Biological Process	GO:0021679	cerebellar molecular layer development	1/2279	2/18866	0.2270118	0.5338711	0.4830655	1	DLL1
Biological Process	GO:0021687	cerebellar molecular layer morphogenesis	1/2279	2/18866	0.2270118	0.5338711	0.4830655	1	DLL1
Biological Process	GO:0021693	cerebellar Purkinje cell layer structural organization	1/2279	2/18866	0.2270118	0.5338711	0.4830655	1	DLL1
Biological Process	GO:0021698	cerebellar cortex structural organization	1/2279	2/18866	0.2270118	0.5338711	0.4830655	1	DLL1

Biological Process	GO:0021730	trigeminal sensory nucleus development	1/2279	2/18866	0.2270118	0.5338711	0.4830655	1	KIRREL3
Biological Process	GO:0021740	principal sensory nucleus of trigeminal nerve development	1/2279	2/18866	0.2270118	0.5338711	0.4830655	1	KIRREL3
Biological Process	GO:0021767	mammillary body development	1/2279	2/18866	0.2270118	0.5338711	0.4830655	1	ZEB2
Biological Process	GO:0021896	forebrain astrocyte differentiation	1/2279	2/18866	0.2270118	0.5338711	0.4830655	1	NF1
Biological Process	GO:0021897	forebrain astrocyte development	1/2279	2/18866	0.2270118	0.5338711	0.4830655	1	NF1
Biological Process	GO:0030389	fructosamine metabolic process	1/2279	2/18866	0.2270118	0.5338711	0.4830655	1	FN3K
Biological Process	GO:0030573	bile acid catabolic process	1/2279	2/18866	0.2270118	0.5338711	0.4830655	1	AKR1D1
Biological Process	GO:0030974	thiamine pyrophosphate transmembrane transport	1/2279	2/18866	0.2270118	0.5338711	0.4830655	1	SLC44A4
Biological Process	GO:0032459	regulation of protein oligomerization	1/2279	2/18866	0.2270118	0.5338711	0.4830655	1	ZDHHC1

Biological Process	GO:0032499	detection of peptidoglycan	1/2279	2/18866	0.2270118	0.5338711	0.4830655	1	NOD2
Biological Process	GO:0032632	interleukin-3 production	1/2279	2/18866	0.2270118	0.5338711	0.4830655	1	SLC11A1
Biological Process	GO:0032699	negative regulation of interleukin-16 production	1/2279	2/18866	0.2270118	0.5338711	0.4830655	1	MIR145
Biological Process	GO:0032796	uropod organization	1/2279	2/18866	0.2270118	0.5338711	0.4830655	1	MYH9
Biological Process	GO:0032877	positive regulation of DNA endoreduplication	1/2279	2/18866	0.2270118	0.5338711	0.4830655	1	E2F7
Biological Process	GO:0032902	nerve growth factor production	1/2279	2/18866	0.2270118	0.5338711	0.4830655	1	PCSK6
Biological Process	GO:0032912	negative regulation of transforming growth factor beta2 production	1/2279	2/18866	0.2270118	0.5338711	0.4830655	1	CDH3
Biological Process	GO:0032915	positive regulation of transforming growth factor beta2 production	1/2279	2/18866	0.2270118	0.5338711	0.4830655	1	WNT11
Biological Process	GO:0032917	polyamine acetylation	1/2279	2/18866	0.2270118	0.5338711	0.4830655	1	SAT2

Biological Process	GO:0032918	spermidine acetylation	1/2279	2/18866	0.2270118	0.5338711	0.4830655	1	SAT2
Biological Process	GO:0033030	negative regulation of neutrophil apoptotic process	1/2279	2/18866	0.2270118	0.5338711	0.4830655	1	ITPKB
Biological Process	GO:0033037	polysaccharide localization	1/2279	2/18866	0.2270118	0.5338711	0.4830655	1	CHST11
Biological Process	GO:0033277	abortive mitotic cell cycle	1/2279	2/18866	0.2270118	0.5338711	0.4830655	1	PTPN6
Biological Process	GO:0033364	mast cell secretory granule organization	1/2279	2/18866	0.2270118	0.5338711	0.4830655	1	LYST
Biological Process	GO:0033505	floor plate morphogenesis	1/2279	2/18866	0.2270118	0.5338711	0.4830655	1	NODAL
Biological Process	GO:0033566	gamma-tubulin complex localization	1/2279	2/18866	0.2270118	0.5338711	0.4830655	1	CEP72
Biological Process	GO:0034146	toll-like receptor 5 signaling pathway	1/2279	2/18866	0.2270118	0.5338711	0.4830655	1	TNFAIP3
Biological Process	GO:0034342	response to type III interferon	1/2279	2/18866	0.2270118	0.5338711	0.4830655	1	SHFL

Biological Process	GO:0034418	urate biosynthetic process	1/2279	2/18866	0.2270118	0.5338711	0.4830655	1	PNP
Biological Process	GO:0034477	U6 snRNA 3'-end processing	1/2279	2/18866	0.2270118	0.5338711	0.4830655	1	TUT1
Biological Process	GO:0035279	mRNA cleavage involved in gene silencing by miRNA	1/2279	2/18866	0.2270118	0.5338711	0.4830655	1	AGO2
Biological Process	GO:0035378	carbon dioxide transmembrane transport	1/2279	2/18866	0.2270118	0.5338711	0.4830655	1	AQP1
Biological Process	GO:0035552	oxidative single-stranded DNA demethylation	1/2279	2/18866	0.2270118	0.5338711	0.4830655	1	FTO
Biological Process	GO:0035566	regulation of metanephros size	1/2279	2/18866	0.2270118	0.5338711	0.4830655	1	PAX2
Biological Process	GO:0035574	histone H4-K20 demethylation	1/2279	2/18866	0.2270118	0.5338711	0.4830655	1	KDM7A
Biological Process	GO:0035600	tRNA methylthiolation	1/2279	2/18866	0.2270118	0.5338711	0.4830655	1	CDKAL1
Biological Process	GO:0035607	fibroblast growth factor receptor signaling pathway involved in orbitofrontal cortex development	1/2279	2/18866	0.2270118	0.5338711	0.4830655	1	FGFR1

Biological Process	GO:0035675	neuromast hair cell development	1/2279	2/18866	0.2270118	0.5338711	0.4830655	1	SLC44A4
Biological Process	GO:0035700	astrocyte chemotaxis	1/2279	2/18866	0.2270118	0.5338711	0.4830655	1	CCR2
Biological Process	GO:0035922	foramen ovale closure	1/2279	2/18866	0.2270118	0.5338711	0.4830655	1	GJA5
Biological Process	GO:0035936	testosterone secretion	1/2279	2/18866	0.2270118	0.5338711	0.4830655	1	MYB
Biological Process	GO:0036115	fatty-acyl-CoA catabolic process	1/2279	2/18866	0.2270118	0.5338711	0.4830655	1	ACOT7
Biological Process	GO:0036292	DNA rewinding	1/2279	2/18866	0.2270118	0.5338711	0.4830655	1	SMARCAL1
Biological Process	GO:0036309	protein localization to M-band	1/2279	2/18866	0.2270118	0.5338711	0.4830655	1	ANK2
Biological Process	GO:0036345	platelet maturation	1/2279	2/18866	0.2270118	0.5338711	0.4830655	1	SLFN14
Biological Process	GO:0036371	protein localization to T-tubule	1/2279	2/18866	0.2270118	0.5338711	0.4830655	1	ANK2

Biological Process	GO:0036413	histone H3-R26 citrullination	1/2279	2/18866	0.2270118	0.5338711	0.4830655	1	PADI2
Biological Process	GO:0036483	neuron intrinsic apoptotic signaling pathway in response to endoplasmic reticulum stress	1/2279	2/18866	0.2270118	0.5338711	0.4830655	1	PRKN
Biological Process	GO:0036525	protein deglycation	1/2279	2/18866	0.2270118	0.5338711	0.4830655	1	FN3K
Biological Process	GO:0038043	interleukin-5-mediated signaling pathway	1/2279	2/18866	0.2270118	0.5338711	0.4830655	1	CSF2RB
Biological Process	GO:0038112	interleukin-8-mediated signaling pathway	1/2279	2/18866	0.2270118	0.5338711	0.4830655	1	CXCR1
Biological Process	GO:0038178	complement component C5a signaling pathway	1/2279	2/18866	0.2270118	0.5338711	0.4830655	1	C5AR2
Biological Process	GO:0039003	pronephric field specification	1/2279	2/18866	0.2270118	0.5338711	0.4830655	1	PAX2
Biological Process	GO:0039017	pattern specification involved in pronephros development	1/2279	2/18866	0.2270118	0.5338711	0.4830655	1	PAX2
Biological Process	GO:0040010	positive regulation of growth rate	1/2279	2/18866	0.2270118	0.5338711	0.4830655	1	GHRL

Biological Process	GO:0042245	RNA repair	1/2279	2/18866	0.2270118	0.5338711	0.4830655	1	FTO
Biological Process	GO:0042264	peptidyl-aspartic acid hydroxylation	1/2279	2/18866	0.2270118	0.5338711	0.4830655	1	ASPH
Biological Process	GO:0042322	negative regulation of circadian sleep/wake cycle, REM sleep	1/2279	2/18866	0.2270118	0.5338711	0.4830655	1	GHRL
Biological Process	GO:0042365	water-soluble vitamin catabolic process	1/2279	2/18866	0.2270118	0.5338711	0.4830655	1	MTHFS
Biological Process	GO:0042710	biofilm formation	1/2279	2/18866	0.2270118	0.5338711	0.4830655	1	LTF
Biological Process	GO:0042823	pyridoxal phosphate biosynthetic process	1/2279	2/18866	0.2270118	0.5338711	0.4830655	1	PDXK
Biological Process	GO:0043007	maintenance of rDNA	1/2279	2/18866	0.2270118	0.5338711	0.4830655	1	RMI2
Biological Process	GO:0043375	CD8-positive, alpha-beta T cell lineage commitment	1/2279	2/18866	0.2270118	0.5338711	0.4830655	1	BCL2
Biological Process	GO:0043377	negative regulation of CD8-positive, alpha-beta T cell differentiation	1/2279	2/18866	0.2270118	0.5338711	0.4830655	1	ZBTB7B

Biological Process	GO:0043387	mycotoxin catabolic process	1/2279	2/18866	0.2270118	0.5338711	0.4830655	1	NFE2L2
Biological Process	GO:0043974	histone H3-K27 acetylation	1/2279	2/18866	0.2270118	0.5338711	0.4830655	1	LIF
Biological Process	GO:0044010	single-species biofilm formation	1/2279	2/18866	0.2270118	0.5338711	0.4830655	1	LTF
Biological Process	GO:0044111	development involved in symbiotic interaction	1/2279	2/18866	0.2270118	0.5338711	0.4830655	1	NEDD4
Biological Process	GO:0044334	canonical Wnt signaling pathway involved in positive regulation of epithelial to mesenchymal transition	1/2279	2/18866	0.2270118	0.5338711	0.4830655	1	TCF7L2
Biological Process	GO:0044407	single-species biofilm formation in or on host organism	1/2279	2/18866	0.2270118	0.5338711	0.4830655	1	LTF
Biological Process	GO:0044501	modulation of signal transduction in other organism	1/2279	2/18866	0.2270118	0.5338711	0.4830655	1	TNIP1
Biological Process	GO:0044778	meiotic DNA integrity checkpoint	1/2279	2/18866	0.2270118	0.5338711	0.4830655	1	HUS1
Biological Process	GO:0045004	DNA replication proofreading	1/2279	2/18866	0.2270118	0.5338711	0.4830655	1	POLE

Biological Process	GO:0045168	cell-cell signaling involved in cell fate commitment	1/2279	2/18866	0.2270118	0.5338711	0.4830655	1	DLL1
Biological Process	GO:0045210	FasL biosynthetic process	1/2279	2/18866	0.2270118	0.5338711	0.4830655	1	PHLDA1
Biological Process	GO:0045643	regulation of eosinophil differentiation	1/2279	2/18866	0.2270118	0.5338711	0.4830655	1	TRIB1
Biological Process	GO:0045645	positive regulation of eosinophil differentiation	1/2279	2/18866	0.2270118	0.5338711	0.4830655	1	TRIB1
Biological Process	GO:0045918	negative regulation of cytolysis	1/2279	2/18866	0.2270118	0.5338711	0.4830655	1	TGFB1
Biological Process	GO:0046223	aflatoxin catabolic process	1/2279	2/18866	0.2270118	0.5338711	0.4830655	1	NFE2L2
Biological Process	GO:0046327	glycerol biosynthetic process from pyruvate	1/2279	2/18866	0.2270118	0.5338711	0.4830655	1	PCK2
Biological Process	GO:0046331	lateral inhibition	1/2279	2/18866	0.2270118	0.5338711	0.4830655	1	DLL1
Biological Process	GO:0046356	acetyl-CoA catabolic process	1/2279	2/18866	0.2270118	0.5338711	0.4830655	1	ACAT1

Biological Process	GO:0046511	sphinganine biosynthetic process	1/2279	2/18866	0.2270118	0.5338711	0.4830655	1	SPTLC2
Biological Process	GO:0046586	regulation of calcium-dependent cell-cell adhesion	1/2279	2/18866	0.2270118	0.5338711	0.4830655	1	KIFAP3
Biological Process	GO:0046618	drug export	1/2279	2/18866	0.2270118	0.5338711	0.4830655	1	ABCC2
Biological Process	GO:0046724	oxalic acid secretion	1/2279	2/18866	0.2270118	0.5338711	0.4830655	1	AGXT
Biological Process	GO:0046878	positive regulation of saliva secretion	1/2279	2/18866	0.2270118	0.5338711	0.4830655	1	AQP1
Biological Process	GO:0048250	iron import into the mitochondrion	1/2279	2/18866	0.2270118	0.5338711	0.4830655	1	SLC25A37
Biological Process	GO:0048320	axial mesoderm formation	1/2279	2/18866	0.2270118	0.5338711	0.4830655	1	NODAL
Biological Process	GO:0048372	lateral mesodermal cell fate commitment	1/2279	2/18866	0.2270118	0.5338711	0.4830655	1	FGFR1
Biological Process	GO:0048377	lateral mesodermal cell fate specification	1/2279	2/18866	0.2270118	0.5338711	0.4830655	1	FGFR1

Biological Process	GO:0048378	regulation of lateral mesodermal cell fate specification	1/2279	2/18866	0.2270118	0.5338711	0.4830655	1	FGFR1
Biological Process	GO:0048817	negative regulation of hair follicle maturation	1/2279	2/18866	0.2270118	0.5338711	0.4830655	1	CDH3
Biological Process	GO:0048882	lateral line development	1/2279	2/18866	0.2270118	0.5338711	0.4830655	1	SLC44A4
Biological Process	GO:0048884	neuromast development	1/2279	2/18866	0.2270118	0.5338711	0.4830655	1	SLC44A4
Biological Process	GO:0048886	neuromast hair cell differentiation	1/2279	2/18866	0.2270118	0.5338711	0.4830655	1	SLC44A4
Biological Process	GO:0050653	chondroitin sulfate proteoglycan biosynthetic process, polysaccharide chain biosynthetic process	1/2279	2/18866	0.2270118	0.5338711	0.4830655	1	CSGALNACT1
Biological Process	GO:0050666	regulation of homocysteine metabolic process	1/2279	2/18866	0.2270118	0.5338711	0.4830655	1	COMT
Biological Process	GO:0050787	detoxification of mercury ion	1/2279	2/18866	0.2270118	0.5338711	0.4830655	1	ABCC2
Biological Process	GO:0050992	dimethylallyl diphosphate biosynthetic process	1/2279	2/18866	0.2270118	0.5338711	0.4830655	1	IDI1

Biological Process	GO:0050993	dimethylallyl diphosphate metabolic process	1/2279	2/18866	0.2270118	0.5338711	0.4830655	1	IDI1
Biological Process	GO:0051134	negative regulation of NK T cell activation	1/2279	2/18866	0.2270118	0.5338711	0.4830655	1	ZBTB7B
Biological Process	GO:0051352	negative regulation of ligase activity	1/2279	2/18866	0.2270118	0.5338711	0.4830655	1	TFIP11
Biological Process	GO:0051389	inactivation of MAPKK activity	1/2279	2/18866	0.2270118	0.5338711	0.4830655	1	IGF1R
Biological Process	GO:0051563	smooth endoplasmic reticulum calcium ion homeostasis	1/2279	2/18866	0.2270118	0.5338711	0.4830655	1	APP
Biological Process	GO:0051958	methotrexate transport	1/2279	2/18866	0.2270118	0.5338711	0.4830655	1	SLC19A1
Biological Process	GO:0051977	lysophospholipid transport	1/2279	2/18866	0.2270118	0.5338711	0.4830655	1	MFSD2A
Biological Process	GO:0052027	modulation by symbiont of host signal transduction pathway	1/2279	2/18866	0.2270118	0.5338711	0.4830655	1	TNIP1
Biological Process	GO:0052314	phytoalexin metabolic process	1/2279	2/18866	0.2270118	0.5338711	0.4830655	1	DDC

Biological Process	GO:0060031	mediolateral intercalation	1/2279	2/18866	0.2270118	0.5338711	0.4830655	1	WNT11
Biological Process	GO:0060057	apoptotic process involved in mammary gland involution	1/2279	2/18866	0.2270118	0.5338711	0.4830655	1	VDR
Biological Process	GO:0060058	positive regulation of apoptotic process involved in mammary gland involution	1/2279	2/18866	0.2270118	0.5338711	0.4830655	1	VDR
Biological Process	GO:0060060	post-embryonic retina morphogenesis in camera-type eye	1/2279	2/18866	0.2270118	0.5338711	0.4830655	1	CRB1
Biological Process	GO:0060279	positive regulation of ovulation	1/2279	2/18866	0.2270118	0.5338711	0.4830655	1	INHBA
Biological Process	GO:0060302	negative regulation of cytokine activity	1/2279	2/18866	0.2270118	0.5338711	0.4830655	1	IL10
Biological Process	GO:0060375	regulation of mast cell differentiation	1/2279	2/18866	0.2270118	0.5338711	0.4830655	1	ZFPM1
Biological Process	GO:0060378	regulation of brood size	1/2279	2/18866	0.2270118	0.5338711	0.4830655	1	SH3PXD2B
Biological Process	GO:0060434	bronchus morphogenesis	1/2279	2/18866	0.2270118	0.5338711	0.4830655	1	TGFBR2

Biological Process	GO:0060459	left lung development	1/2279	2/18866	0.2270118	0.5338711	0.4830655	1	NODAL
Biological Process	GO:0060567	negative regulation of DNA-templated transcription, termination	1/2279	2/18866	0.2270118	0.5338711	0.4830655	1	SCAF8
Biological Process	GO:0060694	regulation of cholesterol transporter activity	1/2279	2/18866	0.2270118	0.5338711	0.4830655	1	PPARG
Biological Process	GO:0060730	regulation of intestinal epithelial structure maintenance	1/2279	2/18866	0.2270118	0.5338711	0.4830655	1	SLC22A5
Biological Process	GO:0060775	planar cell polarity pathway involved in gastrula mediolateral intercalation	1/2279	2/18866	0.2270118	0.5338711	0.4830655	1	WNT11
Biological Process	GO:0060816	random inactivation of X chromosome	1/2279	2/18866	0.2270118	0.5338711	0.4830655	1	CDYL
Biological Process	GO:0060819	inactivation of X chromosome by genetic imprinting	1/2279	2/18866	0.2270118	0.5338711	0.4830655	1	PCGF3
Biological Process	GO:0060922	atrioventricular node cell differentiation	1/2279	2/18866	0.2270118	0.5338711	0.4830655	1	MAML1
Biological Process	GO:0060928	atrioventricular node cell development	1/2279	2/18866	0.2270118	0.5338711	0.4830655	1	MAML1

Biological Process	GO:0061015	snRNA import into nucleus	1/2279	2/18866	0.2270118	0.5338711	0.4830655	1	SNUPN
Biological Process	GO:0061193	taste bud development	1/2279	2/18866	0.2270118	0.5338711	0.4830655	1	NTF4
Biological Process	GO:0061366	behavioral response to chemical pain	1/2279	2/18866	0.2270118	0.5338711	0.4830655	1	NTRK1
Biological Process	GO:0061368	behavioral response to formalin induced pain	1/2279	2/18866	0.2270118	0.5338711	0.4830655	1	NTRK1
Biological Process	GO:0061373	mammillary axonal complex development	1/2279	2/18866	0.2270118	0.5338711	0.4830655	1	ZEB2
Biological Process	GO:0061552	ganglion morphogenesis	1/2279	2/18866	0.2270118	0.5338711	0.4830655	1	NRP2
Biological Process	GO:0061582	intestinal epithelial cell migration	1/2279	2/18866	0.2270118	0.5338711	0.4830655	1	ARSB
Biological Process	GO:0061741	chaperone-mediated protein transport involved in chaperone-mediated autophagy	1/2279	2/18866	0.2270118	0.5338711	0.4830655	1	CLU
Biological Process	GO:0061795	Golgi lumen acidification	1/2279	2/18866	0.2270118	0.5338711	0.4830655	1	UBE3A

Biological Process	GO:0061901	regulation of 1-phosphatidylinositol-3-kinase activity	1/2279	2/18866	0.2270118	0.5338711	0.4830655	1	FPR2
Biological Process	GO:0061903	positive regulation of 1-phosphatidylinositol-3-kinase activity	1/2279	2/18866	0.2270118	0.5338711	0.4830655	1	FPR2
Biological Process	GO:0062111	zinc ion import into organelle	1/2279	2/18866	0.2270118	0.5338711	0.4830655	1	TMEM163
Biological Process	GO:0070165	positive regulation of adiponectin secretion	1/2279	2/18866	0.2270118	0.5338711	0.4830655	1	C1QTNF3
Biological Process	GO:0070453	regulation of heme biosynthetic process	1/2279	2/18866	0.2270118	0.5338711	0.4830655	1	TMEM14C
Biological Process	GO:0070488	neutrophil aggregation	1/2279	2/18866	0.2270118	0.5338711	0.4830655	1	S100A8
Biological Process	GO:0070889	platelet alpha granule organization	1/2279	2/18866	0.2270118	0.5338711	0.4830655	1	ZNF385A
Biological Process	GO:0070898	RNA polymerase III preinitiation complex assembly	1/2279	2/18866	0.2270118	0.5338711	0.4830655	1	BRF1
Biological Process	GO:0070904	transepithelial L-ascorbic acid transport	1/2279	2/18866	0.2270118	0.5338711	0.4830655	1	SLC23A1

Biological Process	GO:0070947	neutrophil-mediated killing of fungus	1/2279	2/18866	0.2270118	0.5338711	0.4830655	1	ELANE
Biological Process	GO:0071031	nuclear mRNA surveillance of mRNA 3'-end processing	1/2279	2/18866	0.2270118	0.5338711	0.4830655	1	EXOSC2
Biological Process	GO:0071049	nuclear retention of pre-mRNA with aberrant 3'-ends at the site of transcription	1/2279	2/18866	0.2270118	0.5338711	0.4830655	1	EXOSC2
Biological Process	GO:0071105	response to interleukin-11	1/2279	2/18866	0.2270118	0.5338711	0.4830655	1	SBNO2
Biological Process	GO:0071306	cellular response to vitamin E	1/2279	2/18866	0.2270118	0.5338711	0.4830655	1	PPARG
Biological Process	GO:0071403	cellular response to high density lipoprotein particle stimulus	1/2279	2/18866	0.2270118	0.5338711	0.4830655	1	ABCG1
Biological Process	GO:0071469	cellular response to alkaline pH	1/2279	2/18866	0.2270118	0.5338711	0.4830655	1	INSRR
Biological Process	GO:0071505	response to mycophenolic acid	1/2279	2/18866	0.2270118	0.5338711	0.4830655	1	PDGFB
Biological Process	GO:0071506	cellular response to mycophenolic acid	1/2279	2/18866	0.2270118	0.5338711	0.4830655	1	PDGFB

Biological Process	GO:0071874	cellular response to norepinephrine stimulus	1/2279	2/18866	0.2270118	0.5338711	0.4830655	1	APP
Biological Process	GO:0071988	protein localization to spindle pole body	1/2279	2/18866	0.2270118	0.5338711	0.4830655	1	NUMA1
Biological Process	GO:0072004	kidney field specification	1/2279	2/18866	0.2270118	0.5338711	0.4830655	1	PAX2
Biological Process	GO:0072209	metanephric mesangial cell differentiation	1/2279	2/18866	0.2270118	0.5338711	0.4830655	1	PDGFB
Biological Process	GO:0072237	metanephric proximal tubule development	1/2279	2/18866	0.2270118	0.5338711	0.4830655	1	ACAT1
Biological Process	GO:0072254	metanephric glomerular mesangial cell differentiation	1/2279	2/18866	0.2270118	0.5338711	0.4830655	1	PDGFB
Biological Process	GO:0072304	regulation of mesenchymal cell apoptotic process involved in metanephric nephron morphogenesis	1/2279	2/18866	0.2270118	0.5338711	0.4830655	1	PAX2
Biological Process	GO:0072305	negative regulation of mesenchymal cell apoptotic process involved in metanephric nephron morphogenesis	1/2279	2/18866	0.2270118	0.5338711	0.4830655	1	PAX2
Biological Process	GO:0072309	mesenchymal stem cell maintenance involved in metanephric nephron morphogenesis	1/2279	2/18866	0.2270118	0.5338711	0.4830655	1	PAX2

Biological Process	GO:0072343	pancreatic stellate cell proliferation	1/2279	2/18866	0.2270118	0.5338711	0.4830655	1	PPARG
Biological Process	GO:0072554	blood vessel lumenization	1/2279	2/18866	0.2270118	0.5338711	0.4830655	1	RBPJ
Biological Process	GO:0072560	type B pancreatic cell maturation	1/2279	2/18866	0.2270118	0.5338711	0.4830655	1	RFX3
Biological Process	GO:0072564	blood microparticle formation	1/2279	2/18866	0.2270118	0.5338711	0.4830655	1	TNF
Biological Process	GO:0072573	tolerance induction to lipopolysaccharide	1/2279	2/18866	0.2270118	0.5338711	0.4830655	1	TNFAIP3
Biological Process	GO:0072720	response to dithiothreitol	1/2279	2/18866	0.2270118	0.5338711	0.4830655	1	KAT7
Biological Process	GO:0072739	response to anisomycin	1/2279	2/18866	0.2270118	0.5338711	0.4830655	1	KAT7
Biological Process	GO:0072752	cellular response to rapamycin	1/2279	2/18866	0.2270118	0.5338711	0.4830655	1	LARP1
Biological Process	GO:0086021	SA node cell to atrial cardiac muscle cell communication by electrical coupling	1/2279	2/18866	0.2270118	0.5338711	0.4830655	1	GJA5

Biological Process	GO:0086053	AV node cell to bundle of His cell communication by electrical coupling	1/2279	2/18866	0.2270118	0.5338711	0.4830655	1	GJA5
Biological Process	GO:0086097	phospholipase C-activating angiotensin-activated signaling pathway	1/2279	2/18866	0.2270118	0.5338711	0.4830655	1	AGTR1
Biological Process	GO:0090149	mitochondrial membrane fission	1/2279	2/18866	0.2270118	0.5338711	0.4830655	1	DNM1L
Biological Process	GO:0090164	asymmetric Golgi ribbon formation	1/2279	2/18866	0.2270118	0.5338711	0.4830655	1	MYO18A
Biological Process	GO:0090182	regulation of secretion of lysosomal enzymes	1/2279	2/18866	0.2270118	0.5338711	0.4830655	1	NR1H2
Biological Process	GO:0090187	positive regulation of pancreatic juice secretion	1/2279	2/18866	0.2270118	0.5338711	0.4830655	1	NR1H2
Biological Process	GO:0090191	negative regulation of branching involved in ureteric bud morphogenesis	1/2279	2/18866	0.2270118	0.5338711	0.4830655	1	TACSTD2
Biological Process	GO:0090265	positive regulation of immune complex clearance by monocytes and macrophages	1/2279	2/18866	0.2270118	0.5338711	0.4830655	1	CCR2
Biological Process	GO:0090291	negative regulation of osteoclast proliferation	1/2279	2/18866	0.2270118	0.5338711	0.4830655	1	TNFAIP3

Biological Process	GO:0090345	cellular organohalogen metabolic process	1/2279	2/18866	0.2270118	0.5338711	0.4830655	1	POR
Biological Process	GO:0090346	cellular organofluorine metabolic process	1/2279	2/18866	0.2270118	0.5338711	0.4830655	1	POR
Biological Process	GO:0090615	mitochondrial mRNA processing	1/2279	2/18866	0.2270118	0.5338711	0.4830655	1	TBRG4
Biological Process	GO:0090675	intermicrovillar adhesion	1/2279	2/18866	0.2270118	0.5338711	0.4830655	1	CDHR2
Biological Process	GO:0097102	endothelial tip cell fate specification	1/2279	2/18866	0.2270118	0.5338711	0.4830655	1	DLL1
Biological Process	GO:0097324	melanocyte migration	1/2279	2/18866	0.2270118	0.5338711	0.4830655	1	ZEB2
Biological Process	GO:0097528	execution phase of necroptosis	1/2279	2/18866	0.2270118	0.5338711	0.4830655	1	RIPK3
Biological Process	GO:0097707	ferroptosis	1/2279	2/18866	0.2270118	0.5338711	0.4830655	1	AIFM2
Biological Process	GO:0097750	endosome membrane tubulation	1/2279	2/18866	0.2270118	0.5338711	0.4830655	1	CORO1C

Biological Process	GO:0098630	aggregation of unicellular organisms	1/2279	2/18866	0.2270118	0.5338711	0.4830655	1	LTF
Biological Process	GO:0098795	mRNA cleavage involved in gene silencing	1/2279	2/18866	0.2270118	0.5338711	0.4830655	1	AGO2
Biological Process	GO:0098886	modification of dendritic spine	1/2279	2/18866	0.2270118	0.5338711	0.4830655	1	SYNPO
Biological Process	GO:0098905	regulation of bundle of His cell action potential	1/2279	2/18866	0.2270118	0.5338711	0.4830655	1	GJA5
Biological Process	GO:0098907	regulation of SA node cell action potential	1/2279	2/18866	0.2270118	0.5338711	0.4830655	1	ANK2
Biological Process	GO:0099178	regulation of retrograde trans-synaptic signaling by endocanabinoid	1/2279	2/18866	0.2270118	0.5338711	0.4830655	1	PLCB1
Biological Process	GO:0099180	zinc ion import into synaptic vesicle	1/2279	2/18866	0.2270118	0.5338711	0.4830655	1	TMEM163
Biological Process	GO:0099403	maintenance of mitotic sister chromatid cohesion, telomeric	1/2279	2/18866	0.2270118	0.5338711	0.4830655	1	TNKS
Biological Process	GO:0099404	mitotic sister chromatid cohesion, telomeric	1/2279	2/18866	0.2270118	0.5338711	0.4830655	1	TNKS

Biological Process	GO:0099642	retrograde axonal protein transport	1/2279	2/18866	0.2270118	0.5338711	0.4830655	1	MAP1A
Biological Process	GO:0110075	regulation of ferroptosis	1/2279	2/18866	0.2270118	0.5338711	0.4830655	1	AIFM2
Biological Process	GO:0110076	negative regulation of ferroptosis	1/2279	2/18866	0.2270118	0.5338711	0.4830655	1	AIFM2
Biological Process	GO:0110091	negative regulation of hippocampal neuron apoptotic process	1/2279	2/18866	0.2270118	0.5338711	0.4830655	1	CX3CR1
Biological Process	GO:0120054	intestinal motility	1/2279	2/18866	0.2270118	0.5338711	0.4830655	1	GHRL
Biological Process	GO:0120055	small intestinal transit	1/2279	2/18866	0.2270118	0.5338711	0.4830655	1	GHRL
Biological Process	GO:0120057	regulation of small intestinal transit	1/2279	2/18866	0.2270118	0.5338711	0.4830655	1	GHRL
Biological Process	GO:0120058	positive regulation of small intestinal transit	1/2279	2/18866	0.2270118	0.5338711	0.4830655	1	GHRL
Biological Process	GO:0120191	negative regulation of termination of RNA polymerase II transcription	1/2279	2/18866	0.2270118	0.5338711	0.4830655	1	SCAF8

Biological Process	GO:0140066	peptidyl-lysine crotonylation	1/2279	2/18866	0.2270118	0.5338711	0.4830655	1	CDYL
Biological Process	GO:1900052	regulation of retinoic acid biosynthetic process	1/2279	2/18866	0.2270118	0.5338711	0.4830655	1	RDH10
Biological Process	GO:1900110	negative regulation of histone H3-K9 dimethylation	1/2279	2/18866	0.2270118	0.5338711	0.4830655	1	PIH1D1
Biological Process	GO:1900169	regulation of glucocorticoid mediated signaling pathway	1/2279	2/18866	0.2270118	0.5338711	0.4830655	1	AKAP13
Biological Process	GO:1900190	regulation of single-species biofilm formation	1/2279	2/18866	0.2270118	0.5338711	0.4830655	1	LTF
Biological Process	GO:1900191	negative regulation of single-species biofilm formation	1/2279	2/18866	0.2270118	0.5338711	0.4830655	1	LTF
Biological Process	GO:1900204	apoptotic process involved in metanephric collecting duct development	1/2279	2/18866	0.2270118	0.5338711	0.4830655	1	PAX2
Biological Process	GO:1900205	apoptotic process involved in metanephric nephron tubule development	1/2279	2/18866	0.2270118	0.5338711	0.4830655	1	PAX2
Biological Process	GO:1900214	regulation of apoptotic process involved in metanephric collecting duct development	1/2279	2/18866	0.2270118	0.5338711	0.4830655	1	PAX2

Biological Process	GO:1900215	negative regulation of apoptotic process involved in metanephric collecting duct development	1/2279	2/18866	0.2270118	0.5338711	0.4830655	1	PAX2
Biological Process	GO:1900217	regulation of apoptotic process involved in metanephric nephron tubule development	1/2279	2/18866	0.2270118	0.5338711	0.4830655	1	PAX2
Biological Process	GO:1900218	negative regulation of apoptotic process involved in metanephric nephron tubule development	1/2279	2/18866	0.2270118	0.5338711	0.4830655	1	PAX2
Biological Process	GO:1900220	semaphorin-plexin signaling pathway involved in bone trabecula morphogenesis	1/2279	2/18866	0.2270118	0.5338711	0.4830655	1	SEMA4D
Biological Process	GO:1900224	positive regulation of nodal signaling pathway involved in determination of lateral mesoderm left/right asymmetry	1/2279	2/18866	0.2270118	0.5338711	0.4830655	1	NODAL
Biological Process	GO:1900228	regulation of single-species biofilm formation in or on host organism	1/2279	2/18866	0.2270118	0.5338711	0.4830655	1	LTF
Biological Process	GO:1900229	negative regulation of single-species biofilm formation in or on host organism	1/2279	2/18866	0.2270118	0.5338711	0.4830655	1	LTF
Biological Process	GO:1900248	negative regulation of cytoplasmic translational elongation	1/2279	2/18866	0.2270118	0.5338711	0.4830655	1	CPEB3
Biological Process	GO:1900533	palmitic acid metabolic process	1/2279	2/18866	0.2270118	0.5338711	0.4830655	1	ACOT7

Biological Process	GO:1900535	palmitic acid biosynthetic process	1/2279	2/18866	0.2270118	0.5338711	0.4830655	1	ACOT7
Biological Process	GO:1901147	mesenchymal cell apoptotic process involved in metanephric nephron morphogenesis	1/2279	2/18866	0.2270118	0.5338711	0.4830655	1	PAX2
Biological Process	GO:1901187	regulation of ephrin receptor signaling pathway	1/2279	2/18866	0.2270118	0.5338711	0.4830655	1	RBPJ
Biological Process	GO:1901255	nucleotide-excision repair involved in interstrand cross-link repair	1/2279	2/18866	0.2270118	0.5338711	0.4830655	1	XPA
Biological Process	GO:1901337	thioester transport	1/2279	2/18866	0.2270118	0.5338711	0.4830655	1	SLC33A1
Biological Process	GO:1901377	organic heteropentacyclic compound catabolic process	1/2279	2/18866	0.2270118	0.5338711	0.4830655	1	NFE2L2
Biological Process	GO:1901383	negative regulation of chorionic trophoblast cell proliferation	1/2279	2/18866	0.2270118	0.5338711	0.4830655	1	NODAL
Biological Process	GO:1901463	regulation of tetrapyrrole biosynthetic process	1/2279	2/18866	0.2270118	0.5338711	0.4830655	1	TMEM14C
Biological Process	GO:1901558	response to metformin	1/2279	2/18866	0.2270118	0.5338711	0.4830655	1	PPARG

Biological Process	GO:1901647	positive regulation of synoviocyte proliferation	1/2279	2/18866	0.2270118	0.5338711	0.4830655	1	TNF
Biological Process	GO:1901674	regulation of histone H3-K27 acetylation	1/2279	2/18866	0.2270118	0.5338711	0.4830655	1	LIF
Biological Process	GO:1901898	negative regulation of relaxation of cardiac muscle	1/2279	2/18866	0.2270118	0.5338711	0.4830655	1	PDE4D
Biological Process	GO:1902283	negative regulation of primary amine oxidase activity	1/2279	2/18866	0.2270118	0.5338711	0.4830655	1	PRKN
Biological Process	GO:1902298	cell cycle DNA replication maintenance of fidelity	1/2279	2/18866	0.2270118	0.5338711	0.4830655	1	BRCA2
Biological Process	GO:1902435	regulation of male mating behavior	1/2279	2/18866	0.2270118	0.5338711	0.4830655	1	HDAC4
Biological Process	GO:1902437	positive regulation of male mating behavior	1/2279	2/18866	0.2270118	0.5338711	0.4830655	1	HDAC4
Biological Process	GO:1902528	regulation of protein linear polyubiquitination	1/2279	2/18866	0.2270118	0.5338711	0.4830655	1	PRKN
Biological Process	GO:1902530	positive regulation of protein linear polyubiquitination	1/2279	2/18866	0.2270118	0.5338711	0.4830655	1	PRKN

Biological Process	GO:1902567	negative regulation of eosinophil activation	1/2279	2/18866	0.2270118	0.5338711	0.4830655	1	CCR2
Biological Process	GO:1902748	positive regulation of lens fiber cell differentiation	1/2279	2/18866	0.2270118	0.5338711	0.4830655	1	ZEB2
Biological Process	GO:1902767	isoprenoid biosynthetic process via mevalonate	1/2279	2/18866	0.2270118	0.5338711	0.4830655	1	HMGCS1
Biological Process	GO:1902908	regulation of melanosome transport	1/2279	2/18866	0.2270118	0.5338711	0.4830655	1	CDH3
Biological Process	GO:1902954	regulation of early endosome to recycling endosome transport	1/2279	2/18866	0.2270118	0.5338711	0.4830655	1	SORL1
Biological Process	GO:1902962	regulation of metalloendopeptidase activity involved in amyloid precursor protein catabolic process	1/2279	2/18866	0.2270118	0.5338711	0.4830655	1	SORL1
Biological Process	GO:1902963	negative regulation of metalloendopeptidase activity involved in amyloid precursor protein catabolic process	1/2279	2/18866	0.2270118	0.5338711	0.4830655	1	SORL1
Biological Process	GO:1903028	positive regulation of opsonization	1/2279	2/18866	0.2270118	0.5338711	0.4830655	1	MYO18A
Biological Process	GO:1903056	regulation of melanosome organization	1/2279	2/18866	0.2270118	0.5338711	0.4830655	1	ZEB2

Biological Process	GO:1903109	positive regulation of mitochondrial transcription	1/2279	2/18866	0.2270118	0.5338711	0.4830655	1	PRKAA1
Biological Process	GO:1903381	regulation of endoplasmic reticulum stress-induced neuron intrinsic apoptotic signaling pathway	1/2279	2/18866	0.2270118	0.5338711	0.4830655	1	PRKN
Biological Process	GO:1903382	negative regulation of endoplasmic reticulum stress-induced neuron intrinsic apoptotic signaling pathway	1/2279	2/18866	0.2270118	0.5338711	0.4830655	1	PRKN
Biological Process	GO:1903393	positive regulation of adherens junction organization	1/2279	2/18866	0.2270118	0.5338711	0.4830655	1	ADD1
Biological Process	GO:1903433	regulation of constitutive secretory pathway	1/2279	2/18866	0.2270118	0.5338711	0.4830655	1	RAB27A
Biological Process	GO:1903464	negative regulation of mitotic cell cycle DNA replication	1/2279	2/18866	0.2270118	0.5338711	0.4830655	1	CHEK2
Biological Process	GO:1903515	calcium ion transport from cytosol to endoplasmic reticulum	1/2279	2/18866	0.2270118	0.5338711	0.4830655	1	ATP2A3
Biological Process	GO:1903519	regulation of mammary gland involution	1/2279	2/18866	0.2270118	0.5338711	0.4830655	1	VDR
Biological Process	GO:1903521	positive regulation of mammary gland involution	1/2279	2/18866	0.2270118	0.5338711	0.4830655	1	VDR

Biological Process	GO:1903537	meiotic cell cycle process involved in oocyte maturation	1/2279	2/18866	0.2270118	0.5338711	0.4830655	1	PPP2R1A
Biological Process	GO:1903538	regulation of meiotic cell cycle process involved in oocyte maturation	1/2279	2/18866	0.2270118	0.5338711	0.4830655	1	PPP2R1A
Biological Process	GO:1903567	regulation of protein localization to ciliary membrane	1/2279	2/18866	0.2270118	0.5338711	0.4830655	1	LZTFL1
Biological Process	GO:1903704	negative regulation of production of siRNA involved in RNA interference	1/2279	2/18866	0.2270118	0.5338711	0.4830655	1	RMRP
Biological Process	GO:1903786	regulation of glutathione biosynthetic process	1/2279	2/18866	0.2270118	0.5338711	0.4830655	1	NFE2L2
Biological Process	GO:1903849	positive regulation of aorta morphogenesis	1/2279	2/18866	0.2270118	0.5338711	0.4830655	1	NOTCH1
Biological Process	GO:1903860	negative regulation of dendrite extension	1/2279	2/18866	0.2270118	0.5338711	0.4830655	1	BCL11A
Biological Process	GO:1903905	positive regulation of establishment of T cell polarity	1/2279	2/18866	0.2270118	0.5338711	0.4830655	1	DOCK8
Biological Process	GO:1903939	regulation of TORC2 signaling	1/2279	2/18866	0.2270118	0.5338711	0.4830655	1	SIK3

Biological Process	GO:1903946	negative regulation of ventricular cardiac muscle cell action potential	1/2279	2/18866	0.2270118	0.5338711	0.4830655	1	BIN1
Biological Process	GO:1903966	monounsaturated fatty acid biosynthetic process	1/2279	2/18866	0.2270118	0.5338711	0.4830655	1	SCD
Biological Process	GO:1904028	positive regulation of collagen fibril organization	1/2279	2/18866	0.2270118	0.5338711	0.4830655	1	RB1
Biological Process	GO:1904049	negative regulation of spontaneous neurotransmitter secretion	1/2279	2/18866	0.2270118	0.5338711	0.4830655	1	PRKN
Biological Process	GO:1904057	negative regulation of sensory perception of pain	1/2279	2/18866	0.2270118	0.5338711	0.4830655	1	IL10
Biological Process	GO:1904155	DN2 thymocyte differentiation	1/2279	2/18866	0.2270118	0.5338711	0.4830655	1	PTPRC
Biological Process	GO:1904161	DNA synthesis involved in UV-damage excision repair	1/2279	2/18866	0.2270118	0.5338711	0.4830655	1	POLD3
Biological Process	GO:1904178	negative regulation of adipose tissue development	1/2279	2/18866	0.2270118	0.5338711	0.4830655	1	KLF7
Biological Process	GO:1904211	membrane protein proteolysis involved in retrograde protein transport, ER to cytosol	1/2279	2/18866	0.2270118	0.5338711	0.4830655	1	HM13

Biological Process	GO:1904245	regulation of polynucleotide adenylyltransferase activity	1/2279	2/18866	0.2270118	0.5338711	0.4830655	1	PABPN1
Biological Process	GO:1904344	regulation of gastric mucosal blood circulation	1/2279	2/18866	0.2270118	0.5338711	0.4830655	1	GHRL
Biological Process	GO:1904346	positive regulation of gastric mucosal blood circulation	1/2279	2/18866	0.2270118	0.5338711	0.4830655	1	GHRL
Biological Process	GO:1904426	positive regulation of GTP binding	1/2279	2/18866	0.2270118	0.5338711	0.4830655	1	CLN5
Biological Process	GO:1904515	positive regulation of TORC2 signaling	1/2279	2/18866	0.2270118	0.5338711	0.4830655	1	SIK3
Biological Process	GO:1904556	L-tryptophan transmembrane transport	1/2279	2/18866	0.2270118	0.5338711	0.4830655	1	SLC7A5
Biological Process	GO:1904576	response to tunicamycin	1/2279	2/18866	0.2270118	0.5338711	0.4830655	1	CLU
Biological Process	GO:1904577	cellular response to tunicamycin	1/2279	2/18866	0.2270118	0.5338711	0.4830655	1	CLU
Biological Process	GO:1904674	positive regulation of somatic stem cell population maintenance	1/2279	2/18866	0.2270118	0.5338711	0.4830655	1	LBH

Biological Process	GO:1904743	negative regulation of telomeric DNA binding	1/2279	2/18866	0.2270118	0.5338711	0.4830655	1	TNKS
Biological Process	GO:1904835	dorsal root ganglion morphogenesis	1/2279	2/18866	0.2270118	0.5338711	0.4830655	1	NRP2
Biological Process	GO:1904875	regulation of DNA ligase activity	1/2279	2/18866	0.2270118	0.5338711	0.4830655	1	TFIP11
Biological Process	GO:1904907	regulation of maintenance of mitotic sister chromatid cohesion, telomeric	1/2279	2/18866	0.2270118	0.5338711	0.4830655	1	TNKS
Biological Process	GO:1904908	negative regulation of maintenance of mitotic sister chromatid cohesion, telomeric	1/2279	2/18866	0.2270118	0.5338711	0.4830655	1	TNKS
Biological Process	GO:1904979	negative regulation of endosome organization	1/2279	2/18866	0.2270118	0.5338711	0.4830655	1	PRKN
Biological Process	GO:1905051	regulation of base-excision repair	1/2279	2/18866	0.2270118	0.5338711	0.4830655	1	FAM168A
Biological Process	GO:1905053	positive regulation of base-excision repair	1/2279	2/18866	0.2270118	0.5338711	0.4830655	1	FAM168A
Biological Process	GO:1905175	negative regulation of vascular associated smooth muscle cell dedifferentiation	1/2279	2/18866	0.2270118	0.5338711	0.4830655	1	MIR145

Biological Process	GO:1905281	positive regulation of retrograde transport, endosome to Golgi	1/2279	2/18866	0.2270118	0.5338711	0.4830655	1	PRKN
Biological Process	GO:1905365	regulation of intralumenal vesicle formation	1/2279	2/18866	0.2270118	0.5338711	0.4830655	1	PRKN
Biological Process	GO:1905366	negative regulation of intralumenal vesicle formation	1/2279	2/18866	0.2270118	0.5338711	0.4830655	1	PRKN
Biological Process	GO:1905371	ceramide phosphoethanolamine metabolic process	1/2279	2/18866	0.2270118	0.5338711	0.4830655	1	SGMS2
Biological Process	GO:1905373	ceramide phosphoethanolamine biosynthetic process	1/2279	2/18866	0.2270118	0.5338711	0.4830655	1	SGMS2
Biological Process	GO:1905524	negative regulation of protein autoubiquitination	1/2279	2/18866	0.2270118	0.5338711	0.4830655	1	MARCHF7
Biological Process	GO:1905526	regulation of Golgi lumen acidification	1/2279	2/18866	0.2270118	0.5338711	0.4830655	1	UBE3A
Biological Process	GO:1905550	regulation of protein localization to endoplasmic reticulum	1/2279	2/18866	0.2270118	0.5338711	0.4830655	1	RTN4
Biological Process	GO:1905552	positive regulation of protein localization to endoplasmic reticulum	1/2279	2/18866	0.2270118	0.5338711	0.4830655	1	RTN4

Biological Process	GO:1905696	regulation of polysome binding	1/2279	2/18866	0.2270118	0.5338711	0.4830655	1	EIF4G1
Biological Process	GO:1905775	negative regulation of DNA helicase activity	1/2279	2/18866	0.2270118	0.5338711	0.4830655	1	MCM2
Biological Process	GO:1905799	regulation of intraciliary retrograde transport	1/2279	2/18866	0.2270118	0.5338711	0.4830655	1	TTC21B
Biological Process	GO:1905863	invadopodium organization	1/2279	2/18866	0.2270118	0.5338711	0.4830655	1	NAV3
Biological Process	GO:1905916	negative regulation of cell differentiation involved in phenotypic switching	1/2279	2/18866	0.2270118	0.5338711	0.4830655	1	DNMT1
Biological Process	GO:1905931	negative regulation of vascular associated smooth muscle cell differentiation involved in phenotypic switching	1/2279	2/18866	0.2270118	0.5338711	0.4830655	1	DNMT1
Biological Process	GO:1990145	maintenance of translational fidelity	1/2279	2/18866	0.2270118	0.5338711	0.4830655	1	CDKAL1
Biological Process	GO:1990267	response to transition metal nanoparticle	1/2279	2/18866	0.2270118	0.5338711	0.4830655	1	MPO
Biological Process	GO:1990402	embryonic liver development	1/2279	2/18866	0.2270118	0.5338711	0.4830655	1	TGFB1

Biological Process	GO:1990426	mitotic recombination-dependent replication fork processing	1/2279	2/18866	0.2270118	0.5338711	0.4830655	1	BRCA2
Biological Process	GO:1990505	mitotic DNA replication maintenance of fidelity	1/2279	2/18866	0.2270118	0.5338711	0.4830655	1	BRCA2
Biological Process	GO:1990768	gastric mucosal blood circulation	1/2279	2/18866	0.2270118	0.5338711	0.4830655	1	GHRL
Biological Process	GO:2000229	regulation of pancreatic stellate cell proliferation	1/2279	2/18866	0.2270118	0.5338711	0.4830655	1	PPARG
Biological Process	GO:2000329	negative regulation of T-helper 17 cell lineage commitment	1/2279	2/18866	0.2270118	0.5338711	0.4830655	1	LOXL3
Biological Process	GO:2000332	regulation of blood microparticle formation	1/2279	2/18866	0.2270118	0.5338711	0.4830655	1	TNF
Biological Process	GO:2000334	positive regulation of blood microparticle formation	1/2279	2/18866	0.2270118	0.5338711	0.4830655	1	TNF
Biological Process	GO:2000349	negative regulation of CD40 signaling pathway	1/2279	2/18866	0.2270118	0.5338711	0.4830655	1	TNFAIP3
Biological Process	GO:2000393	negative regulation of lamellipodium morphogenesis	1/2279	2/18866	0.2270118	0.5338711	0.4830655	1	KANK1

Biological Process	GO:2000422	regulation of eosinophil chemotaxis	1/2279	2/18866	0.2270118	0.5338711	0.4830655	1	DAPK2
Biological Process	GO:2000424	positive regulation of eosinophil chemotaxis	1/2279	2/18866	0.2270118	0.5338711	0.4830655	1	DAPK2
Biological Process	GO:2000458	regulation of astrocyte chemotaxis	1/2279	2/18866	0.2270118	0.5338711	0.4830655	1	CCR2
Biological Process	GO:2000470	positive regulation of peroxidase activity	1/2279	2/18866	0.2270118	0.5338711	0.4830655	1	SIRT3
Biological Process	GO:2000567	regulation of memory T cell activation	1/2279	2/18866	0.2270118	0.5338711	0.4830655	1	CYRIB
Biological Process	GO:2000568	positive regulation of memory T cell activation	1/2279	2/18866	0.2270118	0.5338711	0.4830655	1	CYRIB
Biological Process	GO:2000592	regulation of metanephric DCT cell differentiation	1/2279	2/18866	0.2270118	0.5338711	0.4830655	1	PAX2
Biological Process	GO:2000594	positive regulation of metanephric DCT cell differentiation	1/2279	2/18866	0.2270118	0.5338711	0.4830655	1	PAX2
Biological Process	GO:2000642	negative regulation of early endosome to late endosome transport	1/2279	2/18866	0.2270118	0.5338711	0.4830655	1	SNX3

Biological Process	GO:2000707	positive regulation of dense core granule biogenesis	1/2279	2/18866	0.2270118	0.5338711	0.4830655	1	PRKCA
Biological Process	GO:2000742	regulation of anterior head development	1/2279	2/18866	0.2270118	0.5338711	0.4830655	1	SSBP3
Biological Process	GO:2000744	positive regulation of anterior head development	1/2279	2/18866	0.2270118	0.5338711	0.4830655	1	SSBP3
Biological Process	GO:2000805	negative regulation of termination of RNA polymerase II transcription, poly(A)-coupled	1/2279	2/18866	0.2270118	0.5338711	0.4830655	1	SCAF8
Biological Process	GO:2000828	regulation of parathyroid hormone secretion	1/2279	2/18866	0.2270118	0.5338711	0.4830655	1	FGFR1
Biological Process	GO:2000843	regulation of testosterone secretion	1/2279	2/18866	0.2270118	0.5338711	0.4830655	1	MYB
Biological Process	GO:2000845	positive regulation of testosterone secretion	1/2279	2/18866	0.2270118	0.5338711	0.4830655	1	MYB
Biological Process	GO:2001311	lysobisphosphatidic acid metabolic process	1/2279	2/18866	0.2270118	0.5338711	0.4830655	1	ACP6
Biological Process	GO:0051259	protein complex oligomerization	32/2279	231/18866	0.2289968	0.5380409	0.4868385	32	TRPM2/NLRC4/EHD1/BLM/ACOT13/SLC1A5/KCTD7/TRPM6/HSCB/NACC2/LRRC8C/CRTC3/SLC1A2/ACACB/RNF213/ZDHHC1/NOL3/UPB1/KCTD2/KCTD15/TRPM3/ACACA/TP53BP1/DNM1L/IKZF4/MAT1A/ATL1/KCTD1/ALDH1A2/TK1/KCTD21/STING1

Biological Process	GO:0006497	protein lipidation	14/2279	93/18866	0.2290114	0.5380409	0.4868385	14	ZDHHC18/PIGL/ZDHHC14/PIGU/GPLD1/WIPI2/PIGN/ZDHHC2/ZDHHC7/ABCA1/ZDHHC17/ZDHHC1/PIGQ/SVIP
Biological Process	GO:1903035	negative regulation of response to wounding	14/2279	93/18866	0.2290114	0.5380409	0.4868385	14	PLAU/CD9/SMAD3/TNF/UBASH3B/SERPINB2/THBS1/F12/KREMEN1/PDGFB/CPB2/ADTRP/CERS2/INPP5F
Biological Process	GO:2000117	negative regulation of cysteine-type endopeptidase activity	14/2279	93/18866	0.2290114	0.5380409	0.4868385	14	RPS6KA1/ARRB1/TNF/MICAL1/THBS1/PAX2/AQP1/POR/DNAJB6/NOL3/ARRB2/PIH1D1/LTF/PCID2
Biological Process	GO:0010389	regulation of G2/M transition of mitotic cell cycle	28/2279	200/18866	0.2292285	0.5381517	0.4869387	28	TRIM39/BLM/BRD4/DCTN1/CSNK1D/FOXN3/NDE1/HUS1/DYNC1H1/TUBB4B/CEP135/APP/CDC25A/CEP164/DCTN2/CEP78/CSNK1E/PSMF1/PPP2R1A/TUBA4A/TAOK3/SDCCAG8/ZFYVE19/CLSPN/PSMB7/CEP72/NAE1/PSMD13
Biological Process	GO:0030705	cytoskeleton-dependent intracellular transport	28/2279	200/18866	0.2292285	0.5381517	0.4869387	28	PRKCZ/CCDC88C/NDE1/TNPO1/KIF13A/SPG7/DYNC1H1/APP/ACTN4/WASL/BICD2/CLUAP1/CNIH2/MYO5A/CCDC88B/HTT/KIF5C/HOOK2/IFT140/KIF1B/TTC21B/MAP1A/KIFAP3/MYO1C/AP3D1/FBXW11/NDEL1/DTNBP1
Biological Process	GO:1901654	response to ketone	28/2279	200/18866	0.2292285	0.5381517	0.4869387	28	CALR/PPARG/ADCY2/ABCC2/LARP1/SMYD3/THBS1/FECH/P2RY6/SPHK2/TGFB1/FDX1/AQP1/NCOA1/KIF1B/GNG2/ACACA/SLIT3/PRKCE/UBE3A/PCK2/FOXO1/BCL2L1/NCOA2/SREBF1/RELA/NCOA4/PRKAA1
Biological Process	GO:0030071	regulation of mitotic metaphase/anaphase transition	9/2279	56/18866	0.2300782	0.53948	0.4881407	9	MAD1L1/NSMCE2/CUL3/CDC16/NEK6/RB1/ANAPC7/HECW2/PCID2
Biological Process	GO:0043113	receptor clustering	9/2279	56/18866	0.2300782	0.53948	0.4881407	9	SSH1/GSN/ITGB1BP1/COLQ/ZDHHC2/SYNGAP1/NRXN1/NRXN2/ITGB2
Biological Process	GO:0060563	neuroepithelial cell differentiation	9/2279	56/18866	0.2300782	0.53948	0.4881407	9	DLL1/RBPJ/WNT11/FGFR1/NOTCH1/TMC1/CEBPB/FAM20C/NODAL

Biological Process	GO:0090183	regulation of kidney development	9/2279	56/18866	0.2300782	0.53948	0.4881407	9	TACSTD2/PAX2/PDGFD/WWTR1/IL6R/PDGFB/PDGFRB/LIF/HOXB7
Biological Process	GO:1905515	non-motile cilium assembly	9/2279	56/18866	0.2300782	0.53948	0.4881407	9	DCTN1/CSNK1D/DNM2/DISC1/CEP135/POC1A/IFT140/RPGRIP1/SEPTIN9
Biological Process	GO:0030324	lung development	25/2279	177/18866	0.2307036	0.540813	0.4893468	25	CTSZ/CREB1/CRISPLD2/CHI3L1/TNS3/TNF/LOXL3/SFTPD/TGFBR2/CTSH/RBPJ/WNT11/FGFR1/RDH10/SP3/GRHL2/YWHAZ/NOTCH1/EPAS1/PDGFRB/SREBF1/ALDH1A2/NODAL/LIF/SLC23A1
Biological Process	GO:0032434	regulation of proteasomal ubiquitin-dependent protein catabolic process	19/2279	131/18866	0.2308336	0.5409841	0.4895017	19	TRIM39/RNF144A/FZR1/RNF19A/CSNK1D/CBFA2T3/CLU/SMAD7/CLEC16A/CSNK1E/NFE2L2/RNF144B/MAP1A/GNA12/RNF14/TRIB1/BAG5/SVIP/PRKN
Biological Process	GO:0070482	response to oxygen levels	53/2279	396/18866	0.2309533	0.5411312	0.4896347	53	P4HB/ENO1/PLAU/PPARG/ITPR2/MIR140/TM9SF4/CREB1/MYB/NDRG1/SMAD3/SLC8A1/HIPK2/CBFA2T3/ITPR1/SLC11A2/TGFBR2/THBS1/RBPJ/ACTN4/LIMD1/AQP1/NF1/HK2/ALAS1/AGTRAP/PSMF1/CREBBP/NOL3/CAT/ETS1/NFE2L2/FIS1/PKM/BCL2/PSMB7/NOTCH1/AQP3/DNM1L/EPAS1/PRKCE/NPPC/PDGFRB/SLC2A1/FOXO1/ANKRD1/SLC29A1/BACH1/HSD11B2/ERCC2/PSMD13/MB/PRKAA1
Biological Process	GO:0006354	DNA-templated transcription, elongation	17/2279	116/18866	0.233097	0.5434794	0.4917594	17	BRD4/TAF1C/CTDP1/WDR43/SETD2/POLR2F/RECQL5/ELL2/ENY2/MLLT1/CCNH/SCAF8/TAF1D/KAT7/ERCC2/CDK13/PCID2
Biological Process	GO:0046579	positive regulation of Ras protein signal transduction	11/2279	71/18866	0.2341298	0.5434794	0.4917594	11	AKAP13/ITPKB/ARRB1/LRRC59/SYNPO2L/NTRK1/NOTCH1/F2R/GPR4/PDGFRB/PRAG1
Biological Process	GO:0001771	immunological synapse formation	3/2279	14/18866	0.234562	0.5434794	0.4917594	3	NCK2/DOCK8/DOCK2
Biological Process	GO:0001921	positive regulation of receptor recycling	3/2279	14/18866	0.234562	0.5434794	0.4917594	3	ARAP1/EPS15/INPP5F

Biological Process	GO:0003188	heart valve formation	3/2279	14/18866	0.234562	0.5434794	0.4917594	3	ZFPM1/GJA5/NOTCH1
Biological Process	GO:0003214	cardiac left ventricle morphogenesis	3/2279	14/18866	0.234562	0.5434794	0.4917594	3	TGFBR2/RBPJ/NOTCH1
Biological Process	GO:0010872	regulation of cholesterol esterification	3/2279	14/18866	0.234562	0.5434794	0.4917594	3	ABCG1/MIR27A/AGTR1
Biological Process	GO:0010889	regulation of sequestering of triglyceride	3/2279	14/18866	0.234562	0.5434794	0.4917594	3	PNPLA2/PPARG/ABHD5
Biological Process	GO:0030948	negative regulation of vascular endothelial growth factor receptor signaling pathway	3/2279	14/18866	0.234562	0.5434794	0.4917594	3	EPN2/NEDD4/HHEX
Biological Process	GO:0033169	histone H3-K9 demethylation	3/2279	14/18866	0.234562	0.5434794	0.4917594	3	KDM4B/KDM4C/KDM7A
Biological Process	GO:0034392	negative regulation of smooth muscle cell apoptotic process	3/2279	14/18866	0.234562	0.5434794	0.4917594	3	ARRB2/MIR138-2/DNMT1
Biological Process	GO:0042659	regulation of cell fate specification	3/2279	14/18866	0.234562	0.5434794	0.4917594	3	WNT11/FGFR1/LMO4
Biological Process	GO:0044154	histone H3-K14 acetylation	3/2279	14/18866	0.234562	0.5434794	0.4917594	3	BRD1/KAT7/ING5

Biological Process	GO:0045059	positive thymic T cell selection	3/2279	14/18866	0.234562	0.5434794	0.4917594	3	ITPKB/PTPRC/DOCK2
Biological Process	GO:0045605	negative regulation of epidermal cell differentiation	3/2279	14/18866	0.234562	0.5434794	0.4917594	3	DLL1/GRHL2/NOTCH1
Biological Process	GO:0046462	monoacylglycerol metabolic process	3/2279	14/18866	0.234562	0.5434794	0.4917594	3	MGLL/ABHD16A/ABHD16B
Biological Process	GO:0048681	negative regulation of axon regeneration	3/2279	14/18866	0.234562	0.5434794	0.4917594	3	KREMEN1/CERS2/INPP5F
Biological Process	GO:0051645	Golgi localization	3/2279	14/18866	0.234562	0.5434794	0.4917594	3	UVRAG/YWHAZ/RIPOR1
Biological Process	GO:0051775	response to redox state	3/2279	14/18866	0.234562	0.5434794	0.4917594	3	ARHGDIB/ARNTL/GLRX2
Biological Process	GO:0051917	regulation of fibrinolysis	3/2279	14/18866	0.234562	0.5434794	0.4917594	3	THBS1/F12/CPB2
Biological Process	GO:0052803	imidazole-containing compound metabolic process	3/2279	14/18866	0.234562	0.5434794	0.4917594	3	HAL/MTHFD1/HNMT
Biological Process	GO:0060211	regulation of nuclear-transcribed mRNA poly(A) tail shortening	3/2279	14/18866	0.234562	0.5434794	0.4917594	3	TNRC6B/AGO2/CPEB3

Biological Process	GO:0070431	nucleotide-binding oligomerization domain containing 2 signaling pathway	3/2279	14/18866	0.234562	0.5434794	0.4917594	3	TNFAIP3/NOD2/RELA
Biological Process	GO:0070672	response to interleukin-15	3/2279	14/18866	0.234562	0.5434794	0.4917594	3	STAT5A/JAK1/PLCB1
Biological Process	GO:0070986	left/right axis specification	3/2279	14/18866	0.234562	0.5434794	0.4917594	3	PKD1L1/DLL1/NOTCH1
Biological Process	GO:0071361	cellular response to ethanol	3/2279	14/18866	0.234562	0.5434794	0.4917594	3	ITPR2/PRKCE/PRKAA1
Biological Process	GO:0072425	signal transduction involved in G2 DNA damage checkpoint	3/2279	14/18866	0.234562	0.5434794	0.4917594	3	FZR1/ABRAXAS1/BABAM2
Biological Process	GO:0090161	Golgi ribbon formation	3/2279	14/18866	0.234562	0.5434794	0.4917594	3	VTI1A/MYO18A/NUMA1
Biological Process	GO:0098814	spontaneous synaptic transmission	3/2279	14/18866	0.234562	0.5434794	0.4917594	3	APP/RPH3A/PRKN
Biological Process	GO:0106070	regulation of adenylate cyclase-activating G protein-coupled receptor signaling pathway	3/2279	14/18866	0.234562	0.5434794	0.4917594	3	PRKCA/GNAI2/MRAP
Biological Process	GO:1901626	regulation of postsynaptic membrane organization	3/2279	14/18866	0.234562	0.5434794	0.4917594	3	SSH1/ZMYND8/ZDHHC2

Biological Process	GO:1901722	regulation of cell proliferation involved in kidney development	3/2279	14/18866	0.234562	0.5434794	0.4917594	3	PDGFD/IL6R/PDGFB
Biological Process	GO:1901836	regulation of transcription of nucleolar large rRNA by RNA polymerase I	3/2279	14/18866	0.234562	0.5434794	0.4917594	3	PIH1D1/MARS1/MACROH2A2
Biological Process	GO:1901841	regulation of high voltage-gated calcium channel activity	3/2279	14/18866	0.234562	0.5434794	0.4917594	3	NOS1AP/DYSF/FGF14
Biological Process	GO:1902430	negative regulation of amyloid-beta formation	3/2279	14/18866	0.234562	0.5434794	0.4917594	3	SORL1/BIN1/CLU
Biological Process	GO:1903726	negative regulation of phospholipid metabolic process	3/2279	14/18866	0.234562	0.5434794	0.4917594	3	LPCAT1/PDGFB/MIR138-2
Biological Process	GO:1903799	negative regulation of production of miRNAs involved in gene silencing by miRNA	3/2279	14/18866	0.234562	0.5434794	0.4917594	3	NCOR2/TNF/TGFB1
Biological Process	GO:1904293	negative regulation of ERAD pathway	3/2279	14/18866	0.234562	0.5434794	0.4917594	3	UBAC2/DERL2/SVIP
Biological Process	GO:1904925	positive regulation of autophagy of mitochondrion in response to mitochondrial depolarization	3/2279	14/18866	0.234562	0.5434794	0.4917594	3	SMURF1/HK2/PRKN
Biological Process	GO:2000232	regulation of rRNA processing	3/2279	14/18866	0.234562	0.5434794	0.4917594	3	WDR43/USP36/RIOK1

Biological Process	GO:0002067	glandular epithelial cell differentiation	8/2279	49/18866	0.2348156	0.5434794	0.4917594	8	RXRA/RHEB/RFX3/DLL1/RARA/RARG/ARNTL/NOTCH1
Biological Process	GO:0006654	phosphatidic acid biosynthetic process	8/2279	49/18866	0.2348156	0.5434794	0.4917594	8	LPCAT1/PLD1/ACP6/DGKQ/DGKA/DGKD/ABHD5/AGPAT5
Biological Process	GO:0009395	phospholipid catabolic process	8/2279	49/18866	0.2348156	0.5434794	0.4917594	8	PLA2G15/PLBD2/LIPC/ABHD16A/SMPDL3B/ABHD16B/PLCG1/INPP5F
Biological Process	GO:0015695	organic cation transport	8/2279	49/18866	0.2348156	0.5434794	0.4917594	8	SLC44A4/RALBP1/SEC14L1/ITGB3/SLC22A5/P2RX1/SLC7A8/PDZK1
Biological Process	GO:0018198	peptidyl-cysteine modification	8/2279	49/18866	0.2348156	0.5434794	0.4917594	8	S100A8/ZDHHC18/ZDHHC14/ZDHHC2/NOS1AP/ZDHHC7/ZDHHC1/SNTA1
Biological Process	GO:0035196	production of miRNAs involved in gene silencing by miRNA	8/2279	49/18866	0.2348156	0.5434794	0.4917594	8	NCOR2/ZC3H7A/SMAD3/TNF/AGO1/RIPK1/AGO2/TGFB1
Biological Process	GO:0045058	T cell selection	8/2279	49/18866	0.2348156	0.5434794	0.4917594	8	ZFPM1/ITPKB/LOXL3/LY9/PTPRC/BCL2/CYLD/DOCK2
Biological Process	GO:0048641	regulation of skeletal muscle tissue development	8/2279	49/18866	0.2348156	0.5434794	0.4917594	8	HDAC4/DLL1/TGFB1/ARNTL/BCL2/MEF2C/FLOT1/PRKAA1
Biological Process	GO:0061647	histone H3-K9 modification	8/2279	49/18866	0.2348156	0.5434794	0.4917594	8	MYB/EHMT2/SPHK2/KDM4C/PRDM16/DNMT1/PIH1D1/MECOM

Biological Process	GO:0086009	membrane repolarization	8/2279	49/18866	0.2348156	0.5434794	0.4917594	8	KCNE1/CASQ2/GJA5/NOS1AP/WDR1/SNTA1/KCNQ1/ANK2
Biological Process	GO:0019935	cyclic-nucleotide-mediated signaling	31/2279	224/18866	0.2350229	0.5438266	0.4920737	31	KSR1/PDE4D/MGRN1/ADCY2/AKAP13/PRKAR1B/ADCY9/PRKCA/THBS1/GNAI2/MRAP/CRTC3/RAPGEF2/DGKQ/AQP1/DEFB1/GIPR/ABCA1/CNR2/PDE9A/ADCY4/S1PR2/GNG2/LPAR2/NPPC/S1PR4/GPR4/PDE3B/IRAG1/ADGRG3/ADGRE3
Biological Process	GO:0009791	post-embryonic development	13/2279	86/18866	0.2352595	0.5442416	0.4924491	13	ARID5B/CRB1/PLAGL2/CHST11/ITPR1/AGO2/MORC3/BCL2/STK36/NPPC/ERCC1/ERCC2/ETNK2
Biological Process	GO:0060047	heart contraction	39/2279	287/18866	0.2386561	0.5516087	0.4991152	39	HDAC4/PDE4D/KCNE1/TTN/CASQ2/ITPR2/FXYD2/MAP2K6/CACNA1C/CACNA2D4/FXYD1/SLC8A1/GJA5/ATP2B2/ITPR1/JUP/BIN1/NOS1AP/SMAD7/KCNIP1/SCN1A/KCNK6/CHRM2/ATP2A3/CELF2/SNTA1/KCNQ1/GSTO1/GNAO1/DNM1L/EPAS1/NPPC/CACNA1B/ANK2/ASPH/SREBF1/CASQ1/SPTBN4/GRK2
Biological Process	GO:0001709	cell fate determination	7/2279	42/18866	0.2391272	0.5516087	0.4991152	7	DLL1/IFRD1/PAX2/DSCAML1/MEF2C/NOTCH4/NTF4
Biological Process	GO:0002707	negative regulation of lymphocyte mediated immunity	7/2279	42/18866	0.2391272	0.5516087	0.4991152	7	DUSP22/HLA-F/PTPN6/SMAD7/PTPRC/ARRB2/CD96
Biological Process	GO:0006509	membrane protein ectodomain proteolysis	7/2279	42/18866	0.2391272	0.5516087	0.4991152	7	PRTN3/TIMP2/IL10/TNFRSF1B/GPLD1/TNF/MYH9
Biological Process	GO:0034383	low-density lipoprotein particle clearance	7/2279	42/18866	0.2391272	0.5516087	0.4991152	7	EHD1/AP2A1/MIR199A1/MIR27A/CSK/NPC1/MIR199A2
Biological Process	GO:0048246	macrophage chemotaxis	7/2279	42/18866	0.2391272	0.5516087	0.4991152	7	AZU1/C3AR1/CCL5/CSF1R/SFTPD/THBS1/CXCL17

Biological Process	GO:0071470	cellular response to osmotic stress	7/2279	42/18866	0.2391272	0.5516087	0.4991152	7	MYLK/LRRC8C/AQP1/CAB39/ARHGEF2/SLC2A1/DYSF
Biological Process	GO:0043543	protein acylation	34/2279	248/18866	0.2397141	0.5516087	0.4991152	34	ZDHHC18/SRCAP/BRCA2/ZDHHC14/KANSL1/ARRB1/BRD1/CDYL/EPC1/ZDHHC2/OGDH/RPS6KA4/TAF10/ZDHHC7/KANSL2/SPHK2/CTBP1/POR/NCOA1/ZDHHC17/ZDHHC1/CREBBP/RSF1/ARNTL/PER1/PIH1D1/NAA16/FOXO1/RUVBL1/KAT7/ING5/LIF/PAXIP1/PRKAA1
Biological Process	GO:0050768	negative regulation of neurogenesis	40/2279	295/18866	0.239715	0.5516087	0.4991152	40	SEMA6B/CTSZ/GFI1/SORL1/CALR/KANK1/LSM1/YWHAH/SKI/TNF/BCL11A/SEMA4A/RTN4/PMP22/SEMA4B/DLL1/APP/MIB1/IFRD1/ASAP1/SYNGAP1/KREMEN1/SEMA4D/RAPGEF2/NF1/ITM2C/MEIS1/DNM3/NOTCH1/ZHX2/CDKL3/UBE3A/ARHGEF2/CERS2/INPP5F/BAG5/RAP1GAP2/HES3/PRAG1/SPART
Biological Process	GO:0018958	phenol-containing compound metabolic process	16/2279	109/18866	0.2397186	0.5516087	0.4991152	16	ZEB2/TG/COMT/DDC/FAH/CDH3/RAPGEF2/SULT1A2/MYO5A/CPQ/CTSB/ITGB2/BCL2/EPAS1/CRYM/PRKN
Biological Process	GO:0051926	negative regulation of calcium ion transport	10/2279	64/18866	0.2401269	0.5516087	0.4991152	10	TRIM27/THADA/CASQ2/TLR9/BIN1/BCL2/GSTO1/DYSF/CD33/CBARP
Biological Process	GO:0070265	necrotic cell death	10/2279	64/18866	0.2401269	0.5516087	0.4991152	10	TNF/TRAF2/MIR101-2/RIPK1/RIPK3/DNM1L/ARHGEF2/CYLD/FADD/PELI1
Biological Process	GO:0002449	lymphocyte mediated immunity	49/2279	366/18866	0.2405149	0.5516087	0.4991152	49	PRKCZ/FUT7/IL10/LYST/CD55/INPP5D/SLC11A1/TNFRSF1B/NLRP3/IL1R1/CCR2/PIK3R6/TNF/TRAF2/DUSP22/IGHV6-1/SLAMF7/CTSH/TUBB4B/RAB27A/CD226/HLA-F/PTPN6/CLU/SMAD7/UNG/TGFB1/NCR1/RIPK3/C1S/PTPRC/BCL10/ARRB2/IL18RAP/POU2F2/TP53BP1/IL18R1/ERCC1/TNFSF13/C1QB/CADM1/CD96/BCL3/FADD/PAXIP1/C1RL/CYRIB/NSD2/SLA2
Biological Process	GO:1903522	regulation of blood circulation	41/2279	303/18866	0.2407093	0.5516087	0.4991152	41	HDAC4/PDE4D/KCNE1/CASQ2/ITPR2/FXYD2/CACNA1C/CACNA2D4/FXYD1/SLC8A1/GJA5/ATP2B2/ITPR1/JUP/BIN1/NOS1AP/SMAD7/KCNIP1/KCNK6/HRH1/GHRL/CHRM2/ATP2A3/CELF2/SNTA1/KCNQ1/PER2/GSTO1/GNAO1/P2RX1/EPAS1/F2R/NPPC/AGTR1/CACNA1B/ANK2/ASPH/SREBF1/CASQ1/SPTBN4/GRK2
Biological Process	GO:0008016	regulation of heart contraction	35/2279	256/18866	0.241076	0.5516087	0.4991152	35	HDAC4/PDE4D/KCNE1/CASQ2/ITPR2/FXYD2/CACNA1C/CACNA2D4/FXYD1/SLC8A1/GJA5/ATP2B2/ITPR1/JUP/BIN1/NOS1AP/SMAD7/KCNIP1/KCNK6/CHRM2/ATP2A3/CELF2/SNTA1/KCNQ1/GSTO1/GNAO1/EPAS1/NPPC/CACNA1B/ANK2/ASPH/SREBF1/CASQ1/SPTBN4/GRK2

Biological Process	GO:1904035	regulation of epithelial cell apoptotic process	14/2279	94/18866	0.2413347	0.5516087	0.4991152	14	GSN/ANGPT1/TNF/TNFAIP3/MIR101-2/THBS1/MIR24-2/TCF7L2/PRKCI/NFE2L2/ARRB2/SORT1/ANO6/PLCG1
Biological Process	GO:0021675	nerve development	12/2279	79/18866	0.2417469	0.5516087	0.4991152	12	NRP2/PHOX2A/DRGX/ATP8B1/ILK/LRIG1/PAX2/NTRK1/SLC1A3/NPTX1/HES3/NTF4
Biological Process	GO:0031016	pancreas development	12/2279	79/18866	0.2417469	0.5516087	0.4991152	12	RHEB/RFX3/DLL1/TCF7L2/HNF1A/GIPR/IL6R/MEIS2/ARNTL/FOXO1/ALDH1A2/NR5A2
Biological Process	GO:0070373	negative regulation of ERK1 and ERK2 cascade	12/2279	79/18866	0.2417469	0.5516087	0.4991152	12	SPRED2/ITGB1BP1/ARRB1/PHB/TNIP1/PTPRC/CSK/DUSP3/EZR/LIF/NDRG2/SIRT3
Biological Process	GO:0120192	tight junction assembly	12/2279	79/18866	0.2417469	0.5516087	0.4991152	12	TBCD/RUNX1/TNF/ACTN4/WNT11/PRKCH/ACTG1/GRHL2/PRKCI/MPP7/MYO1C/NPHP4
Biological Process	GO:0051147	regulation of muscle cell differentiation	26/2279	186/18866	0.2418637	0.5516087	0.4991152	26	HDAC4/EHD1/CTDP1/MIR140/MIR145/AKAP13/MAML1/MIR199A1/NFATC2/NFATC1/DLL1/RBPJ/TGFB1/PDGFB/ENG/ARRB2/ANKRD2/BCL2/TCF3/MIR199A2/NOTCH1/DNMT1/MEF2C/FLOT1/SCGB3A1/CEACAM5
Biological Process	GO:0048863	stem cell differentiation	36/2279	264/18866	0.2423494	0.5516087	0.4991152	36	SEMA6B/TACSTD2/NRP2/MYB/RUNX1/SETD2/ANXA6/ZEB2/LBH/SEMA4A/SEMA4B/RBPJ/TCF12/PAX2/SEMA4D/RDH10/KDM4C/PTPRC/RUNX2/SETD1A/PSMF1/NFE2L2/PUS7/ESRRB/TCF3/PSMB7/NOTCH1/CORO1C/MEF2C/TEAD2/ALDH1A2/ERCC2/NTF4/LIF/PSMD13/CDK13
Biological Process	GO:0006369	termination of RNA polymerase II transcription	6/2279	35/18866	0.2426224	0.5516087	0.4991152	6	FIP1L1/CPSF3/SNRPD3/PABPN1/SCAF8/CPSF4
Biological Process	GO:0009112	nucleobase metabolic process	6/2279	35/18866	0.2426224	0.5516087	0.4991152	6	CDA/DPYS/GMPR2/DHODH/AOX1/ACP3

Biological Process	GO:0009262	deoxyribonucleotide metabolic process	6/2279	35/18866	0.2426224	0.5516087	0.4991152	6	RRM2B/UNG/NTHL1/GUK1/AK5/DCTD
Biological Process	GO:0014904	myotube cell development	6/2279	35/18866	0.2426224	0.5516087	0.4991152	6	HDAC4/SKI/NFATC2/SMYD3/BCL2/MYORG
Biological Process	GO:0030262	apoptotic nuclear changes	6/2279	35/18866	0.2426224	0.5516087	0.4991152	6	CECR2/PAM16/DEDD2/DFFB/DNASE1L3/BLCAP
Biological Process	GO:0030947	regulation of vascular endothelial growth factor receptor signaling pathway	6/2279	35/18866	0.2426224	0.5516087	0.4991152	6	GRB10/EPN2/PRKCB/ITGB3/NEDD4/HHEX
Biological Process	GO:0031297	replication fork processing	6/2279	35/18866	0.2426224	0.5516087	0.4991152	6	BLM/BRCA2/RTEL1/EME1/SMARCAL1/FBH1
Biological Process	GO:0032228	regulation of synaptic transmission, GABAergic	6/2279	35/18866	0.2426224	0.5516087	0.4991152	6	SYN3/NISCH/NF1/CNR2/PLCL1/PRKCE
Biological Process	GO:0032633	interleukin-4 production	6/2279	35/18866	0.2426224	0.5516087	0.4991152	6	PRKCZ/ZFPM1/NLRP3/RARA/SLC7A5/CEBPB
Biological Process	GO:0032735	positive regulation of interleukin-12 production	6/2279	35/18866	0.2426224	0.5516087	0.4991152	6	IRF8/IRF5/IL16/TLR9/SCIMP/PLCB1
Biological Process	GO:0032801	receptor catabolic process	6/2279	35/18866	0.2426224	0.5516087	0.4991152	6	AP2A1/UVRAG/TGFB1/SMURF1/NEDD4/MVB12A

Biological Process	GO:0042462	eye photoreceptor cell development	6/2279	35/18866	0.2426224	0.5516087	0.4991152	6	CRB1/GNAT2/PRKCI/RPGRIP1/NRL/CABP4
Biological Process	GO:0045066	regulatory T cell differentiation	6/2279	35/18866	0.2426224	0.5516087	0.4991152	6	FUT7/RUNX1/NFATC2/FANCA/LILRB4/VSIR
Biological Process	GO:0048333	mesodermal cell differentiation	6/2279	35/18866	0.2426224	0.5516087	0.4991152	6	MIR145/PAX2/FGFR1/ITGB3/INHBA/NODAL
Biological Process	GO:0065005	protein-lipid complex assembly	6/2279	35/18866	0.2426224	0.5516087	0.4991152	6	P4HB/MFSD2A/PLAGL2/BIN1/NR1H2/ABCA1
Biological Process	GO:0070306	lens fiber cell differentiation	6/2279	35/18866	0.2426224	0.5516087	0.4991152	6	SPRED2/FZR1/SMAD3/ZEB2/WNT5B/TMOD1
Biological Process	GO:0098801	regulation of renal system process	6/2279	35/18866	0.2426224	0.5516087	0.4991152	6	GJA5/COMT/GNAI2/PDGFB/F2R/AGTR1
Biological Process	GO:0110111	negative regulation of animal organ morphogenesis	6/2279	35/18866	0.2426224	0.5516087	0.4991152	6	TACSTD2/TNF/PAX2/BCL2/NOTCH1/CPB2
Biological Process	GO:1901020	negative regulation of calcium ion transmembrane transporter activity	6/2279	35/18866	0.2426224	0.5516087	0.4991152	6	THADA/CASQ2/TLR9/GSTO1/DYSF/CBARP
Biological Process	GO:1901030	positive regulation of mitochondrial outer membrane permeabilization involved in apoptotic signaling pathway	6/2279	35/18866	0.2426224	0.5516087	0.4991152	6	TFDP1/YWHAH/YWHAZ/BCL2/TP53BP2/YWHAQ

Biological Process	GO:0002363	alpha-beta T cell lineage commitment	4/2279	21/18866	0.2432628	0.5516087	0.4991152	4	ZFPM1/LOXL3/LY9/BCL2
Biological Process	GO:0010829	negative regulation of glucose transmembrane transport	4/2279	21/18866	0.2432628	0.5516087	0.4991152	4	MIR143/TNF/GRB10/PRKCB
Biological Process	GO:0032516	positive regulation of phosphoprotein phosphatase activity	4/2279	21/18866	0.2432628	0.5516087	0.4991152	4	PTPRC/PDGFRB/CD33/PTPA
Biological Process	GO:0043691	reverse cholesterol transport	4/2279	21/18866	0.2432628	0.5516087	0.4991152	4	LIPC/ABCG1/CLU/ABCA1
Biological Process	GO:0046641	positive regulation of alpha-beta T cell proliferation	4/2279	21/18866	0.2432628	0.5516087	0.4991152	4	CD55/CCR2/TGFBR2/PTPRC
Biological Process	GO:0048485	sympathetic nervous system development	4/2279	21/18866	0.2432628	0.5516087	0.4991152	4	NRP2/PHOX2A/NF1/NTRK1
Biological Process	GO:0051797	regulation of hair follicle development	4/2279	21/18866	0.2432628	0.5516087	0.4991152	4	TNF/KRT17/CDH3/NUMA1
Biological Process	GO:0061003	positive regulation of dendritic spine morphogenesis	4/2279	21/18866	0.2432628	0.5516087	0.4991152	4	BAIAP2/DNM1L/CUX2/DBNL
Biological Process	GO:0072111	cell proliferation involved in kidney development	4/2279	21/18866	0.2432628	0.5516087	0.4991152	4	PDGFD/IL6R/PDGFB/PDGFRB

Biological Process	GO:0072574	hepatocyte proliferation	4/2279	21/18866	0.2432628	0.5516087	0.4991152	4	RPS6KA1/TNFAIP3/CPB2/CEBPB
Biological Process	GO:0072575	epithelial cell proliferation involved in liver morphogenesis	4/2279	21/18866	0.2432628	0.5516087	0.4991152	4	RPS6KA1/TNFAIP3/CPB2/CEBPB
Biological Process	GO:0090280	positive regulation of calcium ion import	4/2279	21/18866	0.2432628	0.5516087	0.4991152	4	SPHK2/PDGFB/PDGFRB/TRPV2
Biological Process	GO:0090312	positive regulation of protein deacetylation	4/2279	21/18866	0.2432628	0.5516087	0.4991152	4	C6orf89/ZBTB7B/CTBP1/SREBF1
Biological Process	GO:0090330	regulation of platelet aggregation	4/2279	21/18866	0.2432628	0.5516087	0.4991152	4	CD9/PRKCA/UBASH3B/BLK
Biological Process	GO:0098780	response to mitochondrial depolarisation	4/2279	21/18866	0.2432628	0.5516087	0.4991152	4	SMURF1/ATG14/HK2/PRKN
Biological Process	GO:0099515	actin filament-based transport	4/2279	21/18866	0.2432628	0.5516087	0.4991152	4	ACTN4/WASL/MYO5A/MYO1C
Biological Process	GO:1902307	positive regulation of sodium ion transmembrane transport	4/2279	21/18866	0.2432628	0.5516087	0.4991152	4	FXYD1/DNM2/TESC/ACTN4
Biological Process	GO:1903055	positive regulation of extracellular matrix organization	4/2279	21/18866	0.2432628	0.5516087	0.4991152	4	SMAD3/TGFB1/RB1/CPB2

Biological Process	GO:2000047	regulation of cell-cell adhesion mediated by cadherin	4/2279	21/18866	0.2432628	0.5516087	0.4991152	4	NOTCH1/NOTCH4/PTPRU/FLOT1
Biological Process	GO:0007088	regulation of mitotic nuclear division	23/2279	163/18866	0.2436923	0.5516087	0.4991152	23	TGFA/MAD1L1/TNKS/NSMCE2/RCC1/CUL3/CCDC8/CDC16/TACC3/NUMA1/PDGFB/NEK6/RB1/CDCA5/ANAPC7/PDGFRB/HECW2/CDC14A/AURKAIP1/DAPK3/CDK13/PCID2/SLF1
Biological Process	GO:0031214	biomineral tissue development	23/2279	163/18866	0.2436923	0.5516087	0.4991152	23	SBNO2/ITGB1BP1/SMAD3/SLC8A1/FGR/ALPL/ASGR2/WNT11/TGFB1/BMPR1B/SGMS2/TSPEAR/NOTCH1/MEF2C/LTF/CEBPB/FOXO1/ANO6/FAM20C/ERCC2/TFIP11/RFLNA/NECTIN1
Biological Process	GO:0110148	biomineralization	23/2279	163/18866	0.2436923	0.5516087	0.4991152	23	SBNO2/ITGB1BP1/SMAD3/SLC8A1/FGR/ALPL/ASGR2/WNT11/TGFB1/BMPR1B/SGMS2/TSPEAR/NOTCH1/MEF2C/LTF/CEBPB/FOXO1/ANO6/FAM20C/ERCC2/TFIP11/RFLNA/NECTIN1
Biological Process	GO:1902115	regulation of organelle assembly	27/2279	194/18866	0.2439857	0.5516087	0.4991152	27	GSN/CDKL1/ULK1/AKAP13/DNM2/TNF/RCC1/DYNC1H1/CEP135/PIP4K2A/FEZ2/ASAP1/NRXN1/HTT/NUMA1/IFT140/TBC1D14/SDCCAG8/MEF2C/ARHGEF2/CYLD/SPAG5/EZR/NPRL2/PRKAA1/CEP295NL/SEPTIN9
Biological Process	GO:0045446	endothelial cell differentiation	17/2279	117/18866	0.2441777	0.5516087	0.4991152	17	PDE4D/TJP2/RAP1B/TNF/CCM2/DLL1/RBPJ/TNFRSF1A/RAPGEF2/ENG/ADD1/NOTCH1/S1PR2/NOTCH4/PPP1R16B/EZR/MYADM
Biological Process	GO:0000469	cleavage involved in rRNA processing	5/2279	28/18866	0.2445453	0.5516087	0.4991152	5	EXOSC2/RCL1/NOB1/ERI3/NOP14
Biological Process	GO:0002335	mature B cell differentiation	5/2279	28/18866	0.2445453	0.5516087	0.4991152	5	DLL1/CMTM7/DOCK10/POU2F2/BCL3
Biological Process	GO:0002360	T cell lineage commitment	5/2279	28/18866	0.2445453	0.5516087	0.4991152	5	ZFPM1/LOXL3/LY9/BCL2/CYLD

Biological Process	GO:0003180	aortic valve morphogenesis	5/2279	28/18866	0.2445453	0.5516087	0.4991152	5	NFATC1/TGFB1/RB1/NOTCH1/SLIT3
Biological Process	GO:0006972	hyperosmotic response	5/2279	28/18866	0.2445453	0.5516087	0.4991152	5	AQP1/ARHGEF2/SLC2A1/HNMT/SST
Biological Process	GO:0007205	protein kinase C-activating G protein-coupled receptor signaling pathway	5/2279	28/18866	0.2445453	0.5516087	0.4991152	5	AZU1/DGKQ/DGKA/DGKD/F2R
Biological Process	GO:0008299	isoprenoid biosynthetic process	5/2279	28/18866	0.2445453	0.5516087	0.4991152	5	IDI1/DHRS9/RDH10/HMGCS1/ALDH1A2
Biological Process	GO:0032800	receptor biosynthetic process	5/2279	28/18866	0.2445453	0.5516087	0.4991152	5	IL10/PPARG/TNF/HDAC1/ITGB3
Biological Process	GO:0034311	diol metabolic process	5/2279	28/18866	0.2445453	0.5516087	0.4991152	5	SPTLC2/SPHK2/SGPP1/SGPP2/PLPP3
Biological Process	GO:0045745	positive regulation of G protein-coupled receptor signaling pathway	5/2279	28/18866	0.2445453	0.5516087	0.4991152	5	PRKCA/MRAP/PHB/PDE6H/ACP3
Biological Process	GO:0051647	nucleus localization	5/2279	28/18866	0.2445453	0.5516087	0.4991152	5	DCTN1/DYNC1H1/BIN1/FBXW11/SLIT1
Biological Process	GO:0071168	protein localization to chromatin	5/2279	28/18866	0.2445453	0.5516087	0.4991152	5	SETD2/RB1/PIH1D1/CDCA5/MACROH2A2

Biological Process	GO:0071450	cellular response to oxygen radical	5/2279	28/18866	0.2445453	0.5516087	0.4991152	5	MPO/TNF/NFE2L2/GLRX2/SIRT3
Biological Process	GO:0071451	cellular response to superoxide	5/2279	28/18866	0.2445453	0.5516087	0.4991152	5	MPO/TNF/NFE2L2/GLRX2/SIRT3
Biological Process	GO:0071880	adenylate cyclase-activating adrenergic receptor signaling pathway	5/2279	28/18866	0.2445453	0.5516087	0.4991152	5	PDE4D/AKAP13/ADCY9/GNAI2/RAPGEF2
Biological Process	GO:0000725	recombinational repair	20/2279	140/18866	0.2446402	0.5516087	0.4991152	20	BLM/SPIDR/BRCA2/RMI2/RAD52/SETD2/RTEL1/HUS1/NSMCE2/MCM5/RECQL5/MEIOB/AP5S1/MCM2/RAD21L1/DMC1/TP53BP1/RHNO1/SLX4/FBH1
Biological Process	GO:0048284	organelle fusion	20/2279	140/18866	0.2446402	0.5516087	0.4991152	20	VAT1/ENO1/CALR/STX5/VTI1A/ANKFY1/TGFBRAP1/SPG7/UVRAG/ENO3/VPS18/VCPIP1/FIS1/DNM1L/DYSF/TSNARE1/VAV3/IRAG2/PRKN/RUBCNL
Biological Process	GO:0016042	lipid catabolic process	46/2279	343/18866	0.2448447	0.5516087	0.4991152	46	PNPLA2/PLA2G15/SORL1/MGLL/PLBD2/MFSD2A/HEXB/LIPC/YWHAH/ABHD16A/AMACR/PLD1/GPLD1/GALC/TNF/SMPDL3B/AOAH/ACOXL/AKR1D1/CRTC3/CYP4F3/ACADM/ACACB/MGST2/ABHD16B/FABP6/PLCB1/ACOX2/PLA1A/PRKCE/ABHD5/PCK2/ACAT1/PDE3B/PLCG1/ADTRP/CPT1A/PDXDC1/INPP5F/PLCD3/LPIN1/ASPG/LYPLA2/ACADVL/BSCL2/PRKAA1
Biological Process	GO:0044380	protein localization to cytoskeleton	9/2279	57/18866	0.2462066	0.5516087	0.4991152	9	CSNK1D/CHAMP1/DISC1/DCTN2/NUMA1/KLHL21/MAP1A/SPAG5/CEP72
Biological Process	GO:0046148	pigment biosynthetic process	9/2279	57/18866	0.2462066	0.5516087	0.4991152	9	ZEB2/SLC11A2/FECH/CDH3/RAPGEF2/MYO5A/ALAS1/PPOX/TMEM14C
Biological Process	GO:0061178	regulation of insulin secretion involved in cellular response to glucose stimulus	9/2279	57/18866	0.2462066	0.5516087	0.4991152	9	NADK/LRP5L/ARRB1/GPLD1/ENY2/GHRL/KLF7/PRKCE/C2CD2L

Biological Process	GO:0070936	protein K48-linked ubiquitination	9/2279	57/18866	0.2462066	0.5516087	0.4991152	9	TRIM38/AMFR/TNFAIP3/UBE2Q2/UBE3A/UBE2D2/PELI1/MARCHF6/PRKN
Biological Process	GO:0140056	organelle localization by membrane tethering	24/2279	171/18866	0.2462359	0.5516087	0.4991152	24	DCTN1/STX5/CSNK1D/NDE1/PACS2/DYNC1H1/TUBB4B/CEP135/CEP164/DCTN2/CEP78/VPS18/CTBP2/ATG14/CSNK1E/PPP2R1A/TUBA4A/SDCCAG8/PDZD8/ESYT1/CEP72/TSNARE1/NPHP4/GRAMD2A
Biological Process	GO:0055024	regulation of cardiac muscle tissue development	15/2279	102/18866	0.2466469	0.5516087	0.4991152	15	CTDP1/CREB1/MIR199A1/VGLL4/TGFBR2/DLL1/RIPK1/RBPJ/FGFR1/TGFB1/ARRB2/MEIS1/MIR199A2/NOTCH1/MEF2C
Biological Process	GO:0008637	apoptotic mitochondrial changes	18/2279	125/18866	0.2481577	0.5516087	0.4991152	18	TFDP1/YWHAH/PAM16/BMF/CLU/CIDEB/AIFM2/HK2/NOL3/ARRB2/YWHAZ/FIS1/BCL2/DNM1L/TP53BP2/BCL2L1/YWHAQ/PRKN
Biological Process	GO:0032479	regulation of type I interferon production	18/2279	125/18866	0.2481577	0.5516087	0.4991152	18	IL10/TRIM38/IRF5/SETD2/TNFAIP3/POLR2F/TLR9/TRAIP/CRCP/NLRC5/CREBBP/DDX41/CYLD/RELA/FLOT1/HERC5/STING1/ILRUN
Biological Process	GO:0035270	endocrine system development	18/2279	125/18866	0.2481577	0.5516087	0.4991152	18	ARID5B/CREB1/SMAD3/RHEB/RFX3/TG/DLL1/RBPJ/WNT11/NF1/GIPR/IL6R/ETS1/ARNTL/CDH1/PDGFRB/FOXO1/ALDH1A2
Biological Process	GO:0048593	camera-type eye morphogenesis	18/2279	125/18866	0.2481577	0.5516087	0.4991152	18	MFSD2A/SKI/CRB1/HIPK2/DLL1/GNAT2/PAX2/NF1/SP3/MEIS1/RPGRIP1/ZHX2/SDK2/BCAR3/NRL/CABP4/TSKU/NECTIN1
Biological Process	GO:0006368	transcription elongation from RNA polymerase II promoter	13/2279	87/18866	0.2482701	0.5516087	0.4991152	13	BRD4/CTDP1/WDR43/SETD2/POLR2F/RECQL5/ELL2/ENY2/MLLT1/CCNH/ERCC2/CDK13/PCID2
Biological Process	GO:0032436	positive regulation of proteasomal ubiquitin-dependent protein catabolic process	13/2279	87/18866	0.2482701	0.5516087	0.4991152	13	RNF144A/FZR1/RNF19A/CSNK1D/CBFA2T3/CLU/SMAD7/CSNK1E/NFE2L2/RNF144B/RNF14/TRIB1/PRKN

Biological Process	GO:0051781	positive regulation of cell division	13/2279	87/18866	0.2482701	0.5516087	0.4991152	13	TGFA/ITGB1BP1/LBH/CUL3/TGFB1/SVIL/CSPP1/PDGFD/PDGFB/CAT/PRKCE/PDGFC/CDC14A
Biological Process	GO:0090502	RNA phosphodiester bond hydrolysis, endonucleolytic	13/2279	87/18866	0.2482701	0.5516087	0.4991152	13	PIWIL4/ERN1/CPSF3/SLFN14/RCL1/AGO1/ZC3H12D/AGO2/EDC3/NOB1/SMG6/NOP14/SND1
Biological Process	GO:2001057	reactive nitrogen species metabolic process	13/2279	87/18866	0.2482701	0.5516087	0.4991152	13	IL10/SMAD3/DNM2/MIR199A1/TNF/NOS1AP/CLU/HBB/POR/ITGB2/MIR199A2/CX3CR1/MTARC1
Biological Process	GO:0030072	peptide hormone secretion	35/2279	257/18866	0.2485784	0.5516087	0.4991152	35	NADK/ITPR2/MYRIP/CACNA1C/LRP5L/ARRB1/PRKCA/GPLD1/TNF/CCL5/ITSN1/ITPR1/RFX3/TCF7L2/HNF1A/PTPRN2/IL1RN/CRHBP/ENY2/MYO5A/GIPR/BLK/GHRL/KLF7/ARNTL/PER2/SLC16A1/PRKCE/SLC2A1/SREBF1/CPT1A/RPH3AL/C2CD2L/SIRT3/PRKN
Biological Process	GO:0000480	endonucleolytic cleavage in 5'-ETS of tricistronic rRNA transcript (SSU-rRNA, 5.8S rRNA, LSU-rRNA)	2/2279	8/18866	0.2505201	0.5516087	0.4991152	2	RCL1/NOP14
Biological Process	GO:0000733	DNA strand renaturation	2/2279	8/18866	0.2505201	0.5516087	0.4991152	2	BLM/SMARCAL1
Biological Process	GO:0000738	DNA catabolic process, exonucleolytic	2/2279	8/18866	0.2505201	0.5516087	0.4991152	2	ERI3/ISG20
Biological Process	GO:0001781	neutrophil apoptotic process	2/2279	8/18866	0.2505201	0.5516087	0.4991152	2	PIK3CD/ITPKB
Biological Process	GO:0002118	aggressive behavior	2/2279	8/18866	0.2505201	0.5516087	0.4991152	2	CRHBP/KIRREL3

Biological Process	GO:0002246	wound healing involved in inflammatory response	2/2279	8/18866	0.2505201	0.5516087	0.4991152	2	TGFB1/F2R
Biological Process	GO:0002484	antigen processing and presentation of endogenous peptide antigen via MHC class I via ER pathway	2/2279	8/18866	0.2505201	0.5516087	0.4991152	2	HLA-C/HLA-F
Biological Process	GO:0002486	antigen processing and presentation of endogenous peptide antigen via MHC class I via ER pathway, TAP-independent	2/2279	8/18866	0.2505201	0.5516087	0.4991152	2	HLA-C/HLA-F
Biological Process	GO:0002729	positive regulation of natural killer cell cytokine production	2/2279	8/18866	0.2505201	0.5516087	0.4991152	2	CD226/HLA-F
Biological Process	GO:0002732	positive regulation of dendritic cell cytokine production	2/2279	8/18866	0.2505201	0.5516087	0.4991152	2	SCIMP/NOD2
Biological Process	GO:0003190	atrioventricular valve formation	2/2279	8/18866	0.2505201	0.5516087	0.4991152	2	ZFPM1/NOTCH1
Biological Process	GO:0003339	regulation of mesenchymal to epithelial transition involved in metanephros morphogenesis	2/2279	8/18866	0.2505201	0.5516087	0.4991152	2	PAX2/LIF
Biological Process	GO:0006267	pre-replicative complex assembly involved in nuclear cell cycle DNA replication	2/2279	8/18866	0.2505201	0.5516087	0.4991152	2	MCM5/MCM2
Biological Process	GO:0006384	transcription initiation from RNA polymerase III promoter	2/2279	8/18866	0.2505201	0.5516087	0.4991152	2	CRCP/BRF1

Biological Process	GO:0006548	histidine catabolic process	2/2279	8/18866	0.2505201	0.5516087	0.4991152	2	HAL/HNMT
Biological Process	GO:0006678	glucosylceramide metabolic process	2/2279	8/18866	0.2505201	0.5516087	0.4991152	2	FA2H/PRKAA1
Biological Process	GO:0006924	activation-induced cell death of T cells	2/2279	8/18866	0.2505201	0.5516087	0.4991152	2	RIPK3/FADD
Biological Process	GO:0006975	DNA damage induced protein phosphorylation	2/2279	8/18866	0.2505201	0.5516087	0.4991152	2	MAP2K6/CHEK2
Biological Process	GO:0007217	tachykinin receptor signaling pathway	2/2279	8/18866	0.2505201	0.5516087	0.4991152	2	GRK5/GRK2
Biological Process	GO:0007270	neuron-neuron synaptic transmission	2/2279	8/18866	0.2505201	0.5516087	0.4991152	2	ARID1B/KIF1B
Biological Process	GO:0009120	deoxyribonucleoside metabolic process	2/2279	8/18866	0.2505201	0.5516087	0.4991152	2	TK2/TK1
Biological Process	GO:0009756	carbohydrate mediated signaling	2/2279	8/18866	0.2505201	0.5516087	0.4991152	2	COLEC12/PIH1D1
Biological Process	GO:0010501	RNA secondary structure unwinding	2/2279	8/18866	0.2505201	0.5516087	0.4991152	2	AGO1/AGO2

Biological Process	GO:0010511	regulation of phosphatidylinositol biosynthetic process	2/2279	8/18866	0.2505201	0.5516087	0.4991152	2	FPR2/PDGFB
Biological Process	GO:0010616	negative regulation of cardiac muscle adaptation	2/2279	8/18866	0.2505201	0.5516087	0.4991152	2	SMAD3/FOXO1
Biological Process	GO:0010624	regulation of Schwann cell proliferation	2/2279	8/18866	0.2505201	0.5516087	0.4991152	2	SKI/CERS2
Biological Process	GO:0010871	negative regulation of receptor biosynthetic process	2/2279	8/18866	0.2505201	0.5516087	0.4991152	2	PPARG/ITGB3
Biological Process	GO:0010966	regulation of phosphate transport	2/2279	8/18866	0.2505201	0.5516087	0.4991152	2	FGFR1/CEBPB
Biological Process	GO:0014807	regulation of somitogenesis	2/2279	8/18866	0.2505201	0.5516087	0.4991152	2	DLL1/NOTCH1
Biological Process	GO:0014866	skeletal myofibril assembly	2/2279	8/18866	0.2505201	0.5516087	0.4991152	2	TTN/MYH11
Biological Process	GO:0015014	heparan sulfate proteoglycan biosynthetic process, polysaccharide chain biosynthetic process	2/2279	8/18866	0.2505201	0.5516087	0.4991152	2	CSGALNACT1/NDST1
Biological Process	GO:0015840	urea transport	2/2279	8/18866	0.2505201	0.5516087	0.4991152	2	AQP3/AQP9

Biological Process	GO:0015884	folic acid transport	2/2279	8/18866	0.2505201	0.5516087	0.4991152	2	FOLR3/SLC19A1
Biological Process	GO:0015889	cobalamin transport	2/2279	8/18866	0.2505201	0.5516087	0.4991152	2	ABCC1/CD320
Biological Process	GO:0015911	long-chain fatty acid import across plasma membrane	2/2279	8/18866	0.2505201	0.5516087	0.4991152	2	THBS1/SLC2A1
Biological Process	GO:0031064	negative regulation of histone deacetylation	2/2279	8/18866	0.2505201	0.5516087	0.4991152	2	SKI/SPHK2
Biological Process	GO:0032056	positive regulation of translation in response to stress	2/2279	8/18866	0.2505201	0.5516087	0.4991152	2	NCK2/EIF4G1
Biological Process	GO:0032119	sequestering of zinc ion	2/2279	8/18866	0.2505201	0.5516087	0.4991152	2	S100A8/AP3D1
Biological Process	GO:0032329	serine transport	2/2279	8/18866	0.2505201	0.5516087	0.4991152	2	SLC1A5/SFXN1
Biological Process	GO:0032471	negative regulation of endoplasmic reticulum calcium ion concentration	2/2279	8/18866	0.2505201	0.5516087	0.4991152	2	THADA/FIS1
Biological Process	GO:0032927	positive regulation of activin receptor signaling pathway	2/2279	8/18866	0.2505201	0.5516087	0.4991152	2	ZC3H3/NODAL

Biological Process	GO:0033007	negative regulation of mast cell activation involved in immune response	2/2279	8/18866	0.2505201	0.5516087	0.4991152	2	CD84/FER
Biological Process	GO:0033034	positive regulation of myeloid cell apoptotic process	2/2279	8/18866	0.2505201	0.5516087	0.4991152	2	PIK3CD/MEF2C
Biological Process	GO:0033085	negative regulation of T cell differentiation in thymus	2/2279	8/18866	0.2505201	0.5516087	0.4991152	2	ZC3H8/TMEM131L
Biological Process	GO:0033601	positive regulation of mammary gland epithelial cell proliferation	2/2279	8/18866	0.2505201	0.5516087	0.4991152	2	RREB1/RTN4
Biological Process	GO:0034498	early endosome to Golgi transport	2/2279	8/18866	0.2505201	0.5516087	0.4991152	2	STX5/TRAPPC10
Biological Process	GO:0034983	peptidyl-lysine deacetylation	2/2279	8/18866	0.2505201	0.5516087	0.4991152	2	HDAC4/SIRT3
Biological Process	GO:0035608	protein deglutamylation	2/2279	8/18866	0.2505201	0.5516087	0.4991152	2	AGBL5/AGBL2
Biological Process	GO:0035912	dorsal aorta morphogenesis	2/2279	8/18866	0.2505201	0.5516087	0.4991152	2	RBPJ/ENG
Biological Process	GO:0036155	acylglycerol acyl-chain remodeling	2/2279	8/18866	0.2505201	0.5516087	0.4991152	2	PNPLA2/MGLL

Biological Process	GO:0036388	pre-replicative complex assembly	2/2279	8/18866	0.2505201	0.5516087	0.4991152	2	MCM5/MCM2
Biological Process	GO:0038027	apolipoprotein A-I-mediated signaling pathway	2/2279	8/18866	0.2505201	0.5516087	0.4991152	2	ITGB3/ABCA1
Biological Process	GO:0043320	natural killer cell degranulation	2/2279	8/18866	0.2505201	0.5516087	0.4991152	2	RAB27A/HLA-F
Biological Process	GO:0043615	astrocyte cell migration	2/2279	8/18866	0.2505201	0.5516087	0.4991152	2	HEXB/CCR2
Biological Process	GO:0044771	meiotic cell cycle phase transition	2/2279	8/18866	0.2505201	0.5516087	0.4991152	2	CDC25A/OVOL1
Biological Process	GO:0045008	depyrimidination	2/2279	8/18866	0.2505201	0.5516087	0.4991152	2	UNG/NTHL1
Biological Process	GO:0045607	regulation of inner ear auditory receptor cell differentiation	2/2279	8/18866	0.2505201	0.5516087	0.4991152	2	DLL1/NOTCH1
Biological Process	GO:0045631	regulation of mechanoreceptor differentiation	2/2279	8/18866	0.2505201	0.5516087	0.4991152	2	DLL1/NOTCH1
Biological Process	GO:0045924	regulation of female receptivity	2/2279	8/18866	0.2505201	0.5516087	0.4991152	2	PPP1R1B/NCOA1

Biological Process	GO:0046654	tetrahydrofolate biosynthetic process	2/2279	8/18866	0.2505201	0.5516087	0.4991152	2	MTHFD1L/MTHFD1
Biological Process	GO:0048280	vesicle fusion with Golgi apparatus	2/2279	8/18866	0.2505201	0.5516087	0.4991152	2	STX5/VTI1A
Biological Process	GO:0048549	positive regulation of pinocytosis	2/2279	8/18866	0.2505201	0.5516087	0.4991152	2	ANKFY1/ACTN4
Biological Process	GO:0048733	sebaceous gland development	2/2279	8/18866	0.2505201	0.5516087	0.4991152	2	RBPJ/FA2H
Biological Process	GO:0051045	negative regulation of membrane protein ectodomain proteolysis	2/2279	8/18866	0.2505201	0.5516087	0.4991152	2	TIMP2/IL10
Biological Process	GO:0051126	negative regulation of actin nucleation	2/2279	8/18866	0.2505201	0.5516087	0.4991152	2	GMFB/GMFG
Biological Process	GO:0051683	establishment of Golgi localization	2/2279	8/18866	0.2505201	0.5516087	0.4991152	2	YWHAZ/RIPOR1
Biological Process	GO:0052805	imidazole-containing compound catabolic process	2/2279	8/18866	0.2505201	0.5516087	0.4991152	2	HAL/HNMT
Biological Process	GO:0060137	maternal process involved in parturition	2/2279	8/18866	0.2505201	0.5516087	0.4991152	2	ARNTL/NODAL

Biological Process	GO:0060215	primitive hemopoiesis	2/2279	8/18866	0.2505201	0.5516087	0.4991152	2	ZFPM1/STK3
Biological Process	GO:0060355	positive regulation of cell adhesion molecule production	2/2279	8/18866	0.2505201	0.5516087	0.4991152	2	COLEC12/FLOT1
Biological Process	GO:0060373	regulation of ventricular cardiac muscle cell membrane depolarization	2/2279	8/18866	0.2505201	0.5516087	0.4991152	2	GJA5/SMAD7
Biological Process	GO:0060534	trachea cartilage development	2/2279	8/18866	0.2505201	0.5516087	0.4991152	2	RARA/RARG
Biological Process	GO:0061179	negative regulation of insulin secretion involved in cellular response to glucose stimulus	2/2279	8/18866	0.2505201	0.5516087	0.4991152	2	ENY2/KLF7
Biological Process	GO:0061669	spontaneous neurotransmitter secretion	2/2279	8/18866	0.2505201	0.5516087	0.4991152	2	RPH3A/PRKN
Biological Process	GO:0070212	protein poly-ADP-ribosylation	2/2279	8/18866	0.2505201	0.5516087	0.4991152	2	TNKS/PARP10
Biological Process	GO:0071169	establishment of protein localization to chromatin	2/2279	8/18866	0.2505201	0.5516087	0.4991152	2	PIH1D1/MACROH2A2
Biological Process	GO:0071476	cellular hypotonic response	2/2279	8/18866	0.2505201	0.5516087	0.4991152	2	MYLK/CAB39

Biological Process	GO:0071493	cellular response to UV-B	2/2279	8/18866	0.2505201	0.5516087	0.4991152	2	MFAP4/CRIP1
Biological Process	GO:0071864	positive regulation of cell proliferation in bone marrow	2/2279	8/18866	0.2505201	0.5516087	0.4991152	2	MAP3K3/MEF2C
Biological Process	GO:0072239	metanephric glomerulus vasculature development	2/2279	8/18866	0.2505201	0.5516087	0.4991152	2	PDGFB/PDGFRB
Biological Process	GO:0072386	plus-end-directed organelle transport along microtubule	2/2279	8/18866	0.2505201	0.5516087	0.4991152	2	KIF13A/KIFAP3
Biological Process	GO:0090650	cellular response to oxygen-glucose deprivation	2/2279	8/18866	0.2505201	0.5516087	0.4991152	2	AQP3/DNM1L
Biological Process	GO:0090674	endothelial cell-matrix adhesion via fibronectin	2/2279	8/18866	0.2505201	0.5516087	0.4991152	2	RIN2/CEACAM6
Biological Process	GO:0097049	motor neuron apoptotic process	2/2279	8/18866	0.2505201	0.5516087	0.4991152	2	CRLF1/FADD
Biological Process	GO:0097210	response to gonadotropin-releasing hormone	2/2279	8/18866	0.2505201	0.5516087	0.4991152	2	RAP1B/CRHBP
Biological Process	GO:1900193	regulation of oocyte maturation	2/2279	8/18866	0.2505201	0.5516087	0.4991152	2	PPP2R1A/NPPC

Biological Process	GO:1901213	regulation of transcription from RNA polymerase II promoter involved in heart development	2/2279	8/18866	0.2505201	0.5516087	0.4991152	2	RBPJ/NOTCH1
Biological Process	GO:1901533	negative regulation of hematopoietic progenitor cell differentiation	2/2279	8/18866	0.2505201	0.5516087	0.4991152	2	NFE2L2/NOTCH1
Biological Process	GO:1901993	regulation of meiotic cell cycle phase transition	2/2279	8/18866	0.2505201	0.5516087	0.4991152	2	CDC25A/OVOL1
Biological Process	GO:1902299	pre-replicative complex assembly involved in cell cycle DNA replication	2/2279	8/18866	0.2505201	0.5516087	0.4991152	2	MCM5/MCM2
Biological Process	GO:1902857	positive regulation of non-motile cilium assembly	2/2279	8/18866	0.2505201	0.5516087	0.4991152	2	CEP135/SEPTIN9
Biological Process	GO:1903243	negative regulation of cardiac muscle hypertrophy in response to stress	2/2279	8/18866	0.2505201	0.5516087	0.4991152	2	SMAD3/FOXO1
Biological Process	GO:1903659	regulation of complement-dependent cytotoxicity	2/2279	8/18866	0.2505201	0.5516087	0.4991152	2	CD55/CD59
Biological Process	GO:1903979	negative regulation of microglial cell activation	2/2279	8/18866	0.2505201	0.5516087	0.4991152	2	PTPRC/GRN
Biological Process	GO:1904180	negative regulation of membrane depolarization	2/2279	8/18866	0.2505201	0.5516087	0.4991152	2	HSH2D/BCL2

Biological Process	GO:1904354	negative regulation of telomere capping	2/2279	8/18866	0.2505201	0.5516087	0.4991152	2	SMG6/ERCC1
Biological Process	GO:1904491	protein localization to ciliary transition zone	2/2279	8/18866	0.2505201	0.5516087	0.4991152	2	NPHP4/CPLANE1
Biological Process	GO:1904904	regulation of endothelial cell-matrix adhesion via fibronectin	2/2279	8/18866	0.2505201	0.5516087	0.4991152	2	RIN2/CEACAM6
Biological Process	GO:1905906	regulation of amyloid fibril formation	2/2279	8/18866	0.2505201	0.5516087	0.4991152	2	APP/CLU
Biological Process	GO:1990384	hyaloid vascular plexus regression	2/2279	8/18866	0.2505201	0.5516087	0.4991152	2	NINJ1/FLT1
Biological Process	GO:1990414	replication-born double-strand break repair via sister chromatid exchange	2/2279	8/18866	0.2505201	0.5516087	0.4991152	2	RECQL5/RAD21L1
Biological Process	GO:2000389	regulation of neutrophil extravasation	2/2279	8/18866	0.2505201	0.5516087	0.4991152	2	FUT7/IL1R1
Biological Process	GO:2000617	positive regulation of histone H3-K9 acetylation	2/2279	8/18866	0.2505201	0.5516087	0.4991152	2	SPHK2/PIH1D1
Biological Process	GO:2000698	positive regulation of epithelial cell differentiation involved in kidney development	2/2279	8/18866	0.2505201	0.5516087	0.4991152	2	PAX2/LIF

Biological Process	GO:2000980	regulation of inner ear receptor cell differentiation	2/2279	8/18866	0.2505201	0.5516087	0.4991152	2	DLL1/NOTCH1
Biological Process	GO:2001016	positive regulation of skeletal muscle cell differentiation	2/2279	8/18866	0.2505201	0.5516087	0.4991152	2	ARNTL/MEF2C
Biological Process	GO:2001027	negative regulation of endothelial cell chemotaxis	2/2279	8/18866	0.2505201	0.5516087	0.4991152	2	THBS1/NOTCH1
Biological Process	GO:2001187	positive regulation of CD8-positive, alpha-beta T cell activation	2/2279	8/18866	0.2505201	0.5516087	0.4991152	2	RUNX1/RUNX3
Biological Process	GO:0048592	eye morphogenesis	22/2279	156/18866	0.2506085	0.5516755	0.4991756	22	MFSD2A/SKI/CRB1/HIPK2/DLL1/GNAT2/PAX2/NF1/SP3/RARG/PRKCI/MEIS1/BCL2/RPGRIP1/ZHX2/COL5A1/SDK2/BCAR3/NRL/CABP4/TSKU/NECTIN1
Biological Process	GO:0006289	nucleotide-excision repair	16/2279	110/18866	0.2513237	0.5528656	0.5002524	16	POLE/BRCA2/RAD52/NEIL3/HUS1/POLR2F/COPS5/NTHL1/CCNH/COPS3/ERCC1/SLX4/KAT7/POLD3/XPA/ERCC2
Biological Process	GO:0006664	glycolipid metabolic process	16/2279	110/18866	0.2513237	0.5528656	0.5002524	16	HEXB/PIGL/PIGU/GPLD1/GALC/PIGN/B3GNT5/ST8SIA6/ST3GAL4/B4GALT3/PIGQ/FA2H/ESYT1/CERK/ST3GAL2/PRKAA1
Biological Process	GO:1901800	positive regulation of proteasomal protein catabolic process	16/2279	110/18866	0.2513237	0.5528656	0.5002524	16	RNF144A/FZR1/RNF19A/CSNK1D/CBFA2T3/EDEM1/CLU/SMAD7/EDEM2/CSNK1E/NFE2L2/RNF144B/RNF14/TRIB1/RNF40/PRKN
Biological Process	GO:0042770	signal transduction in response to DNA damage	19/2279	133/18866	0.2517257	0.5536217	0.5009366	19	SPRED2/FZR1/TFDP1/BRCA2/NDRG1/HIPK2/E2F7/PCBP4/ZNF385A/ARID3A/SP100/FOXM1/CHEK2/ANKRD1/BCL3/PAXIP1/MARCHF7/ABRAXAS1/BABAM2

Biological Process	GO:0001774	microglial cell activation	8/2279	50/18866	0.2522951	0.5542325	0.5014892	8	AZU1/TNF/FPR2/APP/CLU/PTPRC/GRN/ITGB2
Biological Process	GO:0002269	leukocyte activation involved in inflammatory response	8/2279	50/18866	0.2522951	0.5542325	0.5014892	8	AZU1/TNF/FPR2/APP/CLU/PTPRC/GRN/ITGB2
Biological Process	GO:0031648	protein destabilization	8/2279	50/18866	0.2522951	0.5542325	0.5014892	8	GSN/CCDC88C/CUL3/CTSH/HTT/CREBBP/FBXW11/PRKN
Biological Process	GO:0045806	negative regulation of endocytosis	8/2279	50/18866	0.2522951	0.5542325	0.5014892	8	MIR199A1/PACSIN2/ANKRD13B/MIR27A/ANKRD13D/NR1H2/ANKRD13A/MIR199A2
Biological Process	GO:2000725	regulation of cardiac muscle cell differentiation	8/2279	50/18866	0.2522951	0.5542325	0.5014892	8	CTDP1/MIR199A1/DLL1/RBPJ/TGFB1/ARRB2/MIR199A2/MEF2C
Biological Process	GO:0050864	regulation of B cell activation	27/2279	195/18866	0.2526811	0.5549521	0.5021404	27	IL10/INPP5D/TNFAIP3/NFATC2/TNFSF13B/TLR9/MNDA/IGHV6-1/PTPN6/TNFRSF21/TGFB1/BLK/PTPRC/CD320/NOD2/BCL2/TP53BP1/MEF2C/BANK1/TNFSF13/CYLD/INHBA/PELI1/PCID2/PAXIP1/VAV3/NSD2
Biological Process	GO:0046165	alcohol biosynthetic process	23/2279	164/18866	0.2531946	0.5559515	0.5030447	23	GFI1/SPTLC2/IDI1/VDR/ITPKB/MBTPS1/TNF/ABCG1/PGP/P2RY6/SPHK2/HRH1/DGKQ/POR/ACACB/IMPA2/ACACA/PCK2/HMGCS1/SCD/SREBF1/PRKAA1/IP6K1
Biological Process	GO:0007589	body fluid secretion	14/2279	95/18866	0.2539102	0.5573938	0.5043497	14	NCOR2/CREB1/VDR/STAT5A/ZBTB7B/AQP1/NR1H2/HK2/NCOA1/PRKCE/USF2/SLC29A1/ATP7B/DHODH
Biological Process	GO:0007569	cell aging	17/2279	118/18866	0.255465	0.5599118	0.5066281	17	CALR/FZR1/MAP3K3/BRCA2/NSMCE2/AKT3/ILK/ZMIZ1/ENG/VASH1/ARNTL/NEK6/MORC3/BCL2/FOXM1/CHEK2/ERCC1

Biological Process	GO:2000736	regulation of stem cell differentiation	17/2279	118/18866	0.255465	0.5599118	0.5066281	17	TACSTD2/MYB/RUNX1/LBH/TCF12/KDM4C/SETD1A/PSMF1/NFE2L2/PUS7/ESRRB/TCF3/PSMB7/NOTCH1/TEAD2/PSMD13/CDK13
Biological Process	GO:0035773	insulin secretion involved in cellular response to glucose stimulus	10/2279	65/18866	0.2554689	0.5599118	0.5066281	10	NADK/LRP5L/ARRB1/GPLD1/PTPRN2/ENY2/GHRL/KLF7/PRKCE/C2CD2L
Biological Process	GO:0060389	pathway-restricted SMAD protein phosphorylation	10/2279	65/18866	0.2554689	0.5599118	0.5066281	10	LDLRAD4/TGFBR2/SMAD7/TGFB1/BMPER/ENG/RBPMS/GDF6/INHBA/NODAL
Biological Process	GO:1905953	negative regulation of lipid localization	10/2279	65/18866	0.2554689	0.5599118	0.5066281	10	PNPLA2/PPARG/TNF/ABCG1/THBS1/MIR27A/NR1H2/ITGB3/ABCA1/ABHD5
Biological Process	GO:0002312	B cell activation involved in immune response	12/2279	80/18866	0.2555287	0.5599118	0.5066281	12	IL10/DLL1/UNG/TGFB1/PTPRC/DOCK10/TP53BP1/ERCC1/TNFSF13/BCL3/PAXIP1/NSD2
Biological Process	GO:0003151	outflow tract morphogenesis	12/2279	80/18866	0.2555287	0.5599118	0.5066281	12	ZFPM1/NRP2/GJA5/TGFBR2/RBPJ/ILK/WNT11/RARA/ENG/NOTCH1/MEF2C/NEDD4
Biological Process	GO:0006110	regulation of glycolytic process	12/2279	80/18866	0.2555287	0.5599118	0.5066281	12	HDAC4/PRKAG2/PFKFB4/RAE1/CBFA2T3/APP/PGAM1/NUP93/SEC13/ESRRB/PFKFB3/PRKAA1
Biological Process	GO:0003007	heart morphogenesis	35/2279	258/18866	0.2561821	0.5612142	0.5078065	35	ZFPM1/RXRA/NRP2/TTN/SMAD3/SUFU/GJA5/MICAL2/RTN4/CCM2L/TGFBR2/SYNPO2L/DLL1/RBPJ/MIB1/ILK/SMAD7/WNT11/ZMIZ1/TGFB1/RARA/GRHL2/ENG/NOTCH1/COL5A1/MEF2C/FHL2/SLIT3/NEDD4/TMED2/TEAD2/ANKRD1/ALDH1A2/NODAL/NSD2
Biological Process	GO:1901796	regulation of signal transduction by p53 class mediator	25/2279	180/18866	0.2577129	0.5643295	0.5106253	25	SPRED2/BLM/PRKAG2/RMI2/HUS1/HIPK2/RRM2B/EHMT2/ZNF385A/PRKAB1/TAF10/HDAC1/DYRK2/MTA2/ANKRD2/BCL2/RHNO1/CHEK2/TP53BP2/ANKRD1/HEXIM1/ING5/PRKAA1/PRKN/MARCHF7

Biological Process	GO:0002823	negative regulation of adaptive immune response based on somatic recombination of immune receptors built from immunoglobulin superfamily domains	7/2279	43/18866	0.2582575	0.5643295	0.5106253	7	LOXL3/DUSP22/HLA-F/PTPN6/SMAD7/ZBTB7B/PTPRC
Biological Process	GO:0006308	DNA catabolic process	7/2279	43/18866	0.2582575	0.5643295	0.5106253	7	CECR2/PAM16/ERI3/DFFB/DNASE1L3/ISG20/FBH1
Biological Process	GO:0015701	bicarbonate transport	7/2279	43/18866	0.2582575	0.5643295	0.5106253	7	SLC26A1/HBB/AQP1/CYB5RL/CA3/CA1/CA12
Biological Process	GO:0015804	neutral amino acid transport	7/2279	43/18866	0.2582575	0.5643295	0.5106253	7	SLC1A5/SLC43A2/SLC7A5/SFXN1/SLC36A3/SLC43A1/SLC7A8
Biological Process	GO:0019432	triglyceride biosynthetic process	7/2279	43/18866	0.2582575	0.5643295	0.5106253	7	ACSL1/MFSD2A/GPLD1/NR1H2/PCK2/SREBF1/LPIN1
Biological Process	GO:0021762	substantia nigra development	7/2279	43/18866	0.2582575	0.5643295	0.5106253	7	YWHAH/PADI2/MBP/SYNGR3/YWHAQ/SYPL2/NDRG2
Biological Process	GO:0030517	negative regulation of axon extension	7/2279	43/18866	0.2582575	0.5643295	0.5106253	7	SEMA6B/SEMA4A/RTN4/SEMA4B/IFRD1/SEMA4D/CDKL3
Biological Process	GO:0045773	positive regulation of axon extension	7/2279	43/18866	0.2582575	0.5643295	0.5106253	7	DISC1/ILK/CDH4/CYFIP1/TRPV2/NDEL1/DBNL
Biological Process	GO:0086004	regulation of cardiac muscle cell contraction	7/2279	43/18866	0.2582575	0.5643295	0.5106253	7	PDE4D/CACNA1C/GJA5/JUP/BIN1/NOS1AP/ANK2

Biological Process	GO:1901659	glycosyl compound biosynthetic process	7/2279	43/18866	0.2582575	0.5643295	0.5106253	7	CDA/TK2/UCK2/GUK1/DHODH/TK1/IMPDH1
Biological Process	GO:0002440	production of molecular mediator of immune response	41/2279	306/18866	0.2616147	0.5715339	0.5171442	41	ELANE/PRKCZ/IGLV3-10/GALNT2/IL10/TRDV2/CD55/SLC11A1/TNFRSF1B/NLRP3/IL1R1/CCR2/TNF/TRAF2/TNFSF13B/TLR9/SPON2/CD226/HLA-F/SMAD7/UNG/SCIMP/SPHK2/TGFB1/PHB/SLC7A5/PTPRC/BCL10/IL18RAP/NOD2/POU2F2/TCF3/TP53BP1/IL18R1/TRAF3IP2/CTNNBL1/ERCC1/TNFSF13/CD96/PAXIP1/NSD2
Biological Process	GO:0071333	cellular response to glucose stimulus	19/2279	134/18866	0.2624452	0.5731226	0.5185817	19	NADK/LRP5L/ERN1/ARRB1/GPLD1/IGF1R/PTPRN2/PAX2/ENY2/GHRL/KLF7/FIS1/PIH1D1/PRKCE/CPB2/PCK2/SLC29A1/C2CD2L/PRKAA1
Biological Process	GO:0002043	blood vessel endothelial cell proliferation involved in sprouting angiogenesis	9/2279	58/18866	0.2627038	0.5731226	0.5185817	9	ITGB1BP1/MIR101-2/THBS1/MIR24-2/MIR27A/MIR23A/BMPER/AGTR1/PPP1R16B
Biological Process	GO:0010656	negative regulation of muscle cell apoptotic process	9/2279	58/18866	0.2627038	0.5731226	0.5185817	9	MIR145/MIR199A1/MIR24-2/ILK/NFE2L2/ARRB2/MIR138-2/MIR199A2/DNMT1
Biological Process	GO:0045851	pH reduction	9/2279	58/18866	0.2627038	0.5731226	0.5185817	9	SLC11A1/ATP6V1B2/GRN/BCL2/ATP6V0B/UBE3A/CLN5/ATP6V0C/ATP6V1C1
Biological Process	GO:1902099	regulation of metaphase/anaphase transition of cell cycle	9/2279	58/18866	0.2627038	0.5731226	0.5185817	9	MAD1L1/NSMCE2/CUL3/CDC16/NEK6/RB1/ANAPC7/HECW2/PCID2
Biological Process	GO:1902373	negative regulation of mRNA catabolic process	9/2279	58/18866	0.2627038	0.5731226	0.5185817	9	SLC11A1/LARP1/PKP1/NBAS/PAIP1/SECISBP2/SYNCRIP/TENT5C/TENT5A
Biological Process	GO:0010611	regulation of cardiac muscle hypertrophy	11/2279	73/18866	0.2631377	0.5734826	0.5189074	11	CTDP1/MIR145/TNFRSF1B/SMAD3/PRKCA/MIR199A1/TNFRSF1A/PDE9A/MIR199A2/NOTCH1/FOXO1

Biological Process	GO:0051145	smooth muscle cell differentiation	11/2279	73/18866	0.2631377	0.5734826	0.5189074	11	MIR140/MIR145/NFATC2/NFATC1/EPC1/PDGFB/ENG/NOTCH1/DNMT1/MEF2C/MRTFA
Biological Process	GO:0035305	negative regulation of dephosphorylation	16/2279	111/18866	0.2631398	0.5734826	0.5189074	16	LMTK2/MGAT5/PPP1R1B/URI1/CABIN1/TNF/CCDC8/GNAI2/ELFN2/PHACTR2/FARP1/SEMA4D/WDR81/PPP1R16B/SPOCD1/PTPA
Biological Process	GO:0051149	positive regulation of muscle cell differentiation	16/2279	111/18866	0.2631398	0.5734826	0.5189074	16	EHD1/MIR140/MIR145/MAML1/MIR199A1/NFATC2/RBPJ/TGFB1/ENG/ARRB2/BCL2/TCF3/MIR199A2/MEF2C/FLOT1/SCGB3A1
Biological Process	GO:0060541	respiratory system development	28/2279	204/18866	0.2631706	0.5734826	0.5189074	28	CTSZ/CREB1/CRISPLD2/CHI3L1/SKI/TNS3/TNF/LOXL3/SFTPD/TGFBR2/CTSH/RBPJ/WNT11/FGFR1/RDH10/RARA/SP3/RARG/GRHL2/YWHAZ/NOTCH1/EPAS1/PDGFRB/SREBF1/ALDH1A2/NODAL/LIF/SLC23A1
Biological Process	GO:0002701	negative regulation of production of molecular mediator of immune response	6/2279	36/18866	0.2638389	0.5738859	0.5192723	6	IL10/TNF/HLA-F/SMAD7/TGFB1/CD96
Biological Process	GO:0003382	epithelial cell morphogenesis	6/2279	36/18866	0.2638389	0.5738859	0.5192723	6	CCDC88C/SPINT2/GRHL2/RILPL1/NOTCH4/FRMD6
Biological Process	GO:0018345	protein palmitoylation	6/2279	36/18866	0.2638389	0.5738859	0.5192723	6	ZDHHC18/ZDHHC14/ZDHHC2/ZDHHC7/ZDHHC17/ZDHHC1
Biological Process	GO:0021955	central nervous system neuron axonogenesis	6/2279	36/18866	0.2638389	0.5738859	0.5192723	6	ZEB2/EPHB3/CDH11/NDEL1/SPTBN4/TSKU
Biological Process	GO:0042554	superoxide anion generation	6/2279	36/18866	0.2638389	0.5738859	0.5192723	6	CD177/FPR2/GNAI2/NCF2/TGFB1/ITGB2

Biological Process	GO:0060323	head morphogenesis	6/2279	36/18866	0.2638389	0.5738859	0.5192723	6	ARID5B/CRISPLD2/SKI/ANKRD11/ASPH/SSBP3
Biological Process	GO:0071108	protein K48-linked deubiquitination	6/2279	36/18866	0.2638389	0.5738859	0.5192723	6	TNFAIP3/OTUB2/VCPIP1/OTUB1/CYLD/MINDY1
Biological Process	GO:2000310	regulation of NMDA receptor activity	6/2279	36/18866	0.2638389	0.5738859	0.5192723	6	CCR2/APP/CRHBP/CNIH2/MAPK8IP2/MEF2C
Biological Process	GO:0072073	kidney epithelium development	20/2279	142/18866	0.2654011	0.5769956	0.5220861	20	TACSTD2/SMAD3/DLL1/ILK/SMAD7/WNT11/PAX2/FGFR1/RARA/MTSS1/BMPER/WWTR1/CAT/BCL2/NOTCH1/MEF2C/ACAT1/CRLF1/LIF/HOXB7
Biological Process	GO:0000045	autophagosome assembly	14/2279	96/18866	0.2667214	0.5769956	0.5220861	14	ULK1/PACS2/WIPI2/PIP4K2A/FEZ2/SMURF1/RUFY4/RAB43/ATG14/TBC1D14/VMP1/UBXN2A/NPRL2/STING1
Biological Process	GO:0035249	synaptic transmission, glutamatergic	14/2279	96/18866	0.2667214	0.5769956	0.5220861	14	CCR2/TNF/DISC1/GRIK4/CNIH2/NF1/NRXN1/MAPK8IP2/NTRK1/P2RX1/MEF2C/SHANK2/GRID1/PRKN
Biological Process	GO:0030323	respiratory tube development	25/2279	181/18866	0.266992	0.5769956	0.5220861	25	CTSZ/CREB1/CRISPLD2/CHI3L1/TNS3/TNF/LOXL3/SFTPD/TGFBR2/CTSH/RBPJ/WNT11/FGFR1/RDH10/SP3/GRHL2/YWHAZ/NOTCH1/EPAS1/PDGFRB/SREBF1/ALDH1A2/NODAL/LIF/SLC23A1
Biological Process	GO:0031503	protein-containing complex localization	39/2279	291/18866	0.2673704	0.5769956	0.5220861	39	PRKCZ/NXF1/FIP1L1/CPSF3/SETD2/RAE1/TNPO1/DNM2/NACC2/SMG7/SMAD7/WASL/PABPN1/ENY2/CLUAP1/CNIH2/NUP93/NUMB/ITGB3/SEC13/KIF5C/SMG6/IFT140/GSG1L/DNM1/KLHL21/TTC21B/DNM3/KIFAP3/EPS15/HHEX/RBM15B/AP3D1/MX1/NOL6/CEP72/CPSF4/EZR/PCID2
Biological Process	GO:0001958	endochondral ossification	5/2279	29/18866	0.2685266	0.5769956	0.5220861	5	CSGALNACT1/MEF2D/ALPL/RUNX2/MEF2C

Biological Process	GO:0007020	microtubule nucleation	5/2279	29/18866	0.2685266	0.5769956	0.5220861	5	DCTN1/CSNK1D/NDE1/TUBGCP2/NDEL1
Biological Process	GO:0019048	modulation by virus of host process	5/2279	29/18866	0.2685266	0.5769956	0.5220861	5	RXRA/HIPK2/PABPN1/BCL2L1/CPSF4
Biological Process	GO:0036075	replacement ossification	5/2279	29/18866	0.2685266	0.5769956	0.5220861	5	CSGALNACT1/MEF2D/ALPL/RUNX2/MEF2C
Biological Process	GO:0048261	negative regulation of receptor-mediated endocytosis	5/2279	29/18866	0.2685266	0.5769956	0.5220861	5	MIR199A1/ANKRD13B/ANKRD13D/ANKRD13A/MIR199A2
Biological Process	GO:0048873	homeostasis of number of cells within a tissue	5/2279	29/18866	0.2685266	0.5769956	0.5220861	5	CCR2/ADD1/BCL2/NOTCH1/F2R
Biological Process	GO:0050690	regulation of defense response to virus by virus	5/2279	29/18866	0.2685266	0.5769956	0.5220861	5	AP2A1/ELMO1/LCK/AP1B1/DOCK2
Biological Process	GO:0061099	negative regulation of protein tyrosine kinase activity	5/2279	29/18866	0.2685266	0.5769956	0.5220861	5	SH3BP5L/SH3BP5/DUSP22/PTPRC/GGNBP2
Biological Process	GO:0070076	histone lysine demethylation	5/2279	29/18866	0.2685266	0.5769956	0.5220861	5	KDM4B/KDM2A/KDM1B/KDM4C/KDM7A
Biological Process	GO:0071624	positive regulation of granulocyte chemotaxis	5/2279	29/18866	0.2685266	0.5769956	0.5220861	5	C3AR1/CAMK1D/DAPK2/DNM1L/DYSF

Biological Process	GO:0090659	walking behavior	5/2279	29/18866	0.2685266	0.5769956	0.5220861	5	HIPK2/SCN1A/ARRB2/NPC1/SPTBN4
Biological Process	GO:0150117	positive regulation of cell-substrate junction organization	5/2279	29/18866	0.2685266	0.5769956	0.5220861	5	ITGB1BP1/SMAD3/PTPRJ/IQSEC1/DUSP3
Biological Process	GO:0001829	trophectodermal cell differentiation	3/2279	15/18866	0.2687921	0.5769956	0.5220861	3	CUL3/SP3/NODAL
Biological Process	GO:0006000	fructose metabolic process	3/2279	15/18866	0.2687921	0.5769956	0.5220861	3	PFKFB4/GLYCTK/PFKFB3
Biological Process	GO:0006646	phosphatidylethanolamine biosynthetic process	3/2279	15/18866	0.2687921	0.5769956	0.5220861	3	CHKB/LPIN1/ETNK2
Biological Process	GO:0009130	pyrimidine nucleoside monophosphate biosynthetic process	3/2279	15/18866	0.2687921	0.5769956	0.5220861	3	UCK2/DCTD/DHODH
Biological Process	GO:0023035	CD40 signaling pathway	3/2279	15/18866	0.2687921	0.5769956	0.5220861	3	TNFAIP3/PHB/FANCA
Biological Process	GO:0032486	Rap protein signal transduction	3/2279	15/18866	0.2687921	0.5769956	0.5220861	3	TIMP2/RAP1B/RAPGEF2
Biological Process	GO:0033540	fatty acid beta-oxidation using acyl-CoA oxidase	3/2279	15/18866	0.2687921	0.5769956	0.5220861	3	AMACR/ACOXL/ACOX2

Biological Process	GO:0033689	negative regulation of osteoblast proliferation	3/2279	15/18866	0.2687921	0.5769956	0.5220861	3	SMAD3/MIR138-2/BCL2
Biological Process	GO:0034111	negative regulation of homotypic cell-cell adhesion	3/2279	15/18866	0.2687921	0.5769956	0.5220861	3	CD9/UBASH3B/CCM2L
Biological Process	GO:0036376	sodium ion export across plasma membrane	3/2279	15/18866	0.2687921	0.5769956	0.5220861	3	FXYD2/FXYD1/SLC8A1
Biological Process	GO:0042559	pteridine-containing compound biosynthetic process	3/2279	15/18866	0.2687921	0.5769956	0.5220861	3	MTHFD1L/MTHFS/MTHFD1
Biological Process	GO:0043101	purine-containing compound salvage	3/2279	15/18866	0.2687921	0.5769956	0.5220861	3	AMPD3/PNP/GMPR2
Biological Process	GO:0043517	positive regulation of DNA damage response, signal transduction by p53 class mediator	3/2279	15/18866	0.2687921	0.5769956	0.5220861	3	SPRED2/ZNF385A/ANKRD1
Biological Process	GO:0044406	adhesion of symbiont to host	3/2279	15/18866	0.2687921	0.5769956	0.5220861	3	SFTPD/LTF/NECTIN1
Biological Process	GO:0044849	estrous cycle	3/2279	15/18866	0.2687921	0.5769956	0.5220861	3	NCOR2/NCOA1/ETS1
Biological Process	GO:0048070	regulation of developmental pigmentation	3/2279	15/18866	0.2687921	0.5769956	0.5220861	3	ZEB2/GNA11/BCL2

Biological Process	GO:0048308	organelle inheritance	3/2279	15/18866	0.2687921	0.5769956	0.5220861	3	STX5/VCPIP1/YWHAZ
Biological Process	GO:0048313	Golgi inheritance	3/2279	15/18866	0.2687921	0.5769956	0.5220861	3	STX5/VCPIP1/YWHAZ
Biological Process	GO:0048368	lateral mesoderm development	3/2279	15/18866	0.2687921	0.5769956	0.5220861	3	FGFR1/TEAD2/NODAL
Biological Process	GO:0050746	regulation of lipoprotein metabolic process	3/2279	15/18866	0.2687921	0.5769956	0.5220861	3	ITGB3/SVIP/ANGPTL8
Biological Process	GO:0051044	positive regulation of membrane protein ectodomain proteolysis	3/2279	15/18866	0.2687921	0.5769956	0.5220861	3	TNFRSF1B/GPLD1/TNF
Biological Process	GO:0051315	attachment of mitotic spindle microtubules to kinetochore	3/2279	15/18866	0.2687921	0.5769956	0.5220861	3	MAD1L1/CHAMP1/MIS12
Biological Process	GO:0051589	negative regulation of neurotransmitter transport	3/2279	15/18866	0.2687921	0.5769956	0.5220861	3	RAP1B/NF1/PRKN
Biological Process	GO:0055057	neuroblast division	3/2279	15/18866	0.2687921	0.5769956	0.5220861	3	FGFR1/NUMB/ARHGEF2
Biological Process	GO:0060099	regulation of phagocytosis, engulfment	3/2279	15/18866	0.2687921	0.5769956	0.5220861	3	PPARG/RAB31/ANO6

Biological Process	GO:0060253	negative regulation of glial cell proliferation	3/2279	15/18866	0.2687921	0.5769956	0.5220861	3	SKI/NOTCH1/CERS2
Biological Process	GO:0060841	venous blood vessel development	3/2279	15/18866	0.2687921	0.5769956	0.5220861	3	CCM2/ENG/NOTCH1
Biological Process	GO:0070365	hepatocyte differentiation	3/2279	15/18866	0.2687921	0.5769956	0.5220861	3	E2F7/TAF10/PCK2
Biological Process	GO:0070571	negative regulation of neuron projection regeneration	3/2279	15/18866	0.2687921	0.5769956	0.5220861	3	KREMEN1/CERS2/INPP5F
Biological Process	GO:0070885	negative regulation of calcineurin-NFAT signaling cascade	3/2279	15/18866	0.2687921	0.5769956	0.5220861	3	RCAN1/DYRK2/FHL2
Biological Process	GO:0071474	cellular hyperosmotic response	3/2279	15/18866	0.2687921	0.5769956	0.5220861	3	AQP1/ARHGEF2/SLC2A1
Biological Process	GO:0072567	chemokine (C-X-C motif) ligand 2 production	3/2279	15/18866	0.2687921	0.5769956	0.5220861	3	FOXP1/TNF/MBP
Biological Process	GO:0099171	presynaptic modulation of chemical synaptic transmission	3/2279	15/18866	0.2687921	0.5769956	0.5220861	3	GIT1/PLEKHG5/PRKCB
Biological Process	GO:0099188	postsynaptic cytoskeleton organization	3/2279	15/18866	0.2687921	0.5769956	0.5220861	3	NOS1AP/ACTG1/DBNL

Biological Process	GO:0106057	negative regulation of calcineurin-mediated signaling	3/2279	15/18866	0.2687921	0.5769956	0.5220861	3	RCAN1/DYRK2/FHL2
Biological Process	GO:1903960	negative regulation of anion transmembrane transport	3/2279	15/18866	0.2687921	0.5769956	0.5220861	3	THBS1/SLC43A2/SLC43A1
Biological Process	GO:1904923	regulation of autophagy of mitochondrion in response to mitochondrial depolarization	3/2279	15/18866	0.2687921	0.5769956	0.5220861	3	SMURF1/HK2/PRKN
Biological Process	GO:1905065	positive regulation of vascular associated smooth muscle cell differentiation	3/2279	15/18866	0.2687921	0.5769956	0.5220861	3	MIR140/MIR145/ENG
Biological Process	GO:2000341	regulation of chemokine (C-X-C motif) ligand 2 production	3/2279	15/18866	0.2687921	0.5769956	0.5220861	3	FOXP1/TNF/MBP
Biological Process	GO:2001044	regulation of integrin-mediated signaling pathway	3/2279	15/18866	0.2687921	0.5769956	0.5220861	3	CD177/ITGB1BP1/LOXL3
Biological Process	GO:0034644	cellular response to UV	12/2279	81/18866	0.2695793	0.5769956	0.5220861	12	MFAP4/PIK3R1/BMF/CDC25A/AQP1/CREBBP/RHNO1/NEDD4/ERCC1/CRIP1/POLD3/XPA
Biological Process	GO:0050829	defense response to Gram-negative bacterium	12/2279	81/18866	0.2695793	0.5769956	0.5220861	12	DEFA4/AZU1/ELANE/CTSG/LYPD8/RNASE3/SLC11A1/TLR9/DEFB1/IL6R/MPEG1/LTF
Biological Process	GO:0021545	cranial nerve development	8/2279	51/18866	0.2701669	0.5769956	0.5220861	8	NRP2/PHOX2A/DRGX/ATP8B1/PAX2/NTRK1/SLC1A3/HES3

Biological Process	GO:0035176	social behavior	8/2279	51/18866	0.2701669	0.5769956	0.5220861	8	ATXN1/NRXN1/NRXN2/MAPK8IP2/SHANK2/CHD8/GRID1/CX3CR1
Biological Process	GO:0038084	vascular endothelial growth factor signaling pathway	8/2279	51/18866	0.2701669	0.5769956	0.5220861	8	NRP2/MIR199A1/DLL1/FLT1/MIR199A2/MYO1C/PDGFRB/ADGRA2
Biological Process	GO:0046473	phosphatidic acid metabolic process	8/2279	51/18866	0.2701669	0.5769956	0.5220861	8	LPCAT1/PLD1/ACP6/DGKQ/DGKA/DGKD/ABHD5/AGPAT5
Biological Process	GO:0050435	amyloid-beta metabolic process	8/2279	51/18866	0.2701669	0.5769956	0.5220861	8	SORL1/TNF/ABCG1/MGAT3/BIN1/APP/CLU/RELA
Biological Process	GO:0072523	purine-containing compound catabolic process	8/2279	51/18866	0.2701669	0.5769956	0.5220861	8	PDE4D/AMPD3/NUDT3/ACOT7/PDE9A/PNP/ACAT1/AOX1
Biological Process	GO:0032606	type I interferon production	18/2279	127/18866	0.2703251	0.5769956	0.5220861	18	IL10/TRIM38/IRF5/SETD2/TNFAIP3/POLR2F/TLR9/TRAIP/CRCP/NLRC5/CREBBP/DDX41/CYLD/RELA/FLOT1/HERC5/STING1/ILRUN
Biological Process	GO:0002562	somatic diversification of immune receptors via germline recombination within a single locus	10/2279	66/18866	0.2711275	0.5769956	0.5220861	10	IL10/UNG/TGFB1/PTPRC/TCF3/TP53BP1/ERCC1/TNFSF13/PAXIP1/NSD2
Biological Process	GO:0016444	somatic cell DNA recombination	10/2279	66/18866	0.2711275	0.5769956	0.5220861	10	IL10/UNG/TGFB1/PTPRC/TCF3/TP53BP1/ERCC1/TNFSF13/PAXIP1/NSD2
Biological Process	GO:0042490	mechanoreceptor differentiation	10/2279	66/18866	0.2711275	0.5769956	0.5220861	10	CDH23/SLC44A4/ATP8B1/DLL1/RBPJ/FGFR1/NTRK1/NOTCH1/TMC1/NTF4

Biological Process	GO:0050922	negative regulation of chemotaxis	10/2279	66/18866	0.2711275	0.5769956	0.5220861	10	ELANE/SEMA6B/SEMA4A/PADI2/THBS1/SEMA4B/C5AR2/SEMA4D/NOTCH1/DUSP3
Biological Process	GO:0150116	regulation of cell-substrate junction organization	10/2279	66/18866	0.2711275	0.5769956	0.5220861	10	ITGB1BP1/SMAD3/DUSP22/THBS1/PTPRJ/IQSEC1/ACTG1/CORO1C/DUSP3/DAPK3
Biological Process	GO:0006067	ethanol metabolic process	4/2279	22/18866	0.2711794	0.5769956	0.5220861	4	ALDH2/ACSS1/SULT1A2/ACSS2
Biological Process	GO:0007398	ectoderm development	4/2279	22/18866	0.2711794	0.5769956	0.5220861	4	MIR145/ZBTB17/ZBTB7B/SMURF1
Biological Process	GO:0009190	cyclic nucleotide biosynthetic process	4/2279	22/18866	0.2711794	0.5769956	0.5220861	4	ADCY2/ADCY9/ADCY4/NPPC
Biological Process	GO:0010560	positive regulation of glycoprotein biosynthetic process	4/2279	22/18866	0.2711794	0.5769956	0.5220861	4	TCF7L2/POMT2/PLCB1/PXYLP1
Biological Process	GO:0032402	melanosome transport	4/2279	22/18866	0.2711794	0.5769956	0.5220861	4	RAB27A/CDH3/DCTN2/MYO5A
Biological Process	GO:0033005	positive regulation of mast cell activation	4/2279	22/18866	0.2711794	0.5769956	0.5220861	4	GAB2/FGR/CD226/SPHK2
Biological Process	GO:0036120	cellular response to platelet-derived growth factor stimulus	4/2279	22/18866	0.2711794	0.5769956	0.5220861	4	CREB1/PDGFD/PDGFRB/MARS1

Biological Process	GO:0042359	vitamin D metabolic process	4/2279	22/18866	0.2711794	0.5769956	0.5220861	4	GFI1/VDR/TNF/FGFR1
Biological Process	GO:0042438	melanin biosynthetic process	4/2279	22/18866	0.2711794	0.5769956	0.5220861	4	ZEB2/CDH3/RAPGEF2/MYO5A
Biological Process	GO:0046058	cAMP metabolic process	4/2279	22/18866	0.2711794	0.5769956	0.5220861	4	PDE4D/ADCY2/ADCY9/ADCY4
Biological Process	GO:0050765	negative regulation of phagocytosis	4/2279	22/18866	0.2711794	0.5769956	0.5220861	4	PRTN3/SNX3/CSK/DYSF
Biological Process	GO:0052652	cyclic purine nucleotide metabolic process	4/2279	22/18866	0.2711794	0.5769956	0.5220861	4	ADCY2/ADCY9/ADCY4/NPPC
Biological Process	GO:0055093	response to hyperoxia	4/2279	22/18866	0.2711794	0.5769956	0.5220861	4	PPARG/CAT/PDGFRB/FOXO1
Biological Process	GO:1901685	glutathione derivative metabolic process	4/2279	22/18866	0.2711794	0.5769956	0.5220861	4	GSTM5/MGST2/GSTO1/GSTO2
Biological Process	GO:1901687	glutathione derivative biosynthetic process	4/2279	22/18866	0.2711794	0.5769956	0.5220861	4	GSTM5/MGST2/GSTO1/GSTO2
Biological Process	GO:1901739	regulation of myoblast fusion	4/2279	22/18866	0.2711794	0.5769956	0.5220861	4	EHD1/NFATC2/FLOT1/SCGB3A1

Biological Process	GO:1901798	positive regulation of signal transduction by p53 class mediator	4/2279	22/18866	0.2711794	0.5769956	0.5220861	4	SPRED2/ZNF385A/ANKRD1/HEXIM1
Biological Process	GO:1902884	positive regulation of response to oxidative stress	4/2279	22/18866	0.2711794	0.5769956	0.5220861	4	TNF/RIPK1/ACOX2/SIRT3
Biological Process	GO:2000316	regulation of T-helper 17 type immune response	4/2279	22/18866	0.2711794	0.5769956	0.5220861	4	NLRP3/LOXL3/SMAD7/ZBTB7B
Biological Process	GO:2000479	regulation of cAMP-dependent protein kinase activity	4/2279	22/18866	0.2711794	0.5769956	0.5220861	4	PRKAG2/PRKAR1B/RAPGEF2/HTT
Biological Process	GO:0000726	non-recombinational repair	15/2279	104/18866	0.2712201	0.5769956	0.5220861	15	RAD52/KDM2A/UVRAG/SMARCAL1/TP53BP1/ERCC1/SLX4/PARP9/PAXIP1/TFIP11/PAXX/NSD2/CYREN/ABRAXAS1/BABAM2
Biological Process	GO:0002027	regulation of heart rate	15/2279	104/18866	0.2712201	0.5769956	0.5220861	15	PDE4D/KCNE1/CASQ2/CACNA1C/SLC8A1/GJA5/JUP/BIN1/NOS1AP/SNTA1/KCNQ1/EPAS1/ANK2/SREBF1/SPTBN4
Biological Process	GO:0006637	acyl-CoA metabolic process	15/2279	104/18866	0.2712201	0.5769956	0.5220861	15	ACSL1/FAR2/ACOT13/OGDH/ACOT7/ACSS1/ACACB/ACSS2/ACACA/ACSF3/HMGCL/ACAT1/HMGCS1/SCD/SLC25A1
Biological Process	GO:0009116	nucleoside metabolic process	15/2279	104/18866	0.2712201	0.5769956	0.5220861	15	CDA/DPYS/AMPD3/TJP2/MACROD1/TK2/UCK2/UPB1/PNP/GUK1/GMPR2/DHODH/TK1/IMPDH1/ACP3
Biological Process	GO:0010923	negative regulation of phosphatase activity	15/2279	104/18866	0.2712201	0.5769956	0.5220861	15	LMTK2/MGAT5/PPP1R1B/URI1/CABIN1/TNF/CCDC8/GNAI2/ELFN2/PHACTR2/FARP1/SEMA4D/WDR81/SPOCD1/PTPA

Biological Process	GO:0035383	thioester metabolic process	15/2279	104/18866	0.2712201	0.5769956	0.5220861	15	ACSL1/FAR2/ACOT13/OGDH/ACOT7/ACSS1/ACACB/ACSS2/ACACA/ACSF3/HMGCL/ACAT1/HMGCS1/SCD/SLC25A1
Biological Process	GO:1901606	alpha-amino acid catabolic process	15/2279	104/18866	0.2712201	0.5769956	0.5220861	15	ACAD8/HAL/HGD/CBS/OGDH/ALDH4A1/FAH/HIBADH/AGXT/HOGA1/MAT1A/HMGCL/CRYM/ACAT1/HNMT
Biological Process	GO:0003158	endothelium development	19/2279	135/18866	0.273334	0.5812324	0.5259197	19	PDE4D/FOXP1/TJP2/RAP1B/TNF/GJA5/CCM2/DLL1/RBPJ/TNFRSF1A/RAPGEF2/ENG/ADD1/NOTCH1/S1PR2/NOTCH4/PPP1R16B/EZR/MYADM
Biological Process	GO:0042157	lipoprotein metabolic process	19/2279	135/18866	0.273334	0.5812324	0.5259197	19	OLR1/ZDHHC18/PIGL/ZDHHC14/PIGU/GPLD1/WIPI2/PIGN/CTSD/ZDHHC2/ZDHHC7/ITGB3/ABCA1/ZDHHC17/ZDHHC1/PIGQ/LYPLA2/SVIP/ANGPTL8
Biological Process	GO:0015718	monocarboxylic acid transport	24/2279	174/18866	0.2746085	0.5838119	0.5282537	24	PRKAG2/RXRA/PPARG/SLC51A/ACSL1/SLC16A3/SLC10A1/MFSD2A/MAP2K6/ABCC2/THBS1/ATP8B1/NF1/SLCO3A1/NCOA1/ACACB/SLC16A5/ABCC3/SLC16A1/ACACA/SLC2A1/NCOA2/CPT1A/AQP9
Biological Process	GO:0043537	negative regulation of blood vessel endothelial cell migration	13/2279	89/18866	0.2750537	0.5846275	0.5289917	13	PPARG/ITGB1BP1/MIR199A1/TNF/MIR101-2/JUP/THBS1/MIR24-2/TGFB1/VASH1/MIR199A2/NOTCH1/MEF2C
Biological Process	GO:0070646	protein modification by small protein removal	40/2279	300/18866	0.2753917	0.5852152	0.5295234	40	FOXK1/WDR20/NLRP3/SMAD3/TRAF2/TNFAIP3/USP3/USP36/SNX3/RIPK1/OTUB2/TAF10/SMAD7/CDC25A/MBD6/ENY2/COPS5/KDM1B/TNIP1/VCPIP1/PSMF1/ARRB2/COPS3/CCNA1/OTUB1/CLSPN/USP49/PSMB7/USP54/MBD5/USP2/CYLD/RUVBL1/UCHL3/PSMD13/USP35/PRKN/MINDY1/ABRAXAS1/BABAM2
Biological Process	GO:0048762	mesenchymal cell differentiation	31/2279	229/18866	0.2757843	0.5859183	0.5301597	31	SEMA6B/SPRED2/NRP2/SMAD3/LDLRAD4/ANXA6/ZEB2/LOXL3/SEMA4A/RTN4/TGFBR2/SEMA4B/RBPJ/TCF7L2/SMAD7/WNT11/PAX2/FGFR1/TGFB1/SEMA4D/RDH10/TGFB1I1/WWTR1/ENG/BCL2/NOTCH1/CORO1C/GCNT2/MEF2C/NOTCH4/ALDH1A2
Biological Process	GO:0010324	membrane invagination	20/2279	143/18866	0.2760244	0.5861662	0.530384	20	PPARG/GSN/ELMO1/RHOH/DOCK1/RHOG/RAB31/IGHV6-1/THBS1/MYH9/SNX3/MARCO/RHOBTB2/SMURF1/ABCA1/SNX18/ARHGAP25/ITGB2/ANO6/ARHGAP12

Biological Process	GO:0072006	nephron development	20/2279	143/18866	0.2760244	0.5861662	0.530384	20	TACSTD2/ANGPT1/DLL1/ILK/WNT11/PAX2/PDGFD/KIRREL3/MTSS1/WWTR1/IL6R/PDGFB/BCL2/NOTCH1/MEF2C/GPR4/PDGFRB/ACAT1/LIF/HOXB7
Biological Process	GO:0001913	T cell mediated cytotoxicity	7/2279	44/18866	0.2778064	0.5892041	0.5331328	7	CTSH/RAB27A/HLA-F/RIPK3/PTPRC/FADD/CYRIB
Biological Process	GO:0006195	purine nucleotide catabolic process	7/2279	44/18866	0.2778064	0.5892041	0.5331328	7	PDE4D/AMPD3/NUDT3/ACOT7/PDE9A/PNP/ACAT1
Biological Process	GO:0032620	interleukin-17 production	7/2279	44/18866	0.2778064	0.5892041	0.5331328	7	LY9/ZBTB7B/TGFB1/PHB/SLC7A5/NOD2/VSIR
Biological Process	GO:0032881	regulation of polysaccharide metabolic process	7/2279	44/18866	0.2778064	0.5892041	0.5331328	7	AP2A1/TGFB1/DYRK2/PDGFB/PPP1CB/ESRRB/RUBCNL
Biological Process	GO:0045005	DNA-dependent DNA replication maintenance of fidelity	7/2279	44/18866	0.2778064	0.5892041	0.5331328	7	BLM/POLE/BRCA2/RTEL1/EME1/SMARCAL1/FBH1
Biological Process	GO:0006520	cellular amino acid metabolic process	45/2279	340/18866	0.277827	0.5892041	0.5331328	45	ACAD8/DPYS/HAL/GADL1/HGD/GGT1/CBS/ODC1/MSRA/COMT/DDC/MTHFS/CARS2/OGDH/ALDH4A1/ENOSF1/FAH/HIBADH/SLC7A7/AGXT/PSMF1/SCLY/UPB1/MTHFD1/ART4/HOGA1/ADO/SLC1A3/BCKDHA/FN3K/PSMB7/MAT1A/HMGCL/GFPT2/CRYM/ADI1/ACAT1/EARS2/ASPG/HNMT/PSMD13/MARS1/GATB/DGLUCY/YARS1
Biological Process	GO:0086003	cardiac muscle cell contraction	11/2279	74/18866	0.2780703	0.5894568	0.5333615	11	PDE4D/KCNE1/CACNA1C/GJA5/JUP/BIN1/NOS1AP/SCN1A/SNTA1/KCNQ1/ANK2
Biological Process	GO:1903051	negative regulation of proteolysis involved in cellular protein catabolic process	11/2279	74/18866	0.2780703	0.5894568	0.5333615	11	TRIM39/SUFU/HIPK2/UBAC2/DERL2/CLEC16A/PSMF1/MAP1A/BAG5/SVIP/MARCHF7

Biological Process	GO:0019731	antibacterial humoral response	9/2279	59/18866	0.279531	0.5902856	0.5341114	9	DEFA4/ELANE/SLPI/CTSG/RNASE3/SFTPD/SPON2/DEFB1/LTF
Biological Process	GO:0031102	neuron projection regeneration	9/2279	59/18866	0.279531	0.5902856	0.5341114	9	ULK1/KREMEN1/STK24/JAM3/GRN/BCL2/CERS2/NDEL1/INPP5F
Biological Process	GO:0042306	regulation of protein import into nucleus	9/2279	59/18866	0.279531	0.5902856	0.5341114	9	ANGPT1/PIK3R1/SMAD3/SUFU/JUP/TGFB1/NF1/CDH1/CABP1
Biological Process	GO:0051058	negative regulation of small GTPase mediated signal transduction	9/2279	59/18866	0.279531	0.5902856	0.5341114	9	TIMP2/KANK1/CUL3/RASA3/SYNGAP1/NF1/ARHGAP25/ARHGAP12/CYRIB
Biological Process	GO:0051289	protein homotetramerization	9/2279	59/18866	0.279531	0.5902856	0.5341114	9	TRPM2/ACOT13/CRTC3/ACACB/UPB1/ACACA/MAT1A/ALDH1A2/TK1
Biological Process	GO:1901799	negative regulation of proteasomal protein catabolic process	9/2279	59/18866	0.279531	0.5902856	0.5341114	9	TRIM39/UBAC2/DERL2/CLEC16A/PSMF1/MAP1A/BAG5/SVIP/MARCHF7
Biological Process	GO:1990823	response to leukemia inhibitory factor	14/2279	97/18866	0.2797511	0.5902856	0.5341114	14	ARID5B/SBNO2/NRP2/SPOCK2/CREB1/PADI2/SMAD7/CTBP2/HK2/RARG/UBXN2A/GFPT2/NR5A2/B3GNT2
Biological Process	GO:1990830	cellular response to leukemia inhibitory factor	14/2279	97/18866	0.2797511	0.5902856	0.5341114	14	ARID5B/SBNO2/NRP2/SPOCK2/CREB1/PADI2/SMAD7/CTBP2/HK2/RARG/UBXN2A/GFPT2/NR5A2/B3GNT2
Biological Process	GO:0031348	negative regulation of defense response	36/2279	269/18866	0.2803268	0.5902856	0.5341114	36	ELANE/ABR/IL10/TRIM38/PPARG/C1QTNF3/MIR140/MIR145/TNFRSF1B/NLRP3/SMAD3/MEFV/SMPDL3B/TNFAIP3/AOAH/FPR2/HLA-F/TNFAIP6/TNFRSF1A/CXCL17/NR1H2/PGLYRP1/SEC14L1/GHRL/PTPRC/GRN/NLRC5/BCR/CNR2/ETS1/ARRB2/MIR138-2/RB1/TYRO3/CD96/ILRUN

Biological Process	GO:0035637	multicellular organismal signaling	28/2279	206/18866	0.2808646	0.5902856	0.5341114	28	PDE4D/KCNE1/CASQ2/ITPR2/FXYD2/CACNA1C/CACNA2D4/FXYD1/SLC8A1/GJA5/ATP2B2/ITPR1/JUP/BIN1/KCNIP1/SCN1A/KCNK6/JAM3/GHRL/ATP2A3/KCNQ1/P2RX1/NPPC/CNTNAP1/ANK2/ASPH/CASQ1/SPTBN4
Biological Process	GO:0046545	development of primary female sexual characteristics	15/2279	105/18866	0.283814	0.5902856	0.5341114	15	ARID5B/BRCA2/ARRB1/EIF2B5/FANCA/BMPR1B/LHFPL2/ARRB2/BCL2/DMC1/SLIT3/UBE3A/CEBPB/BCL2L1/INHBA
Biological Process	GO:0120193	tight junction organization	12/2279	82/18866	0.2838757	0.5902856	0.5341114	12	TBCD/RUNX1/TNF/ACTN4/WNT11/PRKCH/ACTG1/GRHL2/PRKCI/MPP7/MYO1C/NPHP4
Biological Process	GO:0003091	renal water homeostasis	6/2279	37/18866	0.2855032	0.5902856	0.5341114	6	ADCY2/PRKAR1B/ADCY9/AQP1/ADCY4/AQP3
Biological Process	GO:0006921	cellular component disassembly involved in execution phase of apoptosis	6/2279	37/18866	0.2855032	0.5902856	0.5341114	6	CECR2/PAM16/DEDD2/DFFB/DNASE1L3/BLCAP
Biological Process	GO:0010667	negative regulation of cardiac muscle cell apoptotic process	6/2279	37/18866	0.2855032	0.5902856	0.5341114	6	MIR145/MIR199A1/MIR24-2/ILK/NFE2L2/MIR199A2
Biological Process	GO:0032717	negative regulation of interleukin-8 production	6/2279	37/18866	0.2855032	0.5902856	0.5341114	6	ELANE/IL10/ARRB1/TLR9/CD33/BCL3
Biological Process	GO:0045070	positive regulation of viral genome replication	6/2279	37/18866	0.2855032	0.5902856	0.5341114	6	TRIM38/SRPK2/CCL5/LARP1/NOTCH1/NR5A2
Biological Process	GO:0086010	membrane depolarization during action potential	6/2279	37/18866	0.2855032	0.5902856	0.5341114	6	CACNA1C/YWHAH/SLC8A1/GJA5/SCN1A/ANK2

Biological Process	GO:0090050	positive regulation of cell migration involved in sprouting angiogenesis	6/2279	37/18866	0.2855032	0.5902856	0.5341114	6	MAP3K3/HDAC7/MIR101-2/AKT3/MIR27A/MIR23A
Biological Process	GO:0097242	amyloid-beta clearance	6/2279	37/18866	0.2855032	0.5902856	0.5341114	6	TNF/CLU/HDAC1/IGF1R/MARCO/ITGB2
Biological Process	GO:0010212	response to ionizing radiation	20/2279	144/18866	0.2867974	0.5902856	0.5341114	20	BLM/SPIDR/BRCA2/DNM2/HUS1/MTA1/PTPRC/BCL2/DMC1/TP53BP1/RHNO1/CHEK2/ERCC1/BCL2L1/PAXIP1/EEF1D/PRKAA1/MAP3K20/ABRAXAS1/BABAM2
Biological Process	GO:0070613	regulation of protein processing	10/2279	67/18866	0.2870703	0.5902856	0.5341114	10	CTSZ/NLRC4/ENO1/GSN/THBS1/MYH9/F12/NOL3/CPB2/ANGPTL8
Biological Process	GO:0072678	T cell migration	10/2279	67/18866	0.2870703	0.5902856	0.5341114	10	PIK3CD/CCR2/CCL5/APP/DOCK8/RIPK3/CCL20/C10orf99/CCL27/FADD
Biological Process	GO:0031103	axon regeneration	8/2279	52/18866	0.2883817	0.5902856	0.5341114	8	KREMEN1/STK24/JAM3/GRN/BCL2/CERS2/NDEL1/INPP5F
Biological Process	GO:0038066	p38MAPK cascade	8/2279	52/18866	0.2883817	0.5902856	0.5341114	8	MAP3K3/PHLPP1/MIR138-2/PER1/GADD45G/GDF6/CYLD/EZR
Biological Process	GO:0048260	positive regulation of receptor-mediated endocytosis	8/2279	52/18866	0.2883817	0.5902856	0.5341114	8	AP2A1/ANGPT1/ARRB1/DNM2/CLU/WASL/DGKD/ARRB2
Biological Process	GO:0050999	regulation of nitric-oxide synthase activity	8/2279	52/18866	0.2883817	0.5902856	0.5341114	8	DNM2/TNF/NOS1AP/WASL/NOSTRIN/CNR2/MIR138-2/NOSIP

Biological Process	GO:0051445	regulation of meiotic cell cycle	8/2279	52/18866	0.2883817	0.5902856	0.5341114	8	CALR/FZR1/CDC25A/OVOL1/PPP2R1A/PLCB1/NPPC/LIF
Biological Process	GO:0060425	lung morphogenesis	8/2279	52/18866	0.2883817	0.5902856	0.5341114	8	CTSZ/TNF/TGFBR2/CTSH/RDH10/GRHL2/NODAL/LIF
Biological Process	GO:0043470	regulation of carbohydrate catabolic process	13/2279	90/18866	0.288787	0.5902856	0.5341114	13	HDAC4/PRKAG2/PFKFB4/RAE1/CBFA2T3/APP/PGAM1/NUP93/SEC13/PPP1CB/ESRRB/PFKFB3/PRKAA1
Biological Process	GO:0034284	response to monosaccharide	28/2279	207/18866	0.2898745	0.5902856	0.5341114	28	NADK/SLC2A5/LRP5L/ERN1/SLC8A1/ARRB1/GPLD1/EIF2B5/TGFBR2/THBS1/TCF7L2/IGF1R/PTPRN2/PAX2/ENY2/GIPR/GHRL/CAT/KLF7/FIS1/PIH1D1/PRKCE/CPB2/PCK2/SREBF1/SLC29A1/C2CD2L/PRKAA1
Biological Process	GO:0010906	regulation of glucose metabolic process	17/2279	121/18866	0.2904488	0.5902856	0.5341114	17	MAEA/FOXK1/C1QTNF3/GPLD1/PGP/DGKQ/DYRK2/GNB3/ACACB/PPP1CB/ESRRB/FOXO1/NCOA2/SOGA1/PRKN/SDHAF3/RUBCNL
Biological Process	GO:0071496	cellular response to external stimulus	43/2279	326/18866	0.2912925	0.5902856	0.5341114	43	HDAC4/RPTOR/PRKAG2/PPARG/LCN2/MFSD2A/TNKS/ULK1/VDR/CBS/WIPI2/COMT/EHMT2/TNFRSF1A/WNT11/AQP1/STK24/CLEC16A/SLC1A2/ATG14/PTPRC/NCOA1/BCL10/MAP3K14/NFE2L2/ENG/BCL2/AQP3/DNM1L/GLRX2/USF2/CASP1/CD68/SLC2A1/FOXO1/ANKRD1/SREBF1/EIF4G1/FADD/NPRL2/PRKAA1/MARS1/RIPOR1
Biological Process	GO:0010038	response to metal ion	48/2279	366/18866	0.29237	0.5902856	0.5341114	48	TRPM2/S100A8/CPNE6/BSG/CALR/GSN/CPNE2/TTN/CASQ2/CREB1/NDRG1/ITPKB/D2HGDH/SLFN14/GPLD1/CPNE5/EIF2B5/ABCC2/SLC11A2/THBS1/APP/FECH/CPNE1/MTF1/CRHBP/AQP1/ADAMTS13/GIPR/CAT/NFE2L2/NPC1/CDH1/ADD1/BCL2/AQP3/SLC39A13/MEF2C/SYT17/NEDD4/ERCC1/CRIP1/ATP7B/ANXA7/SEC31A/SLC41A1/AQP9/PRKAA1/PRKN
Biological Process	GO:0002832	negative regulation of response to biotic stimulus	14/2279	98/18866	0.2929815	0.5902856	0.5341114	14	TRIM38/PPARG/TNFAIP3/HLA-F/NR1H2/SEC14L1/GRN/NLRC5/ARRB2/LTF/TRIB1/TYRO3/CD96/ILRUN
Biological Process	GO:0006835	dicarboxylic acid transport	14/2279	98/18866	0.2929815	0.5902856	0.5341114	14	SLC1A5/FOLR3/ABCC2/LRRC8C/PPFIA1/SLC19A1/SLC26A1/NF1/SLC1A2/AGXT/SLC1A6/PER2/SLC1A3/SLC13A5

Biological Process	GO:0042158	lipoprotein biosynthetic process	14/2279	98/18866	0.2929815	0.5902856	0.5341114	14	ZDHHC18/PIGL/ZDHHC14/PIGU/GPLD1/WIPI2/PIGN/ZDHHC2/ZDHHC7/ABCA1/ZDHHC17/ZDHHC1/PIGQ/SVIP
Biological Process	GO:0000303	response to superoxide	5/2279	30/18866	0.2929867	0.5902856	0.5341114	5	MPO/TNF/NFE2L2/GLRX2/SIRT3
Biological Process	GO:0006362	transcription elongation from RNA polymerase I promoter	5/2279	30/18866	0.2929867	0.5902856	0.5341114	5	TAF1C/POLR2F/CCNH/TAF1D/ERCC2
Biological Process	GO:0010586	miRNA metabolic process	5/2279	30/18866	0.2929867	0.5902856	0.5341114	5	AGO1/AGO2/DIS3L2/SND1/RELA
Biological Process	GO:0010758	regulation of macrophage chemotaxis	5/2279	30/18866	0.2929867	0.5902856	0.5341114	5	C3AR1/CCL5/CSF1R/THBS1/CXCL17
Biological Process	GO:0016577	histone demethylation	5/2279	30/18866	0.2929867	0.5902856	0.5341114	5	KDM4B/KDM2A/KDM1B/KDM4C/KDM7A
Biological Process	GO:0019934	cGMP-mediated signaling	5/2279	30/18866	0.2929867	0.5902856	0.5341114	5	THBS1/AQP1/PDE9A/NPPC/IRAG1
Biological Process	GO:0030511	positive regulation of transforming growth factor beta receptor signaling pathway	5/2279	30/18866	0.2929867	0.5902856	0.5341114	5	LRG1/HIPK2/THBS1/TGFB1I1/CREBBP
Biological Process	GO:0033137	negative regulation of peptidyl-serine phosphorylation	5/2279	30/18866	0.2929867	0.5902856	0.5341114	5	PDE4D/NCK2/SMAD7/GGNBP2/INPP5F

Biological Process	GO:0044788	modulation by host of viral process	5/2279	30/18866	0.2929867	0.5902856	0.5341114	5	CSF1R/PC/PHB/LTF/PRKN
Biological Process	GO:0060055	angiogenesis involved in wound healing	5/2279	30/18866	0.2929867	0.5902856	0.5341114	5	TNF/TNFAIP3/ITGB3/ETS1/GPR4
Biological Process	GO:1900746	regulation of vascular endothelial growth factor signaling pathway	5/2279	30/18866	0.2929867	0.5902856	0.5341114	5	MIR199A1/DLL1/MIR199A2/MYO1C/ADGRA2
Biological Process	GO:1902186	regulation of viral release from host cell	5/2279	30/18866	0.2929867	0.5902856	0.5341114	5	TRIM27/TRIM8/PC/TRIM5/TRIM26
Biological Process	GO:1903846	positive regulation of cellular response to transforming growth factor beta stimulus	5/2279	30/18866	0.2929867	0.5902856	0.5341114	5	LRG1/HIPK2/THBS1/TGFB1I1/CREBBP
Biological Process	GO:1990126	retrograde transport, endosome to plasma membrane	5/2279	30/18866	0.2929867	0.5902856	0.5341114	5	SNX27/ZDHHC2/AKAP5/MICALL1/VPS35L
Biological Process	GO:0006353	DNA-templated transcription, termination	11/2279	75/18866	0.2932522	0.5902856	0.5341114	11	TAF1C/FIP1L1/CPSF3/SNRPD3/POLR2F/PABPN1/CCNH/SCAF8/TAF1D/CPSF4/ERCC2
Biological Process	GO:0086001	cardiac muscle cell action potential	11/2279	75/18866	0.2932522	0.5902856	0.5341114	11	KCNE1/CACNA1C/SLC8A1/GJA5/JUP/BIN1/NOS1AP/SCN1A/SNTA1/KCNQ1/ANK2
Biological Process	GO:0007187	G protein-coupled receptor signaling pathway, coupled to cyclic nucleotide second messenger	35/2279	263/18866	0.2956013	0.5902856	0.5341114	35	PDE4D/ADCY2/AKAP13/ADCY9/PRKCA/FPR2/FPR1/GNAZ/GNAI2/MRAP/AKAP5/GNAT2/MARCO/RAPGEF2/HRH1/GRK5/GIPR/GNA11/CHRM2/ABCA1/PALM/CNR2/ADCY4/S1PR2/GNG2/GABBR1/GNAO1/LPAR2/GNA12/S1PR4/GPR4/VIPR2/NPY/ADGRG3/ADGRE3

Biological Process	GO:0032414	positive regulation of ion transmembrane transporter activity	15/2279	106/18866	0.2965918	0.5902856	0.5341114	15	CCR2/DNM2/TESC/NOS1AP/ACTN4/P2RY6/HTR3A/HTT/GSTO1/ANK2/PDZK1/CASQ1/FGF14/STIMATE/CRACR2A
Biological Process	GO:0071868	cellular response to monoamine stimulus	15/2279	106/18866	0.2965918	0.5902856	0.5341114	15	PDE4D/KLF16/DNM2/APP/HRH1/HTR3A/GNA11/CHRM2/PALM/KCNQ1/ARRB2/GNG2/GNAO1/DTNBP1/PRKN
Biological Process	GO:0071870	cellular response to catecholamine stimulus	15/2279	106/18866	0.2965918	0.5902856	0.5341114	15	PDE4D/KLF16/DNM2/APP/HRH1/HTR3A/GNA11/CHRM2/PALM/KCNQ1/ARRB2/GNG2/GNAO1/DTNBP1/PRKN
Biological Process	GO:0006949	syncytium formation	9/2279	60/18866	0.2966481	0.5902856	0.5341114	9	EHD1/SBNO2/CD9/TANC1/NFATC2/MYH9/ERCC1/FLOT1/SCGB3A1
Biological Process	GO:0043966	histone H3 acetylation	9/2279	60/18866	0.2966481	0.5902856	0.5341114	9	BRCA2/BRD1/TAF10/SPHK2/PER1/PIH1D1/KAT7/ING5/LIF
Biological Process	GO:0002639	positive regulation of immunoglobulin production	7/2279	45/18866	0.2977097	0.5902856	0.5341114	7	TLR9/TGFB1/PTPRC/TP53BP1/TNFSF13/PAXIP1/NSD2
Biological Process	GO:0006775	fat-soluble vitamin metabolic process	7/2279	45/18866	0.2977097	0.5902856	0.5341114	7	GFI1/VDR/TNF/PLTP/FGFR1/CYP4F3/ALDH1A2
Biological Process	GO:0007520	myoblast fusion	7/2279	45/18866	0.2977097	0.5902856	0.5341114	7	EHD1/CD9/TANC1/NFATC2/MYH9/FLOT1/SCGB3A1
Biological Process	GO:0010677	negative regulation of cellular carbohydrate metabolic process	7/2279	45/18866	0.2977097	0.5902856	0.5341114	7	MAEA/HDAC4/C1QTNF3/CBFA2T3/PGP/SOGA1/PRKN

Biological Process	GO:0031018	endocrine pancreas development	7/2279	45/18866	0.2977097	0.5902856	0.5341114	7	RHEB/RFX3/DLL1/GIPR/IL6R/ARNTL/FOXO1
Biological Process	GO:0034122	negative regulation of toll-like receptor signaling pathway	7/2279	45/18866	0.2977097	0.5902856	0.5341114	7	GRAMD4/MIR140/SMPDL3B/TNFAIP3/TLR9/ARRB2/TYRO3
Biological Process	GO:0035315	hair cell differentiation	7/2279	45/18866	0.2977097	0.5902856	0.5341114	7	SLC44A4/DLL1/RBPJ/FGFR1/NOTCH1/TMC1/ERCC2
Biological Process	GO:0045429	positive regulation of nitric oxide biosynthetic process	7/2279	45/18866	0.2977097	0.5902856	0.5341114	7	SMAD3/DNM2/TNF/NOS1AP/CLU/HBB/ITGB2
Biological Process	GO:0045911	positive regulation of DNA recombination	7/2279	45/18866	0.2977097	0.5902856	0.5341114	7	TGFB1/PTPRC/TP53BP1/TNFSF13/ERCC2/PAXIP1/NSD2
Biological Process	GO:0051204	protein insertion into mitochondrial membrane	7/2279	45/18866	0.2977097	0.5902856	0.5341114	7	TFDP1/YWHAH/BMF/YWHAZ/BCL2/TP53BP2/YWHAQ
Biological Process	GO:0070316	regulation of G0 to G1 transition	7/2279	45/18866	0.2977097	0.5902856	0.5341114	7	TFDP1/MGA/EHMT2/EPC1/PHC3/E2F6/RHNO1
Biological Process	GO:0007612	learning	20/2279	145/18866	0.2977104	0.5902856	0.5341114	20	CREB1/PPP1R1B/TANC1/ATP8A1/ATXN1/COMT/APP/SYNGAP1/HRH1/NF1/NRXN1/HTT/GMFB/MEIS2/NRXN2/MAPK8IP2/MAP1A/SHANK2/SYNPO/PRKN
Biological Process	GO:0000459	exonucleolytic trimming involved in rRNA processing	2/2279	9/18866	0.297936	0.5902856	0.5341114	2	EXOSC2/ERI3

Biological Process	GO:0000467	exonucleolytic trimming to generate mature 3'-end of 5.8S rRNA from tricistronic rRNA transcript (SSU-rRNA, 5.8S rRNA, LSU-rRNA)	2/2279	9/18866	0.297936	0.5902856	0.5341114	2	EXOSC2/ERI3
Biological Process	GO:0000492	box C/D snoRNP assembly	2/2279	9/18866	0.297936	0.5902856	0.5341114	2	PIH1D1/RUVBL1
Biological Process	GO:0001188	RNA polymerase I preinitiation complex assembly	2/2279	9/18866	0.297936	0.5902856	0.5341114	2	TAF1C/PIH1D1
Biological Process	GO:0001514	selenocysteine incorporation	2/2279	9/18866	0.297936	0.5902856	0.5341114	2	TRNAU1AP/SECISBP2
Biological Process	GO:0002578	negative regulation of antigen processing and presentation	2/2279	9/18866	0.297936	0.5902856	0.5341114	2	THBS1/CD68
Biological Process	GO:0002579	positive regulation of antigen processing and presentation	2/2279	9/18866	0.297936	0.5902856	0.5341114	2	SLC11A1/NOD2
Biological Process	GO:0002725	negative regulation of T cell cytokine production	2/2279	9/18866	0.297936	0.5902856	0.5341114	2	HLA-F/SMAD7
Biological Process	GO:0006451	translational readthrough	2/2279	9/18866	0.297936	0.5902856	0.5341114	2	TRNAU1AP/SECISBP2
Biological Process	GO:0006782	protoporphyrinogen IX biosynthetic process	2/2279	9/18866	0.297936	0.5902856	0.5341114	2	ALAS1/PPOX

Biological Process	GO:0007042	lysosomal lumen acidification	2/2279	9/18866	0.297936	0.5902856	0.5341114	2	GRN/CLN5
Biological Process	GO:0010533	regulation of activation of Janus kinase activity	2/2279	9/18866	0.297936	0.5902856	0.5341114	2	CCL5/IL6R
Biological Process	GO:0010898	positive regulation of triglyceride catabolic process	2/2279	9/18866	0.297936	0.5902856	0.5341114	2	PNPLA2/ABHD5
Biological Process	GO:0014010	Schwann cell proliferation	2/2279	9/18866	0.297936	0.5902856	0.5341114	2	SKI/CERS2
Biological Process	GO:0015810	aspartate transmembrane transport	2/2279	9/18866	0.297936	0.5902856	0.5341114	2	LRRC8C/SLC1A6
Biological Process	GO:0015858	nucleoside transport	2/2279	9/18866	0.297936	0.5902856	0.5341114	2	SLC29A3/SLC29A1
Biological Process	GO:0019227	neuronal action potential propagation	2/2279	9/18866	0.297936	0.5902856	0.5341114	2	SCN1A/CNTNAP1
Biological Process	GO:0019317	fucose catabolic process	2/2279	9/18866	0.297936	0.5902856	0.5341114	2	FUT4/FUT7
Biological Process	GO:0021860	pyramidal neuron development	2/2279	9/18866	0.297936	0.5902856	0.5341114	2	DISC1/ZMIZ1

Biological Process	GO:0022417	protein maturation by protein folding	2/2279	9/18866	0.297936	0.5902856	0.5341114	2	CALR/AIP
Biological Process	GO:0030223	neutrophil differentiation	2/2279	9/18866	0.297936	0.5902856	0.5341114	2	INPP5D/TRIB1
Biological Process	GO:0030917	midbrain-hindbrain boundary development	2/2279	9/18866	0.297936	0.5902856	0.5341114	2	SSBP3/HES3
Biological Process	GO:0032959	inositol trisphosphate biosynthetic process	2/2279	9/18866	0.297936	0.5902856	0.5341114	2	P2RY6/HRH1
Biological Process	GO:0033212	iron import into cell	2/2279	9/18866	0.297936	0.5902856	0.5341114	2	SLC11A2/LTF
Biological Process	GO:0034058	endosomal vesicle fusion	2/2279	9/18866	0.297936	0.5902856	0.5341114	2	ANKFY1/TGFBRAP1
Biological Process	GO:0034112	positive regulation of homotypic cell-cell adhesion	2/2279	9/18866	0.297936	0.5902856	0.5341114	2	CCL5/JAK1
Biological Process	GO:0034382	chylomicron remnant clearance	2/2279	9/18866	0.297936	0.5902856	0.5341114	2	LIPC/LMF1
Biological Process	GO:0035973	aggrephagy	2/2279	9/18866	0.297936	0.5902856	0.5341114	2	HTT/WDR81

Biological Process	GO:0036363	transforming growth factor beta activation	2/2279	9/18866	0.297936	0.5902856	0.5341114	2	LTBP1/ITGB6
Biological Process	GO:0038113	interleukin-9-mediated signaling pathway	2/2279	9/18866	0.297936	0.5902856	0.5341114	2	STAT5A/JAK1
Biological Process	GO:0038171	cannabinoid signaling pathway	2/2279	9/18866	0.297936	0.5902856	0.5341114	2	MGLL/CNR2
Biological Process	GO:0042045	epithelial fluid transport	2/2279	9/18866	0.297936	0.5902856	0.5341114	2	ITPR1/AQP1
Biological Process	GO:0042354	L-fucose metabolic process	2/2279	9/18866	0.297936	0.5902856	0.5341114	2	FUT4/FUT7
Biological Process	GO:0042355	L-fucose catabolic process	2/2279	9/18866	0.297936	0.5902856	0.5341114	2	FUT4/FUT7
Biological Process	GO:0043653	mitochondrial fragmentation involved in apoptotic process	2/2279	9/18866	0.297936	0.5902856	0.5341114	2	FIS1/DNM1L
Biological Process	GO:0045003	double-strand break repair via synthesis-dependent strand annealing	2/2279	9/18866	0.297936	0.5902856	0.5341114	2	RAD52/DMC1
Biological Process	GO:0045916	negative regulation of complement activation	2/2279	9/18866	0.297936	0.5902856	0.5341114	2	CD55/CD59

Biological Process	GO:0046645	positive regulation of gamma-delta T cell activation	2/2279	9/18866	0.297936	0.5902856	0.5341114	2	PTPRC/NOD2
Biological Process	GO:0048087	positive regulation of developmental pigmentation	2/2279	9/18866	0.297936	0.5902856	0.5341114	2	ZEB2/BCL2
Biological Process	GO:0048382	mesendoderm development	2/2279	9/18866	0.297936	0.5902856	0.5341114	2	SSBP3/NODAL
Biological Process	GO:0048671	negative regulation of collateral sprouting	2/2279	9/18866	0.297936	0.5902856	0.5341114	2	IFRD1/SPART
Biological Process	GO:0051292	nuclear pore complex assembly	2/2279	9/18866	0.297936	0.5902856	0.5341114	2	RTN4/NUP93
Biological Process	GO:0060124	positive regulation of growth hormone secretion	2/2279	9/18866	0.297936	0.5902856	0.5341114	2	ITSN1/GHRL
Biological Process	GO:0060180	female mating behavior	2/2279	9/18866	0.297936	0.5902856	0.5341114	2	PPP1R1B/NCOA1
Biological Process	GO:0060242	contact inhibition	2/2279	9/18866	0.297936	0.5902856	0.5341114	2	PTPRJ/CDHR2
Biological Process	GO:0060245	detection of cell density	2/2279	9/18866	0.297936	0.5902856	0.5341114	2	PTPRJ/CDHR2

Biological Process	GO:0060297	regulation of sarcomere organization	2/2279	9/18866	0.297936	0.5902856	0.5341114	2	AKAP13/MEF2C
Biological Process	GO:0060484	lung-associated mesenchyme development	2/2279	9/18866	0.297936	0.5902856	0.5341114	2	WNT11/FGFR1
Biological Process	GO:0060586	multicellular organismal iron ion homeostasis	2/2279	9/18866	0.297936	0.5902856	0.5341114	2	SLC11A1/SLC11A2
Biological Process	GO:0060693	regulation of branching involved in salivary gland morphogenesis	2/2279	9/18866	0.297936	0.5902856	0.5341114	2	TNF/FGFR1
Biological Process	GO:0061026	cardiac muscle tissue regeneration	2/2279	9/18866	0.297936	0.5902856	0.5341114	2	MIR199A1/MIR199A2
Biological Process	GO:0061820	telomeric D-loop disassembly	2/2279	9/18866	0.297936	0.5902856	0.5341114	2	BLM/SLX4
Biological Process	GO:0070922	small RNA loading onto RISC	2/2279	9/18866	0.297936	0.5902856	0.5341114	2	AGO1/AGO2
Biological Process	GO:0071225	cellular response to muramyl dipeptide	2/2279	9/18866	0.297936	0.5902856	0.5341114	2	NOD2/ARHGEF2
Biological Process	GO:0071355	cellular response to interleukin-9	2/2279	9/18866	0.297936	0.5902856	0.5341114	2	STAT5A/JAK1

Biological Process	GO:0071374	cellular response to parathyroid hormone stimulus	2/2279	9/18866	0.297936	0.5902856	0.5341114	2	HDAC4/MEF2C
Biological Process	GO:0071421	manganese ion transmembrane transport	2/2279	9/18866	0.297936	0.5902856	0.5341114	2	SLC11A1/SLC11A2
Biological Process	GO:0071830	triglyceride-rich lipoprotein particle clearance	2/2279	9/18866	0.297936	0.5902856	0.5341114	2	LIPC/LMF1
Biological Process	GO:0086015	SA node cell action potential	2/2279	9/18866	0.297936	0.5902856	0.5341114	2	GJA5/ANK2
Biological Process	GO:0086018	SA node cell to atrial cardiac muscle cell signaling	2/2279	9/18866	0.297936	0.5902856	0.5341114	2	GJA5/ANK2
Biological Process	GO:0097084	vascular associated smooth muscle cell development	2/2279	9/18866	0.297936	0.5902856	0.5341114	2	ENG/NOTCH1
Biological Process	GO:0097113	AMPA glutamate receptor clustering	2/2279	9/18866	0.297936	0.5902856	0.5341114	2	SSH1/ZDHHC2
Biological Process	GO:0097688	glutamate receptor clustering	2/2279	9/18866	0.297936	0.5902856	0.5341114	2	SSH1/ZDHHC2
Biological Process	GO:0098870	action potential propagation	2/2279	9/18866	0.297936	0.5902856	0.5341114	2	SCN1A/CNTNAP1

Biological Process	GO:0098976	excitatory chemical synaptic transmission	2/2279	9/18866	0.297936	0.5902856	0.5341114	2	SSH1/PDLIM4
Biological Process	GO:0099641	anterograde axonal protein transport	2/2279	9/18866	0.297936	0.5902856	0.5341114	2	KIF5C/MAP1A
Biological Process	GO:1902459	positive regulation of stem cell population maintenance	2/2279	9/18866	0.297936	0.5902856	0.5341114	2	LBH/ESRRB
Biological Process	GO:1902746	regulation of lens fiber cell differentiation	2/2279	9/18866	0.297936	0.5902856	0.5341114	2	SPRED2/ZEB2
Biological Process	GO:1902959	regulation of aspartic-type endopeptidase activity involved in amyloid precursor protein catabolic process	2/2279	9/18866	0.297936	0.5902856	0.5341114	2	SORL1/BIN1
Biological Process	GO:1903012	positive regulation of bone development	2/2279	9/18866	0.297936	0.5902856	0.5341114	2	GHRL/LTF
Biological Process	GO:1903069	regulation of ER-associated ubiquitin-dependent protein catabolic process	2/2279	9/18866	0.297936	0.5902856	0.5341114	2	NFE2L2/SVIP
Biological Process	GO:1903147	negative regulation of autophagy of mitochondrion	2/2279	9/18866	0.297936	0.5902856	0.5341114	2	CLEC16A/TSC2
Biological Process	GO:2000270	negative regulation of fibroblast apoptotic process	2/2279	9/18866	0.297936	0.5902856	0.5341114	2	MIR24-2/CHD8

Biological Process	GO:2000343	positive regulation of chemokine (C-X-C motif) ligand 2 production	2/2279	9/18866	0.297936	0.5902856	0.5341114	2	TNF/MBP
Biological Process	GO:0014855	striated muscle cell proliferation	12/2279	83/18866	0.2983944	0.5902856	0.5341114	12	RXRA/ANGPT1/MIR199A1/VGLL4/TGFBR2/RIPK1/RBPJ/FGFR1/MEIS1/MIR199A2/NOTCH1/MEF2C
Biological Process	GO:0071277	cellular response to calcium ion	12/2279	83/18866	0.2983944	0.5902856	0.5341114	12	TRPM2/CPNE6/CPNE2/ITPKB/GPLD1/CPNE5/CPNE1/CRHBP/ADD1/MEF2C/SYT17/PRKAA1
Biological Process	GO:0007517	muscle organ development	53/2279	407/18866	0.2993139	0.5902856	0.5341114	53	FOXK1/HDAC4/ARID5B/ZFPM1/RXRA/CTDP1/TTN/CREB1/MEF2D/MIR143/MIR145/SKI/MYLK/SMAD3/MIR199A1/VGLL4/ZFHX3/CCM2L/TGFBR2/MYH15/DLL1/RIPK1/RBPJ/TCF12/SMAD7/FGFR1/PAX7/TGFB1/IGSF8/VAMP5/SVIL/NF1/ENG/ARRB2/ARNTL/UNC45A/MYOM1/ANKRD2/RB1/MEIS1/BCL2/MIR199A2/NOTCH1/MEF2C/HLX/USP2/ANKRD1/FLOT1/CASQ1/SMTN/LIF/PRKAA1/MYORG
Biological Process	GO:0001919	regulation of receptor recycling	4/2279	23/18866	0.2996014	0.5902856	0.5341114	4	TBC1D16/ARAP1/EPS15/INPP5F
Biological Process	GO:0002052	positive regulation of neuroblast proliferation	4/2279	23/18866	0.2996014	0.5902856	0.5341114	4	DISC1/ZNF335/NOTCH1/CX3CR1
Biological Process	GO:0007097	nuclear migration	4/2279	23/18866	0.2996014	0.5902856	0.5341114	4	DCTN1/DYNC1H1/FBXW11/SLIT1
Biological Process	GO:0007413	axonal fasciculation	4/2279	23/18866	0.2996014	0.5902856	0.5341114	4	NCAM2/EPHB3/RTN4/TNFRSF21
Biological Process	GO:0010866	regulation of triglyceride biosynthetic process	4/2279	23/18866	0.2996014	0.5902856	0.5341114	4	MFSD2A/GPLD1/NR1H2/SREBF1

Biological Process	GO:0015813	L-glutamate transmembrane transport	4/2279	23/18866	0.2996014	0.5902856	0.5341114	4	SLC1A2/SLC1A6/PER2/SLC1A3
Biological Process	GO:0022010	central nervous system myelination	4/2279	23/18866	0.2996014	0.5902856	0.5341114	4	CLU/FA2H/CNTNAP1/ERCC2
Biological Process	GO:0032291	axon ensheathment in central nervous system	4/2279	23/18866	0.2996014	0.5902856	0.5341114	4	CLU/FA2H/CNTNAP1/ERCC2
Biological Process	GO:0032401	establishment of melanosome localization	4/2279	23/18866	0.2996014	0.5902856	0.5341114	4	RAB27A/CDH3/DCTN2/MYO5A
Biological Process	GO:0034063	stress granule assembly	4/2279	23/18866	0.2996014	0.5902856	0.5341114	4	DYNC1H1/CSDE1/UBAP2L/PRKAA1
Biological Process	GO:0035458	cellular response to interferon-beta	4/2279	23/18866	0.2996014	0.5902856	0.5341114	4	AIM2/MNDA/DDX41/STING1
Biological Process	GO:0048714	positive regulation of oligodendrocyte differentiation	4/2279	23/18866	0.2996014	0.5902856	0.5341114	4	PPARG/TNFRSF1B/RHEB/HDAC1
Biological Process	GO:0050951	sensory perception of temperature stimulus	4/2279	23/18866	0.2996014	0.5902856	0.5341114	4	WDR47/ARRB2/NTRK1/TRPM3
Biological Process	GO:0051151	negative regulation of smooth muscle cell differentiation	4/2279	23/18866	0.2996014	0.5902856	0.5341114	4	NFATC2/NFATC1/PDGFB/DNMT1

Biological Process	GO:0051457	maintenance of protein location in nucleus	4/2279	23/18866	0.2996014	0.5902856	0.5341114	4	SYNE1/MORC3/SP100/BCL3
Biological Process	GO:0051570	regulation of histone H3-K9 methylation	4/2279	23/18866	0.2996014	0.5902856	0.5341114	4	MYB/KDM4C/DNMT1/PIH1D1
Biological Process	GO:0051904	pigment granule transport	4/2279	23/18866	0.2996014	0.5902856	0.5341114	4	RAB27A/CDH3/DCTN2/MYO5A
Biological Process	GO:0071577	zinc ion transmembrane transport	4/2279	23/18866	0.2996014	0.5902856	0.5341114	4	TRPM2/SLC39A11/TMEM163/SLC39A13
Biological Process	GO:0072170	metanephric tubule development	4/2279	23/18866	0.2996014	0.5902856	0.5341114	4	PAX2/WWTR1/ACAT1/LIF
Biological Process	GO:0072243	metanephric nephron epithelium development	4/2279	23/18866	0.2996014	0.5902856	0.5341114	4	PAX2/WWTR1/ACAT1/LIF
Biological Process	GO:0086012	membrane depolarization during cardiac muscle cell action potential	4/2279	23/18866	0.2996014	0.5902856	0.5341114	4	CACNA1C/SLC8A1/GJA5/ANK2
Biological Process	GO:0106030	neuron projection fasciculation	4/2279	23/18866	0.2996014	0.5902856	0.5341114	4	NCAM2/EPHB3/RTN4/TNFRSF21
Biological Process	GO:1900273	positive regulation of long-term synaptic potentiation	4/2279	23/18866	0.2996014	0.5902856	0.5341114	4	CREB1/ZDHHC2/APP/AKAP5

Biological Process	GO:1903798	regulation of production of miRNAs involved in gene silencing by miRNA	4/2279	23/18866	0.2996014	0.5902856	0.5341114	4	NCOR2/TNF/RIPK1/TGFB1
Biological Process	GO:2000050	regulation of non-canonical Wnt signaling pathway	4/2279	23/18866	0.2996014	0.5902856	0.5341114	4	CSNK1D/CSNK1E/RNF213/ANKRD6
Biological Process	GO:2000726	negative regulation of cardiac muscle cell differentiation	4/2279	23/18866	0.2996014	0.5902856	0.5341114	4	CTDP1/MIR199A1/DLL1/MIR199A2
Biological Process	GO:0016202	regulation of striated muscle tissue development	21/2279	153/18866	0.2996031	0.5902856	0.5341114	21	HDAC4/CTDP1/CREB1/SMAD3/MIR199A1/VGLL4/TGFBR2/DLL1/RIPK1/RBPJ/FGFR1/TGFB1/ARRB2/ARNTL/MEIS1/BCL2/MIR199A2/NOTCH1/MEF2C/FLOT1/PRKAA1
Biological Process	GO:0009062	fatty acid catabolic process	16/2279	114/18866	0.2997191	0.5902856	0.5341114	16	PLA2G15/MFSD2A/ABHD16A/AMACR/ACOXL/CYP4F3/ACADM/ACACB/ACOX2/PCK2/ACAT1/ADTRP/CPT1A/LPIN1/LYPLA2/ACADVL
Biological Process	GO:0043200	response to amino acid	16/2279	114/18866	0.2997191	0.5902856	0.5341114	16	RPTOR/CREB1/TNF/BCL11A/BAIAP2/PDGFD/SLC1A2/CPEB3/SH3BP4/DNMT1/PDGFC/CEBPB/BCL2L1/RELA/DHODH/SST
Biological Process	GO:0019882	antigen processing and presentation	31/2279	232/18866	0.3014741	0.5902856	0.5341114	31	HLA-DMB/DCTN1/CALR/AP2A1/SLC11A1/LNPEP/NCF4/RAB5B/DNM2/DYNC1H1/CTSD/HLA-C/THBS1/CTSH/RAB27A/HLA-F/NCF2/ITGB5/AP1B1/DCTN2/SEC13/PSMF1/SEC24C/NOD2/PSMB7/KIFAP3/CD68/AP3D1/SEC31A/PSMD13/MARCHF8
Biological Process	GO:1901379	regulation of potassium ion transmembrane transport	13/2279	91/18866	0.3027208	0.5902856	0.5341114	13	KCNE1/KCNAB2/CASQ2/WWP2/DNM2/BIN1/NOS1AP/KCNIP1/CAB39/KCNQ1/NEDD4/ANO6/ANK2
Biological Process	GO:0051100	negative regulation of binding	23/2279	169/18866	0.3027889	0.5902856	0.5341114	23	SLPI/CTSZ/ZFPM1/SORL1/IL10/TNKS/ITGB1BP1/CCM2L/DISC1/MIR27A/CPNE1/ATP2A3/CSNK1E/PDGFB/RSF1/ZNF462/ARRB2/PER2/SP100/DTNBP1/TFIP11/ILRUN/FBH1

Biological Process	GO:0002637	regulation of immunoglobulin production	10/2279	68/18866	0.3032641	0.5902856	0.5341114	10	IL10/TNF/TRAF2/TLR9/TGFB1/PTPRC/TP53BP1/TNFSF13/PAXIP1/NSD2
Biological Process	GO:0071479	cellular response to ionizing radiation	10/2279	68/18866	0.3032641	0.5902856	0.5341114	10	BLM/SPIDR/DNM2/HUS1/TP53BP1/RHNO1/CHEK2/BCL2L1/EEF1D/MAP3K20
Biological Process	GO:0099072	regulation of postsynaptic membrane neurotransmitter receptor levels	10/2279	68/18866	0.3032641	0.5902856	0.5341114	10	PRKCZ/DNM2/NUMB/ITGB3/PRKCI/GSG1L/DNM1/DNM3/EPS15/MX1
Biological Process	GO:1904377	positive regulation of protein localization to cell periphery	10/2279	68/18866	0.3032641	0.5902856	0.5341114	10	PIK3R1/RHOG/TNF/ZDHHC2/AKAP5/PRKCH/NUMA1/PRKCI/PRKCE/EZR
Biological Process	GO:0002295	T-helper cell lineage commitment	3/2279	16/18866	0.3035198	0.5902856	0.5341114	3	ZFPM1/LOXL3/LY9
Biological Process	GO:0006206	pyrimidine nucleobase metabolic process	3/2279	16/18866	0.3035198	0.5902856	0.5341114	3	CDA/DPYS/DHODH
Biological Process	GO:0006268	DNA unwinding involved in DNA replication	3/2279	16/18866	0.3035198	0.5902856	0.5341114	3	BLM/RECQL5/MCM2
Biological Process	GO:0007288	sperm axoneme assembly	3/2279	16/18866	0.3035198	0.5902856	0.5341114	3	IQCG/ARMC2/TTLL1
Biological Process	GO:0009129	pyrimidine nucleoside monophosphate metabolic process	3/2279	16/18866	0.3035198	0.5902856	0.5341114	3	UCK2/DCTD/DHODH

Biological Process	GO:0010820	positive regulation of T cell chemotaxis	3/2279	16/18866	0.3035198	0.5902856	0.5341114	3	CCR2/CCL5/CCL27
Biological Process	GO:0017014	protein nitrosylation	3/2279	16/18866	0.3035198	0.5902856	0.5341114	3	S100A8/NOS1AP/SNTA1
Biological Process	GO:0018119	peptidyl-cysteine S-nitrosylation	3/2279	16/18866	0.3035198	0.5902856	0.5341114	3	S100A8/NOS1AP/SNTA1
Biological Process	GO:0031573	intra-S DNA damage checkpoint	3/2279	16/18866	0.3035198	0.5902856	0.5341114	3	HUS1/EME1/CHEK2
Biological Process	GO:0033008	positive regulation of mast cell activation involved in immune response	3/2279	16/18866	0.3035198	0.5902856	0.5341114	3	GAB2/FGR/SPHK2
Biological Process	GO:0042532	negative regulation of tyrosine phosphorylation of STAT protein	3/2279	16/18866	0.3035198	0.5902856	0.5341114	3	PPP2R1A/GGNBP2/INPP5F
Biological Process	GO:0043116	negative regulation of vascular permeability	3/2279	16/18866	0.3035198	0.5902856	0.5341114	3	ANGPT1/MIR23A/PTPRJ
Biological Process	GO:0043306	positive regulation of mast cell degranulation	3/2279	16/18866	0.3035198	0.5902856	0.5341114	3	GAB2/FGR/SPHK2
Biological Process	GO:0043981	histone H4-K5 acetylation	3/2279	16/18866	0.3035198	0.5902856	0.5341114	3	KANSL1/KANSL2/KAT7

Biological Process	GO:0043982	histone H4-K8 acetylation	3/2279	16/18866	0.3035198	0.5902856	0.5341114	3	KANSL1/KANSL2/KAT7
Biological Process	GO:0045342	MHC class II biosynthetic process	3/2279	16/18866	0.3035198	0.5902856	0.5341114	3	AZU1/IL10/SLC11A1
Biological Process	GO:0045989	positive regulation of striated muscle contraction	3/2279	16/18866	0.3035198	0.5902856	0.5341114	3	NOS1AP/KCNQ1/GSTO1
Biological Process	GO:0046325	negative regulation of glucose import	3/2279	16/18866	0.3035198	0.5902856	0.5341114	3	MIR143/TNF/GRB10
Biological Process	GO:0051238	sequestering of metal ion	3/2279	16/18866	0.3035198	0.5902856	0.5341114	3	S100A8/LCN2/AP3D1
Biological Process	GO:0051447	negative regulation of meiotic cell cycle	3/2279	16/18866	0.3035198	0.5902856	0.5341114	3	OVOL1/NPPC/LIF
Biological Process	GO:0051770	positive regulation of nitric-oxide synthase biosynthetic process	3/2279	16/18866	0.3035198	0.5902856	0.5341114	3	MAP2K6/TLR9/NOD2
Biological Process	GO:0060413	atrial septum morphogenesis	3/2279	16/18866	0.3035198	0.5902856	0.5341114	3	ZFPM1/GJA5/NSD2
Biological Process	GO:0060850	regulation of transcription involved in cell fate commitment	3/2279	16/18866	0.3035198	0.5902856	0.5341114	3	PPARG/ZBTB7B/CEBPB

Biological Process	GO:0071391	cellular response to estrogen stimulus	3/2279	16/18866	0.3035198	0.5902856	0.5341114	3	CRHBP/RARA/NCOA4
Biological Process	GO:0072224	metanephric glomerulus development	3/2279	16/18866	0.3035198	0.5902856	0.5341114	3	PAX2/PDGFB/PDGFRB
Biological Process	GO:0086103	G protein-coupled receptor signaling pathway involved in heart process	3/2279	16/18866	0.3035198	0.5902856	0.5341114	3	PDE4D/AKAP13/GNAI2
Biological Process	GO:0090069	regulation of ribosome biogenesis	3/2279	16/18866	0.3035198	0.5902856	0.5341114	3	WDR43/USP36/RIOK1
Biological Process	GO:1901201	regulation of extracellular matrix assembly	3/2279	16/18866	0.3035198	0.5902856	0.5341114	3	SMAD3/TGFB1/NOTCH1
Biological Process	GO:1902166	negative regulation of intrinsic apoptotic signaling pathway in response to DNA damage by p53 class mediator	3/2279	16/18866	0.3035198	0.5902856	0.5341114	3	ZNF385A/BCL2/MARCHF7
Biological Process	GO:1904294	positive regulation of ERAD pathway	3/2279	16/18866	0.3035198	0.5902856	0.5341114	3	EDEM1/EDEM2/NFE2L2
Biological Process	GO:1905153	regulation of membrane invagination	3/2279	16/18866	0.3035198	0.5902856	0.5341114	3	PPARG/RAB31/ANO6
Biological Process	GO:2000651	positive regulation of sodium ion transmembrane transporter activity	3/2279	16/18866	0.3035198	0.5902856	0.5341114	3	DNM2/TESC/ACTN4

Biological Process	GO:2001257	regulation of cation channel activity	25/2279	185/18866	0.3053241	0.5902856	0.5341114	25	PDE4D/KCNE1/CASQ2/CCR2/JSRP1/AHNAK/APP/NOS1AP/P2RY6/CRHBP/CNIH2/MYO5A/HTT/MAPK8IP2/GSG1L/GSTO1/MEF2C/SHANK2/DYSF/ANK2/CASQ1/FGF14/CBARP/STIMATE/CRACR2A
Biological Process	GO:0043161	proteasome-mediated ubiquitin-dependent protein catabolic process	55/2279	424/18866	0.3056633	0.5902856	0.5341114	55	TRIM39/MAEA/RNF144A/FZR1/RNF19A/TRIM38/WWP2/CSNK1D/AMFR/ARRB1/RHBDD2/CBFA2T3/CUL3/EDEM1/CDC16/DERL2/CLU/SMAD7/UBE2J2/PPP2R5C/SMURF1/MTA1/CLEC16A/WWTR1/FBXL19/EDEM2/CSNK1E/PSMF1/FBXL13/UBXN11/KCTD2/NFE2L2/ARRB2/ARNTL/FBXL18/RNF144B/PSMB7/MAP1A/FBXL6/GNA12/UBXN2A/NEDD4/ANAPC7/RNF14/TRIB1/HECW2/FBXW11/BAG5/GID8/UBXN8/PELI1/PSMD13/SVIP/MARCHF6/PRKN
Biological Process	GO:0034612	response to tumor necrosis factor	42/2279	320/18866	0.3057405	0.5902856	0.5341114	42	HDAC4/AIM2/LCN2/CHI3L1/TNFRSF1B/GGT1/TNF/TRAF2/TNFAIP3/CCL5/TRAF1/TNFSF13B/ABCC2/TRAIP/THBS1/RIPK1/ACTN4/MIR24-2/TNFRSF1A/ILK/TNFRSF21/CPNE1/CRHBP/SGMS1/ADAMTS13/CDIP1/PRPF8/CCL20/PSMF1/MAP3K14/NFE2L2/PSMB7/ARHGEF2/CASP1/TNFSF12/TNFSF13/CYLD/ANKRD1/RELA/CCL22/PSMD13/PRKN
Biological Process	GO:0061136	regulation of proteasomal protein catabolic process	26/2279	193/18866	0.3063916	0.5902856	0.5341114	26	TRIM39/RNF144A/FZR1/RNF19A/CSNK1D/CBFA2T3/UBAC2/EDEM1/DERL2/CLU/SMAD7/CLEC16A/EDEM2/CSNK1E/PSMF1/NFE2L2/RNF144B/MAP1A/GNA12/RNF14/TRIB1/RNF40/BAG5/SVIP/PRKN/MARCHF7
Biological Process	GO:0034340	response to type I interferon	14/2279	99/18866	0.3063945	0.5902856	0.5341114	14	IRF8/IRF5/SETD2/HLA-C/HLA-F/PTPN6/JAK1/NLRC5/SP100/MX1/IRF2/ISG20/FADD/SHFL
Biological Process	GO:1905037	autophagosome organization	14/2279	99/18866	0.3063945	0.5902856	0.5341114	14	ULK1/PACS2/WIPI2/PIP4K2A/FEZ2/SMURF1/RUFY4/RAB43/ATG14/TBC1D14/VMP1/UBXN2A/NPRL2/STING1
Biological Process	GO:0045010	actin nucleation	8/2279	53/18866	0.3068892	0.5902856	0.5341114	8	TRIM27/GSN/WHAMM/WASL/GMFB/NCKAP1/CYFIP1/GMFG
Biological Process	GO:0072698	protein localization to microtubule cytoskeleton	8/2279	53/18866	0.3068892	0.5902856	0.5341114	8	CSNK1D/CHAMP1/DISC1/DCTN2/NUMA1/MAP1A/SPAG5/CEP72
Biological Process	GO:0000724	double-strand break repair via homologous recombination	19/2279	138/18866	0.3069143	0.5902856	0.5341114	19	BLM/SPIDR/BRCA2/RMI2/RAD52/SETD2/RTEL1/HUS1/NSMCE2/MCM5/RECQL5/MEIOB/AP5S1/MCM2/RAD21L1/DMC1/TP53BP1/SLX4/FBH1

Biological Process	GO:0098754	detoxification	19/2279	138/18866	0.3069143	0.5902856	0.5341114	19	MPO/LPO/TNF/ABCC2/OSCP1/RALBP1/HBB/MGST2/SLC22A5/CAT/NFE2L2/NXN/GSTO1/PDZK1/TXNRD2/GSTO2/SIRT3/PRXL2B/MTARC1
Biological Process	GO:0003298	physiological muscle hypertrophy	6/2279	38/18866	0.3075287	0.5902856	0.5341114	6	CTDP1/AKAP13/MIR199A1/MIR24-2/MIR23A/MIR199A2
Biological Process	GO:0003301	physiological cardiac muscle hypertrophy	6/2279	38/18866	0.3075287	0.5902856	0.5341114	6	CTDP1/AKAP13/MIR199A1/MIR24-2/MIR23A/MIR199A2
Biological Process	GO:0006084	acetyl-CoA metabolic process	6/2279	38/18866	0.3075287	0.5902856	0.5341114	6	ACSS1/ACACB/ACSS2/ACACA/ACAT1/HMGCS1
Biological Process	GO:0006361	transcription initiation from RNA polymerase I promoter	6/2279	38/18866	0.3075287	0.5902856	0.5341114	6	TAF1C/POLR2F/CCNH/PIH1D1/TAF1D/ERCC2
Biological Process	GO:0032885	regulation of polysaccharide biosynthetic process	6/2279	38/18866	0.3075287	0.5902856	0.5341114	6	AP2A1/TGFB1/DYRK2/PDGFB/PPP1CB/ESRRB
Biological Process	GO:0034661	ncRNA catabolic process	6/2279	38/18866	0.3075287	0.5902856	0.5341114	6	EXOSC2/SLFN14/DEDD2/DIS3L2/SND1/ZCCHC8
Biological Process	GO:0060045	positive regulation of cardiac muscle cell proliferation	6/2279	38/18866	0.3075287	0.5902856	0.5341114	6	MIR199A1/RBPJ/FGFR1/MIR199A2/NOTCH1/MEF2C
Biological Process	GO:0061049	cell growth involved in cardiac muscle cell development	6/2279	38/18866	0.3075287	0.5902856	0.5341114	6	CTDP1/AKAP13/MIR199A1/MIR24-2/MIR23A/MIR199A2

Biological Process	GO:1905898	positive regulation of response to endoplasmic reticulum stress	6/2279	38/18866	0.3075287	0.5902856	0.5341114	6	ERN1/PIK3R1/NCK2/EDEM1/EDEM2/NFE2L2
Biological Process	GO:0014743	regulation of muscle hypertrophy	11/2279	76/18866	0.3086556	0.5902856	0.5341114	11	CTDP1/MIR145/TNFRSF1B/SMAD3/PRKCA/MIR199A1/TNFRSF1A/PDE9A/MIR199A2/NOTCH1/FOXO1
Biological Process	GO:0071229	cellular response to acid chemical	11/2279	76/18866	0.3086556	0.5902856	0.5341114	11	RPTOR/TNF/BCL11A/BAIAP2/PDGFD/CPEB3/SH3BP4/DNMT1/PDGFC/CEBPB/BCL2L1
Biological Process	GO:1901616	organic hydroxy compound catabolic process	11/2279	76/18866	0.3086556	0.5902856	0.5341114	11	SYNJ2/INPP5A/ALDH2/AKR1D1/COMT/NUDT3/FAH/CYP4F3/SULT1A2/INPP5E/IMPA2
Biological Process	GO:0000018	regulation of DNA recombination	15/2279	107/18866	0.3095373	0.5902856	0.5341114	15	BLM/SPIDR/IL10/RMI2/SETD2/RTEL1/RECQL5/TGFB1/PTPRC/TP53BP1/TNFSF13/ERCC2/PAXIP1/NSD2/FBH1
Biological Process	GO:1903426	regulation of reactive oxygen species biosynthetic process	15/2279	107/18866	0.3095373	0.5902856	0.5341114	15	HDAC4/IL10/SMAD3/DNM2/MIR199A1/TNF/RAB27A/NOS1AP/MIR24-2/CLU/SPHK2/HBB/ITGB2/MIR199A2/CX3CR1
Biological Process	GO:0043620	regulation of DNA-templated transcription in response to stress	16/2279	115/18866	0.3122419	0.5902856	0.5341114	16	RPS6KA1/NCK2/RBPJ/LIMD1/PSMF1/CREBBP/NFE2L2/ANKRD2/PSMB7/NOTCH1/EPAS1/NEDD4/CEBPB/BACH1/RELA/PSMD13
Biological Process	GO:0002831	regulation of response to biotic stimulus	53/2279	409/18866	0.3125115	0.5902856	0.5341114	53	IL27/NLRC4/FOXP1/AIM2/TRIM38/PPARG/CD55/CLEC4C/PIK3R6/PRKCA/FGR/TNFAIP3/CCL5/FPR2/MNDA/CD226/HLA-F/PTPN6/MUC12/JAK1/SCIMP/NCR1/LY86/FCN1/TRIM5/MUC20/NR1H2/TREML4/SEC14L1/BCL10/PSMF1/GRN/NLRC5/ZDHHC1/CREBBP/ARRB2/IL18RAP/PSMB7/TRAF3IP2/LTF/TRIB1/TYRO3/CADM1/CD96/RELA/FBXW11/HEXIM1/HERC5/PARP9/FADD/PSMD13/STING1/ILRUN
Biological Process	GO:0014031	mesenchymal cell development	12/2279	84/18866	0.3131111	0.5902856	0.5341114	12	SEMA6B/NRP2/ANXA6/ZEB2/SEMA4A/SEMA4B/SEMA4D/RDH10/BCL2/NOTCH1/CORO1C/ALDH1A2

Biological Process	GO:0045913	positive regulation of carbohydrate metabolic process	12/2279	84/18866	0.3131111	0.5902856	0.5341114	12	PFKFB4/GPLD1/APP/P2RY6/HRH1/DYRK2/PDGFB/ESRRB/PRKCE/FOXO1/PFKFB3/PRKAA1
Biological Process	GO:0046209	nitric oxide metabolic process	12/2279	84/18866	0.3131111	0.5902856	0.5341114	12	IL10/SMAD3/DNM2/MIR199A1/TNF/NOS1AP/CLU/HBB/POR/ITGB2/MIR199A2/CX3CR1
Biological Process	GO:0048864	stem cell development	12/2279	84/18866	0.3131111	0.5902856	0.5341114	12	SEMA6B/NRP2/SETD2/ANXA6/ZEB2/SEMA4A/SEMA4B/SEMA4D/RDH10/PTPRC/CORO1C/ALDH1A2
Biological Process	GO:0010574	regulation of vascular endothelial growth factor production	9/2279	61/18866	0.3140145	0.5902856	0.5341114	9	C3AR1/MIR140/CCR2/MIR199A1/TGFB1/CXCL17/MIR199A2/NODAL/NDRG2
Biological Process	GO:0045843	negative regulation of striated muscle tissue development	9/2279	61/18866	0.3140145	0.5902856	0.5341114	9	CTDP1/MIR199A1/VGLL4/TGFBR2/DLL1/RIPK1/TGFB1/MEIS1/MIR199A2
Biological Process	GO:0046888	negative regulation of hormone secretion	9/2279	61/18866	0.3140145	0.5902856	0.5341114	9	RAB11FIP1/CRHBP/ENY2/GHRL/KLF7/SREBF1/INHBA/LIF/PRKN
Biological Process	GO:0051310	metaphase plate congression	9/2279	61/18866	0.3140145	0.5902856	0.5341114	9	MAD1L1/DYNC1H1/CHAMP1/CUL3/MIS12/DCTN2/NUMA1/CDCA5/SPAG5
Biological Process	GO:1903078	positive regulation of protein localization to plasma membrane	9/2279	61/18866	0.3140145	0.5902856	0.5341114	9	PIK3R1/RHOG/TNF/ZDHHC2/AKAP5/PRKCH/PRKCI/PRKCE/EZR
Biological Process	GO:2000756	regulation of peptidyl-lysine acetylation	9/2279	61/18866	0.3140145	0.5902856	0.5341114	9	ARRB1/RPS6KA4/SPHK2/CTBP1/PIH1D1/KAT7/LIF/PAXIP1/PRKAA1

Biological Process	GO:0051260	protein homooligomerization	24/2279	178/18866	0.3141906	0.5902856	0.5341114	24	TRPM2/NLRC4/EHD1/BLM/ACOT13/SLC1A5/KCTD7/NACC2/CRTC3/SLC1A2/ACACB/RNF213/UPB1/KCTD2/KCTD15/ACACA/TP53BP1/IKZF4/MAT1A/ATL1/KCTD1/ALDH1A2/TK1/KCTD21
Biological Process	GO:0050770	regulation of axonogenesis	25/2279	186/18866	0.3151782	0.5902856	0.5341114	25	SEMA6B/PLXNC1/KIF13B/CTTN/DNM2/ZEB2/EPHB3/SEMA4A/RTN4/DISC1/SEMA4B/IFRD1/ILK/SYNGAP1/SEMA4D/ZSWIM5/CDH4/SIPA1L1/CDKL3/MARK2/CYFIP1/TRPV2/NDEL1/DBNL/SPART
Biological Process	GO:0003206	cardiac chamber morphogenesis	18/2279	131/18866	0.3166103	0.5902856	0.5341114	18	ZFPM1/RXRA/NRP2/GJA5/CCM2L/TGFBR2/RBPJ/SMAD7/WNT11/TGFB1/RARA/GRHL2/ENG/NOTCH1/MEF2C/FHL2/SLIT3/NSD2
Biological Process	GO:0046887	positive regulation of hormone secretion	18/2279	131/18866	0.3166103	0.5902856	0.5341114	18	NADK/C1QTNF3/MYRIP/CREB1/MYB/ARRB1/GPLD1/ITSN1/TCF7L2/FGFR1/GIPR/BLK/GHRL/PRKCE/RPH3AL/INHBA/C2CD2L/SIRT3
Biological Process	GO:0009746	response to hexose	27/2279	202/18866	0.3168171	0.5902856	0.5341114	27	NADK/SLC2A5/LRP5L/ERN1/SLC8A1/ARRB1/GPLD1/EIF2B5/TGFBR2/THBS1/TCF7L2/IGF1R/PTPRN2/PAX2/ENY2/GIPR/GHRL/KLF7/FIS1/PIH1D1/PRKCE/CPB2/PCK2/SREBF1/SLC29A1/C2CD2L/PRKAA1
Biological Process	GO:0006363	termination of RNA polymerase I transcription	5/2279	31/18866	0.3178026	0.5902856	0.5341114	5	TAF1C/POLR2F/CCNH/TAF1D/ERCC2
Biological Process	GO:0009394	2'-deoxyribonucleotide metabolic process	5/2279	31/18866	0.3178026	0.5902856	0.5341114	5	UNG/NTHL1/GUK1/AK5/DCTD
Biological Process	GO:0010644	cell communication by electrical coupling	5/2279	31/18866	0.3178026	0.5902856	0.5341114	5	PDE4D/CASQ2/CACNA1C/SLC8A1/GJA5
Biological Process	GO:0018200	peptidyl-glutamic acid modification	5/2279	31/18866	0.3178026	0.5902856	0.5341114	5	AGBL5/TTLL4/TTLL11/TTLL1/AGBL2

Biological Process	GO:0032770	positive regulation of monooxygenase activity	5/2279	31/18866	0.3178026	0.5902856	0.5341114	5	VDR/TNF/NOS1AP/CDH3/POR
Biological Process	GO:0034368	protein-lipid complex remodeling	5/2279	31/18866	0.3178026	0.5902856	0.5341114	5	MPO/LIPC/ABCG1/PLTP/AGTR1
Biological Process	GO:0034369	plasma lipoprotein particle remodeling	5/2279	31/18866	0.3178026	0.5902856	0.5341114	5	MPO/LIPC/ABCG1/PLTP/AGTR1
Biological Process	GO:0034377	plasma lipoprotein particle assembly	5/2279	31/18866	0.3178026	0.5902856	0.5341114	5	P4HB/MFSD2A/PLAGL2/NR1H2/ABCA1
Biological Process	GO:0045948	positive regulation of translational initiation	5/2279	31/18866	0.3178026	0.5902856	0.5341114	5	RXRA/TNF/CCL5/EIF2B5/LARP1
Biological Process	GO:0046856	phosphatidylinositol dephosphorylation	5/2279	31/18866	0.3178026	0.5902856	0.5341114	5	SYNJ2/INPP5A/INPP5D/INPP5E/INPP5F
Biological Process	GO:0060325	face morphogenesis	5/2279	31/18866	0.3178026	0.5902856	0.5341114	5	ARID5B/CRISPLD2/SKI/ANKRD11/ASPH
Biological Process	GO:0061037	negative regulation of cartilage development	5/2279	31/18866	0.3178026	0.5902856	0.5341114	5	WNT11/RARA/RARG/RFLNA/CCN4
Biological Process	GO:0071539	protein localization to centrosome	5/2279	31/18866	0.3178026	0.5902856	0.5341114	5	CSNK1D/DISC1/DCTN2/SPAG5/CEP72

Biological Process	GO:1902230	negative regulation of intrinsic apoptotic signaling pathway in response to DNA damage	5/2279	31/18866	0.3178026	0.5902856	0.5341114	5	ZNF385A/CLU/BCL2/BCL2L1/MARCHF7
Biological Process	GO:0001754	eye photoreceptor cell differentiation	7/2279	46/18866	0.3179027	0.5902856	0.5341114	7	CRB1/GNAT2/PRKCI/RPGRIP1/SDK2/NRL/CABP4
Biological Process	GO:0032570	response to progesterone	7/2279	46/18866	0.3179027	0.5902856	0.5341114	7	THBS1/TGFB1/NCOA1/UBE3A/NCOA2/SREBF1/RELA
Biological Process	GO:0038202	TORC1 signaling	7/2279	46/18866	0.3179027	0.5902856	0.5341114	7	RPTOR/LARP1/DGKQ/CLEC16A/SIK3/SEC13/PIH1D1
Biological Process	GO:0045023	G0 to G1 transition	7/2279	46/18866	0.3179027	0.5902856	0.5341114	7	TFDP1/MGA/EHMT2/EPC1/PHC3/E2F6/RHNO1
Biological Process	GO:1904407	positive regulation of nitric oxide metabolic process	7/2279	46/18866	0.3179027	0.5902856	0.5341114	7	SMAD3/DNM2/TNF/NOS1AP/CLU/HBB/ITGB2
Biological Process	GO:0006513	protein monoubiquitination	10/2279	69/18866	0.3196753	0.5902856	0.5341114	10	MGRN1/CUL3/UHRF1/UBE2O/ZNF598/PCGF3/NEDD4/RNF40/PRKN/MARCHF7
Biological Process	GO:0042698	ovulation cycle	10/2279	69/18866	0.3196753	0.5902856	0.5341114	10	NCOR2/MAP2K6/ARRB1/BMPR1B/NCOA1/ETS1/ARRB2/SLIT3/TYRO3/INHBA
Biological Process	GO:1903317	regulation of protein maturation	10/2279	69/18866	0.3196753	0.5902856	0.5341114	10	CTSZ/NLRC4/ENO1/GSN/THBS1/MYH9/F12/NOL3/CPB2/ANGPTL8

Biological Process	GO:0051592	response to calcium ion	20/2279	147/18866	0.3199169	0.5902856	0.5341114	20	TRPM2/CPNE6/CPNE2/TTN/CASQ2/ITPKB/GPLD1/CPNE5/THBS1/CPNE1/CRHBP/GIPR/ADD1/AQP3/MEF2C/SYT17/NEDD4/ANXA7/SEC31A/PRKAA1
Biological Process	GO:1904064	positive regulation of cation transmembrane transport	20/2279	147/18866	0.3199169	0.5902856	0.5341114	20	KCNE1/FXYD1/CCR2/DNM2/TESC/NOS1AP/ACTN4/P2RY6/HTT/KCNQ1/GSTO1/F2R/ANO6/ANK2/PLCG1/PDZK1/CASQ1/FGF14/STIMATE/CRACR2A
Biological Process	GO:0008585	female gonad development	14/2279	100/18866	0.3199716	0.5902856	0.5341114	14	ARID5B/BRCA2/ARRB1/EIF2B5/FANCA/BMPR1B/ARRB2/BCL2/DMC1/SLIT3/UBE3A/CEBPB/BCL2L1/INHBA
Biological Process	GO:0036503	ERAD pathway	14/2279	100/18866	0.3199716	0.5902856	0.5341114	14	AMFR/RHBDD2/UBAC2/EDEM1/HM13/DERL2/UBE2J2/POMT2/EDEM2/NFE2L2/UBXN8/SVIP/MARCHF6/PRKN
Biological Process	GO:0000023	maltose metabolic process	1/2279	3/18866	0.3203981	0.5902856	0.5341114	1	MGAM
Biological Process	GO:0000103	sulfate assimilation	1/2279	3/18866	0.3203981	0.5902856	0.5341114	1	PAPSS2
Biological Process	GO:0000430	regulation of transcription from RNA polymerase II promoter by glucose	1/2279	3/18866	0.3203981	0.5902856	0.5341114	1	USF2
Biological Process	GO:0000432	positive regulation of transcription from RNA polymerase II promoter by glucose	1/2279	3/18866	0.3203981	0.5902856	0.5341114	1	USF2
Biological Process	GO:0000494	box C/D snoRNA 3'-end processing	1/2279	3/18866	0.3203981	0.5902856	0.5341114	1	FBL

Biological Process	GO:0000711	meiotic DNA repair synthesis	1/2279	3/18866	0.3203981	0.5902856	0.5341114	1	TEX12
Biological Process	GO:0000962	positive regulation of mitochondrial RNA catabolic process	1/2279	3/18866	0.3203981	0.5902856	0.5341114	1	GRSF1
Biological Process	GO:0001172	transcription, RNA-templated	1/2279	3/18866	0.3203981	0.5902856	0.5341114	1	RMRP
Biological Process	GO:0001550	ovarian cumulus expansion	1/2279	3/18866	0.3203981	0.5902856	0.5341114	1	BMPR1B
Biological Process	GO:0001555	oocyte growth	1/2279	3/18866	0.3203981	0.5902856	0.5341114	1	KMT2D
Biological Process	GO:0001807	regulation of type IV hypersensitivity	1/2279	3/18866	0.3203981	0.5902856	0.5341114	1	FUT7
Biological Process	GO:0001905	activation of membrane attack complex	1/2279	3/18866	0.3203981	0.5902856	0.5341114	1	CD59
Biological Process	GO:0001922	B-1 B cell homeostasis	1/2279	3/18866	0.3203981	0.5902856	0.5341114	1	TNFAIP3
Biological Process	GO:0001951	intestinal D-glucose absorption	1/2279	3/18866	0.3203981	0.5902856	0.5341114	1	EZR

Biological Process	GO:0001969	regulation of activation of membrane attack complex	1/2279	3/18866	0.3203981	0.5902856	0.5341114	1	CD59
Biological Process	GO:0002071	glandular epithelial cell maturation	1/2279	3/18866	0.3203981	0.5902856	0.5341114	1	RFX3
Biological Process	GO:0002191	cap-dependent translational initiation	1/2279	3/18866	0.3203981	0.5902856	0.5341114	1	EIF4G1
Biological Process	GO:0002254	kinin cascade	1/2279	3/18866	0.3203981	0.5902856	0.5341114	1	F12
Biological Process	GO:0002266	follicular dendritic cell activation	1/2279	3/18866	0.3203981	0.5902856	0.5341114	1	BCL3
Biological Process	GO:0002276	basophil activation involved in immune response	1/2279	3/18866	0.3203981	0.5902856	0.5341114	1	PI4K2A
Biological Process	GO:0002277	myeloid dendritic cell activation involved in immune response	1/2279	3/18866	0.3203981	0.5902856	0.5341114	1	DOCK2
Biological Process	GO:0002351	serotonin production involved in inflammatory response	1/2279	3/18866	0.3203981	0.5902856	0.5341114	1	P2RX1
Biological Process	GO:0002353	plasma kallikrein-kinin cascade	1/2279	3/18866	0.3203981	0.5902856	0.5341114	1	F12

Biological Process	GO:0002436	immune complex clearance by monocytes and macrophages	1/2279	3/18866	0.3203981	0.5902856	0.5341114	1	CCR2
Biological Process	GO:0002442	serotonin secretion involved in inflammatory response	1/2279	3/18866	0.3203981	0.5902856	0.5341114	1	P2RX1
Biological Process	GO:0002477	antigen processing and presentation of exogenous peptide antigen via MHC class Ib	1/2279	3/18866	0.3203981	0.5902856	0.5341114	1	HLA-F
Biological Process	GO:0002514	B cell tolerance induction	1/2279	3/18866	0.3203981	0.5902856	0.5341114	1	TGFBR2
Biological Process	GO:0002541	activation of plasma proteins involved in acute inflammatory response	1/2279	3/18866	0.3203981	0.5902856	0.5341114	1	F12
Biological Process	GO:0002554	serotonin secretion by platelet	1/2279	3/18866	0.3203981	0.5902856	0.5341114	1	P2RX1
Biological Process	GO:0002581	negative regulation of antigen processing and presentation of peptide or polysaccharide antigen via MHC class II	1/2279	3/18866	0.3203981	0.5902856	0.5341114	1	THBS1
Biological Process	GO:0002636	positive regulation of germinal center formation	1/2279	3/18866	0.3203981	0.5902856	0.5341114	1	TNFSF13B
Biological Process	GO:0002904	positive regulation of B cell apoptotic process	1/2279	3/18866	0.3203981	0.5902856	0.5341114	1	IL10

Biological Process	GO:0002941	synoviocyte proliferation	1/2279	3/18866	0.3203981	0.5902856	0.5341114	1	TNF
Biological Process	GO:0003249	cell proliferation involved in heart valve morphogenesis	1/2279	3/18866	0.3203981	0.5902856	0.5341114	1	NOTCH1
Biological Process	GO:0003250	regulation of cell proliferation involved in heart valve morphogenesis	1/2279	3/18866	0.3203981	0.5902856	0.5341114	1	NOTCH1
Biological Process	GO:0003285	septum secundum development	1/2279	3/18866	0.3203981	0.5902856	0.5341114	1	NSD2
Biological Process	GO:0003331	positive regulation of extracellular matrix constituent secretion	1/2279	3/18866	0.3203981	0.5902856	0.5341114	1	CPB2
Biological Process	GO:0005988	lactose metabolic process	1/2279	3/18866	0.3203981	0.5902856	0.5341114	1	SLC2A1
Biological Process	GO:0005989	lactose biosynthetic process	1/2279	3/18866	0.3203981	0.5902856	0.5341114	1	SLC2A1
Biological Process	GO:0006021	inositol biosynthetic process	1/2279	3/18866	0.3203981	0.5902856	0.5341114	1	IMPA2
Biological Process	GO:0006046	N-acetylglucosamine catabolic process	1/2279	3/18866	0.3203981	0.5902856	0.5341114	1	AMDHD2

Biological Process	GO:0006208	pyrimidine nucleobase catabolic process	1/2279	3/18866	0.3203981	0.5902856	0.5341114	1	DPYS
Biological Process	GO:0006210	thymine catabolic process	1/2279	3/18866	0.3203981	0.5902856	0.5341114	1	DPYS
Biological Process	GO:0006211	5-methylcytosine catabolic process	1/2279	3/18866	0.3203981	0.5902856	0.5341114	1	TET2
Biological Process	GO:0006226	dUMP biosynthetic process	1/2279	3/18866	0.3203981	0.5902856	0.5341114	1	DCTD
Biological Process	GO:0006231	dTMP biosynthetic process	1/2279	3/18866	0.3203981	0.5902856	0.5341114	1	DCTD
Biological Process	GO:0006311	meiotic gene conversion	1/2279	3/18866	0.3203981	0.5902856	0.5341114	1	RNF212
Biological Process	GO:0006404	RNA import into nucleus	1/2279	3/18866	0.3203981	0.5902856	0.5341114	1	SNUPN
Biological Process	GO:0006667	sphinganine metabolic process	1/2279	3/18866	0.3203981	0.5902856	0.5341114	1	SPTLC2
Biological Process	GO:0006683	galactosylceramide catabolic process	1/2279	3/18866	0.3203981	0.5902856	0.5341114	1	GALC

Biological Process	GO:0006741	NADP biosynthetic process	1/2279	3/18866	0.3203981	0.5902856	0.5341114	1	NADK
Biological Process	GO:0006808	regulation of nitrogen utilization	1/2279	3/18866	0.3203981	0.5902856	0.5341114	1	BCL2
Biological Process	GO:0007207	phospholipase C-activating G protein-coupled acetylcholine receptor signaling pathway	1/2279	3/18866	0.3203981	0.5902856	0.5341114	1	CHRM2
Biological Process	GO:0007228	positive regulation of hh target transcription factor activity	1/2279	3/18866	0.3203981	0.5902856	0.5341114	1	STK36
Biological Process	GO:0007619	courtship behavior	1/2279	3/18866	0.3203981	0.5902856	0.5341114	1	HEXB
Biological Process	GO:0008292	acetylcholine biosynthetic process	1/2279	3/18866	0.3203981	0.5902856	0.5341114	1	SLC44A4
Biological Process	GO:0008315	G2/MI transition of meiotic cell cycle	1/2279	3/18866	0.3203981	0.5902856	0.5341114	1	CDC25A
Biological Process	GO:0009158	ribonucleoside monophosphate catabolic process	1/2279	3/18866	0.3203981	0.5902856	0.5341114	1	AMPD3
Biological Process	GO:0009169	purine ribonucleoside monophosphate catabolic process	1/2279	3/18866	0.3203981	0.5902856	0.5341114	1	AMPD3

Biological Process	GO:0009170	purine deoxyribonucleoside monophosphate metabolic process	1/2279	3/18866	0.3203981	0.5902856	0.5341114	1	GUK1
Biological Process	GO:0009397	folic acid-containing compound catabolic process	1/2279	3/18866	0.3203981	0.5902856	0.5341114	1	MTHFS
Biological Process	GO:0009447	putrescine catabolic process	1/2279	3/18866	0.3203981	0.5902856	0.5341114	1	SAT2
Biological Process	GO:0009449	gamma-aminobutyric acid biosynthetic process	1/2279	3/18866	0.3203981	0.5902856	0.5341114	1	SLC1A3
Biological Process	GO:0009631	cold acclimation	1/2279	3/18866	0.3203981	0.5902856	0.5341114	1	PRKAA1
Biological Process	GO:0009726	detection of endogenous stimulus	1/2279	3/18866	0.3203981	0.5902856	0.5341114	1	PKD1L1
Biological Process	GO:0009946	proximal/distal axis specification	1/2279	3/18866	0.3203981	0.5902856	0.5341114	1	NODAL
Biological Process	GO:0009957	epidermal cell fate specification	1/2279	3/18866	0.3203981	0.5902856	0.5341114	1	RBPJ
Biological Process	GO:0010133	proline catabolic process to glutamate	1/2279	3/18866	0.3203981	0.5902856	0.5341114	1	ALDH4A1

Biological Process	GO:0010446	response to alkaline pH	1/2279	3/18866	0.3203981	0.5902856	0.5341114	1	INSRR
Biological Process	GO:0010621	negative regulation of transcription by transcription factor localization	1/2279	3/18866	0.3203981	0.5902856	0.5341114	1	HHEX
Biological Process	GO:0010643	cell communication by chemical coupling	1/2279	3/18866	0.3203981	0.5902856	0.5341114	1	GJA5
Biological Process	GO:0010751	negative regulation of nitric oxide mediated signal transduction	1/2279	3/18866	0.3203981	0.5902856	0.5341114	1	THBS1
Biological Process	GO:0010956	negative regulation of calcidiol 1-monooxygenase activity	1/2279	3/18866	0.3203981	0.5902856	0.5341114	1	GFI1
Biological Process	GO:0010979	regulation of vitamin D 24-hydroxylase activity	1/2279	3/18866	0.3203981	0.5902856	0.5341114	1	VDR
Biological Process	GO:0010980	positive regulation of vitamin D 24-hydroxylase activity	1/2279	3/18866	0.3203981	0.5902856	0.5341114	1	VDR
Biological Process	GO:0014011	Schwann cell proliferation involved in axon regeneration	1/2279	3/18866	0.3203981	0.5902856	0.5341114	1	CERS2
Biological Process	GO:0014040	positive regulation of Schwann cell differentiation	1/2279	3/18866	0.3203981	0.5902856	0.5341114	1	RELA

Biological Process	GO:0014043	negative regulation of neuron maturation	1/2279	3/18866	0.3203981	0.5902856	0.5341114	1	BCL11A
Biological Process	GO:0015891	siderophore transport	1/2279	3/18866	0.3203981	0.5902856	0.5341114	1	LCN2
Biological Process	GO:0019050	suppression by virus of host apoptotic process	1/2279	3/18866	0.3203981	0.5902856	0.5341114	1	BCL2L1
Biological Process	GO:0019082	viral protein processing	1/2279	3/18866	0.3203981	0.5902856	0.5341114	1	SPCS1
Biological Process	GO:0019249	lactate biosynthetic process	1/2279	3/18866	0.3203981	0.5902856	0.5341114	1	PER2
Biological Process	GO:0019254	carnitine metabolic process, CoA-linked	1/2279	3/18866	0.3203981	0.5902856	0.5341114	1	ACADM
Biological Process	GO:0019276	UDP-N-acetylgalactosamine metabolic process	1/2279	3/18866	0.3203981	0.5902856	0.5341114	1	CSGALNACT1
Biological Process	GO:0019343	cysteine biosynthetic process via cystathionine	1/2279	3/18866	0.3203981	0.5902856	0.5341114	1	CBS
Biological Process	GO:0019857	5-methylcytosine metabolic process	1/2279	3/18866	0.3203981	0.5902856	0.5341114	1	TET2

Biological Process	GO:0019859	thymine metabolic process	1/2279	3/18866	0.3203981	0.5902856	0.5341114	1	DPYS
Biological Process	GO:0019860	uracil metabolic process	1/2279	3/18866	0.3203981	0.5902856	0.5341114	1	DPYS
Biological Process	GO:0021553	olfactory nerve development	1/2279	3/18866	0.3203981	0.5902856	0.5341114	1	NTRK1
Biological Process	GO:0021747	cochlear nucleus development	1/2279	3/18866	0.3203981	0.5902856	0.5341114	1	BCL2
Biological Process	GO:0021775	smoothened signaling pathway involved in ventral spinal cord interneuron specification	1/2279	3/18866	0.3203981	0.5902856	0.5341114	1	SUFU
Biological Process	GO:0021776	smoothened signaling pathway involved in spinal cord motor neuron cell fate specification	1/2279	3/18866	0.3203981	0.5902856	0.5341114	1	SUFU
Biological Process	GO:0021828	gonadotrophin-releasing hormone neuronal migration to the hypothalamus	1/2279	3/18866	0.3203981	0.5902856	0.5341114	1	NRP2
Biological Process	GO:0021847	ventricular zone neuroblast division	1/2279	3/18866	0.3203981	0.5902856	0.5341114	1	FGFR1
Biological Process	GO:0021849	neuroblast division in subventricular zone	1/2279	3/18866	0.3203981	0.5902856	0.5341114	1	NUMB

Biological Process	GO:0021856	hypothalamic tangential migration using cell-axon interactions	1/2279	3/18866	0.3203981	0.5902856	0.5341114	1	NRP2
Biological Process	GO:0030382	sperm mitochondrion organization	1/2279	3/18866	0.3203981	0.5902856	0.5341114	1	MFSD14A
Biological Process	GO:0030846	termination of RNA polymerase II transcription, poly(A)-coupled	1/2279	3/18866	0.3203981	0.5902856	0.5341114	1	SCAF8
Biological Process	GO:0030886	negative regulation of myeloid dendritic cell activation	1/2279	3/18866	0.3203981	0.5902856	0.5341114	1	IL10
Biological Process	GO:0031335	regulation of sulfur amino acid metabolic process	1/2279	3/18866	0.3203981	0.5902856	0.5341114	1	COMT
Biological Process	GO:0032238	adenosine transport	1/2279	3/18866	0.3203981	0.5902856	0.5341114	1	SLC29A1
Biological Process	GO:0032484	Ral protein signal transduction	1/2279	3/18866	0.3203981	0.5902856	0.5341114	1	RALGPS1
Biological Process	GO:0032485	regulation of Ral protein signal transduction	1/2279	3/18866	0.3203981	0.5902856	0.5341114	1	RALGPS1
Biological Process	GO:0032619	interleukin-16 production	1/2279	3/18866	0.3203981	0.5902856	0.5341114	1	MIR145

Biological Process	GO:0032659	regulation of interleukin-16 production	1/2279	3/18866	0.3203981	0.5902856	0.5341114	1	MIR145
Biological Process	GO:0032730	positive regulation of interleukin-1 alpha production	1/2279	3/18866	0.3203981	0.5902856	0.5341114	1	IL16
Biological Process	GO:0032765	positive regulation of mast cell cytokine production	1/2279	3/18866	0.3203981	0.5902856	0.5341114	1	BCL10
Biological Process	GO:0032824	negative regulation of natural killer cell differentiation	1/2279	3/18866	0.3203981	0.5902856	0.5341114	1	PGLYRP1
Biological Process	GO:0032827	negative regulation of natural killer cell differentiation involved in immune response	1/2279	3/18866	0.3203981	0.5902856	0.5341114	1	PGLYRP1
Biological Process	GO:0033076	isoquinoline alkaloid metabolic process	1/2279	3/18866	0.3203981	0.5902856	0.5341114	1	DDC
Biological Process	GO:0033140	negative regulation of peptidyl-serine phosphorylation of STAT protein	1/2279	3/18866	0.3203981	0.5902856	0.5341114	1	GGNBP2
Biological Process	GO:0033227	dsRNA transport	1/2279	3/18866	0.3203981	0.5902856	0.5341114	1	FLOT1
Biological Process	GO:0033387	putrescine biosynthetic process from ornithine	1/2279	3/18866	0.3203981	0.5902856	0.5341114	1	ODC1

Biological Process	GO:0033563	dorsal/ventral axon guidance	1/2279	3/18866	0.3203981	0.5902856	0.5341114	1	SLIT1
Biological Process	GO:0033624	negative regulation of integrin activation	1/2279	3/18866	0.3203981	0.5902856	0.5341114	1	JAM3
Biological Process	GO:0033693	neurofilament bundle assembly	1/2279	3/18866	0.3203981	0.5902856	0.5341114	1	NEFM
Biological Process	GO:0033967	box C/D snoRNA metabolic process	1/2279	3/18866	0.3203981	0.5902856	0.5341114	1	FBL
Biological Process	GO:0034092	negative regulation of maintenance of sister chromatid cohesion	1/2279	3/18866	0.3203981	0.5902856	0.5341114	1	TNKS
Biological Process	GO:0034140	negative regulation of toll-like receptor 3 signaling pathway	1/2279	3/18866	0.3203981	0.5902856	0.5341114	1	TNFAIP3
Biological Process	GO:0034183	negative regulation of maintenance of mitotic sister chromatid cohesion	1/2279	3/18866	0.3203981	0.5902856	0.5341114	1	TNKS
Biological Process	GO:0034963	box C/D snoRNA processing	1/2279	3/18866	0.3203981	0.5902856	0.5341114	1	FBL
Biological Process	GO:0035166	post-embryonic hemopoiesis	1/2279	3/18866	0.3203981	0.5902856	0.5341114	1	ERCC1

Biological Process	GO:0035377	transepithelial water transport	1/2279	3/18866	0.3203981	0.5902856	0.5341114	1	AQP1
Biological Process	GO:0035553	oxidative single-stranded RNA demethylation	1/2279	3/18866	0.3203981	0.5902856	0.5341114	1	FTO
Biological Process	GO:0035668	TRAM-dependent toll-like receptor signaling pathway	1/2279	3/18866	0.3203981	0.5902856	0.5341114	1	PRKCE
Biological Process	GO:0035669	TRAM-dependent toll-like receptor 4 signaling pathway	1/2279	3/18866	0.3203981	0.5902856	0.5341114	1	PRKCE
Biological Process	GO:0035691	macrophage migration inhibitory factor signaling pathway	1/2279	3/18866	0.3203981	0.5902856	0.5341114	1	ANGPT1
Biological Process	GO:0035898	parathyroid hormone secretion	1/2279	3/18866	0.3203981	0.5902856	0.5341114	1	FGFR1
Biological Process	GO:0035983	response to trichostatin A	1/2279	3/18866	0.3203981	0.5902856	0.5341114	1	MEF2C
Biological Process	GO:0035984	cellular response to trichostatin A	1/2279	3/18866	0.3203981	0.5902856	0.5341114	1	MEF2C
Biological Process	GO:0036112	medium-chain fatty-acyl-CoA metabolic process	1/2279	3/18866	0.3203981	0.5902856	0.5341114	1	ACOT7

Biological Process	GO:0036118	hyaluranon cable assembly	1/2279	3/18866	0.3203981	0.5902856	0.5341114	1	HAS3
Biological Process	GO:0036215	response to stem cell factor	1/2279	3/18866	0.3203981	0.5902856	0.5341114	1	FER
Biological Process	GO:0036216	cellular response to stem cell factor stimulus	1/2279	3/18866	0.3203981	0.5902856	0.5341114	1	FER
Biological Process	GO:0036509	trimming of terminal mannose on B branch	1/2279	3/18866	0.3203981	0.5902856	0.5341114	1	EDEM2
Biological Process	GO:0036511	trimming of first mannose on A branch	1/2279	3/18866	0.3203981	0.5902856	0.5341114	1	EDEM2
Biological Process	GO:0036512	trimming of second mannose on A branch	1/2279	3/18866	0.3203981	0.5902856	0.5341114	1	EDEM2
Biological Process	GO:0038086	VEGF-activated platelet-derived growth factor receptor signaling pathway	1/2279	3/18866	0.3203981	0.5902856	0.5341114	1	PDGFRB
Biological Process	GO:0038091	positive regulation of cell proliferation by VEGF-activated platelet derived growth factor receptor signaling pathway	1/2279	3/18866	0.3203981	0.5902856	0.5341114	1	PDGFRB
Biological Process	GO:0038109	Kit signaling pathway	1/2279	3/18866	0.3203981	0.5902856	0.5341114	1	FER

Biological Process	GO:0038129	ERBB3 signaling pathway	1/2279	3/18866	0.3203981	0.5902856	0.5341114	1	RTN4
Biological Process	GO:0038154	interleukin-11-mediated signaling pathway	1/2279	3/18866	0.3203981	0.5902856	0.5341114	1	IL6R
Biological Process	GO:0038183	bile acid signaling pathway	1/2279	3/18866	0.3203981	0.5902856	0.5341114	1	VDR
Biological Process	GO:0042268	regulation of cytolysis	1/2279	3/18866	0.3203981	0.5902856	0.5341114	1	TGFB1
Biological Process	GO:0042271	susceptibility to natural killer cell mediated cytotoxicity	1/2279	3/18866	0.3203981	0.5902856	0.5341114	1	CADM1
Biological Process	GO:0042427	serotonin biosynthetic process	1/2279	3/18866	0.3203981	0.5902856	0.5341114	1	DDC
Biological Process	GO:0042560	pteridine-containing compound catabolic process	1/2279	3/18866	0.3203981	0.5902856	0.5341114	1	MTHFS
Biological Process	GO:0042819	vitamin B6 biosynthetic process	1/2279	3/18866	0.3203981	0.5902856	0.5341114	1	PDXK
Biological Process	GO:0042822	pyridoxal phosphate metabolic process	1/2279	3/18866	0.3203981	0.5902856	0.5341114	1	PDXK

Biological Process	GO:0042942	D-serine transport	1/2279	3/18866	0.3203981	0.5902856	0.5341114	1	SFXN1
Biological Process	GO:0043163	cell envelope organization	1/2279	3/18866	0.3203981	0.5902856	0.5341114	1	TGM3
Biological Process	GO:0043309	regulation of eosinophil degranulation	1/2279	3/18866	0.3203981	0.5902856	0.5341114	1	CCR2
Biological Process	GO:0043316	cytotoxic T cell degranulation	1/2279	3/18866	0.3203981	0.5902856	0.5341114	1	RAB27A
Biological Process	GO:0043321	regulation of natural killer cell degranulation	1/2279	3/18866	0.3203981	0.5902856	0.5341114	1	HLA-F
Biological Process	GO:0043323	positive regulation of natural killer cell degranulation	1/2279	3/18866	0.3203981	0.5902856	0.5341114	1	HLA-F
Biological Process	GO:0043385	mycotoxin metabolic process	1/2279	3/18866	0.3203981	0.5902856	0.5341114	1	NFE2L2
Biological Process	GO:0043402	glucocorticoid mediated signaling pathway	1/2279	3/18866	0.3203981	0.5902856	0.5341114	1	AKAP13
Biological Process	GO:0043418	homocysteine catabolic process	1/2279	3/18866	0.3203981	0.5902856	0.5341114	1	CBS

Biological Process	GO:0043456	regulation of pentose-phosphate shunt	1/2279	3/18866	0.3203981	0.5902856	0.5341114	1	PGAM1
Biological Process	GO:0043578	nuclear matrix organization	1/2279	3/18866	0.3203981	0.5902856	0.5341114	1	SYNE1
Biological Process	GO:0043585	nose morphogenesis	1/2279	3/18866	0.3203981	0.5902856	0.5341114	1	SKI
Biological Process	GO:0043686	co-translational protein modification	1/2279	3/18866	0.3203981	0.5902856	0.5341114	1	PDF
Biological Process	GO:0043969	histone H2B acetylation	1/2279	3/18866	0.3203981	0.5902856	0.5341114	1	SPHK2
Biological Process	GO:0043988	histone H3-S28 phosphorylation	1/2279	3/18866	0.3203981	0.5902856	0.5341114	1	RPS6KA4
Biological Process	GO:0044026	DNA hypermethylation	1/2279	3/18866	0.3203981	0.5902856	0.5341114	1	MORC1
Biological Process	GO:0044036	cell wall macromolecule metabolic process	1/2279	3/18866	0.3203981	0.5902856	0.5341114	1	LYG2
Biological Process	GO:0044110	growth involved in symbiotic interaction	1/2279	3/18866	0.3203981	0.5902856	0.5341114	1	PGLYRP1

Biological Process	GO:0044117	growth of symbiont in host	1/2279	3/18866	0.3203981	0.5902856	0.5341114	1	PGLYRP1
Biological Process	GO:0044205	'de novo' UMP biosynthetic process	1/2279	3/18866	0.3203981	0.5902856	0.5341114	1	DHODH
Biological Process	GO:0044211	CTP salvage	1/2279	3/18866	0.3203981	0.5902856	0.5341114	1	UCK2
Biological Process	GO:0044375	regulation of peroxisome size	1/2279	3/18866	0.3203981	0.5902856	0.5341114	1	PEX11B
Biological Process	GO:0044381	glucose import in response to insulin stimulus	1/2279	3/18866	0.3203981	0.5902856	0.5341114	1	ZDHHC7
Biological Process	GO:0044467	glial cell-derived neurotrophic factor secretion	1/2279	3/18866	0.3203981	0.5902856	0.5341114	1	SORL1
Biological Process	GO:0044830	modulation by host of viral RNA genome replication	1/2279	3/18866	0.3203981	0.5902856	0.5341114	1	PHB
Biological Process	GO:0045007	depurination	1/2279	3/18866	0.3203981	0.5902856	0.5341114	1	MUTYH
Biological Process	GO:0045226	extracellular polysaccharide biosynthetic process	1/2279	3/18866	0.3203981	0.5902856	0.5341114	1	HAS3

Biological Process	GO:0045229	external encapsulating structure organization	1/2279	3/18866	0.3203981	0.5902856	0.5341114	1	TGM3
Biological Process	GO:0045715	negative regulation of low-density lipoprotein particle receptor biosynthetic process	1/2279	3/18866	0.3203981	0.5902856	0.5341114	1	ITGB3
Biological Process	GO:0045764	positive regulation of cellular amino acid metabolic process	1/2279	3/18866	0.3203981	0.5902856	0.5341114	1	COMT
Biological Process	GO:0045925	positive regulation of female receptivity	1/2279	3/18866	0.3203981	0.5902856	0.5341114	1	NCOA1
Biological Process	GO:0045967	negative regulation of growth rate	1/2279	3/18866	0.3203981	0.5902856	0.5341114	1	NOTCH1
Biological Process	GO:0046056	dADP metabolic process	1/2279	3/18866	0.3203981	0.5902856	0.5341114	1	AK5
Biological Process	GO:0046066	dGDP metabolic process	1/2279	3/18866	0.3203981	0.5902856	0.5341114	1	GUK1
Biological Process	GO:0046073	dTMP metabolic process	1/2279	3/18866	0.3203981	0.5902856	0.5341114	1	DCTD
Biological Process	GO:0046110	xanthine metabolic process	1/2279	3/18866	0.3203981	0.5902856	0.5341114	1	AOX1

Biological Process	GO:0046222	aflatoxin metabolic process	1/2279	3/18866	0.3203981	0.5902856	0.5341114	1	NFE2L2
Biological Process	GO:0046338	phosphatidylethanolamine catabolic process	1/2279	3/18866	0.3203981	0.5902856	0.5341114	1	PLA2G15
Biological Process	GO:0046379	extracellular polysaccharide metabolic process	1/2279	3/18866	0.3203981	0.5902856	0.5341114	1	HAS3
Biological Process	GO:0046668	regulation of retinal cell programmed cell death	1/2279	3/18866	0.3203981	0.5902856	0.5341114	1	BCL2
Biological Process	GO:0046882	negative regulation of follicle-stimulating hormone secretion	1/2279	3/18866	0.3203981	0.5902856	0.5341114	1	INHBA
Biological Process	GO:0048165	fused antrum stage	1/2279	3/18866	0.3203981	0.5902856	0.5341114	1	BMPR1B
Biological Process	GO:0048319	axial mesoderm morphogenesis	1/2279	3/18866	0.3203981	0.5902856	0.5341114	1	NODAL
Biological Process	GO:0048371	lateral mesodermal cell differentiation	1/2279	3/18866	0.3203981	0.5902856	0.5341114	1	FGFR1
Biological Process	GO:0048633	positive regulation of skeletal muscle tissue growth	1/2279	3/18866	0.3203981	0.5902856	0.5341114	1	DLL1

Biological Process	GO:0048696	regulation of collateral sprouting in absence of injury	1/2279	3/18866	0.3203981	0.5902856	0.5341114	1	SPART
Biological Process	GO:0048769	sarcomerogenesis	1/2279	3/18866	0.3203981	0.5902856	0.5341114	1	TTN
Biological Process	GO:0048822	enucleate erythrocyte development	1/2279	3/18866	0.3203981	0.5902856	0.5341114	1	MAEA
Biological Process	GO:0048867	stem cell fate determination	1/2279	3/18866	0.3203981	0.5902856	0.5341114	1	NTF4
Biological Process	GO:0048925	lateral line system development	1/2279	3/18866	0.3203981	0.5902856	0.5341114	1	SLC44A4
Biological Process	GO:0050960	detection of temperature stimulus involved in thermoception	1/2279	3/18866	0.3203981	0.5902856	0.5341114	1	WDR47
Biological Process	GO:0051684	maintenance of Golgi location	1/2279	3/18866	0.3203981	0.5902856	0.5341114	1	UVRAG
Biological Process	GO:0051728	cell cycle switching, mitotic to meiotic cell cycle	1/2279	3/18866	0.3203981	0.5902856	0.5341114	1	OVOL1
Biological Process	GO:0051729	germline cell cycle switching, mitotic to meiotic cell cycle	1/2279	3/18866	0.3203981	0.5902856	0.5341114	1	OVOL1

Biological Process	GO:0051941	regulation of amino acid uptake involved in synaptic transmission	1/2279	3/18866	0.3203981	0.5902856	0.5341114	1	PER2
Biological Process	GO:0051946	regulation of glutamate uptake involved in transmission of nerve impulse	1/2279	3/18866	0.3203981	0.5902856	0.5341114	1	PER2
Biological Process	GO:0055011	atrial cardiac muscle cell differentiation	1/2279	3/18866	0.3203981	0.5902856	0.5341114	1	FHL2
Biological Process	GO:0055014	atrial cardiac muscle cell development	1/2279	3/18866	0.3203981	0.5902856	0.5341114	1	FHL2
Biological Process	GO:0055073	cadmium ion homeostasis	1/2279	3/18866	0.3203981	0.5902856	0.5341114	1	SLC11A1
Biological Process	GO:0060025	regulation of synaptic activity	1/2279	3/18866	0.3203981	0.5902856	0.5341114	1	MEF2C
Biological Process	GO:0060168	positive regulation of adenosine receptor signaling pathway	1/2279	3/18866	0.3203981	0.5902856	0.5341114	1	ACP3
Biological Process	GO:0060184	cell cycle switching	1/2279	3/18866	0.3203981	0.5902856	0.5341114	1	OVOL1
Biological Process	GO:0060197	cloacal septation	1/2279	3/18866	0.3203981	0.5902856	0.5341114	1	WNT11

Biological Process	GO:0060278	regulation of ovulation	1/2279	3/18866	0.3203981	0.5902856	0.5341114	1	INHBA
Biological Process	GO:0060448	dichotomous subdivision of terminal units involved in lung branching	1/2279	3/18866	0.3203981	0.5902856	0.5341114	1	CTSH
Biological Process	GO:0060474	positive regulation of flagellated sperm motility involved in capacitation	1/2279	3/18866	0.3203981	0.5902856	0.5341114	1	DEFB1
Biological Process	GO:0060557	positive regulation of vitamin D biosynthetic process	1/2279	3/18866	0.3203981	0.5902856	0.5341114	1	TNF
Biological Process	GO:0060559	positive regulation of calcidiol 1-monooxygenase activity	1/2279	3/18866	0.3203981	0.5902856	0.5341114	1	TNF
Biological Process	GO:0060715	syncytiotrophoblast cell differentiation involved in labyrinthine layer development	1/2279	3/18866	0.3203981	0.5902856	0.5341114	1	DNAJB6
Biological Process	GO:0060741	prostate gland stromal morphogenesis	1/2279	3/18866	0.3203981	0.5902856	0.5341114	1	CRIP1
Biological Process	GO:0060948	cardiac vascular smooth muscle cell development	1/2279	3/18866	0.3203981	0.5902856	0.5341114	1	NOTCH1
Biological Process	GO:0060978	angiogenesis involved in coronary vascular morphogenesis	1/2279	3/18866	0.3203981	0.5902856	0.5341114	1	PDGFRB

Biological Process	GO:0061110	dense core granule biogenesis	1/2279	3/18866	0.3203981	0.5902856	0.5341114	1	PRKCA
Biological Process	GO:0061198	fungiform papilla formation	1/2279	3/18866	0.3203981	0.5902856	0.5341114	1	HDAC1
Biological Process	GO:0061364	apoptotic process involved in luteolysis	1/2279	3/18866	0.3203981	0.5902856	0.5341114	1	SLIT3
Biological Process	GO:0061713	anterior neural tube closure	1/2279	3/18866	0.3203981	0.5902856	0.5341114	1	GRHL2
Biological Process	GO:0061739	protein lipidation involved in autophagosome assembly	1/2279	3/18866	0.3203981	0.5902856	0.5341114	1	WIPI2
Biological Process	GO:0061740	protein targeting to lysosome involved in chaperone-mediated autophagy	1/2279	3/18866	0.3203981	0.5902856	0.5341114	1	CLU
Biological Process	GO:0061819	telomeric DNA-containing double minutes formation	1/2279	3/18866	0.3203981	0.5902856	0.5341114	1	ERCC1
Biological Process	GO:0061909	autophagosome-lysosome fusion	1/2279	3/18866	0.3203981	0.5902856	0.5341114	1	RUBCNL
Biological Process	GO:0070236	negative regulation of activation-induced cell death of T cells	1/2279	3/18866	0.3203981	0.5902856	0.5341114	1	FADD

Biological Process	GO:0070295	renal water absorption	1/2279	3/18866	0.3203981	0.5902856	0.5341114	1	AQP3
Biological Process	GO:0070343	white fat cell proliferation	1/2279	3/18866	0.3203981	0.5902856	0.5341114	1	FTO
Biological Process	GO:0070350	regulation of white fat cell proliferation	1/2279	3/18866	0.3203981	0.5902856	0.5341114	1	FTO
Biological Process	GO:0070425	negative regulation of nucleotide-binding oligomerization domain containing signaling pathway	1/2279	3/18866	0.3203981	0.5902856	0.5341114	1	TNFAIP3
Biological Process	GO:0070428	regulation of nucleotide-binding oligomerization domain containing 1 signaling pathway	1/2279	3/18866	0.3203981	0.5902856	0.5341114	1	TNFAIP3
Biological Process	GO:0070433	negative regulation of nucleotide-binding oligomerization domain containing 2 signaling pathway	1/2279	3/18866	0.3203981	0.5902856	0.5341114	1	TNFAIP3
Biological Process	GO:0070541	response to platinum ion	1/2279	3/18866	0.3203981	0.5902856	0.5341114	1	FECH
Biological Process	GO:0070667	negative regulation of mast cell proliferation	1/2279	3/18866	0.3203981	0.5902856	0.5341114	1	BLK
Biological Process	GO:0070681	glutaminyl-tRNAGln biosynthesis via transamidation	1/2279	3/18866	0.3203981	0.5902856	0.5341114	1	GATB

Biological Process	GO:0071288	cellular response to mercury ion	1/2279	3/18866	0.3203981	0.5902856	0.5341114	1	AQP1
Biological Process	GO:0071314	cellular response to cocaine	1/2279	3/18866	0.3203981	0.5902856	0.5341114	1	CRHBP
Biological Process	GO:0071461	cellular response to redox state	1/2279	3/18866	0.3203981	0.5902856	0.5341114	1	ARHGDIB
Biological Process	GO:0071554	cell wall organization or biogenesis	1/2279	3/18866	0.3203981	0.5902856	0.5341114	1	LYG2
Biological Process	GO:0071630	nuclear protein quality control by the ubiquitin-proteasome system	1/2279	3/18866	0.3203981	0.5902856	0.5341114	1	CUL3
Biological Process	GO:0071641	negative regulation of macrophage inflammatory protein 1 alpha production	1/2279	3/18866	0.3203981	0.5902856	0.5341114	1	MEFV
Biological Process	GO:0071873	response to norepinephrine	1/2279	3/18866	0.3203981	0.5902856	0.5341114	1	APP
Biological Process	GO:0071931	positive regulation of transcription involved in G1/S transition of mitotic cell cycle	1/2279	3/18866	0.3203981	0.5902856	0.5341114	1	ESRRB
Biological Process	GO:0072003	kidney rudiment formation	1/2279	3/18866	0.3203981	0.5902856	0.5341114	1	PAX2

Biological Process	GO:0072069	DCT cell differentiation	1/2279	3/18866	0.3203981	0.5902856	0.5341114	1	PAX2
Biological Process	GO:0072240	metanephric DCT cell differentiation	1/2279	3/18866	0.3203981	0.5902856	0.5341114	1	PAX2
Biological Process	GO:0072262	metanephric glomerular mesangial cell proliferation involved in metanephros development	1/2279	3/18866	0.3203981	0.5902856	0.5341114	1	PDGFRB
Biological Process	GO:0072275	metanephric glomerulus morphogenesis	1/2279	3/18866	0.3203981	0.5902856	0.5341114	1	PDGFRB
Biological Process	GO:0072276	metanephric glomerulus vasculature morphogenesis	1/2279	3/18866	0.3203981	0.5902856	0.5341114	1	PDGFRB
Biological Process	GO:0072277	metanephric glomerular capillary formation	1/2279	3/18866	0.3203981	0.5902856	0.5341114	1	PDGFRB
Biological Process	GO:0072338	cellular lactam metabolic process	1/2279	3/18866	0.3203981	0.5902856	0.5341114	1	ABCC2
Biological Process	GO:0072535	tumor necrosis factor (ligand) superfamily member 11 production	1/2279	3/18866	0.3203981	0.5902856	0.5341114	1	LTF
Biological Process	GO:0072709	cellular response to sorbitol	1/2279	3/18866	0.3203981	0.5902856	0.5341114	1	MAP2K6

Biological Process	GO:0090031	positive regulation of steroid hormone biosynthetic process	1/2279	3/18866	0.3203981	0.5902856	0.5341114	1	POR
Biological Process	GO:0090065	regulation of production of siRNA involved in RNA interference	1/2279	3/18866	0.3203981	0.5902856	0.5341114	1	RMRP
Biological Process	GO:0090264	regulation of immune complex clearance by monocytes and macrophages	1/2279	3/18866	0.3203981	0.5902856	0.5341114	1	CCR2
Biological Process	GO:0090292	nuclear matrix anchoring at nuclear membrane	1/2279	3/18866	0.3203981	0.5902856	0.5341114	1	SYNE1
Biological Process	GO:0090360	platelet-derived growth factor production	1/2279	3/18866	0.3203981	0.5902856	0.5341114	1	NDRG2
Biological Process	GO:0090361	regulation of platelet-derived growth factor production	1/2279	3/18866	0.3203981	0.5902856	0.5341114	1	NDRG2
Biological Process	GO:0090427	activation of meiosis	1/2279	3/18866	0.3203981	0.5902856	0.5341114	1	PLCB1
Biological Process	GO:0097010	eukaryotic translation initiation factor 4F complex assembly	1/2279	3/18866	0.3203981	0.5902856	0.5341114	1	EIF4G1
Biological Process	GO:0097017	renal protein absorption	1/2279	3/18866	0.3203981	0.5902856	0.5341114	1	GSN

Biological Process	GO:0097374	sensory neuron axon guidance	1/2279	3/18866	0.3203981	0.5902856	0.5341114	1	NRP2
Biological Process	GO:0098758	response to interleukin-8	1/2279	3/18866	0.3203981	0.5902856	0.5341114	1	CXCR1
Biological Process	GO:0098759	cellular response to interleukin-8	1/2279	3/18866	0.3203981	0.5902856	0.5341114	1	CXCR1
Biological Process	GO:0098912	membrane depolarization during atrial cardiac muscle cell action potential	1/2279	3/18866	0.3203981	0.5902856	0.5341114	1	CACNA1C
Biological Process	GO:0099074	mitochondrion to lysosome transport	1/2279	3/18866	0.3203981	0.5902856	0.5341114	1	PRKN
Biological Process	GO:0099075	mitochondrion-derived vesicle mediated transport	1/2279	3/18866	0.3203981	0.5902856	0.5341114	1	PRKN
Biological Process	GO:0099552	trans-synaptic signaling by lipid, modulating synaptic transmission	1/2279	3/18866	0.3203981	0.5902856	0.5341114	1	F2R
Biological Process	GO:0099553	trans-synaptic signaling by endocannabinoid, modulating synaptic transmission	1/2279	3/18866	0.3203981	0.5902856	0.5341114	1	F2R
Biological Process	GO:0099575	regulation of protein catabolic process at presynapse, modulating synaptic transmission	1/2279	3/18866	0.3203981	0.5902856	0.5341114	1	PLEKHG5

Biological Process	GO:0099578	regulation of translation at postsynapse, modulating synaptic transmission	1/2279	3/18866	0.3203981	0.5902856	0.5341114	1	CYFIP1
Biological Process	GO:0101010	pulmonary blood vessel remodeling	1/2279	3/18866	0.3203981	0.5902856	0.5341114	1	MIR143
Biological Process	GO:0106135	negative regulation of cardiac muscle cell contraction	1/2279	3/18866	0.3203981	0.5902856	0.5341114	1	BIN1
Biological Process	GO:0110030	regulation of G2/MI transition of meiotic cell cycle	1/2279	3/18866	0.3203981	0.5902856	0.5341114	1	CDC25A
Biological Process	GO:0110032	positive regulation of G2/MI transition of meiotic cell cycle	1/2279	3/18866	0.3203981	0.5902856	0.5341114	1	CDC25A
Biological Process	GO:0140157	ammonium import across plasma membrane	1/2279	3/18866	0.3203981	0.5902856	0.5341114	1	SLC12A7
Biological Process	GO:0140200	adenylate cyclase-activating adrenergic receptor signaling pathway involved in regulation of heart rate	1/2279	3/18866	0.3203981	0.5902856	0.5341114	1	PDE4D
Biological Process	GO:0140245	regulation of translation at postsynapse	1/2279	3/18866	0.3203981	0.5902856	0.5341114	1	CYFIP1
Biological Process	GO:0150007	clathrin-dependent synaptic vesicle endocytosis	1/2279	3/18866	0.3203981	0.5902856	0.5341114	1	ITSN1

Biological Process	GO:0150066	negative regulation of deacetylase activity	1/2279	3/18866	0.3203981	0.5902856	0.5341114	1	SPHK2
Biological Process	GO:1900063	regulation of peroxisome organization	1/2279	3/18866	0.3203981	0.5902856	0.5341114	1	DNM1L
Biological Process	GO:1900104	regulation of hyaluranon cable assembly	1/2279	3/18866	0.3203981	0.5902856	0.5341114	1	HAS3
Biological Process	GO:1900106	positive regulation of hyaluranon cable assembly	1/2279	3/18866	0.3203981	0.5902856	0.5341114	1	HAS3
Biological Process	GO:1900130	regulation of lipid binding	1/2279	3/18866	0.3203981	0.5902856	0.5341114	1	MIR27A
Biological Process	GO:1900131	negative regulation of lipid binding	1/2279	3/18866	0.3203981	0.5902856	0.5341114	1	MIR27A
Biological Process	GO:1900166	regulation of glial cell-derived neurotrophic factor secretion	1/2279	3/18866	0.3203981	0.5902856	0.5341114	1	SORL1
Biological Process	GO:1900168	positive regulation of glial cell-derived neurotrophic factor secretion	1/2279	3/18866	0.3203981	0.5902856	0.5341114	1	SORL1
Biological Process	GO:1900194	negative regulation of oocyte maturation	1/2279	3/18866	0.3203981	0.5902856	0.5341114	1	NPPC

Biological Process	GO:1900200	mesenchymal cell apoptotic process involved in metanephros development	1/2279	3/18866	0.3203981	0.5902856	0.5341114	1	PAX2
Biological Process	GO:1900211	regulation of mesenchymal cell apoptotic process involved in metanephros development	1/2279	3/18866	0.3203981	0.5902856	0.5341114	1	PAX2
Biological Process	GO:1900212	negative regulation of mesenchymal cell apoptotic process involved in metanephros development	1/2279	3/18866	0.3203981	0.5902856	0.5341114	1	PAX2
Biological Process	GO:1900369	negative regulation of RNA interference	1/2279	3/18866	0.3203981	0.5902856	0.5341114	1	RMRP
Biological Process	GO:1900620	acetate ester biosynthetic process	1/2279	3/18866	0.3203981	0.5902856	0.5341114	1	SLC44A4
Biological Process	GO:1901329	regulation of odontoblast differentiation	1/2279	3/18866	0.3203981	0.5902856	0.5341114	1	CEBPB
Biological Process	GO:1901355	response to rapamycin	1/2279	3/18866	0.3203981	0.5902856	0.5341114	1	LARP1
Biological Process	GO:1901376	organic heteropentacyclic compound metabolic process	1/2279	3/18866	0.3203981	0.5902856	0.5341114	1	NFE2L2
Biological Process	GO:1901401	regulation of tetrapyrrole metabolic process	1/2279	3/18866	0.3203981	0.5902856	0.5341114	1	TMEM14C

Biological Process	GO:1901407	regulation of phosphorylation of RNA polymerase II C-terminal domain	1/2279	3/18866	0.3203981	0.5902856	0.5341114	1	BRD4
Biological Process	GO:1901491	negative regulation of lymphangiogenesis	1/2279	3/18866	0.3203981	0.5902856	0.5341114	1	VASH1
Biological Process	GO:1901645	regulation of synoviocyte proliferation	1/2279	3/18866	0.3203981	0.5902856	0.5341114	1	TNF
Biological Process	GO:1901664	regulation of NAD+ ADP-ribosyltransferase activity	1/2279	3/18866	0.3203981	0.5902856	0.5341114	1	TGFB1
Biological Process	GO:1901666	positive regulation of NAD+ ADP-ribosyltransferase activity	1/2279	3/18866	0.3203981	0.5902856	0.5341114	1	TGFB1
Biological Process	GO:1901726	negative regulation of histone deacetylase activity	1/2279	3/18866	0.3203981	0.5902856	0.5341114	1	SPHK2
Biological Process	GO:1901837	negative regulation of transcription of nucleolar large rRNA by RNA polymerase I	1/2279	3/18866	0.3203981	0.5902856	0.5341114	1	MACROH2A2
Biological Process	GO:1901874	negative regulation of post-translational protein modification	1/2279	3/18866	0.3203981	0.5902856	0.5341114	1	P3H1
Biological Process	GO:1901994	negative regulation of meiotic cell cycle phase transition	1/2279	3/18866	0.3203981	0.5902856	0.5341114	1	OVOL1

Biological Process	GO:1902037	negative regulation of hematopoietic stem cell differentiation	1/2279	3/18866	0.3203981	0.5902856	0.5341114	1	NFE2L2
Biological Process	GO:1902162	regulation of DNA damage response, signal transduction by p53 class mediator resulting in transcription of p21 class mediator	1/2279	3/18866	0.3203981	0.5902856	0.5341114	1	ZNF385A
Biological Process	GO:1902164	positive regulation of DNA damage response, signal transduction by p53 class mediator resulting in transcription of p21 class mediator	1/2279	3/18866	0.3203981	0.5902856	0.5341114	1	ZNF385A
Biological Process	GO:1902174	positive regulation of keratinocyte apoptotic process	1/2279	3/18866	0.3203981	0.5902856	0.5341114	1	GSN
Biological Process	GO:1902263	apoptotic process involved in embryonic digit morphogenesis	1/2279	3/18866	0.3203981	0.5902856	0.5341114	1	NOTCH1
Biological Process	GO:1902309	negative regulation of peptidyl-serine dephosphorylation	1/2279	3/18866	0.3203981	0.5902856	0.5341114	1	PPP1R16B
Biological Process	GO:1902499	positive regulation of protein autoubiquitination	1/2279	3/18866	0.3203981	0.5902856	0.5341114	1	MTA1
Biological Process	GO:1902603	carnitine transmembrane transport	1/2279	3/18866	0.3203981	0.5902856	0.5341114	1	SLC22A5
Biological Process	GO:1902659	regulation of glucose mediated signaling pathway	1/2279	3/18866	0.3203981	0.5902856	0.5341114	1	PIH1D1

Biological Process	GO:1902661	positive regulation of glucose mediated signaling pathway	1/2279	3/18866	0.3203981	0.5902856	0.5341114	1	PIH1D1
Biological Process	GO:1902856	negative regulation of non-motile cilium assembly	1/2279	3/18866	0.3203981	0.5902856	0.5341114	1	DNM2
Biological Process	GO:1902896	terminal web assembly	1/2279	3/18866	0.3203981	0.5902856	0.5341114	1	EZR
Biological Process	GO:1902897	regulation of postsynaptic density protein 95 clustering	1/2279	3/18866	0.3203981	0.5902856	0.5341114	1	ZMYND8
Biological Process	GO:1902990	mitotic telomere maintenance via semi-conservative replication	1/2279	3/18866	0.3203981	0.5902856	0.5341114	1	RTEL1
Biological Process	GO:1903070	negative regulation of ER-associated ubiquitin-dependent protein catabolic process	1/2279	3/18866	0.3203981	0.5902856	0.5341114	1	SVIP
Biological Process	GO:1903225	negative regulation of endodermal cell differentiation	1/2279	3/18866	0.3203981	0.5902856	0.5341114	1	COL5A1
Biological Process	GO:1903375	facioacoustic ganglion development	1/2279	3/18866	0.3203981	0.5902856	0.5341114	1	NRP2
Biological Process	GO:1903422	negative regulation of synaptic vesicle recycling	1/2279	3/18866	0.3203981	0.5902856	0.5341114	1	CYFIP1

Biological Process	GO:1903445	protein transport from ciliary membrane to plasma membrane	1/2279	3/18866	0.3203981	0.5902856	0.5341114	1	RILPL1
Biological Process	GO:1903542	negative regulation of exosomal secretion	1/2279	3/18866	0.3203981	0.5902856	0.5341114	1	PRKN
Biological Process	GO:1903650	negative regulation of cytoplasmic transport	1/2279	3/18866	0.3203981	0.5902856	0.5341114	1	SNX3
Biological Process	GO:1903691	positive regulation of wound healing, spreading of epidermal cells	1/2279	3/18866	0.3203981	0.5902856	0.5341114	1	RREB1
Biological Process	GO:1903742	regulation of anterograde synaptic vesicle transport	1/2279	3/18866	0.3203981	0.5902856	0.5341114	1	CNIH2
Biological Process	GO:1903964	monounsaturated fatty acid metabolic process	1/2279	3/18866	0.3203981	0.5902856	0.5341114	1	SCD
Biological Process	GO:1904000	positive regulation of eating behavior	1/2279	3/18866	0.3203981	0.5902856	0.5341114	1	GHRL
Biological Process	GO:1904048	regulation of spontaneous neurotransmitter secretion	1/2279	3/18866	0.3203981	0.5902856	0.5341114	1	PRKN
Biological Process	GO:1904059	regulation of locomotor rhythm	1/2279	3/18866	0.3203981	0.5902856	0.5341114	1	ZFHX3

Biological Process	GO:1904098	regulation of protein O-linked glycosylation	1/2279	3/18866	0.3203981	0.5902856	0.5341114	1	POMT2
Biological Process	GO:1904100	positive regulation of protein O-linked glycosylation	1/2279	3/18866	0.3203981	0.5902856	0.5341114	1	POMT2
Biological Process	GO:1904158	axonemal central apparatus assembly	1/2279	3/18866	0.3203981	0.5902856	0.5341114	1	DNAJB13
Biological Process	GO:1904273	L-alanine import across plasma membrane	1/2279	3/18866	0.3203981	0.5902856	0.5341114	1	SLC7A8
Biological Process	GO:1904347	regulation of small intestine smooth muscle contraction	1/2279	3/18866	0.3203981	0.5902856	0.5341114	1	GHRL
Biological Process	GO:1904349	positive regulation of small intestine smooth muscle contraction	1/2279	3/18866	0.3203981	0.5902856	0.5341114	1	GHRL
Biological Process	GO:1904381	Golgi apparatus mannose trimming	1/2279	3/18866	0.3203981	0.5902856	0.5341114	1	MAN1C1
Biological Process	GO:1904430	negative regulation of t-circle formation	1/2279	3/18866	0.3203981	0.5902856	0.5341114	1	RTEL1
Biological Process	GO:1904533	regulation of telomeric loop disassembly	1/2279	3/18866	0.3203981	0.5902856	0.5341114	1	RTEL1

Biological Process	GO:1904594	regulation of termination of RNA polymerase II transcription	1/2279	3/18866	0.3203981	0.5902856	0.5341114	1	SCAF8
Biological Process	GO:1904761	negative regulation of myofibroblast differentiation	1/2279	3/18866	0.3203981	0.5902856	0.5341114	1	RB1
Biological Process	GO:1904878	negative regulation of calcium ion transmembrane transport via high voltage-gated calcium channel	1/2279	3/18866	0.3203981	0.5902856	0.5341114	1	BIN1
Biological Process	GO:1904891	positive regulation of excitatory synapse assembly	1/2279	3/18866	0.3203981	0.5902856	0.5341114	1	SEMA4A
Biological Process	GO:1905044	regulation of Schwann cell proliferation involved in axon regeneration	1/2279	3/18866	0.3203981	0.5902856	0.5341114	1	CERS2
Biological Process	GO:1905109	regulation of pulmonary blood vessel remodeling	1/2279	3/18866	0.3203981	0.5902856	0.5341114	1	MIR143
Biological Process	GO:1905111	positive regulation of pulmonary blood vessel remodeling	1/2279	3/18866	0.3203981	0.5902856	0.5341114	1	MIR143
Biological Process	GO:1905176	positive regulation of vascular associated smooth muscle cell dedifferentiation	1/2279	3/18866	0.3203981	0.5902856	0.5341114	1	PDGFB
Biological Process	GO:1905289	regulation of CAMKK-AMPK signaling cascade	1/2279	3/18866	0.3203981	0.5902856	0.5341114	1	HTT

Biological Process	GO:1905335	regulation of aggrephagy	1/2279	3/18866	0.3203981	0.5902856	0.5341114	1	HTT
Biological Process	GO:1905337	positive regulation of aggrephagy	1/2279	3/18866	0.3203981	0.5902856	0.5341114	1	HTT
Biological Process	GO:1905457	negative regulation of lymphoid progenitor cell differentiation	1/2279	3/18866	0.3203981	0.5902856	0.5341114	1	NOTCH1
Biological Process	GO:1905463	negative regulation of DNA duplex unwinding	1/2279	3/18866	0.3203981	0.5902856	0.5341114	1	MCM2
Biological Process	GO:1905653	positive regulation of artery morphogenesis	1/2279	3/18866	0.3203981	0.5902856	0.5341114	1	NOTCH1
Biological Process	GO:1905684	regulation of plasma membrane repair	1/2279	3/18866	0.3203981	0.5902856	0.5341114	1	MYH9
Biological Process	GO:1905764	regulation of protection from non-homologous end joining at telomere	1/2279	3/18866	0.3203981	0.5902856	0.5341114	1	ERCC1
Biological Process	GO:1905765	negative regulation of protection from non-homologous end joining at telomere	1/2279	3/18866	0.3203981	0.5902856	0.5341114	1	ERCC1
Biological Process	GO:1905784	regulation of anaphase-promoting complex-dependent catabolic process	1/2279	3/18866	0.3203981	0.5902856	0.5341114	1	FZR1

Biological Process	GO:1905786	positive regulation of anaphase-promoting complex-dependent catabolic process	1/2279	3/18866	0.3203981	0.5902856	0.5341114	1	FZR1
Biological Process	GO:1905917	positive regulation of cell differentiation involved in phenotypic switching	1/2279	3/18866	0.3203981	0.5902856	0.5341114	1	MIR140
Biological Process	GO:1905932	positive regulation of vascular associated smooth muscle cell differentiation involved in phenotypic switching	1/2279	3/18866	0.3203981	0.5902856	0.5341114	1	MIR140
Biological Process	GO:1990074	polyuridylation-dependent mRNA catabolic process	1/2279	3/18866	0.3203981	0.5902856	0.5341114	1	DIS3L2
Biological Process	GO:1990079	cartilage homeostasis	1/2279	3/18866	0.3203981	0.5902856	0.5341114	1	MTF1
Biological Process	GO:1990108	protein linear deubiquitination	1/2279	3/18866	0.3203981	0.5902856	0.5341114	1	CYLD
Biological Process	GO:1990166	protein localization to site of double-strand break	1/2279	3/18866	0.3203981	0.5902856	0.5341114	1	SLF1
Biological Process	GO:1990428	miRNA transport	1/2279	3/18866	0.3203981	0.5902856	0.5341114	1	TGFBR2
Biological Process	GO:1990770	small intestine smooth muscle contraction	1/2279	3/18866	0.3203981	0.5902856	0.5341114	1	GHRL

Biological Process	GO:1990774	tumor necrosis factor secretion	1/2279	3/18866	0.3203981	0.5902856	0.5341114	1	SPHK2
Biological Process	GO:2000078	positive regulation of type B pancreatic cell development	1/2279	3/18866	0.3203981	0.5902856	0.5341114	1	RFX3
Biological Process	GO:2000097	regulation of smooth muscle cell-matrix adhesion	1/2279	3/18866	0.3203981	0.5902856	0.5341114	1	PLAU
Biological Process	GO:2000111	positive regulation of macrophage apoptotic process	1/2279	3/18866	0.3203981	0.5902856	0.5341114	1	MEF2C
Biological Process	GO:2000120	positive regulation of sodium-dependent phosphate transport	1/2279	3/18866	0.3203981	0.5902856	0.5341114	1	CEBPB
Biological Process	GO:2000124	regulation of endocannabinoid signaling pathway	1/2279	3/18866	0.3203981	0.5902856	0.5341114	1	MGLL
Biological Process	GO:2000170	positive regulation of peptidyl-cysteine S-nitrosylation	1/2279	3/18866	0.3203981	0.5902856	0.5341114	1	NOS1AP
Biological Process	GO:2000297	negative regulation of synapse maturation	1/2279	3/18866	0.3203981	0.5902856	0.5341114	1	ARHGEF15
Biological Process	GO:2000307	regulation of tumor necrosis factor (ligand) superfamily member 11 production	1/2279	3/18866	0.3203981	0.5902856	0.5341114	1	LTF

Biological Process	GO:2000412	positive regulation of thymocyte migration	1/2279	3/18866	0.3203981	0.5902856	0.5341114	1	CCR2
Biological Process	GO:2000446	regulation of macrophage migration inhibitory factor signaling pathway	1/2279	3/18866	0.3203981	0.5902856	0.5341114	1	ANGPT1
Biological Process	GO:2000468	regulation of peroxidase activity	1/2279	3/18866	0.3203981	0.5902856	0.5341114	1	SIRT3
Biological Process	GO:2000619	negative regulation of histone H4-K16 acetylation	1/2279	3/18866	0.3203981	0.5902856	0.5341114	1	PIH1D1
Biological Process	GO:2000705	regulation of dense core granule biogenesis	1/2279	3/18866	0.3203981	0.5902856	0.5341114	1	PRKCA
Biological Process	GO:2000804	regulation of termination of RNA polymerase II transcription, poly(A)-coupled	1/2279	3/18866	0.3203981	0.5902856	0.5341114	1	SCAF8
Biological Process	GO:2000974	negative regulation of pro-B cell differentiation	1/2279	3/18866	0.3203981	0.5902856	0.5341114	1	NOTCH1
Biological Process	GO:2001038	regulation of cellular response to drug	1/2279	3/18866	0.3203981	0.5902856	0.5341114	1	NCOA1
Biological Process	GO:2001190	positive regulation of T cell activation via T cell receptor contact with antigen bound to MHC molecule on antigen presenting cell	1/2279	3/18866	0.3203981	0.5902856	0.5341114	1	HLA-DMB

Biological Process	GO:0002200	somatic diversification of immune receptors	11/2279	77/18866	0.3242519	0.5970381	0.5402213	11	IL10/UNG/TGFB1/PTPRC/TCF3/TP53BP1/CTNNBL1/ERCC1/TNFSF13/PAXIP1/NSD2
Biological Process	GO:0032413	negative regulation of ion transmembrane transporter activity	11/2279	77/18866	0.3242519	0.5970381	0.5402213	11	KCNE1/THADA/CASQ2/TLR9/CAB39/GSTO1/PRKCE/NEDD4/DYSF/HECW2/CBARP
Biological Process	GO:0070830	bicellular tight junction assembly	11/2279	77/18866	0.3242519	0.5970381	0.5402213	11	TBCD/RUNX1/TNF/ACTN4/WNT11/PRKCH/GRHL2/PRKCI/MPP7/MYO1C/NPHP4
Biological Process	GO:0046620	regulation of organ growth	16/2279	116/18866	0.3249051	0.598125	0.5412048	16	CTDP1/MIR199A1/VGLL4/TGFBR2/RIPK1/RBPJ/LATS2/FGFR1/POR/STK3/ACACB/MEIS1/MIR199A2/NOTCH1/MEF2C/HLX
Biological Process	GO:0008217	regulation of blood pressure	25/2279	187/18866	0.325127	0.5984176	0.5414695	25	CTSZ/PDE4D/CTSG/SLC2A5/PPARG/LNPEP/SMAD3/MIR199A1/SGK1/GJA5/DLL1/NISCH/KCNK6/HBB/GNB3/AGTRAP/PDGFB/MIR199A2/P2RX1/F2R/AGTR1/CACNA1B/NPY/HSD11B2/AOPEP
Biological Process	GO:1903018	regulation of glycoprotein metabolic process	8/2279	54/18866	0.325639	0.5992438	0.5422171	8	MIR101-2/TCF7L2/AGO2/POMT2/ITM2C/PLCB1/BCL2/PXYLP1
Biological Process	GO:0022612	gland morphogenesis	17/2279	124/18866	0.3268498	0.6013555	0.5441278	17	RXRA/RPS6KA1/LRP5L/VDR/TNF/TNFAIP3/CSF1R/TGFBR2/CUL3/FGFR1/TGFB1/RARG/BCL2/NOTCH1/CPB2/CEBPB/CRIP1
Biological Process	GO:0034502	protein localization to chromosome	12/2279	85/18866	0.3280015	0.6019997	0.5447107	12	SPIDR/TNKS/BRCA2/SETD2/CHAMP1/MIS12/RB1/PIH1D1/CDCA5/CCT6A/MACROH2A2/SLF1
Biological Process	GO:0043154	negative regulation of cysteine-type endopeptidase activity involved in apoptotic process	12/2279	85/18866	0.3280015	0.6019997	0.5447107	12	RPS6KA1/ARRB1/TNF/MICAL1/THBS1/PAX2/AQP1/POR/DNAJB6/NOL3/ARRB2/PIH1D1

Biological Process	GO:0043297	apical junction assembly	12/2279	85/18866	0.3280015	0.6019997	0.5447107	12	TBCD/RUNX1/TNF/ACTN4/WNT11/PRKCH/GRHL2/WDR1/PRKCI/MPP7/MYO1C/NPHP4
Biological Process	GO:0071214	cellular response to abiotic stimulus	43/2279	331/18866	0.3282155	0.6019997	0.5447107	43	HDAC4/BLM/PDE6A/SPIDR/MFAP4/CRB1/PIK3R1/MYLK/BMF/DNM2/GPLD1/HUS1/TNFRSF1A/LRRC8C/WNT11/CDC25A/AQP1/GNA11/CAB39/BCL10/PALM/CREBBP/MAP3K14/ENG/INSRR/TP53BP1/GRK7/RHNO1/CHEK2/ARHGEF2/CASP1/NEDD4/SLC2A1/DYSF/ERCC1/CRIP1/BCL2L1/ANKRD1/POLD3/XPA/FADD/EEF1D/MAP3K20
Biological Process	GO:0104004	cellular response to environmental stimulus	43/2279	331/18866	0.3282155	0.6019997	0.5447107	43	HDAC4/BLM/PDE6A/SPIDR/MFAP4/CRB1/PIK3R1/MYLK/BMF/DNM2/GPLD1/HUS1/TNFRSF1A/LRRC8C/WNT11/CDC25A/AQP1/GNA11/CAB39/BCL10/PALM/CREBBP/MAP3K14/ENG/INSRR/TP53BP1/GRK7/RHNO1/CHEK2/ARHGEF2/CASP1/NEDD4/SLC2A1/DYSF/ERCC1/CRIP1/BCL2L1/ANKRD1/POLD3/XPA/FADD/EEF1D/MAP3K20
Biological Process	GO:0003181	atrioventricular valve morphogenesis	4/2279	24/18866	0.3283404	0.6019997	0.5447107	4	ZFPM1/TGFBR2/NOTCH1/SLIT3
Biological Process	GO:0003272	endocardial cushion formation	4/2279	24/18866	0.3283404	0.6019997	0.5447107	4	TGFBR2/RBPJ/ENG/NOTCH1
Biological Process	GO:0032925	regulation of activin receptor signaling pathway	4/2279	24/18866	0.3283404	0.6019997	0.5447107	4	SKI/SMAD7/ZC3H3/NODAL
Biological Process	GO:0036003	positive regulation of transcription from RNA polymerase II promoter in response to stress	4/2279	24/18866	0.3283404	0.6019997	0.5447107	4	RBPJ/NFE2L2/NOTCH1/CEBPB
Biological Process	GO:0044550	secondary metabolite biosynthetic process	4/2279	24/18866	0.3283404	0.6019997	0.5447107	4	ZEB2/CDH3/RAPGEF2/MYO5A
Biological Process	GO:0045954	positive regulation of natural killer cell mediated cytotoxicity	4/2279	24/18866	0.3283404	0.6019997	0.5447107	4	CD226/HLA-F/IL18RAP/CADM1

Biological Process	GO:0046628	positive regulation of insulin receptor signaling pathway	4/2279	24/18866	0.3283404	0.6019997	0.5447107	4	PRKCZ/SORL1/GNAI2/ZBTB7B
Biological Process	GO:0051905	establishment of pigment granule localization	4/2279	24/18866	0.3283404	0.6019997	0.5447107	4	RAB27A/CDH3/DCTN2/MYO5A
Biological Process	GO:0086064	cell communication by electrical coupling involved in cardiac conduction	4/2279	24/18866	0.3283404	0.6019997	0.5447107	4	PDE4D/CACNA1C/SLC8A1/GJA5
Biological Process	GO:0090169	regulation of spindle assembly	4/2279	24/18866	0.3283404	0.6019997	0.5447107	4	RCC1/DYNC1H1/NUMA1/SPAG5
Biological Process	GO:0090335	regulation of brown fat cell differentiation	4/2279	24/18866	0.3283404	0.6019997	0.5447107	4	RREB1/ZBTB7B/FTO/PRDM16
Biological Process	GO:0098581	detection of external biotic stimulus	4/2279	24/18866	0.3283404	0.6019997	0.5447107	4	NLRC4/PGLYRP1/NOD2/PGLYRP4
Biological Process	GO:1903429	regulation of cell maturation	4/2279	24/18866	0.3283404	0.6019997	0.5447107	4	BCL11A/PPP2R1A/BCL2/NPPC
Biological Process	GO:0001101	response to acid chemical	18/2279	132/18866	0.3285195	0.6022118	0.5449026	18	RPTOR/CD9/CREB1/TNF/BCL11A/BAIAP2/PDGFD/SLC1A2/CPEB3/SH3BP4/NTRK1/DNMT1/PDGFC/CEBPB/BCL2L1/RELA/DHODH/SST
Biological Process	GO:0001662	behavioral fear response	6/2279	39/18866	0.3298295	0.6037977	0.5463376	6	ASIC4/MAPK8IP2/BCL2/MEF2C/MORC1/EIF4G1

Biological Process	GO:0006778	porphyrin-containing compound metabolic process	6/2279	39/18866	0.3298295	0.6037977	0.5463376	6	SLC11A2/FECH/ALAS1/SPTA1/PPOX/TMEM14C
Biological Process	GO:0010453	regulation of cell fate commitment	6/2279	39/18866	0.3298295	0.6037977	0.5463376	6	LOXL3/RBPJ/WNT11/FGFR1/PAX7/LMO4
Biological Process	GO:0010831	positive regulation of myotube differentiation	6/2279	39/18866	0.3298295	0.6037977	0.5463376	6	EHD1/MAML1/NFATC2/BCL2/FLOT1/SCGB3A1
Biological Process	GO:0038179	neurotrophin signaling pathway	6/2279	39/18866	0.3298295	0.6037977	0.5463376	6	CYFIP2/RAPGEF2/SORT1/NTRK1/CYFIP1/NTF4
Biological Process	GO:0055069	zinc ion homeostasis	6/2279	39/18866	0.3298295	0.6037977	0.5463376	6	S100A8/LCK/SLC39A13/AP3D1/ATP7B/PRKN
Biological Process	GO:0072529	pyrimidine-containing compound catabolic process	6/2279	39/18866	0.3298295	0.6037977	0.5463376	6	CDA/DPYS/UNG/NTHL1/UPB1/TET2
Biological Process	GO:0060048	cardiac muscle contraction	19/2279	140/18866	0.3299552	0.6039114	0.5464405	19	HDAC4/PDE4D/KCNE1/TTN/CASQ2/MAP2K6/CACNA1C/SLC8A1/GJA5/JUP/BIN1/NOS1AP/SMAD7/SCN1A/SNTA1/KCNQ1/GSTO1/ANK2/GRK2
Biological Process	GO:0051702	interaction with symbiont	13/2279	93/18866	0.3311051	0.605666	0.5480281	13	AZU1/ELANE/CTSG/CTDP1/CCL5/CSF1R/SFTPD/HDAC1/PC/PHB/AQP1/LTF/PRKN
Biological Process	GO:0061097	regulation of protein tyrosine kinase activity	13/2279	93/18866	0.3311051	0.605666	0.5480281	13	TGFA/SH3BP5L/CCL5/CSF1R/NCK2/SH3BP5/DUSP22/APP/GHRL/PTPRC/PDGFB/CASS4/GGNBP2

Biological Process	GO:0072080	nephron tubule development	13/2279	93/18866	0.3311051	0.605666	0.5480281	13	TACSTD2/DLL1/ILK/WNT11/PAX2/MTSS1/WWTR1/BCL2/NOTCH1/MEF2C/ACAT1/LIF/HOXB7
Biological Process	GO:0007405	neuroblast proliferation	9/2279	62/18866	0.3315896	0.6058524	0.5481968	9	NDE1/DISC1/FGFR1/NF1/NUMB/ZNF335/NOTCH1/ARHGEF2/CX3CR1
Biological Process	GO:0045824	negative regulation of innate immune response	9/2279	62/18866	0.3315896	0.6058524	0.5481968	9	PPARG/TNFAIP3/HLA-F/NR1H2/GRN/NLRC5/ARRB2/TYRO3/CD96
Biological Process	GO:0048635	negative regulation of muscle organ development	9/2279	62/18866	0.3315896	0.6058524	0.5481968	9	CTDP1/MIR199A1/VGLL4/TGFBR2/DLL1/RIPK1/TGFB1/MEIS1/MIR199A2
Biological Process	GO:0055025	positive regulation of cardiac muscle tissue development	9/2279	62/18866	0.3315896	0.6058524	0.5481968	9	CREB1/MIR199A1/RBPJ/FGFR1/TGFB1/ARRB2/MIR199A2/NOTCH1/MEF2C
Biological Process	GO:0060393	regulation of pathway-restricted SMAD protein phosphorylation	9/2279	62/18866	0.3315896	0.6058524	0.5481968	9	LDLRAD4/SMAD7/TGFB1/BMPER/ENG/RBPMS/GDF6/INHBA/NODAL
Biological Process	GO:1904589	regulation of protein import	9/2279	62/18866	0.3315896	0.6058524	0.5481968	9	ANGPT1/PIK3R1/SMAD3/SUFU/JUP/TGFB1/NF1/CDH1/CABP1
Biological Process	GO:0000187	activation of MAPK activity	21/2279	156/18866	0.3322517	0.6068288	0.5490803	21	TGFA/MAP2K6/ERN1/ARRB1/TNF/FPR1/THBS1/RIPK1/UBE2V1/MUC20/CXCL17/GHRL/PTPRC/PDE6H/IRAK2/NOD2/S1PR2/MAPKAPK3/DBNL/PRKAA1/MAP3K20
Biological Process	GO:1901861	regulation of muscle tissue development	21/2279	156/18866	0.3322517	0.6068288	0.5490803	21	HDAC4/CTDP1/CREB1/SMAD3/MIR199A1/VGLL4/TGFBR2/DLL1/RIPK1/RBPJ/FGFR1/TGFB1/ARRB2/ARNTL/MEIS1/BCL2/MIR199A2/NOTCH1/MEF2C/FLOT1/PRKAA1

Biological Process	GO:0018393	internal peptidyl-lysine acetylation	22/2279	164/18866	0.3331624	0.6083752	0.5504794	22	SRCAP/BRCA2/KANSL1/ARRB1/BRD1/EPC1/RPS6KA4/TAF10/KANSL2/SPHK2/CTBP1/POR/NCOA1/CREBBP/RSF1/PER1/PIH1D1/RUVBL1/KAT7/ING5/LIF/PAXIP1
Biological Process	GO:0018394	peptidyl-lysine acetylation	23/2279	172/18866	0.333941	0.6096798	0.55166	23	SRCAP/BRCA2/KANSL1/ARRB1/BRD1/EPC1/RPS6KA4/TAF10/KANSL2/SPHK2/CTBP1/POR/NCOA1/CREBBP/RSF1/PER1/PIH1D1/RUVBL1/KAT7/ING5/LIF/PAXIP1/PRKAA1
Biological Process	GO:0042742	defense response to bacterium	45/2279	348/18866	0.3352567	0.6113644	0.5531842	45	DEFA4/AZU1/ELANE/CEBPE/SLPI/NLRC4/S100A8/CTSG/IRF8/LYPD8/MPO/FOXP1/IL10/LCN2/MIR140/RNASE3/LYST/SLC11A1/NLRP3/LPO/FGR/TNF/SFTPD/FPR2/TLR9/IGHV6-1/LYG2/SPON2/RBPJ/COLEC12/TNFRSF1A/DEFB124/STAB2/DEFB1/PGLYRP1/CCL20/IL6R/C10orf99/GRN/MPEG1/NOD2/LTF/CEBPB/PGLYRP4/BCL3
Biological Process	GO:0072676	lymphocyte migration	16/2279	117/18866	0.3376936	0.6113644	0.5531842	16	FUT7/PIK3CD/CCR2/CCL5/PADI2/APP/MIA3/DOCK8/WASL/RIPK3/CCL20/C10orf99/ADTRP/CCL27/CCL22/FADD
Biological Process	GO:0010569	regulation of double-strand break repair via homologous recombination	7/2279	47/18866	0.3383212	0.6113644	0.5531842	7	SPIDR/RMI2/SETD2/RTEL1/RECQL5/TP53BP1/FBH1
Biological Process	GO:0014003	oligodendrocyte development	7/2279	47/18866	0.3383212	0.6113644	0.5531842	7	CD9/GSN/EIF2B5/CLU/FA2H/CNTNAP1/ERCC2
Biological Process	GO:0014075	response to amine	7/2279	47/18866	0.3383212	0.6113644	0.5531842	7	PPP1R1B/RRM2B/ADAMTS13/RGS17/CNR2/RGS10/HNMT
Biological Process	GO:0032480	negative regulation of type I interferon production	7/2279	47/18866	0.3383212	0.6113644	0.5531842	7	IL10/TNFAIP3/TRAIP/NLRC5/CYLD/HERC5/ILRUN
Biological Process	GO:0045933	positive regulation of muscle contraction	7/2279	47/18866	0.3383212	0.6113644	0.5531842	7	ENO1/CTTN/NOS1AP/GHRL/KCNQ1/GSTO1/F2R

Biological Process	GO:0046677	response to antibiotic	7/2279	47/18866	0.3383212	0.6113644	0.5531842	7	ALPL/JAK1/ZC3H8/SLC1A3/MEF2C/CRIP1/KAT7
Biological Process	GO:0051150	regulation of smooth muscle cell differentiation	7/2279	47/18866	0.3383212	0.6113644	0.5531842	7	MIR140/MIR145/NFATC2/NFATC1/PDGFB/ENG/DNMT1
Biological Process	GO:0060986	endocrine hormone secretion	7/2279	47/18866	0.3383212	0.6113644	0.5531842	7	C1QTNF3/RAB11FIP1/FGFR1/CRHBP/GHRL/AGTR1/INHBA
Biological Process	GO:1903115	regulation of actin filament-based movement	7/2279	47/18866	0.3383212	0.6113644	0.5531842	7	PDE4D/CACNA1C/GJA5/JUP/BIN1/NOS1AP/ANK2
Biological Process	GO:0000076	DNA replication checkpoint	3/2279	17/18866	0.3384164	0.6113644	0.5531842	3	HUS1/CLSPN/NAE1
Biological Process	GO:0002643	regulation of tolerance induction	3/2279	17/18866	0.3384164	0.6113644	0.5531842	3	PHLPP1/TGFBR2/MARCHF7
Biological Process	GO:0002716	negative regulation of natural killer cell mediated immunity	3/2279	17/18866	0.3384164	0.6113644	0.5531842	3	HLA-F/ARRB2/CD96
Biological Process	GO:0003128	heart field specification	3/2279	17/18866	0.3384164	0.6113644	0.5531842	3	RBPJ/WNT11/MEF2C
Biological Process	GO:0003184	pulmonary valve morphogenesis	3/2279	17/18866	0.3384164	0.6113644	0.5531842	3	GJA5/NFATC1/NOTCH1

Biological Process	GO:0006577	amino-acid betaine metabolic process	3/2279	17/18866	0.3384164	0.6113644	0.5531842	3	ACADM/POR/CPT1A
Biological Process	GO:0009048	dosage compensation by inactivation of X chromosome	3/2279	17/18866	0.3384164	0.6113644	0.5531842	3	CDYL/PCGF3/RBM15B
Biological Process	GO:0010819	regulation of T cell chemotaxis	3/2279	17/18866	0.3384164	0.6113644	0.5531842	3	CCR2/CCL5/CCL27
Biological Process	GO:0015732	prostaglandin transport	3/2279	17/18866	0.3384164	0.6113644	0.5531842	3	MAP2K6/ABCC2/SLCO3A1
Biological Process	GO:0016246	RNA interference	3/2279	17/18866	0.3384164	0.6113644	0.5531842	3	AGO2/RMRP/CELF1
Biological Process	GO:0016254	preassembly of GPI anchor in ER membrane	3/2279	17/18866	0.3384164	0.6113644	0.5531842	3	PIGL/PIGN/PIGQ
Biological Process	GO:0021783	preganglionic parasympathetic fiber development	3/2279	17/18866	0.3384164	0.6113644	0.5531842	3	NRP2/PHOX2A/HES3
Biological Process	GO:0030949	positive regulation of vascular endothelial growth factor receptor signaling pathway	3/2279	17/18866	0.3384164	0.6113644	0.5531842	3	GRB10/PRKCB/ITGB3
Biological Process	GO:0032682	negative regulation of chemokine production	3/2279	17/18866	0.3384164	0.6113644	0.5531842	3	IL10/C1QTNF3/MEFV

Biological Process	GO:0033599	regulation of mammary gland epithelial cell proliferation	3/2279	17/18866	0.3384164	0.6113644	0.5531842	3	RREB1/BRCA2/RTN4
Biological Process	GO:0035020	regulation of Rac protein signal transduction	3/2279	17/18866	0.3384164	0.6113644	0.5531842	3	DNM2/NF1/ARHGAP17
Biological Process	GO:0035729	cellular response to hepatocyte growth factor stimulus	3/2279	17/18866	0.3384164	0.6113644	0.5531842	3	IL10/CREB1/RELA
Biological Process	GO:0042753	positive regulation of circadian rhythm	3/2279	17/18866	0.3384164	0.6113644	0.5531842	3	GHRL/ARNTL/FBXW11
Biological Process	GO:0042790	nucleolar large rRNA transcription by RNA polymerase I	3/2279	17/18866	0.3384164	0.6113644	0.5531842	3	PIH1D1/MARS1/MACROH2A2
Biological Process	GO:0043117	positive regulation of vascular permeability	3/2279	17/18866	0.3384164	0.6113644	0.5531842	3	TJP2/ANGPT1/TGFB1
Biological Process	GO:0043968	histone H2A acetylation	3/2279	17/18866	0.3384164	0.6113644	0.5531842	3	EPC1/SPHK2/RUVBL1
Biological Process	GO:0044539	long-chain fatty acid import into cell	3/2279	17/18866	0.3384164	0.6113644	0.5531842	3	ACSL1/THBS1/SLC2A1
Biological Process	GO:0044764	multi-organism cellular process	3/2279	17/18866	0.3384164	0.6113644	0.5531842	3	TPCN2/PI4KA/EPS15

Biological Process	GO:0046459	short-chain fatty acid metabolic process	3/2279	17/18866	0.3384164	0.6113644	0.5531842	3	ACSS1/ACSS2/PCK2
Biological Process	GO:0048532	anatomical structure arrangement	3/2279	17/18866	0.3384164	0.6113644	0.5531842	3	NRP2/DLL1/PAX2
Biological Process	GO:0048535	lymph node development	3/2279	17/18866	0.3384164	0.6113644	0.5531842	3	TGFB1/RIPK3/FADD
Biological Process	GO:0060263	regulation of respiratory burst	3/2279	17/18866	0.3384164	0.6113644	0.5531842	3	CAMK1D/GRN/BCR
Biological Process	GO:0070166	enamel mineralization	3/2279	17/18866	0.3384164	0.6113644	0.5531842	3	FOXO1/FAM20C/NECTIN1
Biological Process	GO:0071360	cellular response to exogenous dsRNA	3/2279	17/18866	0.3384164	0.6113644	0.5531842	3	COLEC12/FLOT1/STING1
Biological Process	GO:0090336	positive regulation of brown fat cell differentiation	3/2279	17/18866	0.3384164	0.6113644	0.5531842	3	RREB1/ZBTB7B/PRDM16
Biological Process	GO:0099558	maintenance of synapse structure	3/2279	17/18866	0.3384164	0.6113644	0.5531842	3	DCTN1/SYNGAP1/CTBP2
Biological Process	GO:0140354	lipid import into cell	3/2279	17/18866	0.3384164	0.6113644	0.5531842	3	ACSL1/THBS1/SLC2A1

Biological Process	GO:1900363	regulation of mRNA polyadenylation	3/2279	17/18866	0.3384164	0.6113644	0.5531842	3	CPEB3/ZC3H3/RNF40
Biological Process	GO:1902992	negative regulation of amyloid precursor protein catabolic process	3/2279	17/18866	0.3384164	0.6113644	0.5531842	3	SORL1/BIN1/CLU
Biological Process	GO:2001267	regulation of cysteine-type endopeptidase activity involved in apoptotic signaling pathway	3/2279	17/18866	0.3384164	0.6113644	0.5531842	3	GSN/SMAD3/PIH1D1
Biological Process	GO:0007368	determination of left/right symmetry	17/2279	125/18866	0.3392365	0.6113644	0.5531842	17	SMAD3/PKD1L1/SUFU/MICAL2/TGFBR2/RFX3/DLL1/MIB1/PCSK6/AP1B1/IFT140/ENG/NOTCH1/MEF2C/TMED2/DNAI2/NODAL
Biological Process	GO:0043500	muscle adaptation	17/2279	125/18866	0.3392365	0.6113644	0.5531842	17	HDAC4/CTDP1/GSN/MIR145/TNFRSF1B/SMAD3/PRKCA/MIR199A1/TNFRSF1A/CAMTA2/PDE9A/MIR199A2/NOTCH1/MEF2C/FOXO1/INPP5F/CASQ1
Biological Process	GO:0000271	polysaccharide biosynthetic process	11/2279	78/18866	0.3400123	0.6113644	0.5531842	11	CSGALNACT1/AP2A1/NDST1/TGFB1/DYRK2/PDGFB/PPP1CB/ESRRB/PER2/HAS3/B3GNT2
Biological Process	GO:0043900	regulation of multi-organism process	11/2279	78/18866	0.3400123	0.6113644	0.5531842	11	HDAC4/CALR/ARHGDIB/RNASE10/AGO2/LHFPL2/PPP2R1A/PLCB1/NPPC/INHBA/NODAL
Biological Process	GO:0050688	regulation of defense response to virus	11/2279	78/18866	0.3400123	0.6113644	0.5531842	11	IL27/AIM2/TRIM38/TNFAIP3/SEC14L1/ZDHHC1/TRAF3IP2/HERC5/PARP9/STING1/ILRUN
Biological Process	GO:0051057	positive regulation of small GTPase mediated signal transduction	11/2279	78/18866	0.3400123	0.6113644	0.5531842	11	AKAP13/ITPKB/ARRB1/LRRC59/SYNPO2L/NTRK1/NOTCH1/F2R/GPR4/PDGFRB/PRAG1

Biological Process	GO:0061045	negative regulation of wound healing	11/2279	78/18866	0.3400123	0.6113644	0.5531842	11	PLAU/CD9/SMAD3/TNF/UBASH3B/SERPINB2/THBS1/F12/PDGFB/CPB2/ADTRP
Biological Process	GO:1903322	positive regulation of protein modification by small protein conjugation or removal	19/2279	141/18866	0.3416384	0.6113644	0.5531842	19	HDAC4/FZR1/TOLLIP/ANGPT1/ARRB1/CUL3/SMAD7/AXIN1/MTA1/TNIP1/BCL10/ARRB2/NOD2/UBE3A/RNF40/PELI1/PAXIP1/PRKN/MARCHF7
Biological Process	GO:0051169	nuclear transport	46/2279	357/18866	0.3423176	0.6113644	0.5531842	46	CALR/NXF1/FIP1L1/AKAP13/ANGPT1/CPSF3/SNRPD3/PIK3R1/SETD2/RAE1/SMAD3/TNPO1/MBTPS1/E2F3/SUFU/IPO13/ATXN1/FAM53B/JUP/XPO7/TCF7L2/SMG7/XPO6/TGFB1/PABPN1/ENY2/SMURF1/BACH2/NF1/NUP93/SEC13/SMG6/ARNTL/CDH1/SNUPN/CABP1/SP100/NEDD4/TSC2/HHEX/RBM15B/NOL6/CPSF4/BANF1/PCID2/GEMIN4
Biological Process	GO:0006482	protein demethylation	5/2279	32/18866	0.342855	0.6113644	0.5531842	5	KDM4B/KDM2A/KDM1B/KDM4C/KDM7A
Biological Process	GO:0006858	extracellular transport	5/2279	32/18866	0.342855	0.6113644	0.5531842	5	WWP2/RFX3/STK36/TTLL1/KATNIP
Biological Process	GO:0008214	protein dealkylation	5/2279	32/18866	0.342855	0.6113644	0.5531842	5	KDM4B/KDM2A/KDM1B/KDM4C/KDM7A
Biological Process	GO:0010165	response to X-ray	5/2279	32/18866	0.342855	0.6113644	0.5531842	5	BLM/BRCA2/DNM2/TP53BP1/ERCC1
Biological Process	GO:0032350	regulation of hormone metabolic process	5/2279	32/18866	0.342855	0.6113644	0.5531842	5	TCF7L2/RDH10/DGKQ/POR/GNB3
Biological Process	GO:0034367	protein-containing complex remodeling	5/2279	32/18866	0.342855	0.6113644	0.5531842	5	MPO/LIPC/ABCG1/PLTP/AGTR1

Biological Process	GO:0035456	response to interferon-beta	5/2279	32/18866	0.342855	0.6113644	0.5531842	5	AIM2/MNDA/DDX41/STING1/SHFL
Biological Process	GO:0042168	heme metabolic process	5/2279	32/18866	0.342855	0.6113644	0.5531842	5	SLC11A2/FECH/ALAS1/PPOX/TMEM14C
Biological Process	GO:0045589	regulation of regulatory T cell differentiation	5/2279	32/18866	0.342855	0.6113644	0.5531842	5	RUNX1/NFATC2/FANCA/LILRB4/VSIR
Biological Process	GO:0046475	glycerophospholipid catabolic process	5/2279	32/18866	0.342855	0.6113644	0.5531842	5	PLA2G15/LIPC/ABHD16A/ABHD16B/INPP5F
Biological Process	GO:0060317	cardiac epithelial to mesenchymal transition	5/2279	32/18866	0.342855	0.6113644	0.5531842	5	RTN4/TGFBR2/RBPJ/ENG/NOTCH1
Biological Process	GO:0071875	adrenergic receptor signaling pathway	5/2279	32/18866	0.342855	0.6113644	0.5531842	5	PDE4D/AKAP13/ADCY9/GNAI2/RAPGEF2
Biological Process	GO:0086019	cell-cell signaling involved in cardiac conduction	5/2279	32/18866	0.342855	0.6113644	0.5531842	5	CASQ2/CACNA1C/GJA5/KCNQ1/ANK2
Biological Process	GO:0097106	postsynaptic density organization	5/2279	32/18866	0.342855	0.6113644	0.5531842	5	ZMYND8/SYNGAP1/NRXN1/NRXN2/SHANK2
Biological Process	GO:0098810	neurotransmitter reuptake	5/2279	32/18866	0.342855	0.6113644	0.5531842	5	SLC1A2/ITGB3/PER2/SLC29A1/PRKN

Biological Process	GO:1902547	regulation of cellular response to vascular endothelial growth factor stimulus	5/2279	32/18866	0.342855	0.6113644	0.5531842	5	MIR199A1/DLL1/MIR199A2/MYO1C/ADGRA2
Biological Process	GO:1903959	regulation of anion transmembrane transport	5/2279	32/18866	0.342855	0.6113644	0.5531842	5	THBS1/RIPK1/SLC43A2/PER2/SLC43A1
Biological Process	GO:0034637	cellular carbohydrate biosynthetic process	12/2279	86/18866	0.3430407	0.6113644	0.5531842	12	CSGALNACT1/PGP/NDST1/DYRK2/PPP1CB/IMPA2/ESRRB/PER2/PCK2/SLC2A1/HAS3/B3GNT2
Biological Process	GO:0048634	regulation of muscle organ development	21/2279	157/18866	0.343337	0.6113644	0.5531842	21	HDAC4/CTDP1/CREB1/SMAD3/MIR199A1/VGLL4/TGFBR2/DLL1/RIPK1/RBPJ/FGFR1/TGFB1/ARRB2/ARNTL/MEIS1/BCL2/MIR199A2/NOTCH1/MEF2C/FLOT1/PRKAA1
Biological Process	GO:0098739	import across plasma membrane	21/2279	157/18866	0.343337	0.6113644	0.5531842	21	TRPM2/KCNJ15/SLC2A5/FXYD2/SLC1A5/SLC8A1/THBS1/SLC43A2/SLC19A1/SLC1A2/SLC7A5/SLC1A6/PER2/SLC1A3/SLC43A1/SLC2A1/SLC7A8/TRPV2/SLC12A1/SLC7A1/SLC12A7
Biological Process	GO:0035065	regulation of histone acetylation	8/2279	55/18866	0.3445806	0.6113644	0.5531842	8	ARRB1/RPS6KA4/SPHK2/CTBP1/PIH1D1/KAT7/LIF/PAXIP1
Biological Process	GO:0046164	alcohol catabolic process	8/2279	55/18866	0.3445806	0.6113644	0.5531842	8	SYNJ2/INPP5A/ALDH2/AKR1D1/NUDT3/SULT1A2/INPP5E/IMPA2
Biological Process	GO:0000338	protein deneddylation	2/2279	10/18866	0.3448376	0.6113644	0.5531842	2	COPS5/COPS3
Biological Process	GO:0000447	endonucleolytic cleavage in ITS1 to separate SSU-rRNA from 5.8S rRNA and LSU-rRNA from tricistronic rRNA transcript (SSU-rRNA, 5.8S rRNA, LSU-rRNA)	2/2279	10/18866	0.3448376	0.6113644	0.5531842	2	RCL1/NOP14

Biological Process	GO:0002664	regulation of T cell tolerance induction	2/2279	10/18866	0.3448376	0.6113644	0.5531842	2	PHLPP1/TGFBR2
Biological Process	GO:0003149	membranous septum morphogenesis	2/2279	10/18866	0.3448376	0.6113644	0.5531842	2	TGFBR2/NSD2
Biological Process	GO:0003183	mitral valve morphogenesis	2/2279	10/18866	0.3448376	0.6113644	0.5531842	2	ZFPM1/NOTCH1
Biological Process	GO:0003211	cardiac ventricle formation	2/2279	10/18866	0.3448376	0.6113644	0.5531842	2	NOTCH1/MEF2C
Biological Process	GO:0003307	regulation of Wnt signaling pathway involved in heart development	2/2279	10/18866	0.3448376	0.6113644	0.5531842	2	RBPJ/WNT11
Biological Process	GO:0006222	UMP biosynthetic process	2/2279	10/18866	0.3448376	0.6113644	0.5531842	2	UCK2/DHODH
Biological Process	GO:0006655	phosphatidylglycerol biosynthetic process	2/2279	10/18866	0.3448376	0.6113644	0.5531842	2	PGS1/CDS2
Biological Process	GO:0006971	hypotonic response	2/2279	10/18866	0.3448376	0.6113644	0.5531842	2	MYLK/CAB39
Biological Process	GO:0007440	foregut morphogenesis	2/2279	10/18866	0.3448376	0.6113644	0.5531842	2	SMAD3/NOTCH1

Biological Process	GO:0009173	pyrimidine ribonucleoside monophosphate metabolic process	2/2279	10/18866	0.3448376	0.6113644	0.5531842	2	UCK2/DHODH
Biological Process	GO:0009174	pyrimidine ribonucleoside monophosphate biosynthetic process	2/2279	10/18866	0.3448376	0.6113644	0.5531842	2	UCK2/DHODH
Biological Process	GO:0010587	miRNA catabolic process	2/2279	10/18866	0.3448376	0.6113644	0.5531842	2	DIS3L2/SND1
Biological Process	GO:0010615	positive regulation of cardiac muscle adaptation	2/2279	10/18866	0.3448376	0.6113644	0.5531842	2	MIR199A1/MIR199A2
Biological Process	GO:0010749	regulation of nitric oxide mediated signal transduction	2/2279	10/18866	0.3448376	0.6113644	0.5531842	2	THBS1/NOS1AP
Biological Process	GO:0014041	regulation of neuron maturation	2/2279	10/18866	0.3448376	0.6113644	0.5531842	2	BCL11A/BCL2
Biological Process	GO:0014877	response to muscle inactivity involved in regulation of muscle adaptation	2/2279	10/18866	0.3448376	0.6113644	0.5531842	2	HDAC4/CASQ1
Biological Process	GO:0014894	response to denervation involved in regulation of muscle adaptation	2/2279	10/18866	0.3448376	0.6113644	0.5531842	2	HDAC4/CASQ1
Biological Process	GO:0015696	ammonium transport	2/2279	10/18866	0.3448376	0.6113644	0.5531842	2	AQP1/SLC12A7

Biological Process	GO:0015808	L-alanine transport	2/2279	10/18866	0.3448376	0.6113644	0.5531842	2	SLC36A3/SLC7A8
Biological Process	GO:0015851	nucleobase transport	2/2279	10/18866	0.3448376	0.6113644	0.5531842	2	SLC23A1/AQP9
Biological Process	GO:0021548	pons development	2/2279	10/18866	0.3448376	0.6113644	0.5531842	2	PHOX2A/BCL2
Biological Process	GO:0031125	rRNA 3'-end processing	2/2279	10/18866	0.3448376	0.6113644	0.5531842	2	EXOSC2/ERI3
Biological Process	GO:0031848	protection from non-homologous end joining at telomere	2/2279	10/18866	0.3448376	0.6113644	0.5531842	2	ERCC1/TFIP11
Biological Process	GO:0034085	establishment of sister chromatid cohesion	2/2279	10/18866	0.3448376	0.6113644	0.5531842	2	RB1/CDCA5
Biological Process	GO:0034427	nuclear-transcribed mRNA catabolic process, exonucleolytic, 3'-5'	2/2279	10/18866	0.3448376	0.6113644	0.5531842	2	EXOSC2/DIS3L2
Biological Process	GO:0035524	proline transmembrane transport	2/2279	10/18866	0.3448376	0.6113644	0.5531842	2	SLC36A3/SLC7A8
Biological Process	GO:0035907	dorsal aorta development	2/2279	10/18866	0.3448376	0.6113644	0.5531842	2	RBPJ/ENG

Biological Process	GO:0036490	regulation of translation in response to endoplasmic reticulum stress	2/2279	10/18866	0.3448376	0.6113644	0.5531842	2	NCK2/EIF4G1
Biological Process	GO:0036500	ATF6-mediated unfolded protein response	2/2279	10/18866	0.3448376	0.6113644	0.5531842	2	CALR/MBTPS1
Biological Process	GO:0042541	hemoglobin biosynthetic process	2/2279	10/18866	0.3448376	0.6113644	0.5531842	2	ALAS1/INHBA
Biological Process	GO:0045348	positive regulation of MHC class II biosynthetic process	2/2279	10/18866	0.3448376	0.6113644	0.5531842	2	AZU1/IL10
Biological Process	GO:0045625	regulation of T-helper 1 cell differentiation	2/2279	10/18866	0.3448376	0.6113644	0.5531842	2	IL27/HLX
Biological Process	GO:0045654	positive regulation of megakaryocyte differentiation	2/2279	10/18866	0.3448376	0.6113644	0.5531842	2	TESC/MTURN
Biological Process	GO:0045713	low-density lipoprotein particle receptor biosynthetic process	2/2279	10/18866	0.3448376	0.6113644	0.5531842	2	PPARG/ITGB3
Biological Process	GO:0045876	positive regulation of sister chromatid cohesion	2/2279	10/18866	0.3448376	0.6113644	0.5531842	2	NSMCE2/SLF1
Biological Process	GO:0045988	negative regulation of striated muscle contraction	2/2279	10/18866	0.3448376	0.6113644	0.5531842	2	BIN1/GRK2

Biological Process	GO:0046049	UMP metabolic process	2/2279	10/18866	0.3448376	0.6113644	0.5531842	2	UCK2/DHODH
Biological Process	GO:0046548	retinal rod cell development	2/2279	10/18866	0.3448376	0.6113644	0.5531842	2	RPGRIP1/NRL
Biological Process	GO:0046598	positive regulation of viral entry into host cell	2/2279	10/18866	0.3448376	0.6113644	0.5531842	2	P4HB/TRIM38
Biological Process	GO:0046710	GDP metabolic process	2/2279	10/18866	0.3448376	0.6113644	0.5531842	2	TJP2/GUK1
Biological Process	GO:0048304	positive regulation of isotype switching to IgG isotypes	2/2279	10/18866	0.3448376	0.6113644	0.5531842	2	PTPRC/PAXIP1
Biological Process	GO:0048340	paraxial mesoderm morphogenesis	2/2279	10/18866	0.3448376	0.6113644	0.5531842	2	SMAD3/WNT11
Biological Process	GO:0048755	branching morphogenesis of a nerve	2/2279	10/18866	0.3448376	0.6113644	0.5531842	2	BCL11A/RTN4
Biological Process	GO:0051231	spindle elongation	2/2279	10/18866	0.3448376	0.6113644	0.5531842	2	NUMA1/PPP2R1A
Biological Process	GO:0051918	negative regulation of fibrinolysis	2/2279	10/18866	0.3448376	0.6113644	0.5531842	2	THBS1/CPB2

Biological Process	GO:0071104	response to interleukin-9	2/2279	10/18866	0.3448376	0.6113644	0.5531842	2	STAT5A/JAK1
Biological Process	GO:0071313	cellular response to caffeine	2/2279	10/18866	0.3448376	0.6113644	0.5531842	2	CASQ2/SLC8A1
Biological Process	GO:0071649	regulation of chemokine (C-C motif) ligand 5 production	2/2279	10/18866	0.3448376	0.6113644	0.5531842	2	IL10/DEFB124
Biological Process	GO:0071670	smooth muscle cell chemotaxis	2/2279	10/18866	0.3448376	0.6113644	0.5531842	2	PDGFD/PDGFRB
Biological Process	GO:0071863	regulation of cell proliferation in bone marrow	2/2279	10/18866	0.3448376	0.6113644	0.5531842	2	MAP3K3/MEF2C
Biological Process	GO:0072488	ammonium transmembrane transport	2/2279	10/18866	0.3448376	0.6113644	0.5531842	2	AQP1/SLC12A7
Biological Process	GO:0072530	purine-containing compound transmembrane transport	2/2279	10/18866	0.3448376	0.6113644	0.5531842	2	SLC29A1/AQP9
Biological Process	GO:0075294	positive regulation by symbiont of entry into host	2/2279	10/18866	0.3448376	0.6113644	0.5531842	2	P4HB/TRIM38
Biological Process	GO:0086070	SA node cell to atrial cardiac muscle cell communication	2/2279	10/18866	0.3448376	0.6113644	0.5531842	2	GJA5/ANK2

Biological Process	GO:0090009	primitive streak formation	2/2279	10/18866	0.3448376	0.6113644	0.5531842	2	NODAL/OTX2
Biological Process	GO:0090037	positive regulation of protein kinase C signaling	2/2279	10/18866	0.3448376	0.6113644	0.5531842	2	WNT11/SPHK2
Biological Process	GO:0090043	regulation of tubulin deacetylation	2/2279	10/18866	0.3448376	0.6113644	0.5531842	2	NEK3/PRKAA1
Biological Process	GO:0090070	positive regulation of ribosome biogenesis	2/2279	10/18866	0.3448376	0.6113644	0.5531842	2	WDR43/RIOK1
Biological Process	GO:0090649	response to oxygen-glucose deprivation	2/2279	10/18866	0.3448376	0.6113644	0.5531842	2	AQP3/DNM1L
Biological Process	GO:0090673	endothelial cell-matrix adhesion	2/2279	10/18866	0.3448376	0.6113644	0.5531842	2	RIN2/CEACAM6
Biological Process	GO:0098722	asymmetric stem cell division	2/2279	10/18866	0.3448376	0.6113644	0.5531842	2	ZBTB16/ARHGEF2
Biological Process	GO:0098779	positive regulation of mitophagy in response to mitochondrial depolarization	2/2279	10/18866	0.3448376	0.6113644	0.5531842	2	SMURF1/PRKN
Biological Process	GO:0099612	protein localization to axon	2/2279	10/18866	0.3448376	0.6113644	0.5531842	2	EPB41L3/CNTNAP1

Biological Process	GO:1901203	positive regulation of extracellular matrix assembly	2/2279	10/18866	0.3448376	0.6113644	0.5531842	2	SMAD3/TGFB1
Biological Process	GO:1901725	regulation of histone deacetylase activity	2/2279	10/18866	0.3448376	0.6113644	0.5531842	2	C6orf89/SPHK2
Biological Process	GO:1901838	positive regulation of transcription of nucleolar large rRNA by RNA polymerase I	2/2279	10/18866	0.3448376	0.6113644	0.5531842	2	PIH1D1/MARS1
Biological Process	GO:1901894	regulation of ATPase-coupled calcium transmembrane transporter activity	2/2279	10/18866	0.3448376	0.6113644	0.5531842	2	THADA/TLR9
Biological Process	GO:1902023	L-arginine transport	2/2279	10/18866	0.3448376	0.6113644	0.5531842	2	SLC11A1/SLC7A1
Biological Process	GO:1903244	positive regulation of cardiac muscle hypertrophy in response to stress	2/2279	10/18866	0.3448376	0.6113644	0.5531842	2	MIR199A1/MIR199A2
Biological Process	GO:1904338	regulation of dopaminergic neuron differentiation	2/2279	10/18866	0.3448376	0.6113644	0.5531842	2	CSNK1D/CSNK1E
Biological Process	GO:1904684	negative regulation of metalloendopeptidase activity	2/2279	10/18866	0.3448376	0.6113644	0.5531842	2	SORL1/MIR24-2
Biological Process	GO:2000048	negative regulation of cell-cell adhesion mediated by cadherin	2/2279	10/18866	0.3448376	0.6113644	0.5531842	2	NOTCH1/NOTCH4

Biological Process	GO:2000169	regulation of peptidyl-cysteine S-nitrosylation	2/2279	10/18866	0.3448376	0.6113644	0.5531842	2	NOS1AP/SNTA1
Biological Process	GO:2000234	positive regulation of rRNA processing	2/2279	10/18866	0.3448376	0.6113644	0.5531842	2	WDR43/RIOK1
Biological Process	GO:2000288	positive regulation of myoblast proliferation	2/2279	10/18866	0.3448376	0.6113644	0.5531842	2	PAX7/MEIS2
Biological Process	GO:2000786	positive regulation of autophagosome assembly	2/2279	10/18866	0.3448376	0.6113644	0.5531842	2	ULK1/PIP4K2A
Biological Process	GO:0034504	protein localization to nucleus	36/2279	277/18866	0.3453081	0.6120844	0.5538357	36	CALR/TRIM8/SYNE1/ANGPT1/PIK3R1/SMAD3/TNPO1/MBTPS1/E2F3/SUFU/IPO13/FAM53B/ZBTB16/JUP/TGFB1/LILRB4/NF1/NUP93/DNAJB6/ARNTL/CDH1/SNUPN/MORC3/CABP1/SP100/TOR1B/TSC2/HHEX/PLRG1/BCL3/KAT7/XPA/CCT6A/PARP9/LIF/ILRUN
Biological Process	GO:0010660	regulation of muscle cell apoptotic process	13/2279	94/18866	0.3455127	0.6121577	0.553902	13	PPARG/MIR140/MIR145/ARRB1/E2F3/MIR199A1/MIR24-2/ILK/NFE2L2/ARRB2/MIR138-2/MIR199A2/DNMT1
Biological Process	GO:1901264	carbohydrate derivative transport	13/2279	94/18866	0.3455127	0.6121577	0.553902	13	SLC25A25/MFSD2A/SLC35C2/SLC29A3/ABCG1/PLTP/RALBP1/SLC37A1/GUK1/SLC33A1/PRKCE/SLC29A1/SLC25A1
Biological Process	GO:0009749	response to glucose	26/2279	197/18866	0.3455428	0.6121577	0.553902	26	NADK/LRP5L/ERN1/SLC8A1/ARRB1/GPLD1/EIF2B5/TGFBR2/THBS1/TCF7L2/IGF1R/PTPRN2/PAX2/ENY2/GIPR/GHRL/KLF7/FIS1/PIH1D1/PRKCE/CPB2/PCK2/SREBF1/SLC29A1/C2CD2L/PRKAA1
Biological Process	GO:0015931	nucleobase-containing compound transport	34/2279	261/18866	0.3456587	0.612249	0.5539846	34	SLC25A25/NXF1/FIP1L1/TNKS/SLC35C2/CPSF3/SETD2/RAE1/SLC29A3/TGFBR2/RIPK1/SMG7/PABPN1/ENY2/BICD2/NUP93/IGF2BP3/SEC13/KIF5C/SMG6/SNUPN/SLC33A1/ABCC11/MYO1C/HHEX/ZC3H3/RBM15B/SLC29A1/NOL6/CPSF4/FLOT1/SLC25A1/PCID2/NCBP3

Biological Process	GO:0031668	cellular response to extracellular stimulus	33/2279	253/18866	0.3457897	0.6123667	0.5540912	33	RPTOR/PRKAG2/PPARG/LCN2/MFSD2A/TNKS/ULK1/VDR/CBS/WIPI2/COMT/EHMT2/STK24/CLEC16A/SLC1A2/ATG14/PTPRC/NCOA1/NFE2L2/BCL2/AQP3/DNM1L/GLRX2/USF2/CD68/SLC2A1/FOXO1/SREBF1/EIF4G1/NPRL2/PRKAA1/MARS1/RIPOR1
Biological Process	GO:0042593	glucose homeostasis	32/2279	245/18866	0.3458871	0.6124085	0.554129	32	FOXK1/NADK/HK1/PPARG/LRP5L/ERN1/PIK3R1/ARRB1/GPLD1/TCF7L2/IGF1R/HNF1A/PTPRN2/PAX2/ENY2/HK2/GHRL/KLF7/SLC16A1/FIS1/PIH1D1/PRKCE/CPB2/PCK2/FOXO1/MBD5/SLC29A1/RPH3AL/ASPSCR1/C2CD2L/PRKAA1/CCN4
Biological Process	GO:0031669	cellular response to nutrient levels	29/2279	221/18866	0.3459422	0.6124085	0.554129	29	RPTOR/PRKAG2/PPARG/LCN2/MFSD2A/TNKS/ULK1/VDR/WIPI2/COMT/EHMT2/STK24/CLEC16A/ATG14/NCOA1/NFE2L2/BCL2/AQP3/DNM1L/USF2/CD68/SLC2A1/FOXO1/SREBF1/EIF4G1/NPRL2/PRKAA1/MARS1/RIPOR1
Biological Process	GO:0000079	regulation of cyclin-dependent protein serine/threonine kinase activity	14/2279	102/18866	0.3475422	0.6151263	0.5565881	14	BLM/TNFAIP3/FBXO7/LATS2/CDC25A/PDGFB/CCNH/CCNA1/HHEX/HEXIM1/CCNY/HERC5/CCNYL1/CCNP
Biological Process	GO:0071867	response to monoamine	15/2279	110/18866	0.3492139	0.6176045	0.5588305	15	PDE4D/KLF16/DNM2/APP/HRH1/HTR3A/GNA11/CHRM2/PALM/KCNQ1/ARRB2/GNG2/GNAO1/DTNBP1/PRKN
Biological Process	GO:0071869	response to catecholamine	15/2279	110/18866	0.3492139	0.6176045	0.5588305	15	PDE4D/KLF16/DNM2/APP/HRH1/HTR3A/GNA11/CHRM2/PALM/KCNQ1/ARRB2/GNG2/GNAO1/DTNBP1/PRKN
Biological Process	GO:1901989	positive regulation of cell cycle phase transition	15/2279	110/18866	0.3492139	0.6176045	0.5588305	15	RPTOR/BRD4/TFDP1/NSMCE2/CUL3/APP/CDC16/CDC25A/TMOD3/PLCB1/RB1/CDCA5/ANAPC7/PLRG1/EIF4G1
Biological Process	GO:0031122	cytoplasmic microtubule organization	9/2279	63/18866	0.3493324	0.6176045	0.5588305	9	DCTN1/LIMK2/CCDC88C/DYNC1H1/CCDC88B/HOOK2/NUMA1/TUBGCP2/EZR
Biological Process	GO:0051893	regulation of focal adhesion assembly	9/2279	63/18866	0.3493324	0.6176045	0.5588305	9	ITGB1BP1/SMAD3/DUSP22/THBS1/PTPRJ/ACTG1/CORO1C/DUSP3/DAPK3

Biological Process	GO:0090109	regulation of cell-substrate junction assembly	9/2279	63/18866	0.3493324	0.6176045	0.5588305	9	ITGB1BP1/SMAD3/DUSP22/THBS1/PTPRJ/ACTG1/CORO1C/DUSP3/DAPK3
Biological Process	GO:0006911	phagocytosis, engulfment	17/2279	126/18866	0.351727	0.6217224	0.5625565	17	PPARG/GSN/ELMO1/RHOH/DOCK1/RHOG/RAB31/IGHV6-1/THBS1/MYH9/MARCO/RHOBTB2/ABCA1/ARHGAP25/ITGB2/ANO6/ARHGAP12
Biological Process	GO:0016236	macroautophagy	40/2279	310/18866	0.3522726	0.6218478	0.5626699	40	RPTOR/PRKAG2/SPTLC2/ULK1/ERN1/VPS13D/RHEB/PACS2/WIPI2/LARP1/USP36/PIP4K2A/VPS26B/ATP6V1B2/FEZ2/PRKAB1/UVRAG/SMURF1/RUFY4/RAB43/CLEC16A/HTT/ATG14/TBC1D14/NPC1/VMP1/ATP6V0B/UBXN2A/NEDD4/TSC2/WDR81/MVB12A/NPRL2/PRKAA1/STING1/PRKN/ATP6V0C/ATP6V1C1/ILRUN/RUBCNL
Biological Process	GO:0002209	behavioral defense response	6/2279	40/18866	0.3523215	0.6218478	0.5626699	6	ASIC4/MAPK8IP2/BCL2/MEF2C/MORC1/EIF4G1
Biological Process	GO:0009187	cyclic nucleotide metabolic process	6/2279	40/18866	0.3523215	0.6218478	0.5626699	6	PDE4D/ADCY2/ADCY9/PDE9A/ADCY4/NPPC
Biological Process	GO:0010664	negative regulation of striated muscle cell apoptotic process	6/2279	40/18866	0.3523215	0.6218478	0.5626699	6	MIR145/MIR199A1/MIR24-2/ILK/NFE2L2/MIR199A2
Biological Process	GO:0019068	virion assembly	6/2279	40/18866	0.3523215	0.6218478	0.5626699	6	PC/RAB43/USP6NL/NEDD4/MVB12A/SPCS1
Biological Process	GO:0051281	positive regulation of release of sequestered calcium ion into cytosol	6/2279	40/18866	0.3523215	0.6218478	0.5626699	6	P2RY6/HTT/GSTO1/F2R/PLCG1/CASQ1
Biological Process	GO:0071526	semaphorin-plexin signaling pathway	6/2279	40/18866	0.3523215	0.6218478	0.5626699	6	SEMA6B/PLXNC1/NRP2/SEMA4A/SEMA4B/SEMA4D

Biological Process	GO:1901381	positive regulation of potassium ion transmembrane transport	6/2279	40/18866	0.3523215	0.6218478	0.5626699	6	KCNE1/DNM2/NOS1AP/KCNQ1/ANO6/ANK2
Biological Process	GO:0009855	determination of bilateral symmetry	18/2279	134/18866	0.3526575	0.6223251	0.5631019	18	SMAD3/PKD1L1/SUFU/MICAL2/TGFBR2/RFX3/DLL1/MIB1/PCSK6/AP1B1/IFT140/ENG/NOTCH1/MEF2C/TMED2/DNAI2/ALDH1A2/NODAL
Biological Process	GO:0042982	amyloid precursor protein metabolic process	10/2279	71/18866	0.3530143	0.6228392	0.563567	10	SORL1/TNF/ABCG1/MIR101-2/BIN1/APP/CLU/AGO2/ITM2C/RELA
Biological Process	GO:0060041	retina development in camera-type eye	20/2279	150/18866	0.3540246	0.6245057	0.565075	20	PDE6A/MFSD2A/LPCAT1/LRP5L/SKI/CRB1/HIPK2/DLL1/GNAT2/PAX2/BMPR1B/RPGRIP1/ZHX2/SDK2/NRL/PDGFRB/ARHGEF15/CABP4/NPHP4/NECTIN1
Biological Process	GO:0033500	carbohydrate homeostasis	32/2279	246/18866	0.3548348	0.6257823	0.5662301	32	FOXK1/NADK/HK1/PPARG/LRP5L/ERN1/PIK3R1/ARRB1/GPLD1/TCF7L2/IGF1R/HNF1A/PTPRN2/PAX2/ENY2/HK2/GHRL/KLF7/SLC16A1/FIS1/PIH1D1/PRKCE/CPB2/PCK2/FOXO1/MBD5/ SLC29A1/RPH3AL/ASPSCR1/C2CD2L/PRKAA1/CCN4
Biological Process	GO:0006475	internal protein amino acid acetylation	22/2279	166/18866	0.35488	0.6257823	0.5662301	22	SRCAP/BRCA2/KANSL1/ARRB1/BRD1/EPC1/RPS6KA4/TAF10/KANSL2/SPHK2/CTBP1/POR/NCOA1/CREBBP/RSF1/PER1/PIH1D1/RUVBL1/KAT7/ING5/LIF/PAXIP1
Biological Process	GO:0006575	cellular modified amino acid metabolic process	25/2279	190/18866	0.3554697	0.6265896	0.5669605	25	OSBPL5/GGT1/ABHD16A/MTHFD1L/TG/CNDP2/MTHFS/ALDH4A1/ACADM/SLC19A1/GSTM5/POR/CPQ/MGST2/ABHD16B/CTSB/OSBPL10/NFE2L2/MTHFD1/HOGA1/PLA1A/GLRX2/CR YM/PTDSS2/CPT1A
Biological Process	GO:0099111	microtubule-based transport	25/2279	190/18866	0.3554697	0.6265896	0.5669605	25	PRKCZ/NDE1/TNPO1/KIF13A/SPG7/DYNC1H1/RFX3/APP/BICD2/CLUAP1/CNIH2/HTT/KIF5C/IFT140/KIF1B/TTC21B/STK36/MAP1A/KIFAP3/AP3D1/FBXW11/NDEL1/DTNBP1/TTLL1/KAT NIP
Biological Process	GO:0006809	nitric oxide biosynthetic process	11/2279	79/18866	0.3559082	0.6267811	0.5671338	11	IL10/SMAD3/DNM2/MIR199A1/TNF/NOS1AP/CLU/HBB/ITGB2/MIR199A2/CX3CR1

Biological Process	GO:0007032	endosome organization	11/2279	79/18866	0.3559082	0.6267811	0.5671338	11	RAB5B/RAB27A/SNX3/VPS18/HOOK2/DNM1/PI4K2A/CORO1C/MVB12A/PHETA1/PRKN
Biological Process	GO:0043407	negative regulation of MAP kinase activity	11/2279	79/18866	0.3559082	0.6267811	0.5671338	11	SPRED2/SORL1/HIPK3/DUSP14/LAX1/PTPN6/PTPRJ/NF1/PPP2R1A/RGS3/DUSP3
Biological Process	GO:0071260	cellular response to mechanical stimulus	11/2279	79/18866	0.3559082	0.6267811	0.5671338	11	HDAC4/TNFRSF1A/WNT11/AQP1/BCL10/MAP3K14/ENG/CASP1/SLC2A1/ANKRD1/FADD
Biological Process	GO:0099601	regulation of neurotransmitter receptor activity	11/2279	79/18866	0.3559082	0.6267811	0.5671338	11	CCR2/APP/DLGAP4/CRHBP/CNIH2/BEGAIN/MAPK8IP2/GSG1L/MEF2C/SHANK2/NPTX1
Biological Process	GO:0000466	maturation of 5.8S rRNA from tricistronic rRNA transcript (SSU-rRNA, 5.8S rRNA, LSU-rRNA)	4/2279	25/18866	0.3572194	0.6279262	0.5681699	4	EXOSC2/RCL1/ERI3/NOP14
Biological Process	GO:0003309	type B pancreatic cell differentiation	4/2279	25/18866	0.3572194	0.6279262	0.5681699	4	RHEB/RFX3/DLL1/ARNTL
Biological Process	GO:0010592	positive regulation of lamellipodium assembly	4/2279	25/18866	0.3572194	0.6279262	0.5681699	4	HDAC4/DNM2/NCKAP1/AVIL
Biological Process	GO:0010738	regulation of protein kinase A signaling	4/2279	25/18866	0.3572194	0.6279262	0.5681699	4	PRKAR1B/AIP/AKAP5/AKAP7
Biological Process	GO:0032400	melanosome localization	4/2279	25/18866	0.3572194	0.6279262	0.5681699	4	RAB27A/CDH3/DCTN2/MYO5A

Biological Process	GO:0032647	regulation of interferon-alpha production	4/2279	25/18866	0.3572194	0.6279262	0.5681699	4	IL10/IRF5/SETD2/TLR9
Biological Process	GO:0035336	long-chain fatty-acyl-CoA metabolic process	4/2279	25/18866	0.3572194	0.6279262	0.5681699	4	ACSL1/FAR2/ACOT7/ACSF3
Biological Process	GO:0048169	regulation of long-term neuronal synaptic plasticity	4/2279	25/18866	0.3572194	0.6279262	0.5681699	4	APP/SYNGAP1/NF1/SYNPO
Biological Process	GO:0051788	response to misfolded protein	4/2279	25/18866	0.3572194	0.6279262	0.5681699	4	CUL3/CLU/F12/POMT2
Biological Process	GO:1905809	negative regulation of synapse organization	4/2279	25/18866	0.3572194	0.6279262	0.5681699	4	DNM3/UBE3A/ARHGEF15/SLIT1
Biological Process	GO:0060420	regulation of heart growth	12/2279	87/18866	0.358204	0.6290752	0.5692095	12	CTDP1/MIR199A1/VGLL4/TGFBR2/RIPK1/RBPJ/FGFR1/ACACB/MEIS1/MIR199A2/NOTCH1/MEF2C
Biological Process	GO:0060761	negative regulation of response to cytokine stimulus	12/2279	87/18866	0.358204	0.6290752	0.5692095	12	PPARG/CCL5/TRAIP/PADI2/MIR24-2/MIR27A/IL1RN/NR1H2/PTPRC/NLRC5/MIR138-2/SLIT3
Biological Process	GO:1903363	negative regulation of cellular protein catabolic process	12/2279	87/18866	0.358204	0.6290752	0.5692095	12	TRIM39/SUFU/HIPK2/UBAC2/MGAT3/DERL2/CLEC16A/PSMF1/MAP1A/BAG5/SVIP/MARCHF7
Biological Process	GO:1904705	regulation of vascular associated smooth muscle cell proliferation	12/2279	87/18866	0.358204	0.6290752	0.5692095	12	IL10/PPARG/MIR140/MEF2D/ERN1/TNF/GNAI2/MIR27A/P2RY6/PDGFB/DNMT1/MEF2C

Biological Process	GO:1990874	vascular associated smooth muscle cell proliferation	12/2279	87/18866	0.358204	0.6290752	0.5692095	12	IL10/PPARG/MIR140/MEF2D/ERN1/TNF/GNAI2/MIR27A/P2RY6/PDGFB/DNMT1/MEF2C
Biological Process	GO:0008089	anterograde axonal transport	7/2279	48/18866	0.358902	0.6296026	0.5696868	7	SPG7/CNIH2/KIF5C/KIF1B/MAP1A/AP3D1/DTNBP1
Biological Process	GO:0008206	bile acid metabolic process	7/2279	48/18866	0.358902	0.6296026	0.5696868	7	AMACR/AKR1D1/ATP8B1/OSBPL6/ACOX2/NPC1/NR5A2
Biological Process	GO:0042220	response to cocaine	7/2279	48/18866	0.358902	0.6296026	0.5696868	7	PPP1R1B/DNM2/CRHBP/HTR3A/UBE3A/HNMT/FADD
Biological Process	GO:0051972	regulation of telomerase activity	7/2279	48/18866	0.358902	0.6296026	0.5696868	7	PPARG/TNKS/HMBOX1/PARP4/GRHL2/SMG6/PARM1
Biological Process	GO:0055010	ventricular cardiac muscle tissue morphogenesis	7/2279	48/18866	0.358902	0.6296026	0.5696868	7	RXRA/CCM2L/RBPJ/SMAD7/TGFB1/ENG/NOTCH1
Biological Process	GO:0061001	regulation of dendritic spine morphogenesis	7/2279	48/18866	0.358902	0.6296026	0.5696868	7	BAIAP2/SIPA1L1/DNM3/DNM1L/UBE3A/CUX2/DBNL
Biological Process	GO:0061326	renal tubule development	13/2279	95/18866	0.360035	0.6313571	0.5712743	13	TACSTD2/DLL1/ILK/WNT11/PAX2/MTSS1/WWTR1/BCL2/NOTCH1/MEF2C/ACAT1/LIF/HOXB7
Biological Process	GO:0097194	execution phase of apoptosis	13/2279	95/18866	0.360035	0.6313571	0.5712743	13	CECR2/PAM16/DEDD2/CIDEB/STK24/DFFB/CASP7/DNASE1L3/DNM1L/CASP1/TP53BP2/BCL2L1/BLCAP

Biological Process	GO:0001823	mesonephros development	14/2279	103/18866	0.3614975	0.6336878	0.5733832	14	TACSTD2/SMAD3/ZBTB16/ILK/SMAD7/WNT11/PAX2/FGFR1/RARA/BMPER/CAT/BCL2/CRLF1/HOXB7
Biological Process	GO:0002042	cell migration involved in sprouting angiogenesis	14/2279	103/18866	0.3614975	0.6336878	0.5733832	14	MAP3K3/HDAC7/ITGB1BP1/MIR199A1/GPLD1/MIR101-2/THBS1/AKT3/MIR27A/MIR23A/MIA3/MIR199A2/NOTCH1/ADTRP
Biological Process	GO:0046660	female sex differentiation	16/2279	119/18866	0.3635856	0.6367813	0.5761823	16	ARID5B/BRCA2/ARRB1/EIF2B5/FANCA/BMPR1B/LHFPL2/ARRB2/BCL2/DMC1/SLIT3/UBE3A/CEBPB/BCL2L1/TYRO3/INHBA
Biological Process	GO:0001755	neural crest cell migration	8/2279	56/18866	0.3636644	0.6367813	0.5761823	8	SEMA6B/NRP2/ANXA6/ZEB2/SEMA4A/SEMA4B/SEMA4D/CORO1C
Biological Process	GO:0060688	regulation of morphogenesis of a branching structure	8/2279	56/18866	0.3636644	0.6367813	0.5761823	8	TACSTD2/RXRA/TNF/BCL11A/RTN4/PAX2/FGFR1/HOXB7
Biological Process	GO:0086065	cell communication involved in cardiac conduction	8/2279	56/18866	0.3636644	0.6367813	0.5761823	8	PDE4D/CASQ2/CACNA1C/SLC8A1/GJA5/JUP/KCNQ1/ANK2
Biological Process	GO:1902017	regulation of cilium assembly	8/2279	56/18866	0.3636644	0.6367813	0.5761823	8	CDKL1/DNM2/CEP135/HTT/IFT140/SDCCAG8/CYLD/SEPTIN9
Biological Process	GO:1903307	positive regulation of regulated secretory pathway	8/2279	56/18866	0.3636644	0.6367813	0.5761823	8	CD177/GAB2/FGR/RAB27A/HLA-F/SPHK2/ITGB2/DNM1L
Biological Process	GO:0009799	specification of symmetry	18/2279	135/18866	0.3648611	0.6385236	0.5777589	18	SMAD3/PKD1L1/SUFU/MICAL2/TGFBR2/RFX3/DLL1/MIB1/PCSK6/AP1B1/IFT140/ENG/NOTCH1/MEF2C/TMED2/DNAI2/ALDH1A2/NODAL

Biological Process	GO:0050853	B cell receptor signaling pathway	18/2279	135/18866	0.3648611	0.6385236	0.5777589	18	FOXP1/PIK3CD/LCK/RUNX1/NFATC2/MNDA/IGHV6-1/PTPN6/KLHL6/PRKCH/PRKCB/CD79A/BLK/PTPRC/BCL2/MEF2C/ELF1/VAV3
Biological Process	GO:0099024	plasma membrane invagination	18/2279	135/18866	0.3648611	0.6385236	0.5777589	18	PPARG/GSN/ELMO1/RHOH/DOCK1/RHOG/RAB31/IGHV6-1/THBS1/MYH9/MARCO/RHOBTB2/ABCA1/SNX18/ARHGAP25/ITGB2/ANO6/ARHGAP12
Biological Process	GO:1901343	negative regulation of vasculature development	27/2279	207/18866	0.3653679	0.6392929	0.5784549	27	PPARG/MIR143/MIR145/ITGB1BP1/CCR2/COL4A2/MIR199A1/TNF/NFATC2/AGO1/SEMA4A/NFATC1/THBS1/MIR24-2/EPN2/NF1/GHRL/PDGFB/VASH1/MIR138-2/MIR199A2/NOTCH1/DNMT1/GPR4/HHEX/PDE3B/CX3CR1
Biological Process	GO:0006261	DNA-dependent DNA replication	20/2279	151/18866	0.3655646	0.6393546	0.5785108	20	BLM/POLE/BRCA2/RTEL1/E2F7/MCM5/WRNIP1/RECQL5/POLA2/EME1/RRM2B/FGFR1/MCM2/SMARCAL1/GINS3/CHEK2/KAT7/POLD3/POLE4/FBH1
Biological Process	GO:0019933	cAMP-mediated signaling	26/2279	199/18866	0.3655693	0.6393546	0.5785108	26	KSR1/PDE4D/MGRN1/ADCY2/AKAP13/PRKAR1B/ADCY9/PRKCA/GNAI2/MRAP/CRTC3/RAPGEF2/DGKQ/DEFB1/GIPR/ABCA1/CNR2/ADCY4/S1PR2/GNG2/LPAR2/S1PR4/GPR4/PDE3B/ADGRG3/ADGRE3
Biological Process	GO:0016573	histone acetylation	21/2279	159/18866	0.3657529	0.6393546	0.5785108	21	SRCAP/BRCA2/KANSL1/ARRB1/BRD1/EPC1/RPS6KA4/TAF10/KANSL2/SPHK2/CTBP1/NCOA1/CREBBP/RSF1/PER1/PIH1D1/RUVBL1/KAT7/ING5/LIF/PAXIP1
Biological Process	GO:1903311	regulation of mRNA metabolic process	44/2279	344/18866	0.3658576	0.6393546	0.5785108	44	RBM19/PRR5L/EXOSC2/SLC11A1/TNRC6B/SRPK2/PABPC4/TNPO1/PRKCA/LARP1/ZC3H12D/PKP1/PCBP4/SAMD4A/AGO2/PABPN1/SF1/CPEB3/DAZAP1/IGF2BP3/SFSWAP/PSMF1/CELF2/FTO/CELF1/NBAS/YWHAZ/PSMB7/PAIP1/RBFOX3/TNFSF13/NSRP1/ZC3H3/RBM15B/SECISBP2/CPSF4/RNF40/SYNCRIP/EIF4G1/PSMD13/PCID2/TBRG4/TENT5C/TENT5A
Biological Process	GO:0007269	neurotransmitter secretion	23/2279	175/18866	0.3658742	0.6393546	0.5785108	23	RAP1B/GIT1/SYN3/PTPRN2/PPFIA1/APBA2/PRKCB/MCTP2/NF1/NRXN1/VPS18/SYN2/CTBP2/CHRM2/NRXN2/RPH3A/P2RX1/DNM1L/MEF2C/SYT17/CACNA1B/DTNBP1/PRKN
Biological Process	GO:0099643	signal release from synapse	23/2279	175/18866	0.3658742	0.6393546	0.5785108	23	RAP1B/GIT1/SYN3/PTPRN2/PPFIA1/APBA2/PRKCB/MCTP2/NF1/NRXN1/VPS18/SYN2/CTBP2/CHRM2/NRXN2/RPH3A/P2RX1/DNM1L/MEF2C/SYT17/CACNA1B/DTNBP1/PRKN

Biological Process	GO:1901568	fatty acid derivative metabolic process	23/2279	175/18866	0.3658742	0.6393546	0.5785108	23	MGLL/ACSL1/ABCC1/FAR2/BDH1/GGT1/ABHD16A/TNFRSF1A/ACOT7/CYP4F3/MGST2/ACACA/EPHX1/ACSF3/HMGCL/DPEP2/ACAT1/SCD/ACSS3/SLC25A1/LYPLA2/ZADH2/PRXL2B
Biological Process	GO:0032507	maintenance of protein location in cell	9/2279	64/18866	0.3672023	0.6412037	0.5801839	9	SORL1/HK1/GSN/SYNE1/VPS13D/HK2/MORC3/SP100/BCL3
Biological Process	GO:0046173	polyol biosynthetic process	9/2279	64/18866	0.3672023	0.6412037	0.5801839	9	SPTLC2/ITPKB/PGP/P2RY6/SPHK2/HRH1/IMPA2/PCK2/IP6K1
Biological Process	GO:1901862	negative regulation of muscle tissue development	9/2279	64/18866	0.3672023	0.6412037	0.5801839	9	CTDP1/MIR199A1/VGLL4/TGFBR2/DLL1/RIPK1/TGFB1/MEIS1/MIR199A2
Biological Process	GO:1905268	negative regulation of chromatin organization	9/2279	64/18866	0.3672023	0.6412037	0.5801839	9	SKI/OTUB2/SPHK2/CTBP1/KDM4C/OTUB1/DNMT1/PIH1D1/TRIP12
Biological Process	GO:0006505	GPI anchor metabolic process	5/2279	33/18866	0.3680293	0.6413241	0.5802928	5	PIGL/PIGU/GPLD1/PIGN/PIGQ
Biological Process	GO:0006536	glutamate metabolic process	5/2279	33/18866	0.3680293	0.6413241	0.5802928	5	HAL/GGT1/MTHFS/ALDH4A1/DGLUCY
Biological Process	GO:0009954	proximal/distal pattern formation	5/2279	33/18866	0.3680293	0.6413241	0.5802928	5	CHSY1/DLL1/HOXA9/ALDH1A2/NODAL
Biological Process	GO:0010984	regulation of lipoprotein particle clearance	5/2279	33/18866	0.3680293	0.6413241	0.5802928	5	MIR199A1/GPLD1/MIR27A/CSK/MIR199A2

Biological Process	GO:0019692	deoxyribose phosphate metabolic process	5/2279	33/18866	0.3680293	0.6413241	0.5802928	5	UNG/NTHL1/GUK1/AK5/DCTD
Biological Process	GO:0045736	negative regulation of cyclin-dependent protein serine/threonine kinase activity	5/2279	33/18866	0.3680293	0.6413241	0.5802928	5	TNFAIP3/FBXO7/LATS2/HHEX/HEXIM1
Biological Process	GO:0045879	negative regulation of smoothened signaling pathway	5/2279	33/18866	0.3680293	0.6413241	0.5802928	5	MGRN1/SUFU/RUNX2/RB1/KCTD21
Biological Process	GO:0048011	neurotrophin TRK receptor signaling pathway	5/2279	33/18866	0.3680293	0.6413241	0.5802928	5	CYFIP2/SORT1/NTRK1/CYFIP1/NTF4
Biological Process	GO:1901797	negative regulation of signal transduction by p53 class mediator	5/2279	33/18866	0.3680293	0.6413241	0.5802928	5	RRM2B/ZNF385A/BCL2/PRKN/MARCHF7
Biological Process	GO:0042440	pigment metabolic process	10/2279	72/18866	0.3698744	0.6413241	0.5802928	10	ZEB2/SLC11A2/FECH/CDH3/RAPGEF2/MYO5A/ALAS1/PPOX/BCL2/TMEM14C
Biological Process	GO:1904427	positive regulation of calcium ion transmembrane transport	10/2279	72/18866	0.3698744	0.6413241	0.5802928	10	P2RY6/HTT/GSTO1/F2R/ANK2/PLCG1/CASQ1/FGF14/STIMATE/CRACR2A
Biological Process	GO:0045861	negative regulation of proteolysis	47/2279	369/18866	0.3713949	0.6413241	0.5802928	47	TRIM39/SLPI/CTSZ/CSTA/TIMP2/SORL1/SERPINA1/SPOCK2/IL10/RPS6KA1/APLP2/SERPINB11/ARRB1/SPINT2/MIR199A1/SUFU/TNF/MICAL1/HIPK2/SERPINB2/UBAC2/BIN1/THBS1/AP P/UBE2O/MIR24-2/DERL2/PAX2/AQP1/POR/CLEC16A/NR1H2/DNAJB6/PSMF1/CRIM1/NOL3/ARRB2/MIR199A2/MAP1A/PIH1D1/CPB2/LTF/COL28A1/BAG5/PCID2/SVIP/MARCHF7
Biological Process	GO:0055021	regulation of cardiac muscle tissue growth	11/2279	80/18866	0.3719107	0.6413241	0.5802928	11	CTDP1/MIR199A1/VGLL4/TGFBR2/RIPK1/RBPJ/FGFR1/MEIS1/MIR199A2/NOTCH1/MEF2C

Biological Process	GO:0000712	resolution of meiotic recombination intermediates	3/2279	18/18866	0.3731904	0.6413241	0.5802928	3	EME1/MEIOB/SLX4
Biological Process	GO:0001710	mesodermal cell fate commitment	3/2279	18/18866	0.3731904	0.6413241	0.5802928	3	PAX2/FGFR1/NODAL
Biological Process	GO:0006349	regulation of gene expression by genetic imprinting	3/2279	18/18866	0.3731904	0.6413241	0.5802928	3	KDM1B/KCNQ1/PCGF3
Biological Process	GO:0006491	N-glycan processing	3/2279	18/18866	0.3731904	0.6413241	0.5802928	3	MGAT4A/ST8SIA6/MAN1C1
Biological Process	GO:0006825	copper ion transport	3/2279	18/18866	0.3731904	0.6413241	0.5802928	3	SLC11A2/ATOX1/ATP7B
Biological Process	GO:0010523	negative regulation of calcium ion transport into cytosol	3/2279	18/18866	0.3731904	0.6413241	0.5802928	3	CASQ2/BCL2/GSTO1
Biological Process	GO:0010766	negative regulation of sodium ion transport	3/2279	18/18866	0.3731904	0.6413241	0.5802928	3	PRKCE/NEDD4/HECW2
Biological Process	GO:0031293	membrane protein intracellular domain proteolysis	3/2279	18/18866	0.3731904	0.6413241	0.5802928	3	MBTPS1/TGFB1/RELA
Biological Process	GO:0032780	negative regulation of ATPase activity	3/2279	18/18866	0.3731904	0.6413241	0.5802928	3	PAM16/MCM2/LTF

Biological Process	GO:0034375	high-density lipoprotein particle remodeling	3/2279	18/18866	0.3731904	0.6413241	0.5802928	3	LIPC/ABCG1/PLTP
Biological Process	GO:0034755	iron ion transmembrane transport	3/2279	18/18866	0.3731904	0.6413241	0.5802928	3	SLC25A37/SLC11A1/SLC11A2
Biological Process	GO:0035330	regulation of hippo signaling	3/2279	18/18866	0.3731904	0.6413241	0.5802928	3	MARK3/VGLL4/LIMD1
Biological Process	GO:0042983	amyloid precursor protein biosynthetic process	3/2279	18/18866	0.3731904	0.6413241	0.5802928	3	MIR101-2/AGO2/ITM2C
Biological Process	GO:0042984	regulation of amyloid precursor protein biosynthetic process	3/2279	18/18866	0.3731904	0.6413241	0.5802928	3	MIR101-2/AGO2/ITM2C
Biological Process	GO:0050961	detection of temperature stimulus involved in sensory perception	3/2279	18/18866	0.3731904	0.6413241	0.5802928	3	WDR47/ARRB2/NTRK1
Biological Process	GO:0051571	positive regulation of histone H3-K4 methylation	3/2279	18/18866	0.3731904	0.6413241	0.5802928	3	MYB/DNMT1/PAXIP1
Biological Process	GO:0060977	coronary vasculature morphogenesis	3/2279	18/18866	0.3731904	0.6413241	0.5802928	3	SETD2/NOTCH1/PDGFRB
Biological Process	GO:0061298	retina vasculature development in camera-type eye	3/2279	18/18866	0.3731904	0.6413241	0.5802928	3	LRP5L/PDGFRB/ARHGEF15

Biological Process	GO:0061577	calcium ion transmembrane transport via high voltage-gated calcium channel	3/2279	18/18866	0.3731904	0.6413241	0.5802928	3	CACNA1C/BIN1/NOS1AP
Biological Process	GO:0070230	positive regulation of lymphocyte apoptotic process	3/2279	18/18866	0.3731904	0.6413241	0.5802928	3	IL10/CCL5/ZC3H8
Biological Process	GO:0071786	endoplasmic reticulum tubular network organization	3/2279	18/18866	0.3731904	0.6413241	0.5802928	3	RTN4/RTN3/ATL1
Biological Process	GO:0072189	ureter development	3/2279	18/18866	0.3731904	0.6413241	0.5802928	3	PAX2/ALDH1A2/NFIA
Biological Process	GO:0097107	postsynaptic density assembly	3/2279	18/18866	0.3731904	0.6413241	0.5802928	3	NRXN1/NRXN2/SHANK2
Biological Process	GO:1900153	positive regulation of nuclear-transcribed mRNA catabolic process, deadenylation-dependent decay	3/2279	18/18866	0.3731904	0.6413241	0.5802928	3	TNRC6B/AGO2/CPEB3
Biological Process	GO:1900221	regulation of amyloid-beta clearance	3/2279	18/18866	0.3731904	0.6413241	0.5802928	3	TNF/CLU/HDAC1
Biological Process	GO:1902165	regulation of intrinsic apoptotic signaling pathway in response to DNA damage by p53 class mediator	3/2279	18/18866	0.3731904	0.6413241	0.5802928	3	ZNF385A/BCL2/MARCHF7
Biological Process	GO:0032410	negative regulation of transporter activity	12/2279	88/18866	0.3734666	0.6413241	0.5802928	12	KCNE1/THADA/CASQ2/WWP2/TLR9/CAB39/GSTO1/PRKCE/NEDD4/DYSF/HECW2/CBARP

Biological Process	GO:0032755	positive regulation of interleukin-6 production	12/2279	88/18866	0.3734666	0.6413241	0.5802928	12	IL16/TNF/TLR9/SPON2/APP/MBP/SCIMP/IL6R/NOD2/POU2F2/F2R/ARHGEF2
Biological Process	GO:0046470	phosphatidylcholine metabolic process	12/2279	88/18866	0.3734666	0.6413241	0.5802928	12	PLA2G15/PCYT1A/MFSD2A/LPCAT1/LIPC/GPLD1/SLC44A4/CHPT1/NR1H2/PCTP/CHKB/LPIN1
Biological Process	GO:1903312	negative regulation of mRNA metabolic process	12/2279	88/18866	0.3734666	0.6413241	0.5802928	12	SLC11A1/LARP1/PKP1/PCBP4/SFSWAP/NBAS/PAIP1/SECISBP2/RNF40/SYNCRIP/TENT5C/TENT5A
Biological Process	GO:0043624	cellular protein complex disassembly	29/2279	224/18866	0.3743838	0.6413241	0.5802928	29	VILL/GSN/MRPL3/CAPG/MICAL1/MICAL2/MICAL3/NAV3/MRPL38/TMOD3/SVIL/SH3GL1/WDR1/SPTA1/ADD1/MRPS21/MRPS26/MAP1A/AVIL/FLII/ARHGEF2/MRPL28/SPTAN1/ASPH/TMOD1/AURKAIP1/SPTBN4/SPEF1/SHFL
Biological Process	GO:0006303	double-strand break repair via nonhomologous end joining	13/2279	96/18866	0.3746504	0.6413241	0.5802928	13	KDM2A/UVRAG/SMARCAL1/TP53BP1/ERCC1/PARP9/PAXIP1/TFIP11/PAXX/NSD2/CYREN/ABRAXAS1/BABAM2
Biological Process	GO:0001941	postsynaptic membrane organization	6/2279	41/18866	0.374923	0.6413241	0.5802928	6	SSH1/ZMYND8/COLQ/ZDHHC2/NRXN1/NRXN2
Biological Process	GO:0042596	fear response	6/2279	41/18866	0.374923	0.6413241	0.5802928	6	ASIC4/MAPK8IP2/BCL2/MEF2C/MORC1/EIF4G1
Biological Process	GO:0045494	photoreceptor cell maintenance	6/2279	41/18866	0.374923	0.6413241	0.5802928	6	CRB1/CDH23/ESRRB/CDHR1/SLC2A1/NPHP4
Biological Process	GO:0070317	negative regulation of G0 to G1 transition	6/2279	41/18866	0.374923	0.6413241	0.5802928	6	TFDP1/MGA/EHMT2/EPC1/PHC3/E2F6

Biological Process	GO:0071548	response to dexamethasone	6/2279	41/18866	0.374923	0.6413241	0.5802928	6	ABCC2/SMYD3/FECH/AQP1/PCK2/FOXO1
Biological Process	GO:0002504	antigen processing and presentation of peptide or polysaccharide antigen via MHC class II	14/2279	104/18866	0.3755407	0.6413241	0.5802928	14	HLA-DMB/DCTN1/AP2A1/DNM2/DYNC1H1/CTSD/THBS1/AP1B1/DCTN2/SEC13/SEC24C/KIFAP3/SEC31A/MARCHF8
Biological Process	GO:0050657	nucleic acid transport	26/2279	200/18866	0.3756711	0.6413241	0.5802928	26	NXF1/FIP1L1/TNKS/CPSF3/SETD2/RAE1/TGFBR2/SMG7/PABPN1/ENY2/BICD2/NUP93/IGF2BP3/SEC13/KIF5C/SMG6/SNUPN/MYO1C/HHEX/ZC3H3/RBM15B/NOL6/CPSF4/FLOT1/PCID2/NCBP3
Biological Process	GO:0050658	RNA transport	26/2279	200/18866	0.3756711	0.6413241	0.5802928	26	NXF1/FIP1L1/TNKS/CPSF3/SETD2/RAE1/TGFBR2/SMG7/PABPN1/ENY2/BICD2/NUP93/IGF2BP3/SEC13/KIF5C/SMG6/SNUPN/MYO1C/HHEX/ZC3H3/RBM15B/NOL6/CPSF4/FLOT1/PCID2/NCBP3
Biological Process	GO:0051588	regulation of neurotransmitter transport	16/2279	120/18866	0.3766583	0.6413241	0.5802928	16	RAP1B/GIT1/APBA2/PRKCB/MCTP2/NF1/VPS18/ITGB3/CHRM2/PER2/P2RX1/DNM1L/MEF2C/FLOT1/DTNBP1/PRKN
Biological Process	GO:0045727	positive regulation of translation	18/2279	136/18866	0.3771375	0.6413241	0.5802928	18	RXRA/PLD1/TNF/CCL5/NCK2/EIF2B5/LARP1/KRT17/THBS1/SAMD4A/CTIF/MIF4GD/CPEB3/PKM/PAIP1/BCL3/EIF4G1/NIBAN1
Biological Process	GO:0006111	regulation of gluconeogenesis	7/2279	49/18866	0.379583	0.6413241	0.5802928	7	MAEA/C1QTNF3/PGP/DGKQ/FOXO1/SOGA1/SDHAF3
Biological Process	GO:0061383	trabecula morphogenesis	7/2279	49/18866	0.379583	0.6413241	0.5802928	7	SBNO2/CCM2L/RBPJ/SEMA4D/ENG/NOTCH1/FHL2
Biological Process	GO:0090151	establishment of protein localization to mitochondrial membrane	7/2279	49/18866	0.379583	0.6413241	0.5802928	7	TFDP1/YWHAH/BMF/YWHAZ/BCL2/TP53BP2/YWHAQ

Biological Process	GO:2000059	negative regulation of ubiquitin-dependent protein catabolic process	7/2279	49/18866	0.379583	0.6413241	0.5802928	7	TRIM39/SUFU/HIPK2/CLEC16A/MAP1A/BAG5/SVIP
Biological Process	GO:0006913	nucleocytoplasmic transport	45/2279	354/18866	0.3804565	0.6413241	0.5802928	45	CALR/NXF1/FIP1L1/AKAP13/ANGPT1/CPSF3/SNRPD3/PIK3R1/SETD2/RAE1/SMAD3/TNPO1/MBTPS1/E2F3/SUFU/IPO13/ATXN1/FAM53B/JUP/XPO7/TCF7L2/SMG7/XPO6/TGFB1/PABPN1/ENY2/SMURF1/BACH2/NF1/NUP93/SEC13/SMG6/ARNTL/CDH1/SNUPN/CABP1/SP100/NEDD4/TSC2/HHEX/RBM15B/NOL6/CPSF4/PCID2/GEMIN4
Biological Process	GO:0019748	secondary metabolic process	8/2279	57/18866	0.3828412	0.6413241	0.5802928	8	ZEB2/ABCC2/DDC/CDH3/RAPGEF2/MYO5A/NFE2L2/BCL2
Biological Process	GO:0061005	cell differentiation involved in kidney development	8/2279	57/18866	0.3828412	0.6413241	0.5802928	8	PAX2/MTSS1/WWTR1/PDGFB/NOTCH1/MEF2C/GPR4/LIF
Biological Process	GO:0098900	regulation of action potential	8/2279	57/18866	0.3828412	0.6413241	0.5802928	8	KCNAB2/CACNA1C/GJA5/JUP/BIN1/NOS1AP/CNR2/ANK2
Biological Process	GO:0048645	animal organ formation	9/2279	65/18866	0.3851595	0.6413241	0.5802928	9	TGFBR2/RBPJ/WNT11/PAX2/FGFR1/RDH10/NOTCH1/MEF2C/NTF4
Biological Process	GO:0000272	polysaccharide catabolic process	4/2279	26/18866	0.3860738	0.6413241	0.5802928	4	AOAH/MGAM/CHIT1/PPP1CB
Biological Process	GO:0001672	regulation of chromatin assembly or disassembly	4/2279	26/18866	0.3860738	0.6413241	0.5802928	4	PADI2/ATF7IP/DNMT1/PARP10
Biological Process	GO:0002227	innate immune response in mucosa	4/2279	26/18866	0.3860738	0.6413241	0.5802928	4	DEFA4/RNASE3/DEFB1/LTF

Biological Process	GO:0006309	apoptotic DNA fragmentation	4/2279	26/18866	0.3860738	0.6413241	0.5802928	4	CECR2/PAM16/DFFB/DNASE1L3
Biological Process	GO:0006688	glycosphingolipid biosynthetic process	4/2279	26/18866	0.3860738	0.6413241	0.5802928	4	ST8SIA6/B4GALT3/FA2H/PRKAA1
Biological Process	GO:0019430	removal of superoxide radicals	4/2279	26/18866	0.3860738	0.6413241	0.5802928	4	MPO/TNF/NFE2L2/SIRT3
Biological Process	GO:0021884	forebrain neuron development	4/2279	26/18866	0.3860738	0.6413241	0.5802928	4	NRP2/DISC1/ZMIZ1/RAPGEF2
Biological Process	GO:0033233	regulation of protein sumoylation	4/2279	26/18866	0.3860738	0.6413241	0.5802928	4	HDAC4/TOLLIP/ZMIZ1/RELA
Biological Process	GO:0033522	histone H2A ubiquitination	4/2279	26/18866	0.3860738	0.6413241	0.5802928	4	OTUB2/OTUB1/PCGF3/TRIP12
Biological Process	GO:0042403	thyroid hormone metabolic process	4/2279	26/18866	0.3860738	0.6413241	0.5802928	4	TG/CPQ/CTSB/CRYM
Biological Process	GO:0048843	negative regulation of axon extension involved in axon guidance	4/2279	26/18866	0.3860738	0.6413241	0.5802928	4	SEMA6B/SEMA4A/SEMA4B/SEMA4D
Biological Process	GO:0051875	pigment granule localization	4/2279	26/18866	0.3860738	0.6413241	0.5802928	4	RAB27A/CDH3/DCTN2/MYO5A

Biological Process	GO:0055083	monovalent inorganic anion homeostasis	4/2279	26/18866	0.3860738	0.6413241	0.5802928	4	ABCC2/SLC12A1/CA12/SLC12A7
Biological Process	GO:0060314	regulation of ryanodine-sensitive calcium-release channel activity	4/2279	26/18866	0.3860738	0.6413241	0.5802928	4	PDE4D/CASQ2/JSRP1/GSTO1
Biological Process	GO:0060384	innervation	4/2279	26/18866	0.3860738	0.6413241	0.5802928	4	LRIG1/NTRK1/NPTX1/NTF4
Biological Process	GO:0071377	cellular response to glucagon stimulus	4/2279	26/18866	0.3860738	0.6413241	0.5802928	4	ADCY2/PRKAR1B/ADCY9/ADCY4
Biological Process	GO:0071425	hematopoietic stem cell proliferation	4/2279	26/18866	0.3860738	0.6413241	0.5802928	4	RUNX1/ETV6/KAT7/MECOM
Biological Process	GO:0072202	cell differentiation involved in metanephros development	4/2279	26/18866	0.3860738	0.6413241	0.5802928	4	PAX2/WWTR1/PDGFB/LIF
Biological Process	GO:1903010	regulation of bone development	4/2279	26/18866	0.3860738	0.6413241	0.5802928	4	POR/GHRL/LTF/RFLNA
Biological Process	GO:1903020	positive regulation of glycoprotein metabolic process	4/2279	26/18866	0.3860738	0.6413241	0.5802928	4	TCF7L2/POMT2/PLCB1/PXYLP1
Biological Process	GO:1903649	regulation of cytoplasmic transport	4/2279	26/18866	0.3860738	0.6413241	0.5802928	4	SORL1/SNX3/EZR/PRKN

Biological Process	GO:1904353	regulation of telomere capping	4/2279	26/18866	0.3860738	0.6413241	0.5802928	4	TNKS/RTEL1/SMG6/ERCC1
Biological Process	GO:1904861	excitatory synapse assembly	4/2279	26/18866	0.3860738	0.6413241	0.5802928	4	SEMA4A/NRXN1/NRXN2/SHANK2
Biological Process	GO:0003208	cardiac ventricle morphogenesis	10/2279	73/18866	0.3868167	0.6413241	0.5802928	10	RXRA/CCM2L/TGFBR2/RBPJ/SMAD7/TGFB1/GRHL2/ENG/NOTCH1/MEF2C
Biological Process	GO:1902036	regulation of hematopoietic stem cell differentiation	10/2279	73/18866	0.3868167	0.6413241	0.5802928	10	MYB/RUNX1/TCF12/SETD1A/PSMF1/NFE2L2/PUS7/TCF3/PSMB7/PSMD13
Biological Process	GO:1902117	positive regulation of organelle assembly	10/2279	73/18866	0.3868167	0.6413241	0.5802928	10	ULK1/TNF/DYNC1H1/CEP135/PIP4K2A/ASAP1/HTT/NUMA1/SPAG5/SEPTIN9
Biological Process	GO:0061025	membrane fusion	22/2279	169/18866	0.3879772	0.6413241	0.5802928	22	CALR/STX5/CD9/VTI1A/ANKFY1/TGFBRAP1/DNM2/FOLR3/UVRAG/VPS18/VCPIP1/DNM1/DNM3/DNM1L/UBXN2A/DYSF/MX1/TSNARE1/ANXA7/VAV3/IRAG2/RUBCNL
Biological Process	GO:0001960	negative regulation of cytokine-mediated signaling pathway	11/2279	81/18866	0.3879914	0.6413241	0.5802928	11	PPARG/CCL5/TRAIP/PADI2/MIR24-2/MIR27A/IL1RN/NR1H2/PTPRC/NLRC5/SLIT3
Biological Process	GO:0016126	sterol biosynthetic process	11/2279	81/18866	0.3879914	0.6413241	0.5802928	11	PRKAG2/IDI1/MBTPS1/ABCG1/POR/ACACB/ACACA/HMGCS1/SCD/SREBF1/PRKAA1
Biological Process	GO:0010970	transport along microtubule	21/2279	161/18866	0.3884303	0.6413241	0.5802928	21	PRKCZ/NDE1/TNPO1/KIF13A/SPG7/DYNC1H1/APP/BICD2/CLUAP1/CNIH2/HTT/KIF5C/IFT140/KIF1B/TTC21B/MAP1A/KIFAP3/AP3D1/FBXW11/NDEL1/DTNBP1

Biological Process	GO:0034767	positive regulation of ion transmembrane transport	21/2279	161/18866	0.3884303	0.6413241	0.5802928	21	KCNE1/FXYD1/CCR2/DNM2/TESC/NOS1AP/ACTN4/P2RY6/HTR3A/HTT/KCNQ1/GSTO1/F2R/ANO6/ANK2/PLCG1/PDZK1/CASQ1/FGF14/STIMATE/CRACR2A
Biological Process	GO:0007260	tyrosine phosphorylation of STAT protein	12/2279	89/18866	0.3888037	0.6413241	0.5802928	12	TNF/CCL5/CSF1R/TNFRSF1A/FER/IL6R/PPP2R1A/GGNBP2/INPP5F/CRLF1/PARP9/LIF
Biological Process	GO:0043506	regulation of JUN kinase activity	12/2279	89/18866	0.3888037	0.6413241	0.5802928	12	HIPK3/ERN1/TNF/ZEB2/TRAF2/TLR9/TNIK/RIPK1/TAOK3/DTNBP1/DBNL/MAP3K20
Biological Process	GO:0043648	dicarboxylic acid metabolic process	13/2279	97/18866	0.3893372	0.6413241	0.5802928	13	HAL/GGT1/D2HGDH/MTHFD1L/MTHFS/OGDH/ALDH4A1/SLC19A1/MTHFD1/HOGA1/ACSF3/SDHAF3/DGLUCY
Biological Process	GO:1901216	positive regulation of neuron death	13/2279	97/18866	0.3893372	0.6413241	0.5802928	13	HDAC4/CTSZ/SSH1/MYB/SRPK2/TNF/CLU/ZNF746/NF1/GRN/ITGB2/FIS1/TP53BP2
Biological Process	GO:0019730	antimicrobial humoral response	18/2279	137/18866	0.3894741	0.6413241	0.5802928	18	DEFA4/AZU1/ELANE/SLPI/PRTN3/S100A8/CTSG/LCN2/RNASE3/SLC11A1/SFTPD/SPON2/CLU/DEFB1/PGLYRP1/LTF/PGLYRP4/BCL3
Biological Process	GO:0062014	negative regulation of small molecule metabolic process	14/2279	105/18866	0.3896525	0.6413241	0.5802928	14	MAEA/HDAC4/GFI1/CDA/AP2A1/C1QTNF3/MFSD2A/CBFA2T3/PGP/TGFB1/ACACB/SOGA1/WDTC1/ACADVL
Biological Process	GO:0006406	mRNA export from nucleus	15/2279	113/18866	0.3897938	0.6413241	0.5802928	15	NXF1/FIP1L1/CPSF3/SETD2/RAE1/SMG7/PABPN1/ENY2/NUP93/SEC13/SMG6/HHEX/RBM15B/CPSF4/PCID2
Biological Process	GO:0071427	mRNA-containing ribonucleoprotein complex export from nucleus	15/2279	113/18866	0.3897938	0.6413241	0.5802928	15	NXF1/FIP1L1/CPSF3/SETD2/RAE1/SMG7/PABPN1/ENY2/NUP93/SEC13/SMG6/HHEX/RBM15B/CPSF4/PCID2

Biological Process	GO:0002065	columnar/cuboidal epithelial cell differentiation	16/2279	121/18866	0.3897949	0.6413241	0.5802928	16	RXRA/RHEB/RFX3/DLL1/RBPJ/WNT11/FGFR1/RARA/RARG/ARNTL/NOTCH1/TMC1/CEBPB/FAM20C/NPY/NODAL
Biological Process	GO:0031398	positive regulation of protein ubiquitination	16/2279	121/18866	0.3897949	0.6413241	0.5802928	16	FZR1/ANGPT1/ARRB1/CUL3/SMAD7/AXIN1/MTA1/BCL10/ARRB2/NOD2/UBE3A/RNF40/PELI1/PAXIP1/PRKN/MARCHF7
Biological Process	GO:0000491	small nucleolar ribonucleoprotein complex assembly	2/2279	11/18866	0.3906575	0.6413241	0.5802928	2	PIH1D1/RUVBL1
Biological Process	GO:0005984	disaccharide metabolic process	2/2279	11/18866	0.3906575	0.6413241	0.5802928	2	MGAM/SLC2A1
Biological Process	GO:0006348	chromatin silencing at telomere	2/2279	11/18866	0.3906575	0.6413241	0.5802928	2	DYDC2/DYDC1
Biological Process	GO:0006558	L-phenylalanine metabolic process	2/2279	11/18866	0.3906575	0.6413241	0.5802928	2	HGD/FAH
Biological Process	GO:0006559	L-phenylalanine catabolic process	2/2279	11/18866	0.3906575	0.6413241	0.5802928	2	HGD/FAH
Biological Process	GO:0006570	tyrosine metabolic process	2/2279	11/18866	0.3906575	0.6413241	0.5802928	2	HGD/FAH
Biological Process	GO:0006857	oligopeptide transport	2/2279	11/18866	0.3906575	0.6413241	0.5802928	2	ABCC1/SLC15A4

Biological Process	GO:0007028	cytoplasm organization	2/2279	11/18866	0.3906575	0.6413241	0.5802928	2	ZMIZ1/ETV6
Biological Process	GO:0007256	activation of JNKK activity	2/2279	11/18866	0.3906575	0.6413241	0.5802928	2	TNIK/RIPK1
Biological Process	GO:0007614	short-term memory	2/2279	11/18866	0.3906575	0.6413241	0.5802928	2	COMT/CUX2
Biological Process	GO:0008611	ether lipid biosynthetic process	2/2279	11/18866	0.3906575	0.6413241	0.5802928	2	CHPT1/PEDS1
Biological Process	GO:0017085	response to insecticide	2/2279	11/18866	0.3906575	0.6413241	0.5802928	2	DDC/FECH
Biological Process	GO:0018401	peptidyl-proline hydroxylation to 4-hydroxy-L-proline	2/2279	11/18866	0.3906575	0.6413241	0.5802928	2	P4HB/P4HA2
Biological Process	GO:0019532	oxalate transport	2/2279	11/18866	0.3906575	0.6413241	0.5802928	2	SLC26A1/AGXT
Biological Process	GO:0021604	cranial nerve structural organization	2/2279	11/18866	0.3906575	0.6413241	0.5802928	2	NRP2/PAX2
Biological Process	GO:0030422	production of siRNA involved in RNA interference	2/2279	11/18866	0.3906575	0.6413241	0.5802928	2	AGO2/RMRP

Biological Process	GO:0033539	fatty acid beta-oxidation using acyl-CoA dehydrogenase	2/2279	11/18866	0.3906575	0.6413241	0.5802928	2	ACADM/ACADVL
Biological Process	GO:0033860	regulation of NAD(P)H oxidase activity	2/2279	11/18866	0.3906575	0.6413241	0.5802928	2	GNAI2/AGTR1
Biological Process	GO:0034378	chylomicron assembly	2/2279	11/18866	0.3906575	0.6413241	0.5802928	2	P4HB/PLAGL2
Biological Process	GO:0035090	maintenance of apical/basal cell polarity	2/2279	11/18866	0.3906575	0.6413241	0.5802928	2	WNT11/WDR1
Biological Process	GO:0035721	intraciliary retrograde transport	2/2279	11/18866	0.3906575	0.6413241	0.5802928	2	IFT140/TTC21B
Biological Process	GO:0035747	natural killer cell chemotaxis	2/2279	11/18866	0.3906575	0.6413241	0.5802928	2	PIK3CD/CCL5
Biological Process	GO:0042670	retinal cone cell differentiation	2/2279	11/18866	0.3906575	0.6413241	0.5802928	2	GNAT2/CABP4
Biological Process	GO:0042758	long-chain fatty acid catabolic process	2/2279	11/18866	0.3906575	0.6413241	0.5802928	2	CYP4F3/ADTRP
Biological Process	GO:0043471	regulation of cellular carbohydrate catabolic process	2/2279	11/18866	0.3906575	0.6413241	0.5802928	2	PGAM1/PPP1CB

Biological Process	GO:0045060	negative thymic T cell selection	2/2279	11/18866	0.3906575	0.6413241	0.5802928	2	PTPRC/DOCK2
Biological Process	GO:0045141	meiotic telomere clustering	2/2279	11/18866	0.3906575	0.6413241	0.5802928	2	RAD21L1/TERB2
Biological Process	GO:0045199	maintenance of epithelial cell apical/basal polarity	2/2279	11/18866	0.3906575	0.6413241	0.5802928	2	WNT11/WDR1
Biological Process	GO:0046504	glycerol ether biosynthetic process	2/2279	11/18866	0.3906575	0.6413241	0.5802928	2	CHPT1/PEDS1
Biological Process	GO:0046549	retinal cone cell development	2/2279	11/18866	0.3906575	0.6413241	0.5802928	2	GNAT2/CABP4
Biological Process	GO:0046642	negative regulation of alpha-beta T cell proliferation	2/2279	11/18866	0.3906575	0.6413241	0.5802928	2	ZBTB7B/VSIR
Biological Process	GO:0046643	regulation of gamma-delta T cell activation	2/2279	11/18866	0.3906575	0.6413241	0.5802928	2	PTPRC/NOD2
Biological Process	GO:0048680	positive regulation of axon regeneration	2/2279	11/18866	0.3906575	0.6413241	0.5802928	2	GRN/NDEL1
Biological Process	GO:0048742	regulation of skeletal muscle fiber development	2/2279	11/18866	0.3906575	0.6413241	0.5802928	2	HDAC4/BCL2

Biological Process	GO:0048853	forebrain morphogenesis	2/2279	11/18866	0.3906575	0.6413241	0.5802928	2	NF1/SLIT1
Biological Process	GO:0060159	regulation of dopamine receptor signaling pathway	2/2279	11/18866	0.3906575	0.6413241	0.5802928	2	PALM/DTNBP1
Biological Process	GO:0060281	regulation of oocyte development	2/2279	11/18866	0.3906575	0.6413241	0.5802928	2	PPP2R1A/NPPC
Biological Process	GO:0060315	negative regulation of ryanodine-sensitive calcium-release channel activity	2/2279	11/18866	0.3906575	0.6413241	0.5802928	2	CASQ2/GSTO1
Biological Process	GO:0060379	cardiac muscle cell myoblast differentiation	2/2279	11/18866	0.3906575	0.6413241	0.5802928	2	RBPJ/NOTCH1
Biological Process	GO:0060525	prostate glandular acinus development	2/2279	11/18866	0.3906575	0.6413241	0.5802928	2	RXRA/NOTCH1
Biological Process	GO:0060947	cardiac vascular smooth muscle cell differentiation	2/2279	11/18866	0.3906575	0.6413241	0.5802928	2	MIR145/NOTCH1
Biological Process	GO:0061299	retina vasculature morphogenesis in camera-type eye	2/2279	11/18866	0.3906575	0.6413241	0.5802928	2	LRP5L/ARHGEF15
Biological Process	GO:0061314	Notch signaling involved in heart development	2/2279	11/18866	0.3906575	0.6413241	0.5802928	2	RBPJ/NOTCH1

Biological Process	GO:0070213	protein auto-ADP-ribosylation	2/2279	11/18866	0.3906575	0.6413241	0.5802928	2	TNKS/PARP10
Biological Process	GO:0070933	histone H4 deacetylation	2/2279	11/18866	0.3906575	0.6413241	0.5802928	2	HDAC4/HDAC1
Biological Process	GO:0071609	chemokine (C-C motif) ligand 5 production	2/2279	11/18866	0.3906575	0.6413241	0.5802928	2	IL10/DEFB124
Biological Process	GO:0071803	positive regulation of podosome assembly	2/2279	11/18866	0.3906575	0.6413241	0.5802928	2	TNF/ASAP1
Biological Process	GO:0071838	cell proliferation in bone marrow	2/2279	11/18866	0.3906575	0.6413241	0.5802928	2	MAP3K3/MEF2C
Biological Process	GO:0072697	protein localization to cell cortex	2/2279	11/18866	0.3906575	0.6413241	0.5802928	2	NUMA1/EZR
Biological Process	GO:0086016	AV node cell action potential	2/2279	11/18866	0.3906575	0.6413241	0.5802928	2	CACNA1C/GJA5
Biological Process	GO:0086027	AV node cell to bundle of His cell signaling	2/2279	11/18866	0.3906575	0.6413241	0.5802928	2	CACNA1C/GJA5
Biological Process	GO:0090030	regulation of steroid hormone biosynthetic process	2/2279	11/18866	0.3906575	0.6413241	0.5802928	2	DGKQ/POR

Biological Process	GO:0090084	negative regulation of inclusion body assembly	2/2279	11/18866	0.3906575	0.6413241	0.5802928	2	SORL1/DNAJB6
Biological Process	GO:0090309	positive regulation of DNA methylation-dependent heterochromatin assembly	2/2279	11/18866	0.3906575	0.6413241	0.5802928	2	ATF7IP/DNMT1
Biological Process	GO:0090557	establishment of endothelial intestinal barrier	2/2279	11/18866	0.3906575	0.6413241	0.5802928	2	TJP2/RAPGEF2
Biological Process	GO:0097048	dendritic cell apoptotic process	2/2279	11/18866	0.3906575	0.6413241	0.5802928	2	RAPGEF2/BLK
Biological Process	GO:0097384	cellular lipid biosynthetic process	2/2279	11/18866	0.3906575	0.6413241	0.5802928	2	CHPT1/PEDS1
Biological Process	GO:0098883	synapse pruning	2/2279	11/18866	0.3906575	0.6413241	0.5802928	2	C1QB/CX3CR1
Biological Process	GO:0106072	negative regulation of adenylate cyclase-activating G protein-coupled receptor signaling pathway	2/2279	11/18866	0.3906575	0.6413241	0.5802928	2	GNAI2/MRAP
Biological Process	GO:0150104	transport across blood-brain barrier	2/2279	11/18866	0.3906575	0.6413241	0.5802928	2	MFSD2A/ABCC2
Biological Process	GO:1900112	regulation of histone H3-K9 trimethylation	2/2279	11/18866	0.3906575	0.6413241	0.5802928	2	KDM4C/PIH1D1

Biological Process	GO:1902093	positive regulation of flagellated sperm motility	2/2279	11/18866	0.3906575	0.6413241	0.5802928	2	RNASE10/DEFB1
Biological Process	GO:1902221	erythrose 4-phosphate/phosphoenolpyruvate family amino acid metabolic process	2/2279	11/18866	0.3906575	0.6413241	0.5802928	2	HGD/FAH
Biological Process	GO:1902222	erythrose 4-phosphate/phosphoenolpyruvate family amino acid catabolic process	2/2279	11/18866	0.3906575	0.6413241	0.5802928	2	HGD/FAH
Biological Process	GO:1903011	negative regulation of bone development	2/2279	11/18866	0.3906575	0.6413241	0.5802928	2	LTF/RFLNA
Biological Process	GO:1903800	positive regulation of production of miRNAs involved in gene silencing by miRNA	2/2279	11/18866	0.3906575	0.6413241	0.5802928	2	RIPK1/TGFB1
Biological Process	GO:1904321	response to forskolin	2/2279	11/18866	0.3906575	0.6413241	0.5802928	2	ADCY2/FDX1
Biological Process	GO:1904322	cellular response to forskolin	2/2279	11/18866	0.3906575	0.6413241	0.5802928	2	ADCY2/FDX1
Biological Process	GO:1904396	regulation of neuromuscular junction development	2/2279	11/18866	0.3906575	0.6413241	0.5802928	2	DCTN1/COLQ
Biological Process	GO:1905383	protein localization to presynapse	2/2279	11/18866	0.3906575	0.6413241	0.5802928	2	KIF5C/MAP1A

Biological Process	GO:1905879	regulation of oogenesis	2/2279	11/18866	0.3906575	0.6413241	0.5802928	2	PPP2R1A/NPPC
Biological Process	GO:2000049	positive regulation of cell-cell adhesion mediated by cadherin	2/2279	11/18866	0.3906575	0.6413241	0.5802928	2	PTPRU/FLOT1
Biological Process	GO:2000155	positive regulation of cilium-dependent cell motility	2/2279	11/18866	0.3906575	0.6413241	0.5802928	2	RNASE10/DEFB1
Biological Process	GO:2000480	negative regulation of cAMP-dependent protein kinase activity	2/2279	11/18866	0.3906575	0.6413241	0.5802928	2	PRKAG2/PRKAR1B
Biological Process	GO:2000551	regulation of T-helper 2 cell cytokine production	2/2279	11/18866	0.3906575	0.6413241	0.5802928	2	PRKCZ/NLRP3
Biological Process	GO:2000615	regulation of histone H3-K9 acetylation	2/2279	11/18866	0.3906575	0.6413241	0.5802928	2	SPHK2/PIH1D1
Biological Process	GO:2000668	regulation of dendritic cell apoptotic process	2/2279	11/18866	0.3906575	0.6413241	0.5802928	2	RAPGEF2/BLK
Biological Process	GO:2001269	positive regulation of cysteine-type endopeptidase activity involved in apoptotic signaling pathway	2/2279	11/18866	0.3906575	0.6413241	0.5802928	2	GSN/SMAD3
Biological Process	GO:0045787	positive regulation of cell cycle	50/2279	396/18866	0.3909818	0.6413241	0.5802928	50	RPTOR/BRD4/CALR/TGFA/IL10/TFDP1/BRCA2/SRPK2/PRKCA/NSMCE2/DYNC1H1/C6orf89/E2F7/CUL3/PCBP4/APP/ZNF385A/CDC16/ZBTB17/POC1A/FGFR1/CDC25A/ARID3A/OVOL1/TMOD3/SVIL/RARA/CSPP1/NUMA1/PDGFB/MEIS2/PLCB1/RB1/MED25/DUSP3/PRKCE/CDCA5/RHNO1/CHEK2/ANAPC7/PDGFRB/USP2/SPAG5/CDC14A/PLRG1/CCNY/CCNYL1/EIF4G1/PCID2/SLF1

Biological Process	GO:0000737	DNA catabolic process, endonucleolytic	5/2279	34/18866	0.3932171	0.6413241	0.5802928	5	CECR2/PAM16/DFFB/DNASE1L3/FBH1
Biological Process	GO:0002888	positive regulation of myeloid leukocyte mediated immunity	5/2279	34/18866	0.3932171	0.6413241	0.5802928	5	CD177/GAB2/FGR/SPHK2/ITGB2
Biological Process	GO:0009156	ribonucleoside monophosphate biosynthetic process	5/2279	34/18866	0.3932171	0.6413241	0.5802928	5	AMPD3/UCK2/LHPP/DHODH/IMPDH1
Biological Process	GO:0016242	negative regulation of macroautophagy	5/2279	34/18866	0.3932171	0.6413241	0.5802928	5	USP36/FEZ2/CLEC16A/NPC1/TSC2
Biological Process	GO:0019076	viral release from host cell	5/2279	34/18866	0.3932171	0.6413241	0.5802928	5	TRIM27/TRIM8/PC/TRIM5/TRIM26
Biological Process	GO:0032148	activation of protein kinase B activity	5/2279	34/18866	0.3932171	0.6413241	0.5802928	5	PRKCZ/MIR143/ITGB1BP1/PRR5/PDGFB
Biological Process	GO:0033198	response to ATP	5/2279	34/18866	0.3932171	0.6413241	0.5802928	5	SSH1/SLC8A1/DGKQ/P2RX1/P2RX5
Biological Process	GO:0035774	positive regulation of insulin secretion involved in cellular response to glucose stimulus	5/2279	34/18866	0.3932171	0.6413241	0.5802928	5	NADK/ARRB1/GPLD1/GHRL/C2CD2L
Biological Process	GO:0035890	exit from host	5/2279	34/18866	0.3932171	0.6413241	0.5802928	5	TRIM27/TRIM8/PC/TRIM5/TRIM26

Biological Process	GO:0035891	exit from host cell	5/2279	34/18866	0.3932171	0.6413241	0.5802928	5	TRIM27/TRIM8/PC/TRIM5/TRIM26
Biological Process	GO:0045648	positive regulation of erythrocyte differentiation	5/2279	34/18866	0.3932171	0.6413241	0.5802928	5	INPP5D/BRD1/ETS1/KAT7/INHBA
Biological Process	GO:0061384	heart trabecula morphogenesis	5/2279	34/18866	0.3932171	0.6413241	0.5802928	5	CCM2L/RBPJ/ENG/NOTCH1/FHL2
Biological Process	GO:0070102	interleukin-6-mediated signaling pathway	5/2279	34/18866	0.3932171	0.6413241	0.5802928	5	GFI1/RIPK1/JAK1/FER/IL6R
Biological Process	GO:0099084	postsynaptic specialization organization	5/2279	34/18866	0.3932171	0.6413241	0.5802928	5	ZMYND8/SYNGAP1/NRXN1/NRXN2/SHANK2
Biological Process	GO:1902692	regulation of neuroblast proliferation	5/2279	34/18866	0.3932171	0.6413241	0.5802928	5	DISC1/NF1/ZNF335/NOTCH1/CX3CR1
Biological Process	GO:1904030	negative regulation of cyclin-dependent protein kinase activity	5/2279	34/18866	0.3932171	0.6413241	0.5802928	5	TNFAIP3/FBXO7/LATS2/HHEX/HEXIM1
Biological Process	GO:0045088	regulation of innate immune response	39/2279	307/18866	0.3936059	0.6413241	0.5802928	39	NLRC4/AIM2/PPARG/CLEC4C/PIK3R6/FGR/TNFAIP3/CCL5/FPR2/MNDA/CD226/HLA-F/PTPN6/MUC12/JAK1/NCR1/FCN1/TRIM5/MUC20/NR1H2/TREML4/BCL10/PSMF1/GRN/NLRC5/CREBBP/ARRB2/IL18RAP/PSMB7/TYRO3/CADM1/CD96/RELA/FBXW11/HEXIM1/PARP9/FADD/PSMD13/STING1
Biological Process	GO:0007601	visual perception	28/2279	218/18866	0.3944045	0.6413241	0.5802928	28	RCVRN/TACSTD2/PDE6A/CACNA2D4/OPA3/CRB1/TGFBI/CDH23/TULP2/CCDC66/GNAT2/PAX2/PITPNA/CDH3/RDH10/NOB1/MYO5A/CRYBB1/PDE6H/RPGRIP1/CABP1/EPAS1/GRK7/NRL/ABLIM1/EYS/KIFC3/CABP4

Biological Process	GO:1901605	alpha-amino acid metabolic process	26/2279	202/18866	0.3960165	0.6413241	0.5802928	26	ACAD8/HAL/HGD/GGT1/CBS/ODC1/MSRA/COMT/MTHFS/OGDH/ALDH4A1/FAH/HIBADH/SLC7A7/AGXT/MTHFD1/ART4/HOGA1/MAT1A/HMGCL/GFPT2/CRYM/ADI1/ACAT1/HNMT/DGLUCY
Biological Process	GO:0009268	response to pH	6/2279	42/18866	0.3975557	0.6413241	0.5802928	6	GPLD1/GNA11/ARSB/INSRR/GPR4/SST
Biological Process	GO:0044058	regulation of digestive system process	6/2279	42/18866	0.3975557	0.6413241	0.5802928	6	SGK1/AQP1/NR1H2/GHRL/SLC22A5/KCNQ1
Biological Process	GO:0051602	response to electrical stimulus	6/2279	42/18866	0.3975557	0.6413241	0.5802928	6	DISC1/BRD1/GHRL/PALM/NTRK1/CYFIP1
Biological Process	GO:0072595	maintenance of protein localization in organelle	6/2279	42/18866	0.3975557	0.6413241	0.5802928	6	HK1/SYNE1/HK2/MORC3/SP100/BCL3
Biological Process	GO:0090278	negative regulation of peptide hormone secretion	6/2279	42/18866	0.3975557	0.6413241	0.5802928	6	CRHBP/ENY2/GHRL/KLF7/SREBF1/PRKN
Biological Process	GO:0090279	regulation of calcium ion import	6/2279	42/18866	0.3975557	0.6413241	0.5802928	6	TRIM27/SPHK2/PDGFB/PDGFRB/DYSF/TRPV2
Biological Process	GO:0098927	vesicle-mediated transport between endosomal compartments	6/2279	42/18866	0.3975557	0.6413241	0.5802928	6	LMTK2/SORL1/SNX3/HOOK2/WDR81/EZR
Biological Process	GO:0043488	regulation of mRNA stability	24/2279	186/18866	0.3975921	0.6413241	0.5802928	24	EXOSC2/SLC11A1/PABPC4/TNPO1/PRKCA/LARP1/ZC3H12D/PCBP4/SAMD4A/CPEB3/IGF2BP3/PSMF1/FTO/YWHAZ/PSMB7/PAIP1/TNFSF13/SYNCRIP/EIF4G1/PSMD13/PCID2/TBRG4/TENT5C/TENT5A

Biological Process	GO:0006941	striated muscle contraction	23/2279	178/18866	0.3983604	0.6413241	0.5802928	23	HDAC4/PDE4D/KCNE1/TTN/CASQ2/MAP2K6/CACNA1C/SLC8A1/GJA5/TNNT3/JUP/BIN1/JSRP1/NOS1AP/SMAD7/SCN1A/SNTA1/KCNQ1/GSTO1/ANK2/CASQ1/MB/GRK2
Biological Process	GO:0019827	stem cell population maintenance	21/2279	162/18866	0.3998423	0.6413241	0.5802928	21	FOXP1/TRIM8/LSM1/MIR145/WDR43/SKI/POLR2F/LBH/DLL1/RBPJ/PAX2/KDM4C/PRDM16/ESRRB/NOTCH1/ZHX2/DIS3L2/FOXO1/NODAL/LIF/ZNF706
Biological Process	GO:0007528	neuromuscular junction development	7/2279	50/18866	0.4003043	0.6413241	0.5802928	7	DCTN1/COLQ/APP/PPFIBP2/SHANK2/F2R/NEDD4
Biological Process	GO:0042987	amyloid precursor protein catabolic process	7/2279	50/18866	0.4003043	0.6413241	0.5802928	7	SORL1/TNF/ABCG1/BIN1/APP/CLU/RELA
Biological Process	GO:0045104	intermediate filament cytoskeleton organization	7/2279	50/18866	0.4003043	0.6413241	0.5802928	7	KRT17/PKP1/NEFM/DNAJB6/GFAP/NDEL1/PPL
Biological Process	GO:0045981	positive regulation of nucleotide metabolic process	7/2279	50/18866	0.4003043	0.6413241	0.5802928	7	RCVRN/ENO1/PFKFB4/APP/ESRRB/PFKFB3/PRKAA1
Biological Process	GO:0048546	digestive tract morphogenesis	7/2279	50/18866	0.4003043	0.6413241	0.5802928	7	SMAD3/EPHB3/WNT11/BCL2/NOTCH1/HLX/NODAL
Biological Process	GO:1900544	positive regulation of purine nucleotide metabolic process	7/2279	50/18866	0.4003043	0.6413241	0.5802928	7	RCVRN/ENO1/PFKFB4/APP/ESRRB/PFKFB3/PRKAA1
Biological Process	GO:0055076	transition metal ion homeostasis	18/2279	138/18866	0.401858	0.6413241	0.5802928	18	S100A8/SLC25A37/LCN2/LCK/SLC11A1/COX19/TTC7A/SLC11A2/APP/ATOX1/SLC39A13/EPAS1/LTF/EPB42/AP3D1/ATP7B/NCOA4/PRKN

Biological Process	GO:0000768	syncytium formation by plasma membrane fusion	8/2279	58/18866	0.4020632	0.6413241	0.5802928	8	EHD1/SBNO2/CD9/TANC1/NFATC2/MYH9/FLOT1/SCGB3A1
Biological Process	GO:0140253	cell-cell fusion	8/2279	58/18866	0.4020632	0.6413241	0.5802928	8	EHD1/SBNO2/CD9/TANC1/NFATC2/MYH9/FLOT1/SCGB3A1
Biological Process	GO:0000706	meiotic DNA double-strand break processing	1/2279	4/18866	0.4025066	0.6413241	0.5802928	1	SLX4
Biological Process	GO:0000720	pyrimidine dimer repair by nucleotide-excision repair	1/2279	4/18866	0.4025066	0.6413241	0.5802928	1	ERCC1
Biological Process	GO:0000821	regulation of arginine metabolic process	1/2279	4/18866	0.4025066	0.6413241	0.5802928	1	SLC7A7
Biological Process	GO:0001806	type IV hypersensitivity	1/2279	4/18866	0.4025066	0.6413241	0.5802928	1	FUT7
Biological Process	GO:0002018	renin-angiotensin regulation of aldosterone production	1/2279	4/18866	0.4025066	0.6413241	0.5802928	1	AGTR1
Biological Process	GO:0002125	maternal aggressive behavior	1/2279	4/18866	0.4025066	0.6413241	0.5802928	1	CRHBP
Biological Process	GO:0002238	response to molecule of fungal origin	1/2279	4/18866	0.4025066	0.6413241	0.5802928	1	SCIMP

Biological Process	GO:0002282	microglial cell activation involved in immune response	1/2279	4/18866	0.4025066	0.6413241	0.5802928	1	GRN
Biological Process	GO:0002309	T cell proliferation involved in immune response	1/2279	4/18866	0.4025066	0.6413241	0.5802928	1	SLC11A1
Biological Process	GO:0002325	natural killer cell differentiation involved in immune response	1/2279	4/18866	0.4025066	0.6413241	0.5802928	1	PGLYRP1
Biological Process	GO:0002399	MHC class II protein complex assembly	1/2279	4/18866	0.4025066	0.6413241	0.5802928	1	HLA-DMB
Biological Process	GO:0002503	peptide antigen assembly with MHC class II protein complex	1/2279	4/18866	0.4025066	0.6413241	0.5802928	1	HLA-DMB
Biological Process	GO:0002678	positive regulation of chronic inflammatory response	1/2279	4/18866	0.4025066	0.6413241	0.5802928	1	TNF
Biological Process	GO:0002901	mature B cell apoptotic process	1/2279	4/18866	0.4025066	0.6413241	0.5802928	1	BCL10
Biological Process	GO:0002905	regulation of mature B cell apoptotic process	1/2279	4/18866	0.4025066	0.6413241	0.5802928	1	BCL10
Biological Process	GO:0002906	negative regulation of mature B cell apoptotic process	1/2279	4/18866	0.4025066	0.6413241	0.5802928	1	BCL10

Biological Process	GO:0002943	tRNA dihydrouridine synthesis	1/2279	4/18866	0.4025066	0.6413241	0.5802928	1	DUS2
Biological Process	GO:0003104	positive regulation of glomerular filtration	1/2279	4/18866	0.4025066	0.6413241	0.5802928	1	PDGFB
Biological Process	GO:0003219	cardiac right ventricle formation	1/2279	4/18866	0.4025066	0.6413241	0.5802928	1	NOTCH1
Biological Process	GO:0003274	endocardial cushion fusion	1/2279	4/18866	0.4025066	0.6413241	0.5802928	1	TGFBR2
Biological Process	GO:0006121	mitochondrial electron transport, succinate to ubiquinone	1/2279	4/18866	0.4025066	0.6413241	0.5802928	1	SDHD
Biological Process	GO:0006145	purine nucleobase catabolic process	1/2279	4/18866	0.4025066	0.6413241	0.5802928	1	AOX1
Biological Process	GO:0006177	GMP biosynthetic process	1/2279	4/18866	0.4025066	0.6413241	0.5802928	1	IMPDH1
Biological Process	GO:0006272	leading strand elongation	1/2279	4/18866	0.4025066	0.6413241	0.5802928	1	POLE
Biological Process	GO:0006545	glycine biosynthetic process	1/2279	4/18866	0.4025066	0.6413241	0.5802928	1	AGXT

Biological Process	GO:0006556	S-adenosylmethionine biosynthetic process	1/2279	4/18866	0.4025066	0.6413241	0.5802928	1	MAT1A
Biological Process	GO:0006562	proline catabolic process	1/2279	4/18866	0.4025066	0.6413241	0.5802928	1	ALDH4A1
Biological Process	GO:0006565	L-serine catabolic process	1/2279	4/18866	0.4025066	0.6413241	0.5802928	1	CBS
Biological Process	GO:0006598	polyamine catabolic process	1/2279	4/18866	0.4025066	0.6413241	0.5802928	1	SAT2
Biological Process	GO:0006642	triglyceride mobilization	1/2279	4/18866	0.4025066	0.6413241	0.5802928	1	LPIN1
Biological Process	GO:0006659	phosphatidylserine biosynthetic process	1/2279	4/18866	0.4025066	0.6413241	0.5802928	1	PTDSS2
Biological Process	GO:0006772	thiamine metabolic process	1/2279	4/18866	0.4025066	0.6413241	0.5802928	1	ACP3
Biological Process	GO:0007227	signal transduction downstream of smoothened	1/2279	4/18866	0.4025066	0.6413241	0.5802928	1	STK36
Biological Process	GO:0007621	negative regulation of female receptivity	1/2279	4/18866	0.4025066	0.6413241	0.5802928	1	PPP1R1B

Biological Process	GO:0008592	regulation of Toll signaling pathway	1/2279	4/18866	0.4025066	0.6413241	0.5802928	1	PELI1
Biological Process	GO:0009073	aromatic amino acid family biosynthetic process	1/2279	4/18866	0.4025066	0.6413241	0.5802928	1	MTHFD1
Biological Process	GO:0009128	purine nucleoside monophosphate catabolic process	1/2279	4/18866	0.4025066	0.6413241	0.5802928	1	AMPD3
Biological Process	GO:0009298	GDP-mannose biosynthetic process	1/2279	4/18866	0.4025066	0.6413241	0.5802928	1	PMM2
Biological Process	GO:0010025	wax biosynthetic process	1/2279	4/18866	0.4025066	0.6413241	0.5802928	1	FAR2
Biological Process	GO:0010046	response to mycotoxin	1/2279	4/18866	0.4025066	0.6413241	0.5802928	1	LCN2
Biological Process	GO:0010166	wax metabolic process	1/2279	4/18866	0.4025066	0.6413241	0.5802928	1	FAR2
Biological Process	GO:0010193	response to ozone	1/2279	4/18866	0.4025066	0.6413241	0.5802928	1	CAT
Biological Process	GO:0010636	positive regulation of mitochondrial fusion	1/2279	4/18866	0.4025066	0.6413241	0.5802928	1	PRKN

Biological Process	GO:0010710	regulation of collagen catabolic process	1/2279	4/18866	0.4025066	0.6413241	0.5802928	1	VSIR
Biological Process	GO:0010724	regulation of definitive erythrocyte differentiation	1/2279	4/18866	0.4025066	0.6413241	0.5802928	1	ZFPM1
Biological Process	GO:0010748	negative regulation of long-chain fatty acid import across plasma membrane	1/2279	4/18866	0.4025066	0.6413241	0.5802928	1	THBS1
Biological Process	GO:0014038	regulation of Schwann cell differentiation	1/2279	4/18866	0.4025066	0.6413241	0.5802928	1	RELA
Biological Process	GO:0015766	disaccharide transport	1/2279	4/18866	0.4025066	0.6413241	0.5802928	1	SLC45A4
Biological Process	GO:0015770	sucrose transport	1/2279	4/18866	0.4025066	0.6413241	0.5802928	1	SLC45A4
Biological Process	GO:0015772	oligosaccharide transport	1/2279	4/18866	0.4025066	0.6413241	0.5802928	1	SLC45A4
Biological Process	GO:0015842	aminergic neurotransmitter loading into synaptic vesicle	1/2279	4/18866	0.4025066	0.6413241	0.5802928	1	DDC
Biological Process	GO:0015860	purine nucleoside transmembrane transport	1/2279	4/18866	0.4025066	0.6413241	0.5802928	1	SLC29A1

Biological Process	GO:0015870	acetylcholine transport	1/2279	4/18866	0.4025066	0.6413241	0.5802928	1	SLC44A4
Biological Process	GO:0015917	aminophospholipid transport	1/2279	4/18866	0.4025066	0.6413241	0.5802928	1	TMEM30A
Biological Process	GO:0016076	snRNA catabolic process	1/2279	4/18866	0.4025066	0.6413241	0.5802928	1	ZCCHC8
Biological Process	GO:0016240	autophagosome membrane docking	1/2279	4/18866	0.4025066	0.6413241	0.5802928	1	ATG14
Biological Process	GO:0018076	N-terminal peptidyl-lysine acetylation	1/2279	4/18866	0.4025066	0.6413241	0.5802928	1	CREBBP
Biological Process	GO:0018197	peptidyl-aspartic acid modification	1/2279	4/18866	0.4025066	0.6413241	0.5802928	1	ASPH
Biological Process	GO:0018201	peptidyl-glycine modification	1/2279	4/18866	0.4025066	0.6413241	0.5802928	1	NTMT1
Biological Process	GO:0018335	protein succinylation	1/2279	4/18866	0.4025066	0.6413241	0.5802928	1	OGDH
Biological Process	GO:0019086	late viral transcription	1/2279	4/18866	0.4025066	0.6413241	0.5802928	1	USF2

Biological Process	GO:0019376	galactolipid catabolic process	1/2279	4/18866	0.4025066	0.6413241	0.5802928	1	GALC
Biological Process	GO:0019556	histidine catabolic process to glutamate and formamide	1/2279	4/18866	0.4025066	0.6413241	0.5802928	1	HAL
Biological Process	GO:0019557	histidine catabolic process to glutamate and formate	1/2279	4/18866	0.4025066	0.6413241	0.5802928	1	HAL
Biological Process	GO:0019740	nitrogen utilization	1/2279	4/18866	0.4025066	0.6413241	0.5802928	1	BCL2
Biological Process	GO:0019747	regulation of isoprenoid metabolic process	1/2279	4/18866	0.4025066	0.6413241	0.5802928	1	RDH10
Biological Process	GO:0021555	midbrain-hindbrain boundary morphogenesis	1/2279	4/18866	0.4025066	0.6413241	0.5802928	1	HES3
Biological Process	GO:0021769	orbitofrontal cortex development	1/2279	4/18866	0.4025066	0.6413241	0.5802928	1	FGFR1
Biological Process	GO:0021773	striatal medium spiny neuron differentiation	1/2279	4/18866	0.4025066	0.6413241	0.5802928	1	INHBA
Biological Process	GO:0021855	hypothalamus cell migration	1/2279	4/18866	0.4025066	0.6413241	0.5802928	1	NRP2

Biological Process	GO:0021886	hypothalamus gonadotrophin-releasing hormone neuron differentiation	1/2279	4/18866	0.4025066	0.6413241	0.5802928	1	NRP2
Biological Process	GO:0021888	hypothalamus gonadotrophin-releasing hormone neuron development	1/2279	4/18866	0.4025066	0.6413241	0.5802928	1	NRP2
Biological Process	GO:0022009	central nervous system vasculogenesis	1/2279	4/18866	0.4025066	0.6413241	0.5802928	1	ENG
Biological Process	GO:0022615	protein to membrane docking	1/2279	4/18866	0.4025066	0.6413241	0.5802928	1	SNX3
Biological Process	GO:0030035	microspike assembly	1/2279	4/18866	0.4025066	0.6413241	0.5802928	1	MTSS1
Biological Process	GO:0030043	actin filament fragmentation	1/2279	4/18866	0.4025066	0.6413241	0.5802928	1	WDR1
Biological Process	GO:0031048	heterochromatin assembly by small RNA	1/2279	4/18866	0.4025066	0.6413241	0.5802928	1	ZNFX1
Biological Process	GO:0031291	Ran protein signal transduction	1/2279	4/18866	0.4025066	0.6413241	0.5802928	1	RCC1
Biological Process	GO:0031444	slow-twitch skeletal muscle fiber contraction	1/2279	4/18866	0.4025066	0.6413241	0.5802928	1	MB

Biological Process	GO:0031536	positive regulation of exit from mitosis	1/2279	4/18866	0.4025066	0.6413241	0.5802928	1	CDCA5
Biological Process	GO:0031554	regulation of DNA-templated transcription, termination	1/2279	4/18866	0.4025066	0.6413241	0.5802928	1	SCAF8
Biological Process	GO:0032264	IMP salvage	1/2279	4/18866	0.4025066	0.6413241	0.5802928	1	AMPD3
Biological Process	GO:0032289	central nervous system myelin formation	1/2279	4/18866	0.4025066	0.6413241	0.5802928	1	ERCC2
Biological Process	GO:0032455	nerve growth factor processing	1/2279	4/18866	0.4025066	0.6413241	0.5802928	1	PCSK6
Biological Process	GO:0032487	regulation of Rap protein signal transduction	1/2279	4/18866	0.4025066	0.6413241	0.5802928	1	TIMP2
Biological Process	GO:0032826	regulation of natural killer cell differentiation involved in immune response	1/2279	4/18866	0.4025066	0.6413241	0.5802928	1	PGLYRP1
Biological Process	GO:0032875	regulation of DNA endoreduplication	1/2279	4/18866	0.4025066	0.6413241	0.5802928	1	E2F7
Biological Process	GO:0032898	neurotrophin production	1/2279	4/18866	0.4025066	0.6413241	0.5802928	1	PCSK6

Biological Process	GO:0033031	positive regulation of neutrophil apoptotic process	1/2279	4/18866	0.4025066	0.6413241	0.5802928	1	PIK3CD
Biological Process	GO:0033088	negative regulation of immature T cell proliferation in thymus	1/2279	4/18866	0.4025066	0.6413241	0.5802928	1	TMEM131L
Biological Process	GO:0033326	cerebrospinal fluid secretion	1/2279	4/18866	0.4025066	0.6413241	0.5802928	1	AQP1
Biological Process	GO:0033590	response to cobalamin	1/2279	4/18866	0.4025066	0.6413241	0.5802928	1	RELA
Biological Process	GO:0033600	negative regulation of mammary gland epithelial cell proliferation	1/2279	4/18866	0.4025066	0.6413241	0.5802928	1	BRCA2
Biological Process	GO:0033668	negative regulation by symbiont of host apoptotic process	1/2279	4/18866	0.4025066	0.6413241	0.5802928	1	BCL2L1
Biological Process	GO:0034157	positive regulation of toll-like receptor 7 signaling pathway	1/2279	4/18866	0.4025066	0.6413241	0.5802928	1	TREML4
Biological Process	GO:0034164	negative regulation of toll-like receptor 9 signaling pathway	1/2279	4/18866	0.4025066	0.6413241	0.5802928	1	GRAMD4
Biological Process	GO:0034165	positive regulation of toll-like receptor 9 signaling pathway	1/2279	4/18866	0.4025066	0.6413241	0.5802928	1	RTN4

Biological Process	GO:0034775	glutathione transmembrane transport	1/2279	4/18866	0.4025066	0.6413241	0.5802928	1	ABCC1
Biological Process	GO:0035021	negative regulation of Rac protein signal transduction	1/2279	4/18866	0.4025066	0.6413241	0.5802928	1	NF1
Biological Process	GO:0035063	nuclear speck organization	1/2279	4/18866	0.4025066	0.6413241	0.5802928	1	SRPK2
Biological Process	GO:0035087	siRNA loading onto RISC involved in RNA interference	1/2279	4/18866	0.4025066	0.6413241	0.5802928	1	AGO2
Biological Process	GO:0035290	trunk segmentation	1/2279	4/18866	0.4025066	0.6413241	0.5802928	1	NRP2
Biological Process	GO:0035583	sequestering of TGFbeta in extracellular matrix	1/2279	4/18866	0.4025066	0.6413241	0.5802928	1	LTBP1
Biological Process	GO:0035585	calcium-mediated signaling using extracellular calcium source	1/2279	4/18866	0.4025066	0.6413241	0.5802928	1	CACNA1C
Biological Process	GO:0035616	histone H2B conserved C-terminal lysine deubiquitination	1/2279	4/18866	0.4025066	0.6413241	0.5802928	1	USP49
Biological Process	GO:0035621	ER to Golgi ceramide transport	1/2279	4/18866	0.4025066	0.6413241	0.5802928	1	SGPP1

Biological Process	GO:0035709	memory T cell activation	1/2279	4/18866	0.4025066	0.6413241	0.5802928	1	CYRIB
Biological Process	GO:0035814	negative regulation of renal sodium excretion	1/2279	4/18866	0.4025066	0.6413241	0.5802928	1	COMT
Biological Process	GO:0035822	gene conversion	1/2279	4/18866	0.4025066	0.6413241	0.5802928	1	RNF212
Biological Process	GO:0035844	cloaca development	1/2279	4/18866	0.4025066	0.6413241	0.5802928	1	WNT11
Biological Process	GO:0035915	pore formation in membrane of other organism	1/2279	4/18866	0.4025066	0.6413241	0.5802928	1	MPEG1
Biological Process	GO:0035995	detection of muscle stretch	1/2279	4/18866	0.4025066	0.6413241	0.5802928	1	TTN
Biological Process	GO:0036438	maintenance of lens transparency	1/2279	4/18866	0.4025066	0.6413241	0.5802928	1	GCNT2
Biological Process	GO:0036484	trunk neural crest cell migration	1/2279	4/18866	0.4025066	0.6413241	0.5802928	1	NRP2
Biological Process	GO:0036486	ventral trunk neural crest cell migration	1/2279	4/18866	0.4025066	0.6413241	0.5802928	1	NRP2

Biological Process	GO:0038028	insulin receptor signaling pathway via phosphatidylinositol 3-kinase	1/2279	4/18866	0.4025066	0.6413241	0.5802928	1	FER
Biological Process	GO:0038123	toll-like receptor TLR1:TLR2 signaling pathway	1/2279	4/18866	0.4025066	0.6413241	0.5802928	1	SCIMP
Biological Process	GO:0038156	interleukin-3-mediated signaling pathway	1/2279	4/18866	0.4025066	0.6413241	0.5802928	1	CSF2RB
Biological Process	GO:0039526	modulation by virus of host apoptotic process	1/2279	4/18866	0.4025066	0.6413241	0.5802928	1	BCL2L1
Biological Process	GO:0042078	germ-line stem cell division	1/2279	4/18866	0.4025066	0.6413241	0.5802928	1	ZBTB16
Biological Process	GO:0042320	regulation of circadian sleep/wake cycle, REM sleep	1/2279	4/18866	0.4025066	0.6413241	0.5802928	1	GHRL
Biological Process	GO:0042360	vitamin E metabolic process	1/2279	4/18866	0.4025066	0.6413241	0.5802928	1	PLTP
Biological Process	GO:0042361	menaquinone catabolic process	1/2279	4/18866	0.4025066	0.6413241	0.5802928	1	CYP4F3
Biological Process	GO:0042377	vitamin K catabolic process	1/2279	4/18866	0.4025066	0.6413241	0.5802928	1	CYP4F3

Biological Process	GO:0042637	catagen	1/2279	4/18866	0.4025066	0.6413241	0.5802928	1	CDH3
Biological Process	GO:0042701	progesterone secretion	1/2279	4/18866	0.4025066	0.6413241	0.5802928	1	INHBA
Biological Process	GO:0042851	L-alanine metabolic process	1/2279	4/18866	0.4025066	0.6413241	0.5802928	1	AGXT
Biological Process	GO:0042853	L-alanine catabolic process	1/2279	4/18866	0.4025066	0.6413241	0.5802928	1	AGXT
Biological Process	GO:0043606	formamide metabolic process	1/2279	4/18866	0.4025066	0.6413241	0.5802928	1	HAL
Biological Process	GO:0043622	cortical microtubule organization	1/2279	4/18866	0.4025066	0.6413241	0.5802928	1	EZR
Biological Process	GO:0044789	modulation by host of viral release from host cell	1/2279	4/18866	0.4025066	0.6413241	0.5802928	1	PC
Biological Process	GO:0044791	positive regulation by host of viral release from host cell	1/2279	4/18866	0.4025066	0.6413241	0.5802928	1	PC
Biological Process	GO:0044878	mitotic cytokinesis checkpoint	1/2279	4/18866	0.4025066	0.6413241	0.5802928	1	ZFYVE19

Biological Process	GO:0045337	farnesyl diphosphate biosynthetic process	1/2279	4/18866	0.4025066	0.6413241	0.5802928	1	HMGCS1
Biological Process	GO:0045575	basophil activation	1/2279	4/18866	0.4025066	0.6413241	0.5802928	1	PI4K2A
Biological Process	GO:0045629	negative regulation of T-helper 2 cell differentiation	1/2279	4/18866	0.4025066	0.6413241	0.5802928	1	HLX
Biological Process	GO:0046010	positive regulation of circadian sleep/wake cycle, non-REM sleep	1/2279	4/18866	0.4025066	0.6413241	0.5802928	1	GHRL
Biological Process	GO:0046016	positive regulation of transcription by glucose	1/2279	4/18866	0.4025066	0.6413241	0.5802928	1	USF2
Biological Process	GO:0046102	inosine metabolic process	1/2279	4/18866	0.4025066	0.6413241	0.5802928	1	PNP
Biological Process	GO:0046398	UDP-glucuronate metabolic process	1/2279	4/18866	0.4025066	0.6413241	0.5802928	1	CSGALNACT1
Biological Process	GO:0046684	response to pyrethroid	1/2279	4/18866	0.4025066	0.6413241	0.5802928	1	DDC
Biological Process	GO:0046898	response to cycloheximide	1/2279	4/18866	0.4025066	0.6413241	0.5802928	1	BCL2L1

Biological Process	GO:0046967	cytosol to endoplasmic reticulum transport	1/2279	4/18866	0.4025066	0.6413241	0.5802928	1	ATP2A3
Biological Process	GO:0048014	Tie signaling pathway	1/2279	4/18866	0.4025066	0.6413241	0.5802928	1	ANGPT1
Biological Process	GO:0048022	negative regulation of melanin biosynthetic process	1/2279	4/18866	0.4025066	0.6413241	0.5802928	1	RAPGEF2
Biological Process	GO:0048104	establishment of body hair or bristle planar orientation	1/2279	4/18866	0.4025066	0.6413241	0.5802928	1	ASTN2
Biological Process	GO:0048105	establishment of body hair planar orientation	1/2279	4/18866	0.4025066	0.6413241	0.5802928	1	ASTN2
Biological Process	GO:0048133	male germ-line stem cell asymmetric division	1/2279	4/18866	0.4025066	0.6413241	0.5802928	1	ZBTB16
Biological Process	GO:0048369	lateral mesoderm morphogenesis	1/2279	4/18866	0.4025066	0.6413241	0.5802928	1	FGFR1
Biological Process	GO:0048370	lateral mesoderm formation	1/2279	4/18866	0.4025066	0.6413241	0.5802928	1	FGFR1
Biological Process	GO:0048499	synaptic vesicle membrane organization	1/2279	4/18866	0.4025066	0.6413241	0.5802928	1	AP3D1

Biological Process	GO:0051030	snRNA transport	1/2279	4/18866	0.4025066	0.6413241	0.5802928	1	SNUPN
Biological Process	GO:0051464	positive regulation of cortisol secretion	1/2279	4/18866	0.4025066	0.6413241	0.5802928	1	GHRL
Biological Process	GO:0051611	regulation of serotonin uptake	1/2279	4/18866	0.4025066	0.6413241	0.5802928	1	ITGB3
Biological Process	GO:0051794	regulation of timing of catagen	1/2279	4/18866	0.4025066	0.6413241	0.5802928	1	CDH3
Biological Process	GO:0051919	positive regulation of fibrinolysis	1/2279	4/18866	0.4025066	0.6413241	0.5802928	1	F12
Biological Process	GO:0051933	amino acid neurotransmitter reuptake	1/2279	4/18866	0.4025066	0.6413241	0.5802928	1	PER2
Biological Process	GO:0051935	glutamate reuptake	1/2279	4/18866	0.4025066	0.6413241	0.5802928	1	PER2
Biological Process	GO:0052041	negative regulation by symbiont of host programmed cell death	1/2279	4/18866	0.4025066	0.6413241	0.5802928	1	BCL2L1
Biological Process	GO:0060005	vestibular reflex	1/2279	4/18866	0.4025066	0.6413241	0.5802928	1	TMC1

Biological Process	GO:0060160	negative regulation of dopamine receptor signaling pathway	1/2279	4/18866	0.4025066	0.6413241	0.5802928	1	PALM
Biological Process	GO:0060164	regulation of timing of neuron differentiation	1/2279	4/18866	0.4025066	0.6413241	0.5802928	1	HES3
Biological Process	GO:0060167	regulation of adenosine receptor signaling pathway	1/2279	4/18866	0.4025066	0.6413241	0.5802928	1	ACP3
Biological Process	GO:0060235	lens induction in camera-type eye	1/2279	4/18866	0.4025066	0.6413241	0.5802928	1	HIPK2
Biological Process	GO:0060283	negative regulation of oocyte development	1/2279	4/18866	0.4025066	0.6413241	0.5802928	1	NPPC
Biological Process	GO:0060300	regulation of cytokine activity	1/2279	4/18866	0.4025066	0.6413241	0.5802928	1	IL10
Biological Process	GO:0060319	primitive erythrocyte differentiation	1/2279	4/18866	0.4025066	0.6413241	0.5802928	1	ZFPM1
Biological Process	GO:0060431	primary lung bud formation	1/2279	4/18866	0.4025066	0.6413241	0.5802928	1	RDH10
Biological Process	GO:0060486	club cell differentiation	1/2279	4/18866	0.4025066	0.6413241	0.5802928	1	RBPJ

Biological Process	GO:0060584	regulation of prostaglandin-endoperoxide synthase activity	1/2279	4/18866	0.4025066	0.6413241	0.5802928	1	NOD2
Biological Process	GO:0060585	positive regulation of prostaglandin-endoperoxide synthase activity	1/2279	4/18866	0.4025066	0.6413241	0.5802928	1	NOD2
Biological Process	GO:0060665	regulation of branching involved in salivary gland morphogenesis by mesenchymal-epithelial signaling	1/2279	4/18866	0.4025066	0.6413241	0.5802928	1	FGFR1
Biological Process	GO:0060800	regulation of cell differentiation involved in embryonic placenta development	1/2279	4/18866	0.4025066	0.6413241	0.5802928	1	STK3
Biological Process	GO:0060926	cardiac pacemaker cell development	1/2279	4/18866	0.4025066	0.6413241	0.5802928	1	MAML1
Biological Process	GO:0060988	lipid tube assembly	1/2279	4/18866	0.4025066	0.6413241	0.5802928	1	BIN1
Biological Process	GO:0061073	ciliary body morphogenesis	1/2279	4/18866	0.4025066	0.6413241	0.5802928	1	TSKU
Biological Process	GO:0061083	regulation of protein refolding	1/2279	4/18866	0.4025066	0.6413241	0.5802928	1	BAG5
Biological Process	GO:0061084	negative regulation of protein refolding	1/2279	4/18866	0.4025066	0.6413241	0.5802928	1	BAG5

Biological Process	GO:0061091	regulation of phospholipid translocation	1/2279	4/18866	0.4025066	0.6413241	0.5802928	1	ATP8A1
Biological Process	GO:0061092	positive regulation of phospholipid translocation	1/2279	4/18866	0.4025066	0.6413241	0.5802928	1	ATP8A1
Biological Process	GO:0061113	pancreas morphogenesis	1/2279	4/18866	0.4025066	0.6413241	0.5802928	1	NR5A2
Biological Process	GO:0061182	negative regulation of chondrocyte development	1/2279	4/18866	0.4025066	0.6413241	0.5802928	1	RFLNA
Biological Process	GO:0061218	negative regulation of mesonephros development	1/2279	4/18866	0.4025066	0.6413241	0.5802928	1	TACSTD2
Biological Process	GO:0061502	early endosome to recycling endosome transport	1/2279	4/18866	0.4025066	0.6413241	0.5802928	1	SORL1
Biological Process	GO:0061526	acetylcholine secretion	1/2279	4/18866	0.4025066	0.6413241	0.5802928	1	SLC44A4
Biological Process	GO:0061535	glutamate secretion, neurotransmission	1/2279	4/18866	0.4025066	0.6413241	0.5802928	1	NF1
Biological Process	GO:0065001	specification of axis polarity	1/2279	4/18866	0.4025066	0.6413241	0.5802928	1	NODAL

Biological Process	GO:0070164	negative regulation of adiponectin secretion	1/2279	4/18866	0.4025066	0.6413241	0.5802928	1	RAB11FIP1
Biological Process	GO:0070294	renal sodium ion absorption	1/2279	4/18866	0.4025066	0.6413241	0.5802928	1	SGK1
Biological Process	GO:0070318	positive regulation of G0 to G1 transition	1/2279	4/18866	0.4025066	0.6413241	0.5802928	1	RHNO1
Biological Process	GO:0070384	Harderian gland development	1/2279	4/18866	0.4025066	0.6413241	0.5802928	1	RARG
Biological Process	GO:0070666	regulation of mast cell proliferation	1/2279	4/18866	0.4025066	0.6413241	0.5802928	1	BLK
Biological Process	GO:0070836	caveola assembly	1/2279	4/18866	0.4025066	0.6413241	0.5802928	1	PACSIN2
Biological Process	GO:0070966	nuclear-transcribed mRNA catabolic process, no-go decay	1/2279	4/18866	0.4025066	0.6413241	0.5802928	1	CSDE1
Biological Process	GO:0070995	NADPH oxidation	1/2279	4/18866	0.4025066	0.6413241	0.5802928	1	KCNAB2
Biological Process	GO:0071034	CUT catabolic process	1/2279	4/18866	0.4025066	0.6413241	0.5802928	1	EXOSC2

Biological Process	GO:0071043	CUT metabolic process	1/2279	4/18866	0.4025066	0.6413241	0.5802928	1	EXOSC2
Biological Process	GO:0071226	cellular response to molecule of fungal origin	1/2279	4/18866	0.4025066	0.6413241	0.5802928	1	SCIMP
Biological Process	GO:0071603	endothelial cell-cell adhesion	1/2279	4/18866	0.4025066	0.6413241	0.5802928	1	JUP
Biological Process	GO:0071623	negative regulation of granulocyte chemotaxis	1/2279	4/18866	0.4025066	0.6413241	0.5802928	1	C5AR2
Biological Process	GO:0071650	negative regulation of chemokine (C-C motif) ligand 5 production	1/2279	4/18866	0.4025066	0.6413241	0.5802928	1	IL10
Biological Process	GO:0071673	positive regulation of smooth muscle cell chemotaxis	1/2279	4/18866	0.4025066	0.6413241	0.5802928	1	PDGFD
Biological Process	GO:0071733	transcriptional activation by promoter-enhancer looping	1/2279	4/18866	0.4025066	0.6413241	0.5802928	1	ZFPM1
Biological Process	GO:0072039	regulation of mesenchymal cell apoptotic process involved in nephron morphogenesis	1/2279	4/18866	0.4025066	0.6413241	0.5802928	1	PAX2
Biological Process	GO:0072040	negative regulation of mesenchymal cell apoptotic process involved in nephron morphogenesis	1/2279	4/18866	0.4025066	0.6413241	0.5802928	1	PAX2

Biological Process	GO:0072278	metanephric comma-shaped body morphogenesis	1/2279	4/18866	0.4025066	0.6413241	0.5802928	1	PDGFRB
Biological Process	GO:0072289	metanephric nephron tubule formation	1/2279	4/18866	0.4025066	0.6413241	0.5802928	1	PAX2
Biological Process	GO:0072716	response to actinomycin D	1/2279	4/18866	0.4025066	0.6413241	0.5802928	1	KAT7
Biological Process	GO:0086017	Purkinje myocyte action potential	1/2279	4/18866	0.4025066	0.6413241	0.5802928	1	GJA5
Biological Process	GO:0090024	negative regulation of neutrophil chemotaxis	1/2279	4/18866	0.4025066	0.6413241	0.5802928	1	C5AR2
Biological Process	GO:0090081	regulation of heart induction by regulation of canonical Wnt signaling pathway	1/2279	4/18866	0.4025066	0.6413241	0.5802928	1	WNT11
Biological Process	GO:0090082	positive regulation of heart induction by negative regulation of canonical Wnt signaling pathway	1/2279	4/18866	0.4025066	0.6413241	0.5802928	1	WNT11
Biological Process	GO:0090116	C-5 methylation of cytosine	1/2279	4/18866	0.4025066	0.6413241	0.5802928	1	DNMT1
Biological Process	GO:0090202	gene looping	1/2279	4/18866	0.4025066	0.6413241	0.5802928	1	ZFPM1

Biological Process	GO:0090210	regulation of establishment of blood-brain barrier	1/2279	4/18866	0.4025066	0.6413241	0.5802928	1	ADGRA2
Biological Process	GO:0090261	positive regulation of inclusion body assembly	1/2279	4/18866	0.4025066	0.6413241	0.5802928	1	CLU
Biological Process	GO:0090403	oxidative stress-induced premature senescence	1/2279	4/18866	0.4025066	0.6413241	0.5802928	1	ARNTL
Biological Process	GO:0090461	glutamate homeostasis	1/2279	4/18866	0.4025066	0.6413241	0.5802928	1	KCTD7
Biological Process	GO:0090521	glomerular visceral epithelial cell migration	1/2279	4/18866	0.4025066	0.6413241	0.5802928	1	KANK1
Biological Process	GO:0090579	dsDNA loop formation	1/2279	4/18866	0.4025066	0.6413241	0.5802928	1	ZFPM1
Biological Process	GO:0097026	dendritic cell dendrite assembly	1/2279	4/18866	0.4025066	0.6413241	0.5802928	1	ARID1B
Biological Process	GO:0097029	mature conventional dendritic cell differentiation	1/2279	4/18866	0.4025066	0.6413241	0.5802928	1	PRTN3
Biological Process	GO:0097252	oligodendrocyte apoptotic process	1/2279	4/18866	0.4025066	0.6413241	0.5802928	1	TNFRSF21

Biological Process	GO:0097296	activation of cysteine-type endopeptidase activity involved in apoptotic signaling pathway	1/2279	4/18866	0.4025066	0.6413241	0.5802928	1	SMAD3
Biological Process	GO:0097490	sympathetic neuron projection extension	1/2279	4/18866	0.4025066	0.6413241	0.5802928	1	NRP2
Biological Process	GO:0097491	sympathetic neuron projection guidance	1/2279	4/18866	0.4025066	0.6413241	0.5802928	1	NRP2
Biological Process	GO:0097676	histone H3-K36 dimethylation	1/2279	4/18866	0.4025066	0.6413241	0.5802928	1	SETD2
Biological Process	GO:0097749	membrane tubulation	1/2279	4/18866	0.4025066	0.6413241	0.5802928	1	CORO1C
Biological Process	GO:0098728	germline stem cell asymmetric division	1/2279	4/18866	0.4025066	0.6413241	0.5802928	1	ZBTB16
Biological Process	GO:0098904	regulation of AV node cell action potential	1/2279	4/18866	0.4025066	0.6413241	0.5802928	1	GJA5
Biological Process	GO:0098971	anterograde dendritic transport of neurotransmitter receptor complex	1/2279	4/18866	0.4025066	0.6413241	0.5802928	1	KIF5C
Biological Process	GO:0099547	regulation of translation at synapse, modulating synaptic transmission	1/2279	4/18866	0.4025066	0.6413241	0.5802928	1	CYFIP1

Biological Process	GO:0106016	positive regulation of inflammatory response to wounding	1/2279	4/18866	0.4025066	0.6413241	0.5802928	1	GRN
Biological Process	GO:0106077	histone succinylation	1/2279	4/18866	0.4025066	0.6413241	0.5802928	1	OGDH
Biological Process	GO:0110024	positive regulation of cardiac muscle myoblast proliferation	1/2279	4/18866	0.4025066	0.6413241	0.5802928	1	MEIS2
Biological Process	GO:0110088	hippocampal neuron apoptotic process	1/2279	4/18866	0.4025066	0.6413241	0.5802928	1	CX3CR1
Biological Process	GO:0110089	regulation of hippocampal neuron apoptotic process	1/2279	4/18866	0.4025066	0.6413241	0.5802928	1	CX3CR1
Biological Process	GO:0140199	negative regulation of adenylate cyclase-activating adrenergic receptor signaling pathway involved in heart process	1/2279	4/18866	0.4025066	0.6413241	0.5802928	1	GNAI2
Biological Process	GO:0140213	negative regulation of long-chain fatty acid import into cell	1/2279	4/18866	0.4025066	0.6413241	0.5802928	1	THBS1
Biological Process	GO:0140243	regulation of translation at synapse	1/2279	4/18866	0.4025066	0.6413241	0.5802928	1	CYFIP1
Biological Process	GO:1900109	regulation of histone H3-K9 dimethylation	1/2279	4/18866	0.4025066	0.6413241	0.5802928	1	PIH1D1

Biological Process	GO:1900127	positive regulation of hyaluronan biosynthetic process	1/2279	4/18866	0.4025066	0.6413241	0.5802928	1	PDGFB
Biological Process	GO:1900365	positive regulation of mRNA polyadenylation	1/2279	4/18866	0.4025066	0.6413241	0.5802928	1	CPEB3
Biological Process	GO:1900377	negative regulation of secondary metabolite biosynthetic process	1/2279	4/18866	0.4025066	0.6413241	0.5802928	1	RAPGEF2
Biological Process	GO:1901145	mesenchymal cell apoptotic process involved in nephron morphogenesis	1/2279	4/18866	0.4025066	0.6413241	0.5802928	1	PAX2
Biological Process	GO:1901295	regulation of canonical Wnt signaling pathway involved in cardiac muscle cell fate commitment	1/2279	4/18866	0.4025066	0.6413241	0.5802928	1	RBPJ
Biological Process	GO:1901624	negative regulation of lymphocyte chemotaxis	1/2279	4/18866	0.4025066	0.6413241	0.5802928	1	PADI2
Biological Process	GO:1901656	glycoside transport	1/2279	4/18866	0.4025066	0.6413241	0.5802928	1	RALBP1
Biological Process	GO:1901842	negative regulation of high voltage-gated calcium channel activity	1/2279	4/18866	0.4025066	0.6413241	0.5802928	1	DYSF
Biological Process	GO:1901873	regulation of post-translational protein modification	1/2279	4/18866	0.4025066	0.6413241	0.5802928	1	P3H1

Biological Process	GO:1902202	regulation of hepatocyte growth factor receptor signaling pathway	1/2279	4/18866	0.4025066	0.6413241	0.5802928	1	ESM1
Biological Process	GO:1902261	positive regulation of delayed rectifier potassium channel activity	1/2279	4/18866	0.4025066	0.6413241	0.5802928	1	NOS1AP
Biological Process	GO:1902511	negative regulation of apoptotic DNA fragmentation	1/2279	4/18866	0.4025066	0.6413241	0.5802928	1	PAM16
Biological Process	GO:1902523	positive regulation of protein K63-linked ubiquitination	1/2279	4/18866	0.4025066	0.6413241	0.5802928	1	NOD2
Biological Process	GO:1902566	regulation of eosinophil activation	1/2279	4/18866	0.4025066	0.6413241	0.5802928	1	CCR2
Biological Process	GO:1902576	negative regulation of nuclear cell cycle DNA replication	1/2279	4/18866	0.4025066	0.6413241	0.5802928	1	CHEK2
Biological Process	GO:1902952	positive regulation of dendritic spine maintenance	1/2279	4/18866	0.4025066	0.6413241	0.5802928	1	ZMYND8
Biological Process	GO:1902953	positive regulation of ER to Golgi vesicle-mediated transport	1/2279	4/18866	0.4025066	0.6413241	0.5802928	1	SORL1
Biological Process	GO:1903027	regulation of opsonization	1/2279	4/18866	0.4025066	0.6413241	0.5802928	1	MYO18A

Biological Process	GO:1903061	positive regulation of protein lipidation	1/2279	4/18866	0.4025066	0.6413241	0.5802928	1	SVIP
Biological Process	GO:1903108	regulation of mitochondrial transcription	1/2279	4/18866	0.4025066	0.6413241	0.5802928	1	PRKAA1
Biological Process	GO:1903276	regulation of sodium ion export across plasma membrane	1/2279	4/18866	0.4025066	0.6413241	0.5802928	1	FXYD1
Biological Process	GO:1903278	positive regulation of sodium ion export across plasma membrane	1/2279	4/18866	0.4025066	0.6413241	0.5802928	1	FXYD1
Biological Process	GO:1903334	positive regulation of protein folding	1/2279	4/18866	0.4025066	0.6413241	0.5802928	1	GRN
Biological Process	GO:1903347	negative regulation of bicellular tight junction assembly	1/2279	4/18866	0.4025066	0.6413241	0.5802928	1	TNF
Biological Process	GO:1903527	positive regulation of membrane tubulation	1/2279	4/18866	0.4025066	0.6413241	0.5802928	1	ASAP1
Biological Process	GO:1903576	response to L-arginine	1/2279	4/18866	0.4025066	0.6413241	0.5802928	1	DHODH
Biological Process	GO:1903762	positive regulation of voltage-gated potassium channel activity involved in ventricular cardiac muscle cell action potential repolarization	1/2279	4/18866	0.4025066	0.6413241	0.5802928	1	NOS1AP

Biological Process	GO:1903766	positive regulation of potassium ion export across plasma membrane	1/2279	4/18866	0.4025066	0.6413241	0.5802928	1	ANO6
Biological Process	GO:1903912	negative regulation of endoplasmic reticulum stress-induced eIF2 alpha phosphorylation	1/2279	4/18866	0.4025066	0.6413241	0.5802928	1	NCK2
Biological Process	GO:1903947	positive regulation of ventricular cardiac muscle cell action potential	1/2279	4/18866	0.4025066	0.6413241	0.5802928	1	NOS1AP
Biological Process	GO:1903999	negative regulation of eating behavior	1/2279	4/18866	0.4025066	0.6413241	0.5802928	1	TTC21B
Biological Process	GO:1904351	negative regulation of protein catabolic process in the vacuole	1/2279	4/18866	0.4025066	0.6413241	0.5802928	1	MGAT3
Biological Process	GO:1904557	L-alanine transmembrane transport	1/2279	4/18866	0.4025066	0.6413241	0.5802928	1	SLC7A8
Biological Process	GO:1904742	regulation of telomeric DNA binding	1/2279	4/18866	0.4025066	0.6413241	0.5802928	1	TNKS
Biological Process	GO:1904823	purine nucleobase transmembrane transport	1/2279	4/18866	0.4025066	0.6413241	0.5802928	1	AQP9
Biological Process	GO:1904849	positive regulation of cell chemotaxis to fibroblast growth factor	1/2279	4/18866	0.4025066	0.6413241	0.5802928	1	FGFR1

Biological Process	GO:1904978	regulation of endosome organization	1/2279	4/18866	0.4025066	0.6413241	0.5802928	1	PRKN
Biological Process	GO:1904999	positive regulation of leukocyte adhesion to arterial endothelial cell	1/2279	4/18866	0.4025066	0.6413241	0.5802928	1	TNF
Biological Process	GO:1905026	positive regulation of membrane repolarization during ventricular cardiac muscle cell action potential	1/2279	4/18866	0.4025066	0.6413241	0.5802928	1	NOS1AP
Biological Process	GO:1905033	positive regulation of membrane repolarization during cardiac muscle cell action potential	1/2279	4/18866	0.4025066	0.6413241	0.5802928	1	NOS1AP
Biological Process	GO:1905066	regulation of canonical Wnt signaling pathway involved in heart development	1/2279	4/18866	0.4025066	0.6413241	0.5802928	1	RBPJ
Biological Process	GO:1905166	negative regulation of lysosomal protein catabolic process	1/2279	4/18866	0.4025066	0.6413241	0.5802928	1	MGAT3
Biological Process	GO:1905598	negative regulation of low-density lipoprotein receptor activity	1/2279	4/18866	0.4025066	0.6413241	0.5802928	1	MIR27A
Biological Process	GO:1905671	regulation of lysosome organization	1/2279	4/18866	0.4025066	0.6413241	0.5802928	1	GRN
Biological Process	GO:1905880	negative regulation of oogenesis	1/2279	4/18866	0.4025066	0.6413241	0.5802928	1	NPPC

Biological Process	GO:1990009	retinal cell apoptotic process	1/2279	4/18866	0.4025066	0.6413241	0.5802928	1	GDF6
Biological Process	GO:1990086	lens fiber cell apoptotic process	1/2279	4/18866	0.4025066	0.6413241	0.5802928	1	TGFBR2
Biological Process	GO:1990168	protein K33-linked deubiquitination	1/2279	4/18866	0.4025066	0.6413241	0.5802928	1	TNFAIP3
Biological Process	GO:1990441	negative regulation of transcription from RNA polymerase II promoter in response to endoplasmic reticulum stress	1/2279	4/18866	0.4025066	0.6413241	0.5802928	1	NCK2
Biological Process	GO:2000137	negative regulation of cell proliferation involved in heart morphogenesis	1/2279	4/18866	0.4025066	0.6413241	0.5802928	1	NOTCH1
Biological Process	GO:2000182	regulation of progesterone biosynthetic process	1/2279	4/18866	0.4025066	0.6413241	0.5802928	1	DGKQ
Biological Process	GO:2000346	negative regulation of hepatocyte proliferation	1/2279	4/18866	0.4025066	0.6413241	0.5802928	1	CPB2
Biological Process	GO:2000410	regulation of thymocyte migration	1/2279	4/18866	0.4025066	0.6413241	0.5802928	1	CCR2
Biological Process	GO:2000418	positive regulation of eosinophil migration	1/2279	4/18866	0.4025066	0.6413241	0.5802928	1	DAPK2

Biological Process	GO:2000546	positive regulation of endothelial cell chemotaxis to fibroblast growth factor	1/2279	4/18866	0.4025066	0.6413241	0.5802928	1	FGFR1
Biological Process	GO:2000672	negative regulation of motor neuron apoptotic process	1/2279	4/18866	0.4025066	0.6413241	0.5802928	1	CRLF1
Biological Process	GO:2000793	cell proliferation involved in heart valve development	1/2279	4/18866	0.4025066	0.6413241	0.5802928	1	NOTCH1
Biological Process	GO:2001168	positive regulation of histone H2B ubiquitination	1/2279	4/18866	0.4025066	0.6413241	0.5802928	1	RNF40
Biological Process	GO:2001288	positive regulation of caveolin-mediated endocytosis	1/2279	4/18866	0.4025066	0.6413241	0.5802928	1	ITSN1
Biological Process	GO:0097237	cellular response to toxic substance	16/2279	122/18866	0.4029802	0.6418342	0.5807544	16	MPO/LPO/TNF/HBB/MGST2/ABTB2/CAT/NFE2L2/FIS1/NXN/GSTO1/TXNRD2/GSTO2/SIRT3/PRXL2B/PRKN
Biological Process	GO:0006360	transcription by RNA polymerase I	9/2279	66/18866	0.4031646	0.6418342	0.5807544	9	TAF1C/WDR43/POLR2F/CCNH/PIH1D1/TAF1D/ERCC2/MARS1/MACROH2A2
Biological Process	GO:0007585	respiratory gaseous exchange by respiratory system	9/2279	66/18866	0.4031646	0.6418342	0.5807544	9	CSF2RB/PHOX2A/SFTPD/CHST11/NDST1/MTG2/FTO/YWHAZ/HNMT
Biological Process	GO:1905207	regulation of cardiocyte differentiation	9/2279	66/18866	0.4031646	0.6418342	0.5807544	9	CTDP1/MIR145/MIR199A1/DLL1/RBPJ/TGFB1/ARRB2/MIR199A2/MEF2C

Biological Process	GO:2000378	negative regulation of reactive oxygen species metabolic process	9/2279	66/18866	0.4031646	0.6418342	0.5807544	9	IL10/MIR199A1/PAX2/HK2/BCR/BCL2/MIR199A2/SIRT3/PRKN
Biological Process	GO:0006305	DNA alkylation	10/2279	74/18866	0.4038083	0.6421147	0.5810082	10	PIWIL4/MOV10L1/GATAD2A/EHMT2/KDM1B/ATF7IP/GRHL2/MTA2/DNMT1/MORC1
Biological Process	GO:0006306	DNA methylation	10/2279	74/18866	0.4038083	0.6421147	0.5810082	10	PIWIL4/MOV10L1/GATAD2A/EHMT2/KDM1B/ATF7IP/GRHL2/MTA2/DNMT1/MORC1
Biological Process	GO:0032481	positive regulation of type I interferon production	10/2279	74/18866	0.4038083	0.6421147	0.5810082	10	IRF5/SETD2/POLR2F/TLR9/CRCP/CREBBP/DDX41/RELA/FLOT1/STING1
Biological Process	GO:0043507	positive regulation of JUN kinase activity	10/2279	74/18866	0.4038083	0.6421147	0.5810082	10	ERN1/TNF/ZEB2/TRAF2/TLR9/TNIK/RIPK1/TAOK3/DBNL/MAP3K20
Biological Process	GO:2001259	positive regulation of cation channel activity	10/2279	74/18866	0.4038083	0.6421147	0.5810082	10	CCR2/NOS1AP/P2RY6/HTT/GSTO1/ANK2/CASQ1/FGF14/STIMATE/CRACR2A
Biological Process	GO:0032091	negative regulation of protein binding	14/2279	106/18866	0.4038139	0.6421147	0.5810082	14	SLPI/CTSZ/ZFPM1/SORL1/IL10/ITGB1BP1/CCM2L/DISC1/ATP2A3/CSNK1E/PDGFB/ARRB2/DTNBP1/TFIP11
Biological Process	GO:1904029	regulation of cyclin-dependent protein kinase activity	14/2279	106/18866	0.4038139	0.6421147	0.5810082	14	BLM/TNFAIP3/FBXO7/LATS2/CDC25A/PDGFB/CCNH/CCNA1/HHEX/HEXIM1/CCNY/HERC5/CCNYL1/CCNP
Biological Process	GO:0001657	ureteric bud development	13/2279	98/18866	0.404074	0.6422822	0.5811598	13	TACSTD2/SMAD3/ILK/SMAD7/WNT11/PAX2/FGFR1/RARA/BMPER/CAT/BCL2/CRLF1/HOXB7

Biological Process	GO:0010657	muscle cell apoptotic process	13/2279	98/18866	0.404074	0.6422822	0.5811598	13	PPARG/MIR140/MIR145/ARRB1/E2F3/MIR199A1/MIR24-2/ILK/NFE2L2/ARRB2/MIR138-2/MIR199A2/DNMT1
Biological Process	GO:0021954	central nervous system neuron development	11/2279	82/18866	0.404122	0.6422822	0.5811598	11	NRP2/ZEB2/EPHB3/DISC1/ZMIZ1/RAPGEF2/CDH11/NDEL1/SPTBN4/NPY/TSKU
Biological Process	GO:1903351	cellular response to dopamine	12/2279	90/18866	0.4041908	0.6422842	0.5811615	12	KLF16/DNM2/HRH1/HTR3A/GNA11/CHRM2/PALM/ARRB2/GNG2/GNAO1/DTNBP1/PRKN
Biological Process	GO:0006473	protein acetylation	26/2279	203/18866	0.406246	0.6434836	0.5822468	26	SRCAP/BRCA2/KANSL1/ARRB1/BRD1/EPC1/RPS6KA4/TAF10/KANSL2/SPHK2/CTBP1/POR/NCOA1/CREBBP/RSF1/ARNTL/PER1/PIH1D1/NAA16/FOXO1/RUVBL1/KAT7/ING5/LIF/PAXIP1/PRKAA1
Biological Process	GO:0051236	establishment of RNA localization	26/2279	203/18866	0.406246	0.6434836	0.5822468	26	NXF1/FIP1L1/TNKS/CPSF3/SETD2/RAE1/TGFBR2/SMG7/PABPN1/ENY2/BICD2/NUP93/IGF2BP3/SEC13/KIF5C/SMG6/SNUPN/MYO1C/HHEX/ZC3H3/RBM15B/NOL6/CPSF4/FLOT1/PCID2/NCBP3
Biological Process	GO:0043487	regulation of RNA stability	25/2279	195/18866	0.4072527	0.6434836	0.5822468	25	EXOSC2/SLC11A1/TNFRSF1B/PABPC4/TNPO1/PRKCA/LARP1/ZC3H12D/PCBP4/SAMD4A/CPEB3/IGF2BP3/PSMF1/FTO/YWHAZ/PSMB7/PAIP1/TNFSF13/SYNCRIP/EIF4G1/PSMD13/PCID2/TBRG4/TENT5C/TENT5A
Biological Process	GO:0001696	gastric acid secretion	3/2279	19/18866	0.407587	0.6434836	0.5822468	3	SGK1/GHRL/KCNQ1
Biological Process	GO:0002922	positive regulation of humoral immune response	3/2279	19/18866	0.407587	0.6434836	0.5822468	3	TNF/PHB/PTPRC
Biological Process	GO:0003159	morphogenesis of an endothelium	3/2279	19/18866	0.407587	0.6434836	0.5822468	3	FOXP1/CCM2/RBPJ

Biological Process	GO:0006271	DNA strand elongation involved in DNA replication	3/2279	19/18866	0.407587	0.6434836	0.5822468	3	POLE/GINS3/POLD3
Biological Process	GO:0009219	pyrimidine deoxyribonucleotide metabolic process	3/2279	19/18866	0.407587	0.6434836	0.5822468	3	UNG/NTHL1/DCTD
Biological Process	GO:0010544	negative regulation of platelet activation	3/2279	19/18866	0.407587	0.6434836	0.5822468	3	CD9/UBASH3B/PDGFB
Biological Process	GO:0010561	negative regulation of glycoprotein biosynthetic process	3/2279	19/18866	0.407587	0.6434836	0.5822468	3	MIR101-2/AGO2/ITM2C
Biological Process	GO:0019054	modulation by virus of host cellular process	3/2279	19/18866	0.407587	0.6434836	0.5822468	3	PABPN1/BCL2L1/CPSF4
Biological Process	GO:0032011	ARF protein signal transduction	3/2279	19/18866	0.407587	0.6434836	0.5822468	3	IQSEC1/CYTH4/FBXO8
Biological Process	GO:0032012	regulation of ARF protein signal transduction	3/2279	19/18866	0.407587	0.6434836	0.5822468	3	IQSEC1/CYTH4/FBXO8
Biological Process	GO:0032026	response to magnesium ion	3/2279	19/18866	0.407587	0.6434836	0.5822468	3	SLFN14/THBS1/SLC41A1
Biological Process	GO:0035728	response to hepatocyte growth factor	3/2279	19/18866	0.407587	0.6434836	0.5822468	3	IL10/CREB1/RELA

Biological Process	GO:0035988	chondrocyte proliferation	3/2279	19/18866	0.407587	0.6434836	0.5822468	3	SMAD7/BMPR1B/LTF
Biological Process	GO:0042053	regulation of dopamine metabolic process	3/2279	19/18866	0.407587	0.6434836	0.5822468	3	COMT/ITGB2/PRKN
Biological Process	GO:0042069	regulation of catecholamine metabolic process	3/2279	19/18866	0.407587	0.6434836	0.5822468	3	COMT/ITGB2/PRKN
Biological Process	GO:0043373	CD4-positive, alpha-beta T cell lineage commitment	3/2279	19/18866	0.407587	0.6434836	0.5822468	3	ZFPM1/LOXL3/LY9
Biological Process	GO:0045947	negative regulation of translational initiation	3/2279	19/18866	0.407587	0.6434836	0.5822468	3	LARP1/AGO2/BANK1
Biological Process	GO:0046653	tetrahydrofolate metabolic process	3/2279	19/18866	0.407587	0.6434836	0.5822468	3	MTHFD1L/MTHFS/MTHFD1
Biological Process	GO:0048245	eosinophil chemotaxis	3/2279	19/18866	0.407587	0.6434836	0.5822468	3	CCL5/HRH1/DAPK2
Biological Process	GO:0048486	parasympathetic nervous system development	3/2279	19/18866	0.407587	0.6434836	0.5822468	3	NRP2/PHOX2A/HES3
Biological Process	GO:0048490	anterograde synaptic vesicle transport	3/2279	19/18866	0.407587	0.6434836	0.5822468	3	CNIH2/AP3D1/DTNBP1

Biological Process	GO:0060438	trachea development	3/2279	19/18866	0.407587	0.6434836	0.5822468	3	TGFBR2/RARA/RARG
Biological Process	GO:0060973	cell migration involved in heart development	3/2279	19/18866	0.407587	0.6434836	0.5822468	3	ENG/NOTCH1/PDGFRB
Biological Process	GO:0061154	endothelial tube morphogenesis	3/2279	19/18866	0.407587	0.6434836	0.5822468	3	FOXP1/CCM2/RBPJ
Biological Process	GO:0071625	vocalization behavior	3/2279	19/18866	0.407587	0.6434836	0.5822468	3	NRXN1/NRXN2/SHANK2
Biological Process	GO:0097623	potassium ion export across plasma membrane	3/2279	19/18866	0.407587	0.6434836	0.5822468	3	KCNE1/KCNQ1/ANO6
Biological Process	GO:0098915	membrane repolarization during ventricular cardiac muscle cell action potential	3/2279	19/18866	0.407587	0.6434836	0.5822468	3	KCNE1/NOS1AP/KCNQ1
Biological Process	GO:0099514	synaptic vesicle cytoskeletal transport	3/2279	19/18866	0.407587	0.6434836	0.5822468	3	CNIH2/AP3D1/DTNBP1
Biological Process	GO:0099517	synaptic vesicle transport along microtubule	3/2279	19/18866	0.407587	0.6434836	0.5822468	3	CNIH2/AP3D1/DTNBP1
Biological Process	GO:0150079	negative regulation of neuroinflammatory response	3/2279	19/18866	0.407587	0.6434836	0.5822468	3	TNFRSF1B/PTPRC/GRN

Biological Process	GO:1900747	negative regulation of vascular endothelial growth factor signaling pathway	3/2279	19/18866	0.407587	0.6434836	0.5822468	3	MIR199A1/MIR199A2/ADGRA2
Biological Process	GO:1903975	regulation of glial cell migration	3/2279	19/18866	0.407587	0.6434836	0.5822468	3	CCR2/CERS2/CX3CR1
Biological Process	GO:1904683	regulation of metalloendopeptidase activity	3/2279	19/18866	0.407587	0.6434836	0.5822468	3	SORL1/MBP/MIR24-2
Biological Process	GO:1990182	exosomal secretion	3/2279	19/18866	0.407587	0.6434836	0.5822468	3	RAB27A/COPS5/PRKN
Biological Process	GO:2000010	positive regulation of protein localization to cell surface	3/2279	19/18866	0.407587	0.6434836	0.5822468	3	TM9SF4/TNF/MAP1A
Biological Process	GO:2000696	regulation of epithelial cell differentiation involved in kidney development	3/2279	19/18866	0.407587	0.6434836	0.5822468	3	PAX2/WWTR1/LIF
Biological Process	GO:0006401	RNA catabolic process	52/2279	415/18866	0.4106195	0.6481635	0.5864813	52	RNH1/PRR5L/RNASE3/LSM1/EXOSC2/SLC11A1/ERN1/TNFRSF1B/TNRC6B/PABPC4/TNPO1/SLFN14/PRKCA/AGO1/DEDD2/LARP1/CSDE1/ZC3H12D/PKP1/PCBP4/SAMD4A/SMG7/CTIF/AGO2/EDC3/LSM7/CPEB3/IGF2BP3/SMG6/PSMF1/PPP2R1A/FTO/NBAS/YWHAZ/PSMB7/ZHX2/PAIP1/DIS3L2/SND1/TNFSF13/SECISBP2/RPL23A/ZCCHC8/SYNCRIP/ISG20/EIF4G1/PSMD13/PCID2/TBRG4/GRSF1/TENT5C/TENT5A
Biological Process	GO:0002433	immune response-regulating cell surface receptor signaling pathway involved in phagocytosis	18/2279	139/18866	0.4142767	0.6525201	0.5904233	18	ELMO1/DOCK1/PIK3R1/WIPF1/FGR/CYFIP2/BAIAP2/WASL/PTPRJ/VAV2/PTPRC/ACTG1/NCKAP1/PRKCE/MYO1C/PLCG1/CYFIP1/VAV3
Biological Process	GO:0038096	Fc-gamma receptor signaling pathway involved in phagocytosis	18/2279	139/18866	0.4142767	0.6525201	0.5904233	18	ELMO1/DOCK1/PIK3R1/WIPF1/FGR/CYFIP2/BAIAP2/WASL/PTPRJ/VAV2/PTPRC/ACTG1/NCKAP1/PRKCE/MYO1C/PLCG1/CYFIP1/VAV3

Biological Process	GO:0001556	oocyte maturation	4/2279	27/18866	0.4147531	0.6525201	0.5904233	4	BRCA2/PPP2R1A/DMC1/NPPC
Biological Process	GO:0001702	gastrulation with mouth forming second	4/2279	27/18866	0.4147531	0.6525201	0.5904233	4	LRP5L/ZBTB17/NODAL/OTX2
Biological Process	GO:0001963	synaptic transmission, dopaminergic	4/2279	27/18866	0.4147531	0.6525201	0.5904233	4	CRHBP/ARRB2/FLOT1/PRKN
Biological Process	GO:0003148	outflow tract septum morphogenesis	4/2279	27/18866	0.4147531	0.6525201	0.5904233	4	NRP2/TGFBR2/RARA/ENG
Biological Process	GO:0006829	zinc ion transport	4/2279	27/18866	0.4147531	0.6525201	0.5904233	4	TRPM2/SLC39A11/TMEM163/SLC39A13
Biological Process	GO:0007214	gamma-aminobutyric acid signaling pathway	4/2279	27/18866	0.4147531	0.6525201	0.5904233	4	GNAI2/PLCL1/GABBR1/GABBR2
Biological Process	GO:0018195	peptidyl-arginine modification	4/2279	27/18866	0.4147531	0.6525201	0.5904233	4	PRMT2/PADI2/ART5/ART4
Biological Process	GO:0032607	interferon-alpha production	4/2279	27/18866	0.4147531	0.6525201	0.5904233	4	IL10/IRF5/SETD2/TLR9
Biological Process	GO:0032786	positive regulation of DNA-templated transcription, elongation	4/2279	27/18866	0.4147531	0.6525201	0.5904233	4	BRD4/SCAF8/KAT7/CDK13

Biological Process	GO:0032897	negative regulation of viral transcription	4/2279	27/18866	0.4147531	0.6525201	0.5904233	4	TRIM27/TRIM8/CCL5/HDAC1
Biological Process	GO:0034243	regulation of transcription elongation from RNA polymerase II promoter	4/2279	27/18866	0.4147531	0.6525201	0.5904233	4	BRD4/WDR43/RECQL5/CDK13
Biological Process	GO:0034698	response to gonadotropin	4/2279	27/18866	0.4147531	0.6525201	0.5904233	4	POR/NOTCH1/HMGCS1/INHBA
Biological Process	GO:0046426	negative regulation of receptor signaling pathway via JAK-STAT	4/2279	27/18866	0.4147531	0.6525201	0.5904233	4	PPP2R1A/GGNBP2/BCL3/INPP5F
Biological Process	GO:0048143	astrocyte activation	4/2279	27/18866	0.4147531	0.6525201	0.5904233	4	TNF/FPR2/APP/GRN
Biological Process	GO:0050996	positive regulation of lipid catabolic process	4/2279	27/18866	0.4147531	0.6525201	0.5904233	4	PNPLA2/PRKCE/ABHD5/CPT1A
Biological Process	GO:0072207	metanephric epithelium development	4/2279	27/18866	0.4147531	0.6525201	0.5904233	4	PAX2/WWTR1/ACAT1/LIF
Biological Process	GO:1901623	regulation of lymphocyte chemotaxis	4/2279	27/18866	0.4147531	0.6525201	0.5904233	4	CCR2/CCL5/PADI2/CCL27
Biological Process	GO:1905208	negative regulation of cardiocyte differentiation	4/2279	27/18866	0.4147531	0.6525201	0.5904233	4	CTDP1/MIR199A1/DLL1/MIR199A2

Biological Process	GO:0007059	chromosome segregation	42/2279	334/18866	0.4148297	0.6525326	0.5904347	42	MAD1L1/TTN/TNKS/NDE1/RMI2/NSMCE2/RCC1/DYNC1H1/CHAMP1/RECQL5/CUL3/EME1/MEIOB/CDC16/MIS12/UVRAG/DCTN2/RNF212/TACC3/STAG1/NUMA1/PPP2R1A/MSTO1/NEK6/RB1/RAD21L1/DMC1/CDCA5/DIS3L2/ANAPC7/SPAG5/HECW2/SLX4/TEX12/NDEL1/NTMT1/BANF1/ERCC2/PCID2/TERB2/ABRAXAS1/SLF1
Biological Process	GO:0006140	regulation of nucleotide metabolic process	16/2279	123/18866	0.4161989	0.6544696	0.5921873	16	HDAC4/PRKAG2/CDA/RCVRN/ENO1/PFKFB4/RAE1/CBFA2T3/APP/PGAM1/SPHK2/NUP93/SEC13/ESRRB/PFKFB3/PRKAA1
Biological Process	GO:0070252	actin-mediated cell contraction	16/2279	123/18866	0.4161989	0.6544696	0.5921873	16	PDE4D/KCNE1/TTN/CCDC88C/CACNA1C/GJA5/TNNT3/JUP/BIN1/NOS1AP/SCN1A/SNTA1/KCNQ1/ANK2/FRMD6/TMOD1
Biological Process	GO:0000819	sister chromatid segregation	25/2279	196/18866	0.4177236	0.6567121	0.5942165	25	MAD1L1/TTN/TNKS/RMI2/NSMCE2/CHAMP1/CUL3/CDC16/MIS12/DCTN2/TACC3/STAG1/NUMA1/PPP2R1A/MSTO1/NEK6/RB1/RAD21L1/CDCA5/DIS3L2/ANAPC7/SPAG5/HECW2/PCID2/SLF1
Biological Process	GO:0010596	negative regulation of endothelial cell migration	14/2279	107/18866	0.418006	0.6567121	0.5942165	14	PPARG/ITGB1BP1/MIR199A1/TNF/MIR101-2/JUP/THBS1/MIR24-2/TGFB1/VASH1/MIR199A2/NOTCH1/MEF2C/SP100
Biological Process	GO:0099175	regulation of postsynapse organization	14/2279	107/18866	0.418006	0.6567121	0.5942165	14	NRP2/SSH1/ZMYND8/BAIAP2/ZDHHC2/NRXN1/GHRL/SIPA1L1/DNM3/DNM1L/UBE3A/CUX2/DBNL/LRFN2
Biological Process	GO:0000291	nuclear-transcribed mRNA catabolic process, exonucleolytic	5/2279	35/18866	0.4183162	0.6567121	0.5942165	5	LSM1/EXOSC2/EDC3/LSM7/DIS3L2
Biological Process	GO:0001914	regulation of T cell mediated cytotoxicity	5/2279	35/18866	0.4183162	0.6567121	0.5942165	5	HLA-F/RIPK3/PTPRC/FADD/CYRIB
Biological Process	GO:0006691	leukotriene metabolic process	5/2279	35/18866	0.4183162	0.6567121	0.5942165	5	ABCC1/GGT1/CYP4F3/MGST2/DPEP2

Biological Process	GO:0010259	multicellular organism aging	5/2279	35/18866	0.4183162	0.6567121	0.5942165	5	INPP5D/DDC/SLC1A2/PRDM2/ERCC1
Biological Process	GO:0060674	placenta blood vessel development	5/2279	35/18866	0.4183162	0.6567121	0.5942165	5	RBPJ/VASH1/GGNBP2/TMED2/PLCD3
Biological Process	GO:0070536	protein K63-linked deubiquitination	5/2279	35/18866	0.4183162	0.6567121	0.5942165	5	TNFAIP3/OTUB2/CYLD/ABRAXAS1/BABAM2
Biological Process	GO:0072525	pyridine-containing compound biosynthetic process	5/2279	35/18866	0.4183162	0.6567121	0.5942165	5	NADK/NMNAT3/PDXK/PARP10/PARP9
Biological Process	GO:0002474	antigen processing and presentation of peptide antigen via MHC class I	13/2279	99/18866	0.4188395	0.6569909	0.5944687	13	CALR/LNPEP/NCF4/HLA-C/HLA-F/NCF2/ITGB5/SEC13/PSMF1/SEC24C/PSMB7/SEC31A/PSMD13
Biological Process	GO:0050830	defense response to Gram-positive bacterium	13/2279	99/18866	0.4188395	0.6569909	0.5944687	13	DEFA4/CTSG/RNASE3/NLRP3/FGR/TNF/LYG2/STAB2/DEFB1/PGLYRP1/C10orf99/MPEG1/PGLYRP4
Biological Process	GO:0072163	mesonephric epithelium development	13/2279	99/18866	0.4188395	0.6569909	0.5944687	13	TACSTD2/SMAD3/ILK/SMAD7/WNT11/PAX2/FGFR1/RARA/BMPER/CAT/BCL2/CRLF1/HOXB7
Biological Process	GO:0072164	mesonephric tubule development	13/2279	99/18866	0.4188395	0.6569909	0.5944687	13	TACSTD2/SMAD3/ILK/SMAD7/WNT11/PAX2/FGFR1/RARA/BMPER/CAT/BCL2/CRLF1/HOXB7
Biological Process	GO:0090277	positive regulation of peptide hormone secretion	13/2279	99/18866	0.4188395	0.6569909	0.5944687	13	NADK/MYRIP/ARRB1/GPLD1/ITSN1/TCF7L2/GIPR/BLK/GHRL/PRKCE/RPH3AL/C2CD2L/SIRT3

Biological Process	GO:0071478	cellular response to radiation	24/2279	188/18866	0.4189591	0.6570701	0.5945403	24	BLM/PDE6A/SPIDR/MFAP4/CRB1/PIK3R1/BMF/DNM2/HUS1/CDC25A/AQP1/CREBBP/TP53BP1/GRK7/RHNO1/CHEK2/NEDD4/ERCC1/CRIP1/BCL2L1/POLD3/XPA/EEF1D/MAP3K20
Biological Process	GO:1903350	response to dopamine	12/2279	91/18866	0.419604	0.6579729	0.5953572	12	KLF16/DNM2/HRH1/HTR3A/GNA11/CHRM2/PALM/ARRB2/GNG2/GNAO1/DTNBP1/PRKN
Biological Process	GO:0001953	negative regulation of cell-matrix adhesion	6/2279	43/18866	0.4201446	0.6582773	0.5956327	6	ITGB1BP1/PIK3R1/DUSP22/THBS1/NF1/CORO1C
Biological Process	GO:0017158	regulation of calcium ion-dependent exocytosis	6/2279	43/18866	0.4201446	0.6582773	0.5956327	6	RAP1B/GNAI2/NOTCH1/SYT17/RPH3AL/CBARP
Biological Process	GO:0046717	acid secretion	6/2279	43/18866	0.4201446	0.6582773	0.5956327	6	SLC51A/SGK1/NF1/AGXT/GHRL/KCNQ1
Biological Process	GO:0071364	cellular response to epidermal growth factor stimulus	6/2279	43/18866	0.4201446	0.6582773	0.5956327	6	DUSP22/BAIAP2/PAX2/DUSP3/PLCG1/MARS1
Biological Process	GO:2000648	positive regulation of stem cell proliferation	6/2279	43/18866	0.4201446	0.6582773	0.5956327	6	DISC1/PTPRC/ZNF335/NOTCH1/KAT7/CX3CR1
Biological Process	GO:0006695	cholesterol biosynthetic process	10/2279	75/18866	0.420817	0.6588672	0.5961664	10	IDI1/MBTPS1/ABCG1/POR/ACACB/ACACA/HMGCS1/SCD/SREBF1/PRKAA1
Biological Process	GO:1901983	regulation of protein acetylation	10/2279	75/18866	0.420817	0.6588672	0.5961664	10	ARRB1/RPS6KA4/SPHK2/CTBP1/ARNTL/PIH1D1/KAT7/LIF/PAXIP1/PRKAA1

Biological Process	GO:1902653	secondary alcohol biosynthetic process	10/2279	75/18866	0.420817	0.6588672	0.5961664	10	IDI1/MBTPS1/ABCG1/POR/ACACB/ACACA/HMGCS1/SCD/SREBF1/PRKAA1
Biological Process	GO:0030490	maturation of SSU-rRNA	7/2279	51/18866	0.421008	0.6588672	0.5961664	7	RCL1/WDR46/NOB1/NOP14/ERCC2/RIOK1/UTP4
Biological Process	GO:0045103	intermediate filament-based process	7/2279	51/18866	0.421008	0.6588672	0.5961664	7	KRT17/PKP1/NEFM/DNAJB6/GFAP/NDEL1/PPL
Biological Process	GO:0048146	positive regulation of fibroblast proliferation	7/2279	51/18866	0.421008	0.6588672	0.5961664	7	MYB/ZMIZ1/AQP1/PDGFD/PDGFB/PDGFC/PDGFRB
Biological Process	GO:1903587	regulation of blood vessel endothelial cell proliferation involved in sprouting angiogenesis	7/2279	51/18866	0.421008	0.6588672	0.5961664	7	MIR101-2/THBS1/MIR24-2/MIR27A/MIR23A/AGTR1/PPP1R16B
Biological Process	GO:0009166	nucleotide catabolic process	9/2279	67/18866	0.4211792	0.6588672	0.5961664	9	PDE4D/AMPD3/NUDT3/ACOT7/UNG/NTHL1/PDE9A/PNP/ACAT1
Biological Process	GO:0050918	positive chemotaxis	9/2279	67/18866	0.4211792	0.6588672	0.5961664	9	AZU1/IL16/ANGPT1/SMAD3/CCL5/FPR2/PTPRJ/PDGFB/TSC2
Biological Process	GO:0043525	positive regulation of neuron apoptotic process	8/2279	59/18866	0.4212839	0.6588672	0.5961664	8	HDAC4/CTSZ/MYB/SRPK2/TNF/NF1/GRN/FIS1
Biological Process	GO:0048008	platelet-derived growth factor receptor signaling pathway	8/2279	59/18866	0.4212839	0.6588672	0.5961664	8	ARID5B/FER/PTPRJ/PDGFD/PDGFB/BCR/PDGFC/PDGFRB

Biological Process	GO:0098727	maintenance of cell number	21/2279	164/18866	0.4227631	0.6610718	0.5981612	21	FOXP1/TRIM8/LSM1/MIR145/WDR43/SKI/POLR2F/LBH/DLL1/RBPJ/PAX2/KDM4C/PRDM16/ESRRB/NOTCH1/ZHX2/DIS3L2/FOXO1/NODAL/LIF/ZNF706
Biological Process	GO:0045165	cell fate commitment	35/2279	278/18866	0.423791	0.6625701	0.599517	35	ZFPM1/PPARG/SUFU/LOXL3/SH3PXD2B/MIAT/MGA/DLL1/RBPJ/TCF7L2/IFRD1/WNT11/PAX2/LY9/FGFR1/ZBTB7B/PAX7/RARA/TGFB1I1/DSCAML1/WNT5B/RUNX2/FEV/BCL2/TCF3/NOTCH1/MEF2C/NOTCH4/EPAS1/ARHGEF2/CEBPB/CYLD/LMO4/NODAL/NTF4
Biological Process	GO:0071241	cellular response to inorganic substance	28/2279	221/18866	0.4239993	0.6627795	0.5997064	28	TRPM2/CPNE6/BLM/CALR/SPIDR/GSN/CPNE2/CREB1/ITPKB/SLFN14/DNM2/GPLD1/CPNE5/TRAF2/APP/CPNE1/MTF1/CRHBP/AQP1/NFE2L2/CDH1/ADD1/MEF2C/SYT17/FOXO1/SLC41A1/PRKAA1/PRKN
Biological Process	GO:0006865	amino acid transport	20/2279	156/18866	0.4240645	0.6627795	0.5997064	20	SLC38A4/SLC38A10/SLC11A1/SLC1A5/SLC15A4/LRRC8C/PPFIA1/SLC43A2/NF1/SLC1A2/SLC7A7/SLC7A5/SFXN1/SLC1A6/PER2/SLC36A3/SLC1A3/SLC43A1/SLC7A8/SLC7A1
Biological Process	GO:0042476	odontogenesis	17/2279	132/18866	0.4280689	0.668928	0.6052698	17	BSG/CSF3R/SLC24A4/ANKRD11/NFIC/ALPL/HDAC1/AQP1/PERP/MYO5A/RUNX2/TSPEAR/AQP3/FOXO1/FAM20C/INHBA/NECTIN1
Biological Process	GO:0010498	proteasomal protein catabolic process	60/2279	483/18866	0.4286975	0.6698002	0.606059	60	TRIM39/MAEA/RNF144A/FZR1/RNF19A/TRIM38/WWP2/CSNK1D/AMFR/ARRB1/RHBDD2/CBFA2T3/UBAC2/CUL3/EDEM1/HM13/CDC16/DERL2/CLU/SMAD7/UBE2J2/PPP2R5C/SMURF1/MTA1/POMT2/CLEC16A/WWTR1/FBXL19/EDEM2/CSNK1E/PSMF1/FBXL13/UBXN11/KCTD2/NFE2L2/ARRB2/ARNTL/FBXL18/RNF144B/PSMB7/MAP1A/FBXL6/GNA12/UBXN2A/NEDD4/ANAPC7/RNF14/TRIB1/HECW2/FBXW11/RNF40/BAG5/GID8/UBXN8/PELI1/PSMD13/SVIP/MARCHF6/PRKN/MARCHF7
Biological Process	GO:0072330	monocarboxylic acid biosynthetic process	31/2279	246/18866	0.4294011	0.6707331	0.6069031	31	PRKAG2/MGLL/DSE/DHRS9/LIPC/AMACR/AKR1D1/OSBPL6/PRKAB1/ACOT7/ACSS1/RDH10/MYO5A/NR1H2/AGXT/ACACB/ACSS2/ACOX2/HOGA1/PER2/SLC1A3/PKM/ACACA/ACSF3/FA2H/SCD/WDTC1/ALDH1A2/ACADVL/PRKAA1/PRXL2B
Biological Process	GO:0010633	negative regulation of epithelial cell migration	16/2279	124/18866	0.4294358	0.6707331	0.6069031	16	TACSTD2/PPARG/ITGB1BP1/MIR199A1/TNF/MIR101-2/JUP/THBS1/MIR24-2/TGFB1/VASH1/MIR199A2/NOTCH1/CORO1C/MEF2C/SP100
Biological Process	GO:0007369	gastrulation	24/2279	189/18866	0.4296761	0.6709981	0.6071429	24	IL10/MIR145/LRP5L/SETD2/SMAD3/COL4A2/TGFBR2/CUL3/MBP/ZBTB17/COL12A1/WNT11/PAX2/ITGB5/FGFR1/IL1RN/ITGB3/ITGB2/COL5A1/LAMB3/MYADM/INHBA/NODAL/OTX2

Biological Process	GO:0034249	negative regulation of cellular amide metabolic process	30/2279	238/18866	0.4308715	0.6715429	0.6076359	30	SORL1/CALR/MIR145/TNRC6B/ORMDL3/MIR101-2/AGO1/LARP1/ZC3H12D/BIN1/SAMD4A/MIR27A/CLU/AGO2/RARA/CPEB3/IGF2BP3/INPP5E/FTO/PUS7/MIR138-2/CELF1/NOTCH4/BANK1/ILF3/SYNCRIP/EIF4G1/DAPK3/PRKAA1/SHFL
Biological Process	GO:0032508	DNA duplex unwinding	14/2279	108/18866	0.4322104	0.6715429	0.6076359	14	BLM/RTEL1/MCM5/WRNIP1/RECQL5/IGHMBP2/MCM2/SMARCAL1/CHD9/CHD8/RUVBL1/XPA/ERCC2/FBH1
Biological Process	GO:0044774	mitotic DNA integrity checkpoint	14/2279	108/18866	0.4322104	0.6715429	0.6076359	14	TRIM39/BLM/TFDP1/FOXN3/HUS1/E2F7/PCBP4/EME1/ZNF385A/ARID3A/TAOK3/CLSPN/CHEK2/NAE1
Biological Process	GO:1902750	negative regulation of cell cycle G2/M phase transition	14/2279	108/18866	0.4322104	0.6715429	0.6076359	14	TRIM39/BLM/FZR1/FOXN3/HUS1/PSMF1/TAOK3/ZFYVE19/CLSPN/PSMB7/NAE1/PSMD13/ABRAXAS1/BABAM2
Biological Process	GO:0050953	sensory perception of light stimulus	28/2279	222/18866	0.4339051	0.6715429	0.6076359	28	RCVRN/TACSTD2/PDE6A/CACNA2D4/OPA3/CRB1/TGFBI/CDH23/TULP2/CCDC66/GNAT2/PAX2/PITPNA/CDH3/RDH10/NOB1/MYO5A/CRYBB1/PDE6H/RPGRIP1/CABP1/EPAS1/GRK7/NRL/ABLIM1/EYS/KIFC3/CABP4
Biological Process	GO:0000727	double-strand break repair via break-induced replication	2/2279	12/18866	0.4349726	0.6715429	0.6076359	2	MCM5/MCM2
Biological Process	GO:0002517	T cell tolerance induction	2/2279	12/18866	0.4349726	0.6715429	0.6076359	2	PHLPP1/TGFBR2
Biological Process	GO:0002730	regulation of dendritic cell cytokine production	2/2279	12/18866	0.4349726	0.6715429	0.6076359	2	SCIMP/NOD2
Biological Process	GO:0003207	cardiac chamber formation	2/2279	12/18866	0.4349726	0.6715429	0.6076359	2	NOTCH1/MEF2C

Biological Process	GO:0003353	positive regulation of cilium movement	2/2279	12/18866	0.4349726	0.6715429	0.6076359	2	RNASE10/DEFB1
Biological Process	GO:0006554	lysine catabolic process	2/2279	12/18866	0.4349726	0.6715429	0.6076359	2	OGDH/CRYM
Biological Process	GO:0009071	serine family amino acid catabolic process	2/2279	12/18866	0.4349726	0.6715429	0.6076359	2	CBS/AGXT
Biological Process	GO:0009086	methionine biosynthetic process	2/2279	12/18866	0.4349726	0.6715429	0.6076359	2	MTHFD1/ADI1
Biological Process	GO:0009151	purine deoxyribonucleotide metabolic process	2/2279	12/18866	0.4349726	0.6715429	0.6076359	2	GUK1/AK5
Biological Process	GO:0010232	vascular transport	2/2279	12/18866	0.4349726	0.6715429	0.6076359	2	MFSD2A/ABCC2
Biological Process	GO:0010960	magnesium ion homeostasis	2/2279	12/18866	0.4349726	0.6715429	0.6076359	2	CNNM2/SLC41A1
Biological Process	GO:0014745	negative regulation of muscle adaptation	2/2279	12/18866	0.4349726	0.6715429	0.6076359	2	SMAD3/FOXO1
Biological Process	GO:0015824	proline transport	2/2279	12/18866	0.4349726	0.6715429	0.6076359	2	SLC36A3/SLC7A8

Biological Process	GO:0021702	cerebellar Purkinje cell differentiation	2/2279	12/18866	0.4349726	0.6715429	0.6076359	2	TTC21B/TTLL1
Biological Process	GO:0021903	rostrocaudal neural tube patterning	2/2279	12/18866	0.4349726	0.6715429	0.6076359	2	SSBP3/HES3
Biological Process	GO:0030157	pancreatic juice secretion	2/2279	12/18866	0.4349726	0.6715429	0.6076359	2	AQP1/NR1H2
Biological Process	GO:0030259	lipid glycosylation	2/2279	12/18866	0.4349726	0.6715429	0.6076359	2	ST3GAL4/ST3GAL2
Biological Process	GO:0031581	hemidesmosome assembly	2/2279	12/18866	0.4349726	0.6715429	0.6076359	2	COL17A1/LAMB3
Biological Process	GO:0033629	negative regulation of cell adhesion mediated by integrin	2/2279	12/18866	0.4349726	0.6715429	0.6076359	2	JAM3/PDE3B
Biological Process	GO:0033700	phospholipid efflux	2/2279	12/18866	0.4349726	0.6715429	0.6076359	2	ABCG1/ABCA1
Biological Process	GO:0034397	telomere localization	2/2279	12/18866	0.4349726	0.6715429	0.6076359	2	RAD21L1/TERB2
Biological Process	GO:0034497	protein localization to phagophore assembly site	2/2279	12/18866	0.4349726	0.6715429	0.6076359	2	PACS2/WIPI2

Biological Process	GO:0035404	histone-serine phosphorylation	2/2279	12/18866	0.4349726	0.6715429	0.6076359	2	RPS6KA4/PRKAA1
Biological Process	GO:0038063	collagen-activated tyrosine kinase receptor signaling pathway	2/2279	12/18866	0.4349726	0.6715429	0.6076359	2	COL4A2/UBASH3B
Biological Process	GO:0038110	interleukin-2-mediated signaling pathway	2/2279	12/18866	0.4349726	0.6715429	0.6076359	2	STAT5A/JAK1
Biological Process	GO:0042340	keratan sulfate catabolic process	2/2279	12/18866	0.4349726	0.6715429	0.6076359	2	HEXB/GALNS
Biological Process	GO:0042492	gamma-delta T cell differentiation	2/2279	12/18866	0.4349726	0.6715429	0.6076359	2	CCR9/PTPRC
Biological Process	GO:0043383	negative T cell selection	2/2279	12/18866	0.4349726	0.6715429	0.6076359	2	PTPRC/DOCK2
Biological Process	GO:0043476	pigment accumulation	2/2279	12/18866	0.4349726	0.6715429	0.6076359	2	SHROOM3/AP3D1
Biological Process	GO:0043482	cellular pigment accumulation	2/2279	12/18866	0.4349726	0.6715429	0.6076359	2	SHROOM3/AP3D1
Biological Process	GO:0044650	adhesion of symbiont to host cell	2/2279	12/18866	0.4349726	0.6715429	0.6076359	2	SFTPD/NECTIN1

Biological Process	GO:0045144	meiotic sister chromatid segregation	2/2279	12/18866	0.4349726	0.6715429	0.6076359	2	PPP2R1A/RAD21L1
Biological Process	GO:0045161	neuronal ion channel clustering	2/2279	12/18866	0.4349726	0.6715429	0.6076359	2	SPTBN4/GLDN
Biological Process	GO:0046185	aldehyde catabolic process	2/2279	12/18866	0.4349726	0.6715429	0.6076359	2	AGXT/HOGA1
Biological Process	GO:0048148	behavioral response to cocaine	2/2279	12/18866	0.4349726	0.6715429	0.6076359	2	PPP1R1B/FADD
Biological Process	GO:0051177	meiotic sister chromatid cohesion	2/2279	12/18866	0.4349726	0.6715429	0.6076359	2	PPP2R1A/RAD21L1
Biological Process	GO:0051340	regulation of ligase activity	2/2279	12/18866	0.4349726	0.6715429	0.6076359	2	RIPK3/TFIP11
Biological Process	GO:0060081	membrane hyperpolarization	2/2279	12/18866	0.4349726	0.6715429	0.6076359	2	PRKCZ/KCTD7
Biological Process	GO:0060394	negative regulation of pathway-restricted SMAD protein phosphorylation	2/2279	12/18866	0.4349726	0.6715429	0.6076359	2	LDLRAD4/SMAD7
Biological Process	GO:0060453	regulation of gastric acid secretion	2/2279	12/18866	0.4349726	0.6715429	0.6076359	2	SGK1/KCNQ1

Biological Process	GO:0060742	epithelial cell differentiation involved in prostate gland development	2/2279	12/18866	0.4349726	0.6715429	0.6076359	2	RXRA/NOTCH1
Biological Process	GO:0061517	macrophage proliferation	2/2279	12/18866	0.4349726	0.6715429	0.6076359	2	CSF1R/CLU
Biological Process	GO:0070202	regulation of establishment of protein localization to chromosome	2/2279	12/18866	0.4349726	0.6715429	0.6076359	2	SPIDR/CCT6A
Biological Process	GO:0070243	regulation of thymocyte apoptotic process	2/2279	12/18866	0.4349726	0.6715429	0.6076359	2	PTCRA/ZC3H8
Biological Process	GO:0070269	pyroptosis	2/2279	12/18866	0.4349726	0.6715429	0.6076359	2	NLRC4/AIM2
Biological Process	GO:0070493	thrombin-activated receptor signaling pathway	2/2279	12/18866	0.4349726	0.6715429	0.6076359	2	DGKQ/F2R
Biological Process	GO:0070572	positive regulation of neuron projection regeneration	2/2279	12/18866	0.4349726	0.6715429	0.6076359	2	GRN/NDEL1
Biological Process	GO:0071107	response to parathyroid hormone	2/2279	12/18866	0.4349726	0.6715429	0.6076359	2	HDAC4/MEF2C
Biological Process	GO:0071481	cellular response to X-ray	2/2279	12/18866	0.4349726	0.6715429	0.6076359	2	DNM2/TP53BP1

Biological Process	GO:0072017	distal tubule development	2/2279	12/18866	0.4349726	0.6715429	0.6076359	2	PAX2/NOTCH1
Biological Process	GO:0072393	microtubule anchoring at microtubule organizing center	2/2279	12/18866	0.4349726	0.6715429	0.6076359	2	DCTN1/BICD2
Biological Process	GO:0072540	T-helper 17 cell lineage commitment	2/2279	12/18866	0.4349726	0.6715429	0.6076359	2	LOXL3/LY9
Biological Process	GO:0072711	cellular response to hydroxyurea	2/2279	12/18866	0.4349726	0.6715429	0.6076359	2	BLM/SPIDR
Biological Process	GO:0086067	AV node cell to bundle of His cell communication	2/2279	12/18866	0.4349726	0.6715429	0.6076359	2	CACNA1C/GJA5
Biological Process	GO:0090209	negative regulation of triglyceride metabolic process	2/2279	12/18866	0.4349726	0.6715429	0.6076359	2	SORL1/GPLD1
Biological Process	GO:0090220	chromosome localization to nuclear envelope involved in homologous chromosome segregation	2/2279	12/18866	0.4349726	0.6715429	0.6076359	2	RAD21L1/TERB2
Biological Process	GO:0090331	negative regulation of platelet aggregation	2/2279	12/18866	0.4349726	0.6715429	0.6076359	2	CD9/UBASH3B
Biological Process	GO:0097104	postsynaptic membrane assembly	2/2279	12/18866	0.4349726	0.6715429	0.6076359	2	NRXN1/NRXN2

Biological Process	GO:0150065	regulation of deacetylase activity	2/2279	12/18866	0.4349726	0.6715429	0.6076359	2	C6orf89/SPHK2
Biological Process	GO:1901503	ether biosynthetic process	2/2279	12/18866	0.4349726	0.6715429	0.6076359	2	CHPT1/PEDS1
Biological Process	GO:1903332	regulation of protein folding	2/2279	12/18866	0.4349726	0.6715429	0.6076359	2	GRN/BAG5
Biological Process	GO:1904465	negative regulation of matrix metallopeptidase secretion	2/2279	12/18866	0.4349726	0.6715429	0.6076359	2	MIR199A1/MIR199A2
Biological Process	GO:1904953	Wnt signaling pathway involved in midbrain dopaminergic neuron differentiation	2/2279	12/18866	0.4349726	0.6715429	0.6076359	2	CSNK1D/CSNK1E
Biological Process	GO:2000105	positive regulation of DNA-dependent DNA replication	2/2279	12/18866	0.4349726	0.6715429	0.6076359	2	E2F7/FGFR1
Biological Process	GO:2001214	positive regulation of vasculogenesis	2/2279	12/18866	0.4349726	0.6715429	0.6076359	2	RIN2/RAPGEF2
Biological Process	GO:0042594	response to starvation	26/2279	206/18866	0.4370763	0.6746811	0.6104754	26	FOXK1/RPTOR/PRKAG2/PPARG/MFSD2A/ULK1/WIPI2/LARP1/COMT/EHMT2/STK24/CLEC16A/ATG14/NFE2L2/BCL2/HMGCL/PCK2/SLC2A1/FOXO1/ACAT1/SREBF1/DHODH/NPRL2/PRKAA1/MARS1/RIPOR1
Biological Process	GO:0008306	associative learning	10/2279	76/18866	0.4378111	0.6755958	0.6113031	10	CREB1/PPP1R1B/TANC1/APP/SYNGAP1/HRH1/NF1/MEIS2/MAP1A/SYNPO

Biological Process	GO:0033555	multicellular organismal response to stress	10/2279	76/18866	0.4378111	0.6755958	0.6113031	10	THBS1/COMT/ASIC4/MAPK8IP2/NTRK1/BCL2/MEF2C/MORC1/CACNA1B/EIF4G1
Biological Process	GO:0002753	cytoplasmic pattern recognition receptor signaling pathway	9/2279	68/18866	0.4391662	0.6759464	0.6116203	9	MAP2K6/TNFAIP3/PHB/UBE2V1/SEC14L1/IRAK2/NOD2/CYLD/RELA
Biological Process	GO:1902369	negative regulation of RNA catabolic process	9/2279	68/18866	0.4391662	0.6759464	0.6116203	9	SLC11A1/LARP1/PKP1/NBAS/PAIP1/SECISBP2/SYNCRIP/TENT5C/TENT5A
Biological Process	GO:0007602	phototransduction	8/2279	60/18866	0.4404586	0.6759464	0.6116203	8	RCVRN/PDE6A/ARRB1/PITPNM1/GNAT2/GNA11/GRK7/CABP4
Biological Process	GO:0018208	peptidyl-proline modification	8/2279	60/18866	0.4404586	0.6759464	0.6116203	8	P4HB/FKBP5/AIP/P4HA2/NTMT1/P3H3/PTPA/P3H1
Biological Process	GO:0032653	regulation of interleukin-10 production	8/2279	60/18866	0.4404586	0.6759464	0.6116203	8	PRKCZ/MIR145/TLR9/DLL1/TNFRSF21/TIGIT/NOD2/VSIR
Biological Process	GO:0043388	positive regulation of DNA binding	8/2279	60/18866	0.4404586	0.6759464	0.6116203	8	PPARG/SKI/HIPK2/TGFB1/RB1/CDCA5/ERCC2/PRKN
Biological Process	GO:0048747	muscle fiber development	8/2279	60/18866	0.4404586	0.6759464	0.6116203	8	HDAC4/MYO18B/TTN/MYH11/SKI/BCL2/DYSF/MYORG
Biological Process	GO:0061900	glial cell activation	8/2279	60/18866	0.4404586	0.6759464	0.6116203	8	AZU1/TNF/FPR2/APP/CLU/PTPRC/GRN/ITGB2

Biological Process	GO:0061951	establishment of protein localization to plasma membrane	8/2279	60/18866	0.4404586	0.6759464	0.6116203	8	KIF13A/RAB31/ZDHHC2/AKAP5/VAMP5/CSK/GORASP2/RILPL1
Biological Process	GO:0001911	negative regulation of leukocyte mediated cytotoxicity	3/2279	20/18866	0.4413878	0.6759464	0.6116203	3	HLA-F/PTPRC/ARRB2
Biological Process	GO:0006144	purine nucleobase metabolic process	3/2279	20/18866	0.4413878	0.6759464	0.6116203	3	GMPR2/AOX1/ACP3
Biological Process	GO:0006346	DNA methylation-dependent heterochromatin assembly	3/2279	20/18866	0.4413878	0.6759464	0.6116203	3	HDAC1/ATF7IP/DNMT1
Biological Process	GO:0006525	arginine metabolic process	3/2279	20/18866	0.4413878	0.6759464	0.6116203	3	FAH/SLC7A7/ART4
Biological Process	GO:0006743	ubiquinone metabolic process	3/2279	20/18866	0.4413878	0.6759464	0.6116203	3	AIFM2/COQ4/COQ8B
Biological Process	GO:0009168	purine ribonucleoside monophosphate biosynthetic process	3/2279	20/18866	0.4413878	0.6759464	0.6116203	3	AMPD3/LHPP/IMPDH1
Biological Process	GO:0015669	gas transport	3/2279	20/18866	0.4413878	0.6759464	0.6116203	3	HBB/AQP1/MB
Biological Process	GO:0031290	retinal ganglion cell axon guidance	3/2279	20/18866	0.4413878	0.6759464	0.6116203	3	EPHB3/BMPR1B/SLIT1

Biological Process	GO:0031498	chromatin disassembly	3/2279	20/18866	0.4413878	0.6759464	0.6116203	3	PADI2/SMARCD1/SMARCD2
Biological Process	GO:0031998	regulation of fatty acid beta-oxidation	3/2279	20/18866	0.4413878	0.6759464	0.6116203	3	MFSD2A/ACACB/CPT1A
Biological Process	GO:0032727	positive regulation of interferon-alpha production	3/2279	20/18866	0.4413878	0.6759464	0.6116203	3	IRF5/SETD2/TLR9
Biological Process	GO:0032793	positive regulation of CREB transcription factor activity	3/2279	20/18866	0.4413878	0.6759464	0.6116203	3	RPS6KA4/CRTC3/CAMK1D
Biological Process	GO:0042089	cytokine biosynthetic process	3/2279	20/18866	0.4413878	0.6759464	0.6116203	3	CEBPE/IGF2BP3/ASB1
Biological Process	GO:0043555	regulation of translation in response to stress	3/2279	20/18866	0.4413878	0.6759464	0.6116203	3	RPS6KA1/NCK2/EIF4G1
Biological Process	GO:0045019	negative regulation of nitric oxide biosynthetic process	3/2279	20/18866	0.4413878	0.6759464	0.6116203	3	IL10/MIR199A1/MIR199A2
Biological Process	GO:0048670	regulation of collateral sprouting	3/2279	20/18866	0.4413878	0.6759464	0.6116203	3	IFRD1/SEMA4D/SPART
Biological Process	GO:0051767	nitric-oxide synthase biosynthetic process	3/2279	20/18866	0.4413878	0.6759464	0.6116203	3	MAP2K6/TLR9/NOD2

Biological Process	GO:0051769	regulation of nitric-oxide synthase biosynthetic process	3/2279	20/18866	0.4413878	0.6759464	0.6116203	3	MAP2K6/TLR9/NOD2
Biological Process	GO:0071071	regulation of phospholipid biosynthetic process	3/2279	20/18866	0.4413878	0.6759464	0.6116203	3	LPCAT1/FPR2/PDGFB
Biological Process	GO:0097734	extracellular exosome biogenesis	3/2279	20/18866	0.4413878	0.6759464	0.6116203	3	RAB27A/COPS5/PRKN
Biological Process	GO:0098703	calcium ion import across plasma membrane	3/2279	20/18866	0.4413878	0.6759464	0.6116203	3	TRPM2/SLC8A1/TRPV2
Biological Process	GO:1900151	regulation of nuclear-transcribed mRNA catabolic process, deadenylation-dependent decay	3/2279	20/18866	0.4413878	0.6759464	0.6116203	3	TNRC6B/AGO2/CPEB3
Biological Process	GO:1900409	positive regulation of cellular response to oxidative stress	3/2279	20/18866	0.4413878	0.6759464	0.6116203	3	TNF/RIPK1/SIRT3
Biological Process	GO:1902236	negative regulation of endoplasmic reticulum stress-induced intrinsic apoptotic signaling pathway	3/2279	20/18866	0.4413878	0.6759464	0.6116203	3	BCL2L1/GRINA/PRKN
Biological Process	GO:1904406	negative regulation of nitric oxide metabolic process	3/2279	20/18866	0.4413878	0.6759464	0.6116203	3	IL10/MIR199A1/MIR199A2
Biological Process	GO:0000186	activation of MAPKK activity	7/2279	52/18866	0.4416388	0.6759464	0.6116203	7	MAP3K3/TNIK/RIPK1/MAP3K14/NTRK1/F2R/MAP3K20

Biological Process	GO:0001912	positive regulation of leukocyte mediated cytotoxicity	7/2279	52/18866	0.4416388	0.6759464	0.6116203	7	CD226/HLA-F/PTPRC/IL18RAP/CADM1/FADD/CYRIB
Biological Process	GO:0060421	positive regulation of heart growth	7/2279	52/18866	0.4416388	0.6759464	0.6116203	7	MIR199A1/RBPJ/FGFR1/ACACB/MIR199A2/NOTCH1/MEF2C
Biological Process	GO:0072132	mesenchyme morphogenesis	7/2279	52/18866	0.4416388	0.6759464	0.6116203	7	SMAD3/TGFBR2/RBPJ/WNT11/FGFR1/ENG/NOTCH1
Biological Process	GO:0045197	establishment or maintenance of epithelial cell apical/basal polarity	6/2279	44/18866	0.442619	0.6759464	0.6116203	6	CRB1/ILK/WNT11/WDR1/PRKCI/MARK2
Biological Process	GO:0045910	negative regulation of DNA recombination	6/2279	44/18866	0.442619	0.6759464	0.6116203	6	BLM/RMI2/RTEL1/RECQL5/TP53BP1/FBH1
Biological Process	GO:0097028	dendritic cell differentiation	6/2279	44/18866	0.442619	0.6759464	0.6116203	6	TRPM2/PRTN3/TGFBR2/RBPJ/BLK/CEBPB
Biological Process	GO:0098813	nuclear chromosome segregation	34/2279	272/18866	0.4431094	0.6759464	0.6116203	34	MAD1L1/TTN/TNKS/RMI2/NSMCE2/DYNC1H1/CHAMP1/CUL3/EME1/MEIOB/CDC16/MIS12/DCTN2/RNF212/TACC3/STAG1/NUMA1/PPP2R1A/MSTO1/NEK6/RB1/RAD21L1/DMC1/CDCA5/DIS3L2/ANAPC7/SPAG5/HECW2/SLX4/TEX12/PCID2/TERB2/ABRAXAS1/SLF1
Biological Process	GO:0001916	positive regulation of T cell mediated cytotoxicity	4/2279	28/18866	0.443121	0.6759464	0.6116203	4	HLA-F/PTPRC/FADD/CYRIB
Biological Process	GO:0006760	folic acid-containing compound metabolic process	4/2279	28/18866	0.443121	0.6759464	0.6116203	4	MTHFD1L/MTHFS/SLC19A1/MTHFD1

Biological Process	GO:0006929	substrate-dependent cell migration	4/2279	28/18866	0.443121	0.6759464	0.6116203	4	ITGB1BP1/CTTN/FMNL1/PTPRC
Biological Process	GO:0009074	aromatic amino acid family catabolic process	4/2279	28/18866	0.443121	0.6759464	0.6116203	4	HAL/HGD/FAH/HNMT
Biological Process	GO:0010575	positive regulation of vascular endothelial growth factor production	4/2279	28/18866	0.443121	0.6759464	0.6116203	4	C3AR1/TGFB1/CXCL17/NODAL
Biological Process	GO:0010669	epithelial structure maintenance	4/2279	28/18866	0.443121	0.6759464	0.6116203	4	PIWIL4/TLR9/SLC22A5/NOD2
Biological Process	GO:0010818	T cell chemotaxis	4/2279	28/18866	0.443121	0.6759464	0.6116203	4	PIK3CD/CCR2/CCL5/CCL27
Biological Process	GO:0021680	cerebellar Purkinje cell layer development	4/2279	28/18866	0.443121	0.6759464	0.6116203	4	DLL1/TTC21B/TTLL1/COQ8B
Biological Process	GO:0021846	cell proliferation in forebrain	4/2279	28/18866	0.443121	0.6759464	0.6116203	4	ZEB2/DISC1/FGFR1/NUMB
Biological Process	GO:0031069	hair follicle morphogenesis	4/2279	28/18866	0.443121	0.6759464	0.6116203	4	KRT17/BCL2/NOTCH1/TGM3
Biological Process	GO:0032958	inositol phosphate biosynthetic process	4/2279	28/18866	0.443121	0.6759464	0.6116203	4	ITPKB/P2RY6/HRH1/IP6K1

Biological Process	GO:0033598	mammary gland epithelial cell proliferation	4/2279	28/18866	0.443121	0.6759464	0.6116203	4	RREB1/BRCA2/RTN4/CEBPB
Biological Process	GO:0035883	enteroendocrine cell differentiation	4/2279	28/18866	0.443121	0.6759464	0.6116203	4	RHEB/RFX3/DLL1/ARNTL
Biological Process	GO:0042044	fluid transport	4/2279	28/18866	0.443121	0.6759464	0.6116203	4	ITPR1/AQP1/AQP3/AQP9
Biological Process	GO:0043901	negative regulation of multi-organism process	4/2279	28/18866	0.443121	0.6759464	0.6116203	4	CALR/ARHGDIB/NPPC/NODAL
Biological Process	GO:0060143	positive regulation of syncytium formation by plasma membrane fusion	4/2279	28/18866	0.443121	0.6759464	0.6116203	4	EHD1/NFATC2/FLOT1/SCGB3A1
Biological Process	GO:0060512	prostate gland morphogenesis	4/2279	28/18866	0.443121	0.6759464	0.6116203	4	RXRA/RARG/NOTCH1/CRIP1
Biological Process	GO:0090383	phagosome acidification	4/2279	28/18866	0.443121	0.6759464	0.6116203	4	ATP6V1B2/ATP6V0B/ATP6V0C/ATP6V1C1
Biological Process	GO:1900101	regulation of endoplasmic reticulum unfolded protein response	4/2279	28/18866	0.443121	0.6759464	0.6116203	4	ERN1/PIK3R1/NCK2/COPS5
Biological Process	GO:1902668	negative regulation of axon guidance	4/2279	28/18866	0.443121	0.6759464	0.6116203	4	SEMA6B/SEMA4A/SEMA4B/SEMA4D

Biological Process	GO:1905563	negative regulation of vascular endothelial cell proliferation	4/2279	28/18866	0.443121	0.6759464	0.6116203	4	PPARG/MIR24-2/FLT1/MEF2C
Biological Process	GO:0010092	specification of animal organ identity	5/2279	36/18866	0.4432319	0.6759464	0.6116203	5	RBPJ/WNT11/PAX2/FGFR1/MEF2C
Biological Process	GO:0010543	regulation of platelet activation	5/2279	36/18866	0.4432319	0.6759464	0.6116203	5	CD9/PRKCA/UBASH3B/BLK/PDGFB
Biological Process	GO:0032205	negative regulation of telomere maintenance	5/2279	36/18866	0.4432319	0.6759464	0.6116203	5	TNKS/RTEL1/SMG6/ERCC1/SLX4
Biological Process	GO:0032435	negative regulation of proteasomal ubiquitin-dependent protein catabolic process	5/2279	36/18866	0.4432319	0.6759464	0.6116203	5	TRIM39/CLEC16A/MAP1A/BAG5/SVIP
Biological Process	GO:0042491	inner ear auditory receptor cell differentiation	5/2279	36/18866	0.4432319	0.6759464	0.6116203	5	DLL1/RBPJ/FGFR1/NOTCH1/TMC1
Biological Process	GO:0045742	positive regulation of epidermal growth factor receptor signaling pathway	5/2279	36/18866	0.4432319	0.6759464	0.6116203	5	TGFA/DOK1/ARAP1/PDE6H/DGKD
Biological Process	GO:0046676	negative regulation of insulin secretion	5/2279	36/18866	0.4432319	0.6759464	0.6116203	5	ENY2/GHRL/KLF7/SREBF1/PRKN
Biological Process	GO:1901099	negative regulation of signal transduction in absence of ligand	5/2279	36/18866	0.4432319	0.6759464	0.6116203	5	TNF/UNC5B/RIPK1/BCL2/BCL2L1

Biological Process	GO:1901385	regulation of voltage-gated calcium channel activity	5/2279	36/18866	0.4432319	0.6759464	0.6116203	5	AHNAK/NOS1AP/DYSF/FGF14/CBARP
Biological Process	GO:2001240	negative regulation of extrinsic apoptotic signaling pathway in absence of ligand	5/2279	36/18866	0.4432319	0.6759464	0.6116203	5	TNF/UNC5B/RIPK1/BCL2/BCL2L1
Biological Process	GO:0090305	nucleic acid phosphodiester bond hydrolysis	39/2279	313/18866	0.4436599	0.6764905	0.6121126	39	PIWIL4/CECR2/RNASE3/FIP1L1/POLE/EXOSC2/ERN1/CPSF3/PAM16/TDP1/SLFN14/RCL1/AGO1/ZC3H12D/EME1/MEIOB/RNASE10/AGO2/EDC3/NOB1/NTHL1/ERI3/SMG6/DFFB/RMRP/DNASE1L3/NOP14/DIS3L2/PLD3/ERCC1/SND1/TOE1/CPSF4/POLD3/XPA/TUT1/ERCC2/ISG20/FBH1
Biological Process	GO:0030282	bone mineralization	15/2279	117/18866	0.4445116	0.6776804	0.6131893	15	SBNO2/SMAD3/SLC8A1/FGR/ASGR2/WNT11/TGFB1/BMPR1B/SGMS2/MEF2C/LTF/ANO6/FAM20C/ERCC2/RFLNA
Biological Process	GO:0048511	rhythmic process	38/2279	305/18866	0.4452766	0.6787378	0.6141461	38	PPARG/NCOR2/CSNK1D/CREB1/MAP2K6/ARRB1/KDM2A/PHLPP1/ZFHX3/DDC/HDAC1/MTA1/GNA11/GHRL/BMPR1B/NCOA1/CSNK1E/CREBBP/PPP1CB/ETS1/ARRB2/ARNTL/PER2/NTRK1/PER1/RAI1/FBXL6/DNM1L/SLIT3/UBE3A/USP2/NCOA2/TYRO3/SREBF1/FBXW11/CPT1A/INHBA/PRKAA1
Biological Process	GO:0007093	mitotic cell cycle checkpoint	21/2279	166/18866	0.4457475	0.6793467	0.614697	21	TRIM39/BLM/TFDP1/MAD1L1/FOXN3/HUS1/E2F7/PCBP4/EME1/ZNF385A/TGFB1/ARID3A/TAOK3/ZFYVE19/CLSPN/RB1/CHEK2/BCL2L1/NAE1/PCID2/MAP3K20
Biological Process	GO:0006939	smooth muscle contraction	14/2279	109/18866	0.4464086	0.6795154	0.6148497	14	PDE4D/MIR143/MIR145/MYH11/MYLK/SLC8A1/CTTN/GHRL/CHRM2/TPCN2/P2RX1/F2R/SMTN/DAPK3
Biological Process	GO:0043618	regulation of transcription from RNA polymerase II promoter in response to stress	14/2279	109/18866	0.4464086	0.6795154	0.6148497	14	NCK2/RBPJ/LIMD1/PSMF1/CREBBP/NFE2L2/ANKRD2/PSMB7/NOTCH1/EPAS1/NEDD4/CEBPB/BACH1/PSMD13
Biological Process	GO:2000278	regulation of DNA biosynthetic process	14/2279	109/18866	0.4464086	0.6795154	0.6148497	14	PPARG/TFDP1/TNKS/HMBOX1/PARP4/GRHL2/SMG6/PDGFB/ARRB2/NPPC/PDGFRB/ANKRD1/PARM1/CCT6A

Biological Process	GO:0002792	negative regulation of peptide secretion	13/2279	101/18866	0.4483736	0.6795154	0.6148497	13	RAB11FIP1/FRMD4A/MIR199A1/RHBDF2/CRHBP/ENY2/GHRL/KLF7/MIR199A2/F2R/SREBF1/ADTRP/PRKN
Biological Process	GO:0031124	mRNA 3'-end processing	13/2279	101/18866	0.4483736	0.6795154	0.6148497	13	FIP1L1/CPSF3/APP/PABPN1/CPEB3/SCAF8/ZC3H3/RPRD1B/CPSF4/RNF40/TUT1/PAPOLG/GRSF1
Biological Process	GO:0042102	positive regulation of T cell proliferation	13/2279	101/18866	0.4483736	0.6795154	0.6148497	13	HLA-DMB/CD55/CCR2/CCL5/NCK2/TNFSF13B/TGFBR2/CCDC88B/PTPRC/SPTA1/PNP/SLC7A1/FADD
Biological Process	GO:0044264	cellular polysaccharide metabolic process	13/2279	101/18866	0.4483736	0.6795154	0.6148497	13	CSGALNACT1/PRKAG2/AOAH/MGAM/NDST1/DYRK2/PPP1CB/ESRRB/PER2/PCDH12/HAS3/B3GNT2/RUBCNL
Biological Process	GO:0044773	mitotic DNA damage checkpoint	13/2279	101/18866	0.4483736	0.6795154	0.6148497	13	TRIM39/BLM/TFDP1/FOXN3/HUS1/E2F7/PCBP4/EME1/ZNF385A/ARID3A/TAOK3/CLSPN/CHEK2
Biological Process	GO:0032984	protein-containing complex disassembly	41/2279	330/18866	0.4486962	0.6795154	0.6148497	41	VILL/GSN/MRPL3/TNF/CAPG/MICAL1/MICAL2/MICAL3/NAV3/MRPL38/UVRAG/IGF1R/TMOD3/AXIN1/SVIL/CLEC16A/ATG14/SH3GL1/WDR1/SPTA1/SMARCD1/FRAT2/SMARCD2/ADD1/MRPS21/MRPS26/MAP1A/AVIL/SCAF8/FLII/ARHGEF2/MRPL28/SPTAN1/ASPH/TMOD1/AURKAIP1/SPTBN4/SPEF1/TFIP11/SHFL/RUBCNL
Biological Process	GO:0045185	maintenance of protein location	12/2279	93/18866	0.4504142	0.6795154	0.6148497	12	LTBP1/SORL1/IL10/HK1/GSN/SYNE1/VPS13D/SUFU/HK2/MORC3/SP100/BCL3
Biological Process	GO:1904894	positive regulation of receptor signaling pathway via STAT	12/2279	93/18866	0.4504142	0.6795154	0.6148497	12	IL10/MGAT5/TNF/CCL5/CSF1R/TNFRSF1A/IL6R/NOTCH1/F2R/CRLF1/PARP9/LIF
Biological Process	GO:0007224	smoothened signaling pathway	18/2279	142/18866	0.4516163	0.6795154	0.6148497	18	MGRN1/CHSY1/KIAA0586/SUFU/HIPK2/TGFBR2/NDST1/POR/DYRK2/RUNX2/IFT140/CREBBP/RB1/TTC21B/STK36/OTX2/KCTD21/TXNDC15

Biological Process	GO:0038094	Fc-gamma receptor signaling pathway	18/2279	142/18866	0.4516163	0.6795154	0.6148497	18	ELMO1/DOCK1/PIK3R1/WIPF1/FGR/CYFIP2/BAIAP2/WASL/PTPRJ/VAV2/PTPRC/ACTG1/NCKAP1/PRKCE/MYO1C/PLCG1/CYFIP1/VAV3
Biological Process	GO:0110110	positive regulation of animal organ morphogenesis	11/2279	85/18866	0.4525395	0.6795154	0.6148497	11	VDR/TNFAIP3/TGFBR2/RBPJ/WNT11/PAX2/FGFR1/ENG/NOTCH1/LIF/HOXB7
Biological Process	GO:0030500	regulation of bone mineralization	10/2279	77/18866	0.4547603	0.6795154	0.6148497	10	SMAD3/SLC8A1/TGFB1/BMPR1B/SGMS2/MEF2C/LTF/ANO6/FAM20C/RFLNA
Biological Process	GO:0046902	regulation of mitochondrial membrane permeability	10/2279	77/18866	0.4547603	0.6795154	0.6148497	10	TFDP1/YWHAH/BMF/SPG7/HK2/YWHAZ/BCL2/TP53BP2/BCL2L1/YWHAQ
Biological Process	GO:0061418	regulation of transcription from RNA polymerase II promoter in response to hypoxia	10/2279	77/18866	0.4547603	0.6795154	0.6148497	10	RBPJ/LIMD1/PSMF1/CREBBP/NFE2L2/PSMB7/NOTCH1/EPAS1/BACH1/PSMD13
Biological Process	GO:0007052	mitotic spindle organization	15/2279	118/18866	0.4581891	0.6795154	0.6148497	15	ARHGEF10/DCTN1/TNKS/RAE1/RCC1/DYNC1H1/POC1A/DCTN2/TACC3/STAG1/NUMA1/MSTO1/CHEK2/PTPA/ABRAXAS1
Biological Process	GO:0033013	tetrapyrrole metabolic process	8/2279	61/18866	0.4595445	0.6795154	0.6148497	8	ABCC1/SLC11A2/FECH/ALAS1/CD320/SPTA1/PPOX/TMEM14C
Biological Process	GO:0042278	purine nucleoside metabolic process	8/2279	61/18866	0.4595445	0.6795154	0.6148497	8	AMPD3/TJP2/MACROD1/PNP/GUK1/GMPR2/IMPDH1/ACP3
Biological Process	GO:1903902	positive regulation of viral life cycle	8/2279	61/18866	0.4595445	0.6795154	0.6148497	8	P4HB/TRIM38/SRPK2/CCL5/LARP1/PC/NOTCH1/NR5A2

Biological Process	GO:0006814	sodium ion transport	30/2279	241/18866	0.4595953	0.6795154	0.6148497	30	TRPM2/SLC38A4/SLC38A10/SLC10A1/FXYD2/YWHAH/SLC24A4/FXYD1/SLC8A1/DNM2/SGK1/TESC/ACTN4/SCN1A/SLC13A4/ASIC4/SLC22A5/SNTA1/PER1/PRKCE/SLC5A10/NEDD4/ANO6/HECW2/SLC13A5/SLC12A1/SPTBN4/SLC23A1/FGF14/SLC41A1
Biological Process	GO:0038093	Fc receptor signaling pathway	30/2279	241/18866	0.4595953	0.6795154	0.6148497	30	LAT/ELMO1/DOCK1/PIK3R1/GAB2/WIPF1/FGR/CYFIP2/GRAP2/NFATC2/NFATC1/BAIAP2/WASL/FER/PTPRJ/VAV2/PTPRC/ACTG1/BCL10/PSMF1/NCKAP1/PSMB7/PRKCE/MYO1C/PLCG1/CYFIP1/RELA/FBXW11/PSMD13/VAV3
Biological Process	GO:0051054	positive regulation of DNA metabolic process	25/2279	200/18866	0.4597379	0.6795154	0.6148497	25	FAM168A/SPIDR/TNKS/HMBOX1/RTEL1/TGFB1/PTPRC/GRHL2/PDGFB/ARRB2/TP53BP1/FOXM1/PDGFRB/ERCC1/TNFSF13/SLX4/PARM1/CCT6A/ERCC2/PARP9/PAXIP1/NSD2/ABRAXAS1/SLF1/BABAM2
Biological Process	GO:0000077	DNA damage checkpoint	19/2279	151/18866	0.4616872	0.6795154	0.6148497	19	TRIM39/BLM/FZR1/TFDP1/FOXN3/HUS1/E2F7/PCBP4/EME1/ZNF385A/ARID3A/TAOK3/CLSPN/TP53BP1/RHNO1/CHEK2/MAP3K20/ABRAXAS1/BABAM2
Biological Process	GO:0043392	negative regulation of DNA binding	7/2279	53/18866	0.4621442	0.6795154	0.6148497	7	TNKS/CPNE1/RSF1/ZNF462/PER2/SP100/ILRUN
Biological Process	GO:0043502	regulation of muscle adaptation	13/2279	102/18866	0.4631018	0.6795154	0.6148497	13	HDAC4/CTDP1/MIR145/TNFRSF1B/SMAD3/PRKCA/MIR199A1/TNFRSF1A/PDE9A/MIR199A2/NOTCH1/FOXO1/CASQ1
Biological Process	GO:0006611	protein export from nucleus	23/2279	184/18866	0.4640666	0.6795154	0.6148497	23	CALR/NXF1/FIP1L1/CPSF3/SETD2/RAE1/XPO7/TCF7L2/SMG7/XPO6/TGFB1/PABPN1/ENY2/SMURF1/NUP93/SEC13/SMG6/SP100/HHEX/RBM15B/NOL6/CPSF4/PCID2
Biological Process	GO:0035094	response to nicotine	6/2279	45/18866	0.4649127	0.6795154	0.6148497	6	CREB1/PPP1R1B/TNF/NTRK1/BCL2/RELA
Biological Process	GO:1901028	regulation of mitochondrial outer membrane permeabilization involved in apoptotic signaling pathway	6/2279	45/18866	0.4649127	0.6795154	0.6148497	6	TFDP1/YWHAH/YWHAZ/BCL2/TP53BP2/YWHAQ

Biological Process	GO:1903214	regulation of protein targeting to mitochondrion	6/2279	45/18866	0.4649127	0.6795154	0.6148497	6	USP36/ABLIM3/UBE2J2/SREBF1/PRKAA1/PRKN
Biological Process	GO:0006296	nucleotide-excision repair, DNA incision, 5'-to lesion	5/2279	37/18866	0.4678769	0.6795154	0.6148497	5	NTHL1/ERCC1/POLD3/XPA/ERCC2
Biological Process	GO:0006882	cellular zinc ion homeostasis	5/2279	37/18866	0.4678769	0.6795154	0.6148497	5	S100A8/LCK/SLC39A13/AP3D1/ATP7B
Biological Process	GO:0043243	positive regulation of protein-containing complex disassembly	5/2279	37/18866	0.4678769	0.6795154	0.6148497	5	TNF/UVRAG/IGF1R/CLEC16A/WDR1
Biological Process	GO:0060969	negative regulation of gene silencing	5/2279	37/18866	0.4678769	0.6795154	0.6148497	5	PPARG/NCOR2/TNF/TGFB1/RMRP
Biological Process	GO:0070897	transcription preinitiation complex assembly	5/2279	37/18866	0.4678769	0.6795154	0.6148497	5	TAF1C/CREB1/ATF7IP/BRF1/PIH1D1
Biological Process	GO:2000785	regulation of autophagosome assembly	5/2279	37/18866	0.4678769	0.6795154	0.6148497	5	ULK1/PIP4K2A/FEZ2/TBC1D14/NPRL2
Biological Process	GO:0007193	adenylate cyclase-inhibiting G protein-coupled receptor signaling pathway	11/2279	86/18866	0.4685993	0.6795154	0.6148497	11	ADCY2/ADCY9/FPR2/GNAZ/GNAI2/AKAP5/MARCO/CHRM2/PALM/ADCY4/GABBR1
Biological Process	GO:0042509	regulation of tyrosine phosphorylation of STAT protein	11/2279	86/18866	0.4685993	0.6795154	0.6148497	11	TNF/CCL5/CSF1R/TNFRSF1A/IL6R/PPP2R1A/GGNBP2/INPP5F/CRLF1/PARP9/LIF

Biological Process	GO:1901532	regulation of hematopoietic progenitor cell differentiation	11/2279	86/18866	0.4685993	0.6795154	0.6148497	11	MYB/RUNX1/TCF12/SETD1A/PSMF1/NFE2L2/PUS7/TCF3/PSMB7/NOTCH1/PSMD13
Biological Process	GO:1900180	regulation of protein localization to nucleus	16/2279	127/18866	0.4691082	0.6795154	0.6148497	16	TRIM8/ANGPT1/PIK3R1/SMAD3/SUFU/JUP/TGFB1/LILRB4/NF1/CDH1/CABP1/KAT7/CCT6A/PARP9/LIF/ILRUN
Biological Process	GO:0001773	myeloid dendritic cell activation	4/2279	29/18866	0.4710561	0.6795154	0.6148497	4	IL10/TGFBR2/RBPJ/DOCK2
Biological Process	GO:0002717	positive regulation of natural killer cell mediated immunity	4/2279	29/18866	0.4710561	0.6795154	0.6148497	4	CD226/HLA-F/IL18RAP/CADM1
Biological Process	GO:0003351	epithelial cilium movement involved in extracellular fluid movement	4/2279	29/18866	0.4710561	0.6795154	0.6148497	4	RFX3/STK36/TTLL1/KATNIP
Biological Process	GO:0021602	cranial nerve morphogenesis	4/2279	29/18866	0.4710561	0.6795154	0.6148497	4	NRP2/PHOX2A/ATP8B1/PAX2
Biological Process	GO:0031365	N-terminal protein amino acid modification	4/2279	29/18866	0.4710561	0.6795154	0.6148497	4	PDF/CREBBP/NAA16/NTMT1
Biological Process	GO:0032373	positive regulation of sterol transport	4/2279	29/18866	0.4710561	0.6795154	0.6148497	4	ABCG1/PLTP/NR1H2/ABCA1
Biological Process	GO:0032376	positive regulation of cholesterol transport	4/2279	29/18866	0.4710561	0.6795154	0.6148497	4	ABCG1/PLTP/NR1H2/ABCA1

Biological Process	GO:0035116	embryonic hindlimb morphogenesis	4/2279	29/18866	0.4710561	0.6795154	0.6148497	4	ZBTB16/RARG/NOTCH1/ALX3
Biological Process	GO:0036296	response to increased oxygen levels	4/2279	29/18866	0.4710561	0.6795154	0.6148497	4	PPARG/CAT/PDGFRB/FOXO1
Biological Process	GO:0045822	negative regulation of heart contraction	4/2279	29/18866	0.4710561	0.6795154	0.6148497	4	PDE4D/BIN1/SPTBN4/GRK2
Biological Process	GO:0060441	epithelial tube branching involved in lung morphogenesis	4/2279	29/18866	0.4710561	0.6795154	0.6148497	4	CTSZ/TNF/CTSH/RDH10
Biological Process	GO:0070979	protein K11-linked ubiquitination	4/2279	29/18866	0.4710561	0.6795154	0.6148497	4	FZR1/CDC16/ANAPC7/PRKN
Biological Process	GO:0090344	negative regulation of cell aging	4/2279	29/18866	0.4710561	0.6795154	0.6148497	4	FZR1/MAP3K3/AKT3/FOXM1
Biological Process	GO:2001032	regulation of double-strand break repair via nonhomologous end joining	4/2279	29/18866	0.4710561	0.6795154	0.6148497	4	PARP9/TFIP11/NSD2/CYREN
Biological Process	GO:0001895	retina homeostasis	10/2279	78/18866	0.471635	0.6795154	0.6148497	10	CRB1/CDH23/CCDC66/CDH3/ACTG1/ESRRB/CDHR1/LTF/SLC2A1/NPHP4
Biological Process	GO:0030433	ubiquitin-dependent ERAD pathway	10/2279	78/18866	0.471635	0.6795154	0.6148497	10	AMFR/RHBDD2/EDEM1/DERL2/UBE2J2/EDEM2/NFE2L2/UBXN8/SVIP/MARCHF6

Biological Process	GO:1903046	meiotic cell cycle process	24/2279	193/18866	0.4725821	0.6795154	0.6148497	24	CALR/FZR1/MOV10L1/BRCA2/HUS1/EME1/MYH9/MEIOB/CDC25A/OVOL1/FANCA/RNF212/PPP2R1A/PLCB1/CCNA1/RAD21L1/DMC1/ERCC1/SLX4/TEX12/LIF/KLHDC3/PSMD13/TERB2
Biological Process	GO:0006293	nucleotide-excision repair, preincision complex stabilization	3/2279	21/18866	0.4744086	0.6795154	0.6148497	3	ERCC1/XPA/ERCC2
Biological Process	GO:0006295	nucleotide-excision repair, DNA incision, 3'-to lesion	3/2279	21/18866	0.4744086	0.6795154	0.6148497	3	ERCC1/XPA/ERCC2
Biological Process	GO:0006833	water transport	3/2279	21/18866	0.4744086	0.6795154	0.6148497	3	AQP1/AQP3/AQP9
Biological Process	GO:0010847	regulation of chromatin assembly	3/2279	21/18866	0.4744086	0.6795154	0.6148497	3	ATF7IP/DNMT1/PARP10
Biological Process	GO:0016075	rRNA catabolic process	3/2279	21/18866	0.4744086	0.6795154	0.6148497	3	EXOSC2/SLFN14/DEDD2
Biological Process	GO:0021854	hypothalamus development	3/2279	21/18866	0.4744086	0.6795154	0.6148497	3	NRP2/NCOA1/ETS1
Biological Process	GO:0030277	maintenance of gastrointestinal epithelium	3/2279	21/18866	0.4744086	0.6795154	0.6148497	3	TLR9/SLC22A5/NOD2
Biological Process	GO:0031061	negative regulation of histone methylation	3/2279	21/18866	0.4744086	0.6795154	0.6148497	3	KDM4C/DNMT1/PIH1D1

Biological Process	GO:0032482	Rab protein signal transduction	3/2279	21/18866	0.4744086	0.6795154	0.6148497	3	DENND1A/DENND3/RAB30
Biological Process	GO:0033630	positive regulation of cell adhesion mediated by integrin	3/2279	21/18866	0.4744086	0.6795154	0.6148497	3	CCL5/PTPN6/LIF
Biological Process	GO:0033962	P-body assembly	3/2279	21/18866	0.4744086	0.6795154	0.6148497	3	DYNC1H1/LIMD1/EDC3
Biological Process	GO:0035357	peroxisome proliferator activated receptor signaling pathway	3/2279	21/18866	0.4744086	0.6795154	0.6148497	3	RXRA/PPARG/ACTN4
Biological Process	GO:0035813	regulation of renal sodium excretion	3/2279	21/18866	0.4744086	0.6795154	0.6148497	3	COMT/GNAI2/AGTR1
Biological Process	GO:0035929	steroid hormone secretion	3/2279	21/18866	0.4744086	0.6795154	0.6148497	3	GHRL/AGTR1/INHBA
Biological Process	GO:0036150	phosphatidylserine acyl-chain remodeling	3/2279	21/18866	0.4744086	0.6795154	0.6148497	3	OSBPL5/OSBPL10/PLA1A
Biological Process	GO:0042107	cytokine metabolic process	3/2279	21/18866	0.4744086	0.6795154	0.6148497	3	CEBPE/IGF2BP3/ASB1
Biological Process	GO:0043984	histone H4-K16 acetylation	3/2279	21/18866	0.4744086	0.6795154	0.6148497	3	KANSL1/KANSL2/PIH1D1

Biological Process	GO:0045943	positive regulation of transcription by RNA polymerase I	3/2279	21/18866	0.4744086	0.6795154	0.6148497	3	WDR43/PIH1D1/MARS1
Biological Process	GO:0071498	cellular response to fluid shear stress	3/2279	21/18866	0.4744086	0.6795154	0.6148497	3	MTSS1/NFE2L2/MEF2C
Biological Process	GO:0098698	postsynaptic specialization assembly	3/2279	21/18866	0.4744086	0.6795154	0.6148497	3	NRXN1/NRXN2/SHANK2
Biological Process	GO:0140131	positive regulation of lymphocyte chemotaxis	3/2279	21/18866	0.4744086	0.6795154	0.6148497	3	CCR2/CCL5/CCL27
Biological Process	GO:1902548	negative regulation of cellular response to vascular endothelial growth factor stimulus	3/2279	21/18866	0.4744086	0.6795154	0.6148497	3	MIR199A1/MIR199A2/ADGRA2
Biological Process	GO:1902656	calcium ion import into cytosol	3/2279	21/18866	0.4744086	0.6795154	0.6148497	3	TRPM2/SLC8A1/TRPV2
Biological Process	GO:1903204	negative regulation of oxidative stress-induced neuron death	3/2279	21/18866	0.4744086	0.6795154	0.6148497	3	IL10/FBXO7/PRKN
Biological Process	GO:2000380	regulation of mesoderm development	3/2279	21/18866	0.4744086	0.6795154	0.6148497	3	FGFR1/PUS7/NODAL
Biological Process	GO:0000472	endonucleolytic cleavage to generate mature 5'-end of SSU-rRNA from (SSU-rRNA, 5.8S rRNA, LSU-rRNA)	1/2279	5/18866	0.4746987	0.6795154	0.6148497	1	NOP14

Biological Process	GO:0000710	meiotic mismatch repair	1/2279	5/18866	0.4746987	0.6795154	0.6148497	1	ERCC1
Biological Process	GO:0000960	regulation of mitochondrial RNA catabolic process	1/2279	5/18866	0.4746987	0.6795154	0.6148497	1	GRSF1
Biological Process	GO:0000967	rRNA 5'-end processing	1/2279	5/18866	0.4746987	0.6795154	0.6148497	1	NOP14
Biological Process	GO:0001757	somite specification	1/2279	5/18866	0.4746987	0.6795154	0.6148497	1	DLL1
Biological Process	GO:0001923	B-1 B cell differentiation	1/2279	5/18866	0.4746987	0.6795154	0.6148497	1	CMTM7
Biological Process	GO:0002034	regulation of blood vessel diameter by renin-angiotensin	1/2279	5/18866	0.4746987	0.6795154	0.6148497	1	AGTR1
Biological Process	GO:0002155	regulation of thyroid hormone mediated signaling pathway	1/2279	5/18866	0.4746987	0.6795154	0.6148497	1	NCOA1
Biological Process	GO:0002249	lymphocyte anergy	1/2279	5/18866	0.4746987	0.6795154	0.6148497	1	PHLPP1
Biological Process	GO:0002318	myeloid progenitor cell differentiation	1/2279	5/18866	0.4746987	0.6795154	0.6148497	1	JAM3

Biological Process	GO:0002580	regulation of antigen processing and presentation of peptide or polysaccharide antigen via MHC class II	1/2279	5/18866	0.4746987	0.6795154	0.6148497	1	THBS1
Biological Process	GO:0002667	regulation of T cell anergy	1/2279	5/18866	0.4746987	0.6795154	0.6148497	1	PHLPP1
Biological Process	GO:0002870	T cell anergy	1/2279	5/18866	0.4746987	0.6795154	0.6148497	1	PHLPP1
Biological Process	GO:0002911	regulation of lymphocyte anergy	1/2279	5/18866	0.4746987	0.6795154	0.6148497	1	PHLPP1
Biological Process	GO:0003072	renal control of peripheral vascular resistance involved in regulation of systemic arterial blood pressure	1/2279	5/18866	0.4746987	0.6795154	0.6148497	1	AGTR1
Biological Process	GO:0003241	growth involved in heart morphogenesis	1/2279	5/18866	0.4746987	0.6795154	0.6148497	1	NOTCH1
Biological Process	GO:0003289	atrial septum primum morphogenesis	1/2279	5/18866	0.4746987	0.6795154	0.6148497	1	NSD2
Biological Process	GO:0006001	fructose catabolic process	1/2279	5/18866	0.4746987	0.6795154	0.6148497	1	GLYCTK
Biological Process	GO:0006104	succinyl-CoA metabolic process	1/2279	5/18866	0.4746987	0.6795154	0.6148497	1	OGDH

Biological Process	GO:0006398	mRNA 3'-end processing by stem-loop binding and cleavage	1/2279	5/18866	0.4746987	0.6795154	0.6148497	1	CPSF3
Biological Process	GO:0006522	alanine metabolic process	1/2279	5/18866	0.4746987	0.6795154	0.6148497	1	AGXT
Biological Process	GO:0006524	alanine catabolic process	1/2279	5/18866	0.4746987	0.6795154	0.6148497	1	AGXT
Biological Process	GO:0006549	isoleucine metabolic process	1/2279	5/18866	0.4746987	0.6795154	0.6148497	1	ACAT1
Biological Process	GO:0006663	platelet activating factor biosynthetic process	1/2279	5/18866	0.4746987	0.6795154	0.6148497	1	CHPT1
Biological Process	GO:0007023	post-chaperonin tubulin folding pathway	1/2279	5/18866	0.4746987	0.6795154	0.6148497	1	TBCD
Biological Process	GO:0007343	egg activation	1/2279	5/18866	0.4746987	0.6795154	0.6148497	1	PLCB1
Biological Process	GO:0008215	spermine metabolic process	1/2279	5/18866	0.4746987	0.6795154	0.6148497	1	SAT2
Biological Process	GO:0008582	regulation of synaptic growth at neuromuscular junction	1/2279	5/18866	0.4746987	0.6795154	0.6148497	1	COLQ

Biological Process	GO:0008628	hormone-mediated apoptotic signaling pathway	1/2279	5/18866	0.4746987	0.6795154	0.6148497	1	SST
Biological Process	GO:0009078	pyruvate family amino acid metabolic process	1/2279	5/18866	0.4746987	0.6795154	0.6148497	1	AGXT
Biological Process	GO:0009080	pyruvate family amino acid catabolic process	1/2279	5/18866	0.4746987	0.6795154	0.6148497	1	AGXT
Biological Process	GO:0009092	homoserine metabolic process	1/2279	5/18866	0.4746987	0.6795154	0.6148497	1	CBS
Biological Process	GO:0009177	pyrimidine deoxyribonucleoside monophosphate biosynthetic process	1/2279	5/18866	0.4746987	0.6795154	0.6148497	1	DCTD
Biological Process	GO:0009233	menaquinone metabolic process	1/2279	5/18866	0.4746987	0.6795154	0.6148497	1	CYP4F3
Biological Process	GO:0009240	isopentenyl diphosphate biosynthetic process	1/2279	5/18866	0.4746987	0.6795154	0.6148497	1	IDI1
Biological Process	GO:0009446	putrescine biosynthetic process	1/2279	5/18866	0.4746987	0.6795154	0.6148497	1	ODC1
Biological Process	GO:0009448	gamma-aminobutyric acid metabolic process	1/2279	5/18866	0.4746987	0.6795154	0.6148497	1	SLC1A3

Biological Process	GO:0009608	response to symbiont	1/2279	5/18866	0.4746987	0.6795154	0.6148497	1	SLC22A5
Biological Process	GO:0009609	response to symbiotic bacterium	1/2279	5/18866	0.4746987	0.6795154	0.6148497	1	SLC22A5
Biological Process	GO:0010138	pyrimidine ribonucleotide salvage	1/2279	5/18866	0.4746987	0.6795154	0.6148497	1	UCK2
Biological Process	GO:0010716	negative regulation of extracellular matrix disassembly	1/2279	5/18866	0.4746987	0.6795154	0.6148497	1	TGFB1
Biological Process	GO:0010746	regulation of long-chain fatty acid import across plasma membrane	1/2279	5/18866	0.4746987	0.6795154	0.6148497	1	THBS1
Biological Process	GO:0010750	positive regulation of nitric oxide mediated signal transduction	1/2279	5/18866	0.4746987	0.6795154	0.6148497	1	NOS1AP
Biological Process	GO:0010936	negative regulation of macrophage cytokine production	1/2279	5/18866	0.4746987	0.6795154	0.6148497	1	TGFB1
Biological Process	GO:0010957	negative regulation of vitamin D biosynthetic process	1/2279	5/18866	0.4746987	0.6795154	0.6148497	1	GFI1
Biological Process	GO:0010961	cellular magnesium ion homeostasis	1/2279	5/18866	0.4746987	0.6795154	0.6148497	1	SLC41A1

Biological Process	GO:0010991	negative regulation of SMAD protein complex assembly	1/2279	5/18866	0.4746987	0.6795154	0.6148497	1	LDLRAD4
Biological Process	GO:0014042	positive regulation of neuron maturation	1/2279	5/18866	0.4746987	0.6795154	0.6148497	1	BCL2
Biological Process	GO:0014045	establishment of endothelial blood-brain barrier	1/2279	5/18866	0.4746987	0.6795154	0.6148497	1	ENG
Biological Process	GO:0014905	myoblast fusion involved in skeletal muscle regeneration	1/2279	5/18866	0.4746987	0.6795154	0.6148497	1	CD9
Biological Process	GO:0015670	carbon dioxide transport	1/2279	5/18866	0.4746987	0.6795154	0.6148497	1	AQP1
Biological Process	GO:0015705	iodide transport	1/2279	5/18866	0.4746987	0.6795154	0.6148497	1	TG
Biological Process	GO:0015755	fructose transmembrane transport	1/2279	5/18866	0.4746987	0.6795154	0.6148497	1	SLC2A5
Biological Process	GO:0015855	pyrimidine nucleobase transport	1/2279	5/18866	0.4746987	0.6795154	0.6148497	1	AQP9
Biological Process	GO:0015862	uridine transport	1/2279	5/18866	0.4746987	0.6795154	0.6148497	1	SLC29A1

Biological Process	GO:0015864	pyrimidine nucleoside transport	1/2279	5/18866	0.4746987	0.6795154	0.6148497	1	SLC29A1
Biological Process	GO:0016191	synaptic vesicle uncoating	1/2279	5/18866	0.4746987	0.6795154	0.6148497	1	SH3GL1
Biological Process	GO:0016199	axon midline choice point recognition	1/2279	5/18866	0.4746987	0.6795154	0.6148497	1	APP
Biological Process	GO:0018101	protein citrullination	1/2279	5/18866	0.4746987	0.6795154	0.6148497	1	PADI2
Biological Process	GO:0019346	transsulfuration	1/2279	5/18866	0.4746987	0.6795154	0.6148497	1	CBS
Biological Process	GO:0019348	dolichol metabolic process	1/2279	5/18866	0.4746987	0.6795154	0.6148497	1	DPAGT1
Biological Process	GO:0019509	L-methionine salvage from methylthioadenosine	1/2279	5/18866	0.4746987	0.6795154	0.6148497	1	ADI1
Biological Process	GO:0019919	peptidyl-arginine methylation, to asymmetrical-dimethyl arginine	1/2279	5/18866	0.4746987	0.6795154	0.6148497	1	PRMT2
Biological Process	GO:0021523	somatic motor neuron differentiation	1/2279	5/18866	0.4746987	0.6795154	0.6148497	1	PHOX2A

Biological Process	GO:0021764	amygdala development	1/2279	5/18866	0.4746987	0.6795154	0.6148497	1	NF1
Biological Process	GO:0021960	anterior commissure morphogenesis	1/2279	5/18866	0.4746987	0.6795154	0.6148497	1	TSKU
Biological Process	GO:0021999	neural plate anterior/posterior regionalization	1/2279	5/18866	0.4746987	0.6795154	0.6148497	1	SSBP3
Biological Process	GO:0022614	membrane to membrane docking	1/2279	5/18866	0.4746987	0.6795154	0.6148497	1	EZR
Biological Process	GO:0030222	eosinophil differentiation	1/2279	5/18866	0.4746987	0.6795154	0.6148497	1	TRIB1
Biological Process	GO:0030263	apoptotic chromosome condensation	1/2279	5/18866	0.4746987	0.6795154	0.6148497	1	DFFB
Biological Process	GO:0030321	transepithelial chloride transport	1/2279	5/18866	0.4746987	0.6795154	0.6148497	1	P2RY6
Biological Process	GO:0030644	cellular chloride ion homeostasis	1/2279	5/18866	0.4746987	0.6795154	0.6148497	1	ABCC2
Biological Process	GO:0030950	establishment or maintenance of actin cytoskeleton polarity	1/2279	5/18866	0.4746987	0.6795154	0.6148497	1	AQP1

Biological Process	GO:0031119	tRNA pseudouridine synthesis	1/2279	5/18866	0.4746987	0.6795154	0.6148497	1	PUS7
Biological Process	GO:0031179	peptide modification	1/2279	5/18866	0.4746987	0.6795154	0.6148497	1	GGT1
Biological Process	GO:0031427	response to methotrexate	1/2279	5/18866	0.4746987	0.6795154	0.6148497	1	ABCC2
Biological Process	GO:0032218	riboflavin transport	1/2279	5/18866	0.4746987	0.6795154	0.6148497	1	SLC52A2
Biological Process	GO:0032262	pyrimidine nucleotide salvage	1/2279	5/18866	0.4746987	0.6795154	0.6148497	1	UCK2
Biological Process	GO:0032687	negative regulation of interferon-alpha production	1/2279	5/18866	0.4746987	0.6795154	0.6148497	1	IL10
Biological Process	GO:0032741	positive regulation of interleukin-18 production	1/2279	5/18866	0.4746987	0.6795154	0.6148497	1	TLR9
Biological Process	GO:0032763	regulation of mast cell cytokine production	1/2279	5/18866	0.4746987	0.6795154	0.6148497	1	BCL10
Biological Process	GO:0032776	DNA methylation on cytosine	1/2279	5/18866	0.4746987	0.6795154	0.6148497	1	DNMT1

Biological Process	GO:0032849	positive regulation of cellular pH reduction	1/2279	5/18866	0.4746987	0.6795154	0.6148497	1	UBE3A
Biological Process	GO:0033087	negative regulation of immature T cell proliferation	1/2279	5/18866	0.4746987	0.6795154	0.6148497	1	TMEM131L
Biological Process	GO:0033132	negative regulation of glucokinase activity	1/2279	5/18866	0.4746987	0.6795154	0.6148497	1	PRKN
Biological Process	GO:0033152	immunoglobulin V(D)J recombination	1/2279	5/18866	0.4746987	0.6795154	0.6148497	1	TCF3
Biological Process	GO:0033184	positive regulation of histone ubiquitination	1/2279	5/18866	0.4746987	0.6795154	0.6148497	1	RNF40
Biological Process	GO:0033578	protein glycosylation in Golgi	1/2279	5/18866	0.4746987	0.6795154	0.6148497	1	LMF1
Biological Process	GO:0033591	response to L-ascorbic acid	1/2279	5/18866	0.4746987	0.6795154	0.6148497	1	CAT
Biological Process	GO:0034136	negative regulation of toll-like receptor 2 signaling pathway	1/2279	5/18866	0.4746987	0.6795154	0.6148497	1	TNFAIP3
Biological Process	GO:0034155	regulation of toll-like receptor 7 signaling pathway	1/2279	5/18866	0.4746987	0.6795154	0.6148497	1	TREML4

Biological Process	GO:0034635	glutathione transport	1/2279	5/18866	0.4746987	0.6795154	0.6148497	1	ABCC1
Biological Process	GO:0035426	extracellular matrix-cell signaling	1/2279	5/18866	0.4746987	0.6795154	0.6148497	1	FER
Biological Process	GO:0035443	tripeptide transmembrane transport	1/2279	5/18866	0.4746987	0.6795154	0.6148497	1	ABCC1
Biological Process	GO:0035483	gastric emptying	1/2279	5/18866	0.4746987	0.6795154	0.6148497	1	GHRL
Biological Process	GO:0035509	negative regulation of myosin-light-chain-phosphatase activity	1/2279	5/18866	0.4746987	0.6795154	0.6148497	1	TNF
Biological Process	GO:0035513	oxidative RNA demethylation	1/2279	5/18866	0.4746987	0.6795154	0.6148497	1	FTO
Biological Process	GO:0035523	protein K29-linked deubiquitination	1/2279	5/18866	0.4746987	0.6795154	0.6148497	1	TNFAIP3
Biological Process	GO:0035564	regulation of kidney size	1/2279	5/18866	0.4746987	0.6795154	0.6148497	1	PAX2
Biological Process	GO:0035610	protein side chain deglutamylation	1/2279	5/18866	0.4746987	0.6795154	0.6148497	1	AGBL2

Biological Process	GO:0036066	protein O-linked fucosylation	1/2279	5/18866	0.4746987	0.6795154	0.6148497	1	SLC35C2
Biological Process	GO:0036091	positive regulation of transcription from RNA polymerase II promoter in response to oxidative stress	1/2279	5/18866	0.4746987	0.6795154	0.6148497	1	NFE2L2
Biological Process	GO:0036100	leukotriene catabolic process	1/2279	5/18866	0.4746987	0.6795154	0.6148497	1	CYP4F3
Biological Process	GO:0036101	leukotriene B4 catabolic process	1/2279	5/18866	0.4746987	0.6795154	0.6148497	1	CYP4F3
Biological Process	GO:0036102	leukotriene B4 metabolic process	1/2279	5/18866	0.4746987	0.6795154	0.6148497	1	CYP4F3
Biological Process	GO:0036135	Schwann cell migration	1/2279	5/18866	0.4746987	0.6795154	0.6148497	1	CERS2
Biological Process	GO:0036301	macrophage colony-stimulating factor production	1/2279	5/18866	0.4746987	0.6795154	0.6148497	1	FOXP1
Biological Process	GO:0036414	histone citrullination	1/2279	5/18866	0.4746987	0.6795154	0.6148497	1	PADI2
Biological Process	GO:0036476	neuron death in response to hydrogen peroxide	1/2279	5/18866	0.4746987	0.6795154	0.6148497	1	IL10

Biological Process	GO:0042023	DNA endoreduplication	1/2279	5/18866	0.4746987	0.6795154	0.6148497	1	E2F7
Biological Process	GO:0042036	negative regulation of cytokine biosynthetic process	1/2279	5/18866	0.4746987	0.6795154	0.6148497	1	ASB1
Biological Process	GO:0042148	strand invasion	1/2279	5/18866	0.4746987	0.6795154	0.6148497	1	DMC1
Biological Process	GO:0042321	negative regulation of circadian sleep/wake cycle, sleep	1/2279	5/18866	0.4746987	0.6795154	0.6148497	1	GHRL
Biological Process	GO:0042374	phylloquinone metabolic process	1/2279	5/18866	0.4746987	0.6795154	0.6148497	1	CYP4F3
Biological Process	GO:0042376	phylloquinone catabolic process	1/2279	5/18866	0.4746987	0.6795154	0.6148497	1	CYP4F3
Biological Process	GO:0042435	indole-containing compound biosynthetic process	1/2279	5/18866	0.4746987	0.6795154	0.6148497	1	DDC
Biological Process	GO:0042997	negative regulation of Golgi to plasma membrane protein transport	1/2279	5/18866	0.4746987	0.6795154	0.6148497	1	CSK
Biological Process	GO:0043400	cortisol secretion	1/2279	5/18866	0.4746987	0.6795154	0.6148497	1	GHRL

Biological Process	GO:0043415	positive regulation of skeletal muscle tissue regeneration	1/2279	5/18866	0.4746987	0.6795154	0.6148497	1	TGFBR2
Biological Process	GO:0043932	ossification involved in bone remodeling	1/2279	5/18866	0.4746987	0.6795154	0.6148497	1	TGFB1
Biological Process	GO:0043972	histone H3-K23 acetylation	1/2279	5/18866	0.4746987	0.6795154	0.6148497	1	BRD1
Biological Process	GO:0043987	histone H3-S10 phosphorylation	1/2279	5/18866	0.4746987	0.6795154	0.6148497	1	RPS6KA4
Biological Process	GO:0044206	UMP salvage	1/2279	5/18866	0.4746987	0.6795154	0.6148497	1	UCK2
Biological Process	GO:0044314	protein K27-linked ubiquitination	1/2279	5/18866	0.4746987	0.6795154	0.6148497	1	PRKN
Biological Process	GO:0044571	[2Fe-2S] cluster assembly	1/2279	5/18866	0.4746987	0.6795154	0.6148497	1	HSCB
Biological Process	GO:0045329	carnitine biosynthetic process	1/2279	5/18866	0.4746987	0.6795154	0.6148497	1	ACADM
Biological Process	GO:0045338	farnesyl diphosphate metabolic process	1/2279	5/18866	0.4746987	0.6795154	0.6148497	1	HMGCS1

Biological Process	GO:0045345	positive regulation of MHC class I biosynthetic process	1/2279	5/18866	0.4746987	0.6795154	0.6148497	1	NLRC5
Biological Process	GO:0045578	negative regulation of B cell differentiation	1/2279	5/18866	0.4746987	0.6795154	0.6148497	1	INHBA
Biological Process	GO:0045794	negative regulation of cell volume	1/2279	5/18866	0.4746987	0.6795154	0.6148497	1	ANO6
Biological Process	GO:0045875	negative regulation of sister chromatid cohesion	1/2279	5/18866	0.4746987	0.6795154	0.6148497	1	TNKS
Biological Process	GO:0046015	regulation of transcription by glucose	1/2279	5/18866	0.4746987	0.6795154	0.6148497	1	USF2
Biological Process	GO:0046060	dATP metabolic process	1/2279	5/18866	0.4746987	0.6795154	0.6148497	1	GUK1
Biological Process	GO:0046069	cGMP catabolic process	1/2279	5/18866	0.4746987	0.6795154	0.6148497	1	PDE9A
Biological Process	GO:0046078	dUMP metabolic process	1/2279	5/18866	0.4746987	0.6795154	0.6148497	1	DCTD
Biological Process	GO:0046219	indolalkylamine biosynthetic process	1/2279	5/18866	0.4746987	0.6795154	0.6148497	1	DDC

Biological Process	GO:0046351	disaccharide biosynthetic process	1/2279	5/18866	0.4746987	0.6795154	0.6148497	1	SLC2A1
Biological Process	GO:0046490	isopentenyl diphosphate metabolic process	1/2279	5/18866	0.4746987	0.6795154	0.6148497	1	IDI1
Biological Process	GO:0046666	retinal cell programmed cell death	1/2279	5/18866	0.4746987	0.6795154	0.6148497	1	BCL2
Biological Process	GO:0048386	positive regulation of retinoic acid receptor signaling pathway	1/2279	5/18866	0.4746987	0.6795154	0.6148497	1	CTBP2
Biological Process	GO:0048550	negative regulation of pinocytosis	1/2279	5/18866	0.4746987	0.6795154	0.6148497	1	NR1H2
Biological Process	GO:0048631	regulation of skeletal muscle tissue growth	1/2279	5/18866	0.4746987	0.6795154	0.6148497	1	DLL1
Biological Process	GO:0048790	maintenance of presynaptic active zone structure	1/2279	5/18866	0.4746987	0.6795154	0.6148497	1	CTBP2
Biological Process	GO:0050747	positive regulation of lipoprotein metabolic process	1/2279	5/18866	0.4746987	0.6795154	0.6148497	1	SVIP
Biological Process	GO:0050748	negative regulation of lipoprotein metabolic process	1/2279	5/18866	0.4746987	0.6795154	0.6148497	1	ITGB3

Biological Process	GO:0050955	thermoception	1/2279	5/18866	0.4746987	0.6795154	0.6148497	1	WDR47
Biological Process	GO:0051097	negative regulation of helicase activity	1/2279	5/18866	0.4746987	0.6795154	0.6148497	1	MCM2
Biological Process	GO:0051140	regulation of NK T cell proliferation	1/2279	5/18866	0.4746987	0.6795154	0.6148497	1	ZBTB7B
Biological Process	GO:0051295	establishment of meiotic spindle localization	1/2279	5/18866	0.4746987	0.6795154	0.6148497	1	MYH9
Biological Process	GO:0051410	detoxification of nitrogen compound	1/2279	5/18866	0.4746987	0.6795154	0.6148497	1	MTARC1
Biological Process	GO:0051414	response to cortisol	1/2279	5/18866	0.4746987	0.6795154	0.6148497	1	SLIT3
Biological Process	GO:0051461	positive regulation of corticotropin secretion	1/2279	5/18866	0.4746987	0.6795154	0.6148497	1	GHRL
Biological Process	GO:0051462	regulation of cortisol secretion	1/2279	5/18866	0.4746987	0.6795154	0.6148497	1	GHRL
Biological Process	GO:0051582	positive regulation of neurotransmitter uptake	1/2279	5/18866	0.4746987	0.6795154	0.6148497	1	PRKN

Biological Process	GO:0051599	response to hydrostatic pressure	1/2279	5/18866	0.4746987	0.6795154	0.6148497	1	NTRK1
Biological Process	GO:0051725	protein de-ADP-ribosylation	1/2279	5/18866	0.4746987	0.6795154	0.6148497	1	MACROD1
Biological Process	GO:0051754	meiotic sister chromatid cohesion, centromeric	1/2279	5/18866	0.4746987	0.6795154	0.6148497	1	PPP2R1A
Biological Process	GO:0051793	medium-chain fatty acid catabolic process	1/2279	5/18866	0.4746987	0.6795154	0.6148497	1	ACADM
Biological Process	GO:0051799	negative regulation of hair follicle development	1/2279	5/18866	0.4746987	0.6795154	0.6148497	1	CDH3
Biological Process	GO:0052405	negative regulation by host of symbiont molecular function	1/2279	5/18866	0.4746987	0.6795154	0.6148497	1	SFTPD
Biological Process	GO:0060266	negative regulation of respiratory burst involved in inflammatory response	1/2279	5/18866	0.4746987	0.6795154	0.6148497	1	GRN
Biological Process	GO:0060509	type I pneumocyte differentiation	1/2279	5/18866	0.4746987	0.6795154	0.6148497	1	CREB1
Biological Process	GO:0060591	chondroblast differentiation	1/2279	5/18866	0.4746987	0.6795154	0.6148497	1	RARA

Biological Process	GO:0060664	epithelial cell proliferation involved in salivary gland morphogenesis	1/2279	5/18866	0.4746987	0.6795154	0.6148497	1	TNF
Biological Process	GO:0060718	chorionic trophoblast cell differentiation	1/2279	5/18866	0.4746987	0.6795154	0.6148497	1	E2F7
Biological Process	GO:0060789	hair follicle placode formation	1/2279	5/18866	0.4746987	0.6795154	0.6148497	1	HDAC1
Biological Process	GO:0060920	cardiac pacemaker cell differentiation	1/2279	5/18866	0.4746987	0.6795154	0.6148497	1	MAML1
Biological Process	GO:0061088	regulation of sequestering of zinc ion	1/2279	5/18866	0.4746987	0.6795154	0.6148497	1	AP3D1
Biological Process	GO:0061197	fungiform papilla morphogenesis	1/2279	5/18866	0.4746987	0.6795154	0.6148497	1	HDAC1
Biological Process	GO:0061302	smooth muscle cell-matrix adhesion	1/2279	5/18866	0.4746987	0.6795154	0.6148497	1	PLAU
Biological Process	GO:0061343	cell adhesion involved in heart morphogenesis	1/2279	5/18866	0.4746987	0.6795154	0.6148497	1	TGFBR2
Biological Process	GO:0061484	hematopoietic stem cell homeostasis	1/2279	5/18866	0.4746987	0.6795154	0.6148497	1	UBAP2L

Biological Process	GO:0061551	trigeminal ganglion development	1/2279	5/18866	0.4746987	0.6795154	0.6148497	1	NRP2
Biological Process	GO:0061624	fructose catabolic process to hydroxyacetone phosphate and glyceraldehyde-3-phosphate	1/2279	5/18866	0.4746987	0.6795154	0.6148497	1	GLYCTK
Biological Process	GO:0062028	regulation of stress granule assembly	1/2279	5/18866	0.4746987	0.6795154	0.6148497	1	PRKAA1
Biological Process	GO:0070072	vacuolar proton-transporting V-type ATPase complex assembly	1/2279	5/18866	0.4746987	0.6795154	0.6148497	1	TM9SF4
Biological Process	GO:0070120	ciliary neurotrophic factor-mediated signaling pathway	1/2279	5/18866	0.4746987	0.6795154	0.6148497	1	IL6R
Biological Process	GO:0070245	positive regulation of thymocyte apoptotic process	1/2279	5/18866	0.4746987	0.6795154	0.6148497	1	ZC3H8
Biological Process	GO:0070427	nucleotide-binding oligomerization domain containing 1 signaling pathway	1/2279	5/18866	0.4746987	0.6795154	0.6148497	1	TNFAIP3
Biological Process	GO:0070640	vitamin D3 metabolic process	1/2279	5/18866	0.4746987	0.6795154	0.6148497	1	FGFR1
Biological Process	GO:0070662	mast cell proliferation	1/2279	5/18866	0.4746987	0.6795154	0.6148497	1	BLK

Biological Process	GO:0070934	CRD-mediated mRNA stabilization	1/2279	5/18866	0.4746987	0.6795154	0.6148497	1	SYNCRIP
Biological Process	GO:0070945	neutrophil-mediated killing of gram-negative bacterium	1/2279	5/18866	0.4746987	0.6795154	0.6148497	1	ELANE
Biological Process	GO:0070948	regulation of neutrophil mediated cytotoxicity	1/2279	5/18866	0.4746987	0.6795154	0.6148497	1	DNASE1L3
Biological Process	GO:0071332	cellular response to fructose stimulus	1/2279	5/18866	0.4746987	0.6795154	0.6148497	1	SLC2A5
Biological Process	GO:0071502	cellular response to temperature stimulus	1/2279	5/18866	0.4746987	0.6795154	0.6148497	1	TRPM2
Biological Process	GO:0071543	diphosphoinositol polyphosphate metabolic process	1/2279	5/18866	0.4746987	0.6795154	0.6148497	1	NUDT3
Biological Process	GO:0071557	histone H3-K27 demethylation	1/2279	5/18866	0.4746987	0.6795154	0.6148497	1	KDM7A
Biological Process	GO:0071878	negative regulation of adenylate cyclase-activating adrenergic receptor signaling pathway	1/2279	5/18866	0.4746987	0.6795154	0.6148497	1	GNAI2
Biological Process	GO:0072025	distal convoluted tubule development	1/2279	5/18866	0.4746987	0.6795154	0.6148497	1	PAX2

Biological Process	GO:0072162	metanephric mesenchymal cell differentiation	1/2279	5/18866	0.4746987	0.6795154	0.6148497	1	PAX2
Biological Process	GO:0072179	nephric duct formation	1/2279	5/18866	0.4746987	0.6795154	0.6148497	1	PAX2
Biological Process	GO:0072221	metanephric distal convoluted tubule development	1/2279	5/18866	0.4746987	0.6795154	0.6148497	1	PAX2
Biological Process	GO:0072300	positive regulation of metanephric glomerulus development	1/2279	5/18866	0.4746987	0.6795154	0.6148497	1	PAX2
Biological Process	GO:0072344	rescue of stalled ribosome	1/2279	5/18866	0.4746987	0.6795154	0.6148497	1	ZNF598
Biological Process	GO:0072733	response to staurosporine	1/2279	5/18866	0.4746987	0.6795154	0.6148497	1	CASP7
Biological Process	GO:0072734	cellular response to staurosporine	1/2279	5/18866	0.4746987	0.6795154	0.6148497	1	CASP7
Biological Process	GO:0080144	amino acid homeostasis	1/2279	5/18866	0.4746987	0.6795154	0.6148497	1	KCTD7
Biological Process	GO:0080184	response to phenylpropanoid	1/2279	5/18866	0.4746987	0.6795154	0.6148497	1	CAT

Biological Process	GO:0086046	membrane depolarization during SA node cell action potential	1/2279	5/18866	0.4746987	0.6795154	0.6148497	1	ANK2
Biological Process	GO:0090063	positive regulation of microtubule nucleation	1/2279	5/18866	0.4746987	0.6795154	0.6148497	1	DCTN1
Biological Process	GO:0090080	positive regulation of MAPKKK cascade by fibroblast growth factor receptor signaling pathway	1/2279	5/18866	0.4746987	0.6795154	0.6148497	1	FGFR1
Biological Process	GO:0090271	positive regulation of fibroblast growth factor production	1/2279	5/18866	0.4746987	0.6795154	0.6148497	1	CCM2L
Biological Process	GO:0090289	regulation of osteoclast proliferation	1/2279	5/18866	0.4746987	0.6795154	0.6148497	1	TNFAIP3
Biological Process	GO:0090660	cerebrospinal fluid circulation	1/2279	5/18866	0.4746987	0.6795154	0.6148497	1	KATNIP
Biological Process	GO:0097039	protein linear polyubiquitination	1/2279	5/18866	0.4746987	0.6795154	0.6148497	1	PRKN
Biological Process	GO:0097176	epoxide metabolic process	1/2279	5/18866	0.4746987	0.6795154	0.6148497	1	EPHX1
Biological Process	GO:0097350	neutrophil clearance	1/2279	5/18866	0.4746987	0.6795154	0.6148497	1	CCR2

Biological Process	GO:0097360	chorionic trophoblast cell proliferation	1/2279	5/18866	0.4746987	0.6795154	0.6148497	1	NODAL
Biological Process	GO:0098708	glucose import across plasma membrane	1/2279	5/18866	0.4746987	0.6795154	0.6148497	1	SLC2A1
Biological Process	GO:0098735	positive regulation of the force of heart contraction	1/2279	5/18866	0.4746987	0.6795154	0.6148497	1	SLC8A1
Biological Process	GO:0098920	retrograde trans-synaptic signaling by lipid	1/2279	5/18866	0.4746987	0.6795154	0.6148497	1	PLCB1
Biological Process	GO:0098921	retrograde trans-synaptic signaling by endocannabinoid	1/2279	5/18866	0.4746987	0.6795154	0.6148497	1	PLCB1
Biological Process	GO:0100012	regulation of heart induction by canonical Wnt signaling pathway	1/2279	5/18866	0.4746987	0.6795154	0.6148497	1	WNT11
Biological Process	GO:0140192	regulation of adenylate cyclase-activating adrenergic receptor signaling pathway involved in heart process	1/2279	5/18866	0.4746987	0.6795154	0.6148497	1	GNAI2
Biological Process	GO:1900145	regulation of nodal signaling pathway involved in determination of left/right asymmetry	1/2279	5/18866	0.4746987	0.6795154	0.6148497	1	NODAL
Biological Process	GO:1900147	regulation of Schwann cell migration	1/2279	5/18866	0.4746987	0.6795154	0.6148497	1	CERS2

Biological Process	GO:1900158	negative regulation of bone mineralization involved in bone maturation	1/2279	5/18866	0.4746987	0.6795154	0.6148497	1	RFLNA
Biological Process	GO:1900175	regulation of nodal signaling pathway involved in determination of lateral mesoderm left/right asymmetry	1/2279	5/18866	0.4746987	0.6795154	0.6148497	1	NODAL
Biological Process	GO:1900226	negative regulation of NLRP3 inflammasome complex assembly	1/2279	5/18866	0.4746987	0.6795154	0.6148497	1	MEFV
Biological Process	GO:1900368	regulation of RNA interference	1/2279	5/18866	0.4746987	0.6795154	0.6148497	1	RMRP
Biological Process	GO:1901078	negative regulation of relaxation of muscle	1/2279	5/18866	0.4746987	0.6795154	0.6148497	1	PDE4D
Biological Process	GO:1901097	negative regulation of autophagosome maturation	1/2279	5/18866	0.4746987	0.6795154	0.6148497	1	CLEC16A
Biological Process	GO:1901162	primary amino compound biosynthetic process	1/2279	5/18866	0.4746987	0.6795154	0.6148497	1	DDC
Biological Process	GO:1901256	regulation of macrophage colony-stimulating factor production	1/2279	5/18866	0.4746987	0.6795154	0.6148497	1	FOXP1
Biological Process	GO:1901382	regulation of chorionic trophoblast cell proliferation	1/2279	5/18866	0.4746987	0.6795154	0.6148497	1	NODAL

Biological Process	GO:1901490	regulation of lymphangiogenesis	1/2279	5/18866	0.4746987	0.6795154	0.6148497	1	VASH1
Biological Process	GO:1901608	regulation of vesicle transport along microtubule	1/2279	5/18866	0.4746987	0.6795154	0.6148497	1	CNIH2
Biological Process	GO:1901662	quinone catabolic process	1/2279	5/18866	0.4746987	0.6795154	0.6148497	1	CYP4F3
Biological Process	GO:1901843	positive regulation of high voltage-gated calcium channel activity	1/2279	5/18866	0.4746987	0.6795154	0.6148497	1	FGF14
Biological Process	GO:1901906	diadenosine pentaphosphate metabolic process	1/2279	5/18866	0.4746987	0.6795154	0.6148497	1	NUDT3
Biological Process	GO:1901907	diadenosine pentaphosphate catabolic process	1/2279	5/18866	0.4746987	0.6795154	0.6148497	1	NUDT3
Biological Process	GO:1901908	diadenosine hexaphosphate metabolic process	1/2279	5/18866	0.4746987	0.6795154	0.6148497	1	NUDT3
Biological Process	GO:1901909	diadenosine hexaphosphate catabolic process	1/2279	5/18866	0.4746987	0.6795154	0.6148497	1	NUDT3
Biological Process	GO:1901910	adenosine 5'-(hexahydrogen pentaphosphate) metabolic process	1/2279	5/18866	0.4746987	0.6795154	0.6148497	1	NUDT3

Biological Process	GO:1901911	adenosine 5'-(hexahydrogen pentaphosphate) catabolic process	1/2279	5/18866	0.4746987	0.6795154	0.6148497	1	NUDT3
Biological Process	GO:1901995	positive regulation of meiotic cell cycle phase transition	1/2279	5/18866	0.4746987	0.6795154	0.6148497	1	CDC25A
Biological Process	GO:1902031	regulation of NADP metabolic process	1/2279	5/18866	0.4746987	0.6795154	0.6148497	1	PGAM1
Biological Process	GO:1902219	negative regulation of intrinsic apoptotic signaling pathway in response to osmotic stress	1/2279	5/18866	0.4746987	0.6795154	0.6148497	1	ARHGEF2
Biological Process	GO:1902292	cell cycle DNA replication initiation	1/2279	5/18866	0.4746987	0.6795154	0.6148497	1	MCM2
Biological Process	GO:1902308	regulation of peptidyl-serine dephosphorylation	1/2279	5/18866	0.4746987	0.6795154	0.6148497	1	PPP1R16B
Biological Process	GO:1902315	nuclear cell cycle DNA replication initiation	1/2279	5/18866	0.4746987	0.6795154	0.6148497	1	MCM2
Biological Process	GO:1902623	negative regulation of neutrophil migration	1/2279	5/18866	0.4746987	0.6795154	0.6148497	1	C5AR2
Biological Process	GO:1902732	positive regulation of chondrocyte proliferation	1/2279	5/18866	0.4746987	0.6795154	0.6148497	1	LTF

Biological Process	GO:1902816	regulation of protein localization to microtubule	1/2279	5/18866	0.4746987	0.6795154	0.6148497	1	MAP1A
Biological Process	GO:1902817	negative regulation of protein localization to microtubule	1/2279	5/18866	0.4746987	0.6795154	0.6148497	1	MAP1A
Biological Process	GO:1902975	mitotic DNA replication initiation	1/2279	5/18866	0.4746987	0.6795154	0.6148497	1	MCM2
Biological Process	GO:1903207	regulation of hydrogen peroxide-induced neuron death	1/2279	5/18866	0.4746987	0.6795154	0.6148497	1	IL10
Biological Process	GO:1903208	negative regulation of hydrogen peroxide-induced neuron death	1/2279	5/18866	0.4746987	0.6795154	0.6148497	1	IL10
Biological Process	GO:1903224	regulation of endodermal cell differentiation	1/2279	5/18866	0.4746987	0.6795154	0.6148497	1	COL5A1
Biological Process	GO:1903300	negative regulation of hexokinase activity	1/2279	5/18866	0.4746987	0.6795154	0.6148497	1	PRKN
Biological Process	GO:1903377	negative regulation of oxidative stress-induced neuron intrinsic apoptotic signaling pathway	1/2279	5/18866	0.4746987	0.6795154	0.6148497	1	PRKN
Biological Process	GO:1903391	regulation of adherens junction organization	1/2279	5/18866	0.4746987	0.6795154	0.6148497	1	ADD1

Biological Process	GO:1903416	response to glycoside	1/2279	5/18866	0.4746987	0.6795154	0.6148497	1	CHEK2
Biological Process	GO:1903625	negative regulation of DNA catabolic process	1/2279	5/18866	0.4746987	0.6795154	0.6148497	1	PAM16
Biological Process	GO:1903760	regulation of voltage-gated potassium channel activity involved in ventricular cardiac muscle cell action potential repolarization	1/2279	5/18866	0.4746987	0.6795154	0.6148497	1	NOS1AP
Biological Process	GO:1903847	regulation of aorta morphogenesis	1/2279	5/18866	0.4746987	0.6795154	0.6148497	1	NOTCH1
Biological Process	GO:1903898	negative regulation of PERK-mediated unfolded protein response	1/2279	5/18866	0.4746987	0.6795154	0.6148497	1	NCK2
Biological Process	GO:1903976	negative regulation of glial cell migration	1/2279	5/18866	0.4746987	0.6795154	0.6148497	1	CERS2
Biological Process	GO:1904398	positive regulation of neuromuscular junction development	1/2279	5/18866	0.4746987	0.6795154	0.6148497	1	DCTN1
Biological Process	GO:1904447	folate import across plasma membrane	1/2279	5/18866	0.4746987	0.6795154	0.6148497	1	SLC19A1
Biological Process	GO:1904760	regulation of myofibroblast differentiation	1/2279	5/18866	0.4746987	0.6795154	0.6148497	1	RB1

Biological Process	GO:1904885	beta-catenin destruction complex assembly	1/2279	5/18866	0.4746987	0.6795154	0.6148497	1	AXIN1
Biological Process	GO:1904970	brush border assembly	1/2279	5/18866	0.4746987	0.6795154	0.6148497	1	CDHR2
Biological Process	GO:1904997	regulation of leukocyte adhesion to arterial endothelial cell	1/2279	5/18866	0.4746987	0.6795154	0.6148497	1	TNF
Biological Process	GO:1905279	regulation of retrograde transport, endosome to Golgi	1/2279	5/18866	0.4746987	0.6795154	0.6148497	1	PRKN
Biological Process	GO:1905333	regulation of gastric motility	1/2279	5/18866	0.4746987	0.6795154	0.6148497	1	GHRL
Biological Process	GO:1905774	regulation of DNA helicase activity	1/2279	5/18866	0.4746987	0.6795154	0.6148497	1	MCM2
Biological Process	GO:1905867	epididymis development	1/2279	5/18866	0.4746987	0.6795154	0.6148497	1	PTPN6
Biological Process	GO:1905907	negative regulation of amyloid fibril formation	1/2279	5/18866	0.4746987	0.6795154	0.6148497	1	CLU
Biological Process	GO:1990049	retrograde neuronal dense core vesicle transport	1/2279	5/18866	0.4746987	0.6795154	0.6148497	1	KIF1B

Biological Process	GO:1990051	activation of protein kinase C activity	1/2279	5/18866	0.4746987	0.6795154	0.6148497	1	PHB
Biological Process	GO:1990481	mRNA pseudouridine synthesis	1/2279	5/18866	0.4746987	0.6795154	0.6148497	1	PUS7
Biological Process	GO:1990962	xenobiotic transport across blood-brain barrier	1/2279	5/18866	0.4746987	0.6795154	0.6148497	1	ABCC2
Biological Process	GO:2000002	negative regulation of DNA damage checkpoint	1/2279	5/18866	0.4746987	0.6795154	0.6148497	1	CHEK2
Biological Process	GO:2000118	regulation of sodium-dependent phosphate transport	1/2279	5/18866	0.4746987	0.6795154	0.6148497	1	CEBPB
Biological Process	GO:2000301	negative regulation of synaptic vesicle exocytosis	1/2279	5/18866	0.4746987	0.6795154	0.6148497	1	RAP1B
Biological Process	GO:2000504	positive regulation of blood vessel remodeling	1/2279	5/18866	0.4746987	0.6795154	0.6148497	1	MIR143
Biological Process	GO:2000671	regulation of motor neuron apoptotic process	1/2279	5/18866	0.4746987	0.6795154	0.6148497	1	CRLF1
Biological Process	GO:2000724	positive regulation of cardiac vascular smooth muscle cell differentiation	1/2279	5/18866	0.4746987	0.6795154	0.6148497	1	MIR145

Biological Process	GO:2000821	regulation of grooming behavior	1/2279	5/18866	0.4746987	0.6795154	0.6148497	1	NRXN1
Biological Process	GO:2000851	positive regulation of glucocorticoid secretion	1/2279	5/18866	0.4746987	0.6795154	0.6148497	1	GHRL
Biological Process	GO:2001023	regulation of response to drug	1/2279	5/18866	0.4746987	0.6795154	0.6148497	1	NCOA1
Biological Process	GO:2001166	regulation of histone H2B ubiquitination	1/2279	5/18866	0.4746987	0.6795154	0.6148497	1	RNF40
Biological Process	GO:2001205	negative regulation of osteoclast development	1/2279	5/18866	0.4746987	0.6795154	0.6148497	1	LTF
Biological Process	GO:2001245	regulation of phosphatidylcholine biosynthetic process	1/2279	5/18866	0.4746987	0.6795154	0.6148497	1	LPCAT1
Biological Process	GO:0009880	embryonic pattern specification	9/2279	70/18866	0.4749157	0.6795154	0.6148497	9	NRP2/SMAD3/ZBTB16/DLL1/PCSK6/MEIS2/MEIS1/TMED2/NODAL
Biological Process	GO:0050771	negative regulation of axonogenesis	9/2279	70/18866	0.4749157	0.6795154	0.6148497	9	SEMA6B/SEMA4A/RTN4/SEMA4B/IFRD1/SYNGAP1/SEMA4D/CDKL3/SPART
Biological Process	GO:0019884	antigen processing and presentation of exogenous antigen	23/2279	185/18866	0.4750141	0.6795154	0.6148497	23	HLA-DMB/DCTN1/CALR/AP2A1/LNPEP/NCF4/DNM2/DYNC1H1/CTSD/HLA-C/HLA-F/NCF2/ITGB5/AP1B1/DCTN2/SEC13/PSMF1/SEC24C/PSMB7/KIFAP3/AP3D1/SEC31A/PSMD13

Biological Process	GO:0030900	forebrain development	48/2279	391/18866	0.4757047	0.6795154	0.6148497	48	NRP2/CREB1/MFSD2A/NDE1/SKI/SETD2/SLC8A1/BTBD3/ZEB2/CSF1R/EIF2B5/EPHB3/RTN4/DISC1/TRAPPC9/NDST1/APP/RBPJ/HDAC1/ZMIZ1/FGFR1/RAPGEF2/AQP1/RARA/NF1/KIRREL3/TACC3/SLC1A2/WDR47/NUMB/NCOA1/PLCB1/ZNF335/ETS1/CDH1/TTC21B/NOTCH1/CORO1C/SLC2A1/TYRO3/SECISBP2/NDEL1/ALDH1A2/SLIT1/NPY/INHBA/OTX2/TSKU
Biological Process	GO:0000212	meiotic spindle organization	2/2279	13/18866	0.477478	0.6795154	0.6148497	2	MYH9/PPP2R1A
Biological Process	GO:0001833	inner cell mass cell proliferation	2/2279	13/18866	0.477478	0.6795154	0.6148497	2	BRCA2/NDEL1
Biological Process	GO:0001967	suckling behavior	2/2279	13/18866	0.477478	0.6795154	0.6148497	2	APP/DERL2
Biological Process	GO:0001973	G protein-coupled adenosine receptor signaling pathway	2/2279	13/18866	0.477478	0.6795154	0.6148497	2	GNAI2/ACP3
Biological Process	GO:0002371	dendritic cell cytokine production	2/2279	13/18866	0.477478	0.6795154	0.6148497	2	SCIMP/NOD2
Biological Process	GO:0002674	negative regulation of acute inflammatory response	2/2279	13/18866	0.477478	0.6795154	0.6148497	2	PPARG/NLRP3
Biological Process	GO:0003337	mesenchymal to epithelial transition involved in metanephros morphogenesis	2/2279	13/18866	0.477478	0.6795154	0.6148497	2	PAX2/LIF
Biological Process	GO:0006068	ethanol catabolic process	2/2279	13/18866	0.477478	0.6795154	0.6148497	2	ALDH2/SULT1A2

Biological Process	GO:0006553	lysine metabolic process	2/2279	13/18866	0.477478	0.6795154	0.6148497	2	OGDH/CRYM
Biological Process	GO:0008228	opsonization	2/2279	13/18866	0.477478	0.6795154	0.6148497	2	MYO18A/SPON2
Biological Process	GO:0009223	pyrimidine deoxyribonucleotide catabolic process	2/2279	13/18866	0.477478	0.6795154	0.6148497	2	UNG/NTHL1
Biological Process	GO:0009313	oligosaccharide catabolic process	2/2279	13/18866	0.477478	0.6795154	0.6148497	2	HEXB/MAN2C1
Biological Process	GO:0010457	centriole-centriole cohesion	2/2279	13/18866	0.477478	0.6795154	0.6148497	2	DCTN1/CEP135
Biological Process	GO:0015697	quaternary ammonium group transport	2/2279	13/18866	0.477478	0.6795154	0.6148497	2	SLC22A5/PDZK1
Biological Process	GO:0015809	arginine transport	2/2279	13/18866	0.477478	0.6795154	0.6148497	2	SLC11A1/SLC7A1
Biological Process	GO:0015816	glycine transport	2/2279	13/18866	0.477478	0.6795154	0.6148497	2	SLC36A3/SLC7A8
Biological Process	GO:0016024	CDP-diacylglycerol biosynthetic process	2/2279	13/18866	0.477478	0.6795154	0.6148497	2	CDS2/AGPAT5

Biological Process	GO:0016322	neuron remodeling	2/2279	13/18866	0.477478	0.6795154	0.6148497	2	BCL11A/APP
Biological Process	GO:0018206	peptidyl-methionine modification	2/2279	13/18866	0.477478	0.6795154	0.6148497	2	PDF/NAA16
Biological Process	GO:0019336	phenol-containing compound catabolic process	2/2279	13/18866	0.477478	0.6795154	0.6148497	2	COMT/FAH
Biological Process	GO:0021694	cerebellar Purkinje cell layer formation	2/2279	13/18866	0.477478	0.6795154	0.6148497	2	TTC21B/TTLL1
Biological Process	GO:0031126	snoRNA 3'-end processing	2/2279	13/18866	0.477478	0.6795154	0.6148497	2	EXOSC2/FBL
Biological Process	GO:0031453	positive regulation of heterochromatin assembly	2/2279	13/18866	0.477478	0.6795154	0.6148497	2	ATF7IP/DNMT1
Biological Process	GO:0031953	negative regulation of protein autophosphorylation	2/2279	13/18866	0.477478	0.6795154	0.6148497	2	PTPRC/ENG
Biological Process	GO:0032352	positive regulation of hormone metabolic process	2/2279	13/18866	0.477478	0.6795154	0.6148497	2	RDH10/POR
Biological Process	GO:0032536	regulation of cell projection size	2/2279	13/18866	0.477478	0.6795154	0.6148497	2	CDHR2/EZR

Biological Process	GO:0032968	positive regulation of transcription elongation from RNA polymerase II promoter	2/2279	13/18866	0.477478	0.6795154	0.6148497	2	BRD4/CDK13
Biological Process	GO:0033147	negative regulation of intracellular estrogen receptor signaling pathway	2/2279	13/18866	0.477478	0.6795154	0.6148497	2	LBH/ZNF366
Biological Process	GO:0033235	positive regulation of protein sumoylation	2/2279	13/18866	0.477478	0.6795154	0.6148497	2	HDAC4/TOLLIP
Biological Process	GO:0034144	negative regulation of toll-like receptor 4 signaling pathway	2/2279	13/18866	0.477478	0.6795154	0.6148497	2	MIR140/TNFAIP3
Biological Process	GO:0034379	very-low-density lipoprotein particle assembly	2/2279	13/18866	0.477478	0.6795154	0.6148497	2	P4HB/MFSD2A
Biological Process	GO:0034982	mitochondrial protein processing	2/2279	13/18866	0.477478	0.6795154	0.6148497	2	SPG7/MIPEP
Biological Process	GO:0035588	G protein-coupled purinergic receptor signaling pathway	2/2279	13/18866	0.477478	0.6795154	0.6148497	2	GNAI2/ACP3
Biological Process	GO:0038007	netrin-activated signaling pathway	2/2279	13/18866	0.477478	0.6795154	0.6148497	2	UNC5B/UNC5D
Biological Process	GO:0042415	norepinephrine metabolic process	2/2279	13/18866	0.477478	0.6795154	0.6148497	2	EPAS1/PRKN

Biological Process	GO:0043568	positive regulation of insulin-like growth factor receptor signaling pathway	2/2279	13/18866	0.477478	0.6795154	0.6148497	2	CDH3/MYORG
Biological Process	GO:0043970	histone H3-K9 acetylation	2/2279	13/18866	0.477478	0.6795154	0.6148497	2	SPHK2/PIH1D1
Biological Process	GO:0045579	positive regulation of B cell differentiation	2/2279	13/18866	0.477478	0.6795154	0.6148497	2	INPP5D/PCID2
Biological Process	GO:0045759	negative regulation of action potential	2/2279	13/18866	0.477478	0.6795154	0.6148497	2	BIN1/CNR2
Biological Process	GO:0048266	behavioral response to pain	2/2279	13/18866	0.477478	0.6795154	0.6148497	2	THBS1/NTRK1
Biological Process	GO:0048302	regulation of isotype switching to IgG isotypes	2/2279	13/18866	0.477478	0.6795154	0.6148497	2	PTPRC/PAXIP1
Biological Process	GO:0048715	negative regulation of oligodendrocyte differentiation	2/2279	13/18866	0.477478	0.6795154	0.6148497	2	NF1/NOTCH1
Biological Process	GO:0048934	peripheral nervous system neuron differentiation	2/2279	13/18866	0.477478	0.6795154	0.6148497	2	RUNX1/RUNX3
Biological Process	GO:0048935	peripheral nervous system neuron development	2/2279	13/18866	0.477478	0.6795154	0.6148497	2	RUNX1/RUNX3

Biological Process	GO:0051386	regulation of neurotrophin TRK receptor signaling pathway	2/2279	13/18866	0.477478	0.6795154	0.6148497	2	CYFIP2/CYFIP1
Biological Process	GO:0051481	negative regulation of cytosolic calcium ion concentration	2/2279	13/18866	0.477478	0.6795154	0.6148497	2	SMAD3/SLC8A1
Biological Process	GO:0051956	negative regulation of amino acid transport	2/2279	13/18866	0.477478	0.6795154	0.6148497	2	SLC43A2/SLC43A1
Biological Process	GO:0055091	phospholipid homeostasis	2/2279	13/18866	0.477478	0.6795154	0.6148497	2	ABCG1/ABCA1
Biological Process	GO:0060123	regulation of growth hormone secretion	2/2279	13/18866	0.477478	0.6795154	0.6148497	2	ITSN1/GHRL
Biological Process	GO:0060221	retinal rod cell differentiation	2/2279	13/18866	0.477478	0.6795154	0.6148497	2	RPGRIP1/NRL
Biological Process	GO:0060340	positive regulation of type I interferon-mediated signaling pathway	2/2279	13/18866	0.477478	0.6795154	0.6148497	2	NLRC5/FADD
Biological Process	GO:0061029	eyelid development in camera-type eye	2/2279	13/18866	0.477478	0.6795154	0.6148497	2	HDAC1/INHBA
Biological Process	GO:0061043	regulation of vascular wound healing	2/2279	13/18866	0.477478	0.6795154	0.6148497	2	TNF/TNFAIP3

Biological Process	GO:0061430	bone trabecula morphogenesis	2/2279	13/18866	0.477478	0.6795154	0.6148497	2	SBNO2/SEMA4D
Biological Process	GO:0071027	nuclear RNA surveillance	2/2279	13/18866	0.477478	0.6795154	0.6148497	2	EXOSC2/PCID2
Biological Process	GO:0071028	nuclear mRNA surveillance	2/2279	13/18866	0.477478	0.6795154	0.6148497	2	EXOSC2/PCID2
Biological Process	GO:0071352	cellular response to interleukin-2	2/2279	13/18866	0.477478	0.6795154	0.6148497	2	STAT5A/JAK1
Biological Process	GO:0071872	cellular response to epinephrine stimulus	2/2279	13/18866	0.477478	0.6795154	0.6148497	2	PDE4D/KCNQ1
Biological Process	GO:0072578	neurotransmitter-gated ion channel clustering	2/2279	13/18866	0.477478	0.6795154	0.6148497	2	SSH1/ZDHHC2
Biological Process	GO:0090042	tubulin deacetylation	2/2279	13/18866	0.477478	0.6795154	0.6148497	2	NEK3/PRKAA1
Biological Process	GO:0097152	mesenchymal cell apoptotic process	2/2279	13/18866	0.477478	0.6795154	0.6148497	2	PAX2/ETV6
Biological Process	GO:0099638	endosome to plasma membrane protein transport	2/2279	13/18866	0.477478	0.6795154	0.6148497	2	ZDHHC2/AKAP5

Biological Process	GO:0099640	axo-dendritic protein transport	2/2279	13/18866	0.477478	0.6795154	0.6148497	2	KIF5C/MAP1A
Biological Process	GO:1900103	positive regulation of endoplasmic reticulum unfolded protein response	2/2279	13/18866	0.477478	0.6795154	0.6148497	2	ERN1/PIK3R1
Biological Process	GO:1900272	negative regulation of long-term synaptic potentiation	2/2279	13/18866	0.477478	0.6795154	0.6148497	2	APP/CX3CR1
Biological Process	GO:1901096	regulation of autophagosome maturation	2/2279	13/18866	0.477478	0.6795154	0.6148497	2	UVRAG/CLEC16A
Biological Process	GO:1902285	semaphorin-plexin signaling pathway involved in neuron projection guidance	2/2279	13/18866	0.477478	0.6795154	0.6148497	2	PLXNC1/NRP2
Biological Process	GO:1903977	positive regulation of glial cell migration	2/2279	13/18866	0.477478	0.6795154	0.6148497	2	CCR2/CX3CR1
Biological Process	GO:1904424	regulation of GTP binding	2/2279	13/18866	0.477478	0.6795154	0.6148497	2	ARHGEF7/CLN5
Biological Process	GO:0002478	antigen processing and presentation of exogenous peptide antigen	22/2279	177/18866	0.4775418	0.6795154	0.6148497	22	HLA-DMB/DCTN1/CALR/AP2A1/LNPEP/NCF4/DNM2/DYNC1H1/CTSD/HLA-C/HLA-F/NCF2/ITGB5/AP1B1/DCTN2/SEC13/PSMF1/SEC24C/PSMB7/KIFAP3/SEC31A/PSMD13
Biological Process	GO:0002495	antigen processing and presentation of peptide antigen via MHC class II	13/2279	103/18866	0.477778	0.6795463	0.6148776	13	HLA-DMB/DCTN1/AP2A1/DNM2/DYNC1H1/CTSD/AP1B1/DCTN2/SEC13/SEC24C/KIFAP3/SEC31A/MARCHF8

Biological Process	GO:0098869	cellular oxidant detoxification	13/2279	103/18866	0.477778	0.6795463	0.6148776	13	MPO/LPO/TNF/HBB/MGST2/CAT/NFE2L2/NXN/GSTO1/TXNRD2/GSTO2/SIRT3/PRXL2B
Biological Process	GO:1901570	fatty acid derivative biosynthetic process	13/2279	103/18866	0.477778	0.6795463	0.6148776	13	ACSL1/FAR2/BDH1/GGT1/MGST2/ACACA/ACSF3/HMGCL/ACAT1/SCD/ACSS3/SLC25A1/PRXL2B
Biological Process	GO:0034764	positive regulation of transmembrane transport	26/2279	210/18866	0.4782535	0.6801206	0.6153973	26	KCNE1/FXYD1/PIK3R1/CCR2/DNM2/TESC/NOS1AP/ACTN4/P2RY6/HTR3A/SLC1A2/HTT/KCNQ1/PRKCI/NFE2L2/GSTO1/F2R/ANO6/ANK2/PLCG1/PDZK1/CASQ1/CLTCL1/FGF14/STIMATE/CRACR2A
Biological Process	GO:0055081	anion homeostasis	8/2279	62/18866	0.4785008	0.6803706	0.6156235	8	ABCG1/ABCC2/KCTD7/ABCA1/SLC12A1/CA12/SLC12A7/PRKAA1
Biological Process	GO:0050709	negative regulation of protein secretion	12/2279	95/18866	0.4810521	0.6834867	0.618443	12	RAB11FIP1/FRMD4A/MIR199A1/RHBDF2/ENY2/GHRL/KLF7/MIR199A2/F2R/SREBF1/ADTRP/PRKN
Biological Process	GO:0060337	type I interferon signaling pathway	12/2279	95/18866	0.4810521	0.6834867	0.618443	12	IRF8/IRF5/HLA-C/HLA-F/PTPN6/JAK1/NLRC5/SP100/MX1/IRF2/ISG20/FADD
Biological Process	GO:0070167	regulation of biomineral tissue development	12/2279	95/18866	0.4810521	0.6834867	0.618443	12	SMAD3/SLC8A1/TGFB1/BMPR1B/SGMS2/NOTCH1/MEF2C/LTF/CEBPB/ANO6/FAM20C/RFLNA
Biological Process	GO:0071357	cellular response to type I interferon	12/2279	95/18866	0.4810521	0.6834867	0.618443	12	IRF8/IRF5/HLA-C/HLA-F/PTPN6/JAK1/NLRC5/SP100/MX1/IRF2/ISG20/FADD
Biological Process	GO:0110149	regulation of biomineralization	12/2279	95/18866	0.4810521	0.6834867	0.618443	12	SMAD3/SLC8A1/TGFB1/BMPR1B/SGMS2/NOTCH1/MEF2C/LTF/CEBPB/ANO6/FAM20C/RFLNA

Biological Process	GO:0046394	carboxylic acid biosynthetic process	45/2279	367/18866	0.4814024	0.6838822	0.6188009	45	PRKAG2/MGLL/AP2A1/DSE/GADL1/DHRS9/LIPC/GGT1/AMACR/MTHFD1L/CBS/AKR1D1/MTHFS/OSBPL6/PRKAB1/ACOT7/ACSS1/TGFB1/RDH10/MYO5A/NR1H2/AGXT/ACACB/MGST2/PDGFB/UPB1/ACSS2/ACOX2/MTHFD1/HOGA1/PER2/SLC1A3/PKM/ACACA/ACSF3/FA2H/ADI1/SCD/WDTC1/ALDH1A2/ASPG/HAS3/ACADVL/PRKAA1/PRXL2B
Biological Process	GO:1903409	reactive oxygen species biosynthetic process	16/2279	128/18866	0.4822718	0.6850148	0.6198257	16	HDAC4/MPO/IL10/SMAD3/DNM2/MIR199A1/TNF/RAB27A/NOS1AP/MIR24-2/CLU/SPHK2/HBB/ITGB2/MIR199A2/CX3CR1
Biological Process	GO:0006754	ATP biosynthetic process	7/2279	54/18866	0.4824747	0.6850982	0.6199012	7	PRKAG2/ENO1/VPS9D1/SPHK2/TGFB1/PKM/ATP5PD
Biological Process	GO:0010524	positive regulation of calcium ion transport into cytosol	7/2279	54/18866	0.4824747	0.6850982	0.6199012	7	P2RY6/HTT/GSTO1/F2R/PLCG1/P2RX5/CASQ1
Biological Process	GO:0031570	DNA integrity checkpoint	20/2279	161/18866	0.4829238	0.6856334	0.6203855	20	TRIM39/BLM/FZR1/TFDP1/FOXN3/HUS1/E2F7/PCBP4/EME1/ZNF385A/ARID3A/TAOK3/CLSPN/TP53BP1/RHNO1/CHEK2/NAE1/MAP3K20/ABRAXAS1/BABAM2
Biological Process	GO:0048002	antigen processing and presentation of peptide antigen	24/2279	194/18866	0.4832793	0.6860357	0.6207494	24	HLA-DMB/DCTN1/CALR/AP2A1/SLC11A1/LNPEP/NCF4/DNM2/DYNC1H1/CTSD/HLA-C/HLA-F/NCF2/ITGB5/AP1B1/DCTN2/SEC13/PSMF1/SEC24C/PSMB7/KIFAP3/SEC31A/PSMD13/MARCHF8
Biological Process	GO:0051262	protein tetramerization	11/2279	87/18866	0.4845776	0.6875704	0.6221381	11	TRPM2/ACOT13/TRPM6/CRTC3/ACACB/UPB1/TRPM3/ACACA/MAT1A/ALDH1A2/TK1
Biological Process	GO:0060218	hematopoietic stem cell differentiation	11/2279	87/18866	0.4845776	0.6875704	0.6221381	11	MYB/RUNX1/TCF12/SETD1A/PSMF1/NFE2L2/PUS7/TCF3/PSMB7/ERCC2/PSMD13
Biological Process	GO:0090049	regulation of cell migration involved in sprouting angiogenesis	11/2279	87/18866	0.4845776	0.6875704	0.6221381	11	MAP3K3/HDAC7/ITGB1BP1/MIR199A1/MIR101-2/THBS1/AKT3/MIR27A/MIR23A/MIR199A2/NOTCH1

Biological Process	GO:0006417	regulation of translation	55/2279	450/18866	0.4847681	0.6877381	0.6222899	55	PIWIL4/RXRA/CALR/RPS6KA1/MIR145/KLHL25/TRNAU1AP/TNRC6B/PLD1/TNF/CCL5/NCK2/MIR101-2/EIF2B5/AGO1/LARP1/KRT17/ZC3H12D/THBS1/APP/SAMD4A/ZNF385A/MIR27A/CTIF/AGO2/MTG2/MIF4GD/RARA/CPEB3/IGF2BP3/MKNK1/INPP5E/FTO/PUS7/HNRNPLL/MIR138-2/CELF1/PER2/PKM/PER1/NOTCH4/PAIP1/ZNF598/BANK1/EIF3H/ILF3/SECISBP2/CYFIP1/BCL3/SYNCRIP/EIF4G1/DAPK3/ZNF706/NIBAN1/SHFL
Biological Process	GO:0006304	DNA modification	15/2279	120/18866	0.4854304	0.6883693	0.622861	15	PIWIL4/MOV10L1/GATAD2A/EHMT2/UNG/NTHL1/KDM1B/ATF7IP/GRHL2/FTO/TET2/MTA2/DNMT1/MORC1/MUTYH
Biological Process	GO:0060147	regulation of posttranscriptional gene silencing	15/2279	120/18866	0.4854304	0.6883693	0.622861	15	PPARG/NCOR2/RAE1/TNF/POLR2F/AGO1/RIPK1/LIMD1/AGO2/TGFB1/NUP93/SEC13/RMRP/EIF4G1/H3C1
Biological Process	GO:0060966	regulation of gene silencing by RNA	15/2279	120/18866	0.4854304	0.6883693	0.622861	15	PPARG/NCOR2/RAE1/TNF/POLR2F/AGO1/RIPK1/LIMD1/AGO2/TGFB1/NUP93/SEC13/RMRP/EIF4G1/H3C1
Biological Process	GO:0006220	pyrimidine nucleotide metabolic process	6/2279	46/18866	0.4869643	0.6900294	0.6243631	6	UNG/UCK2/NTHL1/AK5/DCTD/DHODH
Biological Process	GO:0031641	regulation of myelination	6/2279	46/18866	0.4869643	0.6900294	0.6243631	6	LGI4/TNFRSF1B/TG/TNFRSF21/RARA/RARG
Biological Process	GO:0032007	negative regulation of TOR signaling	6/2279	46/18866	0.4869643	0.6900294	0.6243631	6	SH3BP4/ARNTL/TSC2/TNFAIP8L1/NPRL2/PRKAA1
Biological Process	GO:0048538	thymus development	6/2279	46/18866	0.4869643	0.6900294	0.6243631	6	MAD1L1/EPHB3/RIPK3/BCL2/LMO4/FADD
Biological Process	GO:0050982	detection of mechanical stimulus	6/2279	46/18866	0.4869643	0.6900294	0.6243631	6	TTN/PKD1L1/JUP/SCN1A/NTRK1/TMC1

Biological Process	GO:0043462	regulation of ATPase activity	10/2279	79/18866	0.488407	0.6919706	0.6261195	10	PAM16/TNNT3/UHRF1/HSCB/PFN1/DNAJB6/MCM2/TMEM64/LTF/AHSA2P
Biological Process	GO:0006275	regulation of DNA replication	14/2279	112/18866	0.48879	0.6924099	0.6265171	14	BLM/BRCA2/HUS1/E2F7/WRNIP1/ZBTB38/EHMT2/FGFR1/SMARCAL1/PPP2R1A/CHEK2/BCAR3/KAT7/FBH1
Biological Process	GO:0016053	organic acid biosynthetic process	45/2279	368/18866	0.4891973	0.6928837	0.6269458	45	PRKAG2/MGLL/AP2A1/DSE/GADL1/DHRS9/LIPC/GGT1/AMACR/MTHFD1L/CBS/AKR1D1/MTHFS/OSBPL6/PRKAB1/ACOT7/ACSS1/TGFB1/RDH10/MYO5A/NR1H2/AGXT/ACACB/MGST2/PDGFB/UPB1/ACSS2/ACOX2/MTHFD1/HOGA1/PER2/SLC1A3/PKM/ACACA/ACSF3/FA2H/ADI1/SCD/WDTC1/ALDH1A2/ASPG/HAS3/ACADVL/PRKAA1/PRXL2B
Biological Process	GO:0048562	embryonic organ morphogenesis	37/2279	302/18866	0.4899608	0.6937581	0.627737	37	ABR/SETD2/SMAD3/MTHFD1L/SUFU/HIPK2/MICAL2/CHST11/TGFBR2/SLC44A4/NDST1/DLL1/MIB1/LRIG1/WNT11/PAX2/FGFR1/TTC39C/RDH10/SP3/DSCAML1/RARG/GRHL2/RUNX2/IFT140/BCR/ENG/MTHFD1/HOXA9/NOTCH1/ALX3/MEF2C/HLX/TMED2/TEAD2/NODAL/HOXB7
Biological Process	GO:0090068	positive regulation of cell cycle process	37/2279	302/18866	0.4899608	0.6937581	0.627737	37	RPTOR/BRD4/TGFA/TFDP1/NSMCE2/DYNC1H1/E2F7/CUL3/PCBP4/APP/ZNF385A/CDC16/ZBTB17/POC1A/FGFR1/CDC25A/ARID3A/TMOD3/SVIL/CSPP1/NUMA1/PDGFB/PLCB1/RB1/MED25/PRKCE/CDCA5/RHNO1/CHEK2/ANAPC7/PDGFRB/SPAG5/CDC14A/PLRG1/EIF4G1/PCID2/SLF1
Biological Process	GO:0000462	maturation of SSU-rRNA from tricistronic rRNA transcript (SSU-rRNA, 5.8S rRNA, LSU-rRNA)	5/2279	38/18866	0.4921721	0.6958523	0.6296319	5	RCL1/WDR46/NOP14/ERCC2/UTP4
Biological Process	GO:0010591	regulation of lamellipodium assembly	5/2279	38/18866	0.4921721	0.6958523	0.6296319	5	HDAC4/DNM2/FER/NCKAP1/AVIL
Biological Process	GO:0010661	positive regulation of muscle cell apoptotic process	5/2279	38/18866	0.4921721	0.6958523	0.6296319	5	PPARG/MIR140/ARRB1/E2F3/MIR24-2
Biological Process	GO:0014912	negative regulation of smooth muscle cell migration	5/2279	38/18866	0.4921721	0.6958523	0.6296319	5	MIR140/ILK/NFE2L2/MEF2C/TRIB1

Biological Process	GO:0032733	positive regulation of interleukin-10 production	5/2279	38/18866	0.4921721	0.6958523	0.6296319	5	PRKCZ/MIR145/TLR9/TIGIT/NOD2
Biological Process	GO:0045880	positive regulation of smoothened signaling pathway	5/2279	38/18866	0.4921721	0.6958523	0.6296319	5	CHSY1/NDST1/POR/STK36/TXNDC15
Biological Process	GO:0046466	membrane lipid catabolic process	5/2279	38/18866	0.4921721	0.6958523	0.6296319	5	HEXB/GALC/SMPDL3B/MGST2/PDXDC1
Biological Process	GO:0051930	regulation of sensory perception of pain	5/2279	38/18866	0.4921721	0.6958523	0.6296319	5	MGLL/IL10/COMT/F2R/ACP3
Biological Process	GO:1903432	regulation of TORC1 signaling	5/2279	38/18866	0.4921721	0.6958523	0.6296319	5	DGKQ/CLEC16A/SIK3/SEC13/PIH1D1
Biological Process	GO:1903523	negative regulation of blood circulation	5/2279	38/18866	0.4921721	0.6958523	0.6296319	5	PDE4D/BIN1/F2R/SPTBN4/GRK2
Biological Process	GO:0007200	phospholipase C-activating G protein-coupled receptor signaling pathway	13/2279	104/18866	0.4923834	0.6960474	0.6298084	13	C3AR1/FPR2/FPR1/C5AR2/P2RY6/HRH1/GNA11/CHRM2/F2R/GPR4/AGTR1/LTB4R/CX3CR1
Biological Process	GO:0009201	ribonucleoside triphosphate biosynthetic process	9/2279	71/18866	0.4926111	0.6962657	0.630006	9	PRKAG2/ENO1/VPS9D1/SPHK2/TGFB1/UCK2/PKM/IMPDH1/ATP5PD
Biological Process	GO:0042391	regulation of membrane potential	54/2279	443/18866	0.4937219	0.6977319	0.6313326	54	PRKCZ/SSH1/KCNE1/KCNAB2/CASQ2/WWP2/CACNA1C/YWHAH/FXYD1/ZMYND8/SLC8A1/GJA5/HSH2D/JUP/KCTD7/BIN1/BAIAP2/APP/NOS1AP/SMAD7/GRIK4/SCN1A/KCNK6/CNIH2/HTR3A/NRXN1/GNA11/GHRL/SH3GL1/SLC1A6/WDR1/CNR2/SNTA1/MAPK8IP2/KCNQ1/ARRB2/BCL2/S1PR2/GABBR1/P2RX1/MEF2C/SHANK2/CNTNAP1/NEDD4/CACNA1B/BCL2L1/ANK2/SLC29A1/P2RX5/GRID1/CUX2/FGF14/SPART/PRKN

Biological Process	GO:0050731	positive regulation of peptidyl-tyrosine phosphorylation	24/2279	195/18866	0.4939497	0.6979501	0.63153	24	TGFA/ANGPT1/TNF/CCL5/CSF1R/TNFRSF1A/TGFB1/PTPRJ/SEMA4D/DGKQ/LRRK1/CSF3/ITGB3/GHRL/PTPRC/IL6R/PDGFB/CASS4/ARRB2/BANK1/ARHGEF2/CRLF1/PARP9/LIF
Biological Process	GO:0046034	ATP metabolic process	38/2279	311/18866	0.4962126	0.7010433	0.6343288	38	FOXK1/HDAC4/PRKAG2/AMPD3/ENO1/NADK/SLC25A25/HK1/PFKFB4/RAE1/CBFA2T3/DHTKD1/APP/OGDH/NDUFS2/ATP6V1B2/ADPGK/VPS9D1/PGAM1/SPHK2/TGFB1/NDUFS8/NUP93/ENO3/HK2/SEC13/GUK1/ESRRB/PKM/DNM1L/AK2/AK5/NDUFB6/FAM20C/SDHD/PFKFB3/PRKAA1/ATP5PD
Biological Process	GO:0006081	cellular aldehyde metabolic process	8/2279	63/18866	0.497289	0.702042	0.6352325	8	ALDH4A1/GLYCTK/RDH10/AGXT/PDXK/HOGA1/RELA/ALDH1A2
Biological Process	GO:0007062	sister chromatid cohesion	8/2279	63/18866	0.497289	0.702042	0.6352325	8	TNKS/NSMCE2/STAG1/PPP2R1A/RB1/RAD21L1/CDCA5/SLF1
Biological Process	GO:0032613	interleukin-10 production	8/2279	63/18866	0.497289	0.702042	0.6352325	8	PRKCZ/MIR145/TLR9/DLL1/TNFRSF21/TIGIT/NOD2/VSIR
Biological Process	GO:0046622	positive regulation of organ growth	8/2279	63/18866	0.497289	0.702042	0.6352325	8	MIR199A1/RBPJ/FGFR1/ACACB/MIR199A2/NOTCH1/MEF2C/HLX
Biological Process	GO:1902686	mitochondrial outer membrane permeabilization involved in programmed cell death	8/2279	63/18866	0.497289	0.702042	0.6352325	8	TFDP1/YWHAH/BMF/SPG7/YWHAZ/BCL2/TP53BP2/YWHAQ
Biological Process	GO:0018279	protein N-linked glycosylation via asparagine	4/2279	30/18866	0.4984514	0.7029519	0.6360558	4	RPN1/MGAT5/ASGR2/ST6GAL1
Biological Process	GO:0033688	regulation of osteoblast proliferation	4/2279	30/18866	0.4984514	0.7029519	0.6360558	4	SMAD3/MIR138-2/BCL2/LTF

Biological Process	GO:0042133	neurotransmitter metabolic process	4/2279	30/18866	0.4984514	0.7029519	0.6360558	4	COLQ/COMT/SLC44A4/HNMT
Biological Process	GO:0046337	phosphatidylethanolamine metabolic process	4/2279	30/18866	0.4984514	0.7029519	0.6360558	4	PLA2G15/CHKB/LPIN1/ETNK2
Biological Process	GO:0048265	response to pain	4/2279	30/18866	0.4984514	0.7029519	0.6360558	4	THBS1/COMT/NTRK1/CACNA1B
Biological Process	GO:0051642	centrosome localization	4/2279	30/18866	0.4984514	0.7029519	0.6360558	4	NDE1/BICD2/NDEL1/EZR
Biological Process	GO:0060142	regulation of syncytium formation by plasma membrane fusion	4/2279	30/18866	0.4984514	0.7029519	0.6360558	4	EHD1/NFATC2/FLOT1/SCGB3A1
Biological Process	GO:0007613	memory	15/2279	121/18866	0.4989637	0.7034655	0.6365206	15	PRKCZ/CREB1/PPP1R1B/SGK1/ATXN1/COMT/HRH1/CPEB3/PLCB1/MAP1A/CEBPB/NTAN1/NTF4/CX3CR1/CUX2
Biological Process	GO:1900542	regulation of purine nucleotide metabolic process	15/2279	121/18866	0.4989637	0.7034655	0.6365206	15	HDAC4/PRKAG2/RCVRN/ENO1/PFKFB4/RAE1/CBFA2T3/APP/PGAM1/SPHK2/NUP93/SEC13/ESRRB/PFKFB3/PRKAA1
Biological Process	GO:1901988	negative regulation of cell cycle phase transition	33/2279	270/18866	0.4994366	0.7040278	0.6370293	33	TRIM39/BLM/FZR1/TFDP1/MAD1L1/FOXN3/PRMT2/HUS1/FBXO7/E2F7/CTDSPL/PCBP4/RIPK1/NACC2/ZNF385A/ARID3A/OVOL1/C10orf99/PSMF1/GFI1B/TAOK3/ZFYVE19/MIR138-2/CLSPN/RB1/BCL2/PSMB7/CHEK2/NAE1/PSMD13/PCID2/ABRAXAS1/BABAM2
Biological Process	GO:0070098	chemokine-mediated signaling pathway	11/2279	88/18866	0.5004506	0.7052478	0.6381332	11	CCR2/CCL5/PADI2/ACKR2/CCR9/CCL20/CXCR1/SLIT3/CCL22/CX3CR1/ACKR1

Biological Process	GO:0090559	regulation of membrane permeability	11/2279	88/18866	0.5004506	0.7052478	0.6381332	11	TFDP1/TJP2/YWHAH/BMF/SPG7/HK2/YWHAZ/BCL2/TP53BP2/BCL2L1/YWHAQ
Biological Process	GO:0051168	nuclear export	25/2279	204/18866	0.50161	0.7067768	0.6395168	25	CALR/NXF1/FIP1L1/AKAP13/CPSF3/SETD2/RAE1/ATXN1/XPO7/TCF7L2/SMG7/XPO6/TGFB1/PABPN1/ENY2/SMURF1/NUP93/SEC13/SMG6/SP100/HHEX/RBM15B/NOL6/CPSF4/PCID2
Biological Process	GO:0002931	response to ischemia	7/2279	55/18866	0.5025843	0.7078347	0.640474	7	MAP2K6/CSF1R/ARID1B/HK2/PER2/BCL2/CX3CR1
Biological Process	GO:0003229	ventricular cardiac muscle tissue development	7/2279	55/18866	0.5025843	0.7078347	0.640474	7	RXRA/CCM2L/RBPJ/SMAD7/TGFB1/ENG/NOTCH1
Biological Process	GO:0097345	mitochondrial outer membrane permeabilization	7/2279	55/18866	0.5025843	0.7078347	0.640474	7	TFDP1/YWHAH/BMF/YWHAZ/BCL2/TP53BP2/YWHAQ
Biological Process	GO:0051170	import into nucleus	21/2279	171/18866	0.5029841	0.7081879	0.6407935	21	ANGPT1/SNRPD3/PIK3R1/SMAD3/TNPO1/MBTPS1/E2F3/SUFU/IPO13/FAM53B/JUP/TGFB1/BACH2/NF1/NUP93/ARNTL/CDH1/SNUPN/CABP1/TSC2/GEMIN4
Biological Process	GO:1905952	regulation of lipid localization	21/2279	171/18866	0.5029841	0.7081879	0.6407935	21	PNPLA2/EHD1/PPARG/MYB/MAP2K6/TNF/ABCG1/ATP8A1/PLTP/THBS1/MIR27A/OSBPL6/NR1H2/ITGB3/GHRL/ABCA1/ACACB/FTO/ABHD5/AGTR1/PRKN
Biological Process	GO:0071897	DNA biosynthetic process	24/2279	196/18866	0.5045859	0.7103379	0.642739	24	PPARG/TFDP1/TNKS/POLE/HMBOX1/WRNIP1/TK2/PHB/PARP4/GRHL2/SMG6/PDGFB/ARRB2/NPPC/PDGFRB/ANKRD1/TEX12/PARM1/POLD3/PARP10/CCT6A/POLE4/TK1/POLI
Biological Process	GO:0001508	action potential	17/2279	138/18866	0.5046774	0.7103615	0.6427603	17	KCNE1/KCNAB2/CACNA1C/YWHAH/SLC8A1/GJA5/JUP/BIN1/NOS1AP/SCN1A/GNA11/CNR2/SNTA1/KCNQ1/P2RX1/CNTNAP1/ANK2

Biological Process	GO:0014032	neural crest cell development	10/2279	80/18866	0.5050493	0.7104639	0.642853	10	SEMA6B/NRP2/ANXA6/ZEB2/SEMA4A/SEMA4B/SEMA4D/RDH10/CORO1C/ALDH1A2
Biological Process	GO:0042590	antigen processing and presentation of exogenous peptide antigen via MHC class I	10/2279	80/18866	0.5050493	0.7104639	0.642853	10	CALR/LNPEP/NCF4/HLA-C/HLA-F/NCF2/ITGB5/PSMF1/PSMB7/PSMD13
Biological Process	GO:0048678	response to axon injury	10/2279	80/18866	0.5050493	0.7104639	0.642853	10	KREMEN1/STK24/GIPR/JAM3/GRN/NTRK1/BCL2/CERS2/NDEL1/INPP5F
Biological Process	GO:1900182	positive regulation of protein localization to nucleus	10/2279	80/18866	0.5050493	0.7104639	0.642853	10	TRIM8/PIK3R1/SMAD3/JUP/TGFB1/CDH1/KAT7/CCT6A/PARP9/LIF
Biological Process	GO:0002313	mature B cell differentiation involved in immune response	3/2279	22/18866	0.5064975	0.7105024	0.6428878	3	DLL1/DOCK10/BCL3
Biological Process	GO:0006071	glycerol metabolic process	3/2279	22/18866	0.5064975	0.7105024	0.6428878	3	PGP/PCK2/DYSF
Biological Process	GO:0006085	acetyl-CoA biosynthetic process	3/2279	22/18866	0.5064975	0.7105024	0.6428878	3	ACSS1/ACSS2/ACAT1
Biological Process	GO:0009127	purine nucleoside monophosphate biosynthetic process	3/2279	22/18866	0.5064975	0.7105024	0.6428878	3	AMPD3/LHPP/IMPDH1
Biological Process	GO:0009220	pyrimidine ribonucleotide biosynthetic process	3/2279	22/18866	0.5064975	0.7105024	0.6428878	3	UCK2/AK5/DHODH

Biological Process	GO:0021516	dorsal spinal cord development	3/2279	22/18866	0.5064975	0.7105024	0.6428878	3	DRGX/PAX7/LMO4
Biological Process	GO:0045061	thymic T cell selection	3/2279	22/18866	0.5064975	0.7105024	0.6428878	3	ITPKB/PTPRC/DOCK2
Biological Process	GO:0045663	positive regulation of myoblast differentiation	3/2279	22/18866	0.5064975	0.7105024	0.6428878	3	ILK/PLCB1/MEF2C
Biological Process	GO:0045980	negative regulation of nucleotide metabolic process	3/2279	22/18866	0.5064975	0.7105024	0.6428878	3	HDAC4/CDA/CBFA2T3
Biological Process	GO:0051152	positive regulation of smooth muscle cell differentiation	3/2279	22/18866	0.5064975	0.7105024	0.6428878	3	MIR140/MIR145/ENG
Biological Process	GO:0060487	lung epithelial cell differentiation	3/2279	22/18866	0.5064975	0.7105024	0.6428878	3	CREB1/RBPJ/GRHL2
Biological Process	GO:0060972	left/right pattern formation	3/2279	22/18866	0.5064975	0.7105024	0.6428878	3	PKD1L1/DLL1/NOTCH1
Biological Process	GO:0071359	cellular response to dsRNA	3/2279	22/18866	0.5064975	0.7105024	0.6428878	3	COLEC12/FLOT1/STING1
Biological Process	GO:0071459	protein localization to chromosome, centromeric region	3/2279	22/18866	0.5064975	0.7105024	0.6428878	3	CHAMP1/MIS12/RB1

Biological Process	GO:0071467	cellular response to pH	3/2279	22/18866	0.5064975	0.7105024	0.6428878	3	GPLD1/GNA11/INSRR
Biological Process	GO:0090189	regulation of branching involved in ureteric bud morphogenesis	3/2279	22/18866	0.5064975	0.7105024	0.6428878	3	TACSTD2/PAX2/HOXB7
Biological Process	GO:0140112	extracellular vesicle biogenesis	3/2279	22/18866	0.5064975	0.7105024	0.6428878	3	RAB27A/COPS5/PRKN
Biological Process	GO:1903019	negative regulation of glycoprotein metabolic process	3/2279	22/18866	0.5064975	0.7105024	0.6428878	3	MIR101-2/AGO2/ITM2C
Biological Process	GO:1904753	negative regulation of vascular associated smooth muscle cell migration	3/2279	22/18866	0.5064975	0.7105024	0.6428878	3	MIR140/NFE2L2/MEF2C
Biological Process	GO:1902414	protein localization to cell junction	13/2279	105/18866	0.5068997	0.7108567	0.6432083	13	PRKCZ/BAIAP2/ZDHHC2/ACTN4/CNIH2/NRXN1/JAM3/ACTG1/KIF5C/NRXN2/MPP7/MAP1A/NECTIN1
Biological Process	GO:2001022	positive regulation of response to DNA damage stimulus	13/2279	105/18866	0.5068997	0.7108567	0.6432083	13	FAM168A/SPRED2/SPIDR/NACC2/ZNF385A/FOXM1/ANKRD1/PARP9/PAXIP1/ABRAXAS1/SLF1/FBH1/BABAM2
Biological Process	GO:0071426	ribonucleoprotein complex export from nucleus	16/2279	130/18866	0.5084253	0.7122486	0.6444678	16	NXF1/FIP1L1/CPSF3/SETD2/RAE1/SMG7/PABPN1/ENY2/NUP93/SEC13/SMG6/HHEX/RBM15B/NOL6/CPSF4/PCID2
Biological Process	GO:0006383	transcription by RNA polymerase III	6/2279	47/18866	0.5087169	0.7122486	0.6444678	6	RPTOR/CRCP/ZC3H8/BRF1/CHD8/ZNF76

Biological Process	GO:0007080	mitotic metaphase plate congression	6/2279	47/18866	0.5087169	0.7122486	0.6444678	6	MAD1L1/CHAMP1/CUL3/MIS12/DCTN2/CDCA5
Biological Process	GO:0007140	male meiotic nuclear division	6/2279	47/18866	0.5087169	0.7122486	0.6444678	6	MOV10L1/BRCA2/MEIOB/FANCA/CCNA1/DMC1
Biological Process	GO:0022602	ovulation cycle process	6/2279	47/18866	0.5087169	0.7122486	0.6444678	6	MAP2K6/ARRB1/BMPR1B/ARRB2/SLIT3/INHBA
Biological Process	GO:0043268	positive regulation of potassium ion transport	6/2279	47/18866	0.5087169	0.7122486	0.6444678	6	KCNE1/DNM2/NOS1AP/KCNQ1/ANO6/ANK2
Biological Process	GO:0048483	autonomic nervous system development	6/2279	47/18866	0.5087169	0.7122486	0.6444678	6	NRP2/PHOX2A/NF1/NTRK1/HLX/HES3
Biological Process	GO:0048599	oocyte development	6/2279	47/18866	0.5087169	0.7122486	0.6444678	6	BRCA2/KMT2D/PPP2R1A/BCL2/DMC1/NPPC
Biological Process	GO:0070849	response to epidermal growth factor	6/2279	47/18866	0.5087169	0.7122486	0.6444678	6	DUSP22/BAIAP2/PAX2/DUSP3/PLCG1/MARS1
Biological Process	GO:1904738	vascular associated smooth muscle cell migration	6/2279	47/18866	0.5087169	0.7122486	0.6444678	6	SSH1/MIR140/MIR143/PDGFB/NFE2L2/MEF2C
Biological Process	GO:1904752	regulation of vascular associated smooth muscle cell migration	6/2279	47/18866	0.5087169	0.7122486	0.6444678	6	SSH1/MIR140/MIR143/PDGFB/NFE2L2/MEF2C

Biological Process	GO:0045089	positive regulation of innate immune response	26/2279	213/18866	0.5089501	0.7124702	0.6446683	26	NLRC4/AIM2/CLEC4C/CCL5/FPR2/MNDA/CD226/HLA-F/MUC12/FCN1/TRIM5/MUC20/BCL10/PSMF1/NLRC5/CREBBP/IL18RAP/PSMB7/CADM1/RELA/FBXW11/HEXIM1/PARP9/FADD/PSMD13/STING1
Biological Process	GO:1990868	response to chemokine	12/2279	97/18866	0.5113462	0.7149285	0.6468927	12	CCR2/CCL5/PADI2/ACKR2/DOCK8/CCR9/CCL20/CXCR1/SLIT3/CCL22/CX3CR1/ACKR1
Biological Process	GO:1990869	cellular response to chemokine	12/2279	97/18866	0.5113462	0.7149285	0.6468927	12	CCR2/CCL5/PADI2/ACKR2/DOCK8/CCR9/CCL20/CXCR1/SLIT3/CCL22/CX3CR1/ACKR1
Biological Process	GO:0006457	protein folding	28/2279	230/18866	0.5129966	0.7149285	0.6468927	28	TBCD/P4HB/CALR/FKBP5/AMFR/DNAJB13/GNAZ/AIP/GNAI2/PDIA5/HSPA4L/CLU/CLGN/GNAT2/DNAJB6/GNB3/GRN/UNC45A/GNG2/GNAO1/TOR1B/NPPC/BAG5/CCT6A/PFDN2/PFDN6/AHSA2P/P3H1
Biological Process	GO:0007189	adenylate cyclase-activating G protein-coupled receptor signaling pathway	18/2279	147/18866	0.5134033	0.7149285	0.6468927	18	PDE4D/ADCY2/AKAP13/ADCY9/PRKCA/GNAI2/MRAP/RAPGEF2/ABCA1/CNR2/ADCY4/S1PR2/GNG2/LPAR2/S1PR4/GPR4/ADGRG3/ADGRE3
Biological Process	GO:0015698	inorganic anion transport	21/2279	172/18866	0.5143193	0.7149285	0.6468927	21	CLCN6/SLC11A1/FXYD1/ANO7/TG/LRRC8C/P2RY6/FGFR1/SLC13A4/SLC26A1/SLC22A11/SLC37A1/SLC1A3/LRRC8B/ANO10/CEBPB/ANO6/SLC12A1/ANO8/SLC12A7/PACC1
Biological Process	GO:0050806	positive regulation of synaptic transmission	21/2279	172/18866	0.5143193	0.7149285	0.6468927	21	PRKCZ/SSH1/CREB1/CCR2/TNF/BAIAP2/ZDHHC2/APP/AKAP5/NF1/NRXN1/ARRB2/NTRK1/SLC1A3/DNM1L/SHANK2/PRKCE/FLOT1/DTNBP1/CX3CR1/CUX2
Biological Process	GO:0043687	post-translational protein modification	44/2279	363/18866	0.5148159	0.7149285	0.6468927	44	LTBP1/PNPLA2/P4HB/SERPINA1/APLP2/RAB2A/KLHL25/MBTPS1/GPLD1/MGAT4A/FBXO7/WIPI2/ZBTB16/KCTD7/CUL3/CCDC8/APP/FBXW2/MIA3/SDC2/SPARCL1/FBXO40/LMO7/COPS5/FBXL19/ASB1/DCAF5/PSMF1/FBXL13/FBXL18/COPS3/KLHL21/FN3K/PSMB7/EPAS1/FAM20C/FBXW11/WDTC1/ANO8/NAE1/DCAF17/UCHL3/PSMD13/P3H1
Biological Process	GO:0006694	steroid biosynthetic process	24/2279	197/18866	0.5151807	0.7149285	0.6468927	24	PRKAG2/GFI1/IDI1/VDR/AMACR/MBTPS1/TNF/ABCG1/AKR1D1/HSD17B2/OSBPL6/FDX1/DGKQ/SF1/POR/ACACB/ACBD3/ACOX2/ACACA/HMGCS1/SCD/SREBF1/HSD11B2/PRKAA1

Biological Process	GO:0002066	columnar/cuboidal epithelial cell development	8/2279	64/18866	0.5158728	0.7149285	0.6468927	8	RHEB/RFX3/DLL1/FGFR1/RARA/RARG/ARNTL/TMC1
Biological Process	GO:0002437	inflammatory response to antigenic stimulus	8/2279	64/18866	0.5158728	0.7149285	0.6468927	8	ELANE/FUT7/IL10/TNF/RBPJ/IL1RN/NOTCH1/CD68
Biological Process	GO:0009584	detection of visible light	8/2279	64/18866	0.5158728	0.7149285	0.6468927	8	PDE6A/CACNA2D4/CRB1/CCDC66/GNAT2/GNA11/GRK7/EYS
Biological Process	GO:1900449	regulation of glutamate receptor signaling pathway	8/2279	64/18866	0.5158728	0.7149285	0.6468927	8	CCR2/APP/CRHBP/CNIH2/MAPK8IP2/GSG1L/MEF2C/SHANK2
Biological Process	GO:0001825	blastocyst formation	5/2279	39/18866	0.516046	0.7149285	0.6468927	5	RTN4/CUL3/SP3/KDM4C/NODAL
Biological Process	GO:0007190	activation of adenylate cyclase activity	5/2279	39/18866	0.516046	0.7149285	0.6468927	5	ADCY2/ADCY9/GIPR/ADCY4/VIPR2
Biological Process	GO:0032660	regulation of interleukin-17 production	5/2279	39/18866	0.516046	0.7149285	0.6468927	5	LY9/TGFB1/SLC7A5/NOD2/VSIR
Biological Process	GO:0033146	regulation of intracellular estrogen receptor signaling pathway	5/2279	39/18866	0.516046	0.7149285	0.6468927	5	RUNX1/LBH/ZNF366/AXIN1/KMT2D
Biological Process	GO:0033683	nucleotide-excision repair, DNA incision	5/2279	39/18866	0.516046	0.7149285	0.6468927	5	NTHL1/ERCC1/POLD3/XPA/ERCC2

Biological Process	GO:0045022	early endosome to late endosome transport	5/2279	39/18866	0.516046	0.7149285	0.6468927	5	LMTK2/SNX3/HOOK2/WDR81/EZR
Biological Process	GO:0046326	positive regulation of glucose import	5/2279	39/18866	0.516046	0.7149285	0.6468927	5	PIK3R1/SLC1A2/PRKCI/NFE2L2/CLTCL1
Biological Process	GO:0051931	regulation of sensory perception	5/2279	39/18866	0.516046	0.7149285	0.6468927	5	MGLL/IL10/COMT/F2R/ACP3
Biological Process	GO:0060416	response to growth hormone	5/2279	39/18866	0.516046	0.7149285	0.6468927	5	PIK3R1/STAT5A/PXN/GHRL/MBD5
Biological Process	GO:0071709	membrane assembly	5/2279	39/18866	0.516046	0.7149285	0.6468927	5	PACSIN2/NRXN1/NRXN2/MIR138-2/FLOT1
Biological Process	GO:1901068	guanosine-containing compound metabolic process	5/2279	39/18866	0.516046	0.7149285	0.6468927	5	AMPD3/TJP2/GUK1/GMPR2/IMPDH1
Biological Process	GO:0014033	neural crest cell differentiation	11/2279	89/18866	0.5161956	0.7149285	0.6468927	11	SEMA6B/NRP2/ANXA6/ZEB2/SEMA4A/SEMA4B/SEMA4D/RDH10/CORO1C/MEF2C/ALDH1A2
Biological Process	GO:0061053	somite development	11/2279	89/18866	0.5161956	0.7149285	0.6468927	11	SMAD3/ZEB2/LOXL3/DLL1/RBPJ/MIB1/WNT11/MTHFD1/NOTCH1/TMED2/ALDH1A2
Biological Process	GO:0010466	negative regulation of peptidase activity	33/2279	272/18866	0.5175198	0.7149285	0.6468927	33	SLPI/CSTA/TIMP2/SORL1/SERPINA1/SPOCK2/RPS6KA1/APLP2/SERPINB11/ARRB1/SPINT2/MIR199A1/TNF/MICAL1/SERPINB2/BIN1/THBS1/APP/UBE2O/MIR24-2/PAX2/AQP1/POR/DNAJB6/PSMF1/CRIM1/NOL3/ARRB2/MIR199A2/PIH1D1/LTF/COL28A1/PCID2

Biological Process	GO:0007259	receptor signaling pathway via JAK-STAT	20/2279	164/18866	0.5178804	0.7149285	0.6468927	20	IL10/CCR2/PIGU/TNF/CCL5/CSF1R/STAT5A/TNFRSF1A/FER/PTPRC/IL6R/PPP2R1A/NOTCH1/GGNBP2/F2R/BCL3/INPP5F/CRLF1/PARP9/LIF
Biological Process	GO:0002475	antigen processing and presentation via MHC class Ib	2/2279	14/18866	0.517964	0.7149285	0.6468927	2	HLA-F/AP3D1
Biological Process	GO:0002829	negative regulation of type 2 immune response	2/2279	14/18866	0.517964	0.7149285	0.6468927	2	CCR2/HLX
Biological Process	GO:0003306	Wnt signaling pathway involved in heart development	2/2279	14/18866	0.517964	0.7149285	0.6468927	2	RBPJ/WNT11
Biological Process	GO:0006244	pyrimidine nucleotide catabolic process	2/2279	14/18866	0.517964	0.7149285	0.6468927	2	UNG/NTHL1
Biological Process	GO:0006999	nuclear pore organization	2/2279	14/18866	0.517964	0.7149285	0.6468927	2	RTN4/NUP93
Biological Process	GO:0007100	mitotic centrosome separation	2/2279	14/18866	0.517964	0.7149285	0.6468927	2	NDE1/NDEL1
Biological Process	GO:0007135	meiosis II	2/2279	14/18866	0.517964	0.7149285	0.6468927	2	PPP2R1A/RAD21L1
Biological Process	GO:0009111	vitamin catabolic process	2/2279	14/18866	0.517964	0.7149285	0.6468927	2	MTHFS/CYP4F3

Biological Process	GO:0009214	cyclic nucleotide catabolic process	2/2279	14/18866	0.517964	0.7149285	0.6468927	2	PDE4D/PDE9A
Biological Process	GO:0010752	regulation of cGMP-mediated signaling	2/2279	14/18866	0.517964	0.7149285	0.6468927	2	THBS1/NPPC
Biological Process	GO:0010935	regulation of macrophage cytokine production	2/2279	14/18866	0.517964	0.7149285	0.6468927	2	SPON2/TGFB1
Biological Process	GO:0014819	regulation of skeletal muscle contraction	2/2279	14/18866	0.517964	0.7149285	0.6468927	2	GSTO1/CASQ1
Biological Process	GO:0014874	response to stimulus involved in regulation of muscle adaptation	2/2279	14/18866	0.517964	0.7149285	0.6468927	2	HDAC4/CASQ1
Biological Process	GO:0018410	C-terminal protein amino acid modification	2/2279	14/18866	0.517964	0.7149285	0.6468927	2	GPLD1/WIPI2
Biological Process	GO:0021527	spinal cord association neuron differentiation	2/2279	14/18866	0.517964	0.7149285	0.6468927	2	PAX7/LMO4
Biological Process	GO:0030207	chondroitin sulfate catabolic process	2/2279	14/18866	0.517964	0.7149285	0.6468927	2	HEXB/ARSB
Biological Process	GO:0034356	NAD biosynthesis via nicotinamide riboside salvage pathway	2/2279	14/18866	0.517964	0.7149285	0.6468927	2	PARP10/PARP9

Biological Process	GO:0035627	ceramide transport	2/2279	14/18866	0.517964	0.7149285	0.6468927	2	PLTP/SGPP1
Biological Process	GO:0035751	regulation of lysosomal lumen pH	2/2279	14/18866	0.517964	0.7149285	0.6468927	2	GRN/CLN5
Biological Process	GO:0035791	platelet-derived growth factor receptor-beta signaling pathway	2/2279	14/18866	0.517964	0.7149285	0.6468927	2	PDGFB/PDGFRB
Biological Process	GO:0036092	phosphatidylinositol-3-phosphate biosynthetic process	2/2279	14/18866	0.517964	0.7149285	0.6468927	2	PIK3CD/INPP4A
Biological Process	GO:0036295	cellular response to increased oxygen levels	2/2279	14/18866	0.517964	0.7149285	0.6468927	2	PPARG/FOXO1
Biological Process	GO:0042762	regulation of sulfur metabolic process	2/2279	14/18866	0.517964	0.7149285	0.6468927	2	COMT/NFE2L2
Biological Process	GO:0043173	nucleotide salvage	2/2279	14/18866	0.517964	0.7149285	0.6468927	2	AMPD3/UCK2
Biological Process	GO:0043650	dicarboxylic acid biosynthetic process	2/2279	14/18866	0.517964	0.7149285	0.6468927	2	MTHFD1L/MTHFD1
Biological Process	GO:0043922	negative regulation by host of viral transcription	2/2279	14/18866	0.517964	0.7149285	0.6468927	2	CCL5/HDAC1

Biological Process	GO:0044793	negative regulation by host of viral process	2/2279	14/18866	0.517964	0.7149285	0.6468927	2	LTF/PRKN
Biological Process	GO:0044794	positive regulation by host of viral process	2/2279	14/18866	0.517964	0.7149285	0.6468927	2	CSF1R/PC
Biological Process	GO:0045176	apical protein localization	2/2279	14/18866	0.517964	0.7149285	0.6468927	2	SHROOM3/JAM3
Biological Process	GO:0045591	positive regulation of regulatory T cell differentiation	2/2279	14/18866	0.517964	0.7149285	0.6468927	2	LILRB4/VSIR
Biological Process	GO:0045799	positive regulation of chromatin assembly or disassembly	2/2279	14/18866	0.517964	0.7149285	0.6468927	2	ATF7IP/DNMT1
Biological Process	GO:0045898	regulation of RNA polymerase II transcription preinitiation complex assembly	2/2279	14/18866	0.517964	0.7149285	0.6468927	2	CREB1/ATF7IP
Biological Process	GO:0046321	positive regulation of fatty acid oxidation	2/2279	14/18866	0.517964	0.7149285	0.6468927	2	PPARG/CPT1A
Biological Process	GO:0046341	CDP-diacylglycerol metabolic process	2/2279	14/18866	0.517964	0.7149285	0.6468927	2	CDS2/AGPAT5
Biological Process	GO:0046512	sphingosine biosynthetic process	2/2279	14/18866	0.517964	0.7149285	0.6468927	2	SPTLC2/SPHK2

Biological Process	GO:0046520	sphingoid biosynthetic process	2/2279	14/18866	0.517964	0.7149285	0.6468927	2	SPTLC2/SPHK2
Biological Process	GO:0046940	nucleoside monophosphate phosphorylation	2/2279	14/18866	0.517964	0.7149285	0.6468927	2	AK2/AK5
Biological Process	GO:0047484	regulation of response to osmotic stress	2/2279	14/18866	0.517964	0.7149285	0.6468927	2	MLC1/ARHGEF2
Biological Process	GO:0048291	isotype switching to IgG isotypes	2/2279	14/18866	0.517964	0.7149285	0.6468927	2	PTPRC/PAXIP1
Biological Process	GO:0050667	homocysteine metabolic process	2/2279	14/18866	0.517964	0.7149285	0.6468927	2	CBS/COMT
Biological Process	GO:0050930	induction of positive chemotaxis	2/2279	14/18866	0.517964	0.7149285	0.6468927	2	AZU1/IL16
Biological Process	GO:0050966	detection of mechanical stimulus involved in sensory perception of pain	2/2279	14/18866	0.517964	0.7149285	0.6468927	2	SCN1A/NTRK1
Biological Process	GO:0051280	negative regulation of release of sequestered calcium ion into cytosol	2/2279	14/18866	0.517964	0.7149285	0.6468927	2	CASQ2/GSTO1
Biological Process	GO:0051791	medium-chain fatty acid metabolic process	2/2279	14/18866	0.517964	0.7149285	0.6468927	2	ACOT7/ACADM

Biological Process	GO:0060452	positive regulation of cardiac muscle contraction	2/2279	14/18866	0.517964	0.7149285	0.6468927	2	NOS1AP/KCNQ1
Biological Process	GO:0060707	trophoblast giant cell differentiation	2/2279	14/18866	0.517964	0.7149285	0.6468927	2	E2F7/LIF
Biological Process	GO:0060732	positive regulation of inositol phosphate biosynthetic process	2/2279	14/18866	0.517964	0.7149285	0.6468927	2	P2RY6/HRH1
Biological Process	GO:0061051	positive regulation of cell growth involved in cardiac muscle cell development	2/2279	14/18866	0.517964	0.7149285	0.6468927	2	MIR199A1/MIR199A2
Biological Process	GO:0061983	meiosis II cell cycle process	2/2279	14/18866	0.517964	0.7149285	0.6468927	2	PPP2R1A/RAD21L1
Biological Process	GO:0070234	positive regulation of T cell apoptotic process	2/2279	14/18866	0.517964	0.7149285	0.6468927	2	CCL5/ZC3H8
Biological Process	GO:0070307	lens fiber cell development	2/2279	14/18866	0.517964	0.7149285	0.6468927	2	WNT5B/TMOD1
Biological Process	GO:0070486	leukocyte aggregation	2/2279	14/18866	0.517964	0.7149285	0.6468927	2	S100A8/SEMA4D
Biological Process	GO:0070669	response to interleukin-2	2/2279	14/18866	0.517964	0.7149285	0.6468927	2	STAT5A/JAK1

Biological Process	GO:0071236	cellular response to antibiotic	2/2279	14/18866	0.517964	0.7149285	0.6468927	2	MEF2C/CRIP1
Biological Process	GO:0071285	cellular response to lithium ion	2/2279	14/18866	0.517964	0.7149285	0.6468927	2	CALR/CDH1
Biological Process	GO:0086069	bundle of His cell to Purkinje myocyte communication	2/2279	14/18866	0.517964	0.7149285	0.6468927	2	GJA5/JUP
Biological Process	GO:0090308	regulation of DNA methylation-dependent heterochromatin assembly	2/2279	14/18866	0.517964	0.7149285	0.6468927	2	ATF7IP/DNMT1
Biological Process	GO:0110154	RNA decapping	2/2279	14/18866	0.517964	0.7149285	0.6468927	2	LSM1/EDC3
Biological Process	GO:0110156	methylguanosine-cap decapping	2/2279	14/18866	0.517964	0.7149285	0.6468927	2	LSM1/EDC3
Biological Process	GO:1901070	guanosine-containing compound biosynthetic process	2/2279	14/18866	0.517964	0.7149285	0.6468927	2	GUK1/IMPDH1
Biological Process	GO:1901386	negative regulation of voltage-gated calcium channel activity	2/2279	14/18866	0.517964	0.7149285	0.6468927	2	DYSF/CBARP
Biological Process	GO:1901678	iron coordination entity transport	2/2279	14/18866	0.517964	0.7149285	0.6468927	2	LCN2/LTF

Biological Process	GO:1905205	positive regulation of connective tissue replacement	2/2279	14/18866	0.517964	0.7149285	0.6468927	2	MIR199A1/MIR199A2
Biological Process	GO:1990822	basic amino acid transmembrane transport	2/2279	14/18866	0.517964	0.7149285	0.6468927	2	SLC7A7/SLC7A1
Biological Process	GO:0019395	fatty acid oxidation	13/2279	106/18866	0.5213095	0.7149285	0.6468927	13	PRKAG2/PPARG/MFSD2A/AMACR/ACOXL/ACADM/POR/ACACB/ACOX2/ACAT1/CPT1A/ACADVL/PRKAA1
Biological Process	GO:0046928	regulation of neurotransmitter secretion	13/2279	106/18866	0.5213095	0.7149285	0.6468927	13	RAP1B/GIT1/APBA2/PRKCB/MCTP2/NF1/VPS18/CHRM2/P2RX1/DNM1L/MEF2C/DTNBP1/PRKN
Biological Process	GO:0071166	ribonucleoprotein complex localization	16/2279	131/18866	0.521389	0.7149285	0.6468927	16	NXF1/FIP1L1/CPSF3/SETD2/RAE1/SMG7/PABPN1/ENY2/NUP93/SEC13/SMG6/HHEX/RBM15B/NOL6/CPSF4/PCID2
Biological Process	GO:0010332	response to gamma radiation	7/2279	56/18866	0.52243	0.7149285	0.6468927	7	BRCA2/PTPRC/BCL2/CHEK2/BCL2L1/PRKAA1/MAP3K20
Biological Process	GO:1904645	response to amyloid-beta	7/2279	56/18866	0.52243	0.7149285	0.6468927	7	MIR140/ABCC1/FPR2/APP/IGF1R/DNM1/CACNA1B
Biological Process	GO:0006221	pyrimidine nucleotide biosynthetic process	4/2279	31/18866	0.5252142	0.7149285	0.6468927	4	UCK2/AK5/DCTD/DHODH
Biological Process	GO:0006356	regulation of transcription by RNA polymerase I	4/2279	31/18866	0.5252142	0.7149285	0.6468927	4	WDR43/PIH1D1/MARS1/MACROH2A2

Biological Process	GO:0008045	motor neuron axon guidance	4/2279	31/18866	0.5252142	0.7149285	0.6468927	4	RHOH/RHOG/KIF5C/SLIT1
Biological Process	GO:0014072	response to isoquinoline alkaloid	4/2279	31/18866	0.5252142	0.7149285	0.6468927	4	PPP1R1B/PRKCE/RELA/FADD
Biological Process	GO:0018196	peptidyl-asparagine modification	4/2279	31/18866	0.5252142	0.7149285	0.6468927	4	RPN1/MGAT5/ASGR2/ST6GAL1
Biological Process	GO:0021799	cerebral cortex radially oriented cell migration	4/2279	31/18866	0.5252142	0.7149285	0.6468927	4	RTN4/DISC1/ZMIZ1/NDEL1
Biological Process	GO:0031114	regulation of microtubule depolymerization	4/2279	31/18866	0.5252142	0.7149285	0.6468927	4	NAV3/MAP1A/ARHGEF2/SPEF1
Biological Process	GO:0043278	response to morphine	4/2279	31/18866	0.5252142	0.7149285	0.6468927	4	PPP1R1B/PRKCE/RELA/FADD
Biological Process	GO:0048710	regulation of astrocyte differentiation	4/2279	31/18866	0.5252142	0.7149285	0.6468927	4	BIN1/NF1/NOTCH1/LIF
Biological Process	GO:0060914	heart formation	4/2279	31/18866	0.5252142	0.7149285	0.6468927	4	RBPJ/WNT11/NOTCH1/MEF2C
Biological Process	GO:0061842	microtubule organizing center localization	4/2279	31/18866	0.5252142	0.7149285	0.6468927	4	NDE1/BICD2/NDEL1/EZR

Biological Process	GO:0071466	cellular response to xenobiotic stimulus	15/2279	123/18866	0.5257833	0.7149285	0.6468927	15	NR1I2/ACSL1/GGT1/LPO/ABCC2/AIP/SULT1A2/POR/MGST2/GUK1/RB1/GSTO1/EPHX1/GSTO2/MTARC1
Biological Process	GO:0044728	DNA methylation or demethylation	12/2279	98/18866	0.5263144	0.7149285	0.6468927	12	PIWIL4/MOV10L1/GATAD2A/EHMT2/KDM1B/ATF7IP/GRHL2/FTO/TET2/MTA2/DNMT1/MORC1
Biological Process	GO:0072401	signal transduction involved in DNA integrity checkpoint	9/2279	73/18866	0.527488	0.7149285	0.6468927	9	FZR1/TFDP1/E2F7/PCBP4/ZNF385A/ARID3A/CHEK2/ABRAXAS1/BABAM2
Biological Process	GO:0072422	signal transduction involved in DNA damage checkpoint	9/2279	73/18866	0.527488	0.7149285	0.6468927	9	FZR1/TFDP1/E2F7/PCBP4/ZNF385A/ARID3A/CHEK2/ABRAXAS1/BABAM2
Biological Process	GO:1901292	nucleoside phosphate catabolic process	9/2279	73/18866	0.527488	0.7149285	0.6468927	9	PDE4D/AMPD3/NUDT3/ACOT7/UNG/NTHL1/PDE9A/PNP/ACAT1
Biological Process	GO:0051346	negative regulation of hydrolase activity	57/2279	473/18866	0.5295259	0.7149285	0.6468927	57	SLPI/PRKCZ/LMTK2/CSTA/TIMP2/SORL1/SERPINA1/SPOCK2/RPS6KA1/APLP2/RHOH/MGAT5/PPP1R1B/URI1/SERPINB11/PAM16/ARRB1/CABIN1/SPINT2/MIR199A1/TNF/MICAL1/SERPINB2/AIP/BIN1/THBS1/CCDC8/GNAI2/APP/UBE2O/MIR24-2/ELFN2/PHACTR2/PTPRN2/PAX2/SPHK2/FARP1/SEMA4D/AQP1/POR/DNAJB6/MCM2/SH3BP4/PSMF1/CRIM1/NOL3/ARRB2/SORT1/MIR199A2/PIH1D1/LTF/TMED2/COL28A1/WDR81/SPOCD1/PCID2/PTPA
Biological Process	GO:0060485	mesenchyme development	35/2279	290/18866	0.529641	0.7149285	0.6468927	35	SEMA6B/SPRED2/NRP2/SMAD3/LDLRAD4/ANXA6/ZEB2/LOXL3/SEMA4A/RTN4/TGFBR2/SEMA4B/RBPJ/TCF7L2/SMAD7/WNT11/PAX2/FGFR1/TGFB1/SEMA4D/RDH10/TGFB1I1/WWTR1/ENG/BCL2/NOTCH1/CORO1C/GCNT2/MEF2C/NOTCH4/NEDD4/PDGFRB/TEAD2/ALDH1A2/NODAL
Biological Process	GO:0010862	positive regulation of pathway-restricted SMAD protein phosphorylation	6/2279	48/18866	0.530119	0.7149285	0.6468927	6	TGFB1/ENG/RBPMS/GDF6/INHBA/NODAL
Biological Process	GO:0016574	histone ubiquitination	6/2279	48/18866	0.530119	0.7149285	0.6468927	6	UHRF1/OTUB2/OTUB1/PCGF3/TRIP12/RNF40

Biological Process	GO:0032784	regulation of DNA-templated transcription, elongation	6/2279	48/18866	0.530119	0.7149285	0.6468927	6	BRD4/WDR43/RECQL5/SCAF8/KAT7/CDK13
Biological Process	GO:0035088	establishment or maintenance of apical/basal cell polarity	6/2279	48/18866	0.530119	0.7149285	0.6468927	6	CRB1/ILK/WNT11/WDR1/PRKCI/MARK2
Biological Process	GO:0045601	regulation of endothelial cell differentiation	6/2279	48/18866	0.530119	0.7149285	0.6468927	6	TNF/TNFRSF1A/ADD1/NOTCH1/S1PR2/NOTCH4
Biological Process	GO:0051932	synaptic transmission, GABAergic	6/2279	48/18866	0.530119	0.7149285	0.6468927	6	SYN3/NISCH/NF1/CNR2/PLCL1/PRKCE
Biological Process	GO:0055023	positive regulation of cardiac muscle tissue growth	6/2279	48/18866	0.530119	0.7149285	0.6468927	6	MIR199A1/RBPJ/FGFR1/MIR199A2/NOTCH1/MEF2C
Biological Process	GO:0061245	establishment or maintenance of bipolar cell polarity	6/2279	48/18866	0.530119	0.7149285	0.6468927	6	CRB1/ILK/WNT11/WDR1/PRKCI/MARK2
Biological Process	GO:0090382	phagosome maturation	6/2279	48/18866	0.530119	0.7149285	0.6468927	6	RAB31/ATP6V1B2/RAB43/ATP6V0B/ATP6V0C/ATP6V1C1
Biological Process	GO:1900271	regulation of long-term synaptic potentiation	6/2279	48/18866	0.530119	0.7149285	0.6468927	6	CREB1/ZDHHC2/APP/AKAP5/NF1/CX3CR1
Biological Process	GO:0043044	ATP-dependent chromatin remodeling	11/2279	90/18866	0.5317912	0.7149285	0.6468927	11	CECR2/SRCAP/SMARCA2/HDAC1/ARID1B/RSF1/MTA2/SMARCD2/CHD8/CENPM/RUVBL1

Biological Process	GO:0046427	positive regulation of receptor signaling pathway via JAK-STAT	11/2279	90/18866	0.5317912	0.7149285	0.6468927	11	IL10/TNF/CCL5/CSF1R/TNFRSF1A/IL6R/NOTCH1/F2R/CRLF1/PARP9/LIF
Biological Process	GO:0043271	negative regulation of ion transport	19/2279	157/18866	0.5334403	0.7149285	0.6468927	19	TRIM27/KCNE1/THADA/CASQ2/TLR9/BIN1/THBS1/SLC43A2/CAB39/BCL2/GSTO1/PRKCE/SLC43A1/NEDD4/DYSF/HECW2/YWHAQ/CD33/CBARP
Biological Process	GO:0006687	glycosphingolipid metabolic process	8/2279	65/18866	0.5342185	0.7149285	0.6468927	8	HEXB/GALC/ST8SIA6/B4GALT3/FA2H/ESYT1/CERK/PRKAA1
Biological Process	GO:0006940	regulation of smooth muscle contraction	8/2279	65/18866	0.5342185	0.7149285	0.6468927	8	MIR143/MIR145/CTTN/GHRL/CHRM2/P2RX1/F2R/DAPK3
Biological Process	GO:0009206	purine ribonucleoside triphosphate biosynthetic process	8/2279	65/18866	0.5342185	0.7149285	0.6468927	8	PRKAG2/ENO1/VPS9D1/SPHK2/TGFB1/PKM/IMPDH1/ATP5PD
Biological Process	GO:0017156	calcium-ion regulated exocytosis	8/2279	65/18866	0.5342185	0.7149285	0.6468927	8	RAP1B/GNAI2/SYN2/NOTCH1/SYT17/ARHGAP17/RPH3AL/CBARP
Biological Process	GO:0035794	positive regulation of mitochondrial membrane permeability	8/2279	65/18866	0.5342185	0.7149285	0.6468927	8	TFDP1/YWHAH/BMF/SPG7/YWHAZ/BCL2/TP53BP2/YWHAQ
Biological Process	GO:0048247	lymphocyte chemotaxis	8/2279	65/18866	0.5342185	0.7149285	0.6468927	8	PIK3CD/CCR2/CCL5/PADI2/CCL20/C10orf99/CCL27/CCL22
Biological Process	GO:1902807	negative regulation of cell cycle G1/S phase transition	16/2279	132/18866	0.5342608	0.7149285	0.6468927	16	TFDP1/PRMT2/FBXO7/E2F7/CTDSPL/PCBP4/RIPK1/NACC2/ZNF385A/ARID3A/C10orf99/GFI1B/MIR138-2/RB1/BCL2/CHEK2

Biological Process	GO:0043266	regulation of potassium ion transport	13/2279	107/18866	0.5355962	0.7149285	0.6468927	13	KCNE1/KCNAB2/CASQ2/WWP2/DNM2/BIN1/NOS1AP/KCNIP1/CAB39/KCNQ1/NEDD4/ANO6/ANK2
Biological Process	GO:0051963	regulation of synapse assembly	13/2279	107/18866	0.5355962	0.7149285	0.6468927	13	COLQ/EPHB3/SEMA4A/APP/SEMA4D/NRXN1/GHRL/NTRK1/MEF2C/SLIT1/CUX2/EIF4G1/NECTIN1
Biological Process	GO:0090174	organelle membrane fusion	13/2279	107/18866	0.5355962	0.7149285	0.6468927	13	CALR/STX5/VTI1A/ANKFY1/TGFBRAP1/UVRAG/VPS18/VCPIP1/DYSF/TSNARE1/VAV3/IRAG2/RUBCNL
Biological Process	GO:0035725	sodium ion transmembrane transport	21/2279	174/18866	0.5368079	0.7149285	0.6468927	21	TRPM2/FXYD2/YWHAH/SLC24A4/FXYD1/SLC8A1/DNM2/TESC/ACTN4/SCN1A/ASIC4/SNTA1/PRKCE/SLC5A10/NEDD4/ANO6/HECW2/SLC12A1/SLC23A1/FGF14/SLC41A1
Biological Process	GO:0097696	receptor signaling pathway via STAT	21/2279	174/18866	0.5368079	0.7149285	0.6468927	21	IL10/MGAT5/CCR2/PIGU/TNF/CCL5/CSF1R/STAT5A/TNFRSF1A/FER/PTPRC/IL6R/PPP2R1A/NOTCH1/GGNBP2/F2R/BCL3/INPP5F/CRLF1/PARP9/LIF
Biological Process	GO:0000715	nucleotide-excision repair, DNA damage recognition	3/2279	23/18866	0.5375319	0.7149285	0.6468927	3	COPS5/COPS3/XPA
Biological Process	GO:0006590	thyroid hormone generation	3/2279	23/18866	0.5375319	0.7149285	0.6468927	3	TG/CPQ/CTSB
Biological Process	GO:0007035	vacuolar acidification	3/2279	23/18866	0.5375319	0.7149285	0.6468927	3	SLC11A1/GRN/CLN5
Biological Process	GO:0009068	aspartate family amino acid catabolic process	3/2279	23/18866	0.5375319	0.7149285	0.6468927	3	OGDH/MAT1A/CRYM

Biological Process	GO:0010155	regulation of proton transport	3/2279	23/18866	0.5375319	0.7149285	0.6468927	3	TESC/ACTN4/SPHK2
Biological Process	GO:0010226	response to lithium ion	3/2279	23/18866	0.5375319	0.7149285	0.6468927	3	CALR/EIF2B5/CDH1
Biological Process	GO:0010288	response to lead ion	3/2279	23/18866	0.5375319	0.7149285	0.6468927	3	APP/FECH/CAT
Biological Process	GO:0032693	negative regulation of interleukin-10 production	3/2279	23/18866	0.5375319	0.7149285	0.6468927	3	DLL1/TNFRSF21/VSIR
Biological Process	GO:0032799	low-density lipoprotein receptor particle metabolic process	3/2279	23/18866	0.5375319	0.7149285	0.6468927	3	AP2A1/PPARG/ITGB3
Biological Process	GO:0035162	embryonic hemopoiesis	3/2279	23/18866	0.5375319	0.7149285	0.6468927	3	ZFPM1/TGFBR2/STK3
Biological Process	GO:0035812	renal sodium excretion	3/2279	23/18866	0.5375319	0.7149285	0.6468927	3	COMT/GNAI2/AGTR1
Biological Process	GO:0043576	regulation of respiratory gaseous exchange	3/2279	23/18866	0.5375319	0.7149285	0.6468927	3	PHOX2A/MTG2/FTO
Biological Process	GO:0043586	tongue development	3/2279	23/18866	0.5375319	0.7149285	0.6468927	3	HDAC1/PRDM16/NTF4

Biological Process	GO:0046135	pyrimidine nucleoside catabolic process	3/2279	23/18866	0.5375319	0.7149285	0.6468927	3	CDA/DPYS/UPB1
Biological Process	GO:0060479	lung cell differentiation	3/2279	23/18866	0.5375319	0.7149285	0.6468927	3	CREB1/RBPJ/GRHL2
Biological Process	GO:0072677	eosinophil migration	3/2279	23/18866	0.5375319	0.7149285	0.6468927	3	CCL5/HRH1/DAPK2
Biological Process	GO:0097150	neuronal stem cell population maintenance	3/2279	23/18866	0.5375319	0.7149285	0.6468927	3	DLL1/NOTCH1/FOXO1
Biological Process	GO:2000178	negative regulation of neural precursor cell proliferation	3/2279	23/18866	0.5375319	0.7149285	0.6468927	3	SPINT2/ILK/NF1
Biological Process	GO:0009142	nucleoside triphosphate biosynthetic process	10/2279	82/18866	0.5378438	0.7149285	0.6468927	10	PRKAG2/ENO1/VPS9D1/SPHK2/TGFB1/UCK2/PKM/AK5/IMPDH1/ATP5PD
Biological Process	GO:0000019	regulation of mitotic recombination	1/2279	6/18866	0.5381716	0.7149285	0.6468927	1	ERCC2
Biological Process	GO:0000389	mRNA 3'-splice site recognition	1/2279	6/18866	0.5381716	0.7149285	0.6468927	1	SF1
Biological Process	GO:0000957	mitochondrial RNA catabolic process	1/2279	6/18866	0.5381716	0.7149285	0.6468927	1	GRSF1

Biological Process	GO:0001692	histamine metabolic process	1/2279	6/18866	0.5381716	0.7149285	0.6468927	1	HNMT
Biological Process	GO:0001996	positive regulation of heart rate by epinephrine-norepinephrine	1/2279	6/18866	0.5381716	0.7149285	0.6468927	1	PDE4D
Biological Process	GO:0002036	regulation of L-glutamate import across plasma membrane	1/2279	6/18866	0.5381716	0.7149285	0.6468927	1	PER2
Biological Process	GO:0002278	eosinophil activation involved in immune response	1/2279	6/18866	0.5381716	0.7149285	0.6468927	1	CCR2
Biological Process	GO:0002361	CD4-positive, CD25-positive, alpha-beta regulatory T cell differentiation	1/2279	6/18866	0.5381716	0.7149285	0.6468927	1	FUT7
Biological Process	GO:0002447	eosinophil mediated immunity	1/2279	6/18866	0.5381716	0.7149285	0.6468927	1	CCR2
Biological Process	GO:0002476	antigen processing and presentation of endogenous peptide antigen via MHC class Ib	1/2279	6/18866	0.5381716	0.7149285	0.6468927	1	HLA-F
Biological Process	GO:0002666	positive regulation of T cell tolerance induction	1/2279	6/18866	0.5381716	0.7149285	0.6468927	1	TGFBR2
Biological Process	GO:0002775	antimicrobial peptide production	1/2279	6/18866	0.5381716	0.7149285	0.6468927	1	ELANE

Biological Process	GO:0002778	antibacterial peptide production	1/2279	6/18866	0.5381716	0.7149285	0.6468927	1	ELANE
Biological Process	GO:0002860	positive regulation of natural killer cell mediated cytotoxicity directed against tumor cell target	1/2279	6/18866	0.5381716	0.7149285	0.6468927	1	CD226
Biological Process	GO:0003010	voluntary skeletal muscle contraction	1/2279	6/18866	0.5381716	0.7149285	0.6468927	1	MB
Biological Process	GO:0003057	regulation of the force of heart contraction by chemical signal	1/2279	6/18866	0.5381716	0.7149285	0.6468927	1	GRK2
Biological Process	GO:0003096	renal sodium ion transport	1/2279	6/18866	0.5381716	0.7149285	0.6468927	1	SGK1
Biological Process	GO:0003348	cardiac endothelial cell differentiation	1/2279	6/18866	0.5381716	0.7149285	0.6468927	1	NOTCH1
Biological Process	GO:0003402	planar cell polarity pathway involved in axis elongation	1/2279	6/18866	0.5381716	0.7149285	0.6468927	1	WNT11
Biological Process	GO:0005981	regulation of glycogen catabolic process	1/2279	6/18866	0.5381716	0.7149285	0.6468927	1	PPP1CB
Biological Process	GO:0006105	succinate metabolic process	1/2279	6/18866	0.5381716	0.7149285	0.6468927	1	SDHAF3

Biological Process	GO:0006207	'de novo' pyrimidine nucleobase biosynthetic process	1/2279	6/18866	0.5381716	0.7149285	0.6468927	1	DHODH
Biological Process	GO:0006287	base-excision repair, gap-filling	1/2279	6/18866	0.5381716	0.7149285	0.6468927	1	POLE
Biological Process	GO:0006552	leucine catabolic process	1/2279	6/18866	0.5381716	0.7149285	0.6468927	1	HMGCL
Biological Process	GO:0007354	zygotic determination of anterior/posterior axis, embryo	1/2279	6/18866	0.5381716	0.7149285	0.6468927	1	PCSK6
Biological Process	GO:0009125	nucleoside monophosphate catabolic process	1/2279	6/18866	0.5381716	0.7149285	0.6468927	1	AMPD3
Biological Process	GO:0009176	pyrimidine deoxyribonucleoside monophosphate metabolic process	1/2279	6/18866	0.5381716	0.7149285	0.6468927	1	DCTD
Biological Process	GO:0009744	response to sucrose	1/2279	6/18866	0.5381716	0.7149285	0.6468927	1	ERCC1
Biological Process	GO:0009757	hexose mediated signaling	1/2279	6/18866	0.5381716	0.7149285	0.6468927	1	PIH1D1
Biological Process	GO:0010182	sugar mediated signaling pathway	1/2279	6/18866	0.5381716	0.7149285	0.6468927	1	PIH1D1

Biological Process	GO:0010255	glucose mediated signaling pathway	1/2279	6/18866	0.5381716	0.7149285	0.6468927	1	PIH1D1
Biological Process	GO:0010513	positive regulation of phosphatidylinositol biosynthetic process	1/2279	6/18866	0.5381716	0.7149285	0.6468927	1	FPR2
Biological Process	GO:0010754	negative regulation of cGMP-mediated signaling	1/2279	6/18866	0.5381716	0.7149285	0.6468927	1	THBS1
Biological Process	GO:0010793	regulation of mRNA export from nucleus	1/2279	6/18866	0.5381716	0.7149285	0.6468927	1	SETD2
Biological Process	GO:0010983	positive regulation of high-density lipoprotein particle clearance	1/2279	6/18866	0.5381716	0.7149285	0.6468927	1	GPLD1
Biological Process	GO:0010990	regulation of SMAD protein complex assembly	1/2279	6/18866	0.5381716	0.7149285	0.6468927	1	LDLRAD4
Biological Process	GO:0014721	twitch skeletal muscle contraction	1/2279	6/18866	0.5381716	0.7149285	0.6468927	1	MB
Biological Process	GO:0015015	heparan sulfate proteoglycan biosynthetic process, enzymatic modification	1/2279	6/18866	0.5381716	0.7149285	0.6468927	1	HS3ST3B1
Biological Process	GO:0015677	copper ion import	1/2279	6/18866	0.5381716	0.7149285	0.6468927	1	ATP7B

Biological Process	GO:0015712	hexose phosphate transport	1/2279	6/18866	0.5381716	0.7149285	0.6468927	1	SLC37A1
Biological Process	GO:0015734	taurine transport	1/2279	6/18866	0.5381716	0.7149285	0.6468927	1	LRRC8C
Biological Process	GO:0015747	urate transport	1/2279	6/18866	0.5381716	0.7149285	0.6468927	1	SLC2A9
Biological Process	GO:0015760	glucose-6-phosphate transport	1/2279	6/18866	0.5381716	0.7149285	0.6468927	1	SLC37A1
Biological Process	GO:0015961	diadenosine polyphosphate catabolic process	1/2279	6/18866	0.5381716	0.7149285	0.6468927	1	NUDT3
Biological Process	GO:0016255	attachment of GPI anchor to protein	1/2279	6/18866	0.5381716	0.7149285	0.6468927	1	PIGU
Biological Process	GO:0016479	negative regulation of transcription by RNA polymerase I	1/2279	6/18866	0.5381716	0.7149285	0.6468927	1	MACROH2A2
Biological Process	GO:0021631	optic nerve morphogenesis	1/2279	6/18866	0.5381716	0.7149285	0.6468927	1	PAX2
Biological Process	GO:0021869	forebrain ventricular zone progenitor cell division	1/2279	6/18866	0.5381716	0.7149285	0.6468927	1	FGFR1

Biological Process	GO:0021873	forebrain neuroblast division	1/2279	6/18866	0.5381716	0.7149285	0.6468927	1	FGFR1
Biological Process	GO:0022605	mammalian oogenesis stage	1/2279	6/18866	0.5381716	0.7149285	0.6468927	1	BMPR1B
Biological Process	GO:0030240	skeletal muscle thin filament assembly	1/2279	6/18866	0.5381716	0.7149285	0.6468927	1	TTN
Biological Process	GO:0030885	regulation of myeloid dendritic cell activation	1/2279	6/18866	0.5381716	0.7149285	0.6468927	1	IL10
Biological Process	GO:0031087	deadenylation-independent decapping of nuclear-transcribed mRNA	1/2279	6/18866	0.5381716	0.7149285	0.6468927	1	EDC3
Biological Process	GO:0032020	ISG15-protein conjugation	1/2279	6/18866	0.5381716	0.7149285	0.6468927	1	HERC5
Biological Process	GO:0032696	negative regulation of interleukin-13 production	1/2279	6/18866	0.5381716	0.7149285	0.6468927	1	TNFRSF21
Biological Process	GO:0032792	negative regulation of CREB transcription factor activity	1/2279	6/18866	0.5381716	0.7149285	0.6468927	1	ADGRG3
Biological Process	GO:0033299	secretion of lysosomal enzymes	1/2279	6/18866	0.5381716	0.7149285	0.6468927	1	NR1H2

Biological Process	GO:0033634	positive regulation of cell-cell adhesion mediated by integrin	1/2279	6/18866	0.5381716	0.7149285	0.6468927	1	CCL5
Biological Process	GO:0034201	response to oleic acid	1/2279	6/18866	0.5381716	0.7149285	0.6468927	1	ACSL1
Biological Process	GO:0034285	response to disaccharide	1/2279	6/18866	0.5381716	0.7149285	0.6468927	1	ERCC1
Biological Process	GO:0035511	oxidative DNA demethylation	1/2279	6/18866	0.5381716	0.7149285	0.6468927	1	FTO
Biological Process	GO:0035519	protein K29-linked ubiquitination	1/2279	6/18866	0.5381716	0.7149285	0.6468927	1	PRKN
Biological Process	GO:0035931	mineralocorticoid secretion	1/2279	6/18866	0.5381716	0.7149285	0.6468927	1	AGTR1
Biological Process	GO:0035932	aldosterone secretion	1/2279	6/18866	0.5381716	0.7149285	0.6468927	1	AGTR1
Biological Process	GO:0035964	COPI-coated vesicle budding	1/2279	6/18866	0.5381716	0.7149285	0.6468927	1	TMED2
Biological Process	GO:0036015	response to interleukin-3	1/2279	6/18866	0.5381716	0.7149285	0.6468927	1	CSF2RB

Biological Process	GO:0036016	cellular response to interleukin-3	1/2279	6/18866	0.5381716	0.7149285	0.6468927	1	CSF2RB
Biological Process	GO:0036492	eiF2alpha phosphorylation in response to endoplasmic reticulum stress	1/2279	6/18866	0.5381716	0.7149285	0.6468927	1	NCK2
Biological Process	GO:0042364	water-soluble vitamin biosynthetic process	1/2279	6/18866	0.5381716	0.7149285	0.6468927	1	PDXK
Biological Process	GO:0042636	negative regulation of hair cycle	1/2279	6/18866	0.5381716	0.7149285	0.6468927	1	CDH3
Biological Process	GO:0042661	regulation of mesodermal cell fate specification	1/2279	6/18866	0.5381716	0.7149285	0.6468927	1	FGFR1
Biological Process	GO:0042918	alkanesulfonate transport	1/2279	6/18866	0.5381716	0.7149285	0.6468927	1	LRRC8C
Biological Process	GO:0042939	tripeptide transport	1/2279	6/18866	0.5381716	0.7149285	0.6468927	1	ABCC1
Biological Process	GO:0043305	negative regulation of mast cell degranulation	1/2279	6/18866	0.5381716	0.7149285	0.6468927	1	CD84
Biological Process	GO:0043308	eosinophil degranulation	1/2279	6/18866	0.5381716	0.7149285	0.6468927	1	CCR2

Biological Process	GO:0043553	negative regulation of phosphatidylinositol 3-kinase activity	1/2279	6/18866	0.5381716	0.7149285	0.6468927	1	MIR138-2
Biological Process	GO:0044531	modulation of programmed cell death in other organism	1/2279	6/18866	0.5381716	0.7149285	0.6468927	1	BCL2L1
Biological Process	GO:0044532	modulation of apoptotic process in other organism	1/2279	6/18866	0.5381716	0.7149285	0.6468927	1	BCL2L1
Biological Process	GO:0045046	protein import into peroxisome membrane	1/2279	6/18866	0.5381716	0.7149285	0.6468927	1	PEX26
Biological Process	GO:0045112	integrin biosynthetic process	1/2279	6/18866	0.5381716	0.7149285	0.6468927	1	COL5A1
Biological Process	GO:0045347	negative regulation of MHC class II biosynthetic process	1/2279	6/18866	0.5381716	0.7149285	0.6468927	1	IL10
Biological Process	GO:0045627	positive regulation of T-helper 1 cell differentiation	1/2279	6/18866	0.5381716	0.7149285	0.6468927	1	HLX
Biological Process	GO:0045917	positive regulation of complement activation	1/2279	6/18866	0.5381716	0.7149285	0.6468927	1	PHB
Biological Process	GO:0045938	positive regulation of circadian sleep/wake cycle, sleep	1/2279	6/18866	0.5381716	0.7149285	0.6468927	1	GHRL

Biological Process	GO:0046137	negative regulation of vitamin metabolic process	1/2279	6/18866	0.5381716	0.7149285	0.6468927	1	GFI1
Biological Process	GO:0046826	negative regulation of protein export from nucleus	1/2279	6/18866	0.5381716	0.7149285	0.6468927	1	SP100
Biological Process	GO:0046880	regulation of follicle-stimulating hormone secretion	1/2279	6/18866	0.5381716	0.7149285	0.6468927	1	INHBA
Biological Process	GO:0046881	positive regulation of follicle-stimulating hormone secretion	1/2279	6/18866	0.5381716	0.7149285	0.6468927	1	INHBA
Biological Process	GO:0046958	nonassociative learning	1/2279	6/18866	0.5381716	0.7149285	0.6468927	1	MAPK8IP2
Biological Process	GO:0048200	Golgi transport vesicle coating	1/2279	6/18866	0.5381716	0.7149285	0.6468927	1	TMED2
Biological Process	GO:0048205	COPI coating of Golgi vesicle	1/2279	6/18866	0.5381716	0.7149285	0.6468927	1	TMED2
Biological Process	GO:0050904	diapedesis	1/2279	6/18866	0.5381716	0.7149285	0.6468927	1	FER
Biological Process	GO:0051026	chiasma assembly	1/2279	6/18866	0.5381716	0.7149285	0.6468927	1	RNF212

Biological Process	GO:0051105	regulation of DNA ligation	1/2279	6/18866	0.5381716	0.7149285	0.6468927	1	TFIP11
Biological Process	GO:0051344	negative regulation of cyclic-nucleotide phosphodiesterase activity	1/2279	6/18866	0.5381716	0.7149285	0.6468927	1	AIP
Biological Process	GO:0051415	microtubule nucleation by interphase microtubule organizing center	1/2279	6/18866	0.5381716	0.7149285	0.6468927	1	TUBGCP2
Biological Process	GO:0051454	intracellular pH elevation	1/2279	6/18866	0.5381716	0.7149285	0.6468927	1	BCL2
Biological Process	GO:0051792	medium-chain fatty acid biosynthetic process	1/2279	6/18866	0.5381716	0.7149285	0.6468927	1	ACOT7
Biological Process	GO:0052150	modulation by symbiont of host apoptotic process	1/2279	6/18866	0.5381716	0.7149285	0.6468927	1	BCL2L1
Biological Process	GO:0052248	modulation of programmed cell death in other organism involved in symbiotic interaction	1/2279	6/18866	0.5381716	0.7149285	0.6468927	1	BCL2L1
Biological Process	GO:0052433	modulation by organism of apoptotic process in other organism involved in symbiotic interaction	1/2279	6/18866	0.5381716	0.7149285	0.6468927	1	BCL2L1
Biological Process	GO:0055009	atrial cardiac muscle tissue morphogenesis	1/2279	6/18866	0.5381716	0.7149285	0.6468927	1	ENG

Biological Process	GO:0055059	asymmetric neuroblast division	1/2279	6/18866	0.5381716	0.7149285	0.6468927	1	ARHGEF2
Biological Process	GO:0060264	regulation of respiratory burst involved in inflammatory response	1/2279	6/18866	0.5381716	0.7149285	0.6468927	1	GRN
Biological Process	GO:0060318	definitive erythrocyte differentiation	1/2279	6/18866	0.5381716	0.7149285	0.6468927	1	ZFPM1
Biological Process	GO:0060426	lung vasculature development	1/2279	6/18866	0.5381716	0.7149285	0.6468927	1	LIF
Biological Process	GO:0060440	trachea formation	1/2279	6/18866	0.5381716	0.7149285	0.6468927	1	TGFBR2
Biological Process	GO:0060687	regulation of branching involved in prostate gland morphogenesis	1/2279	6/18866	0.5381716	0.7149285	0.6468927	1	RXRA
Biological Process	GO:0060729	intestinal epithelial structure maintenance	1/2279	6/18866	0.5381716	0.7149285	0.6468927	1	SLC22A5
Biological Process	GO:0060745	mammary gland branching involved in pregnancy	1/2279	6/18866	0.5381716	0.7149285	0.6468927	1	VDR
Biological Process	GO:0060897	neural plate regionalization	1/2279	6/18866	0.5381716	0.7149285	0.6468927	1	SSBP3

Biological Process	GO:0060956	endocardial cell differentiation	1/2279	6/18866	0.5381716	0.7149285	0.6468927	1	NOTCH1
Biological Process	GO:0060979	vasculogenesis involved in coronary vascular morphogenesis	1/2279	6/18866	0.5381716	0.7149285	0.6468927	1	NOTCH1
Biological Process	GO:0061046	regulation of branching involved in lung morphogenesis	1/2279	6/18866	0.5381716	0.7149285	0.6468927	1	TNF
Biological Process	GO:0061196	fungiform papilla development	1/2279	6/18866	0.5381716	0.7149285	0.6468927	1	HDAC1
Biological Process	GO:0061304	retinal blood vessel morphogenesis	1/2279	6/18866	0.5381716	0.7149285	0.6468927	1	LRP5L
Biological Process	GO:0061317	canonical Wnt signaling pathway involved in cardiac muscle cell fate commitment	1/2279	6/18866	0.5381716	0.7149285	0.6468927	1	RBPJ
Biological Process	GO:0061470	T follicular helper cell differentiation	1/2279	6/18866	0.5381716	0.7149285	0.6468927	1	FOXP1
Biological Process	GO:0061550	cranial ganglion development	1/2279	6/18866	0.5381716	0.7149285	0.6468927	1	NRP2
Biological Process	GO:0061757	leukocyte adhesion to arterial endothelial cell	1/2279	6/18866	0.5381716	0.7149285	0.6468927	1	TNF

Biological Process	GO:0070070	proton-transporting V-type ATPase complex assembly	1/2279	6/18866	0.5381716	0.7149285	0.6468927	1	TM9SF4
Biological Process	GO:0070257	positive regulation of mucus secretion	1/2279	6/18866	0.5381716	0.7149285	0.6468927	1	PRKCE
Biological Process	GO:0070432	regulation of nucleotide-binding oligomerization domain containing 2 signaling pathway	1/2279	6/18866	0.5381716	0.7149285	0.6468927	1	TNFAIP3
Biological Process	GO:0070814	hydrogen sulfide biosynthetic process	1/2279	6/18866	0.5381716	0.7149285	0.6468927	1	CBS
Biological Process	GO:0070842	aggresome assembly	1/2279	6/18866	0.5381716	0.7149285	0.6468927	1	PRKN
Biological Process	GO:0071029	nuclear ncRNA surveillance	1/2279	6/18866	0.5381716	0.7149285	0.6468927	1	EXOSC2
Biological Process	GO:0071035	nuclear polyadenylation-dependent rRNA catabolic process	1/2279	6/18866	0.5381716	0.7149285	0.6468927	1	EXOSC2
Biological Process	GO:0071038	nuclear polyadenylation-dependent tRNA catabolic process	1/2279	6/18866	0.5381716	0.7149285	0.6468927	1	EXOSC2
Biological Process	GO:0071046	nuclear polyadenylation-dependent ncRNA catabolic process	1/2279	6/18866	0.5381716	0.7149285	0.6468927	1	EXOSC2

Biological Process	GO:0071321	cellular response to cGMP	1/2279	6/18866	0.5381716	0.7149285	0.6468927	1	RAPGEF2
Biological Process	GO:0071608	macrophage inflammatory protein-1 alpha production	1/2279	6/18866	0.5381716	0.7149285	0.6468927	1	MEFV
Biological Process	GO:0071638	negative regulation of monocyte chemotactic protein-1 production	1/2279	6/18866	0.5381716	0.7149285	0.6468927	1	C1QTNF3
Biological Process	GO:0071640	regulation of macrophage inflammatory protein 1 alpha production	1/2279	6/18866	0.5381716	0.7149285	0.6468927	1	MEFV
Biological Process	GO:0071651	positive regulation of chemokine (C-C motif) ligand 5 production	1/2279	6/18866	0.5381716	0.7149285	0.6468927	1	DEFB124
Biological Process	GO:0071763	nuclear membrane organization	1/2279	6/18866	0.5381716	0.7149285	0.6468927	1	TOR1B
Biological Process	GO:0071896	protein localization to adherens junction	1/2279	6/18866	0.5381716	0.7149285	0.6468927	1	MPP7
Biological Process	GO:0071926	endocannabinoid signaling pathway	1/2279	6/18866	0.5381716	0.7149285	0.6468927	1	MGLL
Biological Process	GO:0072038	mesenchymal stem cell maintenance involved in nephron morphogenesis	1/2279	6/18866	0.5381716	0.7149285	0.6468927	1	PAX2

Biological Process	GO:0072049	comma-shaped body morphogenesis	1/2279	6/18866	0.5381716	0.7149285	0.6468927	1	PDGFRB
Biological Process	GO:0072103	glomerulus vasculature morphogenesis	1/2279	6/18866	0.5381716	0.7149285	0.6468927	1	PDGFRB
Biological Process	GO:0072104	glomerular capillary formation	1/2279	6/18866	0.5381716	0.7149285	0.6468927	1	PDGFRB
Biological Process	GO:0072174	metanephric tubule formation	1/2279	6/18866	0.5381716	0.7149285	0.6468927	1	PAX2
Biological Process	GO:0072284	metanephric S-shaped body morphogenesis	1/2279	6/18866	0.5381716	0.7149285	0.6468927	1	PDGFRB
Biological Process	GO:0072298	regulation of metanephric glomerulus development	1/2279	6/18866	0.5381716	0.7149285	0.6468927	1	PAX2
Biological Process	GO:0072318	clathrin coat disassembly	1/2279	6/18866	0.5381716	0.7149285	0.6468927	1	SH3GL1
Biological Process	GO:0072675	osteoclast fusion	1/2279	6/18866	0.5381716	0.7149285	0.6468927	1	SBNO2
Biological Process	GO:0086045	membrane depolarization during AV node cell action potential	1/2279	6/18866	0.5381716	0.7149285	0.6468927	1	CACNA1C

Biological Process	GO:0086073	bundle of His cell-Purkinje myocyte adhesion involved in cell communication	1/2279	6/18866	0.5381716	0.7149285	0.6468927	1	JUP
Biological Process	GO:0090186	regulation of pancreatic juice secretion	1/2279	6/18866	0.5381716	0.7149285	0.6468927	1	NR1H2
Biological Process	GO:0090230	regulation of centromere complex assembly	1/2279	6/18866	0.5381716	0.7149285	0.6468927	1	RB1
Biological Process	GO:0097021	lymphocyte migration into lymphoid organs	1/2279	6/18866	0.5381716	0.7149285	0.6468927	1	FUT7
Biological Process	GO:0097283	keratinocyte apoptotic process	1/2279	6/18866	0.5381716	0.7149285	0.6468927	1	GSN
Biological Process	GO:0097327	response to antineoplastic agent	1/2279	6/18866	0.5381716	0.7149285	0.6468927	1	ABCC2
Biological Process	GO:0097638	L-arginine import across plasma membrane	1/2279	6/18866	0.5381716	0.7149285	0.6468927	1	SLC7A1
Biological Process	GO:0097680	double-strand break repair via classical nonhomologous end joining	1/2279	6/18866	0.5381716	0.7149285	0.6468927	1	UVRAG
Biological Process	GO:0098838	folate transmembrane transport	1/2279	6/18866	0.5381716	0.7149285	0.6468927	1	SLC19A1

Biological Process	GO:0098880	maintenance of postsynaptic specialization structure	1/2279	6/18866	0.5381716	0.7149285	0.6468927	1		SYNGAP1
Biological Process	GO:0098937	anterograde dendritic transport	1/2279	6/18866	0.5381716	0.7149285	0.6468927	1		KIF5C
Biological Process	GO:0099041	vesicle tethering to Golgi	1/2279	6/18866	0.5381716	0.7149285	0.6468927	1		C17orf75
Biological Process	GO:0106134	positive regulation of cardiac muscle cell contraction	1/2279	6/18866	0.5381716	0.7149285	0.6468927	1		NOS1AP
Biological Process	GO:0110112	regulation of lipid transporter activity	1/2279	6/18866	0.5381716	0.7149285	0.6468927	1		PPARG
Biological Process	GO:1900825	regulation of membrane depolarization during cardiac muscle cell action potential	1/2279	6/18866	0.5381716	0.7149285	0.6468927	1		GJA5
Biological Process	GO:1901166	neural crest cell migration involved in autonomic nervous system development	1/2279	6/18866	0.5381716	0.7149285	0.6468927	1		NRP2
Biological Process	GO:1901374	acetate ester transport	1/2279	6/18866	0.5381716	0.7149285	0.6468927	1		SLC44A4
Biological Process	GO:1901621	negative regulation of smoothened signaling pathway involved in dorsal/ventral neural tube patterning	1/2279	6/18866	0.5381716	0.7149285	0.6468927	1		SUFU

Biological Process	GO:1901727	positive regulation of histone deacetylase activity	1/2279	6/18866	0.5381716	0.7149285	0.6468927	1	C6orf89
Biological Process	GO:1901897	regulation of relaxation of cardiac muscle	1/2279	6/18866	0.5381716	0.7149285	0.6468927	1	PDE4D
Biological Process	GO:1901977	negative regulation of cell cycle checkpoint	1/2279	6/18866	0.5381716	0.7149285	0.6468927	1	CHEK2
Biological Process	GO:1902035	positive regulation of hematopoietic stem cell proliferation	1/2279	6/18866	0.5381716	0.7149285	0.6468927	1	KAT7
Biological Process	GO:1902172	regulation of keratinocyte apoptotic process	1/2279	6/18866	0.5381716	0.7149285	0.6468927	1	GSN
Biological Process	GO:1902218	regulation of intrinsic apoptotic signaling pathway in response to osmotic stress	1/2279	6/18866	0.5381716	0.7149285	0.6468927	1	ARHGEF2
Biological Process	GO:1902338	negative regulation of apoptotic process involved in morphogenesis	1/2279	6/18866	0.5381716	0.7149285	0.6468927	1	PAX2
Biological Process	GO:1902747	negative regulation of lens fiber cell differentiation	1/2279	6/18866	0.5381716	0.7149285	0.6468927	1	SPRED2
Biological Process	GO:1903116	positive regulation of actin filament-based movement	1/2279	6/18866	0.5381716	0.7149285	0.6468927	1	NOS1AP

Biological Process	GO:1903333	negative regulation of protein folding	1/2279	6/18866	0.5381716	0.7149285	0.6468927	1	BAG5
Biological Process	GO:1903348	positive regulation of bicellular tight junction assembly	1/2279	6/18866	0.5381716	0.7149285	0.6468927	1	NPHP4
Biological Process	GO:1903352	L-ornithine transmembrane transport	1/2279	6/18866	0.5381716	0.7149285	0.6468927	1	SLC7A1
Biological Process	GO:1904304	regulation of gastro-intestinal system smooth muscle contraction	1/2279	6/18866	0.5381716	0.7149285	0.6468927	1	GHRL
Biological Process	GO:1904306	positive regulation of gastro-intestinal system smooth muscle contraction	1/2279	6/18866	0.5381716	0.7149285	0.6468927	1	GHRL
Biological Process	GO:1904428	negative regulation of tubulin deacetylation	1/2279	6/18866	0.5381716	0.7149285	0.6468927	1	PRKAA1
Biological Process	GO:1904627	response to phorbol 13-acetate 12-myristate	1/2279	6/18866	0.5381716	0.7149285	0.6468927	1	SPHK2
Biological Process	GO:1904628	cellular response to phorbol 13-acetate 12-myristate	1/2279	6/18866	0.5381716	0.7149285	0.6468927	1	SPHK2
Biological Process	GO:1904746	negative regulation of apoptotic process involved in development	1/2279	6/18866	0.5381716	0.7149285	0.6468927	1	PAX2

Biological Process	GO:1904778	positive regulation of protein localization to cell cortex	1/2279	6/18866	0.5381716	0.7149285	0.6468927	1	NUMA1
Biological Process	GO:1904783	positive regulation of NMDA glutamate receptor activity	1/2279	6/18866	0.5381716	0.7149285	0.6468927	1	CCR2
Biological Process	GO:1904959	regulation of cytochrome-c oxidase activity	1/2279	6/18866	0.5381716	0.7149285	0.6468927	1	SPHK2
Biological Process	GO:1905462	regulation of DNA duplex unwinding	1/2279	6/18866	0.5381716	0.7149285	0.6468927	1	MCM2
Biological Process	GO:1990379	lipid transport across blood-brain barrier	1/2279	6/18866	0.5381716	0.7149285	0.6468927	1	MFSD2A
Biological Process	GO:1990668	vesicle fusion with endoplasmic reticulum-Golgi intermediate compartment (ERGIC) membrane	1/2279	6/18866	0.5381716	0.7149285	0.6468927	1	CALR
Biological Process	GO:2000051	negative regulation of non-canonical Wnt signaling pathway	1/2279	6/18866	0.5381716	0.7149285	0.6468927	1	RNF213
Biological Process	GO:2000138	positive regulation of cell proliferation involved in heart morphogenesis	1/2279	6/18866	0.5381716	0.7149285	0.6468927	1	RBPJ
Biological Process	GO:2000143	negative regulation of DNA-templated transcription, initiation	1/2279	6/18866	0.5381716	0.7149285	0.6468927	1	MORC1

Biological Process	GO:2000328	regulation of T-helper 17 cell lineage commitment	1/2279	6/18866	0.5381716	0.7149285	0.6468927	1	LOXL3
Biological Process	GO:2000371	regulation of DNA topoisomerase (ATP-hydrolyzing) activity	1/2279	6/18866	0.5381716	0.7149285	0.6468927	1	UHRF1
Biological Process	GO:2000373	positive regulation of DNA topoisomerase (ATP-hydrolyzing) activity	1/2279	6/18866	0.5381716	0.7149285	0.6468927	1	UHRF1
Biological Process	GO:2000416	regulation of eosinophil migration	1/2279	6/18866	0.5381716	0.7149285	0.6468927	1	DAPK2
Biological Process	GO:2000481	positive regulation of cAMP-dependent protein kinase activity	1/2279	6/18866	0.5381716	0.7149285	0.6468927	1	RAPGEF2
Biological Process	GO:2000503	positive regulation of natural killer cell chemotaxis	1/2279	6/18866	0.5381716	0.7149285	0.6468927	1	CCL5
Biological Process	GO:2000542	negative regulation of gastrulation	1/2279	6/18866	0.5381716	0.7149285	0.6468927	1	COL5A1
Biological Process	GO:2000618	regulation of histone H4-K16 acetylation	1/2279	6/18866	0.5381716	0.7149285	0.6468927	1	PIH1D1
Biological Process	GO:2000630	positive regulation of miRNA metabolic process	1/2279	6/18866	0.5381716	0.7149285	0.6468927	1	RELA

Biological Process	GO:2000675	negative regulation of type B pancreatic cell apoptotic process	1/2279	6/18866	0.5381716	0.7149285	0.6468927	1	TCF7L2
Biological Process	GO:2000722	regulation of cardiac vascular smooth muscle cell differentiation	1/2279	6/18866	0.5381716	0.7149285	0.6468927	1	MIR145
Biological Process	GO:2000855	regulation of mineralocorticoid secretion	1/2279	6/18866	0.5381716	0.7149285	0.6468927	1	AGTR1
Biological Process	GO:2000858	regulation of aldosterone secretion	1/2279	6/18866	0.5381716	0.7149285	0.6468927	1	AGTR1
Biological Process	GO:2001188	regulation of T cell activation via T cell receptor contact with antigen bound to MHC molecule on antigen presenting cell	1/2279	6/18866	0.5381716	0.7149285	0.6468927	1	HLA-DMB
Biological Process	GO:2001268	negative regulation of cysteine-type endopeptidase activity involved in apoptotic signaling pathway	1/2279	6/18866	0.5381716	0.7149285	0.6468927	1	PIH1D1
Biological Process	GO:0001736	establishment of planar polarity	15/2279	124/18866	0.5390421	0.7154068	0.6473255	15	AP2A1/VANGL1/PRICKLE2/WNT11/SMURF1/PFN1/PSMF1/WDR1/ARRB2/DAAM1/ASTN2/PSMB7/ANKRD6/PSMD13/CPLANE1
Biological Process	GO:0007164	establishment of tissue polarity	15/2279	124/18866	0.5390421	0.7154068	0.6473255	15	AP2A1/VANGL1/PRICKLE2/WNT11/SMURF1/PFN1/PSMF1/WDR1/ARRB2/DAAM1/ASTN2/PSMB7/ANKRD6/PSMD13/CPLANE1
Biological Process	GO:0061982	meiosis I cell cycle process	15/2279	124/18866	0.5390421	0.7154068	0.6473255	15	MOV10L1/BRCA2/EME1/MEIOB/CDC25A/RNF212/CCNA1/RAD21L1/DMC1/ERCC1/SLX4/TEX12/KLHDC3/PSMD13/TERB2

Biological Process	GO:0009072	aromatic amino acid family metabolic process	5/2279	40/18866	0.5394353	0.7154068	0.6473255	5	HAL/HGD/FAH/MTHFD1/HNMT
Biological Process	GO:0016572	histone phosphorylation	5/2279	40/18866	0.5394353	0.7154068	0.6473255	5	BAZ1B/PRKCA/RPS6KA4/PRKCB/PRKAA1
Biological Process	GO:0032467	positive regulation of cytokinesis	5/2279	40/18866	0.5394353	0.7154068	0.6473255	5	CUL3/SVIL/CSPP1/PRKCE/CDC14A
Biological Process	GO:0035735	intraciliary transport involved in cilium assembly	5/2279	40/18866	0.5394353	0.7154068	0.6473255	5	TNPO1/CLUAP1/IFT140/TTC21B/KIFAP3
Biological Process	GO:0042307	positive regulation of protein import into nucleus	5/2279	40/18866	0.5394353	0.7154068	0.6473255	5	PIK3R1/SMAD3/JUP/TGFB1/CDH1
Biological Process	GO:0043902	positive regulation of multi-organism process	5/2279	40/18866	0.5394353	0.7154068	0.6473255	5	HDAC4/AGO2/LHFPL2/PLCB1/INHBA
Biological Process	GO:0045746	negative regulation of Notch signaling pathway	5/2279	40/18866	0.5394353	0.7154068	0.6473255	5	WWP2/ARRB1/EGFL7/DLL1/DLK1
Biological Process	GO:0050892	intestinal absorption	5/2279	40/18866	0.5394353	0.7154068	0.6473255	5	SLC2A5/TJP2/VDR/KCNQ1/EZR
Biological Process	GO:0140353	lipid export from cell	5/2279	40/18866	0.5394353	0.7154068	0.6473255	5	MYB/MAP2K6/GHRL/AGTR1/INHBA

Biological Process	GO:0006626	protein targeting to mitochondrion	12/2279	99/18866	0.5411385	0.7173653	0.6490976	12	PAM16/FBXO7/MIPEP/AIP/USP36/ABLIM3/UBE2J2/FIS1/TIMM44/SREBF1/PRKAA1/PRKN
Biological Process	GO:0010717	regulation of epithelial to mesenchymal transition	12/2279	99/18866	0.5411385	0.7173653	0.6490976	12	SPRED2/SMAD3/LDLRAD4/TGFBR2/TCF7L2/SMAD7/TGFB1/TGFB1I1/WWTR1/ENG/NOTCH1/GCNT2
Biological Process	GO:0019886	antigen processing and presentation of exogenous peptide antigen via MHC class II	12/2279	99/18866	0.5411385	0.7173653	0.6490976	12	HLA-DMB/DCTN1/AP2A1/DNM2/DYNC1H1/CTSD/AP1B1/DCTN2/SEC13/SEC24C/KIFAP3/SEC31A
Biological Process	GO:0010951	negative regulation of endopeptidase activity	31/2279	258/18866	0.5416834	0.7179874	0.6496605	31	SLPI/CSTA/TIMP2/SORL1/SERPINA1/SPOCK2/RPS6KA1/APLP2/SERPINB11/ARRB1/SPINT2/TNF/MICAL1/SERPINB2/BIN1/THBS1/APP/UBE2O/MIR24-2/PAX2/AQP1/POR/DNAJB6/PSMF1/CRIM1/NOL3/ARRB2/PIH1D1/LTF/COL28A1/PCID2
Biological Process	GO:0030520	intracellular estrogen receptor signaling pathway	7/2279	57/18866	0.5419724	0.7182703	0.6499165	7	RUNX1/LBH/PADI2/ZNF366/AXIN1/KMT2D/NCOA4
Biological Process	GO:0009266	response to temperature stimulus	28/2279	233/18866	0.5421935	0.7184631	0.6500909	28	TRPM2/RPTOR/ZNF516/PPARG/RAE1/DRGX/EIF2B5/ABCC2/THBS1/NF1/NUP93/TGFB1I1/WDR47/DNAJB6/SEC13/CREBBP/ARRB2/NTRK1/TRPM3/CPB2/GLRX2/FOXO1/TRPV2/CASQ1/BAG5/SST/ACADVL/PRKAA1
Biological Process	GO:0032392	DNA geometric change	14/2279	116/18866	0.5441829	0.7207977	0.6522034	14	BLM/RTEL1/MCM5/WRNIP1/RECQL5/IGHMBP2/MCM2/SMARCAL1/CHD9/CHD8/RUVBL1/XPA/ERCC2/FBH1
Biological Process	GO:0046916	cellular transition metal ion homeostasis	14/2279	116/18866	0.5441829	0.7207977	0.6522034	14	S100A8/LCN2/LCK/SLC11A1/COX19/TTC7A/SLC11A2/APP/ATOX1/SLC39A13/LTF/AP3D1/ATP7B/NCOA4
Biological Process	GO:0060964	regulation of gene silencing by miRNA	14/2279	116/18866	0.5441829	0.7207977	0.6522034	14	PPARG/NCOR2/RAE1/TNF/POLR2F/AGO1/RIPK1/LIMD1/AGO2/TGFB1/NUP93/SEC13/EIF4G1/H3C1

Biological Process	GO:0006635	fatty acid beta-oxidation	9/2279	74/18866	0.5446128	0.7211661	0.6525367	9	MFSD2A/AMACR/ACOXL/ACADM/ACACB/ACOX2/ACAT1/CPT1A/ACADVL
Biological Process	GO:0072395	signal transduction involved in cell cycle checkpoint	9/2279	74/18866	0.5446128	0.7211661	0.6525367	9	FZR1/TFDP1/E2F7/PCBP4/ZNF385A/ARID3A/CHEK2/ABRAXAS1/BABAM2
Biological Process	GO:0042475	odontogenesis of dentin-containing tooth	11/2279	91/18866	0.547217	0.7243116	0.6553828	11	BSG/CSF3R/SLC24A4/ANKRD11/NFIC/HDAC1/PERP/RUNX2/FOXO1/FAM20C/NECTIN1
Biological Process	GO:0051899	membrane depolarization	11/2279	91/18866	0.547217	0.7243116	0.6553828	11	PRKCZ/CACNA1C/YWHAH/SLC8A1/GJA5/HSH2D/SMAD7/SCN1A/BCL2/CACNA1B/ANK2
Biological Process	GO:0060021	roof of mouth development	11/2279	91/18866	0.547217	0.7243116	0.6553828	11	ARID5B/SKI/LOXL3/EPHB3/TGFBR2/WNT11/PRDM16/MEF2C/ASPH/INHBA/CPLANE1
Biological Process	GO:0006606	protein import into nucleus	18/2279	150/18866	0.5497109	0.7271378	0.6579401	18	ANGPT1/PIK3R1/SMAD3/TNPO1/MBTPS1/E2F3/SUFU/IPO13/FAM53B/JUP/TGFB1/NF1/NUP93/ARNTL/CDH1/SNUPN/CABP1/TSC2
Biological Process	GO:0016525	negative regulation of angiogenesis	23/2279	192/18866	0.550572	0.7271378	0.6579401	23	PPARG/MIR143/MIR145/ITGB1BP1/CCR2/COL4A2/MIR199A1/TNF/AGO1/SEMA4A/THBS1/MIR24-2/EPN2/NF1/GHRL/VASH1/MIR138-2/MIR199A2/NOTCH1/GPR4/HHEX/PDE3B/CX3CR1
Biological Process	GO:0021953	central nervous system neuron differentiation	23/2279	192/18866	0.550572	0.7271378	0.6579401	23	NRP2/CSNK1D/PHOX2A/SUFU/ZEB2/CSF1R/EPHB3/DISC1/ZMIZ1/PAX7/RAPGEF2/CSNK1E/ZNF335/TTC21B/CDH11/NDEL1/LMO4/SPTBN4/NPY/INHBA/TTLL1/TSKU/UNC5D
Biological Process	GO:0021537	telencephalon development	31/2279	259/18866	0.5508478	0.7271378	0.6579401	31	MFSD2A/NDE1/SKI/SLC8A1/BTBD3/ZEB2/CSF1R/EIF2B5/EPHB3/RTN4/DISC1/TRAPPC9/HDAC1/ZMIZ1/AQP1/RARA/NF1/KIRREL3/TACC3/SLC1A2/WDR47/NUMB/NCOA1/PLCB1/CORO1C/SLC2A1/SECISBP2/NDEL1/NPY/INHBA/TSKU

Biological Process	GO:0006402	mRNA catabolic process	45/2279	376/18866	0.5509336	0.7271378	0.6579401	45	RNH1/PRR5L/LSM1/EXOSC2/SLC11A1/ERN1/TNRC6B/PABPC4/TNPO1/SLFN14/PRKCA/AGO1/LARP1/CSDE1/ZC3H12D/PKP1/PCBP4/SAMD4A/SMG7/CTIF/AGO2/EDC3/LSM7/CPEB3/IGF2BP3/SMG6/PSMF1/PPP2R1A/FTO/NBAS/YWHAZ/PSMB7/ZHX2/PAIP1/DIS3L2/TNFSF13/SECISBP2/RPL23A/SYNCRIP/EIF4G1/PSMD13/PCID2/TBRG4/TENT5C/TENT5A
Biological Process	GO:0002673	regulation of acute inflammatory response	6/2279	49/18866	0.551124	0.7271378	0.6579401	6	FUT7/PPARG/NLRP3/TNF/F12/DNASE1L3
Biological Process	GO:0007129	homologous chromosome pairing at meiosis	6/2279	49/18866	0.551124	0.7271378	0.6579401	6	MEIOB/RNF212/RAD21L1/DMC1/TEX12/TERB2
Biological Process	GO:0010171	body morphogenesis	6/2279	49/18866	0.551124	0.7271378	0.6579401	6	ARID5B/CRISPLD2/SKI/ANKRD11/ASPH/SSBP3
Biological Process	GO:0030850	prostate gland development	6/2279	49/18866	0.551124	0.7271378	0.6579401	6	RXRA/RARA/RARG/NOTCH1/UBE3A/CRIP1
Biological Process	GO:0034260	negative regulation of GTPase activity	6/2279	49/18866	0.551124	0.7271378	0.6579401	6	RHOH/ARRB1/PTPRN2/SH3BP4/ARRB2/TMED2
Biological Process	GO:0042059	negative regulation of epidermal growth factor receptor signaling pathway	6/2279	49/18866	0.551124	0.7271378	0.6579401	6	TGFA/ARHGEF7/EPS15L1/PTPRJ/DUSP3/EPS15
Biological Process	GO:0042149	cellular response to glucose starvation	6/2279	49/18866	0.551124	0.7271378	0.6579401	6	PRKAG2/ATG14/NFE2L2/BCL2/SLC2A1/PRKAA1
Biological Process	GO:0072348	sulfur compound transport	6/2279	49/18866	0.551124	0.7271378	0.6579401	6	ABCC1/SLC44A4/LRRC8C/SLC13A4/SLC26A1/SLC33A1

Biological Process	GO:0006506	GPI anchor biosynthetic process	4/2279	32/18866	0.5512655	0.7271378	0.6579401	4	PIGL/PIGU/PIGN/PIGQ
Biological Process	GO:0009164	nucleoside catabolic process	4/2279	32/18866	0.5512655	0.7271378	0.6579401	4	CDA/DPYS/UPB1/PNP
Biological Process	GO:0019359	nicotinamide nucleotide biosynthetic process	4/2279	32/18866	0.5512655	0.7271378	0.6579401	4	NADK/NMNAT3/PARP10/PARP9
Biological Process	GO:0019363	pyridine nucleotide biosynthetic process	4/2279	32/18866	0.5512655	0.7271378	0.6579401	4	NADK/NMNAT3/PARP10/PARP9
Biological Process	GO:0035767	endothelial cell chemotaxis	4/2279	32/18866	0.5512655	0.7271378	0.6579401	4	THBS1/PLEKHG5/FGFR1/NOTCH1
Biological Process	GO:0042744	hydrogen peroxide catabolic process	4/2279	32/18866	0.5512655	0.7271378	0.6579401	4	MPO/LPO/HBB/CAT
Biological Process	GO:0048730	epidermis morphogenesis	4/2279	32/18866	0.5512655	0.7271378	0.6579401	4	KRT17/BCL2/NOTCH1/TGM3
Biological Process	GO:0048741	skeletal muscle fiber development	4/2279	32/18866	0.5512655	0.7271378	0.6579401	4	HDAC4/SKI/BCL2/MYORG
Biological Process	GO:0048841	regulation of axon extension involved in axon guidance	4/2279	32/18866	0.5512655	0.7271378	0.6579401	4	SEMA6B/SEMA4A/SEMA4B/SEMA4D

Biological Process	GO:0051968	positive regulation of synaptic transmission, glutamatergic	4/2279	32/18866	0.5512655	0.7271378	0.6579401	4	CCR2/NRXN1/NTRK1/SHANK2
Biological Process	GO:1902235	regulation of endoplasmic reticulum stress-induced intrinsic apoptotic signaling pathway	4/2279	32/18866	0.5512655	0.7271378	0.6579401	4	NCK2/BCL2L1/GRINA/PRKN
Biological Process	GO:1903955	positive regulation of protein targeting to mitochondrion	4/2279	32/18866	0.5512655	0.7271378	0.6579401	4	USP36/ABLIM3/UBE2J2/PRKAA1
Biological Process	GO:1903578	regulation of ATP metabolic process	15/2279	125/18866	0.552183	0.7281923	0.6588942	15	HDAC4/PRKAG2/ENO1/PFKFB4/RAE1/CBFA2T3/APP/PGAM1/SPHK2/NUP93/SEC13/ESRRB/DNM1L/PFKFB3/PRKAA1
Biological Process	GO:0009145	purine nucleoside triphosphate biosynthetic process	8/2279	66/18866	0.5522949	0.7281923	0.6588942	8	PRKAG2/ENO1/VPS9D1/SPHK2/TGFB1/PKM/IMPDH1/ATP5PD
Biological Process	GO:1901016	regulation of potassium ion transmembrane transporter activity	8/2279	66/18866	0.5522949	0.7281923	0.6588942	8	KCNE1/CASQ2/WWP2/DNM2/NOS1AP/CAB39/NEDD4/ANK2
Biological Process	GO:0007031	peroxisome organization	10/2279	83/18866	0.5539492	0.7287175	0.6593695	10	AMACR/AGXT/CAT/PEX26/ACOX2/FIS1/DNM1L/HMGCL/PEX11B/UBE2D2
Biological Process	GO:0002833	positive regulation of response to biotic stimulus	30/2279	251/18866	0.5542403	0.7287175	0.6593695	30	NLRC4/AIM2/CLEC4C/PRKCA/CCL5/FPR2/MNDA/CD226/HLA-F/MUC12/SCIMP/LY86/FCN1/TRIM5/MUC20/BCL10/PSMF1/GRN/NLRC5/CREBBP/IL18RAP/PSMB7/CADM1/RELA/FBXW11/HEXIM1/PARP9/FADD/PSMD13/STING1
Biological Process	GO:0046425	regulation of receptor signaling pathway via JAK-STAT	17/2279	142/18866	0.5545355	0.7287175	0.6593695	17	IL10/PIGU/TNF/CCL5/CSF1R/TNFRSF1A/PTPRC/IL6R/PPP2R1A/NOTCH1/GGNBP2/F2R/BCL3/INPP5F/CRLF1/PARP9/LIF

Biological Process	GO:0046546	development of primary male sexual characteristics	17/2279	142/18866	0.5545355	0.7287175	0.6593695	17	ARID5B/CSDE1/FANCA/RARA/SF1/NCOA1/LHFPL2/NTRK1/HOXA9/RAD21L1/BCL2/PDGFRB/ERCC1/BCL2L1/HMGCS1/NCOA4/INHBA
Biological Process	GO:0007416	synapse assembly	22/2279	184/18866	0.5546831	0.7287175	0.6593695	22	SPOCK2/COLQ/EPHB3/SEMA4A/APP/FARP1/SEMA4D/NRXN1/KIRREL3/GHRL/NRXN2/NTRK1/CDH1/DNM3/MEF2C/SHANK2/SDK2/SLIT1/CUX2/EIF4G1/DBNL/NECTIN1
Biological Process	GO:0006828	manganese ion transport	2/2279	15/18866	0.5562981	0.7287175	0.6593695	2	SLC11A1/SLC11A2
Biological Process	GO:0009404	toxin metabolic process	2/2279	15/18866	0.5562981	0.7287175	0.6593695	2	DDC/NFE2L2
Biological Process	GO:0009415	response to water	2/2279	15/18866	0.5562981	0.7287175	0.6593695	2	CD9/NTRK1
Biological Process	GO:0015671	oxygen transport	2/2279	15/18866	0.5562981	0.7287175	0.6593695	2	HBB/MB
Biological Process	GO:0018158	protein oxidation	2/2279	15/18866	0.5562981	0.7287175	0.6593695	2	LOXL3/LOXL4
Biological Process	GO:0030238	male sex determination	2/2279	15/18866	0.5562981	0.7287175	0.6593695	2	SF1/INSRR
Biological Process	GO:0031054	pre-miRNA processing	2/2279	15/18866	0.5562981	0.7287175	0.6593695	2	AGO1/AGO2

Biological Process	GO:0032252	secretory granule localization	2/2279	15/18866	0.5562981	0.7287175	0.6593695	2	MYO5A/KIF1B
Biological Process	GO:0032328	alanine transport	2/2279	15/18866	0.5562981	0.7287175	0.6593695	2	SLC36A3/SLC7A8
Biological Process	GO:0032645	regulation of granulocyte macrophage colony-stimulating factor production	2/2279	15/18866	0.5562981	0.7287175	0.6593695	2	CD84/TLR9
Biological Process	GO:0033623	regulation of integrin activation	2/2279	15/18866	0.5562981	0.7287175	0.6593695	2	RAP1B/JAM3
Biological Process	GO:0034134	toll-like receptor 2 signaling pathway	2/2279	15/18866	0.5562981	0.7287175	0.6593695	2	PIK3AP1/TNFAIP3
Biological Process	GO:0034638	phosphatidylcholine catabolic process	2/2279	15/18866	0.5562981	0.7287175	0.6593695	2	PLA2G15/LIPC
Biological Process	GO:0035745	T-helper 2 cell cytokine production	2/2279	15/18866	0.5562981	0.7287175	0.6593695	2	PRKCZ/NLRP3
Biological Process	GO:0038065	collagen-activated signaling pathway	2/2279	15/18866	0.5562981	0.7287175	0.6593695	2	COL4A2/UBASH3B
Biological Process	GO:0042159	lipoprotein catabolic process	2/2279	15/18866	0.5562981	0.7287175	0.6593695	2	CTSD/LYPLA2

Biological Process	GO:0042574	retinal metabolic process	2/2279	15/18866	0.5562981	0.7287175	0.6593695	2	RDH10/ALDH1A2
Biological Process	GO:0042754	negative regulation of circadian rhythm	2/2279	15/18866	0.5562981	0.7287175	0.6593695	2	GHRL/PER2
Biological Process	GO:0042976	activation of Janus kinase activity	2/2279	15/18866	0.5562981	0.7287175	0.6593695	2	CCL5/IL6R
Biological Process	GO:0043144	snoRNA processing	2/2279	15/18866	0.5562981	0.7287175	0.6593695	2	EXOSC2/FBL
Biological Process	GO:0043584	nose development	2/2279	15/18866	0.5562981	0.7287175	0.6593695	2	SKI/RDH10
Biological Process	GO:0045346	regulation of MHC class II biosynthetic process	2/2279	15/18866	0.5562981	0.7287175	0.6593695	2	AZU1/IL10
Biological Process	GO:0045820	negative regulation of glycolytic process	2/2279	15/18866	0.5562981	0.7287175	0.6593695	2	HDAC4/CBFA2T3
Biological Process	GO:0046033	AMP metabolic process	2/2279	15/18866	0.5562981	0.7287175	0.6593695	2	AMPD3/AK2
Biological Process	GO:0046479	glycosphingolipid catabolic process	2/2279	15/18866	0.5562981	0.7287175	0.6593695	2	HEXB/GALC

Biological Process	GO:0048484	enteric nervous system development	2/2279	15/18866	0.5562981	0.7287175	0.6593695	2	PHOX2A/HLX
Biological Process	GO:0050651	dermatan sulfate proteoglycan biosynthetic process	2/2279	15/18866	0.5562981	0.7287175	0.6593695	2	CSGALNACT1/DSE
Biological Process	GO:0051299	centrosome separation	2/2279	15/18866	0.5562981	0.7287175	0.6593695	2	NDE1/NDEL1
Biological Process	GO:0060134	prepulse inhibition	2/2279	15/18866	0.5562981	0.7287175	0.6593695	2	NRXN1/CHD8
Biological Process	GO:0070207	protein homotrimerization	2/2279	15/18866	0.5562981	0.7287175	0.6593695	2	SLC1A5/SLC1A2
Biological Process	GO:0071025	RNA surveillance	2/2279	15/18866	0.5562981	0.7287175	0.6593695	2	EXOSC2/PCID2
Biological Process	GO:0072044	collecting duct development	2/2279	15/18866	0.5562981	0.7287175	0.6593695	2	PAX2/NOTCH1
Biological Process	GO:0072075	metanephric mesenchyme development	2/2279	15/18866	0.5562981	0.7287175	0.6593695	2	PAX2/PDGFRB
Biological Process	GO:1900037	regulation of cellular response to hypoxia	2/2279	15/18866	0.5562981	0.7287175	0.6593695	2	ENO1/NOL3

Biological Process	GO:1900225	regulation of NLRP3 inflammasome complex assembly	2/2279	15/18866	0.5562981	0.7287175	0.6593695	2	MEFV/STMP1
Biological Process	GO:1904464	regulation of matrix metallopeptidase secretion	2/2279	15/18866	0.5562981	0.7287175	0.6593695	2	MIR199A1/MIR199A2
Biological Process	GO:1904874	positive regulation of telomerase RNA localization to Cajal body	2/2279	15/18866	0.5562981	0.7287175	0.6593695	2	RUVBL1/CCT6A
Biological Process	GO:1904889	regulation of excitatory synapse assembly	2/2279	15/18866	0.5562981	0.7287175	0.6593695	2	SEMA4A/NRXN1
Biological Process	GO:1905203	regulation of connective tissue replacement	2/2279	15/18866	0.5562981	0.7287175	0.6593695	2	MIR199A1/MIR199A2
Biological Process	GO:1990773	matrix metallopeptidase secretion	2/2279	15/18866	0.5562981	0.7287175	0.6593695	2	MIR199A1/MIR199A2
Biological Process	GO:2000345	regulation of hepatocyte proliferation	2/2279	15/18866	0.5562981	0.7287175	0.6593695	2	TNFAIP3/CPB2
Biological Process	GO:2000846	regulation of corticosteroid hormone secretion	2/2279	15/18866	0.5562981	0.7287175	0.6593695	2	GHRL/AGTR1
Biological Process	GO:0007623	circadian rhythm	26/2279	218/18866	0.5592427	0.7324738	0.6627683	26	PPARG/CSNK1D/CREB1/KDM2A/PHLPP1/ZFHX3/DDC/HDAC1/MTA1/GNA11/GHRL/CSNK1E/PPP1CB/ARNTL/PER2/NTRK1/PER1/RAI1/FBXL6/UBE3A/USP2/NCOA2/SREBF1/FBXW11/CPT1A/PRKAA1

Biological Process	GO:0006605	protein targeting	53/2279	444/18866	0.5595647	0.7327945	0.6630585	53	SORL1/KCNE1/KIF13B/VTI1A/ZDHHC18/AP4S1/ITGB1BP1/VPS13D/PAM16/ZDHHC14/AMACR/FBXO7/MIPEP/AIP/RABGEF1/RAB27A/USP36/ZDHHC2/CLU/ABLIM3/AKAP5/UBE2J2/ZDHHC7/SMURF1/AGXT/NUMA1/ZDHHC1/CAT/PRKCI/PEX26/PMM2/ITGB2/SORT1/ACOX2/YWHAZ/MICALL1/FIS1/TIMM44/NACAD/HMGCL/NEDD4/MYO1C/MIEF1/AP3D1/SREBF1/RPL23A/YWHAQ/NCOA4/PDZK1/UBE2D2/PRKAA1/SPCS1/PRKN
Biological Process	GO:0045995	regulation of embryonic development	16/2279	134/18866	0.5596809	0.7328457	0.6631048	16	IL10/RBM19/SUFU/DLL1/MBP/FGFR1/IL1RN/STK3/PLCB1/NFE2L2/NOTCH1/COL5A1/TRIP12/MYADM/NODAL/OTX2
Biological Process	GO:0071248	cellular response to metal ion	23/2279	193/18866	0.5611236	0.7342766	0.6643996	23	TRPM2/CPNE6/CALR/GSN/CPNE2/CREB1/ITPKB/SLFN14/GPLD1/CPNE5/APP/CPNE1/MTF1/CRHBP/AQP1/NFE2L2/CDH1/ADD1/MEF2C/SYT17/SLC41A1/PRKAA1/PRKN
Biological Process	GO:0031294	lymphocyte costimulation	7/2279	58/18866	0.5611756	0.7342766	0.6643996	7	LCK/PIK3R1/GRAP2/TNFSF13B/PTPN6/CD320/CSK
Biological Process	GO:0046128	purine ribonucleoside metabolic process	7/2279	58/18866	0.5611756	0.7342766	0.6643996	7	AMPD3/TJP2/PNP/GUK1/GMPR2/IMPDH1/ACP3
Biological Process	GO:0051055	negative regulation of lipid biosynthetic process	7/2279	58/18866	0.5611756	0.7342766	0.6643996	7	GFI1/LPCAT1/ORMDL3/PDGFB/WDTC1/ACADVL/PRKAA1
Biological Process	GO:0002479	antigen processing and presentation of exogenous peptide antigen via MHC class I, TAP-dependent	9/2279	75/18866	0.5614941	0.7342766	0.6643996	9	CALR/NCF4/HLA-C/HLA-F/NCF2/ITGB5/PSMF1/PSMB7/PSMD13
Biological Process	GO:0009064	glutamine family amino acid metabolic process	9/2279	75/18866	0.5614941	0.7342766	0.6643996	9	HAL/GGT1/MTHFS/ALDH4A1/FAH/SLC7A7/ART4/GFPT2/DGLUCY
Biological Process	GO:0015909	long-chain fatty acid transport	9/2279	75/18866	0.5614941	0.7342766	0.6643996	9	PRKAG2/PPARG/ACSL1/MFSD2A/THBS1/ACACB/ACACA/SLC2A1/CPT1A

Biological Process	GO:0031100	animal organ regeneration	9/2279	75/18866	0.5614941	0.7342766	0.6643996	9	IL10/PPARG/TGFBR2/PKM/NOTCH1/CPB2/CEBPB/PTPRU/LPIN1
Biological Process	GO:0035019	somatic stem cell population maintenance	9/2279	75/18866	0.5614941	0.7342766	0.6643996	9	FOXP1/MIR145/SKI/POLR2F/LBH/RBPJ/PAX2/PRDM16/ZHX2
Biological Process	GO:0050871	positive regulation of B cell activation	18/2279	151/18866	0.5616239	0.7342766	0.6643996	18	INPP5D/NFATC2/TNFSF13B/TLR9/IGHV6-1/TGFB1/PTPRC/CD320/NOD2/BCL2/TP53BP1/MEF2C/TNFSF13/PELI1/PCID2/PAXIP1/VAV3/NSD2
Biological Process	GO:1904892	regulation of receptor signaling pathway via STAT	18/2279	151/18866	0.5616239	0.7342766	0.6643996	18	IL10/MGAT5/PIGU/TNF/CCL5/CSF1R/TNFRSF1A/PTPRC/IL6R/PPP2R1A/NOTCH1/GGNBP2/F2R/BCL3/INPP5F/CRLF1/PARP9/LIF
Biological Process	GO:0098742	cell-cell adhesion via plasma-membrane adhesion molecules	33/2279	277/18866	0.5620907	0.7344325	0.6645406	33	BSG/IL10/CADM3/CD177/CRB1/CEACAM6/CDH23/CD84/TGFBR2/MBP/CDH3/SPARCL1/IL1RN/NRXN1/KIRREL3/CDHR2/DSCAML1/CDH4/ITGB2/CDH1/MYOT/CDHR1/SDK2/KIFAP3/PCDH12/CADM1/CDH11/MYADM/UNC5D/CEACAM5/JAML/NECTIN1/NECTIN4
Biological Process	GO:0002251	organ or tissue specific immune response	5/2279	41/18866	0.5622842	0.7344325	0.6645406	5	DEFA4/RNASE3/DEFB1/IL6R/LTF
Biological Process	GO:0021532	neural tube patterning	5/2279	41/18866	0.5622842	0.7344325	0.6645406	5	SUFU/PAX7/IFT140/SSBP3/HES3
Biological Process	GO:0051354	negative regulation of oxidoreductase activity	5/2279	41/18866	0.5622842	0.7344325	0.6645406	5	GFI1/CNR2/MIR138-2/NOSIP/PRKN
Biological Process	GO:0071985	multivesicular body sorting pathway	5/2279	41/18866	0.5622842	0.7344325	0.6645406	5	LYST/RAB27A/UVRAG/SORT1/MVB12A

Biological Process	GO:2000008	regulation of protein localization to cell surface	5/2279	41/18866	0.5622842	0.7344325	0.6645406	5	KCNAB2/TM9SF4/TNF/ASTN2/MAP1A
Biological Process	GO:2000826	regulation of heart morphogenesis	5/2279	41/18866	0.5622842	0.7344325	0.6645406	5	TGFBR2/RBPJ/WNT11/ENG/NOTCH1
Biological Process	GO:1900407	regulation of cellular response to oxidative stress	11/2279	92/18866	0.5624537	0.7345529	0.6646495	11	P4HB/IL10/TNF/FBXO7/RIPK1/AIFM2/NOL3/NFE2L2/BAG5/SIRT3/PRKN
Biological Process	GO:0007156	homophilic cell adhesion via plasma membrane adhesion molecules	20/2279	168/18866	0.5635448	0.7358767	0.6658474	20	BSG/CADM3/CEACAM6/CDH23/CD84/CDH3/KIRREL3/CDHR2/DSCAML1/CDH4/CDH1/MYOT/CDHR1/SDK2/PCDH12/CADM1/CDH11/CEACAM5/NECTIN1/NECTIN4
Biological Process	GO:2000134	negative regulation of G1/S transition of mitotic cell cycle	15/2279	126/18866	0.5651935	0.7379282	0.6677036	15	TFDP1/PRMT2/FBXO7/E2F7/CTDSPL/PCBP4/RIPK1/NACC2/ZNF385A/ARID3A/GFI1B/MIR138-2/RB1/BCL2/CHEK2
Biological Process	GO:0003094	glomerular filtration	3/2279	24/18866	0.5674164	0.7391038	0.6687674	3	GJA5/PDGFB/F2R
Biological Process	GO:0009218	pyrimidine ribonucleotide metabolic process	3/2279	24/18866	0.5674164	0.7391038	0.6687674	3	UCK2/AK5/DHODH
Biological Process	GO:0009309	amine biosynthetic process	3/2279	24/18866	0.5674164	0.7391038	0.6687674	3	ODC1/DDC/SULT1A2
Biological Process	GO:0010869	regulation of receptor biosynthetic process	3/2279	24/18866	0.5674164	0.7391038	0.6687674	3	PPARG/HDAC1/ITGB3

Biological Process	GO:0021697	cerebellar cortex formation	3/2279	24/18866	0.5674164	0.7391038	0.6687674	3	DLL1/TTC21B/TTLL1
Biological Process	GO:0021801	cerebral cortex radial glia guided migration	3/2279	24/18866	0.5674164	0.7391038	0.6687674	3	RTN4/DISC1/ZMIZ1
Biological Process	GO:0022030	telencephalon glial cell migration	3/2279	24/18866	0.5674164	0.7391038	0.6687674	3	RTN4/DISC1/ZMIZ1
Biological Process	GO:0031342	negative regulation of cell killing	3/2279	24/18866	0.5674164	0.7391038	0.6687674	3	HLA-F/PTPRC/ARRB2
Biological Process	GO:0032331	negative regulation of chondrocyte differentiation	3/2279	24/18866	0.5674164	0.7391038	0.6687674	3	RARG/RFLNA/CCN4
Biological Process	GO:0032891	negative regulation of organic acid transport	3/2279	24/18866	0.5674164	0.7391038	0.6687674	3	THBS1/SLC43A2/SLC43A1
Biological Process	GO:0033622	integrin activation	3/2279	24/18866	0.5674164	0.7391038	0.6687674	3	ITGB1BP1/RAP1B/JAM3
Biological Process	GO:0044062	regulation of excretion	3/2279	24/18866	0.5674164	0.7391038	0.6687674	3	COMT/GNAI2/AGTR1
Biological Process	GO:0044247	cellular polysaccharide catabolic process	3/2279	24/18866	0.5674164	0.7391038	0.6687674	3	AOAH/MGAM/PPP1CB

Biological Process	GO:0044346	fibroblast apoptotic process	3/2279	24/18866	0.5674164	0.7391038	0.6687674	3	CUL3/MIR24-2/CHD8
Biological Process	GO:0045724	positive regulation of cilium assembly	3/2279	24/18866	0.5674164	0.7391038	0.6687674	3	CEP135/HTT/SEPTIN9
Biological Process	GO:0051446	positive regulation of meiotic cell cycle	3/2279	24/18866	0.5674164	0.7391038	0.6687674	3	CDC25A/OVOL1/PLCB1
Biological Process	GO:2000291	regulation of myoblast proliferation	3/2279	24/18866	0.5674164	0.7391038	0.6687674	3	PAX7/MEIS2/ANKRD2
Biological Process	GO:0000075	cell cycle checkpoint	26/2279	219/18866	0.5691171	0.7412175	0.6706799	26	TRIM39/BLM/FZR1/TFDP1/MAD1L1/FOXN3/HUS1/E2F7/PCBP4/EME1/ZNF385A/TGFB1/ARID3A/TAOK3/ZFYVE19/CLSPN/RB1/TP53BP1/RHNO1/CHEK2/BCL2L1/NAE1/PCID2/MAP3K20/ABRAXAS1/BABAM2
Biological Process	GO:1905710	positive regulation of membrane permeability	8/2279	67/18866	0.5700733	0.7421291	0.6715047	8	TFDP1/YWHAH/BMF/SPG7/YWHAZ/BCL2/TP53BP2/YWHAQ
Biological Process	GO:0001708	cell fate specification	12/2279	101/18866	0.5702857	0.7421291	0.6715047	12	SUFU/MIAT/MGA/DLL1/RBPJ/WNT11/PAX2/FGFR1/FEV/NOTCH1/LMO4/NODAL
Biological Process	GO:0006906	vesicle fusion	12/2279	101/18866	0.5702857	0.7421291	0.6715047	12	CALR/STX5/VTI1A/ANKFY1/TGFBRAP1/UVRAG/VPS18/DYSF/TSNARE1/VAV3/IRAG2/RUBCNL
Biological Process	GO:0015908	fatty acid transport	12/2279	101/18866	0.5702857	0.7421291	0.6715047	12	PRKAG2/PPARG/ACSL1/MFSD2A/MAP2K6/ABCC2/THBS1/SLCO3A1/ACACB/ACACA/SLC2A1/CPT1A

Biological Process	GO:0042632	cholesterol homeostasis	12/2279	101/18866	0.5702857	0.7421291	0.6715047	12	EHD1/NR1I2/LRP5L/LIPC/VDR/ABCG1/MED13/NR1H2/ABCA1/NPC1/NR5A2/RALY
Biological Process	GO:1902882	regulation of response to oxidative stress	12/2279	101/18866	0.5702857	0.7421291	0.6715047	12	P4HB/IL10/TNF/FBXO7/RIPK1/AIFM2/NOL3/NFE2L2/ACOX2/BAG5/SIRT3/PRKN
Biological Process	GO:0006805	xenobiotic metabolic process	14/2279	118/18866	0.5711303	0.7430246	0.6723151	14	NR1I2/ACSL1/GGT1/LPO/ABCC2/AIP/SULT1A2/POR/MGST2/GUK1/GSTO1/EPHX1/GSTO2/MTARC1
Biological Process	GO:0021987	cerebral cortex development	14/2279	118/18866	0.5711303	0.7430246	0.6723151	14	NDE1/BTBD3/RTN4/DISC1/TRAPPC9/ZMIZ1/NF1/TACC3/WDR47/NCOA1/PLCB1/SLC2A1/NDEL1/NPY
Biological Process	GO:2000181	negative regulation of blood vessel morphogenesis	23/2279	194/18866	0.5715939	0.7431427	0.6724219	23	PPARG/MIR143/MIR145/ITGB1BP1/CCR2/COL4A2/MIR199A1/TNF/AGO1/SEMA4A/THBS1/MIR24-2/EPN2/NF1/GHRL/VASH1/MIR138-2/MIR199A2/NOTCH1/GPR4/HHEX/PDE3B/CX3CR1
Biological Process	GO:0009066	aspartate family amino acid metabolic process	6/2279	50/18866	0.5716904	0.7431427	0.6724219	6	MSRA/OGDH/MTHFD1/MAT1A/CRYM/ADI1
Biological Process	GO:0032608	interferon-beta production	6/2279	50/18866	0.5716904	0.7431427	0.6724219	6	TRIM38/IRF5/TLR9/TRAIP/FLOT1/STING1
Biological Process	GO:0045646	regulation of erythrocyte differentiation	6/2279	50/18866	0.5716904	0.7431427	0.6724219	6	ZFPM1/INPP5D/BRD1/ETS1/KAT7/INHBA
Biological Process	GO:0061014	positive regulation of mRNA catabolic process	6/2279	50/18866	0.5716904	0.7431427	0.6724219	6	PRR5L/TNRC6B/ZC3H12D/AGO2/CPEB3/FTO

Biological Process	GO:1903580	positive regulation of ATP metabolic process	6/2279	50/18866	0.5716904	0.7431427	0.6724219	6	ENO1/PFKFB4/APP/ESRRB/PFKFB3/PRKAA1
Biological Process	GO:0001837	epithelial to mesenchymal transition	18/2279	152/18866	0.5734264	0.7442152	0.6733924	18	SPRED2/SMAD3/LDLRAD4/LOXL3/RTN4/TGFBR2/RBPJ/TCF7L2/SMAD7/WNT11/FGFR1/TGFB1/TGFB1I1/WWTR1/ENG/NOTCH1/GCNT2/NOTCH4
Biological Process	GO:0001975	response to amphetamine	4/2279	33/18866	0.5765395	0.7442152	0.6733924	4	PPP1R1B/RGS17/CNR2/RGS10
Biological Process	GO:0006370	7-methylguanosine mRNA capping	4/2279	33/18866	0.5765395	0.7442152	0.6733924	4	POLR2F/CCNH/ERCC2/NCBP3
Biological Process	GO:0009303	rRNA transcription	4/2279	33/18866	0.5765395	0.7442152	0.6733924	4	BRF1/PIH1D1/MARS1/MACROH2A2
Biological Process	GO:0010955	negative regulation of protein processing	4/2279	33/18866	0.5765395	0.7442152	0.6733924	4	CTSZ/THBS1/NOL3/CPB2
Biological Process	GO:0021696	cerebellar cortex morphogenesis	4/2279	33/18866	0.5765395	0.7442152	0.6733924	4	DLL1/TTC21B/TTLL1/COQ8B
Biological Process	GO:0030149	sphingolipid catabolic process	4/2279	33/18866	0.5765395	0.7442152	0.6733924	4	HEXB/GALC/SMPDL3B/PDXDC1
Biological Process	GO:0040020	regulation of meiotic nuclear division	4/2279	33/18866	0.5765395	0.7442152	0.6733924	4	CALR/FZR1/PLCB1/LIF

Biological Process	GO:0043928	exonucleolytic catabolism of deadenylated mRNA	4/2279	33/18866	0.5765395	0.7442152	0.6733924	4	LSM1/EXOSC2/EDC3/LSM7
Biological Process	GO:0061311	cell surface receptor signaling pathway involved in heart development	4/2279	33/18866	0.5765395	0.7442152	0.6733924	4	RBPJ/WNT11/TGFB1/NOTCH1
Biological Process	GO:1901380	negative regulation of potassium ion transmembrane transport	4/2279	33/18866	0.5765395	0.7442152	0.6733924	4	KCNE1/CASQ2/BIN1/CAB39
Biological Process	GO:1903318	negative regulation of protein maturation	4/2279	33/18866	0.5765395	0.7442152	0.6733924	4	CTSZ/THBS1/NOL3/CPB2
Biological Process	GO:1904893	negative regulation of receptor signaling pathway via STAT	4/2279	33/18866	0.5765395	0.7442152	0.6733924	4	PPP2R1A/GGNBP2/BCL3/INPP5F
Biological Process	GO:0009141	nucleoside triphosphate metabolic process	13/2279	110/18866	0.5775618	0.7442152	0.6733924	13	PRKAG2/AMPD3/ENO1/RRM2B/VPS9D1/SPHK2/TGFB1/UCK2/GUK1/PKM/AK5/IMPDH1/ATP5PD
Biological Process	GO:0072009	nephron epithelium development	13/2279	110/18866	0.5775618	0.7442152	0.6733924	13	TACSTD2/DLL1/ILK/WNT11/PAX2/MTSS1/WWTR1/BCL2/NOTCH1/MEF2C/ACAT1/LIF/HOXB7
Biological Process	GO:0001704	formation of primary germ layer	15/2279	127/18866	0.5780621	0.7442152	0.6733924	15	MIR145/SETD2/SMAD3/COL4A2/COL12A1/WNT11/PAX2/ITGB5/FGFR1/ITGB3/ITGB2/COL5A1/LAMB3/INHBA/NODAL
Biological Process	GO:0033077	T cell differentiation in thymus	9/2279	76/18866	0.5781082	0.7442152	0.6733924	9	ITPKB/RIPK3/ZC3H8/PTPRC/CLPTM1/BCL2/DOCK2/FADD/TMEM131L

Biological Process	GO:0002218	activation of innate immune response	17/2279	144/18866	0.5788296	0.7442152	0.6733924	17	NLRC4/AIM2/CLEC4C/MNDA/MUC12/FCN1/TRIM5/MUC20/BCL10/PSMF1/CREBBP/PSMB7/RELA/FBXW11/HEXIM1/PSMD13/STING1
Biological Process	GO:0000723	telomere maintenance	19/2279	161/18866	0.5797728	0.7442152	0.6733924	19	BLM/TNKS/POLE/BRCA2/HMBOX1/RTEL1/HUS1/NSMCE2/YLPM1/POLA2/SMARCAL1/SMG6/SP100/ERCC1/SLX4/POLD3/CCT6A/POLE4/TFIP11
Biological Process	GO:0060113	inner ear receptor cell differentiation	7/2279	59/18866	0.5800072	0.7442152	0.6733924	7	CDH23/ATP8B1/DLL1/RBPJ/FGFR1/NOTCH1/TMC1
Biological Process	GO:0071385	cellular response to glucocorticoid stimulus	7/2279	59/18866	0.5800072	0.7442152	0.6733924	7	AKAP13/ABCC2/SMYD3/FECH/AQP1/PCK2/FOXO1
Biological Process	GO:0051321	meiotic cell cycle	30/2279	254/18866	0.5817741	0.7442152	0.6733924	30	PIWIL4/CALR/FZR1/MOV10L1/BRCA2/HUS1/EME1/MYH9/MEIOB/STK35/CDC25A/OVOL1/FANCA/RNF212/ZNF318/NUMA1/PPP2R1A/PLCB1/CCNA1/RAD21L1/DMC1/NPPC/ERCC1/SLX4/TEX12/TUBGCP2/LIF/KLHDC3/PSMD13/TERB2
Biological Process	GO:0045137	development of primary sexual characteristics	27/2279	229/18866	0.5844093	0.7442152	0.6733924	27	ARID5B/BRCA2/ARRB1/EIF2B5/CSDE1/FANCA/RDH10/RARA/SF1/BMPR1B/NCOA1/LHFPL2/ARRB2/NTRK1/HOXA9/RAD21L1/BCL2/DMC1/SLIT3/UBE3A/CEBPB/PDGFRB/ERCC1/BCL2L1/HMGCS1/NCOA4/INHBA
Biological Process	GO:0021983	pituitary gland development	5/2279	42/18866	0.5845448	0.7442152	0.6733924	5	CREB1/RBPJ/ETS1/CDH1/ALDH1A2
Biological Process	GO:1904591	positive regulation of protein import	5/2279	42/18866	0.5845448	0.7442152	0.6733924	5	PIK3R1/SMAD3/JUP/TGFB1/CDH1
Biological Process	GO:0022600	digestive system process	12/2279	102/18866	0.5845769	0.7442152	0.6733924	12	SLC2A5/TJP2/VDR/SGK1/TLR9/AQP1/NR1H2/GHRL/SLC22A5/KCNQ1/NOD2/EZR

Biological Process	GO:0055092	sterol homeostasis	12/2279	102/18866	0.5845769	0.7442152	0.6733924	12	EHD1/NR1I2/LRP5L/LIPC/VDR/ABCG1/MED13/NR1H2/ABCA1/NPC1/NR5A2/RALY
Biological Process	GO:0031123	RNA 3'-end processing	18/2279	153/18866	0.5851096	0.7442152	0.6733924	18	FIP1L1/EXOSC2/CPSF3/APP/PABPN1/CPEB3/ERI3/SCAF8/ZC3H3/RPRD1B/TOE1/CPSF4/PTCD1/RNF40/FBL/TUT1/PAPOLG/GRSF1
Biological Process	GO:0009199	ribonucleoside triphosphate metabolic process	10/2279	85/18866	0.58547	0.7442152	0.6733924	10	PRKAG2/AMPD3/ENO1/VPS9D1/SPHK2/TGFB1/UCK2/PKM/IMPDH1/ATP5PD
Biological Process	GO:0070509	calcium ion import	10/2279	85/18866	0.58547	0.7442152	0.6733924	10	TRPM2/TRIM27/CACNA1C/SLC8A1/SPHK2/PDGFB/PDGFRB/DYSF/CACNA1B/TRPV2
Biological Process	GO:0072089	stem cell proliferation	15/2279	128/18866	0.5907776	0.7442152	0.6733924	15	NDE1/RUNX1/DISC1/FGFR1/OVOL1/NF1/NUMB/PTPRC/ETV6/ZNF335/NOTCH1/ARHGEF2/KAT7/MECOM/CX3CR1
Biological Process	GO:0001738	morphogenesis of a polarized epithelium	17/2279	145/18866	0.5907833	0.7442152	0.6733924	17	AP2A1/VANGL1/PRICKLE2/ZDHHC7/WNT11/SMURF1/PFN1/ACTG1/PSMF1/WDR1/ARRB2/DAAM1/ASTN2/PSMB7/ANKRD6/PSMD13/CPLANE1
Biological Process	GO:0002526	acute inflammatory response	13/2279	111/18866	0.5912046	0.7442152	0.6733924	13	ELANE/S100A8/FUT7/SERPINA1/PPARG/TREM1/NLRP3/TNF/F12/IL6R/DNASE1L3/CEBPB/ANO6
Biological Process	GO:0034440	lipid oxidation	13/2279	111/18866	0.5912046	0.7442152	0.6733924	13	PRKAG2/PPARG/MFSD2A/AMACR/ACOXL/ACADM/POR/ACACB/ACOX2/ACAT1/CPT1A/ACADVL/PRKAA1
Biological Process	GO:0021879	forebrain neuron differentiation	6/2279	51/18866	0.5917813	0.7442152	0.6733924	6	NRP2/CSF1R/DISC1/ZMIZ1/RAPGEF2/INHBA

Biological Process	GO:0032206	positive regulation of telomere maintenance	6/2279	51/18866	0.5917813	0.7442152	0.6733924	6	TNKS/HMBOX1/RTEL1/ERCC1/SLX4/CCT6A
Biological Process	GO:0043628	ncRNA 3'-end processing	6/2279	51/18866	0.5917813	0.7442152	0.6733924	6	EXOSC2/ERI3/TOE1/PTCD1/FBL/TUT1
Biological Process	GO:0048255	mRNA stabilization	6/2279	51/18866	0.5917813	0.7442152	0.6733924	6	SLC11A1/LARP1/PAIP1/SYNCRIP/TENT5C/TENT5A
Biological Process	GO:0098876	vesicle-mediated transport to the plasma membrane	11/2279	94/18866	0.5922893	0.7442152	0.6733924	11	OSBPL5/KIF13A/DNM2/RAB31/SNX27/ZDHHC2/AKAP5/VAMP5/CSK/MICALL1/VPS35L
Biological Process	GO:0000394	RNA splicing, via endonucleolytic cleavage and ligation	2/2279	16/18866	0.5924093	0.7442152	0.6733924	2	ERN1/CPSF4
Biological Process	GO:0000478	endonucleolytic cleavage involved in rRNA processing	2/2279	16/18866	0.5924093	0.7442152	0.6733924	2	RCL1/NOP14
Biological Process	GO:0000479	endonucleolytic cleavage of tricistronic rRNA transcript (SSU-rRNA, 5.8S rRNA, LSU-rRNA)	2/2279	16/18866	0.5924093	0.7442152	0.6733924	2	RCL1/NOP14
Biological Process	GO:0001732	formation of cytoplasmic translation initiation complex	2/2279	16/18866	0.5924093	0.7442152	0.6733924	2	EIF3M/EIF3H
Biological Process	GO:0006004	fucose metabolic process	2/2279	16/18866	0.5924093	0.7442152	0.6733924	2	FUT4/FUT7

Biological Process	GO:0006103	2-oxoglutarate metabolic process	2/2279	16/18866	0.5924093	0.7442152	0.6733924	2	D2HGDH/OGDH
Biological Process	GO:0006595	polyamine metabolic process	2/2279	16/18866	0.5924093	0.7442152	0.6733924	2	ODC1/SAT2
Biological Process	GO:0007076	mitotic chromosome condensation	2/2279	16/18866	0.5924093	0.7442152	0.6733924	2	TTN/CDCA5
Biological Process	GO:0007567	parturition	2/2279	16/18866	0.5924093	0.7442152	0.6733924	2	ARNTL/NODAL
Biological Process	GO:0010225	response to UV-C	2/2279	16/18866	0.5924093	0.7442152	0.6733924	2	BRCA2/BCL3
Biological Process	GO:0010919	regulation of inositol phosphate biosynthetic process	2/2279	16/18866	0.5924093	0.7442152	0.6733924	2	P2RY6/HRH1
Biological Process	GO:0010934	macrophage cytokine production	2/2279	16/18866	0.5924093	0.7442152	0.6733924	2	SPON2/TGFB1
Biological Process	GO:0010940	positive regulation of necrotic cell death	2/2279	16/18866	0.5924093	0.7442152	0.6733924	2	RIPK1/RIPK3
Biological Process	GO:0014744	positive regulation of muscle adaptation	2/2279	16/18866	0.5924093	0.7442152	0.6733924	2	MIR199A1/MIR199A2

Biological Process	GO:0030252	growth hormone secretion	2/2279	16/18866	0.5924093	0.7442152	0.6733924	2	ITSN1/GHRL
Biological Process	GO:0032225	regulation of synaptic transmission, dopaminergic	2/2279	16/18866	0.5924093	0.7442152	0.6733924	2	ARRB2/FLOT1
Biological Process	GO:0032604	granulocyte macrophage colony-stimulating factor production	2/2279	16/18866	0.5924093	0.7442152	0.6733924	2	CD84/TLR9
Biological Process	GO:0034310	primary alcohol catabolic process	2/2279	16/18866	0.5924093	0.7442152	0.6733924	2	ALDH2/SULT1A2
Biological Process	GO:0034380	high-density lipoprotein particle assembly	2/2279	16/18866	0.5924093	0.7442152	0.6733924	2	NR1H2/ABCA1
Biological Process	GO:0035067	negative regulation of histone acetylation	2/2279	16/18866	0.5924093	0.7442152	0.6733924	2	CTBP1/PIH1D1
Biological Process	GO:0035864	response to potassium ion	2/2279	16/18866	0.5924093	0.7442152	0.6733924	2	CRHBP/ADAMTS13
Biological Process	GO:0035930	corticosteroid hormone secretion	2/2279	16/18866	0.5924093	0.7442152	0.6733924	2	GHRL/AGTR1
Biological Process	GO:0036124	histone H3-K9 trimethylation	2/2279	16/18866	0.5924093	0.7442152	0.6733924	2	KDM4C/PIH1D1

Biological Process	GO:0042035	regulation of cytokine biosynthetic process	2/2279	16/18866	0.5924093	0.7442152	0.6733924	2	IGF2BP3/ASB1
Biological Process	GO:0042074	cell migration involved in gastrulation	2/2279	16/18866	0.5924093	0.7442152	0.6733924	2	WNT11/NODAL
Biological Process	GO:0042994	cytoplasmic sequestering of transcription factor	2/2279	16/18866	0.5924093	0.7442152	0.6733924	2	IL10/SUFU
Biological Process	GO:0043252	sodium-independent organic anion transport	2/2279	16/18866	0.5924093	0.7442152	0.6733924	2	SLCO3A1/MFSD10
Biological Process	GO:0044065	regulation of respiratory system process	2/2279	16/18866	0.5924093	0.7442152	0.6733924	2	MTG2/FTO
Biological Process	GO:0045725	positive regulation of glycogen biosynthetic process	2/2279	16/18866	0.5924093	0.7442152	0.6733924	2	DYRK2/ESRRB
Biological Process	GO:0045953	negative regulation of natural killer cell mediated cytotoxicity	2/2279	16/18866	0.5924093	0.7442152	0.6733924	2	HLA-F/ARRB2
Biological Process	GO:0048012	hepatocyte growth factor receptor signaling pathway	2/2279	16/18866	0.5924093	0.7442152	0.6733924	2	ESM1/MUC20
Biological Process	GO:0048311	mitochondrion distribution	2/2279	16/18866	0.5924093	0.7442152	0.6733924	2	MSTO1/DNM1L

Biological Process	GO:0048521	negative regulation of behavior	2/2279	16/18866	0.5924093	0.7442152	0.6733924	2	GHRL/TTC21B
Biological Process	GO:0050655	dermatan sulfate proteoglycan metabolic process	2/2279	16/18866	0.5924093	0.7442152	0.6733924	2	CSGALNACT1/DSE
Biological Process	GO:0050884	neuromuscular process controlling posture	2/2279	16/18866	0.5924093	0.7442152	0.6733924	2	SCN1A/CNTNAP1
Biological Process	GO:0050965	detection of temperature stimulus involved in sensory perception of pain	2/2279	16/18866	0.5924093	0.7442152	0.6733924	2	ARRB2/NTRK1
Biological Process	GO:0051284	positive regulation of sequestering of calcium ion	2/2279	16/18866	0.5924093	0.7442152	0.6733924	2	CASQ2/GSTO1
Biological Process	GO:0051444	negative regulation of ubiquitin-protein transferase activity	2/2279	16/18866	0.5924093	0.7442152	0.6733924	2	SMAD7/BAG5
Biological Process	GO:0055119	relaxation of cardiac muscle	2/2279	16/18866	0.5924093	0.7442152	0.6733924	2	PDE4D/SLC8A1
Biological Process	GO:0061684	chaperone-mediated autophagy	2/2279	16/18866	0.5924093	0.7442152	0.6733924	2	CLU/GFAP
Biological Process	GO:0070103	regulation of interleukin-6-mediated signaling pathway	2/2279	16/18866	0.5924093	0.7442152	0.6733924	2	GFI1/RIPK1

Biological Process	GO:0070593	dendrite self-avoidance	2/2279	16/18866	0.5924093	0.7442152	0.6733924	2	BSG/DSCAML1
Biological Process	GO:0071871	response to epinephrine	2/2279	16/18866	0.5924093	0.7442152	0.6733924	2	PDE4D/KCNQ1
Biological Process	GO:0072176	nephric duct development	2/2279	16/18866	0.5924093	0.7442152	0.6733924	2	WNT11/PAX2
Biological Process	GO:0072283	metanephric renal vesicle morphogenesis	2/2279	16/18866	0.5924093	0.7442152	0.6733924	2	PAX2/LIF
Biological Process	GO:0097091	synaptic vesicle clustering	2/2279	16/18866	0.5924093	0.7442152	0.6733924	2	SYN3/SYN2
Biological Process	GO:1900121	negative regulation of receptor binding	2/2279	16/18866	0.5924093	0.7442152	0.6733924	2	IL10/ATP2A3
Biological Process	GO:1902514	regulation of calcium ion transmembrane transport via high voltage-gated calcium channel	2/2279	16/18866	0.5924093	0.7442152	0.6733924	2	BIN1/NOS1AP
Biological Process	GO:1904862	inhibitory synapse assembly	2/2279	16/18866	0.5924093	0.7442152	0.6733924	2	SEMA4A/SEMA4D
Biological Process	GO:1905516	positive regulation of fertilization	2/2279	16/18866	0.5924093	0.7442152	0.6733924	2	LHFPL2/PLCB1

Biological Process	GO:1905874	regulation of postsynaptic density organization	2/2279	16/18866	0.5924093	0.7442152	0.6733924	2	ZMYND8/NRXN1
Biological Process	GO:2001212	regulation of vasculogenesis	2/2279	16/18866	0.5924093	0.7442152	0.6733924	2	RIN2/RAPGEF2
Biological Process	GO:2001224	positive regulation of neuron migration	2/2279	16/18866	0.5924093	0.7442152	0.6733924	2	RAPGEF2/ARHGEF2
Biological Process	GO:0001554	luteolysis	1/2279	7/18866	0.5939779	0.7442152	0.6733924	1	SLIT3
Biological Process	GO:0001842	neural fold formation	1/2279	7/18866	0.5939779	0.7442152	0.6733924	1	NODAL
Biological Process	GO:0001866	NK T cell proliferation	1/2279	7/18866	0.5939779	0.7442152	0.6733924	1	ZBTB7B
Biological Process	GO:0001915	negative regulation of T cell mediated cytotoxicity	1/2279	7/18866	0.5939779	0.7442152	0.6733924	1	PTPRC
Biological Process	GO:0002069	columnar/cuboidal epithelial cell maturation	1/2279	7/18866	0.5939779	0.7442152	0.6733924	1	RFX3
Biological Process	GO:0002154	thyroid hormone mediated signaling pathway	1/2279	7/18866	0.5939779	0.7442152	0.6733924	1	NCOA1

Biological Process	GO:0002428	antigen processing and presentation of peptide antigen via MHC class Ib	1/2279	7/18866	0.5939779	0.7442152	0.6733924	1	HLA-F
Biological Process	GO:0002536	respiratory burst involved in inflammatory response	1/2279	7/18866	0.5939779	0.7442152	0.6733924	1	GRN
Biological Process	GO:0002857	positive regulation of natural killer cell mediated immune response to tumor cell	1/2279	7/18866	0.5939779	0.7442152	0.6733924	1	CD226
Biological Process	GO:0002924	negative regulation of humoral immune response mediated by circulating immunoglobulin	1/2279	7/18866	0.5939779	0.7442152	0.6733924	1	PTPN6
Biological Process	GO:0003228	atrial cardiac muscle tissue development	1/2279	7/18866	0.5939779	0.7442152	0.6733924	1	ENG
Biological Process	GO:0003308	negative regulation of Wnt signaling pathway involved in heart development	1/2279	7/18866	0.5939779	0.7442152	0.6733924	1	WNT11
Biological Process	GO:0003321	positive regulation of blood pressure by epinephrine-norepinephrine	1/2279	7/18866	0.5939779	0.7442152	0.6733924	1	PDE4D
Biological Process	GO:0003357	noradrenergic neuron differentiation	1/2279	7/18866	0.5939779	0.7442152	0.6733924	1	PHOX2A
Biological Process	GO:0006489	dolichyl diphosphate biosynthetic process	1/2279	7/18866	0.5939779	0.7442152	0.6733924	1	DPAGT1

Biological Process	GO:0006578	amino-acid betaine biosynthetic process	1/2279	7/18866	0.5939779	0.7442152	0.6733924	1	ACADM
Biological Process	GO:0006627	protein processing involved in protein targeting to mitochondrion	1/2279	7/18866	0.5939779	0.7442152	0.6733924	1	MIPEP
Biological Process	GO:0006685	sphingomyelin catabolic process	1/2279	7/18866	0.5939779	0.7442152	0.6733924	1	SMPDL3B
Biological Process	GO:0006689	ganglioside catabolic process	1/2279	7/18866	0.5939779	0.7442152	0.6733924	1	HEXB
Biological Process	GO:0006701	progesterone biosynthetic process	1/2279	7/18866	0.5939779	0.7442152	0.6733924	1	DGKQ
Biological Process	GO:0006771	riboflavin metabolic process	1/2279	7/18866	0.5939779	0.7442152	0.6733924	1	SLC52A2
Biological Process	GO:0006824	cobalt ion transport	1/2279	7/18866	0.5939779	0.7442152	0.6733924	1	SLC11A2
Biological Process	GO:0007000	nucleolus organization	1/2279	7/18866	0.5939779	0.7442152	0.6733924	1	USP36
Biological Process	GO:0007406	negative regulation of neuroblast proliferation	1/2279	7/18866	0.5939779	0.7442152	0.6733924	1	NF1

Biological Process	GO:0008063	Toll signaling pathway	1/2279	7/18866	0.5939779	0.7442152	0.6733924	1	PELI1
Biological Process	GO:0009635	response to herbicide	1/2279	7/18866	0.5939779	0.7442152	0.6733924	1	LCN2
Biological Process	GO:0009820	alkaloid metabolic process	1/2279	7/18866	0.5939779	0.7442152	0.6733924	1	DDC
Biological Process	GO:0010571	positive regulation of nuclear cell cycle DNA replication	1/2279	7/18866	0.5939779	0.7442152	0.6733924	1	FGFR1
Biological Process	GO:0010753	positive regulation of cGMP-mediated signaling	1/2279	7/18866	0.5939779	0.7442152	0.6733924	1	NPPC
Biological Process	GO:0010764	negative regulation of fibroblast migration	1/2279	7/18866	0.5939779	0.7442152	0.6733924	1	ITGB1BP1
Biological Process	GO:0010968	regulation of microtubule nucleation	1/2279	7/18866	0.5939779	0.7442152	0.6733924	1	DCTN1
Biological Process	GO:0010982	regulation of high-density lipoprotein particle clearance	1/2279	7/18866	0.5939779	0.7442152	0.6733924	1	GPLD1
Biological Process	GO:0010994	free ubiquitin chain polymerization	1/2279	7/18866	0.5939779	0.7442152	0.6733924	1	PRKN

Biological Process	GO:0014827	intestine smooth muscle contraction	1/2279	7/18866	0.5939779	0.7442152	0.6733924	1	GHRL
Biological Process	GO:0015744	succinate transport	1/2279	7/18866	0.5939779	0.7442152	0.6733924	1	SLC13A5
Biological Process	GO:0015822	ornithine transport	1/2279	7/18866	0.5939779	0.7442152	0.6733924	1	SLC7A1
Biological Process	GO:0016198	axon choice point recognition	1/2279	7/18866	0.5939779	0.7442152	0.6733924	1	APP
Biological Process	GO:0017196	N-terminal peptidyl-methionine acetylation	1/2279	7/18866	0.5939779	0.7442152	0.6733924	1	NAA16
Biological Process	GO:0019530	taurine metabolic process	1/2279	7/18866	0.5939779	0.7442152	0.6733924	1	STAT5A
Biological Process	GO:0019694	alkanesulfonate metabolic process	1/2279	7/18866	0.5939779	0.7442152	0.6733924	1	STAT5A
Biological Process	GO:0021626	central nervous system maturation	1/2279	7/18866	0.5939779	0.7442152	0.6733924	1	CX3CR1
Biological Process	GO:0021798	forebrain dorsal/ventral pattern formation	1/2279	7/18866	0.5939779	0.7442152	0.6733924	1	TTC21B

Biological Process	GO:0021910	smoothened signaling pathway involved in ventral spinal cord patterning	1/2279	7/18866	0.5939779	0.7442152	0.6733924	1	SUFU
Biological Process	GO:0023041	neuronal signal transduction	1/2279	7/18866	0.5939779	0.7442152	0.6733924	1	CLU
Biological Process	GO:0030046	parallel actin filament bundle assembly	1/2279	7/18866	0.5939779	0.7442152	0.6733924	1	ESPN
Biological Process	GO:0031086	nuclear-transcribed mRNA catabolic process, deadenylation-independent decay	1/2279	7/18866	0.5939779	0.7442152	0.6733924	1	EDC3
Biological Process	GO:0031547	brain-derived neurotrophic factor receptor signaling pathway	1/2279	7/18866	0.5939779	0.7442152	0.6733924	1	RAPGEF2
Biological Process	GO:0031622	positive regulation of fever generation	1/2279	7/18866	0.5939779	0.7442152	0.6733924	1	TNF
Biological Process	GO:0032229	negative regulation of synaptic transmission, GABAergic	1/2279	7/18866	0.5939779	0.7442152	0.6733924	1	CNR2
Biological Process	GO:0032261	purine nucleotide salvage	1/2279	7/18866	0.5939779	0.7442152	0.6733924	1	AMPD3
Biological Process	GO:0032277	negative regulation of gonadotropin secretion	1/2279	7/18866	0.5939779	0.7442152	0.6733924	1	INHBA

Biological Process	GO:0032278	positive regulation of gonadotropin secretion	1/2279	7/18866	0.5939779	0.7442152	0.6733924	1	INHBA
Biological Process	GO:0032430	positive regulation of phospholipase A2 activity	1/2279	7/18866	0.5939779	0.7442152	0.6733924	1	AGTR1
Biological Process	GO:0032489	regulation of Cdc42 protein signal transduction	1/2279	7/18866	0.5939779	0.7442152	0.6733924	1	ABCA1
Biological Process	GO:0032625	interleukin-21 production	1/2279	7/18866	0.5939779	0.7442152	0.6733924	1	FOXP1
Biological Process	GO:0032714	negative regulation of interleukin-5 production	1/2279	7/18866	0.5939779	0.7442152	0.6733924	1	TNFRSF21
Biological Process	GO:0032762	mast cell cytokine production	1/2279	7/18866	0.5939779	0.7442152	0.6733924	1	BCL10
Biological Process	GO:0032914	positive regulation of transforming growth factor beta1 production	1/2279	7/18866	0.5939779	0.7442152	0.6733924	1	THBS1
Biological Process	GO:0032988	ribonucleoprotein complex disassembly	1/2279	7/18866	0.5939779	0.7442152	0.6733924	1	TFIP11
Biological Process	GO:0033211	adiponectin-activated signaling pathway	1/2279	7/18866	0.5939779	0.7442152	0.6733924	1	ACSL1

Biological Process	GO:0033313	meiotic cell cycle checkpoint	1/2279	7/18866	0.5939779	0.7442152	0.6733924	1	HUS1
Biological Process	GO:0033564	anterior/posterior axon guidance	1/2279	7/18866	0.5939779	0.7442152	0.6733924	1	UNC5B
Biological Process	GO:0035356	cellular triglyceride homeostasis	1/2279	7/18866	0.5939779	0.7442152	0.6733924	1	C1QTNF3
Biological Process	GO:0035482	gastric motility	1/2279	7/18866	0.5939779	0.7442152	0.6733924	1	GHRL
Biological Process	GO:0035507	regulation of myosin-light-chain-phosphatase activity	1/2279	7/18866	0.5939779	0.7442152	0.6733924	1	TNF
Biological Process	GO:0035726	common myeloid progenitor cell proliferation	1/2279	7/18866	0.5939779	0.7442152	0.6733924	1	ITPKB
Biological Process	GO:0035795	negative regulation of mitochondrial membrane permeability	1/2279	7/18866	0.5939779	0.7442152	0.6733924	1	HK2
Biological Process	GO:0035989	tendon development	1/2279	7/18866	0.5939779	0.7442152	0.6733924	1	COL5A1
Biological Process	GO:0036089	cleavage furrow formation	1/2279	7/18866	0.5939779	0.7442152	0.6733924	1	SNX18

Biological Process	GO:0036123	histone H3-K9 dimethylation	1/2279	7/18866	0.5939779	0.7442152	0.6733924	1	PIH1D1
Biological Process	GO:0036302	atrioventricular canal development	1/2279	7/18866	0.5939779	0.7442152	0.6733924	1	RBPJ
Biological Process	GO:0036491	regulation of translation initiation in response to endoplasmic reticulum stress	1/2279	7/18866	0.5939779	0.7442152	0.6733924	1	NCK2
Biological Process	GO:0038001	paracrine signaling	1/2279	7/18866	0.5939779	0.7442152	0.6733924	1	PDGFB
Biological Process	GO:0038026	reelin-mediated signaling pathway	1/2279	7/18866	0.5939779	0.7442152	0.6733924	1	STAT5A
Biological Process	GO:0038089	positive regulation of cell migration by vascular endothelial growth factor signaling pathway	1/2279	7/18866	0.5939779	0.7442152	0.6733924	1	MYO1C
Biological Process	GO:0038107	nodal signaling pathway involved in determination of left/right asymmetry	1/2279	7/18866	0.5939779	0.7442152	0.6733924	1	NODAL
Biological Process	GO:0042723	thiamine-containing compound metabolic process	1/2279	7/18866	0.5939779	0.7442152	0.6733924	1	ACP3
Biological Process	GO:0042747	circadian sleep/wake cycle, REM sleep	1/2279	7/18866	0.5939779	0.7442152	0.6733924	1	GHRL

Biological Process	GO:0043102	amino acid salvage	1/2279	7/18866	0.5939779	0.7442152	0.6733924	1	ADI1
Biological Process	GO:0043307	eosinophil activation	1/2279	7/18866	0.5939779	0.7442152	0.6733924	1	CCR2
Biological Process	GO:0043634	polyadenylation-dependent ncRNA catabolic process	1/2279	7/18866	0.5939779	0.7442152	0.6733924	1	EXOSC2
Biological Process	GO:0043654	recognition of apoptotic cell	1/2279	7/18866	0.5939779	0.7442152	0.6733924	1	FCN1
Biological Process	GO:0043983	histone H4-K12 acetylation	1/2279	7/18866	0.5939779	0.7442152	0.6733924	1	KAT7
Biological Process	GO:0044341	sodium-dependent phosphate transport	1/2279	7/18866	0.5939779	0.7442152	0.6733924	1	CEBPB
Biological Process	GO:0044362	negative regulation of molecular function in other organism	1/2279	7/18866	0.5939779	0.7442152	0.6733924	1	SFTPD
Biological Process	GO:0044528	regulation of mitochondrial mRNA stability	1/2279	7/18866	0.5939779	0.7442152	0.6733924	1	TBRG4
Biological Process	GO:0044806	G-quadruplex DNA unwinding	1/2279	7/18866	0.5939779	0.7442152	0.6733924	1	BLM

Biological Process	GO:0045054	constitutive secretory pathway	1/2279	7/18866	0.5939779	0.7442152	0.6733924	1	RAB27A
Biological Process	GO:0045341	MHC class I biosynthetic process	1/2279	7/18866	0.5939779	0.7442152	0.6733924	1	NLRC5
Biological Process	GO:0045343	regulation of MHC class I biosynthetic process	1/2279	7/18866	0.5939779	0.7442152	0.6733924	1	NLRC5
Biological Process	GO:0045588	positive regulation of gamma-delta T cell differentiation	1/2279	7/18866	0.5939779	0.7442152	0.6733924	1	PTPRC
Biological Process	GO:0045650	negative regulation of macrophage differentiation	1/2279	7/18866	0.5939779	0.7442152	0.6733924	1	INHBA
Biological Process	GO:0045852	pH elevation	1/2279	7/18866	0.5939779	0.7442152	0.6733924	1	BCL2
Biological Process	GO:0046465	dolichyl diphosphate metabolic process	1/2279	7/18866	0.5939779	0.7442152	0.6733924	1	DPAGT1
Biological Process	GO:0046836	glycolipid transport	1/2279	7/18866	0.5939779	0.7442152	0.6733924	1	PLTP
Biological Process	GO:0046877	regulation of saliva secretion	1/2279	7/18866	0.5939779	0.7442152	0.6733924	1	AQP1

Biological Process	GO:0046884	follicle-stimulating hormone secretion	1/2279	7/18866	0.5939779	0.7442152	0.6733924	1	INHBA
Biological Process	GO:0048003	antigen processing and presentation of lipid antigen via MHC class Ib	1/2279	7/18866	0.5939779	0.7442152	0.6733924	1	AP3D1
Biological Process	GO:0048007	antigen processing and presentation, exogenous lipid antigen via MHC class Ib	1/2279	7/18866	0.5939779	0.7442152	0.6733924	1	AP3D1
Biological Process	GO:0048318	axial mesoderm development	1/2279	7/18866	0.5939779	0.7442152	0.6733924	1	NODAL
Biological Process	GO:0048341	paraxial mesoderm formation	1/2279	7/18866	0.5939779	0.7442152	0.6733924	1	WNT11
Biological Process	GO:0048630	skeletal muscle tissue growth	1/2279	7/18866	0.5939779	0.7442152	0.6733924	1	DLL1
Biological Process	GO:0050957	equilibrioception	1/2279	7/18866	0.5939779	0.7442152	0.6733924	1	CDH23
Biological Process	GO:0051005	negative regulation of lipoprotein lipase activity	1/2279	7/18866	0.5939779	0.7442152	0.6733924	1	SORT1
Biological Process	GO:0052204	negative regulation of molecular function in other organism involved in symbiotic interaction	1/2279	7/18866	0.5939779	0.7442152	0.6733924	1	SFTPD

Biological Process	GO:0052428	modulation by host of symbiont molecular function	1/2279	7/18866	0.5939779	0.7442152	0.6733924	1	SFTPD
Biological Process	GO:0060028	convergent extension involved in axis elongation	1/2279	7/18866	0.5939779	0.7442152	0.6733924	1	WNT11
Biological Process	GO:0060052	neurofilament cytoskeleton organization	1/2279	7/18866	0.5939779	0.7442152	0.6733924	1	NDEL1
Biological Process	GO:0060059	embryonic retina morphogenesis in camera-type eye	1/2279	7/18866	0.5939779	0.7442152	0.6733924	1	HIPK2
Biological Process	GO:0060437	lung growth	1/2279	7/18866	0.5939779	0.7442152	0.6733924	1	PDGFRB
Biological Process	GO:0060449	bud elongation involved in lung branching	1/2279	7/18866	0.5939779	0.7442152	0.6733924	1	RDH10
Biological Process	GO:0060613	fat pad development	1/2279	7/18866	0.5939779	0.7442152	0.6733924	1	ARID5B
Biological Process	GO:0060710	chorio-allantoic fusion	1/2279	7/18866	0.5939779	0.7442152	0.6733924	1	DNAJB6
Biological Process	GO:0061044	negative regulation of vascular wound healing	1/2279	7/18866	0.5939779	0.7442152	0.6733924	1	TNF

Biological Process	GO:0061087	positive regulation of histone H3-K27 methylation	1/2279	7/18866	0.5939779	0.7442152	0.6733924	1	PHF19
Biological Process	GO:0061316	canonical Wnt signaling pathway involved in heart development	1/2279	7/18866	0.5939779	0.7442152	0.6733924	1	RBPJ
Biological Process	GO:0070244	negative regulation of thymocyte apoptotic process	1/2279	7/18866	0.5939779	0.7442152	0.6733924	1	PTCRA
Biological Process	GO:0070305	response to cGMP	1/2279	7/18866	0.5939779	0.7442152	0.6733924	1	RAPGEF2
Biological Process	GO:0071051	polyadenylation-dependent snoRNA 3'-end processing	1/2279	7/18866	0.5939779	0.7442152	0.6733924	1	EXOSC2
Biological Process	GO:0071265	L-methionine biosynthetic process	1/2279	7/18866	0.5939779	0.7442152	0.6733924	1	ADI1
Biological Process	GO:0071267	L-methionine salvage	1/2279	7/18866	0.5939779	0.7442152	0.6733924	1	ADI1
Biological Process	GO:0071362	cellular response to ether	1/2279	7/18866	0.5939779	0.7442152	0.6733924	1	LARP1
Biological Process	GO:0071422	succinate transmembrane transport	1/2279	7/18866	0.5939779	0.7442152	0.6733924	1	SLC13A5

Biological Process	GO:0071694	maintenance of protein location in extracellular region	1/2279	7/18866	0.5939779	0.7442152	0.6733924	1	LTBP1
Biological Process	GO:0071947	protein deubiquitination involved in ubiquitin-dependent protein catabolic process	1/2279	7/18866	0.5939779	0.7442152	0.6733924	1	TNFAIP3
Biological Process	GO:0072235	metanephric distal tubule development	1/2279	7/18866	0.5939779	0.7442152	0.6733924	1	PAX2
Biological Process	GO:0072319	vesicle uncoating	1/2279	7/18866	0.5939779	0.7442152	0.6733924	1	SH3GL1
Biological Process	GO:0072526	pyridine-containing compound catabolic process	1/2279	7/18866	0.5939779	0.7442152	0.6733924	1	PNP
Biological Process	GO:0075044	positive regulation by symbiont of host autophagy	1/2279	7/18866	0.5939779	0.7442152	0.6733924	1	ULK1
Biological Process	GO:0075071	modulation by symbiont of host autophagy	1/2279	7/18866	0.5939779	0.7442152	0.6733924	1	ULK1
Biological Process	GO:0086028	bundle of His cell to Purkinje myocyte signaling	1/2279	7/18866	0.5939779	0.7442152	0.6733924	1	GJA5
Biological Process	GO:0086036	regulation of cardiac muscle cell membrane potential	1/2279	7/18866	0.5939779	0.7442152	0.6733924	1	FXYD1

Biological Process	GO:0086042	cardiac muscle cell-cardiac muscle cell adhesion	1/2279	7/18866	0.5939779	0.7442152	0.6733924	1	JUP
Biological Process	GO:0086043	bundle of His cell action potential	1/2279	7/18866	0.5939779	0.7442152	0.6733924	1	GJA5
Biological Process	GO:0086100	endothelin receptor signaling pathway	1/2279	7/18866	0.5939779	0.7442152	0.6733924	1	BCAR3
Biological Process	GO:0090166	Golgi disassembly	1/2279	7/18866	0.5939779	0.7442152	0.6733924	1	STX5
Biological Process	GO:0090219	negative regulation of lipid kinase activity	1/2279	7/18866	0.5939779	0.7442152	0.6733924	1	MIR138-2
Biological Process	GO:0090232	positive regulation of spindle checkpoint	1/2279	7/18866	0.5939779	0.7442152	0.6733924	1	PCID2
Biological Process	GO:0090267	positive regulation of mitotic cell cycle spindle assembly checkpoint	1/2279	7/18866	0.5939779	0.7442152	0.6733924	1	PCID2
Biological Process	GO:0097065	anterior head development	1/2279	7/18866	0.5939779	0.7442152	0.6733924	1	SSBP3
Biological Process	GO:0097187	dentinogenesis	1/2279	7/18866	0.5939779	0.7442152	0.6733924	1	FAM20C

Biological Process	GO:0097577	sequestering of iron ion	1/2279	7/18866	0.5939779	0.7442152	0.6733924	1	LCN2
Biological Process	GO:0098902	regulation of membrane depolarization during action potential	1/2279	7/18866	0.5939779	0.7442152	0.6733924	1	GJA5
Biological Process	GO:0098914	membrane repolarization during atrial cardiac muscle cell action potential	1/2279	7/18866	0.5939779	0.7442152	0.6733924	1	KCNQ1
Biological Process	GO:0098943	neurotransmitter receptor transport, postsynaptic endosome to lysosome	1/2279	7/18866	0.5939779	0.7442152	0.6733924	1	AP3D1
Biological Process	GO:0106014	regulation of inflammatory response to wounding	1/2279	7/18866	0.5939779	0.7442152	0.6733924	1	GRN
Biological Process	GO:0110021	cardiac muscle myoblast proliferation	1/2279	7/18866	0.5939779	0.7442152	0.6733924	1	MEIS2
Biological Process	GO:0110022	regulation of cardiac muscle myoblast proliferation	1/2279	7/18866	0.5939779	0.7442152	0.6733924	1	MEIS2
Biological Process	GO:0120041	positive regulation of macrophage proliferation	1/2279	7/18866	0.5939779	0.7442152	0.6733924	1	CSF1R
Biological Process	GO:0140212	regulation of long-chain fatty acid import into cell	1/2279	7/18866	0.5939779	0.7442152	0.6733924	1	THBS1

Biological Process	GO:1900094	regulation of transcription from RNA polymerase II promoter involved in determination of left/right symmetry	1/2279	7/18866	0.5939779	0.7442152	0.6733924	1	NODAL
Biological Process	GO:1900107	regulation of nodal signaling pathway	1/2279	7/18866	0.5939779	0.7442152	0.6733924	1	NODAL
Biological Process	GO:1900164	nodal signaling pathway involved in determination of lateral mesoderm left/right asymmetry	1/2279	7/18866	0.5939779	0.7442152	0.6733924	1	NODAL
Biological Process	GO:1900748	positive regulation of vascular endothelial growth factor signaling pathway	1/2279	7/18866	0.5939779	0.7442152	0.6733924	1	MYO1C
Biological Process	GO:1901142	insulin metabolic process	1/2279	7/18866	0.5939779	0.7442152	0.6733924	1	ERN1
Biological Process	GO:1901165	positive regulation of trophoblast cell migration	1/2279	7/18866	0.5939779	0.7442152	0.6733924	1	AGO2
Biological Process	GO:1901321	positive regulation of heart induction	1/2279	7/18866	0.5939779	0.7442152	0.6733924	1	WNT11
Biological Process	GO:1901509	regulation of endothelial tube morphogenesis	1/2279	7/18866	0.5939779	0.7442152	0.6733924	1	FOXP1
Biological Process	GO:1902022	L-lysine transport	1/2279	7/18866	0.5939779	0.7442152	0.6733924	1	SLC7A1

Biological Process	GO:1902083	negative regulation of peptidyl-cysteine S-nitrosylation	1/2279	7/18866	0.5939779	0.7442152	0.6733924	1	SNTA1
Biological Process	GO:1903003	positive regulation of protein deubiquitination	1/2279	7/18866	0.5939779	0.7442152	0.6733924	1	TNIP1
Biological Process	GO:1903059	regulation of protein lipidation	1/2279	7/18866	0.5939779	0.7442152	0.6733924	1	SVIP
Biological Process	GO:1903071	positive regulation of ER-associated ubiquitin-dependent protein catabolic process	1/2279	7/18866	0.5939779	0.7442152	0.6733924	1	NFE2L2
Biological Process	GO:1903367	positive regulation of fear response	1/2279	7/18866	0.5939779	0.7442152	0.6733924	1	MEF2C
Biological Process	GO:1903401	L-lysine transmembrane transport	1/2279	7/18866	0.5939779	0.7442152	0.6733924	1	SLC7A1
Biological Process	GO:1903689	regulation of wound healing, spreading of epidermal cells	1/2279	7/18866	0.5939779	0.7442152	0.6733924	1	RREB1
Biological Process	GO:1904141	positive regulation of microglial cell migration	1/2279	7/18866	0.5939779	0.7442152	0.6733924	1	CX3CR1
Biological Process	GO:1904732	regulation of electron transfer activity	1/2279	7/18866	0.5939779	0.7442152	0.6733924	1	SPHK2

Biological Process	GO:1905150	regulation of voltage-gated sodium channel activity	1/2279	7/18866	0.5939779	0.7442152	0.6733924	1	FGF14
Biological Process	GO:1905206	positive regulation of hydrogen peroxide-induced cell death	1/2279	7/18866	0.5939779	0.7442152	0.6733924	1	RIPK1
Biological Process	GO:1990001	inhibition of cysteine-type endopeptidase activity involved in apoptotic process	1/2279	7/18866	0.5939779	0.7442152	0.6733924	1	NOL3
Biological Process	GO:1990048	anterograde neuronal dense core vesicle transport	1/2279	7/18866	0.5939779	0.7442152	0.6733924	1	KIF1B
Biological Process	GO:1990791	dorsal root ganglion development	1/2279	7/18866	0.5939779	0.7442152	0.6733924	1	NRP2
Biological Process	GO:2000064	regulation of cortisol biosynthetic process	1/2279	7/18866	0.5939779	0.7442152	0.6733924	1	DGKQ
Biological Process	GO:2000197	regulation of ribonucleoprotein complex localization	1/2279	7/18866	0.5939779	0.7442152	0.6733924	1	SETD2
Biological Process	GO:2000210	positive regulation of anoikis	1/2279	7/18866	0.5939779	0.7442152	0.6733924	1	CHEK2
Biological Process	GO:2000391	positive regulation of neutrophil extravasation	1/2279	7/18866	0.5939779	0.7442152	0.6733924	1	IL1R1

Biological Process	GO:2000848	positive regulation of corticosteroid hormone secretion	1/2279	7/18866	0.5939779	0.7442152	0.6733924	1	GHRL
Biological Process	GO:2000973	regulation of pro-B cell differentiation	1/2279	7/18866	0.5939779	0.7442152	0.6733924	1	NOTCH1
Biological Process	GO:2000987	positive regulation of behavioral fear response	1/2279	7/18866	0.5939779	0.7442152	0.6733924	1	MEF2C
Biological Process	GO:2001046	positive regulation of integrin-mediated signaling pathway	1/2279	7/18866	0.5939779	0.7442152	0.6733924	1	LOXL3
Biological Process	GO:2001286	regulation of caveolin-mediated endocytosis	1/2279	7/18866	0.5939779	0.7442152	0.6733924	1	ITSN1
Biological Process	GO:0046785	microtubule polymerization	9/2279	77/18866	0.5944337	0.7444918	0.6736426	9	TBCD/DCTN1/CSNK1D/NDE1/NAV3/NUMA1/TPPP3/TUBGCP2/NDEL1
Biological Process	GO:0048332	mesoderm morphogenesis	9/2279	77/18866	0.5944337	0.7444918	0.6736426	9	MIR145/SETD2/SMAD3/WNT11/PAX2/FGFR1/ITGB3/INHBA/NODAL
Biological Process	GO:0150076	neuroinflammatory response	9/2279	77/18866	0.5944337	0.7444918	0.6736426	9	AZU1/TNFRSF1B/TNF/FPR2/APP/CLU/PTPRC/GRN/ITGB2
Biological Process	GO:0000729	DNA double-strand break processing	3/2279	25/18866	0.5960796	0.7449819	0.6740861	3	BLM/RAD52/SLX4

Biological Process	GO:0002092	positive regulation of receptor internalization	3/2279	25/18866	0.5960796	0.7449819	0.6740861	3	ANGPT1/ARRB1/ARRB2
Biological Process	GO:0008334	histone mRNA metabolic process	3/2279	25/18866	0.5960796	0.7449819	0.6740861	3	LSM1/CPSF3/SNRPD3
Biological Process	GO:0010842	retina layer formation	3/2279	25/18866	0.5960796	0.7449819	0.6740861	3	CRB1/HIPK2/SDK2
Biological Process	GO:0019400	alditol metabolic process	3/2279	25/18866	0.5960796	0.7449819	0.6740861	3	PGP/PCK2/DYSF
Biological Process	GO:0032366	intracellular sterol transport	3/2279	25/18866	0.5960796	0.7449819	0.6740861	3	ABCG1/ABCA1/NPC1
Biological Process	GO:0032367	intracellular cholesterol transport	3/2279	25/18866	0.5960796	0.7449819	0.6740861	3	ABCG1/ABCA1/NPC1
Biological Process	GO:0050687	negative regulation of defense response to virus	3/2279	25/18866	0.5960796	0.7449819	0.6740861	3	TRIM38/SEC14L1/ILRUN
Biological Process	GO:0050927	positive regulation of positive chemotaxis	3/2279	25/18866	0.5960796	0.7449819	0.6740861	3	AZU1/IL16/SMAD3
Biological Process	GO:0051307	meiotic chromosome separation	3/2279	25/18866	0.5960796	0.7449819	0.6740861	3	EME1/MEIOB/SLX4

Biological Process	GO:0051894	positive regulation of focal adhesion assembly	3/2279	25/18866	0.5960796	0.7449819	0.6740861	3	ITGB1BP1/SMAD3/PTPRJ
Biological Process	GO:0061050	regulation of cell growth involved in cardiac muscle cell development	3/2279	25/18866	0.5960796	0.7449819	0.6740861	3	CTDP1/MIR199A1/MIR199A2
Biological Process	GO:0061217	regulation of mesonephros development	3/2279	25/18866	0.5960796	0.7449819	0.6740861	3	TACSTD2/PAX2/HOXB7
Biological Process	GO:0062149	detection of stimulus involved in sensory perception of pain	3/2279	25/18866	0.5960796	0.7449819	0.6740861	3	SCN1A/ARRB2/NTRK1
Biological Process	GO:0097205	renal filtration	3/2279	25/18866	0.5960796	0.7449819	0.6740861	3	GJA5/PDGFB/F2R
Biological Process	GO:2001026	regulation of endothelial cell chemotaxis	3/2279	25/18866	0.5960796	0.7449819	0.6740861	3	THBS1/FGFR1/NOTCH1
Biological Process	GO:0006893	Golgi to plasma membrane transport	7/2279	60/18866	0.5984382	0.7477329	0.6765753	7	OSBPL5/KIF13A/DNM2/RAB31/VAMP5/CSK/VPS35L
Biological Process	GO:0071806	protein transmembrane transport	7/2279	60/18866	0.5984382	0.7477329	0.6765753	7	PAM16/CLU/ABCA1/BCR/PEX26/TIMM44/RTN2
Biological Process	GO:1901890	positive regulation of cell junction assembly	12/2279	103/18866	0.5986602	0.747912	0.6767373	12	ITGB1BP1/SMAD3/EPHB3/SEMA4A/PTPRJ/SEMA4D/NRXN1/GHRL/NTRK1/FLOT1/CUX2/NPHP4

Biological Process	GO:0050772	positive regulation of axonogenesis	10/2279	86/18866	0.6008477	0.7494336	0.6781141	10	PLXNC1/ZEB2/DISC1/ILK/SEMA4D/CDH4/CYFIP1/TRPV2/NDEL1/DBNL
Biological Process	GO:0002756	MyD88-independent toll-like receptor signaling pathway	4/2279	34/18866	0.6009825	0.7494336	0.6781141	4	RIPK1/PRKCE/UBE2D2/FADD
Biological Process	GO:0006409	tRNA export from nucleus	4/2279	34/18866	0.6009825	0.7494336	0.6781141	4	RAE1/NUP93/SEC13/NOL6
Biological Process	GO:0007616	long-term memory	4/2279	34/18866	0.6009825	0.7494336	0.6781141	4	PRKCZ/SGK1/CPEB3/NTF4
Biological Process	GO:0009452	7-methylguanosine RNA capping	4/2279	34/18866	0.6009825	0.7494336	0.6781141	4	POLR2F/CCNH/ERCC2/NCBP3
Biological Process	GO:0010765	positive regulation of sodium ion transport	4/2279	34/18866	0.6009825	0.7494336	0.6781141	4	FXYD1/DNM2/TESC/ACTN4
Biological Process	GO:0015865	purine nucleotide transport	4/2279	34/18866	0.6009825	0.7494336	0.6781141	4	SLC25A25/SLC33A1/ABCC11/SLC25A1
Biological Process	GO:0032814	regulation of natural killer cell activation	4/2279	34/18866	0.6009825	0.7494336	0.6781141	4	FGR/DCAF15/HLA-F/PGLYRP1
Biological Process	GO:0036260	RNA capping	4/2279	34/18866	0.6009825	0.7494336	0.6781141	4	POLR2F/CCNH/ERCC2/NCBP3

Biological Process	GO:0042558	pteridine-containing compound metabolic process	4/2279	34/18866	0.6009825	0.7494336	0.6781141	4	MTHFD1L/MTHFS/SLC19A1/MTHFD1
Biological Process	GO:0043516	regulation of DNA damage response, signal transduction by p53 class mediator	4/2279	34/18866	0.6009825	0.7494336	0.6781141	4	SPRED2/ZNF385A/ANKRD1/MARCHF7
Biological Process	GO:0046685	response to arsenic-containing substance	4/2279	34/18866	0.6009825	0.7494336	0.6781141	4	ABCC2/FECH/GSTO1/GSTO2
Biological Process	GO:0051482	positive regulation of cytosolic calcium ion concentration involved in phospholipase C-activating G protein-coupled signaling pathway	4/2279	34/18866	0.6009825	0.7494336	0.6781141	4	C3AR1/F2R/GPR4/AGTR1
Biological Process	GO:0071431	tRNA-containing ribonucleoprotein complex export from nucleus	4/2279	34/18866	0.6009825	0.7494336	0.6781141	4	RAE1/NUP93/SEC13/NOL6
Biological Process	GO:0071482	cellular response to light stimulus	15/2279	129/18866	0.6033297	0.7521631	0.6805839	15	PDE6A/MFAP4/CRB1/PIK3R1/BMF/CDC25A/AQP1/CREBBP/GRK7/RHNO1/NEDD4/ERCC1/CRIP1/POLD3/XPA
Biological Process	GO:1901657	glycosyl compound metabolic process	15/2279	129/18866	0.6033297	0.7521631	0.6805839	15	CDA/DPYS/AMPD3/TJP2/MACROD1/TK2/UCK2/UPB1/PNP/GUK1/GMPR2/DHODH/TK1/IMPDH1/ACP3
Biological Process	GO:0045930	negative regulation of mitotic cell cycle	40/2279	341/18866	0.6044213	0.7534175	0.6817189	40	TRIM39/BLM/TIMP2/IL10/TFDP1/MAD1L1/FOXN3/TNKS/PRMT2/SMAD3/HUS1/TNF/FBXO7/E2F7/CTDSPL/PCBP4/EME1/RIPK1/NACC2/ZNF385A/TGFB1/ARID3A/OVOL1/PSMF1/GFI1B/TAOK3/ZFYVE19/MIR138-2/CLSPN/RB1/BCL2/PSMB7/CHEK2/BCL2L1/CDC14A/AURKAIP1/NAE1/PSMD13/PCID2/MAP3K20
Biological Process	GO:0035914	skeletal muscle cell differentiation	8/2279	69/18866	0.6046341	0.7534175	0.6817189	8	MEF2D/PAX7/ARNTL/ANKRD2/RB1/NOTCH1/MEF2C/ANKRD1

Biological Process	GO:2000573	positive regulation of DNA biosynthetic process	8/2279	69/18866	0.6046341	0.7534175	0.6817189	8	TNKS/HMBOX1/GRHL2/PDGFB/ARRB2/PDGFRB/PARM1/CCT6A
Biological Process	GO:0046822	regulation of nucleocytoplasmic transport	13/2279	112/18866	0.604653	0.7534175	0.6817189	13	ANGPT1/PIK3R1/SETD2/SMAD3/SUFU/JUP/TCF7L2/TGFB1/NF1/CDH1/CABP1/SP100/NEDD4
Biological Process	GO:0006310	DNA recombination	35/2279	299/18866	0.6061387	0.7550186	0.6831677	35	BLM/SPIDR/IL10/BRCA2/RMI2/RAD52/SETD2/RTEL1/HUS1/NSMCE2/MCM5/RECQL5/EME1/MEIOB/IGHMBP2/UNG/TGFB1/AP5S1/RNF212/PTPRC/MCM2/RAD21L1/TCF3/DMC1/TP53BP1/RHNO1/ERCC1/TNFSF13/SLX4/RUVBL1/ERCC2/KLHDC3/PAXIP1/NSD2/FBH1
Biological Process	GO:0003009	skeletal muscle contraction	5/2279	43/18866	0.6061764	0.7550186	0.6831677	5	TNNT3/JSRP1/GSTO1/CASQ1/MB
Biological Process	GO:1901031	regulation of response to reactive oxygen species	5/2279	43/18866	0.6061764	0.7550186	0.6831677	5	IL10/TNF/RIPK1/NFE2L2/SIRT3
Biological Process	GO:0003073	regulation of systemic arterial blood pressure	11/2279	95/18866	0.6068561	0.7557661	0.683844	11	CTSZ/PDE4D/CTSG/SLC2A5/GJA5/KCNK6/PDGFB/F2R/AGTR1/HSD11B2/AOPEP
Biological Process	GO:0008406	gonad development	26/2279	223/18866	0.6078206	0.7568681	0.6848411	26	ARID5B/BRCA2/ARRB1/EIF2B5/CSDE1/FANCA/RDH10/RARA/SF1/BMPR1B/NCOA1/ARRB2/NTRK1/HOXA9/RAD21L1/BCL2/DMC1/SLIT3/UBE3A/CEBPB/PDGFRB/ERCC1/BCL2L1/HMGCS1/NCOA4/INHBA
Biological Process	GO:0009994	oocyte differentiation	6/2279	52/18866	0.6113652	0.7610823	0.6886543	6	BRCA2/KMT2D/PPP2R1A/BCL2/DMC1/NPPC
Biological Process	GO:0046580	negative regulation of Ras protein signal transduction	6/2279	52/18866	0.6113652	0.7610823	0.6886543	6	TIMP2/KANK1/CUL3/RASA3/SYNGAP1/NF1

Biological Process	GO:1905330	regulation of morphogenesis of an epithelium	21/2279	181/18866	0.6127924	0.7627592	0.6901716	21	RREB1/TACSTD2/RXRA/FOXP1/AP2A1/VANGL1/TNF/PRICKLE2/WNT11/PAX2/FGFR1/SMURF1/PFN1/PSMF1/ARRB2/DAAM1/PSMB7/ANKRD6/LIF/HOXB7/PSMD13
Biological Process	GO:0010948	negative regulation of cell cycle process	42/2279	359/18866	0.6129484	0.7628533	0.6902568	42	TRIM39/BLM/CALR/FZR1/TFDP1/MAD1L1/FOXN3/TNKS/PRMT2/HUS1/FBXO7/E2F7/MGA/CTDSPL/PCBP4/EHMT2/EPC1/RIPK1/NACC2/ZNF385A/ARID3A/OVOL1/PHC3/C10orf99/PSMF1/GFI1B/TAOK3/ZFYVE19/MIR138-2/CLSPN/RB1/BCL2/E2F6/PSMB7/CHEK2/AURKAIP1/LIF/NAE1/PSMD13/PCID2/ABRAXAS1/BABAM2
Biological Process	GO:0031960	response to corticosteroid	19/2279	164/18866	0.6133344	0.7632338	0.690601	19	IL10/AKAP13/TNF/ABCC2/SMYD3/ALPL/FECH/IL1RN/AQP1/AGXT/ENG/BCL2/NOTCH1/SLIT3/PCK2/FOXO1/PTPRU/HSD11B2/HNMT
Biological Process	GO:0051384	response to glucocorticoid	17/2279	147/18866	0.6142598	0.7642853	0.6915525	17	IL10/AKAP13/TNF/ABCC2/SMYD3/ALPL/FECH/IL1RN/AQP1/AGXT/BCL2/SLIT3/PCK2/FOXO1/PTPRU/HSD11B2/HNMT
Biological Process	GO:0009410	response to xenobiotic stimulus	15/2279	130/18866	0.6157085	0.7659842	0.6930897	15	NR1I2/ACSL1/GGT1/LPO/ABCC2/AIP/SULT1A2/POR/MGST2/GUK1/RB1/GSTO1/EPHX1/GSTO2/MTARC1
Biological Process	GO:0006890	retrograde vesicle-mediated transport, Golgi to endoplasmic reticulum	10/2279	87/18866	0.6159478	0.7659842	0.6930897	10	LMAN2/ERGIC1/UVRAG/BICD2/HTT/NBAS/KIFAP3/TMED2/ARF5/PLPP3
Biological Process	GO:0009144	purine nucleoside triphosphate metabolic process	10/2279	87/18866	0.6159478	0.7659842	0.6930897	10	PRKAG2/AMPD3/ENO1/VPS9D1/SPHK2/TGFB1/GUK1/PKM/IMPDH1/ATP5PD
Biological Process	GO:0051453	regulation of intracellular pH	10/2279	87/18866	0.6159478	0.7659842	0.6930897	10	TM9SF4/SLC11A1/ATP6V1B2/GRN/BCL2/ATP6V0B/UBE3A/CLN5/ATP6V0C/ATP6V1C1
Biological Process	GO:0021872	forebrain generation of neurons	7/2279	61/18866	0.6164429	0.7661988	0.6932839	7	NRP2/CSF1R/DISC1/ZMIZ1/FGFR1/RAPGEF2/INHBA

Biological Process	GO:0031343	positive regulation of cell killing	7/2279	61/18866	0.6164429	0.7661988	0.6932839	7	CD226/HLA-F/PTPRC/IL18RAP/CADM1/FADD/CYRIB
Biological Process	GO:0090342	regulation of cell aging	7/2279	61/18866	0.6164429	0.7661988	0.6932839	7	FZR1/MAP3K3/AKT3/VASH1/ARNTL/NEK6/FOXM1
Biological Process	GO:1902110	positive regulation of mitochondrial membrane permeability involved in apoptotic process	7/2279	61/18866	0.6164429	0.7661988	0.6932839	7	TFDP1/YWHAH/BMF/YWHAZ/BCL2/TP53BP2/YWHAQ
Biological Process	GO:0031349	positive regulation of defense response	45/2279	385/18866	0.6178312	0.7678239	0.6947544	45	NLRC4/S100A8/AIM2/IL16/ABCC1/CLEC4C/CCR2/TNF/CCL5/FPR2/TLR9/MNDA/RIPK1/CD226/HLA-F/TNFRSF1A/MUC12/F12/FCN1/TRIM5/MUC20/TNIP1/BCL10/MGST2/PSMF1/GRN/NLRC5/CREBBP/ETS1/IL18RAP/NOD2/PSMB7/MEF2C/GPR4/CEBPB/AGTR1/CADM1/RELA/FBXW11/HEXIM1/PARP9/FADD/PSMD13/STING1/CCN4
Biological Process	GO:0021543	pallium development	20/2279	173/18866	0.6183685	0.7682908	0.6951768	20	MFSD2A/NDE1/BTBD3/ZEB2/EIF2B5/RTN4/DISC1/TRAPPC9/HDAC1/ZMIZ1/RARA/NF1/KIRREL3/TACC3/WDR47/NCOA1/PLCB1/SLC2A1/NDEL1/NPY
Biological Process	GO:0050954	sensory perception of mechanical stimulus	20/2279	173/18866	0.6183685	0.7682908	0.6951768	20	KCNE1/HEXB/DRGX/CDH23/ATP2B2/ATP8B1/MBP/LRIG1/SCN1A/FGFR1/ESPN/WDR1/KCNQ1/NTRK1/TSPEAR/SLC1A3/TMC1/CRYM/CDC14A/SPTBN4
Biological Process	GO:0006959	humoral immune response	44/2279	377/18866	0.6210881	0.7711559	0.6977692	44	DEFA4/AZU1/ELANE/SLPI/PRTN3/S100A8/CTSG/MFAP4/C3AR1/LCN2/RNASE3/CD55/SLC11A1/TREM1/CCR2/TNF/SFTPD/IGHV6-1/SPON2/C5AR2/RBPJ/MASP1/PTPN6/CLU/TNFRSF21/PHB/FCN1/DEFB1/CD59/C1S/PGLYRP1/ST6GAL1/PTPRC/POU2F2/BCL2/NOTCH1/MEF2C/TRAF3IP2/CPB2/LTF/PGLYRP4/C1QB/BCL3/C1RL
Biological Process	GO:0010972	negative regulation of G2/M transition of mitotic cell cycle	11/2279	96/18866	0.6211695	0.7711559	0.6977692	11	TRIM39/BLM/FOXN3/HUS1/PSMF1/TAOK3/ZFYVE19/CLSPN/PSMB7/NAE1/PSMD13
Biological Process	GO:0001756	somitogenesis	8/2279	70/18866	0.621372	0.7711559	0.6977692	8	SMAD3/ZEB2/DLL1/RBPJ/MIB1/NOTCH1/TMED2/ALDH1A2

Biological Process	GO:0032370	positive regulation of lipid transport	8/2279	70/18866	0.621372	0.7711559	0.6977692	8	MYB/MAP2K6/ABCG1/ATP8A1/PLTP/NR1H2/GHRL/ABCA1
Biological Process	GO:0043270	positive regulation of ion transport	33/2279	284/18866	0.6221741	0.7711559	0.6977692	33	KCNE1/MAP2K6/FXYD1/MYLK/CCR2/DNM2/CCL5/ATP8A1/TESC/NOS1AP/ACTN4/P2RY6/SPHK2/HTR3A/HTT/PDGFB/KCNQ1/ARRB2/GSTO1/P2RX1/F2R/CEBPB/PDGFRB/ANO6/ANK2/PLCG1/P2RX5/TRPV2/PDZK1/CASQ1/FGF14/STIMATE/CRACR2A
Biological Process	GO:0008211	glucocorticoid metabolic process	3/2279	26/18866	0.6234717	0.7711559	0.6977692	3	YWHAH/DGKQ/HSD11B2
Biological Process	GO:0010884	positive regulation of lipid storage	3/2279	26/18866	0.6234717	0.7711559	0.6977692	3	EHD1/NR1H2/ACACB
Biological Process	GO:0018904	ether metabolic process	3/2279	26/18866	0.6234717	0.7711559	0.6977692	3	CHPT1/EPHX1/PEDS1
Biological Process	GO:0022616	DNA strand elongation	3/2279	26/18866	0.6234717	0.7711559	0.6977692	3	POLE/GINS3/POLD3
Biological Process	GO:0034453	microtubule anchoring	3/2279	26/18866	0.6234717	0.7711559	0.6977692	3	DCTN1/BICD2/CEP350
Biological Process	GO:0035235	ionotropic glutamate receptor signaling pathway	3/2279	26/18866	0.6234717	0.7711559	0.6977692	3	APP/GRIK4/GRID1
Biological Process	GO:0050926	regulation of positive chemotaxis	3/2279	26/18866	0.6234717	0.7711559	0.6977692	3	AZU1/IL16/SMAD3

Biological Process	GO:0051156	glucose 6-phosphate metabolic process	3/2279	26/18866	0.6234717	0.7711559	0.6977692	3	HK1/PGAM1/HK2
Biological Process	GO:0051873	killing by host of symbiont cells	3/2279	26/18866	0.6234717	0.7711559	0.6977692	3	AZU1/ELANE/CTSG
Biological Process	GO:0060740	prostate gland epithelium morphogenesis	3/2279	26/18866	0.6234717	0.7711559	0.6977692	3	RXRA/RARG/NOTCH1
Biological Process	GO:0070911	global genome nucleotide-excision repair	3/2279	26/18866	0.6234717	0.7711559	0.6977692	3	ERCC1/XPA/ERCC2
Biological Process	GO:0072273	metanephric nephron morphogenesis	3/2279	26/18866	0.6234717	0.7711559	0.6977692	3	PAX2/PDGFRB/LIF
Biological Process	GO:0097066	response to thyroid hormone	3/2279	26/18866	0.6234717	0.7711559	0.6977692	3	CTSH/CAB39/CTSB
Biological Process	GO:1901018	positive regulation of potassium ion transmembrane transporter activity	3/2279	26/18866	0.6234717	0.7711559	0.6977692	3	DNM2/NOS1AP/ANK2
Biological Process	GO:1904754	positive regulation of vascular associated smooth muscle cell migration	3/2279	26/18866	0.6234717	0.7711559	0.6977692	3	SSH1/MIR143/PDGFB
Biological Process	GO:0003203	endocardial cushion morphogenesis	4/2279	35/18866	0.6245523	0.7711559	0.6977692	4	TGFBR2/RBPJ/ENG/NOTCH1

Biological Process	GO:0006739	NADP metabolic process	4/2279	35/18866	0.6245523	0.7711559	0.6977692	4	NADK/KCNAB2/PGAM1/TP53I3
Biological Process	GO:0016486	peptide hormone processing	4/2279	35/18866	0.6245523	0.7711559	0.6977692	4	CTSZ/CTSG/PCSK6/AOPEP
Biological Process	GO:0019362	pyridine nucleotide metabolic process	4/2279	35/18866	0.6245523	0.7711559	0.6977692	4	NADK/NMNAT3/PARP10/PARP9
Biological Process	GO:0033687	osteoblast proliferation	4/2279	35/18866	0.6245523	0.7711559	0.6977692	4	SMAD3/MIR138-2/BCL2/LTF
Biological Process	GO:0045922	negative regulation of fatty acid metabolic process	4/2279	35/18866	0.6245523	0.7711559	0.6977692	4	MFSD2A/ACACB/WDTC1/ACADVL
Biological Process	GO:0046496	nicotinamide nucleotide metabolic process	4/2279	35/18866	0.6245523	0.7711559	0.6977692	4	NADK/NMNAT3/PARP10/PARP9
Biological Process	GO:0050779	RNA destabilization	4/2279	35/18866	0.6245523	0.7711559	0.6977692	4	TNFRSF1B/ZC3H12D/CPEB3/FTO
Biological Process	GO:0140448	signaling receptor ligand precursor processing	4/2279	35/18866	0.6245523	0.7711559	0.6977692	4	CTSZ/CTSG/PCSK6/AOPEP
Biological Process	GO:2001251	negative regulation of chromosome organization	17/2279	148/18866	0.6257665	0.7711559	0.6977692	17	MAD1L1/TNKS/SKI/RTEL1/OTUB2/SPHK2/CTBP1/KDM4C/MCM2/SMG6/OTUB1/DNMT1/PIH1D1/TRIP12/ERCC1/SLX4/PCID2

Biological Process	GO:0009205	purine ribonucleoside triphosphate metabolic process	9/2279	79/18866	0.6261423	0.7711559	0.6977692	9	PRKAG2/AMPD3/ENO1/VPS9D1/SPHK2/TGFB1/PKM/IMPDH1/ATP5PD
Biological Process	GO:0030104	water homeostasis	9/2279	79/18866	0.6261423	0.7711559	0.6977692	9	ADCY2/PRKAR1B/ADCY9/AQP1/ADCY4/AQP3/FA2H/ANXA7/AQP9
Biological Process	GO:0002483	antigen processing and presentation of endogenous peptide antigen	2/2279	17/18866	0.626275	0.7711559	0.6977692	2	HLA-C/HLA-F
Biological Process	GO:0006266	DNA ligation	2/2279	17/18866	0.626275	0.7711559	0.6977692	2	TFIP11/PAXX
Biological Process	GO:0006474	N-terminal protein amino acid acetylation	2/2279	17/18866	0.626275	0.7711559	0.6977692	2	CREBBP/NAA16
Biological Process	GO:0007096	regulation of exit from mitosis	2/2279	17/18866	0.626275	0.7711559	0.6977692	2	CDCA5/CDC14A
Biological Process	GO:0007250	activation of NF-kappaB-inducing kinase activity	2/2279	17/18866	0.626275	0.7711559	0.6977692	2	CHI3L1/TRAF2
Biological Process	GO:0008340	determination of adult lifespan	2/2279	17/18866	0.626275	0.7711559	0.6977692	2	INPP5D/PRDM2
Biological Process	GO:0009299	mRNA transcription	2/2279	17/18866	0.626275	0.7711559	0.6977692	2	HIPK3/SREBF1

Biological Process	GO:0010715	regulation of extracellular matrix disassembly	2/2279	17/18866	0.626275	0.7711559	0.6977692	2	TGFB1/ETS1
Biological Process	GO:0014067	negative regulation of phosphatidylinositol 3-kinase signaling	2/2279	17/18866	0.626275	0.7711559	0.6977692	2	INPP5E/TSC2
Biological Process	GO:0016074	snoRNA metabolic process	2/2279	17/18866	0.626275	0.7711559	0.6977692	2	EXOSC2/FBL
Biological Process	GO:0019377	glycolipid catabolic process	2/2279	17/18866	0.626275	0.7711559	0.6977692	2	HEXB/GALC
Biological Process	GO:0019885	antigen processing and presentation of endogenous peptide antigen via MHC class I	2/2279	17/18866	0.626275	0.7711559	0.6977692	2	HLA-C/HLA-F
Biological Process	GO:0021514	ventral spinal cord interneuron differentiation	2/2279	17/18866	0.626275	0.7711559	0.6977692	2	SUFU/LMO4
Biological Process	GO:0032966	negative regulation of collagen biosynthetic process	2/2279	17/18866	0.626275	0.7711559	0.6977692	2	PPARG/NPPC
Biological Process	GO:0033262	regulation of nuclear cell cycle DNA replication	2/2279	17/18866	0.626275	0.7711559	0.6977692	2	FGFR1/CHEK2
Biological Process	GO:0033631	cell-cell adhesion mediated by integrin	2/2279	17/18866	0.626275	0.7711559	0.6977692	2	CCL5/PLPP3

Biological Process	GO:0034384	high-density lipoprotein particle clearance	2/2279	17/18866	0.626275	0.7711559	0.6977692	2	GPLD1/HDLBP
Biological Process	GO:0035461	vitamin transmembrane transport	2/2279	17/18866	0.626275	0.7711559	0.6977692	2	SLC19A1/SLC23A1
Biological Process	GO:0038092	nodal signaling pathway	2/2279	17/18866	0.626275	0.7711559	0.6977692	2	SMAD3/NODAL
Biological Process	GO:0042448	progesterone metabolic process	2/2279	17/18866	0.626275	0.7711559	0.6977692	2	DHRS9/DGKQ
Biological Process	GO:0043649	dicarboxylic acid catabolic process	2/2279	17/18866	0.626275	0.7711559	0.6977692	2	MTHFS/ACSF3
Biological Process	GO:0046068	cGMP metabolic process	2/2279	17/18866	0.626275	0.7711559	0.6977692	2	PDE9A/NPPC
Biological Process	GO:0048385	regulation of retinoic acid receptor signaling pathway	2/2279	17/18866	0.626275	0.7711559	0.6977692	2	CALR/CTBP2
Biological Process	GO:0060644	mammary gland epithelial cell differentiation	2/2279	17/18866	0.626275	0.7711559	0.6977692	2	LBH/CEBPB
Biological Process	GO:0061158	3'-UTR-mediated mRNA destabilization	2/2279	17/18866	0.626275	0.7711559	0.6977692	2	ZC3H12D/CPEB3

Biological Process	GO:0070206	protein trimerization	2/2279	17/18866	0.626275	0.7711559	0.6977692	2	SLC1A5/SLC1A2
Biological Process	GO:0070242	thymocyte apoptotic process	2/2279	17/18866	0.626275	0.7711559	0.6977692	2	PTCRA/ZC3H8
Biological Process	GO:0070875	positive regulation of glycogen metabolic process	2/2279	17/18866	0.626275	0.7711559	0.6977692	2	DYRK2/ESRRB
Biological Process	GO:0090185	negative regulation of kidney development	2/2279	17/18866	0.626275	0.7711559	0.6977692	2	TACSTD2/PAX2
Biological Process	GO:0097067	cellular response to thyroid hormone stimulus	2/2279	17/18866	0.626275	0.7711559	0.6977692	2	CTSH/CTSB
Biological Process	GO:0099509	regulation of presynaptic cytosolic calcium ion concentration	2/2279	17/18866	0.626275	0.7711559	0.6977692	2	ATP2B2/P2RX1
Biological Process	GO:1901317	regulation of flagellated sperm motility	2/2279	17/18866	0.626275	0.7711559	0.6977692	2	RNASE10/DEFB1
Biological Process	GO:1902358	sulfate transmembrane transport	2/2279	17/18866	0.626275	0.7711559	0.6977692	2	SLC13A4/SLC26A1
Biological Process	GO:1904355	positive regulation of telomere capping	2/2279	17/18866	0.626275	0.7711559	0.6977692	2	TNKS/RTEL1

Biological Process	GO:1904948	midbrain dopaminergic neuron differentiation	2/2279	17/18866	0.626275	0.7711559	0.6977692	2	CSNK1D/CSNK1E
Biological Process	GO:1905331	negative regulation of morphogenesis of an epithelium	2/2279	17/18866	0.626275	0.7711559	0.6977692	2	TACSTD2/TNF
Biological Process	GO:2000136	regulation of cell proliferation involved in heart morphogenesis	2/2279	17/18866	0.626275	0.7711559	0.6977692	2	RBPJ/NOTCH1
Biological Process	GO:0071456	cellular response to hypoxia	24/2279	208/18866	0.6271168	0.7716275	0.698196	24	P4HB/ENO1/MIR140/NDRG1/SLC8A1/HIPK2/RBPJ/LIMD1/AQP1/PSMF1/CREBBP/NOL3/NFE2L2/BCL2/PSMB7/NOTCH1/AQP3/EPAS1/PRKCE/ANKRD1/SLC29A1/BACH1/PSMD13/PRKAA1
Biological Process	GO:0021575	hindbrain morphogenesis	5/2279	44/18866	0.6271453	0.7716275	0.698196	5	DLL1/TTC21B/HES3/TTLL1/COQ8B
Biological Process	GO:0031670	cellular response to nutrient	5/2279	44/18866	0.6271453	0.7716275	0.698196	5	PPARG/TNKS/VDR/NCOA1/USF2
Biological Process	GO:0044091	membrane biogenesis	5/2279	44/18866	0.6271453	0.7716275	0.698196	5	PACSIN2/NRXN1/NRXN2/MIR138-2/FLOT1
Biological Process	GO:0090503	RNA phosphodiester bond hydrolysis, exonucleolytic	5/2279	44/18866	0.6271453	0.7716275	0.698196	5	EXOSC2/ERI3/DIS3L2/TOE1/ISG20
Biological Process	GO:1902667	regulation of axon guidance	5/2279	44/18866	0.6271453	0.7716275	0.698196	5	SEMA6B/SEMA4A/SEMA4B/SEMA4D/ZSWIM5

Biological Process	GO:0048167	regulation of synaptic plasticity	22/2279	191/18866	0.6278705	0.7724197	0.6989128	22	PRKCZ/SSH1/SORCS2/CREB1/YWHAH/BAIAP2/ZDHHC2/APP/AKAP5/SYNGAP1/RAPGEF2/HRH1/RARA/NF1/CPEB3/SIPA1L1/MAP1A/MEF2C/SHANK2/CX3CR1/SYNPO/FGF14
Biological Process	GO:0032200	telomere organization	20/2279	174/18866	0.6289608	0.7736609	0.7000359	20	BLM/TNKS/POLE/BRCA2/HMBOX1/RTEL1/HUS1/NSMCE2/YLPM1/POLA2/SMARCAL1/SMG6/SP100/ERCC1/SLX4/POLD3/CCT6A/POLE4/TFIP11/H3C1
Biological Process	GO:0000731	DNA synthesis involved in DNA repair	6/2279	53/18866	0.6304146	0.7753488	0.7015631	6	POLE/WRNIP1/TEX12/POLD3/PARP10/POLI
Biological Process	GO:1990748	cellular detoxification	13/2279	114/18866	0.6309207	0.7758708	0.7020354	13	MPO/LPO/TNF/HBB/MGST2/CAT/NFE2L2/NXN/GSTO1/TXNRD2/GSTO2/SIRT3/PRXL2B
Biological Process	GO:0010676	positive regulation of cellular carbohydrate metabolic process	7/2279	62/18866	0.6339991	0.775915	0.7020754	7	GPLD1/P2RY6/HRH1/DYRK2/ESRRB/PRKCE/FOXO1
Biological Process	GO:0071384	cellular response to corticosteroid stimulus	7/2279	62/18866	0.6339991	0.775915	0.7020754	7	AKAP13/ABCC2/SMYD3/FECH/AQP1/PCK2/FOXO1
Biological Process	GO:0036473	cell death in response to oxidative stress	11/2279	97/18866	0.6352167	0.775915	0.7020754	11	P4HB/IL10/ZNF622/FBXO7/RIPK1/STK24/NOL3/NFE2L2/BCL2/BAG5/PRKN
Biological Process	GO:1902476	chloride transmembrane transport	11/2279	97/18866	0.6352167	0.775915	0.7020754	11	CLCN6/FXYD1/ANO7/SLC26A1/SLC1A3/ANO10/ANO6/SLC12A1/ANO8/SLC12A7/PACC1
Biological Process	GO:0014823	response to activity	8/2279	71/18866	0.6377226	0.775915	0.7020754	8	SLC25A25/IL10/CREB1/COL4A2/CAB39/CAT/FIS1/PRKAA1

Biological Process	GO:0033692	cellular polysaccharide biosynthetic process	8/2279	71/18866	0.6377226	0.775915	0.7020754	8	CSGALNACT1/NDST1/DYRK2/PPP1CB/ESRRB/PER2/HAS3/B3GNT2
Biological Process	GO:0042531	positive regulation of tyrosine phosphorylation of STAT protein	8/2279	71/18866	0.6377226	0.775915	0.7020754	8	TNF/CCL5/CSF1R/TNFRSF1A/IL6R/CRLF1/PARP9/LIF
Biological Process	GO:0061180	mammary gland epithelium development	8/2279	71/18866	0.6377226	0.775915	0.7020754	8	RREB1/BRCA2/LRP5L/VDR/CSF1R/LBH/RTN4/CEBPB
Biological Process	GO:0007218	neuropeptide signaling pathway	12/2279	106/18866	0.6395352	0.775915	0.7020754	12	SORL1/SORCS2/PNOC/CRCP/LTB4R2/RAPGEF2/SORT1/TYRO3/NPFFR1/NPY/LTB4R/PRLHR
Biological Process	GO:0071453	cellular response to oxygen levels	27/2279	235/18866	0.6400593	0.775915	0.7020754	27	P4HB/ENO1/PPARG/MIR140/NDRG1/SLC8A1/HIPK2/RBPJ/LIMD1/AQP1/PSMF1/CREBBP/NOL3/NFE2L2/BCL2/PSMB7/NOTCH1/AQP3/DNM1L/EPAS1/PRKCE/FOXO1/ANKRD1/SLC29A1/BACH1/PSMD13/PRKAA1
Biological Process	GO:0036294	cellular response to decreased oxygen levels	25/2279	218/18866	0.640937	0.775915	0.7020754	25	P4HB/ENO1/MIR140/NDRG1/SLC8A1/HIPK2/RBPJ/LIMD1/AQP1/PSMF1/CREBBP/NOL3/NFE2L2/BCL2/PSMB7/NOTCH1/AQP3/DNM1L/EPAS1/PRKCE/ANKRD1/SLC29A1/BACH1/PSMD13/PRKAA1
Biological Process	GO:0050729	positive regulation of inflammatory response	18/2279	158/18866	0.641458	0.775915	0.7020754	18	S100A8/IL16/ABCC1/CCR2/TNF/TLR9/RIPK1/TNFRSF1A/F12/TNIP1/MGST2/GRN/ETS1/NOD2/GPR4/CEBPB/AGTR1/CCN4
Biological Process	GO:2000300	regulation of synaptic vesicle exocytosis	9/2279	80/18866	0.6414921	0.775915	0.7020754	9	RAP1B/GIT1/APBA2/PRKCB/VPS18/CHRM2/P2RX1/DNM1L/DTNBP1
Biological Process	GO:0001763	morphogenesis of a branching structure	23/2279	201/18866	0.6421089	0.775915	0.7020754	23	CTSZ/TACSTD2/RXRA/IL10/LRP5L/VDR/SETD2/SPINT2/TNF/BCL11A/RTN4/TGFBR2/CTSH/ILK/PAX2/FGFR1/RDH10/GRHL2/ENG/BCL2/NOTCH1/NOTCH4/HOXB7

Biological Process	GO:0000820	regulation of glutamine family amino acid metabolic process	1/2279	8/18866	0.6430433	0.775915	0.7020754	1	SLC7A7
Biological Process	GO:0001547	antral ovarian follicle growth	1/2279	8/18866	0.6430433	0.775915	0.7020754	1	BMPR1B
Biological Process	GO:0001955	blood vessel maturation	1/2279	8/18866	0.6430433	0.775915	0.7020754	1	LYL1
Biological Process	GO:0002016	regulation of blood volume by renin-angiotensin	1/2279	8/18866	0.6430433	0.775915	0.7020754	1	AGTR1
Biological Process	GO:0002072	optic cup morphogenesis involved in camera-type eye development	1/2279	8/18866	0.6430433	0.775915	0.7020754	1	PAX2
Biological Process	GO:0002420	natural killer cell mediated cytotoxicity directed against tumor cell target	1/2279	8/18866	0.6430433	0.775915	0.7020754	1	CD226
Biological Process	GO:0002765	immune response-inhibiting signal transduction	1/2279	8/18866	0.6430433	0.775915	0.7020754	1	CD33
Biological Process	GO:0002858	regulation of natural killer cell mediated cytotoxicity directed against tumor cell target	1/2279	8/18866	0.6430433	0.775915	0.7020754	1	CD226
Biological Process	GO:0003140	determination of left/right asymmetry in lateral mesoderm	1/2279	8/18866	0.6430433	0.775915	0.7020754	1	NODAL

Biological Process	GO:0006152	purine nucleoside catabolic process	1/2279	8/18866	0.6430433	0.775915	0.7020754	1	PNP
Biological Process	GO:0006551	leucine metabolic process	1/2279	8/18866	0.6430433	0.775915	0.7020754	1	HMGCL
Biological Process	GO:0006776	vitamin A metabolic process	1/2279	8/18866	0.6430433	0.775915	0.7020754	1	ALDH1A2
Biological Process	GO:0006863	purine nucleobase transport	1/2279	8/18866	0.6430433	0.775915	0.7020754	1	AQP9
Biological Process	GO:0007144	female meiosis I	1/2279	8/18866	0.6430433	0.775915	0.7020754	1	MEIOB
Biological Process	GO:0007197	adenylate cyclase-inhibiting G protein-coupled acetylcholine receptor signaling pathway	1/2279	8/18866	0.6430433	0.775915	0.7020754	1	CHRM2
Biological Process	GO:0007253	cytoplasmic sequestering of NF-kappaB	1/2279	8/18866	0.6430433	0.775915	0.7020754	1	IL10
Biological Process	GO:0007341	penetration of zona pellucida	1/2279	8/18866	0.6430433	0.775915	0.7020754	1	HEXB
Biological Process	GO:0008216	spermidine metabolic process	1/2279	8/18866	0.6430433	0.775915	0.7020754	1	SAT2

Biological Process	GO:0008627	intrinsic apoptotic signaling pathway in response to osmotic stress	1/2279	8/18866	0.6430433	0.775915	0.7020754	1	ARHGEF2
Biological Process	GO:0009221	pyrimidine deoxyribonucleotide biosynthetic process	1/2279	8/18866	0.6430433	0.775915	0.7020754	1	DCTD
Biological Process	GO:0009304	tRNA transcription	1/2279	8/18866	0.6430433	0.775915	0.7020754	1	BRF1
Biological Process	GO:0009750	response to fructose	1/2279	8/18866	0.6430433	0.775915	0.7020754	1	SLC2A5
Biological Process	GO:0010668	ectodermal cell differentiation	1/2279	8/18866	0.6430433	0.775915	0.7020754	1	MIR145
Biological Process	GO:0010825	positive regulation of centrosome duplication	1/2279	8/18866	0.6430433	0.775915	0.7020754	1	POC1A
Biological Process	GO:0010886	positive regulation of cholesterol storage	1/2279	8/18866	0.6430433	0.775915	0.7020754	1	EHD1
Biological Process	GO:0014891	striated muscle atrophy	1/2279	8/18866	0.6430433	0.775915	0.7020754	1	GSN
Biological Process	GO:0014908	myotube differentiation involved in skeletal muscle regeneration	1/2279	8/18866	0.6430433	0.775915	0.7020754	1	CD9

Biological Process	GO:0016093	polyprenol metabolic process	1/2279	8/18866	0.6430433	0.775915	0.7020754	1	DPAGT1
Biological Process	GO:0017185	peptidyl-lysine hydroxylation	1/2279	8/18866	0.6430433	0.775915	0.7020754	1	P3H3
Biological Process	GO:0019682	glyceraldehyde-3-phosphate metabolic process	1/2279	8/18866	0.6430433	0.775915	0.7020754	1	GLYCTK
Biological Process	GO:0019856	pyrimidine nucleobase biosynthetic process	1/2279	8/18866	0.6430433	0.775915	0.7020754	1	DHODH
Biological Process	GO:0021979	hypothalamus cell differentiation	1/2279	8/18866	0.6430433	0.775915	0.7020754	1	NRP2
Biological Process	GO:0031620	regulation of fever generation	1/2279	8/18866	0.6430433	0.775915	0.7020754	1	TNF
Biological Process	GO:0031946	regulation of glucocorticoid biosynthetic process	1/2279	8/18866	0.6430433	0.775915	0.7020754	1	DGKQ
Biological Process	GO:0032049	cardiolipin biosynthetic process	1/2279	8/18866	0.6430433	0.775915	0.7020754	1	PGS1
Biological Process	GO:0032594	protein transport within lipid bilayer	1/2279	8/18866	0.6430433	0.775915	0.7020754	1	RILPL1

Biological Process	GO:0032650	regulation of interleukin-1 alpha production	1/2279	8/18866	0.6430433	0.775915	0.7020754	1	IL16
Biological Process	GO:0032713	negative regulation of interleukin-4 production	1/2279	8/18866	0.6430433	0.775915	0.7020754	1	ZFPM1
Biological Process	GO:0033084	regulation of immature T cell proliferation in thymus	1/2279	8/18866	0.6430433	0.775915	0.7020754	1	TMEM131L
Biological Process	GO:0033129	positive regulation of histone phosphorylation	1/2279	8/18866	0.6430433	0.775915	0.7020754	1	RPS6KA4
Biological Process	GO:0033504	floor plate development	1/2279	8/18866	0.6430433	0.775915	0.7020754	1	NODAL
Biological Process	GO:0034214	protein hexamerization	1/2279	8/18866	0.6430433	0.775915	0.7020754	1	LRRC8C
Biological Process	GO:0034475	U4 snRNA 3'-end processing	1/2279	8/18866	0.6430433	0.775915	0.7020754	1	EXOSC2
Biological Process	GO:0035740	CD8-positive, alpha-beta T cell proliferation	1/2279	8/18866	0.6430433	0.775915	0.7020754	1	VSIR
Biological Process	GO:0035754	B cell chemotaxis	1/2279	8/18866	0.6430433	0.775915	0.7020754	1	PIK3CD

Biological Process	GO:0036353	histone H2A-K119 monoubiquitination	1/2279	8/18866	0.6430433	0.775915	0.7020754	1	PCGF3
Biological Process	GO:0036480	neuron intrinsic apoptotic signaling pathway in response to oxidative stress	1/2279	8/18866	0.6430433	0.775915	0.7020754	1	PRKN
Biological Process	GO:0038114	interleukin-21-mediated signaling pathway	1/2279	8/18866	0.6430433	0.775915	0.7020754	1	JAK1
Biological Process	GO:0039536	negative regulation of RIG-I signaling pathway	1/2279	8/18866	0.6430433	0.775915	0.7020754	1	SEC14L1
Biological Process	GO:0042373	vitamin K metabolic process	1/2279	8/18866	0.6430433	0.775915	0.7020754	1	CYP4F3
Biological Process	GO:0042726	flavin-containing compound metabolic process	1/2279	8/18866	0.6430433	0.775915	0.7020754	1	SLC52A2
Biological Process	GO:0042780	tRNA 3'-end processing	1/2279	8/18866	0.6430433	0.775915	0.7020754	1	PTCD1
Biological Process	GO:0042796	snRNA transcription by RNA polymerase III	1/2279	8/18866	0.6430433	0.775915	0.7020754	1	ZC3H8
Biological Process	GO:0043045	DNA methylation involved in embryo development	1/2279	8/18866	0.6430433	0.775915	0.7020754	1	DNMT1

Biological Process	GO:0043416	regulation of skeletal muscle tissue regeneration	1/2279	8/18866	0.6430433	0.775915	0.7020754	1	TGFBR2
Biological Process	GO:0043633	polyadenylation-dependent RNA catabolic process	1/2279	8/18866	0.6430433	0.775915	0.7020754	1	EXOSC2
Biological Process	GO:0044387	negative regulation of protein kinase activity by regulation of protein phosphorylation	1/2279	8/18866	0.6430433	0.775915	0.7020754	1	CORO1C
Biological Process	GO:0044828	negative regulation by host of viral genome replication	1/2279	8/18866	0.6430433	0.775915	0.7020754	1	PRKN
Biological Process	GO:0045188	regulation of circadian sleep/wake cycle, non-REM sleep	1/2279	8/18866	0.6430433	0.775915	0.7020754	1	GHRL
Biological Process	GO:0045617	negative regulation of keratinocyte differentiation	1/2279	8/18866	0.6430433	0.775915	0.7020754	1	GRHL2
Biological Process	GO:0046130	purine ribonucleoside catabolic process	1/2279	8/18866	0.6430433	0.775915	0.7020754	1	PNP
Biological Process	GO:0046469	platelet activating factor metabolic process	1/2279	8/18866	0.6430433	0.775915	0.7020754	1	CHPT1
Biological Process	GO:0046477	glycosylceramide catabolic process	1/2279	8/18866	0.6430433	0.775915	0.7020754	1	GALC

Biological Process	GO:0046533	negative regulation of photoreceptor cell differentiation	1/2279	8/18866	0.6430433	0.775915	0.7020754	1	NOTCH1
Biological Process	GO:0046886	positive regulation of hormone biosynthetic process	1/2279	8/18866	0.6430433	0.775915	0.7020754	1	POR
Biological Process	GO:0048312	intracellular distribution of mitochondria	1/2279	8/18866	0.6430433	0.775915	0.7020754	1	DNM1L
Biological Process	GO:0048743	positive regulation of skeletal muscle fiber development	1/2279	8/18866	0.6430433	0.775915	0.7020754	1	BCL2
Biological Process	GO:0048793	pronephros development	1/2279	8/18866	0.6430433	0.775915	0.7020754	1	PAX2
Biological Process	GO:0048861	leukemia inhibitory factor signaling pathway	1/2279	8/18866	0.6430433	0.775915	0.7020754	1	LIF
Biological Process	GO:0051103	DNA ligation involved in DNA repair	1/2279	8/18866	0.6430433	0.775915	0.7020754	1	PAXX
Biological Process	GO:0051964	negative regulation of synapse assembly	1/2279	8/18866	0.6430433	0.775915	0.7020754	1	SLIT1
Biological Process	GO:0055118	negative regulation of cardiac muscle contraction	1/2279	8/18866	0.6430433	0.775915	0.7020754	1	BIN1

Biological Process	GO:0060020	Bergmann glial cell differentiation	1/2279	8/18866	0.6430433	0.775915	0.7020754	1	TTC21B
Biological Process	GO:0060027	convergent extension involved in gastrulation	1/2279	8/18866	0.6430433	0.775915	0.7020754	1	WNT11
Biological Process	GO:0060056	mammary gland involution	1/2279	8/18866	0.6430433	0.775915	0.7020754	1	VDR
Biological Process	GO:0060158	phospholipase C-activating dopamine receptor signaling pathway	1/2279	8/18866	0.6430433	0.775915	0.7020754	1	GNA11
Biological Process	GO:0060290	transdifferentiation	1/2279	8/18866	0.6430433	0.775915	0.7020754	1	SMAD3
Biological Process	GO:0060316	positive regulation of ryanodine-sensitive calcium-release channel activity	1/2279	8/18866	0.6430433	0.775915	0.7020754	1	GSTO1
Biological Process	GO:0060372	regulation of atrial cardiac muscle cell membrane repolarization	1/2279	8/18866	0.6430433	0.775915	0.7020754	1	KCNQ1
Biological Process	GO:0060430	lung saccule development	1/2279	8/18866	0.6430433	0.775915	0.7020754	1	CREB1
Biological Process	GO:0060638	mesenchymal-epithelial cell signaling	1/2279	8/18866	0.6430433	0.775915	0.7020754	1	FGFR1

Biological Process	GO:0060896	neural plate pattern specification	1/2279	8/18866	0.6430433	0.775915	0.7020754	1	SSBP3
Biological Process	GO:0061004	pattern specification involved in kidney development	1/2279	8/18866	0.6430433	0.775915	0.7020754	1	PAX2
Biological Process	GO:0061101	neuroendocrine cell differentiation	1/2279	8/18866	0.6430433	0.775915	0.7020754	1	WNT11
Biological Process	GO:0061438	renal system vasculature morphogenesis	1/2279	8/18866	0.6430433	0.775915	0.7020754	1	PDGFRB
Biological Process	GO:0061439	kidney vasculature morphogenesis	1/2279	8/18866	0.6430433	0.775915	0.7020754	1	PDGFRB
Biological Process	GO:0070127	tRNA aminoacylation for mitochondrial protein translation	1/2279	8/18866	0.6430433	0.775915	0.7020754	1	EARS2
Biological Process	GO:0070813	hydrogen sulfide metabolic process	1/2279	8/18866	0.6430433	0.775915	0.7020754	1	CBS
Biological Process	GO:0070973	protein localization to endoplasmic reticulum exit site	1/2279	8/18866	0.6430433	0.775915	0.7020754	1	MIA3
Biological Process	GO:0071420	cellular response to histamine	1/2279	8/18866	0.6430433	0.775915	0.7020754	1	HRH1

Biological Process	GO:0071499	cellular response to laminar fluid shear stress	1/2279	8/18866	0.6430433	0.775915	0.7020754	1	NFE2L2
Biological Process	GO:0071671	regulation of smooth muscle cell chemotaxis	1/2279	8/18866	0.6430433	0.775915	0.7020754	1	PDGFD
Biological Process	GO:0071676	negative regulation of mononuclear cell migration	1/2279	8/18866	0.6430433	0.775915	0.7020754	1	PLCB1
Biological Process	GO:0071839	apoptotic process in bone marrow cell	1/2279	8/18866	0.6430433	0.775915	0.7020754	1	BCL2L1
Biological Process	GO:0072048	renal system pattern specification	1/2279	8/18866	0.6430433	0.775915	0.7020754	1	PAX2
Biological Process	GO:0072050	S-shaped body morphogenesis	1/2279	8/18866	0.6430433	0.775915	0.7020754	1	PDGFRB
Biological Process	GO:0072161	mesenchymal cell differentiation involved in kidney development	1/2279	8/18866	0.6430433	0.775915	0.7020754	1	PAX2
Biological Process	GO:0072201	negative regulation of mesenchymal cell proliferation	1/2279	8/18866	0.6430433	0.775915	0.7020754	1	WNT11
Biological Process	GO:0072282	metanephric nephron tubule morphogenesis	1/2279	8/18866	0.6430433	0.775915	0.7020754	1	PAX2

Biological Process	GO:0072674	multinuclear osteoclast differentiation	1/2279	8/18866	0.6430433	0.775915	0.7020754	1	SBNO2
Biological Process	GO:0075713	establishment of integrated proviral latency	1/2279	8/18866	0.6430433	0.775915	0.7020754	1	BANF1
Biological Process	GO:0090045	positive regulation of deacetylase activity	1/2279	8/18866	0.6430433	0.775915	0.7020754	1	C6orf89
Biological Process	GO:0090381	regulation of heart induction	1/2279	8/18866	0.6430433	0.775915	0.7020754	1	WNT11
Biological Process	GO:0090400	stress-induced premature senescence	1/2279	8/18866	0.6430433	0.775915	0.7020754	1	ARNTL
Biological Process	GO:0098700	neurotransmitter loading into synaptic vesicle	1/2279	8/18866	0.6430433	0.775915	0.7020754	1	DDC
Biological Process	GO:0098756	response to interleukin-21	1/2279	8/18866	0.6430433	0.775915	0.7020754	1	JAK1
Biological Process	GO:0098757	cellular response to interleukin-21	1/2279	8/18866	0.6430433	0.775915	0.7020754	1	JAK1
Biological Process	GO:0120040	regulation of macrophage proliferation	1/2279	8/18866	0.6430433	0.775915	0.7020754	1	CSF1R

Biological Process	GO:0150172	regulation of phosphatidylcholine metabolic process	1/2279	8/18866	0.6430433	0.775915	0.7020754	1	LPCAT1
Biological Process	GO:1900364	negative regulation of mRNA polyadenylation	1/2279	8/18866	0.6430433	0.775915	0.7020754	1	RNF40
Biological Process	GO:1901538	changes to DNA methylation involved in embryo development	1/2279	8/18866	0.6430433	0.775915	0.7020754	1	DNMT1
Biological Process	GO:1901620	regulation of smoothened signaling pathway involved in dorsal/ventral neural tube patterning	1/2279	8/18866	0.6430433	0.775915	0.7020754	1	SUFU
Biological Process	GO:1902416	positive regulation of mRNA binding	1/2279	8/18866	0.6430433	0.775915	0.7020754	1	EIF4G1
Biological Process	GO:1902744	negative regulation of lamellipodium organization	1/2279	8/18866	0.6430433	0.775915	0.7020754	1	KANK1
Biological Process	GO:1902949	positive regulation of tau-protein kinase activity	1/2279	8/18866	0.6430433	0.775915	0.7020754	1	CLU
Biological Process	GO:1903376	regulation of oxidative stress-induced neuron intrinsic apoptotic signaling pathway	1/2279	8/18866	0.6430433	0.775915	0.7020754	1	PRKN
Biological Process	GO:1903400	L-arginine transmembrane transport	1/2279	8/18866	0.6430433	0.775915	0.7020754	1	SLC7A1

Biological Process	GO:1903715	regulation of aerobic respiration	1/2279	8/18866	0.6430433	0.775915	0.7020754	1	CBFA2T3
Biological Process	GO:1903764	regulation of potassium ion export across plasma membrane	1/2279	8/18866	0.6430433	0.775915	0.7020754	1	ANO6
Biological Process	GO:1903995	regulation of non-membrane spanning protein tyrosine kinase activity	1/2279	8/18866	0.6430433	0.775915	0.7020754	1	DUSP22
Biological Process	GO:1904124	microglial cell migration	1/2279	8/18866	0.6430433	0.775915	0.7020754	1	CX3CR1
Biological Process	GO:1904139	regulation of microglial cell migration	1/2279	8/18866	0.6430433	0.775915	0.7020754	1	CX3CR1
Biological Process	GO:1904714	regulation of chaperone-mediated autophagy	1/2279	8/18866	0.6430433	0.775915	0.7020754	1	GFAP
Biological Process	GO:1905024	regulation of membrane repolarization during ventricular cardiac muscle cell action potential	1/2279	8/18866	0.6430433	0.775915	0.7020754	1	NOS1AP
Biological Process	GO:1905247	positive regulation of aspartic-type peptidase activity	1/2279	8/18866	0.6430433	0.775915	0.7020754	1	GRN
Biological Process	GO:1905456	regulation of lymphoid progenitor cell differentiation	1/2279	8/18866	0.6430433	0.775915	0.7020754	1	NOTCH1

Biological Process	GO:1990314	cellular response to insulin-like growth factor stimulus	1/2279	8/18866	0.6430433	0.775915	0.7020754	1	CREB1
Biological Process	GO:1990709	presynaptic active zone organization	1/2279	8/18866	0.6430433	0.775915	0.7020754	1	CTBP2
Biological Process	GO:2000192	negative regulation of fatty acid transport	1/2279	8/18866	0.6430433	0.775915	0.7020754	1	THBS1
Biological Process	GO:2000510	positive regulation of dendritic cell chemotaxis	1/2279	8/18866	0.6430433	0.775915	0.7020754	1	CALR
Biological Process	GO:2000564	regulation of CD8-positive, alpha-beta T cell proliferation	1/2279	8/18866	0.6430433	0.775915	0.7020754	1	VSIR
Biological Process	GO:2000643	positive regulation of early endosome to late endosome transport	1/2279	8/18866	0.6430433	0.775915	0.7020754	1	EZR
Biological Process	GO:2000766	negative regulation of cytoplasmic translation	1/2279	8/18866	0.6430433	0.775915	0.7020754	1	CPEB3
Biological Process	GO:2001012	mesenchymal cell differentiation involved in renal system development	1/2279	8/18866	0.6430433	0.775915	0.7020754	1	PAX2
Biological Process	GO:0051225	spindle assembly	13/2279	115/18866	0.6437193	0.776632	0.7027242	13	ARHGEF10/CSNK1D/TNKS/RCC1/DYNC1H1/STAG1/NUMA1/MSTO1/NEK6/CHEK2/SPAG5/TUBGCP2/ABRAXAS1

Biological Process	GO:0007586	digestion	16/2279	141/18866	0.6441659	0.7769734	0.7030332	16	SLC2A5/TJP2/VDR/SGK1/TLR9/MGAM/AKR1D1/CHIT1/AQP1/NR1H2/GHRL/SLC22A5/KCNQ1/NOD2/EZR/SST
Biological Process	GO:0008584	male gonad development	16/2279	141/18866	0.6441659	0.7769734	0.7030332	16	ARID5B/CSDE1/FANCA/RARA/SF1/NCOA1/NTRK1/HOXA9/RAD21L1/BCL2/PDGFRB/ERCC1/BCL2L1/HMGCS1/NCOA4/INHBA
Biological Process	GO:0000289	nuclear-transcribed mRNA poly(A) tail shortening	4/2279	36/18866	0.6472174	0.7796171	0.7054253	4	TNRC6B/SAMD4A/AGO2/CPEB3
Biological Process	GO:0000460	maturation of 5.8S rRNA	4/2279	36/18866	0.6472174	0.7796171	0.7054253	4	EXOSC2/RCL1/ERI3/NOP14
Biological Process	GO:0051973	positive regulation of telomerase activity	4/2279	36/18866	0.6472174	0.7796171	0.7054253	4	TNKS/HMBOX1/GRHL2/PARM1
Biological Process	GO:0070873	regulation of glycogen metabolic process	4/2279	36/18866	0.6472174	0.7796171	0.7054253	4	DYRK2/PPP1CB/ESRRB/RUBCNL
Biological Process	GO:1901661	quinone metabolic process	4/2279	36/18866	0.6472174	0.7796171	0.7054253	4	CYP4F3/AIFM2/COQ4/COQ8B
Biological Process	GO:1905332	positive regulation of morphogenesis of an epithelium	4/2279	36/18866	0.6472174	0.7796171	0.7054253	4	RREB1/PAX2/LIF/HOXB7
Biological Process	GO:2000781	positive regulation of double-strand break repair	4/2279	36/18866	0.6472174	0.7796171	0.7054253	4	SPIDR/FOXM1/PARP9/SLF1

Biological Process	GO:0003197	endocardial cushion development	5/2279	45/18866	0.6474244	0.7796171	0.7054253	5	TGFBR2/RBPJ/ENG/NOTCH1/NEDD4
Biological Process	GO:0010828	positive regulation of glucose transmembrane transport	5/2279	45/18866	0.6474244	0.7796171	0.7054253	5	PIK3R1/SLC1A2/PRKCI/NFE2L2/CLTCL1
Biological Process	GO:0014047	glutamate secretion	5/2279	45/18866	0.6474244	0.7796171	0.7054253	5	PPFIA1/NF1/SLC1A2/SLC1A6/SLC1A3
Biological Process	GO:0032722	positive regulation of chemokine production	5/2279	45/18866	0.6474244	0.7796171	0.7054253	5	TNF/TLR9/MBP/DEFB124/IL6R
Biological Process	GO:0046189	phenol-containing compound biosynthetic process	5/2279	45/18866	0.6474244	0.7796171	0.7054253	5	ZEB2/DDC/CDH3/RAPGEF2/MYO5A
Biological Process	GO:1904646	cellular response to amyloid-beta	5/2279	45/18866	0.6474244	0.7796171	0.7054253	5	MIR140/ABCC1/FPR2/APP/IGF1R
Biological Process	GO:0006584	catecholamine metabolic process	6/2279	54/18866	0.6489071	0.7803115	0.7060536	6	COMT/DDC/SULT1A2/ITGB2/EPAS1/PRKN
Biological Process	GO:0006749	glutathione metabolic process	6/2279	54/18866	0.6489071	0.7803115	0.7060536	6	GGT1/CNDP2/GSTM5/MGST2/NFE2L2/GLRX2
Biological Process	GO:0009712	catechol-containing compound metabolic process	6/2279	54/18866	0.6489071	0.7803115	0.7060536	6	COMT/DDC/SULT1A2/ITGB2/EPAS1/PRKN

Biological Process	GO:0010043	response to zinc ion	6/2279	54/18866	0.6489071	0.7803115	0.7060536	6	S100A8/CREB1/D2HGDH/MTF1/SLC39A13/CRIP1
Biological Process	GO:0030857	negative regulation of epithelial cell differentiation	6/2279	54/18866	0.6489071	0.7803115	0.7060536	6	SPRED2/DLL1/GRHL2/NOTCH1/NOTCH4/NODAL
Biological Process	GO:1901185	negative regulation of ERBB signaling pathway	6/2279	54/18866	0.6489071	0.7803115	0.7060536	6	TGFA/ARHGEF7/EPS15L1/PTPRJ/DUSP3/EPS15
Biological Process	GO:2000179	positive regulation of neural precursor cell proliferation	6/2279	54/18866	0.6489071	0.7803115	0.7060536	6	CTSZ/DISC1/GNAI2/ZNF335/NOTCH1/CX3CR1
Biological Process	GO:0006817	phosphate ion transport	3/2279	27/18866	0.6495615	0.7803115	0.7060536	3	FGFR1/SLC37A1/CEBPB
Biological Process	GO:0007026	negative regulation of microtubule depolymerization	3/2279	27/18866	0.6495615	0.7803115	0.7060536	3	NAV3/ARHGEF2/SPEF1
Biological Process	GO:0009651	response to salt stress	3/2279	27/18866	0.6495615	0.7803115	0.7060536	3	TNF/AQP1/ANXA7
Biological Process	GO:0010971	positive regulation of G2/M transition of mitotic cell cycle	3/2279	27/18866	0.6495615	0.7803115	0.7060536	3	BRD4/APP/CDC25A
Biological Process	GO:0031954	positive regulation of protein autophosphorylation	3/2279	27/18866	0.6495615	0.7803115	0.7060536	3	PDGFD/PDGFB/PDGFC

Biological Process	GO:0033081	regulation of T cell differentiation in thymus	3/2279	27/18866	0.6495615	0.7803115	0.7060536	3	ZC3H8/CLPTM1/TMEM131L
Biological Process	GO:0045603	positive regulation of endothelial cell differentiation	3/2279	27/18866	0.6495615	0.7803115	0.7060536	3	ADD1/NOTCH1/S1PR2
Biological Process	GO:0045672	positive regulation of osteoclast differentiation	3/2279	27/18866	0.6495615	0.7803115	0.7060536	3	CREB1/TNF/TMEM64
Biological Process	GO:0048520	positive regulation of behavior	3/2279	27/18866	0.6495615	0.7803115	0.7060536	3	HDAC4/GHRL/MEF2C
Biological Process	GO:0071514	genetic imprinting	3/2279	27/18866	0.6495615	0.7803115	0.7060536	3	KDM1B/KCNQ1/PCGF3
Biological Process	GO:1900120	regulation of receptor binding	3/2279	27/18866	0.6495615	0.7803115	0.7060536	3	IL10/ATP2A3/PLCL1
Biological Process	GO:1903203	regulation of oxidative stress-induced neuron death	3/2279	27/18866	0.6495615	0.7803115	0.7060536	3	IL10/FBXO7/PRKN
Biological Process	GO:0048524	positive regulation of viral process	12/2279	107/18866	0.6526643	0.7839398	0.7093366	12	P4HB/TRIM38/CTDP1/SRPK2/POLR2F/CCL5/CSF1R/LARP1/PC/RSF1/NOTCH1/NR5A2
Biological Process	GO:0003407	neural retina development	8/2279	72/18866	0.6536698	0.7848498	0.71016	8	CRB1/HIPK2/GNAT2/RPGRIP1/ZHX2/SDK2/NRL/CABP4

Biological Process	GO:0009119	ribonucleoside metabolic process	8/2279	72/18866	0.6536698	0.7848498	0.71016	8	CDA/AMPD3/TJP2/PNP/GUK1/GMPR2/IMPDH1/ACP3
Biological Process	GO:0070988	demethylation	8/2279	72/18866	0.6536698	0.7848498	0.71016	8	KDM4B/KDM2A/POR/KDM1B/KDM4C/FTO/TET2/KDM7A
Biological Process	GO:0006839	mitochondrial transport	31/2279	271/18866	0.6556411	0.786859	0.711978	31	PRKAG2/SLC25A37/TFDP1/YWHAH/PAM16/BMF/SPG7/FBXO7/MIPEP/AIP/USP36/ABLIM3/UBE2J2/HK2/SFXN1/ACACB/YWHAZ/FIS1/BCL2/ACACA/TIMM44/TP53BP2/BCL2L1/SREBF1/YWHAQ/CPT1A/SLC25A1/TMEM14C/PRKAA1/ATP5PD/PRKN
Biological Process	GO:0001937	negative regulation of endothelial cell proliferation	9/2279	81/18866	0.6564866	0.786859	0.711978	9	PPARG/TNF/THBS1/MIR24-2/FLT1/NF1/GHRL/VASH1/MEF2C
Biological Process	GO:2000027	regulation of animal organ morphogenesis	29/2279	254/18866	0.6566068	0.786859	0.711978	29	TACSTD2/RXRA/AP2A1/VDR/VANGL1/TNF/TNFAIP3/TGFBR2/PRICKLE2/RBPJ/WNT11/PAX2/FGFR1/SMURF1/POR/PFN1/RUNX2/PSMF1/ENG/ARRB2/DAAM1/BCL2/PSMB7/NOTCH1/CPB2/ANKRD6/LIF/HOXB7/PSMD13
Biological Process	GO:0001675	acrosome assembly	2/2279	18/18866	0.6579106	0.786859	0.711978	2	ACRBP/MFSD14A
Biological Process	GO:0006337	nucleosome disassembly	2/2279	18/18866	0.6579106	0.786859	0.711978	2	SMARCD1/SMARCD2
Biological Process	GO:0006744	ubiquinone biosynthetic process	2/2279	18/18866	0.6579106	0.786859	0.711978	2	COQ4/COQ8B
Biological Process	GO:0007597	blood coagulation, intrinsic pathway	2/2279	18/18866	0.6579106	0.786859	0.711978	2	GP9/F12

Biological Process	GO:0008272	sulfate transport	2/2279	18/18866	0.6579106	0.786859	0.711978	2	SLC13A4/SLC26A1
Biological Process	GO:0009200	deoxyribonucleoside triphosphate metabolic process	2/2279	18/18866	0.6579106	0.786859	0.711978	2	RRM2B/GUK1
Biological Process	GO:0010713	negative regulation of collagen metabolic process	2/2279	18/18866	0.6579106	0.786859	0.711978	2	PPARG/NPPC
Biological Process	GO:0017121	plasma membrane phospholipid scrambling	2/2279	18/18866	0.6579106	0.786859	0.711978	2	ANO7/ANO6
Biological Process	GO:0021756	striatum development	2/2279	18/18866	0.6579106	0.786859	0.711978	2	SECISBP2/INHBA
Biological Process	GO:0034501	protein localization to kinetochore	2/2279	18/18866	0.6579106	0.786859	0.711978	2	CHAMP1/MIS12
Biological Process	GO:0034587	piRNA metabolic process	2/2279	18/18866	0.6579106	0.786859	0.711978	2	PIWIL4/MOV10L1
Biological Process	GO:0035313	wound healing, spreading of epidermal cells	2/2279	18/18866	0.6579106	0.786859	0.711978	2	RREB1/COL5A1
Biological Process	GO:0035372	protein localization to microtubule	2/2279	18/18866	0.6579106	0.786859	0.711978	2	CHAMP1/MAP1A

Biological Process	GO:0043011	myeloid dendritic cell differentiation	2/2279	18/18866	0.6579106	0.786859	0.711978	2	TGFBR2/RBPJ
Biological Process	GO:0043031	negative regulation of macrophage activation	2/2279	18/18866	0.6579106	0.786859	0.711978	2	PTPRC/GRN
Biological Process	GO:0044827	modulation by host of viral genome replication	2/2279	18/18866	0.6579106	0.786859	0.711978	2	PHB/PRKN
Biological Process	GO:0046885	regulation of hormone biosynthetic process	2/2279	18/18866	0.6579106	0.786859	0.711978	2	DGKQ/POR
Biological Process	GO:0051023	regulation of immunoglobulin secretion	2/2279	18/18866	0.6579106	0.786859	0.711978	2	TNF/TRAF2
Biological Process	GO:0060033	anatomical structure regression	2/2279	18/18866	0.6579106	0.786859	0.711978	2	NINJ1/FLT1
Biological Process	GO:0060192	negative regulation of lipase activity	2/2279	18/18866	0.6579106	0.786859	0.711978	2	POR/SORT1
Biological Process	GO:0061323	cell proliferation involved in heart morphogenesis	2/2279	18/18866	0.6579106	0.786859	0.711978	2	RBPJ/NOTCH1
Biological Process	GO:0070200	establishment of protein localization to telomere	2/2279	18/18866	0.6579106	0.786859	0.711978	2	BRCA2/CCT6A

Biological Process	GO:0090190	positive regulation of branching involved in ureteric bud morphogenesis	2/2279	18/18866	0.6579106	0.786859	0.711978	2	PAX2/HOXB7
Biological Process	GO:1901663	quinone biosynthetic process	2/2279	18/18866	0.6579106	0.786859	0.711978	2	COQ4/COQ8B
Biological Process	GO:1902683	regulation of receptor localization to synapse	2/2279	18/18866	0.6579106	0.786859	0.711978	2	PRKCZ/CNIH2
Biological Process	GO:1904872	regulation of telomerase RNA localization to Cajal body	2/2279	18/18866	0.6579106	0.786859	0.711978	2	RUVBL1/CCT6A
Biological Process	GO:2000641	regulation of early endosome to late endosome transport	2/2279	18/18866	0.6579106	0.786859	0.711978	2	SNX3/EZR
Biological Process	GO:2000831	regulation of steroid hormone secretion	2/2279	18/18866	0.6579106	0.786859	0.711978	2	GHRL/AGTR1
Biological Process	GO:0017148	negative regulation of translation	25/2279	220/18866	0.65933	0.7884574	0.7134243	25	CALR/MIR145/TNRC6B/MIR101-2/AGO1/LARP1/ZC3H12D/SAMD4A/MIR27A/AGO2/RARA/CPEB3/IGF2BP3/INPP5E/FTO/PUS7/MIR138-2/CELF1/NOTCH4/BANK1/ILF3/SYNCRIP/EIF4G1/DAPK3/SHFL
Biological Process	GO:0060071	Wnt signaling pathway, planar cell polarity pathway	12/2279	108/18866	0.6655294	0.7957708	0.7200417	12	AP2A1/VANGL1/PRICKLE2/WNT11/SMURF1/PFN1/PSMF1/ARRB2/DAAM1/PSMB7/ANKRD6/PSMD13
Biological Process	GO:0007131	reciprocal meiotic recombination	6/2279	55/18866	0.6668242	0.7972187	0.7213518	6	EME1/MEIOB/RNF212/DMC1/SLX4/KLHDC3

Biological Process	GO:0010874	regulation of cholesterol efflux	5/2279	46/18866	0.6669927	0.7972195	0.7213525	5	ABCG1/PLTP/MIR27A/NR1H2/ABCA1
Biological Process	GO:0044275	cellular carbohydrate catabolic process	5/2279	46/18866	0.6669927	0.7972195	0.7213525	5	AOAH/MGAM/ENOSF1/PGAM1/PPP1CB
Biological Process	GO:0051898	negative regulation of protein kinase B signaling	7/2279	64/18866	0.6676933	0.7979565	0.7220193	7	MIR145/PHLPP1/PTPRJ/PPP2R5C/ARRB2/BANK1/TSC2
Biological Process	GO:0006699	bile acid biosynthetic process	4/2279	37/18866	0.6689556	0.7987618	0.722748	4	AMACR/AKR1D1/OSBPL6/ACOX2
Biological Process	GO:0033120	positive regulation of RNA splicing	4/2279	37/18866	0.6689556	0.7987618	0.722748	4	ERN1/PIK3R1/DAZAP1/HNRNPLL
Biological Process	GO:0035137	hindlimb morphogenesis	4/2279	37/18866	0.6689556	0.7987618	0.722748	4	ZBTB16/RARG/NOTCH1/ALX3
Biological Process	GO:0048048	embryonic eye morphogenesis	4/2279	37/18866	0.6689556	0.7987618	0.722748	4	HIPK2/PAX2/SP3/RARG
Biological Process	GO:0051031	tRNA transport	4/2279	37/18866	0.6689556	0.7987618	0.722748	4	RAE1/NUP93/SEC13/NOL6
Biological Process	GO:0071542	dopaminergic neuron differentiation	4/2279	37/18866	0.6689556	0.7987618	0.722748	4	CSNK1D/PHOX2A/CSNK1E/OTX2

Biological Process	GO:1904358	positive regulation of telomere maintenance via telomere lengthening	4/2279	37/18866	0.6689556	0.7987618	0.722748	4	TNKS/HMBOX1/RTEL1/CCT6A
Biological Process	GO:0006283	transcription-coupled nucleotide-excision repair	8/2279	73/18866	0.6691999	0.7988526	0.7228302	8	POLR2F/COPS5/CCNH/COPS3/ERCC1/POLD3/XPA/ERCC2
Biological Process	GO:0051966	regulation of synaptic transmission, glutamatergic	8/2279	73/18866	0.6691999	0.7988526	0.7228302	8	CCR2/TNF/DISC1/NRXN1/MAPK8IP2/NTRK1/MEF2C/SHANK2
Biological Process	GO:0007605	sensory perception of sound	17/2279	152/18866	0.6700841	0.7998076	0.7236943	17	KCNE1/HEXB/CDH23/ATP2B2/ATP8B1/MBP/LRIG1/FGFR1/ESPN/WDR1/KCNQ1/TSPEAR/SLC1A3/TMC1/CRYM/CDC14A/SPTBN4
Biological Process	GO:0016925	protein sumoylation	9/2279	82/18866	0.6711138	0.8009361	0.7247154	9	HDAC4/TOLLIP/RAE1/NSMCE2/BCL11A/ZMIZ1/RNF212/NUP93/RELA
Biological Process	GO:0001656	metanephros development	10/2279	91/18866	0.673294	0.8032354	0.7267959	10	CTSH/PAX2/RDH10/NF1/WWTR1/PDGFB/BCL2/PDGFRB/ACAT1/LIF
Biological Process	GO:0033273	response to vitamin	10/2279	91/18866	0.673294	0.8032354	0.7267959	10	PPARG/GSN/VDR/ALPL/RARA/CAT/AQP3/HMGCS1/RELA/ALDH1A2
Biological Process	GO:2000177	regulation of neural precursor cell proliferation	10/2279	91/18866	0.673294	0.8032354	0.7267959	10	CTSZ/SPINT2/DISC1/GNAI2/ILK/NF1/ZNF335/NOTCH1/FOXO1/CX3CR1
Biological Process	GO:0001964	startle response	3/2279	28/18866	0.6743344	0.8033669	0.7269149	3	NRXN1/CHD8/PRKN

Biological Process	GO:0010458	exit from mitosis	3/2279	28/18866	0.6743344	0.8033669	0.7269149	3	CTDP1/CDCA5/CDC14A
Biological Process	GO:0031440	regulation of mRNA 3'-end processing	3/2279	28/18866	0.6743344	0.8033669	0.7269149	3	CPEB3/ZC3H3/RNF40
Biological Process	GO:0031645	negative regulation of nervous system process	3/2279	28/18866	0.6743344	0.8033669	0.7269149	3	IL10/TNFRSF21/S1PR2
Biological Process	GO:0048596	embryonic camera-type eye morphogenesis	3/2279	28/18866	0.6743344	0.8033669	0.7269149	3	HIPK2/PAX2/SP3
Biological Process	GO:0051450	myoblast proliferation	3/2279	28/18866	0.6743344	0.8033669	0.7269149	3	PAX7/MEIS2/ANKRD2
Biological Process	GO:0051590	positive regulation of neurotransmitter transport	3/2279	28/18866	0.6743344	0.8033669	0.7269149	3	DNM1L/DTNBP1/PRKN
Biological Process	GO:0051883	killing of cells in other organism involved in symbiotic interaction	3/2279	28/18866	0.6743344	0.8033669	0.7269149	3	AZU1/ELANE/CTSG
Biological Process	GO:0071280	cellular response to copper ion	3/2279	28/18866	0.6743344	0.8033669	0.7269149	3	APP/AQP1/NFE2L2
Biological Process	GO:0086013	membrane repolarization during cardiac muscle cell action potential	3/2279	28/18866	0.6743344	0.8033669	0.7269149	3	KCNE1/NOS1AP/KCNQ1

Biological Process	GO:0090200	positive regulation of release of cytochrome c from mitochondria	3/2279	28/18866	0.6743344	0.8033669	0.7269149	3	BMF/CIDEB/DNM1L
Biological Process	GO:0032368	regulation of lipid transport	15/2279	135/18866	0.674708	0.803372	0.7269195	15	PPARG/MYB/MAP2K6/ABCG1/ATP8A1/PLTP/THBS1/MIR27A/OSBPL6/NR1H2/ITGB3/GHRL/ABCA1/AGTR1/PRKN
Biological Process	GO:2000241	regulation of reproductive process	19/2279	170/18866	0.676584	0.803372	0.7269195	19	HDAC4/RXRA/CALR/FZR1/ARHGDIB/RNASE10/AGO2/CDC25A/OVOL1/DEFB1/STK3/LHFPL2/PPP2R1A/PLCB1/NOTCH1/NPPC/INHBA/NODAL/LIF
Biological Process	GO:0006405	RNA export from nucleus	16/2279	144/18866	0.6777236	0.803372	0.7269195	16	NXF1/FIP1L1/CPSF3/SETD2/RAE1/SMG7/PABPN1/ENY2/NUP93/SEC13/SMG6/HHEX/RBM15B/NOL6/CPSF4/PCID2
Biological Process	GO:0021510	spinal cord development	12/2279	109/18866	0.6781223	0.803372	0.7269195	12	DCTN1/VIT/PHOX2A/DRGX/SUFU/LOXL3/DLL1/PAX7/NF1/NOTCH1/LMO4/ERCC2
Biological Process	GO:0015918	sterol transport	13/2279	118/18866	0.6806689	0.803372	0.7269195	13	OSBPL5/PPARG/LIPC/ABCG1/PLTP/MIR27A/OSBPL6/CLU/NR1H2/ABCA1/OSBPL10/NPC1/GRAMD1A
Biological Process	GO:0031640	killing of cells of other organism	7/2279	65/18866	0.6838023	0.803372	0.7269195	7	DEFA4/AZU1/ELANE/CTSG/PGLYRP1/LTF/PGLYRP4
Biological Process	GO:0031646	positive regulation of nervous system process	7/2279	65/18866	0.6838023	0.803372	0.7269195	7	PRKCZ/SSH1/TNFRSF1B/BAIAP2/NRXN1/SHANK2/CUX2
Biological Process	GO:0046782	regulation of viral transcription	7/2279	65/18866	0.6838023	0.803372	0.7269195	7	TRIM27/CTDP1/TRIM8/POLR2F/CCL5/HDAC1/RSF1

Biological Process	GO:0051205	protein insertion into membrane	7/2279	65/18866	0.6838023	0.803372	0.7269195	7	TFDP1/YWHAH/BMF/YWHAZ/BCL2/TP53BP2/YWHAQ
Biological Process	GO:0006977	DNA damage response, signal transduction by p53 class mediator resulting in cell cycle arrest	6/2279	56/18866	0.6841515	0.803372	0.7269195	6	TFDP1/E2F7/PCBP4/ZNF385A/ARID3A/CHEK2
Biological Process	GO:0010665	regulation of cardiac muscle cell apoptotic process	6/2279	56/18866	0.6841515	0.803372	0.7269195	6	MIR145/MIR199A1/MIR24-2/ILK/NFE2L2/MIR199A2
Biological Process	GO:0031295	T cell costimulation	6/2279	56/18866	0.6841515	0.803372	0.7269195	6	LCK/PIK3R1/GRAP2/TNFSF13B/PTPN6/CSK
Biological Process	GO:0035825	homologous recombination	6/2279	56/18866	0.6841515	0.803372	0.7269195	6	EME1/MEIOB/RNF212/DMC1/SLX4/KLHDC3
Biological Process	GO:0006073	cellular glucan metabolic process	8/2279	74/18866	0.6843013	0.803372	0.7269195	8	PRKAG2/MGAM/DYRK2/PPP1CB/ESRRB/PER2/PCDH12/RUBCNL
Biological Process	GO:0021536	diencephalon development	8/2279	74/18866	0.6843013	0.803372	0.7269195	8	NRP2/CREB1/ZEB2/RBPJ/NCOA1/ETS1/CDH1/ALDH1A2
Biological Process	GO:0044042	glucan metabolic process	8/2279	74/18866	0.6843013	0.803372	0.7269195	8	PRKAG2/MGAM/DYRK2/PPP1CB/ESRRB/PER2/PCDH12/RUBCNL
Biological Process	GO:2000243	positive regulation of reproductive process	9/2279	83/18866	0.6853635	0.803372	0.7269195	9	HDAC4/RNASE10/AGO2/CDC25A/OVOL1/DEFB1/LHFPL2/PLCB1/INHBA

Biological Process	GO:0048839	inner ear development	22/2279	197/18866	0.6858674	0.803372	0.7269195	22	ABR/CEBPD/CDH23/CCM2/SLC44A4/ATP8B1/DLL1/RBPJ/LRIG1/PAX2/FGFR1/TTC39C/BMPER/LRP10/MCM2/BCR/KCNQ1/ESRRB/NOTCH1/TMC1/PDGFRB/C1QB
Biological Process	GO:0000022	mitotic spindle elongation	1/2279	9/18866	0.6861817	0.803372	0.7269195	1	NUMA1
Biological Process	GO:0001993	regulation of systemic arterial blood pressure by norepinephrine-epinephrine	1/2279	9/18866	0.6861817	0.803372	0.7269195	1	PDE4D
Biological Process	GO:0002084	protein depalmitoylation	1/2279	9/18866	0.6861817	0.803372	0.7269195	1	LYPLA2
Biological Process	GO:0002158	osteoclast proliferation	1/2279	9/18866	0.6861817	0.803372	0.7269195	1	TNFAIP3
Biological Process	GO:0002423	natural killer cell mediated immune response to tumor cell	1/2279	9/18866	0.6861817	0.803372	0.7269195	1	CD226
Biological Process	GO:0002645	positive regulation of tolerance induction	1/2279	9/18866	0.6861817	0.803372	0.7269195	1	TGFBR2
Biological Process	GO:0002855	regulation of natural killer cell mediated immune response to tumor cell	1/2279	9/18866	0.6861817	0.803372	0.7269195	1	CD226
Biological Process	GO:0002883	regulation of hypersensitivity	1/2279	9/18866	0.6861817	0.803372	0.7269195	1	FUT7

Biological Process	GO:0003056	regulation of vascular associated smooth muscle contraction	1/2279	9/18866	0.6861817	0.803372	0.7269195	1	P2RX1
Biological Process	GO:0003266	regulation of secondary heart field cardioblast proliferation	1/2279	9/18866	0.6861817	0.803372	0.7269195	1	NOTCH1
Biological Process	GO:0003344	pericardium morphogenesis	1/2279	9/18866	0.6861817	0.803372	0.7269195	1	NOTCH1
Biological Process	GO:0006198	cAMP catabolic process	1/2279	9/18866	0.6861817	0.803372	0.7269195	1	PDE4D
Biological Process	GO:0006265	DNA topological change	1/2279	9/18866	0.6861817	0.803372	0.7269195	1	TOP1MT
Biological Process	GO:0006290	pyrimidine dimer repair	1/2279	9/18866	0.6861817	0.803372	0.7269195	1	ERCC1
Biological Process	GO:0006591	ornithine metabolic process	1/2279	9/18866	0.6861817	0.803372	0.7269195	1	ODC1
Biological Process	GO:0006768	biotin metabolic process	1/2279	9/18866	0.6861817	0.803372	0.7269195	1	PC
Biological Process	GO:0006868	glutamine transport	1/2279	9/18866	0.6861817	0.803372	0.7269195	1	SLC1A5

Biological Process	GO:0007021	tubulin complex assembly	1/2279	9/18866	0.6861817	0.803372	0.7269195	1	TBCD
Biological Process	GO:0009215	purine deoxyribonucleoside triphosphate metabolic process	1/2279	9/18866	0.6861817	0.803372	0.7269195	1	GUK1
Biological Process	GO:0009414	response to water deprivation	1/2279	9/18866	0.6861817	0.803372	0.7269195	1	CD9
Biological Process	GO:0010873	positive regulation of cholesterol esterification	1/2279	9/18866	0.6861817	0.803372	0.7269195	1	AGTR1
Biological Process	GO:0014012	peripheral nervous system axon regeneration	1/2279	9/18866	0.6861817	0.803372	0.7269195	1	CERS2
Biological Process	GO:0014816	skeletal muscle satellite cell differentiation	1/2279	9/18866	0.6861817	0.803372	0.7269195	1	PAX7
Biological Process	GO:0015959	diadenosine polyphosphate metabolic process	1/2279	9/18866	0.6861817	0.803372	0.7269195	1	NUDT3
Biological Process	GO:0018243	protein O-linked glycosylation via threonine	1/2279	9/18866	0.6861817	0.803372	0.7269195	1	GALNT2
Biological Process	GO:0019043	establishment of viral latency	1/2279	9/18866	0.6861817	0.803372	0.7269195	1	BANF1

Biological Process	GO:0019236	response to pheromone	1/2279	9/18866	0.6861817	0.803372	0.7269195	1	GPR180
Biological Process	GO:0019626	short-chain fatty acid catabolic process	1/2279	9/18866	0.6861817	0.803372	0.7269195	1	PCK2
Biological Process	GO:0021612	facial nerve structural organization	1/2279	9/18866	0.6861817	0.803372	0.7269195	1	NRP2
Biological Process	GO:0030952	establishment or maintenance of cytoskeleton polarity	1/2279	9/18866	0.6861817	0.803372	0.7269195	1	AQP1
Biological Process	GO:0031441	negative regulation of mRNA 3'-end processing	1/2279	9/18866	0.6861817	0.803372	0.7269195	1	RNF40
Biological Process	GO:0031642	negative regulation of myelination	1/2279	9/18866	0.6861817	0.803372	0.7269195	1	TNFRSF21
Biological Process	GO:0032610	interleukin-1 alpha production	1/2279	9/18866	0.6861817	0.803372	0.7269195	1	IL16
Biological Process	GO:0032782	bile acid secretion	1/2279	9/18866	0.6861817	0.803372	0.7269195	1	SLC51A
Biological Process	GO:0033080	immature T cell proliferation in thymus	1/2279	9/18866	0.6861817	0.803372	0.7269195	1	TMEM131L

Biological Process	GO:0033240	positive regulation of cellular amine metabolic process	1/2279	9/18866	0.6861817	0.803372	0.7269195	1	COMT
Biological Process	GO:0033625	positive regulation of integrin activation	1/2279	9/18866	0.6861817	0.803372	0.7269195	1	RAP1B
Biological Process	GO:0034720	histone H3-K4 demethylation	1/2279	9/18866	0.6861817	0.803372	0.7269195	1	KDM1B
Biological Process	GO:0035247	peptidyl-arginine omega-N-methylation	1/2279	9/18866	0.6861817	0.803372	0.7269195	1	PRMT2
Biological Process	GO:0035562	negative regulation of chromatin binding	1/2279	9/18866	0.6861817	0.803372	0.7269195	1	FBH1
Biological Process	GO:0035672	oligopeptide transmembrane transport	1/2279	9/18866	0.6861817	0.803372	0.7269195	1	ABCC1
Biological Process	GO:0038155	interleukin-23-mediated signaling pathway	1/2279	9/18866	0.6861817	0.803372	0.7269195	1	P4HB
Biological Process	GO:0042420	dopamine catabolic process	1/2279	9/18866	0.6861817	0.803372	0.7269195	1	COMT
Biological Process	GO:0042473	outer ear morphogenesis	1/2279	9/18866	0.6861817	0.803372	0.7269195	1	FGFR1

Biological Process	GO:0042748	circadian sleep/wake cycle, non-REM sleep	1/2279	9/18866	0.6861817	0.803372	0.7269195	1	GHRL
Biological Process	GO:0042996	regulation of Golgi to plasma membrane protein transport	1/2279	9/18866	0.6861817	0.803372	0.7269195	1	CSK
Biological Process	GO:0044359	modulation of molecular function in other organism	1/2279	9/18866	0.6861817	0.803372	0.7269195	1	SFTPD
Biological Process	GO:0044851	hair cycle phase	1/2279	9/18866	0.6861817	0.803372	0.7269195	1	CDH3
Biological Process	GO:0045586	regulation of gamma-delta T cell differentiation	1/2279	9/18866	0.6861817	0.803372	0.7269195	1	PTPRC
Biological Process	GO:0045656	negative regulation of monocyte differentiation	1/2279	9/18866	0.6861817	0.803372	0.7269195	1	INPP5D
Biological Process	GO:0045714	regulation of low-density lipoprotein particle receptor biosynthetic process	1/2279	9/18866	0.6861817	0.803372	0.7269195	1	ITGB3
Biological Process	GO:0046085	adenosine metabolic process	1/2279	9/18866	0.6861817	0.803372	0.7269195	1	ACP3
Biological Process	GO:0046532	regulation of photoreceptor cell differentiation	1/2279	9/18866	0.6861817	0.803372	0.7269195	1	NOTCH1

Biological Process	GO:0048149	behavioral response to ethanol	1/2279	9/18866	0.6861817	0.803372	0.7269195	1	CRHBP
Biological Process	GO:0048387	negative regulation of retinoic acid receptor signaling pathway	1/2279	9/18866	0.6861817	0.803372	0.7269195	1	CALR
Biological Process	GO:0048617	embryonic foregut morphogenesis	1/2279	9/18866	0.6861817	0.803372	0.7269195	1	SMAD3
Biological Process	GO:0048819	regulation of hair follicle maturation	1/2279	9/18866	0.6861817	0.803372	0.7269195	1	CDH3
Biological Process	GO:0048865	stem cell fate commitment	1/2279	9/18866	0.6861817	0.803372	0.7269195	1	NTF4
Biological Process	GO:0051133	regulation of NK T cell activation	1/2279	9/18866	0.6861817	0.803372	0.7269195	1	ZBTB7B
Biological Process	GO:0051593	response to folic acid	1/2279	9/18866	0.6861817	0.803372	0.7269195	1	GSN
Biological Process	GO:0051660	establishment of centrosome localization	1/2279	9/18866	0.6861817	0.803372	0.7269195	1	EZR
Biological Process	GO:0052040	modulation by symbiont of host programmed cell death	1/2279	9/18866	0.6861817	0.803372	0.7269195	1	BCL2L1

Biological Process	GO:0052205	modulation of molecular function in other organism involved in symbiotic interaction	1/2279	9/18866	0.6861817	0.803372	0.7269195	1	SFTPD
Biological Process	GO:0060050	positive regulation of protein glycosylation	1/2279	9/18866	0.6861817	0.803372	0.7269195	1	POMT2
Biological Process	GO:0060054	positive regulation of epithelial cell proliferation involved in wound healing	1/2279	9/18866	0.6861817	0.803372	0.7269195	1	JAML
Biological Process	GO:0060075	regulation of resting membrane potential	1/2279	9/18866	0.6861817	0.803372	0.7269195	1	KCNK6
Biological Process	GO:0060267	positive regulation of respiratory burst	1/2279	9/18866	0.6861817	0.803372	0.7269195	1	CAMK1D
Biological Process	GO:0060371	regulation of atrial cardiac muscle cell membrane depolarization	1/2279	9/18866	0.6861817	0.803372	0.7269195	1	GJA5
Biological Process	GO:0060907	positive regulation of macrophage cytokine production	1/2279	9/18866	0.6861817	0.803372	0.7269195	1	SPON2
Biological Process	GO:0060982	coronary artery morphogenesis	1/2279	9/18866	0.6861817	0.803372	0.7269195	1	NOTCH1
Biological Process	GO:0061072	iris morphogenesis	1/2279	9/18866	0.6861817	0.803372	0.7269195	1	HIPK2

Biological Process	GO:0061549	sympathetic ganglion development	1/2279	9/18866	0.6861817	0.803372	0.7269195	1	NRP2
Biological Process	GO:0070255	regulation of mucus secretion	1/2279	9/18866	0.6861817	0.803372	0.7269195	1	PRKCE
Biological Process	GO:0070561	vitamin D receptor signaling pathway	1/2279	9/18866	0.6861817	0.803372	0.7269195	1	VDR
Biological Process	GO:0071635	negative regulation of transforming growth factor beta production	1/2279	9/18866	0.6861817	0.803372	0.7269195	1	CDH3
Biological Process	GO:0072177	mesonephric duct development	1/2279	9/18866	0.6861817	0.803372	0.7269195	1	WNT11
Biological Process	GO:0072217	negative regulation of metanephros development	1/2279	9/18866	0.6861817	0.803372	0.7269195	1	PAX2
Biological Process	GO:0085020	protein K6-linked ubiquitination	1/2279	9/18866	0.6861817	0.803372	0.7269195	1	PRKN
Biological Process	GO:0090129	positive regulation of synapse maturation	1/2279	9/18866	0.6861817	0.803372	0.7269195	1	NRXN1
Biological Process	GO:0090160	Golgi to lysosome transport	1/2279	9/18866	0.6861817	0.803372	0.7269195	1	ANKFY1

Biological Process	GO:0090281	negative regulation of calcium ion import	1/2279	9/18866	0.6861817	0.803372	0.7269195	1	TRIM27
Biological Process	GO:0090647	modulation of age-related behavioral decline	1/2279	9/18866	0.6861817	0.803372	0.7269195	1	APP
Biological Process	GO:0098696	regulation of neurotransmitter receptor localization to postsynaptic specialization membrane	1/2279	9/18866	0.6861817	0.803372	0.7269195	1	PRKCZ
Biological Process	GO:0098792	xenophagy	1/2279	9/18866	0.6861817	0.803372	0.7269195	1	WIPI2
Biological Process	GO:0099022	vesicle tethering	1/2279	9/18866	0.6861817	0.803372	0.7269195	1	C17orf75
Biological Process	GO:0099527	postsynapse to nucleus signaling pathway	1/2279	9/18866	0.6861817	0.803372	0.7269195	1	RELA
Biological Process	GO:1900122	positive regulation of receptor binding	1/2279	9/18866	0.6861817	0.803372	0.7269195	1	PLCL1
Biological Process	GO:1900227	positive regulation of NLRP3 inflammasome complex assembly	1/2279	9/18866	0.6861817	0.803372	0.7269195	1	STMP1
Biological Process	GO:1901844	regulation of cell communication by electrical coupling involved in cardiac conduction	1/2279	9/18866	0.6861817	0.803372	0.7269195	1	PDE4D

Biological Process	GO:1901978	positive regulation of cell cycle checkpoint	1/2279	9/18866	0.6861817	0.803372	0.7269195	1	PCID2
Biological Process	GO:1903044	protein localization to membrane raft	1/2279	9/18866	0.6861817	0.803372	0.7269195	1	FLOT1
Biological Process	GO:1903365	regulation of fear response	1/2279	9/18866	0.6861817	0.803372	0.7269195	1	MEF2C
Biological Process	GO:1904685	positive regulation of metalloendopeptidase activity	1/2279	9/18866	0.6861817	0.803372	0.7269195	1	MBP
Biological Process	GO:1904776	regulation of protein localization to cell cortex	1/2279	9/18866	0.6861817	0.803372	0.7269195	1	NUMA1
Biological Process	GO:1905165	regulation of lysosomal protein catabolic process	1/2279	9/18866	0.6861817	0.803372	0.7269195	1	MGAT3
Biological Process	GO:1905216	positive regulation of RNA binding	1/2279	9/18866	0.6861817	0.803372	0.7269195	1	EIF4G1
Biological Process	GO:1905709	negative regulation of membrane permeability	1/2279	9/18866	0.6861817	0.803372	0.7269195	1	HK2
Biological Process	GO:1905770	regulation of mesodermal cell differentiation	1/2279	9/18866	0.6861817	0.803372	0.7269195	1	FGFR1

Biological Process	GO:1990264	peptidyl-tyrosine dephosphorylation involved in inactivation of protein kinase activity	1/2279	9/18866	0.6861817	0.803372	0.7269195	1	DUSP3
Biological Process	GO:2000096	positive regulation of Wnt signaling pathway, planar cell polarity pathway	1/2279	9/18866	0.6861817	0.803372	0.7269195	1	ANKRD6
Biological Process	GO:2000252	negative regulation of feeding behavior	1/2279	9/18866	0.6861817	0.803372	0.7269195	1	TTC21B
Biological Process	GO:2000253	positive regulation of feeding behavior	1/2279	9/18866	0.6861817	0.803372	0.7269195	1	GHRL
Biological Process	GO:2000321	positive regulation of T-helper 17 cell differentiation	1/2279	9/18866	0.6861817	0.803372	0.7269195	1	NLRP3
Biological Process	GO:2000347	positive regulation of hepatocyte proliferation	1/2279	9/18866	0.6861817	0.803372	0.7269195	1	TNFAIP3
Biological Process	GO:2000501	regulation of natural killer cell chemotaxis	1/2279	9/18866	0.6861817	0.803372	0.7269195	1	CCL5
Biological Process	GO:2000508	regulation of dendritic cell chemotaxis	1/2279	9/18866	0.6861817	0.803372	0.7269195	1	CALR
Biological Process	GO:2000562	negative regulation of CD4-positive, alpha-beta T cell proliferation	1/2279	9/18866	0.6861817	0.803372	0.7269195	1	VSIR

Biological Process	GO:2000674	regulation of type B pancreatic cell apoptotic process	1/2279	9/18866	0.6861817	0.803372	0.7269195	1	TCF7L2
Biological Process	GO:2000822	regulation of behavioral fear response	1/2279	9/18866	0.6861817	0.803372	0.7269195	1	MEF2C
Biological Process	GO:2000833	positive regulation of steroid hormone secretion	1/2279	9/18866	0.6861817	0.803372	0.7269195	1	GHRL
Biological Process	GO:2000849	regulation of glucocorticoid secretion	1/2279	9/18866	0.6861817	0.803372	0.7269195	1	GHRL
Biological Process	GO:0006704	glucocorticoid biosynthetic process	2/2279	19/18866	0.6873604	0.803372	0.7269195	2	DGKQ/HSD11B2
Biological Process	GO:0010744	positive regulation of macrophage derived foam cell differentiation	2/2279	19/18866	0.6873604	0.803372	0.7269195	2	PRKCH/AGTR1
Biological Process	GO:0030011	maintenance of cell polarity	2/2279	19/18866	0.6873604	0.803372	0.7269195	2	WNT11/WDR1
Biological Process	GO:0030150	protein import into mitochondrial matrix	2/2279	19/18866	0.6873604	0.803372	0.7269195	2	PAM16/TIMM44
Biological Process	GO:0031445	regulation of heterochromatin assembly	2/2279	19/18866	0.6873604	0.803372	0.7269195	2	ATF7IP/DNMT1

Biological Process	GO:0032095	regulation of response to food	2/2279	19/18866	0.6873604	0.803372	0.7269195	2	GHRL/NPY
Biological Process	GO:0032239	regulation of nucleobase-containing compound transport	2/2279	19/18866	0.6873604	0.803372	0.7269195	2	SETD2/RIPK1
Biological Process	GO:0035269	protein O-linked mannosylation	2/2279	19/18866	0.6873604	0.803372	0.7269195	2	POMT2/B4GAT1
Biological Process	GO:0035338	long-chain fatty-acyl-CoA biosynthetic process	2/2279	19/18866	0.6873604	0.803372	0.7269195	2	ACSL1/ACSF3
Biological Process	GO:0036342	post-anal tail morphogenesis	2/2279	19/18866	0.6873604	0.803372	0.7269195	2	CHST11/TMED2
Biological Process	GO:0042693	muscle cell fate commitment	2/2279	19/18866	0.6873604	0.803372	0.7269195	2	RBPJ/MEF2C
Biological Process	GO:0046485	ether lipid metabolic process	2/2279	19/18866	0.6873604	0.803372	0.7269195	2	CHPT1/PEDS1
Biological Process	GO:0051546	keratinocyte migration	2/2279	19/18866	0.6873604	0.803372	0.7269195	2	LTB4R2/IQSEC1
Biological Process	GO:0055003	cardiac myofibril assembly	2/2279	19/18866	0.6873604	0.803372	0.7269195	2	TTN/PDGFRB

Biological Process	GO:0061081	positive regulation of myeloid leukocyte cytokine production involved in immune response	2/2279	19/18866	0.6873604	0.803372	0.7269195	2	SPON2/BCL10
Biological Process	GO:0070989	oxidative demethylation	2/2279	19/18866	0.6873604	0.803372	0.7269195	2	FTO/TET2
Biological Process	GO:0072074	kidney mesenchyme development	2/2279	19/18866	0.6873604	0.803372	0.7269195	2	PAX2/PDGFRB
Biological Process	GO:0072077	renal vesicle morphogenesis	2/2279	19/18866	0.6873604	0.803372	0.7269195	2	PAX2/LIF
Biological Process	GO:0090670	RNA localization to Cajal body	2/2279	19/18866	0.6873604	0.803372	0.7269195	2	RUVBL1/CCT6A
Biological Process	GO:0090671	telomerase RNA localization to Cajal body	2/2279	19/18866	0.6873604	0.803372	0.7269195	2	RUVBL1/CCT6A
Biological Process	GO:0090672	telomerase RNA localization	2/2279	19/18866	0.6873604	0.803372	0.7269195	2	RUVBL1/CCT6A
Biological Process	GO:0090685	RNA localization to nucleus	2/2279	19/18866	0.6873604	0.803372	0.7269195	2	RUVBL1/CCT6A
Biological Process	GO:1900424	regulation of defense response to bacterium	2/2279	19/18866	0.6873604	0.803372	0.7269195	2	FOXP1/GRN

Biological Process	GO:2000647	negative regulation of stem cell proliferation	2/2279	19/18866	0.6873604	0.803372	0.7269195	2	OVOL1/NF1
Biological Process	GO:2000757	negative regulation of peptidyl-lysine acetylation	2/2279	19/18866	0.6873604	0.803372	0.7269195	2	CTBP1/PIH1D1
Biological Process	GO:0007215	glutamate receptor signaling pathway	11/2279	101/18866	0.6885282	0.8046379	0.7280649	11	CCR2/APP/GRIK4/CRHBP/CNIH2/PLCB1/MAPK8IP2/GSG1L/MEF2C/SHANK2/GRID1
Biological Process	GO:0002385	mucosal immune response	4/2279	38/18866	0.6897537	0.8055747	0.7289126	4	DEFA4/RNASE3/DEFB1/LTF
Biological Process	GO:0006298	mismatch repair	4/2279	38/18866	0.6897537	0.8055747	0.7289126	4	SETD2/ERCC1/MUTYH/POLD3
Biological Process	GO:0006862	nucleotide transport	4/2279	38/18866	0.6897537	0.8055747	0.7289126	4	SLC25A25/SLC33A1/ABCC11/SLC25A1
Biological Process	GO:0043029	T cell homeostasis	4/2279	38/18866	0.6897537	0.8055747	0.7289126	4	RIPK3/ZC3H8/BCL2/FADD
Biological Process	GO:0097064	ncRNA export from nucleus	4/2279	38/18866	0.6897537	0.8055747	0.7289126	4	RAE1/NUP93/SEC13/NOL6
Biological Process	GO:0090175	regulation of establishment of planar polarity	12/2279	110/18866	0.6904358	0.8062722	0.7295437	12	AP2A1/VANGL1/PRICKLE2/WNT11/SMURF1/PFN1/PSMF1/ARRB2/DAAM1/PSMB7/ANKRD6/PSMD13

Biological Process	GO:0061351	neural precursor cell proliferation	17/2279	154/18866	0.6911343	0.8069887	0.730192	17	CTSZ/NDE1/SPINT2/ZEB2/DISC1/GNAI2/ILK/FGFR1/NF1/WDR47/NUMB/ZNF335/NOTCH1/RPGRIP1/ARHGEF2/FOXO1/CX3CR1
Biological Process	GO:0007127	meiosis I	13/2279	119/18866	0.6924792	0.8084597	0.731523	13	MOV10L1/BRCA2/EME1/MEIOB/RNF212/CCNA1/RAD21L1/DMC1/SLX4/TEX12/KLHDC3/PSMD13/TERB2
Biological Process	GO:0009267	cellular response to starvation	18/2279	163/18866	0.6937533	0.8098476	0.7327789	18	RPTOR/PRKAG2/MFSD2A/WIPI2/COMT/EHMT2/STK24/CLEC16A/ATG14/NFE2L2/BCL2/SLC2A1/FOXO1/SREBF1/NPRL2/PRKAA1/MARS1/RIPOR1
Biological Process	GO:0006294	nucleotide-excision repair, preincision complex assembly	3/2279	29/18866	0.6977895	0.8137595	0.7363185	3	CCNH/XPA/ERCC2
Biological Process	GO:0007263	nitric oxide mediated signal transduction	3/2279	29/18866	0.6977895	0.8137595	0.7363185	3	FPR1/THBS1/NOS1AP
Biological Process	GO:0009435	NAD biosynthetic process	3/2279	29/18866	0.6977895	0.8137595	0.7363185	3	NMNAT3/PARP10/PARP9
Biological Process	GO:0010390	histone monoubiquitination	3/2279	29/18866	0.6977895	0.8137595	0.7363185	3	UHRF1/PCGF3/RNF40
Biological Process	GO:0043032	positive regulation of macrophage activation	3/2279	29/18866	0.6977895	0.8137595	0.7363185	3	IL10/MIR145/THBS1
Biological Process	GO:0055075	potassium ion homeostasis	3/2279	29/18866	0.6977895	0.8137595	0.7363185	3	KCTD7/SLC12A1/SLC12A7

Biological Process	GO:0080154	regulation of fertilization	3/2279	29/18866	0.6977895	0.8137595	0.7363185	3	RNASE10/LHFPL2/PLCB1
Biological Process	GO:1901017	negative regulation of potassium ion transmembrane transporter activity	3/2279	29/18866	0.6977895	0.8137595	0.7363185	3	KCNE1/CASQ2/CAB39
Biological Process	GO:0001707	mesoderm formation	8/2279	75/18866	0.6989649	0.8149301	0.7373777	8	MIR145/SMAD3/WNT11/PAX2/FGFR1/ITGB3/INHBA/NODAL
Biological Process	GO:0050805	negative regulation of synaptic transmission	8/2279	75/18866	0.6989649	0.8149301	0.7373777	8	SORCS2/RAP1B/GNAI2/NF1/CNR2/S1PR2/SHANK2/PRKN
Biological Process	GO:0008652	cellular amino acid biosynthetic process	9/2279	84/18866	0.6992274	0.8151361	0.7375641	9	GADL1/GGT1/CBS/AGXT/UPB1/MTHFD1/SLC1A3/ADI1/ASPG
Biological Process	GO:0046824	positive regulation of nucleocytoplasmic transport	7/2279	66/18866	0.6994049	0.815143	0.7375703	7	PIK3R1/SMAD3/JUP/TCF7L2/TGFB1/CDH1/NEDD4
Biological Process	GO:1902108	regulation of mitochondrial membrane permeability involved in apoptotic process	7/2279	66/18866	0.6994049	0.815143	0.7375703	7	TFDP1/YWHAH/BMF/YWHAZ/BCL2/TP53BP2/YWHAQ
Biological Process	GO:0072431	signal transduction involved in mitotic G1 DNA damage checkpoint	6/2279	57/18866	0.7008785	0.8166601	0.7389431	6	TFDP1/E2F7/PCBP4/ZNF385A/ARID3A/CHEK2
Biological Process	GO:1902400	intracellular signal transduction involved in G1 DNA damage checkpoint	6/2279	57/18866	0.7008785	0.8166601	0.7389431	6	TFDP1/E2F7/PCBP4/ZNF385A/ARID3A/CHEK2

Biological Process	GO:0006821	chloride transport	12/2279	111/18866	0.7024639	0.818407	0.7405237	12	CLCN6/FXYD1/ANO7/P2RY6/SLC26A1/SLC1A3/ANO10/ANO6/SLC12A1/ANO8/SLC12A7/PACC1
Biological Process	GO:1901991	negative regulation of mitotic cell cycle phase transition	28/2279	251/18866	0.7029504	0.8188734	0.7409458	28	TRIM39/BLM/TFDP1/MAD1L1/FOXN3/PRMT2/HUS1/FBXO7/E2F7/CTDSPL/PCBP4/RIPK1/NACC2/ZNF385A/ARID3A/PSMF1/GFI1B/TAOK3/ZFYVE19/MIR138-2/CLSPN/RB1/BCL2/PSMB7/CHEK2/NAE1/PSMD13/PCID2
Biological Process	GO:0046661	male sex differentiation	18/2279	164/18866	0.7036679	0.8196088	0.7416111	18	ARID5B/CSDE1/FANCA/RARA/SF1/ASB1/NCOA1/LHFPL2/NTRK1/HOXA9/RAD21L1/BCL2/PDGFRB/ERCC1/BCL2L1/HMGCS1/NCOA4/INHBA
Biological Process	GO:0009409	response to cold	5/2279	48/18866	0.7039416	0.8196261	0.7416268	5	ZNF516/PPARG/FOXO1/ACADVL/PRKAA1
Biological Process	GO:0032648	regulation of interferon-beta production	5/2279	48/18866	0.7039416	0.8196261	0.7416268	5	TRIM38/IRF5/TLR9/TRAIP/FLOT1
Biological Process	GO:0043039	tRNA aminoacylation	5/2279	48/18866	0.7039416	0.8196261	0.7416268	5	CARS2/EARS2/MARS1/GATB/YARS1
Biological Process	GO:0031111	negative regulation of microtubule polymerization or depolymerization	4/2279	39/18866	0.709606	0.8258166	0.7472282	4	TBCD/NAV3/ARHGEF2/SPEF1
Biological Process	GO:0055090	acylglycerol homeostasis	4/2279	39/18866	0.709606	0.8258166	0.7472282	4	C1QTNF3/LIPC/MED13/ANGPTL8
Biological Process	GO:0070328	triglyceride homeostasis	4/2279	39/18866	0.709606	0.8258166	0.7472282	4	C1QTNF3/LIPC/MED13/ANGPTL8

Biological Process	GO:1904706	negative regulation of vascular associated smooth muscle cell proliferation	4/2279	39/18866	0.709606	0.8258166	0.7472282	4	IL10/PPARG/MIR140/MEF2C
Biological Process	GO:0043484	regulation of RNA splicing	17/2279	156/18866	0.7113942	0.8277962	0.7490194	17	RBM19/ERN1/PIK3R1/SRPK2/PCBP4/AHNAK/CLK3/SF1/DAZAP1/SFSWAP/CELF2/HNRNPLL/CELF1/RBFOX3/NSRP1/RBM15B/GRSF1
Biological Process	GO:0030641	regulation of cellular pH	10/2279	94/18866	0.7128039	0.8290863	0.7501867	10	TM9SF4/SLC11A1/ATP6V1B2/GRN/BCL2/ATP6V0B/UBE3A/CLN5/ATP6V0C/ATP6V1C1
Biological Process	GO:1905954	positive regulation of lipid localization	10/2279	94/18866	0.7128039	0.8290863	0.7501867	10	EHD1/MYB/MAP2K6/ABCG1/ATP8A1/PLTP/NR1H2/GHRL/ABCA1/ACACB
Biological Process	GO:0017038	protein import	22/2279	200/18866	0.7129062	0.8290863	0.7501867	22	ANGPT1/PAM16/PIK3R1/SMAD3/TNPO1/MBTPS1/E2F3/SUFU/IPO13/FAM53B/JUP/CLU/TGFB1/NF1/NUP93/PEX26/ARNTL/CDH1/SNUPN/TIMM44/CABP1/TSC2
Biological Process	GO:0072091	regulation of stem cell proliferation	8/2279	76/18866	0.7131833	0.8290863	0.7501867	8	DISC1/OVOL1/NF1/PTPRC/ZNF335/NOTCH1/KAT7/CX3CR1
Biological Process	GO:0051965	positive regulation of synapse assembly	7/2279	67/18866	0.7144936	0.8290863	0.7501867	7	EPHB3/SEMA4A/SEMA4D/NRXN1/GHRL/NTRK1/CUX2
Biological Process	GO:0001832	blastocyst growth	2/2279	20/18866	0.7146907	0.8290863	0.7501867	2	BRCA2/NDEL1
Biological Process	GO:0002320	lymphoid progenitor cell differentiation	2/2279	20/18866	0.7146907	0.8290863	0.7501867	2	BCL2/NOTCH1

Biological Process	GO:0002689	negative regulation of leukocyte chemotaxis	2/2279	20/18866	0.7146907	0.8290863	0.7501867	2	PADI2/C5AR2
Biological Process	GO:0007350	blastoderm segmentation	2/2279	20/18866	0.7146907	0.8290863	0.7501867	2	NRP2/PCSK6
Biological Process	GO:0008356	asymmetric cell division	2/2279	20/18866	0.7146907	0.8290863	0.7501867	2	ZBTB16/ARHGEF2
Biological Process	GO:0009264	deoxyribonucleotide catabolic process	2/2279	20/18866	0.7146907	0.8290863	0.7501867	2	UNG/NTHL1
Biological Process	GO:0010002	cardioblast differentiation	2/2279	20/18866	0.7146907	0.8290863	0.7501867	2	RBPJ/NOTCH1
Biological Process	GO:0030903	notochord development	2/2279	20/18866	0.7146907	0.8290863	0.7501867	2	WNT11/TEAD2
Biological Process	GO:0032986	protein-DNA complex disassembly	2/2279	20/18866	0.7146907	0.8290863	0.7501867	2	SMARCD1/SMARCD2
Biological Process	GO:0035493	SNARE complex assembly	2/2279	20/18866	0.7146907	0.8290863	0.7501867	2	UVRAG/VPS18
Biological Process	GO:0045056	transcytosis	2/2279	20/18866	0.7146907	0.8290863	0.7501867	2	MFSD2A/IGF1R

Biological Process	GO:0060231	mesenchymal to epithelial transition	2/2279	20/18866	0.7146907	0.8290863	0.7501867	2	PAX2/LIF
Biological Process	GO:0070262	peptidyl-serine dephosphorylation	2/2279	20/18866	0.7146907	0.8290863	0.7501867	2	PPP2R1A/PPP1R16B
Biological Process	GO:0071243	cellular response to arsenic-containing substance	2/2279	20/18866	0.7146907	0.8290863	0.7501867	2	GSTO1/GSTO2
Biological Process	GO:0072087	renal vesicle development	2/2279	20/18866	0.7146907	0.8290863	0.7501867	2	PAX2/LIF
Biological Process	GO:2001169	regulation of ATP biosynthetic process	2/2279	20/18866	0.7146907	0.8290863	0.7501867	2	ENO1/SPHK2
Biological Process	GO:0098661	inorganic anion transmembrane transport	13/2279	121/18866	0.7153056	0.8290863	0.7501867	13	CLCN6/FXYD1/ANO7/SLC13A4/SLC26A1/SLC37A1/SLC1A3/ANO10/ANO6/SLC12A1/ANO8/SLC12A7/PACC1
Biological Process	GO:0010662	regulation of striated muscle cell apoptotic process	6/2279	58/18866	0.7169981	0.8290863	0.7501867	6	MIR145/MIR199A1/MIR24-2/ILK/NFE2L2/MIR199A2
Biological Process	GO:0043489	RNA stabilization	6/2279	58/18866	0.7169981	0.8290863	0.7501867	6	SLC11A1/LARP1/PAIP1/SYNCRIP/TENT5C/TENT5A
Biological Process	GO:1900408	negative regulation of cellular response to oxidative stress	6/2279	58/18866	0.7169981	0.8290863	0.7501867	6	IL10/FBXO7/NOL3/NFE2L2/BAG5/PRKN

Biological Process	GO:1903202	negative regulation of oxidative stress-induced cell death	6/2279	58/18866	0.7169981	0.8290863	0.7501867	6	IL10/FBXO7/NOL3/NFE2L2/BAG5/PRKN
Biological Process	GO:0006338	chromatin remodeling	24/2279	218/18866	0.7174736	0.8290863	0.7501867	24	HDAC4/CECR2/BAZ1B/KDM4B/SRCAP/MYB/SMARCA2/HDAC1/PAX7/ARID1B/ATF7IP/KDM4C/RSF1/MTA2/SMARCD1/SMARCD2/RB1/DNMT1/PIH1D1/CHD8/CENPM/ZNFX1/RUVBL1/H3C1
Biological Process	GO:0071805	potassium ion transmembrane transport	24/2279	218/18866	0.7174736	0.8290863	0.7501867	24	KCNJ15/KCNE1/KCNAB2/CASQ2/WWP2/FXYD2/SLC24A4/DNM2/KCNN1/KCNQ2/BIN1/NOS1AP/KCNIP1/KCNK6/AQP1/CAB39/KCNQ1/SLC1A3/ABCB8/NEDD4/ANO6/ANK2/SLC12A1/SLC12A7
Biological Process	GO:0005979	regulation of glycogen biosynthetic process	3/2279	30/18866	0.7199382	0.8290863	0.7501867	3	DYRK2/PPP1CB/ESRRB
Biological Process	GO:0007176	regulation of epidermal growth factor-activated receptor activity	3/2279	30/18866	0.7199382	0.8290863	0.7501867	3	TGFA/NCK2/APP
Biological Process	GO:0010962	regulation of glucan biosynthetic process	3/2279	30/18866	0.7199382	0.8290863	0.7501867	3	DYRK2/PPP1CB/ESRRB
Biological Process	GO:0031116	positive regulation of microtubule polymerization	3/2279	30/18866	0.7199382	0.8290863	0.7501867	3	DCTN1/NAV3/NUMA1
Biological Process	GO:0032728	positive regulation of interferon-beta production	3/2279	30/18866	0.7199382	0.8290863	0.7501867	3	IRF5/TLR9/FLOT1
Biological Process	GO:0035666	TRIF-dependent toll-like receptor signaling pathway	3/2279	30/18866	0.7199382	0.8290863	0.7501867	3	RIPK1/UBE2D2/FADD

Biological Process	GO:0036475	neuron death in response to oxidative stress	3/2279	30/18866	0.7199382	0.8290863	0.7501867	3	IL10/FBXO7/PRKN
Biological Process	GO:0038111	interleukin-7-mediated signaling pathway	3/2279	30/18866	0.7199382	0.8290863	0.7501867	3	STAT5A/JAK1/H3C1
Biological Process	GO:0044030	regulation of DNA methylation	3/2279	30/18866	0.7199382	0.8290863	0.7501867	3	KDM1B/GRHL2/MORC1
Biological Process	GO:0061082	myeloid leukocyte cytokine production	3/2279	30/18866	0.7199382	0.8290863	0.7501867	3	SPON2/TGFB1/BCL10
Biological Process	GO:0070168	negative regulation of biomineral tissue development	3/2279	30/18866	0.7199382	0.8290863	0.7501867	3	TGFB1/NOTCH1/RFLNA
Biological Process	GO:0070198	protein localization to chromosome, telomeric region	3/2279	30/18866	0.7199382	0.8290863	0.7501867	3	TNKS/BRCA2/CCT6A
Biological Process	GO:0071480	cellular response to gamma radiation	3/2279	30/18866	0.7199382	0.8290863	0.7501867	3	CHEK2/BCL2L1/MAP3K20
Biological Process	GO:0090162	establishment of epithelial cell polarity	3/2279	30/18866	0.7199382	0.8290863	0.7501867	3	FRMD4A/MYO18A/FRMD4B
Biological Process	GO:0110150	negative regulation of biomineralization	3/2279	30/18866	0.7199382	0.8290863	0.7501867	3	TGFB1/NOTCH1/RFLNA

Biological Process	GO:0120033	negative regulation of plasma membrane bounded cell projection assembly	3/2279	30/18866	0.7199382	0.8290863	0.7501867	3	TACSTD2/KANK1/DNM2
Biological Process	GO:1902751	positive regulation of cell cycle G2/M phase transition	3/2279	30/18866	0.7199382	0.8290863	0.7501867	3	BRD4/APP/CDC25A
Biological Process	GO:1903205	regulation of hydrogen peroxide-induced cell death	3/2279	30/18866	0.7199382	0.8290863	0.7501867	3	IL10/RIPK1/NFE2L2
Biological Process	GO:2000765	regulation of cytoplasmic translation	3/2279	30/18866	0.7199382	0.8290863	0.7501867	3	ZNF385A/CPEB3/PKM
Biological Process	GO:0006692	prostanoid metabolic process	5/2279	49/18866	0.7213072	0.8290863	0.7501867	5	ABHD16A/TNFRSF1A/LYPLA2/ZADH2/PRXL2B
Biological Process	GO:0006693	prostaglandin metabolic process	5/2279	49/18866	0.7213072	0.8290863	0.7501867	5	ABHD16A/TNFRSF1A/LYPLA2/ZADH2/PRXL2B
Biological Process	GO:0034656	nucleobase-containing small molecule catabolic process	5/2279	49/18866	0.7213072	0.8290863	0.7501867	5	CDA/DPYS/ENTPD1/UPB1/PNP
Biological Process	GO:0043038	amino acid activation	5/2279	49/18866	0.7213072	0.8290863	0.7501867	5	CARS2/EARS2/MARS1/GATB/YARS1
Biological Process	GO:0048512	circadian behavior	5/2279	49/18866	0.7213072	0.8290863	0.7501867	5	ZFHX3/MTA1/GHRL/USP2/NCOA2

Biological Process	GO:0090051	negative regulation of cell migration involved in sprouting angiogenesis	5/2279	49/18866	0.7213072	0.8290863	0.7501867	5	ITGB1BP1/MIR199A1/THBS1/MIR199A2/NOTCH1
Biological Process	GO:1900087	positive regulation of G1/S transition of mitotic cell cycle	5/2279	49/18866	0.7213072	0.8290863	0.7501867	5	RPTOR/TFDP1/PLCB1/PLRG1/EIF4G1
Biological Process	GO:0000290	deadenylation-dependent decapping of nuclear-transcribed mRNA	1/2279	10/18866	0.7241089	0.8290863	0.7501867	1	LSM1
Biological Process	GO:0001553	luteinization	1/2279	10/18866	0.7241089	0.8290863	0.7501867	1	INHBA
Biological Process	GO:0001660	fever generation	1/2279	10/18866	0.7241089	0.8290863	0.7501867	1	TNF
Biological Process	GO:0002291	T cell activation via T cell receptor contact with antigen bound to MHC molecule on antigen presenting cell	1/2279	10/18866	0.7241089	0.8290863	0.7501867	1	HLA-DMB
Biological Process	GO:0002524	hypersensitivity	1/2279	10/18866	0.7241089	0.8290863	0.7501867	1	FUT7
Biological Process	GO:0003129	heart induction	1/2279	10/18866	0.7241089	0.8290863	0.7501867	1	WNT11
Biological Process	GO:0006188	IMP biosynthetic process	1/2279	10/18866	0.7241089	0.8290863	0.7501867	1	AMPD3

Biological Process	GO:0006307	DNA dealkylation involved in DNA repair	1/2279	10/18866	0.7241089	0.8290863	0.7501867	1	FTO
Biological Process	GO:0006560	proline metabolic process	1/2279	10/18866	0.7241089	0.8290863	0.7501867	1	ALDH4A1
Biological Process	GO:0006930	substrate-dependent cell migration, cell extension	1/2279	10/18866	0.7241089	0.8290863	0.7501867	1	CTTN
Biological Process	GO:0007168	receptor guanylyl cyclase signaling pathway	1/2279	10/18866	0.7241089	0.8290863	0.7501867	1	NPPC
Biological Process	GO:0010269	response to selenium ion	1/2279	10/18866	0.7241089	0.8290863	0.7501867	1	SELENOV
Biological Process	GO:0010986	positive regulation of lipoprotein particle clearance	1/2279	10/18866	0.7241089	0.8290863	0.7501867	1	GPLD1
Biological Process	GO:0010992	ubiquitin recycling	1/2279	10/18866	0.7241089	0.8290863	0.7501867	1	PRKN
Biological Process	GO:0010998	regulation of translational initiation by eIF2 alpha phosphorylation	1/2279	10/18866	0.7241089	0.8290863	0.7501867	1	NCK2
Biological Process	GO:0014051	gamma-aminobutyric acid secretion	1/2279	10/18866	0.7241089	0.8290863	0.7501867	1	NF1

Biological Process	GO:0018242	protein O-linked glycosylation via serine	1/2279	10/18866	0.7241089	0.8290863	0.7501867	1	GALNT2
Biological Process	GO:0019062	virion attachment to host cell	1/2279	10/18866	0.7241089	0.8290863	0.7501867	1	NECTIN1
Biological Process	GO:0019614	catechol-containing compound catabolic process	1/2279	10/18866	0.7241089	0.8290863	0.7501867	1	COMT
Biological Process	GO:0031943	regulation of glucocorticoid metabolic process	1/2279	10/18866	0.7241089	0.8290863	0.7501867	1	DGKQ
Biological Process	GO:0032000	positive regulation of fatty acid beta-oxidation	1/2279	10/18866	0.7241089	0.8290863	0.7501867	1	CPT1A
Biological Process	GO:0032025	response to cobalt ion	1/2279	10/18866	0.7241089	0.8290863	0.7501867	1	D2HGDH
Biological Process	GO:0032308	positive regulation of prostaglandin secretion	1/2279	10/18866	0.7241089	0.8290863	0.7501867	1	MAP2K6
Biological Process	GO:0032926	negative regulation of activin receptor signaling pathway	1/2279	10/18866	0.7241089	0.8290863	0.7501867	1	SKI
Biological Process	GO:0033083	regulation of immature T cell proliferation	1/2279	10/18866	0.7241089	0.8290863	0.7501867	1	TMEM131L

Biological Process	GO:0033131	regulation of glucokinase activity	1/2279	10/18866	0.7241089	0.8290863	0.7501867	1	PRKN
Biological Process	GO:0034135	regulation of toll-like receptor 2 signaling pathway	1/2279	10/18866	0.7241089	0.8290863	0.7501867	1	TNFAIP3
Biological Process	GO:0034454	microtubule anchoring at centrosome	1/2279	10/18866	0.7241089	0.8290863	0.7501867	1	DCTN1
Biological Process	GO:0034651	cortisol biosynthetic process	1/2279	10/18866	0.7241089	0.8290863	0.7501867	1	DGKQ
Biological Process	GO:0034770	histone H4-K20 methylation	1/2279	10/18866	0.7241089	0.8290863	0.7501867	1	NSD2
Biological Process	GO:0034975	protein folding in endoplasmic reticulum	1/2279	10/18866	0.7241089	0.8290863	0.7501867	1	CALR
Biological Process	GO:0035581	sequestering of extracellular ligand from receptor	1/2279	10/18866	0.7241089	0.8290863	0.7501867	1	LTBP1
Biological Process	GO:0035646	endosome to melanosome transport	1/2279	10/18866	0.7241089	0.8290863	0.7501867	1	AP3D1
Biological Process	GO:0035878	nail development	1/2279	10/18866	0.7241089	0.8290863	0.7501867	1	PRKAB1

Biological Process	GO:0035933	glucocorticoid secretion	1/2279	10/18866	0.7241089	0.8290863	0.7501867	1	GHRL
Biological Process	GO:0042424	catecholamine catabolic process	1/2279	10/18866	0.7241089	0.8290863	0.7501867	1	COMT
Biological Process	GO:0042428	serotonin metabolic process	1/2279	10/18866	0.7241089	0.8290863	0.7501867	1	DDC
Biological Process	GO:0042989	sequestering of actin monomers	1/2279	10/18866	0.7241089	0.8290863	0.7501867	1	GSN
Biological Process	GO:0043485	endosome to pigment granule transport	1/2279	10/18866	0.7241089	0.8290863	0.7501867	1	AP3D1
Biological Process	GO:0043587	tongue morphogenesis	1/2279	10/18866	0.7241089	0.8290863	0.7501867	1	HDAC1
Biological Process	GO:0044557	relaxation of smooth muscle	1/2279	10/18866	0.7241089	0.8290863	0.7501867	1	SLC8A1
Biological Process	GO:0048570	notochord morphogenesis	1/2279	10/18866	0.7241089	0.8290863	0.7501867	1	WNT11
Biological Process	GO:0048757	pigment granule maturation	1/2279	10/18866	0.7241089	0.8290863	0.7501867	1	AP3D1

Biological Process	GO:0051342	regulation of cyclic-nucleotide phosphodiesterase activity	1/2279	10/18866	0.7241089	0.8290863	0.7501867	1	AIP
Biological Process	GO:0051351	positive regulation of ligase activity	1/2279	10/18866	0.7241089	0.8290863	0.7501867	1	RIPK3
Biological Process	GO:0051418	microtubule nucleation by microtubule organizing center	1/2279	10/18866	0.7241089	0.8290863	0.7501867	1	TUBGCP2
Biological Process	GO:0052646	alditol phosphate metabolic process	1/2279	10/18866	0.7241089	0.8290863	0.7501867	1	ACP6
Biological Process	GO:0060068	vagina development	1/2279	10/18866	0.7241089	0.8290863	0.7501867	1	TYRO3
Biological Process	GO:0060600	dichotomous subdivision of an epithelial terminal unit	1/2279	10/18866	0.7241089	0.8290863	0.7501867	1	CTSH
Biological Process	GO:0060768	regulation of epithelial cell proliferation involved in prostate gland development	1/2279	10/18866	0.7241089	0.8290863	0.7501867	1	NOTCH1
Biological Process	GO:0060923	cardiac muscle cell fate commitment	1/2279	10/18866	0.7241089	0.8290863	0.7501867	1	RBPJ
Biological Process	GO:0061303	cornea development in camera-type eye	1/2279	10/18866	0.7241089	0.8290863	0.7501867	1	LIMK2

Biological Process	GO:0070391	response to lipoteichoic acid	1/2279	10/18866	0.7241089	0.8290863	0.7501867	1	RELA
Biological Process	GO:0070424	regulation of nucleotide-binding oligomerization domain containing signaling pathway	1/2279	10/18866	0.7241089	0.8290863	0.7501867	1	TNFAIP3
Biological Process	GO:0071223	cellular response to lipoteichoic acid	1/2279	10/18866	0.7241089	0.8290863	0.7501867	1	RELA
Biological Process	GO:0071233	cellular response to leucine	1/2279	10/18866	0.7241089	0.8290863	0.7501867	1	RPTOR
Biological Process	GO:0071394	cellular response to testosterone stimulus	1/2279	10/18866	0.7241089	0.8290863	0.7501867	1	NCOA4
Biological Process	GO:0072070	loop of Henle development	1/2279	10/18866	0.7241089	0.8290863	0.7501867	1	DLL1
Biological Process	GO:0072203	cell proliferation involved in metanephros development	1/2279	10/18866	0.7241089	0.8290863	0.7501867	1	PDGFRB
Biological Process	GO:0097050	type B pancreatic cell apoptotic process	1/2279	10/18866	0.7241089	0.8290863	0.7501867	1	TCF7L2
Biological Process	GO:0097250	mitochondrial respirasome assembly	1/2279	10/18866	0.7241089	0.8290863	0.7501867	1	RAB5IF

Biological Process	GO:0098935	dendritic transport	1/2279	10/18866	0.7241089	0.8290863	0.7501867	1	KIF5C
Biological Process	GO:1900222	negative regulation of amyloid-beta clearance	1/2279	10/18866	0.7241089	0.8290863	0.7501867	1	TNF
Biological Process	GO:1900426	positive regulation of defense response to bacterium	1/2279	10/18866	0.7241089	0.8290863	0.7501867	1	GRN
Biological Process	GO:1901748	leukotriene D4 metabolic process	1/2279	10/18866	0.7241089	0.8290863	0.7501867	1	GGT1
Biological Process	GO:1901750	leukotriene D4 biosynthetic process	1/2279	10/18866	0.7241089	0.8290863	0.7501867	1	GGT1
Biological Process	GO:1902237	positive regulation of endoplasmic reticulum stress-induced intrinsic apoptotic signaling pathway	1/2279	10/18866	0.7241089	0.8290863	0.7501867	1	NCK2
Biological Process	GO:1902415	regulation of mRNA binding	1/2279	10/18866	0.7241089	0.8290863	0.7501867	1	EIF4G1
Biological Process	GO:1902645	tertiary alcohol biosynthetic process	1/2279	10/18866	0.7241089	0.8290863	0.7501867	1	DGKQ
Biological Process	GO:1903431	positive regulation of cell maturation	1/2279	10/18866	0.7241089	0.8290863	0.7501867	1	BCL2

Biological Process	GO:1903826	arginine transmembrane transport	1/2279	10/18866	0.7241089	0.8290863	0.7501867	1	SLC7A1
Biological Process	GO:1903897	regulation of PERK-mediated unfolded protein response	1/2279	10/18866	0.7241089	0.8290863	0.7501867	1	NCK2
Biological Process	GO:1904526	regulation of microtubule binding	1/2279	10/18866	0.7241089	0.8290863	0.7501867	1	MARK2
Biological Process	GO:1904779	regulation of protein localization to centrosome	1/2279	10/18866	0.7241089	0.8290863	0.7501867	1	CEP72
Biological Process	GO:1904851	positive regulation of establishment of protein localization to telomere	1/2279	10/18866	0.7241089	0.8290863	0.7501867	1	CCT6A
Biological Process	GO:1905031	regulation of membrane repolarization during cardiac muscle cell action potential	1/2279	10/18866	0.7241089	0.8290863	0.7501867	1	NOS1AP
Biological Process	GO:1990416	cellular response to brain-derived neurotrophic factor stimulus	1/2279	10/18866	0.7241089	0.8290863	0.7501867	1	UBE3A
Biological Process	GO:2000382	positive regulation of mesoderm development	1/2279	10/18866	0.7241089	0.8290863	0.7501867	1	NODAL
Biological Process	GO:2001054	negative regulation of mesenchymal cell apoptotic process	1/2279	10/18866	0.7241089	0.8290863	0.7501867	1	PAX2

Biological Process	GO:0045471	response to ethanol	13/2279	122/18866	0.726313	0.8315098	0.7523796	13	S100A8/GSN/ITPR2/FECH/CRHBP/RARA/HTR3A/CSF3/CAT/CA3/PRKCE/NPPC/PRKAA1
Biological Process	GO:0061333	renal tubule morphogenesis	8/2279	77/18866	0.7269514	0.8319399	0.7527688	8	TACSTD2/ILK/WNT11/PAX2/MTSS1/BCL2/MEF2C/HOXB7
Biological Process	GO:0072028	nephron morphogenesis	8/2279	77/18866	0.7269514	0.8319399	0.7527688	8	TACSTD2/ILK/WNT11/PAX2/BCL2/PDGFRB/LIF/HOXB7
Biological Process	GO:1903201	regulation of oxidative stress-induced cell death	8/2279	77/18866	0.7269514	0.8319399	0.7527688	8	P4HB/IL10/FBXO7/RIPK1/NOL3/NFE2L2/BAG5/PRKN
Biological Process	GO:0060968	regulation of gene silencing	15/2279	140/18866	0.7282863	0.8325247	0.7532979	15	PPARG/NCOR2/RAE1/TNF/POLR2F/AGO1/RIPK1/LIMD1/AGO2/TGFB1/NUP93/SEC13/RMRP/EIF4G1/H3C1
Biological Process	GO:0007257	activation of JUN kinase activity	4/2279	40/18866	0.7285139	0.8325247	0.7532979	4	ERN1/RIPK1/DBNL/MAP3K20
Biological Process	GO:0008207	C21-steroid hormone metabolic process	4/2279	40/18866	0.7285139	0.8325247	0.7532979	4	DHRS9/AKR1D1/FDX1/DGKQ
Biological Process	GO:0009069	serine family amino acid metabolic process	4/2279	40/18866	0.7285139	0.8325247	0.7532979	4	GGT1/CBS/AGXT/MTHFD1
Biological Process	GO:0010613	positive regulation of cardiac muscle hypertrophy	4/2279	40/18866	0.7285139	0.8325247	0.7532979	4	PRKCA/MIR199A1/PDE9A/MIR199A2

Biological Process	GO:0021587	cerebellum morphogenesis	4/2279	40/18866	0.7285139	0.8325247	0.7532979	4	DLL1/TTC21B/TTLL1/COQ8B
Biological Process	GO:0040036	regulation of fibroblast growth factor receptor signaling pathway	4/2279	40/18866	0.7285139	0.8325247	0.7532979	4	THBS1/RUNX2/FAM20C/OTX2
Biological Process	GO:0042417	dopamine metabolic process	4/2279	40/18866	0.7285139	0.8325247	0.7532979	4	COMT/DDC/ITGB2/PRKN
Biological Process	GO:0043001	Golgi to plasma membrane protein transport	4/2279	40/18866	0.7285139	0.8325247	0.7532979	4	KIF13A/RAB31/VAMP5/CSK
Biological Process	GO:0045740	positive regulation of DNA replication	4/2279	40/18866	0.7285139	0.8325247	0.7532979	4	E2F7/FGFR1/BCAR3/KAT7
Biological Process	GO:0072210	metanephric nephron development	4/2279	40/18866	0.7285139	0.8325247	0.7532979	4	PAX2/PDGFB/PDGFRB/LIF
Biological Process	GO:0097352	autophagosome maturation	4/2279	40/18866	0.7285139	0.8325247	0.7532979	4	UVRAG/CLEC16A/ATG14/RUBCNL
Biological Process	GO:0006625	protein targeting to peroxisome	7/2279	68/18866	0.7290635	0.8325519	0.7533225	7	AMACR/AGXT/CAT/PEX26/ACOX2/HMGCL/UBE2D2
Biological Process	GO:0051298	centrosome duplication	7/2279	68/18866	0.7290635	0.8325519	0.7533225	7	ARHGEF10/NDE1/BRCA2/CEP135/POC1A/CEP72/CEP295NL

Biological Process	GO:0072662	protein localization to peroxisome	7/2279	68/18866	0.7290635	0.8325519	0.7533225	7	AMACR/AGXT/CAT/PEX26/ACOX2/HMGCL/UBE2D2
Biological Process	GO:0072663	establishment of protein localization to peroxisome	7/2279	68/18866	0.7290635	0.8325519	0.7533225	7	AMACR/AGXT/CAT/PEX26/ACOX2/HMGCL/UBE2D2
Biological Process	GO:0098840	protein transport along microtubule	7/2279	68/18866	0.7290635	0.8325519	0.7533225	7	TNPO1/CLUAP1/KIF5C/IFT140/TTC21B/MAP1A/KIFAP3
Biological Process	GO:0099118	microtubule-based protein transport	7/2279	68/18866	0.7290635	0.8325519	0.7533225	7	TNPO1/CLUAP1/KIF5C/IFT140/TTC21B/MAP1A/KIFAP3
Biological Process	GO:0010659	cardiac muscle cell apoptotic process	6/2279	59/18866	0.7325065	0.8359812	0.7564255	6	MIR145/MIR199A1/MIR24-2/ILK/NFE2L2/MIR199A2
Biological Process	GO:0043030	regulation of macrophage activation	6/2279	59/18866	0.7325065	0.8359812	0.7564255	6	IL10/MIR145/MYO18A/THBS1/PTPRC/GRN
Biological Process	GO:0072413	signal transduction involved in mitotic cell cycle checkpoint	6/2279	59/18866	0.7325065	0.8359812	0.7564255	6	TFDP1/E2F7/PCBP4/ZNF385A/ARID3A/CHEK2
Biological Process	GO:1902402	signal transduction involved in mitotic DNA damage checkpoint	6/2279	59/18866	0.7325065	0.8359812	0.7564255	6	TFDP1/E2F7/PCBP4/ZNF385A/ARID3A/CHEK2
Biological Process	GO:1902403	signal transduction involved in mitotic DNA integrity checkpoint	6/2279	59/18866	0.7325065	0.8359812	0.7564255	6	TFDP1/E2F7/PCBP4/ZNF385A/ARID3A/CHEK2

Biological Process	GO:2000045	regulation of G1/S transition of mitotic cell cycle	20/2279	185/18866	0.7352997	0.8390683	0.7592188	20	RPTOR/TFDP1/PRMT2/FBXO7/E2F7/CTDSPL/PCBP4/RIPK1/PTPN6/NACC2/ZNF385A/ARID3A/GFI1B/PLCB1/MIR138-2/RB1/BCL2/CHEK2/PLRG1/EIF4G1
Biological Process	GO:0030004	cellular monovalent inorganic cation homeostasis	12/2279	114/18866	0.7367873	0.8406648	0.7606634	12	TM9SF4/SLC11A1/SLC8A1/KCTD7/ATP6V1B2/GRN/BCL2/ATP6V0B/UBE3A/CLN5/ATP6V0C/ATP6V1C1
Biological Process	GO:0007622	rhythmic behavior	5/2279	50/18866	0.7379313	0.8410031	0.7609695	5	ZFHX3/MTA1/GHRL/USP2/NCOA2
Biological Process	GO:0000380	alternative mRNA splicing, via spliceosome	9/2279	87/18866	0.7384442	0.8410031	0.7609695	9	RBM19/SF1/SFSWAP/CELF2/CELF1/RBFOX3/NSRP1/RBM15B/CDK13
Biological Process	GO:0055072	iron ion homeostasis	9/2279	87/18866	0.7384442	0.8410031	0.7609695	9	SLC25A37/LCN2/SLC11A1/TTC7A/SLC11A2/EPAS1/LTF/EPB42/NCOA4
Biological Process	GO:0006448	regulation of translational elongation	2/2279	21/18866	0.7399835	0.8410031	0.7609695	2	CPEB3/SHFL
Biological Process	GO:0006490	oligosaccharide-lipid intermediate biosynthetic process	2/2279	21/18866	0.7399835	0.8410031	0.7609695	2	ALG11/DPAGT1
Biological Process	GO:0006662	glycerol ether metabolic process	2/2279	21/18866	0.7399835	0.8410031	0.7609695	2	CHPT1/PEDS1
Biological Process	GO:0006837	serotonin transport	2/2279	21/18866	0.7399835	0.8410031	0.7609695	2	ITGB3/P2RX1

Biological Process	GO:0007130	synaptonemal complex assembly	2/2279	21/18866	0.7399835	0.8410031	0.7609695	2	RAD21L1/TEX12
Biological Process	GO:0009067	aspartate family amino acid biosynthetic process	2/2279	21/18866	0.7399835	0.8410031	0.7609695	2	MTHFD1/ADI1
Biological Process	GO:0016540	protein autoprocessing	2/2279	21/18866	0.7399835	0.8410031	0.7609695	2	F12/CASP1
Biological Process	GO:0019370	leukotriene biosynthetic process	2/2279	21/18866	0.7399835	0.8410031	0.7609695	2	GGT1/MGST2
Biological Process	GO:0030728	ovulation	2/2279	21/18866	0.7399835	0.8410031	0.7609695	2	TNFAIP6/INHBA
Biological Process	GO:0030878	thyroid gland development	2/2279	21/18866	0.7399835	0.8410031	0.7609695	2	SMAD3/TG
Biological Process	GO:0036499	PERK-mediated unfolded protein response	2/2279	21/18866	0.7399835	0.8410031	0.7609695	2	NCK2/NFE2L2
Biological Process	GO:0042402	cellular biogenic amine catabolic process	2/2279	21/18866	0.7399835	0.8410031	0.7609695	2	SAT2/HNMT
Biological Process	GO:0045717	negative regulation of fatty acid biosynthetic process	2/2279	21/18866	0.7399835	0.8410031	0.7609695	2	WDTC1/ACADVL

Biological Process	GO:0046386	deoxyribose phosphate catabolic process	2/2279	21/18866	0.7399835	0.8410031	0.7609695	2	UNG/NTHL1
Biological Process	GO:0046514	ceramide catabolic process	2/2279	21/18866	0.7399835	0.8410031	0.7609695	2	HEXB/GALC
Biological Process	GO:0060008	Sertoli cell differentiation	2/2279	21/18866	0.7399835	0.8410031	0.7609695	2	RARA/NTRK1
Biological Process	GO:0060117	auditory receptor cell development	2/2279	21/18866	0.7399835	0.8410031	0.7609695	2	FGFR1/TMC1
Biological Process	GO:0061042	vascular wound healing	2/2279	21/18866	0.7399835	0.8410031	0.7609695	2	TNF/TNFAIP3
Biological Process	GO:0061213	positive regulation of mesonephros development	2/2279	21/18866	0.7399835	0.8410031	0.7609695	2	PAX2/HOXB7
Biological Process	GO:0070734	histone H3-K27 methylation	2/2279	21/18866	0.7399835	0.8410031	0.7609695	2	EHMT2/PHF19
Biological Process	GO:0140467	integrated stress response signaling	2/2279	21/18866	0.7399835	0.8410031	0.7609695	2	NCK2/NFE2L2
Biological Process	GO:1900543	negative regulation of purine nucleotide metabolic process	2/2279	21/18866	0.7399835	0.8410031	0.7609695	2	HDAC4/CBFA2T3

Biological Process	GO:1903978	regulation of microglial cell activation	2/2279	21/18866	0.7399835	0.8410031	0.7609695	2	PTPRC/GRN
Biological Process	GO:1904886	beta-catenin destruction complex disassembly	2/2279	21/18866	0.7399835	0.8410031	0.7609695	2	AXIN1/FRAT2
Biological Process	GO:2000269	regulation of fibroblast apoptotic process	2/2279	21/18866	0.7399835	0.8410031	0.7609695	2	MIR24-2/CHD8
Biological Process	GO:2001014	regulation of skeletal muscle cell differentiation	2/2279	21/18866	0.7399835	0.8410031	0.7609695	2	ARNTL/MEF2C
Biological Process	GO:0055067	monovalent inorganic cation homeostasis	17/2279	159/18866	0.7402413	0.8410031	0.7609695	17	TM9SF4/SLC11A1/SLC8A1/KCTD7/COMT/GNAI2/ATP6V1B2/GRN/BCL2/ATP6V0B/UBE3A/AGTR1/CLN5/SLC12A1/SLC12A7/ATP6V0C/ATP6V1C1
Biological Process	GO:0000288	nuclear-transcribed mRNA catabolic process, deadenylation-dependent decay	8/2279	78/18866	0.7402657	0.8410031	0.7609695	8	LSM1/EXOSC2/TNRC6B/SAMD4A/AGO2/EDC3/LSM7/CPEB3
Biological Process	GO:0002323	natural killer cell activation involved in immune response	3/2279	31/18866	0.7408015	0.8410031	0.7609695	3	RAB27A/HLA-F/PGLYRP1
Biological Process	GO:0034472	snRNA 3'-end processing	3/2279	31/18866	0.7408015	0.8410031	0.7609695	3	EXOSC2/TOE1/TUT1
Biological Process	GO:0045737	positive regulation of cyclin-dependent protein serine/threonine kinase activity	3/2279	31/18866	0.7408015	0.8410031	0.7609695	3	PDGFB/CCNY/CCNYL1

Biological Process	GO:0045987	positive regulation of smooth muscle contraction	3/2279	31/18866	0.7408015	0.8410031	0.7609695	3	CTTN/GHRL/F2R
Biological Process	GO:0050686	negative regulation of mRNA processing	3/2279	31/18866	0.7408015	0.8410031	0.7609695	3	PCBP4/SFSWAP/RNF40
Biological Process	GO:0050974	detection of mechanical stimulus involved in sensory perception	3/2279	31/18866	0.7408015	0.8410031	0.7609695	3	SCN1A/NTRK1/TMC1
Biological Process	GO:0086011	membrane repolarization during action potential	3/2279	31/18866	0.7408015	0.8410031	0.7609695	3	KCNE1/NOS1AP/KCNQ1
Biological Process	GO:0099068	postsynapse assembly	3/2279	31/18866	0.7408015	0.8410031	0.7609695	3	NRXN1/NRXN2/SHANK2
Biological Process	GO:1900745	positive regulation of p38MAPK cascade	3/2279	31/18866	0.7408015	0.8410031	0.7609695	3	MAP3K3/GADD45G/GDF6
Biological Process	GO:1903792	negative regulation of anion transport	3/2279	31/18866	0.7408015	0.8410031	0.7609695	3	THBS1/SLC43A2/SLC43A1
Biological Process	GO:1904046	negative regulation of vascular endothelial growth factor production	3/2279	31/18866	0.7408015	0.8410031	0.7609695	3	MIR140/MIR199A1/MIR199A2
Biological Process	GO:0140013	meiotic nuclear division	19/2279	177/18866	0.7426315	0.8429799	0.7627581	19	CALR/FZR1/MOV10L1/BRCA2/EME1/MEIOB/FANCA/RNF212/PPP2R1A/PLCB1/CCNA1/RAD21L1/DMC1/SLX4/TEX12/LIF/KLHDC3/PSMD13/TERB2

Biological Process	GO:0014742	positive regulation of muscle hypertrophy	4/2279	41/18866	0.7464846	0.846949	0.7663495	4	PRKCA/MIR199A1/PDE9A/MIR199A2
Biological Process	GO:0043267	negative regulation of potassium ion transport	4/2279	41/18866	0.7464846	0.846949	0.7663495	4	KCNE1/CASQ2/BIN1/CAB39
Biological Process	GO:0048286	lung alveolus development	4/2279	41/18866	0.7464846	0.846949	0.7663495	4	CREB1/TNS3/SFTPD/LIF
Biological Process	GO:1901998	toxin transport	4/2279	41/18866	0.7464846	0.846949	0.7663495	4	ABCG1/RAB43/DNM1/SLC7A8
Biological Process	GO:0006631	fatty acid metabolic process	44/2279	396/18866	0.7471195	0.8473837	0.7667428	44	PRKAG2/PLA2G15/MGLL/PPARG/ACSL1/MFSD2A/LIPC/GGT1/ABHD16A/AMACR/AOAH/ACOXL/TNFRSF1A/PRKAB1/ACOT7/ACSS1/CYP4F3/ACADM/POR/MYO5A/NR1H2/ACACB/ACSS2/ACOX2/PER2/ACACA/EPHX1/ACSF3/FA2H/ABHD5/PCK2/ACAT1/SCD/SREBF1/ADTRP/WDTC1/CPT1A/LPIN1/LYPLA2/ZADH2/ACADVL/PRKAA1/PRXL2B/PEDS1
Biological Process	GO:0019229	regulation of vasoconstriction	6/2279	60/18866	0.7474029	0.8473837	0.7667428	6	GJA5/HRH1/PER2/P2RX1/F2R/AGTR1
Biological Process	GO:0061077	chaperone-mediated protein folding	6/2279	60/18866	0.7474029	0.8473837	0.7667428	6	FKBP5/DNAJB13/CLU/UNC45A/TOR1B/P3H1
Biological Process	GO:0061098	positive regulation of protein tyrosine kinase activity	6/2279	60/18866	0.7474029	0.8473837	0.7667428	6	TGFA/CCL5/CSF1R/GHRL/PDGFB/CASS4
Biological Process	GO:1902883	negative regulation of response to oxidative stress	6/2279	60/18866	0.7474029	0.8473837	0.7667428	6	IL10/FBXO7/NOL3/NFE2L2/BAG5/PRKN

Biological Process	GO:2000242	negative regulation of reproductive process	6/2279	60/18866	0.7474029	0.8473837	0.7667428	6	CALR/ARHGDIB/OVOL1/NPPC/NODAL/LIF
Biological Process	GO:0071156	regulation of cell cycle arrest	11/2279	106/18866	0.7481343	0.8481116	0.7674015	11	CALR/TFDP1/E2F7/BIN1/PCBP4/ZNF385A/ZBTB17/ARID3A/MED25/FOXM1/CHEK2
Biological Process	GO:0061138	morphogenesis of a branching epithelium	20/2279	187/18866	0.7524113	0.848352	0.767619	20	CTSZ/TACSTD2/RXRA/IL10/LRP5L/VDR/SPINT2/TNF/TGFBR2/CTSH/ILK/PAX2/FGFR1/RDH10/GRHL2/ENG/BCL2/NOTCH1/NOTCH4/HOXB7
Biological Process	GO:0065002	intracellular protein transmembrane transport	5/2279	51/18866	0.7538172	0.848352	0.767619	5	PAM16/BCR/PEX26/TIMM44/RTN2
Biological Process	GO:0002534	cytokine production involved in inflammatory response	7/2279	70/18866	0.7566386	0.848352	0.767619	7	MIR140/MEFV/TNF/NOD2/PER1/PLD3/CD96
Biological Process	GO:0050795	regulation of behavior	7/2279	70/18866	0.7566386	0.848352	0.767619	7	HDAC4/ZFHX3/NRXN1/GHRL/TTC21B/MEF2C/MBD5
Biological Process	GO:0070192	chromosome organization involved in meiotic cell cycle	7/2279	70/18866	0.7566386	0.848352	0.767619	7	MEIOB/RNF212/PPP2R1A/RAD21L1/DMC1/TEX12/TERB2
Biological Process	GO:0001820	serotonin secretion	1/2279	11/18866	0.757454	0.848352	0.767619	1	P2RX1
Biological Process	GO:0002182	cytoplasmic translational elongation	1/2279	11/18866	0.757454	0.848352	0.767619	1	CPEB3

Biological Process	GO:0002328	pro-B cell differentiation	1/2279	11/18866	0.757454	0.848352	0.767619	1	NOTCH1
Biological Process	GO:0003263	cardioblast proliferation	1/2279	11/18866	0.757454	0.848352	0.767619	1	NOTCH1
Biological Process	GO:0003264	regulation of cardioblast proliferation	1/2279	11/18866	0.757454	0.848352	0.767619	1	NOTCH1
Biological Process	GO:0006020	inositol metabolic process	1/2279	11/18866	0.757454	0.848352	0.767619	1	IMPA2
Biological Process	GO:0006182	cGMP biosynthetic process	1/2279	11/18866	0.757454	0.848352	0.767619	1	NPPC
Biological Process	GO:0006183	GTP biosynthetic process	1/2279	11/18866	0.757454	0.848352	0.767619	1	IMPDH1
Biological Process	GO:0006449	regulation of translational termination	1/2279	11/18866	0.757454	0.848352	0.767619	1	SHFL
Biological Process	GO:0006527	arginine catabolic process	1/2279	11/18866	0.757454	0.848352	0.767619	1	FAH
Biological Process	GO:0006707	cholesterol catabolic process	1/2279	11/18866	0.757454	0.848352	0.767619	1	AKR1D1

Biological Process	GO:0006751	glutathione catabolic process	1/2279	11/18866	0.757454	0.848352	0.767619	1	GGT1
Biological Process	GO:0007191	adenylate cyclase-activating dopamine receptor signaling pathway	1/2279	11/18866	0.757454	0.848352	0.767619	1	GNG2
Biological Process	GO:0009629	response to gravity	1/2279	11/18866	0.757454	0.848352	0.767619	1	PKM
Biological Process	GO:0014820	tonic smooth muscle contraction	1/2279	11/18866	0.757454	0.848352	0.767619	1	MYLK
Biological Process	GO:0014889	muscle atrophy	1/2279	11/18866	0.757454	0.848352	0.767619	1	GSN
Biological Process	GO:0016078	tRNA catabolic process	1/2279	11/18866	0.757454	0.848352	0.767619	1	EXOSC2
Biological Process	GO:0016081	synaptic vesicle docking	1/2279	11/18866	0.757454	0.848352	0.767619	1	CTBP2
Biological Process	GO:0016127	sterol catabolic process	1/2279	11/18866	0.757454	0.848352	0.767619	1	AKR1D1
Biological Process	GO:0019042	viral latency	1/2279	11/18866	0.757454	0.848352	0.767619	1	BANF1

Biological Process	GO:0021521	ventral spinal cord interneuron specification	1/2279	11/18866	0.757454	0.848352	0.767619	1	SUFU
Biological Process	GO:0021561	facial nerve development	1/2279	11/18866	0.757454	0.848352	0.767619	1	NRP2
Biological Process	GO:0021610	facial nerve morphogenesis	1/2279	11/18866	0.757454	0.848352	0.767619	1	NRP2
Biological Process	GO:0031284	positive regulation of guanylate cyclase activity	1/2279	11/18866	0.757454	0.848352	0.767619	1	RCVRN
Biological Process	GO:0031442	positive regulation of mRNA 3'-end processing	1/2279	11/18866	0.757454	0.848352	0.767619	1	CPEB3
Biological Process	GO:0031652	positive regulation of heat generation	1/2279	11/18866	0.757454	0.848352	0.767619	1	TNF
Biological Process	GO:0031915	positive regulation of synaptic plasticity	1/2279	11/18866	0.757454	0.848352	0.767619	1	SSH1
Biological Process	GO:0032253	dense core granule localization	1/2279	11/18866	0.757454	0.848352	0.767619	1	KIF1B
Biological Process	GO:0032276	regulation of gonadotropin secretion	1/2279	11/18866	0.757454	0.848352	0.767619	1	INHBA

Biological Process	GO:0032306	regulation of prostaglandin secretion	1/2279	11/18866	0.757454	0.848352	0.767619	1	MAP2K6
Biological Process	GO:0032908	regulation of transforming growth factor beta1 production	1/2279	11/18866	0.757454	0.848352	0.767619	1	THBS1
Biological Process	GO:0033079	immature T cell proliferation	1/2279	11/18866	0.757454	0.848352	0.767619	1	TMEM131L
Biological Process	GO:0033148	positive regulation of intracellular estrogen receptor signaling pathway	1/2279	11/18866	0.757454	0.848352	0.767619	1	KMT2D
Biological Process	GO:0033327	Leydig cell differentiation	1/2279	11/18866	0.757454	0.848352	0.767619	1	SF1
Biological Process	GO:0033632	regulation of cell-cell adhesion mediated by integrin	1/2279	11/18866	0.757454	0.848352	0.767619	1	CCL5
Biological Process	GO:0034372	very-low-density lipoprotein particle remodeling	1/2279	11/18866	0.757454	0.848352	0.767619	1	LIPC
Biological Process	GO:0034650	cortisol metabolic process	1/2279	11/18866	0.757454	0.848352	0.767619	1	DGKQ
Biological Process	GO:0034776	response to histamine	1/2279	11/18866	0.757454	0.848352	0.767619	1	HRH1

Biological Process	GO:0034969	histone arginine methylation	1/2279	11/18866	0.757454	0.848352	0.767619	1	PRMT2
Biological Process	GO:0035246	peptidyl-arginine N-methylation	1/2279	11/18866	0.757454	0.848352	0.767619	1	PRMT2
Biological Process	GO:0035542	regulation of SNARE complex assembly	1/2279	11/18866	0.757454	0.848352	0.767619	1	VPS18
Biological Process	GO:0035768	endothelial cell chemotaxis to fibroblast growth factor	1/2279	11/18866	0.757454	0.848352	0.767619	1	FGFR1
Biological Process	GO:0035865	cellular response to potassium ion	1/2279	11/18866	0.757454	0.848352	0.767619	1	CRHBP
Biological Process	GO:0039532	negative regulation of viral-induced cytoplasmic pattern recognition receptor signaling pathway	1/2279	11/18866	0.757454	0.848352	0.767619	1	SEC14L1
Biological Process	GO:0040015	negative regulation of multicellular organism growth	1/2279	11/18866	0.757454	0.848352	0.767619	1	RAI1
Biological Process	GO:0042789	mRNA transcription by RNA polymerase II	1/2279	11/18866	0.757454	0.848352	0.767619	1	SREBF1
Biological Process	GO:0044803	multi-organism membrane organization	1/2279	11/18866	0.757454	0.848352	0.767619	1	PI4KA

Biological Process	GO:0044848	biological phase	1/2279	11/18866	0.757454	0.848352	0.767619	1	CDH3
Biological Process	GO:0045835	negative regulation of meiotic nuclear division	1/2279	11/18866	0.757454	0.848352	0.767619	1	LIF
Biological Process	GO:0045899	positive regulation of RNA polymerase II transcription preinitiation complex assembly	1/2279	11/18866	0.757454	0.848352	0.767619	1	CREB1
Biological Process	GO:0046607	positive regulation of centrosome cycle	1/2279	11/18866	0.757454	0.848352	0.767619	1	POC1A
Biological Process	GO:0048642	negative regulation of skeletal muscle tissue development	1/2279	11/18866	0.757454	0.848352	0.767619	1	TGFB1
Biological Process	GO:0048672	positive regulation of collateral sprouting	1/2279	11/18866	0.757454	0.848352	0.767619	1	SEMA4D
Biological Process	GO:0051132	NK T cell activation	1/2279	11/18866	0.757454	0.848352	0.767619	1	ZBTB7B
Biological Process	GO:0051561	positive regulation of mitochondrial calcium ion concentration	1/2279	11/18866	0.757454	0.848352	0.767619	1	FIS1
Biological Process	GO:0051574	positive regulation of histone H3-K9 methylation	1/2279	11/18866	0.757454	0.848352	0.767619	1	MYB

Biological Process	GO:0051610	serotonin uptake	1/2279	11/18866	0.757454	0.848352	0.767619	1	ITGB3
Biological Process	GO:0051974	negative regulation of telomerase activity	1/2279	11/18866	0.757454	0.848352	0.767619	1	PPARG
Biological Process	GO:0060174	limb bud formation	1/2279	11/18866	0.757454	0.848352	0.767619	1	ZNF219
Biological Process	GO:0060287	epithelial cilium movement involved in determination of left/right asymmetry	1/2279	11/18866	0.757454	0.848352	0.767619	1	RFX3
Biological Process	GO:0060433	bronchus development	1/2279	11/18866	0.757454	0.848352	0.767619	1	TGFBR2
Biological Process	GO:0060439	trachea morphogenesis	1/2279	11/18866	0.757454	0.848352	0.767619	1	TGFBR2
Biological Process	GO:0060573	cell fate specification involved in pattern specification	1/2279	11/18866	0.757454	0.848352	0.767619	1	SUFU
Biological Process	GO:0060767	epithelial cell proliferation involved in prostate gland development	1/2279	11/18866	0.757454	0.848352	0.767619	1	NOTCH1
Biological Process	GO:0061085	regulation of histone H3-K27 methylation	1/2279	11/18866	0.757454	0.848352	0.767619	1	PHF19

Biological Process	GO:0061635	regulation of protein complex stability	1/2279	11/18866	0.757454	0.848352	0.767619	1	ATG14
Biological Process	GO:0070203	regulation of establishment of protein localization to telomere	1/2279	11/18866	0.757454	0.848352	0.767619	1	CCT6A
Biological Process	GO:0070254	mucus secretion	1/2279	11/18866	0.757454	0.848352	0.767619	1	PRKCE
Biological Process	GO:0070417	cellular response to cold	1/2279	11/18866	0.757454	0.848352	0.767619	1	FOXO1
Biological Process	GO:0070757	interleukin-35-mediated signaling pathway	1/2279	11/18866	0.757454	0.848352	0.767619	1	JAK1
Biological Process	GO:0072173	metanephric tubule morphogenesis	1/2279	11/18866	0.757454	0.848352	0.767619	1	PAX2
Biological Process	GO:0072205	metanephric collecting duct development	1/2279	11/18866	0.757454	0.848352	0.767619	1	PAX2
Biological Process	GO:0072321	chaperone-mediated protein transport	1/2279	11/18866	0.757454	0.848352	0.767619	1	CLU
Biological Process	GO:0090481	pyrimidine nucleotide-sugar transmembrane transport	1/2279	11/18866	0.757454	0.848352	0.767619	1	SLC35C2

Biological Process	GO:0097396	response to interleukin-17	1/2279	11/18866	0.757454	0.848352	0.767619	1	TRAF3IP2
Biological Process	GO:0097398	cellular response to interleukin-17	1/2279	11/18866	0.757454	0.848352	0.767619	1	TRAF3IP2
Biological Process	GO:0097400	interleukin-17-mediated signaling pathway	1/2279	11/18866	0.757454	0.848352	0.767619	1	TRAF3IP2
Biological Process	GO:0098917	retrograde trans-synaptic signaling	1/2279	11/18866	0.757454	0.848352	0.767619	1	PLCB1
Biological Process	GO:0099519	dense core granule cytoskeletal transport	1/2279	11/18866	0.757454	0.848352	0.767619	1	KIF1B
Biological Process	GO:0099624	atrial cardiac muscle cell membrane repolarization	1/2279	11/18866	0.757454	0.848352	0.767619	1	KCNQ1
Biological Process	GO:0106049	regulation of cellular response to osmotic stress	1/2279	11/18866	0.757454	0.848352	0.767619	1	ARHGEF2
Biological Process	GO:1900247	regulation of cytoplasmic translational elongation	1/2279	11/18866	0.757454	0.848352	0.767619	1	CPEB3
Biological Process	GO:1901077	regulation of relaxation of muscle	1/2279	11/18866	0.757454	0.848352	0.767619	1	PDE4D

Biological Process	GO:1901950	dense core granule transport	1/2279	11/18866	0.757454	0.848352	0.767619	1	KIF1B
Biological Process	GO:1902033	regulation of hematopoietic stem cell proliferation	1/2279	11/18866	0.757454	0.848352	0.767619	1	KAT7
Biological Process	GO:1902513	regulation of organelle transport along microtubule	1/2279	11/18866	0.757454	0.848352	0.767619	1	CNIH2
Biological Process	GO:1903299	regulation of hexokinase activity	1/2279	11/18866	0.757454	0.848352	0.767619	1	PRKN
Biological Process	GO:1904350	regulation of protein catabolic process in the vacuole	1/2279	11/18866	0.757454	0.848352	0.767619	1	MGAT3
Biological Process	GO:1904869	regulation of protein localization to Cajal body	1/2279	11/18866	0.757454	0.848352	0.767619	1	CCT6A
Biological Process	GO:1904871	positive regulation of protein localization to Cajal body	1/2279	11/18866	0.757454	0.848352	0.767619	1	CCT6A
Biological Process	GO:1905902	regulation of mesoderm formation	1/2279	11/18866	0.757454	0.848352	0.767619	1	FGFR1
Biological Process	GO:1990440	positive regulation of transcription from RNA polymerase II promoter in response to endoplasmic reticulum stress	1/2279	11/18866	0.757454	0.848352	0.767619	1	CEBPB

Biological Process	GO:2000318	positive regulation of T-helper 17 type immune response	1/2279	11/18866	0.757454	0.848352	0.767619	1	NLRP3
Biological Process	GO:2000544	regulation of endothelial cell chemotaxis to fibroblast growth factor	1/2279	11/18866	0.757454	0.848352	0.767619	1	FGFR1
Biological Process	GO:2000628	regulation of miRNA metabolic process	1/2279	11/18866	0.757454	0.848352	0.767619	1	RELA
Biological Process	GO:2001138	regulation of phospholipid transport	1/2279	11/18866	0.757454	0.848352	0.767619	1	ATP8A1
Biological Process	GO:2001140	positive regulation of phospholipid transport	1/2279	11/18866	0.757454	0.848352	0.767619	1	ATP8A1
Biological Process	GO:0050684	regulation of mRNA processing	16/2279	152/18866	0.7579217	0.8487758	0.7680025	16	RBM19/SRPK2/PCBP4/PABPN1/SF1/CPEB3/DAZAP1/SFSWAP/CELF2/CELF1/RBFOX3/NSRP1/ZC3H3/RBM15B/CPSF4/RNF40
Biological Process	GO:0003016	respiratory system process	3/2279	32/18866	0.7604087	0.8509591	0.769978	3	MTG2/FTO/YWHAZ
Biological Process	GO:0051503	adenine nucleotide transport	3/2279	32/18866	0.7604087	0.8509591	0.769978	3	SLC25A25/SLC33A1/SLC25A1
Biological Process	GO:0060603	mammary gland duct morphogenesis	3/2279	32/18866	0.7604087	0.8509591	0.769978	3	LRP5L/VDR/CSF1R

Biological Process	GO:0061157	mRNA destabilization	3/2279	32/18866	0.7604087	0.8509591	0.769978	3	ZC3H12D/CPEB3/FTO
Biological Process	GO:0071353	cellular response to interleukin-4	3/2279	32/18866	0.7604087	0.8509591	0.769978	3	RUFY4/ADAMTS13/MCM2
Biological Process	GO:0097421	liver regeneration	3/2279	32/18866	0.7604087	0.8509591	0.769978	3	IL10/CPB2/CEBPB
Biological Process	GO:0010658	striated muscle cell apoptotic process	6/2279	61/18866	0.7616895	0.8521447	0.7710507	6	MIR145/MIR199A1/MIR24-2/ILK/NFE2L2/MIR199A2
Biological Process	GO:1904356	regulation of telomere maintenance via telomere lengthening	6/2279	61/18866	0.7616895	0.8521447	0.7710507	6	TNKS/HMBOX1/RTEL1/SMG6/SLX4/CCT6A
Biological Process	GO:1902806	regulation of cell cycle G1/S phase transition	22/2279	206/18866	0.7626823	0.8521447	0.7710507	22	TRIM39/RPTOR/TFDP1/PRMT2/FBXO7/E2F7/CTDSPL/PCBP4/RIPK1/PTPN6/NACC2/ZNF385A/ARID3A/C10orf99/GFI1B/PLCB1/MIR138-2/RB1/BCL2/CHEK2/PLRG1/EIF4G1
Biological Process	GO:0000717	nucleotide-excision repair, DNA duplex unwinding	2/2279	22/18866	0.7633321	0.8521447	0.7710507	2	XPA/ERCC2
Biological Process	GO:0001573	ganglioside metabolic process	2/2279	22/18866	0.7633321	0.8521447	0.7710507	2	HEXB/ST8SIA6
Biological Process	GO:0007342	fusion of sperm to egg plasma membrane involved in single fertilization	2/2279	22/18866	0.7633321	0.8521447	0.7710507	2	CD9/FOLR3

Biological Process	GO:0009235	cobalamin metabolic process	2/2279	22/18866	0.7633321	0.8521447	0.7710507	2	ABCC1/CD320
Biological Process	GO:0010499	proteasomal ubiquitin-independent protein catabolic process	2/2279	22/18866	0.7633321	0.8521447	0.7710507	2	NFE2L2/PSMB7
Biological Process	GO:0021533	cell differentiation in hindbrain	2/2279	22/18866	0.7633321	0.8521447	0.7710507	2	TTC21B/TTLL1
Biological Process	GO:0034312	diol biosynthetic process	2/2279	22/18866	0.7633321	0.8521447	0.7710507	2	SPTLC2/SPHK2
Biological Process	GO:0035024	negative regulation of Rho protein signal transduction	2/2279	22/18866	0.7633321	0.8521447	0.7710507	2	KANK1/CUL3
Biological Process	GO:0042454	ribonucleoside catabolic process	2/2279	22/18866	0.7633321	0.8521447	0.7710507	2	CDA/PNP
Biological Process	GO:0045655	regulation of monocyte differentiation	2/2279	22/18866	0.7633321	0.8521447	0.7710507	2	FOXP1/INPP5D
Biological Process	GO:0048025	negative regulation of mRNA splicing, via spliceosome	2/2279	22/18866	0.7633321	0.8521447	0.7710507	2	PCBP4/SFSWAP
Biological Process	GO:0051000	positive regulation of nitric-oxide synthase activity	2/2279	22/18866	0.7633321	0.8521447	0.7710507	2	TNF/NOS1AP

Biological Process	GO:0051131	chaperone-mediated protein complex assembly	2/2279	22/18866	0.7633321	0.8521447	0.7710507	2	CLU/PFDN6
Biological Process	GO:0051900	regulation of mitochondrial depolarization	2/2279	22/18866	0.7633321	0.8521447	0.7710507	2	HSH2D/BCL2
Biological Process	GO:0071157	negative regulation of cell cycle arrest	2/2279	22/18866	0.7633321	0.8521447	0.7710507	2	CALR/CHEK2
Biological Process	GO:0010907	positive regulation of glucose metabolic process	4/2279	42/18866	0.7635309	0.8521447	0.7710507	4	GPLD1/DYRK2/ESRRB/FOXO1
Biological Process	GO:0021795	cerebral cortex cell migration	4/2279	42/18866	0.7635309	0.8521447	0.7710507	4	RTN4/DISC1/ZMIZ1/NDEL1
Biological Process	GO:0098760	response to interleukin-7	4/2279	42/18866	0.7635309	0.8521447	0.7710507	4	P4HB/STAT5A/JAK1/H3C1
Biological Process	GO:0098761	cellular response to interleukin-7	4/2279	42/18866	0.7635309	0.8521447	0.7710507	4	P4HB/STAT5A/JAK1/H3C1
Biological Process	GO:1901658	glycosyl compound catabolic process	4/2279	42/18866	0.7635309	0.8521447	0.7710507	4	CDA/DPYS/UPB1/PNP
Biological Process	GO:0043583	ear development	24/2279	224/18866	0.7649198	0.8535944	0.7723626	24	ABR/CEBPD/CDH23/CCM2/SLC44A4/ATP8B1/DLL1/RBPJ/LRIG1/PAX2/FGFR1/TTC39C/RDH10/BMPER/LRP10/MCM2/BCR/KCNQ1/ESRRB/BCL2/NOTCH1/TMC1/PDGFRB/C1QB

Biological Process	GO:0032204	regulation of telomere maintenance	8/2279	80/18866	0.7655283	0.8541732	0.7728862	8	TNKS/HMBOX1/RTEL1/YLPM1/SMG6/ERCC1/SLX4/CCT6A
Biological Process	GO:0048565	digestive tract development	14/2279	135/18866	0.7670886	0.8558136	0.7743706	14	SMAD3/TNF/EPHB3/TGFBR2/WNT11/RB1/BCL2/NOTCH1/PDGFC/HLX/CHD8/ALDH1A2/NPY/NODAL
Biological Process	GO:0043467	regulation of generation of precursor metabolites and energy	17/2279	162/18866	0.7671812	0.8558165	0.7743731	17	HDAC4/PRKAG2/PFKFB4/RAE1/CBFA2T3/APP/PGAM1/SPHK2/NUP93/DYRK2/SEC13/PPP1CB/PRDM16/ESRRB/PFKFB3/PRKAA1/RUBCNL
Biological Process	GO:0006813	potassium ion transport	26/2279	242/18866	0.7673354	0.855888	0.7744378	26	KCNJ15/KCNE1/KCNAB2/CASQ2/WWP2/FXYD2/SLC24A4/FXYD1/DNM2/KCNN1/GJA5/KCNQ2/BIN1/NOS1AP/KCNIP1/KCNK6/AQP1/CAB39/KCNQ1/SLC1A3/ABCB8/NEDD4/ANO6/ANK2/SLC12A1/SLC12A7
Biological Process	GO:0031109	microtubule polymerization or depolymerization	12/2279	117/18866	0.7684068	0.8569824	0.7754281	12	TBCD/DCTN1/CSNK1D/NDE1/NAV3/NUMA1/MAP1A/TPPP3/ARHGEF2/TUBGCP2/NDEL1/SPEF1
Biological Process	GO:0009948	anterior/posterior axis specification	5/2279	52/18866	0.7689716	0.8573104	0.7757249	5	SKI/PCSK6/TMED2/NODAL/OTX2
Biological Process	GO:0055078	sodium ion homeostasis	5/2279	52/18866	0.7689716	0.8573104	0.7757249	5	SLC8A1/COMT/GNAI2/AGTR1/SLC12A1
Biological Process	GO:2001258	negative regulation of cation channel activity	5/2279	52/18866	0.7689716	0.8573104	0.7757249	5	KCNE1/CASQ2/GSTO1/DYSF/CBARP
Biological Process	GO:0015800	acidic amino acid transport	7/2279	71/18866	0.769645	0.8576586	0.77604	7	LRRC8C/PPFIA1/NF1/SLC1A2/SLC1A6/PER2/SLC1A3

Biological Process	GO:0032371	regulation of sterol transport	7/2279	71/18866	0.769645	0.8576586	0.77604	7	PPARG/ABCG1/PLTP/MIR27A/OSBPL6/NR1H2/ABCA1
Biological Process	GO:0045739	positive regulation of DNA repair	7/2279	71/18866	0.769645	0.8576586	0.77604	7	FAM168A/SPIDR/FOXM1/PARP9/ABRAXAS1/SLF1/BABAM2
Biological Process	GO:0050891	multicellular organismal water homeostasis	7/2279	71/18866	0.769645	0.8576586	0.77604	7	ADCY2/PRKAR1B/ADCY9/AQP1/ADCY4/AQP3/FA2H
Biological Process	GO:0090596	sensory organ morphogenesis	29/2279	269/18866	0.7711834	0.8592722	0.7775	29	ABR/MFSD2A/SKI/CRB1/HIPK2/SLC44A4/DLL1/LRIG1/HDAC1/GNAT2/PAX2/FGFR1/TTC39C/NF1/SP3/RARG/BCR/PRKCI/MEIS1/BCL2/RPGRIP1/ZHX2/COL5A1/SDK2/BCAR3/NRL/CABP4/TSKU/NECTIN1
Biological Process	GO:0045143	homologous chromosome segregation	6/2279	62/18866	0.7753706	0.8634754	0.7813032	6	MEIOB/RNF212/RAD21L1/DMC1/TEX12/TERB2
Biological Process	GO:0000910	cytokinesis	18/2279	172/18866	0.7759132	0.8634754	0.7813032	18	CECR2/BRCA2/SETD2/KIF13A/E2F7/CUL3/MYH9/GIT1/UVRAG/SVIL/CSPP1/SNX18/ZFYVE19/KLHL21/PRKCE/BCL2L1/CDC14A/SEPTIN9
Biological Process	GO:0031023	microtubule organizing center organization	14/2279	136/18866	0.7764078	0.8634754	0.7813032	14	ARHGEF10/DCTN1/NDE1/BRCA2/CEP135/UVRAG/POC1A/NUMA1/SDCCAG8/SLC16A1/FBXW11/CEP72/NDEL1/CEP295NL
Biological Process	GO:0003018	vascular process in circulatory system	20/2279	190/18866	0.7767106	0.8634754	0.7813032	20	AZU1/ABR/MFSD2A/TJP2/ANGPT1/ITGB1BP1/SLC8A1/GJA5/ABCC2/MIR23A/TGFB1/PTPRJ/HRH1/BCR/PER2/P2RX1/F2R/NPPC/GPR4/AGTR1
Biological Process	GO:0033238	regulation of cellular amine metabolic process	8/2279	81/18866	0.7774781	0.8634754	0.7813032	8	ODC1/COMT/SLC7A7/PSMF1/ITGB2/PSMB7/PSMD13/PRKN

Biological Process	GO:0071158	positive regulation of cell cycle arrest	8/2279	81/18866	0.7774781	0.8634754	0.7813032	8	TFDP1/E2F7/PCBP4/ZNF385A/ZBTB17/ARID3A/MED25/CHEK2
Biological Process	GO:0002861	regulation of inflammatory response to antigenic stimulus	3/2279	33/18866	0.7787956	0.8634754	0.7813032	3	FUT7/IL10/TNF
Biological Process	GO:0003338	metanephros morphogenesis	3/2279	33/18866	0.7787956	0.8634754	0.7813032	3	PAX2/PDGFRB/LIF
Biological Process	GO:0010464	regulation of mesenchymal cell proliferation	3/2279	33/18866	0.7787956	0.8634754	0.7813032	3	TGFBR2/WNT11/FGFR1
Biological Process	GO:0015868	purine ribonucleotide transport	3/2279	33/18866	0.7787956	0.8634754	0.7813032	3	SLC25A25/SLC33A1/SLC25A1
Biological Process	GO:0042573	retinoic acid metabolic process	3/2279	33/18866	0.7787956	0.8634754	0.7813032	3	DHRS9/RDH10/ALDH1A2
Biological Process	GO:0042755	eating behavior	3/2279	33/18866	0.7787956	0.8634754	0.7813032	3	GHRL/TTC21B/CPT1A
Biological Process	GO:0044319	wound healing, spreading of cells	3/2279	33/18866	0.7787956	0.8634754	0.7813032	3	RREB1/COL5A1/MRTFA
Biological Process	GO:0090505	epiboly involved in wound healing	3/2279	33/18866	0.7787956	0.8634754	0.7813032	3	RREB1/COL5A1/MRTFA

Biological Process	GO:0016339	calcium-dependent cell-cell adhesion via plasma membrane cell adhesion molecules	4/2279	43/18866	0.7796699	0.8634754	0.7813032	4	CDH23/KIFAP3/PCDH12/CDH11
Biological Process	GO:0046688	response to copper ion	4/2279	43/18866	0.7796699	0.8634754	0.7813032	4	APP/AQP1/NFE2L2/ATP7B
Biological Process	GO:0060119	inner ear receptor cell development	4/2279	43/18866	0.7796699	0.8634754	0.7813032	4	CDH23/ATP8B1/FGFR1/TMC1
Biological Process	GO:0150077	regulation of neuroinflammatory response	4/2279	43/18866	0.7796699	0.8634754	0.7813032	4	TNFRSF1B/TNF/PTPRC/GRN
Biological Process	GO:0021761	limbic system development	11/2279	109/18866	0.7799587	0.8634754	0.7813032	11	NRP2/MFSD2A/ZEB2/EIF2B5/HDAC1/FGFR1/RARA/NF1/KIRREL3/NCOA1/ETS1
Biological Process	GO:0043574	peroxisomal transport	7/2279	72/18866	0.7821347	0.8634754	0.7813032	7	AMACR/AGXT/CAT/PEX26/ACOX2/HMGCL/UBE2D2
Biological Process	GO:0035282	segmentation	10/2279	100/18866	0.7821582	0.8634754	0.7813032	10	NRP2/SMAD3/ZEB2/DLL1/RBPJ/MIB1/PCSK6/NOTCH1/TMED2/ALDH1A2
Biological Process	GO:0006336	DNA replication-independent nucleosome assembly	5/2279	53/18866	0.7834046	0.8634754	0.7813032	5	UBN1/CABIN1/RSF1/CENPM/RUVBL1
Biological Process	GO:0042073	intraciliary transport	5/2279	53/18866	0.7834046	0.8634754	0.7813032	5	TNPO1/CLUAP1/IFT140/TTC21B/KIFAP3

Biological Process	GO:0048168	regulation of neuronal synaptic plasticity	5/2279	53/18866	0.7834046	0.8634754	0.7813032	5	ZDHHC2/APP/SYNGAP1/NF1/SYNPO
Biological Process	GO:2000772	regulation of cellular senescence	5/2279	53/18866	0.7834046	0.8634754	0.7813032	5	MAP3K3/AKT3/VASH1/ARNTL/NEK6
Biological Process	GO:0006633	fatty acid biosynthetic process	18/2279	173/18866	0.7840345	0.8634754	0.7813032	18	PRKAG2/MGLL/LIPC/PRKAB1/ACOT7/ACSS1/MYO5A/NR1H2/ACACB/ACSS2/ACACA/ACSF3/FA2H/SCD/WDTC1/ACADVL/PRKAA1/PRXL2B
Biological Process	GO:0001764	neuron migration	17/2279	164/18866	0.7840649	0.8634754	0.7813032	17	NRP2/APBB2/NDE1/DRGX/DISC1/ZMIZ1/FGFR1/RAPGEF2/KIRREL3/SDCCAG8/ASTN2/MEF2C/ARHGEF2/MARK2/NDEL1/UNC5D/NAV1
Biological Process	GO:0000002	mitochondrial genome maintenance	2/2279	23/18866	0.7848369	0.8634754	0.7813032	2	AKT3/MSTO1
Biological Process	GO:0001956	positive regulation of neurotransmitter secretion	2/2279	23/18866	0.7848369	0.8634754	0.7813032	2	DNM1L/DTNBP1
Biological Process	GO:0006297	nucleotide-excision repair, DNA gap filling	2/2279	23/18866	0.7848369	0.8634754	0.7813032	2	POLE/POLD3
Biological Process	GO:0009251	glucan catabolic process	2/2279	23/18866	0.7848369	0.8634754	0.7813032	2	MGAM/PPP1CB
Biological Process	GO:0009310	amine catabolic process	2/2279	23/18866	0.7848369	0.8634754	0.7813032	2	SAT2/HNMT

Biological Process	GO:0032098	regulation of appetite	2/2279	23/18866	0.7848369	0.8634754	0.7813032	2	GHRL/NPY
Biological Process	GO:0033139	regulation of peptidyl-serine phosphorylation of STAT protein	2/2279	23/18866	0.7848369	0.8634754	0.7813032	2	GGNBP2/LIF
Biological Process	GO:0042401	cellular biogenic amine biosynthetic process	2/2279	23/18866	0.7848369	0.8634754	0.7813032	2	ODC1/DDC
Biological Process	GO:0042451	purine nucleoside biosynthetic process	2/2279	23/18866	0.7848369	0.8634754	0.7813032	2	GUK1/IMPDH1
Biological Process	GO:0042455	ribonucleoside biosynthetic process	2/2279	23/18866	0.7848369	0.8634754	0.7813032	2	GUK1/IMPDH1
Biological Process	GO:0043162	ubiquitin-dependent protein catabolic process via the multivesicular body sorting pathway	2/2279	23/18866	0.7848369	0.8634754	0.7813032	2	NEDD4/MVB12A
Biological Process	GO:0046129	purine ribonucleoside biosynthetic process	2/2279	23/18866	0.7848369	0.8634754	0.7813032	2	GUK1/IMPDH1
Biological Process	GO:0051220	cytoplasmic sequestering of protein	2/2279	23/18866	0.7848369	0.8634754	0.7813032	2	IL10/SUFU
Biological Process	GO:0060445	branching involved in salivary gland morphogenesis	2/2279	23/18866	0.7848369	0.8634754	0.7813032	2	TNF/FGFR1

Biological Process	GO:0071218	cellular response to misfolded protein	2/2279	23/18866	0.7848369	0.8634754	0.7813032	2	CUL3/POMT2
Biological Process	GO:1900017	positive regulation of cytokine production involved in inflammatory response	2/2279	23/18866	0.7848369	0.8634754	0.7813032	2	TNF/NOD2
Biological Process	GO:1901032	negative regulation of response to reactive oxygen species	2/2279	23/18866	0.7848369	0.8634754	0.7813032	2	IL10/NFE2L2
Biological Process	GO:1901984	negative regulation of protein acetylation	2/2279	23/18866	0.7848369	0.8634754	0.7813032	2	CTBP1/PIH1D1
Biological Process	GO:1902259	regulation of delayed rectifier potassium channel activity	2/2279	23/18866	0.7848369	0.8634754	0.7813032	2	KCNE1/NOS1AP
Biological Process	GO:1903206	negative regulation of hydrogen peroxide-induced cell death	2/2279	23/18866	0.7848369	0.8634754	0.7813032	2	IL10/NFE2L2
Biological Process	GO:2000773	negative regulation of cellular senescence	2/2279	23/18866	0.7848369	0.8634754	0.7813032	2	MAP3K3/AKT3
Biological Process	GO:0048477	oogenesis	9/2279	91/18866	0.7850931	0.8634754	0.7813032	9	HEXB/BRCA2/KMT2D/BMPR1B/PPP2R1A/BCL2/DMC1/NPPC/ERCC1
Biological Process	GO:0007018	microtubule-based movement	40/2279	368/18866	0.7861975	0.8634754	0.7813032	40	PRKCZ/KIF13B/DNAH3/NDE1/TNPO1/KIF13A/SPG7/DYNC1H1/PLTP/RFX3/APP/RNASE10/BICD2/CLUAP1/CNIH2/KIF21B/DEFB1/HTT/IQCG/KIF5C/IFT140/KIF1B/TTC21B/STK36/MAP1A/ARMC2/KIFAP3/AP3D1/DNAI2/FBXW11/NDEL1/KIFC3/DTNBP1/CCNYL1/TTLL1/NPHP4/CFAP221/KATNIP/CFAP61/CFAP46

Biological Process	GO:0000012	single strand break repair	1/2279	12/18866	0.7867705	0.8634754	0.7813032	1	TDP1
Biological Process	GO:0002024	diet induced thermogenesis	1/2279	12/18866	0.7867705	0.8634754	0.7813032	1	SORL1
Biological Process	GO:0002551	mast cell chemotaxis	1/2279	12/18866	0.7867705	0.8634754	0.7813032	1	PIK3CD
Biological Process	GO:0002713	negative regulation of B cell mediated immunity	1/2279	12/18866	0.7867705	0.8634754	0.7813032	1	PTPN6
Biological Process	GO:0002863	positive regulation of inflammatory response to antigenic stimulus	1/2279	12/18866	0.7867705	0.8634754	0.7813032	1	TNF
Biological Process	GO:0002890	negative regulation of immunoglobulin mediated immune response	1/2279	12/18866	0.7867705	0.8634754	0.7813032	1	PTPN6
Biological Process	GO:0006216	cytidine catabolic process	1/2279	12/18866	0.7867705	0.8634754	0.7813032	1	CDA
Biological Process	GO:0006563	L-serine metabolic process	1/2279	12/18866	0.7867705	0.8634754	0.7813032	1	CBS
Biological Process	GO:0007320	insemination	1/2279	12/18866	0.7867705	0.8634754	0.7813032	1	P2RX1

Biological Process	GO:0009972	cytidine deamination	1/2279	12/18866	0.7867705	0.8634754	0.7813032	1	CDA
Biological Process	GO:0010454	negative regulation of cell fate commitment	1/2279	12/18866	0.7867705	0.8634754	0.7813032	1	LOXL3
Biological Process	GO:0014831	gastro-intestinal system smooth muscle contraction	1/2279	12/18866	0.7867705	0.8634754	0.7813032	1	GHRL
Biological Process	GO:0015780	nucleotide-sugar transmembrane transport	1/2279	12/18866	0.7867705	0.8634754	0.7813032	1	SLC35C2
Biological Process	GO:0016188	synaptic vesicle maturation	1/2279	12/18866	0.7867705	0.8634754	0.7813032	1	ZDHHC2
Biological Process	GO:0016554	cytidine to uridine editing	1/2279	12/18866	0.7867705	0.8634754	0.7813032	1	RBM47
Biological Process	GO:0021559	trigeminal nerve development	1/2279	12/18866	0.7867705	0.8634754	0.7813032	1	DRGX
Biological Process	GO:0030208	dermatan sulfate biosynthetic process	1/2279	12/18866	0.7867705	0.8634754	0.7813032	1	DSE
Biological Process	GO:0030311	poly-N-acetyllactosamine biosynthetic process	1/2279	12/18866	0.7867705	0.8634754	0.7813032	1	B3GNT2

Biological Process	GO:0031987	locomotion involved in locomotory behavior	1/2279	12/18866	0.7867705	0.8634754	0.7813032	1	MYO5A
Biological Process	GO:0032490	detection of molecule of bacterial origin	1/2279	12/18866	0.7867705	0.8634754	0.7813032	1	NOD2
Biological Process	GO:0032525	somite rostral/caudal axis specification	1/2279	12/18866	0.7867705	0.8634754	0.7813032	1	TMED2
Biological Process	GO:0032905	transforming growth factor beta1 production	1/2279	12/18866	0.7867705	0.8634754	0.7813032	1	THBS1
Biological Process	GO:0033234	negative regulation of protein sumoylation	1/2279	12/18866	0.7867705	0.8634754	0.7813032	1	RELA
Biological Process	GO:0033523	histone H2B ubiquitination	1/2279	12/18866	0.7867705	0.8634754	0.7813032	1	RNF40
Biological Process	GO:0033690	positive regulation of osteoblast proliferation	1/2279	12/18866	0.7867705	0.8634754	0.7813032	1	LTF
Biological Process	GO:0035766	cell chemotaxis to fibroblast growth factor	1/2279	12/18866	0.7867705	0.8634754	0.7813032	1	FGFR1
Biological Process	GO:0036462	TRAIL-activated apoptotic signaling pathway	1/2279	12/18866	0.7867705	0.8634754	0.7813032	1	FADD

Biological Process	GO:0042182	ketone catabolic process	1/2279	12/18866	0.7867705	0.8634754	0.7813032	1	CYP4F3
Biological Process	GO:0042363	fat-soluble vitamin catabolic process	1/2279	12/18866	0.7867705	0.8634754	0.7813032	1	CYP4F3
Biological Process	GO:0042416	dopamine biosynthetic process	1/2279	12/18866	0.7867705	0.8634754	0.7813032	1	DDC
Biological Process	GO:0045760	positive regulation of action potential	1/2279	12/18866	0.7867705	0.8634754	0.7813032	1	NOS1AP
Biological Process	GO:0046087	cytidine metabolic process	1/2279	12/18866	0.7867705	0.8634754	0.7813032	1	CDA
Biological Process	GO:0046541	saliva secretion	1/2279	12/18866	0.7867705	0.8634754	0.7813032	1	AQP1
Biological Process	GO:0051013	microtubule severing	1/2279	12/18866	0.7867705	0.8634754	0.7813032	1	TTLL11
Biological Process	GO:0051095	regulation of helicase activity	1/2279	12/18866	0.7867705	0.8634754	0.7813032	1	MCM2
Biological Process	GO:0051549	positive regulation of keratinocyte migration	1/2279	12/18866	0.7867705	0.8634754	0.7813032	1	IQSEC1

Biological Process	GO:0051583	dopamine uptake involved in synaptic transmission	1/2279	12/18866	0.7867705	0.8634754	0.7813032	1	PRKN
Biological Process	GO:0051639	actin filament network formation	1/2279	12/18866	0.7867705	0.8634754	0.7813032	1	ACTN1
Biological Process	GO:0051657	maintenance of organelle location	1/2279	12/18866	0.7867705	0.8634754	0.7813032	1	UVRAG
Biological Process	GO:0051934	catecholamine uptake involved in synaptic transmission	1/2279	12/18866	0.7867705	0.8634754	0.7813032	1	PRKN
Biological Process	GO:0055015	ventricular cardiac muscle cell development	1/2279	12/18866	0.7867705	0.8634754	0.7813032	1	FHL2
Biological Process	GO:0060442	branching involved in prostate gland morphogenesis	1/2279	12/18866	0.7867705	0.8634754	0.7813032	1	RXRA
Biological Process	GO:0060536	cartilage morphogenesis	1/2279	12/18866	0.7867705	0.8634754	0.7813032	1	MEF2C
Biological Process	GO:0060736	prostate gland growth	1/2279	12/18866	0.7867705	0.8634754	0.7813032	1	UBE3A
Biological Process	GO:0060788	ectodermal placode formation	1/2279	12/18866	0.7867705	0.8634754	0.7813032	1	HDAC1

Biological Process	GO:0060900	embryonic camera-type eye formation	1/2279	12/18866	0.7867705	0.8634754	0.7813032	1	PAX2
Biological Process	GO:0061709	reticulophagy	1/2279	12/18866	0.7867705	0.8634754	0.7813032	1	STING1
Biological Process	GO:0070601	centromeric sister chromatid cohesion	1/2279	12/18866	0.7867705	0.8634754	0.7813032	1	PPP2R1A
Biological Process	GO:0071044	histone mRNA catabolic process	1/2279	12/18866	0.7867705	0.8634754	0.7813032	1	LSM1
Biological Process	GO:0071073	positive regulation of phospholipid biosynthetic process	1/2279	12/18866	0.7867705	0.8634754	0.7813032	1	FPR2
Biological Process	GO:0071257	cellular response to electrical stimulus	1/2279	12/18866	0.7867705	0.8634754	0.7813032	1	PALM
Biological Process	GO:0071340	skeletal muscle acetylcholine-gated channel clustering	1/2279	12/18866	0.7867705	0.8634754	0.7813032	1	COLQ
Biological Process	GO:0071472	cellular response to salt stress	1/2279	12/18866	0.7867705	0.8634754	0.7813032	1	AQP1
Biological Process	GO:0071697	ectodermal placode morphogenesis	1/2279	12/18866	0.7867705	0.8634754	0.7813032	1	HDAC1

Biological Process	GO:0071712	ER-associated misfolded protein catabolic process	1/2279	12/18866	0.7867705	0.8634754	0.7813032	1	POMT2
Biological Process	GO:0072178	nephric duct morphogenesis	1/2279	12/18866	0.7867705	0.8634754	0.7813032	1	PAX2
Biological Process	GO:0072520	seminiferous tubule development	1/2279	12/18866	0.7867705	0.8634754	0.7813032	1	RAD21L1
Biological Process	GO:0090085	regulation of protein deubiquitination	1/2279	12/18866	0.7867705	0.8634754	0.7813032	1	TNIP1
Biological Process	GO:0090394	negative regulation of excitatory postsynaptic potential	1/2279	12/18866	0.7867705	0.8634754	0.7813032	1	S1PR2
Biological Process	GO:0097201	negative regulation of transcription from RNA polymerase II promoter in response to stress	1/2279	12/18866	0.7867705	0.8634754	0.7813032	1	NCK2
Biological Process	GO:0097340	inhibition of cysteine-type endopeptidase activity	1/2279	12/18866	0.7867705	0.8634754	0.7813032	1	NOL3
Biological Process	GO:0097341	zymogen inhibition	1/2279	12/18866	0.7867705	0.8634754	0.7813032	1	NOL3
Biological Process	GO:0099151	regulation of postsynaptic density assembly	1/2279	12/18866	0.7867705	0.8634754	0.7813032	1	NRXN1

Biological Process	GO:1900115	extracellular regulation of signal transduction	1/2279	12/18866	0.7867705	0.8634754	0.7813032	1	LTBP1
Biological Process	GO:1900116	extracellular negative regulation of signal transduction	1/2279	12/18866	0.7867705	0.8634754	0.7813032	1	LTBP1
Biological Process	GO:1902018	negative regulation of cilium assembly	1/2279	12/18866	0.7867705	0.8634754	0.7813032	1	DNM2
Biological Process	GO:1902287	semaphorin-plexin signaling pathway involved in axon guidance	1/2279	12/18866	0.7867705	0.8634754	0.7813032	1	PLXNC1
Biological Process	GO:1902510	regulation of apoptotic DNA fragmentation	1/2279	12/18866	0.7867705	0.8634754	0.7813032	1	PAM16
Biological Process	GO:1902950	regulation of dendritic spine maintenance	1/2279	12/18866	0.7867705	0.8634754	0.7813032	1	ZMYND8
Biological Process	GO:1903405	protein localization to nuclear body	1/2279	12/18866	0.7867705	0.8634754	0.7813032	1	CCT6A
Biological Process	GO:1903441	protein localization to ciliary membrane	1/2279	12/18866	0.7867705	0.8634754	0.7813032	1	LZTFL1
Biological Process	GO:1904816	positive regulation of protein localization to chromosome, telomeric region	1/2279	12/18866	0.7867705	0.8634754	0.7813032	1	CCT6A

Biological Process	GO:1904847	regulation of cell chemotaxis to fibroblast growth factor	1/2279	12/18866	0.7867705	0.8634754	0.7813032	1	FGFR1
Biological Process	GO:1904867	protein localization to Cajal body	1/2279	12/18866	0.7867705	0.8634754	0.7813032	1	CCT6A
Biological Process	GO:1905214	regulation of RNA binding	1/2279	12/18866	0.7867705	0.8634754	0.7813032	1	EIF4G1
Biological Process	GO:1905522	negative regulation of macrophage migration	1/2279	12/18866	0.7867705	0.8634754	0.7813032	1	MIR24-2
Biological Process	GO:2000302	positive regulation of synaptic vesicle exocytosis	1/2279	12/18866	0.7867705	0.8634754	0.7813032	1	DNM1L
Biological Process	GO:2000767	positive regulation of cytoplasmic translation	1/2279	12/18866	0.7867705	0.8634754	0.7813032	1	PKM
Biological Process	GO:2001053	regulation of mesenchymal cell apoptotic process	1/2279	12/18866	0.7867705	0.8634754	0.7813032	1	PAX2
Biological Process	GO:2001204	regulation of osteoclast development	1/2279	12/18866	0.7867705	0.8634754	0.7813032	1	LTF
Biological Process	GO:0006521	regulation of cellular amino acid metabolic process	6/2279	63/18866	0.788453	0.865122	0.7827931	6	ODC1/COMT/SLC7A7/PSMF1/PSMB7/PSMD13

Biological Process	GO:0031571	mitotic G1 DNA damage checkpoint	6/2279	63/18866	0.788453	0.865122	0.7827931	6	TFDP1/E2F7/PCBP4/ZNF385A/ARID3A/CHEK2
Biological Process	GO:0022617	extracellular matrix disassembly	8/2279	82/18866	0.7889769	0.865497	0.7831324	8	ELANE/CTSG/TIMP2/BSG/SH3PXD2B/TGFB1/ETS1/FLOT1
Biological Process	GO:1903671	negative regulation of sprouting angiogenesis	8/2279	82/18866	0.7889769	0.865497	0.7831324	8	ITGB1BP1/MIR199A1/THBS1/MIR24-2/EPN2/MIR138-2/MIR199A2/NOTCH1
Biological Process	GO:0001578	microtubule bundle formation	10/2279	101/18866	0.7924473	0.8691033	0.7863955	10	DNAJB13/CCDC66/IQCG/NUMA1/TPPP3/ARMC2/DNAI2/TTLL1/NAV1/CFAP46
Biological Process	GO:1990542	mitochondrial transmembrane transport	10/2279	101/18866	0.7924473	0.8691033	0.7863955	10	PRKAG2/SLC25A37/PAM16/SFXN1/ACACB/ACACA/TIMM44/CPT1A/SLC25A1/ATP5PD
Biological Process	GO:0005977	glycogen metabolic process	7/2279	73/18866	0.7941126	0.8708291	0.7879571	7	PRKAG2/DYRK2/PPP1CB/ESRRB/PER2/PCDH12/RUBCNL
Biological Process	GO:0006099	tricarboxylic acid cycle	3/2279	34/18866	0.7960033	0.8718962	0.7889226	3	DHTKD1/OGDH/SDHD
Biological Process	GO:0031112	positive regulation of microtubule polymerization or depolymerization	3/2279	34/18866	0.7960033	0.8718962	0.7889226	3	DCTN1/NAV3/NUMA1
Biological Process	GO:0032212	positive regulation of telomere maintenance via telomerase	3/2279	34/18866	0.7960033	0.8718962	0.7889226	3	TNKS/HMBOX1/CCT6A

Biological Process	GO:0036474	cell death in response to hydrogen peroxide	3/2279	34/18866	0.7960033	0.8718962	0.7889226	3	IL10/RIPK1/NFE2L2
Biological Process	GO:0045907	positive regulation of vasoconstriction	3/2279	34/18866	0.7960033	0.8718962	0.7889226	3	GJA5/HRH1/F2R
Biological Process	GO:0048566	embryonic digestive tract development	3/2279	34/18866	0.7960033	0.8718962	0.7889226	3	TNF/HLX/ALDH1A2
Biological Process	GO:0048665	neuron fate specification	3/2279	34/18866	0.7960033	0.8718962	0.7889226	3	SUFU/DLL1/FEV
Biological Process	GO:0060795	cell fate commitment involved in formation of primary germ layer	3/2279	34/18866	0.7960033	0.8718962	0.7889226	3	PAX2/FGFR1/NODAL
Biological Process	GO:0090504	epiboly	3/2279	34/18866	0.7960033	0.8718962	0.7889226	3	RREB1/COL5A1/MRTFA
Biological Process	GO:2000352	negative regulation of endothelial cell apoptotic process	3/2279	34/18866	0.7960033	0.8718962	0.7889226	3	ANGPT1/TNFAIP3/NFE2L2
Biological Process	GO:0000387	spliceosomal snRNP assembly	5/2279	54/18866	0.7971289	0.8727266	0.789674	5	SNRPD3/PRPF8/SNUPN/PRPF6/GEMIN4
Biological Process	GO:0006376	mRNA splice site selection	5/2279	54/18866	0.7971289	0.8727266	0.789674	5	SF1/SFSWAP/CELF2/NOL3/CELF1

Biological Process	GO:0034724	DNA replication-independent nucleosome organization	5/2279	54/18866	0.7971289	0.8727266	0.789674	5	UBN1/CABIN1/RSF1/CENPM/RUVBL1
Biological Process	GO:0051438	regulation of ubiquitin-protein transferase activity	5/2279	54/18866	0.7971289	0.8727266	0.789674	5	FZR1/SMAD7/AXIN1/DNM1L/BAG5
Biological Process	GO:0033344	cholesterol efflux	6/2279	64/18866	0.8009454	0.876501	0.7930892	6	ABCG1/PLTP/MIR27A/NR1H2/ABCA1/NPC1
Biological Process	GO:0044783	G1 DNA damage checkpoint	6/2279	64/18866	0.8009454	0.876501	0.7930892	6	TFDP1/E2F7/PCBP4/ZNF385A/ARID3A/CHEK2
Biological Process	GO:0044819	mitotic G1/S transition checkpoint	6/2279	64/18866	0.8009454	0.876501	0.7930892	6	TFDP1/E2F7/PCBP4/ZNF385A/ARID3A/CHEK2
Biological Process	GO:0046794	transport of virus	6/2279	64/18866	0.8009454	0.876501	0.7930892	6	RAE1/NUP93/SEC13/TPCN2/EPS15/MVB12A
Biological Process	GO:0001759	organ induction	2/2279	24/18866	0.8046024	0.8782695	0.7946894	2	WNT11/FGFR1
Biological Process	GO:0002438	acute inflammatory response to antigenic stimulus	2/2279	24/18866	0.8046024	0.8782695	0.7946894	2	ELANE/FUT7
Biological Process	GO:0010528	regulation of transposition	2/2279	24/18866	0.8046024	0.8782695	0.7946894	2	PIWIL4/MORC1

Biological Process	GO:0010529	negative regulation of transposition	2/2279	24/18866	0.8046024	0.8782695	0.7946894	2	PIWIL4/MORC1
Biological Process	GO:0010804	negative regulation of tumor necrosis factor-mediated signaling pathway	2/2279	24/18866	0.8046024	0.8782695	0.7946894	2	TRAIP/MIR24-2
Biological Process	GO:0016556	mRNA modification	2/2279	24/18866	0.8046024	0.8782695	0.7946894	2	PUS7/METTL8
Biological Process	GO:0019883	antigen processing and presentation of endogenous antigen	2/2279	24/18866	0.8046024	0.8782695	0.7946894	2	HLA-C/HLA-F
Biological Process	GO:0021544	subpallium development	2/2279	24/18866	0.8046024	0.8782695	0.7946894	2	SECISBP2/INHBA
Biological Process	GO:0021904	dorsal/ventral neural tube patterning	2/2279	24/18866	0.8046024	0.8782695	0.7946894	2	SUFU/PAX7
Biological Process	GO:0035268	protein mannosylation	2/2279	24/18866	0.8046024	0.8782695	0.7946894	2	POMT2/B4GAT1
Biological Process	GO:0051882	mitochondrial depolarization	2/2279	24/18866	0.8046024	0.8782695	0.7946894	2	HSH2D/BCL2
Biological Process	GO:0060295	regulation of cilium movement involved in cell motility	2/2279	24/18866	0.8046024	0.8782695	0.7946894	2	RNASE10/DEFB1

Biological Process	GO:0060444	branching involved in mammary gland duct morphogenesis	2/2279	24/18866	0.8046024	0.8782695	0.7946894	2	LRP5L/VDR
Biological Process	GO:0062009	secondary palate development	2/2279	24/18866	0.8046024	0.8782695	0.7946894	2	TGFBR2/WNT11
Biological Process	GO:0070193	synaptonemal complex organization	2/2279	24/18866	0.8046024	0.8782695	0.7946894	2	RAD21L1/TEX12
Biological Process	GO:0071294	cellular response to zinc ion	2/2279	24/18866	0.8046024	0.8782695	0.7946894	2	CREB1/MTF1
Biological Process	GO:0098743	cell aggregation	2/2279	24/18866	0.8046024	0.8782695	0.7946894	2	BMPR1B/LTF
Biological Process	GO:1900118	negative regulation of execution phase of apoptosis	2/2279	24/18866	0.8046024	0.8782695	0.7946894	2	PAM16/BCL2L1
Biological Process	GO:1902019	regulation of cilium-dependent cell motility	2/2279	24/18866	0.8046024	0.8782695	0.7946894	2	RNASE10/DEFB1
Biological Process	GO:1904666	regulation of ubiquitin protein ligase activity	2/2279	24/18866	0.8046024	0.8782695	0.7946894	2	FZR1/DNM1L
Biological Process	GO:0002532	production of molecular mediator involved in inflammatory response	9/2279	93/18866	0.806011	0.8782695	0.7946894	9	MIR140/MEFV/TNF/GRN/NOD2/PER1/P2RX1/PLD3/CD96

Biological Process	GO:0045132	meiotic chromosome segregation	9/2279	93/18866	0.806011	0.8782695	0.7946894	9	EME1/MEIOB/RNF212/PPP2R1A/RAD21L1/DMC1/SLX4/TEX12/TERB2
Biological Process	GO:0006418	tRNA aminoacylation for protein translation	4/2279	45/18866	0.8093124	0.8782695	0.7946894	4	CARS2/EARS2/MARS1/YARS1
Biological Process	GO:0060443	mammary gland morphogenesis	4/2279	45/18866	0.8093124	0.8782695	0.7946894	4	LRP5L/VDR/CSF1R/TGFBR2
Biological Process	GO:0016233	telomere capping	5/2279	55/18866	0.8101593	0.8782695	0.7946894	5	TNKS/RTEL1/SMG6/ERCC1/TFIP11
Biological Process	GO:0032781	positive regulation of ATPase activity	5/2279	55/18866	0.8101593	0.8782695	0.7946894	5	UHRF1/HSCB/PFN1/DNAJB6/AHSA2P
Biological Process	GO:0036297	interstrand cross-link repair	5/2279	55/18866	0.8101593	0.8782695	0.7946894	5	EME1/FANCA/ERCC1/SLX4/XPA
Biological Process	GO:0006885	regulation of pH	10/2279	103/18866	0.8119507	0.8782695	0.7946894	10	TM9SF4/SLC11A1/ATP6V1B2/GRN/BCL2/ATP6V0B/UBE3A/CLN5/ATP6V0C/ATP6V1C1
Biological Process	GO:0002183	cytoplasmic translational initiation	3/2279	35/18866	0.8120768	0.8782695	0.7946894	3	EIF3M/EIF3H/EIF4G1
Biological Process	GO:0007435	salivary gland morphogenesis	3/2279	35/18866	0.8120768	0.8782695	0.7946894	3	TNF/FGFR1/TGFB1

Biological Process	GO:0007603	phototransduction, visible light	3/2279	35/18866	0.8120768	0.8782695	0.7946894	3	PDE6A/GNA11/GRK7
Biological Process	GO:0014046	dopamine secretion	3/2279	35/18866	0.8120768	0.8782695	0.7946894	3	SYT17/DTNBP1/PRKN
Biological Process	GO:0014059	regulation of dopamine secretion	3/2279	35/18866	0.8120768	0.8782695	0.7946894	3	SYT17/DTNBP1/PRKN
Biological Process	GO:0021522	spinal cord motor neuron differentiation	3/2279	35/18866	0.8120768	0.8782695	0.7946894	3	PHOX2A/SUFU/LMO4
Biological Process	GO:0032509	endosome transport via multivesicular body sorting pathway	3/2279	35/18866	0.8120768	0.8782695	0.7946894	3	LYST/SORT1/MVB12A
Biological Process	GO:0042407	cristae formation	3/2279	35/18866	0.8120768	0.8782695	0.7946894	3	SPG7/IMMT/ATP5PD
Biological Process	GO:0044743	protein transmembrane import into intracellular organelle	3/2279	35/18866	0.8120768	0.8782695	0.7946894	3	PAM16/PEX26/TIMM44
Biological Process	GO:0046471	phosphatidylglycerol metabolic process	3/2279	35/18866	0.8120768	0.8782695	0.7946894	3	LPCAT1/PGS1/CDS2
Biological Process	GO:0060338	regulation of type I interferon-mediated signaling pathway	3/2279	35/18866	0.8120768	0.8782695	0.7946894	3	PTPN6/NLRC5/FADD

Biological Process	GO:0070670	response to interleukin-4	3/2279	35/18866	0.8120768	0.8782695	0.7946894	3	RUFY4/ADAMTS13/MCM2
Biological Process	GO:1904031	positive regulation of cyclin-dependent protein kinase activity	3/2279	35/18866	0.8120768	0.8782695	0.7946894	3	PDGFB/CCNY/CCNYL1
Biological Process	GO:1905606	regulation of presynapse assembly	3/2279	35/18866	0.8120768	0.8782695	0.7946894	3	APP/NRXN1/EIF4G1
Biological Process	GO:0001574	ganglioside biosynthetic process	1/2279	13/18866	0.8125449	0.8782695	0.7946894	1	ST8SIA6
Biological Process	GO:0002836	positive regulation of response to tumor cell	1/2279	13/18866	0.8125449	0.8782695	0.7946894	1	CD226
Biological Process	GO:0002839	positive regulation of immune response to tumor cell	1/2279	13/18866	0.8125449	0.8782695	0.7946894	1	CD226
Biological Process	GO:0003084	positive regulation of systemic arterial blood pressure	1/2279	13/18866	0.8125449	0.8782695	0.7946894	1	HSD11B2
Biological Process	GO:0005513	detection of calcium ion	1/2279	13/18866	0.8125449	0.8782695	0.7946894	1	CASQ2
Biological Process	GO:0006264	mitochondrial DNA replication	1/2279	13/18866	0.8125449	0.8782695	0.7946894	1	RRM2B

Biological Process	GO:0006596	polyamine biosynthetic process	1/2279	13/18866	0.8125449	0.8782695	0.7946894	1	ODC1
Biological Process	GO:0006703	estrogen biosynthetic process	1/2279	13/18866	0.8125449	0.8782695	0.7946894	1	HSD17B2
Biological Process	GO:0006995	cellular response to nitrogen starvation	1/2279	13/18866	0.8125449	0.8782695	0.7946894	1	NPRL2
Biological Process	GO:0007494	midgut development	1/2279	13/18866	0.8125449	0.8782695	0.7946894	1	ALDH1A2
Biological Process	GO:0009886	post-embryonic animal morphogenesis	1/2279	13/18866	0.8125449	0.8782695	0.7946894	1	CRB1
Biological Process	GO:0010459	negative regulation of heart rate	1/2279	13/18866	0.8125449	0.8782695	0.7946894	1	SPTBN4
Biological Process	GO:0010642	negative regulation of platelet-derived growth factor receptor signaling pathway	1/2279	13/18866	0.8125449	0.8782695	0.7946894	1	PTPRJ
Biological Process	GO:0010870	positive regulation of receptor biosynthetic process	1/2279	13/18866	0.8125449	0.8782695	0.7946894	1	HDAC1
Biological Process	GO:0014842	regulation of skeletal muscle satellite cell proliferation	1/2279	13/18866	0.8125449	0.8782695	0.7946894	1	ANGPT1

Biological Process	GO:0016264	gap junction assembly	1/2279	13/18866	0.8125449	0.8782695	0.7946894	1	GJA5
Biological Process	GO:0016558	protein import into peroxisome matrix	1/2279	13/18866	0.8125449	0.8782695	0.7946894	1	PEX26
Biological Process	GO:0021520	spinal cord motor neuron cell fate specification	1/2279	13/18866	0.8125449	0.8782695	0.7946894	1	SUFU
Biological Process	GO:0021554	optic nerve development	1/2279	13/18866	0.8125449	0.8782695	0.7946894	1	PAX2
Biological Process	GO:0030002	cellular anion homeostasis	1/2279	13/18866	0.8125449	0.8782695	0.7946894	1	ABCC2
Biological Process	GO:0030205	dermatan sulfate metabolic process	1/2279	13/18866	0.8125449	0.8782695	0.7946894	1	DSE
Biological Process	GO:0030309	poly-N-acetyllactosamine metabolic process	1/2279	13/18866	0.8125449	0.8782695	0.7946894	1	B3GNT2
Biological Process	GO:0030320	cellular monovalent inorganic anion homeostasis	1/2279	13/18866	0.8125449	0.8782695	0.7946894	1	ABCC2
Biological Process	GO:0031053	primary miRNA processing	1/2279	13/18866	0.8125449	0.8782695	0.7946894	1	SMAD3

Biological Process	GO:0031115	negative regulation of microtubule polymerization	1/2279	13/18866	0.8125449	0.8782695	0.7946894	1	TBCD
Biological Process	GO:0031274	positive regulation of pseudopodium assembly	1/2279	13/18866	0.8125449	0.8782695	0.7946894	1	CDC42EP1
Biological Process	GO:0031650	regulation of heat generation	1/2279	13/18866	0.8125449	0.8782695	0.7946894	1	TNF
Biological Process	GO:0032230	positive regulation of synaptic transmission, GABAergic	1/2279	13/18866	0.8125449	0.8782695	0.7946894	1	PRKCE
Biological Process	GO:0032429	regulation of phospholipase A2 activity	1/2279	13/18866	0.8125449	0.8782695	0.7946894	1	AGTR1
Biological Process	GO:0032725	positive regulation of granulocyte macrophage colony-stimulating factor production	1/2279	13/18866	0.8125449	0.8782695	0.7946894	1	TLR9
Biological Process	GO:0032823	regulation of natural killer cell differentiation	1/2279	13/18866	0.8125449	0.8782695	0.7946894	1	PGLYRP1
Biological Process	GO:0033127	regulation of histone phosphorylation	1/2279	13/18866	0.8125449	0.8782695	0.7946894	1	RPS6KA4
Biological Process	GO:0033145	positive regulation of intracellular steroid hormone receptor signaling pathway	1/2279	13/18866	0.8125449	0.8782695	0.7946894	1	KMT2D

Biological Process	GO:0033605	positive regulation of catecholamine secretion	1/2279	13/18866	0.8125449	0.8782695	0.7946894	1	GRK2
Biological Process	GO:0034244	negative regulation of transcription elongation from RNA polymerase II promoter	1/2279	13/18866	0.8125449	0.8782695	0.7946894	1	RECQL5
Biological Process	GO:0034309	primary alcohol biosynthetic process	1/2279	13/18866	0.8125449	0.8782695	0.7946894	1	DGKQ
Biological Process	GO:0034370	triglyceride-rich lipoprotein particle remodeling	1/2279	13/18866	0.8125449	0.8782695	0.7946894	1	LIPC
Biological Process	GO:0035641	locomotory exploration behavior	1/2279	13/18866	0.8125449	0.8782695	0.7946894	1	PRKCE
Biological Process	GO:0036109	alpha-linolenic acid metabolic process	1/2279	13/18866	0.8125449	0.8782695	0.7946894	1	ACSL1
Biological Process	GO:0043201	response to leucine	1/2279	13/18866	0.8125449	0.8782695	0.7946894	1	RPTOR
Biological Process	GO:0043248	proteasome assembly	1/2279	13/18866	0.8125449	0.8782695	0.7946894	1	PSMD13
Biological Process	GO:0043562	cellular response to nitrogen levels	1/2279	13/18866	0.8125449	0.8782695	0.7946894	1	NPRL2

Biological Process	GO:0045602	negative regulation of endothelial cell differentiation	1/2279	13/18866	0.8125449	0.8782695	0.7946894	1	NOTCH4
Biological Process	GO:0045741	positive regulation of epidermal growth factor-activated receptor activity	1/2279	13/18866	0.8125449	0.8782695	0.7946894	1	TGFA
Biological Process	GO:0046040	IMP metabolic process	1/2279	13/18866	0.8125449	0.8782695	0.7946894	1	AMPD3
Biological Process	GO:0050910	detection of mechanical stimulus involved in sensory perception of sound	1/2279	13/18866	0.8125449	0.8782695	0.7946894	1	TMC1
Biological Process	GO:0051451	myoblast migration	1/2279	13/18866	0.8125449	0.8782695	0.7946894	1	ITGB1BP1
Biological Process	GO:0051988	regulation of attachment of spindle microtubules to kinetochore	1/2279	13/18866	0.8125449	0.8782695	0.7946894	1	SPAG5
Biological Process	GO:0061469	regulation of type B pancreatic cell proliferation	1/2279	13/18866	0.8125449	0.8782695	0.7946894	1	SGPP2
Biological Process	GO:0070071	proton-transporting two-sector ATPase complex assembly	1/2279	13/18866	0.8125449	0.8782695	0.7946894	1	TM9SF4
Biological Process	GO:0070839	divalent metal ion export	1/2279	13/18866	0.8125449	0.8782695	0.7946894	1	SLC11A1

Biological Process	GO:0071696	ectodermal placode development	1/2279	13/18866	0.8125449	0.8782695	0.7946894	1	HDAC1
Biological Process	GO:0097499	protein localization to non-motile cilium	1/2279	13/18866	0.8125449	0.8782695	0.7946894	1	TTC21B
Biological Process	GO:0097531	mast cell migration	1/2279	13/18866	0.8125449	0.8782695	0.7946894	1	PIK3CD
Biological Process	GO:0098903	regulation of membrane repolarization during action potential	1/2279	13/18866	0.8125449	0.8782695	0.7946894	1	NOS1AP
Biological Process	GO:0099150	regulation of postsynaptic specialization assembly	1/2279	13/18866	0.8125449	0.8782695	0.7946894	1	NRXN1
Biological Process	GO:0150052	regulation of postsynapse assembly	1/2279	13/18866	0.8125449	0.8782695	0.7946894	1	NRXN1
Biological Process	GO:1900102	negative regulation of endoplasmic reticulum unfolded protein response	1/2279	13/18866	0.8125449	0.8782695	0.7946894	1	NCK2
Biological Process	GO:1901387	positive regulation of voltage-gated calcium channel activity	1/2279	13/18866	0.8125449	0.8782695	0.7946894	1	FGF14
Biological Process	GO:1902902	negative regulation of autophagosome assembly	1/2279	13/18866	0.8125449	0.8782695	0.7946894	1	FEZ2

Biological Process	GO:1903025	regulation of RNA polymerase II regulatory region sequence-specific DNA binding	1/2279	13/18866	0.8125449	0.8782695	0.7946894	1	LIF
Biological Process	GO:1905651	regulation of artery morphogenesis	1/2279	13/18866	0.8125449	0.8782695	0.7946894	1	NOTCH1
Biological Process	GO:1990403	embryonic brain development	1/2279	13/18866	0.8125449	0.8782695	0.7946894	1	IFT140
Biological Process	GO:2000009	negative regulation of protein localization to cell surface	1/2279	13/18866	0.8125449	0.8782695	0.7946894	1	ASTN2
Biological Process	GO:2000344	positive regulation of acrosome reaction	1/2279	13/18866	0.8125449	0.8782695	0.7946894	1	PLCB1
Biological Process	GO:2001198	regulation of dendritic cell differentiation	1/2279	13/18866	0.8125449	0.8782695	0.7946894	1	CEBPB
Biological Process	GO:0060675	ureteric bud morphogenesis	6/2279	65/18866	0.8128582	0.8784083	0.794815	6	TACSTD2/ILK/WNT11/PAX2/BCL2/HOXB7
Biological Process	GO:0090307	mitotic spindle assembly	6/2279	65/18866	0.8128582	0.8784083	0.794815	6	ARHGEF10/RCC1/STAG1/MSTO1/CHEK2/ABRAXAS1
Biological Process	GO:0006690	icosanoid metabolic process	12/2279	122/18866	0.8150501	0.8806768	0.7968676	12	MGLL/ABCC1/GGT1/ABHD16A/TNFRSF1A/CYP4F3/MGST2/EPHX1/DPEP2/LYPLA2/ZADH2/PRXL2B

Biological Process	GO:0034308	primary alcohol metabolic process	9/2279	94/18866	0.81588	0.881373	0.7974976	9	PNPLA2/DHRS9/ALDH2/ACSS1/RDH10/SULT1A2/DGKQ/ACSS2/ALDH1A2
Biological Process	GO:0060993	kidney morphogenesis	9/2279	94/18866	0.81588	0.881373	0.7974976	9	TACSTD2/ILK/WNT11/PAX2/WWTR1/BCL2/PDGFRB/LIF/HOXB7
Biological Process	GO:0042274	ribosomal small subunit biogenesis	7/2279	75/18866	0.816561	0.881808	0.7978912	7	RCL1/WDR46/NOB1/NOP14/ERCC2/RIOK1/UTP4
Biological Process	GO:0061844	antimicrobial humoral immune response mediated by antimicrobial peptide	7/2279	75/18866	0.816561	0.881808	0.7978912	7	DEFA4/ELANE/RNASE3/DEFB1/PGLYRP1/LTF/PGLYRP4
Biological Process	GO:0072088	nephron epithelium morphogenesis	7/2279	75/18866	0.816561	0.881808	0.7978912	7	TACSTD2/ILK/WNT11/PAX2/BCL2/LIF/HOXB7
Biological Process	GO:0048024	regulation of mRNA splicing, via spliceosome	11/2279	113/18866	0.8177866	0.8829307	0.798907	11	RBM19/SRPK2/PCBP4/SF1/DAZAP1/SFSWAP/CELF2/CELF1/RBFOX3/NSRP1/RBM15B
Biological Process	GO:0070268	cornification	11/2279	113/18866	0.8177866	0.8829307	0.798907	11	CSTA/TGM5/KRT17/JUP/PKP1/PCSK6/PERP/KRT23/KAZN/KRT7/PPL
Biological Process	GO:0035418	protein localization to synapse	8/2279	85/18866	0.8208154	0.8859991	0.8016834	8	PRKCZ/BAIAP2/ZDHHC2/CNIH2/NRXN1/KIF5C/NRXN2/MAP1A
Biological Process	GO:0034508	centromere complex assembly	5/2279	56/18866	0.822513	0.8859991	0.8016834	5	MIS12/RSF1/RB1/CENPM/RUVBL1

Biological Process	GO:0002053	positive regulation of mesenchymal cell proliferation	2/2279	25/18866	0.8227348	0.8859991	0.8016834	2	TGFBR2/FGFR1
Biological Process	GO:0006851	mitochondrial calcium ion transmembrane transport	2/2279	25/18866	0.8227348	0.8859991	0.8016834	2	SPG7/PHB
Biological Process	GO:0007530	sex determination	2/2279	25/18866	0.8227348	0.8859991	0.8016834	2	SF1/INSRR
Biological Process	GO:0010447	response to acidic pH	2/2279	25/18866	0.8227348	0.8859991	0.8016834	2	GPR4/SST
Biological Process	GO:0014829	vascular associated smooth muscle contraction	2/2279	25/18866	0.8227348	0.8859991	0.8016834	2	SLC8A1/P2RX1
Biological Process	GO:0032104	regulation of response to extracellular stimulus	2/2279	25/18866	0.8227348	0.8859991	0.8016834	2	GHRL/NPY
Biological Process	GO:0032107	regulation of response to nutrient levels	2/2279	25/18866	0.8227348	0.8859991	0.8016834	2	GHRL/NPY
Biological Process	GO:0039529	RIG-I signaling pathway	2/2279	25/18866	0.8227348	0.8859991	0.8016834	2	PHB/SEC14L1
Biological Process	GO:0045992	negative regulation of embryonic development	2/2279	25/18866	0.8227348	0.8859991	0.8016834	2	SUFU/COL5A1

Biological Process	GO:0046039	GTP metabolic process	2/2279	25/18866	0.8227348	0.8859991	0.8016834	2	AMPD3/IMPDH1
Biological Process	GO:0060259	regulation of feeding behavior	2/2279	25/18866	0.8227348	0.8859991	0.8016834	2	GHRL/TTC21B
Biological Process	GO:0060571	morphogenesis of an epithelial fold	2/2279	25/18866	0.8227348	0.8859991	0.8016834	2	RDH10/NODAL
Biological Process	GO:0097164	ammonium ion metabolic process	2/2279	25/18866	0.8227348	0.8859991	0.8016834	2	DDC/HNMT
Biological Process	GO:2000679	positive regulation of transcription regulatory region DNA binding	2/2279	25/18866	0.8227348	0.8859991	0.8016834	2	TGFB1/RB1
Biological Process	GO:0022412	cellular process involved in reproduction in multicellular organism	40/2279	375/18866	0.8227599	0.8859991	0.8016834	40	PIWIL4/CD9/ACRBP/HEXB/MOV10L1/BRCA2/ARRB1/FOLR3/SPAG6/ZBTB16/MEIOB/CDYL/SMARCA2/ZMIZ1/FANCA/KMT2D/RARA/DEFB1/JAM3/KDM1B/IQCG/BMPR1B/ETV6/PPP2R1A/ARRB2/CELF1/CCNA1/BCL2/DMC1/SLIT3/ARMC2/MORC1/NPPC/FSCN3/ERCC1/BCL2L1/FBXW11/INHBA/TTLL1/MFSD14A
Biological Process	GO:0000959	mitochondrial RNA metabolic process	4/2279	46/18866	0.8228666	0.8859991	0.8016834	4	EARS2/TBRG4/PRKAA1/GRSF1
Biological Process	GO:0005978	glycogen biosynthetic process	4/2279	46/18866	0.8228666	0.8859991	0.8016834	4	DYRK2/PPP1CB/ESRRB/PER2
Biological Process	GO:0007019	microtubule depolymerization	4/2279	46/18866	0.8228666	0.8859991	0.8016834	4	NAV3/MAP1A/ARHGEF2/SPEF1

Biological Process	GO:0009250	glucan biosynthetic process	4/2279	46/18866	0.8228666	0.8859991	0.8016834	4	DYRK2/PPP1CB/ESRRB/PER2
Biological Process	GO:0014014	negative regulation of gliogenesis	4/2279	46/18866	0.8228666	0.8859991	0.8016834	4	SKI/NF1/NOTCH1/CERS2
Biological Process	GO:0017144	drug metabolic process	4/2279	46/18866	0.8228666	0.8859991	0.8016834	4	NR1I2/ABCC2/TPMT/AOX1
Biological Process	GO:0051646	mitochondrion localization	4/2279	46/18866	0.8228666	0.8859991	0.8016834	4	MSTO1/KIF1B/DNM1L/MARK2
Biological Process	GO:0044766	multi-organism transport	6/2279	66/18866	0.8242035	0.8871369	0.802713	6	RAE1/NUP93/SEC13/TPCN2/EPS15/MVB12A
Biological Process	GO:0072171	mesonephric tubule morphogenesis	6/2279	66/18866	0.8242035	0.8871369	0.802713	6	TACSTD2/ILK/WNT11/PAX2/BCL2/HOXB7
Biological Process	GO:1902579	multi-organism localization	6/2279	66/18866	0.8242035	0.8871369	0.802713	6	RAE1/NUP93/SEC13/TPCN2/EPS15/MVB12A
Biological Process	GO:0007292	female gamete generation	14/2279	142/18866	0.826872	0.8892085	0.8045874	14	HEXB/BRCA2/MEIOB/TNFAIP6/ZMIZ1/KMT2D/BMPR1B/ETV6/PPP2R1A/BCL2/DMC1/NPPC/ERCC1/INHBA
Biological Process	GO:0000381	regulation of alternative mRNA splicing, via spliceosome	7/2279	76/18866	0.8270486	0.8892085	0.8045874	7	RBM19/SF1/CELF2/CELF1/RBFOX3/NSRP1/RBM15B

Biological Process	GO:1903524	positive regulation of blood circulation	7/2279	76/18866	0.8270486	0.8892085	0.8045874	7	PDE4D/GJA5/NOS1AP/HRH1/GHRL/KCNQ1/F2R
Biological Process	GO:0001569	branching involved in blood vessel morphogenesis	3/2279	36/18866	0.8270641	0.8892085	0.8045874	3	TGFBR2/ENG/NOTCH4
Biological Process	GO:0002755	MyD88-dependent toll-like receptor signaling pathway	3/2279	36/18866	0.8270641	0.8892085	0.8045874	3	TLR9/TNIP1/IRAK2
Biological Process	GO:0016180	snRNA processing	3/2279	36/18866	0.8270641	0.8892085	0.8045874	3	EXOSC2/TOE1/TUT1
Biological Process	GO:0031128	developmental induction	3/2279	36/18866	0.8270641	0.8892085	0.8045874	3	HIPK2/WNT11/FGFR1
Biological Process	GO:0033280	response to vitamin D	3/2279	36/18866	0.8270641	0.8892085	0.8045874	3	VDR/ALPL/AQP3
Biological Process	GO:0051955	regulation of amino acid transport	3/2279	36/18866	0.8270641	0.8892085	0.8045874	3	SLC43A2/PER2/SLC43A1
Biological Process	GO:0099174	regulation of presynapse organization	3/2279	36/18866	0.8270641	0.8892085	0.8045874	3	APP/NRXN1/EIF4G1
Biological Process	GO:0030301	cholesterol transport	10/2279	105/18866	0.830047	0.8922137	0.8073066	10	OSBPL5/LIPC/ABCG1/PLTP/MIR27A/OSBPL6/CLU/NR1H2/ABCA1/NPC1

Biological Process	GO:0044070	regulation of anion transport	10/2279	105/18866	0.830047	0.8922137	0.8073066	10	MAP2K6/ATP8A1/THBS1/RIPK1/FGFR1/SLC43A2/PER2/SLC43A1/CEBPB/PDZK1
Biological Process	GO:0006112	energy reserve metabolic process	8/2279	86/18866	0.8305635	0.8925426	0.8076042	8	PRKAG2/DYRK2/PPP1CB/ESRRB/PER2/GFPT2/PCDH12/RUBCNL
Biological Process	GO:0021515	cell differentiation in spinal cord	5/2279	57/18866	0.8342087	0.8925426	0.8076042	5	PHOX2A/SUFU/PAX7/NOTCH1/LMO4
Biological Process	GO:0050432	catecholamine secretion	5/2279	57/18866	0.8342087	0.8925426	0.8076042	5	NISCH/SYT17/DTNBP1/GRK2/PRKN
Biological Process	GO:0010565	regulation of cellular ketone metabolic process	19/2279	189/18866	0.8342144	0.8925426	0.8076042	19	PRKAG2/PPARG/NCOR2/MFSD2A/ODC1/COMT/RDH10/DGKQ/NR1H2/SLC7A7/ACACB/PSMF1/PSMB7/PRKCE/SREBF1/WDTC1/CPT1A/PSMD13/ACADVL
Biological Process	GO:0048663	neuron fate commitment	6/2279	67/18866	0.8349947	0.8925426	0.8076042	6	SUFU/DLL1/RBPJ/PAX7/FEV/NOTCH1
Biological Process	GO:1900015	regulation of cytokine production involved in inflammatory response	6/2279	67/18866	0.8349947	0.8925426	0.8076042	6	MIR140/MEFV/TNF/NOD2/PER1/PLD3
Biological Process	GO:0001886	endothelial cell morphogenesis	1/2279	14/18866	0.8352049	0.8925426	0.8076042	1	NOTCH4
Biological Process	GO:0006241	CTP biosynthetic process	1/2279	14/18866	0.8352049	0.8925426	0.8076042	1	UCK2

Biological Process	GO:0007512	adult heart development	1/2279	14/18866	0.8352049	0.8925426	0.8076042	1	MEF2D
Biological Process	GO:0010248	establishment or maintenance of transmembrane electrochemical gradient	1/2279	14/18866	0.8352049	0.8925426	0.8076042	1	FXYD2
Biological Process	GO:0010838	positive regulation of keratinocyte proliferation	1/2279	14/18866	0.8352049	0.8925426	0.8076042	1	CDH3
Biological Process	GO:0014841	skeletal muscle satellite cell proliferation	1/2279	14/18866	0.8352049	0.8925426	0.8076042	1	ANGPT1
Biological Process	GO:0014857	regulation of skeletal muscle cell proliferation	1/2279	14/18866	0.8352049	0.8925426	0.8076042	1	ANGPT1
Biological Process	GO:0030007	cellular potassium ion homeostasis	1/2279	14/18866	0.8352049	0.8925426	0.8076042	1	KCTD7
Biological Process	GO:0030836	positive regulation of actin filament depolymerization	1/2279	14/18866	0.8352049	0.8925426	0.8076042	1	WDR1
Biological Process	GO:0031272	regulation of pseudopodium assembly	1/2279	14/18866	0.8352049	0.8925426	0.8076042	1	CDC42EP1
Biological Process	GO:0031282	regulation of guanylate cyclase activity	1/2279	14/18866	0.8352049	0.8925426	0.8076042	1	RCVRN

Biological Process	GO:0032310	prostaglandin secretion	1/2279	14/18866	0.8352049	0.8925426	0.8076042	1	MAP2K6
Biological Process	GO:0032488	Cdc42 protein signal transduction	1/2279	14/18866	0.8352049	0.8925426	0.8076042	1	ABCA1
Biological Process	GO:0032688	negative regulation of interferon-beta production	1/2279	14/18866	0.8352049	0.8925426	0.8076042	1	TRAIP
Biological Process	GO:0032802	low-density lipoprotein particle receptor catabolic process	1/2279	14/18866	0.8352049	0.8925426	0.8076042	1	AP2A1
Biological Process	GO:0035589	G protein-coupled purinergic nucleotide receptor signaling pathway	1/2279	14/18866	0.8352049	0.8925426	0.8076042	1	P2RY6
Biological Process	GO:0035810	positive regulation of urine volume	1/2279	14/18866	0.8352049	0.8925426	0.8076042	1	GNAI2
Biological Process	GO:0035815	positive regulation of renal sodium excretion	1/2279	14/18866	0.8352049	0.8925426	0.8076042	1	GNAI2
Biological Process	GO:0043558	regulation of translational initiation in response to stress	1/2279	14/18866	0.8352049	0.8925426	0.8076042	1	NCK2
Biological Process	GO:0044804	autophagy of nucleus	1/2279	14/18866	0.8352049	0.8925426	0.8076042	1	WIPI2

Biological Process	GO:0045472	response to ether	1/2279	14/18866	0.8352049	0.8925426	0.8076042	1	LARP1
Biological Process	GO:0045836	positive regulation of meiotic nuclear division	1/2279	14/18866	0.8352049	0.8925426	0.8076042	1	PLCB1
Biological Process	GO:0046036	CTP metabolic process	1/2279	14/18866	0.8352049	0.8925426	0.8076042	1	UCK2
Biological Process	GO:0046131	pyrimidine ribonucleoside metabolic process	1/2279	14/18866	0.8352049	0.8925426	0.8076042	1	CDA
Biological Process	GO:0046133	pyrimidine ribonucleoside catabolic process	1/2279	14/18866	0.8352049	0.8925426	0.8076042	1	CDA
Biological Process	GO:0051547	regulation of keratinocyte migration	1/2279	14/18866	0.8352049	0.8925426	0.8076042	1	IQSEC1
Biological Process	GO:0060397	growth hormone receptor signaling pathway via JAK-STAT	1/2279	14/18866	0.8352049	0.8925426	0.8076042	1	STAT5A
Biological Process	GO:0060831	smoothened signaling pathway involved in dorsal/ventral neural tube patterning	1/2279	14/18866	0.8352049	0.8925426	0.8076042	1	SUFU
Biological Process	GO:0060911	cardiac cell fate commitment	1/2279	14/18866	0.8352049	0.8925426	0.8076042	1	RBPJ

Biological Process	GO:0090231	regulation of spindle checkpoint	1/2279	14/18866	0.8352049	0.8925426	0.8076042	1	PCID2
Biological Process	GO:0090266	regulation of mitotic cell cycle spindle assembly checkpoint	1/2279	14/18866	0.8352049	0.8925426	0.8076042	1	PCID2
Biological Process	GO:0090399	replicative senescence	1/2279	14/18866	0.8352049	0.8925426	0.8076042	1	CHEK2
Biological Process	GO:0097154	GABAergic neuron differentiation	1/2279	14/18866	0.8352049	0.8925426	0.8076042	1	INHBA
Biological Process	GO:0099550	trans-synaptic signaling, modulating synaptic transmission	1/2279	14/18866	0.8352049	0.8925426	0.8076042	1	F2R
Biological Process	GO:0150011	regulation of neuron projection arborization	1/2279	14/18866	0.8352049	0.8925426	0.8076042	1	MFSD2A
Biological Process	GO:1901160	primary amino compound metabolic process	1/2279	14/18866	0.8352049	0.8925426	0.8076042	1	DDC
Biological Process	GO:1902188	positive regulation of viral release from host cell	1/2279	14/18866	0.8352049	0.8925426	0.8076042	1	PC
Biological Process	GO:1903504	regulation of mitotic spindle checkpoint	1/2279	14/18866	0.8352049	0.8925426	0.8076042	1	PCID2

Biological Process	GO:1903624	regulation of DNA catabolic process	1/2279	14/18866	0.8352049	0.8925426	0.8076042	1	PAM16
Biological Process	GO:1903894	regulation of IRE1-mediated unfolded protein response	1/2279	14/18866	0.8352049	0.8925426	0.8076042	1	COPS5
Biological Process	GO:1904668	positive regulation of ubiquitin protein ligase activity	1/2279	14/18866	0.8352049	0.8925426	0.8076042	1	FZR1
Biological Process	GO:1904814	regulation of protein localization to chromosome, telomeric region	1/2279	14/18866	0.8352049	0.8925426	0.8076042	1	CCT6A
Biological Process	GO:1990173	protein localization to nucleoplasm	1/2279	14/18866	0.8352049	0.8925426	0.8076042	1	CCT6A
Biological Process	GO:2000543	positive regulation of gastrulation	1/2279	14/18866	0.8352049	0.8925426	0.8076042	1	OTX2
Biological Process	GO:0006953	acute-phase response	4/2279	47/18866	0.8356137	0.8925426	0.8076042	4	SERPINA1/TNF/IL6R/CEBPB
Biological Process	GO:0016073	snRNA metabolic process	4/2279	47/18866	0.8356137	0.8925426	0.8076042	4	EXOSC2/TOE1/ZCCHC8/TUT1
Biological Process	GO:0030261	chromosome condensation	4/2279	47/18866	0.8356137	0.8925426	0.8076042	4	TTN/DFFB/CDCA5/BANF1

Biological Process	GO:0030574	collagen catabolic process	4/2279	47/18866	0.8356137	0.8925426	0.8076042	4	PRTN3/CTSD/CTSB/VSIR
Biological Process	GO:0032369	negative regulation of lipid transport	4/2279	47/18866	0.8356137	0.8925426	0.8076042	4	THBS1/MIR27A/NR1H2/ITGB3
Biological Process	GO:0042572	retinol metabolic process	4/2279	47/18866	0.8356137	0.8925426	0.8076042	4	PNPLA2/DHRS9/RDH10/ALDH1A2
Biological Process	GO:0007498	mesoderm development	13/2279	134/18866	0.8367037	0.8936063	0.8085667	13	MIR145/SETD2/SMAD3/WNT11/PAX2/FGFR1/OVOL1/ITGB3/PUS7/TEAD2/SSBP3/INHBA/NODAL
Biological Process	GO:0007548	sex differentiation	29/2279	280/18866	0.8373284	0.894173	0.8090794	29	ARID5B/BRCA2/ARRB1/EIF2B5/CSDE1/FANCA/RDH10/RARA/SF1/BMPR1B/ASB1/NCOA1/LHFPL2/ARRB2/NTRK1/HOXA9/RAD21L1/BCL2/DMC1/SLIT3/UBE3A/CEBPB/PDGFRB/ERCC1/BCL2L1/HMGCS1/TYRO3/NCOA4/INHBA
Biological Process	GO:0019233	sensory perception of pain	10/2279	106/18866	0.8385796	0.8953149	0.8101127	10	MGLL/IL10/CCR2/COMT/SCN1A/CNR2/ARRB2/NTRK1/F2R/ACP3
Biological Process	GO:0002347	response to tumor cell	2/2279	26/18866	0.8393401	0.8953149	0.8101127	2	CD226/HNMT
Biological Process	GO:0006700	C21-steroid hormone biosynthetic process	2/2279	26/18866	0.8393401	0.8953149	0.8101127	2	FDX1/DGKQ
Biological Process	GO:0030810	positive regulation of nucleotide biosynthetic process	2/2279	26/18866	0.8393401	0.8953149	0.8101127	2	RCVRN/ENO1

Biological Process	GO:0033119	negative regulation of RNA splicing	2/2279	26/18866	0.8393401	0.8953149	0.8101127	2	PCBP4/SFSWAP
Biological Process	GO:0043567	regulation of insulin-like growth factor receptor signaling pathway	2/2279	26/18866	0.8393401	0.8953149	0.8101127	2	CDH3/MYORG
Biological Process	GO:0045026	plasma membrane fusion	2/2279	26/18866	0.8393401	0.8953149	0.8101127	2	CD9/FOLR3
Biological Process	GO:0046755	viral budding	2/2279	26/18866	0.8393401	0.8953149	0.8101127	2	NEDD4/MVB12A
Biological Process	GO:0051123	RNA polymerase II preinitiation complex assembly	2/2279	26/18866	0.8393401	0.8953149	0.8101127	2	CREB1/ATF7IP
Biological Process	GO:1900373	positive regulation of purine nucleotide biosynthetic process	2/2279	26/18866	0.8393401	0.8953149	0.8101127	2	RCVRN/ENO1
Biological Process	GO:0007098	centrosome cycle	12/2279	125/18866	0.839477	0.8953605	0.8101539	12	ARHGEF10/DCTN1/NDE1/BRCA2/CEP135/UVRAG/POC1A/SDCCAG8/SLC16A1/CEP72/NDEL1/CEP295NL
Biological Process	GO:0060291	long-term synaptic potentiation	8/2279	87/18866	0.839892	0.8957025	0.8104634	8	PRKCZ/CREB1/ZDHHC2/APP/AKAP5/NF1/SHANK2/CX3CR1
Biological Process	GO:0007099	centriole replication	3/2279	37/18866	0.8410148	0.8964974	0.8111827	3	CEP135/CEP72/CEP295NL

Biological Process	GO:0032941	secretion by tissue	3/2279	37/18866	0.8410148	0.8964974	0.8111827	3	AQP1/NR1H2/PRKCE
Biological Process	GO:0060428	lung epithelium development	3/2279	37/18866	0.8410148	0.8964974	0.8111827	3	CREB1/RBPJ/GRHL2
Biological Process	GO:0097502	mannosylation	3/2279	37/18866	0.8410148	0.8964974	0.8111827	3	POMT2/ALG11/B4GAT1
Biological Process	GO:0002220	innate immune response activating cell surface receptor signaling pathway	11/2279	116/18866	0.8427973	0.8982967	0.8128108	11	CLEC4C/MUC12/FCN1/MUC20/BCL10/PSMF1/CREBBP/PSMB7/RELA/FBXW11/PSMD13
Biological Process	GO:0002011	morphogenesis of an epithelial sheet	5/2279	58/18866	0.8452663	0.9007262	0.8150091	5	RREB1/NOTCH1/COL5A1/ARHGAP12/MRTFA
Biological Process	GO:0021885	forebrain cell migration	5/2279	58/18866	0.8452663	0.9007262	0.8150091	5	RTN4/DISC1/ZMIZ1/TYRO3/NDEL1
Biological Process	GO:0042310	vasoconstriction	7/2279	78/18866	0.8466013	0.9020477	0.8162048	7	SLC8A1/GJA5/HRH1/PER2/P2RX1/F2R/AGTR1
Biological Process	GO:0060632	regulation of microtubule-based movement	4/2279	48/18866	0.8475839	0.9027909	0.8168773	4	RNASE10/CNIH2/DEFB1/TTC21B
Biological Process	GO:1904036	negative regulation of epithelial cell apoptotic process	4/2279	48/18866	0.8475839	0.9027909	0.8168773	4	ANGPT1/TNFAIP3/TCF7L2/NFE2L2

Biological Process	GO:1990573	potassium ion import across plasma membrane	4/2279	48/18866	0.8475839	0.9027909	0.8168773	4	KCNJ15/FXYD2/SLC12A1/SLC12A7
Biological Process	GO:0009798	axis specification	8/2279	88/18866	0.8488099	0.9039954	0.8179671	8	SKI/PKD1L1/DLL1/PCSK6/NOTCH1/TMED2/NODAL/OTX2
Biological Process	GO:0009952	anterior/posterior pattern specification	22/2279	219/18866	0.8499844	0.9050418	0.8189139	22	LRP5L/SKI/SMAD3/ZEB2/HIPK2/ZBTB16/DLL1/RBPJ/MIB1/PCSK6/RARG/HOXA9/NOTCH1/TMED2/HHEX/SSBP3/ALDH1A2/NODAL/HES3/OTX2/HOXB7/GRSF1
Biological Process	GO:0002758	innate immune response-activating signal transduction	11/2279	117/18866	0.8505171	0.9050418	0.8189139	11	CLEC4C/MUC12/FCN1/MUC20/BCL10/PSMF1/CREBBP/PSMB7/RELA/FBXW11/PSMD13
Biological Process	GO:0002377	immunoglobulin production	21/2279	210/18866	0.8507691	0.9050418	0.8189139	21	IGLV3-10/GALNT2/IL10/TRDV2/TNF/TRAF2/TNFSF13B/TLR9/UNG/TGFB1/PHB/PTPRC/POU2F2/TCF3/TP53BP1/TRAF3IP2/CTNNBL1/ERCC1/TNFSF13/PAXIP1/NSD2
Biological Process	GO:0035278	miRNA mediated inhibition of translation	9/2279	98/18866	0.8515569	0.9050418	0.8189139	9	MIR145/TNRC6B/MIR101-2/AGO1/MIR27A/AGO2/MIR138-2/NOTCH4/EIF4G1
Biological Process	GO:0040033	negative regulation of translation, ncRNA-mediated	9/2279	98/18866	0.8515569	0.9050418	0.8189139	9	MIR145/TNRC6B/MIR101-2/AGO1/MIR27A/AGO2/MIR138-2/NOTCH4/EIF4G1
Biological Process	GO:0045974	regulation of translation, ncRNA-mediated	9/2279	98/18866	0.8515569	0.9050418	0.8189139	9	MIR145/TNRC6B/MIR101-2/AGO1/MIR27A/AGO2/MIR138-2/NOTCH4/EIF4G1
Biological Process	GO:0048754	branching morphogenesis of an epithelial tube	15/2279	155/18866	0.8531388	0.9050418	0.8189139	15	CTSZ/TACSTD2/LRP5L/VDR/TNF/TGFBR2/CTSH/ILK/PAX2/RDH10/ENG/BCL2/NOTCH1/NOTCH4/HOXB7

Biological Process	GO:0003156	regulation of animal organ formation	3/2279	38/18866	0.8539801	0.9050418	0.8189139	3	WNT11/FGFR1/NOTCH1
Biological Process	GO:0045923	positive regulation of fatty acid metabolic process	3/2279	38/18866	0.8539801	0.9050418	0.8189139	3	PPARG/NR1H2/CPT1A
Biological Process	GO:0000244	spliceosomal tri-snRNP complex assembly	2/2279	27/18866	0.8545224	0.9050418	0.8189139	2	PRPF8/PRPF6
Biological Process	GO:0002021	response to dietary excess	2/2279	27/18866	0.8545224	0.9050418	0.8189139	2	SORL1/SLC25A25
Biological Process	GO:0002026	regulation of the force of heart contraction	2/2279	27/18866	0.8545224	0.9050418	0.8189139	2	SLC8A1/GRK2
Biological Process	GO:0006516	glycoprotein catabolic process	2/2279	27/18866	0.8545224	0.9050418	0.8189139	2	EDEM1/EDEM2
Biological Process	GO:0006706	steroid catabolic process	2/2279	27/18866	0.8545224	0.9050418	0.8189139	2	YWHAH/AKR1D1
Biological Process	GO:0009065	glutamine family amino acid catabolic process	2/2279	27/18866	0.8545224	0.9050418	0.8189139	2	ALDH4A1/FAH
Biological Process	GO:0015985	energy coupled proton transport, down electrochemical gradient	2/2279	27/18866	0.8545224	0.9050418	0.8189139	2	VPS9D1/ATP5PD

Biological Process	GO:0015986	ATP synthesis coupled proton transport	2/2279	27/18866	0.8545224	0.9050418	0.8189139	2	VPS9D1/ATP5PD
Biological Process	GO:0022400	regulation of rhodopsin mediated signaling pathway	2/2279	27/18866	0.8545224	0.9050418	0.8189139	2	PDE6A/GRK7
Biological Process	GO:0042501	serine phosphorylation of STAT protein	2/2279	27/18866	0.8545224	0.9050418	0.8189139	2	GGNBP2/LIF
Biological Process	GO:0051953	negative regulation of amine transport	2/2279	27/18866	0.8545224	0.9050418	0.8189139	2	SLC43A2/SLC43A1
Biological Process	GO:1902473	regulation of protein localization to synapse	2/2279	27/18866	0.8545224	0.9050418	0.8189139	2	PRKCZ/CNIH2
Biological Process	GO:1904357	negative regulation of telomere maintenance via telomere lengthening	2/2279	27/18866	0.8545224	0.9050418	0.8189139	2	TNKS/SLX4
Biological Process	GO:0001946	lymphangiogenesis	1/2279	15/18866	0.8551268	0.9050418	0.8189139	1	VASH1
Biological Process	GO:0002864	regulation of acute inflammatory response to antigenic stimulus	1/2279	15/18866	0.8551268	0.9050418	0.8189139	1	FUT7
Biological Process	GO:0006089	lactate metabolic process	1/2279	15/18866	0.8551268	0.9050418	0.8189139	1	PER2

Biological Process	GO:0006388	tRNA splicing, via endonucleolytic cleavage and ligation	1/2279	15/18866	0.8551268	0.9050418	0.8189139	1	CPSF4
Biological Process	GO:0009209	pyrimidine ribonucleoside triphosphate biosynthetic process	1/2279	15/18866	0.8551268	0.9050418	0.8189139	1	UCK2
Biological Process	GO:0010455	positive regulation of cell fate commitment	1/2279	15/18866	0.8551268	0.9050418	0.8189139	1	RBPJ
Biological Process	GO:0014856	skeletal muscle cell proliferation	1/2279	15/18866	0.8551268	0.9050418	0.8189139	1	ANGPT1
Biological Process	GO:0016584	nucleosome positioning	1/2279	15/18866	0.8551268	0.9050418	0.8189139	1	RSF1
Biological Process	GO:0018216	peptidyl-arginine methylation	1/2279	15/18866	0.8551268	0.9050418	0.8189139	1	PRMT2
Biological Process	GO:0019081	viral translation	1/2279	15/18866	0.8551268	0.9050418	0.8189139	1	SHFL
Biological Process	GO:0032274	gonadotropin secretion	1/2279	15/18866	0.8551268	0.9050418	0.8189139	1	INHBA
Biological Process	GO:0032785	negative regulation of DNA-templated transcription, elongation	1/2279	15/18866	0.8551268	0.9050418	0.8189139	1	RECQL5

Biological Process	GO:0033151	V(D)J recombination	1/2279	15/18866	0.8551268	0.9050418	0.8189139	1	TCF3
Biological Process	GO:0035435	phosphate ion transmembrane transport	1/2279	15/18866	0.8551268	0.9050418	0.8189139	1	SLC37A1
Biological Process	GO:0035635	entry of bacterium into host cell	1/2279	15/18866	0.8551268	0.9050418	0.8189139	1	CDH1
Biological Process	GO:0043518	negative regulation of DNA damage response, signal transduction by p53 class mediator	1/2279	15/18866	0.8551268	0.9050418	0.8189139	1	MARCHF7
Biological Process	GO:0046184	aldehyde biosynthetic process	1/2279	15/18866	0.8551268	0.9050418	0.8189139	1	PDXK
Biological Process	GO:0048569	post-embryonic animal organ development	1/2279	15/18866	0.8551268	0.9050418	0.8189139	1	ERCC1
Biological Process	GO:0048712	negative regulation of astrocyte differentiation	1/2279	15/18866	0.8551268	0.9050418	0.8189139	1	NF1
Biological Process	GO:0060009	Sertoli cell development	1/2279	15/18866	0.8551268	0.9050418	0.8189139	1	NTRK1
Biological Process	GO:0060026	convergent extension	1/2279	15/18866	0.8551268	0.9050418	0.8189139	1	WNT11

Biological Process	GO:0060049	regulation of protein glycosylation	1/2279	15/18866	0.8551268	0.9050418	0.8189139	1	POMT2
Biological Process	GO:0060339	negative regulation of type I interferon-mediated signaling pathway	1/2279	15/18866	0.8551268	0.9050418	0.8189139	1	NLRC5
Biological Process	GO:0060347	heart trabecula formation	1/2279	15/18866	0.8551268	0.9050418	0.8189139	1	FHL2
Biological Process	GO:0060579	ventral spinal cord interneuron fate commitment	1/2279	15/18866	0.8551268	0.9050418	0.8189139	1	SUFU
Biological Process	GO:0060581	cell fate commitment involved in pattern specification	1/2279	15/18866	0.8551268	0.9050418	0.8189139	1	SUFU
Biological Process	GO:0070831	basement membrane assembly	1/2279	15/18866	0.8551268	0.9050418	0.8189139	1	LAMB3
Biological Process	GO:0097428	protein maturation by iron-sulfur cluster transfer	1/2279	15/18866	0.8551268	0.9050418	0.8189139	1	HSCB
Biological Process	GO:1900119	positive regulation of execution phase of apoptosis	1/2279	15/18866	0.8551268	0.9050418	0.8189139	1	TP53BP2
Biological Process	GO:1902260	negative regulation of delayed rectifier potassium channel activity	1/2279	15/18866	0.8551268	0.9050418	0.8189139	1	KCNE1

Biological Process	GO:1905146	lysosomal protein catabolic process	1/2279	15/18866	0.8551268	0.9050418	0.8189139	1	MGAT3
Biological Process	GO:2000095	regulation of Wnt signaling pathway, planar cell polarity pathway	1/2279	15/18866	0.8551268	0.9050418	0.8189139	1	ANKRD6
Biological Process	GO:2001028	positive regulation of endothelial cell chemotaxis	1/2279	15/18866	0.8551268	0.9050418	0.8189139	1	FGFR1
Biological Process	GO:2001171	positive regulation of ATP biosynthetic process	1/2279	15/18866	0.8551268	0.9050418	0.8189139	1	ENO1
Biological Process	GO:0010469	regulation of signaling receptor activity	17/2279	174/18866	0.8556866	0.9052524	0.8191046	17	PDE4D/PLAU/TGFA/IL10/CCR2/NCK2/LY6G6D/APP/DLGAP4/CRHBP/CNIH2/BEGAIN/MAPK8IP2/GSG1L/MEF2C/SHANK2/NPTX1
Biological Process	GO:0090398	cellular senescence	7/2279	79/18866	0.8556893	0.9052524	0.8191046	7	CALR/MAP3K3/NSMCE2/AKT3/VASH1/ARNTL/NEK6
Biological Process	GO:1900034	regulation of cellular response to heat	7/2279	79/18866	0.8556893	0.9052524	0.8191046	7	RPTOR/RAE1/NUP93/DNAJB6/SEC13/CREBBP/BAG5
Biological Process	GO:0001658	branching involved in ureteric bud morphogenesis	5/2279	59/18866	0.855707	0.9052524	0.8191046	5	TACSTD2/ILK/PAX2/BCL2/HOXB7
Biological Process	GO:0003254	regulation of membrane depolarization	4/2279	49/18866	0.8588085	0.9083313	0.8218904	4	GJA5/HSH2D/SMAD7/BCL2

Biological Process	GO:0021517	ventral spinal cord development	4/2279	49/18866	0.8588085	0.9083313	0.8218904	4	DCTN1/PHOX2A/SUFU/LMO4
Biological Process	GO:0015837	amine transport	9/2279	99/18866	0.8595619	0.9088406	0.8223512	9	DDC/SLC43A2/PER2/SYT17/SLC43A1/DTNBP1/AQP9/GRK2/PRKN
Biological Process	GO:0003341	cilium movement	15/2279	156/18866	0.8595771	0.9088406	0.8223512	15	PLTP/RFX3/RNASE10/DEFB1/IQCG/STK36/ARMC2/DNAI2/CCNYL1/TTLL1/NPHP4/CFAP221/KATNIP/CFAP61/CFAP46
Biological Process	GO:0034401	chromatin organization involved in regulation of transcription	15/2279	156/18866	0.8595771	0.9088406	0.8223512	15	SERTAD2/PADI2/HDAC1/ZMIZ1/KMT2D/CTBP1/ARID1B/ATF7IP/SMARCD1/DNMT1/ZNFX1/DYDC2/DYDC1/MACROH2A2/H3C1
Biological Process	GO:0019724	B cell mediated immunity	23/2279	230/18866	0.8601477	0.9093428	0.8228056	23	IL10/CD55/INPP5D/TNF/IGHV6-1/CD226/PTPN6/CLU/UNG/TGFB1/C1S/PTPRC/BCL10/POU2F2/TP53BP1/ERCC1/TNFSF13/C1QB/BCL3/PAXIP1/C1RL/NSD2/SLA2
Biological Process	GO:0042445	hormone metabolic process	22/2279	221/18866	0.8609608	0.910101	0.8234917	22	CTSZ/PNPLA2/CTSG/DHRS9/AKR1D1/HSD17B2/TG/COMT/TCF7L2/PCSK6/FDX1/CRHBP/RDH10/DGKQ/POR/CPQ/GNB3/CTSB/CRYM/ALDH1A2/PRLHR/AOPEP
Biological Process	GO:0055123	digestive system development	14/2279	147/18866	0.8620062	0.9111048	0.8244	14	SMAD3/TNF/EPHB3/TGFBR2/WNT11/RB1/BCL2/NOTCH1/PDGFC/HLX/CHD8/ALDH1A2/NPY/NODAL
Biological Process	GO:0007389	pattern specification process	48/2279	455/18866	0.8623066	0.9113209	0.8245955	48	NRP2/SHROOM3/LRP5L/SKI/CHSY1/SMAD3/PKD1L1/SUFU/ZEB2/HIPK2/MICAL2/ZBTB16/TGFBR2/RFX3/DLL1/RBPJ/MIB1/PCSK6/WNT11/PAX2/AP1B1/FGFR1/SYNGAP1/PAX7/DSCAML1/RARG/BMPR1B/IFT140/MEIS2/ENG/HOXA9/MEIS1/TTC21B/NOTCH1/ALX3/MEF2C/TMED2/HHEX/ASPH/DNAI2/SSBP3/ALDH1A2/NODAL/HES3/OTX2/NTF4/HOXB7/GRSF1
Biological Process	GO:0009408	response to heat	16/2279	166/18866	0.8636078	0.9125946	0.825748	16	RPTOR/RAE1/EIF2B5/ABCC2/THBS1/NF1/NUP93/TGFB1I1/DNAJB6/SEC13/CREBBP/CPB2/TRPV2/CASQ1/BAG5/SST

Biological Process	GO:0006879	cellular iron ion homeostasis	6/2279	70/18866	0.864192	0.9128552	0.8259838	6	LCN2/SLC11A1/TTC7A/SLC11A2/LTF/NCOA4
Biological Process	GO:0032374	regulation of cholesterol transport	6/2279	70/18866	0.864192	0.9128552	0.8259838	6	ABCG1/PLTP/MIR27A/OSBPL6/NR1H2/ABCA1
Biological Process	GO:0035082	axoneme assembly	6/2279	70/18866	0.864192	0.9128552	0.8259838	6	DNAJB13/IQCG/ARMC2/DNAI2/TTLL1/CFAP46
Biological Process	GO:0009988	cell-cell recognition	7/2279	80/18866	0.8643349	0.9128552	0.8259838	7	CD9/FOLR3/NCK2/CLGN/DOCK8/UBAP2L/DOCK2
Biological Process	GO:0070828	heterochromatin organization	7/2279	80/18866	0.8643349	0.9128552	0.8259838	7	HDAC1/ATF7IP/PRDM16/DNMT1/ZNFX1/MECOM/H3C1
Biological Process	GO:0051784	negative regulation of nuclear division	5/2279	60/18866	0.8655528	0.913813	0.8268505	5	MAD1L1/TNKS/AURKAIP1/LIF/PCID2
Biological Process	GO:1902808	positive regulation of cell cycle G1/S phase transition	5/2279	60/18866	0.8655528	0.913813	0.8268505	5	RPTOR/TFDP1/PLCB1/PLRG1/EIF4G1
Biological Process	GO:0007210	serotonin receptor signaling pathway	3/2279	39/18866	0.8660112	0.913813	0.8268505	3	HRH1/HTR3A/CHRM2
Biological Process	GO:0007431	salivary gland development	3/2279	39/18866	0.8660112	0.913813	0.8268505	3	TNF/FGFR1/TGFB1

Biological Process	GO:0030049	muscle filament sliding	3/2279	39/18866	0.8660112	0.913813	0.8268505	3	TTN/TNNT3/TMOD1
Biological Process	GO:0033275	actin-myosin filament sliding	3/2279	39/18866	0.8660112	0.913813	0.8268505	3	TTN/TNNT3/TMOD1
Biological Process	GO:1902116	negative regulation of organelle assembly	3/2279	39/18866	0.8660112	0.913813	0.8268505	3	DNM2/FEZ2/ARHGEF2
Biological Process	GO:2000279	negative regulation of DNA biosynthetic process	3/2279	39/18866	0.8660112	0.913813	0.8268505	3	PPARG/NPPC/ANKRD1
Biological Process	GO:0010460	positive regulation of heart rate	2/2279	28/18866	0.868383	0.9151976	0.8281033	2	PDE4D/KCNQ1
Biological Process	GO:0015867	ATP transport	2/2279	28/18866	0.868383	0.9151976	0.8281033	2	SLC25A25/SLC25A1
Biological Process	GO:0045686	negative regulation of glial cell differentiation	2/2279	28/18866	0.868383	0.9151976	0.8281033	2	NF1/NOTCH1
Biological Process	GO:0045932	negative regulation of muscle contraction	2/2279	28/18866	0.868383	0.9151976	0.8281033	2	BIN1/GRK2
Biological Process	GO:0072376	protein activation cascade	2/2279	28/18866	0.868383	0.9151976	0.8281033	2	GP9/F12

Biological Process	GO:0072378	blood coagulation, fibrin clot formation	2/2279	28/18866	0.868383	0.9151976	0.8281033	2	GP9/F12
Biological Process	GO:0080111	DNA demethylation	2/2279	28/18866	0.868383	0.9151976	0.8281033	2	FTO/TET2
Biological Process	GO:0099560	synaptic membrane adhesion	2/2279	28/18866	0.868383	0.9151976	0.8281033	2	SPARCL1/NRXN1
Biological Process	GO:1903579	negative regulation of ATP metabolic process	2/2279	28/18866	0.868383	0.9151976	0.8281033	2	HDAC4/CBFA2T3
Biological Process	GO:1903588	negative regulation of blood vessel endothelial cell proliferation involved in sprouting angiogenesis	2/2279	28/18866	0.868383	0.9151976	0.8281033	2	THBS1/MIR24-2
Biological Process	GO:2000191	regulation of fatty acid transport	2/2279	28/18866	0.868383	0.9151976	0.8281033	2	MAP2K6/THBS1
Biological Process	GO:0021695	cerebellar cortex development	4/2279	50/18866	0.8693196	0.91588	0.8287207	4	DLL1/TTC21B/TTLL1/COQ8B
Biological Process	GO:0071715	icosanoid transport	4/2279	50/18866	0.8693196	0.91588	0.8287207	4	ABCC1/MAP2K6/ABCC2/SLCO3A1
Biological Process	GO:1901571	fatty acid derivative transport	4/2279	50/18866	0.8693196	0.91588	0.8287207	4	ABCC1/MAP2K6/ABCC2/SLCO3A1

Biological Process	GO:0021766	hippocampus development	7/2279	81/18866	0.8725511	0.9165339	0.8293124	7	MFSD2A/ZEB2/EIF2B5/HDAC1/RARA/KIRREL3/NCOA1
Biological Process	GO:0002070	epithelial cell maturation	1/2279	16/18866	0.8726413	0.9165339	0.8293124	1	RFX3
Biological Process	GO:0002834	regulation of response to tumor cell	1/2279	16/18866	0.8726413	0.9165339	0.8293124	1	CD226
Biological Process	GO:0002837	regulation of immune response to tumor cell	1/2279	16/18866	0.8726413	0.9165339	0.8293124	1	CD226
Biological Process	GO:0006098	pentose-phosphate shunt	1/2279	16/18866	0.8726413	0.9165339	0.8293124	1	PGAM1
Biological Process	GO:0006390	mitochondrial transcription	1/2279	16/18866	0.8726413	0.9165339	0.8293124	1	PRKAA1
Biological Process	GO:0006544	glycine metabolic process	1/2279	16/18866	0.8726413	0.9165339	0.8293124	1	AGXT
Biological Process	GO:0007351	tripartite regional subdivision	1/2279	16/18866	0.8726413	0.9165339	0.8293124	1	PCSK6
Biological Process	GO:0007379	segment specification	1/2279	16/18866	0.8726413	0.9165339	0.8293124	1	DLL1

Biological Process	GO:0008595	anterior/posterior axis specification, embryo	1/2279	16/18866	0.8726413	0.9165339	0.8293124	1	PCSK6
Biological Process	GO:0009208	pyrimidine ribonucleoside triphosphate metabolic process	1/2279	16/18866	0.8726413	0.9165339	0.8293124	1	UCK2
Biological Process	GO:0009812	flavonoid metabolic process	1/2279	16/18866	0.8726413	0.9165339	0.8293124	1	POR
Biological Process	GO:0015812	gamma-aminobutyric acid transport	1/2279	16/18866	0.8726413	0.9165339	0.8293124	1	NF1
Biological Process	GO:0016446	somatic hypermutation of immunoglobulin genes	1/2279	16/18866	0.8726413	0.9165339	0.8293124	1	UNG
Biological Process	GO:0031269	pseudopodium assembly	1/2279	16/18866	0.8726413	0.9165339	0.8293124	1	CDC42EP1
Biological Process	GO:0042487	regulation of odontogenesis of dentin-containing tooth	1/2279	16/18866	0.8726413	0.9165339	0.8293124	1	RUNX2
Biological Process	GO:0043508	negative regulation of JUN kinase activity	1/2279	16/18866	0.8726413	0.9165339	0.8293124	1	HIPK3
Biological Process	GO:0045116	protein neddylation	1/2279	16/18866	0.8726413	0.9165339	0.8293124	1	NAE1

Biological Process	GO:0046500	S-adenosylmethionine metabolic process	1/2279	16/18866	0.8726413	0.9165339	0.8293124	1	MAT1A
Biological Process	GO:0046831	regulation of RNA export from nucleus	1/2279	16/18866	0.8726413	0.9165339	0.8293124	1	SETD2
Biological Process	GO:0060572	morphogenesis of an epithelial bud	1/2279	16/18866	0.8726413	0.9165339	0.8293124	1	RDH10
Biological Process	GO:0060628	regulation of ER to Golgi vesicle-mediated transport	1/2279	16/18866	0.8726413	0.9165339	0.8293124	1	SORL1
Biological Process	GO:0061548	ganglion development	1/2279	16/18866	0.8726413	0.9165339	0.8293124	1	NRP2
Biological Process	GO:0080009	mRNA methylation	1/2279	16/18866	0.8726413	0.9165339	0.8293124	1	METTL8
Biological Process	GO:1900451	positive regulation of glutamate receptor signaling pathway	1/2279	16/18866	0.8726413	0.9165339	0.8293124	1	CCR2
Biological Process	GO:1903541	regulation of exosomal secretion	1/2279	16/18866	0.8726413	0.9165339	0.8293124	1	PRKN
Biological Process	GO:1905050	positive regulation of metallopeptidase activity	1/2279	16/18866	0.8726413	0.9165339	0.8293124	1	MBP

Biological Process	GO:2001034	positive regulation of double-strand break repair via nonhomologous end joining	1/2279	16/18866	0.8726413	0.9165339	0.8293124	1	PARP9
Biological Process	GO:0045921	positive regulation of exocytosis	8/2279	91/18866	0.8731982	0.9170174	0.8297499	8	CD177/GAB2/FGR/RAB27A/HLA-F/SPHK2/ITGB2/DNM1L
Biological Process	GO:0019217	regulation of fatty acid metabolic process	9/2279	101/18866	0.8745316	0.9183162	0.8309251	9	PRKAG2/PPARG/MFSD2A/NR1H2/ACACB/SREBF1/WDTC1/CPT1A/ACADVL
Biological Process	GO:0009566	fertilization	19/2279	196/18866	0.8751226	0.9188352	0.8313947	19	CECR2/CD9/ACRBP/HEXB/FOLR3/MEIOB/RNASE10/CLGN/IGSF8/LHFPL2/PLCB1/HOXA9/RAD21L1/UBAP2L/UBE3A/BCL2L1/SPTBN4/UBXN8/IRAG2
Biological Process	GO:0010719	negative regulation of epithelial to mesenchymal transition	3/2279	40/18866	0.8771597	0.9205671	0.8329618	3	SPRED2/LDLRAD4/SMAD7
Biological Process	GO:0030488	tRNA methylation	3/2279	40/18866	0.8771597	0.9205671	0.8329618	3	THADA/METTL6/METTL8
Biological Process	GO:0098534	centriole assembly	3/2279	40/18866	0.8771597	0.9205671	0.8329618	3	CEP135/CEP72/CEP295NL
Biological Process	GO:1903427	negative regulation of reactive oxygen species biosynthetic process	3/2279	40/18866	0.8771597	0.9205671	0.8329618	3	IL10/MIR199A1/MIR199A2
Biological Process	GO:0009308	amine metabolic process	13/2279	140/18866	0.8773812	0.9206979	0.8330801	13	ABCC2/ODC1/COMT/DDC/SULT1A2/SLC7A7/PSMF1/ITGB2/PSMB7/SAT2/HNMT/PSMD13/PRKN

Biological Process	GO:1902600	proton transmembrane transport	15/2279	159/18866	0.8775805	0.9208053	0.8331773	15	SLC11A2/TESC/SLC2A9/ATP6V1B2/ACTN4/VPS9D1/SPHK2/ATP2A3/SLC36A3/SLC33A1/ATP6V0B/NDUFA4L2/ATP5PD/ATP6V0C/ATP6V1C1
Biological Process	GO:0016079	synaptic vesicle exocytosis	11/2279	121/18866	0.8784607	0.9216271	0.8339209	11	RAP1B/GIT1/APBA2/PRKCB/VPS18/CTBP2/CHRM2/P2RX1/DNM1L/SYT17/DTNBP1
Biological Process	GO:0065004	protein-DNA complex assembly	24/2279	243/18866	0.8790346	0.9221274	0.8343736	24	TAF1C/CREB1/UBN1/RAD52/CABIN1/MCM5/SMYD3/MIS12/ATF7IP/MCM2/BRF1/RSF1/CCNH/RB1/DMC1/PIH1D1/CENPM/RUVBL1/CHAF1A/XPA/ERCC2/BRD2/MACROH2A2/H3C1
Biological Process	GO:0031113	regulation of microtubule polymerization	4/2279	51/18866	0.8791496	0.9221462	0.8343906	4	TBCD/DCTN1/NAV3/NUMA1
Biological Process	GO:1903313	positive regulation of mRNA metabolic process	7/2279	82/18866	0.8803513	0.9233048	0.8354389	7	PRR5L/TNRC6B/ZC3H12D/AGO2/CPEB3/DAZAP1/FTO
Biological Process	GO:0038095	Fc-epsilon receptor signaling pathway	16/2279	169/18866	0.8807521	0.923394	0.8355197	16	LAT/PIK3R1/GAB2/GRAP2/NFATC2/NFATC1/FER/VAV2/BCL10/PSMF1/PSMB7/PLCG1/RELA/FBXW11/PSMD13/VAV3
Biological Process	GO:0044106	cellular amine metabolic process	12/2279	131/18866	0.8808068	0.923394	0.8355197	12	ABCC2/ODC1/COMT/DDC/SLC7A7/PSMF1/ITGB2/PSMB7/SAT2/HNMT/PSMD13/PRKN
Biological Process	GO:0006515	protein quality control for misfolded or incompletely synthesized proteins	2/2279	29/18866	0.8810195	0.923394	0.8355197	2	CUL3/POMT2
Biological Process	GO:0008209	androgen metabolic process	2/2279	29/18866	0.8810195	0.923394	0.8355197	2	DHRS9/AKR1D1

Biological Process	GO:0032196	transposition	2/2279	29/18866	0.8810195	0.923394	0.8355197	2	PIWIL4/MORC1
Biological Process	GO:0036151	phosphatidylcholine acyl-chain remodeling	2/2279	29/18866	0.8810195	0.923394	0.8355197	2	LPCAT1/NR1H2
Biological Process	GO:0019226	transmission of nerve impulse	6/2279	72/18866	0.8811726	0.9234526	0.8355727	6	SCN1A/JAM3/GHRL/P2RX1/CNTNAP1/SPTBN4
Biological Process	GO:0032355	response to estradiol	13/2279	141/18866	0.8833001	0.92558	0.8374976	13	CALR/IL10/NCOR2/GGT1/ABCC2/TGFB1/CRHBP/RARA/NCOA1/CAT/ETS1/PDGFRB/ALDH1A2
Biological Process	GO:0001539	cilium or flagellum-dependent cell motility	11/2279	122/18866	0.8847446	0.9268893	0.8386823	11	DNAH3/PLTP/RFX3/RNASE10/DEFB1/IQCG/ARMC2/CCNYL1/TTLL1/NPHP4/CFAP46
Biological Process	GO:0060285	cilium-dependent cell motility	11/2279	122/18866	0.8847446	0.9268893	0.8386823	11	DNAH3/PLTP/RFX3/RNASE10/DEFB1/IQCG/ARMC2/CCNYL1/TTLL1/NPHP4/CFAP46
Biological Process	GO:0008037	cell recognition	22/2279	226/18866	0.8857162	0.9269669	0.8387526	22	BSG/CD9/NCAM2/FOLR3/NCK2/EPHB3/IGHV6-1/RTN4/CD226/APP/COLEC12/TNFRSF21/CLGN/DOCK8/FCN1/DSCAML1/YWHAZ/UBAP2L/PCDH12/DOCK2/CADM1/OPCML
Biological Process	GO:0033574	response to testosterone	3/2279	41/18866	0.8874761	0.9269669	0.8387526	3	CALR/THBS1/NCOA4
Biological Process	GO:0046825	regulation of protein export from nucleus	3/2279	41/18866	0.8874761	0.9269669	0.8387526	3	SETD2/TCF7L2/SP100

Biological Process	GO:0050434	positive regulation of viral transcription	3/2279	41/18866	0.8874761	0.9269669	0.8387526	3	CTDP1/POLR2F/RSF1
Biological Process	GO:1900117	regulation of execution phase of apoptosis	3/2279	41/18866	0.8874761	0.9269669	0.8387526	3	PAM16/TP53BP2/BCL2L1
Biological Process	GO:0031146	SCF-dependent proteasomal ubiquitin-dependent protein catabolic process	8/2279	93/18866	0.887592	0.9269669	0.8387526	8	WWTR1/PSMF1/FBXL13/FBXL18/PSMB7/FBXL6/FBXW11/PSMD13
Biological Process	GO:0031145	anaphase-promoting complex-dependent catabolic process	7/2279	83/18866	0.8877494	0.9269669	0.8387526	7	FZR1/CUL3/CDC16/PSMF1/PSMB7/ANAPC7/PSMD13
Biological Process	GO:0000963	mitochondrial RNA processing	1/2279	17/18866	0.8880392	0.9269669	0.8387526	1	TBRG4
Biological Process	GO:0002566	somatic diversification of immune receptors via somatic mutation	1/2279	17/18866	0.8880392	0.9269669	0.8387526	1	UNG
Biological Process	GO:0006586	indolalkylamine metabolic process	1/2279	17/18866	0.8880392	0.9269669	0.8387526	1	DDC
Biological Process	GO:0007635	chemosensory behavior	1/2279	17/18866	0.8880392	0.9269669	0.8387526	1	NTRK1
Biological Process	GO:0010566	regulation of ketone biosynthetic process	1/2279	17/18866	0.8880392	0.9269669	0.8387526	1	DGKQ

Biological Process	GO:0030322	stabilization of membrane potential	1/2279	17/18866	0.8880392	0.9269669	0.8387526	1	KCNK6
Biological Process	GO:0030540	female genitalia development	1/2279	17/18866	0.8880392	0.9269669	0.8387526	1	TYRO3
Biological Process	GO:0031268	pseudopodium organization	1/2279	17/18866	0.8880392	0.9269669	0.8387526	1	CDC42EP1
Biological Process	GO:0031649	heat generation	1/2279	17/18866	0.8880392	0.9269669	0.8387526	1	TNF
Biological Process	GO:0032305	positive regulation of icosanoid secretion	1/2279	17/18866	0.8880392	0.9269669	0.8387526	1	MAP2K6
Biological Process	GO:0035518	histone H2A monoubiquitination	1/2279	17/18866	0.8880392	0.9269669	0.8387526	1	PCGF3
Biological Process	GO:0043923	positive regulation by host of viral transcription	1/2279	17/18866	0.8880392	0.9269669	0.8387526	1	CTDP1
Biological Process	GO:0045722	positive regulation of gluconeogenesis	1/2279	17/18866	0.8880392	0.9269669	0.8387526	1	FOXO1
Biological Process	GO:0048243	norepinephrine secretion	1/2279	17/18866	0.8880392	0.9269669	0.8387526	1	NISCH

Biological Process	GO:0051382	kinetochore assembly	1/2279	17/18866	0.8880392	0.9269669	0.8387526	1	MIS12
Biological Process	GO:0051969	regulation of transmission of nerve impulse	1/2279	17/18866	0.8880392	0.9269669	0.8387526	1	GHRL
Biological Process	GO:0055089	fatty acid homeostasis	1/2279	17/18866	0.8880392	0.9269669	0.8387526	1	PRKAA1
Biological Process	GO:0061687	detoxification of inorganic compound	1/2279	17/18866	0.8880392	0.9269669	0.8387526	1	ABCC2
Biological Process	GO:0071850	mitotic cell cycle arrest	1/2279	17/18866	0.8880392	0.9269669	0.8387526	1	CDC14A
Biological Process	GO:0090494	dopamine uptake	1/2279	17/18866	0.8880392	0.9269669	0.8387526	1	PRKN
Biological Process	GO:0097094	craniofacial suture morphogenesis	1/2279	17/18866	0.8880392	0.9269669	0.8387526	1	FOXN3
Biological Process	GO:1901673	regulation of mitotic spindle assembly	1/2279	17/18866	0.8880392	0.9269669	0.8387526	1	RCC1
Biological Process	GO:1902931	negative regulation of alcohol biosynthetic process	1/2279	17/18866	0.8880392	0.9269669	0.8387526	1	GFI1

Biological Process	GO:1903209	positive regulation of oxidative stress-induced cell death	1/2279	17/18866	0.8880392	0.9269669	0.8387526	1	RIPK1
Biological Process	GO:1903818	positive regulation of voltage-gated potassium channel activity	1/2279	17/18866	0.8880392	0.9269669	0.8387526	1	NOS1AP
Biological Process	GO:2000001	regulation of DNA damage checkpoint	1/2279	17/18866	0.8880392	0.9269669	0.8387526	1	CHEK2
Biological Process	GO:0006301	postreplication repair	4/2279	52/18866	0.8883311	0.9269669	0.8387526	4	UBE2V1/POLD3/PARP10/POLI
Biological Process	GO:0045839	negative regulation of mitotic nuclear division	4/2279	52/18866	0.8883311	0.9269669	0.8387526	4	MAD1L1/TNKS/AURKAIP1/PCID2
Biological Process	GO:2000677	regulation of transcription regulatory region DNA binding	4/2279	52/18866	0.8883311	0.9269669	0.8387526	4	TGFB1/PER2/RB1/LIF
Biological Process	GO:0072078	nephron tubule morphogenesis	6/2279	73/18866	0.888969	0.9274334	0.8391747	6	TACSTD2/ILK/WNT11/PAX2/BCL2/HOXB7
Biological Process	GO:0071824	protein-DNA complex subunit organization	28/2279	282/18866	0.8889734	0.9274334	0.8391747	28	TAF1C/SRCAP/CREB1/UBN1/RAD52/SETD2/CABIN1/MCM5/SMYD3/MIS12/ATF7IP/MCM2/BRF1/RSF1/CCNH/SMARCD1/SMARCD2/RB1/DMC1/PIH1D1/CENPM/RUVBL1/CHAF1A/XPA/ERCC2/BRD2/MACROH2A2/H3C1
Biological Process	GO:0002223	stimulatory C-type lectin receptor signaling pathway	10/2279	113/18866	0.8892811	0.9276526	0.839373	10	CLEC4C/MUC12/MUC20/BCL10/PSMF1/CREBBP/PSMB7/RELA/FBXW11/PSMD13

Biological Process	GO:0003002	regionalization	36/2279	355/18866	0.8897126	0.9280008	0.8396881	36	NRP2/LRP5L/SKI/CHSY1/SMAD3/PKD1L1/SUFU/ZEB2/HIPK2/ZBTB16/DLL1/RBPJ/MIB1/PCSK6/WNT11/PAX2/FGFR1/PAX7/DSCAML1/RARG/BMPR1B/IFT140/HOXA9/TTC21B/NOTCH1/MEF2C/TMED2/HHEX/SSBP3/ALDH1A2/NODAL/HES3/OTX2/NTF4/HOXB7/GRSF1
Biological Process	GO:0016064	immunoglobulin mediated immune response	22/2279	227/18866	0.8902246	0.9284329	0.8400791	22	IL10/CD55/INPP5D/TNF/IGHV6-1/CD226/PTPN6/CLU/UNG/TGFB1/C1S/PTPRC/BCL10/POU2F2/TP53BP1/ERCC1/TNFSF13/C1QB/BCL3/PAXIP1/C1RL/NSD2
Biological Process	GO:0070507	regulation of microtubule cytoskeleton organization	18/2279	190/18866	0.8918481	0.930024	0.8415188	18	TBCD/DCTN1/RAE1/RCC1/DYNC1H1/NAV3/POC1A/BICD2/TACC3/NUMA1/MAP1A/ARHGEF2/CYLD/SPAG5/MARK2/SPEF1/PRKAA1/CEP295NL
Biological Process	GO:0003401	axis elongation	2/2279	30/18866	0.8925253	0.9302198	0.8416959	2	WNT11/RDH10
Biological Process	GO:0016056	rhodopsin mediated signaling pathway	2/2279	30/18866	0.8925253	0.9302198	0.8416959	2	PDE6A/GRK7
Biological Process	GO:0043457	regulation of cellular respiration	2/2279	30/18866	0.8925253	0.9302198	0.8416959	2	CBFA2T3/PRDM16
Biological Process	GO:0062125	regulation of mitochondrial gene expression	2/2279	30/18866	0.8925253	0.9302198	0.8416959	2	MTG2/PRKAA1
Biological Process	GO:0071711	basement membrane organization	2/2279	30/18866	0.8925253	0.9302198	0.8416959	2	SPINT2/LAMB3
Biological Process	GO:0015844	monoamine transport	7/2279	84/18866	0.8947594	0.9324459	0.8437101	7	NISCH/ITGB3/P2RX1/SYT17/DTNBP1/GRK2/PRKN

Biological Process	GO:0000375	RNA splicing, via transesterification reactions	40/2279	393/18866	0.8960606	0.9336994	0.8448444	40	HNRNPUL1/RBM19/FIP1L1/LSM1/CPSF3/SNRPD3/SF3B3/SRPK2/POLR2F/PCBP4/PABPN1/SF1/LSM7/PRPF8/DAZAP1/SFSWAP/CELF2/NOL3/CELF1/SNRNP35/SNUPN/DHX16/USP49/CTNNBL1/DDX41/RBFOX3/PRPF6/PRPF40B/NSRP1/RBM15B/CHERP/PLRG1/SRRM2/CPSF4/ZCCHC8/SYNCRIP/RALY/CDK13/GEMIN4/TFIP11
Biological Process	GO:0032210	regulation of telomere maintenance via telomerase	4/2279	53/18866	0.8968965	0.9341768	0.8452764	4	TNKS/HMBOX1/SMG6/CCT6A
Biological Process	GO:0043331	response to dsRNA	4/2279	53/18866	0.8968965	0.9341768	0.8452764	4	COLEC12/FLOT1/PELI1/STING1
Biological Process	GO:0019985	translesion synthesis	3/2279	42/18866	0.8970103	0.9341768	0.8452764	3	POLD3/PARP10/POLI
Biological Process	GO:0033048	negative regulation of mitotic sister chromatid segregation	3/2279	42/18866	0.8970103	0.9341768	0.8452764	3	MAD1L1/TNKS/PCID2
Biological Process	GO:1900371	regulation of purine nucleotide biosynthetic process	3/2279	42/18866	0.8970103	0.9341768	0.8452764	3	RCVRN/ENO1/SPHK2
Biological Process	GO:0000956	nuclear-transcribed mRNA catabolic process	20/2279	210/18866	0.8974791	0.9345626	0.8456254	20	LSM1/EXOSC2/TNRC6B/AGO1/CSDE1/SAMD4A/SMG7/CTIF/AGO2/EDC3/LSM7/CPEB3/SMG6/PPP2R1A/NBAS/DIS3L2/SECISBP2/RPL23A/EIF4G1/PCID2
Biological Process	GO:0002920	regulation of humoral immune response	12/2279	134/18866	0.8979979	0.9350004	0.8460215	12	C3AR1/CD55/TNF/C5AR2/PTPN6/CLU/PHB/CD59/C1S/PTPRC/CPB2/C1QB
Biological Process	GO:0046686	response to cadmium ion	5/2279	64/18866	0.8994438	0.9362636	0.8471645	5	GSN/MTF1/CAT/NPC1/ERCC1

Biological Process	GO:0006333	chromatin assembly or disassembly	21/2279	220/18866	0.9002211	0.9362636	0.8471645	21	BAZ1B/UBN1/CABIN1/PADI2/SMYD3/HDAC1/ATF7IP/MCM2/RSF1/MTA2/SMARCD1/SMARCD2/DNMT1/CENPM/ZNFX1/RUVBL1/CHAF1A/PARP10/BRD2/MACROH2A2/H3C1
Biological Process	GO:0000245	spliceosomal complex assembly	7/2279	85/18866	0.9013954	0.9362636	0.8471645	7	SRPK2/SF1/SFSWAP/CELF2/NOL3/CELF1/PRPF6
Biological Process	GO:0031110	regulation of microtubule polymerization or depolymerization	7/2279	85/18866	0.9013954	0.9362636	0.8471645	7	TBCD/DCTN1/NAV3/NUMA1/MAP1A/ARHGEF2/SPEF1
Biological Process	GO:0001522	pseudouridine synthesis	1/2279	18/18866	0.9015762	0.9362636	0.8471645	1	PUS7
Biological Process	GO:0006740	NADPH regeneration	1/2279	18/18866	0.9015762	0.9362636	0.8471645	1	PGAM1
Biological Process	GO:0006883	cellular sodium ion homeostasis	1/2279	18/18866	0.9015762	0.9362636	0.8471645	1	SLC8A1
Biological Process	GO:0010839	negative regulation of keratinocyte proliferation	1/2279	18/18866	0.9015762	0.9362636	0.8471645	1	VDR
Biological Process	GO:0032048	cardiolipin metabolic process	1/2279	18/18866	0.9015762	0.9362636	0.8471645	1	PGS1
Biological Process	GO:0036148	phosphatidylglycerol acyl-chain remodeling	1/2279	18/18866	0.9015762	0.9362636	0.8471645	1	LPCAT1

Biological Process	GO:0042136	neurotransmitter biosynthetic process	1/2279	18/18866	0.9015762	0.9362636	0.8471645	1	SLC44A4
Biological Process	GO:0045187	regulation of circadian sleep/wake cycle, sleep	1/2279	18/18866	0.9015762	0.9362636	0.8471645	1	GHRL
Biological Process	GO:0046112	nucleobase biosynthetic process	1/2279	18/18866	0.9015762	0.9362636	0.8471645	1	DHODH
Biological Process	GO:0048268	clathrin coat assembly	1/2279	18/18866	0.9015762	0.9362636	0.8471645	1	EPS15
Biological Process	GO:0048557	embryonic digestive tract morphogenesis	1/2279	18/18866	0.9015762	0.9362636	0.8471645	1	HLX
Biological Process	GO:0052695	cellular glucuronidation	1/2279	18/18866	0.9015762	0.9362636	0.8471645	1	PRKCE
Biological Process	GO:0060004	reflex	1/2279	18/18866	0.9015762	0.9362636	0.8471645	1	TMC1
Biological Process	GO:0060046	regulation of acrosome reaction	1/2279	18/18866	0.9015762	0.9362636	0.8471645	1	PLCB1
Biological Process	GO:0071318	cellular response to ATP	1/2279	18/18866	0.9015762	0.9362636	0.8471645	1	SSH1

Biological Process	GO:0071605	monocyte chemotactic protein-1 production	1/2279	18/18866	0.9015762	0.9362636	0.8471645	1	C1QTNF3
Biological Process	GO:0071637	regulation of monocyte chemotactic protein-1 production	1/2279	18/18866	0.9015762	0.9362636	0.8471645	1	C1QTNF3
Biological Process	GO:0072079	nephron tubule formation	1/2279	18/18866	0.9015762	0.9362636	0.8471645	1	PAX2
Biological Process	GO:0090493	catecholamine uptake	1/2279	18/18866	0.9015762	0.9362636	0.8471645	1	PRKN
Biological Process	GO:0150078	positive regulation of neuroinflammatory response	1/2279	18/18866	0.9015762	0.9362636	0.8471645	1	TNF
Biological Process	GO:0002675	positive regulation of acute inflammatory response	2/2279	31/18866	0.9029891	0.9370139	0.8478434	2	TNF/F12
Biological Process	GO:0007143	female meiotic nuclear division	2/2279	31/18866	0.9029891	0.9370139	0.8478434	2	MEIOB/PPP2R1A
Biological Process	GO:0021772	olfactory bulb development	2/2279	31/18866	0.9029891	0.9370139	0.8478434	2	SKI/CSF1R
Biological Process	GO:0036257	multivesicular body organization	2/2279	31/18866	0.9029891	0.9370139	0.8478434	2	RAB27A/MVB12A

Biological Process	GO:0039528	cytoplasmic pattern recognition receptor signaling pathway in response to virus	2/2279	31/18866	0.9029891	0.9370139	0.8478434	2	PHB/SEC14L1
Biological Process	GO:0060292	long-term synaptic depression	2/2279	31/18866	0.9029891	0.9370139	0.8478434	2	SORCS2/SHANK2
Biological Process	GO:0090075	relaxation of muscle	2/2279	31/18866	0.9029891	0.9370139	0.8478434	2	PDE4D/SLC8A1
Biological Process	GO:0006414	translational elongation	12/2279	135/18866	0.9032541	0.9371866	0.8479997	12	TRNAU1AP/MRPL3/MRPL38/CPEB3/MRPS21/MRPS26/ZNF598/MRPL28/SECISBP2/AURKAIP1/EEF1D/SHFL
Biological Process	GO:0000413	protein peptidyl-prolyl isomerization	3/2279	43/18866	0.9058109	0.939224	0.8498432	3	FKBP5/AIP/PTPA
Biological Process	GO:0030808	regulation of nucleotide biosynthetic process	3/2279	43/18866	0.9058109	0.939224	0.8498432	3	RCVRN/ENO1/SPHK2
Biological Process	GO:0034080	CENP-A containing nucleosome assembly	3/2279	43/18866	0.9058109	0.939224	0.8498432	3	RSF1/CENPM/RUVBL1
Biological Process	GO:0040019	positive regulation of embryonic development	3/2279	43/18866	0.9058109	0.939224	0.8498432	3	RBM19/PLCB1/OTX2
Biological Process	GO:0045823	positive regulation of heart contraction	3/2279	43/18866	0.9058109	0.939224	0.8498432	3	PDE4D/NOS1AP/KCNQ1

Biological Process	GO:0061641	CENP-A containing chromatin organization	3/2279	43/18866	0.9058109	0.939224	0.8498432	3	RSF1/CENPM/RUVBL1
Biological Process	GO:0030317	flagellated sperm motility	8/2279	96/18866	0.9066106	0.9396934	0.850268	8	PLTP/RNASE10/DEFB1/IQCG/ARMC2/CCNYL1/TTLL1/NPHP4
Biological Process	GO:0097722	sperm motility	8/2279	96/18866	0.9066106	0.9396934	0.850268	8	PLTP/RNASE10/DEFB1/IQCG/ARMC2/CCNYL1/TTLL1/NPHP4
Biological Process	GO:0010257	NADH dehydrogenase complex assembly	5/2279	65/18866	0.9066593	0.9396934	0.850268	5	NDUFAF6/NDUFS2/NDUFS8/NDUFB6/DMAC1
Biological Process	GO:0032981	mitochondrial respiratory chain complex I assembly	5/2279	65/18866	0.9066593	0.9396934	0.850268	5	NDUFAF6/NDUFS2/NDUFS8/NDUFB6/DMAC1
Biological Process	GO:0090150	establishment of protein localization to membrane	34/2279	342/18866	0.9074261	0.9403856	0.8508943	34	KCNE1/TFDP1/ZDHHC18/YWHAH/ITGB1BP1/BMF/ZDHHC14/KIF13A/COLQ/RAB31/RABGEF1/ZDHHC2/AKAP5/ZDHHC7/VAMP5/CSK/ZDHHC1/GORASP2/PRKCI/PEX26/ITGB2/NPC1/RILPL1/YWHAZ/MICALL1/FIS1/BCL2/NACAD/TP53BP2/MYO1C/MIEF1/RPL23A/YWHAQ/PDZK1
Biological Process	GO:0097756	negative regulation of blood vessel diameter	7/2279	86/18866	0.9076716	0.9405375	0.8510317	7	SLC8A1/GJA5/HRH1/PER2/P2RX1/F2R/AGTR1
Biological Process	GO:0050680	negative regulation of epithelial cell proliferation	16/2279	175/18866	0.9098781	0.942721	0.8530074	16	PPARG/BRCA2/VDR/TNF/THBS1/MIR24-2/FLT1/TGFB1/NF1/MTSS1/GHRL/VASH1/RB1/MEF2C/CPB2/RUNX3
Biological Process	GO:0022029	telencephalon cell migration	4/2279	55/18866	0.9123068	0.9442875	0.8544248	4	RTN4/DISC1/ZMIZ1/NDEL1

Biological Process	GO:0042304	regulation of fatty acid biosynthetic process	4/2279	55/18866	0.9123068	0.9442875	0.8544248	4	PRKAG2/NR1H2/WDTC1/ACADVL
Biological Process	GO:0050433	regulation of catecholamine secretion	4/2279	55/18866	0.9123068	0.9442875	0.8544248	4	SYT17/DTNBP1/GRK2/PRKN
Biological Process	GO:0006335	DNA replication-dependent nucleosome assembly	2/2279	32/18866	0.9124944	0.9442875	0.8544248	2	CHAF1A/H3C1
Biological Process	GO:0008210	estrogen metabolic process	2/2279	32/18866	0.9124944	0.9442875	0.8544248	2	HSD17B2/COMT
Biological Process	GO:0034723	DNA replication-dependent nucleosome organization	2/2279	32/18866	0.9124944	0.9442875	0.8544248	2	CHAF1A/H3C1
Biological Process	GO:0038128	ERBB2 signaling pathway	2/2279	32/18866	0.9124944	0.9442875	0.8544248	2	PIK3R1/PRKCA
Biological Process	GO:0051085	chaperone cofactor-dependent protein refolding	2/2279	32/18866	0.9124944	0.9442875	0.8544248	2	DNAJB13/TOR1B
Biological Process	GO:0071295	cellular response to vitamin	2/2279	32/18866	0.9124944	0.9442875	0.8544248	2	PPARG/VDR
Biological Process	GO:1901976	regulation of cell cycle checkpoint	2/2279	32/18866	0.9124944	0.9442875	0.8544248	2	CHEK2/PCID2

Biological Process	GO:1901607	alpha-amino acid biosynthetic process	5/2279	66/18866	0.9134179	0.9442875	0.8544248	5	GGT1/CBS/AGXT/MTHFD1/ADI1
Biological Process	GO:0002862	negative regulation of inflammatory response to antigenic stimulus	1/2279	19/18866	0.9134771	0.9442875	0.8544248	1	IL10
Biological Process	GO:0007039	protein catabolic process in the vacuole	1/2279	19/18866	0.9134771	0.9442875	0.8544248	1	MGAT3
Biological Process	GO:0007625	grooming behavior	1/2279	19/18866	0.9134771	0.9442875	0.8544248	1	NRXN1
Biological Process	GO:0009148	pyrimidine nucleoside triphosphate biosynthetic process	1/2279	19/18866	0.9134771	0.9442875	0.8544248	1	UCK2
Biological Process	GO:0031643	positive regulation of myelination	1/2279	19/18866	0.9134771	0.9442875	0.8544248	1	TNFRSF1B
Biological Process	GO:0035089	establishment of apical/basal cell polarity	1/2279	19/18866	0.9134771	0.9442875	0.8544248	1	PRKCI
Biological Process	GO:0036158	outer dynein arm assembly	1/2279	19/18866	0.9134771	0.9442875	0.8544248	1	DNAI2
Biological Process	GO:0039535	regulation of RIG-I signaling pathway	1/2279	19/18866	0.9134771	0.9442875	0.8544248	1	SEC14L1

Biological Process	GO:0060602	branch elongation of an epithelium	1/2279	19/18866	0.9134771	0.9442875	0.8544248	1	RDH10
Biological Process	GO:0098719	sodium ion import across plasma membrane	1/2279	19/18866	0.9134771	0.9442875	0.8544248	1	SLC8A1
Biological Process	GO:0009060	aerobic respiration	7/2279	87/18866	0.9136024	0.9443142	0.854449	7	CBFA2T3/DHTKD1/OGDH/NDUFS8/CAT/SDHD/SIRT3
Biological Process	GO:0010463	mesenchymal cell proliferation	3/2279	44/18866	0.9139247	0.9443391	0.8544716	3	TGFBR2/WNT11/FGFR1
Biological Process	GO:0033046	negative regulation of sister chromatid segregation	3/2279	44/18866	0.9139247	0.9443391	0.8544716	3	MAD1L1/TNKS/PCID2
Biological Process	GO:0042181	ketone biosynthetic process	3/2279	44/18866	0.9139247	0.9443391	0.8544716	3	DGKQ/COQ4/COQ8B
Biological Process	GO:0000377	RNA splicing, via transesterification reactions with bulged adenosine as nucleophile	39/2279	390/18866	0.9146682	0.9449018	0.8549807	39	HNRNPUL1/RBM19/FIP1L1/CPSF3/SNRPD3/SF3B3/SRPK2/POLR2F/PCBP4/PABPN1/SF1/LSM7/PRPF8/DAZAP1/SFSWAP/CELF2/NOL3/CELF1/SNRNP35/SNUPN/DHX16/USP49/CTNNBL1/DDX41/RBFOX3/PRPF6/PRPF40B/NSRP1/RBM15B/CHERP/PLRG1/SRRM2/CPSF4/ZCCHC8/SYNCRIP/RALY/CDK13/GEMIN4/TFIP11
Biological Process	GO:0000398	mRNA splicing, via spliceosome	39/2279	390/18866	0.9146682	0.9449018	0.8549807	39	HNRNPUL1/RBM19/FIP1L1/CPSF3/SNRPD3/SF3B3/SRPK2/POLR2F/PCBP4/PABPN1/SF1/LSM7/PRPF8/DAZAP1/SFSWAP/CELF2/NOL3/CELF1/SNRNP35/SNUPN/DHX16/USP49/CTNNBL1/DDX41/RBFOX3/PRPF6/PRPF40B/NSRP1/RBM15B/CHERP/PLRG1/SRRM2/CPSF4/ZCCHC8/SYNCRIP/RALY/CDK13/GEMIN4/TFIP11
Biological Process	GO:0033559	unsaturated fatty acid metabolic process	10/2279	118/18866	0.9168206	0.9470224	0.8568995	10	MGLL/ACSL1/ABHD16A/TNFRSF1A/CYP4F3/EPHX1/SCD/LYPLA2/ZADH2/PRXL2B

Biological Process	GO:0032890	regulation of organic acid transport	5/2279	67/18866	0.9197424	0.9499372	0.8595369	5	MAP2K6/THBS1/SLC43A2/PER2/SLC43A1
Biological Process	GO:0003352	regulation of cilium movement	2/2279	33/18866	0.9211201	0.9509467	0.8604503	2	RNASE10/DEFB1
Biological Process	GO:0010039	response to iron ion	2/2279	33/18866	0.9211201	0.9509467	0.8604503	2	SLC11A2/BCL2
Biological Process	GO:0021988	olfactory lobe development	2/2279	33/18866	0.9211201	0.9509467	0.8604503	2	SKI/CSF1R
Biological Process	GO:0050685	positive regulation of mRNA processing	2/2279	33/18866	0.9211201	0.9509467	0.8604503	2	CPEB3/DAZAP1
Biological Process	GO:0051985	negative regulation of chromosome segregation	3/2279	45/18866	0.921397	0.9509472	0.8604508	3	MAD1L1/TNKS/PCID2
Biological Process	GO:2001222	regulation of neuron migration	3/2279	45/18866	0.921397	0.9509472	0.8604508	3	RAPGEF2/ARHGEF2/UNC5D
Biological Process	GO:0009913	epidermal cell differentiation	36/2279	365/18866	0.9214209	0.9509472	0.8604508	36	CSTA/RUNX1/VDR/TGM5/KRT17/JUP/PKP1/SLC44A4/DLL1/RBPJ/PCSK6/HDAC1/CDH3/FGFR1/OVOL1/PRKCH/PERP/GRHL2/NUMA1/BCR/CNFN/KLF7/SGPP1/KRTAP5-9/NOTCH1/AQP3/FA2H/TMC1/TGM3/KRT23/KAZN/ZBED2/KRT7/ERCC2/PPL/MACROH2A2
Biological Process	GO:0098781	ncRNA transcription	9/2279	109/18866	0.9219126	0.9513514	0.8608165	9	POLR2F/ELL2/ZC3H8/BRF1/POU2F2/PIH1D1/RPRD1B/MARS1/MACROH2A2

Biological Process	GO:0002418	immune response to tumor cell	1/2279	20/18866	0.9239395	0.9518922	0.8613058	1	CD226
Biological Process	GO:0003085	negative regulation of systemic arterial blood pressure	1/2279	20/18866	0.9239395	0.9518922	0.8613058	1	KCNK6
Biological Process	GO:0003215	cardiac right ventricle morphogenesis	1/2279	20/18866	0.9239395	0.9518922	0.8613058	1	NOTCH1
Biological Process	GO:0006488	dolichol-linked oligosaccharide biosynthetic process	1/2279	20/18866	0.9239395	0.9518922	0.8613058	1	DPAGT1
Biological Process	GO:0007620	copulation	1/2279	20/18866	0.9239395	0.9518922	0.8613058	1	P2RX1
Biological Process	GO:0015919	peroxisomal membrane transport	1/2279	20/18866	0.9239395	0.9518922	0.8613058	1	PEX26
Biological Process	GO:0023019	signal transduction involved in regulation of gene expression	1/2279	20/18866	0.9239395	0.9518922	0.8613058	1	SMAD3
Biological Process	GO:0032303	regulation of icosanoid secretion	1/2279	20/18866	0.9239395	0.9518922	0.8613058	1	MAP2K6
Biological Process	GO:0032816	positive regulation of natural killer cell activation	1/2279	20/18866	0.9239395	0.9518922	0.8613058	1	HLA-F

Biological Process	GO:0033141	positive regulation of peptidyl-serine phosphorylation of STAT protein	1/2279	20/18866	0.9239395	0.9518922	0.8613058	1	LIF
Biological Process	GO:0036303	lymph vessel morphogenesis	1/2279	20/18866	0.9239395	0.9518922	0.8613058	1	VASH1
Biological Process	GO:0042474	middle ear morphogenesis	1/2279	20/18866	0.9239395	0.9518922	0.8613058	1	FGFR1
Biological Process	GO:1901881	positive regulation of protein depolymerization	1/2279	20/18866	0.9239395	0.9518922	0.8613058	1	WDR1
Biological Process	GO:1902644	tertiary alcohol metabolic process	1/2279	20/18866	0.9239395	0.9518922	0.8613058	1	DGKQ
Biological Process	GO:2000193	positive regulation of fatty acid transport	1/2279	20/18866	0.9239395	0.9518922	0.8613058	1	MAP2K6
Biological Process	GO:0006576	cellular biogenic amine metabolic process	4/2279	57/18866	0.9256296	0.9532458	0.8625307	4	ODC1/DDC/SAT2/HNMT
Biological Process	GO:0042743	hydrogen peroxide metabolic process	4/2279	57/18866	0.9256296	0.9532458	0.8625307	4	MPO/LPO/HBB/CAT
Biological Process	GO:0075733	intracellular transport of virus	4/2279	57/18866	0.9256296	0.9532458	0.8625307	4	RAE1/NUP93/SEC13/MVB12A

Biological Process	GO:0042446	hormone biosynthetic process	5/2279	68/18866	0.9256548	0.9532458	0.8625307	5	HSD17B2/TG/FDX1/DGKQ/POR
Biological Process	GO:0043330	response to exogenous dsRNA	3/2279	46/18866	0.9282712	0.9553863	0.8644674	3	COLEC12/FLOT1/STING1
Biological Process	GO:0007281	germ cell development	26/2279	275/18866	0.9288892	0.9553863	0.8644674	26	ACRBP/HEXB/MOV10L1/BRCA2/SPAG6/ZBTB16/CDYL/SMARCA2/KMT2D/RARA/DEFB1/JAM3/IQCG/BMPR1B/PPP2R1A/CELF1/BCL2/DMC1/ARMC2/NPPC/FSCN3/ERCC1/BCL2L1/FBXW11/TTLL1/MFSD14A
Biological Process	GO:0000038	very long-chain fatty acid metabolic process	2/2279	34/18866	0.9289399	0.9553863	0.8644674	2	ACSL1/ACOX2
Biological Process	GO:0007094	mitotic spindle assembly checkpoint	2/2279	34/18866	0.9289399	0.9553863	0.8644674	2	MAD1L1/PCID2
Biological Process	GO:0019228	neuronal action potential	2/2279	34/18866	0.9289399	0.9553863	0.8644674	2	SCN1A/P2RX1
Biological Process	GO:0030431	sleep	2/2279	34/18866	0.9289399	0.9553863	0.8644674	2	GHRL/SLC29A1
Biological Process	GO:0031577	spindle checkpoint	2/2279	34/18866	0.9289399	0.9553863	0.8644674	2	MAD1L1/PCID2
Biological Process	GO:0035510	DNA dealkylation	2/2279	34/18866	0.9289399	0.9553863	0.8644674	2	FTO/TET2

Biological Process	GO:0042759	long-chain fatty acid biosynthetic process	2/2279	34/18866	0.9289399	0.9553863	0.8644674	2	ACOT7/MYO5A
Biological Process	GO:0071173	spindle assembly checkpoint	2/2279	34/18866	0.9289399	0.9553863	0.8644674	2	MAD1L1/PCID2
Biological Process	GO:0071174	mitotic spindle checkpoint	2/2279	34/18866	0.9289399	0.9553863	0.8644674	2	MAD1L1/PCID2
Biological Process	GO:0098664	G protein-coupled serotonin receptor signaling pathway	2/2279	34/18866	0.9289399	0.9553863	0.8644674	2	HRH1/CHRM2
Biological Process	GO:0008380	RNA splicing	49/2279	487/18866	0.930069	0.9564439	0.8654244	49	HNRNPUL1/RBM19/FIP1L1/LSM1/ERN1/CPSF3/SNRPD3/SF3B3/PIK3R1/SRPK2/POLR2F/PCBP4/AHNAK/PRKRIP1/PABPN1/CLK3/SF1/LSM7/PRPF8/DAZAP1/SFSWAP/CELF2/PPP2R1A/NOL3/HNRNPLL/CELF1/SNRNP35/SNUPN/DHX16/USP49/CTNNBL1/DDX41/RBFOX3/PRPF6/PRPF40B/NSRP1/RBM15B/CHERP/PLRG1/SRRM2/CPSF4/ZCCHC8/SYNCRIP/CLASRP/RALY/CDK13/GEMIN4/TFIP11/GRSF1
Biological Process	GO:0060294	cilium movement involved in cell motility	9/2279	111/18866	0.931025	0.9573234	0.8662202	9	PLTP/RNASE10/DEFB1/IQCG/ARMC2/CCNYL1/TTLL1/NPHP4/CFAP46
Biological Process	GO:0001502	cartilage condensation	1/2279	21/18866	0.9331373	0.9577334	0.8665912	1	BMPR1B
Biological Process	GO:0009713	catechol-containing compound biosynthetic process	1/2279	21/18866	0.9331373	0.9577334	0.8665912	1	DDC
Biological Process	GO:0014821	phasic smooth muscle contraction	1/2279	21/18866	0.9331373	0.9577334	0.8665912	1	GHRL

Biological Process	GO:0016553	base conversion or substitution editing	1/2279	21/18866	0.9331373	0.9577334	0.8665912	1	RBM47
Biological Process	GO:0030502	negative regulation of bone mineralization	1/2279	21/18866	0.9331373	0.9577334	0.8665912	1	RFLNA
Biological Process	GO:0034471	ncRNA 5'-end processing	1/2279	21/18866	0.9331373	0.9577334	0.8665912	1	NOP14
Biological Process	GO:0039702	viral budding via host ESCRT complex	1/2279	21/18866	0.9331373	0.9577334	0.8665912	1	MVB12A
Biological Process	GO:0042276	error-prone translesion synthesis	1/2279	21/18866	0.9331373	0.9577334	0.8665912	1	POLI
Biological Process	GO:0042423	catecholamine biosynthetic process	1/2279	21/18866	0.9331373	0.9577334	0.8665912	1	DDC
Biological Process	GO:0043651	linoleic acid metabolic process	1/2279	21/18866	0.9331373	0.9577334	0.8665912	1	ACSL1
Biological Process	GO:0046827	positive regulation of protein export from nucleus	1/2279	21/18866	0.9331373	0.9577334	0.8665912	1	TCF7L2
Biological Process	GO:0060065	uterus development	1/2279	21/18866	0.9331373	0.9577334	0.8665912	1	HOXA9

Biological Process	GO:0061162	establishment of monopolar cell polarity	1/2279	21/18866	0.9331373	0.9577334	0.8665912	1	PRKCI
Biological Process	GO:0090370	negative regulation of cholesterol efflux	1/2279	21/18866	0.9331373	0.9577334	0.8665912	1	MIR27A
Biological Process	GO:1903817	negative regulation of voltage-gated potassium channel activity	1/2279	21/18866	0.9331373	0.9577334	0.8665912	1	KCNE1
Biological Process	GO:2000678	negative regulation of transcription regulatory region DNA binding	1/2279	21/18866	0.9331373	0.9577334	0.8665912	1	PER2
Biological Process	GO:2000738	positive regulation of stem cell differentiation	1/2279	21/18866	0.9331373	0.9577334	0.8665912	1	TACSTD2
Biological Process	GO:0043588	skin development	42/2279	425/18866	0.9339539	0.958463	0.8672514	42	CSTA/ASPRV1/RUNX1/VDR/SUFU/TNF/TGM5/KRT17/JUP/PKP1/DLL1/RBPJ/PCSK6/HDAC1/CDH3/OVOL1/PRKCH/PERP/MYO5A/NF1/GRHL2/NUMA1/BCR/CNFN/GJB3/SGPP1/KRTAP5-9/BCL2/NOTCH1/AQP3/COL5A1/FA2H/TGM3/KRT23/KAZN/ZBED2/RELA/KRT7/INHBA/ERCC2/PPL/MACROH2A2
Biological Process	GO:0007006	mitochondrial membrane organization	12/2279	142/18866	0.9340807	0.958463	0.8672514	12	TFDP1/YWHAH/BMF/SPG7/HK2/IMMT/YWHAZ/BCL2/TP53BP2/BCL2L1/YWHAQ/ATP5PD
Biological Process	GO:0051952	regulation of amine transport	7/2279	91/18866	0.9341509	0.958463	0.8672514	7	SLC43A2/PER2/SYT17/SLC43A1/DTNBP1/GRK2/PRKN
Biological Process	GO:0031055	chromatin remodeling at centromere	3/2279	47/18866	0.9345888	0.9588087	0.8675642	3	RSF1/CENPM/RUVBL1

Biological Process	GO:0007338	single fertilization	14/2279	162/18866	0.9347907	0.9589124	0.867658	14	CECR2/CD9/HEXB/FOLR3/RNASE10/CLGN/IGSF8/LHFPL2/PLCB1/HOXA9/UBAP2L/UBE3A/UBXN8/IRAG2
Biological Process	GO:0039694	viral RNA genome replication	2/2279	35/18866	0.9360226	0.9598651	0.86852	2	PHB/PI4KA
Biological Process	GO:0051443	positive regulation of ubiquitin-protein transferase activity	2/2279	35/18866	0.9360226	0.9598651	0.86852	2	FZR1/AXIN1
Biological Process	GO:1900016	negative regulation of cytokine production involved in inflammatory response	2/2279	35/18866	0.9360226	0.9598651	0.86852	2	MIR140/MEFV
Biological Process	GO:0033108	mitochondrial respiratory chain complex assembly	8/2279	102/18866	0.936545	0.9602972	0.868911	8	COX19/NDUFAF6/NDUFS2/NDUFS8/NDUFB6/SDHAF1/SDHAF3/DMAC1
Biological Process	GO:0032886	regulation of microtubule-based process	22/2279	240/18866	0.9368873	0.9604523	0.8690513	22	TBCD/DCTN1/RAE1/RCC1/DYNC1H1/NAV3/RNASE10/POC1A/BICD2/CNIH2/DEFB1/TACC3/NUMA1/TTC21B/MAP1A/ARHGEF2/CYLD/SPAG5/MARK2/SPEF1/PRKAA1/CEP295NL
Biological Process	GO:0010833	telomere maintenance via telomere lengthening	6/2279	81/18866	0.9370155	0.9604523	0.8690513	6	TNKS/HMBOX1/RTEL1/SMG6/SLX4/CCT6A
Biological Process	GO:0006413	translational initiation	17/2279	192/18866	0.9370494	0.9604523	0.8690513	17	RXRA/KLHL25/TNF/CCL5/NCK2/EIF2B5/LARP1/CTIF/AGO2/MIF4GD/EIF3M/COPS5/PAIP1/BANK1/EIF3H/RPL23A/EIF4G1
Biological Process	GO:0043486	histone exchange	4/2279	59/18866	0.9371006	0.9604523	0.8690513	4	SRCAP/RSF1/CENPM/RUVBL1

Biological Process	GO:0042180	cellular ketone metabolic process	24/2279	260/18866	0.9395725	0.9628819	0.8712498	24	PRKAG2/PPARG/NCOR2/MFSD2A/DHRS9/ODC1/COMT/CYP4F3/RDH10/DGKQ/AIFM2/NR1H2/SLC7A7/ACACB/PSMF1/PSMB7/PRKCE/SREBF1/WDTC1/CPT1A/COQ4/PSMD13/ACADVL/COQ8B
Biological Process	GO:0062237	protein localization to postsynapse	3/2279	48/18866	0.9403891	0.9630156	0.8713707	3	PRKCZ/ZDHHC2/KIF5C
Biological Process	GO:0005980	glycogen catabolic process	1/2279	22/18866	0.9412233	0.9630156	0.8713707	1	PPP1CB
Biological Process	GO:0021513	spinal cord dorsal/ventral patterning	1/2279	22/18866	0.9412233	0.9630156	0.8713707	1	SUFU
Biological Process	GO:0030859	polarized epithelial cell differentiation	1/2279	22/18866	0.9412233	0.9630156	0.8713707	1	ZDHHC7
Biological Process	GO:0032700	negative regulation of interleukin-17 production	1/2279	22/18866	0.9412233	0.9630156	0.8713707	1	VSIR
Biological Process	GO:0033617	mitochondrial cytochrome c oxidase assembly	1/2279	22/18866	0.9412233	0.9630156	0.8713707	1	COX19
Biological Process	GO:0035809	regulation of urine volume	1/2279	22/18866	0.9412233	0.9630156	0.8713707	1	GNAI2
Biological Process	GO:0039531	regulation of viral-induced cytoplasmic pattern recognition receptor signaling pathway	1/2279	22/18866	0.9412233	0.9630156	0.8713707	1	SEC14L1

Biological Process	GO:0042749	regulation of circadian sleep/wake cycle	1/2279	22/18866	0.9412233	0.9630156	0.8713707	1	GHRL
Biological Process	GO:0044342	type B pancreatic cell proliferation	1/2279	22/18866	0.9412233	0.9630156	0.8713707	1	SGPP2
Biological Process	GO:0050802	circadian sleep/wake cycle, sleep	1/2279	22/18866	0.9412233	0.9630156	0.8713707	1	GHRL
Biological Process	GO:0051383	kinetochore organization	1/2279	22/18866	0.9412233	0.9630156	0.8713707	1	MIS12
Biological Process	GO:0061339	establishment or maintenance of monopolar cell polarity	1/2279	22/18866	0.9412233	0.9630156	0.8713707	1	PRKCI
Biological Process	GO:0099633	protein localization to postsynaptic specialization membrane	1/2279	22/18866	0.9412233	0.9630156	0.8713707	1	PRKCZ
Biological Process	GO:0099645	neurotransmitter receptor localization to postsynaptic specialization membrane	1/2279	22/18866	0.9412233	0.9630156	0.8713707	1	PRKCZ
Biological Process	GO:0035036	sperm-egg recognition	4/2279	60/18866	0.9422112	0.963837	0.8721139	4	CD9/FOLR3/CLGN/UBAP2L
Biological Process	GO:0071103	DNA conformation change	34/2279	355/18866	0.942309	0.963837	0.8721139	34	BLM/TTN/UBN1/TOP1MT/CABIN1/RTEL1/MCM5/WRNIP1/RECQL5/SMYD3/IGHMBP2/HDAC1/ATF7IP/MCM2/SMARCAL1/DFFB/RSF1/CHD9/DNMT1/CDCA5/CHD8/HHEX/CENPM/ZNFX1/RUVBL1/CHAF1A/PARP10/BANF1/XPA/ERCC2/BRD2/MACROH2A2/H3C1/FBH1

Biological Process	GO:0001580	detection of chemical stimulus involved in sensory perception of bitter taste	2/2279	36/18866	0.9424319	0.963837	0.8721139	2	LPO/GNAT2
Biological Process	GO:0045841	negative regulation of mitotic metaphase/anaphase transition	2/2279	36/18866	0.9424319	0.963837	0.8721139	2	MAD1L1/PCID2
Biological Process	GO:0001824	blastocyst development	8/2279	104/18866	0.9444596	0.9658068	0.8738963	8	BRCA2/RTN4/CUL3/SP3/KDM4C/ST8SIA6/NDEL1/NODAL
Biological Process	GO:0031507	heterochromatin assembly	5/2279	72/18866	0.9456079	0.9666688	0.8746762	5	HDAC1/ATF7IP/DNMT1/ZNFX1/H3C1
Biological Process	GO:0051937	catecholamine transport	5/2279	72/18866	0.9456079	0.9666688	0.8746762	5	NISCH/SYT17/DTNBP1/GRK2/PRKN
Biological Process	GO:0015872	dopamine transport	3/2279	49/18866	0.9457095	0.9666688	0.8746762	3	SYT17/DTNBP1/PRKN
Biological Process	GO:0120178	steroid hormone biosynthetic process	3/2279	49/18866	0.9457095	0.9666688	0.8746762	3	HSD17B2/FDX1/DGKQ
Biological Process	GO:0030449	regulation of complement activation	9/2279	115/18866	0.9465174	0.9669565	0.8749365	9	C3AR1/CD55/C5AR2/CLU/PHB/CD59/C1S/CPB2/C1QB
Biological Process	GO:0051084	'de novo' posttranslational protein folding	2/2279	37/18866	0.9482271	0.9669565	0.8749365	2	DNAJB13/TOR1B

Biological Process	GO:1902100	negative regulation of metaphase/anaphase transition of cell cycle	2/2279	37/18866	0.9482271	0.9669565	0.8749365	2	MAD1L1/PCID2
Biological Process	GO:2000273	positive regulation of signaling receptor activity	2/2279	37/18866	0.9482271	0.9669565	0.8749365	2	TGFA/CCR2
Biological Process	GO:0000966	RNA 5'-end processing	1/2279	23/18866	0.9483318	0.9669565	0.8749365	1	NOP14
Biological Process	GO:0006063	uronic acid metabolic process	1/2279	23/18866	0.9483318	0.9669565	0.8749365	1	PRKCE
Biological Process	GO:0006541	glutamine metabolic process	1/2279	23/18866	0.9483318	0.9669565	0.8749365	1	GFPT2
Biological Process	GO:0007274	neuromuscular synaptic transmission	1/2279	23/18866	0.9483318	0.9669565	0.8749365	1	KIF1B
Biological Process	GO:0007289	spermatid nucleus differentiation	1/2279	23/18866	0.9483318	0.9669565	0.8749365	1	MFSD14A
Biological Process	GO:0009147	pyrimidine nucleoside triphosphate metabolic process	1/2279	23/18866	0.9483318	0.9669565	0.8749365	1	UCK2
Biological Process	GO:0016226	iron-sulfur cluster assembly	1/2279	23/18866	0.9483318	0.9669565	0.8749365	1	HSCB

Biological Process	GO:0019585	glucuronate metabolic process	1/2279	23/18866	0.9483318	0.9669565	0.8749365	1	PRKCE
Biological Process	GO:0030539	male genitalia development	1/2279	23/18866	0.9483318	0.9669565	0.8749365	1	ASB1
Biological Process	GO:0031163	metallo-sulfur cluster assembly	1/2279	23/18866	0.9483318	0.9669565	0.8749365	1	HSCB
Biological Process	GO:0034643	establishment of mitochondrion localization, microtubule-mediated	1/2279	23/18866	0.9483318	0.9669565	0.8749365	1	KIF1B
Biological Process	GO:0042026	protein refolding	1/2279	23/18866	0.9483318	0.9669565	0.8749365	1	BAG5
Biological Process	GO:0044458	motile cilium assembly	1/2279	23/18866	0.9483318	0.9669565	0.8749365	1	CFAP221
Biological Process	GO:0045723	positive regulation of fatty acid biosynthetic process	1/2279	23/18866	0.9483318	0.9669565	0.8749365	1	NR1H2
Biological Process	GO:0046599	regulation of centriole replication	1/2279	23/18866	0.9483318	0.9669565	0.8749365	1	CEP295NL
Biological Process	GO:0047497	mitochondrion transport along microtubule	1/2279	23/18866	0.9483318	0.9669565	0.8749365	1	KIF1B

Biological Process	GO:0048026	positive regulation of mRNA splicing, via spliceosome	1/2279	23/18866	0.9483318	0.9669565	0.8749365	1	DAZAP1
Biological Process	GO:0048791	calcium ion-regulated exocytosis of neurotransmitter	1/2279	23/18866	0.9483318	0.9669565	0.8749365	1	SYT17
Biological Process	GO:0071305	cellular response to vitamin D	1/2279	23/18866	0.9483318	0.9669565	0.8749365	1	VDR
Biological Process	GO:0045814	negative regulation of gene expression, epigenetic	10/2279	126/18866	0.9487467	0.9672757	0.8752254	10	TRIM27/EPC1/KMT2D/CTBP1/PHF19/DNMT1/DYDC2/DYDC1/MACROH2A2/H3C1
Biological Process	GO:0042795	snRNA transcription by RNA polymerase II	5/2279	73/18866	0.9497725	0.9682177	0.8760777	5	POLR2F/ELL2/ZC3H8/POU2F2/RPRD1B
Biological Process	GO:0099054	presynapse assembly	3/2279	50/18866	0.9505855	0.9689425	0.8767336	3	APP/NRXN1/EIF4G1
Biological Process	GO:0042769	DNA damage response, detection of DNA damage	2/2279	38/18866	0.9534631	0.9715404	0.8790842	2	MRPS26/POLD3
Biological Process	GO:0045777	positive regulation of blood pressure	2/2279	38/18866	0.9534631	0.9715404	0.8790842	2	PDE4D/HSD11B2
Biological Process	GO:0071392	cellular response to estradiol stimulus	2/2279	38/18866	0.9534631	0.9715404	0.8790842	2	IL10/CRHBP

Biological Process	GO:0009301	snRNA transcription	5/2279	74/18866	0.9536454	0.9715404	0.8790842	5	POLR2F/ELL2/ZC3H8/POU2F2/RPRD1B
Biological Process	GO:0051881	regulation of mitochondrial membrane potential	5/2279	74/18866	0.9536454	0.9715404	0.8790842	5	HSH2D/BCL2/BCL2L1/SPART/PRKN
Biological Process	GO:0010640	regulation of platelet-derived growth factor receptor signaling pathway	1/2279	24/18866	0.9545809	0.9716598	0.8791923	1	PTPRJ
Biological Process	GO:0014850	response to muscle activity	1/2279	24/18866	0.9545809	0.9716598	0.8791923	1	FIS1
Biological Process	GO:0021511	spinal cord patterning	1/2279	24/18866	0.9545809	0.9716598	0.8791923	1	SUFU
Biological Process	GO:0021871	forebrain regionalization	1/2279	24/18866	0.9545809	0.9716598	0.8791923	1	TTC21B
Biological Process	GO:0042430	indole-containing compound metabolic process	1/2279	24/18866	0.9545809	0.9716598	0.8791923	1	DDC
Biological Process	GO:0042738	exogenous drug catabolic process	1/2279	24/18866	0.9545809	0.9716598	0.8791923	1	NR1I2
Biological Process	GO:0042776	mitochondrial ATP synthesis coupled proton transport	1/2279	24/18866	0.9545809	0.9716598	0.8791923	1	ATP5PD

Biological Process	GO:0090103	cochlea morphogenesis	1/2279	24/18866	0.9545809	0.9716598	0.8791923	1	PAX2
Biological Process	GO:0035272	exocrine system development	3/2279	51/18866	0.9550502	0.9720335	0.8795304	3	TNF/FGFR1/TGFB1
Biological Process	GO:0016072	rRNA metabolic process	21/2279	238/18866	0.9556374	0.9725268	0.8799768	21	EXOSC2/WDR43/SLFN14/RCL1/DEDD2/USP36/DDX27/WDR46/NOB1/ERI3/WDR55/RMRP/PIH1D1/NOP14/NOL6/FBL/ERCC2/RIOK1/ISG20/GEMIN4/UTP4
Biological Process	GO:0008543	fibroblast growth factor receptor signaling pathway	9/2279	118/18866	0.9560373	0.9728297	0.8802508	9	SPRED2/POLR2F/FRS3/THBS1/NDST1/FGFR1/RUNX2/FAM20C/OTX2
Biological Process	GO:0098659	inorganic cation import across plasma membrane	7/2279	97/18866	0.9569235	0.9735229	0.880878	7	TRPM2/KCNJ15/FXYD2/SLC8A1/TRPV2/SLC12A1/SLC12A7
Biological Process	GO:0099587	inorganic ion import across plasma membrane	7/2279	97/18866	0.9569235	0.9735229	0.880878	7	TRPM2/KCNJ15/FXYD2/SLC8A1/TRPV2/SLC12A1/SLC12A7
Biological Process	GO:0000578	embryonic axis specification	2/2279	39/18866	0.9581902	0.9743942	0.8816665	2	PCSK6/TMED2
Biological Process	GO:0010837	regulation of keratinocyte proliferation	2/2279	39/18866	0.9581902	0.9743942	0.8816665	2	VDR/CDH3
Biological Process	GO:0032506	cytokinetic process	2/2279	39/18866	0.9581902	0.9743942	0.8816665	2	MYH9/SNX18

Biological Process	GO:2000816	negative regulation of mitotic sister chromatid separation	2/2279	39/18866	0.9581902	0.9743942	0.8816665	2	MAD1L1/PCID2
Biological Process	GO:0006612	protein targeting to membrane	18/2279	210/18866	0.9584311	0.9745349	0.8817937	18	KCNE1/ZDHHC18/ITGB1BP1/ZDHHC14/RABGEF1/ZDHHC2/ZDHHC7/ZDHHC1/PRKCI/PEX26/ITGB2/MICALL1/FIS1/NACAD/MYO1C/MIEF1/RPL23A/PDZK1
Biological Process	GO:0007007	inner mitochondrial membrane organization	3/2279	52/18866	0.9591351	0.9751463	0.882347	3	SPG7/IMMT/ATP5PD
Biological Process	GO:0015874	norepinephrine transport	1/2279	25/18866	0.9600745	0.9754751	0.8826445	1	NISCH
Biological Process	GO:0021895	cerebral cortex neuron differentiation	1/2279	25/18866	0.9600745	0.9754751	0.8826445	1	ZNF335
Biological Process	GO:0022410	circadian sleep/wake cycle process	1/2279	25/18866	0.9600745	0.9754751	0.8826445	1	GHRL
Biological Process	GO:0042537	benzene-containing compound metabolic process	1/2279	25/18866	0.9600745	0.9754751	0.8826445	1	FAH
Biological Process	GO:0060575	intestinal epithelial cell differentiation	1/2279	25/18866	0.9600745	0.9754751	0.8826445	1	NPY
Biological Process	GO:0061436	establishment of skin barrier	1/2279	25/18866	0.9600745	0.9754751	0.8826445	1	FA2H

Biological Process	GO:0008544	epidermis development	46/2279	477/18866	0.9610549	0.9763668	0.8834514	46	CSTA/COL17A1/TFDP1/RUNX1/VDR/TNF/TGM5/KRT17/JUP/PKP1/SLC44A4/DLL1/RBPJ/PCSK6/HDAC1/CDH3/FGFR1/OVOL1/PRKCH/PERP/MYO5A/NF1/GRHL2/NUMA1/BCR/CNFN/KLF7/SGPP1/KRTAP5-9/BCL2/NOTCH1/AQP3/FA2H/TMC1/TGM3/KRT23/KAZN/ZBED2/LAMB3/RELA/KRT7/INHBA/ERCC2/NTF4/PPL/MACROH2A2
Biological Process	GO:1905819	negative regulation of chromosome separation	2/2279	40/18866	0.962455	0.9775356	0.884509	2	MAD1L1/PCID2
Biological Process	GO:0007588	excretion	4/2279	65/18866	0.962514	0.9775356	0.884509	4	COMT/GNAI2/AGTR1/AQP9
Biological Process	GO:0048278	vesicle docking	4/2279	65/18866	0.962514	0.9775356	0.884509	4	STX5/VPS18/CTBP2/TSNARE1
Biological Process	GO:0070125	mitochondrial translational elongation	6/2279	88/18866	0.9628381	0.9776875	0.8846464	6	MRPL3/MRPL38/MRPS21/MRPS26/MRPL28/AURKAIP1
Biological Process	GO:0099172	presynapse organization	3/2279	53/18866	0.9628694	0.9776875	0.8846464	3	APP/NRXN1/EIF4G1
Biological Process	GO:0006342	chromatin silencing	5/2279	77/18866	0.963686	0.9784121	0.885302	5	KMT2D/CTBP1/DYDC2/DYDC1/MACROH2A2
Biological Process	GO:0034728	nucleosome organization	15/2279	183/18866	0.9643489	0.9784984	0.8853801	15	SRCAP/UBN1/SETD2/CABIN1/SMYD3/MCM2/RSF1/SMARCD1/SMARCD2/CENPM/RUVBL1/CHAF1A/BRD2/MACROH2A2/H3C1
Biological Process	GO:0001945	lymph vessel development	1/2279	26/18866	0.9649039	0.9784984	0.8853801	1	VASH1

Biological Process	GO:0008535	respiratory chain complex IV assembly	1/2279	26/18866	0.9649039	0.9784984	0.8853801	1	COX19
Biological Process	GO:0035640	exploration behavior	1/2279	26/18866	0.9649039	0.9784984	0.8853801	1	PRKCE
Biological Process	GO:0040037	negative regulation of fibroblast growth factor receptor signaling pathway	1/2279	26/18866	0.9649039	0.9784984	0.8853801	1	THBS1
Biological Process	GO:0042104	positive regulation of activated T cell proliferation	1/2279	26/18866	0.9649039	0.9784984	0.8853801	1	FADD
Biological Process	GO:0042737	drug catabolic process	1/2279	26/18866	0.9649039	0.9784984	0.8853801	1	NR1I2
Biological Process	GO:0051654	establishment of mitochondrion localization	1/2279	26/18866	0.9649039	0.9784984	0.8853801	1	KIF1B
Biological Process	GO:0060343	trabecula formation	1/2279	26/18866	0.9649039	0.9784984	0.8853801	1	FHL2
Biological Process	GO:0070129	regulation of mitochondrial translation	1/2279	26/18866	0.9649039	0.9784984	0.8853801	1	MTG2
Biological Process	GO:0140058	neuron projection arborization	1/2279	26/18866	0.9649039	0.9784984	0.8853801	1	MFSD2A

Biological Process	GO:0006400	tRNA modification	6/2279	89/18866	0.9656107	0.9790062	0.8858396	6	THADA/METTL6/CDKAL1/PUS7/DUS2/METTL8
Biological Process	GO:0070126	mitochondrial translational termination	6/2279	89/18866	0.9656107	0.9790062	0.8858396	6	MRPL3/MRPL38/MRPS21/MRPS26/MRPL28/AURKAIP1
Biological Process	GO:0022900	electron transport chain	15/2279	184/18866	0.9662992	0.9793916	0.8861883	15	ALDH2/NDUFS2/ALDH4A1/NCF2/FDX1/NDUFS8/POR/AIFM2/NDUFB6/GLRX2/NDUFA4L2/P4HA2/ASPH/SDHD/AOX1
Biological Process	GO:0006458	'de novo' protein folding	2/2279	41/18866	0.9663001	0.9793916	0.8861883	2	DNAJB13/TOR1B
Biological Process	GO:0050913	sensory perception of bitter taste	2/2279	41/18866	0.9663001	0.9793916	0.8861883	2	LPO/GNAT2
Biological Process	GO:0031497	chromatin assembly	16/2279	195/18866	0.9679927	0.9810026	0.8876459	16	UBN1/CABIN1/SMYD3/HDAC1/ATF7IP/MCM2/RSF1/DNMT1/CENPM/ZNFX1/RUVBL1/CHAF1A/PARP10/BRD2/MACROH2A2/H3C1
Biological Process	GO:0042481	regulation of odontogenesis	1/2279	27/18866	0.9691493	0.9820699	0.8886117	1	RUNX2
Biological Process	GO:0006120	mitochondrial electron transport, NADH to ubiquinone	3/2279	55/18866	0.9693945	0.9822137	0.8887418	3	NDUFS2/NDUFS8/NDUFB6
Biological Process	GO:0046006	regulation of activated T cell proliferation	2/2279	42/18866	0.9697645	0.982379	0.8888914	2	RIPK3/FADD

Biological Process	GO:0050912	detection of chemical stimulus involved in sensory perception of taste	2/2279	42/18866	0.9697645	0.982379	0.8888914	2	LPO/GNAT2
Biological Process	GO:0006399	tRNA metabolic process	15/2279	186/18866	0.969918	0.9824297	0.8889373	15	THADA/EXOSC2/CARS2/METTL6/CDKAL1/PUS7/DUS2/EARS2/CPSF4/PTCD1/METTL8/GRSF1/MARS1/GATB/YARS1
Biological Process	GO:0001676	long-chain fatty acid metabolic process	8/2279	113/18866	0.9702575	0.9825898	0.8890821	8	MGLL/ACSL1/ACOT7/CYP4F3/MYO5A/EPHX1/ADTRP/CPT1A
Biological Process	GO:0006334	nucleosome assembly	11/2279	145/18866	0.9702829	0.9825898	0.8890821	11	UBN1/CABIN1/SMYD3/MCM2/RSF1/CENPM/RUVBL1/CHAF1A/BRD2/MACROH2A2/H3C1
Biological Process	GO:0006364	rRNA processing	19/2279	227/18866	0.9712607	0.9834752	0.8898833	19	EXOSC2/WDR43/RCL1/USP36/DDX27/WDR46/NOB1/ERI3/WDR55/RMRP/PIH1D1/NOP14/NOL6/FBL/ERCC2/RIOK1/ISG20/GEMIN4/UTP4
Biological Process	GO:0030216	keratinocyte differentiation	27/2279	306/18866	0.9720907	0.9839656	0.890327	27	CSTA/RUNX1/VDR/TGM5/KRT17/JUP/PKP1/RBPJ/PCSK6/CDH3/PRKCH/PERP/GRHL2/NUMA1/BCR/CNFN/SGPP1/KRTAP5-9/NOTCH1/AQP3/TGM3/KRT23/KAZN/ZBED2/KRT7/PPL/MACROH2A2
Biological Process	GO:0044344	cellular response to fibroblast growth factor stimulus	11/2279	146/18866	0.9721322	0.9839656	0.890327	11	SPRED2/ITGB1BP1/POLR2F/CCL5/FRS3/THBS1/NDST1/FGFR1/RUNX2/FAM20C/OTX2
Biological Process	GO:0022037	metencephalon development	8/2279	114/18866	0.9723176	0.9839656	0.890327	8	PHOX2A/DLL1/NCOA1/BCL2/TTC21B/TTLL1/CPLANE1/COQ8B
Biological Process	GO:0042472	inner ear morphogenesis	7/2279	103/18866	0.9723414	0.9839656	0.890327	7	ABR/SLC44A4/LRIG1/PAX2/FGFR1/TTC39C/BCR

Biological Process	GO:0034605	cellular response to heat	9/2279	125/18866	0.9726256	0.9839656	0.890327	9	RPTOR/RAE1/THBS1/NF1/NUP93/DNAJB6/SEC13/CREBBP/BAG5
Biological Process	GO:0000395	mRNA 5'-splice site recognition	1/2279	28/18866	0.9728814	0.9839656	0.890327	1	SFSWAP
Biological Process	GO:0010894	negative regulation of steroid biosynthetic process	1/2279	28/18866	0.9728814	0.9839656	0.890327	1	GFI1
Biological Process	GO:0031167	rRNA methylation	1/2279	28/18866	0.9728814	0.9839656	0.890327	1	FBL
Biological Process	GO:0033561	regulation of water loss via skin	1/2279	28/18866	0.9728814	0.9839656	0.890327	1	FA2H
Biological Process	GO:0042745	circadian sleep/wake cycle	1/2279	28/18866	0.9728814	0.9839656	0.890327	1	GHRL
Biological Process	GO:0007339	binding of sperm to zona pellucida	2/2279	43/18866	0.9728842	0.9839656	0.890327	2	CLGN/UBAP2L
Biological Process	GO:0006956	complement activation	14/2279	178/18866	0.9735508	0.9845349	0.8908422	14	MFAP4/C3AR1/CD55/IGHV6-1/C5AR2/MASP1/CLU/PHB/FCN1/CD59/C1S/CPB2/C1QB/C1RL
Biological Process	GO:0007004	telomere maintenance via telomerase	4/2279	69/18866	0.9737503	0.9846319	0.8909299	4	TNKS/HMBOX1/SMG6/CCT6A

Biological Process	GO:0045292	mRNA cis splicing, via spliceosome	3/2279	57/18866	0.9748241	0.985508	0.8917226	3	RBM19/SFSWAP/PRPF40B
Biological Process	GO:0097120	receptor localization to synapse	3/2279	57/18866	0.9748241	0.985508	0.8917226	3	PRKCZ/CNIH2/KIF5C
Biological Process	GO:0006323	DNA packaging	20/2279	240/18866	0.9755086	0.9857757	0.8919648	20	TTN/UBN1/CABIN1/SMYD3/HDAC1/ATF7IP/MCM2/DFFB/RSF1/DNMT1/CDCA5/CENPM/ZNFX1/RUVBL1/CHAF1A/PARP10/BANF1/BRD2/MACROH2A2/H3C1
Biological Process	GO:0006904	vesicle docking involved in exocytosis	2/2279	44/18866	0.9756918	0.9857757	0.8919648	2	VPS18/CTBP2
Biological Process	GO:0010824	regulation of centrosome duplication	2/2279	44/18866	0.9756918	0.9857757	0.8919648	2	POC1A/CEP295NL
Biological Process	GO:1903539	protein localization to postsynaptic membrane	2/2279	44/18866	0.9756918	0.9857757	0.8919648	2	PRKCZ/ZDHHC2
Biological Process	GO:0001835	blastocyst hatching	1/2279	29/18866	0.9761622	0.9857757	0.8919648	1	ST8SIA6
Biological Process	GO:0019835	cytolysis	1/2279	29/18866	0.9761622	0.9857757	0.8919648	1	TGFB1
Biological Process	GO:0035188	hatching	1/2279	29/18866	0.9761622	0.9857757	0.8919648	1	ST8SIA6

Biological Process	GO:0048240	sperm capacitation	1/2279	29/18866	0.9761622	0.9857757	0.8919648	1	DEFB1
Biological Process	GO:0071684	organism emergence from protective structure	1/2279	29/18866	0.9761622	0.9857757	0.8919648	1	ST8SIA6
Biological Process	GO:0006415	translational termination	7/2279	105/18866	0.9762302	0.9857757	0.8919648	7	MRPL3/MRPL38/MRPS21/MRPS26/MRPL28/AURKAIP1/SHFL
Biological Process	GO:0007631	feeding behavior	7/2279	105/18866	0.9762302	0.9857757	0.8919648	7	APP/DERL2/GHRL/TTC21B/CPT1A/NPY/PRLHR
Biological Process	GO:0019369	arachidonic acid metabolic process	3/2279	58/18866	0.9771828	0.9865278	0.8926454	3	MGLL/CYP4F3/EPHX1
Biological Process	GO:1903793	positive regulation of anion transport	3/2279	58/18866	0.9771828	0.9865278	0.8926454	3	MAP2K6/ATP8A1/CEBPB
Biological Process	GO:0007286	spermatid development	12/2279	160/18866	0.9777135	0.9869588	0.8930354	12	ACRBP/SPAG6/CDYL/SMARCA2/DEFB1/JAM3/IQCG/DMC1/ARMC2/FSCN3/TTLL1/MFSD14A
Biological Process	GO:0034660	ncRNA metabolic process	46/2279	493/18866	0.9784669	0.9872551	0.8933035	46	PIWIL4/THADA/MOV10L1/EXOSC2/WDR43/SMAD3/SLFN14/RCL1/AGO1/DEDD2/USP36/CARS2/DDX27/AGO2/WDR46/METTL6/NOB1/ERI3/WDR55/CDKAL1/RMRP/PUS7/PIH1D1/NOP14/DIS3L2/DUS2/SND1/EARS2/RELA/TOE1/NOL6/CPSF4/PTCD1/ZCCHC8/FBL/TUT1/ERCC2/RIOK1/ISG20/METTL8/GEMIN4/GRSF1/MARS1/GATB/UTP4/YARS1
Biological Process	GO:0000027	ribosomal large subunit assembly	1/2279	30/18866	0.9790462	0.9872551	0.8933035	1	RPL23A

Biological Process	GO:0001516	prostaglandin biosynthetic process	1/2279	30/18866	0.9790462	0.9872551	0.8933035	1	PRXL2B
Biological Process	GO:0007271	synaptic transmission, cholinergic	1/2279	30/18866	0.9790462	0.9872551	0.8933035	1	COLQ
Biological Process	GO:0032372	negative regulation of sterol transport	1/2279	30/18866	0.9790462	0.9872551	0.8933035	1	MIR27A
Biological Process	GO:0032375	negative regulation of cholesterol transport	1/2279	30/18866	0.9790462	0.9872551	0.8933035	1	MIR27A
Biological Process	GO:0036258	multivesicular body assembly	1/2279	30/18866	0.9790462	0.9872551	0.8933035	1	MVB12A
Biological Process	GO:0045939	negative regulation of steroid metabolic process	1/2279	30/18866	0.9790462	0.9872551	0.8933035	1	GFI1
Biological Process	GO:0046457	prostanoid biosynthetic process	1/2279	30/18866	0.9790462	0.9872551	0.8933035	1	PRXL2B
Biological Process	GO:0060122	inner ear receptor cell stereocilium organization	1/2279	30/18866	0.9790462	0.9872551	0.8933035	1	CDH23
Biological Process	GO:0046456	icosanoid biosynthetic process	3/2279	59/18866	0.97933	0.9874366	0.8934677	3	GGT1/MGST2/PRXL2B

Biological Process	GO:0001510	RNA methylation	5/2279	84/18866	0.9798278	0.9878336	0.8938269	5	THADA/METTL6/RBM15B/FBL/METTL8
Biological Process	GO:0050798	activated T cell proliferation	2/2279	46/18866	0.9804874	0.9883938	0.8943338	2	RIPK3/FADD
Biological Process	GO:0008033	tRNA processing	9/2279	130/18866	0.9807389	0.9885424	0.8944682	9	THADA/METTL6/CDKAL1/PUS7/DUS2/CPSF4/PTCD1/METTL8/GRSF1
Biological Process	GO:0031338	regulation of vesicle fusion	1/2279	31/18866	0.9815814	0.9892866	0.8951417	1	VPS18
Biological Process	GO:0000184	nuclear-transcribed mRNA catabolic process, nonsense-mediated decay	8/2279	120/18866	0.9821704	0.9897752	0.8955838	8	SMG7/CTIF/SMG6/PPP2R1A/NBAS/SECISBP2/RPL23A/EIF4G1
Biological Process	GO:0071774	response to fibroblast growth factor	11/2279	153/18866	0.9824219	0.98982	0.8956243	11	SPRED2/ITGB1BP1/POLR2F/CCL5/FRS3/THBS1/NDST1/FGFR1/RUNX2/FAM20C/OTX2
Biological Process	GO:0045776	negative regulation of blood pressure	2/2279	47/18866	0.9825274	0.98982	0.8956243	2	GJA5/KCNK6
Biological Process	GO:0048806	genitalia development	2/2279	47/18866	0.9825274	0.98982	0.8956243	2	ASB1/TYRO3
Biological Process	GO:0097549	chromatin organization involved in negative regulation of transcription	10/2279	143/18866	0.9833664	0.9905451	0.8962803	10	HDAC1/KMT2D/CTBP1/ATF7IP/DNMT1/ZNFX1/DYDC2/DYDC1/MACROH2A2/H3C1

Biological Process	GO:0120254	olefinic compound metabolic process	8/2279	121/18866	0.9834556	0.9905451	0.8962803	8	MGLL/ACSL1/DHRS9/CYP4F3/RDH10/DGKQ/EPHX1/ALDH1A2
Biological Process	GO:1990928	response to amino acid starvation	2/2279	48/18866	0.9843595	0.9913504	0.897009	2	LARP1/NPRL2
Biological Process	GO:0048515	spermatid differentiation	12/2279	166/18866	0.9849056	0.9917953	0.8974116	12	ACRBP/SPAG6/CDYL/SMARCA2/DEFB1/JAM3/IQCG/DMC1/ARMC2/FSCN3/TTLL1/MFSD14A
Biological Process	GO:0034754	cellular hormone metabolic process	9/2279	134/18866	0.9855671	0.9923331	0.8978982	9	PNPLA2/DHRS9/AKR1D1/HSD17B2/COMT/FDX1/RDH10/DGKQ/ALDH1A2
Biological Process	GO:0070286	axonemal dynein complex assembly	1/2279	33/18866	0.9857691	0.9923331	0.8978982	1	DNAI2
Biological Process	GO:0120255	olefinic compound biosynthetic process	1/2279	33/18866	0.9857691	0.9923331	0.8978982	1	DGKQ
Biological Process	GO:0071826	ribonucleoprotein complex subunit organization	19/2279	240/18866	0.9858575	0.9923331	0.8978982	19	SNRPD3/SRPK2/AGO1/AGO2/SF1/EIF3M/PRPF8/SFSWAP/CELF2/NOL3/CELF1/SNUPN/PIH1D1/PRPF6/EIF3H/RUVBL1/RPL23A/GEMIN4/TFIP11
Biological Process	GO:0006278	RNA-dependent DNA biosynthetic process	4/2279	76/18866	0.9861919	0.9925645	0.8981076	4	TNKS/HMBOX1/SMG6/CCT6A
Biological Process	GO:0032543	mitochondrial translation	9/2279	135/18866	0.9865848	0.9928548	0.8983703	9	MRPL3/MRPL38/MTG2/MRPS21/MRPS26/MRPL28/EARS2/AURKAIP1/GATB

Biological Process	GO:0043616	keratinocyte proliferation	2/2279	50/18866	0.9874799	0.9936504	0.8990901	2	VDR/CDH3
Biological Process	GO:0022618	ribonucleoprotein complex assembly	18/2279	233/18866	0.9882743	0.9943444	0.8997181	18	SNRPD3/SRPK2/AGO1/AGO2/SF1/EIF3M/PRPF8/SFSWAP/CELF2/NOL3/CELF1/SNUPN/PIH1D1/PRPF6/EIF3H/RUVBL1/RPL23A/GEMIN4
Biological Process	GO:0098586	cellular response to virus	3/2279	65/18866	0.9886793	0.9946465	0.8999915	3	PHB/SEC14L1/POU2F2
Biological Process	GO:0090102	cochlea development	2/2279	51/18866	0.9888036	0.9946663	0.9000094	2	PAX2/MCM2
Biological Process	GO:0002181	cytoplasmic translation	6/2279	103/18866	0.9889507	0.9947089	0.9000479	6	ZNF385A/EIF3M/CPEB3/PKM/EIF3H/EIF4G1
Biological Process	GO:0009953	dorsal/ventral pattern formation	5/2279	92/18866	0.9899793	0.9956381	0.9008887	5	SUFU/PAX7/DSCAML1/BMPR1B/TTC21B
Biological Process	GO:0021549	cerebellum development	6/2279	105/18866	0.9906695	0.9962268	0.9014214	6	DLL1/NCOA1/TTC21B/TTLL1/CPLANE1/COQ8B
Biological Process	GO:0019080	viral gene expression	14/2279	195/18866	0.9908329	0.9962857	0.9014747	14	TRIM27/CTDP1/TRIM8/RAE1/POLR2F/CCL5/HDAC1/NUP93/SEC13/RSF1/USF2/RPL23A/SPCS1/SHFL
Biological Process	GO:0017004	cytochrome complex assembly	1/2279	37/18866	0.9915053	0.9968563	0.901991	1	COX19

Biological Process	GO:0034470	ncRNA processing	34/2279	400/18866	0.9916491	0.9968953	0.9020263	34	THADA/EXOSC2/WDR43/SMAD3/RCL1/AGO1/USP36/DDX27/AGO2/WDR46/METTL6/NOB1/ERI3/WDR55/CDKAL1/RMRP/PUS7/PIH1D1/NOP14/DUS2/TOE1/NOL6/CPSF4/PTCD1/ZCCHC8/FBL/TUT1/ERCC2/RIOK1/ISG20/METTL8/GEMIN4/GRSF1/UTP4
Biological Process	GO:0140053	mitochondrial gene expression	11/2279	165/18866	0.9923496	0.9973493	0.9024371	11	MRPL3/MRPL38/MTG2/MRPS21/MRPS26/MRPL28/EARS2/AURKAIP1/TBRG4/PRKAA1/GATB
Biological Process	GO:0051954	positive regulation of amine transport	1/2279	38/18866	0.9925335	0.9973493	0.9024371	1	GRK2
Biological Process	GO:0071276	cellular response to cadmium ion	1/2279	38/18866	0.9925335	0.9973493	0.9024371	1	GSN
Biological Process	GO:0035296	regulation of tube diameter	9/2279	143/18866	0.9926255	0.9973493	0.9024371	9	ITGB1BP1/SLC8A1/GJA5/HRH1/PER2/P2RX1/F2R/NPPC/AGTR1
Biological Process	GO:0097746	regulation of blood vessel diameter	9/2279	143/18866	0.9926255	0.9973493	0.9024371	9	ITGB1BP1/SLC8A1/GJA5/HRH1/PER2/P2RX1/F2R/NPPC/AGTR1
Biological Process	GO:0002455	humoral immune response mediated by circulating immunoglobulin	10/2279	155/18866	0.9929795	0.9975995	0.9026634	10	CD55/TNF/IGHV6-1/PTPN6/CLU/C1S/PTPRC/C1QB/BCL3/C1RL
Biological Process	GO:0035150	regulation of tube size	9/2279	144/18866	0.993168	0.9976816	0.9027377	9	ITGB1BP1/SLC8A1/GJA5/HRH1/PER2/P2RX1/F2R/NPPC/AGTR1
Biological Process	GO:0019083	viral transcription	12/2279	178/18866	0.9933304	0.9976816	0.9027377	12	TRIM27/CTDP1/TRIM8/RAE1/POLR2F/CCL5/HDAC1/NUP93/SEC13/RSF1/USF2/RPL23A

Biological Process	GO:0007340	acrosome reaction	1/2279	39/18866	0.9934373	0.9976816	0.9027377	1	PLCB1
Biological Process	GO:0030902	hindbrain development	10/2279	156/18866	0.9934812	0.9976816	0.9027377	10	PHOX2A/DLL1/NCOA1/BCL2/TTC21B/ALDH1A2/HES3/TTLL1/CPLANE1/COQ8B
Biological Process	GO:0000154	rRNA modification	1/2279	40/18866	0.9942317	0.9981187	0.9031332	1	FBL
Biological Process	GO:0000183	rDNA heterochromatin assembly	1/2279	40/18866	0.9942317	0.9981187	0.9031332	1	H3C1
Biological Process	GO:0032892	positive regulation of organic acid transport	1/2279	40/18866	0.9942317	0.9981187	0.9031332	1	MAP2K6
Biological Process	GO:0042471	ear morphogenesis	7/2279	123/18866	0.9943646	0.9981467	0.9031586	7	ABR/SLC44A4/LRIG1/PAX2/FGFR1/TTC39C/BCR
Biological Process	GO:0015980	energy derivation by oxidation of organic compounds	21/2279	278/18866	0.99482	0.9984923	0.9034713	21	PRKAG2/SLC25A25/CBFA2T3/DHTKD1/OGDH/NDUFS2/NDUFS8/AIFM2/DYRK2/CAT/PPP1CB/PRDM16/ESRRB/PER2/NDUFB6/GFPT2/PCDH12/SDHD/SIRT3/ACADVL/RUBCNL
Biological Process	GO:2000272	negative regulation of signaling receptor activity	2/2279	58/18866	0.9949191	0.9984923	0.9034713	2	LY6G6D/CRHBP
Biological Process	GO:0006636	unsaturated fatty acid biosynthetic process	2/2279	59/18866	0.9954661	0.9988302	0.9037771	2	SCD/PRXL2B

Biological Process	GO:0046605	regulation of centrosome cycle	2/2279	59/18866	0.9954661	0.9988302	0.9037771	2	POC1A/CEP295NL
Biological Process	GO:0097755	positive regulation of blood vessel diameter	2/2279	60/18866	0.9959552	0.9992154	0.9041256	2	GJA5/NPPC
Biological Process	GO:0045333	cellular respiration	12/2279	187/18866	0.9964924	0.9996489	0.9045178	12	SLC25A25/CBFA2T3/DHTKD1/OGDH/NDUFS2/NDUFS8/AIFM2/CAT/PRDM16/NDUFB6/SDHD/SIRT3
Biological Process	GO:0034198	cellular response to amino acid starvation	1/2279	45/18866	0.9969743	0.999987	0.9048237	1	NPRL2
Biological Process	GO:0006720	isoprenoid metabolic process	8/2279	144/18866	0.9973287	0.999987	0.9048237	8	PNPLA2/IDI1/DHRS9/SDC2/RDH10/HMGCS1/ALDH1A2/DPAGT1
Biological Process	GO:0032309	icosanoid secretion	1/2279	46/18866	0.9973407	0.999987	0.9048237	1	MAP2K6
Biological Process	GO:0009451	RNA modification	10/2279	168/18866	0.9973883	0.999987	0.9048237	10	THADA/RBM47/METTL6/FTO/CDKAL1/PUS7/DUS2/RBM15B/FBL/METTL8
Biological Process	GO:0050909	sensory perception of taste	2/2279	64/18866	0.9974434	0.999987	0.9048237	2	LPO/GNAT2
Biological Process	GO:0031424	keratinization	15/2279	225/18866	0.997461	0.999987	0.9048237	15	CSTA/TGM5/KRT17/JUP/PKP1/PCSK6/CDH3/PERP/CNFN/KRTAP5-9/TGM3/KRT23/KAZN/KRT7/PPL

Biological Process	GO:0001523	retinoid metabolic process	5/2279	108/18866	0.9977018	1	0.9048355	5	PNPLA2/DHRS9/SDC2/RDH10/ALDH1A2
Biological Process	GO:0042775	mitochondrial ATP synthesis coupled electron transport	4/2279	97/18866	0.998207	1	0.9048355	4	NDUFS2/NDUFS8/NDUFB6/SDHD
Biological Process	GO:0006721	terpenoid metabolic process	6/2279	124/18866	0.9982563	1	0.9048355	6	PNPLA2/DHRS9/SDC2/RDH10/HMGCS1/ALDH1A2
Biological Process	GO:0042773	ATP synthesis coupled electron transport	4/2279	98/18866	0.9983788	1	0.9048355	4	NDUFS2/NDUFS8/NDUFB6/SDHD
Biological Process	GO:0140029	exocytic process	3/2279	84/18866	0.9984506	1	0.9048355	3	VPS18/CTBP2/P2RX1
Biological Process	GO:0042254	ribosome biogenesis	22/2279	310/18866	0.9986004	1	0.9048355	22	EXOSC2/WDR43/ZNF622/RCL1/USP36/DDX27/MTG2/WDR46/NOB1/ERI3/WDR55/RMRP/PIH1D1/NOP14/RPL23A/NOL6/FBL/ERCC2/RIOK1/ISG20/GEMIN4/UTP4
Biological Process	GO:0016101	diterpenoid metabolic process	5/2279	114/18866	0.9987046	1	0.9048355	5	PNPLA2/DHRS9/SDC2/RDH10/ALDH1A2
Biological Process	GO:0022904	respiratory electron transport chain	5/2279	116/18866	0.9989324	1	0.9048355	5	NDUFS2/NDUFS8/AIFM2/NDUFB6/SDHD
Biological Process	GO:0006910	phagocytosis, recognition	3/2279	88/18866	0.9989934	1	0.9048355	3	IGHV6-1/COLEC12/FCN1

Biological Process	GO:0042273	ribosomal large subunit biogenesis	2/2279	74/18866	0.999199	1	0.9048355	2	ZNF622/RPL23A
Biological Process	GO:0022613	ribonucleoprotein complex biogenesis	37/2279	482/18866	0.9994261	1	0.9048355	37	EXOSC2/WDR43/SNRPD3/SRPK2/ZNF622/RCL1/AGO1/USP36/DDX27/AGO2/MTG2/WDR46/NOB1/SF1/EIF3M/PRPF8/ERI3/SFSWAP/WDR55/CELF2/NOL3/RMRP/CELF1/SNUPN/PIH1D1/NOP14/PRPF6/EIF3H/RUVBL1/RPL23A/NOL6/FBL/ERCC2/RIOK1/ISG20/GEMIN4/UTP4
Biological Process	GO:0070972	protein localization to endoplasmic reticulum	7/2279	152/18866	0.9995589	1	0.9048355	7	UBAC2/RTN4/MIA3/PMM2/ANK2/RPL23A/SPCS1
Biological Process	GO:0006958	complement activation, classical pathway	6/2279	141/18866	0.9996449	1	0.9048355	6	CD55/IGHV6-1/CLU/C1S/C1QB/C1RL
Biological Process	GO:0061640	cytoskeleton-dependent cytokinesis	3/2279	100/18866	0.9997297	1	0.9048355	3	CECR2/SNX18/SEPTIN9
Biological Process	GO:0042255	ribosome assembly	1/2279	67/18866	0.9998234	1	0.9048355	1	RPL23A
Biological Process	GO:0000281	mitotic cytokinesis	1/2279	72/18866	0.9999075	1	0.9048355	1	SNX18
Biological Process	GO:0006119	oxidative phosphorylation	5/2279	149/18866	0.9999615	1	0.9048355	5	NDUFS2/NDUFS8/NDUFB6/SDHD/ATP5PD
Biological Process	GO:0045047	protein targeting to ER	3/2279	120/18866	0.9999714	1	0.9048355	3	PMM2/RPL23A/SPCS1

Biological Process	GO:0072599	establishment of protein localization to endoplasmic reticulum	3/2279	124/18866	0.9999819	1	0.9048355	3	PMM2/RPL23A/SPCS1
Biological Process	GO:0006614	SRP-dependent cotranslational protein targeting to membrane	1/2279	105/18866	0.9999987	1	0.9048355	1	RPL23A
Biological Process	GO:0006613	cotranslational protein targeting to membrane	1/2279	109/18866	0.9999992	1	0.9048355	1	RPL23A
Biological Process	GO:0007608	sensory perception of smell	5/2279	452/18866	1	1	0.9048355	5	SLC24A4/NCAM2/OR52N2/CNGA4/B3GNT2
Biological Process	GO:0050907	detection of chemical stimulus involved in sensory perception	3/2279	472/18866	1	1	0.9048355	3	LPO/GNAT2/OR52N2
Biological Process	GO:0050911	detection of chemical stimulus involved in sensory perception of smell	1/2279	427/18866	1	1	0.9048355	1	OR52N2
Cellular Component	GO:0042581	specific granule	52/2341	160/19559	5.63E-12	6.53E-09	5.85E-09	52	MS4A3/DEFA4/ELANE/LRG1/SLPI/TRPM2/CTSZ/TIMP2/PLAU/SLC2A5/KCNAB2/OLR1/C3AR1/LCN2/TOLLIP/UBR4/CHI3L1/TMEM30A/CD177/TNFRSF1B/TSPAN14/PLD1/ORMDL3/FOLR3/FPR2/ATP8A1/CLEC5A/CTSD/JUP/CHIT1/SLC15A4/ATP11A/RAB27A/PTPN6/PTPRJ/CD59/PGLYRP1/OLFM4/LAIR1/ITGB2/PDXK/ATP8B4/P2RX1/LTF/ANO6/SPTAN1/DOCK2/CYFIP1/CD33/CEACAM3/ADGRG3/CRACR2A
Cellular Component	GO:0030667	secretory granule membrane	76/2341	306/19559	2.95E-10	1.71E-07	1.53E-07	76	MS4A3/AZU1/TRPM2/BSG/PLAU/SLC2A5/KCNAB2/CD9/OLR1/ACRBP/C3AR1/APLP2/ITPR2/UBR4/LPCAT1/SIRPB1/CD55/TMEM30A/CD177/SLC11A1/CLEC4C/TNFRSF1B/DOK3/RAP1B/CEACAM6/RAB5C/RHOG/RAB5B/TSPAN14/PLD1/RAB31/ORMDL3/FPR2/FPR1/ATP8A1/MGAM/CLEC5A/LY6G6F/ITPR1/HLA-C/BRI3/PKP1/SLC15A4/RHOF/ATP11A/RAB27A/ITGA2B/PHACTR2/CPNE1/PTPRN2/PTPRJ/CD59/ITGB3/PTPRC/CXCR1/LHFPL2/LAIR1/ITGB2/ATP8B4/P2RX1/PLA1A/CD68/TMED2/ANO6/CD33/RPH3AL/ANXA7/SVIP/CEACAM3/ADGRG3/STING1/IRAG2/ATP6V0C/ACP3/VPS35L/ADGRE3
Cellular Component	GO:0070820	tertiary granule	45/2341	164/19559	5.42E-08	2.10E-05	1.88E-05	45	LRG1/TRPM2/CDA/TIMP2/PLAU/KCNAB2/OLR1/UBR4/CD55/CD177/SLC11A1/CLEC4C/DOK3/TSPAN14/PLD1/FOLR3/FPR2/FPR1/MGAM/CLEC5A/CTSD/CHIT1/PKP1/ATP11A/CTSH/PTPN6/TNFAIP6/PTPRN2/HBB/CD59/PGLYRP1/OLFM4/LAIR1/ITGB2/ATP8B4/LTF/ANO6/SPTAN1/CYFIP1/CD33/DBNL/SVIP/ATP6V0C/VPS35L/ADGRE3

Cellular Component	GO:0035580	specific granule lumen	23/2341	62/19559	3.12E-07	7.42E-05	6.65E-05	23	DEFA4/ELANE/LRG1/SLPI/CTSZ/TIMP2/LCN2/TOLLIP/CHI3L1/FOLR3/CTSD/JUP/CHIT1/RAB27A/PTPN6/PGLYRP1/OLFM4/PDXK/LTF/SPTAN1/DOCK2/CYFIP1/CRACR2A
Cellular Component	GO:0001726	ruffle	46/2341	179/19559	3.20E-07	7.42E-05	6.65E-05	46	KSR1/GSN/FGD4/KANK1/TLN2/ITGB1BP1/ACAP2/ARHGEF7/WIPF1/CTTN/DNM2/MEFV/FGR/CAPG/FGD5/SPATA13/PACSIN2/TESC/MYH9/BAIAP2/ACTN1/ATP6V1B2/FRMD4B/SCIMP/PTPRJ/SH3YL1/EPS8L3/EPS8L1/MYO5A/MTSS1/SH2D3C/ITGB3/INPP5E/PDE9A/NCKAP1/CORO1C/ARHGEF2/MYO1C/PDGFRB/FSCN3/PLCG1/CYFIP1/EZR/MYADM/DBNL/ARHGAP45
Cellular Component	GO:0035579	specific granule membrane	29/2341	91/19559	4.21E-07	8.14E-05	7.29E-05	29	MS4A3/TRPM2/PLAU/SLC2A5/KCNAB2/OLR1/C3AR1/UBR4/TMEM30A/CD177/TNFRSF1B/TSPAN14/PLD1/ORMDL3/FPR2/ATP8A1/CLEC5A/SLC15A4/ATP11A/PTPRJ/CD59/LAIR1/ITGB2/ATP8B4/P2RX1/ANO6/CD33/CEACAM3/ADGRG3
Cellular Component	GO:0034774	secretory granule lumen	69/2341	322/19559	9.09E-07	0.0001507	0.0001351	69	DEFA4/AZU1/ELANE/LRG1/S100P/SLPI/CTSZ/PRTN3/S100A8/CTSG/VAT1/CDA/TIMP2/MPO/AMPD3/SERPINA1/GSN/LCN2/TOLLIP/RNASE3/HEXB/CRISPLD2/CHI3L1/GALNS/FGR/COTL1/FOLR3/DYNC1H1/MNDA/PADI2/CTSD/JUP/CHIT1/THBS1/CTSH/TUBB4B/RAB27A/ACTN1/APP/PTPN6/ACTN4/CLU/PGAM1/TGFB1/FCN1/PGLYRP1/CREG1/OLFM4/GHRL/CAB39/GRN/PDGFB/CAT/PNP/PDXK/ARSB/PKM/PSMB7/LTF/SPTAN1/DOCK2/CYFIP1/GMFG/PSMD13/IMPDH1/DBNL/ARHGAP45/CYRIB/CRACR2A
Cellular Component	GO:0060205	cytoplasmic vesicle lumen	69/2341	326/19559	1.45E-06	0.0002105	0.0001887	69	DEFA4/AZU1/ELANE/LRG1/S100P/SLPI/CTSZ/PRTN3/S100A8/CTSG/VAT1/CDA/TIMP2/MPO/AMPD3/SERPINA1/GSN/LCN2/TOLLIP/RNASE3/HEXB/CRISPLD2/CHI3L1/GALNS/FGR/COTL1/FOLR3/DYNC1H1/MNDA/PADI2/CTSD/JUP/CHIT1/THBS1/CTSH/TUBB4B/RAB27A/ACTN1/APP/PTPN6/ACTN4/CLU/PGAM1/TGFB1/FCN1/PGLYRP1/CREG1/OLFM4/GHRL/CAB39/GRN/PDGFB/CAT/PNP/PDXK/ARSB/PKM/PSMB7/LTF/SPTAN1/DOCK2/CYFIP1/GMFG/PSMD13/IMPDH1/DBNL/ARHGAP45/CYRIB/CRACR2A
Cellular Component	GO:0031983	vesicle lumen	69/2341	328/19559	1.83E-06	0.0002353	0.0002109	69	DEFA4/AZU1/ELANE/LRG1/S100P/SLPI/CTSZ/PRTN3/S100A8/CTSG/VAT1/CDA/TIMP2/MPO/AMPD3/SERPINA1/GSN/LCN2/TOLLIP/RNASE3/HEXB/CRISPLD2/CHI3L1/GALNS/FGR/COTL1/FOLR3/DYNC1H1/MNDA/PADI2/CTSD/JUP/CHIT1/THBS1/CTSH/TUBB4B/RAB27A/ACTN1/APP/PTPN6/ACTN4/CLU/PGAM1/TGFB1/FCN1/PGLYRP1/CREG1/OLFM4/GHRL/CAB39/GRN/PDGFB/CAT/PNP/PDXK/ARSB/PKM/PSMB7/LTF/SPTAN1/DOCK2/CYFIP1/GMFG/PSMD13/IMPDH1/DBNL/ARHGAP45/CYRIB/CRACR2A
Cellular Component	GO:0005911	cell-cell junction	92/2341	493/19559	8.74E-06	0.0010133	0.0009085	92	PRKCZ/TBCD/LAT/COL17A1/CADM3/TJP2/SHROOM3/YWHAH/UBN1/FXYD1/CRB1/NDRG1/PIK3R1/RAP1B/SLC8A1/FRMD4A/GJA5/ABCC2/PRICKLE4/PACSIN2/JUP/BAIAP2L1/PKP1/CDC42EP1/PDLIM2/MYH9/PMP22/BAIAP2/AHNAK/DLL1/ACTN1/APP/PTPN6/ACTN4/ILK/LIMD1/SMAD7/PXN/FRMD4B/PLEKHG5/SCN1A/CDH3/MLC1/PTPRJ/TMOD3/RAPGEF2/PLEKHA7/VAMP5/PERP/EPB41L3/KIRREL3/JAM3/ITGB3/ACTG1/CSK/GRHL2/WDR1/PRKCI/GJB3/SDCCAG8/CDH1/ADD1/MPP7/NOTCH1/AQP3/ARHGAP17/ARHGEF2/CNTNAP1/PCDH12/SLC2A1/KAZN/PDLIM4/ANK2/CDC42BPB/SHROOM1/FRMD6/PLCG1/CADM1/CDH11/PTPRU/KIFC3/BAIAP2L2/FLOT1/MYADM/SPTBN4/SYNPO/NPHP4/PPL/JAML/NECTIN1/NECTIN4/PLPP3
Cellular Component	GO:0031252	cell leading edge	80/2341	421/19559	1.69E-05	0.001787	0.0016021	80	PRKCZ/KSR1/P4HB/PARVB/SSH1/GSN/FGD4/KANK1/TLN2/APBB2/CD177/PSTPIP1/ITGB1BP1/AAK1/MYLK/ACAP2/ARHGEF7/WIPF1/CTTN/DNM2/MEFV/FGR/CAPG/DUSP22/FGD5/ARHGAP31/ITSN1/SPATA13/PACSIN2/TESC/MYH9/BAIAP2/ACTN1/APP/ATP6V1B2/ILK/ABLIM3/AKAP5/PXN/DOCK8/FRMD4B/SCIMP/PLEKHG5/WASL/FER/PTPRJ/SH3YL1/EPS8L3/EPS8L1/MYO5A/EPB41L3/SLC1A2/MTSS1/SH2D3C/ITGB3/INPP5E/PALM/PDE9A/PRKCI/NCKAP1/CDH1/CORO1C/AVIL/ARHGEF2/MYO1C/PDGFRB/FSCN3/DYSF/PDLIM4/CDC42BPB/PLCG1/CYFIP1/ABLIM1/NDEL1/EZR/FLOT1/LMO4/MYADM/DBNL/ARHGAP45
Cellular Component	GO:0005769	early endosome	73/2341	377/19559	2.03E-05	0.0018184	0.0016303	73	TRIM27/EHD1/LMTK2/SORL1/MGRN1/SORCS2/ANKFY1/TM9SF4/VAC14/LNPEP/TBC1D16/TGFBRAP1/RAB5C/RAB5B/LDLRAD4/RAB31/SNX27/FGD5/RIN3/SLC11A2/PACSIN2/HLA-C/RABGEF1/RUSC1/ACKR2/ANKRD13B/ATP11A/INPP4A/SNX3/VPS26B/APP/HLA-F/OSBPL6/DERL2/UVRAG/TMEM163/NISCH/PHB/MLC1/RABGAP1L/LITAF/MYO5A/VPS18/HTT/NUMB/SH3GL1/KREMEN2/KCNQ1/SORT1/PI4K2A/NTRK1/ASTN2/PLD3/F2R/WDFY1/EPS15/LTF/DYSF/RUFY1/WDR81/HPS6/PDLIM4/RNF11/ANK2/PARM1/INPP5F/FLOT1/ZFYVE9/DBNL/MARCHF3/PHETA1/MARCHF8/ACKR1

Cellular Component	GO:0019898	extrinsic component of membrane	62/2341	306/19559	2.04E-05	0.0018184	0.0016303	62	AZU1/MGLL/KCNAB2/PIK3CD/LCK/ULK1/VAC14/VPS13D/PAM16/AAK1/PIK3R1/PIK3R6/FGR/CDH23/FOLR3/WIPI2/GNAZ/PACSIN2/JUP/SLA/PIK3R5/GNAI2/RGS6/SMAD7/KCNIP1/GNAT2/CDH3/FARP1/FER/FCN1/GNG7/DEFB1/BLK/ATG14/GNA11/NUMB/GNB3/CDH4/NUMA1/CNR2/SNX18/CDH1/MICALL1/GNG2/RPH3A/GNAO1/GNA12/TGM3/WDR81/GRAMD1A/CYLD/EPN3/ESYT1/CDH11/EZR/RNF40/ASPSCR1/COQ4/TEPSIN/GRAMD2A/COQ8B/SLA2
Cellular Component	GO:0070821	tertiary granule membrane	22/2341	73/19559	2.73E-05	0.0022613	0.0020273	22	TRPM2/PLAU/KCNAB2/OLR1/UBR4/CD177/SLC11A1/CLEC4C/TSPAN14/PLD1/FPR2/MGAM/CLEC5A/ATP11A/CD59/LAIR1/ITGB2/ATP8B4/ANO6/CD33/SVIP/ATP6V0C
Cellular Component	GO:0005925	focal adhesion	78/2341	415/19559	3.19E-05	0.0024641	0.0022092	78	P4HB/PARVB/BSG/CALR/PLAU/SNTB2/CD9/GSN/TLN2/TNS3/AFAP1/ARHGAP26/RHOG/ARHGEF7/CTTN/DNM2/ANXA6/ARHGAP31/PACSIN2/JUP/CDC42EP1/TNS1/SLA/MYH9/GIT1/AHNAK/ACTN1/ITGA2B/ACTN4/ILK/LIMD1/SLC9A3R2/PXN/FLT1/ITGB5/PPFIA1/JAK1/LMO7/SVIL/PFN1/TGFB1I1/CD59/NUMB/ITGB3/PTPRC/ACTG1/MPZL1/CAT/PPP1CB/CASS4/SNTB1/ENG/ITGB2/NCKAP1/RALA/YWHAZ/ADD1/PI4KA/CORO1C/PARVG/AVIL/FHL2/GNA12/FLII/ARHGEF2/BCAR3/THSD1/PDGFRB/RSU1/IRF2/CYFIP1/YWHAQ/EZR/LPP/FLOT1/ITGB6/PRAG1/ATP6V0C
Cellular Component	GO:0030055	cell-substrate junction	79/2341	423/19559	3.57E-05	0.00259	0.002322	79	P4HB/PARVB/BSG/CALR/PLAU/SNTB2/CD9/COL17A1/GSN/TLN2/TNS3/AFAP1/ARHGAP26/RHOG/ARHGEF7/CTTN/DNM2/ANXA6/ARHGAP31/PACSIN2/JUP/CDC42EP1/TNS1/SLA/MYH9/GIT1/AHNAK/ACTN1/ITGA2B/ACTN4/ILK/LIMD1/SLC9A3R2/PXN/FLT1/ITGB5/PPFIA1/JAK1/LMO7/SVIL/PFN1/TGFB1I1/CD59/NUMB/ITGB3/PTPRC/ACTG1/MPZL1/CAT/PPP1CB/CASS4/SNTB1/ENG/ITGB2/NCKAP1/RALA/YWHAZ/ADD1/PI4KA/CORO1C/PARVG/AVIL/FHL2/GNA12/FLII/ARHGEF2/BCAR3/THSD1/PDGFRB/RSU1/IRF2/CYFIP1/YWHAQ/EZR/LPP/FLOT1/ITGB6/PRAG1/ATP6V0C
Cellular Component	GO:0005884	actin filament	30/2341	118/19559	4.24E-05	0.0028936	0.0025943	30	GAS7/MYO18B/AKAP13/MYO9B/PSTPIP1/WIPF1/CTTN/COTL1/DUSP22/PRICKLE4/PDLIM2/ACKR2/ACTN1/WASL/MYO5A/VPS18/JAM3/ESPN/ACTG1/NCKAP1/AVIL/MYO1C/PDLIM4/TMOD1/MARK2/EZR/SMTN/SPTBN4/DAPK3/DBNL
Cellular Component	GO:0009898	cytoplasmic side of plasma membrane	37/2341	164/19559	9.52E-05	0.0061321	0.0054978	37	KCNAB2/LCK/FGR/TRAF2/TRAF1/GNAZ/JUP/SLA/GNAI2/RASA3/KCNIP1/AKAP5/GNAT2/FARP1/FER/LITAF/GNG7/BLK/GNA11/PTPRC/GNB3/PALM/CNR2/SNX18/SPTA1/CDH1/GNG2/GNAO1/GNA12/TGM3/GRAMD1A/CYLD/ESYT1/EZR/ASPSCR1/C2CD2L/GRAMD2A
Cellular Component	GO:0030027	lamellipodium	43/2341	201/19559	0.0001006	0.0061444	0.0055088	43	P4HB/PARVB/SSH1/GSN/FGD4/APBB2/CD177/PSTPIP1/ITGB1BP1/MYLK/ARHGEF7/CTTN/DNM2/MEFV/CAPG/DUSP22/FGD5/ARHGAP31/ITSN1/SPATA13/TESC/APP/ILK/ABLIM3/PXN/DOCK8/PLEKHG5/WASL/FER/ITGB3/NCKAP1/CDH1/CORO1C/AVIL/FSCN3/DYSF/PDLIM4/CDC42BPB/PLCG1/CYFIP1/ABLIM1/FLOT1/DBNL
Cellular Component	GO:0098857	membrane microdomain	63/2341	330/19559	0.0001112	0.0062765	0.0056272	63	PRTN3/PRKCZ/LAT/SYNJ2/BSG/KCNE1/HK1/OLR1/ADCY2/LCK/CD55/INPP5D/CD177/ANGPT1/FXYD1/TNFRSF1B/TNF/TRAF2/CTSD/PACSIN2/TGFBR2/UNC5B/AHNAK/DLL1/RIPK1/CD226/GNAI2/APP/NOS1AP/TNFRSF1A/AKAP5/ARID3A/MLC1/CD79A/SLC1A2/PTPRC/CSK/ABCA1/BCL10/KCNQ1/ITGB2/NPC1/PI4K2A/CDH1/ADD1/CORO1C/P2RX1/PPP2R1B/F2R/EFHD2/MYO1C/SLC2A1/DYSF/ANK2/ADTRP/EZR/PDZK1/FLOT1/MYADM/LTB4R/FADD/DAPK3/PLPP3
Cellular Component	GO:0016323	basolateral plasma membrane	50/2341	246/19559	0.0001136	0.0062765	0.0056272	50	TACSTD2/AP2A1/TGFA/SLC51A/ABCC1/SLC16A3/SLC10A1/MAP7/SLC1A5/LPO/ARRB1/TLR9/OSCP1/SLC2A9/C5AR2/P2RY6/MLC1/SLC19A1/SLC26A1/AQP1/MUC20/SLC7A7/SLC7A5/NUMB/IL6R/PDGFB/PALM/KCNQ1/ARRB2/SLC16A1/CNNM2/SLC1A3/NOD2/AQP3/EPS15/MYO1C/SLC2A1/SLC7A8/ANK2/SLC29A1/CADM1/ATP7B/EZR/FLOT1/SLC7A1/SLC23A1/SLC41A1/AQP9/CEACAM5/PLPP3

Cellular Component	GO:0045121	membrane raft	62/2341	329/19559	0.0001825	0.0094172	0.008443	62	PRTN3/PRKCZ/LAT/SYNJ2/BSG/KCNE1/HK1/OLR1/ADCY2/LCK/CD55/INPP5D/CD177/ANGPT1/FXYD1/TNFRSF1B/TNF/TRAF2/CTSD/PACSIN2/TGFBR2/UNC5B/AHNAK/DLL1/RIPK1/CD226/GNAI2/APP/NOS1AP/TNFRSF1A/AKAP5/ARID3A/MLC1/CD79A/SLC1A2/PTPRC/CSK/ABCA1/BCL10/KCNQ1/ITGB2/NPC1/PI4K2A/CDH1/ADD1/CORO1C/P2RX1/PPP2R1B/F2R/EFHD2/MYO1C/SLC2A1/ANK2/ADTRP/EZR/PDZK1/FLOT1/MYADM/LTB4R/FADD/DAPK3/PLPP3
Cellular Component	GO:0098589	membrane region	64/2341	343/19559	0.0001937	0.0094172	0.008443	64	PRTN3/PRKCZ/LAT/SYNJ2/BSG/KCNE1/HK1/OLR1/ADCY2/LCK/CD55/INPP5D/CD177/ANGPT1/FXYD1/TNFRSF1B/TNF/TRAF2/CTSD/PACSIN2/TGFBR2/UNC5B/AHNAK/DLL1/RIPK1/CD226/GNAI2/APP/NOS1AP/TNFRSF1A/AKAP5/ARID3A/MLC1/BICD2/CD79A/SLC1A2/PTPRC/CSK/ABCA1/BCL10/KCNQ1/ITGB2/NPC1/PI4K2A/CDH1/ADD1/CORO1C/P2RX1/PPP2R1B/F2R/EFHD2/MYO1C/SLC2A1/DYSF/ANK2/ADTRP/EZR/PDZK1/FLOT1/MYADM/LTB4R/FADD/DAPK3/PLPP3
Cellular Component	GO:0098562	cytoplasmic side of membrane	40/2341	188/19559	0.0001948	0.0094172	0.008443	40	KCNAB2/THADA/LCK/FGR/TRAF2/TRAF1/GNAZ/JUP/SLA/HM13/GNAI2/RASA3/KCNIP1/AKAP5/GNAT2/FARP1/FER/LITAF/GNG7/CDIP1/BLK/GNA11/PTPRC/GNB3/PALM/CNR2/SNX18/SPTA1/CDH1/GNG2/GNAO1/GNA12/TGM3/GRAMD1A/CYLD/ESYT1/EZR/ASPSCR1/C2CD2L/GRAMD2A
Cellular Component	GO:0005774	vacuolar membrane	76/2341	427/19559	0.0002434	0.0109753	0.0098399	76	AZU1/TRPM2/HLA-DMB/RPTOR/AP2A1/CLCN6/C3AR1/ANKFY1/ULK1/VAC14/RAB2A/LPCAT1/TMEM30A/LNPEP/RAP1B/CEACAM6/RAB5C/SLC29A3/ARRB1/PLD1/GPLD1/RHEB/ANXA6/FPR1/TLR9/ATP8A1/CTSD/SLC11A2/BRI3/SLC15A4/ATP11A/AHNAK/HLA-F/ATP6V1B2/UVRAG/CPNE1/AP1B1/SPHK2/TMEM150B/LITAF/AP5S1/GIMAP5/VPS18/CDIP1/CLEC16A/SLC7A5/ATG14/GNA11/SEC13/ITM2C/GRN/PLEKHM1/TPCN2/SORT1/NPC1/FNIP2/PI4K2A/VMP1/ATP6V0B/PLD3/OSTM1/ABCC11/CD68/WDR81/HPS6/AP3D1/CLN5/FLOT1/NPRL2/MARCHF8/STING1/IRAG2/ATP6V0C/ATP6V1C1/ACP3/RUBCNL
Cellular Component	GO:0055037	recycling endosome	40/2341	190/19559	0.000246	0.0109753	0.0098399	40	EHD1/LMTK2/SORL1/SORCS2/VPS53/ULK1/NDRG1/RAB11FIP1/TNF/ABCG1/ITSN1/SLC11A2/PACSIN2/HLA-C/RABGEF1/TNIK/ACKR2/ATP11A/INPP4A/ZDHHC2/APP/HLA-F/AKAP5/NISCH/MLC1/DENND6B/MYO5A/TBC1D14/NTRK1/MICALL1/LTF/PDLIM4/RNF11/ANK2/AVL9/INPP5F/VPS51/PHETA1/ACKR1/DENND2B
Cellular Component	GO:0101002	ficolin-1-rich granule	29/2341	124/19559	0.0002727	0.0112982	0.0101294	29	LRG1/CTSZ/CDA/TIMP2/AMPD3/SERPINA1/GSN/CRISPLD2/COTL1/MNDA/CTSD/JUP/CTSH/TNFAIP6/PGAM1/HBB/FCN1/CAB39/CTSB/CAT/PNP/ARSB/PKM/PSMB7/GMFG/PSMD13/CDK13/IMPDH1/DBNL
Cellular Component	GO:1904813	ficolin-1-rich granule lumen	29/2341	124/19559	0.0002727	0.0112982	0.0101294	29	LRG1/CTSZ/CDA/TIMP2/AMPD3/SERPINA1/GSN/CRISPLD2/COTL1/MNDA/CTSD/JUP/CTSH/TNFAIP6/PGAM1/HBB/FCN1/CAB39/CTSB/CAT/PNP/ARSB/PKM/PSMB7/GMFG/PSMD13/CDK13/IMPDH1/DBNL
Cellular Component	GO:0005766	primary lysosome	34/2341	155/19559	0.0003139	0.012137	0.0108814	34	DEFA4/AZU1/ELANE/PRTN3/CTSG/VAT1/MPO/C3AR1/TOLLIP/RNASE3/HEXB/LPCAT1/GALNS/TMEM30A/RAP1B/CEACAM6/RAB5C/DYNC1H1/FPR1/ATP8A1/MNDA/PADI2/BRI3/TUBB4B/CPNE1/CREG1/GRN/ARSB/CD68/IMPDH1/ARHGAP45/IRAG2/ATP6V0C/ACP3
Cellular Component	GO:0042582	azurophil granule	34/2341	155/19559	0.0003139	0.012137	0.0108814	34	DEFA4/AZU1/ELANE/PRTN3/CTSG/VAT1/MPO/C3AR1/TOLLIP/RNASE3/HEXB/LPCAT1/GALNS/TMEM30A/RAP1B/CEACAM6/RAB5C/DYNC1H1/FPR1/ATP8A1/MNDA/PADI2/BRI3/TUBB4B/CPNE1/CREG1/GRN/ARSB/CD68/IMPDH1/ARHGAP45/IRAG2/ATP6V0C/ACP3

Cellular Component	GO:0033116	endoplasmic reticulum-Golgi intermediate compartment membrane	20/2341	75/19559	0.0003912	0.0146386	0.0131243	20	CTSZ/CALR/SERPINA1/TGFA/STX5/CSNK1D/SLC35C2/RAB2A/CD55/LMAN2/WHAMM/YIF1A/ERGIC1/CNIH2/CD59/VMP1/TMED2/ASPSCR1/STING1/PLPP3
Cellular Component	GO:0030139	endocytic vesicle	58/2341	313/19559	0.0004515	0.0163667	0.0146736	58	ELANE/CPNE6/EHD1/MPO/CALR/AP2A1/CD9/GSN/SLC11A1/NCF4/RAB5C/RAB5B/RAB11FIP1/HEATR5A/PLD1/DNM2/RAB31/SFTPD/TLR9/ITSN1/HLA-C/SNX3/VPS26B/HLA-F/FMNL1/COLEC12/NCF2/UVRAG/ITGB5/SCIMP/PLEKHG5/WASL/NOSTRIN/MARCO/STAB2/RAPGEF2/EPN2/HBB/RAB43/MTSS1/PGLYRP1/ATG14/ACTG1/WNT5B/ABCA1/MPEG1/ARRB2/RALA/NOD2/LPAR2/ATP6V0B/EPS15/LTF/MYO1C/DYSF/INPP5F/CLTCL1/ATP6V0C
Cellular Component	GO:0031234	extrinsic component of cytoplasmic side of plasma membrane	24/2341	99/19559	0.0005092	0.0171087	0.0153388	24	KCNAB2/LCK/FGR/GNAZ/SLA/GNAI2/KCNIP1/GNAT2/FARP1/FER/GNG7/BLK/GNA11/GNB3/CNR2/SNX18/GNG2/GNAO1/GNA12/TGM3/GRAMD1A/CYLD/ESYT1/GRAMD2A
Cellular Component	GO:1904724	tertiary granule lumen	16/2341	55/19559	0.0005133	0.0171087	0.0153388	16	LRG1/CDA/TIMP2/FOLR3/CTSD/CHIT1/CTSH/PTPN6/TNFAIP6/HBB/PGLYRP1/OLFM4/LTF/SPTAN1/CYFIP1/DBNL
Cellular Component	GO:0005905	clathrin-coated pit	19/2341	71/19559	0.0005162	0.0171087	0.0153388	19	AP2A1/AAK1/ARRB1/CTTN/DNM2/ITSN1/APP/EPS15L1/LRP10/NUMB/SH3BP4/DGKD/ARRB2/SORT1/DNM1L/EPS15/EPN3/INPP5F/CLTCL1
Cellular Component	GO:0019897	extrinsic component of plasma membrane	36/2341	172/19559	0.0005484	0.017672	0.0158439	36	KCNAB2/LCK/AAK1/FGR/CDH23/GNAZ/JUP/SLA/GNAI2/SMAD7/KCNIP1/GNAT2/CDH3/FARP1/FER/FCN1/GNG7/BLK/GNA11/NUMB/GNB3/CDH4/NUMA1/CNR2/SNX18/CDH1/GNG2/GNAO1/GNA12/TGM3/GRAMD1A/CYLD/EPN3/ESYT1/CDH11/GRAMD2A
Cellular Component	GO:0030863	cortical cytoskeleton	25/2341	106/19559	0.000611	0.0191545	0.017173	25	MAEA/GSN/MYRIP/SHROOM3/AKAP13/CTTN/COTL1/MYH9/ACTN4/WASL/PRKCB/NUMA1/WDR1/SPTA1/CDH1/SLC2A1/SPTAN1/SHROOM1/EPB42/TMOD1/EZR/FLOT1/MYADM/SPTBN4/DBNL
Cellular Component	GO:0009925	basal plasma membrane	15/2341	51/19559	0.0006695	0.0204388	0.0183244	15	TACSTD2/ABCC1/SLC1A5/OSCP1/C5AR2/AQP1/MUC20/SLC7A5/SLC16A1/SLC1A3/EPS15/MYO1C/SLC7A8/SLC7A1/SLC23A1
Cellular Component	GO:0005938	cell cortex	56/2341	305/19559	0.0007053	0.0209777	0.0188076	56	MAEA/CTSZ/PRKCZ/DCTN1/ENO1/GSN/ITPR2/MYRIP/RHOH/SHROOM3/AKAP13/MYO9B/RHOG/ARHGEF7/CTTN/COTL1/TRAF2/DYNC1H1/MICAL3/MYH9/RHOF/FMNL1/ACTN4/FRYL/PXN/WASL/FER/RHOBTB2/PRKCB/CTBP1/PFN1/CTBP2/AGTRAP/NUMA1/WDR1/SPTA1/CDH1/ASTN2/CORO1C/CABP1/NEDD4/SLC2A1/SPTAN1/SHROOM1/ASPH/EPB42/TMOD1/EZR/FLOT1/MYADM/SPTBN4/C2CD2L/DBNL/SEPTIN9/STIMATE/DENND2B

Cellular Component	GO:0031094	platelet dense tubular network	6/2341	11/19559	0.0007892	0.0215427	0.0193142	6	EHD1/ITPR2/ITPR1/ATP2A3/F2R/IRAG1
Cellular Component	GO:0042641	actomyosin	20/2341	79/19559	0.0008072	0.0215427	0.0193142	20	HDAC4/PRKCZ/MYLK/MYO18A/PRICKLE4/PDLIM2/MYH9/ACTN1/ACTN4/ILK/ABLIM3/PXN/MYO5A/DAAM1/PDLIM4/CDC42BPB/ABLIM1/LPP/SYNPO/SEPTIN9
Cellular Component	GO:0030016	myofibril	44/2341	227/19559	0.0008116	0.0215427	0.0193142	44	HDAC4/PARVB/ENO1/KCNE1/MYO18B/TTN/CASQ2/SYNE1/CACNA1C/SLC8A1/TNNT3/PRICKLE4/JUP/MYOZ3/BIN1/PDLIM2/MYH15/AHNAK/SYNPO2L/ACTN1/NOS1AP/ACTN4/ILK/SCN1A/TMOD3/SVIL/PDE4DIP/DNAJB6/ACTG1/CAB39/ARHGEF25/MYOM1/ANKRD2/CORO1C/MYOT/FHL2/SLC2A1/PDLIM4/ANK2/ANKRD1/TMOD1/MYL7/CASQ1/SYNPO
Cellular Component	GO:0005765	lysosomal membrane	66/2341	375/19559	0.0008171	0.0215427	0.0193142	66	AZU1/TRPM2/HLA-DMB/RPTOR/AP2A1/CLCN6/C3AR1/ANKFY1/RAB2A/LPCAT1/TMEM30A/LNPEP/RAP1B/CEACAM6/RAB5C/SLC29A3/ARRB1/PLD1/GPLD1/RHEB/ANXA6/FPR1/TLR9/ATP8A1/CTSD/SLC11A2/BRI3/SLC15A4/ATP11A/AHNAK/HLA-F/ATP6V1B2/CPNE1/AP1B1/SPHK2/LITAF/AP5S1/GIMAP5/VPS18/CDIP1/CLEC16A/SLC7A5/GNA11/SEC13/ITM2C/GRN/PLEKHM1/TPCN2/SORT1/NPC1/FNIP2/PI4K2A/PLD3/OSTM1/CD68/WDR81/HPS6/AP3D1/CLN5/FLOT1/NPRL2/MARCHF8/IRAG2/ATP6V0C/ATP6V1C1/ACP3
Cellular Component	GO:0098852	lytic vacuole membrane	66/2341	375/19559	0.0008171	0.0215427	0.0193142	66	AZU1/TRPM2/HLA-DMB/RPTOR/AP2A1/CLCN6/C3AR1/ANKFY1/RAB2A/LPCAT1/TMEM30A/LNPEP/RAP1B/CEACAM6/RAB5C/SLC29A3/ARRB1/PLD1/GPLD1/RHEB/ANXA6/FPR1/TLR9/ATP8A1/CTSD/SLC11A2/BRI3/SLC15A4/ATP11A/AHNAK/HLA-F/ATP6V1B2/CPNE1/AP1B1/SPHK2/LITAF/AP5S1/GIMAP5/VPS18/CDIP1/CLEC16A/SLC7A5/GNA11/SEC13/ITM2C/GRN/PLEKHM1/TPCN2/SORT1/NPC1/FNIP2/PI4K2A/PLD3/OSTM1/CD68/WDR81/HPS6/AP3D1/CLN5/FLOT1/NPRL2/MARCHF8/IRAG2/ATP6V0C/ATP6V1C1/ACP3
Cellular Component	GO:0098844	postsynaptic endocytic zone membrane	4/2341	5/19559	0.0009259	0.0238677	0.0213987	4	DNM2/DNM1/DNM3/MX1
Cellular Component	GO:0030864	cortical actin cytoskeleton	20/2341	80/19559	0.0009571	0.0241359	0.021639	20	MAEA/GSN/MYRIP/SHROOM3/AKAP13/CTTN/COTL1/MYH9/WASL/PRKCB/WDR1/SPTA1/CDH1/SLC2A1/SPTAN1/SHROOM1/FLOT1/MYADM/SPTBN4/DBNL
Cellular Component	GO:0031984	organelle subcompartment	68/2341	392/19559	0.001022	0.0247861	0.022222	68	CSGALNACT1/FUT4/SORL1/GALNT2/FUT7/VTI1A/VPS53/AP4S1/CHSY1/MBTPS1/TGFBI/KIF13A/DNM2/RAB31/MGAT4A/MYO18A/ATP8A1/RTN4/C17orf75/ARAP1/TRAPPC9/ATP11A/ATP8B1/CHST2/APP/CORO7/PITPNM1/AP1B1/XYLT1/COG1/SGMS1/B4GALT7/UXS1/RAB43/ASAP2/COG8/ST6GAL1/VCPIP1/GRN/ST3GAL4/USP6NL/INPP5E/B4GALT3/SORT1/ATP8B4/PI4K2A/CDH1/FUT11/SYT17/RAB30/COG5/TMED2/YIPF1/ATL1/ASPH/ATP7B/RTN2/CASQ1/VPS51/CLTCL1/C2CD2L/LYPLA2/ST3GAL2/PHETA1/TEPSIN/STIMATE/PLPP3/DOP1B
Cellular Component	GO:0045335	phagocytic vesicle	30/2341	140/19559	0.0010256	0.0247861	0.022222	30	ELANE/MPO/CALR/GSN/SLC11A1/NCF4/RAB11FIP1/DNM2/RAB31/TLR9/HLA-C/SNX3/VPS26B/HLA-F/FMNL1/NCF2/UVRAG/ITGB5/SCIMP/RAB43/PGLYRP1/ATG14/ACTG1/ABCA1/MPEG1/NOD2/ATP6V0B/LTF/MYO1C/ATP6V0C

Cellular Component	GO:0005912	adherens junction	34/2341	166/19559	0.0011441	0.0270839	0.0242821	34	TBCD/TJP2/SHROOM3/CRB1/NDRG1/FRMD4A/PRICKLE4/JUP/BAIAP2L1/PKP1/CDC42EP1/PDLIM2/MYH9/BAIAP2/DLL1/LIMD1/SMAD7/FRMD4B/CDH3/TMOD3/PLEKHA7/CDH1/ADD1/MPP7/NOTCH1/PDLIM4/SHROOM1/CDH11/KIFC3/FLOT1/SPTBN4/NECTIN1/NECTIN4/PLPP3
Cellular Component	GO:0043292	contractile fiber	45/2341	238/19559	0.0012166	0.0282247	0.0253049	45	HDAC4/PARVB/ENO1/KCNE1/MYO18B/TTN/CASQ2/MYH11/SYNE1/CACNA1C/SLC8A1/TNNT3/PRICKLE4/JUP/MYOZ3/BIN1/PDLIM2/MYH15/AHNAK/SYNPO2L/ACTN1/NOS1AP/ACTN4/ILK/SCN1A/TMOD3/SVIL/PDE4DIP/DNAJB6/ACTG1/CAB39/ARHGEF25/MYOM1/ANKRD2/CORO1C/MYOT/FHL2/SLC2A1/PDLIM4/ANK2/ANKRD1/TMOD1/MYL7/CASQ1/SYNPO
Cellular Component	GO:0048188	Set1C/COMPASS complex	6/2341	12/19559	0.0014189	0.0321351	0.0288108	6	ASH2L/WDR82/SETD1B/SETD1A/DYDC2/DYDC1
Cellular Component	GO:0031941	filamentous actin	11/2341	34/19559	0.0014546	0.0321351	0.0288108	11	MYO18B/DUSP22/PRICKLE4/PDLIM2/JAM3/ESPN/ACTG1/NCKAP1/MYO1C/PDLIM4/SMTN
Cellular Component	GO:0001772	immunological synapse	12/2341	39/19559	0.0014682	0.0321351	0.0288108	12	LAT/RHOH/LCK/SNX27/MYH9/SCIMP/PTPRJ/BCL10/DUSP3/CD37/EZR/VAV3
Cellular Component	GO:0043197	dendritic spine	35/2341	175/19559	0.0015095	0.0324266	0.0290721	35	ABR/SORCS2/PPP1R1B/ZMYND8/SLC8A1/ARRB1/CTTN/DNM2/ITSN1/SLA/COMT/BAIAP2/APP/AKAP5/ASAP1/APBA2/FARP1/CNIH2/STRN4/BCR/SIPA1L1/ARRB2/DNM1/DOCK10/DNM3/RPH3A/SHANK2/RGS10/NEDD4/PDLIM4/MX1/CYFIP1/MYL7/DTNBP1/SYNPO
Cellular Component	GO:0002193	MAML1-RBP-Jkappa- ICN1 complex	3/2341	3/19559	0.0017127	0.0361218	0.0323851	3	MAML1/RBPJ/NOTCH1
Cellular Component	GO:0098685	Schaffer collateral - CA1 synapse	20/2341	84/19559	0.0018191	0.0376318	0.0337389	20	PRKCZ/ABR/PRKAR1B/ITPR1/BAIAP2/APBA2/NRXN1/SYN2/GHRL/ACTG1/SH3GL1/BCR/PRKCI/GSG1L/GABBR1/PLCG1/CDH11/DTNBP1/SYNPO/LRFN2
Cellular Component	GO:0044309	neuron spine	35/2341	177/19559	0.0018492	0.0376318	0.0337389	35	ABR/SORCS2/PPP1R1B/ZMYND8/SLC8A1/ARRB1/CTTN/DNM2/ITSN1/SLA/COMT/BAIAP2/APP/AKAP5/ASAP1/APBA2/FARP1/CNIH2/STRN4/BCR/SIPA1L1/ARRB2/DNM1/DOCK10/DNM3/RPH3A/SHANK2/RGS10/NEDD4/PDLIM4/MX1/CYFIP1/MYL7/DTNBP1/SYNPO

Cellular Component	GO:0005635	nuclear envelope	78/2341	473/19559	0.0020071	0.0399354	0.0358042	78	PRKCZ/TRIM27/DCTN1/SORL1/CALR/FZR1/VRK2/NXF1/PCYT1A/MAD1L1/SLC16A3/ITPRIP/TNKS/SYNE1/ERN1/RAE1/SMAD3/RCC1/ITSN1/GNAZ/RTN4/ITPR1/NAV3/BIN1/ACKR2/LRRC59/XPO7/EPC1/PRICKLE2/APP/NOS1AP/OSBPL6/LMNB2/CPNE1/CLGN/ENY2/BICD2/LMO7/AQP1/GRK5/MTA1/NUP93/NRXN1/SEC13/MGST2/ATP2A3/PLCB1/TEX2/SORT1/NPC1/MFSD10/SNUPN/BCL2/P2RX1/TOR1B/MYO1C/BCL2L1/RBM15B/MX1/TNRC18/TYRO3/SREBF1/PLRG1/P2RX5/FBXW11/NDEL1/LPIN1/BANF1/RAP1GAP2/ANXA7/PCID2/AGPAT5/TOR3A/TERB2/TEPSIN/LMNTD1/IRAG2/MYORG
Cellular Component	GO:0031095	platelet dense tubular network membrane	5/2341	9/19559	0.0020312	0.0399354	0.0358042	5	EHD1/ITPR2/ITPR1/ATP2A3/IRAG1
Cellular Component	GO:0031674	I band	29/2341	140/19559	0.002125	0.0410831	0.0368331	29	HDAC4/PARVB/KCNE1/MYO18B/TTN/CASQ2/CACNA1C/SLC8A1/PRICKLE4/JUP/MYOZ3/BIN1/PDLIM2/SYNPO2L/ACTN1/NOS1AP/ACTN4/SCN1A/DNAJB6/CAB39/ANKRD2/MYOT/FHL2/SLC2A1/PDLIM4/ANK2/ANKRD1/CASQ1/SYNPO
Cellular Component	GO:0031227	intrinsic component of endoplasmic reticulum membrane	33/2341	166/19559	0.0022413	0.0412417	0.0369753	33	EMC8/CALR/XXYLT1/DHRS9/ERN1/AMFR/PIGU/RHBDD2/RTN4/HLA-C/EDEM1/HM13/HLA-F/DERL2/ERGIC1/SGMS1/SGMS2/SLC37A1/GRAMD1A/SCD/ESYT1/ANKLE2/RTN2/UBXN8/DPAGT1/EMC1/TEX261/BSCL2/SPCS1/MARCHF6/GRAMD2A/STING1/SARAF
Cellular Component	GO:0030018	Z disc	27/2341	128/19559	0.0022617	0.0412417	0.0369753	27	HDAC4/PARVB/KCNE1/MYO18B/TTN/CASQ2/CACNA1C/SLC8A1/PRICKLE4/JUP/MYOZ3/BIN1/PDLIM2/SYNPO2L/ACTN1/NOS1AP/ACTN4/SCN1A/DNAJB6/CAB39/MYOT/FHL2/SLC2A1/PDLIM4/ANK2/CASQ1/SYNPO
Cellular Component	GO:0031092	platelet alpha granule membrane	7/2341	17/19559	0.0022754	0.0412417	0.0369753	7	CD9/APLP2/LY6G6F/ITGA2B/PHACTR2/ITGB3/LHFPL2
Cellular Component	GO:0099092	postsynaptic density, intracellular component	7/2341	17/19559	0.0022754	0.0412417	0.0369753	7	ABR/DNM2/TNIK/BAIAP2/SH3GL1/BCR/ARHGEF2
Cellular Component	GO:0001931	uropod	6/2341	13/19559	0.0023693	0.0416425	0.0373347	6	PIP5K1B/PSTPIP1/MYH9/SCIMP/EZR/FLOT1
Cellular Component	GO:0031254	cell trailing edge	6/2341	13/19559	0.0023693	0.0416425	0.0373347	6	PIP5K1B/PSTPIP1/MYH9/SCIMP/EZR/FLOT1

Cellular Component	GO:0005667	transcription regulator complex	69/2341	413/19559	0.0025362	0.0439101	0.0393677	69	ELANE/HDAC4/ZFPM1/GFI1/RXRA/PPARG/TFDP1/NCOR2/CREB1/RUNX1/SKI/VDR/CORO2A/SMAD3/GATAD2A/E2F3/NFATC2/HIPK2/E2F7/ZFHX3/ZBTB16/NFATC1/RFX3/RBPJ/TCF12/TCF7L2/TAF10/LIMD1/SMAD7/HDAC1/TLE1/MTA1/CTBP1/PHF12/SP3/CREG1/WWTR1/ATF7IP/CTBP2/RARG/RUNX2/BRF1/GFI1B/CREBBP/CCNH/MTA2/ETS1/ARNTL/PRDM16/MED8/HOXA9/RB1/MEIS1/E2F6/TCF3/MED25/SUB1/EPAS1/TAF1D/NAA16/CEBPB/RUNX3/TEAD2/ANKRD1/RELA/SSBP3/NR5A2/LMO4/ERCC2
Cellular Component	GO:0030017	sarcomere	39/2341	207/19559	0.0026425	0.0450774	0.0404142	39	HDAC4/PARVB/ENO1/KCNE1/MYO18B/TTN/CASQ2/SYNE1/CACNA1C/SLC8A1/TNNT3/PRICKLE4/JUP/MYOZ3/BIN1/PDLIM2/SYNPO2L/ACTN1/NOS1AP/ACTN4/ILK/SCN1A/TMOD3/DNAJB6/CAB39/ARHGEF25/MYOM1/ANKRD2/CORO1C/MYOT/FHL2/SLC2A1/PDLIM4/ANK2/ANKRD1/TMOD1/MYL7/CASQ1/SYNPO
Cellular Component	GO:0035577	azurophil granule membrane	15/2341	58/19559	0.0027858	0.0456166	0.0408977	15	AZU1/C3AR1/LPCAT1/TMEM30A/RAP1B/CEACAM6/RAB5C/FPR1/ATP8A1/BRI3/CPNE1/CD68/IRAG2/ATP6V0C/ACP3
Cellular Component	GO:0042470	melanosome	23/2341	105/19559	0.0027921	0.0456166	0.0408977	23	P4HB/BSG/RPN1/MYH11/RAB2A/RAB5C/RAB5B/SLC1A5/ANXA6/CAPG/CTSD/RAB27A/ATP6V1B2/MYO5A/ITGB3/CTSB/YWHAZ/SGSM2/SLC2A1/SND1/TRPV2/FLOT1/DTNBP1
Cellular Component	GO:0048770	pigment granule	23/2341	105/19559	0.0027921	0.0456166	0.0408977	23	P4HB/BSG/RPN1/MYH11/RAB2A/RAB5C/RAB5B/SLC1A5/ANXA6/CAPG/CTSD/RAB27A/ATP6V1B2/MYO5A/ITGB3/CTSB/YWHAZ/SGSM2/SLC2A1/SND1/TRPV2/FLOT1/DTNBP1
Cellular Component	GO:0005874	microtubule	71/2341	431/19559	0.0031829	0.0507822	0.0455288	71	TBCD/DCTN1/SNTB2/KIF13B/KCNAB2/DNAH3/CSNK1D/NDE1/SHROOM3/NDRG1/RASSF3/MAP7/SLC8A1/KIF13A/DNM2/MEFV/DYNC1H1/SPAG6/WHAMM/STAU2/RUSC1/NAV3/CUL3/DISC1/BAIAP2/TUBB4B/EML4/CDC16/CCDC66/CRHBP/DCTN2/KIF21B/SVIL/MTA1/MYO5A/CSPP1/WDR47/PARP4/KIF5C/HOOK2/TTLL4/BCL10/NUMA1/TRIM55/TTLL11/TUBA4B/TUBA4A/TUBA8/DNM1/NEK6/KLHL21/KIF1B/DNM3/MAP1A/DNM1L/TPPP3/ARHGEF2/KIFAP3/DYSF/SHROOM1/CYLD/SPAG5/DNAI2/FBXW11/TUBGCP2/NDEL1/KIFC3/CCT6A/TTLL1/NAV1/SEPTIN9
Cellular Component	GO:0017053	transcription repressor complex	18/2341	76/19559	0.0031958	0.0507822	0.0455288	18	ELANE/HDAC4/ZFPM1/GFI1/NCOR2/SKI/CORO2A/GATAD2A/ZBTB16/RBPJ/HDAC1/MTA1/CTBP1/PHF12/SP3/CTBP2/MTA2/PRDM16
Cellular Component	GO:0098791	Golgi apparatus subcompartment	62/2341	368/19559	0.003339	0.0523418	0.0469271	62	CSGALNACT1/FUT4/SORL1/GALNT2/FUT7/VTI1A/VPS53/AP4S1/CHSY1/MBTPS1/TGFBI/KIF13A/DNM2/RAB31/MGAT4A/MYO18A/ATP8A1/C17orf75/ARAP1/TRAPPC9/ATP11A/ATP8B1/CHST2/APP/CORO7/PITPNM1/AP1B1/XYLT1/COG1/SGMS1/B4GALT7/UXS1/RAB43/ASAP2/COG8/ST6GAL1/VCPIP1/GRN/ST3GAL4/USP6NL/INPP5E/B4GALT3/SORT1/ATP8B4/PI4K2A/CDH1/FUT11/SYT17/RAB30/COG5/TMED2/YIPF1/ATL1/ATP7B/VPS51/CLTCL1/LYPLA2/ST3GAL2/PHETA1/TEPSIN/PLPP3/DOP1B
Cellular Component	GO:0005770	late endosome	49/2341	278/19559	0.0034642	0.0535794	0.0480367	49	HLA-DMB/SORL1/VTI1A/VAC14/SLC11A1/PRKAR1B/SLC29A3/PLD1/ANXA6/SFTPD/SLC11A2/ANKRD13B/CTSH/RAB27A/VPS26B/ANKRD13D/DERL2/UVRAG/CRHBP/LITAF/RAPGEF2/VAMP5/AP5S1/GIMAP5/MYO5A/CD79A/VPS18/CDIP1/CLEC16A/HTT/ANKRD13A/GRN/KCNQ1/NPC1/NTRK1/MICALL1/ASTN2/PLD3/F2R/CD68/DYSF/YIPF1/WDR81/ATP7B/PARM1/MVB12A/CLTCL1/MARCHF8/SLA2

Cellular Component	GO:0016328	lateral plasma membrane	16/2341	65/19559	0.0035129	0.0536172	0.0480706	16	TBCD/TACSTD2/ABCC1/SLC16A3/VANGL1/JUP/AXIN1/NUMA1/PPP2R1A/SNTA1/SLC16A1/CDH1/CORO1C/GNA12/MYO1C/MARK2
Cellular Component	GO:0030176	integral component of endoplasmic reticulum membrane	31/2341	158/19559	0.003677	0.0552641	0.0495471	31	EMC8/CALR/XXYLT1/DHRS9/ERN1/AMFR/PIGU/RHBDD2/RTN4/HLA-C/EDEM1/HM13/HLA-F/DERL2/ERGIC1/SGMS1/SGMS2/SLC37A1/SCD/ESYT1/ANKLE2/RTN2/UBXN8/DPAGT1/EMC1/TEX261/BSCL2/SPCS1/MARCHF6/STING1/SARAF
Cellular Component	GO:0030134	COPII-coated ER to Golgi transport vesicle	21/2341	95/19559	0.003716	0.0552641	0.0495471	21	CTSZ/SERPINA1/TGFA/STX5/VTI1A/SEC23B/LMAN2/YIF1A/HLA-C/APP/HLA-F/ERGIC1/CNIH2/CD59/SEC13/SEC24C/CNIH4/TMED2/SREBF1/SEC31A/TEX261
Cellular Component	GO:0043198	dendritic shaft	11/2341	38/19559	0.0038976	0.0572313	0.0513108	11	ZMYND8/SLC8A1/BAIAP2/APP/ILK/PREX1/SYNGAP1/CNIH2/KIRREL3/MAP1A/ARHGEF2
Cellular Component	GO:0016605	PML body	22/2341	102/19559	0.0041538	0.0602304	0.0539996	22	TRIM27/BLM/TRIM8/HIPK3/SKI/UBN1/HMBOX1/SP140/NSMCE2/HIPK2/ZBTB16/TCF7L2/SP3/ARNTL/ANKRD2/MORC3/RB1/SP100/CHEK2/SPTBN4/ISG20/DAPK3
Cellular Component	GO:0016607	nuclear speck	66/2341	401/19559	0.0044295	0.0634342	0.056872	66	HDAC4/RREB1/MAML3/NXF1/IL16/RBM19/ZNF217/MAML1/RMI2/DOCK1/SRPK2/GATAD2A/MAML2/SGK1/E2F7/YLPM1/ZBTB16/PACSIN2/ZC3H18/BRD1/EHMT2/USP36/CDYL/SYNPO2L/TCF12/PABPN1/CLK3/DGKQ/GRK5/SFMBT2/PRPF8/SETD1B/SETD1A/FAM193B/FEV/PLCB1/FTO/NEK6/UNC45A/MEF2C/EPAS1/TRIP12/KAZN/PRPF6/PRPF40B/RUFY1/PPP1R16B/NSRP1/RBM15B/SDCBP2/PLRG1/SRRM2/TOE1/LHPP/NOC3L/TUT1/MECOM/CDK13/POLI/DAZAP2/PRKAA1/TFIP11/BRD2/TEPSIN/PRKN/NCBP3
Cellular Component	GO:0101003	ficolin-1-rich granule membrane	15/2341	61/19559	0.0046796	0.0661987	0.0593505	15	TRPM2/UBR4/CD55/SLC11A1/CLEC4C/DOK3/FPR2/FPR1/MGAM/PKP1/PTPRN2/ITGB2/ATP6V0C/VPS35L/ADGRE3
Cellular Component	GO:0031901	early endosome membrane	31/2341	161/19559	0.0049139	0.0686764	0.0615719	31	EHD1/SORL1/SORCS2/VAC14/RAB5C/RAB5B/LDLRAD4/RAB31/SNX27/HLA-C/RABGEF1/INPP4A/SNX3/HLA-F/OSBPL6/TMEM163/LITAF/SH3GL1/KREMEN2/PI4K2A/NTRK1/PLD3/EPS15/RUFY1/WDR81/HPS6/PDLIM4/INPP5F/ZFYVE9/MARCHF3/MARCHF8
Cellular Component	GO:0016324	apical plasma membrane	60/2341	361/19559	0.0051965	0.0710824	0.0637291	60	PRKCZ/PDE4D/SLC2A5/AP2A1/KCNE1/CD9/ABCC1/SLC16A3/SHROOM3/TMEM30A/FXYD1/CRB1/CEACAM6/PLD1/TLR9/MGAM/ABCC2/SLC11A2/TNIK/TRPM6/RAB27A/SLC44A4/ATP8B1/SLC2A9/DLL1/ATP6V1B2/SLC9A3R2/P2RY6/MLC1/RAPGEF2/SLC19A1/LMO7/AQP1/SLC22A11/MUC20/CDHR2/SLC7A5/IL6R/SLC22A5/CTSB/PRKCI/SLC16A1/ARHGEF18/NOTCH1/SHANK2/EPS15/PDGFRB/SLC2A1/SLC7A8/ANK2/SHROOM1/SLC29A1/SLC12A1/EZR/PDZK1/SLC7A1/C2CD2L/SLC23A1/PRKAA1/CEACAM5

Cellular Component	GO:0002102	podosome	9/2341	29/19559	0.0052944	0.0710824	0.0637291	9	GSN/CTTN/SH3PXD2B/ASAP1/SVIL/SH3GL1/WDR1/ARHGEF2/DBNL
Cellular Component	GO:0030658	transport vesicle membrane	38/2341	209/19559	0.0055145	0.0710824	0.0637291	38	SPRED2/SORL1/AP2A1/TGFA/SNTB2/STX5/VTI1A/TMEM30A/SV2C/RAB5B/SEC23B/ATP8A1/ITPR1/HLA-C/HLA-F/SYN3/PTPRN2/TMEM163/CNIH2/MCTP2/SYN2/CD59/SEC13/SEC24C/PI4K2A/SYNGR3/AP1G2/RPH3A/DNM1L/TMED2/BCL2L1/SREBF1/RPH3AL/DTNBP1/SYPL2/CLTCL1/SEC31A/CBARP
Cellular Component	GO:0031985	Golgi cisterna	24/2341	117/19559	0.0055611	0.0710824	0.0637291	24	CSGALNACT1/FUT4/SORL1/GALNT2/FUT7/CHSY1/ARAP1/PITPNM1/XYLT1/SGMS1/B4GALT7/UXS1/ASAP2/ST6GAL1/ST3GAL4/INPP5E/B4GALT3/SORT1/FUT11/RAB30/TMED2/YIPF1/ATL1/ST3GAL2
Cellular Component	GO:0001725	stress fiber	16/2341	68/19559	0.0056389	0.0710824	0.0637291	16	PRKCZ/MYLK/PRICKLE4/PDLIM2/MYH9/ACTN1/ACTN4/ILK/ABLIM3/PXN/DAAM1/PDLIM4/ABLIM1/LPP/SYNPO/SEPTIN9
Cellular Component	GO:0044304	main axon	16/2341	68/19559	0.0056389	0.0710824	0.0637291	16	KIF13B/KCNAB2/TNFRSF1B/KCNQ2/BIN1/APP/MBP/SCN1A/CRHBP/EPB41L3/SLC1A2/DNM1/MAP1A/CNTNAP1/SPTBN4/NAV1
Cellular Component	GO:0097517	contractile actin filament bundle	16/2341	68/19559	0.0056389	0.0710824	0.0637291	16	PRKCZ/MYLK/PRICKLE4/PDLIM2/MYH9/ACTN1/ACTN4/ILK/ABLIM3/PXN/DAAM1/PDLIM4/ABLIM1/LPP/SYNPO/SEPTIN9
Cellular Component	GO:0005793	endoplasmic reticulum-Golgi intermediate compartment	26/2341	130/19559	0.0056974	0.0710824	0.0637291	26	CTSZ/P4HB/CALR/SERPINA1/TGFA/STX5/CSNK1D/SLC35C2/RAB2A/CD55/MGAT4A/MYO18A/LMAN2/WHAMM/YIF1A/ERGIC1/CNIH2/CD59/VMP1/CNIH4/TMED2/ASPSCR1/TRAPPC2B/STING1/TRAPPC2/PLPP3
Cellular Component	GO:0016528	sarcoplasm	18/2341	80/19559	0.0056982	0.0710824	0.0637291	18	CALR/GSN/CASQ2/ITPR2/ITPR1/THBS1/JSRP1/NOS1AP/HK2/ATP2A3/NOL3/MEF2C/ASPH/CHERP/RTN2/CASQ1/DTNBP1/IRAG1
Cellular Component	GO:0044291	cell-cell contact zone	17/2341	74/19559	0.0056988	0.0710824	0.0637291	17	TJP2/YWHAH/FXYD1/SLC8A1/GJA5/JUP/AHNAK/ACTN1/SCN1A/VAMP5/JAM3/SLC2A1/ANK2/BAIAP2L2/FLOT1/SPTBN4/NECTIN1

Cellular Component	GO:0002947	tumor necrosis factor receptor superfamily complex	3/2341	4/19559	0.0062364	0.0761499	0.0682724	3	TNFRSF1B/TRAF2/TNFRSF1A
Cellular Component	GO:0097013	phagocytic vesicle lumen	3/2341	4/19559	0.0062364	0.0761499	0.0682724	3	MPO/PGLYRP1/LTF
Cellular Component	GO:0045178	basal part of cell	16/2341	69/19559	0.0065439	0.0790723	0.0708925	16	TACSTD2/ABCC1/SLC1A5/SLC11A2/OSCP1/C5AR2/AQP1/MUC20/SLC7A5/SLC16A1/SLC1A3/EPS15/MYO1C/SLC7A8/SLC7A1/SLC23A1
Cellular Component	GO:0032432	actin filament bundle	17/2341	76/19559	0.0075375	0.0901389	0.0808141	17	PRKCZ/MYLK/PRICKLE4/PDLIM2/MYH9/ACTN1/ACTN4/ILK/ABLIM3/PXN/DAAM1/PDLIM4/ABLIM1/LPP/SYNPO/SEPTIN9/RFLNA
Cellular Component	GO:0098978	glutamatergic synapse	59/2341	361/19559	0.0079203	0.0937503	0.084052	59	PRKCZ/ABR/NRP2/RNF19A/PPP1R1B/PRKAR1B/NDRG1/DNM2/ITSN1/ATP2B2/PACSIN2/TNIK/BAIAP2/ACTN1/SYN3/NOS1AP/ABLIM3/SYNGAP1/SPARCL1/DLGAP4/DGKQ/CNIH2/CTBP1/HTR3A/MYO5A/PFN1/NRXN1/VPS18/SYN2/SLC1A2/CTBP2/NUMB/ITGB3/GHRL/CHRM2/SH3GL1/BCR/SLC1A6/PPP2R1A/PLCB1/PRKCI/GSG1L/DNM1/YWHAZ/CDH1/DNM3/P2RX1/ARHGEF2/EPS15/NEDD4/AP3D1/PLCG1/RELA/CDH11/NPTX1/GRID1/FLOT1/DTNBP1/SYNPO
Cellular Component	GO:0032587	ruffle membrane	20/2341	95/19559	0.0080414	0.0942225	0.0844753	20	KSR1/KANK1/DNM2/FGR/FGD5/SPATA13/PACSIN2/TESC/PTPRJ/SH3YL1/EPS8L3/EPS8L1/SH2D3C/ITGB3/PDE9A/CORO1C/ARHGEF2/MYO1C/EZR/ARHGAP45
Cellular Component	GO:0035097	histone methyltransferase complex	17/2341	77/19559	0.0086209	0.1000023	0.0896572	17	KDM4B/ASH2L/KANSL1/MGA/WDR82/KMT2D/KDM4C/SETD1B/SETD1A/ZNF335/PHF19/E2F6/CHD8/RUVBL1/DYDC2/DYDC1/PAXIP1
Cellular Component	GO:0099091	postsynaptic specialization, intracellular component	7/2341	21/19559	0.0088457	0.1012799	0.0908026	7	ABR/DNM2/TNIK/BAIAP2/SH3GL1/BCR/ARHGEF2
Cellular Component	GO:0031528	microvillus membrane	8/2341	26/19559	0.0089056	0.1012799	0.0908026	8	S100P/MUC20/CDHR2/SLC7A5/ITGB3/SLC7A8/EZR/PDZK1

Cellular Component	GO:0044327	dendritic spine head	5/2341	12/19559	0.0093719	0.1055474	0.0946287	5	PPP1R1B/DNM2/DNM1/DNM3/MX1
Cellular Component	GO:0016234	inclusion body	17/2341	78/19559	0.0098253	0.1095902	0.0982533	17	NXF1/CABIN1/FGR/FBXO7/ATXN1/EDEM1/NEFM/CLU/PABPN1/SFMBT2/HTT/ZDHHC17/PRDM16/EPS15/BAG5/PRKN/SLF1
Cellular Component	GO:0000151	ubiquitin ligase complex	48/2341	287/19559	0.0102445	0.1121503	0.1005486	48	MAEA/RNF144A/FZR1/RNF19A/WWP2/KLHL25/AMFR/RHBDD2/TRAF2/PHC2/FBXO7/CUL3/DCAF15/CDC16/DERL2/UBE2J2/LMO7/ZSWIM5/UBE2V1/DYRK2/FBXL19/ASB1/DCAF5/PHC3/FBXL13/KCTD2/FBXL18/RNF144B/KLHL21/FBXL6/KLHL8/PCGF3/NEDD4/ANAPC7/RNF14/RNF11/PLRG1/FBXW11/WDTC1/RNF40/UBE2D2/GID8/FBXO8/DCAF17/MARCHF6/PRKN/FBH1/BABAM2
Cellular Component	GO:0031253	cell projection membrane	56/2341	344/19559	0.0102482	0.1121503	0.1005486	56	S100P/KSR1/EHD1/PDE6A/IQCE/KANK1/DNM2/PKD1L1/FGR/FGD5/ABCC2/SPATA13/SLC11A2/PACSIN2/TESC/TRPM6/ATP8B1/AKAP5/GNAT2/DOCK8/ASAP1/SCIMP/PTPRJ/SLC19A1/SH3YL1/EPS8L3/AQP1/EPS8L1/EPB41L3/MUC20/CDHR2/SLC1A2/SH2D3C/SLC7A5/CNGA4/ITGB3/PDE6H/SLC22A5/PALM/PDE9A/MFSD10/NCKAP1/TSPEAR/CORO1C/CDHR1/SHANK2/GNA12/ARHGEF2/EPS15/MYO1C/FSCN3/SLC7A8/EZR/PDZK1/TXNDC15/ARHGAP45
Cellular Component	GO:0035578	azurophil granule lumen	19/2341	91/19559	0.0105182	0.1140289	0.1022328	19	AZU1/ELANE/PRTN3/CTSG/VAT1/MPO/TOLLIP/RNASE3/HEXB/GALNS/DYNC1H1/MNDA/PADI2/TUBB4B/CREG1/GRN/ARSB/IMPDH1/ARHGAP45
Cellular Component	GO:0043194	axon initial segment	6/2341	17/19559	0.0112034	0.1203328	0.1078845	6	KCNQ2/BIN1/SCN1A/MAP1A/SPTBN4/NAV1
Cellular Component	GO:0098984	neuron to neuron synapse	59/2341	368/19559	0.0117054	0.1245717	0.111685	59	PRKCZ/ABR/RNF19A/SORCS2/KCNAB2/SLC16A3/CACNA1C/PRKAR1B/SLC8A1/ARRB1/DNM2/RHEB/NCK2/TANC1/BCL11A/RTN4/ATP2B2/ITPR1/RUSC1/TNIK/DISC1/BAIAP2/INPP4A/ZDHHC2/SYN3/MIB1/AKAP5/GRIK4/SYNGAP1/CNIH2/IQSEC1/EPB41L3/SYN2/CPEB3/CHRM2/SH3GL1/BCR/PALM/SIPA1L1/MAPK8IP2/GSG1L/ARRB2/DNM1/YWHAZ/ADD1/SAMD14/DNM3/CABP1/SHANK2/ARHGEF2/TSC2/MX1/GRID1/DTNBP1/AKAP7/SYNPO/DBNL/LRFN2/NECTIN1
Cellular Component	GO:0034399	nuclear periphery	25/2341	131/19559	0.0119915	0.1258021	0.1127881	25	MAEA/PRKCZ/BLM/GFI1/NCOR2/MYB/ATF7/NARF/ATXN1/UHRF1/LMNB2/RGS12/DGKQ/NUP93/STAG1/TGFB1I1/NUMA1/GFI1B/MORC3/SCAF8/ATN1/CEBPB/RUVBL1/SPTBN4/PAXIP1
Cellular Component	GO:0005795	Golgi stack	28/2341	151/19559	0.012038	0.1258021	0.1127881	28	CSGALNACT1/FUT4/SORL1/GALNT2/FUT7/CHSY1/MBTPS1/MGAT4A/ARAP1/PITPNM1/XYLT1/SGMS1/B4GALT7/UXS1/ASAP2/ST6GAL1/VCPIP1/ST3GAL4/INPP5E/B4GALT3/SORT1/FUT11/RAB30/TMED2/YIPF1/ATL1/LYPLA2/ST3GAL2

Cellular Component	GO:0044666	MLL3/4 complex	5/2341	13/19559	0.0137485	0.1395909	0.1251505	5	ASH2L/KMT2D/DYDC2/DYDC1/PAXIP1
Cellular Component	GO:0090724	central region of growth cone	3/2341	5/19559	0.0142022	0.1395909	0.1251505	3	DISC1/CYFIP1/NDEL1
Cellular Component	GO:0097651	phosphatidylinositol 3-kinase complex, class I	3/2341	5/19559	0.0142022	0.1395909	0.1251505	3	PIK3R1/PIK3R6/PIK3R5
Cellular Component	GO:0000308	cytoplasmic cyclin-dependent protein kinase holoenzyme complex	2/2341	2/19559	0.0143201	0.1395909	0.1251505	2	CDK14/CCNY
Cellular Component	GO:0036409	histone H3-K14 acetyltransferase complex	2/2341	2/19559	0.0143201	0.1395909	0.1251505	2	BRD1/KAT7
Cellular Component	GO:0045092	interleukin-18 receptor complex	2/2341	2/19559	0.0143201	0.1395909	0.1251505	2	IL18RAP/IL18R1
Cellular Component	GO:0097632	extrinsic component of phagophore assembly site membrane	2/2341	2/19559	0.0143201	0.1395909	0.1251505	2	ULK1/ATG14
Cellular Component	GO:0098560	cytoplasmic side of late endosome membrane	2/2341	2/19559	0.0143201	0.1395909	0.1251505	2	LITAF/CDIP1
Cellular Component	GO:0030117	membrane coat	19/2341	94/19559	0.0147643	0.1415416	0.1268994	19	AP2A1/AP4S1/SEC23B/EPS15L1/AP1B1/EPN2/AP5S1/VPS18/SEC13/DNM1/SEC24C/AP1G2/EPS15/AP3D1/EPN3/BAIAP2L2/CLTCL1/SEC31A/TEPSIN

Cellular Component	GO:0048475	coated membrane	19/2341	94/19559	0.0147643	0.1415416	0.1268994	19	AP2A1/AP4S1/SEC23B/EPS15L1/AP1B1/EPN2/AP5S1/VPS18/SEC13/DNM1/SEC24C/AP1G2/EPS15/AP3D1/EPN3/BAIAP2L2/CLTCL1/SEC31A/TEPSIN
Cellular Component	GO:0008091	spectrin	4/2341	9/19559	0.0156834	0.1491211	0.1336948	4	PRKCB/SPTA1/SPTAN1/SPTBN4
Cellular Component	GO:0005834	heterotrimeric G-protein complex	9/2341	34/19559	0.0160145	0.1498133	0.1343154	9	GNAZ/GNAI2/GNAT2/GNG7/GNA11/GNB3/GNG2/GNAO1/GNA12
Cellular Component	GO:1905360	GTPase complex	9/2341	34/19559	0.0160145	0.1498133	0.1343154	9	GNAZ/GNAI2/GNAT2/GNG7/GNA11/GNB3/GNG2/GNAO1/GNA12
Cellular Component	GO:0016363	nuclear matrix	21/2341	109/19559	0.0181609	0.1685329	0.1510985	21	MAEA/PRKCZ/BLM/GFI1/NCOR2/MYB/ATXN1/UHRF1/RGS12/DGKQ/STAG1/TGFB1I1/NUMA1/GFI1B/MORC3/SCAF8/ATN1/CEBPB/RUVBL1/SPTBN4/PAXIP1
Cellular Component	GO:0044322	endoplasmic reticulum quality control compartment	7/2341	24/19559	0.0191182	0.176009	0.1578012	7	CALR/AMFR/EDEM1/DERL2/ASGR2/EDEM2/MARCHF6
Cellular Component	GO:0016529	sarcoplasmic reticulum	15/2341	71/19559	0.0193841	0.1770517	0.158736	15	CALR/CASQ2/ITPR2/ITPR1/THBS1/JSRP1/NOS1AP/HK2/ATP2A3/NOL3/ASPH/CHERP/RTN2/CASQ1/IRAG1
Cellular Component	GO:0030135	coated vesicle	48/2341	298/19559	0.0196545	0.1778018	0.1594085	48	CTSZ/CPNE6/SERPINA1/AP2A1/TGFA/STX5/CD9/VTI1A/DENND1A/AAK1/DNM2/SEC23B/SFTPD/LMAN2/YIF1A/HLA-C/STON1/RAB27A/SNX3/APP/HLA-F/ERGIC1/AP1B1/EPN2/CNIH2/VPS18/CD59/NUMB/SEC13/CHRM2/SH3BP4/SNX18/SORT1/SEC24C/AP1G2/ASTN2/CNIH4/EPS15/TMED2/SREBF1/EPN3/INPP5F/CLTCL1/SEC31A/TEX261/DBNL/PHETA1/TEPSIN
Cellular Component	GO:0000159	protein phosphatase type 2A complex	6/2341	19/19559	0.0199261	0.1778018	0.1594085	6	PPP2R5C/PPP2R1A/PPP2R5E/PPP2R5D/PPP2R1B/PTPA

Cellular Component	GO:0016514	SWI/SNF complex	6/2341	19/19559	0.0199261	0.1778018	0.1594085	6	SMARCA2/NCR1/ARID1B/SMARCD1/SMARCD2/RB1
Cellular Component	GO:0098858	actin-based cell projection	37/2341	220/19559	0.0202981	0.1797389	0.1611452	37	S100P/AP2A1/KIF13B/FGD4/ANGPT1/CRB1/CDH23/TBC1D10A/DYNC1H1/LY6G6D/SPATA13/BAIAP2/ATP8B1/APP/ATP6V1B2/SCIMP/TGFB1/FARP1/MYO5A/MUC20/CDHR2/JAM3/ESPN/SLC7A5/ITGB3/PALM/TSPEAR/TMC1/MYO1C/FSCN3/SLC7A8/CDC14A/CYFIP1/EZR/PDZK1/ADGRA2/ACP3
Cellular Component	GO:0034708	methyltransferase complex	20/2341	105/19559	0.0233034	0.2042116	0.1830863	20	KDM4B/ASH2L/SNRPD3/KANSL1/MGA/WDR82/KMT2D/KDM4C/SETD1B/SETD1A/ZNF335/PHF19/E2F6/CHD8/RBM15B/RUVBL1/DYDC2/DYDC1/RIOK1/PAXIP1
Cellular Component	GO:0031301	integral component of organelle membrane	57/2341	367/19559	0.0234904	0.2042116	0.1830863	57	CSGALNACT1/EMC8/CALR/GALNT2/XXYLT1/FAR2/DHRS9/SV2C/ERN1/AMFR/PIGU/RHBDD2/SPG7/ATP8A1/VOPP1/RTN4/YIF1A/HLA-C/EDEM1/HM13/HLA-F/DERL2/ERGIC1/PTPRN2/TMEM163/SGMS1/VAMP5/SFXN1/IMMT/SGMS2/PEX26/SLC37A1/PPOX/NPC1/FIS1/SLC39A13/P2RX1/CASD1/PEX11B/SCD/TPST1/P2RX5/ESYT1/CPT1A/ANKLE2/RTN2/MAN1C1/UBXN8/DPAGT1/EMC1/TEX261/BSCL2/SPCS1/MARCHF6/B4GAT1/STING1/SARAF
Cellular Component	GO:0098843	postsynaptic endocytic zone	4/2341	10/19559	0.0236969	0.2042116	0.1830863	4	DNM2/DNM1/DNM3/MX1
Cellular Component	GO:0030315	T-tubule	12/2341	54/19559	0.023766	0.2042116	0.1830863	12	CACNA1C/FXYD1/SLC8A1/BIN1/AHNAK/NOS1AP/SCN1A/DYSF/ANK2/RTN2/EZR/CASQ1
Cellular Component	GO:0031300	intrinsic component of organelle membrane	61/2341	398/19559	0.0252067	0.2096337	0.1879475	61	CSGALNACT1/EMC8/CALR/GALNT2/XXYLT1/FAR2/DHRS9/SV2C/ERN1/RAB5B/AMFR/PIGU/RHBDD2/SPG7/ATP8A1/VOPP1/RTN4/YIF1A/HLA-C/EDEM1/HM13/CHST2/HLA-F/DERL2/ERGIC1/PTPRN2/TMEM163/SGMS1/VAMP5/SFXN1/IMMT/SGMS2/PEX26/SLC37A1/PPOX/NPC1/FIS1/SLC39A13/P2RX1/CASD1/PEX11B/GRAMD1A/SCD/TPST1/P2RX5/ESYT1/CPT1A/ANKLE2/RTN2/MAN1C1/UBXN8/DPAGT1/EMC1/TEX261/BSCL2/SPCS1/MARCHF6/B4GAT1/GRAMD2A/STING1/SARAF
Cellular Component	GO:0005802	trans-Golgi network	40/2341	245/19559	0.0254845	0.2096337	0.1879475	40	FUT4/SORL1/FUT7/VTI1A/VPS53/AP4S1/TGFBI/KIF13A/DNM2/RAB31/MYO18A/ATP8A1/C17orf75/ARAP1/TRAPPC9/ATP11A/ATP8B1/CHST2/APP/CORO7/AP1B1/COG1/RAB43/COG8/GRN/USP6NL/ATP8B4/PI4K2A/CDH1/SYT17/RAB30/COG5/YIPF1/ATP7B/VPS51/CLTCL1/PHETA1/TEPSIN/PLPP3/DOP1B
Cellular Component	GO:0005903	brush border	20/2341	106/19559	0.0255968	0.2096337	0.1879475	20	ABCC2/SLC11A2/TRPM6/MYH9/ATP8B1/ACTN1/ACTN4/SLC19A1/AQP1/CDHR2/ESPN/SLC22A5/MFSD10/DNM1L/SHANK2/GNA12/FLII/MYO1C/EZR/PDZK1

Cellular Component	GO:0032541	cortical endoplasmic reticulum	3/2341	6/19559	0.0258911	0.2096337	0.1879475	3	ASPH/C2CD2L/STIMATE
Cellular Component	GO:0097427	microtubule bundle	3/2341	6/19559	0.0258911	0.2096337	0.1879475	3	NUMA1/TPPP3/MARK2
Cellular Component	GO:0005902	microvillus	18/2341	93/19559	0.0261506	0.2096337	0.1879475	18	S100P/KIF13B/ANGPT1/CRB1/TBC1D10A/ATP6V1B2/TGFB1/MUC20/CDHR2/JAM3/ESPN/SLC7A5/ITGB3/MYO1C/FSCN3/SLC7A8/EZR/PDZK1
Cellular Component	GO:0032580	Golgi cisterna membrane	18/2341	93/19559	0.0261506	0.2096337	0.1879475	18	CSGALNACT1/FUT4/GALNT2/FUT7/CHSY1/ARAP1/PITPNM1/B4GALT7/UXS1/ASAP2/ST6GAL1/ST3GAL4/INPP5E/B4GALT3/SORT1/FUT11/TMED2/ST3GAL2
Cellular Component	GO:0030008	TRAPP complex	5/2341	15/19559	0.026176	0.2096337	0.1879475	5	TRAPPC10/TRAPPC9/TRAPPC3L/TRAPPC2B/TRAPPC2
Cellular Component	GO:0038201	TOR complex	5/2341	15/19559	0.026176	0.2096337	0.1879475	5	RPTOR/PRR5L/LARP1/PRR5/TTI1
Cellular Component	GO:0090543	Flemming body	8/2341	31/19559	0.0262042	0.2096337	0.1879475	8	IL16/CAPG/MICAL3/CHAMP1/USP3/CCDC66/ZFYVE19/RALA
Cellular Component	GO:0098827	endoplasmic reticulum subcompartment	7/2341	26/19559	0.0293969	0.2335641	0.2094023	7	RTN4/ATL1/ASPH/RTN2/CASQ1/C2CD2L/STIMATE
Cellular Component	GO:0012507	ER to Golgi transport vesicle membrane	13/2341	62/19559	0.0299328	0.2362043	0.2117694	13	TGFA/STX5/VTI1A/SEC23B/HLA-C/HLA-F/CNIH2/CD59/SEC13/SEC24C/TMED2/SREBF1/SEC31A

Cellular Component	GO:0032279	asymmetric synapse	53/2341	343/19559	0.0305536	0.2394742	0.214701	53	PRKCZ/ABR/SORCS2/KCNAB2/SLC16A3/CACNA1C/SLC8A1/ARRB1/DNM2/RHEB/NCK2/TANC1/BCL11A/RTN4/ATP2B2/ITPR1/RUSC1/TNIK/DISC1/BAIAP2/INPP4A/ZDHHC2/SYN3/MIB1/AKAP5/SYNGAP1/CNIH2/IQSEC1/EPB41L3/SYN2/CPEB3/CHRM2/SH3GL1/BCR/PALM/SIPA1L1/MAPK8IP2/GSG1L/ARRB2/DNM1/ADD1/SAMD14/DNM3/CABP1/SHANK2/ARHGEF2/TSC2/MX1/GRID1/DTNBP1/SYNPO/DBNL/LRFN2
Cellular Component	GO:0045177	apical part of cell	65/2341	433/19559	0.0317448	0.2471407	0.2215745	65	PRKCZ/PDE4D/SLC2A5/AP2A1/KCNE1/CD9/ABCC1/SLC16A3/SHROOM3/TMEM30A/FXYD1/CRB1/CEACAM6/PLD1/TLR9/MGAM/ABCC2/SLC11A2/TNIK/TRPM6/RAB27A/SLC44A4/ATP8B1/SLC2A9/DLL1/C5AR2/APP/ATP6V1B2/SLC9A3R2/P2RY6/MLC1/RAPGEF2/SLC19A1/LMO7/AQP1/SLC22A11/MUC20/CDHR2/SLC7A5/NUMB/IL6R/SLC22A5/CTSB/PRKCI/VASH1/SLC16A1/ARHGEF18/NOTCH1/SHANK2/EPS15/PDGFRB/SLC2A1/SLC7A8/ANK2/SHROOM1/SLC29A1/SLC12A1/EZR/PDZK1/SLC7A1/C2CD2L/SLC23A1/PRKAA1/CEACAM5/ATP6V1C1
Cellular Component	GO:0030904	retromer complex	6/2341	21/19559	0.0323751	0.2503675	0.2244674	6	TRIM27/DCTN1/ANKFY1/SNX27/SNX3/VPS26B
Cellular Component	GO:0014069	postsynaptic density	52/2341	337/19559	0.0326338	0.2506967	0.2247626	52	PRKCZ/ABR/SORCS2/KCNAB2/SLC16A3/CACNA1C/SLC8A1/ARRB1/DNM2/RHEB/NCK2/TANC1/BCL11A/RTN4/ATP2B2/ITPR1/RUSC1/TNIK/DISC1/BAIAP2/INPP4A/ZDHHC2/SYN3/MIB1/AKAP5/SYNGAP1/CNIH2/IQSEC1/EPB41L3/SYN2/CPEB3/SH3GL1/BCR/PALM/SIPA1L1/MAPK8IP2/GSG1L/ARRB2/DNM1/ADD1/SAMD14/DNM3/CABP1/SHANK2/ARHGEF2/TSC2/MX1/GRID1/DTNBP1/SYNPO/DBNL/LRFN2
Cellular Component	GO:0043296	apical junction complex	25/2341	143/19559	0.0329182	0.2512182	0.2252301	25	PRKCZ/TBCD/TJP2/SHROOM3/UBN1/FRMD4A/JUP/PMP22/FRMD4B/RAPGEF2/PLEKHA7/JAM3/ACTG1/PRKCI/CDH1/MPP7/ARHGAP17/ARHGEF2/SHROOM1/FRMD6/KIFC3/SYNPO/NPHP4/JAML/NECTIN1
Cellular Component	GO:0099023	vesicle tethering complex	14/2341	70/19559	0.0360251	0.2731314	0.2448765	14	VPS53/TGFBRAP1/TRAPPC10/TRAPPC9/COG1/VPS18/TRAPPC3L/COG8/HOOK2/NBAS/COG5/VPS51/TRAPPC2B/TRAPPC2
Cellular Component	GO:0014704	intercalated disc	11/2341	51/19559	0.0362885	0.2733423	0.2450655	11	YWHAH/FXYD1/SLC8A1/GJA5/JUP/ACTN1/SCN1A/VAMP5/SLC2A1/ANK2/SPTBN4
Cellular Component	GO:0005776	autophagosome	18/2341	97/19559	0.0381842	0.2801785	0.2511945	18	VTI1A/ULK1/MEFV/PIP4K2A/UVRAG/TMEM150B/RUFY4/VPS18/HTT/ATG14/TBC1D14/DAPK2/VMP1/WDR81/GRAMD1A/NCOA4/STING1/RUBCNL
Cellular Component	GO:0000110	nucleotide-excision repair factor 1 complex	2/2341	3/19559	0.039535	0.2801785	0.2511945	2	ERCC1/XPA

Cellular Component	GO:0016513	core-binding factor complex	2/2341	3/19559	0.039535	0.2801785	0.2511945	2	RUNX1/RUNX3
Cellular Component	GO:0030934	anchoring collagen complex	2/2341	3/19559	0.039535	0.2801785	0.2511945	2	COL12A1/COL14A1
Cellular Component	GO:0031905	early endosome lumen	2/2341	3/19559	0.039535	0.2801785	0.2511945	2	LNPEP/PDLIM4
Cellular Component	GO:0035866	alphav-beta3 integrin-PKCalpha complex	2/2341	3/19559	0.039535	0.2801785	0.2511945	2	PRKCA/ITGB3
Cellular Component	GO:0038039	G protein-coupled receptor heterodimeric complex	2/2341	3/19559	0.039535	0.2801785	0.2511945	2	GABBR1/GABBR2
Cellular Component	GO:0097629	extrinsic component of omegasome membrane	2/2341	3/19559	0.039535	0.2801785	0.2511945	2	ULK1/ATG14
Cellular Component	GO:0099073	mitochondrion-derived vesicle	2/2341	3/19559	0.039535	0.2801785	0.2511945	2	DNM1L/PRKN
Cellular Component	GO:0031526	brush border membrane	12/2341	58/19559	0.0396114	0.2801785	0.2511945	12	ABCC2/SLC11A2/TRPM6/ATP8B1/SLC19A1/AQP1/CDHR2/SLC22A5/MFSD10/SHANK2/GNA12/PDZK1
Cellular Component	GO:0005849	mRNA cleavage factor complex	6/2341	22/19559	0.0401441	0.2822255	0.2530298	6	FIP1L1/CPSF3/SCAF8/ZC3H3/CPSF4/TUT1

Cellular Component	GO:0031235	intrinsic component of the cytoplasmic side of the plasma membrane	3/2341	7/19559	0.0413279	0.2887974	0.2589218	3	RASA3/PALM/SPTA1
Cellular Component	GO:0098686	hippocampal mossy fiber to CA3 synapse	8/2341	34/19559	0.0437517	0.3039043	0.2724659	8	RNF19A/PRKAR1B/GRIK4/SH3GL1/YWHAZ/DTNBP1/AKAP7/NECTIN1
Cellular Component	GO:0031902	late endosome membrane	24/2341	140/19559	0.044029	0.3040096	0.2725603	24	HLA-DMB/SORL1/VTI1A/VAC14/SLC11A1/SLC29A3/PLD1/ANXA6/SLC11A2/RAB27A/LITAF/AP5S1/GIMAP5/VPS18/CDIP1/NPC1/NTRK1/MICALL1/PLD3/CD68/YIPF1/WDR81/MVB12A/MARCHF8
Cellular Component	GO:0032153	cell division site	14/2341	72/19559	0.0445183	0.3055693	0.2739587	14	MAEA/SSH1/NDE1/PSTPIP1/MYLK/MYH9/PITPNM1/SVIL/HTR3A/ZFYVE19/RALA/TUBGCP2/PLCD3/SEPTIN9
Cellular Component	GO:0033270	paranode region of axon	4/2341	12/19559	0.0459784	0.3119003	0.2796347	4	KIF13B/EPB41L3/CNTNAP1/SPTBN4
Cellular Component	GO:0042405	nuclear inclusion body	4/2341	12/19559	0.0459784	0.3119003	0.2796347	4	NXF1/ATXN1/PABPN1/SLF1
Cellular Component	GO:0031228	intrinsic component of Golgi membrane	13/2341	66/19559	0.0471529	0.3180079	0.2851106	13	CSGALNACT1/GALNT2/YIF1A/CHST2/ERGIC1/SGMS1/SGMS2/SLC39A13/CASD1/TPST1/MAN1C1/TEX261/B4GAT1
Cellular Component	GO:0030119	AP-type membrane coat adaptor complex	9/2341	41/19559	0.0500621	0.3356766	0.3009514	9	AP2A1/AP4S1/AP1B1/AP5S1/VPS18/AP1G2/EPS15/AP3D1/TEPSIN
Cellular Component	GO:0042383	sarcolemma	23/2341	135/19559	0.0508101	0.3372102	0.3023264	23	BSG/SLC2A5/CACNA1C/FXYD1/SLC8A1/BIN1/AHNAK/NOS1AP/SCN1A/AQP1/SNTA1/PDE9A/SNTB1/CORO1C/MYOT/SLC2A1/DYSF/ANK2/RTN2/EZR/FLOT1/CASQ1/DTNBP1

Cellular Component	GO:0005942	phosphatidylinositol 3-kinase complex	7/2341	29/19559	0.0508722	0.3372102	0.3023264	7	PIK3CD/VAC14/PIK3R1/PIK3R6/PIK3R5/ATG14/SLA2
Cellular Component	GO:0005581	collagen trimer	16/2341	87/19559	0.0521749	0.3437913	0.3082267	16	COL23A1/COL17A1/EMILIN2/C1QTNF3/COL4A2/COLQ/SFTPD/COLEC12/COL12A1/MARCO/FCN1/COL14A1/COL5A1/COL28A1/C1QB/GLDN
Cellular Component	GO:0032154	cleavage furrow	11/2341	54/19559	0.0524578	0.3437913	0.3082267	11	SSH1/NDE1/PSTPIP1/MYLK/MYH9/PITPNM1/SVIL/HTR3A/ZFYVE19/RALA/PLCD3
Cellular Component	GO:0070603	SWI/SNF superfamily-type complex	14/2341	74/19559	0.0543256	0.3540319	0.3174079	14	CECR2/SRCAP/GATAD2A/SMARCA2/HDAC1/NCR1/MTA1/ARID1B/RSF1/MTA2/SMARCD1/SMARCD2/RB1/RUVBL1
Cellular Component	GO:0005847	mRNA cleavage and polyadenylation specificity factor complex	5/2341	18/19559	0.0552203	0.3578523	0.3208331	5	FIP1L1/CPSF3/ZC3H3/CPSF4/TUT1
Cellular Component	GO:0030173	integral component of Golgi membrane	12/2341	61/19559	0.05554	0.3579247	0.320898	12	CSGALNACT1/GALNT2/YIF1A/ERGIC1/SGMS1/SGMS2/SLC39A13/CASD1/TPST1/MAN1C1/TEX261/B4GAT1
Cellular Component	GO:0030133	transport vesicle	60/2341	410/19559	0.0573646	0.3676407	0.3296089	60	SPRED2/SORL1/AP2A1/TGFA/SNTB2/STX5/VTI1A/MYRIP/CRISPLD2/CD55/TMEM30A/SV2C/RAB5B/SEC23B/ATP8A1/ITPR1/HLA-C/STON1/BIN1/DISC1/RAB27A/DDC/APP/HLA-F/SYN3/PTPRN2/TMEM163/APBA2/RAB40C/CNIH2/IQSEC1/MCTP2/COPS5/SYN2/KIRREL3/CD59/SEC13/SORT1/DNM1/SEC24C/PI4K2A/SYNGR3/AP1G2/KIF1B/RPH3A/DNM1L/SYT17/TMED2/BCL2L1/YIPF1/SREBF1/RPH3AL/NDEL1/NPTX1/DTNBP1/SYPL2/CLTCL1/NTF4/SEC31A/CBARP
Cellular Component	GO:0034451	centriolar satellite	17/2341	95/19559	0.0577912	0.3683395	0.3302354	17	ITGB1BP1/PIK3R5/CCDC66/LATS2/PAX2/TACC3/SDCCAG8/FNIP2/NEK6/FLII/AK5/TAF1D/DYSF/SPAG5/KCTD1/CEP72/FLOT1
Cellular Component	GO:0005915	zonula adherens	3/2341	8/19559	0.0603552	0.3764086	0.3374698	3	JUP/PLEKHA7/KIFC3

Cellular Component	GO:0016600	flotillin complex	3/2341	8/19559	0.0603552	0.3764086	0.3374698	3	CDH1/CORO1C/FLOT1
Cellular Component	GO:0031931	TORC1 complex	3/2341	8/19559	0.0603552	0.3764086	0.3374698	3	RPTOR/LARP1/TTI1
Cellular Component	GO:0043203	axon hillock	3/2341	8/19559	0.0603552	0.3764086	0.3374698	3	PRKCZ/NDEL1/SPTBN4
Cellular Component	GO:0005798	Golgi-associated vesicle	29/2341	181/19559	0.0621169	0.3853244	0.3454632	29	CTSZ/SERPINA1/AP2A1/TGFA/STX5/VTI1A/CHIC2/SEC23B/LMAN2/YIF1A/HLA-C/APP/HLA-F/ERGIC1/CNIH2/CD59/CNGA4/SEC13/ZDHHC17/SORT1/SEC24C/AP1G2/PI4KA/CNIH4/TMED2/SREBF1/CLTCL1/SEC31A/TEX261
Cellular Component	GO:0099572	postsynaptic specialization	53/2341	361/19559	0.0671177	0.4009975	0.359515	53	PRKCZ/ABR/SORCS2/KCNAB2/SLC16A3/CACNA1C/SLC8A1/ARRB1/DNM2/RHEB/NCK2/TANC1/BCL11A/RTN4/ATP2B2/ITPR1/RUSC1/TNIK/DISC1/BAIAP2/INPP4A/ZDHHC2/SYN3/MIB1/AKAP5/SYNGAP1/DLGAP4/CNIH2/IQSEC1/EPB41L3/SYN2/CPEB3/SH3GL1/BCR/PALM/SIPA1L1/MAPK8IP2/GSG1L/ARRB2/DNM1/ADD1/SAMD14/DNM3/CABP1/SHANK2/ARHGEF2/TSC2/MX1/GRID1/DTNBP1/SYNPO/DBNL/LRFN2
Cellular Component	GO:1902911	protein kinase complex	18/2341	104/19559	0.0681602	0.4009975	0.359515	18	PRKAG2/ULK1/ERN1/ERC1/CDK14/PRKAB1/UVRAG/IGF1R/CAB39/CCNH/INSRR/CCNA1/RB1/CCNY/ERCC2/CDK13/PRKAA1/CCNP
Cellular Component	GO:0016342	catenin complex	7/2341	31/19559	0.0695553	0.4009975	0.359515	7	CDH23/JUP/SMAD7/CDH3/CDH4/CDH1/CDH11
Cellular Component	GO:0030057	desmosome	6/2341	25/19559	0.0700326	0.4009975	0.359515	6	JUP/PKP1/PERP/JAM3/KAZN/PPL
Cellular Component	GO:0030427	site of polarized growth	30/2341	191/19559	0.0723084	0.4009975	0.359515	30	CTSZ/LMTK2/TIMP2/SSH1/APBB2/AMFR/CTTN/DNM2/DISC1/IGHMBP2/APP/PREX1/FRYL/DCTN2/KIF21B/MYO5A/WDR47/KIF5C/PI4K2A/SHANK2/FSCN3/CYFIP1/TRPV2/NDEL1/DTNBP1/OTX2/NDRG2/DBNL/CBARP/NECTIN1

Cellular Component	GO:0000015	phosphopyruvate hydratase complex	2/2341	4/19559	0.0728335	0.4009975	0.359515	2	ENO1/ENO3
Cellular Component	GO:0000306	extrinsic component of vacuolar membrane	2/2341	4/19559	0.0728335	0.4009975	0.359515	2	ULK1/VAC14
Cellular Component	GO:0005826	actomyosin contractile ring	2/2341	4/19559	0.0728335	0.4009975	0.359515	2	MAEA/MYH9
Cellular Component	GO:0005899	insulin receptor complex	2/2341	4/19559	0.0728335	0.4009975	0.359515	2	IGF1R/INSRR
Cellular Component	GO:0005944	phosphatidylinositol 3-kinase complex, class IB	2/2341	4/19559	0.0728335	0.4009975	0.359515	2	PIK3R6/PIK3R5
Cellular Component	GO:0010009	cytoplasmic side of endosome membrane	2/2341	4/19559	0.0728335	0.4009975	0.359515	2	LITAF/CDIP1
Cellular Component	GO:0014802	terminal cisterna	2/2341	4/19559	0.0728335	0.4009975	0.359515	2	RTN2/CASQ1
Cellular Component	GO:0019034	viral replication complex	2/2341	4/19559	0.0728335	0.4009975	0.359515	2	PI4KA/SHFL
Cellular Component	GO:0030478	actin cap	2/2341	4/19559	0.0728335	0.4009975	0.359515	2	GSN/WASL

Cellular Component	GO:0031379	RNA-directed RNA polymerase complex	2/2341	4/19559	0.0728335	0.4009975	0.359515	2	RMRP/ZNFX1
Cellular Component	GO:0035867	alphav-beta3 integrin-IGF-1-IGF1R complex	2/2341	4/19559	0.0728335	0.4009975	0.359515	2	IGF1R/ITGB3
Cellular Component	GO:0070516	CAK-ERCC2 complex	2/2341	4/19559	0.0728335	0.4009975	0.359515	2	CCNH/ERCC2
Cellular Component	GO:0070522	ERCC4-ERCC1 complex	2/2341	4/19559	0.0728335	0.4009975	0.359515	2	ERCC1/SLX4
Cellular Component	GO:0071664	catenin-TCF7L2 complex	2/2341	4/19559	0.0728335	0.4009975	0.359515	2	JUP/TCF7L2
Cellular Component	GO:0098592	cytoplasmic side of apical plasma membrane	2/2341	4/19559	0.0728335	0.4009975	0.359515	2	EZR/C2CD2L
Cellular Component	GO:1903349	omegasome membrane	2/2341	4/19559	0.0728335	0.4009975	0.359515	2	ULK1/ATG14
Cellular Component	GO:1990604	IRE1-TRAF2-ASK1 complex	2/2341	4/19559	0.0728335	0.4009975	0.359515	2	ERN1/TRAF2
Cellular Component	GO:0016459	myosin complex	11/2341	57/19559	0.0728579	0.4009975	0.359515	11	MYO18B/MYH11/MYO9B/BMF/MYO18A/MYL12A/MYH15/MYH9/MYO5A/MYO1C/MYL7

Cellular Component	GO:0031091	platelet alpha granule	16/2341	91/19559	0.0731695	0.4009975	0.359515	16	SERPINA1/CD9/APLP2/LY6G6F/THBS1/ACTN1/APP/ITGA2B/ACTN4/CLU/PHACTR2/TGFB1/ITGB3/LHFPL2/PDGFB/CYRIB
Cellular Component	GO:0030660	Golgi-associated vesicle membrane	19/2341	112/19559	0.0732858	0.4009975	0.359515	19	AP2A1/TGFA/STX5/VTI1A/SEC23B/HLA-C/HLA-F/CNIH2/CD59/CNGA4/SEC13/ZDHHC17/SEC24C/AP1G2/PI4KA/TMED2/SREBF1/CLTCL1/SEC31A
Cellular Component	GO:0032045	guanyl-nucleotide exchange factor complex	4/2341	14/19559	0.0766981	0.4138128	0.3710045	4	ELMO1/ULK1/DOCK1/PDE3B
Cellular Component	GO:0043218	compact myelin	4/2341	14/19559	0.0766981	0.4138128	0.3710045	4	PMP22/MBP/JAM3/PRKCI
Cellular Component	GO:0099523	presynaptic cytosol	4/2341	14/19559	0.0766981	0.4138128	0.3710045	4	BAIAP2/PRKCB/HTT/CTBP2
Cellular Component	GO:0000421	autophagosome membrane	8/2341	38/19559	0.0771123	0.4141214	0.3712813	8	ULK1/UVRAG/TMEM150B/ATG14/VMP1/WDR81/STING1/RUBCNL
Cellular Component	GO:0005871	kinesin complex	10/2341	51/19559	0.0776271	0.4149651	0.3720376	10	KIF13B/NDE1/KIF13A/DISC1/KIF21B/KIF5C/KIF1B/KIFAP3/NDEL1/KIFC3
Cellular Component	GO:1904949	ATPase complex	14/2341	78/19559	0.0781437	0.4158106	0.3727958	14	CECR2/SRCAP/GATAD2A/SMARCA2/HDAC1/NCR1/MTA1/ARID1B/RSF1/MTA2/SMARCD1/SMARCD2/RB1/RUVBL1
Cellular Component	GO:0071682	endocytic vesicle lumen	5/2341	20/19559	0.0818831	0.4245995	0.3806754	5	MPO/CALR/HBB/PGLYRP1/LTF

Cellular Component	GO:0031588	nucleotide-activated protein kinase complex	3/2341	9/19559	0.0826911	0.4245995	0.3806754	3	PRKAG2/PRKAB1/PRKAA1
Cellular Component	GO:0033018	sarcoplasmic reticulum lumen	3/2341	9/19559	0.0826911	0.4245995	0.3806754	3	CALR/CASQ2/CASQ1
Cellular Component	GO:0043196	varicosity	3/2341	9/19559	0.0826911	0.4245995	0.3806754	3	TNFRSF1B/CRHBP/DNM1
Cellular Component	GO:0060187	cell pole	3/2341	9/19559	0.0826911	0.4245995	0.3806754	3	PI4K2A/ASTN2/EZR
Cellular Component	GO:0070775	H3 histone acetyltransferase complex	3/2341	9/19559	0.0826911	0.4245995	0.3806754	3	BRD1/KAT7/ING5
Cellular Component	GO:0098833	presynaptic endocytic zone	3/2341	9/19559	0.0826911	0.4245995	0.3806754	3	ITSN1/DNM1/DNM1L
Cellular Component	GO:0061695	transferase complex, transferring phosphorus-containing groups	38/2341	253/19559	0.0827237	0.4245995	0.3806754	38	PRKAG2/PIK3CD/POLE/ULK1/VAC14/ERN1/PIK3R1/PIK3R6/POLR2F/RECQL5/POLA2/ERC1/CRCP/PIK3R5/CDK14/PRKAB1/TAF10/UVRAG/IGF1R/ATG14/CAB39/CCNH/RMRP/INSRR/CCNA1/RB1/PKM/ZNFX1/RPRD1B/CCNY/POLD3/ERCC2/POLE4/CDK13/POLR1D/PRKAA1/CCNP/SLA2
Cellular Component	GO:0016235	aggresome	8/2341	39/19559	0.0873158	0.4461955	0.4000374	8	CABIN1/FGR/EDEM1/SFMBT2/ZDHHC17/PRDM16/EPS15/PRKN
Cellular Component	GO:0005775	vacuolar lumen	27/2341	173/19559	0.0897612	0.446429	0.4002467	27	AZU1/ELANE/PRTN3/CTSG/VAT1/MPO/PLBD2/TOLLIP/RNASE3/HEXB/GALNS/GALC/DYNC1H1/MNDA/PADI2/CTSD/TUBB4B/SDC2/CREG1/GRN/CTSB/ARSB/DAPK2/PLD3/PDGFRB/IMPDH1/ARHGAP45

Cellular Component	GO:0005788	endoplasmic reticulum lumen	45/2341	308/19559	0.0908565	0.446429	0.4002467	45	CTSZ/IL27/LTBP1/PNPLA2/P4HB/COL23A1/CALR/SERPINA1/COL17A1/APLP2/CASQ2/LIPC/ARSG/TSPAN14/MBTPS1/COL4A2/MGAT4A/THBS1/APP/PDIA5/COL12A1/CLU/MIA3/SDC2/PTPRN2/SPARCL1/PDGFD/ADAMTS13/GHRL/WNT5B/VCPIP1/EDEM2/PDGFB/COL14A1/ARSB/COL5A1/PDGFC/TOR1B/COL28A1/FAM20C/P4HA2/ANO8/CASQ1/TOR3A/P3H1
Cellular Component	GO:0030118	clathrin coat	9/2341	46/19559	0.0922014	0.446429	0.4002467	9	AP2A1/EPS15L1/AP1B1/EPN2/AP1G2/EPS15/EPN3/BAIAP2L2/CLTCL1
Cellular Component	GO:0150034	distal axon	45/2341	309/19559	0.0945182	0.446429	0.4002467	45	CTSZ/LMTK2/TIMP2/SSH1/KCNAB2/APBB2/AAK1/AMFR/SLC8A1/CTTN/DNM2/MICAL1/TANC1/PNOC/ITSN1/DISC1/GIT1/IGHMBP2/APP/ILK/PREX1/PTPRN2/CRHBP/DCTN2/PRKCB/KIF21B/MYO5A/WDR47/ACTG1/CHRM2/KIF5C/SHANK2/RGS10/FSCN3/AP3D1/CYFIP1/TRPV2/NDEL1/RNF40/DTNBP1/CABP4/OTX2/NDRG2/CBARP/NECTIN1
Cellular Component	GO:0005779	integral component of peroxisomal membrane	4/2341	15/19559	0.0950697	0.446429	0.4002467	4	FAR2/PEX26/FIS1/PEX11B
Cellular Component	GO:0016581	NuRD complex	4/2341	15/19559	0.0950697	0.446429	0.4002467	4	GATAD2A/HDAC1/MTA1/MTA2
Cellular Component	GO:0030127	COPII vesicle coat	4/2341	15/19559	0.0950697	0.446429	0.4002467	4	SEC23B/SEC13/SEC24C/SEC31A
Cellular Component	GO:0031231	intrinsic component of peroxisomal membrane	4/2341	15/19559	0.0950697	0.446429	0.4002467	4	FAR2/PEX26/FIS1/PEX11B
Cellular Component	GO:0033268	node of Ranvier	4/2341	15/19559	0.0950697	0.446429	0.4002467	4	KCNQ2/BIN1/SCN1A/SPTBN4
Cellular Component	GO:0090545	CHD-type complex	4/2341	15/19559	0.0950697	0.446429	0.4002467	4	GATAD2A/HDAC1/MTA1/MTA2

Cellular Component	GO:0030496	midbody	28/2341	182/19559	0.0977386	0.446429	0.4002467	28	SSH1/CTDP1/IL16/KIF13A/DNM2/CAPG/MICAL1/C6orf89/MICAL3/CHAMP1/USP3/GNAI2/PITPNM1/CCDC66/UVRAG/SVIL/AGBL5/ZFYVE19/RALA/SLC2A1/MBD5/CYLD/SPAG5/ZNF330/BCL3/DTNBP1/CEP44/SPART
Cellular Component	GO:0033017	sarcoplasmic reticulum membrane	8/2341	40/19559	0.0982652	0.446429	0.4002467	8	CASQ2/ITPR2/JSRP1/NOS1AP/ATP2A3/ASPH/CHERP/CASQ1
Cellular Component	GO:0031256	leading edge membrane	27/2341	175/19559	0.09977	0.446429	0.4002467	27	KSR1/KANK1/DNM2/FGR/FGD5/SPATA13/PACSIN2/TESC/AKAP5/DOCK8/SCIMP/PTPRJ/SH3YL1/EPS8L3/EPS8L1/EPB41L3/SLC1A2/SH2D3C/ITGB3/PALM/PDE9A/NCKAP1/CORO1C/ARHGEF2/MYO1C/EZR/ARHGAP45
Cellular Component	GO:0008021	synaptic vesicle	31/2341	205/19559	0.1011313	0.446429	0.4002467	31	VTI1A/SV2C/RAB5B/ATP8A1/STON1/BIN1/DISC1/DDC/APP/SYN3/PTPRN2/TMEM163/APBA2/RAB40C/IQSEC1/MCTP2/COPS5/SYN2/KIRREL3/DNM1/PI4K2A/SYNGR3/KIF1B/RPH3A/DNM1L/BCL2L1/NDEL1/DTNBP1/SYPL2/NTF4/CBARP
Cellular Component	GO:0043209	myelin sheath	9/2341	47/19559	0.1025476	0.446429	0.4002467	9	PRKCZ/GSN/PMP22/MBP/JAM3/PRKCI/BCL2/TSPAN2/PRXL2B
Cellular Component	GO:0090575	RNA polymerase II transcription regulator complex	25/2341	161/19559	0.103893	0.446429	0.4002467	25	RXRA/PPARG/TFDP1/CREB1/RUNX1/VDR/SMAD3/E2F3/HIPK2/E2F7/TCF12/TCF7L2/TAF10/SMAD7/TLE1/CCNH/MED8/RB1/E2F6/TCF3/CEBPB/RUNX3/TEAD2/NR5A2/ERCC2
Cellular Component	GO:0000805	X chromosome	3/2341	10/19559	0.1079743	0.446429	0.4002467	3	SIN3B/PCGF3/MACROH2A2
Cellular Component	GO:0000835	ER ubiquitin ligase complex	3/2341	10/19559	0.1079743	0.446429	0.4002467	3	RHBDD2/DERL2/MARCHF6
Cellular Component	GO:0001527	microfibril	3/2341	10/19559	0.1079743	0.446429	0.4002467	3	LTBP1/MFAP4/ADAMTSL5

Cellular Component	GO:0016461	unconventional myosin complex	3/2341	10/19559	0.1079743	0.446429	0.4002467	3	MYO18B/MYO5A/MYO1C
Cellular Component	GO:0042583	chromaffin granule	3/2341	10/19559	0.1079743	0.446429	0.4002467	3	ATP8A1/CLU/ANXA7
Cellular Component	GO:0098981	cholinergic synapse	3/2341	10/19559	0.1079743	0.446429	0.4002467	3	PLD1/DLGAP4/CHRM2
Cellular Component	GO:1990124	messenger ribonucleoprotein complex	3/2341	10/19559	0.1079743	0.446429	0.4002467	3	LSM1/PKP1/CPEB3
Cellular Component	GO:0000938	GARP complex	2/2341	5/19559	0.1119211	0.446429	0.4002467	2	VPS53/VPS51
Cellular Component	GO:0005638	lamin filament	2/2341	5/19559	0.1119211	0.446429	0.4002467	2	NARF/LMNB2
Cellular Component	GO:0005827	polar microtubule	2/2341	5/19559	0.1119211	0.446429	0.4002467	2	CUL3/KLHL21
Cellular Component	GO:0008622	epsilon DNA polymerase complex	2/2341	5/19559	0.1119211	0.446429	0.4002467	2	POLE/POLE4
Cellular Component	GO:0030124	AP-4 adaptor complex	2/2341	5/19559	0.1119211	0.446429	0.4002467	2	AP4S1/TEPSIN

Cellular Component	GO:0033263	CORVET complex	2/2341	5/19559	0.1119211	0.446429	0.4002467	2	TGFBRAP1/VPS18
Cellular Component	GO:0044094	host cell nuclear part	2/2341	5/19559	0.1119211	0.446429	0.4002467	2	PI4KA/SHFL
Cellular Component	GO:0048476	Holliday junction resolvase complex	2/2341	5/19559	0.1119211	0.446429	0.4002467	2	EME1/SLX4
Cellular Component	GO:0065010	extracellular membrane-bounded organelle	2/2341	5/19559	0.1119211	0.446429	0.4002467	2	ALPL/AQP1
Cellular Component	GO:0070390	transcription export complex 2	2/2341	5/19559	0.1119211	0.446429	0.4002467	2	ENY2/PCID2
Cellular Component	GO:0072557	IPAF inflammasome complex	2/2341	5/19559	0.1119211	0.446429	0.4002467	2	NLRC4/CASP1
Cellular Component	GO:0097124	cyclin A2-CDK2 complex	2/2341	5/19559	0.1119211	0.446429	0.4002467	2	CCNA1/CCNP
Cellular Component	GO:0097255	R2TP complex	2/2341	5/19559	0.1119211	0.446429	0.4002467	2	PIH1D1/RUVBL1
Cellular Component	GO:0097418	neurofibrillary tangle	2/2341	5/19559	0.1119211	0.446429	0.4002467	2	NEFM/CLU

Cellular Component	GO:0098574	cytoplasmic side of lysosomal membrane	2/2341	5/19559	0.1119211	0.446429	0.4002467	2	LITAF/CDIP1
Cellular Component	GO:0098826	endoplasmic reticulum tubular network membrane	2/2341	5/19559	0.1119211	0.446429	0.4002467	2	RTN4/ATL1
Cellular Component	GO:1990726	Lsm1-7-Pat1 complex	2/2341	5/19559	0.1119211	0.446429	0.4002467	2	LSM1/LSM7
Cellular Component	GO:1990745	EARP complex	2/2341	5/19559	0.1119211	0.446429	0.4002467	2	VPS53/VPS51
Cellular Component	GO:0042575	DNA polymerase complex	5/2341	22/19559	0.1141808	0.446429	0.4002467	5	POLE/POLA2/CRCP/POLD3/POLE4
Cellular Component	GO:0071782	endoplasmic reticulum tubular network	5/2341	22/19559	0.1141808	0.446429	0.4002467	5	RTN4/ATL1/ASPH/C2CD2L/STIMATE
Cellular Component	GO:0005767	secondary lysosome	4/2341	16/19559	0.1152882	0.446429	0.4002467	4	NCF4/NCF2/CRHBP/NCOA4
Cellular Component	GO:0016442	RISC complex	4/2341	16/19559	0.1152882	0.446429	0.4002467	4	AGO1/LIMD1/AGO2/SND1
Cellular Component	GO:0031332	RNAi effector complex	4/2341	16/19559	0.1152882	0.446429	0.4002467	4	AGO1/LIMD1/AGO2/SND1

Cellular Component	GO:0070382	exocytic vesicle	33/2341	223/19559	0.1158779	0.446429	0.4002467	33	VTI1A/SV2C/RAB5B/ATP8A1/STON1/BIN1/DISC1/RAB27A/DDC/APP/SYN3/PTPRN2/TMEM163/APBA2/RAB40C/IQSEC1/MCTP2/COPS5/SYN2/KIRREL3/DNM1/PI4K2A/SYNGR3/KIF1B/RPH3A/DNM1L/SYT17/BCL2L1/NDEL1/DTNBP1/SYPL2/NTF4/CBARP
Cellular Component	GO:0005595	collagen type XII trimer	1/2341	1/19559	0.1196891	0.446429	0.4002467	1	COL12A1
Cellular Component	GO:0005596	collagen type XIV trimer	1/2341	1/19559	0.1196891	0.446429	0.4002467	1	COL14A1
Cellular Component	GO:0009360	DNA polymerase III complex	1/2341	1/19559	0.1196891	0.446429	0.4002467	1	CRCP
Cellular Component	GO:0014804	terminal cisterna lumen	1/2341	1/19559	0.1196891	0.446429	0.4002467	1	CASQ1
Cellular Component	GO:0030526	granulocyte macrophage colony-stimulating factor receptor complex	1/2341	1/19559	0.1196891	0.446429	0.4002467	1	CSF2RB
Cellular Component	GO:0031257	cell trailing edge membrane	1/2341	1/19559	0.1196891	0.446429	0.4002467	1	SCIMP
Cellular Component	GO:0031259	uropod membrane	1/2341	1/19559	0.1196891	0.446429	0.4002467	1	SCIMP
Cellular Component	GO:0032996	Bcl3-Bcl10 complex	1/2341	1/19559	0.1196891	0.446429	0.4002467	1	BCL3

Cellular Component	GO:0033593	BRCA2-MAGE-D1 complex	1/2341	1/19559	0.1196891	0.446429	0.4002467	1	BRCA2
Cellular Component	GO:0034423	autophagosome lumen	1/2341	1/19559	0.1196891	0.446429	0.4002467	1	DAPK2
Cellular Component	GO:0034777	recycling endosome lumen	1/2341	1/19559	0.1196891	0.446429	0.4002467	1	PDLIM4
Cellular Component	GO:0035525	NF-kappaB p50/p65 complex	1/2341	1/19559	0.1196891	0.446429	0.4002467	1	RELA
Cellular Component	GO:0043509	activin A complex	1/2341	1/19559	0.1196891	0.446429	0.4002467	1	INHBA
Cellular Component	GO:0048180	activin complex	1/2341	1/19559	0.1196891	0.446429	0.4002467	1	INHBA
Cellular Component	GO:0055028	cortical microtubule	1/2341	1/19559	0.1196891	0.446429	0.4002467	1	NUMA1
Cellular Component	GO:0060987	lipid tube	1/2341	1/19559	0.1196891	0.446429	0.4002467	1	BIN1
Cellular Component	GO:0061828	apical tubulobulbar complex	1/2341	1/19559	0.1196891	0.446429	0.4002467	1	DNM3

Cellular Component	GO:0061829	basal tubulobulbar complex	1/2341	1/19559	0.1196891	0.446429	0.4002467	1	DNM3
Cellular Component	GO:0061845	neuron projection branch point	1/2341	1/19559	0.1196891	0.446429	0.4002467	1	BAIAP2
Cellular Component	GO:0061846	dendritic spine cytoplasm	1/2341	1/19559	0.1196891	0.446429	0.4002467	1	BAIAP2
Cellular Component	GO:0070772	PAS complex	1/2341	1/19559	0.1196891	0.446429	0.4002467	1	VAC14
Cellular Component	GO:0070826	paraferritin complex	1/2341	1/19559	0.1196891	0.446429	0.4002467	1	SLC11A2
Cellular Component	GO:0071513	phosphopantothenoylcysteine decarboxylase complex	1/2341	1/19559	0.1196891	0.446429	0.4002467	1	PPCDC
Cellular Component	GO:0071575	integral component of external side of plasma membrane	1/2341	1/19559	0.1196891	0.446429	0.4002467	1	CEACAM5
Cellular Component	GO:0071665	gamma-catenin-TCF7L2 complex	1/2341	1/19559	0.1196891	0.446429	0.4002467	1	JUP
Cellular Component	GO:0097409	glial cytoplasmic inclusion	1/2341	1/19559	0.1196891	0.446429	0.4002467	1	FBXO7

Cellular Component	GO:0097414	classical Lewy body	1/2341	1/19559	0.1196891	0.446429	0.4002467	1	FBXO7
Cellular Component	GO:0097462	Lewy neurite	1/2341	1/19559	0.1196891	0.446429	0.4002467	1	FBXO7
Cellular Component	GO:0098830	presynaptic endosome	1/2341	1/19559	0.1196891	0.446429	0.4002467	1	AP3D1
Cellular Component	GO:0099020	perinuclear endoplasmic reticulum lumen	1/2341	1/19559	0.1196891	0.446429	0.4002467	1	CLU
Cellular Component	GO:1902912	pyruvate kinase complex	1/2341	1/19559	0.1196891	0.446429	0.4002467	1	PKM
Cellular Component	GO:1905720	cytoplasmic microtubule bundle	1/2341	1/19559	0.1196891	0.446429	0.4002467	1	NUMA1
Cellular Component	GO:1990031	pinceau fiber	1/2341	1/19559	0.1196891	0.446429	0.4002467	1	KCNAB2
Cellular Component	GO:1990038	Lewy body corona	1/2341	1/19559	0.1196891	0.446429	0.4002467	1	FBXO7
Cellular Component	GO:1990332	Ire1 complex	1/2341	1/19559	0.1196891	0.446429	0.4002467	1	ERN1

Cellular Component	GO:1990673	intrinsic component of endoplasmic reticulum-Golgi intermediate compartment (ERGIC) membrane	1/2341	1/19559	0.1196891	0.446429	0.4002467	1	STING1
Cellular Component	GO:1990701	integral component of endoplasmic reticulum-Golgi intermediate compartment (ERGIC) membrane	1/2341	1/19559	0.1196891	0.446429	0.4002467	1	STING1
Cellular Component	GO:0030125	clathrin vesicle coat	6/2341	29/19559	0.1252632	0.4627556	0.4148843	6	AP2A1/EPN2/AP1G2/EPS15/EPN3/CLTCL1
Cellular Component	GO:0044665	MLL1/2 complex	6/2341	29/19559	0.1252632	0.4627556	0.4148843	6	ASH2L/KANSL1/MGA/E2F6/CHD8/RUVBL1
Cellular Component	GO:0071339	MLL1 complex	6/2341	29/19559	0.1252632	0.4627556	0.4148843	6	ASH2L/KANSL1/MGA/E2F6/CHD8/RUVBL1
Cellular Component	GO:0000118	histone deacetylase complex	13/2341	77/19559	0.1260085	0.4640314	0.4160281	13	HDAC4/NCOR2/SRCAP/ZNF217/HDAC7/SIN3B/GATAD2A/HDAC1/MTA1/PHF12/MTA2/RUVBL1/MECOM
Cellular Component	GO:0030120	vesicle coat	10/2341	56/19559	0.1265364	0.4645006	0.4164488	10	AP2A1/SEC23B/EPN2/SEC13/SEC24C/AP1G2/EPS15/EPN3/CLTCL1/SEC31A
Cellular Component	GO:0030175	filopodium	17/2341	106/19559	0.1279753	0.4683009	0.419856	17	AP2A1/FGD4/DYNC1H1/LY6G6D/SPATA13/BAIAP2/APP/SCIMP/FARP1/MYO5A/ITGB3/PALM/FSCN3/CYFIP1/EZR/ADGRA2/ACP3
Cellular Component	GO:0008180	COP9 signalosome	7/2341	36/19559	0.1317721	0.4791712	0.4296017	7	LAT/MYH9/COPS5/COPS3/TMOD1/PLCG1/FLOT1

Cellular Component	GO:0098636	protein complex involved in cell adhesion	7/2341	36/19559	0.1317721	0.4791712	0.4296017	7	ITGAE/ITGA2B/ITGB5/JAM3/ITGB3/ITGB2/ITGB6
Cellular Component	GO:0001650	fibrillar center	20/2341	129/19559	0.1356671	0.4902466	0.4395314	20	TRIM27/RREB1/TAF1C/WDR43/SP140/NFIC/POLR2F/SAMD4A/SMAD7/MBD6/MLLT1/MAP3K14/ACACA/ANKRD1/PLRG1/EZR/FBL/EEF1D/IP6K1/UTP4
Cellular Component	GO:0097470	ribbon synapse	3/2341	11/19559	0.1357985	0.4902466	0.4395314	3	CTBP2/DNM1/NPHP4
Cellular Component	GO:0098687	chromosomal region	49/2341	350/19559	0.1367793	0.4902466	0.4395314	49	BLM/DCTN1/BAZ1B/MAD1L1/TNKS/NDE1/BRCA2/HMBOX1/KANSL1/RTEL1/NSMCE2/MCM5/CHAMP1/WRNIP1/MIS12/UVRAG/ZNF276/WDR82/DCTN2/PPP2R5C/STAG1/KDM4C/SEC13/MCM2/SMG6/PPP2R1A/PPP1CB/RAD21L1/DMC1/DNMT1/TP53BP1/CDCA5/SP100/CHEK2/CEBPB/ERCC1/CENPM/SPAG5/ZNF330/SLX4/FBXW11/NDEL1/KAT7/DYDC2/DYDC1/PELI1/TFIP11/TERB2/MACROH2A2
Cellular Component	GO:0042622	photoreceptor outer segment membrane	4/2341	17/19559	0.1371968	0.4902466	0.4395314	4	PDE6A/GNAT2/PDE6H/CDHR1
Cellular Component	GO:0061702	inflammasome complex	4/2341	17/19559	0.1371968	0.4902466	0.4395314	4	NLRC4/AIM2/NLRP3/CASP1
Cellular Component	GO:0098862	cluster of actin-based cell projections	24/2341	159/19559	0.1373536	0.4902466	0.4395314	24	CDH23/ABCC2/SLC11A2/TRPM6/MYH9/ATP8B1/ACTN1/ACTN4/SLC19A1/AQP1/CDHR2/ESPN/SLC22A5/MFSD10/TSPEAR/DNM1L/SHANK2/GNA12/FLII/TMC1/MYO1C/CDC14A/EZR/PDZK1
Cellular Component	GO:0097431	mitotic spindle pole	6/2341	30/19559	0.1416373	0.5039856	0.4518492	6	MAD1L1/TNKS/RAE1/STAG1/NUMA1/SPAG5
Cellular Component	GO:0008287	protein serine/threonine phosphatase complex	9/2341	51/19559	0.1501248	0.5286055	0.4739221	9	ITPR1/WDR82/PPP2R5C/PPP2R1A/PPP1CB/PPP2R5E/PPP2R5D/PPP2R1B/PTPA

Cellular Component	GO:1903293	phosphatase complex	9/2341	51/19559	0.1501248	0.5286055	0.4739221	9	ITPR1/WDR82/PPP2R5C/PPP2R1A/PPP1CB/PPP2R5E/PPP2R5D/PPP2R1B/PTPA
Cellular Component	GO:0032588	trans-Golgi network membrane	15/2341	94/19559	0.1503822	0.5286055	0.4739221	15	VTI1A/VPS53/AP4S1/KIF13A/RAB31/APP/AP1B1/COG1/RAB43/COG8/USP6NL/COG5/ATP7B/VPS51/TEPSIN
Cellular Component	GO:0043601	nuclear replisome	5/2341	24/19559	0.1515853	0.5286055	0.4739221	5	POLA2/SMARCAL1/PLRG1/POLD3/XPA
Cellular Component	GO:0030426	growth cone	27/2341	184/19559	0.153456	0.5286055	0.4739221	27	CTSZ/LMTK2/TIMP2/SSH1/APBB2/AMFR/CTTN/DNM2/DISC1/IGHMBP2/APP/PREX1/DCTN2/KIF21B/MYO5A/WDR47/KIF5C/SHANK2/FSCN3/CYFIP1/TRPV2/NDEL1/DTNBP1/OTX2/NDRG2/CBARP/NECTIN1
Cellular Component	GO:0005593	FACIT collagen trimer	2/2341	6/19559	0.1549361	0.5286055	0.4739221	2	COL12A1/COL14A1
Cellular Component	GO:0032010	phagolysosome	2/2341	6/19559	0.1549361	0.5286055	0.4739221	2	NCF4/NCF2
Cellular Component	GO:0070937	CRD-mediated mRNA stability complex	2/2341	6/19559	0.1549361	0.5286055	0.4739221	2	CSDE1/SYNCRIP
Cellular Component	GO:0097169	AIM2 inflammasome complex	2/2341	6/19559	0.1549361	0.5286055	0.4739221	2	AIM2/CASP1
Cellular Component	GO:0097342	ripoptosome	2/2341	6/19559	0.1549361	0.5286055	0.4739221	2	RIPK1/FADD

Cellular Component	GO:0097433	dense body	2/2341	6/19559	0.1549361	0.5286055	0.4739221	2	ACTG1/SND1
Cellular Component	GO:0097443	sorting endosome	2/2341	6/19559	0.1549361	0.5286055	0.4739221	2	ARHGAP1/CLTCL1
Cellular Component	GO:0150051	postsynaptic Golgi apparatus	2/2341	6/19559	0.1549361	0.5286055	0.4739221	2	MYO18A/FAM234B
Cellular Component	GO:1905347	endodeoxyribonuclease complex	2/2341	6/19559	0.1549361	0.5286055	0.4739221	2	EME1/SLX4
Cellular Component	GO:0008305	integrin complex	6/2341	31/19559	0.158937	0.5406655	0.4847346	6	ITGAE/ITGA2B/ITGB5/ITGB3/ITGB2/ITGB6
Cellular Component	GO:0000109	nucleotide-excision repair complex	3/2341	12/19559	0.1657386	0.5556553	0.4981737	3	ERCC1/SLX4/XPA
Cellular Component	GO:0019908	nuclear cyclin-dependent protein kinase holoenzyme complex	3/2341	12/19559	0.1657386	0.5556553	0.4981737	3	CCNH/RB1/CDK13
Cellular Component	GO:0030128	clathrin coat of endocytic vesicle	3/2341	12/19559	0.1657386	0.5556553	0.4981737	3	AP2A1/EPN2/EPS15
Cellular Component	GO:0031932	TORC2 complex	3/2341	12/19559	0.1657386	0.5556553	0.4981737	3	PRR5L/PRR5/TTI1

Cellular Component	GO:0044615	nuclear pore nuclear basket	3/2341	12/19559	0.1657386	0.5556553	0.4981737	3	MAD1L1/ENY2/PCID2
Cellular Component	GO:0060170	ciliary membrane	12/2341	74/19559	0.169735	0.5659831	0.5074331	12	EHD1/PDE6A/IQCE/PKD1L1/GNAT2/CNGA4/PDE6H/TSPEAR/CDHR1/SHANK2/EPS15/TXNDC15
Cellular Component	GO:0030136	clathrin-coated vesicle	28/2341	194/19559	0.1697949	0.5659831	0.5074331	28	CPNE6/AP2A1/TGFA/CD9/VTI1A/DENND1A/AAK1/DNM2/SFTPD/STON1/RAB27A/SNX3/AP1B1/EPN2/VPS18/NUMB/CHRM2/SH3BP4/SNX18/SORT1/AP1G2/ASTN2/EPS15/EPN3/INPP5F/CLTCL1/DBNL/PHETA1
Cellular Component	GO:0099522	region of cytosol	5/2341	25/19559	0.1719735	0.5716025	0.5124712	5	BAIAP2/PRKCB/HTT/CTBP2/NEDD4
Cellular Component	GO:0030672	synaptic vesicle membrane	16/2341	104/19559	0.1757887	0.5809541	0.5208554	16	SV2C/RAB5B/ATP8A1/SYN3/PTPRN2/TMEM163/MCTP2/SYN2/PI4K2A/SYNGR3/RPH3A/DNM1L/BCL2L1/DTNBP1/SYPL2/CBARP
Cellular Component	GO:0099501	exocytic vesicle membrane	16/2341	104/19559	0.1757887	0.5809541	0.5208554	16	SV2C/RAB5B/ATP8A1/SYN3/PTPRN2/TMEM163/MCTP2/SYN2/PI4K2A/SYNGR3/RPH3A/DNM1L/BCL2L1/DTNBP1/SYPL2/CBARP
Cellular Component	GO:0043034	costamere	4/2341	19/19559	0.1853636	0.5906504	0.5295487	4	AHNAK/ILK/SVIL/ANK2
Cellular Component	GO:0097449	astrocyte projection	4/2341	19/19559	0.1853636	0.5906504	0.5295487	4	APP/MLC1/SLC1A2/EZR
Cellular Component	GO:0001917	photoreceptor inner segment	10/2341	61/19559	0.1886665	0.5906504	0.5295487	10	RCVRN/CRB1/KIAA0586/DNM2/CCDC66/GNAT2/GUK1/DNM1/DNM3/SHANK2

Cellular Component	GO:0030894	replisome	5/2341	26/19559	0.1933426	0.5906504	0.5295487	5	POLA2/SMARCAL1/PLRG1/POLD3/XPA
Cellular Component	GO:0070971	endoplasmic reticulum exit site	6/2341	33/19559	0.1960467	0.5906504	0.5295487	6	SEC23B/MIA3/SEC24C/SEC31A/MIA2/PLPP3
Cellular Component	GO:0005614	interstitial matrix	3/2341	13/19559	0.1973693	0.5906504	0.5295487	3	VIT/EGFLAM/COL14A1
Cellular Component	GO:0016580	Sin3 complex	3/2341	13/19559	0.1973693	0.5906504	0.5295487	3	SIN3B/HDAC1/PHF12
Cellular Component	GO:0017119	Golgi transport complex	3/2341	13/19559	0.1973693	0.5906504	0.5295487	3	COG1/COG8/COG5
Cellular Component	GO:0030130	clathrin coat of trans-Golgi network vesicle	3/2341	13/19559	0.1973693	0.5906504	0.5295487	3	AP2A1/AP1G2/CLTCL1
Cellular Component	GO:0032009	early phagosome	3/2341	13/19559	0.1973693	0.5906504	0.5295487	3	RAB31/TLR9/SNX3
Cellular Component	GO:0032585	multivesicular body membrane	3/2341	13/19559	0.1973693	0.5906504	0.5295487	3	SORL1/RAB27A/GIMAP5
Cellular Component	GO:0033646	host intracellular part	3/2341	13/19559	0.1973693	0.5906504	0.5295487	3	AQP1/PI4KA/SHFL

Cellular Component	GO:0043656	host intracellular region	3/2341	13/19559	0.1973693	0.5906504	0.5295487	3	AQP1/PI4KA/SHFL
Cellular Component	GO:0097730	non-motile cilium	24/2341	167/19559	0.197635	0.5906504	0.5295487	24	RCVRN/PDE6A/PKD1L1/RAB27A/CCDC66/GNAT2/MYO5A/GNA11/PDE6H/TTLL4/IFT140/SDCCAG8/RPGRIP1/CDHR1/SHANK2/GRK7/KIFAP3/CDC14A/HYLS1/EYS/NPFFR1/PRLHR/NPHP4/SEPTIN9
Cellular Component	GO:0016272	prefoldin complex	2/2341	7/19559	0.2003794	0.5906504	0.5295487	2	PFDN2/PFDN6
Cellular Component	GO:0030991	intraciliary transport particle A	2/2341	7/19559	0.2003794	0.5906504	0.5295487	2	IFT140/TTC21B
Cellular Component	GO:0035032	phosphatidylinositol 3-kinase complex, class III	2/2341	7/19559	0.2003794	0.5906504	0.5295487	2	VAC14/ATG14
Cellular Component	GO:0038037	G protein-coupled receptor dimeric complex	2/2341	7/19559	0.2003794	0.5906504	0.5295487	2	GABBR1/GABBR2
Cellular Component	GO:0042025	host cell nucleus	2/2341	7/19559	0.2003794	0.5906504	0.5295487	2	PI4KA/SHFL
Cellular Component	GO:0042584	chromaffin granule membrane	2/2341	7/19559	0.2003794	0.5906504	0.5295487	2	ATP8A1/ANXA7
Cellular Component	GO:0042587	glycogen granule	2/2341	7/19559	0.2003794	0.5906504	0.5295487	2	PCYT1A/PPP1CB

Cellular Component	GO:0044545	NSL complex	2/2341	7/19559	0.2003794	0.5906504	0.5295487	2	KANSL1/KANSL2
Cellular Component	GO:0051286	cell tip	2/2341	7/19559	0.2003794	0.5906504	0.5295487	2	PI4K2A/EZR
Cellular Component	GO:0071541	eukaryotic translation initiation factor 3 complex, eIF3m	2/2341	7/19559	0.2003794	0.5906504	0.5295487	2	EIF3M/EIF3H
Cellular Component	GO:0072357	PTW/PP1 phosphatase complex	2/2341	7/19559	0.2003794	0.5906504	0.5295487	2	WDR82/PPP1CB
Cellular Component	GO:0098684	photoreceptor ribbon synapse	2/2341	7/19559	0.2003794	0.5906504	0.5295487	2	CTBP2/DNM1
Cellular Component	GO:0098835	presynaptic endocytic zone membrane	2/2341	7/19559	0.2003794	0.5906504	0.5295487	2	DNM1/DNM1L
Cellular Component	GO:0043679	axon terminus	19/2341	130/19559	0.208965	0.5906504	0.5295487	19	KCNAB2/AAK1/SLC8A1/MICAL1/TANC1/PNOC/ITSN1/GIT1/ILK/PTPRN2/CRHBP/PRKCB/ACTG1/CHRM2/RGS10/AP3D1/CYFIP1/RNF40/CABP4
Cellular Component	GO:0036019	endolysosome	4/2341	20/19559	0.2112337	0.5906504	0.5295487	4	AP2A1/TLR9/CLEC16A/CTSB
Cellular Component	GO:0001891	phagocytic cup	5/2341	27/19559	0.2155767	0.5906504	0.5295487	5	DNM2/RAB31/TNF/ARHGAP25/ARHGAP12

Cellular Component	GO:1990752	microtubule end	6/2341	34/19559	0.2157093	0.5906504	0.5295487	6	DCTN1/NAV3/SVIL/MYO5A/NUMA1/SPAG5
Cellular Component	GO:0000274	mitochondrial proton-transporting ATP synthase, stator stalk	1/2341	2/19559	0.2250582	0.5906504	0.5295487	1	ATP5PD
Cellular Component	GO:0000806	Y chromosome	1/2341	2/19559	0.2250582	0.5906504	0.5295487	1	SIN3B
Cellular Component	GO:0001651	dense fibrillar component	1/2341	2/19559	0.2250582	0.5906504	0.5295487	1	FBL
Cellular Component	GO:0002096	polkadots	1/2341	2/19559	0.2250582	0.5906504	0.5295487	1	BCL10
Cellular Component	GO:0002945	cyclin K-CDK13 complex	1/2341	2/19559	0.2250582	0.5906504	0.5295487	1	CDK13
Cellular Component	GO:0005602	complement component C1 complex	1/2341	2/19559	0.2250582	0.5906504	0.5295487	1	C1QB
Cellular Component	GO:0005668	RNA polymerase transcription factor SL1 complex	1/2341	2/19559	0.2250582	0.5906504	0.5295487	1	TAF1D
Cellular Component	GO:0005745	m-AAA complex	1/2341	2/19559	0.2250582	0.5906504	0.5295487	1	SPG7

Cellular Component	GO:0016222	procollagen-proline 4-dioxygenase complex	1/2341	2/19559	0.2250582	0.5906504	0.5295487	1	P4HB
Cellular Component	GO:0020005	symbiont-containing vacuole membrane	1/2341	2/19559	0.2250582	0.5906504	0.5295487	1	AQP1
Cellular Component	GO:0030692	Noc4p-Nop14p complex	1/2341	2/19559	0.2250582	0.5906504	0.5295487	1	NOP14
Cellular Component	GO:0030849	autosome	1/2341	2/19559	0.2250582	0.5906504	0.5295487	1	SIN3B
Cellular Component	GO:0031213	RSF complex	1/2341	2/19559	0.2250582	0.5906504	0.5295487	1	RSF1
Cellular Component	GO:0031380	nuclear RNA-directed RNA polymerase complex	1/2341	2/19559	0.2250582	0.5906504	0.5295487	1	ZNFX1
Cellular Component	GO:0032311	angiogenin-PRI complex	1/2341	2/19559	0.2250582	0.5906504	0.5295487	1	RNH1
Cellular Component	GO:0032398	MHC class Ib protein complex	1/2341	2/19559	0.2250582	0.5906504	0.5295487	1	HLA-F
Cellular Component	GO:0033257	Bcl3/NF-kappaB2 complex	1/2341	2/19559	0.2250582	0.5906504	0.5295487	1	BCL3

Cellular Component	GO:0033596	TSC1-TSC2 complex	1/2341	2/19559	0.2250582	0.5906504	0.5295487	1	TSC2
Cellular Component	GO:0034683	integrin alphav-beta3 complex	1/2341	2/19559	0.2250582	0.5906504	0.5295487	1	ITGB3
Cellular Component	GO:0034684	integrin alphav-beta5 complex	1/2341	2/19559	0.2250582	0.5906504	0.5295487	1	ITGB5
Cellular Component	GO:0034685	integrin alphav-beta6 complex	1/2341	2/19559	0.2250582	0.5906504	0.5295487	1	ITGB6
Cellular Component	GO:0034687	integrin alphaL-beta2 complex	1/2341	2/19559	0.2250582	0.5906504	0.5295487	1	ITGB2
Cellular Component	GO:0034688	integrin alphaM-beta2 complex	1/2341	2/19559	0.2250582	0.5906504	0.5295487	1	ITGB2
Cellular Component	GO:0034689	integrin alphaX-beta2 complex	1/2341	2/19559	0.2250582	0.5906504	0.5295487	1	ITGB2
Cellular Component	GO:0035189	Rb-E2F complex	1/2341	2/19559	0.2250582	0.5906504	0.5295487	1	RB1
Cellular Component	GO:0035370	UBC13-UEV1A complex	1/2341	2/19559	0.2250582	0.5906504	0.5295487	1	UBE2V1

Cellular Component	GO:0035632	mitochondrial prohibitin complex	1/2341	2/19559	0.2250582	0.5906504	0.5295487	1	PHB
Cellular Component	GO:0036117	hyaluranon cable	1/2341	2/19559	0.2250582	0.5906504	0.5295487	1	HAS3
Cellular Component	GO:0036186	early phagosome membrane	1/2341	2/19559	0.2250582	0.5906504	0.5295487	1	RAB31
Cellular Component	GO:0036284	tubulobulbar complex	1/2341	2/19559	0.2250582	0.5906504	0.5295487	1	DNM3
Cellular Component	GO:0036488	CHOP-C/EBP complex	1/2341	2/19559	0.2250582	0.5906504	0.5295487	1	CEBPB
Cellular Component	GO:0043511	inhibin complex	1/2341	2/19559	0.2250582	0.5906504	0.5295487	1	INHBA
Cellular Component	GO:0043512	inhibin A complex	1/2341	2/19559	0.2250582	0.5906504	0.5295487	1	INHBA
Cellular Component	GO:0045265	proton-transporting ATP synthase, stator stalk	1/2341	2/19559	0.2250582	0.5906504	0.5295487	1	ATP5PD
Cellular Component	GO:0060053	neurofilament cytoskeleton	1/2341	2/19559	0.2250582	0.5906504	0.5295487	1	NDEL1

Cellular Component	GO:0061851	leading edge of lamellipodium	1/2341	2/19559	0.2250582	0.5906504	0.5295487	1	DUSP22
Cellular Component	GO:0070418	DNA-dependent protein kinase complex	1/2341	2/19559	0.2250582	0.5906504	0.5295487	1	UVRAG
Cellular Component	GO:0071149	TEAD-2-YAP complex	1/2341	2/19559	0.2250582	0.5906504	0.5295487	1	TEAD2
Cellular Component	GO:0071159	NF-kappaB complex	1/2341	2/19559	0.2250582	0.5906504	0.5295487	1	RELA
Cellular Component	GO:0071819	DUBm complex	1/2341	2/19559	0.2250582	0.5906504	0.5295487	1	ENY2
Cellular Component	GO:0090537	CERF complex	1/2341	2/19559	0.2250582	0.5906504	0.5295487	1	CECR2
Cellular Component	GO:0090725	peripheral region of growth cone	1/2341	2/19559	0.2250582	0.5906504	0.5295487	1	CYFIP1
Cellular Component	GO:0097057	TRAF2-GSTP1 complex	1/2341	2/19559	0.2250582	0.5906504	0.5295487	1	TRAF2
Cellular Component	GO:0097058	CRLF-CLCF1 complex	1/2341	2/19559	0.2250582	0.5906504	0.5295487	1	CRLF1

Cellular Component	GO:0097123	cyclin A1-CDK2 complex	1/2341	2/19559	0.2250582	0.5906504	0.5295487	1	CCNA1
Cellular Component	GO:0097574	lateral part of cell	1/2341	2/19559	0.2250582	0.5906504	0.5295487	1	NUMA1
Cellular Component	GO:0097575	lateral cell cortex	1/2341	2/19559	0.2250582	0.5906504	0.5295487	1	NUMA1
Cellular Component	GO:0097635	extrinsic component of autophagosome membrane	1/2341	2/19559	0.2250582	0.5906504	0.5295487	1	ULK1
Cellular Component	GO:0150001	primary dendrite	1/2341	2/19559	0.2250582	0.5906504	0.5295487	1	MAP1A
Cellular Component	GO:1902560	GMP reductase complex	1/2341	2/19559	0.2250582	0.5906504	0.5295487	1	GMPR2
Cellular Component	GO:1904602	serotonin-activated cation-selective channel complex	1/2341	2/19559	0.2250582	0.5906504	0.5295487	1	HTR3A
Cellular Component	GO:1990026	hippocampal mossy fiber expansion	1/2341	2/19559	0.2250582	0.5906504	0.5295487	1	MICAL1
Cellular Component	GO:1990071	TRAPPII protein complex	1/2341	2/19559	0.2250582	0.5906504	0.5295487	1	TRAPPC10

Cellular Component	GO:1990184	amino acid transport complex	1/2341	2/19559	0.2250582	0.5906504	0.5295487	1	SLC7A5
Cellular Component	GO:1990462	omegasome	1/2341	2/19559	0.2250582	0.5906504	0.5295487	1	TRIM5
Cellular Component	GO:1990572	TERT-RMRP complex	1/2341	2/19559	0.2250582	0.5906504	0.5295487	1	RMRP
Cellular Component	GO:1990589	ATF4-CREB1 transcription factor complex	1/2341	2/19559	0.2250582	0.5906504	0.5295487	1	CREB1
Cellular Component	GO:1990597	AIP1-IRE1 complex	1/2341	2/19559	0.2250582	0.5906504	0.5295487	1	ERN1
Cellular Component	GO:1990630	IRE1-RACK1-PP2A complex	1/2341	2/19559	0.2250582	0.5906504	0.5295487	1	ERN1
Cellular Component	GO:1990682	CSF1-CSF1R complex	1/2341	2/19559	0.2250582	0.5906504	0.5295487	1	CSF1R
Cellular Component	GO:0031312	extrinsic component of organelle membrane	8/2341	49/19559	0.2271973	0.5949185	0.5333753	8	ULK1/VAC14/PAM16/ATG14/WDR81/COQ4/TEPSIN/COQ8B
Cellular Component	GO:0000800	lateral element	3/2341	14/19559	0.2302796	0.5962597	0.5345777	3	BLM/BRCA2/RAD21L1

Cellular Component	GO:0031314	extrinsic component of mitochondrial inner membrane	3/2341	14/19559	0.2302796	0.5962597	0.5345777	3	PAM16/COQ4/COQ8B
Cellular Component	GO:0033643	host cell part	3/2341	14/19559	0.2302796	0.5962597	0.5345777	3	AQP1/PI4KA/SHFL
Cellular Component	GO:0034518	RNA cap binding complex	3/2341	14/19559	0.2302796	0.5962597	0.5345777	3	AGO2/CYFIP1/NCBP3
Cellular Component	GO:0036020	endolysosome membrane	3/2341	14/19559	0.2302796	0.5962597	0.5345777	3	AP2A1/TLR9/CLEC16A
Cellular Component	GO:0099738	cell cortex region	7/2341	42/19559	0.231974	0.5993092	0.5373117	7	CTSZ/PRKCZ/DCTN1/ENO1/CTBP1/CTBP2/NUMA1
Cellular Component	GO:0031904	endosome lumen	6/2341	35/19559	0.2360051	0.60805	0.5451483	6	LNPEP/AP4S1/CTSH/APP/CTSB/PDLIM4
Cellular Component	GO:1904115	axon cytoplasm	9/2341	57/19559	0.2378199	0.60805	0.5451483	9	SPG7/DYNC1H1/KIF5C/KIF1B/MAP1A/AP3D1/FBXW11/NDEL1/DTNBP1
Cellular Component	GO:0032806	carboxy-terminal domain protein kinase complex	4/2341	21/19559	0.2380289	0.60805	0.5451483	4	CCNH/RB1/ERCC2/CDK13
Cellular Component	GO:0000781	chromosome, telomeric region	23/2341	164/19559	0.2390722	0.60805	0.5451483	23	BLM/TNKS/BRCA2/HMBOX1/RTEL1/NSMCE2/MCM5/WRNIP1/WDR82/MCM2/SMG6/PPP1CB/DMC1/TP53BP1/SP100/CHEK2/ERCC1/SLX4/DYDC2/DYDC1/TFIP11/TERB2/MACROH2A2

Cellular Component	GO:0044306	neuron projection terminus	21/2341	149/19559	0.2442259	0.60805	0.5451483	21	KCNAB2/VTI1A/AAK1/SLC8A1/MICAL1/TANC1/PNOC/ITSN1/GIT1/BAIAP2/ILK/PTPRN2/CRHBP/PRKCB/ACTG1/CHRM2/RGS10/AP3D1/CYFIP1/RNF40/CABP4
Cellular Component	GO:0000775	chromosome, centromeric region	27/2341	196/19559	0.2458468	0.60805	0.5451483	27	DCTN1/BAZ1B/MAD1L1/NDE1/KANSL1/CHAMP1/MIS12/UVRAG/ZNF276/DCTN2/PPP2R5C/STAG1/KDM4C/SEC13/PPP2R1A/RAD21L1/DNMT1/TP53BP1/CDCA5/CEBPB/CENPM/SPAG5/ZNF330/FBXW11/NDEL1/KAT7/PELI1
Cellular Component	GO:0000839	Hrd1p ubiquitin ligase ERAD-L complex	2/2341	8/19559	0.2470541	0.60805	0.5451483	2	RHBDD2/DERL2
Cellular Component	GO:0008024	cyclin/CDK positive transcription elongation factor complex	2/2341	8/19559	0.2470541	0.60805	0.5451483	2	RB1/CDK13
Cellular Component	GO:0008290	F-actin capping protein complex	2/2341	8/19559	0.2470541	0.60805	0.5451483	2	CAPG/ADD1
Cellular Component	GO:0017059	serine C-palmitoyltransferase complex	2/2341	8/19559	0.2470541	0.60805	0.5451483	2	SPTLC2/ORMDL3
Cellular Component	GO:0031211	endoplasmic reticulum palmitoyltransferase complex	2/2341	8/19559	0.2470541	0.60805	0.5451483	2	SPTLC2/ORMDL3
Cellular Component	GO:0032584	growth cone membrane	2/2341	8/19559	0.2470541	0.60805	0.5451483	2	TRPV2/NECTIN1
Cellular Component	GO:0033010	paranodal junction	2/2341	8/19559	0.2470541	0.60805	0.5451483	2	JAM3/CNTNAP1

Cellular Component	GO:0036449	microtubule minus-end	2/2341	8/19559	0.2470541	0.60805	0.5451483	2	SVIL/NUMA1
Cellular Component	GO:0036513	Derlin-1 retrotranslocation complex	2/2341	8/19559	0.2470541	0.60805	0.5451483	2	AMFR/HM13
Cellular Component	GO:0070531	BRCA1-A complex	2/2341	8/19559	0.2470541	0.60805	0.5451483	2	ABRAXAS1/BABAM2
Cellular Component	GO:0070776	MOZ/MORF histone acetyltransferase complex	2/2341	8/19559	0.2470541	0.60805	0.5451483	2	BRD1/ING5
Cellular Component	GO:0071144	heteromeric SMAD protein complex	2/2341	8/19559	0.2470541	0.60805	0.5451483	2	SMAD3/SMAD7
Cellular Component	GO:0071439	clathrin complex	2/2341	8/19559	0.2470541	0.60805	0.5451483	2	BAIAP2L2/CLTCL1
Cellular Component	GO:0097413	Lewy body	2/2341	8/19559	0.2470541	0.60805	0.5451483	2	FBXO7/PRKN
Cellular Component	GO:0098837	postsynaptic recycling endosome	2/2341	8/19559	0.2470541	0.60805	0.5451483	2	ZDHHC2/AKAP5
Cellular Component	GO:1905961	protein-cysteine S-palmitoyltransferase complex	2/2341	8/19559	0.2470541	0.60805	0.5451483	2	SPTLC2/ORMDL3

Cellular Component	GO:0000794	condensed nuclear chromosome	15/2341	103/19559	0.2474134	0.60805	0.5451483	15	BLM/BRD4/TTN/BRCA2/HUS1/RCC1/MIS12/RGS12/RNF212/RAD21L1/DMC1/KIFAP3/TEX12/NOL6/PELI1
Cellular Component	GO:0000152	nuclear ubiquitin ligase complex	7/2341	43/19559	0.2507393	0.6127345	0.5493482	7	FZR1/PHC2/CDC16/PHC3/PCGF3/ANAPC7/BABAM2
Cellular Component	GO:0000307	cyclin-dependent protein kinase holoenzyme complex	7/2341	43/19559	0.2507393	0.6127345	0.5493482	7	CDK14/CCNH/CCNA1/RB1/CCNY/CDK13/CCNP
Cellular Component	GO:0000932	P-body	13/2341	88/19559	0.2509042	0.6127345	0.5493482	13	LSM1/SYNE1/TNRC6B/AGO1/ZC3H12D/SAMD4A/LIMD1/AGO2/EDC3/TRIM5/RBPMS/DIS3L2/SHFL
Cellular Component	GO:0031965	nuclear membrane	40/2341	301/19559	0.2626989	0.631644	0.5663015	40	TRIM27/FZR1/SLC16A3/ITPRIP/TNKS/SYNE1/ERN1/SMAD3/RCC1/ITPR1/NAV3/ACKR2/EPC1/PRICKLE2/NOS1AP/OSBPL6/LMNB2/CPNE1/AQP1/GRK5/NUP93/NRXN1/ATP2A3/PLCB1/TEX2/SORT1/MFSD10/BCL2/P2RX1/TOR1B/BCL2L1/MX1/TNRC18/PLRG1/P2RX5/LPIN1/RAP1GAP2/TERB2/TEPSIN/MYORG
Cellular Component	GO:0000153	cytoplasmic ubiquitin ligase complex	3/2341	15/19559	0.2640821	0.631644	0.5663015	3	RHBDD2/DERL2/MARCHF6
Cellular Component	GO:0005662	DNA replication factor A complex	3/2341	15/19559	0.2640821	0.631644	0.5663015	3	SMARCAL1/PLRG1/XPA
Cellular Component	GO:0012510	trans-Golgi network transport vesicle membrane	3/2341	15/19559	0.2640821	0.631644	0.5663015	3	AP2A1/AP1G2/CLTCL1
Cellular Component	GO:0030688	preribosome, small subunit precursor	3/2341	15/19559	0.2640821	0.631644	0.5663015	3	NOB1/NOP14/RIOK1

Cellular Component	GO:0035102	PRC1 complex	3/2341	15/19559	0.2640821	0.631644	0.5663015	3	PHC2/PHC3/PCGF3
Cellular Component	GO:0071565	nBAF complex	3/2341	15/19559	0.2640821	0.631644	0.5663015	3	SMARCA2/ARID1B/SMARCD1
Cellular Component	GO:0099059	integral component of presynaptic active zone membrane	3/2341	15/19559	0.2640821	0.631644	0.5663015	3	NRXN1/P2RX1/NECTIN1
Cellular Component	GO:0001750	photoreceptor outer segment	13/2341	89/19559	0.2640925	0.631644	0.5663015	13	RCVRN/PDE6A/RAB27A/CCDC66/GNAT2/MYO5A/GNA11/PDE6H/IFT140/CDHR1/SHANK2/GRK7/EYS
Cellular Component	GO:1902554	serine/threonine protein kinase complex	13/2341	89/19559	0.2640925	0.631644	0.5663015	13	ULK1/ERN1/ERC1/CDK14/UVRAG/CAB39/CCNH/CCNA1/RB1/CCNY/ERCC2/CDK13/CCNP
Cellular Component	GO:0032982	myosin filament	4/2341	22/19559	0.2655507	0.6325231	0.5670897	4	MYH11/MYH15/MYH9/MYOM1
Cellular Component	GO:0097038	perinuclear endoplasmic reticulum	4/2341	22/19559	0.2655507	0.6325231	0.5670897	4	PIK3R1/OSBPL6/CLU/CREB3L2
Cellular Component	GO:0030662	coated vesicle membrane	25/2341	183/19559	0.2700992	0.641585	0.5752141	25	AP2A1/TGFA/STX5/CD9/VTI1A/DENND1A/SEC23B/HLA-C/HLA-F/AP1B1/EPN2/CNIH2/CD59/SEC13/CHRM2/SEC24C/AP1G2/EPS15/TMED2/SREBF1/EPN3/CLTCL1/SEC31A/DBNL/TEPSIN
Cellular Component	GO:0045334	clathrin-coated endocytic vesicle	9/2341	59/19559	0.2704613	0.641585	0.5752141	9	CPNE6/AP2A1/CD9/DNM2/SFTPD/EPN2/EPS15/INPP5F/CLTCL1

Cellular Component	GO:0044853	plasma membrane raft	16/2341	113/19559	0.2747969	0.6480033	0.5809685	16	PRTN3/CD177/FXYD1/PACSIN2/TGFBR2/NOS1AP/MLC1/ITGB2/CDH1/ADD1/CORO1C/F2R/SLC2A1/ADTRP/EZR/FLOT1
Cellular Component	GO:0031672	A band	6/2341	37/19559	0.2781653	0.6480033	0.5809685	6	HDAC4/ENO1/MYOM1/FHL2/ANK2/MYL7
Cellular Component	GO:0005782	peroxisomal matrix	8/2341	52/19559	0.2797209	0.6480033	0.5809685	8	FAR2/AMACR/ACOXL/AGXT/CAT/ACOX2/HMGCL/CRYM
Cellular Component	GO:0031907	microbody lumen	8/2341	52/19559	0.2797209	0.6480033	0.5809685	8	FAR2/AMACR/ACOXL/AGXT/CAT/ACOX2/HMGCL/CRYM
Cellular Component	GO:0000123	histone acetyltransferase complex	12/2341	83/19559	0.287393	0.6480033	0.5809685	12	KANSL1/BRD1/EPC1/TAF10/KANSL2/ENY2/CREBBP/UBAP2L/RUVBL1/KAT7/ING5/POLE4
Cellular Component	GO:0035371	microtubule plus-end	4/2341	23/19559	0.2936061	0.6480033	0.5809685	4	DCTN1/MYO5A/NUMA1/SPAG5
Cellular Component	GO:0000138	Golgi trans cisterna	2/2341	9/19559	0.2940149	0.6480033	0.5809685	2	SGMS1/YIPF1
Cellular Component	GO:0000836	Hrd1p ubiquitin ligase complex	2/2341	9/19559	0.2940149	0.6480033	0.5809685	2	RHBDD2/DERL2
Cellular Component	GO:0005883	neurofilament	2/2341	9/19559	0.2940149	0.6480033	0.5809685	2	NEFM/SHANK2

Cellular Component	GO:0030123	AP-3 adaptor complex	2/2341	9/19559	0.2940149	0.6480033	0.5809685	2	VPS18/AP3D1
Cellular Component	GO:0031264	death-inducing signaling complex	2/2341	9/19559	0.2940149	0.6480033	0.5809685	2	RIPK1/FADD
Cellular Component	GO:0031371	ubiquitin conjugating enzyme complex	2/2341	9/19559	0.2940149	0.6480033	0.5809685	2	UBE2V1/RNF40
Cellular Component	GO:0032059	bleb	2/2341	9/19559	0.2940149	0.6480033	0.5809685	2	FMNL1/PTPRC
Cellular Component	GO:0033180	proton-transporting V-type ATPase, V1 domain	2/2341	9/19559	0.2940149	0.6480033	0.5809685	2	ATP6V1B2/ATP6V1C1
Cellular Component	GO:0033647	host intracellular organelle	2/2341	9/19559	0.2940149	0.6480033	0.5809685	2	PI4KA/SHFL
Cellular Component	GO:0033648	host intracellular membrane-bounded organelle	2/2341	9/19559	0.2940149	0.6480033	0.5809685	2	PI4KA/SHFL
Cellular Component	GO:0042612	MHC class I protein complex	2/2341	9/19559	0.2940149	0.6480033	0.5809685	2	HLA-C/HLA-F
Cellular Component	GO:0070578	RISC-loading complex	2/2341	9/19559	0.2940149	0.6480033	0.5809685	2	AGO1/AGO2

Cellular Component	GO:0071141	SMAD protein complex	2/2341	9/19559	0.2940149	0.6480033	0.5809685	2	SMAD3/SMAD7
Cellular Component	GO:0072559	NLRP3 inflammasome complex	2/2341	9/19559	0.2940149	0.6480033	0.5809685	2	NLRP3/CASP1
Cellular Component	GO:0097224	sperm connecting piece	2/2341	9/19559	0.2940149	0.6480033	0.5809685	2	DNAJB13/PMFBP1
Cellular Component	GO:0097648	G protein-coupled receptor complex	2/2341	9/19559	0.2940149	0.6480033	0.5809685	2	GABBR1/GABBR2
Cellular Component	GO:0048786	presynaptic active zone	11/2341	76/19559	0.2979583	0.6480033	0.5809685	11	APP/EGFLAM/PPFIA1/CTBP1/NRXN1/CTBP2/PPFIBP2/P2RX1/CNTNAP1/FLOT1/NECTIN1
Cellular Component	GO:0030132	clathrin coat of coated pit	3/2341	16/19559	0.2984191	0.6480033	0.5809685	3	AP2A1/EPS15L1/EPS15
Cellular Component	GO:0034045	phagophore assembly site membrane	3/2341	16/19559	0.2984191	0.6480033	0.5809685	3	ULK1/WIPI2/ATG14
Cellular Component	GO:0070822	Sin3-type complex	3/2341	16/19559	0.2984191	0.6480033	0.5809685	3	SIN3B/HDAC1/PHF12
Cellular Component	GO:0097731	9+0 non-motile cilium	19/2341	139/19559	0.3038538	0.6480033	0.5809685	19	RCVRN/PDE6A/RAB27A/CCDC66/GNAT2/MYO5A/GNA11/PDE6H/TTLL4/IFT140/SDCCAG8/RPGRIP1/CDHR1/SHANK2/GRK7/KIFAP3/EYS/NPFFR1/PRLHR

Cellular Component	GO:0005643	nuclear pore	12/2341	85/19559	0.3164303	0.6480033	0.5809685	12	NXF1/MAD1L1/TNKS/RAE1/XPO7/ENY2/BICD2/NUP93/SEC13/SNUPN/MYO1C/PCID2
Cellular Component	GO:0055038	recycling endosome membrane	12/2341	85/19559	0.3164303	0.6480033	0.5809685	12	EHD1/SORL1/SORCS2/NDRG1/PACSIN2/HLA-C/INPP4A/ZDHHC2/HLA-F/NTRK1/MICALL1/PDLIM4
Cellular Component	GO:0000126	transcription factor TFIIIB complex	1/2341	3/19559	0.3178198	0.6480033	0.5809685	1	BRF1
Cellular Component	GO:0000221	vacuolar proton-transporting V-type ATPase, V1 domain	1/2341	3/19559	0.3178198	0.6480033	0.5809685	1	ATP6V1C1
Cellular Component	GO:0000818	nuclear MIS12/MIND complex	1/2341	3/19559	0.3178198	0.6480033	0.5809685	1	MIS12
Cellular Component	GO:0001536	radial spoke stalk	1/2341	3/19559	0.3178198	0.6480033	0.5809685	1	CFAP61
Cellular Component	GO:0005610	laminin-5 complex	1/2341	3/19559	0.3178198	0.6480033	0.5809685	1	LAMB3
Cellular Component	GO:0005863	striated muscle myosin thick filament	1/2341	3/19559	0.3178198	0.6480033	0.5809685	1	MYOM1
Cellular Component	GO:0005896	interleukin-6 receptor complex	1/2341	3/19559	0.3178198	0.6480033	0.5809685	1	IL6R

Cellular Component	GO:0005943	phosphatidylinositol 3-kinase complex, class IA	1/2341	3/19559	0.3178198	0.6480033	0.5809685	1	PIK3R1
Cellular Component	GO:0016939	kinesin II complex	1/2341	3/19559	0.3178198	0.6480033	0.5809685	1	KIFAP3
Cellular Component	GO:0019907	cyclin-dependent protein kinase activating kinase holoenzyme complex	1/2341	3/19559	0.3178198	0.6480033	0.5809685	1	CCNH
Cellular Component	GO:0020003	symbiont-containing vacuole	1/2341	3/19559	0.3178198	0.6480033	0.5809685	1	AQP1
Cellular Component	GO:0020016	ciliary pocket	1/2341	3/19559	0.3178198	0.6480033	0.5809685	1	EHD1
Cellular Component	GO:0020018	ciliary pocket membrane	1/2341	3/19559	0.3178198	0.6480033	0.5809685	1	EHD1
Cellular Component	GO:0030689	Noc complex	1/2341	3/19559	0.3178198	0.6480033	0.5809685	1	NOP14
Cellular Component	GO:0030956	glutamyl-tRNA(Gln) amidotransferase complex	1/2341	3/19559	0.3178198	0.6480033	0.5809685	1	GATB
Cellular Component	GO:0030981	cortical microtubule cytoskeleton	1/2341	3/19559	0.3178198	0.6480033	0.5809685	1	NUMA1

Cellular Component	GO:0031084	BLOC-2 complex	1/2341	3/19559	0.3178198	0.6480033	0.5809685	1	HPS6
Cellular Component	GO:0032545	CURI complex	1/2341	3/19559	0.3178198	0.6480033	0.5809685	1	NOL6
Cellular Component	GO:0033150	cytoskeletal calyx	1/2341	3/19559	0.3178198	0.6480033	0.5809685	1	CCIN
Cellular Component	GO:0033186	CAF-1 complex	1/2341	3/19559	0.3178198	0.6480033	0.5809685	1	CHAF1A
Cellular Component	GO:0033557	Slx1-Slx4 complex	1/2341	3/19559	0.3178198	0.6480033	0.5809685	1	SLX4
Cellular Component	GO:0034455	t-UTP complex	1/2341	3/19559	0.3178198	0.6480033	0.5809685	1	UTP4
Cellular Component	GO:0034456	UTP-C complex	1/2341	3/19559	0.3178198	0.6480033	0.5809685	1	NOL6
Cellular Component	GO:0034751	aryl hydrocarbon receptor complex	1/2341	3/19559	0.3178198	0.6480033	0.5809685	1	AIP
Cellular Component	GO:0034990	nuclear mitotic cohesin complex	1/2341	3/19559	0.3178198	0.6480033	0.5809685	1	RAD21L1

Cellular Component	GO:0035060	brahma complex	1/2341	3/19559	0.3178198	0.6480033	0.5809685	1	ARID1B
Cellular Component	GO:0035838	growing cell tip	1/2341	3/19559	0.3178198	0.6480033	0.5809685	1	PI4K2A
Cellular Component	GO:0035868	alphav-beta3 integrin-HMGB1 complex	1/2341	3/19559	0.3178198	0.6480033	0.5809685	1	ITGB3
Cellular Component	GO:0043564	Ku70:Ku80 complex	1/2341	3/19559	0.3178198	0.6480033	0.5809685	1	PAXX
Cellular Component	GO:0044307	dendritic branch	1/2341	3/19559	0.3178198	0.6480033	0.5809685	1	MAP1A
Cellular Component	GO:0070110	ciliary neurotrophic factor receptor complex	1/2341	3/19559	0.3178198	0.6480033	0.5809685	1	IL6R
Cellular Component	GO:0070209	ASTRA complex	1/2341	3/19559	0.3178198	0.6480033	0.5809685	1	TTI1
Cellular Component	GO:0070369	beta-catenin-TCF7L2 complex	1/2341	3/19559	0.3178198	0.6480033	0.5809685	1	TCF7L2
Cellular Component	GO:0070939	Dsl1/NZR complex	1/2341	3/19559	0.3178198	0.6480033	0.5809685	1	NBAS

Cellular Component	GO:0070985	transcription factor TFIIK complex	1/2341	3/19559	0.3178198	0.6480033	0.5809685	1	CCNH
Cellular Component	GO:0071008	U2-type post-mRNA release spliceosomal complex	1/2341	3/19559	0.3178198	0.6480033	0.5809685	1	TFIP11
Cellular Component	GO:0071062	alphav-beta3 integrin-vitronectin complex	1/2341	3/19559	0.3178198	0.6480033	0.5809685	1	ITGB3
Cellular Component	GO:0072563	endothelial microparticle	1/2341	3/19559	0.3178198	0.6480033	0.5809685	1	ENG
Cellular Component	GO:0090443	FAR/SIN/STRIPAK complex	1/2341	3/19559	0.3178198	0.6480033	0.5809685	1	STRN4
Cellular Component	GO:0097356	perinucleolar compartment	1/2341	3/19559	0.3178198	0.6480033	0.5809685	1	SORL1
Cellular Component	GO:0097454	Schwann cell microvillus	1/2341	3/19559	0.3178198	0.6480033	0.5809685	1	EZR
Cellular Component	GO:0097513	myosin II filament	1/2341	3/19559	0.3178198	0.6480033	0.5809685	1	MYH9
Cellular Component	GO:0098559	cytoplasmic side of early endosome membrane	1/2341	3/19559	0.3178198	0.6480033	0.5809685	1	LITAF

Cellular Component	GO:0099617	matrix side of mitochondrial inner membrane	1/2341	3/19559	0.3178198	0.6480033	0.5809685	1	BDH1
Cellular Component	GO:1901588	dendritic microtubule	1/2341	3/19559	0.3178198	0.6480033	0.5809685	1	MAP1A
Cellular Component	GO:1902636	kinociliary basal body	1/2341	3/19559	0.3178198	0.6480033	0.5809685	1	CDC14A
Cellular Component	GO:1902937	inward rectifier potassium channel complex	1/2341	3/19559	0.3178198	0.6480033	0.5809685	1	NOS1AP
Cellular Component	GO:1990037	Lewy body core	1/2341	3/19559	0.3178198	0.6480033	0.5809685	1	FBXO7
Cellular Component	GO:1990452	Parkin-FBXW7-Cul1 ubiquitin ligase complex	1/2341	3/19559	0.3178198	0.6480033	0.5809685	1	PRKN
Cellular Component	GO:1990761	growth cone lamellipodium	1/2341	3/19559	0.3178198	0.6480033	0.5809685	1	APP
Cellular Component	GO:0005811	lipid droplet	13/2341	93/19559	0.318974	0.6480033	0.5809685	13	PNPLA2/EHD1/TMEM159/LPCAT1/RAP1B/RAB5C/PITPNM1/RDH10/AIFM2/ABHD5/G0S2/BSCL2/SPART
Cellular Component	GO:0031248	protein acetyltransferase complex	13/2341	93/19559	0.318974	0.6480033	0.5809685	13	KANSL1/BRD1/EPC1/TAF10/KANSL2/ENY2/CREBBP/UBAP2L/NAA16/RUVBL1/KAT7/ING5/POLE4

Cellular Component	GO:1902493	acetyltransferase complex	13/2341	93/19559	0.318974	0.6480033	0.5809685	13	KANSL1/BRD1/EPC1/TAF10/KANSL2/ENY2/CREBBP/UBAP2L/NAA16/RUVBL1/KAT7/ING5/POLE4
Cellular Component	GO:0044305	calyx of Held	4/2341	24/19559	0.3220102	0.6530277	0.5854731	4	ITSN1/GIT1/PRKCB/ACTG1
Cellular Component	GO:0097733	photoreceptor cell cilium	16/2341	117/19559	0.3240266	0.6559701	0.5881111	16	RCVRN/PDE6A/RAB27A/CCDC66/GNAT2/MYO5A/GNA11/PDE6H/IFT140/SDCCAG8/RPGRIP1/CDHR1/SHANK2/GRK7/KIFAP3/EYS
Cellular Component	GO:0005852	eukaryotic translation initiation factor 3 complex	3/2341	17/19559	0.3329668	0.6717243	0.6022355	3	EIF3M/COPS5/EIF3H
Cellular Component	GO:0043083	synaptic cleft	3/2341	17/19559	0.3329668	0.6717243	0.6022355	3	COLQ/EGFLAM/DNM3
Cellular Component	GO:0099568	cytoplasmic region	33/2341	254/19559	0.3342312	0.6729287	0.6033154	33	CTSZ/PRKCZ/DCTN1/ENO1/DNAH3/SPG7/DYNC1H1/SPAG6/DNAJB13/BAIAP2/CTBP1/CTBP2/ATG14/CCIN/KIF5C/NUMA1/IFT140/INPP5E/ITGB2/KIF1B/RPGRIP1/MAP1A/KIFAP3/AP3D1/DNAI2/FBXW11/NDEL1/DTNBP1/SPEF1/SEPTIN9/CFAP221/CFAP61/CFAP46
Cellular Component	GO:0000407	phagophore assembly site	5/2341	32/19559	0.335499	0.6729287	0.6033154	5	ULK1/WIPI2/ATG14/VMP1/ILRUN
Cellular Component	GO:0000793	condensed chromosome	29/2341	222/19559	0.3362986	0.6729287	0.6033154	29	BLM/BRD4/BAZ1B/MAD1L1/TTN/NDE1/BRCA2/KANSL1/HUS1/NSMCE2/RCC1/CHAMP1/MIS12/ZNF276/RGS12/RNF212/STAG1/ESRRB/RAD21L1/DMC1/TP53BP1/KIFAP3/CEBPB/CENPM/SPAG5/TEX12/NOL6/NDEL1/PELI1
Cellular Component	GO:0005641	nuclear envelope lumen	2/2341	10/19559	0.3405251	0.6729287	0.6033154	2	SORL1/APP

Cellular Component	GO:0005916	fascia adherens	2/2341	10/19559	0.3405251	0.6729287	0.6033154	2	JUP/ACTN1
Cellular Component	GO:0031209	SCAR complex	2/2341	10/19559	0.3405251	0.6729287	0.6033154	2	NCKAP1/CYFIP1
Cellular Component	GO:0033179	proton-transporting V-type ATPase, V0 domain	2/2341	10/19559	0.3405251	0.6729287	0.6033154	2	ATP6V0B/ATP6V0C
Cellular Component	GO:0044224	juxtaparanode region of axon	2/2341	10/19559	0.3405251	0.6729287	0.6033154	2	KCNAB2/EPB41L3
Cellular Component	GO:0045240	dihydrolipoyl dehydrogenase complex	2/2341	10/19559	0.3405251	0.6729287	0.6033154	2	OGDH/BCKDHA
Cellular Component	GO:0070938	contractile ring	2/2341	10/19559	0.3405251	0.6729287	0.6033154	2	MAEA/MYH9
Cellular Component	GO:0072546	ER membrane protein complex	2/2341	10/19559	0.3405251	0.6729287	0.6033154	2	EMC8/EMC1
Cellular Component	GO:0097197	tetraspanin-enriched microdomain	2/2341	10/19559	0.3405251	0.6729287	0.6033154	2	TSPAN14/SCIMP
Cellular Component	GO:0005657	replication fork	10/2341	71/19559	0.3421255	0.6737955	0.6040925	10	BLM/BAZ1B/POLA2/UHRF1/SMARCAL1/DNMT1/TP53BP1/PLRG1/POLD3/XPA

Cellular Component	GO:0045171	intercellular bridge	10/2341	71/19559	0.3421255	0.6737955	0.6040925	10	DCTN1/MICAL1/MICAL3/MSRA/GSTM5/AGBL5/OLFM4/PRKCI/KCTD1/XPA
Cellular Component	GO:0071013	catalytic step 2 spliceosome	12/2341	87/19559	0.3461265	0.68052	0.6101214	12	SNRPD3/SF3B3/LSM7/PRPF8/DDX41/PRPF6/PLRG1/SRRM2/ZCCHC8/SYNCRIP/RALY/TFIP11
Cellular Component	GO:0030131	clathrin adaptor complex	4/2341	25/19559	0.3505886	0.6869642	0.6158989	4	AP2A1/AP1B1/AP1G2/EPS15
Cellular Component	GO:0031430	M band	4/2341	25/19559	0.3505886	0.6869642	0.6158989	4	ENO1/MYOM1/FHL2/ANK2
Cellular Component	GO:0036464	cytoplasmic ribonucleoprotein granule	30/2341	233/19559	0.3632917	0.7090214	0.6356743	30	PIWIL4/RPTOR/CTSG/NXF1/LSM1/MOV10L1/SYNE1/GABPB1/TNRC6B/PABPC4/AGO1/LARP1/SHB/CSDE1/USP3/ZC3H12D/CTSH/SAMD4A/PITPNM1/LIMD1/AGO2/EDC3/TRIM5/ARNTL/RBPMS/UBAP2L/DIS3L2/EIF4G1/POLI/SHFL
Cellular Component	GO:0043596	nuclear replication fork	6/2341	41/19559	0.3664161	0.7090214	0.6356743	6	BAZ1B/POLA2/SMARCAL1/PLRG1/POLD3/XPA
Cellular Component	GO:0005666	RNA polymerase III complex	3/2341	18/19559	0.3674364	0.7090214	0.6356743	3	POLR2F/CRCP/POLR1D
Cellular Component	GO:0031143	pseudopodium	3/2341	18/19559	0.3674364	0.7090214	0.6356743	3	ARRB1/ACTN1/ACTN4
Cellular Component	GO:0032433	filopodium tip	3/2341	18/19559	0.3674364	0.7090214	0.6356743	3	AP2A1/MYO5A/CYFIP1

Cellular Component	GO:0097440	apical dendrite	3/2341	18/19559	0.3674364	0.7090214	0.6356743	3	ITSN1/CLU/CPEB3
Cellular Component	GO:0099524	postsynaptic cytosol	3/2341	18/19559	0.3674364	0.7090214	0.6356743	3	BAIAP2/HTT/NEDD4
Cellular Component	GO:0005777	peroxisome	18/2341	137/19559	0.3739997	0.7090214	0.6356743	18	ACSL1/IDI1/FAR2/AMACR/ACOXL/MYO5A/AGXT/CAT/PEX26/ACOX2/FIS1/DNM1L/HMGCL/PEX11B/CRYM/CD33/ZADH2/STING1
Cellular Component	GO:0042579	microbody	18/2341	137/19559	0.3739997	0.7090214	0.6356743	18	ACSL1/IDI1/FAR2/AMACR/ACOXL/MYO5A/AGXT/CAT/PEX26/ACOX2/FIS1/DNM1L/HMGCL/PEX11B/CRYM/CD33/ZADH2/STING1
Cellular Component	GO:0036379	myofilament	4/2341	26/19559	0.3791788	0.7090214	0.6356743	4	TNNT3/TMOD3/MYOM1/TMOD1
Cellular Component	GO:0035770	ribonucleoprotein granule	31/2341	243/19559	0.3805557	0.7090214	0.6356743	31	PIWIL4/RPTOR/CTSG/NXF1/LSM1/MOV10L1/SYNE1/GABPB1/TNRC6B/PABPC4/AGO1/LARP1/SHB/CSDE1/USP3/ZC3H12D/CTSH/SAMD4A/PITPNM1/LIMD1/AGO2/EDC3/TRIM5/ARNTL/RBPMS/UBAP2L/DIS3L2/EIF4G1/POLI/GRSF1/SHFL
Cellular Component	GO:0031461	cullin-RING ubiquitin ligase complex	21/2341	162/19559	0.3829149	0.7090214	0.6356743	21	FZR1/KLHL25/FBXO7/CUL3/DCAF15/CDC16/ZSWIM5/FBXL19/DCAF5/FBXL13/KCTD2/FBXL18/KLHL21/FBXL6/KLHL8/ANAPC7/PLRG1/FBXW11/WDTC1/DCAF17/FBH1
Cellular Component	GO:0005790	smooth endoplasmic reticulum	5/2341	34/19559	0.3853481	0.7090214	0.6356743	5	CALR/APP/MYO5A/CASQ1/SVIP
Cellular Component	GO:0097386	glial cell projection	5/2341	34/19559	0.3853481	0.7090214	0.6356743	5	APP/MLC1/SLC1A2/EZR/PRLHR

Cellular Component	GO:0002116	semaphorin receptor complex	2/2341	11/19559	0.3860199	0.7090214	0.6356743	2	PLXNC1/NRP2
Cellular Component	GO:0002178	palmitoyltransferase complex	2/2341	11/19559	0.3860199	0.7090214	0.6356743	2	SPTLC2/ORMDL3
Cellular Component	GO:0005652	nuclear lamina	2/2341	11/19559	0.3860199	0.7090214	0.6356743	2	NARF/LMNB2
Cellular Component	GO:0030122	AP-2 adaptor complex	2/2341	11/19559	0.3860199	0.7090214	0.6356743	2	AP2A1/EPS15
Cellular Component	GO:0031010	ISWI-type complex	2/2341	11/19559	0.3860199	0.7090214	0.6356743	2	CECR2/RSF1
Cellular Component	GO:0035253	ciliary rootlet	2/2341	11/19559	0.3860199	0.7090214	0.6356743	2	APP/KIF5C
Cellular Component	GO:0043220	Schmidt-Lanterman incisure	2/2341	11/19559	0.3860199	0.7090214	0.6356743	2	JAM3/PRKCI
Cellular Component	GO:1990907	beta-catenin-TCF complex	2/2341	11/19559	0.3860199	0.7090214	0.6356743	2	TCF7L2/TLE1
Cellular Component	GO:0005923	bicellular tight junction	16/2341	122/19559	0.3882352	0.7090214	0.6356743	16	PRKCZ/TBCD/TJP2/UBN1/FRMD4A/PMP22/FRMD4B/RAPGEF2/JAM3/PRKCI/MPP7/ARHGAP17/ARHGEF2/SYNPO/NPHP4/JAML

Cellular Component	GO:0000791	euchromatin	6/2341	42/19559	0.3888231	0.7090214	0.6356743	6	CECR2/CREB1/ASH2L/UHRF1/ANKRD2/TCF3
Cellular Component	GO:0071005	U2-type precatalytic spliceosome	7/2341	50/19559	0.3907885	0.7090214	0.6356743	7	SNRPD3/SF3B3/LSM7/PRPF8/DHX16/PRPF6/SRRM2
Cellular Component	GO:0005901	caveola	11/2341	82/19559	0.3919842	0.7090214	0.6356743	11	FXYD1/PACSIN2/TGFBR2/NOS1AP/MLC1/CDH1/CORO1C/F2R/SLC2A1/ADTRP/FLOT1
Cellular Component	GO:0005819	spindle	46/2341	367/19559	0.3920523	0.7090214	0.6356743	46	MAEA/DCTN1/LIMK2/CTDP1/MAD1L1/CSNK1D/TNKS/NDE1/ACOT13/RAE1/CTTN/CAPG/MICAL3/CHAMP1/CUL3/MYH9/APP/EML4/CDC16/POC1A/LATS2/CSPP1/TACC3/STAG1/AGBL5/FAM110A/PARP4/NUMA1/NEK6/KLHL21/RB1/TAF1D/ARHGEF2/KIFAP3/CYLD/SPAG5/HECW2/CDC14A/TUBGCP2/NDEL1/DIDO1/ERCC2/CLTCL1/CEP44/CEP350/IRAG2
Cellular Component	GO:0036064	ciliary basal body	20/2341	155/19559	0.3956079	0.7090214	0.6356743	20	CSNK1D/KIAA0586/DISC1/ERC1/CCDC66/POC1A/CEP78/TTLL4/IFT140/TTLL11/SDCCAG8/DAAM1/RILPL1/KIFAP3/CYLD/CDC14A/EZR/TTLL1/AGBL2/NPHP4
Cellular Component	GO:0000120	RNA polymerase I transcription regulator complex	1/2341	4/19559	0.3994819	0.7090214	0.6356743	1	TAF1D
Cellular Component	GO:0000444	MIS12/MIND type complex	1/2341	4/19559	0.3994819	0.7090214	0.6356743	1	MIS12
Cellular Component	GO:0005588	collagen type V trimer	1/2341	4/19559	0.3994819	0.7090214	0.6356743	1	COL5A1
Cellular Component	GO:0005757	mitochondrial permeability transition pore complex	1/2341	4/19559	0.3994819	0.7090214	0.6356743	1	SPG7

Cellular Component	GO:0005853	eukaryotic translation elongation factor 1 complex	1/2341	4/19559	0.3994819	0.7090214	0.6356743	1	EEF1D
Cellular Component	GO:0008275	gamma-tubulin small complex	1/2341	4/19559	0.3994819	0.7090214	0.6356743	1	TUBGCP2
Cellular Component	GO:0016013	syntrophin complex	1/2341	4/19559	0.3994819	0.7090214	0.6356743	1	SNTA1
Cellular Component	GO:0016035	zeta DNA polymerase complex	1/2341	4/19559	0.3994819	0.7090214	0.6356743	1	POLD3
Cellular Component	GO:0019815	B cell receptor complex	1/2341	4/19559	0.3994819	0.7090214	0.6356743	1	CD79A
Cellular Component	GO:0030892	mitotic cohesin complex	1/2341	4/19559	0.3994819	0.7090214	0.6356743	1	RAD21L1
Cellular Component	GO:0030906	retromer, cargo-selective complex	1/2341	4/19559	0.3994819	0.7090214	0.6356743	1	VPS26B
Cellular Component	GO:0031499	TRAMP complex	1/2341	4/19559	0.3994819	0.7090214	0.6356743	1	ZCCHC8
Cellular Component	GO:0032449	CBM complex	1/2341	4/19559	0.3994819	0.7090214	0.6356743	1	BCL10

Cellular Component	GO:0032777	Piccolo NuA4 histone acetyltransferase complex	1/2341	4/19559	0.3994819	0.7090214	0.6356743	1	EPC1
Cellular Component	GO:0032983	kainate selective glutamate receptor complex	1/2341	4/19559	0.3994819	0.7090214	0.6356743	1	GRIK4
Cellular Component	GO:0033011	perinuclear theca	1/2341	4/19559	0.3994819	0.7090214	0.6356743	1	CCIN
Cellular Component	GO:0033256	I-kappaB/NF-kappaB complex	1/2341	4/19559	0.3994819	0.7090214	0.6356743	1	BCL3
Cellular Component	GO:0033269	internode region of axon	1/2341	4/19559	0.3994819	0.7090214	0.6356743	1	MBP
Cellular Component	GO:0034657	GID complex	1/2341	4/19559	0.3994819	0.7090214	0.6356743	1	MAEA
Cellular Component	GO:0035339	SPOTS complex	1/2341	4/19559	0.3994819	0.7090214	0.6356743	1	ORMDL3
Cellular Component	GO:0035363	histone locus body	1/2341	4/19559	0.3994819	0.7090214	0.6356743	1	ZC3H8
Cellular Component	GO:0043625	delta DNA polymerase complex	1/2341	4/19559	0.3994819	0.7090214	0.6356743	1	POLD3

Cellular Component	GO:0044393	microspike	1/2341	4/19559	0.3994819	0.7090214	0.6356743	1	EZR
Cellular Component	GO:0071953	elastic fiber	1/2341	4/19559	0.3994819	0.7090214	0.6356743	1	MFAP4
Cellular Component	GO:0072558	NLRP1 inflammasome complex	1/2341	4/19559	0.3994819	0.7090214	0.6356743	1	CASP1
Cellular Component	GO:0097444	spine apparatus	1/2341	4/19559	0.3994819	0.7090214	0.6356743	1	SYNPO
Cellular Component	GO:0097452	GAIT complex	1/2341	4/19559	0.3994819	0.7090214	0.6356743	1	SYNCRIP
Cellular Component	GO:0097457	hippocampal mossy fiber	1/2341	4/19559	0.3994819	0.7090214	0.6356743	1	MICAL1
Cellular Component	GO:0150005	enzyme activator complex	1/2341	4/19559	0.3994819	0.7090214	0.6356743	1	CCNH
Cellular Component	GO:1905103	integral component of lysosomal membrane	1/2341	4/19559	0.3994819	0.7090214	0.6356743	1	NPC1
Cellular Component	GO:1990578	perinuclear endoplasmic reticulum membrane	1/2341	4/19559	0.3994819	0.7090214	0.6356743	1	PIK3R1

Cellular Component	GO:1990812	growth cone filopodium	1/2341	4/19559	0.3994819	0.7090214	0.6356743	1	APP
Cellular Component	GO:0016010	dystrophin-associated glycoprotein complex	3/2341	19/19559	0.401575	0.7090214	0.6356743	3	SNTB2/SNTA1/SNTB1
Cellular Component	GO:0016327	apicolateral plasma membrane	3/2341	19/19559	0.401575	0.7090214	0.6356743	3	JUP/PALM/NEDD4
Cellular Component	GO:0032426	stereocilium tip	3/2341	19/19559	0.401575	0.7090214	0.6356743	3	ESPN/TMC1/CDC14A
Cellular Component	GO:0090665	glycoprotein complex	3/2341	19/19559	0.401575	0.7090214	0.6356743	3	SNTB2/SNTA1/SNTB1
Cellular Component	GO:0098945	intrinsic component of presynaptic active zone membrane	3/2341	19/19559	0.401575	0.7090214	0.6356743	3	NRXN1/P2RX1/NECTIN1
Cellular Component	GO:0033176	proton-transporting V-type ATPase complex	4/2341	27/19559	0.4076314	0.716443	0.6423282	4	ATP6V1B2/ATP6V0B/ATP6V0C/ATP6V1C1
Cellular Component	GO:0034364	high-density lipoprotein particle	4/2341	27/19559	0.4076314	0.716443	0.6423282	4	LIPC/HDLBP/PLTP/CLU
Cellular Component	GO:0044232	organelle membrane contact site	4/2341	27/19559	0.4076314	0.716443	0.6423282	4	ATG14/PDZD8/GRAMD1A/GRAMD2A

Cellular Component	GO:0031093	platelet alpha granule lumen	9/2341	67/19559	0.4100906	0.718847	0.6444835	9	SERPINA1/THBS1/ACTN1/APP/ACTN4/CLU/TGFB1/PDGFB/CYRIB
Cellular Component	GO:0080008	Cul4-RING E3 ubiquitin ligase complex	5/2341	35/19559	0.4102386	0.718847	0.6444835	5	DCAF15/DCAF5/PLRG1/WDTC1/DCAF17
Cellular Component	GO:0000922	spindle pole	21/2341	165/19559	0.4168579	0.7293441	0.6538947	21	DCTN1/CTDP1/MAD1L1/TNKS/NDE1/RAE1/CUL3/POC1A/LATS2/CSPP1/TACC3/STAG1/FAM110A/NUMA1/NEK6/KLHL21/SPAG5/CDC14A/TUBGCP2/CEP44/IRAG2
Cellular Component	GO:0072686	mitotic spindle	17/2341	133/19559	0.4252283	0.7380006	0.6616557	17	DCTN1/LIMK2/MAD1L1/TNKS/RAE1/CTTN/CAPG/CUL3/EML4/TACC3/STAG1/AGBL5/NUMA1/TAF1D/SPAG5/HECW2/CDC14A
Cellular Component	GO:0005637	nuclear inner membrane	8/2341	60/19559	0.4298793	0.7380006	0.6616557	8	ERN1/SMAD3/ITPR1/LMNB2/MFSD10/P2RX1/P2RX5/TERB2
Cellular Component	GO:0000439	transcription factor TFIIH core complex	2/2341	12/19559	0.4300762	0.7380006	0.6616557	2	CCNH/ERCC2
Cellular Component	GO:0000812	Swr1 complex	2/2341	12/19559	0.4300762	0.7380006	0.6616557	2	SRCAP/RUVBL1
Cellular Component	GO:0005845	mRNA cap binding complex	2/2341	12/19559	0.4300762	0.7380006	0.6616557	2	AGO2/CYFIP1
Cellular Component	GO:0005890	sodium:potassium-exchanging ATPase complex	2/2341	12/19559	0.4300762	0.7380006	0.6616557	2	FXYD2/FXYD1

Cellular Component	GO:0030897	HOPS complex	2/2341	12/19559	0.4300762	0.7380006	0.6616557	2	VPS18/HOOK2
Cellular Component	GO:0031680	G-protein beta/gamma-subunit complex	2/2341	12/19559	0.4300762	0.7380006	0.6616557	2	GNG7/GNG2
Cellular Component	GO:0042555	MCM complex	2/2341	12/19559	0.4300762	0.7380006	0.6616557	2	MCM5/MCM2
Cellular Component	GO:0043020	NADPH oxidase complex	2/2341	12/19559	0.4300762	0.7380006	0.6616557	2	NCF4/NCF2
Cellular Component	GO:0044214	spanning component of plasma membrane	2/2341	12/19559	0.4300762	0.7380006	0.6616557	2	SLC24A4/CDHR2
Cellular Component	GO:0071564	npBAF complex	2/2341	12/19559	0.4300762	0.7380006	0.6616557	2	SMARCA2/SMARCD1
Cellular Component	GO:1990454	L-type voltage-gated calcium channel complex	2/2341	12/19559	0.4300762	0.7380006	0.6616557	2	CACNA1C/NOS1AP
Cellular Component	GO:0031463	Cul3-RING ubiquitin ligase complex	5/2341	36/19559	0.4349784	0.7412497	0.6645687	5	KLHL25/CUL3/KCTD2/KLHL21/KLHL8
Cellular Component	GO:0097546	ciliary base	5/2341	36/19559	0.4349784	0.7412497	0.6645687	5	PRKAR1B/SUFU/MOK/DISC1/NPHP4

Cellular Component	GO:0098576	lumenal side of membrane	5/2341	36/19559	0.4349784	0.7412497	0.6645687	5	CALR/BDH1/HLA-C/HM13/HLA-F
Cellular Component	GO:0005721	pericentric heterochromatin	3/2341	20/19559	0.4351647	0.7412497	0.6645687	3	BAZ1B/KDM4C/DNMT1
Cellular Component	GO:0031082	BLOC complex	3/2341	20/19559	0.4351647	0.7412497	0.6645687	3	PI4K2A/HPS6/DTNBP1
Cellular Component	GO:0005875	microtubule associated complex	20/2341	159/19559	0.4422029	0.7445223	0.6675027	20	DCTN1/KIF13B/DNAH3/NDE1/MAP7/KIF13A/MEFV/DYNC1H1/DISC1/PXN/DCTN2/KIF21B/KIF5C/KIF1B/MAP1A/KIFAP3/DNAI2/FBXW11/NDEL1/KIFC3
Cellular Component	GO:0030670	phagocytic vesicle membrane	10/2341	77/19559	0.4427562	0.7445223	0.6675027	10	CALR/SLC11A1/RAB11FIP1/DNM2/RAB31/HLA-C/HLA-F/RAB43/ATP6V0B/ATP6V0C
Cellular Component	GO:0071011	precatalytic spliceosome	7/2341	53/19559	0.4521414	0.7445223	0.6675027	7	SNRPD3/SF3B3/LSM7/PRPF8/DHX16/PRPF6/SRRM2
Cellular Component	GO:0001533	cornified envelope	6/2341	45/19559	0.4556771	0.7445223	0.6675027	6	CSTA/JUP/PKP1/CNFN/KAZN/PPL
Cellular Component	GO:1905348	endonuclease complex	5/2341	37/19559	0.4594804	0.7445223	0.6675027	5	ERN1/AGO1/EME1/AGO2/SLX4
Cellular Component	GO:0005689	U12-type spliceosomal complex	4/2341	29/19559	0.4635949	0.7445223	0.6675027	4	SNRPD3/SF3B3/LSM7/SNRNP35

Cellular Component	GO:0044295	axonal growth cone	4/2341	29/19559	0.4635949	0.7445223	0.6675027	4	WDR47/KIF5C/CYFIP1/TRPV2
Cellular Component	GO:0071556	integral component of lumenal side of endoplasmic reticulum membrane	4/2341	29/19559	0.4635949	0.7445223	0.6675027	4	CALR/HLA-C/HM13/HLA-F
Cellular Component	GO:0098553	lumenal side of endoplasmic reticulum membrane	4/2341	29/19559	0.4635949	0.7445223	0.6675027	4	CALR/HLA-C/HM13/HLA-F
Cellular Component	GO:0005771	multivesicular body	8/2341	62/19559	0.4676782	0.7445223	0.6675027	8	SORL1/PRKAR1B/SFTPD/CTSH/RAB27A/CRHBP/GIMAP5/CD79A
Cellular Component	GO:0005697	telomerase holoenzyme complex	3/2341	21/19559	0.4680206	0.7445223	0.6675027	3	SNRPD3/SMG7/SMG6
Cellular Component	GO:0062023	collagen-containing extracellular matrix	52/2341	427/19559	0.4691415	0.7445223	0.6675027	52	ELANE/SLPI/CTSZ/PRTN3/LTBP1/S100A8/CTSG/TIMP2/COL23A1/CALR/SERPINA1/MFAP4/COL17A1/EMILIN2/VIT/ANGPT1/TGFBI/COL4A2/COLQ/ANXA6/EGFL7/CTSD/THBS1/PODNL1/CTSH/EGFLAM/TNXB/PCSK6/COL12A1/CLU/SDC2/F12/SPARCL1/TGFB1/FCN1/TGFB1I1/WNT5B/PDGFB/CTSB/COL14A1/SBSPON/AEBP1/PKM/COL5A1/COL28A1/C1QB/ANGPTL6/LAMB3/ADAMDEC1/EYS/ANXA7/P3H1
Cellular Component	GO:0000125	PCAF complex	1/2341	5/19559	0.4713721	0.7445223	0.6675027	1	TAF10
Cellular Component	GO:0000243	commitment complex	1/2341	5/19559	0.4713721	0.7445223	0.6675027	1	SNRPD3
Cellular Component	GO:0000798	nuclear cohesin complex	1/2341	5/19559	0.4713721	0.7445223	0.6675027	1	RAD21L1

Cellular Component	GO:0000923	equatorial microtubule organizing center	1/2341	5/19559	0.4713721	0.7445223	0.6675027	1	TUBGCP2
Cellular Component	GO:0001405	PAM complex, Tim23 associated import motor	1/2341	5/19559	0.4713721	0.7445223	0.6675027	1	PAM16
Cellular Component	GO:0005658	alpha DNA polymerase:primase complex	1/2341	5/19559	0.4713721	0.7445223	0.6675027	1	POLA2
Cellular Component	GO:0005749	mitochondrial respiratory chain complex II, succinate dehydrogenase complex (ubiquinone)	1/2341	5/19559	0.4713721	0.7445223	0.6675027	1	SDHD
Cellular Component	GO:0005854	nascent polypeptide-associated complex	1/2341	5/19559	0.4713721	0.7445223	0.6675027	1	NACAD
Cellular Component	GO:0005947	mitochondrial alpha-ketoglutarate dehydrogenase complex	1/2341	5/19559	0.4713721	0.7445223	0.6675027	1	BCKDHA
Cellular Component	GO:0005955	calcineurin complex	1/2341	5/19559	0.4713721	0.7445223	0.6675027	1	ITPR1
Cellular Component	GO:0008303	caspase complex	1/2341	5/19559	0.4713721	0.7445223	0.6675027	1	PIGU
Cellular Component	GO:0030896	checkpoint clamp complex	1/2341	5/19559	0.4713721	0.7445223	0.6675027	1	HUS1

Cellular Component	GO:0031415	NatA complex	1/2341	5/19559	0.4713721	0.7445223	0.6675027	1	NAA16
Cellular Component	GO:0032783	super elongation complex	1/2341	5/19559	0.4713721	0.7445223	0.6675027	1	AFF1
Cellular Component	GO:0033093	Weibel-Palade body	1/2341	5/19559	0.4713721	0.7445223	0.6675027	1	RAB27A
Cellular Component	GO:0033106	cis-Golgi network membrane	1/2341	5/19559	0.4713721	0.7445223	0.6675027	1	TRAPPC3L
[truncated: 462,930 more chars]
